# Expression-based segmentation of the Drosophila genome

**DOI:** 10.1186/1471-2164-14-812

**Published:** 2013-11-20

**Authors:** Alan F Rubin, Phil Green

**Affiliations:** 1 Department of Genome Sciences, University of Washington, Seattle, WA 98195, USA

## Abstract

**Background:**

It is generally accepted that gene order in eukaryotes is nonrandom, with adjacent genes often sharing expression patterns across tissues, and that this organization may be important for gene regulation. Here we describe a novel method, based on an explicit probability model instead of correlation analysis, for identifying coordinately expressed gene clusters (‘coexpression segments’), apply it to *Drosophila melanogaster*, and look for epigenetic associations using publicly available data.

**Results:**

We find that two-thirds of *Drosophila* genes fall into multigenic coexpression segments, and that such segments are of two main types, housekeeping and tissue-restricted. Consistent with correlation-based studies, we find that adjacent genes within the same segment tend to be physically closer to each other than to the adjacent genes in different segments, and that tissue-restricted segments are enriched for testis-expressed genes. Our segmentation pattern correlates with Hi-C based physical interaction domains, but segments are generally much smaller than domains. Intersegment regions (including those which do not correspond to physical domain boundaries) are enriched for insulator binding sites.

**Conclusions:**

We describe a novel approach for identifying coexpression clusters that does not require arbitrary cutoff values or heuristics, and find that coexpression of adjacent genes is widespread in the *Drosophila* genome. Coexpression segments appear to reflect a level of regulatory organization related to, but below that of physical interaction domains, and depending in part on insulator binding.

## Background

Many factors contribute to genome organization, but one feature seen broadly across eukaryotes is that genes with similar patterns of expression often are physically clustered [1,2]. The *S. cerevisiae* genome is enriched for pairs and triplets of coexpressed genes, which also often have shared function [3-5]. Essential genes also form clusters in yeast, independently of coexpression clustering [6]. The ordering of coexpressed genes and essential genes in yeast is conserved over large evolutionary distances [6-8]. *Arabidopsis thaliana* also shows evidence of clustering by expression and by function [9,10], but unlike in yeast, *Arabidopsis* clusters can be quite large, including up to 20 genes [10], and up to 10% of *Arabidopsis* genes belong to such clusters [11]. The nematode *C. elegans* has small coexpression clusters of 2–5 genes [12,13] that are not attributable to operons [14]. Unlike other eukaryotes studied, tandem duplicates are heavily represented in *C. elegans* expression clusters [12].

Initial analyses in *Drosophila* described clusters of three or more tissue-specific genes, particularly for testis [15], and large domains of 10–30 coordinately expressed genes [16]. Subsequent statistical analyses indicate that the large domains are actually artifactual aggregates of smaller coexpression clusters, comprised of housekeeping genes and functionally coordinated genes [17], and experiments measuring the effect of chromosomal rearrangements that disrupt the large domains did not support the idea that they are important for controlling gene expression [18]. Evidence for conservation of expression clusters across *Drosophila* species is mixed. Genes within syntenic blocks are more likely to have correlated expression than expected by chance [19], and some regions show evidence of coevolution of expression [20]. However, other studies associate short intergenic distance and coexpression with higher rates of genomic rearrangement [17].

In mammals, housekeeping genes form clusters [21,22], as do low-expression genes that are inactive in most tissues [23]. There is evidence of clustering of testis-specific genes in mouse [24]. In contrast to yeast, there is little evidence for clustering based on gene function in mammals [22]. A screen for mouse essential genes showed that they are enriched in certain chromosomal regions [25], although it is unclear if the genes in these clusters are coordinately expressed. Vertebrate coexpression clusters are thought to arise gradually over evolutionary time, and some are conserved between human and chicken [26], and human and mouse [27]. Clusters that include highly expressed genes are not more likely to be conserved than expected by chance [27], and linkage between highly expressed genes may in fact be deleterious [28].

Functionally coordinated gene clusters, which often overlap with coexpression clusters, are not conserved across eukaryotes, and the genes and functions that cluster differ widely across the species studied [29,30].

The appreciation that genome location affects expression dates back to observations of differential expression of transgene insertions [31], but the mechanisms that maintain coexpression clusters remain unknown. Proposed mechanisms include LCR-mediated activity such as in the β-globin locus [32], sharing of proximal regulatory features [33], or regional enhancers [34]. Analyses in several species have shown that adjacent coexpressed genes tend to be physically closer than the average [7,8,10,35-37], but it is not known if this is required for coexpression. Insulator proteins are thought to help separate genomic regions into domains of activity or inactivity governed by long-range regulatory elements that affect many genes [38]. The insulator protein CTCF has been implicated in the creation and maintenance of chromatin loop domains [39]. Other experiments associate localization to the nuclear pore with increased expression [40], or proximity to the nuclear lamina with repression of transcription [41]. Recent advances in chromatin conformation capture and other methods for interrogating the three-dimensional structure of the nucleus allow characterization of physical contacts between genomic regions [42-46]. These studies provide evidence for interactions among neighboring genes, which may be related to gene coexpression.

Here we describe a novel method for identifying coexpression clusters and apply it to *Drosophila* expression data from a diverse set of tissues. In contrast to previous studies, we use an explicit probability model for segment-dependent gene expression that allows us to find a best-fitting partition of the genome into contiguous segments of coordinately expressed genes. Our approach avoids prior assumptions about segment size, the magnitude of coexpression effects, or other heuristics, and is based on parameters with natural mechanistic interpretations. We identify widespread small clusters of coexpressed genes and explore their properties. In particular we provide evidence for an association with physical interaction domains (contiguous regions that are enriched for internal chromatin contacts) [46] and insulator binding sites.

## Results and discussion

### Expression model

Previous work using correlation-based methods to identify clusters of coordinately expressed genes has had mixed success [4,10,16,47]. Correlation-based results are strongly affected by the choice of arbitrary cutoffs that may over- or under-estimate coexpression and may lead to artifactual clustering [16-18]. We instead use an approach that is based on an explicit probability model for the observed expression data. The model assumes that the genome can be partitioned into contiguous groups of genes (coexpression segments) such that the genes within a segment tend to have similar expression levels across tissues. Specifically, the (tissue dependent) expression value for a gene in a given segment is assumed to be the sum of a segment effect, which represents a regional effect on expression in a given tissue that influences all genes in the segment equally and represents shared regulation, and a gene-specific deviation, which reflects private regulation and ‘noise’ (stochastic or measurement). A segment may consist of one or many genes. We performed our analyses using microarray data, taking the steady-state mRNA abundance measured by these arrays as a proxy for transcriptional activity, however our method is easily adaptable to data from other technologies. Model details, our procedure for finding an optimal segmentation of the genome, and analyses confirming that our approach recovers the correct segmentation from simulated data are described in Methods.

### Properties of Drosophila expression segments

**Figure 1:**
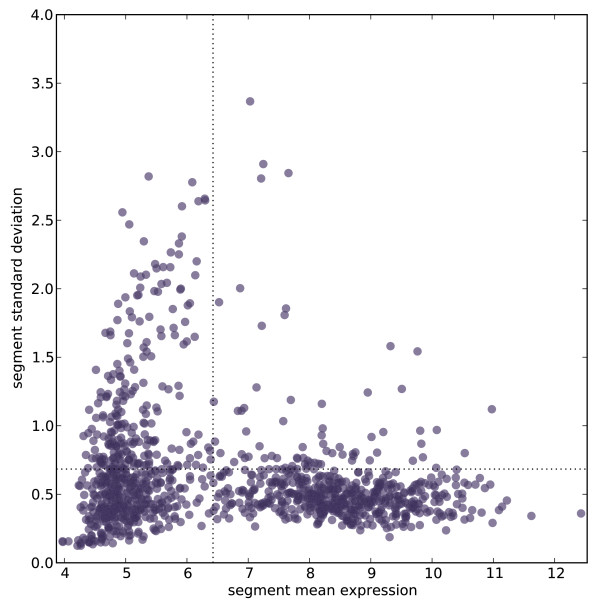
**Scatterplot of across-tissue mean expression vs. across-tissue standard deviation reveals two classes of segments.** Segments with three or more genes are plotted. The tissue-specific expression value of a segment is taken to be the average of its component genes’ values; the mean and standard deviation across tissues of each segment’s values are the coordinates for the plotted point. The horizontal dotted line denotes the cutoff for the top quartile of segments by standard deviation. The vertical dotted line denotes the median gene expression value across all genes and tissues. Segments close to the X-axis have similar expression values for every tissue; those close to the Y-axis have high expression in a minority of tissues.

We analyzed expression data from *Drosophila melanogaster* generated by the FlyAtlas project [48]. The FlyAtlas dataset samples 32 diverse tissues, of which we analyzed 27 after quality filtering, and 11363 genes. Optimal segmentation identification is reasonably robust (see Methods), and we chose the best scoring segmentation for followup analysis. Roughly two thirds of genes fall into multigene segments and thus appear to have coordinated expression with their neighbors across tissues (Additional file 1). Multigenic segments have a mean of 3.1 genes (median 2.0) (Additional file 2: Figure S1A). To examine across-tissue expression patterns, we plotted the across-tissue means and standard deviations of segments with three or more genes. These segments cluster into two classes: one having low mean and high standard deviation (indicating highly variable expression across tissues), and the other having low standard deviation (suggesting “housekeeping” style expression) (Figure 1). The 302 segments in the top quartile for standard deviation value tend to have highly tissue-restricted expression patterns, with mean expression that exceeds the dataset median in only a small number of tissues. For 119 of these, expression is restricted in this sense to a single tissue, and for 106 of the 119, the tissue is testis. This supports previous studies in *Drosophila* and mouse showing that testis-expressed genes often form coexpression clusters [15,24]. In contrast, segments expressed in non-testis tissues are often expressed in at least one other tissue (Additional file 2: Figure S2).

To identify segments with shared function as well as coexpression, we tested each segment for significant enrichment of GO Slim categories associated to its genes. Enriched segments are uncommon but more frequent than expected by chance (based on comparison to shuffled segmentation patterns) with 209 of 2442 multigenic segments having a significantly enriched term (P = 0.00324) (Additional file 3: Table S2).

We then looked for features that may illuminate mechanisms for the formation and maintenance of coexpression segments. Intergenic regions between segments are longer than intergenic regions within segments (P = 1.39e-24 by Kolmogorov-Smirnov test) (Additional file 2: Figure S1B and C). This length difference is consistent with previous work on coexpressed genes in *Drosophila* and other organisms [7,8,10,35-37]. We verified that it is independent of repeats in the intergenic regions (Additional file 2: Figure S1D and E). This suggests that (perhaps not surprisingly) the mechanisms involved in establishing or maintaining coexpression may be less effective over longer distances.

We analyzed gene orientation for adjacent pairs of genes and found that two-gene segments are enriched for “head-to-head” gene pairs, which may be regulated by a bidirectional promoter [49], relative to pairs flanking intersegment regions (P = 1.24e-6) or adjacent pairs within longer (three or more gene) segments (P = 0.0021). 31.6% (390/1236) of all two-gene segments have head-to-head orientation, and 48.8% (1521/3118) of all head-to-head pairs lie within a segment, indicating that while head-to-head orientation may facilitate coexpression, it is neither required nor diagnostic (Additional file 3: Table S3).

Physical interaction domains [46] represent an intriguing candidate mechanism for coexpression regulation. We find a highly significant sharing (P = 2.28e-20) of segment and interaction domain endpoints, with 60.8% (571/939) of interaction domain endpoints also being segment endpoints. However, segments are much smaller than interaction domains (mean sizes 1.8 genes vs. 10.3 genes) and only 49 interaction domains consist of a single segment (not significant, P = 0.248).

**Figure 2:**
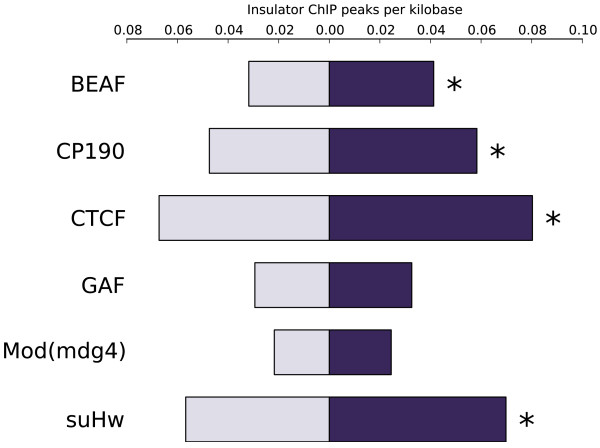
**Insulators are enriched in intersegment regions that do not contain physical domain boundaries.** Insulator ChIP peaks per kilobase in intersegment regions adjacent to long (3+ gene) segments that do not contain a physical interaction domain boundary (dark bars) vs. the rest of the genome (light bars). Asterisk indicates significant enrichment (Bonferroni-corrected P < 0.05) see (Additional file 3: Table S4).

Insulators may play a role in establishing interaction domain boundaries [46]. However, many insulator binding sites do not lie at interaction domain boundaries. We investigated the possibility that insulators may play a broader role in defining segments, using insulator ChIP-seq peak data generated by Nègre *et al.*[50]. We first confirmed that (consistent with the results of Sexton *et al.*) peaks for BEAF-32, CP190, CTCF, GAF, and Mod(mdg4) are significantly enriched (per kilobase) in intersegment regions that do include a physical interaction domain boundary (by enrichment factors of 1.83, 1.54, 1.69, 1.54, and 1.42 respectively), and that this enrichment disappears after masking those peaks that overlap the 2 kb windows centered on interaction domain boundaries as identified by Sexton *et al.* (Additional file 3: Table S4). We then investigated intersegment regions that do not contain an interaction domain boundary. In the set of all such regions, we see no significant enrichment for insulator peaks. However, if we restrict to intersegment regions adjacent to long (three or more gene) segments, we find that the insulators BEAF-32, CP190, CTCF, and Su(Hw) are significantly enriched by factors of 1.30, 1.23, 1.19, and 1.23 respectively (Figure 2) (Additional file 3: Table S4), as compared to the rest of the genome (excluding peaks overlapping the interaction domain windows). Thus it appears that insulators may play a role in defining coexpression segments, beyond their association with physical interaction domains. We also looked for insulator enrichment in intersegment regions adjacent to highly tissue-restricted segments, and found that only Su(Hw) is significantly enriched (by a factor of 1.21 (Additional file 3: Table S4)), consistent with previous findings that Su(Hw) binds in regions where transcription is repressed in most tissues [51].

Finally, we used logistic regression to investigate whether the association between interaction domain and expression segment boundaries is entirely mediated by known insulators. Using a model with interaction domain boundary presence/absence (in a given region) as the dependent variable, and insulator peak counts of various types, region length, and presence/absence of an expression segment boundary as predictors, we find that segment boundaries have significant power to predict physical domain boundaries beyond what can be explained by insulator peak data and region length (Additional file 3: Table S5). This suggests that unknown additional factors are involved in defining both expression segment and interaction domain boundaries.

## Conclusions

We developed a novel method, based on an explicit probability model, for identifying coexpression clusters that in contrast to previous approaches does not rely on arbitrary cutoffs or heuristics. We find that two thirds of *Drosophila* genes fall into multigene coexpression segments, that these segments are of two broad types, housekeeping and tissue restricted, and that clustering of genes expressed in a single tissue is largely confined to testis genes.

Adjacent genes within segments are physically closer to one another than adjacent genes in different segments. Our segmentation pattern is correlated with physical interaction domains [46] and with insulator binding, suggesting that coexpression segments may represent substructure within the interaction domains, and that they may be in part determined by insulator binding. Since coexpression segments are determined from expression data across diverse tissues, their association with physical interaction domains suggests that aspects of the domain structure may be shared between tissues. Although our analyses were confined to *Drosophila*, the observation that coexpression clusters across many eukaryotes tend to have similar properties [2] suggests that an association with insulator binding and physical interaction domains may hold more broadly.

## Methods

### Data sources

Gene models were downloaded from Ensembl release 66 [52] and genomic sequence from dmel release 5 [53]. We performed an all-by-all BLATP search of annotated proteins in FlyBase 5.39 [53] to identify candidate paralogs, and found that 167 genes in the expression dataset have high identity (defined as greater than 50% amino acid identity over a 50 amino acid stretch) with another gene on the same chromosome, comprising 1.5% of all genes. No paralogs were removed from the analysis. Repeats were annotated using RepeatMasker [54].

Raw expression data were downloaded from NCBI GEO accession GSE7763 [48] in CEL format and normalized using RMA [55]. Probes were mapped to genes using the *drosophila*2.db annotation package in Bioconductor [56]. Genes with multiple probesets were assigned a single expression value by taking the median value for the probesets assigned to that gene. We computed the Pearson correlation across genes for each pair of tissue biological replicates and eliminated from further analysis any tissue for which this correlation was less than 0.98 for any biological replicate pair (Additional file 3: Table S6). Tissue-specific expression values for each gene were taken to be the mean of the four biological replicate measurements.

Gene ordering for purposes of assigning to segments or determining gene adjacency was based on the annotated gene transcription start coordinates. The intergenic region between two adjacent genes is defined as the region between their annotated start coordinates; the intersegment region between adjacent segments is the intergenic region between the genes at the proximal segment ends.

### Model implementation

The probability model calculations were implemented as a custom C program that uses parts of the Gnu Scientific Library [57], the R Math Library [58], Argtable2 [59], LibDS [60], Bzip2 [61], and Jansson [62]. Programs for visualization and analysis of the segmentation patterns were implemented in Python and R. Software is available from A. R. by request.

### Model details

Our probability model for expression values involves, for each tissue type, a distribution *f*(s) of segment effects, and a distribution *g*(s) of gene-specific deviations. The expression values we use are normalized microarray fluorescence intensities, but could in principle be derived from RNA-seq or other quantitative assays. The probability for a given segment’s expression data in a single tissue is then:

$$\int_{-\infty }^{\infty }\left(\overset{n}{\underset{i\;=1}{\prod }}\;g\;\left(x_{i}\;-\;s\right)\right)\;f\left(s\right)\text{ds}$$

where the *x*_*i*_ are the tissue-specific expression values for the *n* genes in the segment. We take *f* to be a mixture of two normal distributions, which provides a good fit to gene expression values over all tissues (Additional file 2: Figure S3), and *g* to be a normal distribution with mean 0:

$$\begin{array}{l} f\left(s\right)=\phi N\left(s;\mu_{1},\sigma_{1}\right)+\left(1-\phi \right)N\left(s;\mu_{2},\sigma_{2}\right)\\ \;g\left(x\right)=N\left(x;0,\sigma \right) \end{array}$$

We assume independence of tissues and of segments, so the overall likelihood of a segmentation is a product of probabilities across tissues and segments.

The score (based on BIC) for a segmentation is the log likelihood modified by a parameter penalty that scales with the number of segments [63]:

$$\text{score}=-2\;\ln\left(P\left(x\left|\theta \right)\right)\right)+K\;\text{ln}\left(n\right)$$

where *x* is the set of observed expression values, θ is the set of parameters for *f* and *g*, *K* is the number of estimated parameters (lengths of all segments, and distribution parameters), and *n* is the number of data points in the expression dataset (genes by tissues).

### Model estimation

Finding a best-fitting genome segmentation model for a given expression dataset is challenging, because it requires in principle searching the Cartesian product of the space of all possible genome partitions into segments with the space of parameters for the distributions *f* and *g*. We structure this as a search of the parameter space (carried out using the Simplex algorithm as implemented in the GNU Scientific Library [57]), in which the score associated to each set of parameter values is computed by optimizing over segmentations. Each chromosome arm is analyzed separately. For particular values of *f* and *g* parameters, we search the segmentation space as follows. First, we partition the chromosome arm into segments of random lengths (*i.e.* number of genes), drawn from a geometric distribution having (by default) a mean of 2 genes (in practice, the choice of mean has a negligible impact on the segmentation patterns that the model converges to). We then consider three possible types of “move”: split, which divides a multigenic segment into two segments; merge, which combines two adjacent segments into a single segment; and shift, which changes the boundary between two existing segments by expanding one and shrinking the other, such that at least one gene remains in each segment. Given a segmentation pattern, we evaluate each possible move and select the one that gives the greatest score improvement. The process is iterated until a segmentation is reached for which no moves improve the score. Because this search is strictly downhill, we consider multiple random initial segmentations ("replicates"), generally 1024, and carry out the above search for each of them. This yields a set of 1024 “locally optimal” segmentations; the median of their scores is then taken as the score value assigned to the specified parameter values for purposes of the parameter space search. Our analysis software also supports using the best replicate score or mean replicate score, but exploratory analyses indicated the median gave the most robust results. The best-scoring segmentation that is found with the best-scoring analysis parameters is used for subsequent analysis.

For convenience and computational speed, we made several simplifying assumptions regarding *f* and *g.* First, we assume that a single *f* and a single *g* (per chromosome arm) apply to all tissues, i.e. we do not allow tissue dependent parameter values. Second, we assume that *f* may be estimated as the mixture of normals that best fits the observed distribution of gene expression values over all tissues (Additional file 2: Figure S3). This *f* is found using an EM algorithm implemented in the PyMix package [64], and is fixed during subsequent analysis. Thus only the parameter σ that defines the distribution of gene deviations *g* is estimated iteratively.

### Simulations

We tested our analysis method by simulating 40 datasets each with 2000 genes and 27 tissues (comparable to the FlyAtlas [48] data for a single chromosome arm), using similar distribution parameters to those trained from the real data, and analyzing each simulated dataset. Because each chromosome arm is analyzed independently in our real-data analyses, our simulated datasets each consist of a single simulated chromosome arm. To simulate a dataset with a given number of genes and tissues, we first simulate a segmentation by drawing segment lengths (*i.e.* number of genes) randomly from a geometric distribution with a specified mean until all genes have been assigned. We then simulate expression data for each tissue that conforms to the assumptions of our probability model for a specific choice of *f* and *g*, as follows. For each segment and tissue, a segment effect is drawn randomly from *f*, and for each gene in that segment, a gene-specific deviation is drawn from *g*. These are added to get the gene expression value. For simulations where *g* varies across segments and tissues, an independent σ is drawn for each draw from *f*. Our analysis of simulated datasets used 512 replicates (starting random segmentations) per round of parameter training. In all datasets, the parameter training converged to within 2% of the correct value, and the ‘true’ segmentation pattern used in the simulation was recovered exactly regardless of random starting segmentation. We also simulated data for alternative parameter sets, and found that it is robust to most parameter choices (Additional file 3: Table S1).

### Robustness of real data estimates

The spread of replicate scores for the optimal parameter values for each chromosome arm is much wider for the real data than for the simulated data (Additional file 2: Figure S4), and the best scoring segmentation is only found in one replicate for each chromosome arm. This suggests that the score surface for the real data is more complex than that for the simulated data (where the best scoring segmentation was found repeatedly). However, we find that the best-scoring replicates share 94.3% of their intersegment regions with the second-best replicates, and 91.7% of their intersegment regions with the worst-scoring replicates. Moreover, 84% of segments found in at least one replicate appear in more than half of the replicates. Thus, despite some variability in exact segmentation and score, the replicates are highly similar, implying that our method is reasonably robust to the choice of starting segmentation and that for most of the genome our model finds the same local segmentation regardless of the starting pattern.

### Other analysis procedures

For the promoter-orientation analysis, we counted the number of adjacent gene pairs in the dataset with the same orientation, “head-to-head” opposite orientation, or “tail-to-tail” opposite orientation for three classes: pairs within two-gene segments, pairs within larger multigenic segments, and intersegment pairs. P-values for comparing two classes were calculated using a 2×3 Chi-squared test.

Nègre *et al.*’s [50] insulator peaks were converted from dm3 to dm5 using FlyBase’s coordinate conversion tool [53]. We removed 571 (of a total of 35365) insulator peaks (1.6%) that could not be converted to dm5 coordinates due to assembly incompatibilities. We counted the number of ChIP peaks that overlap regions of a given type using BEDTools [65] and Pybedtools [66], and converted these to peaks per kilobase by dividing by the total size of the regions. Enrichment was calculated as the ratio of the peaks per kilobase values for two specified region types. Significance for comparing two sets of regions was determined by Fisher’s exact test, for a 2×2 table in which the first cell in each row gives the number of peaks overlapping regions of the given type, and the second cell gives the number of “non-peaks” of the same size as peaks, defined as the number of bases in the regions minus the number of peaks, divided by the average peak size. For analyses of intersegment regions for a particular type of segment (e.g. multigenic segments), we consider regions that border a segment of that type as belonging to the analyzed set. Some analyses exclude the subset of peaks that overlaps the 2 kb windows identified by Sexton *et al.*[46] as marking interaction domain boundaries.

Sexton *et al.*’s [46] physical interaction domain coordinates were converted from dm3 to dm5 using FlyBase’s coordinate conversion tool [53]. We removed 12 domains that could not be converted to dm5 coordinates due to assembly incompatibilities. Genes were assigned to interaction domains based on the position of their annotated start site. We tested for significant sharing of endpoints between coexpression segments and interaction domains by performing a Fisher’s exact test on the 2×2 table with cell counts giving the number of intergenic regions (*i.e.* regions between the starts of adjacent genes) that are: (i) segment endpoint and interaction domain endpoint, (ii) segment endpoint only, (iii) interaction domain endpoint only, or (iv) neither segment nor interaction domain endpoint.

Our gene Ontology analysis used generic GO Slim [67]. GO term enrichments were calculated using goatools [68]. Segments in which only one gene was annotated with the enriched term were removed from the list of significant results for that term. We compared the number of segments with one or more enriched terms to the number of segments with one or more enriched terms in 10 shuffled segmentation patterns using Fisher’s exact test. Shuffled segmentations were generated by preserving chromosome gene order while randomly permuting the list of segment lengths and requiring that no segment endpoints were shared between the random segmentation and the real segmentation.

## Competing interests

The authors declare that they have no competing interests.

## Authors’ contributions

AFR and PG designed the study. AFR compiled the data and performed the analysis. AFR and PG wrote the manuscript. Both authors read and approved the final manuscript.

## Supplementary Material

Additional file 1 Detailed information for multigene segments. Click here for file

Additional file 2 Supplemental figures. Click here for file

Additional file 3 Supplemental tables. Click here for file
