# Supplementary material for: Expression-based segmentation of the Drosophila genome
Source: BMC Genomics. 2013 Nov 20;14:812. doi: 10.1186/1471-2164-14-812 (PMC3909303; doi:10.1186/1471-2164-14-812)
Supplement: Additional file 1 — Detailed information for multigene segments. [file 1471-2164-14-812-S1.zip › miniwebsite/chr2L.html]

   ExprSeg Report for chr2L   
 Report for /Users/afrubin/Code/ExprSeg/2012-07-19/output Generated Fri Jun 21 22:59:16 2013 
   Chromosome 2L 
 462 segments 2091 genes 
    Segment 1 
 
   Location   
  Gene key  FBgn0031208-FBgn0051973  
  Heatmap region span   2L:7529..116488   
  Segment span   2L:7529..59242   
  Length (genes)  3  
  Length (bp)  51714  
   Model Scoring   
  BIC  322.427832  
  logL  -155.743299  
  logL ratio  20.384971  
   Expression   
  Mean expression  4.595051  
  Median expression  4.149867  
  Tissue std. dev.  0.560015  
 
  No GO Slim enrichment  
  
   tissue    mean expression   
  5th Passage Drosophila S2 Cells  4.073862  
  Adult Accessory gland  5.230095  
  Adult Brain  4.793694  
  Adult Carcass  4.661796  
  Adult Crop  4.182575  
  Adult Eye  4.893451  
  Adult Fatbody  4.166028  
  Adult Female Spermatheca Mated  4.490786  
  Adult Female Spermatheca Virgin  4.701402  
  Adult Head  4.462715  
  Adult Heart  4.343686  
  Adult Hind Gut  4.205013  
  Adult Male Ejaculatory Duct  4.331499  
  Adult Mid Gut  4.063496  
  Adult Ovary  4.122511  
  Adult Salivary Gland  5.100954  
  Adult Testes  6.529905  
  Adult Thoracoabdominal ganglion  4.869785  
  Adult Whole Fly  4.604792  
  Larvae Wandering Tubules  3.951931  
  Larval Feeding Carcass  5.021301  
  Larval Feeding Central Nevous System  4.562394  
  Larval Feeding Hind Gut  4.166118  
  Larval Feeding Malpighian Tubule  4.028730  
  Larval Feeding Mid Gut  4.172687  
  Larval Feeding Salivary Gland  5.649800  
  Whole Larvae Feeding  4.685377  
 
  
   FlyBase ID    symbol    start    end    strand    length   
   FBgn0031208   CG11023   7529   9484  +  1956  
   FBgn0031209   Ir21a  21919   25151   -  3233  
   FBgn0051973   Cda5  25402   59242   -  33841  
 
 
    Segment 2 
 
   Location   
  Gene key  FBgn0067779-FBgn0031213  
  Heatmap region span   2L:7529..121974   
  Segment span   2L:67044..72388   
  Length (genes)  2  
  Length (bp)  5345  
   Model Scoring   
  BIC  283.175923  
  logL  -136.117344  
  logL ratio  2.357249  
   Expression   
  Mean expression  9.553720  
  Median expression  9.374881  
  Tissue std. dev.  0.491869  
 
  No GO Slim enrichment  
  
   tissue    mean expression   
  5th Passage Drosophila S2 Cells  9.821174  
  Adult Accessory gland  9.374881  
  Adult Brain  9.964306  
  Adult Carcass  9.499892  
  Adult Crop  9.886299  
  Adult Eye  9.594875  
  Adult Fatbody  9.638777  
  Adult Female Spermatheca Mated  9.864188  
  Adult Female Spermatheca Virgin  9.736629  
  Adult Head  9.407912  
  Adult Heart  9.637277  
  Adult Hind Gut  9.656747  
  Adult Male Ejaculatory Duct  10.092087  
  Adult Mid Gut  9.202090  
  Adult Ovary  10.449277  
  Adult Salivary Gland  9.589094  
  Adult Testes  7.508496  
  Adult Thoracoabdominal ganglion  9.940870  
  Adult Whole Fly  9.327381  
  Larvae Wandering Tubules  9.455534  
  Larval Feeding Carcass  9.447596  
  Larval Feeding Central Nevous System  9.762879  
  Larval Feeding Hind Gut  9.565405  
  Larval Feeding Malpighian Tubule  9.310536  
  Larval Feeding Mid Gut  9.259209  
  Larval Feeding Salivary Gland  9.748915  
  Whole Larvae Feeding  9.208120  
 
  
   FlyBase ID    symbol    start    end    strand    length   
   FBgn0067779   dbr   67044   71390  +  4347  
   FBgn0031213   galectin   72388   76211  +  3824  
 
    Segment 3 
 
   Location   
  Gene key  FBgn0031214-FBgn0002931  
  Heatmap region span   2L:7529..132060   
  Segment span   2L:76446..87382   
  Length (genes)  2  
  Length (bp)  10937  
   Model Scoring   
  BIC  219.766206  
  logL  -104.412486  
  logL ratio  2.256944  
   Expression   
  Mean expression  5.145244  
  Median expression  4.744390  
  Tissue std. dev.  0.823580  
 
  No GO Slim enrichment  
  
   tissue    mean expression   
  5th Passage Drosophila S2 Cells  5.318461  
  Adult Accessory gland  4.057325  
  Adult Brain  4.458793  
  Adult Carcass  5.211924  
  Adult Crop  4.970226  
  Adult Eye  4.530187  
  Adult Fatbody  4.823519  
  Adult Female Spermatheca Mated  4.873949  
  Adult Female Spermatheca Virgin  4.250941  
  Adult Head  5.035920  
  Adult Heart  5.268010  
  Adult Hind Gut  5.886997  
  Adult Male Ejaculatory Duct  4.810363  
  Adult Mid Gut  4.779714  
  Adult Ovary  4.134435  
  Adult Salivary Gland  7.466068  
  Adult Testes  5.880294  
  Adult Thoracoabdominal ganglion  4.693625  
  Adult Whole Fly  4.474689  
  Larvae Wandering Tubules  4.727983  
  Larval Feeding Carcass  5.346643  
  Larval Feeding Central Nevous System  5.212768  
  Larval Feeding Hind Gut  5.747898  
  Larval Feeding Malpighian Tubule  5.124736  
  Larval Feeding Mid Gut  5.056874  
  Larval Feeding Salivary Gland  7.639860  
  Whole Larvae Feeding  5.139390  
 
  
   FlyBase ID    symbol    start    end    strand    length   
   FBgn0031214   CG11374   76446   77639  +  1194  
   FBgn0002931   net  82456   87382   -  4927  
 
    Segment 4 
 
   Location   
  Gene key  FBgn0031216-FBgn0026787  
  Heatmap region span   2L:7529..140992   
  Segment span   2L:94752..106718   
  Length (genes)  3  
  Length (bp)  11967  
   Model Scoring   
  BIC  318.570621  
  logL  -153.814693  
  logL ratio  87.607403  
   Expression   
  Mean expression  9.287209  
  Median expression  9.241844  
  Tissue std. dev.  0.640046  
 
  No GO Slim enrichment  
  
   tissue    mean expression   
  5th Passage Drosophila S2 Cells  9.910988  
  Adult Accessory gland  10.363881  
  Adult Brain  9.665101  
  Adult Carcass  8.860069  
  Adult Crop  9.091488  
  Adult Eye  8.568766  
  Adult Fatbody  9.464724  
  Adult Female Spermatheca Mated  9.267955  
  Adult Female Spermatheca Virgin  9.220559  
  Adult Head  8.765862  
  Adult Heart  9.822928  
  Adult Hind Gut  9.076261  
  Adult Male Ejaculatory Duct  10.046375  
  Adult Mid Gut  9.151183  
  Adult Ovary  9.783283  
  Adult Salivary Gland  10.092534  
  Adult Testes  7.822687  
  Adult Thoracoabdominal ganglion  9.660073  
  Adult Whole Fly  8.633064  
  Larvae Wandering Tubules  9.362755  
  Larval Feeding Carcass  8.403112  
  Larval Feeding Central Nevous System  9.823348  
  Larval Feeding Hind Gut  9.010598  
  Larval Feeding Malpighian Tubule  8.985183  
  Larval Feeding Mid Gut  8.928229  
  Larval Feeding Salivary Gland  10.568680  
  Whole Larvae Feeding  8.404968  
 
  
   FlyBase ID    symbol    start    end    strand    length   
   FBgn0031216   Zir   94752   102086  +  7335  
   FBgn0031217   CG11377   102382   104142  +  1761  
   FBgn0026787   Nhe1  103962   106718   -  2757  
 
 
    Segment 5 
 
   Location   
  Gene key  FBgn0025683-FBgn0001142  
  Heatmap region span   2L:76446..158664   
  Segment span   2L:130791..132060   
  Length (genes)  2  
  Length (bp)  1270  
   Model Scoring   
  BIC  309.857817  
  logL  -149.458291  
  logL ratio  41.654353  
   Expression   
  Mean expression  11.263287  
  Median expression  11.678170  
  Tissue std. dev.  1.204378  
 
  No GO Slim enrichment  
  
   tissue    mean expression   
  5th Passage Drosophila S2 Cells  10.663854  
  Adult Accessory gland  7.616996  
  Adult Brain  11.128366  
  Adult Carcass  11.956072  
  Adult Crop  12.581430  
  Adult Eye  11.056287  
  Adult Fatbody  12.570021  
  Adult Female Spermatheca Mated  12.388617  
  Adult Female Spermatheca Virgin  12.282736  
  Adult Head  12.266582  
  Adult Heart  12.327188  
  Adult Hind Gut  11.079239  
  Adult Male Ejaculatory Duct  11.659224  
  Adult Mid Gut  11.441564  
  Adult Ovary  11.029692  
  Adult Salivary Gland  10.023391  
  Adult Testes  8.184402  
  Adult Thoracoabdominal ganglion  11.298463  
  Adult Whole Fly  11.600569  
  Larvae Wandering Tubules  12.760307  
  Larval Feeding Carcass  11.732630  
  Larval Feeding Central Nevous System  10.843543  
  Larval Feeding Hind Gut  10.466135  
  Larval Feeding Malpighian Tubule  12.131882  
  Larval Feeding Mid Gut  11.415705  
  Larval Feeding Salivary Gland  10.029270  
  Whole Larvae Feeding  11.574585  
 
  
   FlyBase ID    symbol    start    end    strand    length   
   FBgn0025683   CG3164  122624   130791   -  8168  
   FBgn0001142   Gs1   132060   134472  +  2413  
 
    Segment 6 
 
   Location   
  Gene key  FBgn0051975-FBgn0051976  
  Heatmap region span   2L:94752..203784   
  Segment span   2L:140992..140992   
  Length (genes)  2  
  Length (bp)  1  
   Model Scoring   
  BIC  200.410876  
  logL  -94.734821  
  logL ratio  16.119717  
   Expression   
  Mean expression  4.948121  
  Median expression  4.652596  
  Tissue std. dev.  0.760043  
 
  No GO Slim enrichment  
  
   tissue    mean expression   
  5th Passage Drosophila S2 Cells  7.426474  
  Adult Accessory gland  4.588242  
  Adult Brain  4.355060  
  Adult Carcass  4.833779  
  Adult Crop  4.646261  
  Adult Eye  4.494865  
  Adult Fatbody  4.770221  
  Adult Female Spermatheca Mated  4.652394  
  Adult Female Spermatheca Virgin  4.869294  
  Adult Head  4.310861  
  Adult Heart  4.472953  
  Adult Hind Gut  5.135129  
  Adult Male Ejaculatory Duct  4.770558  
  Adult Mid Gut  6.160519  
  Adult Ovary  4.526209  
  Adult Salivary Gland  4.931557  
  Adult Testes  4.458880  
  Adult Thoracoabdominal ganglion  4.538018  
  Adult Whole Fly  4.443998  
  Larvae Wandering Tubules  6.208143  
  Larval Feeding Carcass  4.600197  
  Larval Feeding Central Nevous System  4.444579  
  Larval Feeding Hind Gut  4.651415  
  Larval Feeding Malpighian Tubule  6.747513  
  Larval Feeding Mid Gut  5.231587  
  Larval Feeding Salivary Gland  4.695448  
  Whole Larvae Feeding  4.635124  
 
  
   FlyBase ID    symbol    start    end    strand    length   
   FBgn0051975   CG31975  138384   140992   -  2609  
   FBgn0051976   CG31976  138384   140992   -  2609  
 
    Segment 7 
 
   Location   
  Gene key  FBgn0051974-FBgn0031224  
  Heatmap region span   2L:116488..207297   
  Segment span   2L:142978..143309   
  Length (genes)  2  
  Length (bp)  332  
   Model Scoring   
  BIC  271.695000  
  logL  -130.376883  
  logL ratio  6.931687  
   Expression   
  Mean expression  9.253720  
  Median expression  9.326251  
  Tissue std. dev.  1.112841  
 
  No GO Slim enrichment  
  
   tissue    mean expression   
  5th Passage Drosophila S2 Cells  9.437714  
  Adult Accessory gland  7.060529  
  Adult Brain  10.225020  
  Adult Carcass  9.757278  
  Adult Crop  7.750647  
  Adult Eye  9.215823  
  Adult Fatbody  10.134377  
  Adult Female Spermatheca Mated  10.104304  
  Adult Female Spermatheca Virgin  9.981813  
  Adult Head  9.544362  
  Adult Heart  10.489055  
  Adult Hind Gut  9.485660  
  Adult Male Ejaculatory Duct  7.706955  
  Adult Mid Gut  11.034511  
  Adult Ovary  8.031616  
  Adult Salivary Gland  10.502025  
  Adult Testes  7.043529  
  Adult Thoracoabdominal ganglion  10.125539  
  Adult Whole Fly  9.373265  
  Larvae Wandering Tubules  9.586039  
  Larval Feeding Carcass  7.441116  
  Larval Feeding Central Nevous System  8.226467  
  Larval Feeding Hind Gut  8.568719  
  Larval Feeding Malpighian Tubule  10.619768  
  Larval Feeding Mid Gut  9.870019  
  Larval Feeding Salivary Gland  9.284455  
  Whole Larvae Feeding  9.249831  
 
  
   FlyBase ID    symbol    start    end    strand    length   
   FBgn0051974   CG31974  141077   142978   -  1902  
   FBgn0031224   CG11454   143309   144227  +  919  
 
    Segment 8 
 
   Location   
  Gene key  FBgn0031227-FBgn0053635  
  Heatmap region span   2L:130791..232514   
  Segment span   2L:155321..158664   
  Length (genes)  3  
  Length (bp)  3344  
   Model Scoring   
  BIC  315.209484  
  logL  -152.134125  
  logL ratio  67.794089  
   Expression   
  Mean expression  7.902387  
  Median expression  8.048953  
  Tissue std. dev.  0.480987  
 
  No GO Slim enrichment  
  
   tissue    mean expression   
  5th Passage Drosophila S2 Cells  8.681515  
  Adult Accessory gland  8.748178  
  Adult Brain  7.974320  
  Adult Carcass  7.200754  
  Adult Crop  7.632527  
  Adult Eye  7.997549  
  Adult Fatbody  7.694133  
  Adult Female Spermatheca Mated  7.917093  
  Adult Female Spermatheca Virgin  7.791852  
  Adult Head  7.606402  
  Adult Heart  7.705255  
  Adult Hind Gut  7.402359  
  Adult Male Ejaculatory Duct  8.268022  
  Adult Mid Gut  7.766730  
  Adult Ovary  9.091756  
  Adult Salivary Gland  7.741808  
  Adult Testes  6.918391  
  Adult Thoracoabdominal ganglion  7.887489  
  Adult Whole Fly  7.732356  
  Larvae Wandering Tubules  7.900265  
  Larval Feeding Carcass  7.837342  
  Larval Feeding Central Nevous System  8.286791  
  Larval Feeding Hind Gut  7.716921  
  Larval Feeding Malpighian Tubule  7.875141  
  Larval Feeding Mid Gut  7.717591  
  Larval Feeding Salivary Gland  8.840259  
  Whole Larvae Feeding  7.431646  
 
  
   FlyBase ID    symbol    start    end    strand    length   
   FBgn0031227   CG3709  153539   155321   -  1783  
   FBgn0031229   CG3436   156029   157666  +  1638  
   FBgn0053635   CG33635  157836   158664   -  829  
 
 
    Segment 9 
 
   Location   
  Gene key  FBgn0016977-FBgn0031231  
  Heatmap region span   2L:140992..252331   
  Segment span   2L:159034..203784   
  Length (genes)  2  
  Length (bp)  44751  
   Model Scoring   
  BIC  229.123356  
  logL  -109.091061  
  logL ratio  54.174313  
   Expression   
  Mean expression  9.915073  
  Median expression  9.921741  
  Tissue std. dev.  0.531383  
 
  No GO Slim enrichment  
  
   tissue    mean expression   
  5th Passage Drosophila S2 Cells  10.138883  
  Adult Accessory gland  9.323909  
  Adult Brain  11.095550  
  Adult Carcass  9.642566  
  Adult Crop  10.088790  
  Adult Eye  10.322537  
  Adult Fatbody  10.054899  
  Adult Female Spermatheca Mated  9.719422  
  Adult Female Spermatheca Virgin  9.688526  
  Adult Head  10.040637  
  Adult Heart  10.010489  
  Adult Hind Gut  10.333957  
  Adult Male Ejaculatory Duct  9.916638  
  Adult Mid Gut  10.031811  
  Adult Ovary  9.644602  
  Adult Salivary Gland  9.614547  
  Adult Testes  7.947688  
  Adult Thoracoabdominal ganglion  10.587768  
  Adult Whole Fly  9.539870  
  Larvae Wandering Tubules  9.858687  
  Larval Feeding Carcass  9.904094  
  Larval Feeding Central Nevous System  10.575387  
  Larval Feeding Hind Gut  10.120615  
  Larval Feeding Malpighian Tubule  10.098969  
  Larval Feeding Mid Gut  9.837093  
  Larval Feeding Salivary Gland  9.949363  
  Whole Larvae Feeding  9.619686  
 
  
   FlyBase ID    symbol    start    end    strand    length   
   FBgn0016977   spen   159034   203251  +  44218  
   FBgn0031231   mRpL10   203784   204782  +  999  
 
    Segment 10 
 
   Location   
  Gene key  FBgn0086902-FBgn0022246  
  Heatmap region span   2L:159034..297882   
  Segment span   2L:250795..252331   
  Length (genes)  2  
  Length (bp)  1537  
   Model Scoring   
  BIC  275.424094  
  logL  -132.241430  
  logL ratio  -1.007456  
   Expression   
  Mean expression  8.924356  
  Median expression  8.977367  
  Tissue std. dev.  0.691002  
 
  No GO Slim enrichment  
  
   tissue    mean expression   
  5th Passage Drosophila S2 Cells  10.041379  
  Adult Accessory gland  9.254338  
  Adult Brain  9.639703  
  Adult Carcass  8.131491  
  Adult Crop  9.114171  
  Adult Eye  9.133589  
  Adult Fatbody  8.855839  
  Adult Female Spermatheca Mated  9.384075  
  Adult Female Spermatheca Virgin  9.315823  
  Adult Head  8.663801  
  Adult Heart  9.114580  
  Adult Hind Gut  8.651475  
  Adult Male Ejaculatory Duct  8.792699  
  Adult Mid Gut  8.177054  
  Adult Ovary  9.302434  
  Adult Salivary Gland  8.601199  
  Adult Testes  6.733197  
  Adult Thoracoabdominal ganglion  9.226432  
  Adult Whole Fly  8.312865  
  Larvae Wandering Tubules  9.058318  
  Larval Feeding Carcass  8.984231  
  Larval Feeding Central Nevous System  10.614503  
  Larval Feeding Hind Gut  9.005422  
  Larval Feeding Malpighian Tubule  8.801601  
  Larval Feeding Mid Gut  8.289787  
  Larval Feeding Salivary Gland  9.309690  
  Whole Larvae Feeding  8.447906  
 
  
   FlyBase ID    symbol    start    end    strand    length   
   FBgn0086902   kis  210732   250795   -  40064  
   FBgn0022246   Rpp30  251163   252331   -  1169  
 
    Segment 11 
 
   Location   
  Gene key  FBgn0031239-FBgn0031240  
  Heatmap region span   2L:207297..305941   
  Segment span   2L:266688..269088   
  Length (genes)  2  
  Length (bp)  2401  
   Model Scoring   
  BIC  214.357685  
  logL  -101.708225  
  logL ratio  8.778194  
   Expression   
  Mean expression  5.040356  
  Median expression  5.190271  
  Tissue std. dev.  0.845526  
 
  No GO Slim enrichment  
  
   tissue    mean expression   
  5th Passage Drosophila S2 Cells  4.928468  
  Adult Accessory gland  5.043698  
  Adult Brain  5.190271  
  Adult Carcass  4.767831  
  Adult Crop  4.800797  
  Adult Eye  4.642830  
  Adult Fatbody  4.771691  
  Adult Female Spermatheca Mated  4.755503  
  Adult Female Spermatheca Virgin  4.849861  
  Adult Head  4.640721  
  Adult Heart  4.634145  
  Adult Hind Gut  4.734367  
  Adult Male Ejaculatory Duct  4.732524  
  Adult Mid Gut  4.988361  
  Adult Ovary  4.604472  
  Adult Salivary Gland  4.964562  
  Adult Testes  8.983275  
  Adult Thoracoabdominal ganglion  4.741654  
  Adult Whole Fly  6.383788  
  Larvae Wandering Tubules  4.800473  
  Larval Feeding Carcass  4.798269  
  Larval Feeding Central Nevous System  4.599329  
  Larval Feeding Hind Gut  4.719279  
  Larval Feeding Malpighian Tubule  4.821286  
  Larval Feeding Mid Gut  4.909545  
  Larval Feeding Salivary Gland  4.879600  
  Whole Larvae Feeding  5.403016  
 
  
   FlyBase ID    symbol    start    end    strand    length   
   FBgn0031239   CG17075  263782   266688   -  2907  
   FBgn0031240   CG3345   269088   271626  +  2539  
 
    Segment 12 
 
   Location   
  Gene key  FBgn0031238-FBgn0031244  
  Heatmap region span   2L:207399..322101   
  Segment span   2L:271732..283281   
  Length (genes)  6  
  Length (bp)  11550  
   Model Scoring   
  BIC  548.810530  
  logL  -268.934648  
  logL ratio  218.970243  
   Expression   
  Mean expression  8.377097  
  Median expression  8.343855  
  Tissue std. dev.  0.473529  
 
  No GO Slim enrichment  
  
   tissue    mean expression   
  5th Passage Drosophila S2 Cells  8.962295  
  Adult Accessory gland  8.874888  
  Adult Brain  8.133190  
  Adult Carcass  7.922501  
  Adult Crop  8.694392  
  Adult Eye  8.145878  
  Adult Fatbody  8.528624  
  Adult Female Spermatheca Mated  8.636228  
  Adult Female Spermatheca Virgin  8.518773  
  Adult Head  7.935245  
  Adult Heart  8.361371  
  Adult Hind Gut  8.159141  
  Adult Male Ejaculatory Duct  8.330540  
  Adult Mid Gut  7.844049  
  Adult Ovary  9.872795  
  Adult Salivary Gland  8.575069  
  Adult Testes  7.647142  
  Adult Thoracoabdominal ganglion  7.992322  
  Adult Whole Fly  8.657243  
  Larvae Wandering Tubules  8.126420  
  Larval Feeding Carcass  8.375092  
  Larval Feeding Central Nevous System  9.087379  
  Larval Feeding Hind Gut  8.427337  
  Larval Feeding Malpighian Tubule  8.259750  
  Larval Feeding Mid Gut  7.662028  
  Larval Feeding Salivary Gland  8.563518  
  Whole Larvae Feeding  7.888418  
 
  
   FlyBase ID    symbol    start    end    strand    length   
   FBgn0031238   CG3645  252589   271732   -  19144  
   FBgn0086856   CG11555   273955   274766  +  812  
   FBgn0086912   mbm  271912   274096   -  2185  
   FBgn0086855   CG17078  274740   277212   -  2473  
   FBgn0003444   smo   277588   282167  +  4580  
   FBgn0031244   CG11601  281970   283281   -  1312  
 
 
    Segment 13 
 
   Location   
  Gene key  FBgn0031245-FBgn0020622  
  Heatmap region span   2L:250795..357868   
  Segment span   2L:291010..297882   
  Length (genes)  5  
  Length (bp)  6873  
   Model Scoring   
  BIC  591.932865  
  logL  -290.495815  
  logL ratio  120.328739  
   Expression   
  Mean expression  9.797740  
  Median expression  9.371961  
  Tissue std. dev.  0.362254  
 
  No GO Slim enrichment  
  
   tissue    mean expression   
  5th Passage Drosophila S2 Cells  9.909121  
  Adult Accessory gland  9.992531  
  Adult Brain  9.870570  
  Adult Carcass  9.322024  
  Adult Crop  9.692242  
  Adult Eye  9.744488  
  Adult Fatbody  9.938004  
  Adult Female Spermatheca Mated  9.638003  
  Adult Female Spermatheca Virgin  9.549631  
  Adult Head  9.631030  
  Adult Heart  10.134155  
  Adult Hind Gut  9.667612  
  Adult Male Ejaculatory Duct  9.261876  
  Adult Mid Gut  10.027664  
  Adult Ovary  9.880415  
  Adult Salivary Gland  8.539454  
  Adult Testes  9.957484  
  Adult Thoracoabdominal ganglion  9.806999  
  Adult Whole Fly  10.039403  
  Larvae Wandering Tubules  10.019317  
  Larval Feeding Carcass  9.753037  
  Larval Feeding Central Nevous System  10.568587  
  Larval Feeding Hind Gut  10.161823  
  Larval Feeding Malpighian Tubule  10.040789  
  Larval Feeding Mid Gut  10.048452  
  Larval Feeding Salivary Gland  9.535936  
  Whole Larvae Feeding  9.808332  
 
  
   FlyBase ID    symbol    start    end    strand    length   
   FBgn0031245   CG3625  283385   291010   -  7626  
   FBgn0031247   CG11562   292466   293222  +  757  
   FBgn0017457   U2af38  293337   294679   -  1343  
   FBgn0024352   Hop   295109   297413  +  2305  
   FBgn0020622   Pi3K21B   297882   303955  +  6074  
 
 
    Segment 14 
 
   Location   
  Gene key  FBgn0031248-FBgn0053127  
  Heatmap region span   2L:271732..415069   
  Segment span   2L:319254..322101   
  Length (genes)  3  
  Length (bp)  2848  
   Model Scoring   
  BIC  306.620742  
  logL  -147.839753  
  logL ratio  55.344044  
   Expression   
  Mean expression  6.187697  
  Median expression  5.035338  
  Tissue std. dev.  2.638561  
 
  No GO Slim enrichment  
  
   tissue    mean expression   
  5th Passage Drosophila S2 Cells  4.996261  
  Adult Accessory gland  5.096917  
  Adult Brain  4.683572  
  Adult Carcass  5.096057  
  Adult Crop  4.991412  
  Adult Eye  4.657232  
  Adult Fatbody  5.062266  
  Adult Female Spermatheca Mated  4.944608  
  Adult Female Spermatheca Virgin  4.992964  
  Adult Head  4.641309  
  Adult Heart  4.744963  
  Adult Hind Gut  6.919922  
  Adult Male Ejaculatory Duct  5.241000  
  Adult Mid Gut  13.334957  
  Adult Ovary  4.890152  
  Adult Salivary Gland  5.144769  
  Adult Testes  5.345224  
  Adult Thoracoabdominal ganglion  4.814658  
  Adult Whole Fly  10.358277  
  Larvae Wandering Tubules  5.092878  
  Larval Feeding Carcass  5.082695  
  Larval Feeding Central Nevous System  4.842666  
  Larval Feeding Hind Gut  6.494554  
  Larval Feeding Malpighian Tubule  5.046809  
  Larval Feeding Mid Gut  13.417896  
  Larval Feeding Salivary Gland  5.036469  
  Whole Larvae Feeding  12.097335  
 
  
   FlyBase ID    symbol    start    end    strand    length   
   FBgn0031248   CG11912  318362   319254   -  893  
   FBgn0031249   CG11911  320279   321246   -  968  
   FBgn0053127   CG33127   322101   323091  +  991  
 
 
    Segment 15 
 
   Location   
  Gene key  FBgn0051920-FBgn0051921  
  Heatmap region span   2L:288919..419642   
  Segment span   2L:329761..332335   
  Length (genes)  2  
  Length (bp)  2575  
   Model Scoring   
  BIC  183.316041  
  logL  -86.187403  
  logL ratio  36.952140  
   Expression   
  Mean expression  5.281218  
  Median expression  4.954098  
  Tissue std. dev.  1.451120  
 
  No GO Slim enrichment  
  
   tissue    mean expression   
  5th Passage Drosophila S2 Cells  4.878222  
  Adult Accessory gland  5.000649  
  Adult Brain  4.699369  
  Adult Carcass  4.972795  
  Adult Crop  5.047845  
  Adult Eye  4.754893  
  Adult Fatbody  4.879443  
  Adult Female Spermatheca Mated  5.208298  
  Adult Female Spermatheca Virgin  5.077718  
  Adult Head  4.579852  
  Adult Heart  4.909328  
  Adult Hind Gut  4.806380  
  Adult Male Ejaculatory Duct  5.063702  
  Adult Mid Gut  4.727788  
  Adult Ovary  4.756315  
  Adult Salivary Gland  5.251637  
  Adult Testes  12.119933  
  Adult Thoracoabdominal ganglion  4.807087  
  Adult Whole Fly  7.669321  
  Larvae Wandering Tubules  4.891559  
  Larval Feeding Carcass  5.066344  
  Larval Feeding Central Nevous System  4.686929  
  Larval Feeding Hind Gut  4.642900  
  Larval Feeding Malpighian Tubule  4.919441  
  Larval Feeding Mid Gut  4.808501  
  Larval Feeding Salivary Gland  4.931207  
  Whole Larvae Feeding  5.435417  
 
  
   FlyBase ID    symbol    start    end    strand    length   
   FBgn0051920     325520   329761   -  4242  
   FBgn0051921   CG31921  329991   332335   -  2345  
 
    Segment 16 
 
   Location   
  Gene key  FBgn0000061-FBgn0031251  
  Heatmap region span   2L:305941..421407   
  Segment span   2L:378116..402181   
  Length (genes)  2  
  Length (bp)  24066  
   Model Scoring   
  BIC  232.627712  
  logL  -110.843239  
  logL ratio  4.297724  
   Expression   
  Mean expression  6.308852  
  Median expression  6.127824  
  Tissue std. dev.  0.954499  
 
  No GO Slim enrichment  
  
   tissue    mean expression   
  5th Passage Drosophila S2 Cells  5.679937  
  Adult Accessory gland  5.918726  
  Adult Brain  5.098215  
  Adult Carcass  8.156424  
  Adult Crop  7.404825  
  Adult Eye  5.283770  
  Adult Fatbody  5.621175  
  Adult Female Spermatheca Mated  5.634110  
  Adult Female Spermatheca Virgin  5.553154  
  Adult Head  6.093064  
  Adult Heart  6.894240  
  Adult Hind Gut  6.949908  
  Adult Male Ejaculatory Duct  5.929694  
  Adult Mid Gut  5.864610  
  Adult Ovary  5.397380  
  Adult Salivary Gland  5.935467  
  Adult Testes  7.773876  
  Adult Thoracoabdominal ganglion  5.532307  
  Adult Whole Fly  6.599833  
  Larvae Wandering Tubules  5.652056  
  Larval Feeding Carcass  8.741260  
  Larval Feeding Central Nevous System  5.727809  
  Larval Feeding Hind Gut  7.107722  
  Larval Feeding Malpighian Tubule  5.513978  
  Larval Feeding Mid Gut  7.158950  
  Larval Feeding Salivary Gland  5.643770  
  Whole Larvae Feeding  7.472734  
 
  
   FlyBase ID    symbol    start    end    strand    length   
   FBgn0000061   al   378116   387439  +  9324  
   FBgn0031251   CG4213  396595   402181   -  5587  
 
    Segment 17 
 
   Location   
  Gene key  FBgn0003278-FBgn0023444  
  Heatmap region span   2L:319254..423452   
  Segment span   2L:404286..415069   
  Length (genes)  2  
  Length (bp)  10784  
   Model Scoring   
  BIC  255.402980  
  logL  -122.230872  
  logL ratio  12.037710  
   Expression   
  Mean expression  8.621622  
  Median expression  8.719734  
  Tissue std. dev.  0.728990  
 
  No GO Slim enrichment  
  
   tissue    mean expression   
  5th Passage Drosophila S2 Cells  10.060693  
  Adult Accessory gland  9.170483  
  Adult Brain  8.763751  
  Adult Carcass  7.972939  
  Adult Crop  8.548378  
  Adult Eye  7.756220  
  Adult Fatbody  8.391061  
  Adult Female Spermatheca Mated  9.112829  
  Adult Female Spermatheca Virgin  8.810571  
  Adult Head  8.101489  
  Adult Heart  8.128543  
  Adult Hind Gut  8.140706  
  Adult Male Ejaculatory Duct  8.426130  
  Adult Mid Gut  8.717391  
  Adult Ovary  10.187167  
  Adult Salivary Gland  8.523927  
  Adult Testes  6.484445  
  Adult Thoracoabdominal ganglion  8.787496  
  Adult Whole Fly  8.960753  
  Larvae Wandering Tubules  8.131739  
  Larval Feeding Carcass  8.975415  
  Larval Feeding Central Nevous System  9.887904  
  Larval Feeding Hind Gut  8.625922  
  Larval Feeding Malpighian Tubule  8.163313  
  Larval Feeding Mid Gut  8.554351  
  Larval Feeding Salivary Gland  9.082540  
  Whole Larvae Feeding  8.317637  
 
  
   FlyBase ID    symbol    start    end    strand    length   
   FBgn0003278   RpI135   404286   408049  +  3764  
   FBgn0023444      415069   418536  +  3468  
 
    Segment 18 
 
   Location   
  Gene key  FBgn0031254-FBgn0031255  
  Heatmap region span   2L:404286..473021   
  Segment span   2L:421800..423452   
  Length (genes)  2  
  Length (bp)  1653  
   Model Scoring   
  BIC  177.290037  
  logL  -83.174401  
  logL ratio  34.149310  
   Expression   
  Mean expression  5.045562  
  Median expression  5.002776  
  Tissue std. dev.  0.281380  
 
  No GO Slim enrichment  
  
   tissue    mean expression   
  5th Passage Drosophila S2 Cells  6.043866  
  Adult Accessory gland  5.047115  
  Adult Brain  4.612475  
  Adult Carcass  5.123752  
  Adult Crop  4.914089  
  Adult Eye  4.801929  
  Adult Fatbody  4.993217  
  Adult Female Spermatheca Mated  5.004424  
  Adult Female Spermatheca Virgin  5.113197  
  Adult Head  5.161214  
  Adult Heart  4.722563  
  Adult Hind Gut  5.032567  
  Adult Male Ejaculatory Duct  5.153188  
  Adult Mid Gut  5.246210  
  Adult Ovary  4.871664  
  Adult Salivary Gland  5.172729  
  Adult Testes  5.215111  
  Adult Thoracoabdominal ganglion  4.788348  
  Adult Whole Fly  4.399130  
  Larvae Wandering Tubules  5.032198  
  Larval Feeding Carcass  5.109907  
  Larval Feeding Central Nevous System  5.350350  
  Larval Feeding Hind Gut  4.944090  
  Larval Feeding Malpighian Tubule  5.134527  
  Larval Feeding Mid Gut  5.135636  
  Larval Feeding Salivary Gland  5.121177  
  Whole Larvae Feeding  4.985503  
 
  
   FlyBase ID    symbol    start    end    strand    length   
   FBgn0031254   CG13692   421800   422435  +  636  
   FBgn0031255   BBS8   423452   425291  +  1840  
 
    Segment 19 
 
   Location   
  Gene key  FBgn0015924-FBgn0031256  
  Heatmap region span   2L:419931..523467   
  Segment span   2L:453023..453111   
  Length (genes)  2  
  Length (bp)  89  
   Model Scoring   
  BIC  240.721477  
  logL  -114.890121  
  logL ratio  48.354238  
   Expression   
  Mean expression  10.032798  
  Median expression  10.034701  
  Tissue std. dev.  0.575480  
 
  No GO Slim enrichment  
  
   tissue    mean expression   
  5th Passage Drosophila S2 Cells  11.586797  
  Adult Accessory gland  9.917556  
  Adult Brain  9.283596  
  Adult Carcass  9.578521  
  Adult Crop  10.431630  
  Adult Eye  10.088685  
  Adult Fatbody  10.192815  
  Adult Female Spermatheca Mated  10.081662  
  Adult Female Spermatheca Virgin  10.217645  
  Adult Head  10.129182  
  Adult Heart  10.678091  
  Adult Hind Gut  9.890089  
  Adult Male Ejaculatory Duct  10.811469  
  Adult Mid Gut  9.930902  
  Adult Ovary  9.766654  
  Adult Salivary Gland  9.782478  
  Adult Testes  8.592785  
  Adult Thoracoabdominal ganglion  9.589656  
  Adult Whole Fly  9.353574  
  Larvae Wandering Tubules  10.694509  
  Larval Feeding Carcass  9.719781  
  Larval Feeding Central Nevous System  9.384414  
  Larval Feeding Hind Gut  10.152131  
  Larval Feeding Malpighian Tubule  10.132342  
  Larval Feeding Mid Gut  10.697601  
  Larval Feeding Salivary Gland  10.385457  
  Whole Larvae Feeding  9.815527  
 
  
   FlyBase ID    symbol    start    end    strand    length   
   FBgn0015924   crq  448254   453023   -  4770  
   FBgn0031256   CG4164   453111   454654  +  1544  
 
    Segment 20 
 
   Location   
  Gene key  FBgn0031257-FBgn0031258  
  Heatmap region span   2L:421407..544627   
  Segment span   2L:455543..465607   
  Length (genes)  2  
  Length (bp)  10065  
   Model Scoring   
  BIC  236.038235  
  logL  -112.548500  
  logL ratio  -8.145153  
   Expression   
  Mean expression  5.682251  
  Median expression  5.220693  
  Tissue std. dev.  1.081744  
 
  No GO Slim enrichment  
  
   tissue    mean expression   
  5th Passage Drosophila S2 Cells  6.292236  
  Adult Accessory gland  8.467548  
  Adult Brain  8.405943  
  Adult Carcass  5.556888  
  Adult Crop  5.304721  
  Adult Eye  6.547910  
  Adult Fatbody  5.462967  
  Adult Female Spermatheca Mated  4.813130  
  Adult Female Spermatheca Virgin  4.799720  
  Adult Head  6.684303  
  Adult Heart  5.336967  
  Adult Hind Gut  5.202972  
  Adult Male Ejaculatory Duct  5.153448  
  Adult Mid Gut  5.000974  
  Adult Ovary  4.916703  
  Adult Salivary Gland  5.116666  
  Adult Testes  4.921416  
  Adult Thoracoabdominal ganglion  7.718698  
  Adult Whole Fly  5.011131  
  Larvae Wandering Tubules  5.296862  
  Larval Feeding Carcass  4.813516  
  Larval Feeding Central Nevous System  7.122983  
  Larval Feeding Hind Gut  4.711299  
  Larval Feeding Malpighian Tubule  5.383243  
  Larval Feeding Mid Gut  4.830658  
  Larval Feeding Salivary Gland  5.665931  
  Whole Larvae Feeding  4.881939  
 
  
   FlyBase ID    symbol    start    end    strand    length   
   FBgn0031257   CG4133   455543   458745  +  3203  
   FBgn0031258   CG4297  460515   465607   -  5093  
 
    Segment 21 
 
   Location   
  Gene key  FBgn0010602-FBgn0031260  
  Heatmap region span   2L:455543..714968   
  Segment span   2L:542572..544627   
  Length (genes)  2  
  Length (bp)  2056  
   Model Scoring   
  BIC  255.895255  
  logL  -122.477010  
  logL ratio  75.626636  
   Expression   
  Mean expression  11.139190  
  Median expression  10.933336  
  Tissue std. dev.  0.710482  
 
  No GO Slim enrichment  
  
   tissue    mean expression   
  5th Passage Drosophila S2 Cells  12.446683  
  Adult Accessory gland  12.639747  
  Adult Brain  10.529402  
  Adult Carcass  10.473393  
  Adult Crop  10.630035  
  Adult Eye  10.163654  
  Adult Fatbody  11.000616  
  Adult Female Spermatheca Mated  11.746305  
  Adult Female Spermatheca Virgin  11.638527  
  Adult Head  10.611015  
  Adult Heart  10.704301  
  Adult Hind Gut  10.340058  
  Adult Male Ejaculatory Duct  11.879827  
  Adult Mid Gut  10.806390  
  Adult Ovary  11.344684  
  Adult Salivary Gland  12.217235  
  Adult Testes  10.101646  
  Adult Thoracoabdominal ganglion  10.671695  
  Adult Whole Fly  11.014775  
  Larvae Wandering Tubules  10.774513  
  Larval Feeding Carcass  11.269131  
  Larval Feeding Central Nevous System  11.776997  
  Larval Feeding Hind Gut  10.999312  
  Larval Feeding Malpighian Tubule  10.586668  
  Larval Feeding Mid Gut  10.781894  
  Larval Feeding Salivary Gland  12.448464  
  Whole Larvae Feeding  11.161162  
 
  
   FlyBase ID    symbol    start    end    strand    length   
   FBgn0010602   lwr  541235   542572   -  1338  
   FBgn0031260   Spp  542776   544627   -  1852  
 
    Segment 22 
 
   Location   
  Gene key  FBgn0031264-FBgn0031268  
  Heatmap region span   2L:523467..776536   
  Segment span   2L:563303..574486   
  Length (genes)  6  
  Length (bp)  11184  
   Model Scoring   
  BIC  589.973270  
  logL  -289.516018  
  logL ratio  184.315990  
   Expression   
  Mean expression  8.403439  
  Median expression  8.254053  
  Tissue std. dev.  0.429583  
 
  No GO Slim enrichment  
  
   tissue    mean expression   
  5th Passage Drosophila S2 Cells  9.168022  
  Adult Accessory gland  8.681989  
  Adult Brain  7.742984  
  Adult Carcass  8.088262  
  Adult Crop  8.630926  
  Adult Eye  8.207449  
  Adult Fatbody  7.924678  
  Adult Female Spermatheca Mated  8.535167  
  Adult Female Spermatheca Virgin  8.371119  
  Adult Head  7.998931  
  Adult Heart  7.993467  
  Adult Hind Gut  8.525506  
  Adult Male Ejaculatory Duct  8.505874  
  Adult Mid Gut  8.149018  
  Adult Ovary  8.996534  
  Adult Salivary Gland  8.540816  
  Adult Testes  7.935404  
  Adult Thoracoabdominal ganglion  7.757011  
  Adult Whole Fly  8.419757  
  Larvae Wandering Tubules  8.089666  
  Larval Feeding Carcass  9.361503  
  Larval Feeding Central Nevous System  8.790007  
  Larval Feeding Hind Gut  8.989201  
  Larval Feeding Malpighian Tubule  8.211820  
  Larval Feeding Mid Gut  7.941203  
  Larval Feeding Salivary Gland  8.911118  
  Whole Larvae Feeding  8.425423  
 
  
   FlyBase ID    symbol    start    end    strand    length   
   FBgn0031264   CG11835  560794   563303   -  2510  
   FBgn0031265   CG2794   564170   566472  +  2303  
   FBgn0021874   Nle  566366   568162   -  1797  
   FBgn0031266   CG2807   568337   572716  +  4380  
   FBgn0031267   Ipk2  573033   574232   -  1200  
   FBgn0031268   cold   574486   575734  +  1249  
 
 
    Segment 23 
 
   Location   
  Gene key  FBgn0013323-FBgn0000497  
  Heatmap region span   2L:542572..779201   
  Segment span   2L:576896..714968   
  Length (genes)  5  
  Length (bp)  138073  
   Model Scoring   
  BIC  543.145559  
  logL  -266.102162  
  logL ratio  17.729926  
   Expression   
  Mean expression  5.362280  
  Median expression  4.877370  
  Tissue std. dev.  0.463986  
 
  No GO Slim enrichment  
  
   tissue    mean expression   
  5th Passage Drosophila S2 Cells  5.484035  
  Adult Accessory gland  5.157714  
  Adult Brain  5.331875  
  Adult Carcass  5.056612  
  Adult Crop  5.413518  
  Adult Eye  6.821873  
  Adult Fatbody  5.233217  
  Adult Female Spermatheca Mated  5.284154  
  Adult Female Spermatheca Virgin  5.147386  
  Adult Head  5.893253  
  Adult Heart  5.293834  
  Adult Hind Gut  5.481399  
  Adult Male Ejaculatory Duct  5.304682  
  Adult Mid Gut  5.002541  
  Adult Ovary  5.170636  
  Adult Salivary Gland  5.422742  
  Adult Testes  4.721577  
  Adult Thoracoabdominal ganglion  4.981030  
  Adult Whole Fly  4.838390  
  Larvae Wandering Tubules  5.477571  
  Larval Feeding Carcass  5.491507  
  Larval Feeding Central Nevous System  6.759600  
  Larval Feeding Hind Gut  5.303120  
  Larval Feeding Malpighian Tubule  5.247492  
  Larval Feeding Mid Gut  5.077689  
  Larval Feeding Salivary Gland  5.099088  
  Whole Larvae Feeding  5.285032  
 
  
   FlyBase ID    symbol    start    end    strand    length   
   FBgn0013323   Ptth  575711   576896   -  1186  
   FBgn0023489   Pph13   577488   579549  +  2062  
   FBgn0010323   Gsc  583540   594810   -  11271  
   FBgn0031270   CG13689   602813   603562  +  750  
   FBgn0000497   ds  640021   714968   -  74948  
 
 
    Segment 24 
 
   Location   
  Gene key  FBgn0031276-FBgn0040725  
  Heatmap region span   2L:563303..810586   
  Segment span   2L:773575..776536   
  Length (genes)  2  
  Length (bp)  2962  
   Model Scoring   
  BIC  201.634206  
  logL  -95.346486  
  logL ratio  33.743824  
   Expression   
  Mean expression  4.647896  
  Median expression  4.097583  
  Tissue std. dev.  1.675933  
 
  No GO Slim enrichment  
  
   tissue    mean expression   
  5th Passage Drosophila S2 Cells  3.987581  
  Adult Accessory gland  4.249930  
  Adult Brain  3.914743  
  Adult Carcass  4.121963  
  Adult Crop  4.126147  
  Adult Eye  4.053348  
  Adult Fatbody  4.011154  
  Adult Female Spermatheca Mated  4.008151  
  Adult Female Spermatheca Virgin  4.009500  
  Adult Head  3.899798  
  Adult Heart  4.020280  
  Adult Hind Gut  3.993046  
  Adult Male Ejaculatory Duct  3.849371  
  Adult Mid Gut  4.068159  
  Adult Ovary  3.945833  
  Adult Salivary Gland  9.004933  
  Adult Testes  10.636138  
  Adult Thoracoabdominal ganglion  3.930677  
  Adult Whole Fly  5.846917  
  Larvae Wandering Tubules  3.933194  
  Larval Feeding Carcass  4.028954  
  Larval Feeding Central Nevous System  3.822564  
  Larval Feeding Hind Gut  3.954955  
  Larval Feeding Malpighian Tubule  4.033348  
  Larval Feeding Mid Gut  4.096833  
  Larval Feeding Salivary Gland  7.801557  
  Whole Larvae Feeding  4.144134  
 
  
   FlyBase ID    symbol    start    end    strand    length   
   FBgn0031276   CG12506   773575   774020  +  446  
   FBgn0040725   CG13946   776536   776892  +  357  
 
    Segment 25 
 
   Location   
  Gene key  FBgn0041250-FBgn0031279  
  Heatmap region span   2L:728412..818445   
  Segment span   2L:780482..784336   
  Length (genes)  2  
  Length (bp)  3855  
   Model Scoring   
  BIC  192.002434  
  logL  -90.530600  
  logL ratio  22.331325  
   Expression   
  Mean expression  4.639023  
  Median expression  4.473299  
  Tissue std. dev.  0.543444  
 
  No GO Slim enrichment  
  
   tissue    mean expression   
  5th Passage Drosophila S2 Cells  4.650233  
  Adult Accessory gland  4.655861  
  Adult Brain  4.230438  
  Adult Carcass  4.526462  
  Adult Crop  4.493847  
  Adult Eye  4.399976  
  Adult Fatbody  4.455043  
  Adult Female Spermatheca Mated  4.536343  
  Adult Female Spermatheca Virgin  4.523331  
  Adult Head  4.366302  
  Adult Heart  4.389230  
  Adult Hind Gut  4.493319  
  Adult Male Ejaculatory Duct  4.523658  
  Adult Mid Gut  4.760055  
  Adult Ovary  4.434671  
  Adult Salivary Gland  4.923788  
  Adult Testes  7.282106  
  Adult Thoracoabdominal ganglion  4.387059  
  Adult Whole Fly  4.812250  
  Larvae Wandering Tubules  4.753198  
  Larval Feeding Carcass  4.567188  
  Larval Feeding Central Nevous System  4.277655  
  Larval Feeding Hind Gut  4.382891  
  Larval Feeding Malpighian Tubule  4.626267  
  Larval Feeding Mid Gut  4.663411  
  Larval Feeding Salivary Gland  4.719420  
  Whole Larvae Feeding  4.419622  
 
  
   FlyBase ID    symbol    start    end    strand    length   
   FBgn0041250   Gr21a   780482   782885  +  2404  
   FBgn0031279   CG3544   784336   786161  +  1826  
 
    Segment 26 
 
   Location   
  Gene key  FBgn0086130-FBgn0031282  
  Heatmap region span   2L:779201..825964   
  Segment span   2L:811609..816023   
  Length (genes)  3  
  Length (bp)  4415  
   Model Scoring   
  BIC  299.754231  
  logL  -144.406498  
  logL ratio  78.087159  
   Expression   
  Mean expression  7.248409  
  Median expression  7.317671  
  Tissue std. dev.  0.419886  
 
  No GO Slim enrichment  
  
   tissue    mean expression   
  5th Passage Drosophila S2 Cells  7.734062  
  Adult Accessory gland  7.342860  
  Adult Brain  7.180402  
  Adult Carcass  7.033898  
  Adult Crop  7.714531  
  Adult Eye  7.238986  
  Adult Fatbody  7.327539  
  Adult Female Spermatheca Mated  7.384495  
  Adult Female Spermatheca Virgin  7.342780  
  Adult Head  6.977931  
  Adult Heart  7.447948  
  Adult Hind Gut  7.329647  
  Adult Male Ejaculatory Duct  7.536688  
  Adult Mid Gut  7.149055  
  Adult Ovary  8.207911  
  Adult Salivary Gland  6.911503  
  Adult Testes  5.846996  
  Adult Thoracoabdominal ganglion  7.174081  
  Adult Whole Fly  6.886645  
  Larvae Wandering Tubules  7.471866  
  Larval Feeding Carcass  6.822222  
  Larval Feeding Central Nevous System  7.629920  
  Larval Feeding Hind Gut  7.169548  
  Larval Feeding Malpighian Tubule  7.709325  
  Larval Feeding Mid Gut  6.937630  
  Larval Feeding Salivary Gland  7.354088  
  Whole Larvae Feeding  6.844476  
 
  
   FlyBase ID    symbol    start    end    strand    length   
   FBgn0086130   KH1   811609   813380  +  1772  
   FBgn0031281   Saf6  813314   815951   -  2638  
   FBgn0031282   Pex12   816023   817032  +  1010  
 
 
    Segment 27 
 
   Location   
  Gene key  FBgn0031284-FBgn0015621  
  Heatmap region span   2L:790795..860309   
  Segment span   2L:819492..819964   
  Length (genes)  2  
  Length (bp)  473  
   Model Scoring   
  BIC  218.399476  
  logL  -103.729121  
  logL ratio  41.778978  
   Expression   
  Mean expression  7.945429  
  Median expression  7.881592  
  Tissue std. dev.  0.539967  
 
  No GO Slim enrichment  
  
   tissue    mean expression   
  5th Passage Drosophila S2 Cells  8.777925  
  Adult Accessory gland  8.263216  
  Adult Brain  8.524530  
  Adult Carcass  7.071136  
  Adult Crop  7.922984  
  Adult Eye  8.251211  
  Adult Fatbody  7.235256  
  Adult Female Spermatheca Mated  7.971146  
  Adult Female Spermatheca Virgin  7.981267  
  Adult Head  7.621965  
  Adult Heart  8.067704  
  Adult Hind Gut  7.630119  
  Adult Male Ejaculatory Duct  8.099134  
  Adult Mid Gut  7.953168  
  Adult Ovary  9.342033  
  Adult Salivary Gland  7.808199  
  Adult Testes  6.836882  
  Adult Thoracoabdominal ganglion  8.103780  
  Adult Whole Fly  8.155666  
  Larvae Wandering Tubules  8.109780  
  Larval Feeding Carcass  7.726358  
  Larval Feeding Central Nevous System  8.361382  
  Larval Feeding Hind Gut  7.429598  
  Larval Feeding Malpighian Tubule  7.845411  
  Larval Feeding Mid Gut  7.467030  
  Larval Feeding Salivary Gland  8.730463  
  Whole Larvae Feeding  7.239225  
 
  
   FlyBase ID    symbol    start    end    strand    length   
   FBgn0031284   CG3876  818079   819492   -  1414  
   FBgn0015621   Clp   819964   821209  +  1246  
 
    Segment 28 
 
   Location   
  Gene key  FBgn0031286-FBgn0010583  
  Heatmap region span   2L:811609..877293   
  Segment span   2L:825861..825964   
  Length (genes)  2  
  Length (bp)  104  
   Model Scoring   
  BIC  221.572033  
  logL  -105.315399  
  logL ratio  39.403362  
   Expression   
  Mean expression  7.588350  
  Median expression  7.421020  
  Tissue std. dev.  0.819012  
 
  No GO Slim enrichment  
  
   tissue    mean expression   
  5th Passage Drosophila S2 Cells  8.268773  
  Adult Accessory gland  7.080244  
  Adult Brain  9.355264  
  Adult Carcass  6.668068  
  Adult Crop  7.329133  
  Adult Eye  8.437219  
  Adult Fatbody  6.957149  
  Adult Female Spermatheca Mated  7.064933  
  Adult Female Spermatheca Virgin  7.236811  
  Adult Head  7.810467  
  Adult Heart  7.622658  
  Adult Hind Gut  7.210349  
  Adult Male Ejaculatory Duct  7.265529  
  Adult Mid Gut  7.030062  
  Adult Ovary  7.841133  
  Adult Salivary Gland  7.024070  
  Adult Testes  6.255899  
  Adult Thoracoabdominal ganglion  9.457218  
  Adult Whole Fly  6.656762  
  Larvae Wandering Tubules  7.985722  
  Larval Feeding Carcass  7.577768  
  Larval Feeding Central Nevous System  9.599051  
  Larval Feeding Hind Gut  7.627116  
  Larval Feeding Malpighian Tubule  7.806987  
  Larval Feeding Mid Gut  7.204901  
  Larval Feeding Salivary Gland  7.470158  
  Whole Larvae Feeding  7.042017  
 
  
   FlyBase ID    symbol    start    end    strand    length   
   FBgn0031286   CG3862  824329   825861   -  1533  
   FBgn0010583   dock   825964   833245  +  7282  
 
    Segment 29 
 
   Location   
  Gene key  FBgn0020304-FBgn0020545  
  Heatmap region span   2L:818445..885052   
  Segment span   2L:851096..852767   
  Length (genes)  2  
  Length (bp)  1672  
   Model Scoring   
  BIC  241.005384  
  logL  -115.032075  
  logL ratio  54.897290  
   Expression   
  Mean expression  10.201019  
  Median expression  10.167016  
  Tissue std. dev.  0.774857  
 
  No GO Slim enrichment  
  
   tissue    mean expression   
  5th Passage Drosophila S2 Cells  11.572885  
  Adult Accessory gland  9.348645  
  Adult Brain  9.870350  
  Adult Carcass  10.532318  
  Adult Crop  11.887154  
  Adult Eye  10.717927  
  Adult Fatbody  10.541956  
  Adult Female Spermatheca Mated  9.952717  
  Adult Female Spermatheca Virgin  9.978308  
  Adult Head  10.144658  
  Adult Heart  10.786827  
  Adult Hind Gut  11.647125  
  Adult Male Ejaculatory Duct  11.370121  
  Adult Mid Gut  10.061688  
  Adult Ovary  9.323128  
  Adult Salivary Gland  9.052005  
  Adult Testes  9.293743  
  Adult Thoracoabdominal ganglion  10.232918  
  Adult Whole Fly  9.757592  
  Larvae Wandering Tubules  10.571419  
  Larval Feeding Carcass  10.269232  
  Larval Feeding Central Nevous System  9.076686  
  Larval Feeding Hind Gut  10.044785  
  Larval Feeding Malpighian Tubule  10.629110  
  Larval Feeding Mid Gut  9.799800  
  Larval Feeding Salivary Gland  9.093620  
  Whole Larvae Feeding  9.870791  
 
  
   FlyBase ID    symbol    start    end    strand    length   
   FBgn0020304   drongo  833584   851096   -  17513  
   FBgn0020545   kraken   852767   854539  +  1773  
 
    Segment 30 
 
   Location   
  Gene key  FBgn0031288-FBgn0031289  
  Heatmap region span   2L:819492..898647   
  Segment span   2L:855337..860309   
  Length (genes)  2  
  Length (bp)  4973  
   Model Scoring   
  BIC  219.147604  
  logL  -104.103185  
  logL ratio  10.364360  
   Expression   
  Mean expression  4.687115  
  Median expression  4.177394  
  Tissue std. dev.  1.011234  
 
  No GO Slim enrichment  
  
   tissue    mean expression   
  5th Passage Drosophila S2 Cells  4.048593  
  Adult Accessory gland  6.158168  
  Adult Brain  4.093259  
  Adult Carcass  4.157893  
  Adult Crop  3.987645  
  Adult Eye  4.155855  
  Adult Fatbody  4.053701  
  Adult Female Spermatheca Mated  4.705653  
  Adult Female Spermatheca Virgin  4.770501  
  Adult Head  5.314538  
  Adult Heart  4.200693  
  Adult Hind Gut  4.093234  
  Adult Male Ejaculatory Duct  4.805056  
  Adult Mid Gut  4.932630  
  Adult Ovary  4.031710  
  Adult Salivary Gland  6.760556  
  Adult Testes  6.245821  
  Adult Thoracoabdominal ganglion  4.337485  
  Adult Whole Fly  4.197857  
  Larvae Wandering Tubules  4.147002  
  Larval Feeding Carcass  4.140547  
  Larval Feeding Central Nevous System  3.893201  
  Larval Feeding Hind Gut  3.889510  
  Larval Feeding Malpighian Tubule  4.189803  
  Larval Feeding Mid Gut  4.673729  
  Larval Feeding Salivary Gland  8.213210  
  Whole Larvae Feeding  4.354270  
 
  
   FlyBase ID    symbol    start    end    strand    length   
   FBgn0031288   CG13949   855337   856236  +  900  
   FBgn0031289   CG13950   860309   861806  +  1498  
 
    Segment 31 
 
   Location   
  Gene key  FBgn0029095-FBgn0053526  
  Heatmap region span   2L:821418..914090   
  Segment span   2L:868352..870464   
  Length (genes)  3  
  Length (bp)  2113  
   Model Scoring   
  BIC  341.009972  
  logL  -165.034369  
  logL ratio  48.486599  
   Expression   
  Mean expression  8.147857  
  Median expression  8.090490  
  Tissue std. dev.  0.625727  
 
  
   GO ID    description    ratio    P-value   
   GO:0043226   organelle  3/3  0.0325  
 
  
   tissue    mean expression   
  5th Passage Drosophila S2 Cells  9.843807  
  Adult Accessory gland  9.392725  
  Adult Brain  7.925256  
  Adult Carcass  7.962558  
  Adult Crop  9.151479  
  Adult Eye  8.596326  
  Adult Fatbody  7.705183  
  Adult Female Spermatheca Mated  8.268004  
  Adult Female Spermatheca Virgin  8.214530  
  Adult Head  7.975822  
  Adult Heart  7.998681  
  Adult Hind Gut  8.469832  
  Adult Male Ejaculatory Duct  8.634384  
  Adult Mid Gut  7.202964  
  Adult Ovary  8.416583  
  Adult Salivary Gland  8.386569  
  Adult Testes  7.211564  
  Adult Thoracoabdominal ganglion  7.507933  
  Adult Whole Fly  7.648787  
  Larvae Wandering Tubules  7.564217  
  Larval Feeding Carcass  8.143285  
  Larval Feeding Central Nevous System  8.041848  
  Larval Feeding Hind Gut  8.361874  
  Larval Feeding Malpighian Tubule  7.699723  
  Larval Feeding Mid Gut  7.384071  
  Larval Feeding Salivary Gland  8.645537  
  Whole Larvae Feeding  7.638593  
 
  
   FlyBase ID    symbol    start    end    strand    length   
   FBgn0029095   aru  861849   868352   -  6504  
   FBgn0020305   dbe  868675   869911   -  1237  
   FBgn0053526   PNUTS   870464   877050  +  6587  
 
 
    Segment 32 
 
   Location   
  Gene key  FBgn0031295-FBgn0031296  
  Heatmap region span   2L:885052..1129235   
  Segment span   2L:1026897..1037275   
  Length (genes)  2  
  Length (bp)  10379  
   Model Scoring   
  BIC  204.569374  
  logL  -96.814070  
  logL ratio  20.377636  
   Expression   
  Mean expression  4.848903  
  Median expression  4.491421  
  Tissue std. dev.  1.501138  
 
  No GO Slim enrichment  
  
   tissue    mean expression   
  5th Passage Drosophila S2 Cells  4.427684  
  Adult Accessory gland  4.469848  
  Adult Brain  4.101897  
  Adult Carcass  4.574578  
  Adult Crop  4.398314  
  Adult Eye  4.212528  
  Adult Fatbody  4.455106  
  Adult Female Spermatheca Mated  4.282756  
  Adult Female Spermatheca Virgin  4.311743  
  Adult Head  4.233068  
  Adult Heart  4.360173  
  Adult Hind Gut  4.364452  
  Adult Male Ejaculatory Duct  4.422264  
  Adult Mid Gut  4.758135  
  Adult Ovary  4.380705  
  Adult Salivary Gland  4.509314  
  Adult Testes  11.560611  
  Adult Thoracoabdominal ganglion  4.194269  
  Adult Whole Fly  7.665658  
  Larvae Wandering Tubules  4.393284  
  Larval Feeding Carcass  4.380492  
  Larval Feeding Central Nevous System  4.168882  
  Larval Feeding Hind Gut  4.358656  
  Larval Feeding Malpighian Tubule  4.341918  
  Larval Feeding Mid Gut  4.876737  
  Larval Feeding Salivary Gland  4.404420  
  Whole Larvae Feeding  6.312897  
 
  
   FlyBase ID    symbol    start    end    strand    length   
   FBgn0031295   CG4375  1026107   1026897   -  791  
   FBgn0031296   CG4415   1037275   1038685  +  1411  
 
    Segment 33 
 
   Location   
  Gene key  FBgn0031294-FBgn0015905  
  Heatmap region span   2L:898647..1134120   
  Segment span   2L:1048053..1077980   
  Length (genes)  3  
  Length (bp)  29928  
   Model Scoring   
  BIC  347.374770  
  logL  -168.216768  
  logL ratio  18.057000  
   Expression   
  Mean expression  6.770344  
  Median expression  6.662848  
  Tissue std. dev.  0.735343  
 
  
   GO ID    description    ratio    P-value   
   GO:0005886   plasma membrane  2/3  0.0195  
 
  
   tissue    mean expression   
  5th Passage Drosophila S2 Cells  7.261083  
  Adult Accessory gland  7.396335  
  Adult Brain  8.325750  
  Adult Carcass  6.276848  
  Adult Crop  7.022939  
  Adult Eye  6.589352  
  Adult Fatbody  6.224321  
  Adult Female Spermatheca Mated  6.182295  
  Adult Female Spermatheca Virgin  6.038928  
  Adult Head  7.275229  
  Adult Heart  7.098843  
  Adult Hind Gut  6.689714  
  Adult Male Ejaculatory Duct  5.755559  
  Adult Mid Gut  6.833101  
  Adult Ovary  7.959714  
  Adult Salivary Gland  5.970978  
  Adult Testes  6.331008  
  Adult Thoracoabdominal ganglion  7.902409  
  Adult Whole Fly  7.481205  
  Larvae Wandering Tubules  5.690282  
  Larval Feeding Carcass  6.441336  
  Larval Feeding Central Nevous System  8.136190  
  Larval Feeding Hind Gut  6.479354  
  Larval Feeding Malpighian Tubule  5.819337  
  Larval Feeding Mid Gut  6.539046  
  Larval Feeding Salivary Gland  6.927851  
  Whole Larvae Feeding  6.150295  
 
  
   FlyBase ID    symbol    start    end    strand    length   
   FBgn0031294   IA-2  1011421   1048053   -  36633  
   FBgn0003310   S  1050877   1077811   -  26935  
   FBgn0015905   ast   1077980   1080809  +  2830  
 
 
    Segment 34 
 
   Location   
  Gene key  FBgn0031299-FBgn0031302  
  Heatmap region span   2L:922795..1143203   
  Segment span   2L:1102506..1112988   
  Length (genes)  3  
  Length (bp)  10483  
   Model Scoring   
  BIC  314.556532  
  logL  -151.807649  
  logL ratio  14.937665  
   Expression   
  Mean expression  5.317943  
  Median expression  4.903663  
  Tissue std. dev.  0.428684  
 
  No GO Slim enrichment  
  
   tissue    mean expression   
  5th Passage Drosophila S2 Cells  5.956252  
  Adult Accessory gland  5.318670  
  Adult Brain  6.446365  
  Adult Carcass  5.290882  
  Adult Crop  5.008791  
  Adult Eye  5.516396  
  Adult Fatbody  5.329179  
  Adult Female Spermatheca Mated  5.099526  
  Adult Female Spermatheca Virgin  5.213709  
  Adult Head  5.437296  
  Adult Heart  5.270539  
  Adult Hind Gut  5.399724  
  Adult Male Ejaculatory Duct  4.815735  
  Adult Mid Gut  5.337623  
  Adult Ovary  4.902239  
  Adult Salivary Gland  5.362046  
  Adult Testes  6.549399  
  Adult Thoracoabdominal ganglion  5.546912  
  Adult Whole Fly  4.955589  
  Larvae Wandering Tubules  5.371125  
  Larval Feeding Carcass  4.900050  
  Larval Feeding Central Nevous System  5.165170  
  Larval Feeding Hind Gut  4.823534  
  Larval Feeding Malpighian Tubule  5.130790  
  Larval Feeding Mid Gut  5.639969  
  Larval Feeding Salivary Gland  4.900937  
  Whole Larvae Feeding  4.896013  
 
  
   FlyBase ID    symbol    start    end    strand    length   
   FBgn0031299   CG4629  1083090   1102506   -  19417  
   FBgn0031301   CG14339   1108539   1112702  +  4164  
   FBgn0031302   CG14340   1112988   1114140  +  1153  
 
 
    Segment 35 
 
   Location   
  Gene key  FBgn0016926-FBgn0031304  
  Heatmap region span   2L:1026897..1158679   
  Segment span   2L:1128953..1129235   
  Length (genes)  2  
  Length (bp)  283  
   Model Scoring   
  BIC  254.042369  
  logL  -121.550567  
  logL ratio  42.182644  
   Expression   
  Mean expression  10.219455  
  Median expression  10.275597  
  Tissue std. dev.  0.510170  
 
  No GO Slim enrichment  
  
   tissue    mean expression   
  5th Passage Drosophila S2 Cells  10.650699  
  Adult Accessory gland  9.881137  
  Adult Brain  9.978834  
  Adult Carcass  10.714267  
  Adult Crop  10.767003  
  Adult Eye  10.654988  
  Adult Fatbody  10.826377  
  Adult Female Spermatheca Mated  10.071013  
  Adult Female Spermatheca Virgin  9.924618  
  Adult Head  10.462630  
  Adult Heart  10.686172  
  Adult Hind Gut  10.533488  
  Adult Male Ejaculatory Duct  10.244044  
  Adult Mid Gut  10.087338  
  Adult Ovary  10.315041  
  Adult Salivary Gland  11.350617  
  Adult Testes  10.034695  
  Adult Thoracoabdominal ganglion  9.786587  
  Adult Whole Fly  10.059382  
  Larvae Wandering Tubules  10.131125  
  Larval Feeding Carcass  9.504789  
  Larval Feeding Central Nevous System  8.584143  
  Larval Feeding Hind Gut  10.329777  
  Larval Feeding Malpighian Tubule  10.263079  
  Larval Feeding Mid Gut  10.183301  
  Larval Feeding Salivary Gland  10.245572  
  Whole Larvae Feeding  9.654567  
 
  
   FlyBase ID    symbol    start    end    strand    length   
   FBgn0016926   Pino  1114652   1128953   -  14302  
   FBgn0031304   CG4552   1129235   1132413  +  3179  
 
    Segment 36 
 
   Location   
  Gene key  FBgn0031308-FBgn0031312  
  Heatmap region span   2L:1128953..1199204   
  Segment span   2L:1147833..1158679   
  Length (genes)  4  
  Length (bp)  10847  
   Model Scoring   
  BIC  393.457666  
  logL  -191.258216  
  logL ratio  122.426895  
   Expression   
  Mean expression  8.505689  
  Median expression  8.652178  
  Tissue std. dev.  0.447923  
 
  No GO Slim enrichment  
  
   tissue    mean expression   
  5th Passage Drosophila S2 Cells  9.700590  
  Adult Accessory gland  8.923068  
  Adult Brain  8.103357  
  Adult Carcass  7.939991  
  Adult Crop  8.721697  
  Adult Eye  8.243475  
  Adult Fatbody  8.179887  
  Adult Female Spermatheca Mated  8.258874  
  Adult Female Spermatheca Virgin  8.205155  
  Adult Head  7.934318  
  Adult Heart  8.649065  
  Adult Hind Gut  8.411036  
  Adult Male Ejaculatory Duct  8.469318  
  Adult Mid Gut  8.501334  
  Adult Ovary  9.511869  
  Adult Salivary Gland  8.800333  
  Adult Testes  7.792974  
  Adult Thoracoabdominal ganglion  8.117586  
  Adult Whole Fly  8.495695  
  Larvae Wandering Tubules  8.626008  
  Larval Feeding Carcass  8.131681  
  Larval Feeding Central Nevous System  8.729002  
  Larval Feeding Hind Gut  8.631458  
  Larval Feeding Malpighian Tubule  8.889186  
  Larval Feeding Mid Gut  8.397729  
  Larval Feeding Salivary Gland  9.107046  
  Whole Larvae Feeding  8.181883  
 
  
   FlyBase ID    symbol    start    end    strand    length   
   FBgn0031308   CG4749   1147833   1149454  +  1622  
   FBgn0031309   Tfb4  1149445   1150673   -  1229  
   FBgn0031310   CG4764   1150783   1151764  +  982  
   FBgn0031312   Tango14   1158679   1160221  +  1543  
 
 
    Segment 37 
 
   Location   
  Gene key  FBgn0031314-FBgn0031315  
  Heatmap region span   2L:1134702..1219363   
  Segment span   2L:1164470..1166514   
  Length (genes)  2  
  Length (bp)  2045  
   Model Scoring   
  BIC  213.109906  
  logL  -101.084336  
  logL ratio  34.933020  
   Expression   
  Mean expression  6.542724  
  Median expression  6.466903  
  Tissue std. dev.  0.383313  
 
  No GO Slim enrichment  
  
   tissue    mean expression   
  5th Passage Drosophila S2 Cells  7.271834  
  Adult Accessory gland  7.168962  
  Adult Brain  6.529445  
  Adult Carcass  6.015840  
  Adult Crop  6.436872  
  Adult Eye  6.784223  
  Adult Fatbody  6.254555  
  Adult Female Spermatheca Mated  6.464450  
  Adult Female Spermatheca Virgin  6.470401  
  Adult Head  6.272583  
  Adult Heart  6.379730  
  Adult Hind Gut  6.375268  
  Adult Male Ejaculatory Duct  6.967828  
  Adult Mid Gut  6.203574  
  Adult Ovary  7.282336  
  Adult Salivary Gland  6.606027  
  Adult Testes  5.812181  
  Adult Thoracoabdominal ganglion  6.696826  
  Adult Whole Fly  6.332703  
  Larvae Wandering Tubules  6.628355  
  Larval Feeding Carcass  6.532778  
  Larval Feeding Central Nevous System  7.000421  
  Larval Feeding Hind Gut  6.422543  
  Larval Feeding Malpighian Tubule  6.494263  
  Larval Feeding Mid Gut  6.032989  
  Larval Feeding Salivary Gland  7.119925  
  Whole Larvae Feeding  6.096642  
 
  
   FlyBase ID    symbol    start    end    strand    length   
   FBgn0031314   CG4785   1164470   1166432  +  1963  
   FBgn0031315   CG14341   1166514   1167211  +  698  
 
    Segment 38 
 
   Location   
  Gene key  FBgn0024314-FBgn0031322  
  Heatmap region span   2L:1143203..1240038   
  Segment span   2L:1170079..1186256   
  Length (genes)  8  
  Length (bp)  16178  
   Model Scoring   
  BIC  873.487104  
  logL  -431.272935  
  logL ratio  135.358870  
   Expression   
  Mean expression  8.110948  
  Median expression  8.170419  
  Tissue std. dev.  0.348004  
 
  
   GO ID    description    ratio    P-value   
   GO:0003729   mRNA binding  2/8  0.0246  
   GO:0003723   RNA binding  2/8  0.0447  
 
  
   tissue    mean expression   
  5th Passage Drosophila S2 Cells  8.425572  
  Adult Accessory gland  8.700275  
  Adult Brain  8.224741  
  Adult Carcass  7.727866  
  Adult Crop  8.247912  
  Adult Eye  8.063419  
  Adult Fatbody  7.882968  
  Adult Female Spermatheca Mated  8.227942  
  Adult Female Spermatheca Virgin  8.229300  
  Adult Head  7.763416  
  Adult Heart  8.332759  
  Adult Hind Gut  8.020916  
  Adult Male Ejaculatory Duct  8.148507  
  Adult Mid Gut  8.207730  
  Adult Ovary  8.272559  
  Adult Salivary Gland  7.797444  
  Adult Testes  6.982323  
  Adult Thoracoabdominal ganglion  8.090058  
  Adult Whole Fly  7.547957  
  Larvae Wandering Tubules  8.519431  
  Larval Feeding Carcass  8.362708  
  Larval Feeding Central Nevous System  8.506717  
  Larval Feeding Hind Gut  8.121434  
  Larval Feeding Malpighian Tubule  8.418257  
  Larval Feeding Mid Gut  7.938202  
  Larval Feeding Salivary Gland  8.389055  
  Whole Larvae Feeding  7.846121  
 
  
   FlyBase ID    symbol    start    end    strand    length   
   FBgn0024314   Plap  1167172   1170079   -  2908  
   FBgn0051922   CG31922   1170181   1170789  +  609  
   FBgn0031317   CG5118  1170753   1172745   -  1993  
   FBgn0031318   CG4887   1173011   1177500  +  4490  
   FBgn0031319   CG4896   1177706   1182459  +  4754  
   FBgn0031320   CG5126  1182291   1184021   -  1731  
   FBgn0031321   Tgt   1184088   1185698  +  1611  
   FBgn0031322   CG5001   1186256   1196806  +  10551  
 
 
    Segment 39 
 
   Location   
  Gene key  FBgn0031324-FBgn0259229  
  Heatmap region span   2L:1164470..1359784   
  Segment span   2L:1204241..1219363   
  Length (genes)  2  
  Length (bp)  15123  
   Model Scoring   
  BIC  201.512879  
  logL  -95.285822  
  logL ratio  17.486071  
   Expression   
  Mean expression  4.735277  
  Median expression  4.424835  
  Tissue std. dev.  0.561049  
 
  No GO Slim enrichment  
  
   tissue    mean expression   
  5th Passage Drosophila S2 Cells  4.404520  
  Adult Accessory gland  4.501795  
  Adult Brain  4.240127  
  Adult Carcass  5.183986  
  Adult Crop  7.101228  
  Adult Eye  5.044119  
  Adult Fatbody  4.596469  
  Adult Female Spermatheca Mated  4.518578  
  Adult Female Spermatheca Virgin  4.805246  
  Adult Head  5.093587  
  Adult Heart  4.358670  
  Adult Hind Gut  4.701472  
  Adult Male Ejaculatory Duct  4.693138  
  Adult Mid Gut  4.970169  
  Adult Ovary  4.481054  
  Adult Salivary Gland  4.589764  
  Adult Testes  4.280332  
  Adult Thoracoabdominal ganglion  4.334020  
  Adult Whole Fly  4.197742  
  Larvae Wandering Tubules  4.619625  
  Larval Feeding Carcass  4.987967  
  Larval Feeding Central Nevous System  4.186446  
  Larval Feeding Hind Gut  4.659833  
  Larval Feeding Malpighian Tubule  4.446401  
  Larval Feeding Mid Gut  5.471946  
  Larval Feeding Salivary Gland  4.551982  
  Whole Larvae Feeding  4.832261  
 
  
   FlyBase ID    symbol    start    end    strand    length   
   FBgn0031324   CG14342   1204241   1205254  +  1014  
   FBgn0259229   CG42329   1219363   1229471  +  10109  
 
    Segment 40 
 
   Location   
  Gene key  FBgn0041097-FBgn0011294  
  Heatmap region span   2L:1199204..1492295   
  Segment span   2L:1256426..1329161   
  Length (genes)  2  
  Length (bp)  72736  
   Model Scoring   
  BIC  212.850398  
  logL  -100.954582  
  logL ratio  5.014794  
   Expression   
  Mean expression  5.266042  
  Median expression  4.911021  
  Tissue std. dev.  0.849893  
 
  No GO Slim enrichment  
  
   tissue    mean expression   
  5th Passage Drosophila S2 Cells  4.894286  
  Adult Accessory gland  5.075902  
  Adult Brain  6.622445  
  Adult Carcass  4.888381  
  Adult Crop  4.827871  
  Adult Eye  6.216990  
  Adult Fatbody  4.773708  
  Adult Female Spermatheca Mated  4.704064  
  Adult Female Spermatheca Virgin  4.733607  
  Adult Head  8.602974  
  Adult Heart  4.772682  
  Adult Hind Gut  4.706470  
  Adult Male Ejaculatory Duct  5.166360  
  Adult Mid Gut  5.252846  
  Adult Ovary  4.712746  
  Adult Salivary Gland  5.359384  
  Adult Testes  5.017913  
  Adult Thoracoabdominal ganglion  6.413609  
  Adult Whole Fly  5.519331  
  Larvae Wandering Tubules  4.792957  
  Larval Feeding Carcass  4.892267  
  Larval Feeding Central Nevous System  5.926742  
  Larval Feeding Hind Gut  4.664525  
  Larval Feeding Malpighian Tubule  4.743139  
  Larval Feeding Mid Gut  5.362243  
  Larval Feeding Salivary Gland  4.693798  
  Whole Larvae Feeding  4.845897  
 
  
   FlyBase ID    symbol    start    end    strand    length   
   FBgn0041097   robo3   1256426   1294687  +  38262  
   FBgn0011294   a5   1329161   1329904  +  744  
 
    Segment 41 
 
   Location   
  Gene key  FBgn0031331-FBgn0031335  
  Heatmap region span   2L:1200237..1494090   
  Segment span   2L:1334269..1355924   
  Length (genes)  6  
  Length (bp)  21656  
   Model Scoring   
  BIC  441.872457  
  logL  -215.465611  
  logL ratio  188.730385  
   Expression   
  Mean expression  5.113472  
  Median expression  4.849275  
  Tissue std. dev.  1.254398  
 
  No GO Slim enrichment  
  
   tissue    mean expression   
  5th Passage Drosophila S2 Cells  4.824780  
  Adult Accessory gland  4.936597  
  Adult Brain  4.442949  
  Adult Carcass  4.944507  
  Adult Crop  4.791541  
  Adult Eye  4.494428  
  Adult Fatbody  4.885108  
  Adult Female Spermatheca Mated  4.917952  
  Adult Female Spermatheca Virgin  4.868946  
  Adult Head  4.536315  
  Adult Heart  4.614815  
  Adult Hind Gut  4.738164  
  Adult Male Ejaculatory Duct  4.830161  
  Adult Mid Gut  4.912655  
  Adult Ovary  4.636980  
  Adult Salivary Gland  5.169272  
  Adult Testes  11.111067  
  Adult Thoracoabdominal ganglion  4.634209  
  Adult Whole Fly  6.856540  
  Larvae Wandering Tubules  4.863656  
  Larval Feeding Carcass  4.851518  
  Larval Feeding Central Nevous System  4.532059  
  Larval Feeding Hind Gut  4.645546  
  Larval Feeding Malpighian Tubule  4.775543  
  Larval Feeding Mid Gut  4.944034  
  Larval Feeding Salivary Gland  4.919096  
  Whole Larvae Feeding  5.385304  
 
  
   FlyBase ID    symbol    start    end    strand    length   
   FBgn0031331   CG5440   1334269   1334763  +  495  
   FBgn0026141   Cdlc2   1344480   1345144  +  665  
   FBgn0031332   CG5556  1351805   1352869   -  1065  
   FBgn0031333   CG5561  1352970   1354109   -  1140  
   FBgn0051924   CG31924  1354101   1354919   -  819  
   FBgn0031335   CG5565  1354911   1355924   -  1014  
 
 
    Segment 42 
 
   Location   
  Gene key  FBgn0031337-FBgn0051928  
  Heatmap region span   2L:1256426..1651260   
  Segment span   2L:1368102..1492295   
  Length (genes)  3  
  Length (bp)  124194  
   Model Scoring   
  BIC  388.449315  
  logL  -188.754040  
  logL ratio  -55.416141  
   Expression   
  Mean expression  5.570142  
  Median expression  4.738249  
  Tissue std. dev.  0.586942  
 
  No GO Slim enrichment  
  
   tissue    mean expression   
  5th Passage Drosophila S2 Cells  5.689117  
  Adult Accessory gland  4.843685  
  Adult Brain  5.556970  
  Adult Carcass  5.427950  
  Adult Crop  4.933839  
  Adult Eye  4.896376  
  Adult Fatbody  6.088227  
  Adult Female Spermatheca Mated  5.992877  
  Adult Female Spermatheca Virgin  5.906726  
  Adult Head  4.948020  
  Adult Heart  5.737323  
  Adult Hind Gut  5.146078  
  Adult Male Ejaculatory Duct  5.419532  
  Adult Mid Gut  5.539191  
  Adult Ovary  6.393862  
  Adult Salivary Gland  5.026727  
  Adult Testes  7.270024  
  Adult Thoracoabdominal ganglion  5.736401  
  Adult Whole Fly  6.658643  
  Larvae Wandering Tubules  5.517643  
  Larval Feeding Carcass  5.409172  
  Larval Feeding Central Nevous System  6.045285  
  Larval Feeding Hind Gut  5.022873  
  Larval Feeding Malpighian Tubule  4.810101  
  Larval Feeding Mid Gut  5.456744  
  Larval Feeding Salivary Gland  4.982191  
  Whole Larvae Feeding  5.938259  
 
  
   FlyBase ID    symbol    start    end    strand    length   
   FBgn0031337   CG14346   1368102   1369047  +  946  
   FBgn0002543   lea  1380086   1420449   -  40364  
   FBgn0051928   CG31928   1492295   1493645  +  1351  
 
 
    Segment 43 
 
   Location   
  Gene key  FBgn0051926-FBgn0031351  
  Heatmap region span   2L:1359784..1724391   
  Segment span   2L:1495747..1611421   
  Length (genes)  11  
  Length (bp)  115675  
   Model Scoring   
  BIC  1205.382527  
  logL  -597.220646  
  logL ratio  -6.757892  
   Expression   
  Mean expression  5.261540  
  Median expression  4.671315  
  Tissue std. dev.  0.461544  
 
  No GO Slim enrichment  
  
   tissue    mean expression   
  5th Passage Drosophila S2 Cells  5.248433  
  Adult Accessory gland  5.143890  
  Adult Brain  5.125885  
  Adult Carcass  5.149996  
  Adult Crop  5.205847  
  Adult Eye  4.974757  
  Adult Fatbody  5.014426  
  Adult Female Spermatheca Mated  5.092472  
  Adult Female Spermatheca Virgin  5.053073  
  Adult Head  5.052992  
  Adult Heart  4.961798  
  Adult Hind Gut  5.067077  
  Adult Male Ejaculatory Duct  5.122714  
  Adult Mid Gut  5.050591  
  Adult Ovary  6.223052  
  Adult Salivary Gland  5.521637  
  Adult Testes  7.025831  
  Adult Thoracoabdominal ganglion  5.198086  
  Adult Whole Fly  6.108581  
  Larvae Wandering Tubules  5.333271  
  Larval Feeding Carcass  4.862894  
  Larval Feeding Central Nevous System  4.932388  
  Larval Feeding Hind Gut  4.967361  
  Larval Feeding Malpighian Tubule  5.222404  
  Larval Feeding Mid Gut  5.207945  
  Larval Feeding Salivary Gland  5.166193  
  Whole Larvae Feeding  5.027989  
 
  
   FlyBase ID    symbol    start    end    strand    length   
   FBgn0051926   CG31926   1495747   1496979  +  1233  
   FBgn0051661   CG31661   1497254   1498435  +  1182  
   FBgn0031343   CG18131  1498455   1500760   -  2306  
   FBgn0031344   CG7420  1500835   1502903   -  2069  
   FBgn0031345   CG18132  1516790   1517478   -  689  
   FBgn0001174   halo  1517532   1518184   -  653  
   FBgn0026398   Or22a   1520613   1522151  +  1539  
   FBgn0026397   Or22b   1522691   1524259  +  1569  
   FBgn0031347   CG10869  1584864   1587156   -  2293  
   FBgn0051935   CG31935  1595043   1611125   -  16083  
   FBgn0031351   CG14352   1611421   1612579  +  1159  
 
 
    Segment 44 
 
   Location   
  Gene key  FBgn0011570-FBgn0031356  
  Heatmap region span   2L:1494090..1823331   
  Segment span   2L:1704904..1710415   
  Length (genes)  2  
  Length (bp)  5512  
   Model Scoring   
  BIC  228.302225  
  logL  -108.680495  
  logL ratio  66.301538  
   Expression   
  Mean expression  10.162720  
  Median expression  10.066779  
  Tissue std. dev.  0.937165  
 
  No GO Slim enrichment  
  
   tissue    mean expression   
  5th Passage Drosophila S2 Cells  12.671665  
  Adult Accessory gland  10.550466  
  Adult Brain  9.369212  
  Adult Carcass  9.003321  
  Adult Crop  11.108488  
  Adult Eye  9.322548  
  Adult Fatbody  8.880596  
  Adult Female Spermatheca Mated  9.246617  
  Adult Female Spermatheca Virgin  9.149249  
  Adult Head  9.077527  
  Adult Heart  9.474467  
  Adult Hind Gut  10.532252  
  Adult Male Ejaculatory Duct  9.855315  
  Adult Mid Gut  10.671715  
  Adult Ovary  10.069913  
  Adult Salivary Gland  11.058571  
  Adult Testes  9.041708  
  Adult Thoracoabdominal ganglion  9.363529  
  Adult Whole Fly  9.445522  
  Larvae Wandering Tubules  11.321328  
  Larval Feeding Carcass  10.220009  
  Larval Feeding Central Nevous System  10.268190  
  Larval Feeding Hind Gut  10.882720  
  Larval Feeding Malpighian Tubule  11.172338  
  Larval Feeding Mid Gut  11.208521  
  Larval Feeding Salivary Gland  11.049912  
  Whole Larvae Feeding  10.377745  
 
  
   FlyBase ID    symbol    start    end    strand    length   
   FBgn0011570   cpb   1704904   1706962  +  2059  
   FBgn0031356   CG17660  1707132   1710415   -  3284  
 
    Segment 45 
 
   Location   
  Gene key  FBgn0031357-FBgn0031359  
  Heatmap region span   2L:1495747..1826378   
  Segment span   2L:1710466..1724391   
  Length (genes)  3  
  Length (bp)  13926  
   Model Scoring   
  BIC  313.852564  
  logL  -151.455665  
  logL ratio  74.651794  
   Expression   
  Mean expression  8.340710  
  Median expression  8.299081  
  Tissue std. dev.  0.535239  
 
  No GO Slim enrichment  
  
   tissue    mean expression   
  5th Passage Drosophila S2 Cells  8.881263  
  Adult Accessory gland  8.248022  
  Adult Brain  8.279053  
  Adult Carcass  8.649016  
  Adult Crop  8.183909  
  Adult Eye  9.098334  
  Adult Fatbody  8.742445  
  Adult Female Spermatheca Mated  8.280119  
  Adult Female Spermatheca Virgin  8.084321  
  Adult Head  8.430786  
  Adult Heart  9.433828  
  Adult Hind Gut  8.922294  
  Adult Male Ejaculatory Duct  7.880016  
  Adult Mid Gut  8.234491  
  Adult Ovary  8.567574  
  Adult Salivary Gland  8.158666  
  Adult Testes  6.680762  
  Adult Thoracoabdominal ganglion  8.294862  
  Adult Whole Fly  8.153795  
  Larvae Wandering Tubules  8.531682  
  Larval Feeding Carcass  7.783454  
  Larval Feeding Central Nevous System  7.944778  
  Larval Feeding Hind Gut  8.719668  
  Larval Feeding Malpighian Tubule  9.159624  
  Larval Feeding Mid Gut  8.189843  
  Larval Feeding Salivary Gland  7.898060  
  Whole Larvae Feeding  7.768505  
 
  
   FlyBase ID    symbol    start    end    strand    length   
   FBgn0031357   mRpL48   1710466   1711466  +  1001  
   FBgn0086698   frtz  1711299   1715861   -  4563  
   FBgn0031359   CG18317  1716888   1724391   -  7504  
 
 
    Segment 46 
 
   Location   
  Gene key  FBgn0031360-FBgn0027597  
  Heatmap region span   2L:1651260..1869867   
  Segment span   2L:1729946..1752116   
  Length (genes)  4  
  Length (bp)  22171  
   Model Scoring   
  BIC  533.549673  
  logL  -261.304219  
  logL ratio  7.367493  
   Expression   
  Mean expression  8.717814  
  Median expression  8.170265  
  Tissue std. dev.  0.777510  
 
  No GO Slim enrichment  
  
   tissue    mean expression   
  5th Passage Drosophila S2 Cells  7.637729  
  Adult Accessory gland  7.796881  
  Adult Brain  8.574888  
  Adult Carcass  9.372542  
  Adult Crop  9.238250  
  Adult Eye  8.973373  
  Adult Fatbody  9.638848  
  Adult Female Spermatheca Mated  9.640553  
  Adult Female Spermatheca Virgin  9.677557  
  Adult Head  8.990294  
  Adult Heart  9.820331  
  Adult Hind Gut  8.560325  
  Adult Male Ejaculatory Duct  8.802287  
  Adult Mid Gut  7.860167  
  Adult Ovary  8.385438  
  Adult Salivary Gland  9.145238  
  Adult Testes  7.638955  
  Adult Thoracoabdominal ganglion  8.572910  
  Adult Whole Fly  8.698128  
  Larvae Wandering Tubules  10.133465  
  Larval Feeding Carcass  8.318687  
  Larval Feeding Central Nevous System  7.861053  
  Larval Feeding Hind Gut  8.221646  
  Larval Feeding Malpighian Tubule  10.174865  
  Larval Feeding Mid Gut  7.934559  
  Larval Feeding Salivary Gland  7.852377  
  Whole Larvae Feeding  7.859643  
 
  
   FlyBase ID    symbol    start    end    strand    length   
   FBgn0031360   CG31937   1729946   1731070  +  1125  
   FBgn0031361   CG17652  1731358   1732356   -  999  
   FBgn0031362      1732526   1750613  +  18088  
   FBgn0027597   CG17712  1750687   1752116   -  1430  
 
 
    Segment 47 
 
   Location   
  Gene key  FBgn0031364-FBgn0051933  
  Heatmap region span   2L:1704904..1880644   
  Segment span   2L:1752349..1823331   
  Length (genes)  8  
  Length (bp)  70983  
   Model Scoring   
  BIC  604.846414  
  logL  -296.952590  
  logL ratio  231.786515  
   Expression   
  Mean expression  4.634792  
  Median expression  4.585489  
  Tissue std. dev.  0.405096  
 
  No GO Slim enrichment  
  
   tissue    mean expression   
  5th Passage Drosophila S2 Cells  4.516126  
  Adult Accessory gland  4.638518  
  Adult Brain  4.344967  
  Adult Carcass  4.707377  
  Adult Crop  4.544174  
  Adult Eye  4.411080  
  Adult Fatbody  4.545188  
  Adult Female Spermatheca Mated  4.698381  
  Adult Female Spermatheca Virgin  4.711819  
  Adult Head  4.350587  
  Adult Heart  4.466541  
  Adult Hind Gut  4.485299  
  Adult Male Ejaculatory Duct  4.609614  
  Adult Mid Gut  4.684731  
  Adult Ovary  4.459083  
  Adult Salivary Gland  4.841336  
  Adult Testes  6.586359  
  Adult Thoracoabdominal ganglion  4.471269  
  Adult Whole Fly  4.568153  
  Larvae Wandering Tubules  4.767705  
  Larval Feeding Carcass  4.573014  
  Larval Feeding Central Nevous System  4.256345  
  Larval Feeding Hind Gut  4.447974  
  Larval Feeding Malpighian Tubule  4.633631  
  Larval Feeding Mid Gut  4.580901  
  Larval Feeding Salivary Gland  4.607625  
  Whole Larvae Feeding  4.631584  
 
  
   FlyBase ID    symbol    start    end    strand    length   
   FBgn0031364   CG17648   1752349   1752851  +  503  
   FBgn0041249   Gr22f  1755253   1756451   -  1199  
   FBgn0031365   CG17650  1758286   1759078   -  793  
   FBgn0045497   Gr22e   1784062   1785286  +  1225  
   FBgn0045499     1790617   1791823   -  1207  
   FBgn0045500   Gr22b  1792057   1793268   -  1212  
   FBgn0045501   Gr22a  1793668   1794917   -  1250  
   FBgn0051933   CG31933  1821256   1823331   -  2076  
 
 
    Segment 48 
 
   Location   
  Gene key  FBgn0040719-FBgn0053673  
  Heatmap region span   2L:1869867..2006307   
  Segment span   2L:1945709..1947359   
  Length (genes)  2  
  Length (bp)  1651  
   Model Scoring   
  BIC  179.236028  
  logL  -84.147397  
  logL ratio  37.947263  
   Expression   
  Mean expression  4.670801  
  Median expression  4.521700  
  Tissue std. dev.  0.956953  
 
  No GO Slim enrichment  
  
   tissue    mean expression   
  5th Passage Drosophila S2 Cells  4.352368  
  Adult Accessory gland  4.428000  
  Adult Brain  4.181162  
  Adult Carcass  4.578223  
  Adult Crop  4.497896  
  Adult Eye  4.406610  
  Adult Fatbody  4.444424  
  Adult Female Spermatheca Mated  4.559495  
  Adult Female Spermatheca Virgin  4.614453  
  Adult Head  4.213194  
  Adult Heart  4.480531  
  Adult Hind Gut  4.422757  
  Adult Male Ejaculatory Duct  4.376649  
  Adult Mid Gut  4.617731  
  Adult Ovary  4.330254  
  Adult Salivary Gland  4.692851  
  Adult Testes  9.452452  
  Adult Thoracoabdominal ganglion  4.357579  
  Adult Whole Fly  5.012161  
  Larvae Wandering Tubules  4.551645  
  Larval Feeding Carcass  4.430050  
  Larval Feeding Central Nevous System  4.173882  
  Larval Feeding Hind Gut  4.434665  
  Larval Feeding Malpighian Tubule  4.559368  
  Larval Feeding Mid Gut  4.541371  
  Larval Feeding Salivary Gland  4.424582  
  Whole Larvae Feeding  4.977277  
 
  
   FlyBase ID    symbol    start    end    strand    length   
   FBgn0040719   CG15357  1945225   1945709   -  485  
   FBgn0053673   CG33673  1945840   1947359   -  1520  
 
    Segment 49 
 
   Location   
  Gene key  FBgn0031375-FBgn0031377  
  Heatmap region span   2L:1880644..2008460   
  Segment span   2L:1954519..1980755   
  Length (genes)  2  
  Length (bp)  26237  
   Model Scoring   
  BIC  229.615891  
  logL  -109.337328  
  logL ratio  1.081270  
   Expression   
  Mean expression  5.360718  
  Median expression  5.458090  
  Tissue std. dev.  0.892764  
 
  No GO Slim enrichment  
  
   tissue    mean expression   
  5th Passage Drosophila S2 Cells  5.625280  
  Adult Accessory gland  5.125371  
  Adult Brain  7.965660  
  Adult Carcass  4.982973  
  Adult Crop  4.845483  
  Adult Eye  6.884322  
  Adult Fatbody  5.001791  
  Adult Female Spermatheca Mated  4.776544  
  Adult Female Spermatheca Virgin  4.746533  
  Adult Head  6.402895  
  Adult Heart  4.969469  
  Adult Hind Gut  4.822002  
  Adult Male Ejaculatory Duct  5.121997  
  Adult Mid Gut  4.891111  
  Adult Ovary  5.626244  
  Adult Salivary Gland  5.140958  
  Adult Testes  4.958050  
  Adult Thoracoabdominal ganglion  5.086023  
  Adult Whole Fly  4.704563  
  Larvae Wandering Tubules  4.628450  
  Larval Feeding Carcass  5.005159  
  Larval Feeding Central Nevous System  8.019824  
  Larval Feeding Hind Gut  4.820127  
  Larval Feeding Malpighian Tubule  4.883809  
  Larval Feeding Mid Gut  5.064920  
  Larval Feeding Salivary Gland  5.536638  
  Whole Larvae Feeding  5.103196  
 
  
   FlyBase ID    symbol    start    end    strand    length   
   FBgn0031375   erm  1950235   1954519   -  4285  
   FBgn0031377   CG15356  1976236   1980755   -  4520  
 
    Segment 50 
 
   Location   
  Gene key  FBgn0031378-FBgn0031379  
  Heatmap region span   2L:1884056..2109878   
  Segment span   2L:1981310..1982234   
  Length (genes)  2  
  Length (bp)  925  
   Model Scoring   
  BIC  243.081937  
  logL  -116.070351  
  logL ratio  18.712442  
   Expression   
  Mean expression  8.305233  
  Median expression  8.618363  
  Tissue std. dev.  0.684666  
 
  No GO Slim enrichment  
  
   tissue    mean expression   
  5th Passage Drosophila S2 Cells  8.834594  
  Adult Accessory gland  9.570667  
  Adult Brain  7.538295  
  Adult Carcass  7.651970  
  Adult Crop  8.562743  
  Adult Eye  8.155885  
  Adult Fatbody  7.845650  
  Adult Female Spermatheca Mated  8.004306  
  Adult Female Spermatheca Virgin  7.808388  
  Adult Head  7.769884  
  Adult Heart  8.606589  
  Adult Hind Gut  8.100068  
  Adult Male Ejaculatory Duct  8.651938  
  Adult Mid Gut  8.092413  
  Adult Ovary  8.876637  
  Adult Salivary Gland  9.545188  
  Adult Testes  10.233107  
  Adult Thoracoabdominal ganglion  7.871727  
  Adult Whole Fly  8.313527  
  Larvae Wandering Tubules  8.250338  
  Larval Feeding Carcass  7.794923  
  Larval Feeding Central Nevous System  7.553144  
  Larval Feeding Hind Gut  8.047412  
  Larval Feeding Malpighian Tubule  8.054156  
  Larval Feeding Mid Gut  7.727005  
  Larval Feeding Salivary Gland  9.245309  
  Whole Larvae Feeding  7.535418  
 
  
   FlyBase ID    symbol    start    end    strand    length   
   FBgn0031378   CG15362   1981310   1981927  +  618  
   FBgn0031379   CG7289   1982234   1984512  +  2279  
 
    Segment 51 
 
   Location   
  Gene key  FBgn0001125-FBgn0031381  
  Heatmap region span   2L:1884984..2129211   
  Segment span   2L:1987761..1988292   
  Length (genes)  2  
  Length (bp)  532  
   Model Scoring   
  BIC  298.627262  
  logL  -143.843014  
  logL ratio  73.201663  
   Expression   
  Mean expression  11.807466  
  Median expression  11.932970  
  Tissue std. dev.  0.646223  
 
  No GO Slim enrichment  
  
   tissue    mean expression   
  5th Passage Drosophila S2 Cells  11.202316  
  Adult Accessory gland  11.872069  
  Adult Brain  12.314371  
  Adult Carcass  11.690724  
  Adult Crop  11.515360  
  Adult Eye  13.292523  
  Adult Fatbody  11.482954  
  Adult Female Spermatheca Mated  11.016737  
  Adult Female Spermatheca Virgin  11.655331  
  Adult Head  12.740008  
  Adult Heart  12.882863  
  Adult Hind Gut  12.161282  
  Adult Male Ejaculatory Duct  11.248086  
  Adult Mid Gut  11.521329  
  Adult Ovary  12.162092  
  Adult Salivary Gland  12.861714  
  Adult Testes  10.889458  
  Adult Thoracoabdominal ganglion  12.552624  
  Adult Whole Fly  12.011893  
  Larvae Wandering Tubules  11.554886  
  Larval Feeding Carcass  10.976797  
  Larval Feeding Central Nevous System  11.731325  
  Larval Feeding Hind Gut  10.672663  
  Larval Feeding Malpighian Tubule  11.264010  
  Larval Feeding Mid Gut  11.761434  
  Larval Feeding Salivary Gland  11.955741  
  Whole Larvae Feeding  11.810997  
 
  
   FlyBase ID    symbol    start    end    strand    length   
   FBgn0001125   Got2  1984512   1987761   -  3250  
   FBgn0031381   Npc2a   1988292   1988940  +  649  
 
    Segment 52 
 
   Location   
  Gene key  FBgn0043539-FBgn0053543  
  Heatmap region span   2L:1945709..2130815   
  Segment span   2L:1992152..2006307   
  Length (genes)  2  
  Length (bp)  14156  
   Model Scoring   
  BIC  227.092426  
  logL  -108.075596  
  logL ratio  -5.042877  
   Expression   
  Mean expression  4.839128  
  Median expression  4.518714  
  Tissue std. dev.  0.953645  
 
  No GO Slim enrichment  
  
   tissue    mean expression   
  5th Passage Drosophila S2 Cells  4.528335  
  Adult Accessory gland  8.744262  
  Adult Brain  5.417798  
  Adult Carcass  4.673052  
  Adult Crop  4.303799  
  Adult Eye  4.333806  
  Adult Fatbody  4.456223  
  Adult Female Spermatheca Mated  4.522741  
  Adult Female Spermatheca Virgin  4.537498  
  Adult Head  4.619459  
  Adult Heart  4.243303  
  Adult Hind Gut  4.351849  
  Adult Male Ejaculatory Duct  6.920456  
  Adult Mid Gut  4.550081  
  Adult Ovary  4.366449  
  Adult Salivary Gland  4.656923  
  Adult Testes  4.689032  
  Adult Thoracoabdominal ganglion  5.300370  
  Adult Whole Fly  5.850238  
  Larvae Wandering Tubules  4.558319  
  Larval Feeding Carcass  4.388863  
  Larval Feeding Central Nevous System  4.723620  
  Larval Feeding Hind Gut  4.264224  
  Larval Feeding Malpighian Tubule  4.428233  
  Larval Feeding Mid Gut  4.530412  
  Larval Feeding Salivary Gland  4.459920  
  Whole Larvae Feeding  4.237194  
 
  
   FlyBase ID    symbol    start    end    strand    length   
   FBgn0043539   Obp22a  1991705   1992152   -  448  
   FBgn0053543   CG33543  1997102   2006307   -  9206  
 
    Segment 53 
 
   Location   
  Gene key  FBgn0040718-FBgn0040717  
  Heatmap region span   2L:1954519..2192372   
  Segment span   2L:2007193..2008460   
  Length (genes)  2  
  Length (bp)  1268  
   Model Scoring   
  BIC  246.338473  
  logL  -117.698619  
  logL ratio  47.726866  
   Expression   
  Mean expression  8.848872  
  Median expression  8.279046  
  Tissue std. dev.  2.321921  
 
  No GO Slim enrichment  
  
   tissue    mean expression   
  5th Passage Drosophila S2 Cells  5.965521  
  Adult Accessory gland  6.781901  
  Adult Brain  6.111894  
  Adult Carcass  9.134370  
  Adult Crop  7.952128  
  Adult Eye  12.521676  
  Adult Fatbody  8.825459  
  Adult Female Spermatheca Mated  8.328601  
  Adult Female Spermatheca Virgin  8.290886  
  Adult Head  8.778310  
  Adult Heart  9.554796  
  Adult Hind Gut  10.001372  
  Adult Male Ejaculatory Duct  8.231699  
  Adult Mid Gut  7.371776  
  Adult Ovary  6.088762  
  Adult Salivary Gland  9.061996  
  Adult Testes  6.306589  
  Adult Thoracoabdominal ganglion  7.334653  
  Adult Whole Fly  6.708628  
  Larvae Wandering Tubules  6.259437  
  Larval Feeding Carcass  12.864970  
  Larval Feeding Central Nevous System  12.115271  
  Larval Feeding Hind Gut  11.415968  
  Larval Feeding Malpighian Tubule  6.804004  
  Larval Feeding Mid Gut  9.421636  
  Larval Feeding Salivary Gland  13.091046  
  Whole Larvae Feeding  13.596186  
 
  
   FlyBase ID    symbol    start    end    strand    length   
   FBgn0040718   CG15353  2006763   2007193   -  431  
   FBgn0040717   Nplp4   2008460   2008966  +  507  
 
    Segment 54 
 
   Location   
  Gene key  FBgn0031384-FBgn0053516  
  Heatmap region span   2L:1981310..2193010   
  Segment span   2L:2010118..2109878   
  Length (genes)  6  
  Length (bp)  99761  
   Model Scoring   
  BIC  509.840399  
  logL  -249.449582  
  logL ratio  131.149703  
   Expression   
  Mean expression  4.807266  
  Median expression  4.613772  
  Tissue std. dev.  0.371858  
 
  No GO Slim enrichment  
  
   tissue    mean expression   
  5th Passage Drosophila S2 Cells  4.971498  
  Adult Accessory gland  5.712686  
  Adult Brain  4.822168  
  Adult Carcass  4.699953  
  Adult Crop  4.663042  
  Adult Eye  4.484620  
  Adult Fatbody  4.683460  
  Adult Female Spermatheca Mated  4.592740  
  Adult Female Spermatheca Virgin  4.598759  
  Adult Head  4.499613  
  Adult Heart  4.661869  
  Adult Hind Gut  4.808008  
  Adult Male Ejaculatory Duct  4.735609  
  Adult Mid Gut  4.630827  
  Adult Ovary  5.238656  
  Adult Salivary Gland  4.893355  
  Adult Testes  6.229936  
  Adult Thoracoabdominal ganglion  4.901648  
  Adult Whole Fly  4.692287  
  Larvae Wandering Tubules  4.632531  
  Larval Feeding Carcass  4.641800  
  Larval Feeding Central Nevous System  4.829308  
  Larval Feeding Hind Gut  4.754373  
  Larval Feeding Malpighian Tubule  4.544036  
  Larval Feeding Mid Gut  4.565482  
  Larval Feeding Salivary Gland  4.559998  
  Whole Larvae Feeding  4.747914  
 
  
   FlyBase ID    symbol    start    end    strand    length   
   FBgn0031384   CG4238   2010118   2030536  +  20419  
   FBgn0259192   CG42296  2032635   2035936   -  3302  
   FBgn0028952   Kebab   2045525   2048054  +  2530  
   FBgn0026396   Or22c   2054589   2057967  +  3379  
   FBgn0031388   CG12674   2079977   2081286  +  1310  
   FBgn0053516   dpr3  2058859   2109878   -  51020  
 
 
    Segment 55 
 
   Location   
  Gene key  FBgn0031390-FBgn0031395  
  Heatmap region span   2L:2007193..2214179   
  Segment span   2L:2145175..2192372   
  Length (genes)  7  
  Length (bp)  47198  
   Model Scoring   
  BIC  707.246283  
  logL  -348.152524  
  logL ratio  172.699303  
   Expression   
  Mean expression  8.172177  
  Median expression  8.299382  
  Tissue std. dev.  0.386699  
 
  No GO Slim enrichment  
  
   tissue    mean expression   
  5th Passage Drosophila S2 Cells  9.070577  
  Adult Accessory gland  8.171117  
  Adult Brain  8.513827  
  Adult Carcass  7.587318  
  Adult Crop  8.226714  
  Adult Eye  8.034007  
  Adult Fatbody  8.106079  
  Adult Female Spermatheca Mated  8.295555  
  Adult Female Spermatheca Virgin  8.288062  
  Adult Head  7.850707  
  Adult Heart  8.056043  
  Adult Hind Gut  7.885870  
  Adult Male Ejaculatory Duct  7.776468  
  Adult Mid Gut  7.984868  
  Adult Ovary  9.109768  
  Adult Salivary Gland  7.789701  
  Adult Testes  8.393107  
  Adult Thoracoabdominal ganglion  8.663035  
  Adult Whole Fly  8.004891  
  Larvae Wandering Tubules  8.183191  
  Larval Feeding Carcass  8.049502  
  Larval Feeding Central Nevous System  8.920587  
  Larval Feeding Hind Gut  8.075528  
  Larval Feeding Malpighian Tubule  8.169507  
  Larval Feeding Mid Gut  7.865962  
  Larval Feeding Salivary Gland  7.858867  
  Whole Larvae Feeding  7.717926  
 
  
   FlyBase ID    symbol    start    end    strand    length   
   FBgn0031390   tho2  2137350   2145175   -  7826  
   FBgn0031391   CG11723  2145497   2147287   -  1791  
   FBgn0027509   CG7261   2147519   2151664  +  4146  
   FBgn0031392   AIF   2151664   2155390  +  3727  
   FBgn0031393   CG15382   2155760   2156791  +  1032  
   FBgn0000097   aop  2156484   2178749   -  22266  
   FBgn0031395   CG10874  2189913   2192372   -  2460  
 
 
    Segment 56 
 
   Location   
  Gene key  FBgn0051668-FBgn0053124  
  Heatmap region span   2L:2129211..2220767   
  Segment span   2L:2206028..2208906   
  Length (genes)  2  
  Length (bp)  2879  
   Model Scoring   
  BIC  187.725284  
  logL  -88.392025  
  logL ratio  28.109079  
   Expression   
  Mean expression  5.142253  
  Median expression  4.991583  
  Tissue std. dev.  0.383521  
 
  
   GO ID    description    ratio    P-value   
   GO:0055085   transmembrane transport  2/2  0.000497  
   GO:0006810   transport  2/2  0.00239  
 
  
   tissue    mean expression   
  5th Passage Drosophila S2 Cells  5.032571  
  Adult Accessory gland  5.421966  
  Adult Brain  4.685027  
  Adult Carcass  5.005571  
  Adult Crop  4.979924  
  Adult Eye  5.100290  
  Adult Fatbody  5.136182  
  Adult Female Spermatheca Mated  5.122338  
  Adult Female Spermatheca Virgin  5.110486  
  Adult Head  4.936999  
  Adult Heart  5.018580  
  Adult Hind Gut  5.165820  
  Adult Male Ejaculatory Duct  5.098852  
  Adult Mid Gut  5.373960  
  Adult Ovary  4.895855  
  Adult Salivary Gland  5.339925  
  Adult Testes  4.648288  
  Adult Thoracoabdominal ganglion  4.808817  
  Adult Whole Fly  4.600100  
  Larvae Wandering Tubules  5.605299  
  Larval Feeding Carcass  5.635679  
  Larval Feeding Central Nevous System  4.671025  
  Larval Feeding Hind Gut  5.207283  
  Larval Feeding Malpighian Tubule  6.551333  
  Larval Feeding Mid Gut  5.440709  
  Larval Feeding Salivary Gland  5.092588  
  Whole Larvae Feeding  5.155367  
 
  
   FlyBase ID    symbol    start    end    strand    length   
   FBgn0051668   CG31668  2203904   2206028   -  2125  
   FBgn0053124   CG33124  2206796   2208906   -  2111  
 
    Segment 57 
 
   Location   
  Gene key  FBgn0085203-FBgn0031398  
  Heatmap region span   2L:2193010..2255050   
  Segment span   2L:2214305..2216304   
  Length (genes)  2  
  Length (bp)  2000  
   Model Scoring   
  BIC  211.764318  
  logL  -100.411542  
  logL ratio  27.496019  
   Expression   
  Mean expression  6.352359  
  Median expression  6.231118  
  Tissue std. dev.  0.760574  
 
  No GO Slim enrichment  
  
   tissue    mean expression   
  5th Passage Drosophila S2 Cells  5.964773  
  Adult Accessory gland  7.346345  
  Adult Brain  6.431715  
  Adult Carcass  5.464275  
  Adult Crop  6.485040  
  Adult Eye  5.850011  
  Adult Fatbody  5.942025  
  Adult Female Spermatheca Mated  6.763604  
  Adult Female Spermatheca Virgin  6.822252  
  Adult Head  5.643934  
  Adult Heart  6.193412  
  Adult Hind Gut  5.862116  
  Adult Male Ejaculatory Duct  6.400721  
  Adult Mid Gut  5.509666  
  Adult Ovary  8.553788  
  Adult Salivary Gland  5.776543  
  Adult Testes  5.297003  
  Adult Thoracoabdominal ganglion  6.484635  
  Adult Whole Fly  6.730085  
  Larvae Wandering Tubules  7.057709  
  Larval Feeding Carcass  5.979232  
  Larval Feeding Central Nevous System  8.022699  
  Larval Feeding Hind Gut  6.199049  
  Larval Feeding Malpighian Tubule  6.508970  
  Larval Feeding Mid Gut  5.638760  
  Larval Feeding Salivary Gland  6.976183  
  Whole Larvae Feeding  5.609139  
 
  
   FlyBase ID    symbol    start    end    strand    length   
   FBgn0085203   CG34174   2214305   2215167  +  863  
   FBgn0031398   CG10880  2214972   2216304   -  1333  
 
    Segment 58 
 
   Location   
  Gene key  FBgn0031399-FBgn0031401  
  Heatmap region span   2L:2206028..2265321   
  Segment span   2L:2217363..2220767   
  Length (genes)  2  
  Length (bp)  3405  
   Model Scoring   
  BIC  237.662978  
  logL  -113.360872  
  logL ratio  33.645551  
   Expression   
  Mean expression  9.173382  
  Median expression  9.120484  
  Tissue std. dev.  0.449206  
 
  No GO Slim enrichment  
  
   tissue    mean expression   
  5th Passage Drosophila S2 Cells  9.220709  
  Adult Accessory gland  8.717439  
  Adult Brain  8.446611  
  Adult Carcass  9.036897  
  Adult Crop  9.795898  
  Adult Eye  8.845457  
  Adult Fatbody  8.883727  
  Adult Female Spermatheca Mated  9.381083  
  Adult Female Spermatheca Virgin  9.108797  
  Adult Head  8.757829  
  Adult Heart  9.620511  
  Adult Hind Gut  9.754034  
  Adult Male Ejaculatory Duct  9.584060  
  Adult Mid Gut  8.915736  
  Adult Ovary  9.093977  
  Adult Salivary Gland  9.422227  
  Adult Testes  9.911416  
  Adult Thoracoabdominal ganglion  8.551065  
  Adult Whole Fly  8.671065  
  Larvae Wandering Tubules  8.622846  
  Larval Feeding Carcass  9.746550  
  Larval Feeding Central Nevous System  8.585014  
  Larval Feeding Hind Gut  10.065081  
  Larval Feeding Malpighian Tubule  9.184636  
  Larval Feeding Mid Gut  9.297898  
  Larval Feeding Salivary Gland  9.039816  
  Whole Larvae Feeding  9.420936  
 
  
   FlyBase ID    symbol    start    end    strand    length   
   FBgn0031399   mio   2217363   2220625  +  3263  
   FBgn0031401   papi   2220767   2225555  +  4789  
 
    Segment 59 
 
   Location   
  Gene key  FBgn0031403-FBgn0051682  
  Heatmap region span   2L:2212571..2285174   
  Segment span   2L:2226840..2240593   
  Length (genes)  4  
  Length (bp)  13754  
   Model Scoring   
  BIC  329.102978  
  logL  -159.080872  
  logL ratio  90.562422  
   Expression   
  Mean expression  4.983326  
  Median expression  4.726926  
  Tissue std. dev.  0.853203  
 
  
   GO ID    description    ratio    P-value   
   GO:0005739   mitochondrion  3/4  1.81e-05  
   GO:0043226   organelle  3/4  0.0221  
 
  
   tissue    mean expression   
  5th Passage Drosophila S2 Cells  4.826478  
  Adult Accessory gland  4.888451  
  Adult Brain  4.405343  
  Adult Carcass  4.904633  
  Adult Crop  4.787858  
  Adult Eye  4.574776  
  Adult Fatbody  4.831527  
  Adult Female Spermatheca Mated  4.675005  
  Adult Female Spermatheca Virgin  4.659448  
  Adult Head  4.499111  
  Adult Heart  4.622655  
  Adult Hind Gut  4.601752  
  Adult Male Ejaculatory Duct  4.899121  
  Adult Mid Gut  4.976540  
  Adult Ovary  4.868912  
  Adult Salivary Gland  5.208112  
  Adult Testes  9.099711  
  Adult Thoracoabdominal ganglion  4.428987  
  Adult Whole Fly  5.838058  
  Larvae Wandering Tubules  4.976511  
  Larval Feeding Carcass  4.836491  
  Larval Feeding Central Nevous System  4.594055  
  Larval Feeding Hind Gut  4.789594  
  Larval Feeding Malpighian Tubule  4.827569  
  Larval Feeding Mid Gut  4.809512  
  Larval Feeding Salivary Gland  5.139143  
  Whole Larvae Feeding  4.980445  
 
  
   FlyBase ID    symbol    start    end    strand    length   
   FBgn0031403   CG15387  2225549   2226840   -  1292  
   FBgn0051679   Tengl3  2237076   2238251   -  1176  
   FBgn0052463   Tengl2  2238304   2239384   -  1081  
   FBgn0051682   Tengl1  2239414   2240593   -  1180  
 
 
    Segment 60 
 
   Location   
  Gene key  FBgn0051686-FBgn0031406  
  Heatmap region span   2L:2214305..2300103   
  Segment span   2L:2244637..2255050   
  Length (genes)  5  
  Length (bp)  10414  
   Model Scoring   
  BIC  438.143306  
  logL  -213.601036  
  logL ratio  154.579288  
   Expression   
  Mean expression  5.296927  
  Median expression  4.421528  
  Tissue std. dev.  2.345857  
 
  No GO Slim enrichment  
  
   tissue    mean expression   
  5th Passage Drosophila S2 Cells  4.497694  
  Adult Accessory gland  4.466736  
  Adult Brain  4.102063  
  Adult Carcass  5.451562  
  Adult Crop  4.852444  
  Adult Eye  4.216808  
  Adult Fatbody  4.865971  
  Adult Female Spermatheca Mated  13.606355  
  Adult Female Spermatheca Virgin  13.136033  
  Adult Head  4.310388  
  Adult Heart  4.514045  
  Adult Hind Gut  5.346717  
  Adult Male Ejaculatory Duct  4.517434  
  Adult Mid Gut  4.671842  
  Adult Ovary  4.559923  
  Adult Salivary Gland  5.219525  
  Adult Testes  4.234884  
  Adult Thoracoabdominal ganglion  4.253365  
  Adult Whole Fly  6.773969  
  Larvae Wandering Tubules  4.410172  
  Larval Feeding Carcass  4.498801  
  Larval Feeding Central Nevous System  4.178055  
  Larval Feeding Hind Gut  4.631363  
  Larval Feeding Malpighian Tubule  4.370886  
  Larval Feeding Mid Gut  4.574583  
  Larval Feeding Salivary Gland  4.504686  
  Whole Larvae Feeding  4.250723  
 
  
   FlyBase ID    symbol    start    end    strand    length   
   FBgn0051686      2244637   2245352  +  716  
   FBgn0011832   Ser12  2250431   2251275   -  845  
   FBgn0042186   CG17239  2251572   2252341   -  770  
   FBgn0042187   CG17234   2253079   2253834  +  756  
   FBgn0031406   Send1  2254176   2255050   -  875  
 
 
    Segment 61 
 
   Location   
  Gene key  FBgn0031407-FBgn0054049  
  Heatmap region span   2L:2217363..2307948   
  Segment span   2L:2263464..2265321   
  Length (genes)  2  
  Length (bp)  1858  
   Model Scoring   
  BIC  188.307925  
  logL  -88.683345  
  logL ratio  35.366476  
   Expression   
  Mean expression  4.650379  
  Median expression  4.505876  
  Tissue std. dev.  1.191612  
 
  
   GO ID    description    ratio    P-value   
   GO:0005576   extracellular region  2/2  0.00207  
 
  
   tissue    mean expression   
  5th Passage Drosophila S2 Cells  4.296087  
  Adult Accessory gland  4.635461  
  Adult Brain  4.314594  
  Adult Carcass  4.531098  
  Adult Crop  4.302459  
  Adult Eye  4.176676  
  Adult Fatbody  4.535676  
  Adult Female Spermatheca Mated  4.730357  
  Adult Female Spermatheca Virgin  4.632321  
  Adult Head  4.200416  
  Adult Heart  4.270361  
  Adult Hind Gut  4.266940  
  Adult Male Ejaculatory Duct  4.373755  
  Adult Mid Gut  4.200094  
  Adult Ovary  4.251711  
  Adult Salivary Gland  4.723062  
  Adult Testes  10.498410  
  Adult Thoracoabdominal ganglion  4.344085  
  Adult Whole Fly  5.850401  
  Larvae Wandering Tubules  4.341495  
  Larval Feeding Carcass  4.251226  
  Larval Feeding Central Nevous System  4.197968  
  Larval Feeding Hind Gut  4.168859  
  Larval Feeding Malpighian Tubule  4.454143  
  Larval Feeding Mid Gut  4.205699  
  Larval Feeding Salivary Gland  4.397161  
  Whole Larvae Feeding  4.409718  
 
  
   FlyBase ID    symbol    start    end    strand    length   
   FBgn0031407   CG4270   2263464   2264241  +  778  
   FBgn0054049   CG34049  2264231   2265321   -  1091  
 
    Segment 62 
 
   Location   
  Gene key  FBgn0250841-FBgn0031409  
  Heatmap region span   2L:2226840..2357332   
  Segment span   2L:2269119..2285174   
  Length (genes)  2  
  Length (bp)  16056  
   Model Scoring   
  BIC  215.594895  
  logL  -102.326830  
  logL ratio  16.726837  
   Expression   
  Mean expression  5.457338  
  Median expression  4.738024  
  Tissue std. dev.  2.040483  
 
  No GO Slim enrichment  
  
   tissue    mean expression   
  5th Passage Drosophila S2 Cells  4.771978  
  Adult Accessory gland  12.673915  
  Adult Brain  4.347411  
  Adult Carcass  4.812476  
  Adult Crop  4.759688  
  Adult Eye  4.340591  
  Adult Fatbody  4.707160  
  Adult Female Spermatheca Mated  5.491354  
  Adult Female Spermatheca Virgin  5.420507  
  Adult Head  4.331753  
  Adult Heart  4.370006  
  Adult Hind Gut  4.640633  
  Adult Male Ejaculatory Duct  11.463789  
  Adult Mid Gut  5.024845  
  Adult Ovary  4.686619  
  Adult Salivary Gland  5.099408  
  Adult Testes  5.815767  
  Adult Thoracoabdominal ganglion  4.590737  
  Adult Whole Fly  8.587043  
  Larvae Wandering Tubules  4.781497  
  Larval Feeding Carcass  4.814048  
  Larval Feeding Central Nevous System  4.374132  
  Larval Feeding Hind Gut  4.518972  
  Larval Feeding Malpighian Tubule  4.691725  
  Larval Feeding Mid Gut  5.008772  
  Larval Feeding Salivary Gland  4.636720  
  Whole Larvae Feeding  4.586574  
 
  
   FlyBase ID    symbol    start    end    strand    length   
   FBgn0250841   CG17242  2268299   2269119   -  821  
   FBgn0031409   CG4271   2285174   2285926  +  753  
 
    Segment 63 
 
   Location   
  Gene key  FBgn0031410-FBgn0051949  
  Heatmap region span   2L:2244637..2371524   
  Segment span   2L:2289370..2300103   
  Length (genes)  3  
  Length (bp)  10734  
   Model Scoring   
  BIC  281.904663  
  logL  -135.481714  
  logL ratio  56.667551  
   Expression   
  Mean expression  5.698332  
  Median expression  5.387391  
  Tissue std. dev.  1.266269  
 
  No GO Slim enrichment  
  
   tissue    mean expression   
  5th Passage Drosophila S2 Cells  5.271900  
  Adult Accessory gland  5.405074  
  Adult Brain  5.085377  
  Adult Carcass  5.637163  
  Adult Crop  5.317040  
  Adult Eye  5.323525  
  Adult Fatbody  5.545558  
  Adult Female Spermatheca Mated  5.415101  
  Adult Female Spermatheca Virgin  5.436720  
  Adult Head  5.199593  
  Adult Heart  5.455867  
  Adult Hind Gut  5.276161  
  Adult Male Ejaculatory Duct  5.336468  
  Adult Mid Gut  5.512642  
  Adult Ovary  5.215293  
  Adult Salivary Gland  5.667382  
  Adult Testes  11.689702  
  Adult Thoracoabdominal ganglion  5.222605  
  Adult Whole Fly  7.576156  
  Larvae Wandering Tubules  5.364788  
  Larval Feeding Carcass  5.360364  
  Larval Feeding Central Nevous System  5.020232  
  Larval Feeding Hind Gut  5.133732  
  Larval Feeding Malpighian Tubule  5.320614  
  Larval Feeding Mid Gut  5.448736  
  Larval Feeding Salivary Gland  5.350430  
  Whole Larvae Feeding  6.266733  
 
  
   FlyBase ID    symbol    start    end    strand    length   
   FBgn0031410   CG17237  2288503   2289370   -  868  
   FBgn0028570   robl22E  2290224   2290673   -  450  
   FBgn0051949   CG31949   2300103   2300888  +  786  
 
 
    Segment 64 
 
   Location   
  Gene key  FBgn0031414-FBgn0031413  
  Heatmap region span   2L:2269119..2383299   
  Segment span   2L:2311693..2357332   
  Length (genes)  2  
  Length (bp)  45640  
   Model Scoring   
  BIC  242.921820  
  logL  -115.990293  
  logL ratio  -10.469117  
   Expression   
  Mean expression  5.874523  
  Median expression  6.145851  
  Tissue std. dev.  0.222515  
 
  No GO Slim enrichment  
  
   tissue    mean expression   
  5th Passage Drosophila S2 Cells  5.716329  
  Adult Accessory gland  5.864578  
  Adult Brain  5.691383  
  Adult Carcass  5.687983  
  Adult Crop  5.674237  
  Adult Eye  6.369594  
  Adult Fatbody  5.780877  
  Adult Female Spermatheca Mated  5.986780  
  Adult Female Spermatheca Virgin  6.032498  
  Adult Head  5.794139  
  Adult Heart  5.556077  
  Adult Hind Gut  5.959401  
  Adult Male Ejaculatory Duct  6.009234  
  Adult Mid Gut  5.791061  
  Adult Ovary  6.328975  
  Adult Salivary Gland  6.335843  
  Adult Testes  5.783627  
  Adult Thoracoabdominal ganglion  5.678892  
  Adult Whole Fly  5.569018  
  Larvae Wandering Tubules  5.915000  
  Larval Feeding Carcass  6.165444  
  Larval Feeding Central Nevous System  5.671324  
  Larval Feeding Hind Gut  6.036104  
  Larval Feeding Malpighian Tubule  5.887425  
  Larval Feeding Mid Gut  5.835073  
  Larval Feeding Salivary Gland  5.854762  
  Whole Larvae Feeding  5.636467  
 
  
   FlyBase ID    symbol    start    end    strand    length   
   FBgn0031414   eys   2311693   2358181  +  46489  
   FBgn0031413   CG9967  2308758   2357332   -  48575  
 
    Segment 65 
 
   Location   
  Gene key  FBgn0031419-FBgn0031422  
  Heatmap region span   2L:2311693..2573047   
  Segment span   2L:2374689..2383299   
  Length (genes)  4  
  Length (bp)  8611  
   Model Scoring   
  BIC  417.161629  
  logL  -203.110197  
  logL ratio  82.191480  
   Expression   
  Mean expression  7.553787  
  Median expression  7.665801  
  Tissue std. dev.  0.441392  
 
  No GO Slim enrichment  
  
   tissue    mean expression   
  5th Passage Drosophila S2 Cells  7.581399  
  Adult Accessory gland  7.122118  
  Adult Brain  8.005554  
  Adult Carcass  6.938165  
  Adult Crop  7.836169  
  Adult Eye  7.536387  
  Adult Fatbody  7.060298  
  Adult Female Spermatheca Mated  7.440084  
  Adult Female Spermatheca Virgin  7.524906  
  Adult Head  7.310395  
  Adult Heart  7.607272  
  Adult Hind Gut  7.545011  
  Adult Male Ejaculatory Duct  7.229035  
  Adult Mid Gut  6.922584  
  Adult Ovary  8.328731  
  Adult Salivary Gland  7.158366  
  Adult Testes  7.134673  
  Adult Thoracoabdominal ganglion  8.089528  
  Adult Whole Fly  6.886972  
  Larvae Wandering Tubules  7.870181  
  Larval Feeding Carcass  8.066654  
  Larval Feeding Central Nevous System  8.384078  
  Larval Feeding Hind Gut  8.023121  
  Larval Feeding Malpighian Tubule  7.966037  
  Larval Feeding Mid Gut  7.047156  
  Larval Feeding Salivary Gland  7.982300  
  Whole Larvae Feeding  7.355069  
 
  
   FlyBase ID    symbol    start    end    strand    length   
   FBgn0031419   CG15390   2374689   2375697  +  1009  
   FBgn0031420   CG9866   2375894   2380514  +  4621  
   FBgn0031421      2381140   2383077  +  1938  
   FBgn0031422   CG9870   2383299   2384731  +  1433  
 
 
    Segment 66 
 
   Location   
  Gene key  FBgn0031423-FBgn0085477  
  Heatmap region span   2L:2365362..2577671   
  Segment span   2L:2385792..2417965   
  Length (genes)  5  
  Length (bp)  32174  
   Model Scoring   
  BIC  460.845650  
  logL  -224.952208  
  logL ratio  66.867067  
   Expression   
  Mean expression  5.204095  
  Median expression  4.958551  
  Tissue std. dev.  0.722561  
 
  
   GO ID    description    ratio    P-value   
   GO:0006629   lipid metabolic process  2/5  0.00164  
 
  
   tissue    mean expression   
  5th Passage Drosophila S2 Cells  4.850329  
  Adult Accessory gland  5.197758  
  Adult Brain  5.224509  
  Adult Carcass  5.197600  
  Adult Crop  5.140346  
  Adult Eye  4.814037  
  Adult Fatbody  5.063302  
  Adult Female Spermatheca Mated  4.935999  
  Adult Female Spermatheca Virgin  4.993158  
  Adult Head  4.960519  
  Adult Heart  4.873284  
  Adult Hind Gut  5.000850  
  Adult Male Ejaculatory Duct  5.018896  
  Adult Mid Gut  5.021386  
  Adult Ovary  4.877711  
  Adult Salivary Gland  5.097733  
  Adult Testes  8.550492  
  Adult Thoracoabdominal ganglion  5.192094  
  Adult Whole Fly  6.397023  
  Larvae Wandering Tubules  4.948615  
  Larval Feeding Carcass  5.015760  
  Larval Feeding Central Nevous System  4.736883  
  Larval Feeding Hind Gut  4.905902  
  Larval Feeding Malpighian Tubule  4.936859  
  Larval Feeding Mid Gut  5.059577  
  Larval Feeding Salivary Gland  4.969907  
  Whole Larvae Feeding  5.530027  
 
  
   FlyBase ID    symbol    start    end    strand    length   
   FBgn0031423   CG3557  2384761   2385792   -  1032  
   FBgn0031424   VGlut   2400306   2410668  +  10363  
   FBgn0031426   CG18641  2410884   2412129   -  1246  
   FBgn0051948   CG31948  2415434   2416720   -  1287  
   FBgn0085477   CG34448  2416809   2417965   -  1157  
 
 
    Segment 67 
 
   Location   
  Gene key  FBgn0031428-FBgn0011818  
  Heatmap region span   2L:2371524..2585448   
  Segment span   2L:2420808..2492955   
  Length (genes)  4  
  Length (bp)  72148  
   Model Scoring   
  BIC  520.911283  
  logL  -254.985024  
  logL ratio  -5.071460  
   Expression   
  Mean expression  8.093832  
  Median expression  7.830965  
  Tissue std. dev.  0.460381  
 
  No GO Slim enrichment  
  
   tissue    mean expression   
  5th Passage Drosophila S2 Cells  7.382796  
  Adult Accessory gland  8.607469  
  Adult Brain  8.045307  
  Adult Carcass  8.380702  
  Adult Crop  9.006331  
  Adult Eye  8.186130  
  Adult Fatbody  8.926353  
  Adult Female Spermatheca Mated  8.548718  
  Adult Female Spermatheca Virgin  8.640808  
  Adult Head  8.108950  
  Adult Heart  8.847278  
  Adult Hind Gut  7.900351  
  Adult Male Ejaculatory Duct  8.459008  
  Adult Mid Gut  7.891526  
  Adult Ovary  7.633919  
  Adult Salivary Gland  8.062720  
  Adult Testes  7.014381  
  Adult Thoracoabdominal ganglion  8.103547  
  Adult Whole Fly  7.634845  
  Larvae Wandering Tubules  7.928367  
  Larval Feeding Carcass  8.094541  
  Larval Feeding Central Nevous System  8.050750  
  Larval Feeding Hind Gut  7.837850  
  Larval Feeding Malpighian Tubule  7.963154  
  Larval Feeding Mid Gut  7.737323  
  Larval Feeding Salivary Gland  7.693664  
  Whole Larvae Feeding  7.846674  
 
  
   FlyBase ID    symbol    start    end    strand    length   
   FBgn0031428   CG9886   2420808   2422915  +  2108  
   FBgn0000490   dpp   2428454   2459609  +  31156  
   FBgn0015816     2490205   2492666   -  2462  
   FBgn0011818   oaf   2492955   2498846  +  5892  
 
 
    Segment 68 
 
   Location   
  Gene key  FBgn0031430-FBgn0031431  
  Heatmap region span   2L:2374553..2588854   
  Segment span   2L:2518037..2552844   
  Length (genes)  2  
  Length (bp)  34808  
   Model Scoring   
  BIC  176.734422  
  logL  -82.896594  
  logL ratio  36.016278  
   Expression   
  Mean expression  5.015395  
  Median expression  4.817826  
  Tissue std. dev.  0.965157  
 
  No GO Slim enrichment  
  
   tissue    mean expression   
  5th Passage Drosophila S2 Cells  4.801607  
  Adult Accessory gland  4.824758  
  Adult Brain  4.629438  
  Adult Carcass  4.876317  
  Adult Crop  4.782066  
  Adult Eye  4.657801  
  Adult Fatbody  4.809910  
  Adult Female Spermatheca Mated  4.955903  
  Adult Female Spermatheca Virgin  4.823349  
  Adult Head  4.664087  
  Adult Heart  4.899247  
  Adult Hind Gut  4.834683  
  Adult Male Ejaculatory Duct  4.760708  
  Adult Mid Gut  5.052075  
  Adult Ovary  4.796765  
  Adult Salivary Gland  4.964938  
  Adult Testes  9.842493  
  Adult Thoracoabdominal ganglion  4.524928  
  Adult Whole Fly  5.454928  
  Larvae Wandering Tubules  4.861773  
  Larval Feeding Carcass  4.765669  
  Larval Feeding Central Nevous System  4.468095  
  Larval Feeding Hind Gut  4.667708  
  Larval Feeding Malpighian Tubule  4.793057  
  Larval Feeding Mid Gut  4.963563  
  Larval Feeding Salivary Gland  4.807829  
  Whole Larvae Feeding  5.131975  
 
  
   FlyBase ID    symbol    start    end    strand    length   
   FBgn0031430   CG3528  2517102   2518037   -  936  
   FBgn0031431   CG3515  2551144   2552844   -  1701  
 
    Segment 69 
 
   Location   
  Gene key  FBgn0031432-FBgn0041337  
  Heatmap region span   2L:2374689..2696437   
  Segment span   2L:2564848..2573047   
  Length (genes)  2  
  Length (bp)  8200  
   Model Scoring   
  BIC  234.704521  
  logL  -111.881643  
  logL ratio  -0.951998  
   Expression   
  Mean expression  6.302672  
  Median expression  5.436363  
  Tissue std. dev.  1.675272  
 
  No GO Slim enrichment  
  
   tissue    mean expression   
  5th Passage Drosophila S2 Cells  4.776956  
  Adult Accessory gland  4.691060  
  Adult Brain  5.074566  
  Adult Carcass  7.983417  
  Adult Crop  4.765369  
  Adult Eye  7.710305  
  Adult Fatbody  9.545998  
  Adult Female Spermatheca Mated  8.288364  
  Adult Female Spermatheca Virgin  10.067749  
  Adult Head  8.513889  
  Adult Heart  8.915001  
  Adult Hind Gut  5.169505  
  Adult Male Ejaculatory Duct  6.379511  
  Adult Mid Gut  8.005287  
  Adult Ovary  4.821566  
  Adult Salivary Gland  5.182972  
  Adult Testes  6.722719  
  Adult Thoracoabdominal ganglion  5.074174  
  Adult Whole Fly  6.130966  
  Larvae Wandering Tubules  6.542705  
  Larval Feeding Carcass  4.790859  
  Larval Feeding Central Nevous System  4.510872  
  Larval Feeding Hind Gut  4.843642  
  Larval Feeding Malpighian Tubule  5.031752  
  Larval Feeding Mid Gut  6.436875  
  Larval Feeding Salivary Gland  4.742830  
  Whole Larvae Feeding  5.453240  
 
  
   FlyBase ID    symbol    start    end    strand    length   
   FBgn0031432   Cyp309a1  2562885   2564848   -  1964  
   FBgn0041337   Cyp309a2  2564882   2573047   -  8166  
 
    Segment 70 
 
   Location   
  Gene key  FBgn0031434-FBgn0031435  
  Heatmap region span   2L:2385792..2739986   
  Segment span   2L:2577006..2577671   
  Length (genes)  2  
  Length (bp)  666  
   Model Scoring   
  BIC  195.733409  
  logL  -92.396087  
  logL ratio  28.631778  
   Expression   
  Mean expression  5.850177  
  Median expression  5.327446  
  Tissue std. dev.  1.265841  
 
  No GO Slim enrichment  
  
   tissue    mean expression   
  5th Passage Drosophila S2 Cells  5.035201  
  Adult Accessory gland  5.470074  
  Adult Brain  5.763655  
  Adult Carcass  5.216107  
  Adult Crop  5.167896  
  Adult Eye  5.609108  
  Adult Fatbody  5.058997  
  Adult Female Spermatheca Mated  5.188354  
  Adult Female Spermatheca Virgin  5.148921  
  Adult Head  5.195152  
  Adult Heart  5.126053  
  Adult Hind Gut  5.250191  
  Adult Male Ejaculatory Duct  5.176638  
  Adult Mid Gut  5.244623  
  Adult Ovary  10.355875  
  Adult Salivary Gland  5.093883  
  Adult Testes  7.380601  
  Adult Thoracoabdominal ganglion  5.306916  
  Adult Whole Fly  8.414341  
  Larvae Wandering Tubules  7.484020  
  Larval Feeding Carcass  5.124702  
  Larval Feeding Central Nevous System  7.562867  
  Larval Feeding Hind Gut  5.313022  
  Larval Feeding Malpighian Tubule  6.419251  
  Larval Feeding Mid Gut  5.170568  
  Larval Feeding Salivary Gland  5.237593  
  Whole Larvae Feeding  5.440166  
 
  
   FlyBase ID    symbol    start    end    strand    length   
   FBgn0031434   insv  2575276   2577006   -  1731  
   FBgn0031435   CG9883   2577671   2579180  +  1510  
 
    Segment 71 
 
   Location   
  Gene key  FBgn0031436-FBgn0026479  
  Heatmap region span   2L:2420808..2744934   
  Segment span   2L:2580056..2585448   
  Length (genes)  2  
  Length (bp)  5393  
   Model Scoring   
  BIC  261.557123  
  logL  -125.307944  
  logL ratio  55.043071  
   Expression   
  Mean expression  10.801457  
  Median expression  10.575506  
  Tissue std. dev.  0.462234  
 
  No GO Slim enrichment  
  
   tissue    mean expression   
  5th Passage Drosophila S2 Cells  10.106409  
  Adult Accessory gland  10.547562  
  Adult Brain  11.538149  
  Adult Carcass  11.178031  
  Adult Crop  10.859506  
  Adult Eye  10.679157  
  Adult Fatbody  11.018137  
  Adult Female Spermatheca Mated  9.612021  
  Adult Female Spermatheca Virgin  9.701483  
  Adult Head  10.975016  
  Adult Heart  10.934169  
  Adult Hind Gut  11.184875  
  Adult Male Ejaculatory Duct  10.445772  
  Adult Mid Gut  11.028001  
  Adult Ovary  10.379043  
  Adult Salivary Gland  10.662829  
  Adult Testes  11.021169  
  Adult Thoracoabdominal ganglion  11.598327  
  Adult Whole Fly  11.113157  
  Larvae Wandering Tubules  10.992757  
  Larval Feeding Carcass  10.831046  
  Larval Feeding Central Nevous System  10.957192  
  Larval Feeding Hind Gut  11.054094  
  Larval Feeding Malpighian Tubule  11.324180  
  Larval Feeding Mid Gut  10.623157  
  Larval Feeding Salivary Gland  10.712819  
  Whole Larvae Feeding  10.561284  
 
  
   FlyBase ID    symbol    start    end    strand    length   
   FBgn0031436   CG3214  2579217   2580056   -  840  
   FBgn0026479   Drp1  2581437   2585448   -  4012  
 
    Segment 72 
 
   Location   
  Gene key  FBgn0031440-FBgn0031446  
  Heatmap region span   2L:2564848..2752799   
  Segment span   2L:2610146..2696437   
  Length (genes)  9  
  Length (bp)  86292  
   Model Scoring   
  BIC  726.952140  
  logL  -358.005453  
  logL ratio  225.110419  
   Expression   
  Mean expression  4.879031  
  Median expression  4.522531  
  Tissue std. dev.  0.560339  
 
  No GO Slim enrichment  
  
   tissue    mean expression   
  5th Passage Drosophila S2 Cells  4.734973  
  Adult Accessory gland  4.875697  
  Adult Brain  4.794759  
  Adult Carcass  4.954774  
  Adult Crop  4.745048  
  Adult Eye  4.638290  
  Adult Fatbody  4.796149  
  Adult Female Spermatheca Mated  4.845436  
  Adult Female Spermatheca Virgin  4.820277  
  Adult Head  4.641707  
  Adult Heart  4.630911  
  Adult Hind Gut  4.655024  
  Adult Male Ejaculatory Duct  4.829975  
  Adult Mid Gut  4.856081  
  Adult Ovary  4.646503  
  Adult Salivary Gland  4.985097  
  Adult Testes  7.688394  
  Adult Thoracoabdominal ganglion  4.795236  
  Adult Whole Fly  4.939540  
  Larvae Wandering Tubules  4.734122  
  Larval Feeding Carcass  4.705759  
  Larval Feeding Central Nevous System  4.659893  
  Larval Feeding Hind Gut  4.605432  
  Larval Feeding Malpighian Tubule  4.766277  
  Larval Feeding Mid Gut  4.824826  
  Larval Feeding Salivary Gland  4.716570  
  Whole Larvae Feeding  4.847079  
 
  
   FlyBase ID    symbol    start    end    strand    length   
   FBgn0031440   CG15395   2610146   2611285  +  1140  
   FBgn0031441   CG9962  2646577   2647720   -  1144  
   FBgn0053125   CG33125  2648440   2652291   -  3852  
   FBgn0031442   Prosbeta4R1   2653112   2653931  +  820  
   FBgn0026395   Or23a   2654125   2655461  +  1337  
   FBgn0031444   CG9879   2665110   2666195  +  1086  
   FBgn0041248   Gr23a   2669823   2672159  +  2337  
   FBgn0051690   CG31690   2674825   2719431  +  44607  
   FBgn0031446   CG15398  2695375   2696437   -  1063  
 
 
    Segment 73 
 
   Location   
  Gene key  FBgn0031449-FBgn0031450  
  Heatmap region span   2L:2577006..2768166   
  Segment span   2L:2730106..2739986   
  Length (genes)  2  
  Length (bp)  9881  
   Model Scoring   
  BIC  265.703061  
  logL  -127.380913  
  logL ratio  5.378097  
   Expression   
  Mean expression  8.984939  
  Median expression  9.071619  
  Tissue std. dev.  0.801688  
 
  No GO Slim enrichment  
  
   tissue    mean expression   
  5th Passage Drosophila S2 Cells  10.296820  
  Adult Accessory gland  9.062099  
  Adult Brain  8.415866  
  Adult Carcass  9.772060  
  Adult Crop  9.251112  
  Adult Eye  10.319715  
  Adult Fatbody  9.908614  
  Adult Female Spermatheca Mated  9.240413  
  Adult Female Spermatheca Virgin  9.509383  
  Adult Head  10.013123  
  Adult Heart  10.079216  
  Adult Hind Gut  9.365807  
  Adult Male Ejaculatory Duct  8.303655  
  Adult Mid Gut  9.164065  
  Adult Ovary  9.389820  
  Adult Salivary Gland  8.244953  
  Adult Testes  8.445214  
  Adult Thoracoabdominal ganglion  8.708963  
  Adult Whole Fly  8.883228  
  Larvae Wandering Tubules  8.726157  
  Larval Feeding Carcass  9.113853  
  Larval Feeding Central Nevous System  7.186364  
  Larval Feeding Hind Gut  8.991004  
  Larval Feeding Malpighian Tubule  7.841768  
  Larval Feeding Mid Gut  8.707959  
  Larval Feeding Salivary Gland  7.463180  
  Whole Larvae Feeding  8.188935  
 
  
   FlyBase ID    symbol    start    end    strand    length   
   FBgn0031449   CG31689   2730106   2739833  +  9728  
   FBgn0031450   Hrs   2739986   2743377  +  3392  
 
    Segment 74 
 
   Location   
  Gene key  FBgn0250906-FBgn0031453  
  Heatmap region span   2L:2610146..2805518   
  Segment span   2L:2748522..2752799   
  Length (genes)  2  
  Length (bp)  4278  
   Model Scoring   
  BIC  276.223001  
  logL  -132.640883  
  logL ratio  99.380204  
   Expression   
  Mean expression  11.924217  
  Median expression  12.104697  
  Tissue std. dev.  0.799854  
 
  No GO Slim enrichment  
  
   tissue    mean expression   
  5th Passage Drosophila S2 Cells  12.524262  
  Adult Accessory gland  11.419820  
  Adult Brain  12.779086  
  Adult Carcass  12.428966  
  Adult Crop  12.365950  
  Adult Eye  12.315061  
  Adult Fatbody  12.181578  
  Adult Female Spermatheca Mated  12.233812  
  Adult Female Spermatheca Virgin  12.467537  
  Adult Head  12.362312  
  Adult Heart  12.440106  
  Adult Hind Gut  12.266882  
  Adult Male Ejaculatory Duct  11.827119  
  Adult Mid Gut  11.168960  
  Adult Ovary  11.896711  
  Adult Salivary Gland  11.992717  
  Adult Testes  8.665631  
  Adult Thoracoabdominal ganglion  12.301268  
  Adult Whole Fly  12.071040  
  Larvae Wandering Tubules  11.735698  
  Larval Feeding Carcass  12.014063  
  Larval Feeding Central Nevous System  13.140199  
  Larval Feeding Hind Gut  11.568545  
  Larval Feeding Malpighian Tubule  11.627212  
  Larval Feeding Mid Gut  11.067577  
  Larval Feeding Salivary Gland  11.255581  
  Whole Larvae Feeding  11.836153  
 
  
   FlyBase ID    symbol    start    end    strand    length   
   FBgn0250906   Pgk  2746880   2748522   -  1643  
   FBgn0031453   CG9894   2752799   2756606  +  3808  
 
    Segment 75 
 
   Location   
  Gene key  FBgn0031457-FBgn0026324  
  Heatmap region span   2L:2730106..2855749   
  Segment span   2L:2765686..2768166   
  Length (genes)  3  
  Length (bp)  2481  
   Model Scoring   
  BIC  314.708134  
  logL  -151.883450  
  logL ratio  77.784681  
   Expression   
  Mean expression  8.710573  
  Median expression  8.760832  
  Tissue std. dev.  0.503332  
 
  
   GO ID    description    ratio    P-value   
   GO:0005737   cytoplasm  2/3  0.0273  
 
  
   tissue    mean expression   
  5th Passage Drosophila S2 Cells  9.287689  
  Adult Accessory gland  9.283396  
  Adult Brain  9.287085  
  Adult Carcass  8.300851  
  Adult Crop  8.764548  
  Adult Eye  9.172060  
  Adult Fatbody  8.132126  
  Adult Female Spermatheca Mated  7.806423  
  Adult Female Spermatheca Virgin  7.784073  
  Adult Head  8.738140  
  Adult Heart  8.990690  
  Adult Hind Gut  9.010070  
  Adult Male Ejaculatory Duct  8.966690  
  Adult Mid Gut  8.205750  
  Adult Ovary  8.870737  
  Adult Salivary Gland  9.168416  
  Adult Testes  7.997130  
  Adult Thoracoabdominal ganglion  9.175962  
  Adult Whole Fly  8.150223  
  Larvae Wandering Tubules  9.243374  
  Larval Feeding Carcass  8.337858  
  Larval Feeding Central Nevous System  8.960405  
  Larval Feeding Hind Gut  8.612840  
  Larval Feeding Malpighian Tubule  9.120688  
  Larval Feeding Mid Gut  8.314827  
  Larval Feeding Salivary Gland  9.409464  
  Whole Larvae Feeding  8.093946  
 
  
   FlyBase ID    symbol    start    end    strand    length   
   FBgn0031457   CG3077  2764067   2765686   -  1620  
   FBgn0028398   Taf10   2767902   2768612  +  711  
   FBgn0026324   Taf10b  2767281   2768166   -  886  
 
 
    Segment 76 
 
   Location   
  Gene key  FBgn0019830-FBgn0031459  
  Heatmap region span   2L:2744934..2856070   
  Segment span   2L:2770066..2770136   
  Length (genes)  2  
  Length (bp)  71  
   Model Scoring   
  BIC  256.225351  
  logL  -122.642058  
  logL ratio  81.122937  
   Expression   
  Mean expression  11.295778  
  Median expression  11.266113  
  Tissue std. dev.  0.597408  
 
  No GO Slim enrichment  
  
   tissue    mean expression   
  5th Passage Drosophila S2 Cells  11.098471  
  Adult Accessory gland  11.478369  
  Adult Brain  10.217346  
  Adult Carcass  11.831032  
  Adult Crop  11.241823  
  Adult Eye  11.233205  
  Adult Fatbody  12.425359  
  Adult Female Spermatheca Mated  12.436074  
  Adult Female Spermatheca Virgin  12.431037  
  Adult Head  11.759399  
  Adult Heart  11.946656  
  Adult Hind Gut  11.066814  
  Adult Male Ejaculatory Duct  11.826291  
  Adult Mid Gut  11.624408  
  Adult Ovary  10.728053  
  Adult Salivary Gland  11.165582  
  Adult Testes  10.631415  
  Adult Thoracoabdominal ganglion  10.247812  
  Adult Whole Fly  11.241447  
  Larvae Wandering Tubules  10.625896  
  Larval Feeding Carcass  11.186431  
  Larval Feeding Central Nevous System  10.739446  
  Larval Feeding Hind Gut  10.707973  
  Larval Feeding Malpighian Tubule  11.228686  
  Larval Feeding Mid Gut  11.037589  
  Larval Feeding Salivary Gland  11.595118  
  Whole Larvae Feeding  11.234266  
 
  
   FlyBase ID    symbol    start    end    strand    length   
   FBgn0019830   colt  2768590   2770066   -  1477  
   FBgn0031459   CG2862   2770136   2770904  +  769  
 
    Segment 77 
 
   Location   
  Gene key  FBgn0031460-FBgn0004242  
  Heatmap region span   2L:2745102..2857159   
  Segment span   2L:2771776..2799970   
  Length (genes)  2  
  Length (bp)  28195  
   Model Scoring   
  BIC  224.097264  
  logL  -106.578015  
  logL ratio  -2.234514  
   Expression   
  Mean expression  5.555074  
  Median expression  5.192902  
  Tissue std. dev.  0.685323  
 
  No GO Slim enrichment  
  
   tissue    mean expression   
  5th Passage Drosophila S2 Cells  5.574789  
  Adult Accessory gland  5.034999  
  Adult Brain  6.755041  
  Adult Carcass  5.741162  
  Adult Crop  5.023411  
  Adult Eye  6.501399  
  Adult Fatbody  5.789239  
  Adult Female Spermatheca Mated  5.508064  
  Adult Female Spermatheca Virgin  5.185744  
  Adult Head  6.664124  
  Adult Heart  5.515232  
  Adult Hind Gut  4.883980  
  Adult Male Ejaculatory Duct  5.058782  
  Adult Mid Gut  5.081984  
  Adult Ovary  4.823405  
  Adult Salivary Gland  4.935225  
  Adult Testes  6.878293  
  Adult Thoracoabdominal ganglion  7.087701  
  Adult Whole Fly  5.729848  
  Larvae Wandering Tubules  5.238793  
  Larval Feeding Carcass  5.098879  
  Larval Feeding Central Nevous System  6.443421  
  Larval Feeding Hind Gut  4.914473  
  Larval Feeding Malpighian Tubule  5.033112  
  Larval Feeding Mid Gut  4.990198  
  Larval Feeding Salivary Gland  5.055552  
  Whole Larvae Feeding  5.440139  
 
  
   FlyBase ID    symbol    start    end    strand    length   
   FBgn0031460   CG15399  2770884   2771776   -  893  
   FBgn0004242   Syt1  2780741   2799970   -  19230  
 
    Segment 78 
 
   Location   
  Gene key  FBgn0031462-FBgn0031473  
  Heatmap region span   2L:2765686..2878520   
  Segment span   2L:2812437..2855749   
  Length (genes)  12  
  Length (bp)  43313  
   Model Scoring   
  BIC  1155.017938  
  logL  -572.038352  
  logL ratio  121.704318  
   Expression   
  Mean expression  5.308183  
  Median expression  4.839924  
  Tissue std. dev.  0.372120  
 
  
   GO ID    description    ratio    P-value   
   GO:0016757   transferase activity, transferring glycosyl groups  3/12  0  
 
  
   tissue    mean expression   
  5th Passage Drosophila S2 Cells  6.105819  
  Adult Accessory gland  6.200338  
  Adult Brain  5.144138  
  Adult Carcass  5.215059  
  Adult Crop  5.267946  
  Adult Eye  4.984862  
  Adult Fatbody  5.079937  
  Adult Female Spermatheca Mated  5.124076  
  Adult Female Spermatheca Virgin  5.061723  
  Adult Head  5.099078  
  Adult Heart  4.793099  
  Adult Hind Gut  5.103583  
  Adult Male Ejaculatory Duct  5.479372  
  Adult Mid Gut  5.080939  
  Adult Ovary  5.265385  
  Adult Salivary Gland  5.424544  
  Adult Testes  6.229861  
  Adult Thoracoabdominal ganglion  5.093543  
  Adult Whole Fly  5.071599  
  Larvae Wandering Tubules  4.943695  
  Larval Feeding Carcass  5.662721  
  Larval Feeding Central Nevous System  5.109239  
  Larval Feeding Hind Gut  5.677670  
  Larval Feeding Malpighian Tubule  5.084911  
  Larval Feeding Mid Gut  5.101782  
  Larval Feeding Salivary Gland  5.304085  
  Whole Larvae Feeding  5.611947  
 
  
   FlyBase ID    symbol    start    end    strand    length   
   FBgn0031462   CG2964   2812437   2814429  +  1993  
   FBgn0031463   CG15400  2814336   2815760   -  1425  
   FBgn0031465   CG3123  2823546   2825506   -  1961  
   FBgn0031464   Duox  2815970   2830236   -  14267  
   FBgn0031466   CG3119  2834887   2837831   -  2945  
   FBgn0031467   Cpr23B   2838414   2839547  +  1134  
   FBgn0031468   CG2975   2840124   2842638  +  2515  
   FBgn0031469   CG18558  2842589   2844513   -  1925  
   FBgn0031470   CG18557  2844601   2845711   -  1111  
   FBgn0031471   CG3117  2846084   2847197   -  1114  
   FBgn0031472   CG2983   2847632   2848997  +  1366  
   FBgn0031473   CG3104  2849732   2855749   -  6018  
 
 
    Segment 79 
 
   Location   
  Gene key  FBgn0051694-FBgn0051950  
  Heatmap region span   2L:2812437..2992964   
  Segment span   2L:2874518..2878520   
  Length (genes)  4  
  Length (bp)  4003  
   Model Scoring   
  BIC  423.943530  
  logL  -206.501148  
  logL ratio  123.686644  
   Expression   
  Mean expression  9.563585  
  Median expression  9.450895  
  Tissue std. dev.  0.296733  
 
  No GO Slim enrichment  
  
   tissue    mean expression   
  5th Passage Drosophila S2 Cells  9.617622  
  Adult Accessory gland  9.343997  
  Adult Brain  9.135841  
  Adult Carcass  9.352291  
  Adult Crop  9.921347  
  Adult Eye  8.959347  
  Adult Fatbody  9.941572  
  Adult Female Spermatheca Mated  9.386867  
  Adult Female Spermatheca Virgin  9.506404  
  Adult Head  9.231106  
  Adult Heart  9.717502  
  Adult Hind Gut  9.622179  
  Adult Male Ejaculatory Duct  10.317262  
  Adult Mid Gut  9.694640  
  Adult Ovary  9.819146  
  Adult Salivary Gland  9.659039  
  Adult Testes  9.099603  
  Adult Thoracoabdominal ganglion  9.373603  
  Adult Whole Fly  9.339221  
  Larvae Wandering Tubules  9.677062  
  Larval Feeding Carcass  9.512151  
  Larval Feeding Central Nevous System  9.460999  
  Larval Feeding Hind Gut  9.507344  
  Larval Feeding Malpighian Tubule  9.747340  
  Larval Feeding Mid Gut  9.782420  
  Larval Feeding Salivary Gland  10.021243  
  Whole Larvae Feeding  9.469661  
 
  
   FlyBase ID    symbol    start    end    strand    length   
   FBgn0051694   CG31694  2868627   2874518   -  5892  
   FBgn0031478   CG8814   2874872   2877168  +  2297  
   FBgn0031479   Prx6005  2877091   2878232   -  1142  
   FBgn0051950   CG31950   2878520   2879001  +  482  
 
 
    Segment 80 
 
   Location   
  Gene key  FBgn0041111-FBgn0010263  
  Heatmap region span   2L:2857159..3030562   
  Segment span   2L:2885952..2954762   
  Length (genes)  2  
  Length (bp)  68811  
   Model Scoring   
  BIC  241.017627  
  logL  -115.038196  
  logL ratio  -10.187675  
   Expression   
  Mean expression  6.010979  
  Median expression  5.543171  
  Tissue std. dev.  0.573336  
 
  No GO Slim enrichment  
  
   tissue    mean expression   
  5th Passage Drosophila S2 Cells  5.825281  
  Adult Accessory gland  6.276499  
  Adult Brain  5.903481  
  Adult Carcass  6.775621  
  Adult Crop  6.446817  
  Adult Eye  7.213119  
  Adult Fatbody  5.684245  
  Adult Female Spermatheca Mated  5.175327  
  Adult Female Spermatheca Virgin  5.133005  
  Adult Head  6.427003  
  Adult Heart  7.229444  
  Adult Hind Gut  6.178049  
  Adult Male Ejaculatory Duct  6.042656  
  Adult Mid Gut  5.797468  
  Adult Ovary  5.641633  
  Adult Salivary Gland  5.517926  
  Adult Testes  6.352503  
  Adult Thoracoabdominal ganglion  5.802234  
  Adult Whole Fly  5.913530  
  Larvae Wandering Tubules  5.363524  
  Larval Feeding Carcass  6.862970  
  Larval Feeding Central Nevous System  4.982873  
  Larval Feeding Hind Gut  6.268618  
  Larval Feeding Malpighian Tubule  5.529796  
  Larval Feeding Mid Gut  6.109647  
  Larval Feeding Salivary Gland  5.642399  
  Whole Larvae Feeding  6.200777  
 
  
   FlyBase ID    symbol    start    end    strand    length   
   FBgn0041111   lilli   2885952   2953077  +  67126  
   FBgn0010263   Rbp9   2954762   2968434  +  13673  
 
    Segment 81 
 
   Location   
  Gene key  FBgn0004584-FBgn0025681  
  Heatmap region span   2L:2874518..3037403   
  Segment span   2L:2972676..2992964   
  Length (genes)  8  
  Length (bp)  20289  
   Model Scoring   
  BIC  769.916518  
  logL  -379.487642  
  logL ratio  233.963139  
   Expression   
  Mean expression  7.991429  
  Median expression  8.102739  
  Tissue std. dev.  0.536518  
 
  
   GO ID    description    ratio    P-value   
   GO:0003677   DNA binding  3/8  0.0183  
 
  
   tissue    mean expression   
  5th Passage Drosophila S2 Cells  9.232353  
  Adult Accessory gland  7.937683  
  Adult Brain  8.237584  
  Adult Carcass  7.223617  
  Adult Crop  8.172139  
  Adult Eye  8.152106  
  Adult Fatbody  7.412231  
  Adult Female Spermatheca Mated  7.599807  
  Adult Female Spermatheca Virgin  7.555543  
  Adult Head  7.608980  
  Adult Heart  7.923268  
  Adult Hind Gut  7.641477  
  Adult Male Ejaculatory Duct  7.855967  
  Adult Mid Gut  7.503654  
  Adult Ovary  9.522477  
  Adult Salivary Gland  7.744320  
  Adult Testes  8.291699  
  Adult Thoracoabdominal ganglion  8.159266  
  Adult Whole Fly  8.256949  
  Larvae Wandering Tubules  7.777378  
  Larval Feeding Carcass  7.724315  
  Larval Feeding Central Nevous System  8.980982  
  Larval Feeding Hind Gut  7.760902  
  Larval Feeding Malpighian Tubule  7.939141  
  Larval Feeding Mid Gut  7.413621  
  Larval Feeding Salivary Gland  8.401828  
  Whole Larvae Feeding  7.739302  
 
  
   FlyBase ID    symbol    start    end    strand    length   
   FBgn0004584   Rrp1  2969773   2972676   -  2904  
   FBgn0031483   CG9641  2974854   2976859   -  2006  
   FBgn0031484   CG3165   2977127   2978501  +  1375  
   FBgn0031485   CG9643  2978526   2979410   -  885  
   FBgn0250786   Chd1  2979697   2987677   -  7981  
   FBgn0025109   Bem46   2988135   2990094  +  1960  
   FBgn0002989   okr  2989594   2992761   -  3168  
   FBgn0025681   CG3558   2992964   2998614  +  5651  
 
 
    Segment 82 
 
   Location   
  Gene key  FBgn0031489-FBgn0031495  
  Heatmap region span   2L:2885952..3042919   
  Segment span   2L:3018304..3030562   
  Length (genes)  7  
  Length (bp)  12259  
   Model Scoring   
  BIC  753.844986  
  logL  -371.451876  
  logL ratio  135.385969  
   Expression   
  Mean expression  8.184601  
  Median expression  8.301765  
  Tissue std. dev.  0.436749  
 
  No GO Slim enrichment  
  
   tissue    mean expression   
  5th Passage Drosophila S2 Cells  9.597655  
  Adult Accessory gland  8.020618  
  Adult Brain  8.048183  
  Adult Carcass  7.923126  
  Adult Crop  7.985767  
  Adult Eye  8.232071  
  Adult Fatbody  8.560773  
  Adult Female Spermatheca Mated  9.059883  
  Adult Female Spermatheca Virgin  8.923023  
  Adult Head  8.126923  
  Adult Heart  8.780838  
  Adult Hind Gut  8.004563  
  Adult Male Ejaculatory Duct  7.917046  
  Adult Mid Gut  8.179137  
  Adult Ovary  8.328680  
  Adult Salivary Gland  7.980918  
  Adult Testes  7.698876  
  Adult Thoracoabdominal ganglion  8.073151  
  Adult Whole Fly  7.912506  
  Larvae Wandering Tubules  8.147414  
  Larval Feeding Carcass  8.024558  
  Larval Feeding Central Nevous System  8.212416  
  Larval Feeding Hind Gut  7.827655  
  Larval Feeding Malpighian Tubule  8.092353  
  Larval Feeding Mid Gut  7.904931  
  Larval Feeding Salivary Gland  7.766246  
  Whole Larvae Feeding  7.654913  
 
  
   FlyBase ID    symbol    start    end    strand    length   
   FBgn0031489   CG17224  3016591   3018304   -  1714  
   FBgn0031490   CG17264   3018508   3021506  +  2999  
   FBgn0031491   alpha4GT1  3021309   3023169   -  1861  
   FBgn0031492   CG3542   3023235   3026439  +  3205  
   FBgn0031493   CG3605  3026533   3029189   -  2657  
   FBgn0031494   CG17219   3029251   3030616  +  1366  
   FBgn0031495   GABPI   3030562   3032300  +  1739  
 
 
    Segment 83 
 
   Location   
  Gene key  FBgn0031498-FBgn0031500  
  Heatmap region span   2L:3013501..3159630   
  Segment span   2L:3037411..3039976   
  Length (genes)  3  
  Length (bp)  2566  
   Model Scoring   
  BIC  311.705089  
  logL  -150.381927  
  logL ratio  67.324734  
   Expression   
  Mean expression  7.436163  
  Median expression  7.444622  
  Tissue std. dev.  0.398471  
 
  No GO Slim enrichment  
  
   tissue    mean expression   
  5th Passage Drosophila S2 Cells  7.204533  
  Adult Accessory gland  6.994327  
  Adult Brain  7.927995  
  Adult Carcass  7.534555  
  Adult Crop  7.296971  
  Adult Eye  8.524825  
  Adult Fatbody  7.318713  
  Adult Female Spermatheca Mated  7.173193  
  Adult Female Spermatheca Virgin  7.640208  
  Adult Head  7.592724  
  Adult Heart  7.777928  
  Adult Hind Gut  7.232055  
  Adult Male Ejaculatory Duct  7.336549  
  Adult Mid Gut  7.276363  
  Adult Ovary  8.274256  
  Adult Salivary Gland  7.423247  
  Adult Testes  7.487951  
  Adult Thoracoabdominal ganglion  7.782656  
  Adult Whole Fly  7.292753  
  Larvae Wandering Tubules  7.673246  
  Larval Feeding Carcass  7.180987  
  Larval Feeding Central Nevous System  7.404832  
  Larval Feeding Hind Gut  6.916535  
  Larval Feeding Malpighian Tubule  7.654920  
  Larval Feeding Mid Gut  6.772375  
  Larval Feeding Salivary Gland  7.356935  
  Whole Larvae Feeding  6.724779  
 
  
   FlyBase ID    symbol    start    end    strand    length   
   FBgn0031498   CG17260   3037411   3038793  +  1383  
   FBgn0243513   cnir  3038826   3039812   -  987  
   FBgn0031500   CG17221   3039976   3041626  +  1651  
 
 
    Segment 84 
 
   Location   
  Gene key  FBgn0005779-FBgn0031504  
  Heatmap region span   2L:3037403..3172751   
  Segment span   2L:3109672..3128720   
  Length (genes)  2  
  Length (bp)  19049  
   Model Scoring   
  BIC  181.774512  
  logL  -85.416638  
  logL ratio  37.897833  
   Expression   
  Mean expression  4.552319  
  Median expression  4.358962  
  Tissue std. dev.  1.014396  
 
  No GO Slim enrichment  
  
   tissue    mean expression   
  5th Passage Drosophila S2 Cells  4.308108  
  Adult Accessory gland  4.414624  
  Adult Brain  4.029826  
  Adult Carcass  4.333888  
  Adult Crop  4.301127  
  Adult Eye  4.203304  
  Adult Fatbody  4.474401  
  Adult Female Spermatheca Mated  4.548788  
  Adult Female Spermatheca Virgin  4.559691  
  Adult Head  4.106604  
  Adult Heart  4.357995  
  Adult Hind Gut  4.300273  
  Adult Male Ejaculatory Duct  4.358226  
  Adult Mid Gut  4.430243  
  Adult Ovary  4.222217  
  Adult Salivary Gland  4.565176  
  Adult Testes  9.589449  
  Adult Thoracoabdominal ganglion  4.137710  
  Adult Whole Fly  5.219772  
  Larvae Wandering Tubules  4.503245  
  Larval Feeding Carcass  4.373096  
  Larval Feeding Central Nevous System  4.033711  
  Larval Feeding Hind Gut  4.115225  
  Larval Feeding Malpighian Tubule  4.447400  
  Larval Feeding Mid Gut  4.156399  
  Larval Feeding Salivary Gland  4.272018  
  Whole Larvae Feeding  4.550094  
 
  
   FlyBase ID    symbol    start    end    strand    length   
   FBgn0005779   PpD6   3109672   3110998  +  1327  
   FBgn0031504   CG15403   3128720   3129464  +  745  
 
    Segment 85 
 
   Location   
  Gene key  FBgn0015600-FBgn0011648  
  Heatmap region span   2L:3037411..3173989   
  Segment span   2L:3144613..3159630   
  Length (genes)  3  
  Length (bp)  15018  
   Model Scoring   
  BIC  414.636347  
  logL  -201.847556  
  logL ratio  20.530392  
   Expression   
  Mean expression  9.787678  
  Median expression  9.471271  
  Tissue std. dev.  0.577539  
 
  No GO Slim enrichment  
  
   tissue    mean expression   
  5th Passage Drosophila S2 Cells  8.321883  
  Adult Accessory gland  9.935726  
  Adult Brain  10.592126  
  Adult Carcass  9.976111  
  Adult Crop  9.914971  
  Adult Eye  9.833228  
  Adult Fatbody  10.236361  
  Adult Female Spermatheca Mated  9.616268  
  Adult Female Spermatheca Virgin  9.506155  
  Adult Head  10.025921  
  Adult Heart  10.277685  
  Adult Hind Gut  9.958070  
  Adult Male Ejaculatory Duct  10.067334  
  Adult Mid Gut  9.304630  
  Adult Ovary  10.258000  
  Adult Salivary Gland  10.444957  
  Adult Testes  7.916590  
  Adult Thoracoabdominal ganglion  10.454871  
  Adult Whole Fly  9.996588  
  Larvae Wandering Tubules  9.279101  
  Larval Feeding Carcass  10.087011  
  Larval Feeding Central Nevous System  9.959260  
  Larval Feeding Hind Gut  9.746132  
  Larval Feeding Malpighian Tubule  9.429339  
  Larval Feeding Mid Gut  9.817544  
  Larval Feeding Salivary Gland  9.638785  
  Whole Larvae Feeding  9.672664  
 
  
   FlyBase ID    symbol    start    end    strand    length   
   FBgn0015600   toc  3068339   3144613   -  76275  
   FBgn0031505   CG12400  3145125   3145719   -  595  
   FBgn0011648   Mad  3146484   3159630   -  13147  
 
 
    Segment 86 
 
   Location   
  Gene key  FBgn0051698-FBgn0031512  
  Heatmap region span   2L:3144613..3309222   
  Segment span   2L:3173826..3173989   
  Length (genes)  2  
  Length (bp)  164  
   Model Scoring   
  BIC  183.124467  
  logL  -86.091616  
  logL ratio  42.329572  
   Expression   
  Mean expression  5.411901  
  Median expression  4.886496  
  Tissue std. dev.  1.995976  
 
  No GO Slim enrichment  
  
   tissue    mean expression   
  5th Passage Drosophila S2 Cells  4.893419  
  Adult Accessory gland  5.133511  
  Adult Brain  4.532840  
  Adult Carcass  5.087588  
  Adult Crop  4.776705  
  Adult Eye  4.535788  
  Adult Fatbody  5.251285  
  Adult Female Spermatheca Mated  4.964045  
  Adult Female Spermatheca Virgin  4.935135  
  Adult Head  4.696214  
  Adult Heart  4.808768  
  Adult Hind Gut  4.917661  
  Adult Male Ejaculatory Duct  5.108460  
  Adult Mid Gut  5.065710  
  Adult Ovary  4.704667  
  Adult Salivary Gland  4.916620  
  Adult Testes  4.486116  
  Adult Thoracoabdominal ganglion  4.666450  
  Adult Whole Fly  4.439299  
  Larvae Wandering Tubules  4.833041  
  Larval Feeding Carcass  4.911869  
  Larval Feeding Central Nevous System  4.578524  
  Larval Feeding Hind Gut  4.831182  
  Larval Feeding Malpighian Tubule  5.646212  
  Larval Feeding Mid Gut  4.943737  
  Larval Feeding Salivary Gland  13.881205  
  Whole Larvae Feeding  10.575279  
 
  
   FlyBase ID    symbol    start    end    strand    length   
   FBgn0051698   CG31698  3173283   3173826   -  544  
   FBgn0031512   CG15404   3173989   3174442  +  454  
 
    Segment 87 
 
   Location   
  Gene key  FBgn0085422-FBgn0031513  
  Heatmap region span   2L:3162515..3319872   
  Segment span   2L:3199620..3269437   
  Length (genes)  2  
  Length (bp)  69818  
   Model Scoring   
  BIC  190.759737  
  logL  -89.909251  
  logL ratio  23.041606  
   Expression   
  Mean expression  4.997956  
  Median expression  4.756261  
  Tissue std. dev.  0.403883  
 
  No GO Slim enrichment  
  
   tissue    mean expression   
  5th Passage Drosophila S2 Cells  4.849075  
  Adult Accessory gland  4.929214  
  Adult Brain  5.817434  
  Adult Carcass  4.953099  
  Adult Crop  4.884311  
  Adult Eye  4.517011  
  Adult Fatbody  5.129368  
  Adult Female Spermatheca Mated  4.977426  
  Adult Female Spermatheca Virgin  4.878866  
  Adult Head  4.746726  
  Adult Heart  4.748822  
  Adult Hind Gut  4.737384  
  Adult Male Ejaculatory Duct  4.908901  
  Adult Mid Gut  4.977676  
  Adult Ovary  4.836316  
  Adult Salivary Gland  4.860340  
  Adult Testes  6.622893  
  Adult Thoracoabdominal ganglion  5.500182  
  Adult Whole Fly  4.544555  
  Larvae Wandering Tubules  5.081093  
  Larval Feeding Carcass  4.942882  
  Larval Feeding Central Nevous System  4.901788  
  Larval Feeding Hind Gut  4.781035  
  Larval Feeding Malpighian Tubule  5.010213  
  Larval Feeding Mid Gut  4.895658  
  Larval Feeding Salivary Gland  4.992835  
  Whole Larvae Feeding  4.919700  
 
  
   FlyBase ID    symbol    start    end    strand    length   
   FBgn0085422   CG34393  3175112   3199620   -  24509  
   FBgn0031513   CG3347   3269437   3271806  +  2370  
 
    Segment 88 
 
   Location   
  Gene key  FBgn0031515-FBgn0031516  
  Heatmap region span   2L:3172751..3355000   
  Segment span   2L:3294690..3303129   
  Length (genes)  2  
  Length (bp)  8440  
   Model Scoring   
  BIC  240.916192  
  logL  -114.987479  
  logL ratio  12.335574  
   Expression   
  Mean expression  7.475439  
  Median expression  7.699513  
  Tissue std. dev.  1.138361  
 
  No GO Slim enrichment  
  
   tissue    mean expression   
  5th Passage Drosophila S2 Cells  7.311969  
  Adult Accessory gland  5.682917  
  Adult Brain  6.633107  
  Adult Carcass  8.400649  
  Adult Crop  7.569765  
  Adult Eye  7.887190  
  Adult Fatbody  8.717446  
  Adult Female Spermatheca Mated  8.531581  
  Adult Female Spermatheca Virgin  8.977034  
  Adult Head  7.735214  
  Adult Heart  8.247635  
  Adult Hind Gut  6.890127  
  Adult Male Ejaculatory Duct  6.754314  
  Adult Mid Gut  8.686098  
  Adult Ovary  6.216152  
  Adult Salivary Gland  5.726866  
  Adult Testes  7.102529  
  Adult Thoracoabdominal ganglion  6.845831  
  Adult Whole Fly  6.353029  
  Larvae Wandering Tubules  10.015743  
  Larval Feeding Carcass  7.475604  
  Larval Feeding Central Nevous System  6.327516  
  Larval Feeding Hind Gut  6.297668  
  Larval Feeding Malpighian Tubule  8.373881  
  Larval Feeding Mid Gut  9.294413  
  Larval Feeding Salivary Gland  5.968046  
  Whole Larvae Feeding  7.814526  
 
  
   FlyBase ID    symbol    start    end    strand    length   
   FBgn0031515   CG9664  3291245   3294690   -  3446  
   FBgn0031516   CG9663  3294926   3303129   -  8204  
 
    Segment 89 
 
   Location   
  Gene key  FBgn0031518-FBgn0031519  
  Heatmap region span   2L:3199620..3375711   
  Segment span   2L:3313347..3319872   
  Length (genes)  2  
  Length (bp)  6526  
   Model Scoring   
  BIC  209.185086  
  logL  -99.121926  
  logL ratio  10.279568  
   Expression   
  Mean expression  5.120083  
  Median expression  4.891153  
  Tissue std. dev.  0.601497  
 
  No GO Slim enrichment  
  
   tissue    mean expression   
  5th Passage Drosophila S2 Cells  4.946945  
  Adult Accessory gland  4.437531  
  Adult Brain  4.945708  
  Adult Carcass  4.741973  
  Adult Crop  4.557068  
  Adult Eye  4.523331  
  Adult Fatbody  4.583343  
  Adult Female Spermatheca Mated  4.571565  
  Adult Female Spermatheca Virgin  4.677017  
  Adult Head  4.518755  
  Adult Heart  4.763247  
  Adult Hind Gut  5.281265  
  Adult Male Ejaculatory Duct  4.763545  
  Adult Mid Gut  6.788355  
  Adult Ovary  6.333249  
  Adult Salivary Gland  5.703400  
  Adult Testes  5.529400  
  Adult Thoracoabdominal ganglion  4.725341  
  Adult Whole Fly  5.383325  
  Larvae Wandering Tubules  5.565186  
  Larval Feeding Carcass  4.794491  
  Larval Feeding Central Nevous System  5.278086  
  Larval Feeding Hind Gut  5.209739  
  Larval Feeding Malpighian Tubule  5.617223  
  Larval Feeding Mid Gut  6.203787  
  Larval Feeding Salivary Gland  4.621037  
  Whole Larvae Feeding  5.178327  
 
  
   FlyBase ID    symbol    start    end    strand    length   
   FBgn0031518   CG3277   3313347   3317086  +  3740  
   FBgn0031519   CG3326  3317476   3319872   -  2397  
 
    Segment 90 
 
   Location   
  Gene key  FBgn0031520-FBgn0031523  
  Heatmap region span   2L:3280533..3377008   
  Segment span   2L:3320255..3332814   
  Length (genes)  5  
  Length (bp)  12560  
   Model Scoring   
  BIC  468.381004  
  logL  -228.719885  
  logL ratio  93.935399  
   Expression   
  Mean expression  5.768759  
  Median expression  4.977218  
  Tissue std. dev.  1.851759  
 
  
   GO ID    description    ratio    P-value   
   GO:0055085   transmembrane transport  5/5  1.27e-09  
   GO:0006810   transport  5/5  7.74e-08  
   GO:0022857   transmembrane transporter activity  2/5  0.000627  
   GO:0008150   biological_process  5/5  0.000746  
 
  
   tissue    mean expression   
  5th Passage Drosophila S2 Cells  4.727150  
  Adult Accessory gland  5.122827  
  Adult Brain  5.254448  
  Adult Carcass  5.266976  
  Adult Crop  4.815080  
  Adult Eye  5.139060  
  Adult Fatbody  5.374565  
  Adult Female Spermatheca Mated  5.042062  
  Adult Female Spermatheca Virgin  5.074938  
  Adult Head  5.251444  
  Adult Heart  5.481265  
  Adult Hind Gut  5.131505  
  Adult Male Ejaculatory Duct  4.747183  
  Adult Mid Gut  5.062838  
  Adult Ovary  4.690921  
  Adult Salivary Gland  5.215547  
  Adult Testes  5.964622  
  Adult Thoracoabdominal ganglion  5.533708  
  Adult Whole Fly  6.023451  
  Larvae Wandering Tubules  11.896836  
  Larval Feeding Carcass  4.884696  
  Larval Feeding Central Nevous System  4.938374  
  Larval Feeding Hind Gut  5.723970  
  Larval Feeding Malpighian Tubule  12.159217  
  Larval Feeding Mid Gut  4.967719  
  Larval Feeding Salivary Gland  4.829611  
  Whole Larvae Feeding  7.436482  
 
  
   FlyBase ID    symbol    start    end    strand    length   
   FBgn0031520   CG8837   3320255   3323141  +  2887  
   FBgn0053281   CG33281   3324234   3326637  +  2404  
   FBgn0053282   CG33282   3327903   3329792  +  1890  
   FBgn0031522   CG3285   3330930   3332876  +  1947  
   FBgn0031523   CG15408   3332814   3334790  +  1977  
 
 
    Segment 91 
 
   Location   
  Gene key  FBgn0085423-FBgn0031528  
  Heatmap region span   2L:3313347..3461975   
  Segment span   2L:3373047..3375711   
  Length (genes)  2  
  Length (bp)  2665  
   Model Scoring   
  BIC  215.733363  
  logL  -102.396064  
  logL ratio  14.644636  
   Expression   
  Mean expression  5.815825  
  Median expression  5.923350  
  Tissue std. dev.  0.620379  
 
  No GO Slim enrichment  
  
   tissue    mean expression   
  5th Passage Drosophila S2 Cells  5.627466  
  Adult Accessory gland  5.478306  
  Adult Brain  5.739307  
  Adult Carcass  7.724426  
  Adult Crop  6.931779  
  Adult Eye  5.397567  
  Adult Fatbody  5.550722  
  Adult Female Spermatheca Mated  5.382445  
  Adult Female Spermatheca Virgin  5.356629  
  Adult Head  6.182317  
  Adult Heart  7.204835  
  Adult Hind Gut  6.223464  
  Adult Male Ejaculatory Duct  5.534848  
  Adult Mid Gut  5.879575  
  Adult Ovary  5.689989  
  Adult Salivary Gland  5.459716  
  Adult Testes  5.640323  
  Adult Thoracoabdominal ganglion  5.810960  
  Adult Whole Fly  6.239416  
  Larvae Wandering Tubules  5.224751  
  Larval Feeding Carcass  6.402436  
  Larval Feeding Central Nevous System  4.981580  
  Larval Feeding Hind Gut  5.485147  
  Larval Feeding Malpighian Tubule  5.343500  
  Larval Feeding Mid Gut  5.616006  
  Larval Feeding Salivary Gland  5.351978  
  Whole Larvae Feeding  5.567791  
 
  
   FlyBase ID    symbol    start    end    strand    length   
   FBgn0085423   CG34394  3358640   3373047   -  14408  
   FBgn0031528   CG15412  3373567   3375711   -  2145  
 
    Segment 92 
 
   Location   
  Gene key  FBgn0031529-FBgn0016698  
  Heatmap region span   2L:3320255..3466162   
  Segment span   2L:3376686..3377008   
  Length (genes)  2  
  Length (bp)  323  
   Model Scoring   
  BIC  215.237622  
  logL  -102.148194  
  logL ratio  45.402018  
   Expression   
  Mean expression  8.275240  
  Median expression  8.341093  
  Tissue std. dev.  0.447699  
 
  No GO Slim enrichment  
  
   tissue    mean expression   
  5th Passage Drosophila S2 Cells  9.009545  
  Adult Accessory gland  8.361637  
  Adult Brain  8.793041  
  Adult Carcass  8.061493  
  Adult Crop  8.717507  
  Adult Eye  8.177212  
  Adult Fatbody  7.819720  
  Adult Female Spermatheca Mated  8.185716  
  Adult Female Spermatheca Virgin  8.140645  
  Adult Head  8.145404  
  Adult Heart  8.198953  
  Adult Hind Gut  8.384771  
  Adult Male Ejaculatory Duct  8.620468  
  Adult Mid Gut  7.363063  
  Adult Ovary  8.749652  
  Adult Salivary Gland  8.581010  
  Adult Testes  8.527271  
  Adult Thoracoabdominal ganglion  8.904474  
  Adult Whole Fly  7.956790  
  Larvae Wandering Tubules  7.783670  
  Larval Feeding Carcass  8.027958  
  Larval Feeding Central Nevous System  8.823941  
  Larval Feeding Hind Gut  8.251379  
  Larval Feeding Malpighian Tubule  7.776715  
  Larval Feeding Mid Gut  7.396451  
  Larval Feeding Salivary Gland  8.890721  
  Whole Larvae Feeding  7.782280  
 
  
   FlyBase ID    symbol    start    end    strand    length   
   FBgn0031529   CG9662  3375885   3376686   -  802  
   FBgn0016698   Ptpa   3377008   3378586  +  1579  
 
    Segment 93 
 
   Location   
  Gene key  FBgn0085204-FBgn0031533  
  Heatmap region span   2L:3355000..3470182   
  Segment span   2L:3398023..3445997   
  Length (genes)  5  
  Length (bp)  47975  
   Model Scoring   
  BIC  511.521943  
  logL  -250.290354  
  logL ratio  39.234259  
   Expression   
  Mean expression  5.481921  
  Median expression  5.283209  
  Tissue std. dev.  0.894419  
 
  No GO Slim enrichment  
  
   tissue    mean expression   
  5th Passage Drosophila S2 Cells  5.361273  
  Adult Accessory gland  5.131910  
  Adult Brain  5.260910  
  Adult Carcass  4.952716  
  Adult Crop  4.979089  
  Adult Eye  5.089356  
  Adult Fatbody  4.986264  
  Adult Female Spermatheca Mated  5.025088  
  Adult Female Spermatheca Virgin  5.054946  
  Adult Head  5.225506  
  Adult Heart  4.878289  
  Adult Hind Gut  4.969880  
  Adult Male Ejaculatory Duct  4.930461  
  Adult Mid Gut  6.599971  
  Adult Ovary  4.903897  
  Adult Salivary Gland  5.252775  
  Adult Testes  9.208391  
  Adult Thoracoabdominal ganglion  5.250954  
  Adult Whole Fly  6.271721  
  Larvae Wandering Tubules  5.350934  
  Larval Feeding Carcass  5.438406  
  Larval Feeding Central Nevous System  5.046232  
  Larval Feeding Hind Gut  5.075074  
  Larval Feeding Malpighian Tubule  5.541393  
  Larval Feeding Mid Gut  6.602892  
  Larval Feeding Salivary Gland  5.090273  
  Whole Larvae Feeding  6.533278  
 
  
   FlyBase ID    symbol    start    end    strand    length   
   FBgn0085204   CG34175   3398023   3398684  +  662  
   FBgn0031531   CG8840   3410610   3411199  +  590  
   FBgn0051952   CG31952   3416693   3417869  +  1177  
   FBgn0031530   pgant2  3404460   3426642   -  22183  
   FBgn0031533   CG2772   3445997   3447591  +  1595  
 
 
    Segment 94 
 
   Location   
  Gene key  FBgn0031534-FBgn0031537  
  Heatmap region span   2L:3357684..3472735   
  Segment span   2L:3449686..3457329   
  Length (genes)  4  
  Length (bp)  7644  
   Model Scoring   
  BIC  416.755326  
  logL  -202.907045  
  logL ratio  113.408668  
   Expression   
  Mean expression  8.961805  
  Median expression  8.764851  
  Tissue std. dev.  0.405710  
 
  No GO Slim enrichment  
  
   tissue    mean expression   
  5th Passage Drosophila S2 Cells  9.314208  
  Adult Accessory gland  9.581642  
  Adult Brain  8.790199  
  Adult Carcass  8.550277  
  Adult Crop  8.771299  
  Adult Eye  9.187508  
  Adult Fatbody  8.875636  
  Adult Female Spermatheca Mated  8.564817  
  Adult Female Spermatheca Virgin  8.542949  
  Adult Head  8.585511  
  Adult Heart  9.506463  
  Adult Hind Gut  8.576815  
  Adult Male Ejaculatory Duct  9.186298  
  Adult Mid Gut  8.765804  
  Adult Ovary  9.529760  
  Adult Salivary Gland  9.329028  
  Adult Testes  8.695272  
  Adult Thoracoabdominal ganglion  8.687653  
  Adult Whole Fly  8.647241  
  Larvae Wandering Tubules  9.495920  
  Larval Feeding Carcass  8.763931  
  Larval Feeding Central Nevous System  8.409014  
  Larval Feeding Hind Gut  8.881337  
  Larval Feeding Malpighian Tubule  9.180044  
  Larval Feeding Mid Gut  9.095066  
  Larval Feeding Salivary Gland  9.965222  
  Whole Larvae Feeding  8.489826  
 
  
   FlyBase ID    symbol    start    end    strand    length   
   FBgn0031534   Snx1   3449686   3452106  +  2421  
   FBgn0031535   CG12795   3452451   3453596  +  1146  
   FBgn0031536   Cog3  3453697   3457058   -  3362  
   FBgn0031537   sec5   3457329   3460371  +  3043  
 
 
    Segment 95 
 
   Location   
  Gene key  FBgn0031540-FBgn0051776  
  Heatmap region span   2L:3398023..3510082   
  Segment span   2L:3470007..3470182   
  Length (genes)  2  
  Length (bp)  176  
   Model Scoring   
  BIC  216.442251  
  logL  -102.750508  
  logL ratio  1.164654  
   Expression   
  Mean expression  5.209626  
  Median expression  4.779418  
  Tissue std. dev.  0.614222  
 
  No GO Slim enrichment  
  
   tissue    mean expression   
  5th Passage Drosophila S2 Cells  5.559091  
  Adult Accessory gland  4.822618  
  Adult Brain  4.408060  
  Adult Carcass  4.821622  
  Adult Crop  5.077201  
  Adult Eye  4.849197  
  Adult Fatbody  5.095870  
  Adult Female Spermatheca Mated  5.076759  
  Adult Female Spermatheca Virgin  5.136360  
  Adult Head  4.680478  
  Adult Heart  5.036739  
  Adult Hind Gut  4.864457  
  Adult Male Ejaculatory Duct  4.980695  
  Adult Mid Gut  4.969418  
  Adult Ovary  7.190667  
  Adult Salivary Gland  4.964661  
  Adult Testes  6.475298  
  Adult Thoracoabdominal ganglion  4.691999  
  Adult Whole Fly  6.329595  
  Larvae Wandering Tubules  4.894222  
  Larval Feeding Carcass  5.112722  
  Larval Feeding Central Nevous System  6.231522  
  Larval Feeding Hind Gut  5.140835  
  Larval Feeding Malpighian Tubule  4.973263  
  Larval Feeding Mid Gut  4.959649  
  Larval Feeding Salivary Gland  5.223820  
  Whole Larvae Feeding  5.093079  
 
  
   FlyBase ID    symbol    start    end    strand    length   
   FBgn0031540   CG3238  3467416   3470007   -  2592  
   FBgn0051776   CG31776   3470182   3472368  +  2187  
 
    Segment 96 
 
   Location   
  Gene key  FBgn0053123-FBgn0031544  
  Heatmap region span   2L:3472735..3621681   
  Segment span   2L:3514662..3514737   
  Length (genes)  2  
  Length (bp)  76  
   Model Scoring   
  BIC  227.707410  
  logL  -108.383088  
  logL ratio  50.807811  
   Expression   
  Mean expression  9.708524  
  Median expression  9.835674  
  Tissue std. dev.  0.502083  
 
  
   GO ID    description    ratio    P-value   
   GO:0005737   cytoplasm  2/2  0.00471  
 
  
   tissue    mean expression   
  5th Passage Drosophila S2 Cells  10.562848  
  Adult Accessory gland  10.556506  
  Adult Brain  9.213449  
  Adult Carcass  9.518222  
  Adult Crop  9.345828  
  Adult Eye  9.872471  
  Adult Fatbody  10.136473  
  Adult Female Spermatheca Mated  9.900983  
  Adult Female Spermatheca Virgin  9.725580  
  Adult Head  9.259543  
  Adult Heart  10.064239  
  Adult Hind Gut  9.262451  
  Adult Male Ejaculatory Duct  10.598627  
  Adult Mid Gut  9.393658  
  Adult Ovary  10.334462  
  Adult Salivary Gland  9.735496  
  Adult Testes  8.763137  
  Adult Thoracoabdominal ganglion  9.174615  
  Adult Whole Fly  9.886565  
  Larvae Wandering Tubules  9.323393  
  Larval Feeding Carcass  9.878673  
  Larval Feeding Central Nevous System  9.588655  
  Larval Feeding Hind Gut  9.644541  
  Larval Feeding Malpighian Tubule  9.568089  
  Larval Feeding Mid Gut  9.095845  
  Larval Feeding Salivary Gland  10.583013  
  Whole Larvae Feeding  9.142776  
 
  
   FlyBase ID    symbol    start    end    strand    length   
   FBgn0053123   CG33123  3510477   3514662   -  4186  
   FBgn0031544   CG17593   3514737   3517111  +  2375  
 
    Segment 97 
 
   Location   
  Gene key  FBgn0031545-FBgn0031550  
  Heatmap region span   2L:3491048..3656951   
  Segment span   2L:3519697..3530903   
  Length (genes)  5  
  Length (bp)  11207  
   Model Scoring   
  BIC  494.186263  
  logL  -241.622514  
  logL ratio  39.797267  
   Expression   
  Mean expression  5.261494  
  Median expression  4.899167  
  Tissue std. dev.  0.657868  
 
  No GO Slim enrichment  
  
   tissue    mean expression   
  5th Passage Drosophila S2 Cells  5.279531  
  Adult Accessory gland  5.142746  
  Adult Brain  4.488679  
  Adult Carcass  5.296558  
  Adult Crop  5.023979  
  Adult Eye  4.628765  
  Adult Fatbody  5.076689  
  Adult Female Spermatheca Mated  4.911165  
  Adult Female Spermatheca Virgin  4.864549  
  Adult Head  4.810951  
  Adult Heart  5.398441  
  Adult Hind Gut  5.242586  
  Adult Male Ejaculatory Duct  5.044133  
  Adult Mid Gut  5.521672  
  Adult Ovary  5.282973  
  Adult Salivary Gland  4.959920  
  Adult Testes  8.086644  
  Adult Thoracoabdominal ganglion  4.589216  
  Adult Whole Fly  6.210621  
  Larvae Wandering Tubules  5.178613  
  Larval Feeding Carcass  5.561269  
  Larval Feeding Central Nevous System  5.059593  
  Larval Feeding Hind Gut  5.113818  
  Larval Feeding Malpighian Tubule  5.009339  
  Larval Feeding Mid Gut  5.524281  
  Larval Feeding Salivary Gland  5.045144  
  Whole Larvae Feeding  5.708454  
 
  
   FlyBase ID    symbol    start    end    strand    length   
   FBgn0031545   CG3213  3517273   3519697   -  2425  
   FBgn0031546   CG8851   3520262   3522555  +  2294  
   FBgn0031548   CG8852   3525133   3527588  +  2456  
   FBgn0031549   Spindly  3527387   3530317   -  2931  
   FBgn0031550   Che-13   3530903   3532526  +  1624  
 
 
    Segment 98 
 
   Location   
  Gene key  FBgn0024244-FBgn0002985  
  Heatmap region span   2L:3507754..3662745   
  Segment span   2L:3539251..3606748   
  Length (genes)  3  
  Length (bp)  67498  
   Model Scoring   
  BIC  273.928830  
  logL  -131.493798  
  logL ratio  68.225740  
   Expression   
  Mean expression  6.126575  
  Median expression  5.352342  
  Tissue std. dev.  1.649022  
 
  
   GO ID    description    ratio    P-value   
   GO:0005622   intracellular  2/3  0.0165  
 
  
   tissue    mean expression   
  5th Passage Drosophila S2 Cells  4.593504  
  Adult Accessory gland  4.943007  
  Adult Brain  4.757687  
  Adult Carcass  5.322099  
  Adult Crop  4.842757  
  Adult Eye  5.820431  
  Adult Fatbody  4.892211  
  Adult Female Spermatheca Mated  5.002937  
  Adult Female Spermatheca Virgin  4.942955  
  Adult Head  5.214588  
  Adult Heart  7.878798  
  Adult Hind Gut  9.098000  
  Adult Male Ejaculatory Duct  10.609794  
  Adult Mid Gut  8.296910  
  Adult Ovary  4.535983  
  Adult Salivary Gland  5.417612  
  Adult Testes  4.695225  
  Adult Thoracoabdominal ganglion  4.879841  
  Adult Whole Fly  5.687875  
  Larvae Wandering Tubules  7.608683  
  Larval Feeding Carcass  7.148663  
  Larval Feeding Central Nevous System  5.203614  
  Larval Feeding Hind Gut  9.066055  
  Larval Feeding Malpighian Tubule  7.442427  
  Larval Feeding Mid Gut  6.382342  
  Larval Feeding Salivary Gland  4.646828  
  Whole Larvae Feeding  6.486708  
 
  
   FlyBase ID    symbol    start    end    strand    length   
   FBgn0024244   drm   3539251   3548086  +  8836  
   FBgn0004892   sob  3578010   3580766   -  2757  
   FBgn0002985   odd  3604222   3606748   -  2527  
 
 
    Segment 99 
 
   Location   
  Gene key  FBgn0031558-FBgn0031559  
  Heatmap region span   2L:3619191..3703659   
  Segment span   2L:3692588..3693182   
  Length (genes)  2  
  Length (bp)  595  
   Model Scoring   
  BIC  266.618367  
  logL  -127.838566  
  logL ratio  -10.265182  
   Expression   
  Mean expression  7.343925  
  Median expression  6.516393  
  Tissue std. dev.  1.865279  
 
  No GO Slim enrichment  
  
   tissue    mean expression   
  5th Passage Drosophila S2 Cells  5.546852  
  Adult Accessory gland  5.540901  
  Adult Brain  5.990948  
  Adult Carcass  9.359033  
  Adult Crop  6.210056  
  Adult Eye  8.480677  
  Adult Fatbody  9.866348  
  Adult Female Spermatheca Mated  8.898542  
  Adult Female Spermatheca Virgin  9.842140  
  Adult Head  10.047614  
  Adult Heart  8.741796  
  Adult Hind Gut  6.776946  
  Adult Male Ejaculatory Duct  11.119550  
  Adult Mid Gut  5.257236  
  Adult Ovary  5.306987  
  Adult Salivary Gland  5.486195  
  Adult Testes  5.160246  
  Adult Thoracoabdominal ganglion  6.065671  
  Adult Whole Fly  7.407609  
  Larvae Wandering Tubules  6.489156  
  Larval Feeding Carcass  7.076415  
  Larval Feeding Central Nevous System  5.060425  
  Larval Feeding Hind Gut  7.794377  
  Larval Feeding Malpighian Tubule  6.664713  
  Larval Feeding Mid Gut  5.523279  
  Larval Feeding Salivary Gland  7.832083  
  Whole Larvae Feeding  10.740188  
 
  
   FlyBase ID    symbol    start    end    strand    length   
   FBgn0031558   CG16704  3692138   3692588   -  451  
   FBgn0031559   CG3513  3692699   3693182   -  484  
 
    Segment 100 
 
   Location   
  Gene key  FBgn0031560-FBgn0031561  
  Heatmap region span   2L:3621681..3705462   
  Segment span   2L:3695406..3696661   
  Length (genes)  2  
  Length (bp)  1256  
   Model Scoring   
  BIC  325.685253  
  logL  -157.372009  
  logL ratio  8.082738  
   Expression   
  Mean expression  9.552451  
  Median expression  9.883631  
  Tissue std. dev.  3.112349  
 
  No GO Slim enrichment  
  
   tissue    mean expression   
  5th Passage Drosophila S2 Cells  4.963176  
  Adult Accessory gland  7.261099  
  Adult Brain  7.832289  
  Adult Carcass  12.759156  
  Adult Crop  11.077531  
  Adult Eye  12.055744  
  Adult Fatbody  13.311298  
  Adult Female Spermatheca Mated  13.093879  
  Adult Female Spermatheca Virgin  13.243503  
  Adult Head  12.794367  
  Adult Heart  12.623934  
  Adult Hind Gut  13.985778  
  Adult Male Ejaculatory Duct  12.558556  
  Adult Mid Gut  6.829220  
  Adult Ovary  4.849089  
  Adult Salivary Gland  9.294535  
  Adult Testes  5.618610  
  Adult Thoracoabdominal ganglion  8.067373  
  Adult Whole Fly  11.587150  
  Larvae Wandering Tubules  5.598834  
  Larval Feeding Carcass  7.552779  
  Larval Feeding Central Nevous System  5.037315  
  Larval Feeding Hind Gut  12.017918  
  Larval Feeding Malpighian Tubule  7.453719  
  Larval Feeding Mid Gut  5.589396  
  Larval Feeding Salivary Gland  8.802755  
  Whole Larvae Feeding  12.057187  
 
  
   FlyBase ID    symbol    start    end    strand    length   
   FBgn0031560   CG16713  3695032   3695406   -  375  
   FBgn0031561   CG16712  3696240   3696661   -  422  
 
    Segment 101 
 
   Location   
  Gene key  FBgn0003386-FBgn0003430  
  Heatmap region span   2L:3707060..3947160   
  Segment span   2L:3718187..3825680   
  Length (genes)  9  
  Length (bp)  107494  
   Model Scoring   
  BIC  983.385160  
  logL  -486.221963  
  logL ratio  13.553121  
   Expression   
  Mean expression  5.807841  
  Median expression  5.436906  
  Tissue std. dev.  0.321009  
 
  
   GO ID    description    ratio    P-value   
   GO:0055085   transmembrane transport  2/9  0.0451  
 
  
   tissue    mean expression   
  5th Passage Drosophila S2 Cells  5.681635  
  Adult Accessory gland  6.159047  
  Adult Brain  6.400012  
  Adult Carcass  5.507618  
  Adult Crop  6.211065  
  Adult Eye  5.882307  
  Adult Fatbody  5.334417  
  Adult Female Spermatheca Mated  5.533901  
  Adult Female Spermatheca Virgin  5.557461  
  Adult Head  5.767013  
  Adult Heart  5.080585  
  Adult Hind Gut  5.848753  
  Adult Male Ejaculatory Duct  5.742226  
  Adult Mid Gut  5.850412  
  Adult Ovary  5.453331  
  Adult Salivary Gland  6.131849  
  Adult Testes  5.969597  
  Adult Thoracoabdominal ganglion  6.325742  
  Adult Whole Fly  5.279616  
  Larvae Wandering Tubules  5.984616  
  Larval Feeding Carcass  5.730309  
  Larval Feeding Central Nevous System  6.233270  
  Larval Feeding Hind Gut  5.786358  
  Larval Feeding Malpighian Tubule  5.949144  
  Larval Feeding Mid Gut  5.885020  
  Larval Feeding Salivary Gland  5.964627  
  Whole Larvae Feeding  5.561782  
 
  
   FlyBase ID    symbol    start    end    strand    length   
   FBgn0003386   Shaw   3718187   3727587  +  9401  
   FBgn0031568   CG10019   3745692   3752309  +  6618  
   FBgn0051959      3757631   3759212  +  1582  
   FBgn0051772      3760068   3768191  +  8124  
   FBgn0004893   bowl   3771706   3784129  +  12424  
   FBgn0051960   CG31960   3784946   3785515  +  570  
   FBgn0031571   CG3921   3790663   3802245  +  11583  
   FBgn0031573   CG3407  3810799   3813407   -  2609  
   FBgn0003430   slp1   3825680   3827137  +  1458  
 
 
    Segment 102 
 
   Location   
  Gene key  FBgn0004567-FBgn0031574  
  Heatmap region span   2L:3711722..4007690   
  Segment span   2L:3836842..3862681   
  Length (genes)  2  
  Length (bp)  25840  
   Model Scoring   
  BIC  216.135742  
  logL  -102.597254  
  logL ratio  13.869949  
   Expression   
  Mean expression  4.578614  
  Median expression  4.401384  
  Tissue std. dev.  0.784018  
 
  No GO Slim enrichment  
  
   tissue    mean expression   
  5th Passage Drosophila S2 Cells  4.300587  
  Adult Accessory gland  4.147009  
  Adult Brain  4.813468  
  Adult Carcass  4.231718  
  Adult Crop  4.102954  
  Adult Eye  5.486543  
  Adult Fatbody  4.283876  
  Adult Female Spermatheca Mated  4.424262  
  Adult Female Spermatheca Virgin  4.403537  
  Adult Head  4.741492  
  Adult Heart  3.956853  
  Adult Hind Gut  4.062935  
  Adult Male Ejaculatory Duct  4.031016  
  Adult Mid Gut  4.093123  
  Adult Ovary  4.291335  
  Adult Salivary Gland  4.388978  
  Adult Testes  6.621818  
  Adult Thoracoabdominal ganglion  4.963006  
  Adult Whole Fly  4.966258  
  Larvae Wandering Tubules  4.073307  
  Larval Feeding Carcass  4.428899  
  Larval Feeding Central Nevous System  7.445820  
  Larval Feeding Hind Gut  4.180389  
  Larval Feeding Malpighian Tubule  4.131553  
  Larval Feeding Mid Gut  4.142686  
  Larval Feeding Salivary Gland  4.252336  
  Whole Larvae Feeding  4.656812  
 
  
   FlyBase ID    symbol    start    end    strand    length   
   FBgn0004567   slp2   3836842   3839201  +  2360  
   FBgn0031574   CG3964   3862681   3867507  +  4827  
 
    Segment 103 
 
   Location   
  Gene key  FBgn0051957-FBgn0000256  
  Heatmap region span   2L:3713374..4166134   
  Segment span   2L:3872458..3902860   
  Length (genes)  2  
  Length (bp)  30403  
   Model Scoring   
  BIC  240.762331  
  logL  -114.910548  
  logL ratio  22.658181  
   Expression   
  Mean expression  8.267166  
  Median expression  8.091840  
  Tissue std. dev.  0.802763  
 
  No GO Slim enrichment  
  
   tissue    mean expression   
  5th Passage Drosophila S2 Cells  8.882051  
  Adult Accessory gland  8.046425  
  Adult Brain  8.649406  
  Adult Carcass  7.750913  
  Adult Crop  7.699607  
  Adult Eye  8.484158  
  Adult Fatbody  8.543857  
  Adult Female Spermatheca Mated  8.589367  
  Adult Female Spermatheca Virgin  8.569198  
  Adult Head  8.077972  
  Adult Heart  8.203019  
  Adult Hind Gut  8.828920  
  Adult Male Ejaculatory Duct  7.263459  
  Adult Mid Gut  9.029561  
  Adult Ovary  9.141957  
  Adult Salivary Gland  6.801509  
  Adult Testes  10.824970  
  Adult Thoracoabdominal ganglion  8.762747  
  Adult Whole Fly  8.655349  
  Larvae Wandering Tubules  8.366909  
  Larval Feeding Carcass  7.227618  
  Larval Feeding Central Nevous System  7.951632  
  Larval Feeding Hind Gut  8.267005  
  Larval Feeding Malpighian Tubule  7.640464  
  Larval Feeding Mid Gut  8.463673  
  Larval Feeding Salivary Gland  6.790090  
  Whole Larvae Feeding  7.701652  
 
  
   FlyBase ID    symbol    start    end    strand    length   
   FBgn0051957   CG31957  3871480   3872458   -  979  
   FBgn0000256   capu  3872658   3902860   -  30203  
 
    Segment 104 
 
   Location   
  Gene key  FBgn0051773-FBgn0051774  
  Heatmap region span   2L:3718187..4198058   
  Segment span   2L:3914288..3947160   
  Length (genes)  2  
  Length (bp)  32873  
   Model Scoring   
  BIC  216.060319  
  logL  -102.559542  
  logL ratio  3.063459  
   Expression   
  Mean expression  4.925217  
  Median expression  4.631846  
  Tissue std. dev.  0.736095  
 
  No GO Slim enrichment  
  
   tissue    mean expression   
  5th Passage Drosophila S2 Cells  4.599770  
  Adult Accessory gland  4.801030  
  Adult Brain  6.213178  
  Adult Carcass  4.682685  
  Adult Crop  4.496494  
  Adult Eye  5.196945  
  Adult Fatbody  4.660641  
  Adult Female Spermatheca Mated  4.588907  
  Adult Female Spermatheca Virgin  4.563909  
  Adult Head  4.926719  
  Adult Heart  4.434634  
  Adult Hind Gut  4.425090  
  Adult Male Ejaculatory Duct  4.905127  
  Adult Mid Gut  4.536361  
  Adult Ovary  4.584981  
  Adult Salivary Gland  4.763502  
  Adult Testes  7.928230  
  Adult Thoracoabdominal ganglion  5.986593  
  Adult Whole Fly  5.405771  
  Larvae Wandering Tubules  4.568430  
  Larval Feeding Carcass  4.594149  
  Larval Feeding Central Nevous System  5.273421  
  Larval Feeding Hind Gut  4.390819  
  Larval Feeding Malpighian Tubule  4.494646  
  Larval Feeding Mid Gut  4.653766  
  Larval Feeding Salivary Gland  4.589772  
  Whole Larvae Feeding  4.715300  
 
  
   FlyBase ID    symbol    start    end    strand    length   
   FBgn0051773   CG31773  3911790   3914288   -  2499  
   FBgn0051774   fred  3905524   3947160   -  41637  
 
    Segment 105 
 
   Location   
  Gene key  FBgn0085205-FBgn0031581  
  Heatmap region span   2L:3836842..4217852   
  Segment span   2L:4004080..4007690   
  Length (genes)  4  
  Length (bp)  3611  
   Model Scoring   
  BIC  401.108581  
  logL  -195.083673  
  logL ratio  91.750802  
   Expression   
  Mean expression  6.867563  
  Median expression  6.077625  
  Tissue std. dev.  2.003599  
 
  No GO Slim enrichment  
  
   tissue    mean expression   
  5th Passage Drosophila S2 Cells  5.974564  
  Adult Accessory gland  5.839351  
  Adult Brain  5.511652  
  Adult Carcass  5.933149  
  Adult Crop  6.050295  
  Adult Eye  5.469674  
  Adult Fatbody  6.103048  
  Adult Female Spermatheca Mated  6.054151  
  Adult Female Spermatheca Virgin  6.065593  
  Adult Head  5.676291  
  Adult Heart  5.631597  
  Adult Hind Gut  7.935103  
  Adult Male Ejaculatory Duct  6.068626  
  Adult Mid Gut  12.597155  
  Adult Ovary  5.834935  
  Adult Salivary Gland  6.488189  
  Adult Testes  5.928405  
  Adult Thoracoabdominal ganglion  5.486838  
  Adult Whole Fly  8.763796  
  Larvae Wandering Tubules  6.367319  
  Larval Feeding Carcass  5.892442  
  Larval Feeding Central Nevous System  5.597413  
  Larval Feeding Hind Gut  8.204704  
  Larval Feeding Malpighian Tubule  6.237314  
  Larval Feeding Mid Gut  12.423784  
  Larval Feeding Salivary Gland  6.197103  
  Whole Larvae Feeding  11.091723  
 
  
   FlyBase ID    symbol    start    end    strand    length   
   FBgn0085205   CG34176   4004080   4004480  +  401  
   FBgn0031579   CG15422   4004984   4005670  +  687  
   FBgn0031580   CG15423   4006041   4006606  +  566  
   FBgn0031581      4007690   4008999  +  1310  
 
 
    Segment 106 
 
   Location   
  Gene key  FBgn0031585-FBgn0026394  
  Heatmap region span   2L:3872458..4283147   
  Segment span   2L:4149279..4166134   
  Length (genes)  2  
  Length (bp)  16856  
   Model Scoring   
  BIC  221.535551  
  logL  -105.297158  
  logL ratio  13.320529  
   Expression   
  Mean expression  4.189656  
  Median expression  3.968424  
  Tissue std. dev.  0.800342  
 
  No GO Slim enrichment  
  
   tissue    mean expression   
  5th Passage Drosophila S2 Cells  3.901890  
  Adult Accessory gland  4.071336  
  Adult Brain  3.819896  
  Adult Carcass  4.159188  
  Adult Crop  3.930948  
  Adult Eye  3.889937  
  Adult Fatbody  4.005585  
  Adult Female Spermatheca Mated  4.136151  
  Adult Female Spermatheca Virgin  4.231307  
  Adult Head  3.928190  
  Adult Heart  3.983073  
  Adult Hind Gut  3.894389  
  Adult Male Ejaculatory Duct  3.979050  
  Adult Mid Gut  3.912257  
  Adult Ovary  3.866731  
  Adult Salivary Gland  4.082512  
  Adult Testes  7.837007  
  Adult Thoracoabdominal ganglion  3.944705  
  Adult Whole Fly  5.699434  
  Larvae Wandering Tubules  3.937400  
  Larval Feeding Carcass  3.893514  
  Larval Feeding Central Nevous System  3.793924  
  Larval Feeding Hind Gut  3.854299  
  Larval Feeding Malpighian Tubule  3.903034  
  Larval Feeding Mid Gut  3.930405  
  Larval Feeding Salivary Gland  3.946843  
  Whole Larvae Feeding  4.587709  
 
  
   FlyBase ID    symbol    start    end    strand    length   
   FBgn0031585   CG2955   4149279   4151203  +  1925  
   FBgn0026394   Or24a  4164689   4166134   -  1446  
 
    Segment 107 
 
   Location   
  Gene key  FBgn0051961-FBgn0031589  
  Heatmap region span   2L:3914288..4333926   
  Segment span   2L:4187789..4198058   
  Length (genes)  2  
  Length (bp)  10270  
   Model Scoring   
  BIC  272.281914  
  logL  -130.670340  
  logL ratio  0.960048  
   Expression   
  Mean expression  8.775999  
  Median expression  8.504893  
  Tissue std. dev.  0.766301  
 
  No GO Slim enrichment  
  
   tissue    mean expression   
  5th Passage Drosophila S2 Cells  9.015564  
  Adult Accessory gland  7.836389  
  Adult Brain  9.121892  
  Adult Carcass  8.827910  
  Adult Crop  9.745468  
  Adult Eye  8.854186  
  Adult Fatbody  8.758428  
  Adult Female Spermatheca Mated  8.927024  
  Adult Female Spermatheca Virgin  8.890757  
  Adult Head  8.971105  
  Adult Heart  8.837739  
  Adult Hind Gut  8.755419  
  Adult Male Ejaculatory Duct  8.365531  
  Adult Mid Gut  8.389427  
  Adult Ovary  8.845547  
  Adult Salivary Gland  8.698334  
  Adult Testes  6.391751  
  Adult Thoracoabdominal ganglion  9.071765  
  Adult Whole Fly  8.577353  
  Larvae Wandering Tubules  10.640429  
  Larval Feeding Carcass  9.019111  
  Larval Feeding Central Nevous System  8.664233  
  Larval Feeding Hind Gut  8.482590  
  Larval Feeding Malpighian Tubule  10.539141  
  Larval Feeding Mid Gut  8.157631  
  Larval Feeding Salivary Gland  8.189742  
  Whole Larvae Feeding  8.377512  
 
  
   FlyBase ID    symbol    start    end    strand    length   
   FBgn0051961   CG31961  4186245   4187789   -  1545  
   FBgn0031589   CG3714  4189497   4198058   -  8562  
 
    Segment 108 
 
   Location   
  Gene key  FBgn0031590-FBgn0003941  
  Heatmap region span   2L:4031379..4386062   
  Segment span   2L:4227097..4227642   
  Length (genes)  2  
  Length (bp)  546  
   Model Scoring   
  BIC  412.496249  
  logL  -200.777507  
  logL ratio  6.918147  
   Expression   
  Mean expression  12.285807  
  Median expression  11.909947  
  Tissue std. dev.  0.270026  
 
  No GO Slim enrichment  
  
   tissue    mean expression   
  5th Passage Drosophila S2 Cells  12.723479  
  Adult Accessory gland  12.103612  
  Adult Brain  12.261012  
  Adult Carcass  12.024559  
  Adult Crop  11.988441  
  Adult Eye  12.056275  
  Adult Fatbody  12.362017  
  Adult Female Spermatheca Mated  11.964783  
  Adult Female Spermatheca Virgin  11.967747  
  Adult Head  12.094306  
  Adult Heart  12.218275  
  Adult Hind Gut  12.314889  
  Adult Male Ejaculatory Duct  12.586785  
  Adult Mid Gut  12.514495  
  Adult Ovary  12.474117  
  Adult Salivary Gland  12.222787  
  Adult Testes  11.702042  
  Adult Thoracoabdominal ganglion  12.273464  
  Adult Whole Fly  12.391212  
  Larvae Wandering Tubules  12.848150  
  Larval Feeding Carcass  12.161274  
  Larval Feeding Central Nevous System  12.287913  
  Larval Feeding Hind Gut  12.421746  
  Larval Feeding Malpighian Tubule  12.813889  
  Larval Feeding Mid Gut  12.158655  
  Larval Feeding Salivary Gland  12.590853  
  Whole Larvae Feeding  12.190024  
 
  
   FlyBase ID    symbol    start    end    strand    length   
   FBgn0031590   CG3702  4224650   4227097   -  2448  
   FBgn0003941   RpL40   4227642   4228563  +  922  
 
    Segment 109 
 
   Location   
  Gene key  FBgn0031591-FBgn0010473  
  Heatmap region span   2L:4149279..4388321   
  Segment span   2L:4257572..4283147   
  Length (genes)  3  
  Length (bp)  25576  
   Model Scoring   
  BIC  270.184690  
  logL  -129.621728  
  logL ratio  48.679248  
   Expression   
  Mean expression  4.709889  
  Median expression  4.508666  
  Tissue std. dev.  0.631727  
 
  No GO Slim enrichment  
  
   tissue    mean expression   
  5th Passage Drosophila S2 Cells  4.557685  
  Adult Accessory gland  4.698249  
  Adult Brain  5.107206  
  Adult Carcass  4.447849  
  Adult Crop  4.408565  
  Adult Eye  4.484303  
  Adult Fatbody  4.568061  
  Adult Female Spermatheca Mated  4.518728  
  Adult Female Spermatheca Virgin  4.580828  
  Adult Head  4.371274  
  Adult Heart  4.404421  
  Adult Hind Gut  4.399830  
  Adult Male Ejaculatory Duct  4.538594  
  Adult Mid Gut  4.563884  
  Adult Ovary  4.515360  
  Adult Salivary Gland  4.603385  
  Adult Testes  7.723055  
  Adult Thoracoabdominal ganglion  5.055189  
  Adult Whole Fly  5.318886  
  Larvae Wandering Tubules  4.512280  
  Larval Feeding Carcass  4.470203  
  Larval Feeding Central Nevous System  4.525630  
  Larval Feeding Hind Gut  4.333323  
  Larval Feeding Malpighian Tubule  4.537761  
  Larval Feeding Mid Gut  4.595715  
  Larval Feeding Salivary Gland  4.568960  
  Whole Larvae Feeding  4.757786  
 
  
   FlyBase ID    symbol    start    end    strand    length   
   FBgn0031591      4257572   4258425  +  854  
   FBgn0031592   Art2  4277696   4279315   -  1620  
   FBgn0010473   tutl   4283147   4321233  +  38087  
 
 
    Segment 110 
 
   Location   
  Gene key  FBgn0028482-FBgn0020762  
  Heatmap region span   2L:4187789..4393814   
  Segment span   2L:4324573..4333926   
  Length (genes)  2  
  Length (bp)  9354  
   Model Scoring   
  BIC  256.169891  
  logL  -122.614328  
  logL ratio  7.161865  
   Expression   
  Mean expression  7.927841  
  Median expression  7.546255  
  Tissue std. dev.  1.243292  
 
  No GO Slim enrichment  
  
   tissue    mean expression   
  5th Passage Drosophila S2 Cells  7.296312  
  Adult Accessory gland  7.112395  
  Adult Brain  10.477337  
  Adult Carcass  8.509874  
  Adult Crop  8.050785  
  Adult Eye  11.105002  
  Adult Fatbody  8.016189  
  Adult Female Spermatheca Mated  7.902005  
  Adult Female Spermatheca Virgin  7.338260  
  Adult Head  10.097120  
  Adult Heart  8.736657  
  Adult Hind Gut  7.168576  
  Adult Male Ejaculatory Duct  6.575333  
  Adult Mid Gut  7.447616  
  Adult Ovary  6.948247  
  Adult Salivary Gland  7.022880  
  Adult Testes  7.201994  
  Adult Thoracoabdominal ganglion  10.705313  
  Adult Whole Fly  7.292101  
  Larvae Wandering Tubules  7.736131  
  Larval Feeding Carcass  7.144111  
  Larval Feeding Central Nevous System  8.331160  
  Larval Feeding Hind Gut  7.237125  
  Larval Feeding Malpighian Tubule  7.442493  
  Larval Feeding Mid Gut  7.837402  
  Larval Feeding Salivary Gland  6.346221  
  Whole Larvae Feeding  6.973062  
 
  
   FlyBase ID    symbol    start    end    strand    length   
   FBgn0028482   CG16857   4324573   4331837  +  7265  
   FBgn0020762   Atet   4333926   4345776  +  11851  
 
    Segment 111 
 
   Location   
  Gene key  FBgn0031596-FBgn0031597  
  Heatmap region span   2L:4217852..4400953   
  Segment span   2L:4352975..4382719   
  Length (genes)  3  
  Length (bp)  29745  
   Model Scoring   
  BIC  279.334222  
  logL  -134.196493  
  logL ratio  47.752927  
   Expression   
  Mean expression  4.791338  
  Median expression  4.583035  
  Tissue std. dev.  0.532515  
 
  No GO Slim enrichment  
  
   tissue    mean expression   
  5th Passage Drosophila S2 Cells  5.733603  
  Adult Accessory gland  4.349118  
  Adult Brain  4.306537  
  Adult Carcass  4.535839  
  Adult Crop  4.481387  
  Adult Eye  5.042313  
  Adult Fatbody  4.525679  
  Adult Female Spermatheca Mated  4.345157  
  Adult Female Spermatheca Virgin  4.335084  
  Adult Head  4.535753  
  Adult Heart  6.163000  
  Adult Hind Gut  4.723679  
  Adult Male Ejaculatory Duct  4.295849  
  Adult Mid Gut  4.646493  
  Adult Ovary  4.702798  
  Adult Salivary Gland  4.494084  
  Adult Testes  5.899654  
  Adult Thoracoabdominal ganglion  4.323871  
  Adult Whole Fly  4.792782  
  Larvae Wandering Tubules  4.871400  
  Larval Feeding Carcass  4.714880  
  Larval Feeding Central Nevous System  6.005134  
  Larval Feeding Hind Gut  5.164668  
  Larval Feeding Malpighian Tubule  4.493413  
  Larval Feeding Mid Gut  4.444690  
  Larval Feeding Salivary Gland  4.843649  
  Whole Larvae Feeding  4.595599  
 
  
   FlyBase ID    symbol    start    end    strand    length   
   FBgn0031596   CG15429   4352975   4354030  +  1056  
   FBgn0026319   Traf4   4362549   4380725  +  18177  
   FBgn0031597   CG17612  4380508   4382719   -  2212  
 
 
    Segment 112 
 
   Location   
  Gene key  FBgn0031601-FBgn0031607  
  Heatmap region span   2L:4386062..4477024   
  Segment span   2L:4403255..4454457   
  Length (genes)  8  
  Length (bp)  51203  
   Model Scoring   
  BIC  711.274821  
  logL  -350.166793  
  logL ratio  293.045099  
   Expression   
  Mean expression  7.847761  
  Median expression  7.793181  
  Tissue std. dev.  0.390640  
 
  No GO Slim enrichment  
  
   tissue    mean expression   
  5th Passage Drosophila S2 Cells  8.410542  
  Adult Accessory gland  8.258726  
  Adult Brain  8.270030  
  Adult Carcass  7.259586  
  Adult Crop  7.635787  
  Adult Eye  7.820762  
  Adult Fatbody  7.542317  
  Adult Female Spermatheca Mated  7.835874  
  Adult Female Spermatheca Virgin  7.749180  
  Adult Head  7.488308  
  Adult Heart  7.818596  
  Adult Hind Gut  7.414415  
  Adult Male Ejaculatory Duct  8.010660  
  Adult Mid Gut  7.564108  
  Adult Ovary  8.425742  
  Adult Salivary Gland  8.099747  
  Adult Testes  7.579903  
  Adult Thoracoabdominal ganglion  8.261382  
  Adult Whole Fly  7.391924  
  Larvae Wandering Tubules  7.863045  
  Larval Feeding Carcass  7.626243  
  Larval Feeding Central Nevous System  8.429146  
  Larval Feeding Hind Gut  7.587859  
  Larval Feeding Malpighian Tubule  7.792917  
  Larval Feeding Mid Gut  7.418691  
  Larval Feeding Salivary Gland  8.816125  
  Whole Larvae Feeding  7.517929  
 
  
   FlyBase ID    symbol    start    end    strand    length   
   FBgn0031601   Dim1   4403255   4403955  +  701  
   FBgn0031602   CG15431  4406129   4439547   -  33419  
   FBgn0031603   CG15432   4442429   4442985  +  557  
   FBgn0027609   morgue  4442870   4444677   -  1808  
   FBgn0031604   Elp3   4444732   4446765  +  2034  
   FBgn0025684   MFS18  4446662   4448637   -  1976  
   FBgn0031606   CG15439  4449289   4453004   -  3716  
   FBgn0031607   CG15440  4453167   4454457   -  1291  
 
 
    Segment 113 
 
   Location   
  Gene key  FBgn0019982-FBgn0053002  
  Heatmap region span   2L:4393814..4591963   
  Segment span   2L:4456822..4459602   
  Length (genes)  2  
  Length (bp)  2781  
   Model Scoring   
  BIC  217.581139  
  logL  -103.319952  
  logL ratio  54.374303  
   Expression   
  Mean expression  9.422250  
  Median expression  9.566664  
  Tissue std. dev.  0.578846  
 
  No GO Slim enrichment  
  
   tissue    mean expression   
  5th Passage Drosophila S2 Cells  8.975668  
  Adult Accessory gland  9.256158  
  Adult Brain  9.822728  
  Adult Carcass  9.966521  
  Adult Crop  9.812119  
  Adult Eye  10.024347  
  Adult Fatbody  9.916474  
  Adult Female Spermatheca Mated  9.338432  
  Adult Female Spermatheca Virgin  9.599441  
  Adult Head  9.717436  
  Adult Heart  10.197895  
  Adult Hind Gut  10.082869  
  Adult Male Ejaculatory Duct  10.007462  
  Adult Mid Gut  9.622473  
  Adult Ovary  9.430342  
  Adult Salivary Gland  10.018071  
  Adult Testes  7.913227  
  Adult Thoracoabdominal ganglion  10.035275  
  Adult Whole Fly  9.221447  
  Larvae Wandering Tubules  8.549730  
  Larval Feeding Carcass  8.893168  
  Larval Feeding Central Nevous System  9.527505  
  Larval Feeding Hind Gut  8.828978  
  Larval Feeding Malpighian Tubule  9.556736  
  Larval Feeding Mid Gut  8.655735  
  Larval Feeding Salivary Gland  8.705269  
  Whole Larvae Feeding  8.725238  
 
  
   FlyBase ID    symbol    start    end    strand    length   
   FBgn0019982   Gs1l  4455193   4456822   -  1630  
   FBgn0053002   mRpL27  4458980   4459602   -  623  
 
    Segment 114 
 
   Location   
  Gene key  FBgn0031608-FBgn0031611  
  Heatmap region span   2L:4400953..4642998   
  Segment span   2L:4459835..4465200   
  Length (genes)  4  
  Length (bp)  5366  
   Model Scoring   
  BIC  434.852561  
  logL  -211.955663  
  logL ratio  61.607279  
   Expression   
  Mean expression  7.330855  
  Median expression  7.357086  
  Tissue std. dev.  0.473732  
 
  No GO Slim enrichment  
  
   tissue    mean expression   
  5th Passage Drosophila S2 Cells  7.577742  
  Adult Accessory gland  8.247814  
  Adult Brain  7.423265  
  Adult Carcass  6.676001  
  Adult Crop  7.416201  
  Adult Eye  7.605261  
  Adult Fatbody  7.319778  
  Adult Female Spermatheca Mated  7.244414  
  Adult Female Spermatheca Virgin  7.160045  
  Adult Head  6.855656  
  Adult Heart  7.655730  
  Adult Hind Gut  6.891617  
  Adult Male Ejaculatory Duct  7.287720  
  Adult Mid Gut  6.634748  
  Adult Ovary  8.726144  
  Adult Salivary Gland  7.446951  
  Adult Testes  7.647303  
  Adult Thoracoabdominal ganglion  7.348581  
  Adult Whole Fly  7.372605  
  Larvae Wandering Tubules  7.351792  
  Larval Feeding Carcass  6.791796  
  Larval Feeding Central Nevous System  7.898533  
  Larval Feeding Hind Gut  7.023441  
  Larval Feeding Malpighian Tubule  7.283454  
  Larval Feeding Mid Gut  6.693094  
  Larval Feeding Salivary Gland  7.602969  
  Whole Larvae Feeding  6.750444  
 
  
   FlyBase ID    symbol    start    end    strand    length   
   FBgn0031608   CG15435   4459835   4461746  +  1912  
   FBgn0031609   CG15443  4461657   4462994   -  1338  
   FBgn0031610   CG15436   4463642   4465075  +  1434  
   FBgn0031611   CG17840   4465200   4468022  +  2823  
 
 
    Segment 115 
 
   Location   
  Gene key  FBgn0083938-FBgn0031617  
  Heatmap region span   2L:4459835..4813025   
  Segment span   2L:4641296..4642998   
  Length (genes)  2  
  Length (bp)  1703  
   Model Scoring   
  BIC  191.056685  
  logL  -90.057725  
  logL ratio  38.573925  
   Expression   
  Mean expression  5.524343  
  Median expression  5.035187  
  Tissue std. dev.  1.913467  
 
  No GO Slim enrichment  
  
   tissue    mean expression   
  5th Passage Drosophila S2 Cells  4.952798  
  Adult Accessory gland  13.779027  
  Adult Brain  4.594425  
  Adult Carcass  5.949245  
  Adult Crop  5.056618  
  Adult Eye  4.383997  
  Adult Fatbody  4.940627  
  Adult Female Spermatheca Mated  5.073819  
  Adult Female Spermatheca Virgin  4.950277  
  Adult Head  4.769443  
  Adult Heart  4.505020  
  Adult Hind Gut  4.940095  
  Adult Male Ejaculatory Duct  9.326632  
  Adult Mid Gut  4.968241  
  Adult Ovary  4.973384  
  Adult Salivary Gland  5.214385  
  Adult Testes  5.390100  
  Adult Thoracoabdominal ganglion  4.935267  
  Adult Whole Fly  7.836425  
  Larvae Wandering Tubules  5.013329  
  Larval Feeding Carcass  4.925241  
  Larval Feeding Central Nevous System  4.603616  
  Larval Feeding Hind Gut  4.650101  
  Larval Feeding Malpighian Tubule  4.821084  
  Larval Feeding Mid Gut  5.095626  
  Larval Feeding Salivary Gland  4.873646  
  Whole Larvae Feeding  4.634787  
 
  
   FlyBase ID    symbol    start    end    strand    length   
   FBgn0083938   BG642163   4641296   4642747  +  1452  
   FBgn0031617   CG15635   4642998   4646472  +  3475  
 
    Segment 116 
 
   Location   
  Gene key  FBgn0031619-FBgn0031626  
  Heatmap region span   2L:4477024..4821776   
  Segment span   2L:4651403..4734458   
  Length (genes)  8  
  Length (bp)  83056  
   Model Scoring   
  BIC  642.941945  
  logL  -316.000355  
  logL ratio  199.430721  
   Expression   
  Mean expression  4.858949  
  Median expression  4.606730  
  Tissue std. dev.  0.563369  
 
  No GO Slim enrichment  
  
   tissue    mean expression   
  5th Passage Drosophila S2 Cells  6.418303  
  Adult Accessory gland  4.876853  
  Adult Brain  4.550345  
  Adult Carcass  4.639132  
  Adult Crop  4.731775  
  Adult Eye  4.488021  
  Adult Fatbody  4.812157  
  Adult Female Spermatheca Mated  4.746701  
  Adult Female Spermatheca Virgin  4.741898  
  Adult Head  4.559082  
  Adult Heart  4.491560  
  Adult Hind Gut  4.607854  
  Adult Male Ejaculatory Duct  4.849881  
  Adult Mid Gut  4.733872  
  Adult Ovary  4.613703  
  Adult Salivary Gland  4.973494  
  Adult Testes  7.118652  
  Adult Thoracoabdominal ganglion  4.555080  
  Adult Whole Fly  4.842954  
  Larvae Wandering Tubules  4.639782  
  Larval Feeding Carcass  4.844159  
  Larval Feeding Central Nevous System  4.623989  
  Larval Feeding Hind Gut  4.576705  
  Larval Feeding Malpighian Tubule  4.685895  
  Larval Feeding Mid Gut  4.771468  
  Larval Feeding Salivary Gland  4.762673  
  Whole Larvae Feeding  4.935641  
 
  
   FlyBase ID    symbol    start    end    strand    length   
   FBgn0031619   CG3355   4651403   4652877  +  1475  
   FBgn0031620   CG11929   4682677   4687235  +  4559  
   FBgn0000227   Bsg25A  4683050   4684459   -  1410  
   FBgn0031621   CG15634   4687513   4688956  +  1444  
   FBgn0031622   CG3251  4688965   4690702   -  1738  
   FBgn0085380   CG34351   4694721   4722468  +  27748  
   FBgn0031623   Taf12L  4700941   4701588   -  648  
   FBgn0031626   CG15631  4732413   4734458   -  2046  
 
 
    Segment 117 
 
   Location   
  Gene key  FBgn0031627-FBgn0031628  
  Heatmap region span   2L:4578201..4830686   
  Segment span   2L:4794040..4794549   
  Length (genes)  2  
  Length (bp)  510  
   Model Scoring   
  BIC  205.511428  
  logL  -97.285097  
  logL ratio  21.162608  
   Expression   
  Mean expression  5.619563  
  Median expression  5.523512  
  Tissue std. dev.  1.046868  
 
  No GO Slim enrichment  
  
   tissue    mean expression   
  5th Passage Drosophila S2 Cells  5.479653  
  Adult Accessory gland  5.193409  
  Adult Brain  8.300033  
  Adult Carcass  5.063451  
  Adult Crop  5.242278  
  Adult Eye  8.211163  
  Adult Fatbody  4.948054  
  Adult Female Spermatheca Mated  5.000171  
  Adult Female Spermatheca Virgin  5.212139  
  Adult Head  7.283399  
  Adult Heart  5.411656  
  Adult Hind Gut  5.296998  
  Adult Male Ejaculatory Duct  5.191940  
  Adult Mid Gut  5.160911  
  Adult Ovary  5.418781  
  Adult Salivary Gland  5.123933  
  Adult Testes  4.976888  
  Adult Thoracoabdominal ganglion  7.585465  
  Adult Whole Fly  4.987497  
  Larvae Wandering Tubules  5.130126  
  Larval Feeding Carcass  5.180900  
  Larval Feeding Central Nevous System  7.423654  
  Larval Feeding Hind Gut  4.988226  
  Larval Feeding Malpighian Tubule  5.128334  
  Larval Feeding Mid Gut  4.886740  
  Larval Feeding Salivary Gland  5.007708  
  Whole Larvae Feeding  4.894709  
 
  
   FlyBase ID    symbol    start    end    strand    length   
   FBgn0031627   CG15630  4734592   4794040   -  59449  
   FBgn0031628   CG3294   4794549   4796576  +  2028  
 
    Segment 118 
 
   Location   
  Gene key  FBgn0031631-FBgn0031632  
  Heatmap region span   2L:4651403..4852865   
  Segment span   2L:4818210..4821776   
  Length (genes)  2  
  Length (bp)  3567  
   Model Scoring   
  BIC  197.109042  
  logL  -93.083904  
  logL ratio  24.460243  
   Expression   
  Mean expression  5.147144  
  Median expression  4.848580  
  Tissue std. dev.  0.767429  
 
  No GO Slim enrichment  
  
   tissue    mean expression   
  5th Passage Drosophila S2 Cells  5.081228  
  Adult Accessory gland  4.893315  
  Adult Brain  6.914325  
  Adult Carcass  6.079209  
  Adult Crop  4.598878  
  Adult Eye  5.460033  
  Adult Fatbody  4.638796  
  Adult Female Spermatheca Mated  4.729972  
  Adult Female Spermatheca Virgin  4.686899  
  Adult Head  5.638958  
  Adult Heart  4.549455  
  Adult Hind Gut  4.456511  
  Adult Male Ejaculatory Duct  4.804617  
  Adult Mid Gut  4.694169  
  Adult Ovary  5.542959  
  Adult Salivary Gland  4.732895  
  Adult Testes  5.993002  
  Adult Thoracoabdominal ganglion  7.199265  
  Adult Whole Fly  5.591439  
  Larvae Wandering Tubules  4.626762  
  Larval Feeding Carcass  4.592049  
  Larval Feeding Central Nevous System  6.493497  
  Larval Feeding Hind Gut  4.466164  
  Larval Feeding Malpighian Tubule  4.587058  
  Larval Feeding Mid Gut  4.597481  
  Larval Feeding Salivary Gland  4.714366  
  Whole Larvae Feeding  4.609600  
 
  
   FlyBase ID    symbol    start    end    strand    length   
   FBgn0031631   CG3225  4815613   4818210   -  2598  
   FBgn0031632   CG15628   4821776   4828552  +  6777  
 
    Segment 119 
 
   Location   
  Gene key  FBgn0031635-FBgn0031640  
  Heatmap region span   2L:4813025..4892115   
  Segment span   2L:4837996..4850639   
  Length (genes)  6  
  Length (bp)  12644  
   Model Scoring   
  BIC  683.009203  
  logL  -336.033984  
  logL ratio  100.652250  
   Expression   
  Mean expression  8.825995  
  Median expression  8.782450  
  Tissue std. dev.  0.347808  
 
  No GO Slim enrichment  
  
   tissue    mean expression   
  5th Passage Drosophila S2 Cells  8.667341  
  Adult Accessory gland  8.586730  
  Adult Brain  9.296771  
  Adult Carcass  8.557522  
  Adult Crop  8.594760  
  Adult Eye  9.318729  
  Adult Fatbody  8.858287  
  Adult Female Spermatheca Mated  8.229278  
  Adult Female Spermatheca Virgin  8.455275  
  Adult Head  8.966643  
  Adult Heart  9.626528  
  Adult Hind Gut  9.213224  
  Adult Male Ejaculatory Duct  9.251506  
  Adult Mid Gut  9.137169  
  Adult Ovary  8.666390  
  Adult Salivary Gland  8.727750  
  Adult Testes  8.628331  
  Adult Thoracoabdominal ganglion  9.223154  
  Adult Whole Fly  8.396123  
  Larvae Wandering Tubules  8.732054  
  Larval Feeding Carcass  8.464322  
  Larval Feeding Central Nevous System  8.788266  
  Larval Feeding Hind Gut  8.670059  
  Larval Feeding Malpighian Tubule  8.629728  
  Larval Feeding Mid Gut  9.350460  
  Larval Feeding Salivary Gland  8.490570  
  Whole Larvae Feeding  8.774900  
 
  
   FlyBase ID    symbol    start    end    strand    length   
   FBgn0031635   CG15626  4835333   4837996   -  2664  
   FBgn0031636   CG12194   4839219   4841465  +  2247  
   FBgn0031637   CG2950  4842086   4847292   -  5207  
   FBgn0031638   CG11927   4847748   4849499  +  1752  
   FBgn0031639   mRpS2  4849439   4850418   -  980  
   FBgn0031640   CG11926   4850639   4852640  +  2002  
 
 
    Segment 120 
 
   Location   
  Gene key  FBgn0031653-FBgn0020906  
  Heatmap region span   2L:4937409..4981646   
  Segment span   2L:4950763..4955353   
  Length (genes)  3  
  Length (bp)  4591  
   Model Scoring   
  BIC  295.804521  
  logL  -142.431643  
  logL ratio  73.040487  
   Expression   
  Mean expression  6.297440  
  Median expression  5.213541  
  Tissue std. dev.  2.644809  
 
  No GO Slim enrichment  
  
   tissue    mean expression   
  5th Passage Drosophila S2 Cells  5.137349  
  Adult Accessory gland  5.337196  
  Adult Brain  4.701409  
  Adult Carcass  5.473932  
  Adult Crop  5.246130  
  Adult Eye  5.168545  
  Adult Fatbody  5.308569  
  Adult Female Spermatheca Mated  5.310769  
  Adult Female Spermatheca Virgin  5.349015  
  Adult Head  4.948240  
  Adult Heart  5.202914  
  Adult Hind Gut  5.279370  
  Adult Male Ejaculatory Duct  5.365045  
  Adult Mid Gut  13.403787  
  Adult Ovary  5.019092  
  Adult Salivary Gland  5.701303  
  Adult Testes  4.887394  
  Adult Thoracoabdominal ganglion  4.878437  
  Adult Whole Fly  11.532500  
  Larvae Wandering Tubules  5.396308  
  Larval Feeding Carcass  5.132390  
  Larval Feeding Central Nevous System  4.767702  
  Larval Feeding Hind Gut  5.364408  
  Larval Feeding Malpighian Tubule  5.678202  
  Larval Feeding Mid Gut  13.119963  
  Larval Feeding Salivary Gland  5.065887  
  Whole Larvae Feeding  12.255033  
 
  
   FlyBase ID    symbol    start    end    strand    length   
   FBgn0031653   Jon25Biii  4949947   4950763   -  817  
   FBgn0031654   Jon25Bii  4952250   4953134   -  885  
   FBgn0020906   Jon25Bi  4954071   4955353   -  1283  
 
 
    Segment 121 
 
   Location   
  Gene key  FBgn0083961-FBgn0015834  
  Heatmap region span   2L:4945343..5044712   
  Segment span   2L:4965694..4967778   
  Length (genes)  2  
  Length (bp)  2085  
   Model Scoring   
  BIC  332.578486  
  logL  -160.818626  
  logL ratio  4.101763  
   Expression   
  Mean expression  10.953080  
  Median expression  10.570954  
  Tissue std. dev.  0.476889  
 
  No GO Slim enrichment  
  
   tissue    mean expression   
  5th Passage Drosophila S2 Cells  11.658417  
  Adult Accessory gland  11.113494  
  Adult Brain  11.273256  
  Adult Carcass  10.693399  
  Adult Crop  11.248614  
  Adult Eye  11.286968  
  Adult Fatbody  10.910045  
  Adult Female Spermatheca Mated  10.588422  
  Adult Female Spermatheca Virgin  10.674279  
  Adult Head  10.899607  
  Adult Heart  11.041431  
  Adult Hind Gut  11.191360  
  Adult Male Ejaculatory Duct  11.213491  
  Adult Mid Gut  10.460883  
  Adult Ovary  11.171094  
  Adult Salivary Gland  11.328480  
  Adult Testes  9.472015  
  Adult Thoracoabdominal ganglion  11.401549  
  Adult Whole Fly  10.668587  
  Larvae Wandering Tubules  10.785916  
  Larval Feeding Carcass  11.009919  
  Larval Feeding Central Nevous System  11.439605  
  Larval Feeding Hind Gut  11.212698  
  Larval Feeding Malpighian Tubule  10.445156  
  Larval Feeding Mid Gut  10.178191  
  Larval Feeding Salivary Gland  11.772505  
  Whole Larvae Feeding  10.593765  
 
  
   FlyBase ID    symbol    start    end    strand    length   
   FBgn0083961   CG34125   4965694   4966400  +  707  
   FBgn0015834   Trip1  4966367   4967778   -  1412  
 
    Segment 122 
 
   Location   
  Gene key  FBgn0031657-FBgn0031664  
  Heatmap region span   2L:4949751..5050877   
  Segment span   2L:4969004..4981588   
  Length (genes)  7  
  Length (bp)  12585  
   Model Scoring   
  BIC  670.461869  
  logL  -329.760317  
  logL ratio  257.093469  
   Expression   
  Mean expression  9.140252  
  Median expression  9.013287  
  Tissue std. dev.  0.320338  
 
  No GO Slim enrichment  
  
   tissue    mean expression   
  5th Passage Drosophila S2 Cells  9.933733  
  Adult Accessory gland  9.641153  
  Adult Brain  9.054878  
  Adult Carcass  8.527354  
  Adult Crop  8.892214  
  Adult Eye  8.794227  
  Adult Fatbody  8.979454  
  Adult Female Spermatheca Mated  9.122349  
  Adult Female Spermatheca Virgin  9.021995  
  Adult Head  8.693790  
  Adult Heart  9.026202  
  Adult Hind Gut  8.854224  
  Adult Male Ejaculatory Duct  9.412681  
  Adult Mid Gut  9.000796  
  Adult Ovary  9.542690  
  Adult Salivary Gland  8.949321  
  Adult Testes  9.020347  
  Adult Thoracoabdominal ganglion  9.216494  
  Adult Whole Fly  9.016031  
  Larvae Wandering Tubules  9.029735  
  Larval Feeding Carcass  9.044953  
  Larval Feeding Central Nevous System  9.688348  
  Larval Feeding Hind Gut  9.096676  
  Larval Feeding Malpighian Tubule  9.221898  
  Larval Feeding Mid Gut  9.260680  
  Larval Feeding Salivary Gland  9.697469  
  Whole Larvae Feeding  9.047111  
 
  
   FlyBase ID    symbol    start    end    strand    length   
   FBgn0031657   CG3756   4969004   4970216  +  1213  
   FBgn0031659   CG14043   4971704   4973687  +  1984  
   FBgn0031660   mRpL28   4973978   4975284  +  1307  
   FBgn0031661   Gmd  4975300   4977018   -  1719  
   FBgn0031662   CG3792   4977324   4978568  +  1245  
   FBgn0031663   CG8891  4978484   4979377   -  894  
   FBgn0031664   CG8892  4979536   4981588   -  2053  
 
 
    Segment 123 
 
   Location   
  Gene key  FBgn0053113-FBgn0031670  
  Heatmap region span   2L:4955519..5058241   
  Segment span   2L:5009744..5011081   
  Length (genes)  2  
  Length (bp)  1338  
   Model Scoring   
  BIC  280.424734  
  logL  -134.741750  
  logL ratio  77.505476  
   Expression   
  Mean expression  11.653181  
  Median expression  11.609008  
  Tissue std. dev.  0.396463  
 
  No GO Slim enrichment  
  
   tissue    mean expression   
  5th Passage Drosophila S2 Cells  11.708625  
  Adult Accessory gland  11.062645  
  Adult Brain  11.730065  
  Adult Carcass  11.543155  
  Adult Crop  11.713116  
  Adult Eye  12.025021  
  Adult Fatbody  11.585646  
  Adult Female Spermatheca Mated  11.499671  
  Adult Female Spermatheca Virgin  11.204315  
  Adult Head  11.796180  
  Adult Heart  11.609008  
  Adult Hind Gut  11.704467  
  Adult Male Ejaculatory Duct  11.601425  
  Adult Mid Gut  11.615830  
  Adult Ovary  11.920498  
  Adult Salivary Gland  11.355035  
  Adult Testes  10.094596  
  Adult Thoracoabdominal ganglion  11.659807  
  Adult Whole Fly  11.647630  
  Larvae Wandering Tubules  12.119296  
  Larval Feeding Carcass  11.978949  
  Larval Feeding Central Nevous System  11.741378  
  Larval Feeding Hind Gut  12.140360  
  Larval Feeding Malpighian Tubule  11.995458  
  Larval Feeding Mid Gut  11.916528  
  Larval Feeding Salivary Gland  12.012225  
  Whole Larvae Feeding  11.654950  
 
  
   FlyBase ID    symbol    start    end    strand    length   
   FBgn0053113   Rtnl1  4993641   5009744   -  16104  
   FBgn0031670   CG3887   5011081   5012127  +  1047  
 
    Segment 124 
 
   Location   
  Gene key  FBgn0016075-FBgn0031673  
  Heatmap region span   2L:4965694..5094021   
  Segment span   2L:5027412..5044712   
  Length (genes)  3  
  Length (bp)  17301  
   Model Scoring   
  BIC  350.139954  
  logL  -169.599360  
  logL ratio  80.765545  
   Expression   
  Mean expression  9.321237  
  Median expression  9.082935  
  Tissue std. dev.  1.580991  
 
  No GO Slim enrichment  
  
   tissue    mean expression   
  5th Passage Drosophila S2 Cells  12.857981  
  Adult Accessory gland  7.445062  
  Adult Brain  11.136948  
  Adult Carcass  10.597678  
  Adult Crop  8.132958  
  Adult Eye  9.005354  
  Adult Fatbody  10.564027  
  Adult Female Spermatheca Mated  10.437536  
  Adult Female Spermatheca Virgin  10.072058  
  Adult Head  10.204975  
  Adult Heart  11.399750  
  Adult Hind Gut  7.988598  
  Adult Male Ejaculatory Duct  8.336837  
  Adult Mid Gut  6.503318  
  Adult Ovary  9.389349  
  Adult Salivary Gland  7.190907  
  Adult Testes  8.341166  
  Adult Thoracoabdominal ganglion  11.618422  
  Adult Whole Fly  9.664084  
  Larvae Wandering Tubules  7.863820  
  Larval Feeding Carcass  10.081917  
  Larval Feeding Central Nevous System  10.308449  
  Larval Feeding Hind Gut  9.525689  
  Larval Feeding Malpighian Tubule  7.605761  
  Larval Feeding Mid Gut  6.732395  
  Larval Feeding Salivary Gland  8.434969  
  Whole Larvae Feeding  10.233400  
 
  
   FlyBase ID    symbol    start    end    strand    length   
   FBgn0016075   vkg  5012144   5027412   -  15269  
   FBgn0000299   Cg25C   5029615   5037114  +  7500  
   FBgn0031673   CG31650  5040648   5044712   -  4065  
 
 
    Segment 125 
 
   Location   
  Gene key  FBgn0031677-FBgn0031678  
  Heatmap region span   2L:5009744..5207308   
  Segment span   2L:5055004..5058241   
  Length (genes)  2  
  Length (bp)  3238  
   Model Scoring   
  BIC  236.094122  
  logL  -112.576444  
  logL ratio  -9.122257  
   Expression   
  Mean expression  5.672817  
  Median expression  4.957733  
  Tissue std. dev.  1.234240  
 
  No GO Slim enrichment  
  
   tissue    mean expression   
  5th Passage Drosophila S2 Cells  9.110775  
  Adult Accessory gland  4.810752  
  Adult Brain  4.572288  
  Adult Carcass  5.138936  
  Adult Crop  4.622763  
  Adult Eye  4.670865  
  Adult Fatbody  4.960734  
  Adult Female Spermatheca Mated  4.717417  
  Adult Female Spermatheca Virgin  4.760034  
  Adult Head  4.513530  
  Adult Heart  4.765898  
  Adult Hind Gut  4.665188  
  Adult Male Ejaculatory Duct  4.927484  
  Adult Mid Gut  5.029756  
  Adult Ovary  8.843457  
  Adult Salivary Gland  5.015474  
  Adult Testes  7.224065  
  Adult Thoracoabdominal ganglion  4.832944  
  Adult Whole Fly  7.312483  
  Larvae Wandering Tubules  5.882305  
  Larval Feeding Carcass  5.884737  
  Larval Feeding Central Nevous System  6.804841  
  Larval Feeding Hind Gut  5.900533  
  Larval Feeding Malpighian Tubule  6.131571  
  Larval Feeding Mid Gut  5.753049  
  Larval Feeding Salivary Gland  5.867503  
  Whole Larvae Feeding  6.446684  
 
  
   FlyBase ID    symbol    start    end    strand    length   
   FBgn0031677   CG14036  5054579   5055004   -  426  
   FBgn0031678   CG31918  5055982   5058241   -  2260  
 
    Segment 126 
 
   Location   
  Gene key  FBgn0028694-FBgn0031682  
  Heatmap region span   2L:5027412..5210461   
  Segment span   2L:5059816..5094021   
  Length (genes)  4  
  Length (bp)  34206  
   Model Scoring   
  BIC  544.069202  
  logL  -266.563984  
  logL ratio  26.021399  
   Expression   
  Mean expression  9.646148  
  Median expression  9.463199  
  Tissue std. dev.  0.527261  
 
  No GO Slim enrichment  
  
   tissue    mean expression   
  5th Passage Drosophila S2 Cells  9.116035  
  Adult Accessory gland  9.699523  
  Adult Brain  9.884294  
  Adult Carcass  8.917520  
  Adult Crop  8.668509  
  Adult Eye  9.927818  
  Adult Fatbody  9.580752  
  Adult Female Spermatheca Mated  9.177784  
  Adult Female Spermatheca Virgin  9.320512  
  Adult Head  9.557468  
  Adult Heart  9.697997  
  Adult Hind Gut  9.260217  
  Adult Male Ejaculatory Duct  9.983125  
  Adult Mid Gut  9.700859  
  Adult Ovary  9.182731  
  Adult Salivary Gland  10.444820  
  Adult Testes  9.334300  
  Adult Thoracoabdominal ganglion  9.758504  
  Adult Whole Fly  9.278993  
  Larvae Wandering Tubules  10.997963  
  Larval Feeding Carcass  9.023614  
  Larval Feeding Central Nevous System  9.861430  
  Larval Feeding Hind Gut  9.493651  
  Larval Feeding Malpighian Tubule  10.622688  
  Larval Feeding Mid Gut  10.088247  
  Larval Feeding Salivary Gland  10.319267  
  Whole Larvae Feeding  9.547372  
 
  
   FlyBase ID    symbol    start    end    strand    length   
   FBgn0028694   Rpn11  5058404   5059816   -  1413  
   FBgn0028572   qtc   5060832   5070750  +  9919  
   FBgn0031681   pgant5  5072156   5093590   -  21435  
   FBgn0031682   CG5828   5094021   5095439  +  1419  
 
 
    Segment 127 
 
   Location   
  Gene key  FBgn0031690-FBgn0250847  
  Heatmap region span   2L:5096227..5266392   
  Segment span   2L:5214730..5216533   
  Length (genes)  2  
  Length (bp)  1804  
   Model Scoring   
  BIC  214.306521  
  logL  -101.682643  
  logL ratio  13.532746  
   Expression   
  Mean expression  5.226515  
  Median expression  4.502291  
  Tissue std. dev.  1.890429  
 
  No GO Slim enrichment  
  
   tissue    mean expression   
  5th Passage Drosophila S2 Cells  4.646542  
  Adult Accessory gland  12.673265  
  Adult Brain  4.272419  
  Adult Carcass  5.128097  
  Adult Crop  4.403864  
  Adult Eye  4.290733  
  Adult Fatbody  4.458717  
  Adult Female Spermatheca Mated  4.433569  
  Adult Female Spermatheca Virgin  4.536649  
  Adult Head  4.663037  
  Adult Heart  4.445469  
  Adult Hind Gut  4.363459  
  Adult Male Ejaculatory Duct  7.979875  
  Adult Mid Gut  4.537826  
  Adult Ovary  4.471298  
  Adult Salivary Gland  4.623731  
  Adult Testes  8.513531  
  Adult Thoracoabdominal ganglion  4.421829  
  Adult Whole Fly  8.344407  
  Larvae Wandering Tubules  4.597537  
  Larval Feeding Carcass  4.436875  
  Larval Feeding Central Nevous System  4.225976  
  Larval Feeding Hind Gut  4.271792  
  Larval Feeding Malpighian Tubule  4.489788  
  Larval Feeding Mid Gut  4.449865  
  Larval Feeding Salivary Gland  4.490185  
  Whole Larvae Feeding  4.945559  
 
  
   FlyBase ID    symbol    start    end    strand    length   
   FBgn0031690   CG7742  5212395   5214730   -  2336  
   FBgn0250847   CG14034  5215211   5216533   -  1323  
 
    Segment 128 
 
   Location   
  Gene key  FBgn0031695-FBgn0016076  
  Heatmap region span   2L:5218260..5329857   
  Segment span   2L:5268517..5299647   
  Length (genes)  5  
  Length (bp)  31131  
   Model Scoring   
  BIC  577.544370  
  logL  -283.301568  
  logL ratio  83.097742  
   Expression   
  Mean expression  8.924025  
  Median expression  9.117349  
  Tissue std. dev.  0.590231  
 
  No GO Slim enrichment  
  
   tissue    mean expression   
  5th Passage Drosophila S2 Cells  7.805337  
  Adult Accessory gland  9.124251  
  Adult Brain  9.122211  
  Adult Carcass  8.827234  
  Adult Crop  9.630141  
  Adult Eye  9.010812  
  Adult Fatbody  8.724197  
  Adult Female Spermatheca Mated  9.526058  
  Adult Female Spermatheca Virgin  9.479731  
  Adult Head  9.077564  
  Adult Heart  9.477191  
  Adult Hind Gut  9.702432  
  Adult Male Ejaculatory Duct  9.038563  
  Adult Mid Gut  9.421404  
  Adult Ovary  8.469873  
  Adult Salivary Gland  8.186779  
  Adult Testes  7.071697  
  Adult Thoracoabdominal ganglion  8.929156  
  Adult Whole Fly  8.434071  
  Larvae Wandering Tubules  9.226728  
  Larval Feeding Carcass  9.141992  
  Larval Feeding Central Nevous System  8.732222  
  Larval Feeding Hind Gut  9.412212  
  Larval Feeding Malpighian Tubule  8.883836  
  Larval Feeding Mid Gut  9.479929  
  Larval Feeding Salivary Gland  8.225406  
  Whole Larvae Feeding  8.787656  
 
  
   FlyBase ID    symbol    start    end    strand    length   
   FBgn0031695   Cyp4ac3   5268517   5270329  +  1813  
   FBgn0003716   tkv  5218996   5271353   -  52358  
   FBgn0000228   Bsg25D  5271728   5278558   -  6831  
   FBgn0031696   Bub1   5279051   5283002  +  3952  
   FBgn0016076   vri   5299647   5310996  +  11350  
 
 
    Segment 129 
 
   Location   
  Gene key  FBgn0031697-FBgn0031698  
  Heatmap region span   2L:5242707..5338053   
  Segment span   2L:5314885..5324936   
  Length (genes)  2  
  Length (bp)  10052  
   Model Scoring   
  BIC  201.444698  
  logL  -95.251732  
  logL ratio  26.107237  
   Expression   
  Mean expression  5.833635  
  Median expression  5.593568  
  Tissue std. dev.  0.632003  
 
  No GO Slim enrichment  
  
   tissue    mean expression   
  5th Passage Drosophila S2 Cells  6.399031  
  Adult Accessory gland  5.635701  
  Adult Brain  7.987196  
  Adult Carcass  5.441240  
  Adult Crop  5.712778  
  Adult Eye  6.046748  
  Adult Fatbody  5.757701  
  Adult Female Spermatheca Mated  5.276779  
  Adult Female Spermatheca Virgin  5.327241  
  Adult Head  5.921417  
  Adult Heart  5.953741  
  Adult Hind Gut  5.498279  
  Adult Male Ejaculatory Duct  5.298970  
  Adult Mid Gut  5.476367  
  Adult Ovary  6.260158  
  Adult Salivary Gland  5.452364  
  Adult Testes  5.224830  
  Adult Thoracoabdominal ganglion  6.915553  
  Adult Whole Fly  5.076994  
  Larvae Wandering Tubules  5.869300  
  Larval Feeding Carcass  5.491345  
  Larval Feeding Central Nevous System  7.091385  
  Larval Feeding Hind Gut  5.662531  
  Larval Feeding Malpighian Tubule  5.907702  
  Larval Feeding Mid Gut  5.571547  
  Larval Feeding Salivary Gland  5.872755  
  Whole Larvae Feeding  5.378495  
 
  
   FlyBase ID    symbol    start    end    strand    length   
   FBgn0031697   CG14024  5312136   5314885   -  2750  
   FBgn0031698   Ncoa6  5316240   5324936   -  8697  
 
    Segment 130 
 
   Location   
  Gene key  FBgn0016660-FBgn0031707  
  Heatmap region span   2L:5329857..5542527   
  Segment span   2L:5404342..5517823   
  Length (genes)  3  
  Length (bp)  113482  
   Model Scoring   
  BIC  254.534675  
  logL  -121.796720  
  logL ratio  59.552709  
   Expression   
  Mean expression  4.712809  
  Median expression  4.603511  
  Tissue std. dev.  0.331008  
 
  No GO Slim enrichment  
  
   tissue    mean expression   
  5th Passage Drosophila S2 Cells  4.652741  
  Adult Accessory gland  4.749287  
  Adult Brain  4.380830  
  Adult Carcass  4.613967  
  Adult Crop  4.475372  
  Adult Eye  4.549008  
  Adult Fatbody  4.667377  
  Adult Female Spermatheca Mated  4.686410  
  Adult Female Spermatheca Virgin  4.701308  
  Adult Head  4.613404  
  Adult Heart  5.707462  
  Adult Hind Gut  4.639076  
  Adult Male Ejaculatory Duct  4.622482  
  Adult Mid Gut  4.621439  
  Adult Ovary  4.602301  
  Adult Salivary Gland  4.798163  
  Adult Testes  5.904174  
  Adult Thoracoabdominal ganglion  4.796024  
  Adult Whole Fly  4.372993  
  Larvae Wandering Tubules  4.633145  
  Larval Feeding Carcass  4.757605  
  Larval Feeding Central Nevous System  4.486606  
  Larval Feeding Hind Gut  4.442783  
  Larval Feeding Malpighian Tubule  4.673285  
  Larval Feeding Mid Gut  4.592509  
  Larval Feeding Salivary Gland  4.834148  
  Whole Larvae Feeding  4.671951  
 
  
   FlyBase ID    symbol    start    end    strand    length   
   FBgn0016660   H15   5404342   5415928  +  11587  
   FBgn0051647   CR31647  5435582   5439277   -  3696  
   FBgn0031707   CG14020   5517823   5518949  +  1127  
 
 
    Segment 131 
 
   Location   
  Gene key  FBgn0031708-FBgn0000318  
  Heatmap region span   2L:5338053..5547203   
  Segment span   2L:5519889..5520235   
  Length (genes)  2  
  Length (bp)  347  
   Model Scoring   
  BIC  275.031074  
  logL  -132.044920  
  logL ratio  44.968369  
   Expression   
  Mean expression  10.812303  
  Median expression  10.541815  
  Tissue std. dev.  0.501830  
 
  No GO Slim enrichment  
  
   tissue    mean expression   
  5th Passage Drosophila S2 Cells  11.461160  
  Adult Accessory gland  10.026563  
  Adult Brain  10.923227  
  Adult Carcass  10.410597  
  Adult Crop  11.260802  
  Adult Eye  12.023367  
  Adult Fatbody  10.231850  
  Adult Female Spermatheca Mated  9.972055  
  Adult Female Spermatheca Virgin  10.041580  
  Adult Head  10.946359  
  Adult Heart  11.090653  
  Adult Hind Gut  10.962071  
  Adult Male Ejaculatory Duct  10.647470  
  Adult Mid Gut  11.149693  
  Adult Ovary  10.483798  
  Adult Salivary Gland  10.863875  
  Adult Testes  11.006719  
  Adult Thoracoabdominal ganglion  10.675337  
  Adult Whole Fly  10.541253  
  Larvae Wandering Tubules  11.578373  
  Larval Feeding Carcass  10.767093  
  Larval Feeding Central Nevous System  10.494751  
  Larval Feeding Hind Gut  10.937493  
  Larval Feeding Malpighian Tubule  11.751196  
  Larval Feeding Mid Gut  10.627507  
  Larval Feeding Salivary Gland  10.552882  
  Whole Larvae Feeding  10.504460  
 
  
   FlyBase ID    symbol    start    end    strand    length   
   FBgn0031708   CG7382  5518893   5519889   -  997  
   FBgn0000318   cl   5520235   5521382  +  1148  
 
    Segment 132 
 
   Location   
  Gene key  FBgn0031710-FBgn0031713  
  Heatmap region span   2L:5342321..5617747   
  Segment span   2L:5523877..5532195   
  Length (genes)  5  
  Length (bp)  8319  
   Model Scoring   
  BIC  511.615145  
  logL  -250.336955  
  logL ratio  134.451288  
   Expression   
  Mean expression  8.451597  
  Median expression  8.313537  
  Tissue std. dev.  0.387048  
 
  No GO Slim enrichment  
  
   tissue    mean expression   
  5th Passage Drosophila S2 Cells  9.790379  
  Adult Accessory gland  8.466346  
  Adult Brain  8.024047  
  Adult Carcass  8.408380  
  Adult Crop  8.497363  
  Adult Eye  8.136756  
  Adult Fatbody  8.763655  
  Adult Female Spermatheca Mated  8.758463  
  Adult Female Spermatheca Virgin  8.698450  
  Adult Head  8.118323  
  Adult Heart  9.063838  
  Adult Hind Gut  8.220014  
  Adult Male Ejaculatory Duct  8.346812  
  Adult Mid Gut  8.228500  
  Adult Ovary  8.773332  
  Adult Salivary Gland  8.247095  
  Adult Testes  8.159362  
  Adult Thoracoabdominal ganglion  8.207610  
  Adult Whole Fly  8.001552  
  Larvae Wandering Tubules  8.848021  
  Larval Feeding Carcass  8.100468  
  Larval Feeding Central Nevous System  8.391092  
  Larval Feeding Hind Gut  8.349793  
  Larval Feeding Malpighian Tubule  8.803444  
  Larval Feeding Mid Gut  8.085832  
  Larval Feeding Salivary Gland  8.508459  
  Whole Larvae Feeding  8.195726  
 
  
   FlyBase ID    symbol    start    end    strand    length   
   FBgn0031710   CG7371  5521281   5523877   -  2597  
   FBgn0031711   CG6907   5524139   5525809  +  1671  
   FBgn0051648   CG31648   5526962   5527854  +  893  
   FBgn0051915   CG31915   5528087   5530682  +  2596  
   FBgn0031713   CG7277  5530620   5532195   -  1576  
 
 
    Segment 133 
 
   Location   
  Gene key  FBgn0031715-FBgn0031716  
  Heatmap region span   2L:5342349..5658553   
  Segment span   2L:5536726..5539169   
  Length (genes)  2  
  Length (bp)  2444  
   Model Scoring   
  BIC  233.980128  
  logL  -111.519447  
  logL ratio  -5.365655  
   Expression   
  Mean expression  4.752342  
  Median expression  4.487862  
  Tissue std. dev.  0.923312  
 
  No GO Slim enrichment  
  
   tissue    mean expression   
  5th Passage Drosophila S2 Cells  6.221623  
  Adult Accessory gland  4.220587  
  Adult Brain  6.880197  
  Adult Carcass  4.437679  
  Adult Crop  4.100308  
  Adult Eye  5.721212  
  Adult Fatbody  4.089232  
  Adult Female Spermatheca Mated  4.145456  
  Adult Female Spermatheca Virgin  4.194756  
  Adult Head  5.993927  
  Adult Heart  4.174159  
  Adult Hind Gut  4.182796  
  Adult Male Ejaculatory Duct  4.216816  
  Adult Mid Gut  4.262411  
  Adult Ovary  4.279002  
  Adult Salivary Gland  4.308587  
  Adult Testes  5.357335  
  Adult Thoracoabdominal ganglion  7.082021  
  Adult Whole Fly  4.316807  
  Larvae Wandering Tubules  4.392658  
  Larval Feeding Carcass  4.149563  
  Larval Feeding Central Nevous System  6.346557  
  Larval Feeding Hind Gut  4.103563  
  Larval Feeding Malpighian Tubule  4.248202  
  Larval Feeding Mid Gut  4.270019  
  Larval Feeding Salivary Gland  4.318325  
  Whole Larvae Feeding  4.299447  
 
  
   FlyBase ID    symbol    start    end    strand    length   
   FBgn0031715   tomb  5535933   5536726   -  794  
   FBgn0031716   CG14015  5536850   5539169   -  2320  
 
    Segment 134 
 
   Location   
  Gene key  FBgn0014189-FBgn0002525  
  Heatmap region span   2L:5404342..5662849   
  Segment span   2L:5542310..5542527   
  Length (genes)  2  
  Length (bp)  218  
   Model Scoring   
  BIC  264.518646  
  logL  -126.788706  
  logL ratio  24.009671  
   Expression   
  Mean expression  9.651120  
  Median expression  9.675663  
  Tissue std. dev.  0.928644  
 
  No GO Slim enrichment  
  
   tissue    mean expression   
  5th Passage Drosophila S2 Cells  11.618548  
  Adult Accessory gland  10.092465  
  Adult Brain  9.822213  
  Adult Carcass  8.690927  
  Adult Crop  9.436633  
  Adult Eye  9.374928  
  Adult Fatbody  8.928445  
  Adult Female Spermatheca Mated  9.817539  
  Adult Female Spermatheca Virgin  9.702951  
  Adult Head  9.178324  
  Adult Heart  9.310791  
  Adult Hind Gut  8.778607  
  Adult Male Ejaculatory Duct  8.734491  
  Adult Mid Gut  8.879898  
  Adult Ovary  11.118334  
  Adult Salivary Gland  8.409413  
  Adult Testes  9.333442  
  Adult Thoracoabdominal ganglion  9.531120  
  Adult Whole Fly  10.197481  
  Larvae Wandering Tubules  8.959176  
  Larval Feeding Carcass  9.894435  
  Larval Feeding Central Nevous System  12.719514  
  Larval Feeding Hind Gut  9.543361  
  Larval Feeding Malpighian Tubule  9.172290  
  Larval Feeding Mid Gut  9.118982  
  Larval Feeding Salivary Gland  10.272274  
  Whole Larvae Feeding  9.943648  
 
  
   FlyBase ID    symbol    start    end    strand    length   
   FBgn0014189   Hel25E  5539326   5542310   -  2985  
   FBgn0002525   Lam   5542527   5546642  +  4116  
 
    Segment 135 
 
   Location   
  Gene key  FBgn0031718-FBgn0031725  
  Heatmap region span   2L:5523877..5720896   
  Segment span   2L:5550835..5617747   
  Length (genes)  10  
  Length (bp)  66913  
   Model Scoring   
  BIC  871.809415  
  logL  -430.434090  
  logL ratio  186.320713  
   Expression   
  Mean expression  5.125650  
  Median expression  4.758630  
  Tissue std. dev.  0.600199  
 
  No GO Slim enrichment  
  
   tissue    mean expression   
  5th Passage Drosophila S2 Cells  4.972344  
  Adult Accessory gland  4.958884  
  Adult Brain  4.951476  
  Adult Carcass  5.024515  
  Adult Crop  5.034921  
  Adult Eye  4.772018  
  Adult Fatbody  4.898187  
  Adult Female Spermatheca Mated  5.074127  
  Adult Female Spermatheca Virgin  5.005996  
  Adult Head  4.777033  
  Adult Heart  5.244021  
  Adult Hind Gut  5.058516  
  Adult Male Ejaculatory Duct  5.119715  
  Adult Mid Gut  4.994202  
  Adult Ovary  4.803979  
  Adult Salivary Gland  5.125764  
  Adult Testes  8.025638  
  Adult Thoracoabdominal ganglion  4.904009  
  Adult Whole Fly  5.601551  
  Larvae Wandering Tubules  5.088429  
  Larval Feeding Carcass  5.389199  
  Larval Feeding Central Nevous System  4.616586  
  Larval Feeding Hind Gut  4.987253  
  Larval Feeding Malpighian Tubule  4.936381  
  Larval Feeding Mid Gut  4.977779  
  Larval Feeding Salivary Gland  4.853122  
  Whole Larvae Feeding  5.196900  
 
  
   FlyBase ID    symbol    start    end    strand    length   
   FBgn0031718   CG14014  5549707   5550835   -  1129  
   FBgn0031719   CG18269   5552046   5552648  +  603  
   FBgn0031720   CG14013  5552843   5553848   -  1006  
   FBgn0031721   CG14017   5553897   5555137  +  1241  
   FBgn0004620   GluRIIA   5555074   5558994  +  3921  
   FBgn0020429   GluRIIB   5559342   5563502  +  4161  
   FBgn0031722   CG14011  5579580   5581116   -  1537  
   FBgn0031723   CG7251  5587149   5588414   -  1266  
   FBgn0031724   CG18266   5596833   5598636  +  1804  
   FBgn0031725   CG14010  5602958   5617747   -  14790  
 
 
    Segment 136 
 
   Location   
  Gene key  FBgn0031729-FBgn0054011  
  Heatmap region span   2L:5547203..5741244   
  Segment span   2L:5678638..5716819   
  Length (genes)  4  
  Length (bp)  38182  
   Model Scoring   
  BIC  331.591906  
  logL  -160.325336  
  logL ratio  97.941007  
   Expression   
  Mean expression  4.611600  
  Median expression  4.475808  
  Tissue std. dev.  0.356854  
 
  No GO Slim enrichment  
  
   tissue    mean expression   
  5th Passage Drosophila S2 Cells  4.567144  
  Adult Accessory gland  4.599304  
  Adult Brain  4.382405  
  Adult Carcass  4.581110  
  Adult Crop  4.545702  
  Adult Eye  4.648840  
  Adult Fatbody  4.626630  
  Adult Female Spermatheca Mated  4.445083  
  Adult Female Spermatheca Virgin  4.426637  
  Adult Head  4.762033  
  Adult Heart  4.420989  
  Adult Hind Gut  4.525143  
  Adult Male Ejaculatory Duct  4.614008  
  Adult Mid Gut  4.611659  
  Adult Ovary  4.557307  
  Adult Salivary Gland  4.633845  
  Adult Testes  6.355098  
  Adult Thoracoabdominal ganglion  4.461465  
  Adult Whole Fly  4.524035  
  Larvae Wandering Tubules  4.570608  
  Larval Feeding Carcass  4.586356  
  Larval Feeding Central Nevous System  4.322390  
  Larval Feeding Hind Gut  4.446258  
  Larval Feeding Malpighian Tubule  4.567317  
  Larval Feeding Mid Gut  4.672574  
  Larval Feeding Salivary Gland  4.675443  
  Whole Larvae Feeding  4.383817  
 
  
   FlyBase ID    symbol    start    end    strand    length   
   FBgn0031729   CG12511   5678638   5679223  +  586  
   FBgn0031730   CG7236   5708449   5713648  +  5200  
   FBgn0031731   CG14007  5715491   5716650   -  1160  
   FBgn0054011   CG34011   5716819   5718067  +  1249  
 
 
    Segment 137 
 
   Location   
  Gene key  FBgn0024191-FBgn0031736  
  Heatmap region span   2L:5658553..5802255   
  Segment span   2L:5721039..5724658   
  Length (genes)  2  
  Length (bp)  3620  
   Model Scoring   
  BIC  214.336002  
  logL  -101.697384  
  logL ratio  41.205752  
   Expression   
  Mean expression  7.003624  
  Median expression  6.799473  
  Tissue std. dev.  0.466676  
 
  No GO Slim enrichment  
  
   tissue    mean expression   
  5th Passage Drosophila S2 Cells  7.739133  
  Adult Accessory gland  7.012039  
  Adult Brain  6.914343  
  Adult Carcass  6.655247  
  Adult Crop  7.726853  
  Adult Eye  6.662792  
  Adult Fatbody  6.824105  
  Adult Female Spermatheca Mated  6.693786  
  Adult Female Spermatheca Virgin  6.456917  
  Adult Head  6.548630  
  Adult Heart  7.018341  
  Adult Hind Gut  6.778753  
  Adult Male Ejaculatory Duct  7.010280  
  Adult Mid Gut  6.567464  
  Adult Ovary  8.089308  
  Adult Salivary Gland  7.247549  
  Adult Testes  7.981941  
  Adult Thoracoabdominal ganglion  6.627339  
  Adult Whole Fly  6.930368  
  Larvae Wandering Tubules  6.959340  
  Larval Feeding Carcass  6.995762  
  Larval Feeding Central Nevous System  7.468355  
  Larval Feeding Hind Gut  6.841337  
  Larval Feeding Malpighian Tubule  6.812583  
  Larval Feeding Mid Gut  6.409828  
  Larval Feeding Salivary Gland  7.659693  
  Whole Larvae Feeding  6.465758  
 
  
   FlyBase ID    symbol    start    end    strand    length   
   FBgn0024191   sip1   5721039   5723791  +  2753  
   FBgn0031736   CG11030   5724658   5760047  +  35390  
 
    Segment 138 
 
   Location   
  Gene key  FBgn0031735-FBgn0051913  
  Heatmap region span   2L:5720896..5879481   
  Segment span   2L:5746277..5784031   
  Length (genes)  2  
  Length (bp)  37755  
   Model Scoring   
  BIC  221.632435  
  logL  -105.345600  
  logL ratio  13.595923  
   Expression   
  Mean expression  6.086054  
  Median expression  5.936174  
  Tissue std. dev.  0.714997  
 
  No GO Slim enrichment  
  
   tissue    mean expression   
  5th Passage Drosophila S2 Cells  5.895613  
  Adult Accessory gland  6.063890  
  Adult Brain  5.367142  
  Adult Carcass  6.171660  
  Adult Crop  5.902207  
  Adult Eye  5.349908  
  Adult Fatbody  5.779946  
  Adult Female Spermatheca Mated  6.426364  
  Adult Female Spermatheca Virgin  6.410937  
  Adult Head  5.499425  
  Adult Heart  5.418751  
  Adult Hind Gut  5.759546  
  Adult Male Ejaculatory Duct  5.848859  
  Adult Mid Gut  6.846002  
  Adult Ovary  5.732572  
  Adult Salivary Gland  6.182279  
  Adult Testes  9.007064  
  Adult Thoracoabdominal ganglion  5.512974  
  Adult Whole Fly  7.085930  
  Larvae Wandering Tubules  6.035189  
  Larval Feeding Carcass  6.097786  
  Larval Feeding Central Nevous System  5.390911  
  Larval Feeding Hind Gut  5.719628  
  Larval Feeding Malpighian Tubule  6.102332  
  Larval Feeding Mid Gut  6.043420  
  Larval Feeding Salivary Gland  6.137853  
  Whole Larvae Feeding  6.535262  
 
  
   FlyBase ID    symbol    start    end    strand    length   
   FBgn0031735   CG11029   5746277   5747620  +  1344  
   FBgn0051913   CG31913   5784031   5784893  +  863  
 
    Segment 139 
 
   Location   
  Gene key  FBgn0031738-FBgn0031740  
  Heatmap region span   2L:5721039..5893896   
  Segment span   2L:5800204..5802255   
  Length (genes)  3  
  Length (bp)  2052  
   Model Scoring   
  BIC  312.638770  
  logL  -150.848768  
  logL ratio  72.320279  
   Expression   
  Mean expression  7.717865  
  Median expression  7.475117  
  Tissue std. dev.  0.706271  
 
  No GO Slim enrichment  
  
   tissue    mean expression   
  5th Passage Drosophila S2 Cells  8.698392  
  Adult Accessory gland  8.079301  
  Adult Brain  8.375741  
  Adult Carcass  6.498372  
  Adult Crop  7.463046  
  Adult Eye  8.295044  
  Adult Fatbody  6.490967  
  Adult Female Spermatheca Mated  8.160014  
  Adult Female Spermatheca Virgin  8.115696  
  Adult Head  7.330224  
  Adult Heart  7.086643  
  Adult Hind Gut  7.860994  
  Adult Male Ejaculatory Duct  6.565100  
  Adult Mid Gut  7.825739  
  Adult Ovary  8.857403  
  Adult Salivary Gland  8.057822  
  Adult Testes  6.406868  
  Adult Thoracoabdominal ganglion  8.105123  
  Adult Whole Fly  7.316657  
  Larvae Wandering Tubules  8.486800  
  Larval Feeding Carcass  7.136030  
  Larval Feeding Central Nevous System  8.273762  
  Larval Feeding Hind Gut  7.950749  
  Larval Feeding Malpighian Tubule  8.774492  
  Larval Feeding Mid Gut  7.397929  
  Larval Feeding Salivary Gland  7.625812  
  Whole Larvae Feeding  7.147634  
 
  
   FlyBase ID    symbol    start    end    strand    length   
   FBgn0031738   CG9171  5768250   5800204   -  31955  
   FBgn0031739   CG14005  5800517   5801969   -  1453  
   FBgn0031740   CG7239   5802255   5804030  +  1776  
 
 
    Segment 140 
 
   Location   
  Gene key  FBgn0051644-FBgn0085410  
  Heatmap region span   2L:5741244..5907180   
  Segment span   2L:5818393..5824842   
  Length (genes)  2  
  Length (bp)  6450  
   Model Scoring   
  BIC  224.167267  
  logL  -106.613016  
  logL ratio  -1.900242  
   Expression   
  Mean expression  5.108038  
  Median expression  4.923127  
  Tissue std. dev.  0.799284  
 
  No GO Slim enrichment  
  
   tissue    mean expression   
  5th Passage Drosophila S2 Cells  4.775934  
  Adult Accessory gland  5.274061  
  Adult Brain  5.367673  
  Adult Carcass  5.069236  
  Adult Crop  5.428000  
  Adult Eye  4.690657  
  Adult Fatbody  4.750858  
  Adult Female Spermatheca Mated  4.967752  
  Adult Female Spermatheca Virgin  4.992916  
  Adult Head  4.672783  
  Adult Heart  4.857960  
  Adult Hind Gut  4.783552  
  Adult Male Ejaculatory Duct  5.400211  
  Adult Mid Gut  4.699797  
  Adult Ovary  4.711959  
  Adult Salivary Gland  4.795208  
  Adult Testes  8.751085  
  Adult Thoracoabdominal ganglion  5.184759  
  Adult Whole Fly  6.308601  
  Larvae Wandering Tubules  4.714288  
  Larval Feeding Carcass  4.878647  
  Larval Feeding Central Nevous System  4.745724  
  Larval Feeding Hind Gut  4.557137  
  Larval Feeding Malpighian Tubule  4.725417  
  Larval Feeding Mid Gut  4.795573  
  Larval Feeding Salivary Gland  4.767854  
  Whole Larvae Feeding  5.249382  
 
  
   FlyBase ID    symbol    start    end    strand    length   
   FBgn0051644   CG31644  5817892   5818393   -  502  
   FBgn0085410   TrissinR   5824842   5856757  +  31916  
 
    Segment 141 
 
   Location   
  Gene key  FBgn0031746-FBgn0002855  
  Heatmap region span   2L:5800204..5949484   
  Segment span   2L:5886020..5893896   
  Length (genes)  3  
  Length (bp)  7877  
   Model Scoring   
  BIC  282.192831  
  logL  -135.625798  
  logL ratio  86.224015  
   Expression   
  Mean expression  5.058680  
  Median expression  4.089307  
  Tissue std. dev.  2.469347  
 
  
   GO ID    description    ratio    P-value   
   GO:0005615   extracellular space  3/3  2.97e-08  
   GO:0005576   extracellular region  2/3  0.00459  
 
  
   tissue    mean expression   
  5th Passage Drosophila S2 Cells  4.037542  
  Adult Accessory gland  14.312530  
  Adult Brain  3.931710  
  Adult Carcass  5.739734  
  Adult Crop  3.955004  
  Adult Eye  4.469018  
  Adult Fatbody  4.024894  
  Adult Female Spermatheca Mated  4.050585  
  Adult Female Spermatheca Virgin  4.081804  
  Adult Head  3.962341  
  Adult Heart  4.056421  
  Adult Hind Gut  3.957825  
  Adult Male Ejaculatory Duct  9.889018  
  Adult Mid Gut  4.057293  
  Adult Ovary  4.375219  
  Adult Salivary Gland  4.255218  
  Adult Testes  6.296644  
  Adult Thoracoabdominal ganglion  4.048701  
  Adult Whole Fly  10.707956  
  Larvae Wandering Tubules  4.016341  
  Larval Feeding Carcass  4.152762  
  Larval Feeding Central Nevous System  3.914217  
  Larval Feeding Hind Gut  4.024232  
  Larval Feeding Malpighian Tubule  3.991780  
  Larval Feeding Mid Gut  4.229332  
  Larval Feeding Salivary Gland  4.147502  
  Whole Larvae Feeding  3.898725  
 
  
   FlyBase ID    symbol    start    end    strand    length   
   FBgn0031746   CG9029   5886020   5886616  +  597  
   FBgn0002856   Acp26Ab  5892298   5892862   -  565  
   FBgn0002855   Acp26Aa  5892883   5893896   -  1014  
 
 
    Segment 142 
 
   Location   
  Gene key  FBgn0031749-FBgn0015381  
  Heatmap region span   2L:5879481..5969871   
  Segment span   2L:5910289..5925937   
  Length (genes)  3  
  Length (bp)  15649  
   Model Scoring   
  BIC  352.871920  
  logL  -170.965343  
  logL ratio  -5.833305  
   Expression   
  Mean expression  4.631105  
  Median expression  3.977936  
  Tissue std. dev.  1.076125  
 
  No GO Slim enrichment  
  
   tissue    mean expression   
  5th Passage Drosophila S2 Cells  4.228201  
  Adult Accessory gland  4.621627  
  Adult Brain  4.868647  
  Adult Carcass  4.649526  
  Adult Crop  4.118797  
  Adult Eye  4.102348  
  Adult Fatbody  4.156104  
  Adult Female Spermatheca Mated  4.541515  
  Adult Female Spermatheca Virgin  4.332808  
  Adult Head  4.270438  
  Adult Heart  4.262895  
  Adult Hind Gut  4.048908  
  Adult Male Ejaculatory Duct  4.207388  
  Adult Mid Gut  4.105386  
  Adult Ovary  4.168138  
  Adult Salivary Gland  4.484483  
  Adult Testes  9.368589  
  Adult Thoracoabdominal ganglion  4.419279  
  Adult Whole Fly  6.823086  
  Larvae Wandering Tubules  4.239595  
  Larval Feeding Carcass  4.210477  
  Larval Feeding Central Nevous System  5.035278  
  Larval Feeding Hind Gut  4.118112  
  Larval Feeding Malpighian Tubule  4.095733  
  Larval Feeding Mid Gut  4.310003  
  Larval Feeding Salivary Gland  4.153451  
  Whole Larvae Feeding  5.099021  
 
  
   FlyBase ID    symbol    start    end    strand    length   
   FBgn0031749   CG14000  5908906   5910289   -  1384  
   FBgn0031751   CG9016  5922904   5923646   -  743  
   FBgn0015381   dsf   5925937   5938767  +  12831  
 
 
    Segment 143 
 
   Location   
  Gene key  FBgn0001128-FBgn0031752  
  Heatmap region span   2L:5886020..5999483   
  Segment span   2L:5943682..5949484   
  Length (genes)  2  
  Length (bp)  5803  
   Model Scoring   
  BIC  267.983403  
  logL  -128.521084  
  logL ratio  19.570666  
   Expression   
  Mean expression  9.625601  
  Median expression  9.441166  
  Tissue std. dev.  0.830809  
 
  No GO Slim enrichment  
  
   tissue    mean expression   
  5th Passage Drosophila S2 Cells  8.674998  
  Adult Accessory gland  9.707642  
  Adult Brain  9.260566  
  Adult Carcass  10.654256  
  Adult Crop  9.782336  
  Adult Eye  9.127589  
  Adult Fatbody  10.914668  
  Adult Female Spermatheca Mated  11.000920  
  Adult Female Spermatheca Virgin  11.020525  
  Adult Head  10.103459  
  Adult Heart  9.893718  
  Adult Hind Gut  9.500145  
  Adult Male Ejaculatory Duct  9.298223  
  Adult Mid Gut  9.663901  
  Adult Ovary  8.786656  
  Adult Salivary Gland  10.750502  
  Adult Testes  7.378345  
  Adult Thoracoabdominal ganglion  9.043488  
  Adult Whole Fly  9.520665  
  Larvae Wandering Tubules  9.858473  
  Larval Feeding Carcass  9.497380  
  Larval Feeding Central Nevous System  7.962294  
  Larval Feeding Hind Gut  9.922224  
  Larval Feeding Malpighian Tubule  9.742994  
  Larval Feeding Mid Gut  9.699837  
  Larval Feeding Salivary Gland  9.654691  
  Whole Larvae Feeding  9.470745  
 
  
   FlyBase ID    symbol    start    end    strand    length   
   FBgn0001128   Gpdh   5943682   5949092  +  5411  
   FBgn0031752   CG9044   5949484   5955238  +  5755  
 
    Segment 144 
 
   Location   
  Gene key  FBgn0031753-FBgn0040949  
  Heatmap region span   2L:5904674..6000231   
  Segment span   2L:5956466..5956805   
  Length (genes)  2  
  Length (bp)  340  
   Model Scoring   
  BIC  213.729944  
  logL  -101.394355  
  logL ratio  4.090168  
   Expression   
  Mean expression  4.872056  
  Median expression  4.510998  
  Tissue std. dev.  0.695224  
 
  No GO Slim enrichment  
  
   tissue    mean expression   
  5th Passage Drosophila S2 Cells  4.489435  
  Adult Accessory gland  4.467958  
  Adult Brain  6.493625  
  Adult Carcass  4.695050  
  Adult Crop  4.385292  
  Adult Eye  5.329613  
  Adult Fatbody  4.389269  
  Adult Female Spermatheca Mated  4.492859  
  Adult Female Spermatheca Virgin  4.443862  
  Adult Head  5.451151  
  Adult Heart  4.440920  
  Adult Hind Gut  4.368302  
  Adult Male Ejaculatory Duct  4.662465  
  Adult Mid Gut  4.649520  
  Adult Ovary  4.999963  
  Adult Salivary Gland  4.771005  
  Adult Testes  6.929126  
  Adult Thoracoabdominal ganglion  6.369063  
  Adult Whole Fly  4.891528  
  Larvae Wandering Tubules  4.413186  
  Larval Feeding Carcass  4.482475  
  Larval Feeding Central Nevous System  5.585928  
  Larval Feeding Hind Gut  4.349792  
  Larval Feeding Malpighian Tubule  4.456994  
  Larval Feeding Mid Gut  4.513051  
  Larval Feeding Salivary Gland  4.510620  
  Whole Larvae Feeding  4.513471  
 
  
   FlyBase ID    symbol    start    end    strand    length   
   FBgn0031753   CG13999  5955312   5956466   -  1155  
   FBgn0040949   CG13998  5956512   5956805   -  294  
 
    Segment 145 
 
   Location   
  Gene key  FBgn0003980-FBgn0086265  
  Heatmap region span   2L:5907180..6006791   
  Segment span   2L:5957004..5964322   
  Length (genes)  4  
  Length (bp)  7319  
   Model Scoring   
  BIC  436.051803  
  logL  -212.555284  
  logL ratio  30.813661  
   Expression   
  Mean expression  5.946326  
  Median expression  5.482749  
  Tissue std. dev.  1.593851  
 
  No GO Slim enrichment  
  
   tissue    mean expression   
  5th Passage Drosophila S2 Cells  5.426149  
  Adult Accessory gland  5.495300  
  Adult Brain  5.033926  
  Adult Carcass  7.232275  
  Adult Crop  5.444744  
  Adult Eye  5.102416  
  Adult Fatbody  5.863954  
  Adult Female Spermatheca Mated  5.738010  
  Adult Female Spermatheca Virgin  5.927426  
  Adult Head  5.129968  
  Adult Heart  5.235232  
  Adult Hind Gut  5.328193  
  Adult Male Ejaculatory Duct  5.508905  
  Adult Mid Gut  5.771970  
  Adult Ovary  11.656588  
  Adult Salivary Gland  5.826516  
  Adult Testes  5.126144  
  Adult Thoracoabdominal ganglion  5.251051  
  Adult Whole Fly  11.069836  
  Larvae Wandering Tubules  5.402613  
  Larval Feeding Carcass  5.466479  
  Larval Feeding Central Nevous System  5.020137  
  Larval Feeding Hind Gut  5.343986  
  Larval Feeding Malpighian Tubule  5.386414  
  Larval Feeding Mid Gut  6.066176  
  Larval Feeding Salivary Gland  5.452014  
  Whole Larvae Feeding  5.244370  
 
  
   FlyBase ID    symbol    start    end    strand    length   
   FBgn0003980   Vm26Ab   5957004   5957628  +  625  
   FBgn0086266   Vm26Ac  5958005   5958631   -  627  
   FBgn0003979   Vm26Aa  5959700   5960340   -  641  
   FBgn0086265   psd  5963087   5964322   -  1236  
 
 
    Segment 146 
 
   Location   
  Gene key  FBgn0031756-FBgn0031758  
  Heatmap region span   2L:5910289..6034423   
  Segment span   2L:5967632..5969871   
  Length (genes)  3  
  Length (bp)  2240  
   Model Scoring   
  BIC  283.936477  
  logL  -136.497621  
  logL ratio  42.281762  
   Expression   
  Mean expression  5.086224  
  Median expression  4.550690  
  Tissue std. dev.  0.588963  
 
  
   GO ID    description    ratio    P-value   
   GO:0022857   transmembrane transporter activity  2/3  0.000189  
 
  
   tissue    mean expression   
  5th Passage Drosophila S2 Cells  4.976332  
  Adult Accessory gland  4.973649  
  Adult Brain  4.647646  
  Adult Carcass  4.968112  
  Adult Crop  4.868198  
  Adult Eye  4.858091  
  Adult Fatbody  4.943353  
  Adult Female Spermatheca Mated  4.896181  
  Adult Female Spermatheca Virgin  4.908213  
  Adult Head  4.703625  
  Adult Heart  4.920148  
  Adult Hind Gut  4.869499  
  Adult Male Ejaculatory Duct  5.082610  
  Adult Mid Gut  5.039317  
  Adult Ovary  5.821001  
  Adult Salivary Gland  5.321797  
  Adult Testes  7.730828  
  Adult Thoracoabdominal ganglion  4.789862  
  Adult Whole Fly  5.886037  
  Larvae Wandering Tubules  5.052450  
  Larval Feeding Carcass  4.929146  
  Larval Feeding Central Nevous System  4.674109  
  Larval Feeding Hind Gut  4.795489  
  Larval Feeding Malpighian Tubule  4.839335  
  Larval Feeding Mid Gut  4.996303  
  Larval Feeding Salivary Gland  4.943614  
  Whole Larvae Feeding  4.893105  
 
  
   FlyBase ID    symbol    start    end    strand    length   
   FBgn0031756   CG13992  5965519   5967632   -  2114  
   FBgn0031757   Ucp4C   5968597   5969761  +  1165  
   FBgn0031758   Ucp4B   5969871   5971025  +  1155  
 
 
    Segment 147 
 
   Location   
  Gene key  FBgn0000308-FBgn0031759  
  Heatmap region span   2L:5943682..6050829   
  Segment span   2L:5981025..5999483   
  Length (genes)  3  
  Length (bp)  18459  
   Model Scoring   
  BIC  398.989863  
  logL  -194.024314  
  logL ratio  68.429368  
   Expression   
  Mean expression  10.527657  
  Median expression  10.417517  
  Tissue std. dev.  0.584499  
 
  No GO Slim enrichment  
  
   tissue    mean expression   
  5th Passage Drosophila S2 Cells  11.362124  
  Adult Accessory gland  10.555054  
  Adult Brain  10.362605  
  Adult Carcass  9.260380  
  Adult Crop  10.570727  
  Adult Eye  10.360609  
  Adult Fatbody  9.335048  
  Adult Female Spermatheca Mated  10.230690  
  Adult Female Spermatheca Virgin  10.034480  
  Adult Head  9.917125  
  Adult Heart  10.416406  
  Adult Hind Gut  10.746829  
  Adult Male Ejaculatory Duct  10.413154  
  Adult Mid Gut  10.839600  
  Adult Ovary  11.146769  
  Adult Salivary Gland  9.963247  
  Adult Testes  9.595814  
  Adult Thoracoabdominal ganglion  10.343303  
  Adult Whole Fly  10.332778  
  Larvae Wandering Tubules  11.169543  
  Larval Feeding Carcass  10.597917  
  Larval Feeding Central Nevous System  11.538271  
  Larval Feeding Hind Gut  11.204273  
  Larval Feeding Malpighian Tubule  11.210134  
  Larval Feeding Mid Gut  11.131894  
  Larval Feeding Salivary Gland  10.954023  
  Whole Larvae Feeding  10.653936  
 
  
   FlyBase ID    symbol    start    end    strand    length   
   FBgn0000308   chic  5972900   5981025   -  8126  
   FBgn0001941   ifc   5986324   5988858  +  2535  
   FBgn0031759   lid  5990441   5999483   -  9043  
 
 
    Segment 148 
 
   Location   
  Gene key  FBgn0031762-FBgn0031765  
  Heatmap region span   2L:5967632..6066186   
  Segment span   2L:6012338..6034423   
  Length (genes)  4  
  Length (bp)  22086  
   Model Scoring   
  BIC  483.777692  
  logL  -236.418229  
  logL ratio  -22.459147  
   Expression   
  Mean expression  5.890533  
  Median expression  5.797952  
  Tissue std. dev.  0.546588  
 
  No GO Slim enrichment  
  
   tissue    mean expression   
  5th Passage Drosophila S2 Cells  5.803038  
  Adult Accessory gland  5.504826  
  Adult Brain  7.004008  
  Adult Carcass  5.455067  
  Adult Crop  5.364865  
  Adult Eye  5.545229  
  Adult Fatbody  5.711394  
  Adult Female Spermatheca Mated  5.665097  
  Adult Female Spermatheca Virgin  5.567631  
  Adult Head  5.817431  
  Adult Heart  5.260300  
  Adult Hind Gut  5.978949  
  Adult Male Ejaculatory Duct  5.657768  
  Adult Mid Gut  5.614959  
  Adult Ovary  6.157921  
  Adult Salivary Gland  6.914758  
  Adult Testes  5.640332  
  Adult Thoracoabdominal ganglion  6.877174  
  Adult Whole Fly  5.434342  
  Larvae Wandering Tubules  5.633015  
  Larval Feeding Carcass  5.453240  
  Larval Feeding Central Nevous System  7.429641  
  Larval Feeding Hind Gut  5.923272  
  Larval Feeding Malpighian Tubule  5.607546  
  Larval Feeding Mid Gut  6.382875  
  Larval Feeding Salivary Gland  5.947770  
  Whole Larvae Feeding  5.691931  
 
  
   FlyBase ID    symbol    start    end    strand    length   
   FBgn0031762   CG9098  6007364   6012338   -  4975  
   FBgn0031763   CG13996   6020850   6021672  +  823  
   FBgn0031764   CG9107  6023370   6024535   -  1166  
   FBgn0031765   CG9109  6025870   6034423   -  8554  
 
 
    Segment 149 
 
   Location   
  Gene key  FBgn0025742-FBgn0031769  
  Heatmap region span   2L:5981025..6068635   
  Segment span   2L:6036920..6050829   
  Length (genes)  6  
  Length (bp)  13910  
   Model Scoring   
  BIC  645.268370  
  logL  -317.163568  
  logL ratio  117.737321  
   Expression   
  Mean expression  8.127874  
  Median expression  8.158013  
  Tissue std. dev.  0.405994  
 
  No GO Slim enrichment  
  
   tissue    mean expression   
  5th Passage Drosophila S2 Cells  8.345386  
  Adult Accessory gland  8.340951  
  Adult Brain  7.590565  
  Adult Carcass  7.704504  
  Adult Crop  7.844026  
  Adult Eye  7.989152  
  Adult Fatbody  7.914570  
  Adult Female Spermatheca Mated  8.151543  
  Adult Female Spermatheca Virgin  8.240816  
  Adult Head  7.862750  
  Adult Heart  8.163900  
  Adult Hind Gut  7.744391  
  Adult Male Ejaculatory Duct  7.836179  
  Adult Mid Gut  7.796320  
  Adult Ovary  9.231591  
  Adult Salivary Gland  8.374318  
  Adult Testes  7.507483  
  Adult Thoracoabdominal ganglion  7.888117  
  Adult Whole Fly  8.295390  
  Larvae Wandering Tubules  8.831623  
  Larval Feeding Carcass  7.783965  
  Larval Feeding Central Nevous System  8.753977  
  Larval Feeding Hind Gut  8.136966  
  Larval Feeding Malpighian Tubule  8.738505  
  Larval Feeding Mid Gut  8.010128  
  Larval Feeding Salivary Gland  8.478104  
  Whole Larvae Feeding  7.897380  
 
  
   FlyBase ID    symbol    start    end    strand    length   
   FBgn0025742   mtm  6034795   6036920   -  2126  
   FBgn0031766   CG9117  6036990   6037902   -  913  
   FBgn0051643   CG31643   6038161   6041283  +  3123  
   FBgn0000052   ade2  6041178   6045970   -  4793  
   FBgn0031768   CG12393   6047999   6050474  +  2476  
   FBgn0031769   CG9135   6050829   6053498  +  2670  
 
 
    Segment 150 
 
   Location   
  Gene key  FBgn0031772-FBgn0031774  
  Heatmap region span   2L:6012338..6098964   
  Segment span   2L:6063018..6066186   
  Length (genes)  3  
  Length (bp)  3169  
   Model Scoring   
  BIC  334.597342  
  logL  -161.828054  
  logL ratio  53.601822  
   Expression   
  Mean expression  8.242299  
  Median expression  8.230368  
  Tissue std. dev.  0.497120  
 
  No GO Slim enrichment  
  
   tissue    mean expression   
  5th Passage Drosophila S2 Cells  8.019992  
  Adult Accessory gland  7.642131  
  Adult Brain  8.275910  
  Adult Carcass  7.712924  
  Adult Crop  8.139617  
  Adult Eye  8.551598  
  Adult Fatbody  7.910668  
  Adult Female Spermatheca Mated  8.165675  
  Adult Female Spermatheca Virgin  8.188894  
  Adult Head  7.947850  
  Adult Heart  8.304223  
  Adult Hind Gut  8.136982  
  Adult Male Ejaculatory Duct  7.833119  
  Adult Mid Gut  7.775206  
  Adult Ovary  8.790498  
  Adult Salivary Gland  8.236992  
  Adult Testes  10.235118  
  Adult Thoracoabdominal ganglion  8.370404  
  Adult Whole Fly  8.134745  
  Larvae Wandering Tubules  8.817109  
  Larval Feeding Carcass  8.141262  
  Larval Feeding Central Nevous System  8.415495  
  Larval Feeding Hind Gut  8.259590  
  Larval Feeding Malpighian Tubule  8.678111  
  Larval Feeding Mid Gut  7.800927  
  Larval Feeding Salivary Gland  8.275925  
  Whole Larvae Feeding  7.781113  
 
  
   FlyBase ID    symbol    start    end    strand    length   
   FBgn0031772   CG13994   6063018   6063676  +  659  
   FBgn0031773   Fbw5  6063562   6065949   -  2388  
   FBgn0031774   CG9147   6066186   6067635  +  1450  
 
 
    Segment 151 
 
   Location   
  Gene key  FBgn0031776-FBgn0031777  
  Heatmap region span   2L:6054307..6253123   
  Segment span   2L:6069079..6070646   
  Length (genes)  2  
  Length (bp)  1568  
   Model Scoring   
  BIC  245.621379  
  logL  -117.340072  
  logL ratio  22.504265  
   Expression   
  Mean expression  8.760055  
  Median expression  8.802119  
  Tissue std. dev.  0.495048  
 
  No GO Slim enrichment  
  
   tissue    mean expression   
  5th Passage Drosophila S2 Cells  9.742327  
  Adult Accessory gland  9.084668  
  Adult Brain  8.876817  
  Adult Carcass  7.714873  
  Adult Crop  8.939576  
  Adult Eye  8.641984  
  Adult Fatbody  8.380296  
  Adult Female Spermatheca Mated  8.374336  
  Adult Female Spermatheca Virgin  8.255448  
  Adult Head  8.392565  
  Adult Heart  8.682238  
  Adult Hind Gut  8.332109  
  Adult Male Ejaculatory Duct  8.920789  
  Adult Mid Gut  8.630121  
  Adult Ovary  9.342799  
  Adult Salivary Gland  8.294201  
  Adult Testes  8.779055  
  Adult Thoracoabdominal ganglion  8.715860  
  Adult Whole Fly  8.614831  
  Larvae Wandering Tubules  8.572405  
  Larval Feeding Carcass  9.123302  
  Larval Feeding Central Nevous System  9.984993  
  Larval Feeding Hind Gut  8.790594  
  Larval Feeding Malpighian Tubule  8.602892  
  Larval Feeding Mid Gut  8.288248  
  Larval Feeding Salivary Gland  9.749868  
  Whole Larvae Feeding  8.694289  
 
  
   FlyBase ID    symbol    start    end    strand    length   
   FBgn0031776   CG13993   6069079   6069838  +  760  
   FBgn0031777   CG9154  6069790   6070646   -  857  
 
    Segment 152 
 
   Location   
  Gene key  FBgn0051642-FBgn0053531  
  Heatmap region span   2L:6069079..6338772   
  Segment span   2L:6118512..6253123   
  Length (genes)  9  
  Length (bp)  134612  
   Model Scoring   
  BIC  912.748979  
  logL  -450.903872  
  logL ratio  63.964476  
   Expression   
  Mean expression  5.259398  
  Median expression  4.950880  
  Tissue std. dev.  0.545413  
 
  
   GO ID    description    ratio    P-value   
   GO:0007155   cell adhesion  2/9  0.015  
 
  
   tissue    mean expression   
  5th Passage Drosophila S2 Cells  5.664293  
  Adult Accessory gland  5.187904  
  Adult Brain  5.006875  
  Adult Carcass  5.051138  
  Adult Crop  5.067113  
  Adult Eye  4.903223  
  Adult Fatbody  5.000964  
  Adult Female Spermatheca Mated  4.903516  
  Adult Female Spermatheca Virgin  4.932804  
  Adult Head  4.880818  
  Adult Heart  4.918104  
  Adult Hind Gut  4.980641  
  Adult Male Ejaculatory Duct  5.086479  
  Adult Mid Gut  5.462791  
  Adult Ovary  5.129287  
  Adult Salivary Gland  5.173445  
  Adult Testes  7.565373  
  Adult Thoracoabdominal ganglion  5.049469  
  Adult Whole Fly  5.472447  
  Larvae Wandering Tubules  5.146757  
  Larval Feeding Carcass  4.953431  
  Larval Feeding Central Nevous System  4.977516  
  Larval Feeding Hind Gut  4.982686  
  Larval Feeding Malpighian Tubule  5.287673  
  Larval Feeding Mid Gut  6.042161  
  Larval Feeding Salivary Gland  5.164595  
  Whole Larvae Feeding  6.012247  
 
  
   FlyBase ID    symbol    start    end    strand    length   
   FBgn0051642   CG31642   6118512   6120522  +  2011  
   FBgn0031782   WDR79  6125889   6127762   -  1874  
   FBgn0031784   CG9222   6131023   6132484  +  1462  
   FBgn0031785   CG13991   6139461   6145011  +  5551  
   FBgn0040950   Muc26B   6151971   6153701  +  1731  
   FBgn0031786   CG13989   6160444   6161082  +  639  
   FBgn0085409   CG34380   6179514   6209477  +  29964  
   FBgn0026755   Ugt37b1   6225050   6226842  +  1793  
   FBgn0053531   Ddr   6253123   6322251  +  69129  
 
 
    Segment 153 
 
   Location   
  Gene key  FBgn0031792-FBgn0021873  
  Heatmap region span   2L:6098964..6351028   
  Segment span   2L:6264487..6323767   
  Length (genes)  2  
  Length (bp)  59281  
   Model Scoring   
  BIC  184.218599  
  logL  -86.638682  
  logL ratio  29.650376  
   Expression   
  Mean expression  5.066949  
  Median expression  5.006463  
  Tissue std. dev.  0.248811  
 
  No GO Slim enrichment  
  
   tissue    mean expression   
  5th Passage Drosophila S2 Cells  5.800577  
  Adult Accessory gland  4.971622  
  Adult Brain  4.881357  
  Adult Carcass  5.119051  
  Adult Crop  5.261372  
  Adult Eye  4.679119  
  Adult Fatbody  5.173360  
  Adult Female Spermatheca Mated  4.916992  
  Adult Female Spermatheca Virgin  4.994209  
  Adult Head  4.806913  
  Adult Heart  5.084533  
  Adult Hind Gut  4.897708  
  Adult Male Ejaculatory Duct  5.203665  
  Adult Mid Gut  5.016367  
  Adult Ovary  5.593811  
  Adult Salivary Gland  5.117072  
  Adult Testes  5.438204  
  Adult Thoracoabdominal ganglion  5.083950  
  Adult Whole Fly  4.757537  
  Larvae Wandering Tubules  4.988222  
  Larval Feeding Carcass  4.900966  
  Larval Feeding Central Nevous System  5.080834  
  Larval Feeding Hind Gut  4.874030  
  Larval Feeding Malpighian Tubule  5.097262  
  Larval Feeding Mid Gut  5.118479  
  Larval Feeding Salivary Gland  5.236339  
  Whole Larvae Feeding  4.714082  
 
  
   FlyBase ID    symbol    start    end    strand    length   
   FBgn0031792   CG13983  6263557   6264487   -  931  
   FBgn0021873   Gef26   6323767   6331552  +  7786  
 
    Segment 154 
 
   Location   
  Gene key  FBgn0031802-FBgn0043854  
  Heatmap region span   2L:6338493..6412315   
  Segment span   2L:6354331..6372415   
  Length (genes)  5  
  Length (bp)  18085  
   Model Scoring   
  BIC  471.224185  
  logL  -230.141475  
  logL ratio  63.845149  
   Expression   
  Mean expression  4.988169  
  Median expression  4.799016  
  Tissue std. dev.  0.340420  
 
  No GO Slim enrichment  
  
   tissue    mean expression   
  5th Passage Drosophila S2 Cells  5.271572  
  Adult Accessory gland  4.726389  
  Adult Brain  4.450329  
  Adult Carcass  5.321774  
  Adult Crop  4.689176  
  Adult Eye  5.183877  
  Adult Fatbody  5.500041  
  Adult Female Spermatheca Mated  5.425895  
  Adult Female Spermatheca Virgin  5.371388  
  Adult Head  5.022849  
  Adult Heart  5.396431  
  Adult Hind Gut  4.674712  
  Adult Male Ejaculatory Duct  4.901596  
  Adult Mid Gut  4.863669  
  Adult Ovary  5.768887  
  Adult Salivary Gland  4.973261  
  Adult Testes  4.954955  
  Adult Thoracoabdominal ganglion  4.405557  
  Adult Whole Fly  5.062239  
  Larvae Wandering Tubules  4.952977  
  Larval Feeding Carcass  4.838240  
  Larval Feeding Central Nevous System  4.397953  
  Larval Feeding Hind Gut  4.662551  
  Larval Feeding Malpighian Tubule  5.052836  
  Larval Feeding Mid Gut  5.214150  
  Larval Feeding Salivary Gland  4.773857  
  Whole Larvae Feeding  4.823407  
 
  
   FlyBase ID    symbol    start    end    strand    length   
   FBgn0031802   ppk7  6352324   6354331   -  2008  
   FBgn0031803   ppk14   6354956   6356498  +  1543  
   FBgn0031804   CG9500  6357251   6358235   -  985  
   FBgn0031805   CG9505   6367884   6370195  +  2312  
   FBgn0043854   slam   6372415   6377287  +  4873  
 
 
    Segment 155 
 
   Location   
  Gene key  FBgn0259714-FBgn0031811  
  Heatmap region span   2L:6354331..6490828   
  Segment span   2L:6411306..6412315   
  Length (genes)  2  
  Length (bp)  1010  
   Model Scoring   
  BIC  175.420836  
  logL  -82.239801  
  logL ratio  36.033114  
   Expression   
  Mean expression  4.720325  
  Median expression  4.744153  
  Tissue std. dev.  0.175986  
 
  No GO Slim enrichment  
  
   tissue    mean expression   
  5th Passage Drosophila S2 Cells  5.004372  
  Adult Accessory gland  4.781478  
  Adult Brain  5.113433  
  Adult Carcass  4.656773  
  Adult Crop  4.591817  
  Adult Eye  4.706277  
  Adult Fatbody  4.735196  
  Adult Female Spermatheca Mated  4.685921  
  Adult Female Spermatheca Virgin  4.653430  
  Adult Head  4.630940  
  Adult Heart  4.754887  
  Adult Hind Gut  4.755684  
  Adult Male Ejaculatory Duct  4.759099  
  Adult Mid Gut  4.812983  
  Adult Ovary  4.742244  
  Adult Salivary Gland  4.899580  
  Adult Testes  4.548497  
  Adult Thoracoabdominal ganglion  5.127590  
  Adult Whole Fly  4.314723  
  Larvae Wandering Tubules  4.653730  
  Larval Feeding Carcass  4.664728  
  Larval Feeding Central Nevous System  4.495871  
  Larval Feeding Hind Gut  4.576151  
  Larval Feeding Malpighian Tubule  4.699230  
  Larval Feeding Mid Gut  4.861995  
  Larval Feeding Salivary Gland  4.742448  
  Whole Larvae Feeding  4.479709  
 
  
   FlyBase ID    symbol    start    end    strand    length   
   FBgn0259714   CG42368  6377767   6411306   -  33540  
   FBgn0031811   CG13982   6412315   6415344  +  3030  
 
    Segment 156 
 
   Location   
  Gene key  FBgn0011722-FBgn0031817  
  Heatmap region span   2L:6381266..6491122   
  Segment span   2L:6423308..6467700   
  Length (genes)  7  
  Length (bp)  44393  
   Model Scoring   
  BIC  829.862898  
  logL  -409.460832  
  logL ratio  67.964394  
   Expression   
  Mean expression  8.228517  
  Median expression  8.122217  
  Tissue std. dev.  0.538912  
 
  No GO Slim enrichment  
  
   tissue    mean expression   
  5th Passage Drosophila S2 Cells  8.429969  
  Adult Accessory gland  7.393330  
  Adult Brain  8.727365  
  Adult Carcass  8.636008  
  Adult Crop  8.040116  
  Adult Eye  8.315749  
  Adult Fatbody  8.961742  
  Adult Female Spermatheca Mated  8.988281  
  Adult Female Spermatheca Virgin  8.819193  
  Adult Head  8.999508  
  Adult Heart  8.849770  
  Adult Hind Gut  8.404925  
  Adult Male Ejaculatory Duct  8.121636  
  Adult Mid Gut  7.941290  
  Adult Ovary  7.737923  
  Adult Salivary Gland  8.123931  
  Adult Testes  6.960742  
  Adult Thoracoabdominal ganglion  8.987808  
  Adult Whole Fly  8.000533  
  Larvae Wandering Tubules  7.546203  
  Larval Feeding Carcass  7.891873  
  Larval Feeding Central Nevous System  7.621498  
  Larval Feeding Hind Gut  8.142207  
  Larval Feeding Malpighian Tubule  7.562507  
  Larval Feeding Mid Gut  7.995100  
  Larval Feeding Salivary Gland  8.440582  
  Whole Larvae Feeding  8.530178  
 
  
   FlyBase ID    symbol    start    end    strand    length   
   FBgn0011722   Tig  6415257   6423308   -  8052  
   FBgn0031812      6424337   6426373  +  2037  
   FBgn0031813   CG9527  6445070   6448016   -  2947  
   FBgn0031814   retm  6448801   6455934   -  7134  
   FBgn0031815   frj   6456106   6459008  +  2903  
   FBgn0031816   CG16947  6459079   6463843   -  4765  
   FBgn0031817   CG9531  6466217   6467700   -  1484  
 
 
    Segment 157 
 
   Location   
  Gene key  FBgn0259749-FBgn0031820  
  Heatmap region span   2L:6385512..6494527   
  Segment span   2L:6473919..6480956   
  Length (genes)  4  
  Length (bp)  7038  
   Model Scoring   
  BIC  465.440561  
  logL  -227.249663  
  logL ratio  117.331031  
   Expression   
  Mean expression  10.064564  
  Median expression  9.811231  
  Tissue std. dev.  0.494729  
 
  No GO Slim enrichment  
  
   tissue    mean expression   
  5th Passage Drosophila S2 Cells  10.255843  
  Adult Accessory gland  10.960423  
  Adult Brain  9.800122  
  Adult Carcass  9.577059  
  Adult Crop  10.004325  
  Adult Eye  9.367188  
  Adult Fatbody  9.686487  
  Adult Female Spermatheca Mated  10.059922  
  Adult Female Spermatheca Virgin  9.974849  
  Adult Head  9.532025  
  Adult Heart  9.620932  
  Adult Hind Gut  9.836295  
  Adult Male Ejaculatory Duct  10.487376  
  Adult Mid Gut  10.344171  
  Adult Ovary  10.412055  
  Adult Salivary Gland  11.135793  
  Adult Testes  9.399735  
  Adult Thoracoabdominal ganglion  9.746607  
  Adult Whole Fly  9.732461  
  Larvae Wandering Tubules  10.626493  
  Larval Feeding Carcass  10.242683  
  Larval Feeding Central Nevous System  9.554479  
  Larval Feeding Hind Gut  9.780106  
  Larval Feeding Malpighian Tubule  10.530190  
  Larval Feeding Mid Gut  9.988822  
  Larval Feeding Salivary Gland  11.151621  
  Whole Larvae Feeding  9.935163  
 
  
   FlyBase ID    symbol    start    end    strand    length   
   FBgn0259749   mmy  6468538   6473919   -  5382  
   FBgn0031818   CG9536  6474438   6476972   -  2535  
   FBgn0086357   Sec61alpha  6477182   6480489   -  3308  
   FBgn0031820   DLP   6480956   6487585  +  6630  
 
 
    Segment 158 
 
   Location   
  Gene key  FBgn0027496-FBgn0031822  
  Heatmap region span   2L:6411306..6498642   
  Segment span   2L:6489092..6490828   
  Length (genes)  2  
  Length (bp)  1737  
   Model Scoring   
  BIC  239.605026  
  logL  -114.331896  
  logL ratio  48.424094  
   Expression   
  Mean expression  10.018803  
  Median expression  10.031406  
  Tissue std. dev.  0.508387  
 
  No GO Slim enrichment  
  
   tissue    mean expression   
  5th Passage Drosophila S2 Cells  10.575333  
  Adult Accessory gland  10.899496  
  Adult Brain  9.027057  
  Adult Carcass  9.381066  
  Adult Crop  9.818392  
  Adult Eye  9.892881  
  Adult Fatbody  9.712207  
  Adult Female Spermatheca Mated  9.956627  
  Adult Female Spermatheca Virgin  9.778101  
  Adult Head  9.330495  
  Adult Heart  9.877171  
  Adult Hind Gut  9.601781  
  Adult Male Ejaculatory Duct  10.662258  
  Adult Mid Gut  9.925496  
  Adult Ovary  10.457675  
  Adult Salivary Gland  10.378526  
  Adult Testes  10.117454  
  Adult Thoracoabdominal ganglion  9.217786  
  Adult Whole Fly  10.053327  
  Larvae Wandering Tubules  10.057443  
  Larval Feeding Carcass  10.009203  
  Larval Feeding Central Nevous System  10.682919  
  Larval Feeding Hind Gut  9.964251  
  Larval Feeding Malpighian Tubule  10.217482  
  Larval Feeding Mid Gut  9.687425  
  Larval Feeding Salivary Gland  11.293000  
  Whole Larvae Feeding  9.932832  
 
  
   FlyBase ID    symbol    start    end    strand    length   
   FBgn0027496   epsilonCOP   6489092   6490195  +  1104  
   FBgn0031822   CG9548  6490151   6490828   -  678  
 
    Segment 159 
 
   Location   
  Gene key  FBgn0031832-FBgn0026585  
  Heatmap region span   2L:6498642..6649088   
  Segment span   2L:6561660..6562318   
  Length (genes)  2  
  Length (bp)  659  
   Model Scoring   
  BIC  252.757576  
  logL  -120.908171  
  logL ratio  16.423691  
   Expression   
  Mean expression  8.695052  
  Median expression  8.463260  
  Tissue std. dev.  0.808712  
 
  No GO Slim enrichment  
  
   tissue    mean expression   
  5th Passage Drosophila S2 Cells  9.408111  
  Adult Accessory gland  9.335501  
  Adult Brain  7.086249  
  Adult Carcass  8.535094  
  Adult Crop  8.457655  
  Adult Eye  7.649733  
  Adult Fatbody  9.179467  
  Adult Female Spermatheca Mated  9.138143  
  Adult Female Spermatheca Virgin  9.097890  
  Adult Head  7.964123  
  Adult Heart  9.651388  
  Adult Hind Gut  8.573548  
  Adult Male Ejaculatory Duct  9.819534  
  Adult Mid Gut  8.687243  
  Adult Ovary  9.370760  
  Adult Salivary Gland  8.665706  
  Adult Testes  6.442557  
  Adult Thoracoabdominal ganglion  7.303468  
  Adult Whole Fly  8.435647  
  Larvae Wandering Tubules  8.993146  
  Larval Feeding Carcass  8.795893  
  Larval Feeding Central Nevous System  9.976773  
  Larval Feeding Hind Gut  8.556791  
  Larval Feeding Malpighian Tubule  8.955772  
  Larval Feeding Mid Gut  8.756631  
  Larval Feeding Salivary Gland  9.231537  
  Whole Larvae Feeding  8.698049  
 
  
   FlyBase ID    symbol    start    end    strand    length   
   FBgn0031832   CG9596  6560131   6561660   -  1530  
   FBgn0026585      6562318   6564396  +  2079  
 
    Segment 160 
 
   Location   
  Gene key  FBgn0031837-FBgn0051635  
  Heatmap region span   2L:6561660..6658507   
  Segment span   2L:6621700..6649088   
  Length (genes)  4  
  Length (bp)  27389  
   Model Scoring   
  BIC  342.884386  
  logL  -165.971576  
  logL ratio  80.563043  
   Expression   
  Mean expression  5.136320  
  Median expression  4.936797  
  Tissue std. dev.  0.296239  
 
  No GO Slim enrichment  
  
   tissue    mean expression   
  5th Passage Drosophila S2 Cells  5.219520  
  Adult Accessory gland  6.338017  
  Adult Brain  5.019618  
  Adult Carcass  5.215045  
  Adult Crop  5.250661  
  Adult Eye  4.959539  
  Adult Fatbody  5.215524  
  Adult Female Spermatheca Mated  4.922989  
  Adult Female Spermatheca Virgin  5.042777  
  Adult Head  4.916093  
  Adult Heart  5.124622  
  Adult Hind Gut  5.083423  
  Adult Male Ejaculatory Duct  5.175086  
  Adult Mid Gut  5.132826  
  Adult Ovary  5.421867  
  Adult Salivary Gland  5.210231  
  Adult Testes  5.364429  
  Adult Thoracoabdominal ganglion  5.084063  
  Adult Whole Fly  4.799884  
  Larvae Wandering Tubules  5.059212  
  Larval Feeding Carcass  4.982301  
  Larval Feeding Central Nevous System  5.542074  
  Larval Feeding Hind Gut  4.840745  
  Larval Feeding Malpighian Tubule  5.028738  
  Larval Feeding Mid Gut  4.988379  
  Larval Feeding Salivary Gland  4.994934  
  Whole Larvae Feeding  4.748049  
 
  
   FlyBase ID    symbol    start    end    strand    length   
   FBgn0031837   CG11320  6617134   6621700   -  4567  
   FBgn0085374   CG34345   6623123   6625361  +  2239  
   FBgn0051634   Oatp26F  6625128   6632404   -  7277  
   FBgn0051635   CG31635  6634549   6649088   -  14540  
 
 
    Segment 161 
 
   Location   
  Gene key  FBgn0000392-FBgn0028990  
  Heatmap region span   2L:6656315..6716183   
  Segment span   2L:6663965..6673344   
  Length (genes)  3  
  Length (bp)  9380  
   Model Scoring   
  BIC  401.282777  
  logL  -195.170771  
  logL ratio  27.897904  
   Expression   
  Mean expression  9.686105  
  Median expression  9.884203  
  Tissue std. dev.  0.744653  
 
  No GO Slim enrichment  
  
   tissue    mean expression   
  5th Passage Drosophila S2 Cells  9.711909  
  Adult Accessory gland  8.444397  
  Adult Brain  8.486943  
  Adult Carcass  10.594901  
  Adult Crop  10.172925  
  Adult Eye  9.664024  
  Adult Fatbody  10.774991  
  Adult Female Spermatheca Mated  10.468832  
  Adult Female Spermatheca Virgin  10.627483  
  Adult Head  10.093641  
  Adult Heart  10.593498  
  Adult Hind Gut  9.987210  
  Adult Male Ejaculatory Duct  10.427926  
  Adult Mid Gut  8.942795  
  Adult Ovary  9.387155  
  Adult Salivary Gland  10.201141  
  Adult Testes  8.156672  
  Adult Thoracoabdominal ganglion  8.620004  
  Adult Whole Fly  10.154581  
  Larvae Wandering Tubules  9.757144  
  Larval Feeding Carcass  9.663866  
  Larval Feeding Central Nevous System  8.511093  
  Larval Feeding Hind Gut  10.066766  
  Larval Feeding Malpighian Tubule  9.584171  
  Larval Feeding Mid Gut  9.024994  
  Larval Feeding Salivary Gland  9.577504  
  Whole Larvae Feeding  9.828276  
 
  
   FlyBase ID    symbol    start    end    strand    length   
   FBgn0000392   cup   6663965   6674790  +  10826  
   FBgn0031845     6666264   6668031   -  1768  
   FBgn0028990   Spn27A  6671778   6673344   -  1567  
 
 
    Segment 162 
 
   Location   
  Gene key  FBgn0000351-FBgn0031848  
  Heatmap region span   2L:6657879..6723355   
  Segment span   2L:6676635..6677606   
  Length (genes)  2  
  Length (bp)  972  
   Model Scoring   
  BIC  190.053702  
  logL  -89.556234  
  logL ratio  28.261193  
   Expression   
  Mean expression  5.386856  
  Median expression  4.984782  
  Tissue std. dev.  1.044271  
 
  No GO Slim enrichment  
  
   tissue    mean expression   
  5th Passage Drosophila S2 Cells  6.571989  
  Adult Accessory gland  5.293456  
  Adult Brain  4.450356  
  Adult Carcass  4.924025  
  Adult Crop  4.936531  
  Adult Eye  4.637465  
  Adult Fatbody  4.713388  
  Adult Female Spermatheca Mated  4.919509  
  Adult Female Spermatheca Virgin  4.967946  
  Adult Head  4.599642  
  Adult Heart  5.473480  
  Adult Hind Gut  4.845527  
  Adult Male Ejaculatory Duct  5.375191  
  Adult Mid Gut  5.077419  
  Adult Ovary  9.357620  
  Adult Salivary Gland  5.130882  
  Adult Testes  6.105858  
  Adult Thoracoabdominal ganglion  4.931553  
  Adult Whole Fly  7.773653  
  Larvae Wandering Tubules  5.281746  
  Larval Feeding Carcass  5.063261  
  Larval Feeding Central Nevous System  6.301639  
  Larval Feeding Hind Gut  4.791569  
  Larval Feeding Malpighian Tubule  5.198920  
  Larval Feeding Mid Gut  4.776203  
  Larval Feeding Salivary Gland  4.971742  
  Whole Larvae Feeding  4.974533  
 
  
   FlyBase ID    symbol    start    end    strand    length   
   FBgn0000351   cort  6674637   6676635   -  1999  
   FBgn0031848   CG11329  6676729   6677606   -  878  
 
    Segment 163 
 
   Location   
  Gene key  FBgn0031850-FBgn0025595  
  Heatmap region span   2L:6663965..6798864   
  Segment span   2L:6709085..6716183   
  Length (genes)  2  
  Length (bp)  7099  
   Model Scoring   
  BIC  229.372550  
  logL  -109.215658  
  logL ratio  5.539828  
   Expression   
  Mean expression  6.118032  
  Median expression  5.709955  
  Tissue std. dev.  1.372558  
 
  No GO Slim enrichment  
  
   tissue    mean expression   
  5th Passage Drosophila S2 Cells  4.823261  
  Adult Accessory gland  4.807845  
  Adult Brain  5.093275  
  Adult Carcass  8.245363  
  Adult Crop  5.369862  
  Adult Eye  8.445849  
  Adult Fatbody  8.293079  
  Adult Female Spermatheca Mated  8.527140  
  Adult Female Spermatheca Virgin  8.190226  
  Adult Head  8.031034  
  Adult Heart  7.448714  
  Adult Hind Gut  5.372806  
  Adult Male Ejaculatory Duct  7.263191  
  Adult Mid Gut  5.034169  
  Adult Ovary  4.650812  
  Adult Salivary Gland  5.198659  
  Adult Testes  5.619523  
  Adult Thoracoabdominal ganglion  5.085649  
  Adult Whole Fly  6.429875  
  Larvae Wandering Tubules  4.830604  
  Larval Feeding Carcass  6.172627  
  Larval Feeding Central Nevous System  4.513329  
  Larval Feeding Hind Gut  5.565239  
  Larval Feeding Malpighian Tubule  4.883633  
  Larval Feeding Mid Gut  5.100497  
  Larval Feeding Salivary Gland  5.351221  
  Whole Larvae Feeding  6.839381  
 
  
   FlyBase ID    symbol    start    end    strand    length   
   FBgn0031850   Tsp  6686786   6709085   -  22300  
   FBgn0025595   GRHR  6711282   6716183   -  4902  
 
    Segment 164 
 
   Location   
  Gene key  FBgn0031851-FBgn0025777  
  Heatmap region span   2L:6676635..6800834   
  Segment span   2L:6717627..6723355   
  Length (genes)  2  
  Length (bp)  5729  
   Model Scoring   
  BIC  229.410486  
  logL  -109.234626  
  logL ratio  21.204621  
   Expression   
  Mean expression  6.969931  
  Median expression  7.087947  
  Tissue std. dev.  0.680394  
 
  No GO Slim enrichment  
  
   tissue    mean expression   
  5th Passage Drosophila S2 Cells  7.917480  
  Adult Accessory gland  6.723980  
  Adult Brain  7.626062  
  Adult Carcass  6.304693  
  Adult Crop  7.120608  
  Adult Eye  8.035228  
  Adult Fatbody  6.356654  
  Adult Female Spermatheca Mated  6.117564  
  Adult Female Spermatheca Virgin  6.099671  
  Adult Head  7.359996  
  Adult Heart  6.872717  
  Adult Hind Gut  6.726686  
  Adult Male Ejaculatory Duct  6.156931  
  Adult Mid Gut  6.591964  
  Adult Ovary  8.747936  
  Adult Salivary Gland  6.363470  
  Adult Testes  8.029607  
  Adult Thoracoabdominal ganglion  7.402247  
  Adult Whole Fly  7.393479  
  Larvae Wandering Tubules  6.800527  
  Larval Feeding Carcass  6.831317  
  Larval Feeding Central Nevous System  7.561273  
  Larval Feeding Hind Gut  6.751167  
  Larval Feeding Malpighian Tubule  7.016780  
  Larval Feeding Mid Gut  6.126781  
  Larval Feeding Salivary Gland  6.840782  
  Whole Larvae Feeding  6.312523  
 
  
   FlyBase ID    symbol    start    end    strand    length   
   FBgn0031851   CG11188   6717627   6719389  +  1763  
   FBgn0025777   homer  6720395   6723355   -  2961  
 
    Segment 165 
 
   Location   
  Gene key  FBgn0031853-FBgn0031858  
  Heatmap region span   2L:6684357..6802830   
  Segment span   2L:6732866..6774039   
  Length (genes)  7  
  Length (bp)  41174  
   Model Scoring   
  BIC  698.227950  
  logL  -343.643358  
  logL ratio  62.458357  
   Expression   
  Mean expression  5.416032  
  Median expression  5.215129  
  Tissue std. dev.  0.647406  
 
  No GO Slim enrichment  
  
   tissue    mean expression   
  5th Passage Drosophila S2 Cells  4.856372  
  Adult Accessory gland  5.300676  
  Adult Brain  5.864154  
  Adult Carcass  5.574986  
  Adult Crop  5.670486  
  Adult Eye  5.576863  
  Adult Fatbody  4.938782  
  Adult Female Spermatheca Mated  4.972419  
  Adult Female Spermatheca Virgin  4.924168  
  Adult Head  5.914263  
  Adult Heart  5.357604  
  Adult Hind Gut  5.426087  
  Adult Male Ejaculatory Duct  5.128606  
  Adult Mid Gut  5.044647  
  Adult Ovary  4.849217  
  Adult Salivary Gland  5.582350  
  Adult Testes  8.178999  
  Adult Thoracoabdominal ganglion  5.577785  
  Adult Whole Fly  6.007757  
  Larvae Wandering Tubules  4.941881  
  Larval Feeding Carcass  5.763652  
  Larval Feeding Central Nevous System  5.166344  
  Larval Feeding Hind Gut  5.318702  
  Larval Feeding Malpighian Tubule  4.885010  
  Larval Feeding Mid Gut  5.011496  
  Larval Feeding Salivary Gland  4.869038  
  Whole Larvae Feeding  5.530520  
 
  
   FlyBase ID    symbol    start    end    strand    length   
   FBgn0031853   TTLL3B   6732866   6736049  +  3184  
   FBgn0031854   TTLL3A  6736449   6740176   -  3728  
   FBgn0031855   CG11221   6742508   6755686  +  13179  
   FBgn0051910   CG31910  6744656   6745609   -  954  
   FBgn0031856   CG11322  6757864   6759628   -  1765  
   FBgn0031857   CG11321  6760527   6770949   -  10423  
   FBgn0031858   CG17378   6774039   6778456  +  4418  
 
 
    Segment 166 
 
   Location   
  Gene key  FBgn0042092-FBgn0011737  
  Heatmap region span   2L:6805792..6950440   
  Segment span   2L:6907817..6914137   
  Length (genes)  3  
  Length (bp)  6321  
   Model Scoring   
  BIC  320.087084  
  logL  -154.572924  
  logL ratio  65.378481  
   Expression   
  Mean expression  7.603495  
  Median expression  7.407802  
  Tissue std. dev.  0.693503  
 
  No GO Slim enrichment  
  
   tissue    mean expression   
  5th Passage Drosophila S2 Cells  8.246932  
  Adult Accessory gland  7.965297  
  Adult Brain  8.478274  
  Adult Carcass  6.468343  
  Adult Crop  7.604458  
  Adult Eye  7.663306  
  Adult Fatbody  7.272911  
  Adult Female Spermatheca Mated  7.644177  
  Adult Female Spermatheca Virgin  7.494971  
  Adult Head  7.648808  
  Adult Heart  7.313935  
  Adult Hind Gut  7.131008  
  Adult Male Ejaculatory Duct  6.972031  
  Adult Mid Gut  6.881867  
  Adult Ovary  9.710287  
  Adult Salivary Gland  6.932761  
  Adult Testes  7.675380  
  Adult Thoracoabdominal ganglion  8.490294  
  Adult Whole Fly  8.224695  
  Larvae Wandering Tubules  7.098674  
  Larval Feeding Carcass  7.363722  
  Larval Feeding Central Nevous System  8.740882  
  Larval Feeding Hind Gut  7.557189  
  Larval Feeding Malpighian Tubule  7.299103  
  Larval Feeding Mid Gut  6.684435  
  Larval Feeding Salivary Gland  7.757158  
  Whole Larvae Feeding  6.973480  
 
  
   FlyBase ID    symbol    start    end    strand    length   
   FBgn0042092   CG13773  6906918   6907817   -  900  
   FBgn0031868   Rat1   6908087   6911432  +  3346  
   FBgn0011737   wee  6911338   6914137   -  2800  
 
 
    Segment 167 
 
   Location   
  Gene key  FBgn0026196-FBgn0004838  
  Heatmap region span   2L:6870387..6960426   
  Segment span   2L:6918741..6920693   
  Length (genes)  3  
  Length (bp)  1953  
   Model Scoring   
  BIC  397.222041  
  logL  -193.140403  
  logL ratio  42.457462  
   Expression   
  Mean expression  9.806245  
  Median expression  9.850075  
  Tissue std. dev.  0.963975  
 
  
   GO ID    description    ratio    P-value   
   GO:0003729   mRNA binding  2/3  0.00442  
   GO:0003723   RNA binding  2/3  0.00815  
 
  
   tissue    mean expression   
  5th Passage Drosophila S2 Cells  11.429284  
  Adult Accessory gland  9.536249  
  Adult Brain  9.948905  
  Adult Carcass  9.026146  
  Adult Crop  9.437268  
  Adult Eye  9.647406  
  Adult Fatbody  9.810667  
  Adult Female Spermatheca Mated  9.168926  
  Adult Female Spermatheca Virgin  8.880095  
  Adult Head  9.548486  
  Adult Heart  9.813127  
  Adult Hind Gut  9.167132  
  Adult Male Ejaculatory Duct  8.998592  
  Adult Mid Gut  9.275425  
  Adult Ovary  12.390601  
  Adult Salivary Gland  8.351914  
  Adult Testes  8.709596  
  Adult Thoracoabdominal ganglion  9.736413  
  Adult Whole Fly  11.223006  
  Larvae Wandering Tubules  9.019185  
  Larval Feeding Carcass  10.377370  
  Larval Feeding Central Nevous System  11.917723  
  Larval Feeding Hind Gut  10.183659  
  Larval Feeding Malpighian Tubule  8.998494  
  Larval Feeding Mid Gut  9.565398  
  Larval Feeding Salivary Gland  10.585377  
  Whole Larvae Feeding  10.022177  
 
  
   FlyBase ID    symbol    start    end    strand    length   
   FBgn0026196   nop5  6916753   6918741   -  1989  
   FBgn0028554   x16  6914300   6920274   -  5975  
   FBgn0004838   Hrb27C   6920693   6926046  +  5354  
 
 
    Segment 168 
 
   Location   
  Gene key  FBgn0031871-FBgn0020616  
  Heatmap region span   2L:6907817..6967593   
  Segment span   2L:6945284..6950440   
  Length (genes)  4  
  Length (bp)  5157  
   Model Scoring   
  BIC  428.635449  
  logL  -208.847107  
  logL ratio  73.359995  
   Expression   
  Mean expression  7.731377  
  Median expression  7.782005  
  Tissue std. dev.  0.584186  
 
  No GO Slim enrichment  
  
   tissue    mean expression   
  5th Passage Drosophila S2 Cells  8.499256  
  Adult Accessory gland  7.653615  
  Adult Brain  7.974946  
  Adult Carcass  6.882983  
  Adult Crop  7.822830  
  Adult Eye  8.184640  
  Adult Fatbody  6.985694  
  Adult Female Spermatheca Mated  7.552530  
  Adult Female Spermatheca Virgin  7.483177  
  Adult Head  7.515877  
  Adult Heart  7.553215  
  Adult Hind Gut  7.346008  
  Adult Male Ejaculatory Duct  7.493448  
  Adult Mid Gut  7.071757  
  Adult Ovary  9.471158  
  Adult Salivary Gland  7.292470  
  Adult Testes  7.430060  
  Adult Thoracoabdominal ganglion  7.696352  
  Adult Whole Fly  7.951110  
  Larvae Wandering Tubules  7.503025  
  Larval Feeding Carcass  8.336134  
  Larval Feeding Central Nevous System  9.066078  
  Larval Feeding Hind Gut  7.806476  
  Larval Feeding Malpighian Tubule  7.338466  
  Larval Feeding Mid Gut  7.039233  
  Larval Feeding Salivary Gland  7.942096  
  Whole Larvae Feeding  7.854551  
 
  
   FlyBase ID    symbol    start    end    strand    length   
   FBgn0031871   CG10158  6943730   6945284   -  1555  
   FBgn0031872   ihog  6945458   6948778   -  3321  
   FBgn0031873   Gas41  6949310   6950135   -  826  
   FBgn0020616   SA   6950440   6954590  +  4151  
 
 
    Segment 169 
 
   Location   
  Gene key  FBgn0031874-FBgn0031876  
  Heatmap region span   2L:6916387..7012327   
  Segment span   2L:6956872..6960285   
  Length (genes)  3  
  Length (bp)  3414  
   Model Scoring   
  BIC  292.994260  
  logL  -141.026513  
  logL ratio  36.179462  
   Expression   
  Mean expression  5.632770  
  Median expression  5.445237  
  Tissue std. dev.  0.489111  
 
  
   GO ID    description    ratio    P-value   
   GO:0003677   DNA binding  2/3  0.0142  
   GO:0003674   molecular_function  3/3  0.0287  
 
  
   tissue    mean expression   
  5th Passage Drosophila S2 Cells  6.307376  
  Adult Accessory gland  5.688842  
  Adult Brain  5.436956  
  Adult Carcass  5.267448  
  Adult Crop  5.440400  
  Adult Eye  5.304842  
  Adult Fatbody  5.395860  
  Adult Female Spermatheca Mated  5.478342  
  Adult Female Spermatheca Virgin  5.507786  
  Adult Head  5.274109  
  Adult Heart  5.526439  
  Adult Hind Gut  5.293931  
  Adult Male Ejaculatory Duct  4.992687  
  Adult Mid Gut  5.525671  
  Adult Ovary  7.398581  
  Adult Salivary Gland  5.325838  
  Adult Testes  5.394949  
  Adult Thoracoabdominal ganglion  5.381903  
  Adult Whole Fly  6.054157  
  Larvae Wandering Tubules  5.609351  
  Larval Feeding Carcass  5.681558  
  Larval Feeding Central Nevous System  6.640672  
  Larval Feeding Hind Gut  5.510462  
  Larval Feeding Malpighian Tubule  5.723077  
  Larval Feeding Mid Gut  5.448945  
  Larval Feeding Salivary Gland  6.128568  
  Whole Larvae Feeding  5.346041  
 
  
   FlyBase ID    symbol    start    end    strand    length   
   FBgn0031874   CG13775  6954585   6956872   -  2288  
   FBgn0031875   CG3430   6957010   6959104  +  2095  
   FBgn0031876   Atac1  6959099   6960285   -  1187  
 
 
    Segment 170 
 
   Location   
  Gene key  FBgn0021944-FBgn0026170  
  Heatmap region span   2L:6945284..7060851   
  Segment span   2L:6964426..6967593   
  Length (genes)  2  
  Length (bp)  3168  
   Model Scoring   
  BIC  306.993648  
  logL  -148.026206  
  logL ratio  18.621032  
   Expression   
  Mean expression  10.787533  
  Median expression  11.069183  
  Tissue std. dev.  0.538492  
 
  No GO Slim enrichment  
  
   tissue    mean expression   
  5th Passage Drosophila S2 Cells  12.334497  
  Adult Accessory gland  10.736814  
  Adult Brain  10.073723  
  Adult Carcass  10.135634  
  Adult Crop  11.029919  
  Adult Eye  10.429775  
  Adult Fatbody  10.325353  
  Adult Female Spermatheca Mated  10.435215  
  Adult Female Spermatheca Virgin  10.355826  
  Adult Head  10.236603  
  Adult Heart  10.823227  
  Adult Hind Gut  10.992013  
  Adult Male Ejaculatory Duct  10.799629  
  Adult Mid Gut  10.946444  
  Adult Ovary  11.940860  
  Adult Salivary Gland  10.090235  
  Adult Testes  11.265120  
  Adult Thoracoabdominal ganglion  9.995234  
  Adult Whole Fly  11.111192  
  Larvae Wandering Tubules  10.559151  
  Larval Feeding Carcass  10.745569  
  Larval Feeding Central Nevous System  11.425065  
  Larval Feeding Hind Gut  10.923030  
  Larval Feeding Malpighian Tubule  10.767149  
  Larval Feeding Mid Gut  10.724560  
  Larval Feeding Salivary Gland  11.168325  
  Whole Larvae Feeding  10.893225  
 
  
   FlyBase ID    symbol    start    end    strand    length   
   FBgn0021944   Coprox   6964426   6965813  +  1388  
   FBgn0026170     6966776   6967593   -  818  
 
    Segment 171 
 
   Location   
  Gene key  FBgn0031879-FBgn0031880  
  Heatmap region span   2L:6956872..7063985   
  Segment span   2L:7009888..7012327   
  Length (genes)  2  
  Length (bp)  2440  
   Model Scoring   
  BIC  183.276375  
  logL  -86.167570  
  logL ratio  31.285555  
   Expression   
  Mean expression  4.736582  
  Median expression  4.597266  
  Tissue std. dev.  0.326618  
 
  No GO Slim enrichment  
  
   tissue    mean expression   
  5th Passage Drosophila S2 Cells  4.241405  
  Adult Accessory gland  4.469099  
  Adult Brain  4.415116  
  Adult Carcass  4.895558  
  Adult Crop  5.162807  
  Adult Eye  4.851593  
  Adult Fatbody  4.544520  
  Adult Female Spermatheca Mated  4.504329  
  Adult Female Spermatheca Virgin  4.637187  
  Adult Head  4.596656  
  Adult Heart  4.516803  
  Adult Hind Gut  5.135847  
  Adult Male Ejaculatory Duct  4.552353  
  Adult Mid Gut  4.600999  
  Adult Ovary  4.968673  
  Adult Salivary Gland  4.720361  
  Adult Testes  5.637187  
  Adult Thoracoabdominal ganglion  4.560992  
  Adult Whole Fly  4.514602  
  Larvae Wandering Tubules  4.582827  
  Larval Feeding Carcass  5.204776  
  Larval Feeding Central Nevous System  4.480907  
  Larval Feeding Hind Gut  5.444313  
  Larval Feeding Malpighian Tubule  4.624358  
  Larval Feeding Mid Gut  4.550723  
  Larval Feeding Salivary Gland  4.566281  
  Whole Larvae Feeding  4.907426  
 
  
   FlyBase ID    symbol    start    end    strand    length   
   FBgn0031879   uif  6972828   7009888   -  37061  
   FBgn0031880      7012327   7014737  +  2411  
 
    Segment 172 
 
   Location   
  Gene key  FBgn0000053-FBgn0022201  
  Heatmap region span   2L:6964073..7156701   
  Segment span   2L:7023940..7037998   
  Length (genes)  5  
  Length (bp)  14059  
   Model Scoring   
  BIC  566.093012  
  logL  -277.575889  
  logL ratio  97.264017  
   Expression   
  Mean expression  8.911209  
  Median expression  8.864352  
  Tissue std. dev.  0.448607  
 
  No GO Slim enrichment  
  
   tissue    mean expression   
  5th Passage Drosophila S2 Cells  9.074509  
  Adult Accessory gland  8.183959  
  Adult Brain  8.983646  
  Adult Carcass  8.758864  
  Adult Crop  9.187041  
  Adult Eye  9.062597  
  Adult Fatbody  9.125265  
  Adult Female Spermatheca Mated  9.458259  
  Adult Female Spermatheca Virgin  9.708073  
  Adult Head  9.083989  
  Adult Heart  9.050936  
  Adult Hind Gut  9.285215  
  Adult Male Ejaculatory Duct  9.278780  
  Adult Mid Gut  9.054836  
  Adult Ovary  9.432035  
  Adult Salivary Gland  8.905512  
  Adult Testes  7.438759  
  Adult Thoracoabdominal ganglion  8.910504  
  Adult Whole Fly  8.726631  
  Larvae Wandering Tubules  8.918139  
  Larval Feeding Carcass  8.240141  
  Larval Feeding Central Nevous System  9.007427  
  Larval Feeding Hind Gut  8.645166  
  Larval Feeding Malpighian Tubule  9.115703  
  Larval Feeding Mid Gut  8.884244  
  Larval Feeding Salivary Gland  8.770740  
  Whole Larvae Feeding  8.311672  
 
  
   FlyBase ID    symbol    start    end    strand    length   
   FBgn0000053   ade3  7014861   7023940   -  9080  
   FBgn0031881   CG3476   7027594   7028959  +  1366  
   FBgn0031882   Rab30  7030480   7032603   -  2124  
   FBgn0031883   CG11266   7032713   7037620  +  4908  
   FBgn0022201      7037998   7056011  +  18014  
 
 
    Segment 173 
 
   Location   
  Gene key  FBgn0051630-FBgn0051907  
  Heatmap region span   2L:6964426..7182236   
  Segment span   2L:7042467..7060851   
  Length (genes)  3  
  Length (bp)  18385  
   Model Scoring   
  BIC  329.690461  
  logL  -159.374613  
  logL ratio  22.509073  
   Expression   
  Mean expression  4.615085  
  Median expression  3.989027  
  Tissue std. dev.  0.568320  
 
  No GO Slim enrichment  
  
   tissue    mean expression   
  5th Passage Drosophila S2 Cells  4.638773  
  Adult Accessory gland  4.600837  
  Adult Brain  4.941965  
  Adult Carcass  4.381782  
  Adult Crop  4.561197  
  Adult Eye  4.228225  
  Adult Fatbody  4.422778  
  Adult Female Spermatheca Mated  4.314761  
  Adult Female Spermatheca Virgin  4.335052  
  Adult Head  4.457094  
  Adult Heart  4.439454  
  Adult Hind Gut  4.594283  
  Adult Male Ejaculatory Duct  4.425081  
  Adult Mid Gut  4.441887  
  Adult Ovary  4.993704  
  Adult Salivary Gland  4.689638  
  Adult Testes  7.175362  
  Adult Thoracoabdominal ganglion  4.524340  
  Adult Whole Fly  5.415086  
  Larvae Wandering Tubules  4.401893  
  Larval Feeding Carcass  4.346810  
  Larval Feeding Central Nevous System  4.156052  
  Larval Feeding Hind Gut  4.230127  
  Larval Feeding Malpighian Tubule  4.545845  
  Larval Feeding Mid Gut  4.221799  
  Larval Feeding Salivary Gland  4.356204  
  Whole Larvae Feeding  4.767260  
 
  
   FlyBase ID    symbol    start    end    strand    length   
   FBgn0051630      7042467   7047267  +  4801  
   FBgn0031885   Mnn1   7056306   7063406  +  7101  
   FBgn0051907   CG31907  7059318   7060851   -  1534  
 
 
    Segment 174 
 
   Location   
  Gene key  FBgn0031887-FBgn0085407  
  Heatmap region span   2L:7023940..7240500   
  Segment span   2L:7067983..7156701   
  Length (genes)  3  
  Length (bp)  88719  
   Model Scoring   
  BIC  278.198656  
  logL  -133.628711  
  logL ratio  50.156640  
   Expression   
  Mean expression  4.521621  
  Median expression  4.329536  
  Tissue std. dev.  0.571538  
 
  No GO Slim enrichment  
  
   tissue    mean expression   
  5th Passage Drosophila S2 Cells  6.924460  
  Adult Accessory gland  4.320470  
  Adult Brain  4.437956  
  Adult Carcass  4.469016  
  Adult Crop  4.601376  
  Adult Eye  4.144182  
  Adult Fatbody  4.095674  
  Adult Female Spermatheca Mated  4.339692  
  Adult Female Spermatheca Virgin  4.342187  
  Adult Head  4.326557  
  Adult Heart  4.438945  
  Adult Hind Gut  4.358734  
  Adult Male Ejaculatory Duct  5.112462  
  Adult Mid Gut  4.397342  
  Adult Ovary  3.976923  
  Adult Salivary Gland  4.313956  
  Adult Testes  4.046295  
  Adult Thoracoabdominal ganglion  4.190809  
  Adult Whole Fly  3.939232  
  Larvae Wandering Tubules  4.934731  
  Larval Feeding Carcass  4.847029  
  Larval Feeding Central Nevous System  4.600001  
  Larval Feeding Hind Gut  4.367699  
  Larval Feeding Malpighian Tubule  4.186589  
  Larval Feeding Mid Gut  4.616245  
  Larval Feeding Salivary Gland  5.361826  
  Whole Larvae Feeding  4.393366  
 
  
   FlyBase ID    symbol    start    end    strand    length   
   FBgn0031887   CG11289   7067983   7069546  +  1564  
   FBgn0031888   Pvf2  7069578   7084634   -  15057  
   FBgn0085407   Pvf3  7099692   7156701   -  57010  
 
 
    Segment 175 
 
   Location   
  Gene key  FBgn0031894-FBgn0031895  
  Heatmap region span   2L:7063985..7388248   
  Segment span   2L:7188068..7194588   
  Length (genes)  2  
  Length (bp)  6521  
   Model Scoring   
  BIC  206.368236  
  logL  -97.713500  
  logL ratio  18.702019  
   Expression   
  Mean expression  5.244875  
  Median expression  5.378758  
  Tissue std. dev.  0.644183  
 
  No GO Slim enrichment  
  
   tissue    mean expression   
  5th Passage Drosophila S2 Cells  5.779212  
  Adult Accessory gland  6.983592  
  Adult Brain  5.217110  
  Adult Carcass  4.786860  
  Adult Crop  4.981351  
  Adult Eye  5.666648  
  Adult Fatbody  4.656499  
  Adult Female Spermatheca Mated  4.667504  
  Adult Female Spermatheca Virgin  4.727930  
  Adult Head  4.902980  
  Adult Heart  4.853753  
  Adult Hind Gut  4.676137  
  Adult Male Ejaculatory Duct  5.061076  
  Adult Mid Gut  4.699034  
  Adult Ovary  6.931297  
  Adult Salivary Gland  5.019602  
  Adult Testes  6.008714  
  Adult Thoracoabdominal ganglion  5.051265  
  Adult Whole Fly  5.175204  
  Larvae Wandering Tubules  4.928547  
  Larval Feeding Carcass  5.400895  
  Larval Feeding Central Nevous System  5.501666  
  Larval Feeding Hind Gut  4.978643  
  Larval Feeding Malpighian Tubule  4.848396  
  Larval Feeding Mid Gut  4.802329  
  Larval Feeding Salivary Gland  6.393776  
  Whole Larvae Feeding  4.911608  
 
  
   FlyBase ID    symbol    start    end    strand    length   
   FBgn0031894   CG4496  7185461   7188068   -  2608  
   FBgn0031895   CG4497  7190431   7194588   -  4158  
 
    Segment 176 
 
   Location   
  Gene key  FBgn0031897-FBgn0259111  
  Heatmap region span   2L:7067983..7395832   
  Segment span   2L:7220029..7240500   
  Length (genes)  3  
  Length (bp)  20472  
   Model Scoring   
  BIC  346.767966  
  logL  -167.913366  
  logL ratio  24.908332  
   Expression   
  Mean expression  7.220543  
  Median expression  6.995182  
  Tissue std. dev.  0.433479  
 
  No GO Slim enrichment  
  
   tissue    mean expression   
  5th Passage Drosophila S2 Cells  7.423596  
  Adult Accessory gland  6.435612  
  Adult Brain  7.823028  
  Adult Carcass  7.226914  
  Adult Crop  7.232953  
  Adult Eye  7.792766  
  Adult Fatbody  7.686515  
  Adult Female Spermatheca Mated  7.256583  
  Adult Female Spermatheca Virgin  7.099748  
  Adult Head  7.616193  
  Adult Heart  7.610469  
  Adult Hind Gut  7.177953  
  Adult Male Ejaculatory Duct  6.454662  
  Adult Mid Gut  7.037603  
  Adult Ovary  7.384806  
  Adult Salivary Gland  6.576903  
  Adult Testes  7.825348  
  Adult Thoracoabdominal ganglion  7.491914  
  Adult Whole Fly  6.865378  
  Larvae Wandering Tubules  7.385449  
  Larval Feeding Carcass  6.646510  
  Larval Feeding Central Nevous System  6.843444  
  Larval Feeding Hind Gut  7.098156  
  Larval Feeding Malpighian Tubule  7.942972  
  Larval Feeding Mid Gut  7.021061  
  Larval Feeding Salivary Gland  7.435789  
  Whole Larvae Feeding  6.562330  
 
  
   FlyBase ID    symbol    start    end    strand    length   
   FBgn0031897   CG13784  7205250   7220029   -  14780  
   FBgn0243517     7220272   7223079   -  2808  
   FBgn0259111   Ndae1  7224851   7240500   -  15650  
 
 
    Segment 177 
 
   Location   
  Gene key  FBgn0031900-FBgn0002938  
  Heatmap region span   2L:7182236..7398403   
  Segment span   2L:7252772..7377718   
  Length (genes)  7  
  Length (bp)  124947  
   Model Scoring   
  BIC  678.348256  
  logL  -333.703511  
  logL ratio  74.530407  
   Expression   
  Mean expression  5.039213  
  Median expression  4.744341  
  Tissue std. dev.  0.382329  
 
  
   GO ID    description    ratio    P-value   
   GO:0005576   extracellular region  4/7  9.7e-05  
 
  
   tissue    mean expression   
  5th Passage Drosophila S2 Cells  4.578821  
  Adult Accessory gland  4.745970  
  Adult Brain  4.912435  
  Adult Carcass  4.869320  
  Adult Crop  4.743006  
  Adult Eye  6.234066  
  Adult Fatbody  4.811713  
  Adult Female Spermatheca Mated  4.802661  
  Adult Female Spermatheca Virgin  4.798009  
  Adult Head  5.568978  
  Adult Heart  4.691923  
  Adult Hind Gut  5.064979  
  Adult Male Ejaculatory Duct  5.067529  
  Adult Mid Gut  4.891897  
  Adult Ovary  4.580561  
  Adult Salivary Gland  5.410261  
  Adult Testes  5.834081  
  Adult Thoracoabdominal ganglion  4.586369  
  Adult Whole Fly  5.252227  
  Larvae Wandering Tubules  5.153711  
  Larval Feeding Carcass  4.927692  
  Larval Feeding Central Nevous System  5.320314  
  Larval Feeding Hind Gut  5.230174  
  Larval Feeding Malpighian Tubule  5.081349  
  Larval Feeding Mid Gut  4.793668  
  Larval Feeding Salivary Gland  4.874202  
  Whole Larvae Feeding  5.232831  
 
  
   FlyBase ID    symbol    start    end    strand    length   
   FBgn0031900   CG13786  7250719   7252772   -  2054  
   FBgn0051909   CG31909   7257512   7258205  +  694  
   FBgn0010453   Wnt4  7255460   7277168   -  21709  
   FBgn0004009   wg   7307161   7316255  +  9095  
   FBgn0031902   Wnt6   7334801   7352542  +  17742  
   FBgn0031903   Wnt10   7364820   7377528  +  12709  
   FBgn0002938   ninaC   7377718   7384344  +  6627  
 
 
    Segment 178 
 
   Location   
  Gene key  FBgn0041247-FBgn0031923  
  Heatmap region span   2L:7412453..7675078   
  Segment span   2L:7454169..7578559   
  Length (genes)  7  
  Length (bp)  124391  
   Model Scoring   
  BIC  657.180686  
  logL  -323.119726  
  logL ratio  96.967388  
   Expression   
  Mean expression  4.873321  
  Median expression  4.517475  
  Tissue std. dev.  0.365135  
 
  No GO Slim enrichment  
  
   tissue    mean expression   
  5th Passage Drosophila S2 Cells  4.916796  
  Adult Accessory gland  4.775325  
  Adult Brain  4.733703  
  Adult Carcass  4.754045  
  Adult Crop  4.585027  
  Adult Eye  4.864777  
  Adult Fatbody  4.684938  
  Adult Female Spermatheca Mated  4.654299  
  Adult Female Spermatheca Virgin  4.651460  
  Adult Head  5.621763  
  Adult Heart  4.752224  
  Adult Hind Gut  4.952956  
  Adult Male Ejaculatory Duct  4.678216  
  Adult Mid Gut  4.731562  
  Adult Ovary  4.662145  
  Adult Salivary Gland  4.931210  
  Adult Testes  6.356349  
  Adult Thoracoabdominal ganglion  5.033467  
  Adult Whole Fly  5.242343  
  Larvae Wandering Tubules  4.716567  
  Larval Feeding Carcass  4.698933  
  Larval Feeding Central Nevous System  4.543892  
  Larval Feeding Hind Gut  4.701726  
  Larval Feeding Malpighian Tubule  4.685500  
  Larval Feeding Mid Gut  4.756556  
  Larval Feeding Salivary Gland  4.810041  
  Whole Larvae Feeding  5.083848  
 
  
   FlyBase ID    symbol    start    end    strand    length   
   FBgn0041247   Gr28a  7452525   7454169   -  1645  
   FBgn0045495   Gr28b  7454825   7462247   -  7423  
   FBgn0031918   CG6055   7466546   7472493  +  5948  
   FBgn0031920   CG6441   7474704   7476952  +  2249  
   FBgn0011283   Obp28a  7496785   7497360   -  576  
   FBgn0085403     7497805   7576604   -  78800  
   FBgn0031923     7578233   7578559   -  327  
 
 
    Segment 179 
 
   Location   
  Gene key  FBgn0031926-FBgn0040299  
  Heatmap region span   2L:7437506..7702807   
  Segment span   2L:7608390..7666047   
  Length (genes)  3  
  Length (bp)  57658  
   Model Scoring   
  BIC  313.902449  
  logL  -151.480607  
  logL ratio  21.237462  
   Expression   
  Mean expression  5.492507  
  Median expression  5.497269  
  Tissue std. dev.  0.584809  
 
  No GO Slim enrichment  
  
   tissue    mean expression   
  5th Passage Drosophila S2 Cells  5.172259  
  Adult Accessory gland  4.872773  
  Adult Brain  5.387146  
  Adult Carcass  5.412774  
  Adult Crop  5.601097  
  Adult Eye  6.568035  
  Adult Fatbody  4.774095  
  Adult Female Spermatheca Mated  5.623908  
  Adult Female Spermatheca Virgin  5.448091  
  Adult Head  5.982218  
  Adult Heart  5.093911  
  Adult Hind Gut  5.312717  
  Adult Male Ejaculatory Duct  5.153811  
  Adult Mid Gut  6.577362  
  Adult Ovary  4.859174  
  Adult Salivary Gland  5.323667  
  Adult Testes  4.736567  
  Adult Thoracoabdominal ganglion  5.136364  
  Adult Whole Fly  4.832796  
  Larvae Wandering Tubules  4.995975  
  Larval Feeding Carcass  5.666185  
  Larval Feeding Central Nevous System  5.394246  
  Larval Feeding Hind Gut  6.968349  
  Larval Feeding Malpighian Tubule  5.273137  
  Larval Feeding Mid Gut  6.502617  
  Larval Feeding Salivary Gland  5.800551  
  Whole Larvae Feeding  5.827871  
 
  
   FlyBase ID    symbol    start    end    strand    length   
   FBgn0031926   CG6739   7608390   7616528  +  8139  
   FBgn0031927     7643527   7645997   -  2471  
   FBgn0040299   Myo28B1   7666047   7689459  +  23413  
 
 
    Segment 180 
 
   Location   
  Gene key  FBgn0031929-FBgn0031930  
  Heatmap region span   2L:7581932..7719487   
  Segment span   2L:7691057..7692683   
  Length (genes)  2  
  Length (bp)  1627  
   Model Scoring   
  BIC  189.683794  
  logL  -89.371280  
  logL ratio  37.744628  
   Expression   
  Mean expression  5.661852  
  Median expression  4.830548  
  Tissue std. dev.  2.075083  
 
  No GO Slim enrichment  
  
   tissue    mean expression   
  5th Passage Drosophila S2 Cells  4.983269  
  Adult Accessory gland  4.989623  
  Adult Brain  4.498642  
  Adult Carcass  4.981565  
  Adult Crop  4.783964  
  Adult Eye  4.508157  
  Adult Fatbody  4.900569  
  Adult Female Spermatheca Mated  4.883498  
  Adult Female Spermatheca Virgin  4.813795  
  Adult Head  4.488516  
  Adult Heart  4.565014  
  Adult Hind Gut  4.776569  
  Adult Male Ejaculatory Duct  5.906294  
  Adult Mid Gut  11.897113  
  Adult Ovary  4.743669  
  Adult Salivary Gland  4.897697  
  Adult Testes  4.618888  
  Adult Thoracoabdominal ganglion  4.773041  
  Adult Whole Fly  7.363753  
  Larvae Wandering Tubules  5.960909  
  Larval Feeding Carcass  4.680513  
  Larval Feeding Central Nevous System  4.524455  
  Larval Feeding Hind Gut  4.560880  
  Larval Feeding Malpighian Tubule  5.131718  
  Larval Feeding Mid Gut  11.771403  
  Larval Feeding Salivary Gland  4.827271  
  Whole Larvae Feeding  10.039209  
 
  
   FlyBase ID    symbol    start    end    strand    length   
   FBgn0031929   CG18585  7689376   7691057   -  1682  
   FBgn0031930   CG7025  7691109   7692683   -  1575  
 
    Segment 181 
 
   Location   
  Gene key  FBgn0053296-FBgn0031935  
  Heatmap region span   2L:7691057..7753156   
  Segment span   2L:7716645..7719487   
  Length (genes)  2  
  Length (bp)  2843  
   Model Scoring   
  BIC  242.983058  
  logL  -116.020912  
  logL ratio  -5.835042  
   Expression   
  Mean expression  5.162601  
  Median expression  4.218390  
  Tissue std. dev.  1.766906  
 
  No GO Slim enrichment  
  
   tissue    mean expression   
  5th Passage Drosophila S2 Cells  3.927590  
  Adult Accessory gland  4.322938  
  Adult Brain  6.642181  
  Adult Carcass  8.422189  
  Adult Crop  4.079665  
  Adult Eye  4.321962  
  Adult Fatbody  6.923172  
  Adult Female Spermatheca Mated  4.571624  
  Adult Female Spermatheca Virgin  4.854292  
  Adult Head  7.023216  
  Adult Heart  11.072530  
  Adult Hind Gut  6.570018  
  Adult Male Ejaculatory Duct  6.404632  
  Adult Mid Gut  4.418364  
  Adult Ovary  3.948117  
  Adult Salivary Gland  4.749120  
  Adult Testes  3.814226  
  Adult Thoracoabdominal ganglion  7.417369  
  Adult Whole Fly  4.464042  
  Larvae Wandering Tubules  4.197881  
  Larval Feeding Carcass  3.956836  
  Larval Feeding Central Nevous System  3.851341  
  Larval Feeding Hind Gut  3.756907  
  Larval Feeding Malpighian Tubule  4.008307  
  Larval Feeding Mid Gut  3.903629  
  Larval Feeding Salivary Gland  4.051141  
  Whole Larvae Feeding  3.716940  
 
  
   FlyBase ID    symbol    start    end    strand    length   
   FBgn0053296   CG33296  7714069   7716645   -  2577  
   FBgn0031935   CG13793  7716972   7719487   -  2516  
 
    Segment 182 
 
   Location   
  Gene key  FBgn0031936-FBgn0031937  
  Heatmap region span   2L:7701585..7756317   
  Segment span   2L:7722823..7726298   
  Length (genes)  2  
  Length (bp)  3476  
   Model Scoring   
  BIC  234.954153  
  logL  -112.006459  
  logL ratio  12.534349  
   Expression   
  Mean expression  6.845540  
  Median expression  5.950209  
  Tissue std. dev.  2.343480  
 
  No GO Slim enrichment  
  
   tissue    mean expression   
  5th Passage Drosophila S2 Cells  7.557478  
  Adult Accessory gland  4.870544  
  Adult Brain  5.242878  
  Adult Carcass  9.951677  
  Adult Crop  6.020547  
  Adult Eye  8.928497  
  Adult Fatbody  10.878328  
  Adult Female Spermatheca Mated  10.952863  
  Adult Female Spermatheca Virgin  11.411924  
  Adult Head  9.374586  
  Adult Heart  11.815528  
  Adult Hind Gut  6.276131  
  Adult Male Ejaculatory Duct  7.088217  
  Adult Mid Gut  4.768594  
  Adult Ovary  4.256439  
  Adult Salivary Gland  5.297849  
  Adult Testes  4.133992  
  Adult Thoracoabdominal ganglion  6.043153  
  Adult Whole Fly  6.542295  
  Larvae Wandering Tubules  5.500238  
  Larval Feeding Carcass  5.354548  
  Larval Feeding Central Nevous System  4.704649  
  Larval Feeding Hind Gut  5.170203  
  Larval Feeding Malpighian Tubule  5.123511  
  Larval Feeding Mid Gut  4.833012  
  Larval Feeding Salivary Gland  5.744224  
  Whole Larvae Feeding  6.987665  
 
  
   FlyBase ID    symbol    start    end    strand    length   
   FBgn0031936   CG13794  7719847   7722823   -  2977  
   FBgn0031937   CG13795  7723310   7726298   -  2989  
 
    Segment 183 
 
   Location   
  Gene key  FBgn0031939-FBgn0031940  
  Heatmap region span   2L:7702807..7767649   
  Segment span   2L:7732668..7744841   
  Length (genes)  2  
  Length (bp)  12174  
   Model Scoring   
  BIC  242.535451  
  logL  -115.797108  
  logL ratio  -9.129535  
   Expression   
  Mean expression  6.010361  
  Median expression  5.427587  
  Tissue std. dev.  1.348531  
 
  No GO Slim enrichment  
  
   tissue    mean expression   
  5th Passage Drosophila S2 Cells  4.200078  
  Adult Accessory gland  5.155883  
  Adult Brain  4.682072  
  Adult Carcass  8.688557  
  Adult Crop  5.955267  
  Adult Eye  6.462386  
  Adult Fatbody  7.654803  
  Adult Female Spermatheca Mated  6.775254  
  Adult Female Spermatheca Virgin  7.020490  
  Adult Head  5.451925  
  Adult Heart  7.929706  
  Adult Hind Gut  5.936920  
  Adult Male Ejaculatory Duct  8.026449  
  Adult Mid Gut  6.538840  
  Adult Ovary  4.685569  
  Adult Salivary Gland  4.774123  
  Adult Testes  4.395193  
  Adult Thoracoabdominal ganglion  4.883462  
  Adult Whole Fly  5.591333  
  Larvae Wandering Tubules  5.698805  
  Larval Feeding Carcass  6.633325  
  Larval Feeding Central Nevous System  4.551077  
  Larval Feeding Hind Gut  5.529323  
  Larval Feeding Malpighian Tubule  4.867657  
  Larval Feeding Mid Gut  6.542955  
  Larval Feeding Salivary Gland  4.566221  
  Whole Larvae Feeding  9.082085  
 
  
   FlyBase ID    symbol    start    end    strand    length   
   FBgn0031939   CG13796  7727150   7732668   -  5519  
   FBgn0031940   CG7214  7743677   7744841   -  1165  
 
    Segment 184 
 
   Location   
  Gene key  FBgn0031944-FBgn0031943  
  Heatmap region span   2L:7722823..7781503   
  Segment span   2L:7754191..7756317   
  Length (genes)  2  
  Length (bp)  2127  
   Model Scoring   
  BIC  204.527759  
  logL  -96.793262  
  logL ratio  12.577792  
   Expression   
  Mean expression  4.792906  
  Median expression  4.701527  
  Tissue std. dev.  0.712414  
 
  No GO Slim enrichment  
  
   tissue    mean expression   
  5th Passage Drosophila S2 Cells  4.511942  
  Adult Accessory gland  4.694460  
  Adult Brain  4.465357  
  Adult Carcass  4.699993  
  Adult Crop  4.433034  
  Adult Eye  4.608033  
  Adult Fatbody  4.659327  
  Adult Female Spermatheca Mated  4.725311  
  Adult Female Spermatheca Virgin  4.670889  
  Adult Head  4.622857  
  Adult Heart  4.520905  
  Adult Hind Gut  4.330849  
  Adult Male Ejaculatory Duct  4.727904  
  Adult Mid Gut  4.666792  
  Adult Ovary  4.596986  
  Adult Salivary Gland  4.782394  
  Adult Testes  8.204832  
  Adult Thoracoabdominal ganglion  4.820960  
  Adult Whole Fly  5.682699  
  Larvae Wandering Tubules  4.552745  
  Larval Feeding Carcass  4.538898  
  Larval Feeding Central Nevous System  4.467855  
  Larval Feeding Hind Gut  4.402048  
  Larval Feeding Malpighian Tubule  4.620479  
  Larval Feeding Mid Gut  4.662687  
  Larval Feeding Salivary Gland  4.710751  
  Whole Larvae Feeding  5.027489  
 
  
   FlyBase ID    symbol    start    end    strand    length   
   FBgn0031944   CG7196   7754191   7765716  +  11526  
   FBgn0031943   CG14538   7756317   7756934  +  618  
 
    Segment 185 
 
   Location   
  Gene key  FBgn0031947-FBgn0025687  
  Heatmap region span   2L:7767649..7887489   
  Segment span   2L:7782857..7795958   
  Length (genes)  3  
  Length (bp)  13102  
   Model Scoring   
  BIC  375.328144  
  logL  -182.193455  
  logL ratio  14.795016  
   Expression   
  Mean expression  7.969499  
  Median expression  7.589190  
  Tissue std. dev.  0.745523  
 
  No GO Slim enrichment  
  
   tissue    mean expression   
  5th Passage Drosophila S2 Cells  9.183245  
  Adult Accessory gland  6.712241  
  Adult Brain  8.285625  
  Adult Carcass  7.786517  
  Adult Crop  6.883493  
  Adult Eye  8.906292  
  Adult Fatbody  8.158848  
  Adult Female Spermatheca Mated  8.488573  
  Adult Female Spermatheca Virgin  8.447298  
  Adult Head  8.598372  
  Adult Heart  8.231413  
  Adult Hind Gut  7.393590  
  Adult Male Ejaculatory Duct  7.322011  
  Adult Mid Gut  7.966020  
  Adult Ovary  7.623334  
  Adult Salivary Gland  6.768385  
  Adult Testes  7.090200  
  Adult Thoracoabdominal ganglion  8.414963  
  Adult Whole Fly  7.585193  
  Larvae Wandering Tubules  8.547590  
  Larval Feeding Carcass  6.855380  
  Larval Feeding Central Nevous System  8.255386  
  Larval Feeding Hind Gut  7.454836  
  Larval Feeding Malpighian Tubule  9.266126  
  Larval Feeding Mid Gut  9.021819  
  Larval Feeding Salivary Gland  7.407479  
  Whole Larvae Feeding  8.522236  
 
  
   FlyBase ID    symbol    start    end    strand    length   
   FBgn0031947   CG7154   7782857   7786063  +  3207  
   FBgn0031948   CG7149  7786157   7790557   -  4401  
   FBgn0025687   LKR  7791406   7795958   -  4553  
 
 
    Segment 186 
 
   Location   
  Gene key  FBgn0031951-FBgn0027515  
  Heatmap region span   2L:7780085..7994182   
  Segment span   2L:7800167..7821534   
  Length (genes)  3  
  Length (bp)  21368  
   Model Scoring   
  BIC  343.210971  
  logL  -166.134868  
  logL ratio  46.867376  
   Expression   
  Mean expression  8.070016  
  Median expression  7.820846  
  Tissue std. dev.  0.493376  
 
  No GO Slim enrichment  
  
   tissue    mean expression   
  5th Passage Drosophila S2 Cells  7.387220  
  Adult Accessory gland  8.168041  
  Adult Brain  8.509369  
  Adult Carcass  8.009049  
  Adult Crop  8.445973  
  Adult Eye  7.901877  
  Adult Fatbody  7.655611  
  Adult Female Spermatheca Mated  7.710614  
  Adult Female Spermatheca Virgin  7.676023  
  Adult Head  7.986604  
  Adult Heart  8.376050  
  Adult Hind Gut  7.812457  
  Adult Male Ejaculatory Duct  8.304110  
  Adult Mid Gut  7.476926  
  Adult Ovary  9.251481  
  Adult Salivary Gland  7.587141  
  Adult Testes  9.581988  
  Adult Thoracoabdominal ganglion  8.321350  
  Adult Whole Fly  8.327288  
  Larvae Wandering Tubules  8.077622  
  Larval Feeding Carcass  8.137014  
  Larval Feeding Central Nevous System  8.256438  
  Larval Feeding Hind Gut  7.891914  
  Larval Feeding Malpighian Tubule  7.792639  
  Larval Feeding Mid Gut  7.473793  
  Larval Feeding Salivary Gland  8.091897  
  Whole Larvae Feeding  7.679941  
 
  
   FlyBase ID    symbol    start    end    strand    length   
   FBgn0031951   r2d2   7800167   7802008  +  1842  
   FBgn0031952   cdc14  7802415   7810697   -  8283  
   FBgn0027515   CG7115   7821534   7825350  +  3817  
 
 
    Segment 187 
 
   Location   
  Gene key  FBgn0020618-FBgn0004177  
  Heatmap region span   2L:7781503..7998144   
  Segment span   2L:7827252..7827743   
  Length (genes)  2  
  Length (bp)  492  
   Model Scoring   
  BIC  388.642451  
  logL  -188.850608  
  logL ratio  21.900675  
   Expression   
  Mean expression  12.194262  
  Median expression  11.704935  
  Tissue std. dev.  0.503617  
 
  
   GO ID    description    ratio    P-value   
   GO:0005737   cytoplasm  2/2  0.0141  
 
  
   tissue    mean expression   
  5th Passage Drosophila S2 Cells  12.477836  
  Adult Accessory gland  12.183243  
  Adult Brain  12.654464  
  Adult Carcass  12.000504  
  Adult Crop  12.423326  
  Adult Eye  12.505950  
  Adult Fatbody  12.313243  
  Adult Female Spermatheca Mated  12.369077  
  Adult Female Spermatheca Virgin  12.348178  
  Adult Head  12.234743  
  Adult Heart  12.513754  
  Adult Hind Gut  12.280914  
  Adult Male Ejaculatory Duct  11.943218  
  Adult Mid Gut  11.813037  
  Adult Ovary  12.565580  
  Adult Salivary Gland  12.117186  
  Adult Testes  10.114218  
  Adult Thoracoabdominal ganglion  12.513899  
  Adult Whole Fly  12.284690  
  Larvae Wandering Tubules  12.448716  
  Larval Feeding Carcass  11.838041  
  Larval Feeding Central Nevous System  12.977414  
  Larval Feeding Hind Gut  12.401528  
  Larval Feeding Malpighian Tubule  12.356623  
  Larval Feeding Mid Gut  11.602943  
  Larval Feeding Salivary Gland  12.187787  
  Whole Larvae Feeding  11.774949  
 
  
   FlyBase ID    symbol    start    end    strand    length   
   FBgn0020618   Rack1  7825626   7827252   -  1627  
   FBgn0004177   mts   7827743   7832745  +  5003  
 
    Segment 188 
 
   Location   
  Gene key  FBgn0031954-FBgn0031961  
  Heatmap region span   2L:7782857..7998934   
  Segment span   2L:7840481..7887489   
  Length (genes)  6  
  Length (bp)  47009  
   Model Scoring   
  BIC  567.903123  
  logL  -278.480944  
  logL ratio  66.805618  
   Expression   
  Mean expression  5.010619  
  Median expression  4.790554  
  Tissue std. dev.  0.304693  
 
  No GO Slim enrichment  
  
   tissue    mean expression   
  5th Passage Drosophila S2 Cells  4.802204  
  Adult Accessory gland  5.054429  
  Adult Brain  5.399554  
  Adult Carcass  5.149114  
  Adult Crop  5.153054  
  Adult Eye  5.261664  
  Adult Fatbody  4.760401  
  Adult Female Spermatheca Mated  4.849062  
  Adult Female Spermatheca Virgin  4.839464  
  Adult Head  5.244913  
  Adult Heart  4.599081  
  Adult Hind Gut  5.059163  
  Adult Male Ejaculatory Duct  4.859803  
  Adult Mid Gut  4.839696  
  Adult Ovary  4.748029  
  Adult Salivary Gland  4.859879  
  Adult Testes  5.381826  
  Adult Thoracoabdominal ganglion  5.889105  
  Adult Whole Fly  4.790823  
  Larvae Wandering Tubules  4.778059  
  Larval Feeding Carcass  5.171310  
  Larval Feeding Central Nevous System  5.583057  
  Larval Feeding Hind Gut  5.222364  
  Larval Feeding Malpighian Tubule  4.624798  
  Larval Feeding Mid Gut  4.799133  
  Larval Feeding Salivary Gland  4.705548  
  Whole Larvae Feeding  4.861173  
 
  
   FlyBase ID    symbol    start    end    strand    length   
   FBgn0031954   CG14537   7840481   7841262  +  782  
   FBgn0031955   CG14535  7844905   7855255   -  10351  
   FBgn0031957   TwdlE  7857957   7860838   -  2882  
   FBgn0045038   Proct  7864639   7871432   -  6794  
   FBgn0031959   spz3  7873495   7879550   -  6056  
   FBgn0031961   CG7102  7883468   7887489   -  4022  
 
 
    Segment 189 
 
   Location   
  Gene key  FBgn0051902-FBgn0053121  
  Heatmap region span   2L:7799846..8009477   
  Segment span   2L:7889129..7890530   
  Length (genes)  2  
  Length (bp)  1402  
   Model Scoring   
  BIC  196.192280  
  logL  -92.625523  
  logL ratio  37.132344  
   Expression   
  Mean expression  4.593215  
  Median expression  4.241898  
  Tissue std. dev.  1.623160  
 
  No GO Slim enrichment  
  
   tissue    mean expression   
  5th Passage Drosophila S2 Cells  4.137590  
  Adult Accessory gland  11.975543  
  Adult Brain  4.013272  
  Adult Carcass  4.319705  
  Adult Crop  4.112189  
  Adult Eye  4.149968  
  Adult Fatbody  4.134081  
  Adult Female Spermatheca Mated  4.189025  
  Adult Female Spermatheca Virgin  4.253316  
  Adult Head  3.998265  
  Adult Heart  4.104788  
  Adult Hind Gut  4.146199  
  Adult Male Ejaculatory Duct  7.954012  
  Adult Mid Gut  4.285865  
  Adult Ovary  4.078530  
  Adult Salivary Gland  4.530817  
  Adult Testes  4.145004  
  Adult Thoracoabdominal ganglion  4.081435  
  Adult Whole Fly  4.764322  
  Larvae Wandering Tubules  4.211387  
  Larval Feeding Carcass  4.068756  
  Larval Feeding Central Nevous System  3.987128  
  Larval Feeding Hind Gut  3.990546  
  Larval Feeding Malpighian Tubule  4.186997  
  Larval Feeding Mid Gut  4.066567  
  Larval Feeding Salivary Gland  4.127291  
  Whole Larvae Feeding  4.004215  
 
  
   FlyBase ID    symbol    start    end    strand    length   
   FBgn0051902   Spn28Da  7887693   7889129   -  1437  
   FBgn0053121   Spn28Db  7889371   7890530   -  1160  
 
    Segment 190 
 
   Location   
  Gene key  FBgn0031968-FBgn0031969  
  Heatmap region span   2L:7800167..8010635   
  Segment span   2L:7983920..7994182   
  Length (genes)  3  
  Length (bp)  10263  
   Model Scoring   
  BIC  447.182223  
  logL  -218.120494  
  logL ratio  -77.334679  
   Expression   
  Mean expression  7.469741  
  Median expression  7.333656  
  Tissue std. dev.  0.764186  
 
  No GO Slim enrichment  
  
   tissue    mean expression   
  5th Passage Drosophila S2 Cells  8.059413  
  Adult Accessory gland  7.079023  
  Adult Brain  7.770428  
  Adult Carcass  7.732206  
  Adult Crop  7.071180  
  Adult Eye  7.956915  
  Adult Fatbody  8.039824  
  Adult Female Spermatheca Mated  7.603766  
  Adult Female Spermatheca Virgin  7.321819  
  Adult Head  7.713524  
  Adult Heart  8.054719  
  Adult Hind Gut  7.474119  
  Adult Male Ejaculatory Duct  6.503819  
  Adult Mid Gut  9.356672  
  Adult Ovary  7.997425  
  Adult Salivary Gland  7.812253  
  Adult Testes  5.543907  
  Adult Thoracoabdominal ganglion  7.598721  
  Adult Whole Fly  7.772856  
  Larvae Wandering Tubules  7.279428  
  Larval Feeding Carcass  6.366808  
  Larval Feeding Central Nevous System  7.007899  
  Larval Feeding Hind Gut  7.035517  
  Larval Feeding Malpighian Tubule  7.107100  
  Larval Feeding Mid Gut  8.740529  
  Larval Feeding Salivary Gland  6.117578  
  Whole Larvae Feeding  7.565567  
 
  
   FlyBase ID    symbol    start    end    strand    length   
   FBgn0031968   CG7231   7983920   7986786  +  2867  
   FBgn0085450   Snoo  7892316   7984158   -  91843  
   FBgn0031969   pes  7987113   7994182   -  7070  
 
 
    Segment 191 
 
   Location   
  Gene key  FBgn0031972-FBgn0031974  
  Heatmap region span   2L:7889129..8039788   
  Segment span   2L:8004267..8009477   
  Length (genes)  3  
  Length (bp)  5211  
   Model Scoring   
  BIC  359.280955  
  logL  -174.169860  
  logL ratio  17.514169  
   Expression   
  Mean expression  7.800544  
  Median expression  8.077480  
  Tissue std. dev.  0.683888  
 
  No GO Slim enrichment  
  
   tissue    mean expression   
  5th Passage Drosophila S2 Cells  7.450542  
  Adult Accessory gland  7.244703  
  Adult Brain  7.252312  
  Adult Carcass  8.164909  
  Adult Crop  8.844669  
  Adult Eye  8.245604  
  Adult Fatbody  8.541940  
  Adult Female Spermatheca Mated  8.665064  
  Adult Female Spermatheca Virgin  8.743898  
  Adult Head  8.393058  
  Adult Heart  8.588336  
  Adult Hind Gut  8.218709  
  Adult Male Ejaculatory Duct  7.651539  
  Adult Mid Gut  7.347953  
  Adult Ovary  7.441238  
  Adult Salivary Gland  8.914764  
  Adult Testes  5.953617  
  Adult Thoracoabdominal ganglion  7.451660  
  Adult Whole Fly  7.161715  
  Larvae Wandering Tubules  8.016182  
  Larval Feeding Carcass  7.386218  
  Larval Feeding Central Nevous System  7.003637  
  Larval Feeding Hind Gut  7.829643  
  Larval Feeding Malpighian Tubule  7.965784  
  Larval Feeding Mid Gut  7.101315  
  Larval Feeding Salivary Gland  7.327213  
  Whole Larvae Feeding  7.708479  
 
  
   FlyBase ID    symbol    start    end    strand    length   
   FBgn0031972   Wwox  7999670   8004267   -  4598  
   FBgn0031973   Spn28Dc  8004548   8007253   -  2706  
   FBgn0031974   CG12560  8008321   8009477   -  1157  
 
 
    Segment 192 
 
   Location   
  Gene key  FBgn0031977-FBgn0044323  
  Heatmap region span   2L:7998934..8043715   
  Segment span   2L:8029464..8030723   
  Length (genes)  2  
  Length (bp)  1260  
   Model Scoring   
  BIC  249.795842  
  logL  -119.427304  
  logL ratio  35.091248  
   Expression   
  Mean expression  9.780365  
  Median expression  9.926518  
  Tissue std. dev.  0.602268  
 
  
   GO ID    description    ratio    P-value   
   GO:0005634   nucleus  2/2  0.0436  
 
  
   tissue    mean expression   
  5th Passage Drosophila S2 Cells  11.373984  
  Adult Accessory gland  9.512708  
  Adult Brain  10.355354  
  Adult Carcass  9.407871  
  Adult Crop  10.338069  
  Adult Eye  10.046439  
  Adult Fatbody  8.990310  
  Adult Female Spermatheca Mated  9.911901  
  Adult Female Spermatheca Virgin  9.788045  
  Adult Head  9.630892  
  Adult Heart  10.414896  
  Adult Hind Gut  10.199402  
  Adult Male Ejaculatory Duct  9.407027  
  Adult Mid Gut  9.486318  
  Adult Ovary  10.561720  
  Adult Salivary Gland  9.207753  
  Adult Testes  10.025285  
  Adult Thoracoabdominal ganglion  10.445512  
  Adult Whole Fly  9.934173  
  Larvae Wandering Tubules  10.126714  
  Larval Feeding Carcass  9.041489  
  Larval Feeding Central Nevous System  10.153167  
  Larval Feeding Hind Gut  9.703542  
  Larval Feeding Malpighian Tubule  9.266866  
  Larval Feeding Mid Gut  8.706682  
  Larval Feeding Salivary Gland  8.963803  
  Whole Larvae Feeding  9.069930  
 
  
   FlyBase ID    symbol    start    end    strand    length   
   FBgn0031977   baf   8029464   8030150  +  687  
   FBgn0044323   Cka   8030723   8038517  +  7795  
 
    Segment 193 
 
   Location   
  Gene key  FBgn0013531-FBgn0010287  
  Heatmap region span   2L:8041115..8147630   
  Segment span   2L:8072844..8072897   
  Length (genes)  2  
  Length (bp)  54  
   Model Scoring   
  BIC  224.098200  
  logL  -106.578483  
  logL ratio  23.562216  
   Expression   
  Mean expression  6.803506  
  Median expression  6.858709  
  Tissue std. dev.  0.710342  
 
  
   GO ID    description    ratio    P-value   
   GO:0005634   nucleus  2/2  0.0436  
 
  
   tissue    mean expression   
  5th Passage Drosophila S2 Cells  7.768238  
  Adult Accessory gland  7.263666  
  Adult Brain  7.388718  
  Adult Carcass  5.898302  
  Adult Crop  6.793319  
  Adult Eye  6.606127  
  Adult Fatbody  6.031498  
  Adult Female Spermatheca Mated  6.458479  
  Adult Female Spermatheca Virgin  6.316412  
  Adult Head  6.755362  
  Adult Heart  6.583381  
  Adult Hind Gut  6.634742  
  Adult Male Ejaculatory Duct  6.108307  
  Adult Mid Gut  6.778879  
  Adult Ovary  8.608295  
  Adult Salivary Gland  6.272728  
  Adult Testes  8.373789  
  Adult Thoracoabdominal ganglion  7.187472  
  Adult Whole Fly  7.317943  
  Larvae Wandering Tubules  6.865006  
  Larval Feeding Carcass  6.111522  
  Larval Feeding Central Nevous System  7.807076  
  Larval Feeding Hind Gut  6.383437  
  Larval Feeding Malpighian Tubule  6.601478  
  Larval Feeding Mid Gut  5.826127  
  Larval Feeding Salivary Gland  6.963392  
  Whole Larvae Feeding  5.990961  
 
  
   FlyBase ID    symbol    start    end    strand    length   
   FBgn0013531   MED20  8071809   8072844   -  1036  
   FBgn0010287   Trf   8072897   8073949  +  1053  
 
    Segment 194 
 
   Location   
  Gene key  FBgn0020240-FBgn0031985  
  Heatmap region span   2L:8042856..8156827   
  Segment span   2L:8082969..8124001   
  Length (genes)  2  
  Length (bp)  41033  
   Model Scoring   
  BIC  226.757016  
  logL  -107.907891  
  logL ratio  49.599112  
   Expression   
  Mean expression  9.558809  
  Median expression  9.668352  
  Tissue std. dev.  0.650793  
 
  No GO Slim enrichment  
  
   tissue    mean expression   
  5th Passage Drosophila S2 Cells  10.031743  
  Adult Accessory gland  10.565414  
  Adult Brain  9.608175  
  Adult Carcass  8.914363  
  Adult Crop  10.047301  
  Adult Eye  10.081212  
  Adult Fatbody  8.751938  
  Adult Female Spermatheca Mated  10.128356  
  Adult Female Spermatheca Virgin  9.871269  
  Adult Head  9.675638  
  Adult Heart  8.728219  
  Adult Hind Gut  9.733242  
  Adult Male Ejaculatory Duct  9.885521  
  Adult Mid Gut  8.261632  
  Adult Ovary  9.902821  
  Adult Salivary Gland  10.568318  
  Adult Testes  8.655893  
  Adult Thoracoabdominal ganglion  9.475108  
  Adult Whole Fly  8.986146  
  Larvae Wandering Tubules  9.333422  
  Larval Feeding Carcass  9.726723  
  Larval Feeding Central Nevous System  9.663215  
  Larval Feeding Hind Gut  10.171557  
  Larval Feeding Malpighian Tubule  8.859244  
  Larval Feeding Mid Gut  8.559204  
  Larval Feeding Salivary Gland  10.700534  
  Whole Larvae Feeding  9.201640  
 
  
   FlyBase ID    symbol    start    end    strand    length   
   FBgn0020240     8074109   8082969   -  8861  
   FBgn0031985   mon2  8116965   8124001   -  7037  
 
    Segment 195 
 
   Location   
  Gene key  FBgn0031987-FBgn0031988  
  Heatmap region span   2L:8071591..8163298   
  Segment span   2L:8126876..8127024   
  Length (genes)  2  
  Length (bp)  149  
   Model Scoring   
  BIC  251.893143  
  logL  -120.475954  
  logL ratio  25.821692  
   Expression   
  Mean expression  9.407606  
  Median expression  9.453277  
  Tissue std. dev.  0.831991  
 
  No GO Slim enrichment  
  
   tissue    mean expression   
  5th Passage Drosophila S2 Cells  9.692217  
  Adult Accessory gland  6.731141  
  Adult Brain  9.520122  
  Adult Carcass  9.120717  
  Adult Crop  10.222006  
  Adult Eye  9.215686  
  Adult Fatbody  9.083799  
  Adult Female Spermatheca Mated  8.772965  
  Adult Female Spermatheca Virgin  8.859025  
  Adult Head  9.013741  
  Adult Heart  9.466969  
  Adult Hind Gut  9.497462  
  Adult Male Ejaculatory Duct  10.263649  
  Adult Mid Gut  9.863409  
  Adult Ovary  9.652449  
  Adult Salivary Gland  11.576929  
  Adult Testes  8.387998  
  Adult Thoracoabdominal ganglion  9.404581  
  Adult Whole Fly  8.952086  
  Larvae Wandering Tubules  10.251240  
  Larval Feeding Carcass  9.188707  
  Larval Feeding Central Nevous System  8.814034  
  Larval Feeding Hind Gut  9.271646  
  Larval Feeding Malpighian Tubule  10.401220  
  Larval Feeding Mid Gut  10.144396  
  Larval Feeding Salivary Gland  9.468280  
  Whole Larvae Feeding  9.168898  
 
  
   FlyBase ID    symbol    start    end    strand    length   
   FBgn0031987   CG12375  8125668   8126876   -  1209  
   FBgn0031988   CG8668   8127024   8133989  +  6966  
 
    Segment 196 
 
   Location   
  Gene key  FBgn0051607-FBgn0051900  
  Heatmap region span   2L:8082969..8190781   
  Segment span   2L:8148950..8156827   
  Length (genes)  2  
  Length (bp)  7878  
   Model Scoring   
  BIC  245.064772  
  logL  -117.061769  
  logL ratio  -2.239295  
   Expression   
  Mean expression  6.599493  
  Median expression  6.323590  
  Tissue std. dev.  1.403091  
 
  No GO Slim enrichment  
  
   tissue    mean expression   
  5th Passage Drosophila S2 Cells  5.842725  
  Adult Accessory gland  4.835265  
  Adult Brain  4.861616  
  Adult Carcass  7.718322  
  Adult Crop  9.172914  
  Adult Eye  6.204031  
  Adult Fatbody  6.226998  
  Adult Female Spermatheca Mated  5.636986  
  Adult Female Spermatheca Virgin  6.062026  
  Adult Head  7.524791  
  Adult Heart  6.281436  
  Adult Hind Gut  10.327965  
  Adult Male Ejaculatory Duct  8.620352  
  Adult Mid Gut  6.230639  
  Adult Ovary  6.198898  
  Adult Salivary Gland  6.768736  
  Adult Testes  5.436698  
  Adult Thoracoabdominal ganglion  4.979881  
  Adult Whole Fly  6.522909  
  Larvae Wandering Tubules  5.487388  
  Larval Feeding Carcass  7.380349  
  Larval Feeding Central Nevous System  6.123781  
  Larval Feeding Hind Gut  9.274041  
  Larval Feeding Malpighian Tubule  4.893596  
  Larval Feeding Mid Gut  5.542757  
  Larval Feeding Salivary Gland  7.344059  
  Whole Larvae Feeding  6.687144  
 
  
   FlyBase ID    symbol    start    end    strand    length   
   FBgn0051607      8148950   8155603  +  6654  
   FBgn0051900      8156827   8158711  +  1885  
 
    Segment 197 
 
   Location   
  Gene key  FBgn0031990-FBgn0031992  
  Heatmap region span   2L:8124329..8205128   
  Segment span   2L:8159575..8162389   
  Length (genes)  2  
  Length (bp)  2815  
   Model Scoring   
  BIC  226.487908  
  logL  -107.773337  
  logL ratio  60.650608  
   Expression   
  Mean expression  10.124083  
  Median expression  10.096657  
  Tissue std. dev.  0.361334  
 
  No GO Slim enrichment  
  
   tissue    mean expression   
  5th Passage Drosophila S2 Cells  10.066102  
  Adult Accessory gland  10.491425  
  Adult Brain  10.180654  
  Adult Carcass  9.993804  
  Adult Crop  10.130095  
  Adult Eye  9.647036  
  Adult Fatbody  10.437964  
  Adult Female Spermatheca Mated  10.344340  
  Adult Female Spermatheca Virgin  10.460196  
  Adult Head  10.142772  
  Adult Heart  10.344395  
  Adult Hind Gut  9.980746  
  Adult Male Ejaculatory Duct  10.324692  
  Adult Mid Gut  9.981766  
  Adult Ovary  9.936647  
  Adult Salivary Gland  10.449736  
  Adult Testes  9.493016  
  Adult Thoracoabdominal ganglion  9.930427  
  Adult Whole Fly  9.543920  
  Larvae Wandering Tubules  10.322917  
  Larval Feeding Carcass  9.786282  
  Larval Feeding Central Nevous System  9.745700  
  Larval Feeding Hind Gut  10.024918  
  Larval Feeding Malpighian Tubule  10.171917  
  Larval Feeding Mid Gut  10.152898  
  Larval Feeding Salivary Gland  11.342869  
  Whole Larvae Feeding  9.923012  
 
  
   FlyBase ID    symbol    start    end    strand    length   
   FBgn0031990   CG8552  8134247   8159575   -  25329  
   FBgn0031992   CG8498  8161794   8162389   -  596  
 
    Segment 198 
 
   Location   
  Gene key  FBgn0031996-FBgn0031998  
  Heatmap region span   2L:8159575..8218017   
  Segment span   2L:8197196..8205128   
  Length (genes)  3  
  Length (bp)  7933  
   Model Scoring   
  BIC  329.160701  
  logL  -159.109733  
  logL ratio  73.105456  
   Expression   
  Mean expression  9.073241  
  Median expression  8.879854  
  Tissue std. dev.  0.507173  
 
  No GO Slim enrichment  
  
   tissue    mean expression   
  5th Passage Drosophila S2 Cells  8.981963  
  Adult Accessory gland  10.464138  
  Adult Brain  8.922229  
  Adult Carcass  8.506547  
  Adult Crop  8.937975  
  Adult Eye  9.214843  
  Adult Fatbody  9.476954  
  Adult Female Spermatheca Mated  9.627383  
  Adult Female Spermatheca Virgin  9.629725  
  Adult Head  9.006554  
  Adult Heart  9.616099  
  Adult Hind Gut  8.793744  
  Adult Male Ejaculatory Duct  9.924412  
  Adult Mid Gut  9.179207  
  Adult Ovary  9.420003  
  Adult Salivary Gland  8.817892  
  Adult Testes  9.263904  
  Adult Thoracoabdominal ganglion  9.138960  
  Adult Whole Fly  8.740688  
  Larvae Wandering Tubules  8.947126  
  Larval Feeding Carcass  8.331796  
  Larval Feeding Central Nevous System  8.885481  
  Larval Feeding Hind Gut  8.224313  
  Larval Feeding Malpighian Tubule  9.162168  
  Larval Feeding Mid Gut  8.175309  
  Larval Feeding Salivary Gland  9.016246  
  Whole Larvae Feeding  8.571846  
 
  
   FlyBase ID    symbol    start    end    strand    length   
   FBgn0031996   CG8460   8197196   8198813  +  1618  
   FBgn0031997   CG8455   8198959   8200652  +  1694  
   FBgn0031998   CG8451  8200465   8205128   -  4664  
 
 
    Segment 199 
 
   Location   
  Gene key  FBgn0032000-FBgn0032001  
  Heatmap region span   2L:8180222..8241839   
  Segment span   2L:8212881..8213179   
  Length (genes)  2  
  Length (bp)  299  
   Model Scoring   
  BIC  239.865313  
  logL  -114.462039  
  logL ratio  56.969986  
   Expression   
  Mean expression  10.286601  
  Median expression  10.018779  
  Tissue std. dev.  0.662909  
 
  No GO Slim enrichment  
  
   tissue    mean expression   
  5th Passage Drosophila S2 Cells  9.723243  
  Adult Accessory gland  10.427395  
  Adult Brain  9.895252  
  Adult Carcass  10.671330  
  Adult Crop  10.019040  
  Adult Eye  10.750165  
  Adult Fatbody  11.058450  
  Adult Female Spermatheca Mated  10.725274  
  Adult Female Spermatheca Virgin  10.796755  
  Adult Head  10.473314  
  Adult Heart  11.049384  
  Adult Hind Gut  10.456172  
  Adult Male Ejaculatory Duct  10.706837  
  Adult Mid Gut  10.030913  
  Adult Ovary  8.796065  
  Adult Salivary Gland  12.115839  
  Adult Testes  9.700538  
  Adult Thoracoabdominal ganglion  9.801826  
  Adult Whole Fly  9.713506  
  Larvae Wandering Tubules  11.116754  
  Larval Feeding Carcass  9.892132  
  Larval Feeding Central Nevous System  9.340335  
  Larval Feeding Hind Gut  9.846346  
  Larval Feeding Malpighian Tubule  10.205990  
  Larval Feeding Mid Gut  9.880258  
  Larval Feeding Salivary Gland  10.732803  
  Whole Larvae Feeding  9.812304  
 
  
   FlyBase ID    symbol    start    end    strand    length   
   FBgn0032000   CG8372  8212039   8212881   -  843  
   FBgn0032001   CG8360   8213179   8214135  +  957  
 
    Segment 200 
 
   Location   
  Gene key  FBgn0032003-FBgn0032004  
  Heatmap region span   2L:8197196..8303433   
  Segment span   2L:8215915..8218017   
  Length (genes)  2  
  Length (bp)  2103  
   Model Scoring   
  BIC  188.384591  
  logL  -88.721678  
  logL ratio  30.162441  
   Expression   
  Mean expression  5.249600  
  Median expression  4.772417  
  Tissue std. dev.  1.449287  
 
  No GO Slim enrichment  
  
   tissue    mean expression   
  5th Passage Drosophila S2 Cells  7.860141  
  Adult Accessory gland  4.851290  
  Adult Brain  4.634470  
  Adult Carcass  4.917980  
  Adult Crop  4.779585  
  Adult Eye  4.712666  
  Adult Fatbody  4.897844  
  Adult Female Spermatheca Mated  4.905835  
  Adult Female Spermatheca Virgin  4.838468  
  Adult Head  4.666446  
  Adult Heart  5.239412  
  Adult Hind Gut  4.682587  
  Adult Male Ejaculatory Duct  4.927570  
  Adult Mid Gut  4.910647  
  Adult Ovary  4.644514  
  Adult Salivary Gland  4.800873  
  Adult Testes  11.675230  
  Adult Thoracoabdominal ganglion  4.597646  
  Adult Whole Fly  6.991528  
  Larvae Wandering Tubules  4.818186  
  Larval Feeding Carcass  4.751720  
  Larval Feeding Central Nevous System  4.529165  
  Larval Feeding Hind Gut  4.579040  
  Larval Feeding Malpighian Tubule  4.783454  
  Larval Feeding Mid Gut  4.817742  
  Larval Feeding Salivary Gland  4.677504  
  Whole Larvae Feeding  5.247653  
 
  
   FlyBase ID    symbol    start    end    strand    length   
   FBgn0032003   CG8349   8215915   8216901  +  987  
   FBgn0032004   CG8292  8216851   8218017   -  1167  
 
    Segment 201 
 
   Location   
  Gene key  FBgn0032005-FBgn0032006  
  Heatmap region span   2L:8206206..8308188   
  Segment span   2L:8220737..8239875   
  Length (genes)  2  
  Length (bp)  19139  
   Model Scoring   
  BIC  243.178122  
  logL  -116.118444  
  logL ratio  36.197748  
   Expression   
  Mean expression  9.528071  
  Median expression  9.578764  
  Tissue std. dev.  0.735763  
 
  No GO Slim enrichment  
  
   tissue    mean expression   
  5th Passage Drosophila S2 Cells  12.213533  
  Adult Accessory gland  9.749520  
  Adult Brain  8.615552  
  Adult Carcass  9.182901  
  Adult Crop  9.685686  
  Adult Eye  9.151039  
  Adult Fatbody  9.270700  
  Adult Female Spermatheca Mated  9.392297  
  Adult Female Spermatheca Virgin  9.447644  
  Adult Head  9.349443  
  Adult Heart  10.474173  
  Adult Hind Gut  9.563138  
  Adult Male Ejaculatory Duct  9.583689  
  Adult Mid Gut  9.917334  
  Adult Ovary  8.738387  
  Adult Salivary Gland  10.373636  
  Adult Testes  8.860027  
  Adult Thoracoabdominal ganglion  8.930921  
  Adult Whole Fly  8.764826  
  Larvae Wandering Tubules  9.868902  
  Larval Feeding Carcass  9.271783  
  Larval Feeding Central Nevous System  8.269458  
  Larval Feeding Hind Gut  9.788466  
  Larval Feeding Malpighian Tubule  9.683970  
  Larval Feeding Mid Gut  9.690508  
  Larval Feeding Salivary Gland  10.150641  
  Whole Larvae Feeding  9.269749  
 
  
   FlyBase ID    symbol    start    end    strand    length   
   FBgn0032005   Snx6  8218071   8220737   -  2667  
   FBgn0032006   Pvr  8220980   8239875   -  18896  
 
    Segment 202 
 
   Location   
  Gene key  FBgn0032008-FBgn0003502  
  Heatmap region span   2L:8214305..8325299   
  Segment span   2L:8242659..8301072   
  Length (genes)  3  
  Length (bp)  58414  
   Model Scoring   
  BIC  354.378036  
  logL  -171.718401  
  logL ratio  -4.325434  
   Expression   
  Mean expression  6.386667  
  Median expression  5.981061  
  Tissue std. dev.  0.754304  
 
  No GO Slim enrichment  
  
   tissue    mean expression   
  5th Passage Drosophila S2 Cells  5.570917  
  Adult Accessory gland  8.001995  
  Adult Brain  5.955267  
  Adult Carcass  7.430014  
  Adult Crop  6.479850  
  Adult Eye  5.909451  
  Adult Fatbody  6.259895  
  Adult Female Spermatheca Mated  6.526100  
  Adult Female Spermatheca Virgin  6.468079  
  Adult Head  8.382913  
  Adult Heart  5.776691  
  Adult Hind Gut  6.373611  
  Adult Male Ejaculatory Duct  7.240926  
  Adult Mid Gut  5.595238  
  Adult Ovary  6.572900  
  Adult Salivary Gland  5.300910  
  Adult Testes  6.220445  
  Adult Thoracoabdominal ganglion  5.999683  
  Adult Whole Fly  6.951636  
  Larvae Wandering Tubules  6.014068  
  Larval Feeding Carcass  6.827380  
  Larval Feeding Central Nevous System  6.815073  
  Larval Feeding Hind Gut  6.589653  
  Larval Feeding Malpighian Tubule  5.746914  
  Larval Feeding Mid Gut  5.181101  
  Larval Feeding Salivary Gland  5.598778  
  Whole Larvae Feeding  6.650509  
 
  
   FlyBase ID    symbol    start    end    strand    length   
   FBgn0032008   CG14277   8242659   8243426  +  768  
   FBgn0032010   CG8086  8244159   8257502   -  13344  
   FBgn0003502   Btk29A  8259504   8301072   -  41569  
 
 
    Segment 203 
 
   Location   
  Gene key  FBgn0032014-FBgn0032015  
  Heatmap region span   2L:8220737..8338312   
  Segment span   2L:8305590..8308188   
  Length (genes)  2  
  Length (bp)  2599  
   Model Scoring   
  BIC  266.889299  
  logL  -127.974032  
  logL ratio  27.300297  
   Expression   
  Mean expression  9.989361  
  Median expression  9.914403  
  Tissue std. dev.  0.684559  
 
  No GO Slim enrichment  
  
   tissue    mean expression   
  5th Passage Drosophila S2 Cells  10.939751  
  Adult Accessory gland  10.899456  
  Adult Brain  8.613384  
  Adult Carcass  9.393339  
  Adult Crop  9.314606  
  Adult Eye  9.530808  
  Adult Fatbody  9.983723  
  Adult Female Spermatheca Mated  10.349014  
  Adult Female Spermatheca Virgin  10.475478  
  Adult Head  9.177626  
  Adult Heart  9.807588  
  Adult Hind Gut  9.372262  
  Adult Male Ejaculatory Duct  10.667507  
  Adult Mid Gut  10.324717  
  Adult Ovary  11.073136  
  Adult Salivary Gland  10.798167  
  Adult Testes  9.359962  
  Adult Thoracoabdominal ganglion  8.817866  
  Adult Whole Fly  10.621600  
  Larvae Wandering Tubules  10.357536  
  Larval Feeding Carcass  9.769648  
  Larval Feeding Central Nevous System  9.567760  
  Larval Feeding Hind Gut  9.626593  
  Larval Feeding Malpighian Tubule  10.042655  
  Larval Feeding Mid Gut  9.916691  
  Larval Feeding Salivary Gland  11.184850  
  Whole Larvae Feeding  9.727015  
 
  
   FlyBase ID    symbol    start    end    strand    length   
   FBgn0032014   CG7840   8305590   8306743  +  1154  
   FBgn0032015   Ostgamma  8306684   8308188   -  1505  
 
    Segment 204 
 
   Location   
  Gene key  FBgn0032016-FBgn0032020  
  Heatmap region span   2L:8241839..8342764   
  Segment span   2L:8308440..8320324   
  Length (genes)  4  
  Length (bp)  11885  
   Model Scoring   
  BIC  422.944161  
  logL  -206.001463  
  logL ratio  86.520907  
   Expression   
  Mean expression  8.126358  
  Median expression  8.102106  
  Tissue std. dev.  0.493541  
 
  
   GO ID    description    ratio    P-value   
   GO:0006810   transport  2/4  0.0322  
 
  
   tissue    mean expression   
  5th Passage Drosophila S2 Cells  8.943490  
  Adult Accessory gland  7.971218  
  Adult Brain  8.141842  
  Adult Carcass  7.074685  
  Adult Crop  8.167817  
  Adult Eye  8.099173  
  Adult Fatbody  7.461743  
  Adult Female Spermatheca Mated  8.065404  
  Adult Female Spermatheca Virgin  7.982788  
  Adult Head  7.603318  
  Adult Heart  8.103050  
  Adult Hind Gut  8.554077  
  Adult Male Ejaculatory Duct  7.535576  
  Adult Mid Gut  8.084025  
  Adult Ovary  9.027959  
  Adult Salivary Gland  8.681954  
  Adult Testes  7.871661  
  Adult Thoracoabdominal ganglion  8.128256  
  Adult Whole Fly  7.631585  
  Larvae Wandering Tubules  9.058298  
  Larval Feeding Carcass  7.774589  
  Larval Feeding Central Nevous System  8.490372  
  Larval Feeding Hind Gut  8.398137  
  Larval Feeding Malpighian Tubule  8.373357  
  Larval Feeding Mid Gut  7.956797  
  Larval Feeding Salivary Gland  8.740741  
  Whole Larvae Feeding  7.489768  
 
  
   FlyBase ID    symbol    start    end    strand    length   
   FBgn0032016   CG7818   8308440   8309963  +  1524  
   FBgn0032017   CG7810  8309869   8311144   -  1276  
   FBgn0032018   CG7806   8311419   8317017  +  5599  
   FBgn0032020   CG7787   8320324   8321285  +  962  
 
 
    Segment 205 
 
   Location   
  Gene key  FBgn0032029-FBgn0032034  
  Heatmap region span   2L:8346621..8403575   
  Segment span   2L:8370513..8382815   
  Length (genes)  6  
  Length (bp)  12303  
   Model Scoring   
  BIC  596.790567  
  logL  -292.924666  
  logL ratio  180.702876  
   Expression   
  Mean expression  8.470267  
  Median expression  8.303036  
  Tissue std. dev.  0.385910  
 
  No GO Slim enrichment  
  
   tissue    mean expression   
  5th Passage Drosophila S2 Cells  8.761931  
  Adult Accessory gland  8.337885  
  Adult Brain  8.402362  
  Adult Carcass  8.608696  
  Adult Crop  8.691858  
  Adult Eye  8.826702  
  Adult Fatbody  8.833130  
  Adult Female Spermatheca Mated  8.550073  
  Adult Female Spermatheca Virgin  8.554795  
  Adult Head  8.296081  
  Adult Heart  9.307392  
  Adult Hind Gut  8.408893  
  Adult Male Ejaculatory Duct  8.802703  
  Adult Mid Gut  8.361472  
  Adult Ovary  9.232129  
  Adult Salivary Gland  8.411751  
  Adult Testes  7.339675  
  Adult Thoracoabdominal ganglion  8.557111  
  Adult Whole Fly  8.320391  
  Larvae Wandering Tubules  7.889702  
  Larval Feeding Carcass  8.539418  
  Larval Feeding Central Nevous System  8.584042  
  Larval Feeding Hind Gut  8.229962  
  Larval Feeding Malpighian Tubule  8.459958  
  Larval Feeding Mid Gut  7.967569  
  Larval Feeding Salivary Gland  8.320212  
  Whole Larvae Feeding  8.101307  
 
  
   FlyBase ID    symbol    start    end    strand    length   
   FBgn0032029   CG17292   8370513   8374257  +  3745  
   FBgn0032030   Wdr82   8374481   8375549  +  1069  
   FBgn0032031   CG13390  8375501   8376777   -  1277  
   FBgn0032032   CG17294   8377051   8378206  +  1156  
   FBgn0032033   CG13392  8381905   8382723   -  819  
   FBgn0032034   Rcd4   8382815   8383657  +  843  
 
 
    Segment 206 
 
   Location   
  Gene key  FBgn0032035-FBgn0032036  
  Heatmap region span   2L:8352200..8415245   
  Segment span   2L:8384132..8384430   
  Length (genes)  2  
  Length (bp)  299  
   Model Scoring   
  BIC  270.134942  
  logL  -129.596854  
  logL ratio  32.274478  
   Expression   
  Mean expression  10.300445  
  Median expression  10.388972  
  Tissue std. dev.  0.582891  
 
  No GO Slim enrichment  
  
   tissue    mean expression   
  5th Passage Drosophila S2 Cells  12.155846  
  Adult Accessory gland  10.638889  
  Adult Brain  10.783700  
  Adult Carcass  9.726426  
  Adult Crop  9.819847  
  Adult Eye  10.187805  
  Adult Fatbody  9.824513  
  Adult Female Spermatheca Mated  10.195302  
  Adult Female Spermatheca Virgin  10.149358  
  Adult Head  10.196801  
  Adult Heart  10.906356  
  Adult Hind Gut  10.152060  
  Adult Male Ejaculatory Duct  10.614097  
  Adult Mid Gut  9.932629  
  Adult Ovary  10.296042  
  Adult Salivary Gland  11.080794  
  Adult Testes  9.061032  
  Adult Thoracoabdominal ganglion  10.763432  
  Adult Whole Fly  9.861226  
  Larvae Wandering Tubules  10.010255  
  Larval Feeding Carcass  10.080354  
  Larval Feeding Central Nevous System  10.685745  
  Larval Feeding Hind Gut  9.966978  
  Larval Feeding Malpighian Tubule  10.111032  
  Larval Feeding Mid Gut  9.859436  
  Larval Feeding Salivary Gland  11.091299  
  Whole Larvae Feeding  9.960772  
 
  
   FlyBase ID    symbol    start    end    strand    length   
   FBgn0032035     8383612   8384132   -  521  
   FBgn0032036   CG13384   8384430   8388804  +  4375  
 
    Segment 207 
 
   Location   
  Gene key  FBgn0032039-FBgn0001084  
  Heatmap region span   2L:8364609..8437346   
  Segment span   2L:8396713..8403442   
  Length (genes)  4  
  Length (bp)  6730  
   Model Scoring   
  BIC  379.531488  
  logL  -184.295127  
  logL ratio  51.207730  
   Expression   
  Mean expression  5.336603  
  Median expression  5.053975  
  Tissue std. dev.  0.460065  
 
  No GO Slim enrichment  
  
   tissue    mean expression   
  5th Passage Drosophila S2 Cells  5.832259  
  Adult Accessory gland  5.352368  
  Adult Brain  5.231390  
  Adult Carcass  4.931782  
  Adult Crop  5.225981  
  Adult Eye  5.243325  
  Adult Fatbody  5.015553  
  Adult Female Spermatheca Mated  5.184019  
  Adult Female Spermatheca Virgin  5.027571  
  Adult Head  5.078621  
  Adult Heart  4.927197  
  Adult Hind Gut  5.087594  
  Adult Male Ejaculatory Duct  4.864657  
  Adult Mid Gut  5.182550  
  Adult Ovary  6.510592  
  Adult Salivary Gland  5.400358  
  Adult Testes  6.836674  
  Adult Thoracoabdominal ganglion  5.273011  
  Adult Whole Fly  5.822461  
  Larvae Wandering Tubules  5.200289  
  Larval Feeding Carcass  5.147107  
  Larval Feeding Central Nevous System  5.612921  
  Larval Feeding Hind Gut  5.116816  
  Larval Feeding Malpighian Tubule  5.187691  
  Larval Feeding Mid Gut  5.078541  
  Larval Feeding Salivary Gland  5.786851  
  Whole Larvae Feeding  4.930111  
 
  
   FlyBase ID    symbol    start    end    strand    length   
   FBgn0032039   CG13385   8396713   8397886  +  1174  
   FBgn0032040   CG13386   8399044   8399972  +  929  
   FBgn0051898   CG31898   8400457   8401477  +  1021  
   FBgn0001084   fy  8401446   8403442   -  1997  
 
 
    Segment 208 
 
   Location   
  Gene key  FBgn0014417-FBgn0032042  
  Heatmap region span   2L:8384132..8464491   
  Segment span   2L:8411868..8415245   
  Length (genes)  2  
  Length (bp)  3378  
   Model Scoring   
  BIC  247.571348  
  logL  -118.315057  
  logL ratio  6.777414  
   Expression   
  Mean expression  7.769948  
  Median expression  8.115167  
  Tissue std. dev.  0.805506  
 
  No GO Slim enrichment  
  
   tissue    mean expression   
  5th Passage Drosophila S2 Cells  8.853102  
  Adult Accessory gland  7.692392  
  Adult Brain  8.310572  
  Adult Carcass  7.557265  
  Adult Crop  6.747141  
  Adult Eye  7.385488  
  Adult Fatbody  9.128180  
  Adult Female Spermatheca Mated  8.712106  
  Adult Female Spermatheca Virgin  9.088759  
  Adult Head  7.774582  
  Adult Heart  8.213661  
  Adult Hind Gut  7.866998  
  Adult Male Ejaculatory Duct  7.353442  
  Adult Mid Gut  6.931221  
  Adult Ovary  9.470273  
  Adult Salivary Gland  7.751632  
  Adult Testes  5.739138  
  Adult Thoracoabdominal ganglion  7.601147  
  Adult Whole Fly  7.887326  
  Larvae Wandering Tubules  7.929006  
  Larval Feeding Carcass  7.050751  
  Larval Feeding Central Nevous System  8.156659  
  Larval Feeding Hind Gut  7.451785  
  Larval Feeding Malpighian Tubule  7.180203  
  Larval Feeding Mid Gut  6.903859  
  Larval Feeding Salivary Gland  7.221949  
  Whole Larvae Feeding  7.829966  
 
  
   FlyBase ID    symbol    start    end    strand    length   
   FBgn0014417   CG13397  8409051   8411868   -  2818  
   FBgn0032042   CG13398  8412411   8415245   -  2835  
 
    Segment 209 
 
   Location   
  Gene key  FBgn0001137-FBgn0027490  
  Heatmap region span   2L:8396713..8488959   
  Segment span   2L:8433598..8437346   
  Length (genes)  2  
  Length (bp)  3749  
   Model Scoring   
  BIC  242.239212  
  logL  -115.648989  
  logL ratio  7.785039  
   Expression   
  Mean expression  7.037080  
  Median expression  6.753016  
  Tissue std. dev.  0.837401  
 
  No GO Slim enrichment  
  
   tissue    mean expression   
  5th Passage Drosophila S2 Cells  8.365037  
  Adult Accessory gland  7.186384  
  Adult Brain  6.365271  
  Adult Carcass  7.863443  
  Adult Crop  8.501864  
  Adult Eye  6.223725  
  Adult Fatbody  6.172913  
  Adult Female Spermatheca Mated  7.091568  
  Adult Female Spermatheca Virgin  6.845211  
  Adult Head  7.291667  
  Adult Heart  7.988524  
  Adult Hind Gut  7.646611  
  Adult Male Ejaculatory Duct  6.897733  
  Adult Mid Gut  6.363990  
  Adult Ovary  8.937690  
  Adult Salivary Gland  6.059919  
  Adult Testes  5.852958  
  Adult Thoracoabdominal ganglion  6.353850  
  Adult Whole Fly  7.577516  
  Larvae Wandering Tubules  6.387916  
  Larval Feeding Carcass  8.060457  
  Larval Feeding Central Nevous System  7.452859  
  Larval Feeding Hind Gut  7.164908  
  Larval Feeding Malpighian Tubule  6.064348  
  Larval Feeding Mid Gut  6.155677  
  Larval Feeding Salivary Gland  6.406391  
  Whole Larvae Feeding  6.722728  
 
  
   FlyBase ID    symbol    start    end    strand    length   
   FBgn0001137   grk  8431086   8433598   -  2513  
   FBgn0027490   D12  8434058   8437346   -  3289  
 
    Segment 210 
 
   Location   
  Gene key  FBgn0027780-FBgn0029173  
  Heatmap region span   2L:8411868..8506845   
  Segment span   2L:8449571..8464491   
  Length (genes)  3  
  Length (bp)  14921  
   Model Scoring   
  BIC  361.651379  
  logL  -175.355072  
  logL ratio  17.188729  
   Expression   
  Mean expression  7.456559  
  Median expression  7.301289  
  Tissue std. dev.  0.683807  
 
  No GO Slim enrichment  
  
   tissue    mean expression   
  5th Passage Drosophila S2 Cells  8.410340  
  Adult Accessory gland  6.712322  
  Adult Brain  7.866844  
  Adult Carcass  7.221688  
  Adult Crop  7.570708  
  Adult Eye  7.493165  
  Adult Fatbody  7.870916  
  Adult Female Spermatheca Mated  8.135442  
  Adult Female Spermatheca Virgin  8.266903  
  Adult Head  7.633525  
  Adult Heart  7.318952  
  Adult Hind Gut  7.488993  
  Adult Male Ejaculatory Duct  6.471107  
  Adult Mid Gut  8.086935  
  Adult Ovary  9.059849  
  Adult Salivary Gland  6.501180  
  Adult Testes  7.002618  
  Adult Thoracoabdominal ganglion  7.938814  
  Adult Whole Fly  7.747538  
  Larvae Wandering Tubules  7.354456  
  Larval Feeding Carcass  5.941671  
  Larval Feeding Central Nevous System  8.161305  
  Larval Feeding Hind Gut  6.947110  
  Larval Feeding Malpighian Tubule  7.203892  
  Larval Feeding Mid Gut  7.469928  
  Larval Feeding Salivary Gland  6.643865  
  Whole Larvae Feeding  6.807027  
 
  
   FlyBase ID    symbol    start    end    strand    length   
   FBgn0027780   U26  8445861   8449571   -  3711  
   FBgn0026718   fu12  8449691   8463990   -  14300  
   FBgn0029173   fu2   8464491   8466529  +  2039  
 
 
    Segment 211 
 
   Location   
  Gene key  FBgn0032050-FBgn0032054  
  Heatmap region span   2L:8477349..8672043   
  Segment span   2L:8511844..8517446   
  Length (genes)  5  
  Length (bp)  5603  
   Model Scoring   
  BIC  458.205214  
  logL  -223.631990  
  logL ratio  184.083457  
   Expression   
  Mean expression  8.504908  
  Median expression  8.545840  
  Tissue std. dev.  0.407164  
 
  
   GO ID    description    ratio    P-value   
   GO:0003735   structural constituent of ribosome  2/5  0.0205  
   GO:0006412   translation  2/5  0.0205  
   GO:0009058   biosynthetic process  2/5  0.0253  
   GO:0005198   structural molecule activity  2/5  0.0472  
 
  
   tissue    mean expression   
  5th Passage Drosophila S2 Cells  9.300798  
  Adult Accessory gland  8.844912  
  Adult Brain  8.060334  
  Adult Carcass  7.876409  
  Adult Crop  8.152941  
  Adult Eye  8.143380  
  Adult Fatbody  8.471499  
  Adult Female Spermatheca Mated  8.443230  
  Adult Female Spermatheca Virgin  8.313304  
  Adult Head  7.890980  
  Adult Heart  8.535668  
  Adult Hind Gut  8.330006  
  Adult Male Ejaculatory Duct  8.524481  
  Adult Mid Gut  8.448923  
  Adult Ovary  9.489878  
  Adult Salivary Gland  8.316053  
  Adult Testes  8.786705  
  Adult Thoracoabdominal ganglion  8.298115  
  Adult Whole Fly  8.674847  
  Larvae Wandering Tubules  8.270526  
  Larval Feeding Carcass  8.510796  
  Larval Feeding Central Nevous System  9.284395  
  Larval Feeding Hind Gut  8.663763  
  Larval Feeding Malpighian Tubule  8.526722  
  Larval Feeding Mid Gut  8.172301  
  Larval Feeding Salivary Gland  9.113329  
  Whole Larvae Feeding  8.188224  
 
  
   FlyBase ID    symbol    start    end    strand    length   
   FBgn0032050   CG13096  8509251   8511844   -  2594  
   FBgn0032051   CG13097  8512169   8514864   -  2696  
   FBgn0032052   CG13089   8515098   8516756  +  1659  
   FBgn0032053   mRpL51  8516754   8517377   -  624  
   FBgn0032054   CG13090   8517446   8519180  +  1735  
 
 
    Segment 212 
 
   Location   
  Gene key  FBgn0032057-FBgn0032058  
  Heatmap region span   2L:8519690..8752305   
  Segment span   2L:8679782..8684350   
  Length (genes)  2  
  Length (bp)  4569  
   Model Scoring   
  BIC  178.795962  
  logL  -83.927364  
  logL ratio  40.771319  
   Expression   
  Mean expression  4.253029  
  Median expression  4.264681  
  Tissue std. dev.  0.150049  
 
  No GO Slim enrichment  
  
   tissue    mean expression   
  5th Passage Drosophila S2 Cells  4.281867  
  Adult Accessory gland  4.385276  
  Adult Brain  4.074768  
  Adult Carcass  4.159477  
  Adult Crop  4.187511  
  Adult Eye  4.171517  
  Adult Fatbody  4.196188  
  Adult Female Spermatheca Mated  4.256377  
  Adult Female Spermatheca Virgin  4.282525  
  Adult Head  4.151288  
  Adult Heart  4.219087  
  Adult Hind Gut  4.257661  
  Adult Male Ejaculatory Duct  4.432045  
  Adult Mid Gut  4.430366  
  Adult Ovary  4.269265  
  Adult Salivary Gland  4.572088  
  Adult Testes  4.031390  
  Adult Thoracoabdominal ganglion  4.131248  
  Adult Whole Fly  3.938590  
  Larvae Wandering Tubules  4.298754  
  Larval Feeding Carcass  4.376064  
  Larval Feeding Central Nevous System  4.098578  
  Larval Feeding Hind Gut  4.196481  
  Larval Feeding Malpighian Tubule  4.233848  
  Larval Feeding Mid Gut  4.563698  
  Larval Feeding Salivary Gland  4.467077  
  Whole Larvae Feeding  4.168739  
 
  
   FlyBase ID    symbol    start    end    strand    length   
   FBgn0032057   CG9287  8676848   8679782   -  2935  
   FBgn0032058   CG9289  8681403   8684350   -  2948  
 
    Segment 213 
 
   Location   
  Gene key  FBgn0004914-FBgn0003209  
  Heatmap region span   2L:8672043..8898804   
  Segment span   2L:8709038..8740660   
  Length (genes)  2  
  Length (bp)  31623  
   Model Scoring   
  BIC  234.557932  
  logL  -111.808349  
  logL ratio  53.772628  
   Expression   
  Mean expression  9.873294  
  Median expression  9.899118  
  Tissue std. dev.  0.953587  
 
  No GO Slim enrichment  
  
   tissue    mean expression   
  5th Passage Drosophila S2 Cells  9.520007  
  Adult Accessory gland  8.849937  
  Adult Brain  8.980794  
  Adult Carcass  10.942701  
  Adult Crop  11.629167  
  Adult Eye  9.317104  
  Adult Fatbody  11.409341  
  Adult Female Spermatheca Mated  10.975336  
  Adult Female Spermatheca Virgin  10.859880  
  Adult Head  10.399099  
  Adult Heart  11.092450  
  Adult Hind Gut  10.750697  
  Adult Male Ejaculatory Duct  10.301623  
  Adult Mid Gut  10.202230  
  Adult Ovary  10.071935  
  Adult Salivary Gland  9.067388  
  Adult Testes  7.592559  
  Adult Thoracoabdominal ganglion  8.930838  
  Adult Whole Fly  9.899563  
  Larvae Wandering Tubules  8.970563  
  Larval Feeding Carcass  10.323923  
  Larval Feeding Central Nevous System  8.480894  
  Larval Feeding Hind Gut  9.999038  
  Larval Feeding Malpighian Tubule  9.246455  
  Larval Feeding Mid Gut  10.002027  
  Larval Feeding Salivary Gland  9.258080  
  Whole Larvae Feeding  9.505314  
 
  
   FlyBase ID    symbol    start    end    strand    length   
   FBgn0004914   Hnf4  8687281   8709038   -  21758  
   FBgn0003209   raw  8709880   8740660   -  30781  
 
    Segment 214 
 
   Location   
  Gene key  FBgn0032066-FBgn0032069  
  Heatmap region span   2L:8685954..8958977   
  Segment span   2L:8768708..8781251   
  Length (genes)  4  
  Length (bp)  12544  
   Model Scoring   
  BIC  422.324257  
  logL  -205.691511  
  logL ratio  46.723820  
   Expression   
  Mean expression  5.331579  
  Median expression  4.328129  
  Tissue std. dev.  2.102037  
 
  
   GO ID    description    ratio    P-value   
   GO:0005764   lysosome  4/4  8.68e-12  
   GO:0005773   vacuole  4/4  8.68e-12  
   GO:0043226   organelle  4/4  0.00102  
   GO:0005575   cellular_component  4/4  0.0461  
 
  
   tissue    mean expression   
  5th Passage Drosophila S2 Cells  5.080060  
  Adult Accessory gland  4.492342  
  Adult Brain  4.119309  
  Adult Carcass  4.765019  
  Adult Crop  4.386159  
  Adult Eye  4.205667  
  Adult Fatbody  4.419282  
  Adult Female Spermatheca Mated  4.534548  
  Adult Female Spermatheca Virgin  4.486813  
  Adult Head  4.258160  
  Adult Heart  4.185499  
  Adult Hind Gut  4.398748  
  Adult Male Ejaculatory Duct  4.656346  
  Adult Mid Gut  11.915881  
  Adult Ovary  4.318463  
  Adult Salivary Gland  4.702413  
  Adult Testes  5.083177  
  Adult Thoracoabdominal ganglion  4.295138  
  Adult Whole Fly  8.287114  
  Larvae Wandering Tubules  4.498863  
  Larval Feeding Carcass  4.458390  
  Larval Feeding Central Nevous System  4.191457  
  Larval Feeding Hind Gut  4.377779  
  Larval Feeding Malpighian Tubule  4.852398  
  Larval Feeding Mid Gut  10.888543  
  Larval Feeding Salivary Gland  4.468956  
  Whole Larvae Feeding  9.626107  
 
  
   FlyBase ID    symbol    start    end    strand    length   
   FBgn0032066   CG9463  8765361   8768708   -  3348  
   FBgn0032067   CG9465  8769175   8772344   -  3170  
   FBgn0032068   CG9466  8772948   8776257   -  3310  
   FBgn0032069   CG9468  8777661   8781251   -  3591  
 
 
    Segment 215 
 
   Location   
  Gene key  FBgn0052986-FBgn0085427  
  Heatmap region span   2L:8709038..8974976   
  Segment span   2L:8862776..8898804   
  Length (genes)  6  
  Length (bp)  36029  
   Model Scoring   
  BIC  446.650347  
  logL  -217.854556  
  logL ratio  179.246001  
   Expression   
  Mean expression  4.746522  
  Median expression  4.575537  
  Tissue std. dev.  0.687631  
 
  No GO Slim enrichment  
  
   tissue    mean expression   
  5th Passage Drosophila S2 Cells  4.785906  
  Adult Accessory gland  4.658039  
  Adult Brain  4.416026  
  Adult Carcass  4.768185  
  Adult Crop  4.642075  
  Adult Eye  4.494896  
  Adult Fatbody  4.646147  
  Adult Female Spermatheca Mated  4.596068  
  Adult Female Spermatheca Virgin  4.655661  
  Adult Head  4.442276  
  Adult Heart  4.502066  
  Adult Hind Gut  4.637080  
  Adult Male Ejaculatory Duct  4.579049  
  Adult Mid Gut  4.780425  
  Adult Ovary  4.572332  
  Adult Salivary Gland  4.805215  
  Adult Testes  8.184180  
  Adult Thoracoabdominal ganglion  4.402577  
  Adult Whole Fly  4.973013  
  Larvae Wandering Tubules  4.649831  
  Larval Feeding Carcass  4.692146  
  Larval Feeding Central Nevous System  4.355920  
  Larval Feeding Hind Gut  4.501167  
  Larval Feeding Malpighian Tubule  4.660199  
  Larval Feeding Mid Gut  4.625566  
  Larval Feeding Salivary Gland  4.614509  
  Whole Larvae Feeding  4.515534  
 
  
   FlyBase ID    symbol    start    end    strand    length   
   FBgn0052986   CG32986   8862776   8863743  +  968  
   FBgn0052987   CG32987   8863811   8864847  +  1037  
   FBgn0052988   CG32988   8864864   8865692  +  829  
   FBgn0052983   CG32983   8865850   8866659  +  810  
   FBgn0032072   CG9483   8866767   8867686  +  920  
   FBgn0085427   CG34398   8898804   8933382  +  34579  
 
 
    Segment 216 
 
   Location   
  Gene key  FBgn0032074-FBgn0032075  
  Heatmap region span   2L:8752305..8980680   
  Segment span   2L:8933964..8937390   
  Length (genes)  2  
  Length (bp)  3427  
   Model Scoring   
  BIC  321.624475  
  logL  -155.341620  
  logL ratio  -4.349852  
   Expression   
  Mean expression  9.290816  
  Median expression  10.395834  
  Tissue std. dev.  2.940207  
 
  No GO Slim enrichment  
  
   tissue    mean expression   
  5th Passage Drosophila S2 Cells  5.612355  
  Adult Accessory gland  9.205125  
  Adult Brain  4.966285  
  Adult Carcass  7.685112  
  Adult Crop  9.432728  
  Adult Eye  7.707116  
  Adult Fatbody  6.456108  
  Adult Female Spermatheca Mated  10.700444  
  Adult Female Spermatheca Virgin  10.618767  
  Adult Head  8.895709  
  Adult Heart  6.592373  
  Adult Hind Gut  12.698619  
  Adult Male Ejaculatory Duct  9.465809  
  Adult Mid Gut  13.499655  
  Adult Ovary  5.575591  
  Adult Salivary Gland  12.595008  
  Adult Testes  6.691752  
  Adult Thoracoabdominal ganglion  5.568786  
  Adult Whole Fly  11.031258  
  Larvae Wandering Tubules  13.034217  
  Larval Feeding Carcass  5.826671  
  Larval Feeding Central Nevous System  4.801813  
  Larval Feeding Hind Gut  12.133382  
  Larval Feeding Malpighian Tubule  13.045210  
  Larval Feeding Mid Gut  13.506104  
  Larval Feeding Salivary Gland  11.086070  
  Whole Larvae Feeding  12.419963  
 
  
   FlyBase ID    symbol    start    end    strand    length   
   FBgn0032074   Tsp29Fa   8933964   8936096  +  2133  
   FBgn0032075   Tsp29Fb   8937390   8939746  +  2357  
 
    Segment 217 
 
   Location   
  Gene key  FBgn0032078-FBgn0032079  
  Heatmap region span   2L:8768708..8984870   
  Segment span   2L:8951200..8958977   
  Length (genes)  2  
  Length (bp)  7778  
   Model Scoring   
  BIC  266.166384  
  logL  -127.612575  
  logL ratio  7.471060  
   Expression   
  Mean expression  8.766038  
  Median expression  9.017634  
  Tissue std. dev.  1.346547  
 
  No GO Slim enrichment  
  
   tissue    mean expression   
  5th Passage Drosophila S2 Cells  11.366129  
  Adult Accessory gland  6.855069  
  Adult Brain  8.716306  
  Adult Carcass  8.943468  
  Adult Crop  7.684164  
  Adult Eye  8.058988  
  Adult Fatbody  10.073244  
  Adult Female Spermatheca Mated  10.138273  
  Adult Female Spermatheca Virgin  10.163937  
  Adult Head  8.531256  
  Adult Heart  9.910259  
  Adult Hind Gut  9.829801  
  Adult Male Ejaculatory Duct  8.066524  
  Adult Mid Gut  9.285227  
  Adult Ovary  9.580668  
  Adult Salivary Gland  6.990760  
  Adult Testes  6.121032  
  Adult Thoracoabdominal ganglion  8.547863  
  Adult Whole Fly  8.671112  
  Larvae Wandering Tubules  9.917134  
  Larval Feeding Carcass  6.675379  
  Larval Feeding Central Nevous System  8.340397  
  Larval Feeding Hind Gut  9.775022  
  Larval Feeding Malpighian Tubule  10.779898  
  Larval Feeding Mid Gut  9.117860  
  Larval Feeding Salivary Gland  6.368903  
  Whole Larvae Feeding  8.174356  
 
  
   FlyBase ID    symbol    start    end    strand    length   
   FBgn0032078   C1GalTA  8943454   8951200   -  7747  
   FBgn0032079   CG31886  8953481   8958977   -  5497  
 
    Segment 218 
 
   Location   
  Gene key  FBgn0032080-FBgn0052985  
  Heatmap region span   2L:8825625..8989287   
  Segment span   2L:8962354..8962617   
  Length (genes)  2  
  Length (bp)  264  
   Model Scoring   
  BIC  220.497824  
  logL  -104.778295  
  logL ratio  26.759562  
   Expression   
  Mean expression  4.282664  
  Median expression  3.772222  
  Tissue std. dev.  1.577121  
 
  No GO Slim enrichment  
  
   tissue    mean expression   
  5th Passage Drosophila S2 Cells  6.656356  
  Adult Accessory gland  11.528560  
  Adult Brain  3.635320  
  Adult Carcass  3.955405  
  Adult Crop  3.786415  
  Adult Eye  3.754551  
  Adult Fatbody  3.748512  
  Adult Female Spermatheca Mated  3.871393  
  Adult Female Spermatheca Virgin  3.852254  
  Adult Head  3.603453  
  Adult Heart  3.709562  
  Adult Hind Gut  3.700457  
  Adult Male Ejaculatory Duct  5.819951  
  Adult Mid Gut  3.779894  
  Adult Ovary  3.675643  
  Adult Salivary Gland  4.060344  
  Adult Testes  3.836696  
  Adult Thoracoabdominal ganglion  3.744344  
  Adult Whole Fly  4.898081  
  Larvae Wandering Tubules  3.753375  
  Larval Feeding Carcass  3.809538  
  Larval Feeding Central Nevous System  3.664713  
  Larval Feeding Hind Gut  3.710489  
  Larval Feeding Malpighian Tubule  3.798678  
  Larval Feeding Mid Gut  3.776206  
  Larval Feeding Salivary Gland  3.871758  
  Whole Larvae Feeding  3.629977  
 
  
   FlyBase ID    symbol    start    end    strand    length   
   FBgn0032080   CG9525  8960318   8962354   -  2037  
   FBgn0052985   CG32985   8962617   8964628  +  2012  
 
    Segment 219 
 
   Location   
  Gene key  FBgn0052984-FBgn0032083  
  Heatmap region span   2L:8862776..8994463   
  Segment span   2L:8965134..8974976   
  Length (genes)  3  
  Length (bp)  9843  
   Model Scoring   
  BIC  316.346353  
  logL  -152.702559  
  logL ratio  24.448070  
   Expression   
  Mean expression  4.768376  
  Median expression  4.430891  
  Tissue std. dev.  1.065462  
 
  No GO Slim enrichment  
  
   tissue    mean expression   
  5th Passage Drosophila S2 Cells  6.369169  
  Adult Accessory gland  5.229888  
  Adult Brain  4.362377  
  Adult Carcass  5.272275  
  Adult Crop  6.280996  
  Adult Eye  4.033166  
  Adult Fatbody  4.166018  
  Adult Female Spermatheca Mated  5.274025  
  Adult Female Spermatheca Virgin  4.918149  
  Adult Head  4.777247  
  Adult Heart  4.049571  
  Adult Hind Gut  4.232346  
  Adult Male Ejaculatory Duct  4.141524  
  Adult Mid Gut  4.713787  
  Adult Ovary  4.704363  
  Adult Salivary Gland  9.159341  
  Adult Testes  4.181214  
  Adult Thoracoabdominal ganglion  4.476930  
  Adult Whole Fly  4.592774  
  Larvae Wandering Tubules  4.296728  
  Larval Feeding Carcass  4.061300  
  Larval Feeding Central Nevous System  3.979365  
  Larval Feeding Hind Gut  4.031708  
  Larval Feeding Malpighian Tubule  4.093034  
  Larval Feeding Mid Gut  4.960304  
  Larval Feeding Salivary Gland  4.225134  
  Whole Larvae Feeding  4.163405  
 
  
   FlyBase ID    symbol    start    end    strand    length   
   FBgn0052984   CG32984   8965134   8967028  +  1895  
   FBgn0032082   CG18088   8967537   8970259  +  2723  
   FBgn0032083   CG9541  8970801   8974976   -  4176  
 
 
    Segment 220 
 
   Location   
  Gene key  FBgn0032085-FBgn0032086  
  Heatmap region span   2L:8965134..9164235   
  Segment span   2L:8991989..8994463   
  Length (genes)  2  
  Length (bp)  2475  
   Model Scoring   
  BIC  181.786624  
  logL  -85.422695  
  logL ratio  35.462171  
   Expression   
  Mean expression  4.923238  
  Median expression  4.762231  
  Tissue std. dev.  0.857130  
 
  No GO Slim enrichment  
  
   tissue    mean expression   
  5th Passage Drosophila S2 Cells  4.645862  
  Adult Accessory gland  4.774815  
  Adult Brain  4.284067  
  Adult Carcass  4.772272  
  Adult Crop  4.514665  
  Adult Eye  4.601637  
  Adult Fatbody  4.557975  
  Adult Female Spermatheca Mated  4.572146  
  Adult Female Spermatheca Virgin  4.675391  
  Adult Head  4.515963  
  Adult Heart  4.749429  
  Adult Hind Gut  5.321157  
  Adult Male Ejaculatory Duct  4.523027  
  Adult Mid Gut  8.159463  
  Adult Ovary  4.514155  
  Adult Salivary Gland  4.600363  
  Adult Testes  4.561222  
  Adult Thoracoabdominal ganglion  4.504006  
  Adult Whole Fly  4.660896  
  Larvae Wandering Tubules  5.037359  
  Larval Feeding Carcass  4.525023  
  Larval Feeding Central Nevous System  4.348698  
  Larval Feeding Hind Gut  6.298760  
  Larval Feeding Malpighian Tubule  4.809536  
  Larval Feeding Mid Gut  7.046727  
  Larval Feeding Salivary Gland  4.526947  
  Whole Larvae Feeding  4.825867  
 
  
   FlyBase ID    symbol    start    end    strand    length   
   FBgn0032085   CG9555   8991989   8993632  +  1644  
   FBgn0032086   CG17906   8994463   8995546  +  1084  
 
    Segment 221 
 
   Location   
  Gene key  FBgn0040964-FBgn0015316  
  Heatmap region span   2L:8984870..9166802   
  Segment span   2L:8997641..8998537   
  Length (genes)  2  
  Length (bp)  897  
   Model Scoring   
  BIC  237.141571  
  logL  -113.100168  
  logL ratio  -4.129464  
   Expression   
  Mean expression  5.905832  
  Median expression  5.810072  
  Tissue std. dev.  1.030748  
 
  No GO Slim enrichment  
  
   tissue    mean expression   
  5th Passage Drosophila S2 Cells  6.305193  
  Adult Accessory gland  5.133680  
  Adult Brain  7.426513  
  Adult Carcass  5.233849  
  Adult Crop  5.526087  
  Adult Eye  6.650688  
  Adult Fatbody  5.342938  
  Adult Female Spermatheca Mated  5.756562  
  Adult Female Spermatheca Virgin  5.631795  
  Adult Head  6.175465  
  Adult Heart  5.316026  
  Adult Hind Gut  7.790730  
  Adult Male Ejaculatory Duct  4.741993  
  Adult Mid Gut  8.315463  
  Adult Ovary  6.040938  
  Adult Salivary Gland  4.748192  
  Adult Testes  6.645892  
  Adult Thoracoabdominal ganglion  7.512543  
  Adult Whole Fly  6.285243  
  Larvae Wandering Tubules  5.114626  
  Larval Feeding Carcass  4.651392  
  Larval Feeding Central Nevous System  6.791240  
  Larval Feeding Hind Gut  5.074638  
  Larval Feeding Malpighian Tubule  4.493530  
  Larval Feeding Mid Gut  6.765322  
  Larval Feeding Salivary Gland  4.665964  
  Whole Larvae Feeding  5.320963  
 
  
   FlyBase ID    symbol    start    end    strand    length   
   FBgn0040964   CG18661   8997641   8998544  +  904  
   FBgn0015316   Try29F   8998537   8999468  +  932  
 
    Segment 222 
 
   Location   
  Gene key  FBgn0032087-FBgn0032088  
  Heatmap region span   2L:8989287..9251042   
  Segment span   2L:9010586..9012193   
  Length (genes)  2  
  Length (bp)  1608  
   Model Scoring   
  BIC  257.818699  
  logL  -123.438732  
  logL ratio  17.496795  
   Expression   
  Mean expression  7.206658  
  Median expression  5.898017  
  Tissue std. dev.  3.120150  
 
  No GO Slim enrichment  
  
   tissue    mean expression   
  5th Passage Drosophila S2 Cells  5.405782  
  Adult Accessory gland  5.389088  
  Adult Brain  4.832867  
  Adult Carcass  5.506188  
  Adult Crop  5.404272  
  Adult Eye  5.037432  
  Adult Fatbody  5.584627  
  Adult Female Spermatheca Mated  5.368410  
  Adult Female Spermatheca Virgin  5.322075  
  Adult Head  4.917417  
  Adult Heart  4.854344  
  Adult Hind Gut  11.121733  
  Adult Male Ejaculatory Duct  5.433371  
  Adult Mid Gut  13.502517  
  Adult Ovary  5.153716  
  Adult Salivary Gland  5.670979  
  Adult Testes  4.851519  
  Adult Thoracoabdominal ganglion  4.997165  
  Adult Whole Fly  11.230251  
  Larvae Wandering Tubules  10.448476  
  Larval Feeding Carcass  5.225668  
  Larval Feeding Central Nevous System  4.865001  
  Larval Feeding Hind Gut  11.721509  
  Larval Feeding Malpighian Tubule  11.412824  
  Larval Feeding Mid Gut  13.553104  
  Larval Feeding Salivary Gland  5.399878  
  Whole Larvae Feeding  12.369549  
 
  
   FlyBase ID    symbol    start    end    strand    length   
   FBgn0032087   CG9568   9010586   9011342  +  757  
   FBgn0032088   CG13102   9012193   9012890  +  698  
 
    Segment 223 
 
   Location   
  Gene key  FBgn0032089-FBgn0032100  
  Heatmap region span   2L:8991989..9253037   
  Segment span   2L:9014070..9164235   
  Length (genes)  7  
  Length (bp)  150166  
   Model Scoring   
  BIC  635.944791  
  logL  -312.501778  
  logL ratio  132.119748  
   Expression   
  Mean expression  4.787601  
  Median expression  4.554739  
  Tissue std. dev.  0.351082  
 
  No GO Slim enrichment  
  
   tissue    mean expression   
  5th Passage Drosophila S2 Cells  4.601419  
  Adult Accessory gland  4.692185  
  Adult Brain  5.324108  
  Adult Carcass  4.663602  
  Adult Crop  4.806752  
  Adult Eye  4.510011  
  Adult Fatbody  4.750843  
  Adult Female Spermatheca Mated  4.768881  
  Adult Female Spermatheca Virgin  4.745727  
  Adult Head  4.760042  
  Adult Heart  4.519498  
  Adult Hind Gut  4.741731  
  Adult Male Ejaculatory Duct  4.818197  
  Adult Mid Gut  4.656087  
  Adult Ovary  4.411964  
  Adult Salivary Gland  4.849218  
  Adult Testes  6.223166  
  Adult Thoracoabdominal ganglion  5.255481  
  Adult Whole Fly  4.490010  
  Larvae Wandering Tubules  4.615449  
  Larval Feeding Carcass  4.667812  
  Larval Feeding Central Nevous System  5.103159  
  Larval Feeding Hind Gut  4.844919  
  Larval Feeding Malpighian Tubule  4.708831  
  Larval Feeding Mid Gut  4.651623  
  Larval Feeding Salivary Gland  4.573905  
  Whole Larvae Feeding  4.510612  
 
  
   FlyBase ID    symbol    start    end    strand    length   
   FBgn0032089   Rcd-1r  9012885   9014070   -  1186  
   FBgn0032090   CG9582  9024450   9025404   -  955  
   FBgn0051708   CG31708  9051631   9071162   -  19532  
   FBgn0032094   CG12439   9077121   9077988  +  868  
   FBgn0032096   Or30a   9111814   9113285  +  1472  
   FBgn0052982   CG32982   9125751   9163721  +  37971  
   FBgn0032100   CG13108   9164235   9165426  +  1192  
 
 
    Segment 224 
 
   Location   
  Gene key  FBgn0032105-FBgn0010314  
  Heatmap region span   2L:9166543..9397988   
  Segment span   2L:9253116..9330767   
  Length (genes)  3  
  Length (bp)  77652  
   Model Scoring   
  BIC  349.834274  
  logL  -169.446520  
  logL ratio  -6.786891  
   Expression   
  Mean expression  5.997366  
  Median expression  5.333687  
  Tissue std. dev.  1.615428  
 
  No GO Slim enrichment  
  
   tissue    mean expression   
  5th Passage Drosophila S2 Cells  8.139066  
  Adult Accessory gland  4.665210  
  Adult Brain  4.787317  
  Adult Carcass  5.360671  
  Adult Crop  4.893176  
  Adult Eye  4.769590  
  Adult Fatbody  5.392758  
  Adult Female Spermatheca Mated  4.986582  
  Adult Female Spermatheca Virgin  5.304286  
  Adult Head  4.617383  
  Adult Heart  5.532099  
  Adult Hind Gut  4.794874  
  Adult Male Ejaculatory Duct  4.899968  
  Adult Mid Gut  5.061433  
  Adult Ovary  9.778129  
  Adult Salivary Gland  4.988423  
  Adult Testes  9.354164  
  Adult Thoracoabdominal ganglion  4.820893  
  Adult Whole Fly  8.759156  
  Larvae Wandering Tubules  5.385979  
  Larval Feeding Carcass  6.879443  
  Larval Feeding Central Nevous System  9.244663  
  Larval Feeding Hind Gut  6.219763  
  Larval Feeding Malpighian Tubule  4.998597  
  Larval Feeding Mid Gut  5.694658  
  Larval Feeding Salivary Gland  5.001022  
  Whole Larvae Feeding  7.599566  
 
  
   FlyBase ID    symbol    start    end    strand    length   
   FBgn0032105   borr  9251472   9253116   -  1645  
   FBgn0032109   CG17005   9327744   9330067  +  2324  
   FBgn0010314   Cks30A   9330767   9331981  +  1215  
 
 
    Segment 225 
 
   Location   
  Gene key  FBgn0032110-FBgn0085395  
  Heatmap region span   2L:9251042..9426860   
  Segment span   2L:9342832..9384845   
  Length (genes)  4  
  Length (bp)  42014  
   Model Scoring   
  BIC  339.663023  
  logL  -164.360894  
  logL ratio  90.041569  
   Expression   
  Mean expression  4.852217  
  Median expression  4.822325  
  Tissue std. dev.  0.849993  
 
  No GO Slim enrichment  
  
   tissue    mean expression   
  5th Passage Drosophila S2 Cells  4.624821  
  Adult Accessory gland  4.737848  
  Adult Brain  4.513579  
  Adult Carcass  4.711970  
  Adult Crop  4.583510  
  Adult Eye  4.559056  
  Adult Fatbody  4.693632  
  Adult Female Spermatheca Mated  4.700618  
  Adult Female Spermatheca Virgin  4.673420  
  Adult Head  4.508075  
  Adult Heart  4.583044  
  Adult Hind Gut  4.479386  
  Adult Male Ejaculatory Duct  4.868130  
  Adult Mid Gut  4.882231  
  Adult Ovary  4.605639  
  Adult Salivary Gland  4.821966  
  Adult Testes  8.969672  
  Adult Thoracoabdominal ganglion  4.463062  
  Adult Whole Fly  5.844073  
  Larvae Wandering Tubules  4.618249  
  Larval Feeding Carcass  4.690669  
  Larval Feeding Central Nevous System  4.379970  
  Larval Feeding Hind Gut  4.447313  
  Larval Feeding Malpighian Tubule  4.601589  
  Larval Feeding Mid Gut  4.743359  
  Larval Feeding Salivary Gland  4.700994  
  Whole Larvae Feeding  5.003985  
 
  
   FlyBase ID    symbol    start    end    strand    length   
   FBgn0032110   CG3748   9342832   9344491  +  1660  
   FBgn0032111   CG13110   9350470   9350996  +  527  
   FBgn0085210   CG34181   9367640   9368434  +  795  
   FBgn0085395   Shawl  9373025   9384845   -  11821  
 
 
    Segment 226 
 
   Location   
  Gene key  FBgn0032117-FBgn0011232  
  Heatmap region span   2L:9387389..9521214   
  Segment span   2L:9431029..9431403   
  Length (genes)  2  
  Length (bp)  375  
   Model Scoring   
  BIC  253.484293  
  logL  -121.271529  
  logL ratio  11.310731  
   Expression   
  Mean expression  8.011175  
  Median expression  7.711112  
  Tissue std. dev.  0.631822  
 
  No GO Slim enrichment  
  
   tissue    mean expression   
  5th Passage Drosophila S2 Cells  8.247442  
  Adult Accessory gland  8.499359  
  Adult Brain  7.666615  
  Adult Carcass  7.158311  
  Adult Crop  7.739126  
  Adult Eye  7.552412  
  Adult Fatbody  6.994082  
  Adult Female Spermatheca Mated  8.176983  
  Adult Female Spermatheca Virgin  8.049789  
  Adult Head  7.193934  
  Adult Heart  7.512701  
  Adult Hind Gut  7.656165  
  Adult Male Ejaculatory Duct  8.294421  
  Adult Mid Gut  7.424895  
  Adult Ovary  8.072220  
  Adult Salivary Gland  8.823317  
  Adult Testes  9.588407  
  Adult Thoracoabdominal ganglion  7.900435  
  Adult Whole Fly  8.098423  
  Larvae Wandering Tubules  8.566450  
  Larval Feeding Carcass  7.574032  
  Larval Feeding Central Nevous System  7.441080  
  Larval Feeding Hind Gut  7.969349  
  Larval Feeding Malpighian Tubule  8.408796  
  Larval Feeding Mid Gut  7.928681  
  Larval Feeding Salivary Gland  9.638703  
  Whole Larvae Feeding  8.125590  
 
  
   FlyBase ID    symbol    start    end    strand    length   
   FBgn0032117   FucTB  9428771   9431029   -  2259  
   FBgn0011232   scat   9431403   9435341  +  3939  
 
    Segment 227 
 
   Location   
  Gene key  FBgn0002973-FBgn0004868  
  Heatmap region span   2L:9416593..9540631   
  Segment span   2L:9437513..9494927   
  Length (genes)  2  
  Length (bp)  57415  
   Model Scoring   
  BIC  300.543263  
  logL  -144.801014  
  logL ratio  24.181161  
   Expression   
  Mean expression  10.755507  
  Median expression  11.314477  
  Tissue std. dev.  0.730619  
 
  No GO Slim enrichment  
  
   tissue    mean expression   
  5th Passage Drosophila S2 Cells  9.919854  
  Adult Accessory gland  10.370131  
  Adult Brain  10.477032  
  Adult Carcass  9.836455  
  Adult Crop  10.836354  
  Adult Eye  11.199419  
  Adult Fatbody  9.328867  
  Adult Female Spermatheca Mated  10.172548  
  Adult Female Spermatheca Virgin  10.024208  
  Adult Head  10.446241  
  Adult Heart  10.815347  
  Adult Hind Gut  11.218084  
  Adult Male Ejaculatory Duct  10.974184  
  Adult Mid Gut  10.733062  
  Adult Ovary  11.865870  
  Adult Salivary Gland  10.789561  
  Adult Testes  9.453895  
  Adult Thoracoabdominal ganglion  10.130194  
  Adult Whole Fly  10.833146  
  Larvae Wandering Tubules  12.555309  
  Larval Feeding Carcass  11.057949  
  Larval Feeding Central Nevous System  11.328695  
  Larval Feeding Hind Gut  11.582844  
  Larval Feeding Malpighian Tubule  11.943055  
  Larval Feeding Mid Gut  10.922752  
  Larval Feeding Salivary Gland  10.963048  
  Whole Larvae Feeding  10.620584  
 
  
   FlyBase ID    symbol    start    end    strand    length   
   FBgn0002973   numb   9437513   9463293  +  25781  
   FBgn0004868   Gdi  9491918   9494927   -  3010  
 
    Segment 228 
 
   Location   
  Gene key  FBgn0032125-FBgn0032129  
  Heatmap region span   2L:9495472..9634201   
  Segment span   2L:9542878..9566750   
  Length (genes)  4  
  Length (bp)  23873  
   Model Scoring   
  BIC  398.955647  
  logL  -194.007206  
  logL ratio  32.642227  
   Expression   
  Mean expression  5.152588  
  Median expression  4.951083  
  Tissue std. dev.  0.623478  
 
  No GO Slim enrichment  
  
   tissue    mean expression   
  5th Passage Drosophila S2 Cells  4.880438  
  Adult Accessory gland  4.789551  
  Adult Brain  5.553437  
  Adult Carcass  5.839043  
  Adult Crop  5.769719  
  Adult Eye  4.654159  
  Adult Fatbody  4.823415  
  Adult Female Spermatheca Mated  5.033786  
  Adult Female Spermatheca Virgin  4.728985  
  Adult Head  5.134014  
  Adult Heart  5.244787  
  Adult Hind Gut  5.351678  
  Adult Male Ejaculatory Duct  5.040450  
  Adult Mid Gut  4.895941  
  Adult Ovary  7.217714  
  Adult Salivary Gland  4.958131  
  Adult Testes  4.355940  
  Adult Thoracoabdominal ganglion  5.545670  
  Adult Whole Fly  6.623927  
  Larvae Wandering Tubules  4.704655  
  Larval Feeding Carcass  5.458338  
  Larval Feeding Central Nevous System  4.640646  
  Larval Feeding Hind Gut  5.006495  
  Larval Feeding Malpighian Tubule  4.649113  
  Larval Feeding Mid Gut  4.711327  
  Larval Feeding Salivary Gland  4.749886  
  Whole Larvae Feeding  4.758635  
 
  
   FlyBase ID    symbol    start    end    strand    length   
   FBgn0032125   Cpr30B   9542878   9543478  +  601  
   FBgn0032126   CG13113   9544175   9544792  +  618  
   FBgn0032127   CG13114   9545811   9547424  +  1614  
   FBgn0032129   jp  9547720   9566750   -  19031  
 
 
    Segment 229 
 
   Location   
  Gene key  FBgn0051709-FBgn0051882  
  Heatmap region span   2L:9540631..9711269   
  Segment span   2L:9579638..9625285   
  Length (genes)  5  
  Length (bp)  45648  
   Model Scoring   
  BIC  468.064723  
  logL  -228.561744  
  logL ratio  75.121073  
   Expression   
  Mean expression  4.598224  
  Median expression  4.330281  
  Tissue std. dev.  0.729650  
 
  
   GO ID    description    ratio    P-value   
   GO:0008283   cell proliferation  2/5  0.000628  
   GO:0008150   biological_process  4/5  0.0264  
   GO:0003674   molecular_function  4/5  0.0409  
 
  
   tissue    mean expression   
  5th Passage Drosophila S2 Cells  4.263490  
  Adult Accessory gland  4.412429  
  Adult Brain  4.263148  
  Adult Carcass  4.356852  
  Adult Crop  4.278620  
  Adult Eye  4.218498  
  Adult Fatbody  4.558715  
  Adult Female Spermatheca Mated  4.610835  
  Adult Female Spermatheca Virgin  4.685180  
  Adult Head  4.110197  
  Adult Heart  4.348110  
  Adult Hind Gut  4.207257  
  Adult Male Ejaculatory Duct  4.364369  
  Adult Mid Gut  4.333077  
  Adult Ovary  4.220892  
  Adult Salivary Gland  4.567406  
  Adult Testes  7.750894  
  Adult Thoracoabdominal ganglion  4.138264  
  Adult Whole Fly  5.793245  
  Larvae Wandering Tubules  4.369999  
  Larval Feeding Carcass  4.539158  
  Larval Feeding Central Nevous System  5.421915  
  Larval Feeding Hind Gut  4.190621  
  Larval Feeding Malpighian Tubule  4.380540  
  Larval Feeding Mid Gut  4.243455  
  Larval Feeding Salivary Gland  4.330276  
  Whole Larvae Feeding  5.194616  
 
  
   FlyBase ID    symbol    start    end    strand    length   
   FBgn0051709   CG31709  9578536   9579638   -  1103  
   FBgn0014179   gcm  9579449   9581742   -  2294  
   FBgn0032132   CG4382  9598940   9600853   -  1914  
   FBgn0019809   gcm2   9608479   9612710  +  4232  
   FBgn0051882   CG31882  9624452   9625285   -  834  
 
 
    Segment 230 
 
   Location   
  Gene key  FBgn0011207-FBgn0051710  
  Heatmap region span   2L:9576768..9763345   
  Segment span   2L:9700796..9705944   
  Length (genes)  2  
  Length (bp)  5149  
   Model Scoring   
  BIC  251.945001  
  logL  -120.501883  
  logL ratio  5.949296  
   Expression   
  Mean expression  8.024276  
  Median expression  8.209238  
  Tissue std. dev.  0.501661  
 
  No GO Slim enrichment  
  
   tissue    mean expression   
  5th Passage Drosophila S2 Cells  8.214873  
  Adult Accessory gland  8.053127  
  Adult Brain  7.949963  
  Adult Carcass  7.809499  
  Adult Crop  7.749116  
  Adult Eye  8.814259  
  Adult Fatbody  7.538423  
  Adult Female Spermatheca Mated  7.275018  
  Adult Female Spermatheca Virgin  7.152994  
  Adult Head  8.344442  
  Adult Heart  8.186203  
  Adult Hind Gut  7.694865  
  Adult Male Ejaculatory Duct  7.477113  
  Adult Mid Gut  7.927220  
  Adult Ovary  8.103435  
  Adult Salivary Gland  8.048666  
  Adult Testes  7.615593  
  Adult Thoracoabdominal ganglion  7.941656  
  Adult Whole Fly  7.539032  
  Larvae Wandering Tubules  8.038453  
  Larval Feeding Carcass  8.588482  
  Larval Feeding Central Nevous System  7.821927  
  Larval Feeding Hind Gut  8.343651  
  Larval Feeding Malpighian Tubule  8.706104  
  Larval Feeding Mid Gut  8.333623  
  Larval Feeding Salivary Gland  9.579521  
  Whole Larvae Feeding  7.808196  
 
  
   FlyBase ID    symbol    start    end    strand    length   
   FBgn0011207   pelo   9700796   9708883  +  8088  
   FBgn0051710   CG31710  9702964   9705944   -  2981  
 
    Segment 231 
 
   Location   
  Gene key  FBgn0032140-FBgn0022720  
  Heatmap region span   2L:9656600..9773044   
  Segment span   2L:9736645..9758338   
  Length (genes)  4  
  Length (bp)  21694  
   Model Scoring   
  BIC  577.492100  
  logL  -283.275433  
  logL ratio  -80.785021  
   Expression   
  Mean expression  7.662238  
  Median expression  7.812786  
  Tissue std. dev.  0.650025  
 
  No GO Slim enrichment  
  
   tissue    mean expression   
  5th Passage Drosophila S2 Cells  8.217068  
  Adult Accessory gland  7.780713  
  Adult Brain  7.721230  
  Adult Carcass  8.077142  
  Adult Crop  7.735841  
  Adult Eye  9.363969  
  Adult Fatbody  7.999290  
  Adult Female Spermatheca Mated  7.236747  
  Adult Female Spermatheca Virgin  7.435369  
  Adult Head  9.143747  
  Adult Heart  8.147616  
  Adult Hind Gut  7.498368  
  Adult Male Ejaculatory Duct  6.862672  
  Adult Mid Gut  7.102448  
  Adult Ovary  7.076989  
  Adult Salivary Gland  6.959273  
  Adult Testes  6.599691  
  Adult Thoracoabdominal ganglion  7.633197  
  Adult Whole Fly  7.396261  
  Larvae Wandering Tubules  7.779257  
  Larval Feeding Carcass  8.130027  
  Larval Feeding Central Nevous System  7.716897  
  Larval Feeding Hind Gut  7.254607  
  Larval Feeding Malpighian Tubule  8.391751  
  Larval Feeding Mid Gut  6.908849  
  Larval Feeding Salivary Gland  6.842473  
  Whole Larvae Feeding  7.868932  
 
  
   FlyBase ID    symbol    start    end    strand    length   
   FBgn0032140   CG13117   9736645   9737329  +  685  
   FBgn0028704   Nckx30C  9712583   9746483   -  33901  
   FBgn0015035   Cyp4e3  9747839   9750071   -  2233  
   FBgn0022720   zf30C   9758338   9762377  +  4040  
 
 
    Segment 232 
 
   Location   
  Gene key  FBgn0032142-FBgn0032143  
  Heatmap region span   2L:9711269..9790745   
  Segment span   2L:9766912..9767059   
  Length (genes)  2  
  Length (bp)  148  
   Model Scoring   
  BIC  175.531500  
  logL  -82.295133  
  logL ratio  37.341385  
   Expression   
  Mean expression  4.563322  
  Median expression  4.516497  
  Tissue std. dev.  0.146100  
 
  No GO Slim enrichment  
  
   tissue    mean expression   
  5th Passage Drosophila S2 Cells  4.615751  
  Adult Accessory gland  4.802981  
  Adult Brain  4.380567  
  Adult Carcass  4.653433  
  Adult Crop  4.560853  
  Adult Eye  4.616596  
  Adult Fatbody  4.551210  
  Adult Female Spermatheca Mated  4.555355  
  Adult Female Spermatheca Virgin  4.590686  
  Adult Head  4.416339  
  Adult Heart  4.606663  
  Adult Hind Gut  4.602790  
  Adult Male Ejaculatory Duct  4.770078  
  Adult Mid Gut  4.823935  
  Adult Ovary  4.684189  
  Adult Salivary Gland  4.715051  
  Adult Testes  4.433842  
  Adult Thoracoabdominal ganglion  4.397814  
  Adult Whole Fly  4.230279  
  Larvae Wandering Tubules  4.712203  
  Larval Feeding Carcass  4.632867  
  Larval Feeding Central Nevous System  4.273883  
  Larval Feeding Hind Gut  4.491773  
  Larval Feeding Malpighian Tubule  4.566321  
  Larval Feeding Mid Gut  4.547318  
  Larval Feeding Salivary Gland  4.575153  
  Whole Larvae Feeding  4.401774  
 
  
   FlyBase ID    symbol    start    end    strand    length   
   FBgn0032142     9765357   9766912   -  1556  
   FBgn0032143   CG4017   9767059   9768729  +  1671  
 
    Segment 233 
 
   Location   
  Gene key  FBgn0032145-FBgn0065108  
  Heatmap region span   2L:9736645..9888034   
  Segment span   2L:9770996..9773044   
  Length (genes)  2  
  Length (bp)  2049  
   Model Scoring   
  BIC  210.894333  
  logL  -99.976549  
  logL ratio  15.049838  
   Expression   
  Mean expression  5.181048  
  Median expression  5.006106  
  Tissue std. dev.  0.218119  
 
  No GO Slim enrichment  
  
   tissue    mean expression   
  5th Passage Drosophila S2 Cells  5.373147  
  Adult Accessory gland  5.261829  
  Adult Brain  4.822293  
  Adult Carcass  5.455632  
  Adult Crop  5.281477  
  Adult Eye  5.130646  
  Adult Fatbody  5.183872  
  Adult Female Spermatheca Mated  5.164807  
  Adult Female Spermatheca Virgin  5.171530  
  Adult Head  5.046389  
  Adult Heart  4.966164  
  Adult Hind Gut  5.215091  
  Adult Male Ejaculatory Duct  5.350400  
  Adult Mid Gut  5.521178  
  Adult Ovary  5.112686  
  Adult Salivary Gland  5.623390  
  Adult Testes  5.022914  
  Adult Thoracoabdominal ganglion  5.149451  
  Adult Whole Fly  4.638229  
  Larvae Wandering Tubules  5.413864  
  Larval Feeding Carcass  4.991102  
  Larval Feeding Central Nevous System  4.923301  
  Larval Feeding Hind Gut  5.054303  
  Larval Feeding Malpighian Tubule  5.285222  
  Larval Feeding Mid Gut  5.334572  
  Larval Feeding Salivary Gland  5.404117  
  Whole Larvae Feeding  4.990678  
 
  
   FlyBase ID    symbol    start    end    strand    length   
   FBgn0032145     9770395   9770996   -  602  
   FBgn0065108   ppk16  9771155   9773044   -  1890  
 
    Segment 234 
 
   Location   
  Gene key  FBgn0032147-FBgn0032149  
  Heatmap region span   2L:9763345..9895289   
  Segment span   2L:9782390..9790667   
  Length (genes)  2  
  Length (bp)  8278  
   Model Scoring   
  BIC  230.425612  
  logL  -109.742189  
  logL ratio  27.398775  
   Expression   
  Mean expression  8.016282  
  Median expression  8.227498  
  Tissue std. dev.  0.611988  
 
  No GO Slim enrichment  
  
   tissue    mean expression   
  5th Passage Drosophila S2 Cells  8.057859  
  Adult Accessory gland  8.981990  
  Adult Brain  7.045131  
  Adult Carcass  7.581448  
  Adult Crop  8.228658  
  Adult Eye  8.746036  
  Adult Fatbody  8.241969  
  Adult Female Spermatheca Mated  8.568102  
  Adult Female Spermatheca Virgin  8.489705  
  Adult Head  7.488747  
  Adult Heart  8.908238  
  Adult Hind Gut  7.651834  
  Adult Male Ejaculatory Duct  8.268735  
  Adult Mid Gut  7.936685  
  Adult Ovary  7.790362  
  Adult Salivary Gland  8.506362  
  Adult Testes  6.863206  
  Adult Thoracoabdominal ganglion  7.215810  
  Adult Whole Fly  7.277182  
  Larvae Wandering Tubules  8.837080  
  Larval Feeding Carcass  7.749512  
  Larval Feeding Central Nevous System  7.540181  
  Larval Feeding Hind Gut  7.727670  
  Larval Feeding Malpighian Tubule  8.795523  
  Larval Feeding Mid Gut  7.536760  
  Larval Feeding Salivary Gland  8.851514  
  Whole Larvae Feeding  7.553328  
 
  
   FlyBase ID    symbol    start    end    strand    length   
   FBgn0032147   IP3K1   9782390   9788499  +  6110  
   FBgn0032149   CG4036   9790667   9791958  +  1292  
 
    Segment 235 
 
   Location   
  Gene key  FBgn0032153-FBgn0010520  
  Heatmap region span   2L:9782390..9912585   
  Segment span   2L:9890885..9895289   
  Length (genes)  3  
  Length (bp)  4405  
   Model Scoring   
  BIC  347.789864  
  logL  -168.424315  
  logL ratio  21.192420  
   Expression   
  Mean expression  7.078149  
  Median expression  6.827885  
  Tissue std. dev.  0.527358  
 
  No GO Slim enrichment  
  
   tissue    mean expression   
  5th Passage Drosophila S2 Cells  7.550859  
  Adult Accessory gland  7.821848  
  Adult Brain  6.572664  
  Adult Carcass  6.739080  
  Adult Crop  6.882691  
  Adult Eye  6.745245  
  Adult Fatbody  7.279663  
  Adult Female Spermatheca Mated  7.401776  
  Adult Female Spermatheca Virgin  7.113225  
  Adult Head  6.615906  
  Adult Heart  7.419025  
  Adult Hind Gut  6.821668  
  Adult Male Ejaculatory Duct  7.202676  
  Adult Mid Gut  6.986371  
  Adult Ovary  8.732899  
  Adult Salivary Gland  7.267929  
  Adult Testes  6.123687  
  Adult Thoracoabdominal ganglion  6.641250  
  Adult Whole Fly  7.506258  
  Larvae Wandering Tubules  6.739095  
  Larval Feeding Carcass  6.859080  
  Larval Feeding Central Nevous System  7.802273  
  Larval Feeding Hind Gut  6.935068  
  Larval Feeding Malpighian Tubule  6.897182  
  Larval Feeding Mid Gut  6.515518  
  Larval Feeding Salivary Gland  7.471629  
  Whole Larvae Feeding  6.465453  
 
  
   FlyBase ID    symbol    start    end    strand    length   
   FBgn0032153   CG4537   9890885   9891336  +  452  
   FBgn0032154   mtDNA-helicase  9891255   9893419   -  2165  
   FBgn0010520   Bka   9895289   9896104  +  816  
 
 
    Segment 236 
 
   Location   
  Gene key  FBgn0024285-FBgn0032160  
  Heatmap region span   2L:9897542..9963767   
  Segment span   2L:9912907..9918636   
  Length (genes)  4  
  Length (bp)  5730  
   Model Scoring   
  BIC  472.708383  
  logL  -230.883574  
  logL ratio  161.478816  
   Expression   
  Mean expression  10.779297  
  Median expression  10.770999  
  Tissue std. dev.  0.618482  
 
  
   GO ID    description    ratio    P-value   
   GO:0005739   mitochondrion  2/4  0.00656  
   GO:0005811   lipid particle  2/4  0.0158  
 
  
   tissue    mean expression   
  5th Passage Drosophila S2 Cells  11.247443  
  Adult Accessory gland  10.262388  
  Adult Brain  9.403432  
  Adult Carcass  10.823839  
  Adult Crop  11.041052  
  Adult Eye  10.466636  
  Adult Fatbody  11.541981  
  Adult Female Spermatheca Mated  11.323924  
  Adult Female Spermatheca Virgin  11.314276  
  Adult Head  10.798953  
  Adult Heart  11.061912  
  Adult Hind Gut  10.814772  
  Adult Male Ejaculatory Duct  11.189748  
  Adult Mid Gut  10.932575  
  Adult Ovary  10.989748  
  Adult Salivary Gland  10.353853  
  Adult Testes  8.807647  
  Adult Thoracoabdominal ganglion  9.738116  
  Adult Whole Fly  10.668255  
  Larvae Wandering Tubules  11.021491  
  Larval Feeding Carcass  10.991363  
  Larval Feeding Central Nevous System  10.751596  
  Larval Feeding Hind Gut  10.891110  
  Larval Feeding Malpighian Tubule  10.983126  
  Larval Feeding Mid Gut  10.834708  
  Larval Feeding Salivary Gland  11.723112  
  Whole Larvae Feeding  11.063975  
 
  
   FlyBase ID    symbol    start    end    strand    length   
   FBgn0024285   Srp54   9912907   9915044  +  2138  
   FBgn0040064   yip2   9915255   9917221  +  1967  
   FBgn0025700   CG5885  9917221   9918165   -  945  
   FBgn0032160   CG4598   9918636   9919901  +  1266  
 
 
    Segment 237 
 
   Location   
  Gene key  FBgn0032161-FBgn0032162  
  Heatmap region span   2L:9903258..9967312   
  Segment span   2L:9920161..9921639   
  Length (genes)  2  
  Length (bp)  1479  
   Model Scoring   
  BIC  234.410708  
  logL  -111.734737  
  logL ratio  37.522375  
   Expression   
  Mean expression  8.739422  
  Median expression  8.546539  
  Tissue std. dev.  1.291766  
 
  
   GO ID    description    ratio    P-value   
   GO:0005739   mitochondrion  2/2  0.000416  
   GO:0043226   organelle  2/2  0.0483  
 
  
   tissue    mean expression   
  5th Passage Drosophila S2 Cells  7.350409  
  Adult Accessory gland  7.862009  
  Adult Brain  6.134759  
  Adult Carcass  9.719572  
  Adult Crop  8.926781  
  Adult Eye  7.883459  
  Adult Fatbody  9.970563  
  Adult Female Spermatheca Mated  8.454831  
  Adult Female Spermatheca Virgin  8.355168  
  Adult Head  7.994905  
  Adult Heart  10.358197  
  Adult Hind Gut  9.445673  
  Adult Male Ejaculatory Duct  8.799514  
  Adult Mid Gut  8.478472  
  Adult Ovary  8.740273  
  Adult Salivary Gland  10.766996  
  Adult Testes  6.580424  
  Adult Thoracoabdominal ganglion  6.348357  
  Adult Whole Fly  8.443939  
  Larvae Wandering Tubules  9.472900  
  Larval Feeding Carcass  9.034000  
  Larval Feeding Central Nevous System  7.489254  
  Larval Feeding Hind Gut  10.260651  
  Larval Feeding Malpighian Tubule  10.817132  
  Larval Feeding Mid Gut  8.335346  
  Larval Feeding Salivary Gland  10.909233  
  Whole Larvae Feeding  9.031583  
 
  
   FlyBase ID    symbol    start    end    strand    length   
   FBgn0032161   CG4594   9920161   9921459  +  1299  
   FBgn0032162   CG4592   9921639   9922766  +  1128  
 
    Segment 238 
 
   Location   
  Gene key  FBgn0032163-FBgn0259713  
  Heatmap region span   2L:9908649..9967455   
  Segment span   2L:9924860..9930446   
  Length (genes)  3  
  Length (bp)  5587  
   Model Scoring   
  BIC  261.225039  
  logL  -125.141902  
  logL ratio  59.590799  
   Expression   
  Mean expression  4.893078  
  Median expression  5.070788  
  Tissue std. dev.  0.264714  
 
  No GO Slim enrichment  
  
   tissue    mean expression   
  5th Passage Drosophila S2 Cells  4.883322  
  Adult Accessory gland  4.870853  
  Adult Brain  4.422432  
  Adult Carcass  4.846255  
  Adult Crop  4.769724  
  Adult Eye  5.471792  
  Adult Fatbody  5.030810  
  Adult Female Spermatheca Mated  4.818947  
  Adult Female Spermatheca Virgin  4.897081  
  Adult Head  4.881648  
  Adult Heart  4.959431  
  Adult Hind Gut  4.775708  
  Adult Male Ejaculatory Duct  4.810618  
  Adult Mid Gut  5.070224  
  Adult Ovary  4.853515  
  Adult Salivary Gland  5.089756  
  Adult Testes  5.772104  
  Adult Thoracoabdominal ganglion  4.562223  
  Adult Whole Fly  4.565054  
  Larvae Wandering Tubules  4.958832  
  Larval Feeding Carcass  4.841903  
  Larval Feeding Central Nevous System  4.568518  
  Larval Feeding Hind Gut  4.800061  
  Larval Feeding Malpighian Tubule  5.023484  
  Larval Feeding Mid Gut  4.946692  
  Larval Feeding Salivary Gland  4.972831  
  Whole Larvae Feeding  4.649294  
 
  
   FlyBase ID    symbol    start    end    strand    length   
   FBgn0032163   TbCMF46  9922737   9924860   -  2124  
   FBgn0259712   CG42366   9926613   9930378  +  3766  
   FBgn0259713   CG42367   9930446   9930999  +  554  
 
 
    Segment 239 
 
   Location   
  Gene key  FBgn0051876-FBgn0032171  
  Heatmap region span   2L:9912585..9984657   
  Segment span   2L:9933092..9957019   
  Length (genes)  8  
  Length (bp)  23928  
   Model Scoring   
  BIC  883.946463  
  logL  -436.502614  
  logL ratio  118.227556  
   Expression   
  Mean expression  7.756439  
  Median expression  7.667955  
  Tissue std. dev.  0.352538  
 
  No GO Slim enrichment  
  
   tissue    mean expression   
  5th Passage Drosophila S2 Cells  7.627718  
  Adult Accessory gland  7.675370  
  Adult Brain  7.520456  
  Adult Carcass  7.582846  
  Adult Crop  8.034306  
  Adult Eye  8.087465  
  Adult Fatbody  7.999994  
  Adult Female Spermatheca Mated  8.038024  
  Adult Female Spermatheca Virgin  8.108045  
  Adult Head  7.585937  
  Adult Heart  8.107691  
  Adult Hind Gut  8.101245  
  Adult Male Ejaculatory Duct  7.663584  
  Adult Mid Gut  7.863117  
  Adult Ovary  7.944634  
  Adult Salivary Gland  7.619064  
  Adult Testes  6.621974  
  Adult Thoracoabdominal ganglion  7.601840  
  Adult Whole Fly  7.313233  
  Larvae Wandering Tubules  8.376320  
  Larval Feeding Carcass  7.266981  
  Larval Feeding Central Nevous System  7.510215  
  Larval Feeding Hind Gut  7.875382  
  Larval Feeding Malpighian Tubule  8.117274  
  Larval Feeding Mid Gut  7.824432  
  Larval Feeding Salivary Gland  7.900918  
  Whole Larvae Feeding  7.455792  
 
  
   FlyBase ID    symbol    start    end    strand    length   
   FBgn0051876   Cpr30F  9932587   9933092   -  506  
   FBgn0051712   CG31712  9934767   9938142   -  3376  
   FBgn0051713   Apf  9938170   9938787   -  618  
   FBgn0032166   CG4619   9938865   9941099  +  2235  
   FBgn0032167   CG5853  9940769   9947442   -  6674  
   FBgn0032168   CG13126  9952428   9954180   -  1753  
   FBgn0032169   CG4709   9954449   9956179  +  1731  
   FBgn0032171   CG5846  9956148   9957019   -  872  
 
 
    Segment 240 
 
   Location   
  Gene key  FBgn0032170-FBgn0032172  
  Heatmap region span   2L:9912907..10002446   
  Segment span   2L:9957287..9963767   
  Length (genes)  2  
  Length (bp)  6481  
   Model Scoring   
  BIC  229.909933  
  logL  -109.484349  
  logL ratio  48.662784  
   Expression   
  Mean expression  9.654233  
  Median expression  9.660898  
  Tissue std. dev.  0.615758  
 
  No GO Slim enrichment  
  
   tissue    mean expression
[truncated: 374,196 more chars]
