# Supplementary material for: Expression-based segmentation of the Drosophila genome
Source: BMC Genomics. 2013 Nov 20;14:812. doi: 10.1186/1471-2164-14-812 (PMC3909303; doi:10.1186/1471-2164-14-812)
Supplement: Additional file 1 — Detailed information for multigene segments. [file 1471-2164-14-812-S1.zip › miniwebsite/chr2R.html]

   ExprSeg Report for chr2R   
 Report for /Users/afrubin/Code/ExprSeg/2012-07-19/output Generated Fri Jun 21 23:01:11 2013 
   Chromosome 2R 
 466 segments 2312 genes 
    Segment 1 
 
   Location   
  Gene key  FBgn0046692-FBgn0040005  
  Heatmap region span   2R:403961..957715   
  Segment span   2R:434209..559362   
  Length (genes)  3  
  Length (bp)  125154  
   Model Scoring   
  BIC  306.290661  
  logL  -147.624478  
  logL ratio  102.255842  
   Expression   
  Mean expression  9.697515  
  Median expression  9.840835  
  Tissue std. dev.  0.428804  
 
  No GO Slim enrichment  
  
   tissue    mean expression   
  5th Passage Drosophila S2 Cells  9.840045  
  Adult Accessory gland  10.162218  
  Adult Brain  10.384586  
  Adult Carcass  9.077002  
  Adult Crop  9.640533  
  Adult Eye  10.076167  
  Adult Fatbody  9.664332  
  Adult Female Spermatheca Mated  9.531205  
  Adult Female Spermatheca Virgin  9.557190  
  Adult Head  9.638897  
  Adult Heart  9.605873  
  Adult Hind Gut  9.372229  
  Adult Male Ejaculatory Duct  9.822764  
  Adult Mid Gut  9.344036  
  Adult Ovary  10.566725  
  Adult Salivary Gland  9.948405  
  Adult Testes  8.491153  
  Adult Thoracoabdominal ganglion  10.285242  
  Adult Whole Fly  9.635076  
  Larvae Wandering Tubules  10.021863  
  Larval Feeding Carcass  9.393538  
  Larval Feeding Central Nevous System  10.011992  
  Larval Feeding Hind Gut  9.562759  
  Larval Feeding Malpighian Tubule  9.812227  
  Larval Feeding Mid Gut  9.368379  
  Larval Feeding Salivary Gland  9.888513  
  Whole Larvae Feeding  9.129965  
 
  
   FlyBase ID    symbol    start    end    strand    length   
   FBgn0046692   Stlk   434209   452786  +  18578  
   FBgn0032997   CG17486   506534   508338  +  1805  
   FBgn0040005   CG17883   559362   561145  +  1784  
 
 
    Segment 2 
 
   Location   
  Gene key  FBgn0039994-FBgn0033000  
  Heatmap region span   2R:403961..1237998   
  Segment span   2R:631836..716725   
  Length (genes)  3  
  Length (bp)  84890  
   Model Scoring   
  BIC  326.872030  
  logL  -157.915163  
  logL ratio  78.886824  
   Expression   
  Mean expression  9.405560  
  Median expression  9.488173  
  Tissue std. dev.  0.661550  
 
  No GO Slim enrichment  
  
   tissue    mean expression   
  5th Passage Drosophila S2 Cells  8.757556  
  Adult Accessory gland  9.306221  
  Adult Brain  10.589880  
  Adult Carcass  8.560320  
  Adult Crop  9.483823  
  Adult Eye  9.525061  
  Adult Fatbody  8.902639  
  Adult Female Spermatheca Mated  9.061632  
  Adult Female Spermatheca Virgin  9.062218  
  Adult Head  9.453989  
  Adult Heart  9.201167  
  Adult Hind Gut  9.191905  
  Adult Male Ejaculatory Duct  8.905062  
  Adult Mid Gut  9.068945  
  Adult Ovary  11.215471  
  Adult Salivary Gland  9.306878  
  Adult Testes  7.991839  
  Adult Thoracoabdominal ganglion  10.346864  
  Adult Whole Fly  9.980657  
  Larvae Wandering Tubules  9.642458  
  Larval Feeding Carcass  9.474332  
  Larval Feeding Central Nevous System  10.575673  
  Larval Feeding Hind Gut  9.411695  
  Larval Feeding Malpighian Tubule  9.431110  
  Larval Feeding Mid Gut  8.933407  
  Larval Feeding Salivary Gland  9.532457  
  Whole Larvae Feeding  9.036861  
 
  
   FlyBase ID    symbol    start    end    strand    length   
   FBgn0039994   CG17082   631836   645548  +  13713  
   FBgn0250830   CG12547  673803   676143   -  2341  
   FBgn0033000   CG14464   716725   717440  +  716  
 
 
    Segment 3 
 
   Location   
  Gene key  FBgn0033005-FBgn0033017  
  Heatmap region span   2R:619068..1250608   
  Segment span   2R:978092..1228496   
  Length (genes)  5  
  Length (bp)  250405  
   Model Scoring   
  BIC  452.421401  
  logL  -220.689848  
  logL ratio  250.305369  
   Expression   
  Mean expression  10.109358  
  Median expression  10.191268  
  Tissue std. dev.  0.470295  
 
  No GO Slim enrichment  
  
   tissue    mean expression   
  5th Passage Drosophila S2 Cells  10.341247  
  Adult Accessory gland  10.149320  
  Adult Brain  10.779261  
  Adult Carcass  9.295209  
  Adult Crop  10.004021  
  Adult Eye  10.137974  
  Adult Fatbody  9.746767  
  Adult Female Spermatheca Mated  10.121817  
  Adult Female Spermatheca Virgin  10.129811  
  Adult Head  9.996119  
  Adult Heart  9.611919  
  Adult Hind Gut  9.918832  
  Adult Male Ejaculatory Duct  9.948779  
  Adult Mid Gut  9.650558  
  Adult Ovary  11.387678  
  Adult Salivary Gland  10.197835  
  Adult Testes  9.028818  
  Adult Thoracoabdominal ganglion  10.598151  
  Adult Whole Fly  10.264631  
  Larvae Wandering Tubules  10.448556  
  Larval Feeding Carcass  10.120781  
  Larval Feeding Central Nevous System  10.902368  
  Larval Feeding Hind Gut  10.280104  
  Larval Feeding Malpighian Tubule  10.207401  
  Larval Feeding Mid Gut  9.749836  
  Larval Feeding Salivary Gland  10.194510  
  Whole Larvae Feeding  9.740364  
 
  
   FlyBase ID    symbol    start    end    strand    length   
   FBgn0033005   CG3107   978092   982595  +  4504  
   FBgn0026238   gus  1004715   1015817   -  11103  
   FBgn0033010   Atf6   1032202   1042266  +  10065  
   FBgn0033015   d4   1147484   1183475  +  35992  
   FBgn0033017   CG10465  1227207   1228496   -  1290  
 
 
    Segment 4 
 
   Location   
  Gene key  FBgn0039969-FBgn0027507  
  Heatmap region span   2R:1301241..1614335   
  Segment span   2R:1492333..1541144   
  Length (genes)  4  
  Length (bp)  48812  
   Model Scoring   
  BIC  414.744230  
  logL  -201.851262  
  logL ratio  134.088651  
   Expression   
  Mean expression  9.736621  
  Median expression  9.595655  
  Tissue std. dev.  0.373565  
 
  No GO Slim enrichment  
  
   tissue    mean expression   
  5th Passage Drosophila S2 Cells  9.944613  
  Adult Accessory gland  9.731676  
  Adult Brain  10.322278  
  Adult Carcass  9.456391  
  Adult Crop  9.727094  
  Adult Eye  9.542292  
  Adult Fatbody  10.019389  
  Adult Female Spermatheca Mated  9.735368  
  Adult Female Spermatheca Virgin  9.840563  
  Adult Head  9.802015  
  Adult Heart  9.982625  
  Adult Hind Gut  9.650228  
  Adult Male Ejaculatory Duct  9.825582  
  Adult Mid Gut  9.598106  
  Adult Ovary  10.137156  
  Adult Salivary Gland  9.822198  
  Adult Testes  8.488874  
  Adult Thoracoabdominal ganglion  10.124993  
  Adult Whole Fly  9.550756  
  Larvae Wandering Tubules  10.246252  
  Larval Feeding Carcass  9.184539  
  Larval Feeding Central Nevous System  9.957118  
  Larval Feeding Hind Gut  9.762904  
  Larval Feeding Malpighian Tubule  10.137247  
  Larval Feeding Mid Gut  9.703908  
  Larval Feeding Salivary Gland  9.340161  
  Whole Larvae Feeding  9.254429  
 
  
   FlyBase ID    symbol    start    end    strand    length   
   FBgn0039969   Fis1  1491091   1492333   -  1243  
   FBgn0039970   CG17508   1496129   1499094  +  2966  
   FBgn0033028   CG11665  1529160   1540288   -  11129  
   FBgn0027507   CG1344   1541144   1543453  +  2310  
 
 
    Segment 5 
 
   Location   
  Gene key  FBgn0085229-FBgn0033038  
  Heatmap region span   2R:1555898..1854341   
  Segment span   2R:1645198..1647896   
  Length (genes)  2  
  Length (bp)  2699  
   Model Scoring   
  BIC  269.596527  
  logL  -129.277411  
  logL ratio  17.148553  
   Expression   
  Mean expression  9.848523  
  Median expression  9.946202  
  Tissue std. dev.  0.567531  
 
  No GO Slim enrichment  
  
   tissue    mean expression   
  5th Passage Drosophila S2 Cells  12.106420  
  Adult Accessory gland  9.954227  
  Adult Brain  10.222310  
  Adult Carcass  9.813204  
  Adult Crop  9.892164  
  Adult Eye  9.632511  
  Adult Fatbody  9.819914  
  Adult Female Spermatheca Mated  9.988501  
  Adult Female Spermatheca Virgin  9.785031  
  Adult Head  9.825112  
  Adult Heart  9.905816  
  Adult Hind Gut  10.267643  
  Adult Male Ejaculatory Duct  9.719234  
  Adult Mid Gut  9.510552  
  Adult Ovary  9.765833  
  Adult Salivary Gland  9.881234  
  Adult Testes  9.560508  
  Adult Thoracoabdominal ganglion  10.477467  
  Adult Whole Fly  9.481340  
  Larvae Wandering Tubules  8.755788  
  Larval Feeding Carcass  9.274712  
  Larval Feeding Central Nevous System  10.171163  
  Larval Feeding Hind Gut  10.191909  
  Larval Feeding Malpighian Tubule  9.487054  
  Larval Feeding Mid Gut  9.276411  
  Larval Feeding Salivary Gland  9.778569  
  Whole Larvae Feeding  9.365486  
 
  
   FlyBase ID    symbol    start    end    strand    length   
   FBgn0085229   CG34200   1645198   1645572  +  375  
   FBgn0033038   CG7791  1645560   1647896   -  2337  
 
    Segment 6 
 
   Location   
  Gene key  FBgn0033041-FBgn0033046  
  Heatmap region span   2R:1614335..1868785   
  Segment span   2R:1680472..1812362   
  Length (genes)  6  
  Length (bp)  131891  
   Model Scoring   
  BIC  446.584696  
  logL  -217.771496  
  logL ratio  176.798179  
   Expression   
  Mean expression  4.611984  
  Median expression  4.523732  
  Tissue std. dev.  0.169232  
 
  No GO Slim enrichment  
  
   tissue    mean expression   
  5th Passage Drosophila S2 Cells  4.659395  
  Adult Accessory gland  4.508904  
  Adult Brain  4.560670  
  Adult Carcass  4.626259  
  Adult Crop  4.648466  
  Adult Eye  4.564662  
  Adult Fatbody  4.644881  
  Adult Female Spermatheca Mated  4.636424  
  Adult Female Spermatheca Virgin  4.696076  
  Adult Head  4.743598  
  Adult Heart  4.474830  
  Adult Hind Gut  4.497056  
  Adult Male Ejaculatory Duct  4.527649  
  Adult Mid Gut  4.652323  
  Adult Ovary  4.377299  
  Adult Salivary Gland  4.857398  
  Adult Testes  5.271729  
  Adult Thoracoabdominal ganglion  4.510105  
  Adult Whole Fly  4.367964  
  Larvae Wandering Tubules  4.599207  
  Larval Feeding Carcass  4.650470  
  Larval Feeding Central Nevous System  4.428080  
  Larval Feeding Hind Gut  4.747350  
  Larval Feeding Malpighian Tubule  4.556144  
  Larval Feeding Mid Gut  4.602034  
  Larval Feeding Salivary Gland  4.514452  
  Whole Larvae Feeding  4.600153  
 
  
   FlyBase ID    symbol    start    end    strand    length   
   FBgn0033041   Or42a  1678946   1680472   -  1527  
   FBgn0033042   Tsp42A   1682918   1683853  +  936  
   FBgn0033043   Or42b  1684700   1686017   -  1318  
   FBgn0085414   dpr12   1734194   1747693  +  13500  
   FBgn0033045      1808438   1808752  +  315  
   FBgn0033046      1812362   1818868  +  6507  
 
 
    Segment 7 
 
   Location   
  Gene key  FBgn0033047-FBgn0033048  
  Heatmap region span   2R:1645198..1885303   
  Segment span   2R:1845360..1854341   
  Length (genes)  2  
  Length (bp)  8982  
   Model Scoring   
  BIC  232.397305  
  logL  -110.677800  
  logL ratio  4.056865  
   Expression   
  Mean expression  5.511637  
  Median expression  4.352551  
  Tissue std. dev.  2.157441  
 
  
   GO ID    description    ratio    P-value   
   GO:0055085   transmembrane transport  2/2  0.000497  
   GO:0006810   transport  2/2  0.00239  
 
  
   tissue    mean expression   
  5th Passage Drosophila S2 Cells  4.327112  
  Adult Accessory gland  4.228956  
  Adult Brain  3.980276  
  Adult Carcass  4.755125  
  Adult Crop  4.245511  
  Adult Eye  4.050483  
  Adult Fatbody  4.604045  
  Adult Female Spermatheca Mated  4.333029  
  Adult Female Spermatheca Virgin  4.434698  
  Adult Head  5.207271  
  Adult Heart  4.398155  
  Adult Hind Gut  6.003032  
  Adult Male Ejaculatory Duct  4.207790  
  Adult Mid Gut  6.650443  
  Adult Ovary  4.461108  
  Adult Salivary Gland  4.625288  
  Adult Testes  4.110320  
  Adult Thoracoabdominal ganglion  4.104313  
  Adult Whole Fly  7.555066  
  Larvae Wandering Tubules  11.252291  
  Larval Feeding Carcass  4.212379  
  Larval Feeding Central Nevous System  3.961645  
  Larval Feeding Hind Gut  7.086088  
  Larval Feeding Malpighian Tubule  12.063373  
  Larval Feeding Mid Gut  6.879356  
  Larval Feeding Salivary Gland  4.131532  
  Whole Larvae Feeding  8.945503  
 
  
   FlyBase ID    symbol    start    end    strand    length   
   FBgn0033047   CG7882   1845360   1847322  +  1963  
   FBgn0033048   CG7881  1848503   1854341   -  5839  
 
    Segment 8 
 
   Location   
  Gene key  FBgn0033050-FBgn0000043  
  Heatmap region span   2R:1862122..1930160   
  Segment span   2R:1901327..1903190   
  Length (genes)  2  
  Length (bp)  1864  
   Model Scoring   
  BIC  302.677428  
  logL  -145.817862  
  logL ratio  14.382651  
   Expression   
  Mean expression  10.490903  
  Median expression  10.034432  
  Tissue std. dev.  0.959797  
 
  No GO Slim enrichment  
  
   tissue    mean expression   
  5th Passage Drosophila S2 Cells  12.382231  
  Adult Accessory gland  10.920566  
  Adult Brain  9.600389  
  Adult Carcass  9.445435  
  Adult Crop  9.089498  
  Adult Eye  8.898170  
  Adult Fatbody  9.321447  
  Adult Female Spermatheca Mated  10.530179  
  Adult Female Spermatheca Virgin  10.207159  
  Adult Head  9.695030  
  Adult Heart  10.575167  
  Adult Hind Gut  10.095060  
  Adult Male Ejaculatory Duct  9.345046  
  Adult Mid Gut  11.182693  
  Adult Ovary  11.324841  
  Adult Salivary Gland  8.878410  
  Adult Testes  10.948235  
  Adult Thoracoabdominal ganglion  9.721437  
  Adult Whole Fly  10.897268  
  Larvae Wandering Tubules  11.112642  
  Larval Feeding Carcass  11.003761  
  Larval Feeding Central Nevous System  11.875324  
  Larval Feeding Hind Gut  11.687991  
  Larval Feeding Malpighian Tubule  10.450757  
  Larval Feeding Mid Gut  11.695914  
  Larval Feeding Salivary Gland  11.247090  
  Whole Larvae Feeding  11.122642  
 
  
   FlyBase ID    symbol    start    end    strand    length   
   FBgn0033050   Pngl   1901327   1907660  +  6334  
   FBgn0000043   Act42A  1901415   1903190   -  1776  
 
    Segment 9 
 
   Location   
  Gene key  FBgn0085241-FBgn0033056  
  Heatmap region span   2R:1910711..2024808   
  Segment span   2R:1937791..1947063   
  Length (genes)  3  
  Length (bp)  9273  
   Model Scoring   
  BIC  292.940382  
  logL  -140.949339  
  logL ratio  36.019861  
   Expression   
  Mean expression  5.142802  
  Median expression  5.299062  
  Tissue std. dev.  0.492575  
 
  No GO Slim enrichment  
  
   tissue    mean expression   
  5th Passage Drosophila S2 Cells  5.149987  
  Adult Accessory gland  5.009660  
  Adult Brain  4.773129  
  Adult Carcass  5.271370  
  Adult Crop  5.011391  
  Adult Eye  5.109329  
  Adult Fatbody  5.038879  
  Adult Female Spermatheca Mated  5.036410  
  Adult Female Spermatheca Virgin  5.120317  
  Adult Head  4.839678  
  Adult Heart  5.213358  
  Adult Hind Gut  4.823145  
  Adult Male Ejaculatory Duct  4.977509  
  Adult Mid Gut  5.005804  
  Adult Ovary  4.925970  
  Adult Salivary Gland  5.837124  
  Adult Testes  7.428454  
  Adult Thoracoabdominal ganglion  5.013285  
  Adult Whole Fly  5.348984  
  Larvae Wandering Tubules  5.006218  
  Larval Feeding Carcass  5.053321  
  Larval Feeding Central Nevous System  4.849613  
  Larval Feeding Hind Gut  4.827995  
  Larval Feeding Malpighian Tubule  4.933931  
  Larval Feeding Mid Gut  5.060787  
  Larval Feeding Salivary Gland  5.180588  
  Whole Larvae Feeding  5.009408  
 
  
   FlyBase ID    symbol    start    end    strand    length   
   FBgn0085241   CG34212  1937412   1937791   -  380  
   FBgn0033058   CCHa2r   1938477   1956185  +  17709  
   FBgn0033056   CG7856  1944862   1947063   -  2202  
 
 
    Segment 10 
 
   Location   
  Gene key  FBgn0033059-FBgn0033060  
  Heatmap region span   2R:1923922..2056681   
  Segment span   2R:1963882..1964415   
  Length (genes)  2  
  Length (bp)  534  
   Model Scoring   
  BIC  223.488852  
  logL  -106.223574  
  logL ratio  33.164732  
   Expression   
  Mean expression  7.786061  
  Median expression  7.786866  
  Tissue std. dev.  0.489258  
 
  No GO Slim enrichment  
  
   tissue    mean expression   
  5th Passage Drosophila S2 Cells  8.408867  
  Adult Accessory gland  8.162843  
  Adult Brain  8.121959  
  Adult Carcass  7.288386  
  Adult Crop  7.962639  
  Adult Eye  8.126284  
  Adult Fatbody  7.764458  
  Adult Female Spermatheca Mated  7.535609  
  Adult Female Spermatheca Virgin  7.434740  
  Adult Head  7.686175  
  Adult Heart  8.185861  
  Adult Hind Gut  8.093077  
  Adult Male Ejaculatory Duct  7.380777  
  Adult Mid Gut  7.517765  
  Adult Ovary  8.768776  
  Adult Salivary Gland  8.135115  
  Adult Testes  7.034514  
  Adult Thoracoabdominal ganglion  8.225474  
  Adult Whole Fly  7.821623  
  Larvae Wandering Tubules  6.989805  
  Larval Feeding Carcass  7.678743  
  Larval Feeding Central Nevous System  8.458724  
  Larval Feeding Hind Gut  8.096599  
  Larval Feeding Malpighian Tubule  7.441070  
  Larval Feeding Mid Gut  6.878409  
  Larval Feeding Salivary Gland  8.062833  
  Whole Larvae Feeding  6.962516  
 
  
   FlyBase ID    symbol    start    end    strand    length   
   FBgn0033059   CG7845  1961976   1963882   -  1907  
   FBgn0033060   CG7849   1964415   1965593  +  1179  
 
    Segment 11 
 
   Location   
  Gene key  FBgn0040502-FBgn0033067  
  Heatmap region span   2R:1973125..2096813   
  Segment span   2R:2067225..2069131   
  Length (genes)  2  
  Length (bp)  1907  
   Model Scoring   
  BIC  188.582834  
  logL  -88.770565  
  logL ratio  38.820455  
   Expression   
  Mean expression  5.088358  
  Median expression  4.498482  
  Tissue std. dev.  1.977574  
 
  No GO Slim enrichment  
  
   tissue    mean expression   
  5th Passage Drosophila S2 Cells  4.257488  
  Adult Accessory gland  4.246766  
  Adult Brain  4.350511  
  Adult Carcass  5.704316  
  Adult Crop  4.453314  
  Adult Eye  4.386815  
  Adult Fatbody  4.775343  
  Adult Female Spermatheca Mated  4.820559  
  Adult Female Spermatheca Virgin  4.869499  
  Adult Head  13.486470  
  Adult Heart  5.001882  
  Adult Hind Gut  4.372880  
  Adult Male Ejaculatory Duct  4.925436  
  Adult Mid Gut  4.505609  
  Adult Ovary  4.383561  
  Adult Salivary Gland  4.628452  
  Adult Testes  4.294275  
  Adult Thoracoabdominal ganglion  4.739242  
  Adult Whole Fly  10.113681  
  Larvae Wandering Tubules  4.448613  
  Larval Feeding Carcass  4.431973  
  Larval Feeding Central Nevous System  4.278638  
  Larval Feeding Hind Gut  4.338522  
  Larval Feeding Malpighian Tubule  4.401447  
  Larval Feeding Mid Gut  4.452924  
  Larval Feeding Salivary Gland  4.479820  
  Whole Larvae Feeding  4.237642  
 
  
   FlyBase ID    symbol    start    end    strand    length   
   FBgn0040502   CG8343   2067225   2067875  +  651  
   FBgn0033067   CG11211  2068424   2069131   -  708  
 
    Segment 12 
 
   Location   
  Gene key  FBgn0050432-FBgn0033069  
  Heatmap region span   2R:2056681..2488483   
  Segment span   2R:2085847..2088300   
  Length (genes)  2  
  Length (bp)  2454  
   Model Scoring   
  BIC  172.337035  
  logL  -80.647665  
  logL ratio  39.208210  
   Expression   
  Mean expression  4.876503  
  Median expression  4.691847  
  Tissue std. dev.  0.961641  
 
  No GO Slim enrichment  
  
   tissue    mean expression   
  5th Passage Drosophila S2 Cells  4.732278  
  Adult Accessory gland  4.683211  
  Adult Brain  4.334553  
  Adult Carcass  4.745032  
  Adult Crop  4.648232  
  Adult Eye  4.534090  
  Adult Fatbody  4.798625  
  Adult Female Spermatheca Mated  4.722243  
  Adult Female Spermatheca Virgin  4.704471  
  Adult Head  4.530900  
  Adult Heart  4.683298  
  Adult Hind Gut  4.672536  
  Adult Male Ejaculatory Duct  4.691439  
  Adult Mid Gut  4.791846  
  Adult Ovary  4.650692  
  Adult Salivary Gland  4.834264  
  Adult Testes  9.696110  
  Adult Thoracoabdominal ganglion  4.434767  
  Adult Whole Fly  5.195371  
  Larvae Wandering Tubules  4.692165  
  Larval Feeding Carcass  4.766140  
  Larval Feeding Central Nevous System  4.416997  
  Larval Feeding Hind Gut  4.533647  
  Larval Feeding Malpighian Tubule  4.697636  
  Larval Feeding Mid Gut  4.620141  
  Larval Feeding Salivary Gland  4.734298  
  Whole Larvae Feeding  5.120591  
 
  
   FlyBase ID    symbol    start    end    strand    length   
   FBgn0050432   CG30432  2084892   2085847   -  956  
   FBgn0033069   CG8335   2088300   2089461  +  1162  
 
    Segment 13 
 
   Location   
  Gene key  FBgn0033074-FBgn0033076  
  Heatmap region span   2R:2085847..2560850   
  Segment span   2R:2113132..2488483   
  Length (genes)  3  
  Length (bp)  375352  
   Model Scoring   
  BIC  265.852026  
  logL  -127.405160  
  logL ratio  50.218267  
   Expression   
  Mean expression  4.805347  
  Median expression  4.577946  
  Tissue std. dev.  0.768602  
 
  No GO Slim enrichment  
  
   tissue    mean expression   
  5th Passage Drosophila S2 Cells  4.953941  
  Adult Accessory gland  4.639796  
  Adult Brain  4.410033  
  Adult Carcass  4.595113  
  Adult Crop  4.591063  
  Adult Eye  4.523006  
  Adult Fatbody  4.485764  
  Adult Female Spermatheca Mated  4.532917  
  Adult Female Spermatheca Virgin  4.480695  
  Adult Head  4.320189  
  Adult Heart  4.512123  
  Adult Hind Gut  4.507006  
  Adult Male Ejaculatory Duct  4.727449  
  Adult Mid Gut  4.641010  
  Adult Ovary  4.996237  
  Adult Salivary Gland  4.822377  
  Adult Testes  8.556169  
  Adult Thoracoabdominal ganglion  4.533151  
  Adult Whole Fly  5.458167  
  Larvae Wandering Tubules  4.728663  
  Larval Feeding Carcass  4.610233  
  Larval Feeding Central Nevous System  4.762555  
  Larval Feeding Hind Gut  4.461608  
  Larval Feeding Malpighian Tubule  4.699859  
  Larval Feeding Mid Gut  4.573370  
  Larval Feeding Salivary Gland  4.724820  
  Whole Larvae Feeding  4.897068  
 
  
   FlyBase ID    symbol    start    end    strand    length   
   FBgn0033074   tomboy40   2113132   2114389  +  1258  
   FBgn0086655   jing   2389764   2506901  +  117138  
   FBgn0033076   CG15233  2487410   2488483   -  1074  
 
 
    Segment 14 
 
   Location   
  Gene key  FBgn0026761-FBgn0033081  
  Heatmap region span   2R:2090248..2563465   
  Segment span   2R:2523767..2543955   
  Length (genes)  5  
  Length (bp)  20189  
   Model Scoring   
  BIC  460.565006  
  logL  -224.761650  
  logL ratio  182.065967  
   Expression   
  Mean expression  8.824614  
  Median expression  8.724832  
  Tissue std. dev.  0.500377  
 
  No GO Slim enrichment  
  
   tissue    mean expression   
  5th Passage Drosophila S2 Cells  10.043568  
  Adult Accessory gland  8.796068  
  Adult Brain  8.978333  
  Adult Carcass  8.512657  
  Adult Crop  8.716205  
  Adult Eye  8.581035  
  Adult Fatbody  8.405773  
  Adult Female Spermatheca Mated  8.701616  
  Adult Female Spermatheca Virgin  8.647863  
  Adult Head  8.489300  
  Adult Heart  8.724564  
  Adult Hind Gut  8.577338  
  Adult Male Ejaculatory Duct  8.864122  
  Adult Mid Gut  8.135085  
  Adult Ovary  10.394668  
  Adult Salivary Gland  8.706938  
  Adult Testes  8.976780  
  Adult Thoracoabdominal ganglion  8.955632  
  Adult Whole Fly  9.221215  
  Larvae Wandering Tubules  8.502761  
  Larval Feeding Carcass  8.795312  
  Larval Feeding Central Nevous System  9.342126  
  Larval Feeding Hind Gut  8.724235  
  Larval Feeding Malpighian Tubule  8.770843  
  Larval Feeding Mid Gut  8.031898  
  Larval Feeding Salivary Gland  9.338663  
  Whole Larvae Feeding  8.329968  
 
  
   FlyBase ID    symbol    start    end    strand    length   
   FBgn0026761   Trap1  2521152   2523767   -  2616  
   FBgn0042085   Bap170   2524023   2529866  +  5844  
   FBgn0029131   debcl   2536160   2540347  +  4188  
   FBgn0050443   Opbp   2540738   2542772  +  2035  
   FBgn0033081   geminin  2542968   2543955   -  988  
 
 
    Segment 15 
 
   Location   
  Gene key  FBgn0033083-FBgn0000054  
  Heatmap region span   2R:2102078..2580945   
  Segment span   2R:2549623..2549774   
  Length (genes)  2  
  Length (bp)  152  
   Model Scoring   
  BIC  243.114167  
  logL  -116.036231  
  logL ratio  26.962538  
   Expression   
  Mean expression  9.237547  
  Median expression  9.302758  
  Tissue std. dev.  0.525922  
 
  No GO Slim enrichment  
  
   tissue    mean expression   
  5th Passage Drosophila S2 Cells  10.274771  
  Adult Accessory gland  9.634207  
  Adult Brain  9.788587  
  Adult Carcass  8.672497  
  Adult Crop  9.375863  
  Adult Eye  9.616218  
  Adult Fatbody  8.664100  
  Adult Female Spermatheca Mated  9.129020  
  Adult Female Spermatheca Virgin  9.111021  
  Adult Head  9.261833  
  Adult Heart  8.837437  
  Adult Hind Gut  9.131300  
  Adult Male Ejaculatory Duct  9.011669  
  Adult Mid Gut  8.867432  
  Adult Ovary  10.210441  
  Adult Salivary Gland  9.328530  
  Adult Testes  8.231346  
  Adult Thoracoabdominal ganglion  9.386432  
  Adult Whole Fly  9.334805  
  Larvae Wandering Tubules  9.088421  
  Larval Feeding Carcass  9.098705  
  Larval Feeding Central Nevous System  10.560593  
  Larval Feeding Hind Gut  8.956325  
  Larval Feeding Malpighian Tubule  8.969656  
  Larval Feeding Mid Gut  8.627223  
  Larval Feeding Salivary Gland  9.565518  
  Whole Larvae Feeding  8.679816  
 
  
   FlyBase ID    symbol    start    end    strand    length   
   FBgn0033083   Dpit47  2548155   2549623   -  1469  
   FBgn0000054   Adf1   2549774   2555592  +  5819  
 
    Segment 16 
 
   Location   
  Gene key  FBgn0033088-FBgn0033089  
  Heatmap region span   2R:2523767..2603370   
  Segment span   2R:2561114..2563465   
  Length (genes)  3  
  Length (bp)  2352  
   Model Scoring   
  BIC  282.054121  
  logL  -135.506208  
  logL ratio  98.539100  
   Expression   
  Mean expression  7.885007  
  Median expression  7.806730  
  Tissue std. dev.  0.572158  
 
  No GO Slim enrichment  
  
   tissue    mean expression   
  5th Passage Drosophila S2 Cells  8.707834  
  Adult Accessory gland  8.697194  
  Adult Brain  8.114304  
  Adult Carcass  7.040023  
  Adult Crop  7.329551  
  Adult Eye  8.110903  
  Adult Fatbody  7.140066  
  Adult Female Spermatheca Mated  7.523282  
  Adult Female Spermatheca Virgin  7.374028  
  Adult Head  7.670125  
  Adult Heart  7.762657  
  Adult Hind Gut  7.269284  
  Adult Male Ejaculatory Duct  7.850896  
  Adult Mid Gut  7.636817  
  Adult Ovary  9.320539  
  Adult Salivary Gland  7.452563  
  Adult Testes  7.548152  
  Adult Thoracoabdominal ganglion  8.282538  
  Adult Whole Fly  8.084965  
  Larvae Wandering Tubules  8.081959  
  Larval Feeding Carcass  7.693707  
  Larval Feeding Central Nevous System  9.025335  
  Larval Feeding Hind Gut  7.726572  
  Larval Feeding Malpighian Tubule  8.351626  
  Larval Feeding Mid Gut  7.431940  
  Larval Feeding Salivary Gland  8.352760  
  Whole Larvae Feeding  7.315571  
 
  
   FlyBase ID    symbol    start    end    strand    length   
   FBgn0033088   CG3271   2561114   2562566  +  1453  
   FBgn0033092   CG9422   2563332   2580099  +  16768  
   FBgn0033089   CG17266  2562533   2563465   -  933  
 
 
    Segment 17 
 
   Location   
  Gene key  FBgn0033090-FBgn0050446  
  Heatmap region span   2R:2544833..2607038   
  Segment span   2R:2570697..2577467   
  Length (genes)  2  
  Length (bp)  6771  
   Model Scoring   
  BIC  204.079142  
  logL  -96.518719  
  logL ratio  11.836798  
   Expression   
  Mean expression  5.234954  
  Median expression  5.111892  
  Tissue std. dev.  0.478269  
 
  No GO Slim enrichment  
  
   tissue    mean expression   
  5th Passage Drosophila S2 Cells  5.656262  
  Adult Accessory gland  4.832467  
  Adult Brain  5.921176  
  Adult Carcass  5.208620  
  Adult Crop  5.112991  
  Adult Eye  4.644719  
  Adult Fatbody  5.019802  
  Adult Female Spermatheca Mated  4.985406  
  Adult Female Spermatheca Virgin  5.061383  
  Adult Head  5.301020  
  Adult Heart  4.877989  
  Adult Hind Gut  5.237917  
  Adult Male Ejaculatory Duct  5.289567  
  Adult Mid Gut  5.112723  
  Adult Ovary  5.103589  
  Adult Salivary Gland  5.318922  
  Adult Testes  6.908988  
  Adult Thoracoabdominal ganglion  6.366556  
  Adult Whole Fly  4.819393  
  Larvae Wandering Tubules  4.980923  
  Larval Feeding Carcass  5.034357  
  Larval Feeding Central Nevous System  5.383164  
  Larval Feeding Hind Gut  5.154758  
  Larval Feeding Malpighian Tubule  5.223142  
  Larval Feeding Mid Gut  4.942246  
  Larval Feeding Salivary Gland  5.021681  
  Whole Larvae Feeding  4.823996  
 
  
   FlyBase ID    symbol    start    end    strand    length   
   FBgn0033090   CG15909   2570697   2571862  +  1166  
   FBgn0050446   Tdc2  2572250   2577467   -  5218  
 
    Segment 18 
 
   Location   
  Gene key  FBgn0014009-FBgn0033095  
  Heatmap region span   2R:2561114..2684421   
  Segment span   2R:2584227..2603370   
  Length (genes)  2  
  Length (bp)  19144  
   Model Scoring   
  BIC  220.338765  
  logL  -104.648530  
  logL ratio  34.219903  
   Expression   
  Mean expression  7.326630  
  Median expression  7.175552  
  Tissue std. dev.  0.795600  
 
  No GO Slim enrichment  
  
   tissue    mean expression   
  5th Passage Drosophila S2 Cells  6.532839  
  Adult Accessory gland  7.113774  
  Adult Brain  7.303361  
  Adult Carcass  7.883002  
  Adult Crop  7.744293  
  Adult Eye  6.946689  
  Adult Fatbody  8.998656  
  Adult Female Spermatheca Mated  7.938841  
  Adult Female Spermatheca Virgin  7.934124  
  Adult Head  7.292414  
  Adult Heart  8.343633  
  Adult Hind Gut  7.483511  
  Adult Male Ejaculatory Duct  7.225562  
  Adult Mid Gut  7.184154  
  Adult Ovary  7.077180  
  Adult Salivary Gland  6.408117  
  Adult Testes  5.262614  
  Adult Thoracoabdominal ganglion  7.375013  
  Adult Whole Fly  6.708213  
  Larvae Wandering Tubules  9.035640  
  Larval Feeding Carcass  6.858344  
  Larval Feeding Central Nevous System  6.963387  
  Larval Feeding Hind Gut  7.110941  
  Larval Feeding Malpighian Tubule  8.532628  
  Larval Feeding Mid Gut  7.082950  
  Larval Feeding Salivary Gland  6.944069  
  Whole Larvae Feeding  6.535065  
 
  
   FlyBase ID    symbol    start    end    strand    length   
   FBgn0014009   Rab2   2584227   2587441  +  3215  
   FBgn0033095   CG3409  2587200   2603370   -  16171  
 
    Segment 19 
 
   Location   
  Gene key  FBgn0033096-FBgn0033097  
  Heatmap region span   2R:2570697..2695117   
  Segment span   2R:2605312..2607038   
  Length (genes)  2  
  Length (bp)  1727  
   Model Scoring   
  BIC  174.051762  
  logL  -81.505028  
  logL ratio  41.343473  
   Expression   
  Mean expression  4.448272  
  Median expression  4.367705  
  Tissue std. dev.  0.364997  
 
  
   GO ID    description    ratio    P-value   
   GO:0055085   transmembrane transport  2/2  0.000497  
   GO:0006810   transport  2/2  0.00239  
 
  
   tissue    mean expression   
  5th Passage Drosophila S2 Cells  4.305340  
  Adult Accessory gland  4.510688  
  Adult Brain  4.242800  
  Adult Carcass  4.311960  
  Adult Crop  4.314693  
  Adult Eye  4.408218  
  Adult Fatbody  4.350504  
  Adult Female Spermatheca Mated  4.332943  
  Adult Female Spermatheca Virgin  4.350444  
  Adult Head  4.292554  
  Adult Heart  4.404217  
  Adult Hind Gut  4.445768  
  Adult Male Ejaculatory Duct  4.508979  
  Adult Mid Gut  6.157941  
  Adult Ovary  4.357807  
  Adult Salivary Gland  4.623796  
  Adult Testes  4.281909  
  Adult Thoracoabdominal ganglion  4.285815  
  Adult Whole Fly  4.110034  
  Larvae Wandering Tubules  4.589517  
  Larval Feeding Carcass  4.398908  
  Larval Feeding Central Nevous System  4.138160  
  Larval Feeding Hind Gut  4.336190  
  Larval Feeding Malpighian Tubule  4.481497  
  Larval Feeding Mid Gut  4.776186  
  Larval Feeding Salivary Gland  4.557396  
  Whole Larvae Feeding  4.229086  
 
  
   FlyBase ID    symbol    start    end    strand    length   
   FBgn0033096   ZIP1  2603847   2605312   -  1466  
   FBgn0033097   CG9430  2606121   2607038   -  918  
 
    Segment 20 
 
   Location   
  Gene key  FBgn0028579-FBgn0042083  
  Heatmap region span   2R:2580945..2698525   
  Segment span   2R:2629372..2633890   
  Length (genes)  2  
  Length (bp)  4519  
   Model Scoring   
  BIC  239.281706  
  logL  -114.120001  
  logL ratio  19.151384  
   Expression   
  Mean expression  8.544539  
  Median expression  8.506784  
  Tissue std. dev.  0.470590  
 
  No GO Slim enrichment  
  
   tissue    mean expression   
  5th Passage Drosophila S2 Cells  8.374627  
  Adult Accessory gland  7.548961  
  Adult Brain  8.190869  
  Adult Carcass  8.744233  
  Adult Crop  8.563273  
  Adult Eye  8.727586  
  Adult Fatbody  9.483689  
  Adult Female Spermatheca Mated  8.797385  
  Adult Female Spermatheca Virgin  8.948065  
  Adult Head  8.621572  
  Adult Heart  9.527615  
  Adult Hind Gut  8.539444  
  Adult Male Ejaculatory Duct  8.082749  
  Adult Mid Gut  7.872785  
  Adult Ovary  9.270126  
  Adult Salivary Gland  8.400524  
  Adult Testes  7.945989  
  Adult Thoracoabdominal ganglion  8.215629  
  Adult Whole Fly  8.540749  
  Larvae Wandering Tubules  8.168665  
  Larval Feeding Carcass  8.370703  
  Larval Feeding Central Nevous System  8.286549  
  Larval Feeding Hind Gut  9.222768  
  Larval Feeding Malpighian Tubule  8.937123  
  Larval Feeding Mid Gut  8.343151  
  Larval Feeding Salivary Gland  8.714838  
  Whole Larvae Feeding  8.262884  
 
  
   FlyBase ID    symbol    start    end    strand    length   
   FBgn0028579   phtf   2629372   2633649  +  4278  
   FBgn0042083   CG3267   2633890   2636459  +  2570  
 
    Segment 21 
 
   Location   
  Gene key  FBgn0027066-FBgn0033101  
  Heatmap region span   2R:2584130..2726432   
  Segment span   2R:2636697..2646404   
  Length (genes)  3  
  Length (bp)  9708  
   Model Scoring   
  BIC  342.567425  
  logL  -165.762860  
  logL ratio  103.879708  
   Expression   
  Mean expression  10.385959  
  Median expression  10.304007  
  Tissue std. dev.  0.621452  
 
  No GO Slim enrichment  
  
   tissue    mean expression   
  5th Passage Drosophila S2 Cells  10.772713  
  Adult Accessory gland  9.465267  
  Adult Brain  10.048900  
  Adult Carcass  10.350497  
  Adult Crop  10.323997  
  Adult Eye  10.243333  
  Adult Fatbody  10.955654  
  Adult Female Spermatheca Mated  10.468064  
  Adult Female Spermatheca Virgin  10.479726  
  Adult Head  10.654327  
  Adult Heart  10.746709  
  Adult Hind Gut  10.587428  
  Adult Male Ejaculatory Duct  10.287809  
  Adult Mid Gut  10.488474  
  Adult Ovary  10.858925  
  Adult Salivary Gland  9.147494  
  Adult Testes  8.230056  
  Adult Thoracoabdominal ganglion  10.720515  
  Adult Whole Fly  10.523521  
  Larvae Wandering Tubules  11.289540  
  Larval Feeding Carcass  10.588611  
  Larval Feeding Central Nevous System  10.342381  
  Larval Feeding Hind Gut  10.718314  
  Larval Feeding Malpighian Tubule  11.428083  
  Larval Feeding Mid Gut  10.141526  
  Larval Feeding Salivary Gland  10.197016  
  Whole Larvae Feeding  10.361999  
 
  
   FlyBase ID    symbol    start    end    strand    length   
   FBgn0027066   Eb1   2636697   2643542  +  6846  
   FBgn0033100   CG3420  2643801   2644714   -  914  
   FBgn0033101   CG9436  2645106   2646404   -  1299  
 
 
    Segment 22 
 
   Location   
  Gene key  FBgn0000473-FBgn0085421  
  Heatmap region span   2R:2584227..2760274   
  Segment span   2R:2668960..2684421   
  Length (genes)  2  
  Length (bp)  15462  
   Model Scoring   
  BIC  220.271917  
  logL  -104.615106  
  logL ratio  7.800461  
   Expression   
  Mean expression  5.853557  
  Median expression  5.771425  
  Tissue std. dev.  0.734532  
 
  No GO Slim enrichment  
  
   tissue    mean expression   
  5th Passage Drosophila S2 Cells  5.419145  
  Adult Accessory gland  5.594617  
  Adult Brain  6.635229  
  Adult Carcass  6.374752  
  Adult Crop  6.284315  
  Adult Eye  6.607924  
  Adult Fatbody  4.885594  
  Adult Female Spermatheca Mated  5.917532  
  Adult Female Spermatheca Virgin  5.969426  
  Adult Head  7.520572  
  Adult Heart  5.161015  
  Adult Hind Gut  7.274081  
  Adult Male Ejaculatory Duct  5.575043  
  Adult Mid Gut  6.868595  
  Adult Ovary  5.359741  
  Adult Salivary Gland  4.912636  
  Adult Testes  5.421452  
  Adult Thoracoabdominal ganglion  5.790178  
  Adult Whole Fly  5.732342  
  Larvae Wandering Tubules  6.666363  
  Larval Feeding Carcass  4.989696  
  Larval Feeding Central Nevous System  4.984826  
  Larval Feeding Hind Gut  5.515544  
  Larval Feeding Malpighian Tubule  6.339196  
  Larval Feeding Mid Gut  6.140195  
  Larval Feeding Salivary Gland  4.802521  
  Whole Larvae Feeding  5.303510  
 
  
   FlyBase ID    symbol    start    end    strand    length   
   FBgn0000473   Cyp6a2  2667220   2668960   -  1741  
   FBgn0085421   Epac  2649417   2684421   -  35005  
 
    Segment 23 
 
   Location   
  Gene key  FBgn0033104-FBgn0028954  
  Heatmap region span   2R:2629372..2766428   
  Segment span   2R:2697252..2698525   
  Length (genes)  3  
  Length (bp)  1274  
   Model Scoring   
  BIC  312.908803  
  logL  -150.933549  
  logL ratio  82.642940  
   Expression   
  Mean expression  9.185470  
  Median expression  9.284897  
  Tissue std. dev.  0.470331  
 
  No GO Slim enrichment  
  
   tissue    mean expression   
  5th Passage Drosophila S2 Cells  10.194048  
  Adult Accessory gland  9.785500  
  Adult Brain  9.436155  
  Adult Carcass  8.795349  
  Adult Crop  9.526917  
  Adult Eye  9.629693  
  Adult Fatbody  8.808076  
  Adult Female Spermatheca Mated  8.964775  
  Adult Female Spermatheca Virgin  8.811491  
  Adult Head  9.216303  
  Adult Heart  9.125558  
  Adult Hind Gut  8.959105  
  Adult Male Ejaculatory Duct  9.581639  
  Adult Mid Gut  8.621859  
  Adult Ovary  9.918155  
  Adult Salivary Gland  9.048700  
  Adult Testes  8.419476  
  Adult Thoracoabdominal ganglion  9.331462  
  Adult Whole Fly  9.061212  
  Larvae Wandering Tubules  8.857659  
  Larval Feeding Carcass  9.016252  
  Larval Feeding Central Nevous System  10.303380  
  Larval Feeding Hind Gut  9.080364  
  Larval Feeding Malpighian Tubule  8.845438  
  Larval Feeding Mid Gut  8.641285  
  Larval Feeding Salivary Gland  9.295513  
  Whole Larvae Feeding  8.732324  
 
  
   FlyBase ID    symbol    start    end    strand    length   
   FBgn0033104   CG15237  2696549   2697252   -  704  
   FBgn0022224   ubl  2697516   2698019   -  504  
   FBgn0028954      2698525   2702317  +  3793  
 
 
    Segment 24 
 
   Location   
  Gene key  FBgn0033107-FBgn0040674  
  Heatmap region span   2R:2636697..2768220   
  Segment span   2R:2702565..2726432   
  Length (genes)  3  
  Length (bp)  23868  
   Model Scoring   
  BIC  324.087158  
  logL  -156.522726  
  logL ratio  6.802163  
   Expression   
  Mean expression  4.636477  
  Median expression  4.266001  
  Tissue std. dev.  0.773154  
 
  No GO Slim enrichment  
  
   tissue    mean expression   
  5th Passage Drosophila S2 Cells  4.626606  
  Adult Accessory gland  4.577922  
  Adult Brain  6.599723  
  Adult Carcass  4.397337  
  Adult Crop  4.330559  
  Adult Eye  5.640673  
  Adult Fatbody  4.150767  
  Adult Female Spermatheca Mated  4.181494  
  Adult Female Spermatheca Virgin  4.169743  
  Adult Head  5.747934  
  Adult Heart  4.063309  
  Adult Hind Gut  4.139022  
  Adult Male Ejaculatory Duct  4.083731  
  Adult Mid Gut  4.153465  
  Adult Ovary  4.573041  
  Adult Salivary Gland  4.248911  
  Adult Testes  5.868453  
  Adult Thoracoabdominal ganglion  6.133135  
  Adult Whole Fly  4.437264  
  Larvae Wandering Tubules  4.187288  
  Larval Feeding Carcass  3.997250  
  Larval Feeding Central Nevous System  6.159675  
  Larval Feeding Hind Gut  4.125366  
  Larval Feeding Malpighian Tubule  4.136104  
  Larval Feeding Mid Gut  4.073299  
  Larval Feeding Salivary Gland  4.143342  
  Whole Larvae Feeding  4.239477  
 
  
   FlyBase ID    symbol    start    end    strand    length   
   FBgn0033107      2702565   2705704  +  3140  
   FBgn0033108   CG15236   2715863   2725865  +  10003  
   FBgn0040674   CG9445  2725579   2726432   -  854  
 
 
    Segment 25 
 
   Location   
  Gene key  FBgn0066293-FBgn0053558  
  Heatmap region span   2R:2770508..2880191   
  Segment span   2R:2831491..2859139   
  Length (genes)  3  
  Length (bp)  27649  
   Model Scoring   
  BIC  288.691681  
  logL  -138.824988  
  logL ratio  35.953554  
   Expression   
  Mean expression  5.075714  
  Median expression  4.757829  
  Tissue std. dev.  0.306604  
 
  
   GO ID    description    ratio    P-value   
   GO:0043234   protein complex  2/3  0.000144  
 
  
   tissue    mean expression   
  5th Passage Drosophila S2 Cells  5.201953  
  Adult Accessory gland  5.063807  
  Adult Brain  5.110861  
  Adult Carcass  6.261674  
  Adult Crop  5.335683  
  Adult Eye  5.091942  
  Adult Fatbody  4.932601  
  Adult Female Spermatheca Mated  4.914662  
  Adult Female Spermatheca Virgin  4.959534  
  Adult Head  4.848646  
  Adult Heart  4.947267  
  Adult Hind Gut  5.023603  
  Adult Male Ejaculatory Duct  4.957462  
  Adult Mid Gut  5.034225  
  Adult Ovary  5.181472  
  Adult Salivary Gland  5.184002  
  Adult Testes  4.788393  
  Adult Thoracoabdominal ganglion  5.073800  
  Adult Whole Fly  4.952733  
  Larvae Wandering Tubules  5.045876  
  Larval Feeding Carcass  4.838039  
  Larval Feeding Central Nevous System  5.335299  
  Larval Feeding Hind Gut  4.941527  
  Larval Feeding Malpighian Tubule  5.059687  
  Larval Feeding Mid Gut  4.689741  
  Larval Feeding Salivary Gland  5.625043  
  Whole Larvae Feeding  4.644729  
 
  
   FlyBase ID    symbol    start    end    strand    length   
   FBgn0066293   CheB42b  2830675   2831491   -  817  
   FBgn0053349   ppk25   2837890   2839663  +  1774  
   FBgn0053558   mim  2813207   2859139   -  45933  
 
 
    Segment 26 
 
   Location   
  Gene key  FBgn0050157-FBgn0050156  
  Heatmap region span   2R:2779268..2912517   
  Segment span   2R:2862638..2870491   
  Length (genes)  2  
  Length (bp)  7854  
   Model Scoring   
  BIC  208.914578  
  logL  -98.936436  
  logL ratio  17.951284  
   Expression   
  Mean expression  5.815815  
  Median expression  5.594691  
  Tissue std. dev.  0.979925  
 
  No GO Slim enrichment  
  
   tissue    mean expression   
  5th Passage Drosophila S2 Cells  6.317754  
  Adult Accessory gland  6.263215  
  Adult Brain  5.963657  
  Adult Carcass  5.271359  
  Adult Crop  5.174286  
  Adult Eye  5.335761  
  Adult Fatbody  5.471305  
  Adult Female Spermatheca Mated  5.938439  
  Adult Female Spermatheca Virgin  5.658885  
  Adult Head  5.221514  
  Adult Heart  5.303667  
  Adult Hind Gut  5.438533  
  Adult Male Ejaculatory Duct  5.459852  
  Adult Mid Gut  5.419650  
  Adult Ovary  5.327648  
  Adult Salivary Gland  5.675442  
  Adult Testes  10.485159  
  Adult Thoracoabdominal ganglion  5.642364  
  Adult Whole Fly  6.486756  
  Larvae Wandering Tubules  5.828784  
  Larval Feeding Carcass  5.311307  
  Larval Feeding Central Nevous System  5.425528  
  Larval Feeding Hind Gut  5.267507  
  Larval Feeding Malpighian Tubule  5.714948  
  Larval Feeding Mid Gut  5.929879  
  Larval Feeding Salivary Gland  5.989683  
  Whole Larvae Feeding  5.704115  
 
  
   FlyBase ID    symbol    start    end    strand    length   
   FBgn0050157   CG30157  2861543   2862638   -  1096  
   FBgn0050156   CG30156  2868951   2870491   -  1541  
 
    Segment 27 
 
   Location   
  Gene key  FBgn0022960-FBgn0033122  
  Heatmap region span   2R:2810875..2922632   
  Segment span   2R:2873214..2873568   
  Length (genes)  2  
  Length (bp)  355  
   Model Scoring   
  BIC  216.125537  
  logL  -102.541916  
  logL ratio  41.841517  
   Expression   
  Mean expression  8.234979  
  Median expression  8.123455  
  Tissue std. dev.  0.662798  
 
  No GO Slim enrichment  
  
   tissue    mean expression   
  5th Passage Drosophila S2 Cells  9.188519  
  Adult Accessory gland  8.194076  
  Adult Brain  9.320971  
  Adult Carcass  7.261540  
  Adult Crop  8.354940  
  Adult Eye  8.769796  
  Adult Fatbody  7.241083  
  Adult Female Spermatheca Mated  8.221481  
  Adult Female Spermatheca Virgin  8.211301  
  Adult Head  8.193759  
  Adult Heart  7.872067  
  Adult Hind Gut  7.802979  
  Adult Male Ejaculatory Duct  7.856481  
  Adult Mid Gut  7.822993  
  Adult Ovary  8.509165  
  Adult Salivary Gland  8.145023  
  Adult Testes  7.148910  
  Adult Thoracoabdominal ganglion  8.749890  
  Adult Whole Fly  7.467149  
  Larvae Wandering Tubules  8.138650  
  Larval Feeding Carcass  8.188900  
  Larval Feeding Central Nevous System  10.205860  
  Larval Feeding Hind Gut  8.954961  
  Larval Feeding Malpighian Tubule  7.973541  
  Larval Feeding Mid Gut  8.055985  
  Larval Feeding Salivary Gland  8.613878  
  Whole Larvae Feeding  7.880541  
 
  
   FlyBase ID    symbol    start    end    strand    length   
   FBgn0022960   vimar  2862787   2873214   -  10428  
   FBgn0033122   CG17002   2873568   2876427  +  2860  
 
    Segment 28 
 
   Location   
  Gene key  FBgn0029508-FBgn0050159  
  Heatmap region span   2R:2831491..2926737   
  Segment span   2R:2880181..2880191   
  Length (genes)  2  
  Length (bp)  11  
   Model Scoring   
  BIC  256.831655  
  logL  -122.894975  
  logL ratio  42.798271  
   Expression   
  Mean expression  10.196601  
  Median expression  10.371723  
  Tissue std. dev.  0.982511  
 
  No GO Slim enrichment  
  
   tissue    mean expression   
  5th Passage Drosophila S2 Cells  9.324556  
  Adult Accessory gland  10.621298  
  Adult Brain  8.559824  
  Adult Carcass  10.440669  
  Adult Crop  12.125456  
  Adult Eye  11.226848  
  Adult Fatbody  9.631460  
  Adult Female Spermatheca Mated  10.431052  
  Adult Female Spermatheca Virgin  10.362703  
  Adult Head  10.855500  
  Adult Heart  10.073018  
  Adult Hind Gut  11.645366  
  Adult Male Ejaculatory Duct  11.423978  
  Adult Mid Gut  9.633605  
  Adult Ovary  9.911575  
  Adult Salivary Gland  11.629428  
  Adult Testes  8.952514  
  Adult Thoracoabdominal ganglion  9.112186  
  Adult Whole Fly  10.122116  
  Larvae Wandering Tubules  9.022952  
  Larval Feeding Carcass  11.226601  
  Larval Feeding Central Nevous System  8.892663  
  Larval Feeding Hind Gut  11.256622  
  Larval Feeding Malpighian Tubule  9.138017  
  Larval Feeding Mid Gut  8.978027  
  Larval Feeding Salivary Gland  10.508913  
  Whole Larvae Feeding  10.201289  
 
  
   FlyBase ID    symbol    start    end    strand    length   
   FBgn0029508   Tsp42Ea   2880181   2891766  +  11586  
   FBgn0050159   CG30159   2880191   2881811  +  1621  
 
    Segment 29 
 
   Location   
  Gene key  FBgn0029507-FBgn0033127  
  Heatmap region span   2R:2862638..2930426   
  Segment span   2R:2899359..2912517   
  Length (genes)  3  
  Length (bp)  13159  
   Model Scoring   
  BIC  467.606376  
  logL  -228.282336  
  logL ratio  6.710889  
   Expression   
  Mean expression  10.533131  
  Median expression  10.504443  
  Tissue std. dev.  0.800512  
 
  
   GO ID    description    ratio    P-value   
   GO:0008150   biological_process  3/3  0.0102  
   GO:0003674   molecular_function  3/3  0.0144  
 
  
   tissue    mean expression   
  5th Passage Drosophila S2 Cells  12.172126  
  Adult Accessory gland  11.069910  
  Adult Brain  9.652403  
  Adult Carcass  10.043467  
  Adult Crop  11.271619  
  Adult Eye  10.505205  
  Adult Fatbody  10.168150  
  Adult Female Spermatheca Mated  11.037013  
  Adult Female Spermatheca Virgin  10.866047  
  Adult Head  10.694399  
  Adult Heart  10.871658  
  Adult Hind Gut  11.283961  
  Adult Male Ejaculatory Duct  11.017425  
  Adult Mid Gut  10.904068  
  Adult Ovary  8.976383  
  Adult Salivary Gland  10.577517  
  Adult Testes  9.378029  
  Adult Thoracoabdominal ganglion  9.912407  
  Adult Whole Fly  10.199285  
  Larvae Wandering Tubules  11.438498  
  Larval Feeding Carcass  9.940640  
  Larval Feeding Central Nevous System  9.045635  
  Larval Feeding Hind Gut  11.370814  
  Larval Feeding Malpighian Tubule  11.213652  
  Larval Feeding Mid Gut  10.858826  
  Larval Feeding Salivary Gland  9.059885  
  Whole Larvae Feeding  10.865516  
 
  
   FlyBase ID    symbol    start    end    strand    length   
   FBgn0029507   Tsp42Ed  2897365   2899359   -  1995  
   FBgn0029506   Tsp42Ee   2899761   2905869  +  6109  
   FBgn0033127   Tsp42Ef   2912517   2915587  +  3071  
 
 
    Segment 30 
 
   Location   
  Gene key  FBgn0033128-FBgn0033130  
  Heatmap region span   2R:2873214..2937301   
  Segment span   2R:2916418..2922632   
  Length (genes)  3  
  Length (bp)  6215  
   Model Scoring   
  BIC  378.839862  
  logL  -183.899079  
  logL ratio  -4.708093  
   Expression   
  Mean expression  7.593507  
  Median expression  7.833993  
  Tissue std. dev.  1.808091  
 
  
   GO ID    description    ratio    P-value   
   GO:0008150   biological_process  3/3  0.0102  
   GO:0003674   molecular_function  3/3  0.0144  
 
  
   tissue    mean expression   
  5th Passage Drosophila S2 Cells  5.717192  
  Adult Accessory gland  6.516056  
  Adult Brain  4.946304  
  Adult Carcass  9.375695  
  Adult Crop  11.163499  
  Adult Eye  7.288890  
  Adult Fatbody  5.873121  
  Adult Female Spermatheca Mated  7.504952  
  Adult Female Spermatheca Virgin  6.571719  
  Adult Head  8.574572  
  Adult Heart  10.070753  
  Adult Hind Gut  10.450071  
  Adult Male Ejaculatory Duct  8.699604  
  Adult Mid Gut  7.052169  
  Adult Ovary  5.343772  
  Adult Salivary Gland  8.591549  
  Adult Testes  7.367371  
  Adult Thoracoabdominal ganglion  5.310319  
  Adult Whole Fly  7.394733  
  Larvae Wandering Tubules  8.616078  
  Larval Feeding Carcass  10.152495  
  Larval Feeding Central Nevous System  4.914483  
  Larval Feeding Hind Gut  9.373554  
  Larval Feeding Malpighian Tubule  8.682747  
  Larval Feeding Mid Gut  6.197597  
  Larval Feeding Salivary Gland  4.977130  
  Whole Larvae Feeding  8.298270  
 
  
   FlyBase ID    symbol    start    end    strand    length   
   FBgn0033128   Tsp42Eg   2916418   2920738  +  4321  
   FBgn0033129   Tsp42Eh   2921068   2922424  +  1357  
   FBgn0033130   Tsp42Ei   2922632   2924454  +  1823  
 
 
    Segment 31 
 
   Location   
  Gene key  FBgn0033134-FBgn0016032  
  Heatmap region span   2R:2916418..2994447   
  Segment span   2R:2933278..2937301   
  Length (genes)  2  
  Length (bp)  4024  
   Model Scoring   
  BIC  260.714816  
  logL  -124.836555  
  logL ratio  9.207441  
   Expression   
  Mean expression  8.577664  
  Median expression  8.429897  
  Tissue std. dev.  1.424370  
 
  No GO Slim enrichment  
  
   tissue    mean expression   
  5th Passage Drosophila S2 Cells  8.081050  
  Adult Accessory gland  6.509004  
  Adult Brain  6.604676  
  Adult Carcass  9.499933  
  Adult Crop  11.315181  
  Adult Eye  8.650583  
  Adult Fatbody  8.546512  
  Adult Female Spermatheca Mated  9.076061  
  Adult Female Spermatheca Virgin  8.492967  
  Adult Head  9.269306  
  Adult Heart  11.543263  
  Adult Hind Gut  10.562010  
  Adult Male Ejaculatory Duct  9.784412  
  Adult Mid Gut  8.341833  
  Adult Ovary  5.664391  
  Adult Salivary Gland  8.426362  
  Adult Testes  6.436276  
  Adult Thoracoabdominal ganglion  7.634386  
  Adult Whole Fly  7.346953  
  Larvae Wandering Tubules  8.479997  
  Larval Feeding Carcass  10.532750  
  Larval Feeding Central Nevous System  7.794120  
  Larval Feeding Hind Gut  10.060220  
  Larval Feeding Malpighian Tubule  7.824749  
  Larval Feeding Mid Gut  8.040591  
  Larval Feeding Salivary Gland  8.420028  
  Whole Larvae Feeding  8.659301  
 
  
   FlyBase ID    symbol    start    end    strand    length   
   FBgn0033134   Tsp42El   2933278   2936050  +  2773  
   FBgn0016032   lbm   2937301   2939470  +  2170  
 
    Segment 32 
 
   Location   
  Gene key  FBgn0033135-FBgn0033137  
  Heatmap region span   2R:2926737..3017312   
  Segment span   2R:2940279..2945040   
  Length (genes)  3  
  Length (bp)  4762  
   Model Scoring   
  BIC  314.402777  
  logL  -151.680536  
  logL ratio  21.036030  
   Expression   
  Mean expression  5.991818  
  Median expression  5.616941  
  Tissue std. dev.  0.953244  
 
  
   GO ID    description    ratio    P-value   
   GO:0008150   biological_process  3/3  0.0102  
   GO:0003674   molecular_function  3/3  0.0144  
 
  
   tissue    mean expression   
  5th Passage Drosophila S2 Cells  5.201673  
  Adult Accessory gland  5.331664  
  Adult Brain  5.103145  
  Adult Carcass  5.988373  
  Adult Crop  6.685747  
  Adult Eye  5.458605  
  Adult Fatbody  5.394497  
  Adult Female Spermatheca Mated  6.469680  
  Adult Female Spermatheca Virgin  6.234975  
  Adult Head  5.830149  
  Adult Heart  9.235115  
  Adult Hind Gut  6.722822  
  Adult Male Ejaculatory Duct  5.823005  
  Adult Mid Gut  5.498797  
  Adult Ovary  5.191466  
  Adult Salivary Gland  5.371864  
  Adult Testes  5.197189  
  Adult Thoracoabdominal ganglion  5.304475  
  Adult Whole Fly  5.059054  
  Larvae Wandering Tubules  6.485584  
  Larval Feeding Carcass  7.123888  
  Larval Feeding Central Nevous System  5.269497  
  Larval Feeding Hind Gut  6.608193  
  Larval Feeding Malpighian Tubule  5.553278  
  Larval Feeding Mid Gut  5.460606  
  Larval Feeding Salivary Gland  6.187851  
  Whole Larvae Feeding  7.987897  
 
  
   FlyBase ID    symbol    start    end    strand    length   
   FBgn0033135   Tsp42En   2940279   2941548  +  1270  
   FBgn0033136   Tsp42Eo   2942218   2944087  +  1870  
   FBgn0033137   Tsp42Ep  2943902   2945040   -  1139  
 
 
    Segment 33 
 
   Location   
  Gene key  FBgn0033138-FBgn0033139  
  Heatmap region span   2R:2930426..3035203   
  Segment span   2R:2951071..2951921   
  Length (genes)  2  
  Length (bp)  851  
   Model Scoring   
  BIC  209.592422  
  logL  -99.275358  
  logL ratio  42.339003  
   Expression   
  Mean expression  7.272148  
  Median expression  6.446038  
  Tissue std. dev.  2.547280  
 
  No GO Slim enrichment  
  
   tissue    mean expression   
  5th Passage Drosophila S2 Cells  4.504660  
  Adult Accessory gland  4.672920  
  Adult Brain  4.439005  
  Adult Carcass  6.558058  
  Adult Crop  5.549510  
  Adult Eye  5.929544  
  Adult Fatbody  9.136083  
  Adult Female Spermatheca Mated  8.580550  
  Adult Female Spermatheca Virgin  8.566737  
  Adult Head  5.275783  
  Adult Heart  8.440248  
  Adult Hind Gut  9.467243  
  Adult Male Ejaculatory Duct  5.535362  
  Adult Mid Gut  11.924262  
  Adult Ovary  4.734029  
  Adult Salivary Gland  5.072308  
  Adult Testes  6.514917  
  Adult Thoracoabdominal ganglion  4.539347  
  Adult Whole Fly  7.857912  
  Larvae Wandering Tubules  11.333376  
  Larval Feeding Carcass  4.754570  
  Larval Feeding Central Nevous System  4.558338  
  Larval Feeding Hind Gut  10.108496  
  Larval Feeding Malpighian Tubule  11.328549  
  Larval Feeding Mid Gut  11.742919  
  Larval Feeding Salivary Gland  5.244966  
  Whole Larvae Feeding  9.978299  
 
  
   FlyBase ID    symbol    start    end    strand    length   
   FBgn0033138   Tsp42Eq  2949612   2951071   -  1460  
   FBgn0033139   Tsp42Er   2951921   2953618  +  1698  
 
    Segment 34 
 
   Location   
  Gene key  FBgn0033140-FBgn0033142  
  Heatmap region span   2R:2933278..3037142   
  Segment span   2R:2955752..2994447   
  Length (genes)  4  
  Length (bp)  38696  
   Model Scoring   
  BIC  382.560389  
  logL  -185.759342  
  logL ratio  36.764655  
   Expression   
  Mean expression  5.028225  
  Median expression  4.833626  
  Tissue std. dev.  0.429617  
 
  No GO Slim enrichment  
  
   tissue    mean expression   
  5th Passage Drosophila S2 Cells  4.602576  
  Adult Accessory gland  4.639925  
  Adult Brain  5.659862  
  Adult Carcass  4.871844  
  Adult Crop  4.873062  
  Adult Eye  5.197194  
  Adult Fatbody  4.888331  
  Adult Female Spermatheca Mated  4.727508  
  Adult Female Spermatheca Virgin  4.761895  
  Adult Head  5.242467  
  Adult Heart  4.628434  
  Adult Hind Gut  4.567339  
  Adult Male Ejaculatory Duct  4.773471  
  Adult Mid Gut  4.780621  
  Adult Ovary  4.728600  
  Adult Salivary Gland  5.993951  
  Adult Testes  5.801936  
  Adult Thoracoabdominal ganglion  5.675491  
  Adult Whole Fly  4.781671  
  Larvae Wandering Tubules  4.880997  
  Larval Feeding Carcass  5.658311  
  Larval Feeding Central Nevous System  5.734520  
  Larval Feeding Hind Gut  4.595340  
  Larval Feeding Malpighian Tubule  4.673017  
  Larval Feeding Mid Gut  4.837771  
  Larval Feeding Salivary Gland  4.901136  
  Whole Larvae Feeding  5.284804  
 
  
   FlyBase ID    symbol    start    end    strand    length   
   FBgn0033140   CG12836   2955752   2957322  +  1571  
   FBgn0033141   CG12831  2960550   2961621   -  1072  
   FBgn0028642      2973819   3015375  +  41557  
   FBgn0033142      2994447   2995433  +  987  
 
 
    Segment 35 
 
   Location   
  Gene key  FBgn0033149-FBgn0026389  
  Heatmap region span   2R:3038207..3274719   
  Segment span   2R:3063391..3122418   
  Length (genes)  3  
  Length (bp)  59028  
   Model Scoring   
  BIC  304.461019  
  logL  -146.709657  
  logL ratio  34.020501  
   Expression   
  Mean expression  5.066111  
  Median expression  5.273410  
  Tissue std. dev.  0.585618  
 
  No GO Slim enrichment  
  
   tissue    mean expression   
  5th Passage Drosophila S2 Cells  4.909503  
  Adult Accessory gland  5.183253  
  Adult Brain  4.803091  
  Adult Carcass  5.086829  
  Adult Crop  5.055556  
  Adult Eye  4.731736  
  Adult Fatbody  5.034123  
  Adult Female Spermatheca Mated  4.957053  
  Adult Female Spermatheca Virgin  5.026135  
  Adult Head  4.831066  
  Adult Heart  4.726874  
  Adult Hind Gut  4.973342  
  Adult Male Ejaculatory Duct  4.897415  
  Adult Mid Gut  5.131919  
  Adult Ovary  4.822581  
  Adult Salivary Gland  5.348160  
  Adult Testes  7.946208  
  Adult Thoracoabdominal ganglion  4.742721  
  Adult Whole Fly  5.028559  
  Larvae Wandering Tubules  5.104819  
  Larval Feeding Carcass  4.941909  
  Larval Feeding Central Nevous System  4.766374  
  Larval Feeding Hind Gut  4.703179  
  Larval Feeding Malpighian Tubule  5.116207  
  Larval Feeding Mid Gut  5.021962  
  Larval Feeding Salivary Gland  4.982963  
  Whole Larvae Feeding  4.911458  
 
  
   FlyBase ID    symbol    start    end    strand    length   
   FBgn0033149   CG11060   3063391   3065793  +  2403  
   FBgn0050384   CG30384   3070502   3072808  +  2307  
   FBgn0026389   Or43a  3120344   3122418   -  2075  
 
 
    Segment 36 
 
   Location   
  Gene key  FBgn0033153-FBgn0033155  
  Heatmap region span   2R:3042927..3283453   
  Segment span   2R:3136668..3165580   
  Length (genes)  3  
  Length (bp)  28913  
   Model Scoring   
  BIC  376.322180  
  logL  -182.640237  
  logL ratio  -21.227495  
   Expression   
  Mean expression  6.636742  
  Median expression  6.639927  
  Tissue std. dev.  0.395638  
 
  No GO Slim enrichment  
  
   tissue    mean expression   
  5th Passage Drosophila S2 Cells  7.385788  
  Adult Accessory gland  6.376612  
  Adult Brain  6.755628  
  Adult Carcass  6.499371  
  Adult Crop  6.698662  
  Adult Eye  6.567948  
  Adult Fatbody  6.208749  
  Adult Female Spermatheca Mated  5.969557  
  Adult Female Spermatheca Virgin  5.886945  
  Adult Head  6.127197  
  Adult Heart  6.905671  
  Adult Hind Gut  7.016594  
  Adult Male Ejaculatory Duct  6.264574  
  Adult Mid Gut  6.629100  
  Adult Ovary  7.369969  
  Adult Salivary Gland  7.025214  
  Adult Testes  6.371951  
  Adult Thoracoabdominal ganglion  6.542238  
  Adult Whole Fly  6.433035  
  Larvae Wandering Tubules  7.202912  
  Larval Feeding Carcass  7.044892  
  Larval Feeding Central Nevous System  6.463246  
  Larval Feeding Hind Gut  6.864467  
  Larval Feeding Malpighian Tubule  7.044919  
  Larval Feeding Mid Gut  6.691095  
  Larval Feeding Salivary Gland  6.658891  
  Whole Larvae Feeding  6.186803  
 
  
   FlyBase ID    symbol    start    end    strand    length   
   FBgn0033153   Gadd45   3136668   3138160  +  1493  
   FBgn0033154   CG1850  3146372   3148756   -  2385  
   FBgn0033155   Br140  3160037   3165580   -  5544  
 
 
    Segment 37 
 
   Location   
  Gene key  FBgn0003174-FBgn0033159  
  Heatmap region span   2R:3045733..3289373   
  Segment span   2R:3182813..3269374   
  Length (genes)  3  
  Length (bp)  86562  
   Model Scoring   
  BIC  288.072901  
  logL  -138.515598  
  logL ratio  36.668041  
   Expression   
  Mean expression  5.129553  
  Median expression  4.950625  
  Tissue std. dev.  0.345568  
 
  No GO Slim enrichment  
  
   tissue    mean expression   
  5th Passage Drosophila S2 Cells  5.950410  
  Adult Accessory gland  5.597103  
  Adult Brain  5.197314  
  Adult Carcass  5.210372  
  Adult Crop  5.139569  
  Adult Eye  5.402154  
  Adult Fatbody  4.814577  
  Adult Female Spermatheca Mated  4.846276  
  Adult Female Spermatheca Virgin  4.793187  
  Adult Head  5.308730  
  Adult Heart  5.000421  
  Adult Hind Gut  4.995569  
  Adult Male Ejaculatory Duct  5.380544  
  Adult Mid Gut  5.151687  
  Adult Ovary  4.619161  
  Adult Salivary Gland  4.870972  
  Adult Testes  4.706285  
  Adult Thoracoabdominal ganglion  5.245992  
  Adult Whole Fly  4.488084  
  Larvae Wandering Tubules  5.053939  
  Larval Feeding Carcass  5.801549  
  Larval Feeding Central Nevous System  5.340683  
  Larval Feeding Hind Gut  5.626468  
  Larval Feeding Malpighian Tubule  4.939041  
  Larval Feeding Mid Gut  5.011071  
  Larval Feeding Salivary Gland  4.828764  
  Whole Larvae Feeding  5.178011  
 
  
   FlyBase ID    symbol    start    end    strand    length   
   FBgn0003174   pwn   3182813   3187907  +  5095  
   FBgn0033158   CG12164   3198913   3200855  +  1943  
   FBgn0033159   Dscam  3207059   3269374   -  62316  
 
 
    Segment 38 
 
   Location   
  Gene key  FBgn0000352-FBgn0033160  
  Heatmap region span   2R:3063391..3295326   
  Segment span   2R:3274617..3274719   
  Length (genes)  2  
  Length (bp)  103  
   Model Scoring   
  BIC  233.164080  
  logL  -111.061187  
  logL ratio  28.302425  
   Expression   
  Mean expression  8.530589  
  Median expression  8.672645  
  Tissue std. dev.  0.775977  
 
  No GO Slim enrichment  
  
   tissue    mean expression   
  5th Passage Drosophila S2 Cells  9.631716  
  Adult Accessory gland  8.349979  
  Adult Brain  9.856019  
  Adult Carcass  8.063628  
  Adult Crop  8.525949  
  Adult Eye  8.200757  
  Adult Fatbody  8.325590  
  Adult Female Spermatheca Mated  8.200102  
  Adult Female Spermatheca Virgin  8.106248  
  Adult Head  8.651540  
  Adult Heart  8.608628  
  Adult Hind Gut  7.903760  
  Adult Male Ejaculatory Duct  7.640259  
  Adult Mid Gut  7.770207  
  Adult Ovary  10.101409  
  Adult Salivary Gland  8.012075  
  Adult Testes  7.744366  
  Adult Thoracoabdominal ganglion  9.307103  
  Adult Whole Fly  9.043653  
  Larvae Wandering Tubules  7.925250  
  Larval Feeding Carcass  9.006494  
  Larval Feeding Central Nevous System  10.702860  
  Larval Feeding Hind Gut  8.365969  
  Larval Feeding Malpighian Tubule  7.898941  
  Larval Feeding Mid Gut  7.637742  
  Larval Feeding Salivary Gland  8.310178  
  Whole Larvae Feeding  8.435482  
 
  
   FlyBase ID    symbol    start    end    strand    length   
   FBgn0000352   cos  3269669   3274617   -  4949  
   FBgn0033160   CG11107   3274719   3278529  +  3811  
 
    Segment 39 
 
   Location   
  Gene key  FBgn0033164-FBgn0033165  
  Heatmap region span   2R:3182813..3328198   
  Segment span   2R:3288292..3289373   
  Length (genes)  2  
  Length (bp)  1082  
   Model Scoring   
  BIC  195.091635  
  logL  -92.024965  
  logL ratio  34.353696  
   Expression   
  Mean expression  4.931487  
  Median expression  4.646476  
  Tissue std. dev.  1.670718  
 
  No GO Slim enrichment  
  
   tissue    mean expression   
  5th Passage Drosophila S2 Cells  4.427634  
  Adult Accessory gland  12.771145  
  Adult Brain  4.224341  
  Adult Carcass  4.646663  
  Adult Crop  4.497292  
  Adult Eye  4.150868  
  Adult Fatbody  4.694888  
  Adult Female Spermatheca Mated  4.658165  
  Adult Female Spermatheca Virgin  4.674777  
  Adult Head  4.231383  
  Adult Heart  4.266230  
  Adult Hind Gut  4.349336  
  Adult Male Ejaculatory Duct  7.497240  
  Adult Mid Gut  4.581016  
  Adult Ovary  4.410571  
  Adult Salivary Gland  4.793683  
  Adult Testes  4.550078  
  Adult Thoracoabdominal ganglion  4.310925  
  Adult Whole Fly  5.993256  
  Larvae Wandering Tubules  4.535669  
  Larval Feeding Carcass  4.459179  
  Larval Feeding Central Nevous System  4.280195  
  Larval Feeding Hind Gut  4.335755  
  Larval Feeding Malpighian Tubule  4.452488  
  Larval Feeding Mid Gut  4.426116  
  Larval Feeding Salivary Gland  4.655133  
  Whole Larvae Feeding  4.276132  
 
  
   FlyBase ID    symbol    start    end    strand    length   
   FBgn0033164   CG11112   3288292   3288991  +  700  
   FBgn0033165   CG11113   3289373   3289870  +  498  
 
    Segment 40 
 
   Location   
  Gene key  FBgn0003460-FBgn0033168  
  Heatmap region span   2R:3283453..3339056   
  Segment span   2R:3306537..3316098   
  Length (genes)  2  
  Length (bp)  9562  
   Model Scoring   
  BIC  214.724211  
  logL  -101.841253  
  logL ratio  4.385764  
   Expression   
  Mean expression  5.250603  
  Median expression  4.659652  
  Tissue std. dev.  1.050542  
 
  No GO Slim enrichment  
  
   tissue    mean expression   
  5th Passage Drosophila S2 Cells  4.509007  
  Adult Accessory gland  4.959486  
  Adult Brain  4.741309  
  Adult Carcass  5.332633  
  Adult Crop  5.959611  
  Adult Eye  6.785203  
  Adult Fatbody  4.512103  
  Adult Female Spermatheca Mated  4.591397  
  Adult Female Spermatheca Virgin  4.585444  
  Adult Head  5.851510  
  Adult Heart  6.004839  
  Adult Hind Gut  5.244829  
  Adult Male Ejaculatory Duct  4.745966  
  Adult Mid Gut  4.769571  
  Adult Ovary  4.613383  
  Adult Salivary Gland  4.806401  
  Adult Testes  9.600050  
  Adult Thoracoabdominal ganglion  4.384319  
  Adult Whole Fly  5.855963  
  Larvae Wandering Tubules  4.558798  
  Larval Feeding Carcass  5.905388  
  Larval Feeding Central Nevous System  5.351642  
  Larval Feeding Hind Gut  5.170319  
  Larval Feeding Malpighian Tubule  4.383019  
  Larval Feeding Mid Gut  4.709909  
  Larval Feeding Salivary Gland  4.485914  
  Whole Larvae Feeding  5.348265  
 
  
   FlyBase ID    symbol    start    end    strand    length   
   FBgn0003460   so   3306537   3322377  +  15841  
   FBgn0033168   CG11145  3315409   3316098   -  690  
 
    Segment 41 
 
   Location   
  Gene key  FBgn0025885-FBgn0033179  
  Heatmap region span   2R:3328198..3406785   
  Segment span   2R:3346373..3354236   
  Length (genes)  4  
  Length (bp)  7864  
   Model Scoring   
  BIC  529.056883  
  logL  -259.007589  
  logL ratio  3.060066  
   Expression   
  Mean expression  8.983797  
  Median expression  9.030929  
  Tissue std. dev.  0.445378  
 
  No GO Slim enrichment  
  
   tissue    mean expression   
  5th Passage Drosophila S2 Cells  10.089015  
  Adult Accessory gland  8.107384  
  Adult Brain  9.140721  
  Adult Carcass  9.148283  
  Adult Crop  8.895847  
  Adult Eye  9.420647  
  Adult Fatbody  8.899517  
  Adult Female Spermatheca Mated  8.097723  
  Adult Female Spermatheca Virgin  8.218126  
  Adult Head  9.163832  
  Adult Heart  9.696325  
  Adult Hind Gut  8.863272  
  Adult Male Ejaculatory Duct  9.324578  
  Adult Mid Gut  8.644113  
  Adult Ovary  9.260062  
  Adult Salivary Gland  8.699348  
  Adult Testes  9.507327  
  Adult Thoracoabdominal ganglion  9.194812  
  Adult Whole Fly  8.831023  
  Larvae Wandering Tubules  9.000072  
  Larval Feeding Carcass  9.042498  
  Larval Feeding Central Nevous System  9.292053  
  Larval Feeding Hind Gut  9.134753  
  Larval Feeding Malpighian Tubule  8.529704  
  Larval Feeding Mid Gut  8.800656  
  Larval Feeding Salivary Gland  8.844046  
  Whole Larvae Feeding  8.716776  
 
  
   FlyBase ID    symbol    start    end    strand    length   
   FBgn0025885   Inos  3342427   3346373   -  3947  
   FBgn0033178   CG11127   3350876   3352492  +  1617  
   FBgn0033177   CG11141  3347936   3350900   -  2965  
   FBgn0033179   p47  3352523   3354236   -  1714  
 
 
    Segment 42 
 
   Location   
  Gene key  FBgn0033182-FBgn0033184  
  Heatmap region span   2R:3334984..3420487   
  Segment span   2R:3380084..3397959   
  Length (genes)  4  
  Length (bp)  17876  
   Model Scoring   
  BIC  622.805747  
  logL  -305.882021  
  logL ratio  -126.745074  
   Expression   
  Mean expression  8.011068  
  Median expression  8.632618  
  Tissue std. dev.  0.732329  
 
  No GO Slim enrichment  
  
   tissue    mean expression   
  5th Passage Drosophila S2 Cells  9.248831  
  Adult Accessory gland  8.864111  
  Adult Brain  7.220328  
  Adult Carcass  7.526059  
  Adult Crop  7.866416  
  Adult Eye  8.213572  
  Adult Fatbody  7.315266  
  Adult Female Spermatheca Mated  7.638895  
  Adult Female Spermatheca Virgin  7.635211  
  Adult Head  7.613824  
  Adult Heart  7.458196  
  Adult Hind Gut  7.661952  
  Adult Male Ejaculatory Duct  7.608032  
  Adult Mid Gut  7.090790  
  Adult Ovary  9.753195  
  Adult Salivary Gland  7.341805  
  Adult Testes  8.475693  
  Adult Thoracoabdominal ganglion  7.193838  
  Adult Whole Fly  8.935681  
  Larvae Wandering Tubules  7.349801  
  Larval Feeding Carcass  8.754344  
  Larval Feeding Central Nevous System  9.246808  
  Larval Feeding Hind Gut  8.543475  
  Larval Feeding Malpighian Tubule  7.660604  
  Larval Feeding Mid Gut  7.310178  
  Larval Feeding Salivary Gland  8.491940  
  Whole Larvae Feeding  8.279994  
 
  
   FlyBase ID    symbol    start    end    strand    length   
   FBgn0033182     3378341   3380084   -  1744  
   FBgn0033183   CG1620  3381311   3383971   -  2661  
   FBgn0015929   dpa  3384207   3387394   -  3188  
   FBgn0033184   CG12736  3396032   3397959   -  1928  
 
 
    Segment 43 
 
   Location   
  Gene key  FBgn0025185-FBgn0033186  
  Heatmap region span   2R:3339056..3423424   
  Segment span   2R:3400229..3406426   
  Length (genes)  3  
  Length (bp)  6198  
   Model Scoring   
  BIC  272.708540  
  logL  -130.833417  
  logL ratio  82.889125  
   Expression   
  Mean expression  6.384660  
  Median expression  6.184040  
  Tissue std. dev.  0.637048  
 
  
   GO ID    description    ratio    P-value   
   GO:0005622   intracellular  3/3  0.000104  
 
  
   tissue    mean expression   
  5th Passage Drosophila S2 Cells  7.267826  
  Adult Accessory gland  6.685410  
  Adult Brain  7.203982  
  Adult Carcass  5.702344  
  Adult Crop  6.531679  
  Adult Eye  6.595237  
  Adult Fatbody  5.647674  
  Adult Female Spermatheca Mated  6.000084  
  Adult Female Spermatheca Virgin  5.853798  
  Adult Head  6.099559  
  Adult Heart  6.298570  
  Adult Hind Gut  5.970945  
  Adult Male Ejaculatory Duct  5.818075  
  Adult Mid Gut  5.976002  
  Adult Ovary  8.384670  
  Adult Salivary Gland  5.982605  
  Adult Testes  6.756887  
  Adult Thoracoabdominal ganglion  7.014294  
  Adult Whole Fly  6.657984  
  Larvae Wandering Tubules  6.316696  
  Larval Feeding Carcass  5.939370  
  Larval Feeding Central Nevous System  7.479397  
  Larval Feeding Hind Gut  6.076600  
  Larval Feeding Malpighian Tubule  6.126222  
  Larval Feeding Mid Gut  5.702090  
  Larval Feeding Salivary Gland  6.503860  
  Whole Larvae Feeding  5.793970  
 
  
   FlyBase ID    symbol    start    end    strand    length   
   FBgn0025185   az2  3398116   3400229   -  2114  
   FBgn0033185   CG1603  3401575   3403875   -  2301  
   FBgn0033186   CG1602  3404426   3406426   -  2001  
 
 
    Segment 44 
 
   Location   
  Gene key  FBgn0050499-FBgn0033194  
  Heatmap region span   2R:3420487..3510749   
  Segment span   2R:3453254..3454770   
  Length (genes)  3  
  Length (bp)  1517  
   Model Scoring   
  BIC  306.989458  
  logL  -147.973877  
  logL ratio  102.018336  
   Expression   
  Mean expression  9.705212  
  Median expression  9.734842  
  Tissue std. dev.  0.453333  
 
  No GO Slim enrichment  
  
   tissue    mean expression   
  5th Passage Drosophila S2 Cells  10.235784  
  Adult Accessory gland  9.570821  
  Adult Brain  9.033250  
  Adult Carcass  9.915755  
  Adult Crop  10.179502  
  Adult Eye  9.800397  
  Adult Fatbody  10.213456  
  Adult Female Spermatheca Mated  9.659663  
  Adult Female Spermatheca Virgin  9.750813  
  Adult Head  9.558179  
  Adult Heart  10.478535  
  Adult Hind Gut  9.798084  
  Adult Male Ejaculatory Duct  10.687231  
  Adult Mid Gut  9.096372  
  Adult Ovary  9.784678  
  Adult Salivary Gland  9.615550  
  Adult Testes  9.106837  
  Adult Thoracoabdominal ganglion  9.268174  
  Adult Whole Fly  9.481037  
  Larvae Wandering Tubules  9.879528  
  Larval Feeding Carcass  9.644999  
  Larval Feeding Central Nevous System  9.860934  
  Larval Feeding Hind Gut  9.485973  
  Larval Feeding Malpighian Tubule  9.818501  
  Larval Feeding Mid Gut  8.690110  
  Larval Feeding Salivary Gland  10.228143  
  Whole Larvae Feeding  9.198407  
 
  
   FlyBase ID    symbol    start    end    strand    length   
   FBgn0050499   CG30499  3451937   3453254   -  1318  
   FBgn0004132   boca  3453375   3454240   -  866  
   FBgn0033194   Vps13   3454770   3468180  +  13411  
 
 
    Segment 45 
 
   Location   
  Gene key  FBgn0050491-FBgn0033199  
  Heatmap region span   2R:3508216..3558608   
  Segment span   2R:3542633..3542816   
  Length (genes)  2  
  Length (bp)  184  
   Model Scoring   
  BIC  214.270060  
  logL  -101.614177  
  logL ratio  45.162722  
   Expression   
  Mean expression  8.679823  
  Median expression  8.527494  
  Tissue std. dev.  0.498276  
 
  No GO Slim enrichment  
  
   tissue    mean expression   
  5th Passage Drosophila S2 Cells  8.511813  
  Adult Accessory gland  8.662428  
  Adult Brain  9.046314  
  Adult Carcass  8.304130  
  Adult Crop  8.996189  
  Adult Eye  8.263046  
  Adult Fatbody  8.692592  
  Adult Female Spermatheca Mated  8.809978  
  Adult Female Spermatheca Virgin  8.710562  
  Adult Head  8.527216  
  Adult Heart  8.666089  
  Adult Hind Gut  8.775932  
  Adult Male Ejaculatory Duct  8.931123  
  Adult Mid Gut  8.238271  
  Adult Ovary  8.483985  
  Adult Salivary Gland  9.962218  
  Adult Testes  8.701873  
  Adult Thoracoabdominal ganglion  9.118229  
  Adult Whole Fly  8.038030  
  Larvae Wandering Tubules  8.513385  
  Larval Feeding Carcass  8.415699  
  Larval Feeding Central Nevous System  8.164525  
  Larval Feeding Hind Gut  8.395563  
  Larval Feeding Malpighian Tubule  8.529273  
  Larval Feeding Mid Gut  8.257492  
  Larval Feeding Salivary Gland  10.335961  
  Whole Larvae Feeding  8.303316  
 
  
   FlyBase ID    symbol    start    end    strand    length   
   FBgn0050491   CG30491   3542633   3544275  +  1643  
   FBgn0033199   CG17985  3539124   3542816   -  3693  
 
    Segment 46 
 
   Location   
  Gene key  FBgn0033202-FBgn0033203  
  Heatmap region span   2R:3528417..3586395   
  Segment span   2R:3548123..3549517   
  Length (genes)  2  
  Length (bp)  1395  
   Model Scoring   
  BIC  211.611964  
  logL  -100.285129  
  logL ratio  8.740297  
   Expression   
  Mean expression  4.909078  
  Median expression  4.702642  
  Tissue std. dev.  0.475868  
 
  No GO Slim enrichment  
  
   tissue    mean expression   
  5th Passage Drosophila S2 Cells  4.831334  
  Adult Accessory gland  5.111868  
  Adult Brain  4.357291  
  Adult Carcass  6.979143  
  Adult Crop  4.961591  
  Adult Eye  4.597853  
  Adult Fatbody  5.193360  
  Adult Female Spermatheca Mated  4.883621  
  Adult Female Spermatheca Virgin  4.767169  
  Adult Head  4.695567  
  Adult Heart  4.675936  
  Adult Hind Gut  4.891663  
  Adult Male Ejaculatory Duct  4.904516  
  Adult Mid Gut  4.923593  
  Adult Ovary  4.667644  
  Adult Salivary Gland  4.869918  
  Adult Testes  4.704239  
  Adult Thoracoabdominal ganglion  4.719102  
  Adult Whole Fly  4.845903  
  Larvae Wandering Tubules  4.863569  
  Larval Feeding Carcass  5.760348  
  Larval Feeding Central Nevous System  4.473145  
  Larval Feeding Hind Gut  4.660774  
  Larval Feeding Malpighian Tubule  4.853146  
  Larval Feeding Mid Gut  4.844823  
  Larval Feeding Salivary Gland  4.752772  
  Whole Larvae Feeding  4.755223  
 
  
   FlyBase ID    symbol    start    end    strand    length   
   FBgn0033202   CG1339  3546761   3548123   -  1363  
   FBgn0033203   CG2070   3549517   3550764  +  1248  
 
    Segment 47 
 
   Location   
  Gene key  FBgn0033204-FBgn0033206  
  Heatmap region span   2R:3541453..3586786   
  Segment span   2R:3551291..3556534   
  Length (genes)  3  
  Length (bp)  5244  
   Model Scoring   
  BIC  316.701716  
  logL  -152.830006  
  logL ratio  65.861620  
   Expression   
  Mean expression  8.401637  
  Median expression  8.564293  
  Tissue std. dev.  0.671601  
 
  
   GO ID    description    ratio    P-value   
   GO:0016491   oxidoreductase activity  2/3  0.00028  
 
  
   tissue    mean expression   
  5th Passage Drosophila S2 Cells  9.893035  
  Adult Accessory gland  7.197578  
  Adult Brain  7.777338  
  Adult Carcass  8.514603  
  Adult Crop  9.411381  
  Adult Eye  8.088019  
  Adult Fatbody  8.423643  
  Adult Female Spermatheca Mated  8.996547  
  Adult Female Spermatheca Virgin  8.820249  
  Adult Head  8.281845  
  Adult Heart  8.962558  
  Adult Hind Gut  8.954345  
  Adult Male Ejaculatory Duct  8.247678  
  Adult Mid Gut  9.389271  
  Adult Ovary  7.119892  
  Adult Salivary Gland  8.536022  
  Adult Testes  7.506136  
  Adult Thoracoabdominal ganglion  8.419677  
  Adult Whole Fly  7.668456  
  Larvae Wandering Tubules  7.996582  
  Larval Feeding Carcass  8.177389  
  Larval Feeding Central Nevous System  7.493013  
  Larval Feeding Hind Gut  8.559117  
  Larval Feeding Malpighian Tubule  8.464349  
  Larval Feeding Mid Gut  9.099816  
  Larval Feeding Salivary Gland  8.060321  
  Whole Larvae Feeding  8.785333  
 
  
   FlyBase ID    symbol    start    end    strand    length   
   FBgn0033204   CG2065   3551291   3552780  +  1490  
   FBgn0033205   CG2064   3553338   3554848  +  1511  
   FBgn0033206   CG12042  3554753   3556534   -  1782  
 
 
    Segment 48 
 
   Location   
  Gene key  FBgn0033208-FBgn0033209  
  Heatmap region span   2R:3544701..3597379   
  Segment span   2R:3561908..3561951   
  Length (genes)  2  
  Length (bp)  44  
   Model Scoring   
  BIC  220.242592  
  logL  -104.600443  
  logL ratio  53.462773  
   Expression   
  Mean expression  9.734472  
  Median expression  9.770846  
  Tissue std. dev.  0.277766  
 
  No GO Slim enrichment  
  
   tissue    mean expression   
  5th Passage Drosophila S2 Cells  9.641274  
  Adult Accessory gland  9.475121  
  Adult Brain  9.619230  
  Adult Carcass  9.725791  
  Adult Crop  9.738981  
  Adult Eye  9.987578  
  Adult Fatbody  9.949291  
  Adult Female Spermatheca Mated  9.397867  
  Adult Female Spermatheca Virgin  9.369014  
  Adult Head  9.457708  
  Adult Heart  10.099583  
  Adult Hind Gut  9.655285  
  Adult Male Ejaculatory Duct  9.801683  
  Adult Mid Gut  9.735015  
  Adult Ovary  10.566335  
  Adult Salivary Gland  9.872965  
  Adult Testes  9.414265  
  Adult Thoracoabdominal ganglion  9.740355  
  Adult Whole Fly  9.790615  
  Larvae Wandering Tubules  9.756758  
  Larval Feeding Carcass  9.758950  
  Larval Feeding Central Nevous System  9.699795  
  Larval Feeding Hind Gut  9.975591  
  Larval Feeding Malpighian Tubule  9.972247  
  Larval Feeding Mid Gut  9.236510  
  Larval Feeding Salivary Gland  10.036578  
  Whole Larvae Feeding  9.356361  
 
  
   FlyBase ID    symbol    start    end    strand    length   
   FBgn0033208   mRpL52  3561226   3561908   -  683  
   FBgn0033209   CG12107   3561951   3562867  +  917  
 
    Segment 49 
 
   Location   
  Gene key  FBgn0033210-FBgn0033212  
  Heatmap region span   2R:3548123..3602488   
  Segment span   2R:3563857..3586395   
  Length (genes)  2  
  Length (bp)  22539  
   Model Scoring   
  BIC  245.857114  
  logL  -117.407704  
  logL ratio  -5.118826  
   Expression   
  Mean expression  6.492240  
  Median expression  6.630579  
  Tissue std. dev.  0.558020  
 
  No GO Slim enrichment  
  
   tissue    mean expression   
  5th Passage Drosophila S2 Cells  8.009060  
  Adult Accessory gland  6.840592  
  Adult Brain  6.135019  
  Adult Carcass  5.737583  
  Adult Crop  6.292471  
  Adult Eye  5.902776  
  Adult Fatbody  5.989999  
  Adult Female Spermatheca Mated  5.741649  
  Adult Female Spermatheca Virgin  5.755675  
  Adult Head  6.060696  
  Adult Heart  6.570620  
  Adult Hind Gut  6.546041  
  Adult Male Ejaculatory Duct  6.318883  
  Adult Mid Gut  6.763865  
  Adult Ovary  7.496446  
  Adult Salivary Gland  5.952854  
  Adult Testes  6.280509  
  Adult Thoracoabdominal ganglion  5.946706  
  Adult Whole Fly  6.679763  
  Larvae Wandering Tubules  7.254428  
  Larval Feeding Carcass  6.241065  
  Larval Feeding Central Nevous System  7.013098  
  Larval Feeding Hind Gut  6.776643  
  Larval Feeding Malpighian Tubule  6.905893  
  Larval Feeding Mid Gut  6.628539  
  Larval Feeding Salivary Gland  7.093967  
  Whole Larvae Feeding  6.355645  
 
  
   FlyBase ID    symbol    start    end    strand    length   
   FBgn0033210   U2A  3562867   3563857   -  991  
   FBgn0033212   CG1399  3564045   3586395   -  22351  
 
    Segment 50 
 
   Location   
  Gene key  FBgn0033215-FBgn0033216  
  Heatmap region span   2R:3563857..3677896   
  Segment span   2R:3600128..3602488   
  Length (genes)  2  
  Length (bp)  2361  
   Model Scoring   
  BIC  221.438429  
  logL  -105.198362  
  logL ratio  -2.861189  
   Expression   
  Mean expression  5.301780  
  Median expression  4.725729  
  Tissue std. dev.  1.028643  
 
  No GO Slim enrichment  
  
   tissue    mean expression   
  5th Passage Drosophila S2 Cells  4.611546  
  Adult Accessory gland  4.997065  
  Adult Brain  4.178283  
  Adult Carcass  6.138124  
  Adult Crop  4.743576  
  Adult Eye  4.806907  
  Adult Fatbody  6.637004  
  Adult Female Spermatheca Mated  6.221096  
  Adult Female Spermatheca Virgin  5.595511  
  Adult Head  5.336469  
  Adult Heart  6.124270  
  Adult Hind Gut  5.350983  
  Adult Male Ejaculatory Duct  4.857559  
  Adult Mid Gut  9.117048  
  Adult Ovary  4.521731  
  Adult Salivary Gland  4.995722  
  Adult Testes  4.464822  
  Adult Thoracoabdominal ganglion  4.513196  
  Adult Whole Fly  5.192692  
  Larvae Wandering Tubules  4.948242  
  Larval Feeding Carcass  4.661293  
  Larval Feeding Central Nevous System  4.248260  
  Larval Feeding Hind Gut  4.521866  
  Larval Feeding Malpighian Tubule  5.214764  
  Larval Feeding Mid Gut  6.861875  
  Larval Feeding Salivary Gland  4.618515  
  Whole Larvae Feeding  5.669650  
 
  
   FlyBase ID    symbol    start    end    strand    length   
   FBgn0033215   CG1942   3600128   3601695  +  1568  
   FBgn0033216   CG1946   3602488   3604296  +  1809  
 
    Segment 51 
 
   Location   
  Gene key  FBgn0042135-FBgn0050497  
  Heatmap region span   2R:3586786..3680757   
  Segment span   2R:3623124..3670119   
  Length (genes)  2  
  Length (bp)  46996  
   Model Scoring   
  BIC  253.224842  
  logL  -121.091568  
  logL ratio  48.673992  
   Expression   
  Mean expression  10.370302  
  Median expression  10.447855  
  Tissue std. dev.  0.807693  
 
  No GO Slim enrichment  
  
   tissue    mean expression   
  5th Passage Drosophila S2 Cells  10.052148  
  Adult Accessory gland  9.375812  
  Adult Brain  10.224840  
  Adult Carcass  10.282034  
  Adult Crop  11.154221  
  Adult Eye  11.181801  
  Adult Fatbody  10.017948  
  Adult Female Spermatheca Mated  9.350603  
  Adult Female Spermatheca Virgin  9.381089  
  Adult Head  10.696883  
  Adult Heart  10.937489  
  Adult Hind Gut  10.501204  
  Adult Male Ejaculatory Duct  11.381735  
  Adult Mid Gut  10.621645  
  Adult Ovary  10.168834  
  Adult Salivary Gland  11.476527  
  Adult Testes  8.073054  
  Adult Thoracoabdominal ganglion  10.134991  
  Adult Whole Fly  9.944671  
  Larvae Wandering Tubules  10.936600  
  Larval Feeding Carcass  10.914496  
  Larval Feeding Central Nevous System  8.816184  
  Larval Feeding Hind Gut  10.645876  
  Larval Feeding Malpighian Tubule  10.985494  
  Larval Feeding Mid Gut  10.983424  
  Larval Feeding Salivary Gland  11.320183  
  Whole Larvae Feeding  10.438371  
 
  
   FlyBase ID    symbol    start    end    strand    length   
   FBgn0042135   CG18812  3605833   3623124   -  17292  
   FBgn0050497   CG30497  3625273   3670119   -  44847  
 
    Segment 52 
 
   Location   
  Gene key  FBgn0033221-FBgn0033222  
  Heatmap region span   2R:3600128..3711073   
  Segment span   2R:3676719..3677896   
  Length (genes)  2  
  Length (bp)  1178  
   Model Scoring   
  BIC  221.965212  
  logL  -105.461753  
  logL ratio  38.979112  
   Expression   
  Mean expression  7.349598  
  Median expression  5.963049  
  Tissue std. dev.  2.720891  
 
  No GO Slim enrichment  
  
   tissue    mean expression   
  5th Passage Drosophila S2 Cells  5.519367  
  Adult Accessory gland  5.236114  
  Adult Brain  4.875749  
  Adult Carcass  6.314409  
  Adult Crop  5.542483  
  Adult Eye  5.322750  
  Adult Fatbody  6.896556  
  Adult Female Spermatheca Mated  5.840341  
  Adult Female Spermatheca Virgin  6.194373  
  Adult Head  5.397712  
  Adult Heart  9.018847  
  Adult Hind Gut  10.101668  
  Adult Male Ejaculatory Duct  5.521420  
  Adult Mid Gut  11.705614  
  Adult Ovary  4.920343  
  Adult Salivary Gland  5.767277  
  Adult Testes  4.707058  
  Adult Thoracoabdominal ganglion  5.405195  
  Adult Whole Fly  8.428725  
  Larvae Wandering Tubules  12.874805  
  Larval Feeding Carcass  5.679569  
  Larval Feeding Central Nevous System  4.766915  
  Larval Feeding Hind Gut  10.503809  
  Larval Feeding Malpighian Tubule  12.830697  
  Larval Feeding Mid Gut  12.051230  
  Larval Feeding Salivary Gland  6.048433  
  Whole Larvae Feeding  10.967689  
 
  
   FlyBase ID    symbol    start    end    strand    length   
   FBgn0033221   CG12825   3676719   3677496  +  778  
   FBgn0033222   CG12824   3677896   3678676  +  781  
 
    Segment 53 
 
   Location   
  Gene key  FBgn0033224-FBgn0033226  
  Heatmap region span   2R:3673517..3777580   
  Segment span   2R:3693904..3700011   
  Length (genes)  4  
  Length (bp)  6108  
   Model Scoring   
  BIC  396.992501  
  logL  -192.975398  
  logL ratio  140.467465  
   Expression   
  Mean expression  9.564461  
  Median expression  9.466566  
  Tissue std. dev.  0.334901  
 
  No GO Slim enrichment  
  
   tissue    mean expression   
  5th Passage Drosophila S2 Cells  9.828486  
  Adult Accessory gland  9.711779  
  Adult Brain  9.702128  
  Adult Carcass  9.191380  
  Adult Crop  9.721179  
  Adult Eye  9.383136  
  Adult Fatbody  9.753962  
  Adult Female Spermatheca Mated  9.599299  
  Adult Female Spermatheca Virgin  9.653906  
  Adult Head  9.427451  
  Adult Heart  9.687429  
  Adult Hind Gut  10.331963  
  Adult Male Ejaculatory Duct  9.717488  
  Adult Mid Gut  9.726678  
  Adult Ovary  9.948306  
  Adult Salivary Gland  9.403554  
  Adult Testes  8.876064  
  Adult Thoracoabdominal ganglion  9.748149  
  Adult Whole Fly  9.476734  
  Larvae Wandering Tubules  9.669060  
  Larval Feeding Carcass  9.038839  
  Larval Feeding Central Nevous System  9.655258  
  Larval Feeding Hind Gut  10.014304  
  Larval Feeding Malpighian Tubule  9.772685  
  Larval Feeding Mid Gut  9.095133  
  Larval Feeding Salivary Gland  9.176306  
  Whole Larvae Feeding  8.929790  
 
  
   FlyBase ID    symbol    start    end    strand    length   
   FBgn0033224   Nop17l  3688286   3693904   -  5619  
   FBgn0003317   sax   3693983   3697575  +  3593  
   FBgn0033225   CG1550  3697580   3699540   -  1961  
   FBgn0033226   CG1882   3700011   3702250  +  2240  
 
 
    Segment 54 
 
   Location   
  Gene key  FBgn0040780-FBgn0050377  
  Heatmap region span   2R:3693904..3814182   
  Segment span   2R:3719704..3777580   
  Length (genes)  3  
  Length (bp)  57877  
   Model Scoring   
  BIC  315.177410  
  logL  -152.067852  
  logL ratio  24.223288  
   Expression   
  Mean expression  5.990552  
  Median expression  5.787784  
  Tissue std. dev.  0.424683  
 
  No GO Slim enrichment  
  
   tissue    mean expression   
  5th Passage Drosophila S2 Cells  6.183211  
  Adult Accessory gland  6.087971  
  Adult Brain  5.608600  
  Adult Carcass  6.112932  
  Adult Crop  5.730079  
  Adult Eye  5.439407  
  Adult Fatbody  5.866437  
  Adult Female Spermatheca Mated  5.651906  
  Adult Female Spermatheca Virgin  5.834408  
  Adult Head  5.448608  
  Adult Heart  6.647723  
  Adult Hind Gut  6.021996  
  Adult Male Ejaculatory Duct  5.617185  
  Adult Mid Gut  5.674990  
  Adult Ovary  7.154751  
  Adult Salivary Gland  5.672502  
  Adult Testes  6.415313  
  Adult Thoracoabdominal ganglion  5.718540  
  Adult Whole Fly  6.243891  
  Larvae Wandering Tubules  5.908085  
  Larval Feeding Carcass  7.055756  
  Larval Feeding Central Nevous System  5.783314  
  Larval Feeding Hind Gut  6.141820  
  Larval Feeding Malpighian Tubule  6.138328  
  Larval Feeding Mid Gut  5.623572  
  Larval Feeding Salivary Gland  5.856411  
  Whole Larvae Feeding  6.107167  
 
  
   FlyBase ID    symbol    start    end    strand    length   
   FBgn0040780   CG12821   3719704   3723579  +  3876  
   FBgn0085390   Dgk  3723747   3748575   -  24829  
   FBgn0050377   CG30377   3777580   3802537  +  24958  
 
 
    Segment 55 
 
   Location   
  Gene key  FBgn0033232-FBgn0015509  
  Heatmap region span   2R:3711073..3818521   
  Segment span   2R:3803968..3804361   
  Length (genes)  2  
  Length (bp)  394  
   Model Scoring   
  BIC  271.748448  
  logL  -130.353371  
  logL ratio  7.400643  
   Expression   
  Mean expression  9.541428  
  Median expression  9.758417  
  Tissue std. dev.  0.449945  
 
  No GO Slim enrichment  
  
   tissue    mean expression   
  5th Passage Drosophila S2 Cells  10.436032  
  Adult Accessory gland  9.574506  
  Adult Brain  9.941312  
  Adult Carcass  9.477066  
  Adult Crop  9.473727  
  Adult Eye  9.994321  
  Adult Fatbody  9.816670  
  Adult Female Spermatheca Mated  9.429348  
  Adult Female Spermatheca Virgin  9.637911  
  Adult Head  9.573126  
  Adult Heart  9.854913  
  Adult Hind Gut  9.190738  
  Adult Male Ejaculatory Duct  9.542853  
  Adult Mid Gut  8.734272  
  Adult Ovary  10.510748  
  Adult Salivary Gland  9.208029  
  Adult Testes  9.092599  
  Adult Thoracoabdominal ganglion  9.782077  
  Adult Whole Fly  9.547392  
  Larvae Wandering Tubules  10.106787  
  Larval Feeding Carcass  9.173668  
  Larval Feeding Central Nevous System  9.842310  
  Larval Feeding Hind Gut  9.325396  
  Larval Feeding Malpighian Tubule  9.731447  
  Larval Feeding Mid Gut  8.855218  
  Larval Feeding Salivary Gland  9.030488  
  Whole Larvae Feeding  8.735607  
 
  
   FlyBase ID    symbol    start    end    strand    length   
   FBgn0033232   CG12159  3802532   3803968   -  1437  
   FBgn0015509   lin19   3804361   3808560  +  4200  
 
    Segment 56 
 
   Location   
  Gene key  FBgn0023171-FBgn0026722  
  Heatmap region span   2R:3810109..3843655   
  Segment span   2R:3819891..3819931   
  Length (genes)  2  
  Length (bp)  41  
   Model Scoring   
  BIC  215.668559  
  logL  -102.313427  
  logL ratio  40.135275  
   Expression   
  Mean expression  7.738400  
  Median expression  7.740630  
  Tissue std. dev.  0.616041  
 
  No GO Slim enrichment  
  
   tissue    mean expression   
  5th Passage Drosophila S2 Cells  8.489101  
  Adult Accessory gland  8.301707  
  Adult Brain  8.162334  
  Adult Carcass  6.690720  
  Adult Crop  7.988435  
  Adult Eye  7.951125  
  Adult Fatbody  6.663520  
  Adult Female Spermatheca Mated  7.393715  
  Adult Female Spermatheca Virgin  7.217570  
  Adult Head  7.248702  
  Adult Heart  7.535564  
  Adult Hind Gut  7.631114  
  Adult Male Ejaculatory Duct  7.355341  
  Adult Mid Gut  7.552100  
  Adult Ovary  8.648162  
  Adult Salivary Gland  8.859974  
  Adult Testes  7.301320  
  Adult Thoracoabdominal ganglion  8.172494  
  Adult Whole Fly  7.563415  
  Larvae Wandering Tubules  7.935459  
  Larval Feeding Carcass  7.091712  
  Larval Feeding Central Nevous System  8.711623  
  Larval Feeding Hind Gut  7.897553  
  Larval Feeding Malpighian Tubule  8.015166  
  Larval Feeding Mid Gut  6.860560  
  Larval Feeding Salivary Gland  8.612173  
  Whole Larvae Feeding  7.086142  
 
  
   FlyBase ID    symbol    start    end    strand    length   
   FBgn0023171   rnh1  3818559   3819891   -  1333  
   FBgn0026722   drosha   3819931   3824360  +  4430  
 
    Segment 57 
 
   Location   
  Gene key  FBgn0050380-FBgn0033238  
  Heatmap region span   2R:3814182..3849427   
  Segment span   2R:3827144..3837344   
  Length (genes)  4  
  Length (bp)  10201  
   Model Scoring   
  BIC  394.899929  
  logL  -191.929112  
  logL ratio  46.311490  
   Expression   
  Mean expression  4.715634  
  Median expression  4.525214  
  Tissue std. dev.  0.488197  
 
  No GO Slim enrichment  
  
   tissue    mean expression   
  5th Passage Drosophila S2 Cells  4.543132  
  Adult Accessory gland  4.438388  
  Adult Brain  4.246160  
  Adult Carcass  4.783053  
  Adult Crop  4.370386  
  Adult Eye  4.523684  
  Adult Fatbody  4.720023  
  Adult Female Spermatheca Mated  4.873299  
  Adult Female Spermatheca Virgin  4.967013  
  Adult Head  4.749104  
  Adult Heart  4.961683  
  Adult Hind Gut  4.433930  
  Adult Male Ejaculatory Duct  4.443766  
  Adult Mid Gut  4.391067  
  Adult Ovary  4.187964  
  Adult Salivary Gland  4.922979  
  Adult Testes  6.658856  
  Adult Thoracoabdominal ganglion  4.290822  
  Adult Whole Fly  4.800662  
  Larvae Wandering Tubules  4.452932  
  Larval Feeding Carcass  5.180392  
  Larval Feeding Central Nevous System  5.591696  
  Larval Feeding Hind Gut  4.679920  
  Larval Feeding Malpighian Tubule  4.411341  
  Larval Feeding Mid Gut  4.433774  
  Larval Feeding Salivary Gland  4.548120  
  Whole Larvae Feeding  4.717985  
 
  
   FlyBase ID    symbol    start    end    strand    length   
   FBgn0050380   CG30380  3826706   3827144   -  439  
   FBgn0050379   CG30379   3827461   3829375  +  1915  
   FBgn0085460   CG34431   3832343   3834487  +  2145  
   FBgn0033238   CG11165   3837344   3837970  +  627  
 
 
    Segment 58 
 
   Location   
  Gene key  FBgn0027548-FBgn0033244  
  Heatmap region span   2R:3843377..3892308   
  Segment span   2R:3849991..3859554   
  Length (genes)  3  
  Length (bp)  9564  
   Model Scoring   
  BIC  368.115981  
  logL  -178.537138  
  logL ratio  0.517009  
   Expression   
  Mean expression  7.008627  
  Median expression  7.105845  
  Tissue std. dev.  0.411882  
 
  No GO Slim enrichment  
  
   tissue    mean expression   
  5th Passage Drosophila S2 Cells  7.259864  
  Adult Accessory gland  6.793485  
  Adult Brain  7.517313  
  Adult Carcass  6.743276  
  Adult Crop  6.902785  
  Adult Eye  6.918404  
  Adult Fatbody  6.704921  
  Adult Female Spermatheca Mated  6.692808  
  Adult Female Spermatheca Virgin  6.578886  
  Adult Head  6.928471  
  Adult Heart  6.726768  
  Adult Hind Gut  6.863303  
  Adult Male Ejaculatory Duct  6.742786  
  Adult Mid Gut  6.608678  
  Adult Ovary  7.152783  
  Adult Salivary Gland  6.922431  
  Adult Testes  8.517711  
  Adult Thoracoabdominal ganglion  7.478104  
  Adult Whole Fly  7.453690  
  Larvae Wandering Tubules  6.978981  
  Larval Feeding Carcass  6.804879  
  Larval Feeding Central Nevous System  7.633535  
  Larval Feeding Hind Gut  6.808525  
  Larval Feeding Malpighian Tubule  6.905698  
  Larval Feeding Mid Gut  6.561792  
  Larval Feeding Salivary Gland  6.995329  
  Whole Larvae Feeding  7.037723  
 
  
   FlyBase ID    symbol    start    end    strand    length   
   FBgn0027548   nito   3849991   3857320  +  7330  
   FBgn0033243   CG14763  3854283   3855529   -  1247  
   FBgn0033244   CG8726  3855634   3859554   -  3921  
 
 
    Segment 59 
 
   Location   
  Gene key  FBgn0027054-FBgn0033247  
  Heatmap region span   2R:3843655..3894769   
  Segment span   2R:3859680..3875967   
  Length (genes)  3  
  Length (bp)  16288  
   Model Scoring   
  BIC  364.995112  
  logL  -176.976703  
  logL ratio  67.918145  
   Expression   
  Mean expression  10.021490  
  Median expression  9.857870  
  Tissue std. dev.  0.566928  
 
  No GO Slim enrichment  
  
   tissue    mean expression   
  5th Passage Drosophila S2 Cells  10.159812  
  Adult Accessory gland  9.381247  
  Adult Brain  9.621746  
  Adult Carcass  10.848457  
  Adult Crop  10.647343  
  Adult Eye  10.029434  
  Adult Fatbody  11.231533  
  Adult Female Spermatheca Mated  10.664877  
  Adult Female Spermatheca Virgin  10.918098  
  Adult Head  10.174519  
  Adult Heart  11.008558  
  Adult Hind Gut  10.150302  
  Adult Male Ejaculatory Duct  10.392766  
  Adult Mid Gut  9.440759  
  Adult Ovary  10.192944  
  Adult Salivary Gland  9.487663  
  Adult Testes  9.434906  
  Adult Thoracoabdominal ganglion  9.678638  
  Adult Whole Fly  10.289137  
  Larvae Wandering Tubules  9.188563  
  Larval Feeding Carcass  9.592474  
  Larval Feeding Central Nevous System  10.222158  
  Larval Feeding Hind Gut  9.644200  
  Larval Feeding Malpighian Tubule  9.531718  
  Larval Feeding Mid Gut  9.419926  
  Larval Feeding Salivary Gland  9.630155  
  Whole Larvae Feeding  9.598287  
 
  
   FlyBase ID    symbol    start    end    strand    length   
   FBgn0027054   CSN4   3859680   3861604  +  1925  
   FBgn0033246   ACC  3861620   3874729   -  13110  
   FBgn0033247   Nup44A   3875967   3878789  +  2823  
 
 
    Segment 60 
 
   Location   
  Gene key  FBgn0033248-FBgn0027788  
  Heatmap region span   2R:3847532..3901259   
  Segment span   2R:3882473..3886078   
  Length (genes)  2  
  Length (bp)  3606  
   Model Scoring   
  BIC  205.073903  
  logL  -97.016099  
  logL ratio  9.229740  
   Expression   
  Mean expression  5.269026  
  Median expression  5.023470  
  Tissue std. dev.  0.771102  
 
  No GO Slim enrichment  
  
   tissue    mean expression   
  5th Passage Drosophila S2 Cells  4.968674  
  Adult Accessory gland  5.054465  
  Adult Brain  5.029825  
  Adult Carcass  5.137329  
  Adult Crop  5.055440  
  Adult Eye  4.676555  
  Adult Fatbody  5.246855  
  Adult Female Spermatheca Mated  5.035176  
  Adult Female Spermatheca Virgin  5.030261  
  Adult Head  4.761632  
  Adult Heart  4.598035  
  Adult Hind Gut  4.833198  
  Adult Male Ejaculatory Duct  5.216235  
  Adult Mid Gut  4.989903  
  Adult Ovary  5.025942  
  Adult Salivary Gland  5.550892  
  Adult Testes  8.749491  
  Adult Thoracoabdominal ganglion  5.072717  
  Adult Whole Fly  5.806033  
  Larvae Wandering Tubules  5.135292  
  Larval Feeding Carcass  5.089576  
  Larval Feeding Central Nevous System  6.458598  
  Larval Feeding Hind Gut  4.886714  
  Larval Feeding Malpighian Tubule  5.062009  
  Larval Feeding Mid Gut  5.003076  
  Larval Feeding Salivary Gland  5.293581  
  Whole Larvae Feeding  5.496186  
 
  
   FlyBase ID    symbol    start    end    strand    length   
   FBgn0033248   Dic3  3881295   3882473   -  1179  
   FBgn0027788   Hey  3882941   3886078   -  3138  
 
    Segment 61 
 
   Location   
  Gene key  FBgn0025360-FBgn0040778  
  Heatmap region span   2R:3889625..4018938   
  Segment span   2R:3919138..3955157   
  Length (genes)  3  
  Length (bp)  36020  
   Model Scoring   
  BIC  335.644044  
  logL  -162.301169  
  logL ratio  -10.086220  
   Expression   
  Mean expression  5.308769  
  Median expression  4.895757  
  Tissue std. dev.  1.016211  
 
  No GO Slim enrichment  
  
   tissue    mean expression   
  5th Passage Drosophila S2 Cells  5.040011  
  Adult Accessory gland  4.614564  
  Adult Brain  7.600747  
  Adult Carcass  4.759736  
  Adult Crop  4.678763  
  Adult Eye  6.830389  
  Adult Fatbody  4.730628  
  Adult Female Spermatheca Mated  4.608210  
  Adult Female Spermatheca Virgin  4.603833  
  Adult Head  6.486864  
  Adult Heart  4.833249  
  Adult Hind Gut  4.605296  
  Adult Male Ejaculatory Duct  4.684607  
  Adult Mid Gut  4.412371  
  Adult Ovary  5.755946  
  Adult Salivary Gland  4.854338  
  Adult Testes  8.015630  
  Adult Thoracoabdominal ganglion  6.105128  
  Adult Whole Fly  5.407879  
  Larvae Wandering Tubules  4.811142  
  Larval Feeding Carcass  4.710173  
  Larval Feeding Central Nevous System  7.272627  
  Larval Feeding Hind Gut  4.583702  
  Larval Feeding Malpighian Tubule  4.843037  
  Larval Feeding Mid Gut  4.631880  
  Larval Feeding Salivary Gland  4.697232  
  Whole Larvae Feeding  5.158788  
 
  
   FlyBase ID    symbol    start    end    strand    length   
   FBgn0025360   Optix   3919138   3930041  +  10904  
   FBgn0033252   CG12769  3941331   3953056   -  11726  
   FBgn0040778   CG17977  3954114   3955157   -  1044  
 
 
    Segment 62 
 
   Location   
  Gene key  FBgn0020279-FBgn0028563  
  Heatmap region span   2R:3892308..4022588   
  Segment span   2R:3955460..3970483   
  Length (genes)  4  
  Length (bp)  15024  
   Model Scoring   
  BIC  431.845643  
  logL  -210.401969  
  logL ratio  108.666157  
   Expression   
  Mean expression  9.484760  
  Median expression  9.599222  
  Tissue std. dev.  0.557650  
 
  No GO Slim enrichment  
  
   tissue    mean expression   
  5th Passage Drosophila S2 Cells  9.801618  
  Adult Accessory gland  10.630535  
  Adult Brain  8.547164  
  Adult Carcass  9.289050  
  Adult Crop  9.738525  
  Adult Eye  9.256529  
  Adult Fatbody  9.513635  
  Adult Female Spermatheca Mated  9.125166  
  Adult Female Spermatheca Virgin  9.182242  
  Adult Head  8.821561  
  Adult Heart  9.967096  
  Adult Hind Gut  9.784019  
  Adult Male Ejaculatory Duct  10.253385  
  Adult Mid Gut  9.372796  
  Adult Ovary  10.082781  
  Adult Salivary Gland  9.421242  
  Adult Testes  8.050850  
  Adult Thoracoabdominal ganglion  8.578547  
  Adult Whole Fly  9.511785  
  Larvae Wandering Tubules  10.156106  
  Larval Feeding Carcass  9.723103  
  Larval Feeding Central Nevous System  9.007986  
  Larval Feeding Hind Gut  9.407644  
  Larval Feeding Malpighian Tubule  9.781802  
  Larval Feeding Mid Gut  9.678086  
  Larval Feeding Salivary Gland  9.986505  
  Whole Larvae Feeding  9.418750  
 
  
   FlyBase ID    symbol    start    end    strand    length   
   FBgn0020279   lig   3955460   3966320  +  10861  
   FBgn0021814   Vps28  3966742   3967561   -  820  
   FBgn0025469   slv  3968064   3969875   -  1812  
   FBgn0028563   sut1   3970483   3974654  +  4172  
 
 
    Segment 63 
 
   Location   
  Gene key  FBgn0028562-FBgn0033257  
  Heatmap region span   2R:3894769..4044128   
  Segment span   2R:3974921..3979203   
  Length (genes)  3  
  Length (bp)  4283  
   Model Scoring   
  BIC  323.709232  
  logL  -156.333764  
  logL ratio  1.652748  
   Expression   
  Mean expression  5.324418  
  Median expression  5.080767  
  Tissue std. dev.  0.643892  
 
  
   GO ID    description    ratio    P-value   
   GO:0055085   transmembrane transport  2/3  0.00148  
   GO:0006810   transport  2/3  0.00704  
 
  
   tissue    mean expression   
  5th Passage Drosophila S2 Cells  4.901627  
  Adult Accessory gland  5.238143  
  Adult Brain  4.417615  
  Adult Carcass  5.197619  
  Adult Crop  5.393409  
  Adult Eye  4.947863  
  Adult Fatbody  4.710962  
  Adult Female Spermatheca Mated  5.068129  
  Adult Female Spermatheca Virgin  5.054544  
  Adult Head  4.992855  
  Adult Heart  5.915475  
  Adult Hind Gut  5.929815  
  Adult Male Ejaculatory Duct  5.278981  
  Adult Mid Gut  6.515387  
  Adult Ovary  4.945892  
  Adult Salivary Gland  6.885786  
  Adult Testes  6.974916  
  Adult Thoracoabdominal ganglion  4.651973  
  Adult Whole Fly  5.211758  
  Larvae Wandering Tubules  4.962401  
  Larval Feeding Carcass  5.461128  
  Larval Feeding Central Nevous System  4.574755  
  Larval Feeding Hind Gut  4.933867  
  Larval Feeding Malpighian Tubule  4.928915  
  Larval Feeding Mid Gut  6.002027  
  Larval Feeding Salivary Gland  5.408370  
  Whole Larvae Feeding  5.255066  
 
  
   FlyBase ID    symbol    start    end    strand    length   
   FBgn0028562   sut2   3974921   3976802  +  1882  
   FBgn0028561   sut3   3976981   3978802  +  1822  
   FBgn0033257   CG8713   3979203   3984450  +  5248  
 
 
    Segment 64 
 
   Location   
  Gene key  FBgn0033259-FBgn0005648  
  Heatmap region span   2R:3919138..4048739   
  Segment span   2R:3986182..4018938   
  Length (genes)  6  
  Length (bp)  32757  
   Model Scoring   
  BIC  542.173165  
  logL  -265.565730  
  logL ratio  214.076596  
   Expression   
  Mean expression  8.424784  
  Median expression  8.493315  
  Tissue std. dev.  0.482799  
 
  No GO Slim enrichment  
  
   tissue    mean expression   
  5th Passage Drosophila S2 Cells  9.169127  
  Adult Accessory gland  8.164240  
  Adult Brain  8.121477  
  Adult Carcass  7.611066  
  Adult Crop  8.236680  
  Adult Eye  7.961811  
  Adult Fatbody  8.241514  
  Adult Female Spermatheca Mated  8.328309  
  Adult Female Spermatheca Virgin  8.246546  
  Adult Head  7.863271  
  Adult Heart  8.225514  
  Adult Hind Gut  7.966371  
  Adult Male Ejaculatory Duct  8.190779  
  Adult Mid Gut  8.497022  
  Adult Ovary  9.344137  
  Adult Salivary Gland  7.875336  
  Adult Testes  7.894406  
  Adult Thoracoabdominal ganglion  8.133058  
  Adult Whole Fly  8.163330  
  Larvae Wandering Tubules  8.945475  
  Larval Feeding Carcass  8.708875  
  Larval Feeding Central Nevous System  9.400948  
  Larval Feeding Hind Gut  8.780864  
  Larval Feeding Malpighian Tubule  8.817277  
  Larval Feeding Mid Gut  8.921162  
  Larval Feeding Salivary Gland  9.165907  
  Whole Larvae Feeding  8.494667  
 
  
   FlyBase ID    symbol    start    end    strand    length   
   FBgn0033259   CG11210   3986182   3989811  +  3630  
   FBgn0033261   CG18316   3993931   3994878  +  948  
   FBgn0033264   Nup50   4010521   4012563  +  2043  
   FBgn0033265   coil  4012486   4014747   -  2262  
   FBgn0033266   Socs44A   4015136   4016981  +  1846  
   FBgn0005648   Pabp2   4018938   4021879  +  2942  
 
 
    Segment 65 
 
   Location   
  Gene key  FBgn0010504-FBgn0033269  
  Heatmap region span   2R:3974921..4057541   
  Segment span   2R:4044126..4044128   
  Length (genes)  2  
  Length (bp)  3  
   Model Scoring   
  BIC  293.960655  
  logL  -141.459475  
  logL ratio  -7.316964  
   Expression   
  Mean expression  9.357512  
  Median expression  9.334776  
  Tissue std. dev.  1.157555  
 
  No GO Slim enrichment  
  
   tissue    mean expression   
  5th Passage Drosophila S2 Cells  8.402255  
  Adult Accessory gland  7.965291  
  Adult Brain  7.604164  
  Adult Carcass  9.169835  
  Adult Crop  9.460536  
  Adult Eye  9.685379  
  Adult Fatbody  9.085057  
  Adult Female Spermatheca Mated  9.158675  
  Adult Female Spermatheca Virgin  9.669405  
  Adult Head  8.975272  
  Adult Heart  8.967862  
  Adult Hind Gut  10.798038  
  Adult Male Ejaculatory Duct  9.559790  
  Adult Mid Gut  11.725713  
  Adult Ovary  7.959295  
  Adult Salivary Gland  10.368825  
  Adult Testes  11.021946  
  Adult Thoracoabdominal ganglion  7.810425  
  Adult Whole Fly  9.835306  
  Larvae Wandering Tubules  10.668592  
  Larval Feeding Carcass  8.722853  
  Larval Feeding Central Nevous System  7.172136  
  Larval Feeding Hind Gut  9.364824  
  Larval Feeding Malpighian Tubule  10.314456  
  Larval Feeding Mid Gut  11.226833  
  Larval Feeding Salivary Gland  8.004358  
  Whole Larvae Feeding  9.955693  
 
  
   FlyBase ID    symbol    start    end    strand    length   
   FBgn0010504   kermit  4034423   4044126   -  9704  
   FBgn0033269     4024824   4044128   -  19305  
 
    Segment 66 
 
   Location   
  Gene key  FBgn0033272-FBgn0021995  
  Heatmap region span   2R:4022588..4124774   
  Segment span   2R:4051018..4052390   
  Length (genes)  3  
  Length (bp)  1373  
   Model Scoring   
  BIC  304.339754  
  logL  -146.649025  
  logL ratio  87.465442  
   Expression   
  Mean expression  9.094322  
  Median expression  8.937204  
  Tissue std. dev.  0.357343  
 
  No GO Slim enrichment  
  
   tissue    mean expression   
  5th Passage Drosophila S2 Cells  9.152395  
  Adult Accessory gland  9.189198  
  Adult Brain  9.041171  
  Adult Carcass  9.195539  
  Adult Crop  9.062010  
  Adult Eye  9.711980  
  Adult Fatbody  9.319886  
  Adult Female Spermatheca Mated  9.146622  
  Adult Female Spermatheca Virgin  9.086664  
  Adult Head  9.035163  
  Adult Heart  9.668937  
  Adult Hind Gut  9.016969  
  Adult Male Ejaculatory Duct  9.396852  
  Adult Mid Gut  8.757370  
  Adult Ovary  9.440709  
  Adult Salivary Gland  9.576729  
  Adult Testes  7.931686  
  Adult Thoracoabdominal ganglion  9.166541  
  Adult Whole Fly  8.840960  
  Larvae Wandering Tubules  8.876222  
  Larval Feeding Carcass  9.127013  
  Larval Feeding Central Nevous System  9.158269  
  Larval Feeding Hind Gut  8.963145  
  Larval Feeding Malpighian Tubule  9.148516  
  Larval Feeding Mid Gut  8.623955  
  Larval Feeding Salivary Gland  9.365477  
  Whole Larvae Feeding  8.546713  
 
  
   FlyBase ID    symbol    start    end    strand    length   
   FBgn0033272   RagC  4049184   4051018   -  1835  
   FBgn0050373   CG30373  4051453   4052171   -  719  
   FBgn0021995   Rs1   4052390   4055274  +  2885  
 
 
    Segment 67 
 
   Location   
  Gene key  FBgn0033273-FBgn0011659  
  Heatmap region span   2R:4044126..4154787   
  Segment span   2R:4055452..4057541   
  Length (genes)  2  
  Length (bp)  2090  
   Model Scoring   
  BIC  250.291616  
  logL  -119.624955  
  logL ratio  -4.221978  
   Expression   
  Mean expression  6.962122  
  Median expression  6.692616  
  Tissue std. dev.  0.991225  
 
  No GO Slim enrichment  
  
   tissue    mean expression   
  5th Passage Drosophila S2 Cells  8.276143  
  Adult Accessory gland  6.613249  
  Adult Brain  8.142718  
  Adult Carcass  6.539176  
  Adult Crop  6.231318  
  Adult Eye  7.060718  
  Adult Fatbody  6.602619  
  Adult Female Spermatheca Mated  6.582075  
  Adult Female Spermatheca Virgin  6.363590  
  Adult Head  7.358218  
  Adult Heart  6.732399  
  Adult Hind Gut  6.360171  
  Adult Male Ejaculatory Duct  5.969374  
  Adult Mid Gut  5.363908  
  Adult Ovary  9.792679  
  Adult Salivary Gland  6.204597  
  Adult Testes  7.486095  
  Adult Thoracoabdominal ganglion  8.298209  
  Adult Whole Fly  8.291346  
  Larvae Wandering Tubules  6.797847  
  Larval Feeding Carcass  6.314984  
  Larval Feeding Central Nevous System  8.176212  
  Larval Feeding Hind Gut  7.018904  
  Larval Feeding Malpighian Tubule  6.984713  
  Larval Feeding Mid Gut  5.242748  
  Larval Feeding Salivary Gland  6.936952  
  Whole Larvae Feeding  6.236322  
 
  
   FlyBase ID    symbol    start    end    strand    length   
   FBgn0033273   CG2183   4055452   4057596  +  2145  
   FBgn0011659   Mlh1   4057541   4059907  +  2367  
 
    Segment 68 
 
   Location   
  Gene key  FBgn0033275-FBgn0010109  
  Heatmap region span   2R:4048739..4246043   
  Segment span   2R:4093211..4119832   
  Length (genes)  4  
  Length (bp)  26622  
   Model Scoring   
  BIC  315.002543  
  logL  -151.980419  
  logL ratio  101.089517  
   Expression   
  Mean expression  4.724041  
  Median expression  4.604451  
  Tissue std. dev.  0.252186  
 
  No GO Slim enrichment  
  
   tissue    mean expression   
  5th Passage Drosophila S2 Cells  4.585503  
  Adult Accessory gland  4.639062  
  Adult Brain  4.419708  
  Adult Carcass  4.739370  
  Adult Crop  4.615806  
  Adult Eye  4.485048  
  Adult Fatbody  5.173395  
  Adult Female Spermatheca Mated  5.016653  
  Adult Female Spermatheca Virgin  5.000825  
  Adult Head  4.465636  
  Adult Heart  4.956279  
  Adult Hind Gut  4.573690  
  Adult Male Ejaculatory Duct  4.736843  
  Adult Mid Gut  4.838976  
  Adult Ovary  4.592159  
  Adult Salivary Gland  4.861790  
  Adult Testes  4.222599  
  Adult Thoracoabdominal ganglion  4.663683  
  Adult Whole Fly  4.611521  
  Larvae Wandering Tubules  4.674880  
  Larval Feeding Carcass  4.742962  
  Larval Feeding Central Nevous System  5.502400  
  Larval Feeding Hind Gut  4.555789  
  Larval Feeding Malpighian Tubule  4.580303  
  Larval Feeding Mid Gut  4.893966  
  Larval Feeding Salivary Gland  4.790910  
  Whole Larvae Feeding  4.609345  
 
  
   FlyBase ID    symbol    start    end    strand    length   
   FBgn0033275   CG14756  4092393   4093211   -  819  
   FBgn0053087   LRP1   4094023   4113243  +  19221  
   FBgn0085246   CG34217  4115704   4116229   -  526  
   FBgn0010109   dpn  4116476   4119832   -  3357  
 
 
    Segment 69 
 
   Location   
  Gene key  FBgn0033277-FBgn0050375  
  Heatmap region span   2R:4055452..4258578   
  Segment span   2R:4141761..4154787   
  Length (genes)  7  
  Length (bp)  13027  
   Model Scoring   
  BIC  579.804882  
  logL  -284.381589  
  logL ratio  153.407127  
   Expression   
  Mean expression  4.933465  
  Median expression  4.643168  
  Tissue std. dev.  1.012816  
 
  No GO Slim enrichment  
  
   tissue    mean expression   
  5th Passage Drosophila S2 Cells  4.593670  
  Adult Accessory gland  4.589036  
  Adult Brain  4.374586  
  Adult Carcass  4.735671  
  Adult Crop  4.512143  
  Adult Eye  4.522808  
  Adult Fatbody  4.578335  
  Adult Female Spermatheca Mated  4.890831  
  Adult Female Spermatheca Virgin  4.914635  
  Adult Head  4.446449  
  Adult Heart  4.550070  
  Adult Hind Gut  4.539483  
  Adult Male Ejaculatory Duct  4.837852  
  Adult Mid Gut  4.646547  
  Adult Ovary  4.639912  
  Adult Salivary Gland  4.989734  
  Adult Testes  9.727121  
  Adult Thoracoabdominal ganglion  4.439606  
  Adult Whole Fly  6.278130  
  Larvae Wandering Tubules  5.359067  
  Larval Feeding Carcass  4.711102  
  Larval Feeding Central Nevous System  4.324240  
  Larval Feeding Hind Gut  4.513511  
  Larval Feeding Malpighian Tubule  4.984126  
  Larval Feeding Mid Gut  4.742514  
  Larval Feeding Salivary Gland  4.704364  
  Whole Larvae Feeding  5.057997  
 
  
   FlyBase ID    symbol    start    end    strand    length   
   FBgn0033277   CG14760   4141761   4144241  +  2481  
   FBgn0050369   CG30369   4146816   4147289  +  474  
   FBgn0033278   CG14759   4147469   4147988  +  520  
   FBgn0033279   CG2291  4148152   4148968   -  817  
   FBgn0033280   CG12126  4150179   4150783   -  605  
   FBgn0050376   CG30376  4150991   4151931   -  941  
   FBgn0050375   CG30375  4153387   4154787   -  1401  
 
 
    Segment 70 
 
   Location   
  Gene key  FBgn0050362-FBgn0033287  
  Heatmap region span   2R:4141761..4330284   
  Segment span   2R:4254165..4258578   
  Length (genes)  4  
  Length (bp)  4414  
   Model Scoring   
  BIC  307.262888  
  logL  -148.110592  
  logL ratio  133.597555  
   Expression   
  Mean expression  5.526794  
  Median expression  4.952435  
  Tissue std. dev.  1.978225  
 
  No GO Slim enrichment  
  
   tissue    mean expression   
  5th Passage Drosophila S2 Cells  4.942033  
  Adult Accessory gland  5.088973  
  Adult Brain  4.503019  
  Adult Carcass  5.771431  
  Adult Crop  4.871053  
  Adult Eye  4.497719  
  Adult Fatbody  4.908917  
  Adult Female Spermatheca Mated  5.312639  
  Adult Female Spermatheca Virgin  4.980574  
  Adult Head  4.684418  
  Adult Heart  4.605395  
  Adult Hind Gut  4.809470  
  Adult Male Ejaculatory Duct  5.008620  
  Adult Mid Gut  4.881699  
  Adult Ovary  4.687492  
  Adult Salivary Gland  5.213056  
  Adult Testes  13.755169  
  Adult Thoracoabdominal ganglion  4.789232  
  Adult Whole Fly  10.386777  
  Larvae Wandering Tubules  4.916388  
  Larval Feeding Carcass  5.063735  
  Larval Feeding Central Nevous System  4.525268  
  Larval Feeding Hind Gut  4.817483  
  Larval Feeding Malpighian Tubule  4.872017  
  Larval Feeding Mid Gut  5.017798  
  Larval Feeding Salivary Gland  4.973282  
  Whole Larvae Feeding  7.339788  
 
  
   FlyBase ID    symbol    start    end    strand    length   
   FBgn0050362   boly  4253054   4254165   -  1112  
   FBgn0033285   CG18449  4254666   4255432   -  767  
   FBgn0033286   CG2127  4255847   4257543   -  1697  
   FBgn0033287   CG8701   4258578   4259656  +  1079  
 
 
    Segment 71 
 
   Location   
  Gene key  FBgn0002533-FBgn0002534  
  Heatmap region span   2R:4248522..4346329   
  Segment span   2R:4322080..4322949   
  Length (genes)  2  
  Length (bp)  870  
   Model Scoring   
  BIC  225.065051  
  logL  -107.011673  
  logL ratio  34.322055  
   Expression   
  Mean expression  6.475061  
  Median expression  5.618711  
  Tissue std. dev.  2.653878  
 
  No GO Slim enrichment  
  
   tissue    mean expression   
  5th Passage Drosophila S2 Cells  5.544686  
  Adult Accessory gland  5.464475  
  Adult Brain  4.990485  
  Adult Carcass  5.928279  
  Adult Crop  5.381149  
  Adult Eye  6.446924  
  Adult Fatbody  5.676227  
  Adult Female Spermatheca Mated  5.569369  
  Adult Female Spermatheca Virgin  5.521873  
  Adult Head  5.106888  
  Adult Heart  5.075299  
  Adult Hind Gut  5.429245  
  Adult Male Ejaculatory Duct  6.873780  
  Adult Mid Gut  5.541015  
  Adult Ovary  5.232442  
  Adult Salivary Gland  5.958937  
  Adult Testes  5.144901  
  Adult Thoracoabdominal ganglion  5.288526  
  Adult Whole Fly  4.801604  
  Larvae Wandering Tubules  5.570517  
  Larval Feeding Carcass  14.779891  
  Larval Feeding Central Nevous System  5.734834  
  Larval Feeding Hind Gut  12.007491  
  Larval Feeding Malpighian Tubule  5.800664  
  Larval Feeding Mid Gut  5.758752  
  Larval Feeding Salivary Gland  5.611250  
  Whole Larvae Feeding  14.587146  
 
  
   FlyBase ID    symbol    start    end    strand    length   
   FBgn0002533   Lcp2  4321472   4322080   -  609  
   FBgn0002534   Lcp3   4322949   4323523  +  575  
 
    Segment 72 
 
   Location   
  Gene key  FBgn0002570-FBgn0033294  
  Heatmap region span   2R:4322080..4439758   
  Segment span   2R:4339404..4346329   
  Length (genes)  4  
  Length (bp)  6926  
   Model Scoring   
  BIC  355.009075  
  logL  -171.983685  
  logL ratio  98.277920  
   Expression   
  Mean expression  5.728490  
  Median expression  4.888955  
  Tissue std. dev.  2.157043  
 
  
   GO ID    description    ratio    P-value   
   GO:0005975   carbohydrate metabolic process  4/4  4.34e-12  
   GO:0008150   biological_process  4/4  0.00175  
 
  
   tissue    mean expression   
  5th Passage Drosophila S2 Cells  4.639596  
  Adult Accessory gland  4.592748  
  Adult Brain  4.342329  
  Adult Carcass  5.112565  
  Adult Crop  4.719779  
  Adult Eye  5.131934  
  Adult Fatbody  4.695036  
  Adult Female Spermatheca Mated  4.613908  
  Adult Female Spermatheca Virgin  4.710373  
  Adult Head  5.034720  
  Adult Heart  4.624575  
  Adult Hind Gut  7.083327  
  Adult Male Ejaculatory Duct  4.885315  
  Adult Mid Gut  13.004343  
  Adult Ovary  4.698563  
  Adult Salivary Gland  5.306691  
  Adult Testes  4.656802  
  Adult Thoracoabdominal ganglion  4.455844  
  Adult Whole Fly  9.185062  
  Larvae Wandering Tubules  4.764644  
  Larval Feeding Carcass  4.677374  
  Larval Feeding Central Nevous System  4.462066  
  Larval Feeding Hind Gut  5.932562  
  Larval Feeding Malpighian Tubule  4.803630  
  Larval Feeding Mid Gut  11.007094  
  Larval Feeding Salivary Gland  4.709057  
  Whole Larvae Feeding  8.819302  
 
  
   FlyBase ID    symbol    start    end    strand    length   
   FBgn0002570   Mal-A1  4337166   4339404   -  2239  
   FBgn0002569   Mal-A2   4341137   4343291  +  2155  
   FBgn0002571   Mal-A3  4343414   4345390   -  1977  
   FBgn0033294   Mal-A4   4346329   4348389  +  2061  
 
 
    Segment 73 
 
   Location   
  Gene key  FBgn0050360-FBgn0033297  
  Heatmap region span   2R:4332217..4458007   
  Segment span   2R:4352675..4359488   
  Length (genes)  3  
  Length (bp)  6814  
   Model Scoring   
  BIC  257.881678  
  logL  -123.419987  
  logL ratio  75.452320  
   Expression   
  Mean expression  5.468039  
  Median expression  4.801397  
  Tissue std. dev.  1.982814  
 
  
   GO ID    description    ratio    P-value   
   GO:0005975   carbohydrate metabolic process  3/3  7.91e-09  
   GO:0008150   biological_process  3/3  0.0102  
 
  
   tissue    mean expression   
  5th Passage Drosophila S2 Cells  4.625280  
  Adult Accessory gland  4.823704  
  Adult Brain  4.402516  
  Adult Carcass  4.888274  
  Adult Crop  4.645996  
  Adult Eye  4.780941  
  Adult Fatbody  4.762485  
  Adult Female Spermatheca Mated  4.745177  
  Adult Female Spermatheca Virgin  4.920197  
  Adult Head  4.764147  
  Adult Heart  4.657911  
  Adult Hind Gut  4.847591  
  Adult Male Ejaculatory Duct  4.930683  
  Adult Mid Gut  13.374860  
  Adult Ovary  4.668293  
  Adult Salivary Gland  4.697935  
  Adult Testes  5.326693  
  Adult Thoracoabdominal ganglion  4.514851  
  Adult Whole Fly  9.374747  
  Larvae Wandering Tubules  4.783615  
  Larval Feeding Carcass  4.803736  
  Larval Feeding Central Nevous System  4.382878  
  Larval Feeding Hind Gut  4.698302  
  Larval Feeding Malpighian Tubule  4.641947  
  Larval Feeding Mid Gut  9.210286  
  Larval Feeding Salivary Gland  4.717216  
  Whole Larvae Feeding  6.646795  
 
  
   FlyBase ID    symbol    start    end    strand    length   
   FBgn0050360   Mal-A6   4352675   4354701  +  2027  
   FBgn0033296   Mal-A7  4357054   4359154   -  2101  
   FBgn0033297   Mal-A8   4359488   4361466  +  1979  
 
 
    Segment 74 
 
   Location   
  Gene key  FBgn0259226-FBgn0033308  
  Heatmap region span   2R:4419420..4512956   
  Segment span   2R:4471147..4482506   
  Length (genes)  3  
  Length (bp)  11360  
   Model Scoring   
  BIC  316.284300  
  logL  -152.621298  
  logL ratio  21.719501  
   Expression   
  Mean expression  5.637287  
  Median expression  5.677901  
  Tissue std. dev.  0.343989  
 
  No GO Slim enrichment  
  
   tissue    mean expression   
  5th Passage Drosophila S2 Cells  5.705742  
  Adult Accessory gland  6.636972  
  Adult Brain  5.145305  
  Adult Carcass  5.882355  
  Adult Crop  5.518962  
  Adult Eye  6.296035  
  Adult Fatbody  5.668841  
  Adult Female Spermatheca Mated  5.595389  
  Adult Female Spermatheca Virgin  5.565330  
  Adult Head  5.722616  
  Adult Heart  5.714405  
  Adult Hind Gut  5.519094  
  Adult Male Ejaculatory Duct  5.642535  
  Adult Mid Gut  5.715212  
  Adult Ovary  5.311894  
  Adult Salivary Gland  5.799587  
  Adult Testes  5.304158  
  Adult Thoracoabdominal ganglion  5.200383  
  Adult Whole Fly  5.146548  
  Larvae Wandering Tubules  5.477214  
  Larval Feeding Carcass  6.070563  
  Larval Feeding Central Nevous System  5.116556  
  Larval Feeding Hind Gut  5.497127  
  Larval Feeding Malpighian Tubule  5.566894  
  Larval Feeding Mid Gut  5.766355  
  Larval Feeding Salivary Gland  5.546752  
  Whole Larvae Feeding  6.073936  
 
  
   FlyBase ID    symbol    start    end    strand    length   
   FBgn0259226   CG42326  4460868   4471147   -  10280  
   FBgn0033307   CG14752   4478001   4479452  +  1452  
   FBgn0033308   CG8736  4481533   4482506   -  974  
 
 
    Segment 75 
 
   Location   
  Gene key  FBgn0033310-FBgn0033312  
  Heatmap region span   2R:4453481..4519467   
  Segment span   2R:4487938..4501416   
  Length (genes)  2  
  Length (bp)  13479  
   Model Scoring   
  BIC  202.299262  
  logL  -95.628778  
  logL ratio  20.961682  
   Expression   
  Mean expression  5.704296  
  Median expression  5.509953  
  Tissue std. dev.  0.548525  
 
  No GO Slim enrichment  
  
   tissue    mean expression   
  5th Passage Drosophila S2 Cells  5.989335  
  Adult Accessory gland  5.174874  
  Adult Brain  5.588246  
  Adult Carcass  5.151137  
  Adult Crop  5.412703  
  Adult Eye  7.007412  
  Adult Fatbody  5.203060  
  Adult Female Spermatheca Mated  5.530430  
  Adult Female Spermatheca Virgin  5.515257  
  Adult Head  5.857004  
  Adult Heart  6.283773  
  Adult Hind Gut  5.398877  
  Adult Male Ejaculatory Duct  5.521712  
  Adult Mid Gut  5.692864  
  Adult Ovary  5.411916  
  Adult Salivary Gland  6.090020  
  Adult Testes  5.079848  
  Adult Thoracoabdominal ganglion  5.258768  
  Adult Whole Fly  4.771244  
  Larvae Wandering Tubules  6.926081  
  Larval Feeding Carcass  6.454295  
  Larval Feeding Central Nevous System  5.971716  
  Larval Feeding Hind Gut  6.192298  
  Larval Feeding Malpighian Tubule  5.569851  
  Larval Feeding Mid Gut  6.276218  
  Larval Feeding Salivary Gland  5.032988  
  Whole Larvae Feeding  5.654080  
 
  
   FlyBase ID    symbol    start    end    strand    length   
   FBgn0033310   rgr   4487938   4498743  +  10806  
   FBgn0033312   CG8642  4499399   4501416   -  2018  
 
    Segment 76 
 
   Location   
  Gene key  FBgn0033316-FBgn0033317  
  Heatmap region span   2R:4487938..4578955   
  Segment span   2R:4515382..4519467   
  Length (genes)  2  
  Length (bp)  4086  
   Model Scoring   
  BIC  236.429798  
  logL  -112.694046  
  logL ratio  25.442073  
   Expression   
  Mean expression  8.668487  
  Median expression  8.794747  
  Tissue std. dev.  0.444950  
 
  No GO Slim enrichment  
  
   tissue    mean expression   
  5th Passage Drosophila S2 Cells  9.085015  
  Adult Accessory gland  9.137505  
  Adult Brain  8.763361  
  Adult Carcass  8.040896  
  Adult Crop  8.660280  
  Adult Eye  8.514014  
  Adult Fatbody  8.368322  
  Adult Female Spermatheca Mated  8.697048  
  Adult Female Spermatheca Virgin  8.678984  
  Adult Head  8.194062  
  Adult Heart  8.382043  
  Adult Hind Gut  8.233362  
  Adult Male Ejaculatory Duct  8.526778  
  Adult Mid Gut  8.521697  
  Adult Ovary  10.200019  
  Adult Salivary Gland  8.460992  
  Adult Testes  9.114431  
  Adult Thoracoabdominal ganglion  8.730762  
  Adult Whole Fly  9.228599  
  Larvae Wandering Tubules  8.594538  
  Larval Feeding Carcass  8.528092  
  Larval Feeding Central Nevous System  9.036430  
  Larval Feeding Hind Gut  8.342582  
  Larval Feeding Malpighian Tubule  8.703120  
  Larval Feeding Mid Gut  8.124448  
  Larval Feeding Salivary Gland  9.046395  
  Whole Larvae Feeding  8.135372  
 
  
   FlyBase ID    symbol    start    end    strand    length   
   FBgn0033316   CG14749   4515382   4517691  +  2310  
   FBgn0033317   CG8635  4517699   4519467   -  1769  
 
    Segment 77 
 
   Location   
  Gene key  FBgn0050354-FBgn0050355  
  Heatmap region span   2R:4515089..4611342   
  Segment span   2R:4564730..4567453   
  Length (genes)  2  
  Length (bp)  2724  
   Model Scoring   
  BIC  200.824484  
  logL  -94.891389  
  logL ratio  23.575465  
   Expression   
  Mean expression  5.298152  
  Median expression  4.826743  
  Tissue std. dev.  1.362802  
 
  No GO Slim enrichment  
  
   tissue    mean expression   
  5th Passage Drosophila S2 Cells  4.737569  
  Adult Accessory gland  4.892391  
  Adult Brain  4.853346  
  Adult Carcass  4.823523  
  Adult Crop  4.824711  
  Adult Eye  4.609558  
  Adult Fatbody  5.594613  
  Adult Female Spermatheca Mated  5.582605  
  Adult Female Spermatheca Virgin  5.646011  
  Adult Head  4.537332  
  Adult Heart  5.698374  
  Adult Hind Gut  4.850616  
  Adult Male Ejaculatory Duct  4.722840  
  Adult Mid Gut  5.326853  
  Adult Ovary  4.557566  
  Adult Salivary Gland  5.066611  
  Adult Testes  11.514765  
  Adult Thoracoabdominal ganglion  4.555717  
  Adult Whole Fly  7.273290  
  Larvae Wandering Tubules  4.656820  
  Larval Feeding Carcass  4.399273  
  Larval Feeding Central Nevous System  4.484763  
  Larval Feeding Hind Gut  4.471480  
  Larval Feeding Malpighian Tubule  4.938580  
  Larval Feeding Mid Gut  5.545419  
  Larval Feeding Salivary Gland  4.949439  
  Whole Larvae Feeding  5.936041  
 
  
   FlyBase ID    symbol    start    end    strand    length   
   FBgn0050354   CG30354   4564730   4565166  +  437  
   FBgn0050355   CG30355  4566644   4567453   -  810  
 
    Segment 78 
 
   Location   
  Gene key  FBgn0033320-FBgn0033321  
  Heatmap region span   2R:4537140..4617007   
  Segment span   2R:4583301..4583931   
  Length (genes)  2  
  Length (bp)  631  
   Model Scoring   
  BIC  218.314648  
  logL  -103.636471  
  logL ratio  21.951453  
   Expression   
  Mean expression  6.931341  
  Median expression  6.148278  
  Tissue std. dev.  1.877768  
 
  No GO Slim enrichment  
  
   tissue    mean expression   
  5th Passage Drosophila S2 Cells  4.986296  
  Adult Accessory gland  4.772695  
  Adult Brain  5.567937  
  Adult Carcass  9.459446  
  Adult Crop  9.200857  
  Adult Eye  8.898852  
  Adult Fatbody  9.413473  
  Adult Female Spermatheca Mated  9.436896  
  Adult Female Spermatheca Virgin  9.840065  
  Adult Head  9.397184  
  Adult Heart  9.325423  
  Adult Hind Gut  8.314217  
  Adult Male Ejaculatory Duct  8.752491  
  Adult Mid Gut  5.376063  
  Adult Ovary  5.026611  
  Adult Salivary Gland  7.643284  
  Adult Testes  5.082772  
  Adult Thoracoabdominal ganglion  5.458797  
  Adult Whole Fly  6.683879  
  Larvae Wandering Tubules  4.980966  
  Larval Feeding Carcass  6.592586  
  Larval Feeding Central Nevous System  4.476831  
  Larval Feeding Hind Gut  6.036091  
  Larval Feeding Malpighian Tubule  5.051704  
  Larval Feeding Mid Gut  5.465499  
  Larval Feeding Salivary Gland  5.244063  
  Whole Larvae Feeding  6.661224  
 
  
   FlyBase ID    symbol    start    end    strand    length   
   FBgn0033320   CG8586  4581244   4583301   -  2058  
   FBgn0033321   CG8738   4583931   4586088  +  2158  
 
    Segment 79 
 
   Location   
  Gene key  FBgn0033322-FBgn0050357  
  Heatmap region span   2R:4553360..4623324   
  Segment span   2R:4589299..4609129   
  Length (genes)  5  
  Length (bp)  19831  
   Model Scoring   
  BIC  357.256305  
  logL  -173.107300  
  logL ratio  163.654199  
   Expression   
  Mean expression  4.789853  
  Median expression  4.620046  
  Tissue std. dev.  0.879835  
 
  
   GO ID    description    ratio    P-value   
   GO:0055085   transmembrane transport  3/5  0.000118  
   GO:0006810   transport  3/5  0.00126  
   GO:0008150   biological_process  4/5  0.0226  
 
  
   tissue    mean expression   
  5th Passage Drosophila S2 Cells  4.686285  
  Adult Accessory gland  4.713847  
  Adult Brain  4.332416  
  Adult Carcass  4.711397  
  Adult Crop  4.576038  
  Adult Eye  4.501131  
  Adult Fatbody  4.563078  
  Adult Female Spermatheca Mated  4.610359  
  Adult Female Spermatheca Virgin  4.692823  
  Adult Head  4.337417  
  Adult Heart  4.467211  
  Adult Hind Gut  4.565292  
  Adult Male Ejaculatory Duct  4.686448  
  Adult Mid Gut  4.894865  
  Adult Ovary  4.499222  
  Adult Salivary Gland  4.962004  
  Adult Testes  9.180905  
  Adult Thoracoabdominal ganglion  4.457448  
  Adult Whole Fly  5.144358  
  Larvae Wandering Tubules  4.701858  
  Larval Feeding Carcass  4.549063  
  Larval Feeding Central Nevous System  4.337831  
  Larval Feeding Hind Gut  4.465791  
  Larval Feeding Malpighian Tubule  4.666435  
  Larval Feeding Mid Gut  4.724686  
  Larval Feeding Salivary Gland  4.655971  
  Whole Larvae Feeding  4.641845  
 
  
   FlyBase ID    symbol    start    end    strand    length   
   FBgn0033322   CG8584  4588197   4589299   -  1103  
   FBgn0033323   CG12376   4589340   4592007  +  2668  
   FBgn0033324   CG14744  4594583   4596737   -  2155  
   FBgn0033326   CG14743  4597903   4600222   -  2320  
   FBgn0050357   CG30357  4607605   4609129   -  1525  
 
 
    Segment 80 
 
   Location   
  Gene key  FBgn0086784-FBgn0027585  
  Heatmap region span   2R:4589299..4787706   
  Segment span   2R:4621809..4623324   
  Length (genes)  2  
  Length (bp)  1516  
   Model Scoring   
  BIC  251.270119  
  logL  -120.114207  
  logL ratio  12.622945  
   Expression   
  Mean expression  8.467559  
  Median expression  8.409462  
  Tissue std. dev.  1.126190  
 
  No GO Slim enrichment  
  
   tissue    mean expression   
  5th Passage Drosophila S2 Cells  7.050852  
  Adult Accessory gland  7.684807  
  Adult Brain  9.979944  
  Adult Carcass  9.202139  
  Adult Crop  9.676552  
  Adult Eye  10.157722  
  Adult Fatbody  7.848739  
  Adult Female Spermatheca Mated  8.210811  
  Adult Female Spermatheca Virgin  7.982558  
  Adult Head  9.635751  
  Adult Heart  7.968695  
  Adult Hind Gut  10.213202  
  Adult Male Ejaculatory Duct  9.755441  
  Adult Mid Gut  7.457922  
  Adult Ovary  7.370726  
  Adult Salivary Gland  9.892136  
  Adult Testes  7.635146  
  Adult Thoracoabdominal ganglion  10.119463  
  Adult Whole Fly  7.898474  
  Larvae Wandering Tubules  6.580940  
  Larval Feeding Carcass  8.992100  
  Larval Feeding Central Nevous System  7.427686  
  Larval Feeding Hind Gut  9.235333  
  Larval Feeding Malpighian Tubule  6.774574  
  Larval Feeding Mid Gut  7.556954  
  Larval Feeding Salivary Gland  8.364603  
  Whole Larvae Feeding  7.950817  
 
  
   FlyBase ID    symbol    start    end    strand    length   
   FBgn0086784   stmA  4617003   4621809   -  4807  
   FBgn0027585   CG8740   4623324   4647095  +  23772  
 
    Segment 81 
 
   Location   
  Gene key  FBgn0033330-FBgn0050350  
  Heatmap region span   2R:4611342..4795025   
  Segment span   2R:4680138..4713896   
  Length (genes)  3  
  Length (bp)  33759  
   Model Scoring   
  BIC  301.348419  
  logL  -145.153357  
  logL ratio  21.392378  
   Expression   
  Mean expression  5.506557  
  Median expression  5.130859  
  Tissue std. dev.  0.788591  
 
  No GO Slim enrichment  
  
   tissue    mean expression   
  5th Passage Drosophila S2 Cells  5.182943  
  Adult Accessory gland  5.391445  
  Adult Brain  5.876030  
  Adult Carcass  5.431698  
  Adult Crop  5.136669  
  Adult Eye  5.017368  
  Adult Fatbody  5.391912  
  Adult Female Spermatheca Mated  5.154598  
  Adult Female Spermatheca Virgin  5.169296  
  Adult Head  5.095583  
  Adult Heart  5.937040  
  Adult Hind Gut  5.173068  
  Adult Male Ejaculatory Duct  5.360427  
  Adult Mid Gut  5.341474  
  Adult Ovary  5.052551  
  Adult Salivary Gland  5.210215  
  Adult Testes  9.210907  
  Adult Thoracoabdominal ganglion  5.749547  
  Adult Whole Fly  6.436521  
  Larvae Wandering Tubules  5.333132  
  Larval Feeding Carcass  5.311181  
  Larval Feeding Central Nevous System  5.150911  
  Larval Feeding Hind Gut  5.155856  
  Larval Feeding Malpighian Tubule  5.337996  
  Larval Feeding Mid Gut  5.433494  
  Larval Feeding Salivary Gland  5.202013  
  Whole Larvae Feeding  5.433162  
 
  
   FlyBase ID    symbol    start    end    strand    length   
   FBgn0033330   CG8746   4680138   4681282  +  1145  
   FBgn0024189   sns   4686022   4743142  +  57121  
   FBgn0050350   CG30350   4713896   4715268  +  1373  
 
 
    Segment 82 
 
   Location   
  Gene key  FBgn0021825-FBgn0033339  
  Heatmap region span   2R:4617007..4801631   
  Segment span   2R:4779345..4781129   
  Length (genes)  2  
  Length (bp)  1785  
   Model Scoring   
  BIC  230.490863  
  logL  -109.724579  
  logL ratio  61.690628  
   Expression   
  Mean expression  10.283198  
  Median expression  10.176711  
  Tissue std. dev.  0.542280  
 
  No GO Slim enrichment  
  
   tissue    mean expression   
  5th Passage Drosophila S2 Cells  11.357174  
  Adult Accessory gland  11.086044  
  Adult Brain  10.057561  
  Adult Carcass  9.696862  
  Adult Crop  10.062362  
  Adult Eye  9.850020  
  Adult Fatbody  9.927121  
  Adult Female Spermatheca Mated  10.200074  
  Adult Female Spermatheca Virgin  10.053694  
  Adult Head  9.887058  
  Adult Heart  9.843287  
  Adult Hind Gut  10.072494  
  Adult Male Ejaculatory Duct  10.389470  
  Adult Mid Gut  10.161639  
  Adult Ovary  9.918021  
  Adult Salivary Gland  11.230031  
  Adult Testes  9.377336  
  Adult Thoracoabdominal ganglion  10.255801  
  Adult Whole Fly  9.709694  
  Larvae Wandering Tubules  10.329956  
  Larval Feeding Carcass  10.431390  
  Larval Feeding Central Nevous System  10.176711  
  Larval Feeding Hind Gut  10.742870  
  Larval Feeding Malpighian Tubule  10.288218  
  Larval Feeding Mid Gut  10.312548  
  Larval Feeding Salivary Gland  11.853946  
  Whole Larvae Feeding  10.374965  
 
  
   FlyBase ID    symbol    start    end    strand    length   
   FBgn0021825   Dmn   4779345   4780844  +  1500  
   FBgn0033339   sec31   4781129   4786303  +  5175  
 
    Segment 83 
 
   Location   
  Gene key  FBgn0033340-FBgn0050349  
  Heatmap region span   2R:4680138..4810256   
  Segment span   2R:4787746..4795025   
  Length (genes)  5  
  Length (bp)  7280  
   Model Scoring   
  BIC  571.497442  
  logL  -280.227869  
  logL ratio  112.233777  
   Expression   
  Mean expression  9.517771  
  Median expression  9.477628  
  Tissue std. dev.  0.467673  
 
  No GO Slim enrichment  
  
   tissue    mean expression   
  5th Passage Drosophila S2 Cells  10.083593  
  Adult Accessory gland  9.862573  
  Adult Brain  9.971876  
  Adult Carcass  9.266365  
  Adult Crop  9.807045  
  Adult Eye  10.071545  
  Adult Fatbody  9.307902  
  Adult Female Spermatheca Mated  9.161871  
  Adult Female Spermatheca Virgin  9.053230  
  Adult Head  9.934367  
  Adult Heart  9.557314  
  Adult Hind Gut  9.445219  
  Adult Male Ejaculatory Duct  9.424116  
  Adult Mid Gut  9.021476  
  Adult Ovary  9.428173  
  Adult Salivary Gland  9.688051  
  Adult Testes  8.445598  
  Adult Thoracoabdominal ganglion  10.014710  
  Adult Whole Fly  9.819404  
  Larvae Wandering Tubules  9.136933  
  Larval Feeding Carcass  9.391175  
  Larval Feeding Central Nevous System  10.832053  
  Larval Feeding Hind Gut  9.305471  
  Larval Feeding Malpighian Tubule  9.324054  
  Larval Feeding Mid Gut  8.931615  
  Larval Feeding Salivary Gland  9.555899  
  Whole Larvae Feeding  9.138197  
 
  
   FlyBase ID    symbol    start    end    strand    length   
   FBgn0033340   CG13751   4787746   4788122  +  377  
   FBgn0033341   MrgBP  4788071   4788877   -  807  
   FBgn0004921   Ggamma1   4788908   4792296  +  3389  
   FBgn0033342   CG8258  4792347   4794506   -  2160  
   FBgn0050349   CG30349   4795025   4798390  +  3366  
 
 
    Segment 84 
 
   Location   
  Gene key  FBgn0033347-FBgn0033348  
  Heatmap region span   2R:4787746..4839846   
  Segment span   2R:4807871..4810256   
  Length (genes)  2  
  Length (bp)  2386  
   Model Scoring   
  BIC  231.808320  
  logL  -110.383308  
  logL ratio  -9.919699  
   Expression   
  Mean expression  5.472496  
  Median expression  4.861916  
  Tissue std. dev.  1.130698  
 
  No GO Slim enrichment  
  
   tissue    mean expression   
  5th Passage Drosophila S2 Cells  6.291681  
  Adult Accessory gland  4.728407  
  Adult Brain  8.171288  
  Adult Carcass  4.832705  
  Adult Crop  5.013092  
  Adult Eye  7.542560  
  Adult Fatbody  4.476821  
  Adult Female Spermatheca Mated  4.678546  
  Adult Female Spermatheca Virgin  4.533539  
  Adult Head  6.721436  
  Adult Heart  4.977958  
  Adult Hind Gut  4.718126  
  Adult Male Ejaculatory Duct  4.517636  
  Adult Mid Gut  4.598158  
  Adult Ovary  6.638854  
  Adult Salivary Gland  4.917991  
  Adult Testes  4.660911  
  Adult Thoracoabdominal ganglion  7.438363  
  Adult Whole Fly  5.565117  
  Larvae Wandering Tubules  4.885590  
  Larval Feeding Carcass  4.959642  
  Larval Feeding Central Nevous System  7.961832  
  Larval Feeding Hind Gut  5.021662  
  Larval Feeding Malpighian Tubule  5.097690  
  Larval Feeding Mid Gut  4.502169  
  Larval Feeding Salivary Gland  5.323704  
  Whole Larvae Feeding  4.981918  
 
  
   FlyBase ID    symbol    start    end    strand    length   
   FBgn0033347   CG8248  4806445   4807871   -  1427  
   FBgn0033348   CG8247  4807775   4810256   -  2482  
 
    Segment 85 
 
   Location   
  Gene key  FBgn0033349-FBgn0033352  
  Heatmap region span   2R:4797854..4855687   
  Segment span   2R:4810293..4815679   
  Length (genes)  4  
  Length (bp)  5387  
   Model Scoring   
  BIC  361.863129  
  logL  -175.410712  
  logL ratio  182.210519  
   Expression   
  Mean expression  9.778077  
  Median expression  9.772662  
  Tissue std. dev.  0.516847  
 
  No GO Slim enrichment  
  
   tissue    mean expression   
  5th Passage Drosophila S2 Cells  10.259370  
  Adult Accessory gland  10.490129  
  Adult Brain  9.018808  
  Adult Carcass  9.065969  
  Adult Crop  9.684625  
  Adult Eye  9.576929  
  Adult Fatbody  9.423532  
  Adult Female Spermatheca Mated  9.762501  
  Adult Female Spermatheca Virgin  9.809425  
  Adult Head  9.092141  
  Adult Heart  9.660335  
  Adult Hind Gut  9.313804  
  Adult Male Ejaculatory Duct  10.262185  
  Adult Mid Gut  9.633627  
  Adult Ovary  10.143452  
  Adult Salivary Gland  10.545162  
  Adult Testes  9.175289  
  Adult Thoracoabdominal ganglion  9.110755  
  Adult Whole Fly  9.594323  
  Larvae Wandering Tubules  10.469285  
  Larval Feeding Carcass  9.574580  
  Larval Feeding Central Nevous System  9.922297  
  Larval Feeding Hind Gut  9.783533  
  Larval Feeding Malpighian Tubule  10.244942  
  Larval Feeding Mid Gut  9.747359  
  Larval Feeding Salivary Gland  11.141716  
  Whole Larvae Feeding  9.502001  
 
  
   FlyBase ID    symbol    start    end    strand    length   
   FBgn0033349   CG8243   4810293   4812851  +  2559  
   FBgn0033350   CG8237   4813194   4814395  +  1202  
   FBgn0033351   CG8235  4814519   4815605   -  1087  
   FBgn0033352   CG8232   4815679   4820608  +  4930  
 
 
    Segment 86 
 
   Location   
  Gene key  FBgn0033353-FBgn0033355  
  Heatmap region span   2R:4801631..4918546   
  Segment span   2R:4820928..4827920   
  Length (genes)  3  
  Length (bp)  6993  
   Model Scoring   
  BIC  287.558535  
  logL  -138.258415  
  logL ratio  27.724848  
   Expression   
  Mean expression  5.135744  
  Median expression  4.835729  
  Tissue std. dev.  0.583973  
 
  No GO Slim enrichment  
  
   tissue    mean expression   
  5th Passage Drosophila S2 Cells  5.550859  
  Adult Accessory gland  6.726192  
  Adult Brain  4.483844  
  Adult Carcass  4.749171  
  Adult Crop  4.836776  
  Adult Eye  4.717619  
  Adult Fatbody  4.816237  
  Adult Female Spermatheca Mated  4.885264  
  Adult Female Spermatheca Virgin  5.028802  
  Adult Head  4.528547  
  Adult Heart  4.850865  
  Adult Hind Gut  4.708295  
  Adult Male Ejaculatory Duct  5.012066  
  Adult Mid Gut  4.704204  
  Adult Ovary  5.535213  
  Adult Salivary Gland  5.051628  
  Adult Testes  5.373190  
  Adult Thoracoabdominal ganglion  4.621881  
  Adult Whole Fly  4.485284  
  Larvae Wandering Tubules  6.350402  
  Larval Feeding Carcass  5.201959  
  Larval Feeding Central Nevous System  5.360971  
  Larval Feeding Hind Gut  6.594180  
  Larval Feeding Malpighian Tubule  4.978717  
  Larval Feeding Mid Gut  5.369268  
  Larval Feeding Salivary Gland  5.042138  
  Whole Larvae Feeding  5.101503  
 
  
   FlyBase ID    symbol    start    end    strand    length   
   FBgn0033353   CG13749   4820928   4821950  +  1023  
   FBgn0033354   FANCI  4822078   4827299   -  5222  
   FBgn0033355   CG13748   4827920   4828641  +  722  
 
 
    Segment 87 
 
   Location   
  Gene key  FBgn0027607-FBgn0033357  
  Heatmap region span   2R:4802033..4932213   
  Segment span   2R:4834866..4839193   
  Length (genes)  3  
  Length (bp)  4328  
   Model Scoring   
  BIC  417.368200  
  logL  -203.163248  
  logL ratio  32.491390  
   Expression   
  Mean expression  10.240426  
  Median expression  10.189884  
  Tissue std. dev.  0.481063  
 
  No GO Slim enrichment  
  
   tissue    mean expression   
  5th Passage Drosophila S2 Cells  10.217693  
  Adult Accessory gland  10.881345  
  Adult Brain  10.557837  
  Adult Carcass  10.011470  
  Adult Crop  10.542039  
  Adult Eye  10.717205  
  Adult Fatbody  10.564467  
  Adult Female Spermatheca Mated  10.680854  
  Adult Female Spermatheca Virgin  10.800407  
  Adult Head  10.416626  
  Adult Heart  10.256866  
  Adult Hind Gut  10.210118  
  Adult Male Ejaculatory Duct  10.895670  
  Adult Mid Gut  9.414273  
  Adult Ovary  9.566581  
  Adult Salivary Gland  10.391967  
  Adult Testes  9.583210  
  Adult Thoracoabdominal ganglion  10.674276  
  Adult Whole Fly  9.625840  
  Larvae Wandering Tubules  10.737920  
  Larval Feeding Carcass  9.844141  
  Larval Feeding Central Nevous System  9.537853  
  Larval Feeding Hind Gut  9.930203  
  Larval Feeding Malpighian Tubule  10.755518  
  Larval Feeding Mid Gut  9.414961  
  Larval Feeding Salivary Gland  10.421794  
  Whole Larvae Feeding  9.840360  
 
  
   FlyBase ID    symbol    start    end    strand    length   
   FBgn0027607   CG8230  4831801   4834866   -  3066  
   FBgn0033356   CG8229  4835618   4838609   -  2992  
   FBgn0033357   Tom7   4839193   4839694  +  502  
 
 
    Segment 88 
 
   Location   
  Gene key  FBgn0033359-FBgn0033365  
  Heatmap region span   2R:4820928..4956606   
  Segment span   2R:4873063..4918546   
  Length (genes)  6  
  Length (bp)  45484  
   Model Scoring   
  BIC  543.738125  
  logL  -266.348210  
  logL ratio  95.411438  
   Expression   
  Mean expression  4.784243  
  Median expression  4.713858  
  Tissue std. dev.  0.377950  
 
  No GO Slim enrichment  
  
   tissue    mean expression   
  5th Passage Drosophila S2 Cells  4.742604  
  Adult Accessory gland  4.717084  
  Adult Brain  4.338131  
  Adult Carcass  4.620652  
  Adult Crop  4.601714  
  Adult Eye  5.009171  
  Adult Fatbody  4.697913  
  Adult Female Spermatheca Mated  4.642783  
  Adult Female Spermatheca Virgin  4.644316  
  Adult Head  4.497564  
  Adult Heart  4.649028  
  Adult Hind Gut  5.503638  
  Adult Male Ejaculatory Duct  4.619654  
  Adult Mid Gut  4.681781  
  Adult Ovary  4.539717  
  Adult Salivary Gland  4.925644  
  Adult Testes  6.242323  
  Adult Thoracoabdominal ganglion  4.368630  
  Adult Whole Fly  4.992852  
  Larvae Wandering Tubules  4.615755  
  Larval Feeding Carcass  4.829255  
  Larval Feeding Central Nevous System  4.353814  
  Larval Feeding Hind Gut  5.205106  
  Larval Feeding Malpighian Tubule  4.667136  
  Larval Feeding Mid Gut  4.769311  
  Larval Feeding Salivary Gland  4.827317  
  Whole Larvae Feeding  4.871672  
 
  
   FlyBase ID    symbol    start    end    strand    length   
   FBgn0033359   CG8213  4856841   4873063   -  16223  
   FBgn0085379     4881047   4892622   -  11576  
   FBgn0033362   CG8172  4894739   4901013   -  6275  
   FBgn0033363   CG13744  4902011   4904184   -  2174  
   FBgn0033364   CG13747   4905360   4906539  +  1180  
   FBgn0033365   CG8170  4906530   4918546   -  12017  
 
 
    Segment 89 
 
   Location   
  Gene key  FBgn0033371-FBgn0033372  
  Heatmap region span   2R:4947411..5032754   
  Segment span   2R:4969942..4975062   
  Length (genes)  2  
  Length (bp)  5121  
   Model Scoring   
  BIC  203.633745  
  logL  -96.296020  
  logL ratio  14.991922  
   Expression   
  Mean expression  4.791249  
  Median expression  4.553107  
  Tissue std. dev.  0.582181  
 
  No GO Slim enrichment  
  
   tissue    mean expression   
  5th Passage Drosophila S2 Cells  4.987395  
  Adult Accessory gland  4.525703  
  Adult Brain  4.179847  
  Adult Carcass  4.821055  
  Adult Crop  4.683063  
  Adult Eye  4.304740  
  Adult Fatbody  4.559144  
  Adult Female Spermatheca Mated  4.604586  
  Adult Female Spermatheca Virgin  4.551881  
  Adult Head  4.334002  
  Adult Heart  4.361171  
  Adult Hind Gut  4.839479  
  Adult Male Ejaculatory Duct  4.546175  
  Adult Mid Gut  4.829434  
  Adult Ovary  6.126589  
  Adult Salivary Gland  4.343716  
  Adult Testes  6.979027  
  Adult Thoracoabdominal ganglion  4.437204  
  Adult Whole Fly  5.299198  
  Larvae Wandering Tubules  4.847786  
  Larval Feeding Carcass  4.617905  
  Larval Feeding Central Nevous System  5.448477  
  Larval Feeding Hind Gut  4.622068  
  Larval Feeding Malpighian Tubule  4.721051  
  Larval Feeding Mid Gut  4.560774  
  Larval Feeding Salivary Gland  4.459746  
  Whole Larvae Feeding  4.772517  
 
  
   FlyBase ID    symbol    start    end    strand    length   
   FBgn0033371   CNT1   4969942   4973222  +  3281  
   FBgn0033372   CG13742  4972098   4975062   -  2965  
 
    Segment 90 
 
   Location   
  Gene key  FBgn0010220-FBgn0033373  
  Heatmap region span   2R:4949429..5036332   
  Segment span   2R:4975358..4980474   
  Length (genes)  2  
  Length (bp)  5117  
   Model Scoring   
  BIC  252.250562  
  logL  -120.604429  
  logL ratio  12.835869  
   Expression   
  Mean expression  8.715021  
  Median expression  8.372348  
  Tissue std. dev.  0.455230  
 
  No GO Slim enrichment  
  
   tissue    mean expression   
  5th Passage Drosophila S2 Cells  9.015576  
  Adult Accessory gland  8.443088  
  Adult Brain  8.609972  
  Adult Carcass  8.691951  
  Adult Crop  8.277095  
  Adult Eye  8.880063  
  Adult Fatbody  9.324341  
  Adult Female Spermatheca Mated  9.284679  
  Adult Female Spermatheca Virgin  9.589609  
  Adult Head  8.732430  
  Adult Heart  9.210016  
  Adult Hind Gut  8.137579  
  Adult Male Ejaculatory Duct  8.271452  
  Adult Mid Gut  8.715170  
  Adult Ovary  9.120994  
  Adult Salivary Gland  7.805805  
  Adult Testes  8.232581  
  Adult Thoracoabdominal ganglion  8.323989  
  Adult Whole Fly  8.431085  
  Larvae Wandering Tubules  9.513537  
  Larval Feeding Carcass  8.335500  
  Larval Feeding Central Nevous System  8.310090  
  Larval Feeding Hind Gut  8.385527  
  Larval Feeding Malpighian Tubule  9.296715  
  Larval Feeding Mid Gut  9.043882  
  Larval Feeding Salivary Gland  8.657985  
  Whole Larvae Feeding  8.664853  
 
  
   FlyBase ID    symbol    start    end    strand    length   
   FBgn0010220   Dbp45A   4975358   4977304  +  1947  
   FBgn0033373   CG8080  4977279   4980474   -  3196  
 
    Segment 91 
 
   Location   
  Gene key  FBgn0033375-FBgn0027561  
  Heatmap region span   2R:4966232..5055023   
  Segment span   2R:4985972..5019486   
  Length (genes)  9  
  Length (bp)  33515  
   Model Scoring   
  BIC  859.270269  
  logL  -424.114282  
  logL ratio  287.122628  
   Expression   
  Mean expression  8.580781  
  Median expression  8.438991  
  Tissue std. dev.  0.391697  
 
  No GO Slim enrichment  
  
   tissue    mean expression   
  5th Passage Drosophila S2 Cells  9.238997  
  Adult Accessory gland  9.137562  
  Adult Brain  8.590173  
  Adult Carcass  8.022608  
  Adult Crop  8.581605  
  Adult Eye  8.559958  
  Adult Fatbody  8.585376  
  Adult Female Spermatheca Mated  8.606631  
  Adult Female Spermatheca Virgin  8.537687  
  Adult Head  8.090926  
  Adult Heart  8.726072  
  Adult Hind Gut  8.209404  
  Adult Male Ejaculatory Duct  8.647227  
  Adult Mid Gut  7.978659  
  Adult Ovary  9.399451  
  Adult Salivary Gland  8.462445  
  Adult Testes  8.071389  
  Adult Thoracoabdominal ganglion  8.600164  
  Adult Whole Fly  8.385724  
  Larvae Wandering Tubules  8.621636  
  Larval Feeding Carcass  8.577136  
  Larval Feeding Central Nevous System  9.359198  
  Larval Feeding Hind Gut  8.726747  
  Larval Feeding Malpighian Tubule  8.552431  
  Larval Feeding Mid Gut  8.043385  
  Larval Feeding Salivary Gland  9.129569  
  Whole Larvae Feeding  8.238937  
 
  
   FlyBase ID    symbol    start    end    strand    length   
   FBgn0033375   CG8078  4984436   4985972   -  1537  
   FBgn0033376   CG8777   4986075   4990661  +  4587  
   FBgn0015838   Vang   4990749   4994301  +  3553  
   FBgn0033377   Pmm45A  4994647   4997228   -  2582  
   FBgn0033378   tsu   4998306   4999087  +  782  
   FBgn0033379   Mys45A  4999010   5001458   -  2449  
   FBgn0033380   Phax   5001768   5003558  +  1791  
   FBgn0033381   GstE13   5009630   5010814  +  1185  
   FBgn0027561   CG18659  5010809   5019486   -  8678  
 
 
    Segment 92 
 
   Location   
  Gene key  FBgn0033382-FBgn0050344  
  Heatmap region span   2R:4969942..5073866   
  Segment span   2R:5019992..5032754   
  Length (genes)  5  
  Length (bp)  12763  
   Model Scoring   
  BIC  625.160561  
  logL  -307.059428  
  logL ratio  92.757160  
   Expression   
  Mean expression  9.882288  
  Median expression  9.814960  
  Tissue std. dev.  0.580765  
 
  No GO Slim enrichment  
  
   tissue    mean expression   
  5th Passage Drosophila S2 Cells  9.974013  
  Adult Accessory gland  10.104733  
  Adult Brain  9.590821  
  Adult Carcass  10.086964  
  Adult Crop  9.646979  
  Adult Eye  10.341992  
  Adult Fatbody  10.243312  
  Adult Female Spermatheca Mated  10.417442  
  Adult Female Spermatheca Virgin  10.336026  
  Adult Head  10.244885  
  Adult Heart  10.657046  
  Adult Hind Gut  9.910565  
  Adult Male Ejaculatory Duct  10.627641  
  Adult Mid Gut  10.025978  
  Adult Ovary  9.539697  
  Adult Salivary Gland  8.982511  
  Adult Testes  8.810677  
  Adult Thoracoabdominal ganglion  9.723970  
  Adult Whole Fly  9.586015  
  Larvae Wandering Tubules  10.948780  
  Larval Feeding Carcass  9.208871  
  Larval Feeding Central Nevous System  9.030786  
  Larval Feeding Hind Gut  9.655546  
  Larval Feeding Malpighian Tubule  10.517820  
  Larval Feeding Mid Gut  10.373266  
  Larval Feeding Salivary Gland  8.696764  
  Whole Larvae Feeding  9.538681  
 
  
   FlyBase ID    symbol    start    end    strand    length   
   FBgn0033382   Hydr1   5019992   5022958  +  2967  
   FBgn0028955   CG8788   5025979   5029486  +  3508  
   FBgn0086656   shrb  5029651   5031415   -  1765  
   FBgn0050342   Prp38   5031464   5032656  +  1193  
   FBgn0050344   CG30344   5032754   5036019  +  3266  
 
 
    Segment 93 
 
   Location   
  Gene key  FBgn0033388-FBgn0259728  
  Heatmap region span   2R:4985972..5129846   
  Segment span   2R:5052455..5055023   
  Length (genes)  3  
  Length (bp)  2569  
   Model Scoring   
  BIC  314.453936  
  logL  -151.706115  
  logL ratio  21.541388  
   Expression   
  Mean expression  5.190850  
  Median expression  4.790037  
  Tissue std. dev.  0.502604  
 
  No GO Slim enrichment  
  
   tissue    mean expression   
  5th Passage Drosophila S2 Cells  7.325301  
  Adult Accessory gland  5.100039  
  Adult Brain  5.209713  
  Adult Carcass  5.084270  
  Adult Crop  5.030935  
  Adult Eye  5.276974  
  Adult Fatbody  5.027831  
  Adult Female Spermatheca Mated  4.910308  
  Adult Female Spermatheca Virgin  4.873239  
  Adult Head  5.033121  
  Adult Heart  6.082669  
  Adult Hind Gut  5.075325  
  Adult Male Ejaculatory Duct  5.091983  
  Adult Mid Gut  5.080360  
  Adult Ovary  5.586190  
  Adult Salivary Gland  5.068487  
  Adult Testes  4.614794  
  Adult Thoracoabdominal ganglion  5.142665  
  Adult Whole Fly  4.674923  
  Larvae Wandering Tubules  5.173103  
  Larval Feeding Carcass  5.087375  
  Larval Feeding Central Nevous System  5.462666  
  Larval Feeding Hind Gut  4.900057  
  Larval Feeding Malpighian Tubule  5.132446  
  Larval Feeding Mid Gut  4.933258  
  Larval Feeding Salivary Gland  5.330092  
  Whole Larvae Feeding  4.844819  
 
  
   FlyBase ID    symbol    start    end    strand    length   
   FBgn0033388   CG8046  5049542   5052455   -  2914  
   FBgn0033389   Rad51C   5053240   5054371  +  1132  
   FBgn0259728   CG42382  5054071   5055023   -  953  
 
 
    Segment 94 
 
   Location   
  Gene key  FBgn0033391-FBgn0033395  
  Heatmap region span   2R:5036332..5196181   
  Segment span   2R:5079600..5125035   
  Length (genes)  4  
  Length (bp)  45436  
   Model Scoring   
  BIC  434.332675  
  logL  -211.645485  
  logL ratio  14.456648  
   Expression   
  Mean expression  5.896180  
  Median expression  5.842128  
  Tissue std. dev.  0.297186  
 
  No GO Slim enrichment  
  
   tissue    mean expression   
  5th Passage Drosophila S2 Cells  5.865951  
  Adult Accessory gland  5.949623  
  Adult Brain  6.277725  
  Adult Carcass  6.094773  
  Adult Crop  5.697865  
  Adult Eye  5.699551  
  Adult Fatbody  5.941627  
  Adult Female Spermatheca Mated  6.050642  
  Adult Female Spermatheca Virgin  6.058591  
  Adult Head  5.777022  
  Adult Heart  5.705077  
  Adult Hind Gut  6.118846  
  Adult Male Ejaculatory Duct  5.526351  
  Adult Mid Gut  6.264293  
  Adult Ovary  6.353202  
  Adult Salivary Gland  5.819114  
  Adult Testes  5.416328  
  Adult Thoracoabdominal ganglion  6.652761  
  Adult Whole Fly  5.648137  
  Larvae Wandering Tubules  5.957613  
  Larval Feeding Carcass  5.678035  
  Larval Feeding Central Nevous System  6.123781  
  Larval Feeding Hind Gut  5.701250  
  Larval Feeding Malpighian Tubule  6.126023  
  Larval Feeding Mid Gut  5.660456  
  Larval Feeding Salivary Gland  5.710389  
  Whole Larvae Feeding  5.321821  
 
  
   FlyBase ID    symbol    start    end    strand    length   
   FBgn0033391   CG8026  5074175   5079600   -  5426  
   FBgn0015561   unpg   5082719   5087377  +  4659  
   FBgn0033392   CG8027  5091913   5094626   -  2714  
   FBgn0033395   Cyp4p2   5125035   5127338  +  2304  
 
 
    Segment 95 
 
   Location   
  Gene key  FBgn0050343-FBgn0033402  
  Heatmap region span   2R:5079600..5301849   
  Segment span   2R:5139518..5196181   
  Length (genes)  6  
  Length (bp)  56664  
   Model Scoring   
  BIC  578.717091  
  logL  -283.837693  
  logL ratio  181.284573  
   Expression   
  Mean expression  8.569263  
  Median expression  8.566117  
  Tissue std. dev.  0.426725  
 
  
   GO ID    description    ratio    P-value   
   GO:0007165   signal transduction  2/6  0.0141  
 
  
   tissue    mean expression   
  5th Passage Drosophila S2 Cells  8.609096  
  Adult Accessory gland  8.581026  
  Adult Brain  8.505104  
  Adult Carcass  8.118962  
  Adult Crop  8.701972  
  Adult Eye  8.961760  
  Adult Fatbody  8.676488  
  Adult Female Spermatheca Mated  8.455659  
  Adult Female Spermatheca Virgin  8.507628  
  Adult Head  8.690870  
  Adult Heart  8.517935  
  Adult Hind Gut  8.553027  
  Adult Male Ejaculatory Duct  8.357782  
  Adult Mid Gut  8.666661  
  Adult Ovary  9.107874  
  Adult Salivary Gland  9.361684  
  Adult Testes  6.982666  
  Adult Thoracoabdominal ganglion  8.589684  
  Adult Whole Fly  8.171653  
  Larvae Wandering Tubules  8.937630  
  Larval Feeding Carcass  8.774709  
  Larval Feeding Central Nevous System  8.711658  
  Larval Feeding Hind Gut  8.537181  
  Larval Feeding Malpighian Tubule  8.826769  
  Larval Feeding Mid Gut  8.254154  
  Larval Feeding Salivary Gland  9.092538  
  Whole Larvae Feeding  8.117920  
 
  
   FlyBase ID    symbol    start    end    strand    length   
   FBgn0050343   CG30343   5139518   5140760  +  1243  
   FBgn0020621   Pkn  5139815   5172602   -  32788  
   FBgn0033400   CG2063  5172815   5174134   -  1320  
   FBgn0033401   CG1968   5174341   5176531  +  2191  
   FBgn0026326   Mad1  5176462   5179059   -  2598  
   FBgn0033402   Myd88  5191183   5196181   -  4999  
 
 
    Segment 96 
 
   Location   
  Gene key  FBgn0033404-FBgn0033405  
  Heatmap region span   2R:5127548..5311127   
  Segment span   2R:5232927..5261741   
  Length (genes)  4  
  Length (bp)  28815  
   Model Scoring   
  BIC  403.848648  
  logL  -196.403472  
  logL ratio  20.193411  
   Expression   
  Mean expression  4.694082  
  Median expression  4.555112  
  Tissue std. dev.  0.474507  
 
  No GO Slim enrichment  
  
   tissue    mean expression   
  5th Passage Drosophila S2 Cells  4.590605  
  Adult Accessory gland  4.638243  
  Adult Brain  4.589239  
  Adult Carcass  5.710805  
  Adult Crop  4.495101  
  Adult Eye  4.541691  
  Adult Fatbody  4.590222  
  Adult Female Spermatheca Mated  4.433041  
  Adult Female Spermatheca Virgin  4.453115  
  Adult Head  6.582350  
  Adult Heart  4.576416  
  Adult Hind Gut  4.496169  
  Adult Male Ejaculatory Duct  4.733696  
  Adult Mid Gut  4.686222  
  Adult Ovary  4.526702  
  Adult Salivary Gland  4.783678  
  Adult Testes  4.350516  
  Adult Thoracoabdominal ganglion  4.362669  
  Adult Whole Fly  5.440850  
  Larvae Wandering Tubules  4.612450  
  Larval Feeding Carcass  4.630200  
  Larval Feeding Central Nevous System  4.266851  
  Larval Feeding Hind Gut  4.514924  
  Larval Feeding Malpighian Tubule  4.562328  
  Larval Feeding Mid Gut  4.602258  
  Larval Feeding Salivary Gland  4.627166  
  Whole Larvae Feeding  4.342712  
 
  
   FlyBase ID    symbol    start    end    strand    length   
   FBgn0033404   Or45a   5232927   5234117  +  1191  
   FBgn0033403   CG13739  5205046   5239139   -  34094  
   FBgn0040775   CG12158  5250076   5250411   -  336  
   FBgn0033405   CG13954  5257635   5261741   -  4107  
 
 
    Segment 97 
 
   Location   
  Gene key  FBgn0033413-FBgn0017558  
  Heatmap region span   2R:5286255..5449154   
  Segment span   2R:5311576..5313947   
  Length (genes)  2  
  Length (bp)  2372  
   Model Scoring   
  BIC  247.546006  
  logL  -118.252150  
  logL ratio  60.117162  
   Expression   
  Mean expression  10.620079  
  Median expression  10.894872  
  Tissue std. dev.  0.764079  
 
  No GO Slim enrichment  
  
   tissue    mean expression   
  5th Passage Drosophila S2 Cells  9.201933  
  Adult Accessory gland  10.208379  
  Adult Brain  11.140588  
  Adult Carcass  10.905025  
  Adult Crop  11.228367  
  Adult Eye  11.237087  
  Adult Fatbody  11.377842  
  Adult Female Spermatheca Mated  10.909051  
  Adult Female Spermatheca Virgin  11.151678  
  Adult Head  10.975455  
  Adult Heart  11.524335  
  Adult Hind Gut  11.501524  
  Adult Male Ejaculatory Duct  11.244205  
  Adult Mid Gut  10.415654  
  Adult Ovary  10.162054  
  Adult Salivary Gland  10.235881  
  Adult Testes  8.316783  
  Adult Thoracoabdominal ganglion  11.388724  
  Adult Whole Fly  10.167473  
  Larvae Wandering Tubules  10.984409  
  Larval Feeding Carcass  10.120812  
  Larval Feeding Central Nevous System  9.569502  
  Larval Feeding Hind Gut  10.770346  
  Larval Feeding Malpighian Tubule  11.261720  
  Larval Feeding Mid Gut  10.521117  
  Larval Feeding Salivary Gland  9.620755  
  Whole Larvae Feeding  10.601434  
 
  
   FlyBase ID    symbol    start    end    strand    length   
   FBgn0033413   prel   5311576   5313612  +  2037  
   FBgn0017558   Pdk   5313947   5321930  +  7984  
 
    Segment 98 
 
   Location   
  Gene key  FBgn0259234-FBgn0259246  
  Heatmap region span   2R:5300965..5464932   
  Segment span   2R:5333551..5391293   
  Length (genes)  5  
  Length (bp)  57743  
   Model Scoring   
  BIC  369.372222  
  logL  -179.165258  
  logL ratio  162.211997  
   Expression   
  Mean expression  4.462955  
  Median expression  4.353988  
  Tissue std. dev.  0.134999  
 
  No GO Slim enrichment  
  
   tissue    mean expression   
  5th Passage Drosophila S2 Cells  4.605975  
  Adult Accessory gland  4.456826  
  Adult Brain  4.497727  
  Adult Carcass  4.453132  
  Adult Crop  4.384299  
  Adult Eye  4.409413  
  Adult Fatbody  4.466133  
  Adult Female Spermatheca Mated  4.639141  
  Adult Female Spermatheca Virgin  4.539199  
  Adult Head  4.580981  
  Adult Heart  4.284029  
  Adult Hind Gut  4.588842  
  Adult Male Ejaculatory Duct  4.430517  
  Adult Mid Gut  4.381161  
  Adult Ovary  4.387593  
  Adult Salivary Gland  4.558079  
  Adult Testes  4.827296  
  Adult Thoracoabdominal ganglion  4.456356  
  Adult Whole Fly  4.111100  
  Larvae Wandering Tubules  4.449761  
  Larval Feeding Carcass  4.350042  
  Larval Feeding Central Nevous System  4.575389  
  Larval Feeding Hind Gut  4.454694  
  Larval Feeding Malpighian Tubule  4.461803  
  Larval Feeding Mid Gut  4.361467  
  Larval Feeding Salivary Gland  4.532560  
  Whole Larvae Feeding  4.256273  
 
  
   FlyBase ID    symbol    start    end    strand    length   
   FBgn0259234   Camta   5333551   5365595  +  32045  
   FBgn0053758   CG33758  5340914   5341560   -  647  
   FBgn0053757   CG33757  5341802   5342453   -  652  
   FBgn0004360   Wnt2   5381674   5390695  +  9022  
   FBgn0259246   brp   5391293   5424980  +  33688  
 
 
    Segment 99 
 
   Location   
  Gene key  FBgn0085436-FBgn0033427  
  Heatmap region span   2R:5434459..5481588   
  Segment span   2R:5465985..5472050   
  Length (genes)  2  
  Length (bp)  6066  
   Model Scoring   
  BIC  228.471749  
  logL  -108.715022  
  logL ratio  -2.542185  
   Expression   
  Mean expression  5.545094  
  Median expression  5.527947  
  Tissue std. dev.  0.417773  
 
  
   GO ID    description    ratio    P-value   
   GO:0005737   cytoplasm  2/2  0.00941  
 
  
   tissue    mean expression   
  5th Passage Drosophila S2 Cells  6.450665  
  Adult Accessory gland  5.882081  
  Adult Brain  5.348613  
  Adult Carcass  5.067628  
  Adult Crop  5.584447  
  Adult Eye  5.265669  
  Adult Fatbody  5.137859  
  Adult Female Spermatheca Mated  5.356412  
  Adult Female Spermatheca Virgin  5.298681  
  Adult Head  5.031792  
  Adult Heart  5.698906  
  Adult Hind Gut  5.257879  
  Adult Male Ejaculatory Duct  5.401140  
  Adult Mid Gut  5.322128  
  Adult Ovary  6.355840  
  Adult Salivary Gland  5.377529  
  Adult Testes  6.705151  
  Adult Thoracoabdominal ganglion  5.399598  
  Adult Whole Fly  5.506823  
  Larvae Wandering Tubules  5.895018  
  Larval Feeding Carcass  5.215472  
  Larval Feeding Central Nevous System  5.971456  
  Larval Feeding Hind Gut  5.308048  
  Larval Feeding Malpighian Tubule  5.782432  
  Larval Feeding Mid Gut  5.277761  
  Larval Feeding Salivary Gland  5.555631  
  Whole Larvae Feeding  5.262892  
 
  
   FlyBase ID    symbol    start    end    strand    length   
   FBgn0085436   Not1  5453651   5465985   -  12335  
   FBgn0033427   CG1868  5469261   5472050   -  2790  
 
    Segment 100 
 
   Location   
  Gene key  FBgn0033428-FBgn0033429  
  Heatmap region span   2R:5447103..5488716   
  Segment span   2R:5472172..5474180   
  Length (genes)  2  
  Length (bp)  2009  
   Model Scoring   
  BIC  291.731493  
  logL  -140.344894  
  logL ratio  -10.642574  
   Expression   
  Mean expression  9.468795  
  Median expression  9.231598  
  Tissue std. dev.  0.452704  
 
  No GO Slim enrichment  
  
   tissue    mean expression   
  5th Passage Drosophila S2 Cells  9.336451  
  Adult Accessory gland  9.776825  
  Adult Brain  9.159286  
  Adult Carcass  9.560379  
  Adult Crop  9.659781  
  Adult Eye  9.491077  
  Adult Fatbody  9.823031  
  Adult Female Spermatheca Mated  9.458424  
  Adult Female Spermatheca Virgin  9.498179  
  Adult Head  9.090184  
  Adult Heart  10.301039  
  Adult Hind Gut  9.935581  
  Adult Male Ejaculatory Duct  10.524515  
  Adult Mid Gut  9.231930  
  Adult Ovary  9.260715  
  Adult Salivary Gland  10.336638  
  Adult Testes  9.080436  
  Adult Thoracoabdominal ganglion  9.398779  
  Adult Whole Fly  9.015695  
  Larvae Wandering Tubules  9.552906  
  Larval Feeding Carcass  8.806320  
  Larval Feeding Central Nevous System  9.165276  
  Larval Feeding Hind Gut  9.190648  
  Larval Feeding Malpighian Tubule  9.623791  
  Larval Feeding Mid Gut  8.992169  
  Larval Feeding Salivary Gland  9.807472  
  Whole Larvae Feeding  8.579935  
 
  
   FlyBase ID    symbol    start    end    strand    length   
   FBgn0033428   Updo   5472172   5473623  +  1452  
   FBgn0033429   CG12929  5473615   5474180   -  566  
 
    Segment 101 
 
   Location   
  Gene key  FBgn0033431-FBgn0050338  
  Heatmap region span   2R:5464932..5497792   
  Segment span   2R:5476611..5481552   
  Length (genes)  3  
  Length (bp)  4942  
   Model Scoring   
  BIC  382.954598  
  logL  -185.956447  
  logL ratio  0.792582  
   Expression   
  Mean expression  7.883137  
  Median expression  7.582473  
  Tissue std. dev.  0.495730  
 
  No GO Slim enrichment  
  
   tissue    mean expression   
  5th Passage Drosophila S2 Cells  8.621624  
  Adult Accessory gland  7.929162  
  Adult Brain  7.484610  
  Adult Carcass  6.968070  
  Adult Crop  7.181989  
  Adult Eye  7.870515  
  Adult Fatbody  7.497300  
  Adult Female Spermatheca Mated  7.699917  
  Adult Female Spermatheca Virgin  7.775767  
  Adult Head  7.317765  
  Adult Heart  8.265406  
  Adult Hind Gut  7.172581  
  Adult Male Ejaculatory Duct  7.660147  
  Adult Mid Gut  7.420311  
  Adult Ovary  8.555075  
  Adult Salivary Gland  7.592615  
  Adult Testes  8.148432  
  Adult Thoracoabdominal ganglion  7.454188  
  Adult Whole Fly  7.738791  
  Larvae Wandering Tubules  8.870178  
  Larval Feeding Carcass  8.252733  
  Larval Feeding Central Nevous System  8.458795  
  Larval Feeding Hind Gut  7.954109  
  Larval Feeding Malpighian Tubule  8.495970  
  Larval Feeding Mid Gut  7.983809  
  Larval Feeding Salivary Gland  8.608960  
  Whole Larvae Feeding  7.865872  
 
  
   FlyBase ID    symbol    start    end    strand    length   
   FBgn0033431   CG1827   5476611   5477948  +  1338  
   FBgn0010342   Map60   5478331   5480249  +  1919  
   FBgn0050338   CG30338   5481552   5487010  +  5459  
 
 
    Segment 102 
 
   Location   
  Gene key  FBgn0050000-FBgn0050005  
  Heatmap region span   2R:5474688..5593789   
  Segment span   2R:5493495..5494797   
  Length (genes)  2  
  Length (bp)  1303  
   Model Scoring   
  BIC  237.304901  
  logL  -113.131598  
  logL ratio  41.589501  
   Expression   
  Mean expression  9.767455  
  Median expression  9.770878  
  Tissue std. dev.  0.506962  
 
  No GO Slim enrichment  
  
   tissue    mean expression   
  5th Passage Drosophila S2 Cells  10.653697  
  Adult Accessory gland  9.401668  
  Adult Brain  8.794044  
  Adult Carcass  9.986636  
  Adult Crop  10.005968  
  Adult Eye  9.626893  
  Adult Fatbody  10.485212  
  Adult Female Spermatheca Mated  9.824743  
  Adult Female Spermatheca Virgin  9.892375  
  Adult Head  9.408634  
  Adult Heart  10.767526  
  Adult Hind Gut  9.942740  
  Adult Male Ejaculatory Duct  9.621669  
  Adult Mid Gut  10.083683  
  Adult Ovary  10.185472  
  Adult Salivary Gland  9.118576  
  Adult Testes  8.814466  
  Adult Thoracoabdominal ganglion  8.858784  
  Adult Whole Fly  9.636781  
  Larvae Wandering Tubules  9.870431  
  Larval Feeding Carcass  9.777276  
  Larval Feeding Central Nevous System  9.113492  
  Larval Feeding Hind Gut  9.879158  
  Larval Feeding Malpighian Tubule  10.248095  
  Larval Feeding Mid Gut  9.838872  
  Larval Feeding Salivary Gland  10.080291  
  Whole Larvae Feeding  9.804095  
 
  
   FlyBase ID    symbol    start    end    strand    length   
   FBgn0050000   GstT1   5493495   5494689  +  1195  
   FBgn0050005   GstT2   5494797   5495855  +  1059  
 
    Segment 103 
 
   Location   
  Gene key  FBgn0050001-FBgn0033447  
  Heatmap region span   2R:5680848..5733964   
  Segment span   2R:5710539..5714988   
  Length (genes)  2  
  Length (bp)  4450  
   Model Scoring   
  BIC  191.586989  
  logL  -90.272642  
  logL ratio  29.398833  
   Expression   
  Mean expression  4.489655  
  Median expression  4.269974  
  Tissue std. dev.  0.538878  
 
  No GO Slim enrichment  
  
   tissue    mean expression   
  5th Passage Drosophila S2 Cells  5.408796  
  Adult Accessory gland  4.065060  
  Adult Brain  4.194922  
  Adult Carcass  4.195997  
  Adult Crop  4.250216  
  Adult Eye  4.627057  
  Adult Fatbody  3.986532  
  Adult Female Spermatheca Mated  4.166642  
  Adult Female Spermatheca Virgin  4.228023  
  Adult Head  4.210437  
  Adult Heart  4.381470  
  Adult Hind Gut  4.234373  
  Adult Male Ejaculatory Duct  4.147508  
  Adult Mid Gut  4.381597  
  Adult Ovary  5.722998  
  Adult Salivary Gland  4.499484  
  Adult Testes  6.139387  
  Adult Thoracoabdominal ganglion  4.139676  
  Adult Whole Fly  4.632286  
  Larvae Wandering Tubules  4.275593  
  Larval Feeding Carcass  4.281486  
  Larval Feeding Central Nevous System  5.502404  
  Larval Feeding Hind Gut  4.173711  
  Larval Feeding Malpighian Tubule  4.363734  
  Larval Feeding Mid Gut  4.556682  
  Larval Feeding Salivary Gland  4.005631  
  Whole Larvae Feeding  4.448985  
 
  
   FlyBase ID    symbol    start    end    strand    length   
   FBgn0050001   CG30001  5708668   5710539   -  1872  
   FBgn0033447   dila  5710582   5714988   -  4407  
 
    Segment 104 
 
   Location   
  Gene key  FBgn0033449-FBgn0033450  
  Heatmap region span   2R:5707556..5757853   
  Segment span   2R:5724535..5727005   
  Length (genes)  2  
  Length (bp)  2471  
   Model Scoring   
  BIC  213.914722  
  logL  -101.436508  
  logL ratio  22.543493  
   Expression   
  Mean expression  6.188624  
  Median expression  6.027317  
  Tissue std. dev.  0.503806  
 
  No GO Slim enrichment  
  
   tissue    mean expression   
  5th Passage Drosophila S2 Cells  6.369637  
  Adult Accessory gland  5.647382  
  Adult Brain  6.113943  
  Adult Carcass  5.909047  
  Adult Crop  6.067506  
  Adult Eye  5.969132  
  Adult Fatbody  6.153328  
  Adult Female Spermatheca Mated  6.104060  
  Adult Female Spermatheca Virgin  6.163110  
  Adult Head  5.844978  
  Adult Heart  6.477511  
  Adult Hind Gut  5.978936  
  Adult Male Ejaculatory Duct  5.543553  
  Adult Mid Gut  6.108888  
  Adult Ovary  8.136168  
  Adult Salivary Gland  6.346177  
  Adult Testes  6.888845  
  Adult Thoracoabdominal ganglion  6.021713  
  Adult Whole Fly  6.327657  
  Larvae Wandering Tubules  6.781849  
  Larval Feeding Carcass  5.653975  
  Larval Feeding Central Nevous System  6.538557  
  Larval Feeding Hind Gut  5.940147  
  Larval Feeding Malpighian Tubule  6.533958  
  Larval Feeding Mid Gut  5.887715  
  Larval Feeding Salivary Gland  5.831612  
  Whole Larvae Feeding  5.753467  
 
  
   FlyBase ID    symbol    start    end    strand    length   
   FBgn0033449   CG1663   5724535   5725986  +  1452  
   FBgn0033450   Lsm11  5725939   5727005   -  1067  
 
    Segment 105 
 
   Location   
  Gene key  FBgn0033451-FBgn0033453  
  Heatmap region span   2R:5708068..5758977   
  Segment span   2R:5727283..5730192   
  Length (genes)  3  
  Length (bp)  2910  
   Model Scoring   
  BIC  385.541463  
  logL  -187.249879  
  logL ratio  22.356288  
   Expression   
  Mean expression  9.240795  
  Median expression  9.225352  
  Tissue std. dev.  0.667381  
 
  No GO Slim enrichment  
  
   tissue    mean expression   
  5th Passage Drosophila S2 Cells  10.202082  
  Adult Accessory gland  8.758678  
  Adult Brain  8.531231  
  Adult Carcass  8.824754  
  Adult Crop  8.712892  
  Adult Eye  9.125715  
  Adult Fatbody  9.741022  
  Adult Female Spermatheca Mated  9.995829  
  Adult Female Spermatheca Virgin  10.080100  
  Adult Head  9.007616  
  Adult Heart  9.823512  
  Adult Hind Gut  9.231287  
  Adult Male Ejaculatory Duct  8.820618  
  Adult Mid Gut  9.581442  
  Adult Ovary  9.760550  
  Adult Salivary Gland  8.597991  
  Adult Testes  7.235862  
  Adult Thoracoabdominal ganglion  8.634911  
  Adult Whole Fly  8.880161  
  Larvae Wandering Tubules  10.204344  
  Larval Feeding Carcass  9.042945  
  Larval Feeding Central Nevous System  8.742109  
  Larval Feeding Hind Gut  9.458696  
  Larval Feeding Malpighian Tubule  10.097405  
  Larval Feeding Mid Gut  9.857795  
  Larval Feeding Salivary Gland  9.043102  
  Whole Larvae Feeding  9.508807  
 
  
   FlyBase ID    symbol    start    end    strand    length   
   FBgn0033451   CG1665   5727283   5728667  +  1385  
   FBgn0033452   CG1599  5728585   5730014   -  1430  
   FBgn0033453   CG1667   5730192   5733017  +  2826  
 
 
    Segment 106 
 
   Location   
  Gene key  FBgn0033454-FBgn0027580  
  Heatmap region span   2R:5724535..5769858   
  Segment span   2R:5735568..5757853   
  Length (genes)  4  
  Length (bp)  22286  
   Model Scoring   
  BIC  439.084590  
  logL  -214.021443  
  logL ratio  79.827237  
   Expression   
  Mean expression  8.783598  
  Median expression  8.608255  
  Tissue std. dev.  0.303750  
 
  No GO Slim enrichment  
  
   tissue    mean expression   
  5th Passage Drosophila S2 Cells  8.921910  
  Adult Accessory gland  8.056712  
  Adult Brain  8.388383  
  Adult Carcass  8.922960  
  Adult Crop  9.211011  
  Adult Eye  8.476814  
  Adult Fatbody  9.295936  
  Adult Female Spermatheca Mated  8.927367  
  Adult Female Spermatheca Virgin  8.978628  
  Adult Head  8.804623  
  Adult Heart  9.092954  
  Adult Hind Gut  8.703781  
  Adult Male Ejaculatory Duct  8.786991  
  Adult Mid Gut  8.533948  
  Adult Ovary  9.274215  
  Adult Salivary Gland  8.824392  
  Adult Testes  8.125250  
  Adult Thoracoabdominal ganglion  8.727689  
  Adult Whole Fly  8.817593  
  Larvae Wandering Tubules  8.815599  
  Larval Feeding Carcass  8.865122  
  Larval Feeding Central Nevous System  8.875854  
  Larval Feeding Hind Gut  8.831615  
  Larval Feeding Malpighian Tubule  8.980481  
  Larval Feeding Mid Gut  8.346880  
  Larval Feeding Salivary Gland  8.981593  
  Whole Larvae Feeding  8.588852  
 
  
   FlyBase ID    symbol    start    end    strand    length   
   FBgn0033454   CG1671   5735568   5738323  +  2756  
   FBgn0050010   CG30010   5751325   5752081  +  757  
   FBgn0033457   Ntmt   5753154   5754189  +  1036  
   FBgn0027580   CG1516  5738208   5757853   -  19646  
 
 
    Segment 107 
 
   Location   
  Gene key  FBgn0033463-FBgn0050007  
  Heatmap region span   2R:5762789..5848459   
  Segment span   2R:5779183..5779496   
  Length (genes)  2  
  Length (bp)  314  
   Model Scoring   
  BIC  237.294604  
  logL  -113.126449  
  logL ratio  9.237696  
   Expression   
  Mean expression  6.961743  
  Median expression  6.757535  
  Tissue std. dev.  0.771755  
 
  No GO Slim enrichment  
  
   tissue    mean expression   
  5th Passage Drosophila S2 Cells  8.158211  
  Adult Accessory gland  7.287970  
  Adult Brain  8.037700  
  Adult Carcass  6.177487  
  Adult Crop  6.642487  
  Adult Eye  6.499175  
  Adult Fatbody  6.265306  
  Adult Female Spermatheca Mated  5.985727  
  Adult Female Spermatheca Virgin  5.947049  
  Adult Head  6.576460  
  Adult Heart  6.695801  
  Adult Hind Gut  6.670110  
  Adult Male Ejaculatory Duct  5.864113  
  Adult Mid Gut  7.051460  
  Adult Ovary  8.013461  
  Adult Salivary Gland  6.936859  
  Adult Testes  8.584826  
  Adult Thoracoabdominal ganglion  7.706803  
  Adult Whole Fly  6.725973  
  Larvae Wandering Tubules  8.276250  
  Larval Feeding Carcass  6.227613  
  Larval Feeding Central Nevous System  7.345774  
  Larval Feeding Hind Gut  6.665221  
  Larval Feeding Malpighian Tubule  7.782700  
  Larval Feeding Mid Gut  6.664041  
  Larval Feeding Salivary Gland  7.125999  
  Whole Larvae Feeding  6.052475  
 
  
   FlyBase ID    symbol    start    end    strand    length   
   FBgn0033463   CG1513  5770077   5779183   -  9107  
   FBgn0050007   CG30007   5779496   5786018  +  6523  
 
    Segment 108 
 
   Location   
  Gene key  FBgn0011656-FBgn0033467  
  Heatmap region span   2R:5779183..5920393   
  Segment span   2R:5846313..5848459   
  Length (genes)  3  
  Length (bp)  2147  
   Model Scoring   
  BIC  315.268191  
  logL  -152.113243  
  logL ratio  57.547963  
   Expression   
  Mean expression  7.155202  
  Median expression  6.951712  
  Tissue std. dev.  0.424979  
 
  No GO Slim enrichment  
  
   tissue    mean expression   
  5th Passage Drosophila S2 Cells  7.033248  
  Adult Accessory gland  7.567209  
  Adult Brain  6.980490  
  Adult Carcass  6.988103  
  Adult Crop  7.833002  
  Adult Eye  7.498098  
  Adult Fatbody  7.544217  
  Adult Female Spermatheca Mated  7.954503  
  Adult Female Spermatheca Virgin  7.789568  
  Adult Head  7.222798  
  Adult Heart  7.689129  
  Adult Hind Gut  7.167946  
  Adult Male Ejaculatory Duct  7.467369  
  Adult Mid Gut  6.594833  
  Adult Ovary  7.250827  
  Adult Salivary Gland  7.524835  
  Adult Testes  6.678525  
  Adult Thoracoabdominal ganglion  6.978489  
  Adult Whole Fly  6.745873  
  Larvae Wandering Tubules  6.837631  
  Larval Feeding Carcass  6.408375  
  Larval Feeding Central Nevous System  7.243423  
  Larval Feeding Hind Gut  6.930554  
  Larval Feeding Malpighian Tubule  7.338453  
  Larval Feeding Mid Gut  6.686817  
  Larval Feeding Salivary Gland  6.766107  
  Whole Larvae Feeding  6.470038  
 
  
   FlyBase ID    symbol    start    end    strand    length   
   FBgn0011656   Mef2  5801001   5846313   -  45313  
   FBgn0033466   Pal1   5847054   5851358  +  4305  
   FBgn0033467   CG15863  5846624   5848459   -  1836  
 
 
    Segment 109 
 
   Location   
  Gene key  FBgn0033471-FBgn0033474  
  Heatmap region span   2R:5846313..5954154   
  Segment span   2R:5858654..5920393   
  Length (genes)  4  
  Length (bp)  61740  
   Model Scoring   
  BIC  468.384439  
  logL  -228.671367  
  logL ratio  9.256310  
   Expression   
  Mean expression  6.733254  
  Median expression  6.725022  
  Tissue std. dev.  0.433811  
 
  No GO Slim enrichment  
  
   tissue    mean expression   
  5th Passage Drosophila S2 Cells  7.753002  
  Adult Accessory gland  7.705778  
  Adult Brain  6.307023  
  Adult Carcass  6.270526  
  Adult Crop  6.592629  
  Adult Eye  6.753499  
  Adult Fatbody  6.778859  
  Adult Female Spermatheca Mated  6.666212  
  Adult Female Spermatheca Virgin  6.488615  
  Adult Head  6.406039  
  Adult Heart  6.555405  
  Adult Hind Gut  6.254755  
  Adult Male Ejaculatory Duct  6.734064  
  Adult Mid Gut  6.685706  
  Adult Ovary  7.853472  
  Adult Salivary Gland  6.382440  
  Adult Testes  6.895666  
  Adult Thoracoabdominal ganglion  6.339670  
  Adult Whole Fly  6.893296  
  Larvae Wandering Tubules  6.604636  
  Larval Feeding Carcass  6.768368  
  Larval Feeding Central Nevous System  7.246850  
  Larval Feeding Hind Gut  6.651340  
  Larval Feeding Malpighian Tubule  6.675886  
  Larval Feeding Mid Gut  6.453159  
  Larval Feeding Salivary Gland  6.867572  
  Whole Larvae Feeding  6.213379  
 
  
   FlyBase ID    symbol    start    end    strand    length   
   FBgn0033471   CG12134   5858654   5860295  +  1642  
   FBgn0000606   eve   5866746   5868284  +  1539  
   FBgn0033473   CG12128   5914346   5918173  +  3828  
   FBgn0033474   CG1407  5915948   5920393   -  4446  
 
 
    Segment 110 
 
   Location   
  Gene key  FBgn0033475-FBgn0033477  
  Heatmap region span   2R:5852567..5956614   
  Segment span   2R:5920799..5939682   
  Length (genes)  4  
  Length (bp)  18884  
   Model Scoring   
  BIC  510.755268  
  logL  -249.856782  
  logL ratio  46.795551  
   Expression   
  Mean expression  9.579645  
  Median expression  9.056075  
  Tissue std. dev.  0.340448  
 
  No GO Slim enrichment  
  
   tissue    mean expression   
  5th Passage Drosophila S2 Cells  10.193868  
  Adult Accessory gland  9.989027  
  Adult Brain  9.038679  
  Adult Carcass  9.422326  
  Adult Crop  9.228358  
  Adult Eye  9.339509  
  Adult Fatbody  9.871460  
  Adult Female Spermatheca Mated  10.161798  
  Adult Female Spermatheca Virgin  10.132717  
  Adult Head  9.302349  
  Adult Heart  9.538715  
  Adult Hind Gut  8.990095  
  Adult Male Ejaculatory Duct  10.012978  
  Adult Mid Gut  9.472803  
  Adult Ovary  9.499884  
  Adult Salivary Gland  9.212762  
  Adult Testes  9.344115  
  Adult Thoracoabdominal ganglion  9.215350  
  Adult Whole Fly  9.755212  
  Larvae Wandering Tubules  9.802542  
  Larval Feeding Carcass  9.447095  
  Larval Feeding Central Nevous System  9.229701  
  Larval Feeding Hind Gut  9.408252  
  Larval Feeding Malpighian Tubule  9.645848  
  Larval Feeding Mid Gut  9.813673  
  Larval Feeding Salivary Gland  9.748361  
  Whole Larvae Feeding  9.832930  
 
  
   FlyBase ID    symbol    start    end    strand    length   
   FBgn0033475   CG12129   5920799   5922203  +  1405  
   FBgn0033476   oys  5923268   5936123   -  12856  
   FBgn0040773   CoVIIc   5936985   5938379  +  1395  
   FBgn0033477     5938367   5939682   -  1316  
 
 
    Segment 111 
 
   Location   
  Gene key  FBgn0013435-FBgn0033481  
  Heatmap region span   2R:5920799..5975076   
  Segment span   2R:5956206..5956614   
  Length (genes)  2  
  Length (bp)  409  
   Model Scoring   
  BIC  208.195226  
  logL  -98.576760  
  logL ratio  19.244639  
   Expression   
  Mean expression  5.813195  
  Median expression  5.736307  
  Tissue std. dev.  0.271439  
 
  No GO Slim enrichment  
  
   tissue    mean expression   
  5th Passage Drosophila S2 Cells  6.058705  
  Adult Accessory gland  5.945432  
  Adult Brain  5.839590  
  Adult Carcass  5.617446  
  Adult Crop  5.845118  
  Adult Eye  5.964968  
  Adult Fatbody  5.822497  
  Adult Female Spermatheca Mated  5.876821  
  Adult Female Spermatheca Virgin  5.723810  
  Adult Head  5.601068  
  Adult Heart  5.702034  
  Adult Hind Gut  5.773677  
  Adult Male Ejaculatory Duct  5.953858  
  Adult Mid Gut  5.449967  
  Adult Ovary  6.114940  
  Adult Salivary Gland  6.254001  
  Adult Testes  5.364919  
  Adult Thoracoabdominal ganglion  5.825999  
  Adult Whole Fly  5.279687  
  Larvae Wandering Tubules  5.751660  
  Larval Feeding Carcass  5.854273  
  Larval Feeding Central Nevous System  6.225753  
  Larval Feeding Hind Gut  5.754048  
  Larval Feeding Malpighian Tubule  6.121149  
  Larval Feeding Mid Gut  5.404066  
  Larval Feeding Salivary Gland  6.358306  
  Whole Larvae Feeding  5.472476  
 
  
   FlyBase ID    symbol    start    end    strand    length   
   FBgn0013435   cdc2rk  5954722   5956206   -  1485  
   FBgn0033481   CG12920   5956614   5958572  +  1959  
 
    Segment 112 
 
   Location   
  Gene key  FBgn0001291-FBgn0033486  
  Heatmap region span   2R:5963147..6046385   
  Segment span   2R:5983985..6005198   
  Length (genes)  3  
  Length (bp)  21214  
   Model Scoring   
  BIC  341.559028  
  logL  -165.258662  
  logL ratio  72.410025  
   Expression   
  Mean expression  9.684631  
  Median expression  9.557077  
  Tissue std. dev.  0.403956  
 
  No GO Slim enrichment  
  
   tissue    mean expression   
  5th Passage Drosophila S2 Cells  9.875934  
  Adult Accessory gland  9.195232  
  Adult Brain  10.152698  
  Adult Carcass  9.462716  
  Adult Crop  10.243669  
  Adult Eye  10.288746  
  Adult Fatbody  9.122408  
  Adult Female Spermatheca Mated  9.194979  
  Adult Female Spermatheca Virgin  9.228914  
  Adult Head  9.612601  
  Adult Heart  9.836262  
  Adult Hind Gut  10.002991  
  Adult Male Ejaculatory Duct  9.480084  
  Adult Mid Gut  9.056068  
  Adult Ovary  9.964873  
  Adult Salivary Gland  10.023577  
  Adult Testes  10.184999  
  Adult Thoracoabdominal ganglion  10.017894  
  Adult Whole Fly  9.451977  
  Larvae Wandering Tubules  9.642200  
  Larval Feeding Carcass  10.134727  
  Larval Feeding Central Nevous System  10.213852  
  Larval Feeding Hind Gut  9.524268  
  Larval Feeding Malpighian Tubule  9.672944  
  Larval Feeding Mid Gut  8.859700  
  Larval Feeding Salivary Gland  9.530067  
  Whole Larvae Feeding  9.510651  
 
  
   FlyBase ID    symbol    start    end    strand    length   
   FBgn0001291   Jra   5983985   5986061  +  2077  
   FBgn0003071   Pfk   5997245   6004962  +  7718  
   FBgn0033486   CG11866   6005198   6008956  +  3759  
 
 
    Segment 113 
 
   Location   
  Gene key  FBgn0033490-FBgn0033491  
  Heatmap region span   2R:5975076..6128374   
  Segment span   2R:6025161..6029845   
  Length (genes)  3  
  Length (bp)  4685  
   Model Scoring   
  BIC  260.612381  
  logL  -124.785338  
  logL ratio  56.415050  
   Expression   
  Mean expression  4.726746  
  Median expression  4.747179  
  Tissue std. dev.  0.374621  
 
  No GO Slim enrichment  
  
   tissue    mean expression   
  5th Passage Drosophila S2 Cells  4.935928  
  Adult Accessory gland  4.696052  
  Adult Brain  4.537717  
  Adult Carcass  4.476911  
  Adult Crop  4.674912  
  Adult Eye  4.762066  
  Adult Fatbody  4.526287  
  Adult Female Spermatheca Mated  4.587891  
  Adult Female Spermatheca Virgin  4.724986  
  Adult Head  4.404962  
  Adult Heart  4.601125  
  Adult Hind Gut  4.538092  
  Adult Male Ejaculatory Duct  4.618664  
  Adult Mid Gut  4.674292  
  Adult Ovary  5.174029  
  Adult Salivary Gland  4.904358  
  Adult Testes  6.457950  
  Adult Thoracoabdominal ganglion  4.617863  
  Adult Whole Fly  4.714037  
  Larvae Wandering Tubules  4.672191  
  Larval Feeding Carcass  4.672783  
  Larval Feeding Central Nevous System  4.625741  
  Larval Feeding Hind Gut  4.552588  
  Larval Feeding Malpighian Tubule  4.763419  
  Larval Feeding Mid Gut  4.563835  
  Larval Feeding Salivary Gland  4.730720  
  Whole Larvae Feeding  4.412745  
 
  
   FlyBase ID    symbol    start    end    strand    length   
   FBgn0033490   CG12917   6025161   6026772  +  1612  
   FBgn0026388   Or46a   6026769   6029579  +  2811  
   FBgn0033491   CG18011   6029845   6033711  +  3867  
 
 
    Segment 114 
 
   Location   
  Gene key  FBgn0085250-FBgn0000448  
  Heatmap region span   2R:6011793..6144194   
  Segment span   2R:6087196..6124853   
  Length (genes)  3  
  Length (bp)  37658  
   Model Scoring   
  BIC  251.259944  
  logL  -120.109120  
  logL ratio  74.668015  
   Expression   
  Mean expression  4.610286  
  Median expression  4.667658  
  Tissue std. dev.  0.432442  
 
  No GO Slim enrichment  
  
   tissue    mean expression   
  5th Passage Drosophila S2 Cells  4.359274  
  Adult Accessory gland  4.525600  
  Adult Brain  6.226280  
  Adult Carcass  4.314517  
  Adult Crop  4.436488  
  Adult Eye  5.544104  
  Adult Fatbody  4.750439  
  Adult Female Spermatheca Mated  4.599868  
  Adult Female Spermatheca Virgin  4.693500  
  Adult Head  4.558286  
  Adult Heart  4.614791  
  Adult Hind Gut  4.272579  
  Adult Male Ejaculatory Duct  4.516447  
  Adult Mid Gut  4.378993  
  Adult Ovary  4.351440  
  Adult Salivary Gland  4.565534  
  Adult Testes  4.206588  
  Adult Thoracoabdominal ganglion  5.356380  
  Adult Whole Fly  4.186499  
  Larvae Wandering Tubules  4.637616  
  Larval Feeding Carcass  4.514087  
  Larval Feeding Central Nevous System  4.562021  
  Larval Feeding Hind Gut  4.452612  
  Larval Feeding Malpighian Tubule  4.423797  
  Larval Feeding Mid Gut  4.574836  
  Larval Feeding Salivary Gland  4.606281  
  Whole Larvae Feeding  4.248872  
 
  
   FlyBase ID    symbol    start    end    strand    length   
   FBgn0085250   CG34221  6085053   6087196   -  2144  
   FBgn0033497   CG12912  6120971   6122081   -  1111  
   FBgn0000448   Hr46  6092959   6124853   -  31895  
 
 
    Segment 115 
 
   Location   
  Gene key  FBgn0033498-FBgn0033500  
  Heatmap region span   2R:6046385..6156528   
  Segment span   2R:6134977..6136762   
  Length (genes)  3  
  Length (bp)  1786  
   Model Scoring   
  BIC  265.164368  
  logL  -127.061332  
  logL ratio  58.756917  
   Expression   
  Mean expression  4.634963  
  Median expression  4.393372  
  Tissue std. dev.  0.715260  
 
  No GO Slim enrichment  
  
   tissue    mean expression   
  5th Passage Drosophila S2 Cells  4.416549  
  Adult Accessory gland  4.414190  
  Adult Brain  4.942712  
  Adult Carcass  4.573815  
  Adult Crop  4.242495  
  Adult Eye  4.554426  
  Adult Fatbody  4.307742  
  Adult Female Spermatheca Mated  4.418662  
  Adult Female Spermatheca Virgin  4.437721  
  Adult Head  4.444842  
  Adult Heart  4.369584  
  Adult Hind Gut  4.297211  
  Adult Male Ejaculatory Duct  4.551120  
  Adult Mid Gut  4.548016  
  Adult Ovary  4.341767  
  Adult Salivary Gland  4.774670  
  Adult Testes  8.156307  
  Adult Thoracoabdominal ganglion  4.970458  
  Adult Whole Fly  4.835999  
  Larvae Wandering Tubules  4.495030  
  Larval Feeding Carcass  4.409644  
  Larval Feeding Central Nevous System  4.539753  
  Larval Feeding Hind Gut  4.205705  
  Larval Feeding Malpighian Tubule  4.443625  
  Larval Feeding Mid Gut  4.461933  
  Larval Feeding Salivary Gland  4.526451  
  Whole Larvae Feeding  4.463580  
 
  
   FlyBase ID    symbol    start    end    strand    length   
   FBgn0033498   CG12209  6133576   6134977   -  1402  
   FBgn0033499   CG12914   6135319   6136909  +  1591  
   FBgn0033500   CG12913   6136762   6142479  +  5718  
 
 
    Segment 116 
 
   Location   
  Gene key  FBgn0250840-FBgn0033512  
  Heatmap region span   2R:6156528..6324864   
  Segment span   2R:6240873..6243336   
  Length (genes)  2  
  Length (bp)  2464  
   Model Scoring   
  BIC  201.342467  
  logL  -95.150381  
  logL ratio  49.306294  
   Expression   
  Mean expression  4.642607  
  Median expression  4.019865  
  Tissue std. dev.  2.176784  
 
  No GO Slim enrichment  
  
   tissue    mean expression   
  5th Passage Drosophila S2 Cells  3.894830  
  Adult Accessory gland  4.117900  
  Adult Brain  3.900134  
  Adult Carcass  4.669035  
  Adult Crop  3.891056  
  Adult Eye  3.859222  
  Adult Fatbody  3.874731  
  Adult Female Spermatheca Mated  4.075769  
  Adult Female Spermatheca Virgin  3.846389  
  Adult Head  3.743404  
  Adult Heart  3.919023  
  Adult Hind Gut  3.828166  
  Adult Male Ejaculatory Duct  3.970191  
  Adult Mid Gut  3.860476  
  Adult Ovary  3.971346  
  Adult Salivary Gland  3.771838  
  Adult Testes  13.246379  
  Adult Thoracoabdominal ganglion  3.860746  
  Adult Whole Fly  10.410220  
  Larvae Wandering Tubules  4.011660  
  Larval Feeding Carcass  3.996750  
  Larval Feeding Central Nevous System  3.685594  
  Larval Feeding Hind Gut  3.923891  
  Larval Feeding Malpighian Tubule  3.861605  
  Larval Feeding Mid Gut  3.901317  
  Larval Feeding Salivary Gland  3.854194  
  Whole Larvae Feeding  7.404513  
 
  
   FlyBase ID    symbol    start    end    strand    length   
   FBgn0250840   CG12907   6240873   6241889  +  1017  
   FBgn0033512   CG12902  6242225   6243336   -  1112  
 
    Segment 117 
 
   Location   
  Gene key  FBgn0041242-FBgn0053475  
  Heatmap region span   2R:6197629..6346393   
  Segment span   2R:6249810..6298718   
  Length (genes)  6  
  Length (bp)  48909  
   Model Scoring   
  BIC  418.439742  
  logL  -203.699018  
  logL ratio  262.207062  
   Expression   
  Mean expression  3.974946  
  Median expression  3.939456  
  Tissue std. dev.  0.152377  
 
  No GO Slim enrichment  
  
   tissue    mean expression   
  5th Passage Drosophila S2 Cells  4.000542  
  Adult Accessory gland  4.463265  
  Adult Brain  3.845446  
  Adult Carcass  4.289738  
  Adult Crop  3.983518  
  Adult Eye  3.869621  
  Adult Fatbody  4.029125  
  Adult Female Spermatheca Mated  3.986561  
  Adult Female Spermatheca Virgin  3.996670  
  Adult Head  3.838916  
  Adult Heart  3.949442  
  Adult Hind Gut  3.956406  
  Adult Male Ejaculatory Duct  4.050397  
  Adult Mid Gut  4.011434  
  Adult Ovary  4.016265  
  Adult Salivary Gland  4.171254  
  Adult Testes  3.828004  
  Adult Thoracoabdominal ganglion  3.884861  
  Adult Whole Fly  3.713462  
  Larvae Wandering Tubules  3.974715  
  Larval Feeding Carcass  4.008922  
  Larval Feeding Central Nevous System  3.783358  
  Larval Feeding Hind Gut  3.924470  
  Larval Feeding Malpighian Tubule  3.977329  
  Larval Feeding Mid Gut  3.955519  
  Larval Feeding Salivary Gland  4.052731  
  Whole Larvae Feeding  3.761557  
 
  
   FlyBase ID    symbol    start    end    strand    length   
   FBgn0041242   Gr47a   6249810   6251089  +  1280  
   FBgn0033515   Ir47a  6264633   6267614   -  2982  
   FBgn0033516   CG12898  6294480   6294950   -  471  
   FBgn0053477   CG33477  6296660   6297256   -  597  
   FBgn0053476   CG33476  6297490   6298075   -  586  
   FBgn0053475   CG33475  6298275   6298718   -  444  
 
 
    Segment 118 
 
   Location   
  Gene key  FBgn0033527-FBgn0033528  
  Heatmap region span   2R:6308572..6561061   
  Segment span   2R:6356105..6367633   
  Length (genes)  2  
  Length (bp)  11529  
   Model Scoring   
  BIC  230.779497  
  logL  -109.868896  
  logL ratio  30.166375  
   Expression   
  Mean expression  8.481600  
  Median expression  8.340905  
  Tissue std. dev.  0.585586  
 
  No GO Slim enrichment  
  
   tissue    mean expression   
  5th Passage Drosophila S2 Cells  9.356689  
  Adult Accessory gland  8.834675  
  Adult Brain  8.271263  
  Adult Carcass  7.480624  
  Adult Crop  8.460687  
  Adult Eye  8.441916  
  Adult Fatbody  7.478235  
  Adult Female Spermatheca Mated  8.299177  
  Adult Female Spermatheca Virgin  8.180761  
  Adult Head  8.026592  
  Adult Heart  8.361404  
  Adult Hind Gut  8.077780  
  Adult Male Ejaculatory Duct  9.150793  
  Adult Mid Gut  8.041570  
  Adult Ovary  9.521677  
  Adult Salivary Gland  8.054295  
  Adult Testes  8.482719  
  Adult Thoracoabdominal ganglion  8.516533  
  Adult Whole Fly  8.460009  
  Larvae Wandering Tubules  8.744347  
  Larval Feeding Carcass  8.301049  
  Larval Feeding Central Nevous System  10.101923  
  Larval Feeding Hind Gut  8.256621  
  Larval Feeding Malpighian Tubule  8.636671  
  Larval Feeding Mid Gut  7.844062  
  Larval Feeding Salivary Gland  9.296743  
  Whole Larvae Feeding  8.324388  
 
  
   FlyBase ID    symbol    start    end    strand    length   
   FBgn0033527   CG11777   6356105   6357148  +  1044  
   FBgn0033528   trsn  6366516   6367633   -  1118  
 
    Segment 119 
 
   Location   
  Gene key  FBgn0005630-FBgn0033540  
  Heatmap region span   2R:6324864..6616695   
  Segment span   2R:6430794..6533230   
  Length (genes)  5  
  Length (bp)  102437  
   Model Scoring   
  BIC  498.280175  
  logL  -243.619235  
  logL ratio  118.628573  
   Expression   
  Mean expression  7.575301  
  Median expression  7.653439  
  Tissue std. dev.  0.613333  
 
  No GO Slim enrichment  
  
   tissue    mean expression   
  5th Passage Drosophila S2 Cells  8.608306  
  Adult Accessory gland  7.632007  
  Adult Brain  8.304010  
  Adult Carcass  7.375079  
  Adult Crop  7.695338  
  Adult Eye  7.710328  
  Adult Fatbody  7.251377  
  Adult Female Spermatheca Mated  7.178916  
  Adult Female Spermatheca Virgin  7.163750  
  Adult Head  7.578974  
  Adult Heart  7.178061  
  Adult Hind Gut  7.346205  
  Adult Male Ejaculatory Duct  7.417265  
  Adult Mid Gut  6.963852  
  Adult Ovary  9.160089  
  Adult Salivary Gland  7.218154  
  Adult Testes  6.386257  
  Adult Thoracoabdominal ganglion  8.073162  
  Adult Whole Fly  7.757459  
  Larvae Wandering Tubules  7.181053  
  Larval Feeding Carcass  7.981531  
  Larval Feeding Central Nevous System  8.806842  
  Larval Feeding Hind Gut  7.341162  
  Larval Feeding Malpighian Tubule  7.025264  
  Larval Feeding Mid Gut  6.891681  
  Larval Feeding Salivary Gland  8.119399  
  Whole Larvae Feeding  7.187606  
 
  
   FlyBase ID    symbol    start    end    strand    length   
   FBgn0005630   lola  6369712   6430794   -  61083  
   FBgn0004399      6445409   6503753  +  58345  
   FBgn0033538   CG11883   6505169   6528718  +  23550  
   FBgn0033539   Git  6529323   6532925   -  3603  
   FBgn0033540   Elp2   6533230   6536139  +  2910  
 
 
    Segment 120 
 
   Location   
  Gene key  FBgn0033543-FBgn0024956  
  Heatmap region span   2R:6536388..6698599   
  Segment span   2R:6618419..6627339   
  Length (genes)  3  
  Length (bp)  8921  
   Model Scoring   
  BIC  369.311637  
  logL  -179.134966  
  logL ratio  58.345000  
   Expression   
  Mean expression  9.850212  
  Median expression  9.728174  
  Tissue std. dev.  0.769848  
 
  No GO Slim enrichment  
  
   tissue    mean expression   
  5th Passage Drosophila S2 Cells  9.046882  
  Adult Accessory gland  9.467773  
  Adult Brain  9.876015  
  Adult Carcass  9.865743  
  Adult Crop  9.266410  
  Adult Eye  10.851192  
  Adult Fatbody  10.325066  
  Adult Female Spermatheca Mated  10.429443  
  Adult Female Spermatheca Virgin  10.810769  
  Adult Head  10.550322  
  Adult Heart  10.043261  
  Adult Hind Gut  9.820452  
  Adult Male Ejaculatory Duct  9.531353  
  Adult Mid Gut  10.483240  
  Adult Ovary  9.108818  
  Adult Salivary Gland  10.271085  
  Adult Testes  7.813733  
  Adult Thoracoabdominal ganglion  9.978623  
  Adult Whole Fly  9.690066  
  Larvae Wandering Tubules  10.796833  
  Larval Feeding Carcass  8.120506  
  Larval Feeding Central Nevous System  9.096425  
  Larval Feeding Hind Gut  10.488841  
  Larval Feeding Malpighian Tubule  10.375356  
  Larval Feeding Mid Gut  10.721544  
  Larval Feeding Salivary Gland  9.131627  
  Whole Larvae Feeding  9.994334  
 
  
   FlyBase ID    symbol    start    end    strand    length   
   FBgn0033543   CG12338  6617080   6618419   -  1340  
   FBgn0033544   CG7220   6618861   6626116  +  7256  
   FBgn0024956   Mat1  6626122   6627339   -  1218  
 
 
    Segment 121 
 
   Location   
  Gene key  FBgn0053144-FBgn0033547  
  Heatmap region span   2R:6612775..6701056   
  Segment span   2R:6681852..6696898   
  Length (genes)  3  
  Length (bp)  15047  
   Model Scoring   
  BIC  298.814400  
  logL  -143.886347  
  logL ratio  82.631473  
   Expression   
  Mean expression  7.969485  
  Median expression  7.924692  
  Tissue std. dev.  0.467611  
 
  No GO Slim enrichment  
  
   tissue    mean expression   
  5th Passage Drosophila S2 Cells  7.390837  
  Adult Accessory gland  7.727606  
  Adult Brain  9.059998  
  Adult Carcass  7.488745  
  Adult Crop  7.931946  
  Adult Eye  7.931674  
  Adult Fatbody  7.522246  
  Adult Female Spermatheca Mated  7.614469  
  Adult Female Spermatheca Virgin  7.416768  
  Adult Head  7.936890  
  Adult Heart  8.030965  
  Adult Hind Gut  7.868008  
  Adult Male Ejaculatory Duct  8.079585  
  Adult Mid Gut  7.627051  
  Adult Ovary  8.821189  
  Adult Salivary Gland  7.932507  
  Adult Testes  7.652004  
  Adult Thoracoabdominal ganglion  9.094635  
  Adult Whole Fly  7.700878  
  Larvae Wandering Tubules  8.046504  
  Larval Feeding Carcass  7.925439  
  Larval Feeding Central Nevous System  8.746012  
  Larval Feeding Hind Gut  8.254192  
  Larval Feeding Malpighian Tubule  8.315568  
  Larval Feeding Mid Gut  7.657589  
  Larval Feeding Salivary Gland  7.916851  
  Whole Larvae Feeding  7.485939  
 
  
   FlyBase ID    symbol    start    end    strand    length   
   FBgn0053144   CG33144  6627901   6681852   -  53952  
   FBgn0027499   wde  6687924   6696430   -  8507  
   FBgn0033547   CG12935   6696898   6698160  +  1263  
 
 
    Segment 122 
 
   Location   
  Gene key  FBgn0033554-FBgn0033557  
  Heatmap region span   2R:6700835..6782016   
  Segment span   2R:6709145..6711105   
  Length (genes)  3  
  Length (bp)  1961  
   Model Scoring   
  BIC  412.631721  
  logL  -200.795008  
  logL ratio  -38.944656  
   Expression   
  Mean expression  7.545317  
  Median expression  7.143716  
  Tissue std. dev.  0.424610  
 
  No GO Slim enrichment  
  
   tissue    mean expression   
  5th Passage Drosophila S2 Cells  7.824318  
  Adult Accessory gland  7.858945  
  Adult Brain  7.564601  
  Adult Carcass  7.234139  
  Adult Crop  7.849849  
  Adult Eye  7.738684  
  Adult Fatbody  7.591735  
  Adult Female Spermatheca Mated  7.919741  
  Adult Female Spermatheca Virgin  7.637283  
  Adult Head  7.312837  
  Adult Heart  8.021819  
  Adult Hind Gut  7.332160  
  Adult Male Ejaculatory Duct  7.909867  
  Adult Mid Gut  7.107548  
  Adult Ovary  8.856469  
  Adult Salivary Gland  7.056227  
  Adult Testes  7.237148  
  Adult Thoracoabdominal ganglion  7.522283  
  Adult Whole Fly  7.720686  
  Larvae Wandering Tubules  7.245087  
  Larval Feeding Carcass  7.362437  
  Larval Feeding Central Nevous System  7.993042  
  Larval Feeding Hind Gut  7.393471  
  Larval Feeding Malpighian Tubule  7.026279  
  Larval Feeding Mid Gut  6.896040  
  Larval Feeding Salivary Gland  7.686590  
  Whole Larvae Feeding  6.824271  
 
  
   FlyBase ID    symbol    start    end    strand    length   
   FBgn0033554   Lsm10  6708690   6709145   -  456  
   FBgn0033556   CG12343  6709743   6710826   -  1084  
   FBgn0033557   CG12325   6711105   6715149  +  4045  
 
 
    Segment 123 
 
   Location   
  Gene key  FBgn0027525-FBgn0050015  
  Heatmap region span   2R:6707378..7031176   
  Segment span   2R:6726231..6762229   
  Length (genes)  2  
  Length (bp)  35999  
   Model Scoring   
  BIC  300.817244  
  logL  -144.887769  
  logL ratio  12.341959  
   Expression   
  Mean expression  10.539193  
  Median expression  10.632998  
  Tissue std. dev.  0.694771  
 
  No GO Slim enrichment  
  
   tissue    mean expression   
  5th Passage Drosophila S2 Cells  10.637728  
  Adult Accessory gland  10.265485  
  Adult Brain  11.288138  
  Adult Carcass  10.690987  
  Adult Crop  10.658796  
  Adult Eye  11.264468  
  Adult Fatbody  11.285169  
  Adult Female Spermatheca Mated  11.186557  
  Adult Female Spermatheca Virgin  11.392625  
  Adult Head  10.951653  
  Adult Heart  11.043819  
  Adult Hind Gut  10.845190  
  Adult Male Ejaculatory Duct  11.134201  
  Adult Mid Gut  10.588594  
  Adult Ovary  8.581565  
  Adult Salivary Gland  10.952334  
  Adult Testes  9.542516  
  Adult Thoracoabdominal ganglion  11.182176  
  Adult Whole Fly  10.209743  
  Larvae Wandering Tubules  9.314630  
  Larval Feeding Carcass  10.476006  
  Larval Feeding Central Nevous System  10.171975  
  Larval Feeding Hind Gut  10.577304  
  Larval Feeding Malpighian Tubule  9.364736  
  Larval Feeding Mid Gut  10.159152  
  Larval Feeding Salivary Gland  10.907522  
  Whole Larvae Feeding  9.885153  
 
  
   FlyBase ID    symbol    start    end    strand    length   
   FBgn0027525   CG7686  6724511   6726231   -  1721  
   FBgn0050015   CG30015  6726599   6762229   -  35631  
 
    Segment 124 
 
   Location   
  Gene key  FBgn0033562-FBgn0033569  
  Heatmap region span   2R:6709145..7042433   
  Segment span   2R:6763259..6782016   
  Length (genes)  6  
  Length (bp)  18758  
   Model Scoring   
  BIC  581.061645  
  logL  -285.009970  
  logL ratio  146.459770  
   Expression   
  Mean expression  7.106353  
  Median expression  6.804552  
  Tissue std. dev.  0.436623  
 
  No GO Slim enrichment  
  
   tissue    mean expression   
  5th Passage Drosophila S2 Cells  7.645802  
  Adult Accessory gland  7.297341  
  Adult Brain  7.390555  
  Adult Carcass  6.690012  
  Adult Crop  7.328403  
  Adult Eye  6.969551  
  Adult Fatbody  6.794890  
  Adult Female Spermatheca Mated  7.108146  
  Adult Female Spermatheca Virgin  7.049466  
  Adult Head  6.825202  
  Adult Heart  7.091039  
  Adult Hind Gut  6.818286  
  Adult Male Ejaculatory Duct  6.929802  
  Adult Mid Gut  6.683893  
  Adult Ovary  8.481912  
  Adult Salivary Gland  7.051098  
  Adult Testes  6.410452  
  Adult Thoracoabdominal ganglion  7.097073  
  Adult Whole Fly  7.266332  
  Larvae Wandering Tubules  7.139126  
  Larval Feeding Carcass  6.996793  
  Larval Feeding Central Nevous System  8.090772  
  Larval Feeding Hind Gut  6.936666  
  Larval Feeding Malpighian Tubule  6.956867  
  Larval Feeding Mid Gut  6.621685  
  Larval Feeding Salivary Gland  7.519753  
  Whole Larvae Feeding  6.680625  
 
  
   FlyBase ID    symbol    start    end    strand    length   
   FBgn0033562      6763259   6764801  +  1543  
   FBgn0010356   Taf5  6764808   6767175   -  2368  
   FBgn0033566   CG18004   6772524   6773880  +  1357  
   FBgn0050020   CG30020   6774320   6780696  +  6377  
   FBgn0017414   cag  6780528   6781865   -  1338  
   FBgn0033569   CG12942   6782016   6784727  +  2712  
 
 
    Segment 125 
 
   Location   
  Gene key  FBgn0033570-FBgn0033571  
  Heatmap region span   2R:6721741..7061325   
  Segment span   2R:6785388..6786563   
  Length (genes)  2  
  Length (bp)  1176  
   Model Scoring   
  BIC  313.151208  
  logL  -151.054752  
  logL ratio  0.588659  
   Expression   
  Mean expression  10.548974  
  Median expression  10.650434  
  Tissue std. dev.  0.305424  
 
  No GO Slim enrichment  
  
   tissue    mean expression   
  5th Passage Drosophila S2 Cells  10.547062  
  Adult Accessory gland  10.947139  
  Adult Brain  10.476167  
  Adult Carcass  10.841044  
  Adult Crop  10.542376  
  Adult Eye  10.795142  
  Adult Fatbody  10.655378  
  Adult Female Spermatheca Mated  10.517606  
  Adult Female Spermatheca Virgin  10.426422  
  Adult Head  10.666172  
  Adult Heart  10.684521  
  Adult Hind Gut  10.673046  
  Adult Male Ejaculatory Duct  10.857731  
  Adult Mid Gut  10.474649  
  Adult Ovary  10.487507  
  Adult Salivary Gland  10.595968  
  Adult Testes  9.536432  
  Adult Thoracoabdominal ganglion  10.439514  
  Adult Whole Fly  11.013317  
  Larvae Wandering Tubules  9.982341  
  Larval Feeding Carcass  10.555954  
  Larval Feeding Central Nevous System  10.911537  
  Larval Feeding Hind Gut  10.561972  
  Larval Feeding Malpighian Tubule  10.565062  
  Larval Feeding Mid Gut  10.013072  
  Larval Feeding Salivary Gland  10.656534  
  Whole Larvae Feeding  10.398630  
 
  
   FlyBase ID    symbol    start    end    strand    length   
   FBgn0033570   CG7712  6784641   6785388   -  748  
   FBgn0033571   Rpb5  6785747   6786563   -  817  
 
    Segment 126 
 
   Location   
  Gene key  FBgn0033572-FBgn0033579  
  Heatmap region span   2R:6726231..7106718   
  Segment span   2R:6791075..7031176   
  Length (genes)  6  
  Length (bp)  240102  
   Model Scoring   
  BIC  596.044241  
  logL  -292.501268  
  logL ratio  52.484632  
   Expression   
  Mean expression  5.244290  
  Median expression  4.789168  
  Tissue std. dev.  0.387586  
 
  No GO Slim enrichment  
  
   tissue    mean expression   
  5th Passage Drosophila S2 Cells  5.283701  
  Adult Accessory gland  5.168520  
  Adult Brain  5.084091  
  Adult Carcass  5.691681  
  Adult Crop  5.590898  
  Adult Eye  5.741356  
  Adult Fatbody  5.004865  
  Adult Female Spermatheca Mated  5.216489  
  Adult Female Spermatheca Virgin  4.938947  
  Adult Head  5.798212  
  Adult Heart  4.906967  
  Adult Hind Gut  5.341104  
  Adult Male Ejaculatory Duct  5.338779  
  Adult Mid Gut  4.994501  
  Adult Ovary  4.799242  
  Adult Salivary Gland  5.037541  
  Adult Testes  6.666327  
  Adult Thoracoabdominal ganglion  5.085522  
  Adult Whole Fly  4.994179  
  Larvae Wandering Tubules  4.917287  
  Larval Feeding Carcass  5.483039  
  Larval Feeding Central Nevous System  4.913634  
  Larval Feeding Hind Gut  5.227936  
  Larval Feeding Malpighian Tubule  4.980062  
  Larval Feeding Mid Gut  5.009455  
  Larval Feeding Salivary Gland  5.035739  
  Whole Larvae Feeding  5.345767  
 
  
   FlyBase ID    symbol    start    end    strand    length   
   FBgn0033572   CG12943   6791075   6793068  +  1994  
   FBgn0033573   Obp47a   6797428   6797990  +  563  
   FBgn0033574   Spn47C  6827693   6829206   -  1514  
   FBgn0040765   luna  6870281   6987508   -  117228  
   FBgn0033578   BBS4   7023433   7026766  +  3334  
   FBgn0033579   CG13229  7027374   7031176   -  3803  
 
 
    Segment 127 
 
   Location   
  Gene key  FBgn0033580-FBgn0033581  
  Heatmap region span   2R:6762897..7110376   
  Segment span   2R:7033873..7037561   
  Length (genes)  2  
  Length (bp)  3689  
   Model Scoring   
  BIC  218.599359  
  logL  -103.778827  
  logL ratio  37.298583  
   Expression   
  Mean expression  7.371084  
  Median expression  7.330758  
  Tissue std. dev.  0.404550  
 
  No GO Slim enrichment  
  
   tissue    mean expression   
  5th Passage Drosophila S2 Cells  8.109795  
  Adult Accessory gland  7.337410  
  Adult Brain  7.628825  
  Adult Carcass  7.079172  
  Adult Crop  7.383552  
  Adult Eye  6.869960  
  Adult Fatbody  7.492568  
  Adult Female Spermatheca Mated  7.487637  
  Adult Female Spermatheca Virgin  7.545158  
  Adult Head  7.131508  
  Adult Heart  7.126962  
  Adult Hind Gut  7.245405  
  Adult Male Ejaculatory Duct  7.181437  
  Adult Mid Gut  7.228663  
  Adult Ovary  8.315387  
  Adult Salivary Gland  7.226304  
  Adult Testes  6.345279  
  Adult Thoracoabdominal ganglion  7.600855  
  Adult Whole Fly  7.286193  
  Larvae Wandering Tubules  7.404948  
  Larval Feeding Carcass  7.419259  
  Larval Feeding Central Nevous System  8.103341  
  Larval Feeding Hind Gut  7.280144  
  Larval Feeding Malpighian Tubule  7.264596  
  Larval Feeding Mid Gut  7.483066  
  Larval Feeding Salivary Gland  7.759227  
  Whole Larvae Feeding  6.682612  
 
  
   FlyBase ID    symbol    start    end    strand    length   
   FBgn0033580   CG13231   7033873   7034308  +  436  
   FBgn0033581   CG12391  7035102   7037561   -  2460  
 
    Segment 128 
 
   Location   
  Gene key  FBgn0037084-FBgn0033584  
  Heatmap region span   2R:7033873..7132949   
  Segment span   2R:7107046..7110376   
  Length (genes)  2  
  Length (bp)  3331  
   Model Scoring   
  BIC  268.707807  
  logL  -128.833051  
  logL ratio  -5.332534  
   Expression   
  Mean expression  8.563849  
  Median expression  8.851981  
  Tissue std. dev.  0.869027  
 
  No GO Slim enrichment  
  
   tissue    mean expression   
  5th Passage Drosophila S2 Cells  8.605801  
  Adult Accessory gland  7.760459  
  Adult Brain  10.574725  
  Adult Carcass  7.833340  
  Adult Crop  7.679653  
  Adult Eye  8.484635  
  Adult Fatbody  7.629030  
  Adult Female Spermatheca Mated  8.086647  
  Adult Female Spermatheca Virgin  7.871078  
  Adult Head  8.971232  
  Adult Heart  7.588051  
  Adult Hind Gut  7.834172  
  Adult Male Ejaculatory Duct  9.520669  
  Adult Mid Gut  9.366398  
  Adult Ovary  9.863074  
  Adult Salivary Gland  8.103219  
  Adult Testes  8.250418  
  Adult Thoracoabdominal ganglion  10.667807  
  Adult Whole Fly  8.810476  
  Larvae Wandering Tubules  8.037812  
  Larval Feeding Carcass  7.873279  
  Larval Feeding Central Nevous System  9.488380  
  Larval Feeding Hind Gut  8.299677  
  Larval Feeding Malpighian Tubule  8.296040  
  Larval Feeding Mid Gut  9.490384  
  Larval Feeding Salivary Gland  7.899761  
  Whole Larvae Feeding  8.337714  
 
  
   FlyBase ID    symbol    start    end    strand    length   
   FBgn0037084   Syx6   7107046   7109987  +  2942  
   FBgn0033584   CG7737   7110376   7114251  +  3876  
 
    Segment 129 
 
   Location   
  Gene key  FBgn0033587-FBgn0033592  
  Heatmap region span   2R:7061325..7160019   
  Segment span   2R:7121209..7127619   
  Length (genes)  7  
  Length (bp)  6411  
   Model Scoring   
  BIC  560.890400  
  logL  -274.924348  
  logL ratio  170.749238  
   Expression   
  Mean expression  5.034441  
  Median expression  4.826535  
  Tissue std. dev.  0.458721  
 
  No GO Slim enrichment  
  
   tissue    mean expression   
  5th Passage Drosophila S2 Cells  4.874729  
  Adult Accessory gland  5.037265  
  Adult Brain  4.604523  
  Adult Carcass  5.016301  
  Adult Crop  4.903103  
  Adult Eye  4.714555  
  Adult Fatbody  4.970735  
  Adult Female Spermatheca Mated  4.977137  
  Adult Female Spermatheca Virgin  4.983418  
  Adult Head  4.770046  
  Adult Heart  4.827028  
  Adult Hind Gut  5.752831  
  Adult Male Ejaculatory Duct  4.946791  
  Adult Mid Gut  5.027520  
  Adult Ovary  4.822832  
  Adult Salivary Gland  5.170332  
  Adult Testes  4.641623  
  Adult Thoracoabdominal ganglion  4.664210  
  Adult Whole Fly  4.704085  
  Larvae Wandering Tubules  4.949827  
  Larval Feeding Carcass  5.517210  
  Larval Feeding Central Nevous System  4.599581  
  Larval Feeding Hind Gut  6.955545  
  Larval Feeding Malpighian Tubule  5.007360  
  Larval Feeding Mid Gut  5.356941  
  Larval Feeding Salivary Gland  4.983751  
  Whole Larvae Feeding  5.150634  
 
  
   FlyBase ID    symbol    start    end    strand    length   
   FBgn0033587   CG13218  7120769   7121209   -  441  
   FBgn0033588   CG13228   7121508   7121915  +  408  
   FBgn0033589   CG13227   7122759   7123260  +  502  
   FBgn0033590   CG13217  7123708   7124188   -  481  
   FBgn0085253   CG34224  7124677   7125129   -  453  
   FBgn0033591   CG13216  7125613   7126499   -  887  
   FBgn0033592   CG13215  7126999   7127619   -  621  
 
 
    Segment 130 
 
   Location   
  Gene key  FBgn0033594-FBgn0085254  
  Heatmap region span   2R:7107046..7165946   
  Segment span   2R:7131795..7132949   
  Length (genes)  2  
  Length (bp)  1155  
   Model Scoring   
  BIC  171.898957  
  logL  -80.428626  
  logL ratio  42.110497  
   Expression   
  Mean expression  4.399812  
  Median expression  4.412190  
  Tissue std. dev.  0.179125  
 
  No GO Slim enrichment  
  
   tissue    mean expression   
  5th Passage Drosophila S2 Cells  4.386890  
  Adult Accessory gland  4.571120  
  Adult Brain  4.231543  
  Adult Carcass  4.332404  
  Adult Crop  4.346030  
  Adult Eye  4.325653  
  Adult Fatbody  4.387501  
  Adult Female Spermatheca Mated  4.900162  
  Adult Female Spermatheca Virgin  4.592989  
  Adult Head  4.320822  
  Adult Heart  4.398549  
  Adult Hind Gut  4.554685  
  Adult Male Ejaculatory Duct  4.710581  
  Adult Mid Gut  4.489764  
  Adult Ovary  4.309532  
  Adult Salivary Gland  4.491380  
  Adult Testes  4.236506  
  Adult Thoracoabdominal ganglion  4.182119  
  Adult Whole Fly  4.063770  
  Larvae Wandering Tubules  4.370178  
  Larval Feeding Carcass  4.516383  
  Larval Feeding Central Nevous System  4.090067  
  Larval Feeding Hind Gut  4.311915  
  Larval Feeding Malpighian Tubule  4.408520  
  Larval Feeding Mid Gut  4.580338  
  Larval Feeding Salivary Gland  4.420250  
  Whole Larvae Feeding  4.265275  
 
  
   FlyBase ID    symbol    start    end    strand    length   
   FBgn0033594   CG13226   7131795   7132508  +  714  
   FBgn0085254   CG34225   7132949   7133263  +  315  
 
    Segment 131 
 
   Location   
  Gene key  FBgn0026386-FBgn0033603  
  Heatmap region span   2R:7121209..7178541   
  Segment span   2R:7137873..7160019   
  Length (genes)  8  
  Length (bp)  22147  
   Model Scoring   
  BIC  859.506840  
  logL  -424.232568  
  logL ratio  4.539409  
   Expression   
  Mean expression  4.955560  
  Median expression  4.611408  
  Tissue std. dev.  0.478481  
 
  No GO Slim enrichment  
  
   tissue    mean expression   
  5th Passage Drosophila S2 Cells  4.645388  
  Adult Accessory gland  4.756610  
  Adult Brain  4.550405  
  Adult Carcass  5.306171  
  Adult Crop  4.667052  
  Adult Eye  5.713862  
  Adult Fatbody  4.728650  
  Adult Female Spermatheca Mated  4.767544  
  Adult Female Spermatheca Virgin  4.698973  
  Adult Head  5.230582  
  Adult Heart  4.737125  
  Adult Hind Gut  4.641110  
  Adult Male Ejaculatory Duct  5.241185  
  Adult Mid Gut  4.743265  
  Adult Ovary  4.614683  
  Adult Salivary Gland  5.004281  
  Adult Testes  4.956322  
  Adult Thoracoabdominal ganglion  4.662499  
  Adult Whole Fly  4.469220  
  Larvae Wandering Tubules  4.718259  
  Larval Feeding Carcass  6.397134  
  Larval Feeding Central Nevous System  4.361457  
  Larval Feeding Hind Gut  5.722421  
  Larval Feeding Malpighian Tubule  4.684743  
  Larval Feeding Mid Gut  4.735249  
  Larval Feeding Salivary Gland  5.125130  
  Whole Larvae Feeding  5.920809  
 
  
   FlyBase ID    symbol    start    end    strand    length   
   FBgn0026386   Or47a   7137873   7139420  +  1548  
   FBgn0033597   Cpr47Ea  7139256   7140903   -  1648  
   FBgn0033598   Cpr47Eb   7142674   7143488  +  815  
   FBgn0033599   CG13223   7144012   7146272  +  2261  
   FBgn0033600   Cpr47Ec  7146822   7147344   -  523  
   FBgn0033601   Cpr47Ed  7148818   7149295   -  478  
   FBgn0033602   Cpr47Ee   7152631   7154361  +  1731  
   FBgn0033603   Cpr47Ef  7154420   7160019   -  5600  
 
 
    Segment 132 
 
   Location   
  Gene key  FBgn0033605-FBgn0033609  
  Heatmap region span   2R:7135631..7182091   
  Segment span   2R:7167106..7177253   
  Length (genes)  5  
  Length (bp)  10148  
   Model Scoring   
  BIC  585.115671  
  logL  -287.036983  
  logL ratio  69.889775  
   Expression   
  Mean expression  8.943973  
  Median expression  8.989279  
  Tissue std. dev.  0.433869  
 
  No GO Slim enrichment  
  
   tissue    mean expression   
  5th Passage Drosophila S2 Cells  8.755072  
  Adult Accessory gland  9.289949  
  Adult Brain  9.270642  
  Adult Carcass  8.520920  
  Adult Crop  9.237728  
  Adult Eye  9.259510  
  Adult Fatbody  8.807831  
  Adult Female Spermatheca Mated  8.874285  
  Adult Female Spermatheca Virgin  8.968316  
  Adult Head  8.854234  
  Adult Heart  8.885779  
  Adult Hind Gut  9.053221  
  Adult Male Ejaculatory Duct  9.035850  
  Adult Mid Gut  8.702985  
  Adult Ovary  9.601301  
  Adult Salivary Gland  9.039505  
  Adult Testes  7.454696  
  Adult Thoracoabdominal ganglion  9.229264  
  Adult Whole Fly  8.600181  
  Larvae Wandering Tubules  9.321585  
  Larval Feeding Carcass  8.607150  
  Larval Feeding Central Nevous System  9.217634  
  Larval Feeding Hind Gut  8.833530  
  Larval Feeding Malpighian Tubule  9.791576  
  Larval Feeding Mid Gut  8.594629  
  Larval Feeding Salivary Gland  9.212631  
  Whole Larvae Feeding  8.467275  
 
  
   FlyBase ID    symbol    start    end    strand    length   
   FBgn0033605   CG9067  7166398   7167106   -  709  
   FBgn0041174   Vhl   7167231   7168538  +  1308  
   FBgn0033607   CG9062  7167982   7171461   -  3480  
   FBgn0033608   CG13220   7171921   7172605  +  685  
   FBgn0033609   fbl6  7172653   7177253   -  4601  
 
 
    Segment 133 
 
   Location   
  Gene key  FBgn0016047-FBgn0003382  
  Heatmap region span   2R:7189426..7244227   
  Segment span   2R:7206180..7216767   
  Length (genes)  3  
  Length (bp)  10588  
   Model Scoring   
  BIC  230.427170  
  logL  -109.692733  
  logL ratio  81.227722  
   Expression   
  Mean expression  4.607057  
  Median expression  4.626220  
  Tissue std. dev.  0.177295  
 
  No GO Slim enrichment  
  
   tissue    mean expression   
  5th Passage Drosophila S2 Cells  4.582552  
  Adult Accessory gland  4.572861  
  Adult Brain  4.300662  
  Adult Carcass  4.918246  
  Adult Crop  4.512852  
  Adult Eye  4.667861  
  Adult Fatbody  4.812696  
  Adult Female Spermatheca Mated  4.865426  
  Adult Female Spermatheca Virgin  4.870386  
  Adult Head  4.763796  
  Adult Heart  4.765937  
  Adult Hind Gut  4.468065  
  Adult Male Ejaculatory Duct  4.793559  
  Adult Mid Gut  4.595098  
  Adult Ovary  4.544184  
  Adult Salivary Gland  4.731460  
  Adult Testes  4.474937  
  Adult Thoracoabdominal ganglion  4.408329  
  Adult Whole Fly  4.284913  
  Larvae Wandering Tubules  4.813026  
  Larval Feeding Carcass  4.645026  
  Larval Feeding Central Nevous System  4.440754  
  Larval Feeding Hind Gut  4.437295  
  Larval Feeding Malpighian Tubule  4.633596  
  Larval Feeding Mid Gut  4.527660  
  Larval Feeding Salivary Gland  4.623299  
  Whole Larvae Feeding  4.336057  
 
  
   FlyBase ID    symbol    start    end    strand    length   
   FBgn0016047   nompA  7197953   7206180   -  8228  
   FBgn0026385   Or47b  7207215   7208817   -  1603  
   FBgn0003382   sha   7216767   7223482  +  6716  
 
 
    Segment 134 
 
   Location   
  Gene key  FBgn0043470-FBgn0011555  
  Heatmap region span   2R:7190492..7246674   
  Segment span   2R:7224353..7230261   
  Length (genes)  5  
  Length (bp)  5909  
   Model Scoring   
  BIC  451.530429  
  logL  -220.244362  
  logL ratio  121.792545  
   Expression   
  Mean expression  5.915797  
  Median expression  4.838012  
  Tissue std. dev.  2.381009  
 
  
   GO ID    description    ratio    P-value   
   GO:0005576   extracellular region  3/5  0.00022  
 
  
   tissue    mean expression   
  5th Passage Drosophila S2 Cells  4.762672  
  Adult Accessory gland  4.799751  
  Adult Brain  4.449371  
  Adult Carcass  4.865524  
  Adult Crop  4.753399  
  Adult Eye  4.709619  
  Adult Fatbody  4.718308  
  Adult Female Spermatheca Mated  4.949184  
  Adult Female Spermatheca Virgin  4.865862  
  Adult Head  4.557231  
  Adult Heart  4.712931  
  Adult Hind Gut  7.882838  
  Adult Male Ejaculatory Duct  5.036479  
  Adult Mid Gut  12.615198  
  Adult Ovary  4.674212  
  Adult Salivary Gland  4.989445  
  Adult Testes  4.594258  
  Adult Thoracoabdominal ganglion  4.612111  
  Adult Whole Fly  9.483134  
  Larvae Wandering Tubules  4.968817  
  Larval Feeding Carcass  4.798255  
  Larval Feeding Central Nevous System  4.471994  
  Larval Feeding Hind Gut  6.827829  
  Larval Feeding Malpighian Tubule  4.948449  
  Larval Feeding Mid Gut  12.086471  
  Larval Feeding Salivary Gland  4.761408  
  Whole Larvae Feeding  10.831773  
 
  
   FlyBase ID    symbol    start    end    strand    length   
   FBgn0043470   lambdaTry   7224353   7225296  +  944  
   FBgn0043471   kappaTry  7225302   7226282   -  981  
   FBgn0011556   zetaTry  7226691   7227633   -  943  
   FBgn0011554   etaTry  7228018   7228928   -  911  
   FBgn0011555   thetaTry  7229219   7230261   -  1043  
 
 
    Segment 135 
 
   Location   
  Gene key  FBgn0003863-FBgn0010357  
  Heatmap region span   2R:7195070..7248213   
  Segment span   2R:7232620..7233844   
  Length (genes)  3  
  Length (bp)  1225  
   Model Scoring   
  BIC  339.958533  
  logL  -164.458414  
  logL ratio  95.452057  
   Expression   
  Mean expression  7.656564  
  Median expression  6.501560  
  Tissue std. dev.  2.844008  
 
  
   GO ID    description    ratio    P-value   
   GO:0005576   extracellular region  3/3  2.27e-05  
 
  
   tissue    mean expression   
  5th Passage Drosophila S2 Cells  6.020796  
  Adult Accessory gland  6.376114  
  Adult Brain  5.352367  
  Adult Carcass  7.702517  
  Adult Crop  7.095762  
  Adult Eye  6.664927  
  Adult Fatbody  6.180635  
  Adult Female Spermatheca Mated  6.057313  
  Adult Female Spermatheca Virgin  5.974274  
  Adult Head  6.702673  
  Adult Heart  6.121910  
  Adult Hind Gut  8.713457  
  Adult Male Ejaculatory Duct  6.783913  
  Adult Mid Gut  14.625989  
  Adult Ovary  5.826967  
  Adult Salivary Gland  6.531556  
  Adult Testes  6.047597  
  Adult Thoracoabdominal ganglion  5.861303  
  Adult Whole Fly  14.027210  
  Larvae Wandering Tubules  7.119033  
  Larval Feeding Carcass  6.271394  
  Larval Feeding Central Nevous System  5.484481  
  Larval Feeding Hind Gut  8.556080  
  Larval Feeding Malpighian Tubule  6.431952  
  Larval Feeding Mid Gut  14.389575  
  Larval Feeding Salivary Gland  5.998469  
  Whole Larvae Feeding  13.808976  
 
  
   FlyBase ID    symbol    start    end    strand    length   
   FBgn0003863   alphaTry  7231756   7232620   -  865  
   FBgn0010425   epsilonTry  7232734   7233567   -  834  
   FBgn0010357   betaTry   7233844   7234641  +  798  
 
 
    Segment 136 
 
   Location   
  Gene key  FBgn0001276-FBgn0024188  
  Heatmap region span   2R:7232620..7266682   
  Segment span   2R:7247668..7248213   
  Length (genes)  2  
  Length (bp)  546  
   Model Scoring   
  BIC  264.420985  
  logL  -126.689640  
  logL ratio  -5.952162  
   Expression   
  Mean expression  8.145358  
  Median expression  8.358064  
  Tissue std. dev.  0.447796  
 
  No GO Slim enrichment  
  
   tissue    mean expression   
  5th Passage Drosophila S2 Cells  8.658243  
  Adult Accessory gland  8.474071  
  Adult Brain  8.519606  
  Adult Carcass  7.561025  
  Adult Crop  8.037470  
  Adult Eye  8.328481  
  Adult Fatbody  7.989037  
  Adult Female Spermatheca Mated  8.079833  
  Adult Female Spermatheca Virgin  8.202576  
  Adult Head  7.885143  
  Adult Heart  8.258924  
  Adult Hind Gut  7.787184  
  Adult Male Ejaculatory Duct  8.035175  
  Adult Mid Gut  7.629197  
  Adult Ovary  9.531075  
  Adult Salivary Gland  7.936495  
  Adult Testes  7.469858  
  Adult Thoracoabdominal ganglion  8.210308  
  Adult Whole Fly  8.297772  
  Larvae Wandering Tubules  8.225032  
  Larval Feeding Carcass  8.042640  
  Larval Feeding Central Nevous System  8.971802  
  Larval Feeding Hind Gut  8.089795  
  Larval Feeding Malpighian Tubule  8.175161  
  Larval Feeding Mid Gut  7.613646  
  Larval Feeding Salivary Gland  8.453580  
  Whole Larvae Feeding  7.461524  
 
  
   FlyBase ID    symbol    start    end    strand    length   
   FBgn0001276   ix  7246942   7247668   -  727  
   FBgn0024188   san   7248213   7249376  +  1164  
 
    Segment 137 
 
   Location   
  Gene key  FBgn0033628-FBgn0033629  
  Heatmap region span   2R:7247668..7302015   
  Segment span   2R:7261112..7266682   
  Length (genes)  2  
  Length (bp)  5571  
   Model Scoring   
  BIC  173.359129  
  logL  -81.158712  
  logL ratio  35.180274  
   Expression   
  Mean expression  4.887878  
  Median expression  4.836031  
  Tissue std. dev.  0.244710  
 
  No GO Slim enrichment  
  
   tissue    mean expression   
  5th Passage Drosophila S2 Cells  4.788831  
  Adult Accessory gland  4.925830  
  Adult Brain  4.870541  
  Adult Carcass  5.226717  
  Adult Crop  4.708077  
  Adult Eye  4.774484  
  Adult Fatbody  4.788088  
  Adult Female Spermatheca Mated  4.756666  
  Adult Female Spermatheca Virgin  4.822251  
  Adult Head  5.771451  
  Adult Heart  4.861458  
  Adult Hind Gut  4.797002  
  Adult Male Ejaculatory Duct  4.962022  
  Adult Mid Gut  4.914559  
  Adult Ovary  4.768014  
  Adult Salivary Gland  4.945960  
  Adult Testes  5.355425  
  Adult Thoracoabdominal ganglion  4.909811  
  Adult Whole Fly  4.519015  
  Larvae Wandering Tubules  4.812087  
  Larval Feeding Carcass  4.858958  
  Larval Feeding Central Nevous System  5.170879  
  Larval Feeding Hind Gut  4.668611  
  Larval Feeding Malpighian Tubule  4.750583  
  Larval Feeding Mid Gut  4.847474  
  Larval Feeding Salivary Gland  4.794963  
  Whole Larvae Feeding  4.602936  
 
  
   FlyBase ID    symbol    start    end    strand    length   
   FBgn0033628   CG13203   7261112   7264614  +  3503  
   FBgn0033629   Tsp47F  7264967   7266682   -  1716  
 
    Segment 138 
 
   Location   
  Gene key  FBgn0033631-FBgn0050022  
  Heatmap region span   2R:7249405..7317146   
  Segment span   2R:7271613..7276282   
  Length (genes)  2  
  Length (bp)  4670  
   Model Scoring   
  BIC  244.342697  
  logL  -116.650496  
  logL ratio  21.503147  
   Expression   
  Mean expression  8.921104  
  Median expression  8.783333  
  Tissue std. dev.  0.838844  
 
  No GO Slim enrichment  
  
   tissue    mean expression   
  5th Passage Drosophila S2 Cells  7.323846  
  Adult Accessory gland  8.494180  
  Adult Brain  9.503815  
  Adult Carcass  8.716117  
  Adult Crop  9.683461  
  Adult Eye  8.930400  
  Adult Fatbody  7.962581  
  Adult Female Spermatheca Mated  8.667876  
  Adult Female Spermatheca Virgin  8.717603  
  Adult Head  9.485463  
  Adult Heart  8.312102  
  Adult Hind Gut  10.081990  
  Adult Male Ejaculatory Duct  8.724350  
  Adult Mid Gut  9.248129  
  Adult Ovary  9.711464  
  Adult Salivary Gland  8.195343  
  Adult Testes  7.648358  
  Adult Thoracoabdominal ganglion  9.781992  
  Adult Whole Fly  9.037095  
  Larvae Wandering Tubules  9.740433  
  Larval Feeding Carcass  9.491822  
  Larval Feeding Central Nevous System  7.515726  
  Larval Feeding Hind Gut  9.421540  
  Larval Feeding Malpighian Tubule  10.366630  
  Larval Feeding Mid Gut  9.310789  
  Larval Feeding Salivary Gland  7.267183  
  Whole Larvae Feeding  9.529532  
 
  
   FlyBase ID    symbol    start    end    strand    length   
   FBgn0033631   Sod3  7267849   7271613   -  3765  
   FBgn0050022   CG30022  7274663   7276282   -  1620  
 
    Segment 139 
 
   Location   
  Gene key  FBgn0082585-FBgn0033633  
  Heatmap region span   2R:7254216..7323751   
  Segment span   2R:7284935..7293191   
  Length (genes)  2  
  Length (bp)  8257  
   Model Scoring   
  BIC  231.476641  
  logL  -110.217468  
  logL ratio  3.472579  
   Expression   
  Mean expression  6.249792  
  Median expression  6.112292  
  Tissue std. dev.  0.631559  
 
  No GO Slim enrichment  
  
   tissue    mean expression   
  5th Passage Drosophila S2 Cells  6.800857  
  Adult Accessory gland  5.433865  
  Adult Brain  5.610737  
  Adult Carcass  7.392286  
  Adult Crop  7.620593  
  Adult Eye  6.387264  
  Adult Fatbody  5.838581  
  Adult Female Spermatheca Mated  5.824387  
  Adult Female Spermatheca Virgin  5.881432  
  Adult Head  6.448154  
  Adult Heart  7.032201  
  Adult Hind Gut  6.398926  
  Adult Male Ejaculatory Duct  6.168810  
  Adult Mid Gut  6.682984  
  Adult Ovary  5.378139  
  Adult Salivary Gland  6.307457  
  Adult Testes  5.618272  
  Adult Thoracoabdominal ganglion  5.902987  
  Adult Whole Fly  5.573323  
  Larvae Wandering Tubules  6.985865  
  Larval Feeding Carcass  7.289936  
  Larval Feeding Central Nevous System  5.725979  
  Larval Feeding Hind Gut  6.032923  
  Larval Feeding Malpighian Tubule  6.938480  
  Larval Feeding Mid Gut  5.531622  
  Larval Feeding Salivary Gland  6.001803  
  Whole Larvae Feeding  5.936525  
 
  
   FlyBase ID    symbol    start    end    strand    length   
   FBgn0082585   sprt  7272238   7284935   -  12698  
   FBgn0033633   CG7759   7293191   7295584  +  2394  
 
    Segment 140 
 
   Location   
  Gene key  FBgn0054054-FBgn0040503  
  Heatmap region span   2R:7261112..7539382   
  Segment span   2R:7297943..7302015   
  Length (genes)  2  
  Length (bp)  4073  
   Model Scoring   
  BIC  229.565110  
  logL  -109.261702  
  logL ratio  -0.529160  
   Expression   
  Mean expression  5.631236  
  Median expression  4.818054  
  Tissue std. dev.  1.595306  
 
  No GO Slim enrichment  
  
   tissue    mean expression   
  5th Passage Drosophila S2 Cells  4.729427  
  Adult Accessory gland  4.261151  
  Adult Brain  4.277404  
  Adult Carcass  6.146318  
  Adult Crop  4.513811  
  Adult Eye  5.682504  
  Adult Fatbody  9.159254  
  Adult Female Spermatheca Mated  9.083647  
  Adult Female Spermatheca Virgin  9.229782  
  Adult Head  5.368626  
  Adult Heart  8.160660  
  Adult Hind Gut  4.570333  
  Adult Male Ejaculatory Duct  4.780848  
  Adult Mid Gut  4.247165  
  Adult Ovary  4.179019  
  Adult Salivary Gland  4.759287  
  Adult Testes  4.336504  
  Adult Thoracoabdominal ganglion  4.315366  
  Adult Whole Fly  5.003736  
  Larvae Wandering Tubules  5.582504  
  Larval Feeding Carcass  5.049943  
  Larval Feeding Central Nevous System  4.033397  
  Larval Feeding Hind Gut  6.064559  
  Larval Feeding Malpighian Tubule  6.371356  
  Larval Feeding Mid Gut  4.847725  
  Larval Feeding Salivary Gland  5.604996  
  Whole Larvae Feeding  7.684036  
 
  
   FlyBase ID    symbol    start    end    strand    length   
   FBgn0054054   CG34054  7297315   7297943   -  629  
   FBgn0040503   CG7763   7302015   7302842  +  828  
 
    Segment 141 
 
   Location   
  Gene key  FBgn0001269-FBgn0000577  
  Heatmap region span   2R:7296936..7566978   
  Segment span   2R:7361970..7415715   
  Length (genes)  3  
  Length (bp)  53746  
   Model Scoring   
  BIC  281.273199  
  logL  -135.115747  
  logL ratio  41.885629  
   Expression   
  Mean expression  4.869272  
  Median expression  4.544466  
  Tissue std. dev.  0.776651  
 
  No GO Slim enrichment  
  
   tissue    mean expression   
  5th Passage Drosophila S2 Cells  4.430101  
  Adult Accessory gland  4.506329  
  Adult Brain  4.654630  
  Adult Carcass  4.605546  
  Adult Crop  4.411727  
  Adult Eye  5.516158  
  Adult Fatbody  4.558889  
  Adult Female Spermatheca Mated  4.693306  
  Adult Female Spermatheca Virgin  4.753187  
  Adult Head  4.791846  
  Adult Heart  4.307657  
  Adult Hind Gut  4.310099  
  Adult Male Ejaculatory Duct  7.876105  
  Adult Mid Gut  4.409236  
  Adult Ovary  4.312155  
  Adult Salivary Gland  4.768479  
  Adult Testes  4.963013  
  Adult Thoracoabdominal ganglion  5.073383  
  Adult Whole Fly  4.335754  
  Larvae Wandering Tubules  4.353056  
  Larval Feeding Carcass  5.258288  
  Larval Feeding Central Nevous System  5.312029  
  Larval Feeding Hind Gut  6.715182  
  Larval Feeding Malpighian Tubule  4.462354  
  Larval Feeding Mid Gut  4.411688  
  Larval Feeding Salivary Gland  4.578402  
  Whole Larvae Feeding  5.101732  
 
  
   FlyBase ID    symbol    start    end    strand    length   
   FBgn0001269   inv   7361970   7394688  +  32719  
   FBgn0050034   CG30034  7403684   7409553   -  5870  
   FBgn0000577   en  7411509   7415715   -  4207  
 
 
    Segment 142 
 
   Location   
  Gene key  FBgn0033636-FBgn0085257  
  Heatmap region span   2R:7297943..7573937   
  Segment span   2R:7503336..7539382   
  Length (genes)  5  
  Length (bp)  36047  
   Model Scoring   
  BIC  501.996391  
  logL  -245.477343  
  logL ratio  135.810683  
   Expression   
  Mean expression  8.534290  
  Median expression  8.463342  
  Tissue std. dev.  0.422229  
 
  No GO Slim enrichment  
  
   tissue    mean expression   
  5th Passage Drosophila S2 Cells  8.524079  
  Adult Accessory gland  8.066381  
  Adult Brain  9.140683  
  Adult Carcass  8.535620  
  Adult Crop  9.010156  
  Adult Eye  8.694244  
  Adult Fatbody  8.503850  
  Adult Female Spermatheca Mated  8.222784  
  Adult Female Spermatheca Virgin  8.319489  
  Adult Head  8.409809  
  Adult Heart  8.999017  
  Adult Hind Gut  8.944450  
  Adult Male Ejaculatory Duct  8.317216  
  Adult Mid Gut  8.234692  
  Adult Ovary  8.906585  
  Adult Salivary Gland  8.992715  
  Adult Testes  7.418092  
  Adult Thoracoabdominal ganglion  9.205818  
  Adult Whole Fly  8.093102  
  Larvae Wandering Tubules  9.024022  
  Larval Feeding Carcass  8.618672  
  Larval Feeding Central Nevous System  8.695824  
  Larval Feeding Hind Gut  8.760990  
  Larval Feeding Malpighian Tubule  8.561369  
  Larval Feeding Mid Gut  8.325148  
  Larval Feeding Salivary Gland  7.910116  
  Whole Larvae Feeding  7.990907  
 
  
   FlyBase ID    symbol    start    end    strand    length   
   FBgn0033636   tou  7466385   7503336   -  36952  
   FBgn0086712   Egm  7504374   7506515   -  2142  
   FBgn0033638   CG9005  7506984   7520075   -  13092  
   FBgn0033639   CG9003  7529772   7539127   -  9356  
   FBgn0085257   CG34228   7539382   7539844  +  463  
 
 
    Segment 143 
 
   Location   
  Gene key  FBgn0033650-FBgn0033651  
  Heatmap region span   2R:7578380..7732831   
  Segment span   2R:7655958..7676998   
  Length (genes)  3  
  Length (bp)  21041  
   Model Scoring   
  BIC  240.162970  
  logL  -114.560632  
  logL ratio  75.713161  
   Expression   
  Mean expression  4.619066  
  Median expression  4.662822  
  Tissue std. dev.  0.183707  
 
  No GO Slim enrichment  
  
   tissue    mean expression   
  5th Passage Drosophila S2 Cells  4.629575  
  Adult Accessory gland  4.586017  
  Adult Brain  5.005210  
  Adult Carcass  4.473987  
  Adult Crop  4.538574  
  Adult Eye  4.842323  
  Adult Fatbody  4.641781  
  Adult Female Spermatheca Mated  4.632739  
  Adult Female Spermatheca Virgin  4.629864  
  Adult Head  4.623878  
  Adult Heart  4.797455  
  Adult Hind Gut  4.528467  
  Adult Male Ejaculatory Duct  4.705143  
  Adult Mid Gut  4.737202  
  Adult Ovary  4.542910  
  Adult Salivary Gland  4.655443  
  Adult Testes  4.337078  
  Adult Thoracoabdominal ganglion  5.082992  
  Adult Whole Fly  4.233763  
  Larvae Wandering Tubules  4.524299  
  Larval Feeding Carcass  4.577360  
  Larval Feeding Central Nevous System  4.498847  
  Larval Feeding Hind Gut  4.461342  
  Larval Feeding Malpighian Tubule  4.500001  
  Larval Feeding Mid Gut  4.761615  
  Larval Feeding Salivary Gland  4.788250  
  Whole Larvae Feeding  4.378673  
 
  
   FlyBase ID    symbol    start    end    strand    length   
   FBgn0033650   CG13193   7655958   7657066  +  1109  
   FBgn0033652   ths   7658042   7701667  +  43626  
   FBgn0033651   Ir48c  7675313   7676998   -  1686  
 
 
    Segment 144 
 
   Location   
  Gene key  FBgn0259832-FBgn0033656  
  Heatmap region span   2R:7599767..7753888   
  Segment span   2R:7729113..7731743   
  Length (genes)  2  
  Length (bp)  2631  
   Model Scoring   
  BIC  211.292255  
  logL  -100.125275  
  logL ratio  47.166242  
   Expression   
  Mean expression  8.737638  
  Median expression  8.773228  
  Tissue std. dev.  0.341184  
 
  No GO Slim enrichment  
  
   tissue    mean expression   
  5th Passage Drosophila S2 Cells  8.936322  
  Adult Accessory gland  8.827939  
  Adult Brain  8.799943  
  Adult Carcass  8.639297  
  Adult Crop  8.702459  
  Adult Eye  8.938479  
  Adult Fatbody  8.997954  
  Adult Female Spermatheca Mated  9.213259  
  Adult Female Spermatheca Virgin  9.165358  
  Adult Head  8.537304  
  Adult Heart  8.971392  
  Adult Hind Gut  8.378426  
  Adult Male Ejaculatory Duct  9.606558  
  Adult Mid Gut  8.161598  
  Adult Ovary  8.757271  
  Adult Salivary Gland  8.361744  
  Adult Testes  8.650181  
  Adult Thoracoabdominal ganglion  9.174980  
  Adult Whole Fly  8.196905  
  Larvae Wandering Tubules  8.965681  
  Larval Feeding Carcass  8.443577  
  Larval Feeding Central Nevous System  8.829604  
  Larval Feeding Hind Gut  8.502249  
  Larval Feeding Malpighian Tubule  8.924208  
  Larval Feeding Mid Gut  8.356756  
  Larval Feeding Salivary Gland  8.609174  
  Whole Larvae Feeding  8.267616  
 
  
   FlyBase ID    symbol    start    end    strand    length   
   FBgn0259832   CG34229   7729113   7729669  +  557  
   FBgn0033656   S2P  7729608   7731743   -  2136  
 
    Segment 145 
 
   Location   
  Gene key  FBgn0085259-FBgn0033658  
  Heatmap region span   2R:7727035..7787024   
  Segment span   2R:7748815..7750753   
  Length (genes)  2  
  Length (bp)  1939  
   Model Scoring   
  BIC  223.317960  
  logL  -106.138127  
  logL ratio  -1.107528  
   Expression   
  Mean expression  4.889000  
  Median expression  4.425704  
  Tissue std. dev.  0.634831  
 
  No GO Slim enrichment  
  
   tissue    mean expression   
  5th Passage Drosophila S2 Cells  4.504698  
  Adult Accessory gland  4.592757  
  Adult Brain  4.131317  
  Adult Carcass  4.492802  
  Adult Crop  4.595642  
  Adult Eye  5.082731  
  Adult Fatbody  4.787088  
  Adult Female Spermatheca Mated  4.720451  
  Adult Female Spermatheca Virgin  4.687926  
  Adult Head  4.327441  
  Adult Heart  5.002142  
  Adult Hind Gut  4.864534  
  Adult Male Ejaculatory Duct  4.663581  
  Adult Mid Gut  4.791443  
  Adult Ovary  4.421350  
  Adult Salivary Gland  4.752688  
  Adult Testes  7.053589  
  Adult Thoracoabdominal ganglion  4.179485  
  Adult Whole Fly  4.547016  
  Larvae Wandering Tubules  4.384163  
  Larval Feeding Carcass  5.923376  
  Larval Feeding Central Nevous System  4.778094  
  Larval Feeding Hind Gut  5.692527  
  Larval Feeding Malpighian Tubule  4.676160  
  Larval Feeding Mid Gut  5.700784  
  Larval Feeding Salivary Gland  4.727440  
  Whole Larvae Feeding  5.921780  
 
  
   FlyBase ID    symbol    start    end    strand    length   
   FBgn0085259   CG34230   7748815   7749520  +  706  
   FBgn0033658   TwdlBeta  7750042   7750753   -  712  
 
    Segment 146 
 
   Location   
  Gene key  FBgn0033663-FBgn0000556  
  Heatmap region span   2R:7740124..7867990   
  Segment span   2R:7778520..7779597   
  Length (genes)  2  
  Length (bp)  1078  
   Model Scoring   
  BIC  387.167929  
  logL  -188.063112  
  logL ratio  126.464486  
   Expression   
  Mean expression  13.536512  
  Median expression  13.832888  
  Tissue std. dev.  0.396888  
 
  
   GO ID    description    ratio    P-value   
   GO:0005811   lipid particle  2/2  0.00404  
 
  
   tissue    mean expression   
  5th Passage Drosophila S2 Cells  14.226127  
  Adult Accessory gland  14.156386  
  Adult Brain  12.811069  
  Adult Carcass  13.141352  
  Adult Crop  13.257535  
  Adult Eye  12.910341  
  Adult Fatbody  13.368518  
  Adult Female Spermatheca Mated  13.670206  
  Adult Female Spermatheca Virgin  13.576344  
  Adult Head  13.272790  
  Adult Heart  13.362821  
  Adult Hind Gut  13.236088  
  Adult Male Ejaculatory Duct  14.082407  
  Adult Mid Gut  13.265504  
  Adult Ovary  13.735734  
  Adult Salivary Gland  13.833346  
  Adult Testes  12.988466  
  Adult Thoracoabdominal ganglion  12.851812  
  Adult Whole Fly  13.558501  
  Larvae Wandering Tubules  13.865393  
  Larval Feeding Carcass  13.667419  
  Larval Feeding Central Nevous System  13.930457  
  Larval Feeding Hind Gut  13.772202  
  Larval Feeding Malpighian Tubule  13.721066  
  Larval Feeding Mid Gut  13.486997  
  Larval Feeding Salivary Gland  14.134347  
  Whole Larvae Feeding  13.602585  
 
  
   FlyBase ID    symbol    start    end    strand    length   
   FBgn0033663   ERp60  7775073   7778520   -  3448  
   FBgn0000556   Ef1alpha48D   7779597   7783025  +  3429  
 
    Segment 147 
 
   Location   
  Gene key  FBgn0033667-FBgn0033668  
  Heatmap region span   2R:7753888..7872948   
  Segment span   2R:7800280..7826989   
  Length (genes)  2  
  Length (bp)  26710  
   Model Scoring   
  BIC  195.610098  
  logL  -92.284197  
  logL ratio  21.438878  
   Expression   
  Mean expression  5.320009  
  Median expression  5.042625  
  Tissue std. dev.  1.180389  
 
  
   GO ID    description    ratio    P-value   
   GO:0005622   intracellular  2/2  0.00282  
 
  
   tissue    mean expression   
  5th Passage Drosophila S2 Cells  4.707692  
  Adult Accessory gland  4.737461  
  Adult Brain  4.581699  
  Adult Carcass  5.134187  
  Adult Crop  4.807875  
  Adult Eye  5.649780  
  Adult Fatbody  4.955002  
  Adult Female Spermatheca Mated  5.214091  
  Adult Female Spermatheca Virgin  5.129120  
  Adult Head  4.969233  
  Adult Heart  4.845944  
  Adult Hind Gut  5.680020  
  Adult Male Ejaculatory Duct  4.879633  
  Adult Mid Gut  4.740085  
  Adult Ovary  4.813335  
  Adult Salivary Gland  4.981789  
  Adult Testes  4.844470  
  Adult Thoracoabdominal ganglion  4.789330  
  Adult Whole Fly  4.506688  
  Larvae Wandering Tubules  4.917084  
  Larval Feeding Carcass  9.769927  
  Larval Feeding Central Nevous System  4.831520  
  Larval Feeding Hind Gut  7.225674  
  Larval Feeding Malpighian Tubule  4.879886  
  Larval Feeding Mid Gut  4.774875  
  Larval Feeding Salivary Gland  4.988893  
  Whole Larvae Feeding  8.284961  
 
  
   FlyBase ID    symbol    start    end    strand    length   
   FBgn0033667   CG13183  7791828   7800280   -  8453  
   FBgn0033668   CG13188   7826989   7854942  +  27954  
 
    Segment 148 
 
   Location   
  Gene key  FBgn0026573-FBgn0050037  
  Heatmap region span   2R:7778520..7876638   
  Segment span   2R:7858179..7867990   
  Length (genes)  3  
  Length (bp)  9812  
   Model Scoring   
  BIC  262.809359  
  logL  -125.883827  
  logL ratio  50.496505  
   Expression   
  Mean expression  5.115769  
  Median expression  4.904770  
  Tissue std. dev.  0.395713  
 
  No GO Slim enrichment  
  
   tissue    mean expression   
  5th Passage Drosophila S2 Cells  5.362099  
  Adult Accessory gland  4.923550  
  Adult Brain  5.246616  
  Adult Carcass  4.894465  
  Adult Crop  4.978243  
  Adult Eye  4.867353  
  Adult Fatbody  4.762682  
  Adult Female Spermatheca Mated  5.032330  
  Adult Female Spermatheca Virgin  5.009578  
  Adult Head  4.709461  
  Adult Heart  4.841767  
  Adult Hind Gut  4.849404  
  Adult Male Ejaculatory Duct  4.946941  
  Adult Mid Gut  5.269410  
  Adult Ovary  5.726269  
  Adult Salivary Gland  5.215220  
  Adult Testes  6.742726  
  Adult Thoracoabdominal ganglion  5.121876  
  Adult Whole Fly  4.927325  
  Larvae Wandering Tubules  5.156043  
  Larval Feeding Carcass  5.056145  
  Larval Feeding Central Nevous System  5.583995  
  Larval Feeding Hind Gut  4.754181  
  Larval Feeding Malpighian Tubule  5.174161  
  Larval Feeding Mid Gut  5.053054  
  Larval Feeding Salivary Gland  4.987942  
  Whole Larvae Feeding  4.932916  
 
  
   FlyBase ID    symbol    start    end    strand    length   
   FBgn0026573   CG8290   7858179   7865236  +  7058  
   FBgn0050036   CG30036  7865038   7866454   -  1417  
   FBgn0050037   CG30037  7866598   7867990   -  1393  
 
 
    Segment 149 
 
   Location   
  Gene key  FBgn0004839-FBgn0033674  
  Heatmap region span   2R:7870457..7967046   
  Segment span   2R:7907329..7912775   
  Length (genes)  2  
  Length (bp)  5447  
   Model Scoring   
  BIC  207.969918  
  logL  -98.464107  
  logL ratio  22.676369  
   Expression   
  Mean expression  5.610777  
  Median expression  5.727287  
  Tissue std. dev.  0.945756  
 
  No GO Slim enrichment  
  
   tissue    mean expression   
  5th Passage Drosophila S2 Cells  4.904301  
  Adult Accessory gland  4.888102  
  Adult Brain  6.338464  
  Adult Carcass  5.201524  
  Adult Crop  5.585484  
  Adult Eye  4.637283  
  Adult Fatbody  6.103606  
  Adult Female Spermatheca Mated  5.117148  
  Adult Female Spermatheca Virgin  5.252617  
  Adult Head  4.875910  
  Adult Heart  6.303093  
  Adult Hind Gut  6.937017  
  Adult Male Ejaculatory Duct  5.431846  
  Adult Mid Gut  5.346322  
  Adult Ovary  5.238722  
  Adult Salivary Gland  5.368116  
  Adult Testes  4.608053  
  Adult Thoracoabdominal ganglion  6.224480  
  Adult Whole Fly  4.686348  
  Larvae Wandering Tubules  4.913569  
  Larval Feeding Carcass  5.455240  
  Larval Feeding Central Nevous System  9.183408  
  Larval Feeding Hind Gut  6.627563  
  Larval Feeding Malpighian Tubule  4.951710  
  Larval Feeding Mid Gut  6.062637  
  Larval Feeding Salivary Gland  5.072914  
  Whole Larvae Feeding  6.175488  
 
  
   FlyBase ID    symbol    start    end    strand    length   
   FBgn0004839   otk  7888983   7907329   -  18347  
   FBgn0033674   CG8964  7910651   7912775   -  2125  
 
    Segment 150 
 
   Location   
  Gene key  FBgn0033677-FBgn0010339  
  Heatmap region span   2R:7872948..8006377   
  Segment span   2R:7921453..7924811   
  Length (genes)  2  
  Length (bp)  3359  
   Model Scoring   
  BIC  232.173204  
  logL  -110.565749  
  logL ratio  43.725444  
   Expression   
  Mean expression  9.673083  
  Median expression  9.714538  
  Tissue std. dev.  0.576258  
 
  No GO Slim enrichment  
  
   tissue    mean expression   
  5th Passage Drosophila S2 Cells  9.177740  
  Adult Accessory gland  9.602944  
  Adult Brain  9.399349  
  Adult Carcass  9.155958  
  Adult Crop  10.800147  
  Adult Eye  10.293754  
  Adult Fatbody  8.851151  
  Adult Female Spermatheca Mated  9.860374  
  Adult Female Spermatheca Virgin  9.729179  
  Adult Head  9.675194  
  Adult Heart  9.710821  
  Adult Hind Gut  10.391298  
  Adult Male Ejaculatory Duct  9.888388  
  Adult Mid Gut  9.690970  
  Adult Ovary  10.850728  
  Adult Salivary Gland  10.344579  
  Adult Testes  8.281401  
  Adult Thoracoabdominal ganglion  9.539468  
  Adult Whole Fly  9.930246  
  Larvae Wandering Tubules  9.116535  
  Larval Feeding Carcass  8.806030  
  Larval Feeding Central Nevous System  9.795513  
  Larval Feeding Hind Gut  10.020881  
  Larval Feeding Malpighian Tubule  9.636867  
  Larval Feeding Mid Gut  9.574964  
  Larval Feeding Salivary Gland  9.929948  
  Whole Larvae Feeding  9.118823  
 
  
   FlyBase ID    symbol    start    end    strand    length   
   FBgn0033677   CG8321   7921453   7923765  +  2313  
   FBgn0010339   128up   7924811   7926357  +  1547  
 
    Segment 151 
 
   Location   
  Gene key  FBgn0033680-FBgn0086677  
  Heatmap region span   2R:7921453..8032038   
  Segment span   2R:7968781..8006377   
  Length (genes)  2  
  Length (bp)  37597  
   Model Scoring   
  BIC  236.870822  
  logL  -112.914559  
  logL ratio  1.054242  
   Expression   
  Mean expression  4.335907  
  Median expression  4.116756  
  Tissue std. dev.  0.785796  
 
  No GO Slim enrichment  
  
   tissue    mean expression   
  5th Passage Drosophila S2 Cells  3.920668  
  Adult Accessory gland  4.017110  
  Adult Brain  5.850269  
  Adult Carcass  4.270081  
  Adult Crop  4.042098  
  Adult Eye  3.871563  
  Adult Fatbody  4.099344  
  Adult Female Spermatheca Mated  3.973725  
  Adult Female Spermatheca Virgin  3.950951  
  Adult Head  4.317392  
  Adult Heart  4.048191  
  Adult Hind Gut  3.948525  
  Adult Male Ejaculatory Duct  3.944777  
  Adult Mid Gut  3.878439  
  Adult Ovary  3.900475  
  Adult Salivary Gland  4.201362  
  Adult Testes  7.259252  
  Adult Thoracoabdominal ganglion  5.637082  
  Adult Whole Fly  5.219142  
  Larvae Wandering Tubules  3.979675  
  Larval Feeding Carcass  4.187513  
  Larval Feeding Central Nevous System  5.031657  
  Larval Feeding Hind Gut  3.802011  
  Larval Feeding Malpighian Tubule  3.892766  
  Larval Feeding Mid Gut  3.776930  
  Larval Feeding Salivary Gland  3.925533  
  Whole Larvae Feeding  4.122947  
 
  
   FlyBase ID    symbol    start    end    strand    length   
   FBgn0033680   CG13186   7968781   7969562  +  782  
   FBgn0086677   jeb  7978127   8006377   -  28251  
 
    Segment 152 
 
   Location   
  Gene key  FBgn0033686-FBgn0027504  
  Heatmap region span   2R:8026307..8053224   
  Segment span   2R:8033231..8038562   
  Length (genes)  2  
  Length (bp)  5332  
   Model Scoring   
  BIC  251.968457  
  logL  -120.463376  
  logL ratio  5.364114  
   Expression   
  Mean expression  7.986739  
  Median expression  8.132143  
  Tissue std. dev.  0.630855  
 
  No GO Slim enrichment  
  
   tissue    mean expression   
  5th Passage Drosophila S2 Cells  9.012334  
  Adult Accessory gland  8.541978  
  Adult Brain  8.484777  
  Adult Carcass  7.113962  
  Adult Crop  8.068331  
  Adult Eye  8.266232  
  Adult Fatbody  7.571114  
  Adult Female Spermatheca Mated  8.009459  
  Adult Female Spermatheca Virgin  7.853889  
  Adult Head  7.675151  
  Adult Heart  8.177644  
  Adult Hind Gut  7.473141  
  Adult Male Ejaculatory Duct  7.821250  
  Adult Mid Gut  6.707358  
  Adult Ovary  9.382772  
  Adult Salivary Gland  7.859658  
  Adult Testes  8.744158  
  Adult Thoracoabdominal ganglion  8.127797  
  Adult Whole Fly  8.120759  
  Larvae Wandering Tubules  7.899876  
  Larval Feeding Carcass  7.586936  
  Larval Feeding Central Nevous System  9.021078  
  Larval Feeding Hind Gut  7.949249  
  Larval Feeding Malpighian Tubule  8.124253  
  Larval Feeding Mid Gut  6.623035  
  Larval Feeding Salivary Gland  8.024705  
  Whole Larvae Feeding  7.401065  
 
  
   FlyBase ID    symbol    start    end    strand    length   
   FBgn0033686   Hen1   8033231   8040259  +  7029  
   FBgn0027504   CG8878  8033524   8038562   -  5039  
 
    Segment 153 
 
   Location   
  Gene key  FBgn0014184-FBgn0010621  
  Heatmap region span   2R:8050496..8073168   
  Segment span   2R:8061767..8062281   
  Length (genes)  2  
  Length (bp)  515  
   Model Scoring   
  BIC  320.095164  
  logL  -154.526729  
  logL ratio  64.309577  
   Expression   
  Mean expression  11.952600  
  Median expression  12.371602  
  Tissue std. dev.  0.479001  
 
  No GO Slim enrichment  
  
   tissue    mean expression   
  5th Passage Drosophila S2 Cells  11.574113  
  Adult Accessory gland  11.925550  
  Adult Brain  11.443963  
  Adult Carcass  11.566904  
  Adult Crop  12.181935  
  Adult Eye  11.567265  
  Adult Fatbody  11.668071  
  Adult Female Spermatheca Mated  11.955156  
  Adult Female Spermatheca Virgin  11.808941  
  Adult Head  11.613041  
  Adult Heart  11.631487  
  Adult Hind Gut  12.171150  
  Adult Male Ejaculatory Duct  11.709848  
  Adult Mid Gut  12.044970  
  Adult Ovary  12.753615  
  Adult Salivary Gland  11.675957  
  Adult Testes  10.502803  
  Adult Thoracoabdominal ganglion  11.630219  
  Adult Whole Fly  12.587062  
  Larvae Wandering Tubules  12.180709  
  Larval Feeding Carcass  12.362141  
  Larval Feeding Central Nevous System  12.853637  
  Larval Feeding Hind Gut  12.585788  
  Larval Feeding Malpighian Tubule  12.296131  
  Larval Feeding Mid Gut  12.255560  
  Larval Feeding Salivary Gland  12.119438  
  Whole Larvae Feeding  12.054749  
 
  
   FlyBase ID    symbol    start    end    strand    length   
   FBgn0014184   Oda  8054077   8061767   -  7691  
   FBgn0010621   Cct5   8062281   8065039  +  2759  
 
    Segment 154 
 
   Location   
  Gene key  FBgn0033692-FBgn0000426  
  Heatmap region span   2R:8056231..8141497   
  Segment span   2R:8068145..8071778   
  Length (genes)  2  
  Length (bp)  3634  
   Model Scoring   
  BIC  261.825886  
  logL  -125.392090  
  logL ratio  11.836882  
   Expression   
  Mean expression  9.154326  
  Median expression  8.995897  
  Tissue std. dev.  0.695576  
 
  No GO Slim enrichment  
  
   tissue    mean expression   
  5th Passage Drosophila S2 Cells  10.315800  
  Adult Accessory gland  9.470940  
  Adult Brain  8.292284  
  Adult Carcass  8.340407  
  Adult Crop  9.161003  
  Adult Eye  8.657240  
  Adult Fatbody  8.701534  
  Adult Female Spermatheca Mated  9.157989  
  Adult Female Spermatheca Virgin  9.041075  
  Adult Head  8.473190  
  Adult Heart  8.903078  
  Adult Hind Gut  8.725207  
  Adult Male Ejaculatory Duct  9.577440  
  Adult Mid Gut  8.530266  
  Adult Ovary  10.558402  
  Adult Salivary Gland  8.643451  
  Adult Testes  10.888169  
  Adult Thoracoabdominal ganglion  8.278019  
  Adult Whole Fly  9.829083  
  Larvae Wandering Tubules  8.882765  
  Larval Feeding Carcass  9.030069  
  Larval Feeding Central Nevous System  10.099258  
  Larval Feeding Hind Gut  8.964332  
  Larval Feeding Malpighian Tubule  9.063782  
  Larval Feeding Mid Gut  8.654316  
  Larval Feeding Salivary Gland  9.980542  
  Whole Larvae Feeding  8.947170  
 
  
   FlyBase ID    symbol    start    end    strand    length   
   FBgn0033692   wash   8068145   8069878  +  1734  
   FBgn0000426   SmF  8071349   8071778   -  430  
 
    Segment 155 
 
   Location   
  Gene key  FBgn0033696-FBgn0033697  
  Heatmap region span   2R:8066035..8189741   
  Segment span   2R:8077882..8078187   
  Length (genes)  2  
  Length (bp)  306  
   Model Scoring   
  BIC  185.035181  
  logL  -86.996738  
  logL ratio  28.498117  
   Expression   
  Mean expression  5.299029  
  Median expression  5.176970  
  Tissue std. dev.  0.546429  
 
  No GO Slim enrichment  
  
   tissue    mean expression   
  5th Passage Drosophila S2 Cells  5.470496  
  Adult Accessory gland  4.983328  
  Adult Brain  6.322512  
  Adult Carcass  5.480292  
  Adult Crop  5.045264  
  Adult Eye  5.364059  
  Adult Fatbody  5.354039  
  Adult Female Spermatheca Mated  5.186511  
  Adult Female Spermatheca Virgin  5.394139  
  Adult Head  7.655056  
  Adult Heart  5.132320  
  Adult Hind Gut  5.003397  
  Adult Male Ejaculatory Duct  5.128486  
  Adult Mid Gut  5.502416  
  Adult Ovary  5.065911  
  Adult Salivary Gland  5.174730  
  Adult Testes  4.660622  
  Adult Thoracoabdominal ganglion  5.212609  
  Adult Whole Fly  4.833097  
  Larvae Wandering Tubules  5.085943  
  Larval Feeding Carcass  5.265842  
  Larval Feeding Central Nevous System  5.174654  
  Larval Feeding Hind Gut  4.961021  
  Larval Feeding Malpighian Tubule  5.108278  
  Larval Feeding Mid Gut  5.356989  
  Larval Feeding Salivary Gland  5.125953  
  Whole Larvae Feeding  5.025816  
 
  
   FlyBase ID    symbol    start    end    strand    length   
   FBgn0033696   Cyp6g2  8075843   8077882   -  2040  
   FBgn0033697   Cyp6t3   8078187   8079941  +  1755  
 
    Segment 156 
 
   Location   
  Gene key  FBgn0033698-FBgn0033699  
  Heatmap region span   2R:8067392..8193774   
  Segment span   2R:8086343..8088333   
  Length (genes)  2  
  Length (bp)  1991  
   Model Scoring   
  BIC  257.082675  
  logL  -123.020485  
  logL ratio  12.037578  
   Expression   
  Mean expression  8.935463  
  Median expression  8.793376  
  Tissue std. dev.  0.671752  
 
  No GO Slim enrichment  
  
   tissue    mean expression   
  5th Passage Drosophila S2 Cells  10.333106  
  Adult Accessory gland  8.219042  
  Adult Brain  8.789053  
  Adult Carcass  9.377063  
  Adult Crop  8.455886  
  Adult Eye  9.599025  
  Adult Fatbody  9.154187  
  Adult Female Spermatheca Mated  9.129127  
  Adult Female Spermatheca Virgin  9.096780  
  Adult Head  8.808808  
  Adult Heart  9.286925  
  Adult Hind Gut  8.403230  
  Adult Male Ejaculatory Duct  9.206291  
  Adult Mid Gut  8.078106  
  Adult Ovary  9.536331  
  Adult Salivary Gland  8.637497  
  Adult Testes  6.898609  
  Adult Thoracoabdominal ganglion  8.633273  
  Adult Whole Fly  8.742322  
  Larvae Wandering Tubules  9.134060  
  Larval Feeding Carcass  9.306023  
  Larval Feeding Central Nevous System  10.306597  
  Larval Feeding Hind Gut  9.095698  
  Larval Feeding Malpighian Tubule  9.043160  
  Larval Feeding Mid Gut  8.150982  
  Larval Feeding Salivary Gland  9.077020  
  Whole Larvae Feeding  8.759306  
 
  
   FlyBase ID    symbol    start    end    strand    length   
   FBgn0033698   CG8858  8079872   8086343   -  6472  
   FBgn0033699   RpS11  8086524   8088333   -  1810  
 
    Segment 157 
 
   Location   
  Gene key  FBgn0033701-FBgn0033705  
  Heatmap region span   2R:8068145..8194029   
  Segment span   2R:8101509..8141497   
  Length (genes)  5  
  Length (bp)  39989  
   Model Scoring   
  BIC  459.977352  
  logL  -224.467824  
  logL ratio  68.749841  
   Expression   
  Mean expression  4.864347  
  Median expression  4.527579  
  Tissue std. dev.  0.511650  
 
  No GO Slim enrichment  
  
   tissue    mean expression   
  5th Passage Drosophila S2 Cells  4.645161  
  Adult Accessory gland  4.769738  
  Adult Brain  4.500262  
  Adult Carcass  5.124815  
  Adult Crop  5.213744  
  Adult Eye  4.699350  
  Adult Fatbody  4.680767  
  Adult Female Spermatheca Mated  4.588504  
  Adult Female Spermatheca Virgin  4.574006  
  Adult Head  4.781750  
  Adult Heart  4.671128  
  Adult Hind Gut  4.789253  
  Adult Male Ejaculatory Duct  4.639569  
  Adult Mid Gut  4.722709  
  Adult Ovary  4.540886  
  Adult Salivary Gland  4.841188  
  Adult Testes  7.020987  
  Adult Thoracoabdominal ganglion  4.458957  
  Adult Whole Fly  4.982988  
  Larvae Wandering Tubules  4.627460  
  Larval Feeding Carcass  5.140976  
  Larval Feeding Central Nevous System  4.310051  
  Larval Feeding Hind Gut  5.103677  
  Larval Feeding Malpighian Tubule  4.614454  
  Larval Feeding Mid Gut  4.668258  
  Larval Feeding Salivary Gland  5.766725  
  Whole Larvae Feeding  4.860003  
 
  
   FlyBase ID    symbol    start    end    strand    length   
   FBgn0033701   CG13171   8101509   8101900  +  392  
   FBgn0033702   CG8854  8111964   8114351   -  2388  
   FBgn0033703   CG13170   8127914   8128267  +  354  
   FBgn0033704      8128747   8130778  +  2032  
   FBgn0033705   CG13168   8141497   8143197  +  1701  
 
 
    Segment 158 
 
   Location   
  Gene key  FBgn0050203-FBgn0033712  
  Heatmap region span   2R:8146913..8232591   
  Segment span   2R:8198194..8208798   
  Length (genes)  3  
  Length (bp)  10605  
   Model Scoring   
  BIC  306.803070  
  logL  -147.880682  
  logL ratio  21.564208  
   Expression   
  Mean expression  5.514808  
  Median expression  5.339307  
  Tissue std. dev.  0.616639  
 
  No GO Slim enrichment  
  
   tissue    mean expression   
  5th Passage Drosophila S2 Cells  6.609272  
  Adult Accessory gland  4.690260  
  Adult Brain  5.289512  
  Adult Carcass  5.997517  
  Adult Crop  5.012875  
  Adult Eye  6.588944  
  Adult Fatbody  6.471125  
  Adult Female Spermatheca Mated  5.850527  
  Adult Female Spermatheca Virgin  6.205852  
  Adult Head  6.169187  
  Adult Heart  6.687254  
  Adult Hind Gut  4.999060  
  Adult Male Ejaculatory Duct  5.032324  
  Adult Mid Gut  4.956544  
  Adult Ovary  5.968408  
  Adult Salivary Gland  4.884052  
  Adult Testes  5.086399  
  Adult Thoracoabdominal ganglion  5.141636  
  Adult Whole Fly  4.953417  
  Larvae Wandering Tubules  5.790487  
  Larval Feeding Carcass  5.291550  
  Larval Feeding Central Nevous System  5.800938  
  Larval Feeding Hind Gut  5.083508  
  Larval Feeding Malpighian Tubule  5.461340  
  Larval Feeding Mid Gut  4.854322  
  Larval Feeding Salivary Gland  4.991985  
  Whole Larvae Feeding  5.031530  
 
  
   FlyBase ID    symbol    start    end    strand    length   
   FBgn0050203   CG30203   8198194   8201340  +  3147  
   FBgn0050046   CG30046   8201624   8204736  +  3113  
   FBgn0033712   CG13163   8208798   8210017  +  1220  
 
 
    Segment 159 
 
   Location   
  Gene key  FBgn0033713-FBgn0033715  
  Heatmap region span   2R:8189741..8246958   
  Segment span   2R:8213763..8222822   
  Length (genes)  3  
  Length (bp)  9060  
   Model Scoring   
  BIC  359.217991  
  logL  -174.088143  
  logL ratio  11.679049  
   Expression   
  Mean expression  7.625243  
  Median expression  7.884979  
  Tissue std. dev.  0.589145  
 
  No GO Slim enrichment  
  
   tissue    mean expression   
  5th Passage Drosophila S2 Cells  8.906303  
  Adult Accessory gland  8.173565  
  Adult Brain  7.944686  
  Adult Carcass  6.924127  
  Adult Crop  7.241087  
  Adult Eye  7.219539  
  Adult Fatbody  7.490074  
  Adult Female Spermatheca Mated  6.978426  
  Adult Female Spermatheca Virgin  6.848813  
  Adult Head  7.119497  
  Adult Heart  7.387211  
  Adult Hind Gut  7.106932  
  Adult Male Ejaculatory Duct  7.185582  
  Adult Mid Gut  7.031017  
  Adult Ovary  9.018247  
  Adult Salivary Gland  8.148629  
  Adult Testes  7.987751  
  Adult Thoracoabdominal ganglion  7.785979  
  Adult Whole Fly  7.498932  
  Larvae Wandering Tubules  8.015892  
  Larval Feeding Carcass  7.138201  
  Larval Feeding Central Nevous System  8.201684  
  Larval Feeding Hind Gut  7.694570  
  Larval Feeding Malpighian Tubule  8.016319  
  Larval Feeding Mid Gut  7.459857  
  Larval Feeding Salivary Gland  8.406063  
  Whole Larvae Feeding  6.952568  
 
  
   FlyBase ID    symbol    start    end    strand    length   
   FBgn0033713   CG8841  8210012   8213763   -  3752  
   FBgn0033714      8214179   8221324  +  7146  
   FBgn0033715   CG8490   8222822   8223710  +  889  
 
 
    Segment 160 
 
   Location   
  Gene key  FBgn0033716-FBgn0033717  
  Heatmap region span   2R:8194029..8262794   
  Segment span   2R:8225307..8232118   
  Length (genes)  2  
  Length (bp)  6812  
   Model Scoring   
  BIC  285.777712  
  logL  -137.368003  
  logL ratio  6.386680  
   Expression   
  Mean expression  9.966541  
  Median expression  9.703063  
  Tissue std. dev.  0.509219  
 
  No GO Slim enrichment  
  
   tissue    mean expression   
  5th Passage Drosophila S2 Cells  10.094138  
  Adult Accessory gland  9.947817  
  Adult Brain  9.461786  
  Adult Carcass  9.727297  
  Adult Crop  10.270001  
  Adult Eye  9.681795  
  Adult Fatbody  9.688448  
  Adult Female Spermatheca Mated  9.358294  
  Adult Female Spermatheca Virgin  9.444610  
  Adult Head  9.592178  
  Adult Heart  10.057099  
  Adult Hind Gut  10.340269  
  Adult Male Ejaculatory Duct  10.184290  
  Adult Mid Gut  9.835641  
  Adult Ovary  9.118015  
  Adult Salivary Gland  10.543662  
  Adult Testes  10.064656  
  Adult Thoracoabdominal ganglion  9.703063  
  Adult Whole Fly  9.325114  
  Larvae Wandering Tubules  11.409142  
  Larval Feeding Carcass  9.558124  
  Larval Feeding Central Nevous System  10.453684  
  Larval Feeding Hind Gut  10.545794  
  Larval Feeding Malpighian Tubule  10.986330  
  Larval Feeding Mid Gut  9.974072  
  Larval Feeding Salivary Gland  9.830720  
  Whole Larvae Feeding  9.900571  
 
  
   FlyBase ID    symbol    start    end    strand    length   
   FBgn0033716   Den1   8225307   8227050  +  1744  
   FBgn0033717   CG8839  8227115   8232118   -  5004  
 
    Segment 161 
 
   Location   
  Gene key  FBgn0050047-FBgn0050043  
  Heatmap region span   2R:8213763..8274472   
  Segment span   2R:8239298..8246958   
  Length (genes)  3  
  Length (bp)  7661  
   Model Scoring   
  BIC  331.571491  
  logL  -160.264893  
  logL ratio  13.994142  
   Expression   
  Mean expression  5.227665  
  Median expression  4.338861  
  Tissue std. dev.  1.761612  
 
  
   GO ID    description    ratio    P-value   
   GO:0008233   peptidase activity  3/3  1.19e-08  
   GO:0003674   molecular_function  3/3  0.0216  
 
  
   tissue    mean expression   
  5th Passage Drosophila S2 Cells  4.719689  
  Adult Accessory gland  4.342810  
  Adult Brain  3.986238  
  Adult Carcass  4.392475  
  Adult Crop  4.232236  
  Adult Eye  4.022430  
  Adult Fatbody  4.278198  
  Adult Female Spermatheca Mated  4.279436  
  Adult Female Spermatheca Virgin  4.285444  
  Adult Head  4.094907  
  Adult Heart  4.164285  
  Adult Hind Gut  8.347456  
  Adult Male Ejaculatory Duct  4.403332  
  Adult Mid Gut  9.251694  
  Adult Ovary  4.186026  
  Adult Salivary Gland  4.410593  
  Adult Testes  4.129403  
  Adult Thoracoabdominal ganglion  4.190423  
  Adult Whole Fly  5.905735  
  Larvae Wandering Tubules  5.805189  
  Larval Feeding Carcass  4.327573  
  Larval Feeding Central Nevous System  4.235239  
  Larval Feeding Hind Gut  7.408350  
  Larval Feeding Malpighian Tubule  4.875311  
  Larval Feeding Mid Gut  10.021665  
  Larval Feeding Salivary Gland  4.303693  
  Whole Larvae Feeding  8.547120  
 
  
   FlyBase ID    symbol    start    end    strand    length   
   FBgn0050047   CG30047   8239298   8242918  +  3621  
   FBgn0050049   CG30049   8243176   8246592  +  3417  
   FBgn0050043   CG30043   8246958   8250670  +  3713  
 
 
    Segment 162 
 
   Location   
  Gene key  FBgn0053013-FBgn0033720  
  Heatmap region span   2R:8224487..8282447   
  Segment span   2R:8250877..8259101   
  Length (genes)  3  
  Length (bp)  8225  
   Model Scoring   
  BIC  273.099517  
  logL  -131.028906  
  logL ratio  59.580381  
   Expression   
  Mean expression  6.013457  
  Median expression  5.158804  
  Tissue std. dev.  1.878730  
 
  No GO Slim enrichment  
  
   tissue    mean expression   
  5th Passage Drosophila S2 Cells  5.087451  
  Adult Accessory gland  5.213496  
  Adult Brain  4.596079  
  Adult Carcass  5.065759  
  Adult Crop  5.074683  
  Adult Eye  4.640687  
  Adult Fatbody  5.037633  
  Adult Female Spermatheca Mated  5.111010  
  Adult Female Spermatheca Virgin  5.141599  
  Adult Head  4.672114  
  Adult Heart  4.928836  
  Adult Hind Gut  8.576551  
  Adult Male Ejaculatory Duct  5.220392  
  Adult Mid Gut  6.301183  
  Adult Ovary  4.985336  
  Adult Salivary Gland  5.381199  
  Adult Testes  4.686977  
  Adult Thoracoabdominal ganglion  4.851209  
  Adult Whole Fly  5.093574  
  Larvae Wandering Tubules  10.423098  
  Larval Feeding Carcass  5.210688  
  Larval Feeding Central Nevous System  4.688253  
  Larval Feeding Hind Gut  9.808800  
  Larval Feeding Malpighian Tubule  10.230379  
  Larval Feeding Mid Gut  9.295978  
  Larval Feeding Salivary Gland  5.001717  
  Whole Larvae Feeding  8.038673  
 
  
   FlyBase ID    symbol    start    end    strand    length   
   FBgn0053013   CR33013   8250877   8253889  +  3013  
   FBgn0053012   CG33012   8254903   8258338  +  3436  
   FBgn0033720   CG13160   8259101   8262597  +  3497  
 
 
    Segment 163 
 
   Location   
  Gene key  FBgn0050042-FBgn0033723  
  Heatmap region span   2R:8232591..8295341   
  Segment span   2R:8265962..8267931   
  Length (genes)  2  
  Length (bp)  1970  
   Model Scoring   
  BIC  186.550240  
  logL  -87.754267  
  logL ratio  36.306295  
   Expression   
  Mean expression  5.676727  
  Median expression  5.343994  
  Tissue std. dev.  1.463372  
 
  No GO Slim enrichment  
  
   tissue    mean expression   
  5th Passage Drosophila S2 Cells  5.051245  
  Adult Accessory gland  5.195464  
  Adult Brain  4.791934  
  Adult Carcass  11.711073  
  Adult Crop  5.120539  
  Adult Eye  4.917300  
  Adult Fatbody  8.225144  
  Adult Female Spermatheca Mated  5.297192  
  Adult Female Spermatheca Virgin  5.154304  
  Adult Head  5.004521  
  Adult Heart  7.104621  
  Adult Hind Gut  5.014000  
  Adult Male Ejaculatory Duct  5.534354  
  Adult Mid Gut  5.327965  
  Adult Ovary  5.036803  
  Adult Salivary Gland  5.423058  
  Adult Testes  4.919513  
  Adult Thoracoabdominal ganglion  5.276029  
  Adult Whole Fly  7.828712  
  Larvae Wandering Tubules  5.062381  
  Larval Feeding Carcass  6.124707  
  Larval Feeding Central Nevous System  4.738141  
  Larval Feeding Hind Gut  4.929349  
  Larval Feeding Malpighian Tubule  5.085033  
  Larval Feeding Mid Gut  5.163913  
  Larval Feeding Salivary Gland  5.249968  
  Whole Larvae Feeding  4.984356  
 
  
   FlyBase ID    symbol    start    end    strand    length   
   FBgn0050042   Cpr49Ab   8265962   8266901  +  940  
   FBgn0033723   CG13155  8267119   8267931   -  813  
 
    Segment 164 
 
   Location   
  Gene key  FBgn0033724-FBgn0033725  
  Heatmap region span   2R:8239298..8303960   
  Segment span   2R:8271125..8274472   
  Length (genes)  2  
  Length (bp)  3348  
   Model Scoring   
  BIC  226.026900  
  logL  -107.492597  
  logL ratio  6.991881  
   Expression   
  Mean expression  6.161113  
  Median expression  5.320749  
  Tissue std. dev.  1.863942  
 
  No GO Slim enrichment  
  
   tissue    mean expression   
  5th Passage Drosophila S2 Cells  7.374204  
  Adult Accessory gland  4.785993  
  Adult Brain  4.790206  
  Adult Carcass  5.707386  
  Adult Crop  5.053486  
  Adult Eye  4.947743  
  Adult Fatbody  5.138134  
  Adult Female Spermatheca Mated  5.104062  
  Adult Female Spermatheca Virgin  5.269257  
  Adult Head  5.566028  
  Adult Heart  8.456214  
  Adult Hind Gut  5.976966  
  Adult Male Ejaculatory Duct  5.151649  
  Adult Mid Gut  4.989353  
  Adult Ovary  4.882907  
  Adult Salivary Gland  4.777567  
  Adult Testes  4.941121  
  Adult Thoracoabdominal ganglion  4.769630  
  Adult Whole Fly  4.655449  
  Larvae Wandering Tubules  8.261499  
  Larval Feeding Carcass  12.036516  
  Larval Feeding Central Nevous System  5.265168  
  Larval Feeding Hind Gut  8.529827  
  Larval Feeding Malpighian Tubule  6.562775  
  Larval Feeding Mid Gut  5.445771  
  Larval Feeding Salivary Gland  7.581580  
  Whole Larvae Feeding  10.329556  
 
  
   FlyBase ID    symbol    start    end    strand    length   
   FBgn0033724   CG8501   8271125   8272025  +  901  
   FBgn0033725   Cpr49Ac   8274472   8277562  +  3091  
 
    Segment 165 
 
   Location   
  Gene key  FBgn0033726-FBgn0050048  
  Heatmap region span   2R:8250877..8310040   
  Segment span   2R:8279159..8282447   
  Length (genes)  3  
  Length (bp)  3289  
   Model Scoring   
  BIC  276.815260  
  logL  -132.886777  
  logL ratio  55.306478  
   Expression   
  Mean expression  4.316291  
  Median expression  4.135687  
  Tissue std. dev.  0.421009  
 
  No GO Slim enrichment  
  
   tissue    mean expression   
  5th Passage Drosophila S2 Cells  4.169458  
  Adult Accessory gland  4.330953  
  Adult Brain  3.930378  
  Adult Carcass  4.270457  
  Adult Crop  4.111201  
  Adult Eye  4.049891  
  Adult Fatbody  4.278519  
  Adult Female Spermatheca Mated  4.123824  
  Adult Female Spermatheca Virgin  4.190684  
  Adult Head  4.037701  
  Adult Heart  4.049475  
  Adult Hind Gut  4.124686  
  Adult Male Ejaculatory Duct  4.178877  
  Adult Mid Gut  4.289903  
  Adult Ovary  4.082400  
  Adult Salivary Gland  4.486511  
  Adult Testes  5.152312  
  Adult Thoracoabdominal ganglion  4.034512  
  Adult Whole Fly  3.954319  
  Larvae Wandering Tubules  4.384206  
  Larval Feeding Carcass  5.851281  
  Larval Feeding Central Nevous System  4.092856  
  Larval Feeding Hind Gut  4.279844  
  Larval Feeding Malpighian Tubule  4.280125  
  Larval Feeding Mid Gut  4.301167  
  Larval Feeding Salivary Gland  4.275736  
  Whole Larvae Feeding  5.228570  
 
  
   FlyBase ID    symbol    start    end    strand    length   
   FBgn0033726   Cpr49Ad  8278538   8279159   -  622  
   FBgn0033727   Or49a   8280988   8282347  +  1360  
   FBgn0050048   CG30048   8282447   8286611  +  4165  
 
 
    Segment 166 
 
   Location   
  Gene key  FBgn0033729-FBgn0033730  
  Heatmap region span   2R:8265962..8315366   
  Segment span   2R:8293510..8295341   
  Length (genes)  2  
  Length (bp)  1832  
   Model Scoring   
  BIC  224.515300  
  logL  -106.736797  
  logL ratio  9.811850  
   Expression   
  Mean expression  6.193086  
  Median expression  5.228333  
  Tissue std. dev.  2.129215  
 
  No GO Slim enrichment  
  
   tissue    mean expression   
  5th Passage Drosophila S2 Cells  5.016135  
  Adult Accessory gland  5.183628  
  Adult Brain  4.816287  
  Adult Carcass  5.240469  
  Adult Crop  5.256882  
  Adult Eye  11.104783  
  Adult Fatbody  5.145022  
  Adult Female Spermatheca Mated  5.031493  
  Adult Female Spermatheca Virgin  5.002477  
  Adult Head  8.764571  
  Adult Heart  4.869592  
  Adult Hind Gut  5.322366  
  Adult Male Ejaculatory Duct  5.558988  
  Adult Mid Gut  5.588997  
  Adult Ovary  5.070158  
  Adult Salivary Gland  5.381152  
  Adult Testes  4.704332  
  Adult Thoracoabdominal ganglion  4.873809  
  Adult Whole Fly  5.213895  
  Larvae Wandering Tubules  5.342180  
  Larval Feeding Carcass  11.000709  
  Larval Feeding Central Nevous System  4.810980  
  Larval Feeding Hind Gut  10.731034  
  Larval Feeding Malpighian Tubule  5.250424  
  Larval Feeding Mid Gut  6.649576  
  Larval Feeding Salivary Gland  5.262844  
  Whole Larvae Feeding  11.020530  
 
  
   FlyBase ID    symbol    start    end    strand    length   
   FBgn0033729   Cpr49Af   8293510   8293948  +  439  
   FBgn0033730   Cpr49Ag   8295341   8296038  +  698  
 
    Segment 167 
 
   Location   
  Gene key  FBgn0050050-FBgn0033732  
  Heatmap region span   2R:8271125..8324840   
  Segment span   2R:8296459..8303960   
  Length (genes)  3  
  Length (bp)  7502  
   Model Scoring   
  BIC  285.183413  
  logL  -137.070854  
  logL ratio  34.503501  
   Expression   
  Mean expression  4.937958  
  Median expression  4.866374  
  Tissue std. dev.  0.615358  
 
  No GO Slim enrichment  
  
   tissue    mean expression   
  5th Passage Drosophila S2 Cells  4.674163  
  Adult Accessory gland  4.810478  
  Adult Brain  4.504318  
  Adult Carcass  4.788313  
  Adult Crop  4.620072  
  Adult Eye  7.297975  
  Adult Fatbody  4.847928  
  Adult Female Spermatheca Mated  4.797353  
  Adult Female Spermatheca Virgin  4.730946  
  Adult Head  5.891346  
  Adult Heart  4.779179  
  Adult Hind Gut  4.621684  
  Adult Male Ejaculatory Duct  5.136481  
  Adult Mid Gut  4.811317  
  Adult Ovary  4.709819  
  Adult Salivary Gland  4.912343  
  Adult Testes  4.428432  
  Adult Thoracoabdominal ganglion  4.528009  
  Adult Whole Fly  4.458029  
  Larvae Wandering Tubules  4.778091  
  Larval Feeding Carcass  6.281075  
  Larval Feeding Central Nevous System  4.517827  
  Larval Feeding Hind Gut  4.658760  
  Larval Feeding Malpighian Tubule  4.715763  
  Larval Feeding Mid Gut  4.801004  
  Larval Feeding Salivary Gland  4.817847  
  Whole Larvae Feeding  5.406317  
 
  
   FlyBase ID    symbol    start    end    strand    length   
   FBgn0050050   CG30050   8296459   8297218  +  760  
   FBgn0033731   Cpr49Ah   8301467   8302675  +  1209  
   FBgn0033732   CG13157   8303960   8305647  +  1688  
 
 
    Segment 168 
 
   Location   
  Gene key  FBgn0033734-FBgn0033735  
  Heatmap region span   2R:8289720..8356145   
  Segment span   2R:8310490..8312988   
  Length (genes)  2  
  Length (bp)  2499  
   Model Scoring   
  BIC  228.304275  
  logL  -108.631285  
  logL ratio  42.911866  
   Expression   
  Mean expression  9.433880  
  Median expression  9.509467  
  Tissue std. dev.  0.608071  
 
  No GO Slim enrichment  
  
   tissue    mean expression   
  5th Passage Drosophila S2 Cells  9.281637  
  Adult Accessory gland  8.445353  
  Adult Brain  8.234901  
  Adult Carcass  10.303416  
  Adult Crop  9.414155  
  Adult Eye  9.203244  
  Adult Fatbody  8.969879  
  Adult Female Spermatheca Mated  9.166037  
  Adult Female Spermatheca Virgin  9.435282  
  Adult Head  9.504397  
  Adult Heart  9.740254  
  Adult Hind Gut  9.655760  
  Adult Male Ejaculatory Duct  9.511572  
  Adult Mid Gut  9.865773  
  Adult Ovary  9.267250  
  Adult Salivary Gland  9.234574  
  Adult Testes  11.319637  
  Adult Thoracoabdominal ganglion  8.277000  
  Adult Whole Fly  9.730940  
  Larvae Wandering Tubules  9.564069  
  Larval Feeding Carcass  9.482728  
  Larval Feeding Central Nevous System  8.734138  
  Larval Feeding Hind Gut  9.665703  
  Larval Feeding Malpighian Tubule  10.084059  
  Larval Feeding Mid Gut  9.698092  
  Larval Feeding Salivary Gland  9.441128  
  Whole Larvae Feeding  9.483778  
 
  
   FlyBase ID    symbol    start    end    strand    length   
   FBgn0033734   CG8520   8310490   8312964  +  2475  
   FBgn0033735   CG8525   8312988   8314480  +  1493  
 
    Segment 169 
 
   Location   
  Gene key  FBgn0033737-FBgn0033739  
  Heatmap region span   2R:8296459..8359420   
  Segment span   2R:8318600..8324840   
  Length (genes)  4  
  Length (bp)  6241  
   Model Scoring   
  BIC  416.091037  
  logL  -202.524666  
  logL ratio  92.983084  
   Expression   
  Mean expression  8.415590  
  Median expression  8.482427  
  Tissue std. dev.  0.498278  
 
  No GO Slim enrichment  
  
   tissue    mean expression   
  5th Passage Drosophila S2 Cells  8.404770  
  Adult Accessory gland  8.309995  
  Adult Brain  9.157772  
  Adult Carcass  7.540626  
  Adult Crop  8.906658  
  Adult Eye  8.873647  
  Adult Fatbody  7.899092  
  Adult Female Spermatheca Mated  7.998454  
  Adult Female Spermatheca Virgin  7.853488  
  Adult Head  8.217274  
  Adult Heart  8.626239  
  Adult Hind Gut  8.623032  
  Adult Male Ejaculatory Duct  8.396059  
  Adult Mid Gut  7.648294  
  Adult Ovary  8.945491  
  Adult Salivary Gland  8.969903  
  Adult Testes  8.999451  
  Adult Thoracoabdominal ganglion  8.949914  
  Adult Whole Fly  8.186208  
  Larvae Wandering Tubules  8.755994  
  Larval Feeding Carcass  8.063855  
  Larval Feeding Central Nevous System  8.986696  
  Larval Feeding Hind Gut  8.221853  
  Larval Feeding Malpighian Tubule  8.676088  
  Larval Feeding Mid Gut  7.507553  
  Larval Feeding Salivary Gland  8.815017  
  Whole Larvae Feeding  7.687519  
 
  
   FlyBase ID    symbol    start    end    strand    length   
   FBgn0033737   Nup54  8315954   8318600   -  2647  
   FBgn0050051   CG30051   8323602   8324495  +  894  
   FBgn0033738   CG8830  8319407   8324082   -  4676  
   FBgn0033739   Dyb   8324840   8340421  +  15582  
 
 
    Segment 170 
 
   Location   
  Gene key  FBgn0010238-FBgn0033741  
  Heatmap region span   2R:8310490..8374883   
  Segment span   2R:8340696..8356145   
  Length (genes)  2  
  Length (bp)  15450  
   Model Scoring   
  BIC  267.705084  
  logL  -128.331689  
  logL ratio  11.226134  
   Expression   
  Mean expression  9.227805  
  Median expression  9.261444  
  Tissue std. dev.  1.242712  
 
  No GO Slim enrichment  
  
   tissue    mean expression   
  5th Passage Drosophila S2 Cells  10.777348  
  Adult Accessory gland  10.264060  
  Adult Brain  8.698539  
  Adult Carcass  9.230003  
  Adult Crop  10.329489  
  Adult Eye  9.021734  
  Adult Fatbody  8.357397  
  Adult Female Spermatheca Mated  9.416141  
  Adult Female Spermatheca Virgin  9.052325  
  Adult Head  9.504324  
  Adult Heart  8.195973  
  Adult Hind Gut  9.622223  
  Adult Male Ejaculatory Duct  9.701221  
  Adult Mid Gut  7.770223  
  Adult Ovary  10.942652  
  Adult Salivary Gland  9.985881  
  Adult Testes  8.422467  
  Adult Thoracoabdominal ganglion  8.362089  
  Adult Whole Fly  10.042520  
  Larvae Wandering Tubules  6.208635  
  Larval Feeding Carcass  10.156497  
  Larval Feeding Central Nevous System  11.038733  
  Larval Feeding Hind Gut  10.219036  
  Larval Feeding Malpighian Tubule  6.120291  
  Larval Feeding Mid Gut  8.061027  
  Larval Feeding Salivary Gland  10.403028  
  Whole Larvae Feeding  9.246882  
 
  
   FlyBase ID    symbol    start    end    strand    length   
   FBgn0010238   Lac   8340696   8353154  +  12459  
   FBgn0033741   CG8545   8356145   8359227  +  3083  
 
    Segment 171 
 
   Location   
  Gene key  FBgn0033748-FBgn0033749  
  Heatmap region span   2R:8359420..8453692   
  Segment span   2R:8398311..8402556   
  Length (genes)  2  
  Length (bp)  4246  
   Model Scoring   
  BIC  198.783260  
  logL  -93.870778  
  logL ratio  21.013191  
   Expression   
  Mean expression  4.579359  
  Median expression  4.325600  
  Tissue std. dev.  0.719555  
 
  
   GO ID    description    ratio    P-value   
   GO:0005634   nucleus  2/2  0.0218  
   GO:0043226   organelle  2/2  0.0483  
 
  
   tissue    mean expression   
  5th Passage Drosophila S2 Cells  6.216705  
  Adult Accessory gland  5.075906  
  Adult Brain  4.509002  
  Adult Carcass  4.110238  
  Adult Crop  4.267964  
  Adult Eye  4.449679  
  Adult Fatbody  4.360456  
  Adult Female Spermatheca Mated  4.307829  
  Adult Female Spermatheca Virgin  4.315422  
  Adult Head  4.259496  
  Adult Heart  4.234927  
  Adult Hind Gut  4.130116  
  Adult Male Ejaculatory Duct  4.118113  
  Adult Mid Gut  4.193562  
  Adult Ovary  4.515530  
  Adult Salivary Gland  4.351656  
  Adult Testes  7.508833  
  Adult Thoracoabdominal ganglion  4.252619  
  Adult Whole Fly  4.726750  
  Larvae Wandering Tubules  4.235475  
  Larval Feeding Carcass  4.269375  
  Larval Feeding Central Nevous System  5.105906  
  Larval Feeding Hind Gut  4.246349  
  Larval Feeding Malpighian Tubule  4.339865  
  Larval Feeding Mid Gut  4.164122  
  Larval Feeding Salivary Gland  4.448278  
  Whole Larvae Feeding  4.928520  
 
  
   FlyBase ID    symbol    start    end    strand    length   
   FBgn0033748   vis  8395396   8398311   -  2916  
   FBgn0033749   achi  8399035   8402556   -  3522  
 
    Segment 172 
 
   Location   
  Gene key  FBgn0033751-FBgn0033752  
  Heatmap region span   2R:8374883..8457861   
  Segment span   2R:8406423..8406654   
  Length (genes)  2  
  Length (bp)  232  
   Model Scoring   
  BIC  254.295141  
  logL  -121.626718  
  logL ratio  9.645562  
   Expression   
  Mean expression  8.702487  
  Median expression  8.674138  
  Tissue std. dev.  0.310670  
 
  No GO Slim enrichment  
  
   tissue    mean expression   
  5th Passage Drosophila S2 Cells  8.732468  
  Adult Accessory gland  8.952314  
  Adult Brain  9.099039  
  Adult Carcass  8.607337  
  Adult Crop  8.917401  
  Adult Eye  8.841140  
  Adult Fatbody  7.960701  
  Adult Female Spermatheca Mated  8.726336  
  Adult Female Spermatheca Virgin  8.946117  
  Adult Head  8.455400  
  Adult Heart  8.811101  
  Adult Hind Gut  8.896565  
  Adult Male Ejaculatory Duct  8.709869  
  Adult Mid Gut  8.211100  
  Adult Ovary  8.781020  
  Adult Salivary Gland  8.774320  
  Adult Testes  8.373138  
  Adult Thoracoabdominal ganglion  9.031604  
  Adult Whole Fly  8.190637  
  Larvae Wandering Tubules  8.794339  
  Larval Feeding Carcass  8.775360  
  Larval Feeding Central Nevous System  8.925203  
  Larval Feeding Hind Gut  8.793394  
  Larval Feeding Malpighian Tubule  8.933179  
  Larval Feeding Mid Gut  8.308319  
  Larval Feeding Salivary Gland  9.247595  
  Whole Larvae Feeding  8.172156  
 
  
   FlyBase ID    symbol    start    end    strand    length   
   FBgn0033751   CG8818  8404760   8406423   -  1664  
   FBgn0033752   CG8569   8406654   8409131  +  2478  
 
    Segment 173 
 
   Location   
  Gene key  FBgn0050056-FBgn0033753  
  Heatmap region span   2R:8398311..8481088   
  Segment span   2R:8449590..8453692   
  Length (genes)  3  
  Length (bp)  4103  
   Model Scoring   
  BIC  307.660068  
  logL  -148.309181  
  logL ratio  7.550150  
   Expression   
  Mean expression  4.816698  
  Median expression  4.622618  
  Tissue std. dev.  0.563533  
 
  No GO Slim enrichment  
  
   tissue    mean expression   
  5th Passage Drosophila S2 Cells  4.616074  
  Adult Accessory gland  4.513194  
  Adult Brain  4.566093  
  Adult Carcass  4.785353  
  Adult Crop  4.621114  
  Adult Eye  4.558338  
  Adult Fatbody  4.439185  
  Adult Female Spermatheca Mated  4.582742  
  Adult Female Spermatheca Virgin  4.609621  
  Adult Head  4.510236  
  Adult Heart  4.586974  
  Adult Hind Gut  6.493621  
  Adult Male Ejaculatory Duct  4.576493  
  Adult Mid Gut  4.811984  
  Adult Ovary  4.375349  
  Adult Salivary Gland  4.712140  
  Adult Testes  6.397975  
  Adult Thoracoabdominal ganglion  4.419109  
  Adult Whole Fly  5.107746  
  Larvae Wandering Tubules  4.471904  
  Larval Feeding Carcass  4.960349  
  Larval Feeding Central Nevous System  4.474537  
  Larval Feeding Hind Gut  6.098171  
  Larval Feeding Malpighian Tubule  4.428619  
  Larval Feeding Mid Gut  4.716364  
  Larval Feeding Salivary Gland  4.745154  
  Whole Larvae Feeding  4.872411  
 
  
   FlyBase ID    symbol    start    end    strand    length   
   FBgn0050056   CG30056   8449590   8450160  +  571  
   FBgn0053775   CG33775  8451729   8452406   -  678  
   FBgn0033753   Cyp301a1   8453692   8456566  +  2875  
 
 
    Segment 174 
 
   Location   
  Gene key  FBgn0050055-FBgn0022764  
  Heatmap region span   2R:8416561..8513338   
  Segment span   2R:8476808..8480397   
  Length (genes)  2  
  Length (bp)  3590  
   Model Scoring   
  BIC  213.237442  
  logL  -101.097868  
  logL ratio  43.186326  
   Expression   
  Mean expression  7.921158  
  Median expression  7.823059  
  Tissue std. dev.  0.789352  
 
  No GO Slim enrichment  
  
   tissue    mean expression   
  5th Passage Drosophila S2 Cells  8.306897  
  Adult Accessory gland  9.029950  
  Adult Brain  9.167616  
  Adult Carcass  8.021638  
  Adult Crop  8.668251  
  Adult Eye  8.376390  
  Adult Fatbody  8.006401  
  Adult Female Spermatheca Mated  7.561863  
  Adult Female Spermatheca Virgin  7.575730  
  Adult Head  8.178702  
  Adult Heart  8.065796  
  Adult Hind Gut  8.175323  
  Adult Male Ejaculatory Duct  8.084016  
  Adult Mid Gut  7.592078  
  Adult Ovary  9.687206  
  Adult Salivary Gland  8.027522  
  Adult Testes  5.695223  
  Adult Thoracoabdominal ganglion  8.536897  
  Adult Whole Fly  8.252444  
  Larvae Wandering Tubules  7.313778  
  Larval Feeding Carcass  7.384226  
  Larval Feeding Central Nevous System  8.059178  
  Larval Feeding Hind Gut  7.418419  
  Larval Feeding Malpighian Tubule  7.637534  
  Larval Feeding Mid Gut  6.767882  
  Larval Feeding Salivary Gland  7.609212  
  Whole Larvae Feeding  6.671095  
 
  
   FlyBase ID    symbol    start    end    strand    length   
   FBgn0050055   CR30055   8476808   8478387  +  1580  
   FBgn0022764   Sin3A  8462745   8480397   -  17653  
 
    Segment 175 
 
   Location   
  Gene key  FBgn0028683-FBgn0033762  
  Heatmap region span   2R:8500246..8640441   
  Segment span   2R:8521122..8540312   
  Length (genes)  5  
  Length (bp)  19191  
   Model Scoring   
  BIC  611.300371  
  logL  -300.129333  
  logL ratio  34.649031  
   Expression   
  Mean expression  8.901621  
  Median expression  9.093802  
  Tissue std. dev.  0.445622  
 
  No GO Slim enrichment  
  
   tissue    mean expression   
  5th Passage Drosophila S2 Cells  8.740703  
  Adult Accessory gland  8.425632  
  Adult Brain  8.704193  
  Adult Carcass  8.324055  
  Adult Crop  8.276391  
  Adult Eye  8.330148  
  Adult Fatbody  8.199512  
  Adult Female Spermatheca Mated  8.898975  
  Adult Female Spermatheca Virgin  8.845531  
  Adult Head  8.938988  
  Adult Heart  8.241495  
  Adult Hind Gut  8.837927  
  Adult Male Ejaculatory Duct  9.486607  
  Adult Mid Gut  9.450041  
  Adult Ovary  9.303632  
  Adult Salivary Gland  9.790526  
  Adult Testes  8.260817  
  Adult Thoracoabdominal ganglion  8.714232  
  Adult Whole Fly  9.160549  
  Larvae Wandering Tubules  9.209434  
  Larval Feeding Carcass  8.802621  
  Larval Feeding Central Nevous System  9.210502  
  Larval Feeding Hind Gut  9.441861  
  Larval Feeding Malpighian Tubule  9.122204  
  Larval Feeding Mid Gut  9.335266  
  Larval Feeding Salivary Gland  8.894622  
  Whole Larvae Feeding  9.397301  
 
  
   FlyBase ID    symbol    start    end    strand    length   
   FBgn0028683   spt4   8521122   8523546  +  2425  
   FBgn0011604   Iswi   8524123   8528133  +  4011  
   FBgn0033760   CG8785  8528195   8535718   -  7524  
   FBgn0033761   CG8778  8538806   8539987   -  1182  
   FBgn0033762   CG8632   8540312   8543248  +  2937  
 
 
    Segment 176 
 
   Location   
  Gene key  FBgn0033763-FBgn0050053  
  Heatmap region span   2R:8511231..8643915   
  Segment span   2R:8543461..8577381   
  Length (genes)  4  
  Length (bp)  33921  
   Model Scoring   
  BIC  406.058058  
  logL  -197.508176  
  logL ratio  33.573482  
   Expression   
  Mean expression  5.410478  
  Median expression  5.151196  
  Tissue std. dev.  0.429084  
 
  No GO Slim enrichment  
  
   tissue    mean expression   
  5th Passage Drosophila S2 Cells  5.743969  
  Adult Accessory gland  5.433021  
  Adult Brain  6.260874  
  Adult Carcass  5.164740  
  Adult Crop  5.197086  
  Adult Eye  5.569677  
  Adult Fatbody  5.171839  
  Adult Female Spermatheca Mated  5.020555  
  Adult Female Spermatheca Virgin  4.992135  
  Adult Head  5.352781  
  Adult Heart  5.348416  
  Adult Hind Gut  5.250145  
  Adult Male Ejaculatory Duct  5.513933  
  Adult Mid Gut  4.998119  
  Adult Ovary  5.795637  
  Adult Salivary Gland  5.198315  
  Adult Testes  5.487693  
  Adult Thoracoabdominal ganglion  6.384174  
  Adult Whole Fly  5.157662  
  Larvae Wandering Tubules  4.917228  
  Larval Feeding Carcass  5.599096  
  Larval Feeding Central Nevous System  6.609777  
  Larval Feeding Hind Gut  5.387239  
  Larval Feeding Malpighian Tubule  5.070032  
  Larval Feeding Mid Gut  4.956983  
  Larval Feeding Salivary Gland  5.480787  
  Whole Larvae Feeding  5.020992  
 
  
   FlyBase ID    symbol    start    end    strand    length   
   FBgn0033763   CG8646   8543461   8546846  +  3386  
   FBgn0033766   CG8771  8567550   8573670   -  6121  
   FBgn0033767   CG13148   8574114   8576291  +  2178  
   FBgn0050053   CG30053  8576238   8577381   -  1144  
 
 
    Segment 177 
 
   Location   
  Gene key  FBgn0033770-FBgn0033773  
  Heatmap region span   2R:8521122..8694182   
  Segment span   2R:8587586..8640441   
  Length (genes)  2  
  Length (bp)  52856  
   Model Scoring   
  BIC  194.279419  
  logL  -91.618857  
  logL ratio  16.051538  
   Expression   
  Mean expression  4.931588  
  Median expression  4.778918  
  Tissue std. dev.  0.608564  
 
  No GO Slim enrichment  
  
   tissue    mean expression   
  5th Passage Drosophila S2 Cells  4.888447  
  Adult Accessory gland  4.715306  
  Adult Brain  4.389957  
  Adult Carcass  5.089358  
  Adult Crop  4.722255  
  Adult Eye  4.507186  
  Adult Fatbody  4.786098  
  Adult Female Spermatheca Mated  5.025443  
  Adult Female Spermatheca Virgin  5.092258  
  Adult Head  4.350366  
  Adult Heart  4.391099  
  Adult Hind Gut  4.609694  
  Adult Male Ejaculatory Duct  4.760431  
  Adult Mid Gut  4.960939  
  Adult Ovary  7.091907  
  Adult Salivary Gland  4.944265  
  Adult Testes  6.207288  
  Adult Thoracoabdominal ganglion  4.553462  
  Adult Whole Fly  6.222503  
  Larvae Wandering Tubules  4.926832  
  Larval Feeding Carcass  4.791538  
  Larval Feeding Central Nevous System  4.528382  
  Larval Feeding Hind Gut  4.512932  
  Larval Feeding Malpighian Tubule  4.744054  
  Larval Feeding Mid Gut  4.846915  
  Larval Feeding Salivary Gland  4.839582  
  Whole Larvae Feeding  4.654392  
 
  
   FlyBase ID    symbol    start    end    strand    length   
   FBgn0033770   wuc   8587586   8588356  +  771  
   FBgn0033773   mos  8638988   8640441   -  1454  
 
    Segment 178 
 
   Location   
  Gene key  FBgn0026741-FBgn0026619  
  Heatmap region span   2R:8543461..8730063   
  Segment span   2R:8643904..8643915   
  Length (genes)  2  
  Length (bp)  12  
   Model Scoring   
  BIC  214.785924  
  logL  -101.872110  
  logL ratio  47.950502  
   Expression   
  Mean expression  9.075435  
  Median expression  9.130724  
  Tissue std. dev.  0.459384  
 
  No GO Slim enrichment  
  
   tissue    mean expression   
  5th Passage Drosophila S2 Cells  8.718339  
  Adult Accessory gland  8.438627  
  Adult Brain  9.830684  
  Adult Carcass  9.048714  
  Adult Crop  8.587900  
  Adult Eye  9.266033  
  Adult Fatbody  9.383229  
  Adult Female Spermatheca Mated  8.666553  
  Adult Female Spermatheca Virgin  8.803985  
  Adult Head  9.231213  
  Adult Heart  9.251067  
  Adult Hind Gut  9.157207  
  Adult Male Ejaculatory Duct  9.363735  
  Adult Mid Gut  9.811426  
  Adult Ovary  9.710024  
  Adult Salivary Gland  8.134918  
  Adult Testes  9.285250  
  Adult Thoracoabdominal ganglion  9.931302  
  Adult Whole Fly  9.214342  
  Larvae Wandering Tubules  9.115234  
  Larval Feeding Carcass  8.340069  
  Larval Feeding Central Nevous System  8.857957  
  Larval Feeding Hind Gut  8.891118  
  Larval Feeding Malpighian Tubule  9.341482  
  Larval Feeding Mid Gut  9.409319  
  Larval Feeding Salivary Gland  8.629324  
  Whole Larvae Feeding  8.617685  
 
  
   FlyBase ID    symbol    start    end    strand    length   
   FBgn0026741   mRpL18   8643904   8644678  +  775  
   FBgn0026619   Taz  8640531   8643915   -  3385  
 
    Segment 179 
 
   Location   
  Gene key  FBgn0011227-FBgn0086904  
  Heatmap region span   2R:8578460..8730170   
  Segment span   2R:8645028..8648377   
  Length (genes)  3  
  Length (bp)  3350  
   Model Scoring   
  BIC  588.251438  
  logL  -288.604866  
  logL ratio  -17.878196  
   Expression   
  Mean expression  11.620173  
  Median expression  12.315912  
  Tissue std. dev.  0.341799  
 
  No GO Slim enrichment  
  
   tissue    mean expression   
  5th Passage Drosophila S2 Cells  11.379778  
  Adult Accessory gland  11.460761  
  Adult Brain  11.942388  
  Adult Carcass  11.616987  
  Adult Crop  11.844307  
  Adult Eye  12.210550  
  Adult Fatbody  11.481101  
  Adult Female Spermatheca Mated  11.619905  
  Adult Female Spermatheca Virgin  11.570485  
  Adult Head  11.964592  
  Adult Heart  11.669427  
  Adult Hind Gut  11.784069  
  Adult Male Ejaculatory Duct  11.780922  
  Adult Mid Gut  10.933421  
  Adult Ovary  11.406317  
  Adult Salivary Gland  12.046162  
  Adult Testes  11.116554  
  Adult Thoracoabdominal ganglion  11.950996  
  Adult Whole Fly  11.750250  
  Larvae Wandering Tubules  11.591002  
  Larval Feeding Carcass  11.757885  
  Larval Feeding Central Nevous System  11.576448  
  Larval Feeding Hind Gut  11.760927  
  Larval Feeding Malpighian Tubule  11.912003  
  Larval Feeding Mid Gut  10.631354  
  Larval Feeding Salivary Gland  11.773503  
  Whole Larvae Feeding  11.212573  
 
  
   FlyBase ID    symbol    start    end    strand    length   
   FBgn0011227   ox  8644644   8645028   -  385  
   FBgn0020930   Dgkepsilon   8645051   8647226  +  2176  
   FBgn0086904   Nacalpha  8647198   8648377   -  1180  
 
 
    Segment 180 
 
   Location   
  Gene key  FBgn0003326-FBgn0040755  
  Heatmap region span   2R:8587586..8756928   
  Segment span   2R:8668049..8694182   
  Length (genes)  2  
  Length (bp)  26134  
   Model Scoring   
  BIC  199.928205  
  logL  -94.443250  
  logL ratio  15.908377  
   Expression   
  Mean expression  4.740373  
  Median expression  4.526084  
  Tissue std. dev.  0.554131  
 
  No GO Slim enrichment  
  
   tissue    mean expression   
  5th Passage Drosophila S2 Cells  4.407693  
  Adult Accessory gland  4.568919  
  Adult Brain  5.800793  
  Adult Carcass  4.567118  
  Adult Crop  4.479949  
  Adult Eye  4.504046  
  Adult Fatbody  4.834739  
  Adult Female Spermatheca Mated  4.686690  
  Adult Female Spermatheca Virgin  4.769217  
  Adult Head  4.836112  
  Adult Heart  4.457947  
  Adult Hind Gut  4.433248  
  Adult Male Ejaculatory Duct  4.414842  
  Adult Mid Gut  4.338434  
  Adult Ovary  4.181396  
  Adult Salivary Gland  4.723929  
  Adult Testes  6.745951  
  Adult Thoracoabdominal ganglion  5.499756  
  Adult Whole Fly  4.456823  
  Larvae Wandering Tubules  4.363385  
  Larval Feeding Carcass  4.562051  
  Larval Feeding Central Nevous System  5.681680  
  Larval Feeding Hind Gut  4.393489  
  Larval Feeding Malpighian Tubule  4.482015  
  Larval Feeding Mid Gut  4.420057  
  Larval Feeding Salivary Gland  4.513682  
  Whole Larvae Feeding  4.866105  
 
  
   FlyBase ID    symbol    start    end    strand    length   
   FBgn0003326   sca   8668049   8689515  +  21467  
   FBgn0040755   CG17580   8694182   8695316  +  1135  
 
    Segment 181 
 
   Location   
  Gene key  FBgn0250842-FBgn0050486  
  Heatmap region span   2R:8649617..8767328   
  Segment span   2R:8734101..8736481   
  Length (genes)  3  
  Length (bp)  2381  
   Model Scoring   
  BIC  279.360267  
  logL  -134.159281  
  logL ratio  58.457250  
   Expression   
  Mean expression  5.568851  
  Median expression  4.844377  
  Tissue std. dev.  1.702389  
 
  
   GO ID    description    ratio    P-value   
   GO:0005576   extracellular region  3/3  5.68e-05  
 
  
   tissue    mean expression   
  5th Passage Drosophila S2 Cells  5.132861  
  Adult Accessory gland  13.577069  
  Adult Brain  4.836349  
  Adult Carcass  5.681663  
  Adult Crop  4.967841  
  Adult Eye  4.771841  
  Adult Fatbody  5.206745  
  Adult Female Spermatheca Mated  5.098456  
  Adult Female Spermatheca Virgin  5.123841  
  Adult Head  4.883839  
  Adult Heart  5.050501  
  Adult Hind Gut  4.927104  
  Adult Male Ejaculatory Duct  7.396822  
  Adult Mid Gut  5.209001  
  Adult Ovary  5.072596  
  Adult Salivary Gland  5.277296  
  Adult Testes  5.583850  
  Adult Thoracoabdominal ganglion  4.810773  
  Adult Whole Fly  7.503234  
  Larvae Wandering Tubules  5.101987  
  Larval Feeding Carcass  5.059513  
  Larval Feeding Central Nevous System  4.713999  
  Larval Feeding Hind Gut  4.853850  
  Larval Feeding Malpighian Tubule  5.127178  
  Larval Feeding Mid Gut  5.296219  
  Larval Feeding Salivary Gland  5.169886  
  Whole Larvae Feeding  4.924671  
 
  
   FlyBase ID    symbol    start    end    strand    length   
   FBgn0250842   CG17575  8733049   8734101   -  1053  
   FBgn0050488   CG30488  8734353   8735509   -  1157  
   FBgn0050486   CG30486  8735539   8736481   -  943  
 
 
    Segment 182 
 
   Location   
  Gene key  FBgn0010488-FBgn0033781  
  Heatmap region span   2R:8757742..8833850   
  Segment span   2R:8803502..8811409   
  Length (genes)  2  
  Length (bp)  7908  
   Model Scoring   
  BIC  305.240355  
  logL  -147.099325  
  logL ratio  -3.030424  
   Expression   
  Mean expression  10.231400  
  Median expression  10.318307  
  Tissue std. dev.  0.403822  
 
  No GO Slim enrichment  
  
   tissue    mean expression   
  5th Passage Drosophila S2 Cells  10.348602  
  Adult Accessory gland  9.633060  
  Adult Brain  10.087474  
  Adult Carcass  10.179993  
  Adult Crop  10.219641  
  Adult Eye  10.222606  
  Adult Fatbody  10.572225  
  Adult Female Spermatheca Mated  9.912536  
  Adult Female Spermatheca Virgin  9.944413  
  Adult Head  9.844663  
  Adult Heart  10.784692  
  Adult Hind Gut  9.804924  
  Adult Male Ejaculatory Duct  10.286720  
  Adult Mid Gut  9.646979  
  Adult Ovary  10.857045  
  Adult Salivary Gland  9.630579  
  Adult Testes  9.663069  
  Adult Thoracoabdominal ganglion  10.020980  
  Adult Whole Fly  10.200000  
  Larvae Wandering Tubules  11.138705  
  Larval Feeding Carcass  10.719982  
  Larval Feeding Central Nevous System  10.709462  
  Larval Feeding Hind Gut  10.233591  
  Larval Feeding Malpighian Tubule  10.751457  
  Larval Feeding Mid Gut  10.339359  
  Larval Feeding Salivary Gland  10.372958  
  Whole Larvae Feeding  10.122095  
 
  
   FlyBase ID    symbol    start    end    strand    length   
   FBgn0010488   NAT1   8803502   8810745  +  7244  
   FBgn0033781   CG13319   8811409   8812169  +  761  
 
    Segment 183 
 
   Location   
  Gene key  FBgn0033783-FBgn0033784  
  Heatmap region span   2R:8767328..8868494   
  Segment span   2R:8821550..8824032   
  Length (genes)  2  
  Length (bp)  2483  
   Model Scoring   
  BIC  260.645246  
  logL  -124.801771  
  logL ratio  4.778073  
   Expression   
  Mean expression  8.741453  
  Median expression  9.058364  
  Tissue std. dev.  0.394479  
 
  No GO Slim enrichment  
  
   tissue    mean expression   
  5th Passage Drosophila S2 Cells  9.122941  
  Adult Accessory gland  8.393690  
  Adult Brain  9.218418  
  Adult Carcass  8.022252  
  Adult Crop  8.406921  
  Adult Eye  8.538652  
  Adult Fatbody  8.507563  
  Adult Female Spermatheca Mated  8.699832  
  Adult Female Spermatheca Virgin  8.588378  
  Adult Head  8.290347  
  Adult Heart  8.783202  
  Adult Hind Gut  8.271185  
  Adult Male Ejaculatory Duct  8.356780  
  Adult Mid Gut  8.612758  
  Adult Ovary  9.891309  
  Adult Salivary Gland  8.532127  
  Adult Testes  9.298433  
  Adult Thoracoabdominal ganglion  9.079218  
  Adult Whole Fly  8.717398  
  Larvae Wandering Tubules  8.964583  
  Larval Feeding Carcass  8.567406  
  Larval Feeding Central Nevous System  8.940982  
  Larval Feeding Hind Gut  8.771814  
  Larval Feeding Malpighian Tubule  8.677408  
  Larval Feeding Mid Gut  9.336884  
  Larval Feeding Salivary Gland  8.931987  
  Whole Larvae Feeding  8.496752  
 
  
   FlyBase ID    symbol    start    end    strand    length   
   FBgn0033783   CG17019  8818417   8821550   -  3134  
   FBgn0033784   CG13322  8821739   8824032   -  2294  
 
    Segment 184 
 
   Location   
  Gene key  FBgn0033785-FBgn0050487  
  Heatmap region span   2R:8771794..8881402   
  Segment span   2R:8824400..8825848   
  Length (genes)  2  
  Length (bp)  1449  
   Model Scoring   
  BIC  224.782821  
  logL  -106.870558  
  logL ratio  5.230212  
   Expression   
  Mean expression  5.104618  
  Median expression  5.074757  
  Tissue std. dev.  1.013071  
 
  No GO Slim enrichment  
  
   tissue    mean expression   
  5th Passage Drosophila S2 Cells  4.654122  
  Adult Accessory gland  4.627308  
  Adult Brain  4.291200  
  Adult Carcass  4.894951  
  Adult Crop  4.689678  
  Adult Eye  4.776508  
  Adult Fatbody  4.587803  
  Adult Female Spermatheca Mated  5.002724  
  Adult Female Spermatheca Virgin  4.890352  
  Adult Head  4.487423  
  Adult Heart  4.569807  
  Adult Hind Gut  4.832768  
  Adult Male Ejaculatory Duct  4.697076  
  Adult Mid Gut  5.239972  
  Adult Ovary  4.662744  
  Adult Salivary Gland  5.150509  
  Adult Testes  9.626658  
  Adult Thoracoabdominal ganglion  4.471111  
  Adult Whole Fly  6.378966  
  Larvae Wandering Tubules  5.051642  
  Larval Feeding Carcass  5.155512  
  Larval Feeding Central Nevous System  4.395853  
  Larval Feeding Hind Gut  4.959326  
  Larval Feeding Malpighian Tubule  4.982447  
  Larval Feeding Mid Gut  5.887122  
  Larval Feeding Salivary Gland  4.721493  
  Whole Larvae Feeding  6.139606  
 
  
   FlyBase ID    symbol    start    end    strand    length   
   FBgn0033785   Sans   8824400   8827917  +  3518  
   FBgn0050487   CG30487  8825121   8825848   -  728  
 
    Segment 185 
 
   Location   
  Gene key  FBgn0033786-FBgn0033787  
  Heatmap region span   2R:8816368..9003810   
  Segment span   2R:8839791..8841061   
  Length (genes)  2  
  Length (bp)  1271  
   Model Scoring   
  BIC  245.819440  
  logL  -117.388867  
  logL ratio  16.938032  
   Expression   
  Mean expression  7.358541  
  Median expression  6.094364  
  Tissue std. dev.  2.479632  
 
  No GO Slim enrichment  
  
   tissue    mean expression   
  5th Passage Drosophila S2 Cells  7.940611  
  Adult Accessory gland  5.803235  
  Adult Brain  5.095879  
  Adult Carcass  5.671076  
  Adult Crop  5.552482  
  Adult Eye  5.270817  
  Adult Fatbody  5.878483  
  Adult Female Spermatheca Mated  5.858735  
  Adult Female Spermatheca Virgin  5.804487  
  Adult Head  5.305995  
  Adult Heart  6.497578  
  Adult Hind Gut  10.254252  
  Adult Male Ejaculatory Duct  5.871948  
  Adult Mid Gut  12.325461  
  Adult Ovary  6.313050  
  Adult Salivary Gland  5.952408  
  Adult Testes  5.900270  
  Adult Thoracoabdominal ganglion  5.183637  
  Adult Whole Fly  9.032229  
  Larvae Wandering Tubules  11.195299  
  Larval Feeding Carcass  5.766381  
  Larval Feeding Central Nevous System  5.201935  
  Larval Feeding Hind Gut  10.894425  
  Larval Feeding Malpighian Tubule  11.249548  
  Larval Feeding Mid Gut  12.407106  
  Larval Feeding Salivary Gland  5.847682  
  Whole Larvae Feeding  10.605602  
 
  
   FlyBase ID    symbol    start    end    strand    length   
   FBgn0033786     8835853   8839791   -  3939  
   FBgn0033787   CG13321   8841061   8842675  +  1615  
 
    Segment 186 
 
   Location   
  Gene key  FBgn0033788-FBgn0033789  
  Heatmap region span   2R:8833850..9040161   
  Segment span   2R:8934793..8937123   
  Length (genes)  2  
  Length (bp)  2331  
   Model Scoring   
  BIC  220.206706  
  logL  -104.582500  
  logL ratio  30.237786  
   Expression   
  Mean expression  5.907892  
  Median expression  4.400771  
  Tissue std. dev.  2.880028  
 
  No GO Slim enrichment  
  
   tissue    mean expression   
  5th Passage Drosophila S2 Cells  4.106435  
  Adult Accessory gland  4.333699  
  Adult Brain  3.818581  
  Adult Carcass  4.475243  
  Adult Crop  4.369425  
  Adult Eye  4.309574  
  Adult Fatbody  4.770488  
  Adult Female Spermatheca Mated  4.273171  
  Adult Female Spermatheca Virgin  4.338013  
  Adult Head  4.625743  
  Adult Heart  4.137183  
  Adult Hind Gut  4.765237  
  Adult Male Ejaculatory Duct  4.363490  
  Adult Mid Gut  11.928218  
  Adult Ovary  4.073638  
  Adult Salivary Gland  4.348937  
  Adult Testes  5.670533  
  Adult Thoracoabdominal ganglion  4.085987  
  Adult Whole Fly  7.658006  
  Larvae Wandering Tubules  11.073663  
  Larval Feeding Carcass  4.235539  
  Larval Feeding Central Nevous System  3.987610  
  Larval Feeding Hind Gut  6.007165  
  Larval Feeding Malpighian Tubule  12.095135  
  Larval Feeding Mid Gut  12.360347  
  Larval Feeding Salivary Gland  4.162463  
  Whole Larvae Feeding  11.139550  
 
  
   FlyBase ID    symbol    start    end    strand    length   
   FBgn0033788   CG13323  8934238   8934793   -  556  
   FBgn0033789   CG13324  8936647   8937123   -  477  
 
    Segment 187 
 
   Location   
  Gene key  FBgn0033794-FBgn0053007  
  Heatmap region span   2R:8881402..9054111   
  Segment span   2R:9019960..9035785   
  Length (genes)  2  
  Length (bp)  15826  
   Model Scoring   
  BIC  201.542735  
  logL  -95.250515  
  logL ratio  18.068859  
   Expression   
  Mean expression  4.506624  
  Median expression  4.261558  
  Tissue std. dev.  0.623784  
 
  No GO Slim enrichment  
  
   tissue    mean expression   
  5th Passage Drosophila S2 Cells  5.379857  
  Adult Accessory gland  4.289315  
  Adult Brain  5.036224  
  Adult Carcass  4.298658  
  Adult Crop  4.207655  
  Adult Eye  4.413495  
  Adult Fatbody  4.398078  
  Adult Female Spermatheca Mated  4.372213  
  Adult Female Spermatheca Virgin  4.181923  
  Adult Head  4.319375  
  Adult Heart  4.474111  
  Adult Hind Gut  4.035010  
  Adult Male Ejaculatory Duct  4.473735  
  Adult Mid Gut  4.217130  
  Adult Ovary  4.186077  
  Adult Salivary Gland  4.331390  
  Adult Testes  7.336448  
  Adult Thoracoabdominal ganglion  4.631030  
  Adult Whole Fly  4.786249  
  Larvae Wandering Tubules  4.211808  
  Larval Feeding Carcass  4.255399  
  Larval Feeding Central Nevous System  4.609168  
  Larval Feeding Hind Gut  4.081334  
  Larval Feeding Malpighian Tubule  4.187823  
  Larval Feeding Mid Gut  4.260498  
  Larval Feeding Salivary Gland  4.283853  
  Whole Larvae Feeding  4.421000  
 
  
   FlyBase ID    symbol    start    end    strand    length   
   FBgn0033794   CG13326  9017016   9019960   -  2945  
   FBgn0053007     9033718   9035785   -  2068  
 
    Segment 188 
 
   Location   
  Gene key  FBgn0028991-FBgn0033806  
  Heatmap region span   2R:9054111..9122547   
  Segment span   2R:9077579..9094261   
  Length (genes)  3  
  Length (bp)  16683  
   Model Scoring   
  BIC  290.885475  
  logL  -139.921885  
  logL ratio  39.732458  
   Expression   
  Mean expression  5.482323  
  Median expression  5.466024  
  Tissue std. dev.  0.667386  
 
  No GO Slim enrichment  
  
   tissue    mean expression   
  5th Passage Drosophila S2 Cells  5.740408  
  Adult Accessory gland  5.346860  
  Adult Brain  6.775697  
  Adult Carcass  4.804478  
  Adult Crop  5.333107  
  Adult Eye  6.490730  
  Adult Fatbody  4.939514  
  Adult Female Spermatheca Mated  5.143602  
  Adult Female Spermatheca Virgin  5.109915  
  Adult Head  5.132351  
  Adult Heart  5.487116  
  Adult Hind Gut  5.222623  
  Adult Male Ejaculatory Duct  4.835003  
  Adult Mid Gut  5.694542  
  Adult Ovary  5.993127  
  Adult Salivary Gland  4.893680  
  Adult Testes  7.484037  
  Adult Thoracoabdominal ganglion  6.331030  
  Adult Whole Fly  5.189219  
  Larvae Wandering Tubules  5.455249  
  Larval Feeding Carcass  4.823642  
  Larval Feeding Central Nevous System  6.371201  
  Larval Feeding Hind Gut  4.861382  
  Larval Feeding Malpighian Tubule  5.350325  
  Larval Feeding Mid Gut  5.073663  
  Larval Feeding Salivary Gland  5.148925  
  Whole Larvae Feeding  4.991293  
 
  
   FlyBase ID    symbol    start    end    strand    length   
   FBgn0028991   seq  9067187   9077579   -  10393  
   FBgn0053182   Kdm4B  9073242   9085869   -  12628  
   FBgn0033806   FLASH   9094261   9097974  +  3714  
 
 
    Segment 189 
 
   Location   
  Gene key  FBgn0033808-FBgn0033810  
  Heatmap region span   2R:9058906..9123367   
  Segment span   2R:9102238..9107730   
  Length (genes)  3  
  Length (bp)  5493  
   Model Scoring   
  BIC  351.488088  
  logL  -170.223192  
  logL ratio  18.545270  
   Expression   
  Mean expression  7.712059  
  Median expression  8.021439  
  Tissue std. dev.  0.391037  
 
  No GO Slim enrichment  
  
   tissue    mean expression   
  5th Passage Drosophila S2 Cells  8.173522  
  Adult Accessory gland  7.376204  
  Adult Brain  8.145019  
  Adult Carcass  7.728679  
  Adult Crop  8.031789  
  Adult Eye  7.639901  
  Adult Fatbody  7.511823  
  Adult Female Spermatheca Mated  7.433241  
  Adult Female Spermatheca Virgin  7.181701  
  Adult Head  8.038931  
  Adult Heart  7.755944  
  Adult Hind Gut  7.524221  
  Adult Male Ejaculatory Duct  8.797936  
  Adult Mid Gut  7.971443  
  Adult Ovary  7.645852  
  Adult Salivary Gland  7.475577  
  Adult Testes  7.038975  
  Adult Thoracoabdominal ganglion  7.983114  
  Adult Whole Fly  7.193192  
  Larvae Wandering Tubules  7.991479  
  Larval Feeding Carcass  7.505156  
  Larval Feeding Central Nevous System  7.416475  
  Larval Feeding Hind Gut  7.556201  
  Larval Feeding Malpighian Tubule  8.341747  
  Larval Feeding Mid Gut  7.827746  
  Larval Feeding Salivary Gland  7.243709  
  Whole Larvae Feeding  7.696010  
 
  
   FlyBase ID    symbol    start    end    strand    length   
   FBgn0033808   CG4627   9102238   9103265  +  1028  
   FBgn0033809   CG4630  9103159   9106752   -  3594  
   FBgn0033810   CG4646  9106995   9107730   -  736  
 
 
    Segment 190 
 
   Location   
  Gene key  FBgn0040754-FBgn0033812  
  Heatmap region span   2R:9062699..9126972   
  Segment span   2R:9107829..9117733   
  Length (genes)  4  
  Length (bp)  9905  
   Model Scoring   
  BIC  384.804638  
  logL  -186.881466  
  logL ratio  167.984435  
   Expression   
  Mean expression  9.918105  
  Median expression  9.753224  
  Tissue std. dev.  0.367720  
 
  No GO Slim enrichment  
  
   tissue    mean expression   
  5th Passage Drosophila S2 Cells  10.066267  
  Adult Accessory gland  9.996386  
  Adult Brain  10.385644  
  Adult Carcass  9.496209  
  Adult Crop  10.171894  
  Adult Eye  9.925889  
  Adult Fatbody  9.684589  
  Adult Female Spermatheca Mated  9.517200  
  Adult Female Spermatheca Virgin  9.622197  
  Adult Head  9.734521  
  Adult Heart  9.689111  
  Adult Hind Gut  9.790144  
  Adult Male Ejaculatory Duct  9.828565  
  Adult Mid Gut  9.690148  
  Adult Ovary  10.555454  
  Adult Salivary Gland  9.954886  
  Adult Testes  9.163382  
  Adult Thoracoabdominal ganglion  10.202196  
  Adult Whole Fly  9.847338  
  Larvae Wandering Tubules  10.692903  
  Larval Feeding Carcass  9.562570  
  Larval Feeding Central Nevous System  10.315562  
  Larval Feeding Hind Gut  9.749136  
  Larval Feeding Malpighian Tubule  10.649185  
  Larval Feeding Mid Gut  9.673427  
  Larval Feeding Salivary Gland  10.162909  
  Whole Larvae Feeding  9.661127  
 
  
   FlyBase ID    symbol    start    end    strand    length   
   FBgn0040754   CG17059   9107829   9108523  +  695  
   FBgn0011763   Dp   9109902   9115714  +  5813  
   FBgn0043010   Fsn  9108444   9109964   -  1521  
   FBgn0033812   Pex13  9115772   9117733   -  1962  
 
 
    Segment 191 
 
   Location   
  Gene key  FBgn0033818-FBgn0033819  
  Heatmap region span   2R:9123367..9280723   
  Segment span   2R:9139214..9141842   
  Length (genes)  2  
  Length (bp)  2629  
   Model Scoring   
  BIC  174.846665  
  logL  -81.902480  
  logL ratio  43.512768  
   Expression   
  Mean expression  4.840406  
  Median expression  4.458058  
  Tissue std. dev.  1.406690  
 
  No GO Slim enrichment  
  
   tissue    mean expression   
  5th Passage Drosophila S2 Cells  4.511370  
  Adult Accessory gland  4.611636  
  Adult Brain  4.148284  
  Adult Carcass  4.491386  
  Adult Crop  4.459694  
  Adult Eye  4.334110  
  Adult Fatbody  4.707400  
  Adult Female Spermatheca Mated  4.897104  
  Adult Female Spermatheca Virgin  4.787176  
  Adult Head  4.259723  
  Adult Heart  4.489484  
  Adult Hind Gut  4.396158  
  Adult Male Ejaculatory Duct  4.527681  
  Adult Mid Gut  4.471659  
  Adult Ovary  4.407375  
  Adult Salivary Gland  4.610658  
  Adult Testes  11.526266  
  Adult Thoracoabdominal ganglion  4.252731  
  Adult Whole Fly  6.971419  
  Larvae Wandering Tubules  4.448622  
  Larval Feeding Carcass  4.382239  
  Larval Feeding Central Nevous System  4.222563  
  Larval Feeding Hind Gut  4.292638  
  Larval Feeding Malpighian Tubule  4.400061  
  Larval Feeding Mid Gut  4.521769  
  Larval Feeding Salivary Gland  4.486130  
  Whole Larvae Feeding  5.075610  
 
  
   FlyBase ID    symbol    start    end    strand    length   
   FBgn0033818   CG4712  9136399   9139214   -  2816  
   FBgn0033819   CG4714  9139351   9141842   -  2492  
 
    Segment 192 
 
   Location   
  Gene key  FBgn0033820-FBgn0033821  
  Heatmap region span   2R:9126972..9281210   
  Segment span   2R:9144088..9145391   
  Length (genes)  2  
  Length (bp)  1304  
   Model Scoring   
  BIC  271.750808  
  logL  -130.354551  
  logL ratio  34.966624  
   Expression   
  Mean expression  8.773533  
  Median expression  8.114915  
  Tissue std. dev.  3.175433  
 
  No GO Slim enrichment  
  
   tissue    mean expression   
  5th Passage Drosophila S2 Cells  4.649531  
  Adult Accessory gland  5.027029  
  Adult Brain  8.876552  
  Adult Carcass  12.838295  
  Adult Crop  7.846596  
  Adult Eye  12.813201  
  Adult Fatbody  13.481840  
  Adult Female Spermatheca Mated  13.162751  
  Adult Female Spermatheca Virgin  13.292385  
  Adult Head  13.073916  
  Adult Heart  13.145782  
  Adult Hind Gut  9.898411  
  Adult Male Ejaculatory Duct  10.894380  
  Adult Mid Gut  8.101503  
  Adult Ovary  4.823488  
  Adult Salivary Gland  5.518827  
  Adult Testes  5.655888  
  Adult Thoracoabdominal ganglion  8.010187  
  Adult Whole Fly  11.381901  
  Larvae Wandering Tubules  5.155848  
  Larval Feeding Carcass  5.709302  
  Larval Feeding Central Nevous System  4.564706  
  Larval Feeding Hind Gut  7.505509  
  Larval Feeding Malpighian Tubule  5.801659  
  Larval Feeding Mid Gut  8.265031  
  Larval Feeding Salivary Gland  6.744274  
  Whole Larvae Feeding  10.646611  
 
  
   FlyBase ID    symbol    start    end    strand    length   
   FBgn0033820   CG4716   9144088   9144951  +  864  
   FBgn0033821   CG10799   9145391   9145997  +  607  
 
    Segment 193 
 
   Location   
  Gene key  FBgn0033827-FBgn0033832  
  Heatmap region span   2R:9139214..9297458   
  Segment span   2R:9187107..9280723   
  Length (genes)  5  
  Length (bp)  93617  
   Model Scoring   
  BIC  449.445752  
  logL  -219.202023  
  logL ratio  82.512468  
   Expression   
  Mean expression  4.893790  
  Median expression  4.542933  
  Tissue std. dev.  0.455061  
 
  No GO Slim enrichment  
  
   tissue    mean expression   
  5th Passage Drosophila S2 Cells  4.787315  
  Adult Accessory gland  4.793828  
  Adult Brain  4.358623  
  Adult Carcass  4.740613  
  Adult Crop  4.614681  
  Adult Eye  4.759235  
  Adult Fatbody  4.766096  
  Adult Female Spermatheca Mated  4.833366  
  Adult Female Spermatheca Virgin  5.398074  
  Adult Head  4.417775  
  Adult Heart  4.979162  
  Adult Hind Gut  4.575518  
  Adult Male Ejaculatory Duct  4.886679  
  Adult Mid Gut  5.059887  
  Adult Ovary  4.586246  
  Adult Salivary Gland  4.876880  
  Adult Testes  6.726359  
  Adult Thoracoabdominal ganglion  4.533453  
  Adult Whole Fly  4.573704  
  Larvae Wandering Tubules  5.286260  
  Larval Feeding Carcass  4.704302  
  Larval Feeding Central Nevous System  4.406490  
  Larval Feeding Hind Gut  5.361377  
  Larval Feeding Malpighian Tubule  5.303437  
  Larval Feeding Mid Gut  5.001581  
  Larval Feeding Salivary Gland  4.811628  
  Whole Larvae Feeding  4.989769  
 
  
   FlyBase ID    symbol    start    end    strand    length   
   FBgn0033827   CG17047   9187107   9189045  +  1939  
   FBgn0033828   CG17048  9241231   9241973   -  743  
   FBgn0033830   CG10814  9263476   9265219   -  1744  
   FBgn0033831     9277358   9279139   -  1782  
   FBgn0033832     9280035   9280723   -  689  
 
 
    Segment 194 
 
   Location   
  Gene key  FBgn0050060-FBgn0050065  
  Heatmap region span   2R:9281210..9372901   
  Segment span   2R:9311152..9320600   
  Length (genes)  2  
  Length (bp)  9449  
   Model Scoring   
  BIC  180.996332  
  logL  -84.977313  
  logL ratio  43.670218  
   Expression   
  Mean expression  4.536401  
  Median expression  4.268113  
  Tissue std. dev.  1.240103  
 
  No GO Slim enrichment  
  
   tissue    mean expression   
  5th Passage Drosophila S2 Cells  4.208745  
  Adult Accessory gland  4.595508  
  Adult Brain  4.070545  
  Adult Carcass  4.089996  
  Adult Crop  4.268795  
  Adult Eye  4.321425  
  Adult Fatbody  4.125710  
  Adult Female Spermatheca Mated  4.176557  
  Adult Female Spermatheca Virgin  4.140094  
  Adult Head  4.164983  
  Adult Heart  4.209433  
  Adult Hind Gut  4.151425  
  Adult Male Ejaculatory Duct  4.311055  
  Adult Mid Gut  4.294103  
  Adult Ovary  4.234738  
  Adult Salivary Gland  4.227472  
  Adult Testes  10.508802  
  Adult Thoracoabdominal ganglion  4.024214  
  Adult Whole Fly  6.004078  
  Larvae Wandering Tubules  4.197073  
  Larval Feeding Carcass  4.179825  
  Larval Feeding Central Nevous System  4.031404  
  Larval Feeding Hind Gut  4.070275  
  Larval Feeding Malpighian Tubule  4.235387  
  Larval Feeding Mid Gut  4.173015  
  Larval Feeding Salivary Gland  4.172161  
  Whole Larvae Feeding  5.296016  
 
  
   FlyBase ID    symbol    start    end    strand    length   
   FBgn0050060   CG30060  9310483   9311152   -  670  
   FBgn0050065   CG30065   9320600   9321265  +  666  
 
    Segment 195 
 
   Location   
  Gene key  FBgn0013765-FBgn0033842  
  Heatmap region span   2R:9311152..9395063   
  Segment span   2R:9337990..9372901   
  Length (genes)  4  
  Length (bp)  34912  
   Model Scoring   
  BIC  399.197248  
  logL  -194.077771  
  logL ratio  101.084278  
   Expression   
  Mean expression  7.862866  
  Median expression  7.839455  
  Tissue std. dev.  0.589766  
 
  No GO Slim enrichment  
  
   tissue    mean expression   
  5th Passage Drosophila S2 Cells  8.318900  
  Adult Accessory gland  7.072753  
  Adult Brain  8.862492  
  Adult Carcass  7.165485  
  Adult Crop  8.113950  
  Adult Eye  8.179012  
  Adult Fatbody  7.695913  
  Adult Female Spermatheca Mated  7.990270  
  Adult Female Spermatheca Virgin  8.043925  
  Adult Head  7.782356  
  Adult Heart  7.975180  
  Adult Hind Gut  7.727206  
  Adult Male Ejaculatory Duct  7.435070  
  Adult Mid Gut  6.993651  
  Adult Ovary  9.239941  
  Adult Salivary Gland  8.081704  
  Adult Testes  7.317828  
  Adult Thoracoabdominal ganglion  8.668874  
  Adult Whole Fly  7.704285  
  Larvae Wandering Tubules  7.662409  
  Larval Feeding Carcass  7.727026  
  Larval Feeding Central Nevous System  8.976619  
  Larval Feeding Hind Gut  7.470928  
  Larval Feeding Malpighian Tubule  7.419188  
  Larval Feeding Mid Gut  6.949695  
  Larval Feeding Salivary Gland  8.339884  
  Whole Larvae Feeding  7.382848  
 
  
   FlyBase ID    symbol    start    end    strand    length   
   FBgn0013765   cnn  9326819   9337990   -  11172  
   FBgn0086757   cbs   9338169   9340898  +  2730  
   FBgn0000119   arr  9341159   9370723   -  29565  
   FBgn0033842   cbc  9371017   9372901   -  1885  
 
 
    Segment 196 
 
   Location   
  Gene key  FBgn0004638-FBgn0024556  
  Heatmap region span   2R:9337990..9441587   
  Segment span   2R:9390342..9395063   
  Length (genes)  3  
  Length (bp)  4722  
   Model Scoring   
  BIC  429.930580  
  logL  -209.444437  
  logL ratio  -24.571777  
   Expression   
  Mean expression  9.099825  
  Median expression  9.628762  
  Tissue std. dev.  0.587846  
 
  No GO Slim enrichment  
  
   tissue    mean expression   
  5th Passage Drosophila S2 Cells  9.677032  
  Adult Accessory gland  9.473298  
  Adult Brain  9.652192  
  Adult Carcass  8.606549  
  Adult Crop  9.257666  
  Adult Eye  8.698688  
  Adult Fatbody  8.702248  
  Adult Female Spermatheca Mated  9.741825  
  Adult Female Spermatheca Virgin  9.641501  
  Adult Head  8.761215  
  Adult Heart  9.045600  
  Adult Hind Gut  9.064911  
  Adult Male Ejaculatory Duct  8.938938  
  Adult Mid Gut  8.298710  
  Adult Ovary  9.629013  
  Adult Salivary Gland  9.385819  
  Adult Testes  7.458373  
  Adult Thoracoabdominal ganglion  9.432342  
  Adult Whole Fly  8.887010  
  Larvae Wandering Tubules  8.593949  
  Larval Feeding Carcass  9.036794  
  Larval Feeding Central Nevous System  10.427804  
  Larval Feeding Hind Gut  9.337721  
  Larval Feeding Malpighian Tubule  8.979547  
  Larval Feeding Mid Gut  8.490023  
  Larval Feeding Salivary Gland  9.853262  
  Whole Larvae Feeding  8.623257  
 
  
   FlyBase ID    symbol    start    end    strand    length   
   FBgn0004638   drk  9382727   9390342   -  7616  
   FBgn0033846   mip120   9393947   9400617  +  6671  
   FBgn0024556   EfTuM   9395063   9396837  +  1775  
 
 
    Segment 197 
 
   Location   
  Gene key  FBgn0033848-FBgn0033851  
  Heatmap region span   2R:9373106..9446050   
  Segment span   2R:9402764..9412882   
  Length (genes)  3  
  Length (bp)  10119  
   Model Scoring   
  BIC  276.033465  
  logL  -132.495880  
  logL ratio  40.621682  
   Expression   
  Mean expression  4.987230  
  Median expression  4.734294  
  Tissue std. dev.  0.407486  
 
  No GO Slim enrichment  
  
   tissue    mean expression   
  5th Passage Drosophila S2 Cells  4.963067  
  Adult Accessory gland  5.071757  
  Adult Brain  4.777071  
  Adult Carcass  4.881055  
  Adult Crop  4.837803  
  Adult Eye  4.926920  
  Adult Fatbody  4.913532  
  Adult Female Spermatheca Mated  4.830935  
  Adult Female Spermatheca Virgin  4.874303  
  Adult Head  4.784000  
  Adult Heart  4.778318  
  Adult Hind Gut  4.903462  
  Adult Male Ejaculatory Duct  4.947217  
  Adult Mid Gut  4.892074  
  Adult Ovary  4.956948  
  Adult Salivary Gland  5.124602  
  Adult Testes  6.952227  
  Adult Thoracoabdominal ganglion  4.808891  
  Adult Whole Fly  5.341959  
  Larvae Wandering Tubules  4.864388  
  Larval Feeding Carcass  4.908398  
  Larval Feeding Central Nevous System  4.745360  
  Larval Feeding Hind Gut  4.710255  
  Larval Feeding Malpighian Tubule  4.982537  
  Larval Feeding Mid Gut  5.008214  
  Larval Feeding Salivary Gland  5.089499  
  Whole Larvae Feeding  4.780413  
 
  
   FlyBase ID    symbol    start    end    strand    length   
   FBgn0033848   CG13330   9402764   9404951  +  2188  
   FBgn0033850   CG13331   9409269   9410806  +  1538  
   FBgn0033851   CG13332   9412882   9413953  +  1072  
 
 
    Segment 198 
 
   Location   
  Gene key  FBgn0033855-FBgn0033856  
  Heatmap region span   2R:9402764..9496683   
  Segment span   2R:9443398..9446050   
  Length (genes)  2  
  Length (bp)  2653  
   Model Scoring   
  BIC  179.169104  
  logL  -84.063699  
  logL ratio  33.733517  
   Expression   
  Mean expression  4.803608  
  Median expression  4.934553  
  Tissue std. dev.  0.249693  
 
  No GO Slim enrichment  
  
   tissue    mean expression   
  5th Passage Drosophila S2 Cells  4.854771  
  Adult Accessory gland  5.008361  
  Adult Brain  4.537445  
  Adult Carcass  4.815460  
  Adult Crop  4.804148  
  Adult Eye  4.620792  
  Adult Fatbody  4.936956  
  Adult Female Spermatheca Mated  4.754817  
  Adult Female Spermatheca Virgin  4.810940  
  Adult Head  4.627375  
  Adult Heart  4.622300  
  Adult Hind Gut  4.724855  
  Adult Male Ejaculatory Duct  4.924015  
  Adult Mid Gut  4.685104  
  Adult Ovary  4.844775  
  Adult Salivary Gland  5.238117  
  Adult Testes  5.808995  
  Adult Thoracoabdominal ganglion  4.629072  
  Adult Whole Fly  4.702723  
  Larvae Wandering Tubules  4.763758  
  Larval Feeding Carcass  4.808174  
  Larval Feeding Central Nevous System  4.496645  
  Larval Feeding Hind Gut  4.649081  
  Larval Feeding Malpighian Tubule  4.707335  
  Larval Feeding Mid Gut  4.800411  
  Larval Feeding Salivary Gland  4.903460  
  Whole Larvae Feeding  4.617525  
 
  
   FlyBase ID    symbol    start    end    strand    length   
   FBgn0033855   link   9443398   9444847  +  1450  
   FBgn0033856   CG13334   9446050   9447610  +  1561  
 
    Segment 199 
 
   Location   
  Gene key  FBgn0027581-FBgn0033859  
  Heatmap region span   2R:9435082..9510593   
  Segment span   2R:9480841..9481154   
  Length (genes)  2  
  Length (bp)  314  
   Model Scoring   
  BIC  254.333718  
  logL  -121.646006  
  logL ratio  12.189556  
   Expression   
  Mean expression  8.734236  
  Median expression  8.616159  
  Tissue std. dev.  0.654304  
 
  No GO Slim enrichment  
  
   tissue    mean expression   
  5th Passage Drosophila S2 Cells  8.786030  
  Adult Accessory gland  9.110211  
  Adult Brain  9.655756  
  Adult Carcass  8.289513  
  Adult Crop  8.631665  
  Adult Eye  9.870126  
  Adult Fatbody  8.404464  
  Adult Female Spermatheca Mated  8.372723  
  Adult Female Spermatheca Virgin  8.403993  
  Adult Head  8.591323  
  Adult Heart  9.107541  
  Adult Hind Gut  8.308891  
  Adult Male Ejaculatory Duct  8.564334  
  Adult Mid Gut  8.474328  
  Adult Ovary  8.905075  
  Adult Salivary Gland  8.409644  
  Adult Testes  6.972025  
  Adult Thoracoabdominal ganglion  9.392643  
  Adult Whole Fly  8.131239  
  Larvae Wandering Tubules  10.239449  
  Larval Feeding Carcass  8.512448  
  Larval Feeding Central Nevous System  9.045890  
  Larval Feeding Hind Gut  8.783275  
  Larval Feeding Malpighian Tubule  9.842147  
  Larval Feeding Mid Gut  8.281932  
  Larval Feeding Salivary Gland  8.624961  
  Whole Larvae Feeding  8.112733  
 
  
   FlyBase ID    symbol    start    end    strand    length   
   FBgn0027581   CG6191  9468091   9480841   -  12751  
   FBgn0033859   CG6197   9481154   9484068  +  2915  
 
    Segment 200 
 
   Location   
  Gene key  FBgn0033862-FBgn0033867  
  Heatmap region span   2R:9484182..9713880   
  Segment span   2R:9551103..9657420   
  Length (genes)  6  
  Length (bp)  106318  
   Model Scoring   
  BIC  497.226090  
  logL  -243.092192  
  logL ratio  149.616609  
   Expression   
  Mean expression  4.653252  
  Median expression  4.506475  
  Tissue std. dev.  0.714847  
 
  No GO Slim enrichment  
  
   tissue    mean expression   
  5th Passage Drosophila S2 Cells  4.412664  
  Adult Accessory gland  5.219376  
  Adult Brain  4.275959  
  Adult Carcass  4.589530  
  Adult Crop  4.524975  
  Adult Eye  4.319864  
  Adult Fatbody  4.446960  
  Adult Female Spermatheca Mated  4.824118  
  Adult Female Spermatheca Virgin  4.813078  
  Adult Head  4.272521  
  Adult Heart  4.365879  
  Adult Hind Gut  4.574714  
  Adult Male Ejaculatory Duct  4.590385  
  Adult Mid Gut  4.554867  
  Adult Ovary  4.404452  
  Adult Salivary Gland  4.758797  
  Adult Testes  8.090553  
  Adult Thoracoabdominal ganglion  4.331784  
  Adult Whole Fly  5.080161  
  Larvae Wandering Tubules  4.444951  
  Larval Feeding Carcass  4.439476  
  Larval Feeding Central Nevous System  4.180144  
  Larval Feeding Hind Gut  4.240250  
  Larval Feeding Malpighian Tubule  4.388757  
  Larval Feeding Mid Gut  4.482167  
  Larval Feeding Salivary Gland  4.516160  
  Whole Larvae Feeding  4.495262  
 
  
   FlyBase ID    symbol    start    end    strand    length   
   FBgn0033862   CG6209  9549000   9551103   -  2104  
   FBgn0033863   CG13337   9552619   9555184  +  2566  
   FBgn0033864   CG18368   9555241   9558824  +  3584  
   FBgn0033865   CG6220  9579305   9580874   -  1570  
   FBgn0033866   CG6280   9642849   9646362  +  3514  
   FBgn0033867   Cpr50Ca   9657420   9675310  +  17891  
 
 
    Segment 201 
 
   Location   
  Gene key  FBgn0033882-FBgn0033883  
  Heatmap region span   2R:9734358..9867670   
  Segment span   2R:9746462..9749507   
  Length (genes)  2  
  Length (bp)  3046  
   Model Scoring   
  BIC  279.649341  
  logL  -134.303818  
  logL ratio  -4.875323  
   Expression   
  Mean expression  9.249275  
  Median expression  9.286189  
  Tissue std. dev.  0.376403  
 
  No GO Slim enrichment  
  
   tissue    mean expression   
  5th Passage Drosophila S2 Cells  9.035680  
  Adult Accessory gland  8.586381  
  Adult Brain  8.947247  
  Adult Carcass  9.271092  
  Adult Crop  9.338468  
  Adult Eye  9.712335  
  Adult Fatbody  9.855457  
  Adult Female Spermatheca Mated  9.395903  
  Adult Female Spermatheca Virgin  9.570190  
  Adult Head  9.097420  
  Adult Heart  9.984038  
  Adult Hind Gut  9.325852  
  Adult Male Ejaculatory Duct  9.096825  
  Adult Mid Gut  8.768696  
  Adult Ovary  9.425800  
  Adult Salivary Gland  8.859220  
  Adult Testes  9.345340  
  Adult Thoracoabdominal ganglion  9.081444  
  Adult Whole Fly  8.920924  
  Larvae Wandering Tubules  9.749104  
  Larval Feeding Carcass  9.072567  
  Larval Feeding Central Nevous System  9.078173  
  Larval Feeding Hind Gut  9.295174  
  Larval Feeding Malpighian Tubule  10.114304  
  Larval Feeding Mid Gut  8.812499  
  Larval Feeding Salivary Gland  9.027674  
  Whole Larvae Feeding  8.962616  
 
  
   FlyBase ID    symbol    start    end    strand    length   
   FBgn0033882      9746462   9747937  +  1476  
   FBgn0033883   CG16935  9747854   9749507   -  1654  
 
    Segment 202 
 
   Location   
  Gene key  FBgn0013733-FBgn0013770  
  Heatmap region span   2R:9743270..9978536   
  Segment span   2R:9829615..9848164   
  Length (genes)  4  
  Length (bp)  18550  
   Model Scoring   
  BIC  590.452530  
  logL  -289.705413  
  logL ratio  85.382977  
   Expression   
  Mean expression  11.091048  
  Median expression  11.062912  
  Tissue std. dev.  0.384371  
 
  No GO Slim enrichment  
  
   tissue    mean expression   
  5th Passage Drosophila S2 Cells  10.928141  
  Adult Accessory gland  10.774677  
  Adult Brain  11.064617  
  Adult Carcass  11.419071  
  Adult Crop  11.335127  
  Adult Eye  11.309825  
  Adult Fatbody  11.309601  
  Adult Female Spermatheca Mated  10.875618  
  Adult Female Spermatheca Virgin  10.817434  
  Adult Head  11.013198  
  Adult Heart  11.781887  
  Adult Hind Gut  11.125812  
  Adult Male Ejaculatory Duct  11.260333  
  Adult Mid Gut  10.986873  
  Adult Ovary  11.217519  
  Adult Salivary Gland  10.765835  
  Adult Testes  9.743500  
  Adult Thoracoabdominal ganglion  10.828588  
  Adult Whole Fly  11.020206  
  Larvae Wandering Tubules  11.771863  
  Larval Feeding Carcass  11.489896  
  Larval Feeding Central Nevous System  11.203048  
  Larval Feeding Hind Gut  11.423888  
  Larval Feeding Malpighian Tubule  11.282851  
  Larval Feeding Mid Gut  11.091139  
  Larval Feeding Salivary Gland  10.814548  
  Whole Larvae Feeding  10.803188  
 
  
   FlyBase ID    symbol    start    end    strand    length   
   FBgn0013733   shot  9751742   9829615   -  77874  
   FBgn0026611     9834047   9845594   -  11548  
   FBgn0033886   CG13349  9846033   9847909   -  1877  
   FBgn0013770   Cp1   9848164   9855486  +  7323  
 
 
    Segment 203 
 
   Location   
  Gene key  FBgn0033887-FBgn0033888  
  Heatmap region span   2R:9746222..10048754   
  Segment span   2R:9858662..9860336   
  Length (genes)  2  
  Length (bp)  1675  
   Model Scoring   
  BIC  200.401738  
  logL  -94.680016  
  logL ratio  20.827032  
   Expression   
  Mean expression  4.840081  
  Median expression  4.385426  
  Tissue std. dev.  1.182376  
 
  No GO Slim enrichment  
  
   tissue    mean expression   
  5th Passage Drosophila S2 Cells  4.350303  
  Adult Accessory gland  4.630880  
  Adult Brain  4.107729  
  Adult Carcass  4.508403  
  Adult Crop  4.397149  
  Adult Eye  4.200445  
  Adult Fatbody  4.356256  
  Adult Female Spermatheca Mated  4.734369  
  Adult Female Spermatheca Virgin  4.681748  
  Adult Head  4.189255  
  Adult Heart  4.357591  
  Adult Hind Gut  4.369863  
  Adult Male Ejaculatory Duct  4.674010  
  Adult Mid Gut  4.332908  
  Adult Ovary  6.196366  
  Adult Salivary Gland  4.584470  
  Adult Testes  9.613910  
  Adult Thoracoabdominal ganglion  4.070875  
  Adult Whole Fly  7.629314  
  Larvae Wandering Tubules  4.426549  
  Larval Feeding Carcass  5.243382  
  Larval Feeding Central Nevous System  4.149635  
  Larval Feeding Hind Gut  4.395287  
  Larval Feeding Malpighian Tubule  4.392873  
  Larval Feeding Mid Gut  4.399850  
  Larval Feeding Salivary Gland  4.450301  
  Whole Larvae Feeding  5.238454  
 
  
   FlyBase ID    symbol    start    end    strand    length   
   FBgn0033887   St4  9857415   9858662   -  1248  
   FBgn0033888   CG18568  9859106   9860336   -  1231  
 
    Segment 204 
 
   Location   
  Gene key  FBgn0033890-FBgn0002643  
  Heatmap region span   2R:9749517..10056361   
  Segment span   2R:9870979..9878686   
  Length (genes)  3  
  Length (bp)  7708  
   Model Scoring   
  BIC  337.385390  
  logL  -163.171842  
  logL ratio  33.224997  
   Expression   
  Mean expression  7.223590  
  Median expression  7.075908  
  Tissue std. dev.  0.413379  
 
  No GO Slim enrichment  
  
   tissue    mean expression   
  5th Passage Drosophila S2 Cells  7.475946  
  Adult Accessory gland  6.779228  
  Adult Brain  7.476705  
  Adult Carcass  7.099566  
  Adult Crop  7.123473  
  Adult Eye  7.170626  
  Adult Fatbody  7.212657  
  Adult Female Spermatheca Mated  7.248972  
  Adult Female Spermatheca Virgin  7.486031  
  Adult Head  7.095920  
  Adult Heart  7.116936  
  Adult Hind Gut  6.868757  
  Adult Male Ejaculatory Duct  7.095013  
  Adult Mid Gut  6.626755  
  Adult Ovary  8.290394  
  Adult Salivary Gland  7.072077  
  Adult Testes  7.029481  
  Adult Thoracoabdominal ganglion  7.906680  
  Adult Whole Fly  7.262490  
  Larvae Wandering Tubules  6.988034  
  Larval Feeding Carcass  7.292204  
  Larval Feeding Central Nevous System  8.296205  
  Larval Feeding Hind Gut  7.077407  
  Larval Feeding Malpighian Tubule  7.045397  
  Larval Feeding Mid Gut  6.520206  
  Larval Feeding Salivary Gland  7.533946  
  Whole Larvae Feeding  6.845826  
 
  
   FlyBase ID    symbol    start    end    strand    length   
   FBgn0033890   Ctf4  9867867   9870979   -  3113  
   FBgn0033891   CG8067   9871157   9872398  +  1242  
   FBgn0002643   mam   9878686   9947861  +  69176  
 
 
    Segment 205 
 
   Location   
  Gene key  FBgn0033893-FBgn0259184  
  Heatmap region span   2R:9829615..10057971   
  Segment span   2R:9916123..9978536   
  Length (genes)  2  
  Length (bp)  62414  
   Model Scoring   
  BIC  178.790625  
  logL  -83.874460  
  logL ratio  42.488196  
   Expression   
  Mean expression  4.645293  
  Median expression  4.368315  
  Tissue std. dev.  1.236818  
 
  No GO Slim enrichment  
  
   tissue    mean expression   
  5th Passage Drosophila S2 Cells  4.329650  
  Adult Accessory gland  4.412710  
  Adult Brain  4.033562  
  Adult Carcass  4.329930  
  Adult Crop  4.253064  
  Adult Eye  4.493023  
  Adult Fatbody  4.334039  
  Adult Female Spermatheca Mated  4.482444  
  Adult Female Spermatheca Virgin  4.637467  
  Adult Head  4.021798  
  Adult Heart  4.362892  
  Adult Hind Gut  4.209798  
  Adult Male Ejaculatory Duct  4.340750  
  Adult Mid Gut  4.471167  
  Adult Ovary  4.252956  
  Adult Salivary Gland  4.585883  
  Adult Testes  10.585134  
  Adult Thoracoabdominal ganglion  4.161085  
  Adult Whole Fly  6.290757  
  Larvae Wandering Tubules  4.439020  
  Larval Feeding Carcass  4.279457  
  Larval Feeding Central Nevous System  4.005664  
  Larval Feeding Hind Gut  4.151599  
  Larval Feeding Malpighian Tubule  4.367344  
  Larval Feeding Mid Gut  4.338906  
  Larval Feeding Salivary Gland  4.301592  
  Whole Larvae Feeding  4.951229  
 
  
   FlyBase ID    symbol    start    end    strand    length   
   FBgn0033893   CG18371   9916123   9916611  +  489  
   FBgn0259184   CG42288   9978536   9979310  +  775  
 
    Segment 206 
 
   Location   
  Gene key  FBgn0040752-FBgn0033900  
  Heatmap region span   2R:9858662..10073324   
  Segment span   2R:10028403..10048754   
  Length (genes)  6  
  Length (bp)  20352  
   Model Scoring   
  BIC  523.913859  
  logL  -256.436077  
  logL ratio  227.861474  
   Expression   
  Mean expression  8.367622  
  Median expression  8.437618  
  Tissue std. dev.  0.311999  
 
  No GO Slim enrichment  
  
   tissue    mean expression   
  5th Passage Drosophila S2 Cells  8.714052  
  Adult Accessory gland  8.729047  
  Adult Brain  8.710082  
  Adult Carcass  8.112659  
  Adult Crop  8.402179  
  Adult Eye  8.391894  
  Adult Fatbody  7.839115  
  Adult Female Spermatheca Mated  8.432371  
  Adult Female Spermatheca Virgin  8.401859  
  Adult Head  8.120358  
  Adult Heart  8.410246  
  Adult Hind Gut  8.250828  
  Adult Male Ejaculatory Duct  8.486049  
  Adult Mid Gut  8.122079  
  Adult Ovary  9.053709  
  Adult Salivary Gland  8.306984  
  Adult Testes  8.298965  
  Adult Thoracoabdominal ganglion  8.773115  
  Adult Whole Fly  8.259668  
  Larvae Wandering Tubules  8.319010  
  Larval Feeding Carcass  8.232789  
  Larval Feeding Central Nevous System  8.875215  
  Larval Feeding Hind Gut  8.246703  
  Larval Feeding Malpighian Tubule  8.156559  
  Larval Feeding Mid Gut  7.783062  
  Larval Feeding Salivary Gland  8.693223  
  Whole Larvae Feeding  7.803975  
 
  
   FlyBase ID    symbol    start    end    strand    length   
   FBgn0040752   Prosap  9947961   10028403   -  80443  
   FBgn0033897   Rcd1  10035763   10040624   -  4862  
   FBgn0086895   pea   10040661   10044611  +  3951  
   FBgn0040751   CG13018  10044555   10044984   -  430  
   FBgn0033899   CG13016   10045276   10046939  +  1664  
   FBgn0033900   CG8257  10046896   10048754   -  1859  
 
 
    Segment 207 
 
   Location   
  Gene key  FBgn0033903-FBgn0033905  
  Heatmap region span   2R:9870979..10104132   
  Segment span   2R:10052560..10056361   
  Length (genes)  3  
  Length (bp)  3802  
   Model Scoring   
  BIC  353.310231  
  logL  -171.134263  
  logL ratio  26.464457  
   Expression   
  Mean expression  8.198198  
  Median expression  8.439792  
  Tissue std. dev.  0.930131  
 
  
   GO ID    description    ratio    P-value   
   GO:0022857   transmembrane transporter activity  3/3  6.92e-08  
   GO:0003674   molecular_function  3/3  0.0144  
 
  
   tissue    mean expression   
  5th Passage Drosophila S2 Cells  7.680027  
  Adult Accessory gland  7.355449  
  Adult Brain  7.654056  
  Adult Carcass  8.842217  
  Adult Crop  7.860900  
  Adult Eye  8.272203  
  Adult Fatbody  9.664645  
  Adult Female Spermatheca Mated  9.376223  
  Adult Female Spermatheca Virgin  9.093650  
  Adult Head  7.984707  
  Adult Heart  9.072366  
  Adult Hind Gut  8.621503  
  Adult Male Ejaculatory Duct  7.684357  
  Adult Mid Gut  8.833957  
  Adult Ovary  7.070022  
  Adult Salivary Gland  7.899754  
  Adult Testes  6.677294  
  Adult Thoracoabdominal ganglion  7.688881  
  Adult Whole Fly  7.555464  
  Larvae Wandering Tubules  8.794638  
  Larval Feeding Carcass  7.568573  
  Larval Feeding Central Nevous System  6.919463  
  Larval Feeding Hind Gut  8.123464  
  Larval Feeding Malpighian Tubule  11.103811  
  Larval Feeding Mid Gut  8.306961  
  Larval Feeding Salivary Gland  7.627116  
  Whole Larvae Feeding  8.019653  
 
  
   FlyBase ID    symbol    start    end    strand    length   
   FBgn0033903   CG8323   10052560   10054496  +  1937  
   FBgn0033904   CG18327   10054487   10055738  +  1252  
   FBgn0033905   CG18324   10056361   10057738  +  1378  
 
 
    Segment 208 
 
   Location   
  Gene key  FBgn0033907-FBgn0050069  
  Heatmap region span   2R:10028403..10127442   
  Segment span   2R:10061041..10073324   
  Length (genes)  3  
  Length (bp)  12284  
   Model Scoring   
  BIC  384.108265  
  logL  -186.533280  
  logL ratio  16.831147  
   Expression   
  Mean expression  9.058576  
  Median expression  9.401247  
  Tissue std. dev.  0.484713  
 
  No GO Slim enrichment  
  
   tissue    mean expression   
  5th Passage Drosophila S2 Cells  9.027481  
  Adult Accessory gland  8.920636  
  Adult Brain  9.471440  
  Adult Carcass  8.897494  
  Adult Crop  9.741472  
  Adult Eye  9.358348  
  Adult Fatbody  8.883765  
  Adult Female Spermatheca Mated  9.024200  
  Adult Female Spermatheca Virgin  8.865877  
  Adult Head  8.837569  
  Adult Heart  8.769498  
  Adult Hind Gut  9.509340  
  Adult Male Ejaculatory Duct  8.893878  
  Adult Mid Gut  8.577187  
  Adult Ovary  9.302308  
  Adult Salivary Gland  9.107298  
  Adult Testes  7.556768  
  Adult Thoracoabdominal ganglion  9.414024  
  Adult Whole Fly  9.008031  
  Larvae Wandering Tubules  8.412144  
  Larval Feeding Carcass  9.504125  
  Larval Feeding Central Nevous System  9.529204  
  Larval Feeding Hind Gut  9.456902  
  Larval Feeding Malpighian Tubule  8.910212  
  Larval Feeding Mid Gut  8.534239  
  Larval Feeding Salivary Gland  10.132336  
  Whole Larvae Feeding  8.935763  
 
  
   FlyBase ID    symbol    start    end    strand    length   
   FBgn0033907   mRpS16  10060485   10061041   -  557  
   FBgn0000289   cg   10061175   10070345  +  9171  
   FBgn0050069   CG30069   10073324   10085730  +  12407  
 
 
    Segment 209 
 
   Location   
  Gene key  FBgn0033911-FBgn0005613  
  Heatmap region span   2R:10052374..10131192   
  Segment span   2R:10088226..10099914   
  Length (genes)  2  
  Length (bp)  11689  
   Model Scoring   
  BIC  210.350325  
  logL  -99.654310  
  logL ratio  6.604508  
   Expression   
  Mean expression  4.688965  
  Median expression  4.380306  
  Tissue std. dev.  0.733578  
 
  No GO Slim enrichment  
  
   tissue    mean expression   
  5th Passage Drosophila S2 Cells  4.369552  
  Adult Accessory gland  4.489335  
  Adult Brain  6.851571  
  Adult Carcass  4.583557  
  Adult Crop  4.235415  
  Adult Eye  4.876682  
  Adult Fatbody  4.366987  
  Adult Female Spermatheca Mated  4.244398  
  Adult Female Spermatheca Virgin  4.283904  
  Adult Head  5.927033  
  Adult Heart  4.354433  
  Adult Hind Gut  4.265874  
  Adult Male Ejaculatory Duct  4.421276  
  Adult Mid Gut  4.437854  
  Adult Ovary  4.306019  
  Adult Salivary Gland  4.625195  
  Adult Testes  4.096895  
  Adult Thoracoabdominal ganglion  6.729010  
  Adult Whole Fly  4.280008  
  Larvae Wandering Tubules  4.327960  
  Larval Feeding Carcass  4.407488  
  Larval Feeding Central Nevous System  5.937226  
  Larval Feeding Hind Gut  4.198354  
  Larval Feeding Malpighian Tubule  4.424285  
  Larval Feeding Mid Gut  4.680117  
  Larval Feeding Salivary Gland  4.479969  
  Whole Larvae Feeding  4.401645  
 
  
   FlyBase ID    symbol    start    end    strand    length   
   FBgn0033911   VGAT  10086505   10088226   -  1722  
   FBgn0005613   Sox15  10088888   10099914   -  11027  
 
    Segment 210 
 
   Location   
  Gene key  FBgn0033915-FBgn0033916  
  Heatmap region span   2R:10061041..10195667   
  Segment span   2R:10127203..10127442   
  Length (genes)  2  
  Length (bp)  240  
   Model Scoring   
  BIC  219.646059  
  logL  -104.302177  
  logL ratio  40.145291  
   Expression   
  Mean expression  7.984452  
  Median expression  7.714878  
  Tissue std. dev.  0.793453  
 
  No GO Slim enrichment  
  
   tissue    mean expression   
  5th Passage Drosophila S2 Cells  8.170120  
  Adult Accessory gland  7.461469  
  Adult Brain  9.009857  
  Adult Carcass  8.114353  
  Adult Crop  7.759234  
  Adult Eye  7.731187  
  Adult Fatbody  9.520860  
  Adult Female Spermatheca Mated  8.357437  
  Adult Female Spermatheca Virgin  8.555585  
  Adult Head  7.926500  
  Adult Heart  9.036997  
  Adult Hind Gut  7.463216  
  Adult Male Ejaculatory Duct  7.570265  
  Adult Mid Gut  7.185052  
  Adult Ovary  9.442227  
  Adult Salivary Gland  7.415427  
  Adult Testes  9.408575  
  Adult Thoracoabdominal ganglion  8.554014  
  Adult Whole Fly  8.060976  
  Larvae Wandering Tubules  7.258954  
  Larval Feeding Carcass  6.945025  
  Larval Feeding Central Nevous System  8.738245  
  Larval Feeding Hind Gut  7.314865  
  Larval Feeding Malpighian Tubule  7.325601  
  Larval Feeding Mid Gut  7.050533  
  Larval Feeding Salivary Gland  7.412306  
  Whole Larvae Feeding  6.791311  
 
  
   FlyBase ID    symbol    start    end    strand    length   
   FBgn0033915   CG8485  10123286   10127203   -  3918  
   FBgn0033916   CG8494   10127442   10131167  +  3726  
 
    Segment 211 
 
   Location   
  Gene key  FBgn0033918-FBgn0028428  
  Heatmap region span   2R:10107425..10249449   
  Segment span   2R:10143966..10166938   
  Length (genes)  4  
  Length (bp)  22973  
   Model Scoring   
  BIC  572.934885  
  logL  -280.946590  
  logL ratio  -31.727577  
   Expression   
  Mean expression  8.983162  
  Median expression  9.313896  
  Tissue std. dev.  0.747295  
 
  No GO Slim enrichment  
  
   tissue    mean expression   
  5th Passage Drosophila S2 Cells  8.232428  
  Adult Accessory gland  8.631227  
  Adult Brain  9.483429  
  Adult Carcass  9.317898  
  Adult Crop  10.449871  
  Adult Eye  9.603912  
  Adult Fatbody  9.627902  
  Adult Female Spermatheca Mated  9.520615  
  Adult Female Spermatheca Virgin  9.540689  
  Adult Head  9.464921  
  Adult Heart  10.387877  
  Adult Hind Gut  9.343381  
  Adult Male Ejaculatory Duct  9.216683  
  Adult Mid Gut  8.507908  
  Adult Ovary  8.237229  
  Adult Salivary Gland  7.745165  
  Adult Testes  8.349728  
  Adult Thoracoabdominal ganglion  9.694148  
  Adult Whole Fly  8.788418  
  Larvae Wandering Tubules  7.414613  
  Larval Feeding Carcass  9.611530  
  Larval Feeding Central Nevous System  8.869263  
  Larval Feeding Hind Gut  9.049516  
  Larval Feeding Malpighian Tubule  7.917077  
  Larval Feeding Mid Gut  8.250884  
  Larval Feeding Salivary Gland  8.482402  
  Whole Larvae Feeding  8.806657  
 
  
   FlyBase ID    symbol    start    end    strand    length   
   FBgn0033918   CG8531   10143966   10146292  +  2327  
   FBgn0027538   beta4GalNAcTA   10146537   10148655  +  2119  
   FBgn0033919   CG8547  10148711   10155419   -  6709  
   FBgn0028428      10166938   10186413  +  19476  
 
 
    Segment 212 
 
   Location   
  Gene key  FBgn0033921-FBgn0085408  
  Heatmap region span   2R:10127203..10249506   
  Segment span   2R:10189858..10195667   
  Length (genes)  2  
  Length (bp)  5810  
   Model Scoring   
  BIC  203.749812  
  logL  -96.354053  
  logL ratio  10.875436  
   Expression   
  Mean expression  5.016719  
  Median expression  4.889508  
  Tissue std. dev.  0.690757  
 
  No GO Slim enrichment  
  
   tissue    mean expression   
  5th Passage Drosophila S2 Cells  4.721486  
  Adult Accessory gland  6.271422  
  Adult Brain  4.824765  
  Adult Carcass  4.778429  
  Adult Crop  4.601341  
  Adult Eye  4.703769  
  Adult Fatbody  4.770427  
  Adult Female Spermatheca Mated  4.733224  
  Adult Female Spermatheca Virgin  4.884081  
  Adult Head  4.649626  
  Adult Heart  4.748393  
  Adult Hind Gut  4.496702  
  Adult Male Ejaculatory Duct  5.026217  
  Adult Mid Gut  4.659940  
  Adult Ovary  7.547123  
  Adult Salivary Gland  4.862213  
  Adult Testes  5.382245  
  Adult Thoracoabdominal ganglion  5.095804  
  Adult Whole Fly  6.603902  
  Larvae Wandering Tubules  5.223283  
  Larval Feeding Carcass  4.626749  
  Larval Feeding Central Nevous System  4.965257  
  Larval Feeding Hind Gut  4.482748  
  Larval Feeding Malpighian Tubule  4.980077  
  Larval Feeding Mid Gut  4.564268  
  Larval Feeding Salivary Gland  4.699045  
  Whole Larvae Feeding  4.548889  
 
  
   FlyBase ID    symbol    start    end    strand    length   
   FBgn0033921   tej  10187605   10189858   -  2254  
   FBgn0085408   Shroom   10195667   10239312  +  43646  
 
    Segment 213 
 
   Location   
  Gene key  FBgn0033924-FBgn0033925  
  Heatmap region span   2R:10131192..10250556   
  Segment span   2R:10239744..10244789   
  Length (genes)  2  
  Length (bp)  5046  
   Model Scoring   
  BIC  219.649680  
  logL  -104.303987  
  logL ratio  42.861401  
   Expression   
  Mean expression  8.763593  
  Median expression  8.594230  
  Tissue std. dev.  0.665538  
 
  No GO Slim enrichment  
  
   tissue    mean expression   
  5th Passage Drosophila S2 Cells  8.508371  
  Adult Accessory gland  8.894551  
  Adult Brain  10.621968  
  Adult Carcass  8.885669  
  Adult Crop  8.789019  
  Adult Eye  9.280625  
  Adult Fatbody  8.355833  
  Adult Female Spermatheca Mated  8.835719  
  Adult Female Spermatheca Virgin  8.930687  
  Adult Head  9.357280  
  Adult Heart  8.509306  
  Adult Hind Gut  8.398200  
  Adult Male Ejaculatory Duct  9.221326  
  Adult Mid Gut  7.750428  
  Adult Ovary  8.769190  
  Adult Salivary Gland  9.217908  
  Adult Testes  8.714333  
  Adult Thoracoabdominal ganglion  10.317311  
  Adult Whole Fly  8.421226  
  Larvae Wandering Tubules  7.851327  
  Larval Feeding Carcass  8.601945  
  Larval Feeding Central Nevous System  9.078178  
  Larval Feeding Hind Gut  8.223480  
  Larval Feeding Malpighian Tubule  8.048157  
  Larval Feeding Mid Gut  7.737433  
  Larval Feeding Salivary Gland  9.184609  
  Whole Larvae Feeding  8.112929  
 
  
   FlyBase ID    symbol    start    end    strand    length   
   FBgn0033924   CG8613   10239744   10243180  +  3437  
   FBgn0033925   CG8617  10243273   10244789   -  1517  
 
    Segment 214 
 
   Location   
  Gene key  FBgn0085213-FBgn0033933  
  Heatmap region span   2R:10247799..10390978   
  Segment span   2R:10253201..10278291   
  Length (genes)  9  
  Length (bp)  25091  
   Model Scoring   
  BIC  653.505442  
  logL  -321.231869  
  logL ratio  290.194890  
   Expression   
  Mean expression  4.638036  
  Median expression  4.397424  
  Tissue std. dev.  0.387890  
 
  No GO Slim enrichment  
  
   tissue    mean expression   
  5th Passage Drosophila S2 Cells  4.594252  
  Adult Accessory gland  4.722282  
  Adult Brain  4.293719  
  Adult Carcass  4.696986  
  Adult Crop  4.600620  
  Adult Eye  4.334877  
  Adult Fatbody  4.666050  
  Adult Female Spermatheca Mated  4.639593  
  Adult Female Spermatheca Virgin  4.675486  
  Adult Head  4.366463  
  Adult Heart  4.447431  
  Adult Hind Gut  4.531284  
  Adult Male Ejaculatory Duct  4.614822  
  Adult Mid Gut  4.675666  
  Adult Ovary  4.493639  
  Adult Salivary Gland  4.783369  
  Adult Testes  6.490163  
  Adult Thoracoabdominal ganglion  4.380910  
  Adult Whole Fly  4.460858  
  Larvae Wandering Tubules  4.663357  
  Larval Feeding Carcass  4.738039  
  Larval Feeding Central Nevous System  4.318346  
  Larval Feeding Hind Gut  4.494144  
  Larval Feeding Malpighian Tubule  4.660239  
  Larval Feeding Mid Gut  4.674108  
  Larval Feeding Salivary Gland  4.655348  
  Whole Larvae Feeding  4.554925  
 
  
   FlyBase ID    symbol    start    end    strand    length   
   FBgn0085213   CG34184   10253201   10254163  +  963  
   FBgn0085473   CG34444   10256747   10257824  +  1078  
   FBgn0050067   Obp50a  10257836   10258621   -  786  
   FBgn0050072   Obp50c   10258691   10260511  +  1821  
   FBgn0050073   Obp50b   10258691   10260511  +  1821  
   FBgn0050074   Obp50d   10260629   10261264  +  636  
   FBgn0033931   Obp50e  10262077   10262836   -  760  
   FBgn0050075   CG30075   10263426   10264182  +  757  
   FBgn0033933   CG10104   10278291   10279831  +  1541  
 
 
    Segment 215 
 
   Location   
  Gene key  FBgn0033935-FBgn0013725  
  Heatmap region span   2R:10249506..10402713   
  Segment span   2R:10299316..10319840   
  Length (genes)  3  
  Length (bp)  20525  
   Model Scoring   
  BIC  357.104945  
  logL  -173.031620  
  logL ratio  -21.586055  
   Expression   
  Mean expression  5.676556  
  Median expression  5.020841  
  Tissue std. dev.  0.724131  
 
  No GO Slim enrichment  
  
   tissue    mean expression   
  5th Passage Drosophila S2 Cells  5.441259  
  Adult Accessory gland  5.128238  
  Adult Brain  7.490744  
  Adult Carcass  5.373901  
  Adult Crop  5.574630  
  Adult Eye  7.379098  
  Adult Fatbody  5.315417  
  Adult Female Spermatheca Mated  5.201402  
  Adult Female Spermatheca Virgin  5.146604  
  Adult Head  6.536153  
  Adult Heart  5.129353  
  Adult Hind Gut  5.262848  
  Adult Male Ejaculatory Duct  5.396757  
  Adult Mid Gut  5.120882  
  Adult Ovary  5.864620  
  Adult Salivary Gland  5.325294  
  Adult Testes  5.372889  
  Adult Thoracoabdominal ganglion  7.077904  
  Adult Whole Fly  5.402629  
  Larvae Wandering Tubules  5.400284  
  Larval Feeding Carcass  5.988432  
  Larval Feeding Central Nevous System  7.024019  
  Larval Feeding Hind Gut  5.257579  
  Larval Feeding Malpighian Tubule  5.277133  
  Larval Feeding Mid Gut  5.136613  
  Larval Feeding Salivary Gland  5.564163  
  Whole Larvae Feeding  5.078177  
 
  
   FlyBase ID    symbol    start    end    strand    length   
   FBgn0033935   Sin1   10299316   10301356  +  2041  
   FBgn0033936   CG17386   10307846   10312099  +  4254  
   FBgn0013725   phyl  10314841   10319840   -  5000  
 
 
    Segment 216 
 
   Location   
  Gene key  FBgn0019938-FBgn0033945  
  Heatmap region span   2R:10414076..10528131   
  Segment span   2R:10488702..10492840   
  Length (genes)  4  
  Length (bp)  4139  
   Model Scoring   
  BIC  362.023819  
  logL  -175.491057  
  logL ratio  145.468775  
   Expression   
  Mean expression  8.673053  
  Median expression  8.664848  
  Tissue std. dev.  0.283666  
 
  No GO Slim enrichment  
  
   tissue    mean expression   
  5th Passage Drosophila S2 Cells  8.987582  
  Adult Accessory gland  8.996370  
  Adult Brain  8.722003  
  Adult Carcass  8.253623  
  Adult Crop  8.694332  
  Adult Eye  8.816724  
  Adult Fatbody  8.398983  
  Adult Female Spermatheca Mated  8.539343  
  Adult Female Spermatheca Virgin  8.428494  
  Adult Head  8.424411  
  Adult Heart  8.805203  
  Adult Hind Gut  8.422828  
  Adult Male Ejaculatory Duct  8.865896  
  Adult Mid Gut  9.005938  
  Adult Ovary  9.332157  
  Adult Salivary Gland  8.618942  
  Adult Testes  8.003236  
  Adult Thoracoabdominal ganglion  8.750364  
  Adult Whole Fly  8.393994  
  Larvae Wandering Tubules  8.890304  
  Larval Feeding Carcass  8.370799  
  Larval Feeding Central Nevous System  8.941472  
  Larval Feeding Hind Gut  8.680028  
  Larval Feeding Malpighian Tubule  8.867444  
  Larval Feeding Mid Gut  8.833684  
  Larval Feeding Salivary Gland  8.736178  
  Whole Larvae Feeding  8.392097  
 
  
   FlyBase ID    symbol    start    end    strand    length   
   FBgn0019938   RpI1  10483112   10488702   -  5591  
   FBgn0050077   blos1   10488769   10489515  +  747  
   FBgn0003742   tra2  10489509   10491857   -  2349  
   FBgn0033945   CG12868  10492052   10492840   -  789  
 
 
    Segment 217 
 
   Location   
  Gene key  FBgn0053469-FBgn0053468  
  Heatmap region span   2R:10433264..10532776   
  Segment span   2R:10493774..10494466   
  Length (genes)  2  
  Length (bp)  693  
   Model Scoring   
  BIC  179.297632  
  logL  -84.127964  
  logL ratio  35.177364  
   Expression   
  Mean expression  4.773079  
  Median expression  4.619264  
  Tissue std. dev.  0.567613  
 
  No GO Slim enrichment  
  
   tissue    mean expression   
  5th Passage Drosophila S2 Cells  5.366799  
  Adult Accessory gland  4.848410  
  Adult Brain  4.118441  
  Adult Carcass  4.552091  
  Adult Crop  4.453233  
  Adult Eye  4.074665  
  Adult Fatbody  4.535862  
  Adult Female Spermatheca Mated  4.847780  
  Adult Female Spermatheca Virgin  4.685925  
  Adult Head  4.699708  
  Adult Heart  4.375080  
  Adult Hind Gut  4.883771  
  Adult Male Ejaculatory Duct  5.266273  
  Adult Mid Gut  6.587198  
  Adult Ovary  4.227490  
  Adult Salivary Gland  4.654043  
  Adult Testes  4.756776  
  Adult Thoracoabdominal ganglion  4.214279  
  Adult Whole Fly  4.370831  
  Larvae Wandering Tubules  4.714316  
  Larval Feeding Carcass  5.209650  
  Larval Feeding Central Nevous System  4.195746  
  Larval Feeding Hind Gut  4.691700  
  Larval Feeding Malpighian Tubule  4.534410  
  Larval Feeding Mid Gut  5.141628  
  Larval Feeding Salivary Gland  6.177926  
  Whole Larvae Feeding  4.689105  
 
  
   FlyBase ID    symbol    start    end    strand    length   
   FBgn0053469   CG33469  10493157   10493774   -  618  
   FBgn0053468   CG33468  10493812   10494466   -  655  
 
    Segment 218 
 
   Location   
  Gene key  FBgn0033948-FBgn0033949  
  Heatmap region span   2R:10478708..10565638   
  Segment span   2R:10508817..10517418   
  Length (genes)  2  
  Length (bp)  8602  
   Model Scoring   
  BIC  260.642605  
  logL  -124.800450  
  logL ratio  -10.195480  
   Expression   
  Mean expression  7.363094  
  Median expression  7.414350  
  Tissue std. dev.  0.738674  
 
  No GO Slim enrichment  
  
   tissue    mean expression   
  5th Passage Drosophila S2 Cells  8.384385  
  Adult Accessory gland  7.137903  
  Adult Brain  7.182596  
  Adult Carcass  8.201466  
  Adult Crop  6.700319  
  Adult Eye  7.301564  
  Adult Fatbody  7.283307  
  Adult Female Spermatheca Mated  7.109986  
  Adult Female Spermatheca Virgin  7.085670  
  Adult Head  8.080877  
  Adult Heart  9.505434  
  Adult Hind Gut  6.281035  
  Adult Male Ejaculatory Duct  7.350717  
  Adult Mid Gut  6.785550  
  Adult Ovary  7.382105  
  Adult Salivary Gland  6.716069  
  Adult Testes  6.449452  
  Adult Thoracoabdominal ganglion  7.004836  
  Adult Whole Fly  7.094241  
  Larvae Wandering Tubules  8.101803  
  Larval Feeding Carcass  8.455427  
  Larval Feeding Central Nevous System  6.780434  
  Larval Feeding Hind Gut  7.003801  
  Larval Feeding Malpighian Tubule  7.016019  
  Larval Feeding Mid Gut  6.522224  
  Larval Feeding Salivary Gland  7.423127  
  Whole Larvae Feeding  8.463203  
 
  
   FlyBase ID    symbol    start    end    strand    length   
   FBgn0033948   CG12863  10507300   10508817   -  1518  
   FBgn0033949   CG10131   10517418   10518526  +  1109  
 
    Segment 219 
 
   Location   
  Gene key  FBgn0033953-FBgn0033955  
  Heatmap region span   2R:10506232..10640415   
  Segment span   2R:10552856..10555896   
  Length (genes)  3  
  Length (bp)  3041  
   Model Scoring   
  BIC  293.954386  
  logL  -141.456341  
  logL ratio  36.409247  
   Expression   
  Mean expression  5.341711  
  Median expression  4.877011  
  Tissue std. dev.  1.610089  
 
  No GO Slim enrichment  
  
   tissue    mean expression   
  5th Passage Drosophila S2 Cells  4.930302  
  Adult Accessory gland  5.021130  
  Adult Brain  4.451346  
  Adult Carcass  5.599689  
  Adult Crop  4.749016  
  Adult Eye  4.427027  
  Adult Fatbody  4.983495  
  Adult Female Spermatheca Mated  5.090304  
  Adult Female Spermatheca Virgin  4.891317  
  Adult Head  4.574635  
  Adult Heart  4.523459  
  Adult Hind Gut  4.831400  
  Adult Male Ejaculatory Duct  5.019925  
  Adult Mid Gut  5.152584  
  Adult Ovary  4.740030  
  Adult Salivary Gland  5.220107  
  Adult Testes  12.367582  
  Adult Thoracoabdominal ganglion  4.674718  
  Adult Whole Fly  8.811112  
  Larvae Wandering Tubules  4.926233  
  Larval Feeding Carcass  4.958637  
  Larval Feeding Central Nevous System  4.489492  
  Larval Feeding Hind Gut  4.655518  
  Larval Feeding Malpighian Tubule  4.859761  
  Larval Feeding Mid Gut  4.956157  
  Larval Feeding Salivary Gland  4.854084  
  Whole Larvae Feeding  6.467139  
 
  
   FlyBase ID    symbol    start    end    strand    length   
   FBgn0033953   CG12861  10551751   10552856   -  1106  
   FBgn0033954   CG12860  10553146   10554322   -  1177  
   FBgn0033955   CG12866   10555896   10559180  +  3285  
 
 
    Segment 220 
 
   Location   
  Gene key  FBgn0050479-FBgn0050480  
  Heatmap region span   2R:10508817..10643793   
  Segment span   2R:10562558..10565638   
  Length (genes)  2  
  Length (bp)  3081  
   Model Scoring   
  BIC  194.485785  
  logL  -91.722040  
  logL ratio  32.421063  
   Expression   
  Mean expression  5.979448  
  Median expression  5.684140  
  Tissue std. dev.  1.388537  
 
  No GO Slim enrichment  
  
   tissue    mean expression   
  5th Passage Drosophila S2 Cells  5.278587  
  Adult Accessory gland  5.692748  
  Adult Brain  6.226611  
  Adult Carcass  5.480175  
  Adult Crop  5.461412  
  Adult Eye  5.423521  
  Adult Fatbody  5.620710  
  Adult Female Spermatheca Mated  5.646494  
  Adult Female Spermatheca Virgin  5.611197  
  Adult Head  4.937525  
  Adult Heart  5.358526  
  Adult Hind Gut  5.322437  
  Adult Male Ejaculatory Duct  5.466302  
  Adult Mid Gut  9.519216  
  Adult Ovary  5.116210  
  Adult Salivary Gland  6.121386  
  Adult Testes  4.841086  
  Adult Thoracoabdominal ganglion  5.905795  
  Adult Whole Fly  5.515829  
  Larvae Wandering Tubules  5.399813  
  Larval Feeding Carcass  5.433217  
  Larval Feeding Central Nevous System  5.826173  
  Larval Feeding Hind Gut  5.302817  
  Larval Feeding Malpighian Tubule  5.282895  
  Larval Feeding Mid Gut  10.765163  
  Larval Feeding Salivary Gland  5.953736  
  Whole Larvae Feeding  8.935518  
 
  
   FlyBase ID    symbol    start    end    strand    length   
   FBgn0050479   CG30479  10561970   10562558   -  589  
   FBgn0050480   CG30480  10562676   10565638   -  2963  
 
    Segment 221 
 
   Location   
  Gene key  FBgn0020269-FBgn0012042  
  Heatmap region span   2R:10528131..10645334   
  Segment span   2R:10601126..10634867   
  Length (genes)  5  
  Length (bp)  33742  
   Model Scoring   
  BIC  514.025587  
  logL  -251.491941  
  logL ratio  26.895032  
   Expression   
  Mean expression  5.382525  
  Median expression  5.040831  
  Tissue std. dev.  0.738296  
 
  No GO Slim enrichment  
  
   tissue    mean expression   
  5th Passage Drosophila S2 Cells  5.030840  
  Adult Accessory gland  4.932869  
  Adult Brain  7.058512  
  Adult Carcass  5.530333  
  Adult Crop  5.059766  
  Adult Eye  6.854908  
  Adult Fatbody  5.370548  
  Adult Female Spermatheca Mated  5.571743  
  Adult Female Spermatheca Virgin  5.985279  
  Adult Head  7.228627  
  Adult Heart  5.254013  
  Adult Hind Gut  4.995794  
  Adult Male Ejaculatory Duct  4.967444  
  Adult Mid Gut  4.907451  
  Adult Ovary  4.890455  
  Adult Salivary Gland  4.891410  
  Adult Testes  4.197119  
  Adult Thoracoabdominal ganglion  6.554129  
  Adult Whole Fly  5.122126  
  Larvae Wandering Tubules  4.793312  
  Larval Feeding Carcass  5.417376  
  Larval Feeding Central Nevous System  5.933973  
  Larval Feeding Hind Gut  4.980466  
  Larval Feeding Malpighian Tubule  4.980567  
  Larval Feeding Mid Gut  4.819645  
  Larval Feeding Salivary Gland  4.964652  
  Whole Larvae Feeding  5.034824  
 
  
   FlyBase ID    symbol    start    end    strand    length   
   FBgn0020269   mspo  10559440   10601126   -  41687  
   FBgn0033957   CG12865   10626224   10626793  +  570  
   FBgn0033958   CG12858  10627871   10632834   -  4964  
   FBgn0010388   Dro   10633461   10633821  +  361  
   FBgn0012042   AttA   10634867   10635699  +  833  
 
 
    Segment 222 
 
   Location   
  Gene key  FBgn0033961-FBgn0033962  
  Heatmap region span   2R:10601126..10695929   
  Segment span   2R:10644026..10645334   
  Length (genes)  2  
  Length (bp)  1309  
   Model Scoring   
  BIC  284.762019  
  logL  -136.860157  
  logL ratio  11.593071  
   Expression   
  Mean expression  10.147990  
  Median expression  10.112360  
  Tissue std. dev.  0.466684  
 
  No GO Slim enrichment  
  
   tissue    mean expression   
  5th Passage Drosophila S2 Cells  9.200533  
  Adult Accessory gland  10.292606  
  Adult Brain  10.477049  
  Adult Carcass  10.376511  
  Adult Crop  10.055692  
  Adult Eye  10.990440  
  Adult Fatbody  10.158174  
  Adult Female Spermatheca Mated  9.707372  
  Adult Female Spermatheca Virgin  9.896569  
  Adult Head  10.402566  
  Adult Heart  10.374672  
  Adult Hind Gut  10.513361  
  Adult Male Ejaculatory Duct  10.445150  
  Adult Mid Gut  10.035331  
  Adult Ovary  9.642710  
  Adult Salivary Gland  10.336688  
  Adult Testes  8.643790  
  Adult Thoracoabdominal ganglion  10.739172  
  Adult Whole Fly  9.832199  
  Larvae Wandering Tubules  10.275220  
  Larval Feeding Carcass  10.156336  
  Larval Feeding Central Nevous System  10.332976  
  Larval Feeding Hind Gut  10.189729  
  Larval Feeding Malpighian Tubule  10.626366  
  Larval Feeding Mid Gut  9.948750  
  Larval Feeding Salivary Gland  10.446945  
  Whole Larvae Feeding  9.898820  
 
  
   FlyBase ID    symbol    start    end    strand    length   
   FBgn0033961   CG12859   10644026   10644578  +  553  
   FBgn0033962   CG10153  10644566   10645334   -  769  
 
    Segment 223 
 
   Location   
  Gene key  FBgn0259112-FBgn0001319  
  Heatmap region span   2R:10643793..10735864   
  Segment span   2R:10658417..10694566   
  Length (genes)  2  
  Length (bp)  36150  
   Model Scoring   
  BIC  185.319223  
  logL  -87.138759  
  logL ratio  30.013876  
   Expression   
  Mean expression  4.904511  
  Median expression  4.721147  
  Tissue std. dev.  0.520230  
 
  No GO Slim enrichment  
  
   tissue    mean expression   
  5th Passage Drosophila S2 Cells  5.665872  
  Adult Accessory gland  4.433806  
  Adult Brain  5.981312  
  Adult Carcass  4.766870  
  Adult Crop  4.516140  
  Adult Eye  4.896840  
  Adult Fatbody  4.650892  
  Adult Female Spermatheca Mated  4.558441  
  Adult Female Spermatheca Virgin  4.556600  
  Adult Head  5.109730  
  Adult Heart  5.865492  
  Adult Hind Gut  4.534428  
  Adult Male Ejaculatory Duct  4.673052  
  Adult Mid Gut  4.537816  
  Adult Ovary  5.207567  
  Adult Salivary Gland  4.868542  
  Adult Testes  4.809635  
  Adult Thoracoabdominal ganglion  5.950516  
  Adult Whole Fly  4.584224  
  Larvae Wandering Tubules  4.996944  
  Larval Feeding Carcass  4.617976  
  Larval Feeding Central Nevous System  6.031741  
  Larval Feeding Hind Gut  4.243289  
  Larval Feeding Malpighian Tubule  4.644472  
  Larval Feeding Mid Gut  4.467664  
  Larval Feeding Salivary Gland  4.813009  
  Whole Larvae Feeding  4.438915  
 
  
   FlyBase ID    symbol    start    end    strand    length   
   FBgn0259112   CG42254  10657104   10658417   -  1314  
   FBgn0001319   kn  10660152   10694566   -  34415  
 
    Segment 224 
 
   Location   
  Gene key  FBgn0027783-FBgn0028434  
  Heatmap region span   2R:10695929..10763338   
  Segment span   2R:10740155..10740470   
  Length (genes)  2  
  Length (bp)  316  
   Model Scoring   
  BIC  215.059692  
  logL  -102.008994  
  logL ratio  34.583847  
   Expression   
  Mean expression  6.782822  
  Median expression  6.567085  
  Tissue std. dev.  0.871684  
 
  No GO Slim enrichment  
  
   tissue    mean expression   
  5th Passage Drosophila S2 Cells  8.697657  
  Adult Accessory gland  6.453026  
  Adult Brain  6.622655  
  Adult Carcass  5.922947  
  Adult Crop  6.425714  
  Adult Eye  6.398639  
  Adult Fatbody  5.877929  
  Adult Female Spermatheca Mated  6.226838  
  Adult Female Spermatheca Virgin  6.272100  
  Adult Head  6.271448  
  Adult Heart  6.373545  
  Adult Hind Gut  6.289886  
  Adult Male Ejaculatory Duct  6.577018  
  Adult Mid Gut  6.284643  
  Adult Ovary  9.173047  
  Adult Salivary Gland  6.266746  
  Adult Testes  7.140928  
  Adult Thoracoabdominal ganglion  6.697708  
  Adult Whole Fly  7.641482  
  Larvae Wandering Tubules  6.737997  
  Larval Feeding Carcass  6.749371  
  Larval Feeding Central Nevous System  9.153227  
  Larval Feeding Hind Gut  6.587240  
  Larval Feeding Malpighian Tubule  6.723365  
  Larval Feeding Mid Gut  5.875248  
  Larval Feeding Salivary Gland  6.832697  
  Whole Larvae Feeding  6.863084  
 
  
   FlyBase ID    symbol    start    end    strand    length   
   FBgn0027783   SMC2  10736094   10740155   -  4062  
   FBgn0028434   Ercc1   10740470   10741453  +  984  
 
    Segment 225 
 
   Location   
  Gene key  FBgn0033972-FBgn0022063  
  Heatmap region span   2R:10707492..10765293   
  Segment span   2R:10742573..10758575   
  Length (genes)  4  
  Length (bp)  16003  
   Model Scoring   
  BIC  401.783906  
  logL  -195.371101  
  logL ratio  115.660279  
   Expression   
  Mean expression  8.870076  
  Median expression  8.804917  
  Tissue std. dev.  0.359894  
 
  No GO Slim enrichment  
  
   tissue    mean expression   
  5th Passage Drosophila S2 Cells  8.767409  
  Adult Accessory gland  8.970097  
  Adult Brain  9.352429  
  Adult Carcass  8.591330  
  Adult Crop  9.001019  
  Adult Eye  9.039754  
  Adult Fatbody  9.015310  
  Adult Female Spermatheca Mated  8.958101  
  Adult Female Spermatheca Virgin  9.083009  
  Adult Head  8.764696  
  Adult Heart  9.165501  
  Adult Hind Gut  8.856228  
  Adult Male Ejaculatory Duct  8.955458  
  Adult Mid Gut  8.538115  
  Adult Ovary  8.918724  
  Adult Salivary Gland  9.111902  
  Adult Testes  7.775322  
  Adult Thoracoabdominal ganglion  9.104366  
  Adult Whole Fly  8.138624  
  Larvae Wandering Tubules  9.290762  
  Larval Feeding Carcass  8.707112  
  Larval Feeding Central Nevous System  8.944266  
  Larval Feeding Hind Gut  8.885109  
  Larval Feeding Malpighian Tubule  9.326116  
  Larval Feeding Mid Gut  8.550649  
  Larval Feeding Salivary Gland  9.319783  
  Whole Larvae Feeding  8.360860  
 
  
   FlyBase ID    symbol    start    end    strand    length   
   FBgn0033972   Ciao1  10741368   10742573   -  1206  
   FBgn0033973   HPS1   10742947   10745007  +  2061  
   FBgn0053506   CG33506  10744952   10746138   -  1187  
   FBgn0022063     10748661   10758575   -  9915  
 
 
    Segment 226 
 
   Location   
  Gene key  FBgn0033987-FBgn0033989  
  Heatmap region span   2R:10821020..11033003   
  Segment span   2R:10850819..10879728   
  Length (genes)  3  
  Length (bp)  28910  
   Model Scoring   
  BIC  318.733076  
  logL  -153.845686  
  logL ratio  64.160010  
   Expression   
  Mean expression  8.475520  
  Median expression  8.587660  
  Tissue std. dev.  0.584799  
 
  No GO Slim enrichment  
  
   tissue    mean expression   
  5th Passage Drosophila S2 Cells  7.260678  
  Adult Accessory gland  8.964373  
  Adult Brain  9.730111  
  Adult Carcass  8.091156  
  Adult Crop  8.905679  
  Adult Eye  8.575337  
  Adult Fatbody  7.927285  
  Adult Female Spermatheca Mated  8.352939  
  Adult Female Spermatheca Virgin  8.146480  
  Adult Head  8.808010  
  Adult Heart  8.068259  
  Adult Hind Gut  9.271833  
  Adult Male Ejaculatory Duct  8.435367  
  Adult Mid Gut  8.045975  
  Adult Ovary  8.738501  
  Adult Salivary Gland  8.571702  
  Adult Testes  7.801649  
  Adult Thoracoabdominal ganglion  9.420604  
  Adult Whole Fly  8.163210  
  Larvae Wandering Tubules  7.554593  
  Larval Feeding Carcass  8.544546  
  Larval Feeding Central Nevous System  9.036128  
  Larval Feeding Hind Gut  9.263512  
  Larval Feeding Malpighian Tubule  8.508424  
  Larval Feeding Mid Gut  7.798750  
  Larval Feeding Salivary Gland  8.818471  
  Whole Larvae Feeding  8.035479  
 
  
   FlyBase ID    symbol    start    end    strand    length   
   FBgn0033987   ckn   10850819   10868089  +  17271  
   FBgn0033988   pcs   10868782   10878841  +  10060  
   FBgn0033989   CG7639  10875170   10879728   -  4559  
 
 
    Segment 227 
 
   Location   
  Gene key  FBgn0033990-FBgn0029082  
  Heatmap region span   2R:10822366..11035382   
  Segment span   2R:10881336..10898903   
  Length (genes)  2  
  Length (bp)  17568  
   Model Scoring   
  BIC  201.922419  
  logL  -95.440357  
  logL ratio  19.313235  
   Expression   
  Mean expression  5.573106  
  Median expression  5.411893  
  Tissue std. dev.  0.691195  
 
  No GO Slim enrichment  
  
   tissue    mean expression   
  5th Passage Drosophila S2 Cells  5.335602  
  Adult Accessory gland  5.122722  
  Adult Brain  7.235296  
  Adult Carcass  4.835974  
  Adult Crop  5.109084  
  Adult Eye  6.186948  
  Adult Fatbody  5.395867  
  Adult Female Spermatheca Mated  5.661831  
  Adult Female Spermatheca Virgin  5.474625  
  Adult Head  5.917529  
  Adult Heart  4.938227  
  Adult Hind Gut  4.974509  
  Adult Male Ejaculatory Duct  6.576373  
  Adult Mid Gut  4.815787  
  Adult Ovary  5.505732  
  Adult Salivary Gland  5.235019  
  Adult Testes  5.964884  
  Adult Thoracoabdominal ganglion  6.777299  
  Adult Whole Fly  5.246777  
  Larvae Wandering Tubules  5.142471  
  Larval Feeding Carcass  5.555393  
  Larval Feeding Central Nevous System  7.412382  
  Larval Feeding Hind Gut  5.296121  
  Larval Feeding Malpighian Tubule  5.088990  
  Larval Feeding Mid Gut  4.903322  
  Larval Feeding Salivary Gland  5.346156  
  Whole Larvae Feeding  5.418945  
 
  
   FlyBase ID    symbol    start    end    strand    length   
   FBgn0033990   CG10265  10879837   10881336   -  1500  
   FBgn0029082   hbs   10898903   10928901  +  29999  
 
    Segment 228 
 
   Location   
  Gene key  FBgn0033993-FBgn0033994  
  Heatmap region span   2R:10881336..11101137   
  Segment span   2R:11035329..11035382   
  Length (genes)  2  
  Length (bp)  54  
   Model Scoring   
  BIC  202.483606  
  logL  -95.720951  
  logL ratio  24.054554  
   Expression   
  Mean expression  5.698874  
  Median expression  5.663439  
  Tissue std. dev.  0.658109  
 
  No GO Slim enrichment  
  
   tissue    mean expression   
  5th Passage Drosophila S2 Cells  6.291828  
  Adult Accessory gland  6.524215  
  Adult Brain  5.534154  
  Adult Carcass  5.212397  
  Adult Crop  5.841500  
  Adult Eye  5.443758  
  Adult Fatbody  5.485259  
  Adult Female Spermatheca Mated  5.679578  
  Adult Female Spermatheca Virgin  5.507168  
  Adult Head  5.332320  
  Adult Heart  5.635642  
  Adult Hind Gut  5.918374  
  Adult Male Ejaculatory Duct  5.586555  
  Adult Mid Gut  5.192797  
  Adult Ovary  7.918411  
  Adult Salivary Gland  5.840564  
  Adult Testes  4.350632  
  Adult Thoracoabdominal ganglion  5.560445  
  Adult Whole Fly  5.946366  
  Larvae Wandering Tubules  5.816491  
  Larval Feeding Carcass  5.293934  
  Larval Feeding Central Nevous System  6.381592  
  Larval Feeding Hind Gut  5.743708  
  Larval Feeding Malpighian Tubule  5.504428  
  Larval Feeding Mid Gut  4.749588  
  Larval Feeding Salivary Gland  6.603862  
  Whole Larvae Feeding  4.974031  
 
  
   FlyBase ID    symbol    start    end    strand    length   
   FBgn0033993   CG8089  11033330   11035329   -  2000  
   FBgn0033994   CG7544   11035382   11037214  +  1833  
 
    Segment 229 
 
   Location   
  Gene key  FBgn0034000-FBgn0034002  
  Heatmap region span   2R:11089029..11209979   
  Segment span   2R:11103716..11105886   
  Length (genes)  3  
  Length (bp)  2171  
   Model Scoring   
  BIC  350.467667  
  logL  -169.712981  
  logL ratio  28.293233  
   Expression   
  Mean expression  7.808656  
  Median expression  7.708206  
  Tissue std. dev.  0.459634  
 
  No GO Slim enrichment  
  
   tissue    mean expression   
  5th Passage Drosophila S2 Cells  7.881563  
  Adult Accessory gland  7.682183  
  Adult Brain  8.463673  
  Adult Carcass  7.583654  
  Adult Crop  7.695398  
  Adult Eye  7.995645  
  Adult Fatbody  7.749141  
  Adult Female Spermatheca Mated  8.377005  
  Adult Female Spermatheca Virgin  8.504103  
  Adult Head  7.512303  
  Adult Heart  7.879479  
  Adult Hind Gut  7.832595  
  Adult Male Ejaculatory Duct  7.561526  
  Adult Mid Gut  7.397095  
  Adult Ovary  8.682615  
  Adult Salivary Gland  7.377683  
  Adult Testes  7.136316  
  Adult Thoracoabdominal ganglion  8.491281  
  Adult Whole Fly  7.506632  
  Larvae Wandering Tubules  7.802586  
  Larval Feeding Carcass  7.364202  
  Larval Feeding Central Nevous System  8.673320  
  Larval Feeding Hind Gut  7.644261  
  Larval Feeding Malpighian Tubule  7.855441  
  Larval Feeding Mid Gut  7.003503  
  Larval Feeding Salivary Gland  8.005212  
  Whole Larvae Feeding  7.175304  
 
  
   FlyBase ID    symbol    start    end    strand    length   
   FBgn0034000   CG11808   11103716   11104575  +  860  
   FBgn0034001   mRpL41  11104529   11105118   -  590  
   FBgn0034002   CG8079   11105886   11111329  +  5444  
 
 
    Segment 230 
 
   Location   
  Gene key  FBgn0034005-FBgn0034007  
  Heatmap region span   2R:11103191..11259021   
  Segment span   2R:11146272..11185834   
  Length (genes)  4  
  Length (bp)  39563  
   Model Scoring   
  BIC  336.351379  
  logL  -162.654837  
  logL ratio  85.186603  
   Expression   
  Mean expression  5.132797  
  Median expression  5.049760  
  Tissue std. dev.  0.344753  
 
  No GO Slim enrichment  
  
   tissue    mean expression   
  5th Passage Drosophila S2 Cells  5.835221  
  Adult Accessory gland  5.447815  
  Adult Brain  4.932306  
  Adult Carcass  5.090552  
  Adult Crop  5.059097  
  Adult Eye  4.824375  
  Adult Fatbody  5.032676  
  Adult Female Spermatheca Mated  5.006712  
  Adult Female Spermatheca Virgin  4.993998  
  Adult Head  4.741594  
  Adult Heart  5.042303  
  Adult Hind Gut  5.121246  
  Adult Male Ejaculatory Duct  5.171278  
  Adult Mid Gut  5.207979  
  Adult Ovary  5.085796  
  Adult Salivary Gland  5.324546  
  Adult Testes  6.291972  
  Adult Thoracoabdominal ganglion  4.986909  
  Adult Whole Fly  4.734196  
  Larvae Wandering Tubules  5.660293  
  Larval Feeding Carcass  4.947928  
  Larval Feeding Central Nevous System  4.743662  
  Larval Feeding Hind Gut  4.905432  
  Larval Feeding Malpighian Tubule  5.396434  
  Larval Feeding Mid Gut  5.085912  
  Larval Feeding Salivary Gland  5.113805  
  Whole Larvae Feeding  4.801482  
 
  
   FlyBase ID    symbol    start    end    strand    length   
   FBgn0034005   alphaPS4   11146272   11150371  +  4100  
   FBgn0083959     11150309   11180386   -  30078  
   FBgn0085216     11180439   11182593   -  2155  
   FBgn0034007   CG8102  11184269   11185834   -  1566  
 
 
    Segment 231 
 
   Location   
  Gene key  FBgn0034008-FBgn0013750  
  Heatmap region span   2R:11103716..11268223   
  Segment span   2R:11198634..11209979   
  Length (genes)  4  
  Length (bp)  11346  
   Model Scoring   
  BIC  403.982614  
  logL  -196.470455  
  logL ratio  108.561223  
   Expression   
  Mean expression  8.464158  
  Median expression  8.269519  
  Tissue std. dev.  0.534336  
 
  No GO Slim enrichment  
  
   tissue    mean expression   
  5th Passage Drosophila S2 Cells  9.293966  
  Adult Accessory gland  7.926524  
  Adult Brain  9.149256  
  Adult Carcass  7.942521  
  Adult Crop  8.672607  
  Adult Eye  8.348965  
  Adult Fatbody  8.504179  
  Adult Female Spermatheca Mated  8.109205  
  Adult Female Spermatheca Virgin  8.145941  
  Adult Head  8.410940  
  Adult Heart  8.578871  
  Adult Hind Gut  8.188080  
  Adult Male Ejaculatory Duct  8.292292  
  Adult Mid Gut  7.976550  
  Adult Ovary  9.937014  
  Adult Salivary Gland  7.723604  
  Adult Testes  7.885449  
  Adult Thoracoabdominal ganglion  8.885955  
  Adult Whole Fly  8.781406  
  Larvae Wandering Tubules  8.643714  
  Larval Feeding Carcass  8.171352  
  Larval Feeding Central Nevous System  9.483332  
  Larval Feeding Hind Gut  8.909373  
  Larval Feeding Malpighian Tubule  8.532908  
  Larval Feeding Mid Gut  8.178759  
  Larval Feeding Salivary Gland  7.954908  
  Whole Larvae Feeding  7.904586  
 
  
   FlyBase ID    symbol    start    end    strand    length   
   FBgn0034008   CG8152  11197436   11198634   -  1199  
   FBgn0004698   mus210   11198991   11205347  +  6357  
   FBgn0034009   CG8155  11205349   11209674   -  4326  
   FBgn0013750   Arf51F   11209979   11212399  +  2421  
 
 
    Segment 232 
 
   Location   
  Gene key  FBgn0034011-FBgn0034012  
  Heatmap region span   2R:11136290..11296351   
  Segment span   2R:11215770..11219106   
  Length (genes)  2  
  Length (bp)  3337  
   Model Scoring   
  BIC  202.405802  
  logL  -95.682048  
  logL ratio  26.553360  
   Expression   
  Mean expression  4.416331  
  Median expression  4.166737  
  Tissue std. dev.  0.504113  
 
  No GO Slim enrichment  
  
   tissue    mean expression   
  5th Passage Drosophila S2 Cells  4.729100  
  Adult Accessory gland  4.075817  
  Adult Brain  5.129673  
  Adult Carcass  4.073260  
  Adult Crop  4.149929  
  Adult Eye  3.902363  
  Adult Fatbody  4.207491  
  Adult Female Spermatheca Mated  4.405145  
  Adult Female Spermatheca Virgin  4.341105  
  Adult Head  4.159479  
  Adult Heart  4.071751  
  Adult Hind Gut  4.073179  
  Adult Male Ejaculatory Duct  4.039332  
  Adult Mid Gut  4.147213  
  Adult Ovary  3.900850  
  Adult Salivary Gland  4.313497  
  Adult Testes  4.992577  
  Adult Thoracoabdominal ganglion  4.795068  
  Adult Whole Fly  3.977011  
  Larvae Wandering Tubules  4.120076  
  Larval Feeding Carcass  4.131568  
  Larval Feeding Central Nevous System  5.091720  
  Larval Feeding Hind Gut  4.658586  
  Larval Feeding Malpighian Tubule  4.276707  
  Larval Feeding Mid Gut  4.137072  
  Larval Feeding Salivary Gland  5.319389  
  Whole Larvae Feeding  6.021990  
 
  
   FlyBase ID    symbol    start    end    strand    length   
   FBgn0034011   CG8160  11214638   11215770   -  1133  
   FBgn0034012   Hr51   11219106   11226825  +  7720  
 
    Segment 233 
 
   Location   
  Gene key  FBgn0085394-FBgn0050470  
  Heatmap region span   2R:11259021..11443303   
  Segment span   2R:11297677..11348416   
  Length (genes)  3  
  Length (bp)  50740  
   Model Scoring   
  BIC  269.833339  
  logL  -129.395817  
  logL ratio  49.281037  
   Expression   
  Mean expression  4.731125  
  Median expression  4.771884  
  Tissue std. dev.  0.373829  
 
  No GO Slim enrichment  
  
   tissue    mean expression   
  5th Passage Drosophila S2 Cells  4.582785  
  Adult Accessory gland  4.714410  
  Adult Brain  4.475086  
  Adult Carcass  5.573739  
  Adult Crop  5.050428  
  Adult Eye  4.559199  
  Adult Fatbody  4.585234  
  Adult Female Spermatheca Mated  4.559002  
  Adult Female Spermatheca Virgin  4.575228  
  Adult Head  4.539620  
  Adult Heart  4.555704  
  Adult Hind Gut  4.835994  
  Adult Male Ejaculatory Duct  4.522768  
  Adult Mid Gut  4.748697  
  Adult Ovary  4.454391  
  Adult Salivary Gland  4.691764  
  Adult Testes  6.224186  
  Adult Thoracoabdominal ganglion  4.546124  
  Adult Whole Fly  4.899280  
  Larvae Wandering Tubules  4.671850  
  Larval Feeding Carcass  4.696242  
  Larval Feeding Central Nevous System  4.266194  
  Larval Feeding Hind Gut  4.706871  
  Larval Feeding Malpighian Tubule  4.605954  
  Larval Feeding Mid Gut  4.605534  
  Larval Feeding Salivary Gland  4.854689  
  Whole Larvae Feeding  4.639410  
 
  
   FlyBase ID    symbol    start    end    strand    length   
   FBgn0085394      11297677   11351352  +  53676  
   FBgn0085217   CG34188  11314596   11315528   -  933  
   FBgn0050470      11348416   11349063  +  648  
 
 
    Segment 234 
 
   Location   
  Gene key  FBgn0034022-FBgn0050471  
  Heatmap region span   2R:11268465..11458973   
  Segment span   2R:11384712..11422700   
  Length (genes)  6  
  Length (bp)  37989  
   Model Scoring   
  BIC  418.656646  
  logL  -203.807471  
  logL ratio  217.567347  
   Expression   
  Mean expression  4.410519  
  Median expression  4.380901  
  Tissue std. dev.  0.149449  
 
  No GO Slim enrichment  
  
   tissue    mean expression   
  5th Passage Drosophila S2 Cells  4.465193  
  Adult Accessory gland  4.686232  
  Adult Brain  4.195884  
  Adult Carcass  4.480299  
  Adult Crop  4.426476  
  Adult Eye  4.279317  
  Adult Fatbody  4.443690  
  Adult Female Spermatheca Mated  4.470197  
  Adult Female Spermatheca Virgin  4.474815  
  Adult Head  4.248599  
  Adult Heart  4.453143  
  Adult Hind Gut  4.384295  
  Adult Male Ejaculatory Duct  4.439150  
  Adult Mid Gut  4.568895  
  Adult Ovary  4.356966  
  Adult Salivary Gland  4.667605  
  Adult Testes  4.293049  
  Adult Thoracoabdominal ganglion  4.248809  
  Adult Whole Fly  4.063250  
  Larvae Wandering Tubules  4.548808  
  Larval Feeding Carcass  4.496332  
  Larval Feeding Central Nevous System  4.250134  
  Larval Feeding Hind Gut  4.319976  
  Larval Feeding Malpighian Tubule  4.563918  
  Larval Feeding Mid Gut  4.552503  
  Larval Feeding Salivary Gland  4.514550  
  Whole Larvae Feeding  4.191929  
 
  
   FlyBase ID    symbol    start    end    strand    length   
   FBgn0034022   CG12964  11373457   11384712   -  11256  
   FBgn0034023   Ir52a   11387137   11388936  +  1800  
   FBgn0050469   Ir52b   11389325   11391115  +  1791  
   FBgn0050468   Ir52c   11391706   11393505  +  1800  
   FBgn0050464   Ir52d   11394087   11395871  +  1785  
   FBgn0050471   CG30471   11422700   11425155  +  2456  
 
 
    Segment 235 
 
   Location   
  Gene key  FBgn0050467-FBgn0034030  
  Heatmap region span   2R:11296351..11566444   
  Segment span   2R:11425718..11437485   
  Length (genes)  3  
  Length (bp)  11768  
   Model Scoring   
  BIC  315.733373  
  logL  -152.345834  
  logL ratio  42.274307  
   Expression   
  Mean expression  6.493817  
  Median expression  6.434272  
  Tissue std. dev.  0.318488  
 
  
   GO ID    description    ratio    P-value   
   GO:0005576   extracellular region  2/3  0.00306  
 
  
   tissue    mean expression   
  5th Passage Drosophila S2 Cells  6.178542  
  Adult Accessory gland  5.957160  
  Adult Brain  6.932916  
  Adult Carcass  6.673284  
  Adult Crop  6.596848  
  Adult Eye  6.747438  
  Adult Fatbody  6.275081  
  Adult Female Spermatheca Mated  6.455280  
  Adult Female Spermatheca Virgin  6.534149  
  Adult Head  6.332041  
  Adult Heart  6.379288  
  Adult Hind Gut  6.335443  
  Adult Male Ejaculatory Duct  6.514276  
  Adult Mid Gut  6.025114  
  Adult Ovary  7.087421  
  Adult Salivary Gland  6.638010  
  Adult Testes  6.121496  
  Adult Thoracoabdominal ganglion  7.186407  
  Adult Whole Fly  6.197393  
  Larvae Wandering Tubules  6.716569  
  Larval Feeding Carcass  6.686729  
  Larval Feeding Central Nevous System  6.968640  
  Larval Feeding Hind Gut  6.347726  
  Larval Feeding Malpighian Tubule  6.600486  
  Larval Feeding Mid Gut  5.989126  
  Larval Feeding Salivary Gland  6.546965  
  Whole Larvae Feeding  6.309229  
 
  
   FlyBase ID    symbol    start    end    strand    length   
   FBgn0050467   CG30467   11425718   11427520  +  1803  
   FBgn0034027   CG8187  11427057   11428840   -  1784  
   FBgn0034030   CG8192  11431820   11437485   -  5666  
 
 
    Segment 236 
 
   Location   
  Gene key  FBgn0034032-FBgn0013762  
  Heatmap region span   2R:11370338..11570221   
  Segment span   2R:11453291..11458918   
  Length (genes)  3  
  Length (bp)  5628  
   Model Scoring   
  BIC  302.552991  
  logL  -145.755643  
  logL ratio  95.467092  
   Expression   
  Mean expression  9.329071  
  Median expression  9.290634  
  Tissue std. dev.  0.572130  
 
  No GO Slim enrichment  
  
   tissue    mean expression   
  5th Passage Drosophila S2 Cells  9.542604  
  Adult Accessory gland  8.711786  
  Adult Brain  10.314910  
  Adult Carcass  8.608052  
  Adult Crop  9.692633  
  Adult Eye  9.776295  
  Adult Fatbody  9.043433  
  Adult Female Spermatheca Mated  9.558403  
  Adult Female Spermatheca Virgin  9.573175  
  Adult Head  9.596155  
  Adult Heart  9.627173  
  Adult Hind Gut  9.134408  
  Adult Male Ejaculatory Duct  9.456599  
  Adult Mid Gut  8.581719  
  Adult Ovary  9.378982  
  Adult Salivary Gland  9.302874  
  Adult Testes  7.787914  
  Adult Thoracoabdominal ganglion  10.254615  
  Adult Whole Fly  8.664054  
  Larvae Wandering Tubules  9.859913  
  Larval Feeding Carcass  8.965280  
  Larval Feeding Central Nevous System  10.401646  
  Larval Feeding Hind Gut  9.406418  
  Larval Feeding Malpighian Tubule  9.569337  
  Larval Feeding Mid Gut  9.176256  
  Larval Feeding Salivary Gland  8.998851  
  Whole Larvae Feeding  8.901422  
 
  
   FlyBase ID    symbol    start    end    strand    length   
   FBgn0034032   CG8195  11450201   11453291   -  3091  
   FBgn0024754   Flo-1  11453772   11456907   -  3136  
   FBgn0013762   Cdk5  11457035   11458918   -  1884  
 
 
    Segment 237 
 
   Location   
  Gene key  FBgn0034033-FBgn0034035  
  Heatmap region span   2R:11425718..11589199   
  Segment span   2R:11461496..11566444   
  Length (genes)  3  
  Length (bp)  104949  
   Model Scoring   
  BIC  320.552684  
  logL  -154.755490  
  logL ratio  60.051571  
   Expression   
  Mean expression  8.473631  
  Median expression  8.465152  
  Tissue std. dev.  0.615041  
 
  No GO Slim enrichment  
  
   tissue    mean expression   
  5th Passage Drosophila S2 Cells  7.400284  
  Adult Accessory gland  8.672015  
  Adult Brain  6.883499  
  Adult Carcass  8.554946  
  Adult Crop  8.872781  
  Adult Eye  8.544909  
  Adult Fatbody  9.075582  
  Adult Female Spermatheca Mated  8.972243  
  Adult Female Spermatheca Virgin  8.859940  
  Adult Head  8.146689  
  Adult Heart  9.173455  
  Adult Hind Gut  8.512909  
  Adult Male Ejaculatory Duct  9.282018  
  Adult Mid Gut  8.453501  
  Adult Ovary  8.528445  
  Adult Salivary Gland  9.006655  
  Adult Testes  8.342243  
  Adult Thoracoabdominal ganglion  6.951466  
  Adult Whole Fly  8.174690  
  Larvae Wandering Tubules  8.821164  
  Larval Feeding Carcass  8.499152  
  Larval Feeding Central Nevous System  7.679451  
  Larval Feeding Hind Gut  8.461473  
  Larval Feeding Malpighian Tubule  8.794362  
  Larval Feeding Mid Gut  8.419036  
  Larval Feeding Salivary Gland  9.329077  
  Whole Larvae Feeding  8.376067  
 
  
   FlyBase ID    symbol    start    end    strand    length   
   FBgn0034033   CG8204  11460641   11461496   -  856  
   FBgn0023441   fus  11544823   11564198   -  19376  
   FBgn0034035   CG8207  11564723   11566444   -  1722  
 
 
    Segment 238 
 
   Location   
  Gene key  FBgn0034037-FBgn0053462  
  Heatmap region span   2R:11458973..11704314   
  Segment span   2R:11576490..11584309   
  Length (genes)  5  
  Length (bp)  7820  
   Model Scoring   
  BIC  468.995889  
  logL  -228.977092  
  logL ratio  62.512908  
   Expression   
  Mean expression  4.744962  
  Median expression  4.438401  
  Tissue std. dev.  0.485837  
 
  No GO Slim enrichment  
  
   tissue    mean expression   
  5th Passage Drosophila S2 Cells  6.195261  
  Adult Accessory gland  5.689863  
  Adult Brain  4.153254  
  Adult Carcass  4.778526  
  Adult Crop  4.321616  
  Adult Eye  4.873952  
  Adult Fatbody  5.081057  
  Adult Female Spermatheca Mated  4.712772  
  Adult Female Spermatheca Virgin  5.040869  
  Adult Head  4.876131  
  Adult Heart  4.754641  
  Adult Hind Gut  4.465095  
  Adult Male Ejaculatory Duct  4.792059  
  Adult Mid Gut  5.138313  
  Adult Ovary  4.322315  
  Adult Salivary Gland  4.503169  
  Adult Testes  5.663405  
  Adult Thoracoabdominal ganglion  4.234173  
  Adult Whole Fly  4.282338  
  Larvae Wandering Tubules  4.789184  
  Larval Feeding Carcass  4.586905  
  Larval Feeding Central Nevous System  4.157770  
  Larval Feeding Hind Gut  4.511187  
  Larval Feeding Malpighian Tubule  4.447039  
  Larval Feeding Mid Gut  4.471481  
  Larval Feeding Salivary Gland  4.314668  
  Whole Larvae Feeding  4.956926  
 
  
   FlyBase ID    symbol    start    end    strand    length   
   FBgn0034037   CG8214  11574893   11576490   -  1598  
   FBgn0050090   CG30090   11577348   11578519  +  1172  
   FBgn0050088   CG30088   11578657   11579680  +  1024  
   FBgn0050087   CG30087   11579953   11581256  +  1304  
   FBgn0053462   CG33462   11584309   11585515  +  1207  
 
 
    Segment 239 
 
   Location   
  Gene key  FBgn0053465-FBgn0003130  
  Heatmap region span   2R:11589199..11822330   
  Segment span   2R:11708600..11722280   
  Length (genes)  2  
  Length (bp)  13681  
   Model Scoring   
  BIC  173.715245  
  logL  -81.336770  
  logL ratio  40.842545  
   Expression   
  Mean expression  4.466772  
  Median expression  4.399237  
  Tissue std. dev.  0.297713  
 
  No GO Slim enrichment  
  
   tissue    mean expression   
  5th Passage Drosophila S2 Cells  4.583197  
  Adult Accessory gland  4.454985  
  Adult Brain  4.581878  
  Adult Carcass  4.558260  
  Adult Crop  4.335058  
  Adult Eye  4.182412  
  Adult Fatbody  4.609620  
  Adult Female Spermatheca Mated  4.473077  
  Adult Female Spermatheca Virgin  4.294136  
  Adult Head  4.318104  
  Adult Heart  4.263051  
  Adult Hind Gut  4.259775  
  Adult Male Ejaculatory Duct  4.349619  
  Adult Mid Gut  4.381863  
  Adult Ovary  4.291377  
  Adult Salivary Gland  4.523848  
  Adult Testes  4.202615  
  Adult Thoracoabdominal ganglion  4.329056  
  Adult Whole Fly  3.902775  
  Larvae Wandering Tubules  5.287376  
  Larval Feeding Carcass  5.350000  
  Larval Feeding Central Nevous System  4.583083  
  Larval Feeding Hind Gut  4.321143  
  Larval Feeding Malpighian Tubule  4.650126  
  Larval Feeding Mid Gut  4.366573  
  Larval Feeding Salivary Gland  4.367776  
  Whole Larvae Feeding  4.782054  
 
  
   FlyBase ID    symbol    start    end    strand    length   
   FBgn0053465   CG33465  11706619   11708600   -  1982  
   FBgn0003130   Poxn  11714198   11722280   -  8083  
 
    Segment 240 
 
   Location   
  Gene key  FBgn0034045-FBgn0034046  
  Heatmap region span   2R:11600149..11822373   
  Segment span   2R:11734865..11736111   
  Length (genes)  2  
  Length (bp)  1247  
   Model Scoring   
  BIC  252.898499  
  logL  -120.928397  
  logL ratio  -7.283069  
   Expression   
  Mean expression  7.106231  
  Median expression  6.625010  
  Tissue std. dev.  0.931713  
 
  No GO Slim enrichment  
  
   tissue    mean expression   
  5th Passage Drosophila S2 Cells  7.257169  
  Adult Accessory gland  6.238126  
  Adult Brain  6.644213  
  Adult Carcass  7.990662  
  Adult Crop  6.274670  
  Adult Eye  6.484561  
  Adult Fatbody  9.166573  
  Adult Female Spermatheca Mated  9.021677  
  Adult Female Spermatheca Virgin  9.054686  
  Adult Head  7.168499  
  Adult Heart  7.762475  
  Adult Hind Gut  7.470619  
  Adult Male Ejaculatory Duct  6.020904  
  Adult Mid Gut  7.214118  
  Adult Ovary  7.619654  
  Adult Salivary Gland  5.769369  
  Adult Testes  7.251864  
  Adult Thoracoabdominal ganglion  6.513191  
  Adult Whole Fly  7.209835  
  Larvae Wandering Tubules  7.517537  
  Larval Feeding Carcass  7.062749  
  Larval Feeding Central Nevous System  6.190618  
  Larval Feeding Hind Gut  7.266732  
  Larval Feeding Malpighian Tubule  7.550315  
  Larval Feeding Mid Gut  6.138496  
  Larval Feeding Salivary Gland  5.792421  
  Whole Larvae Feeding  6.216496  
 
  
   FlyBase ID    symbol    start    end    strand    length   
   FBgn0034045   CG8249  11731595   11734865   -  3271  
   FBgn0034046   tun   11736111   11741092  +  4982  
 
    Segment 241 
 
   Location   
  Gene key  FBgn0050085-FBgn0034049  
  Heatmap region span   2R:11704314..11828988   
  Segment span   2R:11756650..11819434   
  Length (genes)  2  
  Length (bp)  62785  
   Model Scoring   
  BIC  247.230709  
  logL  -118.094502  
  logL ratio  4.452401  
   Expression   
  Mean expression  7.243788  
  Median expression  7.141824  
  Tissue std. dev.  0.939711  
 
  No GO Slim enrichment  
  
   tissue    mean expression   
  5th Passage Drosophila S2 Cells  10.102004  
  Adult Accessory gland  6.627922  
  Adult Brain  7.003524  
  Adult Carcass  6.724882  
  Adult Crop  7.102556  
  Adult Eye  6.651084  
  Adult Fatbody  6.258168  
  Adult Female Spermatheca Mated  6.455343  
  Adult Female Spermatheca Virgin  6.264143  
  Adult Head  6.772614  
  Adult Heart  6.372630  
  Adult Hind Gut  8.047779  
  Adult Male Ejaculatory Duct  6.871428  
  Adult Mid Gut  6.628810  
  Adult Ovary  9.392653  
  Adult Salivary Gland  5.988748  
  Adult Testes  7.432790  
  Adult Thoracoabdominal ganglion  7.038685  
  Adult Whole Fly  7.975623  
  Larvae Wandering Tubules  6.812628  
  Larval Feeding Carcass  7.804724  
  Larval Feeding Central Nevous System  8.533328  
  Larval Feeding Hind Gut  7.748605  
  Larval Feeding Malpighian Tubule  7.006341  
  Larval Feeding Mid Gut  6.880929  
  Larval Feeding Salivary Gland  7.934677  
  Whole Larvae Feeding  7.149646  
 
  
   FlyBase ID    symbol    start    end    strand    length   
   FBgn0050085   Rif1  11751809   11756650   -  4842  
   FBgn0034049   bdg  11810545   11819434   -  8890  
 
    Segment 242 
 
   Location   
  Gene key  FBgn0034057-FBgn0034060  
  Heatmap region span   2R:11830386..11902285   
  Segment span   2R:11878787..11888628   
  Length (genes)  4  
  Length (bp)  9842  
   Model Scoring   
  BIC  389.398416  
  logL  -189.178356  
  logL ratio  119.783558  
   Expression   
  Mean expression  8.592675  
  Median expression  8.798082
[truncated: 377,205 more chars]
