# Supplementary material for: Expression-based segmentation of the Drosophila genome
Source: BMC Genomics. 2013 Nov 20;14:812. doi: 10.1186/1471-2164-14-812 (PMC3909303; doi:10.1186/1471-2164-14-812)
Supplement: Additional file 1 — Detailed information for multigene segments. [file 1471-2164-14-812-S1.zip › miniwebsite/chr3L.html]

   ExprSeg Report for chr3L   
 Report for /Users/afrubin/Code/ExprSeg/2012-07-19/output Generated Fri Jun 21 23:03:10 2013 
   Chromosome 3L 
 496 segments 2272 genes 
    Segment 1 
 
   Location   
  Gene key  FBgn0035097-FBgn0040688  
  Heatmap region span   3L:22947..176234   
  Segment span   3L:75266..98371   
  Length (genes)  2  
  Length (bp)  23106  
   Model Scoring   
  BIC  216.191098  
  logL  -102.583423  
  logL ratio  15.453858  
   Expression   
  Mean expression  4.229763  
  Median expression  3.951956  
  Tissue std. dev.  0.818746  
 
  No GO Slim enrichment  
  
   tissue    mean expression   
  5th Passage Drosophila S2 Cells  3.961947  
  Adult Accessory gland  4.101485  
  Adult Brain  3.886767  
  Adult Carcass  5.949857  
  Adult Crop  3.975314  
  Adult Eye  3.873305  
  Adult Fatbody  3.962886  
  Adult Female Spermatheca Mated  3.999541  
  Adult Female Spermatheca Virgin  3.950208  
  Adult Head  4.109048  
  Adult Heart  3.958667  
  Adult Hind Gut  3.927961  
  Adult Male Ejaculatory Duct  4.074505  
  Adult Mid Gut  3.914899  
  Adult Ovary  3.933447  
  Adult Salivary Gland  3.942117  
  Adult Testes  7.597617  
  Adult Thoracoabdominal ganglion  3.917306  
  Adult Whole Fly  5.602735  
  Larvae Wandering Tubules  4.044454  
  Larval Feeding Carcass  3.964653  
  Larval Feeding Central Nevous System  3.808329  
  Larval Feeding Hind Gut  3.820561  
  Larval Feeding Malpighian Tubule  3.893559  
  Larval Feeding Mid Gut  3.934324  
  Larval Feeding Salivary Gland  3.932378  
  Whole Larvae Feeding  4.165736  
 
  
   FlyBase ID    symbol    start    end    strand    length   
   FBgn0035097   CG13405  74470   75266   -  797  
   FBgn0040688   CG12483  97619   98371   -  753  
 
    Segment 2 
 
   Location   
  Gene key  FBgn0035099-FBgn0035101  
  Heatmap region span   3L:22947..180294   
  Segment span   3L:146453..150857   
  Length (genes)  2  
  Length (bp)  4405  
   Model Scoring   
  BIC  256.747572  
  logL  -122.861660  
  logL ratio  -1.933928  
   Expression   
  Mean expression  7.831236  
  Median expression  7.778803  
  Tissue std. dev.  0.979962  
 
  No GO Slim enrichment  
  
   tissue    mean expression   
  5th Passage Drosophila S2 Cells  8.647337  
  Adult Accessory gland  6.323801  
  Adult Brain  8.049891  
  Adult Carcass  7.255126  
  Adult Crop  7.297626  
  Adult Eye  7.739266  
  Adult Fatbody  7.509058  
  Adult Female Spermatheca Mated  7.047327  
  Adult Female Spermatheca Virgin  6.866812  
  Adult Head  7.138030  
  Adult Heart  8.166089  
  Adult Hind Gut  8.482585  
  Adult Male Ejaculatory Duct  6.700899  
  Adult Mid Gut  9.247546  
  Adult Ovary  6.808414  
  Adult Salivary Gland  6.647849  
  Adult Testes  6.653699  
  Adult Thoracoabdominal ganglion  8.959899  
  Adult Whole Fly  6.791892  
  Larvae Wandering Tubules  9.429869  
  Larval Feeding Carcass  6.961778  
  Larval Feeding Central Nevous System  9.336184  
  Larval Feeding Hind Gut  8.439415  
  Larval Feeding Malpighian Tubule  9.475452  
  Larval Feeding Mid Gut  8.659004  
  Larval Feeding Salivary Gland  8.712122  
  Whole Larvae Feeding  8.096395  
 
  
   FlyBase ID    symbol    start    end    strand    length   
   FBgn0035099   CG6845   146453   149056  +  2604  
   FBgn0035101   p130CAS   150857   174524  +  23668  
 
    Segment 3 
 
   Location   
  Gene key  FBgn0035107-FBgn0025592  
  Heatmap region span   3L:178629..223069   
  Segment span   3L:200180..205844   
  Length (genes)  3  
  Length (bp)  5665  
   Model Scoring   
  BIC  313.739429  
  logL  -151.357588  
  logL ratio  106.932783  
   Expression   
  Mean expression  9.885049  
  Median expression  9.720297  
  Tissue std. dev.  0.485949  
 
  No GO Slim enrichment  
  
   tissue    mean expression   
  5th Passage Drosophila S2 Cells  9.374084  
  Adult Accessory gland  10.275737  
  Adult Brain  9.335797  
  Adult Carcass  10.362526  
  Adult Crop  10.417250  
  Adult Eye  9.623194  
  Adult Fatbody  10.877624  
  Adult Female Spermatheca Mated  10.404067  
  Adult Female Spermatheca Virgin  10.834639  
  Adult Head  9.894870  
  Adult Heart  10.510988  
  Adult Hind Gut  10.104425  
  Adult Male Ejaculatory Duct  10.399298  
  Adult Mid Gut  9.873836  
  Adult Ovary  9.451927  
  Adult Salivary Gland  10.092210  
  Adult Testes  8.986640  
  Adult Thoracoabdominal ganglion  9.230643  
  Adult Whole Fly  9.641203  
  Larvae Wandering Tubules  9.603000  
  Larval Feeding Carcass  9.334425  
  Larval Feeding Central Nevous System  9.448924  
  Larval Feeding Hind Gut  9.680107  
  Larval Feeding Malpighian Tubule  9.842719  
  Larval Feeding Mid Gut  9.829156  
  Larval Feeding Salivary Gland  9.853360  
  Whole Larvae Feeding  9.613685  
 
  
   FlyBase ID    symbol    start    end    strand    length   
   FBgn0035107   mri   200180   202165  +  1986  
   FBgn0024945   NitFhit   203699   205309  +  1611  
   FBgn0025592   Gyk  202098   205844   -  3747  
 
 
    Segment 4 
 
   Location   
  Gene key  FBgn0027587-FBgn0035110  
  Heatmap region span   3L:184635..247495   
  Segment span   3L:211462..212687   
  Length (genes)  3  
  Length (bp)  1226  
   Model Scoring   
  BIC  270.265465  
  logL  -129.620606  
  logL ratio  114.257184  
   Expression   
  Mean expression  8.990602  
  Median expression  8.938533  
  Tissue std. dev.  0.433667  
 
  No GO Slim enrichment  
  
   tissue    mean expression   
  5th Passage Drosophila S2 Cells  8.778718  
  Adult Accessory gland  9.435280  
  Adult Brain  9.382227  
  Adult Carcass  8.241511  
  Adult Crop  9.161914  
  Adult Eye  8.991691  
  Adult Fatbody  8.835302  
  Adult Female Spermatheca Mated  9.154913  
  Adult Female Spermatheca Virgin  9.027081  
  Adult Head  8.642465  
  Adult Heart  9.075148  
  Adult Hind Gut  8.661536  
  Adult Male Ejaculatory Duct  9.030443  
  Adult Mid Gut  8.376856  
  Adult Ovary  10.283105  
  Adult Salivary Gland  9.048509  
  Adult Testes  8.858065  
  Adult Thoracoabdominal ganglion  9.015234  
  Adult Whole Fly  9.091513  
  Larvae Wandering Tubules  9.009893  
  Larval Feeding Carcass  8.839414  
  Larval Feeding Central Nevous System  9.771552  
  Larval Feeding Hind Gut  8.839683  
  Larval Feeding Malpighian Tubule  8.902200  
  Larval Feeding Mid Gut  8.304939  
  Larval Feeding Salivary Gland  9.535667  
  Whole Larvae Feeding  8.451383  
 
  
   FlyBase ID    symbol    start    end    strand    length   
   FBgn0027587   CG7028  207887   211462   -  3576  
   FBgn0035111   CG16940   212275   216479  +  4205  
   FBgn0035110   thoc7  211636   212687   -  1052  
 
 
    Segment 5 
 
   Location   
  Gene key  FBgn0024806-FBgn0035122  
  Heatmap region span   3L:218067..327987   
  Segment span   3L:257950..264782   
  Length (genes)  3  
  Length (bp)  6833  
   Model Scoring   
  BIC  388.352968  
  logL  -188.664358  
  logL ratio  2.111619  
   Expression   
  Mean expression  8.794950  
  Median expression  9.230932  
  Tissue std. dev.  0.662014  
 
  No GO Slim enrichment  
  
   tissue    mean expression   
  5th Passage Drosophila S2 Cells  9.629305  
  Adult Accessory gland  8.439544  
  Adult Brain  9.471851  
  Adult Carcass  7.917555  
  Adult Crop  8.416129  
  Adult Eye  9.819092  
  Adult Fatbody  8.321325  
  Adult Female Spermatheca Mated  7.756295  
  Adult Female Spermatheca Virgin  7.778325  
  Adult Head  8.895749  
  Adult Heart  8.775364  
  Adult Hind Gut  8.746826  
  Adult Male Ejaculatory Duct  8.004512  
  Adult Mid Gut  8.333920  
  Adult Ovary  10.151748  
  Adult Salivary Gland  8.478625  
  Adult Testes  9.254662  
  Adult Thoracoabdominal ganglion  9.297427  
  Adult Whole Fly  9.177194  
  Larvae Wandering Tubules  8.565930  
  Larval Feeding Carcass  8.629251  
  Larval Feeding Central Nevous System  10.351749  
  Larval Feeding Hind Gut  8.933418  
  Larval Feeding Malpighian Tubule  8.569490  
  Larval Feeding Mid Gut  8.302281  
  Larval Feeding Salivary Gland  8.836841  
  Whole Larvae Feeding  8.609238  
 
  
   FlyBase ID    symbol    start    end    strand    length   
   FBgn0024806   DIP2  250229   257950   -  7722  
   FBgn0035120   wac   258350   259097  +  748  
   FBgn0035122   mRpL17   264782   265599  +  818  
 
 
    Segment 6 
 
   Location   
  Gene key  FBgn0052845-FBgn0085293  
  Heatmap region span   3L:247495..357981   
  Segment span   3L:273370..317084   
  Length (genes)  5  
  Length (bp)  43715  
   Model Scoring   
  BIC  453.346297  
  logL  -221.161022  
  logL ratio  71.634979  
   Expression   
  Mean expression  4.950408  
  Median expression  4.567699  
  Tissue std. dev.  0.621082  
 
  No GO Slim enrichment  
  
   tissue    mean expression   
  5th Passage Drosophila S2 Cells  4.908219  
  Adult Accessory gland  4.791449  
  Adult Brain  4.999469  
  Adult Carcass  4.849489  
  Adult Crop  4.871578  
  Adult Eye  4.687147  
  Adult Fatbody  5.034770  
  Adult Female Spermatheca Mated  5.056411  
  Adult Female Spermatheca Virgin  5.094461  
  Adult Head  4.823282  
  Adult Heart  4.740613  
  Adult Hind Gut  4.631988  
  Adult Male Ejaculatory Duct  4.785983  
  Adult Mid Gut  4.633381  
  Adult Ovary  4.559293  
  Adult Salivary Gland  5.027749  
  Adult Testes  7.956298  
  Adult Thoracoabdominal ganglion  4.983953  
  Adult Whole Fly  5.371582  
  Larvae Wandering Tubules  4.626645  
  Larval Feeding Carcass  4.748795  
  Larval Feeding Central Nevous System  4.949093  
  Larval Feeding Hind Gut  4.579303  
  Larval Feeding Malpighian Tubule  4.608339  
  Larval Feeding Mid Gut  4.612910  
  Larval Feeding Salivary Gland  4.677399  
  Whole Larvae Feeding  5.051419  
 
  
   FlyBase ID    symbol    start    end    strand    length   
   FBgn0052845   CG32845  272210   273370   -  1161  
   FBgn0035124   ttm2   275164   276661  +  1498  
   FBgn0259744      277437   290334  +  12898  
   FBgn0259743      290727   305292  +  14566  
   FBgn0085293   CG34264   317084   317789  +  706  
 
 
    Segment 7 
 
   Location   
  Gene key  FBgn0004373-FBgn0052343  
  Heatmap region span   3L:257950..361478   
  Segment span   3L:319880..327987   
  Length (genes)  3  
  Length (bp)  8108  
   Model Scoring   
  BIC  299.145455  
  logL  -144.060601  
  logL ratio  80.201191  
   Expression   
  Mean expression  8.310089  
  Median expression  8.218767  
  Tissue std. dev.  0.421923  
 
  No GO Slim enrichment  
  
   tissue    mean expression   
  5th Passage Drosophila S2 Cells  8.536836  
  Adult Accessory gland  8.821398  
  Adult Brain  8.644958  
  Adult Carcass  7.613173  
  Adult Crop  8.599965  
  Adult Eye  8.221445  
  Adult Fatbody  8.452464  
  Adult Female Spermatheca Mated  8.504924  
  Adult Female Spermatheca Virgin  8.243517  
  Adult Head  7.929823  
  Adult Heart  8.212966  
  Adult Hind Gut  8.244917  
  Adult Male Ejaculatory Duct  8.249760  
  Adult Mid Gut  7.998325  
  Adult Ovary  9.571842  
  Adult Salivary Gland  8.083763  
  Adult Testes  8.362978  
  Adult Thoracoabdominal ganglion  8.343455  
  Adult Whole Fly  8.348535  
  Larvae Wandering Tubules  7.923440  
  Larval Feeding Carcass  8.040576  
  Larval Feeding Central Nevous System  9.194928  
  Larval Feeding Hind Gut  8.341083  
  Larval Feeding Malpighian Tubule  7.894891  
  Larval Feeding Mid Gut  7.668971  
  Larval Feeding Salivary Gland  8.478000  
  Whole Larvae Feeding  7.845462  
 
  
   FlyBase ID    symbol    start    end    strand    length   
   FBgn0004373   fwd  306207   319880   -  13674  
   FBgn0052344   CG32344  321232   324717   -  3486  
   FBgn0052343   Atac3  324985   327987   -  3003  
 
 
    Segment 8 
 
   Location   
  Gene key  FBgn0035131-FBgn0035132  
  Heatmap region span   3L:267764..433906   
  Segment span   3L:332306..336682   
  Length (genes)  2  
  Length (bp)  4377  
   Model Scoring   
  BIC  239.979451  
  logL  -114.477599  
  logL ratio  -8.666753  
   Expression   
  Mean expression  6.291742  
  Median expression  5.738066  
  Tissue std. dev.  1.258169  
 
  
   GO ID    description    ratio    P-value   
   GO:0006950   response to stress  2/2  0  
 
  
   tissue    mean expression   
  5th Passage Drosophila S2 Cells  6.431041  
  Adult Accessory gland  5.349844  
  Adult Brain  5.068816  
  Adult Carcass  5.518763  
  Adult Crop  7.641739  
  Adult Eye  5.295967  
  Adult Fatbody  5.489752  
  Adult Female Spermatheca Mated  5.791391  
  Adult Female Spermatheca Virgin  5.722331  
  Adult Head  5.472809  
  Adult Heart  5.402931  
  Adult Hind Gut  7.769108  
  Adult Male Ejaculatory Duct  5.443058  
  Adult Mid Gut  6.121498  
  Adult Ovary  5.215542  
  Adult Salivary Gland  5.051578  
  Adult Testes  6.298284  
  Adult Thoracoabdominal ganglion  5.200987  
  Adult Whole Fly  5.080820  
  Larvae Wandering Tubules  8.899570  
  Larval Feeding Carcass  7.965115  
  Larval Feeding Central Nevous System  5.304404  
  Larval Feeding Hind Gut  8.864301  
  Larval Feeding Malpighian Tubule  8.486259  
  Larval Feeding Mid Gut  7.879579  
  Larval Feeding Salivary Gland  5.751406  
  Whole Larvae Feeding  7.360138  
 
  
   FlyBase ID    symbol    start    end    strand    length   
   FBgn0035131   mthl9  329822   332306   -  2485  
   FBgn0035132   mthl10  333475   336682   -  3208  
 
    Segment 9 
 
   Location   
  Gene key  FBgn0035134-FBgn0040291  
  Heatmap region span   3L:273370..540589   
  Segment span   3L:356422..357981   
  Length (genes)  2  
  Length (bp)  1560  
   Model Scoring   
  BIC  235.288170  
  logL  -112.131959  
  logL ratio  -7.573636  
   Expression   
  Mean expression  5.954052  
  Median expression  5.582499  
  Tissue std. dev.  0.609778  
 
  No GO Slim enrichment  
  
   tissue    mean expression   
  5th Passage Drosophila S2 Cells  5.772808  
  Adult Accessory gland  5.805098  
  Adult Brain  6.693112  
  Adult Carcass  5.810750  
  Adult Crop  5.671482  
  Adult Eye  5.890458  
  Adult Fatbody  5.858743  
  Adult Female Spermatheca Mated  5.854129  
  Adult Female Spermatheca Virgin  5.866911  
  Adult Head  5.968599  
  Adult Heart  5.362855  
  Adult Hind Gut  5.676658  
  Adult Male Ejaculatory Duct  5.789184  
  Adult Mid Gut  5.663421  
  Adult Ovary  5.757594  
  Adult Salivary Gland  5.677634  
  Adult Testes  8.625087  
  Adult Thoracoabdominal ganglion  6.839016  
  Adult Whole Fly  6.182113  
  Larvae Wandering Tubules  5.783690  
  Larval Feeding Carcass  5.791268  
  Larval Feeding Central Nevous System  6.249216  
  Larval Feeding Hind Gut  5.642981  
  Larval Feeding Malpighian Tubule  5.740207  
  Larval Feeding Mid Gut  5.509623  
  Larval Feeding Salivary Gland  5.671240  
  Whole Larvae Feeding  5.605514  
 
  
   FlyBase ID    symbol    start    end    strand    length   
   FBgn0035134   CG1231   356422   357664  +  1243  
   FBgn0040291   Roc1b   357981   358486  +  506  
 
    Segment 10 
 
   Location   
  Gene key  FBgn0035136-FBgn0035137  
  Heatmap region span   3L:319880..542916   
  Segment span   3L:361383..361478   
  Length (genes)  2  
  Length (bp)  96  
   Model Scoring   
  BIC  205.549113  
  logL  -97.262430  
  logL ratio  48.420309  
   Expression   
  Mean expression  8.219848  
  Median expression  8.171821  
  Tissue std. dev.  0.398569  
 
  No GO Slim enrichment  
  
   tissue    mean expression   
  5th Passage Drosophila S2 Cells  8.811700  
  Adult Accessory gland  8.367465  
  Adult Brain  8.339517  
  Adult Carcass  7.609916  
  Adult Crop  8.082653  
  Adult Eye  7.848404  
  Adult Fatbody  8.134135  
  Adult Female Spermatheca Mated  8.506350  
  Adult Female Spermatheca Virgin  8.547988  
  Adult Head  7.641668  
  Adult Heart  8.223746  
  Adult Hind Gut  8.129403  
  Adult Male Ejaculatory Duct  8.414755  
  Adult Mid Gut  8.291055  
  Adult Ovary  8.710338  
  Adult Salivary Gland  8.495121  
  Adult Testes  7.227365  
  Adult Thoracoabdominal ganglion  7.961648  
  Adult Whole Fly  7.581732  
  Larvae Wandering Tubules  8.647202  
  Larval Feeding Carcass  8.072689  
  Larval Feeding Central Nevous System  8.725506  
  Larval Feeding Hind Gut  8.318707  
  Larval Feeding Malpighian Tubule  8.748558  
  Larval Feeding Mid Gut  8.374852  
  Larval Feeding Salivary Gland  8.372114  
  Whole Larvae Feeding  7.751298  
 
  
   FlyBase ID    symbol    start    end    strand    length   
   FBgn0035136   CG6905  357851   361383   -  3533  
   FBgn0035137   CG1233   361478   365681  +  4204  
 
    Segment 11 
 
   Location   
  Gene key  FBgn0035138-FBgn0035139  
  Heatmap region span   3L:332306..543070   
  Segment span   3L:366437..433906   
  Length (genes)  2  
  Length (bp)  67470  
   Model Scoring   
  BIC  182.776885  
  logL  -85.876316  
  logL ratio  34.965614  
   Expression   
  Mean expression  4.844983  
  Median expression  4.798956  
  Tissue std. dev.  1.162706  
 
  No GO Slim enrichment  
  
   tissue    mean expression   
  5th Passage Drosophila S2 Cells  4.580213  
  Adult Accessory gland  4.623425  
  Adult Brain  4.499154  
  Adult Carcass  4.566180  
  Adult Crop  4.567541  
  Adult Eye  4.557135  
  Adult Fatbody  4.692705  
  Adult Female Spermatheca Mated  4.610919  
  Adult Female Spermatheca Virgin  4.579322  
  Adult Head  4.376772  
  Adult Heart  4.584994  
  Adult Hind Gut  4.451430  
  Adult Male Ejaculatory Duct  4.605880  
  Adult Mid Gut  4.589216  
  Adult Ovary  4.471510  
  Adult Salivary Gland  5.000821  
  Adult Testes  10.600828  
  Adult Thoracoabdominal ganglion  4.421504  
  Adult Whole Fly  5.893752  
  Larvae Wandering Tubules  4.563464  
  Larval Feeding Carcass  4.525581  
  Larval Feeding Central Nevous System  4.429378  
  Larval Feeding Hind Gut  4.435454  
  Larval Feeding Malpighian Tubule  4.526340  
  Larval Feeding Mid Gut  4.564378  
  Larval Feeding Salivary Gland  4.652811  
  Whole Larvae Feeding  4.843839  
 
  
   FlyBase ID    symbol    start    end    strand    length   
   FBgn0035138   CG13884  365664   366437   -  774  
   FBgn0035139   CG13891  433146   433906   -  761  
 
    Segment 12 
 
   Location   
  Gene key  FBgn0085296-FBgn0085298  
  Heatmap region span   3L:347168..589272   
  Segment span   3L:500446..528339   
  Length (genes)  2  
  Length (bp)  27894  
   Model Scoring   
  BIC  236.919786  
  logL  -112.947767  
  logL ratio  -4.013490  
   Expression   
  Mean expression  6.213312  
  Median expression  6.127013  
  Tissue std. dev.  0.481890  
 
  No GO Slim enrichment  
  
   tissue    mean expression   
  5th Passage Drosophila S2 Cells  6.160195  
  Adult Accessory gland  6.285468  
  Adult Brain  5.500169  
  Adult Carcass  6.353278  
  Adult Crop  6.178053  
  Adult Eye  5.661625  
  Adult Fatbody  6.239410  
  Adult Female Spermatheca Mated  6.145854  
  Adult Female Spermatheca Virgin  6.083497  
  Adult Head  5.748992  
  Adult Heart  5.870637  
  Adult Hind Gut  6.137440  
  Adult Male Ejaculatory Duct  6.472645  
  Adult Mid Gut  6.543755  
  Adult Ovary  5.909677  
  Adult Salivary Gland  6.454952  
  Adult Testes  8.103968  
  Adult Thoracoabdominal ganglion  5.831735  
  Adult Whole Fly  6.147254  
  Larvae Wandering Tubules  6.283164  
  Larval Feeding Carcass  6.978784  
  Larval Feeding Central Nevous System  5.622452  
  Larval Feeding Hind Gut  6.049438  
  Larval Feeding Malpighian Tubule  6.203621  
  Larval Feeding Mid Gut  6.265048  
  Larval Feeding Salivary Gland  6.121136  
  Whole Larvae Feeding  6.407181  
 
  
   FlyBase ID    symbol    start    end    strand    length   
   FBgn0085296   CG34267  500046   500446   -  401  
   FBgn0085298   CG34269   528339   528996  +  658  
 
    Segment 13 
 
   Location   
  Gene key  FBgn0035140-FBgn0035141  
  Heatmap region span   3L:361383..680322   
  Segment span   3L:540851..542916   
  Length (genes)  2  
  Length (bp)  2066  
   Model Scoring   
  BIC  222.395834  
  logL  -105.685791  
  logL ratio  27.721851  
   Expression   
  Mean expression  7.220986  
  Median expression  7.224632  
  Tissue std. dev.  0.532193  
 
  No GO Slim enrichment  
  
   tissue    mean expression   
  5th Passage Drosophila S2 Cells  6.893267  
  Adult Accessory gland  7.108771  
  Adult Brain  7.865940  
  Adult Carcass  6.496723  
  Adult Crop  7.144412  
  Adult Eye  8.430225  
  Adult Fatbody  6.775267  
  Adult Female Spermatheca Mated  6.866019  
  Adult Female Spermatheca Virgin  7.081329  
  Adult Head  7.261470  
  Adult Heart  7.294071  
  Adult Hind Gut  6.829167  
  Adult Male Ejaculatory Duct  7.455805  
  Adult Mid Gut  6.835746  
  Adult Ovary  7.940728  
  Adult Salivary Gland  6.312289  
  Adult Testes  7.886184  
  Adult Thoracoabdominal ganglion  7.830595  
  Adult Whole Fly  6.715135  
  Larvae Wandering Tubules  7.581155  
  Larval Feeding Carcass  6.601306  
  Larval Feeding Central Nevous System  8.204090  
  Larval Feeding Hind Gut  7.117603  
  Larval Feeding Malpighian Tubule  7.391521  
  Larval Feeding Mid Gut  7.061502  
  Larval Feeding Salivary Gland  7.409882  
  Whole Larvae Feeding  6.576414  
 
  
   FlyBase ID    symbol    start    end    strand    length   
   FBgn0035140   CG17180   540851   542147  +  1297  
   FBgn0035141   Cypl  542122   542916   -  795  
 
    Segment 14 
 
   Location   
  Gene key  FBgn0035143-FBgn0035144  
  Heatmap region span   3L:500446..690081   
  Segment span   3L:581337..589272   
  Length (genes)  2  
  Length (bp)  7936  
   Model Scoring   
  BIC  212.519447  
  logL  -100.747597  
  logL ratio  2.731338  
   Expression   
  Mean expression  4.756343  
  Median expression  4.451278  
  Tissue std. dev.  0.836351  
 
  No GO Slim enrichment  
  
   tissue    mean expression   
  5th Passage Drosophila S2 Cells  6.440316  
  Adult Accessory gland  4.487451  
  Adult Brain  4.122758  
  Adult Carcass  5.090860  
  Adult Crop  5.019447  
  Adult Eye  4.313377  
  Adult Fatbody  4.532495  
  Adult Female Spermatheca Mated  4.341290  
  Adult Female Spermatheca Virgin  4.466515  
  Adult Head  4.504957  
  Adult Heart  4.465219  
  Adult Hind Gut  4.474026  
  Adult Male Ejaculatory Duct  4.535411  
  Adult Mid Gut  4.486102  
  Adult Ovary  4.348467  
  Adult Salivary Gland  4.513053  
  Adult Testes  8.115787  
  Adult Thoracoabdominal ganglion  4.094482  
  Adult Whole Fly  5.886833  
  Larvae Wandering Tubules  4.346520  
  Larval Feeding Carcass  4.777726  
  Larval Feeding Central Nevous System  4.038431  
  Larval Feeding Hind Gut  4.318412  
  Larval Feeding Malpighian Tubule  4.446701  
  Larval Feeding Mid Gut  4.686592  
  Larval Feeding Salivary Gland  4.372941  
  Whole Larvae Feeding  5.195085  
 
  
   FlyBase ID    symbol    start    end    strand    length   
   FBgn0035143   Ppm1   581337   582810  +  1474  
   FBgn0035144   CG17181  582824   589272   -  6449  
 
    Segment 15 
 
   Location   
  Gene key  FBgn0035145-FBgn0035149  
  Heatmap region span   3L:540589..739120   
  Segment span   3L:593246..654198   
  Length (genes)  5  
  Length (bp)  60953  
   Model Scoring   
  BIC  567.800120  
  logL  -278.387934  
  logL ratio  90.736813  
   Expression   
  Mean expression  8.910955  
  Median expression  8.682604  
  Tissue std. dev.  0.510374  
 
  No GO Slim enrichment  
  
   tissue    mean expression   
  5th Passage Drosophila S2 Cells  9.911664  
  Adult Accessory gland  9.019727  
  Adult Brain  8.895579  
  Adult Carcass  8.354916  
  Adult Crop  8.541883  
  Adult Eye  8.680284  
  Adult Fatbody  8.089363  
  Adult Female Spermatheca Mated  8.699035  
  Adult Female Spermatheca Virgin  8.741998  
  Adult Head  8.611580  
  Adult Heart  8.604432  
  Adult Hind Gut  8.331543  
  Adult Male Ejaculatory Duct  9.405430  
  Adult Mid Gut  8.877711  
  Adult Ovary  9.066461  
  Adult Salivary Gland  10.050177  
  Adult Testes  8.205985  
  Adult Thoracoabdominal ganglion  8.796814  
  Adult Whole Fly  8.770745  
  Larvae Wandering Tubules  9.223007  
  Larval Feeding Carcass  8.273317  
  Larval Feeding Central Nevous System  8.926072  
  Larval Feeding Hind Gut  9.269070  
  Larval Feeding Malpighian Tubule  8.916330  
  Larval Feeding Mid Gut  9.307447  
  Larval Feeding Salivary Gland  10.079266  
  Whole Larvae Feeding  8.945963  
 
  
   FlyBase ID    symbol    start    end    strand    length   
   FBgn0035145   MED14   593246   599242  +  5997  
   FBgn0035146   CG13893  599094   604001   -  4908  
   FBgn0035147   Gale   648517   651313  +  2797  
   FBgn0035148   CG3402  651538   652834   -  1297  
   FBgn0035149   MED30  652981   654198   -  1218  
 
 
    Segment 16 
 
   Location   
  Gene key  FBgn0035150-FBgn0035151  
  Heatmap region span   3L:540851..749406   
  Segment span   3L:654361..680322   
  Length (genes)  2  
  Length (bp)  25962  
   Model Scoring   
  BIC  205.983087  
  logL  -97.479417  
  logL ratio  34.094399  
   Expression   
  Mean expression  6.471826  
  Median expression  6.283951  
  Tissue std. dev.  0.852588  
 
  No GO Slim enrichment  
  
   tissue    mean expression   
  5th Passage Drosophila S2 Cells  7.807064  
  Adult Accessory gland  6.780954  
  Adult Brain  6.082009  
  Adult Carcass  5.619532  
  Adult Crop  6.595639  
  Adult Eye  6.168448  
  Adult Fatbody  5.733957  
  Adult Female Spermatheca Mated  6.369671  
  Adult Female Spermatheca Virgin  6.291459  
  Adult Head  5.834461  
  Adult Heart  5.817791  
  Adult Hind Gut  6.385012  
  Adult Male Ejaculatory Duct  6.178581  
  Adult Mid Gut  5.835511  
  Adult Ovary  8.758271  
  Adult Salivary Gland  6.540051  
  Adult Testes  9.165052  
  Adult Thoracoabdominal ganglion  6.098178  
  Adult Whole Fly  7.127210  
  Larvae Wandering Tubules  6.030304  
  Larval Feeding Carcass  5.985548  
  Larval Feeding Central Nevous System  6.792972  
  Larval Feeding Hind Gut  6.586724  
  Larval Feeding Malpighian Tubule  6.207861  
  Larval Feeding Mid Gut  5.440206  
  Larval Feeding Salivary Gland  6.325188  
  Whole Larvae Feeding  6.181644  
 
  
   FlyBase ID    symbol    start    end    strand    length   
   FBgn0035150   Rev1   654361   657975  +  3615  
   FBgn0035151   CG17129  677879   680322   -  2444  
 
    Segment 17 
 
   Location   
  Gene key  FBgn0035152-FBgn0035153  
  Heatmap region span   3L:543070..804051   
  Segment span   3L:682193..685545   
  Length (genes)  2  
  Length (bp)  3353  
   Model Scoring   
  BIC  204.026484  
  logL  -96.501116  
  logL ratio  49.325172  
   Expression   
  Mean expression  8.042513  
  Median expression  8.020221  
  Tissue std. dev.  0.597816  
 
  No GO Slim enrichment  
  
   tissue    mean expression   
  5th Passage Drosophila S2 Cells  8.514079  
  Adult Accessory gland  7.977996  
  Adult Brain  8.505529  
  Adult Carcass  7.639452  
  Adult Crop  8.500881  
  Adult Eye  8.562316  
  Adult Fatbody  7.965607  
  Adult Female Spermatheca Mated  8.518828  
  Adult Female Spermatheca Virgin  8.540865  
  Adult Head  8.075990  
  Adult Heart  8.049577  
  Adult Hind Gut  7.903370  
  Adult Male Ejaculatory Duct  8.066465  
  Adult Mid Gut  7.526011  
  Adult Ovary  8.594971  
  Adult Salivary Gland  9.132573  
  Adult Testes  6.452108  
  Adult Thoracoabdominal ganglion  8.416494  
  Adult Whole Fly  7.464968  
  Larvae Wandering Tubules  7.855593  
  Larval Feeding Carcass  6.975518  
  Larval Feeding Central Nevous System  8.684162  
  Larval Feeding Hind Gut  7.719952  
  Larval Feeding Malpighian Tubule  7.802138  
  Larval Feeding Mid Gut  7.567975  
  Larval Feeding Salivary Gland  8.881787  
  Whole Larvae Feeding  7.252650  
 
  
   FlyBase ID    symbol    start    end    strand    length   
   FBgn0035152   CG3386  680648   682193   -  1546  
   FBgn0035153   ebd1  682879   685545   -  2667  
 
    Segment 18 
 
   Location   
  Gene key  FBgn0035154-FBgn0052483  
  Heatmap region span   3L:581337..837514   
  Segment span   3L:687948..690081   
  Length (genes)  2  
  Length (bp)  2134  
   Model Scoring   
  BIC  189.618119  
  logL  -89.296933  
  logL ratio  38.506063  
   Expression   
  Mean expression  5.845458  
  Median expression  4.972395  
  Tissue std. dev.  2.153322  
 
  No GO Slim enrichment  
  
   tissue    mean expression   
  5th Passage Drosophila S2 Cells  4.754904  
  Adult Accessory gland  4.899280  
  Adult Brain  4.376855  
  Adult Carcass  5.038347  
  Adult Crop  4.921970  
  Adult Eye  5.564216  
  Adult Fatbody  5.865178  
  Adult Female Spermatheca Mated  5.594698  
  Adult Female Spermatheca Virgin  5.401991  
  Adult Head  4.749244  
  Adult Heart  4.987178  
  Adult Hind Gut  5.076465  
  Adult Male Ejaculatory Duct  5.071164  
  Adult Mid Gut  11.812866  
  Adult Ovary  4.734537  
  Adult Salivary Gland  5.269634  
  Adult Testes  4.470492  
  Adult Thoracoabdominal ganglion  4.376314  
  Adult Whole Fly  7.606889  
  Larvae Wandering Tubules  4.720154  
  Larval Feeding Carcass  4.736672  
  Larval Feeding Central Nevous System  4.747224  
  Larval Feeding Hind Gut  4.895251  
  Larval Feeding Malpighian Tubule  4.653454  
  Larval Feeding Mid Gut  12.180600  
  Larval Feeding Salivary Gland  6.465523  
  Whole Larvae Feeding  10.856274  
 
  
   FlyBase ID    symbol    start    end    strand    length   
   FBgn0035154   CG3344  685942   687948   -  2007  
   FBgn0052483   CG32483  688523   690081   -  1559  
 
    Segment 19 
 
   Location   
  Gene key  FBgn0035155-FBgn0035160  
  Heatmap region span   3L:593246..837856   
  Segment span   3L:690519..739120   
  Length (genes)  5  
  Length (bp)  48602  
   Model Scoring   
  BIC  482.585026  
  logL  -235.780387  
  logL ratio  117.049500  
   Expression   
  Mean expression  7.011001  
  Median expression  6.940849  
  Tissue std. dev.  0.491225  
 
  
   GO ID    description    ratio    P-value   
   GO:0003677   DNA binding  2/5  0.0339  
 
  
   tissue    mean expression   
  5th Passage Drosophila S2 Cells  7.343104  
  Adult Accessory gland  7.445976  
  Adult Brain  7.564872  
  Adult Carcass  6.557506  
  Adult Crop  7.083320  
  Adult Eye  7.634821  
  Adult Fatbody  6.796444  
  Adult Female Spermatheca Mated  6.946627  
  Adult Female Spermatheca Virgin  6.963252  
  Adult Head  6.886403  
  Adult Heart  7.192785  
  Adult Hind Gut  6.679558  
  Adult Male Ejaculatory Duct  7.164207  
  Adult Mid Gut  6.487218  
  Adult Ovary  7.591210  
  Adult Salivary Gland  6.889880  
  Adult Testes  6.069376  
  Adult Thoracoabdominal ganglion  7.665029  
  Adult Whole Fly  6.403489  
  Larvae Wandering Tubules  7.065530  
  Larval Feeding Carcass  6.857451  
  Larval Feeding Central Nevous System  8.495342  
  Larval Feeding Hind Gut  6.821895  
  Larval Feeding Malpighian Tubule  6.813242  
  Larval Feeding Mid Gut  6.524997  
  Larval Feeding Salivary Gland  6.699197  
  Whole Larvae Feeding  6.654284  
 
  
   FlyBase ID    symbol    start    end    strand    length   
   FBgn0035155   RabX6   690519   691951  +  1433  
   FBgn0035157   CG13894  693088   699143   -  6056  
   FBgn0035158   CG13895  705945   708201   -  2257  
   FBgn0035159   CG13896  710819   711850   -  1032  
   FBgn0035160   CG13897  737677   739120   -  1444  
 
 
    Segment 20 
 
   Location   
  Gene key  FBgn0035162-FBgn0035164  
  Heatmap region span   3L:687948..851319   
  Segment span   3L:833402..837514   
  Length (genes)  2  
  Length (bp)  4113  
   Model Scoring   
  BIC  266.884404  
  logL  -127.930076  
  logL ratio  -2.083970  
   Expression   
  Mean expression  8.561078  
  Median expression  8.477702  
  Tissue std. dev.  0.648847  
 
  No GO Slim enrichment  
  
   tissue    mean expression   
  5th Passage Drosophila S2 Cells  10.141429  
  Adult Accessory gland  8.735139  
  Adult Brain  8.535351  
  Adult Carcass  7.621223  
  Adult Crop  8.426479  
  Adult Eye  8.306649  
  Adult Fatbody  8.139698  
  Adult Female Spermatheca Mated  8.729690  
  Adult Female Spermatheca Virgin  8.355678  
  Adult Head  8.181915  
  Adult Heart  8.258689  
  Adult Hind Gut  8.126616  
  Adult Male Ejaculatory Duct  8.100558  
  Adult Mid Gut  8.198999  
  Adult Ovary  9.826771  
  Adult Salivary Gland  7.979632  
  Adult Testes  9.093654  
  Adult Thoracoabdominal ganglion  8.361866  
  Adult Whole Fly  9.247701  
  Larvae Wandering Tubules  7.618115  
  Larval Feeding Carcass  8.887636  
  Larval Feeding Central Nevous System  10.002363  
  Larval Feeding Hind Gut  8.630919  
  Larval Feeding Malpighian Tubule  7.959775  
  Larval Feeding Mid Gut  8.083350  
  Larval Feeding Salivary Gland  9.212323  
  Whole Larvae Feeding  8.386876  
 
  
   FlyBase ID    symbol    start    end    strand    length   
   FBgn0035162   CG13900  826710   833402   -  6693  
   FBgn0035164   CG13901  836667   837514   -  848  
 
    Segment 21 
 
   Location   
  Gene key  FBgn0035167-FBgn0035168  
  Heatmap region span   3L:804051..897380   
  Segment span   3L:841718..844268   
  Length (genes)  2  
  Length (bp)  2551  
   Model Scoring   
  BIC  179.463507  
  logL  -84.219627  
  logL ratio  26.872966  
   Expression   
  Mean expression  4.976730  
  Median expression  4.855663  
  Tissue std. dev.  0.435788  
 
  No GO Slim enrichment  
  
   tissue    mean expression   
  5th Passage Drosophila S2 Cells  5.848410  
  Adult Accessory gland  4.702009  
  Adult Brain  4.902717  
  Adult Carcass  4.752658  
  Adult Crop  4.574672  
  Adult Eye  4.701531  
  Adult Fatbody  5.090134  
  Adult Female Spermatheca Mated  4.790018  
  Adult Female Spermatheca Virgin  4.951467  
  Adult Head  5.034306  
  Adult Heart  4.887628  
  Adult Hind Gut  4.734834  
  Adult Male Ejaculatory Duct  4.894450  
  Adult Mid Gut  4.836628  
  Adult Ovary  5.234381  
  Adult Salivary Gland  4.901607  
  Adult Testes  6.692386  
  Adult Thoracoabdominal ganglion  5.283950  
  Adult Whole Fly  4.606208  
  Larvae Wandering Tubules  4.842040  
  Larval Feeding Carcass  4.905674  
  Larval Feeding Central Nevous System  5.498039  
  Larval Feeding Hind Gut  4.591670  
  Larval Feeding Malpighian Tubule  4.808891  
  Larval Feeding Mid Gut  4.787642  
  Larval Feeding Salivary Gland  4.811172  
  Whole Larvae Feeding  4.706599  
 
  
   FlyBase ID    symbol    start    end    strand    length   
   FBgn0035167   Gr61a   841718   843411  +  1694  
   FBgn0035168   CG13889   844268   851080  +  6813  
 
    Segment 22 
 
   Location   
  Gene key  FBgn0035170-FBgn0035171  
  Heatmap region span   3L:837856..993988   
  Segment span   3L:857239..865396   
  Length (genes)  2  
  Length (bp)  8158  
   Model Scoring   
  BIC  206.565140  
  logL  -97.770444  
  logL ratio  14.442394  
   Expression   
  Mean expression  5.284743  
  Median expression  5.203850  
  Tissue std. dev.  1.221946  
 
  No GO Slim enrichment  
  
   tissue    mean expression   
  5th Passage Drosophila S2 Cells  4.816276  
  Adult Accessory gland  4.822520  
  Adult Brain  9.346037  
  Adult Carcass  5.141226  
  Adult Crop  4.729866  
  Adult Eye  5.632548  
  Adult Fatbody  4.848705  
  Adult Female Spermatheca Mated  4.959563  
  Adult Female Spermatheca Virgin  4.951980  
  Adult Head  7.031930  
  Adult Heart  4.960722  
  Adult Hind Gut  4.610390  
  Adult Male Ejaculatory Duct  4.655393  
  Adult Mid Gut  4.808430  
  Adult Ovary  4.576051  
  Adult Salivary Gland  4.794812  
  Adult Testes  4.481076  
  Adult Thoracoabdominal ganglion  8.727582  
  Adult Whole Fly  4.788301  
  Larvae Wandering Tubules  4.853987  
  Larval Feeding Carcass  4.736216  
  Larval Feeding Central Nevous System  6.897301  
  Larval Feeding Hind Gut  4.575054  
  Larval Feeding Malpighian Tubule  4.754252  
  Larval Feeding Mid Gut  4.781963  
  Larval Feeding Salivary Gland  4.762423  
  Whole Larvae Feeding  4.643463  
 
  
   FlyBase ID    symbol    start    end    strand    length   
   FBgn0035170   dpr20  852713   857239   -  4527  
   FBgn0035171   CG12502   865396   870895  +  5500  
 
    Segment 23 
 
   Location   
  Gene key  FBgn0052479-FBgn0035173  
  Heatmap region span   3L:841361..1034697   
  Segment span   3L:871903..881873   
  Length (genes)  2  
  Length (bp)  9971  
   Model Scoring   
  BIC  253.181193  
  logL  -121.078470  
  logL ratio  16.042339  
   Expression   
  Mean expression  9.272314  
  Median expression  9.419285  
  Tissue std. dev.  0.734294  
 
  No GO Slim enrichment  
  
   tissue    mean expression   
  5th Passage Drosophila S2 Cells  9.903341  
  Adult Accessory gland  9.458611  
  Adult Brain  9.541296  
  Adult Carcass  9.331583  
  Adult Crop  9.532538  
  Adult Eye  8.778586  
  Adult Fatbody  9.436006  
  Adult Female Spermatheca Mated  9.082385  
  Adult Female Spermatheca Virgin  8.851588  
  Adult Head  9.091618  
  Adult Heart  9.528645  
  Adult Hind Gut  10.131123  
  Adult Male Ejaculatory Duct  9.273259  
  Adult Mid Gut  9.642274  
  Adult Ovary  10.486683  
  Adult Salivary Gland  8.375117  
  Adult Testes  7.076806  
  Adult Thoracoabdominal ganglion  9.804823  
  Adult Whole Fly  9.493592  
  Larvae Wandering Tubules  9.043297  
  Larval Feeding Carcass  9.490954  
  Larval Feeding Central Nevous System  9.937510  
  Larval Feeding Hind Gut  9.699856  
  Larval Feeding Malpighian Tubule  9.340218  
  Larval Feeding Mid Gut  9.834171  
  Larval Feeding Salivary Gland  7.297459  
  Whole Larvae Feeding  8.889134  
 
  
   FlyBase ID    symbol    start    end    strand    length   
   FBgn0052479   CG32479   871903   893534  +  21632  
   FBgn0035173   CG13907  877281   881873   -  4593  
 
    Segment 24 
 
   Location   
  Gene key  FBgn0035178-FBgn0035179  
  Heatmap region span   3L:857239..1177274   
  Segment span   3L:987211..993988   
  Length (genes)  2  
  Length (bp)  6778  
   Model Scoring   
  BIC  183.678806  
  logL  -86.327277  
  logL ratio  38.341193  
   Expression   
  Mean expression  5.128735  
  Median expression  4.363527  
  Tissue std. dev.  1.552501  
 
  No GO Slim enrichment  
  
   tissue    mean expression   
  5th Passage Drosophila S2 Cells  4.064731  
  Adult Accessory gland  4.457878  
  Adult Brain  9.965485  
  Adult Carcass  4.419691  
  Adult Crop  4.454154  
  Adult Eye  5.929006  
  Adult Fatbody  4.986218  
  Adult Female Spermatheca Mated  5.029136  
  Adult Female Spermatheca Virgin  5.018874  
  Adult Head  6.659601  
  Adult Heart  4.307672  
  Adult Hind Gut  4.689339  
  Adult Male Ejaculatory Duct  4.288476  
  Adult Mid Gut  4.182090  
  Adult Ovary  4.248664  
  Adult Salivary Gland  4.642136  
  Adult Testes  4.818352  
  Adult Thoracoabdominal ganglion  9.387659  
  Adult Whole Fly  4.316804  
  Larvae Wandering Tubules  4.527735  
  Larval Feeding Carcass  4.226589  
  Larval Feeding Central Nevous System  8.165234  
  Larval Feeding Hind Gut  4.163081  
  Larval Feeding Malpighian Tubule  4.679284  
  Larval Feeding Mid Gut  4.121957  
  Larval Feeding Salivary Gland  4.476401  
  Whole Larvae Feeding  4.249591  
 
  
   FlyBase ID    symbol    start    end    strand    length   
   FBgn0035178      987211   992473  +  5263  
   FBgn0035179   CG12038   993988   995703  +  1716  
 
    Segment 25 
 
   Location   
  Gene key  FBgn0053966-FBgn0035192  
  Heatmap region span   3L:1204792..1248150   
  Segment span   3L:1234138..1235729   
  Length (genes)  2  
  Length (bp)  1592  
   Model Scoring   
  BIC  221.548953  
  logL  -105.262350  
  logL ratio  -2.522785  
   Expression   
  Mean expression  5.206277  
  Median expression  4.697563  
  Tissue std. dev.  0.983498  
 
  No GO Slim enrichment  
  
   tissue    mean expression   
  5th Passage Drosophila S2 Cells  4.580329  
  Adult Accessory gland  5.864369  
  Adult Brain  3.997415  
  Adult Carcass  4.823771  
  Adult Crop  6.685473  
  Adult Eye  4.161432  
  Adult Fatbody  4.541101  
  Adult Female Spermatheca Mated  4.482302  
  Adult Female Spermatheca Virgin  4.464614  
  Adult Head  4.356279  
  Adult Heart  5.624958  
  Adult Hind Gut  7.226717  
  Adult Male Ejaculatory Duct  5.459674  
  Adult Mid Gut  7.086947  
  Adult Ovary  4.576793  
  Adult Salivary Gland  4.662196  
  Adult Testes  4.205205  
  Adult Thoracoabdominal ganglion  4.353651  
  Adult Whole Fly  4.796090  
  Larvae Wandering Tubules  5.120437  
  Larval Feeding Carcass  6.154196  
  Larval Feeding Central Nevous System  4.506275  
  Larval Feeding Hind Gut  6.673677  
  Larval Feeding Malpighian Tubule  4.713394  
  Larval Feeding Mid Gut  6.678742  
  Larval Feeding Salivary Gland  4.467154  
  Whole Larvae Feeding  6.306279  
 
  
   FlyBase ID    symbol    start    end    strand    length   
   FBgn0053966   CG33966   1234138   1235420  +  1283  
   FBgn0035192   CG9194   1235729   1242025  +  6297  
 
    Segment 26 
 
   Location   
  Gene key  FBgn0035199-FBgn0052333  
  Heatmap region span   3L:1245935..1329806   
  Segment span   3L:1256452..1275972   
  Length (genes)  2  
  Length (bp)  19521  
   Model Scoring   
  BIC  219.580847  
  logL  -104.278297  
  logL ratio  -2.301568  
   Expression   
  Mean expression  5.524980  
  Median expression  4.919522  
  Tissue std. dev.  1.085274  
 
  No GO Slim enrichment  
  
   tissue    mean expression   
  5th Passage Drosophila S2 Cells  4.500077  
  Adult Accessory gland  5.537173  
  Adult Brain  6.821505  
  Adult Carcass  5.493115  
  Adult Crop  6.125348  
  Adult Eye  7.934943  
  Adult Fatbody  4.583374  
  Adult Female Spermatheca Mated  4.748608  
  Adult Female Spermatheca Virgin  4.675139  
  Adult Head  5.802034  
  Adult Heart  4.757077  
  Adult Hind Gut  6.586278  
  Adult Male Ejaculatory Duct  4.618560  
  Adult Mid Gut  5.015138  
  Adult Ovary  4.530140  
  Adult Salivary Gland  4.601840  
  Adult Testes  5.145846  
  Adult Thoracoabdominal ganglion  6.865589  
  Adult Whole Fly  4.688562  
  Larvae Wandering Tubules  4.633102  
  Larval Feeding Carcass  8.467197  
  Larval Feeding Central Nevous System  5.649784  
  Larval Feeding Hind Gut  6.500214  
  Larval Feeding Malpighian Tubule  4.540036  
  Larval Feeding Mid Gut  5.063210  
  Larval Feeding Salivary Gland  4.723122  
  Whole Larvae Feeding  6.567461  
 
  
   FlyBase ID    symbol    start    end    strand    length   
   FBgn0035199   CG9134   1256452   1274498  +  18047  
   FBgn0052333   CG32333   1275972   1294382  +  18411  
 
    Segment 27 
 
   Location   
  Gene key  FBgn0035202-FBgn0035207  
  Heatmap region span   3L:1247817..1331729   
  Segment span   3L:1295742..1313678   
  Length (genes)  8  
  Length (bp)  17937  
   Model Scoring   
  BIC  804.058429  
  logL  -396.517088  
  logL ratio  278.485128  
   Expression   
  Mean expression  9.518907  
  Median expression  9.649673  
  Tissue std. dev.  0.283766  
 
  No GO Slim enrichment  
  
   tissue    mean expression   
  5th Passage Drosophila S2 Cells  9.872759  
  Adult Accessory gland  9.879720  
  Adult Brain  10.000773  
  Adult Carcass  9.111489  
  Adult Crop  9.439306  
  Adult Eye  9.592780  
  Adult Fatbody  9.430956  
  Adult Female Spermatheca Mated  9.426751  
  Adult Female Spermatheca Virgin  9.434444  
  Adult Head  9.324999  
  Adult Heart  9.851468  
  Adult Hind Gut  9.171187  
  Adult Male Ejaculatory Duct  9.645214  
  Adult Mid Gut  8.914708  
  Adult Ovary  9.552010  
  Adult Salivary Gland  9.452096  
  Adult Testes  9.297060  
  Adult Thoracoabdominal ganglion  9.949848  
  Adult Whole Fly  9.117053  
  Larvae Wandering Tubules  9.409833  
  Larval Feeding Carcass  9.687881  
  Larval Feeding Central Nevous System  9.595391  
  Larval Feeding Hind Gut  9.404174  
  Larval Feeding Malpighian Tubule  9.669501  
  Larval Feeding Mid Gut  9.347676  
  Larval Feeding Salivary Gland  10.065264  
  Whole Larvae Feeding  9.366160  
 
  
   FlyBase ID    symbol    start    end    strand    length   
   FBgn0035202      1295742   1298497  +  2756  
   FBgn0025682   scf   1298618   1300389  +  1772  
   FBgn0010333   Rac1  1300879   1302683   -  1805  
   FBgn0035203   CG9149   1302949   1304721  +  1773  
   FBgn0035205   CG2469   1307224   1311812  +  4589  
   FBgn0035204   CG2277  1305275   1307417   -  2143  
   FBgn0035206   CG9186  1311719   1313377   -  1659  
   FBgn0035207   CG9153   1313678   1318328  +  4651  
 
 
    Segment 28 
 
   Location   
  Gene key  FBgn0035210-FBgn0035213  
  Heatmap region span   3L:1318482..1463792   
  Segment span   3L:1333152..1342403   
  Length (genes)  3  
  Length (bp)  9252  
   Model Scoring   
  BIC  313.060471  
  logL  -151.018109  
  logL ratio  56.347396  
   Expression   
  Mean expression  7.267609  
  Median expression  7.188341  
  Tissue std. dev.  0.763272  
 
  No GO Slim enrichment  
  
   tissue    mean expression   
  5th Passage Drosophila S2 Cells  8.492111  
  Adult Accessory gland  7.309619  
  Adult Brain  7.223531  
  Adult Carcass  6.655038  
  Adult Crop  6.825773  
  Adult Eye  7.731453  
  Adult Fatbody  7.146362  
  Adult Female Spermatheca Mated  6.718036  
  Adult Female Spermatheca Virgin  6.850736  
  Adult Head  6.733177  
  Adult Heart  7.587188  
  Adult Hind Gut  6.377220  
  Adult Male Ejaculatory Duct  6.469274  
  Adult Mid Gut  6.925266  
  Adult Ovary  9.598351  
  Adult Salivary Gland  6.236658  
  Adult Testes  7.845003  
  Adult Thoracoabdominal ganglion  7.124967  
  Adult Whole Fly  8.190419  
  Larvae Wandering Tubules  7.213371  
  Larval Feeding Carcass  7.091710  
  Larval Feeding Central Nevous System  8.933256  
  Larval Feeding Hind Gut  7.195286  
  Larval Feeding Malpighian Tubule  7.073276  
  Larval Feeding Mid Gut  6.867097  
  Larval Feeding Salivary Gland  6.762082  
  Whole Larvae Feeding  7.049190  
 
  
   FlyBase ID    symbol    start    end    strand    length   
   FBgn0035210   msd5  1332300   1333152   -  853  
   FBgn0035211   CG2211  1333237   1335303   -  2067  
   FBgn0035213   CG2199  1339457   1342403   -  2947  
 
 
    Segment 29 
 
   Location   
  Gene key  FBgn0029514-FBgn0035216  
  Heatmap region span   3L:1322802..1477619   
  Segment span   3L:1351139..1411088   
  Length (genes)  4  
  Length (bp)  59950  
   Model Scoring   
  BIC  378.579288  
  logL  -183.777518  
  logL ratio  63.796149  
   Expression   
  Mean expression  4.454967  
  Median expression  4.164845  
  Tissue std. dev.  0.537992  
 
  No GO Slim enrichment  
  
   tissue    mean expression   
  5th Passage Drosophila S2 Cells  5.563157  
  Adult Accessory gland  5.995901  
  Adult Brain  5.507520  
  Adult Carcass  4.321595  
  Adult Crop  4.089904  
  Adult Eye  4.896677  
  Adult Fatbody  3.983290  
  Adult Female Spermatheca Mated  4.155233  
  Adult Female Spermatheca Virgin  4.105029  
  Adult Head  4.521168  
  Adult Heart  3.981618  
  Adult Hind Gut  4.054519  
  Adult Male Ejaculatory Duct  4.619761  
  Adult Mid Gut  4.172167  
  Adult Ovary  4.290033  
  Adult Salivary Gland  4.259331  
  Adult Testes  4.524648  
  Adult Thoracoabdominal ganglion  5.252497  
  Adult Whole Fly  4.105248  
  Larvae Wandering Tubules  4.206164  
  Larval Feeding Carcass  4.234167  
  Larval Feeding Central Nevous System  4.938700  
  Larval Feeding Hind Gut  3.992968  
  Larval Feeding Malpighian Tubule  4.120091  
  Larval Feeding Mid Gut  4.139426  
  Larval Feeding Salivary Gland  4.208369  
  Whole Larvae Feeding  4.044920  
 
  
   FlyBase ID    symbol    start    end    strand    length   
   FBgn0029514   312   1351139   1352853  +  1715  
   FBgn0003295   ru   1370620   1387435  +  16816  
   FBgn0052320   CG32320   1398586   1401613  +  3028  
   FBgn0035216   CG9168   1411088   1413089  +  2002  
 
 
    Segment 30 
 
   Location   
  Gene key  FBgn0035217-FBgn0035218  
  Heatmap region span   3L:1329806..1489547   
  Segment span   3L:1416231..1423471   
  Length (genes)  2  
  Length (bp)  7241  
   Model Scoring   
  BIC  168.700251  
  logL  -78.837999  
  logL ratio  41.383164  
   Expression   
  Mean expression  4.975159  
  Median expression  4.665600  
  Tissue std. dev.  1.147377  
 
  No GO Slim enrichment  
  
   tissue    mean expression   
  5th Passage Drosophila S2 Cells  4.810478  
  Adult Accessory gland  4.908540  
  Adult Brain  4.245535  
  Adult Carcass  4.782726  
  Adult Crop  4.766865  
  Adult Eye  4.311274  
  Adult Fatbody  4.712110  
  Adult Female Spermatheca Mated  4.620997  
  Adult Female Spermatheca Virgin  4.614095  
  Adult Head  4.443991  
  Adult Heart  4.397429  
  Adult Hind Gut  4.600036  
  Adult Male Ejaculatory Duct  4.964597  
  Adult Mid Gut  4.849742  
  Adult Ovary  4.883344  
  Adult Salivary Gland  5.129741  
  Adult Testes  10.537182  
  Adult Thoracoabdominal ganglion  4.492704  
  Adult Whole Fly  6.216297  
  Larvae Wandering Tubules  4.737291  
  Larval Feeding Carcass  4.718666  
  Larval Feeding Central Nevous System  4.467277  
  Larval Feeding Hind Gut  4.591297  
  Larval Feeding Malpighian Tubule  4.689656  
  Larval Feeding Mid Gut  4.904982  
  Larval Feeding Salivary Gland  4.909601  
  Whole Larvae Feeding  5.022833  
 
  
   FlyBase ID    symbol    start    end    strand    length   
   FBgn0035217   FucTD   1416231   1417933  +  1703  
   FBgn0035218   CG9173  1421873   1423471   -  1599  
 
    Segment 31 
 
   Location   
  Gene key  FBgn0052319-FBgn0028567  
  Heatmap region span   3L:1351139..1545743   
  Segment span   3L:1472060..1477619   
  Length (genes)  2  
  Length (bp)  5560  
   Model Scoring   
  BIC  201.816178  
  logL  -95.395963  
  logL ratio  19.769507  
   Expression   
  Mean expression  4.676074  
  Median expression  4.307320  
  Tissue std. dev.  1.248804  
 
  No GO Slim enrichment  
  
   tissue    mean expression   
  5th Passage Drosophila S2 Cells  4.150239  
  Adult Accessory gland  4.390071  
  Adult Brain  4.077283  
  Adult Carcass  4.736802  
  Adult Crop  4.341840  
  Adult Eye  4.256738  
  Adult Fatbody  4.360307  
  Adult Female Spermatheca Mated  4.535173  
  Adult Female Spermatheca Virgin  4.436043  
  Adult Head  4.125008  
  Adult Heart  4.328672  
  Adult Hind Gut  4.157104  
  Adult Male Ejaculatory Duct  4.408717  
  Adult Mid Gut  4.415925  
  Adult Ovary  4.188571  
  Adult Salivary Gland  4.847530  
  Adult Testes  10.676015  
  Adult Thoracoabdominal ganglion  4.189457  
  Adult Whole Fly  6.175857  
  Larvae Wandering Tubules  4.414225  
  Larval Feeding Carcass  4.322351  
  Larval Feeding Central Nevous System  4.082952  
  Larval Feeding Hind Gut  4.242684  
  Larval Feeding Malpighian Tubule  4.407381  
  Larval Feeding Mid Gut  4.402973  
  Larval Feeding Salivary Gland  4.328772  
  Whole Larvae Feeding  5.255298  
 
  
   FlyBase ID    symbol    start    end    strand    length   
   FBgn0052319   CG32319  1471268   1472060   -  793  
   FBgn0028567   robl62A   1477619   1478369  +  751  
 
    Segment 32 
 
   Location   
  Gene key  FBgn0028577-FBgn0035229  
  Heatmap region span   3L:1463792..1558454   
  Segment span   3L:1502598..1537149   
  Length (genes)  4  
  Length (bp)  34552  
   Model Scoring   
  BIC  430.098433  
  logL  -209.537090  
  logL ratio  126.003069  
   Expression   
  Mean expression  9.716047  
  Median expression  9.726725  
  Tissue std. dev.  0.565806  
 
  No GO Slim enrichment  
  
   tissue    mean expression   
  5th Passage Drosophila S2 Cells  9.803986  
  Adult Accessory gland  9.589534  
  Adult Brain  10.838726  
  Adult Carcass  10.185545  
  Adult Crop  9.581532  
  Adult Eye  10.026457  
  Adult Fatbody  10.261603  
  Adult Female Spermatheca Mated  9.897455  
  Adult Female Spermatheca Virgin  10.030892  
  Adult Head  9.786617  
  Adult Heart  10.131540  
  Adult Hind Gut  9.988663  
  Adult Male Ejaculatory Duct  9.338900  
  Adult Mid Gut  8.843728  
  Adult Ovary  10.434418  
  Adult Salivary Gland  9.594755  
  Adult Testes  8.516585  
  Adult Thoracoabdominal ganglion  10.780717  
  Adult Whole Fly  9.742999  
  Larvae Wandering Tubules  9.087056  
  Larval Feeding Carcass  9.479515  
  Larval Feeding Central Nevous System  10.193143  
  Larval Feeding Hind Gut  9.481159  
  Larval Feeding Malpighian Tubule  9.402302  
  Larval Feeding Mid Gut  8.758882  
  Larval Feeding Salivary Gland  9.597568  
  Whole Larvae Feeding  8.958980  
 
  
   FlyBase ID    symbol    start    end    strand    length   
   FBgn0028577   pUf68  1496853   1502598   -  5746  
   FBgn0011204   cue  1503226   1517238   -  14013  
   FBgn0035228   CG12091  1534929   1536831   -  1903  
   FBgn0035229   CG7852   1537149   1544826  +  7678  
 
 
    Segment 33 
 
   Location   
  Gene key  FBgn0035233-FBgn0035232  
  Heatmap region span   3L:1502598..1618392   
  Segment span   3L:1558235..1558454   
  Length (genes)  2  
  Length (bp)  220  
   Model Scoring   
  BIC  216.246694  
  logL  -102.611220  
  logL ratio  45.308909  
   Expression   
  Mean expression  8.951079  
  Median expression  8.999818  
  Tissue std. dev.  0.542176  
 
  No GO Slim enrichment  
  
   tissue    mean expression   
  5th Passage Drosophila S2 Cells  9.270772  
  Adult Accessory gland  8.594351  
  Adult Brain  8.474797  
  Adult Carcass  9.368140  
  Adult Crop  10.221461  
  Adult Eye  8.928852  
  Adult Fatbody  9.606558  
  Adult Female Spermatheca Mated  8.638721  
  Adult Female Spermatheca Virgin  8.817758  
  Adult Head  8.731969  
  Adult Heart  9.961393  
  Adult Hind Gut  9.593756  
  Adult Male Ejaculatory Duct  9.363458  
  Adult Mid Gut  8.820662  
  Adult Ovary  8.441481  
  Adult Salivary Gland  9.140232  
  Adult Testes  9.279893  
  Adult Thoracoabdominal ganglion  8.398804  
  Adult Whole Fly  8.481266  
  Larvae Wandering Tubules  9.430953  
  Larval Feeding Carcass  8.280673  
  Larval Feeding Central Nevous System  8.478398  
  Larval Feeding Hind Gut  8.636380  
  Larval Feeding Malpighian Tubule  9.633619  
  Larval Feeding Mid Gut  8.371358  
  Larval Feeding Salivary Gland  8.495169  
  Whole Larvae Feeding  8.218255  
 
  
   FlyBase ID    symbol    start    end    strand    length   
   FBgn0035233   Pex10   1558235   1559863  +  1629  
   FBgn0035232   CG12099  1555363   1558454   -  3092  
 
    Segment 34 
 
   Location   
  Gene key  FBgn0035234-FBgn0035235  
  Heatmap region span   3L:1545743..1628377   
  Segment span   3L:1565392..1568608   
  Length (genes)  2  
  Length (bp)  3217  
   Model Scoring   
  BIC  196.008162  
  logL  -92.491955  
  logL ratio  23.524259  
   Expression   
  Mean expression  5.629565  
  Median expression  5.417205  
  Tissue std. dev.  0.301799  
 
  No GO Slim enrichment  
  
   tissue    mean expression   
  5th Passage Drosophila S2 Cells  5.908386  
  Adult Accessory gland  5.760398  
  Adult Brain  5.997181  
  Adult Carcass  5.408505  
  Adult Crop  5.631610  
  Adult Eye  5.851930  
  Adult Fatbody  5.358243  
  Adult Female Spermatheca Mated  5.709042  
  Adult Female Spermatheca Virgin  5.827744  
  Adult Head  5.537976  
  Adult Heart  5.561634  
  Adult Hind Gut  5.481633  
  Adult Male Ejaculatory Duct  5.342694  
  Adult Mid Gut  5.497815  
  Adult Ovary  5.912299  
  Adult Salivary Gland  6.018191  
  Adult Testes  5.053395  
  Adult Thoracoabdominal ganglion  5.954810  
  Adult Whole Fly  4.895770  
  Larvae Wandering Tubules  5.557865  
  Larval Feeding Carcass  5.679861  
  Larval Feeding Central Nevous System  6.274978  
  Larval Feeding Hind Gut  5.582921  
  Larval Feeding Malpighian Tubule  5.592324  
  Larval Feeding Mid Gut  5.291826  
  Larval Feeding Salivary Gland  5.906484  
  Whole Larvae Feeding  5.402751  
 
  
   FlyBase ID    symbol    start    end    strand    length   
   FBgn0035234   CG12003   1565392   1567802  +  2411  
   FBgn0035235   CG7879  1559800   1568608   -  8809  
 
    Segment 35 
 
   Location   
  Gene key  FBgn0035236-FBgn0035238  
  Heatmap region span   3L:1546081..1629298   
  Segment span   3L:1569200..1602718   
  Length (genes)  3  
  Length (bp)  33519  
   Model Scoring   
  BIC  298.809466  
  logL  -143.892607  
  logL ratio  78.129384  
   Expression   
  Mean expression  8.125585  
  Median expression  8.134714  
  Tissue std. dev.  0.280431  
 
  No GO Slim enrichment  
  
   tissue    mean expression   
  5th Passage Drosophila S2 Cells  8.265213  
  Adult Accessory gland  7.886835  
  Adult Brain  8.676249  
  Adult Carcass  7.999966  
  Adult Crop  8.221004  
  Adult Eye  8.806057  
  Adult Fatbody  7.997333  
  Adult Female Spermatheca Mated  8.062032  
  Adult Female Spermatheca Virgin  8.062072  
  Adult Head  8.162080  
  Adult Heart  8.382625  
  Adult Hind Gut  7.944286  
  Adult Male Ejaculatory Duct  7.987229  
  Adult Mid Gut  7.946464  
  Adult Ovary  8.489503  
  Adult Salivary Gland  7.860760  
  Adult Testes  8.547958  
  Adult Thoracoabdominal ganglion  8.497134  
  Adult Whole Fly  7.837327  
  Larvae Wandering Tubules  8.255704  
  Larval Feeding Carcass  7.919734  
  Larval Feeding Central Nevous System  8.126292  
  Larval Feeding Hind Gut  7.971332  
  Larval Feeding Malpighian Tubule  8.034551  
  Larval Feeding Mid Gut  7.901633  
  Larval Feeding Salivary Gland  7.986567  
  Whole Larvae Feeding  7.562850  
 
  
   FlyBase ID    symbol    start    end    strand    length   
   FBgn0035236   CG12004   1569200   1575621  +  6422  
   FBgn0035237   CG13917   1576699   1600320  +  23622  
   FBgn0035238   CG12104  1600942   1602718   -  1777  
 
 
    Segment 36 
 
   Location   
  Gene key  FBgn0035243-FBgn0035244  
  Heatmap region span   3L:1558235..1645235   
  Segment span   3L:1618170..1618392   
  Length (genes)  2  
  Length (bp)  223  
   Model Scoring   
  BIC  214.238833  
  logL  -101.607290  
  logL ratio  39.946009  
   Expression   
  Mean expression  8.295820  
  Median expression  8.371118  
  Tissue std. dev.  0.274119  
 
  No GO Slim enrichment  
  
   tissue    mean expression   
  5th Passage Drosophila S2 Cells  8.211859  
  Adult Accessory gland  8.391296  
  Adult Brain  8.287354  
  Adult Carcass  7.865662  
  Adult Crop  8.134702  
  Adult Eye  8.239717  
  Adult Fatbody  8.424961  
  Adult Female Spermatheca Mated  8.030236  
  Adult Female Spermatheca Virgin  8.038040  
  Adult Head  7.847691  
  Adult Heart  8.792810  
  Adult Hind Gut  8.009927  
  Adult Male Ejaculatory Duct  8.471699  
  Adult Mid Gut  8.445940  
  Adult Ovary  8.565657  
  Adult Salivary Gland  8.221359  
  Adult Testes  8.168778  
  Adult Thoracoabdominal ganglion  8.177789  
  Adult Whole Fly  7.821711  
  Larvae Wandering Tubules  8.459824  
  Larval Feeding Carcass  8.541127  
  Larval Feeding Central Nevous System  8.374341  
  Larval Feeding Hind Gut  8.383727  
  Larval Feeding Malpighian Tubule  9.048712  
  Larval Feeding Mid Gut  8.529892  
  Larval Feeding Salivary Gland  8.282398  
  Whole Larvae Feeding  8.219930  
 
  
   FlyBase ID    symbol    start    end    strand    length   
   FBgn0035243   CG13926  1617424   1618170   -  747  
   FBgn0035244   ABCB7   1618392   1623413  +  5022  
 
    Segment 37 
 
   Location   
  Gene key  FBgn0035247-FBgn0013753  
  Heatmap region span   3L:1616602..1653005   
  Segment span   3L:1639234..1639687   
  Length (genes)  2  
  Length (bp)  454  
   Model Scoring   
  BIC  253.518642  
  logL  -121.247195  
  logL ratio  -1.969521  
   Expression   
  Mean expression  7.898458  
  Median expression  8.267429  
  Tissue std. dev.  0.839414  
 
  No GO Slim enrichment  
  
   tissue    mean expression   
  5th Passage Drosophila S2 Cells  8.914188  
  Adult Accessory gland  7.245699  
  Adult Brain  9.351872  
  Adult Carcass  7.168967  
  Adult Crop  8.292019  
  Adult Eye  8.850286  
  Adult Fatbody  7.249700  
  Adult Female Spermatheca Mated  7.229894  
  Adult Female Spermatheca Virgin  7.105827  
  Adult Head  8.493350  
  Adult Heart  8.302896  
  Adult Hind Gut  7.713567  
  Adult Male Ejaculatory Duct  7.316345  
  Adult Mid Gut  7.190496  
  Adult Ovary  9.394502  
  Adult Salivary Gland  7.034966  
  Adult Testes  6.626842  
  Adult Thoracoabdominal ganglion  9.105991  
  Adult Whole Fly  8.101200  
  Larvae Wandering Tubules  7.510368  
  Larval Feeding Carcass  7.994400  
  Larval Feeding Central Nevous System  9.756661  
  Larval Feeding Hind Gut  7.688572  
  Larval Feeding Malpighian Tubule  7.443708  
  Larval Feeding Mid Gut  6.992539  
  Larval Feeding Salivary Gland  7.567908  
  Whole Larvae Feeding  7.615614  
 
  
   FlyBase ID    symbol    start    end    strand    length   
   FBgn0035247   metl  1637942   1639234   -  1293  
   FBgn0013753   Bgb   1639687   1640952  +  1266  
 
    Segment 38 
 
   Location   
  Gene key  FBgn0035248-FBgn0035249  
  Heatmap region span   3L:1628377..1668677   
  Segment span   3L:1645467..1647464   
  Length (genes)  2  
  Length (bp)  1998  
   Model Scoring   
  BIC  251.301907  
  logL  -120.138827  
  logL ratio  0.663821  
   Expression   
  Mean expression  7.557413  
  Median expression  7.485861  
  Tissue std. dev.  0.424252  
 
  No GO Slim enrichment  
  
   tissue    mean expression   
  5th Passage Drosophila S2 Cells  7.761752  
  Adult Accessory gland  7.874742  
  Adult Brain  7.473593  
  Adult Carcass  7.372883  
  Adult Crop  7.656577  
  Adult Eye  7.503595  
  Adult Fatbody  7.579547  
  Adult Female Spermatheca Mated  7.706721  
  Adult Female Spermatheca Virgin  7.744726  
  Adult Head  7.077058  
  Adult Heart  7.906831  
  Adult Hind Gut  7.508671  
  Adult Male Ejaculatory Duct  8.084231  
  Adult Mid Gut  7.512619  
  Adult Ovary  7.790059  
  Adult Salivary Gland  8.070168  
  Adult Testes  6.533714  
  Adult Thoracoabdominal ganglion  7.371730  
  Adult Whole Fly  6.702979  
  Larvae Wandering Tubules  8.076231  
  Larval Feeding Carcass  7.210197  
  Larval Feeding Central Nevous System  7.731979  
  Larval Feeding Hind Gut  7.207805  
  Larval Feeding Malpighian Tubule  7.799220  
  Larval Feeding Mid Gut  7.238266  
  Larval Feeding Salivary Gland  8.498933  
  Whole Larvae Feeding  7.055318  
 
  
   FlyBase ID    symbol    start    end    strand    length   
   FBgn0035248   CG13919   1645467   1645983  +  517  
   FBgn0035249   CG17249  1645931   1647464   -  1534  
 
    Segment 39 
 
   Location   
  Gene key  FBgn0035251-FBgn0035252  
  Heatmap region span   3L:1639234..1731093   
  Segment span   3L:1652918..1653005   
  Length (genes)  2  
  Length (bp)  88  
   Model Scoring   
  BIC  237.305069  
  logL  -113.140408  
  logL ratio  60.481115  
   Expression   
  Mean expression  10.249502  
  Median expression  10.213790  
  Tissue std. dev.  0.703516  
 
  No GO Slim enrichment  
  
   tissue    mean expression   
  5th Passage Drosophila S2 Cells  10.442215  
  Adult Accessory gland  10.371196  
  Adult Brain  9.019590  
  Adult Carcass  9.975745  
  Adult Crop  10.936774  
  Adult Eye  10.250261  
  Adult Fatbody  9.761465  
  Adult Female Spermatheca Mated  9.885552  
  Adult Female Spermatheca Virgin  9.769510  
  Adult Head  9.983960  
  Adult Heart  10.373916  
  Adult Hind Gut  10.911845  
  Adult Male Ejaculatory Duct  10.763562  
  Adult Mid Gut  11.405984  
  Adult Ovary  9.870455  
  Adult Salivary Gland  9.807838  
  Adult Testes  8.676762  
  Adult Thoracoabdominal ganglion  9.326770  
  Adult Whole Fly  9.894596  
  Larvae Wandering Tubules  11.279416  
  Larval Feeding Carcass  9.904444  
  Larval Feeding Central Nevous System  9.756174  
  Larval Feeding Hind Gut  10.619232  
  Larval Feeding Malpighian Tubule  11.608438  
  Larval Feeding Mid Gut  11.311673  
  Larval Feeding Salivary Gland  10.145359  
  Whole Larvae Feeding  10.683835  
 
  
   FlyBase ID    symbol    start    end    strand    length   
   FBgn0035251   CG7967  1651432   1652918   -  1487  
   FBgn0035252   CG7970   1653005   1654112  +  1108  
 
    Segment 40 
 
   Location   
  Gene key  FBgn0035253-FBgn0035254  
  Heatmap region span   3L:1645235..1737806   
  Segment span   3L:1665060..1665143   
  Length (genes)  2  
  Length (bp)  84  
   Model Scoring   
  BIC  238.734785  
  logL  -113.855266  
  logL ratio  18.765763  
   Expression   
  Mean expression  8.085861  
  Median expression  8.016605  
  Tissue std. dev.  0.496929  
 
  No GO Slim enrichment  
  
   tissue    mean expression   
  5th Passage Drosophila S2 Cells  7.986877  
  Adult Accessory gland  8.516837  
  Adult Brain  9.070977  
  Adult Carcass  7.468194  
  Adult Crop  7.981712  
  Adult Eye  8.525291  
  Adult Fatbody  7.749352  
  Adult Female Spermatheca Mated  8.242605  
  Adult Female Spermatheca Virgin  8.201906  
  Adult Head  8.292810  
  Adult Heart  7.807487  
  Adult Hind Gut  7.635090  
  Adult Male Ejaculatory Duct  7.792974  
  Adult Mid Gut  7.899247  
  Adult Ovary  8.782601  
  Adult Salivary Gland  8.096420  
  Adult Testes  7.858246  
  Adult Thoracoabdominal ganglion  8.808245  
  Adult Whole Fly  8.019019  
  Larvae Wandering Tubules  8.042376  
  Larval Feeding Carcass  7.702860  
  Larval Feeding Central Nevous System  9.500831  
  Larval Feeding Hind Gut  7.560488  
  Larval Feeding Malpighian Tubule  7.720177  
  Larval Feeding Mid Gut  7.405472  
  Larval Feeding Salivary Gland  8.045773  
  Whole Larvae Feeding  7.604375  
 
  
   FlyBase ID    symbol    start    end    strand    length   
   FBgn0035253   CG7971  1654247   1665060   -  10814  
   FBgn0035254   CG7974   1665143   1666383  +  1241  
 
    Segment 41 
 
   Location   
  Gene key  FBgn0035255-FBgn0035263  
  Heatmap region span   3L:1648943..1746910   
  Segment span   3L:1670084..1724390   
  Length (genes)  7  
  Length (bp)  54307  
   Model Scoring   
  BIC  556.246397  
  logL  -272.611072  
  logL ratio  160.087586  
   Expression   
  Mean expression  5.139682  
  Median expression  4.991797  
  Tissue std. dev.  0.326423  
 
  No GO Slim enrichment  
  
   tissue    mean expression   
  5th Passage Drosophila S2 Cells  5.219100  
  Adult Accessory gland  5.113535  
  Adult Brain  4.926685  
  Adult Carcass  5.053357  
  Adult Crop  4.987884  
  Adult Eye  6.122956  
  Adult Fatbody  5.062091  
  Adult Female Spermatheca Mated  5.015326  
  Adult Female Spermatheca Virgin  5.013250  
  Adult Head  5.261293  
  Adult Heart  4.998538  
  Adult Hind Gut  4.905193  
  Adult Male Ejaculatory Duct  5.144942  
  Adult Mid Gut  5.169643  
  Adult Ovary  5.043605  
  Adult Salivary Gland  5.174120  
  Adult Testes  6.332625  
  Adult Thoracoabdominal ganglion  4.899890  
  Adult Whole Fly  5.078180  
  Larvae Wandering Tubules  5.124724  
  Larval Feeding Carcass  5.076611  
  Larval Feeding Central Nevous System  4.878503  
  Larval Feeding Hind Gut  4.891930  
  Larval Feeding Malpighian Tubule  5.043278  
  Larval Feeding Mid Gut  5.199277  
  Larval Feeding Salivary Gland  5.135301  
  Whole Larvae Feeding  4.899571  
 
  
   FlyBase ID    symbol    start    end    strand    length   
   FBgn0035255   RabX5  1668901   1670084   -  1184  
   FBgn0035256   CG13930  1670472   1672705   -  2234  
   FBgn0035260   CG7991   1674709   1715541  +  40833  
   FBgn0035257   CG12011   1694617   1696412  +  1796  
   FBgn0035258   CG13931  1701356   1702407   -  1052  
   FBgn0035262   CG18171  1720616   1723153   -  2538  
   FBgn0035263   CG12035   1724390   1725647  +  1258  
 
 
    Segment 42 
 
   Location   
  Gene key  FBgn0035264-FBgn0035265  
  Heatmap region span   3L:1665060..1771650   
  Segment span   3L:1735755..1737806   
  Length (genes)  2  
  Length (bp)  2052  
   Model Scoring   
  BIC  201.367060  
  logL  -95.171404  
  logL ratio  18.263053  
   Expression   
  Mean expression  5.251105  
  Median expression  4.904621  
  Tissue std. dev.  1.243093  
 
  No GO Slim enrichment  
  
   tissue    mean expression   
  5th Passage Drosophila S2 Cells  4.603334  
  Adult Accessory gland  4.901137  
  Adult Brain  4.508914  
  Adult Carcass  4.640399  
  Adult Crop  4.551816  
  Adult Eye  4.910362  
  Adult Fatbody  4.455891  
  Adult Female Spermatheca Mated  4.479352  
  Adult Female Spermatheca Virgin  4.530840  
  Adult Head  4.788588  
  Adult Heart  4.594327  
  Adult Hind Gut  5.656217  
  Adult Male Ejaculatory Duct  4.625525  
  Adult Mid Gut  9.725020  
  Adult Ovary  4.735652  
  Adult Salivary Gland  4.658753  
  Adult Testes  6.868610  
  Adult Thoracoabdominal ganglion  4.450361  
  Adult Whole Fly  6.074683  
  Larvae Wandering Tubules  4.624303  
  Larval Feeding Carcass  4.810571  
  Larval Feeding Central Nevous System  5.404336  
  Larval Feeding Hind Gut  4.686145  
  Larval Feeding Malpighian Tubule  4.764102  
  Larval Feeding Mid Gut  8.192487  
  Larval Feeding Salivary Gland  4.825468  
  Whole Larvae Feeding  6.712648  
 
  
   FlyBase ID    symbol    start    end    strand    length   
   FBgn0035264   Oseg4  1730993   1735755   -  4763  
   FBgn0035265   CG18173  1735854   1737806   -  1953  
 
    Segment 43 
 
   Location   
  Gene key  FBgn0022702-FBgn0035268  
  Heatmap region span   3L:1731093..1795426   
  Segment span   3L:1755663..1756159   
  Length (genes)  2  
  Length (bp)  497  
   Model Scoring   
  BIC  263.668087  
  logL  -126.321917  
  logL ratio  -9.763920  
   Expression   
  Mean expression  7.732084  
  Median expression  7.717930  
  Tissue std. dev.  0.945532  
 
  No GO Slim enrichment  
  
   tissue    mean expression   
  5th Passage Drosophila S2 Cells  6.910076  
  Adult Accessory gland  7.407075  
  Adult Brain  8.322843  
  Adult Carcass  7.541085  
  Adult Crop  6.550859  
  Adult Eye  8.222637  
  Adult Fatbody  7.370088  
  Adult Female Spermatheca Mated  6.475655  
  Adult Female Spermatheca Virgin  6.821905  
  Adult Head  7.638323  
  Adult Heart  7.983990  
  Adult Hind Gut  6.802516  
  Adult Male Ejaculatory Duct  9.295240  
  Adult Mid Gut  7.455649  
  Adult Ovary  9.204429  
  Adult Salivary Gland  6.646678  
  Adult Testes  8.677981  
  Adult Thoracoabdominal ganglion  8.172169  
  Adult Whole Fly  8.459126  
  Larvae Wandering Tubules  6.507876  
  Larval Feeding Carcass  9.381692  
  Larval Feeding Central Nevous System  8.916587  
  Larval Feeding Hind Gut  8.940392  
  Larval Feeding Malpighian Tubule  6.508724  
  Larval Feeding Mid Gut  7.099638  
  Larval Feeding Salivary Gland  6.890068  
  Whole Larvae Feeding  8.562957  
 
  
   FlyBase ID    symbol    start    end    strand    length   
   FBgn0022702   Cht2  1752546   1755663   -  3118  
   FBgn0035268   CG8001   1756159   1759208  +  3050  
 
    Segment 44 
 
   Location   
  Gene key  FBgn0035270-FBgn0035272  
  Heatmap region span   3L:1738557..1802835   
  Segment span   3L:1775143..1777441   
  Length (genes)  4  
  Length (bp)  2299  
   Model Scoring   
  BIC  414.588740  
  logL  -201.782244  
  logL ratio  90.165381  
   Expression   
  Mean expression  8.378551  
  Median expression  8.563730  
  Tissue std. dev.  0.425922  
 
  No GO Slim enrichment  
  
   tissue    mean expression   
  5th Passage Drosophila S2 Cells  8.876224  
  Adult Accessory gland  8.249428  
  Adult Brain  8.603511  
  Adult Carcass  7.763389  
  Adult Crop  8.237370  
  Adult Eye  8.165815  
  Adult Fatbody  8.297961  
  Adult Female Spermatheca Mated  8.024464  
  Adult Female Spermatheca Virgin  8.076582  
  Adult Head  8.023472  
  Adult Heart  8.309758  
  Adult Hind Gut  8.109145  
  Adult Male Ejaculatory Duct  8.916679  
  Adult Mid Gut  7.870942  
  Adult Ovary  9.566422  
  Adult Salivary Gland  8.268244  
  Adult Testes  7.871694  
  Adult Thoracoabdominal ganglion  8.605030  
  Adult Whole Fly  8.413474  
  Larvae Wandering Tubules  8.547152  
  Larval Feeding Carcass  8.284094  
  Larval Feeding Central Nevous System  9.305939  
  Larval Feeding Hind Gut  8.509466  
  Larval Feeding Malpighian Tubule  8.683221  
  Larval Feeding Mid Gut  7.874395  
  Larval Feeding Salivary Gland  8.676414  
  Whole Larvae Feeding  8.090592  
 
  
   FlyBase ID    symbol    start    end    strand    length   
   FBgn0035270   CG13933  1771809   1775143   -  3335  
   FBgn0027903   CG12018   1775633   1777024  +  1392  
   FBgn0035271   CG2021  1777008   1777363   -  356  
   FBgn0035272   mRpL46   1777441   1778347  +  907  
 
 
    Segment 45 
 
   Location   
  Gene key  FBgn0067864-FBgn0025820  
  Heatmap region span   3L:1775143..1870019   
  Segment span   3L:1798809..1802835   
  Length (genes)  2  
  Length (bp)  4027  
   Model Scoring   
  BIC  201.324388  
  logL  -95.150067  
  logL ratio  54.039678  
   Expression   
  Mean expression  8.638327  
  Median expression  8.557946  
  Tissue std. dev.  0.417936  
 
  No GO Slim enrichment  
  
   tissue    mean expression   
  5th Passage Drosophila S2 Cells  9.042072  
  Adult Accessory gland  8.951479  
  Adult Brain  8.741647  
  Adult Carcass  8.107309  
  Adult Crop  8.929490  
  Adult Eye  9.696821  
  Adult Fatbody  8.067058  
  Adult Female Spermatheca Mated  8.125898  
  Adult Female Spermatheca Virgin  7.966234  
  Adult Head  8.764238  
  Adult Heart  8.884397  
  Adult Hind Gut  8.878270  
  Adult Male Ejaculatory Duct  9.176718  
  Adult Mid Gut  8.543491  
  Adult Ovary  9.188359  
  Adult Salivary Gland  8.458208  
  Adult Testes  8.390536  
  Adult Thoracoabdominal ganglion  8.689687  
  Adult Whole Fly  8.236755  
  Larvae Wandering Tubules  8.902072  
  Larval Feeding Carcass  8.507012  
  Larval Feeding Central Nevous System  8.523625  
  Larval Feeding Hind Gut  8.584681  
  Larval Feeding Malpighian Tubule  8.671744  
  Larval Feeding Mid Gut  8.215666  
  Larval Feeding Salivary Gland  9.043458  
  Whole Larvae Feeding  7.947894  
 
  
   FlyBase ID    symbol    start    end    strand    length   
   FBgn0067864   Patj   1798809   1802184  +  3376  
   FBgn0025820   JTBR  1802008   1802835   -  828  
 
    Segment 46 
 
   Location   
  Gene key  FBgn0035279-FBgn0035282  
  Heatmap region span   3L:1795426..1883037   
  Segment span   3L:1831783..1855591   
  Length (genes)  4  
  Length (bp)  23809  
   Model Scoring   
  BIC  380.035362  
  logL  -184.505555  
  logL ratio  36.217738  
   Expression   
  Mean expression  5.331710  
  Median expression  5.063358  
  Tissue std. dev.  0.593054  
 
  No GO Slim enrichment  
  
   tissue    mean expression   
  5th Passage Drosophila S2 Cells  5.127831  
  Adult Accessory gland  4.971933  
  Adult Brain  4.842987  
  Adult Carcass  6.436547  
  Adult Crop  4.813034  
  Adult Eye  6.841944  
  Adult Fatbody  5.375406  
  Adult Female Spermatheca Mated  5.892053  
  Adult Female Spermatheca Virgin  5.722013  
  Adult Head  5.960409  
  Adult Heart  5.421219  
  Adult Hind Gut  6.482505  
  Adult Male Ejaculatory Duct  5.360088  
  Adult Mid Gut  4.960667  
  Adult Ovary  4.807937  
  Adult Salivary Gland  5.092992  
  Adult Testes  4.652696  
  Adult Thoracoabdominal ganglion  4.685520  
  Adult Whole Fly  5.177122  
  Larvae Wandering Tubules  4.831584  
  Larval Feeding Carcass  5.379105  
  Larval Feeding Central Nevous System  4.648947  
  Larval Feeding Hind Gut  5.030532  
  Larval Feeding Malpighian Tubule  4.936301  
  Larval Feeding Mid Gut  5.250319  
  Larval Feeding Salivary Gland  5.079128  
  Whole Larvae Feeding  6.175350  
 
  
   FlyBase ID    symbol    start    end    strand    length   
   FBgn0035279   Cpr62Ba  1824127   1831783   -  7657  
   FBgn0035280   Cpr62Bb  1832636   1835166   -  2531  
   FBgn0035281   Cpr62Bc  1840278   1842080   -  1803  
   FBgn0035282   CG13936  1850915   1855591   -  4677  
 
 
    Segment 47 
 
   Location   
  Gene key  FBgn0035283-FBgn0035285  
  Heatmap region span   3L:1797305..1884885   
  Segment span   3L:1857167..1865432   
  Length (genes)  4  
  Length (bp)  8266  
   Model Scoring   
  BIC  462.464015  
  logL  -225.719881  
  logL ratio  94.673661  
   Expression   
  Mean expression  9.669887  
  Median expression  9.527280  
  Tissue std. dev.  0.442712  
 
  
   GO ID    description    ratio    P-value   
   GO:0003924   GTPase activity  2/4  0.00473  
 
  
   tissue    mean expression   
  5th Passage Drosophila S2 Cells  9.656465  
  Adult Accessory gland  9.329421  
  Adult Brain  10.233839  
  Adult Carcass  9.663033  
  Adult Crop  10.034892  
  Adult Eye  10.390541  
  Adult Fatbody  10.131258  
  Adult Female Spermatheca Mated  9.783926  
  Adult Female Spermatheca Virgin  9.702021  
  Adult Head  9.776576  
  Adult Heart  9.942302  
  Adult Hind Gut  9.769131  
  Adult Male Ejaculatory Duct  9.336280  
  Adult Mid Gut  8.561337  
  Adult Ovary  9.656469  
  Adult Salivary Gland  9.219417  
  Adult Testes  9.162253  
  Adult Thoracoabdominal ganglion  10.158888  
  Adult Whole Fly  9.199921  
  Larvae Wandering Tubules  10.202683  
  Larval Feeding Carcass  9.537063  
  Larval Feeding Central Nevous System  9.929535  
  Larval Feeding Hind Gut  9.995833  
  Larval Feeding Malpighian Tubule  9.947952  
  Larval Feeding Mid Gut  8.800107  
  Larval Feeding Salivary Gland  9.831349  
  Whole Larvae Feeding  9.134454  
 
  
   FlyBase ID    symbol    start    end    strand    length   
   FBgn0035283   CG12024   1857167   1859047  +  1881  
   FBgn0004636   R  1859115   1862256   -  3142  
   FBgn0042712   HBS1  1862881   1865309   -  2429  
   FBgn0035285   CG12025   1865432   1867863  +  2432  
 
 
    Segment 48 
 
   Location   
  Gene key  FBgn0035293-FBgn0035294  
  Heatmap region span   3L:1883037..1971662   
  Segment span   3L:1938740..1941062   
  Length (genes)  2  
  Length (bp)  2323  
   Model Scoring   
  BIC  262.197225  
  logL  -125.586486  
  logL ratio  -3.956740  
   Expression   
  Mean expression  7.911206  
  Median expression  7.810351  
  Tissue std. dev.  1.122171  
 
  No GO Slim enrichment  
  
   tissue    mean expression   
  5th Passage Drosophila S2 Cells  7.217497  
  Adult Accessory gland  7.872028  
  Adult Brain  10.460259  
  Adult Carcass  8.686146  
  Adult Crop  7.297237  
  Adult Eye  9.636385  
  Adult Fatbody  6.811257  
  Adult Female Spermatheca Mated  7.500977  
  Adult Female Spermatheca Virgin  7.765685  
  Adult Head  9.538710  
  Adult Heart  7.830998  
  Adult Hind Gut  7.399636  
  Adult Male Ejaculatory Duct  9.040750  
  Adult Mid Gut  6.771132  
  Adult Ovary  7.319560  
  Adult Salivary Gland  7.328942  
  Adult Testes  7.600831  
  Adult Thoracoabdominal ganglion  10.564400  
  Adult Whole Fly  8.056866  
  Larvae Wandering Tubules  6.948258  
  Larval Feeding Carcass  8.246936  
  Larval Feeding Central Nevous System  9.300866  
  Larval Feeding Hind Gut  6.857924  
  Larval Feeding Malpighian Tubule  6.884583  
  Larval Feeding Mid Gut  6.699665  
  Larval Feeding Salivary Gland  6.962524  
  Whole Larvae Feeding  7.002498  
 
  
   FlyBase ID    symbol    start    end    strand    length   
   FBgn0035293   CG5687  1933631   1938740   -  5110  
   FBgn0035294   Mfap1   1941062   1942872  +  1811  
 
    Segment 49 
 
   Location   
  Gene key  FBgn0002872-FBgn0035295  
  Heatmap region span   3L:1884885..2026991   
  Segment span   3L:1947513..1947564   
  Length (genes)  2  
  Length (bp)  52  
   Model Scoring   
  BIC  193.425001  
  logL  -91.200374  
  logL ratio  28.684305  
   Expression   
  Mean expression  5.820965  
  Median expression  5.508805  
  Tissue std. dev.  1.084105  
 
  No GO Slim enrichment  
  
   tissue    mean expression   
  5th Passage Drosophila S2 Cells  7.115927  
  Adult Accessory gland  5.313343  
  Adult Brain  5.610297  
  Adult Carcass  5.027743  
  Adult Crop  5.582608  
  Adult Eye  5.175783  
  Adult Fatbody  4.971161  
  Adult Female Spermatheca Mated  5.189250  
  Adult Female Spermatheca Virgin  5.300900  
  Adult Head  5.188734  
  Adult Heart  5.363810  
  Adult Hind Gut  5.015694  
  Adult Male Ejaculatory Duct  4.881288  
  Adult Mid Gut  5.024470  
  Adult Ovary  8.958240  
  Adult Salivary Gland  4.895513  
  Adult Testes  7.651036  
  Adult Thoracoabdominal ganglion  5.703865  
  Adult Whole Fly  7.137165  
  Larvae Wandering Tubules  5.305051  
  Larval Feeding Carcass  5.995502  
  Larval Feeding Central Nevous System  8.506054  
  Larval Feeding Hind Gut  5.855245  
  Larval Feeding Malpighian Tubule  5.455797  
  Larval Feeding Mid Gut  4.896976  
  Larval Feeding Salivary Gland  6.262328  
  Whole Larvae Feeding  5.782286  
 
  
   FlyBase ID    symbol    start    end    strand    length   
   FBgn0002872   mu2  1942734   1947513   -  4780  
   FBgn0035295   Cnb   1947564   1950262  +  2699  
 
    Segment 50 
 
   Location   
  Gene key  FBgn0035298-FBgn0027547  
  Heatmap region span   3L:1885659..2038183   
  Segment span   3L:1963143..1970075   
  Length (genes)  2  
  Length (bp)  6933  
   Model Scoring   
  BIC  259.863845  
  logL  -124.419796  
  logL ratio  31.306701  
   Expression   
  Mean expression  9.901483  
  Median expression  10.148380  
  Tissue std. dev.  0.826514  
 
  No GO Slim enrichment  
  
   tissue    mean expression   
  5th Passage Drosophila S2 Cells  9.213698  
  Adult Accessory gland  9.307631  
  Adult Brain  8.893620  
  Adult Carcass  10.364479  
  Adult Crop  10.350513  
  Adult Eye  9.941622  
  Adult Fatbody  10.549674  
  Adult Female Spermatheca Mated  9.820688  
  Adult Female Spermatheca Virgin  10.251391  
  Adult Head  10.234879  
  Adult Heart  10.502881  
  Adult Hind Gut  10.923153  
  Adult Male Ejaculatory Duct  10.088029  
  Adult Mid Gut  10.274453  
  Adult Ovary  9.203542  
  Adult Salivary Gland  9.425302  
  Adult Testes  7.533767  
  Adult Thoracoabdominal ganglion  8.863467  
  Adult Whole Fly  9.542406  
  Larvae Wandering Tubules  10.360604  
  Larval Feeding Carcass  10.189860  
  Larval Feeding Central Nevous System  8.852663  
  Larval Feeding Hind Gut  10.973066  
  Larval Feeding Malpighian Tubule  11.698896  
  Larval Feeding Mid Gut  10.175089  
  Larval Feeding Salivary Gland  9.367811  
  Whole Larvae Feeding  10.436857  
 
  
   FlyBase ID    symbol    start    end    strand    length   
   FBgn0035298   CG1140   1963143   1966808  +  3666  
   FBgn0027547   CG1927  1966725   1970075   -  3351  
 
    Segment 51 
 
   Location   
  Gene key  FBgn0259703-FBgn0085288  
  Heatmap region span   3L:1947513..2170941   
  Segment span   3L:2021958..2026991   
  Length (genes)  2  
  Length (bp)  5034  
   Model Scoring   
  BIC  184.938699  
  logL  -86.957223  
  logL ratio  49.472354  
   Expression   
  Mean expression  3.902691  
  Median expression  3.874533  
  Tissue std. dev.  0.139662  
 
  No GO Slim enrichment  
  
   tissue    mean expression   
  5th Passage Drosophila S2 Cells  3.909039  
  Adult Accessory gland  4.014456  
  Adult Brain  3.788012  
  Adult Carcass  3.970387  
  Adult Crop  3.939395  
  Adult Eye  4.025049  
  Adult Fatbody  3.977148  
  Adult Female Spermatheca Mated  4.100457  
  Adult Female Spermatheca Virgin  4.072084  
  Adult Head  3.765891  
  Adult Heart  3.979090  
  Adult Hind Gut  3.882946  
  Adult Male Ejaculatory Duct  3.721191  
  Adult Mid Gut  3.854649  
  Adult Ovary  3.816972  
  Adult Salivary Gland  4.184288  
  Adult Testes  3.693947  
  Adult Thoracoabdominal ganglion  3.795355  
  Adult Whole Fly  3.610764  
  Larvae Wandering Tubules  3.969554  
  Larval Feeding Carcass  3.832836  
  Larval Feeding Central Nevous System  3.958007  
  Larval Feeding Hind Gut  3.912531  
  Larval Feeding Malpighian Tubule  3.962497  
  Larval Feeding Mid Gut  3.918429  
  Larval Feeding Salivary Gland  4.081755  
  Whole Larvae Feeding  3.635919  
 
  
   FlyBase ID    symbol    start    end    strand    length   
   FBgn0259703   CG42357  2020554   2021958   -  1405  
   FBgn0085288   CG34259   2026991   2028326  +  1336  
 
    Segment 52 
 
   Location   
  Gene key  FBgn0052311-FBgn0035308  
  Heatmap region span   3L:1971662..2192180   
  Segment span   3L:2150089..2163860   
  Length (genes)  2  
  Length (bp)  13772  
   Model Scoring   
  BIC  244.152689  
  logL  -116.564218  
  logL ratio  -10.393705  
   Expression   
  Mean expression  6.239933  
  Median expression  5.901935  
  Tissue std. dev.  1.165446  
 
  No GO Slim enrichment  
  
   tissue    mean expression   
  5th Passage Drosophila S2 Cells  4.899491  
  Adult Accessory gland  5.559520  
  Adult Brain  4.658112  
  Adult Carcass  5.836994  
  Adult Crop  6.479412  
  Adult Eye  6.102569  
  Adult Fatbody  4.888852  
  Adult Female Spermatheca Mated  7.005706  
  Adult Female Spermatheca Virgin  6.674682  
  Adult Head  5.999280  
  Adult Heart  4.857653  
  Adult Hind Gut  8.594483  
  Adult Male Ejaculatory Duct  7.860473  
  Adult Mid Gut  5.226914  
  Adult Ovary  5.604044  
  Adult Salivary Gland  8.634298  
  Adult Testes  6.034718  
  Adult Thoracoabdominal ganglion  5.175141  
  Adult Whole Fly  5.489691  
  Larvae Wandering Tubules  5.335508  
  Larval Feeding Carcass  7.823158  
  Larval Feeding Central Nevous System  6.067621  
  Larval Feeding Hind Gut  7.234444  
  Larval Feeding Malpighian Tubule  5.217154  
  Larval Feeding Mid Gut  6.307431  
  Larval Feeding Salivary Gland  8.372492  
  Whole Larvae Feeding  6.538346  
 
  
   FlyBase ID    symbol    start    end    strand    length   
   FBgn0052311   zormin  2117466   2150089   -  32624  
   FBgn0035308   CG15822  2153201   2163860   -  10660  
 
    Segment 53 
 
   Location   
  Gene key  FBgn0035309-FBgn0026570  
  Heatmap region span   3L:2021958..2212503   
  Segment span   3L:2169108..2170941   
  Length (genes)  2  
  Length (bp)  1834  
   Model Scoring   
  BIC  228.447522  
  logL  -108.711635  
  logL ratio  16.209298  
   Expression   
  Mean expression  7.098237  
  Median expression  7.071500  
  Tissue std. dev.  0.901505  
 
  No GO Slim enrichment  
  
   tissue    mean expression   
  5th Passage Drosophila S2 Cells  8.208739  
  Adult Accessory gland  6.807766  
  Adult Brain  5.385958  
  Adult Carcass  7.176584  
  Adult Crop  8.046947  
  Adult Eye  6.524743  
  Adult Fatbody  7.859721  
  Adult Female Spermatheca Mated  7.742377  
  Adult Female Spermatheca Virgin  7.719643  
  Adult Head  6.736766  
  Adult Heart  7.880496  
  Adult Hind Gut  7.734103  
  Adult Male Ejaculatory Duct  7.114024  
  Adult Mid Gut  8.575230  
  Adult Ovary  6.873883  
  Adult Salivary Gland  7.437877  
  Adult Testes  5.301817  
  Adult Thoracoabdominal ganglion  5.533532  
  Adult Whole Fly  6.223204  
  Larvae Wandering Tubules  6.918154  
  Larval Feeding Carcass  6.573875  
  Larval Feeding Central Nevous System  5.836440  
  Larval Feeding Hind Gut  6.973676  
  Larval Feeding Malpighian Tubule  8.250746  
  Larval Feeding Mid Gut  8.183366  
  Larval Feeding Salivary Gland  6.421378  
  Whole Larvae Feeding  7.611357  
 
  
   FlyBase ID    symbol    start    end    strand    length   
   FBgn0035309   CG15879  2167550   2169108   -  1559  
   FBgn0026570   CG5704  2169622   2170941   -  1320  
 
    Segment 54 
 
   Location   
  Gene key  FBgn0035315-FBgn0035316  
  Heatmap region span   3L:2169108..2257409   
  Segment span   3L:2197013..2212503   
  Length (genes)  2  
  Length (bp)  15491  
   Model Scoring   
  BIC  193.142565  
  logL  -91.059156  
  logL ratio  21.583685  
   Expression   
  Mean expression  4.561295  
  Median expression  4.502916  
  Tissue std. dev.  0.560989  
 
  No GO Slim enrichment  
  
   tissue    mean expression   
  5th Passage Drosophila S2 Cells  4.447343  
  Adult Accessory gland  4.524268  
  Adult Brain  4.323618  
  Adult Carcass  4.745473  
  Adult Crop  4.491856  
  Adult Eye  4.144430  
  Adult Fatbody  4.478202  
  Adult Female Spermatheca Mated  4.795692  
  Adult Female Spermatheca Virgin  4.720269  
  Adult Head  4.352157  
  Adult Heart  4.267086  
  Adult Hind Gut  4.490523  
  Adult Male Ejaculatory Duct  4.399714  
  Adult Mid Gut  4.388200  
  Adult Ovary  4.369339  
  Adult Salivary Gland  4.799455  
  Adult Testes  7.246530  
  Adult Thoracoabdominal ganglion  4.437898  
  Adult Whole Fly  4.946952  
  Larvae Wandering Tubules  4.354083  
  Larval Feeding Carcass  4.477284  
  Larval Feeding Central Nevous System  4.183702  
  Larval Feeding Hind Gut  4.226417  
  Larval Feeding Malpighian Tubule  4.256045  
  Larval Feeding Mid Gut  4.322727  
  Larval Feeding Salivary Gland  4.474421  
  Whole Larvae Feeding  4.491268  
 
  
   FlyBase ID    symbol    start    end    strand    length   
   FBgn0035315   CG8960   2197013   2198107  +  1095  
   FBgn0035316   CG15878  2210873   2212503   -  1631  
 
    Segment 55 
 
   Location   
  Gene key  FBgn0052301-FBgn0052305  
  Heatmap region span   3L:2192180..2360557   
  Segment span   3L:2244530..2248635   
  Length (genes)  2  
  Length (bp)  4106  
   Model Scoring   
  BIC  175.689869  
  logL  -82.332808  
  logL ratio  42.836030  
   Expression   
  Mean expression  4.392563  
  Median expression  4.331259  
  Tissue std. dev.  0.634289  
 
  No GO Slim enrichment  
  
   tissue    mean expression   
  5th Passage Drosophila S2 Cells  4.237233  
  Adult Accessory gland  4.353377  
  Adult Brain  4.132963  
  Adult Carcass  4.382947  
  Adult Crop  4.179559  
  Adult Eye  4.239527  
  Adult Fatbody  4.274347  
  Adult Female Spermatheca Mated  4.353919  
  Adult Female Spermatheca Virgin  4.366300  
  Adult Head  4.067596  
  Adult Heart  4.233618  
  Adult Hind Gut  4.052249  
  Adult Male Ejaculatory Duct  4.610997  
  Adult Mid Gut  4.208557  
  Adult Ovary  4.296269  
  Adult Salivary Gland  4.542180  
  Adult Testes  7.560969  
  Adult Thoracoabdominal ganglion  4.244964  
  Adult Whole Fly  4.226710  
  Larvae Wandering Tubules  4.337779  
  Larval Feeding Carcass  4.383353  
  Larval Feeding Central Nevous System  4.073057  
  Larval Feeding Hind Gut  4.111874  
  Larval Feeding Malpighian Tubule  4.268790  
  Larval Feeding Mid Gut  4.272744  
  Larval Feeding Salivary Gland  4.334886  
  Whole Larvae Feeding  4.252429  
 
  
   FlyBase ID    symbol    start    end    strand    length   
   FBgn0052301   CG32301   2244530   2248483  +  3954  
   FBgn0052305   CG32305   2248635   2252729  +  4095  
 
    Segment 56 
 
   Location   
  Gene key  FBgn0015359-FBgn0000543  
  Heatmap region span   3L:2237072..2466903   
  Segment span   3L:2257669..2265811   
  Length (genes)  4  
  Length (bp)  8143  
   Model Scoring   
  BIC  344.482806  
  logL  -166.729276  
  logL ratio  159.182028  
   Expression   
  Mean expression  8.708640  
  Median expression  8.678268  
  Tissue std. dev.  0.317242  
 
  No GO Slim enrichment  
  
   tissue    mean expression   
  5th Passage Drosophila S2 Cells  9.042289  
  Adult Accessory gland  8.282364  
  Adult Brain  8.823524  
  Adult Carcass  8.616620  
  Adult Crop  8.865581  
  Adult Eye  8.923204  
  Adult Fatbody  8.629186  
  Adult Female Spermatheca Mated  8.464002  
  Adult Female Spermatheca Virgin  8.482834  
  Adult Head  8.594048  
  Adult Heart  8.922755  
  Adult Hind Gut  8.373703  
  Adult Male Ejaculatory Duct  8.562124  
  Adult Mid Gut  8.120930  
  Adult Ovary  9.454574  
  Adult Salivary Gland  8.659267  
  Adult Testes  8.876672  
  Adult Thoracoabdominal ganglion  8.655036  
  Adult Whole Fly  8.672165  
  Larvae Wandering Tubules  9.387874  
  Larval Feeding Carcass  8.723616  
  Larval Feeding Central Nevous System  8.916144  
  Larval Feeding Hind Gut  8.481573  
  Larval Feeding Malpighian Tubule  9.037899  
  Larval Feeding Mid Gut  8.275964  
  Larval Feeding Salivary Gland  8.980449  
  Whole Larvae Feeding  8.308888  
 
  
   FlyBase ID    symbol    start    end    strand    length   
   FBgn0015359   CG2034   2257669   2258922  +  1254  
   FBgn0015360   oxt  2258839   2262661   -  3823  
   FBgn0035323   CG13807   2262919   2263615  +  697  
   FBgn0000543   ecd  2263581   2265811   -  2231  
 
 
    Segment 57 
 
   Location   
  Gene key  FBgn0035325-FBgn0052302  
  Heatmap region span   3L:2237610..2475614   
  Segment span   3L:2266394..2268827   
  Length (genes)  2  
  Length (bp)  2434  
   Model Scoring   
  BIC  194.219003  
  logL  -91.597375  
  logL ratio  30.878134  
   Expression   
  Mean expression  5.571275  
  Median expression  4.784223  
  Tissue std. dev.  2.019778  
 
  
   GO ID    description    ratio    P-value   
   GO:0005576   extracellular region  2/2  0.00103  
 
  
   tissue    mean expression   
  5th Passage Drosophila S2 Cells  4.736394  
  Adult Accessory gland  5.105925  
  Adult Brain  4.296145  
  Adult Carcass  4.820204  
  Adult Crop  4.898167  
  Adult Eye  4.379656  
  Adult Fatbody  4.632044  
  Adult Female Spermatheca Mated  4.504430  
  Adult Female Spermatheca Virgin  4.484704  
  Adult Head  4.791185  
  Adult Heart  4.649689  
  Adult Hind Gut  4.769247  
  Adult Male Ejaculatory Duct  4.712143  
  Adult Mid Gut  8.708856  
  Adult Ovary  4.764767  
  Adult Salivary Gland  4.821359  
  Adult Testes  5.401743  
  Adult Thoracoabdominal ganglion  4.424428  
  Adult Whole Fly  5.009247  
  Larvae Wandering Tubules  6.269516  
  Larval Feeding Carcass  4.921448  
  Larval Feeding Central Nevous System  4.544932  
  Larval Feeding Hind Gut  5.356910  
  Larval Feeding Malpighian Tubule  6.910737  
  Larval Feeding Mid Gut  12.703737  
  Larval Feeding Salivary Gland  4.673890  
  Whole Larvae Feeding  11.132926  
 
  
   FlyBase ID    symbol    start    end    strand    length   
   FBgn0035325   CG13806   2266394   2267640  +  1247  
   FBgn0052302   CG32302   2268827   2269903  +  1077  
 
    Segment 58 
 
   Location   
  Gene key  FBgn0035333-FBgn0035335  
  Heatmap region span   3L:2257409..2488819   
  Segment span   3L:2371960..2373678   
  Length (genes)  3  
  Length (bp)  1719  
   Model Scoring   
  BIC  303.953896  
  logL  -146.464822  
  logL ratio  122.983839  
   Expression   
  Mean expression  10.044760  
  Median expression  10.009495  
  Tissue std. dev.  0.495568  
 
  No GO Slim enrichment  
  
   tissue    mean expression   
  5th Passage Drosophila S2 Cells  10.035444  
  Adult Accessory gland  9.808055  
  Adult Brain  11.112173  
  Adult Carcass  10.382604  
  Adult Crop  9.885085  
  Adult Eye  10.339601  
  Adult Fatbody  10.478042  
  Adult Female Spermatheca Mated  9.690350  
  Adult Female Spermatheca Virgin  9.643910  
  Adult Head  10.393344  
  Adult Heart  10.492665  
  Adult Hind Gut  10.448814  
  Adult Male Ejaculatory Duct  10.444787  
  Adult Mid Gut  9.960579  
  Adult Ovary  9.543839  
  Adult Salivary Gland  10.170744  
  Adult Testes  8.669018  
  Adult Thoracoabdominal ganglion  10.997112  
  Adult Whole Fly  9.787108  
  Larvae Wandering Tubules  9.930799  
  Larval Feeding Carcass  9.783285  
  Larval Feeding Central Nevous System  9.885086  
  Larval Feeding Hind Gut  10.110235  
  Larval Feeding Malpighian Tubule  10.467503  
  Larval Feeding Mid Gut  9.524968  
  Larval Feeding Salivary Gland  9.696369  
  Whole Larvae Feeding  9.527012  
 
  
   FlyBase ID    symbol    start    end    strand    length   
   FBgn0035333   CG1317  2368014   2371960   -  3947  
   FBgn0035334   CG8993   2372199   2372876  +  678  
   FBgn0035335   mRpL23  2372844   2373678   -  835  
 
 
    Segment 59 
 
   Location   
  Gene key  FBgn0035336-FBgn0035347  
  Heatmap region span   3L:2257669..2554292   
  Segment span   3L:2373728..2466903   
  Length (genes)  4  
  Length (bp)  93176  
   Model Scoring   
  BIC  364.409716  
  logL  -176.692732  
  logL ratio  122.422066  
   Expression   
  Mean expression  6.906842  
  Median expression  6.863173  
  Tissue std. dev.  0.393646  
 
  No GO Slim enrichment  
  
   tissue    mean expression   
  5th Passage Drosophila S2 Cells  7.421874  
  Adult Accessory gland  7.652726  
  Adult Brain  6.592808  
  Adult Carcass  6.488801  
  Adult Crop  7.397492  
  Adult Eye  7.183369  
  Adult Fatbody  6.314496  
  Adult Female Spermatheca Mated  6.864761  
  Adult Female Spermatheca Virgin  6.698107  
  Adult Head  6.740925  
  Adult Heart  6.700440  
  Adult Hind Gut  6.809638  
  Adult Male Ejaculatory Duct  7.264487  
  Adult Mid Gut  6.820437  
  Adult Ovary  7.821765  
  Adult Salivary Gland  7.140290  
  Adult Testes  6.750864  
  Adult Thoracoabdominal ganglion  6.732219  
  Adult Whole Fly  6.660289  
  Larvae Wandering Tubules  7.494826  
  Larval Feeding Carcass  6.822649  
  Larval Feeding Central Nevous System  7.088815  
  Larval Feeding Hind Gut  6.800441  
  Larval Feeding Malpighian Tubule  6.930675  
  Larval Feeding Mid Gut  6.455609  
  Larval Feeding Salivary Gland  6.610145  
  Whole Larvae Feeding  6.225777  
 
  
   FlyBase ID    symbol    start    end    strand    length   
   FBgn0035336   CG9004   2373728   2376488  +  2761  
   FBgn0035337   CG15877  2376484   2377556   -  1073  
   FBgn0035338   CG13800   2379062   2408752  +  29691  
   FBgn0035347   CG33232   2466903   2499228  +  32326  
 
 
    Segment 60 
 
   Location   
  Gene key  FBgn0035348-FBgn0010905  
  Heatmap region span   3L:2373728..2599541   
  Segment span   3L:2503584..2554292   
  Length (genes)  2  
  Length (bp)  50709  
   Model Scoring   
  BIC  219.580263  
  logL  -104.278005  
  logL ratio  15.557800  
   Expression   
  Mean expression  6.276858  
  Median expression  6.129321  
  Tissue std. dev.  0.515489  
 
  No GO Slim enrichment  
  
   tissue    mean expression   
  5th Passage Drosophila S2 Cells  5.998228  
  Adult Accessory gland  6.039570  
  Adult Brain  6.378853  
  Adult Carcass  5.465124  
  Adult Crop  7.125272  
  Adult Eye  6.902498  
  Adult Fatbody  5.446888  
  Adult Female Spermatheca Mated  5.959174  
  Adult Female Spermatheca Virgin  5.992873  
  Adult Head  5.689899  
  Adult Heart  6.554166  
  Adult Hind Gut  6.188237  
  Adult Male Ejaculatory Duct  7.173564  
  Adult Mid Gut  6.949129  
  Adult Ovary  7.094982  
  Adult Salivary Gland  5.669222  
  Adult Testes  5.868983  
  Adult Thoracoabdominal ganglion  6.558375  
  Adult Whole Fly  6.033031  
  Larvae Wandering Tubules  6.718055  
  Larval Feeding Carcass  5.960365  
  Larval Feeding Central Nevous System  6.175985  
  Larval Feeding Hind Gut  6.330509  
  Larval Feeding Malpighian Tubule  6.686175  
  Larval Feeding Mid Gut  6.894089  
  Larval Feeding Salivary Gland  5.998496  
  Whole Larvae Feeding  5.623415  
 
  
   FlyBase ID    symbol    start    end    strand    length   
   FBgn0035348   CG16758  2498704   2503584   -  4881  
   FBgn0010905   Spn  2505245   2554292   -  49048  
 
    Segment 61 
 
   Location   
  Gene key  FBgn0010909-FBgn0016794  
  Heatmap region span   3L:2475614..2600367   
  Segment span   3L:2586540..2589402   
  Length (genes)  2  
  Length (bp)  2863  
   Model Scoring   
  BIC  213.504246  
  logL  -101.239997  
  logL ratio  46.385199  
   Expression   
  Mean expression  8.604773  
  Median expression  8.619439  
  Tissue std. dev.  0.864943  
 
  No GO Slim enrichment  
  
   tissue    mean expression   
  5th Passage Drosophila S2 Cells  11.149573  
  Adult Accessory gland  8.125623  
  Adult Brain  8.860599  
  Adult Carcass  8.091855  
  Adult Crop  9.321518  
  Adult Eye  8.454376  
  Adult Fatbody  8.607196  
  Adult Female Spermatheca Mated  8.574330  
  Adult Female Spermatheca Virgin  8.673439  
  Adult Head  8.436608  
  Adult Heart  8.801251  
  Adult Hind Gut  8.687833  
  Adult Male Ejaculatory Duct  8.385858  
  Adult Mid Gut  8.616230  
  Adult Ovary  10.539766  
  Adult Salivary Gland  7.884498  
  Adult Testes  7.042293  
  Adult Thoracoabdominal ganglion  8.618214  
  Adult Whole Fly  9.125728  
  Larvae Wandering Tubules  8.167037  
  Larval Feeding Carcass  8.389805  
  Larval Feeding Central Nevous System  9.043838  
  Larval Feeding Hind Gut  8.856963  
  Larval Feeding Malpighian Tubule  7.953492  
  Larval Feeding Mid Gut  9.228051  
  Larval Feeding Salivary Gland  6.621208  
  Whole Larvae Feeding  8.071676  
 
  
   FlyBase ID    symbol    start    end    strand    length   
   FBgn0010909   msn  2555775   2586540   -  30766  
   FBgn0016794   dos   2589402   2595590  +  6189  
 
    Segment 62 
 
   Location   
  Gene key  FBgn0035358-FBgn0035359  
  Heatmap region span   3L:2599302..2739750   
  Segment span   3L:2643424..2645376   
  Length (genes)  2  
  Length (bp)  1953  
   Model Scoring   
  BIC  258.085394  
  logL  -123.530571  
  logL ratio  -10.596622  
   Expression   
  Mean expression  6.353684  
  Median expression  5.340295  
  Tissue std. dev.  2.379843  
 
  No GO Slim enrichment  
  
   tissue    mean expression   
  5th Passage Drosophila S2 Cells  5.061645  
  Adult Accessory gland  4.963532  
  Adult Brain  4.822571  
  Adult Carcass  6.470669  
  Adult Crop  5.796073  
  Adult Eye  5.496117  
  Adult Fatbody  4.825374  
  Adult Female Spermatheca Mated  4.743111  
  Adult Female Spermatheca Virgin  4.767321  
  Adult Head  5.724586  
  Adult Heart  5.410511  
  Adult Hind Gut  13.854078  
  Adult Male Ejaculatory Duct  5.301577  
  Adult Mid Gut  5.228305  
  Adult Ovary  4.700631  
  Adult Salivary Gland  4.765859  
  Adult Testes  5.643899  
  Adult Thoracoabdominal ganglion  4.730310  
  Adult Whole Fly  7.327836  
  Larvae Wandering Tubules  8.919191  
  Larval Feeding Carcass  9.218660  
  Larval Feeding Central Nevous System  4.512325  
  Larval Feeding Hind Gut  12.504165  
  Larval Feeding Malpighian Tubule  8.346877  
  Larval Feeding Mid Gut  4.949996  
  Larval Feeding Salivary Gland  4.865169  
  Whole Larvae Feeding  8.599095  
 
  
   FlyBase ID    symbol    start    end    strand    length   
   FBgn0035358   CG14949  2642184   2643424   -  1241  
   FBgn0035359   CG1143   2645376   2646605  +  1230  
 
    Segment 63 
 
   Location   
  Gene key  FBgn0053233-FBgn0035366  
  Heatmap region span   3L:2600367..2782272   
  Segment span   3L:2659639..2667542   
  Length (genes)  4  
  Length (bp)  7904  
   Model Scoring   
  BIC  375.721825  
  logL  -182.348786  
  logL ratio  52.481610  
   Expression   
  Mean expression  5.248051  
  Median expression  5.076481  
  Tissue std. dev.  0.386903  
 
  
   GO ID    description    ratio    P-value   
   GO:0055085   transmembrane transport  2/4  0.0049  
   GO:0006810   transport  2/4  0.023  
 
  
   tissue    mean expression   
  5th Passage Drosophila S2 Cells  5.138096  
  Adult Accessory gland  5.289638  
  Adult Brain  5.965734  
  Adult Carcass  5.131726  
  Adult Crop  5.093641  
  Adult Eye  5.019042  
  Adult Fatbody  5.209252  
  Adult Female Spermatheca Mated  5.043962  
  Adult Female Spermatheca Virgin  5.009963  
  Adult Head  5.107983  
  Adult Heart  4.957826  
  Adult Hind Gut  5.130315  
  Adult Male Ejaculatory Duct  5.220127  
  Adult Mid Gut  5.493249  
  Adult Ovary  5.128406  
  Adult Salivary Gland  5.353917  
  Adult Testes  6.926024  
  Adult Thoracoabdominal ganglion  5.356806  
  Adult Whole Fly  5.074364  
  Larvae Wandering Tubules  5.147077  
  Larval Feeding Carcass  5.228150  
  Larval Feeding Central Nevous System  4.885079  
  Larval Feeding Hind Gut  5.051425  
  Larval Feeding Malpighian Tubule  5.238680  
  Larval Feeding Mid Gut  5.297553  
  Larval Feeding Salivary Gland  5.229842  
  Whole Larvae Feeding  4.969507  
 
  
   FlyBase ID    symbol    start    end    strand    length   
   FBgn0053233   CG33233   2659639   2661501  +  1863  
   FBgn0035364     2657481   2659656   -  2176  
   FBgn0053234   CG33234   2661622   2663622  +  2001  
   FBgn0035366     2665287   2667542   -  2256  
 
 
    Segment 64 
 
   Location   
  Gene key  FBgn0035370-FBgn0035375  
  Heatmap region span   3L:2659639..3050329   
  Segment span   3L:2766996..2782272   
  Length (genes)  6  
  Length (bp)  15277  
   Model Scoring   
  BIC  652.515286  
  logL  -320.745517  
  logL ratio  147.448130  
   Expression   
  Mean expression  9.301480  
  Median expression  9.314533  
  Tissue std. dev.  0.419059  
 
  No GO Slim enrichment  
  
   tissue    mean expression   
  5th Passage Drosophila S2 Cells  9.832384  
  Adult Accessory gland  9.655155  
  Adult Brain  9.159189  
  Adult Carcass  8.700415  
  Adult Crop  9.110146  
  Adult Eye  8.934495  
  Adult Fatbody  8.909843  
  Adult Female Spermatheca Mated  9.120126  
  Adult Female Spermatheca Virgin  9.248010  
  Adult Head  8.894489  
  Adult Heart  9.160499  
  Adult Hind Gut  9.324317  
  Adult Male Ejaculatory Duct  9.757828  
  Adult Mid Gut  9.319711  
  Adult Ovary  9.399109  
  Adult Salivary Gland  10.263960  
  Adult Testes  8.467306  
  Adult Thoracoabdominal ganglion  9.155100  
  Adult Whole Fly  8.842235  
  Larvae Wandering Tubules  9.472421  
  Larval Feeding Carcass  9.167778  
  Larval Feeding Central Nevous System  9.247192  
  Larval Feeding Hind Gut  9.679200  
  Larval Feeding Malpighian Tubule  9.509972  
  Larval Feeding Mid Gut  9.382599  
  Larval Feeding Salivary Gland  10.326444  
  Whole Larvae Feeding  9.100045  
 
  
   FlyBase ID    symbol    start    end    strand    length   
   FBgn0035370   CG1240   2766996   2768399  +  1404  
   FBgn0035371   CG9977  2769348   2772323   -  2976  
   FBgn0035372   CG12093  2772560   2774001   -  1442  
   FBgn0044452   Atg2   2774379   2780688  +  6310  
   FBgn0035374   mRpS35   2780921   2782105  +  1185  
   FBgn0035375   pgant6   2782272   2786010  +  3739  
 
 
    Segment 65 
 
   Location   
  Gene key  FBgn0035376-FBgn0035382  
  Heatmap region span   3L:2689687..3055102   
  Segment span   3L:2800130..2961534   
  Length (genes)  7  
  Length (bp)  161405  
   Model Scoring   
  BIC  586.118003  
  logL  -287.546875  
  logL ratio  147.195107  
   Expression   
  Mean expression  4.957988  
  Median expression  4.826941  
  Tissue std. dev.  0.323682  
 
  No GO Slim enrichment  
  
   tissue    mean expression   
  5th Passage Drosophila S2 Cells  5.208231  
  Adult Accessory gland  4.779766  
  Adult Brain  5.038308  
  Adult Carcass  5.082635  
  Adult Crop  4.888192  
  Adult Eye  4.981458  
  Adult Fatbody  4.769560  
  Adult Female Spermatheca Mated  4.822386  
  Adult Female Spermatheca Virgin  4.812967  
  Adult Head  4.895012  
  Adult Heart  4.867730  
  Adult Hind Gut  4.705695  
  Adult Male Ejaculatory Duct  5.202225  
  Adult Mid Gut  4.857552  
  Adult Ovary  4.586963  
  Adult Salivary Gland  5.018612  
  Adult Testes  6.451274  
  Adult Thoracoabdominal ganglion  4.916793  
  Adult Whole Fly  4.872929  
  Larvae Wandering Tubules  4.964891  
  Larval Feeding Carcass  4.885548  
  Larval Feeding Central Nevous System  4.850189  
  Larval Feeding Hind Gut  4.739685  
  Larval Feeding Malpighian Tubule  5.077463  
  Larval Feeding Mid Gut  4.850027  
  Larval Feeding Salivary Gland  4.856812  
  Whole Larvae Feeding  4.882765  
 
  
   FlyBase ID    symbol    start    end    strand    length   
   FBgn0035376     2788122   2800130   -  12009  
   FBgn0035378     2848576   2871710   -  23135  
   FBgn0035379   spz5  2883638   2892164   -  8527  
   FBgn0035380   CG9970  2915024   2916654   -  1631  
   FBgn0052846   CG32846  2950174   2951618   -  1445  
   FBgn0054025   CG34025  2959722   2961049   -  1328  
   FBgn0035382   Or63a   2961534   2963788  +  2255  
 
 
    Segment 66 
 
   Location   
  Gene key  FBgn0035384-FBgn0004372  
  Heatmap region span   3L:2766480..3106956   
  Segment span   3L:2995180..3026521   
  Length (genes)  5  
  Length (bp)  31342  
   Model Scoring   
  BIC  398.735166  
  logL  -193.855457  
  logL ratio  125.116008  
   Expression   
  Mean expression  4.572334  
  Median expression  4.352686  
  Tissue std. dev.  0.772271  
 
  No GO Slim enrichment  
  
   tissue    mean expression   
  5th Passage Drosophila S2 Cells  4.365693  
  Adult Accessory gland  4.452152  
  Adult Brain  4.309675  
  Adult Carcass  4.509947  
  Adult Crop  4.269674  
  Adult Eye  4.253977  
  Adult Fatbody  4.401460  
  Adult Female Spermatheca Mated  4.406576  
  Adult Female Spermatheca Virgin  4.398854  
  Adult Head  4.212526  
  Adult Heart  4.383532  
  Adult Hind Gut  4.386754  
  Adult Male Ejaculatory Duct  4.361673  
  Adult Mid Gut  4.534758  
  Adult Ovary  4.287203  
  Adult Salivary Gland  4.640449  
  Adult Testes  8.362487  
  Adult Thoracoabdominal ganglion  4.351190  
  Adult Whole Fly  5.303065  
  Larvae Wandering Tubules  4.416085  
  Larval Feeding Carcass  4.508137  
  Larval Feeding Central Nevous System  4.131304  
  Larval Feeding Hind Gut  4.234719  
  Larval Feeding Malpighian Tubule  4.428237  
  Larval Feeding Mid Gut  4.503013  
  Larval Feeding Salivary Gland  4.368911  
  Whole Larvae Feeding  4.670958  
 
  
   FlyBase ID    symbol    start    end    strand    length   
   FBgn0035384   CG2113   2995180   2996158  +  979  
   FBgn0035385   FR   3002157   3011464  +  9308  
   FBgn0052488   CG32488  3014966   3016069   -  1104  
   FBgn0052487   CG32487  3016150   3017256   -  1107  
   FBgn0004372   aly  3024129   3026521   -  2393  
 
 
    Segment 67 
 
   Location   
  Gene key  FBgn0035388-FBgn0052485  
  Heatmap region span   3L:2766996..3114248   
  Segment span   3L:3035198..3050329   
  Length (genes)  5  
  Length (bp)  15132  
   Model Scoring   
  BIC  478.999478  
  logL  -233.987613  
  logL ratio  176.118695  
   Expression   
  Mean expression  9.261802  
  Median expression  9.303085  
  Tissue std. dev.  0.252831  
 
  No GO Slim enrichment  
  
   tissue    mean expression   
  5th Passage Drosophila S2 Cells  9.588376  
  Adult Accessory gland  9.555653  
  Adult Brain  9.077712  
  Adult Carcass  8.589599  
  Adult Crop  8.942987  
  Adult Eye  9.180711  
  Adult Fatbody  9.218293  
  Adult Female Spermatheca Mated  9.543365  
  Adult Female Spermatheca Virgin  9.656144  
  Adult Head  9.085981  
  Adult Heart  9.361139  
  Adult Hind Gut  9.035552  
  Adult Male Ejaculatory Duct  9.387745  
  Adult Mid Gut  9.081106  
  Adult Ovary  9.678502  
  Adult Salivary Gland  9.279475  
  Adult Testes  9.513619  
  Adult Thoracoabdominal ganglion  8.919271  
  Adult Whole Fly  9.045453  
  Larvae Wandering Tubules  9.329406  
  Larval Feeding Carcass  8.998830  
  Larval Feeding Central Nevous System  9.313344  
  Larval Feeding Hind Gut  9.386586  
  Larval Feeding Malpighian Tubule  9.342566  
  Larval Feeding Mid Gut  9.171253  
  Larval Feeding Salivary Gland  9.491023  
  Whole Larvae Feeding  9.294958  
 
  
   FlyBase ID    symbol    start    end    strand    length   
   FBgn0035388   CG2162   3035198   3040469  +  5272  
   FBgn0040308   Jafrac2   3042375   3044174  +  1800  
   FBgn0035390   scramb2  3044105   3046810   -  2706  
   FBgn0052484   Sk2   3046956   3050161  +  3206  
   FBgn0052485   CG32485   3050329   3051765  +  1437  
 
 
    Segment 68 
 
   Location   
  Gene key  FBgn0035392-FBgn0035393  
  Heatmap region span   3L:2800130..3155721   
  Segment span   3L:3052514..3055102   
  Length (genes)  2  
  Length (bp)  2589  
   Model Scoring   
  BIC  229.958741  
  logL  -109.467244  
  logL ratio  21.668074  
   Expression   
  Mean expression  7.331077  
  Median expression  7.375887  
  Tissue std. dev.  0.449463  
 
  No GO Slim enrichment  
  
   tissue    mean expression   
  5th Passage Drosophila S2 Cells  7.324521  
  Adult Accessory gland  7.601863  
  Adult Brain  7.411160  
  Adult Carcass  6.962593  
  Adult Crop  6.729335  
  Adult Eye  7.705293  
  Adult Fatbody  7.686692  
  Adult Female Spermatheca Mated  7.936827  
  Adult Female Spermatheca Virgin  7.775653  
  Adult Head  7.337496  
  Adult Heart  7.560358  
  Adult Hind Gut  6.771645  
  Adult Male Ejaculatory Duct  7.875101  
  Adult Mid Gut  7.186149  
  Adult Ovary  8.183738  
  Adult Salivary Gland  6.320733  
  Adult Testes  7.684930  
  Adult Thoracoabdominal ganglion  7.497682  
  Adult Whole Fly  7.240001  
  Larvae Wandering Tubules  6.956232  
  Larval Feeding Carcass  6.900035  
  Larval Feeding Central Nevous System  8.023472  
  Larval Feeding Hind Gut  6.737004  
  Larval Feeding Malpighian Tubule  7.002878  
  Larval Feeding Mid Gut  7.152188  
  Larval Feeding Salivary Gland  7.427188  
  Whole Larvae Feeding  6.948326  
 
  
   FlyBase ID    symbol    start    end    strand    length   
   FBgn0035392   CG1271   3052514   3054907  +  2394  
   FBgn0035393   CG16753   3055102   3055844  +  743  
 
    Segment 69 
 
   Location   
  Gene key  FBgn0052486-FBgn0035397  
  Heatmap region span   3L:2981023..3159573   
  Segment span   3L:3070839..3071128   
  Length (genes)  2  
  Length (bp)  290  
   Model Scoring   
  BIC  237.813133  
  logL  -113.394440  
  logL ratio  54.052848  
   Expression   
  Mean expression  10.103128  
  Median expression  9.976748  
  Tissue std. dev.  0.614724  
 
  No GO Slim enrichment  
  
   tissue    mean expression   
  5th Passage Drosophila S2 Cells  9.020577  
  Adult Accessory gland  9.964557  
  Adult Brain  11.685097  
  Adult Carcass  10.247774  
  Adult Crop  10.443114  
  Adult Eye  10.940239  
  Adult Fatbody  9.537499  
  Adult Female Spermatheca Mated  10.169503  
  Adult Female Spermatheca Virgin  10.279428  
  Adult Head  10.230960  
  Adult Heart  10.725347  
  Adult Hind Gut  10.121866  
  Adult Male Ejaculatory Duct  9.798683  
  Adult Mid Gut  10.176317  
  Adult Ovary  9.411839  
  Adult Salivary Gland  10.637833  
  Adult Testes  9.261219  
  Adult Thoracoabdominal ganglion  11.592012  
  Adult Whole Fly  9.771290  
  Larvae Wandering Tubules  9.859476  
  Larval Feeding Carcass  9.816477  
  Larval Feeding Central Nevous System  10.169546  
  Larval Feeding Hind Gut  10.214747  
  Larval Feeding Malpighian Tubule  9.831353  
  Larval Feeding Mid Gut  9.880775  
  Larval Feeding Salivary Gland  9.531177  
  Whole Larvae Feeding  9.465756  
 
  
   FlyBase ID    symbol    start    end    strand    length   
   FBgn0052486   CG32486  3055904   3070839   -  14936  
   FBgn0035397   CG11486   3071128   3088493  +  17366  
 
    Segment 70 
 
   Location   
  Gene key  FBgn0035400-FBgn0035403  
  Heatmap region span   3L:3052514..3166250   
  Segment span   3L:3143454..3155721   
  Length (genes)  5  
  Length (bp)  12268  
   Model Scoring   
  BIC  476.660848  
  logL  -232.818298  
  logL ratio  165.030070  
   Expression   
  Mean expression  8.844775  
  Median expression  8.667706  
  Tissue std. dev.  0.482252  
 
  No GO Slim enrichment  
  
   tissue    mean expression   
  5th Passage Drosophila S2 Cells  9.231888  
  Adult Accessory gland  9.248770  
  Adult Brain  9.008829  
  Adult Carcass  8.353733  
  Adult Crop  8.600673  
  Adult Eye  8.896678  
  Adult Fatbody  8.583728  
  Adult Female Spermatheca Mated  8.875000  
  Adult Female Spermatheca Virgin  8.921221  
  Adult Head  8.443503  
  Adult Heart  8.666887  
  Adult Hind Gut  8.596315  
  Adult Male Ejaculatory Duct  9.145965  
  Adult Mid Gut  8.544287  
  Adult Ovary  9.487175  
  Adult Salivary Gland  9.507357  
  Adult Testes  7.286735  
  Adult Thoracoabdominal ganglion  8.852000  
  Adult Whole Fly  8.486242  
  Larvae Wandering Tubules  9.633392  
  Larval Feeding Carcass  8.849871  
  Larval Feeding Central Nevous System  8.847451  
  Larval Feeding Hind Gut  8.870769  
  Larval Feeding Malpighian Tubule  9.291182  
  Larval Feeding Mid Gut  8.695637  
  Larval Feeding Salivary Gland  9.595606  
  Whole Larvae Feeding  8.288041  
 
  
   FlyBase ID    symbol    start    end    strand    length   
   FBgn0035400   CG11537  3128028   3143454   -  15427  
   FBgn0035401   CG1291   3143810   3145288  +  1479  
   FBgn0035402   CG12082  3145405   3148358   -  2954  
   FBgn0012049      3148675   3154817  +  6143  
   FBgn0035403      3155721   3158408  +  2688  
 
 
    Segment 71 
 
   Location   
  Gene key  FBgn0035405-FBgn0024194  
  Heatmap region span   3L:3106956..3172545   
  Segment span   3L:3159995..3162180   
  Length (genes)  2  
  Length (bp)  2186  
   Model Scoring   
  BIC  232.199229  
  logL  -110.587488  
  logL ratio  20.238712  
   Expression   
  Mean expression  7.583596  
  Median expression  7.489593  
  Tissue std. dev.  0.656679  
 
  No GO Slim enrichment  
  
   tissue    mean expression   
  5th Passage Drosophila S2 Cells  8.632048  
  Adult Accessory gland  8.471398  
  Adult Brain  6.842211  
  Adult Carcass  6.681980  
  Adult Crop  7.180601  
  Adult Eye  7.489593  
  Adult Fatbody  6.929438  
  Adult Female Spermatheca Mated  7.749226  
  Adult Female Spermatheca Virgin  7.656693  
  Adult Head  6.718273  
  Adult Heart  7.577592  
  Adult Hind Gut  7.232606  
  Adult Male Ejaculatory Duct  7.286290  
  Adult Mid Gut  7.405486  
  Adult Ovary  9.420892  
  Adult Salivary Gland  7.694003  
  Adult Testes  6.580642  
  Adult Thoracoabdominal ganglion  6.727611  
  Adult Whole Fly  7.998274  
  Larvae Wandering Tubules  7.931781  
  Larval Feeding Carcass  7.351277  
  Larval Feeding Central Nevous System  8.035866  
  Larval Feeding Hind Gut  7.787570  
  Larval Feeding Malpighian Tubule  8.060569  
  Larval Feeding Mid Gut  7.680259  
  Larval Feeding Salivary Gland  8.410356  
  Whole Larvae Feeding  7.224567  
 
  
   FlyBase ID    symbol    start    end    strand    length   
   FBgn0035405   pfk   3159995   3162024  +  2030  
   FBgn0024194   rasp   3162180   3163852  +  1673  
 
    Segment 72 
 
   Location   
  Gene key  FBgn0052278-FBgn0052281  
  Heatmap region span   3L:3159573..3186664   
  Segment span   3L:3169287..3169375   
  Length (genes)  2  
  Length (bp)  89  
   Model Scoring   
  BIC  258.285361  
  logL  -123.630554  
  logL ratio  7.401974  
   Expression   
  Mean expression  8.701517  
  Median expression  8.599259  
  Tissue std. dev.  0.597765  
 
  No GO Slim enrichment  
  
   tissue    mean expression   
  5th Passage Drosophila S2 Cells  8.509720  
  Adult Accessory gland  9.609921  
  Adult Brain  9.704474  
  Adult Carcass  8.348983  
  Adult Crop  8.463589  
  Adult Eye  10.256180  
  Adult Fatbody  8.336813  
  Adult Female Spermatheca Mated  8.665631  
  Adult Female Spermatheca Virgin  8.456518  
  Adult Head  9.339821  
  Adult Heart  8.602858  
  Adult Hind Gut  8.501754  
  Adult Male Ejaculatory Duct  8.300325  
  Adult Mid Gut  8.629772  
  Adult Ovary  9.387723  
  Adult Salivary Gland  8.496438  
  Adult Testes  8.451868  
  Adult Thoracoabdominal ganglion  9.810892  
  Adult Whole Fly  8.752263  
  Larvae Wandering Tubules  8.335518  
  Larval Feeding Carcass  8.035113  
  Larval Feeding Central Nevous System  8.938308  
  Larval Feeding Hind Gut  8.393805  
  Larval Feeding Malpighian Tubule  8.318424  
  Larval Feeding Mid Gut  7.646105  
  Larval Feeding Salivary Gland  8.699250  
  Whole Larvae Feeding  7.948893  
 
  
   FlyBase ID    symbol    start    end    strand    length   
   FBgn0052278   CG32278   3169287   3171767  +  2481  
   FBgn0052281   CG32281   3169375   3170909  +  1535  
 
    Segment 73 
 
   Location   
  Gene key  FBgn0035412-FBgn0035413  
  Heatmap region span   3L:3172545..3199043   
  Segment span   3L:3187563..3189994   
  Length (genes)  2  
  Length (bp)  2432  
   Model Scoring   
  BIC  246.893105  
  logL  -117.934426  
  logL ratio  -2.104927  
   Expression   
  Mean expression  5.893868  
  Median expression  5.626384  
  Tissue std. dev.  2.032547  
 
  No GO Slim enrichment  
  
   tissue    mean expression   
  5th Passage Drosophila S2 Cells  5.112267  
  Adult Accessory gland  5.037586  
  Adult Brain  4.714639  
  Adult Carcass  5.153573  
  Adult Crop  4.927989  
  Adult Eye  4.814699  
  Adult Fatbody  5.668126  
  Adult Female Spermatheca Mated  4.907452  
  Adult Female Spermatheca Virgin  4.949863  
  Adult Head  4.845897  
  Adult Heart  4.948422  
  Adult Hind Gut  5.582388  
  Adult Male Ejaculatory Duct  5.188225  
  Adult Mid Gut  5.345719  
  Adult Ovary  5.007551  
  Adult Salivary Gland  5.253052  
  Adult Testes  5.598508  
  Adult Thoracoabdominal ganglion  4.890647  
  Adult Whole Fly  5.777238  
  Larvae Wandering Tubules  12.173777  
  Larval Feeding Carcass  5.136673  
  Larval Feeding Central Nevous System  4.628906  
  Larval Feeding Hind Gut  7.919324  
  Larval Feeding Malpighian Tubule  12.689383  
  Larval Feeding Mid Gut  5.604871  
  Larval Feeding Salivary Gland  4.896600  
  Whole Larvae Feeding  8.361051  
 
  
   FlyBase ID    symbol    start    end    strand    length   
   FBgn0035412   CG14957   3187563   3188106  +  544  
   FBgn0035413   CG14958   3189994   3190623  +  630  
 
    Segment 74 
 
   Location   
  Gene key  FBgn0035415-FBgn0035416  
  Heatmap region span   3L:3186664..3206363   
  Segment span   3L:3197520..3199021   
  Length (genes)  2  
  Length (bp)  1502  
   Model Scoring   
  BIC  207.420999  
  logL  -98.198373  
  logL ratio  50.905817  
   Expression   
  Mean expression  8.861440  
  Median expression  8.982477  
  Tissue std. dev.  0.460437  
 
  No GO Slim enrichment  
  
   tissue    mean expression   
  5th Passage Drosophila S2 Cells  9.157834  
  Adult Accessory gland  8.530799  
  Adult Brain  8.055750  
  Adult Carcass  8.152455  
  Adult Crop  8.785640  
  Adult Eye  8.343727  
  Adult Fatbody  8.812746  
  Adult Female Spermatheca Mated  9.041655  
  Adult Female Spermatheca Virgin  9.155980  
  Adult Head  8.318119  
  Adult Heart  9.019686  
  Adult Hind Gut  8.746831  
  Adult Male Ejaculatory Duct  9.448963  
  Adult Mid Gut  8.816847  
  Adult Ovary  9.343265  
  Adult Salivary Gland  8.615771  
  Adult Testes  8.269272  
  Adult Thoracoabdominal ganglion  8.240455  
  Adult Whole Fly  8.387234  
  Larvae Wandering Tubules  9.472465  
  Larval Feeding Carcass  9.414295  
  Larval Feeding Central Nevous System  9.103648  
  Larval Feeding Hind Gut  9.302009  
  Larval Feeding Malpighian Tubule  9.724091  
  Larval Feeding Mid Gut  8.726799  
  Larval Feeding Salivary Gland  9.452806  
  Whole Larvae Feeding  8.819750  
 
  
   FlyBase ID    symbol    start    end    strand    length   
   FBgn0035415   CG14966  3197098   3197520   -  423  
   FBgn0035416   gry   3199021   3211347  +  12327  
 
    Segment 75 
 
   Location   
  Gene key  FBgn0052271-FBgn0053159  
  Heatmap region span   3L:3192468..3223350   
  Segment span   3L:3203162..3204448   
  Length (genes)  2  
  Length (bp)  1287  
   Model Scoring   
  BIC  175.413055  
  logL  -82.194401  
  logL ratio  36.666500  
   Expression   
  Mean expression  4.612507  
  Median expression  4.580340  
  Tissue std. dev.  0.429668  
 
  No GO Slim enrichment  
  
   tissue    mean expression   
  5th Passage Drosophila S2 Cells  4.515225  
  Adult Accessory gland  4.479350  
  Adult Brain  4.285122  
  Adult Carcass  5.019118  
  Adult Crop  4.336279  
  Adult Eye  4.631615  
  Adult Fatbody  4.786058  
  Adult Female Spermatheca Mated  4.956723  
  Adult Female Spermatheca Virgin  4.934011  
  Adult Head  6.510763  
  Adult Heart  4.470892  
  Adult Hind Gut  4.364015  
  Adult Male Ejaculatory Duct  4.706351  
  Adult Mid Gut  4.683020  
  Adult Ovary  4.545074  
  Adult Salivary Gland  4.641568  
  Adult Testes  4.307310  
  Adult Thoracoabdominal ganglion  4.200085  
  Adult Whole Fly  4.462312  
  Larvae Wandering Tubules  4.378118  
  Larval Feeding Carcass  4.467284  
  Larval Feeding Central Nevous System  4.257251  
  Larval Feeding Hind Gut  4.365490  
  Larval Feeding Malpighian Tubule  4.471468  
  Larval Feeding Mid Gut  4.799043  
  Larval Feeding Salivary Gland  4.566099  
  Whole Larvae Feeding  4.398036  
 
  
   FlyBase ID    symbol    start    end    strand    length   
   FBgn0052271   CG32271  3202238   3203162   -  925  
   FBgn0053159   CG33159  3203675   3204448   -  774  
 
    Segment 76 
 
   Location   
  Gene key  FBgn0035420-FBgn0035422  
  Heatmap region span   3L:3199043..3294752   
  Segment span   3L:3218920..3222960   
  Length (genes)  3  
  Length (bp)  4041  
   Model Scoring   
  BIC  284.467277  
  logL  -136.721512  
  logL ratio  117.187884  
   Expression   
  Mean expression  9.571548  
  Median expression  9.628039  
  Tissue std. dev.  0.328205  
 
  No GO Slim enrichment  
  
   tissue    mean expression   
  5th Passage Drosophila S2 Cells  9.784855  
  Adult Accessory gland  9.654446  
  Adult Brain  9.778979  
  Adult Carcass  8.836738  
  Adult Crop  9.187025  
  Adult Eye  9.668613  
  Adult Fatbody  9.342628  
  Adult Female Spermatheca Mated  9.662834  
  Adult Female Spermatheca Virgin  9.663859  
  Adult Head  9.108671  
  Adult Heart  9.588731  
  Adult Hind Gut  9.251160  
  Adult Male Ejaculatory Duct  9.239579  
  Adult Mid Gut  9.413279  
  Adult Ovary  10.463448  
  Adult Salivary Gland  9.593715  
  Adult Testes  9.552515  
  Adult Thoracoabdominal ganglion  9.558778  
  Adult Whole Fly  9.388895  
  Larvae Wandering Tubules  10.041811  
  Larval Feeding Carcass  9.297681  
  Larval Feeding Central Nevous System  10.130929  
  Larval Feeding Hind Gut  9.537215  
  Larval Feeding Malpighian Tubule  9.930069  
  Larval Feeding Mid Gut  9.661544  
  Larval Feeding Salivary Gland  9.636642  
  Whole Larvae Feeding  9.457146  
 
  
   FlyBase ID    symbol    start    end    strand    length   
   FBgn0035420   CG14967  3210992   3218920   -  7929  
   FBgn0035421   CG12034   3219124   3220925  +  1802  
   FBgn0035422   RpL28  3220911   3222960   -  2050  
 
 
    Segment 77 
 
   Location   
  Gene key  FBgn0035426-FBgn0035430  
  Heatmap region span   3L:3218920..3316809   
  Segment span   3L:3244263..3294752   
  Length (genes)  6  
  Length (bp)  50490  
   Model Scoring   
  BIC  528.295216  
  logL  -258.635481  
  logL ratio  79.834401  
   Expression   
  Mean expression  4.906359  
  Median expression  4.669996  
  Tissue std. dev.  0.479416  
 
  
   GO ID    description    ratio    P-value   
   GO:0005576   extracellular region  2/6  0.0293  
 
  
   tissue    mean expression   
  5th Passage Drosophila S2 Cells  4.728357  
  Adult Accessory gland  4.673736  
  Adult Brain  4.359196  
  Adult Carcass  4.922440  
  Adult Crop  4.808892  
  Adult Eye  4.620333  
  Adult Fatbody  4.757697  
  Adult Female Spermatheca Mated  5.203181  
  Adult Female Spermatheca Virgin  5.044414  
  Adult Head  5.219649  
  Adult Heart  4.573355  
  Adult Hind Gut  4.730162  
  Adult Male Ejaculatory Duct  4.675157  
  Adult Mid Gut  4.710718  
  Adult Ovary  4.615328  
  Adult Salivary Gland  4.826302  
  Adult Testes  5.554262  
  Adult Thoracoabdominal ganglion  4.657156  
  Adult Whole Fly  4.758102  
  Larvae Wandering Tubules  4.637597  
  Larval Feeding Carcass  6.709754  
  Larval Feeding Central Nevous System  4.493648  
  Larval Feeding Hind Gut  5.494843  
  Larval Feeding Malpighian Tubule  4.620249  
  Larval Feeding Mid Gut  4.666719  
  Larval Feeding Salivary Gland  4.698363  
  Whole Larvae Feeding  5.712090  
 
  
   FlyBase ID    symbol    start    end    strand    length   
   FBgn0035426   CG12078  3243274   3244263   -  990  
   FBgn0035427   ckd   3258155   3267845  +  9691  
   FBgn0085294   CG34265   3268495   3268994  +  500  
   FBgn0035428   CG14960   3271038   3276259  +  5222  
   FBgn0035429   CG12017   3280247   3290893  +  10647  
   FBgn0035430   CG12009   3294752   3298963  +  4212  
 
 
    Segment 78 
 
   Location   
  Gene key  FBgn0052282-FBgn0035434  
  Heatmap region span   3L:3244263..3334978   
  Segment span   3L:3315637..3316809   
  Length (genes)  2  
  Length (bp)  1173  
   Model Scoring   
  BIC  269.241223  
  logL  -129.108485  
  logL ratio  -0.921194  
   Expression   
  Mean expression  7.442009  
  Median expression  6.774883  
  Tissue std. dev.  2.241050  
 
  No GO Slim enrichment  
  
   tissue    mean expression   
  5th Passage Drosophila S2 Cells  6.144609  
  Adult Accessory gland  6.199368  
  Adult Brain  7.248970  
  Adult Carcass  13.081842  
  Adult Crop  6.204301  
  Adult Eye  11.756071  
  Adult Fatbody  7.477400  
  Adult Female Spermatheca Mated  6.248333  
  Adult Female Spermatheca Virgin  6.520071  
  Adult Head  13.781385  
  Adult Heart  8.112752  
  Adult Hind Gut  6.779251  
  Adult Male Ejaculatory Duct  6.574419  
  Adult Mid Gut  6.405902  
  Adult Ovary  5.771530  
  Adult Salivary Gland  6.520065  
  Adult Testes  6.284007  
  Adult Thoracoabdominal ganglion  7.298849  
  Adult Whole Fly  11.491286  
  Larvae Wandering Tubules  6.263065  
  Larval Feeding Carcass  8.348309  
  Larval Feeding Central Nevous System  5.779581  
  Larval Feeding Hind Gut  6.128115  
  Larval Feeding Malpighian Tubule  6.116902  
  Larval Feeding Mid Gut  6.482560  
  Larval Feeding Salivary Gland  6.068696  
  Whole Larvae Feeding  5.846609  
 
  
   FlyBase ID    symbol    start    end    strand    length   
   FBgn0052282   Drsl4   3315637   3315945  +  309  
   FBgn0035434   Drsl5   3316809   3317144  +  336  
 
    Segment 79 
 
   Location   
  Gene key  FBgn0035436-FBgn0035437  
  Heatmap region span   3L:3303722..3336843   
  Segment span   3L:3319557..3328871   
  Length (genes)  3  
  Length (bp)  9315  
   Model Scoring   
  BIC  462.833962  
  logL  -225.904854  
  logL ratio  14.796208  
   Expression   
  Mean expression  10.496604  
  Median expression  10.134999  
  Tissue std. dev.  0.400828  
 
  No GO Slim enrichment  
  
   tissue    mean expression   
  5th Passage Drosophila S2 Cells  10.342677  
  Adult Accessory gland  10.090162  
  Adult Brain  10.478802  
  Adult Carcass  10.645143  
  Adult Crop  11.169689  
  Adult Eye  10.488874  
  Adult Fatbody  11.112067  
  Adult Female Spermatheca Mated  11.046908  
  Adult Female Spermatheca Virgin  11.334596  
  Adult Head  10.672930  
  Adult Heart  10.975985  
  Adult Hind Gut  10.620324  
  Adult Male Ejaculatory Duct  11.060634  
  Adult Mid Gut  9.998592  
  Adult Ovary  10.570030  
  Adult Salivary Gland  10.356910  
  Adult Testes  9.864107  
  Adult Thoracoabdominal ganglion  10.631677  
  Adult Whole Fly  10.371480  
  Larvae Wandering Tubules  10.293758  
  Larval Feeding Carcass  10.151171  
  Larval Feeding Central Nevous System  10.591625  
  Larval Feeding Hind Gut  10.244300  
  Larval Feeding Malpighian Tubule  10.095453  
  Larval Feeding Mid Gut  9.937416  
  Larval Feeding Salivary Gland  10.218340  
  Whole Larvae Feeding  10.044660  
 
  
   FlyBase ID    symbol    start    end    strand    length   
   FBgn0035436   CG12016   3319557   3322489  +  2933  
   FBgn0035438   PHGPx   3326291   3328626  +  2336  
   FBgn0035437   CG11526  3322484   3328871   -  6388  
 
 
    Segment 80 
 
   Location   
  Gene key  FBgn0014388-FBgn0026259  
  Heatmap region span   3L:3369569..3515685   
  Segment span   3L:3424935..3460602   
  Length (genes)  2  
  Length (bp)  35668  
   Model Scoring   
  BIC  294.435631  
  logL  -141.705689  
  logL ratio  -7.466678  
   Expression   
  Mean expression  9.617436  
  Median expression  9.989230  
  Tissue std. dev.  0.755996  
 
  No GO Slim enrichment  
  
   tissue    mean expression   
  5th Passage Drosophila S2 Cells  12.736892  
  Adult Accessory gland  9.300136  
  Adult Brain  10.178571  
  Adult Carcass  9.278699  
  Adult Crop  9.680801  
  Adult Eye  9.457842  
  Adult Fatbody  9.618229  
  Adult Female Spermatheca Mated  9.502759  
  Adult Female Spermatheca Virgin  9.320025  
  Adult Head  9.320162  
  Adult Heart  9.730418  
  Adult Hind Gut  9.424705  
  Adult Male Ejaculatory Duct  9.096204  
  Adult Mid Gut  10.054083  
  Adult Ovary  8.876437  
  Adult Salivary Gland  9.416746  
  Adult Testes  8.906863  
  Adult Thoracoabdominal ganglion  10.293665  
  Adult Whole Fly  9.130015  
  Larvae Wandering Tubules  8.860173  
  Larval Feeding Carcass  9.902469  
  Larval Feeding Central Nevous System  10.359945  
  Larval Feeding Hind Gut  9.907915  
  Larval Feeding Malpighian Tubule  8.669857  
  Larval Feeding Mid Gut  9.943596  
  Larval Feeding Salivary Gland  9.025185  
  Whole Larvae Feeding  9.678372  
 
  
   FlyBase ID    symbol    start    end    strand    length   
   FBgn0014388   sty  3401137   3424935   -  23799  
   FBgn0026259   eIF5B  3428654   3460602   -  31949  
 
    Segment 81 
 
   Location   
  Gene key  FBgn0041164-FBgn0010317  
  Heatmap region span   3L:3374308..3612194   
  Segment span   3L:3461323..3466414   
  Length (genes)  2  
  Length (bp)  5092  
   Model Scoring   
  BIC  224.907149  
  logL  -106.941448  
  logL ratio  -0.811536  
   Expression   
  Mean expression  5.602634  
  Median expression  5.517150  
  Tissue std. dev.  0.947358  
 
  No GO Slim enrichment  
  
   tissue    mean expression   
  5th Passage Drosophila S2 Cells  6.648957  
  Adult Accessory gland  5.308589  
  Adult Brain  5.749633  
  Adult Carcass  5.188805  
  Adult Crop  4.974305  
  Adult Eye  5.095064  
  Adult Fatbody  5.116155  
  Adult Female Spermatheca Mated  5.121519  
  Adult Female Spermatheca Virgin  5.271956  
  Adult Head  4.935040  
  Adult Heart  4.796423  
  Adult Hind Gut  4.904098  
  Adult Male Ejaculatory Duct  5.266606  
  Adult Mid Gut  5.037073  
  Adult Ovary  9.256752  
  Adult Salivary Gland  5.196207  
  Adult Testes  6.479695  
  Adult Thoracoabdominal ganglion  5.709504  
  Adult Whole Fly  7.601855  
  Larvae Wandering Tubules  5.578744  
  Larval Feeding Carcass  5.346903  
  Larval Feeding Central Nevous System  6.354067  
  Larval Feeding Hind Gut  5.169999  
  Larval Feeding Malpighian Tubule  5.518829  
  Larval Feeding Mid Gut  5.142639  
  Larval Feeding Salivary Gland  5.123296  
  Whole Larvae Feeding  5.378404  
 
  
   FlyBase ID    symbol    start    end    strand    length   
   FBgn0041164   armi   3461323   3466314  +  4992  
   FBgn0010317   CycJ   3466414   3468098  +  1685  
 
    Segment 82 
 
   Location   
  Gene key  FBgn0035449-FBgn0052267  
  Heatmap region span   3L:3376757..3790804   
  Segment span   3L:3471338..3472113   
  Length (genes)  2  
  Length (bp)  776  
   Model Scoring   
  BIC  281.528660  
  logL  -135.252204  
  logL ratio  44.067667  
   Expression   
  Mean expression  10.790720  
  Median expression  11.126845  
  Tissue std. dev.  0.567683  
 
  No GO Slim enrichment  
  
   tissue    mean expression   
  5th Passage Drosophila S2 Cells  10.437527  
  Adult Accessory gland  10.773302  
  Adult Brain  10.358224  
  Adult Carcass  9.747908  
  Adult Crop  10.418314  
  Adult Eye  10.287232  
  Adult Fatbody  10.600540  
  Adult Female Spermatheca Mated  11.105162  
  Adult Female Spermatheca Virgin  11.101352  
  Adult Head  10.073050  
  Adult Heart  10.062436  
  Adult Hind Gut  10.654770  
  Adult Male Ejaculatory Duct  11.131426  
  Adult Mid Gut  10.895861  
  Adult Ovary  11.241668  
  Adult Salivary Gland  12.086868  
  Adult Testes  10.697841  
  Adult Thoracoabdominal ganglion  10.446759  
  Adult Whole Fly  10.302741  
  Larvae Wandering Tubules  11.781977  
  Larval Feeding Carcass  10.482681  
  Larval Feeding Central Nevous System  10.381525  
  Larval Feeding Hind Gut  11.051120  
  Larval Feeding Malpighian Tubule  11.179125  
  Larval Feeding Mid Gut  11.238710  
  Larval Feeding Salivary Gland  12.045104  
  Whole Larvae Feeding  10.766213  
 
  
   FlyBase ID    symbol    start    end    strand    length   
   FBgn0035449   CG14971  3468023   3471338   -  3316  
   FBgn0052267   CG32267  3471555   3472113   -  559  
 
    Segment 83 
 
   Location   
  Gene key  FBgn0259224-FBgn0001254  
  Heatmap region span   3L:3378268..3803454   
  Segment span   3L:3485642..3512677   
  Length (genes)  2  
  Length (bp)  27036  
   Model Scoring   
  BIC  197.691803  
  logL  -93.333775  
  logL ratio  12.957530  
   Expression   
  Mean expression  5.180913  
  Median expression  4.877396  
  Tissue std. dev.  0.580634  
 
  No GO Slim enrichment  
  
   tissue    mean expression   
  5th Passage Drosophila S2 Cells  6.301429  
  Adult Accessory gland  4.884723  
  Adult Brain  4.658957  
  Adult Carcass  5.285504  
  Adult Crop  4.895004  
  Adult Eye  4.664220  
  Adult Fatbody  5.410054  
  Adult Female Spermatheca Mated  5.035483  
  Adult Female Spermatheca Virgin  4.982625  
  Adult Head  4.843881  
  Adult Heart  5.102621  
  Adult Hind Gut  4.838529  
  Adult Male Ejaculatory Duct  4.747332  
  Adult Mid Gut  4.973975  
  Adult Ovary  4.767351  
  Adult Salivary Gland  4.936684  
  Adult Testes  4.446614  
  Adult Thoracoabdominal ganglion  4.630356  
  Adult Whole Fly  5.106165  
  Larvae Wandering Tubules  6.355690  
  Larval Feeding Carcass  6.604857  
  Larval Feeding Central Nevous System  4.585874  
  Larval Feeding Hind Gut  5.441630  
  Larval Feeding Malpighian Tubule  6.030565  
  Larval Feeding Mid Gut  4.929383  
  Larval Feeding Salivary Gland  5.333282  
  Whole Larvae Feeding  6.091857  
 
  
   FlyBase ID    symbol    start    end    strand    length   
   FBgn0259224   CG42324   3485642   3508533  +  22892  
   FBgn0001254   ImpE2  3510974   3512677   -  1704  
 
    Segment 84 
 
   Location   
  Gene key  FBgn0035453-FBgn0052266  
  Heatmap region span   3L:3471338..3811269   
  Segment span   3L:3615382..3790804   
  Length (genes)  6  
  Length (bp)  175423  
   Model Scoring   
  BIC  585.217277  
  logL  -287.096512  
  logL ratio  62.358398  
   Expression   
  Mean expression  4.731990  
  Median expression  4.315968  
  Tissue std. dev.  0.503645  
 
  No GO Slim enrichment  
  
   tissue    mean expression   
  5th Passage Drosophila S2 Cells  4.498908  
  Adult Accessory gland  4.599729  
  Adult Brain  4.307277  
  Adult Carcass  4.761449  
  Adult Crop  4.856130  
  Adult Eye  4.675579  
  Adult Fatbody  4.607100  
  Adult Female Spermatheca Mated  4.471839  
  Adult Female Spermatheca Virgin  4.512653  
  Adult Head  5.196071  
  Adult Heart  4.414407  
  Adult Hind Gut  4.635991  
  Adult Male Ejaculatory Duct  4.553770  
  Adult Mid Gut  4.639747  
  Adult Ovary  4.382660  
  Adult Salivary Gland  4.746832  
  Adult Testes  6.938684  
  Adult Thoracoabdominal ganglion  4.392635  
  Adult Whole Fly  5.485850  
  Larvae Wandering Tubules  4.557582  
  Larval Feeding Carcass  4.694356  
  Larval Feeding Central Nevous System  4.288571  
  Larval Feeding Hind Gut  4.716575  
  Larval Feeding Malpighian Tubule  4.595063  
  Larval Feeding Mid Gut  4.575250  
  Larval Feeding Salivary Gland  4.591010  
  Whole Larvae Feeding  5.068011  
 
  
   FlyBase ID    symbol    start    end    strand    length   
   FBgn0035453   CG10357   3615382   3616682  +  1301  
   FBgn0040694   CG14974  3620432   3621017   -  586  
   FBgn0035454      3626874   3653443  +  26570  
   FBgn0035455   CG10862  3653405   3654771   -  1367  
   FBgn0035458   CG10858  3753693   3755201   -  1509  
   FBgn0052266   CG32266   3790804   3791560  +  757  
 
 
    Segment 85 
 
   Location   
  Gene key  FBgn0035461-FBgn0052262  
  Heatmap region span   3L:3515685..3816354   
  Segment span   3L:3806456..3810181   
  Length (genes)  3  
  Length (bp)  3726  
   Model Scoring   
  BIC  360.244991  
  logL  -174.610369  
  logL ratio  10.468714  
   Expression   
  Mean expression  7.213030  
  Median expression  7.030540  
  Tissue std. dev.  0.676825  
 
  No GO Slim enrichment  
  
   tissue    mean expression   
  5th Passage Drosophila S2 Cells  7.023701  
  Adult Accessory gland  7.119673  
  Adult Brain  7.204471  
  Adult Carcass  7.077143  
  Adult Crop  7.093202  
  Adult Eye  7.202920  
  Adult Fatbody  7.290829  
  Adult Female Spermatheca Mated  6.976995  
  Adult Female Spermatheca Virgin  7.149768  
  Adult Head  6.801862  
  Adult Heart  7.609326  
  Adult Hind Gut  7.107449  
  Adult Male Ejaculatory Duct  7.396712  
  Adult Mid Gut  7.116165  
  Adult Ovary  7.869364  
  Adult Salivary Gland  7.652424  
  Adult Testes  10.182195  
  Adult Thoracoabdominal ganglion  7.147217  
  Adult Whole Fly  7.707100  
  Larvae Wandering Tubules  6.620009  
  Larval Feeding Carcass  6.416174  
  Larval Feeding Central Nevous System  7.031808  
  Larval Feeding Hind Gut  6.544726  
  Larval Feeding Malpighian Tubule  7.075663  
  Larval Feeding Mid Gut  6.961423  
  Larval Feeding Salivary Gland  6.521968  
  Whole Larvae Feeding  6.851518  
 
  
   FlyBase ID    symbol    start    end    strand    length   
   FBgn0035461   ntc  3803834   3806456   -  2623  
   FBgn0035462   IntS10   3806714   3809090  +  2377  
   FBgn0052262   CG32262  3809146   3810181   -  1036  
 
 
    Segment 86 
 
   Location   
  Gene key  FBgn0035469-FBgn0035470  
  Heatmap region span   3L:3818687..3940084   
  Segment span   3L:3896900..3899156   
  Length (genes)  2  
  Length (bp)  2257  
   Model Scoring   
  BIC  275.591705  
  logL  -132.283726  
  logL ratio  -8.313857  
   Expression   
  Mean expression  8.845953  
  Median expression  8.800253  
  Tissue std. dev.  0.437468  
 
  No GO Slim enrichment  
  
   tissue    mean expression   
  5th Passage Drosophila S2 Cells  8.149299  
  Adult Accessory gland  8.939963  
  Adult Brain  8.989947  
  Adult Carcass  8.582733  
  Adult Crop  9.310996  
  Adult Eye  9.338076  
  Adult Fatbody  8.799449  
  Adult Female Spermatheca Mated  9.068295  
  Adult Female Spermatheca Virgin  9.125771  
  Adult Head  8.692499  
  Adult Heart  8.992832  
  Adult Hind Gut  9.185446  
  Adult Male Ejaculatory Duct  9.223351  
  Adult Mid Gut  8.831117  
  Adult Ovary  8.287885  
  Adult Salivary Gland  9.490782  
  Adult Testes  8.237897  
  Adult Thoracoabdominal ganglion  8.940012  
  Adult Whole Fly  7.822427  
  Larvae Wandering Tubules  9.559634  
  Larval Feeding Carcass  8.253650  
  Larval Feeding Central Nevous System  8.550125  
  Larval Feeding Hind Gut  8.892632  
  Larval Feeding Malpighian Tubule  9.229748  
  Larval Feeding Mid Gut  8.879711  
  Larval Feeding Salivary Gland  9.188485  
  Whole Larvae Feeding  8.277959  
 
  
   FlyBase ID    symbol    start    end    strand    length   
   FBgn0035469   CG14977   3896900   3897498  +  599  
   FBgn0035470   CG14980  3897444   3899156   -  1713  
 
    Segment 87 
 
   Location   
  Gene key  FBgn0035475-FBgn0035476  
  Heatmap region span   3L:3896900..3983916   
  Segment span   3L:3937941..3940084   
  Length (genes)  2  
  Length (bp)  2144  
   Model Scoring   
  BIC  230.735608  
  logL  -109.855677  
  logL ratio  11.583322  
   Expression   
  Mean expression  6.584978  
  Median expression  6.452622  
  Tissue std. dev.  0.703166  
 
  No GO Slim enrichment  
  
   tissue    mean expression   
  5th Passage Drosophila S2 Cells  6.264959  
  Adult Accessory gland  6.338389  
  Adult Brain  6.424138  
  Adult Carcass  6.563712  
  Adult Crop  6.495294  
  Adult Eye  6.430703  
  Adult Fatbody  6.967333  
  Adult Female Spermatheca Mated  6.093521  
  Adult Female Spermatheca Virgin  6.300904  
  Adult Head  6.606558  
  Adult Heart  6.698254  
  Adult Hind Gut  7.172800  
  Adult Male Ejaculatory Duct  6.364385  
  Adult Mid Gut  9.240989  
  Adult Ovary  6.687972  
  Adult Salivary Gland  8.066036  
  Adult Testes  6.749839  
  Adult Thoracoabdominal ganglion  6.801976  
  Adult Whole Fly  6.907274  
  Larvae Wandering Tubules  6.091149  
  Larval Feeding Carcass  5.996035  
  Larval Feeding Central Nevous System  5.713690  
  Larval Feeding Hind Gut  5.885393  
  Larval Feeding Malpighian Tubule  6.239851  
  Larval Feeding Mid Gut  6.771041  
  Larval Feeding Salivary Gland  5.811934  
  Whole Larvae Feeding  6.110280  
 
  
   FlyBase ID    symbol    start    end    strand    length   
   FBgn0035475   CG10866  3936597   3937941   -  1345  
   FBgn0035476   CG12766  3938624   3940084   -  1461  
 
    Segment 88 
 
   Location   
  Gene key  FBgn0035477-FBgn0035479  
  Heatmap region span   3L:3905344..4026009   
  Segment span   3L:3942850..3949305   
  Length (genes)  3  
  Length (bp)  6456  
   Model Scoring   
  BIC  252.484999  
  logL  -120.730373  
  logL ratio  54.178441  
   Expression   
  Mean expression  4.968333  
  Median expression  4.854526  
  Tissue std. dev.  0.414577  
 
  No GO Slim enrichment  
  
   tissue    mean expression   
  5th Passage Drosophila S2 Cells  4.861324  
  Adult Accessory gland  4.744376  
  Adult Brain  5.698993  
  Adult Carcass  4.726331  
  Adult Crop  4.620936  
  Adult Eye  4.995770  
  Adult Fatbody  4.847630  
  Adult Female Spermatheca Mated  4.941658  
  Adult Female Spermatheca Virgin  4.889887  
  Adult Head  5.040361  
  Adult Heart  4.565166  
  Adult Hind Gut  4.725835  
  Adult Male Ejaculatory Duct  4.917669  
  Adult Mid Gut  5.028439  
  Adult Ovary  4.649979  
  Adult Salivary Gland  5.152468  
  Adult Testes  6.710289  
  Adult Thoracoabdominal ganglion  5.260236  
  Adult Whole Fly  4.564658  
  Larvae Wandering Tubules  4.797448  
  Larval Feeding Carcass  4.788950  
  Larval Feeding Central Nevous System  4.973493  
  Larval Feeding Hind Gut  4.645997  
  Larval Feeding Malpighian Tubule  4.936249  
  Larval Feeding Mid Gut  5.116720  
  Larval Feeding Salivary Gland  4.923238  
  Whole Larvae Feeding  5.020877  
 
  
   FlyBase ID    symbol    start    end    strand    length   
   FBgn0035477   CG14982   3942850   3946457  +  3608  
   FBgn0035478   CG10853   3947912   3948685  +  774  
   FBgn0035479   CG14983  3948695   3949305   -  611  
 
 
    Segment 89 
 
   Location   
  Gene key  FBgn0035481-FBgn0004880  
  Heatmap region span   3L:3937941..4040534   
  Segment span   3L:3964771..3983916   
  Length (genes)  2  
  Length (bp)  19146  
   Model Scoring   
  BIC  184.277151  
  logL  -86.626449  
  logL ratio  49.386436  
   Expression   
  Mean expression  5.209722  
  Median expression  4.221469  
  Tissue std. dev.  2.209037  
 
  No GO Slim enrichment  
  
   tissue    mean expression   
  5th Passage Drosophila S2 Cells  3.942994  
  Adult Accessory gland  4.079423  
  Adult Brain  11.335186  
  Adult Carcass  4.512016  
  Adult Crop  4.042495  
  Adult Eye  8.439204  
  Adult Fatbody  4.977706  
  Adult Female Spermatheca Mated  4.763980  
  Adult Female Spermatheca Virgin  4.668136  
  Adult Head  8.400154  
  Adult Heart  4.274491  
  Adult Hind Gut  3.808582  
  Adult Male Ejaculatory Duct  3.951941  
  Adult Mid Gut  3.804882  
  Adult Ovary  3.813878  
  Adult Salivary Gland  4.418245  
  Adult Testes  3.806678  
  Adult Thoracoabdominal ganglion  10.788636  
  Adult Whole Fly  4.881782  
  Larvae Wandering Tubules  3.952221  
  Larval Feeding Carcass  4.029655  
  Larval Feeding Central Nevous System  9.349445  
  Larval Feeding Hind Gut  3.978704  
  Larval Feeding Malpighian Tubule  4.056200  
  Larval Feeding Mid Gut  3.900555  
  Larval Feeding Salivary Gland  4.290199  
  Whole Larvae Feeding  4.395103  
 
  
   FlyBase ID    symbol    start    end    strand    length   
   FBgn0035481   CG12605  3956841   3964771   -  7931  
   FBgn0004880   scrt   3983916   3988589  +  4674  
 
    Segment 90 
 
   Location   
  Gene key  FBgn0035482-FBgn0004895  
  Heatmap region span   3L:3942461..4046611   
  Segment span   3L:4003472..4019094   
  Length (genes)  2  
  Length (bp)  15623  
   Model Scoring   
  BIC  175.350246  
  logL  -82.162997  
  logL ratio  34.497047  
   Expression   
  Mean expression  4.868961  
  Median expression  4.832836  
  Tissue std. dev.  0.368783  
 
  No GO Slim enrichment  
  
   tissue    mean expression   
  5th Passage Drosophila S2 Cells  4.841910  
  Adult Accessory gland  4.786371  
  Adult Brain  4.489071  
  Adult Carcass  4.751571  
  Adult Crop  4.738663  
  Adult Eye  4.774848  
  Adult Fatbody  4.700150  
  Adult Female Spermatheca Mated  4.707145  
  Adult Female Spermatheca Virgin  4.865232  
  Adult Head  4.390225  
  Adult Heart  4.682850  
  Adult Hind Gut  5.740056  
  Adult Male Ejaculatory Duct  4.830035  
  Adult Mid Gut  5.461816  
  Adult Ovary  4.600746  
  Adult Salivary Gland  5.056316  
  Adult Testes  4.637904  
  Adult Thoracoabdominal ganglion  4.483987  
  Adult Whole Fly  4.210108  
  Larvae Wandering Tubules  5.106645  
  Larval Feeding Carcass  4.993860  
  Larval Feeding Central Nevous System  4.572195  
  Larval Feeding Hind Gut  5.764142  
  Larval Feeding Malpighian Tubule  5.197684  
  Larval Feeding Mid Gut  5.197727  
  Larval Feeding Salivary Gland  5.184935  
  Whole Larvae Feeding  4.695766  
 
  
   FlyBase ID    symbol    start    end    strand    length   
   FBgn0035482   CG14985   4003472   4006626  +  3155  
   FBgn0004895   fd64A   4019094   4021179  +  2086  
 
    Segment 91 
 
   Location   
  Gene key  FBgn0035483-FBgn0035484  
  Heatmap region span   3L:3942850..4069369   
  Segment span   3L:4021439..4026009   
  Length (genes)  2  
  Length (bp)  4571  
   Model Scoring   
  BIC  266.567361  
  logL  -127.771554  
  logL ratio  -1.718335  
   Expression   
  Mean expression  8.743338  
  Median expression  8.693681  
  Tissue std. dev.  0.930430  
 
  No GO Slim enrichment  
  
   tissue    mean expression   
  5th Passage Drosophila S2 Cells  6.620233  
  Adult Accessory gland  8.070135  
  Adult Brain  7.797518  
  Adult Carcass  9.991005  
  Adult Crop  8.990922  
  Adult Eye  9.118401  
  Adult Fatbody  10.130475  
  Adult Female Spermatheca Mated  9.836202  
  Adult Female Spermatheca Virgin  9.955256  
  Adult Head  9.323055  
  Adult Heart  9.828346  
  Adult Hind Gut  8.965166  
  Adult Male Ejaculatory Duct  9.099684  
  Adult Mid Gut  8.708309  
  Adult Ovary  7.118347  
  Adult Salivary Gland  8.787803  
  Adult Testes  9.002812  
  Adult Thoracoabdominal ganglion  7.682848  
  Adult Whole Fly  8.996541  
  Larvae Wandering Tubules  9.488924  
  Larval Feeding Carcass  8.036270  
  Larval Feeding Central Nevous System  6.924078  
  Larval Feeding Hind Gut  8.001960  
  Larval Feeding Malpighian Tubule  9.514042  
  Larval Feeding Mid Gut  8.646159  
  Larval Feeding Salivary Gland  8.539833  
  Whole Larvae Feeding  8.895812  
 
  
   FlyBase ID    symbol    start    end    strand    length   
   FBgn0035483   Mul1   4021439   4023066  +  1628  
   FBgn0035484   CG11594  4022970   4026009   -  3040  
 
    Segment 92 
 
   Location   
  Gene key  FBgn0045479-FBgn0052255  
  Heatmap region span   3L:3952420..4071713   
  Segment span   3L:4026769..4035799   
  Length (genes)  4  
  Length (bp)  9031  
   Model Scoring   
  BIC  282.364986  
  logL  -135.670367  
  logL ratio  141.828252  
   Expression   
  Mean expression  4.213281  
  Median expression  4.197783  
  Tissue std. dev.  0.124469  
 
  No GO Slim enrichment  
  
   tissue    mean expression   
  5th Passage Drosophila S2 Cells  4.359364  
  Adult Accessory gland  4.306161  
  Adult Brain  3.979150  
  Adult Carcass  4.258819  
  Adult Crop  4.212752  
  Adult Eye  4.082346  
  Adult Fatbody  4.210710  
  Adult Female Spermatheca Mated  4.127731  
  Adult Female Spermatheca Virgin  4.250472  
  Adult Head  4.424851  
  Adult Heart  4.206974  
  Adult Hind Gut  4.189267  
  Adult Male Ejaculatory Duct  4.161703  
  Adult Mid Gut  4.380238  
  Adult Ovary  4.257382  
  Adult Salivary Gland  4.329088  
  Adult Testes  4.354696  
  Adult Thoracoabdominal ganglion  4.155904  
  Adult Whole Fly  3.973261  
  Larvae Wandering Tubules  4.296724  
  Larval Feeding Carcass  4.154248  
  Larval Feeding Central Nevous System  4.034187  
  Larval Feeding Hind Gut  4.141151  
  Larval Feeding Malpighian Tubule  4.297109  
  Larval Feeding Mid Gut  4.376383  
  Larval Feeding Salivary Gland  4.249871  
  Whole Larvae Feeding  3.988054  
 
  
   FlyBase ID    symbol    start    end    strand    length   
   FBgn0045479   Gr64a   4026769   4028483  +  1715  
   FBgn0045478   Gr64b   4028605   4030247  +  1643  
   FBgn0045477   Gr64c   4030264   4032032  +  1769  
   FBgn0052255   Gr64f   4035799   4037539  +  1741  
 
 
    Segment 93 
 
   Location   
  Gene key  FBgn0035488-FBgn0035489  
  Heatmap region span   3L:3964771..4073949   
  Segment span   3L:4040482..4040534   
  Length (genes)  2  
  Length (bp)  53  
   Model Scoring   
  BIC  224.269835  
  logL  -106.622791  
  logL ratio  37.659101  
   Expression   
  Mean expression  8.872235  
  Median expression  8.648708  
  Tissue std. dev.  0.524500  
 
  No GO Slim enrichment  
  
   tissue    mean expression   
  5th Passage Drosophila S2 Cells  9.622836  
  Adult Accessory gland  8.962888  
  Adult Brain  9.752578  
  Adult Carcass  8.325490  
  Adult Crop  9.005617  
  Adult Eye  9.433544  
  Adult Fatbody  8.399340  
  Adult Female Spermatheca Mated  8.824477  
  Adult Female Spermatheca Virgin  8.891191  
  Adult Head  8.838490  
  Adult Heart  8.923044  
  Adult Hind Gut  8.718366  
  Adult Male Ejaculatory Duct  8.913566  
  Adult Mid Gut  7.990679  
  Adult Ovary  10.197927  
  Adult Salivary Gland  8.748397  
  Adult Testes  8.393915  
  Adult Thoracoabdominal ganglion  9.537262  
  Adult Whole Fly  8.978279  
  Larvae Wandering Tubules  8.528122  
  Larval Feeding Carcass  8.636149  
  Larval Feeding Central Nevous System  9.573030  
  Larval Feeding Hind Gut  8.678060  
  Larval Feeding Malpighian Tubule  8.425168  
  Larval Feeding Mid Gut  8.160438  
  Larval Feeding Salivary Gland  8.957448  
  Whole Larvae Feeding  8.134045  
 
  
   FlyBase ID    symbol    start    end    strand    length   
   FBgn0035488   CG11593  4037587   4040482   -  2896  
   FBgn0035489      4040534   4042597  +  2064  
 
    Segment 94 
 
   Location   
  Gene key  FBgn0042179-FBgn0052259  
  Heatmap region span   3L:4021439..4093206   
  Segment span   3L:4059806..4069369   
  Length (genes)  3  
  Length (bp)  9564  
   Model Scoring   
  BIC  315.345248  
  logL  -152.160498  
  logL ratio  9.316933  
   Expression   
  Mean expression  5.217158  
  Median expression  5.055272  
  Tissue std. dev.  0.882626  
 
  No GO Slim enrichment  
  
   tissue    mean expression   
  5th Passage Drosophila S2 Cells  4.766001  
  Adult Accessory gland  4.979250  
  Adult Brain  4.391072  
  Adult Carcass  4.925418  
  Adult Crop  4.682974  
  Adult Eye  5.024516  
  Adult Fatbody  4.843349  
  Adult Female Spermatheca Mated  4.884967  
  Adult Female Spermatheca Virgin  4.818973  
  Adult Head  4.537557  
  Adult Heart  5.083962  
  Adult Hind Gut  5.271413  
  Adult Male Ejaculatory Duct  5.075364  
  Adult Mid Gut  6.367581  
  Adult Ovary  4.870621  
  Adult Salivary Gland  5.043104  
  Adult Testes  8.678050  
  Adult Thoracoabdominal ganglion  4.573173  
  Adult Whole Fly  5.694244  
  Larvae Wandering Tubules  4.868273  
  Larval Feeding Carcass  4.818910  
  Larval Feeding Central Nevous System  4.549803  
  Larval Feeding Hind Gut  5.428757  
  Larval Feeding Malpighian Tubule  4.791034  
  Larval Feeding Mid Gut  6.422290  
  Larval Feeding Salivary Gland  4.804900  
  Whole Larvae Feeding  6.667699  
 
  
   FlyBase ID    symbol    start    end    strand    length   
   FBgn0042179   CG18869   4059806   4061923  +  2118  
   FBgn0000449   dib   4062304   4064302  +  1999  
   FBgn0052259   CG32259   4069369   4070281  +  913  
 
 
    Segment 95 
 
   Location   
  Gene key  FBgn0004516-FBgn0035495  
  Heatmap region span   3L:4046611..4141467   
  Segment span   3L:4088744..4089000   
  Length (genes)  2  
  Length (bp)  257  
   Model Scoring   
  BIC  228.319778  
  logL  -108.647763  
  logL ratio  34.244615  
   Expression   
  Mean expression  5.894251  
  Median expression  4.134125  
  Tissue std. dev.  3.036647  
 
  No GO Slim enrichment  
  
   tissue    mean expression   
  5th Passage Drosophila S2 Cells  4.025118  
  Adult Accessory gland  3.948043  
  Adult Brain  13.262733  
  Adult Carcass  5.858998  
  Adult Crop  4.135113  
  Adult Eye  7.094739  
  Adult Fatbody  4.417542  
  Adult Female Spermatheca Mated  4.087142  
  Adult Female Spermatheca Virgin  4.203581  
  Adult Head  11.208861  
  Adult Heart  3.984321  
  Adult Hind Gut  3.885454  
  Adult Male Ejaculatory Duct  3.909474  
  Adult Mid Gut  5.879209  
  Adult Ovary  3.869042  
  Adult Salivary Gland  4.239952  
  Adult Testes  3.786752  
  Adult Thoracoabdominal ganglion  13.481406  
  Adult Whole Fly  8.118743  
  Larvae Wandering Tubules  3.998367  
  Larval Feeding Carcass  4.044985  
  Larval Feeding Central Nevous System  12.264409  
  Larval Feeding Hind Gut  3.877374  
  Larval Feeding Malpighian Tubule  3.946369  
  Larval Feeding Mid Gut  6.772923  
  Larval Feeding Salivary Gland  3.969204  
  Whole Larvae Feeding  6.874934  
 
  
   FlyBase ID    symbol    start    end    strand    length   
   FBgn0004516   Gad1  4074069   4088744   -  14676  
   FBgn0035495   CG14989   4089000   4091677  +  2678  
 
    Segment 96 
 
   Location   
  Gene key  FBgn0035497-FBgn0028484  
  Heatmap region span   3L:4071713..4148697   
  Segment span   3L:4102697..4108205   
  Length (genes)  2  
  Length (bp)  5509  
   Model Scoring   
  BIC  239.077268  
  logL  -114.026508  
  logL ratio  31.413150  
   Expression   
  Mean expression  8.987089  
  Median expression  8.900560  
  Tissue std. dev.  0.951039  
 
  No GO Slim enrichment  
  
   tissue    mean expression   
  5th Passage Drosophila S2 Cells  8.942556  
  Adult Accessory gland  9.207970  
  Adult Brain  9.719403  
  Adult Carcass  9.329261  
  Adult Crop  10.209965  
  Adult Eye  9.840732  
  Adult Fatbody  8.397987  
  Adult Female Spermatheca Mated  8.692177  
  Adult Female Spermatheca Virgin  8.872884  
  Adult Head  9.348495  
  Adult Heart  8.481691  
  Adult Hind Gut  9.235909  
  Adult Male Ejaculatory Duct  8.884469  
  Adult Mid Gut  8.014007  
  Adult Ovary  8.858883  
  Adult Salivary Gland  8.936543  
  Adult Testes  12.273821  
  Adult Thoracoabdominal ganglion  9.630161  
  Adult Whole Fly  9.839879  
  Larvae Wandering Tubules  7.902703  
  Larval Feeding Carcass  9.032918  
  Larval Feeding Central Nevous System  8.969492  
  Larval Feeding Hind Gut  8.448806  
  Larval Feeding Malpighian Tubule  8.452014  
  Larval Feeding Mid Gut  7.109894  
  Larval Feeding Salivary Gland  7.515558  
  Whole Larvae Feeding  8.503230  
 
  
   FlyBase ID    symbol    start    end    strand    length   
   FBgn0035497   CG14995  4094343   4102697   -  8355  
   FBgn0028484   Ack   4108205   4114161  +  5957  
 
    Segment 97 
 
   Location   
  Gene key  FBgn0035499-FBgn0004574  
  Heatmap region span   3L:4073949..4188530   
  Segment span   3L:4122423..4139201   
  Length (genes)  4  
  Length (bp)  16779  
   Model Scoring   
  BIC  486.106388  
  logL  -237.541068  
  logL ratio  167.258512  
   Expression   
  Mean expression  10.861325  
  Median expression  10.681196  
  Tissue std. dev.  0.545051  
 
  No GO Slim enrichment  
  
   tissue    mean expression   
  5th Passage Drosophila S2 Cells  11.350702  
  Adult Accessory gland  10.860286  
  Adult Brain  11.291893  
  Adult Carcass  10.311535  
  Adult Crop  11.624915  
  Adult Eye  10.963375  
  Adult Fatbody  10.008876  
  Adult Female Spermatheca Mated  10.740454  
  Adult Female Spermatheca Virgin  10.434268  
  Adult Head  10.811187  
  Adult Heart  11.062451  
  Adult Hind Gut  11.570956  
  Adult Male Ejaculatory Duct  10.550058  
  Adult Mid Gut  10.729947  
  Adult Ovary  10.719984  
  Adult Salivary Gland  10.408339  
  Adult Testes  9.224155  
  Adult Thoracoabdominal ganglion  11.462219  
  Adult Whole Fly  10.352279  
  Larvae Wandering Tubules  11.162141  
  Larval Feeding Carcass  11.264866  
  Larval Feeding Central Nevous System  10.481658  
  Larval Feeding Hind Gut  11.791713  
  Larval Feeding Malpighian Tubule  10.942492  
  Larval Feeding Mid Gut  10.904580  
  Larval Feeding Salivary Gland  11.423445  
  Whole Larvae Feeding  10.806994  
 
  
   FlyBase ID    symbol    start    end    strand    length   
   FBgn0035499   Chd64  4114289   4122423   -  8135  
   FBgn0035500     4123548   4134550   -  11003  
   FBgn0003206   Ras64B   4139078   4141398  +  2321  
   FBgn0004574   Rop  4136785   4139201   -  2417  
 
 
    Segment 98 
 
   Location   
  Gene key  FBgn0011653-FBgn0003710  
  Heatmap region span   3L:4122423..4240173   
  Segment span   3L:4167461..4188530   
  Length (genes)  5  
  Length (bp)  21070  
   Model Scoring   
  BIC  434.802111  
  logL  -211.888929  
  logL ratio  106.566233  
   Expression   
  Mean expression  5.129651  
  Median expression  4.730957  
  Tissue std. dev.  0.991855  
 
  No GO Slim enrichment  
  
   tissue    mean expression   
  5th Passage Drosophila S2 Cells  4.622494  
  Adult Accessory gland  4.648322  
  Adult Brain  7.711007  
  Adult Carcass  5.051539  
  Adult Crop  4.684836  
  Adult Eye  6.750071  
  Adult Fatbody  4.677924  
  Adult Female Spermatheca Mated  4.549471  
  Adult Female Spermatheca Virgin  4.585573  
  Adult Head  6.124624  
  Adult Heart  4.736006  
  Adult Hind Gut  4.545453  
  Adult Male Ejaculatory Duct  4.642344  
  Adult Mid Gut  4.668518  
  Adult Ovary  4.583460  
  Adult Salivary Gland  4.773101  
  Adult Testes  4.566692  
  Adult Thoracoabdominal ganglion  7.902223  
  Adult Whole Fly  4.641121  
  Larvae Wandering Tubules  4.601105  
  Larval Feeding Carcass  4.874828  
  Larval Feeding Central Nevous System  7.079588  
  Larval Feeding Hind Gut  4.844934  
  Larval Feeding Malpighian Tubule  4.648061  
  Larval Feeding Mid Gut  4.620525  
  Larval Feeding Salivary Gland  4.579312  
  Whole Larvae Feeding  4.787451  
 
  
   FlyBase ID    symbol    start    end    strand    length   
   FBgn0011653   mas  4161654   4167461   -  5808  
   FBgn0035504   Teh4  4174859   4178270   -  3412  
   FBgn0040697   Teh3  4182645   4184663   -  2019  
   FBgn0035505   Teh2  4179151   4188023   -  8873  
   FBgn0003710   tipE   4188530   4193788  +  5259  
 
 
    Segment 99 
 
   Location   
  Gene key  FBgn0035507-FBgn0035510  
  Heatmap region span   3L:4141467..4242736   
  Segment span   3L:4193993..4209178   
  Length (genes)  4  
  Length (bp)  15186  
   Model Scoring   
  BIC  277.077823  
  logL  -133.026785  
  logL ratio  127.282287  
   Expression   
  Mean expression  4.597389  
  Median expression  4.520473  
  Tissue std. dev.  0.213666  
 
  No GO Slim enrichment  
  
   tissue    mean expression   
  5th Passage Drosophila S2 Cells  5.342439  
  Adult Accessory gland  4.690250  
  Adult Brain  4.365749  
  Adult Carcass  4.751608  
  Adult Crop  4.522171  
  Adult Eye  4.405787  
  Adult Fatbody  4.560724  
  Adult Female Spermatheca Mated  4.527354  
  Adult Female Spermatheca Virgin  4.493455  
  Adult Head  4.357456  
  Adult Heart  4.571631  
  Adult Hind Gut  4.568590  
  Adult Male Ejaculatory Duct  4.643088  
  Adult Mid Gut  4.662058  
  Adult Ovary  4.585720  
  Adult Salivary Gland  4.975206  
  Adult Testes  4.379074  
  Adult Thoracoabdominal ganglion  4.445596  
  Adult Whole Fly  4.243330  
  Larvae Wandering Tubules  4.746117  
  Larval Feeding Carcass  4.748361  
  Larval Feeding Central Nevous System  4.420390  
  Larval Feeding Hind Gut  4.444142  
  Larval Feeding Malpighian Tubule  4.742582  
  Larval Feeding Mid Gut  4.648321  
  Larval Feeding Salivary Gland  4.644686  
  Whole Larvae Feeding  4.643610  
 
  
   FlyBase ID    symbol    start    end    strand    length   
   FBgn0035507      4193993   4200926  +  6934  
   FBgn0035508   CG15005  4200825   4204338   -  3514  
   FBgn0035509      4204569   4205537  +  969  
   FBgn0035510   Cpr64Aa  4207838   4209178   -  1341  
 
 
    Segment 100 
 
   Location   
  Gene key  FBgn0035511-FBgn0035514  
  Heatmap region span   3L:4143092..4254774   
  Segment span   3L:4211540..4216636   
  Length (genes)  4  
  Length (bp)  5097  
   Model Scoring   
  BIC  337.706766  
  logL  -163.341257  
  logL ratio  130.038008  
   Expression   
  Mean expression  6.237708  
  Median expression  6.310987  
  Tissue std. dev.  0.268731  
 
  No GO Slim enrichment  
  
   tissue    mean expression   
  5th Passage Drosophila S2 Cells  6.578481  
  Adult Accessory gland  6.511904  
  Adult Brain  6.253019  
  Adult Carcass  6.301069  
  Adult Crop  6.414506  
  Adult Eye  5.713929  
  Adult Fatbody  6.291964  
  Adult Female Spermatheca Mated  6.099721  
  Adult Female Spermatheca Virgin  6.065674  
  Adult Head  6.201236  
  Adult Heart  5.865092  
  Adult Hind Gut  6.313261  
  Adult Male Ejaculatory Duct  6.257090  
  Adult Mid Gut  6.587185  
  Adult Ovary  6.380144  
  Adult Salivary Gland  6.637342  
  Adult Testes  5.685117  
  Adult Thoracoabdominal ganglion  6.338600  
  Adult Whole Fly  5.782021  
  Larvae Wandering Tubules  6.571130  
  Larval Feeding Carcass  6.305916  
  Larval Feeding Central Nevous System  5.883405  
  Larval Feeding Hind Gut  6.279964  
  Larval Feeding Malpighian Tubule  6.532319  
  Larval Feeding Mid Gut  6.251185  
  Larval Feeding Salivary Gland  6.361944  
  Whole Larvae Feeding  5.954899  
 
  
   FlyBase ID    symbol    start    end    strand    length   
   FBgn0035511   Cpr64Ab  4210880   4211540   -  661  
   FBgn0035512   Cpr64Ac  4211964   4212915   -  952  
   FBgn0035513   Cpr64Ad   4215387   4216414  +  1028  
   FBgn0035514      4216636   4224004  +  7369  
 
 
    Segment 101 
 
   Location   
  Gene key  FBgn0001257-FBgn0035515  
  Heatmap region span   3L:4167461..4259419   
  Segment span   3L:4236147..4240173   
  Length (genes)  2  
  Length (bp)  4027  
   Model Scoring   
  BIC  267.325121  
  logL  -128.150434  
  logL ratio  25.075199  
   Expression   
  Mean expression  9.874978  
  Median expression  9.941007  
  Tissue std. dev.  0.892481  
 
  No GO Slim enrichment  
  
   tissue    mean expression   
  5th Passage Drosophila S2 Cells  11.676245  
  Adult Accessory gland  8.232821  
  Adult Brain  9.580037  
  Adult Carcass  10.643083  
  Adult Crop  9.336228  
  Adult Eye  9.571357  
  Adult Fatbody  10.784144  
  Adult Female Spermatheca Mated  10.051206  
  Adult Female Spermatheca Virgin  10.013010  
  Adult Head  9.942961  
  Adult Heart  11.218886  
  Adult Hind Gut  10.376077  
  Adult Male Ejaculatory Duct  9.341866  
  Adult Mid Gut  9.739192  
  Adult Ovary  9.197091  
  Adult Salivary Gland  8.069193  
  Adult Testes  8.203156  
  Adult Thoracoabdominal ganglion  10.434115  
  Adult Whole Fly  9.873738  
  Larvae Wandering Tubules  9.907333  
  Larval Feeding Carcass  10.967593  
  Larval Feeding Central Nevous System  9.736436  
  Larval Feeding Hind Gut  10.828384  
  Larval Feeding Malpighian Tubule  9.821107  
  Larval Feeding Mid Gut  10.231136  
  Larval Feeding Salivary Gland  8.481469  
  Whole Larvae Feeding  10.366545  
 
  
   FlyBase ID    symbol    start    end    strand    length   
   FBgn0001257   ImpL2  4224699   4236147   -  11449  
   FBgn0035515   CG14997   4240173   4242603  +  2431  
 
    Segment 102 
 
   Location   
  Gene key  FBgn0041171-FBgn0035517  
  Heatmap region span   3L:4211540..4263721   
  Segment span   3L:4254309..4254774   
  Length (genes)  2  
  Length (bp)  466  
   Model Scoring   
  BIC  269.322093  
  logL  -129.148920  
  logL ratio  8.962567  
   Expression   
  Mean expression  9.423961  
  Median expression  9.473202  
  Tissue std. dev.  0.875149  
 
  No GO Slim enrichment  
  
   tissue    mean expression   
  5th Passage Drosophila S2 Cells  10.178999  
  Adult Accessory gland  7.656139  
  Adult Brain  9.880388  
  Adult Carcass  9.755676  
  Adult Crop  9.065279  
  Adult Eye  9.824926  
  Adult Fatbody  8.873428  
  Adult Female Spermatheca Mated  8.862072  
  Adult Female Spermatheca Virgin  8.807099  
  Adult Head  9.422921  
  Adult Heart  9.381576  
  Adult Hind Gut  10.582860  
  Adult Male Ejaculatory Duct  8.131944  
  Adult Mid Gut  10.261230  
  Adult Ovary  9.621226  
  Adult Salivary Gland  7.744906  
  Adult Testes  9.403192  
  Adult Thoracoabdominal ganglion  9.997604  
  Adult Whole Fly  9.497912  
  Larvae Wandering Tubules  8.902372  
  Larval Feeding Carcass  11.030379  
  Larval Feeding Central Nevous System  9.946181  
  Larval Feeding Hind Gut  10.341396  
  Larval Feeding Malpighian Tubule  8.408714  
  Larval Feeding Mid Gut  10.251251  
  Larval Feeding Salivary Gland  8.134016  
  Whole Larvae Feeding  10.483274  
 
  
   FlyBase ID    symbol    start    end    strand    length   
   FBgn0041171   ago  4246326   4254309   -  7984  
   FBgn0035517   CG1265   4254774   4256055  +  1282  
 
    Segment 103 
 
   Location   
  Gene key  FBgn0035523-FBgn0015829  
  Heatmap region span   3L:4259152..4365467   
  Segment span   3L:4277964..4287038   
  Length (genes)  4  
  Length (bp)  9075  
   Model Scoring   
  BIC  398.858008  
  logL  -193.916878  
  logL ratio  116.321753  
   Expression   
  Mean expression  8.821288  
  Median expression  8.740316  
  Tissue std. dev.  0.467830  
 
  No GO Slim enrichment  
  
   tissue    mean expression   
  5th Passage Drosophila S2 Cells  9.713032  
  Adult Accessory gland  10.000144  
  Adult Brain  9.009880  
  Adult Carcass  8.342505  
  Adult Crop  8.994655  
  Adult Eye  8.885201  
  Adult Fatbody  8.581701  
  Adult Female Spermatheca Mated  8.452181  
  Adult Female Spermatheca Virgin  8.275132  
  Adult Head  8.591854  
  Adult Heart  8.707256  
  Adult Hind Gut  8.795567  
  Adult Male Ejaculatory Duct  8.788089  
  Adult Mid Gut  8.382234  
  Adult Ovary  9.432856  
  Adult Salivary Gland  8.807570  
  Adult Testes  8.623255  
  Adult Thoracoabdominal ganglion  8.826224  
  Adult Whole Fly  8.786026  
  Larvae Wandering Tubules  9.022064  
  Larval Feeding Carcass  8.506319  
  Larval Feeding Central Nevous System  9.713922  
  Larval Feeding Hind Gut  8.700474  
  Larval Feeding Malpighian Tubule  8.934998  
  Larval Feeding Mid Gut  7.946154  
  Larval Feeding Salivary Gland  9.171605  
  Whole Larvae Feeding  8.183884  
 
  
   FlyBase ID    symbol    start    end    strand    length   
   FBgn0035523   CG1311   4277964   4281618  +  3655  
   FBgn0035524   CG11583  4281504   4282798   -  1295  
   FBgn0035526   CG1316   4283855   4286865  +  3011  
   FBgn0015829   TfIIEbeta   4287038   4288349  +  1312  
 
 
    Segment 104 
 
   Location   
  Gene key  FBgn0041630-FBgn0035529  
  Heatmap region span   3L:4259419..4367458   
  Segment span   3L:4288642..4292497   
  Length (genes)  3  
  Length (bp)  3856  
   Model Scoring   
  BIC  355.896040  
  logL  -172.435894  
  logL ratio  104.139031  
   Expression   
  Mean expression  10.493676  
  Median expression  10.480753  
  Tissue std. dev.  0.469200  
 
  No GO Slim enrichment  
  
   tissue    mean expression   
  5th Passage Drosophila S2 Cells  10.197681  
  Adult Accessory gland  9.887835  
  Adult Brain  10.337433  
  Adult Carcass  10.246978  
  Adult Crop  11.116042  
  Adult Eye  10.922102  
  Adult Fatbody  10.263250  
  Adult Female Spermatheca Mated  10.458105  
  Adult Female Spermatheca Virgin  10.680989  
  Adult Head  10.665467  
  Adult Heart  10.345337  
  Adult Hind Gut  11.027188  
  Adult Male Ejaculatory Duct  10.587012  
  Adult Mid Gut  11.279632  
  Adult Ovary  10.072334  
  Adult Salivary Gland  10.190673  
  Adult Testes  8.994496  
  Adult Thoracoabdominal ganglion  10.661301  
  Adult Whole Fly  10.129696  
  Larvae Wandering Tubules  10.926913  
  Larval Feeding Carcass  10.731283  
  Larval Feeding Central Nevous System  10.016061  
  Larval Feeding Hind Gut  10.896471  
  Larval Feeding Malpighian Tubule  10.759946  
  Larval Feeding Mid Gut  11.098720  
  Larval Feeding Salivary Gland  10.316142  
  Whole Larvae Feeding  10.520180  
 
  
   FlyBase ID    symbol    start    end    strand    length   
   FBgn0041630   Hexo1   4288642   4291211  +  2570  
   FBgn0035528   CG15012  4291208   4292389   -  1182  
   FBgn0035529   CG1319   4292497   4293432  +  936  
 
 
    Segment 105 
 
   Location   
  Gene key  FBgn0066365-FBgn0052251  
  Heatmap region span   3L:4262461..4380376   
  Segment span   3L:4307711..4313635   
  Length (genes)  2  
  Length (bp)  5925  
   Model Scoring   
  BIC  227.291727  
  logL  -108.133737  
  logL ratio  -7.934714  
   Expression   
  Mean expression  5.010605  
  Median expression  4.563194  
  Tissue std. dev.  0.582980  
 
  No GO Slim enrichment  
  
   tissue    mean expression   
  5th Passage Drosophila S2 Cells  5.745706  
  Adult Accessory gland  4.589051  
  Adult Brain  4.301601  
  Adult Carcass  4.936691  
  Adult Crop  4.578534  
  Adult Eye  4.619211  
  Adult Fatbody  4.761352  
  Adult Female Spermatheca Mated  4.861305  
  Adult Female Spermatheca Virgin  4.849289  
  Adult Head  4.639636  
  Adult Heart  4.388311  
  Adult Hind Gut  4.501475  
  Adult Male Ejaculatory Duct  4.740503  
  Adult Mid Gut  4.831801  
  Adult Ovary  6.883821  
  Adult Salivary Gland  4.935824  
  Adult Testes  4.697719  
  Adult Thoracoabdominal ganglion  4.457702  
  Adult Whole Fly  5.894356  
  Larvae Wandering Tubules  4.794392  
  Larval Feeding Carcass  5.272462  
  Larval Feeding Central Nevous System  5.793072  
  Larval Feeding Hind Gut  5.884265  
  Larval Feeding Malpighian Tubule  4.911737  
  Larval Feeding Mid Gut  4.798207  
  Larval Feeding Salivary Gland  5.615483  
  Whole Larvae Feeding  5.002835  
 
  
   FlyBase ID    symbol    start    end    strand    length   
   FBgn0066365   dyl  4293995   4307711   -  13717  
   FBgn0052251   Claspin   4313635   4318473  +  4839  
 
    Segment 106 
 
   Location   
  Gene key  FBgn0052250-FBgn0035534  
  Heatmap region span   3L:4263721..4381564   
  Segment span   3L:4318757..4364555   
  Length (genes)  4  
  Length (bp)  45799  
   Model Scoring   
  BIC  365.524781  
  logL  -177.250264  
  logL ratio  141.232826  
   Expression   
  Mean expression  8.688198  
  Median expression  8.682049  
  Tissue std. dev.  0.482017  
 
  No GO Slim enrichment  
  
   tissue    mean expression   
  5th Passage Drosophila S2 Cells  8.726066  
  Adult Accessory gland  8.636926  
  Adult Brain  9.048758  
  Adult Carcass  8.274535  
  Adult Crop  9.601562  
  Adult Eye  8.828834  
  Adult Fatbody  8.498865  
  Adult Female Spermatheca Mated  7.883998  
  Adult Female Spermatheca Virgin  7.694478  
  Adult Head  8.490022  
  Adult Heart  8.839657  
  Adult Hind Gut  9.047076  
  Adult Male Ejaculatory Duct  9.184218  
  Adult Mid Gut  9.007243  
  Adult Ovary  9.796672  
  Adult Salivary Gland  8.323636  
  Adult Testes  9.137685  
  Adult Thoracoabdominal ganglion  8.922100  
  Adult Whole Fly  8.925079  
  Larvae Wandering Tubules  8.157527  
  Larval Feeding Carcass  8.083746  
  Larval Feeding Central Nevous System  9.174470  
  Larval Feeding Hind Gut  8.366330  
  Larval Feeding Malpighian Tubule  8.381612  
  Larval Feeding Mid Gut  8.591439  
  Larval Feeding Salivary Gland  8.642218  
  Whole Larvae Feeding  8.316578  
 
  
   FlyBase ID    symbol    start    end    strand    length   
   FBgn0052250   CG32250   4318757   4321109  +  2353  
   FBgn0035532   CG15014  4321105   4322230   -  1126  
   FBgn0035533   Cip4  4323530   4364120   -  40591  
   FBgn0035534   mRpS6   4364555   4365243  +  689  
 
 
    Segment 107 
 
   Location   
  Gene key  FBgn0035540-FBgn0035542  
  Heatmap region span   3L:4367458..4461339   
  Segment span   3L:4403500..4406948   
  Length (genes)  3  
  Length (bp)  3449  
   Model Scoring   
  BIC  332.571622  
  logL  -160.773684  
  logL ratio  73.750809  
   Expression   
  Mean expression  9.429572  
  Median expression  9.483830  
  Tissue std. dev.  0.486510  
 
  No GO Slim enrichment  
  
   tissue    mean expression   
  5th Passage Drosophila S2 Cells  9.055657  
  Adult Accessory gland  9.994677  
  Adult Brain  9.641837  
  Adult Carcass  9.934214  
  Adult Crop  10.060545  
  Adult Eye  9.711254  
  Adult Fatbody  10.041148  
  Adult Female Spermatheca Mated  10.029839  
  Adult Female Spermatheca Virgin  9.969147  
  Adult Head  9.579119  
  Adult Heart  10.247805  
  Adult Hind Gut  9.583388  
  Adult Male Ejaculatory Duct  9.678181  
  Adult Mid Gut  9.038034  
  Adult Ovary  9.200772  
  Adult Salivary Gland  9.438695  
  Adult Testes  8.437934  
  Adult Thoracoabdominal ganglion  9.649942  
  Adult Whole Fly  9.312147  
  Larvae Wandering Tubules  9.231478  
  Larval Feeding Carcass  9.402157  
  Larval Feeding Central Nevous System  8.770302  
  Larval Feeding Hind Gut  9.158759  
  Larval Feeding Malpighian Tubule  8.792297  
  Larval Feeding Mid Gut  9.128528  
  Larval Feeding Salivary Gland  8.485045  
  Whole Larvae Feeding  9.025538  
 
  
   FlyBase ID    symbol    start    end    strand    length   
   FBgn0035540   Syx17   4403500   4405169  +  1670  
   FBgn0035541   CG15019  4405127   4406059   -  933  
   FBgn0035542   DOR   4406948   4420266  +  13319  
 
 
    Segment 108 
 
   Location   
  Gene key  FBgn0035545-FBgn0035547  
  Heatmap region span   3L:4384455..4482809   
  Segment span   3L:4446575..4460313   
  Length (genes)  3  
  Length (bp)  13739  
   Model Scoring   
  BIC  303.510716  
  logL  -146.243232  
  logL ratio  55.047293  
   Expression   
  Mean expression  6.639141  
  Median expression  6.720289  
  Tissue std. dev.  0.509056  
 
  No GO Slim enrichment  
  
   tissue    mean expression   
  5th Passage Drosophila S2 Cells  6.572640  
  Adult Accessory gland  6.865315  
  Adult Brain  6.193417  
  Adult Carcass  6.796007  
  Adult Crop  6.534064  
  Adult Eye  6.022679  
  Adult Fatbody  6.979825  
  Adult Female Spermatheca Mated  6.824012  
  Adult Female Spermatheca Virgin  6.827860  
  Adult Head  6.251239  
  Adult Heart  6.302431  
  Adult Hind Gut  6.805995  
  Adult Male Ejaculatory Duct  6.644850  
  Adult Mid Gut  6.610362  
  Adult Ovary  6.221676  
  Adult Salivary Gland  6.967410  
  Adult Testes  6.157804  
  Adult Thoracoabdominal ganglion  6.236808  
  Adult Whole Fly  5.902282  
  Larvae Wandering Tubules  6.416567  
  Larval Feeding Carcass  8.482281  
  Larval Feeding Central Nevous System  6.233974  
  Larval Feeding Hind Gut  7.092435  
  Larval Feeding Malpighian Tubule  6.425574  
  Larval Feeding Mid Gut  6.573027  
  Larval Feeding Salivary Gland  6.822874  
  Whole Larvae Feeding  7.493406  
 
  
   FlyBase ID    symbol    start    end    strand    length   
   FBgn0035545   CG12607   4446575   4448064  +  1490  
   FBgn0035546   CG11345  4457320   4458191   -  872  
   FBgn0035547   CG15022  4459192   4460313   -  1122  
 
 
    Segment 109 
 
   Location   
  Gene key  FBgn0052241-FBgn0052249  
  Heatmap region span   3L:4422246..4500537   
  Segment span   3L:4466630..4470001   
  Length (genes)  2  
  Length (bp)  3372  
   Model Scoring   
  BIC  220.764885  
  logL  -104.870316  
  logL ratio  33.541111  
   Expression   
  Mean expression  6.522491  
  Median expression  5.993754  
  Tissue std. dev.  2.334407  
 
  No GO Slim enrichment  
  
   tissue    mean expression   
  5th Passage Drosophila S2 Cells  5.758052  
  Adult Accessory gland  5.718374  
  Adult Brain  5.465504  
  Adult Carcass  5.887752  
  Adult Crop  5.681061  
  Adult Eye  6.489022  
  Adult Fatbody  5.846082  
  Adult Female Spermatheca Mated  5.574024  
  Adult Female Spermatheca Virgin  5.639157  
  Adult Head  5.437520  
  Adult Heart  5.500644  
  Adult Hind Gut  5.674846  
  Adult Male Ejaculatory Duct  5.947199  
  Adult Mid Gut  6.022826  
  Adult Ovary  5.594027  
  Adult Salivary Gland  5.962026  
  Adult Testes  5.220859  
  Adult Thoracoabdominal ganglion  5.596573  
  Adult Whole Fly  5.254099  
  Larvae Wandering Tubules  5.686781  
  Larval Feeding Carcass  14.262362  
  Larval Feeding Central Nevous System  5.516051  
  Larval Feeding Hind Gut  11.008346  
  Larval Feeding Malpighian Tubule  5.814679  
  Larval Feeding Mid Gut  6.056167  
  Larval Feeding Salivary Gland  5.910431  
  Whole Larvae Feeding  13.582788  
 
  
   FlyBase ID    symbol    start    end    strand    length   
   FBgn0052241   CG32241  4465226   4466630   -  1405  
   FBgn0052249   CG32249   4470001   4471032  +  1032  
 
    Segment 110 
 
   Location   
  Gene key  FBgn0052248-FBgn0035551  
  Heatmap region span   3L:4436721..4532145   
  Segment span   3L:4471758..4480282   
  Length (genes)  3  
  Length (bp)  8525  
   Model Scoring   
  BIC  304.779702  
  logL  -146.877724  
  logL ratio  28.971720  
   Expression   
  Mean expression  5.521196  
  Median expression  5.513195  
  Tissue std. dev.  1.105315  
 
  No GO Slim enrichment  
  
   tissue    mean expression   
  5th Passage Drosophila S2 Cells  5.315584  
  Adult Accessory gland  5.289607  
  Adult Brain  4.884988  
  Adult Carcass  5.358427  
  Adult Crop  5.314239  
  Adult Eye  4.958716  
  Adult Fatbody  5.255530  
  Adult Female Spermatheca Mated  5.276812  
  Adult Female Spermatheca Virgin  5.160084  
  Adult Head  4.956986  
  Adult Heart  4.851211  
  Adult Hind Gut  5.240195  
  Adult Male Ejaculatory Duct  5.527728  
  Adult Mid Gut  5.387615  
  Adult Ovary  5.269342  
  Adult Salivary Gland  5.366643  
  Adult Testes  4.970156  
  Adult Thoracoabdominal ganglion  5.045605  
  Adult Whole Fly  4.821248  
  Larvae Wandering Tubules  5.299830  
  Larval Feeding Carcass  9.385170  
  Larval Feeding Central Nevous System  5.373443  
  Larval Feeding Hind Gut  5.423911  
  Larval Feeding Malpighian Tubule  5.259991  
  Larval Feeding Mid Gut  5.488512  
  Larval Feeding Salivary Gland  5.234659  
  Whole Larvae Feeding  9.356068  
 
  
   FlyBase ID    symbol    start    end    strand    length   
   FBgn0052248   CG32248   4471758   4472384  +  627  
   FBgn0035550   CG11349   4473068   4475095  +  2028  
   FBgn0035551   CG7465   4480282   4481487  +  1206  
 
 
    Segment 111 
 
   Location   
  Gene key  FBgn0035554-FBgn0035555  
  Heatmap region span   3L:4466630..4621265   
  Segment span   3L:4497658..4500537   
  Length (genes)  2  
  Length (bp)  2880  
   Model Scoring   
  BIC  169.847315  
  logL  -79.411531  
  logL ratio  41.139371  
   Expression   
  Mean expression  4.432623  
  Median expression  4.390252  
  Tissue std. dev.  0.157624  
 
  No GO Slim enrichment  
  
   tissue    mean expression   
  5th Passage Drosophila S2 Cells  4.431068  
  Adult Accessory gland  4.521277  
  Adult Brain  4.223936  
  Adult Carcass  4.451621  
  Adult Crop  4.583467  
  Adult Eye  4.385854  
  Adult Fatbody  4.438681  
  Adult Female Spermatheca Mated  4.537287  
  Adult Female Spermatheca Virgin  4.556199  
  Adult Head  4.244613  
  Adult Heart  4.399370  
  Adult Hind Gut  4.362145  
  Adult Male Ejaculatory Duct  4.581021  
  Adult Mid Gut  4.452656  
  Adult Ovary  4.383992  
  Adult Salivary Gland  4.751006  
  Adult Testes  4.188678  
  Adult Thoracoabdominal ganglion  4.287076  
  Adult Whole Fly  4.110759  
  Larvae Wandering Tubules  4.461391  
  Larval Feeding Carcass  4.727529  
  Larval Feeding Central Nevous System  4.167093  
  Larval Feeding Hind Gut  4.317603  
  Larval Feeding Malpighian Tubule  4.585806  
  Larval Feeding Mid Gut  4.509582  
  Larval Feeding Salivary Gland  4.548735  
  Whole Larvae Feeding  4.472373  
 
  
   FlyBase ID    symbol    start    end    strand    length   
   FBgn0035554   CG13721  4497208   4497658   -  451  
   FBgn0035555   CG13720  4498975   4500537   -  1563  
 
    Segment 112 
 
   Location   
  Gene key  FBgn0052243-FBgn0035558  
  Heatmap region span   3L:4488013..4690070   
  Segment span   3L:4541238..4542051   
  Length (genes)  2  
  Length (bp)  814  
   Model Scoring   
  BIC  228.400418  
  logL  -108.688083  
  logL ratio  43.729419  
   Expression   
  Mean expression  9.177709  
  Median expression  9.100519  
  Tissue std. dev.  1.001506  
 
  No GO Slim enrichment  
  
   tissue    mean expression   
  5th Passage Drosophila S2 Cells  11.845356  
  Adult Accessory gland  7.287740  
  Adult Brain  10.222225  
  Adult Carcass  7.674106  
  Adult Crop  8.156629  
  Adult Eye  8.796257  
  Adult Fatbody  9.055220  
  Adult Female Spermatheca Mated  9.349678  
  Adult Female Spermatheca Virgin  9.082597  
  Adult Head  8.890429  
  Adult Heart  9.209353  
  Adult Hind Gut  8.692235  
  Adult Male Ejaculatory Duct  8.503614  
  Adult Mid Gut  8.451028  
  Adult Ovary  10.485889  
  Adult Salivary Gland  7.648655  
  Adult Testes  8.594796  
  Adult Thoracoabdominal ganglion  9.638075  
  Adult Whole Fly  9.217429  
  Larvae Wandering Tubules  10.727834  
  Larval Feeding Carcass  8.858363  
  Larval Feeding Central Nevous System  10.715684  
  Larval Feeding Hind Gut  9.778097  
  Larval Feeding Malpighian Tubule  9.911536  
  Larval Feeding Mid Gut  9.150078  
  Larval Feeding Salivary Gland  8.920037  
  Whole Larvae Feeding  8.935191  
 
  
   FlyBase ID    symbol    start    end    strand    length   
   FBgn0052243   CG32243  4539302   4541238   -  1937  
   FBgn0035558   CG11357   4542051   4553457  +  11407  
 
    Segment 113 
 
   Location   
  Gene key  FBgn0035563-FBgn0035568  
  Heatmap region span   3L:4541238..4861304   
  Segment span   3L:4634789..4690070   
  Length (genes)  6  
  Length (bp)  55282  
   Model Scoring   
  BIC  494.937064  
  logL  -241.956405  
  logL ratio  130.570124  
   Expression   
  Mean expression  4.728528  
  Median expression  4.452947  
  Tissue std. dev.  0.857564  
 
  
   GO ID    description    ratio    P-value   
   GO:0022857   transmembrane transporter activity  2/6  0.000752  
 
  
   tissue    mean expression   
  5th Passage Drosophila S2 Cells  4.426575  
  Adult Accessory gland  4.535130  
  Adult Brain  4.324775  
  Adult Carcass  4.668962  
  Adult Crop  4.391389  
  Adult Eye  4.281483  
  Adult Fatbody  4.532112  
  Adult Female Spermatheca Mated  4.745727  
  Adult Female Spermatheca Virgin  4.698077  
  Adult Head  4.230258  
  Adult Heart  4.317639  
  Adult Hind Gut  4.987989  
  Adult Male Ejaculatory Duct  4.478630  
  Adult Mid Gut  4.530495  
  Adult Ovary  4.285132  
  Adult Salivary Gland  4.842609  
  Adult Testes  8.825339  
  Adult Thoracoabdominal ganglion  4.506305  
  Adult Whole Fly  5.787017  
  Larvae Wandering Tubules  4.573774  
  Larval Feeding Carcass  4.476812  
  Larval Feeding Central Nevous System  4.305506  
  Larval Feeding Hind Gut  4.478904  
  Larval Feeding Malpighian Tubule  4.603329  
  Larval Feeding Mid Gut  4.515316  
  Larval Feeding Salivary Gland  4.488093  
  Whole Larvae Feeding  4.832893  
 
  
   FlyBase ID    symbol    start    end    strand    length   
   FBgn0035563   CG13716  4634436   4634789   -  354  
   FBgn0052238   CG32238   4648276   4650306  +  2031  
   FBgn0046793   CG32236  4665927   4667389   -  1463  
   FBgn0085295   CG34266   4677851   4678117  +  267  
   FBgn0035567   CG7514  4687587   4688774   -  1188  
   FBgn0035568   CG18418  4688929   4690070   -  1142  
 
 
    Segment 114 
 
   Location   
  Gene key  FBgn0035569-FBgn0035577  
  Heatmap region span   3L:4626196..4898900   
  Segment span   3L:4694823..4833074   
  Length (genes)  8  
  Length (bp)  138252  
   Model Scoring   
  BIC  686.714756  
  logL  -337.845252  
  logL ratio  139.881301  
   Expression   
  Mean expression  4.746441  
  Median expression  4.527149  
  Tissue std. dev.  0.414576  
 
  No GO Slim enrichment  
  
   tissue    mean expression   
  5th Passage Drosophila S2 Cells  4.611104  
  Adult Accessory gland  4.730229  
  Adult Brain  4.929677  
  Adult Carcass  4.551412  
  Adult Crop  4.460097  
  Adult Eye  4.820658  
  Adult Fatbody  4.542783  
  Adult Female Spermatheca Mated  4.610580  
  Adult Female Spermatheca Virgin  4.592309  
  Adult Head  4.795438  
  Adult Heart  4.385603  
  Adult Hind Gut  4.855106  
  Adult Male Ejaculatory Duct  4.699613  
  Adult Mid Gut  5.025003  
  Adult Ovary  4.510553  
  Adult Salivary Gland  4.832419  
  Adult Testes  6.659781  
  Adult Thoracoabdominal ganglion  4.896843  
  Adult Whole Fly  4.842507  
  Larvae Wandering Tubules  4.848752  
  Larval Feeding Carcass  4.592621  
  Larval Feeding Central Nevous System  4.354579  
  Larval Feeding Hind Gut  4.390370  
  Larval Feeding Malpighian Tubule  4.657645  
  Larval Feeding Mid Gut  4.566167  
  Larval Feeding Salivary Gland  4.555843  
  Whole Larvae Feeding  4.836199  
 
  
   FlyBase ID    symbol    start    end    strand    length   
   FBgn0035569   CG15876  4694205   4694823   -  619  
   FBgn0042199   CG13713   4697679   4697978  +  300  
   FBgn0035570   CG13712  4709503   4709856   -  354  
   FBgn0035571   CG12493  4752393   4753718   -  1326  
   FBgn0035572   CG13711   4758029   4758412  +  384  
   FBgn0035575   CG7509  4803077   4805274   -  2198  
   FBgn0042131   CG18808  4806138   4807406   -  1269  
   FBgn0035577   CG13708  4829092   4833074   -  3983  
 
 
    Segment 115 
 
   Location   
  Gene key  FBgn0052237-FBgn0035581  
  Heatmap region span   3L:4634789..4976172   
  Segment span   3L:4852455..4861304   
  Length (genes)  3  
  Length (bp)  8850  
   Model Scoring   
  BIC  230.237846  
  logL  -109.606797  
  logL ratio  91.001378  
   Expression   
  Mean expression  4.226293  
  Median expression  4.202940  
  Tissue std. dev.  0.164456  
 
  No GO Slim enrichment  
  
   tissue    mean expression   
  5th Passage Drosophila S2 Cells  4.277770  
  Adult Accessory gland  4.216526  
  Adult Brain  4.110306  
  Adult Carcass  4.121537  
  Adult Crop  4.136479  
  Adult Eye  4.147127  
  Adult Fatbody  4.097100  
  Adult Female Spermatheca Mated  4.102314  
  Adult Female Spermatheca Virgin  4.090790  
  Adult Head  4.065875  
  Adult Heart  4.320868  
  Adult Hind Gut  4.192315  
  Adult Male Ejaculatory Duct  4.245194  
  Adult Mid Gut  4.229642  
  Adult Ovary  4.282870  
  Adult Salivary Gland  4.229080  
  Adult Testes  4.347850  
  Adult Thoracoabdominal ganglion  4.226748  
  Adult Whole Fly  3.976145  
  Larvae Wandering Tubules  4.778669  
  Larval Feeding Carcass  4.233151  
  Larval Feeding Central Nevous System  4.198647  
  Larval Feeding Hind Gut  4.179273  
  Larval Feeding Malpighian Tubule  4.659440  
  Larval Feeding Mid Gut  4.241525  
  Larval Feeding Salivary Gland  4.311581  
  Whole Larvae Feeding  4.091082  
 
  
   FlyBase ID    symbol    start    end    strand    length   
   FBgn0052237   CG32237  4849435   4852455   -  3021  
   FBgn0047330   CG32235  4859809   4860470   -  662  
   FBgn0035581   CG17150   4861304   4895458  +  34155  
 
 
    Segment 116 
 
   Location   
  Gene key  FBgn0035584-FBgn0035594  
  Heatmap region span   3L:4896858..5132495   
  Segment span   3L:4980769..5122774   
  Length (genes)  5  
  Length (bp)  142006  
   Model Scoring   
  BIC  416.104315  
  logL  -202.540031  
  logL ratio  126.569337  
   Expression   
  Mean expression  4.338101  
  Median expression  4.178974  
  Tissue std. dev.  0.659926  
 
  No GO Slim enrichment  
  
   tissue    mean expression   
  5th Passage Drosophila S2 Cells  4.198499  
  Adult Accessory gland  4.189946  
  Adult Brain  4.378307  
  Adult Carcass  4.276224  
  Adult Crop  4.338054  
  Adult Eye  4.010903  
  Adult Fatbody  4.203297  
  Adult Female Spermatheca Mated  4.150318  
  Adult Female Spermatheca Virgin  4.189912  
  Adult Head  3.973115  
  Adult Heart  4.140496  
  Adult Hind Gut  4.032139  
  Adult Male Ejaculatory Duct  4.143810  
  Adult Mid Gut  4.188103  
  Adult Ovary  4.057324  
  Adult Salivary Gland  4.426900  
  Adult Testes  7.573313  
  Adult Thoracoabdominal ganglion  4.222019  
  Adult Whole Fly  4.946649  
  Larvae Wandering Tubules  4.171081  
  Larval Feeding Carcass  4.168748  
  Larval Feeding Central Nevous System  4.090564  
  Larval Feeding Hind Gut  4.065390  
  Larval Feeding Malpighian Tubule  4.194923  
  Larval Feeding Mid Gut  4.133573  
  Larval Feeding Salivary Gland  4.257191  
  Whole Larvae Feeding  4.407922  
 
  
   FlyBase ID    symbol    start    end    strand    length   
   FBgn0035584   CG17030  4979981   4980769   -  789  
   FBgn0052232   CG32232  4993950   4996877   -  2928  
   FBgn0035585   CG12027  5011508   5012114   -  607  
   FBgn0054047   CR34047  5098968   5099735   -  768  
   FBgn0035594   CG4597   5122774   5123340  +  567  
 
 
    Segment 117 
 
   Location   
  Gene key  FBgn0035593-FBgn0035592  
  Heatmap region span   3L:4976172..5139248   
  Segment span   3L:5127321..5129392   
  Length (genes)  2  
  Length (bp)  2072  
   Model Scoring   
  BIC  230.204069  
  logL  -109.589908  
  logL ratio  66.166259  
   Expression   
  Mean expression  10.350301  
  Median expression  10.244231  
  Tissue std. dev.  0.264063  
 
  No GO Slim enrichment  
  
   tissue    mean expression   
  5th Passage Drosophila S2 Cells  10.372407  
  Adult Accessory gland  10.454111  
  Adult Brain  10.035856  
  Adult Carcass  10.236670  
  Adult Crop  10.120476  
  Adult Eye  11.053906  
  Adult Fatbody  10.204593  
  Adult Female Spermatheca Mated  10.387574  
  Adult Female Spermatheca Virgin  10.438901  
  Adult Head  10.291652  
  Adult Heart  10.310488  
  Adult Hind Gut  10.195876  
  Adult Male Ejaculatory Duct  10.654291  
  Adult Mid Gut  10.254689  
  Adult Ovary  10.624762  
  Adult Salivary Gland  10.265880  
  Adult Testes  10.493588  
  Adult Thoracoabdominal ganglion  10.081527  
  Adult Whole Fly  10.294908  
  Larvae Wandering Tubules  10.832220  
  Larval Feeding Carcass  10.291703  
  Larval Feeding Central Nevous System  10.154153  
  Larval Feeding Hind Gut  10.121986  
  Larval Feeding Malpighian Tubule  10.697992  
  Larval Feeding Mid Gut  10.037305  
  Larval Feeding Salivary Gland  10.672921  
  Whole Larvae Feeding  9.877699  
 
  
   FlyBase ID    symbol    start    end    strand    length   
   FBgn0035593   CG4603   5127321   5128770  +  1450  
   FBgn0035592   CG10674  5128848   5129392   -  545  
 
    Segment 118 
 
   Location   
  Gene key  FBgn0035591-FBgn0035590  
  Heatmap region span   3L:4980769..5145447   
  Segment span   3L:5129484..5132495   
  Length (genes)  2  
  Length (bp)  3012  
   Model Scoring   
  BIC  208.823583  
  logL  -98.899665  
  logL ratio  44.497685  
   Expression   
  Mean expression  7.719529  
  Median expression  7.742916  
  Tissue std. dev.  0.318901  
 
  No GO Slim enrichment  
  
   tissue    mean expression   
  5th Passage Drosophila S2 Cells  7.994172  
  Adult Accessory gland  8.096908  
  Adult Brain  7.628268  
  Adult Carcass  6.922409  
  Adult Crop  7.766738  
  Adult Eye  7.375845  
  Adult Fatbody  7.534021  
  Adult Female Spermatheca Mated  8.002851  
  Adult Female Spermatheca Virgin  8.004395  
  Adult Head  7.334958  
  Adult Heart  7.615075  
  Adult Hind Gut  7.506120  
  Adult Male Ejaculatory Duct  7.807483  
  Adult Mid Gut  7.538868  
  Adult Ovary  8.239581  
  Adult Salivary Gland  7.784352  
  Adult Testes  7.534835  
  Adult Thoracoabdominal ganglion  7.977451  
  Adult Whole Fly  7.247437  
  Larvae Wandering Tubules  7.663632  
  Larval Feeding Carcass  7.689332  
  Larval Feeding Central Nevous System  8.257116  
  Larval Feeding Hind Gut  7.870908  
  Larval Feeding Malpighian Tubule  7.906023  
  Larval Feeding Mid Gut  7.519841  
  Larval Feeding Salivary Gland  8.204469  
  Whole Larvae Feeding  7.404200  
 
  
   FlyBase ID    symbol    start    end    strand    length   
   FBgn0035591   CG4611   5129484   5131779  +  2296  
   FBgn0035590   CG10673  5131752   5132495   -  744  
 
    Segment 119 
 
   Location   
  Gene key  FBgn0035589-FBgn0035588  
  Heatmap region span   3L:5124650..5145669   
  Segment span   3L:5132773..5134754   
  Length (genes)  2  
  Length (bp)  1982  
   Model Scoring   
  BIC  249.308848  
  logL  -119.142298  
  logL ratio  31.776334  
   Expression   
  Mean expression  9.616729  
  Median expression  9.337972  
  Tissue std. dev.  0.730944  
 
  No GO Slim enrichment  
  
   tissue    mean expression   
  5th Passage Drosophila S2 Cells  9.363470  
  Adult Accessory gland  9.031383  
  Adult Brain  8.987414  
  Adult Carcass  9.836403  
  Adult Crop  10.881090  
  Adult Eye  9.526383  
  Adult Fatbody  10.068044  
  Adult Female Spermatheca Mated  10.197161  
  Adult Female Spermatheca Virgin  10.132286  
  Adult Head  9.558891  
  Adult Heart  10.197254  
  Adult Hind Gut  10.444164  
  Adult Male Ejaculatory Duct  10.600365  
  Adult Mid Gut  10.124116  
  Adult Ovary  8.971491  
  Adult Salivary Gland  9.608913  
  Adult Testes  7.590507  
  Adult Thoracoabdominal ganglion  9.214511  
  Adult Whole Fly  9.111522  
  Larvae Wandering Tubules  9.796782  
  Larval Feeding Carcass  8.827164  
  Larval Feeding Central Nevous System  8.422865  
  Larval Feeding Hind Gut  9.186336  
  Larval Feeding Malpighian Tubule  10.687046  
  Larval Feeding Mid Gut  10.303559  
  Larval Feeding Salivary Gland  9.322214  
  Whole Larvae Feeding  9.660339  
 
  
   FlyBase ID    symbol    start    end    strand    length   
   FBgn0035589   CHMP2B   5132773   5133573  +  801  
   FBgn0035588   CG10672  5133540   5134754   -  1215  
 
    Segment 120 
 
   Location   
  Gene key  FBgn0016031-FBgn0004380  
  Heatmap region span   3L:5145447..5472950   
  Segment span   3L:5349023..5352354   
  Length (genes)  2  
  Length (bp)  3332  
   Model Scoring   
  BIC  261.998413  
  logL  -125.487080  
  logL ratio  7.292445  
   Expression   
  Mean expression  9.045908  
  Median expression  9.070297  
  Tissue std. dev.  0.510841  
 
  No GO Slim enrichment  
  
   tissue    mean expression   
  5th Passage Drosophila S2 Cells  8.960575  
  Adult Accessory gland  8.107517  
  Adult Brain  9.678358  
  Adult Carcass  9.173077  
  Adult Crop  9.792900  
  Adult Eye  9.644167  
  Adult Fatbody  8.980887  
  Adult Female Spermatheca Mated  9.087166  
  Adult Female Spermatheca Virgin  9.072041  
  Adult Head  9.471366  
  Adult Heart  9.285808  
  Adult Hind Gut  8.911135  
  Adult Male Ejaculatory Duct  9.097371  
  Adult Mid Gut  8.569973  
  Adult Ovary  9.419527  
  Adult Salivary Gland  9.069083  
  Adult Testes  7.472428  
  Adult Thoracoabdominal ganglion  9.390399  
  Adult Whole Fly  8.570100  
  Larvae Wandering Tubules  9.092702  
  Larval Feeding Carcass  9.247466  
  Larval Feeding Central Nevous System  8.701354  
  Larval Feeding Hind Gut  8.523433  
  Larval Feeding Malpighian Tubule  9.578150  
  Larval Feeding Mid Gut  9.199842  
  Larval Feeding Salivary Gland  9.632430  
  Whole Larvae Feeding  8.510267  
 
  
   FlyBase ID    symbol    start    end    strand    length   
   FBgn0016031   lama  5336715   5349023   -  12309  
   FBgn0004380   Klp64D  5350321   5352354   -  2034  
 
    Segment 121 
 
   Location   
  Gene key  FBgn0035600-FBgn0035601  
  Heatmap region span   3L:5145669..5492058   
  Segment span   3L:5353290..5359325   
  Length (genes)  2  
  Length (bp)  6036  
   Model Scoring   
  BIC  301.361589  
  logL  -145.168668  
  logL ratio  109.660225  
   Expression   
  Mean expression  12.191761  
  Median expression  12.134622  
  Tissue std. dev.  0.598048  
 
  No GO Slim enrichment  
  
   tissue    mean expression   
  5th Passage Drosophila S2 Cells  11.541090  
  Adult Accessory gland  11.617078  
  Adult Brain  12.119710  
  Adult Carcass  12.466541  
  Adult Crop  12.348634  
  Adult Eye  13.060204  
  Adult Fatbody  12.287627  
  Adult Female Spermatheca Mated  12.379748  
  Adult Female Spermatheca Virgin  12.385662  
  Adult Head  12.514725  
  Adult Heart  12.613733  
  Adult Hind Gut  12.662412  
  Adult Male Ejaculatory Duct  12.098593  
  Adult Mid Gut  11.999879  
  Adult Ovary  12.430357  
  Adult Salivary Gland  12.223686  
  Adult Testes  9.663726  
  Adult Thoracoabdominal ganglion  12.235636  
  Adult Whole Fly  12.657100  
  Larvae Wandering Tubules  11.869153  
  Larval Feeding Carcass  12.508081  
  Larval Feeding Central Nevous System  12.272240  
  Larval Feeding Hind Gut  12.601057  
  Larval Feeding Malpighian Tubule  12.660957  
  Larval Feeding Mid Gut  11.997505  
  Larval Feeding Salivary Gland  11.880771  
  Whole Larvae Feeding  12.081630  
 
  
   FlyBase ID    symbol    start    end    strand    length   
   FBgn0035600   CG4769   5353290   5355898  +  2609  
   FBgn0035601   Uev1A  5356252   5359325   -  3074  
 
    Segment 122 
 
   Location   
  Gene key  FBgn0035604-FBgn0085420  
  Heatmap region span   3L:5271044..5522441   
  Segment span   3L:5375921..5424732   
  Length (genes)  2  
  Length (bp)  48812  
   Model Scoring   
  BIC  182.414729  
  logL  -85.695238  
  logL ratio  31.726496  
   Expression   
  Mean expression  5.413598  
  Median expression  5.350537  
  Tissue std. dev.  0.248546  
 
  No GO Slim enrichment  
  
   tissue    mean expression   
  5th Passage Drosophila S2 Cells  5.361530  
  Adult Accessory gland  5.482219  
  Adult Brain  5.438181  
  Adult Carcass  5.509606  
  Adult Crop  5.350900  
  Adult Eye  5.093419  
  Adult Fatbody  5.687793  
  Adult Female Spermatheca Mated  5.792169  
  Adult Female Spermatheca Virgin  5.853455  
  Adult Head  5.359033  
  Adult Heart  4.970071  
  Adult Hind Gut  5.233821  
  Adult Male Ejaculatory Duct  5.479671  
  Adult Mid Gut  5.466082  
  Adult Ovary  5.283139  
  Adult Salivary Gland  5.770271  
  Adult Testes  5.752093  
  Adult Thoracoabdominal ganglion  5.743574  
  Adult Whole Fly  4.807676  
  Larvae Wandering Tubules  5.405435  
  Larval Feeding Carcass  5.388177  
  Larval Feeding Central Nevous System  5.306310  
  Larval Feeding Hind Gut  5.181162  
  Larval Feeding Malpighian Tubule  5.386852  
  Larval Feeding Mid Gut  5.377379  
  Larval Feeding Salivary Gland  5.543819  
  Whole Larvae Feeding  5.143311  
 
  
   FlyBase ID    symbol    start    end    strand    length   
   FBgn0035604   Ir64a  5371886   5375921   -  4036  
   FBgn0085420   CG34391   5424732   5433143  +  8412  
 
    Segment 123 
 
   Location   
  Gene key  FBgn0010894-FBgn0040239  
  Heatmap region span   3L:5503920..5597075   
  Segment span   3L:5554697..5558334   
  Length (genes)  4  
  Length (bp)  3638  
   Model Scoring   
  BIC  404.290968  
  logL  -196.633358  
  logL ratio  119.691443  
   Expression   
  Mean expression  9.171180  
  Median expression  9.171935  
  Tissue std. dev.  0.574568  
 
  No GO Slim enrichment  
  
   tissue    mean expression   
  5th Passage Drosophila S2 Cells  9.943558  
  Adult Accessory gland  9.048149  
  Adult Brain  8.712435  
  Adult Carcass  8.916061  
  Adult Crop  9.960688  
  Adult Eye  8.690581  
  Adult Fatbody  8.583075  
  Adult Female Spermatheca Mated  8.207247  
  Adult Female Spermatheca Virgin  7.912146  
  Adult Head  8.623515  
  Adult Heart  9.033162  
  Adult Hind Gut  9.817483  
  Adult Male Ejaculatory Duct  9.433834  
  Adult Mid Gut  9.264388  
  Adult Ovary  9.523341  
  Adult Salivary Gland  9.767146  
  Adult Testes  8.625529  
  Adult Thoracoabdominal ganglion  8.556971  
  Adult Whole Fly  8.802942  
  Larvae Wandering Tubules  9.886821  
  Larval Feeding Carcass  9.129404  
  Larval Feeding Central Nevous System  8.822426  
  Larval Feeding Hind Gut  9.813420  
  Larval Feeding Malpighian Tubule  9.852625  
  Larval Feeding Mid Gut  9.339770  
  Larval Feeding Salivary Gland  9.954987  
  Whole Larvae Feeding  9.400168  
 
  
   FlyBase ID    symbol    start    end    strand    length   
   FBgn0010894   sinu  5552570   5554697   -  2128  
   FBgn0029121   Sras   5556780   5558203  +  1424  
   FBgn0029118   Sucb  5554799   5556784   -  1986  
   FBgn0040239   bc10   5558334   5559867  +  1534  
 
 
    Segment 124 
 
   Location   
  Gene key  FBgn0035619-FBgn0035620  
  Heatmap region span   3L:5528859..5601418   
  Segment span   3L:5588372..5588646   
  Length (genes)  2  
  Length (bp)  275  
   Model Scoring   
  BIC  193.506493  
  logL  -91.241120  
  logL ratio  37.282956  
   Expression   
  Mean expression  5.384040  
  Median expression  4.579321  
  Tissue std. dev.  2.033699  
 
  No GO Slim enrichment  
  
   tissue    mean expression   
  5th Passage Drosophila S2 Cells  5.807195  
  Adult Accessory gland  4.510903  
  Adult Brain  4.063320  
  Adult Carcass  4.840471  
  Adult Crop  4.416415  
  Adult Eye  4.250342  
  Adult Fatbody  4.558857  
  Adult Female Spermatheca Mated  4.615704  
  Adult Female Spermatheca Virgin  4.577322  
  Adult Head  4.370483  
  Adult Heart  4.315710  
  Adult Hind Gut  4.525396  
  Adult Male Ejaculatory Duct  4.576685  
  Adult Mid Gut  12.342099  
  Adult Ovary  4.314115  
  Adult Salivary Gland  4.869794  
  Adult Testes  4.285139  
  Adult Thoracoabdominal ganglion  4.236385  
  Adult Whole Fly  8.736789  
  Larvae Wandering Tubules  4.587934  
  Larval Feeding Carcass  4.532793  
  Larval Feeding Central Nevous System  4.140424  
  Larval Feeding Hind Gut  6.318891  
  Larval Feeding Malpighian Tubule  4.471935  
  Larval Feeding Mid Gut  10.274829  
  Larval Feeding Salivary Gland  4.578350  
  Whole Larvae Feeding  8.250810  
 
  
   FlyBase ID    symbol    start    end    strand    length   
   FBgn0035619   CG10592  5585841   5588372   -  2532  
   FBgn0035620   CG5150   5588646   5590452  +  1807  
 
    Segment 125 
 
   Location   
  Gene key  FBgn0035627-FBgn0085447  
  Heatmap region span   3L:5592789..5804073   
  Segment span   3L:5655341..5660920   
  Length (genes)  2  
  Length (bp)  5580  
   Model Scoring   
  BIC  222.672855  
  logL  -105.824301  
  logL ratio  -7.680523  
   Expression   
  Mean expression  5.396447  
  Median expression  4.881009  
  Tissue std. dev.  0.772045  
 
  No GO Slim enrichment  
  
   tissue    mean expression   
  5th Passage Drosophila S2 Cells  5.918325  
  Adult Accessory gland  5.115941  
  Adult Brain  6.523857  
  Adult Carcass  5.041963  
  Adult Crop  4.787546  
  Adult Eye  6.546297  
  Adult Fatbody  4.946685  
  Adult Female Spermatheca Mated  4.867749  
  Adult Female Spermatheca Virgin  4.850537  
  Adult Head  6.003891  
  Adult Heart  5.041493  
  Adult Hind Gut  4.960011  
  Adult Male Ejaculatory Duct  5.172194  
  Adult Mid Gut  4.752958  
  Adult Ovary  6.797201  
  Adult Salivary Gland  4.872941  
  Adult Testes  5.447130  
  Adult Thoracoabdominal ganglion  6.547409  
  Adult Whole Fly  6.404570  
  Larvae Wandering Tubules  4.790005  
  Larval Feeding Carcass  4.944979  
  Larval Feeding Central Nevous System  7.314768  
  Larval Feeding Hind Gut  5.051645  
  Larval Feeding Malpighian Tubule  4.633657  
  Larval Feeding Mid Gut  4.855670  
  Larval Feeding Salivary Gland  4.555245  
  Whole Larvae Feeding  4.959395  
 
  
   FlyBase ID    symbol    start    end    strand    length   
   FBgn0035627   Sse  5652794   5655341   -  2548  
   FBgn0085447   sif   5660920   5741139  +  80220  
 
    Segment 126 
 
   Location   
  Gene key  FBgn0016756-FBgn0035631  
  Heatmap region span   3L:5597075..5806183   
  Segment span   3L:5746086..5758947   
  Length (genes)  3  
  Length (bp)  12862  
   Model Scoring   
  BIC  325.633082  
  logL  -157.304415  
  logL ratio  123.561631  
   Expression   
  Mean expression  10.404822  
  Median expression  10.440573  
  Tissue std. dev.  0.427205  
 
  No GO Slim enrichment  
  
   tissue    mean expression   
  5th Passage Drosophila S2 Cells  10.817880  
  Adult Accessory gland  10.990581  
  Adult Brain  9.592216  
  Adult Carcass  10.298533  
  Adult Crop  10.896534  
  Adult Eye  9.795547  
  Adult Fatbody  10.265267  
  Adult Female Spermatheca Mated  9.981230  
  Adult Female Spermatheca Virgin  9.854976  
  Adult Head  9.881507  
  Adult Heart  10.439818  
  Adult Hind Gut  10.430956  
  Adult Male Ejaculatory Duct  10.531473  
  Adult Mid Gut  10.154125  
  Adult Ovary  10.850675  
  Adult Salivary Gland  10.469282  
  Adult Testes  10.774772  
  Adult Thoracoabdominal ganglion  9.544134  
  Adult Whole Fly  10.561049  
  Larvae Wandering Tubules  10.534147  
  Larval Feeding Carcass  11.236501  
  Larval Feeding Central Nevous System  10.570375  
  Larval Feeding Hind Gut  10.734588  
  Larval Feeding Malpighian Tubule  10.284098  
  Larval Feeding Mid Gut  10.213925  
  Larval Feeding Salivary Gland  10.918057  
  Whole Larvae Feeding  10.307944  
 
  
   FlyBase ID    symbol    start    end    strand    length   
   FBgn0016756   Ubp64E   5746086   5754602  +  8517  
   FBgn0035630   CG10576  5755070   5758806   -  3737  
   FBgn0035631   Txl   5758947   5760184  +  1238  
 
 
    Segment 127 
 
   Location   
  Gene key  FBgn0040298-FBgn0086694  
  Heatmap region span   3L:5597888..5890738   
  Segment span   3L:5772687..5787291   
  Length (genes)  4  
  Length (bp)  14605  
   Model Scoring   
  BIC  546.266108  
  logL  -267.620928  
  logL ratio  -73.831776  
   Expression   
  Mean expression  7.008119  
  Median expression  7.375313  
  Tissue std. dev.  0.439896  
 
  No GO Slim enrichment  
  
   tissue    mean expression   
  5th Passage Drosophila S2 Cells  7.709273  
  Adult Accessory gland  6.791014  
  Adult Brain  7.131534  
  Adult Carcass  6.598947  
  Adult Crop  8.271049  
  Adult Eye  6.911932  
  Adult Fatbody  6.624380  
  Adult Female Spermatheca Mated  6.863641  
  Adult Female Spermatheca Virgin  6.932824  
  Adult Head  7.255273  
  Adult Heart  6.870470  
  Adult Hind Gut  6.862613  
  Adult Male Ejaculatory Duct  6.747565  
  Adult Mid Gut  7.031268  
  Adult Ovary  7.890782  
  Adult Salivary Gland  6.893188  
  Adult Testes  6.234342  
  Adult Thoracoabdominal ganglion  7.337502  
  Adult Whole Fly  6.850735  
  Larvae Wandering Tubules  7.329347  
  Larval Feeding Carcass  6.933791  
  Larval Feeding Central Nevous System  7.232483  
  Larval Feeding Hind Gut  6.829201  
  Larval Feeding Malpighian Tubule  7.372925  
  Larval Feeding Mid Gut  6.345051  
  Larval Feeding Salivary Gland  6.853601  
  Whole Larvae Feeding  6.514483  
 
  
   FlyBase ID    symbol    start    end    strand    length   
   FBgn0040298   Myt1  5770572   5772687   -  2116  
   FBgn0044419   Pmi   5773145   5777785  +  4641  
   FBgn0035636   Cralbp  5776581   5779741   -  3161  
   FBgn0086694   Bre1  5783274   5787291   -  4018  
 
 
    Segment 128 
 
   Location   
  Gene key  FBgn0015806-FBgn0035639  
  Heatmap region span   3L:5655341..5900505   
  Segment span   3L:5804057..5804073   
  Length (genes)  2  
  Length (bp)  17  
   Model Scoring   
  BIC  246.677407  
  logL  -117.826577  
  logL ratio  34.000915  
   Expression   
  Mean expression  9.713277  
  Median expression  9.912738  
  Tissue std. dev.  0.720999  
 
  No GO Slim enrichment  
  
   tissue    mean expression   
  5th Passage Drosophila S2 Cells  10.409620  
  Adult Accessory gland  10.088266  
  Adult Brain  9.149405  
  Adult Carcass  9.658400  
  Adult Crop  10.234545  
  Adult Eye  9.325380  
  Adult Fatbody  10.795060  
  Adult Female Spermatheca Mated  10.538923  
  Adult Female Spermatheca Virgin  10.479580  
  Adult Head  9.141523  
  Adult Heart  10.616167  
  Adult Hind Gut  10.106540  
  Adult Male Ejaculatory Duct  9.981816  
  Adult Mid Gut  9.909065  
  Adult Ovary  9.357470  
  Adult Salivary Gland  9.530669  
  Adult Testes  7.063284  
  Adult Thoracoabdominal ganglion  8.952893  
  Adult Whole Fly  9.101852  
  Larvae Wandering Tubules  9.805398  
  Larval Feeding Carcass  9.473700  
  Larval Feeding Central Nevous System  9.388911  
  Larval Feeding Hind Gut  10.264930  
  Larval Feeding Malpighian Tubule  9.629266  
  Larval Feeding Mid Gut  9.774427  
  Larval Feeding Salivary Gland  10.055729  
  Whole Larvae Feeding  9.425669  
 
  
   FlyBase ID    symbol    start    end    strand    length   
   FBgn0015806   S6k  5792049   5804057   -  12009  
   FBgn0035639   CG5537   5804073   5805328  +  1256  
 
    Segment 129 
 
   Location   
  Gene key  FBgn0003984-FBgn0035645  
  Heatmap region span   3L:5772687..5917155   
  Segment span   3L:5838764..5890738   
  Length (genes)  5  
  Length (bp)  51975  
   Model Scoring   
  BIC  502.351043  
  logL  -245.663395  
  logL ratio  31.991745  
   Expression   
  Mean expression  5.033063  
  Median expression  4.761439  
  Tissue std. dev.  0.461447  
 
  No GO Slim enrichment  
  
   tissue    mean expression   
  5th Passage Drosophila S2 Cells  5.802511  
  Adult Accessory gland  4.679811  
  Adult Brain  4.470027  
  Adult Carcass  4.690372  
  Adult Crop  5.049195  
  Adult Eye  5.141607  
  Adult Fatbody  4.742472  
  Adult Female Spermatheca Mated  4.732491  
  Adult Female Spermatheca Virgin  4.641798  
  Adult Head  4.630329  
  Adult Heart  5.168444  
  Adult Hind Gut  4.693941  
  Adult Male Ejaculatory Duct  4.693064  
  Adult Mid Gut  4.648545  
  Adult Ovary  6.521323  
  Adult Salivary Gland  4.593995  
  Adult Testes  5.461841  
  Adult Thoracoabdominal ganglion  4.891004  
  Adult Whole Fly  5.553365  
  Larvae Wandering Tubules  5.152281  
  Larval Feeding Carcass  5.269014  
  Larval Feeding Central Nevous System  5.817268  
  Larval Feeding Hind Gut  5.052736  
  Larval Feeding Malpighian Tubule  4.999822  
  Larval Feeding Mid Gut  4.808993  
  Larval Feeding Salivary Gland  5.189243  
  Whole Larvae Feeding  4.797221  
 
  
   FlyBase ID    symbol    start    end    strand    length   
   FBgn0003984   vn  5806438   5838764   -  32327  
   FBgn0035641   CG5568   5864602   5866618  +  2017  
   FBgn0035643   CG13287   5875507   5876892  +  1386  
   FBgn0035644   Pole2  5888785   5890479   -  1695  
   FBgn0035645   CG5592   5890738   5892495  +  1758  
 
 
    Segment 130 
 
   Location   
  Gene key  FBgn0052409-FBgn0052412  
  Heatmap region span   3L:5796133..5925873   
  Segment span   3L:5892642..5895550   
  Length (genes)  2  
  Length (bp)  2909  
   Model Scoring   
  BIC  235.994055  
  logL  -112.484901  
  logL ratio  25.673239  
   Expression   
  Mean expression  8.364007  
  Median expression  8.092946  
  Tissue std. dev.  0.982183  
 
  No GO Slim enrichment  
  
   tissue    mean expression   
  5th Passage Drosophila S2 Cells  9.781121  
  Adult Accessory gland  9.313004  
  Adult Brain  7.057068  
  Adult Carcass  8.094416  
  Adult Crop  9.466566  
  Adult Eye  8.697465  
  Adult Fatbody  8.326368  
  Adult Female Spermatheca Mated  8.703696  
  Adult Female Spermatheca Virgin  8.343913  
  Adult Head  8.494961  
  Adult Heart  8.266202  
  Adult Hind Gut  8.496264  
  Adult Male Ejaculatory Duct  9.169394  
  Adult Mid Gut  7.332310  
  Adult Ovary  10.634811  
  Adult Salivary Gland  9.155615  
  Adult Testes  8.303543  
  Adult Thoracoabdominal ganglion  7.324163  
  Adult Whole Fly  9.508208  
  Larvae Wandering Tubules  6.384935  
  Larval Feeding Carcass  8.280190  
  Larval Feeding Central Nevous System  8.532956  
  Larval Feeding Hind Gut  8.700704  
  Larval Feeding Malpighian Tubule  6.330048  
  Larval Feeding Mid Gut  7.190277  
  Larval Feeding Salivary Gland  8.179127  
  Whole Larvae Feeding  7.760868  
 
  
   FlyBase ID    symbol    start    end    strand    length   
   FBgn0052409   CG32409   5892642   5894036  +  1395  
   FBgn0052412   QC  5893935   5895550   -  1616  
 
    Segment 131 
 
   Location   
  Gene key  FBgn0052413-FBgn0035648  
  Heatmap region span   3L:5804057..5943755   
  Segment span   3L:5897152..5900505   
  Length (genes)  3  
  Length (bp)  3354  
   Model Scoring   
  BIC  266.686394  
  logL  -127.831071  
  logL ratio  59.120423  
   Expression   
  Mean expression  4.636339  
  Median expression  4.632389  
  Tissue std. dev.  0.635428  
 
  No GO Slim enrichment  
  
   tissue    mean expression   
  5th Passage Drosophila S2 Cells  4.516068  
  Adult Accessory gland  7.766584  
  Adult Brain  4.534542  
  Adult Carcass  4.605656  
  Adult Crop  4.434860  
  Adult Eye  4.273205  
  Adult Fatbody  4.466508  
  Adult Female Spermatheca Mated  4.378200  
  Adult Female Spermatheca Virgin  4.439152  
  Adult Head  4.395045  
  Adult Heart  4.320209  
  Adult Hind Gut  4.560187  
  Adult Male Ejaculatory Duct  4.678106  
  Adult Mid Gut  4.727894  
  Adult Ovary  4.472809  
  Adult Salivary Gland  4.517538  
  Adult Testes  4.927157  
  Adult Thoracoabdominal ganglion  4.800930  
  Adult Whole Fly  4.234567  
  Larvae Wandering Tubules  4.719214  
  Larval Feeding Carcass  4.444986  
  Larval Feeding Central Nevous System  4.462446  
  Larval Feeding Hind Gut  4.342144  
  Larval Feeding Malpighian Tubule  4.636085  
  Larval Feeding Mid Gut  4.577678  
  Larval Feeding Salivary Gland  4.657358  
  Whole Larvae Feeding  4.292023  
 
  
   FlyBase ID    symbol    start    end    strand    length   
   FBgn0052413   CG32413  5895986   5897152   -  1167  
   FBgn0035647   CG10486  5897307   5899799   -  2493  
   FBgn0035648   CG13288   5900505   5910650  +  10146  
 
 
    Segment 132 
 
   Location   
  Gene key  FBgn0052407-FBgn0053523  
  Heatmap region span   3L:5838764..5990314   
  Segment span   3L:5915155..5917155   
  Length (genes)  2  
  Length (bp)  2001  
   Model Scoring   
  BIC  248.643325  
  logL  -118.809536  
  logL ratio  12.677213  
   Expression   
  Mean expression  8.148235  
  Median expression  7.859630  
  Tissue std. dev.  1.322479  
 
  No GO Slim enrichment  
  
   tissue    mean expression   
  5th Passage Drosophila S2 Cells  7.489083  
  Adult Accessory gland  6.612198  
  Adult Brain  8.315361  
  Adult Carcass  8.322490  
  Adult Crop  11.022220  
  Adult Eye  9.633910  
  Adult Fatbody  7.076996  
  Adult Female Spermatheca Mated  7.595022  
  Adult Female Spermatheca Virgin  7.372043  
  Adult Head  9.091509  
  Adult Heart  8.357457  
  Adult Hind Gut  9.944074  
  Adult Male Ejaculatory Duct  6.607831  
  Adult Mid Gut  10.303553  
  Adult Ovary  6.061962  
  Adult Salivary Gland  8.897233  
  Adult Testes  9.096845  
  Adult Thoracoabdominal ganglion  8.537252  
  Adult Whole Fly  7.929087  
  Larvae Wandering Tubules  7.554745  
  Larval Feeding Carcass  6.750169  
  Larval Feeding Central Nevous System  6.748711  
  Larval Feeding Hind Gut  7.508051  
  Larval Feeding Malpighian Tubule  8.367244  
  Larval Feeding Mid Gut  9.899191  
  Larval Feeding Salivary Gland  5.735927  
  Whole Larvae Feeding  9.172188  
 
  
   FlyBase ID    symbol    start    end    strand    length   
   FBgn0052407   CG32407   5915155   5916667  +  1513  
   FBgn0053523   CG33523   5917155   5923220  +  6066  
 
    Segment 133 
 
   Location   
  Gene key  FBgn0250815-FBgn0035665  
  Heatmap region span   3L:6004121..6056006   
  Segment span   3L:6032257..6043874   
  Length (genes)  2  
  Length (bp)  11618  
   Model Scoring   
  BIC  226.256983  
  logL  -107.616365  
  logL ratio  71.624668  
   Expression   
  Mean expression  7.554258  
  Median expression  6.256508  
  Tissue std. dev.  3.222172  
 
  No GO Slim enrichment  
  
   tissue    mean expression   
  5th Passage Drosophila S2 Cells  5.076655  
  Adult Accessory gland  5.394647  
  Adult Brain  4.720169  
  Adult Carcass  8.137484  
  Adult Crop  7.387651  
  Adult Eye  6.761478  
  Adult Fatbody  5.894809  
  Adult Female Spermatheca Mated  6.308836  
  Adult Female Spermatheca Virgin  5.833168  
  Adult Head  6.773707  
  Adult Heart  6.128188  
  Adult Hind Gut  10.632650  
  Adult Male Ejaculatory Duct  7.839441  
  Adult Mid Gut  14.869339  
  Adult Ovary  5.134183  
  Adult Salivary Gland  6.236306  
  Adult Testes  5.490451  
  Adult Thoracoabdominal ganglion  5.344903  
  Adult Whole Fly  14.412976  
  Larvae Wandering Tubules  5.313975  
  Larval Feeding Carcass  6.261867  
  Larval Feeding Central Nevous System  5.299316  
  Larval Feeding Hind Gut  7.590804  
  Larval Feeding Malpighian Tubule  5.813256  
  Larval Feeding Mid Gut  14.926968  
  Larval Feeding Salivary Gland  5.681277  
  Whole Larvae Feeding  14.700446  
 
  
   FlyBase ID    symbol    start    end    strand    length   
   FBgn0250815   Jon65Aiv   6032257   6033248  +  992  
   FBgn0035665   Jon65Aiii   6043874   6044800  +  927  
 
    Segment 134 
 
   Location   
  Gene key  FBgn0035666-FBgn0035667  
  Heatmap region span   3L:6027711..6065244   
  Segment span   3L:6045450..6047526   
  Length (genes)  2  
  Length (bp)  2077  
   Model Scoring   
  BIC  194.124106  
  logL  -91.549927  
  logL ratio  54.497514  
   Expression   
  Mean expression  6.179776  
  Median expression  5.048722  
  Tissue std. dev.  2.893068  
 
  No GO Slim enrichment  
  
   tissue    mean expression   
  5th Passage Drosophila S2 Cells  5.043412  
  Adult Accessory gland  5.067118  
  Adult Brain  4.655366  
  Adult Carcass  4.928147  
  Adult Crop  4.796217  
  Adult Eye  5.059720  
  Adult Fatbody  4.971057  
  Adult Female Spermatheca Mated  4.846333  
  Adult Female Spermatheca Virgin  4.885613  
  Adult Head  4.775013  
  Adult Heart  4.761720  
  Adult Hind Gut  4.869601  
  Adult Male Ejaculatory Duct  5.860745  
  Adult Mid Gut  13.304104  
  Adult Ovary  5.132062  
  Adult Salivary Gland  5.043489  
  Adult Testes  4.741698  
  Adult Thoracoabdominal ganglion  4.802976  
  Adult Whole Fly  10.611391  
  Larvae Wandering Tubules  5.071496  
  Larval Feeding Carcass  5.264483  
  Larval Feeding Central Nevous System  5.025963  
  Larval Feeding Hind Gut  5.354783  
  Larval Feeding Malpighian Tubule  5.018052  
  Larval Feeding Mid Gut  14.204184  
  Larval Feeding Salivary Gland  4.984343  
  Whole Larvae Feeding  13.774862  
 
  
   FlyBase ID    symbol    start    end    strand    length   
   FBgn0035666   Jon65Aii   6045450   6046291  +  842  
   FBgn0035667   Jon65Ai  6046684   6047526   -  843  
 
    Segment 135 
 
   Location   
  Gene key  FBgn0259164-FBgn0035673  
  Heatmap region span   3L:6045450..6088093   
  Segment span   3L:6059595..6065244   
  Length (genes)  2  
  Length (bp)  5650  
   Model Scoring   
  BIC  194.683241  
  logL  -91.829494  
  logL ratio  19.769284  
   Expression   
  Mean expression  5.480613  
  Median expression  5.267966  
  Tissue std. dev.  0.575587  
 
  No GO Slim enrichment  
  
   tissue    mean expression   
  5th Passage Drosophila S2 Cells  5.233931  
  Adult Accessory gland  5.472874  
  Adult Brain  4.816244  
  Adult Carcass  5.524391  
  Adult Crop  5.484694  
  Adult Eye  4.796516  
  Adult Fatbody  5.201163  
  Adult Female Spermatheca Mated  5.217108  
  Adult Female Spermatheca Virgin  5.336021  
  Adult Head  5.031691  
  Adult Heart  5.901459  
  Adult Hind Gut  7.810315  
  Adult Male Ejaculatory Duct  5.325240  
  Adult Mid Gut  5.269800  
  Adult Ovary  5.112602  
  Adult Salivary Gland  5.437361  
  Adult Testes  5.436061  
  Adult Thoracoabdominal ganglion  5.251583  
  Adult Whole Fly  6.235825  
  Larvae Wandering Tubules  5.747423  
  Larval Feeding Carcass  6.141110  
  Larval Feeding Central Nevous System  5.286256  
  Larval Feeding Hind Gut  5.472398  
  Larval Feeding Malpighian Tubule  6.006270  
  Larval Feeding Mid Gut  5.179826  
  Larval Feeding Salivary Gland  5.151463  
  Whole Larvae Feeding  5.096915  
 
  
   FlyBase ID    symbol    start    end    strand    length   
   FBgn0259164   CG42269   6059595   6064645  +  5051  
   FBgn0035673   CG6602   6065244   6066639  +  1396  
 
    Segment 136 
 
   Location   
  Gene key  FBgn0035674-FBgn0035675  
  Heatmap region span   3L:6048278..6091222   
  Segment span   3L:6068340..6068637   
  Length (genes)  2  
  Length (bp)  298  
   Model Scoring   
  BIC  212.701153  
  logL  -100.838450  
  logL ratio  42.796894  
   Expression   
  Mean expression  8.489194  
  Median expression  8.395295  
  Tissue std. dev.  0.467204  
 
  No GO Slim enrichment  
  
   tissue    mean expression   
  5th Passage Drosophila S2 Cells  8.737565  
  Adult Accessory gland  8.618940  
  Adult Brain  8.099437  
  Adult Carcass  7.945835  
  Adult Crop  8.602166  
  Adult Eye  8.207804  
  Adult Fatbody  8.333247  
  Adult Female Spermatheca Mated  8.427665  
  Adult Female Spermatheca Virgin  8.486410  
  Adult Head  8.273155  
  Adult Heart  8.768579  
  Adult Hind Gut  8.851240  
  Adult Male Ejaculatory Duct  8.289976  
  Adult Mid Gut  8.365811  
  Adult Ovary  9.162596  
  Adult Salivary Gland  8.562191  
  Adult Testes  6.860991  
  Adult Thoracoabdominal ganglion  8.107564  
  Adult Whole Fly  8.580392  
  Larvae Wandering Tubules  8.747750  
  Larval Feeding Carcass  8.378100  
  Larval Feeding Central Nevous System  8.994144  
  Larval Feeding Hind Gut  9.126728  
  Larval Feeding Malpighian Tubule  9.404480  
  Larval Feeding Mid Gut  8.342044  
  Larval Feeding Salivary Gland  8.656817  
  Whole Larvae Feeding  8.276604  
 
  
   FlyBase ID    symbol    start    end    strand    length   
   FBgn0035674   CG13295  6066589   6068340   -  1752  
   FBgn0035675   CG6610   6068637   6069358  +  722  
 
    Segment 137 
 
   Location   
  Gene key  FBgn0035676-FBgn0035677  
  Heatmap region span   3L:6053046..6124086   
  Segment span   3L:6069822..6080369   
  Length (genes)  2  
  Length (bp)  10548  
   Model Scoring   
  BIC  171.445225  
  logL  -80.210486  
  logL ratio  37.032350  
   Expression   
  Mean expression  4.912699  
  Median expression  4.711114  
  Tissue std. dev.  0.443605  
 
  No GO Slim enrichment  
  
   tissue    mean expression   
  5th Passage Drosophila S2 Cells  4.626724  
  Adult Accessory gland  4.619531  
  Adult Brain  6.256636  
  Adult Carcass  4.898352  
  Adult Crop  4.641053  
  Adult Eye  5.650771  
  Adult Fatbody  4.632851  
  Adult Female Spermatheca Mated  4.568892  
  Adult Female Spermatheca Virgin  4.667812  
  Adult Head  5.000336  
  Adult Heart  4.433118  
  Adult Hind Gut  4.574898  
  Adult Male Ejaculatory Duct  4.978306  
  Adult Mid Gut  4.963386  
  Adult Ovary  4.739063  
  Adult Salivary Gland  5.288582  
  Adult Testes  4.592178  
  Adult Thoracoabdominal ganglion  5.697608  
  Adult Whole Fly  4.310190  
  Larvae Wandering Tubules  5.152054  
  Larval Feeding Carcass  4.799568  
  Larval Feeding Central Nevous System  5.555046  
  Larval Feeding Hind Gut  4.537519  
  Larval Feeding Malpighian Tubule  5.149093  
  Larval Feeding Mid Gut  5.036607  
  Larval Feeding Salivary Gland  4.684904  
  Whole Larvae Feeding  4.587802  
 
  
   FlyBase ID    symbol    start    end    strand    length   
   FBgn0035676   CG6619   6069822   6074636  +  4815  
   FBgn0035677   CG13293   6080369   6083134  +  2766  
 
    Segment 138 
 
   Location   
  Gene key  FBgn0035679-FBgn0061492  
  Heatmap region span   3L:6059595..6139771   
  Segment span   3L:6087836..6088093   
  Length (genes)  2  
  Length (bp)  258  
   Model Scoring   
  BIC  262.889944  
  logL  -125.932846  
  logL ratio  37.130960  
   Expression   
  Mean expression  10.045338  
  Median expression  9.946608  
  Tissue std. dev.  1.043778  
 
  No GO Slim enrichment  
  
   tissue    mean expression   
  5th Passage Drosophila S2 Cells  9.910592  
  Adult Accessory gland  10.893872  
  Adult Brain  8.076127  
  Adult Carcass  9.625668  
  Adult Crop  9.864647  
  Adult Eye  9.209105  
  Adult Fatbody  10.396971  
  Adult Female Spermatheca Mated  10.509245  
  Adult Female Spermatheca Virgin  10.578551  
  Adult Head  9.560681  
  Adult Heart  10.403040  
  Adult Hind Gut  10.583433  
  Adult Male Ejaculatory Duct  10.291744  
  Adult Mid Gut  11.251612  
  Adult Ovary  9.331095  
  Adult Salivary Gland  10.514734  
  Adult Testes  7.471966  
  Adult Thoracoabdominal ganglion  8.123977  
  Adult Whole Fly  9.812998  
  Larvae Wandering Tubules  11.840570  
  Larval Feeding Carcass  10.392730  
  Larval Feeding Central Nevous System  8.715758  
  Larval Feeding Hind Gut  9.868252  
  Larval Feeding Malpighian Tubule  11.652662  
  Larval Feeding Mid Gut  10.167814  
  Larval Feeding Salivary Gland  11.295478  
  Whole Larvae Feeding  10.880798  
 
  
   FlyBase ID    symbol    start    end    strand    length   
   FBgn0035679   CG10467  6086304   6087836   -  1533  
   FBgn0061492   loj   6088093   6089617  +  1525  
 
    Segment 139 
 
   Location   
  Gene key  FBgn0020637-FBgn0020643  
  Heatmap region span   3L:6087836..6167348   
  Segment span   3L:6126700..6139771   
  Length (genes)  8  
  Length (bp)  13072  
   Model Scoring   
  BIC  804.466659  
  logL  -396.721203  
  logL ratio  83.559428  
   Expression   
  Mean expression  5.287906  
  Median expression  4.790996  
  Tissue std. dev.  1.503228  
 
  No GO Slim enrichment  
  
   tissue    mean expression   
  5th Passage Drosophila S2 Cells  4.739620  
  Adult Accessory gland  4.845965  
  Adult Brain  4.389296  
  Adult Carcass  4.938372  
  Adult Crop  4.883997  
  Adult Eye  4.898740  
  Adult Fatbody  4.790377  
  Adult Female Spermatheca Mated  4.829508  
  Adult Female Spermatheca Virgin  4.807674  
  Adult Head  4.916893  
  Adult Heart  4.678327  
  Adult Hind Gut  4.721293  
  Adult Male Ejaculatory Duct  4.838240  
  Adult Mid Gut  4.891817  
  Adult Ovary  4.603406  
  Adult Salivary Gland  5.051466  
  Adult Testes  4.477804  
  Adult Thoracoabdominal ganglion  4.544134  
  Adult Whole Fly  4.764424  
  Larvae Wandering Tubules  4.728222  
  Larval Feeding Carcass  10.926619  
  Larval Feeding Central Nevous System  4.600121  
  Larval Feeding Hind Gut  7.643784  
  Larval Feeding Malpighian Tubule  4.765844  
  Larval Feeding Mid Gut  5.209676  
  Larval Feeding Salivary Gland  4.898249  
  Whole Larvae Feeding  9.389603  
 
  
   FlyBase ID    symbol    start    end    strand    length   
   FBgn0020637   Lcp65Ag2  6126089   6126700   -  612  
   FBgn0020639   Lcp65Af  6129404   6129871   -  468  
   FBgn0020640   Lcp65Ae  6130819   6131178   -  360  
   FBgn0052405   Cpr65Av  6132323   6133073   -  751  
   FBgn0052404   Cpr65Aw  6135065   6135483   -  419  
   FBgn0020641   Lcp65Ad   6136507   6137109  +  603  
   FBgn0020642   Lcp65Ac   6137924   6138493  +  570  
   FBgn0020643   Lcp65Ab2   6139771   6140189  +  419  
 
 
    Segment 140 
 
   Location   
  Gene key  FBgn0020645-FBgn0020765  
  Heatmap region span   3L:6091222..6182857   
  Segment span   3L:6144404..6145845   
  Length (genes)  2  
  Length (bp)  1442  
   Model Scoring   
  BIC  229.294300  
  logL  -109.135024  
  logL ratio  20.862062  
   Expression   
  Mean expression  6.783838  
  Median expression  6.512882  
  Tissue std. dev.  0.937067  
 
  No GO Slim enrichment  
  
   tissue    mean expression   
  5th Passage Drosophila S2 Cells  6.557139  
  Adult Accessory gland  6.415703  
  Adult Brain  5.948511  
  Adult Carcass  7.680895  
  Adult Crop  6.656982  
  Adult Eye  6.206796  
  Adult Fatbody  6.884497  
  Adult Female Spermatheca Mated  6.592885  
  Adult Female Spermatheca Virgin  6.596699  
  Adult Head  6.300546  
  Adult Heart  6.898964  
  Adult Hind Gut  6.370246  
  Adult Male Ejaculatory Duct  6.327044  
  Adult Mid Gut  6.745857  
  Adult Ovary  6.235723  
  Adult Salivary Gland  6.866007  
  Adult Testes  6.020947  
  Adult Thoracoabdominal ganglion  6.064483  
  Adult Whole Fly  6.085533  
  Larvae Wandering Tubules  6.576780  
  Larval Feeding Carcass  10.122067  
  Larval Feeding Central Nevous System  5.953175  
  Larval Feeding Hind Gut  8.941854  
  Larval Feeding Malpighian Tubule  6.534974  
  Larval Feeding Mid Gut  6.821592  
  Larval Feeding Salivary Gland  6.440501  
  Whole Larvae Feeding  8.317233  
 
  
   FlyBase ID    symbol    start    end    strand    length   
   FBgn0020645   Lcp65Aa   6144404   6144741  +  338  
   FBgn0020765   Acp65Aa  6145150   6145845   -  696  
 
    Segment 141 
 
   Location   
  Gene key  FBgn0035685-FBgn0035687  
  Heatmap region span   3L:6124086..6186584   
  Segment span   3L:6150473..6156257   
  Length (genes)  3  
  Length (bp)  5785  
   Model Scoring   
  BIC  219.087320  
  logL  -104.031533  
  logL ratio  82.867775  
   Expression   
  Mean expression  4.669748  
  Median expression  4.713235  
  Tissue std. dev.  0.150424  
 
  No GO Slim enrichment  
  
   tissue    mean expression   
  5th Passage Drosophila S2 Cells  4.661563  
  Adult Accessory gland  4.859605  
  Adult Brain  4.480054  
  Adult Carcass  4.732217  
  Adult Crop  4.557673  
  Adult Eye  4.561987  
  Adult Fatbody  4.860061  
  Adult Female Spermatheca Mated  4.694102  
  Adult Female Spermatheca Virgin  4.785904  
  Adult Head  4.578794  
  Adult Heart  4.574054  
  Adult Hind Gut  4.597814  
  Adult Male Ejaculatory Duct  4.837267  
  Adult Mid Gut  4.755014  
  Adult Ovary  4.744001  
  Adult Salivary Gland  4.842929  
  Adult Testes  4.457737  
  Adult Thoracoabdominal ganglion  4.524041  
  Adult Whole Fly  4.223509  
  Larvae Wandering Tubules  4.699576  
  Larval Feeding Carcass  4.891802  
  Larval Feeding Central Nevous System  4.643262  
  Larval Feeding Hind Gut  4.602842  
  Larval Feeding Malpighian Tubule  4.729932  
  Larval Feeding Mid Gut  4.738104  
  Larval Feeding Salivary Gland  4.851186  
  Whole Larvae Feeding  4.598163  
 
  
   FlyBase ID    symbol    start    end    strand    length   
   FBgn0035685   CG13297  6149695   6150473   -  779  
   FBgn0035686   Cpr65Az   6153914   6154689  +  776  
   FBgn0035687   CG13296   6156257   6157782  +  1526  
 
 
    Segment 142 
 
   Location   
  Gene key  FBgn0035690-FBgn0035691  
  Heatmap region span   3L:6144404..6225461   
  Segment span   3L:6174883..6182857   
  Length (genes)  4  
  Length (bp)  7975  
   Model Scoring   
  BIC  388.569954  
  logL  -188.772851  
  logL ratio  94.838915  
   Expression   
  Mean expression  7.020947  
  Median expression  7.046888  
  Tissue std. dev.  0.484783  
 
  
   GO ID    description    ratio    P-value   
   GO:0005634   nucleus  4/4  0.000155  
   GO:0043226   organelle  4/4  0.000766  
   GO:0005575   cellular_component  4/4  0.0346  
 
  
   tissue    mean expression   
  5th Passage Drosophila S2 Cells  7.201721  
  Adult Accessory gland  7.437528  
  Adult Brain  7.744238  
  Adult Carcass  6.355146  
  Adult Crop  6.953912  
  Adult Eye  6.934875  
  Adult Fatbody  6.742842  
  Adult Female Spermatheca Mated  6.951663  
  Adult Female Spermatheca Virgin  6.907565  
  Adult Head  6.732633  
  Adult Heart  6.658490  
  Adult Hind Gut  6.810237  
  Adult Male Ejaculatory Duct  7.249801  
  Adult Mid Gut  6.575354  
  Adult Ovary  8.529159  
  Adult Salivary Gland  7.137471  
  Adult Testes  6.839874  
  Adult Thoracoabdominal ganglion  7.483414  
  Adult Whole Fly  6.685406  
  Larvae Wandering Tubules  6.782021  
  Larval Feeding Carcass  6.699482  
  Larval Feeding Central Nevous System  7.977231  
  Larval Feeding Hind Gut  6.837092  
  Larval Feeding Malpighian Tubule  6.821555  
  Larval Feeding Mid Gut  6.546749  
  Larval Feeding Salivary Gland  7.501087  
  Whole Larvae Feeding  6.469020  
 
  
   FlyBase ID    symbol    start    end    strand    length   
   FBgn0035690   CG10274  6171779   6174883   -  3105  
   FBgn0022699   D19B  6175274   6178138   -  2865  
   FBgn0022935   D19A  6178935   6182201   -  3267  
   FBgn0035691   CG7386   6182857   6185150  +  2294  
 
 
    Segment 143 
 
   Location   
  Gene key  FBgn0035692-FBgn0024921  
  Heatmap region span   3L:6150473..6226481   
  Segment span   3L:6186286..6186584   
  Length (genes)  2  
  Length (bp)  299  
   Model Scoring   
  BIC  232.601923  
  logL  -110.788835  
  logL ratio  77.884810  
   Expression   
  Mean expression  10.647356  
  Median expression  10.530828  
  Tissue std. dev.  0.518305  
 
  No GO Slim enrichment  
  
   tissue    mean expression   
  5th Passage Drosophila S2 Cells  10.800406  
  Adult Accessory gland  10.737438  
  Adult Brain  10.869657  
  Adult Carcass  9.918423  
  Adult Crop  10.684373  
  Adult Eye  10.945192  
  Adult Fatbody  10.420098  
  Adult Female Spermatheca Mated  10.162178  
  Adult Female Spermatheca Virgin  10.059371  
  Adult Head  10.359150  
  Adult Heart  10.473530  
  Adult Hind Gut  10.531275  
  Adult Male Ejaculatory Duct  10.487902  
  Adult Mid Gut  10.385421  
  Adult Ovary  11.580766  
  Adult Salivary Gland  10.326519  
  Adult Testes  9.601395  
  Adult Thoracoabdominal ganglion  10.506436  
  Adult Whole Fly  10.479491  
  Larvae Wandering Tubules  11.364873  
  Larval Feeding Carcass  10.876935  
  Larval Feeding Central Nevous System  12.264638  
  Larval Feeding Hind Gut  10.846270  
  Larval Feeding Malpighian Tubule  10.561396  
  Larval Feeding Mid Gut  10.623003  
  Larval Feeding Salivary Gland  11.193482  
  Whole Larvae Feeding  10.418979  
 
  
   FlyBase ID    symbol    start    end    strand    length   
   FBgn0035692   CG13298  6185218   6186286   -  1069  
   FBgn0024921   Trn   6186584   6192105  +  5522  
 
    Segment 144 
 
   Location   
  Gene key  FBgn0035695-FBgn0004513  
  Heatmap region span   3L:6193573..6320708   
  Segment span   3L:6232689..6238836   
  Length (genes)  2  
  Length (bp)  6148  
   Model Scoring   
  BIC  208.824300  
  logL  -98.900024  
  logL ratio  23.447710  
   Expression   
  Mean expression  6.488155  
  Median expression  5.679927  
  Tissue std. dev.  1.742983  
 
  
   GO ID    description    ratio    P-value   
   GO:0055085   transmembrane transport  2/2  0.000829  
   GO:0006810   transport  2/2  0.00398  
 
  
   tissue    mean expression   
  5th Passage Drosophila S2 Cells  4.919840  
  Adult Accessory gland  7.308253  
  Adult Brain  10.493107  
  Adult Carcass  6.208649  
  Adult Crop  5.838293  
  Adult Eye  9.175497  
  Adult Fatbody  5.093682  
  Adult Female Spermatheca Mated  5.531998  
  Adult Female Spermatheca Virgin  5.449407  
  Adult Head  9.038274  
  Adult Heart  5.531997  
  Adult Hind Gut  8.154954  
  Adult Male Ejaculatory Duct  5.404141  
  Adult Mid Gut  5.345894  
  Adult Ovary  5.104482  
  Adult Salivary Gland  5.157433  
  Adult Testes  5.520001  
  Adult Thoracoabdominal ganglion  10.503056  
  Adult Whole Fly  6.017842  
  Larvae Wandering Tubules  5.431454  
  Larval Feeding Carcass  6.592719  
  Larval Feeding Central Nevous System  9.805619  
  Larval Feeding Hind Gut  5.534415  
  Larval Feeding Malpighian Tubule  5.773394  
  Larval Feeding Mid Gut  5.025289  
  Larval Feeding Salivary Gland  5.210185  
  Whole Larvae Feeding  6.010300  
 
  
   FlyBase ID    symbol    start    end    strand    length   
   FBgn0035695   CG10226  6227417   6232689   -  5273  
   FBgn0004513   Mdr65  6233600   6238836   -  5237  
 
    Segment 145 
 
   Location   
  Gene key  FBgn0041625-FBgn0041623  
  Heatmap region span   3L:6232689..6577454   
  Segment span   3L:6313711..6320708   
  Length (genes)  3  
  Length (bp)  6998  
   Model Scoring   
  BIC  225.196203  
  logL  -107.085975  
  logL ratio  85.913298  
   Expression   
  Mean expression  4.440088  
  Median expression  4.373566  
  Tissue std. dev.  0.196806  
 
  No GO Slim enrichment  
  
   tissue    mean expression   
  5th Passage Drosophila S2 Cells  4.426945  
  Adult Accessory gland  4.980798  
  Adult Brain  4.144785  
  Adult Carcass  4.651993  
  Adult Crop  4.458262  
  Adult Eye  4.332642  
  Adult Fatbody  4.457767  
  Adult Female Spermatheca Mated  4.512192  
  Adult Female Spermatheca Virgin  4.466372  
  Adult Head  4.732334  
  Adult Heart  4.325741  
  Adult Hind Gut  4.427335  
  Adult Male Ejaculatory Duct  4.647809  
  Adult Mid Gut  4.682707  
  Adult Ovary  4.341679  
  Adult Salivary Gland  4.707138  
  Adult Testes  4.206857  
  Adult Thoracoabdominal ganglion  4.380791  
  Adult Whole Fly  4.102039  
  Larvae Wandering Tubules  4.462286  
  Larval Feeding Carcass  4.468269  
  Larval Feeding Central Nevous System  4.131753  
  Larval Feeding Hind Gut  4.285153  
  Larval Feeding Malpighian Tubule  4.451578  
  Larval Feeding Mid Gut  4.448917  
  Larval Feeding Salivary Gland  4.353604  
  Whole Larvae Feeding  4.294634  
 
  
   FlyBase ID    symbol    start    end    strand    length   
   FBgn0041625   Or65a   6313711   6315198  +  1488  
   FBgn0041624   Or65b   6318712   6320149  +  1438  
   FBgn0041623   Or65c   6320708   6322169  +  1462  
 
 
    Segment 146 
 
   Location   
  Gene key  FBgn0035699-FBgn0020251  
  Heatmap region span   3L:6249766..6596466   
  Segment span   3L:6345490..6488780   
  Length (genes)  5  
  Length (bp)  143291  
   Model Scoring   
  BIC  472.215851  
  logL  -230.595799  
  logL ratio  117.780063  
   Expression   
  Mean expression  6.688855  
  Median expression  6.725279  
  Tissue std. dev.  0.323475  
 
  No GO Slim enrichment  
  
   tissue    mean expression   
  5th Passage Drosophila S2 Cells  6.682729  
  Adult Accessory gland  6.494735  
  Adult Brain  7.015637  
  Adult Carcass  6.259690  
  Adult Crop  7.008038  
  Adult Eye  6.838634  
  Adult Fatbody  6.771269  
  Adult Female Spermatheca Mated  6.836233  
  Adult Female Spermatheca Virgin  6.722192  
  Adult Head  6.390683  
  Adult Heart  7.364311  
  Adult Hind Gut  6.520346  
  Adult Male Ejaculatory Duct  6.395658  
  Adult Mid Gut  6.761798  
  Adult Ovary  7.261096  
  Adult Salivary Gland  6.587252  
  Adult Testes  6.398381  
  Adult Thoracoabdominal ganglion  6.691504  
  Adult Whole Fly  6.215279  
  Larvae Wandering Tubules  7.163810  
  Larval Feeding Carcass  6.380276  
  Larval Feeding Central Nevous System  6.869063  
  Larval Feeding Hind Gut  6.540871  
  Larval Feeding Malpighian Tubule  7.205382  
  Larval Feeding Mid Gut  6.524578  
  Larval Feeding Salivary Gland  6.607144  
  Whole Larvae Feeding  6.092501  
 
  
   FlyBase ID    symbol    start    end    strand    length   
   FBgn0035699   CG13300  6323846   6345490   -  21645  
   FBgn0035702   CG10147  6479697   6481524   -  1828  
   FBgn0035703   CG8270   6481776   6484310  +  2535  
   FBgn0035704   CG10144  6484330   6488472   -  4143  
   FBgn0020251   sfl   6488780   6542333  +  53554  
 
 
    Segment 147 
 
   Location   
  Gene key  FBgn0035713-FBgn0035715  
  Heatmap region span   3L:6660916..6936056   
  Segment span   3L:6745386..6749619   
  Length (genes)  3  
  Length (bp)  4234  
   Model Scoring   
  BIC  303.040202  
  logL  -146.007975  
  logL ratio  85.618466  
   Expression   
  Mean expression  9.027348  
  Median expression  9.090937  
  Tissue std. dev.  0.272532  
 
  No GO Slim enrichment  
  
   tissue    mean expression   
  5th Passage Drosophila S2 Cells  9.517586  
  Adult Accessory gland  9.447269  
  Adult Brain  8.832444  
  Adult Carcass  8.799315  
  Adult Crop  9.008611  
  Adult Eye  8.748823  
  Adult Fatbody  8.962334  
  Adult Female Spermatheca Mated  9.082682  
  Adult Female Spermatheca Virgin  9.248506  
  Adult Head  8.690264  
  Adult Heart  9.073015  
  Adult Hind Gut  8.864035  
  Adult Male Ejaculatory Duct  8.956315  
  Adult Mid Gut  9.095742  
  Adult Ovary  9.522793  
  Adult Salivary Gland  8.727499  
  Adult Testes  9.502219  
  Adult Thoracoabdominal ganglion  8.735797  
  Adult Whole Fly  8.939913  
  Larvae Wandering Tubules  9.356725  
  Larval Feeding Carcass  8.921300  
  Larval Feeding Central Nevous System  9.036307  
  Larval Feeding Hind Gut  8.941950  
  Larval Feeding Malpighian Tubule  9.275753  
  Larval Feeding Mid Gut  8.670951  
  Larval Feeding Salivary Gland  9.210645  
  Whole Larvae Feeding  8.569591  
 
  
   FlyBase ID    symbol    start    end    strand    length   
   FBgn0035713   velo  6737526   6745386   -  7861  
   FBgn0035714   CG8549   6746013   6747137  +  1125  
   FBgn0035715   CG10103  6747116   6749619   -  2504  
 
 
    Segment 148 
 
   Location   
  Gene key  FBgn0052392-FBgn0086680  
  Heatmap region span   3L:6696619..6949004   
  Segment span   3L:6756346..6783252   
  Length (genes)  2  
  Length (bp)  26907  
   Model Scoring   
  BIC  226.280118  
  logL  -107.627933  
  logL ratio  -1.592035  
   Expression   
  Mean expression  5.761452  
  Median expression  5.519149  
  Tissue std. dev.  0.824674  
 
  No GO Slim enrichment  
  
   tissue    mean expression   
  5th Passage Drosophila S2 Cells  4.878144  
  Adult Accessory gland  5.175839  
  Adult Brain  6.812256  
  Adult Carcass  6.759235  
  Adult Crop  6.015271  
  Adult Eye  5.454942  
  Adult Fatbody  6.842454  
  Adult Female Spermatheca Mated  5.086048  
  Adult Female Spermatheca Virgin  5.051188  
  Adult Head  6.132607  
  Adult Heart  7.053821  
  Adult Hind Gut  5.359094  
  Adult Male Ejaculatory Duct  6.528253  
  Adult Mid Gut  5.017600  
  Adult Ovary  4.687672  
  Adult Salivary Gland  5.066990  
  Adult Testes  7.761656  
  Adult Thoracoabdominal ganglion  6.673712  
  Adult Whole Fly  6.137949  
  Larvae Wandering Tubules  4.978034  
  Larval Feeding Carcass  6.127056  
  Larval Feeding Central Nevous System  6.088762  
  Larval Feeding Hind Gut  5.374757  
  Larval Feeding Malpighian Tubule  4.933489  
  Larval Feeding Mid Gut  4.837743  
  Larval Feeding Salivary Gland  4.995467  
  Whole Larvae Feeding  5.729166  
 
  
   FlyBase ID    symbol    start    end    strand    length   
   FBgn0052392   CG32392  6749723   6756346   -  6624  
   FBgn0086680   vvl   6783252   6787637  +  4386  
 
    Segment 149 
 
   Location   
  Gene key  FBgn0035722-FBgn0029117  
  Heatmap region span   3L:6926030..7019881   
  Segment span   3L:6951214..6951446   
  Length (genes)  2  
  Length (bp)  233  
   Model Scoring   
  BIC  212.346754  
  logL  -100.661251  
  logL ratio  46.964069  
   Expression   
  Mean expression  8.931765  
  Median expression  8.862330  
  Tissue std. dev.  0.337922  
 
  No GO Slim enrichment  
  
   tissue    mean expression   
  5th Passage Drosophila S2 Cells  8.197819  
  Adult Accessory gland  8.601624  
  Adult Brain  9.126241  
  Adult Carcass  9.448878  
  Adult Crop  9.233031  
  Adult Eye  9.043522  
  Adult Fatbody  9.094477  
  Adult Female Spermatheca Mated  8.832704  
  Adult Female Spermatheca Virgin  9.158383  
  Adult Head  8.943838  
  Adult Heart  9.480896  
  Adult Hind Gut  9.031247  
  Adult Male Ejaculatory Duct  8.837654  
  Adult Mid Gut  8.765659  
  Adult Ovary  9.523430  
  Adult Salivary Gland  8.645366  
  Adult Testes  8.741445  
  Adult Thoracoabdominal ganglion  9.375390  
  Adult Whole Fly  8.990394  
  Larvae Wandering Tubules  8.960863  
  Larval Feeding Carcass  8.819730  
  Larval Feeding Central Nevous System  8.327318  
  Larval Feeding Hind Gut  8.911499  
  Larval Feeding Malpighian Tubule  9.331156  
  Larval Feeding Mid Gut  8.594125  
  Larval Feeding Salivary Gland  8.637755  
  Whole Larvae Feeding  8.503210  
 
  
   FlyBase ID    symbol    start    end    strand    length   
   FBgn0035722   CG10075  6950046   6951214   -  1169  
   FBgn0029117   Surf1   6951446   6952686  +  1241  
 
    Segment 150 
 
   Location   
  Gene key  FBgn0035727-FBgn0035730  
  Heatmap region span   3L:6951214..7122003   
  Segment span   3L:6965521..7019881   
  Length (genes)  4  
  Length (bp)  54361  
   Model Scoring   
  BIC  360.905047  
  logL  -174.940397  
  logL ratio  63.665640  
   Expression   
  Mean expression  4.877617  
  Median expression  4.426597  
  Tissue std. dev.  1.118849  
 
  No GO Slim enrichment  
  
   tissue    mean expression   
  5th Passage Drosophila S2 Cells  4.705048  
  Adult Accessory gland  4.629295  
  Adult Brain  4.238697  
  Adult Carcass  4.845793  
  Adult Crop  5.032402  
  Adult Eye  4.366191  
  Adult Fatbody  4.477761  
  Adult Female Spermatheca Mated  4.641700  
  Adult Female Spermatheca Virgin  4.616556  
  Adult Head  4.336431  
  Adult Heart  4.452236  
  Adult Hind Gut  4.717128  
  Adult Male Ejaculatory Duct  4.692337  
  Adult Mid Gut  4.720210  
  Adult Ovary  4.438321  
  Adult Salivary Gland  4.740101  
  Adult Testes  10.047782  
  Adult Thoracoabdominal ganglion  4.387876  
  Adult Whole Fly  6.704650  
  Larvae Wandering Tubules  4.479641  
  Larval Feeding Carcass  4.496510  
  Larval Feeding Central Nevous System  4.181833  
  Larval Feeding Hind Gut  4.530875  
  Larval Feeding Malpighian Tubule  4.463230  
  Larval Feeding Mid Gut  4.707557  
  Larval Feeding Salivary Gland  4.522999  
  Whole Larvae Feeding  5.522493  
 
  
   FlyBase ID    symbol    start    end    strand    length   
   FBgn0035727   CG10063  6964675   6965521   -  847  
   FBgn0250849   CG32388  6976572   6977672   -  1101  
   FBgn0045759   bin   6983722   6987544  +  3823  
   FBgn0035730   CG17744  7018901   7019881   -  981  
 
 
    Segment 151 
 
   Location   
  Gene key  FBgn0052391-FBgn0054030  
  Heatmap region span   3L:6962142..7151199   
  Segment span   3L:7062297..7068347   
  Length (genes)  2  
  Length (bp)  6051  
   Model Scoring   
  BIC  186.773870  
  logL  -87.874809  
  logL ratio  41.359060  
   Expression   
  Mean expression  4.098489  
  Median expression  3.983612  
  Tissue std. dev.  0.530353  
 
  No GO Slim enrichment  
  
   tissue    mean expression   
  5th Passage Drosophila S2 Cells  3.933228  
  Adult Accessory gland  4.039894  
  Adult Brain  3.843260  
  Adult Carcass  4.130956  
  Adult Crop  3.994695  
  Adult Eye  4.280731  
  Adult Fatbody  3.981536  
  Adult Female Spermatheca Mated  4.009142  
  Adult Female Spermatheca Virgin  4.208604  
  Adult Head  3.871230  
  Adult Heart  4.046708  
  Adult Hind Gut  3.927357  
  Adult Male Ejaculatory Duct  4.001341  
  Adult Mid Gut  4.165003  
  Adult Ovary  3.833746  
  Adult Salivary Gland  4.179821  
  Adult Testes  6.712820  
  Adult Thoracoabdominal ganglion  3.930382  
  Adult Whole Fly  3.829857  
  Larvae Wandering Tubules  4.166674  
  Larval Feeding Carcass  3.884492  
  Larval Feeding Central Nevous System  3.727543  
  Larval Feeding Hind Gut  3.820852  
  Larval Feeding Malpighian Tubule  4.039123  
  Larval Feeding Mid Gut  4.172382  
  Larval Feeding Salivary Gland  3.960229  
  Whole Larvae Feeding  3.967605  
 
  
   FlyBase ID    symbol    start    end    strand    length   
   FBgn0052391   CG32391  7061311   7062297   -  987  
   FBgn0054030   CG34030  7066576   7068347   -  1772  
 
    Segment 152 
 
   Location   
  Gene key  FBgn0035733-FBgn0035741  
  Heatmap region span   3L:6962711..7231199   
  Segment span   3L:7070562..7118731   
  Length (genes)  6  
  Length (bp)  48170  
   Model Scoring   
  BIC  540.988333  
  logL  -264.982040  
  logL ratio  105.117960  
   Expression   
  Mean expression  5.494968  
  Median expression  5.401127  
  Tissue std. dev.  0.231433  
 
  No GO Slim enrichment  
  
   tissue    mean expression   
  5th Passage Drosophila S2 Cells  5.238618  
  Adult Accessory gland  5.487531  
  Adult Brain  5.451487  
  Adult Carcass  5.813901  
  Adult Crop  5.678266  
  Adult Eye  5.276447  
  Adult Fatbody  5.685160  
  Adult Female Spermatheca Mated  5.462284  
  Adult Female Spermatheca Virgin  5.472548  
  Adult Head  5.414376  
  Adult Heart  5.373317  
  Adult Hind Gut  5.681722  
  Adult Male Ejaculatory Duct  5.535984  
  Adult Mid Gut  5.525264  
  Adult Ovary  5.119596  
  Adult Salivary Gland  6.019607  
  Adult Testes  5.428252  
  Adult Thoracoabdominal ganglion  5.410242  
  Adult Whole Fly  4.971336  
  Larvae Wandering Tubules  5.322959  
  Larval Feeding Carcass  6.016070  
  Larval Feeding Central Nevous System  5.311234  
  Larval Feeding Hind Gut  5.571029  
  Larval Feeding Malpighian Tubule  5.401030  
  Larval Feeding Mid Gut  5.595443  
  Larval Feeding Salivary Gland  5.675194  
  Whole Larvae Feeding  5.425225  
 
  
   FlyBase ID    symbol    start    end    strand    length   
   FBgn0035733   CG8641  7053006   7070562   -  17557  
   FBgn0035735   Cpr65Ea  7079156   7079706   -  551  
   FBgn0035736   Cpr65Eb  7080807   7081885   -  1079  
   FBgn0035737   Cpr65Ec   7086309   7086943  +  635  
   FBgn0053556   form3   7087918   7116397  +  28480  
   FBgn0035741   BBS1   7118731   7120988  +  2258  
 
 
    Segment 153 
 
   Location   
  Gene key  FBgn0035742-FBgn0035743  
  Heatmap region span   3L:6965521..7234449   
  Segment span   3L:7121360..7122003   
  Length (genes)  2  
  Length (bp)  644  
   Model Scoring   
  BIC  244.532327  
  logL  -116.754037  
  logL ratio  -0.781236  
   Expression   
  Mean expression  6.230603  
  Median expression  5.345325  
  Tissue std. dev.  2.296666  
 
  No GO Slim enrichment  
  
   tissue    mean expression   
  5th Passage Drosophila S2 Cells  5.189395  
  Adult Accessory gland  5.244655  
  Adult Brain  4.814542  
  Adult Carcass  5.588399  
  Adult Crop  5.195018  
  Adult Eye  4.910552  
  Adult Fatbody  5.269008  
  Adult Female Spermatheca Mated  5.184582  
  Adult Female Spermatheca Virgin  5.134913  
  Adult Head  5.062170  
  Adult Heart  4.747782  
  Adult Hind Gut  6.416296  
  Adult Male Ejaculatory Duct  6.087559  
  Adult Mid Gut  8.927124  
  Adult Ovary  5.324845  
  Adult Salivary Gland  5.447404  
  Adult Testes  5.333057  
  Adult Thoracoabdominal ganglion  4.971044  
  Adult Whole Fly  6.899817  
  Larvae Wandering Tubules  5.496538  
  Larval Feeding Carcass  5.406246  
  Larval Feeding Central Nevous System  5.034285  
  Larval Feeding Hind Gut  8.373739  
  Larval Feeding Malpighian Tubule  5.902240  
  Larval Feeding Mid Gut  13.998957  
  Larval Feeding Salivary Gland  5.120722  
  Whole Larvae Feeding  13.145405  
 
  
   FlyBase ID    symbol    start    end    strand    length   
   FBgn0035742   CG8629  7121013   7121360   -  348  
   FBgn0035743   CG15829   7122003   7122480  +  478  
 
    Segment 154 
 
   Location   
  Gene key  FBgn0023001-FBgn0040837  
  Heatmap region span   3L:7062297..7257017   
  Segment span   3L:7127618..7151199   
  Length (genes)  3  
  Length (bp)  23582  
   Model Scoring   
  BIC  304.126378  
  logL  -146.551062  
  logL ratio  35.160529  
   Expression   
  Mean expression  6.183356  
  Median expression  5.747699  
  Tissue std. dev.  0.786593  
 
  No GO Slim enrichment  
  
   tissue    mean expression   
  5th Passage Drosophila S2 Cells  5.660824  
  Adult Accessory gland  5.642133  
  Adult Brain  6.449301  
  Adult Carcass  5.348964  
  Adult Crop  6.389316  
  Adult Eye  6.475850  
  Adult Fatbody  5.142979  
  Adult Female Spermatheca Mated  5.367870  
  Adult Female Spermatheca Virgin  5.449892  
  Adult Head  5.853997  
  Adult Heart  5.750835  
  Adult Hind Gut  6.736095  
  Adult Male Ejaculatory Duct  5.952016  
  Adult Mid Gut  7.003600  
  Adult Ovary  5.240897  
  Adult Salivary Gland  5.533217  
  Adult Testes  5.282201  
  Adult Thoracoabdominal ganglion  6.251159  
  Adult Whole Fly  5.382199  
  Larvae Wandering Tubules  7.881747  
  Larval Feeding Carcass  6.855367  
  Larval Feeding Central Nevous System  6.779538  
  Larval Feeding Hind Gut  6.926234  
  Larval Feeding Malpighian Tubule  8.009329  
  Larval Feeding Mid Gut  7.096359  
  Larval Feeding Salivary Gland  5.773192  
  Whole Larvae Feeding  6.715496  
 
  
   FlyBase ID    symbol    start    end    strand    length   
   FBgn0023001   melt   7127618   7136174  +  8557  
   FBgn0259173   corn  7136086   7150173   -  14088  
   FBgn0040837   CG8620   7151199   7152010  +  812  
 
 
    Segment 155 
 
   Location   
  Gene key  FBgn0035746-FBgn0035751  
  Heatmap region span   3L:7070562..7316509   
  Segment span   3L:7155414..7231199   
  Length (genes)  4  
  Length (bp)  75786  
   Model Scoring   
  BIC  315.538910  
  logL  -152.257329  
  logL ratio  102.553178  
   Expression   
  Mean expression  4.655013  
  Median expression  4.378118  
  Tissue std. dev.  0.328085  
 
  No GO Slim enrichment  
  
   tissue    mean expression   
  5th Passage Drosophila S2 Cells  4.631741  
  Adult Accessory gland  4.607916  
  Adult Brain  5.205570  
  Adult Carcass  4.554578  
  Adult Crop  4.487123  
  Adult Eye  4.392190  
  Adult Fatbody  4.545931  
  Adult Female Spermatheca Mated  4.581338  
  Adult Female Spermatheca Virgin  4.633075  
  Adult Head  4.457186  
  Adult Heart  4.413849  
  Adult Hind Gut  5.123357  
  Adult Male Ejaculatory Duct  4.576350  
  Adult Mid Gut  4.591267  
  Adult Ovary  4.444275  
  Adult Salivary Gland  4.655704  
  Adult Testes  4.849823  
  Adult Thoracoabdominal ganglion  4.843752  
  Adult Whole Fly  4.230365  
  Larvae Wandering Tubules  4.652424  
  Larval Feeding Carcass  4.599050  
  Larval Feeding Central Nevous System  4.435915  
  Larval Feeding Hind Gut  5.972891  
  Larval Feeding Malpighian Tubule  4.598179  
  Larval Feeding Mid Gut  4.624791  
  Larval Feeding Salivary Gland  4.523012  
  Whole Larvae Feeding  4.453689  
 
  
   FlyBase ID    symbol    start    end    strand    length   
   FBgn0035746     7154457   7155414   -  958  
   FBgn0259141     7155528   7183017   -  27490  
   FBgn0035750   CG14826   7227612   7228765  +  1154  
   FBgn0035751   CG14829  7230474   7231199   -  726  
 
 
    Segment 156 
 
   Location   
  Gene key  FBgn0024542-FBgn0035754  
  Heatmap region span   3L:7124965..7340223   
  Segment span   3L:7234792..7243623   
  Length (genes)  4  
  Length (bp)  8832  
   Model Scoring   
  BIC  372.818208  
  logL  -180.896978  
  logL ratio  121.850200  
   Expression   
  Mean expression  7.665407  
  Median expression  7.579189  
  Tissue std. dev.  0.509880  
 
  No GO Slim enrichment  
  
   tissue    mean expression   
  5th Passage Drosophila S2 Cells  7.919797  
  Adult Accessory gland  7.493848  
  Adult Brain  8.221573  
  Adult Carcass  6.991447  
  Adult Crop  7.692782  
  Adult Eye  7.746746  
  Adult Fatbody  7.262101  
  Adult Female Spermatheca Mated  7.254246  
  Adult Female Spermatheca Virgin  7.214799  
  Adult Head  7.357137  
  Adult Heart  7.701774  
  Adult Hind Gut  7.519966  
  Adult Male Ejaculatory Duct  7.533866  
  Adult Mid Gut  7.030050  
  Adult Ovary  8.934353  
  Adult Salivary Gland  7.493556  
  Adult Testes  8.798182  
  Adult Thoracoabdominal ganglion  8.028838  
  Adult Whole Fly  7.768638  
  Larvae Wandering Tubules  7.600063  
  Larval Feeding Carcass  7.357337  
  Larval Feeding Central Nevous System  8.710844  
  Larval Feeding Hind Gut  7.527677  
  Larval Feeding Malpighian Tubule  7.808185  
  Larval Feeding Mid Gut  6.967368  
  Larval Feeding Salivary Gland  7.870144  
  Whole Larvae Feeding  7.160663  
 
  
   FlyBase ID    symbol    start    end    strand    length   
   FBgn0024542   Neos   7234792   7236239  +  1448  
   FBgn0028648   mRpL50  7236174   7237032   -  859  
   FBgn0012058   Cdc27  7238856   7242439   -  3584  
   FBgn0035754   MED4  7242629   7243623   -  995  
 
 
    Segment 157 
 
   Location   
  Gene key  FBgn0035760-FBgn0035761  
  Heatmap region span   3L:7155414..7341415   
  Segment span   3L:7313170..7316509   
  Length (genes)  2  
  Length (bp)  3340  
   Model Scoring   
  BIC  237.541206  
  logL  -113.258477  
  logL ratio  -3.021132  
   Expression   
  Mean expression  6.310601  
  Median expression  6.371344  
  Tissue std. dev.  0.745088  
 
  No GO Slim enrichment  
  
   tissue    mean expression   
  5th Passage Drosophila S2 Cells  7.594626  
  Adult Accessory gland  6.479295  
  Adult Brain  5.614149  
  Adult Carcass  5.528034  
  Adult Crop  6.088311  
  Adult Eye  6.173861  
  Adult Fatbody  6.004889  
  Adult Female Spermatheca Mated  6.016380  
  Adult Female Spermatheca Virgin  6.024571  
  Adult Head  5.700506  
  Adult Heart  6.017266  
  Adult Hind Gut  6.115581  
  Adult Male Ejaculatory Duct  5.797734  
  Adult Mid Gut  6.035775  
  Adult Ovary  8.554512  
  Adult Salivary Gland  6.072185  
  Adult Testes  5.325773  
  Adult Thoracoabdominal ganglion  5.804063  
  Adult Whole Fly  7.060258  
  Larvae Wandering Tubules  5.896071  
  Larval Feeding Carcass  6.507776  
  Larval Feeding Central Nevous System  8.243348  
  Larval Feeding Hind Gut  6.610990  
  Larval Feeding Malpighian Tubule  6.254874  
  Larval Feeding Mid Gut  6.101734  
  Larval Feeding Salivary Gland  6.186885  
  Whole Larvae Feeding  6.576788  
 
  
   FlyBase ID    symbol    start    end    strand    length   
   FBgn0035760   CG8607   7313170   7314248  +  1079  
   FBgn0035761   RhoGEF4  7314198   7316509   -  2312  
 
    Segment 158 
 
   Location   
  Gene key  FBgn0035762-FBgn0035767  
  Heatmap region span   3L:7234449..7349796   
  Segment span   3L:7316729..7338281   
  Length (genes)  6  
  Length (bp)  21553  
   Model Scoring   
  BIC  740.057540  
  logL  -364.516644  
  logL ratio  96.483088  
   Expression   
  Mean expression  9.565899  
  Median expression  9.698172  
  Tissue std. dev.  0.439743  
 
  
   GO ID    description    ratio    P-value   
   GO:0055085   transmembrane transport  2/6  0.0313  
 
  
   tissue    mean expression   
  5th Passage Drosophila S2 Cells  10.198916  
  Adult Accessory gland  10.116709  
  Adult Brain  8.666963  
  Adult Carcass  9.438434  
  Adult Crop  10.024543  
  Adult Eye  9.615968  
  Adult Fatbody  9.522781  
  Adult Female Spermatheca Mated  9.771700  
  Adult Female Spermatheca Virgin  9.729323  
  Adult Head  9.223634  
  Adult Heart  9.796990  
  Adult Hind Gut  9.604623  
  Adult Male Ejaculatory Duct  9.943499  
  Adult Mid Gut  8.892223  
  Adult Ovary  10.030562  
  Adult Salivary Gland  9.878839  
  Adult Testes  8.747259  
  Adult Thoracoabdominal ganglion  8.588420  
  Adult Whole Fly  9.553672  
  Larvae Wandering Tubules  9.695918  
  Larval Feeding Carcass  9.899407  
  Larval Feeding Central Nevous System  9.320215  
  Larval Feeding Hind Gut  9.844667  
  Larval Feeding Malpighian Tubule  9.499683  
  Larval Feeding Mid Gut  9.216228  
  Larval Feeding Salivary Gland  10.085895  
  Whole Larvae Feeding  9.372203  
 
  
   FlyBase ID    symbol    start    end    strand    length   
   FBgn0035762   CG8605   7316729   7319358  +  2630  
   FBgn0035763   CG8602  7319373   7321958   -  2586  
   FBgn0035765     7326638   7328343   -  1706  
   FBgn0035766   eco  7328435   7332088   -  3654  
   FBgn0011640   lark   7332366   7334996  +  2631  
   FBgn0035767   CG8596  7334983   7338281   -  3299  
 
 
    Segment 159 
 
   Location   
  Gene key  FBgn0035770-FBgn0082598  
  Heatmap region span   3L:7340223..7397281   
  Segment span   3L:7353363..7362900   
  Length (genes)  4  
  Length (bp)  9538  
   Model Scoring   
  BIC  478.148153  
  logL  -233.561950  
  logL ratio  209.774109  
   Expression   
  Mean expression  11.220410  
  Median expression  11.023232  
  Tissue std. dev.  0.454492  
 
  No GO Slim enrichment  
  
   tissue    mean expression   
  5th Passage Drosophila S2 Cells  11.882973  
  Adult Accessory gland  11.880755  
  Adult Brain  10.244708  
  Adult Carcass  10.908599  
  Adult Crop  11.250870  
  Adult Eye  11.105115  
  Adult Fatbody  11.477795  
  Adult Female Spermatheca Mated  11.408146  
  Adult Female Spermatheca Virgin  11.304033  
  Adult Head  10.995371  
  Adult Heart  11.348314  
  Adult Hind Gut  11.011678  
  Adult Male Ejaculatory Duct  11.682490  
  Adult Mid Gut  11.482559  
  Adult Ovary  11.222902  
  Adult Salivary Gland  11.350143  
  Adult Testes  10.174468  
  Adult Thoracoabdominal ganglion  10.463260  
  Adult Whole Fly  11.194847  
  Larvae Wandering Tubules  11.243401  
  Larval Feeding Carcass  11.013462  
  Larval Feeding Central Nevous System  10.768944  
  Larval Feeding Hind Gut  11.605301  
  Larval Feeding Malpighian Tubule  10.780966  
  Larval Feeding Mid Gut  11.755889  
  Larval Feeding Salivary Gland  11.945168  
  Whole Larvae Feeding  11.448925  
 
  
   FlyBase ID    symbol    start    end    strand    length   
   FBgn0035770   pst  7350375   7353363   -  2989  
   FBgn0035771   sec63   7353663   7357466  +  3804  
   FBgn0035772   Sh3beta  7357900   7362357   -  4458  
   FBgn0082598   akirin   7362900   7366958  +  4059  
 
 
    Segment 160 
 
   Location   
  Gene key  FBgn0052380-FBgn0016983  
  Heatmap region span   3L:7341125..7428279   
  Segment span   3L:7374617..7374953   
  Length (genes)  2  
  Length (bp)  337  
   Model Scoring   
  BIC  208.317351  
  logL  -98.646549  
  logL ratio  50.439705  
   Expression   
  Mean expression  8.803605  
  Median expression  8.739783  
  Tissue std. dev.  0.522673  
 
  No GO Slim enrichment  
  
   tissue    mean expression   
  5th Passage Drosophila S2 Cells  9.786698  
  Adult Accessory gland  9.183430  
  Adult Brain  9.289146  
  Adult Carcass  8.245317  
  Adult Crop  8.787940  
  Adult Eye  8.483793  
  Adult Fatbody  8.519955  
  Adult Female Spermatheca Mated  8.714696  
  Adult Female Spermatheca Virgin  8.378991  
  Adult Head  8.491026  
  Adult Heart  8.411873  
  Adult Hind Gut  8.590136  
  Adult Male Ejaculatory Duct  8.519424  
  Adult Mid Gut  8.264410  
  Adult Ovary  10.437055  
  Adult Salivary Gland  8.857756  
  Adult Testes  8.276538  
  Adult Thoracoabdominal ganglion  9.232589  
  Adult Whole Fly  9.202721  
  Larvae Wandering Tubules  8.809686  
  Larval Feeding Carcass  8.482503  
  Larval Feeding Central Nevous System  9.715061  
  Larval Feeding Hind Gut  8.782818  
  Larval Feeding Malpighian Tubule  8.853991  
  Larval Feeding Mid Gut  8.480284  
  Larval Feeding Salivary Gland  8.667027  
  Whole Larvae Feeding  8.232465  
 
  
   FlyBase ID    symbol    start    end    strand    length   
   FBgn0052380   SMSr  7367142   7374617   -  7476  
   FBgn0016983   smid   7374953   7378742  +  3790  
 
    Segment 161 
 
   Location   
  Gene key  FBgn0035777-FBgn0052379  
  Heatmap region span   3L:7349796..7433833   
  Segment span   3L:7384179..7389455   
  Length (genes)  2  
  Length (bp)  5277  
   Model Scoring   
  BIC  172.452906  
  logL  -80.714327  
  logL ratio  35.713195  
   Expression   
  Mean expression  4.810763  
  Median expression  4.692127  
  Tissue std. dev.  0.381692  
 
  No GO Slim enrichment  
  
   tissue    mean expression   
  5th Passage Drosophila S2 Cells  4.924084  
  Adult Accessory gland  4.724988  
  Adult Brain  4.410431  
  Adult Carcass  4.643045  
  Adult Crop  4.937487  
  Adult Eye  4.406071  
  Adult Fatbody  4.757324  
  Adult Female Spermatheca Mated  4.523668  
  Adult Female Spermatheca Virgin  4.658082  
  Adult Head  4.633309  
  Adult Heart  4.577449  
  Adult Hind Gut  4.996541  
  Adult Male Ejaculatory Duct  4.822677  
  Adult Mid Gut  5.838323  
  Adult Ovary  4.742530  
  Adult Salivary Gland  4.954859  
  Adult Testes  4.574045  
  Adult Thoracoabdominal ganglion  4.309369  
  Adult Whole Fly  4.514282  
  Larvae Wandering Tubules  4.820544  
  Larval Feeding Carcass  5.334540  
  Larval Feeding Central Nevous System  4.402469  
  Larval Feeding Hind Gut  4.759531  
  Larval Feeding Malpighian Tubule  4.749485  
  Larval Feeding Mid Gut  5.903415  
  Larval Feeding Salivary Gland  4.724757  
  Whole Larvae Feeding  5.247287  
 
  
   FlyBase ID    symbol    start    end    strand    length   
   FBgn0035777   CG8563   7384179   7389116  +  4938  
   FBgn0052379   CG32379   7389455   7391309  +  1855  
 
    Segment 162 
 
   Location   
  Gene key  FBgn0035779-FBgn0035781  
  Heatmap region span   3L:7353363..7458609   
  Segment span   3L:7391388..7397281   
  Length (genes)  3  
  Length (bp)  5894  
   Model Scoring   
  BIC  341.154065  
  logL  -165.064906  
  logL ratio  6.798898  
   Expression   
  Mean expression  6.158590  
  Median expression  5.234387  
  Tissue std. dev.  2.200101  
 
  No GO Slim enrichment  
  
   tissue    mean expression   
  5th Passage Drosophila S2 Cells  5.108153  
  Adult Accessory gland  5.065736  
  Adult Brain  4.647709  
  Adult Carcass  5.078457  
  Adult Crop  5.017110  
  Adult Eye  4.592821  
  Adult Fatbody  5.057548  
  Adult Female Spermatheca Mated  5.231599  
  Adult Female Spermatheca Virgin  5.145860  
  Adult Head  4.837608  
  Adult Heart  4.853874  
  Adult Hind Gut  7.789305  
  Adult Male Ejaculatory Duct  5.144642  
  Adult Mid Gut  11.305956  
  Adult Ovary  4.844998  
  Adult Salivary Gland  5.153749  
  Adult Testes  6.764500  
  Adult Thoracoabdominal ganglion  4.634926  
  Adult Whole Fly  8.052048  
  Larvae Wandering Tubules  5.458341  
  Larval Feeding Carcass  5.256401  
  Larval Feeding Central Nevous System  4.818293  
  Larval Feeding Hind Gut  8.454545  
  Larval Feeding Malpighian Tubule  5.304928  
  Larval Feeding Mid Gut  12.341615  
  Larval Feeding Salivary Gland  4.989861  
  Whole Larvae Feeding  11.331333  
 
  
   FlyBase ID    symbol    start    end    strand    length   
   FBgn0035779   CG8562   7391388   7393012  +  1625  
   FBgn0035780   CG18417   7393177   7394774  +  1598  
   FBgn0035781   CG8560  7395430   7397281   -  1852  
 
 
    Segment 163 
 
   Location   
  Gene key  FBgn0052383-FBgn0052382  
  Heatmap region span   3L:7384179..7489279   
  Segment span   3L:7432505..7433833   
  Length (genes)  2  
  Length (bp)  1329  
   Model Scoring   
  BIC  171.819410  
  logL  -80.397579  
  logL ratio  43.624768  
   Expression   
  Mean expression  4.992332  
  Median expression  4.613466  
  Tissue std. dev.  1.470002  
 
  No GO Slim enrichment  
  
   tissue    mean expression   
  5th Passage Drosophila S2 Cells  4.450186  
  Adult Accessory gland  12.151565  
  Adult Brain  4.370065  
  Adult Carcass  4.972727  
  Adult Crop  4.654447  
  Adult Eye  4.260708  
  Adult Fatbody  4.860616  
  Adult Female Spermatheca Mated  5.099496  
  Adult Female Spermatheca Virgin  5.081475  
  Adult Head  4.502298  
  Adult Heart  4.431721  
  Adult Hind Gut  4.521543  
  Adult Male Ejaculatory Duct  6.437593  
  Adult Mid Gut  4.806636  
  Adult Ovary  4.542352  
  Adult Salivary Gland  4.895662  
  Adult Testes  4.379443  
  Adult Thoracoabdominal ganglion  4.420030  
  Adult Whole Fly  5.323318  
  Larvae Wandering Tubules  4.672548  
  Larval Feeding Carcass  4.609569  
  Larval Feeding Central Nevous System  4.304791  
  Larval Feeding Hind Gut  4.447237  
  Larval Feeding Malpighian Tubule  4.676544  
  Larval Feeding Mid Gut  4.631177  
  Larval Feeding Salivary Gland  5.036829  
  Whole Larvae Feeding  4.252379  
 
  
   FlyBase ID    symbol    start    end    strand    length   
   FBgn0052383   sphinx1  7431521   7432505   -  985  
   FBgn0052382   sphinx2  7432799   7433833   -  1035  
 
    Segment 164 
 
   Location   
  Gene key  FBgn0053278-FBgn0035786  
  Heatmap region span   3L:7391388..7491771   
  Segment span   3L:7443953..7458609   
  Length (genes)  3  
  Length (bp)  14657  
   Model Scoring   
  BIC  260.996802  
  logL  -124.986274  
  logL ratio  61.657131  
   Expression   
  Mean expression  4.454665  
  Median expression  4.221666  
  Tissue std. dev.  0.762340  
 
  No GO Slim enrichment  
  
   tissue    mean expression   
  5th Passage Drosophila S2 Cells  4.205631  
  Adult Accessory gland  4.330166  
  Adult Brain  4.064922  
  Adult Carcass  4.480644  
  Adult Crop  4.231874  
  Adult Eye  4.256689  
  Adult Fatbody  4.355440  
  Adult Female Spermatheca Mated  4.395918  
  Adult Female Spermatheca Virgin  4.347507  
  Adult Head  4.116869  
  Adult Heart  4.352935  
  Adult Hind Gut  4.228380  
  Adult Male Ejaculatory Duct  4.272115  
  Adult Mid Gut  4.260980  
  Adult Ovary  4.132535  
  Adult Salivary Gland  4.513882  
  Adult Testes  8.238431  
  Adult Thoracoabdominal ganglion  4.372400  
  Adult Whole Fly  4.918395  
  Larvae Wandering Tubules  4.337507  
  Larval Feeding Carcass  4.501232  
  Larval Feeding Central Nevous System  3.961254  
  Larval Feeding Hind Gut  4.185322  
  Larval Feeding Malpighian Tubule  4.276511  
  Larval Feeding Mid Gut  4.256134  
  Larval Feeding Salivary Gland  4.226283  
  Whole Larvae Feeding  4.455990  
 
  
   FlyBase ID    symbol    start    end    strand    length   
   FBgn0053278   CG33278   7443953   7445259  +  1307  
   FBgn0035785   CG8546   7449129   7451453  +  2325  
   FBgn0035786   Tsp66A   7458609   7461908  +  3300  
 
 
    Segment 165 
 
   Location   
  Gene key  FBgn0035788-FBgn0035790  
  Heatmap region span   3L:7430566..7504090   
  Segment span   3L:7464710..7484964   
  Length (genes)  3  
  Length (bp)  20255  
   Model Scoring   
  BIC  285.348584  
  logL  -137.162166  
  logL ratio  47.369310  
   Expression   
  Mean expression  4.697140  
  Median expression  4.277177  
  Tissue std. dev.  0.395509  
 
  No GO Slim enrichment  
  
   tissue    mean expression   
  5th Passage Drosophila S2 Cells  4.568113  
  Adult Accessory gland  4.749364  
  Adult Brain  4.250797  
  Adult Carcass  4.660679  
  Adult Crop  4.520776  
  Adult Eye  4.370533  
  Adult Fatbody  4.612494  
  Adult Female Spermatheca Mated  4.509919  
  Adult Female Spermatheca Virgin  4.562166  
  Adult Head  4.309226  
  Adult Heart  4.513586  
  Adult Hind Gut  4.510738  
  Adult Male Ejaculatory Duct  4.533222  
  Adult Mid Gut  4.712673  
  Adult Ovary  4.575413  
  Adult Salivary Gland  4.798004  
  Adult Testes  4.543647  
  Adult Thoracoabdominal ganglion  4.514588  
  Adult Whole Fly  4.240272  
  Larvae Wandering Tubules  5.764090  
  Larval Feeding Carcass  5.439542  
  Larval Feeding Central Nevous System  4.385870  
  Larval Feeding Hind Gut  4.588306  
  Larval Feeding Malpighian Tubule  5.477198  
  Larval Feeding Mid Gut  4.954510  
  Larval Feeding Salivary Gland  4.576107  
  Whole Larvae Feeding  5.580948  
 
  
   FlyBase ID    symbol    start    end    strand    length   
   FBgn0035788   CG8541   7464710   7465537  +  828  
   FBgn0035789   mthl6   7479170   7480943  +  1774  
   FBgn0035790   Cyp316a1   7484964   7486584  +  1621  
 
 
    Segment 166 
 
   Location   
  Gene key  FBgn0035795-FBgn0035797  
  Heatmap region span   3L:7499317..7643683   
  Segment span   3L:7532936..7552922   
  Length (genes)  4  
  Length (bp)  19987  
   Model Scoring   
  BIC  283.137357  
  logL  -136.056552  
  logL ratio  131.878655  
   Expression   
  Mean expression  4.396511  
  Median expression  4.361281  
  Tissue std. dev.  0.151302  
 
  No GO Slim enrichment  
  
   tissue    mean expression   
  5th Passage Drosophila S2 Cells  4.469218  
  Adult Accessory gland  4.541836  
  Adult Brain  4.189044  
  Adult Carcass  4.437607  
  Adult Crop  4.405124  
  Adult Eye  4.352599  
  Adult Fatbody  4.448107  
  Adult Female Spermatheca Mated  4.557111  
  Adult Female Spermatheca Virgin  4.519817  
  Adult Head  4.235802  
  Adult Heart  4.322424  
  Adult Hind Gut  4.341729  
  Adult Male Ejaculatory Duct  4.604363  
  Adult Mid Gut  4.601527  
  Adult Ovary  4.413062  
  Adult Salivary Gland  4.635077  
  Adult Testes  4.195068  
  Adult Thoracoabdominal ganglion  4.224085  
  Adult Whole Fly  4.046865  
  Larvae Wandering Tubules  4.405739  
  Larval Feeding Carcass  4.389123  
  Larval Feeding Central Nevous System  4.239979  
  Larval Feeding Hind Gut  4.292580  
  Larval Feeding Malpighian Tubule  4.438400  
  Larval Feeding Mid Gut  4.606757  
  Larval Feeding Salivary Gland  4.555426  
  Whole Larvae Feeding  4.237340  
 
  
   FlyBase ID    symbol    start    end    strand    length   
   FBgn0035795   CG16998  7532160   7532936   -  777  
   FBgn0052374   CG32374  7533369   7534268   -  900  
   FBgn0052376   CG32376  7534682   7535557   -  876  
   FBgn0035797   CG14837  7548968   7552922   -  3955  
 
 
    Segment 167 
 
   Location   
  Gene key  FBgn0035799-FBgn0035802  
  Heatmap region span   3L:7512588..7712461   
  Segment span   3L:7576554..7586206   
  Length (genes)  3  
  Length (bp)  9653  
   Model Scoring   
  BIC  247.525162  
  logL  -118.250455  
  logL ratio  59.312461  
   Expression   
  Mean expression  4.801863  
  Median expression  4.630518  
  Tissue std. dev.  0.647783  
 
  No GO Slim enrichment  
  
   tissue    mean expression   
  5th Passage Drosophila S2 Cells  4.620153  
  Adult Accessory gland  4.729247  
  Adult Brain  4.450659  
  Adult Carcass  4.794265  
  Adult Crop  4.612686  
  Adult Eye  4.573564  
  Adult Fatbody  4.701616  
  Adult Female Spermatheca Mated  4.626103  
  Adult Female Spermatheca Virgin  4.609828  
  Adult Head  4.537409  
  Adult Heart  4.655954  
  Adult Hind Gut  4.667287  
  Adult Male Ejaculatory Duct  4.767539  
  Adult Mid Gut  4.766389  
  Adult Ovary  4.620274  
  Adult Salivary Gland  5.030042  
  Adult Testes  7.985821  
  Adult Thoracoabdominal ganglion  4.519987  
  Adult Whole Fly  5.277736  
  Larvae Wandering Tubules  4.579949  
  Larval Feeding Carcass  4.691452  
  Larval Feeding Central Nevous System  4.375478  
  Larval Feeding Hind Gut  4.527796  
  Larval Feeding Malpighian Tubule  4.597484  
  Larval Feeding Mid Gut  4.785936  
  Larval Feeding Salivary Gland  4.769338  
  Whole Larvae Feeding  4.776317  
 
  
   FlyBase ID    symbol    start    end    strand    length   
   FBgn0035799   CG14838   7576554   7580348  +  3795  
   FBgn0035800   CG7716  7581708   7584098   -  2391  
   FBgn0035802   CG33275   7586206   7632681  +  46476  
 
 
    Segment 168 
 
   Location   
  Gene key  FBgn0053276-FBgn0035805  
  Heatmap region span   3L:7521904..7719542   
  Segment span   3L:7633546..7634746   
  Length (genes)  2  
  Length (bp)  1201  
   Model Scoring   
  BIC  221.744824  
  logL  -105.360286  
  logL ratio  31.361428  
   Expression   
  Mean expression  7.949226  
  Median expression  7.997799  
  Tissue std. dev.  0.408650  
 
  No GO Slim enrichment  
  
   tissue    mean expression   
  5th Passage Drosophila S2 Cells  7.803511  
  Adult Accessory gland  8.481041  
  Adult Brain  8.141068  
  Adult Carcass  7.650211  
  Adult Crop  7.795854  
  Adult Eye  8.157172  
  Adult Fatbody  8.132920  
  Adult Female Spermatheca Mated  8.059676  
  Adult Female Spermatheca Virgin  8.254164  
  Adult Head  7.864185  
  Adult Heart  8.190739  
  Adult Hind Gut  7.768144  
  Adult Male Ejaculatory Duct  8.047066  
  Adult Mid Gut  7.738272  
  Adult Ovary  8.080278  
  Adult Salivary Gland  7.926889  
  Adult Testes  6.411429  
  Adult Thoracoabdominal ganglion  8.556377  
  Adult Whole Fly  7.248443  
  Larvae Wandering Tubules  7.986735  
  Larval Feeding Carcass  7.930039  
  Larval Feeding Central Nevous System  8.378417  
  Larval Feeding Hind Gut  8.040837  
  Larval Feeding Malpighian Tubule  8.272004  
  Larval Feeding Mid Gut  7.718491  
  Larval Feeding Salivary Gland  8.244321  
  Whole Larvae Feeding  7.750831  
 
  
   FlyBase ID    symbol    start    end    strand    length   
   FBgn0053276   CG33276  7632911   7633546   -  636  
   FBgn0035805   CG7506  7633898   7634746   -  849  
 
    Segment 169 
 
   Location   
  Gene key  FBgn0035813-FBgn0035816  
  Heatmap region span   3L:7643683..7768703   
  Segment span   3L:7725087..7740717   
  Length (genes)  3  
  Length (bp)  15631  
   Model Scoring   
  BIC  329.164361  
  logL  -159.070054  
  logL ratio  -3.183952  
   Expression   
  Mean expression  5.196199  
  Median expression  4.792114  
  Tissue std. dev.  0.722287  
 
  No GO Slim enrichment  
  
   tissue    mean expression   
  5th Passage Drosophila S2 Cells  4.757473  
  Adult Accessory gland  4.860705  
  Adult Brain  5.381139  
  Adult Carcass  5.274367  
  Adult Crop  7.499738  
  Adult Eye  4.593976  
  Adult Fatbody  4.937197  
  Adult Female Spermatheca Mated  4.925361  
  Adult Female Spermatheca Virgin  4.785369  
  Adult Head  5.361147  
  Adult Heart  6.007001  
  Adult Hind Gut  6.591856  
  Adult Male Ejaculatory Duct  4.905026  
  Adult Mid Gut  4.866821  
  Adult Ovary  4.536525  
  Adult Salivary Gland  5.719426  
  Adult Testes  4.513831  
  Adult Thoracoabdominal ganglion  5.168194  
  Adult Whole Fly  4.514619  
  Larvae Wandering Tubules  4.724430  
  Larval Feeding Carcass  6.567309  
  Larval Feeding Central Nevous System  4.616386  
  Larval Feeding Hind Gut  5.709223  
  Larval Feeding Malpighian Tubule  4.681053  
  Larval Feeding Mid Gut  4.807794  
  Larval Feeding Salivary Gland  4.774122  
  Whole Larvae Feeding  5.217284  
 
  
   FlyBase ID    symbol    start    end    strand    length   
   FBgn0035813   CG8492  7721264   7725087   -  3824  
   FBgn0035815   Snmp2  7726350   7735785   -  9436  
   FBgn0035816   CG13685   7740717   7741466  +  750  
 
 
    Segment 170 
 
   Location   
  Gene key  FBgn0052369-FBgn0024187  
  Heatmap region span   3L:7747509..8084912   
  Segment span   3L:7802925..7929063   
  Length (genes)  8  
  Length (bp)  126139  
   Model Scoring   
  BIC  1063.632423  
  logL  -526.304085  
  logL ratio  -101.724631  
   Expression   
  Mean expression  7.327487  
  Median expression  7.337877  
  Tissue std. dev.  0.436080  
 
  No GO Slim enrichment  
  
   tissue    mean expression   
  5th Passage Drosophila S2 Cells  8.349333  
  Adult Accessory gland  7.937124  
  Adult Brain  7.865365  
  Adult Carcass  7.025385  
  Adult Crop  7.281023  
  Adult Eye  7.605373  
  Adult Fatbody  6.887344  
  Adult Female Spermatheca Mated  7.087161  
  Adult Female Spermatheca Virgin  7.016729  
  Adult Head  7.269750  
  Adult Heart  7.577222  
  Adult Hind Gut  7.989446  
  Adult Male Ejaculatory Duct  7.277826  
  Adult Mid Gut  7.671756  
  Adult Ovary  7.392640  
  Adult Salivary Gland  7.633257  
  Adult Testes  6.383380  
  Adult Thoracoabdominal ganglion  7.661292  
  Adult Whole Fly  7.211787  
  Larvae Wandering Tubules  7.071920  
  Larval Feeding Carcass  6.777314  
  Larval Feeding Central Nevous System  6.944496  
  Larval Feeding Hind Gut  7.648998  
  Larval Feeding Malpighian Tubule  7.390856  
  Larval Feeding Mid Gut  7.235416  
  Larval Feeding Salivary Gland  6.959691  
  Whole Larvae Feeding  6.690266  
 
  
   FlyBase ID    symbol    start    end    strand    length   
   FBgn0052369   CG32369  7770952   7802925   -  31974  
   FBgn0016694   Pdp1  7807437   7860472   -  53036  
   FBgn0052365   CG32365   7862144   7887914  +  25771  
   FBgn0003041   pbl  7889214   7905051   -  15838  
   FBgn0035824   CG8281   7905563   7906863  +  1301  
   FBgn0035825   CG8111  7906764   7908218   -  1455  
   FBgn0052368   CG32368   7908537   7908946  +  410  
   FBgn0024187   syd  7919125   7929063   -  9939  
 
 
    Segment 171 
 
   Location   
  Gene key  FBgn0010406-FBgn0026263  
  Heatmap region span   3L:7768703..8117631   
  Segment span   3L:7963693..8051811   
  Length (genes)  7  
  Length (bp)  88119  
   Model Scoring   
  BIC  798.975311  
  logL  -393.975529  
  logL ratio  98.455810  
   Expression   
  Mean expression  8.512373  
  Median expression  8.468864  
  Tissue std. dev.  0.447266  
 
  
   GO ID    description    ratio    P-value   
   GO:0003723   RNA binding  2/7  0.0296  
 
  
   tissue    mean expression   
  5th Passage Drosophila S2 Cells  9.056259  
  Adult Accessory gland  8.677018  
  Adult Brain  8.841598  
  Adult Carcass  8.119145  
  Adult Crop  8.403212  
  Adult Eye  8.698042  
  Adult Fatbody  8.419278  
  Adult Female Spermatheca Mated  8.101512  
  Adult Female Spermatheca Virgin  8.086368  
  Adult Head  8.295084  
  Adult Heart  8.538134  
  Adult Hind Gut  8.157638  
  Adult Male Ejaculatory Duct  8.661999  
  Adult Mid Gut  7.687959  
  Adult Ovary  9.571733  
  Adult Salivary Gland  8.268133  
  Adult Testes  9.057899  
  Adult Thoracoabdominal ganglion  8.775677  
  Adult Whole Fly  8.750331  
  Larvae Wandering Tubules  8.623449  
  Larval Feeding Carcass  8.357516  
  Larval Feeding Central Nevous System  9.557026  
  Larval Feeding Hind Gut  8.379634  
  Larval Feeding Malpighian Tubule  8.317689  
  Larval Feeding Mid Gut  7.779392  
  Larval Feeding Salivary Gland  8.589828  
  Whole Larvae Feeding  8.062512  
 
  
   FlyBase ID    symbol    start    end    strand    length   
   FBgn0010406   RNaseX25   7963693   7965549  +  1857  
   FBgn0027554   CG8042  7965549   7968108   -  2560  
   FBgn0035829   HP4  7968538   7969257   -  720  
   FBgn0035830   CG8209   7969403   7970949  +  1547  
   FBgn0035831   CG8038  7970905   7971739   -  835  
   FBgn0011817   nmo   7972149   8043433  +  71285  
   FBgn0026263   bip1  8047433   8051811   -  4379  
 
 
    Segment 172 
 
   Location   
  Gene key  FBgn0027549-FBgn0026252  
  Heatmap region span   3L:7941291..8144908   
  Segment span   3L:8103363..8109278   
  Length (genes)  3  
  Length (bp)  5916  
   Model Scoring   
  BIC  329.479152  
  logL  -159.227450  
  logL ratio  122.610881  
   Expression   
  Mean expression  10.425682  
  Median expression  10.337255  
  Tissue std. dev.  0.502358  
 
  No GO Slim enrichment  
  
   tissue    mean expression   
  5th Passage Drosophila S2 Cells  11.471111  
  Adult Accessory gland  11.003368  
  Adult Brain  9.752384  
  Adult Carcass  9.608134  
  Adult Crop  10.563361  
  Adult Eye  9.530001  
  Adult Fatbody  10.123636  
  Adult Female Spermatheca Mated  10.258272  
  Adult Female Spermatheca Virgin  9.998741  
  Adult Head  9.618480  
  Adult Heart  10.090855  
  Adult Hind Gut  10.373297  
  Adult Male Ejaculatory Duct  10.345330  
  Adult Mid Gut  10.865603  
  Adult Ovary  10.996756  
  Adult Salivary Gland  10.372837  
  Adult Testes  9.978441  
  Adult Thoracoabdominal ganglion  9.922485  
  Adult Whole Fly  10.495879  
  Larvae Wandering Tubules  10.442923  
  Larval Feeding Carcass  10.798201  
  Larval Feeding Central Nevous System  11.022192  
  Larval Feeding Hind Gut  10.972803  
  Larval Feeding Malpighian Tubule  10.447995  
  Larval Feeding Mid Gut  11.019927  
  Larval Feeding Salivary Gland  10.909530  
  Whole Larvae Feeding  10.510869  
 
  
   FlyBase ID    symbol    start    end    strand    length   
   FBgn0027549   CG7927   8103363   8106427  +  3065  
   FBgn0011744     8106588   8109000   -  2413  
   FBgn0026252   msk   8109278   8114681  +  5404  
 
 
    Segment 173 
 
   Location   
  Gene key  FBgn0035838-FBgn0035839  
  Heatmap region span   3L:7963693..8168998   
  Segment span   3L:8114601..8117631   
  Length (genes)  2  
  Length (bp)  3031  
   Model Scoring   
  BIC  240.035507  
  logL  -114.505627  
  logL ratio  14.018691  
   Expression   
  Mean expression  7.731420  
  Median expression  7.855619  
  Tissue std. dev.  0.519466  
 
  No GO Slim enrichment  
  
   tissue    mean expression   
  5th Passage Drosophila S2 Cells  7.593622  
  Adult Accessory gland  7.754736  
  Adult Brain  7.363179  
  Adult Carcass  7.240579  
  Adult Crop  7.761102  
  Adult Eye  7.292817  
  Adult Fatbody  7.291918  
  Adult Female Spermatheca Mated  7.556026  
  Adult Female Spermatheca Virgin  7.512530  
  Adult Head  7.111185  
  Adult Heart  7.536124  
  Adult Hind Gut  7.558037  
  Adult Male Ejaculatory Duct  8.009436  
  Adult Mid Gut  7.398424  
  Adult Ovary  8.669612  
  Adult Salivary Gland  7.540227  
  Adult Testes  7.734402  
  Adult Thoracoabdominal ganglion  7.507675  
  Adult Whole Fly  7.285733  
  Larvae Wandering Tubules  9.465725  
  Larval Feeding Carcass  7.847159  
  Larval Feeding Central Nevous System  8.000287  
  Larval Feeding Hind Gut  7.580341  
  Larval Feeding Malpighian Tubule  8.807326  
  Larval Feeding Mid Gut  7.743452  
  Larval Feeding Salivary Gland  8.203475  
  Whole Larvae Feeding  7.383216  
 
  
   FlyBase ID    symbol    start    end    strand    length   
   FBgn0035838   ldbr   8114601   8116725  +  2125  
   FBgn0035839   CG7550  8116711   8117631   -  921  
 
    Segment 174 
 
   Location   
  Gene key  FBgn0042112-FBgn0010350  
  Heatmap region span   3L:8084912..8171510   
  Segment span   3L:8118213..8121152   
  Length (genes)  3  
  Length (bp)  2940  
   Model Scoring   
  BIC  357.512776  
  logL  -173.244262  
  logL ratio  75.104859  
   Expression   
  Mean expression  9.950305  
  Median expression  9.587607  
  Tissue std. dev.  0.567247  
 
  No GO Slim enrichment  
  
   tissue    mean expression   
  5th Passage Drosophila S2 Cells  10.564774  
  Adult Accessory gland  9.066174  
  Adult Brain  10.606497  
  Adult Carcass  9.354083  
  Adult Crop  10.281327  
  Adult Eye  10.717942  
  Adult Fatbody  9.839484  
  Adult Female Spermatheca Mated  9.441883  
  Adult Female Spermatheca Virgin  9.373289  
  Adult Head  10.374559  
  Adult Heart  10.036179  
  Adult Hind Gut  10.049931  
  Adult Male Ejaculatory Duct  9.719000  
  Adult Mid Gut  9.504021  
  Adult Ovary  10.577996  
  Adult Salivary Gland  9.090156  
  Adult Testes  8.779146  
  Adult Thoracoabdominal ganglion  10.479651  
  Adult Whole Fly  10.208955  
  Larvae Wandering Tubules  10.237577  
  Larval Feeding Carcass  9.757952  
  Larval Feeding Central Nevous System  11.239601  
  Larval Feeding Hind Gut  9.911076  
  Larval Feeding Malpighian Tubule  10.029762  
  Larval Feeding Mid Gut  9.456851  
  Larval Feeding Salivary Gland  10.206446  
  Whole Larvae Feeding  9.753923  
 
  
   FlyBase ID    symbol    start    end    strand    length   
   FBgn0042112   mRpL36  8117684   8118213   -  530  
   FBgn0029113   Uba2  8118423   8120919   -  2497  
   FBgn0010350   CdsA   8121152   8124775  +  3624  
 
 
    Segment 175 
 
   Location   
  Gene key  FBgn0035842-FBgn0002899  
  Heatmap region span   3L:8102232..8173614   
  Segment span   3L:8130313..8130914   
  Length (genes)  2  
  Length (bp)  602  
   Model Scoring   
  BIC  190.827125  
  logL  -89.901436  
  logL ratio  32.677579  
   Expression   
  Mean expression  5.841254  
  Median expression  5.603431  
  Tissue std. dev.  0.901554  
 
  No GO Slim enrichment  
  
   tissue    mean expression   
  5th Passage Drosophila S2 Cells  7.559770  
  Adult Accessory gland  5.554517  
  Adult Brain  5.676818  
  Adult Carcass  5.088471  
  Adult Crop  5.654338  
  Adult Eye  5.377436  
  Adult Fatbody  5.313930  
  Adult Female Spermatheca Mated  5.238592  
  Adult Female Spermatheca Virgin  5.275633  
  Adult Head  5.198088  
  Adult Heart  5.306199  
  Adult Hind Gut  5.280917  
  Adult Male Ejaculatory Duct  5.171133  
  Adult Mid Gut  5.122887  
  Adult Ovary  8.924326  
  Adult Salivary Gland  5.665536  
  Adult Testes  6.758254  
  Adult Thoracoabdominal ganglion  5.807024  
  Adult Whole Fly  6.921192  
  Larvae Wandering Tubules  5.802954  
  Larval Feeding Carcass  5.511859  
  Larval Feeding Central Nevous System  7.457826  
  Larval Feeding Hind Gut  5.637289  
  Larval Feeding Malpighian Tubule  5.972290  
  Larval Feeding Mid Gut  5.143790  
  Larval Feeding Salivary Gland  5.990699  
  Whole Larvae Feeding  5.302098  
 
  
   FlyBase ID    symbol    start    end    strand    length   
   FBgn0035842   CG7504  8124542   8130313   -  5772  
   FBgn0002899   mus301   8130914   8135114  +  4201  
 
    Segment 176 
 
   Location   
  Gene key  FBgn0035845-FBgn0040290  
  Heatmap region span   3L:8114601..8179750   
  Segment span   3L:8157552..8168998   
  Length (genes)  2  
  Length (bp)  11447  
   Model Scoring   
  BIC  205.459115  
  logL  -97.217431  
  logL ratio  8.417790  
   Expression   
  Mean expression  4.802602  
  Median expression  4.509988  
  Tissue std. dev.  0.604671  
 
  No GO Slim enrichment  
  
   tissue    mean expression   
  5th Passage Drosophila S2 Cells  5.893272  
  Adult Accessory gland  4.481341  
  Adult Brain  4.222645  
  Adult Carcass  4.422101  
  Adult Crop  4.484485  
  Adult Eye  4.540401  
  Adult Fatbody  4.719861  
  Adult Female Spermatheca Mated  4.504839  
  Adult Female Spermatheca Virgin  4.542166  
  Adult Head  4.421811  
  Adult Heart  4.509988  
  Adult Hind Gut  4.382168  
  Adult Male Ejaculatory Duct  4.390044  
  Adult Mid Gut  4.377126  
  Adult Ovary  6.739334  
  Adult Salivary Gland  4.572990  
  Adult Testes  4.429607  
  Adult Thoracoabdominal ganglion  4.189533  
  Adult Whole Fly  5.743326  
  Larvae Wandering Tubules  4.572455  
  Larval Feeding Carcass  5.173675  
  Larval Feeding Central Nevous System  5.779432  
  Larval Feeding Hind Gut  4.976802  
  Larval Feeding Malpighian Tubule  4.875314  
  Larval Feeding Mid Gut  4.531562  
  Larval Feeding Salivary Gland  5.530137  
  Whole Larvae Feeding  4.663836  
 
  
   FlyBase ID    symbol    start    end    strand    length   
   FBgn0035845   CG13675  8153415   8157552   -  4138  
   FBgn0040290   RecQ4  8163919   8168998   -  5080  
 
    Segment 177 
 
   Location   
  Gene key  FBgn0035855-FBgn0035857  
  Heatmap region span   3L:8184372..8282957   
  Segment span   3L:8195496..8204990   
  Length (genes)  4  
  Length (bp)  9495  
   Model Scoring   
  BIC  321.816660  
  logL  -155.396204  
  logL ratio  107.475297  
   Expression   
  Mean expression  4.497689  
  Median expression  4.329302  
  Tissue std. dev.  0.823248  
 
  No GO Slim enrichment  
  
   tissue    mean expression   
  5th Passage Drosophila S2 Cells  4.428497  
  Adult Accessory gland  4.250441  
  Adult Brain  4.593462  
  Adult Carcass  4.268065  
  Adult Crop  4.515981  
  Adult Eye  4.108275  
  Adult Fatbody  4.201237  
  Adult Female Spermatheca Mated  4.347914  
  Adult Female Spermatheca Virgin  4.345931  
  Adult Head  4.183020  
  Adult Heart  4.031971  
  Adult Hind Gut  4.161006  
  Adult Male Ejaculatory Duct  4.339040  
  Adult Mid Gut  4.214088  
  Adult Ovary  4.907663  
  Adult Salivary Gland  4.501564  
  Adult Testes  8.501661  
  Adult Thoracoabdominal ganglion  4.371766  
  Adult Whole Fly  5.222493  
  Larvae Wandering Tubules  4.278161  
  Larval Feeding Carcass  4.289843  
  Larval Feeding Central Nevous System  4.150856  
  Larval Feeding Hind Gut  4.181389  
  Larval Feeding Malpighian Tubule  4.160891  
  Larval Feeding Mid Gut  4.171934  
  Larval Feeding Salivary Gland  4.217521  
  Whole Larvae Feeding  4.492928  
 
  
   FlyBase ID    symbol    start    end    strand    length   
   FBgn0035855   CG7366  8194173   8195496   -  1324  
   FBgn0035856   CG13679   8195740   8196446  +  707  
   FBgn0052364   CG32364  8202677   8203771   -  1095  
   FBgn0035857   CG8006   8204990   8206722  +  1733  
 
 
    Segment 178 
 
   Location   
  Gene key  FBgn0259232-FBgn0035861  
  Heatmap region span   3L:8191309..8300080   
  Segment span   3L:8231747..8233701   
  Length (genes)  2  
  Length (bp)  1955  
   Model Scoring   
  BIC  178.934236  
  logL  -83.954992  
  logL ratio  31.409955  
   Expression   
  Mean expression  4.942842  
  Median expression  4.937974  
  Tissue std. dev.  0.211324  
 
  No GO Slim enrichment  
  
   tissue    mean expression   
  5th Passage Drosophila S2 Cells  4.926602  
  Adult Accessory gland  4.943550  
  Adult Brain  5.232498  
  Adult Carcass  4.860397  
  Adult Crop  4.809359  
  Adult Eye  5.159630  
  Adult Fatbody  5.136011  
  Adult Female Spermatheca Mated  4.952605  
  Adult Female Spermatheca Virgin  5.152541  
  Adult Head  4.936500  
  Adult Heart  4.811204  
  Adult Hind Gut  4.677379  
  Adult Male Ejaculatory Duct  5.089695  
  Adult Mid Gut  4.890780  
  Adult Ovary  4.821489  
  Adult Salivary Gland  5.417332  
  Adult Testes  5.211704  
  Adult Thoracoabdominal ganglion  4.937974  
  Adult Whole Fly  4.335598  
  Larvae Wandering Tubules  4.759047  
  Larval Feeding Carcass  5.017491  
  Larval Feeding Central Nevous System  4.973790  
  Larval Feeding Hind Gut  4.766781  
  Larval Feeding Malpighian Tubule  4.839625  
  Larval Feeding Mid Gut  4.923960  
  Larval Feeding Salivary Gland  5.152422  
  Whole Larvae Feeding  4.720764  
 
  
   FlyBase ID    symbol    start    end    strand    length   
   FBgn0259232      8231747   8274251  +  42505  
   FBgn0035861   CG7213  8224914   8233701   -  8788  
 
    Segment 179 
 
   Location   
  Gene key  FBgn0035867-FBgn0087039  
  Heatmap region span   3L:8215243..8340263   
  Segment span   3L:8292791..8297260   
  Length (genes)  4  
  Length (bp)  4470  
   Model Scoring   
  BIC  464.864249  
  logL  -226.919998  
  logL ratio  25.218624  
   Expression   
  Mean expression  7.881280  
  Median expression  8.225479  
  Tissue std. dev.  0.467508  
 
  No GO Slim enrichment  
  
   tissue    mean expression   
  5th Passage Drosophila S2 Cells  8.219272  
  Adult Accessory gland  8.643593  
  Adult Brain  7.691416  
  Adult Carcass  7.601484  
  Adult Crop  7.617033  
  Adult Eye  7.969459  
  Adult Fatbody  7.841001  
  Adult Female Spermatheca Mated  8.214067  
  Adult Female Spermatheca Virgin  8.474078  
  Adult Head  7.735494  
  Adult Heart  8.847974  
  Adult Hind Gut  8.155903  
  Adult Male Ejaculatory Duct  7.375375  
  Adult Mid Gut  8.612539  
  Adult Ovary  8.830151  
  Adult Salivary Gland  7.301215  
  Adult Testes  7.386173  
  Adult Thoracoabdominal ganglion  7.508375  
  Adult Whole Fly  8.028156  
  Larvae Wandering Tubules  7.953980  
  Larval Feeding Carcass  7.588642  
  Larval Feeding Central Nevous System  7.814309  
  Larval Feeding Hind Gut  7.512958  
  Larval Feeding Malpighian Tubule  7.611161  
  Larval Feeding Mid Gut  7.722260  
  Larval Feeding Salivary Gland  7.209202  
  Whole Larvae Feeding  7.329278  
 
  
   FlyBase ID    symbol    start    end    strand    length   
   FBgn0035867   CG13671   8292791   8295446  +  2656  
   FBgn0035866   CG7197  8291595   8292897   -  1303  
   FBgn0035868   CG7194  8295464   8297159   -  1696  
   FBgn0087039   Sbp2   8297260   8298433  +  1174  
 
 
    Segment 180 
 
   Location   
  Gene key  FBgn0035871-FBgn0035872  
  Heatmap region span   3L:8282957..8354628   
  Segment span   3L:8302080..8308602   
  Length (genes)  2  
  Length (bp)  6523  
   Model Scoring   
  BIC  253.157637  
  logL  -121.066692  
  logL ratio  17.535948  
   Expression   
  Mean expression  9.190418  
  Median expression  9.156727  
  Tissue std. dev.  0.497017  
 
  No GO Slim enrichment  
  
   tissue    mean expression   
  5th Passage Drosophila S2 Cells  9.681853  
  Adult Accessory gland  9.631892  
  Adult Brain  9.281769  
  Adult Carcass  8.640066  
  Adult Crop  9.075940  
  Adult Eye  9.041434  
  Adult Fatbody  9.070290  
  Adult Female Spermatheca Mated  8.991162  
  Adult Female Spermatheca Virgin  8.965013  
  Adult Head  8.722943  
  Adult Heart  9.144812  
  Adult Hind Gut  8.961755  
  Adult Male Ejaculatory Duct  8.985157  
  Adult Mid Gut  8.563624  
  Adult Ovary  9.618403  
  Adult Salivary Gland  9.092299  
  Adult Testes  10.783316  
  Adult Thoracoabdominal ganglion  9.182272  
  Adult Whole Fly  10.054570  
  Larvae Wandering Tubules  9.348548  
  Larval Feeding Carcass  8.850006  
  Larval Feeding Central Nevous System  9.950932  
  Larval Feeding Hind Gut  8.975389  
  Larval Feeding Malpighian Tubule  9.106027  
  Larval Feeding Mid Gut  8.391928  
  Larval Feeding Salivary Gland  9.265136  
  Whole Larvae Feeding  8.764762  
 
  
   FlyBase ID    symbol    start    end    strand    length   
   FBgn0035871   BI-1  8300245   8302080   -  1836  
   FBgn0035872   CG7185  8303593   8308602   -  5010  
 
    Segment 181 
 
   Location   
  Gene key  FBgn0035873-FBgn0035875  
  Heatmap region span   3L:8288422..8365509   
  Segment span   3L:8313863..8329772   
  Length (genes)  3  
  Length (bp)  15910  
   Model Scoring   
  BIC  319.217124  
  logL  -154.096435  
  logL ratio  4.034214  
   Expression   
  Mean expression  5.334969  
  Median expression  4.773507  
  Tissue std. dev.  0.499934  
 
  No GO Slim enrichment  
  
   tissue    mean expression   
  5th Passage Drosophila S2 Cells  5.219284  
  Adult Accessory gland  5.255792  
  Adult Brain  4.768038  
  Adult Carcass  5.207870  
  Adult Crop  5.037885  
  Adult Eye  6.251618  
  Adult Fatbody  5.295427  
  Adult Female Spermatheca Mated  5.295144  
  Adult Female Spermatheca Virgin  5.325498  
  Adult Head  5.581162  
  Adult Heart  4.804569  
  Adult Hind Gut  7.394764  
  Adult Male Ejaculatory Duct  5.400136  
  Adult Mid Gut  5.343330  
  Adult Ovary  5.100300  
  Adult Salivary Gland  5.299128  
  Adult Testes  5.442758  
  Adult Thoracoabdominal ganglion  4.925897  
  Adult Whole Fly  4.868945  
  Larvae Wandering Tubules  5.227304  
  Larval Feeding Carcass  5.518554  
  Larval Feeding Central Nevous System  4.922708  
  Larval Feeding Hind Gut  5.619507  
  Larval Feeding Malpighian Tubule  5.149212  
  Larval Feeding Mid Gut  5.463757  
  Larval Feeding Salivary Gland  5.237492  
  Whole Larvae Feeding  5.088083  
 
  
   FlyBase ID    symbol    start    end    strand    length   
   FBgn0035873   CG13670   8313863   8315383  +  1521  
   FBgn0085491   CG34462   8321780   8323216  +  1437  
   FBgn0035875   Cpr66Cb   8329772   8331695  +  1924  
 
 
    Segment 182 
 
   Location   
  Gene key  FBgn0035876-FBgn0035879  
  Heatmap region span   3L:8292791..8386749   
  Segment span   3L:8332153..8340263   
  Length (genes)  4  
  Length (bp)  8111  
   Model Scoring   
  BIC  392.951500  
  logL  -190.963624  
  logL ratio  107.476034  
   Expression   
  Mean expression  8.242352  
  Median expression  8.289102  
  Tissue std. dev.  0.403526  
 
  No GO Slim enrichment  
  
   tissue    mean expression   
  5th Passage Drosophila S2 Cells  8.368635  
  Adult Accessory gland  8.260293  
  Adult Brain  7.746707  
  Adult Carcass  8.352213  
  Adult Crop  8.877778  
  Adult Eye  8.016120  
  Adult Fatbody  8.588191  
  Adult Female Spermatheca Mated  8.435343  
  Adult Female Spermatheca Virgin  8.542143  
  Adult Head  8.025588  
  Adult Heart  8.943267  
  Adult Hind Gut  8.398754  
  Adult Male Ejaculatory Duct  8.884200  
  Adult Mid Gut  8.217795  
  Adult Ovary  8.950475  
  Adult Salivary Gland  8.429251  
  Adult Testes  7.334164  
  Adult Thoracoabdominal ganglion  7.617192  
  Adult Whole Fly  8.090215  
  Larvae Wandering Tubules  8.216353  
  Larval Feeding Carcass  8.064860  
  Larval Feeding Central Nevous System  7.934968  
  Larval Feeding Hind Gut  8.195562  
  Larval Feeding Malpighian Tubule  8.052926  
  Larval Feeding Mid Gut  8.072122  
  Larval Feeding Salivary Gland  8.365379  
  Whole Larvae Feeding  7.563000  
 
  
   FlyBase ID    symbol    start    end    strand    length   
   FBgn0035876   Pex2   8332153   8333376  +  1224  
   FBgn0035877   CG7083   8333885   8335976  +  2092  
   FBgn0035878   CG7182  8337521   8339430   -  1910  
   FBgn0035879   GapcenA   8340263   8345379  +  5117  
 
 
    Segment 183 
 
   Location   
  Gene key  FBgn0035886-FBgn0035887  
  Heatmap region span   3L:8332153..8443416   
  Segment span   3L:8384642..8386749   
  Length (genes)  2  
  Length (bp)  2108  
   Model Scoring   
  BIC  221.031137  
  logL  -105.003442  
  logL ratio  34.016469  
   Expression   
  Mean expression  6.517283  
  Median expression  5.770284  
  Tissue std. dev.  2.461613  
 
  No GO Slim enrichment  
  
   tissue    mean expression   
  5th Passage Drosophila S2 Cells  5.699498  
  Adult Accessory gland  5.705970  
  Adult Brain  5.131438  
  Adult Carcass  5.752683  
  Adult Crop  5.526412  
  Adult Eye  5.560555  
  Adult Fatbody  5.773753  
  Adult Female Spermatheca Mated  5.661226  
  Adult Female Spermatheca Virgin  5.629182  
  Adult Head  5.276164  
  Adult Heart  5.301654  
  Adult Hind Gut  5.593844  
  Adult Male Ejaculatory Duct  6.023001  
  Adult Mid Gut  11.380115  
  Adult Ovary  5.471031  
  Adult Salivary Gland  5.638168  
  Adult Testes  5.040935  
  Adult Thoracoabdominal ganglion  5.200788  
  Adult Whole Fly  7.493505  
  Larvae Wandering Tubules  5.671649  
  Larval Feeding Carcass  5.908533  
  Larval Feeding Central Nevous System  5.675523  
  Larval Feeding Hind Gut  6.111933  
  Larval Feeding Malpighian Tubule  5.658708  
  Larval Feeding Mid Gut  14.517789  
  Larval Feeding Salivary Gland  5.701033  
  Whole Larvae Feeding  13.861544  
 
  
   FlyBase ID    symbol    start    end    strand    length   
   FBgn0035886   Jon66Ci   8384642   8385479  +  838  
   FBgn0035887   Jon66Cii  8385958   8386749   -  792  
 
    Segment 184 
 
   Location   
  Gene key  FBgn0035888-FBgn0035890  
  Heatmap region span   3L:8349396..8507991   
  Segment span   3L:8392269..8398532   
  Length (genes)  2  
  Length (bp)  6264  
   Model Scoring   
  BIC  232.576833  
  logL  -110.776290  
  logL ratio  17.514814  
   Expression   
  Mean expression  7.245863  
  Median expression  7.393431  
  Tissue std. dev.  0.599314  
 
  No GO Slim enrichment  
  
   tissue    mean expression   
  5th Passage Drosophila S2 Cells  7.850435  
  Adult Accessory gland  6.983344  
  Adult Brain  7.658247  
  Adult Carcass  6.937576  
  Adult Crop  6.948344  
  Adult Eye  6.893142  
  Adult Fatbody  6.955194  
  Adult Female Spermatheca Mated  7.001122  
  Adult Female Spermatheca Virgin  6.927708  
  Adult Head  7.166503  
  Adult Heart  7.862656  
  Adult Hind Gut  7.736671  
  Adult Male Ejaculatory Duct  8.107122  
  Adult Mid Gut  6.978440  
  Adult Ovary  7.469396  
  Adult Salivary Gland  6.653217  
  Adult Testes  9.522959  
  Adult Thoracoabdominal ganglion  7.506438  
  Adult Whole Fly  7.015733  
  Larvae Wandering Tubules  6.839427  
  Larval Feeding Carcass  6.578110  
  Larval Feeding Central Nevous System  7.196696  
  Larval Feeding Hind Gut  7.108130  
  Larval Feeding Malpighian Tubule  6.806052  
  Larval Feeding Mid Gut  7.421570  
  Larval Feeding Salivary Gland  6.925713  
  Whole Larvae Feeding  6.588345  
 
  
   FlyBase ID    symbol    start    end    strand    length   
   FBgn0035888   CG7120   8392269   8395395  +  3127  
   FBgn0035890   CG13667   8398532   8400455  +  1924  
 
    Segment 185 
 
   Location   
  Gene key  FBgn0035891-FBgn0010431  
  Heatmap region span   3L:8354628..8511988   
  Segment span   3L:8404675..8406115   
  Length (genes)  2  
  Length (bp)  1441  
   Model Scoring   
  BIC  219.411490  
  logL  -104.193619  
  logL ratio  3.360210  
   Expression   
  Mean expression  5.558490  
  Median expression  5.007240  
  Tissue std. dev.  1.403111  
 
  No GO Slim enrichment  
  
   tissue    mean expression   
  5th Passage Drosophila S2 Cells  4.790544  
  Adult Accessory gland  5.449672  
  Adult Brain  5.162586  
  Adult Carcass  5.852785  
  Adult Crop  5.225764  
  Adult Eye  5.626689  
  Adult Fatbody  5.149100  
  Adult Female Spermatheca Mated  5.231331  
  Adult Female Spermatheca Virgin  5.649610  
  Adult Head  5.134965  
  Adult Heart  4.608137  
  Adult Hind Gut  4.975557  
  Adult Male Ejaculatory Duct  4.915105  
  Adult Mid Gut  4.713420  
  Adult Ovary  10.760655  
  Adult Salivary Gland  5.632910  
  Adult Testes  7.355206  
  Adult Thoracoabdominal ganglion  5.392313  
  Adult Whole Fly  9.321597  
  Larvae Wandering Tubules  4.940246  
  Larval Feeding Carcass  4.677163  
  Larval Feeding Central Nevous System  5.939547  
  Larval Feeding Hind Gut  4.699155  
  Larval Feeding Malpighian Tubule  4.772365  
  Larval Feeding Mid Gut  4.625309  
  Larval Feeding Salivary Gland  4.711058  
  Whole Larvae Feeding  4.766439  
 
  
   FlyBase ID    symbol    start    end    strand    length   
   FBgn0035891     8400369   8404675   -  4307  
   FBgn0010431   mtrm   8406115   8407203  +  1089  
 
    Segment 186 
 
   Location   
  Gene key  FBgn0020224-FBgn0010825  
  Heatmap region span   3L:8384642..8569173   
  Segment span   3L:8418045..8443416   
  Length (genes)  4  
  Length (bp)  25372  
   Model Scoring   
  BIC  381.146278  
  logL  -185.061013  
  logL ratio  119.458355  
   Expression   
  Mean expression  8.083966  
  Median expression  8.114224  
  Tissue std. dev.  0.729767  
 
  No GO Slim enrichment  
  
   tissue    mean expression   
  5th Passage Drosophila S2 Cells  8.329868  
  Adult Accessory gland  7.668126  
  Adult Brain  9.302362  
  Adult Carcass  7.514769  
  Adult Crop  9.125101  
  Adult Eye  9.403507  
  Adult Fatbody  7.310629  
  Adult Female Spermatheca Mated  8.042265  
  Adult Female Spermatheca Virgin  7.944009  
  Adult Head  8.539209  
  Adult Heart  8.373089  
  Adult Hind Gut  8.226936  
  Adult Male Ejaculatory Duct  7.297709  
  Adult Mid Gut  7.584105  
  Adult Ovary  8.585383  
  Adult Salivary Gland  7.553987  
  Adult Testes  6.396909  
  Adult Thoracoabdominal ganglion  9.013408  
  Adult Whole Fly  7.805394  
  Larvae Wandering Tubules  8.488049  
  Larval Feeding Carcass  7.987270  
  Larval Feeding Central Nevous System  9.452846  
  Larval Feeding Hind Gut  8.055617  
  Larval Feeding Malpighian Tubule  8.019359  
  Larval Feeding Mid Gut  7.435837  
  Larval Feeding Salivary Gland  7.435657  
  Whole Larvae Feeding  7.375688  
 
  
   FlyBase ID    symbol    start    end    strand    length   
   FBgn0020224   Cbl   8418045   8424324  +  6280  
   FBgn0035895     8427149   8431786   -  4638  
   FBgn0035896   CG6983  8436345   8442472   -  6128  
   FBgn0010825   Gug   8443416   8465769  +  22354  
 
 
    Segment 187 
 
   Location   
  Gene key  FBgn0035898-FBgn0035902  
  Heatmap region span   3L:8392269..8594102   
  Segment span   3L:8474162..8507991   
  Length (genes)  6  
  Length (bp)  33830  
   Model Scoring   
  BIC  598.972755  
  logL  -293.974251  
  logL ratio  60.251029  
   Expression   
  Mean expression  5.439705  
  Median expression  5.408110  
  Tissue std. dev.  0.426697  
 
  No GO Slim enrichment  
  
   tissue    mean expression   
  5th Passage Drosophila S2 Cells  5.703249  
  Adult Accessory gland  5.415447  
  Adult Brain  6.312710  
  Adult Carcass  5.104463  
  Adult Crop  5.559030  
  Adult Eye  6.002222  
  Adult Fatbody  5.113073  
  Adult Female Spermatheca Mated  5.248539  
  Adult Female Spermatheca Virgin  5.140658  
  Adult Head  5.638453  
  Adult Heart  5.084169  
  Adult Hind Gut  5.219687  
  Adult Male Ejaculatory Duct  5.165058  
  Adult Mid Gut  5.091925  
  Adult Ovary  6.000667  
  Adult Salivary Gland  5.475713  
  Adult Testes  5.024429  
  Adult Thoracoabdominal ganglion  6.050907  
  Adult Whole Fly  5.154875  
  Larvae Wandering Tubules  5.208560  
  Larval Feeding Carcass  5.433044  
  Larval Feeding Central Nevous System  6.703913  
  Larval Feeding Hind Gut  5.352989  
  Larval Feeding Malpighian Tubule  5.161340  
  Larval Feeding Mid Gut  4.986088  
  Larval Feeding Salivary Gland  5.414391  
  Whole Larvae Feeding  5.106438  
 
  
   FlyBase ID    symbol    start    end    strand    length   
   FBgn0035898     8466107   8474162   -  8056  
   FBgn0035899     8484214   8487411   -  3198  
   FBgn0035900   ZC3H3  8500569   8502847   -  2279  
   FBgn0035901   CG6745   8503138   8506104  +  2967  
   FBgn0035903   CG6765   8505866   8511708  +  5843  
   FBgn0035902   CG6683  8506969   8507991   -  1023  
 
 
    Segment 188 
 
   Location   
  Gene key  FBgn0035906-FBgn0035911  
  Heatmap region span   3L:8418045..8612988   
  Segment span   3L:8516600..8569173   
  Length (genes)  5  
  Length (bp)  52574  
   Model Scoring   
  BIC  695.353148  
  logL  -342.164448  
  logL ratio  -8.763754  
   Expression   
  Mean expression  9.340050  
  Median expression  9.473550  
  Tissue std. dev.  0.544062  
 
  No GO Slim enrichment  
  
   tissue    mean expression   
  5th Passage Drosophila S2 Cells  9.659273  
  Adult Accessory gland  9.916053  
  Adult Brain  8.020164  
  Adult Carcass  9.380824  
  Adult Crop  9.472813  
  Adult Eye  9.394574  
  Adult Fatbody  10.080036  
  Adult Female Spermatheca Mated  9.899082  
  Adult Female Spermatheca Virgin  9.795897  
  Adult Head  9.305011  
  Adult Heart  10.005707  
  Adult Hind Gut  9.302812  
  Adult Male Ejaculatory Duct  9.478784  
  Adult Mid Gut  9.362589  
  Adult Ovary  9.976465  
  Adult Salivary Gland  9.311664  
  Adult Testes  7.948680  
  Adult Thoracoabdominal ganglion  8.316812  
  Adult Whole Fly  9.401432  
  Larvae Wandering Tubules  8.786909  
  Larval Feeding Carcass  9.925496  
  Larval Feeding Central Nevous System  9.103922  
  Larval Feeding Hind Gut  9.461787  
  Larval Feeding Malpighian Tubule  9.175598  
  Larval Feeding Mid Gut  9.000566  
  Larval Feeding Salivary Gland  9.292586  
  Whole Larvae Feeding  9.405829  
 
  
   FlyBase ID    symbol    start    end    strand    length   
   FBgn0035906   GstO2  8514539   8516600   -  2062  
   FBgn0035907   GstO1  8516842   8517961   -  1120  
   FBgn0024236   foi   8518344   8527084  +  8741  
   FBgn0035909   ergic53   8528052   8531118  +  3067  
   FBgn0035911   CG6638  8567507   8569173   -  1667  
 
 
    Segment 189 
 
   Location   
  Gene key  FBgn0035915-FBgn0052351  
  Heatmap region span   3L:8516600..8640245   
  Segment span   3L:8610335..8612988   
  Length (genes)  2  
  Length (bp)  2654  
   Model Scoring   
  BIC  190.165599  
  logL  -89.570673  
  logL ratio  44.130210  
   Expression   
  Mean expression  5.458685  
  Median expression  4.529894  
  Tissue std. dev.  2.365280  
 
  
   GO ID    description    ratio    P-value   
   GO:0005737   cytoplasm  2/2  0.00471  
 
  
   tissue    mean expression   
  5th Passage Drosophila S2 Cells  4.518342  
  Adult Accessory gland  4.627197  
  Adult Brain  5.093437  
  Adult Carcass  5.668476  
  Adult Crop  4.432881  
  Adult Eye  4.798056  
  Adult Fatbody  4.610455  
  Adult Female Spermatheca Mated  5.059404  
  Adult Female Spermatheca Virgin  4.729200  
  Adult Head  4.652793  
  Adult Heart  4.529894  
  Adult Hind Gut  4.305540  
  Adult Male Ejaculatory Duct  4.446775  
  Adult Mid Gut  4.244457  
  Adult Ovary  4.437794  
  Adult Salivary Gland  4.817813  
  Adult Testes  14.449713  
  Adult Thoracoabdominal ganglion  5.029803  
  Adult Whole Fly  11.924228  
  Larvae Wandering Tubules  4.460248  
  Larval Feeding Carcass  4.763889  
  Larval Feeding Central Nevous System  4.685321  
  Larval Feeding Hind Gut  4.327275  
  Larval Feeding Malpighian Tubule  4.601187  
  Larval Feeding Mid Gut  4.760905  
  Larval Feeding Salivary Gland  4.572092  
  Whole Larvae Feeding  8.837310  
 
  
   FlyBase ID    symbol    start    end    strand    length   
   FBgn0035915   S-Lap1   8610335   8612574  +  2240  
   FBgn0052351   S-Lap2   8612988   8615191  +  2204  
 
    Segment 190 
 
   Location   
  Gene key  FBgn0052029-FBgn0035922  
  Heatmap region span   3L:8610335..8714602   
  Segment span   3L:8633574..8640245   
  Length (genes)  3  
  Length (bp)  6672  
   Model Scoring   
  BIC  260.686075  
  logL  -124.830911  
  logL ratio  49.742169  
   Expression   
  Mean expression  5.013110  
  Median expression  4.730731  
  Tissue std. dev.  0.640311  
 
  No GO Slim enrichment  
  
   tissue    mean expression   
  5th Passage Drosophila S2 Cells  4.645040  
  Adult Accessory gland  4.779227  
  Adult Brain  4.451370  
  Adult Carcass  5.657921  
  Adult Crop  5.576719  
  Adult Eye  6.638552  
  Adult Fatbody  4.631850  
  Adult Female Spermatheca Mated  4.640580  
  Adult Female Spermatheca Virgin  4.584623  
  Adult Head  5.305509  
  Adult Heart  5.225228  
  Adult Hind Gut  4.984651  
  Adult Male Ejaculatory Duct  5.227894  
  Adult Mid Gut  4.673468  
  Adult Ovary  4.975773  
  Adult Salivary Gland  4.866761  
  Adult Testes  4.381870  
  Adult Thoracoabdominal ganglion  4.682027  
  Adult Whole Fly  4.687129  
  Larvae Wandering Tubules  4.641814  
  Larval Feeding Carcass  5.355186  
  Larval Feeding Central Nevous System  7.247377  
  Larval Feeding Hind Gut  4.610408  
  Larval Feeding Malpighian Tubule  4.609278  
  Larval Feeding Mid Gut  4.663271  
  Larval Feeding Salivary Gland  4.734006  
  Whole Larvae Feeding  4.876447  
 
  
   FlyBase ID    symbol    start    end    strand    length   
   FBgn0052029   Cpr66D   8633574   8636684  +  3111  
   FBgn0035921   CG13305  8638012   8639918   -  1907  
   FBgn0035922   Pex7   8640245   8644111  +  3867  
 
 
    Segment 191 
 
   Location   
  Gene key  FBgn0011509-FBgn0052022  
  Heatmap region span   3L:8627181..8749089   
  Segment span   3L:8713317..8714372   
  Length (genes)  2  
  Length (bp)  1056  
   Model Scoring   
  BIC  221.760428  
  logL  -105.368088  
  logL ratio  40.139612  
   Expression   
  Mean expression  8.771994  
  Median expression  8.554483  
  Tissue std. dev.  0.716533  
 
  No GO Slim enrichment  
  
   tissue    mean expression   
  5th Passage Drosophila S2 Cells  11.044721  
  Adult Accessory gland  9.952337  
  Adult Brain  8.653605  
  Adult Carcass  8.165964  
  Adult Crop  8.290871  
  Adult Eye  8.635700  
  Adult Fatbody  8.871951  
  Adult Female Spermatheca Mated  8.445792  
  Adult Female Spermatheca Virgin  8.129599  
  Adult Head  8.195199  
  Adult Heart  9.060782  
  Adult Hind Gut  8.050950  
  Adult Male Ejaculatory Duct  10.104107  
  Adult Mid Gut  8.115441  
  Adult Ovary  9.138976  
  Adult Salivary Gland  9.511541  
  Adult Testes  8.663799  
  Adult Thoracoabdominal ganglion  8.329866  
  Adult Whole Fly  8.494467  
  Larvae Wandering Tubules  8.307327  
  Larval Feeding Carcass  8.690630  
  Larval Feeding Central Nevous System  8.443452  
  Larval Feeding Hind Gut  8.463530  
  Larval Feeding Malpighian Tubule  8.373398  
  Larval Feeding Mid Gut  8.299713  
  Larval Feeding Salivary Gland  9.858272  
  Whole Larvae Feeding  8.551851  
 
  
   FlyBase ID    symbol    start    end    strand    length   
   FBgn0011509   SrpRbeta  8712198   8713317   -  1120  
   FBgn0052022   CG32022  8713380   8714372   -  993  
 
    Segment 192 
 
   Location   
  Gene key  FBgn0000357-FBgn0000356  
  Heatmap region span   3L:8642036..8793572   
  Segment span   3L:8719891..8724842   
  Length (genes)  4  
  Length (bp)  4952  
   Model Scoring   
  BIC  324.124047  
  logL  -156.549897  
  logL ratio  147.917434  
   Expression   
  Mean expression  6.135546  
  Median expression  5.518776  
  Tissue std. dev.  2.098427  
 
  No GO Slim enrichment  
  
   tissue    mean expression   
  5th Passage Drosophila S2 Cells  5.382962  
  Adult Accessory gland  5.461732  
  Adult Brain  5.066101  
  Adult Carcass  6.377102  
  Adult Crop  5.425050  
  Adult Eye  5.636732  
  Adult Fatbody  6.293532  
  Adult Female Spermatheca Mated  5.287489  
  Adult Female Spermatheca Virgin  5.821112  
  Adult Head  5.171798  
  Adult Heart  5.624311  
  Adult Hind Gut  5.310595  
  Adult Male Ejaculatory Duct  6.007593  
  Adult Mid Gut  5.523055  
  Adult Ovary  13.996094  
  Adult Salivary Gland  6.064324  
  Adult Testes  5.564063  
  Adult Thoracoabdominal ganglion  5.341237  
  Adult Whole Fly  12.875908  
  Larvae Wandering Tubules  5.297615  
  Larval Feeding Carcass  5.648950  
  Larval Feeding Central Nevous System  4.981007  
  Larval Feeding Hind Gut  5.346224  
  Larval Feeding Malpighian Tubule  5.366560  
  Larval Feeding Mid Gut  6.019027  
  Larval Feeding Salivary Gland  5.385543  
  Whole Larvae Feeding  5.384023  
 
  
   FlyBase ID    symbol    start    end    strand    length   
   FBgn0000357   Cp18   8719891   8720713  +  823  
   FBgn0000355   Cp15   8721581   8722166  +  586  
   FBgn0000358   Cp19   8722965   8723704  +  740  
   FBgn0000356   Cp16   8724842   8725699  +  858  
 
 
    Segment 193 
 
   Location   
  Gene key  FBgn0040828-FBgn0035924  
  Heatmap region span   3L:8713317..8803495   
  Segment span   3L:8742826..8749089   
  Length (genes)  2  
  Length (bp)  6264  
   Model Scoring   
  BIC  207.477574  
  logL  -98.226661  
  logL ratio  7.140108  
   Expression   
  Mean expression  5.474235  
  Median expression  5.327524  
  Tissue std. dev.  0.636276  
 
  No GO Slim enrichment  
  
   tissue    mean expression   
  5th Passage Drosophila S2 Cells  5.162566  
  Adult Accessory gland  5.642966  
  Adult Brain  4.707358  
  Adult Carcass  6.603451  
  Adult Crop  6.469787  
  Adult Eye  5.006563  
  Adult Fatbody  5.134111  
  Adult Female Spermatheca Mated  5.134156  
  Adult Female Spermatheca Virgin  5.182555  
  Adult Head  5.356993  
  Adult Heart  6.321051  
  Adult Hind Gut  5.595387  
  Adult Male Ejaculatory Duct  5.473375  
  Adult Mid Gut  5.321091  
  Adult Ovary  5.110786  
  Adult Salivary Gland  5.314985  
  Adult Testes  7.656987  
  Adult Thoracoabdominal ganglion  4.820520  
  Adult Whole Fly  5.822482  
  Larvae Wandering Tubules  5.154492  
  Larval Feeding Carcass  5.924114  
  Larval Feeding Central Nevous System  4.717920  
  Larval Feeding Hind Gut  5.225240  
  Larval Feeding Malpighian Tubule  5.152890  
  Larval Feeding Mid Gut  5.288199  
  Larval Feeding Salivary Gland  5.276519  
  Whole Larvae Feeding  5.227802  
 
  
   FlyBase ID    symbol    start    end    strand    length   
   FBgn0040828   CG13306  8742237   8742826   -  590  
   FBgn0035924   CG6576   8749089   8750267  +  1179  
 
    Segment 194 
 
   Location   
  Gene key  FBgn0035929-FBgn0035931  
  Heatmap region span   3L:8738447..8884710   
  Segment span   3L:8795262..8800313   
  Length (genes)  4  
  Length (bp)  5052  
   Model Scoring   
  BIC  389.996931  
  logL  -189.486339  
  logL ratio  57.868004  
   Expression   
  Mean expression  5.582606  
  Median expression  4.884043  
  Tissue std. dev.  1.654749  
 
  No GO Slim enrichment  
  
   tissue    mean expression   
  5th Passage Drosophila S2 Cells  5.015574  
  Adult Accessory gland  5.092659  
  Adult Brain  4.548566  
  Adult Carcass  5.163578  
  Adult Crop  4.855648  
  Adult Eye  4.659365  
  Adult Fatbody  5.358856  
  Adult Female Spermatheca Mated  5.132799  
  Adult Female Spermatheca Virgin  5.277806  
  Adult Head  4.607026  
  Adult Heart  4.941623  
  Adult Hind Gut  4.870120  
  Adult Male Ejaculatory Duct  5.152755  
  Adult Mid Gut  5.237620  
  Adult Ovary  4.779713  
  Adult Salivary Gland  5.190656  
  Adult Testes  4.845356  
  Adult Thoracoabdominal ganglion  4.838144  
  Adult Whole Fly  5.722844  
  Larvae Wandering Tubules  10.328196  
  Larval Feeding Carcass  5.059647  
  Larval Feeding Central Nevous System  4.658362  
  Larval Feeding Hind Gut  5.969869  
  Larval Feeding Malpighian Tubule  11.798717  
  Larval Feeding Mid Gut  5.122537  
  Larval Feeding Salivary Gland  5.075365  
  Whole Larvae Feeding  7.426963  
 
  
   FlyBase ID    symbol    start    end    strand    length   
   FBgn0035929   CG13311  8794640   8795262   -  623  
   FBgn0052023   CG32023  8796702   8797282   -  581  
   FBgn0085456   CG34427   8797864   8798872  +  1009  
   FBgn0035931   CG13312  8798911   8800313   -  1403  
 
 
    Segment 195 
 
   Location   
  Gene key  FBgn0052024-FBgn0035933  
  Heatmap region span   3L:8742826..8896780   
  Segment span   3L:8800725..8803495   
  Length (genes)  3  
  Length (bp)  2771  
   Model Scoring   
  BIC  247.607962  
  logL  -118.291855  
  logL ratio  70.131051  
   Expression   
  Mean expression  4.546514  
  Median expression  4.386695  
  Tissue std. dev.  0.603841  
 
  No GO Slim enrichment  
  
   tissue    mean expression   
  5th Passage Drosophila S2 Cells  4.430927  
  Adult Accessory gland  4.661132  
  Adult Brain  4.127176  
  Adult Carcass  4.618667  
  Adult Crop  4.331431  
  Adult Eye  4.166658  
  Adult Fatbody  4.486635  
  Adult Female Spermatheca Mated  4.325450  
  Adult Female Spermatheca Virgin  4.493599  
  Adult Head  4.154992  
  Adult Heart  4.654711  
  Adult Hind Gut  6.522775  
  Adult Male Ejaculatory Duct  4.361460  
  Adult Mid Gut  4.691534  
  Adult Ovary  4.320989  
  Adult Salivary Gland  4.734992  
  Adult Testes  4.100897  
  Adult Thoracoabdominal ganglion  4.173561  
  Adult Whole Fly  6.606402  
  Larvae Wandering Tubules  4.578261  
  Larval Feeding Carcass  4.293723  
  Larval Feeding Central Nevous System  4.122618  
  Larval Feeding Hind Gut  4.178822  
  Larval Feeding Malpighian Tubule  4.494922  
  Larval Feeding Mid Gut  4.633779  
  Larval Feeding Salivary Gland  4.359731  
  Whole Larvae Feeding  4.130030  
 
  
   FlyBase ID    symbol    start    end    strand    length   
   FBgn0052024   CG32024   8800725   8801507  +  783  
   FBgn0035932   CG13308   8801644   8802407  +  764  
   FBgn0035933   CG13309   8803495   8804365  +  871  
 
 
    Segment 196 
 
   Location   
  Gene key  FBgn0035942-FBgn0035943  
  Heatmap region span   3L:8905771..8974895   
  Segment span   3L:8957763..8959474   
  Length (genes)  2  
  Length (bp)  1712  
   Model Scoring   
  BIC  234.526224  
  logL  -111.750986  
  logL ratio  -2.614340  
   Expression   
  Mean expression  6.197347  
  Median expression  6.274715  
  Tissue std. dev.  0.780003  
 
  No GO Slim enrichment  
  
   tissue    mean expression   
  5th Passage Drosophila S2 Cells  6.131427  
  Adult Accessory gland  6.052897  
  Adult Brain  6.282129  
  Adult Carcass  5.790829  
  Adult Crop  5.723949  
  Adult Eye  6.570025  
  Adult Fatbody  5.872485  
  Adult Female Spermatheca Mated  6.146778  
  Adult Female Spermatheca Virgin  6.300427  
  Adult Head  5.906380  
  Adult Heart  5.608557  
  Adult Hind Gut  5.754445  
  Adult Male Ejaculatory Duct  5.891329  
  Adult Mid Gut  5.899444  
  Adult Ovary  6.693715  
  Adult Salivary Gland  6.292573  
  Adult Testes  9.921350  
  Adult Thoracoabdominal ganglion  6.468891  
  Adult Whole Fly  6.529947  
  Larvae Wandering Tubules  6.011520  
  Larval Feeding Carcass  5.782645  
  Larval Feeding Central Nevous System  6.061373  
  Larval Feeding Hind Gut  5.790974  
  Larval Feeding Malpighian Tubule  6.051258  
  Larval Feeding Mid Gut  5.873689  
  Larval Feeding Salivary Gland  5.974215  
  Whole Larvae Feeding  5.945113  
 
  
   FlyBase ID    symbol    start    end    strand    length   
   FBgn0035942   CG5660  8954375   8957763   -  3389  
   FBgn0035943   CG5653  8958011   8959474   -  1464  
 
    Segment 197 
 
   Location   
  Gene key  FBgn0086706-FBgn0035947  
  Heatmap region span   3L:8951476..8983328   
  Segment span   3L:8967854..8968148   
  Length (genes)  2  
  Length (bp)  295  
   Model Scoring   
  BIC  282.018856  
  logL  -135.497302  
  logL ratio  64.646769  
   Expression   
  Mean expression  11.204242  
  Median expression  11.540965  
  Tissue std. dev.  0.625273  
 
  No GO Slim enrichment  
  
   tissue    mean expression   
  5th Passage Drosophila S2 Cells  12.125724  
  Adult Accessory gland  12.285217  
  Adult Brain  10.364252  
  Adult Carcass  10.979102  
  Adult Crop  11.114129  
  Adult Eye  10.438738  
  Adult Fatbody  11.615041  
  Adult Female Spermatheca Mated  11.524787  
  Adult Female Spermatheca Virgin  11.441213  
  Adult Head  10.877963  
  Adult Heart  11.057600  
  Adult Hind Gut  10.893377  
  Adult Male Ejaculatory Duct  11.969402  
  Adult Mid Gut  10.886609  
  Adult Ovary  11.384888  
  Adult Salivary Gland  12.512929  
  Adult Testes  9.873212  
  Adult Thoracoabdominal ganglion  10.438678  
  Adult Whole Fly  11.088940  
  Larvae Wandering Tubules  10.909368  
  Larval Feeding Carcass  11.405952  
  Larval Feeding Central Nevous System  11.109530  
  Larval Feeding Hind Gut  11.252255  
  Larval Feeding Malpighian Tubule  10.940770  
  Larval Feeding Mid Gut  10.692408  
  Larval Feeding Salivary Gland  12.350316  
  Whole Larvae Feeding  10.982129  
 
  
   FlyBase ID    symbol    start    end    strand    length   
   FBgn0086706   pix  8965160   8967854   -  2695  
   FBgn0035947   Srp68   8968148   8970510  +  2363  
 
    Segment 198 
 
   Location   
  Gene key  FBgn0035948-FBgn0035949  
  Heatmap region span   3L:8957763..8991286   
  Segment span   3L:8972980..8974895   
  Length (genes)  2  
  Length (bp)  1916  
   Model Scoring   
  BIC  216.694736  
  logL  -102.835242  
  logL ratio  4.853588  
   Expression   
  Mean expression  5.780135  
  Median expression  5.670585  
  Tissue std. dev.  0.664407  
 
  No GO Slim enrichment  
  
   tissue    mean expression   
  5th Passage Drosophila S2 Cells  5.839196  
  Adult Accessory gland  5.288657  
  Adult Brain  6.964058  
  Adult Carcass  5.869115  
  Adult Crop  5.495109  
  Adult Eye  7.760295  
  Adult Fatbody  5.349685  
  Adult Female Spermatheca Mated  5.404343  
  Adult Female Spermatheca Virgin  5.519030  
  Adult Head  6.546776  
  Adult Heart  6.167565  
  Adult Hind Gut  5.597549  
  Adult Male Ejaculatory Duct  5.784349  
  Adult Mid Gut  5.426590  
  Adult Ovary  5.717848  
  Adult Salivary Gland  5.518634  
  Adult Testes  5.316692  
  Adult Thoracoabdominal ganglion  7.325317  
  Adult Whole Fly  5.259232  
  Larvae Wandering Tubules  5.395045  
  Larval Feeding Carcass  6.532647  
  Larval Feeding Central Nevous System  5.057482  
  Larval Feeding Hind Gut  5.327658  
  Larval Feeding Malpighian Tubule  5.353471  
  Larval Feeding Mid Gut  5.454790  
  Larval Feeding Salivary Gland  5.417409  
  Whole Larvae Feeding  5.375093  
 
  
   FlyBase ID    symbol    start    end    strand    length   
   FBgn0035948   CG5644  8970495   8972980   -  2486  
   FBgn0035949   CG13314   8974895   8976312  +  1418  
 
    Segment 199 
 
   Location   
  Gene key  FBgn0016070-FBgn0035953  
  Heatmap region span   3L:8972980..9086154   
  Segment span   3L:8991089..8991286   
  Length (genes)  2  
  Length (bp)  198  
   Model Scoring   
  BIC  232.507774  
  logL  -110.741761  
  logL ratio  26.335740  
   Expression   
  Mean expression  8.238835  
  Median expression  8.200199  
  Tissue std. dev.  0.861869  
 
  No GO Slim enrichment  
  
   tissue    mean expression   
  5th Passage Drosophila S2 Cells  8.030577  
  Adult Accessory gland  7.474946  
  Adult Brain  8.910998  
  Adult Carcass  8.309140  
  Adult Crop  8.378528  
  Adult Eye  8.301144  
  Adult Fatbody  8.648181  
  Adult Female Spermatheca Mated  8.307982  
  Adult Female Spermatheca Virgin  8.631146  
  Adult Head  8.055171  
  Adult Heart  8.385408  
  Adult Hind Gut  8.099751  
  Adult Male Ejaculatory Duct  7.392402  
  Adult Mid Gut  7.258917  
  Adult Ovary  11.117673  
  Adult Salivary Gland  7.634661  
  Adult Testes  6.333035  
  Adult Thoracoabdominal ganglion  8.694100  
  Adult Whole Fly  9.592228  
  Larvae Wandering Tubules  8.187090  
  Larval Feeding Carcass  7.732466  
  Larval Feeding Central Nevous System  8.716019  
  Larval Feeding Hind Gut  9.088842  
  Larval Feeding Malpighian Tubule  8.263867  
  Larval Feeding Mid Gut  7.861562  
  Larval Feeding Salivary Gland  7.809401  
  Whole Larvae Feeding  7.233299  
 
  
   FlyBase ID    symbol    start    end    strand    length   
   FBgn0016070   smg  8983637   8991089   -  7453  
   FBgn0035953   CG5087   8991286   8995666  +  4381  
 
    Segment 200 
 
   Location   
  Gene key  FBgn0035954-FBgn0035957  
  Heatmap region span   3L:8979189..9089976   
  Segment span   3L:8998298..9040540   
  Length (genes)  5  
  Length (bp)  42243  
   Model Scoring   
  BIC  493.590345  
  logL  -241.283046  
  logL ratio  37.584884  
   Expression   
  Mean expression  5.361144  
  Median expression  5.035258  
  Tissue std. dev.  0.896550  
 
  
   GO ID    description    ratio    P-value   
   GO:0005634   nucleus  3/5  0.0162  
 
  
   tissue    mean expression   
  5th Passage Drosophila S2 Cells  4.745530  
  Adult Accessory gland  4.805704  
  Adult Brain  4.425825  
  Adult Carcass  5.064082  
  Adult Crop  4.764785  
  Adult Eye  4.927718  
  Adult Fatbody  4.778866  
  Adult Female Spermatheca Mated  4.795268  
  Adult Female Spermatheca Virgin  4.892949  
  Adult Head  4.641696  
  Adult Heart  5.188266  
  Adult Hind Gut  6.246323  
  Adult Male Ejaculatory Duct  4.805531  
  Adult Mid Gut  4.719920  
  Adult Ovary  6.091256  
  Adult Salivary Gland  4.986287  
  Adult Testes  7.902404  
  Adult Thoracoabdominal ganglion  4.558379  
  Adult Whole Fly  5.915673  
  Larvae Wandering Tubules  6.972742  
  Larval Feeding Carcass  5.327597  
  Larval Feeding Central Nevous System  4.941306  
  Larval Feeding Hind Gut  6.810962  
  Larval Feeding Malpighian Tubule  7.076964  
  Larval Feeding Mid Gut  4.773262  
  Larval Feeding Salivary Gland  4.895240  
  Whole Larvae Feeding  5.696366  
 
  
   FlyBase ID    symbol    start    end    strand    length   
   FBgn0035954   Doc3   8998298   9000351  +  2054  
   FBgn0035955   CG5194  9001724   9002951   -  1228  
   FBgn0035956   Doc2  9005786   9012340   -  6555  
   FBgn0028789   Doc1   9034477   9038144  +  3668  
   FBgn0035957   CG5144   9040540   9041833  +  1294  
 
 
    Segment 201 
 
   Location   
  Gene key  FBgn0035959-FBgn0035960  
  Heatmap region span   3L:8983328..9130203   
  Segment span   3L:9065829..9066228   
  Length (genes)  2  
  Length (bp)  400  
   Model Scoring   
  BIC  238.044349  
  logL  -113.510048  
  logL ratio  -2.271390  
   Expression   
  Mean expression  6.176612  
  Median expression  6.327084  
  Tissue std. dev.  0.759711  
 
  No GO Slim enrichment  
  
   tissue    mean expression   
  5th Passage Drosophila S2 Cells  6.733018  
  Adult Accessory gland  6.043423  
  Adult Brain  6.665754  
  Adult Carcass  5.861256  
  Adult Crop  6.483288  
  Adult Eye  5.044583  
  Adult Fatbody  5.583613  
  Adult Female Spermatheca Mated  5.988304  
  Adult Female Spermatheca Virgin  6.097758  
  Adult Head  5.710730  
  Adult Heart  5.866315  
  Adult Hind Gut  6.466095  
  Adult Male Ejaculatory Duct  5.480104  
  Adult Mid Gut  5.858705  
  Adult Ovary  9.090701  
  Adult Salivary Gland  5.808091  
  Adult Testes  5.826237  
  Adult Thoracoabdominal ganglion  6.513704  
  Adult Whole Fly  7.364190  
  Larvae Wandering Tubules  6.456065  
  Larval Feeding Carcass  5.885319  
  Larval Feeding Central Nevous System  5.732952  
  Larval Feeding Hind Gut  6.173511  
  Larval Feeding Malpighian Tubule  6.498134  
  Larval Feeding Mid Gut  5.805692  
  Larval Feeding Salivary Gland  6.605887  
  Whole Larvae Feeding  5.125109  
 
  
   FlyBase ID    symbol    start    end    strand    length   
   FBgn0035959   CG4911  9062749   9065829   -  3081  
   FBgn0035960   CG4942   9066228   9067567  +  1340  
 
    Segment 202 
 
   Location   
  Gene key  FBgn0023479-FBgn0043806  
  Heatmap region span   3L:8991089..9170704   
  Segment span   3L:9067745..9086154   
  Length (genes)  2  
  Length (bp)  18410  
   Model Scoring   
  BIC  251.092261  
  logL  -120.034004  
  logL ratio  4.963273  
   Expression   
  Mean expression  7.826961  
  Median expression  7.340753  
  Tissue std. dev.  1.950609  
 
  No GO Slim enrichment  
  
   tissue    mean expression   
  5th Passage Drosophila S2 Cells  10.899753  
  Adult Accessory gland  6.268890  
  Adult Brain  9.782622  
  Adult Carcass  9.820699  
  Adult Crop  5.737508  
  Adult Eye  10.096201  
  Adult Fatbody  10.516630  
  Adult Female Spermatheca Mated  9.842381  
  Adult Female Spermatheca Virgin  10.265673  
  Adult Head  10.176183  
  Adult Heart  9.907730  
  Adult Hind Gut  7.844878  
  Adult Male Ejaculatory Duct  7.422324  
  Adult Mid Gut  5.291418  
  Adult Ovary  5.245007  
  Adult Salivary Gland  5.669659  
  Adult Testes  6.047247  
  Adult Thoracoabdominal ganglion  9.890307  
  Adult Whole Fly  6.982746  
  Larvae Wandering Tubules  5.744637  
  Larval Feeding Carcass  6.845703  
  Larval Feeding Central Nevous System  8.130866  
  Larval Feeding Hind Gut  8.258906  
  Larval Feeding Malpighian Tubule  5.558077  
  Larval Feeding Mid Gut  5.324241  
  Larval Feeding Salivary Gland  5.979659  
  Whole Larvae Feeding  7.778013  
 
  
   FlyBase ID    symbol    start    end    strand    length   
   FBgn0023479   Tequila   9067745   9085231  +  17487  
   FBgn0043806   CG32032   9086154   9087956  +  1803  
 
    Segment 203 
 
   Location   
  Gene key  FBgn0035964-FBgn0035965  
  Heatmap region span   3L:9065829..9352031   
  Segment span   3L:9127661..9130203   
  Length (genes)  2  
  Length (bp)  2543  
   Model Scoring   
  BIC  254.937520  
  logL  -121.956634  
  logL ratio  7.593297  
   Expression   
  Mean expression  8.588077  
  Median expression  8.564421  
  Tissue std. dev.  0.471132  
 
  No GO Slim enrichment  
  
   tissue    mean expression   
  5th Passage Drosophila S2 Cells  8.515325  
  Adult Accessory gland  8.337180  
  Adult Brain  8.414022  
  Adult Carcass  8.562535  
  Adult Crop  8.662531  
  Adult Eye  8.632117  
  Adult Fatbody  8.491509  
  Adult Female Spermatheca Mated  8.942861  
  Adult Female Spermatheca Virgin  8.988211  
  Adult Head  8.858871  
  Adult Heart  8.488185  
  Adult Hind Gut  8.148345  
  Adult Male Ejaculatory Duct  8.864049  
  Adult Mid Gut  8.111080  
  Adult Ovary  9.005456  
  Adult Salivary Gland  8.583526  
  Adult Testes  7.148651  
  Adult Thoracoabdominal ganglion  8.606946  
  Adult Whole Fly  8.144693  
  Larvae Wandering Tubules  8.619392  
  Larval Feeding Carcass  9.948676  
  Larval Feeding Central Nevous System  8.825226  
  Larval Feeding Hind Gut  8.652596  
  Larval Feeding Malpighian Tubule  8.590623  
  Larval Feeding Mid Gut  8.044500  
  Larval Feeding Salivary Gland  9.182375  
  Whole Larvae Feeding  8.508608  
 
  
   FlyBase ID    symbol    start    end    strand    length   
   FBgn0035964   Dhpr   9127661   9129016  +  1356  
   FBgn0035965   Use1  9128805   9130203   -  1399  
 
    Segment 204 
 
   Location   
  Gene key  FBgn0035966-FBgn0004244  
  Heatmap region span   3L:9067745..9352082   
  Segment span   3L:9131777..9170704   
  Length (genes)  3  
  Length (bp)  38928  
   Model Scoring   
  BIC  313.152821  
  logL  -151.064284  
  logL ratio  19.553920  
   Expression   
  Mean expression  5.802554  
  Median expression  5.106486  
  Tissue std. dev.  1.661400  
 
  No GO Slim enrichment  
  
   tissue    mean expression   
  5th Passage Drosophila S2 Cells  4.843338  
  Adult Accessory gland  4.956746  
  Adult Brain  10.977503  
  Adult Carcass  5.319259  
  Adult Crop  4.911079  
  Adult Eye  8.053522  
  Adult Fatbody  5.294879  
  Adult Female Spermatheca Mated  5.314932  
  Adult Female Spermatheca Virgin  5.337580  
  Adult Head  7.637340  
  Adult Heart  4.836198  
  Adult Hind Gut  4.984220  
  Adult Male Ejaculatory Duct  4.928102  
  Adult Mid Gut  5.585705  
  Adult Ovary  4.791984  
  Adult Salivary Gland  5.322554  
  Adult Testes  4.388973  
  Adult Thoracoabdominal ganglion  10.384267  
  Adult Whole Fly  5.350255  
  Larvae Wandering Tubules  4.913174  
  Larval Feeding Carcass  4.993796  
  Larval Feeding Central Nevous System  7.981016  
  Larval Feeding Hind Gut  4.866213  
  Larval Feeding Malpighian Tubule  4.930031  
  Larval Feeding Mid Gut  5.726315  
  Larval Feeding Salivary Gland  4.928666  
  Whole Larvae Feeding  5.111302  
 
  
   FlyBase ID    symbol    start    end    strand    length   
   FBgn0035966      9131777   9134677  +  2901  
   FBgn0035967      9135881   9138809  +  2929  
   FBgn0004244   Rdl  9143695   9170704   -  27010  
 
 
    Segment 205 
 
   Location   
  Gene key  FBgn0035970-FBgn0035978  
  Heatmap region span   3L:9127661..9360040   
  Segment span   3L:9229748..9352031   
  Length (genes)  4  
  Length (bp)  122284  
   Model Scoring   
  BIC  362.191062  
  logL  -175.583405  
  logL ratio  52.800481  
   Expression   
  Mean expression  5.210848  
  Median expression  5.028094  
  Tissue std. dev.  0.545992  
 
  No GO Slim enrichment  
  
   tissue    mean expression   
  5th Passage Drosophila S2 Cells  5.384011  
  Adult Accessory gland  4.888750  
  Adult Brain  4.637729  
  Adult Carcass  5.460698  
  Adult Crop  5.234628  
  Adult Eye  4.904498  
  Adult Fatbody  6.027529  
  Adult Female Spermatheca Mated  5.950807  
  Adult Female Spermatheca Virgin  6.006801  
  Adult Head  5.419284  
  Adult Heart  5.306427  
  Adult Hind Gut  5.153506  
  Adult Male Ejaculatory Duct  5.134403  
  Adult Mid Gut  4.987826  
  Adult Ovary  4.653686  
  Adult Salivary Gland  5.133882  
  Adult Testes  7.180887  
  Adult Thoracoabdominal ganglion  4.849331  
  Adult Whole Fly  5.284940  
  Larvae Wandering Tubules  4.927118  
  Larval Feeding Carcass  4.965384  
  Larval Feeding Central Nevous System  4.653628  
  Larval Feeding Hind Gut  4.765990  
  Larval Feeding Malpighian Tubule  5.083223  
  Larval Feeding Mid Gut  4.630501  
  Larval Feeding Salivary Gland  5.223140  
  Whole Larvae Feeding  4.844284  
 
  
   FlyBase ID    symbol    start    end    strand    length   
   FBgn0035970   CG4483  9228250   9229748   -  1499  
   FBgn0035971   CG4477   9243325   9244438  +  1114  
   FBgn0052040   CG32040   9347133   9348557  +  1425  
   FBgn0035978   UGP  9343967   9352031   -  8065  
 
 
    Segment 206 
 
   Location   
  Gene key  FBgn0035981-FBgn0011769  
  Heatmap region span   3L:9352082..9372634   
  Segment span   3L:9360484..9364338   
  Length (genes)  2  
  Length (bp)  3855  
   Model Scoring   
  BIC  237.317949  
  logL  -113.146848  
  logL ratio  30.338896  
   Expression   
  Mean expression  9.250893  
  Median expression  9.155201  
  Tissue std. dev.  0.268867  
 
  No GO Slim enrichment  
  
   tissue    mean expression   
  5th Passage Drosophila S2 Cells  9.195283  
  Adult Accessory gland  9.237602  
  Adult Brain  9.344155  
  Adult Carcass  9.017398  
  Adult Crop  9.724187  
  Adult Eye  8.806522  
  Adult Fatbody  9.383411  
  Adult Female Spermatheca Mated  9.267712  
  Adult Female Spermatheca Virgin  9.225098  
  Adult Head  8.987373  
  Adult Heart  9.096294  
  Adult Hind Gut  9.469100  
  Adult Male Ejaculatory Duct  9.252633  
  Adult Mid Gut  9.108593  
  Adult Ovary  9.637811  
  Adult Salivary Gland  9.190308  
  Adult Testes  9.268881  
  Adult Thoracoabdominal ganglion  9.226961  
  Adult Whole Fly  8.965953  
  Larvae Wandering Tubules  9.889155  
  Larval Feeding Carcass  9.106973  
  Larval Feeding Central Nevous System  9.332396  
  Larval Feeding Hind Gut  9.343062  
  Larval Feeding Malpighian Tubule  9.700983  
  Larval Feeding Mid Gut  8.919441  
  Larval Feeding Salivary Gland  9.356766  
  Whole Larvae Feeding  8.720051  
 
  
   FlyBase ID    symbol    start    end    strand    length   
   FBgn0035981   CG4452   9360484   9363400  +  2917  
   FBgn0011769   Fdxh  9363295   9364338   -  1044  
 
    Segment 207 
 
   Location   
  Gene key  FBgn0001224-FBgn0001226  
  Heatmap region span   3L:9365583..9410488   
  Segment span   3L:9374984..9377165   
  Length (genes)  2  
  Length (bp)  2182  
   Model Scoring   
  BIC  281.970129  
  logL  -135.472938  
  logL ratio  27.804737  
   Expression   
  Mean expression  9.165757  
  Median expression  8.774738  
  Tissue std. dev.  2.465158  
 
  No GO Slim enrichment  
  
   tissue    mean expression   
  5th Passage Drosophila S2 Cells  10.652190  
  Adult Accessory gland  6.136649  
  Adult Brain  8.726736  
  Adult Carcass  6.430328  
  Adult Crop  8.724508  
  Adult Eye  6.646153  
  Adult Fatbody  6.783821  
  Adult Female Spermatheca Mated  8.609395  
  Adult Female Spermatheca Virgin  6.971628  
  Adult Head  7.562976  
  Adult Heart  8.169528  
  Adult Hind Gut  5.629576  
  Adult Male Ejaculatory Duct  5.559021  
  Adult Mid Gut  7.737516  
  Adult Ovary  11.143325  
  Adult Salivary Gland  6.015667  
  Adult Testes  11.798104  
  Adult Thoracoabdominal ganglion  8.891225  
  Adult Whole Fly  10.461009  
  Larvae Wandering Tubules  13.926402  
  Larval Feeding Carcass  11.866739  
  Larval Feeding Central Nevous System  13.630395  
  Larval Feeding Hind Gut  10.333031  
  Larval Feeding Malpighian Tubule  12.109053  
  Larval Feeding Mid Gut  9.417742  
  Larval Feeding Salivary Gland  12.265486  
  Whole Larvae Feeding  11.277252  
 
  
   FlyBase ID    symbol    start    end    strand    length   
   FBgn0001224   Hsp23   9374984   9375867  +  884  
   FBgn0001226   Hsp27   9377165   9378384  +  1220  
 
    Segment 208 
 
   Location   
  Gene key  FBgn0035986-FBgn0035987  
  Heatmap region span   3L:9372634..9422917   
  Segment span   3L:9403931..9404159   
  Length (genes)  2  
  Length (bp)  229  
   Model Scoring   
  BIC  228.409375  
  logL  -108.692561  
  logL ratio  30.931771  
   Expression   
  Mean expression  8.414309  
  Median expression  8.254831  
  Tissue std. dev.  0.653828  
 
  No GO Slim enrichment  
  
   tissue    mean expression   
  5th Passage Drosophila S2 Cells  8.613821  
  Adult Accessory gland  8.643540  
  Adult Brain  9.678211  
  Adult Carcass  7.403207  
  Adult Crop  8.651193  
  Adult Eye  8.579425  
  Adult Fatbody  7.875368  
  Adult Female Spermatheca Mated  7.965094  
  Adult Female Spermatheca Virgin  8.104607  
  Adult Head  8.090704  
  Adult Heart  7.918537  
  Adult Hind Gut  8.113950  
  Adult Male Ejaculatory Duct  7.722692  
  Adult Mid Gut  7.843461  
  Adult Ovary  9.791694  
  Adult Salivary Gland  8.145630  
  Adult Testes  7.924027  
  Adult Thoracoabdominal ganglion  9.360338  
  Adult Whole Fly  8.310474  
  Larvae Wandering Tubules  8.861051  
  Larval Feeding Carcass  8.141196  
  Larval Feeding Central Nevous System  10.182054  
  Larval Feeding Hind Gut  8.229200  
  Larval Feeding Malpighian Tubule  8.494214  
  Larval Feeding Mid Gut  7.923920  
  Larval Feeding Salivary Gland  8.528346  
  Whole Larvae Feeding  8.090380  
 
  
   FlyBase ID    symbol    start    end    strand    length   
   FBgn0035986   CG4022  9401272   9403931   -  2660  
   FBgn0035987   CG3689   9404159   9406735  +  2577  
 
    Segment 209 
 
   Location   
  Gene key  FBgn0035989-FBgn0015296  
  Heatmap region span   3L:9397895..9465970   
  Segment span   3L:9419777..9420100   
  Length (genes)  2  
  Length (bp)  324  
   Model Scoring   
  BIC  229.626435  
  logL  -109.301091  
  logL ratio  26.857135  
   Expression   
  Mean expression  8.122672  
  Median expression  8.072691  
  Tissue std. dev.  0.553793  
 
  No GO Slim enrichment  
  
   tissue    mean expression   
  5th Passage Drosophila S2 Cells  9.463146  
  Adult Accessory gland  7.963155  
  Adult Brain  8.886533  
  Adult Carcass  7.515979  
  Adult Crop  8.462259  
  Adult Eye  8.482725  
  Adult Fatbody  7.372545  
  Adult Female Spermatheca Mated  7.567998  
  Adult Female Spermatheca Virgin  7.485774  
  Adult Head  8.198312  
  Adult Heart  8.267046  
  Adult Hind Gut  7.916065  
  Adult Male Ejaculatory Duct  7.903055  
  Adult Mid Gut  7.390570  
  Adult Ovary  8.474724  
  Adult Salivary Gland  7.488046  
  Adult Testes  7.909342  
  Adult Thoracoabdominal ganglion  8.678635  
  Adult Whole Fly  7.706921  
  Larvae Wandering Tubules  8.439614  
  Larval Feeding Carcass  8.414314  
  Larval Feeding Central Nevous System  9.487931  
  Larval Feeding Hind Gut  8.211613  
  Larval Feeding Malpighian Tubule  8.063969  
  Larval Feeding Mid Gut  7.867989  
  Larval Feeding Salivary Gland  7.861211  
  Whole Larvae Feeding  7.832664  
 
  
   FlyBase ID    symbol    start    end    strand    length   
   FBgn0035989   CG3967  9406696   9419777   -  13082  
   FBgn0015296   Shc   9420100   9421779  +  1680  
 
    Segment 210 
 
   Location   
  Gene key  FBgn0035995-FBgn0036000  
  Heatmap region span   3L:9410488..9485294   
  Segment span   3L:9439975..9453874   
  Length (genes)  7  
  Length (bp)  13900  
   Model Scoring   
  BIC  742.768117  
  logL  -365.871932  
  logL ratio  154.890350  
   Expression   
  Mean expression  8.655544  
  Median expression  8.567431  
  Tissue std. dev.  0.359770  
 
  No GO Slim enrichment  
  
   tissue    mean expression   
  5th Passage Drosophila S2 Cells  9.080426  
  Adult Accessory gland  9.233818  
  Adult Brain  8.726511  
  Adult Carcass  8.103057  
  Adult Crop  8.619291  
  Adult Eye  8.951816  
  Adult Fatbody  8.393371  
  Adult Female Spermatheca Mated  8.397992  
  Adult Female Spermatheca Virgin  8.383804  
  Adult Head  8.353653  
  Adult Heart  8.817565  
  Adult Hind Gut  8.907956  
  Adult Male Ejaculatory Duct  8.559387  
  Adult Mid Gut  8.809769  
  Adult Ovary  9.365091  
  Adult Salivary Gland  8.665676  
  Adult Testes  8.486880  
  Adult Thoracoabdominal ganglion  8.558884  
  Adult Whole Fly  8.642724  
  Larvae Wandering Tubules  9.110656  
  Larval Feeding Carcass  8.322014  
  Larval Feeding Central Nevous System  8.902583  
  Larval Feeding Hind Gut  8.403224  
  Larval Feeding Malpighian Tubule  8.769044  
  Larval Feeding Mid Gut  8.156940  
  Larval Feeding Salivary Gland  9.142886  
  Whole Larvae Feeding  7.834670  
 
  
   FlyBase ID    symbol    start    end    strand    length   
   FBgn0035995   CG3529   9439975   9443886  +  3912  
   FBgn0035996   CG3448  9443642   9444658   -  1017  
   FBgn0052038   CG32038   9444960   9445890  +  931  
   FBgn0035997   phol  9446148   9449356   -  3209  
   FBgn0035999   CG3552   9449454   9452470  +  3017  
   FBgn0035998   CG3437  9449442   9450761   -  1320  
   FBgn0036000   CG3434  9452469   9453874   -  1406  
 
 
    Segment 211 
 
   Location   
  Gene key  FBgn0036004-FBgn0036005  
  Heatmap region span   3L:9422917..9504642   
  Segment span   3L:9470150..9480867   
  Length (genes)  2  
  Length (bp)  10718  
   Model Scoring   
  BIC  231.719239  
  logL  -110.347493  
  logL ratio  29.822367  
   Expression   
  Mean expression  8.780032  
  Median expression  8.788448  
  Tissue std. dev.  0.513585  
 
  No GO Slim enrichment  
  
   tissue    mean expression   
  5th Passage Drosophila S2 Cells  9.093208  
  Adult Accessory gland  9.109005  
  Adult Brain  9.350073  
  Adult Carcass  8.428703  
  Adult Crop  8.841511  
  Adult Eye  8.991286  
  Adult Fatbody  8.646400  
  Adult Female Spermatheca Mated  8.756560  
  Adult Female Spermatheca Virgin  8.726192  
  Adult Head  8.339595  
  Adult Heart  8.893764  
  Adult Hind Gut  8.610120  
  Adult Male Ejaculatory Duct  8.478654  
  Adult Mid Gut  8.170335  
  Adult Ovary  10.133132  
  Adult Salivary Gland  8.936187  
  Adult Testes  7.469235  
  Adult Thoracoabdominal ganglion  9.087397  
  Adult Whole Fly  8.299330  
  Larvae Wandering Tubules  9.366629  
  Larval Feeding Carcass  8.570080  
  Larval Feeding Central Nevous System  9.005088  
  Larval Feeding Hind Gut  8.819922  
  Larval Feeding Malpighian Tubule  9.251262  
  Larval Feeding Mid Gut  8.332057  
  Larval Feeding Salivary Gland  9.354292  
  Whole Larvae Feeding  8.000860  
 
  
   FlyBase ID    symbol    start    end    strand    length   
   FBgn0036004   Jarid2   9470150   9479632  +  9483  
   FBgn0036005   pall  9479548   9480867   -  1320  
 
    Segment 212 
 
   Location   
  Gene key  FBgn0052036-FBgn0052037  
  Heatmap region span   3L:9439975..9590954   
  Segment span   3L:9482234..9485294   
  Length (genes)  2  
  Length (bp)  3061  
   Model Scoring   
  BIC  171.629590  
  logL  -80.302668  
  logL ratio  36.191290  
   Expression   
  Mean expression  4.835210  
  Median expression  4.799217  
  Tissue std. dev.  0.247148  
 
  No GO Slim enrichment  
  
   tissue    mean expression   
  5th Passage Drosophila S2 Cells  5.223729  
  Adult Accessory gland  5.274021  
  Adult Brain  4.384058  
  Adult Carcass  4.833479  
  Adult Crop  4.937783  
  Adult Eye  4.751692  
  Adult Fatbody  5.129147  
  Adult Female Spermatheca Mated  5.027361  
  Adult Female Spermatheca Virgin  5.079523  
  Adult Head  4.933328  
  Adult Heart  5.089757  
  Adult Hind Gut  4.793793  
  Adult Male Ejaculatory Duct  4.816802  
  Adult Mid Gut  4.748139  
  Adult Ovary  4.817836  
  Adult Salivary Gland  5.138819  
  Adult Testes  4.578053  
  Adult Thoracoabdominal ganglion  4.449855  
  Adult Whole Fly  4.307683  
  Larvae Wandering Tubules  4.857781  
  Larval Feeding Carcass  4.840263  
  Larval Feeding Central Nevous System  4.654692  
  Larval Feeding Hind Gut  4.766765  
  Larval Feeding Malpighian Tubule  5.005759  
  Larval Feeding Mid Gut  4.772811  
  Larval Feeding Salivary Gland  4.899195  
  Whole Larvae Feeding  4.438545  
 
  
   FlyBase ID    symbol    start    end    strand    length   
   FBgn0052036   CG32036  9481238   9482234   -  997  
   FBgn0052037   CG32037  9482898   9485294   -  2397  
 
    Segment 213 
 
   Location   
  Gene key  FBgn0053703-FBgn0036013  
  Heatmap region span   3L:9482234..9621776   
  Segment span   3L:9505760..9590954   
  Length (genes)  4  
  Length (bp)  85195  
   Model Scoring   
  BIC  298.721998  
  logL  -143.848873  
  logL ratio  132.300863  
   Expression   
  Mean expression  4.268184  
  Median expression  4.204287  
  Tissue std. dev.  0.124927  
 
  No GO Slim enrichment  
  
   tissue    mean expression   
  5th Passage Drosophila S2 Cells  4.192317  
  Adult Accessory gland  4.475748  
  Adult Brain  4.139633  
  Adult Carcass  4.343871  
  Adult Crop  4.289488  
  Adult Eye  4.296895  
  Adult Fatbody  4.411321  
  Adult Female Spermatheca Mated  4.324882  
  Adult Female Spermatheca Virgin  4.338485  
  Adult Head  4.283369  
  Adult Heart  4.234984  
  Adult Hind Gut  4.242352  
  Adult Male Ejaculatory Duct  4.360144  
  Adult Mid Gut  4.408109  
  Adult Ovary  4.247757  
  Adult Salivary Gland  4.522053  
  Adult Testes  4.256393  
  Adult Thoracoabdominal ganglion  4.130520  
  Adult Whole Fly  3.987817  
  Larvae Wandering Tubules  4.255283  
  Larval Feeding Carcass  4.198316  
  Larval Feeding Central Nevous System  4.043633  
  Larval Feeding Hind Gut  4.188835  
  Larval Feeding Malpighian Tubule  4.246308  
  Larval Feeding Mid Gut  4.319365  
  Larval Feeding Salivary Gland  4.428103  
  Whole Larvae Feeding  4.074982  
 
  
   FlyBase ID    symbol    start    end    strand    length   
   FBgn0053703   CG33703  9505108   9505760   -  653  
   FBgn0036009   Or67a   9523021   9524436  +  1416  
   FBgn0036010   Ir67a  9535340   9537099   -  1760  
   FBgn0036013   CG14177   9590954   9591295  +  342  
 
 
    Segment 214 
 
   Location   
  Gene key  FBgn0036016-FBgn0036017  
  Heatmap region span   3L:9504642..9634116   
  Segment span   3L:9594623..9595689   
  Length (genes)  2  
  Length (bp)  1067  
   Model Scoring   
  BIC  198.576281  
  logL  -93.776014  
  logL ratio  15.491590  
   Expression   
  Mean expression  5.127140  
  Median expression  5.269725  
  Tissue std. dev.  0.656480  
 
  No GO Slim enrichment  
  
   tissue    mean expression   
  5th Passage Drosophila S2 Cells  4.974951  
  Adult Accessory gland  5.061298  
  Adult Brain  4.896993  
  Adult Carcass  4.924665  
  Adult Crop  5.046077  
  Adult Eye  4.924646  
  Adult Fatbody  4.963795  
  Adult Female Spermatheca Mated  4.774871  
  Adult Female Spermatheca Virgin  4.826684  
  Adult Head  5.377560  
  Adult Heart  4.943851  
  Adult Hind Gut  4.964118  
  Adult Male Ejaculatory Duct  5.207018  
  Adult Mid Gut  4.943815  
  Adult Ovary  5.004379  
  Adult Salivary Gland  5.139879  
  Adult Testes  8.339909  
  Adult Thoracoabdominal ganglion  4.850573  
  Adult Whole Fly  5.639122  
  Larvae Wandering Tubules  5.032602  
  Larval Feeding Carcass  4.943829  
  Larval Feeding Central Nevous System  4.708941  
  Larval Feeding Hind Gut  4.810553  
  Larval Feeding Malpighian Tubule  4.905774  
  Larval Feeding Mid Gut  4.985656  
  Larval Feeding Salivary Gland  5.138202  
  Whole Larvae Feeding  5.103013  
 
  
   FlyBase ID    symbol    start    end    strand    length   
   FBgn0036016   CG3306   9594623   9595648  +  1026  
   FBgn0036017   CG3280   9595689   9606904  +  11216  
 
    Segment 215 
 
   Location   
  Gene key  FBgn0002528-FBgn0036020  
  Heatmap region span   3L:9505760..9637209   
  Segment span   3L:9608019..9621776   
  Length (genes)  3  
  Length (bp)  13758  
   Model Scoring   
  BIC  367.629609  
  logL  -178.302678  
  logL ratio  23.154263  
   Expression   
  Mean expression  8.410405  
  Median expression  8.178196  
  Tissue std. dev.  0.625764  
 
  No GO Slim enrichment  
  
   tissue    mean expression   
  5th Passage Drosophila S2 Cells  10.045226  
  Adult Accessory gland  9.284658  
  Adult Brain  7.576728  
  Adult Carcass  7.950715  
  Adult Crop  9.370153  
  Adult Eye  7.977932  
  Adult Fatbody  8.386654  
  Adult Female Spermatheca Mated  7.925447  
  Adult Female Spermatheca Virgin  7.505827  
  Adult Head  7.841042  
  Adult Heart  8.882025  
  Adult Hind Gut  8.649171  
  Adult Male Ejaculatory Duct  8.644629  
  Adult Mid Gut  7.537747  
  Adult Ovary  8.732006  
  Adult Salivary Gland  8.196641  
  Adult Testes  8.100215  
  Adult Thoracoabdominal ganglion  7.747636  
  Adult Whole Fly  7.980614  
  Larvae Wandering Tubules  8.645351  
  Larval Feeding Carcass  8.406032  
  Larval Feeding Central Nevous System  8.617912  
  Larval Feeding Hind Gut  9.430986  
  Larval Feeding Malpighian Tubule  8.798466  
  Larval Feeding Mid Gut  7.845491  
  Larval Feeding Salivary Gland  8.776475  
  Whole Larvae Feeding  8.225151  
 
  
   FlyBase ID    symbol    start    end    strand    length   
   FBgn0002528   LanB2   9608019   9617080  +  9062  
   FBgn0036018   CG3335   9617383   9620425  +  3043  
   FBgn0036020   CG8336   9621776   9624895  +  3120  
 
 
    Segment 216 
 
   Location   
  Gene key  FBgn0036023-FBgn0036024  
  Heatmap region span   3L:9594623..9678271   
  Segment span   3L:9632373..9634116   
  Length (genes)  2  
  Length (bp)  1744  
   Model Scoring   
  BIC  215.455225  
  logL  -102.215486  
  logL ratio  34.312895  
   Expression   
  Mean expression  6.147659  
  Median expression  4.966641  
  Tissue std. dev.  2.793741  
 
  No GO Slim enrichment  
  
   tissue    mean expression   
  5th Passage Drosophila S2 Cells  4.728523  
  Adult Accessory gland  4.836661  
  Adult Brain  4.322049  
  Adult Carcass  6.151713  
  Adult Crop  5.475531  
  Adult Eye  4.851436  
  Adult Fatbody  4.903467  
  Adult Female Spermatheca Mated  4.708369  
  Adult Female Spermatheca Virgin  4.794861  
  Adult Head  5.756116  
  Adult Heart  4.781719  
  Adult Hind Gut  5.114638  
  Adult Male Ejaculatory Duct  5.490373  
  Adult Mid Gut  14.194760  
  Adult Ovary  4.635652  
  Adult Salivary Gland  5.173763  
  Adult Testes  4.461775  
  Adult Thoracoabdominal ganglion  4.590996  
  Adult Whole Fly  11.316797  
  Larvae Wandering Tubules  4.853817  
  Larval Feeding Carcass  5.095038  
  Larval Feeding Central Nevous System  5.084843  
  Larval Feeding Hind Gut  5.849182  
  Larval Feeding Malpighian Tubule  4.734711  
  Larval Feeding Mid Gut  13.222133  
  Larval Feeding Salivary Gland  4.813711  
  Whole Larvae Feeding  12.044165  
 
  
   FlyBase ID    symbol    start    end    strand    length   
   FBgn0036023   CG18179   9632373   9633240  +  868  
   FBgn0036024   CG18180   9634116   9635013  +  898  
 
    Segment 217 
 
   Location   
  Gene key  FBgn0016081-FBgn0036028  
  Heatmap region span   3L:9622293..9697770   
  Segment span   3L:9672203..9672329   
  Length (genes)  2  
  Length (bp)  127  
   Model Scoring   
  BIC  217.149975  
  logL  -103.062861  
  logL ratio  36.905790  
   Expression   
  Mean expression  7.676452  
  Median expression  7.616231  
  Tissue std. dev.  0.426527  
 
  No GO Slim enrichment  
  
   tissue    mean expression   
  5th Passage Drosophila S2 Cells  8.399162  
  Adult Accessory gland  7.222953  
  Adult Brain  8.334708  
  Adult Carcass  7.386224  
  Adult Crop  8.174745  
  Adult Eye  8.006200  
  Adult Fatbody  7.001998  
  Adult Female Spermatheca Mated  6.941658  
  Adult Female Spermatheca Virgin  6.878045  
  Adult Head  7.606082  
  Adult Heart  7.863568  
  Adult Hind Gut  7.806166  
  Adult Male Ejaculatory Duct  7.500565  
  Adult Mid Gut  7.668202  
  Adult Ovary  7.785654  
  Adult Salivary Gland  7.556145  
  Adult Testes  8.206233  
  Adult Thoracoabdominal ganglion  8.167474  
  Adult Whole Fly  7.212409  
  Larvae Wandering Tubules  7.641220  
  Larval Feeding Carcass  7.844293  
  Larval Feeding Central Nevous System  7.860833  
  Larval Feeding Hind Gut  7.470526  
  Larval Feeding Malpighian Tubule  8.367773  
  Larval Feeding Mid Gut  7.701492  
  Larval Feeding Salivary Gland  7.303721  
  Whole Larvae Feeding  7.356159  
 
  
   FlyBase ID    symbol    start    end    strand    length   
   FBgn0016081   fry  9624934   9672203   -  47270  
   FBgn0036028   CG16717   9672329   9674160  +  1832  
 
    Segment 218 
 
   Location   
  Gene key  FBgn0036030-FBgn0015321  
  Heatmap region span   3L:9637209..9716527   
  Segment span   3L:9689793..9691442   
  Length (genes)  2  
  Length (bp)  1650  
   Model Scoring   
  BIC  264.064208  
  logL  -126.519978  
  logL ratio  32.415500  
   Expression   
  Mean expression  10.132374  
  Median expression  9.888044  
  Tissue std. dev.  0.540109  
 
  No GO Slim enrichment  
  
   tissue    mean expression   
  5th Passage Drosophila S2 Cells  10.446665  
  Adult Accessory gland  10.177015  
  Adult Brain  10.166383  
  Adult Carcass  10.069344  
  Adult Crop  10.112436  
  Adult Eye  9.845544  
  Adult Fatbody  10.991518  
  Adult Female Spermatheca Mated  10.975995  
  Adult Female Spermatheca Virgin  11.214419  
  Adult Head  10.203754  
  Adult Heart  10.169759  
  Adult Hind Gut  9.798664  
  Adult Male Ejaculatory Duct  9.975807  
  Adult Mid Gut  9.246862  
  Adult Ovary  11.020326  
  Adult Salivary Gland  9.813684  
  Adult Testes  8.747526  
  Adult Thoracoabdominal ganglion  10.061581  
  Adult Whole Fly  10.442743  
  Larvae Wandering Tubules  10.047576  
  Larval Feeding Carcass  9.863477  
  Larval Feeding Central Nevous System  10.802470  
  Larval Feeding Hind Gut  9.784201  
  Larval Feeding Malpighian Tubule  10.403742  
  Larval Feeding Mid Gut  9.617908  
  Larval Feeding Salivary Gland  9.735633  
  Whole Larvae Feeding  9.839059  
 
  
   FlyBase ID    symbol    start    end    strand    length   
   FBgn0036030   CG6767  9676676   9689793   -  13118  
   FBgn0015321   UbcD4   9691442   9693581  +  2140  
 
    Segment 219 
 
   Location   
  Gene key  FBgn0036031-FBgn0036032  
  Heatmap region span   3L:9672203..9720004   
  Segment span   3L:9696695..9697770   
  Length (genes)  2  
  Length (bp)  1076  
   Model Scoring   
  BIC  235.777185  
  logL  -112.376466  
  logL ratio  -10.290771  
   Expression   
  Mean expression  5.941398  
  Median expression  5.654165  
  Tissue std. dev.  0.847918  
 
  No GO Slim enrichment  
  
   tissue    mean expression   
  5th Passage Drosophila S2 Cells  6.094743  
  Adult Accessory gland  5.905643  
  Adult Brain  6.781661  
  Adult Carcass  5.408214  
  Adult Crop  5.477108  
  Adult Eye  6.510830  
  Adult Fatbody  5.471614  
  Adult Female Spermatheca Mated  5.781372  
  Adult Female Spermatheca Virgin  5.797279  
  Adult Head  5.875584  
  Adult Heart  6.099009  
  Adult Hind Gut  5.431805  
  Adult Male Ejaculatory Duct  5.587281  
  Adult Mid Gut  5.474152  
  Adult Ovary  5.932686  
  Adult Salivary Gland  5.717823  
  Adult Testes  9.803179  
  Adult Thoracoabdominal ganglion  6.680662  
  Adult Whole Fly  6.034040  
  Larvae Wandering Tubules  5.419847  
  Larval Feeding Carcass  5.456677  
  Larval Feeding Central Nevous System  5.941075  
  Larval Feeding Hind Gut  5.490182  
  Larval Feeding Malpighian Tubule  5.350942  
  Larval Feeding Mid Gut  5.465314  
  Larval Feeding Salivary Gland  5.872868  
  Whole Larvae Feeding  5.556156  
 
  
   FlyBase ID    symbol    start    end    strand    length   
   FBgn0036031   CG6761  9693617   9696695   -  3079  
   FBgn0036032   CG16711   9697770   9702809  +  5040  
 
    Segment 220 
 
   Location   
  Gene key  FBgn0036035-FBgn0036038  
  Heatmap region span   3L:9689793..9756652   
  Segment span   3L:9712244..9716527   
  Length (genes)  3  
  Length (bp)  4284  
   Model Scoring   
  BIC  331.612151  
  logL  -160.293949  
  logL ratio  31.291218  
   Expression   
  Mean expression  7.042873  
  Median expression  7.054452  
  Tissue std. dev.  0.587939  
 
  No GO Slim enrichment  
  
   tissue    mean expression   
  5th Passage Drosophila S2 Cells  8.096744  
  Adult Accessory gland  7.603763  
  Adult Brain  6.582547  
  Adult Carcass  6.482869  
  Adult Crop  7.365798  
  Adult Eye  6.838352  
  Adult Fatbody  7.086994  
  Adult Female Spermatheca Mated  7.388649  
  Adult Female Spermatheca Virgin  7.200346  
  Adult Head  6.658867  
  Adult Heart  7.064214  
  Adult Hind Gut  6.602687  
  Adult Male Ejaculatory Duct  6.770186  
  Adult Mid Gut  6.536185  
  Adult Ovary  8.464496  
  Adult Salivary Gland  6.499189  
  Adult Testes  8.309487  
  Adult Thoracoabdominal ganglion  6.656313  
  Adult Whole Fly  7.190360  
  Larvae Wandering Tubules  6.670509  
  Larval Feeding Carcass  6.969247  
  Larval Feeding Central Nevous System  7.850283  
  Larval Feeding Hind Gut  6.704063  
  Larval Feeding Malpighian Tubule  6.649738  
  Larval Feeding Mid Gut  6.141229  
  Larval Feeding Salivary Gland  7.333527  
  Whole Larvae Feeding  6.440925  
 
  
   FlyBase ID    symbol    start    end    strand    length   
   FBgn0036035   CG18178  9710886   9712244   -  1359  
   FBgn0036036   CG14174   9712428   9713564  +  1137  
   FBgn0036038   defl   9716527   9719806  +  3280  
 
 
    Segment 221 
 
   Location   
  Gene key  FBgn0036044-FBgn0044051  
  Heatmap region span   3L:9720004..9816997   
  Segment span   3L:9790626..9791534   
  Length (genes)  2  
  Length (bp)  909  
   Model Scoring   
  BIC  165.915093  
  logL  -77.445420  
  logL ratio  42.181341  
   Expression   
  Mean expression  4.452233  
  Median expression  4.437892  
  Tissue std. dev.  0.135741  
 
  No GO Slim enrichment  
  
   tissue    mean expression   
  5th Passage Drosophila S2 Cells  4.490145  
  Adult Accessory gland  4.624780  
  Adult Brain  4.395357  
  Adult Carcass  4.894108  
  Adult Crop  4.463226  
  Adult Eye  4.424838  
  Adult Fatbody  4.547066  
  Adult Female Spermatheca Mated  4.373371  
  Adult Female Spermatheca Virgin  4.412572  
  Adult Head  4.414335  
  Adult Heart  4.386708  
  Adult Hind Gut  4.419790  
  Adult Male Ejaculatory Duct  4.577247  
  Adult Mid Gut  4.482647  
  Adult Ovary  4.567263  
  Adult Salivary Gland  4.577701  
  Adult Testes  4.294855  
  Adult Thoracoabdominal ganglion  4.347834  
  Adult Whole Fly  4.177822  
  Larvae Wandering Tubules  4.449476  
  Larval Feeding Carcass  4.535992  
  Larval Feeding Central Nevous System  4.319306  
  Larval Feeding Hind Gut  4.355489  
  Larval Feeding Malpighian Tubule  4.442322  
  Larval Feeding Mid Gut  4.423476  
  Larval Feeding Salivary Gland  4.560633  
  Whole Larvae Feeding  4.251915  
 
  
   FlyBase ID    symbol    start    end    strand    length   
   FBgn0036044   CG14168  9781676   9790626   -  8951  
   FBgn0044051   Ilp1   9791534   9791998  +  465  
 
    Segment 222 
 
   Location   
  Gene key  FBgn0036046-FBgn0044050  
  Heatmap region span   3L:9726907..9818799   
  Segment span   3L:9792798..9795220   
  Length (genes)  2  
  Length (bp)  2423  
   Model Scoring   
  BIC  201.146245  
  logL  -95.060996  
  logL ratio  17.846363  
   Expression   
  Mean expression  5.632391  
  Median expression  4.964228  
  Tissue std. dev.  1.588806  
 
  
   GO ID    description    ratio    P-value   
   GO:0005576   extracellular region  2/2  0.00207  
 
  
   tissue    mean expression   
  5th Passage Drosophila S2 Cells  4.943526  
  Adult Accessory gland  4.992032  
  Adult Brain  11.099335  
  Adult Carcass  4.828100  
  Adult Crop  5.257623  
  Adult Eye  5.145826  
  Adult Fatbody  4.997063  
  Adult Female Spermatheca Mated  4.903428  
  Adult Female Spermatheca Virgin  4.954227  
  Adult Head  9.027294  
  Adult Heart  4.659270  
  Adult Hind Gut  4.861629  
  Adult Male Ejaculatory Duct  5.111457  
  Adult Mid Gut  6.724725  
  Adult Ovary  4.705301  
  Adult Salivary Gland  4.947141  
  Adult Testes  4.680894  
  Adult Thoracoabdominal ganglion  4.903328  
  Adult Whole Fly  5.405052  
  Larvae Wandering Tubules  4.799000  
  Larval Feeding Carcass  4.890102  
  Larval Feeding Central Nevous System  9.189618  
  Larval Feeding Hind Gut  4.559424  
  Larval Feeding Malpighian Tubule  4.890539  
  Larval Feeding Mid Gut  6.738781  
  Larval Feeding Salivary Gland  4.834316  
  Whole Larvae Feeding  6.025523  
 
  
   FlyBase ID    symbol    start    end    strand    length   
   FBgn0036046   Ilp2   9792798   9793545  +  748  
   FBgn0044050   Ilp3  9794398   9795220   -  823  
 
    Segment 223 
 
   Location   
  Gene key  FBgn0044049-FBgn0044328  
  Heatmap region span   3L:9753495..9848379   
  Segment span   3L:9797378..9797560   
  Length (genes)  2  
  Length (bp)  183  
   Model Scoring   
  BIC  217.214598  
  logL  -103.095173  
  logL ratio  10.569008  
   Expression   
  Mean expression  4.404285  
  Median expression  4.198363  
  Tissue std. dev.  0.719210  
 
  No GO Slim enrichment  
  
   tissue    mean expression   
  5th Passage Drosophila S2 Cells  3.964172  
  Adult Accessory gland  4.155589  
  Adult Brain  6.568163  
  Adult Carcass  4.029191  
  Adult Crop  4.197243  
  Adult Eye  5.403354  
  Adult Fatbody  4.143294  
  Adult Female Spermatheca Mated  4.246357  
  Adult Female Spermatheca Virgin  4.248680  
  Adult Head  5.579452  
  Adult Heart  4.308523  
  Adult Hind Gut  3.912373  
  Adult Male Ejaculatory Duct  4.098526  
  Adult Mid Gut  4.025246  
  Adult Ovary  3.920398  
  Adult Salivary Gland  4.430218  
  Adult Testes  3.816688  
  Adult Thoracoabdominal ganglion  6.316676  
  Adult Whole Fly  4.083538  
  Larvae Wandering Tubules  4.007967  
  Larval Feeding Carcass  4.114942  
  Larval Feeding Central Nevous System  5.142912  
  Larval Feeding Hind Gut  3.996560  
  Larval Feeding Malpighian Tubule  4.042919  
  Larval Feeding Mid Gut  4.038694  
  Larval Feeding Salivary Gland  4.247102  
  Whole Larvae Feeding  3.876914  
 
  
   FlyBase ID    symbol    start    end    strand    length   
   FBgn0044049   Ilp4  9796491   9797378   -  888  
   FBgn0044328   CG32052   9797560   9801813  +  4254  
 
    Segment 224 
 
   Location   
  Gene key  FBgn0015618-FBgn0004390  
  Heatmap region span   3L:9797378..9892329   
  Segment span   3L:9831750..9848379   
  Length (genes)  2  
  Length (bp)  16630  
   Model Scoring   
  BIC  205.596279  
  logL  -97.286013  
  logL ratio  37.093572  
   Expression   
  Mean expression  6.513018  
  Median expression  6.367369  
  Tissue std. dev.  0.690886  
 
  No GO Slim enrichment  
  
   tissue    mean expression   
  5th Passage Drosophila S2 Cells  7.983724  
  Adult Accessory gland  6.495124  
  Adult Brain  7.101460  
  Adult Carcass  5.668235  
  Adult Crop  6.662309  
  Adult Eye  6.607983  
  Adult Fatbody  6.044569  
  Adult Female Spermatheca Mated  6.393288  
  Adult Female Spermatheca Virgin  6.511411  
  Adult Head  6.214175  
  Adult Heart  6.331135  
  Adult Hind Gut  6.343706  
  Adult Male Ejaculatory Duct  6.100995  
  Adult Mid Gut  6.418052  
  Adult Ovary  8.487934  
  Adult Salivary Gland  6.329090  
  Adult Testes  4.760787  
  Adult Thoracoabdominal ganglion  6.710257  
  Adult Whole Fly  6.483315  
  Larvae Wandering Tubules  6.873640  
  Larval Feeding Carcass  6.237311  
  Larval Feeding Central Nevous System  7.717628  
  Larval Feeding Hind Gut  6.378320  
  Larval Feeding Malpighian Tubule  6.353161  
  Larval Feeding Mid Gut  6.255049  
  Larval Feeding Salivary Gland  6.360231  
  Whole Larvae Feeding  6.028611  
 
  
   FlyBase ID    symbol    start    end    strand    length   
   FBgn0015618   Cdk8  9830185   9831750   -  1566  
   FBgn0004390   Gap1  9832195   9848379   -  16185  
 
    Segment 225 
 
   Location   
  Gene key  FBgn0036052-FBgn0027567  
  Heatmap region span   3L:9809170..9936247   
  Segment span   3L:9852989..9857706   
  Length (genes)  2  
  Length (bp)  4718  
   Model Scoring   
  BIC  240.432530  
  logL  -114.704139  
  logL ratio  23.480053  
   Expression   
  Mean expression  8.769824  
  Median expression  8.748144  
  Tissue std. dev.  0.658528  
 
  No GO Slim enrichment  
  
   tissue    mean expression   
  5th Passage Drosophila S2 Cells  9.404288  
  Adult Accessory gland  8.803743  
  Adult Brain  9.965372  
  Adult Carcass  7.990531  
  Adult Crop  8.565770  
  Adult Eye  8.831940  
  Adult Fatbody  8.570538  
  Adult Female Spermatheca Mated  8.529922  
  Adult Female Spermatheca Virgin  8.689101  
  Adult Head  8.470777  
  Adult Heart  8.588573  
  Adult Hind Gut  8.310923  
  Adult Male Ejaculatory Duct  7.787364  
  Adult Mid Gut  7.998177  
  Adult Ovary  10.194556  
  Adult Salivary Gland  8.162554  
  Adult Testes  9.473322  
  Adult Thoracoabdominal ganglion  9.673384  
  Adult Whole Fly  8.861189  
  Larvae Wandering Tubules  8.573498  
  Larval Feeding Carcass  8.446426  
  Larval Feeding Central Nevous System  10.421951  
  Larval Feeding Hind Gut  8.668440  
  Larval Feeding Malpighian Tubule  8.373524  
  Larval Feeding Mid Gut  8.204168  
  Larval Feeding Salivary Gland  8.833932  
  Whole Larvae Feeding  8.391288  
 
  
   FlyBase ID    symbol    start    end    strand    length   
   FBgn0036052   CG10809  9850688   9852989   -  2302  
   FBgn0027567   CG8108   9857706   9863774  +  6069  
 
    Segment 226 
 
   Location   
  Gene key  FBgn0025866-FBgn0036059  
  Heatmap region span   3L:9831750..10087838   
  Segment span   3L:9881862..9892329   
  Length (genes)  3  
  Length (bp)  10468  
   Model Scoring   
  BIC  284.249059  
  logL  -136.612403  
  logL ratio  129.421141  
   Expression   
  Mean expression  9.821744  
  Median expression  9.803880  
  Tissue std. dev.  0.525630  
 
  No GO Slim enrichment  
  
   tissue    mean expression   
  5th Passage Drosophila S2 Cells  10.582541  
  Adult Accessory gland  9.312848  
  Adult Brain  10.549496  
  Adult Carcass  9.457007  
  Adult Crop  10.477271  
  Adult Eye  9.868341  
  Adult Fatbody  9.281301  
  Adult Female Spermatheca Mated  9.501248  
  Adult Female Spermatheca Virgin  9.380157  
  Adult Head  9.880356  
  Adult Heart  9.960420  
  Adult Hind Gut  10.018193  
  Adult Male Ejaculatory Duct  9.619390  
  Adult Mid Gut  9.217122  
  Adult Ovary  10.238456  
  Adult Salivary Gland  10.142587  
  Adult Testes  8.332032  
  Adult Thoracoabdominal ganglion  10.551922  
  Adult Whole Fly  9.222349  
  Larvae Wandering Tubules  9.879150  
  Larval Feeding Carcass  10.173855  
  Larval Feeding Central Nevous System  10.079733  
  Larval Feeding Hind Gut  10.453123  
  Larval Feeding Malpighian Tubule  10.197062  
  Larval Feeding Mid Gut  9.969525  
  Larval Feeding Salivary Gland  9.371288  
  Whole Larvae Feeding  9.470319  
 
  
   FlyBase ID    symbol    start    end    strand    length   
   FBgn0025866   CalpB   9881862   9886419  +  4558  
   FBgn0036058   CG6707  9887915   9891693   -  3779  
   FBgn0036059   nudE   9892329   9896066  +  3738  
 
 
    Segment 227 
 
   Location   
  Gene key  FBgn0036062-FBgn0036063  
  Heatmap region span   3L:9872085..10213264   
  Segment span   3L:9961401..9965150   
  Length (genes)  3  
  Length (bp)  3750  
   Model Scoring   
  BIC  294.780489  
  logL  -141.878118  
  logL ratio  76.325625  
   Expression   
  Mean expression  7.189195  
  Median expression  7.008738  
  Tissue std. dev.  0.625729  
 
  No GO Slim enrichment  
  
   tissue    mean expression   
  5th Passage Drosophila S2 Cells  7.817498  
  Adult Accessory gland  6.729113  
  Adult Brain  7.568938  
  Adult Carcass  6.384821  
  Adult Crop  6.883964  
  Adult Eye  7.091493  
  Adult Fatbody  6.525819  
  Adult Female Spermatheca Mated  6.589941  
  Adult Female Spermatheca Virgin  6.633865  
  Adult Head  6.765215  
  Adult Heart  7.294044  
  Adult Hind Gut  6.710105  
  Adult Male Ejaculatory Duct  6.365235  
  Adult Mid Gut  6.845171  
  Adult Ovary  9.088937  
  Adult Salivary Gland  6.979045  
  Adult Testes  8.315643  
  Adult Thoracoabdominal ganglion  7.128776  
  Adult Whole Fly  7.763626  
  Larvae Wandering Tubules  7.881812  
  Larval Feeding Carcass  7.261628  
  Larval Feeding Central Nevous System  8.033013  
  Larval Feeding Hind Gut  7.252399  
  Larval Feeding Malpighian Tubule  7.431139  
  Larval Feeding Mid Gut  7.017563  
  Larval Feeding Salivary Gland  6.832472  
  Whole Larvae Feeding  6.916995  
 
  
   FlyBase ID    symbol    start    end    strand    length   
   FBgn0036062   CG6685  9960308   9961401   -  1094  
   FBgn0026404   Nc   9961543   9964102  +  2560  
   FBgn0036063   CG6674  9964251   9965150   -  900  
 
 
    Segment 228 
 
   Location   
  Gene key  FBgn0040823-FBgn0052054  
  Heatmap region span   3L:9881862..10427205   
  Segment span   3L:9967719..10087838   
  Length (genes)  4  
  Length (bp)  120120  
   Model Scoring   
  BIC  345.705609  
  logL  -167.340678  
  logL ratio  74.667159  
   Expression   
  Mean expression  5.140877  
  Median expression  4.769640  
  Tissue std. dev.  0.878670  
 
  
   GO ID    description    ratio    P-value   
   GO:0022857   transmembrane transporter activity  3/4  6.91e-07  
   GO:0055085   transmembrane transport  3/4  3.97e-05  
   GO:0006810   transport  3/4  0.000428  
 
  
   tissue    mean expression   
  5th Passage Drosophila S2 Cells  4.640866  
  Adult Accessory gland  4.790464  
  Adult Brain  4.848146  
  Adult Carcass  4.792509  
  Adult Crop  4.645776  
  Adult Eye  4.734890  
  Adult Fatbody  4.784409  
  Adult Female Spermatheca Mated  4.763445  
  Adult Female Spermatheca Virgin  4.764330  
  Adult Head  4.549290  
  Adult Heart  4.544029  
  Adult Hind Gut  6.095247  
  Adult Male Ejaculatory Duct  4.606016  
  Adult Mid Gut  8.200534  
  Adult Ovary  4.789126  
  Adult Salivary Gland  5.009592  
  Adult Testes  5.101959  
  Adult Thoracoabdominal ganglion  4.912501  
  Adult Whole Fly  4.932622  
  Larvae Wandering Tubules  5.120790  
  Larval Feeding Carcass  4.578535  
  Larval Feeding Central Nevous System  4.506073  
  Larval Feeding Hind Gut  5.775123  
  Larval Feeding Malpighian Tubule  5.531308  
  Larval Feeding Mid Gut  7.658312  
  Larval Feeding Salivary Gland  4.664953  
  Whole Larvae Feeding  5.462824  
 
  
   FlyBase ID    symbol    start    end    strand    length   
   FBgn0040823   dpr6   9967719   10034408  +  66690  
   FBgn0036066   CG14160  10079847   10082500   -  2654  
   FBgn0052053   CG32053  10083270   10085439   -  2170  
   FBgn0052054   CG32054  10085868   10087838   -  1971  
 
 
    Segment 229 
 
   Location   
  Gene key  FBgn0036070-FBgn0036072  
  Heatmap region span   3L:9936247..10456199   
  Segment span   3L:10145064..10160123   
  Length (genes)  2  
  Length (bp)  15060  
   Model Scoring   
  BIC  183.160197  
  logL  -86.067972  
  logL ratio  38.016090  
   Expression   
  Mean expression  4.585256  
  Median expression  4.213270  
  Tissue std. dev.  1.321660  
 
  
   GO ID    description    ratio    P-value   
   GO:0005576   extracellular region  2/2  0.00207  
 
  
   tissue    mean expression   
  5th Passage Drosophila S2 Cells  4.238044  
  Adult Accessory gland  4.291251  
  Adult Brain  4.092101  
  Adult Carcass  4.634838  
  Adult Crop  4.152161  
  Adult Eye  4.060768  
  Adult Fatbody  4.457120  
  Adult Female Spermatheca Mated  4.341959  
  Adult Female Spermatheca Virgin  4.287336  
  Adult Head  4.268346  
  Adult Heart  4.094065  
  Adult Hind Gut  4.321734  
  Adult Male Ejaculatory Duct  4.229380  
  Adult Mid Gut  4.334141  
  Adult Ovary  4.149645  
  Adult Salivary Gland  4.933285  
  Adult Testes  10.913916  
  Adult Thoracoabdominal ganglion  4.179586  
  Adult Whole Fly  6.467502  
  Larvae Wandering Tubules  4.208993  
  Larval Feeding Carcass  4.158449  
  Larval Feeding Central Nevous System  4.043916  
  Larval Feeding Hind Gut  4.176211  
  Larval Feeding Malpighian Tubule  4.263405  
  Larval Feeding Mid Gut  4.170854  
  Larval Feeding Salivary Gland  4.133409  
  Whole Larvae Feeding  4.199499  
 
  
   FlyBase ID    symbol    start    end    strand    length   
   FBgn0036070   CG8072   10145064   10145856  +  793  
   FBgn0036072   CG6628  10158909   10160123   -  1215  
 
    Segment 230 
 
   Location   
  Gene key  FBgn0052057-FBgn0052055  
  Heatmap region span   3L:9961401..10621584   
  Segment span   3L:10179205..10213264   
  Length (genes)  4  
  Length (bp)  34060  
   Model Scoring   
  BIC  363.270749  
  logL  -176.123248  
  logL ratio  71.679840  
   Expression   
  Mean expression  4.532250  
  Median expression  4.239930  
  Tissue std. dev.  0.470555  
 
  No GO Slim enrichment  
  
   tissue    mean expression   
  5th Passage Drosophila S2 Cells  4.143878  
  Adult Accessory gland  4.452439  
  Adult Brain  6.029604  
  Adult Carcass  4.329715  
  Adult Crop  4.219869  
  Adult Eye  4.456055  
  Adult Fatbody  4.273981  
  Adult Female Spermatheca Mated  4.283399  
  Adult Female Spermatheca Virgin  4.311693  
  Adult Head  4.924219  
  Adult Heart  4.839646  
  Adult Hind Gut  5.121281  
  Adult Male Ejaculatory Duct  4.205975  
  Adult Mid Gut  4.257889  
  Adult Ovary  4.190956  
  Adult Salivary Gland  4.408145  
  Adult Testes  4.469508  
  Adult Thoracoabdominal ganglion  5.720842  
  Adult Whole Fly  4.173404  
  Larvae Wandering Tubules  4.344530  
  Larval Feeding Carcass  4.520201  
  Larval Feeding Central Nevous System  5.190124  
  Larval Feeding Hind Gut  4.517969  
  Larval Feeding Malpighian Tubule  4.304342  
  Larval Feeding Mid Gut  4.265765  
  Larval Feeding Salivary Gland  4.232455  
  Whole Larvae Feeding  4.182875  
 
  
   FlyBase ID    symbol    start    end    strand    length   
   FBgn0052057   dpr10  10140553   10179205   -  38653  
   FBgn0000451   ect  10183376   10194934   -  11559  
   FBgn0036075   CG8065   10208765   10209799  +  1035  
   FBgn0052055   CG32055  10211604   10213264   -  1661  
 
 
    Segment 231 
 
   Location   
  Gene key  FBgn0036078-FBgn0052058  
  Heatmap region span   3L:9967719..10631253   
  Segment span   3L:10237806..10427205   
  Length (genes)  6  
  Length (bp)  189400  
   Model Scoring   
  BIC  485.251655  
  logL  -237.113701  
  logL ratio  171.312343  
   Expression   
  Mean expression  4.265551  
  Median expression  4.029521  
  Tissue std. dev.  0.404777  
 
  No GO Slim enrichment  
  
   tissue    mean expression   
  5th Passage Drosophila S2 Cells  4.169696  
  Adult Accessory gland  4.353821  
  Adult Brain  4.067083  
  Adult Carcass  4.304551  
  Adult Crop  4.139540  
  Adult Eye  4.101617  
  Adult Fatbody  4.220184  
  Adult Female Spermatheca Mated  4.346222  
  Adult Female Spermatheca Virgin  4.317287  
  Adult Head  4.126892  
  Adult Heart  4.091722  
  Adult Hind Gut  4.115711  
  Adult Male Ejaculatory Duct  4.126400  
  Adult Mid Gut  4.151228  
  Adult Ovary  4.031840  
  Adult Salivary Gland  4.432468  
  Adult Testes  6.247015  
  Adult Thoracoabdominal ganglion  4.128757  
  Adult Whole Fly  4.475517  
  Larvae Wandering Tubules  4.198065  
  Larval Feeding Carcass  4.093064  
  Larval Feeding Central Nevous System  4.255898  
  Larval Feeding Hind Gut  4.034226  
  Larval Feeding Malpighian Tubule  4.162588  
  Larval Feeding Mid Gut  4.129456  
  Larval Feeding Salivary Gland  4.209831  
  Whole Larvae Feeding  4.139199  
 
  
   FlyBase ID    symbol    start    end    strand    length   
   FBgn0036078   Or67c  10236420   10237806   -  1387  
   FBgn0011569   can  10257007   10260211   -  3205  
   FBgn0036080   Or67d   10266304   10267724  +  1421  
   FBgn0036082   CG12362   10405916   10407877  +  1962  
   FBgn0036083   Ir67b  10422789   10425083   -  2295  
   FBgn0052058   Ir67c  10425484   10427205   -  1722  
 
 
    Segment 232 
 
   Location   
  Gene key  FBgn0052061-FBgn0036089  
  Heatmap region span   3L:10179205..10636633   
  Segment span   3L:10456916..10621584   
  Length (genes)  5  
  Length (bp)  164669  
   Model Scoring   
  BIC  399.216014  
  logL  -194.095881  
  logL ratio  143.122470  
   Expression   
  Mean expression  5.053822  
  Median expression  4.668836  
  Tissue std. dev.  1.674811  
 
  No GO Slim enrichment  
  
   tissue    mean expression   
  5th Passage Drosophila S2 Cells  4.494918  
  Adult Accessory gland  4.651163  
  Adult Brain  4.266240  
  Adult Carcass  4.910030  
  Adult Crop  4.445496  
  Adult Eye  4.366785  
  Adult Fatbody  4.622447  
  Adult Female Spermatheca Mated  4.666016  
  Adult Female Spermatheca Virgin  4.687437  
  Adult Head  4.280345  
  Adult Heart  4.595549  
  Adult Hind Gut  4.493659  
  Adult Male Ejaculatory Duct  4.540317  
  Adult Mid Gut  4.531970  
  Adult Ovary  4.289418  
  Adult Salivary Gland  4.931368  
  Adult Testes  12.473300  
  Adult Thoracoabdominal ganglion  4.343276  
  Adult Whole Fly  8.502471  
  Larvae Wandering Tubules  4.541328  
  Larval Feeding Carcass  4.640040  
  Larval Feeding Central Nevous System  4.298096  
  Larval Feeding Hind Gut  4.412647  
  Larval Feeding Malpighian Tubule  4.617021  
  Larval Feeding Mid Gut  4.803815  
  Larval Feeding Salivary Gland  4.660634  
  Whole Larvae Feeding  6.387419  
 
  
   FlyBase ID    symbol    start    end    strand    length   
   FBgn0052061   CG32061   10456916   10457735  +  820  
   FBgn0045770   S-Lap3   10458287   10461580  +  3294  
   FBgn0052064   S-Lap4   10483961   10486034  +  2074  
   FBgn0036085   CG6527  10551265   10552182   -  918  
   FBgn0036089   CG14151  10620780   10621584   -  805  
 
 
    Segment 233 
 
   Location   
  Gene key  FBgn0001179-FBgn0036090  
  Heatmap region span   3L:10237806..10656489   
  Segment span   3L:10624227..10631253   
  Length (genes)  2  
  Length (bp)  7027  
   Model Scoring   
  BIC  220.595681  
  logL  -104.785714  
  logL ratio  37.122490  
   Expression   
  Mean expression  8.676701  
  Median expression  8.749460  
  Tissue std. dev.  0.295670  
 
  No GO Slim enrichment  
  
   tissue    mean expression   
  5th Passage Drosophila S2 Cells  9.020129  
  Adult Accessory gland  8.706454  
  Adult Brain  9.126646  
  Adult Carcass  8.300409  
  Adult Crop  8.667699  
  Adult Eye  8.794111  
  Adult Fatbody  8.513070  
  Adult Female Spermatheca Mated  8.521593  
  Adult Female Spermatheca Virgin  8.546622  
  Adult Head  8.424932  
  Adult Heart  8.465516  
  Adult Hind Gut  8.363372  
  Adult Male Ejaculatory Duct  8.749901  
  Adult Mid Gut  9.021274  
  Adult Ovary  9.352951  
  Adult Salivary Gland  8.459073  
  Adult Testes  8.598823  
  Adult Thoracoabdominal ganglion  8.955954  
  Adult Whole Fly  8.324448  
  Larvae Wandering Tubules  8.899975  
  Larval Feeding Carcass  8.377182  
  Larval Feeding Central Nevous System  9.313913  
  Larval Feeding Hind Gut  8.353140  
  Larval Feeding Malpighian Tubule  8.627095  
  Larval Feeding Mid Gut  8.762425  
  Larval Feeding Salivary Gland  8.683185  
  Whole Larvae Feeding  8.341045  
 
  
   FlyBase ID    symbol    start    end    strand    length   
   FBgn0001179   hay   10624227   10627069  +  2843  
   FBgn0036090   CG8009   10631253   10632731  +  1479  
 
    Segment 234 
 
   Location   
  Gene key  FBgn0036093-FBgn0036094  
  Heatmap region span   3L:10456916..10684117   
  Segment span   3L:10635016..10636633   
  Length (genes)  2  
  Length (bp)  1618  
   Model Scoring   
  BIC  223.169063  
  logL  -106.072405  
  logL ratio  -8.238945  
   Expression   
  Mean expression  5.100420  
  Median expression  4.615989  
  Tissue std. dev.  0.871341  
 
  No GO Slim enrichment  
  
   tissue    mean expression   
  5th Passage Drosophila S2 Cells  4.460114  
  Adult Accessory gland  4.748439  
  Adult Brain  5.465423  
  Adult Carcass  6.007968  
  Adult Crop  4.526155  
  Adult Eye  5.441681  
  Adult Fatbody  5.043533  
  Adult Female Spermatheca Mated  4.700648  
  Adult Female Spermatheca Virgin  4.936663  
  Adult Head  6.636239  
  Adult Heart  6.265443  
  Adult Hind Gut  4.384936  
  Adult Male Ejaculatory Duct  6.474067  
  Adult Mid Gut  4.376119  
  Adult Ovary  4.480291  
  Adult Salivary Gland  4.809323  
  Adult Testes  7.912085  
  Adult Thoracoabdominal ganglion  5.126063  
  Adult Whole Fly  5.785799  
  Larvae Wandering Tubules  4.471713  
  Larval Feeding Carcass  4.425696  
  Larval Feeding Central Nevous System  4.394980  
  Larval Feeding Hind Gut  4.406136  
  Larval Feeding Malpighian Tubule  4.492960  
  Larval Feeding Mid Gut  4.434412  
  Larval Feeding Salivary Gland  4.788462  
  Whole Larvae Feeding  4.715999  
 
  
   FlyBase ID    symbol    start    end    strand    length   
   FBgn0036093   CG14154   10635016   10636014  +  999  
   FBgn0036094   CG14153   10636633   10637715  +  1083  
 
    Segment 235 
 
   Location   
  Gene key  FBgn0052066-FBgn0036096  
  Heatmap region span   3L:10624199..10689974   
  Segment span   3L:10654113..10654579   
  Length (genes)  2  
  Length (bp)  467  
   Model Scoring   
  BIC  241.886942  
  logL  -115.431345  
  logL ratio  23.425430  
   Expression   
  Mean expression  8.931950  
  Median expression  8.724106  
  Tissue std. dev.  0.544866  
 
  No GO Slim enrichment  
  
   tissue    mean expression   
  5th Passage Drosophila S2 Cells  10.018223  
  Adult Accessory gland  8.254029  
  Adult Brain  8.813747  
  Adult Carcass  8.164332  
  Adult Crop  9.572783  
  Adult Eye  8.374892  
  Adult Fatbody  9.209998  
  Adult Female Spermatheca Mated  9.340635  
  Adult Female Spermatheca Virgin  9.388305  
  Adult Head  8.444121  
  Adult Heart  9.146233  
  Adult Hind Gut  8.787496  
  Adult Male Ejaculatory Duct  8.531023  
  Adult Mid Gut  9.262116  
  Adult Ovary  9.799945  
  Adult Salivary Gland  8.074967  
  Adult Testes  8.428275  
  Adult Thoracoabdominal ganglion  8.512697  
  Adult Whole Fly  8.866979  
  Larvae Wandering Tubules  9.699096  
  Larval Feeding Carcass  8.557072  
  Larval Feeding Central Nevous System  9.769707  
  Larval Feeding Hind Gut  9.000241  
  Larval Feeding Malpighian Tubule  9.274787  
  Larval Feeding Mid Gut  8.983849  
  Larval Feeding Salivary Gland  8.255785  
  Whole Larvae Feeding  8.631318  
 
  
   FlyBase ID    symbol    start    end    strand    length   
   FBgn0052066   CG32066  10633493   10654113   -  20621  
   FBgn0036096   CG8003   10654579   10656077  +  1499  
 
    Segment 236 
 
   Location   
  Gene key  FBgn0053493-FBgn0036101  
  Heatmap region span   3L:10654113..10875151   
  Segment span   3L:10686235..10689974   
  Length (genes)  2  
  Length (bp)  3740  
   Model Scoring   
  BIC  267.664764  
  logL  -128.320256  
  logL ratio  -10.585661  
   Expression   
  Mean expression  7.881264  
  Median expression  7.398710  
  Tissue std. dev.  1.729455  
 
  No GO Slim enrichment  
  
   tissue    mean expression   
  5th Passage Drosophila S2 Cells  7.103403  
  Adult Accessory gland  5.391367  
  Adult Brain  6.441742  
  Adult Carcass  10.192823  
  Adult Crop  7.810185  
  Adult Eye  8.977172  
  Adult Fatbody  10.691448  
  Adult Female Spermatheca Mated  10.196115  
  Adult Female Spermatheca Virgin  10.455499  
  Adult Head  9.831073  
  Adult Heart  10.658136  
  Adult Hind Gut  8.939584  
  Adult Male Ejaculatory Duct  8.674945  
  Adult Mid Gut  6.581910  
  Adult Ovary  5.754065  
  Adult Salivary Gland  5.381761  
  Adult Testes  5.385540  
  Adult Thoracoabdominal ganglion  7.124282  
  Adult Whole Fly  7.763891  
  Larvae Wandering Tubules  6.538955  
  Larval Feeding Carcass  8.455866  
  Larval Feeding Central Nevous System  6.605035  
  Larval Feeding Hind Gut  8.511377  
  Larval Feeding Malpighian Tubule  6.574908  
  Larval Feeding Mid Gut  7.349979  
  Larval Feeding Salivary Gland  5.871424  
  Whole Larvae Feeding  9.531655  
 
  
   FlyBase ID    symbol    start    end    strand    length   
   FBgn0053493   CG33493  10685915   10686235   -  321  
   FBgn0036101   NijA  10687457   10689974   -  2518  
 
    Segment 237 
 
   Location   
  Gene key  FBgn0026160-FBgn0036104  
  Heatmap region span   3L:10657843..10884843   
  Segment span   3L:10828628..10872135   
  Length (genes)  2  
  Length (bp)  43508  
   Model Scoring   
  BIC  233.778792  
  logL  -111.377270  
  logL ratio  35.155177  
   Expression   
  Mean expression  9.208702  
  Median expression  9.042419  
  Tissue std. dev.  0.694909  
 
  No GO Slim enrichment  
  
   tissue    mean expression   
  5th Passage Drosophila S2 Cells  9.795786  
  Adult Accessory gland  9.247111  
  Adult Brain  10.451673  
  Adult Carcass  8.557663  
  Adult Crop  9.738103  
  Adult Eye  10.028963  
  Adult Fatbody  8.730167  
  Adult Female Spermatheca Mated  8.881251  
  Adult Female Spermatheca Virgin  9.056860  
  Adult Head  9.228656  
  Adult Heart  9.130161  
  Adult Hind Gut  9.681798  
  Adult Male Ejaculatory Duct  9.527395  
  Adult Mid Gut  8.492300  
  Adult Ovary  7.877598  
  Adult Salivary Gland  9.072145  
  Adult Testes  10.154734  
  Adult Thoracoabdominal ganglion  10.166822  
  Adult Whole Fly  8.549497  
  Larvae Wandering Tubules  9.075295  
  Larval Feeding Carcass  8.793058  
  Larval Feeding Central Nevous System  10.769856  
  Larval Feeding Hind Gut  9.473501  
  Larval Feeding Malpighian Tubule  8.609328  
  Larval Feeding Mid Gut  8.434670  
  Larval Feeding Salivary Gland  8.648985  
  Whole Larvae Feeding  8.461571  
 
  
   FlyBase ID    symbol    start    end    strand    length   
   FBgn0026160   tna   10828628   10867746  +  39119  
   FBgn0036104   CG6418  10869415   10872135   -  2721  
 
    Segment 238 
 
   Location   
  Gene key  FBgn0036107-FBgn0027615  
  Heatmap region span   3L:10794292..11001924   
  Segment span   3L:10876063..10879571   
  Length (genes)  2  
  Length (bp)  3509  
   Model Scoring   
  BIC  224.199950  
  logL  -106.587848  
  logL ratio  40.500852  
   Expression   
  Mean expression  9.181246  
  Median expression  9.232753  
  Tissue std. dev.  0.340504  
 
  No GO Slim enrichment  
  
   tissue    mean expression   
  5th Passage Drosophila S2 Cells  9.114255  
  Adult Accessory gland  9.137308  
  Adult Brain  9.218003  
  Adult Carcass  9.130384  
  Adult Crop  9.064370  
  Adult Eye  8.698963  
  Adult Fatbody  9.189097  
  Adult Female Spermatheca Mated  9.065777  
  Adult Female Spermatheca Virgin  9.082725  
  Adult Head  8.908501  
  Adult Heart  9.134812  
  Adult Hind Gut  9.014636  
  Adult Male Ejaculatory Duct  9.052637  
  Adult Mid Gut  9.100763  
  Adult Ovary  10.425334  
  Adult Salivary Gland  9.137586  
  Adult Testes  9.842221  
  Adult Thoracoabdominal ganglion  9.544394  
  Adult Whole Fly  9.571461  
  Larvae Wandering Tubules  9.080192  
  Larval Feeding Carcass  8.907383  
  Larval Feeding Central Nevous System  9.152378  
  Larval Feeding Hind Gut  9.217427  
  Larval Feeding Malpighian Tubule  9.350744  
  Larval Feeding Mid Gut  8.844529  
  Larval Feeding Salivary Gland  9.198697  
  Whole Larvae Feeding  8.709064  
 
  
   FlyBase ID    symbol    start    end    strand    length   
   FBgn0036107   CG7949   10876063   10876588  +  526  
   FBgn0027615   CG6404  10877361   10879571   -  2211  
 
    Segment 239 
 
   Location   
  Gene key  FBgn0036108-FBgn0036109  
  Heatmap region span   3L:10828628..11016635   
  Segment span   3L:10883097..10884843   
  Length (genes)  2  
  Length (bp)  1747  
   Model Scoring   
  BIC  179.845856  
  logL  -84.410801  
  logL ratio  42.646495  
   Expression   
  Mean expression  5.732484  
  Median expression  5.013749  
  Tissue std. dev.  1.889202  
 
  No GO Slim enrichment  
  
   tissue    mean expression   
  5th Passage Drosophila S2 Cells  5.888063  
  Adult Accessory gland  5.111519  
  Adult Brain  4.705948  
  Adult Carcass  5.455502  
  Adult Crop  4.942132  
  Adult Eye  4.855429  
  Adult Fatbody  5.406610  
  Adult Female Spermatheca Mated  5.642837  
  Adult Female Spermatheca Virgin  5.471359  
  Adult Head  4.713330  
  Adult Heart  4.725796  
  Adult Hind Gut  4.925391  
  Adult Male Ejaculatory Duct  5.335171  
  Adult Mid Gut  4.879567  
  Adult Ovary  4.903400  
  Adult Salivary Gland  5.665666  
  Adult Testes  4.758068  
  Adult Thoracoabdominal ganglion  5.378225  
  Adult Whole Fly  4.746479  
  Larvae Wandering Tubules  4.936765  
  Larval Feeding Carcass  12.473137  
  Larval Feeding Central Nevous System  4.969657  
  Larval Feeding Hind Gut  8.590495  
  Larval Feeding Malpighian Tubule  5.040477  
  Larval Feeding Mid Gut  4.956362  
  Larval Feeding Salivary Gland  5.040769  
  Whole Larvae Feeding  11.258910  
 
  
   FlyBase ID    symbol    start    end    strand    length   
   FBgn0036108   Cpr67Fa1   10883097   10883800  +  704  
   FBgn0036109   Cpr67Fa2   10884843   10885616  +  774  
 
    Segment 240 
 
   Location   
  Gene key  FBgn0054050-FBgn0013469  
  Heatmap region span   3L:10876063..11047137   
  Segment span   3L:10937556..11001924   
  Length (genes)  3  
  Length (bp)  64369  
   Model Scoring   
  BIC  235.968783  
  logL  -112.472265  
  logL ratio  70.448311  
   Expression   
  Mean expression  4.952226  
  Median expression  4.843549  
  Tissue std. dev.  0.229706  
 
  No GO Slim enrichment  
  
   tissue    mean expression   
  5th Passage Drosophila S2 Cells  4.857842  
  Adult Accessory gland  4.936584  
  Adult Brain  4.689602  
  Adult Carcass  5.184870  
  Adult Crop  4.846862  
  Adult Eye  4.902882  
  Adult Fatbody  5.281979  
  Adult Female Spermatheca Mated  5.075843  
  Adult Female Spermatheca Virgin  5.174753  
  Adult Head  4.667150  
  Adult Heart  5.422386  
  Adult Hind Gut  4.902073  
  Adult Male Ejaculatory Duct  5.330491  
  Adult Mid Gut  5.103393  
  Adult Ovary  4.851192  
  Adult Salivary Gland  5.040181  
  Adult Testes  4.456915  
  Adult Thoracoabdominal ganglion  5.051632  
  Adult Whole Fly  4.507425  
  Larvae Wandering Tubules  4.823578  
  Larval Feeding Carcass  5.004471  
  Larval Feeding Central Nevous System  5.236062  
  Larval Feeding Hind Gut  4.974693  
  Larval Feeding Malpighian Tubule  4.794174  
  Larval Feeding Mid Gut  4.903240  
  Larval Feeding Salivary Gland  4.924877  
  Whole Larvae Feeding  4.764943  
 
  
   FlyBase ID    symbol    start    end    strand    length   
   FBgn0054050   CG34050   10937556   10938200  +  645  
   FBgn0036112   CG14147   10965754   10966582  +  829  
   FBgn0013469   klu  10974826   11001924   -  27099  
 
 
    Segment 241 
 
   Location   
  Gene key  FBgn0052079-FBgn0052081  
  Heatmap region span   3L:10887273..11067871   
  Segment span   3L:11018661..11020679   
  Length (genes)  2  
  Length (bp)  2019  
   Model Scoring   
  BIC  213.379093  
  logL  -101.177420  
  logL ratio  4.957778  
   Expression   
  Mean expression  4.750036  
  Median expression  4.461317  
  Tissue std. dev.  1.016216  
 
  No GO Slim enrichment  
  
   tissue    mean expression   
  5th Passage Drosophila S2 Cells  4.349011  
  Adult Accessory gland  4.508114  
  Adult Brain  4.365866  
  Adult Carcass  4.801937  
  Adult Crop  4.382446  
  Adult Eye  4.141426  
  Adult Fatbody  4.585712  
  Adult Female Spermatheca Mated  5.108308  
  Adult Female Spermatheca Virgin  4.963503  
  Adult Head  4.214844  
  Adult Heart  4.272646  
  Adult Hind Gut  4.506239  
  Adult Male Ejaculatory Duct  4.442997  
  Adult Mid Gut  4.630123  
  Adult Ovary  4.260493  
  Adult Salivary Gland  4.633422  
  Adult Testes  9.531934  
  Adult Thoracoabdominal ganglion  4.303838  
  Adult Whole Fly  6.166921  
  Larvae Wandering Tubules  4.559973  
  Larval Feeding Carcass  4.375350  
  Larval Feeding Central Nevous System  4.246978  
  Larval Feeding Hind Gut  4.300990  
  Larval Feeding Malpighian Tubule  4.605617  
  Larval Feeding Mid Gut  4.540442  
  Larval Feeding Salivary Gland  4.501606  
  Whole Larvae Feeding  4.950227  
 
  
   FlyBase ID    symbol    start    end    strand    length   
   FBgn0052079   CG32079   11018661   11020432  +  1772  
   FBgn0052081   CG32081   11020679   11022545  +  1867  
 
    Segment 242 
 
   Location   
  Gene key  FBgn0036117-FBgn0052069  
  Heatmap region span   3L:11016635..11096123   
  Segment span   3L:11054319..11055714   
  Length (genes)  3  
  Length (bp)  1396  
   Model Scoring   
  BIC  346.234323  
  logL  -167.605035  
  logL ratio  41.719081  
   Expression   
  Mean expression  8.651182  
  Median expression  8.782330  
  Tissue std. dev.  0.539229  
 
  No GO Slim enrichment  
  
   tissue    mean expression   
  5th Passage Drosophila S2 Cells  8.518135  
  Adult Accessory gland  8.771665  
  Adult Brain  7.807073  
  Adult Carcass  8.371681  
  Adult Crop  8.496587  
  Adult Eye  8.571863  
  Adult Fatbody  9.045924  
  Adult Female Spermatheca Mated  8.926114  
  Adult Female Spermatheca Virgin  8.880987  
  Adult Head  8.451917  
  Adult Heart  9.225785  
  Adult Hind Gut  8.377665  
  Adult Male Ejaculatory Duct  10.150274  
  Adult Mid Gut  8.684916  
  Adult Ovary  8.923451  
  Adult Salivary Gland  8.848964  
  Adult Testes  7.775513  
  Adult Thoracoabdominal ganglion  7.968799  
  Adult Whole Fly  8.274853  
  Larvae Wandering Tubules  9.094599  
  Larval Feeding Carcass  8.213502  
  Larval Feeding Central Nevous System  8.084512  
  Larval Feeding Hind Gut  8.357134  
  Larval Feeding Malpighian Tubule  9.842312  
  Larval Feeding Mid Gut  8.508180  
  Larval Feeding Salivary Gland  9.087046  
  Whole Larvae Feeding  8.322464  
 
  
   FlyBase ID    symbol    start    end    strand    length   
   FBgn0036117   CG6321  11052537   11054319   -  1783  
   FBgn0036118   blos2   11054671   11055315  +  645  
   FBgn0052069   CG32069  11055231   11055714   -  484  
 
 
    Segment 243 
 
   Location   
  Gene key  FBgn0052075-FBgn0036124  
  Heatmap region span   3L:11018661..11100476   
  Segment span   3L:11058037..11067871   
  Length (genes)  5  
  Length (bp)  9835  
   Model Scoring   
  BIC  475.229507  
  logL  -232.102627  
  logL ratio  139.812495  
   Expression   
  Mean expression  7.471946  
  Median expression  7.435126  
  Tissue std. dev.  0.498818  
 
  No GO Slim enrichment  
  
   tissue    mean expression   
  5th Passage Drosophila S2 Cells  8.753592  
  Adult Accessory gland  7.612869  
  Adult Brain  7.321572  
  Adult Carcass  6.967773  
  Adult Crop  7.849170  
  Adult Eye  7.154009  
  Adult Fatbody  7.453488  
  Adult Female Spermatheca Mated  7.423409  
  Adult Female Spermatheca Virgin  7.347047  
  Adult Head  7.463154  
  Adult Heart  7.799652  
  Adult Hind Gut  6.957551  
  Adult Male Ejaculatory Duct  6.863594  
  Adult Mid Gut  7.531068  
  Adult Ovary  8.880601  
  Adult Salivary Gland  6.969519  
  Adult Testes  6.486287  
  Adult Thoracoabdominal ganglion  7.375399  
  Adult Whole Fly  7.362295  
  Larvae Wandering Tubules  7.313247
[truncated: 424,171 more chars]
