# Supplementary material for: Expression-based segmentation of the Drosophila genome
Source: BMC Genomics. 2013 Nov 20;14:812. doi: 10.1186/1471-2164-14-812 (PMC3909303; doi:10.1186/1471-2164-14-812)
Supplement: Additional file 1 — Detailed information for multigene segments. [file 1471-2164-14-812-S1.zip › miniwebsite/chr3R.html]

   ExprSeg Report for chr3R   
 Report for /Users/afrubin/Code/ExprSeg/2012-07-19/output Generated Fri Jun 21 23:05:05 2013 
   Chromosome 3R 
 608 segments 2863 genes 
    Segment 1 
 
   Location   
  Gene key  FBgn0037215-FBgn0037218  
  Heatmap region span   3R:380..60868   
  Segment span   3R:23029..37505   
  Length (genes)  2  
  Length (bp)  14477  
   Model Scoring   
  BIC  244.709808  
  logL  -116.727173  
  logL ratio  43.862182  
   Expression   
  Mean expression  9.941448  
  Median expression  10.149960  
  Tissue std. dev.  0.632550  
 
  No GO Slim enrichment  
  
   tissue    mean expression   
  5th Passage Drosophila S2 Cells  10.638683  
  Adult Accessory gland  9.890666  
  Adult Brain  9.704300  
  Adult Carcass  9.439256  
  Adult Crop  9.346345  
  Adult Eye  10.098842  
  Adult Fatbody  10.039669  
  Adult Female Spermatheca Mated  10.439437  
  Adult Female Spermatheca Virgin  10.491412  
  Adult Head  9.728760  
  Adult Heart  10.774165  
  Adult Hind Gut  9.864773  
  Adult Male Ejaculatory Duct  9.275672  
  Adult Mid Gut  10.907501  
  Adult Ovary  9.948141  
  Adult Salivary Gland  10.520809  
  Adult Testes  7.979570  
  Adult Thoracoabdominal ganglion  9.704677  
  Adult Whole Fly  9.471129  
  Larvae Wandering Tubules  10.883508  
  Larval Feeding Carcass  9.450472  
  Larval Feeding Central Nevous System  9.296214  
  Larval Feeding Hind Gut  9.998938  
  Larval Feeding Malpighian Tubule  10.779235  
  Larval Feeding Mid Gut  10.294778  
  Larval Feeding Salivary Gland  9.816671  
  Whole Larvae Feeding  9.635474  
 
  
   FlyBase ID    symbol    start    end    strand    length   
   FBgn0037215   CG12582   23029   30295  +  7267  
   FBgn0037218   aux   37505   53244  +  15740  
 
    Segment 2 
 
   Location   
  Gene key  FBgn0037220-FBgn0015331  
  Heatmap region span   3R:16170..92676   
  Segment span   3R:58077..58765   
  Length (genes)  2  
  Length (bp)  689  
   Model Scoring   
  BIC  271.976614  
  logL  -130.360576  
  logL ratio  -0.612159  
   Expression   
  Mean expression  9.067254  
  Median expression  9.675393  
  Tissue std. dev.  0.415968  
 
  No GO Slim enrichment  
  
   tissue    mean expression   
  5th Passage Drosophila S2 Cells  9.832952  
  Adult Accessory gland  8.866308  
  Adult Brain  8.933309  
  Adult Carcass  8.345560  
  Adult Crop  9.040625  
  Adult Eye  8.758152  
  Adult Fatbody  8.812960  
  Adult Female Spermatheca Mated  9.139321  
  Adult Female Spermatheca Virgin  9.269585  
  Adult Head  8.616725  
  Adult Heart  8.840642  
  Adult Hind Gut  8.594059  
  Adult Male Ejaculatory Duct  8.853789  
  Adult Mid Gut  8.711785  
  Adult Ovary  9.686685  
  Adult Salivary Gland  8.642565  
  Adult Testes  9.956529  
  Adult Thoracoabdominal ganglion  8.709861  
  Adult Whole Fly  8.956426  
  Larvae Wandering Tubules  9.600407  
  Larval Feeding Carcass  8.990872  
  Larval Feeding Central Nevous System  9.781419  
  Larval Feeding Hind Gut  9.167431  
  Larval Feeding Malpighian Tubule  9.184448  
  Larval Feeding Mid Gut  8.997146  
  Larval Feeding Salivary Gland  9.615780  
  Whole Larvae Feeding  8.910528  
 
  
   FlyBase ID    symbol    start    end    strand    length   
   FBgn0037220   CG14641  56475   58077   -  1603  
   FBgn0015331   abs   58765   60763  +  1999  
 
    Segment 3 
 
   Location   
  Gene key  FBgn0037224-FBgn0041621  
  Heatmap region span   3R:45852..120558   
  Segment span   3R:72744..82694   
  Length (genes)  5  
  Length (bp)  9951  
   Model Scoring   
  BIC  461.449572  
  logL  -225.097055  
  logL ratio  53.971273  
   Expression   
  Mean expression  4.962119  
  Median expression  4.664881  
  Tissue std. dev.  0.355616  
 
  
   GO ID    description    ratio    P-value   
   GO:0005578   proteinaceous extracellular matrix  3/5  2.36e-06  
 
  
   tissue    mean expression   
  5th Passage Drosophila S2 Cells  4.745369  
  Adult Accessory gland  4.791138  
  Adult Brain  5.516597  
  Adult Carcass  4.877452  
  Adult Crop  4.767690  
  Adult Eye  4.533476  
  Adult Fatbody  5.019242  
  Adult Female Spermatheca Mated  4.993865  
  Adult Female Spermatheca Virgin  5.034438  
  Adult Head  4.976231  
  Adult Heart  4.745429  
  Adult Hind Gut  4.724535  
  Adult Male Ejaculatory Duct  4.715914  
  Adult Mid Gut  4.744553  
  Adult Ovary  4.684906  
  Adult Salivary Gland  4.841668  
  Adult Testes  5.838351  
  Adult Thoracoabdominal ganglion  5.852335  
  Adult Whole Fly  5.029589  
  Larvae Wandering Tubules  4.916842  
  Larval Feeding Carcass  5.095189  
  Larval Feeding Central Nevous System  4.503754  
  Larval Feeding Hind Gut  4.664900  
  Larval Feeding Malpighian Tubule  5.701974  
  Larval Feeding Mid Gut  4.983370  
  Larval Feeding Salivary Gland  4.719498  
  Whole Larvae Feeding  4.958921  
 
  
   FlyBase ID    symbol    start    end    strand    length   
   FBgn0037224   TwdlF   72744   74040  +  1297  
   FBgn0037225   TwdlG  74439   76518   -  2080  
   FBgn0250821   CG14644  77614   78765   -  1152  
   FBgn0037227   TwdlV   80502   81316  +  815  
   FBgn0041621   Or82a   82694   84166  +  1473  
 
 
    Segment 4 
 
   Location   
  Gene key  FBgn0037234-FBgn0037236  
  Heatmap region span   3R:120558..228771   
  Segment span   3R:145412..165287   
  Length (genes)  4  
  Length (bp)  19876  
   Model Scoring   
  BIC  410.504577  
  logL  -199.624557  
  logL ratio  111.761361  
   Expression   
  Mean expression  8.971145  
  Median expression  8.983000  
  Tissue std. dev.  0.634446  
 
  No GO Slim enrichment  
  
   tissue    mean expression   
  5th Passage Drosophila S2 Cells  10.236228  
  Adult Accessory gland  8.784103  
  Adult Brain  8.575430  
  Adult Carcass  8.297567  
  Adult Crop  8.747829  
  Adult Eye  8.774307  
  Adult Fatbody  8.289847  
  Adult Female Spermatheca Mated  8.952132  
  Adult Female Spermatheca Virgin  8.926266  
  Adult Head  8.240334  
  Adult Heart  8.793444  
  Adult Hind Gut  8.482845  
  Adult Male Ejaculatory Duct  8.431705  
  Adult Mid Gut  8.337491  
  Adult Ovary  10.765019  
  Adult Salivary Gland  8.503998  
  Adult Testes  8.841128  
  Adult Thoracoabdominal ganglion  8.609553  
  Adult Whole Fly  9.656800  
  Larvae Wandering Tubules  9.421657  
  Larval Feeding Carcass  9.507921  
  Larval Feeding Central Nevous System  9.865950  
  Larval Feeding Hind Gut  9.177322  
  Larval Feeding Malpighian Tubule  9.082462  
  Larval Feeding Mid Gut  8.295665  
  Larval Feeding Salivary Gland  9.802542  
  Whole Larvae Feeding  8.821366  
 
  
   FlyBase ID    symbol    start    end    strand    length   
   FBgn0037234   CG9795   145412   152096  +  6685  
   FBgn0027866   CG9776  153527   159727   -  6201  
   FBgn0037235   CG1103   163482   165640  +  2159  
   FBgn0037236   Skp2  161143   165287   -  4145  
 
 
    Segment 5 
 
   Location   
  Gene key  FBgn0037239-FBgn0037241  
  Heatmap region span   3R:136667..263102   
  Segment span   3R:204386..216097   
  Length (genes)  4  
  Length (bp)  11712  
   Model Scoring   
  BIC  465.695660  
  logL  -227.220099  
  logL ratio  74.008161  
   Expression   
  Mean expression  9.307681  
  Median expression  9.217803  
  Tissue std. dev.  0.490282  
 
  No GO Slim enrichment  
  
   tissue    mean expression   
  5th Passage Drosophila S2 Cells  9.599620  
  Adult Accessory gland  8.500457  
  Adult Brain  9.069975  
  Adult Carcass  9.466594  
  Adult Crop  9.689675  
  Adult Eye  9.817081  
  Adult Fatbody  9.765102  
  Adult Female Spermatheca Mated  10.216215  
  Adult Female Spermatheca Virgin  10.071424  
  Adult Head  9.659242  
  Adult Heart  9.625740  
  Adult Hind Gut  9.212068  
  Adult Male Ejaculatory Duct  9.263922  
  Adult Mid Gut  8.709858  
  Adult Ovary  9.821494  
  Adult Salivary Gland  8.682629  
  Adult Testes  8.956760  
  Adult Thoracoabdominal ganglion  8.815437  
  Adult Whole Fly  9.160137  
  Larvae Wandering Tubules  8.478460  
  Larval Feeding Carcass  9.394135  
  Larval Feeding Central Nevous System  9.570924  
  Larval Feeding Hind Gut  9.665260  
  Larval Feeding Malpighian Tubule  8.334994  
  Larval Feeding Mid Gut  9.049462  
  Larval Feeding Salivary Gland  9.127884  
  Whole Larvae Feeding  9.582847  
 
  
   FlyBase ID    symbol    start    end    strand    length   
   FBgn0037239   CG11739   204386   206932  +  2547  
   FBgn0037240   Cont   207032   212741  +  5710  
   FBgn0003882   tub   212967   215535  +  2569  
   FBgn0037241   CG14646   216097   217833  +  1737  
 
 
    Segment 6 
 
   Location   
  Gene key  FBgn0037242-FBgn0037245  
  Heatmap region span   3R:145412..304828   
  Segment span   3R:223609..228771   
  Length (genes)  4  
  Length (bp)  5163  
   Model Scoring   
  BIC  459.353877  
  logL  -224.049207  
  logL ratio  132.986960  
   Expression   
  Mean expression  10.227953  
  Median expression  9.839727  
  Tissue std. dev.  0.236768  
 
  No GO Slim enrichment  
  
   tissue    mean expression   
  5th Passage Drosophila S2 Cells  10.378431  
  Adult Accessory gland  10.451686  
  Adult Brain  10.178485  
  Adult Carcass  9.701841  
  Adult Crop  10.342349  
  Adult Eye  10.062019  
  Adult Fatbody  10.114669  
  Adult Female Spermatheca Mated  10.344613  
  Adult Female Spermatheca Virgin  10.340779  
  Adult Head  9.930708  
  Adult Heart  10.190592  
  Adult Hind Gut  10.183457  
  Adult Male Ejaculatory Duct  10.283539  
  Adult Mid Gut  9.991783  
  Adult Ovary  10.969461  
  Adult Salivary Gland  10.182845  
  Adult Testes  10.564074  
  Adult Thoracoabdominal ganglion  10.105249  
  Adult Whole Fly  10.329717  
  Larvae Wandering Tubules  10.264900  
  Larval Feeding Carcass  10.104874  
  Larval Feeding Central Nevous System  10.350651  
  Larval Feeding Hind Gut  10.215279  
  Larval Feeding Malpighian Tubule  10.329600  
  Larval Feeding Mid Gut  9.985231  
  Larval Feeding Salivary Gland  10.374334  
  Whole Larvae Feeding  9.883559  
 
  
   FlyBase ID    symbol    start    end    strand    length   
   FBgn0037242   CG9855  221886   223609   -  1724  
   FBgn0037244   CG14647   226212   227739  +  1528  
   FBgn0086605   CG9853  224280   227749   -  3470  
   FBgn0037245      228771   232914  +  4144  
 
 
    Segment 7 
 
   Location   
  Gene key  FBgn0052944-FBgn0051525  
  Heatmap region span   3R:185510..306534   
  Segment span   3R:241835..252752   
  Length (genes)  3  
  Length (bp)  10918  
   Model Scoring   
  BIC  284.627494  
  logL  -136.686016  
  logL ratio  35.494993  
   Expression   
  Mean expression  4.863155  
  Median expression  4.456718  
  Tissue std. dev.  1.005608  
 
  No GO Slim enrichment  
  
   tissue    mean expression   
  5th Passage Drosophila S2 Cells  4.438666  
  Adult Accessory gland  4.622716  
  Adult Brain  5.552596  
  Adult Carcass  4.561469  
  Adult Crop  4.373879  
  Adult Eye  4.386900  
  Adult Fatbody  4.406930  
  Adult Female Spermatheca Mated  4.593608  
  Adult Female Spermatheca Virgin  4.585599  
  Adult Head  4.594022  
  Adult Heart  4.193401  
  Adult Hind Gut  4.286036  
  Adult Male Ejaculatory Duct  4.585013  
  Adult Mid Gut  4.524428  
  Adult Ovary  4.327209  
  Adult Salivary Gland  4.860031  
  Adult Testes  9.494983  
  Adult Thoracoabdominal ganglion  5.672060  
  Adult Whole Fly  5.664364  
  Larvae Wandering Tubules  4.453794  
  Larval Feeding Carcass  4.520735  
  Larval Feeding Central Nevous System  5.152686  
  Larval Feeding Hind Gut  4.278774  
  Larval Feeding Malpighian Tubule  4.514196  
  Larval Feeding Mid Gut  4.543216  
  Larval Feeding Salivary Gland  4.520043  
  Whole Larvae Feeding  5.597825  
 
  
   FlyBase ID    symbol    start    end    strand    length   
   FBgn0052944   CG32944  233159   241835   -  8677  
   FBgn0051528   CG31528   242153   243389  +  1237  
   FBgn0051525   CG31525   252752   253927  +  1176  
 
 
    Segment 8 
 
   Location   
  Gene key  FBgn0037248-FBgn0037252  
  Heatmap region span   3R:204386..310763   
  Segment span   3R:255051..263102   
  Length (genes)  4  
  Length (bp)  8052  
   Model Scoring   
  BIC  369.468427  
  logL  -179.106483  
  logL ratio  133.035007  
   Expression   
  Mean expression  8.502900  
  Median expression  8.623410  
  Tissue std. dev.  0.584901  
 
  No GO Slim enrichment  
  
   tissue    mean expression   
  5th Passage Drosophila S2 Cells  9.055689  
  Adult Accessory gland  9.251135  
  Adult Brain  8.713110  
  Adult Carcass  7.806755  
  Adult Crop  8.488311  
  Adult Eye  8.101840  
  Adult Fatbody  8.479685  
  Adult Female Spermatheca Mated  8.931827  
  Adult Female Spermatheca Virgin  8.577500  
  Adult Head  8.181137  
  Adult Heart  8.345168  
  Adult Hind Gut  8.195758  
  Adult Male Ejaculatory Duct  8.577883  
  Adult Mid Gut  8.056172  
  Adult Ovary  9.842795  
  Adult Salivary Gland  8.619376  
  Adult Testes  7.398404  
  Adult Thoracoabdominal ganglion  8.614858  
  Adult Whole Fly  8.614238  
  Larvae Wandering Tubules  7.669870  
  Larval Feeding Carcass  8.867189  
  Larval Feeding Central Nevous System  9.503361  
  Larval Feeding Hind Gut  8.552089  
  Larval Feeding Malpighian Tubule  7.961526  
  Larval Feeding Mid Gut  7.622335  
  Larval Feeding Salivary Gland  9.469122  
  Whole Larvae Feeding  8.081176  
 
  
   FlyBase ID    symbol    start    end    strand    length   
   FBgn0037248   Spargel  248205   255051   -  6847  
   FBgn0037250   CG1074   259715   261897  +  2183  
   FBgn0037251   CG9804  261944   263051   -  1108  
   FBgn0037252   CG14650   263102   267050  +  3949  
 
 
    Segment 9 
 
   Location   
  Gene key  FBgn0051526-FBgn0052945  
  Heatmap region span   3R:279036..471653   
  Segment span   3R:359017..404366   
  Length (genes)  4  
  Length (bp)  45350  
   Model Scoring   
  BIC  395.672723  
  logL  -192.208631  
  logL ratio  41.262365  
   Expression   
  Mean expression  4.575428  
  Median expression  4.523084  
  Tissue std. dev.  0.655586  
 
  No GO Slim enrichment  
  
   tissue    mean expression   
  5th Passage Drosophila S2 Cells  4.428306  
  Adult Accessory gland  6.383942  
  Adult Brain  4.243214  
  Adult Carcass  4.495020  
  Adult Crop  4.291776  
  Adult Eye  4.135016  
  Adult Fatbody  4.524097  
  Adult Female Spermatheca Mated  4.388675  
  Adult Female Spermatheca Virgin  4.391293  
  Adult Head  4.250160  
  Adult Heart  4.255040  
  Adult Hind Gut  4.273350  
  Adult Male Ejaculatory Duct  4.583806  
  Adult Mid Gut  4.445100  
  Adult Ovary  4.304856  
  Adult Salivary Gland  4.588862  
  Adult Testes  7.166491  
  Adult Thoracoabdominal ganglion  4.284211  
  Adult Whole Fly  5.083908  
  Larvae Wandering Tubules  4.446476  
  Larval Feeding Carcass  4.367647  
  Larval Feeding Central Nevous System  4.201265  
  Larval Feeding Hind Gut  4.242858  
  Larval Feeding Malpighian Tubule  4.368301  
  Larval Feeding Mid Gut  4.415850  
  Larval Feeding Salivary Gland  4.553292  
  Whole Larvae Feeding  4.423733  
 
  
   FlyBase ID    symbol    start    end    strand    length   
   FBgn0051526   CG31526   359017   359662  +  646  
   FBgn0037260   Mur82C   367078   368937  +  1860  
   FBgn0085386   CG34357  319126   383789   -  64664  
   FBgn0052945   CG32945  403661   404366   -  706  
 
 
    Segment 10 
 
   Location   
  Gene key  FBgn0037261-FBgn0037262  
  Heatmap region span   3R:304828..474945   
  Segment span   3R:467317..467696   
  Length (genes)  2  
  Length (bp)  380  
   Model Scoring   
  BIC  264.591477  
  logL  -126.668007  
  logL ratio  22.827781  
   Expression   
  Mean expression  9.823554  
  Median expression  9.797263  
  Tissue std. dev.  0.472102  
 
  No GO Slim enrichment  
  
   tissue    mean expression   
  5th Passage Drosophila S2 Cells  10.140227  
  Adult Accessory gland  10.323050  
  Adult Brain  10.614460  
  Adult Carcass  9.723580  
  Adult Crop  9.966009  
  Adult Eye  10.294676  
  Adult Fatbody  9.330761  
  Adult Female Spermatheca Mated  9.145907  
  Adult Female Spermatheca Virgin  9.332601  
  Adult Head  9.841746  
  Adult Heart  9.709035  
  Adult Hind Gut  9.690831  
  Adult Male Ejaculatory Duct  9.876520  
  Adult Mid Gut  9.168760  
  Adult Ovary  10.435756  
  Adult Salivary Gland  10.329104  
  Adult Testes  9.575631  
  Adult Thoracoabdominal ganglion  10.194282  
  Adult Whole Fly  9.795980  
  Larvae Wandering Tubules  9.933606  
  Larval Feeding Carcass  9.408150  
  Larval Feeding Central Nevous System  10.709525  
  Larval Feeding Hind Gut  9.515901  
  Larval Feeding Malpighian Tubule  9.771055  
  Larval Feeding Mid Gut  8.839164  
  Larval Feeding Salivary Gland  10.292472  
  Whole Larvae Feeding  9.277163  
 
  
   FlyBase ID    symbol    start    end    strand    length   
   FBgn0037261   CG9775  463732   467317   -  3586  
   FBgn0037262   MED31   467696   469014  +  1319  
 
    Segment 11 
 
   Location   
  Gene key  FBgn0037265-FBgn0051531  
  Heatmap region span   3R:467696..601323   
  Segment span   3R:480550..485305   
  Length (genes)  2  
  Length (bp)  4756  
   Model Scoring   
  BIC  224.194982  
  logL  -106.469760  
  logL ratio  30.114015  
   Expression   
  Mean expression  7.832580  
  Median expression  7.791971  
  Tissue std. dev.  0.783982  
 
  No GO Slim enrichment  
  
   tissue    mean expression   
  5th Passage Drosophila S2 Cells  6.803193  
  Adult Accessory gland  7.327595  
  Adult Brain  7.179654  
  Adult Carcass  8.501337  
  Adult Crop  9.858140  
  Adult Eye  8.295763  
  Adult Fatbody  8.521280  
  Adult Female Spermatheca Mated  8.260446  
  Adult Female Spermatheca Virgin  8.455690  
  Adult Head  8.169047  
  Adult Heart  8.844797  
  Adult Hind Gut  8.897644  
  Adult Male Ejaculatory Duct  8.419428  
  Adult Mid Gut  7.047210  
  Adult Ovary  7.452182  
  Adult Salivary Gland  8.289505  
  Adult Testes  6.446522  
  Adult Thoracoabdominal ganglion  7.297036  
  Adult Whole Fly  7.150996  
  Larvae Wandering Tubules  7.949923  
  Larval Feeding Carcass  8.219852  
  Larval Feeding Central Nevous System  6.730453  
  Larval Feeding Hind Gut  8.055062  
  Larval Feeding Malpighian Tubule  7.805430  
  Larval Feeding Mid Gut  7.188890  
  Larval Feeding Salivary Gland  7.041030  
  Whole Larvae Feeding  7.271548  
 
  
   FlyBase ID    symbol    start    end    strand    length   
   FBgn0037265   CG12001   480550   483707  +  3158  
   FBgn0051531      485305   530979  +  45675  
 
    Segment 12 
 
   Location   
  Gene key  FBgn0051534-FBgn0026620  
  Heatmap region span   3R:471316..606053   
  Segment span   3R:531458..574777   
  Length (genes)  4  
  Length (bp)  43320  
   Model Scoring   
  BIC  565.026801  
  logL  -276.885669  
  logL ratio  31.589452  
   Expression   
  Mean expression  10.067347  
  Median expression  10.230066  
  Tissue std. dev.  0.692976  
 
  No GO Slim enrichment  
  
   tissue    mean expression   
  5th Passage Drosophila S2 Cells  9.472013  
  Adult Accessory gland  9.304427  
  Adult Brain  10.624384  
  Adult Carcass  9.817545  
  Adult Crop  9.963040  
  Adult Eye  10.414055  
  Adult Fatbody  8.950163  
  Adult Female Spermatheca Mated  10.632953  
  Adult Female Spermatheca Virgin  10.513971  
  Adult Head  10.547356  
  Adult Heart  9.132710  
  Adult Hind Gut  10.502213  
  Adult Male Ejaculatory Duct  10.115662  
  Adult Mid Gut  9.497856  
  Adult Ovary  9.769512  
  Adult Salivary Gland  11.477016  
  Adult Testes  8.411998  
  Adult Thoracoabdominal ganglion  10.794381  
  Adult Whole Fly  9.827605  
  Larvae Wandering Tubules  10.684361  
  Larval Feeding Carcass  10.798191  
  Larval Feeding Central Nevous System  9.564431  
  Larval Feeding Hind Gut  10.798702  
  Larval Feeding Malpighian Tubule  10.485374  
  Larval Feeding Mid Gut  9.356409  
  Larval Feeding Salivary Gland  10.585755  
  Whole Larvae Feeding  9.776294  
 
  
   FlyBase ID    symbol    start    end    strand    length   
   FBgn0051534      531458   537915  +  6458  
   FBgn0037270   CG9769  538861   539918   -  1058  
   FBgn0027570   Nep2  545520   557598   -  12079  
   FBgn0026620   tacc  560134   574777   -  14644  
 
 
    Segment 13 
 
   Location   
  Gene key  FBgn0037283-FBgn0020280  
  Heatmap region span   3R:610444..787070   
  Segment span   3R:656235..704255   
  Length (genes)  4  
  Length (bp)  48021  
   Model Scoring   
  BIC  378.975185  
  logL  -183.859861  
  logL ratio  42.235929  
   Expression   
  Mean expression  5.181622  
  Median expression  5.077421  
  Tissue std. dev.  0.645227  
 
  No GO Slim enrichment  
  
   tissue    mean expression   
  5th Passage Drosophila S2 Cells  4.883443  
  Adult Accessory gland  4.868873  
  Adult Brain  5.461658  
  Adult Carcass  5.111972  
  Adult Crop  4.792017  
  Adult Eye  4.992237  
  Adult Fatbody  5.000353  
  Adult Female Spermatheca Mated  4.949441  
  Adult Female Spermatheca Virgin  4.983777  
  Adult Head  5.105640  
  Adult Heart  4.837192  
  Adult Hind Gut  4.750713  
  Adult Male Ejaculatory Duct  4.775013  
  Adult Mid Gut  4.963572  
  Adult Ovary  4.822003  
  Adult Salivary Gland  5.302633  
  Adult Testes  8.154663  
  Adult Thoracoabdominal ganglion  5.357280  
  Adult Whole Fly  5.202209  
  Larvae Wandering Tubules  5.020182  
  Larval Feeding Carcass  5.399185  
  Larval Feeding Central Nevous System  6.028259  
  Larval Feeding Hind Gut  4.749777  
  Larval Feeding Malpighian Tubule  4.931151  
  Larval Feeding Mid Gut  5.096535  
  Larval Feeding Salivary Gland  4.990715  
  Whole Larvae Feeding  5.373296  
 
  
   FlyBase ID    symbol    start    end    strand    length   
   FBgn0037283   CG14658  655633   656235   -  603  
   FBgn0037284   CG14659  656571   657019   -  449  
   FBgn0003002   opa   678535   695709  +  17175  
   FBgn0020280   laf  701727   704255   -  2529  
 
 
    Segment 14 
 
   Location   
  Gene key  FBgn0004913-FBgn0051536  
  Heatmap region span   3R:639058..791169   
  Segment span   3R:732348..736278   
  Length (genes)  2  
  Length (bp)  3931  
   Model Scoring   
  BIC  238.347368  
  logL  -113.545953  
  logL ratio  16.664683  
   Expression   
  Mean expression  7.889908  
  Median expression  7.792281  
  Tissue std. dev.  0.367840  
 
  No GO Slim enrichment  
  
   tissue    mean expression   
  5th Passage Drosophila S2 Cells  8.994167  
  Adult Accessory gland  7.974648  
  Adult Brain  7.718322  
  Adult Carcass  7.509942  
  Adult Crop  7.979253  
  Adult Eye  7.802098  
  Adult Fatbody  7.529311  
  Adult Female Spermatheca Mated  7.532940  
  Adult Female Spermatheca Virgin  7.536913  
  Adult Head  7.478025  
  Adult Heart  7.822113  
  Adult Hind Gut  7.773768  
  Adult Male Ejaculatory Duct  7.766959  
  Adult Mid Gut  7.659072  
  Adult Ovary  8.259960  
  Adult Salivary Gland  8.105750  
  Adult Testes  7.975824  
  Adult Thoracoabdominal ganglion  7.709349  
  Adult Whole Fly  7.834966  
  Larvae Wandering Tubules  8.499273  
  Larval Feeding Carcass  7.729558  
  Larval Feeding Central Nevous System  8.453243  
  Larval Feeding Hind Gut  7.887757  
  Larval Feeding Malpighian Tubule  8.341407  
  Larval Feeding Mid Gut  7.415029  
  Larval Feeding Salivary Gland  8.206456  
  Whole Larvae Feeding  7.531408  
 
  
   FlyBase ID    symbol    start    end    strand    length   
   FBgn0004913   Gnf1   732348   735803  +  3456  
   FBgn0051536      736278   771298  +  35021  
 
    Segment 15 
 
   Location   
  Gene key  FBgn0037291-FBgn0037292  
  Heatmap region span   3R:778406..1063037   
  Segment span   3R:809954..836753   
  Length (genes)  2  
  Length (bp)  26800  
   Model Scoring   
  BIC  194.691357  
  logL  -91.717947  
  logL ratio  18.188604  
   Expression   
  Mean expression  5.287016  
  Median expression  5.062834  
  Tissue std. dev.  0.730631  
 
  No GO Slim enrichment  
  
   tissue    mean expression   
  5th Passage Drosophila S2 Cells  4.864482  
  Adult Accessory gland  5.260159  
  Adult Brain  4.693333  
  Adult Carcass  8.232111  
  Adult Crop  5.166440  
  Adult Eye  4.942920  
  Adult Fatbody  5.067997  
  Adult Female Spermatheca Mated  4.851049  
  Adult Female Spermatheca Virgin  4.781662  
  Adult Head  5.902221  
  Adult Heart  6.085342  
  Adult Hind Gut  4.905277  
  Adult Male Ejaculatory Duct  5.062124  
  Adult Mid Gut  5.381096  
  Adult Ovary  4.780663  
  Adult Salivary Gland  5.359320  
  Adult Testes  6.355752  
  Adult Thoracoabdominal ganglion  4.945236  
  Adult Whole Fly  6.232012  
  Larvae Wandering Tubules  5.013749  
  Larval Feeding Carcass  4.911698  
  Larval Feeding Central Nevous System  4.789035  
  Larval Feeding Hind Gut  4.929384  
  Larval Feeding Malpighian Tubule  4.998287  
  Larval Feeding Mid Gut  5.380070  
  Larval Feeding Salivary Gland  4.869228  
  Whole Larvae Feeding  4.988799  
 
  
   FlyBase ID    symbol    start    end    strand    length   
   FBgn0037291   CG14662  807721   809954   -  2234  
   FBgn0037292   CG2022  812222   836753   -  24532  
 
    Segment 16 
 
   Location   
  Gene key  FBgn0010313-FBgn0037293  
  Heatmap region span   3R:780974..1066457   
  Segment span   3R:912408..953810   
  Length (genes)  2  
  Length (bp)  41403  
   Model Scoring   
  BIC  245.850229  
  logL  -117.297383  
  logL ratio  35.343439  
   Expression   
  Mean expression  9.687686  
  Median expression  9.655452  
  Tissue std. dev.  0.706821  
 
  No GO Slim enrichment  
  
   tissue    mean expression   
  5th Passage Drosophila S2 Cells  7.817085  
  Adult Accessory gland  8.615488  
  Adult Brain  9.852690  
  Adult Carcass  9.106737  
  Adult Crop  10.457568  
  Adult Eye  10.361657  
  Adult Fatbody  10.082185  
  Adult Female Spermatheca Mated  9.892385  
  Adult Female Spermatheca Virgin  9.969328  
  Adult Head  9.622630  
  Adult Heart  10.118005  
  Adult Hind Gut  9.967418  
  Adult Male Ejaculatory Duct  9.633195  
  Adult Mid Gut  9.375039  
  Adult Ovary  10.396536  
  Adult Salivary Gland  10.418559  
  Adult Testes  7.621204  
  Adult Thoracoabdominal ganglion  9.634248  
  Adult Whole Fly  9.315399  
  Larvae Wandering Tubules  10.190668  
  Larval Feeding Carcass  9.600095  
  Larval Feeding Central Nevous System  10.305343  
  Larval Feeding Hind Gut  10.143217  
  Larval Feeding Malpighian Tubule  9.785627  
  Larval Feeding Mid Gut  9.808086  
  Larval Feeding Salivary Gland  10.216088  
  Whole Larvae Feeding  9.261042  
 
  
   FlyBase ID    symbol    start    end    strand    length   
   FBgn0010313   corto  909343   912408   -  3066  
   FBgn0037293   CG12007   953810   955665  +  1856  
 
    Segment 17 
 
   Location   
  Gene key  FBgn0040684-FBgn0037296  
  Heatmap region span   3R:787070..1079070   
  Segment span   3R:963295..1045168   
  Length (genes)  5  
  Length (bp)  81874  
   Model Scoring   
  BIC  420.969822  
  logL  -204.857180  
  logL ratio  106.858245  
   Expression   
  Mean expression  4.771367  
  Median expression  4.587958  
  Tissue std. dev.  0.433080  
 
  No GO Slim enrichment  
  
   tissue    mean expression   
  5th Passage Drosophila S2 Cells  4.721320  
  Adult Accessory gland  4.649130  
  Adult Brain  4.927742  
  Adult Carcass  4.724721  
  Adult Crop  4.670783  
  Adult Eye  4.639437  
  Adult Fatbody  4.577098  
  Adult Female Spermatheca Mated  4.559070  
  Adult Female Spermatheca Virgin  4.587289  
  Adult Head  4.629215  
  Adult Heart  4.900868  
  Adult Hind Gut  4.542191  
  Adult Male Ejaculatory Duct  4.640394  
  Adult Mid Gut  4.633721  
  Adult Ovary  4.921017  
  Adult Salivary Gland  4.826515  
  Adult Testes  6.886235  
  Adult Thoracoabdominal ganglion  4.896247  
  Adult Whole Fly  4.748092  
  Larvae Wandering Tubules  4.715247  
  Larval Feeding Carcass  4.583846  
  Larval Feeding Central Nevous System  4.749378  
  Larval Feeding Hind Gut  4.405885  
  Larval Feeding Malpighian Tubule  4.723842  
  Larval Feeding Mid Gut  4.556651  
  Larval Feeding Salivary Gland  4.762634  
  Whole Larvae Feeding  4.648342  
 
  
   FlyBase ID    symbol    start    end    strand    length   
   FBgn0040684   CG12589  962779   963295   -  517  
   FBgn0037295   dpr16   976630   995849  +  19220  
   FBgn0037294   CG12590   985334   986627  +  1294  
   FBgn0259212   cno  998392   1043147   -  44756  
   FBgn0037296   Prosbeta2R2  1043473   1045168   -  1696  
 
 
    Segment 18 
 
   Location   
  Gene key  FBgn0037297-FBgn0037301  
  Heatmap region span   3R:791169..1082763   
  Segment span   3R:1045390..1058268   
  Length (genes)  4  
  Length (bp)  12879  
   Model Scoring   
  BIC  469.685933  
  logL  -229.215235  
  logL ratio  64.699485  
   Expression   
  Mean expression  9.078515  
  Median expression  9.114574  
  Tissue std. dev.  0.373301  
 
  No GO Slim enrichment  
  
   tissue    mean expression   
  5th Passage Drosophila S2 Cells  8.987329  
  Adult Accessory gland  9.298567  
  Adult Brain  8.782509  
  Adult Carcass  9.033167  
  Adult Crop  10.135136  
  Adult Eye  9.251038  
  Adult Fatbody  9.105387  
  Adult Female Spermatheca Mated  8.821143  
  Adult Female Spermatheca Virgin  8.827616  
  Adult Head  9.216236  
  Adult Heart  9.128911  
  Adult Hind Gut  9.636481  
  Adult Male Ejaculatory Duct  10.150098  
  Adult Mid Gut  8.727418  
  Adult Ovary  9.067605  
  Adult Salivary Gland  8.612443  
  Adult Testes  8.886787  
  Adult Thoracoabdominal ganglion  8.672493  
  Adult Whole Fly  8.811136  
  Larvae Wandering Tubules  9.094579  
  Larval Feeding Carcass  8.814526  
  Larval Feeding Central Nevous System  9.034856  
  Larval Feeding Hind Gut  8.903831  
  Larval Feeding Malpighian Tubule  8.888811  
  Larval Feeding Mid Gut  9.128875  
  Larval Feeding Salivary Gland  9.278198  
  Whole Larvae Feeding  8.824730  
 
  
   FlyBase ID    symbol    start    end    strand    length   
   FBgn0037297   CG1116   1045390   1047270  +  1881  
   FBgn0037298   CG2604  1047354   1049197   -  1844  
   FBgn0037299   CG1115   1051882   1053248  +  1367  
   FBgn0037301   Mms19   1058268   1062011  +  3744  
 
 
    Segment 19 
 
   Location   
  Gene key  FBgn0037305-FBgn0086689  
  Heatmap region span   3R:1045390..1195262   
  Segment span   3R:1082423..1082763   
  Length (genes)  2  
  Length (bp)  341  
   Model Scoring   
  BIC  237.670365  
  logL  -113.207451  
  logL ratio  60.139458  
   Expression   
  Mean expression  10.217394  
  Median expression  9.908279  
  Tissue std. dev.  0.714329  
 
  No GO Slim enrichment  
  
   tissue    mean expression   
  5th Passage Drosophila S2 Cells  10.655067  
  Adult Accessory gland  10.093377  
  Adult Brain  9.652881  
  Adult Carcass  9.248559  
  Adult Crop  10.858697  
  Adult Eye  9.488999  
  Adult Fatbody  9.765707  
  Adult Female Spermatheca Mated  10.378059  
  Adult Female Spermatheca Virgin  10.498278  
  Adult Head  9.415725  
  Adult Heart  9.660239  
  Adult Hind Gut  11.559335  
  Adult Male Ejaculatory Duct  10.162769  
  Adult Mid Gut  10.822578  
  Adult Ovary  10.960307  
  Adult Salivary Gland  9.694173  
  Adult Testes  9.110136  
  Adult Thoracoabdominal ganglion  9.556442  
  Adult Whole Fly  10.274630  
  Larvae Wandering Tubules  11.409988  
  Larval Feeding Carcass  9.582733  
  Larval Feeding Central Nevous System  9.917832  
  Larval Feeding Hind Gut  10.693234  
  Larval Feeding Malpighian Tubule  12.000415  
  Larval Feeding Mid Gut  10.319231  
  Larval Feeding Salivary Gland  9.875880  
  Whole Larvae Feeding  10.214359  
 
  
   FlyBase ID    symbol    start    end    strand    length   
   FBgn0037305   CG12173  1081166   1082423   -  1258  
   FBgn0086689      1082763   1094197  +  11435  
 
    Segment 20 
 
   Location   
  Gene key  FBgn0051538-FBgn0037310  
  Heatmap region span   3R:1066457..1200353   
  Segment span   3R:1103557..1125867   
  Length (genes)  2  
  Length (bp)  22311  
   Model Scoring   
  BIC  200.487030  
  logL  -94.615784  
  logL ratio  22.354442  
   Expression   
  Mean expression  5.392243  
  Median expression  4.776848  
  Tissue std. dev.  1.609230  
 
  No GO Slim enrichment  
  
   tissue    mean expression   
  5th Passage Drosophila S2 Cells  4.694565  
  Adult Accessory gland  5.208884  
  Adult Brain  4.621265  
  Adult Carcass  5.056557  
  Adult Crop  4.968203  
  Adult Eye  4.428650  
  Adult Fatbody  4.998988  
  Adult Female Spermatheca Mated  5.185216  
  Adult Female Spermatheca Virgin  5.176550  
  Adult Head  4.493576  
  Adult Heart  4.558840  
  Adult Hind Gut  4.906185  
  Adult Male Ejaculatory Duct  5.775695  
  Adult Mid Gut  4.817145  
  Adult Ovary  4.588208  
  Adult Salivary Gland  6.069382  
  Adult Testes  12.489019  
  Adult Thoracoabdominal ganglion  4.553965  
  Adult Whole Fly  8.428736  
  Larvae Wandering Tubules  5.003665  
  Larval Feeding Carcass  4.801751  
  Larval Feeding Central Nevous System  4.693944  
  Larval Feeding Hind Gut  4.649276  
  Larval Feeding Malpighian Tubule  4.973382  
  Larval Feeding Mid Gut  4.889969  
  Larval Feeding Salivary Gland  4.923723  
  Whole Larvae Feeding  6.635223  
 
  
   FlyBase ID    symbol    start    end    strand    length   
   FBgn0051538   CG31538  1101394   1103557   -  2164  
   FBgn0037310   Tim17b1   1125867   1126911  +  1045  
 
    Segment 21 
 
   Location   
  Gene key  FBgn0037312-FBgn0037315  
  Heatmap region span   3R:1079070..1200792   
  Segment span   3R:1180469..1194027   
  Length (genes)  4  
  Length (bp)  13559  
   Model Scoring   
  BIC  502.772603  
  logL  -245.758571  
  logL ratio  130.156355  
   Expression   
  Mean expression  10.619668  
  Median expression  10.862298  
  Tissue std. dev.  0.326906  
 
  No GO Slim enrichment  
  
   tissue    mean expression   
  5th Passage Drosophila S2 Cells  10.985612  
  Adult Accessory gland  10.979336  
  Adult Brain  10.258565  
  Adult Carcass  10.654892  
  Adult Crop  10.761632  
  Adult Eye  10.680337  
  Adult Fatbody  10.685512  
  Adult Female Spermatheca Mated  10.430133  
  Adult Female Spermatheca Virgin  10.425043  
  Adult Head  10.357634  
  Adult Heart  11.027482  
  Adult Hind Gut  10.701068  
  Adult Male Ejaculatory Duct  11.406900  
  Adult Mid Gut  10.411573  
  Adult Ovary  10.549185  
  Adult Salivary Gland  10.773213  
  Adult Testes  9.982255  
  Adult Thoracoabdominal ganglion  10.415693  
  Adult Whole Fly  10.281672  
  Larvae Wandering Tubules  11.083818  
  Larval Feeding Carcass  10.595607  
  Larval Feeding Central Nevous System  10.137612  
  Larval Feeding Hind Gut  10.810605  
  Larval Feeding Malpighian Tubule  10.516141  
  Larval Feeding Mid Gut  10.679309  
  Larval Feeding Salivary Gland  11.011217  
  Whole Larvae Feeding  10.128981  
 
  
   FlyBase ID    symbol    start    end    strand    length   
   FBgn0037312   CG11999  1179439   1180469   -  1031  
   FBgn0037313   CG1161   1180822   1182467  +  1646  
   FBgn0250746   Prosbeta7  1182734   1184086   -  1353  
   FBgn0037315   Cerk  1184585   1194027   -  9443  
 
 
    Segment 22 
 
   Location   
  Gene key  FBgn0037322-FBgn0037325  
  Heatmap region span   3R:1202476..1328424   
  Segment span   3R:1224918..1252979   
  Length (genes)  5  
  Length (bp)  28062  
   Model Scoring   
  BIC  450.189915  
  logL  -219.467226  
  logL ratio  67.276841  
   Expression   
  Mean expression  4.659552  
  Median expression  4.492880  
  Tissue std. dev.  0.368831  
 
  No GO Slim enrichment  
  
   tissue    mean expression   
  5th Passage Drosophila S2 Cells  4.506684  
  Adult Accessory gland  4.654526  
  Adult Brain  4.568382  
  Adult Carcass  4.725809  
  Adult Crop  4.455796  
  Adult Eye  4.648113  
  Adult Fatbody  4.541370  
  Adult Female Spermatheca Mated  4.467930  
  Adult Female Spermatheca Virgin  4.523061  
  Adult Head  5.250914  
  Adult Heart  4.470759  
  Adult Hind Gut  4.510700  
  Adult Male Ejaculatory Duct  6.183919  
  Adult Mid Gut  4.733546  
  Adult Ovary  4.492378  
  Adult Salivary Gland  4.787476  
  Adult Testes  5.250155  
  Adult Thoracoabdominal ganglion  4.542419  
  Adult Whole Fly  4.786780  
  Larvae Wandering Tubules  4.535317  
  Larval Feeding Carcass  4.546457  
  Larval Feeding Central Nevous System  4.319553  
  Larval Feeding Hind Gut  4.388919  
  Larval Feeding Malpighian Tubule  4.495182  
  Larval Feeding Mid Gut  4.550044  
  Larval Feeding Salivary Gland  4.506756  
  Whole Larvae Feeding  4.364947  
 
  
   FlyBase ID    symbol    start    end    strand    length   
   FBgn0037322   Or83a  1223365   1224918   -  1554  
   FBgn0037323   CG2663  1226316   1229851   -  3536  
   FBgn0037324   Orco  1232902   1238128   -  5227  
   FBgn0037326   CG14669   1240275   1283322  +  43048  
   FBgn0037325   CG12147  1251091   1252979   -  1889  
 
 
    Segment 23 
 
   Location   
  Gene key  FBgn0037329-FBgn0051550  
  Heatmap region span   3R:1219650..1376889   
  Segment span   3R:1293515..1311254   
  Length (genes)  7  
  Length (bp)  17740  
   Model Scoring   
  BIC  625.414178  
  logL  -307.079358  
  logL ratio  261.837455  
   Expression   
  Mean expression  8.755266  
  Median expression  8.914553  
  Tissue std. dev.  0.381455  
 
  No GO Slim enrichment  
  
   tissue    mean expression   
  5th Passage Drosophila S2 Cells  9.000978  
  Adult Accessory gland  8.640134  
  Adult Brain  9.568356  
  Adult Carcass  8.575827  
  Adult Crop  8.807966  
  Adult Eye  9.024445  
  Adult Fatbody  8.521492  
  Adult Female Spermatheca Mated  8.729680  
  Adult Female Spermatheca Virgin  8.682301  
  Adult Head  8.622301  
  Adult Heart  8.774294  
  Adult Hind Gut  8.576508  
  Adult Male Ejaculatory Duct  8.524350  
  Adult Mid Gut  8.221348  
  Adult Ovary  9.616563  
  Adult Salivary Gland  8.459151  
  Adult Testes  8.441545  
  Adult Thoracoabdominal ganglion  9.451041  
  Adult Whole Fly  8.761666  
  Larvae Wandering Tubules  8.497943  
  Larval Feeding Carcass  8.573760  
  Larval Feeding Central Nevous System  9.588230  
  Larval Feeding Hind Gut  8.686727  
  Larval Feeding Malpighian Tubule  8.729294  
  Larval Feeding Mid Gut  8.333138  
  Larval Feeding Salivary Gland  8.725893  
  Whole Larvae Feeding  8.257244  
 
  
   FlyBase ID    symbol    start    end    strand    length   
   FBgn0037329   CG12162   1293515   1295288  +  1774  
   FBgn0037330   mRpL44  1295185   1296382   -  1198  
   FBgn0011715   Snr1   1296639   1298476  +  1838  
   FBgn0025825   Hdac3  1298532   1300320   -  1789  
   FBgn0037332   Hcs   1300624   1311115  +  10492  
   FBgn0015402   ksr  1305871   1309607   -  3737  
   FBgn0051550   CG31550   1311254   1315211  +  3958  
 
 
    Segment 24 
 
   Location   
  Gene key  FBgn0037339-FBgn0041191  
  Heatmap region span   3R:1373490..1449844   
  Segment span   3R:1389347..1394618   
  Length (genes)  5  
  Length (bp)  5272  
   Model Scoring   
  BIC  542.523833  
  logL  -265.634185  
  logL ratio  143.283028  
   Expression   
  Mean expression  9.551479  
  Median expression  9.424028  
  Tissue std. dev.  0.358106  
 
  No GO Slim enrichment  
  
   tissue    mean expression   
  5th Passage Drosophila S2 Cells  9.446473  
  Adult Accessory gland  9.907042  
  Adult Brain  9.514251  
  Adult Carcass  9.259787  
  Adult Crop  10.265156  
  Adult Eye  9.557002  
  Adult Fatbody  9.548196  
  Adult Female Spermatheca Mated  9.576441  
  Adult Female Spermatheca Virgin  9.648253  
  Adult Head  9.332958  
  Adult Heart  9.738454  
  Adult Hind Gut  9.813629  
  Adult Male Ejaculatory Duct  10.313727  
  Adult Mid Gut  9.613707  
  Adult Ovary  9.654343  
  Adult Salivary Gland  9.668167  
  Adult Testes  8.576774  
  Adult Thoracoabdominal ganglion  9.586817  
  Adult Whole Fly  9.236599  
  Larvae Wandering Tubules  9.756777  
  Larval Feeding Carcass  9.012896  
  Larval Feeding Central Nevous System  9.240637  
  Larval Feeding Hind Gut  9.478167  
  Larval Feeding Malpighian Tubule  9.884269  
  Larval Feeding Mid Gut  9.304448  
  Larval Feeding Salivary Gland  9.859111  
  Whole Larvae Feeding  9.095849  
 
  
   FlyBase ID    symbol    start    end    strand    length   
   FBgn0037339   Pi4KIIalpha  1383385   1389347   -  5963  
   FBgn0037340   CG14671  1389844   1390537   -  694  
   FBgn0037341   CG12746   1390593   1393157  +  2565  
   FBgn0037342   CG2931  1393224   1394332   -  1109  
   FBgn0041191   Rheb   1394618   1396213  +  1596  
 
 
    Segment 25 
 
   Location   
  Gene key  FBgn0037344-FBgn0014366  
  Heatmap region span   3R:1380033..1453574   
  Segment span   3R:1414097..1417180   
  Length (genes)  3  
  Length (bp)  3084  
   Model Scoring   
  BIC  293.037978  
  logL  -140.891258  
  logL ratio  89.484223  
   Expression   
  Mean expression  8.656688  
  Median expression  8.640299  
  Tissue std. dev.  0.424188  
 
  No GO Slim enrichment  
  
   tissue    mean expression   
  5th Passage Drosophila S2 Cells  9.475394  
  Adult Accessory gland  8.981244  
  Adult Brain  8.978019  
  Adult Carcass  8.149864  
  Adult Crop  8.760940  
  Adult Eye  8.253059  
  Adult Fatbody  8.528486  
  Adult Female Spermatheca Mated  8.164056  
  Adult Female Spermatheca Virgin  8.047227  
  Adult Head  8.359698  
  Adult Heart  8.479699  
  Adult Hind Gut  8.760103  
  Adult Male Ejaculatory Duct  8.746232  
  Adult Mid Gut  8.316483  
  Adult Ovary  9.416998  
  Adult Salivary Gland  8.716007  
  Adult Testes  8.798028  
  Adult Thoracoabdominal ganglion  8.744464  
  Adult Whole Fly  8.321298  
  Larvae Wandering Tubules  9.016498  
  Larval Feeding Carcass  8.350930  
  Larval Feeding Central Nevous System  9.372752  
  Larval Feeding Hind Gut  8.772014  
  Larval Feeding Malpighian Tubule  9.029873  
  Larval Feeding Mid Gut  7.955136  
  Larval Feeding Salivary Gland  9.146710  
  Whole Larvae Feeding  8.089352  
 
  
   FlyBase ID    symbol    start    end    strand    length   
   FBgn0037344   CG2926  1405023   1414097   -  9075  
   FBgn0037345   rev7   1414595   1415425  +  831  
   FBgn0014366   noi  1415323   1417180   -  1858  
 
 
    Segment 26 
 
   Location   
  Gene key  FBgn0015324-FBgn0250753  
  Heatmap region span   3R:1380812..1459346   
  Segment span   3R:1417453..1426891   
  Length (genes)  2  
  Length (bp)  9439  
   Model Scoring   
  BIC  337.279441  
  logL  -163.011990  
  logL ratio  78.146564  
   Expression   
  Mean expression  12.166310  
  Median expression  11.992095  
  Tissue std. dev.  0.434126  
 
  No GO Slim enrichment  
  
   tissue    mean expression   
  5th Passage Drosophila S2 Cells  12.430386  
  Adult Accessory gland  11.578851  
  Adult Brain  12.615695  
  Adult Carcass  11.556022  
  Adult Crop  11.978275  
  Adult Eye  12.115267  
  Adult Fatbody  11.532958  
  Adult Female Spermatheca Mated  11.602006  
  Adult Female Spermatheca Virgin  11.427611  
  Adult Head  12.088182  
  Adult Heart  11.671654  
  Adult Hind Gut  12.657346  
  Adult Male Ejaculatory Duct  12.128981  
  Adult Mid Gut  11.925853  
  Adult Ovary  12.392392  
  Adult Salivary Gland  12.432552  
  Adult Testes  11.562854  
  Adult Thoracoabdominal ganglion  12.381756  
  Adult Whole Fly  12.247189  
  Larvae Wandering Tubules  12.703957  
  Larval Feeding Carcass  12.539454  
  Larval Feeding Central Nevous System  12.833498  
  Larval Feeding Hind Gut  12.922293  
  Larval Feeding Malpighian Tubule  12.672704  
  Larval Feeding Mid Gut  12.208601  
  Larval Feeding Salivary Gland  12.169607  
  Whole Larvae Feeding  12.114417  
 
  
   FlyBase ID    symbol    start    end    strand    length   
   FBgn0015324   Vha26   1417453   1420429  +  2977  
   FBgn0250753   exba  1421086   1426891   -  5806  
 
    Segment 27 
 
   Location   
  Gene key  FBgn0037347-FBgn0044823  
  Heatmap region span   3R:1389347..1461346   
  Segment span   3R:1427155..1449844   
  Length (genes)  5  
  Length (bp)  22690  
   Model Scoring   
  BIC  504.053250  
  logL  -246.398894  
  logL ratio  129.064195  
   Expression   
  Mean expression  8.379018  
  Median expression  8.316671  
  Tissue std. dev.  0.437624  
 
  No GO Slim enrichment  
  
   tissue    mean expression   
  5th Passage Drosophila S2 Cells  8.854044  
  Adult Accessory gland  8.380875  
  Adult Brain  8.604588  
  Adult Carcass  7.563008  
  Adult Crop  9.096184  
  Adult Eye  8.358185  
  Adult Fatbody  7.944910  
  Adult Female Spermatheca Mated  8.109508  
  Adult Female Spermatheca Virgin  8.056260  
  Adult Head  7.930459  
  Adult Heart  8.736550  
  Adult Hind Gut  8.616888  
  Adult Male Ejaculatory Duct  8.207175  
  Adult Mid Gut  7.758070  
  Adult Ovary  9.096342  
  Adult Salivary Gland  8.706126  
  Adult Testes  7.723157  
  Adult Thoracoabdominal ganglion  8.494079  
  Adult Whole Fly  8.073964  
  Larvae Wandering Tubules  8.914391  
  Larval Feeding Carcass  8.327737  
  Larval Feeding Central Nevous System  8.856971  
  Larval Feeding Hind Gut  8.746652  
  Larval Feeding Malpighian Tubule  8.616299  
  Larval Feeding Mid Gut  7.902122  
  Larval Feeding Salivary Gland  8.744949  
  Whole Larvae Feeding  7.813985  
 
  
   FlyBase ID    symbol    start    end    strand    length   
   FBgn0037347   CG1427   1427155   1428909  +  1755  
   FBgn0017550   Rga  1433065   1438830   -  5766  
   FBgn0019637   Atu   1439086   1441664  +  2579  
   FBgn0037350   CG2911  1447834   1449810   -  1977  
   FBgn0044823   Spec2  1442368   1449844   -  7477  
 
 
    Segment 28 
 
   Location   
  Gene key  FBgn0037352-FBgn0051551  
  Heatmap region span   3R:1414097..1495051   
  Segment span   3R:1453049..1453574   
  Length (genes)  2  
  Length (bp)  526  
   Model Scoring   
  BIC  179.870922  
  logL  -84.307730  
  logL ratio  39.061076  
   Expression   
  Mean expression  4.351690  
  Median expression  4.427625  
  Tissue std. dev.  0.273119  
 
  No GO Slim enrichment  
  
   tissue    mean expression   
  5th Passage Drosophila S2 Cells  4.459811  
  Adult Accessory gland  4.412288  
  Adult Brain  4.113288  
  Adult Carcass  4.511171  
  Adult Crop  4.350190  
  Adult Eye  4.198344  
  Adult Fatbody  4.297294  
  Adult Female Spermatheca Mated  4.370189  
  Adult Female Spermatheca Virgin  4.253132  
  Adult Head  4.170615  
  Adult Heart  4.142082  
  Adult Hind Gut  4.332068  
  Adult Male Ejaculatory Duct  4.543068  
  Adult Mid Gut  4.397701  
  Adult Ovary  4.386763  
  Adult Salivary Gland  4.744271  
  Adult Testes  5.490998  
  Adult Thoracoabdominal ganglion  4.173466  
  Adult Whole Fly  4.112656  
  Larvae Wandering Tubules  4.424511  
  Larval Feeding Carcass  4.278709  
  Larval Feeding Central Nevous System  4.008608  
  Larval Feeding Hind Gut  4.147485  
  Larval Feeding Malpighian Tubule  4.384024  
  Larval Feeding Mid Gut  4.303907  
  Larval Feeding Salivary Gland  4.357185  
  Whole Larvae Feeding  4.131797  
 
  
   FlyBase ID    symbol    start    end    strand    length   
   FBgn0037352   CG14673  1452216   1453049   -  834  
   FBgn0051551   CG31551   1453574   1457755  +  4182  
 
    Segment 29 
 
   Location   
  Gene key  FBgn0051549-FBgn0037354  
  Heatmap region span   3R:1417453..1519008   
  Segment span   3R:1457914..1459346   
  Length (genes)  2  
  Length (bp)  1433  
   Model Scoring   
  BIC  233.036548  
  logL  -110.890543  
  logL ratio  61.964646  
   Expression   
  Mean expression  10.229164  
  Median expression  10.286029  
  Tissue std. dev.  0.550054  
 
  No GO Slim enrichment  
  
   tissue    mean expression   
  5th Passage Drosophila S2 Cells  10.728593  
  Adult Accessory gland  10.585475  
  Adult Brain  9.181737  
  Adult Carcass  10.117261  
  Adult Crop  10.206305  
  Adult Eye  9.882238  
  Adult Fatbody  10.383087  
  Adult Female Spermatheca Mated  10.287802  
  Adult Female Spermatheca Virgin  10.350465  
  Adult Head  9.830525  
  Adult Heart  10.495622  
  Adult Hind Gut  10.266210  
  Adult Male Ejaculatory Duct  10.143934  
  Adult Mid Gut  11.426348  
  Adult Ovary  10.490534  
  Adult Salivary Gland  10.493589  
  Adult Testes  8.473593  
  Adult Thoracoabdominal ganglion  9.258821  
  Adult Whole Fly  10.316401  
  Larvae Wandering Tubules  10.157638  
  Larval Feeding Carcass  10.624740  
  Larval Feeding Central Nevous System  10.168632  
  Larval Feeding Hind Gut  10.077233  
  Larval Feeding Malpighian Tubule  10.672437  
  Larval Feeding Mid Gut  10.520100  
  Larval Feeding Salivary Gland  10.657705  
  Whole Larvae Feeding  10.390411  
 
  
   FlyBase ID    symbol    start    end    strand    length   
   FBgn0051549   CG31549   1457914   1459043  +  1130  
   FBgn0037354   CG12171   1459346   1460362  +  1017  
 
    Segment 30 
 
   Location   
  Gene key  FBgn0051548-FBgn0037358  
  Heatmap region span   3R:1450186..1605031   
  Segment span   3R:1462567..1480613   
  Length (genes)  3  
  Length (bp)  18047  
   Model Scoring   
  BIC  337.380943  
  logL  -163.062740  
  logL ratio  108.386345  
   Expression   
  Mean expression  10.237609  
  Median expression  10.321131  
  Tissue std. dev.  0.612820  
 
  No GO Slim enrichment  
  
   tissue    mean expression   
  5th Passage Drosophila S2 Cells  9.787338  
  Adult Accessory gland  9.691591  
  Adult Brain  9.573186  
  Adult Carcass  10.533056  
  Adult Crop  10.489689  
  Adult Eye  10.145127  
  Adult Fatbody  10.903848  
  Adult Female Spermatheca Mated  10.024682  
  Adult Female Spermatheca Virgin  10.094635  
  Adult Head  10.473022  
  Adult Heart  10.907570  
  Adult Hind Gut  11.105567  
  Adult Male Ejaculatory Duct  10.476273  
  Adult Mid Gut  10.500598  
  Adult Ovary  9.927212  
  Adult Salivary Gland  10.970329  
  Adult Testes  8.576899  
  Adult Thoracoabdominal ganglion  9.650298  
  Adult Whole Fly  10.034371  
  Larvae Wandering Tubules  10.407675  
  Larval Feeding Carcass  9.626081  
  Larval Feeding Central Nevous System  9.410255  
  Larval Feeding Hind Gut  11.398052  
  Larval Feeding Malpighian Tubule  11.187962  
  Larval Feeding Mid Gut  10.261495  
  Larval Feeding Salivary Gland  10.184211  
  Whole Larvae Feeding  10.074417  
 
  
   FlyBase ID    symbol    start    end    strand    length   
   FBgn0051548   CG31548  1461622   1462567   -  946  
   FBgn0037356   CG12170   1462959   1464379  +  1421  
   FBgn0037358   elm  1478555   1480613   -  2059  
 
 
    Segment 31 
 
   Location   
  Gene key  FBgn0037359-FBgn0010772  
  Heatmap region span   3R:1453049..1614796   
  Segment span   3R:1480867..1495051   
  Length (genes)  4  
  Length (bp)  14185  
   Model Scoring   
  BIC  473.280869  
  logL  -231.012703  
  logL ratio  39.401206  
   Expression   
  Mean expression  8.386546  
  Median expression  8.368608  
  Tissue std. dev.  0.390025  
 
  No GO Slim enrichment  
  
   tissue    mean expression   
  5th Passage Drosophila S2 Cells  8.727746  
  Adult Accessory gland  8.586322  
  Adult Brain  8.946263  
  Adult Carcass  7.743756  
  Adult Crop  8.536593  
  Adult Eye  8.332129  
  Adult Fatbody  7.978519  
  Adult Female Spermatheca Mated  8.365176  
  Adult Female Spermatheca Virgin  8.333073  
  Adult Head  7.815302  
  Adult Heart  8.334144  
  Adult Hind Gut  8.188211  
  Adult Male Ejaculatory Duct  8.197184  
  Adult Mid Gut  7.888294  
  Adult Ovary  9.260481  
  Adult Salivary Gland  8.389014  
  Adult Testes  8.357551  
  Adult Thoracoabdominal ganglion  8.634284  
  Adult Whole Fly  8.179827  
  Larvae Wandering Tubules  8.687132  
  Larval Feeding Carcass  8.350726  
  Larval Feeding Central Nevous System  9.200736  
  Larval Feeding Hind Gut  8.362529  
  Larval Feeding Malpighian Tubule  8.486914  
  Larval Feeding Mid Gut  7.802144  
  Larval Feeding Salivary Gland  8.824112  
  Whole Larvae Feeding  7.928569  
 
  
   FlyBase ID    symbol    start    end    strand    length   
   FBgn0037359   MED27   1480867   1482058  +  1192  
   FBgn0037360   CG2182  1481952   1485631   -  3680  
   FBgn0046222   CG1109   1486359   1491168  +  4810  
   FBgn0010772   Xe7  1490861   1495051   -  4191  
 
 
    Segment 32 
 
   Location   
  Gene key  FBgn0037363-FBgn0037365  
  Heatmap region span   3R:1457914..1615211   
  Segment span   3R:1495435..1519008   
  Length (genes)  3  
  Length (bp)  23574  
   Model Scoring   
  BIC  315.130858  
  logL  -151.937698  
  logL ratio  14.875953  
   Expression   
  Mean expression  5.882380  
  Median expression  5.511719  
  Tissue std. dev.  0.541261  
 
  No GO Slim enrichment  
  
   tissue    mean expression   
  5th Passage Drosophila S2 Cells  5.996924  
  Adult Accessory gland  5.651597  
  Adult Brain  5.663259  
  Adult Carcass  5.938879  
  Adult Crop  5.880057  
  Adult Eye  6.803628  
  Adult Fatbody  6.010388  
  Adult Female Spermatheca Mated  5.069912  
  Adult Female Spermatheca Virgin  5.071520  
  Adult Head  5.790578  
  Adult Heart  6.277743  
  Adult Hind Gut  5.719877  
  Adult Male Ejaculatory Duct  6.126771  
  Adult Mid Gut  5.501892  
  Adult Ovary  7.713999  
  Adult Salivary Gland  5.757329  
  Adult Testes  6.319427  
  Adult Thoracoabdominal ganglion  5.368384  
  Adult Whole Fly  6.671667  
  Larvae Wandering Tubules  5.860690  
  Larval Feeding Carcass  6.111851  
  Larval Feeding Central Nevous System  5.407894  
  Larval Feeding Hind Gut  5.820832  
  Larval Feeding Malpighian Tubule  5.738652  
  Larval Feeding Mid Gut  5.317131  
  Larval Feeding Salivary Gland  5.648641  
  Whole Larvae Feeding  5.584739  
 
  
   FlyBase ID    symbol    start    end    strand    length   
   FBgn0037363   CG1347   1495435   1505652  +  10218  
   FBgn0037364   Rab23  1505776   1511148   -  5373  
   FBgn0037365   CG2104  1517547   1519008   -  1462  
 
 
    Segment 33 
 
   Location   
  Gene key  FBgn0037368-FBgn0037374  
  Heatmap region span   3R:1462567..1620426   
  Segment span   3R:1559263..1605031   
  Length (genes)  7  
  Length (bp)  45769  
   Model Scoring   
  BIC  634.592981  
  logL  -311.668760  
  logL ratio  240.112050  
   Expression   
  Mean expression  8.273831  
  Median expression  8.225814  
  Tissue std. dev.  0.406038  
 
  No GO Slim enrichment  
  
   tissue    mean expression   
  5th Passage Drosophila S2 Cells  8.642761  
  Adult Accessory gland  8.547830  
  Adult Brain  8.197285  
  Adult Carcass  7.669535  
  Adult Crop  8.566696  
  Adult Eye  7.946036  
  Adult Fatbody  7.952847  
  Adult Female Spermatheca Mated  8.137535  
  Adult Female Spermatheca Virgin  8.086607  
  Adult Head  7.715809  
  Adult Heart  8.341418  
  Adult Hind Gut  7.952206  
  Adult Male Ejaculatory Duct  8.266880  
  Adult Mid Gut  7.728095  
  Adult Ovary  9.530754  
  Adult Salivary Gland  8.465950  
  Adult Testes  7.996189  
  Adult Thoracoabdominal ganglion  8.211303  
  Adult Whole Fly  8.458631  
  Larvae Wandering Tubules  8.501944  
  Larval Feeding Carcass  8.288069  
  Larval Feeding Central Nevous System  8.784897  
  Larval Feeding Hind Gut  8.363985  
  Larval Feeding Malpighian Tubule  8.337305  
  Larval Feeding Mid Gut  7.670354  
  Larval Feeding Salivary Gland  8.878762  
  Whole Larvae Feeding  8.153764  
 
  
   FlyBase ID    symbol    start    end    strand    length   
   FBgn0037368   CG1239   1559263   1560424  +  1162  
   FBgn0037369   CG2100  1560420   1562118   -  1699  
   FBgn0037370   CG1236   1562466   1563996  +  1531  
   FBgn0037371   Sym  1563900   1568168   -  4269  
   FBgn0027497   Madm   1568214   1571789  +  3576  
   FBgn0037372   CG2091  1579040   1580495   -  1456  
   FBgn0037374   jagn   1605031   1607030  +  2000  
 
 
    Segment 34 
 
   Location   
  Gene key  FBgn0027608-FBgn0037376  
  Heatmap region span   3R:1480867..1628050   
  Segment span   3R:1606798..1614796   
  Length (genes)  2  
  Length (bp)  7999  
   Model Scoring   
  BIC  224.347034  
  logL  -106.545786  
  logL ratio  -3.336959  
   Expression   
  Mean expression  5.771274  
  Median expression  5.338814  
  Tissue std. dev.  0.922782  
 
  No GO Slim enrichment  
  
   tissue    mean expression   
  5th Passage Drosophila S2 Cells  5.644139  
  Adult Accessory gland  4.876184  
  Adult Brain  7.936046  
  Adult Carcass  6.165850  
  Adult Crop  5.055993  
  Adult Eye  7.232774  
  Adult Fatbody  5.008307  
  Adult Female Spermatheca Mated  4.703817  
  Adult Female Spermatheca Virgin  4.649485  
  Adult Head  6.723490  
  Adult Heart  5.245513  
  Adult Hind Gut  5.320432  
  Adult Male Ejaculatory Duct  4.958182  
  Adult Mid Gut  5.171689  
  Adult Ovary  5.615834  
  Adult Salivary Gland  5.268624  
  Adult Testes  7.637044  
  Adult Thoracoabdominal ganglion  7.461381  
  Adult Whole Fly  6.434289  
  Larvae Wandering Tubules  5.549620  
  Larval Feeding Carcass  6.376472  
  Larval Feeding Central Nevous System  6.423151  
  Larval Feeding Hind Gut  5.612233  
  Larval Feeding Malpighian Tubule  5.209131  
  Larval Feeding Mid Gut  5.075701  
  Larval Feeding Salivary Gland  5.205611  
  Whole Larvae Feeding  5.263418  
 
  
   FlyBase ID    symbol    start    end    strand    length   
   FBgn0027608   CG2082  1584992   1606798   -  21807  
   FBgn0037376   CG2051  1613059   1614796   -  1738  
 
    Segment 35 
 
   Location   
  Gene key  FBgn0037377-FBgn0037378  
  Heatmap region span   3R:1543022..1650327   
  Segment span   3R:1617608..1620053   
  Length (genes)  2  
  Length (bp)  2446  
   Model Scoring   
  BIC  262.007685  
  logL  -125.376112  
  logL ratio  4.547004  
   Expression   
  Mean expression  8.671241  
  Median expression  8.369411  
  Tissue std. dev.  0.627470  
 
  No GO Slim enrichment  
  
   tissue    mean expression   
  5th Passage Drosophila S2 Cells  8.969062  
  Adult Accessory gland  8.249660  
  Adult Brain  8.057463  
  Adult Carcass  9.014527  
  Adult Crop  8.711838  
  Adult Eye  7.786166  
  Adult Fatbody  9.441161  
  Adult Female Spermatheca Mated  7.797755  
  Adult Female Spermatheca Virgin  7.587489  
  Adult Head  7.963275  
  Adult Heart  9.832147  
  Adult Hind Gut  8.441921  
  Adult Male Ejaculatory Duct  9.542751  
  Adult Mid Gut  8.369568  
  Adult Ovary  9.923805  
  Adult Salivary Gland  7.822235  
  Adult Testes  8.969793  
  Adult Thoracoabdominal ganglion  7.912089  
  Adult Whole Fly  9.195562  
  Larvae Wandering Tubules  9.093360  
  Larval Feeding Carcass  8.914800  
  Larval Feeding Central Nevous System  9.174017  
  Larval Feeding Hind Gut  8.633843  
  Larval Feeding Malpighian Tubule  8.738674  
  Larval Feeding Mid Gut  8.661615  
  Larval Feeding Salivary Gland  8.853644  
  Whole Larvae Feeding  8.465293  
 
  
   FlyBase ID    symbol    start    end    strand    length   
   FBgn0037377   CG1218   1617608   1619105  +  1498  
   FBgn0037378   CG2046  1619030   1620053   -  1024  
 
    Segment 36 
 
   Location   
  Gene key  FBgn0037382-FBgn0037384  
  Heatmap region span   3R:1617608..1674675   
  Segment span   3R:1647149..1650327   
  Length (genes)  3  
  Length (bp)  3179  
   Model Scoring   
  BIC  292.552145  
  logL  -140.648341  
  logL ratio  85.322521  
   Expression   
  Mean expression  8.187982  
  Median expression  8.294780  
  Tissue std. dev.  0.485749  
 
  No GO Slim enrichment  
  
   tissue    mean expression   
  5th Passage Drosophila S2 Cells  8.729215  
  Adult Accessory gland  8.592448  
  Adult Brain  8.473703  
  Adult Carcass  7.593758  
  Adult Crop  7.982893  
  Adult Eye  8.017072  
  Adult Fatbody  7.996349  
  Adult Female Spermatheca Mated  8.019993  
  Adult Female Spermatheca Virgin  7.922098  
  Adult Head  7.778177  
  Adult Heart  8.112004  
  Adult Hind Gut  7.764891  
  Adult Male Ejaculatory Duct  7.976271  
  Adult Mid Gut  7.902712  
  Adult Ovary  9.614481  
  Adult Salivary Gland  8.466596  
  Adult Testes  9.063258  
  Adult Thoracoabdominal ganglion  8.392718  
  Adult Whole Fly  8.445715  
  Larvae Wandering Tubules  7.986180  
  Larval Feeding Carcass  7.779183  
  Larval Feeding Central Nevous System  8.483042  
  Larval Feeding Hind Gut  7.898700  
  Larval Feeding Malpighian Tubule  7.810924  
  Larval Feeding Mid Gut  7.576313  
  Larval Feeding Salivary Gland  9.004259  
  Whole Larvae Feeding  7.692573  
 
  
   FlyBase ID    symbol    start    end    strand    length   
   FBgn0037382   Hpr1  1644516   1647149   -  2634  
   FBgn0037383   CG2023  1649252   1650226   -  975  
   FBgn0037384   dgrn   1650327   1652298  +  1972  
 
 
    Segment 37 
 
   Location   
  Gene key  FBgn0037388-FBgn0037389  
  Heatmap region span   3R:1647149..1834301   
  Segment span   3R:1672942..1674675   
  Length (genes)  2  
  Length (bp)  1734  
   Model Scoring   
  BIC  194.746460  
  logL  -91.745499  
  logL ratio  21.194647  
   Expression   
  Mean expression  4.930736  
  Median expression  4.980401  
  Tissue std. dev.  0.666109  
 
  No GO Slim enrichment  
  
   tissue    mean expression   
  5th Passage Drosophila S2 Cells  4.840191  
  Adult Accessory gland  4.831641  
  Adult Brain  4.402577  
  Adult Carcass  4.785777  
  Adult Crop  4.942819  
  Adult Eye  4.476672  
  Adult Fatbody  4.892746  
  Adult Female Spermatheca Mated  4.928157  
  Adult Female Spermatheca Virgin  4.818519  
  Adult Head  4.531352  
  Adult Heart  4.655433  
  Adult Hind Gut  4.752892  
  Adult Male Ejaculatory Duct  5.046059  
  Adult Mid Gut  4.809156  
  Adult Ovary  4.794936  
  Adult Salivary Gland  5.320218  
  Adult Testes  8.125711  
  Adult Thoracoabdominal ganglion  4.628524  
  Adult Whole Fly  5.493861  
  Larvae Wandering Tubules  4.811464  
  Larval Feeding Carcass  4.853450  
  Larval Feeding Central Nevous System  4.567435  
  Larval Feeding Hind Gut  4.695171  
  Larval Feeding Malpighian Tubule  4.657170  
  Larval Feeding Mid Gut  4.655123  
  Larval Feeding Salivary Gland  4.891106  
  Whole Larvae Feeding  4.921724  
 
  
   FlyBase ID    symbol    start    end    strand    length   
   FBgn0037388   CG14676   1672942   1673899  +  958  
   FBgn0037389   CR10991   1674675   1675175  +  501  
 
    Segment 38 
 
   Location   
  Gene key  FBgn0029088-FBgn0037391  
  Heatmap region span   3R:1655784..1837654   
  Segment span   3R:1682033..1688133   
  Length (genes)  3  
  Length (bp)  6101  
   Model Scoring   
  BIC  347.940403  
  logL  -168.342470  
  logL ratio  38.744997  
   Expression   
  Mean expression  8.467718  
  Median expression  8.208258  
  Tissue std. dev.  0.442697  
 
  No GO Slim enrichment  
  
   tissue    mean expression   
  5th Passage Drosophila S2 Cells  8.982836  
  Adult Accessory gland  8.373072  
  Adult Brain  9.549509  
  Adult Carcass  8.482375  
  Adult Crop  8.631096  
  Adult Eye  8.678377  
  Adult Fatbody  8.145253  
  Adult Female Spermatheca Mated  8.024606  
  Adult Female Spermatheca Virgin  8.215222  
  Adult Head  8.377019  
  Adult Heart  8.734702  
  Adult Hind Gut  8.560734  
  Adult Male Ejaculatory Duct  8.560969  
  Adult Mid Gut  7.966902  
  Adult Ovary  8.386879  
  Adult Salivary Gland  8.300463  
  Adult Testes  7.536330  
  Adult Thoracoabdominal ganglion  9.552010  
  Adult Whole Fly  7.907097  
  Larvae Wandering Tubules  8.699496  
  Larval Feeding Carcass  8.479527  
  Larval Feeding Central Nevous System  8.612859  
  Larval Feeding Hind Gut  8.825710  
  Larval Feeding Malpighian Tubule  8.694601  
  Larval Feeding Mid Gut  8.292467  
  Larval Feeding Salivary Gland  7.998291  
  Whole Larvae Feeding  8.059980  
 
  
   FlyBase ID    symbol    start    end    strand    length   
   FBgn0029088   disp  1675416   1682033   -  6618  
   FBgn0028436   ECSIT   1682307   1683636  +  1330  
   FBgn0037391   CG2017  1683799   1688133   -  4335  
 
 
    Segment 39 
 
   Location   
  Gene key  FBgn0083949-FBgn0040679  
  Heatmap region span   3R:1657350..1852962   
  Segment span   3R:1765245..1786707   
  Length (genes)  2  
  Length (bp)  21463  
   Model Scoring   
  BIC  212.822440  
  logL  -100.783489  
  logL ratio  -0.261846  
   Expression   
  Mean expression  4.786148  
  Median expression  4.540454  
  Tissue std. dev.  0.805915  
 
  No GO Slim enrichment  
  
   tissue    mean expression   
  5th Passage Drosophila S2 Cells  4.606839  
  Adult Accessory gland  4.511020  
  Adult Brain  4.399546  
  Adult Carcass  4.780613  
  Adult Crop  4.499009  
  Adult Eye  4.381853  
  Adult Fatbody  4.628777  
  Adult Female Spermatheca Mated  4.572239  
  Adult Female Spermatheca Virgin  4.510036  
  Adult Head  4.349612  
  Adult Heart  4.464094  
  Adult Hind Gut  4.438720  
  Adult Male Ejaculatory Duct  4.705754  
  Adult Mid Gut  4.571896  
  Adult Ovary  4.508088  
  Adult Salivary Gland  4.804336  
  Adult Testes  8.385671  
  Adult Thoracoabdominal ganglion  4.470762  
  Adult Whole Fly  6.346108  
  Larvae Wandering Tubules  4.555419  
  Larval Feeding Carcass  4.669648  
  Larval Feeding Central Nevous System  4.360629  
  Larval Feeding Hind Gut  4.432315  
  Larval Feeding Malpighian Tubule  4.514425  
  Larval Feeding Mid Gut  4.640468  
  Larval Feeding Salivary Gland  4.716357  
  Whole Larvae Feeding  5.401752  
 
  
   FlyBase ID    symbol    start    end    strand    length   
   FBgn0083949   CG34113  1688587   1765245   -  76659  
   FBgn0040679   CG11373  1786045   1786707   -  663  
 
    Segment 40 
 
   Location   
  Gene key  FBgn0037397-FBgn0037399  
  Heatmap region span   3R:1800967..2168724   
  Segment span   3R:1853489..1904005   
  Length (genes)  3  
  Length (bp)  50517  
   Model Scoring   
  BIC  247.106307  
  logL  -117.925423  
  logL ratio  60.133099  
   Expression   
  Mean expression  4.670929  
  Median expression  4.590304  
  Tissue std. dev.  0.410897  
 
  No GO Slim enrichment  
  
   tissue    mean expression   
  5th Passage Drosophila S2 Cells  4.596577  
  Adult Accessory gland  4.758441  
  Adult Brain  4.354363  
  Adult Carcass  4.787424  
  Adult Crop  4.479308  
  Adult Eye  4.299481  
  Adult Fatbody  4.798651  
  Adult Female Spermatheca Mated  5.006017  
  Adult Female Spermatheca Virgin  5.039532  
  Adult Head  4.292281  
  Adult Heart  4.386325  
  Adult Hind Gut  4.564276  
  Adult Male Ejaculatory Duct  4.684388  
  Adult Mid Gut  4.689101  
  Adult Ovary  4.402136  
  Adult Salivary Gland  5.243552  
  Adult Testes  6.416291  
  Adult Thoracoabdominal ganglion  4.538065  
  Adult Whole Fly  4.396989  
  Larvae Wandering Tubules  4.640152  
  Larval Feeding Carcass  4.537255  
  Larval Feeding Central Nevous System  4.352354  
  Larval Feeding Hind Gut  4.441204  
  Larval Feeding Malpighian Tubule  4.618702  
  Larval Feeding Mid Gut  4.569779  
  Larval Feeding Salivary Gland  4.720512  
  Whole Larvae Feeding  4.501923  
 
  
   FlyBase ID    symbol    start    end    strand    length   
   FBgn0037397   CR1075   1853489   1854480  +  992  
   FBgn0037398   CG15580  1880432   1883259   -  2828  
   FBgn0037399   Or83c  1902526   1904005   -  1480  
 
 
    Segment 41 
 
   Location   
  Gene key  FBgn0046878-FBgn0046876  
  Heatmap region span   3R:1837654..2174965   
  Segment span   3R:1933856..1936740   
  Length (genes)  2  
  Length (bp)  2885  
   Model Scoring   
  BIC  192.399222  
  logL  -90.571880  
  logL ratio  25.380919  
   Expression   
  Mean expression  5.685124  
  Median expression  4.979408  
  Tissue std. dev.  1.391282  
 
  No GO Slim enrichment  
  
   tissue    mean expression   
  5th Passage Drosophila S2 Cells  4.708990  
  Adult Accessory gland  4.753849  
  Adult Brain  4.931684  
  Adult Carcass  7.409574  
  Adult Crop  4.882181  
  Adult Eye  7.856447  
  Adult Fatbody  8.098347  
  Adult Female Spermatheca Mated  8.254319  
  Adult Female Spermatheca Virgin  7.994385  
  Adult Head  7.237762  
  Adult Heart  9.003946  
  Adult Hind Gut  4.806404  
  Adult Male Ejaculatory Duct  5.236211  
  Adult Mid Gut  4.850884  
  Adult Ovary  4.728692  
  Adult Salivary Gland  5.036898  
  Adult Testes  4.974708  
  Adult Thoracoabdominal ganglion  4.823005  
  Adult Whole Fly  5.026960  
  Larvae Wandering Tubules  5.001400  
  Larval Feeding Carcass  4.958454  
  Larval Feeding Central Nevous System  4.476097  
  Larval Feeding Hind Gut  4.893984  
  Larval Feeding Malpighian Tubule  4.895709  
  Larval Feeding Mid Gut  4.792305  
  Larval Feeding Salivary Gland  4.794892  
  Whole Larvae Feeding  5.070256  
 
  
   FlyBase ID    symbol    start    end    strand    length   
   FBgn0046878   Obp83cd   1933856   1935457  +  1602  
   FBgn0046876   Obp83ef  1935402   1936740   -  1339  
 
    Segment 42 
 
   Location   
  Gene key  FBgn0051559-FBgn0037432  
  Heatmap region span   3R:1853489..2206050   
  Segment span   3R:1982949..2168724   
  Length (genes)  31  
  Length (bp)  185776  
   Model Scoring   
  BIC  2067.549657  
  logL  -1028.147097  
  logL ratio  1068.439046  
   Expression   
  Mean expression  4.789414  
  Median expression  4.665769  
  Tissue std. dev.  0.142431  
 
  
   GO ID    description    ratio    P-value   
   GO:0003674   molecular_function  20/31  1.05e-07  
   GO:0008150   biological_process  19/31  1.12e-07  
   GO:0005575   cellular_component  2/31  0.00234  
 
  
   tissue    mean expression   
  5th Passage Drosophila S2 Cells  4.661544  
  Adult Accessory gland  4.937121  
  Adult Brain  4.538929  
  Adult Carcass  4.766936  
  Adult Crop  4.894337  
  Adult Eye  4.735542  
  Adult Fatbody  4.818006  
  Adult Female Spermatheca Mated  4.877579  
  Adult Female Spermatheca Virgin  4.861232  
  Adult Head  4.657143  
  Adult Heart  4.581613  
  Adult Hind Gut  4.776294  
  Adult Male Ejaculatory Duct  4.794861  
  Adult Mid Gut  4.805015  
  Adult Ovary  4.780840  
  Adult Salivary Gland  4.993459  
  Adult Testes  5.231021  
  Adult Thoracoabdominal ganglion  4.627193  
  Adult Whole Fly  4.776332  
  Larvae Wandering Tubules  4.751594  
  Larval Feeding Carcass  4.966101  
  Larval Feeding Central Nevous System  4.573834  
  Larval Feeding Hind Gut  4.836920  
  Larval Feeding Malpighian Tubule  4.771666  
  Larval Feeding Mid Gut  4.838003  
  Larval Feeding Salivary Gland  4.771424  
  Whole Larvae Feeding  4.689648  
 
  
   FlyBase ID    symbol    start    end    strand    length   
   FBgn0051559   CG31559  1975838   1982949   -  7112  
   FBgn0037405   CG1077   1993240   1996032  +  2793  
   FBgn0037406   Osi1   1999367   2000555  +  1189  
   FBgn0051562   CG31562   2010177   2011160  +  984  
   FBgn0037408   NPFR1   2013122   2021136  +  8015  
   FBgn0037409   Osi24   2024535   2033403  +  8869  
   FBgn0037410   Osi2   2036721   2039214  +  2494  
   FBgn0037411   Osi3   2041558   2043675  +  2118  
   FBgn0037412   Osi4  2044040   2047503   -  3464  
   FBgn0037413   Osi5   2053117   2054550  +  1434  
   FBgn0027527   Osi6   2060431   2062603  +  2173  
   FBgn0037414   Osi7   2074714   2077405  +  2692  
   FBgn0037415   Osi8   2080997   2082114  +  1118  
   FBgn0037416   Osi9   2085935   2087832  +  1898  
   FBgn0037417   Osi10   2089581   2093117  +  3537  
   FBgn0037418   Osi11  2093502   2094410   -  909  
   FBgn0037419   Osi12   2104253   2108217  +  3965  
   FBgn0037420   CG15597  2110181   2110922   -  742  
   FBgn0037421   CG15594   2113457   2116719  +  3263  
   FBgn0037422   Osi13   2117624   2118491  +  868  
   FBgn0040279   Osi14   2124854   2126472  +  1619  
   FBgn0037424   Osi15   2127528   2129375  +  1848  
   FBgn0051561   Osi16   2130992   2131887  +  896  
   FBgn0051556   CG31556  2132967   2135018   -  2052  
   FBgn0051560   CG31560   2135718   2136612  +  895  
   FBgn0037427   Osi17   2141020   2153033  +  12014  
   FBgn0037428   Osi18   2155972   2157536  +  1565  
   FBgn0037429   Osi19   2161086   2162918  +  1833  
   FBgn0037430   Osi20   2165819   2167146  +  1328  
   FBgn0037431   CG17917   2167690   2168595  +  906  
   FBgn0037432   CG10298   2168724   2169521  +  798  
 
 
    Segment 43 
 
   Location   
  Gene key  FBgn0037435-FBgn0037436  
  Heatmap region span   3R:1933856..2282165   
  Segment span   3R:2171838..2174965   
  Length (genes)  2  
  Length (bp)  3128  
   Model Scoring   
  BIC  213.755821  
  logL  -101.250180  
  logL ratio  5.579466  
   Expression   
  Mean expression  5.303455  
  Median expression  4.848985  
  Tissue std. dev.  0.363487  
 
  No GO Slim enrichment  
  
   tissue    mean expression   
  5th Passage Drosophila S2 Cells  5.604318  
  Adult Accessory gland  5.729560  
  Adult Brain  5.662242  
  Adult Carcass  4.710707  
  Adult Crop  5.222848  
  Adult Eye  5.468328  
  Adult Fatbody  4.622803  
  Adult Female Spermatheca Mated  5.100835  
  Adult Female Spermatheca Virgin  4.987037  
  Adult Head  5.106806  
  Adult Heart  5.143439  
  Adult Hind Gut  5.422638  
  Adult Male Ejaculatory Duct  5.413295  
  Adult Mid Gut  5.191289  
  Adult Ovary  6.375601  
  Adult Salivary Gland  5.618201  
  Adult Testes  4.754084  
  Adult Thoracoabdominal ganglion  5.407437  
  Adult Whole Fly  5.274983  
  Larvae Wandering Tubules  5.180020  
  Larval Feeding Carcass  4.985299  
  Larval Feeding Central Nevous System  5.565527  
  Larval Feeding Hind Gut  5.568467  
  Larval Feeding Malpighian Tubule  5.514032  
  Larval Feeding Mid Gut  5.113741  
  Larval Feeding Salivary Gland  5.511086  
  Whole Larvae Feeding  4.938674  
 
  
   FlyBase ID    symbol    start    end    strand    length   
   FBgn0037435   CG18048   2171838   2173247  +  1410  
   FBgn0037436   Hr83   2174965   2176234  +  1270  
 
    Segment 44 
 
   Location   
  Gene key  FBgn0014001-FBgn0037440  
  Heatmap region span   3R:1938019..2282850   
  Segment span   3R:2176620..2199747   
  Length (genes)  3  
  Length (bp)  23128  
   Model Scoring   
  BIC  316.616290  
  logL  -152.680414  
  logL ratio  74.869671  
   Expression   
  Mean expression  8.796601  
  Median expression  8.611032  
  Tissue std. dev.  0.659093  
 
  No GO Slim enrichment  
  
   tissue    mean expression   
  5th Passage Drosophila S2 Cells  9.175152  
  Adult Accessory gland  9.577071  
  Adult Brain  7.484694  
  Adult Carcass  8.212719  
  Adult Crop  8.711603  
  Adult Eye  7.926274  
  Adult Fatbody  8.780270  
  Adult Female Spermatheca Mated  9.263674  
  Adult Female Spermatheca Virgin  9.173767  
  Adult Head  8.247018  
  Adult Heart  8.674301  
  Adult Hind Gut  8.704823  
  Adult Male Ejaculatory Duct  8.836364  
  Adult Mid Gut  9.345125  
  Adult Ovary  9.088133  
  Adult Salivary Gland  9.195107  
  Adult Testes  7.007972  
  Adult Thoracoabdominal ganglion  7.790267  
  Adult Whole Fly  8.336218  
  Larvae Wandering Tubules  8.699588  
  Larval Feeding Carcass  9.414039  
  Larval Feeding Central Nevous System  8.898283  
  Larval Feeding Hind Gut  9.369101  
  Larval Feeding Malpighian Tubule  9.052606  
  Larval Feeding Mid Gut  9.696741  
  Larval Feeding Salivary Gland  9.650192  
  Whole Larvae Feeding  9.197114  
 
  
   FlyBase ID    symbol    start    end    strand    length   
   FBgn0014001   Pak   2176620   2185974  +  9355  
   FBgn0037439   CG10286   2194676   2196837  +  2162  
   FBgn0037440   CG1041  2196748   2199747   -  3000  
 
 
    Segment 45 
 
   Location   
  Gene key  FBgn0010282-FBgn0037443  
  Heatmap region span   3R:2169753..2308518   
  Segment span   3R:2206181..2231613   
  Length (genes)  3  
  Length (bp)  25433  
   Model Scoring   
  BIC  300.874750  
  logL  -144.809644  
  logL ratio  99.396468  
   Expression   
  Mean expression  9.402566  
  Median expression  9.379879  
  Tissue std. dev.  0.388312  
 
  No GO Slim enrichment  
  
   tissue    mean expression   
  5th Passage Drosophila S2 Cells  9.285338  
  Adult Accessory gland  9.498056  
  Adult Brain  9.904969  
  Adult Carcass  9.330504  
  Adult Crop  9.456420  
  Adult Eye  9.267650  
  Adult Fatbody  10.160765  
  Adult Female Spermatheca Mated  9.845662  
  Adult Female Spermatheca Virgin  9.911515  
  Adult Head  9.461414  
  Adult Heart  9.622239  
  Adult Hind Gut  8.934414  
  Adult Male Ejaculatory Duct  9.316708  
  Adult Mid Gut  9.079014  
  Adult Ovary  9.775120  
  Adult Salivary Gland  9.250334  
  Adult Testes  8.342073  
  Adult Thoracoabdominal ganglion  9.523507  
  Adult Whole Fly  8.987729  
  Larvae Wandering Tubules  9.834376  
  Larval Feeding Carcass  9.188002  
  Larval Feeding Central Nevous System  9.761607  
  Larval Feeding Hind Gut  9.484443  
  Larval Feeding Malpighian Tubule  9.479631  
  Larval Feeding Mid Gut  9.259840  
  Larval Feeding Salivary Gland  9.161643  
  Whole Larvae Feeding  8.746303  
 
  
   FlyBase ID    symbol    start    end    strand    length   
   FBgn0010282   TfIIFalpha   2206181   2208326  +  2146  
   FBgn0037442   CG10277   2208633   2214026  +  5394  
   FBgn0037443   CG1021  2214884   2231613   -  16730  
 
 
    Segment 46 
 
   Location   
  Gene key  FBgn0037449-FBgn0051286  
  Heatmap region span   3R:2282165..2512450   
  Segment span   3R:2353251..2466954   
  Length (genes)  12  
  Length (bp)  113704  
   Model Scoring   
  BIC  894.137326  
  logL  -441.440932  
  logL ratio  387.702084  
   Expression   
  Mean expression  4.711807  
  Median expression  4.314763  
  Tissue std. dev.  1.232005  
 
  No GO Slim enrichment  
  
   tissue    mean expression   
  5th Passage Drosophila S2 Cells  4.293957  
  Adult Accessory gland  4.481973  
  Adult Brain  4.462112  
  Adult Carcass  4.469303  
  Adult Crop  4.305100  
  Adult Eye  4.170866  
  Adult Fatbody  4.457732  
  Adult Female Spermatheca Mated  4.470267  
  Adult Female Spermatheca Virgin  4.496211  
  Adult Head  4.253336  
  Adult Heart  4.248276  
  Adult Hind Gut  4.295148  
  Adult Male Ejaculatory Duct  4.354458  
  Adult Mid Gut  4.370581  
  Adult Ovary  4.248223  
  Adult Salivary Gland  4.650371  
  Adult Testes  10.562086  
  Adult Thoracoabdominal ganglion  4.518659  
  Adult Whole Fly  6.600391  
  Larvae Wandering Tubules  4.407593  
  Larval Feeding Carcass  4.292610  
  Larval Feeding Central Nevous System  4.393203  
  Larval Feeding Hind Gut  4.214692  
  Larval Feeding Malpighian Tubule  4.365202  
  Larval Feeding Mid Gut  4.362561  
  Larval Feeding Salivary Gland  4.380544  
  Whole Larvae Feeding  5.093332  
 
  
   FlyBase ID    symbol    start    end    strand    length   
   FBgn0037449   CG15185   2353251   2354279  +  1029  
   FBgn0046873   Pif2   2374758   2375705  +  948  
   FBgn0037454   CG1137  2384337   2386073   -  1737  
   FBgn0037455   CG2336   2406506   2407993  +  1488  
   FBgn0053202   dpr11  2356021   2419052   -  63032  
   FBgn0037456   CG1138  2422786   2423744   -  959  
   FBgn0051482   CG31482   2427032   2427855  +  824  
   FBgn0250846   glob2  2433635   2434636   -  1002  
   FBgn0037460   sowi  2452652   2453622   -  971  
   FBgn0037461   CG15177  2453922   2455043   -  1122  
   FBgn0037462   sunz   2455892   2456907  +  1016  
   FBgn0051286   CG31286   2466954   2469068  +  2115  
 
 
    Segment 47 
 
   Location   
  Gene key  FBgn0010355-FBgn0026566  
  Heatmap region span   3R:2294134..2585199   
  Segment span   3R:2472611..2483208   
  Length (genes)  2  
  Length (bp)  10598  
   Model Scoring   
  BIC  231.000930  
  logL  -109.872734  
  logL ratio  39.181380  
   Expression   
  Mean expression  9.371747  
  Median expression  9.384849  
  Tissue std. dev.  0.372933  
 
  No GO Slim enrichment  
  
   tissue    mean expression   
  5th Passage Drosophila S2 Cells  9.471779  
  Adult Accessory gland  9.874783  
  Adult Brain  9.509484  
  Adult Carcass  8.954897  
  Adult Crop  9.383577  
  Adult Eye  9.579026  
  Adult Fatbody  9.414112  
  Adult Female Spermatheca Mated  9.558947  
  Adult Female Spermatheca Virgin  9.573473  
  Adult Head  9.122075  
  Adult Heart  9.487955  
  Adult Hind Gut  9.009624  
  Adult Male Ejaculatory Duct  9.619799  
  Adult Mid Gut  8.822678  
  Adult Ovary  10.230512  
  Adult Salivary Gland  9.610274  
  Adult Testes  9.118582  
  Adult Thoracoabdominal ganglion  9.482010  
  Adult Whole Fly  9.313552  
  Larvae Wandering Tubules  8.986154  
  Larval Feeding Carcass  9.198922  
  Larval Feeding Central Nevous System  10.211676  
  Larval Feeding Hind Gut  9.125924  
  Larval Feeding Malpighian Tubule  8.974214  
  Larval Feeding Mid Gut  8.858348  
  Larval Feeding Salivary Gland  9.690071  
  Whole Larvae Feeding  8.854729  
 
  
   FlyBase ID    symbol    start    end    strand    length   
   FBgn0010355   Taf1   2472611   2481770  +  9160  
   FBgn0026566   CG1307  2482012   2483208   -  1197  
 
    Segment 48 
 
   Location   
  Gene key  FBgn0002522-FBgn0004777  
  Heatmap region span   3R:2353251..2690046   
  Segment span   3R:2504312..2512450   
  Length (genes)  3  
  Length (bp)  8139  
   Model Scoring   
  BIC  258.677271  
  logL  -123.710905  
  logL ratio  60.302545  
   Expression   
  Mean expression  4.473880  
  Median expression  4.308224  
  Tissue std. dev.  0.368732  
 
  No GO Slim enrichment  
  
   tissue    mean expression   
  5th Passage Drosophila S2 Cells  4.362907  
  Adult Accessory gland  4.440276  
  Adult Brain  4.425959  
  Adult Carcass  4.404070  
  Adult Crop  4.336828  
  Adult Eye  4.669398  
  Adult Fatbody  4.464364  
  Adult Female Spermatheca Mated  4.318116  
  Adult Female Spermatheca Virgin  4.341203  
  Adult Head  4.495607  
  Adult Heart  4.243378  
  Adult Hind Gut  4.185601  
  Adult Male Ejaculatory Duct  4.351492  
  Adult Mid Gut  5.416079  
  Adult Ovary  4.199176  
  Adult Salivary Gland  4.472079  
  Adult Testes  4.370983  
  Adult Thoracoabdominal ganglion  4.237646  
  Adult Whole Fly  4.122576  
  Larvae Wandering Tubules  4.272767  
  Larval Feeding Carcass  4.324888  
  Larval Feeding Central Nevous System  4.628467  
  Larval Feeding Hind Gut  4.236932  
  Larval Feeding Malpighian Tubule  4.312643  
  Larval Feeding Mid Gut  5.837232  
  Larval Feeding Salivary Gland  4.389555  
  Whole Larvae Feeding  4.934545  
 
  
   FlyBase ID    symbol    start    end    strand    length   
   FBgn0002522   lab  2487140   2504312   -  17173  
   FBgn0000552   Edg84A   2510172   2510855  +  684  
   FBgn0004777   Ccp84Ag   2512450   2513761  +  1312  
 
 
    Segment 49 
 
   Location   
  Gene key  FBgn0004778-FBgn0004781  
  Heatmap region span   3R:2470156..2887897   
  Segment span   3R:2515961..2521993   
  Length (genes)  3  
  Length (bp)  6033  
   Model Scoring   
  BIC  274.054222  
  logL  -131.399380  
  logL ratio  82.537065  
   Expression   
  Mean expression  6.358687  
  Median expression  6.419435  
  Tissue std. dev.  0.360141  
 
  No GO Slim enrichment  
  
   tissue    mean expression   
  5th Passage Drosophila S2 Cells  6.512394  
  Adult Accessory gland  6.753815  
  Adult Brain  5.875529  
  Adult Carcass  6.628596  
  Adult Crop  6.337171  
  Adult Eye  5.542229  
  Adult Fatbody  6.680621  
  Adult Female Spermatheca Mated  6.371734  
  Adult Female Spermatheca Virgin  6.423160  
  Adult Head  6.056811  
  Adult Heart  5.911708  
  Adult Hind Gut  6.404251  
  Adult Male Ejaculatory Duct  6.467599  
  Adult Mid Gut  6.882125  
  Adult Ovary  6.207290  
  Adult Salivary Gland  6.964129  
  Adult Testes  5.908137  
  Adult Thoracoabdominal ganglion  6.067977  
  Adult Whole Fly  5.979494  
  Larvae Wandering Tubules  6.509300  
  Larval Feeding Carcass  6.741221  
  Larval Feeding Central Nevous System  5.846916  
  Larval Feeding Hind Gut  6.269696  
  Larval Feeding Malpighian Tubule  6.401512  
  Larval Feeding Mid Gut  6.950151  
  Larval Feeding Salivary Gland  6.577026  
  Whole Larvae Feeding  6.413957  
 
  
   FlyBase ID    symbol    start    end    strand    length   
   FBgn0004778   Ccp84Af  2515324   2515961   -  638  
   FBgn0004779   Ccp84Ae  2516609   2517480   -  872  
   FBgn0004781   Ccp84Ac  2520870   2521993   -  1124  
 
 
    Segment 50 
 
   Location   
  Gene key  FBgn0051481-FBgn0000166  
  Heatmap region span   3R:2472611..2911811   
  Segment span   3R:2567785..2585199   
  Length (genes)  5  
  Length (bp)  17415  
   Model Scoring   
  BIC  391.680197  
  logL  -190.212367  
  logL ratio  139.437921  
   Expression   
  Mean expression  4.398312  
  Median expression  4.213276  
  Tissue std. dev.  0.244138  
 
  
   GO ID    description    ratio    P-value   
   GO:0005634   nucleus  4/5  0.000721  
   GO:0043226   organelle  4/5  0.00345  
 
  
   tissue    mean expression   
  5th Passage Drosophila S2 Cells  4.287337  
  Adult Accessory gland  4.403014  
  Adult Brain  4.197599  
  Adult Carcass  4.450562  
  Adult Crop  4.332592  
  Adult Eye  4.224067  
  Adult Fatbody  4.489004  
  Adult Female Spermatheca Mated  4.385121  
  Adult Female Spermatheca Virgin  4.374347  
  Adult Head  4.277039  
  Adult Heart  4.275066  
  Adult Hind Gut  4.286583  
  Adult Male Ejaculatory Duct  4.401774  
  Adult Mid Gut  4.438182  
  Adult Ovary  5.532848  
  Adult Salivary Gland  4.557174  
  Adult Testes  4.321566  
  Adult Thoracoabdominal ganglion  4.431821  
  Adult Whole Fly  4.576487  
  Larvae Wandering Tubules  4.358223  
  Larval Feeding Carcass  4.406338  
  Larval Feeding Central Nevous System  4.252160  
  Larval Feeding Hind Gut  4.201303  
  Larval Feeding Malpighian Tubule  4.310381  
  Larval Feeding Mid Gut  4.389220  
  Larval Feeding Salivary Gland  4.409269  
  Whole Larvae Feeding  4.185348  
 
  
   FlyBase ID    symbol    start    end    strand    length   
   FBgn0051481   pb  2533518   2567785   -  34268  
   FBgn0004054   zen2  2568854   2569862   -  1009  
   FBgn0085326   CG34297  2572814   2573685   -  872  
   FBgn0004053   zen  2578586   2582059   -  3474  
   FBgn0000166   bcd  2581564   2585199   -  3636  
 
 
    Segment 51 
 
   Location   
  Gene key  FBgn0000439-FBgn0001077  
  Heatmap region span   3R:2504312..2943453   
  Segment span   3R:2617558..2690046   
  Length (genes)  3  
  Length (bp)  72489  
   Model Scoring   
  BIC  258.191175  
  logL  -123.467856  
  logL ratio  60.845243  
   Expression   
  Mean expression  4.660801  
  Median expression  4.492292  
  Tissue std. dev.  0.502057  
 
  
   GO ID    description    ratio    P-value   
   GO:0005634   nucleus  3/3  0.00368  
   GO:0043226   organelle  3/3  0.0122  
 
  
   tissue    mean expression   
  5th Passage Drosophila S2 Cells  4.377629  
  Adult Accessory gland  4.381414  
  Adult Brain  5.330337  
  Adult Carcass  4.919252  
  Adult Crop  4.359332  
  Adult Eye  4.395542  
  Adult Fatbody  4.733813  
  Adult Female Spermatheca Mated  4.597033  
  Adult Female Spermatheca Virgin  4.543672  
  Adult Head  6.479161  
  Adult Heart  4.426163  
  Adult Hind Gut  4.288234  
  Adult Male Ejaculatory Duct  4.347865  
  Adult Mid Gut  4.292356  
  Adult Ovary  4.364334  
  Adult Salivary Gland  5.017069  
  Adult Testes  4.969130  
  Adult Thoracoabdominal ganglion  4.410040  
  Adult Whole Fly  4.430211  
  Larvae Wandering Tubules  4.339475  
  Larval Feeding Carcass  4.522640  
  Larval Feeding Central Nevous System  5.736435  
  Larval Feeding Hind Gut  4.293381  
  Larval Feeding Malpighian Tubule  4.382151  
  Larval Feeding Mid Gut  4.365912  
  Larval Feeding Salivary Gland  5.074072  
  Whole Larvae Feeding  4.464972  
 
  
   FlyBase ID    symbol    start    end    strand    length   
   FBgn0000439   Dfd   2617558   2628153  +  10596  
   FBgn0003339   Scr  2648842   2675703   -  26862  
   FBgn0001077   ftz   2690046   2691950  +  1905  
 
 
    Segment 52 
 
   Location   
  Gene key  FBgn0026563-FBgn0037464  
  Heatmap region span   3R:2515961..2950272   
  Segment span   3R:2880308..2887897   
  Length (genes)  4  
  Length (bp)  7590  
   Model Scoring   
  BIC  377.864233  
  logL  -183.304386  
  logL ratio  68.900613  
   Expression   
  Mean expression  5.038845  
  Median expression  4.490689  
  Tissue std. dev.  1.490157  
 
  No GO Slim enrichment  
  
   tissue    mean expression   
  5th Passage Drosophila S2 Cells  4.221860  
  Adult Accessory gland  4.508481  
  Adult Brain  4.346902  
  Adult Carcass  4.967246  
  Adult Crop  4.505356  
  Adult Eye  4.420957  
  Adult Fatbody  5.663587  
  Adult Female Spermatheca Mated  5.764386  
  Adult Female Spermatheca Virgin  6.107769  
  Adult Head  4.476774  
  Adult Heart  5.239390  
  Adult Hind Gut  4.710812  
  Adult Male Ejaculatory Duct  4.464947  
  Adult Mid Gut  4.334194  
  Adult Ovary  4.191962  
  Adult Salivary Gland  4.654779  
  Adult Testes  11.699418  
  Adult Thoracoabdominal ganglion  4.351224  
  Adult Whole Fly  7.485015  
  Larvae Wandering Tubules  4.513944  
  Larval Feeding Carcass  4.277360  
  Larval Feeding Central Nevous System  4.353749  
  Larval Feeding Hind Gut  4.489280  
  Larval Feeding Malpighian Tubule  4.391714  
  Larval Feeding Mid Gut  4.367479  
  Larval Feeding Salivary Gland  4.468339  
  Whole Larvae Feeding  5.071902  
 
  
   FlyBase ID    symbol    start    end    strand    length   
   FBgn0026563   CG1979   2880308   2884393  +  4086  
   FBgn0019828   dj   2885037   2886238  +  1202  
   FBgn0037463   djl   2886489   2887641  +  1153  
   FBgn0037464   CG1988   2887897   2890092  +  2196  
 
 
    Segment 53 
 
   Location   
  Gene key  FBgn0037465-FBgn0037470  
  Heatmap region span   3R:2567785..2953751   
  Segment span   3R:2892854..2911811   
  Length (genes)  7  
  Length (bp)  18958  
   Model Scoring   
  BIC  751.587798  
  logL  -370.166168  
  logL ratio  191.618354  
   Expression   
  Mean expression  9.315318  
  Median expression  9.149519  
  Tissue std. dev.  0.436601  
 
  No GO Slim enrichment  
  
   tissue    mean expression   
  5th Passage Drosophila S2 Cells  10.068006  
  Adult Accessory gland  9.425205  
  Adult Brain  9.650445  
  Adult Carcass  8.517270  
  Adult Crop  9.495916  
  Adult Eye  9.442370  
  Adult Fatbody  8.576673  
  Adult Female Spermatheca Mated  8.600104  
  Adult Female Spermatheca Virgin  8.692538  
  Adult Head  9.028149  
  Adult Heart  9.105852  
  Adult Hind Gut  9.544294  
  Adult Male Ejaculatory Duct  9.455472  
  Adult Mid Gut  8.908372  
  Adult Ovary  9.913889  
  Adult Salivary Gland  9.330094  
  Adult Testes  9.164795  
  Adult Thoracoabdominal ganglion  9.442896  
  Adult Whole Fly  9.116130  
  Larvae Wandering Tubules  9.898344  
  Larval Feeding Carcass  9.300104  
  Larval Feeding Central Nevous System  9.880474  
  Larval Feeding Hind Gut  9.485276  
  Larval Feeding Malpighian Tubule  9.570943  
  Larval Feeding Mid Gut  8.818152  
  Larval Feeding Salivary Gland  9.999891  
  Whole Larvae Feeding  9.081922  
 
  
   FlyBase ID    symbol    start    end    strand    length   
   FBgn0037465   CG1105  2889908   2892854   -  2947  
   FBgn0037466   CG1965   2893134   2897346  +  4213  
   FBgn0037467   CG1104  2897343   2900172   -  2830  
   FBgn0037468   CG1943   2900559   2903509  +  2951  
   FBgn0010774   Ref1  2903618   2906126   -  2509  
   FBgn0037469   Dpck   2907911   2908973  +  1063  
   FBgn0037470   CG1091  2908945   2911811   -  2867  
 
 
    Segment 54 
 
   Location   
  Gene key  FBgn0051493-FBgn0037477  
  Heatmap region span   3R:2943453..3042805   
  Segment span   3R:2957282..2969978   
  Length (genes)  3  
  Length (bp)  12697  
   Model Scoring   
  BIC  277.849116  
  logL  -133.296827  
  logL ratio  34.537280  
   Expression   
  Mean expression  4.943295  
  Median expression  4.658746  
  Tissue std. dev.  0.696921  
 
  No GO Slim enrichment  
  
   tissue    mean expression   
  5th Passage Drosophila S2 Cells  4.706287  
  Adult Accessory gland  8.138074  
  Adult Brain  5.778510  
  Adult Carcass  4.836503  
  Adult Crop  4.613748  
  Adult Eye  4.608414  
  Adult Fatbody  4.704374  
  Adult Female Spermatheca Mated  4.595883  
  Adult Female Spermatheca Virgin  4.624140  
  Adult Head  5.289625  
  Adult Heart  4.649073  
  Adult Hind Gut  4.592537  
  Adult Male Ejaculatory Duct  5.539323  
  Adult Mid Gut  4.848354  
  Adult Ovary  4.617413  
  Adult Salivary Gland  4.843344  
  Adult Testes  4.894940  
  Adult Thoracoabdominal ganglion  4.741021  
  Adult Whole Fly  4.721700  
  Larvae Wandering Tubules  4.717034  
  Larval Feeding Carcass  4.730016  
  Larval Feeding Central Nevous System  5.384362  
  Larval Feeding Hind Gut  4.577326  
  Larval Feeding Malpighian Tubule  4.738489  
  Larval Feeding Mid Gut  4.769725  
  Larval Feeding Salivary Gland  4.683068  
  Whole Larvae Feeding  4.525679  
 
  
   FlyBase ID    symbol    start    end    strand    length   
   FBgn0051493   CG31493   2957282   2958417  +  1136  
   FBgn0037475   Fer1   2961930   2967694  +  5765  
   FBgn0037477   CG14610  2969481   2969978   -  498  
 
 
    Segment 55 
 
   Location   
  Gene key  FBgn0037478-FBgn0037482  
  Heatmap region span   3R:2950272..3067482   
  Segment span   3R:2970465..3012375   
  Length (genes)  5  
  Length (bp)  41911  
   Model Scoring   
  BIC  541.107432  
  logL  -264.925985  
  logL ratio  69.393729  
   Expression   
  Mean expression  7.817402  
  Median expression  8.035767  
  Tissue std. dev.  0.499194  
 
  No GO Slim enrichment  
  
   tissue    mean expression   
  5th Passage Drosophila S2 Cells  7.792906  
  Adult Accessory gland  8.400108  
  Adult Brain  7.239730  
  Adult Carcass  7.732076  
  Adult Crop  8.533969  
  Adult Eye  8.020447  
  Adult Fatbody  7.428751  
  Adult Female Spermatheca Mated  7.980059  
  Adult Female Spermatheca Virgin  7.938773  
  Adult Head  7.880743  
  Adult Heart  7.674950  
  Adult Hind Gut  8.093774  
  Adult Male Ejaculatory Duct  8.434996  
  Adult Mid Gut  6.942574  
  Adult Ovary  8.359023  
  Adult Salivary Gland  7.881348  
  Adult Testes  8.502924  
  Adult Thoracoabdominal ganglion  7.061491  
  Adult Whole Fly  7.728794  
  Larvae Wandering Tubules  7.168812  
  Larval Feeding Carcass  8.402752  
  Larval Feeding Central Nevous System  8.231294  
  Larval Feeding Hind Gut  8.260208  
  Larval Feeding Malpighian Tubule  7.284132  
  Larval Feeding Mid Gut  6.694036  
  Larval Feeding Salivary Gland  7.730734  
  Whole Larvae Feeding  7.670449  
 
  
   FlyBase ID    symbol    start    end    strand    length   
   FBgn0037478   CG2656   2970465   2977001  +  6537  
   FBgn0250732   gfzf  2971596   2976661   -  5066  
   FBgn0037481   MAGE  2979960   2980898   -  939  
   FBgn0002306   sas   2988551   3009424  +  20874  
   FBgn0037482   CG10055  3009823   3012375   -  2553  
 
 
    Segment 56 
 
   Location   
  Gene key  FBgn0037483-FBgn0250845  
  Heatmap region span   3R:2955042..3077679   
  Segment span   3R:3026692..3034137   
  Length (genes)  2  
  Length (bp)  7446  
   Model Scoring   
  BIC  197.633275  
  logL  -93.188906  
  logL ratio  24.399633  
   Expression   
  Mean expression  5.648865  
  Median expression  5.188244  
  Tissue std. dev.  1.525455  
 
  No GO Slim enrichment  
  
   tissue    mean expression   
  5th Passage Drosophila S2 Cells  4.943475  
  Adult Accessory gland  5.111551  
  Adult Brain  5.186364  
  Adult Carcass  5.652568  
  Adult Crop  5.037711  
  Adult Eye  4.669596  
  Adult Fatbody  5.457525  
  Adult Female Spermatheca Mated  5.770452  
  Adult Female Spermatheca Virgin  5.641960  
  Adult Head  4.601734  
  Adult Heart  4.780259  
  Adult Hind Gut  5.050762  
  Adult Male Ejaculatory Duct  5.214519  
  Adult Mid Gut  5.086684  
  Adult Ovary  4.989454  
  Adult Salivary Gland  5.529889  
  Adult Testes  12.502498  
  Adult Thoracoabdominal ganglion  5.261954  
  Adult Whole Fly  8.458761  
  Larvae Wandering Tubules  5.217202  
  Larval Feeding Carcass  5.257257  
  Larval Feeding Central Nevous System  5.607937  
  Larval Feeding Hind Gut  5.126034  
  Larval Feeding Malpighian Tubule  5.170619  
  Larval Feeding Mid Gut  5.122396  
  Larval Feeding Salivary Gland  5.384573  
  Whole Larvae Feeding  6.685619  
 
  
   FlyBase ID    symbol    start    end    strand    length   
   FBgn0037483   CG14609   3026692   3029084  +  2393  
   FBgn0250845   CG1288  3033095   3034137   -  1043  
 
    Segment 57 
 
   Location   
  Gene key  FBgn0037486-FBgn0037488  
  Heatmap region span   3R:2970465..3091783   
  Segment span   3R:3045427..3067482   
  Length (genes)  3  
  Length (bp)  22056  
   Model Scoring   
  BIC  322.417469  
  logL  -155.581004  
  logL ratio  14.955021  
   Expression   
  Mean expression  4.837345  
  Median expression  4.706340  
  Tissue std. dev.  0.438963  
 
  No GO Slim enrichment  
  
   tissue    mean expression   
  5th Passage Drosophila S2 Cells  4.731283  
  Adult Accessory gland  4.767931  
  Adult Brain  4.462499  
  Adult Carcass  5.705677  
  Adult Crop  4.798906  
  Adult Eye  4.472899  
  Adult Fatbody  4.886820  
  Adult Female Spermatheca Mated  4.792861  
  Adult Female Spermatheca Virgin  4.757932  
  Adult Head  4.569869  
  Adult Heart  4.739201  
  Adult Hind Gut  4.644037  
  Adult Male Ejaculatory Duct  4.699317  
  Adult Mid Gut  4.866371  
  Adult Ovary  4.667435  
  Adult Salivary Gland  4.838184  
  Adult Testes  6.693177  
  Adult Thoracoabdominal ganglion  4.434902  
  Adult Whole Fly  5.197487  
  Larvae Wandering Tubules  4.711501  
  Larval Feeding Carcass  4.908295  
  Larval Feeding Central Nevous System  4.448651  
  Larval Feeding Hind Gut  4.664267  
  Larval Feeding Malpighian Tubule  4.726178  
  Larval Feeding Mid Gut  4.936723  
  Larval Feeding Salivary Gland  4.711824  
  Whole Larvae Feeding  4.774088  
 
  
   FlyBase ID    symbol    start    end    strand    length   
   FBgn0037486   CG14605  3043171   3045427   -  2257  
   FBgn0037487   CG14608   3050810   3066860  +  16051  
   FBgn0037488   CG14607   3067482   3069728  +  2247  
 
 
    Segment 58 
 
   Location   
  Gene key  FBgn0037489-FBgn0037491  
  Heatmap region span   3R:3012719..3135119   
  Segment span   3R:3072717..3075457   
  Length (genes)  3  
  Length (bp)  2741  
   Model Scoring   
  BIC  303.582408  
  logL  -146.163473  
  logL ratio  71.370569  
   Expression   
  Mean expression  7.816639  
  Median expression  7.822231  
  Tissue std. dev.  0.457113  
 
  No GO Slim enrichment  
  
   tissue    mean expression   
  5th Passage Drosophila S2 Cells  7.825793  
  Adult Accessory gland  8.090280  
  Adult Brain  7.471778  
  Adult Carcass  7.065328  
  Adult Crop  7.945911  
  Adult Eye  7.452866  
  Adult Fatbody  7.696841  
  Adult Female Spermatheca Mated  8.101714  
  Adult Female Spermatheca Virgin  7.978948  
  Adult Head  7.358955  
  Adult Heart  7.707635  
  Adult Hind Gut  7.630281  
  Adult Male Ejaculatory Duct  7.642466  
  Adult Mid Gut  7.740002  
  Adult Ovary  9.016275  
  Adult Salivary Gland  8.336299  
  Adult Testes  8.038298  
  Adult Thoracoabdominal ganglion  7.388930  
  Adult Whole Fly  7.882157  
  Larvae Wandering Tubules  8.034285  
  Larval Feeding Carcass  7.411501  
  Larval Feeding Central Nevous System  8.433881  
  Larval Feeding Hind Gut  7.779536  
  Larval Feeding Malpighian Tubule  8.407900  
  Larval Feeding Mid Gut  7.035962  
  Larval Feeding Salivary Gland  8.428002  
  Whole Larvae Feeding  7.147437  
 
  
   FlyBase ID    symbol    start    end    strand    length   
   FBgn0037489   CG1234  3069896   3072717   -  2822  
   FBgn0037490   CG10053   3072963   3073862  +  900  
   FBgn0037491   CG1227  3073873   3075457   -  1585  
 
 
    Segment 59 
 
   Location   
  Gene key  FBgn0037492-FBgn0011282  
  Heatmap region span   3R:3026692..3170791   
  Segment span   3R:3075521..3077679   
  Length (genes)  2  
  Length (bp)  2159  
   Model Scoring   
  BIC  199.220215  
  logL  -93.982377  
  logL ratio  18.520435  
   Expression   
  Mean expression  4.749972  
  Median expression  4.526013  
  Tissue std. dev.  0.689177  
 
  No GO Slim enrichment  
  
   tissue    mean expression   
  5th Passage Drosophila S2 Cells  4.785235  
  Adult Accessory gland  4.481537  
  Adult Brain  4.854755  
  Adult Carcass  4.391421  
  Adult Crop  4.640796  
  Adult Eye  5.557654  
  Adult Fatbody  4.439194  
  Adult Female Spermatheca Mated  4.428761  
  Adult Female Spermatheca Virgin  4.350422  
  Adult Head  7.679793  
  Adult Heart  4.376073  
  Adult Hind Gut  4.330067  
  Adult Male Ejaculatory Duct  4.500124  
  Adult Mid Gut  4.462390  
  Adult Ovary  5.687943  
  Adult Salivary Gland  4.521226  
  Adult Testes  4.131963  
  Adult Thoracoabdominal ganglion  4.994609  
  Adult Whole Fly  5.230564  
  Larvae Wandering Tubules  4.483071  
  Larval Feeding Carcass  4.672354  
  Larval Feeding Central Nevous System  5.144049  
  Larval Feeding Hind Gut  4.388095  
  Larval Feeding Malpighian Tubule  4.602667  
  Larval Feeding Mid Gut  4.281462  
  Larval Feeding Salivary Gland  4.651492  
  Whole Larvae Feeding  4.181511  
 
  
   FlyBase ID    symbol    start    end    strand    length   
   FBgn0037492   CG10050   3075521   3077470  +  1950  
   FBgn0011282   Obp84a  3076531   3077679   -  1149  
 
    Segment 60 
 
   Location   
  Gene key  FBgn0037493-FBgn0259172  
  Heatmap region span   3R:3072717..3299499   
  Segment span   3R:3097314..3135119   
  Length (genes)  3  
  Length (bp)  37806  
   Model Scoring   
  BIC  274.830944  
  logL  -131.787741  
  logL ratio  48.200278  
   Expression   
  Mean expression  4.837173  
  Median expression  4.503107  
  Tissue std. dev.  0.332506  
 
  No GO Slim enrichment  
  
   tissue    mean expression   
  5th Passage Drosophila S2 Cells  4.919638  
  Adult Accessory gland  5.003683  
  Adult Brain  4.638180  
  Adult Carcass  4.872429  
  Adult Crop  4.731633  
  Adult Eye  4.495020  
  Adult Fatbody  4.845559  
  Adult Female Spermatheca Mated  4.730975  
  Adult Female Spermatheca Virgin  4.757745  
  Adult Head  4.579315  
  Adult Heart  4.590675  
  Adult Hind Gut  4.729993  
  Adult Male Ejaculatory Duct  4.915875  
  Adult Mid Gut  4.826045  
  Adult Ovary  4.738591  
  Adult Salivary Gland  5.140788  
  Adult Testes  6.357752  
  Adult Thoracoabdominal ganglion  4.760275  
  Adult Whole Fly  4.509024  
  Larvae Wandering Tubules  4.783619  
  Larval Feeding Carcass  4.838163  
  Larval Feeding Central Nevous System  4.705260  
  Larval Feeding Hind Gut  4.719183  
  Larval Feeding Malpighian Tubule  4.807532  
  Larval Feeding Mid Gut  4.840881  
  Larval Feeding Salivary Gland  5.044019  
  Whole Larvae Feeding  4.721808  
 
  
   FlyBase ID    symbol    start    end    strand    length   
   FBgn0037493   CG10032   3097314   3098231  +  918  
   FBgn0045843   RacGAP84C   3110655   3113354  +  2700  
   FBgn0259172   rn  3099460   3135119   -  35660  
 
 
    Segment 61 
 
   Location   
  Gene key  FBgn0037498-FBgn0051496  
  Heatmap region span   3R:3075521..3548139   
  Segment span   3R:3142929..3170791   
  Length (genes)  2  
  Length (bp)  27863  
   Model Scoring   
  BIC  228.827205  
  logL  -108.785872  
  logL ratio  -1.882709  
   Expression   
  Mean expression  5.639670  
  Median expression  4.971811  
  Tissue std. dev.  1.932999  
 
  No GO Slim enrichment  
  
   tissue    mean expression   
  5th Passage Drosophila S2 Cells  4.991734  
  Adult Accessory gland  13.527966  
  Adult Brain  4.590675  
  Adult Carcass  5.043815  
  Adult Crop  4.902632  
  Adult Eye  4.582188  
  Adult Fatbody  4.951707  
  Adult Female Spermatheca Mated  4.633013  
  Adult Female Spermatheca Virgin  4.663639  
  Adult Head  4.559950  
  Adult Heart  4.577979  
  Adult Hind Gut  4.937141  
  Adult Male Ejaculatory Duct  8.314996  
  Adult Mid Gut  5.061998  
  Adult Ovary  5.038538  
  Adult Salivary Gland  5.087456  
  Adult Testes  5.075801  
  Adult Thoracoabdominal ganglion  4.749094  
  Adult Whole Fly  6.883715  
  Larvae Wandering Tubules  4.931331  
  Larval Feeding Carcass  4.967812  
  Larval Feeding Central Nevous System  4.660602  
  Larval Feeding Hind Gut  4.914157  
  Larval Feeding Malpighian Tubule  5.002609  
  Larval Feeding Mid Gut  5.025310  
  Larval Feeding Salivary Gland  9.227804  
  Whole Larvae Feeding  7.367430  
 
  
   FlyBase ID    symbol    start    end    strand    length   
   FBgn0037498   CG10029  3141446   3142929   -  1484  
   FBgn0051496   CG31496   3170791   3172076  +  1286  
 
    Segment 62 
 
   Location   
  Gene key  FBgn0051501-FBgn0004172  
  Heatmap region span   3R:3091423..3575798   
  Segment span   3R:3172555..3191572   
  Length (genes)  6  
  Length (bp)  19018  
   Model Scoring   
  BIC  481.074635  
  logL  -234.909586  
  logL ratio  180.875943  
   Expression   
  Mean expression  5.102077  
  Median expression  4.601630  
  Tissue std. dev.  1.791776  
 
  No GO Slim enrichment  
  
   tissue    mean expression   
  5th Passage Drosophila S2 Cells  4.460623  
  Adult Accessory gland  4.624345  
  Adult Brain  4.166125  
  Adult Carcass  5.254006  
  Adult Crop  4.495476  
  Adult Eye  4.474068  
  Adult Fatbody  4.596339  
  Adult Female Spermatheca Mated  4.913106  
  Adult Female Spermatheca Virgin  4.542120  
  Adult Head  4.343028  
  Adult Heart  4.577048  
  Adult Hind Gut  4.432512  
  Adult Male Ejaculatory Duct  4.606755  
  Adult Mid Gut  4.483375  
  Adult Ovary  4.341732  
  Adult Salivary Gland  4.788346  
  Adult Testes  12.705310  
  Adult Thoracoabdominal ganglion  4.376333  
  Adult Whole Fly  9.303085  
  Larvae Wandering Tubules  4.518082  
  Larval Feeding Carcass  4.686042  
  Larval Feeding Central Nevous System  4.168163  
  Larval Feeding Hind Gut  4.469519  
  Larval Feeding Malpighian Tubule  4.511619  
  Larval Feeding Mid Gut  4.717960  
  Larval Feeding Salivary Gland  4.503283  
  Whole Larvae Feeding  6.697669  
 
  
   FlyBase ID    symbol    start    end    strand    length   
   FBgn0051501   nxf4   3172555   3173558  +  1004  
   FBgn0037500   CG17944  3187102   3187845   -  744  
   FBgn0004175   Mst84Dd  3189017   3189495   -  479  
   FBgn0004174   Mst84Dc  3189975   3190395   -  421  
   FBgn0004173   Mst84Db  3190880   3191349   -  470  
   FBgn0004172   Mst84Da   3191572   3191985  +  414  
 
 
    Segment 63 
 
   Location   
  Gene key  FBgn0015770-FBgn0037503  
  Heatmap region span   3R:3091783..3583893   
  Segment span   3R:3195027..3265488   
  Length (genes)  5  
  Length (bp)  70462  
   Model Scoring   
  BIC  380.597414  
  logL  -184.670976  
  logL ratio  143.955476  
   Expression   
  Mean expression  4.434200  
  Median expression  4.361296  
  Tissue std. dev.  0.281608  
 
  
   GO ID    description    ratio    P-value   
   GO:0005886   plasma membrane  2/5  0.0419  
 
  
   tissue    mean expression   
  5th Passage Drosophila S2 Cells  4.340001  
  Adult Accessory gland  4.700487  
  Adult Brain  4.154047  
  Adult Carcass  4.526148  
  Adult Crop  4.347216  
  Adult Eye  4.201655  
  Adult Fatbody  4.489446  
  Adult Female Spermatheca Mated  4.462459  
  Adult Female Spermatheca Virgin  4.478393  
  Adult Head  4.225452  
  Adult Heart  4.272253  
  Adult Hind Gut  4.312519  
  Adult Male Ejaculatory Duct  4.385396  
  Adult Mid Gut  4.473479  
  Adult Ovary  4.172083  
  Adult Salivary Gland  4.693388  
  Adult Testes  4.196917  
  Adult Thoracoabdominal ganglion  4.200737  
  Adult Whole Fly  4.033350  
  Larvae Wandering Tubules  4.561160  
  Larval Feeding Carcass  5.513342  
  Larval Feeding Central Nevous System  4.545631  
  Larval Feeding Hind Gut  4.274915  
  Larval Feeding Malpighian Tubule  4.440118  
  Larval Feeding Mid Gut  4.428747  
  Larval Feeding Salivary Gland  4.421222  
  Whole Larvae Feeding  4.872855  
 
  
   FlyBase ID    symbol    start    end    strand    length   
   FBgn0015770   MstProx  3191661   3195027   -  3367  
   FBgn0037501   Ir84a   3232504   3235106  +  2603  
   FBgn0051544   CG31544   3244720   3245854  +  1135  
   FBgn0054023   CG34023   3245990   3246420  +  431  
   FBgn0037503   CG14598   3265488   3267714  +  2227  
 
 
    Segment 64 
 
   Location   
  Gene key  FBgn0037506-FBgn0037512  
  Heatmap region span   3R:3142929..3597469   
  Segment span   3R:3328098..3548139   
  Length (genes)  7  
  Length (bp)  220042  
   Model Scoring   
  BIC  564.373462  
  logL  -276.559000  
  logL ratio  160.112818  
   Expression   
  Mean expression  4.846147  
  Median expression  4.566295  
  Tissue std. dev.  0.670581  
 
  No GO Slim enrichment  
  
   tissue    mean expression   
  5th Passage Drosophila S2 Cells  4.720839  
  Adult Accessory gland  4.736043  
  Adult Brain  4.655223  
  Adult Carcass  4.841403  
  Adult Crop  5.199129  
  Adult Eye  4.569447  
  Adult Fatbody  4.716096  
  Adult Female Spermatheca Mated  4.700560  
  Adult Female Spermatheca Virgin  4.678075  
  Adult Head  4.608309  
  Adult Heart  4.763031  
  Adult Hind Gut  4.656956  
  Adult Male Ejaculatory Duct  4.580688  
  Adult Mid Gut  4.665276  
  Adult Ovary  4.572574  
  Adult Salivary Gland  4.728182  
  Adult Testes  8.084353  
  Adult Thoracoabdominal ganglion  4.648948  
  Adult Whole Fly  5.636571  
  Larvae Wandering Tubules  4.631455  
  Larval Feeding Carcass  4.654528  
  Larval Feeding Central Nevous System  4.615379  
  Larval Feeding Hind Gut  4.602536  
  Larval Feeding Malpighian Tubule  4.618374  
  Larval Feeding Mid Gut  4.598643  
  Larval Feeding Salivary Gland  4.626842  
  Whole Larvae Feeding  4.736497  
 
  
   FlyBase ID    symbol    start    end    strand    length   
   FBgn0037506   CG1287  3326673   3328098   -  1426  
   FBgn0017456   Ubc84D  3344542   3345178   -  637  
   FBgn0015831   Rtnl2  3355004   3355478   -  475  
   FBgn0003885   alphaTub84D   3356377   3358873  +  2497  
   FBgn0083963   CG34127  3397848   3465156   -  67309  
   FBgn0051146   Nlg1   3483971   3520040  +  36070  
   FBgn0037512   CG2616   3548139   3550140  +  2002  
 
 
    Segment 65 
 
   Location   
  Gene key  FBgn0042104-FBgn0037517  
  Heatmap region span   3R:3575798..3712441   
  Segment span   3R:3609123..3616363   
  Length (genes)  5  
  Length (bp)  7241  
   Model Scoring   
  BIC  452.323170  
  logL  -220.533854  
  logL ratio  100.810979  
   Expression   
  Mean expression  5.969827  
  Median expression  5.158527  
  Tissue std. dev.  1.757831  
 
  
   GO ID    description    ratio    P-value   
   GO:0007165   signal transduction  2/5  0.00761  
 
  
   tissue    mean expression   
  5th Passage Drosophila S2 Cells  4.924793  
  Adult Accessory gland  4.946252  
  Adult Brain  4.511947  
  Adult Carcass  4.948437  
  Adult Crop  4.825391  
  Adult Eye  4.631919  
  Adult Fatbody  5.090025  
  Adult Female Spermatheca Mated  5.146555  
  Adult Female Spermatheca Virgin  5.072046  
  Adult Head  4.664565  
  Adult Heart  5.300855  
  Adult Hind Gut  7.501000  
  Adult Male Ejaculatory Duct  4.942048  
  Adult Mid Gut  10.221705  
  Adult Ovary  4.716987  
  Adult Salivary Gland  5.072859  
  Adult Testes  5.606086  
  Adult Thoracoabdominal ganglion  4.628710  
  Adult Whole Fly  5.941788  
  Larvae Wandering Tubules  8.735069  
  Larval Feeding Carcass  5.073471  
  Larval Feeding Central Nevous System  4.709189  
  Larval Feeding Hind Gut  7.800286  
  Larval Feeding Malpighian Tubule  9.032167  
  Larval Feeding Mid Gut  10.028767  
  Larval Feeding Salivary Gland  4.991543  
  Whole Larvae Feeding  8.120871  
 
  
   FlyBase ID    symbol    start    end    strand    length   
   FBgn0042104   CG18747  3606712   3609123   -  2412  
   FBgn0042102   CG18745  3609563   3611841   -  2279  
   FBgn0042105   CG18748  3611892   3613645   -  1754  
   FBgn0042103   CG18746  3614001   3615904   -  1904  
   FBgn0037517   CG10086   3616363   3619221  +  2859  
 
 
    Segment 66 
 
   Location   
  Gene key  FBgn0042101-FBgn0037521  
  Heatmap region span   3R:3585508..3728384   
  Segment span   3R:3624493..3635710   
  Length (genes)  2  
  Length (bp)  11218  
   Model Scoring   
  BIC  224.378777  
  logL  -106.561658  
  logL ratio  -7.826948  
   Expression   
  Mean expression  4.913855  
  Median expression  4.660903  
  Tissue std. dev.  0.702025  
 
  No GO Slim enrichment  
  
   tissue    mean expression   
  5th Passage Drosophila S2 Cells  4.356255  
  Adult Accessory gland  4.595628  
  Adult Brain  6.456713  
  Adult Carcass  4.490403  
  Adult Crop  4.536045  
  Adult Eye  4.339810  
  Adult Fatbody  4.749517  
  Adult Female Spermatheca Mated  4.534747  
  Adult Female Spermatheca Virgin  4.630027  
  Adult Head  4.798390  
  Adult Heart  4.347097  
  Adult Hind Gut  4.893056  
  Adult Male Ejaculatory Duct  4.263929  
  Adult Mid Gut  6.427340  
  Adult Ovary  4.727387  
  Adult Salivary Gland  4.715639  
  Adult Testes  4.085045  
  Adult Thoracoabdominal ganglion  6.266894  
  Adult Whole Fly  4.643016  
  Larvae Wandering Tubules  5.097869  
  Larval Feeding Carcass  4.332642  
  Larval Feeding Central Nevous System  5.481826  
  Larval Feeding Hind Gut  4.731703  
  Larval Feeding Malpighian Tubule  4.892112  
  Larval Feeding Mid Gut  6.600233  
  Larval Feeding Salivary Gland  4.443193  
  Whole Larvae Feeding  5.237585  
 
  
   FlyBase ID    symbol    start    end    strand    length   
   FBgn0042101   CG18744  3622798   3624493   -  1696  
   FBgn0037521   CG2993  3631717   3635710   -  3994  
 
    Segment 67 
 
   Location   
  Gene key  FBgn0051473-FBgn0037525  
  Heatmap region span   3R:3609123..3738999   
  Segment span   3R:3637536..3712441   
  Length (genes)  3  
  Length (bp)  74906  
   Model Scoring   
  BIC  320.413417  
  logL  -154.578977  
  logL ratio  2.546754  
   Expression   
  Mean expression  5.124341  
  Median expression  4.906830  
  Tissue std. dev.  0.624438  
 
  No GO Slim enrichment  
  
   tissue    mean expression   
  5th Passage Drosophila S2 Cells  4.627357  
  Adult Accessory gland  4.540782  
  Adult Brain  6.150750  
  Adult Carcass  4.714955  
  Adult Crop  5.241277  
  Adult Eye  5.320788  
  Adult Fatbody  5.018023  
  Adult Female Spermatheca Mated  5.048763  
  Adult Female Spermatheca Virgin  5.220076  
  Adult Head  4.925587  
  Adult Heart  4.745699  
  Adult Hind Gut  4.752980  
  Adult Male Ejaculatory Duct  4.720936  
  Adult Mid Gut  4.794534  
  Adult Ovary  5.124418  
  Adult Salivary Gland  5.093737  
  Adult Testes  7.441378  
  Adult Thoracoabdominal ganglion  6.089750  
  Adult Whole Fly  5.518903  
  Larvae Wandering Tubules  4.862543  
  Larval Feeding Carcass  4.843447  
  Larval Feeding Central Nevous System  5.891347  
  Larval Feeding Hind Gut  4.493082  
  Larval Feeding Malpighian Tubule  4.808028  
  Larval Feeding Mid Gut  4.676033  
  Larval Feeding Salivary Gland  4.822942  
  Whole Larvae Feeding  4.869081  
 
  
   FlyBase ID    symbol    start    end    strand    length   
   FBgn0051473   CG31473   3637536   3638731  +  1196  
   FBgn0085413   CG34384   3640083   3680507  +  40425  
   FBgn0037525   CG17816  3683896   3712441   -  28546  
 
 
    Segment 68 
 
   Location   
  Gene key  FBgn0037530-FBgn0014930  
  Heatmap region span   3R:3637536..3803488   
  Segment span   3R:3733088..3738999   
  Length (genes)  3  
  Length (bp)  5912  
   Model Scoring   
  BIC  360.115919  
  logL  -174.430228  
  logL ratio  49.031098  
   Expression   
  Mean expression  9.364732  
  Median expression  9.586855  
  Tissue std. dev.  0.579339  
 
  No GO Slim enrichment  
  
   tissue    mean expression   
  5th Passage Drosophila S2 Cells  10.121341  
  Adult Accessory gland  10.160521  
  Adult Brain  8.302856  
  Adult Carcass  9.228284  
  Adult Crop  9.654692  
  Adult Eye  9.787747  
  Adult Fatbody  9.518842  
  Adult Female Spermatheca Mated  9.155060  
  Adult Female Spermatheca Virgin  8.966321  
  Adult Head  9.130311  
  Adult Heart  9.690256  
  Adult Hind Gut  9.493809  
  Adult Male Ejaculatory Duct  10.399000  
  Adult Mid Gut  9.453012  
  Adult Ovary  9.974034  
  Adult Salivary Gland  10.081278  
  Adult Testes  8.058684  
  Adult Thoracoabdominal ganglion  8.487833  
  Adult Whole Fly  9.465062  
  Larvae Wandering Tubules  8.694817  
  Larval Feeding Carcass  9.334252  
  Larval Feeding Central Nevous System  9.301570  
  Larval Feeding Hind Gut  9.738565  
  Larval Feeding Malpighian Tubule  9.114189  
  Larval Feeding Mid Gut  8.797346  
  Larval Feeding Salivary Gland  9.924904  
  Whole Larvae Feeding  8.813187  
 
  
   FlyBase ID    symbol    start    end    strand    length   
   FBgn0037530   CG2943  3728975   3733088   -  4114  
   FBgn0004901   Prat  3735320   3737501   -  2182  
   FBgn0014930   CG2846  3737739   3738999   -  1261  
 
 
    Segment 69 
 
   Location   
  Gene key  FBgn0014931-FBgn0037531  
  Heatmap region span   3R:3712806..3821568   
  Segment span   3R:3739579..3746021   
  Length (genes)  2  
  Length (bp)  6443  
   Model Scoring   
  BIC  197.410853  
  logL  -93.077695  
  logL ratio  20.384392  
   Expression   
  Mean expression  4.960692  
  Median expression  4.501627  
  Tissue std. dev.  0.910529  
 
  
   GO ID    description    ratio    P-value   
   GO:0005634   nucleus  2/2  0.0436  
 
  
   tissue    mean expression   
  5th Passage Drosophila S2 Cells  7.090647  
  Adult Accessory gland  4.367845  
  Adult Brain  4.139248  
  Adult Carcass  4.702447  
  Adult Crop  4.949307  
  Adult Eye  4.320184  
  Adult Fatbody  5.748601  
  Adult Female Spermatheca Mated  5.707298  
  Adult Female Spermatheca Virgin  5.771991  
  Adult Head  4.466975  
  Adult Heart  5.154276  
  Adult Hind Gut  4.910342  
  Adult Male Ejaculatory Duct  4.228084  
  Adult Mid Gut  4.611166  
  Adult Ovary  8.085638  
  Adult Salivary Gland  4.517853  
  Adult Testes  4.296394  
  Adult Thoracoabdominal ganglion  4.365403  
  Adult Whole Fly  5.861967  
  Larvae Wandering Tubules  4.726598  
  Larval Feeding Carcass  4.156133  
  Larval Feeding Central Nevous System  4.637062  
  Larval Feeding Hind Gut  5.253580  
  Larval Feeding Malpighian Tubule  4.776630  
  Larval Feeding Mid Gut  4.321623  
  Larval Feeding Salivary Gland  4.392076  
  Whole Larvae Feeding  4.379312  
 
  
   FlyBase ID    symbol    start    end    strand    length   
   FBgn0014931   CG2678   3739579   3742272  +  2694  
   FBgn0037531   CG10445  3742321   3746021   -  3701  
 
    Segment 70 
 
   Location   
  Gene key  FBgn0037536-FBgn0037537  
  Heatmap region span   3R:3793130..3859027   
  Segment span   3R:3827282..3833841   
  Length (genes)  2  
  Length (bp)  6560  
   Model Scoring   
  BIC  246.827495  
  logL  -117.786016  
  logL ratio  47.264503  
   Expression   
  Mean expression  10.041017  
  Median expression  10.073500  
  Tissue std. dev.  0.766456  
 
  No GO Slim enrichment  
  
   tissue    mean expression   
  5th Passage Drosophila S2 Cells  10.329604  
  Adult Accessory gland  10.216112  
  Adult Brain  9.466321  
  Adult Carcass  10.085310  
  Adult Crop  11.574573  
  Adult Eye  12.016849  
  Adult Fatbody  10.354460  
  Adult Female Spermatheca Mated  10.224291  
  Adult Female Spermatheca Virgin  10.089061  
  Adult Head  10.946222  
  Adult Heart  10.306584  
  Adult Hind Gut  10.374488  
  Adult Male Ejaculatory Duct  10.363749  
  Adult Mid Gut  8.811340  
  Adult Ovary  9.873842  
  Adult Salivary Gland  9.703239  
  Adult Testes  8.709407  
  Adult Thoracoabdominal ganglion  9.481198  
  Adult Whole Fly  9.484576  
  Larvae Wandering Tubules  9.229379  
  Larval Feeding Carcass  10.900814  
  Larval Feeding Central Nevous System  9.941695  
  Larval Feeding Hind Gut  10.232765  
  Larval Feeding Malpighian Tubule  9.312163  
  Larval Feeding Mid Gut  8.683737  
  Larval Feeding Salivary Gland  10.163473  
  Whole Larvae Feeding  10.232196  
 
  
   FlyBase ID    symbol    start    end    strand    length   
   FBgn0037536   CG2698   3827282   3831432  +  4151  
   FBgn0037537   CG2767  3831646   3833841   -  2196  
 
    Segment 71 
 
   Location   
  Gene key  FBgn0037538-FBgn0037539  
  Heatmap region span   3R:3821568..3912974   
  Segment span   3R:3835093..3838920   
  Length (genes)  2  
  Length (bp)  3828  
   Model Scoring   
  BIC  210.970582  
  logL  -99.857560  
  logL ratio  44.309147  
   Expression   
  Mean expression  8.338076  
  Median expression  8.201581  
  Tissue std. dev.  0.415975  
 
  No GO Slim enrichment  
  
   tissue    mean expression   
  5th Passage Drosophila S2 Cells  8.651603  
  Adult Accessory gland  8.190961  
  Adult Brain  8.306630  
  Adult Carcass  7.854539  
  Adult Crop  8.243173  
  Adult Eye  8.420961  
  Adult Fatbody  8.228143  
  Adult Female Spermatheca Mated  7.957917  
  Adult Female Spermatheca Virgin  8.146861  
  Adult Head  7.788443  
  Adult Heart  8.395813  
  Adult Hind Gut  7.990073  
  Adult Male Ejaculatory Duct  8.244667  
  Adult Mid Gut  7.841314  
  Adult Ovary  9.047959  
  Adult Salivary Gland  8.090654  
  Adult Testes  8.679827  
  Adult Thoracoabdominal ganglion  8.128459  
  Adult Whole Fly  7.936853  
  Larvae Wandering Tubules  9.571058  
  Larval Feeding Carcass  8.721761  
  Larval Feeding Central Nevous System  8.825023  
  Larval Feeding Hind Gut  8.461578  
  Larval Feeding Malpighian Tubule  8.833604  
  Larval Feeding Mid Gut  8.494410  
  Larval Feeding Salivary Gland  8.328781  
  Whole Larvae Feeding  7.746997  
 
  
   FlyBase ID    symbol    start    end    strand    length   
   FBgn0037538   CG3223   3835093   3836740  +  1648  
   FBgn0037539   CG10435  3836631   3838920   -  2290  
 
    Segment 72 
 
   Location   
  Gene key  FBgn0037540-FBgn0001255  
  Heatmap region span   3R:3825592..3952336   
  Segment span   3R:3839546..3856167   
  Length (genes)  2  
  Length (bp)  16622  
   Model Scoring   
  BIC  229.224444  
  logL  -108.984491  
  logL ratio  -0.971468  
   Expression   
  Mean expression  5.947185  
  Median expression  5.934264  
  Tissue std. dev.  0.582656  
 
  No GO Slim enrichment  
  
   tissue    mean expression   
  5th Passage Drosophila S2 Cells  5.782383  
  Adult Accessory gland  5.401359  
  Adult Brain  5.766170  
  Adult Carcass  5.170870  
  Adult Crop  5.972473  
  Adult Eye  5.960890  
  Adult Fatbody  5.540249  
  Adult Female Spermatheca Mated  6.013984  
  Adult Female Spermatheca Virgin  6.024983  
  Adult Head  5.302874  
  Adult Heart  5.642758  
  Adult Hind Gut  6.257259  
  Adult Male Ejaculatory Duct  5.701214  
  Adult Mid Gut  5.487570  
  Adult Ovary  7.081290  
  Adult Salivary Gland  6.589360  
  Adult Testes  5.027215  
  Adult Thoracoabdominal ganglion  5.897362  
  Adult Whole Fly  5.443270  
  Larvae Wandering Tubules  6.973567  
  Larval Feeding Carcass  7.640713  
  Larval Feeding Central Nevous System  6.327452  
  Larval Feeding Hind Gut  5.968833  
  Larval Feeding Malpighian Tubule  6.173173  
  Larval Feeding Mid Gut  5.519395  
  Larval Feeding Salivary Gland  6.113277  
  Whole Larvae Feeding  5.794048  
 
  
   FlyBase ID    symbol    start    end    strand    length   
   FBgn0037540   Pbp95   3839546   3842332  +  2787  
   FBgn0001255   ImpE3  3853932   3856167   -  2236  
 
    Segment 73 
 
   Location   
  Gene key  FBgn0037541-FBgn0010812  
  Heatmap region span   3R:3827282..4007164   
  Segment span   3R:3856505..3859027   
  Length (genes)  4  
  Length (bp)  2523  
   Model Scoring   
  BIC  374.002592  
  logL  -181.373565  
  logL ratio  124.422455  
   Expression   
  Mean expression  8.148267  
  Median expression  8.172563  
  Tissue std. dev.  0.301001  
 
  No GO Slim enrichment  
  
   tissue    mean expression   
  5th Passage Drosophila S2 Cells  8.324955  
  Adult Accessory gland  7.800543  
  Adult Brain  8.505935  
  Adult Carcass  8.263694  
  Adult Crop  8.479110  
  Adult Eye  8.251768  
  Adult Fatbody  7.949867  
  Adult Female Spermatheca Mated  7.884246  
  Adult Female Spermatheca Virgin  7.770303  
  Adult Head  8.050842  
  Adult Heart  8.398818  
  Adult Hind Gut  8.419847  
  Adult Male Ejaculatory Duct  8.429484  
  Adult Mid Gut  7.830642  
  Adult Ovary  8.352923  
  Adult Salivary Gland  8.056326  
  Adult Testes  7.886359  
  Adult Thoracoabdominal ganglion  8.623298  
  Adult Whole Fly  7.650878  
  Larvae Wandering Tubules  8.243396  
  Larval Feeding Carcass  8.396985  
  Larval Feeding Central Nevous System  8.073566  
  Larval Feeding Hind Gut  8.213479  
  Larval Feeding Malpighian Tubule  8.264399  
  Larval Feeding Mid Gut  7.737382  
  Larval Feeding Salivary Gland  8.614353  
  Whole Larvae Feeding  7.529797  
 
  
   FlyBase ID    symbol    start    end    strand    length   
   FBgn0037541   CG2747  3841932   3856505   -  14574  
   FBgn0037543   CG10903   3856551   3857867  +  1317  
   FBgn0037544   CG11035  3857861   3858669   -  809  
   FBgn0010812   unc-45   3859027   3862374  +  3348  
 
 
    Segment 74 
 
   Location   
  Gene key  FBgn0037548-FBgn0037551  
  Heatmap region span   3R:3839546..4069514   
  Segment span   3R:3919846..3952336   
  Length (genes)  5  
  Length (bp)  32491  
   Model Scoring   
  BIC  548.753971  
  logL  -268.749254  
  logL ratio  94.297631  
   Expression   
  Mean expression  8.742394  
  Median expression  8.848196  
  Tissue std. dev.  0.452767  
 
  No GO Slim enrichment  
  
   tissue    mean expression   
  5th Passage Drosophila S2 Cells  9.248277  
  Adult Accessory gland  8.301732  
  Adult Brain  8.174461  
  Adult Carcass  8.488445  
  Adult Crop  9.221010  
  Adult Eye  8.729758  
  Adult Fatbody  8.953270  
  Adult Female Spermatheca Mated  8.976539  
  Adult Female Spermatheca Virgin  8.974895  
  Adult Head  8.836607  
  Adult Heart  9.152693  
  Adult Hind Gut  9.360399  
  Adult Male Ejaculatory Duct  9.209403  
  Adult Mid Gut  9.068569  
  Adult Ovary  8.830234  
  Adult Salivary Gland  8.869258  
  Adult Testes  7.153239  
  Adult Thoracoabdominal ganglion  8.056345  
  Adult Whole Fly  8.288137  
  Larvae Wandering Tubules  8.855576  
  Larval Feeding Carcass  8.684373  
  Larval Feeding Central Nevous System  8.823124  
  Larval Feeding Hind Gut  8.790316  
  Larval Feeding Malpighian Tubule  9.006012  
  Larval Feeding Mid Gut  8.787229  
  Larval Feeding Salivary Gland  8.788977  
  Whole Larvae Feeding  8.415770  
 
  
   FlyBase ID    symbol    start    end    strand    length   
   FBgn0037548   CG7900   3919846   3930805  +  10960  
   FBgn0243512   puc   3931057   3948023  +  16967  
   FBgn0037549   CG7878   3948421   3950995  +  2575  
   FBgn0037550   CG9667  3951009   3952055   -  1047  
   FBgn0037551   Gie   3952336   3954568  +  2233  
 
 
    Segment 75 
 
   Location   
  Gene key  FBgn0037555-FBgn0037556  
  Heatmap region span   3R:3919846..4103514   
  Segment span   3R:4066234..4069514   
  Length (genes)  2  
  Length (bp)  3281  
   Model Scoring   
  BIC  225.953542  
  logL  -107.349040  
  logL ratio  34.850427  
   Expression   
  Mean expression  8.483959  
  Median expression  8.199264  
  Tissue std. dev.  0.686692  
 
  No GO Slim enrichment  
  
   tissue    mean expression   
  5th Passage Drosophila S2 Cells  8.973029  
  Adult Accessory gland  8.827941  
  Adult Brain  9.583203  
  Adult Carcass  7.628028  
  Adult Crop  8.037975  
  Adult Eye  8.529386  
  Adult Fatbody  7.945042  
  Adult Female Spermatheca Mated  8.110518  
  Adult Female Spermatheca Virgin  8.249760  
  Adult Head  8.608847  
  Adult Heart  7.979617  
  Adult Hind Gut  7.814070  
  Adult Male Ejaculatory Duct  8.300752  
  Adult Mid Gut  7.541517  
  Adult Ovary  10.353521  
  Adult Salivary Gland  8.399682  
  Adult Testes  7.664910  
  Adult Thoracoabdominal ganglion  9.192490  
  Adult Whole Fly  8.947438  
  Larvae Wandering Tubules  8.584664  
  Larval Feeding Carcass  8.639584  
  Larval Feeding Central Nevous System  9.932071  
  Larval Feeding Hind Gut  8.227663  
  Larval Feeding Malpighian Tubule  8.345512  
  Larval Feeding Mid Gut  7.718457  
  Larval Feeding Salivary Gland  8.920662  
  Whole Larvae Feeding  8.010563  
 
  
   FlyBase ID    symbol    start    end    strand    length   
   FBgn0037555   Ada2b  4062838   4066234   -  3397  
   FBgn0037556   CG9636  4066466   4069514   -  3049  
 
    Segment 76 
 
   Location   
  Gene key  FBgn0037561-FBgn0010433  
  Heatmap region span   3R:4066234..4134344   
  Segment span   3R:4095604..4103514   
  Length (genes)  2  
  Length (bp)  7911  
   Model Scoring   
  BIC  217.912828  
  logL  -103.328683  
  logL ratio  12.635226  
   Expression   
  Mean expression  6.041319  
  Median expression  5.859432  
  Tissue std. dev.  0.355242  
 
  No GO Slim enrichment  
  
   tissue    mean expression   
  5th Passage Drosophila S2 Cells  6.455281  
  Adult Accessory gland  6.708780  
  Adult Brain  5.947542  
  Adult Carcass  5.859432  
  Adult Crop  6.144350  
  Adult Eye  5.575978  
  Adult Fatbody  6.102877  
  Adult Female Spermatheca Mated  6.293117  
  Adult Female Spermatheca Virgin  6.189466  
  Adult Head  5.799825  
  Adult Heart  5.925113  
  Adult Hind Gut  6.009839  
  Adult Male Ejaculatory Duct  6.020866  
  Adult Mid Gut  5.898376  
  Adult Ovary  6.658786  
  Adult Salivary Gland  6.115011  
  Adult Testes  5.108270  
  Adult Thoracoabdominal ganglion  5.736728  
  Adult Whole Fly  5.907293  
  Larvae Wandering Tubules  5.833823  
  Larval Feeding Carcass  6.177217  
  Larval Feeding Central Nevous System  6.849671  
  Larval Feeding Hind Gut  6.164229  
  Larval Feeding Malpighian Tubule  5.833427  
  Larval Feeding Mid Gut  5.870997  
  Larval Feeding Salivary Gland  6.244436  
  Whole Larvae Feeding  5.684876  
 
  
   FlyBase ID    symbol    start    end    strand    length   
   FBgn0037561   CG9630  4093657   4095604   -  1948  
   FBgn0010433   ato   4103514   4104996  +  1483  
 
    Segment 77 
 
   Location   
  Gene key  FBgn0037563-FBgn0051463  
  Heatmap region span   3R:4087620..4138936   
  Segment span   3R:4116287..4118073   
  Length (genes)  3  
  Length (bp)  1787  
   Model Scoring   
  BIC  277.805813  
  logL  -133.275175  
  logL ratio  48.575917  
   Expression   
  Mean expression  5.026414  
  Median expression  4.476738  
  Tissue std. dev.  1.602716  
 
  No GO Slim enrichment  
  
   tissue    mean expression   
  5th Passage Drosophila S2 Cells  4.475992  
  Adult Accessory gland  4.437646  
  Adult Brain  4.226090  
  Adult Carcass  4.353084  
  Adult Crop  4.621264  
  Adult Eye  4.427255  
  Adult Fatbody  4.380041  
  Adult Female Spermatheca Mated  4.493766  
  Adult Female Spermatheca Virgin  4.501909  
  Adult Head  4.640682  
  Adult Heart  4.339308  
  Adult Hind Gut  4.275216  
  Adult Male Ejaculatory Duct  4.531174  
  Adult Mid Gut  8.924310  
  Adult Ovary  4.409848  
  Adult Salivary Gland  4.511453  
  Adult Testes  4.276613  
  Adult Thoracoabdominal ganglion  4.230851  
  Adult Whole Fly  6.009641  
  Larvae Wandering Tubules  4.404535  
  Larval Feeding Carcass  4.473392  
  Larval Feeding Central Nevous System  4.187098  
  Larval Feeding Hind Gut  4.250214  
  Larval Feeding Malpighian Tubule  4.510241  
  Larval Feeding Mid Gut  10.331284  
  Larval Feeding Salivary Gland  4.468647  
  Whole Larvae Feeding  9.021617  
 
  
   FlyBase ID    symbol    start    end    strand    length   
   FBgn0037563   CG11672  4115128   4116287   -  1160  
   FBgn0051464   CG31464   4116974   4117726  +  753  
   FBgn0051463   CG31463   4118073   4119331  +  1259  
 
 
    Segment 78 
 
   Location   
  Gene key  FBgn0037566-FBgn0037569  
  Heatmap region span   3R:4095604..4147469   
  Segment span   3R:4127164..4134344   
  Length (genes)  3  
  Length (bp)  7181  
   Model Scoring   
  BIC  319.979764  
  logL  -154.362151  
  logL ratio  57.776752  
   Expression   
  Mean expression  8.075987  
  Median expression  8.149091  
  Tissue std. dev.  0.478588  
 
  No GO Slim enrichment  
  
   tissue    mean expression   
  5th Passage Drosophila S2 Cells  8.752309  
  Adult Accessory gland  8.841546  
  Adult Brain  7.798854  
  Adult Carcass  7.515068  
  Adult Crop  7.721398  
  Adult Eye  7.554657  
  Adult Fatbody  7.995947  
  Adult Female Spermatheca Mated  8.087605  
  Adult Female Spermatheca Virgin  8.153110  
  Adult Head  7.481794  
  Adult Heart  8.433317  
  Adult Hind Gut  7.769052  
  Adult Male Ejaculatory Duct  8.278140  
  Adult Mid Gut  8.161123  
  Adult Ovary  8.775133  
  Adult Salivary Gland  7.972431  
  Adult Testes  6.792329  
  Adult Thoracoabdominal ganglion  8.044464  
  Adult Whole Fly  7.796599  
  Larvae Wandering Tubules  8.055111  
  Larval Feeding Carcass  7.955606  
  Larval Feeding Central Nevous System  8.637198  
  Larval Feeding Hind Gut  8.219513  
  Larval Feeding Malpighian Tubule  8.390663  
  Larval Feeding Mid Gut  7.926636  
  Larval Feeding Salivary Gland  9.065144  
  Whole Larvae Feeding  7.876896  
 
  
   FlyBase ID    symbol    start    end    strand    length   
   FBgn0037566   mRpL1   4127164   4128492  +  1329  
   FBgn0037567     4128408   4129733   -  1326  
   FBgn0037569   tex  4133195   4134344   -  1150  
 
 
    Segment 79 
 
   Location   
  Gene key  FBgn0037570-FBgn0037572  
  Heatmap region span   3R:4113661..4150057   
  Segment span   3R:4135656..4137945   
  Length (genes)  3  
  Length (bp)  2290  
   Model Scoring   
  BIC  255.915933  
  logL  -122.330235  
  logL ratio  57.280858  
   Expression   
  Mean expression  5.145454  
  Median expression  4.876027  
  Tissue std. dev.  0.494851  
 
  No GO Slim enrichment  
  
   tissue    mean expression   
  5th Passage Drosophila S2 Cells  5.236621  
  Adult Accessory gland  5.197332  
  Adult Brain  4.762353  
  Adult Carcass  4.982180  
  Adult Crop  4.977709  
  Adult Eye  4.927424  
  Adult Fatbody  5.206090  
  Adult Female Spermatheca Mated  5.053281  
  Adult Female Spermatheca Virgin  5.077617  
  Adult Head  4.880980  
  Adult Heart  5.174517  
  Adult Hind Gut  4.950886  
  Adult Male Ejaculatory Duct  5.205966  
  Adult Mid Gut  5.153218  
  Adult Ovary  4.990414  
  Adult Salivary Gland  5.343673  
  Adult Testes  7.530329  
  Adult Thoracoabdominal ganglion  4.743836  
  Adult Whole Fly  4.911644  
  Larvae Wandering Tubules  4.996127  
  Larval Feeding Carcass  5.186336  
  Larval Feeding Central Nevous System  4.741012  
  Larval Feeding Hind Gut  4.973427  
  Larval Feeding Malpighian Tubule  5.142243  
  Larval Feeding Mid Gut  5.212371  
  Larval Feeding Salivary Gland  5.308906  
  Whole Larvae Feeding  5.060755  
 
  
   FlyBase ID    symbol    start    end    strand    length   
   FBgn0037570   CG11693  4134700   4135656   -  957  
   FBgn0037571   CG11694  4136925   4137807   -  883  
   FBgn0037572   CG11698   4137945   4138819  +  875  
 
 
    Segment 80 
 
   Location   
  Gene key  FBgn0062412-FBgn0037576  
  Heatmap region span   3R:4127164..4169219   
  Segment span   3R:4143337..4147469   
  Length (genes)  2  
  Length (bp)  4133  
   Model Scoring   
  BIC  190.643530  
  logL  -89.694034  
  logL ratio  46.089632  
   Expression   
  Mean expression  3.927906  
  Median expression  3.788863  
  Tissue std. dev.  0.247230  
 
  No GO Slim enrichment  
  
   tissue    mean expression   
  5th Passage Drosophila S2 Cells  3.678994  
  Adult Accessory gland  3.942669  
  Adult Brain  3.613026  
  Adult Carcass  3.883937  
  Adult Crop  4.862162  
  Adult Eye  3.834013  
  Adult Fatbody  4.126411  
  Adult Female Spermatheca Mated  4.051095  
  Adult Female Spermatheca Virgin  4.172817  
  Adult Head  4.098458  
  Adult Heart  3.996686  
  Adult Hind Gut  3.835371  
  Adult Male Ejaculatory Duct  4.049518  
  Adult Mid Gut  3.893465  
  Adult Ovary  3.777693  
  Adult Salivary Gland  3.895920  
  Adult Testes  3.718148  
  Adult Thoracoabdominal ganglion  3.748482  
  Adult Whole Fly  3.718318  
  Larvae Wandering Tubules  4.145966  
  Larval Feeding Carcass  3.710726  
  Larval Feeding Central Nevous System  3.589819  
  Larval Feeding Hind Gut  4.120984  
  Larval Feeding Malpighian Tubule  3.938964  
  Larval Feeding Mid Gut  4.034694  
  Larval Feeding Salivary Gland  3.775986  
  Whole Larvae Feeding  3.839144  
 
  
   FlyBase ID    symbol    start    end    strand    length   
   FBgn0062412   Ctr1B   4143337   4144927  +  1591  
   FBgn0037576   Or85a   4147469   4148776  +  1308  
 
    Segment 81 
 
   Location   
  Gene key  FBgn0004908-FBgn0037578  
  Heatmap region span   3R:4143337..4338744   
  Segment span   3R:4166822..4169219   
  Length (genes)  2  
  Length (bp)  2398  
   Model Scoring   
  BIC  205.370712  
  logL  -97.057625  
  logL ratio  50.263095  
   Expression   
  Mean expression  8.405407  
  Median expression  8.348495  
  Tissue std. dev.  0.553592  
 
  No GO Slim enrichment  
  
   tissue    mean expression   
  5th Passage Drosophila S2 Cells  9.127947  
  Adult Accessory gland  7.981409  
  Adult Brain  8.994843  
  Adult Carcass  7.327949  
  Adult Crop  8.377527  
  Adult Eye  9.084121  
  Adult Fatbody  7.592861  
  Adult Female Spermatheca Mated  8.116515  
  Adult Female Spermatheca Virgin  8.310389  
  Adult Head  8.250331  
  Adult Heart  8.323653  
  Adult Hind Gut  7.984229  
  Adult Male Ejaculatory Duct  8.242897  
  Adult Mid Gut  7.907477  
  Adult Ovary  9.218198  
  Adult Salivary Gland  7.789014  
  Adult Testes  9.181818  
  Adult Thoracoabdominal ganglion  9.352938  
  Adult Whole Fly  8.329090  
  Larvae Wandering Tubules  8.566009  
  Larval Feeding Carcass  8.259965  
  Larval Feeding Central Nevous System  9.502555  
  Larval Feeding Hind Gut  8.222281  
  Larval Feeding Malpighian Tubule  8.524883  
  Larval Feeding Mid Gut  7.810108  
  Larval Feeding Salivary Gland  8.546260  
  Whole Larvae Feeding  8.020725  
 
  
   FlyBase ID    symbol    start    end    strand    length   
   FBgn0004908   Arf84F   4166822   4167608  +  787  
   FBgn0037578   CG9601  4167383   4169219   -  1837  
 
    Segment 82 
 
   Location   
  Gene key  FBgn0037579-FBgn0037581  
  Heatmap region span   3R:4150057..4343586   
  Segment span   3R:4169402..4173301   
  Length (genes)  2  
  Length (bp)  3900  
   Model Scoring   
  BIC  198.818951  
  logL  -93.781745  
  logL ratio  25.671339  
   Expression   
  Mean expression  4.829792  
  Median expression  4.579533  
  Tissue std. dev.  1.338458  
 
  No GO Slim enrichment  
  
   tissue    mean expression   
  5th Passage Drosophila S2 Cells  4.917745  
  Adult Accessory gland  4.300335  
  Adult Brain  4.510451  
  Adult Carcass  4.393393  
  Adult Crop  4.130608  
  Adult Eye  4.513792  
  Adult Fatbody  4.285029  
  Adult Female Spermatheca Mated  4.279896  
  Adult Female Spermatheca Virgin  4.215302  
  Adult Head  4.244720  
  Adult Heart  4.375440  
  Adult Hind Gut  4.108545  
  Adult Male Ejaculatory Duct  4.521048  
  Adult Mid Gut  4.224258  
  Adult Ovary  5.140771  
  Adult Salivary Gland  4.433107  
  Adult Testes  11.034679  
  Adult Thoracoabdominal ganglion  4.907853  
  Adult Whole Fly  6.745541  
  Larvae Wandering Tubules  4.306407  
  Larval Feeding Carcass  4.538527  
  Larval Feeding Central Nevous System  5.534287  
  Larval Feeding Hind Gut  4.171827  
  Larval Feeding Malpighian Tubule  4.272729  
  Larval Feeding Mid Gut  4.455436  
  Larval Feeding Salivary Gland  4.418013  
  Whole Larvae Feeding  5.424656  
 
  
   FlyBase ID    symbol    start    end    strand    length   
   FBgn0037579   CG18193   4169402   4170032  +  631  
   FBgn0037581   CG7352   4173301   4175018  +  1718  
 
    Segment 83 
 
   Location   
  Gene key  FBgn0037584-FBgn0037591  
  Heatmap region span   3R:4166822..4378330   
  Segment span   3R:4188548..4338744   
  Length (genes)  6  
  Length (bp)  150197  
   Model Scoring   
  BIC  436.061098  
  logL  -212.402818  
  logL ratio  185.494334  
   Expression   
  Mean expression  4.507355  
  Median expression  4.513905  
  Tissue std. dev.  0.156091  
 
  No GO Slim enrichment  
  
   tissue    mean expression   
  5th Passage Drosophila S2 Cells  4.540329  
  Adult Accessory gland  4.533892  
  Adult Brain  4.976047  
  Adult Carcass  4.483642  
  Adult Crop  4.449359  
  Adult Eye  4.429166  
  Adult Fatbody  4.478943  
  Adult Female Spermatheca Mated  4.463823  
  Adult Female Spermatheca Virgin  4.495470  
  Adult Head  4.564980  
  Adult Heart  4.385052  
  Adult Hind Gut  4.399351  
  Adult Male Ejaculatory Duct  4.546587  
  Adult Mid Gut  4.570221  
  Adult Ovary  4.500990  
  Adult Salivary Gland  4.602713  
  Adult Testes  4.321333  
  Adult Thoracoabdominal ganglion  4.898252  
  Adult Whole Fly  4.177830  
  Larvae Wandering Tubules  4.592149  
  Larval Feeding Carcass  4.449267  
  Larval Feeding Central Nevous System  4.512606  
  Larval Feeding Hind Gut  4.354918  
  Larval Feeding Malpighian Tubule  4.514745  
  Larval Feeding Mid Gut  4.567470  
  Larval Feeding Salivary Gland  4.581822  
  Whole Larvae Feeding  4.307625  
 
  
   FlyBase ID    symbol    start    end    strand    length   
   FBgn0037584   CG7963   4188548   4189616  +  1069  
   FBgn0259182     4190813   4192494   -  1682  
   FBgn0051462   CG31462  4210766   4213924   -  3159  
   FBgn0037589   Obp85a  4245544   4246319   -  776  
   FBgn0037590   Or85b  4335799   4337080   -  1282  
   FBgn0037591   Or85c  4337456   4338744   -  1289  
 
 
    Segment 84 
 
   Location   
  Gene key  FBgn0051454-FBgn0051259  
  Heatmap region span   3R:4182689..4388574   
  Segment span   3R:4347748..4349668   
  Length (genes)  2  
  Length (bp)  1921  
   Model Scoring   
  BIC  192.441588  
  logL  -90.593063  
  logL ratio  29.678607  
   Expression   
  Mean expression  5.511523  
  Median expression  4.881087  
  Tissue std. dev.  1.460702  
 
  No GO Slim enrichment  
  
   tissue    mean expression   
  5th Passage Drosophila S2 Cells  5.168947  
  Adult Accessory gland  4.593846  
  Adult Brain  4.264513  
  Adult Carcass  5.015327  
  Adult Crop  4.627220  
  Adult Eye  4.581422  
  Adult Fatbody  5.507971  
  Adult Female Spermatheca Mated  5.600148  
  Adult Female Spermatheca Virgin  5.671677  
  Adult Head  4.520632  
  Adult Heart  5.099260  
  Adult Hind Gut  5.351395  
  Adult Male Ejaculatory Duct  4.436111  
  Adult Mid Gut  9.686608  
  Adult Ovary  4.395236  
  Adult Salivary Gland  4.963192  
  Adult Testes  4.886167  
  Adult Thoracoabdominal ganglion  4.394769  
  Adult Whole Fly  5.872017  
  Larvae Wandering Tubules  6.139417  
  Larval Feeding Carcass  4.458111  
  Larval Feeding Central Nevous System  4.557013  
  Larval Feeding Hind Gut  6.261441  
  Larval Feeding Malpighian Tubule  6.295402  
  Larval Feeding Mid Gut  10.076752  
  Larval Feeding Salivary Gland  4.656327  
  Whole Larvae Feeding  7.730191  
 
  
   FlyBase ID    symbol    start    end    strand    length   
   FBgn0051454   CG31454   4347748   4349242  +  1495  
   FBgn0051259   CG31259   4349668   4351846  +  2179  
 
    Segment 85 
 
   Location   
  Gene key  FBgn0051450-FBgn0051460  
  Heatmap region span   3R:4347748..4503865   
  Segment span   3R:4388305..4388574   
  Length (genes)  2  
  Length (bp)  270  
   Model Scoring   
  BIC  218.816777  
  logL  -103.780657  
  logL ratio  45.214361  
   Expression   
  Mean expression  9.064461  
  Median expression  8.984707  
  Tissue std. dev.  0.535895  
 
  No GO Slim enrichment  
  
   tissue    mean expression   
  5th Passage Drosophila S2 Cells  9.585040  
  Adult Accessory gland  9.190956  
  Adult Brain  9.382041  
  Adult Carcass  8.537793  
  Adult Crop  8.693873  
  Adult Eye  8.701166  
  Adult Fatbody  8.698895  
  Adult Female Spermatheca Mated  8.957051  
  Adult Female Spermatheca Virgin  8.918129  
  Adult Head  8.691353  
  Adult Heart  9.314470  
  Adult Hind Gut  8.889233  
  Adult Male Ejaculatory Duct  9.161134  
  Adult Mid Gut  8.463156  
  Adult Ovary  10.234542  
  Adult Salivary Gland  9.238380  
  Adult Testes  9.233888  
  Adult Thoracoabdominal ganglion  9.494352  
  Adult Whole Fly  9.380366  
  Larvae Wandering Tubules  8.106839  
  Larval Feeding Carcass  8.866326  
  Larval Feeding Central Nevous System  10.266011  
  Larval Feeding Hind Gut  9.084733  
  Larval Feeding Malpighian Tubule  8.639112  
  Larval Feeding Mid Gut  8.162286  
  Larval Feeding Salivary Gland  10.008636  
  Whole Larvae Feeding  8.840676  
 
  
   FlyBase ID    symbol    start    end    strand    length   
   FBgn0051450   mRpS18A  4387628   4388305   -  678  
   FBgn0051460   CG31460   4388574   4389170  +  597  
 
    Segment 86 
 
   Location   
  Gene key  FBgn0024326-FBgn0037606  
  Heatmap region span   3R:4378330..4523540   
  Segment span   3R:4476516..4490085   
  Length (genes)  7  
  Length (bp)  13570  
   Model Scoring   
  BIC  664.680807  
  logL  -326.712673  
  logL ratio  250.900802  
   Expression   
  Mean expression  9.178473  
  Median expression  9.242394  
  Tissue std. dev.  0.307535  
 
  No GO Slim enrichment  
  
   tissue    mean expression   
  5th Passage Drosophila S2 Cells  9.403936  
  Adult Accessory gland  9.508153  
  Adult Brain  9.532106  
  Adult Carcass  8.797697  
  Adult Crop  9.119860  
  Adult Eye  9.435344  
  Adult Fatbody  9.172630  
  Adult Female Spermatheca Mated  9.194233  
  Adult Female Spermatheca Virgin  9.174014  
  Adult Head  9.133286  
  Adult Heart  9.510393  
  Adult Hind Gut  8.962876  
  Adult Male Ejaculatory Duct  9.479352  
  Adult Mid Gut  8.905896  
  Adult Ovary  9.124575  
  Adult Salivary Gland  9.241495  
  Adult Testes  8.696576  
  Adult Thoracoabdominal ganglion  9.553224  
  Adult Whole Fly  8.697535  
  Larvae Wandering Tubules  9.313669  
  Larval Feeding Carcass  8.826128  
  Larval Feeding Central Nevous System  9.451944  
  Larval Feeding Hind Gut  8.951564  
  Larval Feeding Malpighian Tubule  9.500447  
  Larval Feeding Mid Gut  8.712861  
  Larval Feeding Salivary Gland  9.723711  
  Whole Larvae Feeding  8.695251  
 
  
   FlyBase ID    symbol    start    end    strand    length   
   FBgn0024326   Mkk4  4473306   4476516   -  3211  
   FBgn0037602   CG8021   4476806   4477215  +  410  
   FBgn0037603   CG11753   4478738   4479569  +  832  
   FBgn0000447   Dhod  4477113   4478992   -  1880  
   FBgn0000171     4481309   4486483   -  5175  
   FBgn0086679   p  4486625   4489607   -  2983  
   FBgn0037606   CG8032   4490085   4493076  +  2992  
 
 
    Segment 87 
 
   Location   
  Gene key  FBgn0037608-FBgn0037611  
  Heatmap region span   3R:4388305..4547093   
  Segment span   3R:4499839..4503865   
  Length (genes)  4  
  Length (bp)  4027  
   Model Scoring   
  BIC  395.858673  
  logL  -192.301605  
  logL ratio  106.314193  
   Expression   
  Mean expression  8.201595  
  Median expression  8.234074  
  Tissue std. dev.  0.394964  
 
  No GO Slim enrichment  
  
   tissue    mean expression   
  5th Passage Drosophila S2 Cells  8.676766  
  Adult Accessory gland  8.330535  
  Adult Brain  8.255541  
  Adult Carcass  7.898661  
  Adult Crop  7.959061  
  Adult Eye  8.059879  
  Adult Fatbody  8.083020  
  Adult Female Spermatheca Mated  8.286544  
  Adult Female Spermatheca Virgin  8.176713  
  Adult Head  7.803782  
  Adult Heart  8.513114  
  Adult Hind Gut  7.994125  
  Adult Male Ejaculatory Duct  8.050558  
  Adult Mid Gut  7.895869  
  Adult Ovary  9.206853  
  Adult Salivary Gland  8.468124  
  Adult Testes  9.099112  
  Adult Thoracoabdominal ganglion  8.374379  
  Adult Whole Fly  8.564429  
  Larvae Wandering Tubules  8.075565  
  Larval Feeding Carcass  7.706687  
  Larval Feeding Central Nevous System  8.212675  
  Larval Feeding Hind Gut  8.017825  
  Larval Feeding Malpighian Tubule  8.305886  
  Larval Feeding Mid Gut  7.546826  
  Larval Feeding Salivary Gland  8.409249  
  Whole Larvae Feeding  7.471288  
 
  
   FlyBase ID    symbol    start    end    strand    length   
   FBgn0037608   mRpL19   4499839   4500918  +  1080  
   FBgn0037609   CG9773  4501005   4502349   -  1345  
   FBgn0037610   CG8043   4502639   4503955  +  1317  
   FBgn0037611   CG11755   4503865   4504677  +  813  
 
 
    Segment 88 
 
   Location   
  Gene key  FBgn0037612-FBgn0037615  
  Heatmap region span   3R:4493726..4557819   
  Segment span   3R:4526704..4544920   
  Length (genes)  4  
  Length (bp)  18217  
   Model Scoring   
  BIC  453.616929  
  logL  -221.180733  
  logL ratio  48.123125  
   Expression   
  Mean expression  8.073554  
  Median expression  8.096911  
  Tissue std. dev.  0.682733  
 
  No GO Slim enrichment  
  
   tissue    mean expression   
  5th Passage Drosophila S2 Cells  9.016746  
  Adult Accessory gland  6.873392  
  Adult Brain  8.701036  
  Adult Carcass  8.829589  
  Adult Crop  7.788038  
  Adult Eye  8.558628  
  Adult Fatbody  8.776640  
  Adult Female Spermatheca Mated  8.059296  
  Adult Female Spermatheca Virgin  7.537786  
  Adult Head  8.351539  
  Adult Heart  9.211394  
  Adult Hind Gut  7.781635  
  Adult Male Ejaculatory Duct  6.989688  
  Adult Mid Gut  7.834566  
  Adult Ovary  9.413100  
  Adult Salivary Gland  7.431110  
  Adult Testes  7.308024  
  Adult Thoracoabdominal ganglion  8.540881  
  Adult Whole Fly  8.740422  
  Larvae Wandering Tubules  8.574188  
  Larval Feeding Carcass  8.106733  
  Larval Feeding Central Nevous System  7.910484  
  Larval Feeding Hind Gut  7.384370  
  Larval Feeding Malpighian Tubule  7.998893  
  Larval Feeding Mid Gut  7.337786  
  Larval Feeding Salivary Gland  7.413038  
  Whole Larvae Feeding  7.516945  
 
  
   FlyBase ID    symbol    start    end    strand    length   
   FBgn0037612   CG8112   4526704   4540990  +  14287  
   FBgn0037613   Cks85A  4540922   4541600   -  679  
   FBgn0037614   CG8116   4541956   4547050  +  5095  
   FBgn0037615   CG11760   4544920   4545867  +  948  
 
 
    Segment 89 
 
   Location   
  Gene key  FBgn0037617-FBgn0037620  
  Heatmap region span   3R:4506052..4598408   
  Segment span   3R:4548895..4555415   
  Length (genes)  4  
  Length (bp)  6521  
   Model Scoring   
  BIC  436.417577  
  logL  -212.581058  
  logL ratio  29.338405  
   Expression   
  Mean expression  6.532589  
  Median expression  6.420910  
  Tissue std. dev.  0.628787  
 
  
   GO ID    description    ratio    P-value   
   GO:0005634   nucleus  3/4  0.0069  
   GO:0043226   organelle  3/4  0.0221  
 
  
   tissue    mean expression   
  5th Passage Drosophila S2 Cells  6.717389  
  Adult Accessory gland  6.149433  
  Adult Brain  6.756660  
  Adult Carcass  5.910585  
  Adult Crop  6.301873  
  Adult Eye  6.693056  
  Adult Fatbody  6.162131  
  Adult Female Spermatheca Mated  6.450175  
  Adult Female Spermatheca Virgin  6.421747  
  Adult Head  6.129117  
  Adult Heart  6.333971  
  Adult Hind Gut  6.198707  
  Adult Male Ejaculatory Duct  6.118148  
  Adult Mid Gut  6.683414  
  Adult Ovary  7.312324  
  Adult Salivary Gland  6.442496  
  Adult Testes  6.186997  
  Adult Thoracoabdominal ganglion  6.472367  
  Adult Whole Fly  6.100834  
  Larvae Wandering Tubules  8.549406  
  Larval Feeding Carcass  5.956740  
  Larval Feeding Central Nevous System  6.749974  
  Larval Feeding Hind Gut  6.246544  
  Larval Feeding Malpighian Tubule  8.370293  
  Larval Feeding Mid Gut  6.111473  
  Larval Feeding Salivary Gland  6.803115  
  Whole Larvae Feeding  6.050923  
 
  
   FlyBase ID    symbol    start    end    strand    length   
   FBgn0037617   CG8145   4548895   4550434  +  1540  
   FBgn0037618   CG11762   4551201   4552271  +  1071  
   FBgn0037619   CG8159   4552576   4554092  +  1517  
   FBgn0037620   ranshi  4554089   4555415   -  1327  
 
 
    Segment 90 
 
   Location   
  Gene key  FBgn0037623-FBgn0037624  
  Heatmap region span   3R:4547093..4618269   
  Segment span   3R:4573372..4573528   
  Length (genes)  2  
  Length (bp)  157  
   Model Scoring   
  BIC  259.015920  
  logL  -123.880229  
  logL ratio  13.724234  
   Expression   
  Mean expression  9.153837  
  Median expression  9.163256  
  Tissue std. dev.  0.948109  
 
  No GO Slim enrichment  
  
   tissue    mean expression   
  5th Passage Drosophila S2 Cells  7.659949  
  Adult Accessory gland  9.181667  
  Adult Brain  9.844995  
  Adult Carcass  8.223798  
  Adult Crop  9.798764  
  Adult Eye  8.940694  
  Adult Fatbody  8.118702  
  Adult Female Spermatheca Mated  8.674206  
  Adult Female Spermatheca Virgin  8.640583  
  Adult Head  9.065002  
  Adult Heart  8.423811  
  Adult Hind Gut  9.471428  
  Adult Male Ejaculatory Duct  9.839572  
  Adult Mid Gut  7.500267  
  Adult Ovary  10.170195  
  Adult Salivary Gland  8.684159  
  Adult Testes  7.256544  
  Adult Thoracoabdominal ganglion  9.680993  
  Adult Whole Fly  9.131482  
  Larvae Wandering Tubules  11.016891  
  Larval Feeding Carcass  9.062852  
  Larval Feeding Central Nevous System  11.008900  
  Larval Feeding Hind Gut  9.765308  
  Larval Feeding Malpighian Tubule  10.109449  
  Larval Feeding Mid Gut  9.075938  
  Larval Feeding Salivary Gland  10.226101  
  Whole Larvae Feeding  8.581338  
 
  
   FlyBase ID    symbol    start    end    strand    length   
   FBgn0037623   CG9801  4561601   4573372   -  11772  
   FBgn0037624   CG8223   4573528   4579827  +  6300  
 
    Segment 91 
 
   Location   
  Gene key  FBgn0083971-FBgn0037627  
  Heatmap region span   3R:4548895..4620406   
  Segment span   3R:4578463..4598408   
  Length (genes)  4  
  Length (bp)  19946  
   Model Scoring   
  BIC  406.408561  
  logL  -197.576549  
  logL ratio  20.879964  
   Expression   
  Mean expression  5.477194  
  Median expression  5.353216  
  Tissue std. dev.  0.384180  
 
  No GO Slim enrichment  
  
   tissue    mean expression   
  5th Passage Drosophila S2 Cells  5.291635  
  Adult Accessory gland  5.190985  
  Adult Brain  5.704639  
  Adult Carcass  5.368650  
  Adult Crop  5.245537  
  Adult Eye  5.931361  
  Adult Fatbody  5.218078  
  Adult Female Spermatheca Mated  6.394590  
  Adult Female Spermatheca Virgin  6.138232  
  Adult Head  5.907703  
  Adult Heart  5.033728  
  Adult Hind Gut  5.249379  
  Adult Male Ejaculatory Duct  5.195309  
  Adult Mid Gut  5.327584  
  Adult Ovary  5.009651  
  Adult Salivary Gland  5.388530  
  Adult Testes  6.282499  
  Adult Thoracoabdominal ganglion  6.111746  
  Adult Whole Fly  5.349967  
  Larvae Wandering Tubules  5.278652  
  Larval Feeding Carcass  5.468129  
  Larval Feeding Central Nevous System  5.060480  
  Larval Feeding Hind Gut  5.146331  
  Larval Feeding Malpighian Tubule  5.325438  
  Larval Feeding Mid Gut  5.512516  
  Larval Feeding Salivary Gland  5.339267  
  Whole Larvae Feeding  5.413627  
 
  
   FlyBase ID    symbol    start    end    strand    length   
   FBgn0083971   CG34135  4574928   4578463   -  3536  
   FBgn0037625   CG11768  4579760   4593922   -  14163  
   FBgn0037626   CG8236   4595116   4596875  +  1760  
   FBgn0037627   CG13318  4596969   4598408   -  1440  
 
 
    Segment 92 
 
   Location   
  Gene key  FBgn0053191-FBgn0053189  
  Heatmap region span   3R:4557819..4635588   
  Segment span   3R:4605645..4606511   
  Length (genes)  2  
  Length (bp)  867  
   Model Scoring   
  BIC  172.882527  
  logL  -80.813532  
  logL ratio  40.913275  
   Expression   
  Mean expression  5.216776  
  Median expression  4.835539  
  Tissue std. dev.  1.403908  
 
  No GO Slim enrichment  
  
   tissue    mean expression   
  5th Passage Drosophila S2 Cells  5.011769  
  Adult Accessory gland  5.018242  
  Adult Brain  4.546801  
  Adult Carcass  5.149486  
  Adult Crop  4.773651  
  Adult Eye  4.555228  
  Adult Fatbody  4.893938  
  Adult Female Spermatheca Mated  4.784766  
  Adult Female Spermatheca Virgin  4.910309  
  Adult Head  4.653360  
  Adult Heart  4.679524  
  Adult Hind Gut  4.669583  
  Adult Male Ejaculatory Duct  4.905759  
  Adult Mid Gut  5.151137  
  Adult Ovary  4.729719  
  Adult Salivary Gland  5.117413  
  Adult Testes  11.850958  
  Adult Thoracoabdominal ganglion  4.654298  
  Adult Whole Fly  7.339294  
  Larvae Wandering Tubules  4.877108  
  Larval Feeding Carcass  4.929727  
  Larval Feeding Central Nevous System  4.476175  
  Larval Feeding Hind Gut  4.638681  
  Larval Feeding Malpighian Tubule  4.874149  
  Larval Feeding Mid Gut  5.143510  
  Larval Feeding Salivary Gland  4.830274  
  Whole Larvae Feeding  5.688098  
 
  
   FlyBase ID    symbol    start    end    strand    length   
   FBgn0053191   CG33191  4604099   4605645   -  1547  
   FBgn0053189   CG33189  4605835   4606511   -  677  
 
    Segment 93 
 
   Location   
  Gene key  FBgn0037633-FBgn0037634  
  Heatmap region span   3R:4599157..4688504   
  Segment span   3R:4631352..4632046   
  Length (genes)  2  
  Length (bp)  695  
   Model Scoring   
  BIC  218.997919  
  logL  -103.871229  
  logL ratio  31.576159  
   Expression   
  Mean expression  7.091342  
  Median expression  6.868736  
  Tissue std. dev.  0.675063  
 
  No GO Slim enrichment  
  
   tissue    mean expression   
  5th Passage Drosophila S2 Cells  7.922942  
  Adult Accessory gland  6.987412  
  Adult Brain  7.596570  
  Adult Carcass  6.250288  
  Adult Crop  7.435420  
  Adult Eye  7.410750  
  Adult Fatbody  6.348552  
  Adult Female Spermatheca Mated  6.919958  
  Adult Female Spermatheca Virgin  6.776008  
  Adult Head  6.899760  
  Adult Heart  6.838189  
  Adult Hind Gut  6.795445  
  Adult Male Ejaculatory Duct  6.384974  
  Adult Mid Gut  6.429474  
  Adult Ovary  8.915085  
  Adult Salivary Gland  6.627243  
  Adult Testes  7.244877  
  Adult Thoracoabdominal ganglion  7.657159  
  Adult Whole Fly  7.468310  
  Larvae Wandering Tubules  6.816391  
  Larval Feeding Carcass  6.719095  
  Larval Feeding Central Nevous System  8.896880  
  Larval Feeding Hind Gut  6.844177  
  Larval Feeding Malpighian Tubule  6.939473  
  Larval Feeding Mid Gut  6.218187  
  Larval Feeding Salivary Gland  7.424573  
  Whole Larvae Feeding  6.699052  
 
  
   FlyBase ID    symbol    start    end    strand    length   
   FBgn0037633   CG9839  4629193   4631352   -  2160  
   FBgn0037634   CG8359   4632046   4633303  +  1258  
 
    Segment 94 
 
   Location   
  Gene key  FBgn0037636-FBgn0037637  
  Heatmap region span   3R:4618269..4760853   
  Segment span   3R:4646386..4649895   
  Length (genes)  3  
  Length (bp)  3510  
   Model Scoring   
  BIC  407.772971  
  logL  -198.258754  
  logL ratio  110.156433  
   Expression   
  Mean expression  10.974993  
  Median expression  11.124308  
  Tissue std. dev.  1.120655  
 
  No GO Slim enrichment  
  
   tissue    mean expression   
  5th Passage Drosophila S2 Cells  10.351722  
  Adult Accessory gland  10.872572  
  Adult Brain  13.408368  
  Adult Carcass  11.598956  
  Adult Crop  10.836351  
  Adult Eye  12.338297  
  Adult Fatbody  11.100976  
  Adult Female Spermatheca Mated  11.258729  
  Adult Female Spermatheca Virgin  11.540989  
  Adult Head  12.105587  
  Adult Heart  11.599745  
  Adult Hind Gut  11.835748  
  Adult Male Ejaculatory Duct  11.705659  
  Adult Mid Gut  9.627219  
  Adult Ovary  9.298666  
  Adult Salivary Gland  10.851142  
  Adult Testes  9.424000  
  Adult Thoracoabdominal ganglion  13.281084  
  Adult Whole Fly  11.063870  
  Larvae Wandering Tubules  9.968561  
  Larval Feeding Carcass  9.861340  
  Larval Feeding Central Nevous System  11.508947  
  Larval Feeding Hind Gut  11.452469  
  Larval Feeding Malpighian Tubule  9.398222  
  Larval Feeding Mid Gut  9.026676  
  Larval Feeding Salivary Gland  10.285017  
  Whole Larvae Feeding  10.723887  
 
  
   FlyBase ID    symbol    start    end    strand    length   
   FBgn0037636   CG9821  4640407   4646386   -  5980  
   FBgn0040532   CG8369   4647730   4648582  +  853  
   FBgn0037637   CG9836  4648571   4649895   -  1325  
 
 
    Segment 95 
 
   Location   
  Gene key  FBgn0037646-FBgn0037648  
  Heatmap region span   3R:4760853..4834157   
  Segment span   3R:4806643..4818979   
  Length (genes)  4  
  Length (bp)  12337  
   Model Scoring   
  BIC  491.331794  
  logL  -240.038166  
  logL ratio  20.764718  
   Expression   
  Mean expression  8.518069  
  Median expression  8.751372  
  Tissue std. dev.  0.460164  
 
  No GO Slim enrichment  
  
   tissue    mean expression   
  5th Passage Drosophila S2 Cells  8.863273  
  Adult Accessory gland  7.875093  
  Adult Brain  8.407858  
  Adult Carcass  8.445062  
  Adult Crop  8.665963  
  Adult Eye  8.532473  
  Adult Fatbody  9.218967  
  Adult Female Spermatheca Mated  8.866206  
  Adult Female Spermatheca Virgin  8.984974  
  Adult Head  8.615911  
  Adult Heart  8.982143  
  Adult Hind Gut  8.584344  
  Adult Male Ejaculatory Duct  8.544598  
  Adult Mid Gut  8.453919  
  Adult Ovary  9.179830  
  Adult Salivary Gland  8.759427  
  Adult Testes  6.923835  
  Adult Thoracoabdominal ganglion  8.287036  
  Adult Whole Fly  8.201068  
  Larvae Wandering Tubules  8.814401  
  Larval Feeding Carcass  7.969504  
  Larval Feeding Central Nevous System  8.213600  
  Larval Feeding Hind Gut  8.718725  
  Larval Feeding Malpighian Tubule  8.917375  
  Larval Feeding Mid Gut  8.565950  
  Larval Feeding Salivary Gland  8.178275  
  Whole Larvae Feeding  8.218064  
 
  
   FlyBase ID    symbol    start    end    strand    length   
   FBgn0037646   CAHbeta   4806643   4808553  +  1911  
   FBgn0037647   RagA  4808446   4810029   -  1584  
   FBgn0027503   CG11970   4810654   4816371  +  5718  
   FBgn0037648   CG11975   4818979   4820561  +  1583  
 
 
    Segment 96 
 
   Location   
  Gene key  FBgn0037652-FBgn0037653  
  Heatmap region span   3R:4788683..4864001   
  Segment span   3R:4829740..4832174   
  Length (genes)  3  
  Length (bp)  2435  
   Model Scoring   
  BIC  439.212393  
  logL  -213.978465  
  logL ratio  23.881191  
   Expression   
  Mean expression  10.276816  
  Median expression  9.613250  
  Tissue std. dev.  0.369666  
 
  No GO Slim enrichment  
  
   tissue    mean expression   
  5th Passage Drosophila S2 Cells  10.230116  
  Adult Accessory gland  10.605096  
  Adult Brain  9.719247  
  Adult Carcass  10.163723  
  Adult Crop  10.402354  
  Adult Eye  10.179820  
  Adult Fatbody  10.410186  
  Adult Female Spermatheca Mated  9.726126  
  Adult Female Spermatheca Virgin  9.807614  
  Adult Head  9.867522  
  Adult Heart  10.564265  
  Adult Hind Gut  10.163328  
  Adult Male Ejaculatory Duct  10.585080  
  Adult Mid Gut  9.927434  
  Adult Ovary  11.070693  
  Adult Salivary Gland  10.219573  
  Adult Testes  10.649363  
  Adult Thoracoabdominal ganglion  9.591715  
  Adult Whole Fly  10.624864  
  Larvae Wandering Tubules  10.709147  
  Larval Feeding Carcass  10.413333  
  Larval Feeding Central Nevous System  10.717119  
  Larval Feeding Hind Gut  10.376167  
  Larval Feeding Malpighian Tubule  10.435916  
  Larval Feeding Mid Gut  9.905954  
  Larval Feeding Salivary Gland  10.567457  
  Whole Larvae Feeding  9.840813  
 
  
   FlyBase ID    symbol    start    end    strand    length   
   FBgn0037652   CG11980   4829740   4831053  +  1314  
   FBgn0026380   Prosbeta3  4831035   4831948   -  914  
   FBgn0037653   CG11982   4832174   4833752  +  1579  
 
 
    Segment 97 
 
   Location   
  Gene key  FBgn0037655-FBgn0040534  
  Heatmap region span   3R:4823419..4878240   
  Segment span   3R:4840631..4841005   
  Length (genes)  2  
  Length (bp)  375  
   Model Scoring   
  BIC  219.833421  
  logL  -104.288979  
  logL ratio  58.017551  
   Expression   
  Mean expression  9.792422  
  Median expression  9.773101  
  Tissue std. dev.  0.343466  
 
  No GO Slim enrichment  
  
   tissue    mean expression   
  5th Passage Drosophila S2 Cells  9.614506  
  Adult Accessory gland  9.713188  
  Adult Brain  10.321504  
  Adult Carcass  9.789691  
  Adult Crop  9.977718  
  Adult Eye  9.961209  
  Adult Fatbody  9.590668  
  Adult Female Spermatheca Mated  9.605619  
  Adult Female Spermatheca Virgin  9.633482  
  Adult Head  9.890805  
  Adult Heart  9.728758  
  Adult Hind Gut  9.619540  
  Adult Male Ejaculatory Duct  9.760624  
  Adult Mid Gut  9.269527  
  Adult Ovary  10.737168  
  Adult Salivary Gland  9.607491  
  Adult Testes  9.920250  
  Adult Thoracoabdominal ganglion  10.078582  
  Adult Whole Fly  10.070539  
  Larvae Wandering Tubules  9.886848  
  Larval Feeding Carcass  9.484786  
  Larval Feeding Central Nevous System  10.570982  
  Larval Feeding Hind Gut  9.482074  
  Larval Feeding Malpighian Tubule  9.599317  
  Larval Feeding Mid Gut  9.291297  
  Larval Feeding Salivary Gland  9.829717  
  Whole Larvae Feeding  9.359519  
 
  
   FlyBase ID    symbol    start    end    strand    length   
   FBgn0037655   CG11984  4836004   4840631   -  4628  
   FBgn0040534   CG11985  4840694   4841005   -  312  
 
    Segment 98 
 
   Location   
  Gene key  FBgn0015014-FBgn0037657  
  Heatmap region span   3R:4829740..5066376   
  Segment span   3R:4845739..4864001   
  Length (genes)  2  
  Length (bp)  18263  
   Model Scoring   
  BIC  207.562272  
  logL  -98.153405  
  logL ratio  53.621160  
   Expression   
  Mean expression  9.005422  
  Median expression  8.939202  
  Tissue std. dev.  0.542830  
 
  No GO Slim enrichment  
  
   tissue    mean expression   
  5th Passage Drosophila S2 Cells  9.640826  
  Adult Accessory gland  9.514900  
  Adult Brain  9.466545  
  Adult Carcass  8.362236  
  Adult Crop  9.325152  
  Adult Eye  8.659498  
  Adult Fatbody  8.428504  
  Adult Female Spermatheca Mated  9.199527  
  Adult Female Spermatheca Virgin  9.147851  
  Adult Head  8.859943  
  Adult Heart  8.514308  
  Adult Hind Gut  8.766333  
  Adult Male Ejaculatory Duct  9.354947  
  Adult Mid Gut  8.394445  
  Adult Ovary  9.877558  
  Adult Salivary Gland  9.313796  
  Adult Testes  7.718039  
  Adult Thoracoabdominal ganglion  9.194353  
  Adult Whole Fly  9.010808  
  Larvae Wandering Tubules  9.035871  
  Larval Feeding Carcass  8.969756  
  Larval Feeding Central Nevous System  10.229893  
  Larval Feeding Hind Gut  8.953724  
  Larval Feeding Malpighian Tubule  8.805543  
  Larval Feeding Mid Gut  8.294926  
  Larval Feeding Salivary Gland  9.617589  
  Whole Larvae Feeding  8.489528  
 
  
   FlyBase ID    symbol    start    end    strand    length   
   FBgn0015014     4842492   4845739   -  3248  
   FBgn0037657   hyx   4864001   4868072  +  4072  
 
    Segment 99 
 
   Location   
  Gene key  FBgn0014018-FBgn0037659  
  Heatmap region span   3R:4840631..5079267   
  Segment span   3R:4873699..4878240   
  Length (genes)  4  
  Length (bp)  4542  
   Model Scoring   
  BIC  421.296009  
  logL  -205.020274  
  logL ratio  109.174108  
   Expression   
  Mean expression  9.147416  
  Median expression  8.889347  
  Tissue std. dev.  0.559743  
 
  No GO Slim enrichment  
  
   tissue    mean expression   
  5th Passage Drosophila S2 Cells  9.873642  
  Adult Accessory gland  9.091718  
  Adult Brain  9.001456  
  Adult Carcass  8.770788  
  Adult Crop  9.675048  
  Adult Eye  9.346279  
  Adult Fatbody  9.455713  
  Adult Female Spermatheca Mated  9.590271  
  Adult Female Spermatheca Virgin  10.053635  
  Adult Head  9.265848  
  Adult Heart  9.549526  
  Adult Hind Gut  9.503999  
  Adult Male Ejaculatory Duct  9.316743  
  Adult Mid Gut  8.268246  
  Adult Ovary  9.503262  
  Adult Salivary Gland  8.669561  
  Adult Testes  7.233375  
  Adult Thoracoabdominal ganglion  8.892408  
  Adult Whole Fly  8.928498  
  Larvae Wandering Tubules  9.110644  
  Larval Feeding Carcass  9.677851  
  Larval Feeding Central Nevous System  9.411059  
  Larval Feeding Hind Gut  9.601002  
  Larval Feeding Malpighian Tubule  8.702618  
  Larval Feeding Mid Gut  8.758380  
  Larval Feeding Salivary Gland  8.990906  
  Whole Larvae Feeding  8.737748  
 
  
   FlyBase ID    symbol    start    end    strand    length   
   FBgn0014018   Rel  4869900   4873699   -  3800  
   FBgn0010222   Nmdmc  4868226   4876271   -  8046  
   FBgn0028708   Mst85C   4876546   4877953  +  1408  
   FBgn0037659   Kdm2   4878240   4888967  +  10728  
 
 
    Segment 100 
 
   Location   
  Gene key  FBgn0037660-FBgn0003165  
  Heatmap region span   3R:4841168..5080774   
  Segment span   3R:4890819..5063417   
  Length (genes)  4  
  Length (bp)  172599  
   Model Scoring   
  BIC  494.996971  
  logL  -241.870754  
  logL ratio  -33.825706  
   Expression   
  Mean expression  6.203591  
  Median expression  6.217510  
  Tissue std. dev.  0.469035  
 
  No GO Slim enrichment  
  
   tissue    mean expression   
  5th Passage Drosophila S2 Cells  7.147489  
  Adult Accessory gland  6.041490  
  Adult Brain  6.421216  
  Adult Carcass  5.700999  
  Adult Crop  5.941047  
  Adult Eye  6.534570  
  Adult Fatbody  5.873665  
  Adult Female Spermatheca Mated  5.831068  
  Adult Female Spermatheca Virgin  5.915145  
  Adult Head  5.949683  
  Adult Heart  6.471922  
  Adult Hind Gut  5.853558  
  Adult Male Ejaculatory Duct  5.898848  
  Adult Mid Gut  5.793918  
  Adult Ovary  7.731052  
  Adult Salivary Gland  5.809403  
  Adult Testes  6.325799  
  Adult Thoracoabdominal ganglion  6.039084  
  Adult Whole Fly  6.484933  
  Larvae Wandering Tubules  6.066298  
  Larval Feeding Carcass  6.451104  
  Larval Feeding Central Nevous System  6.858632  
  Larval Feeding Hind Gut  6.116554  
  Larval Feeding Malpighian Tubule  6.368711  
  Larval Feeding Mid Gut  5.692079  
  Larval Feeding Salivary Gland  6.444447  
  Whole Larvae Feeding  5.734238  
 
  
   FlyBase ID    symbol    start    end    strand    length   
   FBgn0037660   beag  4888854   4890819   -  1966  
   FBgn0037661   Ada   4890901   4892025  +  1125  
   FBgn0037662   CG11997   5003239   5004627  +  1389  
   FBgn0003165   pum  4893300   5063417   -  170118  
 
 
    Segment 101 
 
   Location   
  Gene key  FBgn0037665-FBgn0037666  
  Heatmap region span   3R:4873699..5089464   
  Segment span   3R:5077575..5079267   
  Length (genes)  2  
  Length (bp)  1693  
   Model Scoring   
  BIC  174.138118  
  logL  -81.441328  
  logL ratio  43.244476  
   Expression   
  Mean expression  4.228606  
  Median expression  4.203158  
  Tissue std. dev.  0.161055  
 
  No GO Slim enrichment  
  
   tissue    mean expression   
  5th Passage Drosophila S2 Cells  4.180638  
  Adult Accessory gland  4.632453  
  Adult Brain  4.017506  
  Adult Carcass  4.108071  
  Adult Crop  4.192031  
  Adult Eye  4.401624  
  Adult Fatbody  4.442899  
  Adult Female Spermatheca Mated  4.427219  
  Adult Female Spermatheca Virgin  4.214603  
  Adult Head  4.070279  
  Adult Heart  4.203641  
  Adult Hind Gut  4.094271  
  Adult Male Ejaculatory Duct  4.475359  
  Adult Mid Gut  4.239577  
  Adult Ovary  4.213237  
  Adult Salivary Gland  4.311988  
  Adult Testes  4.048568  
  Adult Thoracoabdominal ganglion  4.066679  
  Adult Whole Fly  3.933748  
  Larvae Wandering Tubules  4.325960  
  Larval Feeding Carcass  4.431639  
  Larval Feeding Central Nevous System  4.100863  
  Larval Feeding Hind Gut  4.069384  
  Larval Feeding Malpighian Tubule  4.211569  
  Larval Feeding Mid Gut  4.271961  
  Larval Feeding Salivary Gland  4.186101  
  Whole Larvae Feeding  4.300482  
 
  
   FlyBase ID    symbol    start    end    strand    length   
   FBgn0037665   St2   5077575   5078687  +  1113  
   FBgn0037666   CR16735   5079267   5080405  +  1139  
 
    Segment 102 
 
   Location   
  Gene key  FBgn0037669-FBgn0037670  
  Heatmap region span   3R:5073173..5156047   
  Segment span   3R:5084223..5084691   
  Length (genes)  2  
  Length (bp)  469  
   Model Scoring   
  BIC  210.644933  
  logL  -99.694735  
  logL ratio  45.479170  
   Expression   
  Mean expression  8.250243  
  Median expression  8.170168  
  Tissue std. dev.  0.565597  
 
  No GO Slim enrichment  
  
   tissue    mean expression   
  5th Passage Drosophila S2 Cells  8.712750  
  Adult Accessory gland  8.274520  
  Adult Brain  8.334718  
  Adult Carcass  7.593623  
  Adult Crop  8.232658  
  Adult Eye  8.285685  
  Adult Fatbody  7.750854  
  Adult Female Spermatheca Mated  8.306273  
  Adult Female Spermatheca Virgin  8.070123  
  Adult Head  7.976407  
  Adult Heart  8.352432  
  Adult Hind Gut  7.846523  
  Adult Male Ejaculatory Duct  8.451872  
  Adult Mid Gut  7.476846  
  Adult Ovary  9.556958  
  Adult Salivary Gland  7.714879  
  Adult Testes  8.612103  
  Adult Thoracoabdominal ganglion  7.943054  
  Adult Whole Fly  8.499745  
  Larvae Wandering Tubules  7.906387  
  Larval Feeding Carcass  8.107889  
  Larval Feeding Central Nevous System  10.056850  
  Larval Feeding Hind Gut  8.106641  
  Larval Feeding Malpighian Tubule  8.087739  
  Larval Feeding Mid Gut  7.393033  
  Larval Feeding Salivary Gland  8.837232  
  Whole Larvae Feeding  8.268767  
 
  
   FlyBase ID    symbol    start    end    strand    length   
   FBgn0037669   CG9740  5083449   5084223   -  775  
   FBgn0037670   CG8436   5084691   5086122  +  1432  
 
    Segment 103 
 
   Location   
  Gene key  FBgn0037680-FBgn0053936  
  Heatmap region span   3R:5154227..5220265   
  Segment span   3R:5160897..5167592   
  Length (genes)  2  
  Length (bp)  6696  
   Model Scoring   
  BIC  285.056815  
  logL  -136.900677  
  logL ratio  5.010115  
   Expression   
  Mean expression  9.802206  
  Median expression  10.325073  
  Tissue std. dev.  0.778676  
 
  No GO Slim enrichment  
  
   tissue    mean expression   
  5th Passage Drosophila S2 Cells  9.904051  
  Adult Accessory gland  10.424115  
  Adult Brain  9.933719  
  Adult Carcass  9.890344  
  Adult Crop  10.925947  
  Adult Eye  10.260048  
  Adult Fatbody  9.260419  
  Adult Female Spermatheca Mated  10.322418  
  Adult Female Spermatheca Virgin  10.188247  
  Adult Head  9.943503  
  Adult Heart  9.468653  
  Adult Hind Gut  10.490011  
  Adult Male Ejaculatory Duct  10.240257  
  Adult Mid Gut  8.482249  
  Adult Ovary  10.609375  
  Adult Salivary Gland  10.361813  
  Adult Testes  7.717320  
  Adult Thoracoabdominal ganglion  9.664877  
  Adult Whole Fly  9.783415  
  Larvae Wandering Tubules  8.727868  
  Larval Feeding Carcass  10.522998  
  Larval Feeding Central Nevous System  9.862001  
  Larval Feeding Hind Gut  10.747947  
  Larval Feeding Malpighian Tubule  8.322694  
  Larval Feeding Mid Gut  8.808051  
  Larval Feeding Salivary Gland  10.150151  
  Whole Larvae Feeding  9.647070  
 
  
   FlyBase ID    symbol    start    end    strand    length   
   FBgn0037680   CG8121   5160897   5162987  +  2091  
   FBgn0053936      5167592   5185154  +  17563  
 
    Segment 104 
 
   Location   
  Gene key  FBgn0037687-FBgn0037689  
  Heatmap region span   3R:5220265..5320525   
  Segment span   3R:5227242..5230097   
  Length (genes)  3  
  Length (bp)  2856  
   Model Scoring   
  BIC  314.357572  
  logL  -151.551055  
  logL ratio  72.223277  
   Expression   
  Mean expression  8.729217  
  Median expression  8.610999  
  Tissue std. dev.  0.385109  
 
  No GO Slim enrichment  
  
   tissue    mean expression   
  5th Passage Drosophila S2 Cells  8.611324  
  Adult Accessory gland  9.577982  
  Adult Brain  8.692509  
  Adult Carcass  8.443449  
  Adult Crop  8.263399  
  Adult Eye  9.041826  
  Adult Fatbody  8.744961  
  Adult Female Spermatheca Mated  8.739561  
  Adult Female Spermatheca Virgin  8.853574  
  Adult Head  8.691039  
  Adult Heart  8.804778  
  Adult Hind Gut  8.455005  
  Adult Male Ejaculatory Duct  9.115574  
  Adult Mid Gut  8.628469  
  Adult Ovary  8.730290  
  Adult Salivary Gland  8.897963  
  Adult Testes  8.157854  
  Adult Thoracoabdominal ganglion  8.489834  
  Adult Whole Fly  8.144695  
  Larvae Wandering Tubules  9.232476  
  Larval Feeding Carcass  8.424553  
  Larval Feeding Central Nevous System  8.413926  
  Larval Feeding Hind Gut  8.470157  
  Larval Feeding Malpighian Tubule  9.510875  
  Larval Feeding Mid Gut  8.612589  
  Larval Feeding Salivary Gland  9.521265  
  Whole Larvae Feeding  8.418939  
 
  
   FlyBase ID    symbol    start    end    strand    length   
   FBgn0037687   CG8132   5227242   5228340  +  1099  
   FBgn0037688   CG9356  5228286   5229991   -  1706  
   FBgn0037689   CG8135   5230097   5233579  +  3483  
 
 
    Segment 105 
 
   Location   
  Gene key  FBgn0037697-FBgn0043791  
  Heatmap region span   3R:5227242..5391946   
  Segment span   3R:5286362..5320525   
  Length (genes)  3  
  Length (bp)  34164  
   Model Scoring   
  BIC  343.224050  
  logL  -165.984294  
  logL ratio  -16.555707  
   Expression   
  Mean expression  5.132854  
  Median expression  4.779335  
  Tissue std. dev.  0.703614  
 
  No GO Slim enrichment  
  
   tissue    mean expression   
  5th Passage Drosophila S2 Cells  4.598965  
  Adult Accessory gland  4.474965  
  Adult Brain  5.323022  
  Adult Carcass  6.453130  
  Adult Crop  4.488330  
  Adult Eye  6.727988  
  Adult Fatbody  5.464410  
  Adult Female Spermatheca Mated  5.253205  
  Adult Female Spermatheca Virgin  5.370763  
  Adult Head  6.644494  
  Adult Heart  5.786129  
  Adult Hind Gut  4.468691  
  Adult Male Ejaculatory Duct  4.795727  
  Adult Mid Gut  4.598020  
  Adult Ovary  4.578337  
  Adult Salivary Gland  4.719310  
  Adult Testes  4.287794  
  Adult Thoracoabdominal ganglion  5.036323  
  Adult Whole Fly  4.856361  
  Larvae Wandering Tubules  4.671758  
  Larval Feeding Carcass  5.690399  
  Larval Feeding Central Nevous System  6.051459  
  Larval Feeding Hind Gut  4.413232  
  Larval Feeding Malpighian Tubule  5.708072  
  Larval Feeding Mid Gut  4.677611  
  Larval Feeding Salivary Gland  4.458981  
  Whole Larvae Feeding  4.989587  
 
  
   FlyBase ID    symbol    start    end    strand    length   
   FBgn0037697   GstZ2  5283369   5286362   -  2994  
   FBgn0037698   CG16779  5292212   5301505   -  9294  
   FBgn0043791   CG8147   5320525   5322452  +  1928  
 
 
    Segment 106 
 
   Location   
  Gene key  FBgn0014380-FBgn0003205  
  Heatmap region span   3R:5236004..5395936   
  Segment span   3R:5328299..5338809   
  Length (genes)  3  
  Length (bp)  10511  
   Model Scoring   
  BIC  353.592909  
  logL  -171.168723  
  logL ratio  83.797429  
   Expression   
  Mean expression  10.042683  
  Median expression  9.979463  
  Tissue std. dev.  0.554260  
 
  
   GO ID    description    ratio    P-value   
   GO:0003924   GTPase activity  2/3  0.00272  
 
  
   tissue    mean expression   
  5th Passage Drosophila S2 Cells  10.961555  
  Adult Accessory gland  10.143912  
  Adult Brain  9.699569  
  Adult Carcass  9.421215  
  Adult Crop  10.621419  
  Adult Eye  9.558075  
  Adult Fatbody  9.482241  
  Adult Female Spermatheca Mated  9.230594  
  Adult Female Spermatheca Virgin  9.107334  
  Adult Head  9.727054  
  Adult Heart  10.177030  
  Adult Hind Gut  10.097872  
  Adult Male Ejaculatory Duct  9.666474  
  Adult Mid Gut  10.619901  
  Adult Ovary  9.752795  
  Adult Salivary Gland  10.159072  
  Adult Testes  8.842926  
  Adult Thoracoabdominal ganglion  9.851739  
  Adult Whole Fly  10.033133  
  Larvae Wandering Tubules  11.069262  
  Larval Feeding Carcass  10.097608  
  Larval Feeding Central Nevous System  10.445879  
  Larval Feeding Hind Gut  10.534534  
  Larval Feeding Malpighian Tubule  10.731148  
  Larval Feeding Mid Gut  10.604083  
  Larval Feeding Salivary Gland  10.205505  
  Whole Larvae Feeding  10.310522  
 
  
   FlyBase ID    symbol    start    end    strand    length   
   FBgn0014380   RhoL  5322917   5328299   -  5383  
   FBgn0037700   CG8149   5330383   5331587  +  1205  
   FBgn0003205   Ras85D  5336283   5338809   -  2527  
 
 
    Segment 107 
 
   Location   
  Gene key  FBgn0014022-FBgn0037703  
  Heatmap region span   3R:5238015..5397345   
  Segment span   3R:5339061..5341731   
  Length (genes)  2  
  Length (bp)  2671  
   Model Scoring   
  BIC  211.357541  
  logL  -100.051040  
  logL ratio  36.845829  
   Expression   
  Mean expression  6.905188  
  Median expression  6.752555  
  Tissue std. dev.  0.601725  
 
  No GO Slim enrichment  
  
   tissue    mean expression   
  5th Passage Drosophila S2 Cells  7.613718  
  Adult Accessory gland  6.809173  
  Adult Brain  7.884129  
  Adult Carcass  6.127026  
  Adult Crop  6.869290  
  Adult Eye  6.982765  
  Adult Fatbody  6.458827  
  Adult Female Spermatheca Mated  6.694214  
  Adult Female Spermatheca Virgin  6.613351  
  Adult Head  6.879367  
  Adult Heart  7.199078  
  Adult Hind Gut  6.769043  
  Adult Male Ejaculatory Duct  6.347052  
  Adult Mid Gut  6.450001  
  Adult Ovary  7.900958  
  Adult Salivary Gland  6.367480  
  Adult Testes  5.712848  
  Adult Thoracoabdominal ganglion  7.916116  
  Adult Whole Fly  6.632782  
  Larvae Wandering Tubules  7.348033  
  Larval Feeding Carcass  6.858322  
  Larval Feeding Central Nevous System  8.286604  
  Larval Feeding Hind Gut  6.871531  
  Larval Feeding Malpighian Tubule  7.293664  
  Larval Feeding Mid Gut  6.421942  
  Larval Feeding Salivary Gland  6.898093  
  Whole Larvae Feeding  6.234675  
 
  
   FlyBase ID    symbol    start    end    strand    length   
   FBgn0014022   Rlb1   5339061   5340549  +  1489  
   FBgn0037703   JHDM2   5341731   5346660  +  4930  
 
    Segment 108 
 
   Location   
  Gene key  FBgn0014023-FBgn0037710  
  Heatmap region span   3R:5282264..5399927   
  Segment span   3R:5341829..5389392   
  Length (genes)  7  
  Length (bp)  47564  
   Model Scoring   
  BIC  668.269677  
  logL  -328.507107  
  logL ratio  215.310390  
   Expression   
  Mean expression  8.608250  
  Median expression  8.490541  
  Tissue std. dev.  0.306711  
 
  No GO Slim enrichment  
  
   tissue    mean expression   
  5th Passage Drosophila S2 Cells  8.350879  
  Adult Accessory gland  8.471025  
  Adult Brain  8.499038  
  Adult Carcass  8.360735  
  Adult Crop  8.889648  
  Adult Eye  8.383196  
  Adult Fatbody  8.843396  
  Adult Female Spermatheca Mated  8.856331  
  Adult Female Spermatheca Virgin  8.843134  
  Adult Head  8.411197  
  Adult Heart  9.090498  
  Adult Hind Gut  8.615740  
  Adult Male Ejaculatory Duct  8.456094  
  Adult Mid Gut  8.375274  
  Adult Ovary  8.977241  
  Adult Salivary Gland  8.574736  
  Adult Testes  7.788686  
  Adult Thoracoabdominal ganglion  8.524583  
  Adult Whole Fly  8.374956  
  Larvae Wandering Tubules  8.598208  
  Larval Feeding Carcass  8.816309  
  Larval Feeding Central Nevous System  8.812006  
  Larval Feeding Hind Gut  9.034107  
  Larval Feeding Malpighian Tubule  8.907973  
  Larval Feeding Mid Gut  8.262777  
  Larval Feeding Salivary Gland  9.089070  
  Whole Larvae Feeding  8.215910  
 
  
   FlyBase ID    symbol    start    end    strand    length   
   FBgn0014023   mRpL47  5340538   5341829   -  1292  
   FBgn0000244   by  5353960   5358734   -  4775  
   FBgn0037705   mura  5363876   5377528   -  13653  
   FBgn0037707   RnpS1   5377765   5379469  +  1705  
   FBgn0037708   CG9386  5379418   5381111   -  1694  
   FBgn0037709   CG8199   5381517   5383509  +  1993  
   FBgn0037710   CG9393  5387302   5389392   -  2091  
 
 
    Segment 109 
 
   Location   
  Gene key  FBgn0037718-FBgn0037720  
  Heatmap region span   3R:5397345..5457361   
  Segment span   3R:5412661..5417033   
  Length (genes)  3  
  Length (bp)  4373  
   Model Scoring   
  BIC  365.580138  
  logL  -177.162338  
  logL ratio  47.994493  
   Expression   
  Mean expression  9.448530  
  Median expression  9.452793  
  Tissue std. dev.  0.462440  
 
  No GO Slim enrichment  
  
   tissue    mean expression   
  5th Passage Drosophila S2 Cells  9.205237  
  Adult Accessory gland  9.932059  
  Adult Brain  8.591577  
  Adult Carcass  9.405154  
  Adult Crop  9.586714  
  Adult Eye  9.056124  
  Adult Fatbody  9.005182  
  Adult Female Spermatheca Mated  9.592998  
  Adult Female Spermatheca Virgin  9.598202  
  Adult Head  9.285988  
  Adult Heart  9.315817  
  Adult Hind Gut  9.475222  
  Adult Male Ejaculatory Duct  10.281471  
  Adult Mid Gut  9.456151  
  Adult Ovary  9.499613  
  Adult Salivary Gland  10.491288  
  Adult Testes  9.025325  
  Adult Thoracoabdominal ganglion  8.983281  
  Adult Whole Fly  9.090970  
  Larvae Wandering Tubules  9.653208  
  Larval Feeding Carcass  9.666505  
  Larval Feeding Central Nevous System  8.420881  
  Larval Feeding Hind Gut  9.880209  
  Larval Feeding Malpighian Tubule  10.082137  
  Larval Feeding Mid Gut  9.904260  
  Larval Feeding Salivary Gland  9.342521  
  Whole Larvae Feeding  9.282220  
 
  
   FlyBase ID    symbol    start    end    strand    length   
   FBgn0037718   P58IPK   5412661   5415303  +  2643  
   FBgn0037719   bocksbeutel  5415190   5416709   -  1520  
   FBgn0037720   CG8312   5417033   5450798  +  33766  
 
 
    Segment 110 
 
   Location   
  Gene key  FBgn0069242-FBgn0037731  
  Heatmap region span   3R:5499539..5566644   
  Segment span   3R:5511253..5511481   
  Length (genes)  2  
  Length (bp)  229  
   Model Scoring   
  BIC  297.618853  
  logL  -143.181695  
  logL ratio  28.981091  
   Expression   
  Mean expression  10.686606  
  Median expression  10.717922  
  Tissue std. dev.  0.701866  
 
  No GO Slim enrichment  
  
   tissue    mean expression   
  5th Passage Drosophila S2 Cells  11.161959  
  Adult Accessory gland  11.267510  
  Adult Brain  10.900988  
  Adult Carcass  10.241682  
  Adult Crop  10.478546  
  Adult Eye  10.819244  
  Adult Fatbody  10.896992  
  Adult Female Spermatheca Mated  11.547811  
  Adult Female Spermatheca Virgin  11.483494  
  Adult Head  10.619249  
  Adult Heart  10.564004  
  Adult Hind Gut  10.286398  
  Adult Male Ejaculatory Duct  11.896672  
  Adult Mid Gut  9.540582  
  Adult Ovary  10.597448  
  Adult Salivary Gland  11.852671  
  Adult Testes  9.022813  
  Adult Thoracoabdominal ganglion  11.170141  
  Adult Whole Fly  9.966800  
  Larvae Wandering Tubules  10.458500  
  Larval Feeding Carcass  10.354313  
  Larval Feeding Central Nevous System  10.620071  
  Larval Feeding Hind Gut  10.745585  
  Larval Feeding Malpighian Tubule  10.711228  
  Larval Feeding Mid Gut  9.367845  
  Larval Feeding Salivary Gland  11.717961  
  Whole Larvae Feeding  10.247854  
 
  
   FlyBase ID    symbol    start    end    strand    length   
   FBgn0069242   eca  5510191   5511253   -  1063  
   FBgn0037731   CG18542   5511481   5512863  +  1383  
 
    Segment 111 
 
   Location   
  Gene key  FBgn0051352-FBgn0016792  
  Heatmap region span   3R:5501245..5571207   
  Segment span   3R:5531165..5536862   
  Length (genes)  3  
  Length (bp)  5698  
   Model Scoring   
  BIC  342.553328  
  logL  -165.648933  
  logL ratio  36.845805  
   Expression   
  Mean expression  8.142468  
  Median expression  8.218299  
  Tissue std. dev.  0.286901  
 
  No GO Slim enrichment  
  
   tissue    mean expression   
  5th Passage Drosophila S2 Cells  8.146914  
  Adult Accessory gland  7.888606  
  Adult Brain  8.113875  
  Adult Carcass  8.001810  
  Adult Crop  8.452178  
  Adult Eye  8.262222  
  Adult Fatbody  8.212712  
  Adult Female Spermatheca Mated  8.020599  
  Adult Female Spermatheca Virgin  8.048019  
  Adult Head  7.841451  
  Adult Heart  8.225949  
  Adult Hind Gut  8.315759  
  Adult Male Ejaculatory Duct  8.204261  
  Adult Mid Gut  7.645096  
  Adult Ovary  8.461704  
  Adult Salivary Gland  8.340235  
  Adult Testes  8.051452  
  Adult Thoracoabdominal ganglion  8.043900  
  Adult Whole Fly  8.043170  
  Larvae Wandering Tubules  8.183955  
  Larval Feeding Carcass  7.947185  
  Larval Feeding Central Nevous System  8.964507  
  Larval Feeding Hind Gut  8.418031  
  Larval Feeding Malpighian Tubule  8.159132  
  Larval Feeding Mid Gut  7.866710  
  Larval Feeding Salivary Gland  8.512507  
  Whole Larvae Feeding  7.474706  
 
  
   FlyBase ID    symbol    start    end    strand    length   
   FBgn0051352   Unc-115a  5520444   5531165   -  10722  
   FBgn0037734   trbd  5531512   5536754   -  5243  
   FBgn0016792   dmt   5536862   5540397  +  3536  
 
 
    Segment 112 
 
   Location   
  Gene key  FBgn0003886-FBgn0037736  
  Heatmap region span   3R:5509744..5580303   
  Segment span   3R:5558987..5565651   
  Length (genes)  2  
  Length (bp)  6665  
   Model Scoring   
  BIC  232.349424  
  logL  -110.546981  
  logL ratio  -9.380427  
   Expression   
  Mean expression  5.879913  
  Median expression  5.591748  
  Tissue std. dev.  0.792891  
 
  No GO Slim enrichment  
  
   tissue    mean expression   
  5th Passage Drosophila S2 Cells  5.613682  
  Adult Accessory gland  5.422823  
  Adult Brain  7.004424  
  Adult Carcass  6.165738  
  Adult Crop  5.495110  
  Adult Eye  5.569584  
  Adult Fatbody  5.512270  
  Adult Female Spermatheca Mated  5.271740  
  Adult Female Spermatheca Virgin  5.317068  
  Adult Head  5.972145  
  Adult Heart  5.041148  
  Adult Hind Gut  5.399251  
  Adult Male Ejaculatory Duct  5.503037  
  Adult Mid Gut  5.241635  
  Adult Ovary  5.233787  
  Adult Salivary Gland  5.475003  
  Adult Testes  8.321669  
  Adult Thoracoabdominal ganglion  6.824113  
  Adult Whole Fly  5.405197  
  Larvae Wandering Tubules  5.533913  
  Larval Feeding Carcass  7.712392  
  Larval Feeding Central Nevous System  6.669892  
  Larval Feeding Hind Gut  5.628589  
  Larval Feeding Malpighian Tubule  5.526886  
  Larval Feeding Mid Gut  5.359159  
  Larval Feeding Salivary Gland  5.904006  
  Whole Larvae Feeding  6.633385  
 
  
   FlyBase ID    symbol    start    end    strand    length   
   FBgn0003886   alphaTub85E  5556943   5558987   -  2045  
   FBgn0037736   CG12950  5560560   5565651   -  5092  
 
    Segment 113 
 
   Location   
  Gene key  FBgn0051415-FBgn0085438  
  Heatmap region span   3R:5531165..5588636   
  Segment span   3R:5569239..5571207   
  Length (genes)  2  
  Length (bp)  1969  
   Model Scoring   
  BIC  196.459356  
  logL  -92.601947  
  logL ratio  18.055113  
   Expression   
  Mean expression  5.495195  
  Median expression  5.330555  
  Tissue std. dev.  0.679341  
 
  No GO Slim enrichment  
  
   tissue    mean expression   
  5th Passage Drosophila S2 Cells  5.494115  
  Adult Accessory gland  5.138356  
  Adult Brain  5.370416  
  Adult Carcass  5.563023  
  Adult Crop  5.236034  
  Adult Eye  5.008415  
  Adult Fatbody  5.376643  
  Adult Female Spermatheca Mated  5.494382  
  Adult Female Spermatheca Virgin  5.523294  
  Adult Head  5.227227  
  Adult Heart  5.191192  
  Adult Hind Gut  5.267281  
  Adult Male Ejaculatory Duct  5.369211  
  Adult Mid Gut  5.595014  
  Adult Ovary  5.260654  
  Adult Salivary Gland  5.467690  
  Adult Testes  8.763331  
  Adult Thoracoabdominal ganglion  5.162265  
  Adult Whole Fly  6.202525  
  Larvae Wandering Tubules  5.473122  
  Larval Feeding Carcass  5.426436  
  Larval Feeding Central Nevous System  5.244260  
  Larval Feeding Hind Gut  5.086966  
  Larval Feeding Malpighian Tubule  5.290602  
  Larval Feeding Mid Gut  5.620356  
  Larval Feeding Salivary Gland  5.246200  
  Whole Larvae Feeding  5.271262  
 
  
   FlyBase ID    symbol    start    end    strand    length   
   FBgn0051415   CG31415   5569239   5570386  +  1148  
   FBgn0085438   CG34409  5568598   5571207   -  2610  
 
    Segment 114 
 
   Location   
  Gene key  FBgn0037739-FBgn0005777  
  Heatmap region span   3R:5551333..5589372   
  Segment span   3R:5573254..5573597   
  Length (genes)  2  
  Length (bp)  344  
   Model Scoring   
  BIC  245.923060  
  logL  -117.333799  
  logL ratio  18.589376  
   Expression   
  Mean expression  8.845668  
  Median expression  8.901184  
  Tissue std. dev.  0.640791  
 
  No GO Slim enrichment  
  
   tissue    mean expression   
  5th Passage Drosophila S2 Cells  9.695857  
  Adult Accessory gland  8.286615  
  Adult Brain  8.735703  
  Adult Carcass  8.410248  
  Adult Crop  9.400243  
  Adult Eye  8.488918  
  Adult Fatbody  8.610057  
  Adult Female Spermatheca Mated  9.237313  
  Adult Female Spermatheca Virgin  9.164367  
  Adult Head  8.482106  
  Adult Heart  8.497264  
  Adult Hind Gut  8.566899  
  Adult Male Ejaculatory Duct  8.671796  
  Adult Mid Gut  8.041909  
  Adult Ovary  10.642911  
  Adult Salivary Gland  8.056840  
  Adult Testes  7.920286  
  Adult Thoracoabdominal ganglion  8.829641  
  Adult Whole Fly  9.257289  
  Larvae Wandering Tubules  8.665603  
  Larval Feeding Carcass  9.430285  
  Larval Feeding Central Nevous System  10.108000  
  Larval Feeding Hind Gut  9.077269  
  Larval Feeding Malpighian Tubule  8.460669  
  Larval Feeding Mid Gut  8.001431  
  Larval Feeding Salivary Gland  9.403945  
  Whole Larvae Feeding  8.689571  
 
  
   FlyBase ID    symbol    start    end    strand    length   
   FBgn0037739   CG12948  5571889   5573254   -  1366  
   FBgn0005777   PpD3   5573597   5576822  +  3226  
 
    Segment 115 
 
   Location   
  Gene key  FBgn0037743-FBgn0028401  
  Heatmap region span   3R:5569239..5605786   
  Segment span   3R:5582215..5588636   
  Length (genes)  3  
  Length (bp)  6422  
   Model Scoring   
  BIC  426.883379  
  logL  -207.813958  
  logL ratio  -3.439883  
   Expression   
  Mean expression  9.456215  
  Median expression  9.253506  
  Tissue std. dev.  0.486876  
 
  No GO Slim enrichment  
  
   tissue    mean expression   
  5th Passage Drosophila S2 Cells  9.707417  
  Adult Accessory gland  9.186824  
  Adult Brain  9.703968  
  Adult Carcass  9.493088  
  Adult Crop  10.193937  
  Adult Eye  9.667157  
  Adult Fatbody  9.554694  
  Adult Female Spermatheca Mated  8.973648  
  Adult Female Spermatheca Virgin  9.186505  
  Adult Head  9.769913  
  Adult Heart  9.756410  
  Adult Hind Gut  10.249305  
  Adult Male Ejaculatory Duct  9.323702  
  Adult Mid Gut  9.121483  
  Adult Ovary  8.728746  
  Adult Salivary Gland  9.952457  
  Adult Testes  8.322665  
  Adult Thoracoabdominal ganglion  9.507166  
  Adult Whole Fly  8.729667  
  Larvae Wandering Tubules  9.991736  
  Larval Feeding Carcass  10.001073  
  Larval Feeding Central Nevous System  9.098758  
  Larval Feeding Hind Gut  9.111158  
  Larval Feeding Malpighian Tubule  10.116364  
  Larval Feeding Mid Gut  8.855567  
  Larval Feeding Salivary Gland  9.759760  
  Whole Larvae Feeding  9.254633  
 
  
   FlyBase ID    symbol    start    end    strand    length   
   FBgn0037743   CG8412   5582215   5585035  +  2821  
   FBgn0037744   CG8417   5585319   5586930  +  1612  
   FBgn0028401   Snap24  5587688   5588636   -  949  
 
 
    Segment 116 
 
   Location   
  Gene key  FBgn0037747-FBgn0037749  
  Heatmap region span   3R:5580303..5610408   
  Segment span   3R:5593573..5597264   
  Length (genes)  3  
  Length (bp)  3692  
   Model Scoring   
  BIC  370.440179  
  logL  -179.592358  
  logL ratio  32.315806  
   Expression   
  Mean expression  9.131169  
  Median expression  9.390693  
  Tissue std. dev.  0.362628  
 
  No GO Slim enrichment  
  
   tissue    mean expression   
  5th Passage Drosophila S2 Cells  8.960037  
  Adult Accessory gland  9.642380  
  Adult Brain  9.787682  
  Adult Carcass  9.214056  
  Adult Crop  9.167390  
  Adult Eye  9.293042  
  Adult Fatbody  8.817276  
  Adult Female Spermatheca Mated  9.019099  
  Adult Female Spermatheca Virgin  9.003310  
  Adult Head  9.028201  
  Adult Heart  9.534660  
  Adult Hind Gut  9.267681  
  Adult Male Ejaculatory Duct  8.788048  
  Adult Mid Gut  9.070928  
  Adult Ovary  9.683325  
  Adult Salivary Gland  8.300502  
  Adult Testes  8.970292  
  Adult Thoracoabdominal ganglion  9.944416  
  Adult Whole Fly  9.099702  
  Larvae Wandering Tubules  8.993706  
  Larval Feeding Carcass  8.833589  
  Larval Feeding Central Nevous System  9.512693  
  Larval Feeding Hind Gut  9.087350  
  Larval Feeding Malpighian Tubule  9.189802  
  Larval Feeding Mid Gut  8.793538  
  Larval Feeding Salivary Gland  8.634490  
  Whole Larvae Feeding  8.904377  
 
  
   FlyBase ID    symbol    start    end    strand    length   
   FBgn0037747   CG8481   5593573   5595749  +  2177  
   FBgn0024330   MED6  5591753   5594706   -  2954  
   FBgn0037749   CG9471  5596254   5597264   -  1011  
 
 
    Segment 117 
 
   Location   
  Gene key  FBgn0037751-FBgn0037753  
  Heatmap region span   3R:5582215..5617192   
  Segment span   3R:5600843..5605786   
  Length (genes)  2  
  Length (bp)  4944  
   Model Scoring   
  BIC  202.677808  
  logL  -95.711173  
  logL ratio  15.885335  
   Expression   
  Mean expression  4.805734  
  Median expression  4.736457  
  Tissue std. dev.  0.490464  
 
  No GO Slim enrichment  
  
   tissue    mean expression   
  5th Passage Drosophila S2 Cells  5.498202  
  Adult Accessory gland  4.588407  
  Adult Brain  4.523156  
  Adult Carcass  4.539350  
  Adult Crop  5.258831  
  Adult Eye  5.020711  
  Adult Fatbody  4.640826  
  Adult Female Spermatheca Mated  4.426784  
  Adult Female Spermatheca Virgin  4.639161  
  Adult Head  4.631353  
  Adult Heart  4.834668  
  Adult Hind Gut  4.671177  
  Adult Male Ejaculatory Duct  5.669673  
  Adult Mid Gut  4.652900  
  Adult Ovary  4.960838  
  Adult Salivary Gland  4.435277  
  Adult Testes  6.761890  
  Adult Thoracoabdominal ganglion  4.579633  
  Adult Whole Fly  4.486528  
  Larvae Wandering Tubules  4.540571  
  Larval Feeding Carcass  4.705301  
  Larval Feeding Central Nevous System  4.652681  
  Larval Feeding Hind Gut  4.888821  
  Larval Feeding Malpighian Tubule  4.613094  
  Larval Feeding Mid Gut  4.513960  
  Larval Feeding Salivary Gland  4.518408  
  Whole Larvae Feeding  4.502607  
 
  
   FlyBase ID    symbol    start    end    strand    length   
   FBgn0037751   topi   5600843   5603665  +  2823  
   FBgn0037753   CG12947   5605786   5608779  +  2994  
 
    Segment 118 
 
   Location   
  Gene key  FBgn0037759-FBgn0037760  
  Heatmap region span   3R:5616939..5874889   
  Segment span   3R:5627008..5635085   
  Length (genes)  2  
  Length (bp)  8078  
   Model Scoring   
  BIC  222.898241  
  logL  -105.821389  
  logL ratio  26.306397  
   Expression   
  Mean expression  7.199535  
  Median expression  7.199920  
  Tissue std. dev.  0.605828  
 
  No GO Slim enrichment  
  
   tissue    mean expression   
  5th Passage Drosophila S2 Cells  7.083540  
  Adult Accessory gland  6.675897  
  Adult Brain  7.878630  
  Adult Carcass  6.148124  
  Adult Crop  7.826536  
  Adult Eye  7.455305  
  Adult Fatbody  7.283321  
  Adult Female Spermatheca Mated  7.016279  
  Adult Female Spermatheca Virgin  6.838158  
  Adult Head  6.482198  
  Adult Heart  7.772728  
  Adult Hind Gut  7.172287  
  Adult Male Ejaculatory Duct  6.480849  
  Adult Mid Gut  6.673893  
  Adult Ovary  8.497715  
  Adult Salivary Gland  7.102412  
  Adult Testes  6.663640  
  Adult Thoracoabdominal ganglion  7.591626  
  Adult Whole Fly  6.295108  
  Larvae Wandering Tubules  8.136918  
  Larval Feeding Carcass  7.097912  
  Larval Feeding Central Nevous System  8.131991  
  Larval Feeding Hind Gut  7.559200  
  Larval Feeding Malpighian Tubule  7.268714  
  Larval Feeding Mid Gut  6.826557  
  Larval Feeding Salivary Gland  7.847696  
  Whole Larvae Feeding  6.580209  
 
  
   FlyBase ID    symbol    start    end    strand    length   
   FBgn0037759   CG8526   5627008   5629183  +  2176  
   FBgn0037760   FBX011  5630494   5635085   -  4592  
 
    Segment 119 
 
   Location   
  Gene key  FBgn0037762-FBgn0037765  
  Heatmap region span   3R:5619030..5886019   
  Segment span   3R:5639883..5645599   
  Length (genes)  4  
  Length (bp)  5717  
   Model Scoring   
  BIC  356.069299  
  logL  -172.406918  
  logL ratio  130.107837  
   Expression   
  Mean expression  6.086790  
  Median expression  4.933725  
  Tissue std. dev.  2.777087  
 
  No GO Slim enrichment  
  
   tissue    mean expression   
  5th Passage Drosophila S2 Cells  4.670659  
  Adult Accessory gland  5.022306  
  Adult Brain  4.267205  
  Adult Carcass  12.948089  
  Adult Crop  4.999822  
  Adult Eye  4.436111  
  Adult Fatbody  13.305772  
  Adult Female Spermatheca Mated  5.014812  
  Adult Female Spermatheca Virgin  4.804062  
  Adult Head  4.471141  
  Adult Heart  13.409931  
  Adult Hind Gut  5.257647  
  Adult Male Ejaculatory Duct  4.743829  
  Adult Mid Gut  4.824302  
  Adult Ovary  4.702900  
  Adult Salivary Gland  4.963219  
  Adult Testes  5.519554  
  Adult Thoracoabdominal ganglion  5.269878  
  Adult Whole Fly  10.252019  
  Larvae Wandering Tubules  4.920516  
  Larval Feeding Carcass  7.587507  
  Larval Feeding Central Nevous System  4.368248  
  Larval Feeding Hind Gut  4.555925  
  Larval Feeding Malpighian Tubule  4.766992  
  Larval Feeding Mid Gut  4.840490  
  Larval Feeding Salivary Gland  4.817965  
  Whole Larvae Feeding  5.602428  
 
  
   FlyBase ID    symbol    start    end    strand    length   
   FBgn0037762   eloF   5639883   5640880  +  998  
   FBgn0037763   CG16904  5641650   5642708   -  1059  
   FBgn0037764   CG9459  5643150   5644117   -  968  
   FBgn0037765   CG9458  5644636   5645599   -  964  
 
 
    Segment 120 
 
   Location   
  Gene key  FBgn0085331-FBgn0053208  
  Heatmap region span   3R:5626773..5893627   
  Segment span   3R:5649545..5868543   
  Length (genes)  10  
  Length (bp)  218999  
   Model Scoring   
  BIC  1020.473777  
  logL  -504.609158  
  logL ratio  35.388223  
   Expression   
  Mean expression  5.327325  
  Median expression  5.009953  
  Tissue std. dev.  0.334086  
 
  No GO Slim enrichment  
  
   tissue    mean expression   
  5th Passage Drosophila S2 Cells  5.539490  
  Adult Accessory gland  5.054055  
  Adult Brain  5.716452  
  Adult Carcass  5.032150  
  Adult Crop  5.278861  
  Adult Eye  5.273751  
  Adult Fatbody  5.126024  
  Adult Female Spermatheca Mated  4.982598  
  Adult Female Spermatheca Virgin  5.070200  
  Adult Head  5.103349  
  Adult Heart  5.021601  
  Adult Hind Gut  5.088950  
  Adult Male Ejaculatory Duct  4.975896  
  Adult Mid Gut  5.186878  
  Adult Ovary  6.050566  
  Adult Salivary Gland  5.075920  
  Adult Testes  6.292199  
  Adult Thoracoabdominal ganglion  5.555353  
  Adult Whole Fly  5.812150  
  Larvae Wandering Tubules  5.464822  
  Larval Feeding Carcass  5.257399  
  Larval Feeding Central Nevous System  5.694726  
  Larval Feeding Hind Gut  5.127057  
  Larval Feeding Malpighian Tubule  5.315793  
  Larval Feeding Mid Gut  5.126691  
  Larval Feeding Salivary Gland  5.442646  
  Whole Larvae Feeding  5.172209  
 
  
   FlyBase ID    symbol    start    end    strand    length   
   FBgn0085331   CG34302  5648721   5649545   -  825  
   FBgn0037766   Teh1  5653580   5693842   -  40263  
   FBgn0250818      5700403   5821086  +  120684  
   FBgn0037769   CG12419   5806775   5807898  +  1124  
   FBgn0037770   Art4  5808908   5811777   -  2870  
   FBgn0045473   Gr85a  5821266   5822511   -  1246  
   FBgn0037772   Spn85F   5823261   5826036  +  2776  
   FBgn0037773   CG5359  5825491   5826998   -  1508  
   FBgn0051407   CG31407  5848568   5849330   -  763  
   FBgn0053208   Mical  5828033   5868543   -  40511  
 
 
    Segment 121 
 
   Location   
  Gene key  FBgn0027524-FBgn0037780  
  Heatmap region span   3R:5627008..5901088   
  Segment span   3R:5868980..5874889   
  Length (genes)  5  
  Length (bp)  5910  
   Model Scoring   
  BIC  486.790500  
  logL  -237.767519  
  logL ratio  149.180019  
   Expression   
  Mean expression  8.615219  
  Median expression  8.522969  
  Tissue std. dev.  0.305039  
 
  No GO Slim enrichment  
  
   tissue    mean expression   
  5th Passage Drosophila S2 Cells  8.320492  
  Adult Accessory gland  8.623363  
  Adult Brain  8.474395  
  Adult Carcass  8.527561  
  Adult Crop  8.709254  
  Adult Eye  8.722651  
  Adult Fatbody  9.003953  
  Adult Female Spermatheca Mated  9.040927  
  Adult Female Spermatheca Virgin  9.026289  
  Adult Head  8.610240  
  Adult Heart  8.898572  
  Adult Hind Gut  8.439235  
  Adult Male Ejaculatory Duct  8.849993  
  Adult Mid Gut  7.966826  
  Adult Ovary  9.096945  
  Adult Salivary Gland  8.793502  
  Adult Testes  8.561788  
  Adult Thoracoabdominal ganglion  8.660656  
  Adult Whole Fly  8.435680  
  Larvae Wandering Tubules  8.652937  
  Larval Feeding Carcass  8.518221  
  Larval Feeding Central Nevous System  8.551239  
  Larval Feeding Hind Gut  8.549534  
  Larval Feeding Malpighian Tubule  8.781645  
  Larval Feeding Mid Gut  7.676934  
  Larval Feeding Salivary Gland  8.724454  
  Whole Larvae Feeding  8.393619  
 
  
   FlyBase ID    symbol    start    end    strand    length   
   FBgn0027524   CG3909   5868980   5870328  +  1349  
   FBgn0037777   CG11722  5870318   5871245   -  928  
   FBgn0037778   mtTFB2   5871298   5872982  +  1685  
   FBgn0037779   CG12811  5872974   5874289   -  1316  
   FBgn0037780   CG3925   5874889   5877249  +  2361  
 
 
    Segment 122 
 
   Location   
  Gene key  FBgn0051410-FBgn0037783  
  Heatmap region span   3R:5639883..5938268   
  Segment span   3R:5879120..5886019   
  Length (genes)  3  
  Length (bp)  6900  
   Model Scoring   
  BIC  307.069054  
  logL  -147.906796  
  logL ratio  30.057431  
   Expression   
  Mean expression  5.612405  
  Median expression  4.659311  
  Tissue std. dev.  2.157852  
 
  No GO Slim enrichment  
  
   tissue    mean expression   
  5th Passage Drosophila S2 Cells  4.623782  
  Adult Accessory gland  4.596394  
  Adult Brain  4.363260  
  Adult Carcass  4.894795  
  Adult Crop  4.614278  
  Adult Eye  4.401842  
  Adult Fatbody  4.544554  
  Adult Female Spermatheca Mated  4.586182  
  Adult Female Spermatheca Virgin  4.814262  
  Adult Head  4.343093  
  Adult Heart  4.587618  
  Adult Hind Gut  6.038086  
  Adult Male Ejaculatory Duct  4.939565  
  Adult Mid Gut  11.663480  
  Adult Ovary  4.578948  
  Adult Salivary Gland  4.655974  
  Adult Testes  4.513891  
  Adult Thoracoabdominal ganglion  4.524462  
  Adult Whole Fly  7.619665  
  Larvae Wandering Tubules  5.420358  
  Larval Feeding Carcass  4.595756  
  Larval Feeding Central Nevous System  4.349329  
  Larval Feeding Hind Gut  4.594256  
  Larval Feeding Malpighian Tubule  6.582721  
  Larval Feeding Mid Gut  11.909414  
  Larval Feeding Salivary Gland  4.768420  
  Whole Larvae Feeding  10.410538  
 
  
   FlyBase ID    symbol    start    end    strand    length   
   FBgn0051410   Npc2e   5879120   5879982  +  863  
   FBgn0037782   Npc2d  5881078   5881805   -  728  
   FBgn0037783   Npc2c   5886019   5886858  +  840  
 
 
    Segment 123 
 
   Location   
  Gene key  FBgn0053631-FBgn0053784  
  Heatmap region span   3R:5649545..5947122   
  Segment span   3R:5889729..5893627   
  Length (genes)  2  
  Length (bp)  3899  
   Model Scoring   
  BIC  184.317750  
  logL  -86.531144  
  logL ratio  31.095974  
   Expression   
  Mean expression  4.703195  
  Median expression  4.603560  
  Tissue std. dev.  0.324457  
 
  No GO Slim enrichment  
  
   tissue    mean expression   
  5th Passage Drosophila S2 Cells  4.620793  
  Adult Accessory gland  4.999152  
  Adult Brain  4.358842  
  Adult Carcass  4.744186  
  Adult Crop  4.667423  
  Adult Eye  5.862464  
  Adult Fatbody  5.045019  
  Adult Female Spermatheca Mated  4.977030  
  Adult Female Spermatheca Virgin  4.972250  
  Adult Head  5.103359  
  Adult Heart  4.616110  
  Adult Hind Gut  4.622835  
  Adult Male Ejaculatory Duct  4.485249  
  Adult Mid Gut  4.650343  
  Adult Ovary  4.571753  
  Adult Salivary Gland  4.707994  
  Adult Testes  4.265279  
  Adult Thoracoabdominal ganglion  4.377733  
  Adult Whole Fly  4.195189  
  Larvae Wandering Tubules  4.790827  
  Larval Feeding Carcass  4.622349  
  Larval Feeding Central Nevous System  4.449000  
  Larval Feeding Hind Gut  4.584181  
  Larval Feeding Malpighian Tubule  4.783099  
  Larval Feeding Mid Gut  4.849588  
  Larval Feeding Salivary Gland  4.676014  
  Whole Larvae Feeding  4.388191  
 
  
   FlyBase ID    symbol    start    end    strand    length   
   FBgn0053631   CG33631   5889729   5890367  +  639  
   FBgn0053784   CG33784   5893627   5894294  +  668  
 
    Segment 124 
 
   Location   
  Gene key  FBgn0051477-FBgn0001321  
  Heatmap region span   3R:5901088..6013742   
  Segment span   3R:5947523..5951383   
  Length (genes)  2  
  Length (bp)  3861  
   Model Scoring   
  BIC  223.247885  
  logL  -105.996211  
  logL ratio  8.016396  
   Expression   
  Mean expression  6.218445  
  Median expression  6.015223  
  Tissue std. dev.  0.916258  
 
  No GO Slim enrichment  
  
   tissue    mean expression   
  5th Passage Drosophila S2 Cells  5.837457  
  Adult Accessory gland  5.539637  
  Adult Brain  5.191117  
  Adult Carcass  6.163344  
  Adult Crop  6.825201  
  Adult Eye  5.695362  
  Adult Fatbody  5.512504  
  Adult Female Spermatheca Mated  5.987469  
  Adult Female Spermatheca Virgin  5.648059  
  Adult Head  6.184041  
  Adult Heart  5.300957  
  Adult Hind Gut  7.851433  
  Adult Male Ejaculatory Duct  7.969380  
  Adult Mid Gut  5.996109  
  Adult Ovary  5.555337  
  Adult Salivary Gland  5.675676  
  Adult Testes  7.616699  
  Adult Thoracoabdominal ganglion  5.393761  
  Adult Whole Fly  5.639470  
  Larvae Wandering Tubules  5.730396  
  Larval Feeding Carcass  7.728054  
  Larval Feeding Central Nevous System  5.654614  
  Larval Feeding Hind Gut  8.379239  
  Larval Feeding Malpighian Tubule  5.743158  
  Larval Feeding Mid Gut  5.986245  
  Larval Feeding Salivary Gland  5.786625  
  Whole Larvae Feeding  7.306658  
 
  
   FlyBase ID    symbol    start    end    strand    length   
   FBgn0051477   CG31477   5947523   5948042  +  520  
   FBgn0001321   knk  5948087   5951383   -  3297  
 
    Segment 125 
 
   Location   
  Gene key  FBgn0037792-FBgn0037794  
  Heatmap region span   3R:5938268..6038710   
  Segment span   3R:5969896..5987050   
  Length (genes)  3  
  Length (bp)  17155  
   Model Scoring   
  BIC  316.564867  
  logL  -152.654703  
  logL ratio  45.644216  
   Expression   
  Mean expression  6.894300  
  Median expression  6.690645  
  Tissue std. dev.  0.792220  
 
  No GO Slim enrichment  
  
   tissue    mean expression   
  5th Passage Drosophila S2 Cells  7.790882  
  Adult Accessory gland  6.133114  
  Adult Brain  8.489664  
  Adult Carcass  6.836594  
  Adult Crop  6.344053  
  Adult Eye  7.105127  
  Adult Fatbody  7.496819  
  Adult Female Spermatheca Mated  6.525266  
  Adult Female Spermatheca Virgin  6.510909  
  Adult Head  7.314861  
  Adult Heart  7.439323  
  Adult Hind Gut  6.276250  
  Adult Male Ejaculatory Duct  6.608041  
  Adult Mid Gut  5.962197  
  Adult Ovary  8.329332  
  Adult Salivary Gland  6.468302  
  Adult Testes  6.566461  
  Adult Thoracoabdominal ganglion  8.382846  
  Adult Whole Fly  7.028123  
  Larvae Wandering Tubules  6.727358  
  Larval Feeding Carcass  6.595179  
  Larval Feeding Central Nevous System  8.442160  
  Larval Feeding Hind Gut  6.124521  
  Larval Feeding Malpighian Tubule  6.638617  
  Larval Feeding Mid Gut  5.833825  
  Larval Feeding Salivary Gland  6.222672  
  Whole Larvae Feeding  5.953608  
 
  
   FlyBase ID    symbol    start    end    strand    length   
   FBgn0037792   CG6241  5966283   5969896   -  3614  
   FBgn0086359   Invadolysin   5970421   5984858  +  14438  
   FBgn0037794   CG6254  5984858   5987050   -  2193  
 
 
    Segment 126 
 
   Location   
  Gene key  FBgn0037797-FBgn0037802  
  Heatmap region span   3R:5996207..6137317   
  Segment span   3R:6048435..6087462   
  Length (genes)  4  
  Length (bp)  39028  
   Model Scoring   
  BIC  438.848888  
  logL  -213.796713  
  logL ratio  -16.434791  
   Expression   
  Mean expression  5.588917  
  Median expression  5.106945  
  Tissue std. dev.  0.441214  
 
  No GO Slim enrichment  
  
   tissue    mean expression   
  5th Passage Drosophila S2 Cells  5.377341  
  Adult Accessory gland  5.063004  
  Adult Brain  6.229766  
  Adult Carcass  5.657075  
  Adult Crop  5.200996  
  Adult Eye  5.979942  
  Adult Fatbody  6.287672  
  Adult Female Spermatheca Mated  6.034411  
  Adult Female Spermatheca Virgin  6.133162  
  Adult Head  6.147412  
  Adult Heart  5.688191  
  Adult Hind Gut  5.112445  
  Adult Male Ejaculatory Duct  5.330613  
  Adult Mid Gut  5.117779  
  Adult Ovary  5.658864  
  Adult Salivary Gland  5.149601  
  Adult Testes  5.213137  
  Adult Thoracoabdominal ganglion  6.298309  
  Adult Whole Fly  5.387691  
  Larvae Wandering Tubules  5.333302  
  Larval Feeding Carcass  5.166346  
  Larval Feeding Central Nevous System  6.323632  
  Larval Feeding Hind Gut  5.394269  
  Larval Feeding Malpighian Tubule  5.176856  
  Larval Feeding Mid Gut  4.935779  
  Larval Feeding Salivary Gland  5.606900  
  Whole Larvae Feeding  5.896270  
 
  
   FlyBase ID    symbol    start    end    strand    length   
   FBgn0037797   CG12420   6048435   6049467  +  1033  
   FBgn0037798   CG12817  6049422   6050432   -  1011  
   FBgn0037801   CG3999   6077240   6081002  +  3763  
   FBgn0037802   Sirt6  6086509   6087462   -  954  
 
 
    Segment 127 
 
   Location   
  Gene key  FBgn0037808-FBgn0037809  
  Heatmap region span   3R:6087834..6239023   
  Segment span   3R:6157071..6157303   
  Length (genes)  2  
  Length (bp)  233  
   Model Scoring   
  BIC  236.148672  
  logL  -112.446605  
  logL ratio  21.128623  
   Expression   
  Mean expression  8.271993  
  Median expression  8.114480  
  Tissue std. dev.  0.385434  
 
  No GO Slim enrichment  
  
   tissue    mean expression   
  5th Passage Drosophila S2 Cells  8.764831  
  Adult Accessory gland  8.773858  
  Adult Brain  8.561927  
  Adult Carcass  8.223733  
  Adult Crop  8.543928  
  Adult Eye  7.998925  
  Adult Fatbody  8.251418  
  Adult Female Spermatheca Mated  7.619852  
  Adult Female Spermatheca Virgin  7.743080  
  Adult Head  8.055939  
  Adult Heart  8.563657  
  Adult Hind Gut  8.462152  
  Adult Male Ejaculatory Duct  8.264091  
  Adult Mid Gut  8.228655  
  Adult Ovary  8.984334  
  Adult Salivary Gland  8.364659  
  Adult Testes  8.713588  
  Adult Thoracoabdominal ganglion  8.281829  
  Adult Whole Fly  7.908473  
  Larvae Wandering Tubules  8.641300  
  Larval Feeding Carcass  7.882414  
  Larval Feeding Central Nevous System  8.200135  
  Larval Feeding Hind Gut  8.012786  
  Larval Feeding Malpighian Tubule  8.676699  
  Larval Feeding Mid Gut  7.581635  
  Larval Feeding Salivary Gland  8.486294  
  Whole Larvae Feeding  7.553634  
 
  
   FlyBase ID    symbol    start    end    strand    length   
   FBgn0037808   Bruce  6138366   6157071   -  18706  
   FBgn0037809   CG12818   6157303   6158417  +  1115  
 
    Segment 128 
 
   Location   
  Gene key  FBgn0037812-FBgn0051406  
  Heatmap region span   3R:6090028..6241766   
  Segment span   3R:6166671..6174554   
  Length (genes)  2  
  Length (bp)  7884  
   Model Scoring   
  BIC  176.978071  
  logL  -82.861304  
  logL ratio  43.379867  
   Expression   
  Mean expression  4.459433  
  Median expression  4.191767  
  Tissue std. dev.  1.069390  
 
  No GO Slim enrichment  
  
   tissue    mean expression   
  5th Passage Drosophila S2 Cells  4.647138  
  Adult Accessory gland  4.459419  
  Adult Brain  3.923803  
  Adult Carcass  4.316769  
  Adult Crop  4.228433  
  Adult Eye  4.057301  
  Adult Fatbody  4.195522  
  Adult Female Spermatheca Mated  4.223659  
  Adult Female Spermatheca Virgin  4.288576  
  Adult Head  3.975439  
  Adult Heart  4.236161  
  Adult Hind Gut  4.116402  
  Adult Male Ejaculatory Duct  4.318124  
  Adult Mid Gut  4.170765  
  Adult Ovary  4.145072  
  Adult Salivary Gland  4.480595  
  Adult Testes  9.787005  
  Adult Thoracoabdominal ganglion  4.027289  
  Adult Whole Fly  4.900040  
  Larvae Wandering Tubules  4.233822  
  Larval Feeding Carcass  4.174510  
  Larval Feeding Central Nevous System  4.017281  
  Larval Feeding Hind Gut  4.088908  
  Larval Feeding Malpighian Tubule  4.226811  
  Larval Feeding Mid Gut  4.104128  
  Larval Feeding Salivary Gland  4.264935  
  Whole Larvae Feeding  4.796771  
 
  
   FlyBase ID    symbol    start    end    strand    length   
   FBgn0037812   CG18545   6166671   6167390  +  720  
   FBgn0051406   CG31406   6174554   6175727  +  1174  
 
    Segment 129 
 
   Location   
  Gene key  FBgn0020379-FBgn0045443  
  Heatmap region span   3R:6137317..6464386   
  Segment span   3R:6203790..6226754   
  Length (genes)  2  
  Length (bp)  22965  
   Model Scoring   
  BIC  199.720099  
  logL  -94.232318  
  logL ratio  17.783963  
   Expression   
  Mean expression  4.837419  
  Median expression  4.590218  
  Tissue std. dev.  0.331865  
 
  No GO Slim enrichment  
  
   tissue    mean expression   
  5th Passage Drosophila S2 Cells  4.820428  
  Adult Accessory gland  4.787495  
  Adult Brain  5.637203  
  Adult Carcass  5.204812  
  Adult Crop  4.544747  
  Adult Eye  5.107821  
  Adult Fatbody  4.635086  
  Adult Female Spermatheca Mated  4.513563  
  Adult Female Spermatheca Virgin  4.555301  
  Adult Head  4.959484  
  Adult Heart  4.765157  
  Adult Hind Gut  4.653116  
  Adult Male Ejaculatory Duct  4.824150  
  Adult Mid Gut  4.534914  
  Adult Ovary  5.498829  
  Adult Salivary Gland  4.910067  
  Adult Testes  5.408343  
  Adult Thoracoabdominal ganglion  5.043799  
  Adult Whole Fly  4.544052  
  Larvae Wandering Tubules  4.894806  
  Larval Feeding Carcass  4.556395  
  Larval Feeding Central Nevous System  5.363320  
  Larval Feeding Hind Gut  4.483332  
  Larval Feeding Malpighian Tubule  4.732842  
  Larval Feeding Mid Gut  4.546245  
  Larval Feeding Salivary Gland  4.629143  
  Whole Larvae Feeding  4.455864  
 
  
   FlyBase ID    symbol    start    end    strand    length   
   FBgn0020379   Rfx  6188233   6203790   -  15558  
   FBgn0045443   mthl11   6226754   6228551  +  1798  
 
    Segment 130 
 
   Location   
  Gene key  FBgn0037814-FBgn0037815  
  Heatmap region span   3R:6157071..6482168   
  Segment span   3R:6233006..6239023   
  Length (genes)  2  
  Length (bp)  6018  
   Model Scoring   
  BIC  213.016205  
  logL  -100.880371  
  logL ratio  43.089209  
   Expression   
  Mean expression  8.210879  
  Median expression  8.065784  
  Tissue std. dev.  0.624343  
 
  No GO Slim enrichment  
  
   tissue    mean expression   
  5th Passage Drosophila S2 Cells  9.178545  
  Adult Accessory gland  8.853829  
  Adult Brain  8.054495  
  Adult Carcass  7.499681  
  Adult Crop  8.058082  
  Adult Eye  7.752225  
  Adult Fatbody  8.288891  
  Adult Female Spermatheca Mated  8.584340  
  Adult Female Spermatheca Virgin  8.487419  
  Adult Head  7.614985  
  Adult Heart  7.990433  
  Adult Hind Gut  7.638435  
  Adult Male Ejaculatory Duct  8.109452  
  Adult Mid Gut  7.748980  
  Adult Ovary  9.727158  
  Adult Salivary Gland  7.823889  
  Adult Testes  6.932670  
  Adult Thoracoabdominal ganglion  8.410844  
  Adult Whole Fly  8.350843  
  Larvae Wandering Tubules  7.798329  
  Larval Feeding Carcass  8.425755  
  Larval Feeding Central Nevous System  9.649432  
  Larval Feeding Hind Gut  8.158891  
  Larval Feeding Malpighian Tubule  8.001980  
  Larval Feeding Mid Gut  7.586671  
  Larval Feeding Salivary Gland  8.816152  
  Whole Larvae Feeding  8.151339  
 
  
   FlyBase ID    symbol    start    end    strand    length   
   FBgn0037814   CG6325  6228683   6233006   -  4324  
   FBgn0037815   Rrp46   6239023   6239968  +  946  
 
    Segment 131 
 
   Location   
  Gene key  FBgn0020385-FBgn0037822  
  Heatmap region span   3R:6482168..6581356   
  Segment span   3R:6522240..6522365   
  Length (genes)  2  
  Length (bp)  126  
   Model Scoring   
  BIC  281.951476  
  logL  -135.348007  
  logL ratio  1.522122  
   Expression   
  Mean expression  9.323799  
  Median expression  9.175434  
  Tissue std. dev.  0.964270  
 
  No GO Slim enrichment  
  
   tissue    mean expression   
  5th Passage Drosophila S2 Cells  7.943155  
  Adult Accessory gland  7.732231  
  Adult Brain  8.503395  
  Adult Carcass  10.540858  
  Adult Crop  8.535818  
  Adult Eye  10.007886  
  Adult Fatbody  10.695220  
  Adult Female Spermatheca Mated  10.809583  
  Adult Female Spermatheca Virgin  11.142568  
  Adult Head  10.597848  
  Adult Heart  10.341527  
  Adult Hind Gut  9.260756  
  Adult Male Ejaculatory Duct  9.724310  
  Adult Mid Gut  8.997466  
  Adult Ovary  9.185196  
  Adult Salivary Gland  8.316095  
  Adult Testes  8.088152  
  Adult Thoracoabdominal ganglion  8.879775  
  Adult Whole Fly  9.699126  
  Larvae Wandering Tubules  8.066564  
  Larval Feeding Carcass  8.544956  
  Larval Feeding Central Nevous System  8.466869  
  Larval Feeding Hind Gut  10.005427  
  Larval Feeding Malpighian Tubule  9.589588  
  Larval Feeding Mid Gut  9.501310  
  Larval Feeding Salivary Gland  8.689580  
  Whole Larvae Feeding  9.877326  
 
  
   FlyBase ID    symbol    start    end    strand    length   
   FBgn0020385   pug   6522240   6529011  +  6772  
   FBgn0037822   CG14683   6522365   6524698  +  2334  
 
    Segment 132 
 
   Location   
  Gene key  FBgn0051391-FBgn0037824  
  Heatmap region span   3R:6502892..6585113   
  Segment span   3R:6530797..6532696   
  Length (genes)  3  
  Length (bp)  1900  
   Model Scoring   
  BIC  231.923442  
  logL  -110.333990  
  logL ratio  79.811807  
   Expression   
  Mean expression  4.833464  
  Median expression  4.537418  
  Tissue std. dev.  1.089892  
 
  No GO Slim enrichment  
  
   tissue    mean expression   
  5th Passage Drosophila S2 Cells  4.629360  
  Adult Accessory gland  4.567700  
  Adult Brain  4.645973  
  Adult Carcass  4.535718  
  Adult Crop  4.358288  
  Adult Eye  4.454190  
  Adult Fatbody  4.499760  
  Adult Female Spermatheca Mated  4.777226  
  Adult Female Spermatheca Virgin  4.777501  
  Adult Head  4.472311  
  Adult Heart  4.682516  
  Adult Hind Gut  4.410143  
  Adult Male Ejaculatory Duct  4.531715  
  Adult Mid Gut  4.421206  
  Adult Ovary  4.462469  
  Adult Salivary Gland  4.905328  
  Adult Testes  10.262186  
  Adult Thoracoabdominal ganglion  4.705510  
  Adult Whole Fly  5.559217  
  Larvae Wandering Tubules  4.594635  
  Larval Feeding Carcass  4.451958  
  Larval Feeding Central Nevous System  4.835674  
  Larval Feeding Hind Gut  4.435919  
  Larval Feeding Malpighian Tubule  4.653241  
  Larval Feeding Mid Gut  4.439658  
  Larval Feeding Salivary Gland  4.609117  
  Whole Larvae Feeding  4.825013  
 
  
   FlyBase ID    symbol    start    end    strand    length   
   FBgn0051391   CG31391  6529090   6530797   -  1708  
   FBgn0051467   CG31467  6530889   6532585   -  1697  
   FBgn0037824   CG14684   6532696   6533182  +  487  
 
 
    Segment 133 
 
   Location   
  Gene key  FBgn0037826-FBgn0037829  
  Heatmap region span   3R:6522240..6605297   
  Segment span   3R:6536970..6581356   
  Length (genes)  5  
  Length (bp)  44387  
   Model Scoring   
  BIC  427.127777  
  logL  -207.936157  
  logL ratio  113.554930  
   Expression   
  Mean expression  4.633381  
  Median expression  4.268844  
  Tissue std. dev.  1.061837  
 
  No GO Slim enrichment  
  
   tissue    mean expression   
  5th Passage Drosophila S2 Cells  4.260223  
  Adult Accessory gland  4.429112  
  Adult Brain  4.630705  
  Adult Carcass  4.412189  
  Adult Crop  4.241291  
  Adult Eye  4.253924  
  Adult Fatbody  4.302266  
  Adult Female Spermatheca Mated  4.265681  
  Adult Female Spermatheca Virgin  4.302415  
  Adult Head  4.310387  
  Adult Heart  4.167809  
  Adult Hind Gut  4.297286  
  Adult Male Ejaculatory Duct  4.296850  
  Adult Mid Gut  4.351458  
  Adult Ovary  4.254634  
  Adult Salivary Gland  4.475159  
  Adult Testes  9.768507  
  Adult Thoracoabdominal ganglion  4.675324  
  Adult Whole Fly  5.959586  
  Larvae Wandering Tubules  4.507422  
  Larval Feeding Carcass  4.289085  
  Larval Feeding Central Nevous System  4.562660  
  Larval Feeding Hind Gut  4.166409  
  Larval Feeding Malpighian Tubule  4.528942  
  Larval Feeding Mid Gut  4.289568  
  Larval Feeding Salivary Gland  4.291408  
  Whole Larvae Feeding  4.810979  
 
  
   FlyBase ID    symbol    start    end    strand    length   
   FBgn0037826   CG14689  6535307   6536970   -  1664  
   FBgn0037827   CG4073   6540520   6541621  +  1102  
   FBgn0037828   tomboy20  6561068   6561679   -  612  
   FBgn0004841   Takr86C  6563314   6576950   -  13637  
   FBgn0037829   CG14691  6579052   6581356   -  2305  
 
 
    Segment 134 
 
   Location   
  Gene key  FBgn0051390-FBgn0010421  
  Heatmap region span   3R:6534048..6614250   
  Segment span   3R:6586754..6593668   
  Length (genes)  2  
  Length (bp)  6915  
   Model Scoring   
  BIC  207.271768  
  logL  -98.008153  
  logL ratio  49.899637  
   Expression   
  Mean expression  8.649437  
  Median expression  8.637172  
  Tissue std. dev.  0.494184  
 
  
   GO ID    description    ratio    P-value   
   GO:0005634   nucleus  2/2  0.0218  
   GO:0043226   organelle  2/2  0.0483  
 
  
   tissue    mean expression   
  5th Passage Drosophila S2 Cells  8.930802  
  Adult Accessory gland  9.182126  
  Adult Brain  8.978518  
  Adult Carcass  7.951819  
  Adult Crop  8.417363  
  Adult Eye  9.268214  
  Adult Fatbody  8.347489  
  Adult Female Spermatheca Mated  8.070322  
  Adult Female Spermatheca Virgin  8.106837  
  Adult Head  8.435869  
  Adult Heart  8.628940  
  Adult Hind Gut  8.050607  
  Adult Male Ejaculatory Duct  8.608921  
  Adult Mid Gut  8.238090  
  Adult Ovary  9.993889  
  Adult Salivary Gland  8.376053  
  Adult Testes  8.751168  
  Adult Thoracoabdominal ganglion  8.596414  
  Adult Whole Fly  8.960289  
  Larvae Wandering Tubules  8.863950  
  Larval Feeding Carcass  8.612280  
  Larval Feeding Central Nevous System  9.572263  
  Larval Feeding Hind Gut  8.333855  
  Larval Feeding Malpighian Tubule  8.538205  
  Larval Feeding Mid Gut  8.164504  
  Larval Feeding Salivary Gland  9.339942  
  Whole Larvae Feeding  8.216075  
 
  
   FlyBase ID    symbol    start    end    strand    length   
   FBgn0051390   MED7  6585793   6586754   -  962  
   FBgn0010421   TfIIFbeta  6592344   6593668   -  1325  
 
    Segment 135 
 
   Location   
  Gene key  FBgn0037836-FBgn0037837  
  Heatmap region span   3R:6605297..6680221   
  Segment span   3R:6629631..6640769   
  Length (genes)  2  
  Length (bp)  11139  
   Model Scoring   
  BIC  179.445807  
  logL  -84.095173  
  logL ratio  35.273115  
   Expression   
  Mean expression  4.359364  
  Median expression  4.283285  
  Tissue std. dev.  0.381155  
 
  No GO Slim enrichment  
  
   tissue    mean expression   
  5th Passage Drosophila S2 Cells  4.206687  
  Adult Accessory gland  4.316793  
  Adult Brain  4.066745  
  Adult Carcass  4.373431  
  Adult Crop  4.187241  
  Adult Eye  4.263136  
  Adult Fatbody  4.192413  
  Adult Female Spermatheca Mated  4.493004  
  Adult Female Spermatheca Virgin  4.418056  
  Adult Head  4.532296  
  Adult Heart  4.290685  
  Adult Hind Gut  4.177515  
  Adult Male Ejaculatory Duct  4.286416  
  Adult Mid Gut  4.357709  
  Adult Ovary  4.187418  
  Adult Salivary Gland  4.579339  
  Adult Testes  6.160766  
  Adult Thoracoabdominal ganglion  4.221203  
  Adult Whole Fly  4.093850  
  Larvae Wandering Tubules  4.355288  
  Larval Feeding Carcass  4.317788  
  Larval Feeding Central Nevous System  4.046717  
  Larval Feeding Hind Gut  4.115823  
  Larval Feeding Malpighian Tubule  4.310352  
  Larval Feeding Mid Gut  4.440510  
  Larval Feeding Salivary Gland  4.543053  
  Whole Larvae Feeding  4.168586  
 
  
   FlyBase ID    symbol    start    end    strand    length   
   FBgn0037836   CG14692  6619849   6629631   -  9783  
   FBgn0037837   CG14693  6637303   6640769   -  3467  
 
    Segment 136 
 
   Location   
  Gene key  FBgn0037842-FBgn0037843  
  Heatmap region span   3R:6629631..6698102   
  Segment span   3R:6680144..6680221   
  Length (genes)  2  
  Length (bp)  78  
   Model Scoring   
  BIC  230.424198  
  logL  -109.584368  
  logL ratio  30.922616  
   Expression   
  Mean expression  8.783769  
  Median expression  8.767907  
  Tissue std. dev.  0.414400  
 
  No GO Slim enrichment  
  
   tissue    mean expression   
  5th Passage Drosophila S2 Cells  8.898132  
  Adult Accessory gland  8.178149  
  Adult Brain  8.134563  
  Adult Carcass  8.626514  
  Adult Crop  9.091151  
  Adult Eye  8.424899  
  Adult Fatbody  9.255792  
  Adult Female Spermatheca Mated  8.758547  
  Adult Female Spermatheca Virgin  8.774457  
  Adult Head  8.419016  
  Adult Heart  9.223324  
  Adult Hind Gut  8.320201  
  Adult Male Ejaculatory Duct  8.763167  
  Adult Mid Gut  8.775866  
  Adult Ovary  9.562373  
  Adult Salivary Gland  7.990096  
  Adult Testes  9.128420  
  Adult Thoracoabdominal ganglion  7.915540  
  Adult Whole Fly  8.781347  
  Larvae Wandering Tubules  8.865941  
  Larval Feeding Carcass  8.914347  
  Larval Feeding Central Nevous System  8.979686  
  Larval Feeding Hind Gut  9.078852  
  Larval Feeding Malpighian Tubule  9.061147  
  Larval Feeding Mid Gut  8.875498  
  Larval Feeding Salivary Gland  9.466110  
  Whole Larvae Feeding  8.898627  
 
  
   FlyBase ID    symbol    start    end    strand    length   
   FBgn0037842   CG6567  6676815   6680144   -  3330  
   FBgn0037843   CG4511   6680221   6682134  +  1914  
 
    Segment 137 
 
   Location   
  Gene key  FBgn0037846-FBgn0037848  
  Heatmap region span   3R:6678446..6719252   
  Segment span   3R:6688710..6694434   
  Length (genes)  3  
  Length (bp)  5725  
   Model Scoring   
  BIC  375.910931  
  logL  -182.327735  
  logL ratio  7.160262  
   Expression   
  Mean expression  7.946447  
  Median expression  7.665425  
  Tissue std. dev.  0.686760  
 
  No GO Slim enrichment  
  
   tissue    mean expression   
  5th Passage Drosophila S2 Cells  8.131994  
  Adult Accessory gland  7.559442  
  Adult Brain  8.789490  
  Adult Carcass  7.743492  
  Adult Crop  7.770261  
  Adult Eye  8.318735  
  Adult Fatbody  7.201490  
  Adult Female Spermatheca Mated  7.002969  
  Adult Female Spermatheca Virgin  7.076325  
  Adult Head  7.842649  
  Adult Heart  8.232324  
  Adult Hind Gut  8.199449  
  Adult Male Ejaculatory Duct  7.315587  
  Adult Mid Gut  8.119807  
  Adult Ovary  7.869935  
  Adult Salivary Gland  8.132803  
  Adult Testes  6.797388  
  Adult Thoracoabdominal ganglion  8.630151  
  Adult Whole Fly  7.228384  
  Larvae Wandering Tubules  9.419482  
  Larval Feeding Carcass  7.661187  
  Larval Feeding Central Nevous System  7.791893  
  Larval Feeding Hind Gut  7.750922  
  Larval Feeding Malpighian Tubule  9.937076  
  Larval Feeding Mid Gut  8.237391  
  Larval Feeding Salivary Gland  8.153844  
  Whole Larvae Feeding  7.639608  
 
  
   FlyBase ID    symbol    start    end    strand    length   
   FBgn0037846   CG6574  6686251   6688710   -  2460  
   FBgn0037847   SelR  6689071   6694379   -  5309  
   FBgn0037848   Tsp86D   6694434   6696232  +  1799  
 
 
    Segment 138 
 
   Location   
  Gene key  FBgn0037852-FBgn0037855  
  Heatmap region span   3R:6688710..6982822   
  Segment span   3R:6707909..6719252   
  Length (genes)  5  
  Length (bp)  11344  
   Model Scoring   
  BIC  566.717872  
  logL  -277.731205  
  logL ratio  54.866561  
   Expression   
  Mean expression  8.009727  
  Median expression  8.127784  
  Tissue std. dev.  0.376780  
 
  No GO Slim enrichment  
  
   tissue    mean expression   
  5th Passage Drosophila S2 Cells  9.142720  
  Adult Accessory gland  7.488573  
  Adult Brain  8.544479  
  Adult Carcass  7.878593  
  Adult Crop  8.226049  
  Adult Eye  7.856999  
  Adult Fatbody  7.825028  
  Adult Female Spermatheca Mated  7.426604  
  Adult Female Spermatheca Virgin  7.455394  
  Adult Head  7.745572  
  Adult Heart  8.143380  
  Adult Hind Gut  7.606657  
  Adult Male Ejaculatory Duct  7.759018  
  Adult Mid Gut  7.963537  
  Adult Ovary  8.218880  
  Adult Salivary Gland  8.187248  
  Adult Testes  8.018748  
  Adult Thoracoabdominal ganglion  8.536748  
  Adult Whole Fly  7.622695  
  Larvae Wandering Tubules  8.013134  
  Larval Feeding Carcass  8.128846  
  Larval Feeding Central Nevous System  8.614883  
  Larval Feeding Hind Gut  7.893486  
  Larval Feeding Malpighian Tubule  8.067403  
  Larval Feeding Mid Gut  7.974191  
  Larval Feeding Salivary Gland  7.993122  
  Whole Larvae Feeding  7.930644  
 
  
   FlyBase ID    symbol    start    end    strand    length   
   FBgn0037852   Tpc1  6705294   6707909   -  2616  
   FBgn0037853   CG14696  6708155   6710723   -  2569  
   FBgn0037856   CG4674   6711269   6722140  +  10872  
   FBgn0042094   Adk3  6713288   6715310   -  2023  
   FBgn0037855   CG6621  6715490   6719252   -  3763  
 
 
    Segment 139 
 
   Location   
  Gene key  FBgn0037857-FBgn0037860  
  Heatmap region span   3R:6698102..6986924   
  Segment span   3R:6776006..6941428   
  Length (genes)  3  
  Length (bp)  165423  
   Model Scoring   
  BIC  271.570203  
  logL  -130.157370  
  logL ratio  48.900507  
   Expression   
  Mean expression  4.718893  
  Median expression  4.623660  
  Tissue std. dev.  0.866683  
 
  No GO Slim enrichment  
  
   tissue    mean expression   
  5th Passage Drosophila S2 Cells  4.482994  
  Adult Accessory gland  4.494015  
  Adult Brain  4.898358  
  Adult Carcass  4.598871  
  Adult Crop  4.415596  
  Adult Eye  4.365302  
  Adult Fatbody  4.594598  
  Adult Female Spermatheca Mated  4.526180  
  Adult Female Spermatheca Virgin  4.503637  
  Adult Head  4.384180  
  Adult Heart  4.314786  
  Adult Hind Gut  4.330151  
  Adult Male Ejaculatory Duct  4.659970  
  Adult Mid Gut  4.373132  
  Adult Ovary  4.483541  
  Adult Salivary Gland  4.799466  
  Adult Testes  8.889954  
  Adult Thoracoabdominal ganglion  4.651068  
  Adult Whole Fly  5.816019  
  Larvae Wandering Tubules  4.425694  
  Larval Feeding Carcass  4.529514  
  Larval Feeding Central Nevous System  4.387604  
  Larval Feeding Hind Gut  4.321835  
  Larval Feeding Malpighian Tubule  4.452471  
  Larval Feeding Mid Gut  4.437010  
  Larval Feeding Salivary Gland  4.492964  
  Whole Larvae Feeding  4.781195  
 
  
   FlyBase ID    symbol    start    end    strand    length   
   FBgn0037857   Tengl4   6776006   6777519  +  1514  
   FBgn0083950   CG34114   6819205   6875525  +  56321  
   FBgn0037860   CG6629  6940393   6941428   -  1036  
 
 
    Segment 140 
 
   Location   
  Gene key  FBgn0037862-FBgn0040257  
  Heatmap region span   3R:6705041..6995955   
  Segment span   3R:6962899..6980377   
  Length (genes)  3  
  Length (bp)  17479  
   Model Scoring   
  BIC  285.207045  
  logL  -136.975791  
  logL ratio  27.405531  
   Expression   
  Mean expression  4.819229  
  Median expression  4.578873  
  Tissue std. dev.  0.472156  
 
  No GO Slim enrichment  
  
   tissue    mean expression   
  5th Passage Drosophila S2 Cells  4.475643  
  Adult Accessory gland  5.583824  
  Adult Brain  4.839769  
  Adult Carcass  4.674417  
  Adult Crop  4.432283  
  Adult Eye  4.396470  
  Adult Fatbody  4.837397  
  Adult Female Spermatheca Mated  4.565354  
  Adult Female Spermatheca Virgin  4.886513  
  Adult Head  4.773676  
  Adult Heart  4.479617  
  Adult Hind Gut  4.564455  
  Adult Male Ejaculatory Duct  4.653195  
  Adult Mid Gut  6.309387  
  Adult Ovary  4.466351  
  Adult Salivary Gland  4.758278  
  Adult Testes  6.123600  
  Adult Thoracoabdominal ganglion  4.766511  
  Adult Whole Fly  4.978965  
  Larvae Wandering Tubules  4.517760  
  Larval Feeding Carcass  4.741033  
  Larval Feeding Central Nevous System  5.277248  
  Larval Feeding Hind Gut  4.465170  
  Larval Feeding Malpighian Tubule  4.614699  
  Larval Feeding Mid Gut  4.691923  
  Larval Feeding Salivary Gland  4.478546  
  Whole Larvae Feeding  4.767112  
 
  
   FlyBase ID    symbol    start    end    strand    length   
   FBgn0037862   CG4706   6962899   6965467  +  2569  
   FBgn0040251   Ugt86Di  6977629   6979571   -  1943  
   FBgn0040257   Ugt86Dc   6980377   6982235  +  1859  
 
 
    Segment 141 
 
   Location   
  Gene key  FBgn0040253-FBgn0040255  
  Heatmap region span   3R:6954035..7042037   
  Segment span   3R:6991796..6993699   
  Length (genes)  2  
  Length (bp)  1904  
   Model Scoring   
  BIC  175.961719  
  logL  -82.353128  
  logL ratio  35.533577  
   Expression   
  Mean expression  4.743490  
  Median expression  4.665797  
  Tissue std. dev.  0.364788  
 
  No GO Slim enrichment  
  
   tissue    mean expression   
  5th Passage Drosophila S2 Cells  4.583902  
  Adult Accessory gland  5.182370  
  Adult Brain  4.806237  
  Adult Carcass  4.622486  
  Adult Crop  4.508482  
  Adult Eye  4.623708  
  Adult Fatbody  4.571440  
  Adult Female Spermatheca Mated  4.748876  
  Adult Female Spermatheca Virgin  4.711021  
  Adult Head  4.499986  
  Adult Heart  4.621018  
  Adult Hind Gut  4.580965  
  Adult Male Ejaculatory Duct  4.891854  
  Adult Mid Gut  5.156425  
  Adult Ovary  4.497418  
  Adult Salivary Gland  4.819771  
  Adult Testes  4.358650  
  Adult Thoracoabdominal ganglion  5.051682  
  Adult Whole Fly  4.297724  
  Larvae Wandering Tubules  4.717634  
  Larval Feeding Carcass  4.659026  
  Larval Feeding Central Nevous System  4.466215  
  Larval Feeding Hind Gut  4.470244  
  Larval Feeding Malpighian Tubule  6.187027  
  Larval Feeding Mid Gut  5.175547  
  Larval Feeding Salivary Gland  4.633972  
  Whole Larvae Feeding  4.630540  
 
  
   FlyBase ID    symbol    start    end    strand    length   
   FBgn0040253   Ugt86Dg  6990145   6991796   -  1652  
   FBgn0040255   Ugt86De  6992020   6993699   -  1680  
 
    Segment 142 
 
   Location   
  Gene key  FBgn0040250-FBgn0037870  
  Heatmap region span   3R:6986924..7070006   
  Segment span   3R:7001130..7021826   
  Length (genes)  3  
  Length (bp)  20697  
   Model Scoring   
  BIC  274.130386  
  logL  -131.437462  
  logL ratio  55.324576  
   Expression   
  Mean expression  5.074115  
  Median expression  4.433076  
  Tissue std. dev.  1.462422  
 
  No GO Slim enrichment  
  
   tissue    mean expression   
  5th Passage Drosophila S2 Cells  4.302834  
  Adult Accessory gland  4.479354  
  Adult Brain  4.152409  
  Adult Carcass  4.434497  
  Adult Crop  4.244904  
  Adult Eye  4.100667  
  Adult Fatbody  4.340306  
  Adult Female Spermatheca Mated  4.449255  
  Adult Female Spermatheca Virgin  4.345552  
  Adult Head  4.087603  
  Adult Heart  4.337604  
  Adult Hind Gut  6.692667  
  Adult Male Ejaculatory Duct  4.344213  
  Adult Mid Gut  9.613946  
  Adult Ovary  4.141436  
  Adult Salivary Gland  4.432358  
  Adult Testes  4.026523  
  Adult Thoracoabdominal ganglion  4.293271  
  Adult Whole Fly  4.961823  
  Larvae Wandering Tubules  6.284648  
  Larval Feeding Carcass  4.168500  
  Larval Feeding Central Nevous System  4.142317  
  Larval Feeding Hind Gut  6.383669  
  Larval Feeding Malpighian Tubule  6.509303  
  Larval Feeding Mid Gut  8.820811  
  Larval Feeding Salivary Gland  4.250006  
  Whole Larvae Feeding  6.660638  
 
  
   FlyBase ID    symbol    start    end    strand    length   
   FBgn0040250   Ugt86Dj  6999163   7001130   -  1968  
   FBgn0040252   Ugt86Dh   7001251   7003373  +  2123  
   FBgn0037870   CG18577   7021826   7023007  +  1182  
 
 
    Segment 143 
 
   Location   
  Gene key  FBgn0037873-FBgn0086472  
  Heatmap region span   3R:6991796..7131330   
  Segment span   3R:7035571..7042037   
  Length (genes)  4  
  Length (bp)  6467  
   Model Scoring   
  BIC  834.086716  
  logL  -411.415627  
  logL ratio  82.985884  
   Expression   
  Mean expression  12.430534  
  Median expression  12.937154  
  Tissue std. dev.  0.359998  
 
  No GO Slim enrichment  
  
   tissue    mean expression   
  5th Passage Drosophila S2 Cells  12.685173  
  Adult Accessory gland  12.701869  
  Adult Brain  12.197954  
  Adult Carcass  12.471859  
  Adult Crop  12.395209  
  Adult Eye  12.347935  
  Adult Fatbody  12.569541  
  Adult Female Spermatheca Mated  12.655862  
  Adult Female Spermatheca Virgin  12.564061  
  Adult Head  12.356399  
  Adult Heart  12.455104  
  Adult Hind Gut  12.515484  
  Adult Male Ejaculatory Duct  12.743315  
  Adult Mid Gut  12.413845  
  Adult Ovary  12.308340  
  Adult Salivary Gland  12.826745  
  Adult Testes  10.804970  
  Adult Thoracoabdominal ganglion  12.248950  
  Adult Whole Fly  12.446106  
  Larvae Wandering Tubules  12.419313  
  Larval Feeding Carcass  12.681816  
  Larval Feeding Central Nevous System  12.339021  
  Larval Feeding Hind Gut  12.656209  
  Larval Feeding Malpighian Tubule  12.757643  
  Larval Feeding Mid Gut  12.311712  
  Larval Feeding Salivary Gland  12.378250  
  Whole Larvae Feeding  12.371729  
 
  
   FlyBase ID    symbol    start    end    strand    length   
   FBgn0037873   SdhC  7034856   7035571   -  716  
   FBgn0037874   Tctp   7035975   7037006  +  1032  
   FBgn0037875   CG6672  7037962   7040239   -  2278  
   FBgn0086472   RpS25  7040942   7042037   -  1096  
 
 
    Segment 144 
 
   Location   
  Gene key  FBgn0037876-FBgn0037884  
  Heatmap region span   3R:6995955..7155624   
  Segment span   3R:7042333..7060907   
  Length (genes)  10  
  Length (bp)  18575  
   Model Scoring   
  BIC  883.500956  
  logL  -436.122747  
  logL ratio  347.801103  
   Expression   
  Mean expression  7.966416  
  Median expression  7.840133  
  Tissue std. dev.  0.353150  
 
  
   GO ID    description    ratio    P-value   
   GO:0051082   unfolded protein binding  2/10  0.0105  
   GO:0006457   protein folding  2/10  0.0225  
 
  
   tissue    mean expression   
  5th Passage Drosophila S2 Cells  8.689340  
  Adult Accessory gland  8.293795  
  Adult Brain  8.320011  
  Adult Carcass  7.649406  
  Adult Crop  8.106282  
  Adult Eye  8.165672  
  Adult Fatbody  7.710346  
  Adult Female Spermatheca Mated  7.848937  
  Adult Female Spermatheca Virgin  7.735275  
  Adult Head  7.838866  
  Adult Heart  8.045254  
  Adult Hind Gut  7.682117  
  Adult Male Ejaculatory Duct  8.307356  
  Adult Mid Gut  7.510397  
  Adult Ovary  8.744553  
  Adult Salivary Gland  7.912994  
  Adult Testes  7.526356  
  Adult Thoracoabdominal ganglion  8.190212  
  Adult Whole Fly  7.765412  
  Larvae Wandering Tubules  7.775221  
  Larval Feeding Carcass  7.966280  
  Larval Feeding Central Nevous System  8.295802  
  Larval Feeding Hind Gut  7.856170  
  Larval Feeding Malpighian Tubule  8.002287  
  Larval Feeding Mid Gut  7.369602  
  Larval Feeding Salivary Gland  8.379647  
  Whole Larvae Feeding  7.405631  
 
  
   FlyBase ID    symbol    start    end    strand    length   
   FBgn0037876   CG4820   7042333   7043620  +  1288  
   FBgn0037877   CG6689  7043620   7045766   -  2147  
   FBgn0037878   CG6693  7045990   7047361   -  1372  
   FBgn0020910   RpL3   7047616   7050666  +  3051  
   FBgn0037880   CG17726   7052486   7053949  +  1464  
   FBgn0037881   CG10703  7053880   7056291   -  2412  
   FBgn0037882   CG17187  7056339   7057578   -  1240  
   FBgn0037883   CG14701   7057634   7058261  +  628  
   FBgn0037885   CG17721   7060862   7061799  +  938  
   FBgn0037884   CG17184  7058151   7060907   -  2757  
 
 
    Segment 145 
 
   Location   
  Gene key  FBgn0051441-FBgn0051388  
  Heatmap region span   3R:6998386..7178853   
  Segment span   3R:7062008..7063537   
  Length (genes)  2  
  Length (bp)  1530  
   Model Scoring   
  BIC  211.872118  
  logL  -100.308328  
  logL ratio  31.006623  
   Expression   
  Mean expression  6.638517  
  Median expression  6.482484  
  Tissue std. dev.  0.739951  
 
  
   GO ID    description    ratio    P-value   
   GO:0005634   nucleus  2/2  0.0218  
   GO:0043226   organelle  2/2  0.0483  
 
  
   tissue    mean expression   
  5th Passage Drosophila S2 Cells  7.056950  
  Adult Accessory gland  5.685370  
  Adult Brain  8.171869  
  Adult Carcass  6.372937  
  Adult Crop  7.294979  
  Adult Eye  7.725523  
  Adult Fatbody  6.336956  
  Adult Female Spermatheca Mated  6.576157  
  Adult Female Spermatheca Virgin  6.634114  
  Adult Head  7.236734  
  Adult Heart  6.736441  
  Adult Hind Gut  6.448711  
  Adult Male Ejaculatory Duct  6.369479  
  Adult Mid Gut  5.768640  
  Adult Ovary  7.750694  
  Adult Salivary Gland  6.567255  
  Adult Testes  5.712160  
  Adult Thoracoabdominal ganglion  8.052295  
  Adult Whole Fly  6.256884  
  Larvae Wandering Tubules  6.615457  
  Larval Feeding Carcass  6.170981  
  Larval Feeding Central Nevous System  7.593570  
  Larval Feeding Hind Gut  6.215115  
  Larval Feeding Malpighian Tubule  6.839047  
  Larval Feeding Mid Gut  5.628640  
  Larval Feeding Salivary Gland  5.757106  
  Whole Larvae Feeding  5.665893  
 
  
   FlyBase ID    symbol    start    end    strand    length   
   FBgn0051441   CG31441   7062008   7063346  +  1339  
   FBgn0051388   CG31388   7063537   7065836  +  2300  
 
    Segment 146 
 
   Location   
  Gene key  FBgn0037890-FBgn0037891  
  Heatmap region span   3R:7001130..7238244   
  Segment span   3R:7069700..7070006   
  Length (genes)  2  
  Length (bp)  307  
   Model Scoring   
  BIC  263.998499  
  logL  -126.371518  
  logL ratio  64.748974  
   Expression   
  Mean expression  10.867141  
  Median expression  11.068132  
  Tissue std. dev.  0.693451  
 
  No GO Slim enrichment  
  
   tissue    mean expression   
  5th Passage Drosophila S2 Cells  9.677938  
  Adult Accessory gland  9.542459  
  Adult Brain  11.697042  
  Adult Carcass  11.892989  
  Adult Crop  11.210379  
  Adult Eye  11.205373  
  Adult Fatbody  11.182351  
  Adult Female Spermatheca Mated  10.748994  
  Adult Female Spermatheca Virgin  10.830026  
  Adult Head  11.313529  
  Adult Heart  11.121271  
  Adult Hind Gut  11.781360  
  Adult Male Ejaculatory Duct  10.122370  
  Adult Mid Gut  10.144848  
  Adult Ovary  9.759610  
  Adult Salivary Gland  11.284537  
  Adult Testes  9.836109  
  Adult Thoracoabdominal ganglion  11.938632  
  Adult Whole Fly  10.944616  
  Larvae Wandering Tubules  10.158279  
  Larval Feeding Carcass  11.152682  
  Larval Feeding Central Nevous System  10.990832  
  Larval Feeding Hind Gut  11.420378  
  Larval Feeding Malpighian Tubule  11.607522  
  Larval Feeding Mid Gut  10.634650  
  Larval Feeding Salivary Gland  10.226439  
  Whole Larvae Feeding  10.987580  
 
  
   FlyBase ID    symbol    start    end    strand    length   
   FBgn0037890   CG17734  7067930   7069700   -  1771  
   FBgn0037891   CG5214   7070006   7072586  +  2581  
 
    Segment 147 
 
   Location   
  Gene key  FBgn0026064-FBgn0026063  
  Heatmap region span   3R:7062008..7263507   
  Segment span   3R:7178853..7178853   
  Length (genes)  2  
  Length (bp)  1  
   Model Scoring   
  BIC  195.598211  
  logL  -92.171374  
  logL ratio  16.566433  
   Expression   
  Mean expression  4.889912  
  Median expression  4.643565  
  Tissue std. dev.  0.705188  
 
  No GO Slim enrichment  
  
   tissue    mean expression   
  5th Passage Drosophila S2 Cells  5.845468  
  Adult Accessory gland  4.837834  
  Adult Brain  4.203959  
  Adult Carcass  4.757273  
  Adult Crop  4.467111  
  Adult Eye  4.335350  
  Adult Fatbody  4.576448  
  Adult Female Spermatheca Mated  4.509709  
  Adult Female Spermatheca Virgin  4.578199  
  Adult Head  4.388609  
  Adult Heart  4.437328  
  Adult Hind Gut  4.374116  
  Adult Male Ejaculatory Duct  4.529575  
  Adult Mid Gut  4.877635  
  Adult Ovary  4.502850  
  Adult Salivary Gland  4.872423  
  Adult Testes  7.143850  
  Adult Thoracoabdominal ganglion  4.356763  
  Adult Whole Fly  4.639045  
  Larvae Wandering Tubules  4.658652  
  Larval Feeding Carcass  5.175720  
  Larval Feeding Central Nevous System  6.811232  
  Larval Feeding Hind Gut  5.748746  
  Larval Feeding Malpighian Tubule  4.522817  
  Larval Feeding Mid Gut  4.809933  
  Larval Feeding Salivary Gland  4.932721  
  Whole Larvae Feeding  5.134253  
 
  
   FlyBase ID    symbol    start    end    strand    length   
   FBgn0026064   KP78a  7172014   7178853   -  6840  
   FBgn0026063   KP78b  7172014   7178853   -  6840  
 
    Segment 148 
 
   Location   
  Gene key  FBgn0037892-FBgn0037894  
  Heatmap region span   3R:7069700..7276872   
  Segment span   3R:7233619..7238244   
  Length (genes)  4  
  Length (bp)  4626  
   Model Scoring   
  BIC  486.910227  
  logL  -237.827382  
  logL ratio  26.946079  
   Expression   
  Mean expression  8.431858  
  Median expression  8.288853  
  Tissue std. dev.  0.466810  
 
  
   GO ID    description    ratio    P-value   
   GO:0003674   molecular_function  4/4  0.0166  
 
  
   tissue    mean expression   
  5th Passage Drosophila S2 Cells  9.195967  
  Adult Accessory gland  8.550925  
  Adult Brain  8.041253  
  Adult Carcass  7.829141  
  Adult Crop  8.389418  
  Adult Eye  8.189298  
  Adult Fatbody  8.266880  
  Adult Female Spermatheca Mated  8.050322  
  Adult Female Spermatheca Virgin  8.012785  
  Adult Head  7.876196  
  Adult Heart  8.466815  
  Adult Hind Gut  8.365004  
  Adult Male Ejaculatory Duct  8.381980  
  Adult Mid Gut  8.312910  
  Adult Ovary  9.923603  
  Adult Salivary Gland  7.953998  
  Adult Testes  8.427387  
  Adult Thoracoabdominal ganglion  8.258986  
  Adult Whole Fly  8.933607  
  Larvae Wandering Tubules  8.405371  
  Larval Feeding Carcass  8.557202  
  Larval Feeding Central Nevous System  9.273868  
  Larval Feeding Hind Gut  8.686676  
  Larval Feeding Malpighian Tubule  8.473996  
  Larval Feeding Mid Gut  7.994043  
  Larval Feeding Salivary Gland  8.815136  
  Whole Larvae Feeding  8.027394  
 
  
   FlyBase ID    symbol    start    end    strand    length   
   FBgn0037892   mRpL40   7233619   7234478  +  860  
   FBgn0037893     7234514   7235609   -  1096  
   FBgn0011774   Irbp   7235770   7238162  +  2393  
   FBgn0037894   Ranbp9   7238244   7241864  +  3621  
 
 
    Segment 149 
 
   Location   
  Gene key  FBgn0037895-FBgn0037896  
  Heatmap region span   3R:7131330..7368671   
  Segment span   3R:7244389..7247415   
  Length (genes)  2  
  Length (bp)  3027  
   Model Scoring   
  BIC  233.091676  
  logL  -110.918107  
  logL ratio  -8.542696  
   Expression   
  Mean expression  5.789999  
  Median expression  5.305518  
  Tissue std. dev.  1.471415  
 
  
   GO ID    description    ratio    P-value   
   GO:0006810   transport  2/2  0.00478  
 
  
   tissue    mean expression   
  5th Passage Drosophila S2 Cells  5.037180  
  Adult Accessory gland  5.092892  
  Adult Brain  8.374923  
  Adult Carcass  6.519535  
  Adult Crop  4.953052  
  Adult Eye  9.864767  
  Adult Fatbody  4.971896  
  Adult Female Spermatheca Mated  5.105213  
  Adult Female Spermatheca Virgin  5.072211  
  Adult Head  8.784952  
  Adult Heart  6.509683  
  Adult Hind Gut  4.729354  
  Adult Male Ejaculatory Duct  5.050852  
  Adult Mid Gut  4.987925  
  Adult Ovary  4.760528  
  Adult Salivary Gland  5.431826  
  Adult Testes  4.634823  
  Adult Thoracoabdominal ganglion  8.830033  
  Adult Whole Fly  5.222364  
  Larvae Wandering Tubules  5.183586  
  Larval Feeding Carcass  5.033556  
  Larval Feeding Central Nevous System  7.445234  
  Larval Feeding Hind Gut  4.718203  
  Larval Feeding Malpighian Tubule  5.031810  
  Larval Feeding Mid Gut  4.844312  
  Larval Feeding Salivary Gland  5.132423  
  Whole Larvae Feeding  5.006838  
 
  
   FlyBase ID    symbol    start    end    strand    length   
   FBgn0037895   CG6723  7241940   7244389   -  2450  
   FBgn0037896   ninaG  7245098   7247415   -  2318  
 
    Segment 150 
 
   Location   
  Gene key  FBgn0037897-FBgn0037901  
  Heatmap region span   3R:7155624..7379867   
  Segment span   3R:7247814..7261425   
  Length (genes)  4  
  Length (bp)  13612  
   Model Scoring   
  BIC  434.783632  
  logL  -211.764085  
  logL ratio  73.696374  
   Expression   
  Mean expression  8.360554  
  Median expression  8.340806  
  Tissue std. dev.  0.394128  
 
  No GO Slim enrichment  
  
   tissue    mean expression   
  5th Passage Drosophila S2 Cells  8.479433  
  Adult Accessory gland  8.711567  
  Adult Brain  8.070904  
  Adult Carcass  8.031959  
  Adult Crop  8.035266  
  Adult Eye  8.030891  
  Adult Fatbody  8.271171  
  Adult Female Spermatheca Mated  8.124688  
  Adult Female Spermatheca Virgin  8.067690  
  Adult Head  7.808407  
  Adult Heart  8.557660  
  Adult Hind Gut  7.823451  
  Adult Male Ejaculatory Duct  8.218103  
  Adult Mid Gut  8.151415  
  Adult Ovary  8.933406  
  Adult Salivary Gland  8.730621  
  Adult Testes  8.693396  
  Adult Thoracoabdominal ganglion  8.024664  
  Adult Whole Fly  7.930461  
  Larvae Wandering Tubules  9.146291  
  Larval Feeding Carcass  8.390915  
  Larval Feeding Central Nevous System  8.073044  
  Larval Feeding Hind Gut  8.623798  
  Larval Feeding Malpighian Tubule  9.044446  
  Larval Feeding Mid Gut  8.612972  
  Larval Feeding Salivary Gland  9.092883  
  Whole Larvae Feeding  8.055443  
 
  
   FlyBase ID    symbol    start    end    strand    length   
   FBgn0037897   CG5270   7247814   7255691  +  7878  
   FBgn0037898   CG18643   7255783   7256484  +  702  
   FBgn0037900   CG5276   7257460   7259265  +  1806  
   FBgn0037901   CG6744  7259210   7261425   -  2216  
 
 
    Segment 151 
 
   Location   
  Gene key  FBgn0037902-FBgn0046225  
  Heatmap region span   3R:7178853..7383451   
  Segment span   3R:7261572..7263507   
  Length (genes)  2  
  Length (bp)  1936  
   Model Scoring   
  BIC  204.983254  
  logL  -96.863896  
  logL ratio  20.805634  
   Expression   
  Mean expression  6.121168  
  Median expression  5.249976  
  Tissue std. dev.  1.515467  
 
  No GO Slim enrichment  
  
   tissue    mean expression   
  5th Passage Drosophila S2 Cells  7.022874  
  Adult Accessory gland  5.872675  
  Adult Brain  9.542334  
  Adult Carcass  5.089893  
  Adult Crop  5.121971  
  Adult Eye  8.225088  
  Adult Fatbody  4.695242  
  Adult Female Spermatheca Mated  4.804146  
  Adult Female Spermatheca Virgin  4.791614  
  Adult Head  7.482653  
  Adult Heart  5.056985  
  Adult Hind Gut  5.518608  
  Adult Male Ejaculatory Duct  4.933685  
  Adult Mid Gut  5.256508  
  Adult Ovary  6.469836  
  Adult Salivary Gland  4.926847  
  Adult Testes  9.542634  
  Adult Thoracoabdominal ganglion  9.358279  
  Adult Whole Fly  6.503036  
  Larvae Wandering Tubules  5.311903  
  Larval Feeding Carcass  5.360966  
  Larval Feeding Central Nevous System  7.821552  
  Larval Feeding Hind Gut  5.515046  
  Larval Feeding Malpighian Tubule  5.268461  
  Larval Feeding Mid Gut  5.021805  
  Larval Feeding Salivary Gland  4.883669  
  Whole Larvae Feeding  5.873226  
 
  
   FlyBase ID    symbol    start    end    strand    length   
   FBgn0037902   CG5281   7261572   7263245  +  1674  
   FBgn0046225   CG17230   7263507   7291360  +  27854  
 
    Segment 152 
 
   Location   
  Gene key  FBgn0037908-FBgn0053512  
  Heatmap region span   3R:7244389..7404548   
  Segment span   3R:7325706..7368671   
  Length (genes)  2  
  Length (bp)  42966  
   Model Scoring   
  BIC  188.316592  
  logL  -88.530565  
  logL ratio  29.647924  
   Expression   
  Mean expression  5.533342  
  Median expression  5.350889  
  Tissue std. dev.  0.976248  
 
  No GO Slim enrichment  
  
   tissue    mean expression   
  5th Passage Drosophila S2 Cells  4.937995  
  Adult Accessory gland  5.203430  
  Adult Brain  8.724177  
  Adult Carcass  5.201519  
  Adult Crop  4.896528  
  Adult Eye  5.799250  
  Adult Fatbody  5.518938  
  Adult Female Spermatheca Mated  5.125799  
  Adult Female Spermatheca Virgin  5.145116  
  Adult Head  6.518459  
  Adult Heart  5.055988  
  Adult Hind Gut  4.927977  
  Adult Male Ejaculatory Duct  5.310474  
  Adult Mid Gut  5.080720  
  Adult Ovary  4.841142  
  Adult Salivary Gland  5.409220  
  Adult Testes  5.949072  
  Adult Thoracoabdominal ganglion  8.190882  
  Adult Whole Fly  4.835055  
  Larvae Wandering Tubules  4.986750  
  Larval Feeding Carcass  5.068644  
  Larval Feeding Central Nevous System  7.151461  
  Larval Feeding Hind Gut  5.086852  
  Larval Feeding Malpighian Tubule  5.226007  
  Larval Feeding Mid Gut  5.088923  
  Larval Feeding Salivary Gland  5.253747  
  Whole Larvae Feeding  4.866103  
 
  
   FlyBase ID    symbol    start    end    strand    length   
   FBgn0037908   dpr5   7325706   7331677  +  5972  
   FBgn0053512   dpr4  7359205   7368671   -  9467  
 
    Segment 153 
 
   Location   
  Gene key  FBgn0025701-FBgn0037916  
  Heatmap region span   3R:7325706..7503835   
  Segment span   3R:7394972..7404548   
  Length (genes)  3  
  Length (bp)  9577  
   Model Scoring   
  BIC  247.317562  
  logL  -118.031050  
  logL ratio  63.584368  
   Expression   
  Mean expression  4.905907  
  Median expression  4.807626  
  Tissue std. dev.  0.804341  
 
  No GO Slim enrichment  
  
   tissue    mean expression   
  5th Passage Drosophila S2 Cells  4.936892  
  Adult Accessory gland  4.971312  
  Adult Brain  4.425228  
  Adult Carcass  4.863126  
  Adult Crop  4.850059  
  Adult Eye  4.401583  
  Adult Fatbody  4.776961  
  Adult Female Spermatheca Mated  4.712140  
  Adult Female Spermatheca Virgin  4.771940  
  Adult Head  4.511569  
  Adult Heart  4.825359  
  Adult Hind Gut  4.695423  
  Adult Male Ejaculatory Duct  4.549927  
  Adult Mid Gut  4.802512  
  Adult Ovary  4.533385  
  Adult Salivary Gland  4.743789  
  Adult Testes  8.829702  
  Adult Thoracoabdominal ganglion  4.561122  
  Adult Whole Fly  5.578642  
  Larvae Wandering Tubules  4.953451  
  Larval Feeding Carcass  4.704195  
  Larval Feeding Central Nevous System  4.534005  
  Larval Feeding Hind Gut  4.685455  
  Larval Feeding Malpighian Tubule  4.709631  
  Larval Feeding Mid Gut  4.740068  
  Larval Feeding Salivary Gland  4.643156  
  Whole Larvae Feeding  5.148862  
 
  
   FlyBase ID    symbol    start    end    strand    length   
   FBgn0025701      7394972   7401659  +  6688  
   FBgn0037915   CG6790  7401623   7404467   -  2845  
   FBgn0037916   CG5342   7404548   7407198  +  2651  
 
 
    Segment 154 
 
   Location   
  Gene key  FBgn0037918-FBgn0037922  
  Heatmap region span   3R:7383451..7510062   
  Segment span   3R:7415864..7449096   
  Length (genes)  4  
  Length (bp)  33233  
   Model Scoring   
  BIC  423.437466  
  logL  -206.091002  
  logL ratio  62.908576  
   Expression   
  Mean expression  7.198055  
  Median expression  7.283143  
  Tissue std. dev.  0.577247  
 
  
   GO ID    description    ratio    P-value   
   GO:0005634   nucleus  3/4  0.0161  
 
  
   tissue    mean expression   
  5th Passage Drosophila S2 Cells  7.734348  
  Adult Accessory gland  6.996528  
  Adult Brain  8.136218  
  Adult Carcass  6.689430  
  Adult Crop  6.985363  
  Adult Eye  7.358734  
  Adult Fatbody  6.674116  
  Adult Female Spermatheca Mated  6.774700  
  Adult Female Spermatheca Virgin  6.754937  
  Adult Head  6.854806  
  Adult Heart  6.998233  
  Adult Hind Gut  6.621524  
  Adult Male Ejaculatory Duct  6.952665  
  Adult Mid Gut  6.677699  
  Adult Ovary  8.246555  
  Adult Salivary Gland  7.309565  
  Adult Testes  8.831463  
  Adult Thoracoabdominal ganglion  7.644572  
  Adult Whole Fly  7.745919  
  Larvae Wandering Tubules  6.956161  
  Larval Feeding Carcass  6.996330  
  Larval Feeding Central Nevous System  8.062614  
  Larval Feeding Hind Gut  6.663795  
  Larval Feeding Malpighian Tubule  6.823857  
  Larval Feeding Mid Gut  6.541939  
  Larval Feeding Salivary Gland  7.191102  
  Whole Larvae Feeding  7.124319  
 
  
   FlyBase ID    symbol    start    end    strand    length   
   FBgn0037918   CG6791  7411076   7415864   -  4789  
   FBgn0051363   Jupiter  7416108   7445656   -  29549  
   FBgn0037920   CG14710   7445814   7447450  +  1637  
   FBgn0037922   CG14711   7449096   7450670  +  1575  
 
 
    Segment 155 
 
   Location   
  Gene key  FBgn0037921-FBgn0037924  
  Heatmap region span   3R:7392771..7515745   
  Segment span   3R:7451366..7454483   
  Length (genes)  4  
  Length (bp)  3118  
   Model Scoring   
  BIC  356.042011  
  logL  -172.393275  
  logL ratio  87.620197  
   Expression   
  Mean expression  5.744687  
  Median expression  5.888706  
  Tissue std. dev.  0.523109  
 
  
   GO ID    description    ratio    P-value   
   GO:0005634   nucleus  3/4  0.0092  
   GO:0043226   organelle  3/4  0.0294  
   GO:0005575   cellular_component  4/4  0.0461  
 
  
   tissue    mean expression   
  5th Passage Drosophila S2 Cells  6.078379  
  Adult Accessory gland  5.680556  
  Adult Brain  6.386804  
  Adult Carcass  5.162341  
  Adult Crop  6.235612  
  Adult Eye  6.707813  
  Adult Fatbody  5.438052  
  Adult Female Spermatheca Mated  5.605709  
  Adult Female Spermatheca Virgin  5.503790  
  Adult Head  5.686782  
  Adult Heart  5.843364  
  Adult Hind Gut  5.602380  
  Adult Male Ejaculatory Duct  5.363492  
  Adult Mid Gut  5.141702  
  Adult Ovary  7.066806  
  Adult Salivary Gland  5.509554  
  Adult Testes  5.592456  
  Adult Thoracoabdominal ganglion  6.153472  
  Adult Whole Fly  5.337387  
  Larvae Wandering Tubules  5.605229  
  Larval Feeding Carcass  5.446646  
  Larval Feeding Central Nevous System  6.912009  
  Larval Feeding Hind Gut  5.518690  
  Larval Feeding Malpighian Tubule  5.551103  
  Larval Feeding Mid Gut  5.110184  
  Larval Feeding Salivary Gland  5.835328  
  Whole Larvae Feeding  5.030905  
 
  
   FlyBase ID    symbol    start    end    strand    length   
   FBgn0037921   CG6808  7447188   7451366   -  4179  
   FBgn0037923   CG6813  7450991   7452360   -  1370  
   FBgn0042205   CG18764   7452654   7454185  +  1532  
   FBgn0037924   CG14712   7454483   7459426  +  4944  
 
 
    Segment 156 
 
   Location   
  Gene key  FBgn0037926-FBgn0037931  
  Heatmap region span   3R:7394972..7547526   
  Segment span   3R:7476710..7503835   
  Length (genes)  4  
  Length (bp)  27126  
   Model Scoring   
  BIC  419.276205  
  logL  -204.010371  
  logL ratio  72.764681  
   Expression   
  Mean expression  7.533736  
  Median expression  7.558466  
  Tissue std. dev.  0.375787  
 
  No GO Slim enrichment  
  
   tissue    mean expression   
  5th Passage Drosophila S2 Cells  8.559612  
  Adult Accessory gland  7.594853  
  Adult Brain  7.806501  
  Adult Carcass  7.525325  
  Adult Crop  7.437601  
  Adult Eye  7.487084  
  Adult Fatbody  7.523134  
  Adult Female Spermatheca Mated  7.424796  
  Adult Female Spermatheca Virgin  7.378362  
  Adult Head  7.483619  
  Adult Heart  8.317422  
  Adult Hind Gut  7.454518  
  Adult Male Ejaculatory Duct  7.593479  
  Adult Mid Gut  7.482427  
  Adult Ovary  8.010229  
  Adult Salivary Gland  7.501384  
  Adult Testes  6.473642  
  Adult Thoracoabdominal ganglion  7.753794  
  Adult Whole Fly  7.095986  
  Larvae Wandering Tubules  7.547205  
  Larval Feeding Carcass  7.264636  
  Larval Feeding Central Nevous System  7.813283  
  Larval Feeding Hind Gut  7.347218  
  Larval Feeding Malpighian Tubule  7.633826  
  Larval Feeding Mid Gut  7.154900  
  Larval Feeding Salivary Gland  7.479868  
  Whole Larvae Feeding  7.266173  
 
  
   FlyBase ID    symbol    start    end    strand    length   
   FBgn0037926   Elp1   7476710   7481062  +  4353  
   FBgn0259227   CG42327   7481875   7499697  +  17823  
   FBgn0037930   CG14715   7499941   7500645  +  705  
   FBgn0037931   CG18476  7500642   7503835   -  3194  
 
 
    Segment 157 
 
   Location   
  Gene key  FBgn0011290-FBgn0037933  
  Heatmap region span   3R:7407301..7561169   
  Segment span   3R:7505408..7505481   
  Length (genes)  2  
  Length (bp)  74  
   Model Scoring   
  BIC  249.479146  
  logL  -119.111842  
  logL ratio  30.378183  
   Expression   
  Mean expression  9.646884  
  Median expression  9.701819  
  Tissue std. dev.  0.433059  
 
  No GO Slim enrichment  
  
   tissue    mean expression   
  5th Passage Drosophila S2 Cells  9.431985  
  Adult Accessory gland  9.652901  
  Adult Brain  10.680793  
  Adult Carcass  9.268598  
  Adult Crop  9.410514  
  Adult Eye  10.018804  
  Adult Fatbody  9.392826  
  Adult Female Spermatheca Mated  9.337495  
  Adult Female Spermatheca Virgin  9.537630  
  Adult Head  9.864580  
  Adult Heart  9.773518  
  Adult Hind Gut  9.411817  
  Adult Male Ejaculatory Duct  9.958764  
  Adult Mid Gut  8.901855  
  Adult Ovary  10.075433  
  Adult Salivary Gland  9.450362  
  Adult Testes  9.134209  
  Adult Thoracoabdominal ganglion  10.516129  
  Adult Whole Fly  9.467785  
  Larvae Wandering Tubules  10.526922  
  Larval Feeding Carcass  9.854903  
  Larval Feeding Central Nevous System  9.635427  
  Larval Feeding Hind Gut  9.388182  
  Larval Feeding Malpighian Tubule  9.819191  
  Larval Feeding Mid Gut  9.630008  
  Larval Feeding Salivary Gland  9.087457  
  Whole Larvae Feeding  9.237780  
 
  
   FlyBase ID    symbol    start    end    strand    length   
   FBgn0011290   Taf12  7504099   7505408   -  1310  
   FBgn0037933   Ho   7505481   7506698  +  1218  
 
    Segment 158 
 
   Location   
  Gene key  FBgn0037935-FBgn0037936  
  Heatmap region span   3R:7451366..7575186   
  Segment span   3R:7513852..7515745   
  Length (genes)  2  
  Length (bp)  1894  
   Model Scoring   
  BIC  250.209243  
  logL  -119.476890  
  logL ratio  9.587752  
   Expression   
  Mean expression  8.178167  
  Median expression  8.496770  
  Tissue std. dev.  1.813975  
 
  No GO Slim enrichment  
  
   tissue    mean expression   
  5th Passage Drosophila S2 Cells  4.795720  
  Adult Accessory gland  7.988600  
  Adult Brain  7.753664  
  Adult Carcass  9.490413  
  Adult Crop  5.668334  
  Adult Eye  9.977836  
  Adult Fatbody  9.713680  
  Adult Female Spermatheca Mated  9.243324  
  Adult Female Spermatheca Virgin  10.025007  
  Adult Head  10.313446  
  Adult Heart  8.913342  
  Adult Hind Gut  9.187963  
  Adult Male Ejaculatory Duct  9.202462  
  Adult Mid Gut  10.718396  
  Adult Ovary  5.864988  
  Adult Salivary Gland  5.138537  
  Adult Testes  5.492561  
  Adult Thoracoabdominal ganglion  7.986793  
  Adult Whole Fly  7.894145  
  Larvae Wandering Tubules  9.517254  
  Larval Feeding Carcass  6.254873  
  Larval Feeding Central Nevous System  6.809222  
  Larval Feeding Hind Gut  8.988368  
  Larval Feeding Malpighian Tubule  8.550298  
  Larval Feeding Mid Gut  10.501649  
  Larval Feeding Salivary Gland  5.319184  
  Whole Larvae Feeding  9.500444  
 
  
   FlyBase ID    symbol    start    end    strand    length   
   FBgn0037935   CG6834  7510391   7513852   -  3462  
   FBgn0037936   CG6908  7514099   7515745   -  1647  
 
    Segment 159 
 
   Location   
  Gene key  FBgn0042110-FBgn0037941  
  Heatmap region span   3R:7476710..7590179   
  Segment span   3R:7517521..7547526   
  Length (genes)  6  
  Length (bp)  30006  
   Model Scoring   
  BIC  568.905720  
  logL  -278.825129  
  logL ratio  66.873075  
   Expression   
  Mean expression  4.581925  
  Median expression  4.316493  
  Tissue std. dev.  0.491591  
 
  No GO Slim enrichment  
  
   tissue    mean expression   
  5th Passage Drosophila S2 Cells  4.356402  
  Adult Accessory gland  4.407153  
  Adult Brain  4.895391  
  Adult Carcass  4.375950  
  Adult Crop  4.295462  
  Adult Eye  4.341308  
  Adult Fatbody  4.290038  
  Adult Female Spermatheca Mated  4.366297  
  Adult Female Spermatheca Virgin  4.317101  
  Adult Head  4.561927  
  Adult Heart  4.345503  
  Adult Hind Gut  4.316558  
  Adult Male Ejaculatory Duct  4.353756  
  Adult Mid Gut  4.716660  
  Adult Ovary  4.220796  
  Adult Salivary Gland  4.398053  
  Adult Testes  6.679879  
  Adult Thoracoabdominal ganglion  4.999580  
  Adult Whole Fly  5.321066  
  Larvae Wandering Tubules  4.282410  
  Larval Feeding Carcass  4.329809  
  Larval Feeding Central Nevous System  4.935925  
  Larval Feeding Hind Gut  4.735278  
  Larval Feeding Malpighian Tubule  4.289627  
  Larval Feeding Mid Gut  4.558673  
  Larval Feeding Salivary Gland  4.311401  
  Whole Larvae Feeding  4.709968  
 
  
   FlyBase ID    symbol    start    end    strand    length   
   FBgn0042110   CG18765  7516107   7517521   -  1415  
   FBgn0037937   Fer3  7519132   7519999   -  868  
   FBgn0037938      7522231   7523384  +  1154  
   FBgn0037939   CG14718   7526336   7528442  +  2107  
   FBgn0037940   CG14720   7537501   7538076  +  576  
   FBgn0037941   CG12594   7547526   7558201  +  10676  
 
 
    Segment 160 
 
   Location   
  Gene key  FBgn0037943-FBgn0037944  
  Heatmap region span   3R:7513852..7622157   
  Segment span   3R:7567584..7575186   
  Length (genes)  2  
  Length (bp)  7603  
   Model Scoring   
  BIC  242.722302  
  logL  -115.733420  
  logL ratio  8.081726  
   Expression   
  Mean expression  7.571520  
  Median expression  7.761247  
  Tissue std. dev.  0.567509  
 
  No GO Slim enrichment  
  
   tissue    mean expression   
  5th Passage Drosophila S2 Cells  8.021720  
  Adult Accessory gland  8.247611  
  Adult Brain  7.973075  
  Adult Carcass  7.050294  
  Adult Crop  7.509037  
  Adult Eye  7.488487  
  Adult Fatbody  7.311280  
  Adult Female Spermatheca Mated  7.117412  
  Adult Female Spermatheca Virgin  7.041730  
  Adult Head  7.173520  
  Adult Heart  7.351849  
  Adult Hind Gut  7.069448  
  Adult Male Ejaculatory Duct  7.208317  
  Adult Mid Gut  7.033544  
  Adult Ovary  9.012422  
  Adult Salivary Gland  8.137937  
  Adult Testes  9.039966  
  Adult Thoracoabdominal ganglion  7.783110  
  Adult Whole Fly  7.628278  
  Larvae Wandering Tubules  7.530061  
  Larval Feeding Carcass  7.232148  
  Larval Feeding Central Nevous System  8.020157  
  Larval Feeding Hind Gut  7.166028  
  Larval Feeding Malpighian Tubule  7.578999  
  Larval Feeding Mid Gut  6.920526  
  Larval Feeding Salivary Gland  7.933058  
  Whole Larvae Feeding  6.851024  
 
  
   FlyBase ID    symbol    start    end    strand    length   
   FBgn0037943   CG14722   7567584   7569749  +  2166  
   FBgn0037944   CG6923  7570205   7575186   -  4982  
 
    Segment 161 
 
   Location   
  Gene key  FBgn0259139-FBgn0037955  
  Heatmap region span   3R:7612736..7719019   
  Segment span   3R:7645142..7648210   
  Length (genes)  2  
  Length (bp)  3069  
   Model Scoring   
  BIC  236.654938  
  logL  -112.699738  
  logL ratio  50.778381  
   Expression   
  Mean expression  10.009992  
  Median expression  9.978151  
  Tissue std. dev.  0.378181  
 
  No GO Slim enrichment  
  
   tissue    mean expression   
  5th Passage Drosophila S2 Cells  9.939788  
  Adult Accessory gland  9.880180  
  Adult Brain  10.254708  
  Adult Carcass  10.056055  
  Adult Crop  9.746438  
  Adult Eye  9.836820  
  Adult Fatbody  10.224913  
  Adult Female Spermatheca Mated  9.992836  
  Adult Female Spermatheca Virgin  10.087955  
  Adult Head  10.154716  
  Adult Heart  10.111788  
  Adult Hind Gut  9.929436  
  Adult Male Ejaculatory Duct  10.228223  
  Adult Mid Gut  9.819268  
  Adult Ovary  10.251943  
  Adult Salivary Gland  9.924522  
  Adult Testes  8.629058  
  Adult Thoracoabdominal ganglion  9.933736  
  Adult Whole Fly  9.983342  
  Larvae Wandering Tubules  10.304833  
  Larval Feeding Carcass  10.110318  
  Larval Feeding Central Nevous System  10.598653  
  Larval Feeding Hind Gut  9.964825  
  Larval Feeding Malpighian Tubule  10.866812  
  Larval Feeding Mid Gut  9.483833  
  Larval Feeding Salivary Gland  10.158969  
  Whole Larvae Feeding  9.795807  
 
  
   FlyBase ID    symbol    start    end    strand    length   
   FBgn0259139   glo  7640928   7645142   -  4215  
   FBgn0037955   CG6950  7646103   7648210   -  2108  
 
    Segment 162 
 
   Location   
  Gene key  FBgn0037958-FBgn0037960  
  Heatmap region span   3R:7645081..7775402   
  Segment span   3R:7708257..7712887   
  Length (genes)  3  
  Length (bp)  4631  
   Model Scoring   
  BIC  301.315347  
  logL  -145.029942  
  logL ratio  66.047114  
   Expression   
  Mean expression  7.062856  
  Median expression  7.100558  
  Tissue std. dev.  0.641119  
 
  No GO Slim enrichment  
  
   tissue    mean expression   
  5th Passage Drosophila S2 Cells  8.184752  
  Adult Accessory gland  7.493086  
  Adult Brain  6.528762  
  Adult Carcass  6.496176  
  Adult Crop  7.312418  
  Adult Eye  7.216482  
  Adult Fatbody  6.791195  
  Adult Female Spermatheca Mated  7.420432  
  Adult Female Spermatheca Virgin  7.460741  
  Adult Head  6.634826  
  Adult Heart  6.978400  
  Adult Hind Gut  6.611907  
  Adult Male Ejaculatory Duct  7.306749  
  Adult Mid Gut  6.051001  
  Adult Ovary  8.379314  
  Adult Salivary Gland  6.959571  
  Adult Testes  6.795222  
  Adult Thoracoabdominal ganglion  6.631615  
  Adult Whole Fly  6.842306  
  Larvae Wandering Tubules  6.305765  
  Larval Feeding Carcass  7.365417  
  Larval Feeding Central Nevous System  8.580501  
  Larval Feeding Hind Gut  7.142855  
  Larval Feeding Malpighian Tubule  6.382224  
  Larval Feeding Mid Gut  6.187694  
  Larval Feeding Salivary Gland  7.927247  
  Whole Larvae Feeding  6.710454  
 
  
   FlyBase ID    symbol    start    end    strand    length   
   FBgn0037958   CG6962  7705424   7708257   -  2834  
   FBgn0051368   CG31368   7708517   7718104  +  9588  
   FBgn0037960   mthl5  7709660   7712887   -  3228  
 
 
    Segment 163 
 
   Location   
  Gene key  FBgn0000147-FBgn0037970  
  Heatmap region span   3R:7719019..7810263   
  Segment span   3R:7788852..7797416   
  Length (genes)  2  
  Length (bp)  8565  
   Model Scoring   
  BIC  225.810034  
  logL  -107.277286  
  logL ratio  -4.836211  
   Expression   
  Mean expression  5.631626  
  Median expression  5.241625  
  Tissue std. dev.  1.237321  
 
  No GO Slim enrichment  
  
   tissue    mean expression   
  5th Passage Drosophila S2 Cells  6.512661  
  Adult Accessory gland  6.758297  
  Adult Brain  5.237089  
  Adult Carcass  4.875298  
  Adult Crop  4.936540  
  Adult Eye  5.465023  
  Adult Fatbody  4.832586  
  Adult Female Spermatheca Mated  4.989543  
  Adult Female Spermatheca Virgin  4.982771  
  Adult Head  4.855710  
  Adult Heart  5.028976  
  Adult Hind Gut  4.850293  
  Adult Male Ejaculatory Duct  5.257319  
  Adult Mid Gut  5.241575  
  Adult Ovary  7.232902  
  Adult Salivary Gland  5.240593  
  Adult Testes  10.694734  
  Adult Thoracoabdominal ganglion  5.226304  
  Adult Whole Fly  7.584830  
  Larvae Wandering Tubules  4.894923  
  Larval Feeding Carcass  5.154122  
  Larval Feeding Central Nevous System  6.254222  
  Larval Feeding Hind Gut  5.029418  
  Larval Feeding Malpighian Tubule  4.939769  
  Larval Feeding Mid Gut  5.195621  
  Larval Feeding Salivary Gland  5.197083  
  Whole Larvae Feeding  5.585697  
 
  
   FlyBase ID    symbol    start    end    strand    length   
   FBgn0000147   aur   7788852   7790550  +  1699  
   FBgn0037970   CG12201   7797416   7798627  +  1212  
 
    Segment 164 
 
   Location   
  Gene key  FBgn0037978-FBgn0037981  
  Heatmap region span   3R:7806112..8023663   
  Segment span   3R:7836152..7842822   
  Length (genes)  4  
  Length (bp)  6671  
   Model Scoring   
  BIC  360.384923  
  logL  -174.564731  
  logL ratio  135.337936  
   Expression   
  Mean expression  7.569320  
  Median expression  7.492294  
  Tissue std. dev.  0.347316  
 
  No GO Slim enrichment  
  
   tissue    mean expression   
  5th Passage Drosophila S2 Cells  7.763624  
  Adult Accessory gland  7.778689  
  Adult Brain  8.010015  
  Adult Carcass  7.101402  
  Adult Crop  7.405733  
  Adult Eye  7.564000  
  Adult Fatbody  7.512860  
  Adult Female Spermatheca Mated  7.137448  
  Adult Female Spermatheca Virgin  7.100672  
  Adult Head  7.332873  
  Adult Heart  7.598425  
  Adult Hind Gut  7.146907  
  Adult Male Ejaculatory Duct  7.289563  
  Adult Mid Gut  7.228248  
  Adult Ovary  8.332361  
  Adult Salivary Gland  7.682524  
  Adult Testes  8.308122  
  Adult Thoracoabdominal ganglion  7.938770  
  Adult Whole Fly  7.230395  
  Larvae Wandering Tubules  7.975691  
  Larval Feeding Carcass  7.673896  
  Larval Feeding Central Nevous System  7.835483  
  Larval Feeding Hind Gut  7.319945  
  Larval Feeding Malpighian Tubule  7.788618  
  Larval Feeding Mid Gut  7.394054  
  Larval Feeding Salivary Gland  7.675786  
  Whole Larvae Feeding  7.245546  
 
  
   FlyBase ID    symbol    start    end    strand    length   
   FBgn0037978   KLHL18  7833832   7836152   -  2321  
   FBgn0037979   CG3532   7836504   7840784  +  4281  
   FBgn0037980   CG3313  7840665   7842753   -  2089  
   FBgn0037981   Spt3   7842822   7844272  +  1451  
 
 
    Segment 165 
 
   Location   
  Gene key  FBgn0051358-FBgn0037988  
  Heatmap region span   3R:7807743..8036079   
  Segment span   3R:7857479..7888666   
  Length (genes)  5  
  Length (bp)  31188  
   Model Scoring   
  BIC  397.274571  
  logL  -193.009554  
  logL ratio  121.462313  
   Expression   
  Mean expression  5.026456  
  Median expression  4.710475  
  Tissue std. dev.  0.988694  
 
  No GO Slim enrichment  
  
   tissue    mean expression   
  5th Passage Drosophila S2 Cells  4.787667  
  Adult Accessory gland  4.900770  
  Adult Brain  4.425894  
  Adult Carcass  4.969625  
  Adult Crop  4.739468  
  Adult Eye  4.476746  
  Adult Fatbody  4.764492  
  Adult Female Spermatheca Mated  4.915267  
  Adult Female Spermatheca Virgin  4.802729  
  Adult Head  4.523184  
  Adult Heart  4.623506  
  Adult Hind Gut  4.675829  
  Adult Male Ejaculatory Duct  4.809719  
  Adult Mid Gut  4.971088  
  Adult Ovary  4.748261  
  Adult Salivary Gland  5.115334  
  Adult Testes  9.675584  
  Adult Thoracoabdominal ganglion  4.610771  
  Adult Whole Fly  6.534440  
  Larvae Wandering Tubules  4.908329  
  Larval Feeding Carcass  4.837571  
  Larval Feeding Central Nevous System  4.443919  
  Larval Feeding Hind Gut  4.663411  
  Larval Feeding Malpighian Tubule  4.865774  
  Larval Feeding Mid Gut  4.856303  
  Larval Feeding Salivary Gland  4.779524  
  Whole Larvae Feeding  5.289097  
 
  
   FlyBase ID    symbol    start    end    strand    length   
   FBgn0051358   CG31358  7853792   7857479   -  3688  
   FBgn0037985   ssp5   7858864   7861247  +  2384  
   FBgn0037986   CG14736   7866250   7869175  +  2926  
   FBgn0037987   CG14739  7873975   7874935   -  961  
   FBgn0037988   CG14740  7886792   7888666   -  1875  
 
 
    Segment 166 
 
   Location   
  Gene key  FBgn0037989-FBgn0051361  
  Heatmap region span   3R:7810263..8047185   
  Segment span   3R:7903198..7920600   
  Length (genes)  2  
  Length (bp)  17403  
   Model Scoring   
  BIC  228.585662  
  logL  -108.665100  
  logL ratio  2.872880  
   Expression   
  Mean expression  6.231542  
  Median expression  5.778077  
  Tissue std. dev.  1.226327  
 
  No GO Slim enrichment  
  
   tissue    mean expression   
  5th Passage Drosophila S2 Cells  10.179884  
  Adult Accessory gland  5.551398  
  Adult Brain  8.438225  
  Adult Carcass  5.702163  
  Adult Crop  5.457885  
  Adult Eye  5.691572  
  Adult Fatbody  5.409637  
  Adult Female Spermatheca Mated  5.425563  
  Adult Female Spermatheca Virgin  5.365329  
  Adult Head  6.559430  
  Adult Heart  5.184719  
  Adult Hind Gut  6.083097  
  Adult Male Ejaculatory Duct  5.946891  
  Adult Mid Gut  5.906140  
  Adult Ovary  5.090413  
  Adult Salivary Gland  5.882664  
  Adult Testes  8.957149  
  Adult Thoracoabdominal ganglion  8.235571  
  Adult Whole Fly  6.179362  
  Larvae Wandering Tubules  5.729330  
  Larval Feeding Carcass  5.796267  
  Larval Feeding Central Nevous System  6.604693  
  Larval Feeding Hind Gut  5.733835  
  Larval Feeding Malpighian Tubule  6.337713  
  Larval Feeding Mid Gut  5.642150  
  Larval Feeding Salivary Gland  5.337452  
  Whole Larvae Feeding  5.823111  
 
  
   FlyBase ID    symbol    start    end    strand    length   
   FBgn0037989   CG14741  7894964   7903198   -  8235  
   FBgn0051361   dpr17   7920600   7937144  +  16545  
 
    Segment 167 
 
   Location   
  Gene key  FBgn0037992-FBgn0037993  
  Heatmap region span   3R:7831194..8050741   
  Segment span   3R:7953919..7984317   
  Length (genes)  2  
  Length (bp)  30399  
   Model Scoring   
  BIC  221.519968  
  logL  -105.132253  
  logL ratio  10.301365  
   Expression   
  Mean expression  4.732316  
  Median expression  4.898915  
  Tissue std. dev.  0.686658  
 
  No GO Slim enrichment  
  
   tissue    mean expression   
  5th Passage Drosophila S2 Cells  4.386703  
  Adult Accessory gland  4.383245  
  Adult Brain  6.852603  
  Adult Carcass  4.367765  
  Adult Crop  4.518972  
  Adult Eye  4.517235  
  Adult Fatbody  4.502515  
  Adult Female Spermatheca Mated  4.285626  
  Adult Female Spermatheca Virgin  4.419724  
  Adult Head  5.240733  
  Adult Heart  4.266514  
  Adult Hind Gut  4.571853  
  Adult Male Ejaculatory Duct  4.273417  
  Adult Mid Gut  4.588394  
  Adult Ovary  4.183083  
  Adult Salivary Gland  4.698086  
  Adult Testes  4.699861  
  Adult Thoracoabdominal ganglion  6.865406  
  Adult Whole Fly  4.766316  
  Larvae Wandering Tubules  4.452076  
  Larval Feeding Carcass  4.508557  
  Larval Feeding Central Nevous System  5.887135  
  Larval Feeding Hind Gut  4.783131  
  Larval Feeding Malpighian Tubule  4.482563  
  Larval Feeding Mid Gut  4.533588  
  Larval Feeding Salivary Gland  4.359997  
  Whole Larvae Feeding  4.377423  
 
  
   FlyBase ID    symbol    start    end    strand    length   
   FBgn0037992   CG4702  7947299   7953919   -  6621  
   FBgn0037993   dpr15  7964555   7984317   -  19763  
 
    Segment 168 
 
   Location   
  Gene key  FBgn0037994-FBgn0037995  
  Heatmap region span   3R:7836152..8052061   
  Segment span   3R:8020733..8023663   
  Length (genes)  2  
  Length (bp)  2931  
   Model Scoring   
  BIC  172.442953  
  logL  -80.593745  
  logL ratio  41.208519  
   Expression   
  Mean expression  5.002973  
  Median expression  4.687038  
  Tissue std. dev.  1.367459  
 
  No GO Slim enrichment  
  
   tissue    mean expression   
  5th Passage Drosophila S2 Cells  4.694546  
  Adult Accessory gland  4.613020  
  Adult Brain  4.418238  
  Adult Carcass  4.705451  
  Adult Crop  4.624234  
  Adult Eye  4.769272  
  Adult Fatbody  4.667945  
  Adult Female Spermatheca Mated  4.632775  
  Adult Female Spermatheca Virgin  4.658012  
  Adult Head  4.385153  
  Adult Heart  4.853309  
  Adult Hind Gut  4.602018  
  Adult Male Ejaculatory Duct  4.784781  
  Adult Mid Gut  4.679724  
  Adult Ovary  4.530407  
  Adult Salivary Gland  4.988180  
  Adult Testes  11.659622  
  Adult Thoracoabdominal ganglion  4.475657  
  Adult Whole Fly  6.612452  
  Larvae Wandering Tubules  4.549627  
  Larval Feeding Carcass  4.844504  
  Larval Feeding Central Nevous System  4.304450  
  Larval Feeding Hind Gut  4.587862  
  Larval Feeding Malpighian Tubule  4.720195  
  Larval Feeding Mid Gut  4.767613  
  Larval Feeding Salivary Gland  4.755603  
  Whole Larvae Feeding  5.195621  
 
  
   FlyBase ID    symbol    start    end    strand    length   
   FBgn0037994   CG4810  8018425   8020733   -  2309  
   FBgn0037995   CG3809   8023663   8025196  +  1534  
 
    Segment 169 
 
   Location   
  Gene key  FBgn0037998-FBgn0037999  
  Heatmap region span   3R:7903198..8096612   
  Segment span   3R:8045412..8047185   
  Length (genes)  2  
  Length (bp)  1774  
   Model Scoring   
  BIC  223.064615  
  logL  -105.904577  
  logL ratio  30.054460  
   Expression   
  Mean expression  8.048111  
  Median expression  8.217199  
  Tissue std. dev.  0.545142  
 
  No GO Slim enrichment  
  
   tissue    mean expression   
  5th Passage Drosophila S2 Cells  9.238871  
  Adult Accessory gland  7.839249  
  Adult Brain  6.963849  
  Adult Carcass  8.076736  
  Adult Crop  8.406755  
  Adult Eye  7.440366  
  Adult Fatbody  8.563248  
  Adult Female Spermatheca Mated  8.059498  
  Adult Female Spermatheca Virgin  8.128054  
  Adult Head  7.348771  
  Adult Heart  8.492687  
  Adult Hind Gut  7.778892  
  Adult Male Ejaculatory Duct  7.949038  
  Adult Mid Gut  7.997028  
  Adult Ovary  8.335213  
  Adult Salivary Gland  8.316840  
  Adult Testes  7.446304  
  Adult Thoracoabdominal ganglion  7.127854  
  Adult Whole Fly  7.369483  
  Larvae Wandering Tubules  8.172282  
  Larval Feeding Carcass  8.672691  
  Larval Feeding Central Nevous System  7.798887  
  Larval Feeding Hind Gut  8.451137  
  Larval Feeding Malpighian Tubule  8.044840  
  Larval Feeding Mid Gut  8.198518  
  Larval Feeding Salivary Gland  9.204915  
  Whole Larvae Feeding  7.876990  
 
  
   FlyBase ID    symbol    start    end    strand    length   
   FBgn0037998   CG4848  8041377   8045412   -  4036  
   FBgn0037999   CG4860  8045603   8047185   -  1583  
 
    Segment 170 
 
   Location   
  Gene key  FBgn0038000-FBgn0038001  
  Heatmap region span   3R:7953919..8149245   
  Segment span   3R:8048109..8050741   
  Length (genes)  2  
  Length (bp)  2633  
   Model Scoring   
  BIC  203.815723  
  logL  -96.280131  
  logL ratio  10.428685  
   Expression   
  Mean expression  4.668115  
  Median expression  4.479029  
  Tissue std. dev.  0.600987  
 
  No GO Slim enrichment  
  
   tissue    mean expression   
  5th Passage Drosophila S2 Cells  4.497470  
  Adult Accessory gland  4.744692  
  Adult Brain  4.344518  
  Adult Carcass  4.725726  
  Adult Crop  4.462399  
  Adult Eye  4.307182  
  Adult Fatbody  4.694666  
  Adult Female Spermatheca Mated  4.444369  
  Adult Female Spermatheca Virgin  4.404348  
  Adult Head  4.262256  
  Adult Heart  4.392248  
  Adult Hind Gut  4.431446  
  Adult Male Ejaculatory Duct  4.686679  
  Adult Mid Gut  4.754582  
  Adult Ovary  4.398642  
  Adult Salivary Gland  4.940724  
  Adult Testes  7.496660  
  Adult Thoracoabdominal ganglion  4.409123  
  Adult Whole Fly  5.296984  
  Larvae Wandering Tubules  4.571862  
  Larval Feeding Carcass  4.708661  
  Larval Feeding Central Nevous System  4.259981  
  Larval Feeding Hind Gut  4.287826  
  Larval Feeding Malpighian Tubule  4.408966  
  Larval Feeding Mid Gut  4.697997  
  Larval Feeding Salivary Gland  4.639569  
  Whole Larvae Feeding  4.769522  
 
  
   FlyBase ID    symbol    start    end    strand    length   
   FBgn0038000   CG10014   8048109   8049770  +  1662  
   FBgn0038001   CG17404  8049713   8050741   -  1029  
 
    Segment 171 
 
   Location   
  Gene key  FBgn0038003-FBgn0003651  
  Heatmap region span   3R:8036079..8185733   
  Segment span   3R:8055178..8084471   
  Length (genes)  7  
  Length (bp)  29294  
   Model Scoring   
  BIC  570.659215  
  logL  -279.701877  
  logL ratio  153.675823  
   Expression   
  Mean expression  4.800326  
  Median expression  4.643826  
  Tissue std. dev.  0.404324  
 
  No GO Slim enrichment  
  
   tissue    mean expression   
  5th Passage Drosophila S2 Cells  4.413270  
  Adult Accessory gland  4.512310  
  Adult Brain  4.442911  
  Adult Carcass  5.067618  
  Adult Crop  4.780880  
  Adult Eye  4.441866  
  Adult Fatbody  5.016297  
  Adult Female Spermatheca Mated  5.192346  
  Adult Female Spermatheca Virgin  5.066855  
  Adult Head  4.554608  
  Adult Heart  4.720129  
  Adult Hind Gut  4.655826  
  Adult Male Ejaculatory Duct  5.852839  
  Adult Mid Gut  4.619708  
  Adult Ovary  4.354214  
  Adult Salivary Gland  4.708956  
  Adult Testes  5.900014  
  Adult Thoracoabdominal ganglion  4.646624  
  Adult Whole Fly  4.765205  
  Larvae Wandering Tubules  4.790578  
  Larval Feeding Carcass  4.720305  
  Larval Feeding Central Nevous System  4.511135  
  Larval Feeding Hind Gut  4.454045  
  Larval Feeding Malpighian Tubule  4.653566  
  Larval Feeding Mid Gut  4.570589  
  Larval Feeding Salivary Gland  4.623486  
  Whole Larvae Feeding  5.572633  
 
  
   FlyBase ID    symbol    start    end    strand    length   
   FBgn0038003   CG3916   8055178   8056041  +  864  
   FBgn0011270   Pglym87  8057049   8058475   -  1427  
   FBgn0038005   Cyp313a5  8058575   8060406   -  1832  
   FBgn0038006   Cyp313a2  8060792   8062621   -  1830  
   FBgn0038007   Cyp313a3  8063001   8064831   -  1831  
   FBgn0038008   CG3942   8079909   8082314  +  2406  
   FBgn0003651   svp   8084471   8128509  +  44039  
 
 
    Segment 172 
 
   Location   
  Gene key  FBgn0038011-FBgn0038012  
  Heatmap region span   3R:8048109..8191440   
  Segment span   3R:8137075..8149245   
  Length (genes)  2  
  Length (bp)  12171  
   Model Scoring   
  BIC  195.357436  
  logL  -92.050987  
  logL ratio  27.912005  
   Expression   
  Mean expression  4.273083  
  Median expression  4.065062  
  Tissue std. dev.  0.538180  
 
  No GO Slim enrichment  
  
   tissue    mean expression   
  5th Passage Drosophila S2 Cells  4.161627  
  Adult Accessory gland  4.134485  
  Adult Brain  3.949539  
  Adult Carcass  4.330006  
  Adult Crop  4.131580  
  Adult Eye  3.859859  
  Adult Fatbody  4.162800  
  Adult Female Spermatheca Mated  4.127643  
  Adult Female Spermatheca Virgin  4.160278  
  Adult Head  3.912975  
  Adult Heart  3.960841  
  Adult Hind Gut  3.980741  
  Adult Male Ejaculatory Duct  4.206605  
  Adult Mid Gut  4.018298  
  Adult Ovary  5.935586  
  Adult Salivary Gland  4.363518  
  Adult Testes  6.224760  
  Adult Thoracoabdominal ganglion  4.094143  
  Adult Whole Fly  4.599894  
  Larvae Wandering Tubules  4.145512  
  Larval Feeding Carcass  4.170332  
  Larval Feeding Central Nevous System  3.959487  
  Larval Feeding Hind Gut  3.952547  
  Larval Feeding Malpighian Tubule  4.213684  
  Larval Feeding Mid Gut  3.991646  
  Larval Feeding Salivary Gland  4.193796  
  Whole Larvae Feeding  4.431073  
 
  
   FlyBase ID    symbol    start    end    strand    length   
   FBgn0038011   CG4066   8137075   8138841  +  1767  
   FBgn0038012   CG10013   8149245   8150936  +  1692  
 
    Segment 173 
 
   Location   
  Gene key  FBgn0010040-FBgn0053098  
  Heatmap region span   3R:8193286..8248151   
  Segment span   3R:8199824..8213287   
  Length (genes)  9  
  Length (bp)  13464  
   Model Scoring   
  BIC  748.005017  
  logL  -368.374777  
  logL ratio  176.461255  
   Expression   
  Mean expression  5.239669  
  Median expression  5.055714  
  Tissue std. dev.  0.397701  
 
  No GO Slim enrichment  
  
   tissue    mean expression   
  5th Passage Drosophila S2 Cells  6.018373  
  Adult Accessory gland  5.222776  
  Adult Brain  5.081521  
  Adult Carcass  5.253174  
  Adult Crop  4.815693  
  Adult Eye  4.846672  
  Adult Fatbody  4.908807  
  Adult Female Spermatheca Mated  5.071327  
  Adult Female Spermatheca Virgin  4.936767  
  Adult Head  5.382787  
  Adult Heart  5.239321  
  Adult Hind Gut  5.437416  
  Adult Male Ejaculatory Duct  5.116923  
  Adult Mid Gut  6.132150  
  Adult Ovary  4.851761  
  Adult Salivary Gland  5.201330  
  Adult Testes  5.373630  
  Adult Thoracoabdominal ganglion  4.803868  
  Adult Whole Fly  4.675576  
  Larvae Wandering Tubules  5.203020  
  Larval Feeding Carcass  5.103590  
  Larval Feeding Central Nevous System  4.945401  
  Larval Feeding Hind Gut  5.307946  
  Larval Feeding Malpighian Tubule  5.462002  
  Larval Feeding Mid Gut  6.329349  
  Larval Feeding Salivary Gland  5.109292  
  Whole Larvae Feeding  5.640594  
 
  
   FlyBase ID    symbol    start    end    strand    length   
   FBgn0010040   GstD4   8199824   8200546  +  723  
   FBgn0010041   GstD5   8201486   8202136  +  651  
   FBgn0010042   GstD6   8202894   8203629  +  736  
   FBgn0010043   GstD7   8204261   8204977  +  717  
   FBgn0010044   GstD8   8205745   8206537  +  793  
   FBgn0038028   CG10035  8207373   8208412   -  1040  
   FBgn0038029   GstD11   8209083   8211348  +  2266  
   FBgn0085431   CG34402   8211971   8228627  +  16657  
   FBgn0053098   CG33098   8213287   8214519  +  1233  
 
 
    Segment 174 
 
   Location   
  Gene key  FBgn0038037-FBgn0038039  
  Heatmap region span   3R:8197693..8252300   
  Segment span   3R:8231809..8234044   
  Length (genes)  3  
  Length (bp)  2236  
   Model Scoring   
  BIC  385.981605  
  logL  -187.363071  
  logL ratio  65.683196  
   Expression   
  Mean expression  10.076617  
  Median expression  10.038781  
  Tissue std. dev.  0.969069  
 
  No GO Slim enrichment  
  
   tissue    mean expression   
  5th Passage Drosophila S2 Cells  10.273720  
  Adult Accessory gland  9.418348  
  Adult Brain  9.468289  
  Adult Carcass  10.474154  
  Adult Crop  11.365537  
  Adult Eye  10.498005  
  Adult Fatbody  10.641616  
  Adult Female Spermatheca Mated  9.954361  
  Adult Female Spermatheca Virgin  9.946295  
  Adult Head  10.411644  
  Adult Heart  10.960533  
  Adult Hind Gut  11.035847  
  Adult Male Ejaculatory Duct  10.154673  
  Adult Mid Gut  11.265352  
  Adult Ovary  8.383009  
  Adult Salivary Gland  10.531627  
  Adult Testes  7.144654  
  Adult Thoracoabdominal ganglion  9.668839  
  Adult Whole Fly  9.549329  
  Larvae Wandering Tubules  10.667060  
  Larval Feeding Carcass  9.166668  
  Larval Feeding Central Nevous System  9.165856  
  Larval Feeding Hind Gut  10.260489  
  Larval Feeding Malpighian Tubule  11.043222  
  Larval Feeding Mid Gut  11.331508  
  Larval Feeding Salivary Gland  8.671124  
  Whole Larvae Feeding  10.616891  
 
  
   FlyBase ID    symbol    start    end    strand    length   
   FBgn0038037   Cyp9f2  8229057   8231809   -  2753  
   FBgn0038038   CG5167   8232254   8233824  +  1571  
   FBgn0038039   CG5196   8234044   8237380  +  3337  
 
 
    Segment 175 
 
   Location   
  Gene key  FBgn0086134-FBgn0038049  
  Heatmap region span   3R:8248490..8291020   
  Segment span   3R:8257047..8259824   
  Length (genes)  3  
  Length (bp)  2778  
   Model Scoring   
  BIC  388.321661  
  logL  -188.533100  
  logL ratio  63.147828  
   Expression   
  Mean expression  10.201153  
  Median expression  9.715017  
  Tissue std. dev.  0.504978  
 
  No GO Slim enrichment  
  
   tissue    mean expression   
  5th Passage Drosophila S2 Cells  10.801438  
  Adult Accessory gland  10.141220  
  Adult Brain  9.716017  
  Adult Carcass  9.933044  
  Adult Crop  9.927079  
  Adult Eye  9.723826  
  Adult Fatbody  10.277278  
  Adult Female Spermatheca Mated  9.734004  
  Adult Female Spermatheca Virgin  9.717545  
  Adult Head  9.751371  
  Adult Heart  10.401508  
  Adult Hind Gut  9.765003  
  Adult Male Ejaculatory Duct  10.311545  
  Adult Mid Gut  9.821257  
  Adult Ovary  11.465901  
  Adult Salivary Gland  9.392072  
  Adult Testes  10.525237  
  Adult Thoracoabdominal ganglion  9.669938  
  Adult Whole Fly  10.788633  
  Larvae Wandering Tubules  10.531717  
  Larval Feeding Carcass  10.457191  
  Larval Feeding Central Nevous System  11.363933  
  Larval Feeding Hind Gut  10.215970  
  Larval Feeding Malpighian Tubule  10.315021  
  Larval Feeding Mid Gut  9.871110  
  Larval Feeding Salivary Gland  10.674731  
  Whole Larvae Feeding  10.137541  
 
  
   FlyBase ID    symbol    start    end    strand    length   
   FBgn0086134   Pros25   8257047   8258279  +  1233  
   FBgn0029512   Aos1  8258255   8259626   -  1372  
   FBgn0038049   CG5844   8259824   8261365  +  1542  
 
 
    Segment 176 
 
   Location   
  Gene key  FBgn0043043-FBgn0038051  
  Heatmap region span   3R:8252300..8334798   
  Segment span   3R:8261970..8264861   
  Length (genes)  2  
  Length (bp)  2892  
   Model Scoring   
  BIC  190.908320  
  logL  -89.826429  
  logL ratio  22.750415  
   Expression   
  Mean expression  5.317706  
  Median expression  5.324301  
  Tissue std. dev.  0.388075  
 
  No GO Slim enrichment  
  
   tissue    mean expression   
  5th Passage Drosophila S2 Cells  5.602604  
  Adult Accessory gland  5.406983  
  Adult Brain  4.938383  
  Adult Carcass  5.536936  
  Adult Crop  5.156116  
  Adult Eye  4.802496  
  Adult Fatbody  5.499175  
  Adult Female Spermatheca Mated  5.268647  
  Adult Female Spermatheca Virgin  5.504683  
  Adult Head  5.000934  
  Adult Heart  5.194973  
  Adult Hind Gut  5.268821  
  Adult Male Ejaculatory Duct  5.431192  
  Adult Mid Gut  5.548292  
  Adult Ovary  5.117622  
  Adult Salivary Gland  5.388342  
  Adult Testes  6.993987  
  Adult Thoracoabdominal ganglion  5.108100  
  Adult Whole Fly  5.021181  
  Larvae Wandering Tubules  5.259617  
  Larval Feeding Carcass  5.243000  
  Larval Feeding Central Nevous System  4.951448  
  Larval Feeding Hind Gut  5.095000  
  Larval Feeding Malpighian Tubule  5.193885  
  Larval Feeding Mid Gut  5.480435  
  Larval Feeding Salivary Gland  5.194009  
  Whole Larvae Feeding  5.371192  
 
  
   FlyBase ID    symbol    start    end    strand    length   
   FBgn0043043   desat2   8261970   8263582  +  1613  
   FBgn0038051   CG17207  8263536   8264861   -  1326  
 
    Segment 177 
 
   Location   
  Gene key  FBgn0038053-FBgn0038058  
  Heatmap region span   3R:8257047..8453763   
  Segment span   3R:8275018..8291020   
  Length (genes)  5  
  Length (bp)  16003  
   Model Scoring   
  BIC  514.299786  
  logL  -251.522162  
  logL ratio  112.582527  
   Expression   
  Mean expression  7.963974  
  Median expression  7.822845  
  Tissue std. dev.  0.595033  
 
  No GO Slim enrichment  
  
   tissue    mean expression   
  5th Passage Drosophila S2 Cells  8.702645  
  Adult Accessory gland  8.293037  
  Adult Brain  8.877543  
  Adult Carcass  7.654296  
  Adult Crop  7.774509  
  Adult Eye  8.310866  
  Adult Fatbody  7.803090  
  Adult Female Spermatheca Mated  8.331508  
  Adult Female Spermatheca Virgin  8.320950  
  Adult Head  8.197087  
  Adult Heart  8.119038  
  Adult Hind Gut  7.759004  
  Adult Male Ejaculatory Duct  8.073632  
  Adult Mid Gut  7.753809  
  Adult Ovary  8.612647  
  Adult Salivary Gland  8.013673  
  Adult Testes  6.124632  
  Adult Thoracoabdominal ganglion  9.037753  
  Adult Whole Fly  7.663992  
  Larvae Wandering Tubules  8.313602  
  Larval Feeding Carcass  7.881701  
  Larval Feeding Central Nevous System  8.066994  
  Larval Feeding Hind Gut  7.318532  
  Larval Feeding Malpighian Tubule  7.875964  
  Larval Feeding Mid Gut  7.006358  
  Larval Feeding Salivary Gland  8.076966  
  Whole Larvae Feeding  7.063475  
 
  
   FlyBase ID    symbol    start    end    strand    length   
   FBgn0038053   CG18549   8275018   8282084  +  7067  
   FBgn0038055   trus   8282256   8284166  +  1911  
   FBgn0038056   CG5961  8284143   8286016   -  1874  
   FBgn0038057   CG12267   8286136   8288140  +  2005  
   FBgn0038058   CG5608  8288284   8291020   -  2737  
 
 
    Segment 178 
 
   Location   
  Gene key  FBgn0250910-FBgn0038063  
  Heatmap region span   3R:8273375..8480299   
  Segment span   3R:8373951..8421447   
  Length (genes)  2  
  Length (bp)  47497  
   Model Scoring   
  BIC  166.945464  
  logL  -77.845001  
  logL ratio  40.178237  
   Expression   
  Mean expression  4.544506  
  Median expression  4.530128  
  Tissue std. dev.  0.171546  
 
  No GO Slim enrichment  
  
   tissue    mean expression   
  5th Passage Drosophila S2 Cells  4.554860  
  Adult Accessory gland  4.615941  
  Adult Brain  4.923377  
  Adult Carcass  4.629264  
  Adult Crop  4.624949  
  Adult Eye  4.500634  
  Adult Fatbody  4.464201  
  Adult Female Spermatheca Mated  4.478032  
  Adult Female Spermatheca Virgin  4.521292  
  Adult Head  4.490318  
  Adult Heart  4.576811  
  Adult Hind Gut  4.443851  
  Adult Male Ejaculatory Duct  4.574020  
  Adult Mid Gut  4.540882  
  Adult Ovary  4.457275  
  Adult Salivary Gland  4.680266  
  Adult Testes  4.403375  
  Adult Thoracoabdominal ganglion  4.714406  
  Adult Whole Fly  4.098667  
  Larvae Wandering Tubules  4.527462  
  Larval Feeding Carcass  5.051623  
  Larval Feeding Central Nevous System  4.362907  
  Larval Feeding Hind Gut  4.388955  
  Larval Feeding Malpighian Tubule  4.520566  
  Larval Feeding Mid Gut  4.604256  
  Larval Feeding Salivary Gland  4.510371  
  Whole Larvae Feeding  4.443109  
 
  
   FlyBase ID    symbol    start    end    strand    length   
   FBgn0250910   Octbeta3R  8337292   8373951   -  36660  
   FBgn0038063   Octbeta2R  8378211   8421447   -  43237  
 
    Segment 179 
 
   Location   
  Gene key  FBgn0005671-FBgn0038065  
  Heatmap region span   3R:8275018..8489943   
  Segment span   3R:8453551..8453763   
  Length (genes)  2  
  Length (bp)  213  
   Model Scoring   
  BIC  302.258569  
  logL  -145.501553  
  logL ratio  78.007431  
   Expression   
  Mean expression  11.715772  
  Median expression  11.470142  
  Tissue std. dev.  0.550595  
 
  No GO Slim enrichment  
  
   tissue    mean expression   
  5th Passage Drosophila S2 Cells  12.055066  
  Adult Accessory gland  11.085814  
  Adult Brain  12.147412  
  Adult Carcass  11.220227  
  Adult Crop  11.429872  
  Adult Eye  12.137844  
  Adult Fatbody  10.875152  
  Adult Female Spermatheca Mated  11.181289  
  Adult Female Spermatheca Virgin  11.038047  
  Adult Head  11.889767  
  Adult Heart  11.672878  
  Adult Hind Gut  12.185596  
  Adult Male Ejaculatory Duct  11.663204  
  Adult Mid Gut  11.356994  
  Adult Ovary  11.751262  
  Adult Salivary Gland  11.787689  
  Adult Testes  10.220201  
  Adult Thoracoabdominal ganglion  12.173392  
  Adult Whole Fly  11.580616  
  Larvae Wandering Tubules  12.652938  
  Larval Feeding Carcass  11.813836  
  Larval Feeding Central Nevous System  11.898339  
  Larval Feeding Hind Gut  12.592050  
  Larval Feeding Malpighian Tubule  12.596730  
  Larval Feeding Mid Gut  12.108295  
  Larval Feeding Salivary Gland  11.480901  
  Whole Larvae Feeding  11.730442  
 
  
   FlyBase ID    symbol    start    end    strand    length   
   FBgn0005671   Vha55  8449484   8453551   -  4068  
   FBgn0038065   Snx3   8453763   8455783  +  2021  
 
    Segment 180 
 
   Location   
  Gene key  FBgn0042207-FBgn0038070  
  Heatmap region span   3R:8334798..8500331   
  Segment span   3R:8459081..8468295   
  Length (genes)  5  
  Length (bp)  9215  
   Model Scoring   
  BIC  418.162142  
  logL  -203.453340  
  logL ratio  106.917677  
   Expression   
  Mean expression  4.814970  
  Median expression  4.427002  
  Tissue std. dev.  0.965122  
 
  
   GO ID    description    ratio    P-value   
   GO:0006629   lipid metabolic process  5/5  5.51e-12  
   GO:0008150   biological_process  5/5  0.000298  
 
  
   tissue    mean expression   
  5th Passage Drosophila S2 Cells  4.669626  
  Adult Accessory gland  9.204088  
  Adult Brain  4.213324  
  Adult Carcass  4.721546  
  Adult Crop  4.529380  
  Adult Eye  4.575106  
  Adult Fatbody  4.521335  
  Adult Female Spermatheca Mated  4.575477  
  Adult Female Spermatheca Virgin  4.544873  
  Adult Head  4.387513  
  Adult Heart  4.419532  
  Adult Hind Gut  4.424113  
  Adult Male Ejaculatory Duct  6.422322  
  Adult Mid Gut  4.585716  
  Adult Ovary  4.456573  
  Adult Salivary Gland  5.785111  
  Adult Testes  4.454884  
  Adult Thoracoabdominal ganglion  4.377454  
  Adult Whole Fly  4.540822  
  Larvae Wandering Tubules  4.545896  
  Larval Feeding Carcass  4.735469  
  Larval Feeding Central Nevous System  4.423497  
  Larval Feeding Hind Gut  4.577523  
  Larval Feeding Malpighian Tubule  4.509670  
  Larval Feeding Mid Gut  4.675328  
  Larval Feeding Salivary Gland  4.655483  
  Whole Larvae Feeding  4.472532  
 
  
   FlyBase ID    symbol    start    end    strand    length   
   FBgn0042207   CG18530   8459081   8460374  +  1294  
   FBgn0038067   CG11598   8460639   8462005  +  1367  
   FBgn0038068   CG11600   8462275   8463602  +  1328  
   FBgn0038069   CG11608   8464203   8465669  +  1467  
   FBgn0038070   CG6753  8465885   8468295   -  2411  
 
 
    Segment 181 
 
   Location   
  Gene key  FBgn0026207-FBgn0038076  
  Heatmap region span   3R:8473782..8530816   
  Segment span   3R:8504766..8507097   
  Length (genes)  2  
  Length (bp)  2332  
   Model Scoring   
  BIC  231.286704  
  logL  -110.015621  
  logL ratio  1.823016  
   Expression   
  Mean expression  6.142843  
  Median expression  6.127990  
  Tissue std. dev.  0.414127  
 
  No GO Slim enrichment  
  
   tissue    mean expression   
  5th Passage Drosophila S2 Cells  6.211157  
  Adult Accessory gland  5.904319  
  Adult Brain  5.410998  
  Adult Carcass  6.451748  
  Adult Crop  6.114800  
  Adult Eye  5.893347  
  Adult Fatbody  7.059300  
  Adult Female Spermatheca Mated  6.234243  
  Adult Female Spermatheca Virgin  6.093628  
  Adult Head  5.973923  
  Adult Heart  7.097921  
  Adult Hind Gut  6.646282  
  Adult Male Ejaculatory Duct  5.755806  
  Adult Mid Gut  6.272054  
  Adult Ovary  6.933736  
  Adult Salivary Gland  5.936282  
  Adult Testes  5.829648  
  Adult Thoracoabdominal ganglion  5.644559  
  Adult Whole Fly  5.979254  
  Larvae Wandering Tubules  5.866103  
  Larval Feeding Carcass  6.173222  
  Larval Feeding Central Nevous System  6.688601  
  Larval Feeding Hind Gut  6.091727  
  Larval Feeding Malpighian Tubule  5.904220  
  Larval Feeding Mid Gut  5.885407  
  Larval Feeding Salivary Gland  5.953743  
  Whole Larvae Feeding  5.850731  
 
  
   FlyBase ID    symbol    start    end    strand    length   
   FBgn0026207   mbo  8502208   8504766   -  2559  
   FBgn0038076   Cyp313a4  8505001   8507097   -  2097  
 
    Segment 182 
 
   Location   
  Gene key  FBgn0051347-FBgn0038079  
  Heatmap region span   3R:8500331..8565384   
  Segment span   3R:8518319..8520868   
  Length (genes)  3  
  Length (bp)  2550  
   Model Scoring   
  BIC  278.496338  
  logL  -133.620438  
  logL ratio  37.091412  
   Expression   
  Mean expression  4.924224  
  Median expression  4.606820  
  Tissue std. dev.  0.870665  
 
  No GO Slim enrichment  
  
   tissue    mean expression   
  5th Passage Drosophila S2 Cells  4.520524  
  Adult Accessory gland  4.571644  
  Adult Brain  4.299606  
  Adult Carcass  4.735891  
  Adult Crop  4.838019  
  Adult Eye  4.697661  
  Adult Fatbody  4.630584  
  Adult Female Spermatheca Mated  4.638181  
  Adult Female Spermatheca Virgin  4.715476  
  Adult Head  4.734179  
  Adult Heart  4.875455  
  Adult Hind Gut  4.604297  
  Adult Male Ejaculatory Duct  4.605710  
  Adult Mid Gut  5.500757  
  Adult Ovary  4.475605  
  Adult Salivary Gland  4.819142  
  Adult Testes  8.905574  
  Adult Thoracoabdominal ganglion  4.416659  
  Adult Whole Fly  5.976469  
  Larvae Wandering Tubules  4.564327  
  Larval Feeding Carcass  4.935102  
  Larval Feeding Central Nevous System  4.266269  
  Larval Feeding Hind Gut  4.460360  
  Larval Feeding Malpighian Tubule  4.607955  
  Larval Feeding Mid Gut  5.535871  
  Larval Feeding Salivary Gland  4.684081  
  Whole Larvae Feeding  5.338657  
 
  
   FlyBase ID    symbol    start    end    strand    length   
   FBgn0051347   CG31347  8517455   8518319   -  865  
   FBgn0038078   CG14391  8519945   8520564   -  620  
   FBgn0038079   NijC   8520868   8523265  +  2398  
 
 
    Segment 183 
 
   Location   
  Gene key  FBgn0016693-FBgn0038080  
  Heatmap region span   3R:8504766..8569402   
  Segment span   3R:8523044..8530816   
  Length (genes)  2  
  Length (bp)  7773  
   Model Scoring   
  BIC  241.616695  
  logL  -115.180617  
  logL ratio  51.148207  
   Expression   
  Mean expression  10.118078  
  Median expression  9.984708  
  Tissue std. dev.  0.498188  
 
  No GO Slim enrichment  
  
   tissue    mean expression   
  5th Passage Drosophila S2 Cells  11.350386  
  Adult Accessory gland  9.730370  
  Adult Brain  9.863187  
  Adult Carcass  9.547300  
  Adult Crop  10.322299  
  Adult Eye  9.548732  
  Adult Fatbody  9.957037  
  Adult Female Spermatheca Mated  9.938129  
  Adult Female Spermatheca Virgin  9.708394  
  Adult Head  9.705766  
  Adult Heart  10.193525  
  Adult Hind Gut  9.988214  
  Adult Male Ejaculatory Duct  10.297463  
  Adult Mid Gut  9.874669  
  Adult Ovary  9.439801  
  Adult Salivary Gland  9.893221  
  Adult Testes  11.019800  
  Adult Thoracoabdominal ganglion  10.600831  
  Adult Whole Fly  9.546491  
  Larvae Wandering Tubules  10.094487  
  Larval Feeding Carcass  10.043570  
  Larval Feeding Central Nevous System  9.883260  
  Larval Feeding Hind Gut  10.590039  
  Larval Feeding Malpighian Tubule  11.015093  
  Larval Feeding Mid Gut  10.017697  
  Larval Feeding Salivary Gland  10.997740  
  Whole Larvae Feeding  10.020599  
 
  
   FlyBase ID    symbol    start    end    strand    length   
   FBgn0016693   Past1   8523044   8529871  +  6828  
   FBgn0038080   CG12279   8530816   8531541  +  726  
 
    Segment 184 
 
   Location   
  Gene key  FBgn0038090-FBgn0038097  
  Heatmap region span   3R:8565384..8814233   
  Segment span   3R:8737014..8792442   
  Length (genes)  4  
  Length (bp)  55429  
   Model Scoring   
  BIC  439.207272  
  logL  -213.975905  
  logL ratio  -15.789119  
   Expression   
  Mean expression  5.121034  
  Median expression  4.712815  
  Tissue std. dev.  0.766439  
 
  No GO Slim enrichment  
  
   tissue    mean expression   
  5th Passage Drosophila S2 Cells  5.737194  
  Adult Accessory gland  4.666178  
  Adult Brain  4.364744  
  Adult Carcass  4.902659  
  Adult Crop  4.637923  
  Adult Eye  4.328667  
  Adult Fatbody  5.406902  
  Adult Female Spermatheca Mated  5.591235  
  Adult Female Spermatheca Virgin  5.082415  
  Adult Head  4.416618  
  Adult Heart  4.715292  
  Adult Hind Gut  4.582326  
  Adult Male Ejaculatory Duct  4.676112  
  Adult Mid Gut  4.715977  
  Adult Ovary  5.927560  
  Adult Salivary Gland  4.755718  
  Adult Testes  7.913165  
  Adult Thoracoabdominal ganglion  4.496014  
  Adult Whole Fly  6.047265  
  Larvae Wandering Tubules  4.736496  
  Larval Feeding Carcass  4.952178  
  Larval Feeding Central Nevous System  5.631642  
  Larval Feeding Hind Gut  4.810817  
  Larval Feeding Malpighian Tubule  4.606662  
  Larval Feeding Mid Gut  5.775944  
  Larval Feeding Salivary Gland  4.698618  
  Whole Larvae Feeding  6.091598  
 
  
   FlyBase ID    symbol    start    end    strand    length   
   FBgn0038090   CG10909  8735842   8737014   -  1173  
   FBgn0087021   Spc25   8787474   8788975  +  1502  
   FBgn0038095   Cyp304a1   8789519   8791652  +  2134  
   FBgn0038097   CG14384  8791660   8792442   -  783  
 
 
    Segment 185 
 
   Location   
  Gene key  FBgn0038100-FBgn0051342  
  Heatmap region span   3R:8684939..8856372   
  Segment span   3R:8806768..8811786   
  Length (genes)  2  
  Length (bp)  5019  
   Model Scoring   
  BIC  231.836007  
  logL  -110.290272  
  logL ratio  41.425788  
   Expression   
  Mean expression  9.377873  
  Median expression  9.355242  
  Tissue std. dev.  0.695387  
 
  No GO Slim enrichment  
  
   tissue    mean expression   
  5th Passage Drosophila S2 Cells  10.321015  
  Adult Accessory gland  9.028222  
  Adult Brain  9.913185  
  Adult Carcass  8.977813  
  Adult Crop  9.585629  
  Adult Eye  10.125115  
  Adult Fatbody  8.142275  
  Adult Female Spermatheca Mated  8.934958  
  Adult Female Spermatheca Virgin  9.198147  
  Adult Head  9.119544  
  Adult Heart  9.670967  
  Adult Hind Gut  9.655103  
  Adult Male Ejaculatory Duct  8.815134  
  Adult Mid Gut  8.750254  
  Adult Ovary  10.819954  
  Adult Salivary Gland  8.187798  
  Adult Testes  11.011658  
  Adult Thoracoabdominal ganglion  9.341402  
  Adult Whole Fly  10.043035  
  Larvae Wandering Tubules  9.833759  
  Larval Feeding Carcass  9.311671  
  Larval Feeding Central Nevous System  9.199039  
  Larval Feeding Hind Gut  9.616539  
  Larval Feeding Malpighian Tubule  9.321098  
  Larval Feeding Mid Gut  8.369588  
  Larval Feeding Salivary Gland  9.036078  
  Whole Larvae Feeding  8.873597  
 
  
   FlyBase ID    symbol    start    end    strand    length   
   FBgn0038100   Paip2   8806768   8810345  +  3578  
   FBgn0051342   CG31342   8811786   8817265  +  5480  
 
    Segment 186 
 
   Location   
  Gene key  FBgn0038106-FBgn0038108  
  Heatmap region span   3R:8803193..8868492   
  Segment span   3R:8821369..8824307   
  Length (genes)  3  
  Length (bp)  2939  
   Model Scoring   
  BIC  271.230880  
  logL  -129.987709  
  logL ratio  111.848050  
   Expression   
  Mean expression  8.874862  
  Median expression  8.861011  
  Tissue std. dev.  0.471805  
 
  No GO Slim enrichment  
  
   tissue    mean expression   
  5th Passage Drosophila S2 Cells  10.118164  
  Adult Accessory gland  8.661320  
  Adult Brain  9.659882  
  Adult Carcass  8.734248  
  Adult Crop  9.173271  
  Adult Eye  9.272953  
  Adult Fatbody  8.472147  
  Adult Female Spermatheca Mated  8.250852  
  Adult Female Spermatheca Virgin  8.321795  
  Adult Head  8.859993  
  Adult Heart  8.930875  
  Adult Hind Gut  9.070132  
  Adult Male Ejaculatory Duct  8.795769  
  Adult Mid Gut  8.388533  
  Adult Ovary  9.285837  
  Adult Salivary Gland  8.792165  
  Adult Testes  8.120124  
  Adult Thoracoabdominal ganglion  9.592877  
  Adult Whole Fly  8.590044  
  Larvae Wandering Tubules  8.734833  
  Larval Feeding Carcass  8.888287  
  Larval Feeding Central Nevous System  9.446234  
  Larval Feeding Hind Gut  8.972153  
  Larval Feeding Malpighian Tubule  8.912166  
  Larval Feeding Mid Gut  8.286330  
  Larval Feeding Salivary Gland  8.993334  
  Whole Larvae Feeding  8.296946  
 
  
   FlyBase ID    symbol    start    end    strand    length   
   FBgn0038106   CG7488   8821369   8822647  +  1279  
   FBgn0038107   CG17327  8822590   8823404   -  815  
   FBgn0038108   CG7518   8824307   8834126  +  9820  
 
 
    Segment 187 
 
   Location   
  Gene key  FBgn0038110-FBgn0038111  
  Heatmap region span   3R:8806768..8877172   
  Segment span   3R:8837010..8856372   
  Length (genes)  3  
  Length (bp)  19363  
   Model Scoring   
  BIC  320.485826  
  logL  -154.615182  
  logL ratio  75.535106  
   Expression   
  Mean expression  9.063695  
  Median expression  8.769465  
  Tissue std. dev.  0.516412  
 
  No GO Slim enrichment  
  
   tissue    mean expression   
  5th Passage Drosophila S2 Cells  10.190363  
  Adult Accessory gland  9.111357  
  Adult Brain  8.743634  
  Adult Carcass  8.520558  
  Adult Crop  8.701173  
  Adult Eye  8.654848  
  Adult Fatbody  9.027039  
  Adult Female Spermatheca Mated  8.741434  
  Adult Female Spermatheca Virgin  8.722881  
  Adult Head  8.408834  
  Adult Heart  9.027931  
  Adult Hind Gut  8.372214  
  Adult Male Ejaculatory Duct  8.560910  
  Adult Mid Gut  9.352412  
  Adult Ovary  9.489914  
  Adult Salivary Gland  9.183634  
  Adult Testes  8.967925  
  Adult Thoracoabdominal ganglion  8.581282  
  Adult Whole Fly  8.828416  
  Larvae Wandering Tubules  10.435047  
  Larval Feeding Carcass  8.873261  
  Larval Feeding Central Nevous System  9.003264  
  Larval Feeding Hind Gut  9.116304  
  Larval Feeding Malpighian Tubule  9.953007  
  Larval Feeding Mid Gut  9.509666  
  Larval Feeding Salivary Gland  9.658827  
  Whole Larvae Feeding  8.983637  
 
  
   FlyBase ID    symbol    start    end    strand    length   
   FBgn0038110   CG8031  8834106   8837010   -  2905  
   FBgn0020496   CtBP   8837388   8851905  +  14518  
   FBgn0038111   CG12360  8852747   8856372   -  3626  
 
 
    Segment 188 
 
   Location   
  Gene key  FBgn0038113-FBgn0003450  
  Heatmap region span   3R:8821369..9046300   
  Segment span   3R:8864135..8868492   
  Length (genes)  3  
  Length (bp)  4358  
   Model Scoring   
  BIC  281.133176  
  logL  -134.938857  
  logL ratio  38.912286  
   Expression   
  Mean expression  5.075354  
  Median expression  4.824986  
  Tissue std. dev.  0.536620  
 
  No GO Slim enrichment  
  
   tissue    mean expression   
  5th Passage Drosophila S2 Cells  4.939454  
  Adult Accessory gland  4.746580  
  Adult Brain  5.247168  
  Adult Carcass  5.054560  
  Adult Crop  4.970111  
  Adult Eye  4.873359  
  Adult Fatbody  4.886474  
  Adult Female Spermatheca Mated  4.935840  
  Adult Female Spermatheca Virgin  4.933853  
  Adult Head  5.287882  
  Adult Heart  4.744047  
  Adult Hind Gut  4.962002  
  Adult Male Ejaculatory Duct  4.894518  
  Adult Mid Gut  4.781296  
  Adult Ovary  5.256087  
  Adult Salivary Gland  4.867454  
  Adult Testes  4.393387  
  Adult Thoracoabdominal ganglion  4.997913  
  Adult Whole Fly  4.497468  
  Larvae Wandering Tubules  5.068879  
  Larval Feeding Carcass  7.384134  
  Larval Feeding Central Nevous System  5.407797  
  Larval Feeding Hind Gut  5.502902  
  Larval Feeding Malpighian Tubule  5.223825  
  Larval Feeding Mid Gut  4.680904  
  Larval Feeding Salivary Gland  4.767590  
  Whole Larvae Feeding  5.729070  
 
  
   FlyBase ID    symbol    start    end    strand    length   
   FBgn0038113   CG11668   8864135   8865906  +  1772  
   FBgn0038114   CG11670   8867790   8870123  +  2334  
   FBgn0003450   snk  8865887   8868492   -  2606  
 
 
    Segment 189 
 
   Location   
  Gene key  FBgn0004666-FBgn0038118  
  Heatmap region span   3R:8858259..9105445   
  Segment span   3R:8883481..8914373   
  Length (genes)  2  
  Length (bp)  30893  
   Model Scoring   
  BIC  211.086668  
  logL  -99.915603  
  logL ratio  14.646823  
   Expression   
  Mean expression  5.034136  
  Median expression  5.015216  
  Tissue std. dev.  0.856142  
 
  No GO Slim enrichment  
  
   tissue    mean expression   
  5th Passage Drosophila S2 Cells  5.178937  
  Adult Accessory gland  4.894502  
  Adult Brain  5.084170  
  Adult Carcass  4.577997  
  Adult Crop  4.424828  
  Adult Eye  4.780927  
  Adult Fatbody  4.436214  
  Adult Female Spermatheca Mated  4.617577  
  Adult Female Spermatheca Virgin  4.702103  
  Adult Head  4.551069  
  Adult Heart  4.405338  
  Adult Hind Gut  4.377806  
  Adult Male Ejaculatory Duct  6.641675  
  Adult Mid Gut  4.412137  
  Adult Ovary  7.940186  
  Adult Salivary Gland  4.630515  
  Adult Testes  5.473172  
  Adult Thoracoabdominal ganglion  4.384447  
  Adult Whole Fly  6.254198  
  Larvae Wandering Tubules  4.582222  
  Larval Feeding Carcass  4.962157  
  Larval Feeding Central Nevous System  6.683489  
  Larval Feeding Hind Gut  4.827492  
  Larval Feeding Malpighian Tubule  4.665048  
  Larval Feeding Mid Gut  4.444137  
  Larval Feeding Salivary Gland  5.355799  
  Whole Larvae Feeding  4.633541  
 
  
   FlyBase ID    symbol    start    end    strand    length   
   FBgn0004666   sim   8883481   8903950  +  20470  
   FBgn0038118   timeout   8914373   8989598  +  75226  
 
    Segment 190 
 
   Location   
  Gene key  FBgn0038122-FBgn0038127  
  Heatmap region span   3R:8864135..9118920   
  Segment span   3R:9007026..9046300   
  Length (genes)  6  
  Length (bp)  39275  
   Model Scoring   
  BIC  549.318241  
  logL  -269.031389  
  logL ratio  74.373042  
   Expression   
  Mean expression  5.292466  
  Median expression  4.879239  
  Tissue std. dev.  1.021735  
 
  No GO Slim enrichment  
  
   tissue    mean expression   
  5th Passage Drosophila S2 Cells  4.789870  
  Adult Accessory gland  4.917075  
  Adult Brain  4.522256  
  Adult Carcass  5.430566  
  Adult Crop  5.557144  
  Adult Eye  4.783641  
  Adult Fatbody  4.985425  
  Adult Female Spermatheca Mated  4.972814  
  Adult Female Spermatheca Virgin  4.914756  
  Adult Head  4.669374  
  Adult Heart  4.593805  
  Adult Hind Gut  5.047507  
  Adult Male Ejaculatory Duct  5.034663  
  Adult Mid Gut  5.114753  
  Adult Ovary  4.683628  
  Adult Salivary Gland  5.986269  
  Adult Testes  9.881658  
  Adult Thoracoabdominal ganglion  4.769014  
  Adult Whole Fly  6.466794  
  Larvae Wandering Tubules  4.837370  
  Larval Feeding Carcass  5.881658  
  Larval Feeding Central Nevous System  4.619391  
  Larval Feeding Hind Gut  5.430535  
  Larval Feeding Malpighian Tubule  4.839949  
  Larval Feeding Mid Gut  5.123573  
  Larval Feeding Salivary Gland  4.998225  
  Whole Larvae Feeding  6.044876  
 
  
   FlyBase ID    symbol    start    end    strand    length   
   FBgn0038122   CG8138   9007026   9007876  +  851  
   FBgn0038123   CG8508  9011248   9012232   -  985  
   FBgn0038124   CG14380   9014287   9015290  +  1004  
   FBgn0038125   CG8141   9017423   9018231  +  809  
   FBgn0038126   CG8483  9034470   9040187   -  5718  
   FBgn0038127   CG8476  9045362   9046300   -  939  
 
 
    Segment 191 
 
   Location   
  Gene key  FBgn0040551-FBgn0038129  
  Heatmap region span   3R:8877172..9128375   
  Segment span   3R:9086079..9097748   
  Length (genes)  3  
  Length (bp)  11670  
   Model Scoring   
  BIC  389.396697  
  logL  -189.070617  
  logL ratio  3.978161  
   Expression   
  Mean expression  8.525317  
  Median expression  8.415270  
  Tissue std. dev.  0.476339  
 
  No GO Slim enrichment  
  
   tissue    mean expression   
  5th Passage Drosophila S2 Cells  7.943359  
  Adult Accessory gland  8.061051  
  Adult Brain  8.033785  
  Adult Carcass  8.958987  
  Adult Crop  8.744652  
  Adult Eye  8.449976  
  Adult Fatbody  9.062423  
  Adult Female Spermatheca Mated  9.350857  
  Adult Female Spermatheca Virgin  9.338629  
  Adult Head  8.656691  
  Adult Heart  9.366057  
  Adult Hind Gut  8.473341  
  Adult Male Ejaculatory Duct  8.670129  
  Adult Mid Gut  8.121084  
  Adult Ovary  8.317555  
  Adult Salivary Gland  7.915449  
  Adult Testes  7.751138  
  Adult Thoracoabdominal ganglion  8.254182  
  Adult Whole Fly  8.015855  
  Larvae Wandering Tubules  9.142996  
  Larval Feeding Carcass  8.307389  
  Larval Feeding Central Nevous System  7.850480  
  Larval Feeding Hind Gut  8.894327  
  Larval Feeding Malpighian Tubule  8.910544  
  Larval Feeding Mid Gut  8.661375  
  Larval Feeding Salivary Gland  8.509100  
  Whole Larvae Feeding  8.422153  
 
  
   FlyBase ID    symbol    start    end    strand    length   
   FBgn0040551   CG11686   9086079   9087949  +  1871  
   FBgn0038128   Ravus  9088454   9089755   -  1302  
   FBgn0038129   CG8449  9094859   9097748   -  2890  
 
 
    Segment 192 
 
   Location   
  Gene key  FBgn0038131-FBgn0038134  
  Heatmap region span   3R:9007026..9192629   
  Segment span   3R:9112251..9118920   
  Length (genes)  4  
  Length (bp)  6670  
   Model Scoring   
  BIC  300.758611  
  logL  -144.751574  
  logL ratio  110.657185  
   Expression   
  Mean expression  4.723458  
  Median expression  4.615616  
  Tissue std. dev.  0.243260  
 
  No GO Slim enrichment  
  
   tissue    mean expression   
  5th Passage Drosophila S2 Cells  4.664684  
  Adult Accessory gland  4.657996  
  Adult Brain  4.363139  
  Adult Carcass  4.772736  
  Adult Crop  4.608989  
  Adult Eye  5.379858  
  Adult Fatbody  4.796601  
  Adult Female Spermatheca Mated  4.806080  
  Adult Female Spermatheca Virgin  4.739908  
  Adult Head  4.753892  
  Adult Heart  4.669476  
  Adult Hind Gut  5.082923  
  Adult Male Ejaculatory Duct  4.961535  
  Adult Mid Gut  5.022720  
  Adult Ovary  4.676039  
  Adult Salivary Gland  4.863486  
  Adult Testes  4.308504  
  Adult Thoracoabdominal ganglion  4.462410  
  Adult Whole Fly  4.405169  
  Larvae Wandering Tubules  4.618823  
  Larval Feeding Carcass  4.676376  
  Larval Feeding Central Nevous System  4.369718  
  Larval Feeding Hind Gut  4.597719  
  Larval Feeding Malpighian Tubule  4.708709  
  Larval Feeding Mid Gut  5.142976  
  Larval Feeding Salivary Gland  4.829622  
  Whole Larvae Feeding  4.593265  
 
  
   FlyBase ID    symbol    start    end    strand    length   
   FBgn0038131   CG15888  9110671   9112251   -  1581  
   FBgn0038132   CG15887  9113371   9114532   -  1162  
   FBgn0038133   Osi22   9116438   9117568  +  1131  
   FBgn0038134   wntD  9117774   9118920   -  1147  
 
 
    Segment 193 
 
   Location   
  Gene key  FBgn0038135-FBgn0038136  
  Heatmap region span   3R:9085240..9198352   
  Segment span   3R:9120472..9124569   
  Length (genes)  2  
  Length (bp)  4098  
   Model Scoring   
  BIC  255.028070  
  logL  -121.886304  
  logL ratio  -5.255851  
   Expression   
  Mean expression  6.609145  
  Median expression  5.224925  
  Tissue std. dev.  2.639018  
 
  No GO Slim enrichment  
  
   tissue    mean expression   
  5th Passage Drosophila S2 Cells  4.915100  
  Adult Accessory gland  8.272274  
  Adult Brain  4.552095  
  Adult Carcass  5.086956  
  Adult Crop  4.800301  
  Adult Eye  4.593029  
  Adult Fatbody  4.981818  
  Adult Female Spermatheca Mated  4.973672  
  Adult Female Spermatheca Virgin  4.983554  
  Adult Head  4.598114  
  Adult Heart  4.555274  
  Adult Hind Gut  10.746109  
  Adult Male Ejaculatory Duct  5.943869  
  Adult Mid Gut  11.973062  
  Adult Ovary  4.786017  
  Adult Salivary Gland  5.410617  
  Adult Testes  4.750943  
  Adult Thoracoabdominal ganglion  4.710399  
  Adult Whole Fly  8.631443  
  Larvae Wandering Tubules  7.644089  
  Larval Feeding Carcass  4.989537  
  Larval Feeding Central Nevous System  4.576221  
  Larval Feeding Hind Gut  10.920045  
  Larval Feeding Malpighian Tubule  8.263122  
  Larval Feeding Mid Gut  12.507109  
  Larval Feeding Salivary Gland  4.867916  
  Whole Larvae Feeding  11.414240  
 
  
   FlyBase ID    symbol    start    end    strand    length   
   FBgn0038135   CG8773   9120472   9124010  +  3539  
   FBgn0038136   CG8774   9124569   9128056  +  3488  
 
    Segment 194 
 
   Location   
  Gene key  FBgn0038139-FBgn0045442  
  Heatmap region span   3R:9105445..9205862   
  Segment span   3R:9159885..9179395   
  Length (genes)  3  
  Length (bp)  19511  
   Model Scoring   
  BIC  280.315701  
  logL  -134.530119  
  logL ratio  50.136918  
   Expression   
  Mean expression  4.673370  
  Median expression  4.433257  
  Tissue std. dev.  0.362179  
 
  No GO Slim enrichment  
  
   tissue    mean expression   
  5th Passage Drosophila S2 Cells  4.497724  
  Adult Accessory gland  5.135929  
  Adult Brain  5.109985  
  Adult Carcass  4.554577  
  Adult Crop  5.538182  
  Adult Eye  4.450453  
  Adult Fatbody  4.576957  
  Adult Female Spermatheca Mated  4.500880  
  Adult Female Spermatheca Virgin  4.473924  
  Adult Head  4.377495  
  Adult Heart  4.532092  
  Adult Hind Gut  4.353876  
  Adult Male Ejaculatory Duct  4.437581  
  Adult Mid Gut  4.549666  
  Adult Ovary  4.434611  
  Adult Salivary Gland  4.714630  
  Adult Testes  4.382508  
  Adult Thoracoabdominal ganglion  5.135749  
  Adult Whole Fly  4.204181  
  Larvae Wandering Tubules  4.482559  
  Larval Feeding Carcass  5.782426  
  Larval Feeding Central Nevous System  4.887527  
  Larval Feeding Hind Gut  4.570331  
  Larval Feeding Malpighian Tubule  4.504928  
  Larval Feeding Mid Gut  4.641877  
  Larval Feeding Salivary Gland  4.728725  
  Whole Larvae Feeding  4.621616  
 
  
   FlyBase ID    symbol    start    end    strand    length   
   FBgn0038139   CG8795  9154787   9159885   -  5099  
   FBgn0038140   CG8784   9165892   9170508  +  4617  
   FBgn0045442   mthl12   9179395   9181135  +  1741  
 
 
    Segment 195 
 
   Location   
  Gene key  FBgn0027610-FBgn0086371  
  Heatmap region span   3R:9112251..9207799   
  Segment span   3R:9190661..9192629   
  Length (genes)  2  
  Length (bp)  1969  
   Model Scoring   
  BIC  260.142086  
  logL  -124.443312  
  logL ratio  42.888359  
   Expression   
  Mean expression  10.299546  
  Median expression  10.147018  
  Tissue std. dev.  0.565686  
 
  No GO Slim enrichment  
  
   tissue    mean expression   
  5th Passage Drosophila S2 Cells  9.814822  
  Adult Accessory gland  9.034029  
  Adult Brain  10.145336  
  Adult Carcass  10.520369  
  Adult Crop  10.374531  
  Adult Eye  10.390805  
  Adult Fatbody  10.847254  
  Adult Female Spermatheca Mated  10.953967  
  Adult Female Spermatheca Virgin  11.060343  
  Adult Head  10.338266  
  Adult Heart  10.770449  
  Adult Hind Gut  11.176567  
  Adult Male Ejaculatory Duct  11.239527  
  Adult Mid Gut  9.932301  
  Adult Ovary  9.675795  
  Adult Salivary Gland  10.720866  
  Adult Testes  9.794496  
  Adult Thoracoabdominal ganglion  10.189339  
  Adult Whole Fly  10.157407  
  Larvae Wandering Tubules  9.885690  
  Larval Feeding Carcass  9.593371  
  Larval Feeding Central Nevous System  10.144966  
  Larval Feeding Hind Gut  11.088360  
  Larval Feeding Malpighian Tubule  10.845185  
  Larval Feeding Mid Gut  9.997383  
  Larval Feeding Salivary Gland  9.456626  
  Whole Larvae Feeding  9.939704  
 
  
   FlyBase ID    symbol    start    end    strand    length   
   FBgn0027610   Dic1   9190661   9194813  +  4153  
   FBgn0086371   poly  9187538   9192629   -  5092  
 
    Segment 196 
 
   Location   
  Gene key  FBgn0038142-FBgn0259831  
  Heatmap region span   3R:9120472..9214436   
  Segment span   3R:9195391..9198352   
  Length (genes)  3  
  Length (bp)  2962  
   Model Scoring   
  BIC  280.643904  
  logL  -134.694221  
  logL ratio  43.958618  
   Expression   
  Mean expression  4.488621  
  Median expression  4.342585  
  Tissue std. dev.  0.593358  
 
  No GO Slim enrichment  
  
   tissue    mean expression   
  5th Passage Drosophila S2 Cells  4.484134  
  Adult Accessory gland  4.354284  
  Adult Brain  4.002637  
  Adult Carcass  6.204901  
  Adult Crop  4.193064  
  Adult Eye  4.336044  
  Adult Fatbody  4.304478  
  Adult Female Spermatheca Mated  4.215013  
  Adult Female Spermatheca Virgin  4.205040  
  Adult Head  6.421414  
  Adult Heart  4.176254  
  Adult Hind Gut  4.266743  
  Adult Male Ejaculatory Duct  4.372352  
  Adult Mid Gut  5.181373  
  Adult Ovary  4.193794  
  Adult Salivary Gland  4.319237  
  Adult Testes  4.170897  
  Adult Thoracoabdominal ganglion  4.197004  
  Adult Whole Fly  5.330575  
  Larvae Wandering Tubules  4.302385  
  Larval Feeding Carcass  4.209607  
  Larval Feeding Central Nevous System  3.973947  
  Larval Feeding Hind Gut  4.206950  
  Larval Feeding Malpighian Tubule  4.278938  
  Larval Feeding Mid Gut  4.691290  
  Larval Feeding Salivary Gland  4.240688  
  Whole Larvae Feeding  4.359731  
 
  
   FlyBase ID    symbol    start    end    strand    length   
   FBgn0038142   CheA87a   9195391   9196068  +  678  
   FBgn0023495   Lip3  9195960   9197626   -  1667  
   FBgn0259831   CG34309   9198352   9199049  +  698  
 
 
    Segment 197 
 
   Location   
  Gene key  FBgn0044511-FBgn0038145  
  Heatmap region span   3R:9190661..9245995   
  Segment span   3R:9207514..9207799   
  Length (genes)  2  
  Length (bp)  286  
   Model Scoring   
  BIC  303.978563  
  logL  -146.361550  
  logL ratio  56.785213  
   Expression   
  Mean expression  11.408799  
  Median expression  11.348647  
  Tissue std. dev.  0.358766  
 
  No GO Slim enrichment  
  
   tissue    mean expression   
  5th Passage Drosophila S2 Cells  11.036034  
  Adult Accessory gland  11.339663  
  Adult Brain  11.815690  
  Adult Carcass  11.409200  
  Adult Crop  11.408278  
  Adult Eye  11.882972  
  Adult Fatbody  11.438018  
  Adult Female Spermatheca Mated  11.505926  
  Adult Female Spermatheca Virgin  11.349650  
  Adult Head  11.708917  
  Adult Heart  11.604145  
  Adult Hind Gut  11.450337  
  Adult Male Ejaculatory Duct  11.622510  
  Adult Mid Gut  11.018030  
  Adult Ovary  12.166504  
  Adult Salivary Gland  11.046456  
  Adult Testes  11.682841  
  Adult Thoracoabdominal ganglion  11.801569  
  Adult Whole Fly  11.829292  
  Larvae Wandering Tubules  10.982832  
  Larval Feeding Carcass  11.122684  
  Larval Feeding Central Nevous System  11.855508  
  Larval Feeding Hind Gut  11.182182  
  Larval Feeding Malpighian Tubule  11.102089  
  Larval Feeding Mid Gut  10.716674  
  Larval Feeding Salivary Gland  11.106213  
  Whole Larvae Feeding  10.853352  
 
  
   FlyBase ID    symbol    start    end    strand    length   
   FBgn0044511   mRpS21  9207051   9207514   -  464  
   FBgn0038145   Droj2   9207799   9211013  +  3215  
 
    Segment 198 
 
   Location   
  Gene key  FBgn0038156-FBgn0038160  
  Heatmap region span   3R:9227580..9482116   
  Segment span   3R:9276312..9434968   
  Length (genes)  7  
  Length (bp)  158657  
   Model Scoring   
  BIC  527.014213  
  logL  -257.879375  
  logL ratio  197.794038  
   Expression   
  Mean expression  4.634493  
  Median expression  4.420836  
  Tissue std. dev.  0.194507  
 
  No GO Slim enrichment  
  
   tissue    mean expression   
  5th Passage Drosophila S2 Cells  4.597497  
  Adult Accessory gland  4.532481  
  Adult Brain  4.728161  
  Adult Carcass  4.466663  
  Adult Crop  4.526638  
  Adult Eye  4.533148  
  Adult Fatbody  4.642222  
  Adult Female Spermatheca Mated  4.717939  
  Adult Female Spermatheca Virgin  4.654538  
  Adult Head  4.473207  
  Adult Heart  4.553248  
  Adult Hind Gut  4.390223  
  Adult Male Ejaculatory Duct  4.568469  
  Adult Mid Gut  4.576878  
  Adult Ovary  4.469949  
  Adult Salivary Gland  4.695038  
  Adult Testes  5.150251  
  Adult Thoracoabdominal ganglion  4.814200  
  Adult Whole Fly  4.313422  
  Larvae Wandering Tubules  4.896171  
  Larval Feeding Carcass  5.064541  
  Larval Feeding Central Nevous System  4.398743  
  Larval Feeding Hind Gut  4.531962  
  Larval Feeding Malpighian Tubule  4.731970  
  Larval Feeding Mid Gut  4.643294  
  Larval Feeding Salivary Gland  4.551434  
  Whole Larvae Feeding  4.909032  
 
  
   FlyBase ID    symbol    start    end    strand    length   
   FBgn0038156   CG14372   9276312   9288887  +  12576  
   FBgn0040554   CR17025  9284057   9291828   -  7772  
   FBgn0038157   CG12538  9348673   9349257   -  585  
   FBgn0051337   CG31337  9369688   9371106   -  1419  
   FBgn0038158   CG14370   9413719   9414447  +  729  
   FBgn0038159   CG14369  9417998   9418336   -  339  
   FBgn0038160   CG9759  9434475   9434968   -  494  
 
 
    Segment 199 
 
   Location   
  Gene key  FBgn0003060-FBgn0038161  
  Heatmap region span   3R:9245995..9486241   
  Segment span   3R:9438151..9441726   
  Length (genes)  2  
  Length (bp)  3576  
   Model Scoring   
  BIC  211.162410  
  logL  -99.953474  
  logL ratio  13.211257  
   Expression   
  Mean expression  5.351255  
  Median expression  4.674571  
  Tissue std. dev.  1.826075  
 
  No GO Slim enrichment  
  
   tissue    mean expression   
  5th Passage Drosophila S2 Cells  4.501458  
  Adult Accessory gland  5.411265  
  Adult Brain  4.424767  
  Adult Carcass  4.589601  
  Adult Crop  4.631188  
  Adult Eye  4.979503  
  Adult Fatbody  4.837903  
  Adult Female Spermatheca Mated  5.005512  
  Adult Female Spermatheca Virgin  5.064353  
  Adult Head  4.869208  
  Adult Heart  4.524593  
  Adult Hind Gut  4.516962  
  Adult Male Ejaculatory Duct  4.869048  
  Adult Mid Gut  4.570911  
  Adult Ovary  4.519358  
  Adult Salivary Gland  5.117476  
  Adult Testes  4.225409  
  Adult Thoracoabdominal ganglion  4.412911  
  Adult Whole Fly  4.276526  
  Larvae Wandering Tubules  4.936421  
  Larval Feeding Carcass  10.340955  
  Larval Feeding Central Nevous System  4.709542  
  Larval Feeding Hind Gut  10.937448  
  Larval Feeding Malpighian Tubule  4.757595  
  Larval Feeding Mid Gut  4.553305  
  Larval Feeding Salivary Gland  4.827784  
  Whole Larvae Feeding  10.072879  
 
  
   FlyBase ID    symbol    start    end    strand    length   
   FBgn0003060   CG9757  9437588   9438151   -  564  
   FBgn0038161   CG9269   9441726   9442166  +  441  
 
    Segment 200 
 
   Location   
  Gene key  FBgn0086897-FBgn0015778  
  Heatmap region span   3R:9260704..9509680   
  Segment span   3R:9472026..9472687   
  Length (genes)  2  
  Length (bp)  662  
   Model Scoring   
  BIC  231.907160  
  logL  -110.325849  
  logL ratio  43.928164  
   Expression   
  Mean expression  9.530174  
  Median expression  9.574907  
  Tissue std. dev.  0.617018  
 
  No GO Slim enrichment  
  
   tissue    mean expression   
  5th Passage Drosophila S2 Cells  10.649541  
  Adult Accessory gland  9.719393  
  Adult Brain  9.750151  
  Adult Carcass  8.505436  
  Adult Crop  9.351066  
  Adult Eye  9.726529  
  Adult Fatbody  9.460265  
  Adult Female Spermatheca Mated  9.048500  
  Adult Female Spermatheca Virgin  8.960504  
  Adult Head  8.749793  
  Adult Heart  9.980535  
  Adult Hind Gut  9.436820  
  Adult Male Ejaculatory Duct  9.059320  
  Adult Mid Gut  9.100789  
  Adult Ovary  11.039606  
  Adult Salivary Gland  8.953552  
  Adult Testes  8.834454  
  Adult Thoracoabdominal ganglion  9.396971  
  Adult Whole Fly  9.634095  
  Larvae Wandering Tubules  9.577789  
  Larval Feeding Carcass  9.896060  
  Larval Feeding Central Nevous System  10.916362  
  Larval Feeding Hind Gut  9.762465  
  Larval Feeding Malpighian Tubule  9.521207  
  Larval Feeding Mid Gut  8.860717  
  Larval Feeding Salivary Gland  10.088413  
  Whole Larvae Feeding  9.334355  
 
  
   FlyBase ID    symbol    start    end    strand    length   
   FBgn0086897     9461214   9472026   -  10813  
   FBgn0015778   rin   9472687   9478761  +  6075  
 
    Segment 201 
 
   Location   
  Gene key  FBgn0004587-FBgn0038167  
  Heatmap region span   3R:9457664..9535661   
  Segment span   3R:9487022..9502271   
  Length (genes)  3  
  Length (bp)  15250  
   Model Scoring   
  BIC  340.305659  
  logL  -164.525098  
  logL ratio  -4.235412  
   Expression   
  Mean expression  5.770672  
  Median expression  5.804216  
  Tissue std. dev.  0.682334  
 
  No GO Slim enrichment  
  
   tissue    mean expression   
  5th Passage Drosophila S2 Cells  5.817979  
  Adult Accessory gland  6.033852  
  Adult Brain  7.793464  
  Adult Carcass  5.544376  
  Adult Crop  5.472018  
  Adult Eye  6.559490  
  Adult Fatbody  5.342123  
  Adult Female Spermatheca Mated  5.196513  
  Adult Female Spermatheca Virgin  5.110252  
  Adult Head  6.358678  
  Adult Heart  5.362986  
  Adult Hind Gut  5.747556  
  Adult Male Ejaculatory Duct  5.390795  
  Adult Mid Gut  5.280625  
  Adult Ovary  5.972049  
  Adult Salivary Gland  5.514942  
  Adult Testes  6.257980  
  Adult Thoracoabdominal ganglion  7.074759  
  Adult Whole Fly  5.290464  
  Larvae Wandering Tubules  5.461361  
  Larval Feeding Carcass  5.229589  
  Larval Feeding Central Nevous System  7.209278  
  Larval Feeding Hind Gut  5.565917  
  Larval Feeding Malpighian Tubule  5.492665  
  Larval Feeding Mid Gut  5.174402  
  Larval Feeding Salivary Gland  5.504578  
  Whole Larvae Feeding  5.049448  
 
  
   FlyBase ID    symbol    start    end    strand    length   
   FBgn0004587   B52   9487022   9492613  +  5592  
   FBgn0038165   Task6  9494313   9497823   -  3511  
   FBgn0038167   lkb1   9502271   9505152  +  2882  
 
 
    Segment 202 
 
   Location   
  Gene key  FBgn0038166-FBgn0024555  
  Heatmap region span   3R:9472026..9540945   
  Segment span   3R:9502398..9509680   
  Length (genes)  2  
  Length (bp)  7283  
   Model Scoring   
  BIC  243.392429  
  logL  -116.068483  
  logL ratio  32.343138  
   Expression   
  Mean expression  9.477973  
  Median expression  9.509686  
  Tissue std. dev.  0.540739  
 
  No GO Slim enrichment  
  
   tissue    mean expression   
  5th Passage Drosophila S2 Cells  9.938021  
  Adult Accessory gland  9.533976  
  Adult Brain  9.875734  
  Adult Carcass  9.117857  
  Adult Crop  10.026010  
  Adult Eye  9.573561  
  Adult Fatbody  9.705033  
  Adult Female Spermatheca Mated  9.562152  
  Adult Female Spermatheca Virgin  9.510103  
  Adult Head  9.332021  
  Adult Heart  10.181584  
  Adult Hind Gut  9.693410  
  Adult Male Ejaculatory Duct  9.694879  
  Adult Mid Gut  9.307383  
  Adult Ovary  9.015221  
  Adult Salivary Gland  9.387098  
  Adult Testes  7.596587  
  Adult Thoracoabdominal ganglion  9.480493  
  Adult Whole Fly  8.577803  
  Larvae Wandering Tubules  9.815831  
  Larval Feeding Carcass  9.748837  
  Larval Feeding Central Nevous System  10.646819  
  Larval Feeding Hind Gut  9.500258  
  Larval Feeding Malpighian Tubule  9.411060  
  Larval Feeding Mid Gut  9.129492  
  Larval Feeding Salivary Gland  9.424838  
  Whole Larvae Feeding  9.119198  
 
  
   FlyBase ID    symbol    start    end    strand    length   
   FBgn0038166   CG9588  9500814   9502398   -  1585  
   FBgn0024555   flfl   9509680   9519629  +  9950  
 
    Segment 203 
 
   Location   
  Gene key  FBgn0038170-FBgn0002937  
  Heatmap region span   3R:9486241..9591497   
  Segment span   3R:9519775..9526308   
  Length (genes)  2  
  Length (bp)  6534  
   Model Scoring   
  BIC  216.668532  
  logL  -102.706535  
  logL ratio  5.787955  
   Expression   
  Mean expression  5.837730  
  Median expression  5.327031  
  Tissue std. dev.  1.320326  
 
  No GO Slim enrichment  
  
   tissue    mean expression   
  5th Passage Drosophila S2 Cells  4.863563  
  Adult Accessory gland  5.347889  
  Adult Brain  8.370272  
  Adult Carcass  5.857959  
  Adult Crop  5.308945  
  Adult Eye  9.902702  
  Adult Fatbody  5.544947  
  Adult Female Spermatheca Mated  5.257903  
  Adult Female Spermatheca Virgin  5.367077  
  Adult Head  8.753624  
  Adult Heart  5.610390  
  Adult Hind Gut  5.292344  
  Adult Male Ejaculatory Duct  5.327416  
  Adult Mid Gut  5.223327  
  Adult Ovary  5.352500  
  Adult Salivary Gland  5.302635  
  Adult Testes  5.676693  
  Adult Thoracoabdominal ganglion  8.601356  
  Adult Whole Fly  5.485772  
  Larvae Wandering Tubules  5.453285  
  Larval Feeding Carcass  5.301165  
  Larval Feeding Central Nevous System  5.224912  
  Larval Feeding Hind Gut  4.865644  
  Larval Feeding Malpighian Tubule  5.376606  
  Larval Feeding Mid Gut  5.056086  
  Larval Feeding Salivary Gland  5.108153  
  Whole Larvae Feeding  4.785548  
 
  
   FlyBase ID    symbol    start    end    strand    length   
   FBgn0038170   CG14367   9519775   9521484  +  1710  
   FBgn0002937   ninaB   9526308   9528844  +  2537  
 
    Segment 204 
 
   Location   
  Gene key  FBgn0259721-FBgn0038183  
  Heatmap region span   3R:9543898..9784473   
  Segment span   3R:9605303..9606714   
  Length (genes)  2  
  Length (bp)  1412  
   Model Scoring   
  BIC  209.058542  
  logL  -98.901540  
  logL ratio  48.154476  
   Expression   
  Mean expression  8.805994  
  Median expression  8.784606  
  Tissue std. dev.  0.221368  
 
  No GO Slim enrichment  
  
   tissue    mean expression   
  5th Passage Drosophila S2 Cells  8.593727  
  Adult Accessory gland  8.736691  
  Adult Brain  8.421572  
  Adult Carcass  8.791085  
  Adult Crop  8.960380  
  Adult Eye  9.263449  
  Adult Fatbody  9.105251  
  Adult Female Spermatheca Mated  8.880749  
  Adult Female Spermatheca Virgin  8.978354  
  Adult Head  8.725734  
  Adult Heart  9.164352  
  Adult Hind Gut  8.760759  
  Adult Male Ejaculatory Duct  8.555841  
  Adult Mid Gut  8.664151  
  Adult Ovary  9.039708  
  Adult Salivary Gland  8.838633  
  Adult Testes  8.923237  
  Adult Thoracoabdominal ganglion  8.628932  
  Adult Whole Fly  8.582228  
  Larvae Wandering Tubules  8.429300  
  Larval Feeding Carcass  8.876558  
  Larval Feeding Central Nevous System  9.134400  
  Larval Feeding Hind Gut  8.840768  
  Larval Feeding Malpighian Tubule  8.733741  
  Larval Feeding Mid Gut  8.661345  
  Larval Feeding Salivary Gland  8.964347  
  Whole Larvae Feeding  8.506540  
 
  
   FlyBase ID    symbol    start    end    strand    length   
   FBgn0259721   CG42375   9605303   9606517  +  1215  
   FBgn0038183   CG9286   9606714   9607808  +  1095  
 
    Segment 205 
 
   Location   
  Gene key  FBgn0013998-FBgn0051495  
  Heatmap region span   3R:9593273..9854952   
  Segment span   3R:9663708..9665652   
  Length (genes)  2  
  Length (bp)  1945  
   Model Scoring   
  BIC  224.121001  
  logL  -106.432769  
  logL ratio  31.691364  
   Expression   
  Mean expression  8.183827  
  Median expression  8.211533  
  Tissue std. dev.  0.482368  
 
  No GO Slim enrichment  
  
   tissue    mean expression   
  5th Passage Drosophila S2 Cells  8.236959  
  Adult Accessory gland  8.344542  
  Adult Brain  7.266372  
  Adult Carcass  8.092152  
  Adult Crop  8.255577  
  Adult Eye  7.840885  
  Adult Fatbody  8.504572  
  Adult Female Spermatheca Mated  8.236463  
  Adult Female Spermatheca Virgin  8.101496  
  Adult Head  7.569548  
  Adult Heart  8.738287  
  Adult Hind Gut  8.346944  
  Adult Male Ejaculatory Duct  8.680407  
  Adult Mid Gut  7.995463  
  Adult Ovary  8.401740  
  Adult Salivary Gland  8.777313  
  Adult Testes  7.460732  
  Adult Thoracoabdominal ganglion  7.437779  
  Adult Whole Fly  7.673662  
  Larvae Wandering Tubules  9.136940  
  Larval Feeding Carcass  8.175700  
  Larval Feeding Central Nevous System  7.604584  
  Larval Feeding Hind Gut  8.268968  
  Larval Feeding Malpighian Tubule  9.095564  
  Larval Feeding Mid Gut  8.258679  
  Larval Feeding Salivary Gland  8.645066  
  Whole Larvae Feeding  7.816925  
 
  
   FlyBase ID    symbol    start    end    strand    length   
   FBgn0013998   Nsf2  9660195   9663708   -  3514  
   FBgn0051495   CG31495  9663960   9665652   -  1693  
 
    Segment 206 
 
   Location   
  Gene key  FBgn0038186-FBgn0038190  
  Heatmap region span   3R:9597320..9857777   
  Segment span   3R:9681176..9776860   
  Length (genes)  7  
  Length (bp)  95685  
   Model Scoring   
  BIC  538.805633  
  logL  -263.775085  
  logL ratio  180.384533  
   Expression   
  Mean expression  4.848029  
  Median expression  4.623360  
  Tissue std. dev.  0.389851  
 
  No GO Slim enrichment  
  
   tissue    mean expression   
  5th Passage Drosophila S2 Cells  4.602102  
  Adult Accessory gland  4.784912  
  Adult Brain  4.461757  
  Adult Carcass  4.885982  
  Adult Crop  4.556724  
  Adult Eye  4.665094  
  Adult Fatbody  4.746232  
  Adult Female Spermatheca Mated  4.770071  
  Adult Female Spermatheca Virgin  4.811156  
  Adult Head  4.597434  
  Adult Heart  5.332223  
  Adult Hind Gut  4.523277  
  Adult Male Ejaculatory Duct  4.706023  
  Adult Mid Gut  4.669424  
  Adult Ovary  5.658753  
  Adult Salivary Gland  4.833419  
  Adult Testes  6.354584  
  Adult Thoracoabdominal ganglion  4.696351  
  Adult Whole Fly  5.245934  
  Larvae Wandering Tubules  4.686964  
  Larval Feeding Carcass  4.941428  
  Larval Feeding Central Nevous System  4.714318  
  Larval Feeding Hind Gut  4.573452  
  Larval Feeding Malpighian Tubule  4.617365  
  Larval Feeding Mid Gut  4.770026  
  Larval Feeding Salivary Gland  4.815940  
  Whole Larvae Feeding  4.875844  
 
  
   FlyBase ID    symbol    start    end    strand    length   
   FBgn0038186   CG14362  9680321   9681176   -  856  
   FBgn0008646   E5  9693067   9700638   -  7572  
   FBgn0000576   ems   9727581   9730346  +  2766  
   FBgn0038188   Art9  9767271   9769124   -  1854  
   FBgn0038189   Art6  9770376   9771534   -  1159  
   FBgn0047199   CG31517   9774157   9775052  +  896  
   FBgn0038190   CG9926  9775946   9776860   -  915  
 
 
    Segment 207 
 
   Location   
  Gene key  FBgn0038191-FBgn0015270  
  Heatmap region span   3R:9653432..9882690   
  Segment span   3R:9789891..9790639   
  Length (genes)  2  
  Length (bp)  749  
   Model Scoring   
  BIC  203.163104  
  logL  -95.953821  
  logL ratio  22.306105  
   Expression   
  Mean expression  4.930588  
  Median expression  4.497606  
  Tissue std. dev.  1.291199  
 
  No GO Slim enrichment  
  
   tissue    mean expression   
  5th Passage Drosophila S2 Cells  5.583901  
  Adult Accessory gland  4.585708  
  Adult Brain  4.070041  
  Adult Carcass  4.689686  
  Adult Crop  4.374663  
  Adult Eye  4.141738  
  Adult Fatbody  4.406921  
  Adult Female Spermatheca Mated  4.472411  
  Adult Female Spermatheca Virgin  4.568215  
  Adult Head  4.115925  
  Adult Heart  4.163013  
  Adult Hind Gut  4.188777  
  Adult Male Ejaculatory Duct  4.225778  
  Adult Mid Gut  4.607992  
  Adult Ovary  10.069596  
  Adult Salivary Gland  4.364398  
  Adult Testes  5.946181  
  Adult Thoracoabdominal ganglion  4.226774  
  Adult Whole Fly  8.112395  
  Larvae Wandering Tubules  4.496158  
  Larval Feeding Carcass  4.675526  
  Larval Feeding Central Nevous System  5.583019  
  Larval Feeding Hind Gut  4.660600  
  Larval Feeding Malpighian Tubule  4.586141  
  Larval Feeding Mid Gut  4.494148  
  Larval Feeding Salivary Gland  5.223164  
  Whole Larvae Feeding  4.493009  
 
  
   FlyBase ID    symbol    start    end    strand    length   
   FBgn0038191   CG9925  9786432   9789891   -  3460  
   FBgn0015270   Orc2   9790639   9792803  +  2165  
 
    Segment 208 
 
   Location   
  Gene key  FBgn0038195-FBgn0038196  
  Heatmap region span   3R:9784473..9923802   
  Segment span   3R:9858089..9861887   
  Length (genes)  2  
  Length (bp)  3799  
   Model Scoring   
  BIC  284.875387  
  logL  -136.809962  
  logL ratio  42.477846  
   Expression   
  Mean expression  10.826855  
  Median expression  10.740312  
  Tissue std. dev.  0.339454  
 
  No GO Slim enrichment  
  
   tissue    mean expression   
  5th Passage Drosophila S2 Cells  11.319399  
  Adult Accessory gland  11.447433  
  Adult Brain  10.441475  
  Adult Carcass  10.487152  
  Adult Crop  10.893458  
  Adult Eye  10.734006  
  Adult Fatbody  11.055060  
  Adult Female Spermatheca Mated  11.241120  
  Adult Female Spermatheca Virgin  11.087221  
  Adult Head  10.528496  
  Adult Heart  10.976793  
  Adult Hind Gut  10.386956  
  Adult Male Ejaculatory Duct  11.276689  
  Adult Mid Gut  10.327033  
  Adult Ovary  11.030037  
  Adult Salivary Gland  10.766390  
  Adult Testes  10.829028  
  Adult Thoracoabdominal ganglion  10.641731  
  Adult Whole Fly  10.706978  
  Larvae Wandering Tubules  10.848025  
  Larval Feeding Carcass  10.658635  
  Larval Feeding Central Nevous System  11.162845  
  Larval Feeding Hind Gut  10.744328  
  Larval Feeding Malpighian Tubule  10.826669  
  Larval Feeding Mid Gut  10.170734  
  Larval Feeding Salivary Gland  11.359521  
  Whole Larvae Feeding  10.377885  
 
  
   FlyBase ID    symbol    start    end    strand    length   
   FBgn0038195   CG3061   9858089   9860764  +  2676  
   FBgn0038196   CG9922  9860738   9861887   -  1150  
 
    Segment 209 
 
   Location   
  Gene key  FBgn0038201-FBgn0038205  
  Heatmap region span   3R:9882690..10040517   
  Segment span   3R:9928562..9944121   
  Length (genes)  5  
  Length (bp)  15560  
   Model Scoring   
  BIC  380.459399  
  logL  -184.601969  
  logL ratio  137.107988  
   Expression   
  Mean expression  4.606856  
  Median expression  4.519749  
  Tissue std. dev.  0.218948  
 
  No GO Slim enrichment  
  
   tissue    mean expression   
  5th Passage Drosophila S2 Cells  4.402712  
  Adult Accessory gland  4.472340  
  Adult Brain  5.062897  
  Adult Carcass  4.835218  
  Adult Crop  4.720292  
  Adult Eye  4.585564  
  Adult Fatbody  4.687189  
  Adult Female Spermatheca Mated  4.550945  
  Adult Female Spermatheca Virgin  4.610610  
  Adult Head  4.816400  
  Adult Heart  4.645850  
  Adult Hind Gut  4.502286  
  Adult Male Ejaculatory Duct  4.644035  
  Adult Mid Gut  4.535178  
  Adult Ovary  4.265365  
  Adult Salivary Gland  4.872007  
  Adult Testes  4.895476  
  Adult Thoracoabdominal ganglion  4.984859  
  Adult Whole Fly  4.225279  
  Larvae Wandering Tubules  4.434046  
  Larval Feeding Carcass  4.460244  
  Larval Feeding Central Nevous System  4.965332  
  Larval Feeding Hind Gut  4.387412  
  Larval Feeding Malpighian Tubule  4.450103  
  Larval Feeding Mid Gut  4.513459  
  Larval Feeding Salivary Gland  4.485116  
  Whole Larvae Feeding  4.374912  
 
  
   FlyBase ID    symbol    start    end    strand    length   
   FBgn0038201   Pk1r  9926126   9928562   -  2437  
   FBgn0038202   CG12402  9930595   9933878   -  3284  
   FBgn0038203   Or88a   9935167   9936545  +  1379  
   FBgn0038204   CG14357  9936873   9937888   -  1016  
   FBgn0038205   Kif19A  9938345   9944121   -  5777  
 
 
    Segment 210 
 
   Location   
  Gene key  FBgn0020510-FBgn0003276  
  Heatmap region span   3R:9915245..10045927   
  Segment span   3R:9948142..9951515   
  Length (genes)  4  
  Length (bp)  3374  
   Model Scoring   
  BIC  392.731817  
  logL  -190.738177  
  logL ratio  148.472536  
   Expression   
  Mean expression  9.570910  
  Median expression  9.696359  
  Tissue std. dev.  0.358044  
 
  No GO Slim enrichment  
  
   tissue    mean expression   
  5th Passage Drosophila S2 Cells  10.256201  
  Adult Accessory gland  9.523997  
  Adult Brain  9.554804  
  Adult Carcass  8.948862  
  Adult Crop  9.418054  
  Adult Eye  9.493108  
  Adult Fatbody  9.213376  
  Adult Female Spermatheca Mated  9.405959  
  Adult Female Spermatheca Virgin  9.421962  
  Adult Head  9.093092  
  Adult Heart  9.427291  
  Adult Hind Gut  9.455695  
  Adult Male Ejaculatory Duct  9.259574  
  Adult Mid Gut  9.494285  
  Adult Ovary  10.170110  
  Adult Salivary Gland  9.601315  
  Adult Testes  8.955497  
  Adult Thoracoabdominal ganglion  9.381352  
  Adult Whole Fly  9.383141  
  Larvae Wandering Tubules  10.214914  
  Larval Feeding Carcass  9.620691  
  Larval Feeding Central Nevous System  10.019707  
  Larval Feeding Hind Gut  9.905480  
  Larval Feeding Malpighian Tubule  10.058624  
  Larval Feeding Mid Gut  9.730360  
  Larval Feeding Salivary Gland  10.013119  
  Whole Larvae Feeding  9.393989  
 
  
   FlyBase ID    symbol    start    end    strand    length   
   FBgn0020510   Abi  9944982   9948142   -  3161  
   FBgn0038206   twf   9948380   9950199  +  1820  
   FBgn0010340   140up  9949940   9951252   -  1313  
   FBgn0003276      9951515   9955548  +  4034  
 
 
    Segment 211 
 
   Location   
  Gene key  FBgn0038208-FBgn0038210  
  Heatmap region span   3R:9923802..10052468   
  Segment span   3R:9977721..9988441   
  Length (genes)  5  
  Length (bp)  10721  
   Model Scoring   
  BIC  359.807775  
  logL  -174.276156  
  logL ratio  159.483046  
   Expression   
  Mean expression  4.993040  
  Median expression  4.631257  
  Tissue std. dev.  1.308332  
 
  No GO Slim enrichment  
  
   tissue    mean expression   
  5th Passage Drosophila S2 Cells  4.606878  
  Adult Accessory gland  4.809970  
  Adult Brain  4.383830  
  Adult Carcass  4.880533  
  Adult Crop  4.619576  
  Adult Eye  4.282458  
  Adult Fatbody  4.603983  
  Adult Female Spermatheca Mated  4.602214  
  Adult Female Spermatheca Virgin  4.588698  
  Adult Head  4.462152  
  Adult Heart  4.392168  
  Adult Hind Gut  4.626589  
  Adult Male Ejaculatory Duct  4.939726  
  Adult Mid Gut  4.831297  
  Adult Ovary  4.594249  
  Adult Salivary Gland  4.832785  
  Adult Testes  11.227340  
  Adult Thoracoabdominal ganglion  4.448683  
  Adult Whole Fly  6.784544  
  Larvae Wandering Tubules  4.660266  
  Larval Feeding Carcass  5.056855  
  Larval Feeding Central Nevous System  4.332902  
  Larval Feeding Hind Gut  4.505342  
  Larval Feeding Malpighian Tubule  4.680440  
  Larval Feeding Mid Gut  4.821447  
  Larval Feeding Salivary Gland  4.780908  
  Whole Larvae Feeding  5.456251  
 
  
   FlyBase ID    symbol    start    end    strand    length   
   FBgn0038208   CG14355  9974230   9977721   -  3492  
   FBgn0038209   CG9722  9978607   9979551   -  945  
   FBgn0051533   CG31533  9980450   9983153   -  2704  
   FBgn0051327   CG31327  9983282   9985904   -  2623  
   FBgn0038210   CG3199   9988441   9989347  +  907  
 
 
    Segment 212 
 
   Location   
  Gene key  FBgn0038211-FBgn0051326  
  Heatmap region span   3R:9948142..10071193   
  Segment span   3R:10043588..10045927   
  Length (genes)  2  
  Length (bp)  2340  
   Model Scoring   
  BIC  236.866816  
  logL  -112.805677  
  logL ratio  24.744601  
   Expression   
  Mean expression  7.476744  
  Median expression  5.884110  
  Tissue std. dev.  2.712481  
 
  No GO Slim enrichment  
  
   tissue    mean expression   
  5th Passage Drosophila S2 Cells  5.017241  
  Adult Accessory gland  5.068281  
  Adult Brain  6.145725  
  Adult Carcass  11.203323  
  Adult Crop  10.510367  
  Adult Eye  10.577923  
  Adult Fatbody  12.148211  
  Adult Female Spermatheca Mated  11.636283  
  Adult Female Spermatheca Virgin  11.932976  
  Adult Head  10.721468  
  Adult Heart  11.443409  
  Adult Hind Gut  8.065359  
  Adult Male Ejaculatory Duct  9.134836  
  Adult Mid Gut  5.192881  
  Adult Ovary  5.184534  
  Adult Salivary Gland  7.364666  
  Adult Testes  5.027000  
  Adult Thoracoabdominal ganglion  5.968328  
  Adult Whole Fly  8.462281  
  Larvae Wandering Tubules  4.966660  
  Larval Feeding Carcass  5.455928  
  Larval Feeding Central Nevous System  4.747340  
  Larval Feeding Hind Gut  5.179705  
  Larval Feeding Malpighian Tubule  5.079399  
  Larval Feeding Mid Gut  5.261350  
  Larval Feeding Salivary Gland  5.104073  
  Whole Larvae Feeding  5.272557  
 
  
   FlyBase ID    symbol    start    end    strand    length   
   FBgn0038211   CG9649  10041181   10043588   -  2408  
   FBgn0051326   CG31326  10043858   10045927   -  2070  
 
    Segment 213 
 
   Location   
  Gene key  FBgn0053329-FBgn0027563  
  Heatmap region span   3R:9977721..10114794   
  Segment span   3R:10049877..10052468   
  Length (genes)  2  
  Length (bp)  2592  
   Model Scoring   
  BIC  227.769663  
  logL  -108.257100  
  logL ratio  13.004232  
   Expression   
  Mean expression  6.987982  
  Median expression  6.174470  
  Tissue std. dev.  1.567416  
 
  No GO Slim enrichment  
  
   tissue    mean expression   
  5th Passage Drosophila S2 Cells  6.480091  
  Adult Accessory gland  5.638917  
  Adult Brain  5.411174  
  Adult Carcass  9.195829  
  Adult Crop  9.755593  
  Adult Eye  8.445301  
  Adult Fatbody  9.090989  
  Adult Female Spermatheca Mated  8.549201  
  Adult Female Spermatheca Virgin  8.882148  
  Adult Head  8.909672  
  Adult Heart  8.595868  
  Adult Hind Gut  7.589169  
  Adult Male Ejaculatory Duct  6.520620  
  Adult Mid Gut  5.631630  
  Adult Ovary  5.574998  
  Adult Salivary Gland  9.794039  
  Adult Testes  5.470833  
  Adult Thoracoabdominal ganglion  5.361131  
  Adult Whole Fly  6.494827  
  Larvae Wandering Tubules  5.599657  
  Larval Feeding Carcass  7.345245  
  Larval Feeding Central Nevous System  5.090399  
  Larval Feeding Hind Gut  6.369781  
  Larval Feeding Malpighian Tubule  5.527172  
  Larval Feeding Mid Gut  5.682336  
  Larval Feeding Salivary Gland  5.407011  
  Whole Larvae Feeding  6.261883  
 
  
   FlyBase ID    symbol    start    end    strand    length   
   FBgn0053329   Sp212  10047796   10049877   -  2082  
   FBgn0027563   CG9631  10050820   10052468   -  1649  
 
    Segment 214 
 
   Location   
  Gene key  FBgn0038217-FBgn0038218  
  Heatmap region span   3R:10047301..10136089   
  Segment span   3R:10086935..10088140   
  Length (genes)  3  
  Length (bp)  1206  
   Model Scoring   
  BIC  228.017195  
  logL  -108.380866  
  logL ratio  82.036416  
   Expression   
  Mean expression  4.768756  
  Median expression  4.512231  
  Tissue std. dev.  0.911459  
 
  No GO Slim enrichment  
  
   tissue    mean expression   
  5th Passage Drosophila S2 Cells  4.556389  
  Adult Accessory gland  4.728646  
  Adult Brain  4.260603  
  Adult Carcass  4.620752  
  Adult Crop  4.480160  
  Adult Eye  4.637271  
  Adult Fatbody  4.547867  
  Adult Female Spermatheca Mated  4.541491  
  Adult Female Spermatheca Virgin  4.554333  
  Adult Head  4.310622  
  Adult Heart  4.645927  
  Adult Hind Gut  4.494775  
  Adult Male Ejaculatory Duct  4.813719  
  Adult Mid Gut  4.621413  
  Adult Ovary  4.566406  
  Adult Salivary Gland  4.938710  
  Adult Testes  9.329293  
  Adult Thoracoabdominal ganglion  4.280458  
  Adult Whole Fly  4.941740  
  Larvae Wandering Tubules  4.462521  
  Larval Feeding Carcass  4.673147  
  Larval Feeding Central Nevous System  4.341293  
  Larval Feeding Hind Gut  4.515129  
  Larval Feeding Malpighian Tubule  4.590282  
  Larval Feeding Mid Gut  4.736233  
  Larval Feeding Salivary Gland  4.703470  
  Whole Larvae Feeding  4.863753  
 
  
   FlyBase ID    symbol    start    end    strand    length   
   FBgn0038217   CG14840  10085930   10086935   -  1006  
   FBgn0038219   CG14839   10088109   10089094  +  986  
   FBgn0038218   CG14841  10087113   10088140   -  1028  
 
 
    Segment 215 
 
   Location   
  Gene key  FBgn0003862-FBgn0038220  
  Heatmap region span   3R:10049877..10152404   
  Segment span   3R:10112623..10114794   
  Length (genes)  2  
  Length (bp)  2172  
   Model Scoring   
  BIC  237.217422  
  logL  -112.980980  
  logL ratio  40.010383  
   Expression   
  Mean expression  9.610719  
  Median expression  9.605929  
  Tissue std. dev.  0.586051  
 
  No GO Slim enrichment  
  
   tissue    mean expression   
  5th Passage Drosophila S2 Cells  9.830274  
  Adult Accessory gland  8.918146  
  Adult Brain  10.433954  
  Adult Carcass  9.421194  
  Adult Crop  10.210577  
  Adult Eye  10.030728  
  Adult Fatbody  8.686308  
  Adult Female Spermatheca Mated  9.365222  
  Adult Female Spermatheca Virgin  9.340196  
  Adult Head  9.631601  
  Adult Heart  9.047198  
  Adult Hind Gut  9.960601  
  Adult Male Ejaculatory Duct  9.845708  
  Adult Mid Gut  9.894906  
  Adult Ovary  9.907485  
  Adult Salivary Gland  10.168299  
  Adult Testes  7.564277  
  Adult Thoracoabdominal ganglion  10.031021  
  Adult Whole Fly  9.056865  
  Larvae Wandering Tubules  9.890961  
  Larval Feeding Carcass  9.411765  
  Larval Feeding Central Nevous System  10.073994  
  Larval Feeding Hind Gut  10.170299  
  Larval Feeding Malpighian Tubule  9.666602  
  Larval Feeding Mid Gut  10.014495  
  Larval Feeding Salivary Gland  9.423389  
  Whole Larvae Feeding  9.493343  
 
  
   FlyBase ID    symbol    start    end    strand    length   
   FBgn0003862   trx  10089080   10112623   -  23544  
   FBgn0038220   CG12207   10114794   10127995  +  13202  
 
    Segment 216 
 
   Location   
  Gene key  FBgn0013767-FBgn0038223  
  Heatmap region span   3R:10112623..10342764   
  Segment span   3R:10140658..10152404   
  Length (genes)  2  
  Length (bp)  11747  
   Model Scoring   
  BIC  243.180911  
  logL  -115.962724  
  logL ratio  -10.572442  
   Expression   
  Mean expression  6.195110  
  Median expression  5.756778  
  Tissue std. dev.  1.025371  
 
  No GO Slim enrichment  
  
   tissue    mean expression   
  5th Passage Drosophila S2 Cells  6.226955  
  Adult Accessory gland  5.869039  
  Adult Brain  9.835700  
  Adult Carcass  5.498936  
  Adult Crop  5.852118  
  Adult Eye  6.028308  
  Adult Fatbody  5.601550  
  Adult Female Spermatheca Mated  5.467220  
  Adult Female Spermatheca Virgin  5.426132  
  Adult Head  7.888415  
  Adult Heart  5.271507  
  Adult Hind Gut  5.629453  
  Adult Male Ejaculatory Duct  5.629397  
  Adult Mid Gut  5.762066  
  Adult Ovary  6.248582  
  Adult Salivary Gland  6.313560  
  Adult Testes  6.961218  
  Adult Thoracoabdominal ganglion  6.571592  
  Adult Whole Fly  5.727152  
  Larvae Wandering Tubules  5.924753  
  Larval Feeding Carcass  5.773276  
  Larval Feeding Central Nevous System  8.636753  
  Larval Feeding Hind Gut  5.584700  
  Larval Feeding Malpighian Tubule  5.923090  
  Larval Feeding Mid Gut  5.453959  
  Larval Feeding Salivary Gland  6.377675  
  Whole Larvae Feeding  5.784875  
 
  
   FlyBase ID    symbol    start    end    strand    length   
   FBgn0013767   Crz   10140658   10141439  +  782  
   FBgn0038223   Afti  10147101   10152404   -  5304  
 
    Segment 217 
 
   Location   
  Gene key  FBgn0051320-FBgn0038233  
  Heatmap region span   3R:10134816..10355513   
  Segment span   3R:10187461..10337414   
  Length (genes)  4  
  Length (bp)  149954  
   Model Scoring   
  BIC  427.241591  
  logL  -207.993064  
  logL ratio  16.105735  
   Expression   
  Mean expression  5.717020  
  Median expression  5.848941  
  Tissue std. dev.  0.602854  
 
  No GO Slim enrichment  
  
   tissue    mean expression   
  5th Passage Drosophila S2 Cells  5.945261  
  Adult Accessory gland  5.415766  
  Adult Brain  6.193967  
  Adult Carcass  5.561187  
  Adult Crop  5.538063  
  Adult Eye  5.138109  
  Adult Fatbody  5.610301  
  Adult Female Spermatheca Mated  5.745236  
  Adult Female Spermatheca Virgin  5.690222  
  Adult Head  5.710202  
  Adult Heart  5.381509  
  Adult Hind Gut  5.468410  
  Adult Male Ejaculatory Duct  5.188359  
  Adult Mid Gut  5.583842  
  Adult Ovary  6.029407  
  Adult Salivary Gland  5.909931  
  Adult Testes  8.466746  
  Adult Thoracoabdominal ganglion  5.370308  
  Adult Whole Fly  6.120226  
  Larvae Wandering Tubules  5.526032  
  Larval Feeding Carcass  5.316072  
  Larval Feeding Central Nevous System  5.932302  
  Larval Feeding Hind Gut  5.361202  
  Larval Feeding Malpighian Tubule  5.604379  
  Larval Feeding Mid Gut  5.313834  
  Larval Feeding Salivary Gland  5.511491  
  Whole Larvae Feeding  5.727166  
 
  
   FlyBase ID    symbol    start    end    strand    length   
   FBgn0051320   CG31320  10184078   10187461   -  3384  
   FBgn0038225   soti  10206105   10207421   -  1317  
   FBgn0085417   CG34388   10244315   10247587  +  3273  
   FBgn0038233   HtrA2  10335616   10337414   -  1799  
 
 
    Segment 218 
 
   Location   
  Gene key  FBgn0038234-FBgn0038235  
  Heatmap region span   3R:10136089..10388084   
  Segment span   3R:10337590..10339327   
  Length (genes)  2  
  Length (bp)  1738  
   Model Scoring   
  BIC  203.449174  
  logL  -96.096856  
  logL ratio  50.028327  
   Expression   
  Mean expression  7.891441  
  Median expression  7.974971  
  Tissue std. dev.  0.424772  
 
  No GO Slim enrichment  
  
   tissue    mean expression   
  5th Passage Drosophila S2 Cells  7.912069  
  Adult Accessory gland  7.972818  
  Adult Brain  8.338218  
  Adult Carcass  7.227650  
  Adult Crop  7.740803  
  Adult Eye  8.174062  
  Adult Fatbody  7.786941  
  Adult Female Spermatheca Mated  8.249229  
  Adult Female Spermatheca Virgin  8.216119  
  Adult Head  7.802584  
  Adult Heart  8.033235  
  Adult Hind Gut  7.793980  
  Adult Male Ejaculatory Duct  7.725206  
  Adult Mid Gut  7.599075  
  Adult Ovary  9.056681  
  Adult Salivary Gland  7.944669  
  Adult Testes  7.797833  
  Adult Thoracoabdominal ganglion  8.477113  
  Adult Whole Fly  7.801081  
  Larvae Wandering Tubules  7.534552  
  Larval Feeding Carcass  7.562911  
  Larval Feeding Central Nevous System  8.580782  
  Larval Feeding Hind Gut  7.830128  
  Larval Feeding Malpighian Tubule  7.910889  
  Larval Feeding Mid Gut  7.028077  
  Larval Feeding Salivary Gland  7.816002  
  Whole Larvae Feeding  7.156205  
 
  
   FlyBase ID    symbol    start    end    strand    length   
   FBgn0038234   mRpL11   10337590   10338415  +  826  
   FBgn0038235   CG8461  10338358   10339327   -  970  
 
    Segment 219 
 
   Location   
  Gene key  FBgn0038238-FBgn0038240  
  Heatmap region span   3R:10337590..10395363   
  Segment span   3R:10385776..10388084   
  Length (genes)  3  
  Length (bp)  2309  
   Model Scoring   
  BIC  287.199408  
  logL  -137.971973  
  logL ratio  27.699113  
   Expression   
  Mean expression  5.078965  
  Median expression  4.919944  
  Tissue std. dev.  0.617030  
 
  No GO Slim enrichment  
  
   tissue    mean expression   
  5th Passage Drosophila S2 Cells  4.822439  
  Adult Accessory gland  5.974533  
  Adult Brain  4.563946  
  Adult Carcass  5.112567  
  Adult Crop  5.182330  
  Adult Eye  4.883460  
  Adult Fatbody  5.073277  
  Adult Female Spermatheca Mated  5.074897  
  Adult Female Spermatheca Virgin  5.012811  
  Adult Head  4.838577  
  Adult Heart  5.036692  
  Adult Hind Gut  4.890326  
  Adult Male Ejaculatory Duct  6.126652  
  Adult Mid Gut  4.877390  
  Adult Ovary  4.828737  
  Adult Salivary Gland  5.094938  
  Adult Testes  4.541219  
  Adult Thoracoabdominal ganglion  4.406896  
  Adult Whole Fly  4.452479  
  Larvae Wandering Tubules  4.662839  
  Larval Feeding Carcass  5.224481  
  Larval Feeding Central Nevous System  4.675303  
  Larval Feeding Hind Gut  4.697241  
  Larval Feeding Malpighian Tubule  4.948663  
  Larval Feeding Mid Gut  5.025801  
  Larval Feeding Salivary Gland  7.500795  
  Whole Larvae Feeding  5.602753  
 
  
   FlyBase ID    symbol    start    end    strand    length   
   FBgn0038238   CG14854  10384886   10385776   -  891  
   FBgn0038239   CG14850   10386704   10387243  +  540  
   FBgn0038240   CG14851   10388084   10388542  +  459  
 
 
    Segment 220 
 
   Location   
  Gene key  FBgn0038241-FBgn0038242  
  Heatmap region span   3R:10342764..10402941   
  Segment span   3R:10390504..10391668   
  Length (genes)  2  
  Length (bp)  1165  
   Model Scoring   
  BIC  180.861118  
  logL  -84.802828  
  logL ratio  43.700960  
   Expression   
  Mean expression  5.348565  
  Median expression  4.768482  
  Tissue std. dev.  2.085356  
 
  No GO Slim enrichment  
  
   tissue    mean expression   
  5th Passage Drosophila S2 Cells  4.799336  
  Adult Accessory gland  4.814183  
  Adult Brain  4.407623  
  Adult Carcass  5.055930  
  Adult Crop  4.739159  
  Adult Eye  4.487935  
  Adult Fatbody  4.842251  
  Adult Female Spermatheca Mated  5.071817  
  Adult Female Spermatheca Virgin  4.974431  
  Adult Head  4.477079  
  Adult Heart  4.569501  
  Adult Hind Gut  4.670825  
  Adult Male Ejaculatory Duct  5.295805  
  Adult Mid Gut  4.849571  
  Adult Ovary  4.693330  
  Adult Salivary Gland  5.357267  
  Adult Testes  4.594245  
  Adult Thoracoabdominal ganglion  4.584745  
  Adult Whole Fly  4.225598  
  Larvae Wandering Tubules  4.799477  
  Larval Feeding Carcass  4.922119  
  Larval Feeding Central Nevous System  4.500984  
  Larval Feeding Hind Gut  4.642017  
  Larval Feeding Malpighian Tubule  5.043230  
  Larval Feeding Mid Gut  4.917193  
  Larval Feeding Salivary Gland  13.952181  
  Whole Larvae Feeding  11.123430  
 
  
   FlyBase ID    symbol    start    end    strand    length   
   FBgn0038241   CG8087  10390044   10390504   -  461  
   FBgn0038242   CG14852   10391668   10392192  +  525  
 
    Segment 221 
 
   Location   
  Gene key  FBgn0038244-FBgn0020299  
  Heatmap region span   3R:10390504..10483179   
  Segment span   3R:10401570..10402941   
  Length (genes)  2  
  Length (bp)  1372  
   Model Scoring   
  BIC  255.752079  
  logL  -122.248308  
  logL ratio  -0.415842  
   Expression   
  Mean expression  8.060539  
  Median expression  8.234642  
  Tissue std. dev.  0.977849  
 
  
   GO ID    description    ratio    P-value   
   GO:0005622   intracellular  2/2  0.00423  
 
  
   tissue    mean expression   
  5th Passage Drosophila S2 Cells  6.192128  
  Adult Accessory gland  8.216090  
  Adult Brain  9.759462  
  Adult Carcass  8.389890  
  Adult Crop  8.963318  
  Adult Eye  8.588806  
  Adult Fatbody  7.895043  
  Adult Female Spermatheca Mated  7.726171  
  Adult Female Spermatheca Virgin  7.751084  
  Adult Head  8.844123  
  Adult Heart  8.552037  
  Adult Hind Gut  8.335339  
  Adult Male Ejaculatory Duct  7.046240  
  Adult Mid Gut  7.742127  
  Adult Ovary  7.662384  
  Adult Salivary Gland  9.886149  
  Adult Testes  7.479256  
  Adult Thoracoabdominal ganglion  9.996224  
  Adult Whole Fly  7.747684  
  Larvae Wandering Tubules  6.331239  
  Larval Feeding Carcass  8.399472  
  Larval Feeding Central Nevous System  9.076387  
  Larval Feeding Hind Gut  8.278861  
  Larval Feeding Malpighian Tubule  6.468924  
  Larval Feeding Mid Gut  7.644197  
  Larval Feeding Salivary Gland  6.952369  
  Whole Larvae Feeding  7.709552  
 
  
   FlyBase ID    symbol    start    end    strand    length   
   FBgn0038244   CG7987  10396194   10401570   -  5377  
   FBgn0020299   stumps   10402941   10433804  +  30864  
 
    Segment 222 
 
   Location   
  Gene key  FBgn0003169-FBgn0013981  
  Heatmap region span   3R:10394532..10523057   
  Segment span   3R:10451421..10451461   
  Length (genes)  2  
  Length (bp)  41  
   Model Scoring   
  BIC  327.019415  
  logL  -157.881976  
  logL ratio  14.127269  
   Expression   
  Mean expression  10.926119  
  Median expression  10.381396  
  Tissue std. dev.  0.534016  
 
  No GO Slim enrichment  
  
   tissue    mean expression   
  5th Passage Drosophila S2 Cells  11.370280  
  Adult Accessory gland  11.106107  
  Adult Brain  10.777859  
  Adult Carcass  10.649805  
  Adult Crop  11.156535  
  Adult Eye  10.938158  
  Adult Fatbody  11.105839  
  Adult Female Spermatheca Mated  10.135160  
  Adult Female Spermatheca Virgin  10.258794  
  Adult Head  10.956575  
  Adult Heart  10.773855  
  Adult Hind Gut  11.041066  
  Adult Male Ejaculatory Duct  11.202699  
  Adult Mid Gut  10.716763  
  Adult Ovary  11.811338  
  Adult Salivary Gland  11.348436  
  Adult Testes  9.002232  
  Adult Thoracoabdominal ganglion  10.507742  
  Adult Whole Fly  10.959962  
  Larvae Wandering Tubules  11.455538  
  Larval Feeding Carcass  11.081492  
  Larval Feeding Central Nevous System  11.299996  
  Larval Feeding Hind Gut  11.276604  
  Larval Feeding Malpighian Tubule  11.644499  
  Larval Feeding Mid Gut  10.685685  
  Larval Feeding Salivary Gland  11.059595  
  Whole Larvae Feeding  10.682602  
 
  
   FlyBase ID    symbol    start    end    strand    length   
   FBgn0003169   put  10446052   10451421   -  5370  
   FBgn0013981   His4r   10451461   10452336  +  876  
 
    Segment 223 
 
   Location   
  Gene key  FBgn0038247-FBgn0038248  
  Heatmap region span   3R:10395363..10566933   
  Segment span   3R:10460475..10468285   
  Length (genes)  2  
  Length (bp)  7811  
   Model Scoring   
  BIC  216.749347  
  logL  -102.746943  
  logL ratio  -1.648337  
   Expression   
  Mean expression  4.702980  
  Median expression  4.454964  
  Tissue std. dev.  0.829538  
 
  No GO Slim enrichment  
  
   tissue    mean expression   
  5th Passage Drosophila S2 Cells  4.497162  
  Adult Accessory gland  4.641070  
  Adult Brain  4.200584  
  Adult Carcass  4.656324  
  Adult Crop  4.327156  
  Adult Eye  4.359082  
  Adult Fatbody  4.361574  
  Adult Female Spermatheca Mated  4.383128  
  Adult Female Spermatheca Virgin  4.419303  
  Adult Head  4.271811  
  Adult Heart  4.261125  
  Adult Hind Gut  4.454125  
  Adult Male Ejaculatory Duct  4.617301  
  Adult Mid Gut  4.695939  
  Adult Ovary  4.831101  
  Adult Salivary Gland  4.605873  
  Adult Testes  8.434644  
  Adult Thoracoabdominal ganglion  4.342866  
  Adult Whole Fly  6.270278  
  Larvae Wandering Tubules  4.641108  
  Larval Feeding Carcass  4.567308  
  Larval Feeding Central Nevous System  4.202385  
  Larval Feeding Hind Gut  4.378345  
  Larval Feeding Malpighian Tubule  4.500891  
  Larval Feeding Mid Gut  4.444132  
  Larval Feeding Salivary Gland  4.473114  
  Whole Larvae Feeding  5.142725  
 
  
   FlyBase ID    symbol    start    end    strand    length   
   FBgn0038247   Cad88C   10460475   10467334  +  6860  
   FBgn0038248   CG7886  10452395   10468285   -  15891  
 
    Segment 224 
 
   Location   
  Gene key  FBgn0025808-FBgn0051314  
  Heatmap region span   3R:10451421..10705163   
  Segment span   3R:10488914..10523057   
  Length (genes)  3  
  Length (bp)  34144  
   Model Scoring   
  BIC  328.867485  
  logL  -158.806011  
  logL ratio  4.836441  
   Expression   
  Mean expression  5.876456  
  Median expression  5.663353  
  Tissue std. dev.  0.824040  
 
  No GO Slim enrichment  
  
   tissue    mean expression   
  5th Passage Drosophila S2 Cells  7.757510  
  Adult Accessory gland  5.720105  
  Adult Brain  5.706486  
  Adult Carcass  5.570328  
  Adult Crop  5.436073  
  Adult Eye  5.266361  
  Adult Fatbody  5.329422  
  Adult Female Spermatheca Mated  5.397302  
  Adult Female Spermatheca Virgin  5.482508  
  Adult Head  5.301340  
  Adult Heart  5.530661  
  Adult Hind Gut  5.281810  
  Adult Male Ejaculatory Duct  5.317629  
  Adult Mid Gut  5.429627  
  Adult Ovary  8.634554  
  Adult Salivary Gland  5.897303  
  Adult Testes  7.012084  
  Adult Thoracoabdominal ganglion  5.612555  
  Adult Whole Fly  7.437617  
  Larvae Wandering Tubules  5.816467  
  Larval Feeding Carcass  5.709889  
  Larval Feeding Central Nevous System  5.920334  
  Larval Feeding Hind Gut  5.388237  
  Larval Feeding Malpighian Tubule  5.684969  
  Larval Feeding Mid Gut  5.423797  
  Larval Feeding Salivary Gland  6.076812  
  Whole Larvae Feeding  5.522522  
 
  
   FlyBase ID    symbol    start    end    strand    length   
   FBgn0025808   Rad17  10486898   10488914   -  2017  
   FBgn0038252   CG3509   10489165   10490544  +  1380  
   FBgn0051314     10491559   10523057   -  31499  
 
 
    Segment 225 
 
   Location   
  Gene key  FBgn0038256-FBgn0011217  
  Heatmap region span   3R:10460475..10712202   
  Segment span   3R:10557231..10566933   
  Length (genes)  2  
  Length (bp)  9703  
   Model Scoring   
  BIC  335.701654  
  logL  -162.223096  
  logL ratio  10.873494  
   Expression   
  Mean expression  10.968324  
  Median expression  11.597316  
  Tissue std. dev.  0.799532  
 
  No GO Slim enrichment  
  
   tissue    mean expression   
  5th Passage Drosophila S2 Cells  11.376750  
  Adult Accessory gland  9.692284  
  Adult Brain  11.194364  
  Adult Carcass  11.658176  
  Adult Crop  12.498735  
  Adult Eye  11.243305  
  Adult Fatbody  11.793171  
  Adult Female Spermatheca Mated  11.472848  
  Adult Female Spermatheca Virgin  11.445958  
  Adult Head  11.296743  
  Adult Heart  11.874348  
  Adult Hind Gut  11.231386  
  Adult Male Ejaculatory Duct  11.453877  
  Adult Mid Gut  10.038361  
  Adult Ovary  12.035326  
  Adult Salivary Gland  10.109441  
  Adult Testes  10.608303  
  Adult Thoracoabdominal ganglion  11.173137  
  Adult Whole Fly  11.453387  
  Larvae Wandering Tubules  10.132517  
  Larval Feeding Carcass  10.658120  
  Larval Feeding Central Nevous System  11.143327  
  Larval Feeding Hind Gut  10.763975  
  Larval Feeding Malpighian Tubule  10.360115  
  Larval Feeding Mid Gut  8.809014  
  Larval Feeding Salivary Gland  10.644809  
  Whole Larvae Feeding  9.982964  
 
  
   FlyBase ID    symbol    start    end    strand    length   
   FBgn0038256   CG7530  10550472   10557231   -  6760  
   FBgn0011217   eff  10558412   10566933   -  8522  
 
    Segment 226 
 
   Location   
  Gene key  FBgn0038260-FBgn0038268  
  Heatmap region span   3R:10488914..10720506   
  Segment span   3R:10622029..10705163   
  Length (genes)  7  
  Length (bp)  83135  
   Model Scoring   
  BIC  651.981051  
  logL  -320.362794  
  logL ratio  95.001643  
   Expression   
  Mean expression  5.700338  
  Median expression  5.297479  
  Tissue std. dev.  0.401143  
 
  
   GO ID    description    ratio    P-value   
   GO:0055085   transmembrane transport  3/7  0.000203  
   GO:0006810   transport  3/7  0.00212  
 
  
   tissue    mean expression   
  5th Passage Drosophila S2 Cells  5.485692  
  Adult Accessory gland  5.491294  
  Adult Brain  5.591615  
  Adult Carcass  5.602752  
  Adult Crop  5.436629  
  Adult Eye  5.399289  
  Adult Fatbody  5.434415  
  Adult Female Spermatheca Mated  5.483949  
  Adult Female Spermatheca Virgin  5.404925  
  Adult Head  5.545292  
  Adult Heart  5.839757  
  Adult Hind Gut  5.677227  
  Adult Male Ejaculatory Duct  5.419984  
  Adult Mid Gut  5.775308  
  Adult Ovary  5.598285  
  Adult Salivary Gland  5.919572  
  Adult Testes  5.595654  
  Adult Thoracoabdominal ganglion  5.523764  
  Adult Whole Fly  5.263758  
  Larvae Wandering Tubules  6.945702  
  Larval Feeding Carcass  5.971002  
  Larval Feeding Central Nevous System  5.674462  
  Larval Feeding Hind Gut  5.778192  
  Larval Feeding Malpighian Tubule  7.015719  
  Larval Feeding Mid Gut  5.770055  
  Larval Feeding Salivary Gland  5.824701  
  Whole Larvae Feeding  5.440140  
 
  
   FlyBase ID    symbol    start    end    strand    length   
   FBgn0038260   CG14855   10622029   10624193  +  2165  
   FBgn0038261   CG14856   10624712   10626887  +  2176  
   FBgn0038262   CG14857   10627512   10630067  +  2556  
   FBgn0038266   CG3610   10670765   10672459  +  1695  
   FBgn0053555     10629811   10679626   -  49816  
   FBgn0038267     10685027   10685560   -  534  
   FBgn0038268   CG3631   10705163   10708348  +  3186  
 
 
    Segment 227 
 
   Location   
  Gene key  FBgn0038269-FBgn0067629  
  Heatmap region span   3R:10557231..10727310   
  Segment span   3R:10711918..10712202   
  Length (genes)  3  
  Length (bp)  285  
   Model Scoring   
  BIC  313.704987  
  logL  -151.224763  
  logL ratio  56.588894  
   Expression   
  Mean expression  7.380238  
  Median expression  7.447754  
  Tissue std. dev.  0.459522  
 
  No GO Slim enrichment  
  
   tissue    mean expression   
  5th Passage Drosophila S2 Cells  7.636864  
  Adult Accessory gland  7.530355  
  Adult Brain  7.386079  
  Adult Carcass  6.743719  
  Adult Crop  7.332686  
  Adult Eye  7.237735  
  Adult Fatbody  7.317768  
  Adult Female Spermatheca Mated  7.297017  
  Adult Female Spermatheca Virgin  7.380825  
  Adult Head  6.934647  
  Adult Heart  7.545972  
  Adult Hind Gut  7.060953  
  Adult Male Ejaculatory Duct  7.202895  
  Adult Mid Gut  7.126809  
  Adult Ovary  9.180759  
  Adult Salivary Gland  6.964219  
  Adult Testes  7.294291  
  Adult Thoracoabdominal ganglion  7.460748  
  Adult Whole Fly  7.540496  
  Larvae Wandering Tubules  7.487716  
  Larval Feeding Carcass  7.120368  
  Larval Feeding Central Nevous System  8.021497  
  Larval Feeding Hind Gut  7.369053  
  Larval Feeding Malpighian Tubule  7.608256  
  Larval Feeding Mid Gut  6.943352  
  Larval Feeding Salivary Gland  7.789772  
  Whole Larvae Feeding  6.751571  
 
  
   FlyBase ID    symbol    start    end    strand    length   
   FBgn0038269   Rrp6  10708105   10711918   -  3814  
   FBgn0067628   CG33331   10712198   10713461  +  1264  
   FBgn0067629   CG33332   10712202   10714868  +  2667  
 
 
    Segment 228 
 
   Location   
  Gene key  FBgn0004597-FBgn0038272  
  Heatmap region span   3R:10619489..10905007   
  Segment span   3R:10715915..10719675   
  Length (genes)  2  
  Length (bp)  3761  
   Model Scoring   
  BIC  213.279635  
  logL  -101.012086  
  logL ratio  45.848932  
   Expression   
  Mean expression  8.797147  
  Median expression  8.688379  
  Tissue std. dev.  0.447616  
 
  No GO Slim enrichment  
  
   tissue    mean expression   
  5th Passage Drosophila S2 Cells  9.217790  
  Adult Accessory gland  9.093981  
  Adult Brain  8.483092  
  Adult Carcass  8.116107  
  Adult Crop  9.012005  
  Adult Eye  8.788058  
  Adult Fatbody  8.364850  
  Adult Female Spermatheca Mated  8.284749  
  Adult Female Spermatheca Virgin  8.446851  
  Adult Head  8.168504  
  Adult Heart  8.721822  
  Adult Hind Gut  8.976647  
  Adult Male Ejaculatory Duct  8.950477  
  Adult Mid Gut  8.960711  
  Adult Ovary  8.995791  
  Adult Salivary Gland  9.312871  
  Adult Testes  7.819221  
  Adult Thoracoabdominal ganglion  8.395686  
  Adult Whole Fly  8.335289  
  Larvae Wandering Tubules  9.747992  
  Larval Feeding Carcass  8.745696  
  Larval Feeding Central Nevous System  9.326040  
  Larval Feeding Hind Gut  9.021625  
  Larval Feeding Malpighian Tubule  9.459200  
  Larval Feeding Mid Gut  8.992663  
  Larval Feeding Salivary Gland  9.129900  
  Whole Larvae Feeding  8.655362  
 
  
   FlyBase ID    symbol    start    end    strand    length   
   FBgn0004597   CycC  10714726   10715915   -  1190  
   FBgn0038272   CG7265  10718114   10719675   -  1562  
 
    Segment 229 
 
   Location   
  Gene key  FBgn0038274-FBgn0038277  
  Heatmap region span   3R:10711918..10962884   
  Segment span   3R:10724090..10727310   
  Length (genes)  3  
  Length (bp)  3221  
   Model Scoring   
  BIC  330.833954  
  logL  -159.789246  
  logL ratio  50.147323  
   Expression   
  Mean expression  7.184765  
  Median expression  6.947722  
  Tissue std. dev.  0.850527  
 
  No GO Slim enrichment  
  
   tissue    mean expression   
  5th Passage Drosophila S2 Cells  7.986469  
  Adult Accessory gland  7.133655  
  Adult Brain  6.548838  
  Adult Carcass  7.036552  
  Adult Crop  6.951763  
  Adult Eye  6.518291  
  Adult Fatbody  7.240124  
  Adult Female Spermatheca Mated  6.893544  
  Adult Female Spermatheca Virgin  7.035990  
  Adult Head  6.587282  
  Adult Heart  6.718977  
  Adult Hind Gut  6.703780  
  Adult Male Ejaculatory Duct  6.762945  
  Adult Mid Gut  6.647956  
  Adult Ovary  10.228822  
  Adult Salivary Gland  6.758933  
  Adult Testes  8.235706  
  Adult Thoracoabdominal ganglion  6.523951  
  Adult Whole Fly  9.181429  
  Larvae Wandering Tubules  6.617691  
  Larval Feeding Carcass  6.943951  
  Larval Feeding Central Nevous System  8.042926  
  Larval Feeding Hind Gut  6.906774  
  Larval Feeding Malpighian Tubule  6.742083  
  Larval Feeding Mid Gut  6.752823  
  Larval Feeding Salivary Gland  7.256559  
  Whole Larvae Feeding  7.030857  
 
  
   FlyBase ID    symbol    start    end    strand    length   
   FBgn0038274   CG7262  10721425   10724090   -  2666  
   FBgn0038275   CG3817   10724435   10725339  +  905  
   FBgn0038277   RpS5b  10726154   10727310   -  1157  
 
 
    Segment 230 
 
   Location   
  Gene key  FBgn0038280-FBgn0038285  
  Heatmap region span   3R:10715915..11006143   
  Segment span   3R:10840492..10905007   
  Length (genes)  4  
  Length (bp)  64516  
   Model Scoring   
  BIC  355.878613  
  logL  -172.311575  
  logL ratio  61.357851  
   Expression   
  Mean expression  4.807733  
  Median expression  4.520489  
  Tissue std. dev.  0.804269  
 
  No GO Slim enrichment  
  
   tissue    mean expression   
  5th Passage Drosophila S2 Cells  4.568600  
  Adult Accessory gland  4.576303  
  Adult Brain  5.064387  
  Adult Carcass  4.653862  
  Adult Crop  4.522472  
  Adult Eye  4.675892  
  Adult Fatbody  4.610187  
  Adult Female Spermatheca Mated  4.638974  
  Adult Female Spermatheca Virgin  4.566233  
  Adult Head  4.721670  
  Adult Heart  4.331145  
  Adult Hind Gut  4.374334  
  Adult Male Ejaculatory Duct  4.485199  
  Adult Mid Gut  4.545293  
  Adult Ovary  4.411894  
  Adult Salivary Gland  4.897984  
  Adult Testes  8.630437  
  Adult Thoracoabdominal ganglion  5.029300  
  Adult Whole Fly  5.787876  
  Larvae Wandering Tubules  4.563109  
  Larval Feeding Carcass  4.438313  
  Larval Feeding Central Nevous System  4.827857  
  Larval Feeding Hind Gut  4.318508  
  Larval Feeding Malpighian Tubule  4.545160  
  Larval Feeding Mid Gut  4.565220  
  Larval Feeding Salivary Gland  4.528093  
  Whole Larvae Feeding  4.930495  
 
  
   FlyBase ID    symbol    start    end    strand    length   
   FBgn0038280   CG14861   10840492   10841759  +  1268  
   FBgn0038281   RpL10Aa   10863088   10863872  +  785  
   FBgn0038282   dpr9  10882195   10902927   -  20733  
   FBgn0038285   CG6974  10904252   10905007   -  756  
 
 
    Segment 231 
 
   Location   
  Gene key  FBgn0038290-FBgn0038291  
  Heatmap region span   3R:10724090..11034484   
  Segment span   3R:10962702..10962884   
  Length (genes)  2  
  Length (bp)  183  
   Model Scoring   
  BIC  245.695233  
  logL  -117.219886  
  logL ratio  17.128482  
   Expression   
  Mean expression  7.871082  
  Median expression  7.120514  
  Tissue std. dev.  1.687278  
 
  No GO Slim enrichment  
  
   tissue    mean expression   
  5th Passage Drosophila S2 Cells  11.951203  
  Adult Accessory gland  6.698958  
  Adult Brain  8.404860  
  Adult Carcass  10.141233  
  Adult Crop  7.336388  
  Adult Eye  6.143512  
  Adult Fatbody  10.923343  
  Adult Female Spermatheca Mated  10.344171  
  Adult Female Spermatheca Virgin  10.132316  
  Adult Head  8.640504  
  Adult Heart  9.357817  
  Adult Hind Gut  7.616264  
  Adult Male Ejaculatory Duct  7.058931  
  Adult Mid Gut  6.857897  
  Adult Ovary  6.198815  
  Adult Salivary Gland  6.722476  
  Adult Testes  5.616922  
  Adult Thoracoabdominal ganglion  8.593682  
  Adult Whole Fly  7.071433  
  Larvae Wandering Tubules  6.006663  
  Larval Feeding Carcass  6.961118  
  Larval Feeding Central Nevous System  6.226781  
  Larval Feeding Hind Gut  7.694716  
  Larval Feeding Malpighian Tubule  6.336844  
  Larval Feeding Mid Gut  6.435948  
  Larval Feeding Salivary Gland  7.433336  
  Whole Larvae Feeding  9.613083  
 
  
   FlyBase ID    symbol    start    end    strand    length   
   FBgn0038290   CG6912  10960860   10962702   -  1843  
   FBgn0038291   CG3984   10962884   10964626  +  1743  
 
    Segment 232 
 
   Location   
  Gene key  FBgn0038293-FBgn0038294  
  Heatmap region span   3R:10840492..11039696   
  Segment span   3R:10973329..11006143   
  Length (genes)  2  
  Length (bp)  32815  
   Model Scoring   
  BIC  359.252183  
  logL  -173.998361  
  logL ratio  1.062453  
   Expression   
  Mean expression  10.890125  
  Median expression  11.035506  
  Tissue std. dev.  1.544073  
 
  No GO Slim enrichment  
  
   tissue    mean expression   
  5th Passage Drosophila S2 Cells  8.328004  
  Adult Accessory gland  10.606662  
  Adult Brain  9.694809  
  Adult Carcass  13.354205  
  Adult Crop  13.086783  
  Adult Eye  10.347894  
  Adult Fatbody  11.151355  
  Adult Female Spermatheca Mated  11.072909  
  Adult Female Spermatheca Virgin  11.022582  
  Adult Head  12.305573  
  Adult Heart  12.433189  
  Adult Hind Gut  12.272917  
  Adult Male Ejaculatory Duct  10.995103  
  Adult Mid Gut  10.736543  
  Adult Ovary  10.325040  
  Adult Salivary Gland  9.136479  
  Adult Testes  8.128871  
  Adult Thoracoabdominal ganglion  10.488865  
  Adult Whole Fly  12.835169  
  Larvae Wandering Tubules  9.429395  
  Larval Feeding Carcass  13.479475  
  Larval Feeding Central Nevous System  9.548824  
  Larval Feeding Hind Gut  12.088597  
  Larval Feeding Malpighian Tubule  9.445697  
  Larval Feeding Mid Gut  10.696240  
  Larval Feeding Salivary Gland  8.365312  
  Whole Larvae Feeding  12.656884  
 
  
   FlyBase ID    symbol    start    end    strand    length   
   FBgn0038293   CG6904  10967562   10973329   -  5768  
   FBgn0038294   Mf  10996347   11006143   -  9797  
 
    Segment 233 
 
   Location   
  Gene key  FBgn0038295-FBgn0038296  
  Heatmap region span   3R:10921333..11051289   
  Segment span   3R:11009908..11021932   
  Length (genes)  2  
  Length (bp)  12025  
   Model Scoring   
  BIC  180.303882  
  logL  -84.524210  
  logL ratio  38.995348  
   Expression   
  Mean expression  4.429167  
  Median expression  4.494429  
  Tissue std. dev.  0.430403  
 
  No GO Slim enrichment  
  
   tissue    mean expression   
  5th Passage Drosophila S2 Cells  4.342002  
  Adult Accessory gland  4.208722  
  Adult Brain  5.719010  
  Adult Carcass  4.237830  
  Adult Crop  4.368672  
  Adult Eye  4.459195  
  Adult Fatbody  4.306839  
  Adult Female Spermatheca Mated  4.218387  
  Adult Female Spermatheca Virgin  4.264020  
  Adult Head  4.682396  
  Adult Heart  4.274622  
  Adult Hind Gut  4.201242  
  Adult Male Ejaculatory Duct  4.134932  
  Adult Mid Gut  4.290384  
  Adult Ovary  4.300769  
  Adult Salivary Gland  4.415565  
  Adult Testes  4.329381  
  Adult Thoracoabdominal ganglion  5.717024  
  Adult Whole Fly  4.124790  
  Larvae Wandering Tubules  4.326291  
  Larval Feeding Carcass  4.389007  
  Larval Feeding Central Nevous System  5.321617  
  Larval Feeding Hind Gut  4.085304  
  Larval Feeding Malpighian Tubule  4.199822  
  Larval Feeding Mid Gut  4.301722  
  Larval Feeding Salivary Gland  4.268584  
  Whole Larvae Feeding  4.099374  
 
  
   FlyBase ID    symbol    start    end    strand    length   
   FBgn0038295   Gyc88E   11009908   11016964  +  7057  
   FBgn0038296   CG6752  11016272   11021932   -  5661  
 
    Segment 234 
 
   Location   
  Gene key  FBgn0028984-FBgn0038299  
  Heatmap region span   3R:10962702..11067958   
  Segment span   3R:11032168..11034484   
  Length (genes)  2  
  Length (bp)  2317  
   Model Scoring   
  BIC  288.421351  
  logL  -138.582944  
  logL ratio  -8.677146  
   Expression   
  Mean expression  8.757282  
  Median expression  8.691492  
  Tissue std. dev.  1.644394  
 
  No GO Slim enrichment  
  
   tissue    mean expression   
  5th Passage Drosophila S2 Cells  10.131741  
  Adult Accessory gland  7.564436  
  Adult Brain  8.036204  
  Adult Carcass  10.413464  
  Adult Crop  8.321058  
  Adult Eye  9.768076  
  Adult Fatbody  10.248967  
  Adult Female Spermatheca Mated  11.768860  
  Adult Female Spermatheca Virgin  11.758265  
  Adult Head  10.474680  
  Adult Heart  10.699921  
  Adult Hind Gut  9.005439  
  Adult Male Ejaculatory Duct  10.489396  
  Adult Mid Gut  6.261129  
  Adult Ovary  7.880779  
  Adult Salivary Gland  7.016841  
  Adult Testes  6.618369  
  Adult Thoracoabdominal ganglion  8.373695  
  Adult Whole Fly  9.195184  
  Larvae Wandering Tubules  6.360128  
  Larval Feeding Carcass  8.678119  
  Larval Feeding Central Nevous System  7.414075  
  Larval Feeding Hind Gut  9.283034  
  Larval Feeding Malpighian Tubule  6.291621  
  Larval Feeding Mid Gut  6.581548  
  Larval Feeding Salivary Gland  8.038739  
  Whole Larvae Feeding  9.772841  
 
  
   FlyBase ID    symbol    start    end    strand    length   
   FBgn0028984   Spn88Ea  11028791   11032168   -  3378  
   FBgn0038299   Spn88Eb  11032418   11034484   -  2067  
 
    Segment 235 
 
   Location   
  Gene key  FBgn0038300-FBgn0038301  
  Heatmap region span   3R:10965707..11074859   
  Segment span   3R:11034928..11039322   
  Length (genes)  2  
  Length (bp)  4395  
   Model Scoring   
  BIC  207.542345  
  logL  -98.143441  
  logL ratio  45.864378  
   Expression   
  Mean expression  7.344926  
  Median expression  7.228660  
  Tissue std. dev.  0.629807  
 
  No GO Slim enrichment  
  
   tissue    mean expression   
  5th Passage Drosophila S2 Cells  8.052865  
  Adult Accessory gland  7.142755  
  Adult Brain  8.604223  
  Adult Carcass  7.021159  
  Adult Crop  7.397130  
  Adult Eye  7.209740  
  Adult Fatbody  7.137296  
  Adult Female Spermatheca Mated  7.148663  
  Adult Female Spermatheca Virgin  7.116863  
  Adult Head  7.203259  
  Adult Heart  7.430784  
  Adult Hind Gut  6.977820  
  Adult Male Ejaculatory Duct  7.323499  
  Adult Mid Gut  6.776508  
  Adult Ovary  8.819711  
  Adult Salivary Gland  7.234058  
  Adult Testes  5.581160  
  Adult Thoracoabdominal ganglion  8.269719  
  Adult Whole Fly  7.395546  
  Larvae Wandering Tubules  7.388763  
  Larval Feeding Carcass  7.110305  
  Larval Feeding Central Nevous System  8.346497  
  Larval Feeding Hind Gut  7.232551  
  Larval Feeding Malpighian Tubule  7.227142  
  Larval Feeding Mid Gut  6.980573  
  Larval Feeding Salivary Gland  7.500599  
  Whole Larvae Feeding  6.683824  
 
  
   FlyBase ID    symbol    start    end    strand    length   
   FBgn0038300   CG4203   11034928   11037071  +  2144  
   FBgn0038301   CG6654  11036999   11039322   -  2324  
 
    Segment 236 
 
   Location   
  Gene key  FBgn0038303-FBgn0015610  
  Heatmap region span   3R:11009908..11089257   
  Segment span   3R:11044596..11051289   
  Length (genes)  5  
  Length (bp)  6694  
   Model Scoring   
  BIC  498.022297  
  logL  -243.383417  
  logL ratio  155.329379  
   Expression   
  Mean expression  8.997028  
  Median expression  8.884328  
  Tissue std. dev.  0.468029  
 
  No GO Slim enrichment  
  
   tissue    mean expression   
  5th Passage Drosophila S2 Cells  9.764626  
  Adult Accessory gland  8.996619  
  Adult Brain  9.474094  
  Adult Carcass  8.252408  
  Adult Crop  8.705109  
  Adult Eye  9.445490  
  Adult Fatbody  8.315337  
  Adult Female Spermatheca Mated  8.696927  
  Adult Female Spermatheca Virgin  8.595023  
  Adult Head  8.980280  
  Adult Heart  8.711942  
  Adult Hind Gut  8.647453  
  Adult Male Ejaculatory Duct  8.531671  
  Adult Mid Gut  8.551648  
  Adult Ovary  10.086676  
  Adult Salivary Gland  9.007508  
  Adult Testes  8.551504  
  Adult Thoracoabdominal ganglion  9.474367  
  Adult Whole Fly  9.107648  
  Larvae Wandering Tubules  9.108711  
  Larval Feeding Carcass  8.981357  
  Larval Feeding Central Nevous System  9.761555  
  Larval Feeding Hind Gut  9.229223  
  Larval Feeding Malpighian Tubule  9.063684  
  Larval Feeding Mid Gut  8.657562  
  Larval Feeding Salivary Gland  9.610196  
  Whole Larvae Feeding  8.611129  
 
  
   FlyBase ID    symbol    start    end    strand    length   
   FBgn0038303   SIDL  11040471   11044596   -  4126  
   FBgn0038304   CG12241   11044984   11048093  +  3110  
   FBgn0051155   Rpb7  11047971   11048849   -  879  
   FBgn0051344   CG31344  11049091   11051144   -  2054  
   FBgn0015610      11051289   11053286  +  1998  
 
 
    Segment 237 
 
   Location   
  Gene key  FBgn0038306-FBgn0045035  
  Heatmap region span   3R:11032168..11091298   
  Segment span   3R:11055161..11067958   
  Length (genes)  2  
  Length (bp)  12798  
   Model Scoring   
  BIC  214.880522  
  logL  -101.812530  
  logL ratio  36.373688  
   Expression   
  Mean expression  7.261589  
  Median expression  6.975886  
  Tissue std. dev.  0.825197  
 
  No GO Slim enrichment  
  
   tissue    mean expression   
  5th Passage Drosophila S2 Cells  8.925185  
  Adult Accessory gland  9.055694  
  Adult Brain  6.725332  
  Adult Carcass  6.401664  
  Adult Crop  7.544685  
  Adult Eye  6.775100  
  Adult Fatbody  7.035363  
  Adult Female Spermatheca Mated  7.053204  
  Adult Female Spermatheca Virgin  6.688299  
  Adult Head  6.239319  
  Adult Heart  7.117968  
  Adult Hind Gut  6.879596  
  Adult Male Ejaculatory Duct  7.729738  
  Adult Mid Gut  6.369233  
  Adult Ovary  8.609107  
  Adult Salivary Gland  7.390453  
  Adult Testes  8.110791  
  Adult Thoracoabdominal ganglion  6.830471  
  Adult Whole Fly  7.117351  
  Larvae Wandering Tubules  6.267447  
  Larval Feeding Carcass  7.189787  
  Larval Feeding Central Nevous System  8.618424  
  Larval Feeding Hind Gut  7.447658  
  Larval Feeding Malpighian Tubule  6.655858  
  Larval Feeding Mid Gut  6.217719  
  Larval Feeding Salivary Gland  8.298944  
  Whole Larvae Feeding  6.768505  
 
  
   FlyBase ID    symbol    start    end    strand    length   
   FBgn0038306   Art3  11053327   11055161   -  1835  
   FBgn0045035   tefu  11057934   11067958   -  10025  
 
    Segment 238 
 
   Location   
  Gene key  FBgn0038312-FBgn0027378  
  Heatmap region span   3R:11077403..11168978   
  Segment span   3R:11094405..11103831   
  Length (genes)  4  
  Length (bp)  9427  
   Model Scoring   
  BIC  446.391789  
  logL  -217.568163  
  logL ratio  76.824235  
   Expression   
  Mean expression  8.845149  
  Median expression  8.719811  
  Tissue std. dev.  0.532326  
 
  No GO Slim enrichment  
  
   tissue    mean expression   
  5th Passage Drosophila S2 Cells  9.412955  
  Adult Accessory gland  8.914544  
  Adult Brain  8.749617  
  Adult Carcass  8.515876  
  Adult Crop  8.705724  
  Adult Eye  8.589565  
  Adult Fatbody  8.799730  
  Adult Female Spermatheca Mated  8.889949  
  Adult Female Spermatheca Virgin  8.830984  
  Adult Head  8.510205  
  Adult Heart  8.907750  
  Adult Hind Gut  8.608559  
  Adult Male Ejaculatory Duct  8.637335  
  Adult Mid Gut  8.653229  
  Adult Ovary  10.001371  
  Adult Salivary Gland  8.589342  
  Adult Testes  7.555689  
  Adult Thoracoabdominal ganglion  8.527174  
  Adult Whole Fly  8.879643  
  Larvae Wandering Tubules  9.372166  
  Larval Feeding Carcass  8.512450  
  Larval Feeding Central Nevous System  9.219826  
  Larval Feeding Hind Gut  8.608602  
  Larval Feeding Malpighian Tubule  9.480043  
  Larval Feeding Mid Gut  8.386184  
  Larval Feeding Salivary Gland  10.400203  
  Whole Larvae Feeding  8.560313  
 
  
   FlyBase ID    symbol    start    end    strand    length   
   FBgn0038312   CG4334   11094405   11096254  +  1850  
   FBgn0000283   Cp190  11096146   11100927   -  4782  
   FBgn0038313   CG4338   11101258   11102196  +  939  
   FBgn0027378   MRG15  11102162   11103831   -  1670  
 
 
    Segment 239 
 
   Location   
  Gene key  FBgn0038316-FBgn0038321  
  Heatmap region span   3R:11094405..11182967   
  Segment span   3R:11145768..11168978   
  Length (genes)  6  
  Length (bp)  23211  
   Model Scoring   
  BIC  652.217363  
  logL  -320.480950  
  logL ratio  95.183375  
   Expression   
  Mean expression  8.170036  
  Median expression  8.378596  
  Tissue std. dev.  0.439793  
 
  No GO Slim enrichment  
  
   tissue    mean expression   
  5th Passage Drosophila S2 Cells  8.100775  
  Adult Accessory gland  7.834427  
  Adult Brain  8.768516  
  Adult Carcass  7.612252  
  Adult Crop  8.315811  
  Adult Eye  8.362910  
  Adult Fatbody  7.508436  
  Adult Female Spermatheca Mated  7.817743  
  Adult Female Spermatheca Virgin  7.778647  
  Adult Head  8.402036  
  Adult Heart  7.775833  
  Adult Hind Gut  8.186406  
  Adult Male Ejaculatory Duct  8.108155  
  Adult Mid Gut  7.862187  
  Adult Ovary  9.113578  
  Adult Salivary Gland  7.989853  
  Adult Testes  7.134708  
  Adult Thoracoabdominal ganglion  8.908990  
  Adult Whole Fly  7.973026  
  Larvae Wandering Tubules  8.463353  
  Larval Feeding Carcass  8.318646  
  Larval Feeding Central Nevous System  8.769648  
  Larval Feeding Hind Gut  8.648408  
  Larval Feeding Malpighian Tubule  8.258465  
  Larval Feeding Mid Gut  8.371876  
  Larval Feeding Salivary Gland  8.204404  
  Whole Larvae Feeding  8.001893  
 
  
   FlyBase ID    symbol    start    end    strand    length   
   FBgn0038316   CG6276  11143924   11145768   -  1845  
   FBgn0026441   ear   11146484   11150197  +  3714  
   FBgn0038318   CG6236  11151039   11154000   -  2962  
   FBgn0000533   ea   11154452   11156135  +  1684  
   FBgn0038319   mRpL9   11156462   11157314  +  853  
   FBgn0038321   CG6218  11163630   11168978   -  5349  
 
 
    Segment 240 
 
   Location
[truncated: 617,376 more chars]
