# Supplementary material for: Expression-based segmentation of the Drosophila genome
Source: BMC Genomics. 2013 Nov 20;14:812. doi: 10.1186/1471-2164-14-812 (PMC3909303; doi:10.1186/1471-2164-14-812)
Supplement: Additional file 1 — Detailed information for multigene segments. [file 1471-2164-14-812-S1.zip › miniwebsite/chrX.html]

   ExprSeg Report for chrX   
 Report for /Users/afrubin/Code/ExprSeg/2012-07-19/output Generated Fri Jun 21 23:07:39 2013 
   Chromosome X 
 410 segments 1825 genes 
    Segment 1 
 
   Location   
  Gene key  FBgn0025835-FBgn0024989  
  Heatmap region span   X:23076..371180   
  Segment span   X:44813..173736   
  Length (genes)  6  
  Length (bp)  128924  
   Model Scoring   
  BIC  647.908049  
  logL  -318.551439  
  logL ratio  9.535223  
   Expression   
  Mean expression  6.051699  
  Median expression  5.685600  
  Tissue std. dev.  0.606819  
 
  No GO Slim enrichment  
  
   tissue    mean expression   
  5th Passage Drosophila S2 Cells  5.898780  
  Adult Accessory gland  5.635391  
  Adult Brain  6.449766  
  Adult Carcass  5.455788  
  Adult Crop  5.865377  
  Adult Eye  6.160200  
  Adult Fatbody  5.541832  
  Adult Female Spermatheca Mated  5.759327  
  Adult Female Spermatheca Virgin  5.732455  
  Adult Head  5.665904  
  Adult Heart  5.750020  
  Adult Hind Gut  6.282791  
  Adult Male Ejaculatory Duct  5.504708  
  Adult Mid Gut  5.563692  
  Adult Ovary  7.127998  
  Adult Salivary Gland  5.588863  
  Adult Testes  4.837299  
  Adult Thoracoabdominal ganglion  6.090414  
  Adult Whole Fly  5.974917  
  Larvae Wandering Tubules  6.002627  
  Larval Feeding Carcass  7.147151  
  Larval Feeding Central Nevous System  7.252606  
  Larval Feeding Hind Gut  7.441977  
  Larval Feeding Malpighian Tubule  5.955591  
  Larval Feeding Mid Gut  5.825521  
  Larval Feeding Salivary Gland  6.208499  
  Whole Larvae Feeding  6.676374  
 
  
   FlyBase ID    symbol    start    end    strand    length   
   FBgn0025835   CG17707  39388   44813   -  5426  
   FBgn0029128   tyn   72457   96056  +  23600  
   FBgn0040373   CG3038  138083   139875   -  1793  
   FBgn0040372   G9a   140040   148058  +  8019  
   FBgn0005427   ewg  162554   173667   -  11114  
   FBgn0024989   CG3777   173736   244237  +  70502  
 
 
    Segment 2 
 
   Location   
  Gene key  FBgn0040370-FBgn0000137  
  Heatmap region span   X:23076..373342   
  Segment span   X:188995..354533   
  Length (genes)  8  
  Length (bp)  165539  
   Model Scoring   
  BIC  836.218405  
  logL  -412.706616  
  logL ratio  46.177215  
   Expression   
  Mean expression  4.588131  
  Median expression  4.255710  
  Tissue std. dev.  0.395202  
 
  
   GO ID    description    ratio    P-value   
   GO:0003677   DNA binding  3/8  0.0203  
 
  
   tissue    mean expression   
  5th Passage Drosophila S2 Cells  5.041293  
  Adult Accessory gland  4.434325  
  Adult Brain  4.382343  
  Adult Carcass  4.395500  
  Adult Crop  4.261604  
  Adult Eye  4.097395  
  Adult Fatbody  4.353640  
  Adult Female Spermatheca Mated  4.544704  
  Adult Female Spermatheca Virgin  4.470028  
  Adult Head  4.215824  
  Adult Heart  4.412300  
  Adult Hind Gut  4.355780  
  Adult Male Ejaculatory Duct  4.730558  
  Adult Mid Gut  5.088407  
  Adult Ovary  4.223315  
  Adult Salivary Gland  4.513396  
  Adult Testes  5.245145  
  Adult Thoracoabdominal ganglion  4.252402  
  Adult Whole Fly  4.948366  
  Larvae Wandering Tubules  4.268535  
  Larval Feeding Carcass  4.321984  
  Larval Feeding Central Nevous System  5.328595  
  Larval Feeding Hind Gut  4.195089  
  Larval Feeding Malpighian Tubule  4.248497  
  Larval Feeding Mid Gut  5.103819  
  Larval Feeding Salivary Gland  4.979066  
  Whole Larvae Feeding  5.467612  
 
  
   FlyBase ID    symbol    start    end    strand    length   
   FBgn0040370   CG13375  180434   188995   -  8562  
   FBgn0040371   CG12470   201717   202975  +  1259  
   FBgn0029521   Or1a   209156   210590  +  1435  
   FBgn0004034   y   250542   255278  +  4737  
   FBgn0000022   ac   264064   264980  +  917  
   FBgn0004170   sc   290093   291530  +  1438  
   FBgn0011822   pcl  330172   331475   -  1304  
   FBgn0000137   ase   354533   357209  +  2677  
 
 
    Segment 3 
 
   Location   
  Gene key  FBgn0001337-FBgn0029522  
  Heatmap region span   X:44813..388933   
  Segment span   X:370625..371180   
  Length (genes)  2  
  Length (bp)  556  
   Model Scoring   
  BIC  260.485689  
  logL  -124.840258  
  logL ratio  -8.969428  
   Expression   
  Mean expression  7.956780  
  Median expression  7.916288  
  Tissue std. dev.  0.632321  
 
  No GO Slim enrichment  
  
   tissue    mean expression   
  5th Passage Drosophila S2 Cells  8.491347  
  Adult Accessory gland  8.240239  
  Adult Brain  7.601227  
  Adult Carcass  7.310256  
  Adult Crop  7.533095  
  Adult Eye  7.522046  
  Adult Fatbody  7.668754  
  Adult Female Spermatheca Mated  8.110015  
  Adult Female Spermatheca Virgin  8.095781  
  Adult Head  7.172116  
  Adult Heart  7.663575  
  Adult Hind Gut  7.421813  
  Adult Male Ejaculatory Duct  7.403835  
  Adult Mid Gut  7.741850  
  Adult Ovary  10.155722  
  Adult Salivary Gland  7.600943  
  Adult Testes  7.156707  
  Adult Thoracoabdominal ganglion  7.465070  
  Adult Whole Fly  8.776164  
  Larvae Wandering Tubules  8.620217  
  Larval Feeding Carcass  7.852882  
  Larval Feeding Central Nevous System  8.867894  
  Larval Feeding Hind Gut  8.074369  
  Larval Feeding Malpighian Tubule  8.320651  
  Larval Feeding Mid Gut  8.087226  
  Larval Feeding Salivary Gland  8.152538  
  Whole Larvae Feeding  7.726717  
 
  
   FlyBase ID    symbol    start    end    strand    length   
   FBgn0001337   Exp6  365085   370625   -  5541  
   FBgn0029522   CG13373   371180   372224  +  1045  
 
    Segment 4 
 
   Location   
  Gene key  FBgn0029525-FBgn0023536  
  Heatmap region span   X:363366..403451   
  Segment span   X:381587..381972   
  Length (genes)  2  
  Length (bp)  386  
   Model Scoring   
  BIC  207.868201  
  logL  -98.531514  
  logL ratio  40.829160  
   Expression   
  Mean expression  7.912885  
  Median expression  7.883364  
  Tissue std. dev.  0.359783  
 
  No GO Slim enrichment  
  
   tissue    mean expression   
  5th Passage Drosophila S2 Cells  8.037514  
  Adult Accessory gland  7.904355  
  Adult Brain  7.843871  
  Adult Carcass  7.761079  
  Adult Crop  7.917380  
  Adult Eye  7.695207  
  Adult Fatbody  8.217033  
  Adult Female Spermatheca Mated  7.976456  
  Adult Female Spermatheca Virgin  7.698121  
  Adult Head  7.516740  
  Adult Heart  8.226582  
  Adult Hind Gut  7.543171  
  Adult Male Ejaculatory Duct  8.166156  
  Adult Mid Gut  7.640104  
  Adult Ovary  9.163303  
  Adult Salivary Gland  7.792205  
  Adult Testes  8.166158  
  Adult Thoracoabdominal ganglion  7.848060  
  Adult Whole Fly  8.161070  
  Larvae Wandering Tubules  7.561715  
  Larval Feeding Carcass  7.829491  
  Larval Feeding Central Nevous System  8.277405  
  Larval Feeding Hind Gut  7.850575  
  Larval Feeding Malpighian Tubule  7.917457  
  Larval Feeding Mid Gut  7.449834  
  Larval Feeding Salivary Gland  8.235344  
  Whole Larvae Feeding  7.251520  
 
  
   FlyBase ID    symbol    start    end    strand    length   
   FBgn0029525   CG18273  376810   381587   -  4778  
   FBgn0023536   CG3156   381972   384767  +  2796  
 
    Segment 5 
 
   Location   
  Gene key  FBgn0025633-FBgn0026879  
  Heatmap region span   X:390504..550105   
  Segment span   X:514228..519234   
  Length (genes)  4  
  Length (bp)  5007  
   Model Scoring   
  BIC  454.763992  
  logL  -221.979410  
  logL ratio  98.916133  
   Expression   
  Mean expression  9.681651  
  Median expression  9.732697  
  Tissue std. dev.  0.542712  
 
  No GO Slim enrichment  
  
   tissue    mean expression   
  5th Passage Drosophila S2 Cells  10.072887  
  Adult Accessory gland  10.036965  
  Adult Brain  9.731556  
  Adult Carcass  9.371670  
  Adult Crop  9.542898  
  Adult Eye  10.239828  
  Adult Fatbody  9.199924  
  Adult Female Spermatheca Mated  9.131118  
  Adult Female Spermatheca Virgin  9.050789  
  Adult Head  9.625255  
  Adult Heart  9.645195  
  Adult Hind Gut  9.753468  
  Adult Male Ejaculatory Duct  9.788120  
  Adult Mid Gut  9.779659  
  Adult Ovary  9.912388  
  Adult Salivary Gland  10.218545  
  Adult Testes  7.536025  
  Adult Thoracoabdominal ganglion  9.726367  
  Adult Whole Fly  9.605651  
  Larvae Wandering Tubules  9.844587  
  Larval Feeding Carcass  9.671604  
  Larval Feeding Central Nevous System  9.913434  
  Larval Feeding Hind Gut  9.950950  
  Larval Feeding Malpighian Tubule  10.434950  
  Larval Feeding Mid Gut  9.710304  
  Larval Feeding Salivary Gland  10.432816  
  Whole Larvae Feeding  9.477623  
 
  
   FlyBase ID    symbol    start    end    strand    length   
   FBgn0025633   CG13366  504579   514228   -  9650  
   FBgn0029529   CG13365  514748   515241   -  494  
   FBgn0025640   CG13369   517500   518798  +  1299  
   FBgn0026879   CG13364  518791   519234   -  444  
 
 
    Segment 6 
 
   Location   
  Gene key  FBgn0020381-FBgn0025638  
  Heatmap region span   X:425652..653628   
  Segment span   X:529760..541320   
  Length (genes)  3  
  Length (bp)  11561  
   Model Scoring   
  BIC  297.349475  
  logL  -143.272152  
  logL ratio  82.368130  
   Expression   
  Mean expression  8.805510  
  Median expression  8.678106  
  Tissue std. dev.  0.419146  
 
  No GO Slim enrichment  
  
   tissue    mean expression   
  5th Passage Drosophila S2 Cells  9.001395  
  Adult Accessory gland  8.558603  
  Adult Brain  8.925785  
  Adult Carcass  8.766834  
  Adult Crop  9.320272  
  Adult Eye  9.229253  
  Adult Fatbody  8.923361  
  Adult Female Spermatheca Mated  9.009331  
  Adult Female Spermatheca Virgin  9.064174  
  Adult Head  8.731366  
  Adult Heart  9.406440  
  Adult Hind Gut  9.028358  
  Adult Male Ejaculatory Duct  8.671237  
  Adult Mid Gut  8.650516  
  Adult Ovary  8.662528  
  Adult Salivary Gland  8.876055  
  Adult Testes  7.298745  
  Adult Thoracoabdominal ganglion  8.872359  
  Adult Whole Fly  8.174664  
  Larvae Wandering Tubules  9.355005  
  Larval Feeding Carcass  8.862800  
  Larval Feeding Central Nevous System  8.990788  
  Larval Feeding Hind Gut  8.788546  
  Larval Feeding Malpighian Tubule  9.130541  
  Larval Feeding Mid Gut  8.438616  
  Larval Feeding Salivary Gland  8.774619  
  Whole Larvae Feeding  8.236572  
 
  
   FlyBase ID    symbol    start    end    strand    length   
   FBgn0020381   Dredd  527553   529760   -  2208  
   FBgn0025634   CG13367   538794   540419  +  1626  
   FBgn0025638   Roc1a   541320   542636  +  1317  
 
 
    Segment 7 
 
   Location   
  Gene key  FBgn0021764-FBgn0029532  
  Heatmap region span   X:519588..664061   
  Segment span   X:580873..647686   
  Length (genes)  3  
  Length (bp)  66814  
   Model Scoring   
  BIC  309.804182  
  logL  -149.499505  
  logL ratio  19.782065  
   Expression   
  Mean expression  5.689248  
  Median expression  5.661885  
  Tissue std. dev.  0.396697  
 
  No GO Slim enrichment  
  
   tissue    mean expression   
  5th Passage Drosophila S2 Cells  5.464753  
  Adult Accessory gland  6.578632  
  Adult Brain  5.620538  
  Adult Carcass  5.640822  
  Adult Crop  5.758605  
  Adult Eye  5.658730  
  Adult Fatbody  5.343708  
  Adult Female Spermatheca Mated  5.165994  
  Adult Female Spermatheca Virgin  5.114383  
  Adult Head  5.320952  
  Adult Heart  5.637111  
  Adult Hind Gut  5.587936  
  Adult Male Ejaculatory Duct  5.590255  
  Adult Mid Gut  5.447293  
  Adult Ovary  6.531736  
  Adult Salivary Gland  5.732949  
  Adult Testes  4.999518  
  Adult Thoracoabdominal ganglion  5.537376  
  Adult Whole Fly  5.406518  
  Larvae Wandering Tubules  5.807606  
  Larval Feeding Carcass  6.006887  
  Larval Feeding Central Nevous System  6.423633  
  Larval Feeding Hind Gut  5.739365  
  Larval Feeding Malpighian Tubule  5.668234  
  Larval Feeding Mid Gut  5.774495  
  Larval Feeding Salivary Gland  6.395067  
  Whole Larvae Feeding  5.656606  
 
  
   FlyBase ID    symbol    start    end    strand    length   
   FBgn0021764   sdk   580873   643529  +  62657  
   FBgn0029531   CG13362  643274   645330   -  2057  
   FBgn0029532   CG13361  646044   647686   -  1643  
 
 
    Segment 8 
 
   Location   
  Gene key  FBgn0025621-FBgn0015799  
  Heatmap region span   X:659172..829265   
  Segment span   X:687201..695490   
  Length (genes)  2  
  Length (bp)  8290  
   Model Scoring   
  BIC  229.878366  
  logL  -109.536597  
  logL ratio  20.450836  
   Expression   
  Mean expression  8.078691  
  Median expression  8.246582  
  Tissue std. dev.  0.404121  
 
  No GO Slim enrichment  
  
   tissue    mean expression   
  5th Passage Drosophila S2 Cells  8.450300  
  Adult Accessory gland  8.570016  
  Adult Brain  8.030147  
  Adult Carcass  7.770005  
  Adult Crop  7.764902  
  Adult Eye  7.843682  
  Adult Fatbody  7.984522  
  Adult Female Spermatheca Mated  7.627599  
  Adult Female Spermatheca Virgin  7.652013  
  Adult Head  7.710589  
  Adult Heart  8.068141  
  Adult Hind Gut  7.803334  
  Adult Male Ejaculatory Duct  7.855836  
  Adult Mid Gut  7.760616  
  Adult Ovary  9.281122  
  Adult Salivary Gland  8.403970  
  Adult Testes  7.926780  
  Adult Thoracoabdominal ganglion  7.702307  
  Adult Whole Fly  8.232010  
  Larvae Wandering Tubules  7.684421  
  Larval Feeding Carcass  8.310505  
  Larval Feeding Central Nevous System  8.509891  
  Larval Feeding Hind Gut  8.431055  
  Larval Feeding Malpighian Tubule  7.985178  
  Larval Feeding Mid Gut  8.046070  
  Larval Feeding Salivary Gland  8.905217  
  Whole Larvae Feeding  7.814431  
 
  
   FlyBase ID    symbol    start    end    strand    length   
   FBgn0025621   CG16989   687201   690404  +  3204  
   FBgn0015799   Rbf  691121   695490   -  4370  
 
    Segment 9 
 
   Location   
  Gene key  FBgn0025616-FBgn0029538  
  Heatmap region span   X:664061..835090   
  Segment span   X:697503..802854   
  Length (genes)  9  
  Length (bp)  105352  
   Model Scoring   
  BIC  745.066761  
  logL  -367.130795  
  logL ratio  214.463039  
   Expression   
  Mean expression  4.874347  
  Median expression  4.592893  
  Tissue std. dev.  0.425548  
 
  No GO Slim enrichment  
  
   tissue    mean expression   
  5th Passage Drosophila S2 Cells  4.899210  
  Adult Accessory gland  5.405357  
  Adult Brain  4.641807  
  Adult Carcass  4.829632  
  Adult Crop  4.676333  
  Adult Eye  4.849060  
  Adult Fatbody  4.880374  
  Adult Female Spermatheca Mated  4.845173  
  Adult Female Spermatheca Virgin  4.861230  
  Adult Head  4.625676  
  Adult Heart  4.628249  
  Adult Hind Gut  4.635089  
  Adult Male Ejaculatory Duct  4.960869  
  Adult Mid Gut  4.789053  
  Adult Ovary  4.767811  
  Adult Salivary Gland  5.090299  
  Adult Testes  6.863366  
  Adult Thoracoabdominal ganglion  4.572125  
  Adult Whole Fly  4.928468  
  Larvae Wandering Tubules  4.751724  
  Larval Feeding Carcass  4.836479  
  Larval Feeding Central Nevous System  4.605711  
  Larval Feeding Hind Gut  4.655355  
  Larval Feeding Malpighian Tubule  4.743960  
  Larval Feeding Mid Gut  4.757381  
  Larval Feeding Salivary Gland  4.826884  
  Whole Larvae Feeding  4.680706  
 
  
   FlyBase ID    symbol    start    end    strand    length   
   FBgn0025616   CG13359  695701   697503   -  1803  
   FBgn0026874   CG13358  697739   698954   -  1216  
   FBgn0029535   CG14635   738870   739229  +  360  
   FBgn0040343   CG3713  740673   741201   -  529  
   FBgn0040892   CG14634   756641   757234  +  594  
   FBgn0040341   CG11664   769765   770529  +  765  
   FBgn0259100     702250   778497   -  76248  
   FBgn0029539   CG11663   788515   789240  +  726  
   FBgn0029538     785380   802854   -  17475  
 
 
    Segment 10 
 
   Location   
  Gene key  FBgn0040344-FBgn0040339  
  Heatmap region span   X:678824..839805   
  Segment span   X:821596..822170   
  Length (genes)  2  
  Length (bp)  575  
   Model Scoring   
  BIC  215.131036  
  logL  -102.162932  
  logL ratio  41.002930  
   Expression   
  Mean expression  8.671798  
  Median expression  8.704607  
  Tissue std. dev.  0.677603  
 
  
   GO ID    description    ratio    P-value   
   GO:0005634   nucleus  2/2  0.0363  
 
  
   tissue    mean expression   
  5th Passage Drosophila S2 Cells  8.851267  
  Adult Accessory gland  8.850225  
  Adult Brain  10.213109  
  Adult Carcass  7.908927  
  Adult Crop  8.693521  
  Adult Eye  9.306516  
  Adult Fatbody  7.872487  
  Adult Female Spermatheca Mated  8.325401  
  Adult Female Spermatheca Virgin  8.428193  
  Adult Head  9.123938  
  Adult Heart  8.421506  
  Adult Hind Gut  8.184503  
  Adult Male Ejaculatory Duct  8.727270  
  Adult Mid Gut  7.855180  
  Adult Ovary  9.808931  
  Adult Salivary Gland  8.963428  
  Adult Testes  7.874810  
  Adult Thoracoabdominal ganglion  10.093214  
  Adult Whole Fly  8.528363  
  Larvae Wandering Tubules  8.528821  
  Larval Feeding Carcass  8.119857  
  Larval Feeding Central Nevous System  9.744659  
  Larval Feeding Hind Gut  8.336752  
  Larval Feeding Malpighian Tubule  8.352125  
  Larval Feeding Mid Gut  7.869505  
  Larval Feeding Salivary Gland  9.113329  
  Whole Larvae Feeding  8.042702  
 
  
   FlyBase ID    symbol    start    end    strand    length   
   FBgn0040344   CG3711  817200   821596   -  4397  
   FBgn0040339   MED22   822170   822893  +  724  
 
    Segment 11 
 
   Location   
  Gene key  FBgn0040345-FBgn0040342  
  Heatmap region span   X:684122..841517   
  Segment span   X:824886..826704   
  Length (genes)  3  
  Length (bp)  1819  
   Model Scoring   
  BIC  284.008750  
  logL  -136.601789  
  logL ratio  30.566027  
   Expression   
  Mean expression  5.221099  
  Median expression  4.933659  
  Tissue std. dev.  0.515965  
 
  No GO Slim enrichment  
  
   tissue    mean expression   
  5th Passage Drosophila S2 Cells  5.026316  
  Adult Accessory gland  5.053651  
  Adult Brain  5.341063  
  Adult Carcass  5.032752  
  Adult Crop  4.821638  
  Adult Eye  4.928692  
  Adult Fatbody  5.069809  
  Adult Female Spermatheca Mated  5.132670  
  Adult Female Spermatheca Virgin  5.057291  
  Adult Head  4.828548  
  Adult Heart  5.193723  
  Adult Hind Gut  4.755573  
  Adult Male Ejaculatory Duct  5.090152  
  Adult Mid Gut  6.742180  
  Adult Ovary  4.826336  
  Adult Salivary Gland  5.225054  
  Adult Testes  6.366959  
  Adult Thoracoabdominal ganglion  5.144308  
  Adult Whole Fly  5.437600  
  Larvae Wandering Tubules  4.909482  
  Larval Feeding Carcass  5.059585  
  Larval Feeding Central Nevous System  4.813021  
  Larval Feeding Hind Gut  4.785155  
  Larval Feeding Malpighian Tubule  4.988947  
  Larval Feeding Mid Gut  6.604455  
  Larval Feeding Salivary Gland  5.201006  
  Whole Larvae Feeding  5.533712  
 
  
   FlyBase ID    symbol    start    end    strand    length   
   FBgn0040345   CG3708  822825   824886   -  2062  
   FBgn0052815   CG32815   826499   828792  +  2294  
   FBgn0040342   CG3706  824964   826704   -  1741  
 
 
    Segment 12 
 
   Location   
  Gene key  FBgn0040346-FBgn0040347  
  Heatmap region span   X:697503..906068   
  Segment span   X:834834..835090   
  Length (genes)  2  
  Length (bp)  257  
   Model Scoring   
  BIC  254.302189  
  logL  -121.748508  
  logL ratio  -8.613260  
   Expression   
  Mean expression  7.604257  
  Median expression  8.013568  
  Tissue std. dev.  0.773177  
 
  No GO Slim enrichment  
  
   tissue    mean expression   
  5th Passage Drosophila S2 Cells  7.891341  
  Adult Accessory gland  7.879138  
  Adult Brain  7.428418  
  Adult Carcass  7.288437  
  Adult Crop  7.540544  
  Adult Eye  7.554880  
  Adult Fatbody  7.545089  
  Adult Female Spermatheca Mated  8.237066  
  Adult Female Spermatheca Virgin  8.101948  
  Adult Head  7.147434  
  Adult Heart  7.548698  
  Adult Hind Gut  7.110660  
  Adult Male Ejaculatory Duct  7.325171  
  Adult Mid Gut  7.296401  
  Adult Ovary  10.334252  
  Adult Salivary Gland  7.336580  
  Adult Testes  5.809801  
  Adult Thoracoabdominal ganglion  7.771031  
  Adult Whole Fly  8.791602  
  Larvae Wandering Tubules  7.206703  
  Larval Feeding Carcass  7.331897  
  Larval Feeding Central Nevous System  8.730744  
  Larval Feeding Hind Gut  7.170132  
  Larval Feeding Malpighian Tubule  7.410377  
  Larval Feeding Mid Gut  7.122974  
  Larval Feeding Salivary Gland  7.426304  
  Whole Larvae Feeding  6.977315  
 
  
   FlyBase ID    symbol    start    end    strand    length   
   FBgn0040346   CG3704  833202   834834   -  1633  
   FBgn0040347   mus81   835090   837262  +  2173  
 
    Segment 13 
 
   Location   
  Gene key  FBgn0042146-FBgn0029552  
  Heatmap region span   X:834834..1058065   
  Segment span   X:884615..906068   
  Length (genes)  2  
  Length (bp)  21454  
   Model Scoring   
  BIC  179.628886  
  logL  -84.411857  
  logL ratio  30.831064  
   Expression   
  Mean expression  4.983549  
  Median expression  4.817433  
  Tissue std. dev.  0.582884  
 
  No GO Slim enrichment  
  
   tissue    mean expression   
  5th Passage Drosophila S2 Cells  4.737758  
  Adult Accessory gland  4.823493  
  Adult Brain  4.589943  
  Adult Carcass  4.973740  
  Adult Crop  4.887455  
  Adult Eye  4.642648  
  Adult Fatbody  4.936118  
  Adult Female Spermatheca Mated  5.007678  
  Adult Female Spermatheca Virgin  5.036134  
  Adult Head  4.706150  
  Adult Heart  4.956676  
  Adult Hind Gut  4.870790  
  Adult Male Ejaculatory Duct  5.098190  
  Adult Mid Gut  4.946944  
  Adult Ovary  4.848766  
  Adult Salivary Gland  5.345188  
  Adult Testes  7.823180  
  Adult Thoracoabdominal ganglion  4.565493  
  Adult Whole Fly  4.957024  
  Larvae Wandering Tubules  4.854311  
  Larval Feeding Carcass  4.928714  
  Larval Feeding Central Nevous System  4.522315  
  Larval Feeding Hind Gut  4.749873  
  Larval Feeding Malpighian Tubule  4.907291  
  Larval Feeding Mid Gut  4.960149  
  Larval Feeding Salivary Gland  4.864911  
  Whole Larvae Feeding  5.014899  
 
  
   FlyBase ID    symbol    start    end    strand    length   
   FBgn0042146   CG18823   884615   884935  +  321  
   FBgn0029552   CG14631   906068   906657  +  590  
 
    Segment 14 
 
   Location   
  Gene key  FBgn0040337-FBgn0026143  
  Heatmap region span   X:841517..1104130   
  Segment span   X:920867..924606   
  Length (genes)  2  
  Length (bp)  3740  
   Model Scoring   
  BIC  253.638022  
  logL  -121.416425  
  logL ratio  -3.710151  
   Expression   
  Mean expression  8.001318  
  Median expression  8.419054  
  Tissue std. dev.  0.529854  
 
  
   GO ID    description    ratio    P-value   
   GO:0005737   cytoplasm  2/2  0.0141  
 
  
   tissue    mean expression   
  5th Passage Drosophila S2 Cells  8.233080  
  Adult Accessory gland  8.569647  
  Adult Brain  7.979091  
  Adult Carcass  7.192317  
  Adult Crop  7.440693  
  Adult Eye  8.120629  
  Adult Fatbody  7.599274  
  Adult Female Spermatheca Mated  8.246711  
  Adult Female Spermatheca Virgin  8.072524  
  Adult Head  7.479886  
  Adult Heart  8.135711  
  Adult Hind Gut  7.351356  
  Adult Male Ejaculatory Duct  7.553610  
  Adult Mid Gut  7.472349  
  Adult Ovary  9.543355  
  Adult Salivary Gland  8.286854  
  Adult Testes  7.845929  
  Adult Thoracoabdominal ganglion  8.072703  
  Adult Whole Fly  8.554342  
  Larvae Wandering Tubules  7.472296  
  Larval Feeding Carcass  8.042003  
  Larval Feeding Central Nevous System  8.602937  
  Larval Feeding Hind Gut  8.039008  
  Larval Feeding Malpighian Tubule  8.069097  
  Larval Feeding Mid Gut  7.501174  
  Larval Feeding Salivary Gland  8.969224  
  Whole Larvae Feeding  7.589781  
 
  
   FlyBase ID    symbol    start    end    strand    length   
   FBgn0040337   CG3021   920867   922323  +  1457  
   FBgn0026143   CDC45L  922324   924606   -  2283  
 
    Segment 15 
 
   Location   
  Gene key  FBgn0040397-FBgn0085349  
  Heatmap region span   X:884615..1168682   
  Segment span   X:1028547..1058065   
  Length (genes)  3  
  Length (bp)  29519  
   Model Scoring   
  BIC  257.735836  
  logL  -123.465332  
  logL ratio  57.760500  
   Expression   
  Mean expression  4.734075  
  Median expression  4.648674  
  Tissue std. dev.  0.372741  
 
  No GO Slim enrichment  
  
   tissue    mean expression   
  5th Passage Drosophila S2 Cells  4.566459  
  Adult Accessory gland  4.693609  
  Adult Brain  4.461452  
  Adult Carcass  4.791267  
  Adult Crop  4.789295  
  Adult Eye  4.373858  
  Adult Fatbody  4.746245  
  Adult Female Spermatheca Mated  4.754519  
  Adult Female Spermatheca Virgin  4.655379  
  Adult Head  4.520040  
  Adult Heart  4.505584  
  Adult Hind Gut  4.685377  
  Adult Male Ejaculatory Duct  4.672922  
  Adult Mid Gut  4.776741  
  Adult Ovary  4.825538  
  Adult Salivary Gland  4.980249  
  Adult Testes  6.488741  
  Adult Thoracoabdominal ganglion  4.611169  
  Adult Whole Fly  4.736274  
  Larvae Wandering Tubules  4.758463  
  Larval Feeding Carcass  4.748425  
  Larval Feeding Central Nevous System  4.375504  
  Larval Feeding Hind Gut  4.622435  
  Larval Feeding Malpighian Tubule  4.764834  
  Larval Feeding Mid Gut  4.533805  
  Larval Feeding Salivary Gland  4.534592  
  Whole Larvae Feeding  4.847251  
 
  
   FlyBase ID    symbol    start    end    strand    length   
   FBgn0040397   CG3655  967931   1028547   -  60617  
   FBgn0040365   CG14628   1039677   1040167  +  491  
   FBgn0085349   CG34320  1057653   1058065   -  413  
 
 
    Segment 16 
 
   Location   
  Gene key  FBgn0040363-FBgn0040367  
  Heatmap region span   X:920867..1216427   
  Segment span   X:1070055..1104130   
  Length (genes)  9  
  Length (bp)  34076  
   Model Scoring   
  BIC  694.486012  
  logL  -341.840420  
  logL ratio  246.392683  
   Expression   
  Mean expression  4.725308  
  Median expression  4.520604  
  Tissue std. dev.  0.256351  
 
  No GO Slim enrichment  
  
   tissue    mean expression   
  5th Passage Drosophila S2 Cells  4.630017  
  Adult Accessory gland  4.703831  
  Adult Brain  4.271196  
  Adult Carcass  4.816175  
  Adult Crop  4.569962  
  Adult Eye  4.491173  
  Adult Fatbody  4.661772  
  Adult Female Spermatheca Mated  4.549425  
  Adult Female Spermatheca Virgin  4.585053  
  Adult Head  4.447834  
  Adult Heart  4.548920  
  Adult Hind Gut  4.742915  
  Adult Male Ejaculatory Duct  4.782951  
  Adult Mid Gut  4.974512  
  Adult Ovary  5.541263  
  Adult Salivary Gland  4.858309  
  Adult Testes  5.115242  
  Adult Thoracoabdominal ganglion  4.470755  
  Adult Whole Fly  5.053675  
  Larvae Wandering Tubules  4.659136  
  Larval Feeding Carcass  4.743042  
  Larval Feeding Central Nevous System  4.370808  
  Larval Feeding Hind Gut  4.847794  
  Larval Feeding Malpighian Tubule  4.790492  
  Larval Feeding Mid Gut  5.001036  
  Larval Feeding Salivary Gland  4.726117  
  Whole Larvae Feeding  4.629914  
 
  
   FlyBase ID    symbol    start    end    strand    length   
   FBgn0040363   CG11384  1067908   1070055   -  2148  
   FBgn0040362   CG11379   1074198   1074980  +  783  
   FBgn0040361   CG14627   1075720   1077382  +  1663  
   FBgn0040360   CG14626   1078815   1082623  +  3809  
   FBgn0040359   CG11380   1085444   1090705  +  5262  
   FBgn0040358   CG14625   1093651   1096954  +  3304  
   FBgn0029568   CG11381   1097812   1099468  +  1657  
   FBgn0040357   CG14624   1100802   1101494  +  693  
   FBgn0040367   CG11382   1104130   1105746  +  1617  
 
 
    Segment 17 
 
   Location   
  Gene key  FBgn0040366-FBgn0026876  
  Heatmap region span   X:945570..1227729   
  Segment span   X:1109221..1133925   
  Length (genes)  2  
  Length (bp)  24705  
   Model Scoring   
  BIC  202.121090  
  logL  -95.657959  
  logL ratio  27.242561  
   Expression   
  Mean expression  6.255103  
  Median expression  5.965137  
  Tissue std. dev.  0.847108  
 
  No GO Slim enrichment  
  
   tissue    mean expression   
  5th Passage Drosophila S2 Cells  7.202389  
  Adult Accessory gland  5.682116  
  Adult Brain  6.382432  
  Adult Carcass  5.489914  
  Adult Crop  6.187672  
  Adult Eye  6.560797  
  Adult Fatbody  5.622606  
  Adult Female Spermatheca Mated  6.307576  
  Adult Female Spermatheca Virgin  6.160159  
  Adult Head  5.813319  
  Adult Heart  6.169055  
  Adult Hind Gut  5.946115  
  Adult Male Ejaculatory Duct  5.907259  
  Adult Mid Gut  5.576214  
  Adult Ovary  9.420589  
  Adult Salivary Gland  5.903512  
  Adult Testes  5.456396  
  Adult Thoracoabdominal ganglion  6.543756  
  Adult Whole Fly  7.491557  
  Larvae Wandering Tubules  6.105486  
  Larval Feeding Carcass  5.962989  
  Larval Feeding Central Nevous System  7.873034  
  Larval Feeding Hind Gut  5.947518  
  Larval Feeding Malpighian Tubule  5.898205  
  Larval Feeding Mid Gut  5.535755  
  Larval Feeding Salivary Gland  5.829212  
  Whole Larvae Feeding  5.912152  
 
  
   FlyBase ID    symbol    start    end    strand    length   
   FBgn0040366   CG11398  1107553   1109221   -  1669  
   FBgn0026876   CG11403  1130906   1133925   -  3020  
 
    Segment 18 
 
   Location   
  Gene key  FBgn0024360-FBgn0024364  
  Heatmap region span   X:1216427..1346736   
  Segment span   X:1243390..1248076   
  Length (genes)  3  
  Length (bp)  4687  
   Model Scoring   
  BIC  305.952769  
  logL  -147.573798  
  logL ratio  65.108583  
   Expression   
  Mean expression  7.863190  
  Median expression  7.710722  
  Tissue std. dev.  0.627610  
 
  No GO Slim enrichment  
  
   tissue    mean expression   
  5th Passage Drosophila S2 Cells  8.543457  
  Adult Accessory gland  8.484368  
  Adult Brain  7.374688  
  Adult Carcass  7.135980  
  Adult Crop  7.611185  
  Adult Eye  7.042671  
  Adult Fatbody  7.666275  
  Adult Female Spermatheca Mated  7.414707  
  Adult Female Spermatheca Virgin  7.260021  
  Adult Head  6.959415  
  Adult Heart  7.500196  
  Adult Hind Gut  7.552277  
  Adult Male Ejaculatory Duct  7.517739  
  Adult Mid Gut  7.999663  
  Adult Ovary  9.423882  
  Adult Salivary Gland  7.708709  
  Adult Testes  6.995341  
  Adult Thoracoabdominal ganglion  7.462315  
  Adult Whole Fly  8.086844  
  Larvae Wandering Tubules  9.062775  
  Larval Feeding Carcass  7.918541  
  Larval Feeding Central Nevous System  8.183572  
  Larval Feeding Hind Gut  8.528388  
  Larval Feeding Malpighian Tubule  8.690975  
  Larval Feeding Mid Gut  8.424119  
  Larval Feeding Salivary Gland  8.084292  
  Whole Larvae Feeding  7.673730  
 
  
   FlyBase ID    symbol    start    end    strand    length   
   FBgn0024360   CG11418  1238905   1243390   -  4486  
   FBgn0024365   CG12773  1243920   1248009   -  4090  
   FBgn0024364   CG11417   1248076   1250513  +  2438  
 
 
    Segment 19 
 
   Location   
  Gene key  FBgn0000826-FBgn0029573  
  Heatmap region span   X:1227729..1355700   
  Segment span   X:1251646..1263311   
  Length (genes)  2  
  Length (bp)  11666  
   Model Scoring   
  BIC  204.336408  
  logL  -96.765618  
  logL ratio  17.437034  
   Expression   
  Mean expression  5.769414  
  Median expression  5.700351  
  Tissue std. dev.  0.499999  
 
  No GO Slim enrichment  
  
   tissue    mean expression   
  5th Passage Drosophila S2 Cells  6.432444  
  Adult Accessory gland  5.929620  
  Adult Brain  5.310983  
  Adult Carcass  5.564752  
  Adult Crop  5.621258  
  Adult Eye  5.469513  
  Adult Fatbody  5.734633  
  Adult Female Spermatheca Mated  5.376276  
  Adult Female Spermatheca Virgin  5.438919  
  Adult Head  5.393347  
  Adult Heart  5.577939  
  Adult Hind Gut  5.616713  
  Adult Male Ejaculatory Duct  6.107490  
  Adult Mid Gut  5.962563  
  Adult Ovary  7.643502  
  Adult Salivary Gland  5.607835  
  Adult Testes  5.396900  
  Adult Thoracoabdominal ganglion  5.232046  
  Adult Whole Fly  6.778709  
  Larvae Wandering Tubules  5.566370  
  Larval Feeding Carcass  5.783024  
  Larval Feeding Central Nevous System  5.476491  
  Larval Feeding Hind Gut  5.652494  
  Larval Feeding Malpighian Tubule  5.681301  
  Larval Feeding Mid Gut  6.045463  
  Larval Feeding Salivary Gland  5.886465  
  Whole Larvae Feeding  5.487118  
 
  
   FlyBase ID    symbol    start    end    strand    length   
   FBgn0000826   png  1250521   1251646   -  1126  
   FBgn0029573   CG14770   1263311   1264209  +  899  
 
    Segment 20 
 
   Location   
  Gene key  FBgn0024987-FBgn0023169  
  Heatmap region span   X:1236118..1360786   
  Segment span   X:1264581..1269580   
  Length (genes)  2  
  Length (bp)  5000  
   Model Scoring   
  BIC  267.445334  
  logL  -128.320081  
  logL ratio  39.203113  
   Expression   
  Mean expression  10.418883  
  Median expression  10.592828  
  Tissue std. dev.  0.482394  
 
  No GO Slim enrichment  
  
   tissue    mean expression   
  5th Passage Drosophila S2 Cells  10.721059  
  Adult Accessory gland  10.554826  
  Adult Brain  11.333447  
  Adult Carcass  10.115293  
  Adult Crop  10.471431  
  Adult Eye  11.105122  
  Adult Fatbody  10.049252  
  Adult Female Spermatheca Mated  10.304548  
  Adult Female Spermatheca Virgin  10.376997  
  Adult Head  10.576098  
  Adult Heart  10.307871  
  Adult Hind Gut  10.547128  
  Adult Male Ejaculatory Duct  10.646515  
  Adult Mid Gut  10.051849  
  Adult Ovary  10.458880  
  Adult Salivary Gland  10.973975  
  Adult Testes  8.784161  
  Adult Thoracoabdominal ganglion  10.937041  
  Adult Whole Fly  9.993856  
  Larvae Wandering Tubules  10.491607  
  Larval Feeding Carcass  10.195754  
  Larval Feeding Central Nevous System  11.055401  
  Larval Feeding Hind Gut  10.640598  
  Larval Feeding Malpighian Tubule  10.385379  
  Larval Feeding Mid Gut  10.084509  
  Larval Feeding Salivary Gland  10.282853  
  Whole Larvae Feeding  9.864380  
 
  
   FlyBase ID    symbol    start    end    strand    length   
   FBgn0024987   ssx   1264581   1268717  +  4137  
   FBgn0023169   SNF1A   1269580   1272890  +  3311  
 
    Segment 21 
 
   Location   
  Gene key  FBgn0024986-FBgn0024985  
  Heatmap region span   X:1240584..1363679   
  Segment span   X:1274509..1287098   
  Length (genes)  3  
  Length (bp)  12590  
   Model Scoring   
  BIC  296.644650  
  logL  -142.919739  
  logL ratio  81.421342  
   Expression   
  Mean expression  8.538557  
  Median expression  8.633947  
  Tissue std. dev.  0.789595  
 
  No GO Slim enrichment  
  
   tissue    mean expression   
  5th Passage Drosophila S2 Cells  8.015531  
  Adult Accessory gland  9.025161  
  Adult Brain  9.855224  
  Adult Carcass  8.492765  
  Adult Crop  9.462104  
  Adult Eye  8.510125  
  Adult Fatbody  7.463648  
  Adult Female Spermatheca Mated  8.485560  
  Adult Female Spermatheca Virgin  8.408109  
  Adult Head  8.451448  
  Adult Heart  8.948114  
  Adult Hind Gut  8.999449  
  Adult Male Ejaculatory Duct  8.819042  
  Adult Mid Gut  9.170829  
  Adult Ovary  7.072387  
  Adult Salivary Gland  9.149029  
  Adult Testes  6.149348  
  Adult Thoracoabdominal ganglion  9.753557  
  Adult Whole Fly  7.524695  
  Larvae Wandering Tubules  8.729223  
  Larval Feeding Carcass  8.261633  
  Larval Feeding Central Nevous System  8.579066  
  Larval Feeding Hind Gut  8.506872  
  Larval Feeding Malpighian Tubule  8.907572  
  Larval Feeding Mid Gut  9.123362  
  Larval Feeding Salivary Gland  8.715115  
  Whole Larvae Feeding  7.962059  
 
  
   FlyBase ID    symbol    start    end    strand    length   
   FBgn0024986   CG3719  1272741   1274509   -  1769  
   FBgn0052813   CG32813   1274949   1286884  +  11936  
   FBgn0024985   CG11448   1287098   1291371  +  4274  
 
 
    Segment 22 
 
   Location   
  Gene key  FBgn0259108-FBgn0027796  
  Heatmap region span   X:1243390..1364699   
  Segment span   X:1307911..1346736   
  Length (genes)  2  
  Length (bp)  38826  
   Model Scoring   
  BIC  170.971083  
  logL  -80.082955  
  logL ratio  38.227744  
   Expression   
  Mean expression  4.916979  
  Median expression  4.973581  
  Tissue std. dev.  0.213485  
 
  No GO Slim enrichment  
  
   tissue    mean expression   
  5th Passage Drosophila S2 Cells  5.014184  
  Adult Accessory gland  5.003551  
  Adult Brain  4.874211  
  Adult Carcass  5.180582  
  Adult Crop  4.996691  
  Adult Eye  4.860983  
  Adult Fatbody  5.090455  
  Adult Female Spermatheca Mated  4.995337  
  Adult Female Spermatheca Virgin  4.993806  
  Adult Head  4.711953  
  Adult Heart  4.706816  
  Adult Hind Gut  4.771024  
  Adult Male Ejaculatory Duct  5.125804  
  Adult Mid Gut  5.145763  
  Adult Ovary  4.822915  
  Adult Salivary Gland  5.393563  
  Adult Testes  4.626880  
  Adult Thoracoabdominal ganglion  4.827031  
  Adult Whole Fly  4.373413  
  Larvae Wandering Tubules  5.023253  
  Larval Feeding Carcass  4.979093  
  Larval Feeding Central Nevous System  4.576441  
  Larval Feeding Hind Gut  4.702728  
  Larval Feeding Malpighian Tubule  5.004499  
  Larval Feeding Mid Gut  5.154986  
  Larval Feeding Salivary Gland  5.005362  
  Whole Larvae Feeding  4.797104  
 
  
   FlyBase ID    symbol    start    end    strand    length   
   FBgn0259108   futsch   1307911   1345416  +  37506  
   FBgn0027796      1346736   1348137  +  1402  
 
    Segment 23 
 
   Location   
  Gene key  FBgn0027795-FBgn0027793  
  Heatmap region span   X:1251646..1370963   
  Segment span   X:1351087..1355700   
  Length (genes)  3  
  Length (bp)  4614  
   Model Scoring   
  BIC  336.815480  
  logL  -163.005154  
  logL ratio  14.595637  
   Expression   
  Mean expression  6.623399  
  Median expression  6.329995  
  Tissue std. dev.  0.453680  
 
  No GO Slim enrichment  
  
   tissue    mean expression   
  5th Passage Drosophila S2 Cells  6.711195  
  Adult Accessory gland  6.477459  
  Adult Brain  6.275757  
  Adult Carcass  6.416763  
  Adult Crop  6.343976  
  Adult Eye  6.151901  
  Adult Fatbody  6.582312  
  Adult Female Spermatheca Mated  6.635353  
  Adult Female Spermatheca Virgin  6.820494  
  Adult Head  6.200992  
  Adult Heart  6.761983  
  Adult Hind Gut  6.435054  
  Adult Male Ejaculatory Duct  6.301918  
  Adult Mid Gut  6.411115  
  Adult Ovary  6.380193  
  Adult Salivary Gland  6.557383  
  Adult Testes  7.150488  
  Adult Thoracoabdominal ganglion  6.608394  
  Adult Whole Fly  6.168004  
  Larvae Wandering Tubules  7.330618  
  Larval Feeding Carcass  6.253266  
  Larval Feeding Central Nevous System  7.511457  
  Larval Feeding Hind Gut  6.415496  
  Larval Feeding Malpighian Tubule  8.207434  
  Larval Feeding Mid Gut  6.587002  
  Larval Feeding Salivary Gland  6.336018  
  Whole Larvae Feeding  6.799758  
 
  
   FlyBase ID    symbol    start    end    strand    length   
   FBgn0027795   CG14785  1349248   1351087   -  1840  
   FBgn0027794   CG14786  1351208   1354591   -  3384  
   FBgn0027793   CG14787  1354840   1355700   -  861  
 
 
    Segment 24 
 
   Location   
  Gene key  FBgn0028274-FBgn0026872  
  Heatmap region span   X:1264581..1372585   
  Segment span   X:1358245..1360786   
  Length (genes)  2  
  Length (bp)  2542  
   Model Scoring   
  BIC  250.675202  
  logL  -119.935015  
  logL ratio  18.081943  
   Expression   
  Mean expression  9.202342  
  Median expression  9.028140  
  Tissue std. dev.  0.697603  
 
  No GO Slim enrichment  
  
   tissue    mean expression   
  5th Passage Drosophila S2 Cells  9.299776  
  Adult Accessory gland  9.353098  
  Adult Brain  9.476556  
  Adult Carcass  9.400733  
  Adult Crop  9.395995  
  Adult Eye  9.676880  
  Adult Fatbody  9.800888  
  Adult Female Spermatheca Mated  9.649270  
  Adult Female Spermatheca Virgin  9.302111  
  Adult Head  9.310264  
  Adult Heart  9.660381  
  Adult Hind Gut  9.616887  
  Adult Male Ejaculatory Duct  9.499585  
  Adult Mid Gut  9.040253  
  Adult Ovary  8.554692  
  Adult Salivary Gland  10.272144  
  Adult Testes  6.304309  
  Adult Thoracoabdominal ganglion  9.667773  
  Adult Whole Fly  9.028140  
  Larvae Wandering Tubules  9.040778  
  Larval Feeding Carcass  8.690552  
  Larval Feeding Central Nevous System  8.676914  
  Larval Feeding Hind Gut  9.405978  
  Larval Feeding Malpighian Tubule  9.780845  
  Larval Feeding Mid Gut  9.125562  
  Larval Feeding Salivary Gland  8.831295  
  Whole Larvae Feeding  8.601579  
 
  
   FlyBase ID    symbol    start    end    strand    length   
   FBgn0028274   ns3  1356013   1358245   -  2233  
   FBgn0026872   CG14777   1360786   1362725  +  1940  
 
    Segment 25 
 
   Location   
  Gene key  FBgn0025383-FBgn0025382  
  Heatmap region span   X:1351087..1563312   
  Segment span   X:1366558..1370963   
  Length (genes)  2  
  Length (bp)  4406  
   Model Scoring   
  BIC  212.903771  
  logL  -101.049299  
  logL ratio  14.423397  
   Expression   
  Mean expression  6.136377  
  Median expression  5.890446  
  Tissue std. dev.  0.544106  
 
  No GO Slim enrichment  
  
   tissue    mean expression   
  5th Passage Drosophila S2 Cells  7.401535  
  Adult Accessory gland  5.937561  
  Adult Brain  7.469291  
  Adult Carcass  5.856655  
  Adult Crop  5.775709  
  Adult Eye  5.561443  
  Adult Fatbody  5.709699  
  Adult Female Spermatheca Mated  5.695415  
  Adult Female Spermatheca Virgin  5.749786  
  Adult Head  6.391331  
  Adult Heart  5.509284  
  Adult Hind Gut  5.687591  
  Adult Male Ejaculatory Duct  5.679034  
  Adult Mid Gut  6.793062  
  Adult Ovary  6.557500  
  Adult Salivary Gland  5.993830  
  Adult Testes  5.811722  
  Adult Thoracoabdominal ganglion  6.504358  
  Adult Whole Fly  5.907335  
  Larvae Wandering Tubules  6.057823  
  Larval Feeding Carcass  6.078078  
  Larval Feeding Central Nevous System  6.636617  
  Larval Feeding Hind Gut  5.709264  
  Larval Feeding Malpighian Tubule  5.932974  
  Larval Feeding Mid Gut  7.067521  
  Larval Feeding Salivary Gland  5.674933  
  Whole Larvae Feeding  6.532831  
 
  
   FlyBase ID    symbol    start    end    strand    length   
   FBgn0025383   CG14780   1366558   1367760  +  1203  
   FBgn0025382   Rab27  1367917   1370963   -  3047  
 
    Segment 26 
 
   Location   
  Gene key  FBgn0053513-FBgn0025393  
  Heatmap region span   X:1364699..1589922   
  Segment span   X:1401842..1406832   
  Length (genes)  2  
  Length (bp)  4991  
   Model Scoring   
  BIC  226.127092  
  logL  -107.660960  
  logL ratio  -9.958606  
   Expression   
  Mean expression  5.199381  
  Median expression  4.880375  
  Tissue std. dev.  0.801532  
 
  No GO Slim enrichment  
  
   tissue    mean expression   
  5th Passage Drosophila S2 Cells  7.128143  
  Adult Accessory gland  4.916099  
  Adult Brain  5.002963  
  Adult Carcass  5.044015  
  Adult Crop  4.743689  
  Adult Eye  4.693979  
  Adult Fatbody  5.042295  
  Adult Female Spermatheca Mated  4.899323  
  Adult Female Spermatheca Virgin  4.723217  
  Adult Head  4.942713  
  Adult Heart  4.641803  
  Adult Hind Gut  4.877838  
  Adult Male Ejaculatory Duct  5.257509  
  Adult Mid Gut  5.149641  
  Adult Ovary  4.886295  
  Adult Salivary Gland  5.142071  
  Adult Testes  4.657074  
  Adult Thoracoabdominal ganglion  4.834397  
  Adult Whole Fly  4.569636  
  Larvae Wandering Tubules  4.918266  
  Larval Feeding Carcass  8.073173  
  Larval Feeding Central Nevous System  5.149505  
  Larval Feeding Hind Gut  5.355784  
  Larval Feeding Malpighian Tubule  4.923217  
  Larval Feeding Mid Gut  5.190307  
  Larval Feeding Salivary Gland  4.790291  
  Whole Larvae Feeding  6.830045  
 
  
   FlyBase ID    symbol    start    end    strand    length   
   FBgn0053513   Nmdar2  1380259   1401842   -  21584  
   FBgn0025393   CG14795  1406260   1406832   -  573  
 
    Segment 27 
 
   Location   
  Gene key  FBgn0025394-FBgn0023530  
  Heatmap region span   X:1366558..1716183   
  Segment span   X:1407092..1563312   
  Length (genes)  5  
  Length (bp)  156221  
   Model Scoring   
  BIC  566.727268  
  logL  -277.961048  
  logL ratio  4.637166  
   Expression   
  Mean expression  6.707400  
  Median expression  6.598431  
  Tissue std. dev.  0.420850  
 
  No GO Slim enrichment  
  
   tissue    mean expression   
  5th Passage Drosophila S2 Cells  6.732717  
  Adult Accessory gland  6.927644  
  Adult Brain  6.433356  
  Adult Carcass  6.415123  
  Adult Crop  6.852205  
  Adult Eye  6.392303  
  Adult Fatbody  6.554165  
  Adult Female Spermatheca Mated  6.487414  
  Adult Female Spermatheca Virgin  6.481514  
  Adult Head  6.259101  
  Adult Heart  6.777358  
  Adult Hind Gut  6.621248  
  Adult Male Ejaculatory Duct  6.712976  
  Adult Mid Gut  6.515115  
  Adult Ovary  7.259356  
  Adult Salivary Gland  6.651345  
  Adult Testes  5.541018  
  Adult Thoracoabdominal ganglion  6.457748  
  Adult Whole Fly  6.443909  
  Larvae Wandering Tubules  7.645377  
  Larval Feeding Carcass  7.042050  
  Larval Feeding Central Nevous System  7.235752  
  Larval Feeding Hind Gut  6.808350  
  Larval Feeding Malpighian Tubule  7.193374  
  Larval Feeding Mid Gut  6.715759  
  Larval Feeding Salivary Gland  7.529354  
  Whole Larvae Feeding  6.414178  
 
  
   FlyBase ID    symbol    start    end    strand    length   
   FBgn0025394   inc   1407092   1411653  +  4562  
   FBgn0000210   br   1469165   1553962  +  84798  
   FBgn0025390   Mur2B  1417933   1512188   -  94256  
   FBgn0000482   dor   1559088   1562487  +  3400  
   FBgn0023530   CG3740  1562033   1563312   -  1280  
 
 
    Segment 28 
 
   Location   
  Gene key  FBgn0029587-FBgn0029588  
  Heatmap region span   X:1377446..1759868   
  Segment span   X:1577778..1586869   
  Length (genes)  4  
  Length (bp)  9092  
   Model Scoring   
  BIC  333.048064  
  logL  -161.121446  
  logL ratio  91.370574  
   Expression   
  Mean expression  4.894545  
  Median expression  4.830288  
  Tissue std. dev.  0.396565  
 
  No GO Slim enrichment  
  
   tissue    mean expression   
  5th Passage Drosophila S2 Cells  4.841423  
  Adult Accessory gland  4.840187  
  Adult Brain  5.970458  
  Adult Carcass  4.747482  
  Adult Crop  4.549564  
  Adult Eye  5.544855  
  Adult Fatbody  4.755505  
  Adult Female Spermatheca Mated  4.690964  
  Adult Female Spermatheca Virgin  4.719182  
  Adult Head  5.011857  
  Adult Heart  4.958222  
  Adult Hind Gut  4.571865  
  Adult Male Ejaculatory Duct  4.739166  
  Adult Mid Gut  4.680764  
  Adult Ovary  5.555532  
  Adult Salivary Gland  4.913111  
  Adult Testes  4.674061  
  Adult Thoracoabdominal ganglion  5.791062  
  Adult Whole Fly  4.577063  
  Larvae Wandering Tubules  4.635609  
  Larval Feeding Carcass  4.954470  
  Larval Feeding Central Nevous System  5.422723  
  Larval Feeding Hind Gut  4.476664  
  Larval Feeding Malpighian Tubule  4.574418  
  Larval Feeding Mid Gut  4.639322  
  Larval Feeding Salivary Gland  4.804013  
  Whole Larvae Feeding  4.513170  
 
  
   FlyBase ID    symbol    start    end    strand    length   
   FBgn0029587   CG14797   1577778   1578489  +  712  
   FBgn0024897   b6   1580659   1584220  +  3562  
   FBgn0023531   CG32809  1569113   1586580   -  17468  
   FBgn0029588   CG14798   1586869   1587876  +  1008  
 
 
    Segment 29 
 
   Location   
  Gene key  FBgn0025378-FBgn0029590  
  Heatmap region span   X:1407092..1762821   
  Segment span   X:1604196..1716183   
  Length (genes)  6  
  Length (bp)  111988  
   Model Scoring   
  BIC  481.920026  
  logL  -235.557427  
  logL ratio  150.687647  
   Expression   
  Mean expression  5.240572  
  Median expression  5.222384  
  Tissue std. dev.  0.537416  
 
  No GO Slim enrichment  
  
   tissue    mean expression   
  5th Passage Drosophila S2 Cells  5.213959  
  Adult Accessory gland  5.355918  
  Adult Brain  5.320714  
  Adult Carcass  5.542942  
  Adult Crop  5.150950  
  Adult Eye  5.010124  
  Adult Fatbody  4.988414  
  Adult Female Spermatheca Mated  5.057320  
  Adult Female Spermatheca Virgin  5.016225  
  Adult Head  4.965176  
  Adult Heart  4.912556  
  Adult Hind Gut  5.073589  
  Adult Male Ejaculatory Duct  5.138107  
  Adult Mid Gut  5.140853  
  Adult Ovary  5.087371  
  Adult Salivary Gland  5.391841  
  Adult Testes  7.870393  
  Adult Thoracoabdominal ganglion  5.227582  
  Adult Whole Fly  5.235265  
  Larvae Wandering Tubules  5.252228  
  Larval Feeding Carcass  5.279088  
  Larval Feeding Central Nevous System  5.019490  
  Larval Feeding Hind Gut  4.963756  
  Larval Feeding Malpighian Tubule  5.145946  
  Larval Feeding Mid Gut  4.976250  
  Larval Feeding Salivary Gland  4.966659  
  Whole Larvae Feeding  5.192732  
 
  
   FlyBase ID    symbol    start    end    strand    length   
   FBgn0025378   CG3795  1602971   1604196   -  1226  
   FBgn0025391   Scgdelta  1639909   1648096   -  8188  
   FBgn0026086   Adar   1667783   1678034  +  10252  
   FBgn0052806   CG32806   1683623   1684449  +  827  
   FBgn0029589   CG14810  1713528   1714374   -  847  
   FBgn0029590   CG14811  1715533   1716183   -  651  
 
 
    Segment 30 
 
   Location   
  Gene key  FBgn0026090-FBgn0028969  
  Heatmap region span   X:1563626..1774330   
  Segment span   X:1752984..1755825   
  Length (genes)  2  
  Length (bp)  2842  
   Model Scoring   
  BIC  246.603923  
  logL  -117.899375  
  logL ratio  58.889588  
   Expression   
  Mean expression  10.425575  
  Median expression  10.339355  
  Tissue std. dev.  0.711980  
 
  No GO Slim enrichment  
  
   tissue    mean expression   
  5th Passage Drosophila S2 Cells  11.429091  
  Adult Accessory gland  11.299100  
  Adult Brain  10.128269  
  Adult Carcass  9.879990  
  Adult Crop  10.146065  
  Adult Eye  10.505895  
  Adult Fatbody  10.162781  
  Adult Female Spermatheca Mated  9.990987  
  Adult Female Spermatheca Virgin  10.022007  
  Adult Head  10.113664  
  Adult Heart  10.113899  
  Adult Hind Gut  10.156606  
  Adult Male Ejaculatory Duct  11.008229  
  Adult Mid Gut  10.347317  
  Adult Ovary  9.700354  
  Adult Salivary Gland  11.222634  
  Adult Testes  8.641619  
  Adult Thoracoabdominal ganglion  10.189201  
  Adult Whole Fly  9.592567  
  Larvae Wandering Tubules  11.218893  
  Larval Feeding Carcass  10.339357  
  Larval Feeding Central Nevous System  10.206745  
  Larval Feeding Hind Gut  10.530686  
  Larval Feeding Malpighian Tubule  10.912244  
  Larval Feeding Mid Gut  10.615939  
  Larval Feeding Salivary Gland  12.509787  
  Whole Larvae Feeding  10.506587  
 
  
   FlyBase ID    symbol    start    end    strand    length   
   FBgn0026090   CG14812  1752004   1752984   -  981  
   FBgn0028969   deltaCOP  1753174   1755825   -  2652  
 
    Segment 31 
 
   Location   
  Gene key  FBgn0023516-FBgn0029594  
  Heatmap region span   X:1752984..1834269   
  Segment span   X:1763094..1774330   
  Length (genes)  7  
  Length (bp)  11237  
   Model Scoring   
  BIC  622.230211  
  logL  -305.712520  
  logL ratio  364.260361  
   Expression   
  Mean expression  10.069898  
  Median expression  10.096752  
  Tissue std. dev.  0.313783  
 
  No GO Slim enrichment  
  
   tissue    mean expression   
  5th Passage Drosophila S2 Cells  10.000455  
  Adult Accessory gland  10.103844  
  Adult Brain  10.087813  
  Adult Carcass  9.881451  
  Adult Crop  10.284046  
  Adult Eye  10.278387  
  Adult Fatbody  10.107563  
  Adult Female Spermatheca Mated  9.979402  
  Adult Female Spermatheca Virgin  9.984592  
  Adult Head  9.993129  
  Adult Heart  10.308851  
  Adult Hind Gut  10.244233  
  Adult Male Ejaculatory Duct  10.455940  
  Adult Mid Gut  9.933015  
  Adult Ovary  10.361140  
  Adult Salivary Gland  10.124743  
  Adult Testes  8.767073  
  Adult Thoracoabdominal ganglion  10.071807  
  Adult Whole Fly  9.892718  
  Larvae Wandering Tubules  10.207976  
  Larval Feeding Carcass  9.877341  
  Larval Feeding Central Nevous System  10.045686  
  Larval Feeding Hind Gut  10.242239  
  Larval Feeding Malpighian Tubule  10.471430  
  Larval Feeding Mid Gut  9.968720  
  Larval Feeding Salivary Gland  10.400121  
  Whole Larvae Feeding  9.813524  
 
  
   FlyBase ID    symbol    start    end    strand    length   
   FBgn0023516   Pex5  1760865   1763094   -  2230  
   FBgn0023517   Pgam5  1766435   1767570   -  1136  
   FBgn0014411   Vps26   1767896   1770514  +  2619  
   FBgn0026089   CG14817  1770738   1771231   -  494  
   FBgn0023514   CG14805   1771352   1773114  +  1763  
   FBgn0026088   CG14818  1773044   1773615   -  572  
   FBgn0029594   CG14806   1774330   1775467  +  1138  
 
 
    Segment 32 
 
   Location   
  Gene key  FBgn0023519-FBgn0052803  
  Heatmap region span   X:1759942..1936344   
  Segment span   X:1784332..1795971   
  Length (genes)  3  
  Length (bp)  11640  
   Model Scoring   
  BIC  381.504208  
  logL  -185.349518  
  logL ratio  60.623512  
   Expression   
  Mean expression  10.123756  
  Median expression  9.723337  
  Tissue std. dev.  0.361454  
 
  No GO Slim enrichment  
  
   tissue    mean expression   
  5th Passage Drosophila S2 Cells  10.958867  
  Adult Accessory gland  10.049695  
  Adult Brain  10.355194  
  Adult Carcass  9.738819  
  Adult Crop  10.386328  
  Adult Eye  9.992130  
  Adult Fatbody  9.697253  
  Adult Female Spermatheca Mated  9.511011  
  Adult Female Spermatheca Virgin  9.507051  
  Adult Head  9.945935  
  Adult Heart  10.048564  
  Adult Hind Gut  10.100206  
  Adult Male Ejaculatory Duct  10.364872  
  Adult Mid Gut  9.817423  
  Adult Ovary  10.545837  
  Adult Salivary Gland  10.368595  
  Adult Testes  9.454242  
  Adult Thoracoabdominal ganglion  10.219902  
  Adult Whole Fly  9.969551  
  Larvae Wandering Tubules  10.313456  
  Larval Feeding Carcass  10.125040  
  Larval Feeding Central Nevous System  10.859132  
  Larval Feeding Hind Gut  10.196134  
  Larval Feeding Malpighian Tubule  10.311912  
  Larval Feeding Mid Gut  10.044975  
  Larval Feeding Salivary Gland  10.377507  
  Whole Larvae Feeding  10.081790  
 
  
   FlyBase ID    symbol    start    end    strand    length   
   FBgn0023519   mRpL16   1784332   1785313  +  982  
   FBgn0000117   arm  1785434   1794673   -  9240  
   FBgn0052803   CG32803  1794721   1795971   -  1251  
 
 
    Segment 33 
 
   Location   
  Gene key  FBgn0023509-FBgn0023521  
  Heatmap region span   X:1763094..1943620   
  Segment span   X:1802246..1834269   
  Length (genes)  6  
  Length (bp)  32024  
   Model Scoring   
  BIC  615.904651  
  logL  -302.549739  
  logL ratio  113.417679  
   Expression   
  Mean expression  7.921084  
  Median expression  7.850298  
  Tissue std. dev.  0.412779  
 
  No GO Slim enrichment  
  
   tissue    mean expression   
  5th Passage Drosophila S2 Cells  8.406662  
  Adult Accessory gland  8.606447  
  Adult Brain  8.348220  
  Adult Carcass  7.473977  
  Adult Crop  8.008114  
  Adult Eye  8.405997  
  Adult Fatbody  7.776342  
  Adult Female Spermatheca Mated  7.662599  
  Adult Female Spermatheca Virgin  7.652230  
  Adult Head  7.884378  
  Adult Heart  7.952061  
  Adult Hind Gut  7.657133  
  Adult Male Ejaculatory Duct  7.775433  
  Adult Mid Gut  7.466991  
  Adult Ovary  8.677248  
  Adult Salivary Gland  8.634517  
  Adult Testes  7.340624  
  Adult Thoracoabdominal ganglion  7.943325  
  Adult Whole Fly  7.621333  
  Larvae Wandering Tubules  7.972334  
  Larval Feeding Carcass  7.708041  
  Larval Feeding Central Nevous System  8.373153  
  Larval Feeding Hind Gut  7.564270  
  Larval Feeding Malpighian Tubule  7.924106  
  Larval Feeding Mid Gut  7.447780  
  Larval Feeding Salivary Gland  8.369960  
  Whole Larvae Feeding  7.215986  
 
  
   FlyBase ID    symbol    start    end    strand    length   
   FBgn0023509   mip130   1802246   1814351  +  12106  
   FBgn0023511   Edem1  1797737   1802860   -  5124  
   FBgn0023508   Ocrl   1817038   1821555  +  4518  
   FBgn0023522   CG11596   1821713   1824552  +  2840  
   FBgn0023520   CG3857  1831731   1834040   -  2310  
   FBgn0023521   CG3587   1834269   1835574  +  1306  
 
 
    Segment 34 
 
   Location   
  Gene key  FBgn0026087-FBgn0023546  
  Heatmap region span   X:1783808..1955948   
  Segment span   X:1836776..1891620   
  Length (genes)  2  
  Length (bp)  54845  
   Model Scoring   
  BIC  172.160979  
  logL  -80.677903  
  logL ratio  37.484471  
   Expression   
  Mean expression  4.897234  
  Median expression  4.948851  
  Tissue std. dev.  0.173310  
 
  No GO Slim enrichment  
  
   tissue    mean expression   
  5th Passage Drosophila S2 Cells  4.919114  
  Adult Accessory gland  4.894882  
  Adult Brain  4.748664  
  Adult Carcass  4.863936  
  Adult Crop  4.911461  
  Adult Eye  4.829669  
  Adult Fatbody  5.161262  
  Adult Female Spermatheca Mated  5.001356  
  Adult Female Spermatheca Virgin  5.013549  
  Adult Head  4.702628  
  Adult Heart  4.912325  
  Adult Hind Gut  4.813012  
  Adult Male Ejaculatory Duct  4.906086  
  Adult Mid Gut  4.886422  
  Adult Ovary  5.132693  
  Adult Salivary Gland  5.005439  
  Adult Testes  4.660937  
  Adult Thoracoabdominal ganglion  4.760706  
  Adult Whole Fly  4.478168  
  Larvae Wandering Tubules  5.305606  
  Larval Feeding Carcass  5.123556  
  Larval Feeding Central Nevous System  4.732473  
  Larval Feeding Hind Gut  4.935137  
  Larval Feeding Malpighian Tubule  4.870895  
  Larval Feeding Mid Gut  5.001964  
  Larval Feeding Salivary Gland  4.996457  
  Whole Larvae Feeding  4.656910  
 
  
   FlyBase ID    symbol    start    end    strand    length   
   FBgn0026087      1836776   1888227  +  51452  
   FBgn0023546      1891620   1898128  +  6509  
 
    Segment 35 
 
   Location   
  Gene key  FBgn0023545-FBgn0000667  
  Heatmap region span   X:1784332..1959169   
  Segment span   X:1903376..1936344   
  Length (genes)  3  
  Length (bp)  32969  
   Model Scoring   
  BIC  342.089448  
  logL  -165.642138  
  logL ratio  56.967919  
   Expression   
  Mean expression  9.199475  
  Median expression  9.013770  
  Tissue std. dev.  0.619265  
 
  No GO Slim enrichment  
  
   tissue    mean expression   
  5th Passage Drosophila S2 Cells  9.131924  
  Adult Accessory gland  9.603280  
  Adult Brain  9.331109  
  Adult Carcass  9.356923  
  Adult Crop  9.980816  
  Adult Eye  9.063762  
  Adult Fatbody  8.623051  
  Adult Female Spermatheca Mated  8.499149  
  Adult Female Spermatheca Virgin  8.490838  
  Adult Head  9.195915  
  Adult Heart  9.637947  
  Adult Hind Gut  9.741664  
  Adult Male Ejaculatory Duct  9.437278  
  Adult Mid Gut  8.673035  
  Adult Ovary  9.619397  
  Adult Salivary Gland  8.985755  
  Adult Testes  7.283435  
  Adult Thoracoabdominal ganglion  8.947017  
  Adult Whole Fly  9.299365  
  Larvae Wandering Tubules  8.741965  
  Larval Feeding Carcass  10.256801  
  Larval Feeding Central Nevous System  9.397782  
  Larval Feeding Hind Gut  9.968712  
  Larval Feeding Malpighian Tubule  8.522057  
  Larval Feeding Mid Gut  8.922770  
  Larval Feeding Salivary Gland  10.057331  
  Whole Larvae Feeding  9.616736  
 
  
   FlyBase ID    symbol    start    end    strand    length   
   FBgn0023545   CG4406  1901937   1903376   -  1440  
   FBgn0003964   usp  1932831   1935343   -  2513  
   FBgn0000667   Actn  1919149   1936344   -  17196  
 
 
    Segment 36 
 
   Location   
  Gene key  FBgn0025632-FBgn0025625  
  Heatmap region span   X:1836776..2043633   
  Segment span   X:1946650..1955948   
  Length (genes)  2  
  Length (bp)  9299  
   Model Scoring   
  BIC  204.187811  
  logL  -96.691320  
  logL ratio  19.306469  
   Expression   
  Mean expression  5.846421  
  Median expression  5.568051  
  Tissue std. dev.  0.792268  
 
  No GO Slim enrichment  
  
   tissue    mean expression   
  5th Passage Drosophila S2 Cells  5.551182  
  Adult Accessory gland  5.989830  
  Adult Brain  7.864373  
  Adult Carcass  5.778763  
  Adult Crop  6.396206  
  Adult Eye  7.265225  
  Adult Fatbody  5.824915  
  Adult Female Spermatheca Mated  5.193357  
  Adult Female Spermatheca Virgin  5.199884  
  Adult Head  6.488153  
  Adult Heart  6.079910  
  Adult Hind Gut  6.121719  
  Adult Male Ejaculatory Duct  5.687281  
  Adult Mid Gut  5.550254  
  Adult Ovary  5.588754  
  Adult Salivary Gland  5.547427  
  Adult Testes  5.043824  
  Adult Thoracoabdominal ganglion  7.735504  
  Adult Whole Fly  4.955955  
  Larvae Wandering Tubules  5.238525  
  Larval Feeding Carcass  5.221955  
  Larval Feeding Central Nevous System  6.976271  
  Larval Feeding Hind Gut  5.655222  
  Larval Feeding Malpighian Tubule  5.056730  
  Larval Feeding Mid Gut  5.574666  
  Larval Feeding Salivary Gland  5.146597  
  Whole Larvae Feeding  5.120875  
 
  
   FlyBase ID    symbol    start    end    strand    length   
   FBgn0025632   CG4313   1946650   1949447  +  2798  
   FBgn0025625   Sik2  1949343   1955948   -  6606  
 
    Segment 37 
 
   Location   
  Gene key  FBgn0029596-FBgn0025627  
  Heatmap region span   X:1937851..2056160   
  Segment span   X:1960392..1962627   
  Length (genes)  2  
  Length (bp)  2236  
   Model Scoring   
  BIC  183.029711  
  logL  -86.112269  
  logL ratio  30.380815  
   Expression   
  Mean expression  4.688462  
  Median expression  4.581495  
  Tissue std. dev.  0.441549  
 
  No GO Slim enrichment  
  
   tissue    mean expression   
  5th Passage Drosophila S2 Cells  4.827468  
  Adult Accessory gland  4.904530  
  Adult Brain  4.197608  
  Adult Carcass  4.779850  
  Adult Crop  4.630892  
  Adult Eye  4.312319  
  Adult Fatbody  4.589899  
  Adult Female Spermatheca Mated  4.654269  
  Adult Female Spermatheca Virgin  4.612936  
  Adult Head  4.344685  
  Adult Heart  4.322048  
  Adult Hind Gut  4.788895  
  Adult Male Ejaculatory Duct  4.647224  
  Adult Mid Gut  4.905938  
  Adult Ovary  4.556447  
  Adult Salivary Gland  5.105280  
  Adult Testes  6.670089  
  Adult Thoracoabdominal ganglion  4.360727  
  Adult Whole Fly  4.673787  
  Larvae Wandering Tubules  4.629753  
  Larval Feeding Carcass  4.676171  
  Larval Feeding Central Nevous System  4.330278  
  Larval Feeding Hind Gut  4.436694  
  Larval Feeding Malpighian Tubule  4.528496  
  Larval Feeding Mid Gut  4.803285  
  Larval Feeding Salivary Gland  4.730446  
  Whole Larvae Feeding  4.568456  
 
  
   FlyBase ID    symbol    start    end    strand    length   
   FBgn0029596   CG14054  1959306   1960392   -  1087  
   FBgn0025627   CG4194   1962627   1963721  +  1095  
 
    Segment 38 
 
   Location   
  Gene key  FBgn0025628-FBgn0013432  
  Heatmap region span   X:1946650..2066561   
  Segment span   X:1967511..2043633   
  Length (genes)  8  
  Length (bp)  76123  
   Model Scoring   
  BIC  849.590478  
  logL  -419.392653  
  logL ratio  241.858592  
   Expression   
  Mean expression  9.543450  
  Median expression  9.414517  
  Tissue std. dev.  0.507041  
 
  No GO Slim enrichment  
  
   tissue    mean expression   
  5th Passage Drosophila S2 Cells  10.226102  
  Adult Accessory gland  9.425463  
  Adult Brain  9.074872  
  Adult Carcass  9.764560  
  Adult Crop  9.862285  
  Adult Eye  9.552220  
  Adult Fatbody  10.079147  
  Adult Female Spermatheca Mated  9.839251  
  Adult Female Spermatheca Virgin  9.966497  
  Adult Head  9.643549  
  Adult Heart  9.980922  
  Adult Hind Gut  10.096976  
  Adult Male Ejaculatory Duct  9.816635  
  Adult Mid Gut  9.706125  
  Adult Ovary  9.331549  
  Adult Salivary Gland  9.506472  
  Adult Testes  7.642580  
  Adult Thoracoabdominal ganglion  9.029845  
  Adult Whole Fly  9.294783  
  Larvae Wandering Tubules  9.620094  
  Larval Feeding Carcass  9.567224  
  Larval Feeding Central Nevous System  8.794899  
  Larval Feeding Hind Gut  9.777490  
  Larval Feeding Malpighian Tubule  9.972345  
  Larval Feeding Mid Gut  9.509584  
  Larval Feeding Salivary Gland  9.210614  
  Whole Larvae Feeding  9.381058  
 
  
   FlyBase ID    symbol    start    end    strand    length   
   FBgn0025628   CG4199  1960468   1967511   -  7044  
   FBgn0025629   CG4045  1968828   1969933   -  1106  
   FBgn0025624   CG4025   1971015   1972488  +  1474  
   FBgn0040394   CG16903  1972371   1974416   -  2046  
   FBgn0000382   csw   1988269   2007698  +  19430  
   FBgn0023507   CG3835   2035521   2038972  +  3452  
   FBgn0004654   Pgd   2039169   2042599  +  3431  
   FBgn0013432   bcn92  2042480   2043633   -  1154  
 
 
    Segment 39 
 
   Location   
  Gene key  FBgn0023541-FBgn0015036  
  Heatmap region span   X:2053016..2107808   
  Segment span   X:2069346..2072917   
  Length (genes)  3  
  Length (bp)  3572  
   Model Scoring   
  BIC  384.746812  
  logL  -186.970820  
  logL ratio  -33.135237  
   Expression   
  Mean expression  6.933440  
  Median expression  6.314049  
  Tissue std. dev.  1.129599  
 
  No GO Slim enrichment  
  
   tissue    mean expression   
  5th Passage Drosophila S2 Cells  5.553180  
  Adult Accessory gland  6.061328  
  Adult Brain  6.008206  
  Adult Carcass  7.396984  
  Adult Crop  6.753398  
  Adult Eye  7.168320  
  Adult Fatbody  7.478310  
  Adult Female Spermatheca Mated  6.709433  
  Adult Female Spermatheca Virgin  7.001483  
  Adult Head  6.887783  
  Adult Heart  7.411231  
  Adult Hind Gut  8.410622  
  Adult Male Ejaculatory Duct  7.461051  
  Adult Mid Gut  9.507805  
  Adult Ovary  5.422998  
  Adult Salivary Gland  6.637297  
  Adult Testes  5.722107  
  Adult Thoracoabdominal ganglion  6.213573  
  Adult Whole Fly  6.571895  
  Larvae Wandering Tubules  7.611438  
  Larval Feeding Carcass  5.510673  
  Larval Feeding Central Nevous System  5.143571  
  Larval Feeding Hind Gut  7.418728  
  Larval Feeding Malpighian Tubule  8.519465  
  Larval Feeding Mid Gut  9.129616  
  Larval Feeding Salivary Gland  5.493095  
  Whole Larvae Feeding  7.999290  
 
  
   FlyBase ID    symbol    start    end    strand    length   
   FBgn0023541   Cyp4d14  2067448   2069346   -  1899  
   FBgn0011576   Cyp4d2   2070924   2072761  +  1838  
   FBgn0015036   Cyp4ae1   2072917   2075034  +  2118  
 
 
    Segment 40 
 
   Location   
  Gene key  FBgn0003116-FBgn0040899  
  Heatmap region span   X:2056160..2116492   
  Segment span   X:2075127..2078973   
  Length (genes)  3  
  Length (bp)  3847  
   Model Scoring   
  BIC  356.317003  
  logL  -172.755916  
  logL ratio  48.749340  
   Expression   
  Mean expression  9.384009  
  Median expression  9.708569  
  Tissue std. dev.  0.498641  
 
  No GO Slim enrichment  
  
   tissue    mean expression   
  5th Passage Drosophila S2 Cells  9.088569  
  Adult Accessory gland  9.657228  
  Adult Brain  9.149812  
  Adult Carcass  9.834187  
  Adult Crop  9.094072  
  Adult Eye  9.757522  
  Adult Fatbody  10.372863  
  Adult Female Spermatheca Mated  10.127380  
  Adult Female Spermatheca Virgin  10.212345  
  Adult Head  9.472555  
  Adult Heart  10.185648  
  Adult Hind Gut  9.071226  
  Adult Male Ejaculatory Duct  9.714163  
  Adult Mid Gut  9.126461  
  Adult Ovary  9.836767  
  Adult Salivary Gland  9.392062  
  Adult Testes  8.251078  
  Adult Thoracoabdominal ganglion  9.400327  
  Adult Whole Fly  9.459397  
  Larvae Wandering Tubules  9.110870  
  Larval Feeding Carcass  8.996506  
  Larval Feeding Central Nevous System  9.184768  
  Larval Feeding Hind Gut  9.020828  
  Larval Feeding Malpighian Tubule  9.279520  
  Larval Feeding Mid Gut  8.632360  
  Larval Feeding Salivary Gland  9.215134  
  Whole Larvae Feeding  8.724593  
 
  
   FlyBase ID    symbol    start    end    strand    length   
   FBgn0003116   pn   2075127   2076972  +  1846  
   FBgn0023542   Nmd3  2076851   2078683   -  1833  
   FBgn0040899   CG17776   2078973   2079295  +  323  
 
 
    Segment 41 
 
   Location   
  Gene key  FBgn0040900-FBgn0029603  
  Heatmap region span   X:2060848..2117884   
  Segment span   X:2080888..2087092   
  Length (genes)  3  
  Length (bp)  6205  
   Model Scoring   
  BIC  303.369449  
  logL  -146.282138  
  logL ratio  26.357364  
   Expression   
  Mean expression  5.810851  
  Median expression  5.662492  
  Tissue std. dev.  0.491155  
 
  No GO Slim enrichment  
  
   tissue    mean expression   
  5th Passage Drosophila S2 Cells  6.158967  
  Adult Accessory gland  5.713292  
  Adult Brain  5.639288  
  Adult Carcass  6.270323  
  Adult Crop  5.494945  
  Adult Eye  6.136252  
  Adult Fatbody  5.861297  
  Adult Female Spermatheca Mated  5.648981  
  Adult Female Spermatheca Virgin  5.657155  
  Adult Head  5.552136  
  Adult Heart  5.479687  
  Adult Hind Gut  5.533780  
  Adult Male Ejaculatory Duct  5.982949  
  Adult Mid Gut  5.673496  
  Adult Ovary  6.859417  
  Adult Salivary Gland  5.698996  
  Adult Testes  7.713887  
  Adult Thoracoabdominal ganglion  5.332455  
  Adult Whole Fly  5.671017  
  Larvae Wandering Tubules  5.592398  
  Larval Feeding Carcass  5.859889  
  Larval Feeding Central Nevous System  5.214394  
  Larval Feeding Hind Gut  5.601885  
  Larval Feeding Malpighian Tubule  5.584196  
  Larval Feeding Mid Gut  5.778146  
  Larval Feeding Salivary Gland  5.558020  
  Whole Larvae Feeding  5.625729  
 
  
   FlyBase ID    symbol    start    end    strand    length   
   FBgn0040900   CG17777   2080888   2081501  +  614  
   FBgn0024984   CG3457   2081855   2082748  +  894  
   FBgn0029603   CG14053  2085978   2087092   -  1115  
 
 
    Segment 42 
 
   Location   
  Gene key  FBgn0023550-FBgn0053548  
  Heatmap region span   X:2069346..2140264   
  Segment span   X:2097837..2107808   
  Length (genes)  2  
  Length (bp)  9972  
   Model Scoring   
  BIC  233.458098  
  logL  -111.326463  
  logL ratio  -8.089746  
   Expression   
  Mean expression  5.922956  
  Median expression  5.014653  
  Tissue std. dev.  1.459704  
 
  No GO Slim enrichment  
  
   tissue    mean expression   
  5th Passage Drosophila S2 Cells  4.859332  
  Adult Accessory gland  5.054481  
  Adult Brain  4.925053  
  Adult Carcass  8.519805  
  Adult Crop  7.046997  
  Adult Eye  7.259370  
  Adult Fatbody  8.515386  
  Adult Female Spermatheca Mated  4.738153  
  Adult Female Spermatheca Virgin  4.650601  
  Adult Head  6.360292  
  Adult Heart  9.092160  
  Adult Hind Gut  5.679435  
  Adult Male Ejaculatory Duct  5.064014  
  Adult Mid Gut  5.184503  
  Adult Ovary  4.814159  
  Adult Salivary Gland  4.936549  
  Adult Testes  4.715511  
  Adult Thoracoabdominal ganglion  4.862998  
  Adult Whole Fly  6.426838  
  Larvae Wandering Tubules  4.954638  
  Larval Feeding Carcass  9.298239  
  Larval Feeding Central Nevous System  4.591183  
  Larval Feeding Hind Gut  6.625300  
  Larval Feeding Malpighian Tubule  4.918639  
  Larval Feeding Mid Gut  5.081278  
  Larval Feeding Salivary Gland  4.829008  
  Whole Larvae Feeding  6.915893  
 
  
   FlyBase ID    symbol    start    end    strand    length   
   FBgn0023550   CG18031  2095852   2097837   -  1986  
   FBgn0053548   msta  2099883   2107808   -  7926  
 
    Segment 43 
 
   Location   
  Gene key  FBgn0003048-FBgn0001330  
  Heatmap region span   X:2088616..2186779   
  Segment span   X:2119744..2136071   
  Length (genes)  2  
  Length (bp)  16328  
   Model Scoring   
  BIC  230.251469  
  logL  -109.723149  
  logL ratio  9.830810  
   Expression   
  Mean expression  6.991030  
  Median expression  7.015835  
  Tissue std. dev.  0.556627  
 
  No GO Slim enrichment  
  
   tissue    mean expression   
  5th Passage Drosophila S2 Cells  7.739998  
  Adult Accessory gland  7.330414  
  Adult Brain  7.289694  
  Adult Carcass  6.421155  
  Adult Crop  7.124063  
  Adult Eye  6.915209  
  Adult Fatbody  7.136414  
  Adult Female Spermatheca Mated  6.970038  
  Adult Female Spermatheca Virgin  6.585441  
  Adult Head  6.496529  
  Adult Heart  7.172951  
  Adult Hind Gut  6.573217  
  Adult Male Ejaculatory Duct  6.707940  
  Adult Mid Gut  6.604458  
  Adult Ovary  8.739610  
  Adult Salivary Gland  6.742660  
  Adult Testes  5.763468  
  Adult Thoracoabdominal ganglion  7.213672  
  Adult Whole Fly  6.768412  
  Larvae Wandering Tubules  6.807128  
  Larval Feeding Carcass  7.275785  
  Larval Feeding Central Nevous System  7.803613  
  Larval Feeding Hind Gut  7.086737  
  Larval Feeding Malpighian Tubule  6.667219  
  Larval Feeding Mid Gut  6.883160  
  Larval Feeding Salivary Gland  7.584680  
  Whole Larvae Feeding  6.354157  
 
  
   FlyBase ID    symbol    start    end    strand    length   
   FBgn0003048   pcx   2119744   2132089  +  12346  
   FBgn0001330   kz  2131956   2136071   -  4116  
 
    Segment 44 
 
   Location   
  Gene key  FBgn0000377-FBgn0029608  
  Heatmap region span   X:2116492..2238670   
  Segment span   X:2144014..2148163   
  Length (genes)  3  
  Length (bp)  4150  
   Model Scoring   
  BIC  391.806346  
  logL  -190.500587  
  logL ratio  -8.772772  
   Expression   
  Mean expression  8.492033  
  Median expression  8.859273  
  Tissue std. dev.  0.844737  
 
  
   GO ID    description    ratio    P-value   
   GO:0006810   transport  2/3  0.0141  
   GO:0005622   intracellular  2/3  0.0247  
 
  
   tissue    mean expression   
  5th Passage Drosophila S2 Cells  8.724980  
  Adult Accessory gland  7.925742  
  Adult Brain  8.966625  
  Adult Carcass  9.123277  
  Adult Crop  8.698726  
  Adult Eye  9.590537  
  Adult Fatbody  9.360242  
  Adult Female Spermatheca Mated  9.078202  
  Adult Female Spermatheca Virgin  9.089735  
  Adult Head  9.333695  
  Adult Heart  9.622196  
  Adult Hind Gut  9.480301  
  Adult Male Ejaculatory Duct  8.722044  
  Adult Mid Gut  9.435703  
  Adult Ovary  6.912974  
  Adult Salivary Gland  8.261891  
  Adult Testes  7.428378  
  Adult Thoracoabdominal ganglion  9.163360  
  Adult Whole Fly  8.041413  
  Larvae Wandering Tubules  7.152204  
  Larval Feeding Carcass  8.730249  
  Larval Feeding Central Nevous System  7.678974  
  Larval Feeding Hind Gut  8.010300  
  Larval Feeding Malpighian Tubule  7.623025  
  Larval Feeding Mid Gut  8.298821  
  Larval Feeding Salivary Gland  6.557751  
  Whole Larvae Feeding  8.273548  
 
  
   FlyBase ID    symbol    start    end    strand    length   
   FBgn0000377   crn  2141610   2144014   -  2405  
   FBgn0023525   CG3191  2144172   2146017   -  1846  
   FBgn0029608   CG3091  2146365   2148163   -  1799  
 
 
    Segment 45 
 
   Location   
  Gene key  FBgn0023527-FBgn0023526  
  Heatmap region span   X:2119744..2322996   
  Segment span   X:2156873..2186779   
  Length (genes)  4  
  Length (bp)  29907  
   Model Scoring   
  BIC  451.552233  
  logL  -220.373531  
  logL ratio  74.430157  
   Expression   
  Mean expression  9.066832  
  Median expression  8.741102  
  Tissue std. dev.  0.492581  
 
  No GO Slim enrichment  
  
   tissue    mean expression   
  5th Passage Drosophila S2 Cells  9.430487  
  Adult Accessory gland  9.781117  
  Adult Brain  9.591210  
  Adult Carcass  8.502631  
  Adult Crop  8.768946  
  Adult Eye  8.913631  
  Adult Fatbody  9.123801  
  Adult Female Spermatheca Mated  8.946656  
  Adult Female Spermatheca Virgin  8.783574  
  Adult Head  8.734295  
  Adult Heart  9.168076  
  Adult Hind Gut  8.664271  
  Adult Male Ejaculatory Duct  9.214967  
  Adult Mid Gut  8.958291  
  Adult Ovary  10.439567  
  Adult Salivary Gland  9.377344  
  Adult Testes  7.837227  
  Adult Thoracoabdominal ganglion  9.413713  
  Adult Whole Fly  9.411081  
  Larvae Wandering Tubules  9.173283  
  Larval Feeding Carcass  9.080257  
  Larval Feeding Central Nevous System  9.634474  
  Larval Feeding Hind Gut  8.871140  
  Larval Feeding Malpighian Tubule  8.892876  
  Larval Feeding Mid Gut  8.606474  
  Larval Feeding Salivary Gland  9.083529  
  Whole Larvae Feeding  8.401543  
 
  
   FlyBase ID    symbol    start    end    strand    length   
   FBgn0023527   CG3071   2156873   2158858  +  1986  
   FBgn0023528   CG2924  2159024   2164789   -  5766  
   FBgn0023529   CG2918  2165318   2169111   -  3794  
   FBgn0023526   CG2865  2175545   2186779   -  11235  
 
 
    Segment 46 
 
   Location   
  Gene key  FBgn0040392-FBgn0053218  
  Heatmap region span   X:2140264..2341802   
  Segment span   X:2219723..2224192   
  Length (genes)  2  
  Length (bp)  4470  
   Model Scoring   
  BIC  225.023078  
  logL  -107.108953  
  logL ratio  1.301298  
   Expression   
  Mean expression  5.177028  
  Median expression  5.351895  
  Tissue std. dev.  0.645209  
 
  No GO Slim enrichment  
  
   tissue    mean expression   
  5th Passage Drosophila S2 Cells  5.521779  
  Adult Accessory gland  5.020455  
  Adult Brain  4.676671  
  Adult Carcass  4.972647  
  Adult Crop  5.053079  
  Adult Eye  4.934893  
  Adult Fatbody  4.901417  
  Adult Female Spermatheca Mated  5.036117  
  Adult Female Spermatheca Virgin  5.112297  
  Adult Head  4.844225  
  Adult Heart  4.939801  
  Adult Hind Gut  4.874968  
  Adult Male Ejaculatory Duct  5.147960  
  Adult Mid Gut  4.996171  
  Adult Ovary  4.906486  
  Adult Salivary Gland  5.280400  
  Adult Testes  8.242498  
  Adult Thoracoabdominal ganglion  4.935897  
  Adult Whole Fly  5.775049  
  Larvae Wandering Tubules  4.954865  
  Larval Feeding Carcass  5.040439  
  Larval Feeding Central Nevous System  5.122730  
  Larval Feeding Hind Gut  4.713056  
  Larval Feeding Malpighian Tubule  4.892572  
  Larval Feeding Mid Gut  5.152505  
  Larval Feeding Salivary Gland  5.405143  
  Whole Larvae Feeding  5.325642  
 
  
   FlyBase ID    symbol    start    end    strand    length   
   FBgn0040392   CG14050  2218859   2219723   -  865  
   FBgn0053218   CR33218   2224192   2224859  +  668  
 
    Segment 47 
 
   Location   
  Gene key  FBgn0044047-FBgn0040389  
  Heatmap region span   X:2144014..2364036   
  Segment span   X:2228691..2238670   
  Length (genes)  3  
  Length (bp)  9980  
   Model Scoring   
  BIC  333.486663  
  logL  -161.340745  
  logL ratio  37.449424  
   Expression   
  Mean expression  8.170323  
  Median expression  8.182970  
  Tissue std. dev.  0.635874  
 
  No GO Slim enrichment  
  
   tissue    mean expression   
  5th Passage Drosophila S2 Cells  9.660729  
  Adult Accessory gland  7.367233  
  Adult Brain  9.232039  
  Adult Carcass  7.969923  
  Adult Crop  7.680210  
  Adult Eye  8.493832  
  Adult Fatbody  8.441254  
  Adult Female Spermatheca Mated  8.069015  
  Adult Female Spermatheca Virgin  8.240833  
  Adult Head  8.821184  
  Adult Heart  8.490143  
  Adult Hind Gut  7.674909  
  Adult Male Ejaculatory Duct  7.629233  
  Adult Mid Gut  7.547251  
  Adult Ovary  8.836045  
  Adult Salivary Gland  7.742543  
  Adult Testes  7.140642  
  Adult Thoracoabdominal ganglion  9.088804  
  Adult Whole Fly  7.992256  
  Larvae Wandering Tubules  7.667707  
  Larval Feeding Carcass  7.698236  
  Larval Feeding Central Nevous System  8.962811  
  Larval Feeding Hind Gut  7.797072  
  Larval Feeding Malpighian Tubule  7.691759  
  Larval Feeding Mid Gut  7.527478  
  Larval Feeding Salivary Gland  8.910396  
  Whole Larvae Feeding  8.225191  
 
  
   FlyBase ID    symbol    start    end    strand    length   
   FBgn0044047   Ilp6  2225526   2228691   -  3166  
   FBgn0003079   phl   2234159   2237904  +  3746  
   FBgn0040389   mRpL14  2238030   2238670   -  641  
 
 
    Segment 48 
 
   Location   
  Gene key  FBgn0040390-FBgn0001150  
  Heatmap region span   X:2156873..2479732   
  Segment span   X:2269805..2322996   
  Length (genes)  5  
  Length (bp)  53192  
   Model Scoring   
  BIC  415.843274  
  logL  -202.519051  
  logL ratio  131.894442  
   Expression   
  Mean expression  4.666265  
  Median expression  4.420227  
  Tissue std. dev.  0.354417  
 
  No GO Slim enrichment  
  
   tissue    mean expression   
  5th Passage Drosophila S2 Cells  4.553594  
  Adult Accessory gland  4.668847  
  Adult Brain  5.635168  
  Adult Carcass  4.621397  
  Adult Crop  4.446789  
  Adult Eye  5.159552  
  Adult Fatbody  4.561878  
  Adult Female Spermatheca Mated  4.712434  
  Adult Female Spermatheca Virgin  4.761335  
  Adult Head  4.744054  
  Adult Heart  4.524107  
  Adult Hind Gut  4.384315  
  Adult Male Ejaculatory Duct  4.591553  
  Adult Mid Gut  4.464616  
  Adult Ovary  4.353105  
  Adult Salivary Gland  5.164489  
  Adult Testes  4.361113  
  Adult Thoracoabdominal ganglion  5.534632  
  Adult Whole Fly  4.121047  
  Larvae Wandering Tubules  4.521869  
  Larval Feeding Carcass  4.529090  
  Larval Feeding Central Nevous System  5.127028  
  Larval Feeding Hind Gut  4.333613  
  Larval Feeding Malpighian Tubule  4.485329  
  Larval Feeding Mid Gut  4.594193  
  Larval Feeding Salivary Gland  4.652284  
  Whole Larvae Feeding  4.381722  
 
  
   FlyBase ID    symbol    start    end    strand    length   
   FBgn0040390     2262591   2269805   -  7215  
   FBgn0040387     2272151   2281697   -  9547  
   FBgn0040385   CG12496  2303390   2304932   -  1543  
   FBgn0052797   CG32797  2319130   2319738   -  609  
   FBgn0001150   gt  2321140   2322996   -  1857  
 
 
    Segment 49 
 
   Location   
  Gene key  FBgn0003714-FBgn0004050  
  Heatmap region span   X:2219723..2482534   
  Segment span   X:2338009..2341802   
  Length (genes)  2  
  Length (bp)  3794  
   Model Scoring   
  BIC  247.889286  
  logL  -118.542057  
  logL ratio  13.055044  
   Expression   
  Mean expression  8.839026  
  Median expression  9.126692  
  Tissue std. dev.  0.407452  
 
  No GO Slim enrichment  
  
   tissue    mean expression   
  5th Passage Drosophila S2 Cells  9.079896  
  Adult Accessory gland  8.807416  
  Adult Brain  9.442766  
  Adult Carcass  8.353769  
  Adult Crop  9.007082  
  Adult Eye  8.896532  
  Adult Fatbody  8.488010  
  Adult Female Spermatheca Mated  8.560624  
  Adult Female Spermatheca Virgin  8.367812  
  Adult Head  8.704178  
  Adult Heart  8.665145  
  Adult Hind Gut  8.964334  
  Adult Male Ejaculatory Duct  8.839944  
  Adult Mid Gut  8.680434  
  Adult Ovary  9.774144  
  Adult Salivary Gland  8.952705  
  Adult Testes  8.037586  
  Adult Thoracoabdominal ganglion  9.335529  
  Adult Whole Fly  8.907698  
  Larvae Wandering Tubules  8.509806  
  Larval Feeding Carcass  8.682218  
  Larval Feeding Central Nevous System  9.808802  
  Larval Feeding Hind Gut  8.826301  
  Larval Feeding Malpighian Tubule  8.961531  
  Larval Feeding Mid Gut  8.359794  
  Larval Feeding Salivary Gland  9.119805  
  Whole Larvae Feeding  8.519849  
 
  
   FlyBase ID    symbol    start    end    strand    length   
   FBgn0003714   tko  2336346   2338009   -  1664  
   FBgn0004050   z   2341802   2344583  +  2782  
 
    Segment 50 
 
   Location   
  Gene key  FBgn0040066-FBgn0001404  
  Heatmap region span   X:2338009..2513771   
  Segment span   X:2480010..2482534   
  Length (genes)  2  
  Length (bp)  2525  
   Model Scoring   
  BIC  255.833685  
  logL  -122.514256  
  logL ratio  2.135917  
   Expression   
  Mean expression  8.355127  
  Median expression  8.205866  
  Tissue std. dev.  0.676106  
 
  No GO Slim enrichment  
  
   tissue    mean expression   
  5th Passage Drosophila S2 Cells  10.021417  
  Adult Accessory gland  9.139233  
  Adult Brain  8.708327  
  Adult Carcass  7.596451  
  Adult Crop  8.046131  
  Adult Eye  8.521748  
  Adult Fatbody  7.675654  
  Adult Female Spermatheca Mated  7.789188  
  Adult Female Spermatheca Virgin  7.447656  
  Adult Head  7.587789  
  Adult Heart  8.639820  
  Adult Hind Gut  8.555646  
  Adult Male Ejaculatory Duct  8.039372  
  Adult Mid Gut  8.843688  
  Adult Ovary  9.110360  
  Adult Salivary Gland  7.233082  
  Adult Testes  7.171596  
  Adult Thoracoabdominal ganglion  8.378111  
  Adult Whole Fly  8.127773  
  Larvae Wandering Tubules  9.363238  
  Larval Feeding Carcass  8.172950  
  Larval Feeding Central Nevous System  9.010658  
  Larval Feeding Hind Gut  8.836510  
  Larval Feeding Malpighian Tubule  8.836362  
  Larval Feeding Mid Gut  8.619252  
  Larval Feeding Salivary Gland  8.023362  
  Whole Larvae Feeding  8.093055  
 
  
   FlyBase ID    symbol    start    end    strand    length   
   FBgn0040066   wds   2480010   2481926  +  1917  
   FBgn0001404   egh   2482534   2492638  +  10105  
 
    Segment 51 
 
   Location   
  Gene key  FBgn0040333-FBgn0011276  
  Heatmap region span   X:2502700..2597809   
  Segment span   X:2518608..2575756   
  Length (genes)  3  
  Length (bp)  57149  
   Model Scoring   
  BIC  309.262463  
  logL  -149.228646  
  logL ratio  11.856300  
   Expression   
  Mean expression  4.956725  
  Median expression  4.653786  
  Tissue std. dev.  0.690439  
 
  No GO Slim enrichment  
  
   tissue    mean expression   
  5th Passage Drosophila S2 Cells  4.593889  
  Adult Accessory gland  5.121561  
  Adult Brain  6.435922  
  Adult Carcass  4.670436  
  Adult Crop  4.440805  
  Adult Eye  6.341988  
  Adult Fatbody  4.669416  
  Adult Female Spermatheca Mated  4.524331  
  Adult Female Spermatheca Virgin  4.572651  
  Adult Head  5.991331  
  Adult Heart  4.751256  
  Adult Hind Gut  4.455726  
  Adult Male Ejaculatory Duct  4.729978  
  Adult Mid Gut  4.484107  
  Adult Ovary  4.442294  
  Adult Salivary Gland  4.483369  
  Adult Testes  5.760455  
  Adult Thoracoabdominal ganglion  6.607840  
  Adult Whole Fly  4.598831  
  Larvae Wandering Tubules  4.594906  
  Larval Feeding Carcass  4.785031  
  Larval Feeding Central Nevous System  6.034873  
  Larval Feeding Hind Gut  4.484571  
  Larval Feeding Malpighian Tubule  4.454728  
  Larval Feeding Mid Gut  4.462181  
  Larval Feeding Salivary Gland  4.722037  
  Whole Larvae Feeding  4.617052  
 
  
   FlyBase ID    symbol    start    end    strand    length   
   FBgn0040333   brv3  2516102   2518608   -  2507  
   FBgn0003371   sgg   2527983   2571879  +  43897  
   FBgn0011276   HLH3B  2574232   2575756   -  1525  
 
 
    Segment 52 
 
   Location   
  Gene key  FBgn0025838-FBgn0000092  
  Heatmap region span   X:2504395..2601450   
  Segment span   X:2576965..2587919   
  Length (genes)  3  
  Length (bp)  10955  
   Model Scoring   
  BIC  343.829622  
  logL  -166.512225  
  logL ratio  -5.224191  
   Expression   
  Mean expression  6.289162  
  Median expression  5.961272  
  Tissue std. dev.  0.556938  
 
  No GO Slim enrichment  
  
   tissue    mean expression   
  5th Passage Drosophila S2 Cells  5.972574  
  Adult Accessory gland  6.010408  
  Adult Brain  6.514667  
  Adult Carcass  6.413605  
  Adult Crop  6.389541  
  Adult Eye  7.648717  
  Adult Fatbody  6.197070  
  Adult Female Spermatheca Mated  5.975110  
  Adult Female Spermatheca Virgin  6.130461  
  Adult Head  7.464402  
  Adult Heart  6.221123  
  Adult Hind Gut  7.507254  
  Adult Male Ejaculatory Duct  6.580638  
  Adult Mid Gut  6.245772  
  Adult Ovary  5.808575  
  Adult Salivary Gland  6.427070  
  Adult Testes  7.139534  
  Adult Thoracoabdominal ganglion  6.651400  
  Adult Whole Fly  6.080774  
  Larvae Wandering Tubules  5.809644  
  Larval Feeding Carcass  5.940438  
  Larval Feeding Central Nevous System  5.785045  
  Larval Feeding Hind Gut  5.551158  
  Larval Feeding Malpighian Tubule  5.904980  
  Larval Feeding Mid Gut  5.984010  
  Larval Feeding Salivary Gland  5.803810  
  Whole Larvae Feeding  5.649590  
 
  
   FlyBase ID    symbol    start    end    strand    length   
   FBgn0025838   CG2652  2576020   2576965   -  946  
   FBgn0003068   per   2579613   2586813  +  7201  
   FBgn0000092   CG2650  2586764   2587919   -  1156  
 
 
    Segment 53 
 
   Location   
  Gene key  FBgn0024998-FBgn0024973  
  Heatmap region span   X:2592737..2777666   
  Segment span   X:2607990..2614358   
  Length (genes)  3  
  Length (bp)  6369  
   Model Scoring   
  BIC  309.404375  
  logL  -149.299601  
  logL ratio  59.883304  
   Expression   
  Mean expression  7.930401  
  Median expression  7.839885  
  Tissue std. dev.  0.404987  
 
  No GO Slim enrichment  
  
   tissue    mean expression   
  5th Passage Drosophila S2 Cells  8.201173  
  Adult Accessory gland  8.325164  
  Adult Brain  8.001259  
  Adult Carcass  7.421658  
  Adult Crop  7.855877  
  Adult Eye  7.992315  
  Adult Fatbody  7.683711  
  Adult Female Spermatheca Mated  7.893382  
  Adult Female Spermatheca Virgin  7.802138  
  Adult Head  7.354034  
  Adult Heart  7.894444  
  Adult Hind Gut  7.663895  
  Adult Male Ejaculatory Duct  8.171520  
  Adult Mid Gut  7.402961  
  Adult Ovary  9.318168  
  Adult Salivary Gland  8.033709  
  Adult Testes  8.015588  
  Adult Thoracoabdominal ganglion  7.930441  
  Adult Whole Fly  8.153910  
  Larvae Wandering Tubules  7.930639  
  Larval Feeding Carcass  7.828003  
  Larval Feeding Central Nevous System  8.553430  
  Larval Feeding Hind Gut  7.878619  
  Larval Feeding Malpighian Tubule  7.819436  
  Larval Feeding Mid Gut  7.341546  
  Larval Feeding Salivary Gland  8.233653  
  Whole Larvae Feeding  7.420166  
 
  
   FlyBase ID    symbol    start    end    strand    length   
   FBgn0024998   CG2685  2605781   2607990   -  2210  
   FBgn0024991   CG2694   2608114   2611688  +  3575  
   FBgn0024973   CG2701  2611673   2614358   -  2686  
 
 
    Segment 54 
 
   Location   
  Gene key  FBgn0000520-FBgn0040384  
  Heatmap region span   X:2601450..2841138   
  Segment span   X:2621733..2683975   
  Length (genes)  6  
  Length (bp)  62243  
   Model Scoring   
  BIC  669.446327  
  logL  -329.320577  
  logL ratio  46.955859  
   Expression   
  Mean expression  7.661612  
  Median expression  7.664558  
  Tissue std. dev.  0.327441  
 
  
   GO ID    description    ratio    P-value   
   GO:0043226   organelle  4/6  0.0372  
 
  
   tissue    mean expression   
  5th Passage Drosophila S2 Cells  7.723205  
  Adult Accessory gland  7.291832  
  Adult Brain  8.202887  
  Adult Carcass  7.434525  
  Adult Crop  7.261055  
  Adult Eye  7.850538  
  Adult Fatbody  7.817091  
  Adult Female Spermatheca Mated  7.579008  
  Adult Female Spermatheca Virgin  7.632513  
  Adult Head  7.532452  
  Adult Heart  7.517031  
  Adult Hind Gut  8.025635  
  Adult Male Ejaculatory Duct  7.623506  
  Adult Mid Gut  7.856451  
  Adult Ovary  8.515733  
  Adult Salivary Gland  7.679734  
  Adult Testes  6.939314  
  Adult Thoracoabdominal ganglion  7.897346  
  Adult Whole Fly  7.658997  
  Larvae Wandering Tubules  7.470388  
  Larval Feeding Carcass  7.222981  
  Larval Feeding Central Nevous System  7.764455  
  Larval Feeding Hind Gut  7.766315  
  Larval Feeding Malpighian Tubule  8.106677  
  Larval Feeding Mid Gut  7.797997  
  Larval Feeding Salivary Gland  7.372164  
  Whole Larvae Feeding  7.323702  
 
  
   FlyBase ID    symbol    start    end    strand    length   
   FBgn0000520   dwg   2621733   2624092  +  2360  
   FBgn0250874   ttm50  2624252   2626218   -  1967  
   FBgn0024975   CG2712   2626411   2628442  +  2032  
   FBgn0000376   crm  2628277   2632810   -  4534  
   FBgn0024980   Syx4  2633120   2638099   -  4980  
   FBgn0040384   CG32795  2676939   2683975   -  7037  
 
 
    Segment 55 
 
   Location   
  Gene key  FBgn0040356-FBgn0040354  
  Heatmap region span   X:2607990..3028905   
  Segment span   X:2703914..2777666   
  Length (genes)  4  
  Length (bp)  73753  
   Model Scoring   
  BIC  332.320383  
  logL  -160.757605  
  logL ratio  90.088301  
   Expression   
  Mean expression  5.036281  
  Median expression  5.161046  
  Tissue std. dev.  0.263759  
 
  No GO Slim enrichment  
  
   tissue    mean expression   
  5th Passage Drosophila S2 Cells  5.091498  
  Adult Accessory gland  5.123373  
  Adult Brain  4.743124  
  Adult Carcass  4.849028  
  Adult Crop  4.955298  
  Adult Eye  4.897471  
  Adult Fatbody  5.036898  
  Adult Female Spermatheca Mated  5.038083  
  Adult Female Spermatheca Virgin  4.998018  
  Adult Head  4.817003  
  Adult Heart  4.892128  
  Adult Hind Gut  4.813832  
  Adult Male Ejaculatory Duct  5.155850  
  Adult Mid Gut  5.115631  
  Adult Ovary  5.021709  
  Adult Salivary Gland  5.358273  
  Adult Testes  6.163381  
  Adult Thoracoabdominal ganglion  4.875051  
  Adult Whole Fly  4.891389  
  Larvae Wandering Tubules  5.024352  
  Larval Feeding Carcass  5.129936  
  Larval Feeding Central Nevous System  4.783451  
  Larval Feeding Hind Gut  4.903797  
  Larval Feeding Malpighian Tubule  4.992887  
  Larval Feeding Mid Gut  5.148041  
  Larval Feeding Salivary Gland  5.239199  
  Whole Larvae Feeding  4.920879  
 
  
   FlyBase ID    symbol    start    end    strand    length   
   FBgn0040356   CG12498   2703914   2704879  +  966  
   FBgn0040352   CG14416  2766023   2766854   -  832  
   FBgn0040353   CG14417  2769564   2770556   -  993  
   FBgn0040354   CG14418  2776599   2777666   -  1068  
 
 
    Segment 56 
 
   Location   
  Gene key  FBgn0040355-FBgn0029647  
  Heatmap region span   X:2621733..3069631   
  Segment span   X:2784801..2841138   
  Length (genes)  11  
  Length (bp)  56338  
   Model Scoring   
  BIC  753.970782  
  logL  -371.582805  
  logL ratio  370.746229  
   Expression   
  Mean expression  4.918265  
  Median expression  4.803028  
  Tissue std. dev.  0.194184  
 
  No GO Slim enrichment  
  
   tissue    mean expression   
  5th Passage Drosophila S2 Cells  4.833879  
  Adult Accessory gland  4.983721  
  Adult Brain  4.571813  
  Adult Carcass  4.897890  
  Adult Crop  4.946307  
  Adult Eye  4.692980  
  Adult Fatbody  4.933962  
  Adult Female Spermatheca Mated  4.867958  
  Adult Female Spermatheca Virgin  4.953643  
  Adult Head  4.972239  
  Adult Heart  4.833957  
  Adult Hind Gut  4.956006  
  Adult Male Ejaculatory Duct  5.038944  
  Adult Mid Gut  4.968024  
  Adult Ovary  4.838965  
  Adult Salivary Gland  5.160610  
  Adult Testes  5.051548  
  Adult Thoracoabdominal ganglion  4.590387  
  Adult Whole Fly  4.512475  
  Larvae Wandering Tubules  4.930958  
  Larval Feeding Carcass  5.394187  
  Larval Feeding Central Nevous System  4.616357  
  Larval Feeding Hind Gut  5.117953  
  Larval Feeding Malpighian Tubule  4.905367  
  Larval Feeding Mid Gut  5.171714  
  Larval Feeding Salivary Gland  4.993294  
  Whole Larvae Feeding  5.058009  
 
  
   FlyBase ID    symbol    start    end    strand    length   
   FBgn0040355   CG3526   2784801   2788265  +  3465  
   FBgn0025643   CG3588   2813803   2818416  +  4614  
   FBgn0025644   CG14424  2820082   2821202   -  1121  
   FBgn0052793   CG32793   2823829   2826575  +  2747  
   FBgn0029642   CG3592   2829203   2829952  +  750  
   FBgn0025645   CG3598   2831161   2832106  +  946  
   FBgn0029643   CG14420   2832889   2834560  +  1672  
   FBgn0029644   CG14421   2835515   2836303  +  789  
   FBgn0029645   CG14422   2837434   2838602  +  1169  
   FBgn0029646   CG14423   2839436   2840622  +  1187  
   FBgn0029647   CG17959   2841138   2841584  +  447  
 
 
    Segment 57 
 
   Location   
  Gene key  FBgn0028369-FBgn0004647  
  Heatmap region span   X:2703914..3143534   
  Segment span   X:2862308..3028905   
  Length (genes)  4  
  Length (bp)  166598  
   Model Scoring   
  BIC  415.546698  
  logL  -202.370763  
  logL ratio  22.483767  
   Expression   
  Mean expression  6.063265  
  Median expression  5.582226  
  Tissue std. dev.  0.868731  
 
  
   GO ID    description    ratio    P-value   
   GO:0005886   plasma membrane  3/4  0.000608  
 
  
   tissue    mean expression   
  5th Passage Drosophila S2 Cells  5.917952  
  Adult Accessory gland  5.055777  
  Adult Brain  7.252002  
  Adult Carcass  5.845363  
  Adult Crop  5.794298  
  Adult Eye  7.850073  
  Adult Fatbody  5.962371  
  Adult Female Spermatheca Mated  6.142983  
  Adult Female Spermatheca Virgin  6.037349  
  Adult Head  6.471110  
  Adult Heart  7.366092  
  Adult Hind Gut  5.631694  
  Adult Male Ejaculatory Duct  5.420116  
  Adult Mid Gut  5.229418  
  Adult Ovary  6.773955  
  Adult Salivary Gland  5.618829  
  Adult Testes  4.768342  
  Adult Thoracoabdominal ganglion  7.454866  
  Adult Whole Fly  5.509097  
  Larvae Wandering Tubules  5.094485  
  Larval Feeding Carcass  6.844298  
  Larval Feeding Central Nevous System  7.853197  
  Larval Feeding Hind Gut  5.584894  
  Larval Feeding Malpighian Tubule  5.080190  
  Larval Feeding Mid Gut  5.412479  
  Larval Feeding Salivary Gland  5.439101  
  Whole Larvae Feeding  6.297832  
 
  
   FlyBase ID    symbol    start    end    strand    length   
   FBgn0028369   kirre   2862308   3026836  +  164529  
   FBgn0003285   rst  2846240   2867865   -  21626  
   FBgn0029649   CG4116  2960801   2961595   -  795  
   FBgn0004647   N   3028905   3066295  +  37391  
 
 
    Segment 58 
 
   Location   
  Gene key  FBgn0028746-FBgn0040396  
  Heatmap region span   X:2779944..3210435   
  Segment span   X:3067172..3069152   
  Length (genes)  2  
  Length (bp)  1981  
   Model Scoring   
  BIC  232.955365  
  logL  -111.075097  
  logL ratio  44.959665  
   Expression   
  Mean expression  9.734763  
  Median expression  9.869343  
  Tissue std. dev.  0.527648  
 
  No GO Slim enrichment  
  
   tissue    mean expression   
  5th Passage Drosophila S2 Cells  9.299913  
  Adult Accessory gland  9.601394  
  Adult Brain  9.036306  
  Adult Carcass  9.095390  
  Adult Crop  9.872155  
  Adult Eye  9.716187  
  Adult Fatbody  9.640363  
  Adult Female Spermatheca Mated  9.314042  
  Adult Female Spermatheca Virgin  9.302163  
  Adult Head  9.347016  
  Adult Heart  9.943473  
  Adult Hind Gut  9.797062  
  Adult Male Ejaculatory Duct  10.333562  
  Adult Mid Gut  9.970332  
  Adult Ovary  9.809801  
  Adult Salivary Gland  10.602915  
  Adult Testes  10.367275  
  Adult Thoracoabdominal ganglion  9.067300  
  Adult Whole Fly  9.361016  
  Larvae Wandering Tubules  10.990286  
  Larval Feeding Carcass  9.082183  
  Larval Feeding Central Nevous System  9.464436  
  Larval Feeding Hind Gut  9.515400  
  Larval Feeding Malpighian Tubule  10.886349  
  Larval Feeding Mid Gut  9.709564  
  Larval Feeding Salivary Gland  10.160617  
  Whole Larvae Feeding  9.552098  
 
  
   FlyBase ID    symbol    start    end    strand    length   
   FBgn0028746   CG18508  3066487   3067172   -  686  
   FBgn0040396   CG3939   3069152   3070256  +  1105  
 
    Segment 59 
 
   Location   
  Gene key  FBgn0040393-FBgn0003374  
  Heatmap region span   X:2862308..3376355   
  Segment span   X:3130715..3143534   
  Length (genes)  7  
  Length (bp)  12820  
   Model Scoring   
  BIC  609.530126  
  logL  -299.362477  
  logL ratio  196.575924  
   Expression   
  Mean expression  5.189113  
  Median expression  4.697169  
  Tissue std. dev.  1.951687  
 
  
   GO ID    description    ratio    P-value   
   GO:0005198   structural molecule activity  5/7  4.54e-07  
   GO:0005576   extracellular region  5/7  5.14e-07  
   GO:0003674   molecular_function  6/7  0.00148  
   GO:0005575   cellular_component  6/7  0.031  
 
  
   tissue    mean expression   
  5th Passage Drosophila S2 Cells  4.642131  
  Adult Accessory gland  4.719105  
  Adult Brain  4.378152  
  Adult Carcass  4.665088  
  Adult Crop  4.601517  
  Adult Eye  4.545734  
  Adult Fatbody  4.815659  
  Adult Female Spermatheca Mated  4.696486  
  Adult Female Spermatheca Virgin  4.773589  
  Adult Head  4.383681  
  Adult Heart  4.527201  
  Adult Hind Gut  4.540320  
  Adult Male Ejaculatory Duct  4.891752  
  Adult Mid Gut  4.711848  
  Adult Ovary  4.509689  
  Adult Salivary Gland  5.056973  
  Adult Testes  4.431965  
  Adult Thoracoabdominal ganglion  4.525802  
  Adult Whole Fly  4.184851  
  Larvae Wandering Tubules  4.634888  
  Larval Feeding Carcass  4.835091  
  Larval Feeding Central Nevous System  4.499276  
  Larval Feeding Hind Gut  4.563997  
  Larval Feeding Malpighian Tubule  5.107453  
  Larval Feeding Mid Gut  4.966088  
  Larval Feeding Salivary Gland  13.175650  
  Whole Larvae Feeding  10.722073  
 
  
   FlyBase ID    symbol    start    end    strand    length   
   FBgn0040393   CG14265  3130157   3130715   -  559  
   FBgn0010294   ng2  3134058   3134604   -  547  
   FBgn0010295   ng3  3135524   3136117   -  594  
   FBgn0002933   ng1   3136445   3136967  +  523  
   FBgn0010296   ng4  3137984   3138221   -  238  
   FBgn0003086   Pig1  3142628   3143322   -  695  
   FBgn0003374   Sgs4   3143534   3145073  +  1540  
 
 
    Segment 60 
 
   Location   
  Gene key  FBgn0029658-FBgn0029662  
  Heatmap region span   X:3070475..3467653   
  Segment span   X:3329840..3368553   
  Length (genes)  6  
  Length (bp)  38714  
   Model Scoring   
  BIC  462.223551  
  logL  -225.709189  
  logL ratio  179.410339  
   Expression   
  Mean expression  4.864009  
  Median expression  4.612280  
  Tissue std. dev.  1.068886  
 
  No GO Slim enrichment  
  
   tissue    mean expression   
  5th Passage Drosophila S2 Cells  5.424636  
  Adult Accessory gland  4.630945  
  Adult Brain  4.244614  
  Adult Carcass  4.782360  
  Adult Crop  4.523879  
  Adult Eye  4.328822  
  Adult Fatbody  4.602771  
  Adult Female Spermatheca Mated  4.657830  
  Adult Female Spermatheca Virgin  4.616257  
  Adult Head  4.437044  
  Adult Heart  4.371842  
  Adult Hind Gut  4.482402  
  Adult Male Ejaculatory Duct  4.658925  
  Adult Mid Gut  4.618902  
  Adult Ovary  4.505944  
  Adult Salivary Gland  4.676587  
  Adult Testes  9.968170  
  Adult Thoracoabdominal ganglion  4.327612  
  Adult Whole Fly  5.850042  
  Larvae Wandering Tubules  4.452679  
  Larval Feeding Carcass  5.383833  
  Larval Feeding Central Nevous System  4.387222  
  Larval Feeding Hind Gut  4.622022  
  Larval Feeding Malpighian Tubule  4.480693  
  Larval Feeding Mid Gut  4.523555  
  Larval Feeding Salivary Gland  4.449742  
  Whole Larvae Feeding  5.318917  
 
  
   FlyBase ID    symbol    start    end    strand    length   
   FBgn0029658   CG14269  3329006   3329840   -  835  
   FBgn0029657   CG12535  3286519   3342976   -  56458  
   FBgn0029659   CG16782  3353049   3354273   -  1225  
   FBgn0029660   CG10801   3354661   3356533  +  1873  
   FBgn0029661   CG16781  3356507   3358077   -  1571  
   FBgn0029662   CG12206  3359770   3368553   -  8784  
 
 
    Segment 61 
 
   Location   
  Gene key  FBgn0029664-FBgn0029666  
  Heatmap region span   X:3210435..3565834   
  Segment span   X:3377002..3379686   
  Length (genes)  3  
  Length (bp)  2685  
   Model Scoring   
  BIC  303.827719  
  logL  -146.511273  
  logL ratio  69.764562  
   Expression   
  Mean expression  8.306629  
  Median expression  8.369250  
  Tissue std. dev.  0.528529  
 
  No GO Slim enrichment  
  
   tissue    mean expression   
  5th Passage Drosophila S2 Cells  8.348641  
  Adult Accessory gland  8.224944  
  Adult Brain  9.058804  
  Adult Carcass  8.095458  
  Adult Crop  8.253157  
  Adult Eye  8.860138  
  Adult Fatbody  7.648443  
  Adult Female Spermatheca Mated  7.985764  
  Adult Female Spermatheca Virgin  8.209208  
  Adult Head  8.137509  
  Adult Heart  8.404214  
  Adult Hind Gut  7.831927  
  Adult Male Ejaculatory Duct  6.987305  
  Adult Mid Gut  7.981463  
  Adult Ovary  9.467916  
  Adult Salivary Gland  8.244756  
  Adult Testes  8.417227  
  Adult Thoracoabdominal ganglion  9.161534  
  Adult Whole Fly  8.596676  
  Larvae Wandering Tubules  8.101179  
  Larval Feeding Carcass  8.236659  
  Larval Feeding Central Nevous System  9.288320  
  Larval Feeding Hind Gut  8.202346  
  Larval Feeding Malpighian Tubule  8.521777  
  Larval Feeding Mid Gut  7.780405  
  Larval Feeding Salivary Gland  8.542056  
  Whole Larvae Feeding  7.691167  
 
  
   FlyBase ID    symbol    start    end    strand    length   
   FBgn0029664   CG10802   3377002   3378593  +  1592  
   FBgn0029665   CG14270  3378633   3379407   -  775  
   FBgn0029666   CG10803   3379686   3382941  +  3256  
 
 
    Segment 62 
 
   Location   
  Gene key  FBgn0029667-FBgn0052792  
  Heatmap region span   X:3267216..3613665   
  Segment span   X:3398884..3451845   
  Length (genes)  4  
  Length (bp)  52962  
   Model Scoring   
  BIC  419.137403  
  logL  -204.166116  
  logL ratio  28.715321  
   Expression   
  Mean expression  4.821436  
  Median expression  4.275013  
  Tissue std. dev.  0.408728  
 
  No GO Slim enrichment  
  
   tissue    mean expression   
  5th Passage Drosophila S2 Cells  4.807521  
  Adult Accessory gland  4.693702  
  Adult Brain  5.357604  
  Adult Carcass  4.604640  
  Adult Crop  4.577154  
  Adult Eye  4.851916  
  Adult Fatbody  4.921513  
  Adult Female Spermatheca Mated  4.985623  
  Adult Female Spermatheca Virgin  4.897735  
  Adult Head  4.679804  
  Adult Heart  4.763632  
  Adult Hind Gut  4.556598  
  Adult Male Ejaculatory Duct  4.575019  
  Adult Mid Gut  4.571649  
  Adult Ovary  4.622027  
  Adult Salivary Gland  4.932454  
  Adult Testes  6.491479  
  Adult Thoracoabdominal ganglion  5.457766  
  Adult Whole Fly  4.458620  
  Larvae Wandering Tubules  4.614488  
  Larval Feeding Carcass  4.631353  
  Larval Feeding Central Nevous System  5.117826  
  Larval Feeding Hind Gut  4.539927  
  Larval Feeding Malpighian Tubule  4.665450  
  Larval Feeding Mid Gut  4.562934  
  Larval Feeding Salivary Gland  4.767875  
  Whole Larvae Feeding  4.472466  
 
  
   FlyBase ID    symbol    start    end    strand    length   
   FBgn0029667   Gas8  3385042   3398884   -  13843  
   FBgn0029669   CG13021   3408349   3410093  +  1745  
   FBgn0052791   CG32791  3405354   3424695   -  19342  
   FBgn0052792   CG32792   3451845   3466033  +  14189  
 
 
    Segment 63 
 
   Location   
  Gene key  FBgn0028961-FBgn0044046  
  Heatmap region span   X:3376355..3762359   
  Segment span   X:3468571..3562077   
  Length (genes)  2  
  Length (bp)  93507  
   Model Scoring   
  BIC  204.763648  
  logL  -96.979238  
  logL ratio  23.348740  
   Expression   
  Mean expression  4.737896  
  Median expression  4.260141  
  Tissue std. dev.  1.075569  
 
  No GO Slim enrichment  
  
   tissue    mean expression   
  5th Passage Drosophila S2 Cells  4.171377  
  Adult Accessory gland  4.181508  
  Adult Brain  6.727138  
  Adult Carcass  4.073054  
  Adult Crop  3.983042  
  Adult Eye  6.515504  
  Adult Fatbody  4.484649  
  Adult Female Spermatheca Mated  4.708522  
  Adult Female Spermatheca Virgin  4.576977  
  Adult Head  5.442670  
  Adult Heart  4.293487  
  Adult Hind Gut  4.055524  
  Adult Male Ejaculatory Duct  4.266269  
  Adult Mid Gut  4.065542  
  Adult Ovary  5.138740  
  Adult Salivary Gland  4.491256  
  Adult Testes  4.277236  
  Adult Thoracoabdominal ganglion  8.434747  
  Adult Whole Fly  4.223250  
  Larvae Wandering Tubules  4.121559  
  Larval Feeding Carcass  4.112054  
  Larval Feeding Central Nevous System  6.690131  
  Larval Feeding Hind Gut  4.178096  
  Larval Feeding Malpighian Tubule  4.243885  
  Larval Feeding Mid Gut  4.198416  
  Larval Feeding Salivary Gland  4.246455  
  Whole Larvae Feeding  4.022106  
 
  
   FlyBase ID    symbol    start    end    strand    length   
   FBgn0028961   AlstR   3468571   3560759  +  92189  
   FBgn0044046   Ilp7   3562077   3562737  +  661  
 
    Segment 64 
 
   Location   
  Gene key  FBgn0023216-FBgn0023215  
  Heatmap region span   X:3377002..3763797   
  Segment span   X:3565602..3565834   
  Length (genes)  2  
  Length (bp)  233  
   Model Scoring   
  BIC  225.092689  
  logL  -107.143758  
  logL ratio  20.023294  
   Expression   
  Mean expression  7.278523  
  Median expression  7.136261  
  Tissue std. dev.  0.426099  
 
  No GO Slim enrichment  
  
   tissue    mean expression   
  5th Passage Drosophila S2 Cells  7.282402  
  Adult Accessory gland  7.048192  
  Adult Brain  8.102635  
  Adult Carcass  6.811165  
  Adult Crop  7.445957  
  Adult Eye  7.674116  
  Adult Fatbody  7.274247  
  Adult Female Spermatheca Mated  7.266882  
  Adult Female Spermatheca Virgin  7.280399  
  Adult Head  6.938932  
  Adult Heart  7.801227  
  Adult Hind Gut  7.153343  
  Adult Male Ejaculatory Duct  6.786216  
  Adult Mid Gut  6.899864  
  Adult Ovary  8.351834  
  Adult Salivary Gland  7.013723  
  Adult Testes  6.838953  
  Adult Thoracoabdominal ganglion  7.748327  
  Adult Whole Fly  6.955329  
  Larvae Wandering Tubules  7.766423  
  Larval Feeding Carcass  6.982261  
  Larval Feeding Central Nevous System  7.540402  
  Larval Feeding Hind Gut  7.151974  
  Larval Feeding Malpighian Tubule  7.346531  
  Larval Feeding Mid Gut  6.872305  
  Larval Feeding Salivary Gland  7.635288  
  Whole Larvae Feeding  6.551201  
 
  
   FlyBase ID    symbol    start    end    strand    length   
   FBgn0023216   Parg  3562754   3565602   -  2849  
   FBgn0023215   Mnt   3565834   3594813  +  28980  
 
    Segment 65 
 
   Location   
  Gene key  FBgn0086899-FBgn0000542  
  Heatmap region span   X:3467653..3778205   
  Segment span   X:3614054..3705976   
  Length (genes)  5  
  Length (bp)  91923  
   Model Scoring   
  BIC  382.596234  
  logL  -185.895531  
  logL ratio  169.059631  
   Expression   
  Mean expression  4.405795  
  Median expression  4.359381  
  Tissue std. dev.  0.148211  
 
  No GO Slim enrichment  
  
   tissue    mean expression   
  5th Passage Drosophila S2 Cells  4.500636  
  Adult Accessory gland  4.621510  
  Adult Brain  4.189233  
  Adult Carcass  4.474113  
  Adult Crop  4.351843  
  Adult Eye  4.367947  
  Adult Fatbody  4.504696  
  Adult Female Spermatheca Mated  4.445991  
  Adult Female Spermatheca Virgin  4.442483  
  Adult Head  4.265333  
  Adult Heart  4.369626  
  Adult Hind Gut  4.381682  
  Adult Male Ejaculatory Duct  4.427438  
  Adult Mid Gut  4.427673  
  Adult Ovary  4.345071  
  Adult Salivary Gland  4.600461  
  Adult Testes  4.754506  
  Adult Thoracoabdominal ganglion  4.188156  
  Adult Whole Fly  4.087663  
  Larvae Wandering Tubules  4.374540  
  Larval Feeding Carcass  4.421266  
  Larval Feeding Central Nevous System  4.244229  
  Larval Feeding Hind Gut  4.373535  
  Larval Feeding Malpighian Tubule  4.437230  
  Larval Feeding Mid Gut  4.438639  
  Larval Feeding Salivary Gland  4.682048  
  Whole Larvae Feeding  4.238914  
 
  
   FlyBase ID    symbol    start    end    strand    length   
   FBgn0086899   tlk   3614054   3682851  +  68798  
   FBgn0053221   CR33221  3691530   3692928   -  1399  
   FBgn0061173      3696349   3704546  +  8198  
   FBgn0052779      3696590   3697786  +  1197  
   FBgn0000542   ec   3705976   3751658  +  45683  
 
 
    Segment 66 
 
   Location   
  Gene key  FBgn0029685-FBgn0029689  
  Heatmap region span   X:3762359..4011053   
  Segment span   X:3830284..3862597   
  Length (genes)  4  
  Length (bp)  32314  
   Model Scoring   
  BIC  435.077789  
  logL  -212.136308  
  logL ratio  54.819818  
   Expression   
  Mean expression  7.974344  
  Median expression  7.997766  
  Tissue std. dev.  0.527077  
 
  No GO Slim enrichment  
  
   tissue    mean expression   
  5th Passage Drosophila S2 Cells  8.472316  
  Adult Accessory gland  8.136755  
  Adult Brain  8.123563  
  Adult Carcass  7.571366  
  Adult Crop  7.788610  
  Adult Eye  7.399511  
  Adult Fatbody  7.948980  
  Adult Female Spermatheca Mated  7.963596  
  Adult Female Spermatheca Virgin  7.999841  
  Adult Head  7.384174  
  Adult Heart  8.130279  
  Adult Hind Gut  8.069179  
  Adult Male Ejaculatory Duct  7.931299  
  Adult Mid Gut  7.684883  
  Adult Ovary  8.958901  
  Adult Salivary Gland  8.477194  
  Adult Testes  6.320117  
  Adult Thoracoabdominal ganglion  8.268200  
  Adult Whole Fly  7.515850  
  Larvae Wandering Tubules  8.445032  
  Larval Feeding Carcass  7.499881  
  Larval Feeding Central Nevous System  7.948692  
  Larval Feeding Hind Gut  8.592942  
  Larval Feeding Malpighian Tubule  8.653966  
  Larval Feeding Mid Gut  7.983801  
  Larval Feeding Salivary Gland  8.592344  
  Whole Larvae Feeding  7.446007  
 
  
   FlyBase ID    symbol    start    end    strand    length   
   FBgn0029685   CG2938  3826189   3830284   -  4096  
   FBgn0029686   CG2941   3830553   3834039  +  3487  
   FBgn0029688   lva  3849578   3858787   -  9210  
   FBgn0029689   CG6428  3859459   3862597   -  3139  
 
 
    Segment 67 
 
   Location   
  Gene key  FBgn0029690-FBgn0029694  
  Heatmap region span   X:3763797..4100123   
  Segment span   X:3865381..3995441   
  Length (genes)  6  
  Length (bp)  130061  
   Model Scoring   
  BIC  628.098994  
  logL  -308.646911  
  logL ratio  23.574985  
   Expression   
  Mean expression  5.575563  
  Median expression  5.437055  
  Tissue std. dev.  0.246435  
 
  No GO Slim enrichment  
  
   tissue    mean expression   
  5th Passage Drosophila S2 Cells  5.679539  
  Adult Accessory gland  5.623549  
  Adult Brain  5.955290  
  Adult Carcass  5.422303  
  Adult Crop  5.469070  
  Adult Eye  5.337687  
  Adult Fatbody  5.570820  
  Adult Female Spermatheca Mated  5.435617  
  Adult Female Spermatheca Virgin  5.524131  
  Adult Head  5.315939  
  Adult Heart  5.299764  
  Adult Hind Gut  5.424628  
  Adult Male Ejaculatory Duct  5.602083  
  Adult Mid Gut  5.455529  
  Adult Ovary  5.593798  
  Adult Salivary Gland  5.881555  
  Adult Testes  5.338064  
  Adult Thoracoabdominal ganglion  5.877510  
  Adult Whole Fly  5.018407  
  Larvae Wandering Tubules  5.508178  
  Larval Feeding Carcass  6.240266  
  Larval Feeding Central Nevous System  5.893673  
  Larval Feeding Hind Gut  5.458604  
  Larval Feeding Malpighian Tubule  5.529460  
  Larval Feeding Mid Gut  5.583656  
  Larval Feeding Salivary Gland  5.677226  
  Whole Larvae Feeding  5.823856  
 
  
   FlyBase ID    symbol    start    end    strand    length   
   FBgn0029690   CG6414  3863031   3865381   -  2351  
   FBgn0052790      3971908   3973247  +  1340  
   FBgn0052778      3974170   3980830  +  6661  
   FBgn0029692      3982070   3984759  +  2690  
   FBgn0029693   CG6379  3991948   3995068   -  3121  
   FBgn0029694   CG15375   3995441   3996588  +  1148  
 
 
    Segment 68 
 
   Location   
  Gene key  FBgn0004832-FBgn0000221  
  Heatmap region span   X:3766454..4139802   
  Segment span   X:3998230..3998377   
  Length (genes)  2  
  Length (bp)  148  
   Model Scoring   
  BIC  204.909113  
  logL  -97.051971  
  logL ratio  40.863478  
   Expression   
  Mean expression  7.134833  
  Median expression  6.946029  
  Tissue std. dev.  0.502074  
 
  No GO Slim enrichment  
  
   tissue    mean expression   
  5th Passage Drosophila S2 Cells  8.044550  
  Adult Accessory gland  7.549727  
  Adult Brain  7.682591  
  Adult Carcass  6.505026  
  Adult Crop  6.685447  
  Adult Eye  7.759115  
  Adult Fatbody  6.388064  
  Adult Female Spermatheca Mated  6.759609  
  Adult Female Spermatheca Virgin  6.703815  
  Adult Head  6.955515  
  Adult Heart  7.154746  
  Adult Hind Gut  6.672705  
  Adult Male Ejaculatory Duct  7.049969  
  Adult Mid Gut  6.732560  
  Adult Ovary  8.221526  
  Adult Salivary Gland  7.050204  
  Adult Testes  7.898155  
  Adult Thoracoabdominal ganglion  7.868244  
  Adult Whole Fly  7.047783  
  Larvae Wandering Tubules  7.018127  
  Larval Feeding Carcass  6.738089  
  Larval Feeding Central Nevous System  7.669609  
  Larval Feeding Hind Gut  6.704804  
  Larval Feeding Malpighian Tubule  7.139456  
  Larval Feeding Mid Gut  6.798250  
  Larval Feeding Salivary Gland  7.088894  
  Whole Larvae Feeding  6.753913  
 
  
   FlyBase ID    symbol    start    end    strand    length   
   FBgn0004832   Xpac  3997047   3998230   -  1184  
   FBgn0000221   brn   3998377   3999830  +  1454  
 
    Segment 69 
 
   Location   
  Gene key  FBgn0026079-FBgn0026083  
  Heatmap region span   X:3830284..4216234   
  Segment span   X:4007767..4011053   
  Length (genes)  3  
  Length (bp)  3287  
   Model Scoring   
  BIC  322.242118  
  logL  -155.718473  
  logL ratio  24.085841  
   Expression   
  Mean expression  6.643142  
  Median expression  6.454749  
  Tissue std. dev.  0.674496  
 
  No GO Slim enrichment  
  
   tissue    mean expression   
  5th Passage Drosophila S2 Cells  7.837939  
  Adult Accessory gland  6.823657  
  Adult Brain  5.909007  
  Adult Carcass  6.195354  
  Adult Crop  6.608154  
  Adult Eye  5.893140  
  Adult Fatbody  6.680992  
  Adult Female Spermatheca Mated  6.748359  
  Adult Female Spermatheca Virgin  6.639426  
  Adult Head  5.994836  
  Adult Heart  6.468680  
  Adult Hind Gut  6.373976  
  Adult Male Ejaculatory Duct  6.184440  
  Adult Mid Gut  6.326363  
  Adult Ovary  9.032772  
  Adult Salivary Gland  6.115638  
  Adult Testes  6.507263  
  Adult Thoracoabdominal ganglion  6.018367  
  Adult Whole Fly  7.525139  
  Larvae Wandering Tubules  6.579048  
  Larval Feeding Carcass  6.766136  
  Larval Feeding Central Nevous System  7.479674  
  Larval Feeding Hind Gut  6.660073  
  Larval Feeding Malpighian Tubule  6.594993  
  Larval Feeding Mid Gut  6.141440  
  Larval Feeding Salivary Gland  7.142201  
  Whole Larvae Feeding  6.117774  
 
  
   FlyBase ID    symbol    start    end    strand    length   
   FBgn0026079   Nsun2  4005189   4007767   -  2579  
   FBgn0026085   dgt4   4008192   4008903  +  712  
   FBgn0026083   tyf   4011053   4022980  +  11928  
 
 
    Segment 70 
 
   Location   
  Gene key  FBgn0026080-FBgn0000635  
  Heatmap region span   X:3865381..4216659   
  Segment span   X:4011475..4100123   
  Length (genes)  2  
  Length (bp)  88649  
   Model Scoring   
  BIC  209.621994  
  logL  -99.408411  
  logL ratio  39.521082  
   Expression   
  Mean expression  8.001254  
  Median expression  7.945648  
  Tissue std. dev.  0.574169  
 
  No GO Slim enrichment  
  
   tissue    mean expression   
  5th Passage Drosophila S2 Cells  7.378241  
  Adult Accessory gland  7.718338  
  Adult Brain  8.932309  
  Adult Carcass  7.945684  
  Adult Crop  8.170286  
  Adult Eye  8.819755  
  Adult Fatbody  7.339771  
  Adult Female Spermatheca Mated  7.826460  
  Adult Female Spermatheca Virgin  7.684773  
  Adult Head  7.996655  
  Adult Heart  7.879151  
  Adult Hind Gut  8.064367  
  Adult Male Ejaculatory Duct  7.657115  
  Adult Mid Gut  7.478931  
  Adult Ovary  7.807970  
  Adult Salivary Gland  7.986017  
  Adult Testes  6.684144  
  Adult Thoracoabdominal ganglion  9.131903  
  Adult Whole Fly  7.662396  
  Larvae Wandering Tubules  8.447732  
  Larval Feeding Carcass  7.955248  
  Larval Feeding Central Nevous System  9.104316  
  Larval Feeding Hind Gut  8.035595  
  Larval Feeding Malpighian Tubule  8.990423  
  Larval Feeding Mid Gut  7.471249  
  Larval Feeding Salivary Gland  8.059461  
  Whole Larvae Feeding  7.805569  
 
  
   FlyBase ID    symbol    start    end    strand    length   
   FBgn0026080   Tip60  4008833   4011475   -  2643  
   FBgn0000635   Fas2  4028405   4100123   -  71719  
 
    Segment 71 
 
   Location   
  Gene key  FBgn0029696-FBgn0052774  
  Heatmap region span   X:3998230..4223336   
  Segment span   X:4111386..4139802   
  Length (genes)  4  
  Length (bp)  28417  
   Model Scoring   
  BIC  303.499990  
  logL  -146.347409  
  logL ratio  135.249613  
   Expression   
  Mean expression  4.686926  
  Median expression  4.231412  
  Tissue std. dev.  1.209234  
 
  No GO Slim enrichment  
  
   tissue    mean expression   
  5th Passage Drosophila S2 Cells  4.358808  
  Adult Accessory gland  4.469749  
  Adult Brain  4.092507  
  Adult Carcass  4.588590  
  Adult Crop  4.326373  
  Adult Eye  4.209449  
  Adult Fatbody  4.449550  
  Adult Female Spermatheca Mated  4.394794  
  Adult Female Spermatheca Virgin  4.451410  
  Adult Head  4.167747  
  Adult Heart  4.362260  
  Adult Hind Gut  4.205197  
  Adult Male Ejaculatory Duct  4.513588  
  Adult Mid Gut  4.458059  
  Adult Ovary  9.803101  
  Adult Salivary Gland  4.460736  
  Adult Testes  4.637682  
  Adult Thoracoabdominal ganglion  4.167904  
  Adult Whole Fly  7.854147  
  Larvae Wandering Tubules  4.316875  
  Larval Feeding Carcass  4.388709  
  Larval Feeding Central Nevous System  4.131126  
  Larval Feeding Hind Gut  4.228974  
  Larval Feeding Malpighian Tubule  4.301469  
  Larval Feeding Mid Gut  4.505161  
  Larval Feeding Salivary Gland  4.488697  
  Whole Larvae Feeding  4.214343  
 
  
   FlyBase ID    symbol    start    end    strand    length   
   FBgn0029696   CG15571  4110928   4111386   -  459  
   FBgn0029697   CG15570   4112259   4117216  +  4958  
   FBgn0041252   Femcoat  4135999   4136840   -  842  
   FBgn0052774   Muc4B  4138254   4139802   -  1549  
 
 
    Segment 72 
 
   Location   
  Gene key  FBgn0029700-FBgn0040906  
  Heatmap region span   X:4000148..4260452   
  Segment span   X:4146730..4205832   
  Length (genes)  7  
  Length (bp)  59103  
   Model Scoring   
  BIC  505.250443  
  logL  -247.222636  
  logL ratio  247.753770  
   Expression   
  Mean expression  4.848985  
  Median expression  4.478739  
  Tissue std. dev.  1.153486  
 
  No GO Slim enrichment  
  
   tissue    mean expression   
  5th Passage Drosophila S2 Cells  4.622546  
  Adult Accessory gland  4.685269  
  Adult Brain  4.289703  
  Adult Carcass  4.700957  
  Adult Crop  4.498579  
  Adult Eye  4.364017  
  Adult Fatbody  4.587233  
  Adult Female Spermatheca Mated  4.593479  
  Adult Female Spermatheca Virgin  4.551537  
  Adult Head  4.374022  
  Adult Heart  4.511396  
  Adult Hind Gut  4.429623  
  Adult Male Ejaculatory Duct  4.763617  
  Adult Mid Gut  4.781785  
  Adult Ovary  4.490305  
  Adult Salivary Gland  4.688850  
  Adult Testes  10.498110  
  Adult Thoracoabdominal ganglion  4.387370  
  Adult Whole Fly  6.025542  
  Larvae Wandering Tubules  4.644684  
  Larval Feeding Carcass  4.638028  
  Larval Feeding Central Nevous System  4.344665  
  Larval Feeding Hind Gut  4.417373  
  Larval Feeding Malpighian Tubule  4.578195  
  Larval Feeding Mid Gut  4.677392  
  Larval Feeding Salivary Gland  4.657962  
  Whole Larvae Feeding  5.120362  
 
  
   FlyBase ID    symbol    start    end    strand    length   
   FBgn0029700   CG15576  4145908   4146730   -  823  
   FBgn0029701   CG12691   4160130   4160702  +  573  
   FBgn0029702   CG15572   4162734   4165116  +  2383  
   FBgn0040904   CG15577  4170526   4171033   -  508  
   FBgn0040905   CG15578  4171580   4171994   -  415  
   FBgn0029703   CG12692  4194185   4196379   -  2195  
   FBgn0040906   CG15579  4205397   4205832   -  436  
 
 
    Segment 73 
 
   Location   
  Gene key  FBgn0029704-FBgn0040907  
  Heatmap region span   X:4007767..4282284   
  Segment span   X:4213473..4216234   
  Length (genes)  2  
  Length (bp)  2762  
   Model Scoring   
  BIC  244.247842  
  logL  -116.721335  
  logL ratio  46.149586  
   Expression   
  Mean expression  10.086237  
  Median expression  10.226797  
  Tissue std. dev.  0.505035  
 
  No GO Slim enrichment  
  
   tissue    mean expression   
  5th Passage Drosophila S2 Cells  10.300863  
  Adult Accessory gland  10.038535  
  Adult Brain  9.488948  
  Adult Carcass  9.652500  
  Adult Crop  9.570953  
  Adult Eye  9.492674  
  Adult Fatbody  10.456312  
  Adult Female Spermatheca Mated  10.062599  
  Adult Female Spermatheca Virgin  9.937916  
  Adult Head  9.630515  
  Adult Heart  10.174618  
  Adult Hind Gut  9.816292  
  Adult Male Ejaculatory Duct  10.033658  
  Adult Mid Gut  10.242388  
  Adult Ovary  11.541709  
  Adult Salivary Gland  9.971599  
  Adult Testes  8.829800  
  Adult Thoracoabdominal ganglion  9.686046  
  Adult Whole Fly  10.663294  
  Larvae Wandering Tubules  10.252747  
  Larval Feeding Carcass  10.267055  
  Larval Feeding Central Nevous System  10.908648  
  Larval Feeding Hind Gut  10.497618  
  Larval Feeding Malpighian Tubule  10.299957  
  Larval Feeding Mid Gut  10.190967  
  Larval Feeding Salivary Gland  10.268821  
  Whole Larvae Feeding  10.051364  
 
  
   FlyBase ID    symbol    start    end    strand    length   
   FBgn0029704   CG2982   4213473   4215778  +  2306  
   FBgn0040907   mRpL33  4215797   4216234   -  438  
 
    Segment 74 
 
   Location   
  Gene key  FBgn0029707-FBgn0052773  
  Heatmap region span   X:4213473..4448378   
  Segment span   X:4275505..4282284   
  Length (genes)  2  
  Length (bp)  6780  
   Model Scoring   
  BIC  173.616697  
  logL  -81.405762  
  logL ratio  39.583463  
   Expression   
  Mean expression  4.641961  
  Median expression  4.588770  
  Tissue std. dev.  0.260608  
 
  No GO Slim enrichment  
  
   tissue    mean expression   
  5th Passage Drosophila S2 Cells  4.702922  
  Adult Accessory gland  5.240999  
  Adult Brain  4.360141  
  Adult Carcass  4.531449  
  Adult Crop  4.480755  
  Adult Eye  4.494402  
  Adult Fatbody  4.616891  
  Adult Female Spermatheca Mated  4.734435  
  Adult Female Spermatheca Virgin  4.688249  
  Adult Head  4.389855  
  Adult Heart  4.587582  
  Adult Hind Gut  4.459098  
  Adult Male Ejaculatory Duct  4.750177  
  Adult Mid Gut  4.677441  
  Adult Ovary  5.473493  
  Adult Salivary Gland  4.942924  
  Adult Testes  4.467119  
  Adult Thoracoabdominal ganglion  4.313936  
  Adult Whole Fly  4.298402  
  Larvae Wandering Tubules  4.649027  
  Larval Feeding Carcass  4.641584  
  Larval Feeding Central Nevous System  4.542406  
  Larval Feeding Hind Gut  4.538784  
  Larval Feeding Malpighian Tubule  4.596282  
  Larval Feeding Mid Gut  4.813399  
  Larval Feeding Salivary Gland  4.910996  
  Whole Larvae Feeding  4.430190  
 
  
   FlyBase ID    symbol    start    end    strand    length   
   FBgn0029707   CG12688   4275505   4277202  +  1698  
   FBgn0052773   CG32773  4281865   4282284   -  420  
 
    Segment 75 
 
   Location   
  Gene key  FBgn0000179-FBgn0029708  
  Heatmap region span   X:4216659..4465962   
  Segment span   X:4306889..4424264   
  Length (genes)  2  
  Length (bp)  117376  
   Model Scoring   
  BIC  207.468761  
  logL  -98.331795  
  logL ratio  17.874769  
   Expression   
  Mean expression  5.819369  
  Median expression  5.117760  
  Tissue std. dev.  1.666754  
 
  No GO Slim enrichment  
  
   tissue    mean expression   
  5th Passage Drosophila S2 Cells  4.665439  
  Adult Accessory gland  5.071320  
  Adult Brain  9.880419  
  Adult Carcass  5.813339  
  Adult Crop  4.841204  
  Adult Eye  9.012716  
  Adult Fatbody  5.360486  
  Adult Female Spermatheca Mated  5.458339  
  Adult Female Spermatheca Virgin  5.351578  
  Adult Head  7.746749  
  Adult Heart  4.838213  
  Adult Hind Gut  4.829328  
  Adult Male Ejaculatory Duct  4.625314  
  Adult Mid Gut  4.784764  
  Adult Ovary  4.459154  
  Adult Salivary Gland  5.347784  
  Adult Testes  5.019733  
  Adult Thoracoabdominal ganglion  9.422452  
  Adult Whole Fly  5.117962  
  Larvae Wandering Tubules  5.500282  
  Larval Feeding Carcass  5.290250  
  Larval Feeding Central Nevous System  9.843019  
  Larval Feeding Hind Gut  4.667204  
  Larval Feeding Malpighian Tubule  5.133681  
  Larval Feeding Mid Gut  4.712459  
  Larval Feeding Salivary Gland  4.920580  
  Whole Larvae Feeding  5.409180  
 
  
   FlyBase ID    symbol    start    end    strand    length   
   FBgn0000179   bi   4306889   4378485  +  71597  
   FBgn0029708   CG3556   4424264   4427872  +  3609  
 
    Segment 76 
 
   Location   
  Gene key  FBgn0003210-FBgn0029709  
  Heatmap region span   X:4223336..4511602   
  Segment span   X:4434669..4434943   
  Length (genes)  2  
  Length (bp)  275  
   Model Scoring   
  BIC  306.168660  
  logL  -147.681744  
  logL ratio  -2.956964  
   Expression   
  Mean expression  10.098526  
  Median expression  9.704988  
  Tissue std. dev.  0.623973  
 
  No GO Slim enrichment  
  
   tissue    mean expression   
  5th Passage Drosophila S2 Cells  10.272734  
  Adult Accessory gland  10.643861  
  Adult Brain  9.839659  
  Adult Carcass  9.887659  
  Adult Crop  10.264760  
  Adult Eye  10.627248  
  Adult Fatbody  10.258524  
  Adult Female Spermatheca Mated  10.693094  
  Adult Female Spermatheca Virgin  10.584456  
  Adult Head  10.006382  
  Adult Heart  10.095914  
  Adult Hind Gut  9.849616  
  Adult Male Ejaculatory Duct  10.901281  
  Adult Mid Gut  9.625684  
  Adult Ovary  9.592386  
  Adult Salivary Gland  10.898968  
  Adult Testes  7.974228  
  Adult Thoracoabdominal ganglion  9.878262  
  Adult Whole Fly  9.361414  
  Larvae Wandering Tubules  9.979944  
  Larval Feeding Carcass  10.411618  
  Larval Feeding Central Nevous System  9.957762  
  Larval Feeding Hind Gut  10.074639  
  Larval Feeding Malpighian Tubule  9.836915  
  Larval Feeding Mid Gut  9.752341  
  Larval Feeding Salivary Gland  11.462713  
  Whole Larvae Feeding  9.928152  
 
  
   FlyBase ID    symbol    start    end    strand    length   
   FBgn0003210   rb  4429668   4434669   -  5002  
   FBgn0029709   CHOp24   4434943   4436471  +  1529  
 
    Segment 77 
 
   Location   
  Gene key  FBgn0029710-FBgn0029713  
  Heatmap region span   X:4260452..4541447   
  Segment span   X:4436663..4445913   
  Length (genes)  4  
  Length (bp)  9251  
   Model Scoring   
  BIC  358.373056  
  logL  -173.783942  
  logL ratio  106.348110  
   Expression   
  Mean expression  6.615010  
  Median expression  6.537900  
  Tissue std. dev.  0.521214  
 
  No GO Slim enrichment  
  
   tissue    mean expression   
  5th Passage Drosophila S2 Cells  6.311933  
  Adult Accessory gland  6.705921  
  Adult Brain  7.923374  
  Adult Carcass  6.073240  
  Adult Crop  6.324881  
  Adult Eye  6.749718  
  Adult Fatbody  6.304715  
  Adult Female Spermatheca Mated  6.189597  
  Adult Female Spermatheca Virgin  6.120041  
  Adult Head  6.573588  
  Adult Heart  6.973907  
  Adult Hind Gut  6.358705  
  Adult Male Ejaculatory Duct  6.269303  
  Adult Mid Gut  6.298098  
  Adult Ovary  7.106605  
  Adult Salivary Gland  6.632923  
  Adult Testes  6.272922  
  Adult Thoracoabdominal ganglion  7.721638  
  Adult Whole Fly  5.883248  
  Larvae Wandering Tubules  6.527900  
  Larval Feeding Carcass  6.494008  
  Larval Feeding Central Nevous System  7.768790  
  Larval Feeding Hind Gut  6.461207  
  Larval Feeding Malpighian Tubule  6.719727  
  Larval Feeding Mid Gut  6.278507  
  Larval Feeding Salivary Gland  7.287162  
  Whole Larvae Feeding  6.273615  
 
  
   FlyBase ID    symbol    start    end    strand    length   
   FBgn0029710   CG3568   4436663   4438530  +  1868  
   FBgn0029711   Usf  4439313   4442802   -  3490  
   FBgn0029712   CG15912   4443103   4443990  +  888  
   FBgn0029713   CG11436  4443916   4445913   -  1998  
 
 
    Segment 78 
 
   Location   
  Gene key  FBgn0029714-FBgn0029715  
  Heatmap region span   X:4275505..4543865   
  Segment span   X:4446000..4448378   
  Length (genes)  2  
  Length (bp)  2379  
   Model Scoring   
  BIC  247.453365  
  logL  -118.324096  
  logL ratio  23.787305  
   Expression   
  Mean expression  9.392083  
  Median expression  9.423617  
  Tissue std. dev.  0.436417  
 
  No GO Slim enrichment  
  
   tissue    mean expression   
  5th Passage Drosophila S2 Cells  9.565182  
  Adult Accessory gland  10.117786  
  Adult Brain  9.117592  
  Adult Carcass  9.026996  
  Adult Crop  9.504931  
  Adult Eye  9.348234  
  Adult Fatbody  9.485117  
  Adult Female Spermatheca Mated  9.407793  
  Adult Female Spermatheca Virgin  9.199053  
  Adult Head  9.210684  
  Adult Heart  9.351758  
  Adult Hind Gut  9.207817  
  Adult Male Ejaculatory Duct  9.414485  
  Adult Mid Gut  9.247173  
  Adult Ovary  10.642771  
  Adult Salivary Gland  9.277280  
  Adult Testes  8.761472  
  Adult Thoracoabdominal ganglion  9.103900  
  Adult Whole Fly  9.836468  
  Larvae Wandering Tubules  9.189408  
  Larval Feeding Carcass  9.480932  
  Larval Feeding Central Nevous System  9.987109  
  Larval Feeding Hind Gut  9.300442  
  Larval Feeding Malpighian Tubule  9.066733  
  Larval Feeding Mid Gut  8.576552  
  Larval Feeding Salivary Gland  10.178584  
  Whole Larvae Feeding  8.979991  
 
  
   FlyBase ID    symbol    start    end    strand    length   
   FBgn0029714   CG3527   4446000   4447039  +  1040  
   FBgn0029715   CG11444  4447150   4448378   -  1229  
 
    Segment 79 
 
   Location   
  Gene key  FBgn0029716-FBgn0029717  
  Heatmap region span   X:4306889..4560730   
  Segment span   X:4452105..4465962   
  Length (genes)  2  
  Length (bp)  13858  
   Model Scoring   
  BIC  191.726015  
  logL  -90.460422  
  logL ratio  19.854433  
   Expression   
  Mean expression  4.916068  
  Median expression  4.793545  
  Tissue std. dev.  0.538387  
 
  No GO Slim enrichment  
  
   tissue    mean expression   
  5th Passage Drosophila S2 Cells  4.827371  
  Adult Accessory gland  4.928686  
  Adult Brain  4.561507  
  Adult Carcass  4.879976  
  Adult Crop  4.767383  
  Adult Eye  4.552369  
  Adult Fatbody  4.899176  
  Adult Female Spermatheca Mated  4.814874  
  Adult Female Spermatheca Virgin  4.773020  
  Adult Head  4.757937  
  Adult Heart  4.584626  
  Adult Hind Gut  4.788861  
  Adult Male Ejaculatory Duct  4.848466  
  Adult Mid Gut  5.060795  
  Adult Ovary  4.688620  
  Adult Salivary Gland  5.100618  
  Adult Testes  7.470378  
  Adult Thoracoabdominal ganglion  4.680556  
  Adult Whole Fly  5.469838  
  Larvae Wandering Tubules  4.869996  
  Larval Feeding Carcass  4.907513  
  Larval Feeding Central Nevous System  4.425799  
  Larval Feeding Hind Gut  4.614472  
  Larval Feeding Malpighian Tubule  4.804511  
  Larval Feeding Mid Gut  4.820204  
  Larval Feeding Salivary Gland  4.947235  
  Whole Larvae Feeding  4.889038  
 
  
   FlyBase ID    symbol    start    end    strand    length   
   FBgn0029716   CG3546   4452105   4454035  +  1931  
   FBgn0029717   CG12684  4465024   4465962   -  939  
 
    Segment 80 
 
   Location   
  Gene key  FBgn0025387-FBgn0011277  
  Heatmap region span   X:4436663..4581834   
  Segment span   X:4524969..4541447   
  Length (genes)  6  
  Length (bp)  16479  
   Model Scoring   
  BIC  552.195848  
  logL  -270.695338  
  logL ratio  93.912247  
   Expression   
  Mean expression  4.853842  
  Median expression  4.440717  
  Tissue std. dev.  0.606525  
 
  No GO Slim enrichment  
  
   tissue    mean expression   
  5th Passage Drosophila S2 Cells  5.166942  
  Adult Accessory gland  4.699353  
  Adult Brain  4.711477  
  Adult Carcass  4.444676  
  Adult Crop  4.580469  
  Adult Eye  4.464445  
  Adult Fatbody  4.579687  
  Adult Female Spermatheca Mated  4.644618  
  Adult Female Spermatheca Virgin  4.671788  
  Adult Head  4.370337  
  Adult Heart  4.519647  
  Adult Hind Gut  4.492512  
  Adult Male Ejaculatory Duct  4.484530  
  Adult Mid Gut  4.517074  
  Adult Ovary  5.666966  
  Adult Salivary Gland  4.706484  
  Adult Testes  7.019875  
  Adult Thoracoabdominal ganglion  5.164295  
  Adult Whole Fly  5.051596  
  Larvae Wandering Tubules  4.513918  
  Larval Feeding Carcass  4.725298  
  Larval Feeding Central Nevous System  6.474998  
  Larval Feeding Hind Gut  4.582749  
  Larval Feeding Malpighian Tubule  4.519973  
  Larval Feeding Mid Gut  4.670072  
  Larval Feeding Salivary Gland  4.693332  
  Whole Larvae Feeding  4.916611  
 
  
   FlyBase ID    symbol    start    end    strand    length   
   FBgn0025387   CG12184  4521413   4524969   -  3557  
   FBgn0025388   CG12179  4525085   4530028   -  4944  
   FBgn0025739   pon   4530363   4533145  +  2783  
   FBgn0025613   CG3081  4533358   4535109   -  1752  
   FBgn0025612   CG3062  4535300   4538901   -  3602  
   FBgn0011277   HLH4C  4539160   4541447   -  2288  
 
 
    Segment 81 
 
   Location   
  Gene key  FBgn0029718-FBgn0025615  
  Heatmap region span   X:4446000..4769703   
  Segment span   X:4541910..4543865   
  Length (genes)  2  
  Length (bp)  1956  
   Model Scoring   
  BIC  223.923319  
  logL  -106.559074  
  logL ratio  57.601609  
   Expression   
  Mean expression  9.939342  
  Median expression  10.010700  
  Tissue std. dev.  0.467751  
 
  No GO Slim enrichment  
  
   tissue    mean expression   
  5th Passage Drosophila S2 Cells  10.263051  
  Adult Accessory gland  10.274922  
  Adult Brain  9.678213  
  Adult Carcass  9.859828  
  Adult Crop  9.769372  
  Adult Eye  10.142964  
  Adult Fatbody  10.313615  
  Adult Female Spermatheca Mated  9.823719  
  Adult Female Spermatheca Virgin  9.814731  
  Adult Head  9.753142  
  Adult Heart  10.421716  
  Adult Hind Gut  9.858244  
  Adult Male Ejaculatory Duct  10.586865  
  Adult Mid Gut  9.644938  
  Adult Ovary  10.423934  
  Adult Salivary Gland  9.823303  
  Adult Testes  8.188690  
  Adult Thoracoabdominal ganglion  9.916514  
  Adult Whole Fly  9.825303  
  Larvae Wandering Tubules  9.967992  
  Larval Feeding Carcass  9.840870  
  Larval Feeding Central Nevous System  10.507316  
  Larval Feeding Hind Gut  10.120047  
  Larval Feeding Malpighian Tubule  10.277109  
  Larval Feeding Mid Gut  9.268581  
  Larval Feeding Salivary Gland  10.406555  
  Whole Larvae Feeding  9.590699  
 
  
   FlyBase ID    symbol    start    end    strand    length   
   FBgn0029718   mRpL30   4541910   4542682  +  773  
   FBgn0025615   Torsin  4542623   4543865   -  1243  
 
    Segment 82 
 
   Location   
  Gene key  FBgn0022942-FBgn0029720  
  Heatmap region span   X:4452105..4800945   
  Segment span   X:4544054..4560730   
  Length (genes)  3  
  Length (bp)  16677  
   Model Scoring   
  BIC  338.828542  
  logL  -164.011685  
  logL ratio  -6.382225  
   Expression   
  Mean expression  5.862360  
  Median expression  5.233799  
  Tissue std. dev.  0.704416  
 
  No GO Slim enrichment  
  
   tissue    mean expression   
  5th Passage Drosophila S2 Cells  5.603378  
  Adult Accessory gland  5.464863  
  Adult Brain  7.836130  
  Adult Carcass  5.601225  
  Adult Crop  6.274839  
  Adult Eye  6.191490  
  Adult Fatbody  5.523825  
  Adult Female Spermatheca Mated  5.391084  
  Adult Female Spermatheca Virgin  5.407185  
  Adult Head  6.670735  
  Adult Heart  5.345631  
  Adult Hind Gut  5.541104  
  Adult Male Ejaculatory Duct  5.464351  
  Adult Mid Gut  5.362569  
  Adult Ovary  5.768782  
  Adult Salivary Gland  5.505216  
  Adult Testes  4.541937  
  Adult Thoracoabdominal ganglion  7.524473  
  Adult Whole Fly  5.441021  
  Larvae Wandering Tubules  6.252217  
  Larval Feeding Carcass  5.790335  
  Larval Feeding Central Nevous System  6.990229  
  Larval Feeding Hind Gut  6.350591  
  Larval Feeding Malpighian Tubule  5.596394  
  Larval Feeding Mid Gut  5.362242  
  Larval Feeding Salivary Gland  5.982017  
  Whole Larvae Feeding  5.499869  
 
  
   FlyBase ID    symbol    start    end    strand    length   
   FBgn0022942   Cbp80   4544054   4552292  +  8239  
   FBgn0029719   CG15473  4546086   4548481   -  2396  
   FBgn0029720   CG3009  4552191   4560730   -  8540  
 
 
    Segment 83 
 
   Location   
  Gene key  FBgn0003200-FBgn0022768  
  Heatmap region span   X:4511602..4832077   
  Segment span   X:4573619..4580904   
  Length (genes)  2  
  Length (bp)  7286  
   Model Scoring   
  BIC  235.170594  
  logL  -112.182711  
  logL ratio  18.550910  
   Expression   
  Mean expression  8.197846  
  Median expression  8.097163  
  Tissue std. dev.  0.584793  
 
  No GO Slim enrichment  
  
   tissue    mean expression   
  5th Passage Drosophila S2 Cells  8.026241  
  Adult Accessory gland  7.745782  
  Adult Brain  9.217882  
  Adult Carcass  7.664036  
  Adult Crop  8.232077  
  Adult Eye  7.762036  
  Adult Fatbody  7.604417  
  Adult Female Spermatheca Mated  7.950228  
  Adult Female Spermatheca Virgin  8.098539  
  Adult Head  8.021578  
  Adult Heart  7.943494  
  Adult Hind Gut  8.441389  
  Adult Male Ejaculatory Duct  8.095637  
  Adult Mid Gut  7.556337  
  Adult Ovary  9.984235  
  Adult Salivary Gland  8.457809  
  Adult Testes  8.595792  
  Adult Thoracoabdominal ganglion  8.975398  
  Adult Whole Fly  8.351921  
  Larvae Wandering Tubules  7.740480  
  Larval Feeding Carcass  7.892682  
  Larval Feeding Central Nevous System  9.329846  
  Larval Feeding Hind Gut  8.101782  
  Larval Feeding Malpighian Tubule  7.686827  
  Larval Feeding Mid Gut  7.802765  
  Larval Feeding Salivary Gland  8.462415  
  Whole Larvae Feeding  7.600206  
 
  
   FlyBase ID    symbol    start    end    strand    length   
   FBgn0003200     4561315   4573619   -  12305  
   FBgn0022768   Pp2C1  4574433   4580904   -  6472  
 
    Segment 84 
 
   Location   
  Gene key  FBgn0029722-FBgn0029730  
  Heatmap region span   X:4541910..4891572   
  Segment span   X:4599342..4769703   
  Length (genes)  9  
  Length (bp)  170362  
   Model Scoring   
  BIC  652.829169  
  logL  -321.011998  
  logL ratio  293.008245  
   Expression   
  Mean expression  4.795984  
  Median expression  4.602076  
  Tissue std. dev.  0.761166  
 
  No GO Slim enrichment  
  
   tissue    mean expression   
  5th Passage Drosophila S2 Cells  4.547158  
  Adult Accessory gland  4.598523  
  Adult Brain  4.635393  
  Adult Carcass  4.694604  
  Adult Crop  4.520202  
  Adult Eye  4.387500  
  Adult Fatbody  4.729419  
  Adult Female Spermatheca Mated  4.758377  
  Adult Female Spermatheca Virgin  4.731852  
  Adult Head  4.378127  
  Adult Heart  4.959486  
  Adult Hind Gut  4.478482  
  Adult Male Ejaculatory Duct  4.641067  
  Adult Mid Gut  4.660825  
  Adult Ovary  4.464649  
  Adult Salivary Gland  4.898656  
  Adult Testes  8.550253  
  Adult Thoracoabdominal ganglion  4.663991  
  Adult Whole Fly  5.302801  
  Larvae Wandering Tubules  4.639494  
  Larval Feeding Carcass  4.590098  
  Larval Feeding Central Nevous System  4.496555  
  Larval Feeding Hind Gut  4.459044  
  Larval Feeding Malpighian Tubule  4.622731  
  Larval Feeding Mid Gut  4.608553  
  Larval Feeding Salivary Gland  4.540053  
  Whole Larvae Feeding  4.933662  
 
  
   FlyBase ID    symbol    start    end    strand    length   
   FBgn0029722   CG7024   4599342   4601537  +  2196  
   FBgn0029724   CG15472  4663138   4664116   -  979  
   FBgn0025679   Bteb2  4664589   4665550   -  962  
   FBgn0029725   CG2871  4669991   4671408   -  1418  
   FBgn0029726   CG15471  4676233   4676809   -  577  
   FBgn0029727   CG6978   4678231   4679670  +  1440  
   FBgn0029728   CG2861  4691394   4698895   -  7502  
   FBgn0029729   CG12682   4720007   4720866  +  860  
   FBgn0029730   CG12681   4769703   4771296  +  1594  
 
 
    Segment 85 
 
   Location   
  Gene key  FBgn0052772-FBgn0029738  
  Heatmap region span   X:4573619..4979866   
  Segment span   X:4803181..4832077   
  Length (genes)  4  
  Length (bp)  28897  
   Model Scoring   
  BIC  435.623117  
  logL  -212.408973  
  logL ratio  67.515793  
   Expression   
  Mean expression  8.475251  
  Median expression  8.501853  
  Tissue std. dev.  0.427447  
 
  
   GO ID    description    ratio    P-value   
   GO:0005622   intracellular  2/4  0.0322  
 
  
   tissue    mean expression   
  5th Passage Drosophila S2 Cells  9.235788  
  Adult Accessory gland  8.813813  
  Adult Brain  9.202322  
  Adult Carcass  8.507289  
  Adult Crop  8.591407  
  Adult Eye  8.476665  
  Adult Fatbody  8.398964  
  Adult Female Spermatheca Mated  8.278772  
  Adult Female Spermatheca Virgin  8.297736  
  Adult Head  8.342306  
  Adult Heart  8.765662  
  Adult Hind Gut  8.433774  
  Adult Male Ejaculatory Duct  8.607482  
  Adult Mid Gut  7.601800  
  Adult Ovary  9.150862  
  Adult Salivary Gland  8.488618  
  Adult Testes  8.339817  
  Adult Thoracoabdominal ganglion  8.815607  
  Adult Whole Fly  8.245448  
  Larvae Wandering Tubules  8.220622  
  Larval Feeding Carcass  8.336205  
  Larval Feeding Central Nevous System  9.102180  
  Larval Feeding Hind Gut  8.006190  
  Larval Feeding Malpighian Tubule  7.976426  
  Larval Feeding Mid Gut  7.783627  
  Larval Feeding Salivary Gland  8.980375  
  Whole Larvae Feeding  7.832018  
 
  
   FlyBase ID    symbol    start    end    strand    length   
   FBgn0052772   CG32772   4803181   4808994  +  5814  
   FBgn0029736   CG4041  4809313   4812478   -  3166  
   FBgn0029737   CG6903   4812763   4815681  +  2919  
   FBgn0029738   CG4068  4820694   4832077   -  11384  
 
 
    Segment 86 
 
   Location   
  Gene key  FBgn0061196-FBgn0029740  
  Heatmap region span   X:4599342..5203280   
  Segment span   X:4855033..4891572   
  Length (genes)  2  
  Length (bp)  36540  
   Model Scoring   
  BIC  189.581173  
  logL  -89.388001  
  logL ratio  28.262071  
   Expression   
  Mean expression  5.161076  
  Median expression  4.819712  
  Tissue std. dev.  0.988765  
 
  No GO Slim enrichment  
  
   tissue    mean expression   
  5th Passage Drosophila S2 Cells  4.897521  
  Adult Accessory gland  5.758182  
  Adult Brain  4.485156  
  Adult Carcass  4.822482  
  Adult Crop  4.910111  
  Adult Eye  4.582628  
  Adult Fatbody  5.215883  
  Adult Female Spermatheca Mated  5.876143  
  Adult Female Spermatheca Virgin  5.856337  
  Adult Head  4.584975  
  Adult Heart  4.483213  
  Adult Hind Gut  4.827303  
  Adult Male Ejaculatory Duct  4.976885  
  Adult Mid Gut  4.761100  
  Adult Ovary  4.970496  
  Adult Salivary Gland  5.542964  
  Adult Testes  9.765967  
  Adult Thoracoabdominal ganglion  4.502263  
  Adult Whole Fly  5.681394  
  Larvae Wandering Tubules  4.974626  
  Larval Feeding Carcass  4.827778  
  Larval Feeding Central Nevous System  4.895510  
  Larval Feeding Hind Gut  4.643174  
  Larval Feeding Malpighian Tubule  4.836853  
  Larval Feeding Mid Gut  4.887424  
  Larval Feeding Salivary Gland  4.840771  
  Whole Larvae Feeding  4.941904  
 
  
   FlyBase ID    symbol    start    end    strand    length   
   FBgn0061196   SIP3  4853959   4855033   -  1075  
   FBgn0029740   CG12680   4891572   4892219  +  648  
 
    Segment 87 
 
   Location   
  Gene key  FBgn0052767-FBgn0086911  
  Heatmap region span   X:4803181..5206406   
  Segment span   X:4978760..4979866   
  Length (genes)  2  
  Length (bp)  1107  
   Model Scoring   
  BIC  208.349932  
  logL  -98.772380  
  logL ratio  25.720517  
   Expression   
  Mean expression  6.406360  
  Median expression  6.268448  
  Tissue std. dev.  0.531297  
 
  No GO Slim enrichment  
  
   tissue    mean expression   
  5th Passage Drosophila S2 Cells  6.223555  
  Adult Accessory gland  6.608811  
  Adult Brain  7.574860  
  Adult Carcass  5.661166  
  Adult Crop  6.204658  
  Adult Eye  6.909507  
  Adult Fatbody  5.706827  
  Adult Female Spermatheca Mated  6.276779  
  Adult Female Spermatheca Virgin  6.108263  
  Adult Head  6.105326  
  Adult Heart  5.865862  
  Adult Hind Gut  6.557101  
  Adult Male Ejaculatory Duct  6.577359  
  Adult Mid Gut  6.424900  
  Adult Ovary  6.899196  
  Adult Salivary Gland  6.091698  
  Adult Testes  5.257713  
  Adult Thoracoabdominal ganglion  7.195246  
  Adult Whole Fly  5.731886  
  Larvae Wandering Tubules  6.967759  
  Larval Feeding Carcass  6.257140  
  Larval Feeding Central Nevous System  7.248633  
  Larval Feeding Hind Gut  6.461492  
  Larval Feeding Malpighian Tubule  6.828784  
  Larval Feeding Mid Gut  6.344151  
  Larval Feeding Salivary Gland  6.847242  
  Whole Larvae Feeding  6.035796  
 
  
   FlyBase ID    symbol    start    end    strand    length   
   FBgn0052767   CG32767  4969028   4978760   -  9733  
   FBgn0086911   rg   4979866   5145183  +  165318  
 
    Segment 88 
 
   Location   
  Gene key  FBgn0029745-FBgn0029751  
  Heatmap region span   X:4833165..5211328   
  Segment span   X:5025931..5201450   
  Length (genes)  6  
  Length (bp)  175520  
   Model Scoring   
  BIC  527.111109  
  logL  -258.152969  
  logL ratio  112.046634  
   Expression   
  Mean expression  5.132304  
  Median expression  4.922142  
  Tissue std. dev.  0.731032  
 
  No GO Slim enrichment  
  
   tissue    mean expression   
  5th Passage Drosophila S2 Cells  4.733125  
  Adult Accessory gland  5.105808  
  Adult Brain  5.776172  
  Adult Carcass  4.716724  
  Adult Crop  4.638939  
  Adult Eye  5.283920  
  Adult Fatbody  4.873721  
  Adult Female Spermatheca Mated  4.937094  
  Adult Female Spermatheca Virgin  4.940184  
  Adult Head  4.839884  
  Adult Heart  4.901086  
  Adult Hind Gut  4.790737  
  Adult Male Ejaculatory Duct  4.943625  
  Adult Mid Gut  4.907237  
  Adult Ovary  4.765671  
  Adult Salivary Gland  5.018721  
  Adult Testes  8.608699  
  Adult Thoracoabdominal ganglion  5.445195  
  Adult Whole Fly  5.482180  
  Larvae Wandering Tubules  4.967355  
  Larval Feeding Carcass  4.825648  
  Larval Feeding Central Nevous System  5.383729  
  Larval Feeding Hind Gut  4.716915  
  Larval Feeding Malpighian Tubule  4.903620  
  Larval Feeding Mid Gut  4.909272  
  Larval Feeding Salivary Gland  5.161243  
  Whole Larvae Feeding  4.995697  
 
  
   FlyBase ID    symbol    start    end    strand    length   
   FBgn0029745   CG6789   5025931   5027022  +  1092  
   FBgn0029746   CG15465   5059364   5070541  +  11178  
   FBgn0029747   CG5062  5074214   5076402   -  2189  
   FBgn0029748   CG15464   5081005   5081534  +  530  
   FBgn0029750   CG3323  5197021   5199751   -  2731  
   FBgn0029751   CG17764  5200294   5201450   -  1157  
 
 
    Segment 89 
 
   Location   
  Gene key  FBgn0015617-FBgn0003449  
  Heatmap region span   X:4855033..5216759   
  Segment span   X:5202970..5203280   
  Length (genes)  2  
  Length (bp)  311  
   Model Scoring   
  BIC  256.588669  
  logL  -122.891748  
  logL ratio  10.244177  
   Expression   
  Mean expression  9.095933  
  Median expression  9.300593  
  Tissue std. dev.  0.416629  
 
  No GO Slim enrichment  
  
   tissue    mean expression   
  5th Passage Drosophila S2 Cells  9.316183  
  Adult Accessory gland  9.509881  
  Adult Brain  9.389323  
  Adult Carcass  8.496295  
  Adult Crop  9.057374  
  Adult Eye  9.198574  
  Adult Fatbody  8.776179  
  Adult Female Spermatheca Mated  8.929199  
  Adult Female Spermatheca Virgin  8.947682  
  Adult Head  8.944572  
  Adult Heart  9.103396  
  Adult Hind Gut  8.899991  
  Adult Male Ejaculatory Duct  9.027478  
  Adult Mid Gut  8.647321  
  Adult Ovary  10.061082  
  Adult Salivary Gland  9.145050  
  Adult Testes  8.382836  
  Adult Thoracoabdominal ganglion  9.532110  
  Adult Whole Fly  9.270759  
  Larvae Wandering Tubules  9.145476  
  Larval Feeding Carcass  8.939265  
  Larval Feeding Central Nevous System  10.279159  
  Larval Feeding Hind Gut  8.958498  
  Larval Feeding Malpighian Tubule  9.100523  
  Larval Feeding Mid Gut  8.470537  
  Larval Feeding Salivary Gland  9.231965  
  Whole Larvae Feeding  8.829484  
 
  
   FlyBase ID    symbol    start    end    strand    length   
   FBgn0015617     5201498   5202970   -  1473  
   FBgn0003449   snf   5203280   5204534  +  1255  
 
    Segment 90 
 
   Location   
  Gene key  FBgn0029753-FBgn0029754  
  Heatmap region span   X:5025931..5226778   
  Segment span   X:5207299..5211328   
  Length (genes)  2  
  Length (bp)  4030  
   Model Scoring   
  BIC  190.311960  
  logL  -89.753394  
  logL ratio  25.307305  
   Expression   
  Mean expression  5.385665  
  Median expression  5.228228  
  Tissue std. dev.  0.865087  
 
  No GO Slim enrichment  
  
   tissue    mean expression   
  5th Passage Drosophila S2 Cells  5.195170  
  Adult Accessory gland  5.230937  
  Adult Brain  4.773052  
  Adult Carcass  5.150177  
  Adult Crop  5.068379  
  Adult Eye  4.649617  
  Adult Fatbody  5.538538  
  Adult Female Spermatheca Mated  5.582082  
  Adult Female Spermatheca Virgin  5.669725  
  Adult Head  4.875151  
  Adult Heart  4.972575  
  Adult Hind Gut  5.135460  
  Adult Male Ejaculatory Duct  5.156801  
  Adult Mid Gut  5.154779  
  Adult Ovary  5.819548  
  Adult Salivary Gland  5.338525  
  Adult Testes  9.467105  
  Adult Thoracoabdominal ganglion  4.869781  
  Adult Whole Fly  6.249137  
  Larvae Wandering Tubules  5.218677  
  Larval Feeding Carcass  5.084543  
  Larval Feeding Central Nevous System  5.024630  
  Larval Feeding Hind Gut  5.111168  
  Larval Feeding Malpighian Tubule  5.182726  
  Larval Feeding Mid Gut  5.064749  
  Larval Feeding Salivary Gland  5.418892  
  Whole Larvae Feeding  5.411036  
 
  
   FlyBase ID    symbol    start    end    strand    length   
   FBgn0029753   CG4198   5207299   5209268  +  1970  
   FBgn0029754   CG15930  5209163   5211328   -  2166  
 
    Segment 91 
 
   Location   
  Gene key  FBgn0029755-FBgn0014024  
  Heatmap region span   X:5202970..5234271   
  Segment span   X:5211462..5216759   
  Length (genes)  2  
  Length (bp)  5298  
   Model Scoring   
  BIC  220.092061  
  logL  -104.643444  
  logL ratio  30.624295  
   Expression   
  Mean expression  8.212929  
  Median expression  8.327796  
  Tissue std. dev.  0.662942  
 
  No GO Slim enrichment  
  
   tissue    mean expression   
  5th Passage Drosophila S2 Cells  8.202639  
  Adult Accessory gland  8.338425  
  Adult Brain  8.571652  
  Adult Carcass  7.595694  
  Adult Crop  8.729076  
  Adult Eye  8.268075  
  Adult Fatbody  8.280537  
  Adult Female Spermatheca Mated  8.842102  
  Adult Female Spermatheca Virgin  8.594479  
  Adult Head  7.849364  
  Adult Heart  7.972568  
  Adult Hind Gut  7.959194  
  Adult Male Ejaculatory Duct  7.527695  
  Adult Mid Gut  8.068031  
  Adult Ovary  9.937948  
  Adult Salivary Gland  8.134269  
  Adult Testes  6.395088  
  Adult Thoracoabdominal ganglion  8.437432  
  Adult Whole Fly  8.406928  
  Larvae Wandering Tubules  7.832547  
  Larval Feeding Carcass  8.200014  
  Larval Feeding Central Nevous System  9.709710  
  Larval Feeding Hind Gut  8.222144  
  Larval Feeding Malpighian Tubule  7.671688  
  Larval Feeding Mid Gut  7.749496  
  Larval Feeding Salivary Gland  8.625097  
  Whole Larvae Feeding  7.627192  
 
  
   FlyBase ID    symbol    start    end    strand    length   
   FBgn0029755   Sas10   5211462   5213104  +  1643  
   FBgn0014024   Rnp4F  5212961   5216759   -  3799  
 
    Segment 92 
 
   Location   
  Gene key  FBgn0029756-FBgn0026751  
  Heatmap region span   X:5206406..5315563   
  Segment span   X:5223383..5223944   
  Length (genes)  2  
  Length (bp)  562  
   Model Scoring   
  BIC  197.635044  
  logL  -93.414936  
  logL ratio  22.268685  
   Expression   
  Mean expression  5.677207  
  Median expression  5.515467  
  Tissue std. dev.  0.606965  
 
  No GO Slim enrichment  
  
   tissue    mean expression   
  5th Passage Drosophila S2 Cells  6.580929  
  Adult Accessory gland  5.583300  
  Adult Brain  6.097507  
  Adult Carcass  5.095857  
  Adult Crop  5.746779  
  Adult Eye  5.623559  
  Adult Fatbody  5.224972  
  Adult Female Spermatheca Mated  5.430668  
  Adult Female Spermatheca Virgin  5.298077  
  Adult Head  5.460292  
  Adult Heart  5.382975  
  Adult Hind Gut  5.317003  
  Adult Male Ejaculatory Duct  5.310192  
  Adult Mid Gut  4.960245  
  Adult Ovary  7.655342  
  Adult Salivary Gland  5.142558  
  Adult Testes  5.916908  
  Adult Thoracoabdominal ganglion  5.784808  
  Adult Whole Fly  5.856334  
  Larvae Wandering Tubules  6.808813  
  Larval Feeding Carcass  5.507962  
  Larval Feeding Central Nevous System  6.393306  
  Larval Feeding Hind Gut  5.365258  
  Larval Feeding Malpighian Tubule  6.007748  
  Larval Feeding Mid Gut  4.961317  
  Larval Feeding Salivary Gland  5.751497  
  Whole Larvae Feeding  5.020378  
 
  
   FlyBase ID    symbol    start    end    strand    length   
   FBgn0029756   CG3309  5220708   5223383   -  2676  
   FBgn0026751   XRCC1   5223944   5226468  +  2525  
 
    Segment 93 
 
   Location   
  Gene key  FBgn0029764-FBgn0029763  
  Heatmap region span   X:5223383..5390082   
  Segment span   X:5314896..5315563   
  Length (genes)  2  
  Length (bp)  668  
   Model Scoring   
  BIC  272.395585  
  logL  -130.795207  
  logL ratio  3.857812  
   Expression   
  Mean expression  9.346293  
  Median expression  9.556494  
  Tissue std. dev.  0.667902  
 
  No GO Slim enrichment  
  
   tissue    mean expression   
  5th Passage Drosophila S2 Cells  9.846424  
  Adult Accessory gland  9.069124  
  Adult Brain  8.645589  
  Adult Carcass  9.745420  
  Adult Crop  8.967184  
  Adult Eye  8.685097  
  Adult Fatbody  11.090765  
  Adult Female Spermatheca Mated  9.825374  
  Adult Female Spermatheca Virgin  9.822633  
  Adult Head  8.874256  
  Adult Heart  10.157255  
  Adult Hind Gut  8.971643  
  Adult Male Ejaculatory Duct  8.989612  
  Adult Mid Gut  8.807282  
  Adult Ovary  11.015380  
  Adult Salivary Gland  8.705704  
  Adult Testes  9.353748  
  Adult Thoracoabdominal ganglion  8.695765  
  Adult Whole Fly  9.832619  
  Larvae Wandering Tubules  8.454151  
  Larval Feeding Carcass  9.269825  
  Larval Feeding Central Nevous System  9.727259  
  Larval Feeding Hind Gut  9.437148  
  Larval Feeding Malpighian Tubule  8.609511  
  Larval Feeding Mid Gut  9.279018  
  Larval Feeding Salivary Gland  9.523604  
  Whole Larvae Feeding  8.948512  
 
  
   FlyBase ID    symbol    start    end    strand    length   
   FBgn0029764     5307840   5314896   -  7057  
   FBgn0029763   CG4165   5315563   5320941  +  5379  
 
    Segment 94 
 
   Location   
  Gene key  FBgn0052762-FBgn0029770  
  Heatmap region span   X:5321550..5556085   
  Segment span   X:5395219..5395865   
  Length (genes)  2  
  Length (bp)  647  
   Model Scoring   
  BIC  191.898382  
  logL  -90.546605  
  logL ratio  25.569376  
   Expression   
  Mean expression  5.070307  
  Median expression  4.944353  
  Tissue std. dev.  0.214100  
 
  No GO Slim enrichment  
  
   tissue    mean expression   
  5th Passage Drosophila S2 Cells  5.208067  
  Adult Accessory gland  5.223088  
  Adult Brain  4.734511  
  Adult Carcass  5.255556  
  Adult Crop  5.036144  
  Adult Eye  4.761356  
  Adult Fatbody  5.155517  
  Adult Female Spermatheca Mated  5.252486  
  Adult Female Spermatheca Virgin  5.193435  
  Adult Head  4.848904  
  Adult Heart  4.906110  
  Adult Hind Gut  5.003744  
  Adult Male Ejaculatory Duct  5.483896  
  Adult Mid Gut  5.349801  
  Adult Ovary  5.127014  
  Adult Salivary Gland  5.225797  
  Adult Testes  4.928127  
  Adult Thoracoabdominal ganglion  4.822179  
  Adult Whole Fly  4.666493  
  Larvae Wandering Tubules  5.132661  
  Larval Feeding Carcass  5.192794  
  Larval Feeding Central Nevous System  4.821749  
  Larval Feeding Hind Gut  4.860034  
  Larval Feeding Malpighian Tubule  5.036124  
  Larval Feeding Mid Gut  5.306774  
  Larval Feeding Salivary Gland  5.391346  
  Whole Larvae Feeding  4.974576  
 
  
   FlyBase ID    symbol    start    end    strand    length   
   FBgn0052762   CG32762  5394298   5395219   -  922  
   FBgn0029770   CG4151   5395865   5396608  +  744  
 
    Segment 95 
 
   Location   
  Gene key  FBgn0053980-FBgn0250904  
  Heatmap region span   X:5340694..5566475   
  Segment span   X:5454216..5510403   
  Length (genes)  4  
  Length (bp)  56188  
   Model Scoring   
  BIC  341.183904  
  logL  -165.189366  
  logL ratio  86.927596  
   Expression   
  Mean expression  4.889268  
  Median expression  4.930963  
  Tissue std. dev.  0.430472  
 
  No GO Slim enrichment  
  
   tissue    mean expression   
  5th Passage Drosophila S2 Cells  4.733318  
  Adult Accessory gland  4.813313  
  Adult Brain  5.418681  
  Adult Carcass  4.653543  
  Adult Crop  4.670635  
  Adult Eye  4.555484  
  Adult Fatbody  4.850595  
  Adult Female Spermatheca Mated  4.816929  
  Adult Female Spermatheca Virgin  4.825801  
  Adult Head  4.735087  
  Adult Heart  4.500456  
  Adult Hind Gut  4.632990  
  Adult Male Ejaculatory Duct  4.886318  
  Adult Mid Gut  4.800492  
  Adult Ovary  4.803382  
  Adult Salivary Gland  5.003040  
  Adult Testes  6.828332  
  Adult Thoracoabdominal ganglion  4.698076  
  Adult Whole Fly  4.869387  
  Larvae Wandering Tubules  4.692713  
  Larval Feeding Carcass  4.779747  
  Larval Feeding Central Nevous System  5.368648  
  Larval Feeding Hind Gut  4.636291  
  Larval Feeding Malpighian Tubule  4.664814  
  Larval Feeding Mid Gut  4.882199  
  Larval Feeding Salivary Gland  4.928451  
  Whole Larvae Feeding  4.961510  
 
  
   FlyBase ID    symbol    start    end    strand    length   
   FBgn0053980     5422960   5454216   -  31257  
   FBgn0029775      5488306   5506266  +  17961  
   FBgn0085464   CG34435  5507470   5508428   -  959  
   FBgn0250904   CG34434  5508587   5510403   -  1817  
 
 
    Segment 96 
 
   Location   
  Gene key  FBgn0029783-FBgn0028474  
  Heatmap region span   X:5411294..5589239   
  Segment span   X:5560002..5560127   
  Length (genes)  2  
  Length (bp)  126  
   Model Scoring   
  BIC  212.154069  
  logL  -100.674449  
  logL ratio  37.191156  
   Expression   
  Mean expression  8.141458  
  Median expression  8.191413  
  Tissue std. dev.  0.379440  
 
  No GO Slim enrichment  
  
   tissue    mean expression   
  5th Passage Drosophila S2 Cells  8.066047  
  Adult Accessory gland  7.366094  
  Adult Brain  8.446580  
  Adult Carcass  8.049311  
  Adult Crop  8.288466  
  Adult Eye  8.518712  
  Adult Fatbody  8.191014  
  Adult Female Spermatheca Mated  8.178861  
  Adult Female Spermatheca Virgin  8.374035  
  Adult Head  7.887674  
  Adult Heart  8.617036  
  Adult Hind Gut  8.154131  
  Adult Male Ejaculatory Duct  7.617738  
  Adult Mid Gut  7.636033  
  Adult Ovary  8.690680  
  Adult Salivary Gland  7.842259  
  Adult Testes  7.435747  
  Adult Thoracoabdominal ganglion  8.418302  
  Adult Whole Fly  7.654210  
  Larvae Wandering Tubules  8.180510  
  Larval Feeding Carcass  8.649760  
  Larval Feeding Central Nevous System  8.412434  
  Larval Feeding Hind Gut  8.551654  
  Larval Feeding Malpighian Tubule  8.433479  
  Larval Feeding Mid Gut  7.838831  
  Larval Feeding Salivary Gland  8.516347  
  Whole Larvae Feeding  7.803430  
 
  
   FlyBase ID    symbol    start    end    strand    length   
   FBgn0029783   Sirt4  5558661   5560002   -  1342  
   FBgn0028474   CG4119   5560127   5564586  +  4460  
 
    Segment 97 
 
   Location   
  Gene key  FBgn0029789-FBgn0027564  
  Heatmap region span   X:5560002..5641782   
  Segment span   X:5583504..5589239   
  Length (genes)  3  
  Length (bp)  5736  
   Model Scoring   
  BIC  343.403698  
  logL  -166.299263  
  logL ratio  32.575902  
   Expression   
  Mean expression  8.250406  
  Median expression  8.255324  
  Tissue std. dev.  0.381172  
 
  No GO Slim enrichment  
  
   tissue    mean expression   
  5th Passage Drosophila S2 Cells  8.799407  
  Adult Accessory gland  8.316048  
  Adult Brain  8.406001  
  Adult Carcass  8.257716  
  Adult Crop  8.948976  
  Adult Eye  8.522773  
  Adult Fatbody  8.246751  
  Adult Female Spermatheca Mated  8.043364  
  Adult Female Spermatheca Virgin  7.992995  
  Adult Head  8.563098  
  Adult Heart  8.395251  
  Adult Hind Gut  8.829375  
  Adult Male Ejaculatory Duct  8.388695  
  Adult Mid Gut  7.994371  
  Adult Ovary  8.462796  
  Adult Salivary Gland  8.132375  
  Adult Testes  7.040192  
  Adult Thoracoabdominal ganglion  8.351222  
  Adult Whole Fly  7.546442  
  Larvae Wandering Tubules  8.276497  
  Larval Feeding Carcass  8.329613  
  Larval Feeding Central Nevous System  8.136323  
  Larval Feeding Hind Gut  8.532784  
  Larval Feeding Malpighian Tubule  8.211807  
  Larval Feeding Mid Gut  8.043285  
  Larval Feeding Salivary Gland  8.196917  
  Whole Larvae Feeding  7.795876  
 
  
   FlyBase ID    symbol    start    end    strand    length   
   FBgn0029789   CG3160  5579470   5583504   -  4035  
   FBgn0000394   cv   5584029   5587117  +  3089  
   FBgn0027564   CG3149  5586978   5589239   -  2262  
 
 
    Segment 98 
 
   Location   
  Gene key  FBgn0052758-FBgn0029798  
  Heatmap region span   X:5583504..5683520   
  Segment span   X:5632072..5641782   
  Length (genes)  2  
  Length (bp)  9711  
   Model Scoring   
  BIC  210.984001  
  logL  -100.089415  
  logL ratio  31.293436  
   Expression   
  Mean expression  6.826017  
  Median expression  6.667696  
  Tissue std. dev.  0.584108  
 
  No GO Slim enrichment  
  
   tissue    mean expression   
  5th Passage Drosophila S2 Cells  7.727930  
  Adult Accessory gland  6.217711  
  Adult Brain  7.706857  
  Adult Carcass  6.542967  
  Adult Crop  6.680959  
  Adult Eye  7.279532  
  Adult Fatbody  7.221059  
  Adult Female Spermatheca Mated  6.644231  
  Adult Female Spermatheca Virgin  6.573232  
  Adult Head  6.930675  
  Adult Heart  7.356756  
  Adult Hind Gut  6.619490  
  Adult Male Ejaculatory Duct  6.475079  
  Adult Mid Gut  6.376594  
  Adult Ovary  8.183108  
  Adult Salivary Gland  6.923616  
  Adult Testes  5.232297  
  Adult Thoracoabdominal ganglion  7.219418  
  Adult Whole Fly  6.683340  
  Larvae Wandering Tubules  6.560011  
  Larval Feeding Carcass  6.636169  
  Larval Feeding Central Nevous System  7.759230  
  Larval Feeding Hind Gut  6.589884  
  Larval Feeding Malpighian Tubule  6.800009  
  Larval Feeding Mid Gut  6.431194  
  Larval Feeding Salivary Gland  6.555395  
  Whole Larvae Feeding  6.375723  
 
  
   FlyBase ID    symbol    start    end    strand    length   
   FBgn0052758   CG32758  5613768   5632072   -  18305  
   FBgn0029798   CG4078   5641782   5645815  +  4034  
 
    Segment 99 
 
   Location   
  Gene key  FBgn0259149-FBgn0029809  
  Heatmap region span   X:5632072..5770884   
  Segment span   X:5670612..5683520   
  Length (genes)  3  
  Length (bp)  12909  
   Model Scoring   
  BIC  245.545749  
  logL  -117.370288  
  logL ratio  73.300553  
   Expression   
  Mean expression  4.537640  
  Median expression  4.480154  
  Tissue std. dev.  0.273453  
 
  No GO Slim enrichment  
  
   tissue    mean expression   
  5th Passage Drosophila S2 Cells  4.558394  
  Adult Accessory gland  4.493788  
  Adult Brain  4.376468  
  Adult Carcass  4.410589  
  Adult Crop  4.447208  
  Adult Eye  4.365887  
  Adult Fatbody  4.565111  
  Adult Female Spermatheca Mated  4.591594  
  Adult Female Spermatheca Virgin  4.554762  
  Adult Head  4.375839  
  Adult Heart  4.448627  
  Adult Hind Gut  4.447663  
  Adult Male Ejaculatory Duct  4.536333  
  Adult Mid Gut  4.634169  
  Adult Ovary  4.515565  
  Adult Salivary Gland  4.617080  
  Adult Testes  5.803873  
  Adult Thoracoabdominal ganglion  4.316873  
  Adult Whole Fly  4.394806  
  Larvae Wandering Tubules  4.512514  
  Larval Feeding Carcass  4.643060  
  Larval Feeding Central Nevous System  4.275693  
  Larval Feeding Hind Gut  4.319534  
  Larval Feeding Malpighian Tubule  4.536009  
  Larval Feeding Mid Gut  4.625897  
  Larval Feeding Salivary Gland  4.752824  
  Whole Larvae Feeding  4.396130  
 
  
   FlyBase ID    symbol    start    end    strand    length   
   FBgn0259149   CG42264  5667170   5670612   -  3443  
   FBgn0029807   CG3108  5671228   5675649   -  4422  
   FBgn0029809   CG15767   5683520   5684952  +  1433  
 
 
    Segment 100 
 
   Location   
  Gene key  FBgn0029811-FBgn0029817  
  Heatmap region span   X:5649671..5794897   
  Segment span   X:5698710..5763128   
  Length (genes)  6  
  Length (bp)  64419  
   Model Scoring   
  BIC  563.977083  
  logL  -276.585956  
  logL ratio  74.460202  
   Expression   
  Mean expression  5.257770  
  Median expression  5.058153  
  Tissue std. dev.  0.467958  
 
  No GO Slim enrichment  
  
   tissue    mean expression   
  5th Passage Drosophila S2 Cells  5.203337  
  Adult Accessory gland  5.050731  
  Adult Brain  5.492753  
  Adult Carcass  5.239058  
  Adult Crop  5.068802  
  Adult Eye  4.848621  
  Adult Fatbody  5.279504  
  Adult Female Spermatheca Mated  5.056582  
  Adult Female Spermatheca Virgin  5.090770  
  Adult Head  5.172550  
  Adult Heart  5.184943  
  Adult Hind Gut  5.046957  
  Adult Male Ejaculatory Duct  5.179067  
  Adult Mid Gut  5.316865  
  Adult Ovary  5.118387  
  Adult Salivary Gland  5.387755  
  Adult Testes  7.541906  
  Adult Thoracoabdominal ganglion  5.268317  
  Adult Whole Fly  5.424760  
  Larvae Wandering Tubules  5.060346  
  Larval Feeding Carcass  5.164817  
  Larval Feeding Central Nevous System  5.105493  
  Larval Feeding Hind Gut  4.998432  
  Larval Feeding Malpighian Tubule  5.088051  
  Larval Feeding Mid Gut  5.273450  
  Larval Feeding Salivary Gland  5.142704  
  Whole Larvae Feeding  5.154832  
 
  
   FlyBase ID    symbol    start    end    strand    length   
   FBgn0029811   Cpr5C   5698710   5699377  +  668  
   FBgn0029812   Prosbeta2R1   5703036   5704191  +  1156  
   FBgn0029813   CG15766  5708897   5709853   -  957  
   FBgn0029814   CG15765   5712597   5733606  +  21010  
   FBgn0029816   CG12729   5739313   5740139  +  827  
   FBgn0029817   CG15764   5763128   5764141  +  1014  
 
 
    Segment 101 
 
   Location   
  Gene key  FBgn0029819-FBgn0029820  
  Heatmap region span   X:5686180..5810826   
  Segment span   X:5773866..5773891   
  Length (genes)  2  
  Length (bp)  26  
   Model Scoring   
  BIC  223.131401  
  logL  -106.163115  
  logL ratio  60.559568  
   Expression   
  Mean expression  9.991275  
  Median expression  10.001366  
  Tissue std. dev.  0.530284  
 
  No GO Slim enrichment  
  
   tissue    mean expression   
  5th Passage Drosophila S2 Cells  9.339593  
  Adult Accessory gland  9.617600  
  Adult Brain  10.756564  
  Adult Carcass  9.960174  
  Adult Crop  10.171550  
  Adult Eye  10.479772  
  Adult Fatbody  10.015084  
  Adult Female Spermatheca Mated  10.197230  
  Adult Female Spermatheca Virgin  10.389976  
  Adult Head  10.258609  
  Adult Heart  9.967709  
  Adult Hind Gut  10.396342  
  Adult Male Ejaculatory Duct  10.129762  
  Adult Mid Gut  9.276905  
  Adult Ovary  9.545123  
  Adult Salivary Gland  9.787590  
  Adult Testes  11.292732  
  Adult Thoracoabdominal ganglion  10.743648  
  Adult Whole Fly  9.783926  
  Larvae Wandering Tubules  10.754240  
  Larval Feeding Carcass  9.633118  
  Larval Feeding Central Nevous System  9.614698  
  Larval Feeding Hind Gut  9.857076  
  Larval Feeding Malpighian Tubule  10.091073  
  Larval Feeding Mid Gut  9.431166  
  Larval Feeding Salivary Gland  9.089043  
  Whole Larvae Feeding  9.184127  
 
  
   FlyBase ID    symbol    start    end    strand    length   
   FBgn0029819   CG3016  5771029   5773866   -  2838  
   FBgn0029820   CG16721   5773891   5777433  +  3543  
 
    Segment 102 
 
   Location   
  Gene key  FBgn0003302-FBgn0029833  
  Heatmap region span   X:5849663..5971515   
  Segment span   X:5931209..5931918   
  Length (genes)  2  
  Length (bp)  710  
   Model Scoring   
  BIC  233.515390  
  logL  -111.355109  
  logL ratio  11.528326  
   Expression   
  Mean expression  7.255215  
  Median expression  7.077996  
  Tissue std. dev.  0.666635  
 
  No GO Slim enrichment  
  
   tissue    mean expression   
  5th Passage Drosophila S2 Cells  7.784588  
  Adult Accessory gland  6.722216  
  Adult Brain  8.533586  
  Adult Carcass  6.320366  
  Adult Crop  7.420444  
  Adult Eye  7.901093  
  Adult Fatbody  6.647447  
  Adult Female Spermatheca Mated  7.465514  
  Adult Female Spermatheca Virgin  7.482168  
  Adult Head  7.395874  
  Adult Heart  7.330153  
  Adult Hind Gut  6.707201  
  Adult Male Ejaculatory Duct  6.411183  
  Adult Mid Gut  6.408526  
  Adult Ovary  8.633471  
  Adult Salivary Gland  6.872670  
  Adult Testes  8.029545  
  Adult Thoracoabdominal ganglion  7.828984  
  Adult Whole Fly  7.375912  
  Larvae Wandering Tubules  7.070072  
  Larval Feeding Carcass  6.804768  
  Larval Feeding Central Nevous System  8.567721  
  Larval Feeding Hind Gut  6.953601  
  Larval Feeding Malpighian Tubule  7.071582  
  Larval Feeding Mid Gut  6.422896  
  Larval Feeding Salivary Gland  7.172881  
  Whole Larvae Feeding  6.556348  
 
  
   FlyBase ID    symbol    start    end    strand    length   
   FBgn0003302   rux  5929778   5931209   -  1432  
   FBgn0029833   CG5941   5931918   5933611  +  1694  
 
    Segment 103 
 
   Location   
  Gene key  FBgn0029834-FBgn0029835  
  Heatmap region span   X:5860314..5985803   
  Segment span   X:5934735..5953943   
  Length (genes)  2  
  Length (bp)  19209  
   Model Scoring   
  BIC  225.703110  
  logL  -107.448969  
  logL ratio  -9.668007  
   Expression   
  Mean expression  5.350525  
  Median expression  4.873569  
  Tissue std. dev.  0.679178  
 
  No GO Slim enrichment  
  
   tissue    mean expression   
  5th Passage Drosophila S2 Cells  4.883749  
  Adult Accessory gland  4.976805  
  Adult Brain  5.093918  
  Adult Carcass  5.044114  
  Adult Crop  5.020752  
  Adult Eye  5.447899  
  Adult Fatbody  4.995811  
  Adult Female Spermatheca Mated  4.930840  
  Adult Female Spermatheca Virgin  5.069866  
  Adult Head  5.091920  
  Adult Heart  4.915060  
  Adult Hind Gut  6.031558  
  Adult Male Ejaculatory Duct  5.219266  
  Adult Mid Gut  6.934281  
  Adult Ovary  5.000255  
  Adult Salivary Gland  5.154262  
  Adult Testes  4.503395  
  Adult Thoracoabdominal ganglion  4.807505  
  Adult Whole Fly  5.064490  
  Larvae Wandering Tubules  6.710218  
  Larval Feeding Carcass  4.904097  
  Larval Feeding Central Nevous System  4.640868  
  Larval Feeding Hind Gut  6.077727  
  Larval Feeding Malpighian Tubule  6.718828  
  Larval Feeding Mid Gut  6.712258  
  Larval Feeding Salivary Gland  5.019970  
  Whole Larvae Feeding  5.494464  
 
  
   FlyBase ID    symbol    start    end    strand    length   
   FBgn0029834   CG5937   5934735   5942543  +  7809  
   FBgn0029835   CG5921   5953943   5958324  +  4382  
 
    Segment 104 
 
   Location   
  Gene key  FBgn0029839-FBgn0029843  
  Heatmap region span   X:5934735..6131350   
  Segment span   X:5975400..5985803   
  Length (genes)  3  
  Length (bp)  10404  
   Model Scoring   
  BIC  356.284360  
  logL  -172.739594  
  logL ratio  -15.357274  
   Expression   
  Mean expression  6.102850  
  Median expression  5.917099  
  Tissue std. dev.  0.884116  
 
  No GO Slim enrichment  
  
   tissue    mean expression   
  5th Passage Drosophila S2 Cells  5.508033  
  Adult Accessory gland  5.134449  
  Adult Brain  7.838674  
  Adult Carcass  5.892923  
  Adult Crop  6.809454  
  Adult Eye  8.062822  
  Adult Fatbody  5.367365  
  Adult Female Spermatheca Mated  5.740152  
  Adult Female Spermatheca Virgin  5.726759  
  Adult Head  7.215864  
  Adult Heart  5.422106  
  Adult Hind Gut  7.758919  
  Adult Male Ejaculatory Duct  5.246511  
  Adult Mid Gut  6.958039  
  Adult Ovary  5.763501  
  Adult Salivary Gland  5.529637  
  Adult Testes  5.527716  
  Adult Thoracoabdominal ganglion  7.139792  
  Adult Whole Fly  5.598706  
  Larvae Wandering Tubules  5.370349  
  Larval Feeding Carcass  6.505616  
  Larval Feeding Central Nevous System  6.998537  
  Larval Feeding Hind Gut  5.186681  
  Larval Feeding Malpighian Tubule  5.348769  
  Larval Feeding Mid Gut  6.074135  
  Larval Feeding Salivary Gland  5.448345  
  Whole Larvae Feeding  5.603091  
 
  
   FlyBase ID    symbol    start    end    strand    length   
   FBgn0029839   CG4660  5972744   5975400   -  2657  
   FBgn0029840   raptor  5978807   5983739   -  4933  
   FBgn0029843   Nep1   5985803   5998006  +  12204  
 
 
    Segment 105 
 
   Location   
  Gene key  FBgn0052750-FBgn0052751  
  Heatmap region span   X:5961993..6185276   
  Segment span   X:6090450..6092687   
  Length (genes)  2  
  Length (bp)  2238  
   Model Scoring   
  BIC  215.204812  
  logL  -102.199820  
  logL ratio  14.834701  
   Expression   
  Mean expression  6.065075  
  Median expression  5.597149  
  Tissue std. dev.  1.623870  
 
  No GO Slim enrichment  
  
   tissue    mean expression   
  5th Passage Drosophila S2 Cells  5.015632  
  Adult Accessory gland  5.032509  
  Adult Brain  4.756699  
  Adult Carcass  5.237013  
  Adult Crop  5.243474  
  Adult Eye  4.992417  
  Adult Fatbody  5.315625  
  Adult Female Spermatheca Mated  9.513307  
  Adult Female Spermatheca Virgin  9.731445  
  Adult Head  5.096502  
  Adult Heart  5.265852  
  Adult Hind Gut  5.442680  
  Adult Male Ejaculatory Duct  5.291116  
  Adult Mid Gut  8.539284  
  Adult Ovary  5.149189  
  Adult Salivary Gland  5.294959  
  Adult Testes  4.878798  
  Adult Thoracoabdominal ganglion  4.757113  
  Adult Whole Fly  5.575636  
  Larvae Wandering Tubules  6.413075  
  Larval Feeding Carcass  5.138631  
  Larval Feeding Central Nevous System  4.738487  
  Larval Feeding Hind Gut  6.638863  
  Larval Feeding Malpighian Tubule  7.859432  
  Larval Feeding Mid Gut  9.879905  
  Larval Feeding Salivary Gland  5.021380  
  Whole Larvae Feeding  7.938014  
 
  
   FlyBase ID    symbol    start    end    strand    length   
   FBgn0052750   CG32750  6088821   6090450   -  1630  
   FBgn0052751   CG32751  6090757   6092687   -  1931  
 
    Segment 106 
 
   Location   
  Gene key  FBgn0029849-FBgn0003514  
  Heatmap region span   X:5971515..6198023   
  Segment span   X:6113957..6119196   
  Length (genes)  2  
  Length (bp)  5240  
   Model Scoring   
  BIC  266.796629  
  logL  -127.995728  
  logL ratio  53.640673  
   Expression   
  Mean expression  10.725048  
  Median expression  10.475848  
  Tissue std. dev.  0.582406  
 
  No GO Slim enrichment  
  
   tissue    mean expression   
  5th Passage Drosophila S2 Cells  11.623310  
  Adult Accessory gland  10.141003  
  Adult Brain  10.484061  
  Adult Carcass  10.323368  
  Adult Crop  11.109831  
  Adult Eye  11.134556  
  Adult Fatbody  10.024858  
  Adult Female Spermatheca Mated  9.958690  
  Adult Female Spermatheca Virgin  10.002240  
  Adult Head  10.627816  
  Adult Heart  10.711962  
  Adult Hind Gut  11.296489  
  Adult Male Ejaculatory Duct  10.999166  
  Adult Mid Gut  11.231707  
  Adult Ovary  10.033213  
  Adult Salivary Gland  11.581121  
  Adult Testes  9.616032  
  Adult Thoracoabdominal ganglion  10.238233  
  Adult Whole Fly  10.140389  
  Larvae Wandering Tubules  11.587936  
  Larval Feeding Carcass  10.768449  
  Larval Feeding Central Nevous System  10.461460  
  Larval Feeding Hind Gut  11.025593  
  Larval Feeding Malpighian Tubule  11.630899  
  Larval Feeding Mid Gut  10.880635  
  Larval Feeding Salivary Gland  11.368009  
  Whole Larvae Feeding  10.575257  
 
  
   FlyBase ID    symbol    start    end    strand    length   
   FBgn0029849   Efr   6113957   6116753  +  2797  
   FBgn0003514   sqh  6117094   6119196   -  2103  
 
    Segment 107 
 
   Location   
  Gene key  FBgn0029850-FBgn0029851  
  Heatmap region span   X:5975400..6210131   
  Segment span   X:6119969..6131350   
  Length (genes)  2  
  Length (bp)  11382  
   Model Scoring   
  BIC  197.850883  
  logL  -93.522856  
  logL ratio  17.500371  
   Expression   
  Mean expression  5.180900  
  Median expression  4.859650  
  Tissue std. dev.  0.688503  
 
  No GO Slim enrichment  
  
   tissue    mean expression   
  5th Passage Drosophila S2 Cells  5.456838  
  Adult Accessory gland  4.796824  
  Adult Brain  6.271448  
  Adult Carcass  6.292266  
  Adult Crop  4.864412  
  Adult Eye  5.110618  
  Adult Fatbody  4.826674  
  Adult Female Spermatheca Mated  4.560558  
  Adult Female Spermatheca Virgin  4.666163  
  Adult Head  6.922239  
  Adult Heart  4.493367  
  Adult Hind Gut  4.717469  
  Adult Male Ejaculatory Duct  4.814747  
  Adult Mid Gut  4.683684  
  Adult Ovary  5.593744  
  Adult Salivary Gland  4.974031  
  Adult Testes  4.637591  
  Adult Thoracoabdominal ganglion  6.862104  
  Adult Whole Fly  5.026642  
  Larvae Wandering Tubules  4.814448  
  Larval Feeding Carcass  6.117313  
  Larval Feeding Central Nevous System  5.379781  
  Larval Feeding Hind Gut  4.672383  
  Larval Feeding Malpighian Tubule  4.873872  
  Larval Feeding Mid Gut  4.732568  
  Larval Feeding Salivary Gland  4.892265  
  Whole Larvae Feeding  4.830236  
 
  
   FlyBase ID    symbol    start    end    strand    length   
   FBgn0029850      6119969   6127199  +  7231  
   FBgn0029851   CG14445   6131350   6134591  +  3242  
 
    Segment 108 
 
   Location   
  Gene key  FBgn0015818-FBgn0028685  
  Heatmap region span   X:6090450..6224891   
  Segment span   X:6149841..6185276   
  Length (genes)  8  
  Length (bp)  35436  
   Model Scoring   
  BIC  908.555468  
  logL  -448.875148  
  logL ratio  156.862226  
   Expression   
  Mean expression  9.176964  
  Median expression  8.992276  
  Tissue std. dev.  0.405082  
 
  No GO Slim enrichment  
  
   tissue    mean expression   
  5th Passage Drosophila S2 Cells  9.604303  
  Adult Accessory gland  9.613038  
  Adult Brain  8.973354  
  Adult Carcass  8.676798  
  Adult Crop  9.061435  
  Adult Eye  8.928712  
  Adult Fatbody  9.423528  
  Adult Female Spermatheca Mated  8.963584  
  Adult Female Spermatheca Virgin  9.022975  
  Adult Head  8.782557  
  Adult Heart  9.367654  
  Adult Hind Gut  8.853641  
  Adult Male Ejaculatory Duct  9.421330  
  Adult Mid Gut  8.916728  
  Adult Ovary  9.729278  
  Adult Salivary Gland  9.039323  
  Adult Testes  7.986777  
  Adult Thoracoabdominal ganglion  8.951810  
  Adult Whole Fly  8.949268  
  Larvae Wandering Tubules  9.800944  
  Larval Feeding Carcass  9.226521  
  Larval Feeding Central Nevous System  9.717651  
  Larval Feeding Hind Gut  9.431404  
  Larval Feeding Malpighian Tubule  9.582665  
  Larval Feeding Mid Gut  9.117135  
  Larval Feeding Salivary Gland  9.728499  
  Whole Larvae Feeding  8.907121  
 
  
   FlyBase ID    symbol    start    end    strand    length   
   FBgn0015818   Spx   6149841   6151629  +  1789  
   FBgn0029853   CG3781   6155202   6156152  +  951  
   FBgn0040918   schlank  6151701   6161260   -  9560  
   FBgn0028982   Spt6   6161847   6169019  +  7173  
   FBgn0029854   CG3566  6169003   6170711   -  1709  
   FBgn0026015   Top3beta  6178171   6181997   -  3827  
   FBgn0029857   wuho   6182156   6183591  +  1436  
   FBgn0028685   Rpt4  6183501   6185276   -  1776  
 
 
    Segment 109 
 
   Location   
  Gene key  FBgn0029858-FBgn0043796  
  Heatmap region span   X:6113327..6233560   
  Segment span   X:6185383..6195303   
  Length (genes)  3  
  Length (bp)  9921  
   Model Scoring   
  BIC  273.109237  
  logL  -131.152033  
  logL ratio  91.233787  
   Expression   
  Mean expression  7.269558  
  Median expression  7.302101  
  Tissue std. dev.  0.441821  
 
  No GO Slim enrichment  
  
   tissue    mean expression   
  5th Passage Drosophila S2 Cells  7.536148  
  Adult Accessory gland  7.319829  
  Adult Brain  7.562907  
  Adult Carcass  6.758984  
  Adult Crop  7.357457  
  Adult Eye  7.253111  
  Adult Fatbody  6.775475  
  Adult Female Spermatheca Mated  6.975914  
  Adult Female Spermatheca Virgin  6.955432  
  Adult Head  6.850588  
  Adult Heart  7.108850  
  Adult Hind Gut  7.112702  
  Adult Male Ejaculatory Duct  7.045498  
  Adult Mid Gut  6.874841  
  Adult Ovary  8.991862  
  Adult Salivary Gland  7.454048  
  Adult Testes  7.203094  
  Adult Thoracoabdominal ganglion  7.235111  
  Adult Whole Fly  7.517372  
  Larvae Wandering Tubules  7.235677  
  Larval Feeding Carcass  7.227914  
  Larval Feeding Central Nevous System  7.809629  
  Larval Feeding Hind Gut  7.289421  
  Larval Feeding Malpighian Tubule  7.264227  
  Larval Feeding Mid Gut  6.922260  
  Larval Feeding Salivary Gland  7.816450  
  Whole Larvae Feeding  6.823272  
 
  
   FlyBase ID    symbol    start    end    strand    length   
   FBgn0029858   CG15896   6185383   6187198  +  1816  
   FBgn0029861   CG3815   6189424   6193006  +  3583  
   FBgn0043796   CG12219  6193084   6195303   -  2220  
 
 
    Segment 110 
 
   Location   
  Gene key  FBgn0029867-FBgn0029868  
  Heatmap region span   X:6198023..6421894   
  Segment span   X:6241136..6244432   
  Length (genes)  2  
  Length (bp)  3297  
   Model Scoring   
  BIC  304.626740  
  logL  -146.910784  
  logL ratio  20.050977  
   Expression   
  Mean expression  10.692976  
  Median expression  10.660634  
  Tissue std. dev.  0.456511  
 
  No GO Slim enrichment  
  
   tissue    mean expression   
  5th Passage Drosophila S2 Cells  10.464455  
  Adult Accessory gland  10.436056  
  Adult Brain  11.184555  
  Adult Carcass  10.861778  
  Adult Crop  10.801939  
  Adult Eye  10.703489  
  Adult Fatbody  10.920016  
  Adult Female Spermatheca Mated  10.881333  
  Adult Female Spermatheca Virgin  10.870071  
  Adult Head  10.716825  
  Adult Heart  10.804761  
  Adult Hind Gut  10.807629  
  Adult Male Ejaculatory Duct  10.627101  
  Adult Mid Gut  10.375294  
  Adult Ovary  10.686933  
  Adult Salivary Gland  11.066551  
  Adult Testes  8.652470  
  Adult Thoracoabdominal ganglion  10.910930  
  Adult Whole Fly  10.804426  
  Larvae Wandering Tubules  10.467887  
  Larval Feeding Carcass  10.963900  
  Larval Feeding Central Nevous System  11.220534  
  Larval Feeding Hind Gut  10.881151  
  Larval Feeding Malpighian Tubule  10.868097  
  Larval Feeding Mid Gut  10.488634  
  Larval Feeding Salivary Gland  10.837532  
  Whole Larvae Feeding  10.406007  
 
  
   FlyBase ID    symbol    start    end    strand    length   
   FBgn0029867   CG3847   6241136   6243273  +  2138  
   FBgn0029868   CG3446  6243356   6244432   -  1077  
 
    Segment 111 
 
   Location   
  Gene key  FBgn0029870-FBgn0003139  
  Heatmap region span   X:6224891..6427318   
  Segment span   X:6259982..6266874   
  Length (genes)  2  
  Length (bp)  6893  
   Model Scoring   
  BIC  263.504966  
  logL  -126.349897  
  logL ratio  25.748868  
   Expression   
  Mean expression  9.934531  
  Median expression  9.871851  
  Tissue std. dev.  0.585747  
 
  No GO Slim enrichment  
  
   tissue    mean expression   
  5th Passage Drosophila S2 Cells  9.596419  
  Adult Accessory gland  9.856685  
  Adult Brain  10.201857  
  Adult Carcass  9.969895  
  Adult Crop  10.190717  
  Adult Eye  10.064690  
  Adult Fatbody  10.189782  
  Adult Female Spermatheca Mated  10.121547  
  Adult Female Spermatheca Virgin  10.133458  
  Adult Head  9.796256  
  Adult Heart  10.220557  
  Adult Hind Gut  10.174982  
  Adult Male Ejaculatory Duct  10.026785  
  Adult Mid Gut  9.884514  
  Adult Ovary  10.266925  
  Adult Salivary Gland  9.917157  
  Adult Testes  7.091380  
  Adult Thoracoabdominal ganglion  10.039879  
  Adult Whole Fly  9.884655  
  Larvae Wandering Tubules  10.070212  
  Larval Feeding Carcass  10.191730  
  Larval Feeding Central Nevous System  9.960126  
  Larval Feeding Hind Gut  10.420374  
  Larval Feeding Malpighian Tubule  10.277218  
  Larval Feeding Mid Gut  9.924268  
  Larval Feeding Salivary Gland  9.994086  
  Whole Larvae Feeding  9.766193  
 
  
   FlyBase ID    symbol    start    end    strand    length   
   FBgn0029870   Marf   6259982   6264960  +  4979  
   FBgn0003139   PpV  6265092   6266874   -  1783  
 
    Segment 112 
 
   Location   
  Gene key  FBgn0029873-FBgn0029874  
  Heatmap region span   X:6241136..6434804   
  Segment span   X:6418506..6421894   
  Length (genes)  2  
  Length (bp)  3389  
   Model Scoring   
  BIC  261.903821  
  logL  -125.549324  
  logL ratio  -7.268561  
   Expression   
  Mean expression  8.272761  
  Median expression  8.315248  
  Tissue std. dev.  0.486543  
 
  No GO Slim enrichment  
  
   tissue    mean expression   
  5th Passage Drosophila S2 Cells  8.837984  
  Adult Accessory gland  8.495055  
  Adult Brain  8.167692  
  Adult Carcass  7.469730  
  Adult Crop  8.272723  
  Adult Eye  7.754602  
  Adult Fatbody  7.846926  
  Adult Female Spermatheca Mated  8.393559  
  Adult Female Spermatheca Virgin  8.552869  
  Adult Head  7.578854  
  Adult Heart  7.967083  
  Adult Hind Gut  8.070906  
  Adult Male Ejaculatory Duct  7.939999  
  Adult Mid Gut  8.386562  
  Adult Ovary  9.516830  
  Adult Salivary Gland  8.308867  
  Adult Testes  7.080437  
  Adult Thoracoabdominal ganglion  8.146904  
  Adult Whole Fly  8.181338  
  Larvae Wandering Tubules  8.636482  
  Larval Feeding Carcass  8.594300  
  Larval Feeding Central Nevous System  8.908235  
  Larval Feeding Hind Gut  8.536566  
  Larval Feeding Malpighian Tubule  8.718511  
  Larval Feeding Mid Gut  8.529303  
  Larval Feeding Salivary Gland  8.474212  
  Whole Larvae Feeding  7.998020  
 
  
   FlyBase ID    symbol    start    end    strand    length   
   FBgn0029873   CG3918   6418506   6420375  +  1870  
   FBgn0029874   CG3342  6420139   6421894   -  1756  
 
    Segment 113 
 
   Location   
  Gene key  FBgn0029882-FBgn0029885  
  Heatmap region span   X:6484811..6577831   
  Segment span   X:6548219..6555480   
  Length (genes)  2  
  Length (bp)  7262  
   Model Scoring   
  BIC  229.559031  
  logL  -109.376930  
  logL ratio  38.653150  
   Expression   
  Mean expression  9.387909  
  Median expression  9.355500  
  Tissue std. dev.  0.452531  
 
  No GO Slim enrichment  
  
   tissue    mean expression   
  5th Passage Drosophila S2 Cells  9.848547  
  Adult Accessory gland  10.301077  
  Adult Brain  9.297770  
  Adult Carcass  8.957500  
  Adult Crop  9.289024  
  Adult Eye  9.228887  
  Adult Fatbody  9.154466  
  Adult Female Spermatheca Mated  9.172515  
  Adult Female Spermatheca Virgin  8.984004  
  Adult Head  9.168711  
  Adult Heart  9.296921  
  Adult Hind Gut  9.256339  
  Adult Male Ejaculatory Duct  9.549116  
  Adult Mid Gut  9.182632  
  Adult Ovary  10.717857  
  Adult Salivary Gland  9.253497  
  Adult Testes  9.066176  
  Adult Thoracoabdominal ganglion  9.132122  
  Adult Whole Fly  9.998812  
  Larvae Wandering Tubules  9.205582  
  Larval Feeding Carcass  9.160699  
  Larval Feeding Central Nevous System  10.108590  
  Larval Feeding Hind Gut  9.218654  
  Larval Feeding Malpighian Tubule  9.368885  
  Larval Feeding Mid Gut  8.742681  
  Larval Feeding Salivary Gland  9.890834  
  Whole Larvae Feeding  8.921654  
 
  
   FlyBase ID    symbol    start    end    strand    length   
   FBgn0029882   CG3226  6547139   6548219   -  1081  
   FBgn0029885   CG3224  6554586   6555480   -  895  
 
    Segment 114 
 
   Location   
  Gene key  FBgn0062413-FBgn0025815  
  Heatmap region span   X:6491078..6592536   
  Segment span   X:6555680..6568422   
  Length (genes)  3  
  Length (bp)  12743  
   Model Scoring   
  BIC  334.792718  
  logL  -161.993773  
  logL ratio  34.290055  
   Expression   
  Mean expression  7.549620  
  Median expression  7.389858  
  Tissue std. dev.  0.681275  
 
  No GO Slim enrichment  
  
   tissue    mean expression   
  5th Passage Drosophila S2 Cells  8.993038  
  Adult Accessory gland  7.664133  
  Adult Brain  7.725887  
  Adult Carcass  7.187534  
  Adult Crop  7.285147  
  Adult Eye  7.405433  
  Adult Fatbody  6.954679  
  Adult Female Spermatheca Mated  6.878119  
  Adult Female Spermatheca Virgin  6.805721  
  Adult Head  6.997703  
  Adult Heart  7.390129  
  Adult Hind Gut  7.214613  
  Adult Male Ejaculatory Duct  6.686324  
  Adult Mid Gut  6.986828  
  Adult Ovary  8.980354  
  Adult Salivary Gland  7.093820  
  Adult Testes  6.716157  
  Adult Thoracoabdominal ganglion  7.600529  
  Adult Whole Fly  8.068709  
  Larvae Wandering Tubules  8.368442  
  Larval Feeding Carcass  7.779603  
  Larval Feeding Central Nevous System  8.784886  
  Larval Feeding Hind Gut  7.319573  
  Larval Feeding Malpighian Tubule  8.636910  
  Larval Feeding Mid Gut  6.829577  
  Larval Feeding Salivary Gland  7.844412  
  Whole Larvae Feeding  7.641483  
 
  
   FlyBase ID    symbol    start    end    strand    length   
   FBgn0062413   Ctr1A   6555680   6562300  +  6621  
   FBgn0029887   CG3198  6565230   6568002   -  2773  
   FBgn0025815   Mcm6   6568422   6571205  +  2784  
 
 
    Segment 115 
 
   Location   
  Gene key  FBgn0029892-FBgn0029894  
  Heatmap region span   X:6555680..6641582   
  Segment span   X:6582803..6592536   
  Length (genes)  3  
  Length (bp)  9734  
   Model Scoring   
  BIC  315.912605  
  logL  -152.553717  
  logL ratio  44.397328  
   Expression   
  Mean expression  7.435730  
  Median expression  7.668604  
  Tissue std. dev.  0.592228  
 
  No GO Slim enrichment  
  
   tissue    mean expression   
  5th Passage Drosophila S2 Cells  7.370882  
  Adult Accessory gland  7.412690  
  Adult Brain  8.500974  
  Adult Carcass  6.800027  
  Adult Crop  7.181321  
  Adult Eye  8.661415  
  Adult Fatbody  7.358100  
  Adult Female Spermatheca Mated  7.752150  
  Adult Female Spermatheca Virgin  7.764864  
  Adult Head  7.445627  
  Adult Heart  7.896500  
  Adult Hind Gut  7.224755  
  Adult Male Ejaculatory Duct  7.141761  
  Adult Mid Gut  7.100108  
  Adult Ovary  8.236034  
  Adult Salivary Gland  7.237483  
  Adult Testes  5.701383  
  Adult Thoracoabdominal ganglion  8.106363  
  Adult Whole Fly  6.959335  
  Larvae Wandering Tubules  7.360702  
  Larval Feeding Carcass  7.219010  
  Larval Feeding Central Nevous System  8.193571  
  Larval Feeding Hind Gut  7.344522  
  Larval Feeding Malpighian Tubule  7.369402  
  Larval Feeding Mid Gut  7.084298  
  Larval Feeding Salivary Gland  7.629336  
  Whole Larvae Feeding  6.712093  
 
  
   FlyBase ID    symbol    start    end    strand    length   
   FBgn0029892   CG3184  6581629   6582803   -  1175  
   FBgn0029893   CG14442  6583329   6589034   -  5706  
   FBgn0029894   CG14440  6590736   6592536   -  1801  
 
 
    Segment 116 
 
   Location   
  Gene key  FBgn0029911-FBgn0029915  
  Heatmap region span   X:6697720..6892696   
  Segment span   X:6737953..6746671   
  Length (genes)  5  
  Length (bp)  8719  
   Model Scoring   
  BIC  478.841958  
  logL  -234.018393  
  logL ratio  124.148282  
   Expression   
  Mean expression  7.548003  
  Median expression  7.355837  
  Tissue std. dev.  0.451688  
 
  No GO Slim enrichment  
  
   tissue    mean expression   
  5th Passage Drosophila S2 Cells  8.070882  
  Adult Accessory gland  7.952314  
  Adult Brain  7.714859  
  Adult Carcass  7.024577  
  Adult Crop  7.321528  
  Adult Eye  7.443592  
  Adult Fatbody  7.377815  
  Adult Female Spermatheca Mated  7.242347  
  Adult Female Spermatheca Virgin  7.199786  
  Adult Head  7.098885  
  Adult Heart  7.798215  
  Adult Hind Gut  7.139881  
  Adult Male Ejaculatory Duct  7.441607  
  Adult Mid Gut  7.480288  
  Adult Ovary  8.461815  
  Adult Salivary Gland  7.599490  
  Adult Testes  6.353014  
  Adult Thoracoabdominal ganglion  7.755356  
  Adult Whole Fly  7.300131  
  Larvae Wandering Tubules  8.524014  
  Larval Feeding Carcass  7.426828  
  Larval Feeding Central Nevous System  7.897156  
  Larval Feeding Hind Gut  7.482385  
  Larval Feeding Malpighian Tubule  7.966490  
  Larval Feeding Mid Gut  7.777152  
  Larval Feeding Salivary Gland  7.869510  
  Whole Larvae Feeding  7.076152  
 
  
   FlyBase ID    symbol    start    end    strand    length   
   FBgn0029911   CG14435  6733455   6737953   -  4499  
   FBgn0029912   CG4557   6738376   6741880  +  3505  
   FBgn0029913   Cht11  6741910   6743520   -  1611  
   FBgn0029914   CG4558   6743806   6745917  +  2112  
   FBgn0029915   CG14434  6745942   6746671   -  730  
 
 
    Segment 117 
 
   Location   
  Gene key  FBgn0029925-FBgn0027108  
  Heatmap region span   X:6737953..6907936   
  Segment span   X:6867443..6892696   
  Length (genes)  3  
  Length (bp)  25254  
   Model Scoring   
  BIC  360.593778  
  logL  -174.894303  
  logL ratio  42.312923  
   Expression   
  Mean expression  9.203714  
  Median expression  9.198915  
  Tissue std. dev.  0.954165  
 
  No GO Slim enrichment  
  
   tissue    mean expression   
  5th Passage Drosophila S2 Cells  6.892603  
  Adult Accessory gland  9.339322  
  Adult Brain  10.524347  
  Adult Carcass  9.159093  
  Adult Crop  9.469878  
  Adult Eye  10.401157  
  Adult Fatbody  10.035263  
  Adult Female Spermatheca Mated  8.882265  
  Adult Female Spermatheca Virgin  8.986710  
  Adult Head  10.027466  
  Adult Heart  9.356320  
  Adult Hind Gut  9.369518  
  Adult Male Ejaculatory Duct  8.545041  
  Adult Mid Gut  8.558585  
  Adult Ovary  9.246230  
  Adult Salivary Gland  6.856661  
  Adult Testes  7.845628  
  Adult Thoracoabdominal ganglion  10.799519  
  Adult Whole Fly  9.159568  
  Larvae Wandering Tubules  8.863369  
  Larval Feeding Carcass  10.013407  
  Larval Feeding Central Nevous System  10.752725  
  Larval Feeding Hind Gut  9.360987  
  Larval Feeding Malpighian Tubule  8.836163  
  Larval Feeding Mid Gut  8.710311  
  Larval Feeding Salivary Gland  9.070576  
  Whole Larvae Feeding  9.437561  
 
  
   FlyBase ID    symbol    start    end    strand    length   
   FBgn0029925   CG3040  6866357   6867443   -  1087  
   FBgn0004646   ogre  6868049   6875731   -  7683  
   FBgn0027108   Inx2   6892696   6898182  +  5487  
 
 
    Segment 118 
 
   Location   
  Gene key  FBgn0029931-FBgn0029930  
  Heatmap region span   X:6903902..7218251   
  Segment span   X:6934641..6960085   
  Length (genes)  3  
  Length (bp)  25445  
   Model Scoring   
  BIC  262.379507  
  logL  -125.787168  
  logL ratio  54.277313  
   Expression   
  Mean expression  5.205963  
  Median expression  5.227922  
  Tissue std. dev.  0.297654  
 
  No GO Slim enrichment  
  
   tissue    mean expression   
  5th Passage Drosophila S2 Cells  5.107542  
  Adult Accessory gland  5.368420  
  Adult Brain  5.420876  
  Adult Carcass  5.168275  
  Adult Crop  6.268148  
  Adult Eye  5.602362  
  Adult Fatbody  5.164265  
  Adult Female Spermatheca Mated  5.144525  
  Adult Female Spermatheca Virgin  5.174094  
  Adult Head  5.012837  
  Adult Heart  4.975781  
  Adult Hind Gut  5.090539  
  Adult Male Ejaculatory Duct  5.491984  
  Adult Mid Gut  5.284732  
  Adult Ovary  5.195294  
  Adult Salivary Gland  5.552010  
  Adult Testes  4.866972  
  Adult Thoracoabdominal ganglion  5.354244  
  Adult Whole Fly  4.642084  
  Larvae Wandering Tubules  5.006385  
  Larval Feeding Carcass  5.152620  
  Larval Feeding Central Nevous System  5.189118  
  Larval Feeding Hind Gut  4.962613  
  Larval Feeding Malpighian Tubule  5.103007  
  Larval Feeding Mid Gut  5.098677  
  Larval Feeding Salivary Gland  5.299857  
  Whole Larvae Feeding  4.863750  
 
  
   FlyBase ID    symbol    start    end    strand    length   
   FBgn0029931   CG14427   6934641   6935802  +  1162  
   FBgn0004143   nullo   6943511   6944354  +  844  
   FBgn0029930   CG12541  6916418   6960085   -  43668  
 
 
    Segment 119 
 
   Location   
  Gene key  FBgn0003659-FBgn0027342  
  Heatmap region span   X:6907695..7231403   
  Segment span   X:6992086..7004325   
  Length (genes)  5  
  Length (bp)  12240  
   Model Scoring   
  BIC  503.196932  
  logL  -246.195880  
  logL ratio  98.078358  
   Expression   
  Mean expression  7.688694  
  Median expression  7.745724  
  Tissue std. dev.  0.637804  
 
  No GO Slim enrichment  
  
   tissue    mean expression   
  5th Passage Drosophila S2 Cells  8.128626  
  Adult Accessory gland  8.678833  
  Adult Brain  8.619399  
  Adult Carcass  6.860151  
  Adult Crop  7.714404  
  Adult Eye  8.811483  
  Adult Fatbody  7.232125  
  Adult Female Spermatheca Mated  7.157324  
  Adult Female Spermatheca Virgin  6.909112  
  Adult Head  7.677918  
  Adult Heart  7.913842  
  Adult Hind Gut  7.374996  
  Adult Male Ejaculatory Duct  7.368858  
  Adult Mid Gut  7.501809  
  Adult Ovary  8.707029  
  Adult Salivary Gland  7.364824  
  Adult Testes  6.020189  
  Adult Thoracoabdominal ganglion  8.227945  
  Adult Whole Fly  7.498237  
  Larvae Wandering Tubules  7.614876  
  Larval Feeding Carcass  7.456927  
  Larval Feeding Central Nevous System  8.665397  
  Larval Feeding Hind Gut  7.575212  
  Larval Feeding Malpighian Tubule  7.490751  
  Larval Feeding Mid Gut  7.567442  
  Larval Feeding Salivary Gland  8.099047  
  Whole Larvae Feeding  7.357975  
 
  
   FlyBase ID    symbol    start    end    strand    length   
   FBgn0003659     6969698   6992086   -  22389  
   FBgn0029935   CG4615  6992611   6996330   -  3720  
   FBgn0029936   CG4617  6996583   6998531   -  1949  
   FBgn0029937   CG8300   6998933   7000378  +  1446  
   FBgn0027342   fz4  7000305   7004325   -  4021  
 
 
    Segment 120 
 
   Location   
  Gene key  FBgn0029939-FBgn0029940  
  Heatmap region span   X:6907936..7297207   
  Segment span   X:7087109..7160937   
  Length (genes)  2  
  Length (bp)  73829  
   Model Scoring   
  BIC  231.703952  
  logL  -110.449390  
  logL ratio  -7.616624  
   Expression   
  Mean expression  5.482286  
  Median expression  5.426301  
  Tissue std. dev.  0.914551  
 
  No GO Slim enrichment  
  
   tissue    mean expression   
  5th Passage Drosophila S2 Cells  5.153524  
  Adult Accessory gland  5.177276  
  Adult Brain  4.992037  
  Adult Carcass  5.050956  
  Adult Crop  4.951383  
  Adult Eye  4.897972  
  Adult Fatbody  5.783916  
  Adult Female Spermatheca Mated  6.365447  
  Adult Female Spermatheca Virgin  6.264457  
  Adult Head  4.798203  
  Adult Heart  4.934273  
  Adult Hind Gut  4.883664  
  Adult Male Ejaculatory Duct  4.922946  
  Adult Mid Gut  5.082376  
  Adult Ovary  4.887168  
  Adult Salivary Gland  5.555285  
  Adult Testes  9.202258  
  Adult Thoracoabdominal ganglion  4.878152  
  Adult Whole Fly  6.932348  
  Larvae Wandering Tubules  5.222252  
  Larval Feeding Carcass  5.050532  
  Larval Feeding Central Nevous System  6.255301  
  Larval Feeding Hind Gut  5.030729  
  Larval Feeding Malpighian Tubule  5.098838  
  Larval Feeding Mid Gut  5.145138  
  Larval Feeding Salivary Gland  5.505446  
  Whole Larvae Feeding  5.999852  
 
  
   FlyBase ID    symbol    start    end    strand    length   
   FBgn0029939   CG9650   7087109   7134453  +  47345  
   FBgn0029940   CG1958   7160937   7161914  +  978  
 
    Segment 121 
 
   Location   
  Gene key  FBgn0029942-FBgn0029944  
  Heatmap region span   X:6934641..7299793   
  Segment span   X:7180391..7218251   
  Length (genes)  3  
  Length (bp)  37861  
   Model Scoring   
  BIC  316.399743  
  logL  -152.797286  
  logL ratio  60.724142  
   Expression   
  Mean expression  8.557848  
  Median expression  8.583564  
  Tissue std. dev.  0.472698  
 
  No GO Slim enrichment  
  
   tissue    mean expression   
  5th Passage Drosophila S2 Cells  8.997012  
  Adult Accessory gland  8.246086  
  Adult Brain  8.336668  
  Adult Carcass  8.244760  
  Adult Crop  8.835572  
  Adult Eye  8.370038  
  Adult Fatbody  8.863965  
  Adult Female Spermatheca Mated  8.518356  
  Adult Female Spermatheca Virgin  8.320139  
  Adult Head  8.281467  
  Adult Heart  8.652450  
  Adult Hind Gut  9.016625  
  Adult Male Ejaculatory Duct  8.082892  
  Adult Mid Gut  8.077627  
  Adult Ovary  9.659832  
  Adult Salivary Gland  8.790925  
  Adult Testes  7.184457  
  Adult Thoracoabdominal ganglion  8.088185  
  Adult Whole Fly  8.457433  
  Larvae Wandering Tubules  9.099189  
  Larval Feeding Carcass  8.781701  
  Larval Feeding Central Nevous System  8.765412  
  Larval Feeding Hind Gut  9.225723  
  Larval Feeding Malpighian Tubule  9.018925  
  Larval Feeding Mid Gut  8.412772  
  Larval Feeding Salivary Gland  8.533173  
  Whole Larvae Feeding  8.200518  
 
  
   FlyBase ID    symbol    start    end    strand    length   
   FBgn0029942   CG2059   7180391   7182150  +  1760  
   FBgn0029943   Atg5  7208655   7217798   -  9144  
   FBgn0029944   Dok   7218251   7222839  +  4589  
 
 
    Segment 122 
 
   Location   
  Gene key  FBgn0029946-FBgn0029949  
  Heatmap region span   X:7087109..7346241   
  Segment span   X:7241539..7297207   
  Length (genes)  4  
  Length (bp)  55669  
   Model Scoring   
  BIC  386.146651  
  logL  -187.670739  
  logL ratio  67.815936  
   Expression   
  Mean expression  4.591747  
  Median expression  4.158565  
  Tissue std. dev.  1.244662  
 
  No GO Slim enrichment  
  
   tissue    mean expression   
  5th Passage Drosophila S2 Cells  4.193368  
  Adult Accessory gland  4.226375  
  Adult Brain  3.922207  
  Adult Carcass  6.122605  
  Adult Crop  4.160105  
  Adult Eye  4.078701  
  Adult Fatbody  4.182948  
  Adult Female Spermatheca Mated  4.318845  
  Adult Female Spermatheca Virgin  4.206247  
  Adult Head  3.990903  
  Adult Heart  4.093923  
  Adult Hind Gut  4.152671  
  Adult Male Ejaculatory Duct  4.182607  
  Adult Mid Gut  4.225866  
  Adult Ovary  4.091457  
  Adult Salivary Gland  4.351538  
  Adult Testes  9.717286  
  Adult Thoracoabdominal ganglion  4.262689  
  Adult Whole Fly  7.535776  
  Larvae Wandering Tubules  4.158975  
  Larval Feeding Carcass  4.184001  
  Larval Feeding Central Nevous System  4.083087  
  Larval Feeding Hind Gut  4.095909  
  Larval Feeding Malpighian Tubule  4.236860  
  Larval Feeding Mid Gut  4.185686  
  Larval Feeding Salivary Gland  4.131645  
  Whole Larvae Feeding  4.884883  
 
  
   FlyBase ID    symbol    start    end    strand    length   
   FBgn0029946   CG15034  7240746   7241539   -  794  
   FBgn0029947   CG1999   7252829   7254091  +  1263  
   FBgn0029948   CheA7a   7272173   7272986  +  814  
   FBgn0029949   CG15035  7295731   7297207   -  1477  
 
 
    Segment 123 
 
   Location   
  Gene key  FBgn0085366-FBgn0040923  
  Heatmap region span   X:7241539..7630922   
  Segment span   X:7311286..7346241   
  Length (genes)  5  
  Length (bp)  34956  
   Model Scoring   
  BIC  556.834293  
  logL  -273.014561  
  logL ratio  -1.554315  
   Expression   
  Mean expression  4.754112  
  Median expression  4.401950  
  Tissue std. dev.  0.869229  
 
  No GO Slim enrichment  
  
   tissue    mean expression   
  5th Passage Drosophila S2 Cells  4.316903  
  Adult Accessory gland  4.451955  
  Adult Brain  6.460080  
  Adult Carcass  5.414875  
  Adult Crop  4.253142  
  Adult Eye  4.747212  
  Adult Fatbody  4.363315  
  Adult Female Spermatheca Mated  4.340424  
  Adult Female Spermatheca Virgin  4.285324  
  Adult Head  8.188991  
  Adult Heart  4.282262  
  Adult Hind Gut  4.317857  
  Adult Male Ejaculatory Duct  4.239612  
  Adult Mid Gut  4.416105  
  Adult Ovary  4.264930  
  Adult Salivary Gland  4.536298  
  Adult Testes  4.161055  
  Adult Thoracoabdominal ganglion  5.757436  
  Adult Whole Fly  5.291133  
  Larvae Wandering Tubules  4.364587  
  Larval Feeding Carcass  4.332852  
  Larval Feeding Central Nevous System  5.179829  
  Larval Feeding Hind Gut  4.169076  
  Larval Feeding Malpighian Tubule  4.377792  
  Larval Feeding Mid Gut  4.393858  
  Larval Feeding Salivary Gland  4.322881  
  Whole Larvae Feeding  5.131229  
 
  
   FlyBase ID    symbol    start    end    strand    length   
   FBgn0085366   CG34337   7311286   7311726  +  441  
   FBgn0029950   CG9657   7312628   7315672  +  3045  
   FBgn0040922   CG15036  7318560   7318875   -  316  
   FBgn0052726   CG32726  7319253   7319776   -  524  
   FBgn0040923   CG11368   7346241   7346696  +  456  
 
 
    Segment 124 
 
   Location   
  Gene key  FBgn0052719-FBgn0029952  
  Heatmap region span   X:7299793..7778693   
  Segment span   X:7406935..7480164   
  Length (genes)  4  
  Length (bp)  73230  
   Model Scoring   
  BIC  339.828032  
  logL  -164.511430  
  logL ratio  84.839985  
   Expression   
  Mean expression  4.880903  
  Median expression  4.772819  
  Tissue std. dev.  0.897817  
 
  No GO Slim enrichment  
  
   tissue    mean expression   
  5th Passage Drosophila S2 Cells  4.639690  
  Adult Accessory gland  4.922707  
  Adult Brain  4.476136  
  Adult Carcass  4.853835  
  Adult Crop  4.670689  
  Adult Eye  4.464285  
  Adult Fatbody  4.802498  
  Adult Female Spermatheca Mated  4.682264  
  Adult Female Spermatheca Virgin  4.710956  
  Adult Head  4.459233  
  Adult Heart  4.479390  
  Adult Hind Gut  4.654946  
  Adult Male Ejaculatory Duct  4.768273  
  Adult Mid Gut  4.795923  
  Adult Ovary  4.653695  
  Adult Salivary Gland  4.801824  
  Adult Testes  9.241832  
  Adult Thoracoabdominal ganglion  4.476673  
  Adult Whole Fly  5.843914  
  Larvae Wandering Tubules  4.766186  
  Larval Feeding Carcass  4.668045  
  Larval Feeding Central Nevous System  4.367492  
  Larval Feeding Hind Gut  4.501263  
  Larval Feeding Malpighian Tubule  4.665769  
  Larval Feeding Mid Gut  4.662031  
  Larval Feeding Salivary Gland  4.642344  
  Whole Larvae Feeding  5.112490  
 
  
   FlyBase ID    symbol    start    end    strand    length   
   FBgn0052719   CG32719  7402684   7406935   -  4252  
   FBgn0052720   CG32720  7429912   7430922   -  1011  
   FBgn0029951   CG11369   7468323   7470800  +  2478  
   FBgn0029952   CG12689  7479336   7480164   -  829  
 
 
    Segment 125 
 
   Location   
  Gene key  FBgn0004198-FBgn0029955  
  Heatmap region span   X:7301241..7782785   
  Segment span   X:7503181..7611961   
  Length (genes)  2  
  Length (bp)  108781  
   Model Scoring   
  BIC  216.540526  
  logL  -102.867677  
  logL ratio  3.021478  
   Expression   
  Mean expression  5.393030  
  Median expression  4.815066  
  Tissue std. dev.  0.937185  
 
  No GO Slim enrichment  
  
   tissue    mean expression   
  5th Passage Drosophila S2 Cells  5.826135  
  Adult Accessory gland  4.868745  
  Adult Brain  7.662189  
  Adult Carcass  4.974538  
  Adult Crop  4.701548  
  Adult Eye  5.788286  
  Adult Fatbody  5.037354  
  Adult Female Spermatheca Mated  5.456646  
  Adult Female Spermatheca Virgin  5.422206  
  Adult Head  5.180351  
  Adult Heart  4.976701  
  Adult Hind Gut  4.716024  
  Adult Male Ejaculatory Duct  4.634163  
  Adult Mid Gut  4.768572  
  Adult Ovary  4.830059  
  Adult Salivary Gland  5.109379  
  Adult Testes  4.231126  
  Adult Thoracoabdominal ganglion  6.501527  
  Adult Whole Fly  4.389339  
  Larvae Wandering Tubules  6.598984  
  Larval Feeding Carcass  5.151277  
  Larval Feeding Central Nevous System  7.853400  
  Larval Feeding Hind Gut  4.660167  
  Larval Feeding Malpighian Tubule  6.595077  
  Larval Feeding Mid Gut  4.490367  
  Larval Feeding Salivary Gland  6.332798  
  Whole Larvae Feeding  4.854857  
 
  
   FlyBase ID    symbol    start    end    strand    length   
   FBgn0004198   ct   7503181   7572292  +  69112  
   FBgn0029955   CG15478  7606816   7611961   -  5146  
 
    Segment 126 
 
   Location   
  Gene key  FBgn0022786-FBgn0029957  
  Heatmap region span   X:7305939..7785605   
  Segment span   X:7616167..7622792   
  Length (genes)  2  
  Length (bp)  6626  
   Model Scoring   
  BIC  208.144812  
  logL  -98.669820  
  logL ratio  41.104090  
   Expression   
  Mean expression  7.940543  
  Median expression  7.907657  
  Tissue std. dev.  0.571927  
 
  No GO Slim enrichment  
  
   tissue    mean expression   
  5th Passage Drosophila S2 Cells  8.629506  
  Adult Accessory gland  8.269905  
  Adult Brain  8.337143  
  Adult Carcass  7.278684  
  Adult Crop  7.824943  
  Adult Eye  7.348446  
  Adult Fatbody  7.516252  
  Adult Female Spermatheca Mated  8.012424  
  Adult Female Spermatheca Virgin  7.947313  
  Adult Head  7.485028  
  Adult Heart  7.296708  
  Adult Hind Gut  7.493889  
  Adult Male Ejaculatory Duct  7.496288  
  Adult Mid Gut  7.299641  
  Adult Ovary  9.668148  
  Adult Salivary Gland  7.772657  
  Adult Testes  7.475314  
  Adult Thoracoabdominal ganglion  7.984229  
  Adult Whole Fly  8.246531  
  Larvae Wandering Tubules  8.244049  
  Larval Feeding Carcass  7.819447  
  Larval Feeding Central Nevous System  9.008594  
  Larval Feeding Hind Gut  7.794090  
  Larval Feeding Malpighian Tubule  8.541491  
  Larval Feeding Mid Gut  7.516730  
  Larval Feeding Salivary Gland  8.552851  
  Whole Larvae Feeding  7.534347  
 
  
   FlyBase ID    symbol    start    end    strand    length   
   FBgn0022786   Hira   7616167   7619942  +  3776  
   FBgn0029957   CG12155   7622792   7625762  +  2971  
 
    Segment 127 
 
   Location   
  Gene key  FBgn0029958-FBgn0016041  
  Heatmap region span   X:7311286..7787829   
  Segment span   X:7628435..7630922   
  Length (genes)  3  
  Length (bp)  2488  
   Model Scoring   
  BIC  296.222876  
  logL  -142.708852  
  logL ratio  112.953474  
   Expression   
  Mean expression  9.760111  
  Median expression  9.824324  
  Tissue std. dev.  0.389500  
 
  No GO Slim enrichment  
  
   tissue    mean expression   
  5th Passage Drosophila S2 Cells  9.764413  
  Adult Accessory gland  9.494579  
  Adult Brain  10.030050  
  Adult Carcass  9.626675  
  Adult Crop  9.885101  
  Adult Eye  9.769481  
  Adult Fatbody  10.156957  
  Adult Female Spermatheca Mated  9.888262  
  Adult Female Spermatheca Virgin  10.159658  
  Adult Head  9.664499  
  Adult Heart  10.062899  
  Adult Hind Gut  9.927275  
  Adult Male Ejaculatory Duct  9.416870  
  Adult Mid Gut  9.283972  
  Adult Ovary  10.571046  
  Adult Salivary Gland  9.301394  
  Adult Testes  8.516465  
  Adult Thoracoabdominal ganglion  9.953623  
  Adult Whole Fly  9.791836  
  Larvae Wandering Tubules  10.012020  
  Larval Feeding Carcass  9.796358  
  Larval Feeding Central Nevous System  9.886040  
  Larval Feeding Hind Gut  9.933859  
  Larval Feeding Malpighian Tubule  10.268293  
  Larval Feeding Mid Gut  9.526636  
  Larval Feeding Salivary Gland  9.397858  
  Whole Larvae Feeding  9.436878  
 
  
   FlyBase ID    symbol    start    end    strand    length   
   FBgn0029958   Pdp  7625809   7628435   -  2627  
   FBgn0029959   Rab39   7628959   7630510  +  1552  
   FBgn0016041   Tom40   7630922   7633622  +  2701  
 
 
    Segment 128 
 
   Location   
  Gene key  FBgn0029961-FBgn0029968  
  Heatmap region span   X:7406935..7788070   
  Segment span   X:7659015..7778693   
  Length (genes)  10  
  Length (bp)  119679  
   Model Scoring   
  BIC  767.521403  
  logL  -378.358116  
  logL ratio  296.089524  
   Expression   
  Mean expression  4.525152  
  Median expression  4.369357  
  Tissue std. dev.  0.339672  
 
  No GO Slim enrichment  
  
   tissue    mean expression   
  5th Passage Drosophila S2 Cells  5.101479  
  Adult Accessory gland  4.417231  
  Adult Brain  4.523100  
  Adult Carcass  4.513700  
  Adult Crop  4.295809  
  Adult Eye  4.249246  
  Adult Fatbody  4.472450  
  Adult Female Spermatheca Mated  4.519243  
  Adult Female Spermatheca Virgin  4.463306  
  Adult Head  4.288417  
  Adult Heart  4.297293  
  Adult Hind Gut  4.338994  
  Adult Male Ejaculatory Duct  4.480541  
  Adult Mid Gut  4.418597  
  Adult Ovary  4.521248  
  Adult Salivary Gland  4.778406  
  Adult Testes  6.025895  
  Adult Thoracoabdominal ganglion  4.684343  
  Adult Whole Fly  4.497451  
  Larvae Wandering Tubules  4.401721  
  Larval Feeding Carcass  4.377312  
  Larval Feeding Central Nevous System  4.508769  
  Larval Feeding Hind Gut  4.275398  
  Larval Feeding Malpighian Tubule  4.423544  
  Larval Feeding Mid Gut  4.397011  
  Larval Feeding Salivary Gland  4.432982  
  Whole Larvae Feeding  4.475614  
 
  
   FlyBase ID    symbol    start    end    strand    length   
   FBgn0029961   Ir7a   7659015   7660859  +  1845  
   FBgn0085367   CG34338   7662810   7663142  +  333  
   FBgn0052718   CG32718   7672238   7673404  +  1167  
   FBgn0029962   CG1402   7720474   7734718  +  14245  
   FBgn0029963   CG10920  7746535   7748246   -  1712  
   FBgn0029964   CG1409   7752206   7752827  +  622  
   FBgn0029965   Ir7b  7758623   7760950   -  2328  
   FBgn0029966   Ir7c   7768859   7770832  +  1974  
   FBgn0259190   Ir7d  7770996   7772869   -  1874  
   FBgn0029968   Ir7g   7778693   7781009  +  2317  
 
 
    Segment 129 
 
   Location   
  Gene key  FBgn0029976-FBgn0029977  
  Heatmap region span   X:7795335..7858057   
  Segment span   X:7806122..7814164   
  Length (genes)  2  
  Length (bp)  8043  
   Model Scoring   
  BIC  233.051121  
  logL  -111.122975  
  logL ratio  -7.489779  
   Expression   
  Mean expression  5.305557  
  Median expression  5.175161  
  Tissue std. dev.  0.525760  
 
  No GO Slim enrichment  
  
   tissue    mean expression   
  5th Passage Drosophila S2 Cells  7.310158  
  Adult Accessory gland  5.253951  
  Adult Brain  5.904735  
  Adult Carcass  5.324244  
  Adult Crop  5.251296  
  Adult Eye  5.076148  
  Adult Fatbody  5.107896  
  Adult Female Spermatheca Mated  4.713230  
  Adult Female Spermatheca Virgin  4.769377  
  Adult Head  5.249709  
  Adult Heart  5.592650  
  Adult Hind Gut  5.261057  
  Adult Male Ejaculatory Duct  5.046939  
  Adult Mid Gut  5.218352  
  Adult Ovary  5.805942  
  Adult Salivary Gland  5.230982  
  Adult Testes  5.583280  
  Adult Thoracoabdominal ganglion  5.866228  
  Adult Whole Fly  4.910706  
  Larvae Wandering Tubules  4.897819  
  Larval Feeding Carcass  4.880895  
  Larval Feeding Central Nevous System  5.500467  
  Larval Feeding Hind Gut  5.171755  
  Larval Feeding Malpighian Tubule  4.802307  
  Larval Feeding Mid Gut  5.844649  
  Larval Feeding Salivary Gland  4.851422  
  Whole Larvae Feeding  4.823839  
 
  
   FlyBase ID    symbol    start    end    strand    length   
   FBgn0029976   snz   7806122   7811478  +  5357  
   FBgn0029977   hdm  7812550   7814164   -  1615  
 
    Segment 130 
 
   Location   
  Gene key  FBgn0029979-FBgn0029980  
  Heatmap region span   X:7795935..7862269   
  Segment span   X:7825388..7827313   
  Length (genes)  2  
  Length (bp)  1926  
   Model Scoring   
  BIC  222.067065  
  logL  -105.630946  
  logL ratio  28.469665  
   Expression   
  Mean expression  8.261540  
  Median expression  8.294870  
  Tissue std. dev.  0.561144  
 
  No GO Slim enrichment  
  
   tissue    mean expression   
  5th Passage Drosophila S2 Cells  8.192602  
  Adult Accessory gland  8.695494  
  Adult Brain  8.599669  
  Adult Carcass  7.722390  
  Adult Crop  8.563961  
  Adult Eye  8.203317  
  Adult Fatbody  8.670798  
  Adult Female Spermatheca Mated  8.434109  
  Adult Female Spermatheca Virgin  8.460370  
  Adult Head  7.929168  
  Adult Heart  8.506418  
  Adult Hind Gut  8.466547  
  Adult Male Ejaculatory Duct  8.504812  
  Adult Mid Gut  7.853819  
  Adult Ovary  8.591196  
  Adult Salivary Gland  8.341214  
  Adult Testes  6.056573  
  Adult Thoracoabdominal ganglion  8.618864  
  Adult Whole Fly  7.357490  
  Larvae Wandering Tubules  8.440123  
  Larval Feeding Carcass  8.163892  
  Larval Feeding Central Nevous System  8.946028  
  Larval Feeding Hind Gut  8.787974  
  Larval Feeding Malpighian Tubule  8.426013  
  Larval Feeding Mid Gut  8.237569  
  Larval Feeding Salivary Gland  8.576121  
  Whole Larvae Feeding  7.715037  
 
  
   FlyBase ID    symbol    start    end    strand    length   
   FBgn0029979   CG10777  7818151   7825388   -  7238  
   FBgn0029980   CG10778  7825762   7827313   -  1552  
 
    Segment 131 
 
   Location   
  Gene key  FBgn0004403-FBgn0004404  
  Heatmap region span   X:7801814..7915297   
  Segment span   X:7827779..7828937   
  Length (genes)  2  
  Length (bp)  1159  
   Model Scoring   
  BIC  364.561918  
  logL  -176.878373  
  logL ratio  105.351515  
   Expression   
  Mean expression  12.797921  
  Median expression  12.423610  
  Tissue std. dev.  0.513309  
 
  
   GO ID    description    ratio    P-value   
   GO:0005840   ribosome  2/2  0.000397  
   GO:0003735   structural constituent of ribosome  2/2  0.00211  
   GO:0006412   translation  2/2  0.00211  
   GO:0009058   biosynthetic process  2/2  0.00261  
   GO:0005198   structural molecule activity  2/2  0.00493  
 
  
   tissue    mean expression   
  5th Passage Drosophila S2 Cells  13.316725  
  Adult Accessory gland  13.029742  
  Adult Brain  12.813624  
  Adult Carcass  12.706421  
  Adult Crop  12.920591  
  Adult Eye  13.044004  
  Adult Fatbody  13.206174  
  Adult Female Spermatheca Mated  12.714517  
  Adult Female Spermatheca Virgin  12.686877  
  Adult Head  12.975247  
  Adult Heart  13.079745  
  Adult Hind Gut  13.004584  
  Adult Male Ejaculatory Duct  12.970813  
  Adult Mid Gut  12.560818  
  Adult Ovary  12.805614  
  Adult Salivary Gland  12.897006  
  Adult Testes  10.365253  
  Adult Thoracoabdominal ganglion  12.907407  
  Adult Whole Fly  12.827932  
  Larvae Wandering Tubules  12.865689  
  Larval Feeding Carcass  12.836451  
  Larval Feeding Central Nevous System  13.196523  
  Larval Feeding Hind Gut  12.995794  
  Larval Feeding Malpighian Tubule  12.872362  
  Larval Feeding Mid Gut  12.484653  
  Larval Feeding Salivary Gland  12.838268  
  Whole Larvae Feeding  12.621041  
 
  
   FlyBase ID    symbol    start    end    strand    length   
   FBgn0004403   RpS14a   7827779   7828713  +  935  
   FBgn0004404   RpS14b   7828937   7829832  +  896  
 
    Segment 132 
 
   Location   
  Gene key  FBgn0029986-FBgn0029987  
  Heatmap region span   X:7802925..7931533   
  Segment span   X:7850023..7850097   
  Length (genes)  2  
  Length (bp)  75  
   Model Scoring   
  BIC  174.401925  
  logL  -81.798376  
  logL ratio  39.748567  
   Expression   
  Mean expression  4.763028  
  Median expression  4.577173  
  Tissue std. dev.  0.691282  
 
  No GO Slim enrichment  
  
   tissue    mean expression   
  5th Passage Drosophila S2 Cells  4.773852  
  Adult Accessory gland  4.681526  
  Adult Brain  4.406142  
  Adult Carcass  4.839583  
  Adult Crop  4.585608  
  Adult Eye  4.290715  
  Adult Fatbody  4.831989  
  Adult Female Spermatheca Mated  4.880458  
  Adult Female Spermatheca Virgin  5.032818  
  Adult Head  4.429903  
  Adult Heart  4.376990  
  Adult Hind Gut  4.514679  
  Adult Male Ejaculatory Duct  4.746214  
  Adult Mid Gut  4.705445  
  Adult Ovary  4.392075  
  Adult Salivary Gland  4.975951  
  Adult Testes  8.154906  
  Adult Thoracoabdominal ganglion  4.529420  
  Adult Whole Fly  4.457600  
  Larvae Wandering Tubules  4.620542  
  Larval Feeding Carcass  4.658931  
  Larval Feeding Central Nevous System  4.437800  
  Larval Feeding Hind Gut  4.460433  
  Larval Feeding Malpighian Tubule  4.644837  
  Larval Feeding Mid Gut  4.655236  
  Larval Feeding Salivary Gland  4.787551  
  Whole Larvae Feeding  4.730557  
 
  
   FlyBase ID    symbol    start    end    strand    length   
   FBgn0029986   CG15332  7847885   7850023   -  2139  
   FBgn0029987   CG15330   7850097   7850735  +  639  
 
    Segment 133 
 
   Location   
  Gene key  FBgn0010329-FBgn0029989  
  Heatmap region span   X:7827779..7975635   
  Segment span   X:7889730..7915297   
  Length (genes)  2  
  Length (bp)  25568  
   Model Scoring   
  BIC  204.193444  
  logL  -96.694136  
  logL ratio  12.083775  
   Expression   
  Mean expression  4.893880  
  Median expression  4.642273  
  Tissue std. dev.  0.595623  
 
  No GO Slim enrichment  
  
   tissue    mean expression   
  5th Passage Drosophila S2 Cells  5.308641  
  Adult Accessory gland  4.636328  
  Adult Brain  6.061377  
  Adult Carcass  4.628168  
  Adult Crop  4.660559  
  Adult Eye  4.425038  
  Adult Fatbody  4.937159  
  Adult Female Spermatheca Mated  4.873400  
  Adult Female Spermatheca Virgin  4.991484  
  Adult Head  4.895287  
  Adult Heart  4.522216  
  Adult Hind Gut  4.558727  
  Adult Male Ejaculatory Duct  4.650675  
  Adult Mid Gut  4.485441  
  Adult Ovary  4.444991  
  Adult Salivary Gland  4.718862  
  Adult Testes  6.908696  
  Adult Thoracoabdominal ganglion  6.267596  
  Adult Whole Fly  4.503551  
  Larvae Wandering Tubules  4.592001  
  Larval Feeding Carcass  4.571850  
  Larval Feeding Central Nevous System  5.356948  
  Larval Feeding Hind Gut  4.521587  
  Larval Feeding Malpighian Tubule  4.748945  
  Larval Feeding Mid Gut  4.543959  
  Larval Feeding Salivary Gland  4.741197  
  Whole Larvae Feeding  4.580070  
 
  
   FlyBase ID    symbol    start    end    strand    length   
   FBgn0010329   Tbh   7889730   7920567  +  30838  
   FBgn0029989   CG15333  7913553   7915297   -  1745  
 
    Segment 134 
 
   Location   
  Gene key  FBgn0029992-FBgn0029993  
  Heatmap region span   X:7862269..8010177   
  Segment span   X:7969508..7969910   
  Length (genes)  2  
  Length (bp)  403  
   Model Scoring   
  BIC  225.324296  
  logL  -107.259562  
  logL ratio  2.729452  
   Expression   
  Mean expression  5.270504  
  Median expression  5.307743  
  Tissue std. dev.  0.649586  
 
  No GO Slim enrichment  
  
   tissue    mean expression   
  5th Passage Drosophila S2 Cells  5.765099  
  Adult Accessory gland  5.351733  
  Adult Brain  5.070264  
  Adult Carcass  4.999078  
  Adult Crop  5.351673  
  Adult Eye  4.902893  
  Adult Fatbody  5.195376  
  Adult Female Spermatheca Mated  5.045451  
  Adult Female Spermatheca Virgin  4.925611  
  Adult Head  5.098505  
  Adult Heart  4.961354  
  Adult Hind Gut  4.980272  
  Adult Male Ejaculatory Duct  5.169437  
  Adult Mid Gut  4.815793  
  Adult Ovary  5.686421  
  Adult Salivary Gland  5.216213  
  Adult Testes  8.336930  
  Adult Thoracoabdominal ganglion  4.697236  
  Adult Whole Fly  5.217990  
  Larvae Wandering Tubules  5.284066  
  Larval Feeding Carcass  5.222549  
  Larval Feeding Central Nevous System  5.381187  
  Larval Feeding Hind Gut  5.130519  
  Larval Feeding Malpighian Tubule  5.405820  
  Larval Feeding Mid Gut  4.899070  
  Larval Feeding Salivary Gland  5.344718  
  Whole Larvae Feeding  4.848340  
 
  
   FlyBase ID    symbol    start    end    strand    length   
   FBgn0029992   Upf2  7964277   7969508   -  5232  
   FBgn0029993   CG1571   7969910   7972273  +  2364  
 
    Segment 135 
 
   Location   
  Gene key  FBgn0029999-FBgn0030004  
  Heatmap region span   X:7975635..8046981   
  Segment span   X:8011096..8026439   
  Length (genes)  4  
  Length (bp)  15344  
   Model Scoring   
  BIC  446.291446  
  logL  -217.743137  
  logL ratio  24.640856  
   Expression   
  Mean expression  7.027663  
  Median expression  6.570949  
  Tissue std. dev.  0.332077  
 
  No GO Slim enrichment  
  
   tissue    mean expression   
  5th Passage Drosophila S2 Cells  7.507543  
  Adult Accessory gland  7.368122  
  Adult Brain  7.041754  
  Adult Carcass  6.545263  
  Adult Crop  6.984699  
  Adult Eye  6.699679  
  Adult Fatbody  7.016463  
  Adult Female Spermatheca Mated  7.243736  
  Adult Female Spermatheca Virgin  7.117402  
  Adult Head  6.595986  
  Adult Heart  6.928845  
  Adult Hind Gut  6.691819  
  Adult Male Ejaculatory Duct  6.762239  
  Adult Mid Gut  6.817975  
  Adult Ovary  7.952925  
  Adult Salivary Gland  6.848565  
  Adult Testes  7.265679  
  Adult Thoracoabdominal ganglion  7.004806  
  Adult Whole Fly  7.054654  
  Larvae Wandering Tubules  6.821948  
  Larval Feeding Carcass  7.121468  
  Larval Feeding Central Nevous System  7.467084  
  Larval Feeding Hind Gut  6.962685  
  Larval Feeding Malpighian Tubule  6.890273  
  Larval Feeding Mid Gut  6.747006  
  Larval Feeding Salivary Gland  7.639831  
  Whole Larvae Feeding  6.648448  
 
  
   FlyBase ID    symbol    start    end    strand    length   
   FBgn0029999   CG1575   8011096   8013166  +  2071  
   FBgn0030000   CG2260  8013111   8015153   -  2043  
   FBgn0030003   CG2116   8023773   8025816  +  2044  
   FBgn0030004   CG10958   8026439   8029000  +  2562  
 
 
    Segment 136 
 
   Location   
  Gene key  FBgn0030005-FBgn0030001  
  Heatmap region span   X:7981246..8051687   
  Segment span   X:8029357..8029746   
  Length (genes)  2  
  Length (bp)  390  
   Model Scoring   
  BIC  213.755156  
  logL  -101.474992  
  logL ratio  1.339451  
   Expression   
  Mean expression  5.202613  
  Median expression  4.914450  
  Tissue std. dev.  0.760223  
 
  No GO Slim enrichment  
  
   tissue    mean expression   
  5th Passage Drosophila S2 Cells  4.797644  
  Adult Accessory gland  4.736100  
  Adult Brain  4.654557  
  Adult Carcass  4.797607  
  Adult Crop  7.182523  
  Adult Eye  6.043139  
  Adult Fatbody  4.935428  
  Adult Female Spermatheca Mated  4.857376  
  Adult Female Spermatheca Virgin  4.854800  
  Adult Head  5.323201  
  Adult Heart  4.652261  
  Adult Hind Gut  6.266034  
  Adult Male Ejaculatory Duct  4.550323  
  Adult Mid Gut  7.029070  
  Adult Ovary  4.651896  
  Adult Salivary Gland  4.964431  
  Adult Testes  4.586278  
  Adult Thoracoabdominal ganglion  4.740244  
  Adult Whole Fly  5.020760  
  Larvae Wandering Tubules  5.038868  
  Larval Feeding Carcass  4.688401  
  Larval Feeding Central Nevous System  4.623333  
  Larval Feeding Hind Gut  4.864097  
  Larval Feeding Malpighian Tubule  5.147393  
  Larval Feeding Mid Gut  6.815613  
  Larval Feeding Salivary Gland  4.972575  
  Whole Larvae Feeding  5.676591  
 
  
   FlyBase ID    symbol    start    end    strand    length   
   FBgn0030005   CG2120   8029357   8031079  +  1723  
   FBgn0030001   cyr  8015309   8029746   -  14438  
 
    Segment 137 
 
   Location   
  Gene key  FBgn0025800-FBgn0030007  
  Heatmap region span   X:7996087..8063955   
  Segment span   X:8036301..8039634   
  Length (genes)  3  
  Length (bp)  3334  
   Model Scoring   
  BIC  299.849347  
  logL  -144.522087  
  logL ratio  74.006537  
   Expression   
  Mean expression  8.363142  
  Median expression  8.255037  
  Tissue std. dev.  0.410314  
 
  No GO Slim enrichment  
  
   tissue    mean expression   
  5th Passage Drosophila S2 Cells  8.252564  
  Adult Accessory gland  8.396187  
  Adult Brain  8.146694  
  Adult Carcass  7.643032  
  Adult Crop  8.425430  
  Adult Eye  8.720744  
  Adult Fatbody  8.483413  
  Adult Female Spermatheca Mated  8.780517  
  Adult Female Spermatheca Virgin  8.874876  
  Adult Head  8.004252  
  Adult Heart  8.862611  
  Adult Hind Gut  8.240025  
  Adult Male Ejaculatory Duct  8.570577  
  Adult Mid Gut  8.172956  
  Adult Ovary  8.363725  
  Adult Salivary Gland  8.113554  
  Adult Testes  7.518866  
  Adult Thoracoabdominal ganglion  7.953473  
  Adult Whole Fly  7.475818  
  Larvae Wandering Tubules  8.651184  
  Larval Feeding Carcass  8.328717  
  Larval Feeding Central Nevous System  9.062531  
  Larval Feeding Hind Gut  8.573619  
  Larval Feeding Malpighian Tubule  8.822971  
  Larval Feeding Mid Gut  8.583845  
  Larval Feeding Salivary Gland  8.780175  
  Whole Larvae Feeding  8.002489  
 
  
   FlyBase ID    symbol    start    end    strand    length   
   FBgn0025800   Smox  8032424   8036301   -  3878  
   FBgn0030006   CG17982  8036583   8037638   -  1056  
   FBgn0030007   CG2263  8037937   8039634   -  1698  
 
 
    Segment 138 
 
   Location   
  Gene key  FBgn0030008-FBgn0030009  
  Heatmap region span   X:8010177..8145699   
  Segment span   X:8039887..8041982   
  Length (genes)  2  
  Length (bp)  2096  
   Model Scoring   
  BIC  199.275465  
  logL  -94.235146  
  logL ratio  17.806536  
   Expression   
  Mean expression  5.212643  
  Median expression  5.116511  
  Tissue std. dev.  0.430141  
 
  
   GO ID    description    ratio    P-value   
   GO:0005622   intracellular  2/2  0.00282  
 
  
   tissue    mean expression   
  5th Passage Drosophila S2 Cells  5.422910  
  Adult Accessory gland  5.254535  
  Adult Brain  4.949129  
  Adult Carcass  4.774649  
  Adult Crop  5.115122  
  Adult Eye  5.018136  
  Adult Fatbody  4.900396  
  Adult Female Spermatheca Mated  5.429508  
  Adult Female Spermatheca Virgin  5.458318  
  Adult Head  4.848363  
  Adult Heart  5.291347  
  Adult Hind Gut  4.732973  
  Adult Male Ejaculatory Duct  5.113934  
  Adult Mid Gut  4.844220  
  Adult Ovary  6.579724  
  Adult Salivary Gland  5.240860  
  Adult Testes  5.164818  
  Adult Thoracoabdominal ganglion  4.832838  
  Adult Whole Fly  5.239963  
  Larvae Wandering Tubules  5.125395  
  Larval Feeding Carcass  4.952463  
  Larval Feeding Central Nevous System  5.334415  
  Larval Feeding Hind Gut  4.888314  
  Larval Feeding Malpighian Tubule  4.913214  
  Larval Feeding Mid Gut  6.436371  
  Larval Feeding Salivary Gland  5.537265  
  Whole Larvae Feeding  5.342177  
 
  
   FlyBase ID    symbol    start    end    strand    length   
   FBgn0030008   CG2129   8039887   8041888  +  2002  
   FBgn0030009   CG15336   8041982   8042529  +  548  
 
    Segment 139 
 
   Location   
  Gene key  FBgn0030010-FBgn0030012  
  Heatmap region span   X:8011096..8147264   
  Segment span   X:8042837..8046981   
  Length (genes)  3  
  Length (bp)  4145  
   Model Scoring   
  BIC  342.424110  
  logL  -165.809469  
  logL ratio  6.018848  
   Expression   
  Mean expression  6.546404  
  Median expression  6.288357  
  Tissue std. dev.  0.800475  
 
  
   GO ID    description    ratio    P-value   
   GO:0005622   intracellular  2/3  0.0165  
 
  
   tissue    mean expression   
  5th Passage Drosophila S2 Cells  6.009500  
  Adult Accessory gland  6.236042  
  Adult Brain  8.248027  
  Adult Carcass  5.890468  
  Adult Crop  7.157724  
  Adult Eye  7.311850  
  Adult Fatbody  5.770329  
  Adult Female Spermatheca Mated  5.549929  
  Adult Female Spermatheca Virgin  5.691827  
  Adult Head  7.003614  
  Adult Heart  6.525064  
  Adult Hind Gut  6.390235  
  Adult Male Ejaculatory Duct  5.972357  
  Adult Mid Gut  6.086220  
  Adult Ovary  7.807939  
  Adult Salivary Gland  5.841098  
  Adult Testes  6.721498  
  Adult Thoracoabdominal ganglion  7.959878  
  Adult Whole Fly  6.595037  
  Larvae Wandering Tubules  6.238124  
  Larval Feeding Carcass  5.600309  
  Larval Feeding Central Nevous System  8.156987  
  Larval Feeding Hind Gut  6.212850  
  Larval Feeding Malpighian Tubule  5.806872  
  Larval Feeding Mid Gut  7.324646  
  Larval Feeding Salivary Gland  5.841870  
  Whole Larvae Feeding  6.802625  
 
  
   FlyBase ID    symbol    start    end    strand    length   
   FBgn0030010   CG10959   8042837   8044719  +  1883  
   FBgn0030011   Gbeta5  8044764   8046311   -  1548  
   FBgn0030012   CG18262   8046981   8048566  +  1586  
 
 
    Segment 140 
 
   Location   
  Gene key  FBgn0026318-FBgn0030013  
  Heatmap region span   X:8029357..8165437   
  Segment span   X:8051399..8051687   
  Length (genes)  2  
  Length (bp)  289  
   Model Scoring   
  BIC  251.585191  
  logL  -120.390009  
  logL ratio  24.680557  
   Expression   
  Mean expression  9.407608  
  Median expression  9.227241  
  Tissue std. dev.  0.873669  
 
  No GO Slim enrichment  
  
   tissue    mean expression   
  5th Passage Drosophila S2 Cells  8.485272  
  Adult Accessory gland  8.963681  
  Adult Brain  9.806824  
  Adult Carcass  9.368369  
  Adult Crop  11.611581  
  Adult Eye  10.074588  
  Adult Fatbody  9.983894  
  Adult Female Spermatheca Mated  10.399307  
  Adult Female Spermatheca Virgin  10.255508  
  Adult Head  9.940673  
  Adult Heart  9.892868  
  Adult Hind Gut  9.730410  
  Adult Male Ejaculatory Duct  9.017537  
  Adult Mid Gut  8.472597  
  Adult Ovary  10.166756  
  Adult Salivary Gland  8.796607  
  Adult Testes  6.568521  
  Adult Thoracoabdominal ganglion  9.729280  
  Adult Whole Fly  9.306935  
  Larvae Wandering Tubules  9.060122  
  Larval Feeding Carcass  9.000188  
  Larval Feeding Central Nevous System  9.432900  
  Larval Feeding Hind Gut  9.064868  
  Larval Feeding Malpighian Tubule  8.938668  
  Larval Feeding Mid Gut  8.664296  
  Larval Feeding Salivary Gland  9.575492  
  Whole Larvae Feeding  9.697688  
 
  
   FlyBase ID    symbol    start    end    strand    length   
   FBgn0026318   Traf6  8048632   8051399   -  2768  
   FBgn0030013   GIIIspla2   8051687   8053050  +  1364  
 
    Segment 141 
 
   Location   
  Gene key  FBgn0030014-FBgn0030017  
  Heatmap region span   X:8036301..8167839   
  Segment span   X:8054146..8063955   
  Length (genes)  4  
  Length (bp)  9810  
   Model Scoring   
  BIC  340.291464  
  logL  -164.743146  
  logL ratio  85.785507  
   Expression   
  Mean expression  4.837448  
  Median expression  4.779287  
  Tissue std. dev.  0.484501  
 
  No GO Slim enrichment  
  
   tissue    mean expression   
  5th Passage Drosophila S2 Cells  4.806705  
  Adult Accessory gland  4.898758  
  Adult Brain  4.557887  
  Adult Carcass  4.702841  
  Adult Crop  4.996663  
  Adult Eye  4.641432  
  Adult Fatbody  4.809230  
  Adult Female Spermatheca Mated  4.759594  
  Adult Female Spermatheca Virgin  4.817064  
  Adult Head  4.702021  
  Adult Heart  4.727917  
  Adult Hind Gut  4.724166  
  Adult Male Ejaculatory Duct  4.885952  
  Adult Mid Gut  4.799659  
  Adult Ovary  4.637013  
  Adult Salivary Gland  4.885855  
  Adult Testes  7.245124  
  Adult Thoracoabdominal ganglion  4.635050  
  Adult Whole Fly  4.857098  
  Larvae Wandering Tubules  4.725154  
  Larval Feeding Carcass  4.705220  
  Larval Feeding Central Nevous System  4.475484  
  Larval Feeding Hind Gut  4.637828  
  Larval Feeding Malpighian Tubule  4.733014  
  Larval Feeding Mid Gut  4.735867  
  Larval Feeding Salivary Gland  4.787723  
  Whole Larvae Feeding  4.720784  
 
  
   FlyBase ID    symbol    start    end    strand    length   
   FBgn0030014   CG15337   8054146   8055586  +  1441  
   FBgn0030015   CG10761  8055568   8056825   -  1258  
   FBgn0030016   Or7a  8058299   8059741   -  1443  
   FBgn0030017   CG2278  8061822   8063955   -  2134  
 
 
    Segment 142 
 
   Location   
  Gene key  FBgn0030018-FBgn0017566  
  Heatmap region span   X:8039887..8292294   
  Segment span   X:8071187..8145699   
  Length (genes)  4  
  Length (bp)  74513  
   Model Scoring   
  BIC  451.676787  
  logL  -220.435808  
  logL ratio  82.326953  
   Expression   
  Mean expression  9.328467  
  Median expression  9.190777  
  Tissue std. dev.  0.260863  
 
  No GO Slim enrichment  
  
   tissue    mean expression   
  5th Passage Drosophila S2 Cells  9.183028  
  Adult Accessory gland  9.675188  
  Adult Brain  8.924302  
  Adult Carcass  9.391462  
  Adult Crop  9.319592  
  Adult Eye  9.282020  
  Adult Fatbody  9.606789  
  Adult Female Spermatheca Mated  9.257375  
  Adult Female Spermatheca Virgin  9.408417  
  Adult Head  8.952051  
  Adult Heart  9.568821  
  Adult Hind Gut  9.408571  
  Adult Male Ejaculatory Duct  9.622924  
  Adult Mid Gut  9.252120  
  Adult Ovary  9.470442  
  Adult Salivary Gland  9.524612  
  Adult Testes  8.555470  
  Adult Thoracoabdominal ganglion  9.023439  
  Adult Whole Fly  9.280058  
  Larvae Wandering Tubules  9.325128  
  Larval Feeding Carcass  9.422577  
  Larval Feeding Central Nevous System  9.075938  
  Larval Feeding Hind Gut  9.575995  
  Larval Feeding Malpighian Tubule  9.752146  
  Larval Feeding Mid Gut  9.521538  
  Larval Feeding Salivary Gland  9.350860  
  Whole Larvae Feeding  9.137754  
 
  
   FlyBase ID    symbol    start    end    strand    length   
   FBgn0030018   slpr  8064408   8071187   -  6780  
   FBgn0030025   CG2147   8134717   8135519  +  803  
   FBgn0030026   sni  8135531   8137337   -  1807  
   FBgn0017566   ND75  8142684   8145699   -  3016  
 
 
    Segment 143 
 
   Location   
  Gene key  FBgn0030030-FBgn0030029  
  Heatmap region span   X:8054146..8336374   
  Segment span   X:8167450..8167839   
  Length (genes)  2  
  Length (bp)  390  
   Model Scoring   
  BIC  222.007325  
  logL  -105.601077  
  logL ratio  21.200832  
   Expression   
  Mean expression  7.210820  
  Median expression  7.232983  
  Tissue std. dev.  0.601862  
 
  No GO Slim enrichment  
  
   tissue    mean expression   
  5th Passage Drosophila S2 Cells  7.191667  
  Adult Accessory gland  7.860962  
  Adult Brain  7.724486  
  Adult Carcass  8.021480  
  Adult Crop  6.673203  
  Adult Eye  8.401418  
  Adult Fatbody  7.559040  
  Adult Female Spermatheca Mated  6.820555  
  Adult Female Spermatheca Virgin  6.755325  
  Adult Head  7.675895  
  Adult Heart  7.438486  
  Adult Hind Gut  6.999826  
  Adult Male Ejaculatory Duct  7.672775  
  Adult Mid Gut  6.595451  
  Adult Ovary  6.987704  
  Adult Salivary Gland  8.408094  
  Adult Testes  6.459239  
  Adult Thoracoabdominal ganglion  7.594734  
  Adult Whole Fly  6.737870  
  Larvae Wandering Tubules  6.634402  
  Larval Feeding Carcass  6.978267  
  Larval Feeding Central Nevous System  6.697143  
  Larval Feeding Hind Gut  6.783546  
  Larval Feeding Malpighian Tubule  6.678812  
  Larval Feeding Mid Gut  6.258890  
  Larval Feeding Salivary Gland  8.126350  
  Whole Larvae Feeding  6.956507  
 
  
   FlyBase ID    symbol    start    end    strand    length   
   FBgn0030030   CG1636   8167450   8170234  +  2785  
   FBgn0030029   CG15343  8166357   8167839   -  1483  
 
    Segment 144 
 
   Location   
  Gene key  FBgn0030033-FBgn0040928  
  Heatmap region span   X:8071187..8354927   
  Segment span   X:8279814..8292294   
  Length (genes)  2  
  Length (bp)  12481  
   Model Scoring   
  BIC  211.326774  
  logL  -100.260801  
  logL ratio  4.873343  
   Expression   
  Mean expression  5.368193  
  Median expression  5.000289  
  Tissue std. dev.  0.606101  
 
  No GO Slim enrichment  
  
   tissue    mean expression   
  5th Passage Drosophila S2 Cells  5.208499  
  Adult Accessory gland  5.221310  
  Adult Brain  5.783231  
  Adult Carcass  4.625549  
  Adult Crop  5.558274  
  Adult Eye  5.517022  
  Adult Fatbody  4.777805  
  Adult Female Spermatheca Mated  5.143527  
  Adult Female Spermatheca Virgin  5.139120  
  Adult Head  4.957930  
  Adult Heart  4.941663  
  Adult Hind Gut  5.091181  
  Adult Male Ejaculatory Duct  4.747027  
  Adult Mid Gut  4.887491  
  Adult Ovary  6.918943  
  Adult Salivary Gland  5.442381  
  Adult Testes  7.302850  
  Adult Thoracoabdominal ganglion  5.803114  
  Adult Whole Fly  6.066410  
  Larvae Wandering Tubules  5.412913  
  Larval Feeding Carcass  5.142067  
  Larval Feeding Central Nevous System  5.824903  
  Larval Feeding Hind Gut  5.194662  
  Larval Feeding Malpighian Tubule  5.231005  
  Larval Feeding Mid Gut  4.867027  
  Larval Feeding Salivary Gland  5.171960  
  Whole Larvae Feeding  4.963340  
 
  
   FlyBase ID    symbol    start    end    strand    length   
   FBgn0030033   CG1387   8279814   8283537  +  3724  
   FBgn0040928   CG15345  8291189   8292294   -  1106  
 
    Segment 145 
 
   Location   
  Gene key  FBgn0030034-FBgn0052711  
  Heatmap region span   X:8147264..8359030   
  Segment span   X:8292973..8304128   
  Length (genes)  3  
  Length (bp)  11156  
   Model Scoring   
  BIC  292.869375  
  logL  -141.032102  
  logL ratio  79.253043  
   Expression   
  Mean expression  8.335442  
  Median expression  8.314954  
  Tissue std. dev.  0.632217  
 
  No GO Slim enrichment  
  
   tissue    mean expression   
  5th Passage Drosophila S2 Cells  8.822767  
  Adult Accessory gland  8.972753  
  Adult Brain  9.064340  
  Adult Carcass  7.500568  
  Adult Crop  8.762577  
  Adult Eye  8.803362  
  Adult Fatbody  7.794396  
  Adult Female Spermatheca Mated  7.891753  
  Adult Female Spermatheca Virgin  7.915169  
  Adult Head  8.080655  
  Adult Heart  8.556241  
  Adult Hind Gut  8.301399  
  Adult Male Ejaculatory Duct  8.063791  
  Adult Mid Gut  7.991433  
  Adult Ovary  9.577885  
  Adult Salivary Gland  7.961075  
  Adult Testes  6.550953  
  Adult Thoracoabdominal ganglion  9.074689  
  Adult Whole Fly  8.194525  
  Larvae Wandering Tubules  8.654989  
  Larval Feeding Carcass  8.139480  
  Larval Feeding Central Nevous System  9.459074  
  Larval Feeding Hind Gut  8.266925  
  Larval Feeding Malpighian Tubule  8.572310  
  Larval Feeding Mid Gut  7.898706  
  Larval Feeding Salivary Gland  8.503761  
  Whole Larvae Feeding  7.681357  
 
  
   FlyBase ID    symbol    start    end    strand    length   
   FBgn0030034   CG10555   8292973   8297975  +  5003  
   FBgn0030035   CG11190  8298570   8301319   -  2750  
   FBgn0052711     8302041   8304128   -  2088  
 
 
    Segment 146 
 
   Location   
  Gene key  FBgn0030037-FBgn0030039  
  Heatmap region span   X:8167450..8375870   
  Segment span   X:8330833..8336374   
  Length (genes)  3  
  Length (bp)  5542  
   Model Scoring   
  BIC  388.154810  
  logL  -188.674819  
  logL ratio  4.669984  
   Expression   
  Mean expression  8.745185  
  Median expression  8.672516  
  Tissue std. dev.  0.534732  
 
  No GO Slim enrichment  
  
   tissue    mean expression   
  5th Passage Drosophila S2 Cells  8.898377  
  Adult Accessory gland  8.391207  
  Adult Brain  9.499166  
  Adult Carcass  8.303148  
  Adult Crop  8.740026  
  Adult Eye  8.347962  
  Adult Fatbody  8.999873  
  Adult Female Spermatheca Mated  8.481064  
  Adult Female Spermatheca Virgin  8.424601  
  Adult Head  8.526107  
  Adult Heart  9.090287  
  Adult Hind Gut  8.397455  
  Adult Male Ejaculatory Duct  8.189395  
  Adult Mid Gut  8.070306  
  Adult Ovary  10.265649  
  Adult Salivary Gland  8.312700  
  Adult Testes  9.311074  
  Adult Thoracoabdominal ganglion  9.292289  
  Adult Whole Fly  9.229270  
  Larvae Wandering Tubules  9.195665  
  Larval Feeding Carcass  8.439741  
  Larval Feeding Central Nevous System  8.964390  
  Larval Feeding Hind Gut  8.273646  
  Larval Feeding Malpighian Tubule  9.627656  
  Larval Feeding Mid Gut  8.349581  
  Larval Feeding Salivary Gland  8.334625  
  Whole Larvae Feeding  8.164740  
 
  
   FlyBase ID    symbol    start    end    strand    length   
   FBgn0030037   CG12125  8327950   8330833   -  2884  
   FBgn0030038   CG1440   8331113   8334969  +  3857  
   FBgn0030039   CG12123  8335280   8336374   -  1095  
 
 
    Segment 147 
 
   Location   
  Gene key  FBgn0023506-FBgn0014032  
  Heatmap region span   X:8292973..8383553   
  Segment span   X:8355509..8359030   
  Length (genes)  2  
  Length (bp)  3522  
   Model Scoring   
  BIC  227.382254  
  logL  -108.288541  
  logL ratio  27.359427  
   Expression   
  Mean expression  8.432408  
  Median expression  8.344141  
  Tissue std. dev.  0.553067  
 
  No GO Slim enrichment  
  
   tissue    mean expression   
  5th Passage Drosophila S2 Cells  7.767397  
  Adult Accessory gland  8.128670  
  Adult Brain  8.564258  
  Adult Carcass  8.172322  
  Adult Crop  9.512981  
  Adult Eye  8.832659  
  Adult Fatbody  7.982671  
  Adult Female Spermatheca Mated  8.651095  
  Adult Female Spermatheca Virgin  8.765427  
  Adult Head  8.550812  
  Adult Heart  8.204988  
  Adult Hind Gut  9.044511  
  Adult Male Ejaculatory Duct  8.358661  
  Adult Mid Gut  8.055557  
  Adult Ovary  9.680152  
  Adult Salivary Gland  9.539050  
  Adult Testes  8.227971  
  Adult Thoracoabdominal ganglion  8.352923  
  Adult Whole Fly  8.583997  
  Larvae Wandering Tubules  7.704144  
  Larval Feeding Carcass  8.525417  
  Larval Feeding Central Nevous System  8.356846  
  Larval Feeding Hind Gut  8.393822  
  Larval Feeding Malpighian Tubule  7.902760  
  Larval Feeding Mid Gut  7.355014  
  Larval Feeding Salivary Gland  8.699997  
  Whole Larvae Feeding  7.760921  
 
  
   FlyBase ID    symbol    start    end    strand    length   
   FBgn0023506   Es2   8355509   8357382  +  1874  
   FBgn0014032   Sptr  8357338   8359030   -  1693  
 
    Segment 148 
 
   Location   
  Gene key  FBgn0014464-FBgn0000360  
  Heatmap region span   X:8330833..8423731   
  Segment span   X:8364921..8375870   
  Length (genes)  5  
  Length (bp)  10950  
   Model Scoring   
  BIC  433.738710  
  logL  -211.466769  
  logL ratio  125.402608  
   Expression   
  Mean expression  5.782122  
  Median expression  5.214379  
  Tissue std. dev.  1.714391  
 
  No GO Slim enrichment  
  
   tissue    mean expression   
  5th Passage Drosophila S2 Cells  5.119153  
  Adult Accessory gland  5.259747  
  Adult Brain  5.068661  
  Adult Carcass  6.643160  
  Adult Crop  5.143033  
  Adult Eye  4.965116  
  Adult Fatbody  5.654459  
  Adult Female Spermatheca Mated  5.697019  
  Adult Female Spermatheca Virgin  5.697170  
  Adult Head  4.982566  
  Adult Heart  5.134158  
  Adult Hind Gut  5.317597  
  Adult Male Ejaculatory Duct  5.407045  
  Adult Mid Gut  5.345963  
  Adult Ovary  12.193898  
  Adult Salivary Gland  5.490207  
  Adult Testes  4.939244  
  Adult Thoracoabdominal ganglion  5.105024  
  Adult Whole Fly  11.222299  
  Larvae Wandering Tubules  5.230363  
  Larval Feeding Carcass  5.306585  
  Larval Feeding Central Nevous System  5.003079  
  Larval Feeding Hind Gut  5.135131  
  Larval Feeding Malpighian Tubule  5.195980  
  Larval Feeding Mid Gut  5.478830  
  Larval Feeding Salivary Gland  5.313674  
  Whole Larvae Feeding  5.068143  
 
  
   FlyBase ID    symbol    start    end    strand    length   
   FBgn0014464   Cp7Fa   8364921   8367120  +  2200  
   FBgn0014465   Cp7Fb   8367238   8370104  +  2867  
   FBgn0014466   Cp7Fc   8371150   8372353  +  1204  
   FBgn0000359   Cp36   8373390   8374333  +  944  
   FBgn0000360   Cp38   8375870   8377344  +  1475  
 
 
    Segment 149 
 
   Location   
  Gene key  FBgn0030051-FBgn0030052  
  Heatmap region span   X:8411406..8571096   
  Segment span   X:8467614..8468337   
  Length (genes)  2  
  Length (bp)  724  
   Model Scoring   
  BIC  212.331413  
  logL  -100.763120  
  logL ratio  13.044139  
   Expression   
  Mean expression  5.996585  
  Median expression  5.838153  
  Tissue std. dev.  0.553884  
 
  No GO Slim enrichment  
  
   tissue    mean expression   
  5th Passage Drosophila S2 Cells  7.198719  
  Adult Accessory gland  5.364026  
  Adult Brain  5.476809  
  Adult Carcass  6.505764  
  Adult Crop  6.975835  
  Adult Eye  6.381570  
  Adult Fatbody  5.991727  
  Adult Female Spermatheca Mated  5.919787  
  Adult Female Spermatheca Virgin  5.998246  
  Adult Head  6.220904  
  Adult Heart  5.819034  
  Adult Hind Gut  6.915987  
  Adult Male Ejaculatory Duct  5.625767  
  Adult Mid Gut  5.986323  
  Adult Ovary  5.516113  
  Adult Salivary Gland  5.586995  
  Adult Testes  5.187713  
  Adult Thoracoabdominal ganglion  5.666989  
  Adult Whole Fly  5.124118  
  Larvae Wandering Tubules  5.644718  
  Larval Feeding Carcass  6.843590  
  Larval Feeding Central Nevous System  5.299845  
  Larval Feeding Hind Gut  6.550579  
  Larval Feeding Malpighian Tubule  5.957574  
  Larval Feeding Mid Gut  6.263469  
  Larval Feeding Salivary Gland  5.723816  
  Whole Larvae Feeding  6.161789  
 
  
   FlyBase ID    symbol    start    end    strand    length   
   FBgn0030051   spirit  8465132   8467614   -  2483  
   FBgn0030052   CG12065   8468337   8482561  +  14225  
 
    Segment 150 
 
   Location   
  Gene key  FBgn0030053-FBgn0025864  
  Heatmap region span   X:8423731..8575615   
  Segment span   X:8482857..8486086   
  Length (genes)  2  
  Length (bp)  3230  
   Model Scoring   
  BIC  218.948514  
  logL  -104.071671  
  logL ratio  34.529711  
   Expression   
  Mean expression  8.483843  
  Median expression  8.358092  
  Tissue std. dev.  0.498145  
 
  No GO Slim enrichment  
  
   tissue    mean expression   
  5th Passage Drosophila S2 Cells  8.765387  
  Adult Accessory gland  8.391792  
  Adult Brain  8.135082  
  Adult Carcass  8.040082  
  Adult Crop  8.605518  
  Adult Eye  8.194199  
  Adult Fatbody  8.605182  
  Adult Female Spermatheca Mated  8.192622  
  Adult Female Spermatheca Virgin  8.157284  
  Adult Head  7.721792  
  Adult Heart  8.530812  
  Adult Hind Gut  9.490551  
  Adult Male Ejaculatory Duct  8.048645  
  Adult Mid Gut  8.364883  
  Adult Ovary  9.149698  
  Adult Salivary Gland  8.747440  
  Adult Testes  7.257353  
  Adult Thoracoabdominal ganglion  8.341653  
  Adult Whole Fly  7.813359  
  Larvae Wandering Tubules  8.749991  
  Larval Feeding Carcass  8.459412  
  Larval Feeding Central Nevous System  8.751177  
  Larval Feeding Hind Gut  9.444678  
  Larval Feeding Malpighian Tubule  8.496806  
  Larval Feeding Mid Gut  8.945372  
  Larval Feeding Salivary Gland  9.132327  
  Whole Larvae Feeding  8.530658  
 
  
   FlyBase ID    symbol    start    end    strand    length   
   FBgn0030053   CG12081   8482857   8484743  +  1887  
   FBgn0025864   Crag   8486086   8495264  +  9179  
 
    Segment 151 
 
   Location   
  Gene key  FBgn0030057-FBgn0027864  
  Heatmap region span   X:8486423..8792343   
  Segment span   X:8584077..8584363   
  Length (genes)  2  
  Length (bp)  287  
   Model Scoring   
  BIC  209.961062  
  logL  -99.577945  
  logL ratio  39.959335  
   Expression   
  Mean expression  8.030612  
  Median expression  7.916670  
  Tissue std. dev.  0.592498  
 
  No GO Slim enrichment  
  
   tissue    mean expression   
  5th Passage Drosophila S2 Cells  9.433096  
  Adult Accessory gland  8.661763  
  Adult Brain  7.655343  
  Adult Carcass  7.643895  
  Adult Crop  7.977317  
  Adult Eye  7.859732  
  Adult Fatbody  8.513462  
  Adult Female Spermatheca Mated  8.639062  
  Adult Female Spermatheca Virgin  8.594014  
  Adult Head  7.560208  
  Adult Heart  8.959208  
  Adult Hind Gut  7.685805  
  Adult Male Ejaculatory Duct  7.727976  
  Adult Mid Gut  7.431093  
  Adult Ovary  8.726001  
  Adult Salivary Gland  8.113585  
  Adult Testes  6.828307  
  Adult Thoracoabdominal ganglion  7.821156  
  Adult Whole Fly  7.515631  
  Larvae Wandering Tubules  8.819175  
  Larval Feeding Carcass  7.523333  
  Larval Feeding Central Nevous System  7.990587  
  Larval Feeding Hind Gut  8.223771  
  Larval Feeding Malpighian Tubule  8.373670  
  Larval Feeding Mid Gut  7.447715  
  Larval Feeding Salivary Gland  7.811056  
  Whole Larvae Feeding  7.290557  
 
  
   FlyBase ID    symbol    start    end    strand    length   
   FBgn0030057   Ppt1  8582660   8584077   -  1418  
   FBgn0027864   Ogg1   8584363   8585844  +  1482  
 
    Segment 152 
 
   Location   
  Gene key  FBgn0041629-FBgn0030063  
  Heatmap region span   X:8571096..8878216   
  Segment span   X:8603687..8614590   
  Length (genes)  4  
  Length (bp)  10904  
   Model Scoring   
  BIC  466.001099  
  logL  -227.597963  
  logL ratio  60.992525  
   Expression   
  Mean expression  8.959371  
  Median expression  8.710652  
  Tissue std. dev.  0.661414  
 
  No GO Slim enrichment  
  
   tissue    mean expression   
  5th Passage Drosophila S2 Cells  10.209477  
  Adult Accessory gland  10.529274  
  Adult Brain  9.066513  
  Adult Carcass  9.009813  
  Adult Crop  9.613088  
  Adult Eye  8.865983  
  Adult Fatbody  9.300587  
  Adult Female Spermatheca Mated  9.068542  
  Adult Female Spermatheca Virgin  9.122131  
  Adult Head  9.073991  
  Adult Heart  9.659932  
  Adult Hind Gut  8.520974  
  Adult Male Ejaculatory Duct  9.879767  
  Adult Mid Gut  8.389906  
  Adult Ovary  9.358746  
  Adult Salivary Gland  8.533124  
  Adult Testes  7.583309  
  Adult Thoracoabdominal ganglion  9.086412  
  Adult Whole Fly  8.861187  
  Larvae Wandering Tubules  8.130613  
  Larval Feeding Carcass  8.864993  
  Larval Feeding Central Nevous System  9.335116  
  Larval Feeding Hind Gut  8.479739  
  Larval Feeding Malpighian Tubule  7.967262  
  Larval Feeding Mid Gut  8.117684  
  Larval Feeding Salivary Gland  8.842786  
  Whole Larvae Feeding  8.432080  
 
  
   FlyBase ID    symbol    start    end    strand    length   
   FBgn0041629   Hexo2   8603687   8606515  +  2829  
   FBgn0030060   CG2004  8606497   8608665   -  2169  
   FBgn0030061   CG1785   8608984   8611098  +  2115  
   FBgn0030063   CG1789   8614590   8615534  +  945  
 
 
    Segment 153 
 
   Location   
  Gene key  FBgn0026411-FBgn0030065  
  Heatmap region span   X:8575615..8943020   
  Segment span   X:8699808..8758146   
  Length (genes)  2  
  Length (bp)  58339  
   Model Scoring   
  BIC  229.461373  
  logL  -109.328100  
  logL ratio  -2.086859  
   Expression   
  Mean expression  5.868999  
  Median expression  5.856633  
  Tissue std. dev.  0.786285  
 
  No GO Slim enrichment  
  
   tissue    mean expression   
  5th Passage Drosophila S2 Cells  5.204500  
  Adult Accessory gland  5.618895  
  Adult Brain  7.128208  
  Adult Carcass  5.646534  
  Adult Crop  5.364626  
  Adult Eye  5.863895  
  Adult Fatbody  5.420972  
  Adult Female Spermatheca Mated  6.971296  
  Adult Female Spermatheca Virgin  6.793454  
  Adult Head  6.297214  
  Adult Heart  4.892440  
  Adult Hind Gut  5.384855  
  Adult Male Ejaculatory Duct  8.002930  
  Adult Mid Gut  5.144481  
  Adult Ovary  5.294812  
  Adult Salivary Gland  5.847973  
  Adult Testes  6.298418  
  Adult Thoracoabdominal ganglion  7.001889  
  Adult Whole Fly  5.213351  
  Larvae Wandering Tubules  5.146283  
  Larval Feeding Carcass  6.269239  
  Larval Feeding Central Nevous System  6.933186  
  Larval Feeding Hind Gut  5.522019  
  Larval Feeding Malpighian Tubule  5.159868  
  Larval Feeding Mid Gut  5.143005  
  Larval Feeding Salivary Gland  5.320658  
  Whole Larvae Feeding  5.577957  
 
  
   FlyBase ID    symbol    start    end    strand    length   
   FBgn0026411   Lim1  8651268   8699808   -  48541  
   FBgn0030065   CG12075   8758146   8766168  +  8023  
 
    Segment 154 
 
   Location   
  Gene key  FBgn0030066-FBgn0030067  
  Heatmap region span   X:8587587..8972528   
  Segment span   X:8794211..8795722   
  Length (genes)  2  
  Length (bp)  1512  
   Model Scoring   
  BIC  231.318143  
  logL  -110.256485  
  logL ratio  13.086059  
   Expression   
  Mean expression  7.165550  
  Median expression  6.882758  
  Tissue std. dev.  0.447986  
 
  No GO Slim enrichment  
  
   tissue    mean expression   
  5th Passage Drosophila S2 Cells  7.083772  
  Adult Accessory gland  7.942652  
  Adult Brain  6.784313  
  Adult Carcass  7.253546  
  Adult Crop  7.601297  
  Adult Eye  7.095301  
  Adult Fatbody  7.461313  
  Adult Female Spermatheca Mated  7.269560  
  Adult Female Spermatheca Virgin  7.063520  
  Adult Head  6.845616  
  Adult Heart  7.356925  
  Adult Hind Gut  7.138345  
  Adult Male Ejaculatory Duct  7.806364  
  Adult Mid Gut  6.593924  
  Adult Ovary  8.379014  
  Adult Salivary Gland  6.989791  
  Adult Testes  7.103136  
  Adult Thoracoabdominal ganglion  6.890859  
  Adult Whole Fly  7.209727  
  Larvae Wandering Tubules  6.649986  
  Larval Feeding Carcass  7.283751  
  Larval Feeding Central Nevous System  7.686025  
  Larval Feeding Hind Gut  7.041111  
  Larval Feeding Malpighian Tubule  6.815395  
  Larval Feeding Mid Gut  6.407694  
  Larval Feeding Salivary Gland  7.326403  
  Whole Larvae Feeding  6.390505  
 
  
   FlyBase ID    symbol    start    end    strand    length   
   FBgn0030066   CG1885  8793065   8794211   -  1147  
   FBgn0030067   Rbm13   8795722   8797511  +  1790  
 
    Segment 155 
 
   Location   
  Gene key  FBgn0030071-FBgn0030074  
  Heatmap region span   X:8603687..8979970   
  Segment span   X:8817651..8878216   
  Length (genes)  2  
  Length (bp)  60566  
   Model Scoring   
  BIC  184.789456  
  logL  -86.992142  
  logL ratio  28.507325  
   Expression   
  Mean expression  4.933995  
  Median expression  5.015225  
  Tissue std. dev.  0.380219  
 
  No GO Slim enrichment  
  
   tissue    mean expression   
  5th Passage Drosophila S2 Cells  4.921304  
  Adult Accessory gland  4.834125  
  Adult Brain  4.560858  
  Adult Carcass  4.949029  
  Adult Crop  4.794260  
  Adult Eye  4.581250  
  Adult Fatbody  4.946531  
  Adult Female Spermatheca Mated  5.110094  
  Adult Female Spermatheca Virgin  5.054590  
  Adult Head  4.712300  
  Adult Heart  4.707935  
  Adult Hind Gut  4.913979  
  Adult Male Ejaculatory Duct  5.146444  
  Adult Mid Gut  4.812557  
  Adult Ovary  5.130507  
  Adult Salivary Gland  5.313538  
  Adult Testes  6.658231  
  Adult Thoracoabdominal ganglion  4.833564  
  Adult Whole Fly  4.690560  
  Larvae Wandering Tubules  4.942502  
  Larval Feeding Carcass  4.851491  
  Larval Feeding Central Nevous System  4.598012  
  Larval Feeding Hind Gut  4.814862  
  Larval Feeding Malpighian Tubule  4.864198  
  Larval Feeding Mid Gut  4.868904  
  Larval Feeding Salivary Gland  4.827276  
  Whole Larvae Feeding  4.778964  
 
  
   FlyBase ID    symbol    start    end    strand    length   
   FBgn0030071   CG12661  8817256   8817651   -  396  
   FBgn0030074   CG12662  8877701   8878216   -  516  
 
    Segment 156 
 
   Location   
  Gene key  FBgn0030077-FBgn0030080  
  Heatmap region span   X:8792343..9043092   
  Segment span   X:8962016..8967293   
  Length (genes)  3  
  Length (bp)  5278  
   Model Scoring   
  BIC  259.575952  
  logL  -124.385390  
  logL ratio  56.592976  
   Expression   
  Mean expression  5.177339  
  Median expression  5.066304  
  Tissue std. dev.  0.343502  
 
  No GO Slim enrichment  
  
   tissue    mean expression   
  5th Passage Drosophila S2 Cells  4.903375  
  Adult Accessory gland  4.832480  
  Adult Brain  4.878252  
  Adult Carcass  4.830351  
  Adult Crop  5.927212  
  Adult Eye  4.968496  
  Adult Fatbody  5.187927  
  Adult Female Spermatheca Mated  5.224500  
  Adult Female Spermatheca Virgin  5.254460  
  Adult Head  4.687272  
  Adult Heart  5.394467  
  Adult Hind Gut  5.564645  
  Adult Male Ejaculatory Duct  5.010201  
  Adult Mid Gut  5.820195  
  Adult Ovary  4.897051  
  Adult Salivary Gland  5.074225  
  Adult Testes  6.062163  
  Adult Thoracoabdominal ganglion  5.266008  
  Adult Whole Fly  4.755265  
  Larvae Wandering Tubules  5.434914  
  Larval Feeding Carcass  5.076412  
  Larval Feeding Central Nevous System  5.007888  
  Larval Feeding Hind Gut  5.154088  
  Larval Feeding Malpighian Tubule  5.318428  
  Larval Feeding Mid Gut  5.239025  
  Larval Feeding Salivary Gland  5.159867  
  Whole Larvae Feeding  4.858991  
 
  
   FlyBase ID    symbol    start    end    strand    length   
   FBgn0030077   CG15365  8948731   8962016   -  13286  
   FBgn0030078   CG10970  8963632   8965441   -  1810  
   FBgn0030080     8966940   8967293   -  354  
 
 
    Segment 157 
 
   Location   
  Gene key  FBgn0030081-FBgn0052708  
  Heatmap region span   X:8817651..9085706   
  Segment span   X:8975091..8979970   
  Length (genes)  4  
  Length (bp)  4880  
   Model Scoring   
  BIC  395.417088  
  logL  -192.305958  
  logL ratio  90.457225  
   Expression   
  Mean expression  7.708363  
  Median expression  7.739218  
  Tissue std. dev.  0.506334  
 
  No GO Slim enrichment  
  
   tissue    mean expression   
  5th Passage Drosophila S2 Cells  8.595507  
  Adult Accessory gland  8.278463  
  Adult Brain  7.807007  
  Adult Carcass  7.071152  
  Adult Crop  7.626311  
  Adult Eye  7.878870  
  Adult Fatbody  7.338867  
  Adult Female Spermatheca Mated  8.000280  
  Adult Female Spermatheca Virgin  7.812491  
  Adult Head  7.346807  
  Adult Heart  7.514557  
  Adult Hind Gut  7.072105  
  Adult Male Ejaculatory Duct  7.429983  
  Adult Mid Gut  7.334498  
  Adult Ovary  9.223909  
  Adult Salivary Gland  7.450705  
  Adult Testes  7.540819  
  Adult Thoracoabdominal ganglion  7.818395  
  Adult Whole Fly  7.808853  
  Larvae Wandering Tubules  7.437907  
  Larval Feeding Carcass  7.679671  
  Larval Feeding Central Nevous System  8.751165  
  Larval Feeding Hind Gut  7.441671  
  Larval Feeding Malpighian Tubule  7.385664  
  Larval Feeding Mid Gut  7.194677  
  Larval Feeding Salivary Gland  8.038230  
  Whole Larvae Feeding  7.247249  
 
  
   FlyBase ID    symbol    start    end    strand    length   
   FBgn0030081   CG7246  8972856   8975091   -  2236  
   FBgn0030082   HP1b   8975096   8976391  +  1296  
   FBgn0052707   APC4   8976668   8979900  +  3233  
   FBgn0052708   CG32708   8979970   8980899  +  930  
 
 
    Segment 158 
 
   Location   
  Gene key  FBgn0030086-FBgn0004856  
  Heatmap region span   X:8943020..9087975   
  Segment span   X:8983361..9000635   
  Length (genes)  3  
  Length (bp)  17275  
   Model Scoring   
  BIC  375.227707  
  logL  -182.211268  
  logL ratio  88.677864  
   Expression   
  Mean expression  10.490578  
  Median expression  10.498971  
  Tissue std. dev.  0.504603  
 
  No GO Slim enrichment  
  
   tissue    mean expression   
  5th Passage Drosophila S2 Cells  10.919410  
  Adult Accessory gland  10.552228  
  Adult Brain  10.587982  
  Adult Carcass  10.479860  
  Adult Crop  10.716199  
  Adult Eye  10.116153  
  Adult Fatbody  10.682297  
  Adult Female Spermatheca Mated  10.458055  
  Adult Female Spermatheca Virgin  10.510103  
  Adult Head  10.245757  
  Adult Heart  10.506334  
  Adult Hind Gut  10.667300  
  Adult Male Ejaculatory Duct  10.247090  
  Adult Mid Gut  10.173703  
  Adult Ovary  11.391020  
  Adult Salivary Gland  10.512301  
  Adult Testes  8.645997  
  Adult Thoracoabdominal ganglion  10.826034  
  Adult Whole Fly  10.980025  
  Larvae Wandering Tubules  9.890653  
  Larval Feeding Carcass  10.971647  
  Larval Feeding Central Nevous System  11.344492  
  Larval Feeding Hind Gut  10.617312  
  Larval Feeding Malpighian Tubule  10.160277  
  Larval Feeding Mid Gut  10.116154  
  Larval Feeding Salivary Gland  10.611909  
  Whole Larvae Feeding  10.315313  
 
  
   FlyBase ID    symbol    start    end    strand    length   
   FBgn0030086   CG7033   8983361   8986866  +  3506  
   FBgn0030087   CG7766  8987639   8997756   -  10118  
   FBgn0004856   Bx42  8998351   9000635   -  2285  
 
 
    Segment 159 
 
   Location   
  Gene key  FBgn0030088-FBgn0030092  
  Heatmap region span   X:8962016..9090494   
  Segment span   X:9001032..9043092   
  Length (genes)  4  
  Length (bp)  42061  
   Model Scoring   
  BIC  392.793700  
  logL  -190.994264  
  logL ratio  86.844998  
   Expression   
  Mean expression  7.449983  
  Median expression  7.452125  
  Tissue std. dev.  0.622729  
 
  No GO Slim enrichment  
  
   tissue    mean expression   
  5th Passage Drosophila S2 Cells  7.330973  
  Adult Accessory gland  7.466516  
  Adult Brain  8.834802  
  Adult Carcass  6.938537  
  Adult Crop  7.053124  
  Adult Eye  8.047642  
  Adult Fatbody  7.233951  
  Adult Female Spermatheca Mated  7.309039  
  Adult Female Spermatheca Virgin  7.353364  
  Adult Head  7.547715  
  Adult Heart  7.870980  
  Adult Hind Gut  6.907331  
  Adult Male Ejaculatory Duct  7.326322  
  Adult Mid Gut  6.627545  
  Adult Ovary  8.651372  
  Adult Salivary Gland  7.177568  
  Adult Testes  7.508972  
  Adult Thoracoabdominal ganglion  8.906931  
  Adult Whole Fly  7.258494  
  Larvae Wandering Tubules  7.513958  
  Larval Feeding Carcass  6.690563  
  Larval Feeding Central Nevous System  8.367964  
  Larval Feeding Hind Gut  6.948043  
  Larval Feeding Malpighian Tubule  7.472384  
  Larval Feeding Mid Gut  6.645150  
  Larval Feeding Salivary Gland  7.423278  
  Whole Larvae Feeding  6.737019  
 
  
   FlyBase ID    symbol    start    end    strand    length   
   FBgn0030088   CG7039   9001032   9002391  +  1360  
   FBgn0030090   fend  9019165   9029051   -  9887  
   FBgn0030091   CG7065   9036585   9041763  +  5179  
   FBgn0030092   fh  9042127   9043092   -  966  
 
 
    Segment 160 
 
   Location   
  Gene key  FBgn0030093-FBgn0030096  
  Heatmap region span   X:8972528..9104580   
  Segment span   X:9043358..9056074   
  Length (genes)  5  
  Length (bp)  12717  
   Model Scoring   
  BIC  613.075176  
  logL  -301.135002  
  logL ratio  69.611764  
   Expression   
  Mean expression  9.410940  
  Median expression  9.435390  
  Tissue std. dev.  0.338529  
 
  No GO Slim enrichment  
  
   tissue    mean expression   
  5th Passage Drosophila S2 Cells  9.753890  
  Adult Accessory gland  9.971064  
  Adult Brain  9.126279  
  Adult Carcass  8.889680  
  Adult Crop  9.041249  
  Adult Eye  9.258307  
  Adult Fatbody  9.322779  
  Adult Female Spermatheca Mated  9.416061  
  Adult Female Spermatheca Virgin  9.351516  
  Adult Head  9.034500  
  Adult Heart  9.353910  
  Adult Hind Gut  9.098233  
  Adult Male Ejaculatory Duct  9.937015  
  Adult Mid Gut  9.180989  
  Adult Ovary  9.834138  
  Adult Salivary Gland  9.625496  
  Adult Testes  9.252581  
  Adult Thoracoabdominal ganglion  9.169688  
  Adult Whole Fly  9.435495  
  Larvae Wandering Tubules  9.155846  
  Larval Feeding Carcass  9.365512  
  Larval Feeding Central Nevous System  10.123005  
  Larval Feeding Hind Gut  9.420885  
  Larval Feeding Malpighian Tubule  9.442081  
  Larval Feeding Mid Gut  9.111209  
  Larval Feeding Salivary Gland  10.165376  
  Whole Larvae Feeding  9.258598  
 
  
   FlyBase ID    symbol    start    end    strand    length   
   FBgn0030093   dalao   9043358   9046011  +  2654  
   FBgn0014868   Ost48  9050955   9052544   -  1590  
   FBgn0040467   Dip1   9052684   9053405  +  722  
   FBgn0040931   CG9034  9053541   9054018   -  478  
   FBgn0030096   Zpr1  9054150   9056074   -  1925  
 
 
    Segment 161 
 
   Location   
  Gene key  FBgn0030097-FBgn0030098  
  Heatmap region span   X:8975091..9108929   
  Segment span   X:9084614..9085706   
  Length (genes)  2  
  Length (bp)  1093  
   Model Scoring   
  BIC  242.380449  
  logL  -115.787638  
  logL ratio  -3.980444  
   Expression   
  Mean expression  6.116275  
  Median expression  5.306038  
  Tissue std. dev.  2.084531  
 
  No GO Slim enrichment  
  
   tissue    mean expression   
  5th Passage Drosophila S2 Cells  5.383432  
  Adult Accessory gland  5.344640  
  Adult Brain  4.804282  
  Adult Carcass  5.250675  
  Adult Crop  5.201676  
  Adult Eye  4.882481  
  Adult Fatbody  5.504278  
  Adult Female Spermatheca Mated  5.548998  
  Adult Female Spermatheca Virgin  5.649735  
  Adult Head  4.983302  
  Adult Heart  5.004143  
  Adult Hind Gut  5.413724  
  Adult Male Ejaculatory Duct  5.688934  
  Adult Mid Gut  9.912246  
  Adult Ovary  5.285077  
  Adult Salivary Gland  5.481521  
  Adult Testes  4.853982  
  Adult Thoracoabdominal ganglion  4.898233  
  Adult Whole Fly  8.199744  
  Larvae Wandering Tubules  5.441458  
  Larval Feeding Carcass  5.800875  
  Larval Feeding Central Nevous System  5.058772  
  Larval Feeding Hind Gut  5.433974  
  Larval Feeding Malpighian Tubule  5.789535  
  Larval Feeding Mid Gut  12.925756  
  Larval Feeding Salivary Gland  5.354292  
  Whole Larvae Feeding  12.043641  
 
  
   FlyBase ID    symbol    start    end    strand    length   
   FBgn0030097   CG12115  9083356   9084614   -  1259  
   FBgn0030098   CG12057   9085706   9086582  +  877  
 
    Segment 162 
 
   Location   
  Gene key  FBgn0030099-FBgn0030100  
  Heatmap region span   X:8983361..9109912   
  Segment span   X:9086702..9087975   
  Length (genes)  2  
  Length (bp)  1274  
   Model Scoring   
  BIC  212.108189  
  logL  -100.651508  
  logL ratio  34.254391  
   Expression   
  Mean expression  7.287011  
  Median expression  7.074304  
  Tissue std. dev.  0.583824  
 
  No GO Slim enrichment  
  
   tissue    mean expression   
  5th Passage Drosophila S2 Cells  7.873269  
  Adult Accessory gland  7.954564  
  Adult Brain  7.926100  
  Adult Carcass  6.999996  
  Adult Crop  7.830528  
  Adult Eye  8.659264  
  Adult Fatbody  6.750832  
  Adult Female Spermatheca Mated  6.931285  
  Adult Female Spermatheca Virgin  6.745414  
  Adult Head  7.364407  
  Adult Heart  7.516236  
  Adult Hind Gut  7.157763  
  Adult Male Ejaculatory Duct  8.046284  
  Adult Mid Gut  6.868054  
  Adult Ovary  7.634608  
  Adult Salivary Gland  7.538829  
  Adult Testes  6.234923  
  Adult Thoracoabdominal ganglion  8.021735  
  Adult Whole Fly  6.449880  
  Larvae Wandering Tubules  7.209250  
  Larval Feeding Carcass  7.067117  
  Larval Feeding Central Nevous System  7.303159  
  Larval Feeding Hind Gut  6.935386  
  Larval Feeding Malpighian Tubule  7.260771  
  Larval Feeding Mid Gut  6.469554  
  Larval Feeding Salivary Gland  7.577137  
  Whole Larvae Feeding  6.422953  
 
  
   FlyBase ID    symbol    start    end    strand    length   
   FBgn0030099   CG12056   9086702   9087660  +  959  
   FBgn0030100   CG12106   9087975   9089382  +  1408  
 
    Segment 163 
 
   Location   
  Gene key  FBgn0030107-FBgn0030108  
  Heatmap region span   X:9108929..9163958   
  Segment span   X:9119489..9121170   
  Length (genes)  2  
  Length (bp)  1682  
   Model Scoring   
  BIC  186.181474  
  logL  -87.688151  
  logL ratio  34.152391  
   Expression   
  Mean expression  4.694433  
  Median expression  4.605474  
  Tissue std. dev.  0.729231  
 
  No GO Slim enrichment  
  
   tissue    mean expression   
  5th Passage Drosophila S2 Cells  4.582882  
  Adult Accessory gland  4.554701  
  Adult Brain  4.226584  
  Adult Carcass  4.868596  
  Adult Crop  4.557272  
  Adult Eye  4.596466  
  Adult Fatbody  4.660239  
  Adult Female Spermatheca Mated  4.605130  
  Adult Female Spermatheca Virgin  4.542121  
  Adult Head  4.498217  
  Adult Heart  4.508019  
  Adult Hind Gut  4.504045  
  Adult Male Ejaculatory Duct  8.304721  
  Adult Mid Gut  4.783582  
  Adult Ovary  4.619932  
  Adult Salivary Gland  4.937969  
  Adult Testes  4.338774  
  Adult Thoracoabdominal ganglion  4.391369  
  Adult Whole Fly  4.241199  
  Larvae Wandering Tubules  4.672654  
  Larval Feeding Carcass  4.738911  
  Larval Feeding Central Nevous System  4.259233  
  Larval Feeding Hind Gut  4.538265  
  Larval Feeding Malpighian Tubule  4.650647  
  Larval Feeding Mid Gut  4.666833  
  Larval Feeding Salivary Gland  4.588627  
  Whole Larvae Feeding  4.312702  
 
  
   FlyBase ID    symbol    start    end    strand    length   
   FBgn0030107   CG15370   9119489   9120601  +  1113  
   FBgn0030108   Gr8a   9121170   9122864  +  1695  
 
    Segment 164 
 
   Location   
  Gene key  FBgn0000077-FBgn0028292  
  Heatmap region span   X:9117290..9427851   
  Segment span   X:9141369..9150210   
  Length (genes)  4  
  Length (bp)  8842  
   Model Scoring   
  BIC  371.249955  
  logL  -180.222392  
  logL ratio  118.767012  
   Expression   
  Mean expression  8.100468  
  Median expression  8.144592  
  Tissue std. dev.  0.467247  
 
  No GO Slim enrichment  
  
   tissue    mean expression   
  5th Passage Drosophila S2 Cells  8.405773  
  Adult Accessory gland  8.162786  
  Adult Brain  8.449716  
  Adult Carcass  7.409216  
  Adult Crop  7.869795  
  Adult Eye  7.973633  
  Adult Fatbody  7.915549  
  Adult Female Spermatheca Mated  8.002921  
  Adult Female Spermatheca Virgin  8.007583  
  Adult Head  7.659091  
  Adult Heart  8.240317  
  Adult Hind Gut  7.909030  
  Adult Male Ejaculatory Duct  8.070053  
  Adult Mid Gut  8.019638  
  Adult Ovary  8.755768  
  Adult Salivary Gland  8.099017  
  Adult Testes  6.765828  
  Adult Thoracoabdominal ganglion  8.126536  
  Adult Whole Fly  7.747558  
  Larvae Wandering Tubules  9.226437  
  Larval Feeding Carcass  7.881632  
  Larval Feeding Central Nevous System  8.113681  
  Larval Feeding Hind Gut  8.439661  
  Larval Feeding Malpighian Tubule  8.849221  
  Larval Feeding Mid Gut  8.625139  
  Larval Feeding Salivary Gland  8.303365  
  Whole Larvae Feeding  7.683690  
 
  
   FlyBase ID    symbol    start    end    strand    length   
   FBgn0000077   amx  9139948   9141369   -  1422  
   FBgn0010269   Dsor1   9141828   9144070  +  2243  
   FBgn0030114   CG17754   9144460   9149853  +  5394  
   FBgn0028292   ric8a   9150210   9152703  +  2494  
 
 
    Segment 165 
 
   Location   
  Gene key  FBgn0052703-FBgn0030120  
  Heatmap region span   X:9119489..9445262   
  Segment span   X:9161731..9163958   
  Length (genes)  2  
  Length (bp)  2228  
   Model Scoring   
  BIC  188.194996  
  logL  -88.694912  
  logL ratio  37.263169  
   Expression   
  Mean expression  4.622235  
  Median expression  4.433416  
  Tissue std. dev.  0.956468  
 
  No GO Slim enrichment  
  
   tissue    mean expression   
  5th Passage Drosophila S2 Cells  4.498805  
  Adult Accessory gland  4.584507  
  Adult Brain  4.190929  
  Adult Carcass  4.501805  
  Adult Crop  4.406196  
  Adult Eye  4.204676  
  Adult Fatbody  4.338965  
  Adult Female Spermatheca Mated  4.522266  
  Adult Female Spermatheca Virgin  4.482609  
  Adult Head  4.276460  
  Adult Heart  4.304848  
  Adult Hind Gut  4.216240  
  Adult Male Ejaculatory Duct  4.656203  
  Adult Mid Gut  4.463206  
  Adult Ovary  4.419299  
  Adult Salivary Gland  4.736074  
  Adult Testes  9.408568  
  Adult Thoracoabdominal ganglion  4.306869  
  Adult Whole Fly  4.873926  
  Larvae Wandering Tubules  4.354738  
  Larval Feeding Carcass  4.392952  
  Larval Feeding Central Nevous System  4.181211  
  Larval Feeding Hind Gut  4.228561  
  Larval Feeding Malpighian Tubule  4.348154  
  Larval Feeding Mid Gut  4.504314  
  Larval Feeding Salivary Gland  4.556568  
  Whole Larvae Feeding  4.841410  
 
  
   FlyBase ID    symbol    start    end    strand    length   
   FBgn0052703   Erk7  9155409   9161731   -  6323  
   FBgn0030120   CG17440  9162471   9163958   -  1488  
 
    Segment 166 
 
   Location   
  Gene key  FBgn0030121-FBgn0030122  
  Heatmap region span   X:9126406..9448655   
  Segment span   X:9166922..9167450   
  Length (genes)  2  
  Length (bp)  529  
   Model Scoring   
  BIC  209.394684  
  logL  -99.294756  
  logL ratio  36.480709  
   Expression   
  Mean expression  7.233849  
  Median expression  7.072582  
  Tissue std. dev.  0.583182  
 
  No GO Slim enrichment  
  
   tissue    mean expression   
  5th Passage Drosophila S2 Cells  8.077089  
  Adult Accessory gland  7.235891  
  Adult Brain  7.603934  
  Adult Carcass  6.643096  
  Adult Crop  6.776634  
  Adult Eye  7.359058  
  Adult Fatbody  6.906887  
  Adult Female Spermatheca Mated  6.792073  
  Adult Female Spermatheca Virgin  6.950537  
  Adult Head  6.959943  
  Adult Heart  7.086704  
  Adult Hind Gut  6.731587  
  Adult Male Ejaculatory Duct  6.694376  
  Adult Mid Gut  6.630591  
  Adult Ovary  9.129463  
  Adult Salivary Gland  6.461180  
  Adult Testes  7.133908  
  Adult Thoracoabdominal ganglion  7.598413  
  Adult Whole Fly  7.940388  
  Larvae Wandering Tubules  7.634626  
  Larval Feeding Carcass  7.146152  
  Larval Feeding Central Nevous System  8.088426  
  Larval Feeding Hind Gut  7.243246  
  Larval Feeding Malpighian Tubule  7.419096  
  Larval Feeding Mid Gut  6.722444  
  Larval Feeding Salivary Gland  7.586770  
  Whole Larvae Feeding  6.761406  
 
  
   FlyBase ID    symbol    start    end    strand    length   
   FBgn0030121   Cfp1  9164634   9166922   -  2289  
   FBgn0030122   CG16892   9167450   9169387  +  1938  
 
    Segment 167 
 
   Location   
  Gene key  FBgn0002576-FBgn0054028  
  Heatmap region span   X:9130618..9465341   
  Segment span   X:9178682..9405557   
  Length (genes)  3  
  Length (bp)  226876  
   Model Scoring   
  BIC  351.687413  
  logL  -170.441120  
  logL ratio  -1.269454  
   Expression   
  Mean expression  5.001317  
  Median expression  4.224547  
  Tissue std. dev.  0.388328  
 
  No GO Slim enrichment  
  
   tissue    mean expression   
  5th Passage Drosophila S2 Cells  5.556361  
  Adult Accessory gland  4.729599  
  Adult Brain  4.953724  
  Adult Carcass  4.961417  
  Adult Crop  4.791119  
  Adult Eye  6.071151  
  Adult Fatbody  4.912665  
  Adult Female Spermatheca Mated  5.977190  
  Adult Female Spermatheca Virgin  5.677414  
  Adult Head  5.119173  
  Adult Heart  5.026804  
  Adult Hind Gut  4.703498  
  Adult Male Ejaculatory Duct  4.578299  
  Adult Mid Gut  4.610952  
  Adult Ovary  5.423448  
  Adult Salivary Gland  4.826097  
  Adult Testes  4.791745  
  Adult Thoracoabdominal ganglion  4.685280  
  Adult Whole Fly  4.820429  
  Larvae Wandering Tubules  4.770554  
  Larval Feeding Carcass  5.044424  
  Larval Feeding Central Nevous System  4.944581  
  Larval Feeding Hind Gut  4.859637  
  Larval Feeding Malpighian Tubule  4.795871  
  Larval Feeding Mid Gut  4.773905  
  Larval Feeding Salivary Gland  4.910328  
  Whole Larvae Feeding  4.719900  
 
  
   FlyBase ID    symbol    start    end    strand    length   
   FBgn0002576   lz   9178682   9197670  +  18989  
   FBgn0085478   CG34449  9220691   9248599   -  27909  
   FBgn0054028   CG34028   9405557   9406195  +  639  
 
 
    Segment 168 
 
   Location   
  Gene key  FBgn0030137-FBgn0052702  
  Heatmap region span   X:9178682..9556789   
  Segment span   X:9450577..9465341   
  Length (genes)  2  
  Length (bp)  14765  
   Model Scoring   
  BIC  235.397891  
  logL  -112.296359  
  logL ratio  8.419687  
   Expression   
  Mean expression  7.007281  
  Median expression  6.872846  
  Tissue std. dev.  0.845967  
 
  No GO Slim enrichment  
  
   tissue    mean expression   
  5th Passage Drosophila S2 Cells  7.693760  
  Adult Accessory gland  7.565131  
  Adult Brain  6.812156  
  Adult Carcass  7.406592  
  Adult Crop  6.256593  
  Adult Eye  6.735594  
  Adult Fatbody  7.217880  
  Adult Female Spermatheca Mated  6.104621  
  Adult Female Spermatheca Virgin  6.181987  
  Adult Head  6.133736  
  Adult Heart  9.857012  
  Adult Hind Gut  6.624094  
  Adult Male Ejaculatory Duct  6.330314  
  Adult Mid Gut  7.134927  
  Adult Ovary  7.133629  
  Adult Salivary Gland  7.194743  
  Adult Testes  5.164215  
  Adult Thoracoabdominal ganglion  6.886461  
  Adult Whole Fly  6.189931  
  Larvae Wandering Tubules  7.984069  
  Larval Feeding Carcass  7.625561  
  Larval Feeding Central Nevous System  7.005098  
  Larval Feeding Hind Gut  6.673494  
  Larval Feeding Malpighian Tubule  7.629437  
  Larval Feeding Mid Gut  7.395999  
  Larval Feeding Salivary Gland  7.636297  
  Whole Larvae Feeding  6.623259  
 
  
   FlyBase ID    symbol    start    end    strand    length   
   FBgn0030137   CG15317  9449640   9450577   -  938  
   FBgn0052702   CG32702  9452698   9465341   -  12644  
 
    Segment 169 
 
   Location   
  Gene key  FBgn0030141-FBgn0030142  
  Heatmap region span   X:9448655..9759251   
  Segment span   X:9495067..9499999   
  Length (genes)  2  
  Length (bp)  4933  
   Model Scoring   
  BIC  212.236072  
  logL  -100.715450  
  logL ratio  41.780384  
   Expression   
  Mean expression  8.702855  
  Median expression  8.680034  
  Tissue std. dev.  0.546686  
 
  No GO Slim enrichment  
  
   tissue    mean expression   
  5th Passage Drosophila S2 Cells  9.044598  
  Adult Accessory gland  8.763047  
  Adult Brain  8.175579  
  Adult Carcass  7.872236  
  Adult Crop  8.999567  
  Adult Eye  8.559589  
  Adult Fatbody  8.524966  
  Adult Female Spermatheca Mated  8.591428  
  Adult Female Spermatheca Virgin  8.525762  
  Adult Head  8.221120  
  Adult Heart  8.451023  
  Adult Hind Gut  8.585981  
  Adult Male Ejaculatory Duct  9.055913  
  Adult Mid Gut  8.891622  
  Adult Ovary  9.597962  
  Adult Salivary Gland  8.635753  
  Adult Testes  7.015569  
  Adult Thoracoabdominal ganglion  8.130833  
  Adult Whole Fly  8.519797  
  Larvae Wandering Tubules  9.163040  
  Larval Feeding Carcass  8.761277  
  Larval Feeding Central Nevous System  9.705361  
  Larval Feeding Hind Gut  9.042494  
  Larval Feeding Malpighian Tubule  9.283617  
  Larval Feeding Mid Gut  8.838895  
  Larval Feeding Salivary Gland  9.475950  
  Whole Larvae Feeding  8.544093  
 
  
   FlyBase ID    symbol    start    end    strand    length   
   FBgn0030141   Gga   9495067   9498313  +  3247  
   FBgn0030142     9498187   9499999   -  1813  
 
    Segment 170 
 
   Location   
  Gene key  FBgn0000233-FBgn0020378  
  Heatmap region span   X:9470234..9770705   
  Segment span   X:9588221..9623708   
  Length (genes)  2  
  Length (bp)  35488  
   Model Scoring   
  BIC  180.162448  
  logL  -84.678638  
  logL ratio  31.903290  
   Expression   
  Mean expression  4.919154  
  Median expression  4.898140  
  Tissue std. dev.  0.398677  
 
  
   GO ID    description    ratio    P-value   
   GO:0005634   nucleus  2/2  0.0436  
 
  
   tissue    mean expression   
  5th Passage Drosophila S2 Cells  4.769892  
  Adult Accessory gland  5.008363  
  Adult Brain  5.074285  
  Adult Carcass  5.284542  
  Adult Crop  4.772421  
  Adult Eye  4.824879  
  Adult Fatbody  4.966650  
  Adult Female Spermatheca Mated  4.827812  
  Adult Female Spermatheca Virgin  4.770067  
  Adult Head  4.967644  
  Adult Heart  4.738824  
  Adult Hind Gut  4.636114  
  Adult Male Ejaculatory Duct  4.699766  
  Adult Mid Gut  4.746178  
  Adult Ovary  4.609530  
  Adult Salivary Gland  5.084661  
  Adult Testes  4.300811  
  Adult Thoracoabdominal ganglion  5.648930  
  Adult Whole Fly  4.456572  
  Larvae Wandering Tubules  4.723199  
  Larval Feeding Carcass  5.230135  
  Larval Feeding Central Nevous System  6.465689  
  Larval Feeding Hind Gut  4.774553  
  Larval Feeding Malpighian Tubule  4.735926  
  Larval Feeding Mid Gut  4.808774  
  Larval Feeding Salivary Gland  4.957354  
  Whole Larvae Feeding  4.933592  
 
  
   FlyBase ID    symbol    start    end    strand    length   
   FBgn0000233   btd   9588221   9591606  +  3386  
   FBgn0020378   Sp1   9623708   9649831  +  26124  
 
    Segment 171 
 
   Location   
  Gene key  FBgn0052698-FBgn0019929  
  Heatmap region span   X:9495067..9773902   
  Segment span   X:9682515..9759251   
  Length (genes)  2  
  Length (bp)  76737  
   Model Scoring   
  BIC  225.428893  
  logL  -107.311860  
  logL ratio  12.344172  
   Expression   
  Mean expression  6.782294  
  Median expression  5.807634  
  Tissue std. dev.  1.870715  
 
  No GO Slim enrichment  
  
   tissue    mean expression   
  5th Passage Drosophila S2 Cells  5.307861  
  Adult Accessory gland  5.451249  
  Adult Brain  5.929056  
  Adult Carcass  9.873356  
  Adult Crop  5.672890  
  Adult Eye  9.179430  
  Adult Fatbody  10.570587  
  Adult Female Spermatheca Mated  9.828788  
  Adult Female Spermatheca Virgin  9.552003  
  Adult Head  9.455738  
  Adult Heart  10.024510  
  Adult Hind Gut  6.286654  
  Adult Male Ejaculatory Duct  7.387327  
  Adult Mid Gut  5.333658  
  Adult Ovary  5.309552  
  Adult Salivary Gland  5.379864  
  Adult Testes  5.288841  
  Adult Thoracoabdominal ganglion  5.558446  
  Adult Whole Fly  7.233531  
  Larvae Wandering Tubules  5.231802  
  Larval Feeding Carcass  5.336123  
  Larval Feeding Central Nevous System  5.101463  
  Larval Feeding Hind Gut  5.459829  
  Larval Feeding Malpighian Tubule  5.420379  
  Larval Feeding Mid Gut  5.406982  
  Larval Feeding Salivary Gland  5.951818  
  Whole Larvae Feeding  6.590198  
 
  
   FlyBase ID    symbol    start    end    strand    length   
   FBgn0052698   CG32698   9682515   9743263  +  60749  
   FBgn0019929   Ser7   9759251   9761103  +  1853  
 
    Segment 172 
 
   Location   
  Gene key  FBgn0030161-FBgn0030162  
  Heatmap region span   X:9772455..9947885   
  Segment span   X:9783193..9805159   
  Length (genes)  2  
  Length (bp)  21967  
   Model Scoring   
  BIC  170.528408  
  logL  -79.861618  
  logL ratio  41.662002  
   Expression   
  Mean expression  4.583848  
  Median expression  4.573607  
  Tissue std. dev.  0.181407  
 
  No GO Slim enrichment  
  
   tissue    mean expression   
  5th Passage Drosophila S2 Cells  4.746828  
  Adult Accessory gland  4.659613  
  Adult Brain  4.373644  
  Adult Carcass  4.651046  
  Adult Crop  4.464240  
  Adult Eye  4.653926  
  Adult Fatbody  4.498831  
  Adult Female Spermatheca Mated  4.721910  
  Adult Female Spermatheca Virgin  4.671231  
  Adult Head  4.364427  
  Adult Heart  4.567878  
  Adult Hind Gut  4.641898  
  Adult Male Ejaculatory Duct  4.853810  
  Adult Mid Gut  4.588515  
  Adult Ovary  4.559006  
  Adult Salivary Gland  5.079254  
  Adult Testes  4.504369  
  Adult Thoracoabdominal ganglion  4.403814  
  Adult Whole Fly  4.142500  
  Larvae Wandering Tubules  4.653376  
  Larval Feeding Carcass  4.765104  
  Larval Feeding Central Nevous System  4.331162  
  Larval Feeding Hind Gut  4.515245  
  Larval Feeding Malpighian Tubule  4.604544  
  Larval Feeding Mid Gut  4.551243  
  Larval Feeding Salivary Gland  4.743154  
  Whole Larvae Feeding  4.453316  
 
  
   FlyBase ID    symbol    start    end    strand    length   
   FBgn0030161   CG15249  9782323   9783193   -  871  
   FBgn0030162   CG1986  9803425   9805159   -  1735  
 
    Segment 173 
 
   Location   
  Gene key  FBgn0030163-FBgn0030164  
  Heatmap region span   X:9776016..9964899   
  Segment span   X:9858911..9859274   
  Length (genes)  2  
  Length (bp)  364  
   Model Scoring   
  BIC  185.570249  
  logL  -87.382538  
  logL ratio  44.088312  
   Expression   
  Mean expression  4.976105  
  Median expression  4.248109  
  Tissue std. dev.  1.560192  
 
  
   GO ID    description    ratio    P-value   
   GO:0007165   signal transduction  2/2  0.000391  
 
  
   tissue    mean expression   
  5th Passage Drosophila S2 Cells  4.200452  
  Adult Accessory gland  4.303869  
  Adult Brain  3.948487  
  Adult Carcass  8.503186  
  Adult Crop  7.877396  
  Adult Eye  8.823281  
  Adult Fatbody  4.542165  
  Adult Female Spermatheca Mated  4.379812  
  Adult Female Spermatheca Virgin  4.401014  
  Adult Head  8.615939  
  Adult Heart  4.978025  
  Adult Hind Gut  6.902636  
  Adult Male Ejaculatory Duct  4.222010  
  Adult Mid Gut  4.208249  
  Adult Ovary  4.073959  
  Adult Salivary Gland  4.408521  
  Adult Testes  4.091926  
  Adult Thoracoabdominal ganglion  4.084931  
  Adult Whole Fly  4.980202  
  Larvae Wandering Tubules  4.091252  
  Larval Feeding Carcass  4.053658  
  Larval Feeding Central Nevous System  3.978658  
  Larval Feeding Hind Gut  4.025915  
  Larval Feeding Malpighian Tubule  4.225209  
  Larval Feeding Mid Gut  4.302211  
  Larval Feeding Salivary Gland  4.214312  
  Whole Larvae Feeding  3.917557  
 
  
   FlyBase ID    symbol    start    end    strand    length   
   FBgn0030163   CG1791  9857486   9858911   -  1426  
   FBgn0030164   CG1889   9859274   9860995  +  1722  
 
    Segment 174 
 
   Location   
  Gene key  FBgn0030165-FBgn0030173  
  Heatmap region span   X:9778475..9980204   
  Segment span   X:9861152..9943758   
  Length (genes)  9  
  Length (bp)  82607  
   Model Scoring   
  BIC  783.803216  
  logL  -386.499022  
  logL ratio  162.727766  
   Expression   
  Mean expression  5.159687  
  Median expression  4.972570  
  Tissue std. dev.  0.392135  
 
  No GO Slim enrichment  
  
   tissue    mean expression   
  5th Passage Drosophila S2 Cells  5.012133  
  Adult Accessory gland  4.880761  
  Adult Brain  4.705693  
  Adult Carcass  5.187348  
  Adult Crop  5.624433  
  Adult Eye  4.925412  
  Adult Fatbody  5.162099  
  Adult Female Spermatheca Mated  5.312112  
  Adult Female Spermatheca Virgin  5.198211  
  Adult Head  4.821035  
  Adult Heart  4.857642  
  Adult Hind Gut  5.494898  
  Adult Male Ejaculatory Duct  5.587625  
  Adult Mid Gut  4.928611  
  Adult Ovary  5.276500  
  Adult Salivary Gland  5.237070  
  Adult Testes  4.737315  
  Adult Thoracoabdominal ganglion  4.683373  
  Adult Whole Fly  4.710582  
  Larvae Wandering Tubules  4.871532  
  Larval Feeding Carcass  6.404129  
  Larval Feeding Central Nevous System  4.980794  
  Larval Feeding Hind Gut  5.656161  
  Larval Feeding Malpighian Tubule  4.938428  
  Larval Feeding Mid Gut  5.076319  
  Larval Feeding Salivary Gland  5.254537  
  Whole Larvae Feeding  5.786795  
 
  
   FlyBase ID    symbol    start    end    strand    length   
   FBgn0030165     9860744   9861152   -  409  
   FBgn0030166     9861355   9864791   -  3437  
   FBgn0030167   CG15252  9872238   9874660   -  2423  
   FBgn0052694   CG32694   9883728   9900918  +  17191  
   FBgn0053557   CG33557   9903268   9903886  +  619  
   FBgn0030170   CG2990   9904536   9908312  +  3777  
   FBgn0030171      9924145   9938633  +  14489  
   FBgn0030172      9941963   9942538  +  576  
   FBgn0030173      9943758   9944448  +  691  
 
 
    Segment 175 
 
   Location   
  Gene key  FBgn0005391-FBgn0004045  
  Heatmap region span   X:9783193..10080484   
  Segment span   X:9946669..9947885   
  Length (genes)  2  
  Length (bp)  1217  
   Model Scoring   
  BIC  286.597512  
  logL  -137.896170  
  logL ratio  116.912413  
   Expression   
  Mean expression  9.939900  
  Median expression  9.733418  
  Tissue std. dev.  4.106702  
 
  
   GO ID    description    ratio    P-value   
   GO:0006629   lipid metabolic process  2/2  0.000198  
   GO:0005811   lipid particle  2/2  0.00202  
   GO:0005198   structural molecule activity  2/2  0.00296  
 
  
   tissue    mean expression   
  5th Passage Drosophila S2 Cells  5.511493  
  Adult Accessory gland  6.314735  
  Adult Brain  12.937781  
  Adult Carcass  14.697926  
  Adult Crop  12.389267  
  Adult Eye  14.108480  
  Adult Fatbody  14.746588  
  Adult Female Spermatheca Mated  14.710336  
  Adult Female Spermatheca Virgin  14.750928  
  Adult Head  14.607563  
  Adult Heart  14.683295  
  Adult Hind Gut  13.495052  
  Adult Male Ejaculatory Duct  5.770772  
  Adult Mid Gut  7.910074  
  Adult Ovary  13.880969  
  Adult Salivary Gland  9.733418  
  Adult Testes  4.936443  
  Adult Thoracoabdominal ganglion  13.252893  
  Adult Whole Fly  14.406150  
  Larvae Wandering Tubules  5.117431  
  Larval Feeding Carcass  5.816231  
  Larval Feeding Central Nevous System  5.333549  
  Larval Feeding Hind Gut  5.697579  
  Larval Feeding Malpighian Tubule  5.394452  
  Larval Feeding Mid Gut  7.414282  
  Larval Feeding Salivary Gland  5.893060  
  Whole Larvae Feeding  4.866549  
 
  
   FlyBase ID    symbol    start    end    strand    length   
   FBgn0005391   Yp2  9945049   9946669   -  1621  
   FBgn0004045   Yp1   9947885   9949531  +  1647  
 
    Segment 176 
 
   Location   
  Gene key  FBgn0052685-FBgn0030178  
  Heatmap region span   X:9861152..10114143   
  Segment span   X:9976515..9980204   
  Length (genes)  3  
  Length (bp)  3690  
   Model Scoring   
  BIC  284.861025  
  logL  -137.027927  
  logL ratio  85.001059  
   Expression   
  Mean expression  8.216717  
  Median expression  8.249780  
  Tissue std. dev.  0.356103  
 
  No GO Slim enrichment  
  
   tissue    mean expression   
  5th Passage Drosophila S2 Cells  8.284357  
  Adult Accessory gland  8.512583  
  Adult Brain  7.888481  
  Adult Carcass  7.693678  
  Adult Crop  8.466746  
  Adult Eye  8.166288  
  Adult Fatbody  8.428575  
  Adult Female Spermatheca Mated  8.735251  
  Adult Female Spermatheca Virgin  8.634034  
  Adult Head  7.854433  
  Adult Heart  8.380721  
  Adult Hind Gut  8.083254  
  Adult Male Ejaculatory Duct  8.247520  
  Adult Mid Gut  7.862513  
  Adult Ovary  9.061288  
  Adult Salivary Gland  8.092757  
  Adult Testes  7.791922  
  Adult Thoracoabdominal ganglion  7.856250  
  Adult Whole Fly  7.926650  
  Larvae Wandering Tubules  8.335607  
  Larval Feeding Carcass  8.106617  
  Larval Feeding Central Nevous System  8.873096  
  Larval Feeding Hind Gut  8.241564  
  Larval Feeding Malpighian Tubule  8.327399  
  Larval Feeding Mid Gut  7.818845  
  Larval Feeding Salivary Gland  8.485287  
  Whole Larvae Feeding  7.695651  
 
  
   FlyBase ID    symbol    start    end    strand    length   
   FBgn0052685   ZAP3  9966179   9976515   -  10337  
   FBgn0030177   CG2972   9977203   9979083  +  1881  
   FBgn0030178   CG2974  9978930   9980204   -  1275  
 
 
    Segment 177 
 
   Location   
  Gene key  FBgn0083940-FBgn0030182  
  Heatmap region span   X:9946669..10126428   
  Segment span   X:9980566..10080484   
  Length (genes)  4  
  Length (bp)  99919  
   Model Scoring   
  BIC  301.957898  
  logL  -145.576363  
  logL ratio  111.067934  
   Expression   
  Mean expression  4.845695  
  Median expression  4.759571  
  Tissue std. dev.  0.230148  
 
  No GO Slim enrichment  
  
   tissue    mean expression   
  5th Passage Drosophila S2 Cells  4.765324  
  Adult Accessory gland  4.851500  
  Adult Brain  5.464526  
  Adult Carcass  4.923624  
  Adult Crop  4.767374  
  Adult Eye  4.956691  
  Adult Fatbody  4.785856  
  Adult Female Spermatheca Mated  4.791729  
  Adult Female Spermatheca Virgin  4.780563  
  Adult Head  4.799510  
  Adult Heart  4.545984  
  Adult Hind Gut  4.760116  
  Adult Male Ejaculatory Duct  4.960809  
  Adult Mid Gut  4.760258  
  Adult Ovary  4.701246  
  Adult Salivary Gland  5.340358  
  Adult Testes  5.131859  
  Adult Thoracoabdominal ganglion  5.203460  
  Adult Whole Fly  4.346369  
  Larvae Wandering Tubules  4.902569  
  Larval Feeding Carcass  4.854999  
  Larval Feeding Central Nevous System  4.838611  
  Larval Feeding Hind Gut  4.614609  
  Larval Feeding Malpighian Tubule  4.790516  
  Larval Feeding Mid Gut  4.720375  
  Larval Feeding Salivary Gland  4.913344  
  Whole Larvae Feeding  4.561574  
 
  
   FlyBase ID    symbol    start    end    strand    length   
   FBgn0083940   CG34104   9980566   10042911  +  62346  
   FBgn0030181   CG12645   10047645   10049044  +  1400  
   FBgn0040940      10052843   10053124  +  282  
   FBgn0030182      10080484   10086952  +  6469  
 
 
    Segment 178 
 
   Location   
  Gene key  FBgn0030186-FBgn0085437  
  Heatmap region span   X:9976515..10223882   
  Segment span   X:10110670..10114143   
  Length (genes)  3  
  Length (bp)  3474  
   Model Scoring   
  BIC  304.722358  
  logL  -146.958593  
  logL ratio  59.724281  
   Expression   
  Mean expression  7.099116  
  Median expression  6.903285  
  Tissue std. dev.  0.532432  
 
  No GO Slim enrichment  
  
   tissue    mean expression   
  5th Passage Drosophila S2 Cells  7.809314  
  Adult Accessory gland  7.213775  
  Adult Brain  6.749175  
  Adult Carcass  7.076907  
  Adult Crop  7.118660  
  Adult Eye  6.374391  
  Adult Fatbody  7.268787  
  Adult Female Spermatheca Mated  7.095933  
  Adult Female Spermatheca Virgin  7.135786  
  Adult Head  6.741589  
  Adult Heart  7.103242  
  Adult Hind Gut  7.027166  
  Adult Male Ejaculatory Duct  7.147251  
  Adult Mid Gut  6.963427  
  Adult Ovary  6.764525  
  Adult Salivary Gland  7.249315  
  Adult Testes  6.197067  
  Adult Thoracoabdominal ganglion  6.817738  
  Adult Whole Fly  6.711984  
  Larvae Wandering Tubules  7.025780  
  Larval Feeding Carcass  9.248655  
  Larval Feeding Central Nevous System  6.615105  
  Larval Feeding Hind Gut  7.415722  
  Larval Feeding Malpighian Tubule  7.056242  
  Larval Feeding Mid Gut  7.016675  
  Larval Feeding Salivary Gland  7.453686  
  Whole Larvae Feeding  7.278242  
 
  
   FlyBase ID    symbol    start    end    strand    length   
   FBgn0030186   CG2962  10108891   10110670   -  1780  
   FBgn0040941   CG15308  10112092   10113153   -  1062  
   FBgn0085437   CG34408   10114143   10123948  +  9806  
 
 
    Segment 179 
 
   Location   
  Gene key  FBgn0001203-FBgn0085348  
  Heatmap region span   X:10098433..10261494   
  Segment span   X:10148337..10150208   
  Length (genes)  2  
  Length (bp)  1872  
   Model Scoring   
  BIC  238.897026  
  logL  -114.045927  
  logL ratio  -7.668809  
   Expression   
  Mean expression  6.218787  
  Median expression  5.881200  
  Tissue std. dev.  1.078601  
 
  No GO Slim enrichment  
  
   tissue    mean expression   
  5th Passage Drosophila S2 Cells  7.413627  
  Adult Accessory gland  5.661257  
  Adult Brain  9.027534  
  Adult Carcass  6.470161  
  Adult Crop  6.154914  
  Adult Eye  8.397965  
  Adult Fatbody  5.704601  
  Adult Female Spermatheca Mated  5.927756  
  Adult Female Spermatheca Virgin  5.711055  
  Adult Head  7.462410  
  Adult Heart  6.303292  
  Adult Hind Gut  6.064756  
  Adult Male Ejaculatory Duct  5.728665  
  Adult Mid Gut  5.800059  
  Adult Ovary  5.245406  
  Adult Salivary Gland  5.911165  
  Adult Testes  4.993139  
  Adult Thoracoabdominal ganglion  8.963653  
  Adult Whole Fly  5.686756  
  Larvae Wandering Tubules  5.358562  
  Larval Feeding Carcass  6.066259  
  Larval Feeding Central Nevous System  6.622706  
  Larval Feeding Hind Gut  5.701750  
  Larval Feeding Malpighian Tubule  5.378347  
  Larval Feeding Mid Gut  5.460527  
  Larval Feeding Salivary Gland  5.398714  
  Whole Larvae Feeding  5.292210  
 
  
   FlyBase ID    symbol    start    end    strand    length   
   FBgn0001203     10132277   10148337   -  16061  
   FBgn0085348     10148738   10150208   -  1471  
 
    Segment 180 
 
   Location   
  Gene key  FBgn0040942-FBgn0030189  
  Heatmap region span   X:10106379..10262084   
  Segment span   X:10159290..10220504   
  Length (genes)  2  
  Length (bp)  61215  
   Model Scoring   
  BIC  249.510306  
  logL  -119.352567  
  logL ratio  -0.020026  
   Expression   
  Mean expression  7.955236  
  Median expression  8.089308  
  Tissue std. dev.  1.104010  
 
  No GO Slim enrichment  
  
   tissue    mean expression   
  5th Passage Drosophila S2 Cells  9.185951  
  Adult Accessory gland  7.115099  
  Adult Brain  6.693599  
  Adult Carcass  7.727776  
  Adult Crop  6.669215  
  Adult Eye  7.579404  
  Adult Fatbody  9.066835  
  Adult Female Spermatheca Mated  8.589755  
  Adult Female Spermatheca Virgin  8.934637  
  Adult Head  7.786443  
  Adult Heart  8.798201  
  Adult Hind Gut  6.995959  
  Adult Male Ejaculatory Duct  6.294370  
  Adult Mid Gut  9.508108  
  Adult Ovary  6.243883  
  Adult Salivary Gland  6.652243  
  Adult Testes  6.401406  
  Adult Thoracoabdominal ganglion  6.963026  
  Adult Whole Fly  6.580249  
  Larvae Wandering Tubules  8.989355  
  Larval Feeding Carcass  8.377350  
  Larval Feeding Central Nevous System  9.441392  
  Larval Feeding Hind Gut  8.394906  
  Larval Feeding Malpighian Tubule  8.791437  
  Larval Feeding Mid Gut  9.387312  
  Larval Feeding Salivary Gland  8.670079  
  Whole Larvae Feeding  8.953378  
 
  
   FlyBase ID    symbol    start    end    strand    length   
   FBgn0040942   CG12643  10158257   10159290   -  1034  
   FBgn0030189   CG2909   10220504   10222979  +  2476  
 
    Segment 181 
 
   Location   
  Gene key  FBgn0030192-FBgn0052679  
  Heatmap region span   X:10148337..10279966   
  Segment span   X:10229917..10261494   
  Length (genes)  3  
  Length (bp)  31578  
   Model Scoring   
  BIC  289.465919  
  logL  -139.330374  
  logL ratio  39.019048  
   Expression   
  Mean expression  5.494675  
  Median expression  5.674901  
  Tissue std. dev.  0.386012  
 
  No GO Slim enrichment  
  
   tissue    mean expression   
  5th Passage Drosophila S2 Cells  5.350502  
  Adult Accessory gland  5.346642  
  Adult Brain  6.725835  
  Adult Carcass  5.598403  
  Adult Crop  5.201645  
  Adult Eye  5.081803  
  Adult Fatbody  5.449558  
  Adult Female Spermatheca Mated  5.397162  
  Adult Female Spermatheca Virgin  5.421731  
  Adult Head  5.365250  
  Adult Heart  5.760852  
  Adult Hind Gut  5.254724  
  Adult Male Ejaculatory Duct  5.460126  
  Adult Mid Gut  5.689698  
  Adult Ovary  5.231102  
  Adult Salivary Gland  5.552366  
  Adult Testes  5.576289  
  Adult Thoracoabdominal ganglion  6.048255  
  Adult Whole Fly  4.914902  
  Larvae Wandering Tubules  5.296838  
  Larval Feeding Carcass  5.391321  
  Larval Feeding Central Nevous System  5.251920  
  Larval Feeding Hind Gut  5.178185  
  Larval Feeding Malpighian Tubule  5.349597  
  Larval Feeding Mid Gut  5.713337  
  Larval Feeding Salivary Gland  6.469315  
  Whole Larvae Feeding  5.278860  
 
  
   FlyBase ID    symbol    start    end    strand    length   
   FBgn0030192      10229917   10241069  +  11153  
   FBgn0030193      10240438   10241827  +  1390  
   FBgn0052679   CG32679  10260543   10261494   -  952  
 
 
    Segment 182 
 
   Location   
  Gene key  FBgn0052686-FBgn0052681  
  Heatmap region span   X:10223882..10367971   
  Segment span   X:10269349..10275289   
  Length (genes)  3  
  Length (bp)  5941  
   Model Scoring   
  BIC  240.421044  
  logL  -114.807936  
  logL ratio  81.833518  
   Expression   
  Mean expression  4.721717  
  Median expression  4.531019  
  Tissue std. dev.  0.909040  
 
  No GO Slim enrichment  
  
   tissue    mean expression   
  5th Passage Drosophila S2 Cells  4.670250  
  Adult Accessory gland  4.847765  
  Adult Brain  4.203225  
  Adult Carcass  4.536012  
  Adult Crop  4.349476  
  Adult Eye  4.319786  
  Adult Fatbody  4.481999  
  Adult Female Spermatheca Mated  4.607836  
  Adult Female Spermatheca Virgin  4.670805  
  Adult Head  4.301878  
  Adult Heart  4.540368  
  Adult Hind Gut  4.375487  
  Adult Male Ejaculatory Duct  4.973947  
  Adult Mid Gut  4.544598  
  Adult Ovary  4.477896  
  Adult Salivary Gland  4.853961  
  Adult Testes  9.219862  
  Adult Thoracoabdominal ganglion  4.317845  
  Adult Whole Fly  5.181148  
  Larvae Wandering Tubules  4.488318  
  Larval Feeding Carcass  4.582030  
  Larval Feeding Central Nevous System  4.288585  
  Larval Feeding Hind Gut  4.362653  
  Larval Feeding Malpighian Tubule  4.495779  
  Larval Feeding Mid Gut  4.528993  
  Larval Feeding Salivary Gland  4.715411  
  Whole Larvae Feeding  4.550440  
 
  
   FlyBase ID    symbol    start    end    strand    length   
   FBgn0052686   CG32686  10265515   10269349   -  3835  
   FBgn0030195   CG2898   10269407   10270661  +  1255  
   FBgn0052681   CG32681  10270769   10275289   -  4521  
 
 
    Segment 183 
 
   Location   
  Gene key  FBgn0030200-FBgn0030204  
  Heatmap region span   X:10262084..10380660   
  Segment span   X:10311518..10353709   
  Length (genes)  4  
  Length (bp)  42192  
   Model Scoring   
  BIC  345.943481  
  logL  -167.569155  
  logL ratio  74.649086  
   Expression   
  Mean expression  4.922435  
  Median expression  4.765842  
  Tissue std. dev.  0.332061  
 
  No GO Slim enrichment  
  
   tissue    mean expression   
  5th Passage Drosophila S2 Cells  4.737872  
  Adult Accessory gland  4.955679  
  Adult Brain  5.934995  
  Adult Carcass  4.860019  
  Adult Crop  4.767165  
  Adult Eye  4.625436  
  Adult Fatbody  4.903918  
  Adult Female Spermatheca Mated  5.006717  
  Adult Female Spermatheca Virgin  4.990742  
  Adult Head  5.317035  
  Adult Heart  4.511750  
  Adult Hind Gut  4.711886  
  Adult Male Ejaculatory Duct  4.740229  
  Adult Mid Gut  4.961490  
  Adult Ovary  4.701973  
  Adult Salivary Gland  5.059488  
  Adult Testes  5.173312  
  Adult Thoracoabdominal ganglion  5.633309  
  Adult Whole Fly  4.391990  
  Larvae Wandering Tubules  4.895709  
  Larval Feeding Carcass  4.754288  
  Larval Feeding Central Nevous System  5.405865  
  Larval Feeding Hind Gut  4.617102  
  Larval Feeding Malpighian Tubule  4.785947  
  Larval Feeding Mid Gut  4.928172  
  Larval Feeding Salivary Gland  4.916661  
  Whole Larvae Feeding  4.617003  
 
  
   FlyBase ID    symbol    start    end    strand    length   
   FBgn0030200   RabX2   10311518   10312252  +  735  
   FBgn0030202   CG12640  10326498   10326845   -  348  
   FBgn0052683   CG32683  10304420   10348928   -  44509  
   FBgn0030204   Or9a  10352219   10353709   -  1491  
 
 
    Segment 184 
 
   Location   
  Gene key  FBgn0030215-FBgn0085443  
  Heatmap region span   X:10367971..10651699   
  Segment span   X:10460532..10479056   
  Length (genes)  2  
  Length (bp)  18525  
   Model Scoring   
  BIC  207.536442  
  logL  -98.365635  
  logL ratio  14.484787  
   Expression   
  Mean expression  4.713653  
  Median expression  4.629130  
  Tissue std. dev.  0.718557  
 
  No GO Slim enrichment  
  
   tissue    mean expression   
  5th Passage Drosophila S2 Cells  5.930795  
  Adult Accessory gland  4.800579  
  Adult Brain  4.427691  
  Adult Carcass  4.444759  
  Adult Crop  4.425453  
  Adult Eye  4.422589  
  Adult Fatbody  4.555027  
  Adult Female Spermatheca Mated  4.521521  
  Adult Female Spermatheca Virgin  4.450798  
  Adult Head  4.234566  
  Adult Heart  4.481193  
  Adult Hind Gut  4.496593  
  Adult Male Ejaculatory Duct  4.488343  
  Adult Mid Gut  4.433555  
  Adult Ovary  4.901437  
  Adult Salivary Gland  4.495000  
  Adult Testes  7.967691  
  Adult Thoracoabdominal ganglion  4.408784  
  Adult Whole Fly  5.214828  
  Larvae Wandering Tubules  4.566592  
  Larval Feeding Carcass  4.425315  
  Larval Feeding Central Nevous System  4.328953  
  Larval Feeding Hind Gut  4.336709  
  Larval Feeding Malpighian Tubule  4.805957  
  Larval Feeding Mid Gut  4.375167  
  Larval Feeding Salivary Gland  4.670500  
  Whole Larvae Feeding  4.658231  
 
  
   FlyBase ID    symbol    start    end    strand    length   
   FBgn0030215   CG15296  10459868   10460532   -  665  
   FBgn0085443   spri  10384098   10479056   -  94959  
 
    Segment 185 
 
   Location   
  Gene key  FBgn0001206-FBgn0030218  
  Heatmap region span   X:10372526..10658269   
  Segment span   X:10489849..10496747   
  Length (genes)  3  
  Length (bp)  6899  
   Model Scoring   
  BIC  318.444557  
  logL  -153.819692  
  logL ratio  31.301883  
   Expression   
  Mean expression  6.669432  
  Median expression  6.353578  
  Tissue std. dev.  0.543911  
 
  No GO Slim enrichment  
  
   tissue    mean expression   
  5th Passage Drosophila S2 Cells  6.935517  
  Adult Accessory gland  6.377118  
  Adult Brain  6.866855  
  Adult Carcass  6.685944  
  Adult Crop  6.183502  
  Adult Eye  7.082281  
  Adult Fatbody  6.950524  
  Adult Female Spermatheca Mated  7.443525  
  Adult Female Spermatheca Virgin  7.419602  
  Adult Head  7.278688  
  Adult Heart  6.999855  
  Adult Hind Gut  5.904281  
  Adult Male Ejaculatory Duct  5.843286  
  Adult Mid Gut  6.109068  
  Adult Ovary  7.879787  
  Adult Salivary Gland  6.191921  
  Adult Testes  6.960522  
  Adult Thoracoabdominal ganglion  6.927934  
  Adult Whole Fly  6.809332  
  Larvae Wandering Tubules  5.799544  
  Larval Feeding Carcass  6.231759  
  Larval Feeding Central Nevous System  7.441205  
  Larval Feeding Hind Gut  6.426754  
  Larval Feeding Malpighian Tubule  6.616189  
  Larval Feeding Mid Gut  6.271059  
  Larval Feeding Salivary Gland  6.375183  
  Whole Larvae Feeding  6.063429  
 
  
   FlyBase ID    symbol    start    end    strand    length   
   FBgn0001206   Hmr  10484952   10489849   -  4898  
   FBgn0030217   CG2124   10490110   10493200  +  3091  
   FBgn0030218   CG1628  10492176   10496747   -  4572  
 
 
    Segment 186 
 
   Location   
  Gene key  FBgn0030219-FBgn0030224  
  Heatmap region span   X:10372866..10662368   
  Segment span   X:10507048..10567499   
  Length (genes)  6  
  Length (bp)  60452  
   Model Scoring   
  BIC  529.287556  
  logL  -259.241192  
  logL ratio  98.760944  
   Expression   
  Mean expression  4.839773  
  Median expression  4.619158  
  Tissue std. dev.  0.516630  
 
  No GO Slim enrichment  
  
   tissue    mean expression   
  5th Passage Drosophila S2 Cells  4.621949  
  Adult Accessory gland  7.019724  
  Adult Brain  5.141110  
  Adult Carcass  4.730194  
  Adult Crop  4.538175  
  Adult Eye  4.704102  
  Adult Fatbody  4.721057  
  Adult Female Spermatheca Mated  4.720316  
  Adult Female Spermatheca Virgin  4.730810  
  Adult Head  4.593910  
  Adult Heart  4.530612  
  Adult Hind Gut  4.519812  
  Adult Male Ejaculatory Duct  5.057067  
  Adult Mid Gut  4.631418  
  Adult Ovary  4.625951  
  Adult Salivary Gland  4.886460  
  Adult Testes  5.993851  
  Adult Thoracoabdominal ganglion  4.933007  
  Adult Whole Fly  4.689293  
  Larvae Wandering Tubules  4.658038  
  Larval Feeding Carcass  4.677345  
  Larval Feeding Central Nevous System  4.953793  
  Larval Feeding Hind Gut  4.491902  
  Larval Feeding Malpighian Tubule  4.619142  
  Larval Feeding Mid Gut  4.608563  
  Larval Feeding Salivary Gland  4.731387  
  Whole Larvae Feeding  4.544876  
 
  
   FlyBase ID    symbol    start    end    strand    length   
   FBgn0030219     10497970   10507048   -  9079  
   FBgn0030220     10510488   10512898   -  2411  
   FBgn0030222   CG9806  10517758   10521145   -  3388  
   FBgn0052677   X11Lbeta   10523348   10603712  +  80365  
   FBgn0030223   CG2111   10551722   10554704  +  2983  
   FBgn0030224   CG12637  10566466   10567499   -  1034  
 
 
    Segment 187 
 
   Location   
  Gene key  FBgn0003204-FBgn0052676  
  Heatmap region span   X:10380660..10666577   
  Segment span   X:10638539..10638938   
  Length (genes)  2  
  Length (bp)  400  
   Model Scoring   
  BIC  283.832669  
  logL  -136.513748  
  logL ratio  -0.249925  
   Expression   
  Mean expression  9.571228  
  Median expression  9.651817  
  Tissue std. dev.  0.740549  
 
  No GO Slim enrichment  
  
   tissue    mean expression   
  5th Passage Drosophila S2 Cells  8.985420  
  Adult Accessory gland  10.014515  
  Adult Brain  8.802343  
  Adult Carcass  9.390036  
  Adult Crop  9.383749  
  Adult Eye  9.941558  
  Adult Fatbody  9.990414  
  Adult Female Spermatheca Mated  9.777081  
  Adult Female Spermatheca Virgin  9.803170  
  Adult Head  9.295280  
  Adult Heart  9.445763  
  Adult Hind Gut  9.362690  
  Adult Male Ejaculatory Duct  9.428344  
  Adult Mid Gut  9.780559  
  Adult Ovary  10.818274  
  Adult Salivary Gland  9.956569  
  Adult Testes  6.618777  
  Adult Thoracoabdominal ganglion  9.026419  
  Adult Whole Fly  9.787679  
  Larvae Wandering Tubules  10.442755  
  Larval Feeding Carcass  10.354450  
  Larval Feeding Central Nevous System  9.787880  
  Larval Feeding Hind Gut  9.748181  
  Larval Feeding Malpighian Tubule  9.596471  
  Larval Feeding Mid Gut  9.224412  
  Larval Feeding Salivary Gland  10.344544  
  Whole Larvae Feeding  9.315832  
 
  
   FlyBase ID    symbol    start    end    strand    length   
   FBgn0003204   ras   10638539   10642839  +  4301  
   FBgn0052676   CG32676  10607151   10638938   -  31788  
 
    Segment 188 
 
   Location   
  Gene key  FBgn0030228-FBgn0030234  
  Heatmap region span   X:10638539..10733263   
  Segment span   X:10662570..10666577   
  Length (genes)  2  
  Length (bp)  4008  
   Model Scoring   
  BIC  257.050078  
  logL  -123.122453  
  logL ratio  1.113301  
   Expression   
  Mean expression  8.386184  
  Median expression  8.150486  
  Tissue std. dev.  0.709627  
 
  No GO Slim enrichment  
  
   tissue    mean expression   
  5th Passage Drosophila S2 Cells  8.547924  
  Adult Accessory gland  7.359647  
  Adult Brain  7.666176  
  Adult Carcass  8.409168  
  Adult Crop  9.063434  
  Adult Eye  9.544054  
  Adult Fatbody  7.011794  
  Adult Female Spermatheca Mated  8.319330  
  Adult Female Spermatheca Virgin  7.924067  
  Adult Head  8.768173  
  Adult Heart  8.222932  
  Adult Hind Gut  8.976012  
  Adult Male Ejaculatory Duct  8.532220  
  Adult Mid Gut  9.570720  
  Adult Ovary  7.592174  
  Adult Salivary Gland  8.028267  
  Adult Testes  8.807726  
  Adult Thoracoabdominal ganglion  8.119839  
  Adult Whole Fly  8.365946  
  Larvae Wandering Tubules  8.144888  
  Larval Feeding Carcass  9.055737  
  Larval Feeding Central Nevous System  7.745856  
  Larval Feeding Hind Gut  8.947258  
  Larval Feeding Malpighian Tubule  7.924713  
  Larval Feeding Mid Gut  9.808439  
  Larval Feeding Salivary Gland  7.163629  
  Whole Larvae Feeding  8.806844  
 
  
   FlyBase ID    symbol    start    end    strand    length   
   FBgn0030228   BTBD9   10662570   10665736  +  3167  
   FBgn0030234   CG15211   10666577   10673014  +  6438  
 
    Segment 189 
 
   Location   
  Gene key  FBgn0003321-FBgn0030239  
  Heatmap region span   X:10716815..10784957   
  Segment span   X:10741125..10741529   
  Length (genes)  2  
  Length (bp)  405  
   Model Scoring   
  BIC  239.197715  
  logL  -114.196272  
  logL ratio  37.906379  
   Expression   
  Mean expression  9.679281  
  Median expression  9.569544  
  Tissue std. dev.  0.536255  
 
  No GO Slim enrichment  
  
   tissue    mean expression   
  5th Passage Drosophila S2 Cells  10.169182  
  Adult Accessory gland  9.648474  
  Adult Brain  9.045573  
  Adult Carcass  10.184623  
  Adult Crop  10.308720  
  Adult Eye  9.841925  
  Adult Fatbody  10.510597  
  Adult Female Spermatheca Mated  9.944762  
  Adult Female Spermatheca Virgin  10.063738  
  Adult Head  9.922611  
  Adult Heart  10.514108  
  Adult Hind Gut  10.033983  
  Adult Male Ejaculatory Duct  9.758719  
  Adult Mid Gut  9.895063  
  Adult Ovary  10.210752  
  Adult Salivary Gland  9.355921  
  Adult Testes  8.086569  
  Adult Thoracoabdominal ganglion  9.008337  
  Adult Whole Fly  9.614348  
  Larvae Wandering Tubules  9.670281  
  Larval Feeding Carcass  8.981996  
  Larval Feeding Central Nevous System  9.206768  
  Larval Feeding Hind Gut  9.405189  
  Larval Feeding Malpighian Tubule  9.900276  
  Larval Feeding Mid Gut  9.270450  
  Larval Feeding Salivary Gland  9.563866  
  Whole Larvae Feeding  9.223761  
 
  
   FlyBase ID    symbol    start    end    strand    length   
   FBgn0003321   sbr  10726785   10741125   -  14341  
   FBgn0030239   CG17333   10741529   10742567  +  1039  
 
    Segment 190 
 
   Location   
  Gene key  FBgn0030240-FBgn0030243  
  Heatmap region span   X:10733263..10793335   
  Segment span   X:10742795..10751690   
  Length (genes)  4  
  Length (bp)  8896  
   Model Scoring   
  BIC  472.129341  
  logL  -230.662085  
  logL ratio  -0.599871  
   Expression   
  Mean expression  6.830956  
  Median expression  6.910600  
  Tissue std. dev.  1.108465  
 
  No GO Slim enrichment  
  
   tissue    mean expression   
  5th Passage Drosophila S2 Cells  8.429784  
  Adult Accessory gland  6.023487  
  Adult Brain  6.090517  
  Adult Carcass  6.225125  
  Adult Crop  6.384749  
  Adult Eye  6.793070  
  Adult Fatbody  6.091119  
  Adult Female Spermatheca Mated  5.865773  
  Adult Female Spermatheca Virgin  6.028515  
  Adult Head  5.989127  
  Adult Heart  6.046122  
  Adult Hind Gut  6.141670  
  Adult Male Ejaculatory Duct  5.942597  
  Adult Mid Gut  6.237673  
  Adult Ovary  10.415343  
  Adult Salivary Gland  6.224916  
  Adult Testes  8.475407  
  Adult Thoracoabdominal ganglion  6.195437  
  Adult Whole Fly  8.657827  
  Larvae Wandering Tubules  6.662097  
  Larval Feeding Carcass  6.958107  
  Larval Feeding Central Nevous System  8.778885  
  Larval Feeding Hind Gut  6.794385  
  Larval Feeding Malpighian Tubule  6.676188  
  Larval Feeding Mid Gut  6.383654  
  Larval Feeding Salivary Gland  6.845134  
  Whole Larvae Feeding  7.079112  
 
  
   FlyBase ID    symbol    start    end    strand    length   
   FBgn0030240   CG2202   10742795   10746360  +  3566  
   FBgn0030241   feo  10746478   10748907   -  2430  
   FBgn0030242   sofe  10749174   10751415   -  2242  
   FBgn0030243   CG2186   10751690   10757613  +  5924  
 
 
    Segment 191 
 
   Location   
  Gene key  FBgn0030247-FBgn0030249  
  Heatmap region span   X:10763320..10938791   
  Segment span   X:10794393..10806367   
  Length (genes)  3  
  Length (bp)  11975  
   Model Scoring   
  BIC  283.156621  
  logL  -136.175724  
  logL ratio  43.428494  
   Expression   
  Mean expression  4.708485  
  Median expression  4.666427  
  Tissue std. dev.  0.518437  
 
  No GO Slim enrichment  
  
   tissue    mean expression   
  5th Passage Drosophila S2 Cells  4.596063  
  Adult Accessory gland  4.665488  
  Adult Brain  4.515133  
  Adult Carcass  4.625957  
  Adult Crop  4.543674  
  Adult Eye  4.470519  
  Adult Fatbody  4.554985  
  Adult Female Spermatheca Mated  4.540218  
  Adult Female Spermatheca Virgin  4.521889  
  Adult Head  4.475292  
  Adult Heart  4.440202  
  Adult Hind Gut  4.595989  
  Adult Male Ejaculatory Duct  4.696820  
  Adult Mid Gut  4.637292  
  Adult Ovary  4.560291  
  Adult Salivary Gland  4.657542  
  Adult Testes  7.166847  
  Adult Thoracoabdominal ganglion  4.516472  
  Adult Whole Fly  5.397745  
  Larvae Wandering Tubules  4.541572  
  Larval Feeding Carcass  4.598832  
  Larval Feeding Central Nevous System  4.350680  
  Larval Feeding Hind Gut  4.544779  
  Larval Feeding Malpighian Tubule  4.609648  
  Larval Feeding Mid Gut  4.730103  
  Larval Feeding Salivary Gland  4.580048  
  Whole Larvae Feeding  4.995006  
 
  
   FlyBase ID    symbol    start    end    strand    length   
   FBgn0030247   CG15208   10794393   10795771  +  1379  
   FBgn0030248      10800117   10803230  +  3114  
   FBgn0030249      10806367   10814329  +  7963  
 
 
    Segment 192 
 
   Location   
  Gene key  FBgn0052670-FBgn0030257  
  Heatmap region span   X:10794393..10984414   
  Segment span   X:10846519..10938791   
  Length (genes)  5  
  Length (bp)  92273  
   Model Scoring   
  BIC  430.937040  
  logL  -210.065934  
  logL ratio  100.596828  
   Expression   
  Mean expression  4.854800  
  Median expression  4.549778  
  Tissue std. dev.  0.267712  
 
  No GO Slim enrichment  
  
   tissue    mean expression   
  5th Passage Drosophila S2 Cells  4.607236  
  Adult Accessory gland  4.802527  
  Adult Brain  4.963034  
  Adult Carcass  5.143201  
  Adult Crop  5.516138  
  Adult Eye  4.579885  
  Adult Fatbody  4.663778  
  Adult Female Spermatheca Mated  4.715429  
  Adult Female Spermatheca Virgin  4.617868  
  Adult Head  4.740079  
  Adult Heart  5.229068  
  Adult Hind Gut  5.155411  
  Adult Male Ejaculatory Duct  5.048081  
  Adult Mid Gut  4.625900  
  Adult Ovary  4.697747  
  Adult Salivary Gland  4.666252  
  Adult Testes  5.285449  
  Adult Thoracoabdominal ganglion  4.724572  
  Adult Whole Fly  4.565060  
  Larvae Wandering Tubules  4.556470  
  Larval Feeding Carcass  5.309638  
  Larval Feeding Central Nevous System  5.065501  
  Larval Feeding Hind Gut  4.981482  
  Larval Feeding Malpighian Tubule  4.578382  
  Larval Feeding Mid Gut  4.701633  
  Larval Feeding Salivary Gland  4.752212  
  Whole Larvae Feeding  4.787562  
 
  
   FlyBase ID    symbol    start    end    strand    length   
   FBgn0052670   Rab9Fb  10845642   10846519   -  878  
   FBgn0030254      10849620   10850756  +  1137  
   FBgn0021742   C901  10858240   10861165   -  2926  
   FBgn0259241   CG42339  10861752   10931844   -  70093  
   FBgn0030257   CG11160   10938791   10943578  +  4788  
 
 
    Segment 193 
 
   Location   
  Gene key  FBgn0030258-FBgn0030260  
  Heatmap region span   X:10818023..10986526   
  Segment span   X:10947549..10957118   
  Length (genes)  3  
  Length (bp)  9570  
   Model Scoring   
  BIC  288.714706  
  logL  -138.954767  
  logL ratio  60.203252  
   Expression   
  Mean expression  5.503712  
  Median expression  4.443283  
  Tissue std. dev.  2.148562  
 
  No GO Slim enrichment  
  
   tissue    mean expression   
  5th Passage Drosophila S2 Cells  4.230531  
  Adult Accessory gland  4.479966  
  Adult Brain  11.189371  
  Adult Carcass  4.734721  
  Adult Crop  6.518585  
  Adult Eye  6.274447  
  Adult Fatbody  4.396424  
  Adult Female Spermatheca Mated  4.328011  
  Adult Female Spermatheca Virgin  4.354635  
  Adult Head  9.177101  
  Adult Heart  5.239859  
  Adult Hind Gut  4.700261  
  Adult Male Ejaculatory Duct  4.263469  
  Adult Mid Gut  4.477865  
  Adult Ovary  4.198426  
  Adult Salivary Gland  4.356687  
  Adult Testes  3.953739  
  Adult Thoracoabdominal ganglion  11.654029  
  Adult Whole Fly  5.090974  
  Larvae Wandering Tubules  4.368992  
  Larval Feeding Carcass  4.545513  
  Larval Feeding Central Nevous System  9.496300  
  Larval Feeding Hind Gut  4.630876  
  Larval Feeding Malpighian Tubule  4.394012  
  Larval Feeding Mid Gut  4.384774  
  Larval Feeding Salivary Gland  4.275713  
  Whole Larvae Feeding  4.884950  
 
  
   FlyBase ID    symbol    start    end    strand    length   
   FBgn0030258   CG1552  10946369   10947549   -  1181  
   FBgn0030259   CG1545  10951578   10953652   -  2075  
   FBgn0030260   CG1537  10956054   10957118   -  1065  
 
 
    Segment 194 
 
   Location   
  Gene key  FBgn0030261-FBgn0003366  
  Heatmap region span   X:10821432..10990306   
  Segment span   X:10960613..10980332   
  Length (genes)  2  
  Length (bp)  19720  
   Model Scoring   
  BIC  211.471388  
  logL  -100.333108  
  logL ratio  20.612651  
   Expression   
  Mean expression  6.697374  
  Median expression  6.271690  
  Tissue std. dev.  1.475113  
 
  No GO Slim enrichment  
  
   tissue    mean expression   
  5th Passage Drosophila S2 Cells  4.839396  
  Adult Accessory gland  5.611954  
  Adult Brain  8.064872  
  Adult Carcass  9.029210  
  Adult Crop  6.752360  
  Adult Eye  7.580965  
  Adult Fatbody  9.550733  
  Adult Female Spermatheca Mated  6.929353  
  Adult Female Spermatheca Virgin  6.841363  
  Adult Head  8.323745  
  Adult Heart  9.492853  
  Adult Hind Gut  6.351520  
  Adult Male Ejaculatory Duct  7.303786  
  Adult Mid Gut  5.042801  
  Adult Ovary  5.066028  
  Adult Salivary Gland  5.123123  
  Adult Testes  5.788824  
  Adult Thoracoabdominal ganglion  8.752830  
  Adult Whole Fly  6.202081  
  Larvae Wandering Tubules  4.961362  
  Larval Feeding Carcass  7.509819  
  Larval Feeding Central Nevous System  7.855028  
  Larval Feeding Hind Gut  5.939565  
  Larval Feeding Malpighian Tubule  4.981891  
  Larval Feeding Mid Gut  4.949774  
  Larval Feeding Salivary Gland  5.246053  
  Whole Larvae Feeding  6.737811  
 
  
   FlyBase ID    symbol    start    end    strand    length   
   FBgn0030261   CG15203   10960613   10962114  +  1502  
   FBgn0003366   sev  10965474   10980332   -  14859  
 
    Segment 195 
 
   Location   
  Gene key  FBgn0030268-FBgn0030269  
  Heatmap region span   X:10990306..11038153   
  Segment span   X:11031056..11031915   
  Length (genes)  2  
  Length (bp)  860  
   Model Scoring   
  BIC  271.113583  
  logL  -130.154205  
  logL ratio  -9.342662  
   Expression   
  Mean expression  8.634208  
  Median expression  9.097872  
  Tissue std. dev.  0.695833  
 
  No GO Slim enrichment  
  
   tissue    mean expression   
  5th Passage Drosophila S2 Cells  10.058927  
  Adult Accessory gland  7.239934  
  Adult Brain  8.731746  
  Adult Carcass  8.000156  
  Adult Crop  8.784747  
  Adult Eye  8.396948  
  Adult Fatbody  7.942582  
  Adult Female Spermatheca Mated  8.360862  
  Adult Female Spermatheca Virgin  8.326384  
  Adult Head  7.950211  
  Adult Heart  8.588140  
  Adult Hind Gut  8.432113  
  Adult Male Ejaculatory Duct  7.977296  
  Adult Mid Gut  8.350867  
  Adult Ovary  10.667676  
  Adult Salivary Gland  8.300356  
  Adult Testes  8.871564  
  Adult Thoracoabdominal ganglion  8.444490  
  Adult Whole Fly  9.508786  
  Larvae Wandering Tubules  8.939201  
  Larval Feeding Carcass  8.596650  
  Larval Feeding Central Nevous System  9.665052  
  Larval Feeding Hind Gut  8.707710  
  Larval Feeding Malpighian Tubule  8.774672  
  Larval Feeding Mid Gut  8.119200  
  Larval Feeding Salivary Gland  9.129553  
  Whole Larvae Feeding  8.257779  
 
  
   FlyBase ID    symbol    start    end    strand    length   
   FBgn0030268   Klp10A  11024269   11031056   -  6788  
   FBgn0030269   CG18292   11031915   11033726  +  1812  
 
    Segment 196 
 
   Location   
  Gene key  FBgn0020255-FBgn0030276  
  Heatmap region span   X:11034296..11234117   
  Segment span   X:11042622..11048434   
  Length (genes)  3  
  Length (bp)  5813  
   Model Scoring   
  BIC  401.064902  
  logL  -195.129865  
  logL ratio  42.716382  
   Expression   
  Mean expression  10.087329  
  Median expression  10.095083  
  Tissue std. dev.  0.373897  
 
  No GO Slim enrichment  
  
   tissue    mean expression   
  5th Passage Drosophila S2 Cells  9.849535  
  Adult Accessory gland  9.739702  
  Adult Brain  10.208005  
  Adult Carcass  9.622972  
  Adult Crop  10.283420  
  Adult Eye  9.907488  
  Adult Fatbody  9.922282  
  Adult Female Spermatheca Mated  10.304827  
  Adult Female Spermatheca Virgin  10.237587  
  Adult Head  9.673237  
  Adult Heart  9.979379  
  Adult Hind Gut  9.975284  
  Adult Male Ejaculatory Duct  9.879013  
  Adult Mid Gut  9.560274  
  Adult Ovary  11.106465  
  Adult Salivary Gland  9.651169  
  Adult Testes  9.495642  
  Adult Thoracoabdominal ganglion  10.305440  
  Adult Whole Fly  10.295275  
  Larvae Wandering Tubules  10.488371  
  Larval Feeding Carcass  10.350989  
  Larval Feeding Central Nevous System  10.763123  
  Larval Feeding Hind Gut  10.533102  
  Larval Feeding Malpighian Tubule  10.101256  
  Larval Feeding Mid Gut  10.018659  
  Larval Feeding Salivary Gland  10.296972  
  Whole Larvae Feeding  9.808407  
 
  
   FlyBase ID    symbol    start    end    strand    length   
   FBgn0020255   ran  11038895   11042622   -  3728  
   FBgn0030274   Lint-1   11043482   11046625  +  3144  
   FBgn0030276   Dlic   11048434   11057245  +  8812  
 
 
    Segment 197 
 
   Location   
  Gene key  FBgn0052668-FBgn0026268  
  Heatmap region span   X:11035868..11234485   
  Segment span   X:11053793..11063091   
  Length (genes)  2  
  Length (bp)  9299  
   Model Scoring   
  BIC  214.044905  
  logL  -101.619867  
  logL ratio  2.295682  
   Expression   
  Mean expression  4.976547  
  Median expression  4.710539  
  Tissue std. dev.  0.760048  
 
  No GO Slim enrichment  
  
   tissue    mean expression   
  5th Passage Drosophila S2 Cells  4.697401  
  Adult Accessory gland  4.777092  
  Adult Brain  4.566256  
  Adult Carcass  5.315694  
  Adult Crop  4.756086  
  Adult Eye  6.503444  
  Adult Fatbody  4.898288  
  Adult Female Spermatheca Mated  4.523734  
  Adult Female Spermatheca Virgin  4.593114  
  Adult Head  8.133158  
  Adult Heart  4.733904  
  Adult Hind Gut  5.827774  
  Adult Male Ejaculatory Duct  4.916881  
  Adult Mid Gut  4.979285  
  Adult Ovary  4.819168  
  Adult Salivary Gland  4.672052  
  Adult Testes  4.425422  
  Adult Thoracoabdominal ganglion  4.630793  
  Adult Whole Fly  5.452542  
  Larvae Wandering Tubules  4.632022  
  Larval Feeding Carcass  4.641106  
  Larval Feeding Central Nevous System  4.462200  
  Larval Feeding Hind Gut  4.637167  
  Larval Feeding Malpighian Tubule  4.714611  
  Larval Feeding Mid Gut  4.722646  
  Larval Feeding Salivary Gland  4.713491  
  Whole Larvae Feeding  4.621440  
 
  
   FlyBase ID    symbol    start    end    strand    length   
   FBgn0052668   CG32668  11050793   11053793   -  3001  
   FBgn0026268   antdh  11062048   11063091   -  1044  
 
    Segment 198 
 
   Location   
  Gene key  FBgn0030277-FBgn0030283  
  Heatmap region span   X:11036713..11242033   
  Segment span   X:11064681..11142269   
  Length (genes)  4  
  Length (bp)  77589  
   Model Scoring   
  BIC  353.636003  
  logL  -171.415415  
  logL ratio  105.298179  
   Expression   
  Mean expression  5.385507  
  Median expression  4.560331  
  Tissue std. dev.  1.794913  
 
  No GO Slim enrichment  
  
   tissue    mean expression   
  5th Passage Drosophila S2 Cells  4.849661  
  Adult Accessory gland  5.002069  
  Adult Brain  4.517894  
  Adult Carcass  5.149995  
  Adult Crop  4.805663  
  Adult Eye  4.627508  
  Adult Fatbody  4.827511  
  Adult Female Spermatheca Mated  4.931823  
  Adult Female Spermatheca Virgin  4.828548  
  Adult Head  4.626575  
  Adult Heart  4.623357  
  Adult Hind Gut  4.793488  
  Adult Male Ejaculatory Duct  4.964663  
  Adult Mid Gut  4.859334  
  Adult Ovary  4.861274  
  Adult Salivary Gland  5.031833  
  Adult Testes  13.193159  
  Adult Thoracoabdominal ganglion  4.608399  
  Adult Whole Fly  9.221957  
  Larvae Wandering Tubules  4.842203  
  Larval Feeding Carcass  4.913923  
  Larval Feeding Central Nevous System  4.544643  
  Larval Feeding Hind Gut  4.783565  
  Larval Feeding Malpighian Tubule  4.856825  
  Larval Feeding Mid Gut  5.058432  
  Larval Feeding Salivary Gland  4.918155  
  Whole Larvae Feeding  7.166238  
 
  
   FlyBase ID    symbol    start    end    strand    length   
   FBgn0030277   CG1394  11063855   11064681   -  827  
   FBgn0030278   CG15200   11076964   11077520  +  557  
   FBgn0030280   CG11106   11096302   11096840  +  539  
   FBgn0030283   CG15198   11142269   11142918  +  650  
 
 
    Segment 199 
 
   Location   
  Gene key  FBgn0083980-FBgn0003411  
  Heatmap region span   X:11038153..11243087   
  Segment span   X:11196682..11216379   
  Length (genes)  3  
  Length (bp)  19698  
   Model Scoring   
  BIC  242.112921  
  logL  -115.653874  
  logL ratio  87.792378  
   Expression   
  Mean expression  4.341610  
  Median expression  4.348501  
  Tissue std. dev.  0.168660  
 
  No GO Slim enrichment  
  
   tissue    mean expression   
  5th Passage Drosophila S2 Cells  4.314390  
  Adult Accessory gland  4.429999  
  Adult Brain  4.067852  
  Adult Carcass  4.299505  
  Adult Crop  4.773013  
  Adult Eye  4.159350  
  Adult Fatbody  4.426533  
  Adult Female Spermatheca Mated  4.576744  
  Adult Female Spermatheca Virgin  4.598606  
  Adult Head  4.189509  
  Adult Heart  4.205124  
  Adult Hind Gut  4.418165  
  Adult Male Ejaculatory Duct  4.325527  
  Adult Mid Gut  4.373868  
  Adult Ovary  4.436678  
  Adult Salivary Gland  4.620821  
  Adult Testes  4.549070  
  Adult Thoracoabdominal ganglion  4.118697  
  Adult Whole Fly  4.135574  
  Larvae Wandering Tubules  4.343217  
  Larval Feeding Carcass  4.252081  
  Larval Feeding Central Nevous System  4.285510  
  Larval Feeding Hind Gut  4.216942  
  Larval Feeding Malpighian Tubule  4.350138  
  Larval Feeding Mid Gut  4.300807  
  Larval Feeding Salivary Gland  4.296745  
  Whole Larvae Feeding  4.158998  
 
  
   FlyBase ID    symbol    start    end    strand    length   
   FBgn0083980   CG34144  11191336   11196682   -  5347  
   FBgn0083979   Ir10a  11202566   11205241   -  2676  
   FBgn0003411   sisA  11215612   11216379   -  768  
 
 
    Segment 200 
 
   Location   
  Gene key  FBgn0030286-FBgn0028421  
  Heatmap region span   X:11042622..11262494   
  Segment span   X:11219495..11234117   
  Length (genes)  2  
  Length (bp)  14623  
   Model Scoring   
  BIC  208.731929  
  logL  -98.963378  
  logL ratio  44.121373  
   Expression   
  Mean expression  8.369236  
  Median expression  8.259680  
  Tissue std. dev.  0.884559  
 
  No GO Slim enrichment  
  
   tissue    mean expression   
  5th Passage Drosophila S2 Cells  8.177625  
  Adult Accessory gland  8.500651  
  Adult Brain  10.581515  
  Adult Carcass  8.439179  
  Adult Crop  8.124768  
  Adult Eye  9.754189  
  Adult Fatbody  7.974684  
  Adult Female Spermatheca Mated  8.003959  
  Adult Female Spermatheca Virgin  7.965740  
  Adult Head  9.084537  
  Adult Heart  8.764888  
  Adult Hind Gut  8.576815  
  Adult Male Ejaculatory Duct  8.479003  
  Adult Mid Gut  7.738465  
  Adult Ovary  9.283966  
  Adult Salivary Gland  8.609015  
  Adult Testes  6.379317  
  Adult Thoracoabdominal ganglion  10.176168  
  Adult Whole Fly  8.154153  
  Larvae Wandering Tubules  8.225085  
  Larval Feeding Carcass  7.569809  
  Larval Feeding Central Nevous System  8.946167  
  Larval Feeding Hind Gut  8.045406  
  Larval Feeding Malpighian Tubule  8.155641  
  Larval Feeding Mid Gut  8.054125  
  Larval Feeding Salivary Gland  7.145399  
  Whole Larvae Feeding  7.059112  
 
  
   FlyBase ID    symbol    start    end    strand    length   
   FBgn0030286   CG1657   11219495   11228107  +  8613  
   FBgn0028421   Kap3  11228220   11234117   -  5898  
 
    Segment 201 
 
   Location   
  Gene key  FBgn0030290-FBgn0030291  
  Heatmap region span   X:11196682..11316888   
  Segment span   X:11242153..11243087   
  Length (genes)  2  
  Length (bp)  935  
   Model Scoring   
  BIC  220.554835  
  logL  -104.874831  
  logL ratio  5.542430  
   Expression   
  Mean expression  6.070731  
  Median expression  5.807336  
  Tissue std. dev.  0.584762  
 
  No GO Slim enrichment  
  
   tissue    mean expression   
  5th Passage Drosophila S2 Cells  6.415324  
  Adult Accessory gland  6.244877  
  Adult Brain  6.125132  
  Adult Carcass  5.533479  
  Adult Crop  5.723855  
  Adult Eye  5.765134  
  Adult Fatbody  5.653939  
  Adult Female Spermatheca Mated  5.959908  
  Adult Female Spermatheca Virgin  5.849733  
  Adult Head  5.811042  
  Adult Heart  5.865453  
  Adult Hind Gut  5.702514  
  Adult Male Ejaculatory Duct  5.840383  
  Adult Mid Gut  5.944385  
  Adult Ovary  7.291377  
  Adult Salivary Gland  6.028733  
  Adult Testes  8.265068  
  Adult Thoracoabdominal ganglion  6.083880  
  Adult Whole Fly  6.710166  
  Larvae Wandering Tubules  5.698110  
  Larval Feeding Carcass  5.973287  
  Larval Feeding Central Nevous System  6.681421  
  Larval Feeding Hind Gut  5.700947  
  Larval Feeding Malpighian Tubule  5.614598  
  Larval Feeding Mid Gut  5.515742  
  Larval Feeding Salivary Gland  6.192965  
  Whole Larvae Feeding  5.718283  
 
  
   FlyBase ID    symbol    start    end    strand    length   
   FBgn0030290   CG11756   11242153   11242899  +  747  
   FBgn0030291   CG1738   11243087   11243881  +  795  
 
    Segment 202 
 
   Location   
  Gene key  FBgn0030292-FBgn0027359  
  Heatmap region span   X:11219495..11336574   
  Segment span   X:11244032..11262494   
  Length (genes)  6  
  Length (bp)  18463  
   Model Scoring   
  BIC  668.776137  
  logL  -328.985482  
  logL ratio  118.670473  
   Expression   
  Mean expression  9.085439  
  Median expression  9.028562  
  Tissue std. dev.  0.521881  
 
  No GO Slim enrichment  
  
   tissue    mean expression   
  5th Passage Drosophila S2 Cells  9.123057  
  Adult Accessory gland  9.142899  
  Adult Brain  9.880504  
  Adult Carcass  8.759179  
  Adult Crop  9.027674  
  Adult Eye  9.299671  
  Adult Fatbody  8.903028  
  Adult Female Spermatheca Mated  8.989265  
  Adult Female Spermatheca Virgin  8.928646  
  Adult Head  9.060318  
  Adult Heart  8.999132  
  Adult Hind Gut  8.936291  
  Adult Male Ejaculatory Duct  8.925226  
  Adult Mid Gut  8.629270  
  Adult Ovary  10.097147  
  Adult Salivary Gland  9.135225  
  Adult Testes  7.232370  
  Adult Thoracoabdominal ganglion  9.478473  
  Adult Whole Fly  9.219670  
  Larvae Wandering Tubules  8.800911  
  Larval Feeding Carcass  9.093832  
  Larval Feeding Central Nevous System  9.999042  
  Larval Feeding Hind Gut  9.241369  
  Larval Feeding Malpighian Tubule  9.043397  
  Larval Feeding Mid Gut  8.791808  
  Larval Feeding Salivary Gland  9.779790  
  Whole Larvae Feeding  8.789653  
 
  
   FlyBase ID    symbol    start    end    strand    length   
   FBgn0030292   CG11752   11244032   11244527  +  496  
   FBgn0030293   CG1737   11244667   11249331  +  4665  
   FBgn0000499   dsh   11249914   11252757  +  2844  
   FBgn0030294   Pa1   11253270   11254962  +  1693  
   FBgn0004864   hop  11254932   11262130   -  7199  
   FBgn0027359   Tim8   11262494   11263216  +  723  
 
 
    Segment 203 
 
   Location   
  Gene key  FBgn0030296-FBgn0030298  
  Heatmap region span   X:11242033..11364809   
  Segment span   X:11303980..11311436   
  Length (genes)  4  
  Length (bp)  7457  
   Model Scoring   
  BIC  337.970645  
  logL  -163.582737  
  logL ratio  103.937140  
   Expression   
  Mean expression  4.353466  
  Median expression  4.134794  
  Tissue std. dev.  0.314786  
 
  No GO Slim enrichment  
  
   tissue    mean expression   
  5th Passage Drosophila S2 Cells  4.296600  
  Adult Accessory gland  4.549924  
  Adult Brain  4.120240  
  Adult Carcass  4.165485  
  Adult Crop  4.318049  
  Adult Eye  4.166237  
  Adult Fatbody  4.465539  
  Adult Female Spermatheca Mated  4.565203  
  Adult Female Spermatheca Virgin  4.552601  
  Adult Head  4.220894  
  Adult Heart  4.191511  
  Adult Hind Gut  4.289251  
  Adult Male Ejaculatory Duct  4.155781  
  Adult Mid Gut  4.317492  
  Adult Ovary  4.198495  
  Adult Salivary Gland  4.382812  
  Adult Testes  5.811884  
  Adult Thoracoabdominal ganglion  4.240079  
  Adult Whole Fly  4.480951  
  Larvae Wandering Tubules  4.320018  
  Larval Feeding Carcass  4.172341  
  Larval Feeding Central Nevous System  4.188312  
  Larval Feeding Hind Gut  4.154730  
  Larval Feeding Malpighian Tubule  4.360169  
  Larval Feeding Mid Gut  4.408456  
  Larval Feeding Salivary Gland  4.273273  
  Whole Larvae Feeding  4.177245  
 
  
   FlyBase ID    symbol    start    end    strand    length   
   FBgn0030296   CG15196   11303980   11305700  +  1721  
   FBgn0030297   Gr10b  11305945   11307223   -  1279  
   FBgn0045502   Gr10a  11307533   11309602   -  2070  
   FBgn0030298   Or10a  11309922   11311436   -  1515  
 
 
    Segment 204 
 
   Location   
  Gene key  FBgn0030299-FBgn0030301  
  Heatmap region span   X:11263670..11385556   
  Segment span   X:11348335..11348824   
  Length (genes)  2  
  Length (bp)  490  
   Model Scoring   
  BIC  207.445925  
  logL  -98.320377  
  logL ratio  21.577239  
   Expression   
  Mean expression  6.165497  
  Median expression  6.078456  
  Tissue std. dev.  0.643022  
 
  No GO Slim enrichment  
  
   tissue    mean expression   
  5th Passage Drosophila S2 Cells  6.899180  
  Adult Accessory gland  5.978333  
  Adult Brain  7.682654  
  Adult Carcass  5.742256  
  Adult Crop  5.882166  
  Adult Eye  6.031405  
  Adult Fatbody  5.577186  
  Adult Female Spermatheca Mated  5.415655  
  Adult Female Spermatheca Virgin  5.355490  
  Adult Head  6.027274  
  Adult Heart  5.902348  
  Adult Hind Gut  5.832880  
  Adult Male Ejaculatory Duct  6.027750  
  Adult Mid Gut  5.739259  
  Adult Ovary  7.646311  
  Adult Salivary Gland  6.660421  
  Adult Testes  6.463135  
  Adult Thoracoabdominal ganglion  7.191954  
  Adult Whole Fly  6.231048  
  Larvae Wandering Tubules  5.930276  
  Larval Feeding Carcass  6.019398  
  Larval Feeding Central Nevous System  7.347015  
  Larval Feeding Hind Gut  5.776308  
  Larval Feeding Malpighian Tubule  5.759819  
  Larval Feeding Mid Gut  5.673600  
  Larval Feeding Salivary Gland  6.167343  
  Whole Larvae Feeding  5.507966  
 
  
   FlyBase ID    symbol    start    end    strand    length   
   FBgn0030299     11324559   11348335   -  23777  
   FBgn0030301   HP5   11348824   11352345  +  3522  
 
    Segment 205 
 
   Location   
  Gene key  FBgn0030302-FBgn0030303  
  Heatmap region span   X:11303980..11387203   
  Segment span   X:11353915..11364809   
  Length (genes)  2  
  Length (bp)  10895  
   Model Scoring   
  BIC  171.573770  
  logL  -80.384299  
  logL ratio  39.553010  
   Expression   
  Mean expression  4.739985  
  Median expression  4.796917  
  Tissue std. dev.  0.248727  
 
  No GO Slim enrichment  
  
   tissue    mean expression   
  5th Passage Drosophila S2 Cells  4.711159  
  Adult Accessory gland  5.048514  
  Adult Brain  4.785823  
  Adult Carcass  4.701877  
  Adult Crop  5.386268  
  Adult Eye  4.432728  
  Adult Fatbody  4.816940  
  Adult Female Spermatheca Mated  4.959929  
  Adult Female Spermatheca Virgin  4.796720  
  Adult Head  4.599635  
  Adult Heart  5.144680  
  Adult Hind Gut  4.884308  
  Adult Male Ejaculatory Duct  4.803659  
  Adult Mid Gut  4.687311  
  Adult Ovary  4.647752  
  Adult Salivary Gland  4.861185  
  Adult Testes  4.299908  
  Adult Thoracoabdominal ganglion  5.021408  
  Adult Whole Fly  4.145447  
  Larvae Wandering Tubules  4.563617  
  Larval Feeding Carcass  4.740213  
  Larval Feeding Central Nevous System  4.761883  
  Larval Feeding Hind Gut  4.638618  
  Larval Feeding Malpighian Tubule  4.705056  
  Larval Feeding Mid Gut  4.714454  
  Larval Feeding Salivary Gland  4.687079  
  Whole Larvae Feeding  4.433433  
 
  
   FlyBase ID    symbol    start    end    strand    length   
   FBgn0030302      11353915   11354832  +  918  
   FBgn0030303      11364809   11370301  +  5493  
 
    Segment 206 
 
   Location   
  Gene key  FBgn0030305-FBgn0030306  
  Heatmap region span   X:11336574..11466475   
  Segment span   X:11377540..11379285   
  Length (genes)  2  
  Length (bp)  1746  
   Model Scoring   
  BIC  266.634947  
  logL  -127.914888  
  logL ratio  35.093445  
   Expression   
  Mean expression  10.103273  
  Median expression  10.249233  
  Tissue std. dev.  1.001478  
 
  No GO Slim enrichment  
  
   tissue    mean expression   
  5th Passage Drosophila S2 Cells  10.430044  
  Adult Accessory gland  12.176800  
  Adult Brain  8.699633  
  Adult Carcass  9.290105  
  Adult Crop  9.496975  
  Adult Eye  9.114798  
  Adult Fatbody  9.942421  
  Adult Female Spermatheca Mated  10.639208  
  Adult Female Spermatheca Virgin  10.393822  
  Adult Head  9.401094  
  Adult Heart  9.462522  
  Adult Hind Gut  9.164544  
  Adult Male Ejaculatory Duct  11.733795  
  Adult Mid Gut  9.859431  
  Adult Ovary  10.059988  
  Adult Salivary Gland  12.024795  
  Adult Testes  8.494870  
  Adult Thoracoabdominal ganglion  8.802882  
  Adult Whole Fly  9.860657  
  Larvae Wandering Tubules  10.318828  
  Larval Feeding Carcass  10.514123  
  Larval Feeding Central Nevous System  10.108284  
  Larval Feeding Hind Gut  10.010915  
  Larval Feeding Malpighian Tubule  10.220374  
  Larval Feeding Mid Gut  9.810306  
  Larval Feeding Salivary Gland  12.424978  
  Whole Larvae Feeding  10.332166  
 
  
   FlyBase ID    symbol    start    end    strand    length   
   FBgn0030305   CG1749   11377540   11379097  +  1558  
   FBgn0030306   Spase25   11379285   11380302  +  1018  
 
    Segment 207 
 
   Location   
  Gene key  FBgn0030309-FBgn0030311  
  Heatmap region span   X:11371114..11478193   
  Segment span   X:11452687..11464657   
  Length (genes)  3  
  Length (bp)  11971  
   Model Scoring   
  BIC  320.979630  
  logL  -155.087229  
  logL ratio  130.206688  
   Expression   
  Mean expression  10.400778  
  Median expression  10.373905  
  Tissue std. dev.  0.679482  
 
  No GO Slim enrichment  
  
   tissue    mean expression   
  5th Passage Drosophila S2 Cells  11.392417  
  Adult Accessory gland  10.339072  
  Adult Brain  9.349215  
  Adult Carcass  10.242034  
  Adult Crop  11.233870  
  Adult Eye  9.749653  
  Adult Fatbody  10.269014  
  Adult Female Spermatheca Mated  10.091137  
  Adult Female Spermatheca Virgin  9.846730  
  Adult Head  10.025018  
  Adult Heart  10.886667  
  Adult Hind Gut  10.491743  
  Adult Male Ejaculatory Duct  10.831525  
  Adult Mid Gut  9.988102  
  Adult Ovary  10.011541  
  Adult Salivary Gland  10.735453  
  Adult Testes  8.475216  
  Adult Thoracoabdominal ganglion  9.787889  
  Adult Whole Fly  9.877784  
  Larvae Wandering Tubules  10.523062  
  Larval Feeding Carcass  11.482994  
  Larval Feeding Central Nevous System  10.828598  
  Larval Feeding Hind Gut  11.352758  
  Larval Feeding Malpighian Tubule  10.279266  
  Larval Feeding Mid Gut  10.520011  
  Larval Feeding Salivary Gland  11.379864  
  Whole Larvae Feeding  10.830374  
 
  
   FlyBase ID    symbol    start    end    strand    length   
   FBgn0030309   CG1572  11449318   11452687   -  3370  
   FBgn0003277   RpII215  11456806   11464355   -  7550  
   FBgn0030311   CG11699   11464657   11465445  +  789  
 
 
    Segment 208 
 
   Location   
  Gene key  FBgn0030314-FBgn0002948  
  Heatmap region span   X:11387203..11572584   
  Segment span   X:11467834..11474851   
  Length (genes)  3  
  Length (bp)  7018  
   Model Scoring   
  BIC  282.926947  
  logL  -136.060887  
  logL ratio  79.871258  
   Expression   
  Mean expression  7.232081  
  Median expression  7.174729  
  Tissue std. dev.  0.431168  
 
  
   GO ID    description    ratio    P-value   
   GO:0003677   DNA binding  2/3  0.0178  
 
  
   tissue    mean expression   
  5th Passage Drosophila S2 Cells  7.650038  
  Adult Accessory gland  7.703676  
  Adult Brain  7.875732  
  Adult Carcass  6.866667  
  Adult Crop  7.424190  
  Adult Eye  7.494353  
  Adult Fatbody  6.993526  
  Adult Female Spermatheca Mated  6.785548  
  Adult Female Spermatheca Virgin  6.969740  
  Adult Head  7.130488  
  Adult Heart  6.999633  
  Adult Hind Gut  7.024529  
  Adult Male Ejaculatory Duct  7.420316  
  Adult Mid Gut  6.795767  
  Adult Ovary  8.469957  
  Adult Salivary Gland  7.196987  
  Adult Testes  6.966015  
  Adult Thoracoabdominal ganglion  7.699961  
  Adult Whole Fly  7.525787  
  Larvae Wandering Tubules  6.974551  
  Larval Feeding Carcass  6.867722  
  Larval Feeding Central Nevous System  7.898796  
  Larval Feeding Hind Gut  7.134565  
  Larval Feeding Malpighian Tubule  7.019225  
  Larval Feeding Mid Gut  6.669707  
  Larval Feeding Salivary Gland  7.079018  
  Whole Larvae Feeding  6.629689  
 
  
   FlyBase ID    symbol    start    end    strand    length   
   FBgn0030314   CG11696   11467834   11470183  +  2350  
   FBgn0030316   CG11695   11470916   11472839  +  1924  
   FBgn0002948   nod   11474851   11479787  +  4937  
 
 
    Segment 209 
 
   Location   
  Gene key  FBgn0030320-FBgn0030321  
  Heatmap region span   X:11466740..11614268   
  Segment span   X:11505679..11506050   
  Length (genes)  2  
  Length (bp)  372  
   Model Scoring   
  BIC  253.753016  
  logL  -121.473922  
  logL ratio  13.453269  
   Expression   
  Mean expression  9.071103  
  Median expression  9.399993  
  Tissue std. dev.  0.623379  
 
  No GO Slim enrichment  
  
   tissue    mean expression   
  5th Passage Drosophila S2 Cells  9.584300  
  Adult Accessory gland  10.052354  
  Adult Brain  8.569309  
  Adult Carcass  8.319875  
  Adult Crop  9.253591  
  Adult Eye  8.344180  
  Adult Fatbody  8.891882  
  Adult Female Spermatheca Mated  9.434645  
  Adult Female Spermatheca Virgin  9.352509  
  Adult Head  8.357667  
  Adult Heart  8.869799  
  Adult Hind Gut  8.341221  
  Adult Male Ejaculatory Duct  8.908675  
  Adult Mid Gut  8.413177  
  Adult Ovary  10.669594  
  Adult Salivary Gland  8.724430  
  Adult Testes  9.626443  
  Adult Thoracoabdominal ganglion  8.476287  
  Adult Whole Fly  9.439885  
  Larvae Wandering Tubules  8.856993  
  Larval Feeding Carcass  9.260744  
  Larval Feeding Central Nevous System  10.067066  
  Larval Feeding Hind Gut  8.909088  
  Larval Feeding Malpighian Tubule  8.628678  
  Larval Feeding Mid Gut  8.626619  
  Larval Feeding Salivary Gland  10.109702  
  Whole Larvae Feeding  8.831056  
 
  
   FlyBase ID    symbol    start    end    strand    length   
   FBgn0030320   CG2247  11500322   11505679   -  5358  
   FBgn0030321   CG1703   11506050   11509161  +  3112  
 
    Segment 210 
 
   Location   
  Gene key  FBgn0030322-FBgn0030323  
  Heatmap region span   X:11467834..11648306   
  Segment span   X:11509873..11572584   
  Length (genes)  4  
  Length (bp)  62712  
   Model Scoring   
  BIC  410.707343  
  logL  -199.951085  
  logL ratio  55.430722  
   Expression   
  Mean expression  6.708812  
  Median expression  6.696618  
  Tissue std. dev.  0.499882  
 
  No GO Slim enrichment  
  
   tissue    mean expression   
  5th Passage Drosophila S2 Cells  7.279253  
  Adult Accessory gland  6.396494  
  Adult Brain  7.248766  
  Adult Carcass  6.185975  
  Adult Crop  7.035655  
  Adult Eye  6.911465  
  Adult Fatbody  6.356122  
  Adult Female Spermatheca Mated  6.177676  
  Adult Female Spermatheca Virgin  6.048240  
  Adult Head  6.599118  
  Adult Heart  6.686668  
  Adult Hind Gut  6.780831  
  Adult Male Ejaculatory Duct  6.440022  
  Adult Mid Gut  6.085799  
  Adult Ovary  8.203370  
  Adult Salivary Gland  6.051339  
  Adult Testes  6.716356  
  Adult Thoracoabdominal ganglion  6.997041  
  Adult Whole Fly  6.956825  
  Larvae Wandering Tubules  6.843761  
  Larval Feeding Carcass  6.690393  
  Larval Feeding Central Nevous System  7.447850  
  Larval Feeding Hind Gut  6.865943  
  Larval Feeding Malpighian Tubule  7.001785  
  Larval Feeding Mid Gut  5.947541  
  Larval Feeding Salivary Gland  6.905921  
  Whole Larvae Feeding  6.277700  
 
  
   FlyBase ID    symbol    start    end    strand    length   
   FBgn0030322   CG15220  11509262   11509873   -  612  
   FBgn0002723   Met   11510159   11513981  +  3823  
   FBgn0004370   Ptp10D   11516061   11571371  +  55311  
   FBgn0030323   CG2371  11571376   11572584   -  1209  
 
 
    Segment 211 
 
   Location   
  Gene key  FBgn0014133-FBgn0011754  
  Heatmap region span   X:11478193..11658621   
  Segment span   X:11573028..11589145   
  Length (genes)  2  
  Length (bp)  16118  
   Model Scoring   
  BIC  249.502521  
  logL  -119.348674  
  logL ratio  30.499936  
   Expression   
  Mean expression  9.727706  
  Median expression  9.844916  
  Tissue std. dev.  0.477556  
 
  No GO Slim enrichment  
  
   tissue    mean expression   
  5th Passage Drosophila S2 Cells  10.306433  
  Adult Accessory gland  9.652627  
  Adult Brain  10.247777  
  Adult Carcass  9.459542  
  Adult Crop  9.530379  
  Adult Eye  10.697880  
  Adult Fatbody  9.034833  
  Adult Female Spermatheca Mated  8.977659  
  Adult Female Spermatheca Virgin  9.260932  
  Adult Head  9.868178  
  Adult Heart  9.511887  
  Adult Hind Gut  9.583439  
  Adult Male Ejaculatory Duct  8.735473  
  Adult Mid Gut  9.691693  
  Adult Ovary  9.996847  
  Adult Salivary Gland  9.913342  
  Adult Testes  8.927716  
  Adult Thoracoabdominal ganglion  10.278186  
  Adult Whole Fly  9.586572  
  Larvae Wandering Tubules  10.310067  
  Larval Feeding Carcass  9.665534  
  Larval Feeding Central Nevous System  10.428811  
  Larval Feeding Hind Gut  9.424851  
  Larval Feeding Malpighian Tubule  10.068693  
  Larval Feeding Mid Gut  9.923713  
  Larval Feeding Salivary Gland  9.736622  
  Whole Larvae Feeding  9.828373  
 
  
   FlyBase ID    symbol    start    end    strand    length   
   FBgn0014133   bif   11573028   11588774  +  15747  
   FBgn0011754   PhKgamma   11589145   11598427  +  9283  
 
    Segment 212 
 
   Location   
  Gene key  FBgn0030327-FBgn0030330  
  Heatmap region span   X:11505679..11682879   
  Segment span   X:11603983..11614268   
  Length (genes)  4  
  Length (bp)  10286  
   Model Scoring   
  BIC  420.738118  
  logL  -204.966473  
  logL ratio  120.730457  
   Expression   
  Mean expression  9.548048  
  Median expression  9.443575  
  Tissue std. dev.  0.513328  
 
  No GO Slim enrichment  
  
   tissue    mean expression   
  5th Passage Drosophila S2 Cells  9.933405  
  Adult Accessory gland  10.632190  
  Adult Brain  10.300757  
  Adult Carcass  9.017620  
  Adult Crop  9.053688  
  Adult Eye  9.641699  
  Adult Fatbody  9.351017  
  Adult Female Spermatheca Mated  9.668491  
  Adult Female Spermatheca Virgin  9.572126  
  Adult Head  9.328818  
  Adult Heart  9.668468  
  Adult Hind Gut  9.118593  
  Adult Male Ejaculatory Duct  9.538130  
  Adult Mid Gut  8.680528  
  Adult Ovary  9.979329  
  Adult Salivary Gland  9.652073  
  Adult Testes  8.260234  
  Adult Thoracoabdominal ganglion  10.155346  
  Adult Whole Fly  9.151198  
  Larvae Wandering Tubules  9.573005  
  Larval Feeding Carcass  9.359573  
  Larval Feeding Central Nevous System  10.284296  
  Larval Feeding Hind Gut  10.052550  
  Larval Feeding Malpighian Tubule  9.398628  
  Larval Feeding Mid Gut  9.314330  
  Larval Feeding Salivary Gland  9.970776  
  Whole Larvae Feeding  9.140439  
 
  
   FlyBase ID    symbol    start    end    strand    length   
   FBgn0030327   FucT6  11600608   11603983   -  3376  
   FBgn0030328   Amun  11604153   11609063   -  4911  
   FBgn0030329   prtp   11610821   11613927  +  3107  
   FBgn0030330   Tango10   11614268   11617887  +  3620  
 
 
    Segment 213 
 
   Location   
  Gene key  FBgn0030331-FBgn0002577  
  Heatmap region span   X:11509873..11723451   
  Segment span   X:11618373..11648306   
  Length (genes)  2  
  Length (bp)  29934  
   Model Scoring   
  BIC  217.388646  
  logL  -103.291737  
  logL ratio  1.658546  
   Expression   
  Mean expression  4.884292  
  Median expression  4.572608  
  Tissue std. dev.  0.781740  
 
  No GO Slim enrichment  
  
   tissue    mean expression   
  5th Passage Drosophila S2 Cells  4.509512  
  Adult Accessory gland  4.370531  
  Adult Brain  4.414468  
  Adult Carcass  4.712433  
  Adult Crop  6.135471  
  Adult Eye  4.598752  
  Adult Fatbody  5.095096  
  Adult Female Spermatheca Mated  4.491795  
  Adult Female Spermatheca Virgin  4.524815  
  Adult Head  4.312963  
  Adult Heart  4.642493  
  Adult Hind Gut  5.392459  
  Adult Male Ejaculatory Duct  4.588927  
  Adult Mid Gut  4.579562  
  Adult Ovary  4.764682  
  Adult Salivary Gland  5.485669  
  Adult Testes  4.191740  
  Adult Thoracoabdominal ganglion  4.321739  
  Adult Whole Fly  4.441349  
  Larvae Wandering Tubules  6.536440  
  Larval Feeding Carcass  4.868366  
  Larval Feeding Central Nevous System  4.230422  
  Larval Feeding Hind Gut  4.641450  
  Larval Feeding Malpighian Tubule  7.774097  
  Larval Feeding Mid Gut  4.555160  
  Larval Feeding Salivary Gland  4.869801  
  Whole Larvae Feeding  4.825686  
 
  
   FlyBase ID    symbol    start    end    strand    length   
   FBgn0030331   CG15221   11618373   11630650  +  12278  
   FBgn0002577   m   11648306   11663548  +  15243  
 
    Segment 214 
 
   Location   
  Gene key  FBgn0000259-FBgn0052662  
  Heatmap region span   X:11618373..11770989   
  Segment span   X:11686569..11723451   
  Length (genes)  4  
  Length (bp)  36883  
   Model Scoring   
  BIC  401.825679  
  logL  -195.510253  
  logL ratio  90.418110  
   Expression   
  Mean expression  8.184697  
  Median expression  8.226469  
  Tissue std. dev.  0.508057  
 
  No GO Slim enrichment  
  
   tissue    mean expression   
  5th Passage Drosophila S2 Cells  8.255072  
  Adult Accessory gland  8.002380  
  Adult Brain  8.821449  
  Adult Carcass  7.970881  
  Adult Crop  8.144083  
  Adult Eye  8.648210  
  Adult Fatbody  8.385358  
  Adult Female Spermatheca Mated  8.439468  
  Adult Female Spermatheca Virgin  8.498668  
  Adult Head  7.680411  
  Adult Heart  8.976566  
  Adult Hind Gut  7.753639  
  Adult Male Ejaculatory Duct  7.489453  
  Adult Mid Gut  7.824406  
  Adult Ovary  8.906199  
  Adult Salivary Gland  8.382931  
  Adult Testes  6.515633  
  Adult Thoracoabdominal ganglion  8.579769  
  Adult Whole Fly  7.783997  
  Larvae Wandering Tubules  8.692291  
  Larval Feeding Carcass  8.157452  
  Larval Feeding Central Nevous System  8.441638  
  Larval Feeding Hind Gut  8.093941  
  Larval Feeding Malpighian Tubule  8.212157  
  Larval Feeding Mid Gut  8.498662  
  Larval Feeding Salivary Gland  8.181315  
  Whole Larvae Feeding  7.650782  
 
  
   FlyBase ID    symbol    start    end    strand    length   
   FBgn0000259   CkIIbeta   11686569   11694097  +  7529  
   FBgn0030336   CG1578  11703388   11710565   -  7178  
   FBgn0052663      11711791   11729759  +  17969  
   FBgn0052662      11723451   11727536  +  4086  
 
 
    Segment 215 
 
   Location   
  Gene key  FBgn0030338-FBgn0030340  
  Heatmap region span   X:11658621..11781313   
  Segment span   X:11732043..11740132   
  Length (genes)  3  
  Length (bp)  8090  
   Model Scoring   
  BIC  324.514974  
  logL  -156.854901  
  logL ratio  1.489718  
   Expression   
  Mean expression  5.380413  
  Median expression  5.013809  
  Tissue std. dev.  0.718130  
 
  No GO Slim enrichment  
  
   tissue    mean expression   
  5th Passage Drosophila S2 Cells  5.380971  
  Adult Accessory gland  7.556871  
  Adult Brain  5.692975  
  Adult Carcass  4.965854  
  Adult Crop  5.058329  
  Adult Eye  4.918175  
  Adult Fatbody  5.496441  
  Adult Female Spermatheca Mated  5.146760  
  Adult Female Spermatheca Virgin  5.154364  
  Adult Head  5.649279  
  Adult Heart  5.077846  
  Adult Hind Gut  4.961069  
  Adult Male Ejaculatory Duct  5.400461  
  Adult Mid Gut  5.256337  
  Adult Ovary  5.012179  
  Adult Salivary Gland  5.456741  
  Adult Testes  4.604691  
  Adult Thoracoabdominal ganglion  5.606253  
  Adult Whole Fly  4.831681  
  Larvae Wandering Tubules  4.985478  
  Larval Feeding Carcass  5.279792  
  Larval Feeding Central Nevous System  5.189735  
  Larval Feeding Hind Gut  5.033954  
  Larval Feeding Malpighian Tubule  5.059602  
  Larval Feeding Mid Gut  5.363274  
  Larval Feeding Salivary Gland  7.943212  
  Whole Larvae Feeding  5.188819  
 
  
   FlyBase ID    symbol    start    end    strand    length   
   FBgn0030338   CG15741  11731636   11732043   -  408  
   FBgn0030339   Cyp28c1   11733641   11735414  +  1774  
   FBgn0030340   CG15740  11736695   11740132   -  3438  
 
 
    Segment 216 
 
   Location   
  Gene key  FBgn0030342-FBgn0030346  
  Heatmap region span   X:11672709..11799373   
  Segment span   X:11748913..11766811   
  Length (genes)  5  
  Length (bp)  17899  
   Model Scoring   
  BIC  452.549174  
  logL  -220.872001  
  logL ratio  165.300541  
   Expression   
  Mean expression  8.528602  
  Median expression  8.635787  
  Tissue std. dev.  0.343393  
 
  No GO Slim enrichment  
  
   tissue    mean expression   
  5th Passage Drosophila S2 Cells  8.527264  
  Adult Accessory gland  8.897670  
  Adult Brain  8.845861  
  Adult Carcass  7.987417  
  Adult Crop  8.432893  
  Adult Eye  8.896835  
  Adult Fatbody  8.274329  
  Adult Female Spermatheca Mated  8.212871  
  Adult Female Spermatheca Virgin  8.199123  
  Adult Head  8.413869  
  Adult Heart  8.592722  
  Adult Hind Gut  8.305177  
  Adult Male Ejaculatory Duct  8.463596  
  Adult Mid Gut  8.360118  
  Adult Ovary  9.337460  
  Adult Salivary Gland  8.146108  
  Adult Testes  9.184727  
  Adult Thoracoabdominal ganglion  8.895345  
  Adult Whole Fly  8.369977  
  Larvae Wandering Tubules  8.801860  
  Larval Feeding Carcass  8.040350  
  Larval Feeding Central Nevous System  8.794762  
  Larval Feeding Hind Gut  8.474891  
  Larval Feeding Malpighian Tubule  8.813389  
  Larval Feeding Mid Gut  8.459217  
  Larval Feeding Salivary Gland  8.538376  
  Whole Larvae Feeding  8.006035  
 
  
   FlyBase ID    symbol    start    end    strand    length   
   FBgn0030342   CG10347  11746725   11748913   -  2189  
   FBgn0030343   ATP7   11749066   11757861  +  8796  
   FBgn0030344   CG2025  11758682   11762917   -  4236  
   FBgn0030345   CG1847   11763221   11765199  +  1979  
   FBgn0030346   CG11802  11765332   11766811   -  1480  
 
 
    Segment 217 
 
   Location   
  Gene key  FBgn0030349-FBgn0030350  
  Heatmap region span   X:11732043..11877936   
  Segment span   X:11781192..11781313   
  Length (genes)  2  
  Length (bp)  122  
   Model Scoring   
  BIC  276.445738  
  logL  -132.820283  
  logL ratio  35.769800  
   Expression   
  Mean expression  10.430945  
  Median expression  10.432846  
  Tissue std. dev.  0.788197  
 
  No GO Slim enrichment  
  
   tissue    mean expression   
  5th Passage Drosophila S2 Cells  10.563513  
  Adult Accessory gland  10.492804  
  Adult Brain  10.496036  
  Adult Carcass  10.760369  
  Adult Crop  12.461296  
  Adult Eye  11.103124  
  Adult Fatbody  10.627828  
  Adult Female Spermatheca Mated  10.180170  
  Adult Female Spermatheca Virgin  10.212792  
  Adult Head  10.719957  
  Adult Heart  10.965312  
  Adult Hind Gut  11.441270  
  Adult Male Ejaculatory Duct  10.809855  
  Adult Mid Gut  10.011838  
  Adult Ovary  8.886501  
  Adult Salivary Gland  11.225440  
  Adult Testes  8.223002  
  Adult Thoracoabdominal ganglion  10.306369  
  Adult Whole Fly  9.570391  
  Larvae Wandering Tubules  10.207364  
  Larval Feeding Carcass  10.765625  
  Larval Feeding Central Nevous System  9.796885  
  Larval Feeding Hind Gut  10.728939  
  Larval Feeding Malpighian Tubule  10.121586  
  Larval Feeding Mid Gut  10.006848  
  Larval Feeding Salivary Gland  11.035274  
  Whole Larvae Feeding  9.915128  
 
  
   FlyBase ID    symbol    start    end    strand    length   
   FBgn0030349   CG10353  11771772   11781192   -  9421  
   FBgn0030350   SelG   11781313   11782277  +  965  
 
    Segment 218 
 
   Location   
  Gene key  FBgn0030351-FBgn0030354  
  Heatmap region span   X:11748913..11882946   
  Segment span   X:11782391..11799373   
  Length (genes)  3  
  Length (bp)  16983  
   Model Scoring   
  BIC  269.249669  
  logL  -129.222248  
  logL ratio  98.576615  
   Expression   
  Mean expression  8.174926  
  Median expression  8.245743  
  Tissue std. dev.  0.381587  
 
  No GO Slim enrichment  
  
   tissue    mean expression   
  5th Passage Drosophila S2 Cells  8.456987  
  Adult Accessory gland  8.785020  
  Adult Brain  8.401315  
  Adult Carcass  7.823006  
  Adult Crop  8.192804  
  Adult Eye  8.511524  
  Adult Fatbody  8.032096  
  Adult Female Spermatheca Mated  7.825877  
  Adult Female Spermatheca Virgin  7.574167  
  Adult Head  7.822234  
  Adult Heart  8.827624  
  Adult Hind Gut  8.120008  
  Adult Male Ejaculatory Duct  8.226123  
  Adult Mid Gut  8.109327  
  Adult Ovary  8.624315  
  Adult Salivary Gland  8.435194  
  Adult Testes  8.596104  
  Adult Thoracoabdominal ganglion  8.502999  
  Adult Whole Fly  7.731756  
  Larvae Wandering Tubules  8.390104  
  Larval Feeding Carcass  7.902601  
  Larval Feeding Central Nevous System  8.339858  
  Larval Feeding Hind Gut  7.805507  
  Larval Feeding Malpighian Tubule  8.135067  
  Larval Feeding Mid Gut  7.532191  
  Larval Feeding Salivary Gland  8.567643  
  Whole Larvae Feeding  7.451549  
 
  
   FlyBase ID    symbol    start    end    strand    length   
   FBgn0030351   CG1840   11782391   11783222  +  832  
   FBgn0030352   CG15738   11786043   11787782  +  1740  
   FBgn0030354   Upf1  11794470   11799373   -  4904  
 
 
    Segment 219 
 
   Location   
  Gene key  FBgn0030358-FBgn0005563  
  Heatmap region span   X:11781192..11906467   
  Segment span   X:11824883..11877936   
  Length (genes)  2  
  Length (bp)  53054  
   Model Scoring   
  BIC  190.037468  
  logL  -89.616148  
  logL ratio  41.173149  
   Expression   
  Mean expression  5.333768  
  Median expression  4.365287  
  Tissue std. dev.  2.004707  
 
  No GO Slim enrichment  
  
   tissue    mean expression   
  5th Passage Drosophila S2 Cells  4.067149  
  Adult Accessory gland  4.200846  
  Adult Brain  10.492079  
  Adult Carcass  4.617532  
  Adult Crop  4.320439  
  Adult Eye  9.310821  
  Adult Fatbody  5.180222  
  Adult Female Spermatheca Mated  4.990152  
  Adult Female Spermatheca Virgin  5.072931  
  Adult Head  7.981688  
  Adult Heart  4.515258  
  Adult Hind Gut  4.226449  
  Adult Male Ejaculatory Duct  4.318591  
  Adult Mid Gut  4.146642  
  Adult Ovary  4.373210  
  Adult Salivary Gland  4.531773  
  Adult Testes  4.057792  
  Adult Thoracoabdominal ganglion  10.412617  
  Adult Whole Fly  4.637365  
  Larvae Wandering Tubules  4.347978  
  Larval Feeding Carcass  4.238078  
  Larval Feeding Central Nevous System  8.836720  
  Larval Feeding Hind Gut  4.061535  
  Larval Feeding Malpighian Tubule  4.191581  
  Larval Feeding Mid Gut  4.314251  
  Larval Feeding Salivary Gland  4.195049  
  Whole Larvae Feeding  4.372987  
 
  
   FlyBase ID    symbol    start    end    strand    length   
   FBgn0030358   CG10362  11820443   11824883   -  4441  
   FBgn0005563     11827738   11877936   -  50199  
 
    Segment 220 
 
   Location   
  Gene key  FBgn0000808-FBgn0003865  
  Heatmap region span   X:11782391..11923061   
  Segment span   X:11881780..11882946   
  Length (genes)  2  
  Length (bp)  1167  
   Model Scoring   
  BIC  171.953870  
  logL  -80.574349  
  logL ratio  36.816997  
   Expression   
  Mean expression  5.047629  
  Median expression  5.007500  
  Tissue std. dev.  0.235226  
 
  
   GO ID    description    ratio    P-value   
   GO:0005576   extracellular region  2/2  0.00207  
 
  
   tissue    mean expression   
  5th Passage Drosophila S2 Cells  5.003922  
  Adult Accessory gland  5.082542  
  Adult Brain  4.648953  
  Adult Carcass  5.100163  
  Adult Crop  5.007382  
  Adult Eye  4.799315  
  Adult Fatbody  5.164665  
  Adult Female Spermatheca Mated  5.296513  
  Adult Female Spermatheca Virgin  5.303630  
  Adult Head  4.880591  
  Adult Heart  5.111777  
  Adult Hind Gut  4.908603  
  Adult Male Ejaculatory Duct  5.157324  
  Adult Mid Gut  5.209396  
  Adult Ovary  5.813517  
  Adult Salivary Gland  5.471079  
  Adult Testes  4.820918  
  Adult Thoracoabdominal ganglion  4.772890  
  Adult Whole Fly  4.864783  
  Larvae Wandering Tubules  5.046739  
  Larval Feeding Carcass  5.034574  
  Larval Feeding Central Nevous System  4.987842  
  Larval Feeding Hind Gut  4.909120  
  Larval Feeding Malpighian Tubule  4.981127  
  Larval Feeding Mid Gut  5.020214  
  Larval Feeding Salivary Gland  5.116548  
  Whole Larvae Feeding  4.771858  
 
  
   FlyBase ID    symbol    start    end    strand    length   
   FBgn0000808   gd  11879567   11881780   -  2214  
   FBgn0003865   tsg  11882196   11882946   -  751  
 
    Segment 221 
 
   Location   
  Gene key  FBgn0030359-FBgn0030360  
  Heatmap region span   X:11811266..12351793   
  Segment span   X:11886801..11901211   
  Length (genes)  3  
  Length (bp)  14411  
   Model Scoring   
  BIC  331.608002  
  logL  -160.401415  
  logL ratio  11.588139  
   Expression   
  Mean expression  6.348015  
  Median expression  6.215389  
  Tissue std. dev.  0.841369  
 
  No GO Slim enrichment  
  
   tissue    mean expression   
  5th Passage Drosophila S2 Cells  5.314066  
  Adult Accessory gland  6.286378  
  Adult Brain  5.202710  
  Adult Carcass  6.565041  
  Adult Crop  6.909453  
  Adult Eye  7.986516  
  Adult Fatbody  5.865564  
  Adult Female Spermatheca Mated  7.171734  
  Adult Female Spermatheca Virgin  6.968153  
  Adult Head  6.801240  
  Adult Heart  5.625514  
  Adult Hind Gut  6.239378  
  Adult Male Ejaculatory Duct  6.077296  
  Adult Mid Gut  5.549500  
  Adult Ovary  5.410912  
  Adult Salivary Gland  7.028019  
  Adult Testes  6.318463  
  Adult Thoracoabdominal ganglion  5.349631  
  Adult Whole Fly  5.545540  
  Larvae Wandering Tubules  5.964888  
  Larval Feeding Carcass  8.178499  
  Larval Feeding Central Nevous System  6.724835  
  Larval Feeding Hind Gut  7.907594  
  Larval Feeding Malpighian Tubule  5.577253  
  Larval Feeding Mid Gut  5.611083  
  Larval Feeding Salivary Gland  6.056776  
  Whole Larvae Feeding  7.160358  
 
  
   FlyBase ID    symbol    start    end    strand    length   
   FBgn0030359   CG18130  11884151   11886801   -  2651  
   FBgn0001083   fw  11886948   11897943   -  10996  
   FBgn0030360   CG1806   11901211   11903940  +  2730  
 
 
    Segment 222 
 
   Location   
  Gene key  FBgn0030364-FBgn0030366  
  Heatmap region span   X:11881780..12384416   
  Segment span   X:11912677..11923061   
  Length (genes)  3  
  Length (bp)  10385  
   Model Scoring   
  BIC  327.784812  
  logL  -158.489820  
  logL ratio  55.810173  
   Expression   
  Mean expression  8.667187  
  Median expression  8.383375  
  Tissue std. dev.  0.527734  
 
  No GO Slim enrichment  
  
   tissue    mean expression   
  5th Passage Drosophila S2 Cells  8.994806  
  Adult Accessory gland  8.804071  
  Adult Brain  9.221763  
  Adult Carcass  7.988510  
  Adult Crop  8.750889  
  Adult Eye  8.896501  
  Adult Fatbody  8.583774  
  Adult Female Spermatheca Mated  8.432811  
  Adult Female Spermatheca Virgin  8.541967  
  Adult Head  8.588237  
  Adult Heart  8.544485  
  Adult Hind Gut  8.422998  
  Adult Male Ejaculatory Duct  8.566500  
  Adult Mid Gut  8.123056  
  Adult Ovary  9.636188  
  Adult Salivary Gland  8.356099  
  Adult Testes  6.970845  
  Adult Thoracoabdominal ganglion  9.039687  
  Adult Whole Fly  8.753661  
  Larvae Wandering Tubules  8.996939  
  Larval Feeding Carcass  8.736482  
  Larval Feeding Central Nevous System  9.894915  
  Larval Feeding Hind Gut  8.744195  
  Larval Feeding Malpighian Tubule  8.799534  
  Larval Feeding Mid Gut  8.328921  
  Larval Feeding Salivary Gland  9.023655  
  Whole Larvae Feeding  8.272561  
 
  
   FlyBase ID    symbol    start    end    strand    length   
   FBgn0030364   CG15735   11912677   11914764  +  2088  
   FBgn0030365   Tango4   11915097   11916781  +  1685  
   FBgn0030366   Usp7  11916861   11923061   -  6201  
 
 
    Segment 223 
 
   Location   
  Gene key  FBgn0030367-FBgn0030390  
  Heatmap region span   X:11886801..12390630   
  Segment span   X:11925498..12351793   
  Length (genes)  18  
  Length (bp)  426296  
   Model Scoring   
  BIC  1439.387670  
  logL  -714.291249  
  logL ratio  498.113078  
   Expression   
  Mean expression  4.696352  
  Median expression  4.430002  
  Tissue std. dev.  0.455942  
 
  No GO Slim enrichment  
  
   tissue    mean expression   
  5th Passage Drosophila S2 Cells  4.761521  
  Adult Accessory gland  4.638078  
  Adult Brain  4.262705  
  Adult Carcass  4.852099  
  Adult Crop  4.654968  
  Adult Eye  4.523144  
  Adult Fatbody  4.672155  
  Adult Female Spermatheca Mated  4.713276  
  Adult Female Spermatheca Virgin  4.655728  
  Adult Head  4.413815  
  Adult Heart  4.716964  
  Adult Hind Gut  4.670391  
  Adult Male Ejaculatory Duct  4.598006  
  Adult Mid Gut  4.567925  
  Adult Ovary  4.589033  
  Adult Salivary Gland  4.705802  
  Adult Testes  6.878363  
  Adult Thoracoabdominal ganglion  4.346246  
  Adult Whole Fly  5.053430  
  Larvae Wandering Tubules  4.543180  
  Larval Feeding Carcass  4.625762  
  Larval Feeding Central Nevous System  4.331379  
  Larval Feeding Hind Gut  4.542611  
  Larval Feeding Malpighian Tubule  4.525257  
  Larval Feeding Mid Gut  4.709191  
  Larval Feeding Salivary Gland  4.581310  
  Whole Larvae Feeding  4.669174  
 
  
   FlyBase ID    symbol    start    end    strand    length   
   FBgn0030367   Cyp311a1  11923317   11925498   -  2182  
   FBgn0030369   Cyp318a1   11926665   11929316  +  2652  
   FBgn0030370   CG1950  11949371   11950668   -  1298  
   FBgn0085352   CG34323   11964470   11965296  +  827  
   FBgn0030373   CG12721  11966041   11966859   -  819  
   FBgn0030374   CG15734  11980502   11981058   -  557  
   FBgn0030375   CG11356  11996977   11997843   -  867  
   FBgn0030376   CG2750   12001233   12007390  +  6158  
   FBgn0030377   CG1924  12070928   12072640   -  1713  
   FBgn0052655   CG32655  12128704   12129762   -  1059  
   FBgn0030384   CG2577   12267244   12268484  +  1241  
   FBgn0030385   Ir11a  12269629   12271788   -  2160  
   FBgn0052651   CG32651  12300582   12302438   -  1857  
   FBgn0030386   CG2574   12302922   12303864  +  943  
   FBgn0052652   CG32652  12305093   12306214   -  1122  
   FBgn0259680   Pkcdelta   12326259   12347683  +  21425  
   FBgn0030389   CG15927   12348902   12349471  +  570  
   FBgn0030390   CG15731  12350437   12351793   -  1357  
 
 
    Segment 224 
 
   Location   
  Gene key  FBgn0030391-FBgn0259143  
  Heatmap region span   X:11906242..12391194   
  Segment span   X:12357141..12358132   
  Length (genes)  2  
  Length (bp)  992  
   Model Scoring   
  BIC  249.768748  
  logL  -119.481788  
  logL ratio  -7.366365  
   Expression   
  Mean expression  7.335747  
  Median expression  7.503802  
  Tissue std. dev.  0.414457  
 
  No GO Slim enrichment  
  
   tissue    mean expression   
  5th Passage Drosophila S2 Cells  6.986125  
  Adult Accessory gland  7.080782  
  Adult Brain  7.698970  
  Adult Carcass  7.155147  
  Adult Crop  8.228044  
  Adult Eye  7.291838  
  Adult Fatbody  7.256747  
  Adult Female Spermatheca Mated  7.069386  
  Adult Female Spermatheca Virgin  6.977431  
  Adult Head  7.200658  
  Adult Heart  7.874059  
  Adult Hind Gut  7.921348  
  Adult Male Ejaculatory Duct  7.643680  
  Adult Mid Gut  7.037479  
  Adult Ovary  8.175595  
  Adult Salivary Gland  7.141485  
  Adult Testes  6.487796  
  Adult Thoracoabdominal ganglion  7.600127  
  Adult Whole Fly  7.012979  
  Larvae Wandering Tubules  7.189421  
  Larval Feeding Carcass  7.359602  
  Larval Feeding Central Nevous System  7.464742  
  Larval Feeding Hind Gut  7.815576  
  Larval Feeding Malpighian Tubule  7.391552  
  Larval Feeding Mid Gut  7.368946  
  Larval Feeding Salivary Gland  6.849057  
  Whole Larvae Feeding  6.786612  
 
  
   FlyBase ID    symbol    start    end    strand    length   
   FBgn0030391   Rab40  12353823   12357141   -  3319  
   FBgn0259143   CG42258   12358132   12371539  +  13408  
 
    Segment 225 
 
   Location   
  Gene key  FBgn0259109-FBgn0030399  
  Heatmap region span   X:12381006..12553395   
  Segment span   X:12425266..12459484   
  Length (genes)  3  
  Length (bp)  34219  
   Model Scoring   
  BIC  264.571001  
  logL  -126.882914  
  logL ratio  58.938876  
   Expression   
  Mean expression  4.827794  
  Median expression  4.755353  
  Tissue std. dev.  0.383978  
 
  No GO Slim enrichment  
  
   tissue    mean expression   
  5th Passage Drosophila S2 Cells  4.648536  
  Adult Accessory gland  4.606425  
  Adult Brain  5.410341  
  Adult Carcass  4.638488  
  Adult Crop  4.739701  
  Adult Eye  5.009528  
  Adult Fatbody  4.626713  
  Adult Female Spermatheca Mated  4.648352  
  Adult Female Spermatheca Virgin  4.703991  
  Adult Head  5.710341  
  Adult Heart  4.925629  
  Adult Hind Gut  4.528788  
  Adult Male Ejaculatory Duct  4.962741  
  Adult Mid Gut  4.571849  
  Adult Ovary  4.693901  
  Adult Salivary Gland  4.971530  
  Adult Testes  4.388104  
  Adult Thoracoabdominal ganglion  4.902074  
  Adult Whole Fly  4.215598  
  Larvae Wandering Tubules  4.610618  
  Larval Feeding Carcass  5.168337  
  Larval Feeding Central Nevous System  4.984218  
  Larval Feeding Hind Gut  4.488849  
  Larval Feeding Malpighian Tubule  4.642703  
  Larval Feeding Mid Gut  4.748261  
  Larval Feeding Salivary Gland  4.762671  
  Whole Larvae Feeding  6.042150  
 
  
   FlyBase ID    symbol    start    end    strand    length   
   FBgn0259109     12413012   12425266   -  12255  
   FBgn0030398   Cpr11B   12447163   12448274  +  1112  
   FBgn0030399     12457076   12459484   -  2409  
 
 
    Segment 226 
 
   Location   
  Gene key  FBgn0030400-FBgn0030407  
  Heatmap region span   X:12384416..12600231   
  Segment span   X:12477215..12505947   
  Length (genes)  6  
  Length (bp)  28733  
   Model Scoring   
  BIC  606.246894  
  logL  -297.720861  
  logL ratio  127.411829  
   Expression   
  Mean expression  8.008160  
  Median expression  7.891656  
  Tissue std. dev.  0.508288  
 
  No GO Slim enrichment  
  
   tissue    mean expression   
  5th Passage Drosophila S2 Cells  7.895441  
  Adult Accessory gland  8.070430  
  Adult Brain  8.532681  
  Adult Carcass  7.591781  
  Adult Crop  7.948203  
  Adult Eye  8.306507  
  Adult Fatbody  8.342496  
  Adult Female Spermatheca Mated  7.994886  
  Adult Female Spermatheca Virgin  7.969701  
  Adult Head  7.833480  
  Adult Heart  8.129979  
  Adult Hind Gut  7.944349  
  Adult Male Ejaculatory Duct  7.863806  
  Adult Mid Gut  8.228949  
  Adult Ovary  8.470223  
  Adult Salivary Gland  8.497758  
  Adult Testes  6.016710  
  Adult Thoracoabdominal ganglion  8.435268  
  Adult Whole Fly  7.135616  
  Larvae Wandering Tubules  7.874649  
  Larval Feeding Carcass  7.839899  
  Larval Feeding Central Nevous System  8.399378  
  Larval Feeding Hind Gut  8.146885  
  Larval Feeding Malpighian Tubule  8.279806  
  Larval Feeding Mid Gut  8.317654  
  Larval Feeding Salivary Gland  8.537984  
  Whole Larvae Feeding  7.615813  
 
  
   FlyBase ID    symbol    start    end    strand    length   
   FBgn0030400   CG11138  12465243   12477215   -  11973  
   FBgn0041203   LIMK1  12478418   12486226   -  7809  
   FBgn0030403   CG1824  12486588   12489668   -  3081  
   FBgn0052654   Sec16   12490106   12503943  +  13838  
   FBgn0030406   CG1463  12503590   12505632   -  2043  
   FBgn0030407   CG2543   12505947   12509563  +  3617  
 
 
    Segment 227 
 
   Location   
  Gene key  FBgn0030408-FBgn0030409  
  Heatmap region span   X:12390630..12636476   
  Segment span   X:12510349..12521126   
  Length (genes)  2  
  Length (bp)  10778  
   Model Scoring   
  BIC  191.019440  
  logL  -90.107134  
  logL ratio  24.485906  
   Expression   
  Mean expression  4.652751  
  Median expression  4.560768  
  Tissue std. dev.  0.479249  
 
  No GO Slim enrichment  
  
   tissue    mean expression   
  5th Passage Drosophila S2 Cells  4.587532  
  Adult Accessory gland  4.450031  
  Adult Brain  5.075231  
  Adult Carcass  4.578533  
  Adult Crop  4.296983  
  Adult Eye  4.556079  
  Adult Fatbody  4.737266  
  Adult Female Spermatheca Mated  4.564226  
  Adult Female Spermatheca Virgin  4.545697  
  Adult Head  4.569647  
  Adult Heart  4.601638  
  Adult Hind Gut  4.389743  
  Adult Male Ejaculatory Duct  4.783622  
  Adult Mid Gut  4.591770  
  Adult Ovary  4.663698  
  Adult Salivary Gland  4.556038  
  Adult Testes  6.965097  
  Adult Thoracoabdominal ganglion  4.213745  
  Adult Whole Fly  4.643352  
  Larvae Wandering Tubules  4.481610  
  Larval Feeding Carcass  4.486750  
  Larval Feeding Central Nevous System  4.654981  
  Larval Feeding Hind Gut  4.430242  
  Larval Feeding Malpighian Tubule  4.544702  
  Larval Feeding Mid Gut  4.555151  
  Larval Feeding Salivary Gland  4.629179  
  Whole Larvae Feeding  4.471740  
 
  
   FlyBase ID    symbol    start    end    strand    length   
   FBgn0030408   CG11085   12510349   12515251  +  4903  
   FBgn0030409   CG15728   12521126   12522458  +  1333  
 
    Segment 228 
 
   Location   
  Gene key  FBgn0030410-FBgn0030411  
  Heatmap region span   X:12391194..12638499   
  Segment span   X:12523631..12523943   
  Length (genes)  2  
  Length (bp)  313  
   Model Scoring   
  BIC  205.714374  
  logL  -97.454601  
  logL ratio  41.986478  
   Expression   
  Mean expression  7.678882  
  Median expression  7.607465  
  Tissue std. dev.  0.452580  
 
  No GO Slim enrichment  
  
   tissue    mean expression   
  5th Passage Drosophila S2 Cells  7.470082  
  Adult Accessory gland  7.538610  
  Adult Brain  7.925493  
  Adult Carcass  6.703963  
  Adult Crop  7.450091  
  Adult Eye  8.716812  
  Adult Fatbody  7.500739  
  Adult Female Spermatheca Mated  7.750825  
  Adult Female Spermatheca Virgin  7.833946  
  Adult Head  7.652319  
  Adult Heart  7.565999  
  Adult Hind Gut  7.607782  
  Adult Male Ejaculatory Duct  7.753936  
  Adult Mid Gut  7.738923  
  Adult Ovary  8.809350  
  Adult Salivary Gland  7.676797  
  Adult Testes  7.125172  
  Adult Thoracoabdominal ganglion  8.314304  
  Adult Whole Fly  7.474774  
  Larvae Wandering Tubules  7.856087  
  Larval Feeding Carcass  6.939344  
  Larval Feeding Central Nevous System  7.475841  
  Larval Feeding Hind Gut  7.355725  
  Larval Feeding Malpighian Tubule  8.069980  
  Larval Feeding Mid Gut  8.064097  
  Larval Feeding Salivary Gland  7.705910  
  Whole Larvae Feeding  7.252920  
 
  
   FlyBase ID    symbol    start    end    strand    length   
   FBgn0030410   Aven  12522403   12523631   -  1229  
   FBgn0030411   CG2540   12523943   12525152  +  1210  
 
    Segment 229 
 
   Location   
  Gene key  FBgn0030412-FBgn0011837  
  Heatmap region span   X:12425266..12654651   
  Segment span   X:12525653..12553395   
  Length (genes)  3  
  Length (bp)  27743  
   Model Scoring   
  BIC  321.298321  
  logL  -155.246574  
  logL ratio  94.068473  
   Expression   
  Mean expression  9.748582  
  Median expression  9.735598  
  Tissue std. dev.  0.572699  
 
  No GO Slim enrichment  
  
   tissue    mean expression   
  5th Passage Drosophila S2 Cells  9.433756  
  Adult Accessory gland  10.184345  
  Adult Brain  10.610050  
  Adult Carcass  9.178949  
  Adult Crop  9.906164  
  Adult Eye  10.573843  
  Adult Fatbody  9.881736  
  Adult Female Spermatheca Mated  10.248561  
  Adult Female Spermatheca Virgin  10.378145  
  Adult Head  10.000211  
  Adult Heart  10.372636  
  Adult Hind Gut  9.507419  
  Adult Male Ejaculatory Duct  9.256714  
  Adult Mid Gut  9.329007  
  Adult Ovary  10.176269  
  Adult Salivary Gland  9.351081  
  Adult Testes  7.887514  
  Adult Thoracoabdominal ganglion  10.448373  
  Adult Whole Fly  9.373367  
  Larvae Wandering Tubules  10.067369  
  Larval Feeding Carcass  9.575385  
  Larval Feeding Central Nevous System  9.979830  
  Larval Feeding Hind Gut  9.882865  
  Larval Feeding Malpighian Tubule  9.622966  
  Larval Feeding Mid Gut  9.408909  
  Larval Feeding Salivary Gland  9.350666  
  Whole Larvae Feeding  9.225599  
 
  
   FlyBase ID    symbol    start    end    strand    length   
   FBgn0030412   tomosyn   12525653   12547377  +  21725  
   FBgn0015024   CkIalpha   12547816   12551354  +  3539  
   FBgn0011837   Tis11   12553395   12573736  +  20342  
 
 
    Segment 230 
 
   Location   
  Gene key  FBgn0030420-FBgn0030421  
  Heatmap region span   X:12600231..12797550   
  Segment span   X:12657638..12659301   
  Length (genes)  2  
  Length (bp)  1664  
   Model Scoring   
  BIC  194.787655  
  logL  -91.991242  
  logL ratio  31.262518  
   Expression   
  Mean expression  4.962266  
  Median expression  4.743162  
  Tissue std. dev.  0.917437  
 
  No GO Slim enrichment  
  
   tissue    mean expression   
  5th Passage Drosophila S2 Cells  5.886935  
  Adult Accessory gland  4.739661  
  Adult Brain  6.534455  
  Adult Carcass  4.366639  
  Adult Crop  4.674768  
  Adult Eye  5.593953  
  Adult Fatbody  4.120194  
  Adult Female Spermatheca Mated  4.279420  
  Adult Female Spermatheca Virgin  4.293960  
  Adult Head  5.425657  
  Adult Heart  4.351843  
  Adult Hind Gut  4.699770  
  Adult Male Ejaculatory Duct  4.433262  
  Adult Mid Gut  4.305531  
  Adult Ovary  8.165695  
  Adult Salivary Gland  4.568575  
  Adult Testes  5.019488  
  Adult Thoracoabdominal ganglion  5.714523  
  Adult Whole Fly  5.776709  
  Larvae Wandering Tubules  4.542879  
  Larval Feeding Carcass  4.249531  
  Larval Feeding Central Nevous System  6.184260  
  Larval Feeding Hind Gut  4.400558  
  Larval Feeding Malpighian Tubule  4.603671  
  Larval Feeding Mid Gut  4.105155  
  Larval Feeding Salivary Gland  4.608968  
  Whole Larvae Feeding  4.335119  
 
  
   FlyBase ID    symbol    start    end    strand    length   
   FBgn0030420   CG12717  12654911   12657638   -  2728  
   FBgn0030421   CG3812   12659301   12665016  +  5716  
 
    Segment 231 
 
   Location   
  Gene key  FBgn0259171-FBgn0052650  
  Heatmap region span   X:12654651..12832568   
  Segment span   X:12725669..12792702   
  Length (genes)  3  
  Length (bp)  67034  
   Model Scoring   
  BIC  301.151178  
  logL  -145.173003  
  logL ratio  25.911917  
   Expression   
  Mean expression  5.587442  
  Median expression  5.399693  
  Tissue std. dev.  0.682704  
 
  No GO Slim enrichment  
  
   tissue    mean expression   
  5th Passage Drosophila S2 Cells  5.754491  
  Adult Accessory gland  5.298756  
  Adult Brain  6.042012  
  Adult Carcass  5.339683  
  Adult Crop  5.281123  
  Adult Eye  5.539396  
  Adult Fatbody  5.629922  
  Adult Female Spermatheca Mated  5.320549  
  Adult Female Spermatheca Virgin  5.457358  
  Adult Head  5.301999  
  Adult Heart  5.640563  
  Adult Hind Gut  5.097357  
  Adult Male Ejaculatory Duct  5.292181  
  Adult Mid Gut  5.509677  
  Adult Ovary  5.097583  
  Adult Salivary Gland  5.277133  
  Adult Testes  8.880065  
  Adult Thoracoabdominal ganglion  5.775695  
  Adult Whole Fly  5.770839  
  Larvae Wandering Tubules  5.663756  
  Larval Feeding Carcass  5.223180  
  Larval Feeding Central Nevous System  5.272843  
  Larval Feeding Hind Gut  5.320310  
  Larval Feeding Malpighian Tubule  5.625996  
  Larval Feeding Mid Gut  5.429332  
  Larval Feeding Salivary Gland  5.569003  
  Whole Larvae Feeding  5.450118  
 
  
   FlyBase ID    symbol    start    end    strand    length   
   FBgn0259171   Pde9  12685497   12725669   -  40173  
   FBgn0030429   CG4661  12749791   12751755   -  1965  
   FBgn0052650   CG32650  12791536   12792702   -  1167  
 
 
    Segment 232 
 
   Location   
  Gene key  FBgn0052649-FBgn0030431  
  Heatmap region span   X:12657638..12870264   
  Segment span   X:12795109..12797550   
  Length (genes)  2  
  Length (bp)  2442  
   Model Scoring   
  BIC  214.332409  
  logL  -101.763618  
  logL ratio  40.270200  
   Expression   
  Mean expression  8.605180  
  Median expression  8.566650  
  Tissue std. dev.  0.691750  
 
  No GO Slim enrichment  
  
   tissue    mean expression   
  5th Passage Drosophila S2 Cells  8.293024  
  Adult Accessory gland  8.079918  
  Adult Brain  8.455739  
  Adult Carcass  9.444212  
  Adult Crop  9.617760  
  Adult Eye  8.162952  
  Adult Fatbody  9.158653  
  Adult Female Spermatheca Mated  8.148373  
  Adult Female Spermatheca Virgin  8.269433  
  Adult Head  8.712073  
  Adult Heart  9.091147  
  Adult Hind Gut  9.835164  
  Adult Male Ejaculatory Duct  9.882540  
  Adult Mid Gut  8.858052  
  Adult Ovary  9.167372  
  Adult Salivary Gland  8.426603  
  Adult Testes  6.956385  
  Adult Thoracoabdominal ganglion  8.759459  
  Adult Whole Fly  8.609026  
  Larvae Wandering Tubules  8.084135  
  Larval Feeding Carcass  8.754871  
  Larval Feeding Central Nevous System  7.620462  
  Larval Feeding Hind Gut  9.069327  
  Larval Feeding Malpighian Tubule  8.992118  
  Larval Feeding Mid Gut  8.472998  
  Larval Feeding Salivary Gland  7.414918  
  Whole Larvae Feeding  8.003135  
 
  
   FlyBase ID    symbol    start    end    strand    length   
   FBgn0052649   CG32649  12792801   12795109   -  2309  
   FBgn0030431   CG4407  12795386   12797550   -  2165  
 
    Segment 233 
 
   Location   
  Gene key  FBgn0030433-FBgn0030435  
  Heatmap region span   X:12695741..12949325   
  Segment span   X:12801139..12803986   
  Length (genes)  3  
  Length (bp)  2848  
   Model Scoring   
  BIC  302.690541  
  logL  -145.942685  
  logL ratio  93.851417  
   Expression   
  Mean expression  9.394850  
  Median expression  9.465055  
  Tissue std. dev.  0.400625  
 
  No GO Slim enrichment  
  
   tissue    mean expression   
  5th Passage Drosophila S2 Cells  9.237287  
  Adult Accessory gland  9.969661  
  Adult Brain  9.560323  
  Adult Carcass  9.157271  
  Adult Crop  9.320581  
  Adult Eye  9.719479  
  Adult Fatbody  9.182458  
  Adult Female Spermatheca Mated  9.304405  
  Adult Female Spermatheca Virgin  9.366360  
  Adult Head  9.195567  
  Adult Heart  9.438149  
  Adult Hind Gut  9.381771  
  Adult Male Ejaculatory Duct  9.608614  
  Adult Mid Gut  9.094778  
  Adult Ovary  10.209783  
  Adult Salivary Gland  9.883445  
  Adult Testes  8.307633  
  Adult Thoracoabdominal ganglion  9.566034  
  Adult Whole Fly  9.389391  
  Larvae Wandering Tubules  9.194126  
  Larval Feeding Carcass  9.174372  
  Larval Feeding Central Nevous System  9.614228  
  Larval Feeding Hind Gut  9.475075  
  Larval Feeding Malpighian Tubule  9.435920  
  Larval Feeding Mid Gut  8.864968  
  Larval Feeding Salivary Gland  10.207381  
  Whole Larvae Feeding  8.801897  
 
  
   FlyBase ID    symbol    start    end    strand    length   
   FBgn0030433   mRpL49   12801139   12801877  +  739  
   FBgn0030434   CG4400  12802017   12803631   -  1615  
   FBgn0030435   CG4645   12803986   12805948  +  1963  
 
 
    Segment 234 
 
   Location   
  Gene key  FBgn0030437-FBgn0030439  
  Heatmap region span   X:12795109..13009170   
  Segment span   X:12851468..12870264   
  Length (genes)  5  
  Length (bp)  18797  
   Model Scoring   
  BIC  376.784068  
  logL  -182.989448  
  logL ratio  166.555312  
   Expression   
  Mean expression  4.515405  
  Median expression  4.330498  
  Tissue std. dev.  0.762109  
 
  No GO Slim enrichment  
  
   tissue    mean expression   
  5th Passage Drosophila S2 Cells  4.271946  
  Adult Accessory gland  4.463133  
  Adult Brain  4.358515  
  Adult Carcass  4.390807  
  Adult Crop  4.334276  
  Adult Eye  4.012841  
  Adult Fatbody  4.516545  
  Adult Female Spermatheca Mated  4.467568  
  Adult Female Spermatheca Virgin  4.495875  
  Adult Head  4.067293  
  Adult Heart  4.028254  
  Adult Hind Gut  4.252296  
  Adult Male Ejaculatory Duct  4.278877  
  Adult Mid Gut  4.393147  
  Adult Ovary  7.819676  
  Adult Salivary Gland  4.769958  
  Adult Testes  4.091646  
  Adult Thoracoabdominal ganglion  4.292622  
  Adult Whole Fly  6.250249  
  Larvae Wandering Tubules  4.408459  
  Larval Feeding Carcass  4.308468  
  Larval Feeding Central Nevous System  4.224643  
  Larval Feeding Hind Gut  4.206019  
  Larval Feeding Malpighian Tubule  4.315012  
  Larval Feeding Mid Gut  4.328307  
  Larval Feeding Salivary Gland  4.478992  
  Whole Larvae Feeding  4.090498  
 
  
   FlyBase ID    symbol    start    end    strand    length   
   FBgn0030437   hec  12845337   12851468   -  6132  
   FBgn0052642   CG32642   12853315   12854543  +  1229  
   FBgn0052644   Mur11Da   12861942   12863649  +  1708  
   FBgn0030438   CG15721   12864761   12868521  +  3761  
   FBgn0030439   CG12716  12868818   12870264   -  1447  
 
 
    Segment 235 
 
   Location   
  Gene key  FBgn0052643-FBgn0030441  
  Heatmap region span   X:12800068..13025571   
  Segment span   X:12878590..12916231   
  Length (genes)  3  
  Length (bp)  37642  
   Model Scoring   
  BIC  258.522351  
  logL  -123.858589  
  logL ratio  84.978799  
   Expression   
  Mean expression  4.550661  
  Median expression  4.025393  
  Tissue std. dev.  1.164395  
 
  No GO Slim enrichment  
  
   tissue    mean expression   
  5th Passage Drosophila S2 Cells  4.218735  
  Adult Accessory gland  4.284538  
  Adult Brain  4.456245  
  Adult Carcass  4.377171  
  Adult Crop  4.286986  
  Adult Eye  4.076248  
  Adult Fatbody  4.214597  
  Adult Female Spermatheca Mated  4.192080  
  Adult Female Spermatheca Virgin  4.181645  
  Adult Head  4.097653  
  Adult Heart  4.197890  
  Adult Hind Gut  4.320215  
  Adult Male Ejaculatory Duct  4.332144  
  Adult Mid Gut  4.294943  
  Adult Ovary  4.187147  
  Adult Salivary Gland  4.647696  
  Adult Testes  10.340087  
  Adult Thoracoabdominal ganglion  4.387495  
  Adult Whole Fly  5.482589  
  Larvae Wandering Tubules  4.385511  
  Larval Feeding Carcass  4.221470  
  Larval Feeding Central Nevous System  4.154737  
  Larval Feeding Hind Gut  4.114972  
  Larval Feeding Malpighian Tubule  4.334300  
  Larval Feeding Mid Gut  4.255274  
  Larval Feeding Salivary Gland  4.340700  
  Whole Larvae Feeding  4.484786  
 
  
   FlyBase ID    symbol    start    end    strand    length   
   FBgn0052643   CG32643   12878590   12879238  +  649  
   FBgn0030440   CG15719  12897988   12898777   -  790  
   FBgn0030441   CG2209   12916231   12917225  +  995  
 
 
    Segment 236 
 
   Location   
  Gene key  FBgn0010303-FBgn0030448  
  Heatmap region span   X:12832568..13048957   
  Segment span   X:12984466..12995964   
  Length (genes)  3  
  Length (bp)  11499  
   Model Scoring   
  BIC  344.548844  
  logL  -166.871836  
  logL ratio  32.146872  
   Expression   
  Mean expression  8.322823  
  Median expression  8.304297  
  Tissue std. dev.  0.600265  
 
  No GO Slim enrichment  
  
   tissue    mean expression   
  5th Passage Drosophila S2 Cells  8.065169  
  Adult Accessory gland  7.821920  
  Adult Brain  8.141153  
  Adult Carcass  7.972826  
  Adult Crop  7.960585  
  Adult Eye  8.330451  
  Adult Fatbody  8.702857  
  Adult Female Spermatheca Mated  8.634884  
  Adult Female Spermatheca Virgin  8.523644  
  Adult Head  7.729879  
  Adult Heart  8.868684  
  Adult Hind Gut  8.346278  
  Adult Male Ejaculatory Duct  8.295701  
  Adult Mid Gut  8.530537  
  Adult Ovary  8.965865  
  Adult Salivary Gland  9.181277  
  Adult Testes  6.116325  
  Adult Thoracoabdominal ganglion  8.379443  
  Adult Whole Fly  7.754136  
  Larvae Wandering Tubules  8.643165  
  Larval Feeding Carcass  8.261219  
  Larval Feeding Central Nevous System  7.867493  
  Larval Feeding Hind Gut  8.921232  
  Larval Feeding Malpighian Tubule  8.530191  
  Larval Feeding Mid Gut  9.065567  
  Larval Feeding Salivary Gland  9.016750  
  Whole Larvae Feeding  8.089001  
 
  
   FlyBase ID    symbol    start    end    strand    length   
   FBgn0010303   hep  12973240   12984466   -  11227  
   FBgn0030447   CG2200   12988547   12989770  +  1224  
   FBgn0030448     12990125   12995964   -  5840  
 
 
    Segment 237 
 
   Location   
  Gene key  FBgn0030449-FBgn0250862  
  Heatmap region span   X:12851468..13053739   
  Segment span   X:13002227..13009170   
  Length (genes)  3  
  Length (bp)  6944  
   Model Scoring   
  BIC  306.748000  
  logL  -147.971414  
  logL ratio  19.511053  
   Expression   
  Mean expression  4.826910  
  Median expression  4.491075  
  Tissue std. dev.  0.616338  
 
  No GO Slim enrichment  
  
   tissue    mean expression   
  5th Passage Drosophila S2 Cells  4.585742  
  Adult Accessory gland  4.453985  
  Adult Brain  4.349761  
  Adult Carcass  4.800305  
  Adult Crop  4.601775  
  Adult Eye  4.499746  
  Adult Fatbody  4.667627  
  Adult Female Spermatheca Mated  4.656377  
  Adult Female Spermatheca Virgin  4.524674  
  Adult Head  4.581644  
  Adult Heart  4.638855  
  Adult Hind Gut  4.959189  
  Adult Male Ejaculatory Duct  7.003845  
  Adult Mid Gut  4.623767  
  Adult Ovary  4.802937  
  Adult Salivary Gland  4.874310  
  Adult Testes  6.790758  
  Adult Thoracoabdominal ganglion  4.451202  
  Adult Whole Fly  5.165430  
  Larvae Wandering Tubules  4.657683  
  Larval Feeding Carcass  4.782768  
  Larval Feeding Central Nevous System  4.297930  
  Larval Feeding Hind Gut  5.022460  
  Larval Feeding Malpighian Tubule  4.693630  
  Larval Feeding Mid Gut  4.667418  
  Larval Feeding Salivary Gland  4.661385  
  Whole Larvae Feeding  4.511380  
 
  
   FlyBase ID    symbol    start    end    strand    length   
   FBgn0030449   Fer3HCH  13001415   13002227   -  813  
   FBgn0040862     13005393   13008469   -  3077  
   FBgn0250862   CG42237   13009170   13017100  +  7931  
 
 
    Segment 238 
 
   Location   
  Gene key  FBgn0052638-FBgn0030452  
  Heatmap region span   X:12878590..13085915   
  Segment span   X:13022827..13025571   
  Length (genes)  3  
  Length (bp)  2745  
   Model Scoring   
  BIC  359.738249  
  logL  -174.466538  
  logL ratio  38.277667  
   Expression   
  Mean expression  9.102640  
  Median expression  9.027542  
  Tissue std. dev.  0.804039  
 
  No GO Slim enrichment  
  
   tissue    mean expression   
  5th Passage Drosophila S2 Cells  10.119902  
  Adult Accessory gland  9.933531  
  Adult Brain  7.929833  
  Adult Carcass  9.057045  
  Adult Crop  8.859511  
  Adult Eye  9.362411  
  Adult Fatbody  9.114669  
  Adult Female Spermatheca Mated  8.705281  
  Adult Female Spermatheca Virgin  8.751269  
  Adult Head  9.109668  
  Adult Heart  10.466380  
  Adult Hind Gut  9.135286  
  Adult Male Ejaculatory Duct  9.563838  
  Adult Mid Gut  9.341964  
  Adult Ovary  8.436844  
  Adult Salivary Gland  9.849795  
  Adult Testes  6.856014  
  Adult Thoracoabdominal ganglion  7.897727  
  Adult Whole Fly  8.268352  
  Larvae Wandering Tubules  10.393145  
  Larval Feeding Carcass  9.177114  
  Larval Feeding Central Nevous System  8.136873  
  Larval Feeding Hind Gut  9.507848  
  Larval Feeding Malpighian Tubule  9.902572  
  Larval Feeding Mid Gut  9.489809  
  Larval Feeding Salivary Gland  9.341910  
  Whole Larvae Feeding  9.062687  
 
  
   FlyBase ID    symbol    start    end    strand    length   
   FBgn0052638   CG32638  13016909   13022827   -  5919  
   FBgn0030451   CG15717   13023053   13024324  +  1272  
   FBgn0030452   MFS10   13025571   13028598  +  3028  
 
 
    Segment 239 
 
   Location   
  Gene key  FBgn0030456-FBgn0030457  
  Heatmap region span   X:13002227..13130599   
  Segment span   X:13049073..13053739   
  Length (genes)  3  
  Length (bp)  4667  
   Model Scoring   
  BIC  285.475207  
  logL  -137.335017  
  logL ratio  99.183314  
   Expression   
  Mean expression  9.039255  
  Median expression  9.126369  
  Tissue std. dev.  0.514642  
 
  No GO Slim enrichment  
  
   tissue    mean expression   
  5th Passage Drosophila S2 Cells  9.367798  
  Adult Accessory gland  9.861963  
  Adult Brain  8.529457  
  Adult Carcass  8.428448  
  Adult Crop  9.027216  
  Adult Eye  8.485957  
  Adult Fatbody  9.048599  
  Adult Female Spermatheca Mated  9.046347  
  Adult Female Spermatheca Virgin  9.051447  
  Adult Head  8.473739  
  Adult Heart  9.055853  
  Adult Hind Gut  8.695892  
  Adult Male Ejaculatory Duct  9.529138  
  Adult Mid Gut  8.336804  
  Adult Ovary  10.027542  
  Adult Salivary Gland  9.144787  
  Adult Testes  8.327568  
  Adult Thoracoabdominal ganglion  8.435178  
  Adult Whole Fly  9.206541  
  Larvae Wandering Tubules  9.297145  
  Larval Feeding Carcass  9.197583  
  Larval Feeding Central Nevous System  9.941371  
  Larval Feeding Hind Gut  9.093995  
  Larval Feeding Malpighian Tubule  9.101154  
  Larval Feeding Mid Gut  8.447940  
  Larval Feeding Salivary Gland  10.070089  
  Whole Larvae Feeding  8.830340  
 
  
   FlyBase ID    symbol    start    end    strand    length   
   FBgn0030456   CG4332   13049073   13051405  +  2333  
   FBgn0025463   Bap60  13051478   13053611   -  2134  
   FBgn0030457   CG12096   13053739   13055732  +  1994  
 
 
    Segment 240 
 
   Location   
  Gene key  FBgn0085440-FBgn0030459  
  Heatmap region span   X:13022827..13152523   
  Segment span   X:13083197..13085915   
  Length (genes)  2  
  Length (bp)  2719  
   Model Scoring   
  BIC  194.206482  
  logL  -91.700655  
  logL ratio  23.942715  
   Expression   
  Mean expression  5.324160  
  Median expression  5.473980  
  Tissue std. dev.  0.238490  
 
  No GO Slim enrichment  
  
   tissue    mean expression   
  5th Passage Drosophila S2 Cells  5.272420  
  Adult Accessory gland  5.387428  
  Adult Brain  5.499690  
  Adult Carcass  5.419199  
  Adult Crop  5.863874  
  Adult Eye  4.827617  
  Adult Fatbody  5.412652  
  Adult Female Spermatheca Mated  5.189040  
  Adult Female Spermatheca Virgin  5.366270  
  Adult Head  5.177640  
  Adult Heart  5.236280  
  Adult Hind Gut  5.422654  
  Adult Male Ejaculatory Duct  5.367923  
  Adult Mid Gut  5.660567  
  Adult Ovary  5.237447  
  Adult Salivary Gland  5.577054  
  Adult Testes  5.130269  
  Adult Thoracoabdominal ganglion  5.705329  
  Adult Whole Fly  4.843125  
  Larvae Wandering Tubules  5.386830  
  Larval Feeding Carcass  5.205946  
  Larval Feeding Central Nevous System  5.072292  
  Larval Feeding Hind Gut  5.203575  
  Larval Feeding Malpighian Tubule  5.300605  
  Larval Feeding Mid Gut  5.507169  
  Larval Feeding Salivary Gland  5.503849  
  Whole Larvae Feeding  4.975569  
 
  
   FlyBase ID    symbol    start    end    strand    length   
   FBgn0085440   CG34411  13055747   13083197   -  27451  
   FBgn0030459   CG12723  13083676   13085915   -  2240  
 
    Segment 241 
 
   Location   
  Gene key  FBgn0030460-FBgn0005410  
  Heatmap region span   X:13033239..13152528   
  Segment span   X:13088406..13105699   
  Length (genes)  2  
  Length (bp)  17294  
   Model Scoring   
  BIC  231.192034  
  logL  -110.193431  
  logL ratio  10.164667
[truncated: 282,240 more chars]
