# Supplementary material for: Expression-based segmentation of the Drosophila genome
Source: BMC Genomics. 2013 Nov 20;14:812. doi: 10.1186/1471-2164-14-812 (PMC3909303; doi:10.1186/1471-2164-14-812)
Supplement: Additional file 1 — Detailed information for multigene segments. [file 1471-2164-14-812-S1.zip › miniwebsite/genes.html]

ExprSeg Report Gene List

Report for /Users/afrubin/Code/ExprSeg/2012-07-19/output  
Generated Fri Jun 21 23:09:31 2013

FBgn0000008  
*FBgn0000017*  
*FBgn0000018*  
FBgn0000022  
*FBgn0000024*  
FBgn0000028  
*FBgn0000042*  
FBgn0000043  
*FBgn0000044*  
*FBgn0000045*  
*FBgn0000046*  
*FBgn0000047*  
FBgn0000052  
FBgn0000053  
FBgn0000054  
FBgn0000057  
FBgn0000061  
*FBgn0000063*  
*FBgn0000064*  
*FBgn0000071*  
FBgn0000075  
FBgn0000077  
*FBgn0000083*  
*FBgn0000084*  
FBgn0000092  
*FBgn0000094*  
FBgn0000097  
*FBgn0000099*  
*FBgn0000100*  
*FBgn0000108*  
*FBgn0000109*  
*FBgn0000114*  
FBgn0000115  
*FBgn0000116*  
FBgn0000117  
FBgn0000119  
*FBgn0000120*  
*FBgn0000121*  
FBgn0000137  
FBgn0000139  
*FBgn0000140*  
FBgn0000146  
FBgn0000147  
*FBgn0000150*  
FBgn0000152  
FBgn0000153  
FBgn0000157  
*FBgn0000158*  
*FBgn0000163*  
FBgn0000166  
FBgn0000171  
*FBgn0000173*  
FBgn0000179  
FBgn0000180  
*FBgn0000181*  
FBgn0000182  
FBgn0000183  
*FBgn0000206*  
FBgn0000210  
FBgn0000212  
FBgn0000216  
FBgn0000221  
FBgn0000227  
FBgn0000228  
FBgn0000229  
FBgn0000233  
*FBgn0000239*  
FBgn0000241  
FBgn0000242  
FBgn0000244  
FBgn0000247  
FBgn0000250  
*FBgn0000251*  
*FBgn0000253*  
FBgn0000256  
FBgn0000257  
FBgn0000258  
FBgn0000259  
*FBgn0000261*  
FBgn0000278  
FBgn0000279  
FBgn0000283  
FBgn0000287  
FBgn0000289  
FBgn0000299  
FBgn0000303  
FBgn0000307  
FBgn0000308  
*FBgn0000313*  
*FBgn0000317*  
FBgn0000318  
*FBgn0000319*  
*FBgn0000320*  
FBgn0000326  
*FBgn0000330*  
*FBgn0000337*  
*FBgn0000338*  
FBgn0000339  
*FBgn0000346*  
FBgn0000351  
FBgn0000352  
FBgn0000355  
FBgn0000356  
FBgn0000357  
FBgn0000358  
FBgn0000359  
FBgn0000360  
*FBgn0000363*  
FBgn0000370  
FBgn0000376  
FBgn0000377  
FBgn0000382  
FBgn0000384  
FBgn0000392  
FBgn0000394  
*FBgn0000404*  
*FBgn0000405*  
*FBgn0000411*  
*FBgn0000412*  
FBgn0000413  
*FBgn0000414*  
FBgn0000422  
FBgn0000426  
FBgn0000439  
*FBgn0000442*  
FBgn0000447  
FBgn0000448  
FBgn0000449  
FBgn0000451  
FBgn0000459  
FBgn0000463  
FBgn0000464  
*FBgn0000472*  
FBgn0000473  
*FBgn0000477*  
*FBgn0000479*  
FBgn0000482  
FBgn0000490  
*FBgn0000492*  
FBgn0000497  
FBgn0000499  
*FBgn0000500*  
*FBgn0000504*  
FBgn0000520  
*FBgn0000524*  
*FBgn0000527*  
FBgn0000529  
FBgn0000533  
FBgn0000535  
FBgn0000536  
FBgn0000542  
FBgn0000543  
*FBgn0000546*  
*FBgn0000547*  
FBgn0000551  
FBgn0000552  
FBgn0000556  
*FBgn0000557*  
*FBgn0000559*  
FBgn0000560  
*FBgn0000562*  
FBgn0000564  
*FBgn0000565*  
FBgn0000566  
*FBgn0000567*  
*FBgn0000568*  
*FBgn0000575*  
FBgn0000576  
FBgn0000577  
FBgn0000578  
*FBgn0000579*  
*FBgn0000588*  
FBgn0000606  
FBgn0000611  
*FBgn0000615*  
FBgn0000625  
*FBgn0000633*  
*FBgn0000634*  
FBgn0000635  
*FBgn0000636*  
*FBgn0000639*  
*FBgn0000640*  
*FBgn0000644*  
FBgn0000658  
*FBgn0000659*  
FBgn0000667  
FBgn0000709  
*FBgn0000711*  
*FBgn0000715*  
*FBgn0000719*  
*FBgn0000721*  
*FBgn0000723*  
FBgn0000808  
FBgn0000826  
*FBgn0000964*  
*FBgn0000996*  
*FBgn0001075*  
FBgn0001077  
*FBgn0001079*  
FBgn0001083  
FBgn0001084  
*FBgn0001085*  
*FBgn0001086*  
*FBgn0001087*  
*FBgn0001089*  
*FBgn0001090*  
*FBgn0001091*  
FBgn0001092  
*FBgn0001098*  
FBgn0001105  
FBgn0001108  
*FBgn0001112*  
*FBgn0001114*  
*FBgn0001120*  
FBgn0001124  
FBgn0001125  
FBgn0001128  
FBgn0001133  
FBgn0001134  
FBgn0001137  
*FBgn0001138*  
FBgn0001139  
FBgn0001142  
*FBgn0001145*  
*FBgn0001148*  
*FBgn0001149*  
FBgn0001150  
*FBgn0001168*  
FBgn0001169  
FBgn0001174  
FBgn0001179  
*FBgn0001180*  
FBgn0001185  
*FBgn0001189*  
*FBgn0001197*  
FBgn0001202  
FBgn0001203  
FBgn0001205  
FBgn0001206  
*FBgn0001208*  
FBgn0001215  
FBgn0001222  
FBgn0001224  
*FBgn0001225*  
FBgn0001226  
*FBgn0001227*  
*FBgn0001229*  
FBgn0001230  
*FBgn0001233*  
*FBgn0001235*  
FBgn0001247  
*FBgn0001248*  
FBgn0001250  
*FBgn0001253*  
FBgn0001254  
FBgn0001255  
FBgn0001256  
FBgn0001257  
*FBgn0001258*  
FBgn0001259  
*FBgn0001263*  
FBgn0001269  
FBgn0001276  
*FBgn0001280*  
FBgn0001281  
*FBgn0001285*  
FBgn0001291  
*FBgn0001296*  
FBgn0001297  
*FBgn0001301*  
FBgn0001308  
*FBgn0001311*  
*FBgn0001316*  
FBgn0001319  
FBgn0001320  
FBgn0001321  
FBgn0001323  
FBgn0001324  
FBgn0001325  
FBgn0001330  
*FBgn0001332*  
FBgn0001337  
FBgn0001404  
FBgn0001565  
*FBgn0001624*  
FBgn0001941  
FBgn0001961  
FBgn0001965  
*FBgn0001967*  
FBgn0001970  
FBgn0001978  
FBgn0001981  
FBgn0001983  
FBgn0001987  
FBgn0001990  
FBgn0001992  
FBgn0001994  
FBgn0001995  
FBgn0002022  
FBgn0002023  
FBgn0002036  
*FBgn0002044*  
*FBgn0002183*  
*FBgn0002284*  
FBgn0002306  
*FBgn0002413*  
*FBgn0002431*  
*FBgn0002466*  
FBgn0002522  
*FBgn0002524*  
FBgn0002525  
*FBgn0002526*  
FBgn0002528  
FBgn0002533  
FBgn0002534  
*FBgn0002542*  
FBgn0002543  
*FBgn0002552*  
*FBgn0002562*  
*FBgn0002563*  
*FBgn0002564*  
*FBgn0002565*  
FBgn0002566  
*FBgn0002567*  
FBgn0002569  
FBgn0002570  
FBgn0002571  
*FBgn0002573*  
FBgn0002576  
FBgn0002577  
*FBgn0002578*  
*FBgn0002579*  
*FBgn0002590*  
FBgn0002592  
*FBgn0002593*  
*FBgn0002607*  
FBgn0002609  
*FBgn0002626*  
FBgn0002629  
FBgn0002631  
FBgn0002632  
FBgn0002633  
*FBgn0002638*  
FBgn0002641  
FBgn0002643  
FBgn0002645  
FBgn0002652  
FBgn0002673  
*FBgn0002719*  
FBgn0002723  
*FBgn0002732*  
*FBgn0002733*  
FBgn0002734  
FBgn0002735  
FBgn0002736  
*FBgn0002772*  
*FBgn0002773*  
*FBgn0002774*  
FBgn0002778  
FBgn0002780  
FBgn0002783  
*FBgn0002787*  
*FBgn0002789*  
FBgn0002791  
FBgn0002842  
FBgn0002855  
FBgn0002856  
*FBgn0002862*  
FBgn0002863  
FBgn0002865  
*FBgn0002868*  
*FBgn0002869*  
FBgn0002872  
*FBgn0002873*  
*FBgn0002878*  
*FBgn0002891*  
FBgn0002899  
FBgn0002901  
*FBgn0002905*  
*FBgn0002906*  
FBgn0002914  
FBgn0002917  
*FBgn0002921*  
FBgn0002922  
FBgn0002924  
*FBgn0002926*  
*FBgn0002930*  
FBgn0002931  
*FBgn0002932*  
FBgn0002933  
*FBgn0002936*  
FBgn0002937  
FBgn0002938  
*FBgn0002939*  
*FBgn0002940*  
FBgn0002941  
FBgn0002945  
FBgn0002948  
*FBgn0002962*  
*FBgn0002968*  
FBgn0002973  
FBgn0002985  
FBgn0002989  
FBgn0003002  
FBgn0003008  
FBgn0003009  
FBgn0003011  
*FBgn0003015*  
*FBgn0003016*  
*FBgn0003022*  
*FBgn0003023*  
*FBgn0003028*  
*FBgn0003034*  
FBgn0003041  
FBgn0003042  
FBgn0003044  
*FBgn0003046*  
FBgn0003048  
*FBgn0003053*  
FBgn0003057  
FBgn0003060  
FBgn0003062  
*FBgn0003065*  
*FBgn0003067*  
FBgn0003068  
FBgn0003071  
*FBgn0003074*  
FBgn0003076  
FBgn0003079  
*FBgn0003082*  
FBgn0003086  
*FBgn0003087*  
FBgn0003089  
*FBgn0003090*  
*FBgn0003091*  
FBgn0003093  
*FBgn0003114*  
FBgn0003116  
*FBgn0003117*  
*FBgn0003118*  
*FBgn0003124*  
*FBgn0003129*  
FBgn0003130  
*FBgn0003137*  
*FBgn0003138*  
FBgn0003139  
*FBgn0003141*  
FBgn0003145  
*FBgn0003149*  
*FBgn0003159*  
*FBgn0003162*  
FBgn0003165  
FBgn0003169  
FBgn0003174  
*FBgn0003175*  
*FBgn0003177*  
*FBgn0003178*  
*FBgn0003187*  
FBgn0003189  
FBgn0003200  
FBgn0003204  
FBgn0003205  
FBgn0003206  
FBgn0003209  
FBgn0003210  
*FBgn0003218*  
*FBgn0003227*  
FBgn0003248  
*FBgn0003249*  
*FBgn0003250*  
FBgn0003254  
FBgn0003255  
*FBgn0003261*  
*FBgn0003267*  
*FBgn0003268*  
*FBgn0003270*  
*FBgn0003274*  
*FBgn0003275*  
FBgn0003276  
FBgn0003277  
FBgn0003278  
*FBgn0003279*  
FBgn0003285  
FBgn0003292  
FBgn0003295  
FBgn0003300  
FBgn0003301  
FBgn0003302  
*FBgn0003308*  
FBgn0003310  
*FBgn0003312*  
FBgn0003313  
FBgn0003317  
*FBgn0003319*  
FBgn0003321  
FBgn0003326  
*FBgn0003328*  
FBgn0003330  
*FBgn0003334*  
FBgn0003339  
FBgn0003345  
FBgn0003346  
*FBgn0003353*  
*FBgn0003358*  
*FBgn0003360*  
FBgn0003366  
FBgn0003371  
*FBgn0003372*  
*FBgn0003373*  
FBgn0003374  
FBgn0003375  
FBgn0003377  
FBgn0003378  
FBgn0003380  
FBgn0003382  
FBgn0003386  
*FBgn0003388*  
*FBgn0003390*  
*FBgn0003391*  
FBgn0003392  
*FBgn0003396*  
FBgn0003401  
*FBgn0003410*  
FBgn0003411  
FBgn0003415  
FBgn0003416  
FBgn0003423  
*FBgn0003429*  
FBgn0003430  
*FBgn0003435*  
FBgn0003444  
*FBgn0003447*  
FBgn0003448  
FBgn0003449  
FBgn0003450  
FBgn0003459  
FBgn0003460  
*FBgn0003462*  
FBgn0003463  
FBgn0003464  
FBgn0003475  
*FBgn0003486*  
*FBgn0003495*  
FBgn0003499  
*FBgn0003501*  
FBgn0003502  
*FBgn0003507*  
FBgn0003513  
FBgn0003514  
*FBgn0003515*  
*FBgn0003517*  
FBgn0003520  
*FBgn0003525*  
*FBgn0003527*  
FBgn0003545  
FBgn0003651  
*FBgn0003655*  
*FBgn0003656*  
FBgn0003659  
*FBgn0003660*  
*FBgn0003687*  
FBgn0003701  
FBgn0003710  
FBgn0003714  
FBgn0003715  
FBgn0003716  
*FBgn0003719*  
FBgn0003720  
*FBgn0003721*  
*FBgn0003731*  
FBgn0003732  
*FBgn0003733*  
FBgn0003741  
FBgn0003742  
FBgn0003744  
*FBgn0003747*  
FBgn0003748  
*FBgn0003751*  
*FBgn0003861*  
FBgn0003862  
FBgn0003863  
FBgn0003865  
*FBgn0003866*  
*FBgn0003867*  
FBgn0003870  
FBgn0003882  
*FBgn0003884*  
FBgn0003885  
FBgn0003886  
*FBgn0003887*  
*FBgn0003888*  
*FBgn0003889*  
FBgn0003890  
*FBgn0003891*  
*FBgn0003892*  
*FBgn0003896*  
FBgn0003900  
FBgn0003941  
*FBgn0003944*  
FBgn0003950  
*FBgn0003961*  
*FBgn0003963*  
FBgn0003964  
*FBgn0003965*  
FBgn0003969  
*FBgn0003975*  
FBgn0003977  
FBgn0003978  
FBgn0003979  
FBgn0003980  
FBgn0003983  
FBgn0003984  
*FBgn0003996*  
FBgn0003997  
*FBgn0004003*  
FBgn0004009  
*FBgn0004028*  
FBgn0004034  
FBgn0004045  
*FBgn0004047*  
*FBgn0004049*  
FBgn0004050  
*FBgn0004052*  
FBgn0004053  
FBgn0004054  
*FBgn0004055*  
*FBgn0004057*  
FBgn0004087  
FBgn0004101  
*FBgn0004102*  
*FBgn0004106*  
*FBgn0004107*  
FBgn0004108  
FBgn0004110  
*FBgn0004117*  
FBgn0004132  
*FBgn0004133*  
FBgn0004143  
*FBgn0004167*  
*FBgn0004169*  
FBgn0004170  
FBgn0004171  
FBgn0004172  
FBgn0004173  
FBgn0004174  
FBgn0004175  
FBgn0004177  
*FBgn0004179*  
FBgn0004181  
FBgn0004197  
FBgn0004198  
FBgn0004227  
*FBgn0004228*  
*FBgn0004237*  
*FBgn0004240*  
FBgn0004242  
FBgn0004244  
FBgn0004359  
FBgn0004360  
*FBgn0004362*  
*FBgn0004363*  
*FBgn0004364*  
FBgn0004366  
*FBgn0004368*  
FBgn0004369  
FBgn0004370  
FBgn0004372  
FBgn0004373  
*FBgn0004374*  
FBgn0004377  
*FBgn0004378*  
*FBgn0004379*  
FBgn0004380  
FBgn0004381  
FBgn0004387  
FBgn0004389  
FBgn0004390  
FBgn0004391  
FBgn0004394  
*FBgn0004395*  
*FBgn0004396*  
*FBgn0004397*  
FBgn0004399  
FBgn0004400  
FBgn0004401  
FBgn0004403  
FBgn0004404  
FBgn0004406  
*FBgn0004414*  
FBgn0004419  
*FBgn0004429*  
*FBgn0004430*  
*FBgn0004431*  
FBgn0004432  
*FBgn0004435*  
*FBgn0004436*  
*FBgn0004456*  
FBgn0004462  
*FBgn0004463*  
*FBgn0004507*  
FBgn0004509  
FBgn0004510  
*FBgn0004511*  
*FBgn0004512*  
FBgn0004513  
*FBgn0004514*  
FBgn0004516  
*FBgn0004552*  
FBgn0004554  
*FBgn0004556*  
FBgn0004567  
FBgn0004569  
FBgn0004574  
*FBgn0004575*  
*FBgn0004577*  
*FBgn0004580*  
*FBgn0004581*  
*FBgn0004583*  
FBgn0004584  
FBgn0004587  
FBgn0004588  
FBgn0004589  
FBgn0004590  
FBgn0004591  
*FBgn0004592*  
FBgn0004593  
FBgn0004594  
*FBgn0004595*  
FBgn0004597  
FBgn0004598  
*FBgn0004603*  
*FBgn0004606*  
*FBgn0004611*  
*FBgn0004618*  
FBgn0004620  
FBgn0004622  
*FBgn0004623*  
*FBgn0004625*  
*FBgn0004629*  
*FBgn0004635*  
FBgn0004636  
FBgn0004638  
*FBgn0004644*  
FBgn0004646  
FBgn0004647  
*FBgn0004648*  
*FBgn0004649*  
FBgn0004652  
FBgn0004654  
*FBgn0004655*  
*FBgn0004657*  
FBgn0004666  
FBgn0004698  
FBgn0004777  
FBgn0004778  
FBgn0004779  
FBgn0004781  
*FBgn0004784*  
*FBgn0004795*  
*FBgn0004797*  
FBgn0004832  
FBgn0004838  
FBgn0004839  
FBgn0004841  
FBgn0004842  
*FBgn0004852*  
*FBgn0004855*  
FBgn0004856  
FBgn0004858  
FBgn0004862  
FBgn0004863  
FBgn0004864  
*FBgn0004865*  
*FBgn0004867*  
FBgn0004868  
*FBgn0004870*  
*FBgn0004872*  
*FBgn0004873*  
*FBgn0004875*  
*FBgn0004876*  
*FBgn0004878*  
FBgn0004880  
*FBgn0004882*  
*FBgn0004885*  
*FBgn0004888*  
*FBgn0004889*  
FBgn0004892  
FBgn0004893  
FBgn0004895  
FBgn0004896  
FBgn0004897  
FBgn0004898  
FBgn0004901  
FBgn0004903  
FBgn0004908  
FBgn0004913  
FBgn0004914  
FBgn0004915  
FBgn0004919  
FBgn0004921  
FBgn0004924  
FBgn0004956  
FBgn0004957  
FBgn0004959  
FBgn0005198  
FBgn0005322  
FBgn0005386  
FBgn0005391  
FBgn0005410  
FBgn0005411  
FBgn0005427  
*FBgn0005533*  
*FBgn0005536*  
FBgn0005563  
*FBgn0005564*  
*FBgn0005585*  
*FBgn0005586*  
*FBgn0005590*  
FBgn0005592  
*FBgn0005593*  
FBgn0005596  
FBgn0005612  
FBgn0005613  
*FBgn0005614*  
*FBgn0005619*  
*FBgn0005624*  
*FBgn0005626*  
FBgn0005630  
FBgn0005631  
FBgn0005632  
*FBgn0005633*  
*FBgn0005634*  
*FBgn0005636*  
*FBgn0005638*  
*FBgn0005640*  
FBgn0005642  
FBgn0005648  
FBgn0005649  
*FBgn0005655*  
*FBgn0005658*  
FBgn0005659  
*FBgn0005660*  
FBgn0005664  
*FBgn0005670*  
FBgn0005671  
*FBgn0005672*  
*FBgn0005677*  
FBgn0005683  
FBgn0005694  
*FBgn0005695*  
*FBgn0005771*  
*FBgn0005775*  
FBgn0005777  
FBgn0005778  
FBgn0005779  
*FBgn0008635*  
*FBgn0008636*  
FBgn0008646  
FBgn0008649  
FBgn0008651  
*FBgn0010014*  
*FBgn0010015*  
*FBgn0010019*  
*FBgn0010038*  
*FBgn0010039*  
FBgn0010040  
FBgn0010041  
FBgn0010042  
FBgn0010043  
FBgn0010044  
FBgn0010052  
FBgn0010053  
*FBgn0010078*  
FBgn0010097  
*FBgn0010100*  
FBgn0010105  
FBgn0010109  
*FBgn0010113*  
*FBgn0010114*  
FBgn0010194  
FBgn0010197  
*FBgn0010213*  
FBgn0010220  
FBgn0010222  
*FBgn0010223*  
*FBgn0010225*  
*FBgn0010226*  
*FBgn0010228*  
FBgn0010235  
FBgn0010238  
FBgn0010240  
*FBgn0010241*  
*FBgn0010246*  
*FBgn0010256*  
*FBgn0010258*  
FBgn0010263  
*FBgn0010265*  
FBgn0010269  
FBgn0010278  
FBgn0010280  
FBgn0010282  
FBgn0010287  
*FBgn0010288*  
*FBgn0010292*  
FBgn0010294  
FBgn0010295  
FBgn0010296  
FBgn0010300  
FBgn0010303  
FBgn0010309  
FBgn0010313  
FBgn0010314  
FBgn0010315  
*FBgn0010316*  
FBgn0010317  
FBgn0010323  
*FBgn0010328*  
FBgn0010329  
FBgn0010333  
FBgn0010339  
FBgn0010340  
FBgn0010341  
FBgn0010342  
*FBgn0010348*  
FBgn0010350  
*FBgn0010352*  
FBgn0010355  
FBgn0010356  
FBgn0010357  
*FBgn0010379*  
*FBgn0010380*  
*FBgn0010381*  
*FBgn0010382*  
*FBgn0010383*  
*FBgn0010385*  
*FBgn0010387*  
FBgn0010388  
*FBgn0010389*  
*FBgn0010397*  
*FBgn0010398*  
*FBgn0010399*  
FBgn0010406  
*FBgn0010407*  
*FBgn0010408*  
*FBgn0010409*  
*FBgn0010411*  
*FBgn0010412*  
*FBgn0010414*  
FBgn0010415  
FBgn0010416  
FBgn0010417  
FBgn0010421  
FBgn0010422  
*FBgn0010423*  
*FBgn0010424*  
FBgn0010425  
FBgn0010431  
FBgn0010433  
FBgn0010434  
*FBgn0010435*  
FBgn0010438  
*FBgn0010441*  
*FBgn0010452*  
FBgn0010453  
*FBgn0010470*  
FBgn0010473  
FBgn0010488  
FBgn0010504  
*FBgn0010516*  
FBgn0010520  
*FBgn0010531*  
*FBgn0010575*  
FBgn0010583  
*FBgn0010590*  
*FBgn0010591*  
FBgn0010602  
*FBgn0010609*  
*FBgn0010611*  
*FBgn0010620*  
FBgn0010621  
*FBgn0010638*  
*FBgn0010660*  
FBgn0010709  
*FBgn0010747*  
*FBgn0010750*  
*FBgn0010762*  
FBgn0010768  
*FBgn0010770*  
FBgn0010772  
FBgn0010774  
FBgn0010812  
FBgn0010825  
FBgn0010894  
FBgn0010905  
FBgn0010909  
FBgn0011016  
FBgn0011202  
FBgn0011204  
FBgn0011205  
*FBgn0011206*  
FBgn0011207  
*FBgn0011211*  
FBgn0011217  
FBgn0011224  
FBgn0011225  
FBgn0011227  
*FBgn0011230*  
FBgn0011232  
FBgn0011236  
*FBgn0011241*  
*FBgn0011244*  
FBgn0011270  
*FBgn0011272*  
FBgn0011273  
FBgn0011274  
FBgn0011276  
FBgn0011277  
FBgn0011278  
*FBgn0011279*  
*FBgn0011280*  
*FBgn0011281*  
FBgn0011282  
FBgn0011283  
*FBgn0011284*  
FBgn0011285  
*FBgn0011288*  
FBgn0011290  
*FBgn0011293*  
FBgn0011294  
*FBgn0011300*  
FBgn0011305  
*FBgn0011336*  
*FBgn0011361*  
*FBgn0011481*  
FBgn0011509  
FBgn0011554  
FBgn0011555  
FBgn0011556  
*FBgn0011559*  
*FBgn0011566*  
FBgn0011569  
FBgn0011570  
FBgn0011571  
*FBgn0011573*  
FBgn0011576  
*FBgn0011577*  
*FBgn0011581*  
*FBgn0011582*  
FBgn0011584  
*FBgn0011589*  
*FBgn0011591*  
*FBgn0011592*  
*FBgn0011596*  
*FBgn0011603*  
FBgn0011604  
*FBgn0011606*  
FBgn0011638  
FBgn0011640  
FBgn0011648  
FBgn0011653  
*FBgn0011655*  
FBgn0011656  
FBgn0011659  
*FBgn0011660*  
*FBgn0011661*  
*FBgn0011666*  
FBgn0011668  
FBgn0011670  
FBgn0011672  
*FBgn0011674*  
FBgn0011676  
*FBgn0011692*  
*FBgn0011693*  
FBgn0011694  
*FBgn0011695*  
*FBgn0011701*  
*FBgn0011703*  
*FBgn0011704*  
*FBgn0011705*  
FBgn0011706  
FBgn0011708  
FBgn0011710  
FBgn0011715  
FBgn0011722  
FBgn0011723  
*FBgn0011725*  
*FBgn0011726*  
FBgn0011737  
*FBgn0011739*  
FBgn0011741  
FBgn0011742  
FBgn0011743  
FBgn0011744  
*FBgn0011745*  
*FBgn0011746*  
FBgn0011754  
*FBgn0011760*  
*FBgn0011761*  
FBgn0011763  
FBgn0011764  
*FBgn0011768*  
FBgn0011769  
*FBgn0011770*  
*FBgn0011771*  
FBgn0011774  
FBgn0011785  
*FBgn0011787*  
*FBgn0011802*  
FBgn0011817  
FBgn0011818  
FBgn0011822  
*FBgn0011823*  
FBgn0011824  
*FBgn0011828*  
FBgn0011829  
FBgn0011832  
*FBgn0011834*  
*FBgn0011836*  
FBgn0011837  
FBgn0012034  
*FBgn0012036*  
FBgn0012037  
FBgn0012042  
FBgn0012049  
FBgn0012051  
FBgn0012058  
*FBgn0012344*  
FBgn0013263  
FBgn0013272  
*FBgn0013279*  
FBgn0013300  
FBgn0013301  
*FBgn0013303*  
*FBgn0013305*  
*FBgn0013307*  
*FBgn0013308*  
FBgn0013323  
*FBgn0013325*  
FBgn0013334  
*FBgn0013348*  
FBgn0013432  
FBgn0013435  
*FBgn0013442*  
*FBgn0013467*  
FBgn0013469  
FBgn0013531  
FBgn0013563  
*FBgn0013717*  
*FBgn0013718*  
*FBgn0013720*  
FBgn0013725  
*FBgn0013726*  
*FBgn0013732*  
FBgn0013733  
*FBgn0013745*  
*FBgn0013746*  
FBgn0013750  
*FBgn0013751*  
FBgn0013753  
*FBgn0013756*  
*FBgn0013759*  
FBgn0013762  
*FBgn0013763*  
FBgn0013764  
FBgn0013765  
FBgn0013767  
FBgn0013770  
*FBgn0013771*  
*FBgn0013772*  
*FBgn0013773*  
FBgn0013799  
FBgn0013809  
FBgn0013810  
*FBgn0013811*  
FBgn0013812  
FBgn0013813  
*FBgn0013948*  
FBgn0013949  
*FBgn0013953*  
*FBgn0013955*  
FBgn0013972  
FBgn0013973  
FBgn0013981  
FBgn0013983  
FBgn0013984  
*FBgn0013995*  
FBgn0013998  
*FBgn0014000*  
FBgn0014001  
FBgn0014002  
FBgn0014006  
FBgn0014007  
FBgn0014009  
*FBgn0014010*  
*FBgn0014011*  
FBgn0014018  
*FBgn0014019*  
FBgn0014020  
FBgn0014022  
FBgn0014023  
FBgn0014024  
*FBgn0014026*  
*FBgn0014028*  
FBgn0014029  
*FBgn0014031*  
FBgn0014032  
*FBgn0014073*  
*FBgn0014075*  
*FBgn0014076*  
FBgn0014092  
FBgn0014127  
FBgn0014133  
FBgn0014135  
*FBgn0014141*  
FBgn0014143  
*FBgn0014163*  
FBgn0014179  
FBgn0014184  
FBgn0014189  
FBgn0014269  
*FBgn0014340*  
*FBgn0014342*  
FBgn0014343  
FBgn0014362  
FBgn0014366  
FBgn0014368  
FBgn0014380  
FBgn0014388  
*FBgn0014391*  
*FBgn0014395*  
*FBgn0014396*  
FBgn0014411  
FBgn0014417  
*FBgn0014427*  
*FBgn0014455*  
FBgn0014464  
FBgn0014465  
FBgn0014466  
*FBgn0014469*  
FBgn0014848  
FBgn0014849  
FBgn0014850  
FBgn0014851  
FBgn0014859  
*FBgn0014861*  
*FBgn0014863*  
*FBgn0014865*  
FBgn0014868  
FBgn0014869  
*FBgn0014870*  
*FBgn0014877*  
*FBgn0014879*  
*FBgn0014906*  
FBgn0014930  
FBgn0014931  
*FBgn0014949*  
*FBgn0015001*  
FBgn0015008  
*FBgn0015010*  
*FBgn0015011*  
FBgn0015014  
FBgn0015019  
FBgn0015024  
*FBgn0015031*  
FBgn0015032  
*FBgn0015033*  
*FBgn0015034*  
FBgn0015035  
FBgn0015036  
*FBgn0015037*  
*FBgn0015038*  
*FBgn0015039*  
*FBgn0015040*  
FBgn0015075  
FBgn0015129  
FBgn0015221  
FBgn0015222  
*FBgn0015229*  
FBgn0015230  
FBgn0015239  
FBgn0015240  
*FBgn0015245*  
*FBgn0015247*  
*FBgn0015268*  
FBgn0015269  
FBgn0015270  
FBgn0015271  
*FBgn0015277*  
FBgn0015278  
FBgn0015279  
FBgn0015283  
*FBgn0015286*  
*FBgn0015288*  
FBgn0015295  
FBgn0015296  
*FBgn0015298*  
FBgn0015300  
FBgn0015316  
FBgn0015320  
FBgn0015321  
FBgn0015323  
FBgn0015324  
FBgn0015331  
FBgn0015336  
FBgn0015338  
FBgn0015359  
FBgn0015360  
*FBgn0015371*  
*FBgn0015372*  
FBgn0015374  
*FBgn0015376*  
FBgn0015379  
*FBgn0015380*  
FBgn0015381  
FBgn0015391  
*FBgn0015396*  
FBgn0015399  
FBgn0015400  
FBgn0015402  
FBgn0015509  
FBgn0015513  
*FBgn0015521*  
FBgn0015524  
*FBgn0015541*  
FBgn0015542  
FBgn0015544  
FBgn0015546  
*FBgn0015550*  
FBgn0015553  
*FBgn0015558*  
FBgn0015561  
FBgn0015562  
*FBgn0015565*  
*FBgn0015582*  
*FBgn0015583*  
FBgn0015584  
*FBgn0015585*  
*FBgn0015586*  
FBgn0015589  
*FBgn0015591*  
FBgn0015600  
FBgn0015609  
FBgn0015610  
*FBgn0015614*  
FBgn0015615  
FBgn0015617  
FBgn0015618  
FBgn0015621  
*FBgn0015622*  
*FBgn0015623*  
*FBgn0015625*  
*FBgn0015663*  
*FBgn0015664*  
*FBgn0015714*  
FBgn0015737  
*FBgn0015756*  
FBgn0015765  
FBgn0015770  
FBgn0015772  
*FBgn0015773*  
*FBgn0015774*  
*FBgn0015776*  
*FBgn0015777*  
FBgn0015778  
FBgn0015781  
*FBgn0015789*  
*FBgn0015790*  
FBgn0015791  
*FBgn0015795*  
*FBgn0015797*  
FBgn0015799  
FBgn0015803  
*FBgn0015805*  
FBgn0015806  
FBgn0015808  
FBgn0015816  
FBgn0015818  
FBgn0015828  
FBgn0015829  
FBgn0015831  
FBgn0015834  
FBgn0015838  
*FBgn0015872*  
FBgn0015903  
FBgn0015904  
FBgn0015905  
*FBgn0015907*  
FBgn0015919  
FBgn0015924  
FBgn0015925  
*FBgn0015926*  
FBgn0015929  
FBgn0015946  
*FBgn0015949*  
*FBgn0016013*  
FBgn0016031  
FBgn0016032  
FBgn0016034  
FBgn0016041  
FBgn0016047  
FBgn0016061  
FBgn0016070  
FBgn0016075  
FBgn0016076  
*FBgn0016078*  
FBgn0016081  
*FBgn0016122*  
*FBgn0016131*  
*FBgn0016650*  
FBgn0016660  
*FBgn0016672*  
*FBgn0016685*  
*FBgn0016691*  
FBgn0016693  
FBgn0016694  
FBgn0016696  
*FBgn0016697*  
FBgn0016698  
FBgn0016700  
FBgn0016701  
*FBgn0016726*  
*FBgn0016754*  
FBgn0016756  
*FBgn0016762*  
FBgn0016792  
FBgn0016794  
FBgn0016797  
*FBgn0016917*  
*FBgn0016919*  
*FBgn0016920*  
FBgn0016926  
*FBgn0016930*  
FBgn0016977  
FBgn0016983  
*FBgn0016984*  
*FBgn0017397*  
FBgn0017414  
*FBgn0017429*  
FBgn0017448  
FBgn0017453  
FBgn0017456  
FBgn0017457  
*FBgn0017482*  
FBgn0017549  
FBgn0017550  
*FBgn0017551*  
FBgn0017558  
*FBgn0017561*  
FBgn0017566  
*FBgn0017567*  
FBgn0017572  
*FBgn0017577*  
FBgn0017578  
*FBgn0017579*  
*FBgn0017581*  
*FBgn0017590*  
*FBgn0019624*  
FBgn0019637  
FBgn0019643  
*FBgn0019662*  
FBgn0019686  
FBgn0019809  
FBgn0019828  
FBgn0019830  
*FBgn0019886*  
FBgn0019890  
FBgn0019925  
*FBgn0019928*  
FBgn0019929  
FBgn0019932  
FBgn0019938  
FBgn0019940  
FBgn0019947  
*FBgn0019948*  
FBgn0019949  
FBgn0019952  
FBgn0019957  
FBgn0019960  
FBgn0019972  
FBgn0019982  
FBgn0019990  
FBgn0020018  
FBgn0020224  
*FBgn0020236*  
FBgn0020240  
*FBgn0020245*  
*FBgn0020248*  
*FBgn0020249*  
FBgn0020251  
FBgn0020255  
FBgn0020257  
FBgn0020258  
FBgn0020261  
FBgn0020269  
FBgn0020270  
*FBgn0020272*  
*FBgn0020277*  
FBgn0020278  
FBgn0020279  
FBgn0020280  
*FBgn0020294*  
FBgn0020299  
*FBgn0020300*  
*FBgn0020303*  
FBgn0020304  
FBgn0020305  
FBgn0020306  
*FBgn0020307*  
FBgn0020309  
FBgn0020312  
*FBgn0020369*  
*FBgn0020370*  
FBgn0020371  
FBgn0020372  
FBgn0020378  
FBgn0020379  
FBgn0020381  
FBgn0020385  
*FBgn0020386*  
FBgn0020388  
FBgn0020389  
*FBgn0020391*  
*FBgn0020392*  
*FBgn0020399*  
*FBgn0020407*  
FBgn0020414  
FBgn0020415  
FBgn0020416  
FBgn0020429  
*FBgn0020439*  
FBgn0020440  
FBgn0020443  
*FBgn0020445*  
FBgn0020493  
FBgn0020496  
*FBgn0020497*  
FBgn0020506  
*FBgn0020508*  
*FBgn0020509*  
FBgn0020510  
*FBgn0020513*  
FBgn0020521  
FBgn0020545  
FBgn0020616  
FBgn0020617  
FBgn0020618  
FBgn0020621  
FBgn0020622  
*FBgn0020623*  
FBgn0020626  
*FBgn0020633*  
FBgn0020637  
FBgn0020639  
FBgn0020640  
FBgn0020641  
FBgn0020642  
FBgn0020643  
FBgn0020645  
FBgn0020647  
FBgn0020655  
FBgn0020762  
*FBgn0020764*  
FBgn0020765  
*FBgn0020767*  
*FBgn0020880*  
FBgn0020906  
*FBgn0020909*  
FBgn0020910  
*FBgn0020912*  
FBgn0020930  
FBgn0021742  
*FBgn0021760*  
FBgn0021761  
FBgn0021764  
*FBgn0021765*  
FBgn0021768  
FBgn0021776  
*FBgn0021795*  
FBgn0021796  
FBgn0021814  
FBgn0021818  
FBgn0021825  
*FBgn0021872*  
FBgn0021873  
FBgn0021874  
FBgn0021875  
FBgn0021895  
*FBgn0021906*  
FBgn0021944  
*FBgn0021953*  
*FBgn0021967*  
FBgn0021995  
*FBgn0022027*  
FBgn0022063  
FBgn0022085  
FBgn0022201  
*FBgn0022213*  
FBgn0022224  
FBgn0022238  
FBgn0022246  
FBgn0022268  
FBgn0022338  
FBgn0022343  
*FBgn0022344*  
*FBgn0022349*  
*FBgn0022355*  
FBgn0022699  
FBgn0022700  
FBgn0022702  
*FBgn0022708*  
*FBgn0022709*  
FBgn0022710  
FBgn0022720  
FBgn0022724  
*FBgn0022740*  
FBgn0022764  
FBgn0022768  
*FBgn0022772*  
FBgn0022774  
FBgn0022786  
FBgn0022787  
*FBgn0022800*  
FBgn0022893  
FBgn0022935  
FBgn0022936  
FBgn0022942  
FBgn0022943  
*FBgn0022959*  
FBgn0022960  
*FBgn0022981*  
FBgn0022987  
*FBgn0023000*  
FBgn0023001  
*FBgn0023023*  
*FBgn0023076*  
FBgn0023081  
FBgn0023083  
FBgn0023090  
FBgn0023091  
FBgn0023094  
*FBgn0023095*  
FBgn0023096  
FBgn0023097  
*FBgn0023129*  
*FBgn0023130*  
*FBgn0023143*  
*FBgn0023167*  
FBgn0023169  
*FBgn0023170*  
FBgn0023171  
*FBgn0023172*  
*FBgn0023174*  
*FBgn0023175*  
*FBgn0023178*  
*FBgn0023179*  
*FBgn0023180*  
FBgn0023181  
*FBgn0023197*  
*FBgn0023214*  
FBgn0023215  
FBgn0023216  
FBgn0023388  
*FBgn0023395*  
*FBgn0023407*  
FBgn0023415  
FBgn0023416  
FBgn0023423  
FBgn0023441  
FBgn0023444  
*FBgn0023477*  
FBgn0023479  
FBgn0023489  
FBgn0023495  
FBgn0023496  
FBgn0023506  
FBgn0023507  
FBgn0023508  
FBgn0023509  
FBgn0023511  
*FBgn0023513*  
FBgn0023514  
*FBgn0023515*  
FBgn0023516  
FBgn0023517  
*FBgn0023518*  
FBgn0023519  
FBgn0023520  
FBgn0023521  
FBgn0023522  
*FBgn0023523*  
*FBgn0023524*  
FBgn0023525  
FBgn0023526  
FBgn0023527  
FBgn0023528  
FBgn0023529  
FBgn0023530  
FBgn0023531  
*FBgn0023534*  
*FBgn0023535*  
FBgn0023536  
*FBgn0023537*  
*FBgn0023540*  
FBgn0023541  
FBgn0023542  
FBgn0023545  
FBgn0023546  
*FBgn0023549*  
FBgn0023550  
*FBgn0024150*  
*FBgn0024177*  
*FBgn0024179*  
FBgn0024183  
FBgn0024187  
FBgn0024188  
FBgn0024189  
FBgn0024191  
FBgn0024194  
*FBgn0024196*  
FBgn0024222  
*FBgn0024227*  
FBgn0024230  
FBgn0024232  
FBgn0024234  
FBgn0024236  
FBgn0024238  
FBgn0024244  
*FBgn0024245*  
FBgn0024248  
FBgn0024249  
*FBgn0024252*  
FBgn0024273  
*FBgn0024277*  
FBgn0024285  
FBgn0024288  
*FBgn0024290*  
FBgn0024291  
*FBgn0024293*  
*FBgn0024294*  
*FBgn0024308*  
FBgn0024314  
*FBgn0024315*  
FBgn0024319  
FBgn0024320  
FBgn0024326  
FBgn0024329  
FBgn0024330  
*FBgn0024332*  
FBgn0024352  
FBgn0024360  
*FBgn0024361*  
*FBgn0024362*  
FBgn0024364  
FBgn0024365  
*FBgn0024366*  
FBgn0024371  
*FBgn0024432*  
FBgn0024491  
*FBgn0024509*  
*FBgn0024510*  
FBgn0024542  
FBgn0024555  
FBgn0024556  
FBgn0024558  
FBgn0024689  
*FBgn0024698*  
FBgn0024733  
FBgn0024740  
FBgn0024754  
FBgn0024806  
FBgn0024807  
*FBgn0024814*  
*FBgn0024836*  
*FBgn0024841*  
FBgn0024846  
FBgn0024887  
FBgn0024891  
FBgn0024897  
*FBgn0024909*  
*FBgn0024912*  
*FBgn0024920*  
FBgn0024921  
*FBgn0024941*  
*FBgn0024943*  
FBgn0024944  
FBgn0024945  
*FBgn0024947*  
FBgn0024956  
FBgn0024963  
FBgn0024973  
FBgn0024975  
*FBgn0024977*  
FBgn0024980  
*FBgn0024983*  
FBgn0024984  
FBgn0024985  
FBgn0024986  
FBgn0024987  
FBgn0024989  
FBgn0024991  
*FBgn0024992*  
*FBgn0024993*  
*FBgn0024994*  
*FBgn0024995*  
*FBgn0024997*  
FBgn0024998  
FBgn0025109  
*FBgn0025111*  
FBgn0025115  
*FBgn0025117*  
FBgn0025140  
FBgn0025185  
*FBgn0025286*  
*FBgn0025334*  
FBgn0025335  
FBgn0025336  
FBgn0025352  
FBgn0025355  
FBgn0025360  
*FBgn0025366*  
*FBgn0025373*  
FBgn0025378  
*FBgn0025381*  
FBgn0025382  
FBgn0025383  
FBgn0025387  
FBgn0025388  
FBgn0025390  
FBgn0025391  
FBgn0025393  
FBgn0025394  
*FBgn0025454*  
*FBgn0025455*  
FBgn0025456  
*FBgn0025457*  
*FBgn0025458*  
FBgn0025463  
FBgn0025469  
FBgn0025519  
*FBgn0025525*  
FBgn0025558  
FBgn0025571  
FBgn0025573  
*FBgn0025574*  
*FBgn0025578*  
FBgn0025582  
FBgn0025583  
FBgn0025592  
FBgn0025595  
FBgn0025608  
FBgn0025612  
FBgn0025613  
FBgn0025615  
FBgn0025616  
*FBgn0025620*  
FBgn0025621  
FBgn0025624  
FBgn0025625  
*FBgn0025626*  
FBgn0025627  
FBgn0025628  
FBgn0025629  
*FBgn0025630*  
*FBgn0025631*  
FBgn0025632  
FBgn0025633  
FBgn0025634  
*FBgn0025635*  
*FBgn0025637*  
FBgn0025638  
FBgn0025640  
*FBgn0025641*  
*FBgn0025642*  
FBgn0025643  
FBgn0025644  
FBgn0025645  
FBgn0025674  
*FBgn0025678*  
FBgn0025679  
*FBgn0025680*  
FBgn0025681  
FBgn0025682  
FBgn0025683  
FBgn0025684  
*FBgn0025686*  
FBgn0025687  
*FBgn0025693*  
FBgn0025700  
FBgn0025701  
*FBgn0025702*  
*FBgn0025709*  
*FBgn0025712*  
FBgn0025716  
FBgn0025720  
*FBgn0025725*  
FBgn0025739  
FBgn0025742  
FBgn0025743  
FBgn0025776  
FBgn0025777  
*FBgn0025781*  
*FBgn0025790*  
FBgn0025800  
*FBgn0025802*  
FBgn0025803  
FBgn0025806  
*FBgn0025807*  
FBgn0025808  
*FBgn0025814*  
FBgn0025815  
FBgn0025820  
FBgn0025825  
FBgn0025827  
FBgn0025830  
FBgn0025832  
*FBgn0025833*  
FBgn0025835  
*FBgn0025836*  
*FBgn0025837*  
FBgn0025838  
*FBgn0025839*  
FBgn0025864  
FBgn0025865  
FBgn0025866  
FBgn0025874  
*FBgn0025878*  
*FBgn0025879*  
FBgn0025885  
FBgn0026015  
FBgn0026056  
FBgn0026058  
FBgn0026059  
*FBgn0026061*  
FBgn0026063  
FBgn0026064  
*FBgn0026076*  
*FBgn0026077*  
FBgn0026079  
FBgn0026080  
FBgn0026083  
*FBgn0026084*  
FBgn0026085  
FBgn0026086  
FBgn0026087  
FBgn0026088  
FBgn0026089  
FBgn0026090  
FBgn0026136  
FBgn0026141  
FBgn0026143  
*FBgn0026144*  
FBgn0026147  
FBgn0026148  
*FBgn0026150*  
FBgn0026160  
FBgn0026170  
FBgn0026174  
*FBgn0026176*  
FBgn0026179  
*FBgn0026181*  
FBgn0026190  
FBgn0026196  
*FBgn0026197*  
FBgn0026207  
*FBgn0026208*  
FBgn0026238  
FBgn0026239  
FBgn0026252  
FBgn0026255  
FBgn0026257  
FBgn0026259  
FBgn0026261  
FBgn0026263  
FBgn0026268  
FBgn0026309  
FBgn0026313  
*FBgn0026314*  
*FBgn0026315*  
FBgn0026316  
FBgn0026317  
FBgn0026318  
FBgn0026319  
FBgn0026320  
FBgn0026323  
FBgn0026324  
FBgn0026326  
*FBgn0026361*  
FBgn0026369  
*FBgn0026370*  
*FBgn0026371*  
*FBgn0026372*  
FBgn0026373  
*FBgn0026374*  
FBgn0026375  
FBgn0026376  
*FBgn0026378*  
FBgn0026379  
FBgn0026380  
FBgn0026384  
FBgn0026385  
FBgn0026386  
FBgn0026388  
FBgn0026389  
FBgn0026390  
FBgn0026391  
FBgn0026392  
*FBgn0026393*  
FBgn0026394  
FBgn0026395  
FBgn0026396  
FBgn0026397  
FBgn0026398  
FBgn0026400  
FBgn0026402  
*FBgn0026403*  
FBgn0026404  
*FBgn0026409*  
FBgn0026411  
*FBgn0026415*  
*FBgn0026418*  
*FBgn0026428*  
FBgn0026430  
FBgn0026431  
*FBgn0026432*  
FBgn0026433  
*FBgn0026438*  
*FBgn0026439*  
FBgn0026441  
FBgn0026479  
*FBgn0026533*  
FBgn0026563  
*FBgn0026565*  
FBgn0026566  
FBgn0026570  
FBgn0026573  
FBgn0026575  
FBgn0026576  
FBgn0026577  
*FBgn0026582*  
FBgn0026585  
*FBgn0026592*  
*FBgn0026593*  
FBgn0026597  
*FBgn0026598*  
*FBgn0026602*  
FBgn0026611  
FBgn0026619  
FBgn0026620  
FBgn0026630  
FBgn0026634  
FBgn0026718  
FBgn0026722  
FBgn0026737  
FBgn0026738  
FBgn0026741  
FBgn0026749  
FBgn0026751  
*FBgn0026753*  
*FBgn0026754*  
FBgn0026755  
FBgn0026756  
FBgn0026760  
FBgn0026761  
FBgn0026787  
FBgn0026872  
*FBgn0026873*  
FBgn0026874  
FBgn0026876  
*FBgn0026878*  
FBgn0026879  
FBgn0027052  
FBgn0027053  
FBgn0027054  
FBgn0027055  
FBgn0027057  
FBgn0027066  
FBgn0027070  
FBgn0027073  
*FBgn0027074*  
FBgn0027095  
FBgn0027103  
*FBgn0027106*  
FBgn0027107  
FBgn0027108  
FBgn0027109  
*FBgn0027111*  
*FBgn0027259*  
FBgn0027287  
FBgn0027335  
*FBgn0027339*  
FBgn0027342  
*FBgn0027343*  
*FBgn0027348*  
*FBgn0027356*  
FBgn0027359  
FBgn0027360  
*FBgn0027363*  
FBgn0027364  
FBgn0027375  
FBgn0027376  
FBgn0027378  
*FBgn0027453*  
FBgn0027490  
*FBgn0027491*  
FBgn0027492  
FBgn0027493  
FBgn0027494  
*FBgn0027495*  
FBgn0027496  
FBgn0027497  
*FBgn0027498*  
FBgn0027499  
FBgn0027503  
FBgn0027504  
*FBgn0027506*  
FBgn0027507  
FBgn0027508  
FBgn0027509  
*FBgn0027512*  
*FBgn0027513*  
*FBgn0027514*  
FBgn0027515  
FBgn0027518  
*FBgn0027521*  
FBgn0027524  
FBgn0027525  
FBgn0027526  
FBgn0027527  
FBgn0027528  
FBgn0027529  
FBgn0027532  
*FBgn0027534*  
FBgn0027535  
FBgn0027537  
FBgn0027538  
*FBgn0027539*  
*FBgn0027544*  
*FBgn0027546*  
FBgn0027547  
FBgn0027548  
FBgn0027549  
FBgn0027550  
*FBgn0027552*  
FBgn0027554  
*FBgn0027556*  
*FBgn0027558*  
*FBgn0027560*  
FBgn0027561  
FBgn0027562  
FBgn0027563  
FBgn0027564  
FBgn0027565  
FBgn0027567  
FBgn0027568  
*FBgn0027569*  
FBgn0027570  
*FBgn0027571*  
FBgn0027572  
FBgn0027574  
*FBgn0027578*  
*FBgn0027579*  
FBgn0027580  
FBgn0027581  
*FBgn0027582*  
FBgn0027583  
*FBgn0027584*  
FBgn0027585  
*FBgn0027586*  
FBgn0027587  
FBgn0027588  
*FBgn0027589*  
*FBgn0027590*  
*FBgn0027592*  
*FBgn0027594*  
*FBgn0027596*  
FBgn0027597  
*FBgn0027598*  
FBgn0027599  
*FBgn0027601*  
FBgn0027602  
FBgn0027603  
*FBgn0027604*  
FBgn0027605  
FBgn0027607  
FBgn0027608  
FBgn0027609  
FBgn0027610  
*FBgn0027611*  
FBgn0027615  
FBgn0027617  
*FBgn0027619*  
FBgn0027620  
FBgn0027621  
FBgn0027654  
*FBgn0027655*  
*FBgn0027657*  
FBgn0027660  
*FBgn0027779*  
FBgn0027780  
FBgn0027783  
FBgn0027784  
*FBgn0027786*  
FBgn0027788  
*FBgn0027790*  
FBgn0027793  
FBgn0027794  
FBgn0027795  
FBgn0027796  
FBgn0027835  
*FBgn0027843*  
FBgn0027844  
FBgn0027864  
FBgn0027865  
FBgn0027866  
FBgn0027868  
*FBgn0027872*  
FBgn0027873  
FBgn0027885  
FBgn0027889  
FBgn0027903  
*FBgn0027914*  
FBgn0027929  
*FBgn0027930*  
*FBgn0027932*  
FBgn0027936  
FBgn0027945  
*FBgn0027948*  
*FBgn0027949*  
FBgn0028274  
FBgn0028292  
FBgn0028369  
FBgn0028370  
*FBgn0028371*  
*FBgn0028373*  
*FBgn0028374*  
FBgn0028375  
*FBgn0028379*  
*FBgn0028380*  
*FBgn0028381*  
*FBgn0028382*  
*FBgn0028387*  
*FBgn0028394*  
*FBgn0028396*  
*FBgn0028397*  
FBgn0028398  
*FBgn0028399*  
*FBgn0028400*  
FBgn0028401  
FBgn0028402  
FBgn0028410  
FBgn0028411  
FBgn0028412  
FBgn0028418  
FBgn0028421  
FBgn0028422  
FBgn0028427  
FBgn0028428  
FBgn0028430  
*FBgn0028433*  
FBgn0028434  
FBgn0028436  
*FBgn0028467*  
FBgn0028468  
FBgn0028471  
*FBgn0028473*  
FBgn0028474  
FBgn0028475  
*FBgn0028476*  
*FBgn0028479*  
*FBgn0028480*  
*FBgn0028481*  
FBgn0028482  
FBgn0028484  
*FBgn0028490*  
*FBgn0028491*  
*FBgn0028494*  
*FBgn0028496*  
FBgn0028497  
FBgn0028499  
FBgn0028500  
*FBgn0028504*  
FBgn0028506  
FBgn0028507  
FBgn0028509  
*FBgn0028513*  
FBgn0028514  
*FBgn0028515*  
*FBgn0028516*  
FBgn0028518  
FBgn0028519  
FBgn0028520  
FBgn0028523  
*FBgn0028526*  
*FBgn0028527*  
FBgn0028530  
FBgn0028531  
FBgn0028532  
FBgn0028533  
FBgn0028534  
*FBgn0028537*  
FBgn0028538  
FBgn0028539  
FBgn0028540  
FBgn0028541  
FBgn0028542  
FBgn0028543  
FBgn0028544  
*FBgn0028546*  
*FBgn0028550*  
*FBgn0028552*  
FBgn0028554  
*FBgn0028560*  
FBgn0028561  
FBgn0028562  
FBgn0028563  
FBgn0028567  
FBgn0028569  
FBgn0028570  
FBgn0028572  
FBgn0028573  
FBgn0028577  
FBgn0028579  
*FBgn0028582*  
FBgn0028642  
*FBgn0028646*  
FBgn0028647  
FBgn0028648  
*FBgn0028658*  
FBgn0028675  
FBgn0028683  
FBgn0028685  
*FBgn0028686*  
*FBgn0028687*  
FBgn0028688  
*FBgn0028689*  
*FBgn0028690*  
FBgn0028691  
*FBgn0028692*  
FBgn0028693  
FBgn0028694  
FBgn0028695  
*FBgn0028699*  
FBgn0028700  
*FBgn0028703*  
FBgn0028704  
FBgn0028707  
FBgn0028708  
FBgn0028717  
*FBgn0028734*  
*FBgn0028737*  
FBgn0028738  
FBgn0028740  
*FBgn0028743*  
FBgn0028744  
FBgn0028746  
FBgn0028789  
*FBgn0028836*  
FBgn0028837  
FBgn0028838  
*FBgn0028841*  
FBgn0028844  
FBgn0028847  
FBgn0028848  
FBgn0028850  
FBgn0028852  
FBgn0028853  
FBgn0028855  
FBgn0028856  
FBgn0028857  
FBgn0028858  
*FBgn0028862*  
*FBgn0028863*  
FBgn0028866  
FBgn0028868  
FBgn0028870  
FBgn0028871  
FBgn0028872  
*FBgn0028878*  
*FBgn0028879*  
FBgn0028880  
FBgn0028884  
*FBgn0028886*  
FBgn0028887  
FBgn0028888  
FBgn0028892  
FBgn0028893  
FBgn0028894  
FBgn0028895  
FBgn0028896  
FBgn0028897  
FBgn0028899  
FBgn0028900  
FBgn0028901  
FBgn0028903  
FBgn0028904  
FBgn0028905  
FBgn0028906  
FBgn0028913  
FBgn0028916  
*FBgn0028917*  
FBgn0028919  
FBgn0028920  
FBgn0028926  
*FBgn0028931*  
FBgn0028932  
FBgn0028935  
FBgn0028936  
FBgn0028938  
FBgn0028939  
*FBgn0028940*  
FBgn0028941  
FBgn0028942  
FBgn0028943  
*FBgn0028944*  
FBgn0028945  
FBgn0028946  
FBgn0028947  
FBgn0028948  
FBgn0028949  
*FBgn0028950*  
FBgn0028952  
FBgn0028953  
FBgn0028954  
FBgn0028955  
FBgn0028956  
FBgn0028961  
*FBgn0028963*  
FBgn0028965  
*FBgn0028968*  
FBgn0028969  
FBgn0028978  
FBgn0028979  
*FBgn0028980*  
FBgn0028982  
FBgn0028983  
FBgn0028984  
*FBgn0028985*  
*FBgn0028986*  
*FBgn0028987*  
*FBgn0028988*  
FBgn0028990  
FBgn0028991  
FBgn0028992  
*FBgn0029002*  
*FBgn0029006*  
FBgn0029079  
FBgn0029082  
FBgn0029084  
FBgn0029088  
FBgn0029090  
*FBgn0029093*  
*FBgn0029094*  
FBgn0029095  
FBgn0029113  
*FBgn0029114*  
FBgn0029117  
FBgn0029118  
FBgn0029121  
*FBgn0029123*  
FBgn0029128  
FBgn0029131  
*FBgn0029133*  
*FBgn0029134*  
FBgn0029137  
*FBgn0029147*  
FBgn0029148  
FBgn0029152  
FBgn0029155  
FBgn0029157  
FBgn0029158  
*FBgn0029167*  
FBgn0029170  
*FBgn0029172*  
FBgn0029173  
*FBgn0029174*  
FBgn0029176  
FBgn0029501  
*FBgn0029502*  
FBgn0029503  
FBgn0029506  
FBgn0029507  
FBgn0029508  
FBgn0029512  
FBgn0029514  
FBgn0029521  
FBgn0029522  
*FBgn0029523*  
FBgn0029525  
FBgn0029529  
FBgn0029531  
FBgn0029532  
FBgn0029535  
FBgn0029538  
FBgn0029539  
FBgn0029552  
FBgn0029568  
FBgn0029573  
FBgn0029587  
FBgn0029588  
FBgn0029589  
FBgn0029590  
FBgn0029594  
FBgn0029596  
FBgn0029603  
*FBgn0029606*  
FBgn0029608  
*FBgn0029629*  
*FBgn0029639*  
FBgn0029642  
FBgn0029643  
FBgn0029644  
FBgn0029645  
FBgn0029646  
FBgn0029647  
*FBgn0029648*  
FBgn0029649  
*FBgn0029656*  
FBgn0029657  
FBgn0029658  
FBgn0029659  
FBgn0029660  
FBgn0029661  
FBgn0029662  
*FBgn0029663*  
FBgn0029664  
FBgn0029665  
FBgn0029666  
FBgn0029667  
FBgn0029669  
*FBgn0029672*  
*FBgn0029681*  
FBgn0029685  
FBgn0029686  
FBgn0029688  
FBgn0029689  
FBgn0029690  
FBgn0029692  
FBgn0029693  
FBgn0029694  
FBgn0029696  
FBgn0029697  
FBgn0029700  
FBgn0029701  
FBgn0029702  
FBgn0029703  
FBgn0029704  
*FBgn0029705*  
*FBgn0029706*  
FBgn0029707  
FBgn0029708  
FBgn0029709  
FBgn0029710  
FBgn0029711  
FBgn0029712  
FBgn0029713  
FBgn0029714  
FBgn0029715  
FBgn0029716  
FBgn0029717  
FBgn0029718  
FBgn0029719  
FBgn0029720  
FBgn0029722  
FBgn0029724  
FBgn0029725  
FBgn0029726  
FBgn0029727  
FBgn0029728  
FBgn0029729  
FBgn0029730  
*FBgn0029733*  
FBgn0029736  
FBgn0029737  
FBgn0029738  
FBgn0029740  
FBgn0029745  
FBgn0029746  
FBgn0029747  
FBgn0029748  
FBgn0029750  
FBgn0029751  
*FBgn0029752*  
FBgn0029753  
FBgn0029754  
FBgn0029755  
FBgn0029756  
*FBgn0029761*  
*FBgn0029762*  
FBgn0029763  
FBgn0029764  
*FBgn0029765*  
*FBgn0029766*  
*FBgn0029768*  
*FBgn0029769*  
FBgn0029770  
*FBgn0029771*  
FBgn0029775  
*FBgn0029778*  
FBgn0029783  
*FBgn0029785*  
FBgn0029789  
*FBgn0029791*  
*FBgn0029795*  
FBgn0029798  
*FBgn0029799*  
*FBgn0029801*  
*FBgn0029804*  
FBgn0029807  
FBgn0029809  
*FBgn0029810*  
FBgn0029811  
FBgn0029812  
FBgn0029813  
FBgn0029814  
FBgn0029816  
FBgn0029817  
*FBgn0029818*  
FBgn0029819  
FBgn0029820  
*FBgn0029821*  
*FBgn0029822*  
*FBgn0029823*  
*FBgn0029824*  
*FBgn0029825*  
*FBgn0029826*  
*FBgn0029827*  
*FBgn0029828*  
*FBgn0029830*  
*FBgn0029831*  
FBgn0029833  
FBgn0029834  
FBgn0029835  
*FBgn0029836*  
*FBgn0029837*  
*FBgn0029838*  
FBgn0029839  
FBgn0029840  
FBgn0029843  
*FBgn0029848*  
FBgn0029849  
FBgn0029850  
FBgn0029851  
FBgn0029853  
FBgn0029854  
FBgn0029857  
FBgn0029858  
FBgn0029861  
*FBgn0029863*  
*FBgn0029864*  
*FBgn0029866*  
FBgn0029867  
FBgn0029868  
FBgn0029870  
FBgn0029873  
FBgn0029874  
*FBgn0029877*  
*FBgn0029878*  
*FBgn0029879*  
*FBgn0029880*  
*FBgn0029881*  
FBgn0029882  
FBgn0029885  
FBgn0029887  
*FBgn0029888*  
*FBgn0029890*  
*FBgn0029891*  
FBgn0029892  
FBgn0029893  
FBgn0029894  
*FBgn0029895*  
*FBgn0029896*  
*FBgn0029897*  
*FBgn0029898*  
*FBgn0029899*  
*FBgn0029903*  
*FBgn0029909*  
FBgn0029911  
FBgn0029912  
FBgn0029913  
FBgn0029914  
FBgn0029915  
*FBgn0029922*  
*FBgn0029924*  
FBgn0029925  
*FBgn0029928*  
*FBgn0029929*  
FBgn0029930  
FBgn0029931  
*FBgn0029932*  
FBgn0029935  
FBgn0029936  
FBgn0029937  
FBgn0029939  
FBgn0029940  
*FBgn0029941*  
FBgn0029942  
FBgn0029943  
FBgn0029944  
*FBgn0029945*  
FBgn0029946  
FBgn0029947  
FBgn0029948  
FBgn0029949  
FBgn0029950  
FBgn0029951  
FBgn0029952  
FBgn0029955  
FBgn0029957  
FBgn0029958  
FBgn0029959  
FBgn0029961  
FBgn0029962  
FBgn0029963  
FBgn0029964  
FBgn0029965  
FBgn0029966  
FBgn0029968  
*FBgn0029969*  
*FBgn0029970*  
*FBgn0029971*  
*FBgn0029974*  
*FBgn0029975*  
FBgn0029976  
FBgn0029977  
FBgn0029979  
FBgn0029980  
FBgn0029986  
FBgn0029987  
FBgn0029989  
*FBgn0029990*  
FBgn0029992  
FBgn0029993  
*FBgn0029994*  
*FBgn0029995*  
*FBgn0029997*  
FBgn0029999  
FBgn0030000  
FBgn0030001  
FBgn0030003  
FBgn0030004  
FBgn0030005  
FBgn0030006  
FBgn0030007  
FBgn0030008  
FBgn0030009  
FBgn0030010  
FBgn0030011  
FBgn0030012  
FBgn0030013  
FBgn0030014  
FBgn0030015  
FBgn0030016  
FBgn0030017  
FBgn0030018  
FBgn0030025  
FBgn0030026  
*FBgn0030027*  
*FBgn0030028*  
FBgn0030029  
FBgn0030030  
FBgn0030033  
FBgn0030034  
FBgn0030035  
FBgn0030037  
FBgn0030038  
FBgn0030039  
*FBgn0030040*  
*FBgn0030041*  
*FBgn0030048*  
*FBgn0030050*  
FBgn0030051  
FBgn0030052  
FBgn0030053  
*FBgn0030055*  
*FBgn0030056*  
FBgn0030057  
*FBgn0030058*  
FBgn0030060  
FBgn0030061  
FBgn0030063  
FBgn0030065  
FBgn0030066  
FBgn0030067  
FBgn0030071  
*FBgn0030073*  
FBgn0030074  
FBgn0030077  
FBgn0030078  
*FBgn0030079*  
FBgn0030080  
FBgn0030081  
FBgn0030082  
FBgn0030086  
FBgn0030087  
FBgn0030088  
FBgn0030090  
FBgn0030091  
FBgn0030092  
FBgn0030093  
FBgn0030096  
FBgn0030097  
FBgn0030098  
FBgn0030099  
FBgn0030100  
*FBgn0030101*  
*FBgn0030102*  
*FBgn0030103*  
*FBgn0030104*  
*FBgn0030105*  
FBgn0030107  
FBgn0030108  
*FBgn0030109*  
FBgn0030114  
FBgn0030120  
FBgn0030121  
FBgn0030122  
*FBgn0030136*  
FBgn0030137  
FBgn0030141  
FBgn0030142  
*FBgn0030148*  
*FBgn0030151*  
*FBgn0030156*  
*FBgn0030157*  
*FBgn0030158*  
*FBgn0030159*  
*FBgn0030160*  
FBgn0030161  
FBgn0030162  
FBgn0030163  
FBgn0030164  
FBgn0030165  
FBgn0030166  
FBgn0030167  
FBgn0030170  
FBgn0030171  
FBgn0030172  
FBgn0030173  
*FBgn0030174*  
FBgn0030177  
FBgn0030178  
FBgn0030181  
FBgn0030182  
*FBgn0030183*  
FBgn0030186  
*FBgn0030187*  
FBgn0030189  
*FBgn0030191*  
FBgn0030192  
FBgn0030193  
FBgn0030195  
*FBgn0030196*  
FBgn0030200  
FBgn0030202  
FBgn0030204  
*FBgn0030206*  
*FBgn0030207*  
*FBgn0030208*  
FBgn0030215  
FBgn0030217  
FBgn0030218  
FBgn0030219  
FBgn0030220  
FBgn0030222  
FBgn0030223  
FBgn0030224  
FBgn0030228  
*FBgn0030230*  
FBgn0030234  
*FBgn0030235*  
*FBgn0030237*  
FBgn0030239  
FBgn0030240  
FBgn0030241  
FBgn0030242  
FBgn0030243  
*FBgn0030244*  
*FBgn0030245*  
*FBgn0030246*  
FBgn0030247  
FBgn0030248  
FBgn0030249  
*FBgn0030251*  
*FBgn0030252*  
FBgn0030254  
FBgn0030257  
FBgn0030258  
FBgn0030259  
FBgn0030260  
FBgn0030261  
*FBgn0030262*  
*FBgn0030263*  
*FBgn0030266*  
FBgn0030268  
FBgn0030269  
*FBgn0030270*  
*FBgn0030271*  
*FBgn0030272*  
FBgn0030274  
FBgn0030276  
FBgn0030277  
FBgn0030278  
FBgn0030280  
FBgn0030283  
FBgn0030286  
*FBgn0030289*  
FBgn0030290  
FBgn0030291  
FBgn0030292  
FBgn0030293  
FBgn0030294  
FBgn0030296  
FBgn0030297  
FBgn0030298  
FBgn0030299  
*FBgn0030300*  
FBgn0030301  
FBgn0030302  
FBgn0030303  
*FBgn0030304*  
FBgn0030305  
FBgn0030306  
*FBgn0030307*  
FBgn0030309  
FBgn0030311  
*FBgn0030313*  
FBgn0030314  
FBgn0030316  
*FBgn0030317*  
*FBgn0030319*  
FBgn0030320  
FBgn0030321  
FBgn0030322  
FBgn0030323  
*FBgn0030326*  
FBgn0030327  
FBgn0030328  
FBgn0030329  
FBgn0030330  
FBgn0030331  
*FBgn0030332*  
*FBgn0030334*  
FBgn0030336  
FBgn0030338  
FBgn0030339  
FBgn0030340  
FBgn0030342  
FBgn0030343  
FBgn0030344  
FBgn0030345  
FBgn0030346  
*FBgn0030347*  
*FBgn0030348*  
FBgn0030349  
FBgn0030350  
FBgn0030351  
FBgn0030352  
FBgn0030354  
*FBgn0030357*  
FBgn0030358  
FBgn0030359  
FBgn0030360  
*FBgn0030361*  
*FBgn0030362*  
FBgn0030364  
FBgn0030365  
FBgn0030366  
FBgn0030367  
FBgn0030369  
FBgn0030370  
FBgn0030373  
FBgn0030374  
FBgn0030375  
FBgn0030376  
FBgn0030377  
FBgn0030384  
FBgn0030385  
FBgn0030386  
FBgn0030389  
FBgn0030390  
FBgn0030391  
*FBgn0030394*  
*FBgn0030395*  
*FBgn0030396*  
FBgn0030398  
FBgn0030399  
FBgn0030400  
FBgn0030403  
FBgn0030406  
FBgn0030407  
FBgn0030408  
FBgn0030409  
FBgn0030410  
FBgn0030411  
FBgn0030412  
*FBgn0030417*  
*FBgn0030418*  
FBgn0030420  
FBgn0030421  
*FBgn0030425*  
FBgn0030429  
FBgn0030431  
*FBgn0030432*  
FBgn0030433  
FBgn0030434  
FBgn0030435  
FBgn0030437  
FBgn0030438  
FBgn0030439  
FBgn0030440  
FBgn0030441  
*FBgn0030443*  
FBgn0030447  
FBgn0030448  
FBgn0030449  
FBgn0030451  
FBgn0030452  
*FBgn0030455*  
FBgn0030456  
FBgn0030457  
FBgn0030459  
FBgn0030460  
*FBgn0030462*  
FBgn0030465  
FBgn0030466  
FBgn0030467  
FBgn0030468  
FBgn0030469  
*FBgn0030474*  
*FBgn0030477*  
*FBgn0030478*  
FBgn0030480  
FBgn0030481  
*FBgn0030482*  
*FBgn0030483*  
*FBgn0030484*  
FBgn0030485  
FBgn0030486  
FBgn0030492  
FBgn0030493  
FBgn0030494  
FBgn0030495  
*FBgn0030499*  
FBgn0030500  
FBgn0030501  
FBgn0030502  
FBgn0030503  
FBgn0030504  
*FBgn0030505*  
FBgn0030506  
FBgn0030507  
FBgn0030508  
FBgn0030509  
FBgn0030510  
FBgn0030511  
FBgn0030512  
FBgn0030514  
FBgn0030518  
FBgn0030519  
FBgn0030520  
*FBgn0030521*  
*FBgn0030522*  
FBgn0030524  
FBgn0030525  
FBgn0030528  
FBgn0030529  
FBgn0030530  
FBgn0030532  
FBgn0030536  
FBgn0030537  
FBgn0030538  
*FBgn0030539*  
FBgn0030540  
FBgn0030541  
FBgn0030542  
FBgn0030543  
FBgn0030544  
*FBgn0030545*  
*FBgn0030551*  
FBgn0030552  
FBgn0030554  
FBgn0030555  
*FBgn0030558*  
FBgn0030559  
FBgn0030562  
FBgn0030563  
FBgn0030566  
FBgn0030569  
FBgn0030570  
FBgn0030571  
FBgn0030572  
*FBgn0030574*  
*FBgn0030575*  
*FBgn0030576*  
FBgn0030577  
FBgn0030578  
FBgn0030581  
*FBgn0030582*  
*FBgn0030583*  
*FBgn0030584*  
FBgn0030586  
FBgn0030587  
FBgn0030588  
*FBgn0030589*  
FBgn0030590  
FBgn0030591  
FBgn0030592  
*FBgn0030593*  
*FBgn0030594*  
FBgn0030595  
FBgn0030596  
FBgn0030597  
FBgn0030598  
FBgn0030599  
*FBgn0030600*  
*FBgn0030603*  
FBgn0030605  
FBgn0030606  
*FBgn0030607*  
FBgn0030610  
FBgn0030611  
FBgn0030612  
*FBgn0030614*  
*FBgn0030615*  
*FBgn0030616*  
*FBgn0030617*  
FBgn0030618  
FBgn0030619  
FBgn0030620  
FBgn0030622  
FBgn0030623  
FBgn0030624  
FBgn0030625  
FBgn0030628  
FBgn0030631  
*FBgn0030634*  
FBgn0030636  
FBgn0030638  
FBgn0030640  
*FBgn0030641*  
FBgn0030642  
FBgn0030643  
FBgn0030645  
FBgn0030646  
FBgn0030647  
FBgn0030648  
*FBgn0030653*  
FBgn0030654  
FBgn0030655  
FBgn0030657  
*FBgn0030658*  
FBgn0030659  
FBgn0030660  
FBgn0030661  
FBgn0030662  
FBgn0030663  
FBgn0030664  
FBgn0030665  
FBgn0030666  
FBgn0030667  
FBgn0030668  
FBgn0030669  
FBgn0030670  
FBgn0030671  
FBgn0030672  
FBgn0030673  
FBgn0030674  
FBgn0030675  
FBgn0030676  
FBgn0030678  
FBgn0030679  
FBgn0030680  
FBgn0030683  
*FBgn0030684*  
FBgn0030685  
FBgn0030686  
FBgn0030687  
*FBgn0030688*  
*FBgn0030691*  
*FBgn0030692*  
*FBgn0030693*  
FBgn0030694  
*FBgn0030696*  
FBgn0030697  
*FBgn0030699*  
*FBgn0030700*  
*FBgn0030701*  
*FBgn0030703*  
FBgn0030704  
FBgn0030706  
FBgn0030707  
*FBgn0030710*  
*FBgn0030711*  
FBgn0030715  
*FBgn0030716*  
*FBgn0030717*  
FBgn0030718  
FBgn0030719  
FBgn0030720  
FBgn0030721  
FBgn0030722  
*FBgn0030723*  
*FBgn0030724*  
*FBgn0030725*  
FBgn0030729  
*FBgn0030731*  
FBgn0030733  
FBgn0030734  
FBgn0030735  
*FBgn0030737*  
*FBgn0030738*  
*FBgn0030739*  
FBgn0030740  
*FBgn0030742*  
FBgn0030743  
FBgn0030744  
FBgn0030745  
FBgn0030746  
FBgn0030747  
FBgn0030749  
FBgn0030752  
FBgn0030753  
FBgn0030755  
FBgn0030756  
FBgn0030757  
FBgn0030759  
FBgn0030761  
FBgn0030763  
FBgn0030764  
FBgn0030766  
FBgn0030768  
FBgn0030769  
FBgn0030770  
FBgn0030774  
FBgn0030775  
FBgn0030776  
FBgn0030777  
*FBgn0030778*  
FBgn0030780  
FBgn0030786  
FBgn0030787  
FBgn0030788  
FBgn0030789  
FBgn0030790  
FBgn0030791  
FBgn0030792  
*FBgn0030793*  
FBgn0030794  
FBgn0030795  
*FBgn0030796*  
*FBgn0030797*  
*FBgn0030798*  
FBgn0030799  
FBgn0030800  
*FBgn0030802*  
*FBgn0030803*  
FBgn0030804  
FBgn0030805  
FBgn0030806  
FBgn0030807  
FBgn0030808  
FBgn0030809  
FBgn0030810  
FBgn0030811  
FBgn0030812  
FBgn0030813  
FBgn0030814  
FBgn0030815  
*FBgn0030816*  
*FBgn0030817*  
*FBgn0030823*  
FBgn0030824  
FBgn0030826  
*FBgn0030827*  
*FBgn0030828*  
FBgn0030829  
FBgn0030830  
FBgn0030832  
FBgn0030833  
FBgn0030834  
*FBgn0030837*  
FBgn0030838  
FBgn0030839  
*FBgn0030841*  
*FBgn0030842*  
FBgn0030843  
FBgn0030844  
*FBgn0030846*  
*FBgn0030847*  
FBgn0030850  
FBgn0030851  
*FBgn0030852*  
FBgn0030853  
FBgn0030854  
FBgn0030855  
FBgn0030858  
*FBgn0030859*  
FBgn0030863  
FBgn0030864  
FBgn0030868  
FBgn0030869  
FBgn0030870  
FBgn0030871  
FBgn0030872  
*FBgn0030873*  
FBgn0030874  
FBgn0030876  
FBgn0030877  
FBgn0030878  
FBgn0030881  
FBgn0030882  
FBgn0030883  
FBgn0030884  
FBgn0030886  
FBgn0030887  
FBgn0030889  
FBgn0030890  
FBgn0030891  
FBgn0030892  
FBgn0030893  
FBgn0030894  
*FBgn0030895*  
*FBgn0030897*  
FBgn0030898  
FBgn0030899  
FBgn0030900  
FBgn0030904  
FBgn0030905  
*FBgn0030912*  
*FBgn0030913*  
FBgn0030914  
FBgn0030915  
FBgn0030918  
FBgn0030921  
FBgn0030925  
FBgn0030926  
FBgn0030927  
FBgn0030928  
FBgn0030929  
FBgn0030931  
*FBgn0030932*  
FBgn0030933  
FBgn0030936  
FBgn0030937  
FBgn0030938  
*FBgn0030940*  
FBgn0030941  
FBgn0030943  
FBgn0030944  
FBgn0030945  
FBgn0030946  
FBgn0030947  
FBgn0030949  
FBgn0030951  
FBgn0030952  
*FBgn0030955*  
*FBgn0030956*  
*FBgn0030958*  
FBgn0030959  
FBgn0030960  
*FBgn0030961*  
FBgn0030963  
FBgn0030964  
FBgn0030966  
*FBgn0030968*  
*FBgn0030969*  
FBgn0030970  
FBgn0030973  
FBgn0030974  
*FBgn0030975*  
*FBgn0030976*  
FBgn0030979  
FBgn0030980  
FBgn0030981  
FBgn0030982  
FBgn0030983  
FBgn0030984  
*FBgn0030985*  
*FBgn0030989*  
FBgn0030990  
FBgn0030991  
*FBgn0030992*  
*FBgn0030993*  
*FBgn0030994*  
FBgn0030995  
FBgn0030996  
FBgn0030997  
FBgn0030998  
FBgn0030999  
*FBgn0031000*  
*FBgn0031001*  
FBgn0031002  
FBgn0031003  
*FBgn0031004*  
FBgn0031006  
FBgn0031008  
FBgn0031010  
FBgn0031011  
FBgn0031012  
*FBgn0031016*  
*FBgn0031018*  
FBgn0031020  
FBgn0031021  
*FBgn0031022*  
FBgn0031023  
FBgn0031026  
FBgn0031031  
FBgn0031032  
FBgn0031033  
FBgn0031034  
*FBgn0031036*  
*FBgn0031037*  
*FBgn0031040*  
*FBgn0031042*  
*FBgn0031043*  
*FBgn0031045*  
FBgn0031047  
FBgn0031048  
FBgn0031049  
FBgn0031050  
FBgn0031051  
FBgn0031052  
*FBgn0031053*  
*FBgn0031054*  
*FBgn0031055*  
FBgn0031057  
*FBgn0031058*  
FBgn0031059  
FBgn0031060  
FBgn0031061  
FBgn0031062  
FBgn0031064  
*FBgn0031065*  
FBgn0031068  
FBgn0031069  
FBgn0031070  
FBgn0031074  
FBgn0031077  
FBgn0031078  
*FBgn0031080*  
*FBgn0031081*  
FBgn0031082  
FBgn0031085  
FBgn0031086  
*FBgn0031088*  
FBgn0031089  
FBgn0031090  
*FBgn0031091*  
FBgn0031092  
FBgn0031093  
FBgn0031094  
FBgn0031098  
FBgn0031099  
FBgn0031100  
FBgn0031101  
FBgn0031103  
FBgn0031104  
FBgn0031106  
FBgn0031107  
FBgn0031108  
FBgn0031109  
FBgn0031110  
FBgn0031111  
FBgn0031114  
FBgn0031115  
FBgn0031116  
*FBgn0031117*  
FBgn0031118  
FBgn0031119  
*FBgn0031126*  
FBgn0031127  
FBgn0031128  
FBgn0031129  
FBgn0031130  
FBgn0031132  
FBgn0031134  
FBgn0031135  
FBgn0031139  
FBgn0031141  
*FBgn0031143*  
*FBgn0031144*  
*FBgn0031146*  
*FBgn0031148*  
FBgn0031149  
*FBgn0031150*  
FBgn0031155  
FBgn0031157  
*FBgn0031161*  
FBgn0031163  
FBgn0031164  
FBgn0031168  
*FBgn0031169*  
*FBgn0031170*  
*FBgn0031171*  
*FBgn0031174*  
*FBgn0031176*  
*FBgn0031178*  
FBgn0031181  
*FBgn0031182*  
*FBgn0031183*  
*FBgn0031184*  
FBgn0031187  
FBgn0031188  
FBgn0031189  
FBgn0031190  
*FBgn0031191*  
FBgn0031194  
FBgn0031195  
FBgn0031196  
FBgn0031197  
*FBgn0031201*  
FBgn0031208  
FBgn0031209  
FBgn0031213  
FBgn0031214  
FBgn0031216  
FBgn0031217  
*FBgn0031219*  
*FBgn0031220*  
FBgn0031224  
FBgn0031227  
FBgn0031229  
FBgn0031231  
*FBgn0031232*  
*FBgn0031233*  
*FBgn0031235*  
FBgn0031238  
FBgn0031239  
FBgn0031240  
FBgn0031244  
FBgn0031245  
FBgn0031247  
FBgn0031248  
FBgn0031249  
*FBgn0031250*  
FBgn0031251  
*FBgn0031252*  
*FBgn0031253*  
FBgn0031254  
FBgn0031255  
FBgn0031256  
FBgn0031257  
FBgn0031258  
FBgn0031260  
*FBgn0031263*  
FBgn0031264  
FBgn0031265  
FBgn0031266  
FBgn0031267  
FBgn0031268  
FBgn0031270  
FBgn0031276  
*FBgn0031277*  
FBgn0031279  
FBgn0031281  
FBgn0031282  
*FBgn0031283*  
FBgn0031284  
*FBgn0031285*  
FBgn0031286  
FBgn0031288  
FBgn0031289  
*FBgn0031292*  
FBgn0031294  
FBgn0031295  
FBgn0031296  
*FBgn0031298*  
FBgn0031299  
FBgn0031301  
FBgn0031302  
FBgn0031304  
*FBgn0031305*  
*FBgn0031306*  
*FBgn0031307*  
FBgn0031308  
FBgn0031309  
FBgn0031310  
FBgn0031312  
*FBgn0031313*  
FBgn0031314  
FBgn0031315  
FBgn0031317  
FBgn0031318  
FBgn0031319  
FBgn0031320  
FBgn0031321  
FBgn0031322  
*FBgn0031323*  
FBgn0031324  
*FBgn0031327*  
FBgn0031331  
FBgn0031332  
FBgn0031333  
FBgn0031335  
FBgn0031337  
FBgn0031343  
FBgn0031344  
FBgn0031345  
FBgn0031347  
FBgn0031351  
FBgn0031356  
FBgn0031357  
FBgn0031359  
FBgn0031360  
FBgn0031361  
FBgn0031362  
FBgn0031364  
FBgn0031365  
*FBgn0031372*  
*FBgn0031373*  
*FBgn0031374*  
FBgn0031375  
FBgn0031377  
FBgn0031378  
FBgn0031379  
FBgn0031381  
FBgn0031384  
FBgn0031388  
*FBgn0031389*  
FBgn0031390  
FBgn0031391  
FBgn0031392  
FBgn0031393  
FBgn0031395  
*FBgn0031397*  
FBgn0031398  
FBgn0031399  
FBgn0031401  
FBgn0031403  
*FBgn0031405*  
FBgn0031406  
FBgn0031407  
FBgn0031409  
FBgn0031410  
*FBgn0031412*  
FBgn0031413  
FBgn0031414  
*FBgn0031417*  
*FBgn0031418*  
FBgn0031419  
FBgn0031420  
FBgn0031421  
FBgn0031422  
FBgn0031423  
FBgn0031424  
FBgn0031426  
FBgn0031428  
FBgn0031430  
FBgn0031431  
FBgn0031432  
FBgn0031434  
FBgn0031435  
FBgn0031436  
FBgn0031440  
FBgn0031441  
FBgn0031442  
FBgn0031444  
FBgn0031446  
FBgn0031449  
FBgn0031450  
*FBgn0031451*  
*FBgn0031452*  
FBgn0031453  
FBgn0031457  
FBgn0031459  
FBgn0031460  
*FBgn0031461*  
FBgn0031462  
FBgn0031463  
FBgn0031464  
FBgn0031465  
FBgn0031466  
FBgn0031467  
FBgn0031468  
FBgn0031469  
FBgn0031470  
FBgn0031471  
FBgn0031472  
FBgn0031473  
*FBgn0031474*  
*FBgn0031476*  
FBgn0031478  
FBgn0031479  
FBgn0031483  
FBgn0031484  
FBgn0031485  
*FBgn0031488*  
FBgn0031489  
FBgn0031490  
FBgn0031491  
FBgn0031492  
FBgn0031493  
FBgn0031494  
FBgn0031495  
*FBgn0031496*  
*FBgn0031497*  
FBgn0031498  
FBgn0031500  
*FBgn0031501*  
FBgn0031504  
FBgn0031505  
FBgn0031512  
FBgn0031513  
*FBgn0031514*  
FBgn0031515  
FBgn0031516  
*FBgn0031517*  
FBgn0031518  
FBgn0031519  
FBgn0031520  
FBgn0031522  
FBgn0031523  
*FBgn0031526*  
FBgn0031528  
FBgn0031529  
FBgn0031530  
FBgn0031531  
FBgn0031533  
FBgn0031534  
FBgn0031535  
FBgn0031536  
FBgn0031537  
*FBgn0031538*  
FBgn0031540  
*FBgn0031542*  
FBgn0031544  
FBgn0031545  
FBgn0031546  
FBgn0031548  
FBgn0031549  
FBgn0031550  
*FBgn0031554*  
FBgn0031558  
FBgn0031559  
FBgn0031560  
FBgn0031561  
*FBgn0031562*  
*FBgn0031563*  
*FBgn0031564*  
*FBgn0031566*  
FBgn0031568  
FBgn0031571  
FBgn0031573  
FBgn0031574  
*FBgn0031575*  
FBgn0031579  
FBgn0031580  
FBgn0031581  
FBgn0031585  
FBgn0031589  
FBgn0031590  
FBgn0031591  
FBgn0031592  
FBgn0031596  
FBgn0031597  
*FBgn0031598*  
*FBgn0031600*  
FBgn0031601  
FBgn0031602  
FBgn0031603  
FBgn0031604  
FBgn0031606  
FBgn0031607  
FBgn0031608  
FBgn0031609  
FBgn0031610  
FBgn0031611  
*FBgn0031613*  
FBgn0031617  
FBgn0031619  
FBgn0031620  
FBgn0031621  
FBgn0031622  
FBgn0031623  
FBgn0031626  
FBgn0031627  
FBgn0031628  
*FBgn0031629*  
*FBgn0031630*  
FBgn0031631  
FBgn0031632  
*FBgn0031633*  
*FBgn0031634*  
FBgn0031635  
FBgn0031636  
FBgn0031637  
FBgn0031638  
FBgn0031639  
FBgn0031640  
*FBgn0031643*  
*FBgn0031644*  
*FBgn0031645*  
*FBgn0031646*  
*FBgn0031649*  
*FBgn0031650*  
*FBgn0031651*  
*FBgn0031652*  
FBgn0031653  
FBgn0031654  
*FBgn0031655*  
FBgn0031657  
FBgn0031659  
FBgn0031660  
FBgn0031661  
FBgn0031662  
FBgn0031663  
FBgn0031664  
FBgn0031670  
FBgn0031673  
*FBgn0031675*  
*FBgn0031676*  
FBgn0031677  
FBgn0031678  
FBgn0031681  
FBgn0031682  
*FBgn0031683*  
*FBgn0031684*  
*FBgn0031688*  
*FBgn0031689*  
FBgn0031690  
*FBgn0031692*  
*FBgn0031693*  
*FBgn0031694*  
FBgn0031695  
FBgn0031696  
FBgn0031697  
FBgn0031698  
*FBgn0031700*  
*FBgn0031701*  
*FBgn0031702*  
*FBgn0031703*  
FBgn0031707  
FBgn0031708  
FBgn0031710  
FBgn0031711  
FBgn0031713  
FBgn0031715  
FBgn0031716  
*FBgn0031717*  
FBgn0031718  
FBgn0031719  
FBgn0031720  
FBgn0031721  
FBgn0031722  
FBgn0031723  
FBgn0031724  
FBgn0031725  
*FBgn0031728*  
FBgn0031729  
FBgn0031730  
FBgn0031731  
*FBgn0031732*  
*FBgn0031733*  
*FBgn0031734*  
FBgn0031735  
FBgn0031736  
FBgn0031738  
FBgn0031739  
FBgn0031740  
*FBgn0031741*  
*FBgn0031745*  
FBgn0031746  
*FBgn0031747*  
FBgn0031749  
FBgn0031751  
FBgn0031752  
FBgn0031753  
FBgn0031756  
FBgn0031757  
FBgn0031758  
FBgn0031759  
*FBgn0031760*  
FBgn0031762  
FBgn0031763  
FBgn0031764  
FBgn0031765  
FBgn0031766  
FBgn0031768  
FBgn0031769  
*FBgn0031770*  
*FBgn0031771*  
FBgn0031772  
FBgn0031773  
FBgn0031774  
*FBgn0031775*  
FBgn0031776  
FBgn0031777  
*FBgn0031778*  
*FBgn0031779*  
FBgn0031782  
FBgn0031784  
FBgn0031785  
FBgn0031786  
*FBgn0031791*  
FBgn0031792  
*FBgn0031799*  
*FBgn0031800*  
*FBgn0031801*  
FBgn0031802  
FBgn0031803  
FBgn0031804  
FBgn0031805  
*FBgn0031808*  
FBgn0031811  
FBgn0031812  
FBgn0031813  
FBgn0031814  
FBgn0031815  
FBgn0031816  
FBgn0031817  
FBgn0031818  
FBgn0031820  
*FBgn0031821*  
FBgn0031822  
*FBgn0031824*  
*FBgn0031826*  
*FBgn0031830*  
*FBgn0031831*  
FBgn0031832  
*FBgn0031834*  
*FBgn0031835*  
*FBgn0031836*  
FBgn0031837  
*FBgn0031842*  
*FBgn0031844*  
FBgn0031845  
FBgn0031848  
*FBgn0031849*  
FBgn0031850  
FBgn0031851  
FBgn0031853  
FBgn0031854  
FBgn0031855  
FBgn0031856  
FBgn0031857  
FBgn0031858  
*FBgn0031859*  
*FBgn0031860*  
*FBgn0031861*  
*FBgn0031865*  
*FBgn0031866*  
FBgn0031868  
*FBgn0031869*  
FBgn0031871  
FBgn0031872  
FBgn0031873  
FBgn0031874  
FBgn0031875  
FBgn0031876  
*FBgn0031877*  
*FBgn0031878*  
FBgn0031879  
FBgn0031880  
FBgn0031881  
FBgn0031882  
FBgn0031883  
FBgn0031885  
*FBgn0031886*  
FBgn0031887  
FBgn0031888  
*FBgn0031893*  
FBgn0031894  
FBgn0031895  
*FBgn0031896*  
FBgn0031897  
FBgn0031900  
FBgn0031902  
FBgn0031903  
*FBgn0031904*  
*FBgn0031905*  
*FBgn0031906*  
*FBgn0031907*  
*FBgn0031908*  
*FBgn0031909*  
*FBgn0031910*  
*FBgn0031912*  
*FBgn0031913*  
*FBgn0031914*  
FBgn0031918  
FBgn0031920  
FBgn0031923  
*FBgn0031925*  
FBgn0031926  
FBgn0031927  
FBgn0031929  
FBgn0031930  
*FBgn0031934*  
FBgn0031935  
FBgn0031936  
FBgn0031937  
FBgn0031939  
FBgn0031940  
*FBgn0031941*  
*FBgn0031942*  
FBgn0031943  
FBgn0031944  
*FBgn0031945*  
*FBgn0031946*  
FBgn0031947  
FBgn0031948  
*FBgn0031950*  
FBgn0031951  
FBgn0031952  
FBgn0031954  
FBgn0031955  
FBgn0031957  
FBgn0031959  
FBgn0031961  
FBgn0031968  
FBgn0031969  
*FBgn0031970*  
*FBgn0031971*  
FBgn0031972  
FBgn0031973  
FBgn0031974  
*FBgn0031975*  
*FBgn0031976*  
FBgn0031977  
*FBgn0031979*  
*FBgn0031980*  
*FBgn0031981*  
FBgn0031985  
*FBgn0031986*  
FBgn0031987  
FBgn0031988  
FBgn0031990  
FBgn0031992  
*FBgn0031993*  
FBgn0031996  
FBgn0031997  
FBgn0031998  
*FBgn0031999*  
FBgn0032000  
FBgn0032001  
*FBgn0032002*  
FBgn0032003  
FBgn0032004  
FBgn0032005  
FBgn0032006  
FBgn0032008  
FBgn0032010  
*FBgn0032013*  
FBgn0032014  
FBgn0032015  
FBgn0032016  
FBgn0032017  
FBgn0032018  
FBgn0032020  
*FBgn0032021*  
*FBgn0032022*  
*FBgn0032023*  
*FBgn0032024*  
*FBgn0032025*  
*FBgn0032026*  
FBgn0032029  
FBgn0032030  
FBgn0032031  
FBgn0032032  
FBgn0032033  
FBgn0032034  
FBgn0032035  
FBgn0032036  
FBgn0032039  
FBgn0032040  
FBgn0032042  
*FBgn0032047*  
*FBgn0032048*  
*FBgn0032049*  
FBgn0032050  
FBgn0032051  
FBgn0032052  
FBgn0032053  
FBgn0032054  
*FBgn0032055*  
FBgn0032057  
FBgn0032058  
*FBgn0032059*  
*FBgn0032061*  
*FBgn0032065*  
FBgn0032066  
FBgn0032067  
FBgn0032068  
FBgn0032069  
FBgn0032072  
FBgn0032074  
FBgn0032075  
FBgn0032078  
FBgn0032079  
FBgn0032080  
FBgn0032082  
FBgn0032083  
*FBgn0032084*  
FBgn0032085  
FBgn0032086  
FBgn0032087  
FBgn0032088  
FBgn0032089  
FBgn0032090  
FBgn0032094  
FBgn0032096  
FBgn0032100  
*FBgn0032101*  
*FBgn0032104*  
FBgn0032105  
FBgn0032109  
FBgn0032110  
FBgn0032111  
*FBgn0032115*  
*FBgn0032116*  
FBgn0032117  
*FBgn0032119*  
*FBgn0032120*  
*FBgn0032122*  
*FBgn0032123*  
*FBgn0032124*  
FBgn0032125  
FBgn0032126  
FBgn0032127  
FBgn0032129  
*FBgn0032130*  
FBgn0032132  
*FBgn0032136*  
*FBgn0032138*  
*FBgn0032139*  
FBgn0032140  
FBgn0032142  
FBgn0032143  
*FBgn0032144*  
FBgn0032145  
FBgn0032147  
FBgn0032149  
*FBgn0032150*  
FBgn0032153  
FBgn0032154  
*FBgn0032156*  
*FBgn0032157*  
FBgn0032160  
FBgn0032161  
FBgn0032162  
FBgn0032163  
FBgn0032166  
FBgn0032167  
FBgn0032168  
FBgn0032169  
FBgn0032170  
FBgn0032171  
FBgn0032172  
FBgn0032175  
FBgn0032176  
FBgn0032178  
FBgn0032180  
FBgn0032181  
FBgn0032184  
*FBgn0032187*  
FBgn0032188  
FBgn0032189  
FBgn0032191  
*FBgn0032192*  
FBgn0032193  
FBgn0032194  
FBgn0032195  
FBgn0032196  
FBgn0032197  
*FBgn0032198*  
FBgn0032200  
FBgn0032202  
FBgn0032204  
FBgn0032205  
*FBgn0032208*  
*FBgn0032209*  
*FBgn0032210*  
*FBgn0032211*  
FBgn0032213  
FBgn0032214  
FBgn0032215  
FBgn0032216  
FBgn0032217  
FBgn0032218  
*FBgn0032219*  
*FBgn0032221*  
FBgn0032222  
FBgn0032223  
FBgn0032224  
FBgn0032225  
FBgn0032228  
FBgn0032229  
*FBgn0032230*  
*FBgn0032231*  
*FBgn0032233*  
*FBgn0032234*  
FBgn0032236  
FBgn0032237  
FBgn0032242  
FBgn0032243  
FBgn0032244  
FBgn0032246  
FBgn0032247  
FBgn0032248  
FBgn0032249  
FBgn0032250  
FBgn0032251  
*FBgn0032252*  
*FBgn0032253*  
FBgn0032258  
FBgn0032259  
*FBgn0032260*  
FBgn0032261  
*FBgn0032262*  
*FBgn0032264*  
*FBgn0032265*  
FBgn0032266  
FBgn0032268  
FBgn0032271  
FBgn0032275  
FBgn0032276  
FBgn0032280  
FBgn0032281  
FBgn0032282  
*FBgn0032283*  
*FBgn0032284*  
*FBgn0032285*  
FBgn0032286  
*FBgn0032287*  
FBgn0032289  
FBgn0032291  
FBgn0032292  
FBgn0032295  
FBgn0032296  
FBgn0032297  
FBgn0032298  
*FBgn0032299*  
FBgn0032303  
FBgn0032304  
*FBgn0032305*  
FBgn0032311  
FBgn0032312  
FBgn0032313  
FBgn0032314  
FBgn0032315  
*FBgn0032318*  
*FBgn0032322*  
FBgn0032329  
FBgn0032330  
FBgn0032331  
FBgn0032335  
*FBgn0032338*  
FBgn0032339  
FBgn0032341  
FBgn0032343  
*FBgn0032345*  
FBgn0032346  
FBgn0032348  
*FBgn0032350*  
*FBgn0032354*  
FBgn0032358  
FBgn0032359  
FBgn0032360  
FBgn0032361  
FBgn0032362  
FBgn0032363  
*FBgn0032366*  
FBgn0032368  
FBgn0032369  
FBgn0032370  
FBgn0032371  
FBgn0032372  
FBgn0032374  
FBgn0032375  
FBgn0032376  
FBgn0032377  
FBgn0032378  
*FBgn0032381*  
*FBgn0032382*  
FBgn0032385  
*FBgn0032387*  
*FBgn0032388*  
FBgn0032390  
FBgn0032391  
FBgn0032393  
FBgn0032394  
*FBgn0032395*  
*FBgn0032397*  
*FBgn0032398*  
*FBgn0032399*  
*FBgn0032400*  
*FBgn0032401*  
*FBgn0032402*  
*FBgn0032405*  
FBgn0032406  
FBgn0032407  
FBgn0032408  
FBgn0032412  
FBgn0032413  
FBgn0032414  
FBgn0032416  
FBgn0032419  
FBgn0032420  
FBgn0032421  
FBgn0032422  
FBgn0032424  
FBgn0032428  
FBgn0032429  
FBgn0032430  
*FBgn0032431*  
*FBgn0032433*  
*FBgn0032434*  
*FBgn0032435*  
FBgn0032436  
FBgn0032437  
FBgn0032439  
FBgn0032442  
FBgn0032444  
FBgn0032445  
FBgn0032446  
FBgn0032447  
FBgn0032448  
FBgn0032449  
FBgn0032450  
*FBgn0032451*  
*FBgn0032452*  
FBgn0032453  
FBgn0032454  
FBgn0032455  
FBgn0032456  
FBgn0032457  
FBgn0032462  
FBgn0032465  
FBgn0032467  
FBgn0032470  
FBgn0032471  
*FBgn0032472*  
FBgn0032473  
FBgn0032475  
FBgn0032476  
FBgn0032477  
*FBgn0032478*  
*FBgn0032479*  
*FBgn0032480*  
*FBgn0032481*  
*FBgn0032482*  
*FBgn0032483*  
*FBgn0032484*  
FBgn0032485  
FBgn0032486  
FBgn0032487  
FBgn0032488  
FBgn0032489  
FBgn0032490  
FBgn0032491  
FBgn0032492  
FBgn0032493  
*FBgn0032494*  
*FBgn0032495*  
*FBgn0032497*  
FBgn0032498  
FBgn0032499  
FBgn0032502  
FBgn0032503  
FBgn0032504  
*FBgn0032505*  
*FBgn0032506*  
*FBgn0032507*  
FBgn0032509  
*FBgn0032511*  
FBgn0032512  
FBgn0032513  
FBgn0032514  
FBgn0032515  
FBgn0032516  
*FBgn0032517*  
*FBgn0032518*  
FBgn0032519  
FBgn0032520  
FBgn0032521  
FBgn0032522  
FBgn0032523  
*FBgn0032524*  
FBgn0032525  
FBgn0032533  
FBgn0032538  
FBgn0032546  
FBgn0032549  
FBgn0032551  
FBgn0032553  
FBgn0032554  
FBgn0032563  
FBgn0032577  
FBgn0032582  
*FBgn0032585*  
FBgn0032586  
FBgn0032587  
FBgn0032588  
FBgn0032590  
FBgn0032593  
FBgn0032595  
FBgn0032596  
FBgn0032597  
FBgn0032598  
*FBgn0032600*  
*FBgn0032602*  
*FBgn0032603*  
*FBgn0032609*  
*FBgn0032612*  
*FBgn0032613*  
*FBgn0032614*  
FBgn0032615  
FBgn0032620  
FBgn0032624  
FBgn0032625  
FBgn0032626  
FBgn0032631  
FBgn0032632  
*FBgn0032633*  
FBgn0032634  
FBgn0032635  
FBgn0032636  
FBgn0032637  
FBgn0032639  
FBgn0032640  
FBgn0032642  
FBgn0032643  
FBgn0032644  
*FBgn0032645*  
*FBgn0032646*  
FBgn0032647  
FBgn0032648  
FBgn0032649  
FBgn0032650  
*FBgn0032651*  
*FBgn0032652*  
FBgn0032654  
*FBgn0032656*  
FBgn0032658  
FBgn0032660  
FBgn0032663  
FBgn0032664  
*FBgn0032665*  
*FBgn0032666*  
*FBgn0032668*  
*FBgn0032669*  
FBgn0032670  
FBgn0032671  
*FBgn0032673*  
FBgn0032677  
FBgn0032679  
FBgn0032681  
FBgn0032682  
FBgn0032683  
*FBgn0032684*  
*FBgn0032685*  
*FBgn0032688*  
*FBgn0032689*  
FBgn0032690  
FBgn0032691  
*FBgn0032692*  
FBgn0032693  
*FBgn0032694*  
FBgn0032698  
FBgn0032699  
FBgn0032700  
FBgn0032701  
FBgn0032702  
FBgn0032703  
FBgn0032704  
*FBgn0032705*  
*FBgn0032706*  
FBgn0032707  
FBgn0032709  
FBgn0032713  
*FBgn0032717*  
*FBgn0032719*  
FBgn0032720  
FBgn0032721  
*FBgn0032723*  
FBgn0032724  
FBgn0032725  
FBgn0032726  
FBgn0032727  
FBgn0032728  
FBgn0032729  
*FBgn0032730*  
FBgn0032731  
FBgn0032732  
FBgn0032733  
FBgn0032734  
FBgn0032740  
*FBgn0032741*  
FBgn0032744  
FBgn0032746  
FBgn0032748  
FBgn0032749  
FBgn0032750  
FBgn0032751  
FBgn0032752  
FBgn0032753  
FBgn0032754  
FBgn0032755  
FBgn0032763  
FBgn0032768  
FBgn0032769  
*FBgn0032770*  
FBgn0032771  
FBgn0032772  
FBgn0032773  
FBgn0032774  
FBgn0032775  
FBgn0032779  
*FBgn0032780*  
FBgn0032781  
FBgn0032782  
*FBgn0032783*  
*FBgn0032785*  
*FBgn0032787*  
FBgn0032788  
FBgn0032789  
FBgn0032790  
FBgn0032791  
FBgn0032793  
FBgn0032796  
FBgn0032797  
FBgn0032798  
*FBgn0032799*  
*FBgn0032800*  
FBgn0032801  
FBgn0032803  
FBgn0032804  
*FBgn0032805*  
FBgn0032808  
FBgn0032809  
*FBgn0032810*  
FBgn0032811  
FBgn0032812  
FBgn0032813  
FBgn0032814  
FBgn0032815  
FBgn0032817  
FBgn0032818  
FBgn0032819  
*FBgn0032820*  
FBgn0032821  
*FBgn0032822*  
FBgn0032824  
FBgn0032825  
FBgn0032827  
*FBgn0032833*  
FBgn0032834  
FBgn0032835  
FBgn0032836  
FBgn0032838  
*FBgn0032839*  
*FBgn0032840*  
*FBgn0032843*  
FBgn0032845  
FBgn0032846  
FBgn0032847  
FBgn0032848  
*FBgn0032849*  
FBgn0032850  
FBgn0032851  
FBgn0032853  
FBgn0032856  
FBgn0032857  
FBgn0032858  
FBgn0032860  
FBgn0032863  
*FBgn0032864*  
FBgn0032867  
FBgn0032868  
*FBgn0032869*  
*FBgn0032870*  
FBgn0032871  
FBgn0032873  
FBgn0032876  
FBgn0032877  
FBgn0032878  
FBgn0032879  
FBgn0032880  
FBgn0032881  
FBgn0032882  
FBgn0032883  
*FBgn0032884*  
FBgn0032886  
FBgn0032888  
FBgn0032889  
*FBgn0032891*  
*FBgn0032894*  
*FBgn0032895*  
*FBgn0032896*  
FBgn0032897  
FBgn0032899  
FBgn0032900  
FBgn0032901  
FBgn0032904  
FBgn0032906  
FBgn0032907  
*FBgn0032908*  
*FBgn0032910*  
*FBgn0032911*  
*FBgn0032913*  
FBgn0032915  
FBgn0032916  
FBgn0032919  
FBgn0032921  
FBgn0032922  
FBgn0032923  
FBgn0032924  
FBgn0032925  
FBgn0032926  
FBgn0032929  
FBgn0032934  
*FBgn0032935*  
FBgn0032938  
FBgn0032940  
FBgn0032943  
FBgn0032945  
*FBgn0032946*  
*FBgn0032949*  
FBgn0032955  
FBgn0032957  
*FBgn0032961*  
FBgn0032964  
FBgn0032965  
FBgn0032966  
FBgn0032967  
FBgn0032968  
FBgn0032969  
FBgn0032971  
FBgn0032972  
*FBgn0032973*  
FBgn0032974  
FBgn0032979  
FBgn0032981  
*FBgn0032986*  
*FBgn0032987*  
FBgn0032997  
FBgn0033000  
FBgn0033005  
FBgn0033010  
FBgn0033015  
FBgn0033017  
*FBgn0033019*  
*FBgn0033020*  
*FBgn0033021*  
*FBgn0033027*  
FBgn0033028  
*FBgn0033031*  
*FBgn0033032*  
*FBgn0033033*  
FBgn0033038  
*FBgn0033039*  
FBgn0033041  
FBgn0033042  
FBgn0033043  
FBgn0033045  
FBgn0033046  
FBgn0033047  
FBgn0033048  
*FBgn0033049*  
FBgn0033050  
*FBgn0033051*  
*FBgn0033052*  
*FBgn0033054*  
*FBgn0033055*  
FBgn0033056  
FBgn0033058  
FBgn0033059  
FBgn0033060  
*FBgn0033061*  
*FBgn0033062*  
*FBgn0033063*  
*FBgn0033065*  
FBgn0033067  
*FBgn0033068*  
FBgn0033069  
*FBgn0033072*  
*FBgn0033073*  
FBgn0033074  
FBgn0033076  
FBgn0033081  
FBgn0033083  
*FBgn0033087*  
FBgn0033088  
FBgn0033089  
FBgn0033090  
FBgn0033092  
*FBgn0033093*  
FBgn0033095  
FBgn0033096  
FBgn0033097  
FBgn0033100  
FBgn0033101  
FBgn0033104  
FBgn0033107  
FBgn0033108  
*FBgn0033109*  
*FBgn0033110*  
*FBgn0033112*  
*FBgn0033113*  
*FBgn0033115*  
*FBgn0033117*  
*FBgn0033121*  
FBgn0033122  
*FBgn0033124*  
FBgn0033127  
FBgn0033128  
FBgn0033129  
FBgn0033130  
*FBgn0033131*  
*FBgn0033132*  
*FBgn0033133*  
FBgn0033134  
FBgn0033135  
FBgn0033136  
FBgn0033137  
FBgn0033138  
FBgn0033139  
FBgn0033140  
FBgn0033141  
FBgn0033142  
*FBgn0033145*  
FBgn0033149  
FBgn0033153  
FBgn0033154  
FBgn0033155  
FBgn0033158  
FBgn0033159  
FBgn0033160  
*FBgn0033162*  
FBgn0033164  
FBgn0033165  
*FBgn0033166*  
*FBgn0033167*  
FBgn0033168  
*FBgn0033169*  
*FBgn0033170*  
*FBgn0033174*  
FBgn0033177  
FBgn0033178  
FBgn0033179  
FBgn0033182  
FBgn0033183  
FBgn0033184  
FBgn0033185  
FBgn0033186  
*FBgn0033187*  
*FBgn0033188*  
*FBgn0033190*  
*FBgn0033191*  
*FBgn0033192*  
FBgn0033194  
*FBgn0033195*  
*FBgn0033196*  
FBgn0033199  
FBgn0033202  
FBgn0033203  
FBgn0033204  
FBgn0033205  
FBgn0033206  
*FBgn0033207*  
FBgn0033208  
FBgn0033209  
FBgn0033210  
FBgn0033212  
*FBgn0033214*  
FBgn0033215  
FBgn0033216  
FBgn0033221  
FBgn0033222  
FBgn0033224  
FBgn0033225  
FBgn0033226  
FBgn0033232  
*FBgn0033233*  
*FBgn0033234*  
*FBgn0033235*  
*FBgn0033236*  
FBgn0033238  
*FBgn0033240*  
*FBgn0033241*  
FBgn0033243  
FBgn0033244  
FBgn0033246  
FBgn0033247  
FBgn0033248  
*FBgn0033249*  
*FBgn0033250*  
FBgn0033252  
FBgn0033257  
*FBgn0033258*  
FBgn0033259  
FBgn0033261  
FBgn0033264  
FBgn0033265  
FBgn0033266  
*FBgn0033268*  
FBgn0033269  
*FBgn0033271*  
FBgn0033272  
FBgn0033273  
*FBgn0033274*  
FBgn0033275  
FBgn0033277  
FBgn0033278  
FBgn0033279  
FBgn0033280  
*FBgn0033283*  
FBgn0033285  
FBgn0033286  
FBgn0033287  
*FBgn0033289*  
*FBgn0033292*  
FBgn0033294  
FBgn0033296  
FBgn0033297  
*FBgn0033301*  
*FBgn0033302*  
*FBgn0033304*  
FBgn0033307  
FBgn0033308  
*FBgn0033309*  
FBgn0033310  
FBgn0033312  
*FBgn0033313*  
*FBgn0033315*  
FBgn0033316  
FBgn0033317  
FBgn0033320  
FBgn0033321  
FBgn0033322  
FBgn0033323  
FBgn0033324  
FBgn0033326  
FBgn0033330  
*FBgn0033337*  
FBgn0033339  
FBgn0033340  
FBgn0033341  
FBgn0033342  
*FBgn0033344*  
FBgn0033347  
FBgn0033348  
FBgn0033349  
FBgn0033350  
FBgn0033351  
FBgn0033352  
FBgn0033353  
FBgn0033354  
FBgn0033355  
FBgn0033356  
FBgn0033357  
*FBgn0033358*  
FBgn0033359  
FBgn0033362  
FBgn0033363  
FBgn0033364  
FBgn0033365  
*FBgn0033367*  
*FBgn0033368*  
*FBgn0033369*  
FBgn0033371  
FBgn0033372  
FBgn0033373  
*FBgn0033374*  
FBgn0033375  
FBgn0033376  
FBgn0033377  
FBgn0033378  
FBgn0033379  
FBgn0033380  
FBgn0033381  
FBgn0033382  
*FBgn0033387*  
FBgn0033388  
FBgn0033389  
FBgn0033391  
FBgn0033392  
FBgn0033395  
*FBgn0033397*  
FBgn0033400  
FBgn0033401  
FBgn0033402  
FBgn0033403  
FBgn0033404  
FBgn0033405  
*FBgn0033412*  
FBgn0033413  
*FBgn0033421*  
*FBgn0033422*  
*FBgn0033423*  
*FBgn0033426*  
FBgn0033427  
FBgn0033428  
FBgn0033429  
FBgn0033431  
*FBgn0033434*  
*FBgn0033437*  
*FBgn0033438*  
*FBgn0033439*  
*FBgn0033440*  
*FBgn0033442*  
*FBgn0033443*  
*FBgn0033446*  
FBgn0033447  
*FBgn0033448*  
FBgn0033449  
FBgn0033450  
FBgn0033451  
FBgn0033452  
FBgn0033453  
FBgn0033454  
FBgn0033457  
*FBgn0033458*  
*FBgn0033459*  
*FBgn0033460*  
*FBgn0033461*  
FBgn0033463  
*FBgn0033464*  
*FBgn0033465*  
FBgn0033466  
FBgn0033467  
*FBgn0033468*  
*FBgn0033469*  
FBgn0033471  
FBgn0033473  
FBgn0033474  
FBgn0033475  
FBgn0033476  
FBgn0033477  
*FBgn0033479*  
*FBgn0033480*  
FBgn0033481  
*FBgn0033482*  
*FBgn0033483*  
*FBgn0033484*  
FBgn0033486  
FBgn0033490  
FBgn0033491  
*FBgn0033494*  
*FBgn0033495*  
FBgn0033497  
FBgn0033498  
FBgn0033499  
FBgn0033500  
*FBgn0033501*  
*FBgn0033502*  
*FBgn0033504*  
*FBgn0033507*  
*FBgn0033508*  
FBgn0033512  
FBgn0033515  
FBgn0033516  
*FBgn0033519*  
*FBgn0033523*  
*FBgn0033524*  
FBgn0033527  
FBgn0033528  
*FBgn0033529*  
FBgn0033538  
FBgn0033539  
FBgn0033540  
*FBgn0033541*  
*FBgn0033542*  
FBgn0033543  
FBgn0033544  
FBgn0033547  
*FBgn0033548*  
*FBgn0033549*  
*FBgn0033550*  
*FBgn0033551*  
FBgn0033554  
FBgn0033556  
FBgn0033557  
*FBgn0033558*  
FBgn0033562  
FBgn0033566  
FBgn0033569  
FBgn0033570  
FBgn0033571  
FBgn0033572  
FBgn0033573  
FBgn0033574  
FBgn0033578  
FBgn0033579  
FBgn0033580  
FBgn0033581  
*FBgn0033582*  
FBgn0033584  
FBgn0033587  
FBgn0033588  
FBgn0033589  
FBgn0033590  
FBgn0033591  
FBgn0033592  
*FBgn0033593*  
FBgn0033594  
FBgn0033597  
FBgn0033598  
FBgn0033599  
FBgn0033600  
FBgn0033601  
FBgn0033602  
FBgn0033603  
FBgn0033605  
FBgn0033607  
FBgn0033608  
FBgn0033609  
*FBgn0033610*  
*FBgn0033614*  
*FBgn0033615*  
*FBgn0033616*  
*FBgn0033623*  
*FBgn0033624*  
*FBgn0033627*  
FBgn0033628  
FBgn0033629  
FBgn0033631  
FBgn0033633  
*FBgn0033635*  
FBgn0033636  
FBgn0033638  
FBgn0033639  
*FBgn0033640*  
*FBgn0033645*  
*FBgn0033648*  
*FBgn0033649*  
FBgn0033650  
FBgn0033651  
FBgn0033652  
*FBgn0033653*  
*FBgn0033654*  
FBgn0033656  
*FBgn0033657*  
FBgn0033658  
*FBgn0033659*  
*FBgn0033661*  
FBgn0033663  
*FBgn0033665*  
FBgn0033667  
FBgn0033668  
*FBgn0033669*  
*FBgn0033673*  
FBgn0033674  
FBgn0033677  
*FBgn0033679*  
FBgn0033680  
*FBgn0033683*  
*FBgn0033685*  
FBgn0033686  
*FBgn0033687*  
*FBgn0033688*  
*FBgn0033690*  
*FBgn0033691*  
FBgn0033692  
FBgn0033696  
FBgn0033697  
FBgn0033698  
FBgn0033699  
FBgn0033701  
FBgn0033702  
FBgn0033703  
FBgn0033704  
FBgn0033705  
*FBgn0033708*  
*FBgn0033710*  
FBgn0033712  
FBgn0033713  
FBgn0033714  
FBgn0033715  
FBgn0033716  
FBgn0033717  
*FBgn0033718*  
FBgn0033720  
*FBgn0033721*  
FBgn0033723  
FBgn0033724  
FBgn0033725  
FBgn0033726  
FBgn0033727  
*FBgn0033728*  
FBgn0033729  
FBgn0033730  
FBgn0033731  
FBgn0033732  
*FBgn0033733*  
FBgn0033734  
FBgn0033735  
*FBgn0033736*  
FBgn0033737  
FBgn0033738  
FBgn0033739  
*FBgn0033740*  
FBgn0033741  
*FBgn0033742*  
*FBgn0033744*  
FBgn0033748  
FBgn0033749  
*FBgn0033750*  
FBgn0033751  
FBgn0033752  
FBgn0033753  
*FBgn0033754*  
*FBgn0033756*  
*FBgn0033757*  
FBgn0033760  
FBgn0033761  
FBgn0033762  
FBgn0033763  
FBgn0033766  
FBgn0033767  
*FBgn0033769*  
FBgn0033770  
FBgn0033773  
*FBgn0033774*  
*FBgn0033775*  
*FBgn0033777*  
*FBgn0033778*  
FBgn0033781  
*FBgn0033782*  
FBgn0033783  
FBgn0033784  
FBgn0033785  
FBgn0033786  
FBgn0033787  
FBgn0033788  
FBgn0033789  
*FBgn0033792*  
*FBgn0033793*  
FBgn0033794  
*FBgn0033799*  
*FBgn0033802*  
FBgn0033806  
FBgn0033808  
FBgn0033809  
FBgn0033810  
FBgn0033812  
*FBgn0033813*  
*FBgn0033814*  
*FBgn0033815*  
*FBgn0033816*  
*FBgn0033817*  
FBgn0033818  
FBgn0033819  
FBgn0033820  
FBgn0033821  
*FBgn0033826*  
FBgn0033827  
FBgn0033828  
FBgn0033830  
FBgn0033831  
FBgn0033832  
*FBgn0033834*  
*FBgn0033835*  
*FBgn0033836*  
FBgn0033842  
*FBgn0033844*  
*FBgn0033845*  
FBgn0033846  
FBgn0033848  
FBgn0033850  
FBgn0033851  
*FBgn0033853*  
FBgn0033855  
FBgn0033856  
FBgn0033859  
*FBgn0033860*  
*FBgn0033861*  
FBgn0033862  
FBgn0033863  
FBgn0033864  
FBgn0033865  
FBgn0033866  
FBgn0033867  
*FBgn0033868*  
*FBgn0033869*  
*FBgn0033871*  
*FBgn0033872*  
*FBgn0033873*  
*FBgn0033874*  
*FBgn0033875*  
*FBgn0033876*  
*FBgn0033879*  
*FBgn0033880*  
FBgn0033882  
FBgn0033883  
*FBgn0033884*  
FBgn0033886  
FBgn0033887  
FBgn0033888  
*FBgn0033889*  
FBgn0033890  
FBgn0033891  
FBgn0033893  
FBgn0033897  
FBgn0033899  
FBgn0033900  
*FBgn0033902*  
FBgn0033903  
FBgn0033904  
FBgn0033905  
*FBgn0033906*  
FBgn0033907  
FBgn0033911  
*FBgn0033912*  
*FBgn0033913*  
FBgn0033915  
FBgn0033916  
*FBgn0033917*  
FBgn0033918  
FBgn0033919  
FBgn0033921  
FBgn0033924  
FBgn0033925  
*FBgn0033926*  
*FBgn0033927*  
*FBgn0033928*  
*FBgn0033929*  
FBgn0033931  
FBgn0033933  
*FBgn0033934*  
FBgn0033935  
FBgn0033936  
*FBgn0033942*  
*FBgn0033943*  
FBgn0033945  
FBgn0033948  
FBgn0033949  
*FBgn0033950*  
*FBgn0033951*  
FBgn0033953  
FBgn0033954  
FBgn0033955  
FBgn0033957  
FBgn0033958  
*FBgn0033960*  
FBgn0033961  
FBgn0033962  
*FBgn0033963*  
*FBgn0033968*  
*FBgn0033969*  
*FBgn0033970*  
*FBgn0033971*  
FBgn0033972  
FBgn0033973  
*FBgn0033978*  
*FBgn0033979*  
*FBgn0033980*  
*FBgn0033981*  
*FBgn0033982*  
*FBgn0033983*  
*FBgn0033984*  
*FBgn0033985*  
FBgn0033987  
FBgn0033988  
FBgn0033989  
FBgn0033990  
FBgn0033993  
FBgn0033994  
*FBgn0033996*  
*FBgn0033998*  
*FBgn0033999*  
FBgn0034000  
FBgn0034001  
FBgn0034002  
FBgn0034005  
FBgn0034007  
FBgn0034008  
FBgn0034009  
*FBgn0034010*  
FBgn0034011  
FBgn0034012  
*FBgn0034021*  
FBgn0034022  
FBgn0034023  
FBgn0034027  
FBgn0034030  
*FBgn0034031*  
FBgn0034032  
FBgn0034033  
FBgn0034035  
FBgn0034037  
FBgn0034045  
FBgn0034046  
*FBgn0034047*  
FBgn0034049  
*FBgn0034050*  
*FBgn0034051*  
*FBgn0034052*  
*FBgn0034053*  
*FBgn0034054*  
*FBgn0034056*  
FBgn0034057  
FBgn0034058  
FBgn0034059  
FBgn0034060  
FBgn0034061  
*FBgn0034062*  
FBgn0034063  
FBgn0034065  
FBgn0034067  
*FBgn0034068*  
*FBgn0034069*  
FBgn0034070  
FBgn0034071  
FBgn0034072  
FBgn0034073  
*FBgn0034075*  
FBgn0034076  
*FBgn0034082*  
FBgn0034083  
FBgn0034084  
FBgn0034085  
FBgn0034086  
FBgn0034087  
FBgn0034089  
FBgn0034091  
FBgn0034092  
*FBgn0034093*  
FBgn0034094  
FBgn0034095  
FBgn0034096  
FBgn0034097  
FBgn0034098  
FBgn0034099  
*FBgn0034103*  
FBgn0034104  
FBgn0034105  
FBgn0034106  
FBgn0034109  
FBgn0034110  
FBgn0034113  
*FBgn0034114*  
FBgn0034117  
FBgn0034118  
FBgn0034120  
FBgn0034121  
FBgn0034122  
*FBgn0034126*  
*FBgn0034127*  
*FBgn0034128*  
FBgn0034129  
FBgn0034131  
FBgn0034132  
FBgn0034133  
FBgn0034135  
FBgn0034136  
*FBgn0034137*  
*FBgn0034138*  
*FBgn0034139*  
*FBgn0034140*  
*FBgn0034141*  
*FBgn0034142*  
*FBgn0034143*  
*FBgn0034144*  
*FBgn0034145*  
*FBgn0034151*  
FBgn0034152  
FBgn0034153  
FBgn0034154  
FBgn0034156  
FBgn0034157  
*FBgn0034158*  
FBgn0034159  
*FBgn0034160*  
*FBgn0034162*  
FBgn0034165  
*FBgn0034166*  
*FBgn0034168*  
FBgn0034172  
*FBgn0034173*  
*FBgn0034175*  
*FBgn0034176*  
FBgn0034177  
FBgn0034179  
FBgn0034180  
FBgn0034181  
*FBgn0034182*  
*FBgn0034183*  
*FBgn0034184*  
FBgn0034186  
FBgn0034187  
*FBgn0034191*  
FBgn0034194  
*FBgn0034195*  
*FBgn0034196*  
*FBgn0034197*  
FBgn0034198  
FBgn0034199  
*FBgn0034200*  
FBgn0034201  
FBgn0034202  
FBgn0034204  
FBgn0034205  
FBgn0034206  
*FBgn0034210*  
FBgn0034214  
FBgn0034215  
FBgn0034217  
FBgn0034218  
FBgn0034219  
FBgn0034221  
FBgn0034223  
*FBgn0034224*  
FBgn0034225  
*FBgn0034229*  
FBgn0034230  
FBgn0034231  
FBgn0034232  
FBgn0034238  
FBgn0034239  
FBgn0034240  
FBgn0034242  
FBgn0034243  
FBgn0034245  
*FBgn0034247*  
*FBgn0034248*  
*FBgn0034249*  
FBgn0034251  
FBgn0034253  
FBgn0034255  
FBgn0034259  
FBgn0034261  
*FBgn0034262*  
*FBgn0034263*  
*FBgn0034264*  
FBgn0034265  
FBgn0034269  
FBgn0034270  
FBgn0034271  
FBgn0034272  
FBgn0034274  
*FBgn0034275*  
*FBgn0034276*  
FBgn0034277  
*FBgn0034278*  
FBgn0034279  
FBgn0034281  
*FBgn0034282*  
FBgn0034283  
FBgn0034284  
FBgn0034286  
*FBgn0034288*  
*FBgn0034289*  
*FBgn0034290*  
FBgn0034291  
FBgn0034292  
FBgn0034293  
FBgn0034294  
FBgn0034295  
FBgn0034296  
FBgn0034299  
FBgn0034300  
FBgn0034301  
FBgn0034304  
FBgn0034307  
FBgn0034308  
FBgn0034310  
FBgn0034312  
FBgn0034313  
*FBgn0034314*  
*FBgn0034315*  
*FBgn0034318*  
FBgn0034321  
FBgn0034322  
FBgn0034323  
FBgn0034324  
FBgn0034325  
FBgn0034326  
FBgn0034327  
*FBgn0034328*  
FBgn0034329  
FBgn0034330  
FBgn0034331  
*FBgn0034335*  
FBgn0034345  
FBgn0034346  
FBgn0034351  
*FBgn0034352*  
*FBgn0034354*  
*FBgn0034356*  
FBgn0034360  
FBgn0034361  
FBgn0034362  
*FBgn0034363*  
*FBgn0034364*  
FBgn0034366  
FBgn0034367  
FBgn0034368  
FBgn0034371  
FBgn0034374  
FBgn0034377  
FBgn0034379  
FBgn0034380  
*FBgn0034381*  
*FBgn0034382*  
*FBgn0034383*  
FBgn0034387  
*FBgn0034389*  
*FBgn0034390*  
*FBgn0034391*  
*FBgn0034392*  
*FBgn0034394*  
FBgn0034396  
FBgn0034397  
*FBgn0034398*  
FBgn0034399  
FBgn0034400  
FBgn0034401  
*FBgn0034402*  
*FBgn0034403*  
FBgn0034405  
*FBgn0034406*  
*FBgn0034407*  
*FBgn0034408*  
FBgn0034410  
*FBgn0034411*  
*FBgn0034412*  
FBgn0034413  
*FBgn0034415*  
FBgn0034416  
*FBgn0034417*  
FBgn0034418  
FBgn0034419  
FBgn0034420  
FBgn0034421  
FBgn0034422  
FBgn0034423  
*FBgn0034425*  
FBgn0034426  
FBgn0034427  
*FBgn0034429*  
FBgn0034430  
*FBgn0034432*  
FBgn0034433  
FBgn0034434  
*FBgn0034435*  
*FBgn0034436*  
*FBgn0034437*  
*FBgn0034438*  
*FBgn0034439*  
FBgn0034440  
FBgn0034441  
*FBgn0034442*  
*FBgn0034443*  
*FBgn0034446*  
*FBgn0034447*  
*FBgn0034451*  
*FBgn0034452*  
*FBgn0034454*  
*FBgn0034455*  
FBgn0034456  
FBgn0034457  
FBgn0034458  
FBgn0034459  
FBgn0034460  
FBgn0034461  
FBgn0034462  
FBgn0034463  
FBgn0034464  
*FBgn0034467*  
*FBgn0034468*  
*FBgn0034470*  
*FBgn0034471*  
*FBgn0034472*  
*FBgn0034473*  
*FBgn0034474*  
FBgn0034475  
FBgn0034477  
FBgn0034478  
*FBgn0034479*  
*FBgn0034480*  
FBgn0034481  
FBgn0034483  
FBgn0034484  
*FBgn0034485*  
FBgn0034486  
FBgn0034487  
*FBgn0034488*  
*FBgn0034489*  
*FBgn0034490*  
FBgn0034491  
*FBgn0034493*  
*FBgn0034494*  
FBgn0034495  
FBgn0034496  
*FBgn0034497*  
*FBgn0034498*  
FBgn0034499  
FBgn0034500  
FBgn0034501  
FBgn0034502  
FBgn0034503  
FBgn0034504  
FBgn0034505  
FBgn0034506  
FBgn0034507  
FBgn0034509  
*FBgn0034510*  
*FBgn0034512*  
FBgn0034513  
FBgn0034514  
FBgn0034515  
FBgn0034520  
*FBgn0034521*  
*FBgn0034523*  
FBgn0034527  
FBgn0034528  
FBgn0034529  
*FBgn0034530*  
*FBgn0034532*  
FBgn0034535  
FBgn0034537  
*FBgn0034538*  
FBgn0034539  
*FBgn0034540*  
FBgn0034541  
*FBgn0034543*  
FBgn0034545  
FBgn0034546  
FBgn0034548  
FBgn0034550  
FBgn0034551  
FBgn0034552  
FBgn0034553  
*FBgn0034554*  
FBgn0034558  
FBgn0034560  
FBgn0034563  
*FBgn0034564*  
FBgn0034565  
FBgn0034566  
*FBgn0034567*  
FBgn0034568  
FBgn0034569  
FBgn0034570  
FBgn0034572  
FBgn0034573  
*FBgn0034576*  
*FBgn0034577*  
FBgn0034578  
FBgn0034579  
*FBgn0034580*  
FBgn0034582  
FBgn0034583  
FBgn0034585  
FBgn0034588  
*FBgn0034590*  
*FBgn0034592*  
*FBgn0034595*  
FBgn0034598  
FBgn0034599  
*FBgn0034601*  
FBgn0034602  
FBgn0034603  
*FBgn0034605*  
*FBgn0034606*  
*FBgn0034611*  
*FBgn0034612*  
*FBgn0034614*  
FBgn0034617  
FBgn0034618  
FBgn0034622  
FBgn0034623  
FBgn0034624  
*FBgn0034626*  
*FBgn0034627*  
FBgn0034631  
FBgn0034634  
FBgn0034636  
*FBgn0034638*  
FBgn0034639  
FBgn0034641  
*FBgn0034642*  
FBgn0034643  
FBgn0034644  
FBgn0034646  
FBgn0034647  
FBgn0034650  
FBgn0034651  
*FBgn0034654*  
*FBgn0034655*  
*FBgn0034656*  
*FBgn0034657*  
FBgn0034659  
FBgn0034660  
FBgn0034661  
*FBgn0034662*  
FBgn0034663  
FBgn0034664  
FBgn0034665  
FBgn0034666  
FBgn0034667  
FBgn0034670  
FBgn0034671  
*FBgn0034674*  
FBgn0034683  
FBgn0034684  
*FBgn0034687*  
FBgn0034688  
FBgn0034691  
*FBgn0034692*  
*FBgn0034693*  
*FBgn0034694*  
FBgn0034697  
FBgn0034700  
FBgn0034703  
FBgn0034704  
FBgn0034705  
*FBgn0034706*  
FBgn0034707  
FBgn0034708  
FBgn0034709  
FBgn0034710  
FBgn0034711  
FBgn0034712  
*FBgn0034713*  
*FBgn0034716*  
*FBgn0034717*  
FBgn0034718  
*FBgn0034720*  
*FBgn0034721*  
*FBgn0034722*  
*FBgn0034723*  
*FBgn0034724*  
*FBgn0034725*  
FBgn0034726  
FBgn0034727  
FBgn0034728  
*FBgn0034729*  
FBgn0034730  
FBgn0034731  
FBgn0034732  
*FBgn0034733*  
FBgn0034734  
FBgn0034735  
FBgn0034736  
FBgn0034737  
FBgn0034739  
*FBgn0034740*  
*FBgn0034742*  
*FBgn0034743*  
*FBgn0034744*  
*FBgn0034745*  
FBgn0034748  
FBgn0034750  
*FBgn0034753*  
FBgn0034755  
FBgn0034756  
FBgn0034759  
*FBgn0034761*  
FBgn0034763  
FBgn0034766  
FBgn0034768  
FBgn0034769  
FBgn0034770  
FBgn0034774  
FBgn0034776  
FBgn0034782  
FBgn0034783  
FBgn0034784  
FBgn0034785  
*FBgn0034786*  
FBgn0034788  
FBgn0034789  
FBgn0034791  
FBgn0034792  
FBgn0034793  
FBgn0034794  
*FBgn0034797*  
*FBgn0034800*  
FBgn0034802  
FBgn0034803  
*FBgn0034804*  
*FBgn0034807*  
*FBgn0034808*  
FBgn0034814  
*FBgn0034816*  
*FBgn0034817*  
*FBgn0034819*  
FBgn0034820  
FBgn0034821  
FBgn0034822  
FBgn0034824  
FBgn0034825  
FBgn0034826  
FBgn0034827  
*FBgn0034828*  
FBgn0034829  
FBgn0034833  
FBgn0034834  
FBgn0034835  
FBgn0034838  
FBgn0034839  
FBgn0034840  
FBgn0034841  
FBgn0034842  
FBgn0034844  
FBgn0034846  
FBgn0034849  
*FBgn0034850*  
FBgn0034853  
FBgn0034854  
FBgn0034859  
*FBgn0034860*  
*FBgn0034861*  
FBgn0034862  
FBgn0034863  
FBgn0034865  
FBgn0034866  
FBgn0034867  
FBgn0034869  
FBgn0034870  
*FBgn0034871*  
FBgn0034876  
*FBgn0034877*  
FBgn0034878  
FBgn0034879  
*FBgn0034880*  
FBgn0034882  
FBgn0034883  
FBgn0034884  
*FBgn0034885*  
*FBgn0034886*  
*FBgn0034887*  
*FBgn0034888*  
*FBgn0034889*  
FBgn0034893  
FBgn0034894  
FBgn0034897  
*FBgn0034898*  
*FBgn0034901*  
*FBgn0034902*  
FBgn0034903  
FBgn0034904  
FBgn0034906  
FBgn0034907  
FBgn0034908  
*FBgn0034909*  
FBgn0034910  
FBgn0034911  
FBgn0034913  
FBgn0034914  
FBgn0034915  
FBgn0034918  
FBgn0034919  
*FBgn0034920*  
*FBgn0034921*  
FBgn0034922  
FBgn0034923  
FBgn0034924  
*FBgn0034925*  
*FBgn0034926*  
*FBgn0034928*  
FBgn0034931  
FBgn0034933  
*FBgn0034935*  
*FBgn0034936*  
*FBgn0034937*  
FBgn0034938  
FBgn0034939  
FBgn0034940  
*FBgn0034942*  
FBgn0034945  
FBgn0034946  
FBgn0034948  
*FBgn0034950*  
*FBgn0034951*  
FBgn0034956  
FBgn0034957  
FBgn0034958  
FBgn0034959  
FBgn0034961  
FBgn0034962  
FBgn0034963  
FBgn0034964  
FBgn0034965  
FBgn0034966  
*FBgn0034968*  
FBgn0034970  
FBgn0034971  
FBgn0034972  
FBgn0034973  
FBgn0034974  
FBgn0034975  
FBgn0034976  
FBgn0034978  
*FBgn0034979*  
FBgn0034982  
*FBgn0034985*  
FBgn0034986  
FBgn0034987  
FBgn0034988  
FBgn0034989  
FBgn0034990  
FBgn0034994  
FBgn0034996  
*FBgn0034997*  
*FBgn0034998*  
*FBgn0034999*  
FBgn0035000  
FBgn0035001  
FBgn0035002  
FBgn0035003  
FBgn0035004  
FBgn0035005  
FBgn0035006  
FBgn0035007  
FBgn0035008  
FBgn0035009  
FBgn0035010  
FBgn0035011  
FBgn0035012  
FBgn0035014  
FBgn0035016  
*FBgn0035019*  
*FBgn0035020*  
FBgn0035021  
*FBgn0035022*  
FBgn0035023  
FBgn0035024  
FBgn0035025  
FBgn0035026  
FBgn0035027  
FBgn0035028  
FBgn0035031  
*FBgn0035033*  
*FBgn0035034*  
FBgn0035035  
FBgn0035036  
*FBgn0035039*  
*FBgn0035040*  
*FBgn0035041*  
*FBgn0035042*  
*FBgn0035043*  
*FBgn0035044*  
*FBgn0035046*  
*FBgn0035047*  
*FBgn0035049*  
FBgn0035050  
FBgn0035056  
FBgn0035057  
FBgn0035059  
FBgn0035063  
FBgn0035064  
FBgn0035065  
FBgn0035068  
FBgn0035069  
FBgn0035070  
FBgn0035073  
FBgn0035077  
FBgn0035078  
FBgn0035082  
*FBgn0035084*  
FBgn0035085  
*FBgn0035086*  
*FBgn0035087*  
*FBgn0035088*  
*FBgn0035090*  
*FBgn0035091*  
*FBgn0035092*  
*FBgn0035094*  
FBgn0035097  
FBgn0035099  
*FBgn0035100*  
FBgn0035101  
*FBgn0035102*  
*FBgn0035103*  
*FBgn0035104*  
*FBgn0035106*  
FBgn0035107  
*FBgn0035109*  
FBgn0035110  
FBgn0035111  
*FBgn0035112*  
*FBgn0035113*  
FBgn0035120  
FBgn0035122  
FBgn0035124  
FBgn0035131  
FBgn0035132  
FBgn0035134  
FBgn0035136  
FBgn0035137  
FBgn0035138  
FBgn0035139  
FBgn0035140  
FBgn0035141  
*FBgn0035142*  
FBgn0035143  
FBgn0035144  
FBgn0035145  
FBgn0035146  
FBgn0035147  
FBgn0035148  
FBgn0035149  
FBgn0035150  
FBgn0035151  
FBgn0035152  
FBgn0035153  
FBgn0035154  
FBgn0035155  
FBgn0035157  
FBgn0035158  
FBgn0035159  
FBgn0035160  
*FBgn0035161*  
FBgn0035162  
FBgn0035164  
*FBgn0035165*  
*FBgn0035166*  
FBgn0035167  
FBgn0035168  
*FBgn0035169*  
FBgn0035170  
FBgn0035171  
FBgn0035173  
*FBgn0035176*  
FBgn0035178  
FBgn0035179  
*FBgn0035181*  
*FBgn0035186*  
*FBgn0035187*  
*FBgn0035189*  
FBgn0035192  
*FBgn0035193*  
*FBgn0035194*  
*FBgn0035195*  
*FBgn0035196*  
FBgn0035199  
FBgn0035202  
FBgn0035203  
FBgn0035204  
FBgn0035205  
FBgn0035206  
FBgn0035207  
*FBgn0035208*  
*FBgn0035209*  
FBgn0035210  
FBgn0035211  
FBgn0035213  
FBgn0035216  
FBgn0035217  
FBgn0035218  
FBgn0035228  
FBgn0035229  
*FBgn0035231*  
FBgn0035232  
FBgn0035233  
FBgn0035234  
FBgn0035235  
FBgn0035236  
FBgn0035237  
FBgn0035238  
*FBgn0035241*  
FBgn0035243  
FBgn0035244  
*FBgn0035245*  
*FBgn0035246*  
FBgn0035247  
FBgn0035248  
FBgn0035249  
FBgn0035251  
FBgn0035252  
FBgn0035253  
FBgn0035254  
FBgn0035255  
FBgn0035256  
FBgn0035257  
FBgn0035258  
FBgn0035260  
FBgn0035262  
FBgn0035263  
FBgn0035264  
FBgn0035265  
*FBgn0035266*  
*FBgn0035267*  
FBgn0035268  
FBgn0035270  
FBgn0035271  
FBgn0035272  
*FBgn0035273*  
FBgn0035279  
FBgn0035280  
FBgn0035281  
FBgn0035282  
FBgn0035283  
FBgn0035285  
*FBgn0035286*  
*FBgn0035287*  
*FBgn0035289*  
*FBgn0035290*  
FBgn0035293  
FBgn0035294  
FBgn0035295  
FBgn0035298  
*FBgn0035299*  
*FBgn0035300*  
FBgn0035308  
FBgn0035309  
*FBgn0035312*  
*FBgn0035313*  
FBgn0035315  
FBgn0035316  
*FBgn0035318*  
*FBgn0035321*  
FBgn0035323  
FBgn0035325  
*FBgn0035332*  
FBgn0035333  
FBgn0035334  
FBgn0035335  
FBgn0035336  
FBgn0035337  
FBgn0035338  
*FBgn0035343*  
*FBgn0035344*  
*FBgn0035346*  
FBgn0035347  
FBgn0035348  
*FBgn0035355*  
*FBgn0035356*  
FBgn0035358  
FBgn0035359  
*FBgn0035360*  
FBgn0035364  
FBgn0035366  
*FBgn0035367*  
FBgn0035370  
FBgn0035371  
FBgn0035372  
FBgn0035374  
FBgn0035375  
FBgn0035376  
FBgn0035378  
FBgn0035379  
FBgn0035380  
FBgn0035382  
*FBgn0035383*  
FBgn0035384  
FBgn0035385  
FBgn0035388  
FBgn0035390  
FBgn0035392  
FBgn0035393  
FBgn0035397  
*FBgn0035398*  
*FBgn0035399*  
FBgn0035400  
FBgn0035401  
FBgn0035402  
FBgn0035403  
*FBgn0035404*  
FBgn0035405  
*FBgn0035407*  
*FBgn0035409*  
*FBgn0035410*  
*FBgn0035411*  
FBgn0035412  
FBgn0035413  
*FBgn0035414*  
FBgn0035415  
FBgn0035416  
FBgn0035420  
FBgn0035421  
FBgn0035422  
*FBgn0035423*  
*FBgn0035424*  
*FBgn0035425*  
FBgn0035426  
FBgn0035427  
FBgn0035428  
FBgn0035429  
FBgn0035430  
*FBgn0035431*  
*FBgn0035432*  
FBgn0035434  
*FBgn0035435*  
FBgn0035436  
FBgn0035437  
FBgn0035438  
*FBgn0035439*  
*FBgn0035440*  
*FBgn0035443*  
*FBgn0035444*  
*FBgn0035445*  
FBgn0035449  
*FBgn0035452*  
FBgn0035453  
FBgn0035454  
FBgn0035455  
FBgn0035458  
FBgn0035461  
FBgn0035462  
*FBgn0035464*  
*FBgn0035468*  
FBgn0035469  
FBgn0035470  
*FBgn0035471*  
*FBgn0035473*  
FBgn0035475  
FBgn0035476  
FBgn0035477  
FBgn0035478  
FBgn0035479  
*FBgn0035480*  
FBgn0035481  
FBgn0035482  
FBgn0035483  
FBgn0035484  
FBgn0035488  
FBgn0035489  
*FBgn0035490*  
FBgn0035495  
*FBgn0035496*  
FBgn0035497  
FBgn0035499  
FBgn0035500  
*FBgn0035501*  
FBgn0035504  
FBgn0035505  
FBgn0035507  
FBgn0035508  
FBgn0035509  
FBgn0035510  
FBgn0035511  
FBgn0035512  
FBgn0035513  
FBgn0035514  
FBgn0035515  
FBgn0035517  
*FBgn0035518*  
*FBgn0035519*  
*FBgn0035520*  
*FBgn0035522*  
FBgn0035523  
FBgn0035524  
FBgn0035526  
FBgn0035528  
FBgn0035529  
FBgn0035532  
FBgn0035533  
FBgn0035534  
*FBgn0035537*  
*FBgn0035538*  
*FBgn0035539*  
FBgn0035540  
FBgn0035541  
FBgn0035542  
*FBgn0035543*  
*FBgn0035544*  
FBgn0035545  
FBgn0035546  
FBgn0035547  
*FBgn0035548*  
FBgn0035550  
FBgn0035551  
*FBgn0035552*  
*FBgn0035553*  
FBgn0035554  
FBgn0035555  
*FBgn0035557*  
FBgn0035558  
FBgn0035563  
FBgn0035567  
FBgn0035568  
FBgn0035569  
FBgn0035570  
FBgn0035571  
FBgn0035572  
*FBgn0035574*  
FBgn0035575  
FBgn0035577  
*FBgn0035578*  
FBgn0035581  
*FBgn0035582*  
*FBgn0035583*  
FBgn0035584  
FBgn0035585  
*FBgn0035586*  
*FBgn0035587*  
FBgn0035588  
FBgn0035589  
FBgn0035590  
FBgn0035591  
FBgn0035592  
FBgn0035593  
FBgn0035594  
*FBgn0035598*  
FBgn0035600  
FBgn0035601  
*FBgn0035603*  
FBgn0035604  
*FBgn0035607*  
*FBgn0035608*  
*FBgn0035610*  
*FBgn0035611*  
*FBgn0035612*  
FBgn0035619  
FBgn0035620  
*FBgn0035621*  
*FBgn0035622*  
*FBgn0035623*  
*FBgn0035624*  
FBgn0035627  
FBgn0035630  
FBgn0035631  
FBgn0035636  
FBgn0035639  
*FBgn0035640*  
FBgn0035641  
FBgn0035643  
FBgn0035644  
FBgn0035645  
FBgn0035647  
FBgn0035648  
*FBgn0035649*  
*FBgn0035656*  
*FBgn0035657*  
*FBgn0035661*  
*FBgn0035663*  
FBgn0035665  
FBgn0035666  
FBgn0035667  
*FBgn0035669*  
*FBgn0035670*  
FBgn0035673  
FBgn0035674  
FBgn0035675  
FBgn0035676  
FBgn0035677  
*FBgn0035678*  
FBgn0035679  
FBgn0035685  
FBgn0035686  
FBgn0035687  
*FBgn0035688*  
*FBgn0035689*  
FBgn0035690  
FBgn0035691  
FBgn0035692  
*FBgn0035693*  
*FBgn0035694*  
FBgn0035695  
*FBgn0035696*  
*FBgn0035697*  
FBgn0035699  
FBgn0035702  
FBgn0035703  
FBgn0035704  
*FBgn0035707*  
*FBgn0035708*  
*FBgn0035710*  
*FBgn0035711*  
FBgn0035713  
FBgn0035714  
FBgn0035715  
*FBgn0035718*  
*FBgn0035719*  
*FBgn0035720*  
*FBgn0035721*  
FBgn0035722  
*FBgn0035724*  
*FBgn0035725*  
*FBgn0035726*  
FBgn0035727  
FBgn0035730  
FBgn0035733  
*FBgn0035734*  
FBgn0035735  
FBgn0035736  
FBgn0035737  
FBgn0035741  
FBgn0035742  
FBgn0035743  
FBgn0035746  
FBgn0035750  
FBgn0035751  
*FBgn0035753*  
FBgn0035754  
*FBgn0035755*  
FBgn0035760  
FBgn0035761  
FBgn0035762  
FBgn0035763  
FBgn0035765  
FBgn0035766  
FBgn0035767  
*FBgn0035768*  
*FBgn0035769*  
FBgn0035770  
FBgn0035771  
FBgn0035772  
*FBgn0035776*  
FBgn0035777  
FBgn0035779  
FBgn0035780  
FBgn0035781  
*FBgn0035782*  
FBgn0035785  
FBgn0035786  
*FBgn0035787*  
FBgn0035788  
FBgn0035789  
FBgn0035790  
*FBgn0035791*  
*FBgn0035792*  
*FBgn0035793*  
FBgn0035795  
FBgn0035797  
*FBgn0035798*  
FBgn0035799  
FBgn0035800  
FBgn0035802  
FBgn0035805  
*FBgn0035807*  
*FBgn0035811*  
*FBgn0035812*  
FBgn0035813  
FBgn0035815  
FBgn0035816  
*FBgn0035817*  
FBgn0035824  
FBgn0035825  
*FBgn0035827*  
FBgn0035829  
FBgn0035830  
FBgn0035831  
*FBgn0035833*  
FBgn0035838  
FBgn0035839  
FBgn0035842  
*FBgn0035844*  
FBgn0035845  
*FBgn0035847*  
*FBgn0035848*  
*FBgn0035849*  
*FBgn0035850*  
*FBgn0035851*  
*FBgn0035852*  
*FBgn0035853*  
*FBgn0035854*  
FBgn0035855  
FBgn0035856  
FBgn0035857  
*FBgn0035859*  
FBgn0035861  
*FBgn0035865*  
FBgn0035866  
FBgn0035867  
FBgn0035868  
*FBgn0035870*  
FBgn0035871  
FBgn0035872  
FBgn0035873  
FBgn0035875  
FBgn0035876  
FBgn0035877  
FBgn0035878  
FBgn0035879  
*FBgn0035880*  
FBgn0035886  
FBgn0035887  
FBgn0035888  
FBgn0035890  
FBgn0035891  
FBgn0035895  
FBgn0035896  
FBgn0035898  
FBgn0035899  
FBgn0035900  
FBgn0035901  
FBgn0035902  
FBgn0035903  
*FBgn0035904*  
FBgn0035906  
FBgn0035907  
FBgn0035909  
FBgn0035911  
*FBgn0035914*  
FBgn0035915  
*FBgn0035916*  
*FBgn0035917*  
*FBgn0035918*  
FBgn0035921  
FBgn0035922  
*FBgn0035923*  
FBgn0035924  
*FBgn0035926*  
*FBgn0035928*  
FBgn0035929  
FBgn0035931  
FBgn0035932  
FBgn0035933  
*FBgn0035934*  
*FBgn0035935*  
*FBgn0035936*  
*FBgn0035941*  
FBgn0035942  
FBgn0035943  
*FBgn0035944*  
*FBgn0035945*  
FBgn0035947  
FBgn0035948  
FBgn0035949  
*FBgn0035950*  
*FBgn0035951*  
*FBgn0035952*  
FBgn0035953  
FBgn0035954  
FBgn0035955  
FBgn0035956  
FBgn0035957  
FBgn0035959  
FBgn0035960  
FBgn0035964  
FBgn0035965  
FBgn0035966  
FBgn0035967  
*FBgn0035968*  
*FBgn0035969*  
FBgn0035970  
FBgn0035971  
FBgn0035978  
*FBgn0035980*  
FBgn0035981  
*FBgn0035982*  
*FBgn0035983*  
*FBgn0035985*  
FBgn0035986  
FBgn0035987  
*FBgn0035988*  
FBgn0035989  
FBgn0035995  
FBgn0035996  
FBgn0035997  
FBgn0035998  
FBgn0035999  
FBgn0036000  
*FBgn0036003*  
FBgn0036004  
FBgn0036005  
*FBgn0036007*  
*FBgn0036008*  
FBgn0036009  
FBgn0036010  
FBgn0036013  
*FBgn0036014*  
*FBgn0036015*  
FBgn0036016  
FBgn0036017  
FBgn0036018  
*FBgn0036019*  
FBgn0036020  
*FBgn0036022*  
FBgn0036023  
FBgn0036024  
FBgn0036028  
*FBgn0036029*  
FBgn0036030  
FBgn0036031  
FBgn0036032  
FBgn0036035  
FBgn0036036  
FBgn0036038  
*FBgn0036039*  
*FBgn0036040*  
*FBgn0036043*  
FBgn0036044  
FBgn0036046  
FBgn0036052  
FBgn0036058  
FBgn0036059  
FBgn0036062  
FBgn0036063  
*FBgn0036064*  
FBgn0036066  
FBgn0036070  
FBgn0036072  
FBgn0036075  
FBgn0036078  
FBgn0036080  
FBgn0036082  
FBgn0036083  
FBgn0036085  
FBgn0036089  
FBgn0036090  
*FBgn0036091*  
FBgn0036093  
FBgn0036094  
FBgn0036096  
*FBgn0036099*  
FBgn0036101  
*FBgn0036102*  
FBgn0036104  
*FBgn0036105*  
*FBgn0036106*  
FBgn0036107  
FBgn0036108  
FBgn0036109  
*FBgn0036110*  
*FBgn0036111*  
FBgn0036112  
*FBgn0036115*  
*FBgn0036116*  
FBgn0036117  
FBgn0036118  
FBgn0036121  
FBgn0036122  
FBgn0036124  
*FBgn0036125*  
*FBgn0036126*  
FBgn0036128  
FBgn0036131  
*FBgn0036133*  
FBgn0036134  
FBgn0036135  
FBgn0036139  
FBgn0036141  
FBgn0036142  
*FBgn0036143*  
FBgn0036145  
FBgn0036146  
FBgn0036147  
*FBgn0036150*  
FBgn0036152  
FBgn0036153  
*FBgn0036154*  
FBgn0036155  
FBgn0036156  
*FBgn0036157*  
*FBgn0036158*  
FBgn0036159  
FBgn0036160  
FBgn0036161  
FBgn0036162  
*FBgn0036165*  
*FBgn0036168*  
*FBgn0036173*  
FBgn0036179  
*FBgn0036180*  
*FBgn0036181*  
*FBgn0036183*  
*FBgn0036184*  
*FBgn0036186*  
FBgn0036187  
FBgn0036188  
FBgn0036191  
FBgn0036192  
FBgn0036194  
*FBgn0036195*  
FBgn0036196  
FBgn0036198  
FBgn0036199  
*FBgn0036202*  
*FBgn0036203*  
*FBgn0036204*  
FBgn0036205  
*FBgn0036206*  
FBgn0036207  
*FBgn0036208*  
*FBgn0036210*  
*FBgn0036211*  
*FBgn0036212*  
*FBgn0036213*  
*FBgn0036214*  
FBgn0036217  
FBgn0036218  
FBgn0036219  
FBgn0036220  
FBgn0036221  
FBgn0036222  
FBgn0036223  
FBgn0036224  
FBgn0036225  
FBgn0036226  
FBgn0036227  
FBgn0036229  
FBgn0036230  
FBgn0036232  
FBgn0036233  
FBgn0036234  
FBgn0036235  
FBgn0036236  
*FBgn0036237*  
*FBgn0036239*  
*FBgn0036240*  
*FBgn0036242*  
*FBgn0036246*  
FBgn0036248  
FBgn0036249  
*FBgn0036250*  
FBgn0036254  
FBgn0036255  
FBgn0036257  
*FBgn0036258*  
FBgn0036259  
FBgn0036260  
*FBgn0036262*  
FBgn0036263  
FBgn0036264  
FBgn0036266  
FBgn0036271  
FBgn0036272  
*FBgn0036273*  
FBgn0036274  
*FBgn0036277*  
*FBgn0036278*  
*FBgn0036282*  
FBgn0036285  
*FBgn0036286*  
FBgn0036287  
*FBgn0036288*  
*FBgn0036289*  
FBgn0036290  
FBgn0036291  
FBgn0036292  
*FBgn0036294*  
FBgn0036298  
FBgn0036299  
FBgn0036300  
FBgn0036301  
FBgn0036302  
FBgn0036305  
FBgn0036306  
FBgn0036309  
FBgn0036310  
FBgn0036311  
*FBgn0036314*  
*FBgn0036316*  
*FBgn0036317*  
*FBgn0036318*  
*FBgn0036319*  
FBgn0036320  
FBgn0036321  
FBgn0036322  
FBgn0036323  
FBgn0036324  
FBgn0036325  
FBgn0036327  
FBgn0036328  
FBgn0036329  
FBgn0036330  
FBgn0036331  
FBgn0036332  
*FBgn0036334*  
FBgn0036335  
FBgn0036336  
FBgn0036337  
FBgn0036338  
*FBgn0036340*  
FBgn0036341  
FBgn0036342  
FBgn0036343  
FBgn0036345  
FBgn0036346  
FBgn0036348  
FBgn0036349  
FBgn0036350  
FBgn0036351  
FBgn0036352  
*FBgn0036353*  
*FBgn0036354*  
*FBgn0036356*  
FBgn0036359  
FBgn0036360  
FBgn0036361  
FBgn0036362  
FBgn0036363  
FBgn0036364  
FBgn0036365  
FBgn0036366  
*FBgn0036367*  
*FBgn0036368*  
*FBgn0036369*  
FBgn0036372  
FBgn0036373  
FBgn0036374  
FBgn0036376  
FBgn0036377  
FBgn0036380  
*FBgn0036381*  
FBgn0036382  
FBgn0036386  
FBgn0036389  
FBgn0036390  
FBgn0036391  
FBgn0036393  
FBgn0036395  
FBgn0036396  
FBgn0036397  
FBgn0036398  
FBgn0036402  
*FBgn0036403*  
FBgn0036405  
FBgn0036406  
FBgn0036410  
FBgn0036411  
*FBgn0036414*  
*FBgn0036415*  
FBgn0036416  
FBgn0036417  
*FBgn0036419*  
*FBgn0036421*  
*FBgn0036422*  
FBgn0036423  
*FBgn0036426*  
*FBgn0036427*  
FBgn0036428  
*FBgn0036433*  
*FBgn0036436*  
FBgn0036437  
FBgn0036438  
FBgn0036439  
FBgn0036440  
FBgn0036441  
FBgn0036442  
FBgn0036443  
FBgn0036446  
*FBgn0036447*  
FBgn0036448  
FBgn0036449  
FBgn0036450  
FBgn0036451  
*FBgn0036454*  
FBgn0036459  
FBgn0036460  
*FBgn0036461*  
*FBgn0036462*  
*FBgn0036463*  
FBgn0036465  
FBgn0036466  
FBgn0036467  
FBgn0036468  
FBgn0036469  
*FBgn0036470*  
*FBgn0036471*  
FBgn0036474  
FBgn0036476  
FBgn0036478  
FBgn0036479  
FBgn0036480  
FBgn0036481  
FBgn0036482  
FBgn0036483  
FBgn0036484  
*FBgn0036485*  
*FBgn0036486*  
FBgn0036487  
FBgn0036488  
FBgn0036489  
*FBgn0036490*  
FBgn0036491  
FBgn0036492  
*FBgn0036493*  
*FBgn0036495*  
FBgn0036496  
FBgn0036498  
*FBgn0036499*  
FBgn0036500  
FBgn0036501  
FBgn0036502  
*FBgn0036503*  
*FBgn0036505*  
FBgn0036509  
FBgn0036510  
FBgn0036511  
FBgn0036512  
FBgn0036514  
FBgn0036515  
FBgn0036516  
FBgn0036518  
FBgn0036519  
FBgn0036520  
FBgn0036522  
FBgn0036527  
FBgn0036528  
*FBgn0036529*  
FBgn0036531  
FBgn0036532  
FBgn0036534  
*FBgn0036536*  
FBgn0036537  
FBgn0036538  
FBgn0036541  
FBgn0036542  
FBgn0036544  
FBgn0036545  
FBgn0036546  
FBgn0036547  
FBgn0036548  
FBgn0036549  
*FBgn0036550*  
*FBgn0036551*  
FBgn0036552  
FBgn0036553  
FBgn0036556  
FBgn0036557  
FBgn0036558  
*FBgn0036560*  
*FBgn0036563*  
FBgn0036564  
FBgn0036565  
FBgn0036567  
FBgn0036568  
FBgn0036569  
FBgn0036570  
FBgn0036571  
*FBgn0036573*  
*FBgn0036574*  
FBgn0036575  
*FBgn0036576*  
FBgn0036577  
FBgn0036578  
FBgn0036579  
FBgn0036581  
FBgn0036583  
FBgn0036584  
FBgn0036585  
FBgn0036586  
*FBgn0036587*  
FBgn0036588  
FBgn0036589  
FBgn0036590  
FBgn0036591  
FBgn0036592  
FBgn0036593  
FBgn0036594  
FBgn0036595  
FBgn0036596  
*FBgn0036597*  
*FBgn0036599*  
*FBgn0036600*  
FBgn0036601  
FBgn0036602  
FBgn0036603  
FBgn0036605  
FBgn0036606  
FBgn0036607  
FBgn0036608  
FBgn0036609  
FBgn0036610  
FBgn0036612  
FBgn0036614  
FBgn0036615  
*FBgn0036616*  
FBgn0036617  
FBgn0036618  
FBgn0036619  
FBgn0036620  
*FBgn0036621*  
*FBgn0036622*  
*FBgn0036623*  
*FBgn0036624*  
FBgn0036626  
FBgn0036627  
FBgn0036628  
FBgn0036629  
FBgn0036637  
FBgn0036638  
FBgn0036639  
*FBgn0036640*  
FBgn0036641  
FBgn0036643  
FBgn0036652  
FBgn0036654  
FBgn0036655  
FBgn0036656  
*FBgn0036659*  
FBgn0036660  
FBgn0036661  
FBgn0036662  
FBgn0036663  
*FBgn0036665*  
FBgn0036666  
FBgn0036667  
FBgn0036668  
FBgn0036670  
*FBgn0036671*  
FBgn0036676  
FBgn0036677  
FBgn0036678  
FBgn0036679  
FBgn0036680  
FBgn0036681  
FBgn0036684  
FBgn0036685  
FBgn0036686  
*FBgn0036687*  
*FBgn0036688*  
FBgn0036689  
FBgn0036690  
FBgn0036691  
FBgn0036695  
FBgn0036696  
*FBgn0036697*  
*FBgn0036698*  
*FBgn0036702*  
FBgn0036703  
FBgn0036704  
FBgn0036705  
FBgn0036706  
FBgn0036707  
FBgn0036708  
FBgn0036709  
*FBgn0036710*  
FBgn0036711  
FBgn0036712  
*FBgn0036713*  
*FBgn0036714*  
*FBgn0036715*  
*FBgn0036716*  
*FBgn0036717*  
*FBgn0036725*  
*FBgn0036726*  
*FBgn0036727*  
*FBgn0036728*  
FBgn0036729  
FBgn0036730  
FBgn0036731  
*FBgn0036732*  
FBgn0036734  
FBgn0036735  
*FBgn0036738*  
*FBgn0036740*  
FBgn0036741  
FBgn0036742  
FBgn0036745  
FBgn0036746  
FBgn0036747  
FBgn0036749  
FBgn0036750  
FBgn0036754  
*FBgn0036756*  
FBgn0036757  
FBgn0036759  
FBgn0036760  
FBgn0036761  
*FBgn0036762*  
FBgn0036763  
FBgn0036764  
*FBgn0036765*  
FBgn0036766  
FBgn0036767  
*FBgn0036768*  
*FBgn0036769*  
*FBgn0036770*  
FBgn0036771  
FBgn0036772  
FBgn0036773  
FBgn0036774  
FBgn0036775  
*FBgn0036777*  
*FBgn0036778*  
*FBgn0036780*  
FBgn0036781  
FBgn0036782  
FBgn0036783  
FBgn0036784  
FBgn0036785  
FBgn0036786  
*FBgn0036787*  
FBgn0036789  
FBgn0036790  
FBgn0036794  
FBgn0036795  
FBgn0036796  
FBgn0036804  
FBgn0036805  
*FBgn0036806*  
FBgn0036807  
FBgn0036808  
FBgn0036809  
FBgn0036810  
FBgn0036811  
FBgn0036812  
FBgn0036813  
FBgn0036814  
FBgn0036815  
*FBgn0036816*  
*FBgn0036817*  
FBgn0036818  
FBgn0036819  
*FBgn0036820*  
*FBgn0036821*  
*FBgn0036822*  
FBgn0036824  
FBgn0036825  
FBgn0036826  
FBgn0036827  
FBgn0036828  
*FBgn0036831*  
*FBgn0036832*  
*FBgn0036833*  
*FBgn0036834*  
*FBgn0036835*  
FBgn0036836  
*FBgn0036837*  
FBgn0036838  
FBgn0036839  
FBgn0036842  
FBgn0036843  
*FBgn0036844*  
FBgn0036846  
FBgn0036847  
FBgn0036848  
*FBgn0036849*  
*FBgn0036850*  
FBgn0036851  
FBgn0036853  
*FBgn0036857*  
FBgn0036858  
FBgn0036859  
FBgn0036860  
FBgn0036861  
FBgn0036862  
FBgn0036868  
*FBgn0036870*  
FBgn0036871  
FBgn0036874  
FBgn0036875  
FBgn0036876  
*FBgn0036877*  
FBgn0036878  
FBgn0036879  
FBgn0036880  
FBgn0036881  
*FBgn0036882*  
*FBgn0036886*  
FBgn0036887  
FBgn0036888  
FBgn0036889  
*FBgn0036890*  
*FBgn0036891*  
FBgn0036892  
FBgn0036893  
*FBgn0036895*  
FBgn0036896  
FBgn0036897  
*FBgn0036899*  
*FBgn0036900*  
*FBgn0036905*  
*FBgn0036906*  
*FBgn0036909*  
*FBgn0036910*  
FBgn0036911  
FBgn0036913  
FBgn0036915  
FBgn0036916  
FBgn0036918  
FBgn0036919  
FBgn0036920  
FBgn0036921  
*FBgn0036922*  
FBgn0036923  
FBgn0036924  
FBgn0036925  
*FBgn0036926*  
*FBgn0036927*  
FBgn0036928  
FBgn0036929  
*FBgn0036931*  
*FBgn0036932*  
*FBgn0036935*  
FBgn0036936  
FBgn0036937  
FBgn0036938  
*FBgn0036939*  
FBgn0036941  
FBgn0036942  
*FBgn0036945*  
*FBgn0036948*  
FBgn0036949  
FBgn0036950  
FBgn0036951  
FBgn0036952  
FBgn0036953  
*FBgn0036956*  
FBgn0036958  
FBgn0036959  
FBgn0036960  
FBgn0036962  
FBgn0036964  
FBgn0036967  
FBgn0036969  
FBgn0036970  
FBgn0036972  
*FBgn0036973*  
*FBgn0036974*  
FBgn0036975  
FBgn0036977  
FBgn0036979  
FBgn0036980  
*FBgn0036984*  
*FBgn0036985*  
*FBgn0036986*  
FBgn0036987  
FBgn0036988  
FBgn0036990  
FBgn0036991  
*FBgn0036992*  
*FBgn0036993*  
*FBgn0036994*  
*FBgn0036995*  
*FBgn0036996*  
*FBgn0036997*  
FBgn0036998  
FBgn0036999  
*FBgn0037000*  
*FBgn0037001*  
FBgn0037003  
FBgn0037004  
FBgn0037005  
*FBgn0037007*  
FBgn0037008  
FBgn0037009  
FBgn0037010  
FBgn0037011  
*FBgn0037012*  
*FBgn0037013*  
FBgn0037014  
FBgn0037015  
*FBgn0037016*  
FBgn0037017  
FBgn0037018  
FBgn0037019  
FBgn0037020  
FBgn0037021  
FBgn0037022  
FBgn0037023  
*FBgn0037024*  
*FBgn0037025*  
FBgn0037026  
FBgn0037027  
FBgn0037028  
*FBgn0037030*  
*FBgn0037031*  
*FBgn0037035*  
FBgn0037036  
FBgn0037037  
FBgn0037038  
FBgn0037039  
FBgn0037040  
FBgn0037042  
FBgn0037044  
FBgn0037045  
FBgn0037046  
*FBgn0037050*  
*FBgn0037051*  
FBgn0037057  
FBgn0037059  
FBgn0037060  
FBgn0037061  
*FBgn0037063*  
*FBgn0037064*  
*FBgn0037065*  
FBgn0037067  
FBgn0037068  
FBgn0037069  
FBgn0037070  
FBgn0037071  
*FBgn0037073*  
FBgn0037074  
FBgn0037076  
FBgn0037078  
FBgn0037081  
FBgn0037082  
*FBgn0037083*  
FBgn0037084  
FBgn0037085  
FBgn0037086  
FBgn0037087  
FBgn0037092  
*FBgn0037093*  
*FBgn0037094*  
FBgn0037097  
*FBgn0037098*  
FBgn0037099  
FBgn0037100  
FBgn0037101  
*FBgn0037102*  
FBgn0037105  
FBgn0037106  
*FBgn0037107*  
*FBgn0037108*  
*FBgn0037109*  
*FBgn0037110*  
*FBgn0037114*  
*FBgn0037115*  
FBgn0037116  
FBgn0037117  
FBgn0037120  
FBgn0037121  
FBgn0037122  
FBgn0037123  
FBgn0037124  
FBgn0037125  
*FBgn0037126*  
FBgn0037127  
FBgn0037128  
FBgn0037129  
FBgn0037130  
FBgn0037131  
FBgn0037133  
FBgn0037134  
*FBgn0037135*  
FBgn0037137  
FBgn0037138  
*FBgn0037139*  
FBgn0037140  
FBgn0037142  
*FBgn0037143*  
*FBgn0037144*  
*FBgn0037146*  
*FBgn0037147*  
FBgn0037149  
FBgn0037150  
FBgn0037151  
*FBgn0037153*  
FBgn0037156  
FBgn0037162  
*FBgn0037163*  
*FBgn0037164*  
*FBgn0037165*  
*FBgn0037166*  
*FBgn0037167*  
FBgn0037168  
FBgn0037169  
FBgn0037171  
FBgn0037172  
FBgn0037174  
FBgn0037175  
FBgn0037176  
FBgn0037177  
FBgn0037179  
*FBgn0037181*  
*FBgn0037182*  
FBgn0037183  
FBgn0037184  
FBgn0037185  
FBgn0037186  
*FBgn0037191*  
FBgn0037195  
FBgn0037197  
FBgn0037199  
*FBgn0037200*  
FBgn0037202  
*FBgn0037203*  
*FBgn0037204*  
FBgn0037205  
FBgn0037206  
FBgn0037207  
*FBgn0037213*  
FBgn0037215  
*FBgn0037217*  
FBgn0037218  
FBgn0037220  
*FBgn0037222*  
FBgn0037224  
FBgn0037225  
FBgn0037227  
*FBgn0037228*  
*FBgn0037229*  
*FBgn0037230*  
*FBgn0037231*  
*FBgn0037232*  
FBgn0037234  
FBgn0037235  
FBgn0037236  
*FBgn0037238*  
FBgn0037239  
FBgn0037240  
FBgn0037241  
FBgn0037242  
FBgn0037244  
FBgn0037245  
FBgn0037248  
FBgn0037250  
FBgn0037251  
FBgn0037252  
*FBgn0037254*  
*FBgn0037255*  
FBgn0037260  
FBgn0037261  
FBgn0037262  
*FBgn0037263*  
FBgn0037265  
FBgn0037270  
*FBgn0037273*  
*FBgn0037275*  
*FBgn0037276*  
*FBgn0037279*  
*FBgn0037280*  
*FBgn0037282*  
FBgn0037283  
FBgn0037284  
*FBgn0037288*  
*FBgn0037290*  
FBgn0037291  
FBgn0037292  
FBgn0037293  
FBgn0037294  
FBgn0037295  
FBgn0037296  
FBgn0037297  
FBgn0037298  
FBgn0037299  
FBgn0037301  
*FBgn0037304*  
FBgn0037305  
*FBgn0037307*  
FBgn0037310  
FBgn0037312  
FBgn0037313  
FBgn0037315  
*FBgn0037317*  
*FBgn0037320*  
*FBgn0037321*  
FBgn0037322  
FBgn0037323  
FBgn0037324  
FBgn0037325  
FBgn0037326  
*FBgn0037327*  
*FBgn0037328*  
FBgn0037329  
FBgn0037330  
FBgn0037332  
*FBgn0037336*  
*FBgn0037338*  
FBgn0037339  
FBgn0037340  
FBgn0037341  
FBgn0037342  
FBgn0037344  
FBgn0037345  
FBgn0037347  
FBgn0037350  
*FBgn0037351*  
FBgn0037352  
FBgn0037354  
FBgn0037356  
FBgn0037358  
FBgn0037359  
FBgn0037360  
FBgn0037363  
FBgn0037364  
FBgn0037365  
FBgn0037368  
FBgn0037369  
FBgn0037370  
FBgn0037371  
FBgn0037372  
FBgn0037374  
FBgn0037376  
FBgn0037377  
FBgn0037378  
*FBgn0037379*  
*FBgn0037380*  
FBgn0037382  
FBgn0037383  
FBgn0037384  
*FBgn0037385*  
*FBgn0037386*  
*FBgn0037387*  
FBgn0037388  
FBgn0037389  
FBgn0037391  
*FBgn0037395*  
*FBgn0037396*  
FBgn0037397  
FBgn0037398  
FBgn0037399  
FBgn0037405  
FBgn0037406  
FBgn0037408  
FBgn0037409  
FBgn0037410  
FBgn0037411  
FBgn0037412  
FBgn0037413  
FBgn0037414  
FBgn0037415  
FBgn0037416  
FBgn0037417  
FBgn0037418  
FBgn0037419  
FBgn0037420  
FBgn0037421  
FBgn0037422  
FBgn0037424  
FBgn0037427  
FBgn0037428  
FBgn0037429  
FBgn0037430  
FBgn0037431  
FBgn0037432  
*FBgn0037433*  
FBgn0037435  
FBgn0037436  
FBgn0037439  
FBgn0037440  
FBgn0037442  
FBgn0037443  
*FBgn0037445*  
*FBgn0037446*  
*FBgn0037447*  
*FBgn0037448*  
FBgn0037449  
FBgn0037454  
FBgn0037455  
FBgn0037456  
FBgn0037460  
FBgn0037461  
FBgn0037462  
FBgn0037463  
FBgn0037464  
FBgn0037465  
FBgn0037466  
FBgn0037467  
FBgn0037468  
FBgn0037469  
FBgn0037470  
*FBgn0037472*  
*FBgn0037473*  
FBgn0037475  
FBgn0037477  
FBgn0037478  
FBgn0037481  
FBgn0037482  
FBgn0037483  
*FBgn0037485*  
FBgn0037486  
FBgn0037487  
FBgn0037488  
FBgn0037489  
FBgn0037490  
FBgn0037491  
FBgn0037492  
FBgn0037493  
FBgn0037498  
FBgn0037500  
FBgn0037501  
FBgn0037503  
*FBgn0037504*  
FBgn0037506  
FBgn0037512  
*FBgn0037513*  
*FBgn0037514*  
*FBgn0037515*  
*FBgn0037516*  
FBgn0037517  
*FBgn0037518*  
FBgn0037521  
FBgn0037525  
*FBgn0037526*  
*FBgn0037529*  
FBgn0037530  
FBgn0037531  
*FBgn0037533*  
*FBgn0037534*  
*FBgn0037535*  
FBgn0037536  
FBgn0037537  
FBgn0037538  
FBgn0037539  
FBgn0037540  
FBgn0037541  
FBgn0037543  
FBgn0037544  
*FBgn0037546*  
*FBgn0037547*  
FBgn0037548  
FBgn0037549  
FBgn0037550  
FBgn0037551  
*FBgn0037552*  
*FBgn0037553*  
FBgn0037555  
FBgn0037556  
*FBgn0037560*  
FBgn0037561  
*FBgn0037562*  
FBgn0037563  
*FBgn0037565*  
FBgn0037566  
FBgn0037567  
FBgn0037569  
FBgn0037570  
FBgn0037571  
FBgn0037572  
*FBgn0037573*  
*FBgn0037574*  
FBgn0037576  
*FBgn0037577*  
FBgn0037578  
FBgn0037579  
FBgn0037581  
*FBgn0037583*  
FBgn0037584  
FBgn0037589  
FBgn0037590  
FBgn0037591  
*FBgn0037592*  
*FBgn0037594*  
*FBgn0037601*  
FBgn0037602  
FBgn0037603  
FBgn0037606  
*FBgn0037607*  
FBgn0037608  
FBgn0037609  
FBgn0037610  
FBgn0037611  
FBgn0037612  
FBgn0037613  
FBgn0037614  
FBgn0037615  
*FBgn0037616*  
FBgn0037617  
FBgn0037618  
FBgn0037619  
FBgn0037620  
*FBgn0037621*  
*FBgn0037622*  
FBgn0037623  
FBgn0037624  
FBgn0037625  
FBgn0037626  
FBgn0037627  
*FBgn0037630*  
FBgn0037633  
FBgn0037634  
*FBgn0037635*  
FBgn0037636  
FBgn0037637  
*FBgn0037638*  
*FBgn0037640*  
*FBgn0037643*  
*FBgn0037644*  
*FBgn0037645*  
FBgn0037646  
FBgn0037647  
FBgn0037648  
*FBgn0037650*  
FBgn0037652  
FBgn0037653  
*FBgn0037654*  
FBgn0037655  
*FBgn0037656*  
FBgn0037657  
FBgn0037659  
FBgn0037660  
FBgn0037661  
FBgn0037662  
*FBgn0037664*  
FBgn0037665  
FBgn0037666  
*FBgn0037667*  
*FBgn0037668*  
FBgn0037669  
FBgn0037670  
*FBgn0037672*  
*FBgn0037675*  
*FBgn0037676*  
*FBgn0037677*  
*FBgn0037678*  
*FBgn0037679*  
FBgn0037680  
*FBgn0037683*  
*FBgn0037684*  
*FBgn0037685*  
*FBgn0037686*  
FBgn0037687  
FBgn0037688  
FBgn0037689  
*FBgn0037690*  
*FBgn0037696*  
FBgn0037697  
FBgn0037698  
FBgn0037700  
FBgn0037703  
FBgn0037705  
FBgn0037707  
FBgn0037708  
FBgn0037709  
FBgn0037710  
*FBgn0037712*  
*FBgn0037713*  
*FBgn0037714*  
*FBgn0037715*  
*FBgn0037716*  
*FBgn0037717*  
FBgn0037718  
FBgn0037719  
FBgn0037720  
*FBgn0037721*  
*FBgn0037722*  
*FBgn0037723*  
*FBgn0037726*  
*FBgn0037727*  
*FBgn0037728*  
*FBgn0037730*  
FBgn0037731  
FBgn0037734  
FBgn0037736  
*FBgn0037737*  
FBgn0037739  
*FBgn0037741*  
*FBgn0037742*  
FBgn0037743  
FBgn0037744  
*FBgn0037746*  
FBgn0037747  
FBgn0037749  
*FBgn0037750*  
FBgn0037751  
FBgn0037753  
*FBgn0037754*  
*FBgn0037755*  
*FBgn0037756*  
*FBgn0037757*  
*FBgn0037758*  
FBgn0037759  
FBgn0037760  
*FBgn0037761*  
FBgn0037762  
FBgn0037763  
FBgn0037764  
FBgn0037765  
FBgn0037766  
FBgn0037769  
FBgn0037770  
FBgn0037772  
FBgn0037773  
FBgn0037777  
FBgn0037778  
FBgn0037779  
FBgn0037780  
*FBgn0037781*  
FBgn0037782  
FBgn0037783  
*FBgn0037786*  
*FBgn0037788*  
*FBgn0037789*  
FBgn0037792  
FBgn0037794  
*FBgn0037796*  
FBgn0037797  
FBgn0037798  
FBgn0037801  
FBgn0037802  
*FBgn0037804*  
*FBgn0037806*  
*FBgn0037807*  
FBgn0037808  
FBgn0037809  
FBgn0037812  
FBgn0037814  
FBgn0037815  
*FBgn0037816*  
*FBgn0037817*  
*FBgn0037818*  
*FBgn0037819*  
*FBgn0037820*  
FBgn0037822  
FBgn0037824  
FBgn0037826  
FBgn0037827  
FBgn0037828  
FBgn0037829  
*FBgn0037832*  
*FBgn0037834*  
*FBgn0037835*  
FBgn0037836  
FBgn0037837  
*FBgn0037838*  
*FBgn0037841*  
FBgn0037842  
FBgn0037843  
*FBgn0037844*  
*FBgn0037845*  
FBgn0037846  
FBgn0037847  
FBgn0037848  
*FBgn0037849*  
*FBgn0037850*  
FBgn0037852  
FBgn0037853  
FBgn0037855  
FBgn0037856  
FBgn0037857  
FBgn0037860  
FBgn0037862  
FBgn0037870  
FBgn0037873  
FBgn0037874  
FBgn0037875  
FBgn0037876  
FBgn0037877  
FBgn0037878  
FBgn0037880  
FBgn0037881  
FBgn0037882  
FBgn0037883  
FBgn0037884  
FBgn0037885  
FBgn0037890  
FBgn0037891  
FBgn0037892  
FBgn0037893  
FBgn0037894  
FBgn0037895  
FBgn0037896  
FBgn0037897  
FBgn0037898  
FBgn0037900  
FBgn0037901  
FBgn0037902  
FBgn0037908  
*FBgn0037910*  
*FBgn0037911*  
*FBgn0037913*  
FBgn0037915  
FBgn0037916  
*FBgn0037917*  
FBgn0037918  
FBgn0037920  
FBgn0037921  
FBgn0037922  
FBgn0037923  
FBgn0037924  
FBgn0037926  
FBgn0037930  
FBgn0037931  
FBgn0037933  
*FBgn0037934*  
FBgn0037935  
FBgn0037936  
FBgn0037937  
FBgn0037938  
FBgn0037939  
FBgn0037940  
FBgn0037941  
*FBgn0037942*  
FBgn0037943  
FBgn0037944  
*FBgn0037949*  
*FBgn0037950*  
FBgn0037955  
*FBgn0037956*  
FBgn0037958  
FBgn0037960  
*FBgn0037962*  
*FBgn0037963*  
*FBgn0037964*  
FBgn0037970  
*FBgn0037972*  
*FBgn0037973*  
*FBgn0037974*  
*FBgn0037975*  
*FBgn0037976*  
FBgn0037978  
FBgn0037979  
FBgn0037980  
FBgn0037981  
FBgn0037985  
FBgn0037986  
FBgn0037987  
FBgn0037988  
FBgn0037989  
FBgn0037992  
FBgn0037993  
FBgn0037994  
FBgn0037995  
*FBgn0037996*  
FBgn0037998  
FBgn0037999  
FBgn0038000  
FBgn0038001  
*FBgn0038002*  
FBgn0038003  
FBgn0038005  
FBgn0038006  
FBgn0038007  
FBgn0038008  
*FBgn0038009*  
FBgn0038011  
FBgn0038012  
*FBgn0038014*  
*FBgn0038017*  
*FBgn0038018*  
*FBgn0038020*  
FBgn0038028  
FBgn0038029  
*FBgn0038035*  
FBgn0038037  
FBgn0038038  
FBgn0038039  
*FBgn0038042*  
*FBgn0038043*  
*FBgn0038045*  
*FBgn0038046*  
*FBgn0038047*  
FBgn0038049  
FBgn0038051  
*FBgn0038052*  
FBgn0038053  
FBgn0038055  
FBgn0038056  
FBgn0038057  
FBgn0038058  
FBgn0038063  
FBgn0038065  
FBgn0038067  
FBgn0038068  
FBgn0038069  
FBgn0038070  
*FBgn0038071*  
*FBgn0038072*  
*FBgn0038073*  
*FBgn0038074*  
FBgn0038076  
FBgn0038078  
FBgn0038079  
FBgn0038080  
*FBgn0038082*  
*FBgn0038083*  
*FBgn0038088*  
FBgn0038090  
FBgn0038095  
FBgn0038097  
*FBgn0038098*  
*FBgn0038099*  
FBgn0038100  
*FBgn0038102*  
FBgn0038106  
FBgn0038107  
FBgn0038108  
*FBgn0038109*  
FBgn0038110  
FBgn0038111  
FBgn0038113  
FBgn0038114  
*FBgn0038115*  
FBgn0038118  
FBgn0038122  
FBgn0038123  
FBgn0038124  
FBgn0038125  
FBgn0038126  
FBgn0038127  
FBgn0038128  
FBgn0038129  
*FBgn0038130*  
FBgn0038131  
FBgn0038132  
FBgn0038133  
FBgn0038134  
FBgn0038135  
FBgn0038136  
FBgn0038139  
FBgn0038140  
FBgn0038142  
*FBgn0038143*  
*FBgn0038144*  
FBgn0038145  
*FBgn0038146*  
*FBgn0038147*  
*FBgn0038149*  
*FBgn0038153*  
FBgn0038156  
FBgn0038157  
FBgn0038158  
FBgn0038159  
FBgn0038160  
FBgn0038161  
*FBgn0038163*  
FBgn0038165  
FBgn0038166  
FBgn0038167  
*FBgn0038168*  
FBgn0038170  
*FBgn0038175*  
*FBgn0038179*  
*FBgn0038180*  
*FBgn0038181*  
FBgn0038183  
FBgn0038186  
FBgn0038188  
FBgn0038189  
FBgn0038190  
FBgn0038191  
*FBgn0038194*  
FBgn0038195  
FBgn0038196  
*FBgn0038197*  
*FBgn0038198*  
*FBgn0038199*  
*FBgn0038200*  
FBgn0038201  
FBgn0038202  
FBgn0038203  
FBgn0038204  
FBgn0038205  
FBgn0038206  
*FBgn0038207*  
FBgn0038208  
FBgn0038209  
FBgn0038210  
FBgn0038211  
*FBgn0038213*  
*FBgn0038214*  
FBgn0038217  
FBgn0038218  
FBgn0038219  
FBgn0038220  
*FBgn0038221*  
FBgn0038223  
*FBgn0038224*  
FBgn0038225  
FBgn0038233  
FBgn0038234  
FBgn0038235  
*FBgn0038236*  
*FBgn0038237*  
FBgn0038238  
FBgn0038239  
FBgn0038240  
FBgn0038241  
FBgn0038242  
*FBgn0038243*  
FBgn0038244  
*FBgn0038246*  
FBgn0038247  
FBgn0038248  
*FBgn0038250*  
*FBgn0038251*  
FBgn0038252  
FBgn0038256  
*FBgn0038258*  
FBgn0038260  
FBgn0038261  
FBgn0038262  
FBgn0038266  
FBgn0038267  
FBgn0038268  
FBgn0038269  
*FBgn0038271*  
FBgn0038272  
*FBgn0038273*  
FBgn0038274  
FBgn0038275  
FBgn0038277  
*FBgn0038279*  
FBgn0038280  
FBgn0038281  
FBgn0038282  
FBgn0038285  
*FBgn0038286*  
FBgn0038290  
FBgn0038291  
*FBgn0038292*  
FBgn0038293  
FBgn0038294  
FBgn0038295  
FBgn0038296  
FBgn0038299  
FBgn0038300  
FBgn0038301  
*FBgn0038302*  
FBgn0038303  
FBgn0038304  
FBgn0038306  
*FBgn0038309*  
*FBgn0038311*  
FBgn0038312  
FBgn0038313  
*FBgn0038315*  
FBgn0038316  
FBgn0038318  
FBgn0038319  
FBgn0038321  
*FBgn0038323*  
*FBgn0038324*  
FBgn0038325  
FBgn0038326  
FBgn0038327  
FBgn0038330  
FBgn0038331  
*FBgn0038332*  
*FBgn0038337*  
*FBgn0038339*  
FBgn0038341  
FBgn0038342  
*FBgn0038343*  
*FBgn0038344*  
*FBgn0038345*  
*FBgn0038346*  
*FBgn0038347*  
*FBgn0038348*  
*FBgn0038349*  
FBgn0038350  
FBgn0038351  
*FBgn0038353*  
*FBgn0038354*  
FBgn0038355  
FBgn0038356  
FBgn0038357  
FBgn0038358  
FBgn0038359  
*FBgn0038360*  
*FBgn0038361*  
*FBgn0038363*  
FBgn0038365  
FBgn0038366  
*FBgn0038368*  
*FBgn0038369*  
*FBgn0038371*  
*FBgn0038373*  
*FBgn0038377*  
*FBgn0038380*  
*FBgn0038381*  
FBgn0038385  
FBgn0038386  
FBgn0038387  
FBgn0038388  
FBgn0038389  
*FBgn0038390*  
*FBgn0038391*  
*FBgn0038394*  
FBgn0038395  
FBgn0038396  
FBgn0038397  
FBgn0038398  
*FBgn0038400*  
*FBgn0038401*  
FBgn0038402  
FBgn0038404  
FBgn0038405  
FBgn0038407  
*FBgn0038412*  
FBgn0038414  
FBgn0038415  
FBgn0038416  
*FBgn0038418*  
*FBgn0038419*  
FBgn0038420  
FBgn0038421  
*FBgn0038422*  
FBgn0038423  
FBgn0038424  
FBgn0038425  
FBgn0038426  
FBgn0038427  
FBgn0038428  
*FBgn0038429*  
*FBgn0038431*  
*FBgn0038432*  
*FBgn0038437*  
FBgn0038439  
FBgn0038440  
FBgn0038445  
*FBgn0038446*  
FBgn0038447  
FBgn0038448  
FBgn0038449  
FBgn0038450  
FBgn0038451  
FBgn0038452  
FBgn0038453  
FBgn0038454  
*FBgn0038460*  
FBgn0038462  
FBgn0038463  
*FBgn0038464*  
*FBgn0038465*  
*FBgn0038466*  
*FBgn0038467*  
FBgn0038468  
FBgn0038469  
*FBgn0038470*  
FBgn0038471  
FBgn0038472  
FBgn0038473  
FBgn0038474  
FBgn0038475  
FBgn0038476  
FBgn0038478  
FBgn0038479  
FBgn0038481  
FBgn0038482  
*FBgn0038484*  
FBgn0038485  
FBgn0038486  
FBgn0038487  
FBgn0038490  
FBgn0038491  
FBgn0038492  
FBgn0038499  
FBgn0038500  
FBgn0038501  
FBgn0038505  
FBgn0038506  
FBgn0038507  
FBgn0038508  
FBgn0038509  
FBgn0038510  
FBgn0038511  
FBgn0038512  
*FBgn0038515*  
*FBgn0038516*  
FBgn0038519  
FBgn0038523  
*FBgn0038524*  
FBgn0038525  
FBgn0038526  
FBgn0038527  
FBgn0038528  
FBgn0038529  
*FBgn0038530*  
*FBgn0038531*  
*FBgn0038532*  
*FBgn0038533*  
FBgn0038535  
FBgn0038536  
*FBgn0038539*  
FBgn0038540  
FBgn0038541  
FBgn0038542  
FBgn0038545  
FBgn0038546  
FBgn0038547  
FBgn0038548  
FBgn0038549  
FBgn0038550  
FBgn0038551  
FBgn0038552  
FBgn0038558  
*FBgn0038564*  
*FBgn0038565*  
FBgn0038566  
FBgn0038567  
FBgn0038568  
FBgn0038569  
*FBgn0038575*  
FBgn0038576  
*FBgn0038577*  
*FBgn0038578*  
*FBgn0038579*  
FBgn0038581  
FBgn0038582  
FBgn0038583  
FBgn0038584  
FBgn0038585  
FBgn0038586  
FBgn0038588  
FBgn0038589  
FBgn0038590  
*FBgn0038592*  
*FBgn0038593*  
*FBgn0038595*  
*FBgn0038596*  
*FBgn0038597*  
*FBgn0038598*  
*FBgn0038601*  
*FBgn0038602*  
*FBgn0038603*  
FBgn0038606  
FBgn0038607  
*FBgn0038608*  
*FBgn0038609*  
*FBgn0038610*  
FBgn0038611  
FBgn0038612  
FBgn0038617  
FBgn0038619  
*FBgn0038621*  
FBgn0038626  
FBgn0038627  
FBgn0038628  
FBgn0038629  
FBgn0038630  
*FBgn0038631*  
FBgn0038632  
FBgn0038633  
FBgn0038638  
FBgn0038639  
FBgn0038640  
*FBgn0038641*  
*FBgn0038642*  
FBgn0038643  
FBgn0038645  
FBgn0038646  
FBgn0038647  
FBgn0038649  
FBgn0038651  
FBgn0038652  
*FBgn0038653*  
FBgn0038654  
FBgn0038655  
FBgn0038656  
FBgn0038658  
*FBgn0038659*  
*FBgn0038660*  
*FBgn0038662*  
*FBgn0038665*  
FBgn0038666  
FBgn0038672  
FBgn0038673  
*FBgn0038674*  
FBgn0038675  
*FBgn0038676*  
*FBgn0038678*  
*FBgn0038679*  
FBgn0038680  
FBgn0038681  
FBgn0038683  
FBgn0038685  
*FBgn0038686*  
FBgn0038690  
FBgn0038691  
*FBgn0038692*  
*FBgn0038693*  
FBgn0038694  
FBgn0038695  
FBgn0038697  
FBgn0038700  
FBgn0038701  
FBgn0038702  
FBgn0038704  
FBgn0038705  
FBgn0038706  
FBgn0038708  
FBgn0038709  
FBgn0038714  
FBgn0038715  
FBgn0038716  
FBgn0038717  
FBgn0038718  
FBgn0038719  
FBgn0038720  
FBgn0038721  
*FBgn0038722*  
*FBgn0038723*  
FBgn0038725  
FBgn0038727  
FBgn0038730  
FBgn0038732  
*FBgn0038733*  
*FBgn0038734*  
FBgn0038735  
FBgn0038737  
FBgn0038738  
FBgn0038739  
FBgn0038740  
FBgn0038741  
FBgn0038742  
*FBgn0038744*  
*FBgn0038745*  
FBgn0038746  
FBgn0038747  
*FBgn0038749*  
*FBgn0038750*  
*FBgn0038751*  
*FBgn0038752*  
FBgn0038753  
FBgn0038755  
*FBgn0038756*  
FBgn0038760  
FBgn0038761  
*FBgn0038762*  
FBgn0038763  
FBgn0038765  
FBgn0038766  
FBgn0038767  
FBgn0038768  
FBgn0038769  
*FBgn0038771*  
*FBgn0038772*  
FBgn0038773  
*FBgn0038774*  
FBgn0038775  
FBgn0038783  
FBgn0038784  
FBgn0038787  
FBgn0038788  
*FBgn0038789*  
*FBgn0038790*  
*FBgn0038795*  
FBgn0038796  
FBgn0038797  
*FBgn0038798*  
*FBgn0038799*  
FBgn0038803  
*FBgn0038804*  
FBgn0038805  
*FBgn0038806*  
FBgn0038808  
FBgn0038809  
FBgn0038810  
FBgn0038811  
*FBgn0038814*  
*FBgn0038815*  
*FBgn0038816*  
*FBgn0038818*  
*FBgn0038819*  
*FBgn0038820*  
FBgn0038821  
*FBgn0038826*  
FBgn0038827  
*FBgn0038828*  
FBgn0038829  
FBgn0038830  
*FBgn0038832*  
*FBgn0038833*  
*FBgn0038834*  
FBgn0038837  
FBgn0038838  
*FBgn0038839*  
*FBgn0038840*  
*FBgn0038842*  
*FBgn0038845*  
*FBgn0038846*  
FBgn0038847  
FBgn0038849  
FBgn0038850  
FBgn0038851  
FBgn0038852  
*FBgn0038853*  
FBgn0038854  
FBgn0038855  
FBgn0038856  
FBgn0038857  
FBgn0038858  
*FBgn0038859*  
*FBgn0038860*  
FBgn0038861  
FBgn0038862  
*FBgn0038865*  
*FBgn0038866*  
FBgn0038868  
FBgn0038869  
FBgn0038870  
FBgn0038871  
FBgn0038873  
FBgn0038874  
FBgn0038876  
FBgn0038877  
*FBgn0038878*  
FBgn0038879  
FBgn0038880  
*FBgn0038881*  
FBgn0038886  
FBgn0038887  
FBgn0038888  
FBgn0038889  
FBgn0038890  
FBgn0038891  
FBgn0038892  
FBgn0038893  
FBgn0038894  
*FBgn0038897*  
FBgn0038901  
FBgn0038902  
FBgn0038903  
FBgn0038909  
*FBgn0038912*  
*FBgn0038914*  
FBgn0038915  
*FBgn0038916*  
FBgn0038917  
FBgn0038918  
FBgn0038919  
*FBgn0038921*  
FBgn0038922  
FBgn0038923  
FBgn0038924  
FBgn0038925  
*FBgn0038926*  
FBgn0038927  
FBgn0038928  
*FBgn0038929*  
*FBgn0038930*  
*FBgn0038931*  
FBgn0038934  
*FBgn0038938*  
FBgn0038941  
FBgn0038942  
*FBgn0038943*  
FBgn0038944  
FBgn0038945  
*FBgn0038946*  
*FBgn0038947*  
FBgn0038948  
FBgn0038950  
FBgn0038951  
*FBgn0038952*  
FBgn0038953  
*FBgn0038956*  
*FBgn0038957*  
FBgn0038958  
FBgn0038959  
FBgn0038960  
FBgn0038961  
FBgn0038964  
FBgn0038965  
*FBgn0038966*  
*FBgn0038967*  
FBgn0038968  
FBgn0038972  
*FBgn0038973*  
*FBgn0038974*  
*FBgn0038976*  
*FBgn0038977*  
FBgn0038978  
FBgn0038979  
*FBgn0038980*  
*FBgn0038981*  
*FBgn0038983*  
*FBgn0038984*  
*FBgn0038986*  
FBgn0038989  
*FBgn0038993*  
FBgn0038996  
FBgn0039000  
*FBgn0039002*  
FBgn0039003  
FBgn0039004  
*FBgn0039005*  
*FBgn0039006*  
FBgn0039007  
*FBgn0039008*  
FBgn0039009  
FBgn0039010  
FBgn0039013  
*FBgn0039015*  
FBgn0039017  
FBgn0039018  
*FBgn0039019*  
FBgn0039020  
FBgn0039022  
FBgn0039023  
*FBgn0039024*  
FBgn0039025  
FBgn0039026  
FBgn0039027  
FBgn0039028  
FBgn0039029  
*FBgn0039030*  
*FBgn0039031*  
FBgn0039033  
FBgn0039034  
*FBgn0039038*  
*FBgn0039039*  
*FBgn0039040*  
FBgn0039041  
FBgn0039042  
*FBgn0039043*  
FBgn0039044  
FBgn0039045  
*FBgn0039048*  
*FBgn0039049*  
*FBgn0039050*  
FBgn0039051  
FBgn0039052  
*FBgn0039053*  
*FBgn0039054*  
FBgn0039055  
FBgn0039056  
FBgn0039059  
FBgn0039060  
*FBgn0039061*  
*FBgn0039064*  
FBgn0039065  
FBgn0039066  
FBgn0039067  
*FBgn0039068*  
*FBgn0039069*  
FBgn0039070  
FBgn0039071  
*FBgn0039073*  
FBgn0039075  
FBgn0039077  
FBgn0039078  
FBgn0039079  
FBgn0039080  
*FBgn0039081*  
*FBgn0039083*  
*FBgn0039084*  
*FBgn0039085*  
FBgn0039086  
FBgn0039087  
FBgn0039088  
FBgn0039091  
FBgn0039092  
FBgn0039094  
*FBgn0039098*  
*FBgn0039099*  
FBgn0039101  
*FBgn0039102*  
FBgn0039104  
FBgn0039106  
*FBgn0039107*  
FBgn0039108  
FBgn0039109  
FBgn0039110  
FBgn0039111  
FBgn0039112  
FBgn0039113  
FBgn0039115  
FBgn0039116  
FBgn0039117  
FBgn0039118  
FBgn0039120  
*FBgn0039124*  
FBgn0039125  
FBgn0039126  
*FBgn0039127*  
FBgn0039128  
FBgn0039129  
*FBgn0039130*  
*FBgn0039131*  
FBgn0039135  
FBgn0039136  
*FBgn0039137*  
*FBgn0039139*  
FBgn0039140  
FBgn0039141  
FBgn0039145  
*FBgn0039147*  
FBgn0039149  
FBgn0039150  
FBgn0039151  
FBgn0039152  
FBgn0039153  
*FBgn0039154*  
FBgn0039156  
*FBgn0039157*  
FBgn0039158  
FBgn0039159  
*FBgn0039160*  
*FBgn0039161*  
FBgn0039163  
FBgn0039164  
FBgn0039165  
FBgn0039167  
FBgn0039169  
*FBgn0039170*  
FBgn0039175  
*FBgn0039177*  
*FBgn0039178*  
*FBgn0039179*  
*FBgn0039180*  
FBgn0039182  
*FBgn0039183*  
*FBgn0039184*  
FBgn0039186  
FBgn0039187  
FBgn0039188  
*FBgn0039189*  
FBgn0039190  
FBgn0039192  
FBgn0039193  
FBgn0039194  
FBgn0039195  
FBgn0039197  
FBgn0039198  
FBgn0039199  
FBgn0039200  
FBgn0039201  
FBgn0039202  
*FBgn0039203*  
FBgn0039204  
FBgn0039205  
FBgn0039207  
FBgn0039208  
FBgn0039209  
FBgn0039210  
*FBgn0039212*  
*FBgn0039213*  
FBgn0039214  
FBgn0039215  
*FBgn0039217*  
*FBgn0039218*  
*FBgn0039223*  
FBgn0039224  
FBgn0039225  
*FBgn0039226*  
*FBgn0039227*  
*FBgn0039228*  
*FBgn0039232*  
FBgn0039233  
FBgn0039234  
FBgn0039235  
FBgn0039237  
FBgn0039238  
FBgn0039239  
*FBgn0039240*  
*FBgn0039241*  
FBgn0039244  
FBgn0039246  
FBgn0039249  
*FBgn0039250*  
FBgn0039252  
FBgn0039254  
*FBgn0039255*  
FBgn0039258  
FBgn0039259  
FBgn0039260  
*FBgn0039261*  
FBgn0039264  
FBgn0039265  
FBgn0039266  
FBgn0039269  
FBgn0039270  
FBgn0039271  
FBgn0039272  
FBgn0039273  
FBgn0039274  
*FBgn0039277*  
*FBgn0039280*  
FBgn0039282  
FBgn0039283  
FBgn0039286  
FBgn0039288  
FBgn0039290  
*FBgn0039291*  
*FBgn0039293*  
*FBgn0039294*  
*FBgn0039296*  
*FBgn0039297*  
*FBgn0039298*  
*FBgn0039299*  
*FBgn0039300*  
*FBgn0039301*  
FBgn0039302  
FBgn0039303  
FBgn0039304  
FBgn0039305  
FBgn0039306  
*FBgn0039307*  
*FBgn0039310*  
FBgn0039311  
FBgn0039312  
FBgn0039313  
FBgn0039315  
FBgn0039316  
FBgn0039319  
*FBgn0039321*  
*FBgn0039323*  
FBgn0039324  
FBgn0039325  
*FBgn0039326*  
FBgn0039328  
FBgn0039329  
FBgn0039331  
*FBgn0039332*  
FBgn0039335  
FBgn0039336  
FBgn0039337  
FBgn0039338  
*FBgn0039339*  
*FBgn0039341*  
*FBgn0039342*  
*FBgn0039343*  
FBgn0039344  
FBgn0039346  
FBgn0039347  
FBgn0039348  
FBgn0039349  
FBgn0039350  
FBgn0039354  
FBgn0039355  
FBgn0039356  
FBgn0039357  
FBgn0039358  
FBgn0039359  
FBgn0039360  
FBgn0039366  
FBgn0039367  
FBgn0039368  
FBgn0039369  
FBgn0039370  
FBgn0039371  
FBgn0039373  
FBgn0039374  
FBgn0039376  
FBgn0039378  
*FBgn0039379*  
*FBgn0039380*  
FBgn0039381  
FBgn0039385  
FBgn0039386  
FBgn0039387  
FBgn0039395  
FBgn0039396  
FBgn0039398  
FBgn0039402  
FBgn0039404  
FBgn0039405  
*FBgn0039406*  
*FBgn0039407*  
FBgn0039408  
FBgn0039411  
FBgn0039413  
FBgn0039415  
*FBgn0039417*  
*FBgn0039419*  
*FBgn0039420*  
FBgn0039421  
FBgn0039424  
*FBgn0039427*  
*FBgn0039428*  
*FBgn0039429*  
*FBgn0039430*  
*FBgn0039431*  
FBgn0039434  
FBgn0039435  
FBgn0039436  
FBgn0039438  
FBgn0039439  
FBgn0039441  
FBgn0039443  
FBgn0039444  
FBgn0039448  
*FBgn0039449*  
*FBgn0039450*  
FBgn0039451  
FBgn0039453  
FBgn0039454  
FBgn0039459  
FBgn0039461  
*FBgn0039462*  
FBgn0039464  
FBgn0039465  
FBgn0039466  
*FBgn0039467*  
FBgn0039469  
FBgn0039470  
FBgn0039471  
FBgn0039472  
FBgn0039473  
FBgn0039474  
FBgn0039476  
FBgn0039478  
FBgn0039479  
FBgn0039480  
FBgn0039481  
*FBgn0039482*  
FBgn0039483  
FBgn0039485  
*FBgn0039486*  
FBgn0039487  
FBgn0039489  
FBgn0039490  
FBgn0039491  
FBgn0039492  
*FBgn0039494*  
FBgn0039495  
FBgn0039498  
FBgn0039500  
FBgn0039501  
FBgn0039503  
FBgn0039504  
FBgn0039505  
FBgn0039507  
FBgn0039508  
FBgn0039509  
FBgn0039510  
FBgn0039511  
FBgn0039518  
FBgn0039519  
FBgn0039520  
*FBgn0039521*  
FBgn0039522  
FBgn0039523  
FBgn0039525  
*FBgn0039527*  
*FBgn0039528*  
*FBgn0039529*  
*FBgn0039530*  
FBgn0039531  
FBgn0039532  
*FBgn0039536*  
*FBgn0039537*  
FBgn0039538  
FBgn0039540  
FBgn0039543  
FBgn0039544  
FBgn0039551  
FBgn0039552  
*FBgn0039553*  
FBgn0039554  
FBgn0039555  
FBgn0039557  
FBgn0039558  
*FBgn0039560*  
FBgn0039561  
FBgn0039562  
FBgn0039563  
FBgn0039564  
FBgn0039565  
FBgn0039566  
FBgn0039568  
FBgn0039576  
FBgn0039577  
*FBgn0039580*  
FBgn0039582  
FBgn0039585  
*FBgn0039588*  
FBgn0039589  
*FBgn0039590*  
FBgn0039591  
FBgn0039592  
*FBgn0039593*  
*FBgn0039594*  
FBgn0039596  
FBgn0039597  
FBgn0039598  
FBgn0039599  
FBgn0039600  
FBgn0039601  
*FBgn0039602*  
*FBgn0039609*  
FBgn0039611  
FBgn0039612  
FBgn0039613  
*FBgn0039616*  
*FBgn0039617*  
*FBgn0039620*  
*FBgn0039621*  
FBgn0039623  
FBgn0039625  
FBgn0039626  
FBgn0039627  
FBgn0039628  
FBgn0039629  
FBgn0039630  
FBgn0039631  
FBgn0039633  
FBgn0039634  
FBgn0039635  
FBgn0039636  
FBgn0039637  
*FBgn0039638*  
FBgn0039639  
FBgn0039640  
FBgn0039641  
FBgn0039642  
*FBgn0039644*  
FBgn0039645  
FBgn0039647  
FBgn0039648  
*FBgn0039650*  
FBgn0039651  
*FBgn0039654*  
*FBgn0039655*  
FBgn0039656  
*FBgn0039659*  
FBgn0039663  
FBgn0039664  
FBgn0039665  
*FBgn0039666*  
*FBgn0039667*  
*FBgn0039668*  
*FBgn0039669*  
FBgn0039670  
FBgn0039671  
*FBgn0039673*  
*FBgn0039674*  
FBgn0039675  
FBgn0039676  
FBgn0039677  
*FBgn0039678*  
*FBgn0039679*  
FBgn0039681  
*FBgn0039682*  
*FBgn0039683*  
*FBgn0039684*  
*FBgn0039685*  
FBgn0039686  
FBgn0039687  
FBgn0039688  
FBgn0039689  
FBgn0039690  
FBgn0039691  
FBgn0039694  
FBgn0039696  
FBgn0039697  
FBgn0039698  
FBgn0039702  
FBgn0039703  
FBgn0039704  
*FBgn0039705*  
*FBgn0039707*  
*FBgn0039709*  
FBgn0039710  
FBgn0039711  
FBgn0039712  
*FBgn0039713*  
*FBgn0039714*  
FBgn0039718  
FBgn0039719  
*FBgn0039722*  
*FBgn0039723*  
FBgn0039727  
FBgn0039728  
FBgn0039730  
FBgn0039733  
*FBgn0039734*  
*FBgn0039736*  
FBgn0039737  
FBgn0039738  
*FBgn0039739*  
FBgn0039740  
FBgn0039741  
FBgn0039742  
FBgn0039743  
*FBgn0039744*  
FBgn0039747  
FBgn0039748  
FBgn0039749  
*FBgn0039751*  
*FBgn0039752*  
*FBgn0039754*  
*FBgn0039755*  
FBgn0039756  
FBgn0039757  
FBgn0039758  
FBgn0039759  
FBgn0039760  
FBgn0039761  
FBgn0039764  
FBgn0039765  
FBgn0039766  
FBgn0039767  
FBgn0039768  
FBgn0039769  
*FBgn0039770*  
*FBgn0039771*  
*FBgn0039773*  
*FBgn0039774*  
*FBgn0039776*  
*FBgn0039779*  
FBgn0039780  
FBgn0039782  
FBgn0039783  
FBgn0039784  
*FBgn0039787*  
*FBgn0039788*  
*FBgn0039789*  
*FBgn0039790*  
FBgn0039792  
FBgn0039795  
FBgn0039796  
FBgn0039797  
FBgn0039798  
FBgn0039799  
FBgn0039800  
FBgn0039801  
FBgn0039804  
FBgn0039805  
FBgn0039806  
FBgn0039807  
*FBgn0039808*  
*FBgn0039809*  
FBgn0039810  
FBgn0039811  
FBgn0039812  
FBgn0039816  
FBgn0039817  
FBgn0039818  
FBgn0039820  
FBgn0039821  
*FBgn0039827*  
FBgn0039828  
FBgn0039829  
*FBgn0039830*  
*FBgn0039831*  
FBgn0039832  
FBgn0039833  
FBgn0039835  
FBgn0039836  
FBgn0039838  
FBgn0039839  
FBgn0039840  
*FBgn0039844*  
FBgn0039846  
FBgn0039848  
FBgn0039849  
FBgn0039850  
FBgn0039851  
FBgn0039852  
FBgn0039854  
*FBgn0039855*  
*FBgn0039856*  
*FBgn0039857*  
*FBgn0039858*  
FBgn0039859  
FBgn0039860  
FBgn0039861  
*FBgn0039862*  
*FBgn0039863*  
*FBgn0039864*  
FBgn0039868  
FBgn0039869  
FBgn0039870  
FBgn0039872  
FBgn0039873  
FBgn0039874  
FBgn0039875  
FBgn0039876  
FBgn0039877  
FBgn0039879  
*FBgn0039881*  
FBgn0039882  
*FBgn0039883*  
FBgn0039886  
*FBgn0039887*  
FBgn0039965  
FBgn0039966  
FBgn0039969  
FBgn0039970  
*FBgn0039972*  
*FBgn0039977*  
*FBgn0039979*  
FBgn0039994  
FBgn0040005  
*FBgn0040007*  
*FBgn0040009*  
FBgn0040010  
FBgn0040011  
FBgn0040045  
*FBgn0040056*  
*FBgn0040060*  
FBgn0040063  
FBgn0040064  
FBgn0040066  
FBgn0040068  
FBgn0040071  
*FBgn0040074*  
FBgn0040075  
*FBgn0040078*  
FBgn0040079  
FBgn0040080  
*FBgn0040087*  
FBgn0040089  
*FBgn0040091*  
*FBgn0040206*  
*FBgn0040207*  
*FBgn0040211*  
FBgn0040230  
*FBgn0040232*  
FBgn0040237  
*FBgn0040238*  
FBgn0040239  
FBgn0040250  
FBgn0040251  
FBgn0040252  
FBgn0040253  
FBgn0040255  
*FBgn0040256*  
FBgn0040257  
*FBgn0040259*  
*FBgn0040260*  
FBgn0040261  
FBgn0040262  
FBgn0040268  
*FBgn0040271*  
FBgn0040273  
FBgn0040279  
*FBgn0040281*  
*FBgn0040282*  
FBgn0040283  
*FBgn0040284*  
*FBgn0040285*  
FBgn0040286  
FBgn0040290  
FBgn0040291  
FBgn0040294  
FBgn0040296  
FBgn0040297  
FBgn0040298  
FBgn0040299  
FBgn0040308  
*FBgn0040309*  
FBgn0040318  
*FBgn0040319*  
*FBgn0040321*  
FBgn0040322  
FBgn0040323  
FBgn0040333  
*FBgn0040334*  
*FBgn0040336*  
FBgn0040337  
FBgn0040339  
*FBgn0040340*  
FBgn0040341  
FBgn0040342  
FBgn0040343  
FBgn0040344  
FBgn0040345  
FBgn0040346  
FBgn0040347  
*FBgn0040348*  
*FBgn0040349*  
*FBgn0040350*  
*FBgn0040351*  
FBgn0040352  
FBgn0040353  
FBgn0040354  
FBgn0040355  
FBgn0040356  
FBgn0040357  
FBgn0040358  
FBgn0040359  
FBgn0040360  
FBgn0040361  
FBgn0040362  
FBgn0040363  
*FBgn0040364*  
FBgn0040365  
FBgn0040366  
FBgn0040367  
FBgn0040370  
FBgn0040371  
FBgn0040372  
FBgn0040373  
*FBgn0040375*  
*FBgn0040376*  
*FBgn0040382*  
*FBgn0040383*  
FBgn0040384  
FBgn0040385  
FBgn0040387  
*FBgn0040388*  
FBgn0040389  
FBgn0040390  
FBgn0040392  
FBgn0040393  
FBgn0040394  
FBgn0040396  
FBgn0040397  
*FBgn0040398*  
*FBgn0040465*  
*FBgn0040466*  
FBgn0040467  
*FBgn0040475*  
*FBgn0040477*  
FBgn0040487  
*FBgn0040491*  
FBgn0040496  
FBgn0040502  
FBgn0040503  
*FBgn0040505*  
FBgn0040506  
*FBgn0040507*  
FBgn0040508  
FBgn0040509  
FBgn0040510  
*FBgn0040512*  
FBgn0040519  
*FBgn0040524*  
*FBgn0040528*  
*FBgn0040529*  
*FBgn0040531*  
FBgn0040532  
FBgn0040534  
FBgn0040551  
FBgn0040554  
*FBgn0040571*  
FBgn0040575  
*FBgn0040582*  
FBgn0040585  
*FBgn0040590*  
*FBgn0040600*  
FBgn0040601  
*FBgn0040602*  
*FBgn0040606*  
FBgn0040607  
FBgn0040609  
FBgn0040623  
FBgn0040625  
FBgn0040628  
FBgn0040629  
FBgn0040634  
FBgn0040636  
FBgn0040637  
FBgn0040648  
*FBgn0040649*  
FBgn0040650  
FBgn0040651  
*FBgn0040653*  
FBgn0040658  
FBgn0040660  
FBgn0040666  
FBgn0040674  
FBgn0040679  
FBgn0040684  
FBgn0040688  
FBgn0040694  
FBgn0040697  
*FBgn0040705*  
FBgn0040717  
FBgn0040718  
FBgn0040719  
*FBgn0040723*  
FBgn0040725  
FBgn0040726  
FBgn0040730  
*FBgn0040732*  
FBgn0040733  
FBgn0040734  
FBgn0040735  
FBgn0040743  
*FBgn0040747*  
FBgn0040751  
FBgn0040752  
FBgn0040754  
FBgn0040755  
*FBgn0040759*  
*FBgn0040763*  
*FBgn0040764*  
FBgn0040765  
FBgn0040773  
FBgn0040775  
*FBgn0040777*  
FBgn0040778  
FBgn0040780  
FBgn0040786  
*FBgn0040793*  
FBgn0040794  
FBgn0040795  
FBgn0040796  
FBgn0040797  
*FBgn0040798*  
FBgn0040801  
*FBgn0040805*  
FBgn0040812  
*FBgn0040813*  
FBgn0040814  
FBgn0040816  
FBgn0040817  
FBgn0040823  
*FBgn0040827*  
FBgn0040828  
*FBgn0040832*  
FBgn0040837  
*FBgn0040842*  
*FBgn0040843*  
*FBgn0040849*  
*FBgn0040850*  
FBgn0040862  
FBgn0040871  
FBgn0040877  
FBgn0040890  
FBgn0040892  
FBgn0040899  
FBgn0040900  
FBgn0040904  
FBgn0040905  
FBgn0040906  
FBgn0040907  
FBgn0040918  
FBgn0040922  
FBgn0040923  
FBgn0040928  
*FBgn0040929*  
FBgn0040931  
FBgn0040940  
FBgn0040941  
FBgn0040942  
FBgn0040949  
FBgn0040950  
*FBgn0040954*  
*FBgn0040963*  
FBgn0040964  
FBgn0040965  
FBgn0040967  
*FBgn0040968*  
FBgn0040972  
FBgn0040984  
*FBgn0040985*  
FBgn0040989  
*FBgn0040992*  
FBgn0040994  
FBgn0040995  
FBgn0040996  
FBgn0040998  
FBgn0041004  
*FBgn0041087*  
*FBgn0041092*  
*FBgn0041094*  
*FBgn0041096*  
FBgn0041097  
*FBgn0041100*  
FBgn0041102  
FBgn0041103  
FBgn0041111  
*FBgn0041147*  
*FBgn0041150*  
*FBgn0041156*  
FBgn0041160  
FBgn0041161  
FBgn0041164  
FBgn0041171  
FBgn0041174  
*FBgn0041180*  
*FBgn0041181*  
*FBgn0041182*  
FBgn0041183  
*FBgn0041184*  
FBgn0041186  
*FBgn0041188*  
FBgn0041191  
*FBgn0041194*  
FBgn0041195  
FBgn0041203  
FBgn0041205  
*FBgn0041210*  
FBgn0041224  
*FBgn0041225*  
FBgn0041229  
FBgn0041231  
*FBgn0041232*  
FBgn0041233  
FBgn0041234  
FBgn0041235  
FBgn0041236  
FBgn0041237  
FBgn0041238  
FBgn0041239  
FBgn0041240  
*FBgn0041241*  
FBgn0041242  
*FBgn0041243*  
FBgn0041244  
FBgn0041245  
*FBgn0041246*  
FBgn0041247  
FBgn0041248  
FBgn0041249  
FBgn0041250  
FBgn0041252  
FBgn0041337  
*FBgn0041342*  
*FBgn0041579*  
FBgn0041582  
FBgn0041588  
FBgn0041604  
*FBgn0041605*  
FBgn0041621  
FBgn0041622  
FBgn0041623  
FBgn0041624  
FBgn0041625  
FBgn0041627  
FBgn0041629  
FBgn0041630  
FBgn0041702  
FBgn0041706  
*FBgn0041707*  
FBgn0041775  
FBgn0041780  
FBgn0041789  
FBgn0042083  
FBgn0042085  
FBgn0042092  
FBgn0042094  
FBgn0042098  
FBgn0042101  
FBgn0042102  
FBgn0042103  
FBgn0042104  
FBgn0042105  
FBgn0042106  
FBgn0042110  
*FBgn0042111*  
FBgn0042112  
*FBgn0042119*  
*FBgn0042126*  
*FBgn0042129*  
FBgn0042131  
*FBgn0042132*  
*FBgn0042133*  
*FBgn0042134*  
FBgn0042135  
*FBgn0042138*  
FBgn0042146  
*FBgn0042174*  
FBgn0042179  
FBgn0042180  
*FBgn0042185*  
FBgn0042186  
FBgn0042187  
*FBgn0042189*  
FBgn0042198  
FBgn0042199  
*FBgn0042201*  
FBgn0042205  
*FBgn0042206*  
FBgn0042207  
FBgn0042213  
FBgn0042630  
FBgn0042712  
FBgn0043005  
FBgn0043010  
FBgn0043025  
FBgn0043043  
FBgn0043070  
*FBgn0043362*  
*FBgn0043364*  
FBgn0043455  
FBgn0043456  
*FBgn0043457*  
*FBgn0043458*  
FBgn0043470  
FBgn0043471  
*FBgn0043530*  
FBgn0043532  
*FBgn0043533*  
FBgn0043535  
FBgn0043539  
FBgn0043550  
*FBgn0043783*  
FBgn0043791  
FBgn0043792  
FBgn0043796  
FBgn0043806  
FBgn0043825  
FBgn0043854  
FBgn0043884  
FBgn0043900  
FBgn0043903  
*FBgn0044011*  
*FBgn0044020*  
*FBgn0044028*  
FBgn0044030  
FBgn0044046  
FBgn0044047  
*FBgn0044048*  
FBgn0044049  
FBgn0044050  
FBgn0044051  
FBgn0044323  
FBgn0044324  
FBgn0044328  
FBgn0044419  
FBgn0044452  
*FBgn0044510*  
FBgn0044511  
FBgn0044809  
*FBgn0044810*  
FBgn0044811  
*FBgn0044812*  
FBgn0044823  
*FBgn0044826*  
FBgn0044871  
*FBgn0044872*  
FBgn0045035  
FBgn0045038  
*FBgn0045063*  
FBgn0045064  
FBgn0045073  
FBgn0045442  
FBgn0045443  
FBgn0045468  
FBgn0045469  
FBgn0045471  
FBgn0045473  
*FBgn0045474*  
FBgn0045477  
FBgn0045478  
FBgn0045479  
FBgn0045482  
FBgn0045483  
FBgn0045485  
FBgn0045486  
FBgn0045487  
FBgn0045495  
FBgn0045497  
FBgn0045499  
FBgn0045500  
FBgn0045501  
FBgn0045502  
FBgn0045759  
FBgn0045761  
FBgn0045770  
*FBgn0045823*  
FBgn0045827  
*FBgn0045842*  
FBgn0045843  
FBgn0045852  
FBgn0045862  
*FBgn0045866*  
FBgn0045980  
*FBgn0046113*  
FBgn0046114  
*FBgn0046214*  
FBgn0046222  
FBgn0046225  
FBgn0046247  
FBgn0046253  
FBgn0046258  
*FBgn0046294*  
FBgn0046296  
*FBgn0046297*  
FBgn0046301  
*FBgn0046302*  
FBgn0046322  
FBgn0046332  
FBgn0046685  
*FBgn0046687*  
FBgn0046689  
FBgn0046692  
FBgn0046763  
*FBgn0046776*  
FBgn0046793  
FBgn0046873  
*FBgn0046874*  
*FBgn0046875*  
FBgn0046876  
FBgn0046878  
FBgn0046879  
FBgn0046880  
FBgn0046885  
FBgn0046886  
FBgn0046887  
FBgn0046888  
FBgn0046999  
*FBgn0047114*  
*FBgn0047135*  
FBgn0047178  
FBgn0047199  
FBgn0047330  
*FBgn0047334*  
FBgn0047338  
FBgn0047351  
FBgn0050000  
FBgn0050001  
FBgn0050005  
FBgn0050007  
*FBgn0050008*  
FBgn0050010  
*FBgn0050011*  
FBgn0050015  
*FBgn0050016*  
*FBgn0050018*  
FBgn0050020  
*FBgn0050021*  
FBgn0050022  
*FBgn0050026*  
*FBgn0050033*  
FBgn0050034  
FBgn0050036  
FBgn0050037  
*FBgn0050039*  
FBgn0050042  
FBgn0050043  
FBgn0050046  
FBgn0050047  
FBgn0050048  
FBgn0050049  
FBgn0050050  
FBgn0050051  
*FBgn0050052*  
FBgn0050053  
*FBgn0050054*  
FBgn0050055  
FBgn0050056  
FBgn0050060  
*FBgn0050062*  
FBgn0050065  
FBgn0050067  
FBgn0050069  
FBgn0050072  
FBgn0050073  
FBgn0050074  
FBgn0050075  
*FBgn0050076*  
FBgn0050077  
*FBgn0050080*  
*FBgn0050081*  
*FBgn0050083*  
FBgn0050085  
FBgn0050087  
FBgn0050088  
*FBgn0050089*  
FBgn0050090  
*FBgn0050091*  
*FBgn0050093*  
FBgn0050094  
*FBgn0050095*  
FBgn0050096  
FBgn0050098  
FBgn0050099  
*FBgn0050101*  
*FBgn0050103*  
FBgn0050104  
FBgn0050105  
FBgn0050106  
*FBgn0050109*  
FBgn0050110  
FBgn0050114  
FBgn0050115  
FBgn0050118  
FBgn0050121  
FBgn0050122  
FBgn0050125  
FBgn0050126  
FBgn0050127  
FBgn0050145  
*FBgn0050147*  
*FBgn0050148*  
FBgn0050151  
FBgn0050154  
FBgn0050156  
FBgn0050157  
*FBgn0050158*  
FBgn0050159  
*FBgn0050161*  
*FBgn0050163*  
FBgn0050169  
FBgn0050172  
FBgn0050177  
*FBgn0050178*  
FBgn0050181  
*FBgn0050183*  
FBgn0050184  
FBgn0050185  
*FBgn0050187*  
FBgn0050192  
FBgn0050194  
FBgn0050195  
FBgn0050196  
*FBgn0050197*  
FBgn0050203  
FBgn0050217  
FBgn0050222  
FBgn0050259  
FBgn0050263  
FBgn0050265  
FBgn0050268  
*FBgn0050269*  
FBgn0050271  
FBgn0050272  
*FBgn0050273*  
FBgn0050274  
FBgn0050275  
*FBgn0050277*  
*FBgn0050279*  
FBgn0050280  
FBgn0050281  
*FBgn0050284*  
FBgn0050286  
FBgn0050287  
FBgn0050288  
FBgn0050289  
FBgn0050290  
*FBgn0050293*  
FBgn0050295  
FBgn0050323  
FBgn0050324  
FBgn0050325  
*FBgn0050334*  
FBgn0050338  
*FBgn0050340*  
FBgn0050342  
FBgn0050343  
FBgn0050344  
*FBgn0050345*  
FBgn0050349  
FBgn0050350  
FBgn0050354  
FBgn0050355  
*FBgn0050356*  
FBgn0050357  
*FBgn0050359*  
FBgn0050360  
*FBgn0050361*  
FBgn0050362  
*FBgn0050365*  
FBgn0050369  
*FBgn0050371*  
FBgn0050373  
*FBgn0050374*  
FBgn0050375  
FBgn0050376  
FBgn0050377  
FBgn0050379  
FBgn0050380  
*FBgn0050381*  
*FBgn0050383*  
FBgn0050384  
FBgn0050389  
*FBgn0050390*  
FBgn0050391  
*FBgn0050392*  
FBgn0050393  
FBgn0050394  
FBgn0050395  
*FBgn0050398*  
FBgn0050401  
*FBgn0050403*  
*FBgn0050404*  
FBgn0050409  
FBgn0050410  
*FBgn0050411*  
FBgn0050412  
FBgn0050413  
*FBgn0050414*  
FBgn0050417  
FBgn0050418  
FBgn0050419  
*FBgn0050421*  
*FBgn0050423*  
*FBgn0050424*  
*FBgn0050428*  
FBgn0050429  
*FBgn0050430*  
*FBgn0050431*  
FBgn0050432  
*FBgn0050438*  
*FBgn0050440*  
*FBgn0050441*  
FBgn0050443  
FBgn0050446  
FBgn0050447  
FBgn0050456  
FBgn0050457  
FBgn0050458  
*FBgn0050459*  
FBgn0050460  
*FBgn0050463*  
FBgn0050464  
*FBgn0050466*  
FBgn0050467  
FBgn0050468  
FBgn0050469  
FBgn0050470  
FBgn0050471  
*FBgn0050476*  
FBgn0050479  
FBgn0050480  
*FBgn0050484*  
FBgn0050486  
FBgn0050487  
FBgn0050488  
FBgn0050491  
*FBgn0050492*  
*FBgn0050493*  
*FBgn0050495*  
FBgn0050497  
FBgn0050499  
*FBgn0050502*  
FBgn0051002  
*FBgn0051004*  
FBgn0051005  
FBgn0051007  
FBgn0051008  
FBgn0051010  
FBgn0051013  
FBgn0051014  
FBgn0051015  
FBgn0051016  
FBgn0051017  
*FBgn0051019*  
FBgn0051021  
FBgn0051025  
*FBgn0051028*  
FBgn0051029  
*FBgn0051030*  
FBgn0051031  
FBgn0051036  
FBgn0051038  
FBgn0051040  
FBgn0051041  
FBgn0051044  
*FBgn0051048*  
FBgn0051051  
*FBgn0051052*  
*FBgn0051053*  
FBgn0051055  
*FBgn0051057*  
FBgn0051058  
FBgn0051064  
FBgn0051065  
FBgn0051068  
*FBgn0051072*  
*FBgn0051075*  
*FBgn0051076*  
FBgn0051077  
FBgn0051080  
FBgn0051081  
*FBgn0051082*  
FBgn0051084  
*FBgn0051086*  
*FBgn0051087*  
*FBgn0051088*  
FBgn0051089  
FBgn0051091  
FBgn0051092  
FBgn0051093  
FBgn0051097  
*FBgn0051098*  
*FBgn0051099*  
*FBgn0051100*  
FBgn0051102  
*FBgn0051103*  
FBgn0051104  
FBgn0051105  
*FBgn0051106*  
FBgn0051108  
FBgn0051109  
FBgn0051111  
*FBgn0051115*  
FBgn0051118  
*FBgn0051119*  
FBgn0051120  
FBgn0051121  
*FBgn0051122*  
FBgn0051125  
FBgn0051126  
FBgn0051128  
FBgn0051133  
FBgn0051139  
FBgn0051140  
*FBgn0051141*  
FBgn0051145  
FBgn0051146  
*FBgn0051148*  
*FBgn0051150*  
FBgn0051151  
FBgn0051155  
FBgn0051156  
*FBgn0051157*  
*FBgn0051158*  
FBgn0051159  
FBgn0051161  
FBgn0051163  
FBgn0051164  
FBgn0051169  
FBgn0051174  
FBgn0051176  
*FBgn0051183*  
FBgn0051184  
FBgn0051189  
*FBgn0051191*  
*FBgn0051195*  
FBgn0051198  
FBgn0051199  
FBgn0051200  
FBgn0051201  
*FBgn0051202*  
FBgn0051204  
FBgn0051205  
FBgn0051206  
FBgn0051207  
*FBgn0051210*  
*FBgn0051211*  
*FBgn0051213*  
FBgn0051216  
*FBgn0051217*  
FBgn0051219  
*FBgn0051220*  
*FBgn0051221*  
*FBgn0051223*  
FBgn0051224  
FBgn0051225  
*FBgn0051226*  
FBgn0051229  
*FBgn0051230*  
*FBgn0051231*  
FBgn0051232  
FBgn0051233  
FBgn0051235  
FBgn0051244  
FBgn0051245  
*FBgn0051248*  
FBgn0051249  
FBgn0051251  
FBgn0051259  
FBgn0051262  
FBgn0051265  
FBgn0051266  
FBgn0051267  
FBgn0051268  
FBgn0051269  
*FBgn0051272*  
*FBgn0051278*  
FBgn0051279  
*FBgn0051281*  
FBgn0051286  
FBgn0051288  
FBgn0051291  
FBgn0051294  
*FBgn0051296*  
*FBgn0051300*  
FBgn0051301  
*FBgn0051313*  
FBgn0051314  
FBgn0051320  
*FBgn0051321*  
FBgn0051323  
*FBgn0051324*  
FBgn0051326  
FBgn0051327  
FBgn0051337  
FBgn0051342  
FBgn0051343  
FBgn0051344  
*FBgn0051345*  
FBgn0051347  
FBgn0051352  
FBgn0051357  
FBgn0051358  
FBgn0051360  
FBgn0051361  
FBgn0051363  
FBgn0051365  
FBgn0051368  
FBgn0051370  
FBgn0051371  
*FBgn0051373*  
*FBgn0051374*  
*FBgn0051380*  
*FBgn0051386*  
FBgn0051388  
FBgn0051390  
FBgn0051391  
FBgn0051404  
FBgn0051406  
FBgn0051407  
FBgn0051410  
FBgn0051413  
FBgn0051414  
FBgn0051415  
FBgn0051418  
FBgn0051419  
FBgn0051423  
FBgn0051424  
FBgn0051427  
*FBgn0051431*  
FBgn0051436  
*FBgn0051437*  
FBgn0051438  
*FBgn0051439*  
FBgn0051441  
*FBgn0051445*  
*FBgn0051446*  
FBgn0051450  
FBgn0051451  
*FBgn0051453*  
FBgn0051454  
FBgn0051457  
FBgn0051459  
FBgn0051460  
FBgn0051462  
FBgn0051463  
FBgn0051464  
FBgn0051465  
FBgn0051467  
*FBgn0051469*  
*FBgn0051472*  
FBgn0051473  
*FBgn0051475*  
FBgn0051477  
FBgn0051481  
FBgn0051482  
FBgn0051493  
FBgn0051495  
FBgn0051496  
FBgn0051501  
FBgn0051510  
FBgn0051515  
*FBgn0051516*  
*FBgn0051522*  
*FBgn0051523*  
FBgn0051524  
FBgn0051525  
FBgn0051526  
FBgn0051528  
*FBgn0051530*  
FBgn0051531  
FBgn0051533  
FBgn0051534  
FBgn0051536  
FBgn0051538  
*FBgn0051542*  
FBgn0051544  
*FBgn0051546*  
*FBgn0051547*  
FBgn0051548  
FBgn0051549  
FBgn0051550  
FBgn0051551  
*FBgn0051555*  
FBgn0051556  
FBgn0051559  
FBgn0051560  
FBgn0051561  
FBgn0051562  
FBgn0051600  
FBgn0051601  
*FBgn0051606*  
FBgn0051607  
FBgn0051612  
*FBgn0051619*  
FBgn0051624  
FBgn0051626  
*FBgn0051627*  
FBgn0051630  
*FBgn0051633*  
FBgn0051634  
FBgn0051635  
*FBgn0051636*  
*FBgn0051637*  
*FBgn0051638*  
*FBgn0051641*  
FBgn0051642  
FBgn0051643  
FBgn0051644  
*FBgn0051646*  
FBgn0051647  
FBgn0051648  
*FBgn0051658*  
*FBgn0051659*  
*FBgn0051660*  
FBgn0051661  
*FBgn0051663*  
*FBgn0051664*  
*FBgn0051665*  
FBgn0051668  
FBgn0051673  
*FBgn0051674*  
FBgn0051675  
*FBgn0051676*  
FBgn0051677  
FBgn0051678  
FBgn0051679  
FBgn0051680  
*FBgn0051681*  
FBgn0051682  
FBgn0051683  
FBgn0051686  
FBgn0051688  
FBgn0051690  
FBgn0051693  
FBgn0051694  
FBgn0051697  
FBgn0051698  
FBgn0051700  
*FBgn0051704*  
*FBgn0051706*  
FBgn0051708  
FBgn0051709  
FBgn0051710  
FBgn0051712  
FBgn0051713  
FBgn0051715  
FBgn0051716  
FBgn0051717  
*FBgn0051718*  
*FBgn0051720*  
FBgn0051721  
FBgn0051728  
FBgn0051729  
FBgn0051730  
FBgn0051735  
*FBgn0051740*  
FBgn0051741  
FBgn0051742  
*FBgn0051752*  
FBgn0051755  
*FBgn0051759*  
*FBgn0051760*  
*FBgn0051769*  
FBgn0051772  
FBgn0051773  
FBgn0051774  
FBgn0051776  
*FBgn0051777*  
*FBgn0051778*  
*FBgn0051781*  
FBgn0051782  
FBgn0051784  
*FBgn0051785*  
FBgn0051787  
FBgn0051788  
*FBgn0051789*  
FBgn0051790  
*FBgn0051792*  
*FBgn0051793*  
FBgn0051797  
*FBgn0051798*  
FBgn0051800  
FBgn0051802  
FBgn0051803  
FBgn0051804  
FBgn0051805  
FBgn0051806  
FBgn0051807  
FBgn0051809  
*FBgn0051810*  
FBgn0051812  
FBgn0051813  
FBgn0051814  
FBgn0051815  
FBgn0051816  
FBgn0051820  
FBgn0051821  
FBgn0051822  
FBgn0051823  
FBgn0051824  
*FBgn0051832*  
*FBgn0051835*  
*FBgn0051845*  
FBgn0051846  
FBgn0051848  
*FBgn0051849*  
FBgn0051851  
FBgn0051852  
FBgn0051855  
FBgn0051860  
FBgn0051862  
FBgn0051867  
FBgn0051869  
*FBgn0051870*  
FBgn0051871  
FBgn0051872  
FBgn0051874  
FBgn0051875  
FBgn0051876  
FBgn0051882  
*FBgn0051897*  
FBgn0051898  
FBgn0051900  
*FBgn0051901*  
FBgn0051902  
FBgn0051907  
FBgn0051909  
FBgn0051910  
FBgn0051913  
FBgn0051915  
FBgn0051920  
FBgn0051921  
FBgn0051922  
FBgn0051924  
FBgn0051926  
FBgn0051928  
FBgn0051933  
FBgn0051935  
FBgn0051948  
FBgn0051949  
FBgn0051950  
FBgn0051952  
*FBgn0051953*  
*FBgn0051954*  
*FBgn0051955*  
*FBgn0051956*  
FBgn0051957  
FBgn0051959  
FBgn0051960  
FBgn0051961  
FBgn0051973  
FBgn0051974  
FBgn0051975  
FBgn0051976  
FBgn0051988  
FBgn0052022  
FBgn0052023  
FBgn0052024  
*FBgn0052026*  
*FBgn0052027*  
FBgn0052029  
FBgn0052036  
FBgn0052037  
FBgn0052038  
*FBgn0052039*  
FBgn0052040  
*FBgn0052043*  
*FBgn0052050*  
FBgn0052053  
FBgn0052054  
FBgn0052055  
*FBgn0052056*  
FBgn0052057  
FBgn0052058  
FBgn0052061  
FBgn0052064  
FBgn0052066  
*FBgn0052068*  
FBgn0052069  
FBgn0052071  
FBgn0052072  
FBgn0052073  
FBgn0052074  
FBgn0052075  
FBgn0052076  
FBgn0052079  
FBgn0052081  
FBgn0052082  
FBgn0052085  
FBgn0052086  
FBgn0052087  
FBgn0052088  
*FBgn0052091*  
*FBgn0052095*  
*FBgn0052099*  
FBgn0052100  
*FBgn0052102*  
FBgn0052103  
*FBgn0052104*  
FBgn0052105  
FBgn0052106  
*FBgn0052107*  
FBgn0052109  
*FBgn0052110*  
FBgn0052111  
FBgn0052112  
FBgn0052113  
*FBgn0052115*  
FBgn0052117  
*FBgn0052119*  
FBgn0052121  
FBgn0052132  
FBgn0052133  
FBgn0052135  
FBgn0052137  
FBgn0052138  
*FBgn0052141*  
FBgn0052150  
FBgn0052152  
FBgn0052154  
FBgn0052155  
FBgn0052160  
*FBgn0052161*  
FBgn0052163  
*FBgn0052170*  
FBgn0052176  
FBgn0052177  
FBgn0052179  
FBgn0052181  
FBgn0052182  
*FBgn0052183*  
FBgn0052185  
FBgn0052187  
FBgn0052189  
*FBgn0052190*  
*FBgn0052191*  
*FBgn0052192*  
*FBgn0052195*  
*FBgn0052196*  
FBgn0052198  
FBgn0052199  
FBgn0052201  
FBgn0052202  
FBgn0052203  
*FBgn0052204*  
*FBgn0052206*  
FBgn0052207  
*FBgn0052212*  
*FBgn0052219*  
*FBgn0052220*  
FBgn0052221  
*FBgn0052225*  
FBgn0052226  
FBgn0052227  
FBgn0052228  
*FBgn0052230*  
FBgn0052232  
FBgn0052237  
FBgn0052238  
*FBgn0052240*  
FBgn0052241  
FBgn0052243  
*FBgn0052246*  
FBgn0052248  
FBgn0052249  
FBgn0052250  
FBgn0052251  
*FBgn0052252*  
FBgn0052255  
FBgn0052259  
*FBgn0052260*  
FBgn0052262  
*FBgn0052263*  
*FBgn0052264*  
FBgn0052266  
FBgn0052267  
*FBgn0052268*  
FBgn0052271  
*FBgn0052277*  
FBgn0052278  
*FBgn0052280*  
FBgn0052281  
FBgn0052282  
*FBgn0052283*  
*FBgn0052284*  
*FBgn0052296*  
FBgn0052301  
FBgn0052302  
FBgn0052305  
FBgn0052311  
*FBgn0052313*  
FBgn0052319  
FBgn0052320  
FBgn0052333  
FBgn0052343  
FBgn0052344  
*FBgn0052350*  
FBgn0052351  
*FBgn0052354*  
FBgn0052364  
FBgn0052365  
FBgn0052368  
FBgn0052369  
*FBgn0052371*  
*FBgn0052372*  
*FBgn0052373*  
FBgn0052374  
FBgn0052376  
FBgn0052379  
FBgn0052380  
FBgn0052382  
FBgn0052383  
FBgn0052391  
FBgn0052392  
*FBgn0052396*  
*FBgn0052397*  
FBgn0052404  
FBgn0052405  
*FBgn0052406*  
FBgn0052407  
FBgn0052409  
FBgn0052412  
FBgn0052413  
*FBgn0052423*  
*FBgn0052425*  
FBgn0052428  
FBgn0052432  
FBgn0052436  
FBgn0052437  
*FBgn0052438*  
FBgn0052440  
*FBgn0052441*  
FBgn0052442  
*FBgn0052446*  
*FBgn0052447*  
FBgn0052448  
FBgn0052450  
FBgn0052451  
FBgn0052452  
FBgn0052459  
FBgn0052461  
FBgn0052462  
FBgn0052463  
*FBgn0052473*  
*FBgn0052475*  
*FBgn0052476*  
FBgn0052479  
FBgn0052483  
FBgn0052484  
FBgn0052485  
FBgn0052486  
FBgn0052487  
FBgn0052488  
*FBgn0052499*  
FBgn0052506  
FBgn0052511  
FBgn0052512  
FBgn0052521  
FBgn0052523  
FBgn0052528  
FBgn0052529  
FBgn0052532  
FBgn0052537  
FBgn0052541  
*FBgn0052544*  
FBgn0052547  
FBgn0052548  
*FBgn0052549*  
FBgn0052554  
*FBgn0052556*  
FBgn0052557  
FBgn0052563  
FBgn0052564  
FBgn0052568  
FBgn0052569  
FBgn0052570  
*FBgn0052571*  
FBgn0052572  
FBgn0052573  
FBgn0052574  
*FBgn0052576*  
*FBgn0052579*  
FBgn0052580  
FBgn0052582  
FBgn0052588  
*FBgn0052590*  
FBgn0052591  
*FBgn0052594*  
*FBgn0052595*  
FBgn0052600  
FBgn0052602  
*FBgn0052603*  
FBgn0052606  
*FBgn0052625*  
*FBgn0052626*  
*FBgn0052627*  
*FBgn0052628*  
FBgn0052631  
*FBgn0052633*  
*FBgn0052635*  
FBgn0052638  
*FBgn0052639*  
FBgn0052642  
FBgn0052643  
FBgn0052644  
*FBgn0052645*  
*FBgn0052647*  
FBgn0052649  
FBgn0052650  
FBgn0052651  
FBgn0052652  
FBgn0052654  
FBgn0052655  
FBgn0052662  
FBgn0052663  
*FBgn0052666*  
*FBgn0052667*  
FBgn0052668  
*FBgn0052669*  
FBgn0052670  
*FBgn0052672*  
*FBgn0052675*  
FBgn0052676  
FBgn0052677  
FBgn0052679  
FBgn0052681  
FBgn0052683  
FBgn0052685  
FBgn0052686  
*FBgn0052687*  
*FBgn0052690*  
*FBgn0052693*  
FBgn0052694  
*FBgn0052695*  
FBgn0052698  
*FBgn0052699*  
*FBgn0052700*  
FBgn0052702  
FBgn0052703  
*FBgn0052704*  
FBgn0052707  
FBgn0052708  
FBgn0052711  
*FBgn0052712*  
FBgn0052718  
FBgn0052719  
FBgn0052720  
FBgn0052726  
*FBgn0052727*  
*FBgn0052732*  
*FBgn0052743*  
FBgn0052750  
FBgn0052751  
*FBgn0052755*  
*FBgn0052756*  
FBgn0052758  
FBgn0052762  
FBgn0052767  
FBgn0052772  
FBgn0052773  
FBgn0052774  
FBgn0052778  
FBgn0052779  
FBgn0052790  
FBgn0052791  
FBgn0052792  
FBgn0052793  
FBgn0052797  
*FBgn0052801*  
FBgn0052803  
FBgn0052806  
*FBgn0052808*  
FBgn0052813  
FBgn0052815  
*FBgn0052816*  
FBgn0052821  
FBgn0052823  
*FBgn0052829*  
FBgn0052830  
*FBgn0052832*  
*FBgn0052833*  
*FBgn0052834*  
*FBgn0052835*  
FBgn0052845  
FBgn0052846  
FBgn0052847  
FBgn0052855  
FBgn0052856  
FBgn0052944  
FBgn0052945  
FBgn0052971  
FBgn0052982  
FBgn0052983  
FBgn0052984  
FBgn0052985  
FBgn0052986  
FBgn0052987  
FBgn0052988  
FBgn0053002  
*FBgn0053003*  
FBgn0053007  
FBgn0053012  
FBgn0053013  
FBgn0053017  
FBgn0053051  
FBgn0053054  
FBgn0053056  
FBgn0053060  
FBgn0053061  
*FBgn0053080*  
FBgn0053087  
FBgn0053092  
*FBgn0053093*  
*FBgn0053094*  
*FBgn0053095*  
*FBgn0053096*  
FBgn0053098  
*FBgn0053099*  
FBgn0053108  
*FBgn0053109*  
*FBgn0053110*  
FBgn0053111  
FBgn0053113  
FBgn0053116  
FBgn0053117  
*FBgn0053120*  
FBgn0053121  
FBgn0053123  
FBgn0053124  
FBgn0053125  
*FBgn0053126*  
FBgn0053127  
*FBgn0053128*  
*FBgn0053129*  
*FBgn0053136*  
*FBgn0053137*  
*FBgn0053138*  
*FBgn0053139*  
*FBgn0053143*  
FBgn0053144  
*FBgn0053145*  
*FBgn0053156*  
FBgn0053158  
FBgn0053159  
*FBgn0053160*  
*FBgn0053169*  
FBgn0053170  
FBgn0053172  
FBgn0053173  
*FBgn0053177*  
*FBgn0053178*  
FBgn0053180  
*FBgn0053181*  
FBgn0053182  
FBgn0053189  
FBgn0053191  
*FBgn0053192*  
FBgn0053193  
*FBgn0053194*  
*FBgn0053196*  
FBgn0053202  
FBgn0053203  
*FBgn0053205*  
FBgn0053207  
FBgn0053208  
FBgn0053213  
FBgn0053214  
FBgn0053218  
FBgn0053221  
*FBgn0053222*  
FBgn0053225  
*FBgn0053229*  
FBgn0053233  
FBgn0053234  
FBgn0053252  
FBgn0053257  
*FBgn0053258*  
FBgn0053262  
FBgn0053263  
FBgn0053265  
*FBgn0053272*  
FBgn0053276  
FBgn0053278  
FBgn0053281  
FBgn0053282  
FBgn0053283  
*FBgn0053284*  
FBgn0053287  
FBgn0053288  
*FBgn0053289*  
*FBgn0053290*  
*FBgn0053291*  
*FBgn0053293*  
FBgn0053296  
*FBgn0053299*  
*FBgn0053300*  
FBgn0053302  
*FBgn0053307*  
FBgn0053309  
FBgn0053322  
*FBgn0053325*  
FBgn0053329  
*FBgn0053330*  
FBgn0053333  
FBgn0053337  
FBgn0053340  
FBgn0053341  
*FBgn0053346*  
FBgn0053349  
FBgn0053453  
FBgn0053454  
FBgn0053458  
*FBgn0053459*  
FBgn0053462  
FBgn0053465  
FBgn0053468  
FBgn0053469  
*FBgn0053474*  
FBgn0053475  
FBgn0053476  
FBgn0053477  
FBgn0053483  
FBgn0053489  
FBgn0053490  
FBgn0053493  
*FBgn0053494*  
FBgn0053506  
*FBgn0053510*  
FBgn0053512  
FBgn0053513  
*FBgn0053514*  
FBgn0053516  
FBgn0053517  
FBgn0053520  
FBgn0053523  
FBgn0053526  
FBgn0053527  
FBgn0053530  
FBgn0053531  
FBgn0053542  
FBgn0053543  
FBgn0053544  
FBgn0053547  
FBgn0053548  
FBgn0053555  
FBgn0053556  
FBgn0053557  
FBgn0053558  
FBgn0053631  
FBgn0053635  
FBgn0053639  
FBgn0053640  
FBgn0053642  
*FBgn0053658*  
FBgn0053673  
*FBgn0053679*  
*FBgn0053696*  
FBgn0053703  
FBgn0053725  
FBgn0053757  
FBgn0053758  
FBgn0053771  
FBgn0053773  
FBgn0053775  
FBgn0053784  
FBgn0053795  
*FBgn0053798*  
FBgn0053928  
FBgn0053936  
*FBgn0053937*  
FBgn0053958  
FBgn0053960  
FBgn0053966  
FBgn0053969  
*FBgn0053970*  
FBgn0053980  
*FBgn0053986*  
FBgn0053988  
*FBgn0053993*  
FBgn0053998  
*FBgn0054001*  
*FBgn0054002*  
FBgn0054003  
FBgn0054005  
FBgn0054006  
FBgn0054007  
*FBgn0054008*  
*FBgn0054010*  
FBgn0054011  
FBgn0054012  
FBgn0054015  
*FBgn0054021*  
*FBgn0054022*  
FBgn0054023  
FBgn0054024  
FBgn0054025  
*FBgn0054026*  
*FBgn0054027*  
FBgn0054028  
FBgn0054029  
FBgn0054030  
*FBgn0054031*  
FBgn0054032  
*FBgn0054033*  
FBgn0054034  
FBgn0054039  
FBgn0054040  
FBgn0054041  
*FBgn0054043*  
FBgn0054045  
*FBgn0054046*  
FBgn0054047  
FBgn0054049  
FBgn0054050  
FBgn0054051  
FBgn0054054  
*FBgn0054057*  
*FBgn0058006*  
*FBgn0058045*  
FBgn0058053  
*FBgn0058298*  
FBgn0058439  
FBgn0058470  
FBgn0060296  
FBgn0061173  
FBgn0061196  
*FBgn0061197*  
FBgn0061200  
FBgn0061435  
FBgn0061469  
*FBgn0061476*  
FBgn0061492  
*FBgn0061515*  
FBgn0062411  
FBgn0062412  
FBgn0062413  
FBgn0062440  
FBgn0062442  
*FBgn0062449*  
*FBgn0062517*  
FBgn0063261  
*FBgn0063485*  
FBgn0063491  
FBgn0063493  
FBgn0063494  
*FBgn0063495*  
*FBgn0063496*  
*FBgn0063497*  
*FBgn0063498*  
*FBgn0063499*  
FBgn0063649  
*FBgn0063667*  
*FBgn0064119*  
*FBgn0064225*  
FBgn0064237  
FBgn0064766  
*FBgn0064912*  
*FBgn0065032*  
FBgn0065035  
FBgn0065108  
*FBgn0065110*  
*FBgn0066084*  
*FBgn0066101*  
FBgn0066293  
FBgn0066365  
FBgn0067317  
FBgn0067628  
FBgn0067629  
FBgn0067779  
FBgn0067861  
FBgn0067864  
FBgn0067903  
FBgn0069242  
*FBgn0069354*  
FBgn0069913  
FBgn0069938  
FBgn0069972  
*FBgn0069973*  
FBgn0082582  
FBgn0082585  
FBgn0082598  
*FBgn0082831*  
*FBgn0083077*  
*FBgn0083141*  
FBgn0083228  
*FBgn0083919*  
FBgn0083938  
FBgn0083940  
FBgn0083945  
FBgn0083946  
FBgn0083949  
FBgn0083950  
FBgn0083951  
FBgn0083952  
*FBgn0083953*  
FBgn0083956  
FBgn0083959  
FBgn0083961  
*FBgn0083962*  
FBgn0083963  
*FBgn0083966*  
FBgn0083967  
FBgn0083969  
*FBgn0083970*  
FBgn0083971  
*FBgn0083972*  
FBgn0083975  
FBgn0083978  
FBgn0083979  
FBgn0083980  
FBgn0083981  
FBgn0083986  
FBgn0084001  
FBgn0085188  
FBgn0085190  
FBgn0085194  
FBgn0085196  
FBgn0085197  
FBgn0085198  
*FBgn0085200*  
*FBgn0085201*  
FBgn0085203  
FBgn0085204  
FBgn0085205  
FBgn0085210  
FBgn0085213  
*FBgn0085215*  
FBgn0085216  
FBgn0085217  
FBgn0085221  
FBgn0085224  
FBgn0085225  
FBgn0085227  
FBgn0085228  
FBgn0085229  
FBgn0085233  
FBgn0085234  
FBgn0085239  
FBgn0085241  
*FBgn0085243*  
FBgn0085246  
FBgn0085250  
FBgn0085253  
FBgn0085254  
*FBgn0085256*  
FBgn0085257  
FBgn0085259  
*FBgn0085260*  
*FBgn0085261*  
*FBgn0085263*  
*FBgn0085265*  
FBgn0085272  
FBgn0085274  
FBgn0085275  
FBgn0085276  
FBgn0085277  
FBgn0085278  
*FBgn0085280*  
*FBgn0085281*  
*FBgn0085282*  
FBgn0085285  
FBgn0085288  
FBgn0085289  
FBgn0085290  
FBgn0085293  
FBgn0085294  
FBgn0085295  
FBgn0085296  
FBgn0085298  
*FBgn0085302*  
FBgn0085303  
FBgn0085305  
*FBgn0085307*  
*FBgn0085308*  
FBgn0085310  
FBgn0085311  
*FBgn0085312*  
FBgn0085313  
FBgn0085315  
FBgn0085317  
FBgn0085318  
*FBgn0085319*  
FBgn0085321  
FBgn0085324  
FBgn0085325  
FBgn0085326  
*FBgn0085329*  
*FBgn0085330*  
FBgn0085331  
*FBgn0085333*  
FBgn0085346  
FBgn0085348  
FBgn0085349  
FBgn0085352  
*FBgn0085353*  
*FBgn0085354*  
*FBgn0085357*  
*FBgn0085359*  
*FBgn0085360*  
*FBgn0085365*  
FBgn0085366  
FBgn0085367  
*FBgn0085369*  
*FBgn0085370*  
*FBgn0085371*  
FBgn0085374  
FBgn0085375  
FBgn0085376  
*FBgn0085377*  
FBgn0085378  
FBgn0085379  
FBgn0085380  
FBgn0085382  
FBgn0085383  
*FBgn0085384*  
*FBgn0085385*  
FBgn0085386  
FBgn0085387  
FBgn0085388  
FBgn0085390  
FBgn0085391  
FBgn0085394  
FBgn0085395  
FBgn0085396  
FBgn0085397  
FBgn0085398  
FBgn0085399  
FBgn0085400  
FBgn0085401  
FBgn0085403  
*FBgn0085404*  
FBgn0085405  
FBgn0085406  
FBgn0085407  
FBgn0085408  
FBgn0085409  
FBgn0085410  
*FBgn0085411*  
*FBgn0085412*  
FBgn0085413  
FBgn0085414  
*FBgn0085415*  
FBgn0085417  
*FBgn0085419*  
FBgn0085420  
FBgn0085421  
FBgn0085422  
FBgn0085423  
FBgn0085424  
*FBgn0085425*  
FBgn0085426  
FBgn0085427  
FBgn0085428  
FBgn0085429  
FBgn0085430  
FBgn0085431  
FBgn0085433  
FBgn0085434  
*FBgn0085435*  
FBgn0085436  
FBgn0085437  
FBgn0085438  
FBgn0085440  
*FBgn0085442*  
FBgn0085443  
FBgn0085444  
*FBgn0085446*  
FBgn0085447  
*FBgn0085448*  
FBgn0085449  
FBgn0085450  
FBgn0085451  
FBgn0085453  
FBgn0085456  
FBgn0085458  
FBgn0085460  
FBgn0085464  
FBgn0085473  
FBgn0085474  
FBgn0085477  
FBgn0085478  
FBgn0085480  
FBgn0085481  
*FBgn0085485*  
*FBgn0085487*  
*FBgn0085489*  
FBgn0085491  
FBgn0086129  
FBgn0086130  
FBgn0086134  
*FBgn0086251*  
FBgn0086253  
*FBgn0086254*  
FBgn0086265  
FBgn0086266  
*FBgn0086346*  
FBgn0086347  
*FBgn0086348*  
FBgn0086350  
*FBgn0086356*  
FBgn0086357  
FBgn0086358  
FBgn0086359  
*FBgn0086361*  
*FBgn0086364*  
FBgn0086365  
*FBgn0086367*  
*FBgn0086368*  
FBgn0086370  
FBgn0086371  
*FBgn0086372*  
*FBgn0086377*  
*FBgn0086384*  
FBgn0086408  
FBgn0086441  
FBgn0086442  
FBgn0086448  
FBgn0086472  
FBgn0086475  
*FBgn0086519*  
FBgn0086604  
FBgn0086605  
*FBgn0086608*  
FBgn0086610  
*FBgn0086611*  
*FBgn0086613*  
FBgn0086655  
FBgn0086656  
FBgn0086657  
*FBgn0086673*  
*FBgn0086674*  
*FBgn0086675*  
FBgn0086676  
FBgn0086677  
FBgn0086679  
FBgn0086680  
FBgn0086681  
*FBgn0086683*  
*FBgn0086687*  
FBgn0086689  
FBgn0086690  
*FBgn0086691*  
*FBgn0086693*  
FBgn0086694  
*FBgn0086695*  
FBgn0086698  
FBgn0086704  
FBgn0086706  
FBgn0086707  
FBgn0086708  
*FBgn0086710*  
FBgn0086711  
FBgn0086712  
FBgn0086736  
FBgn0086757  
*FBgn0086758*  
*FBgn0086768*  
*FBgn0086778*  
*FBgn0086779*  
FBgn0086782  
*FBgn0086783*  
FBgn0086784  
FBgn0086785  
FBgn0086855  
FBgn0086856  
FBgn0086895  
FBgn0086897  
*FBgn0086898*  
FBgn0086899  
FBgn0086902  
FBgn0086904  
*FBgn0086906*  
*FBgn0086908*  
*FBgn0086909*  
FBgn0086911  
FBgn0086912  
*FBgn0086913*  
*FBgn0086915*  
FBgn0086916  
*FBgn0087005*  
*FBgn0087007*  
*FBgn0087013*  
FBgn0087021  
FBgn0087035  
FBgn0087039  
*FBgn0087040*  
FBgn0243486  
FBgn0243511  
FBgn0243512  
FBgn0243513  
FBgn0243514  
FBgn0243516  
FBgn0243517  
FBgn0243586  
FBgn0250732  
FBgn0250746  
FBgn0250753  
*FBgn0250754*  
FBgn0250755  
*FBgn0250757*  
FBgn0250785  
FBgn0250786  
*FBgn0250791*  
FBgn0250814  
FBgn0250815  
*FBgn0250816*  
FBgn0250818  
FBgn0250820  
FBgn0250821  
*FBgn0250823*  
FBgn0250830  
*FBgn0250832*  
FBgn0250834  
*FBgn0250835*  
FBgn0250837  
*FBgn0250838*  
*FBgn0250839*  
FBgn0250840  
FBgn0250841  
FBgn0250842  
FBgn0250843  
FBgn0250844  
FBgn0250845  
FBgn0250846  
FBgn0250847  
FBgn0250849  
FBgn0250850  
FBgn0250862  
*FBgn0250869*  
*FBgn0250871*  
FBgn0250874  
FBgn0250904  
FBgn0250906  
*FBgn0250907*  
FBgn0250910  
*FBgn0259098*  
FBgn0259099  
FBgn0259100  
*FBgn0259101*  
FBgn0259108  
FBgn0259109  
FBgn0259110  
FBgn0259111  
FBgn0259112  
FBgn0259139  
*FBgn0259140*  
FBgn0259141  
*FBgn0259142*  
FBgn0259143  
FBgn0259145  
FBgn0259146  
FBgn0259149  
*FBgn0259150*  
FBgn0259151  
*FBgn0259152*  
FBgn0259162  
*FBgn0259163*  
FBgn0259164  
FBgn0259166  
*FBgn0259167*  
FBgn0259168  
FBgn0259171  
FBgn0259172  
FBgn0259173  
FBgn0259174  
FBgn0259175  
*FBgn0259176*  
FBgn0259178  
FBgn0259179  
FBgn0259182  
FBgn0259184  
*FBgn0259187*  
FBgn0259190  
FBgn0259192  
FBgn0259194  
FBgn0259209  
FBgn0259210  
FBgn0259211  
FBgn0259212  
FBgn0259213  
FBgn0259215  
*FBgn0259219*  
FBgn0259220  
*FBgn0259221*  
FBgn0259222  
FBgn0259223  
FBgn0259224  
FBgn0259225  
FBgn0259226  
FBgn0259227  
*FBgn0259228*  
FBgn0259229  
FBgn0259232  
*FBgn0259233*  
FBgn0259234  
*FBgn0259236*  
FBgn0259237  
*FBgn0259238*  
FBgn0259239  
FBgn0259241  
*FBgn0259242*  
FBgn0259244  
FBgn0259245  
FBgn0259246  
*FBgn0259247*  
*FBgn0259481*  
FBgn0259482  
*FBgn0259483*  
*FBgn0259678*  
FBgn0259680  
FBgn0259682  
FBgn0259683  
FBgn0259685  
FBgn0259697  
*FBgn0259701*  
FBgn0259703  
FBgn0259704  
*FBgn0259707*  
FBgn0259711  
FBgn0259712  
FBgn0259713  
FBgn0259714  
*FBgn0259715*  
*FBgn0259716*  
FBgn0259721  
FBgn0259728  
FBgn0259729  
FBgn0259735  
*FBgn0259736*  
*FBgn0259740*  
*FBgn0259741*  
FBgn0259742  
FBgn0259743  
FBgn0259744  
*FBgn0259745*  
FBgn0259749  
FBgn0259750  
FBgn0259784  
*FBgn0259785*  
FBgn0259789  
*FBgn0259791*  
FBgn0259794  
*FBgn0259818*  
*FBgn0259823*  
FBgn0259824  
FBgn0259831  
FBgn0259832  
FBgn0259834  
*FBgn0259878*  
FBgn0259896  
