# Supplementary figures and images for: Expression-based segmentation of the Drosophila genome (part 2 of 2)
Source: BMC Genomics. 2013 Nov 20;14:812. doi: 10.1186/1471-2164-14-812 (PMC3909303; doi:10.1186/1471-2164-14-812)

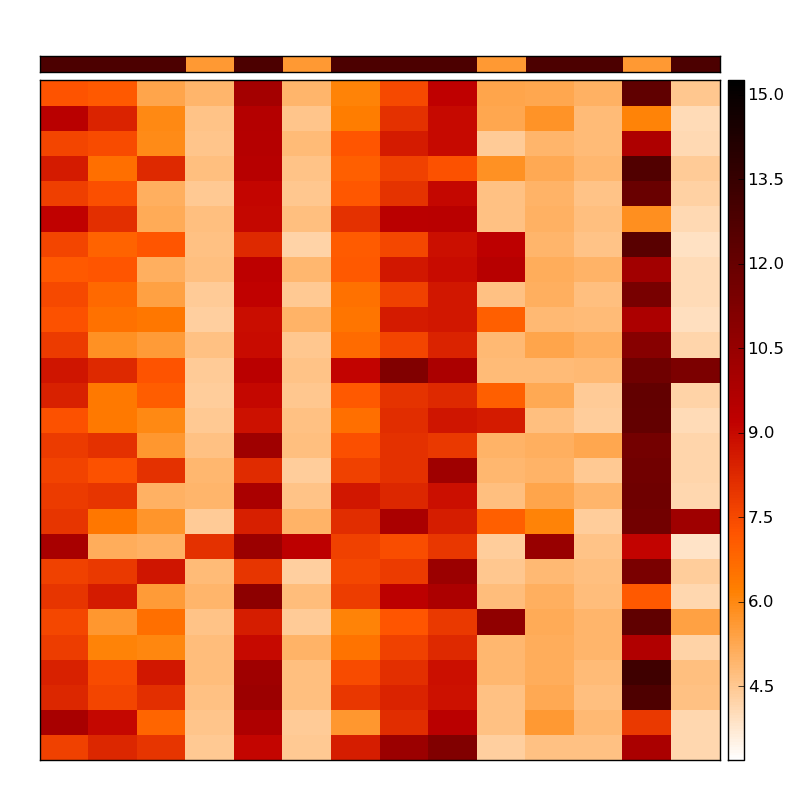

Supplement: Additional file 1 — Detailed information for multigene segments. [file 1471-2164-14-812-S1.zip › miniwebsite/heatmaps/chr2L_heatmap280.png]

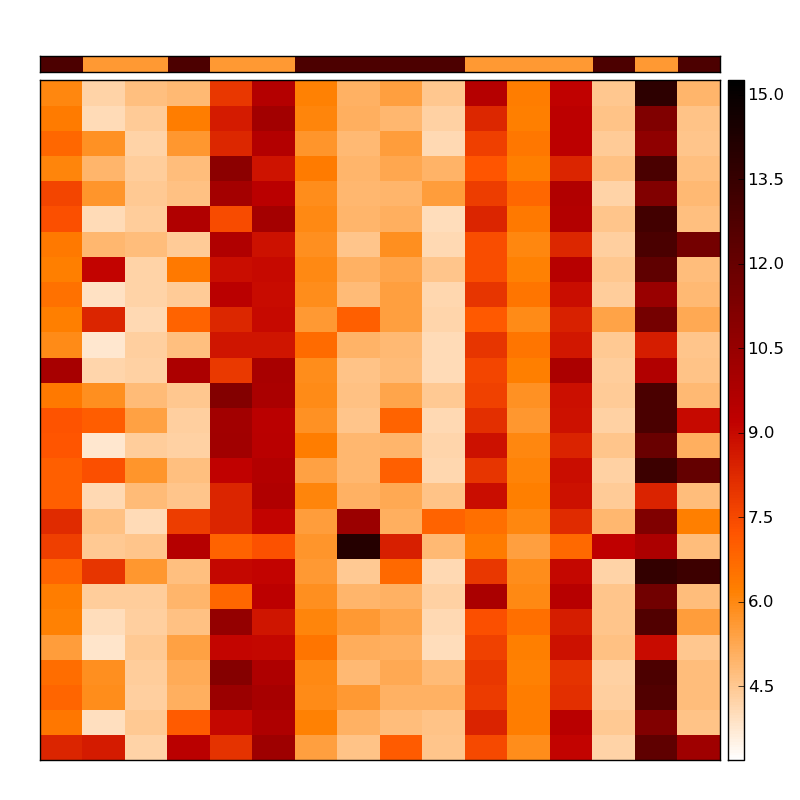

Supplement: Additional file 1 — Detailed information for multigene segments. [file 1471-2164-14-812-S1.zip › miniwebsite/heatmaps/chr2L_heatmap284.png]

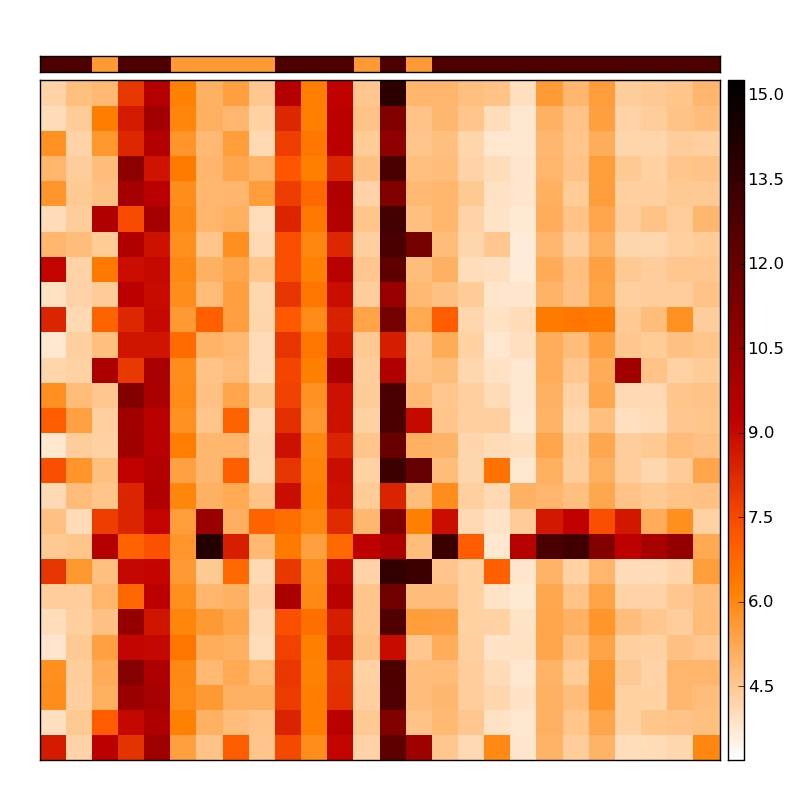

Supplement: Additional file 1 — Detailed information for multigene segments. [file 1471-2164-14-812-S1.zip › miniwebsite/heatmaps/chr2L_heatmap285.png]

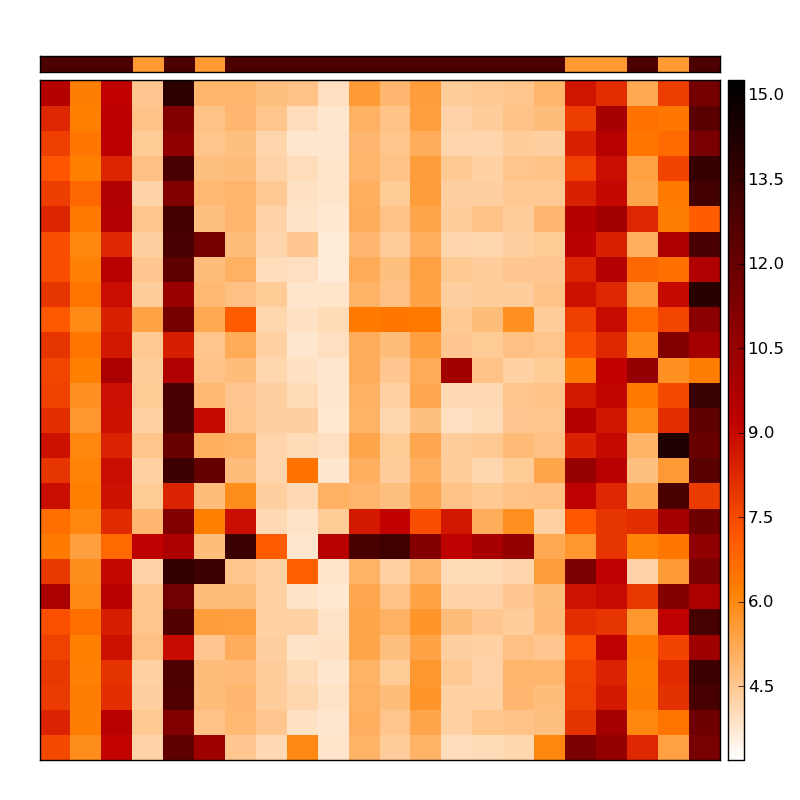

Supplement: Additional file 1 — Detailed information for multigene segments. [file 1471-2164-14-812-S1.zip › miniwebsite/heatmaps/chr2L_heatmap286.png]

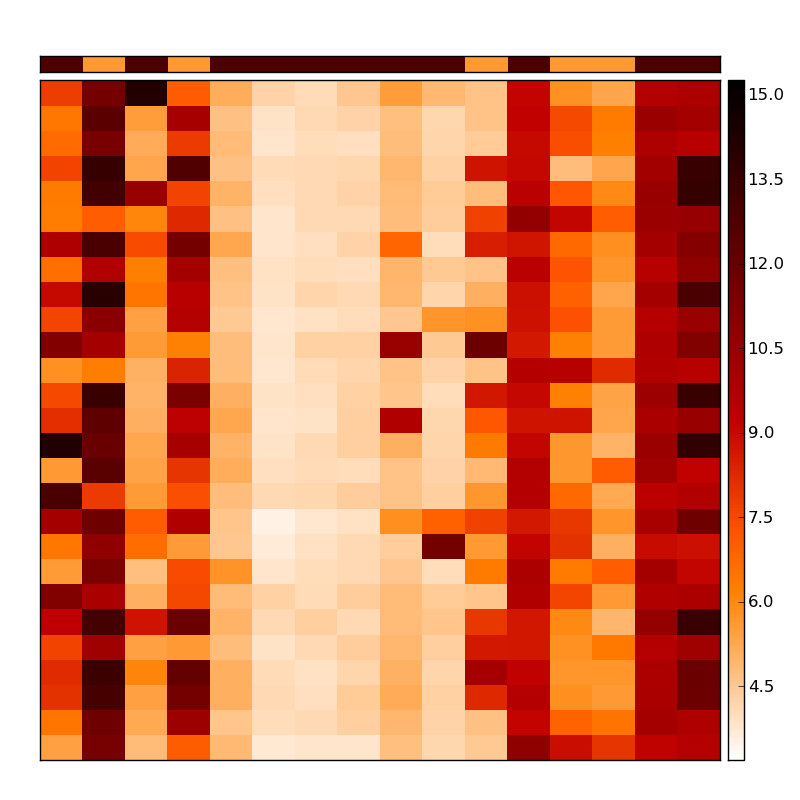

Supplement: Additional file 1 — Detailed information for multigene segments. [file 1471-2164-14-812-S1.zip › miniwebsite/heatmaps/chr2L_heatmap288.png]

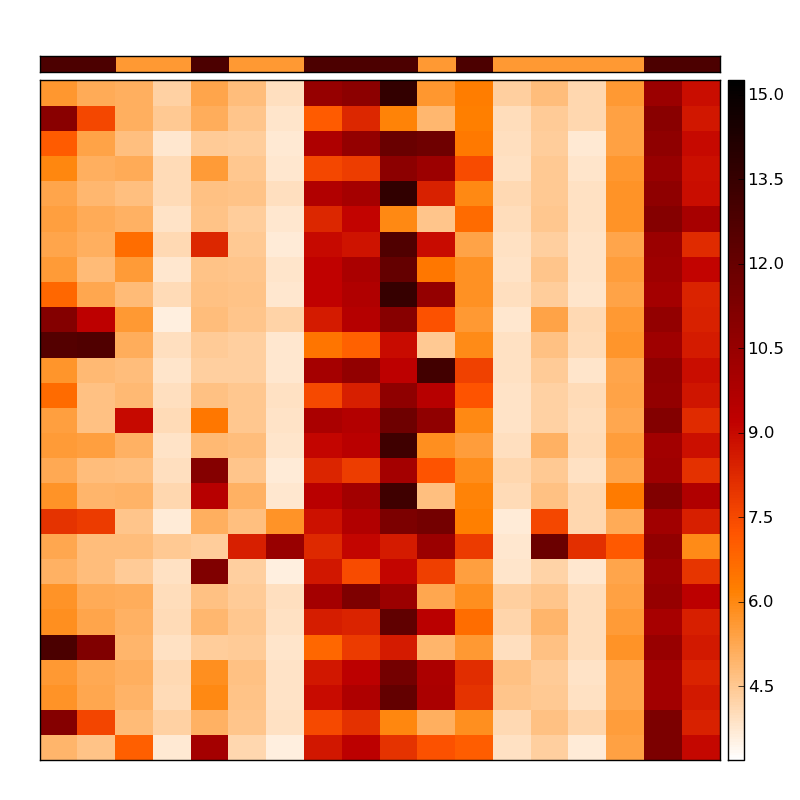

Supplement: Additional file 1 — Detailed information for multigene segments. [file 1471-2164-14-812-S1.zip › miniwebsite/heatmaps/chr2L_heatmap297.png]

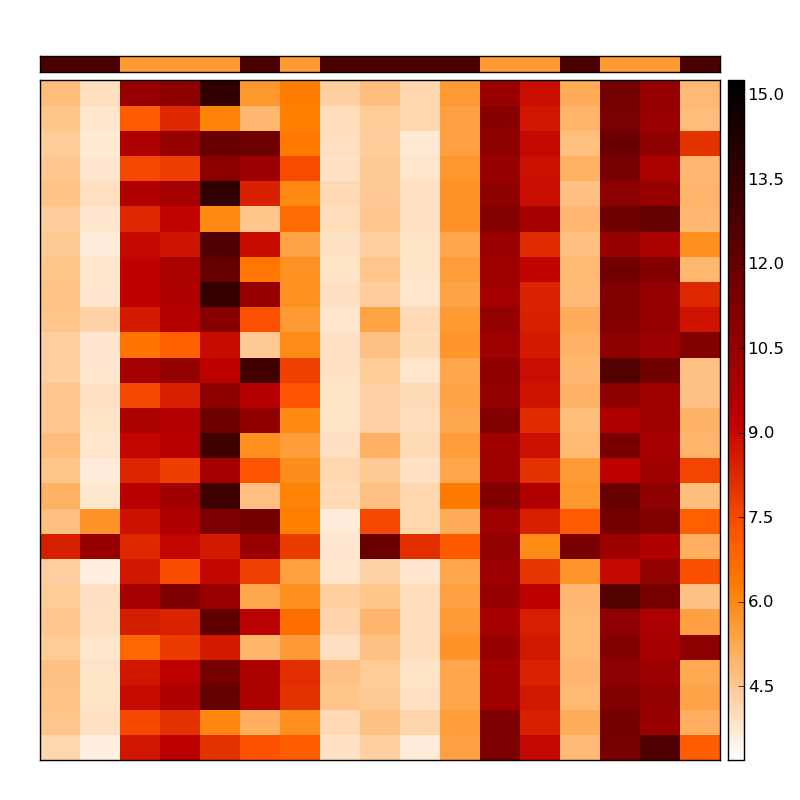

Supplement: Additional file 1 — Detailed information for multigene segments. [file 1471-2164-14-812-S1.zip › miniwebsite/heatmaps/chr2L_heatmap298.png]

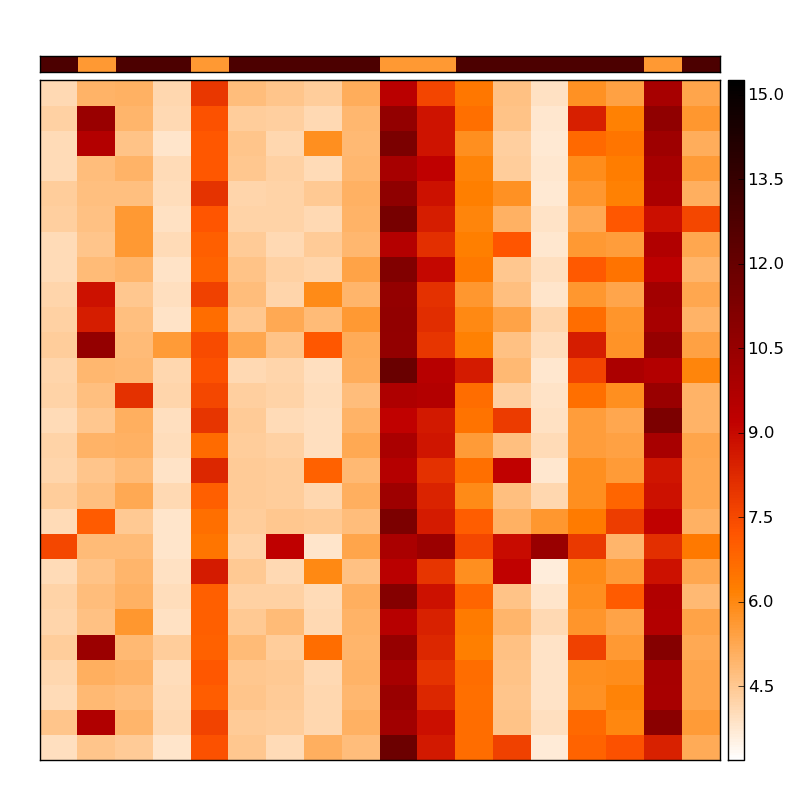

Supplement: Additional file 1 — Detailed information for multigene segments. [file 1471-2164-14-812-S1.zip › miniwebsite/heatmaps/chr2L_heatmap302.png]

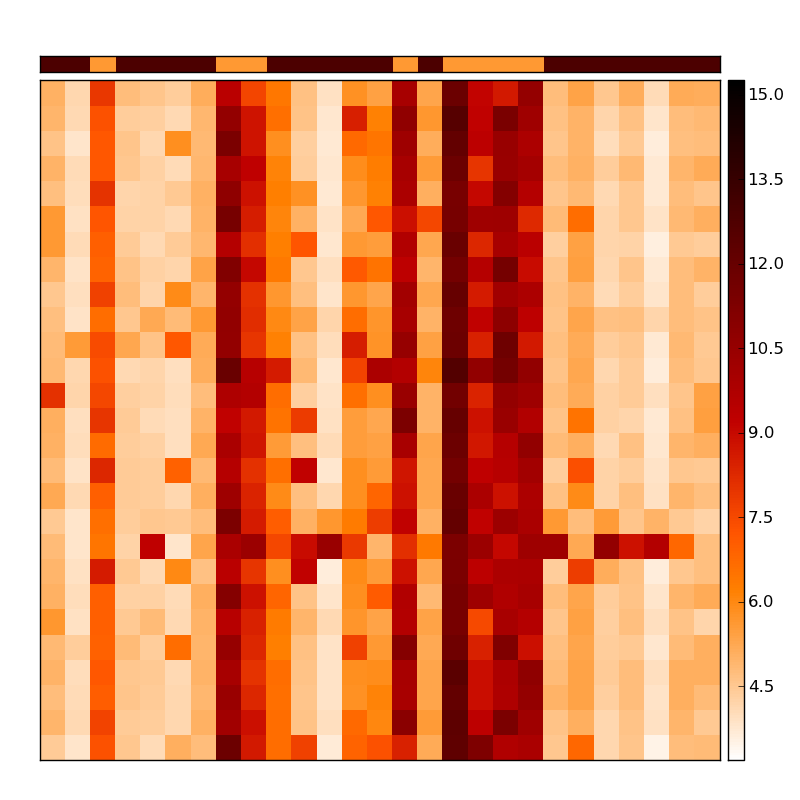

Supplement: Additional file 1 — Detailed information for multigene segments. [file 1471-2164-14-812-S1.zip › miniwebsite/heatmaps/chr2L_heatmap304.png]

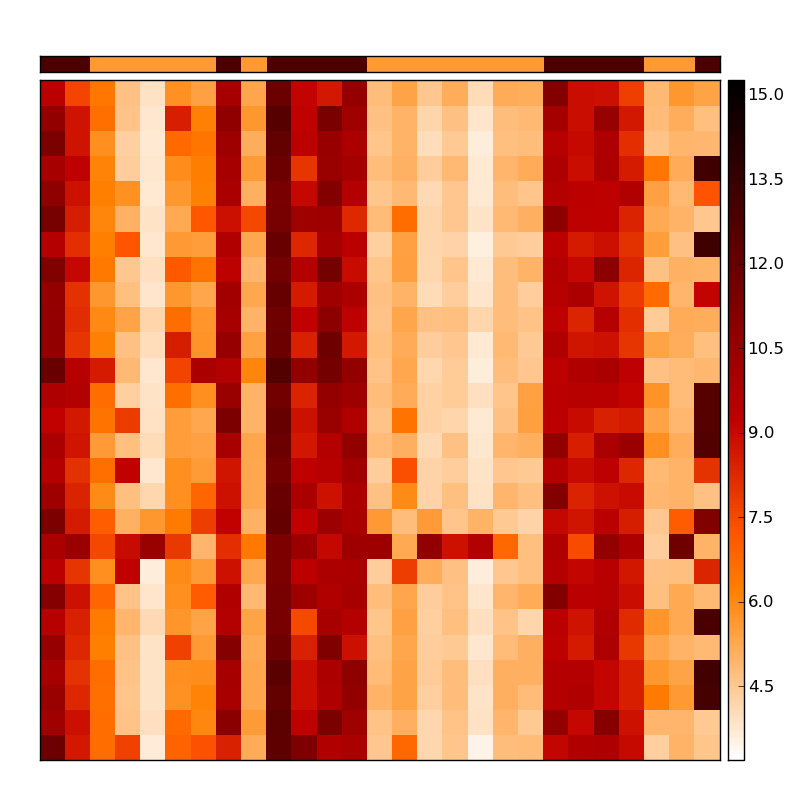

Supplement: Additional file 1 — Detailed information for multigene segments. [file 1471-2164-14-812-S1.zip › miniwebsite/heatmaps/chr2L_heatmap305.png]

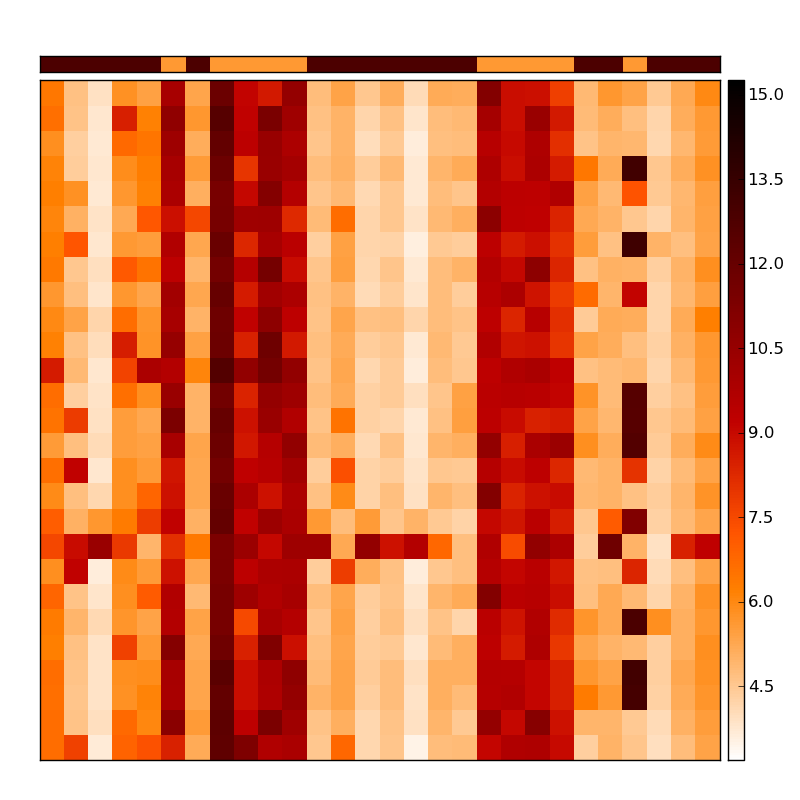

Supplement: Additional file 1 — Detailed information for multigene segments. [file 1471-2164-14-812-S1.zip › miniwebsite/heatmaps/chr2L_heatmap306.png]

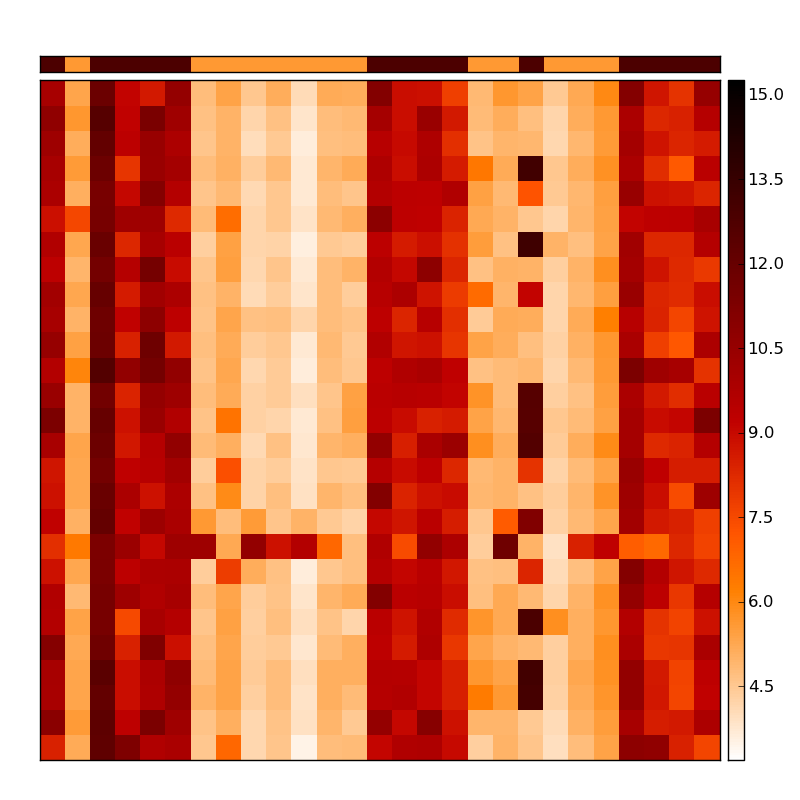

Supplement: Additional file 1 — Detailed information for multigene segments. [file 1471-2164-14-812-S1.zip › miniwebsite/heatmaps/chr2L_heatmap307.png]

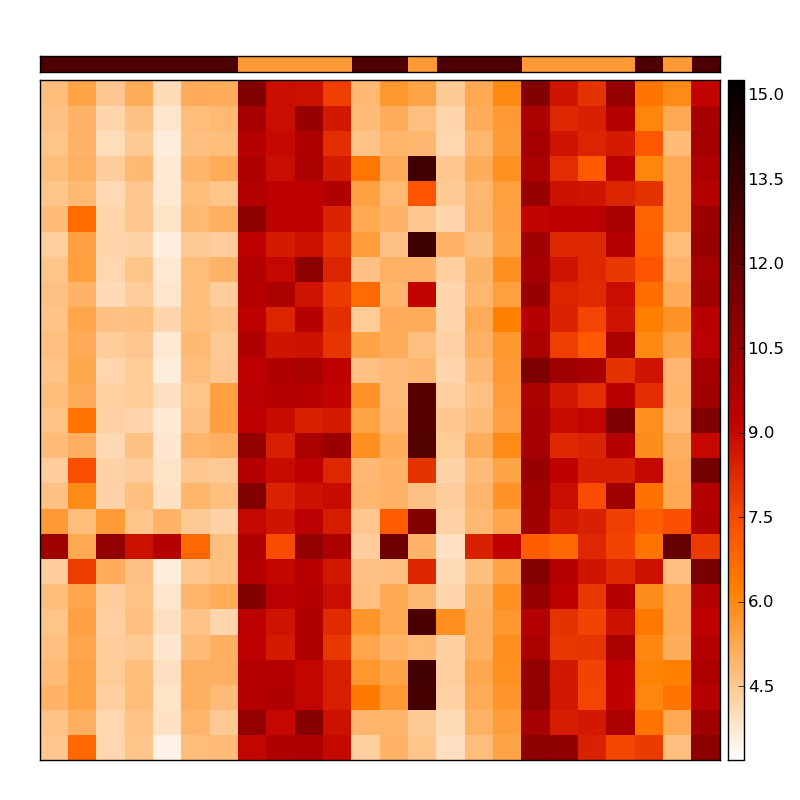

Supplement: Additional file 1 — Detailed information for multigene segments. [file 1471-2164-14-812-S1.zip › miniwebsite/heatmaps/chr2L_heatmap309.png]

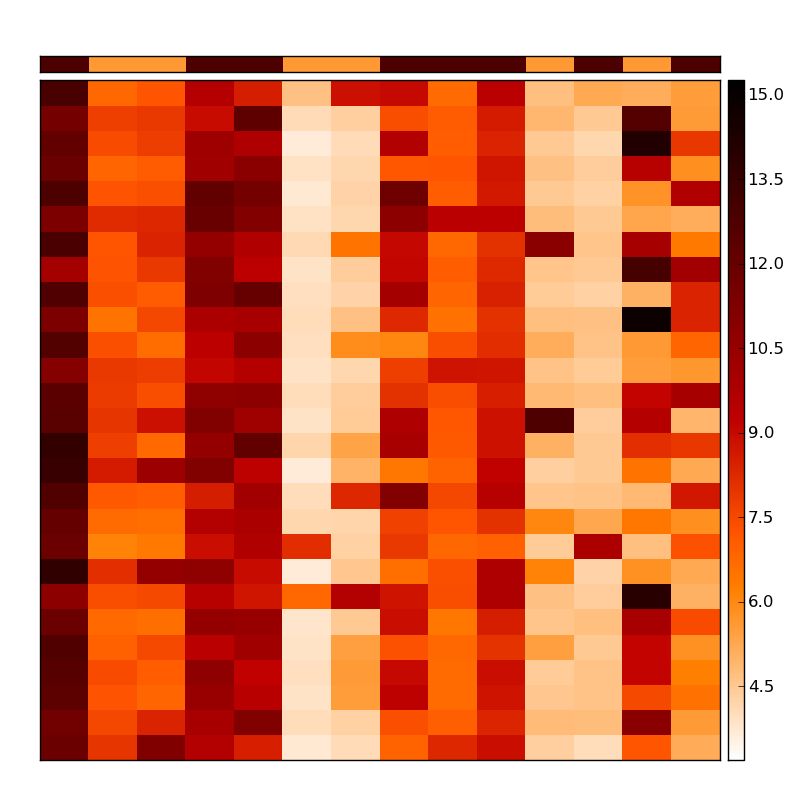

Supplement: Additional file 1 — Detailed information for multigene segments. [file 1471-2164-14-812-S1.zip › miniwebsite/heatmaps/chr2L_heatmap31.png]

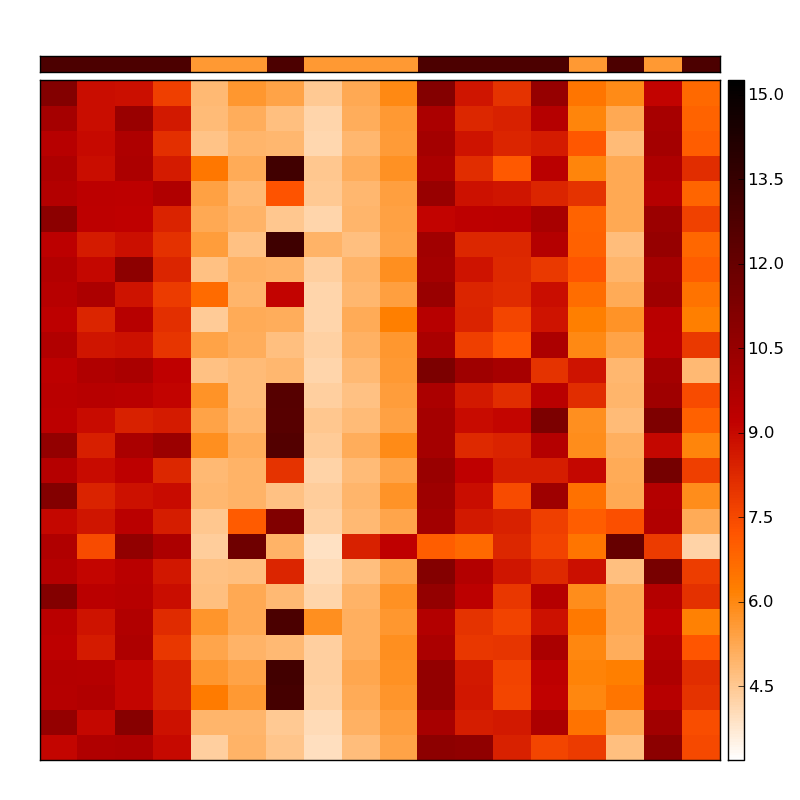

Supplement: Additional file 1 — Detailed information for multigene segments. [file 1471-2164-14-812-S1.zip › miniwebsite/heatmaps/chr2L_heatmap310.png]

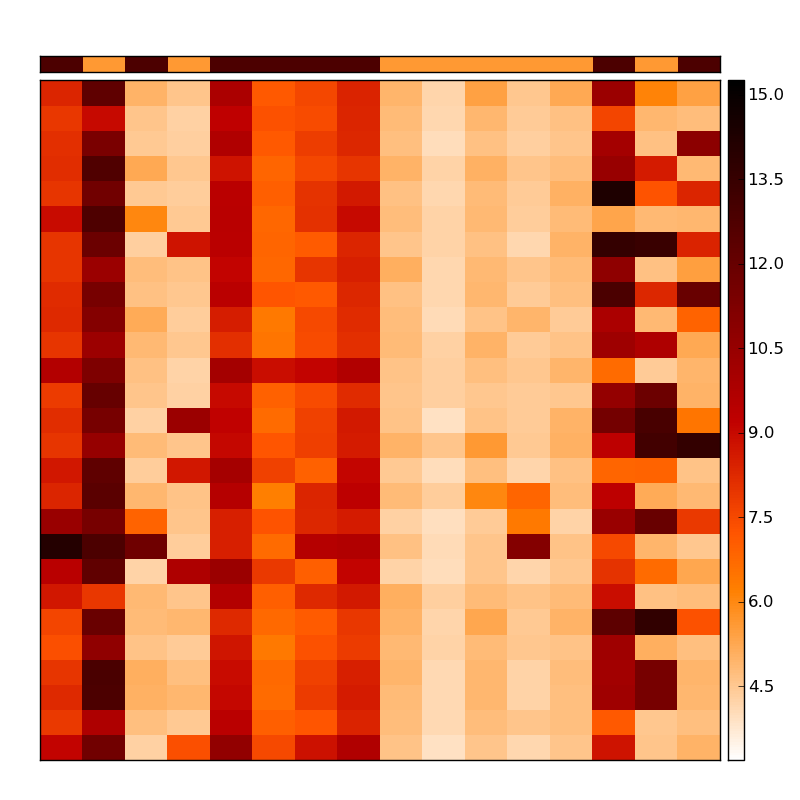

Supplement: Additional file 1 — Detailed information for multigene segments. [file 1471-2164-14-812-S1.zip › miniwebsite/heatmaps/chr2L_heatmap311.png]

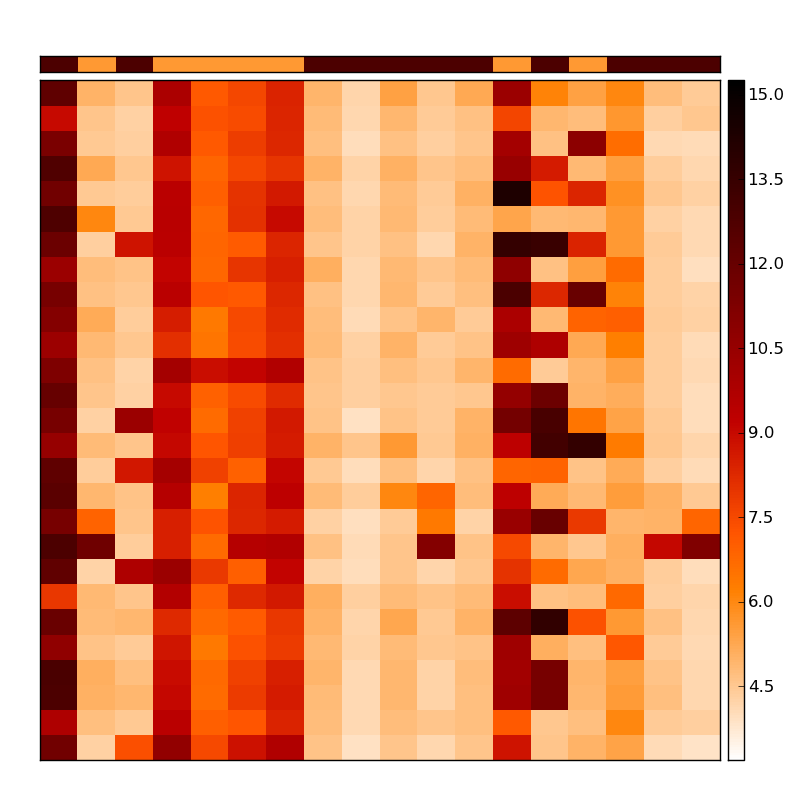

Supplement: Additional file 1 — Detailed information for multigene segments. [file 1471-2164-14-812-S1.zip › miniwebsite/heatmaps/chr2L_heatmap312.png]

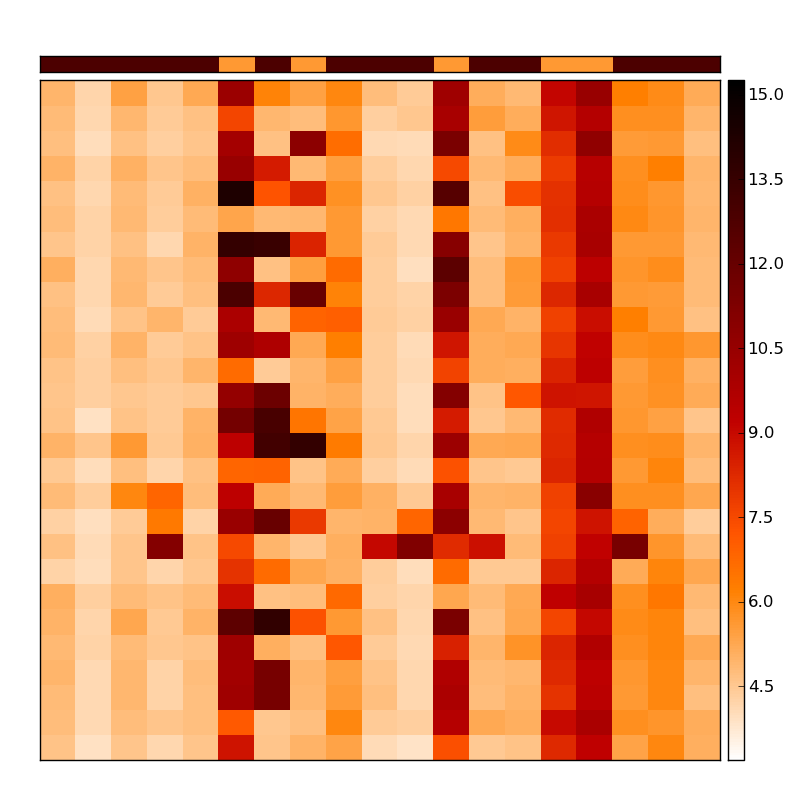

Supplement: Additional file 1 — Detailed information for multigene segments. [file 1471-2164-14-812-S1.zip › miniwebsite/heatmaps/chr2L_heatmap313.png]

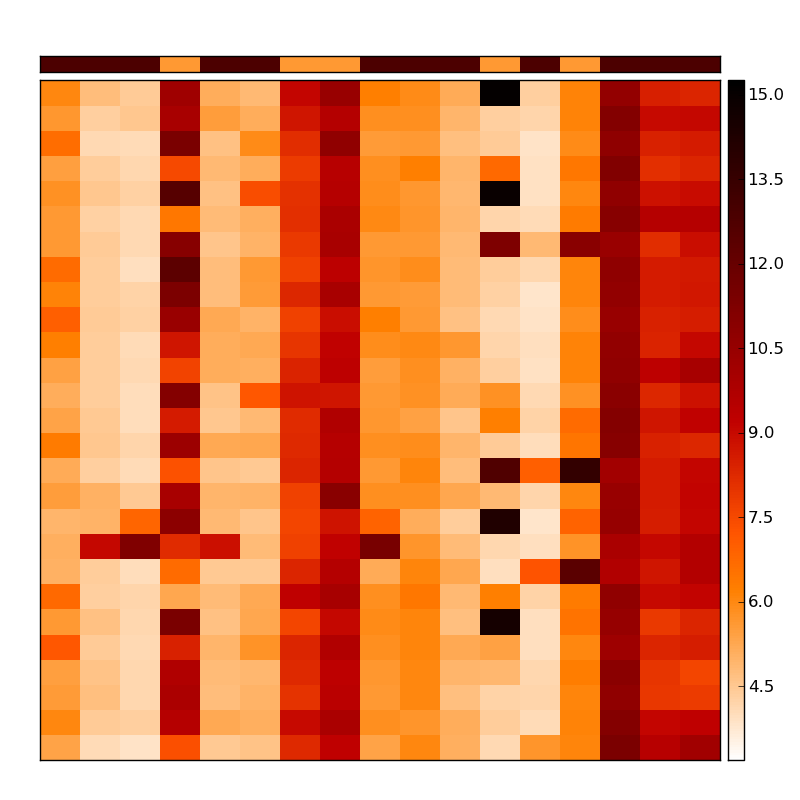

Supplement: Additional file 1 — Detailed information for multigene segments. [file 1471-2164-14-812-S1.zip › miniwebsite/heatmaps/chr2L_heatmap316.png]

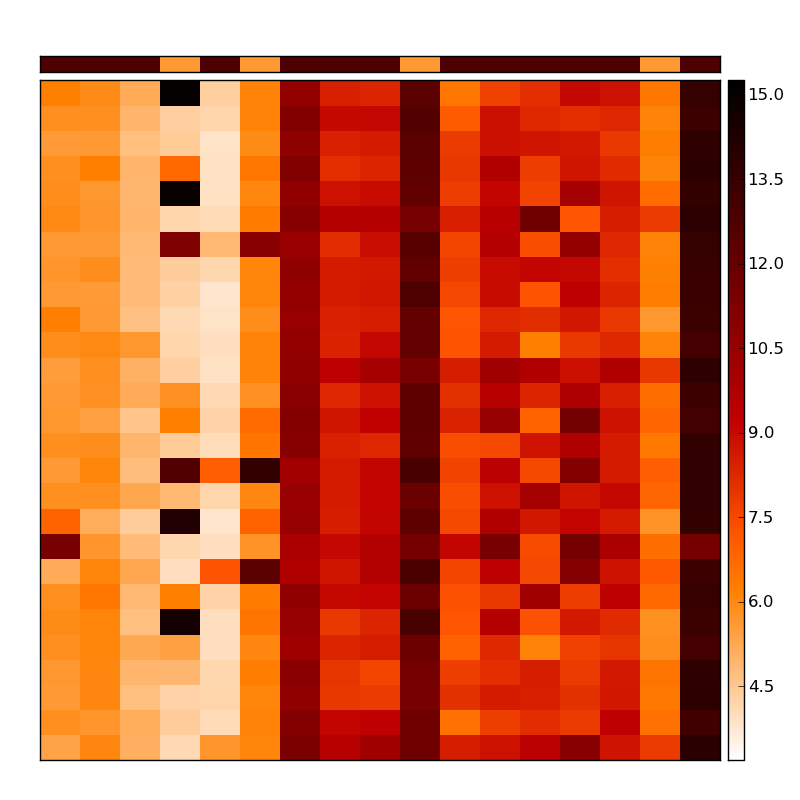

Supplement: Additional file 1 — Detailed information for multigene segments. [file 1471-2164-14-812-S1.zip › miniwebsite/heatmaps/chr2L_heatmap317.png]

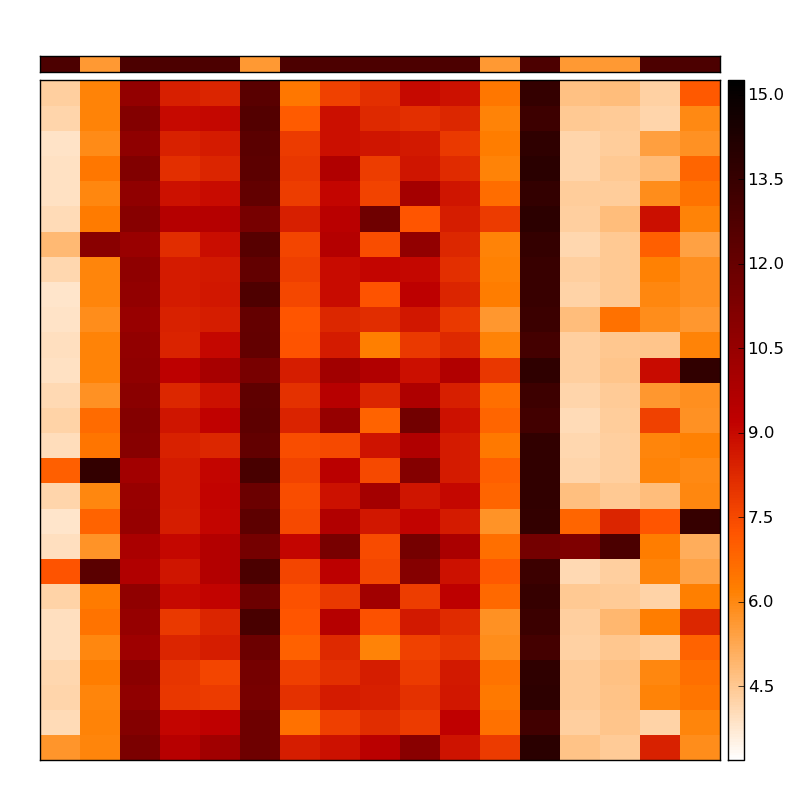

Supplement: Additional file 1 — Detailed information for multigene segments. [file 1471-2164-14-812-S1.zip › miniwebsite/heatmaps/chr2L_heatmap318.png]

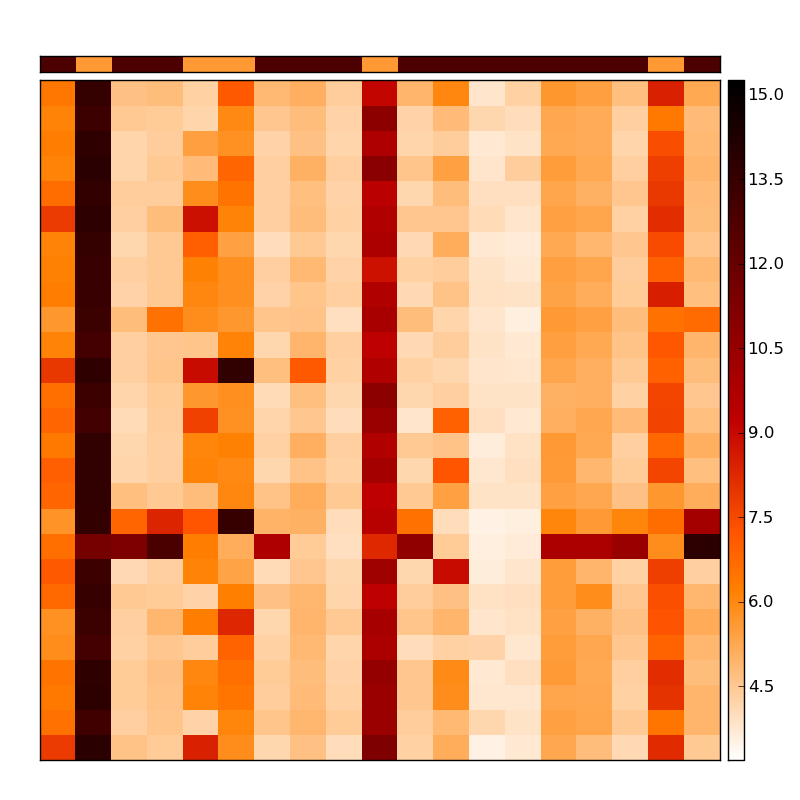

Supplement: Additional file 1 — Detailed information for multigene segments. [file 1471-2164-14-812-S1.zip › miniwebsite/heatmaps/chr2L_heatmap321.png]

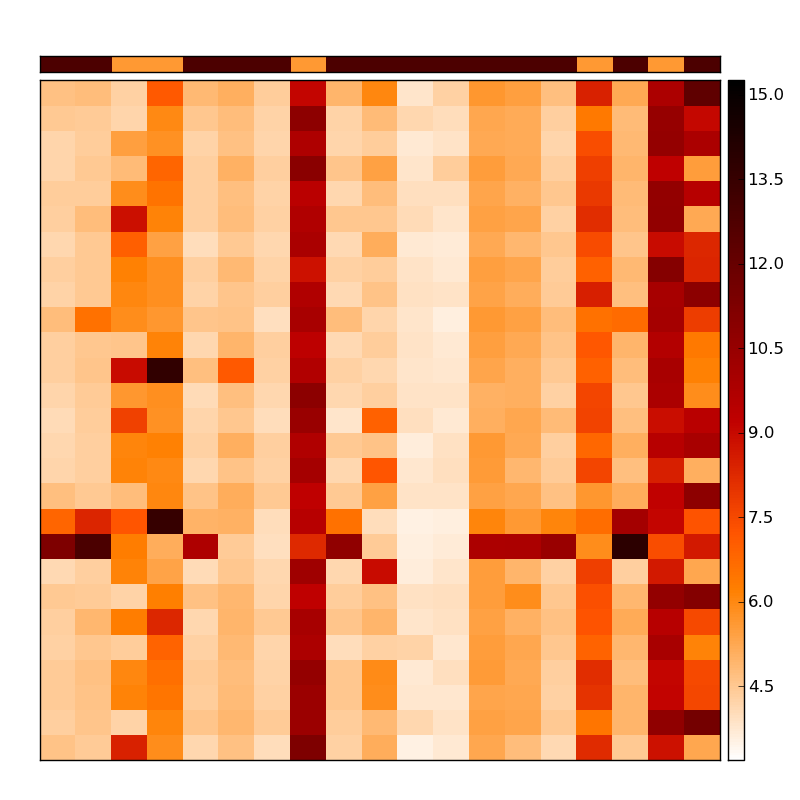

Supplement: Additional file 1 — Detailed information for multigene segments. [file 1471-2164-14-812-S1.zip › miniwebsite/heatmaps/chr2L_heatmap322.png]

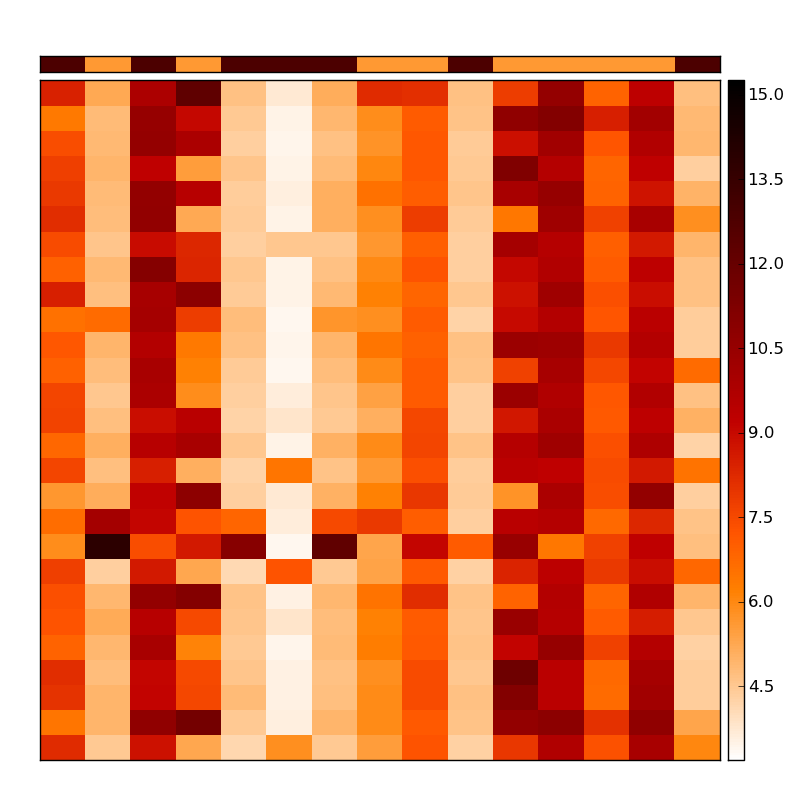

Supplement: Additional file 1 — Detailed information for multigene segments. [file 1471-2164-14-812-S1.zip › miniwebsite/heatmaps/chr2L_heatmap323.png]

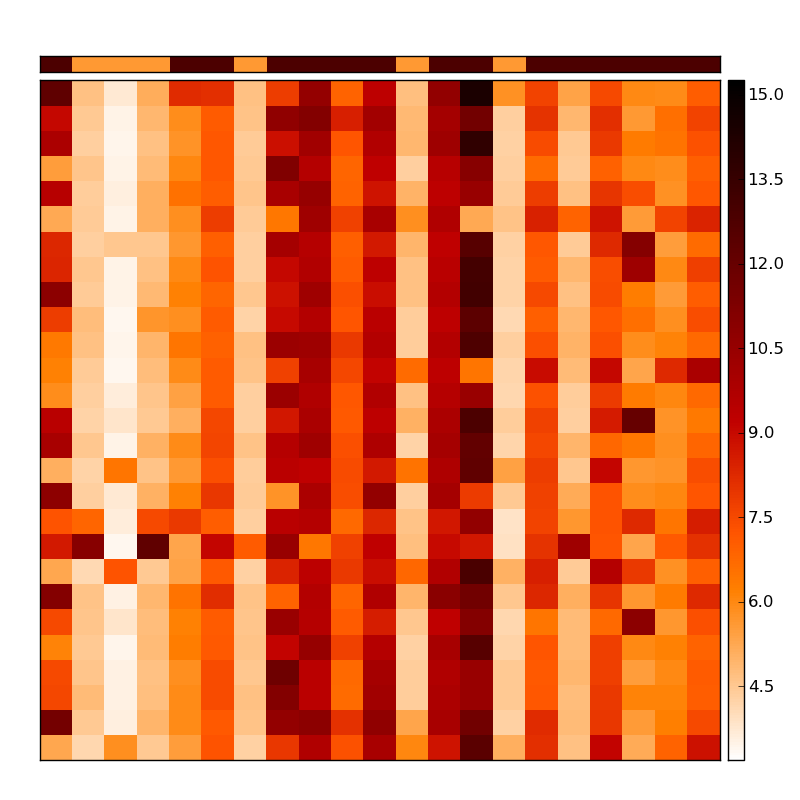

Supplement: Additional file 1 — Detailed information for multigene segments. [file 1471-2164-14-812-S1.zip › miniwebsite/heatmaps/chr2L_heatmap325.png]

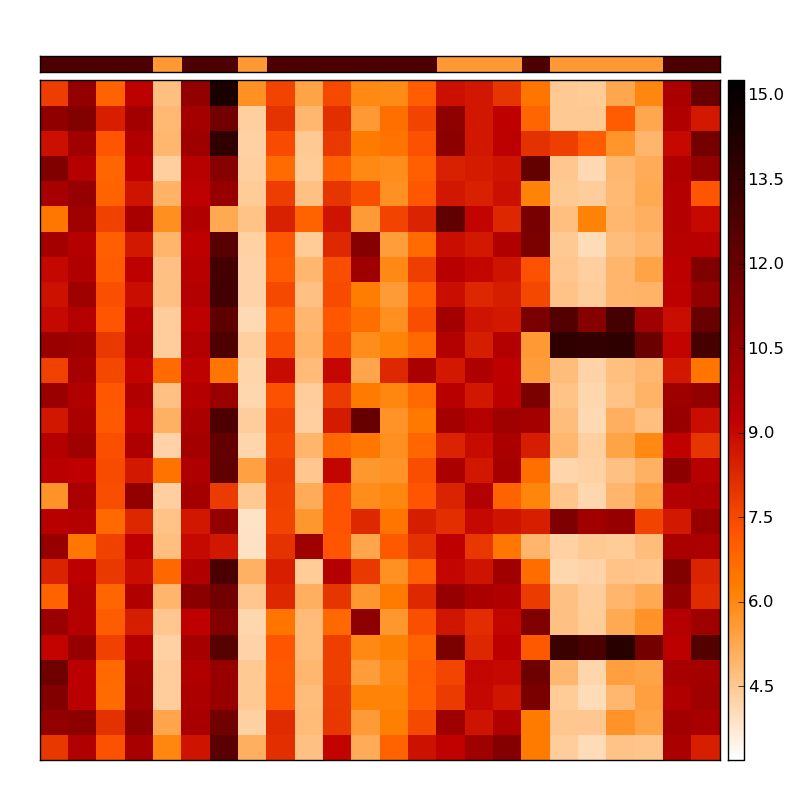

Supplement: Additional file 1 — Detailed information for multigene segments. [file 1471-2164-14-812-S1.zip › miniwebsite/heatmaps/chr2L_heatmap327.png]

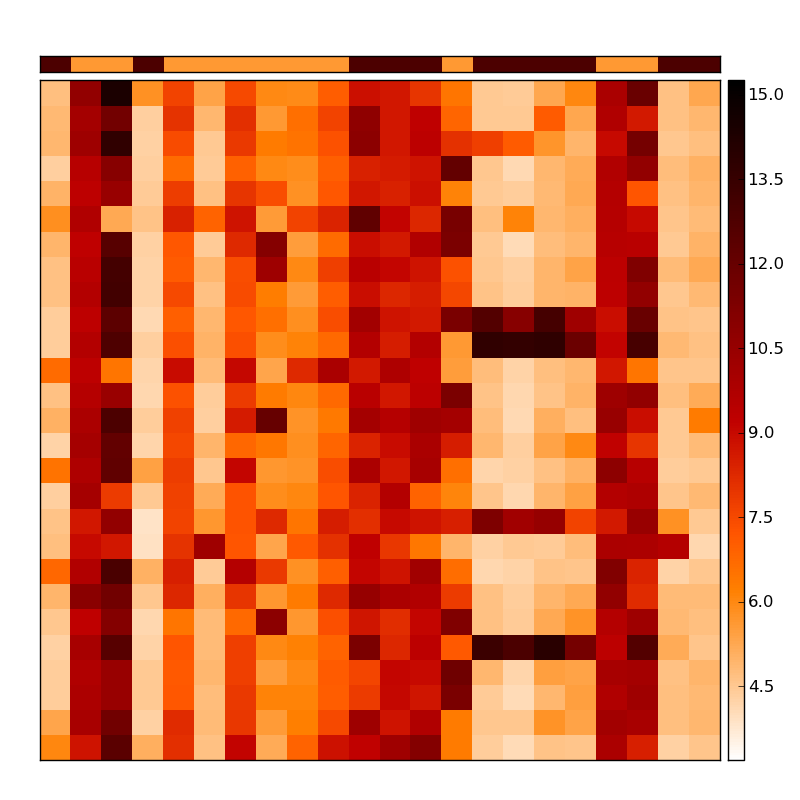

Supplement: Additional file 1 — Detailed information for multigene segments. [file 1471-2164-14-812-S1.zip › miniwebsite/heatmaps/chr2L_heatmap328.png]

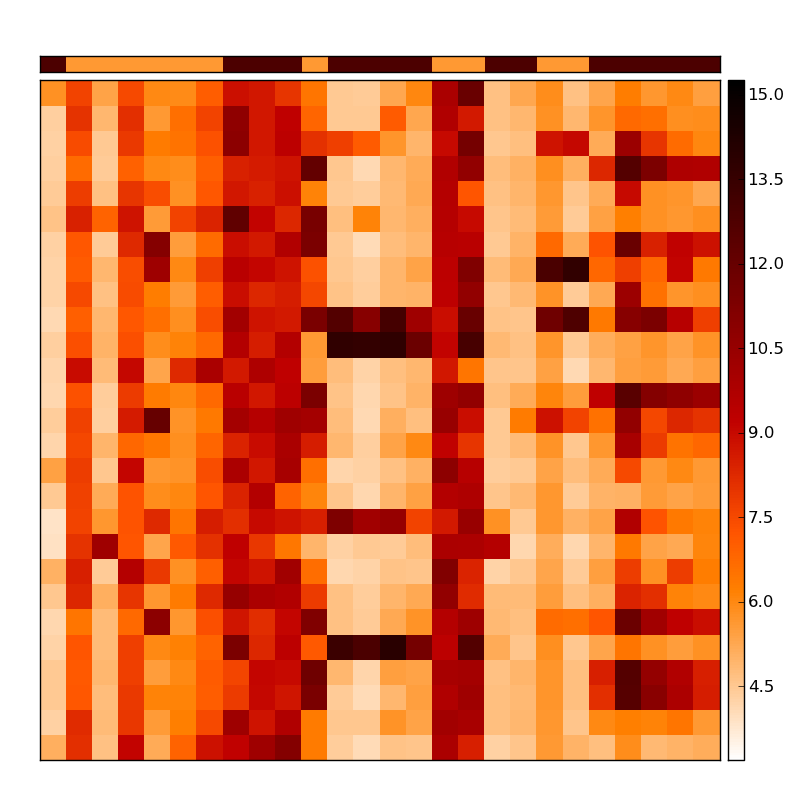

Supplement: Additional file 1 — Detailed information for multigene segments. [file 1471-2164-14-812-S1.zip › miniwebsite/heatmaps/chr2L_heatmap329.png]

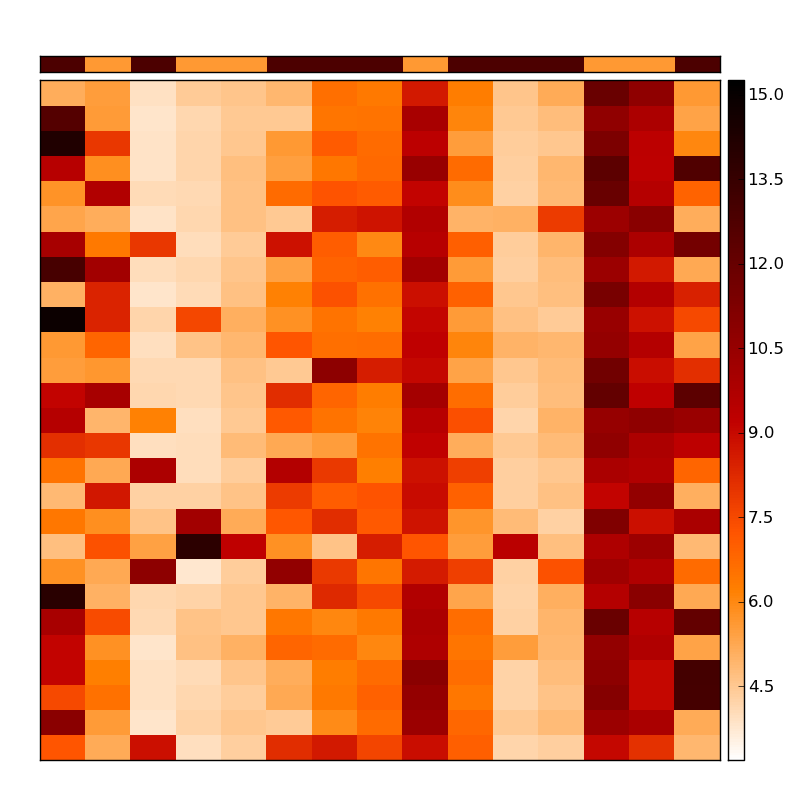

Supplement: Additional file 1 — Detailed information for multigene segments. [file 1471-2164-14-812-S1.zip › miniwebsite/heatmaps/chr2L_heatmap33.png]

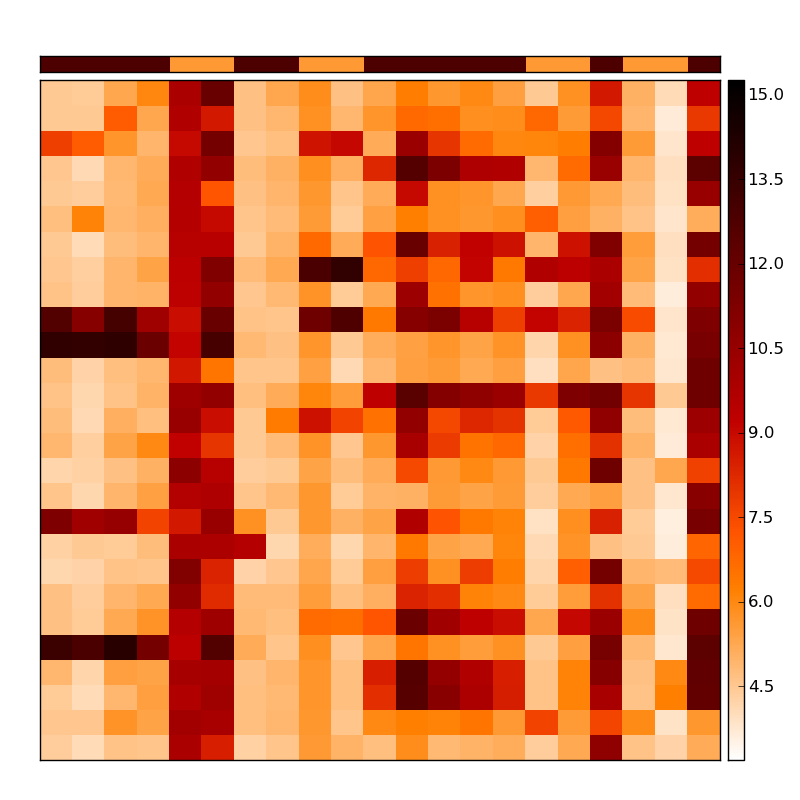

Supplement: Additional file 1 — Detailed information for multigene segments. [file 1471-2164-14-812-S1.zip › miniwebsite/heatmaps/chr2L_heatmap333.png]

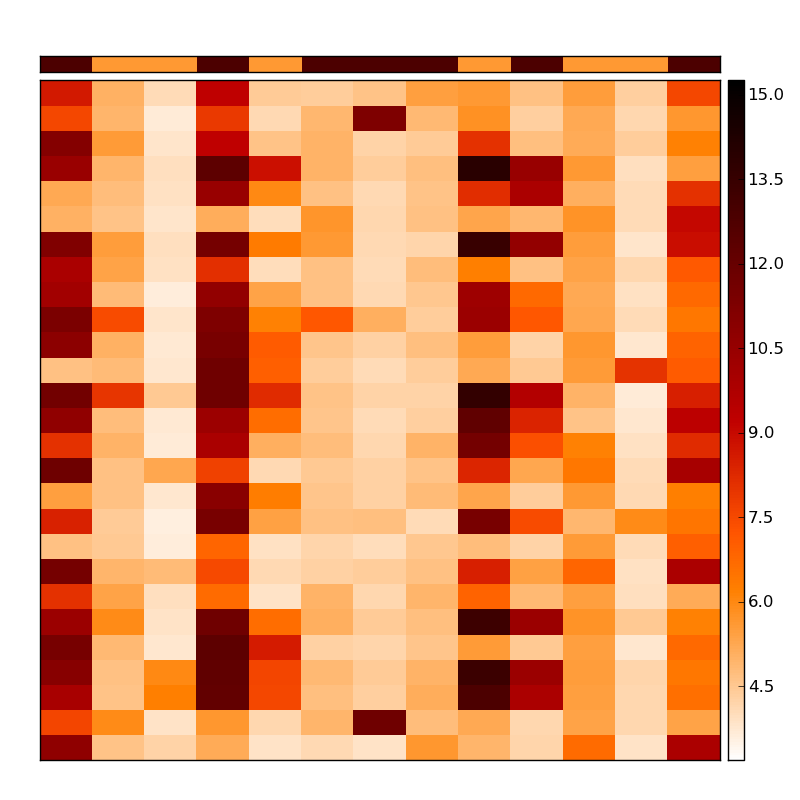

Supplement: Additional file 1 — Detailed information for multigene segments. [file 1471-2164-14-812-S1.zip › miniwebsite/heatmaps/chr2L_heatmap336.png]

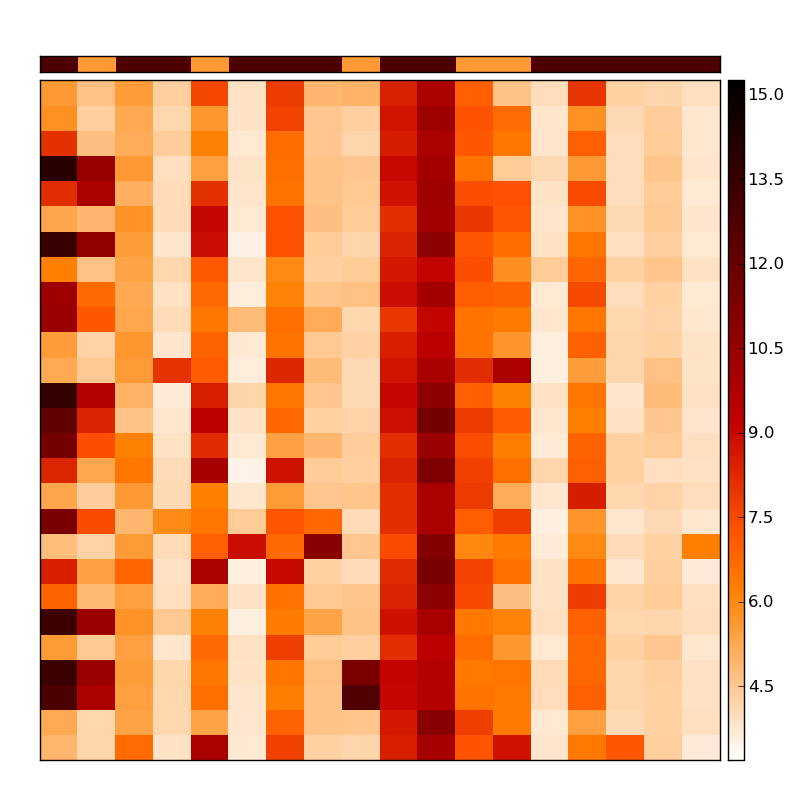

Supplement: Additional file 1 — Detailed information for multigene segments. [file 1471-2164-14-812-S1.zip › miniwebsite/heatmaps/chr2L_heatmap338.png]

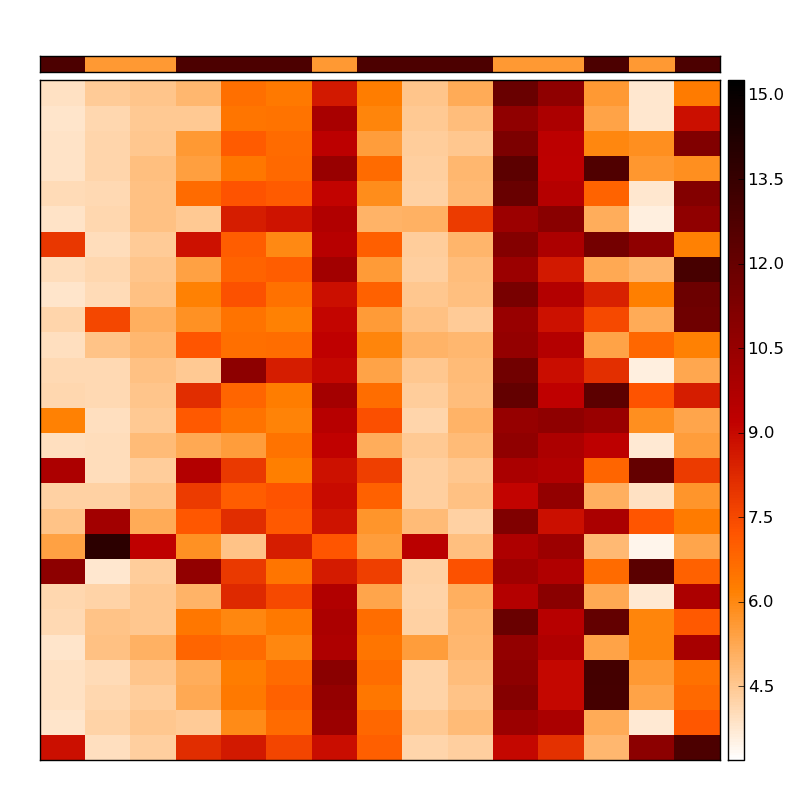

Supplement: Additional file 1 — Detailed information for multigene segments. [file 1471-2164-14-812-S1.zip › miniwebsite/heatmaps/chr2L_heatmap34.png]

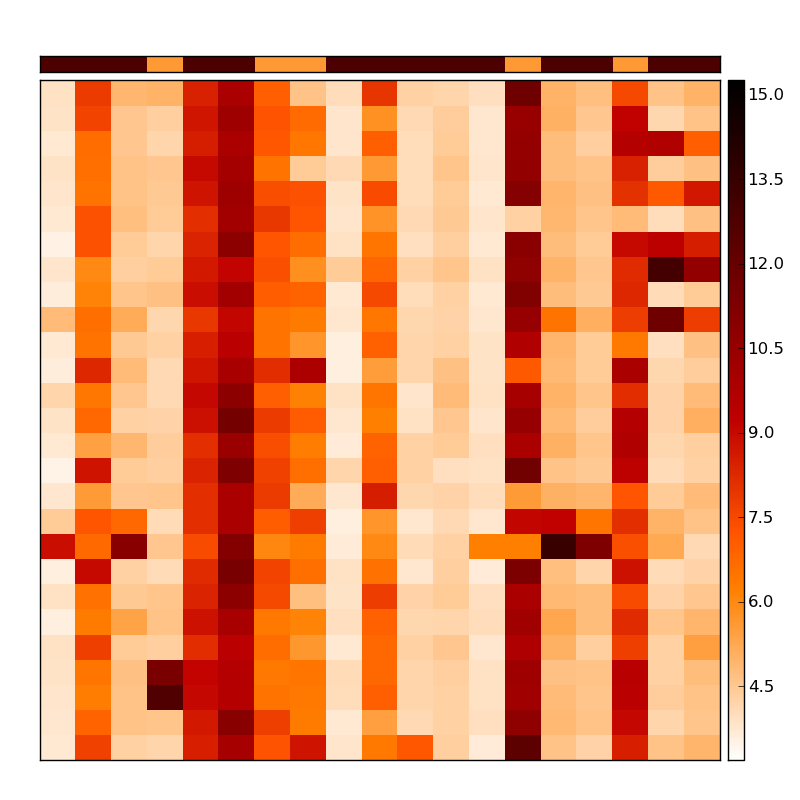

Supplement: Additional file 1 — Detailed information for multigene segments. [file 1471-2164-14-812-S1.zip › miniwebsite/heatmaps/chr2L_heatmap341.png]

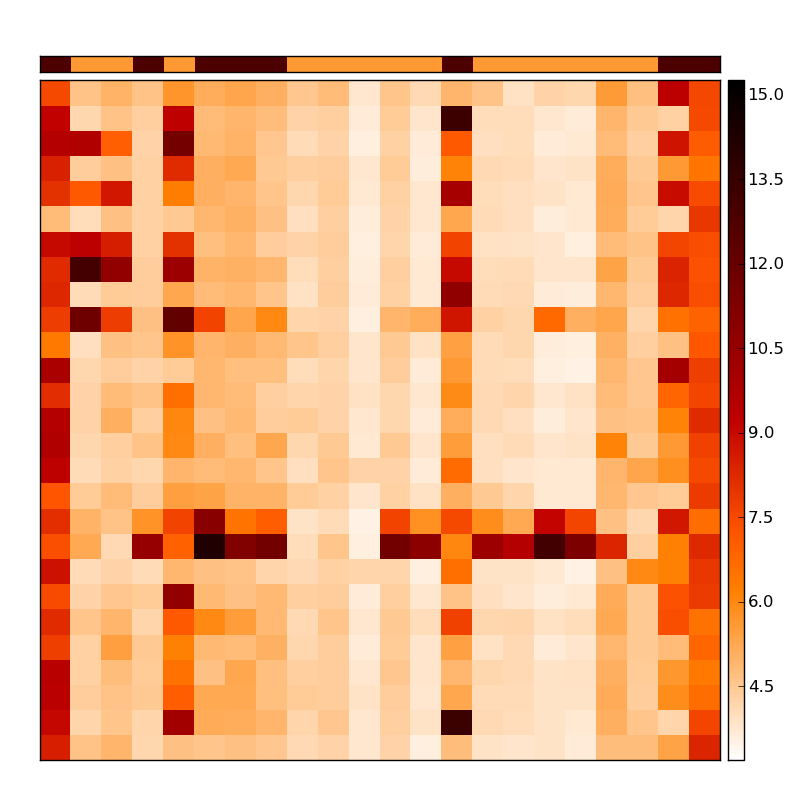

Supplement: Additional file 1 — Detailed information for multigene segments. [file 1471-2164-14-812-S1.zip › miniwebsite/heatmaps/chr2L_heatmap344.png]

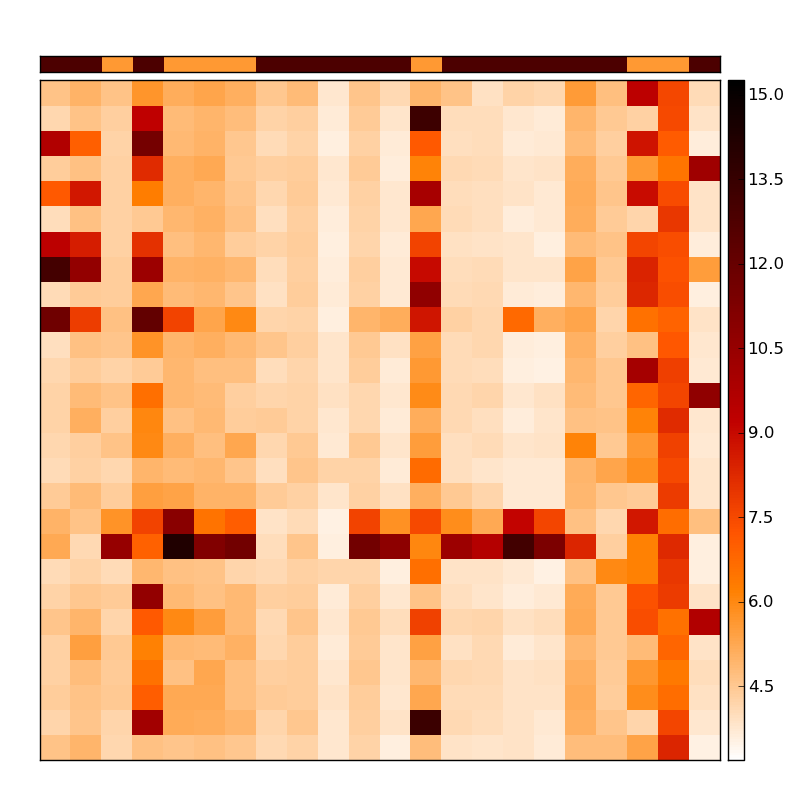

Supplement: Additional file 1 — Detailed information for multigene segments. [file 1471-2164-14-812-S1.zip › miniwebsite/heatmaps/chr2L_heatmap345.png]

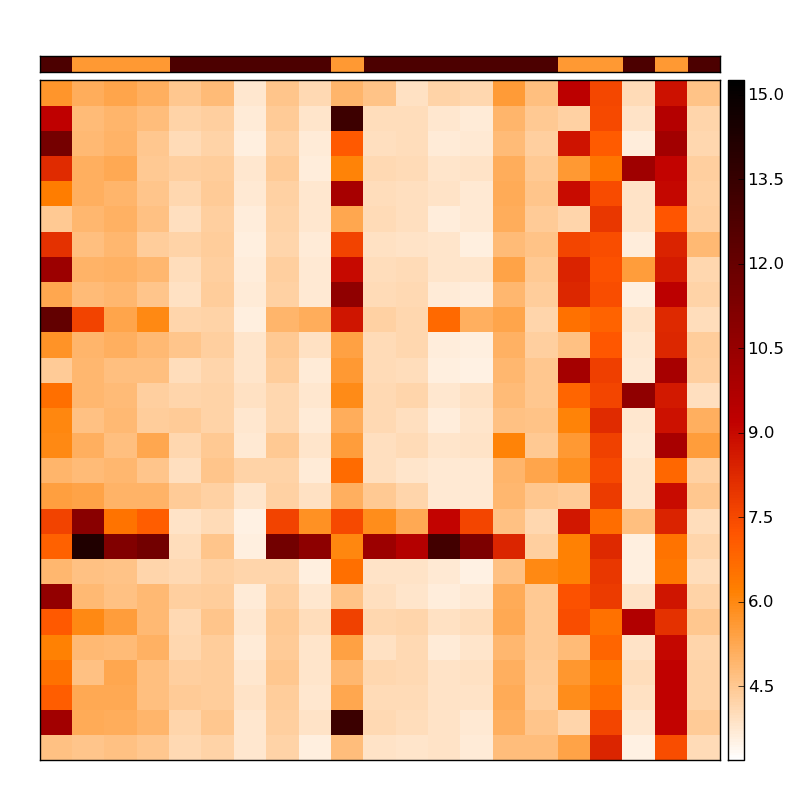

Supplement: Additional file 1 — Detailed information for multigene segments. [file 1471-2164-14-812-S1.zip › miniwebsite/heatmaps/chr2L_heatmap346.png]

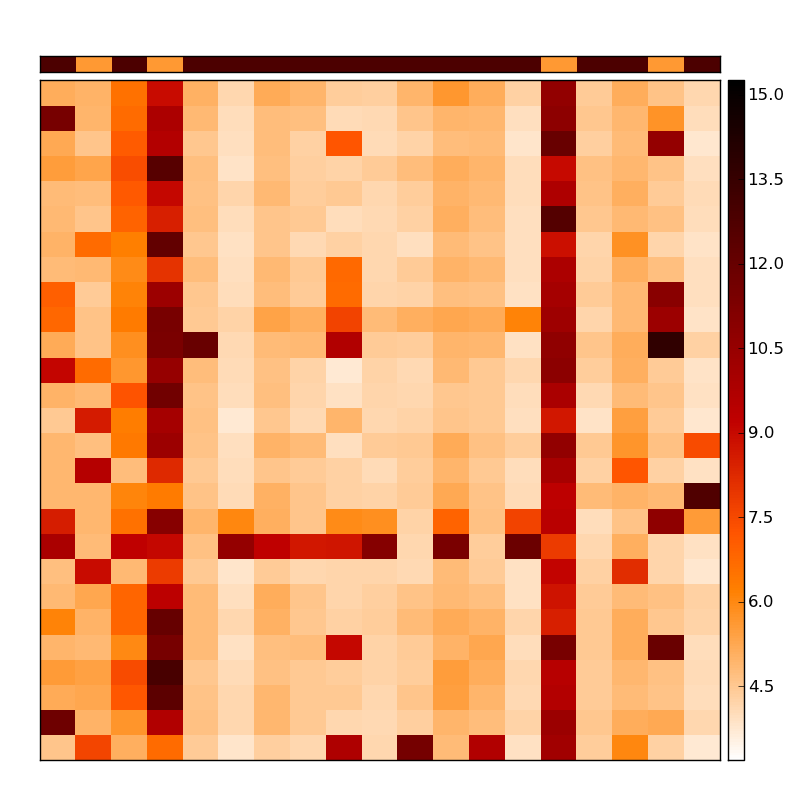

Supplement: Additional file 1 — Detailed information for multigene segments. [file 1471-2164-14-812-S1.zip › miniwebsite/heatmaps/chr2L_heatmap350.png]

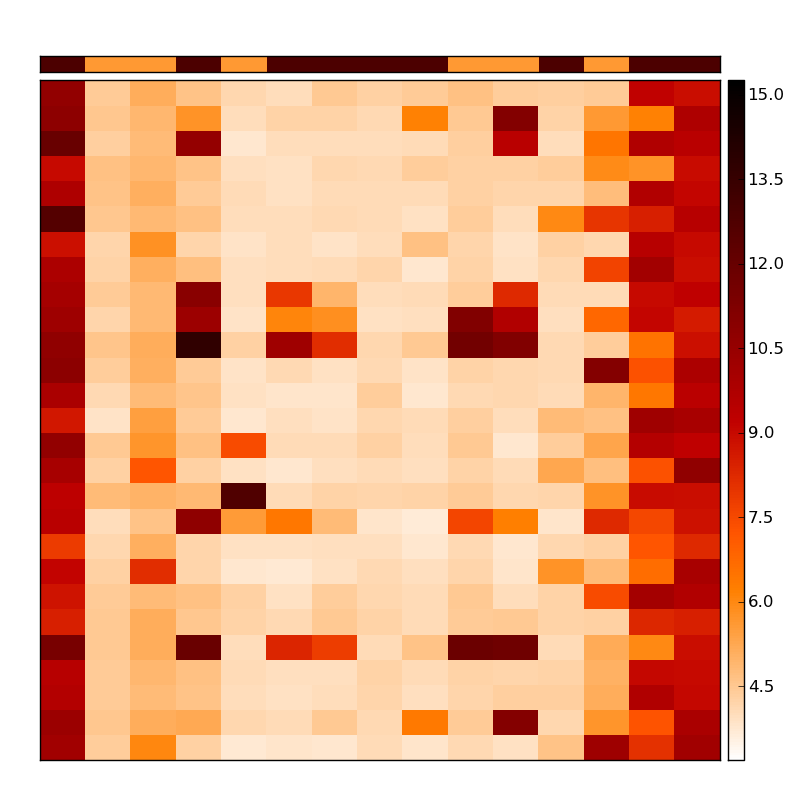

Supplement: Additional file 1 — Detailed information for multigene segments. [file 1471-2164-14-812-S1.zip › miniwebsite/heatmaps/chr2L_heatmap352.png]

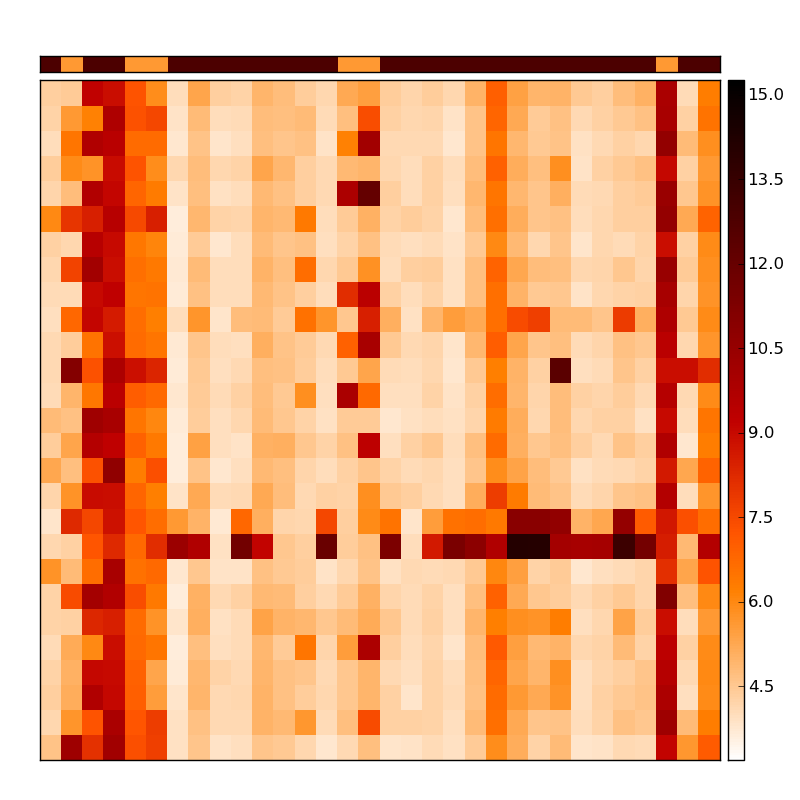

Supplement: Additional file 1 — Detailed information for multigene segments. [file 1471-2164-14-812-S1.zip › miniwebsite/heatmaps/chr2L_heatmap356.png]

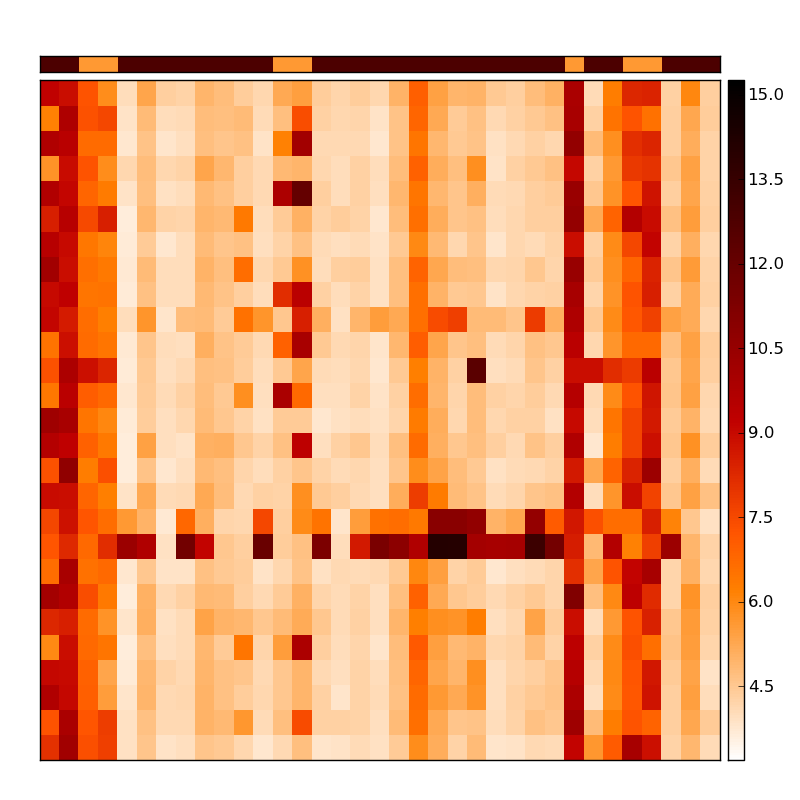

Supplement: Additional file 1 — Detailed information for multigene segments. [file 1471-2164-14-812-S1.zip › miniwebsite/heatmaps/chr2L_heatmap358.png]

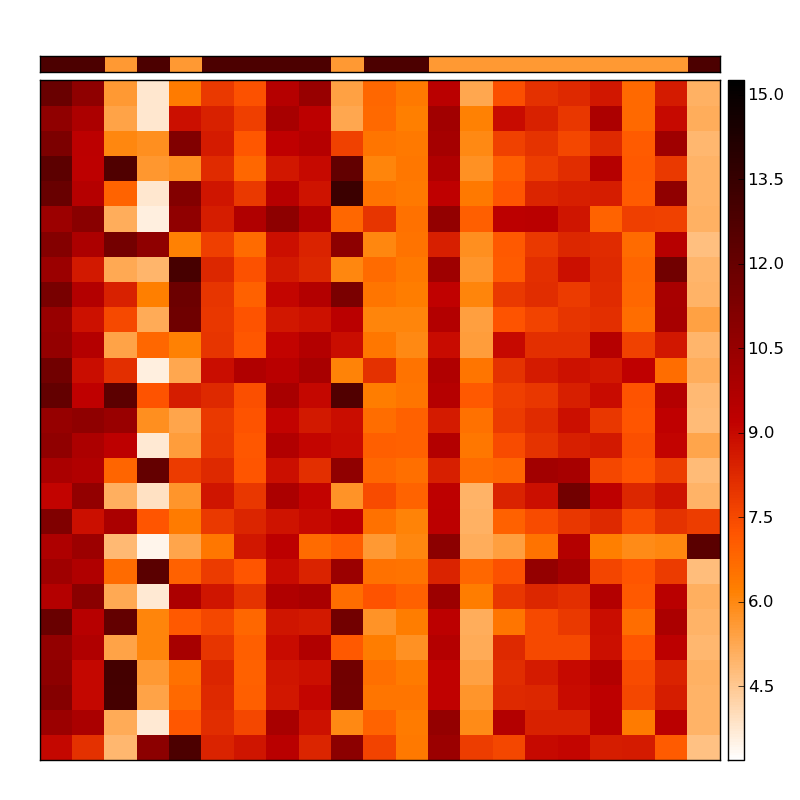

Supplement: Additional file 1 — Detailed information for multigene segments. [file 1471-2164-14-812-S1.zip › miniwebsite/heatmaps/chr2L_heatmap36.png]

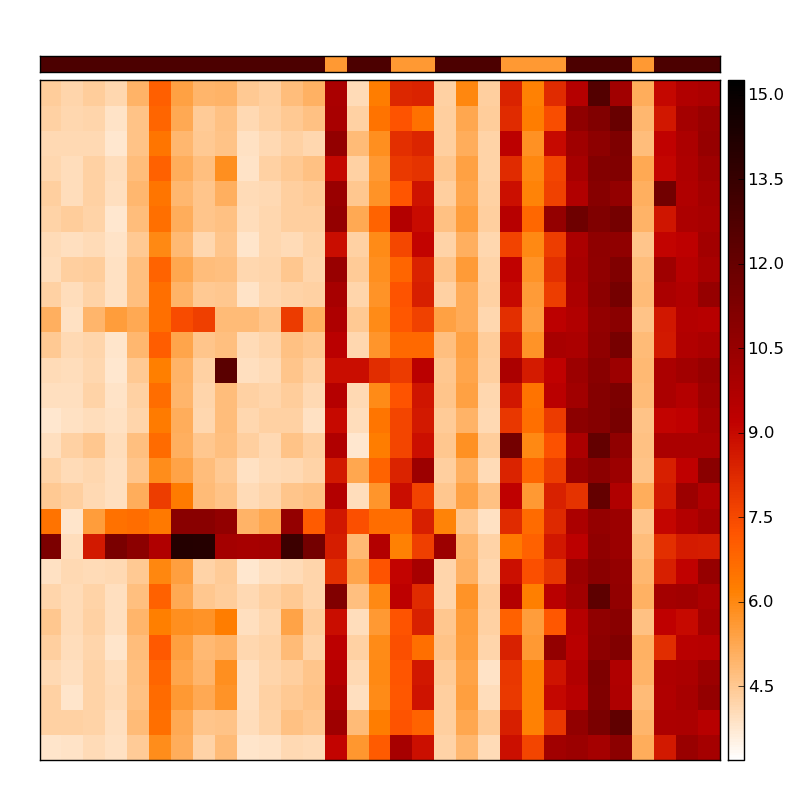

Supplement: Additional file 1 — Detailed information for multigene segments. [file 1471-2164-14-812-S1.zip › miniwebsite/heatmaps/chr2L_heatmap361.png]

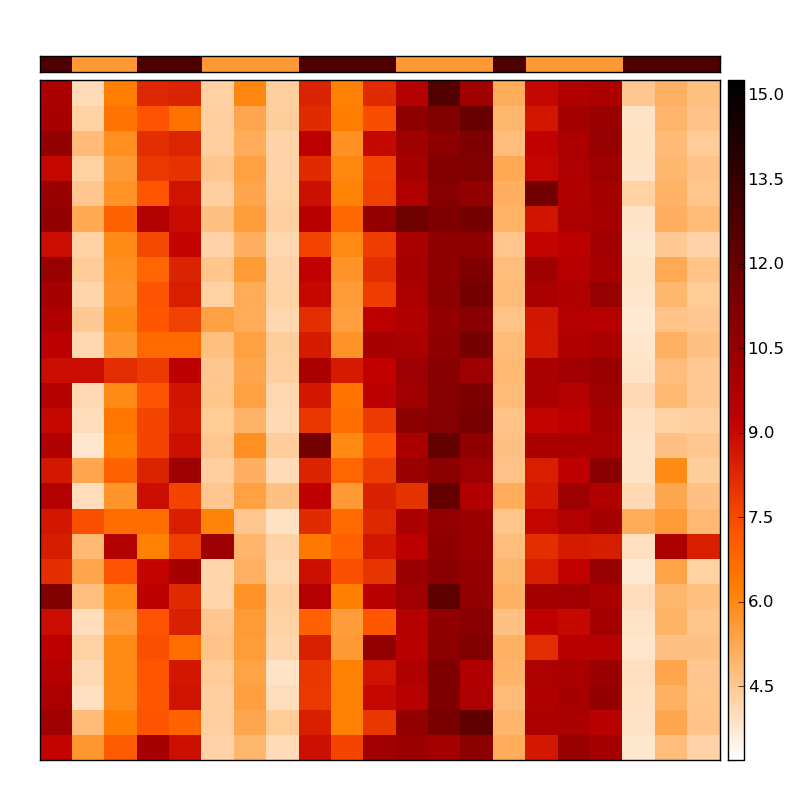

Supplement: Additional file 1 — Detailed information for multigene segments. [file 1471-2164-14-812-S1.zip › miniwebsite/heatmaps/chr2L_heatmap362.png]

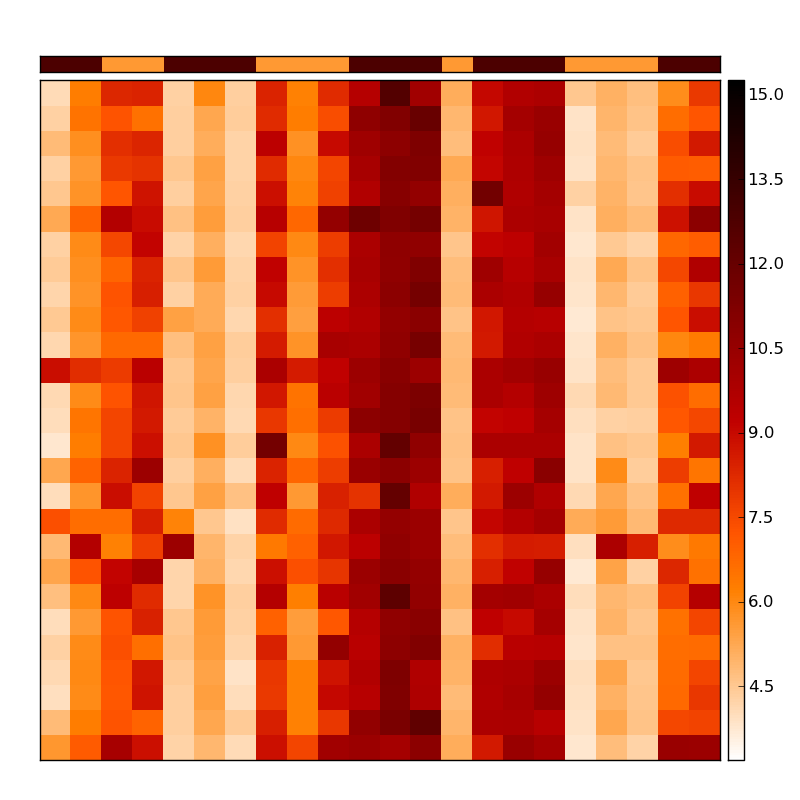

Supplement: Additional file 1 — Detailed information for multigene segments. [file 1471-2164-14-812-S1.zip › miniwebsite/heatmaps/chr2L_heatmap363.png]

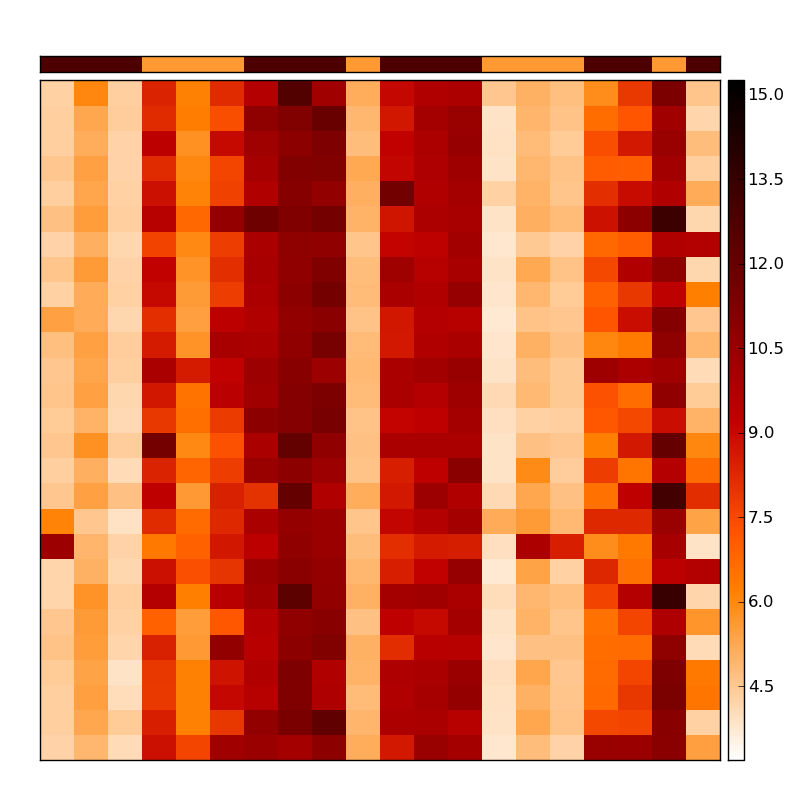

Supplement: Additional file 1 — Detailed information for multigene segments. [file 1471-2164-14-812-S1.zip › miniwebsite/heatmaps/chr2L_heatmap364.png]

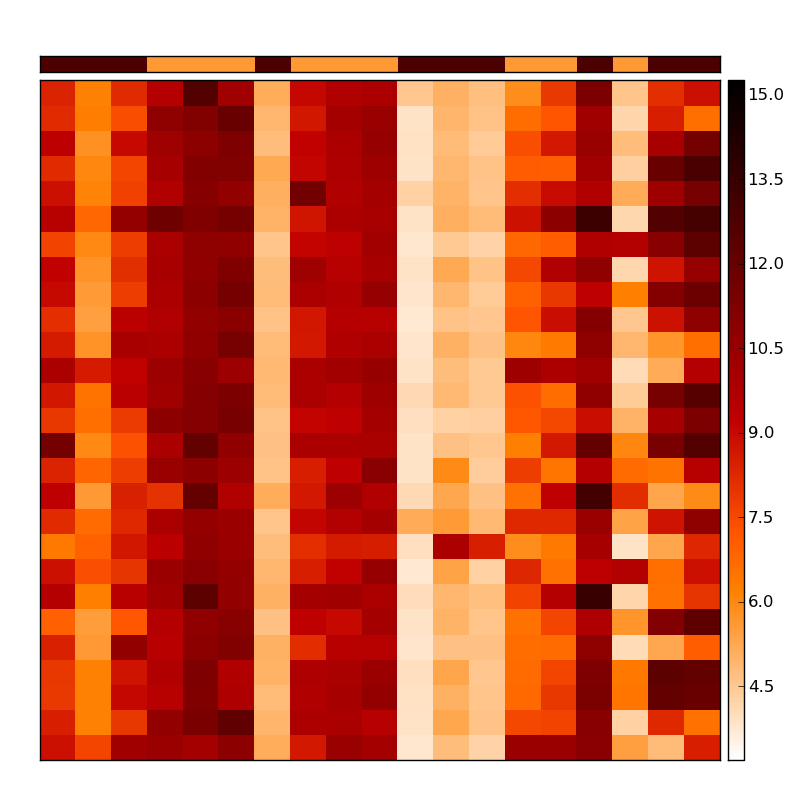

Supplement: Additional file 1 — Detailed information for multigene segments. [file 1471-2164-14-812-S1.zip › miniwebsite/heatmaps/chr2L_heatmap365.png]

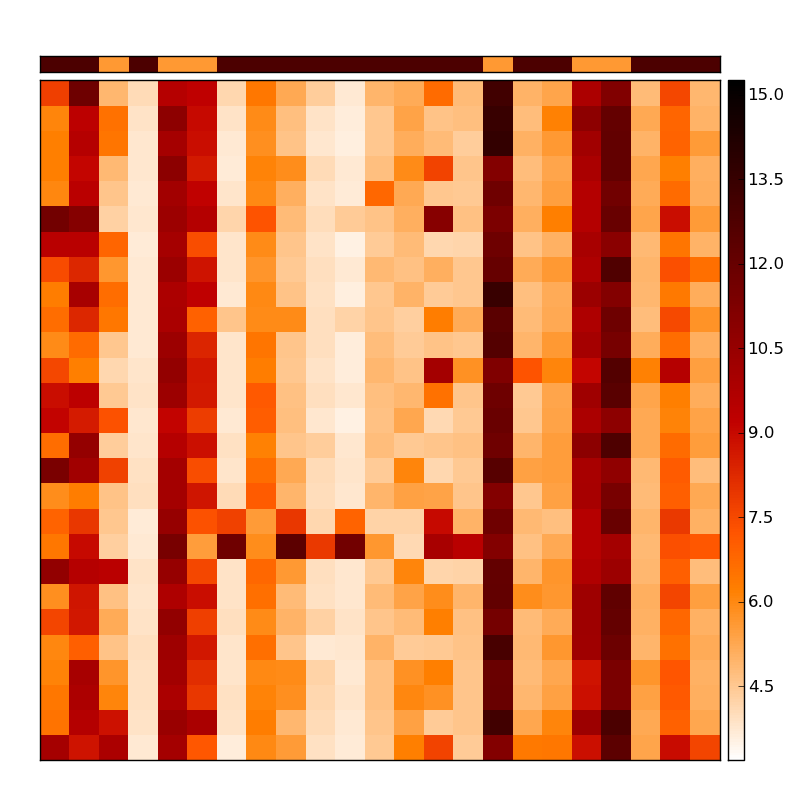

Supplement: Additional file 1 — Detailed information for multigene segments. [file 1471-2164-14-812-S1.zip › miniwebsite/heatmaps/chr2L_heatmap370.png]

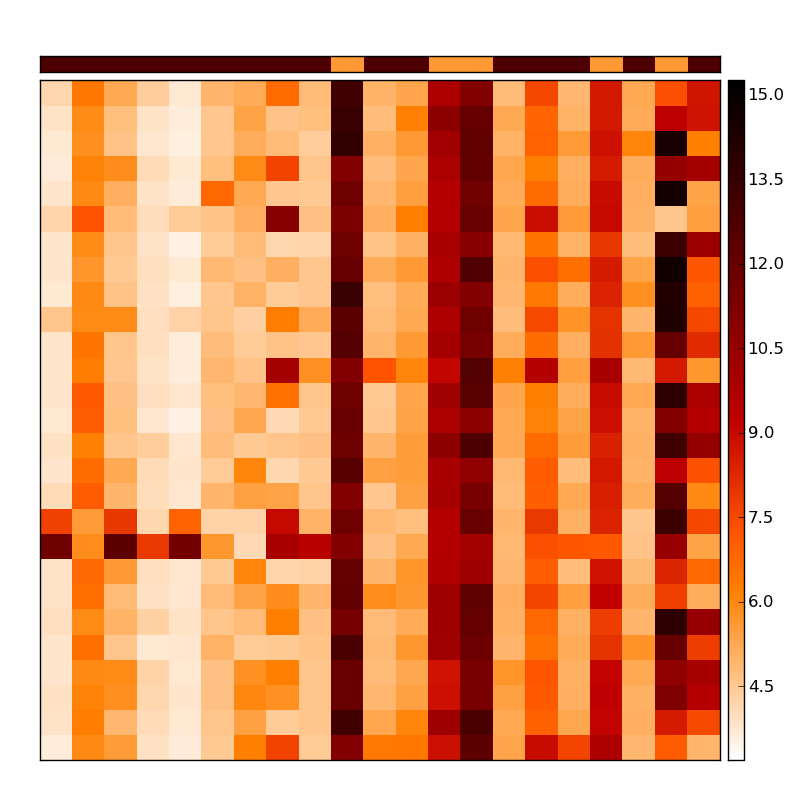

Supplement: Additional file 1 — Detailed information for multigene segments. [file 1471-2164-14-812-S1.zip › miniwebsite/heatmaps/chr2L_heatmap373.png]

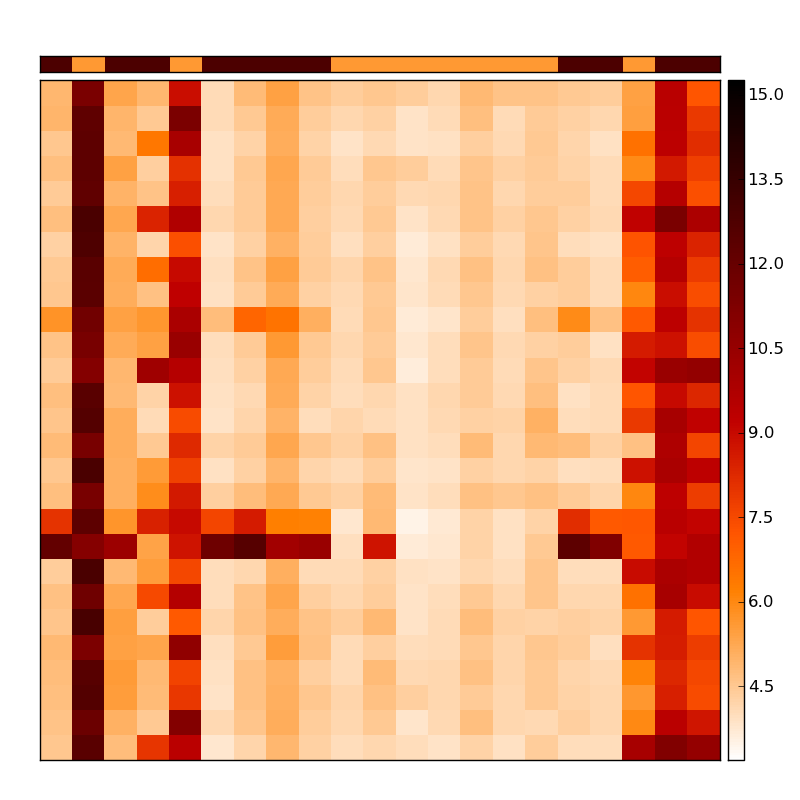

Supplement: Additional file 1 — Detailed information for multigene segments. [file 1471-2164-14-812-S1.zip › miniwebsite/heatmaps/chr2L_heatmap377.png]

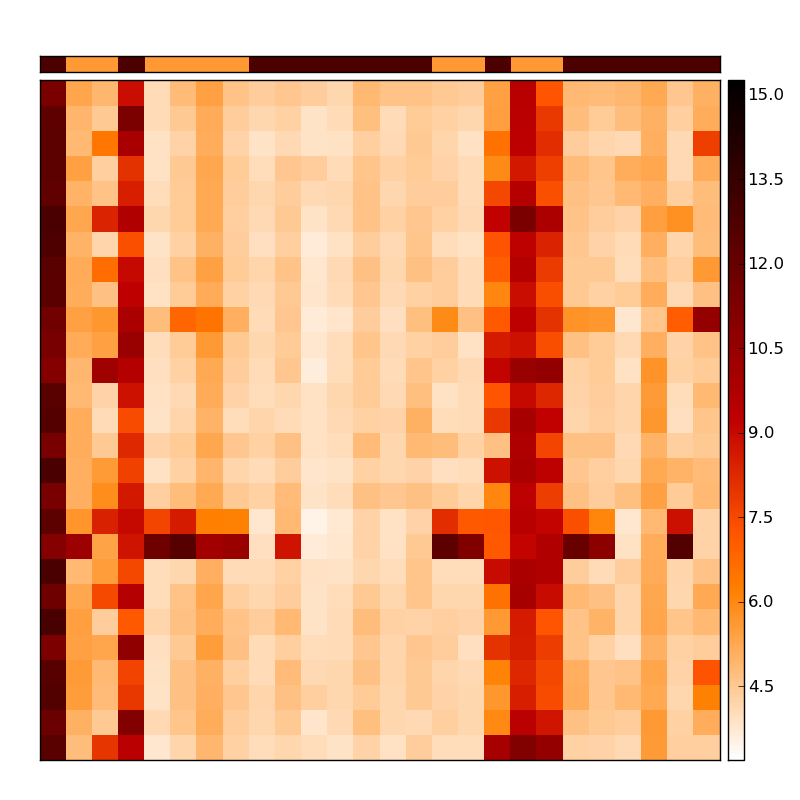

Supplement: Additional file 1 — Detailed information for multigene segments. [file 1471-2164-14-812-S1.zip › miniwebsite/heatmaps/chr2L_heatmap378.png]

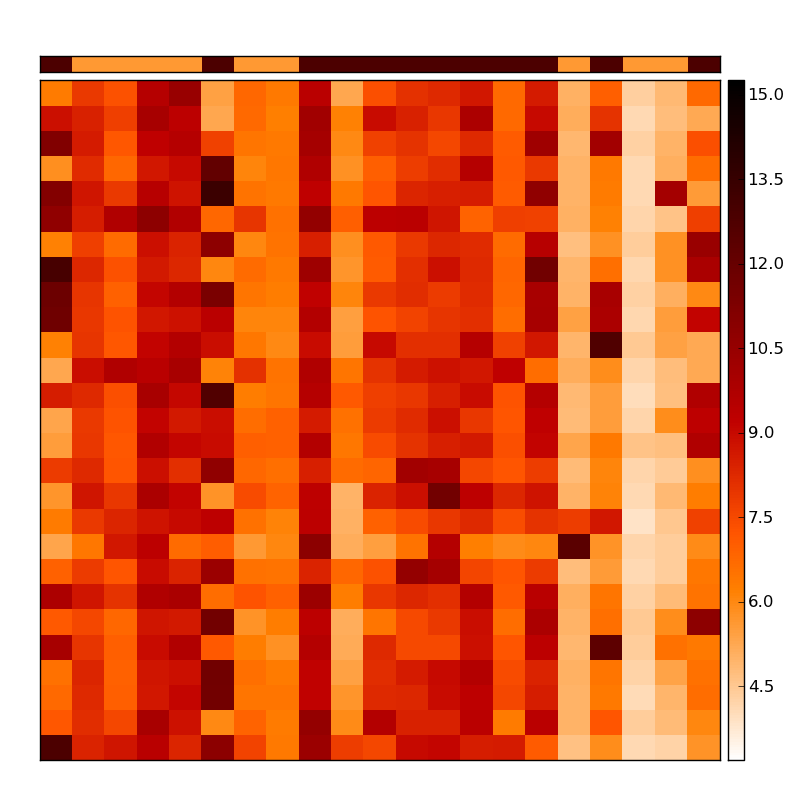

Supplement: Additional file 1 — Detailed information for multigene segments. [file 1471-2164-14-812-S1.zip › miniwebsite/heatmaps/chr2L_heatmap38.png]

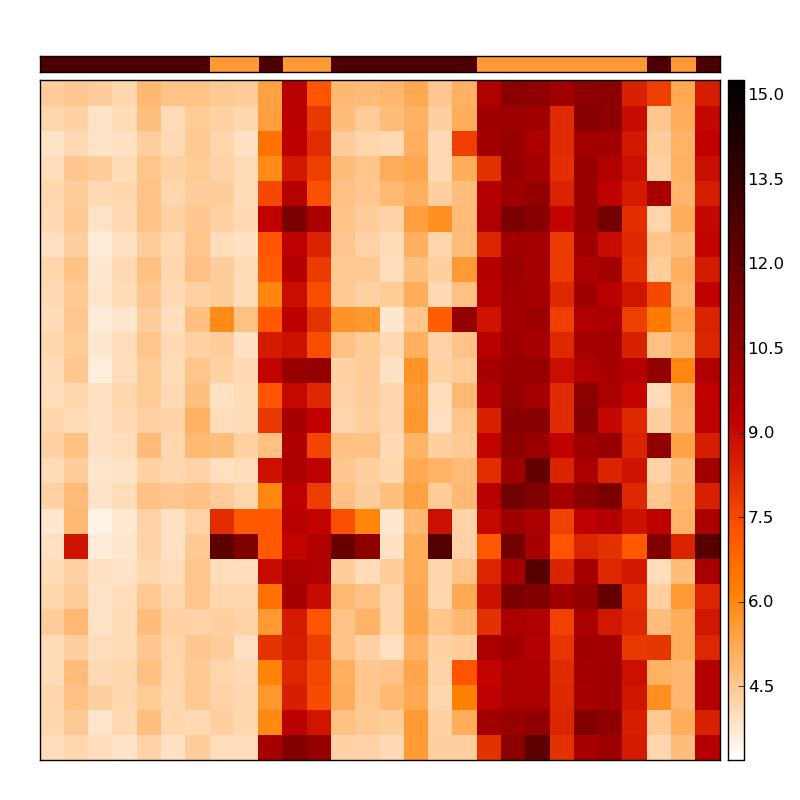

Supplement: Additional file 1 — Detailed information for multigene segments. [file 1471-2164-14-812-S1.zip › miniwebsite/heatmaps/chr2L_heatmap381.png]

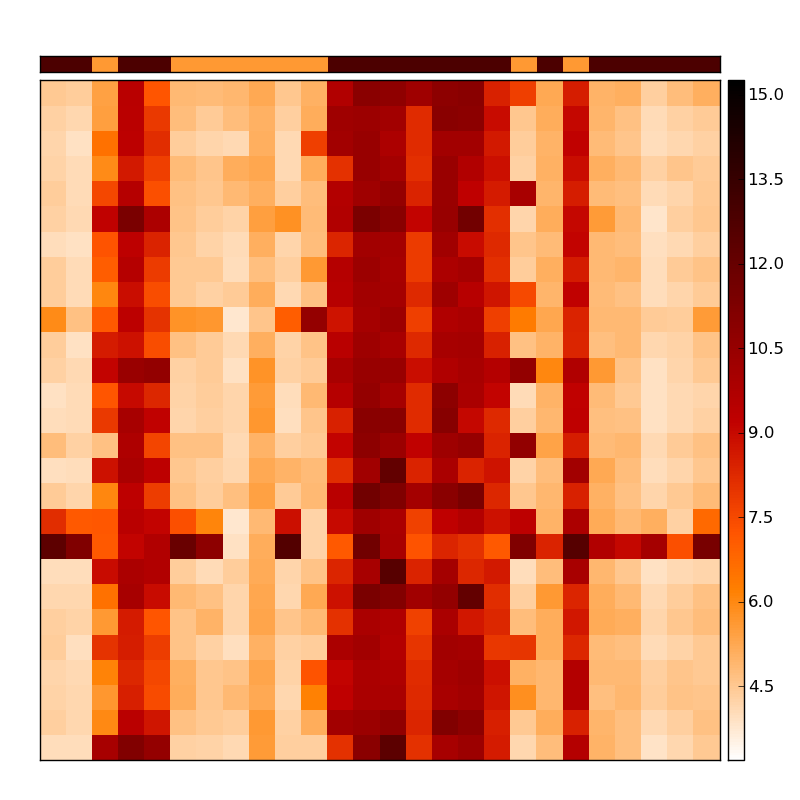

Supplement: Additional file 1 — Detailed information for multigene segments. [file 1471-2164-14-812-S1.zip › miniwebsite/heatmaps/chr2L_heatmap382.png]

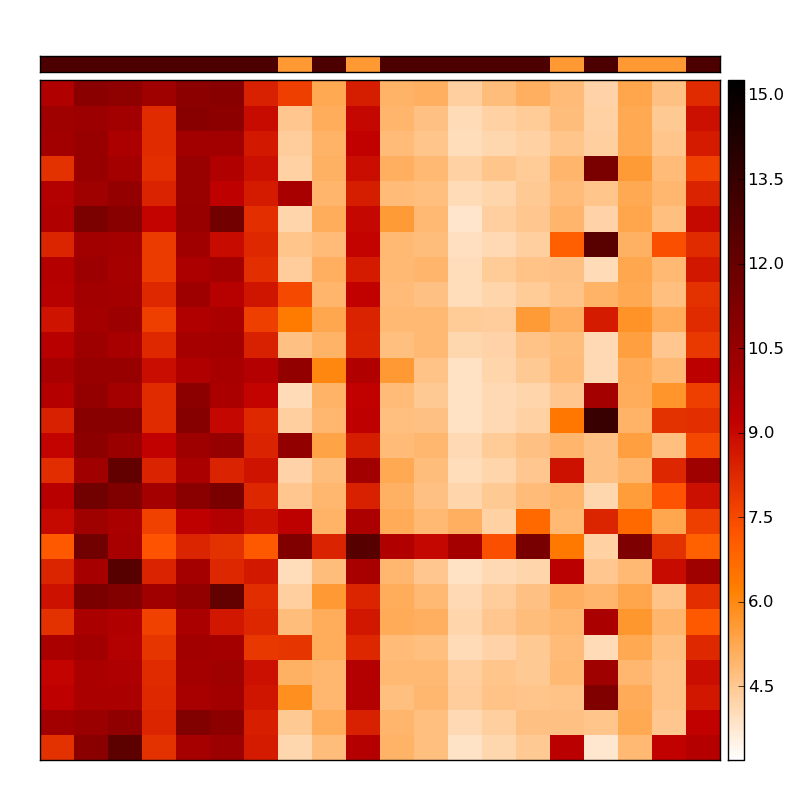

Supplement: Additional file 1 — Detailed information for multigene segments. [file 1471-2164-14-812-S1.zip › miniwebsite/heatmaps/chr2L_heatmap383.png]

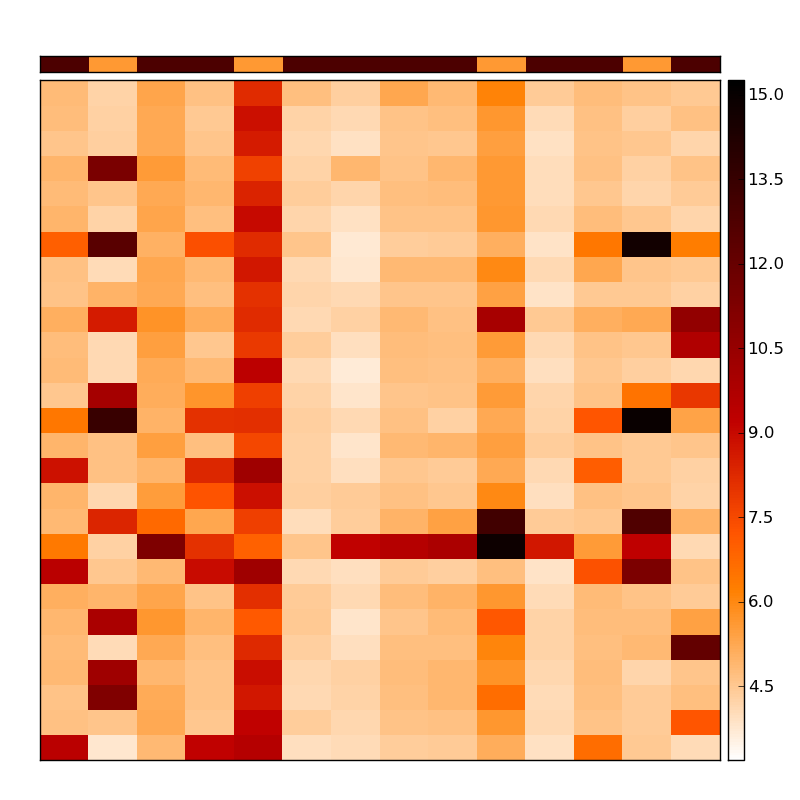

Supplement: Additional file 1 — Detailed information for multigene segments. [file 1471-2164-14-812-S1.zip › miniwebsite/heatmaps/chr2L_heatmap385.png]

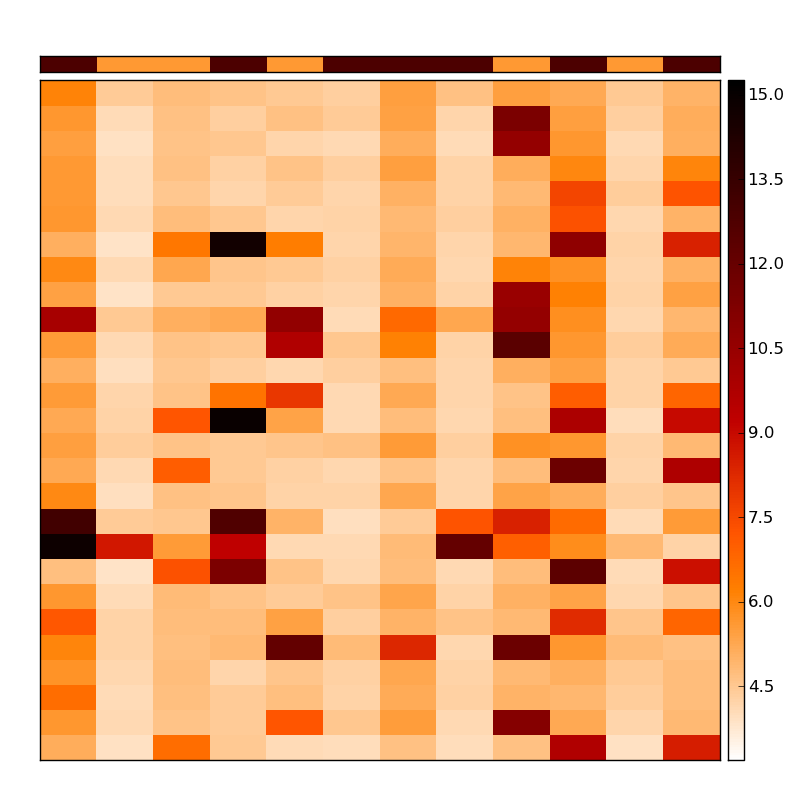

Supplement: Additional file 1 — Detailed information for multigene segments. [file 1471-2164-14-812-S1.zip › miniwebsite/heatmaps/chr2L_heatmap387.png]

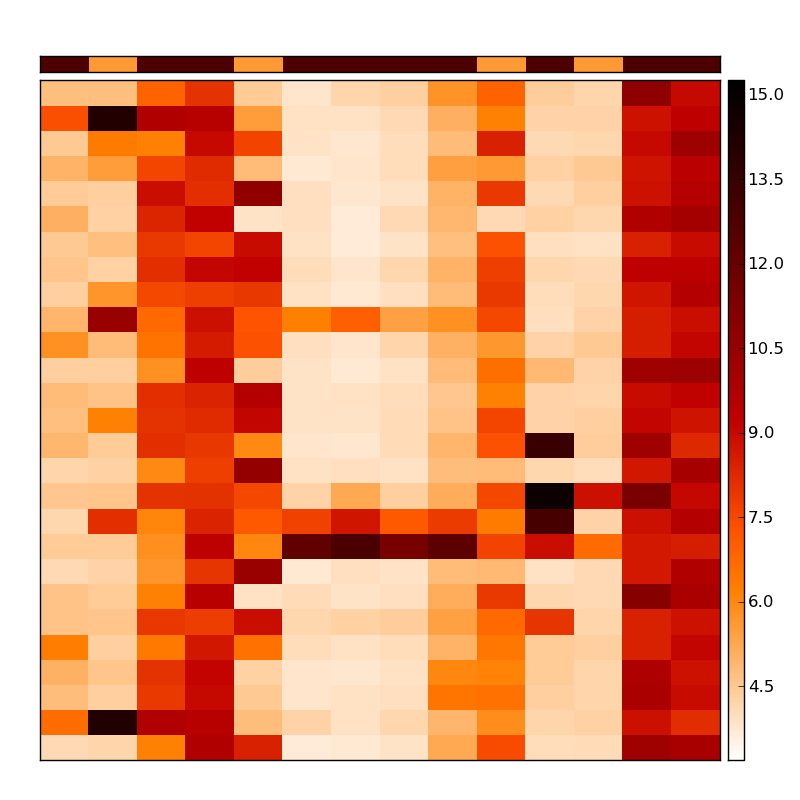

Supplement: Additional file 1 — Detailed information for multigene segments. [file 1471-2164-14-812-S1.zip › miniwebsite/heatmaps/chr2L_heatmap389.png]

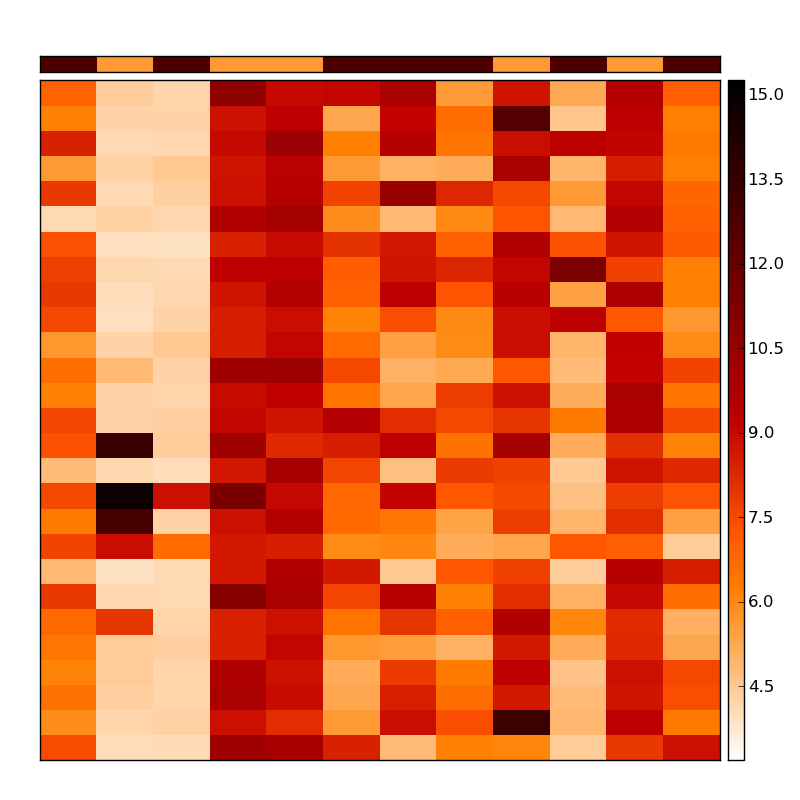

Supplement: Additional file 1 — Detailed information for multigene segments. [file 1471-2164-14-812-S1.zip › miniwebsite/heatmaps/chr2L_heatmap391.png]

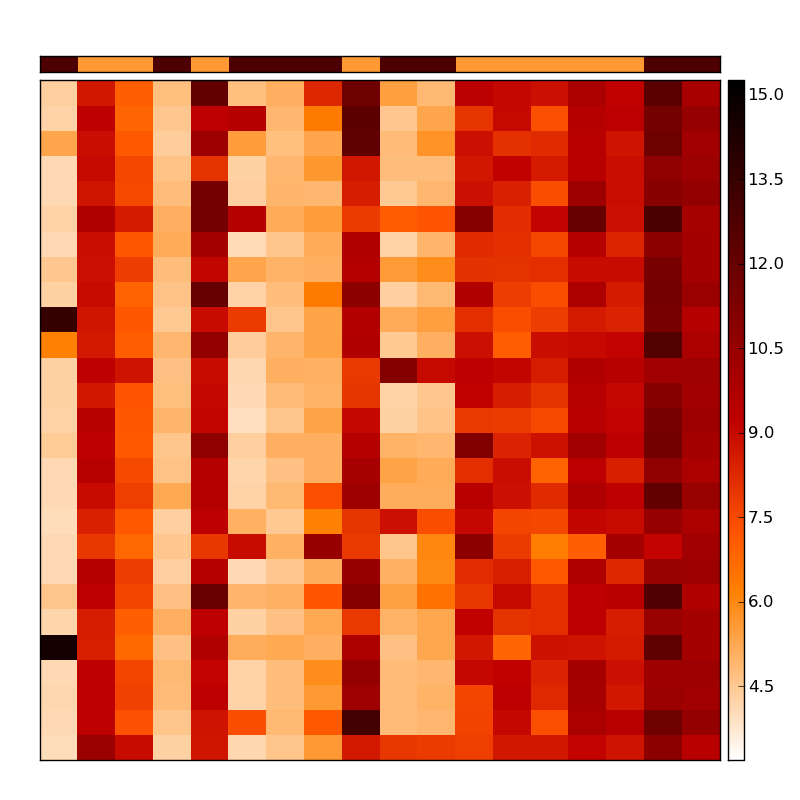

Supplement: Additional file 1 — Detailed information for multigene segments. [file 1471-2164-14-812-S1.zip › miniwebsite/heatmaps/chr2L_heatmap393.png]

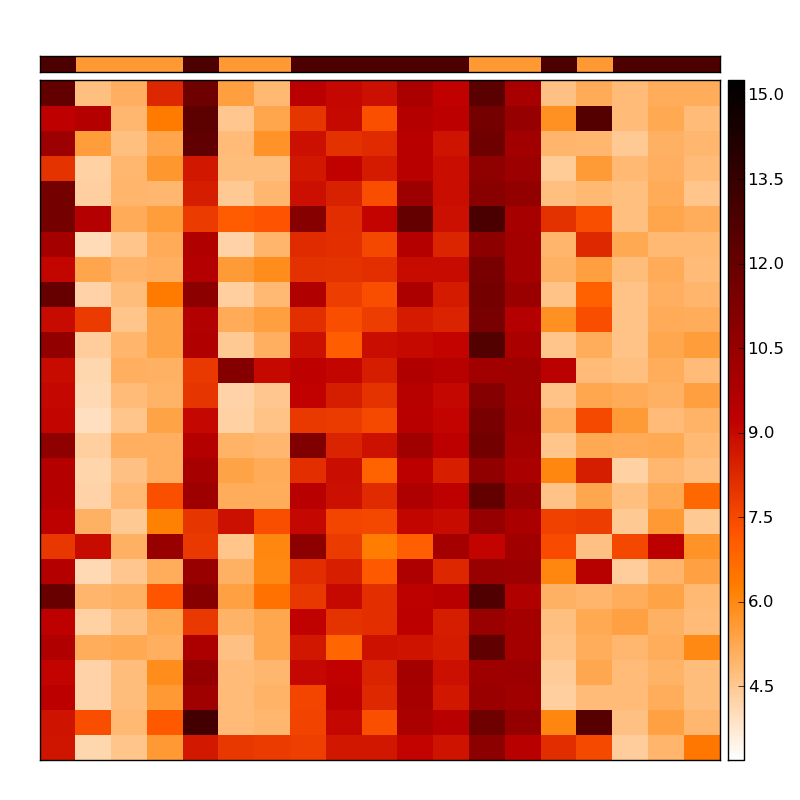

Supplement: Additional file 1 — Detailed information for multigene segments. [file 1471-2164-14-812-S1.zip › miniwebsite/heatmaps/chr2L_heatmap395.png]

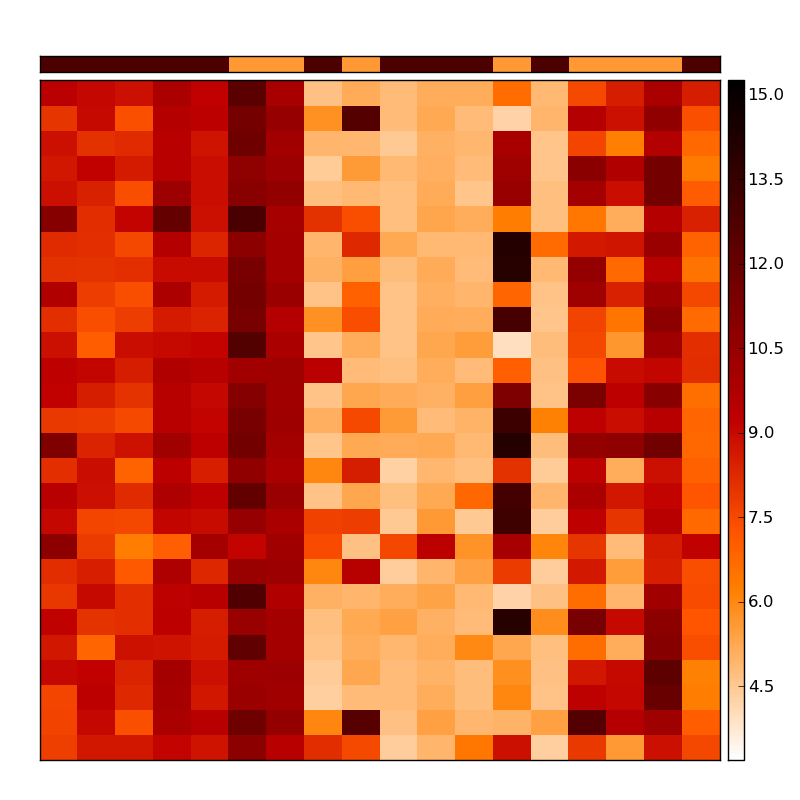

Supplement: Additional file 1 — Detailed information for multigene segments. [file 1471-2164-14-812-S1.zip › miniwebsite/heatmaps/chr2L_heatmap397.png]

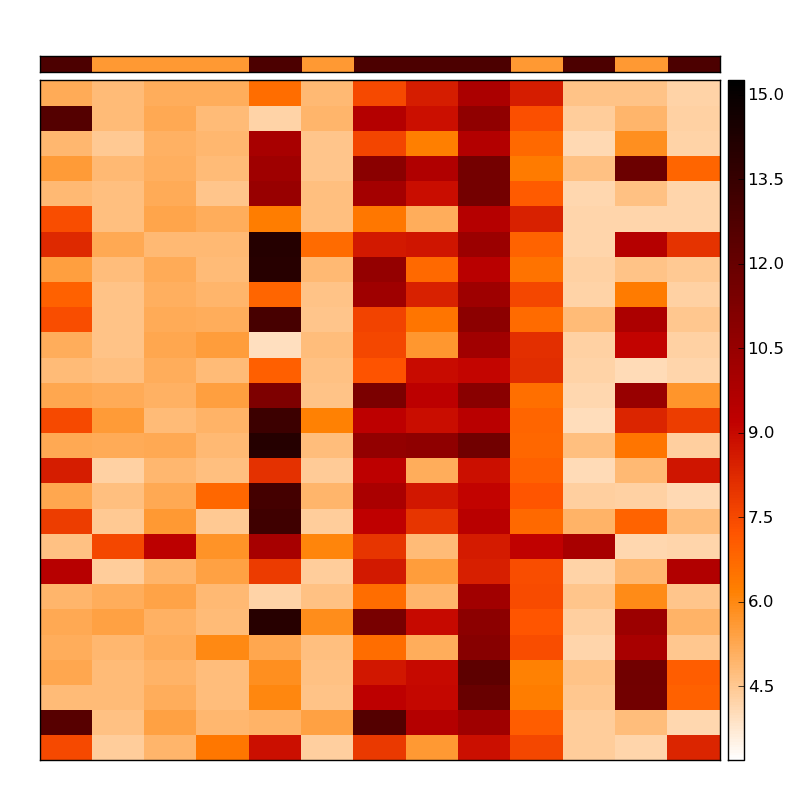

Supplement: Additional file 1 — Detailed information for multigene segments. [file 1471-2164-14-812-S1.zip › miniwebsite/heatmaps/chr2L_heatmap398.png]

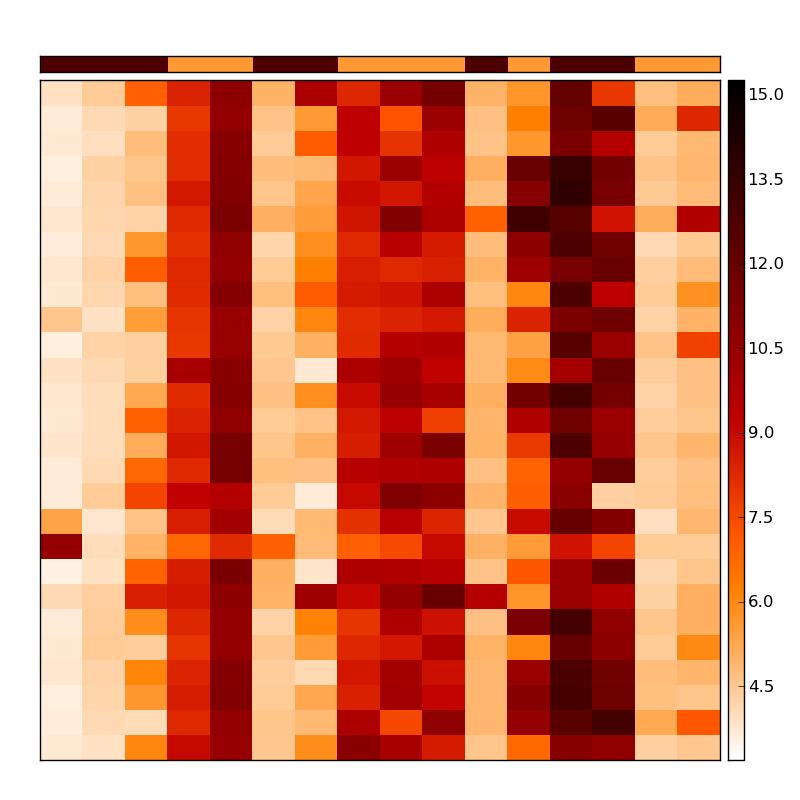

Supplement: Additional file 1 — Detailed information for multigene segments. [file 1471-2164-14-812-S1.zip › miniwebsite/heatmaps/chr2L_heatmap4.png]

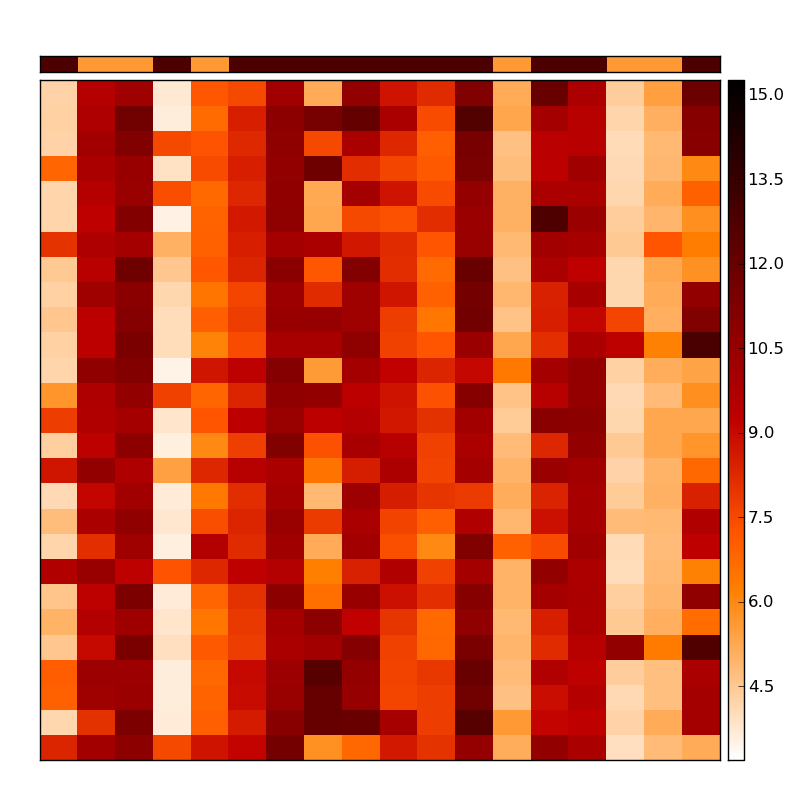

Supplement: Additional file 1 — Detailed information for multigene segments. [file 1471-2164-14-812-S1.zip › miniwebsite/heatmaps/chr2L_heatmap400.png]

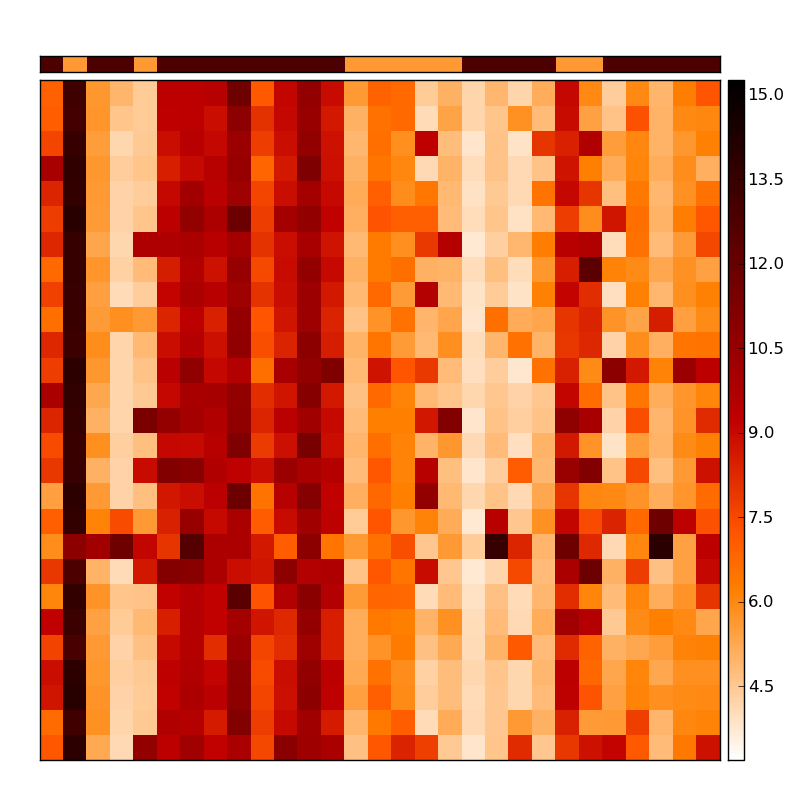

Supplement: Additional file 1 — Detailed information for multigene segments. [file 1471-2164-14-812-S1.zip › miniwebsite/heatmaps/chr2L_heatmap404.png]

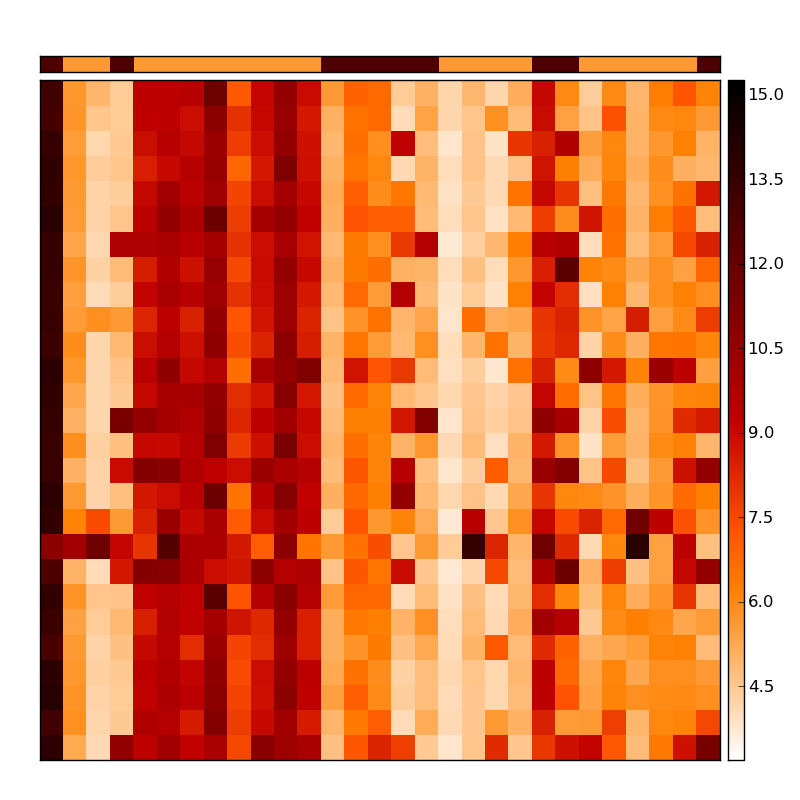

Supplement: Additional file 1 — Detailed information for multigene segments. [file 1471-2164-14-812-S1.zip › miniwebsite/heatmaps/chr2L_heatmap405.png]

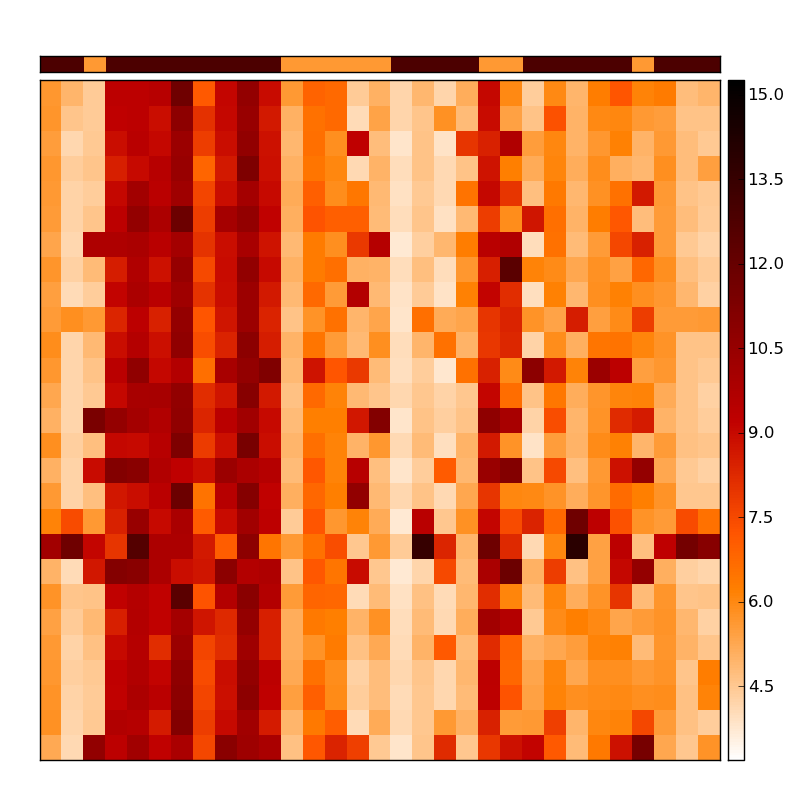

Supplement: Additional file 1 — Detailed information for multigene segments. [file 1471-2164-14-812-S1.zip › miniwebsite/heatmaps/chr2L_heatmap406.png]

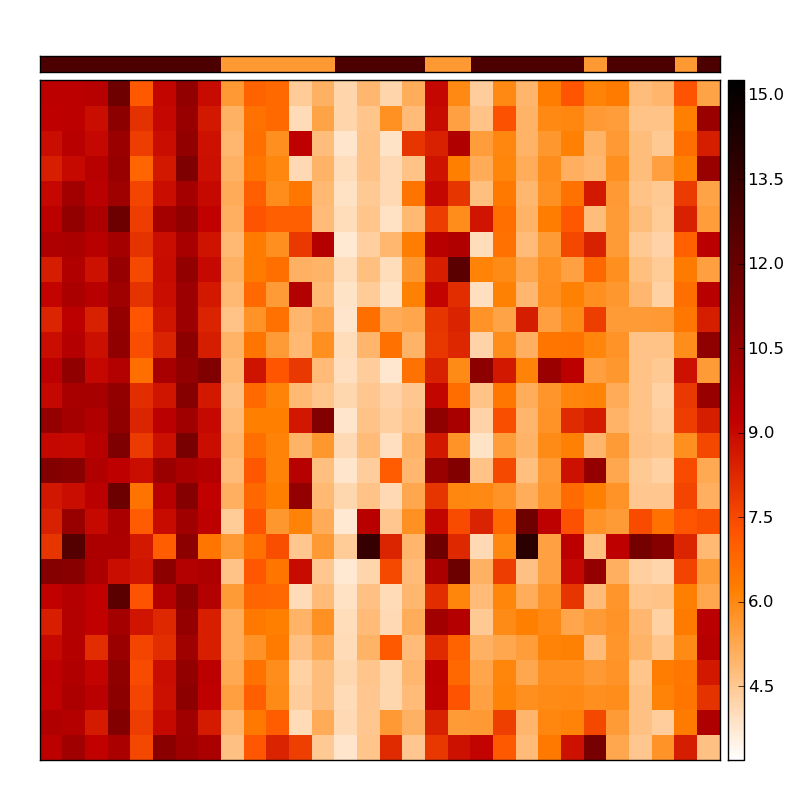

Supplement: Additional file 1 — Detailed information for multigene segments. [file 1471-2164-14-812-S1.zip › miniwebsite/heatmaps/chr2L_heatmap408.png]

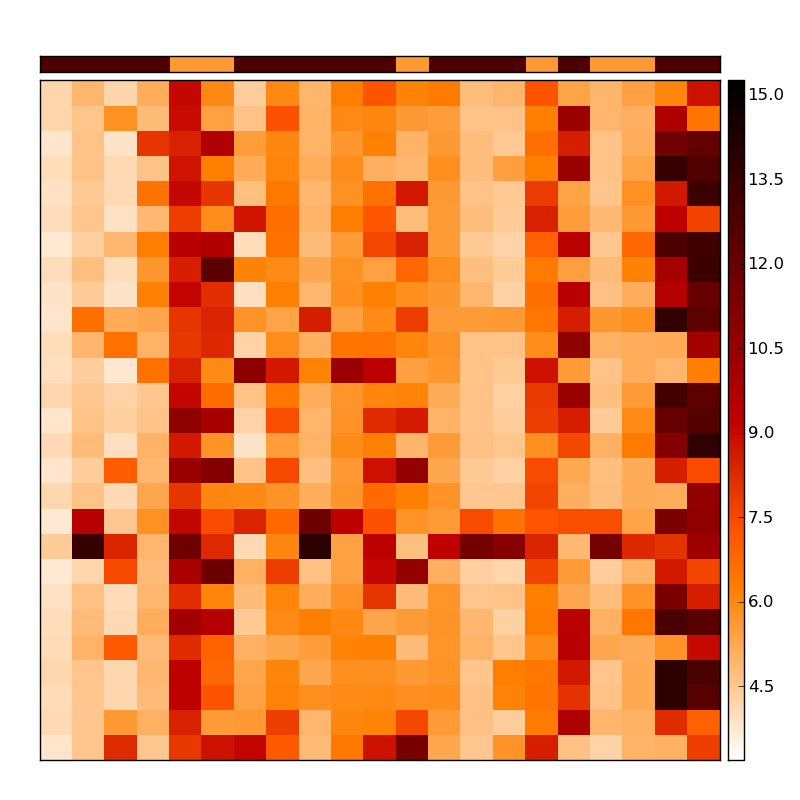

Supplement: Additional file 1 — Detailed information for multigene segments. [file 1471-2164-14-812-S1.zip › miniwebsite/heatmaps/chr2L_heatmap409.png]

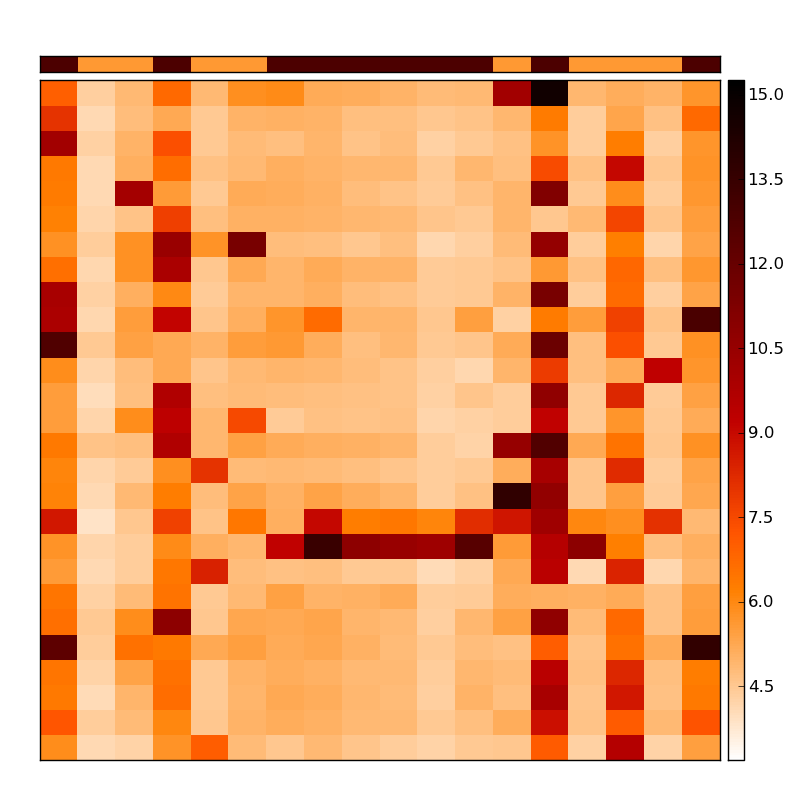

Supplement: Additional file 1 — Detailed information for multigene segments. [file 1471-2164-14-812-S1.zip › miniwebsite/heatmaps/chr2L_heatmap41.png]

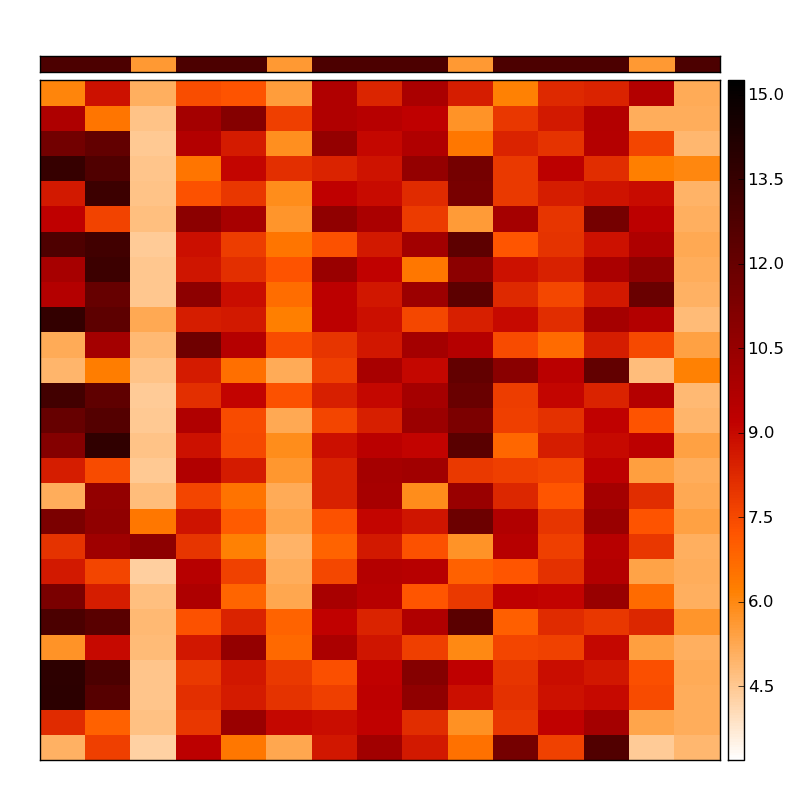

Supplement: Additional file 1 — Detailed information for multigene segments. [file 1471-2164-14-812-S1.zip › miniwebsite/heatmaps/chr2L_heatmap413.png]

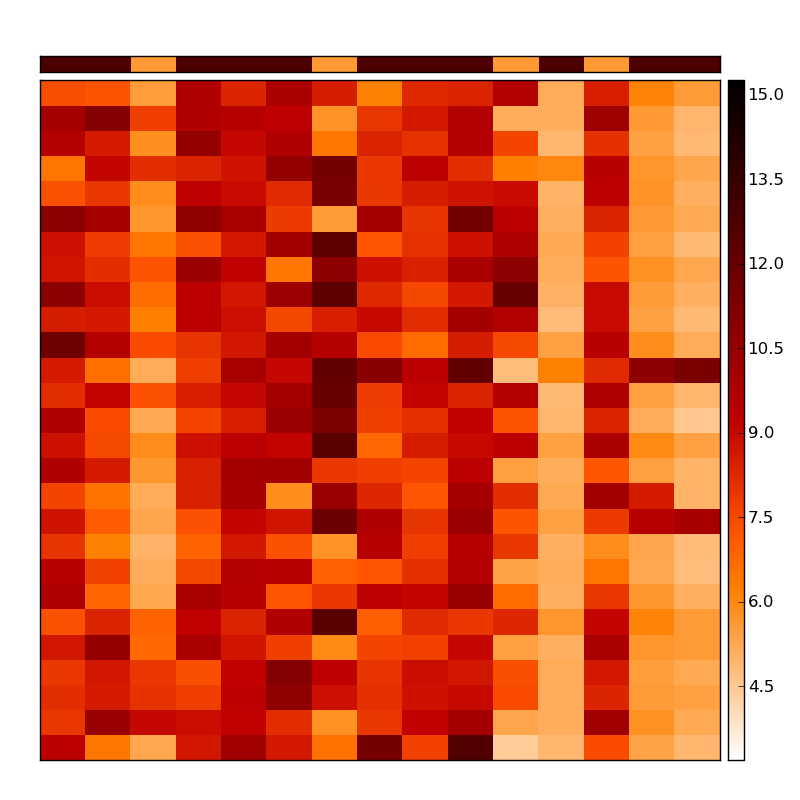

Supplement: Additional file 1 — Detailed information for multigene segments. [file 1471-2164-14-812-S1.zip › miniwebsite/heatmaps/chr2L_heatmap414.png]

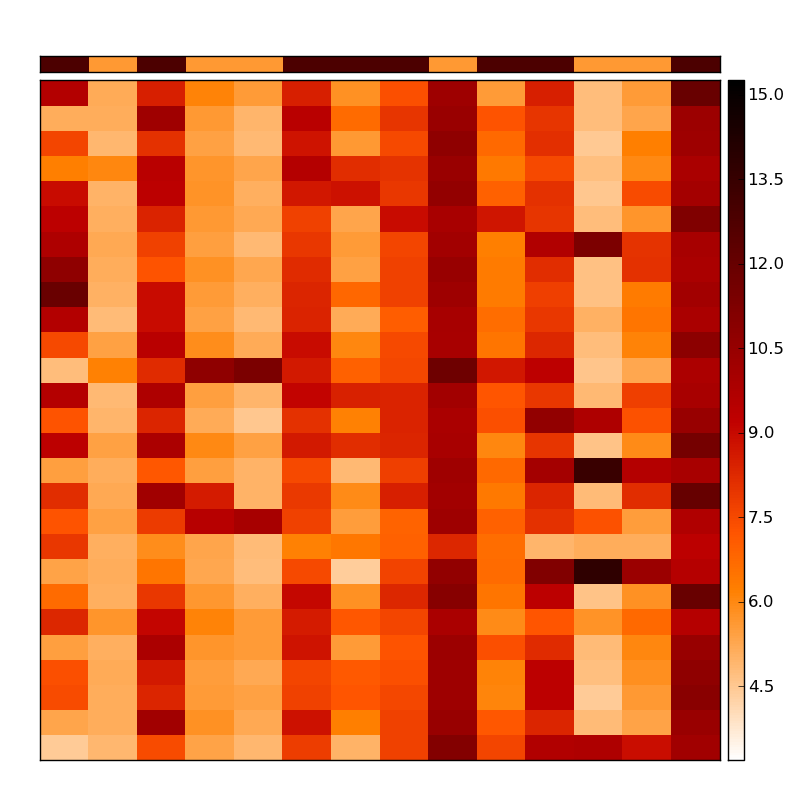

Supplement: Additional file 1 — Detailed information for multigene segments. [file 1471-2164-14-812-S1.zip › miniwebsite/heatmaps/chr2L_heatmap416.png]

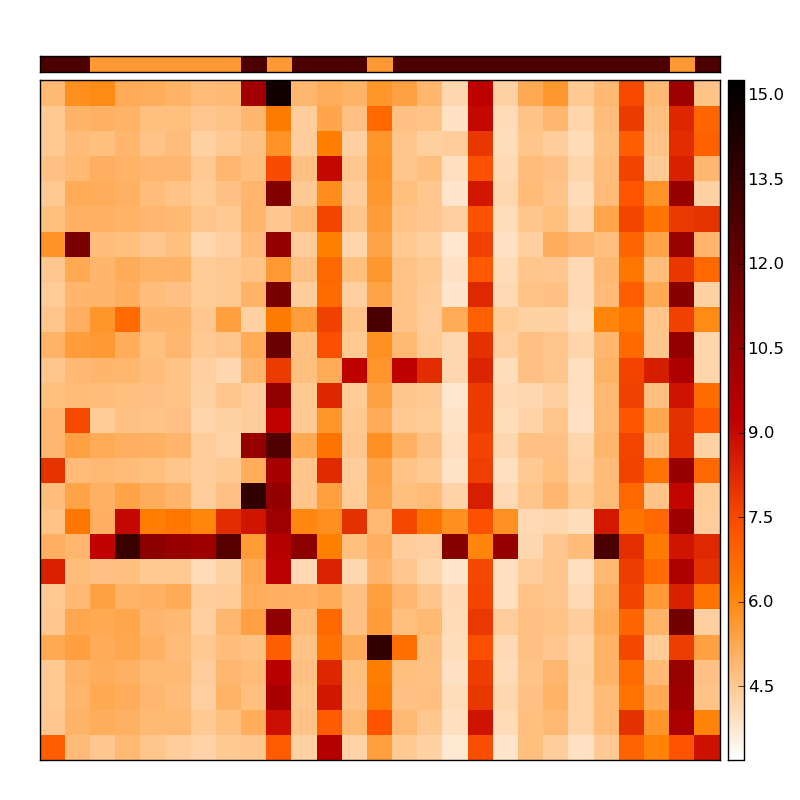

Supplement: Additional file 1 — Detailed information for multigene segments. [file 1471-2164-14-812-S1.zip › miniwebsite/heatmaps/chr2L_heatmap42.png]

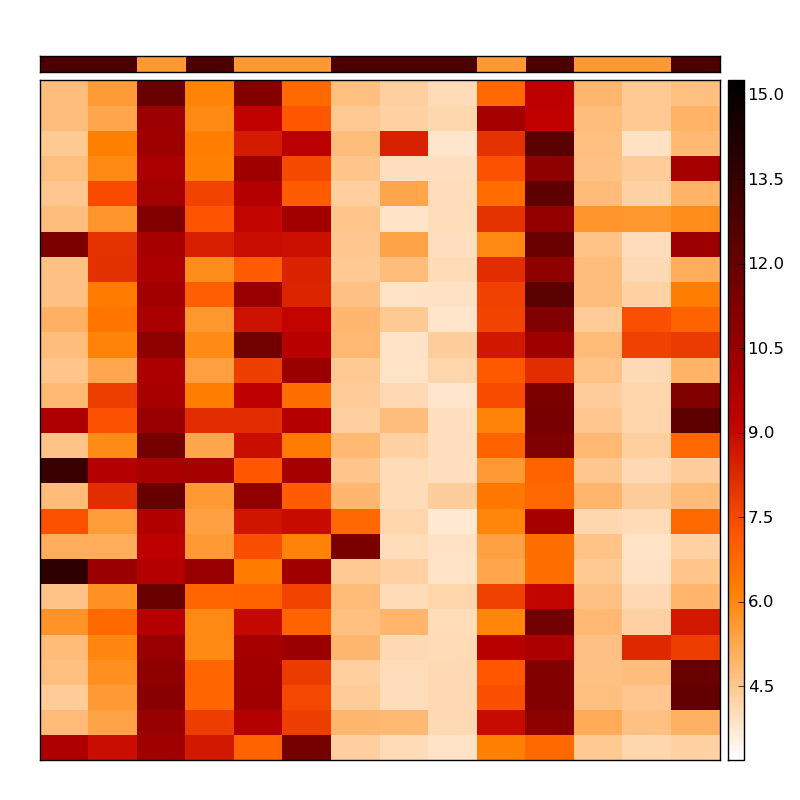

Supplement: Additional file 1 — Detailed information for multigene segments. [file 1471-2164-14-812-S1.zip › miniwebsite/heatmaps/chr2L_heatmap420.png]

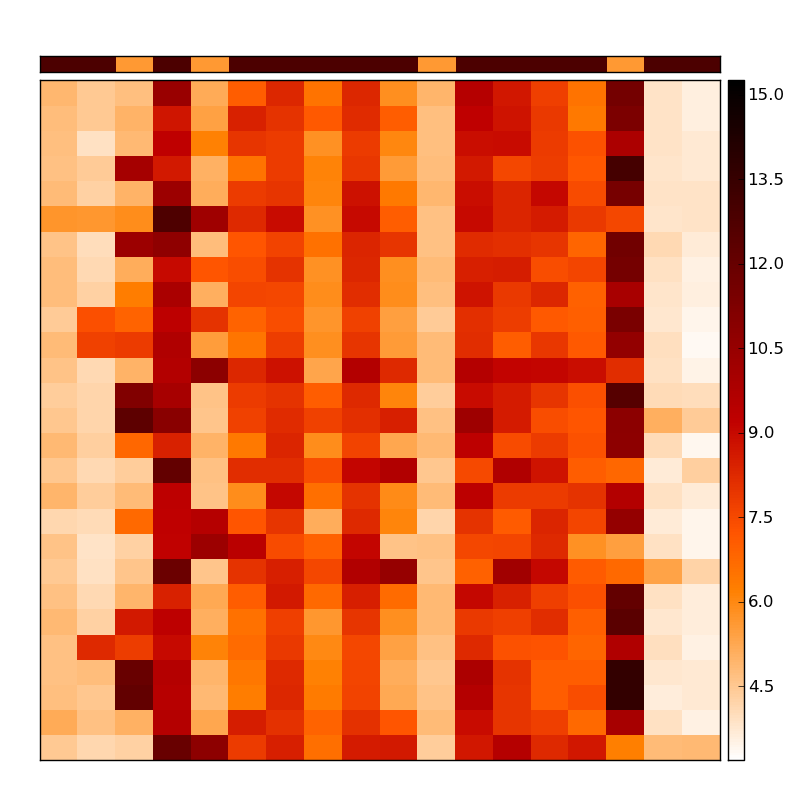

Supplement: Additional file 1 — Detailed information for multigene segments. [file 1471-2164-14-812-S1.zip › miniwebsite/heatmaps/chr2L_heatmap422.png]

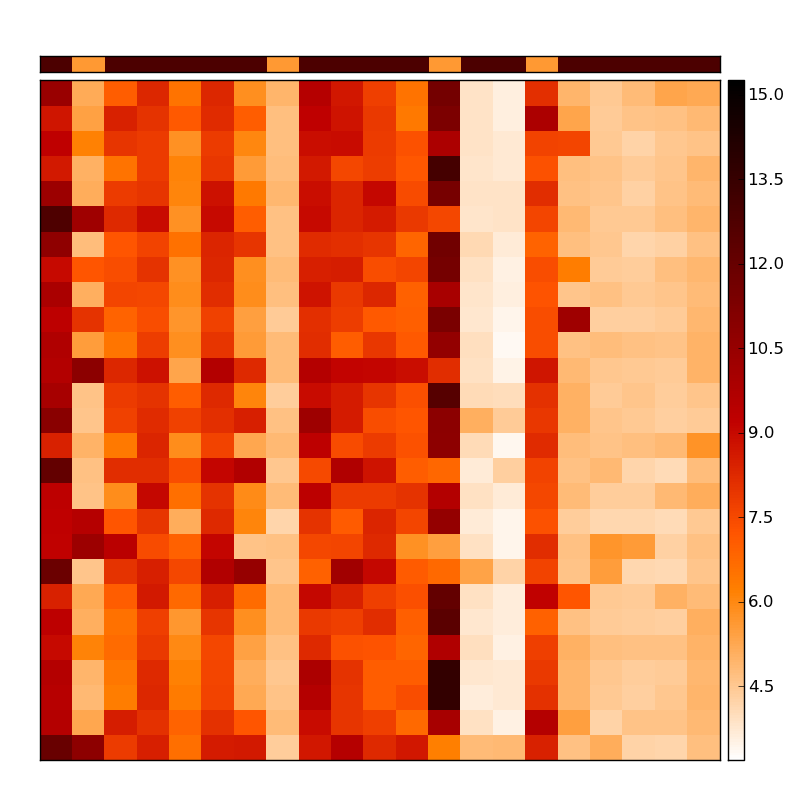

Supplement: Additional file 1 — Detailed information for multigene segments. [file 1471-2164-14-812-S1.zip › miniwebsite/heatmaps/chr2L_heatmap423.png]

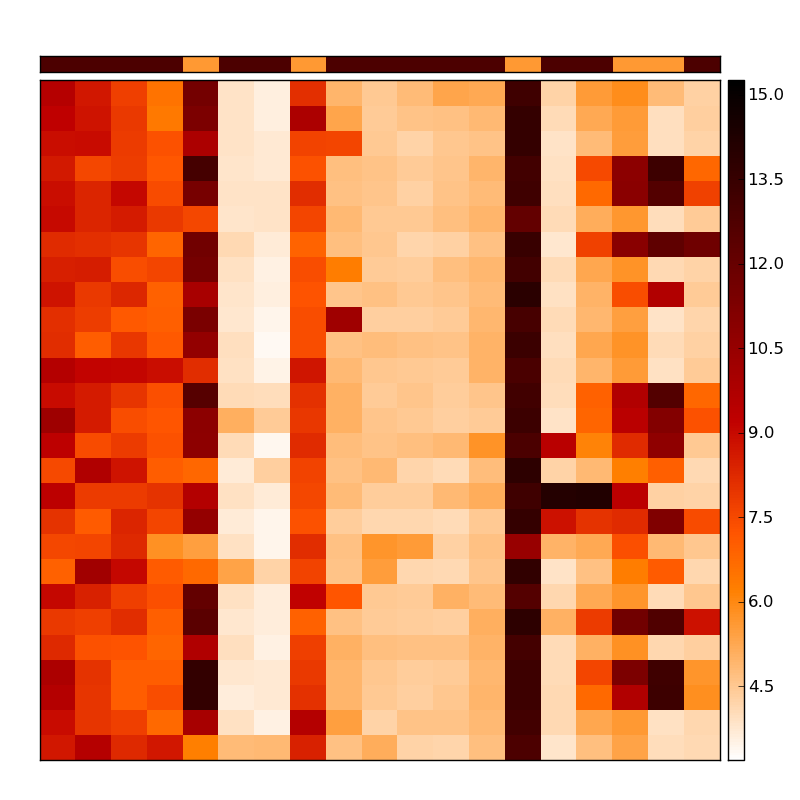

Supplement: Additional file 1 — Detailed information for multigene segments. [file 1471-2164-14-812-S1.zip › miniwebsite/heatmaps/chr2L_heatmap425.png]

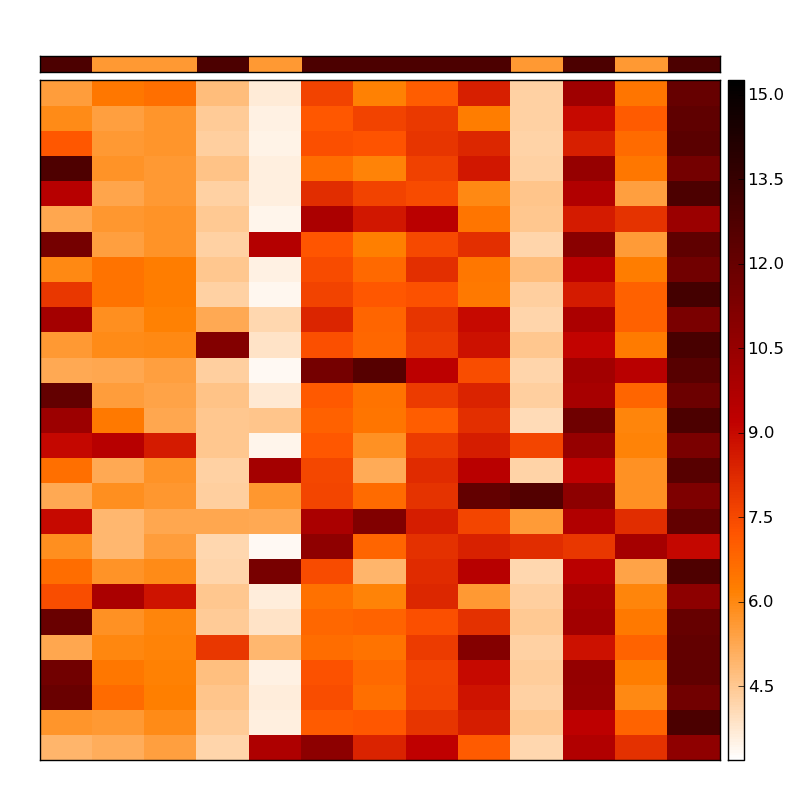

Supplement: Additional file 1 — Detailed information for multigene segments. [file 1471-2164-14-812-S1.zip › miniwebsite/heatmaps/chr2L_heatmap429.png]

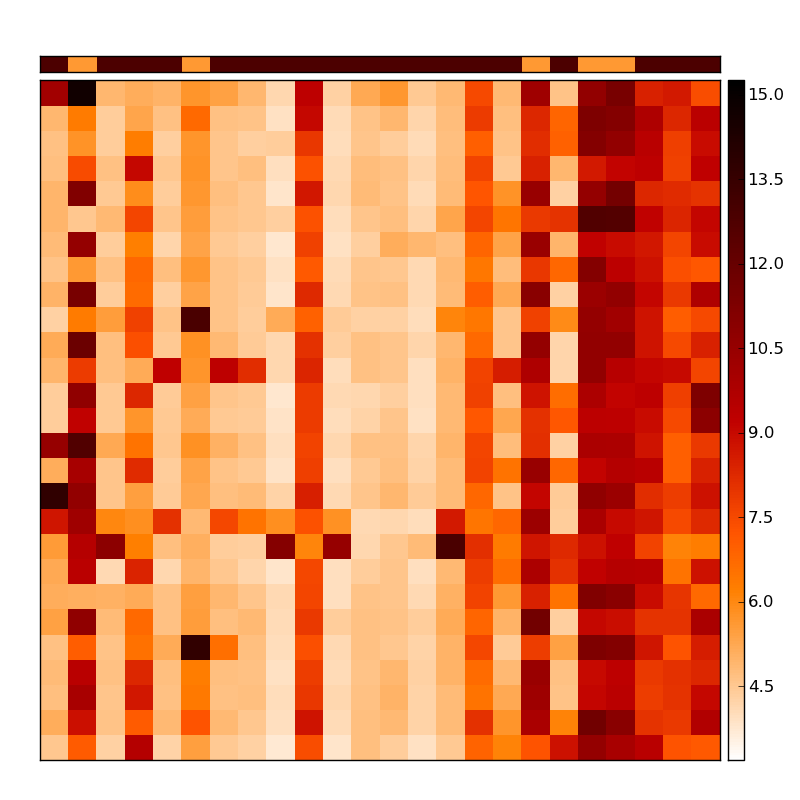

Supplement: Additional file 1 — Detailed information for multigene segments. [file 1471-2164-14-812-S1.zip › miniwebsite/heatmaps/chr2L_heatmap43.png]

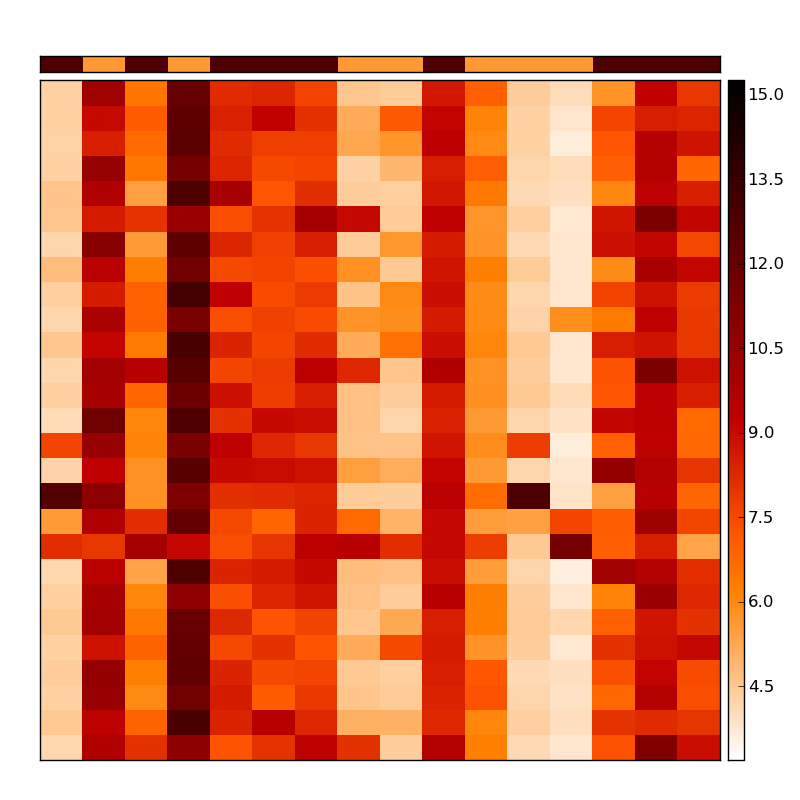

Supplement: Additional file 1 — Detailed information for multigene segments. [file 1471-2164-14-812-S1.zip › miniwebsite/heatmaps/chr2L_heatmap430.png]

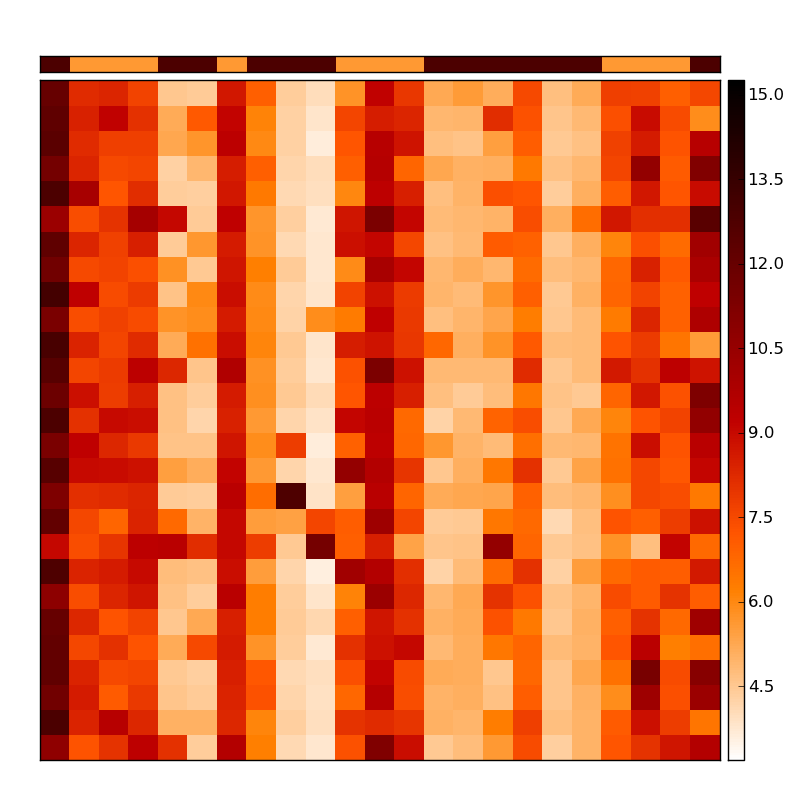

Supplement: Additional file 1 — Detailed information for multigene segments. [file 1471-2164-14-812-S1.zip › miniwebsite/heatmaps/chr2L_heatmap432.png]

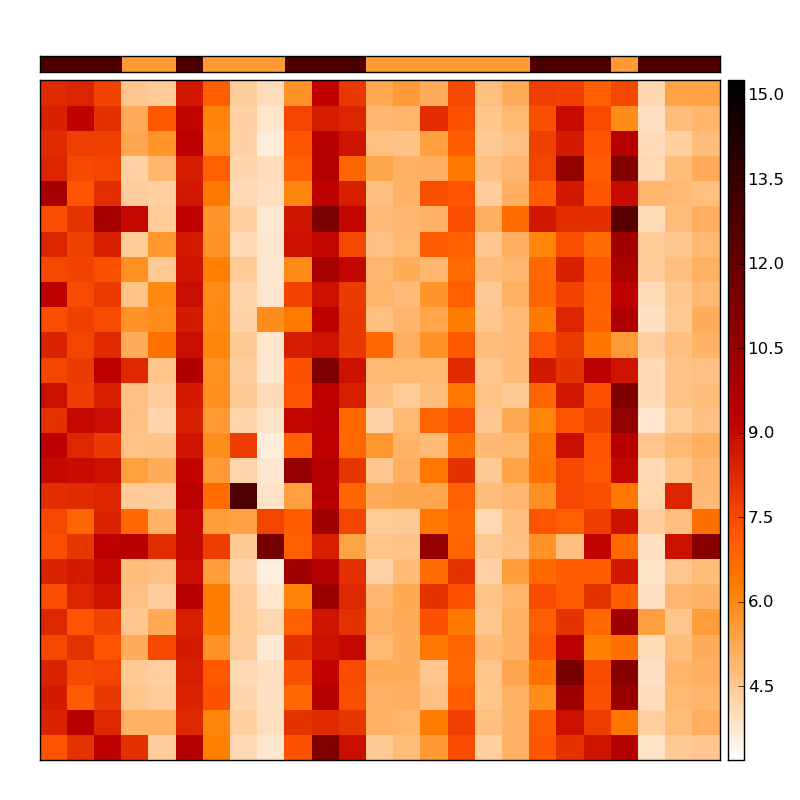

Supplement: Additional file 1 — Detailed information for multigene segments. [file 1471-2164-14-812-S1.zip › miniwebsite/heatmaps/chr2L_heatmap433.png]

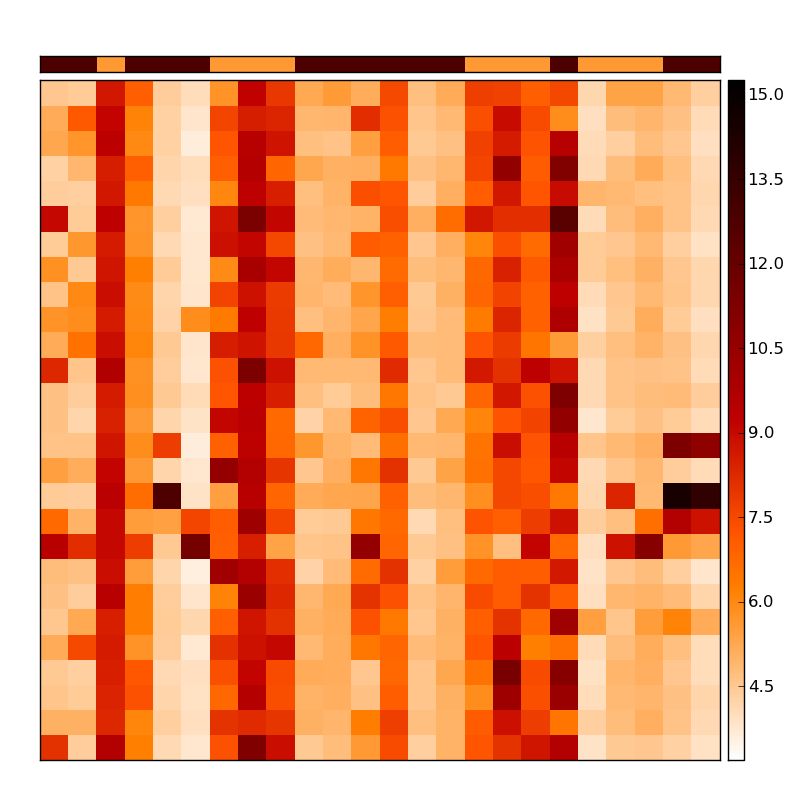

Supplement: Additional file 1 — Detailed information for multigene segments. [file 1471-2164-14-812-S1.zip › miniwebsite/heatmaps/chr2L_heatmap434.png]

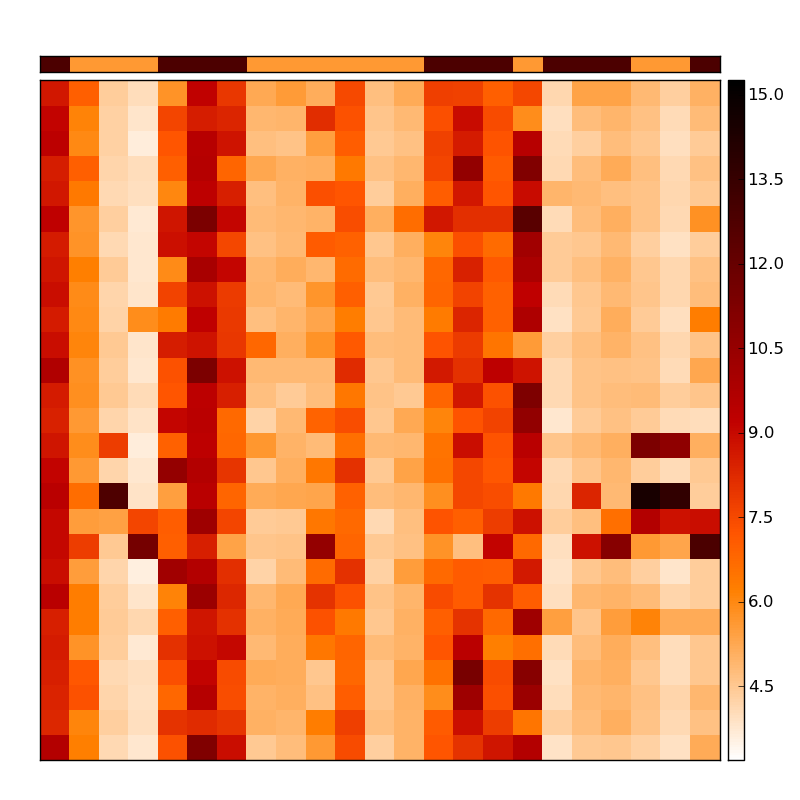

Supplement: Additional file 1 — Detailed information for multigene segments. [file 1471-2164-14-812-S1.zip › miniwebsite/heatmaps/chr2L_heatmap435.png]

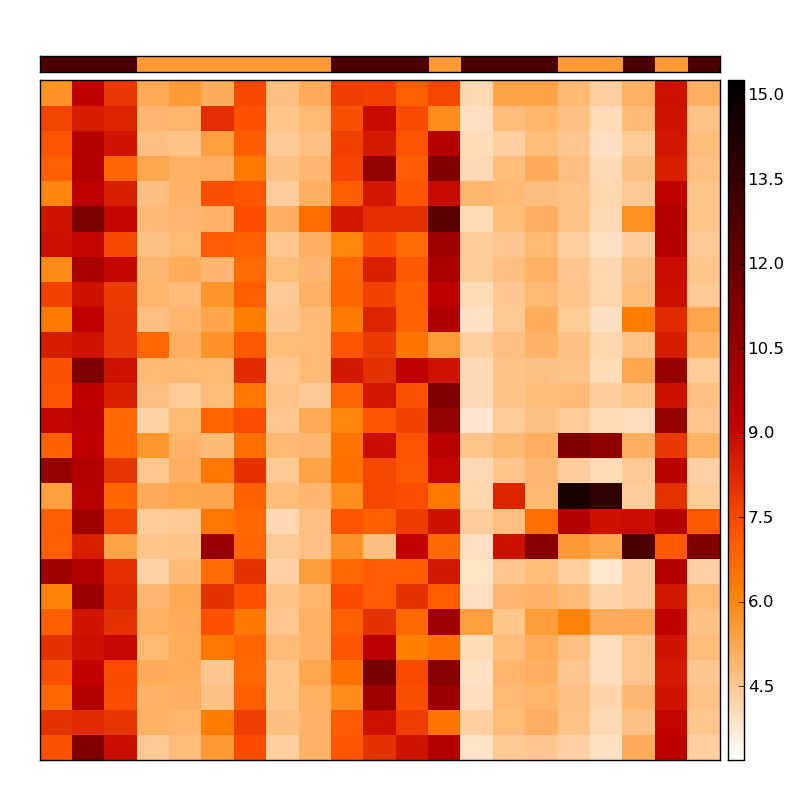

Supplement: Additional file 1 — Detailed information for multigene segments. [file 1471-2164-14-812-S1.zip › miniwebsite/heatmaps/chr2L_heatmap436.png]

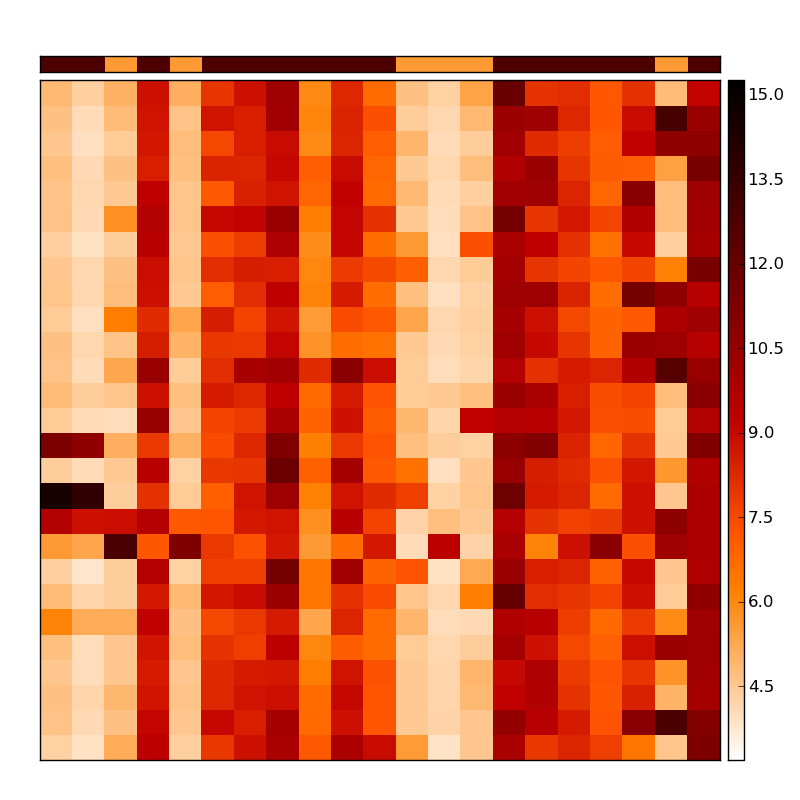

Supplement: Additional file 1 — Detailed information for multigene segments. [file 1471-2164-14-812-S1.zip › miniwebsite/heatmaps/chr2L_heatmap438.png]

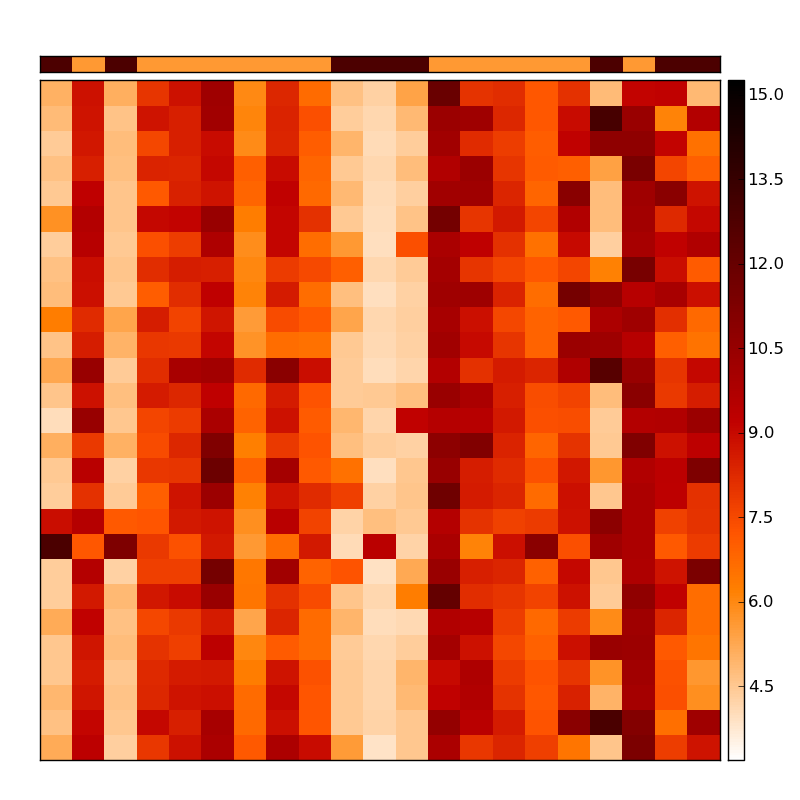

Supplement: Additional file 1 — Detailed information for multigene segments. [file 1471-2164-14-812-S1.zip › miniwebsite/heatmaps/chr2L_heatmap439.png]

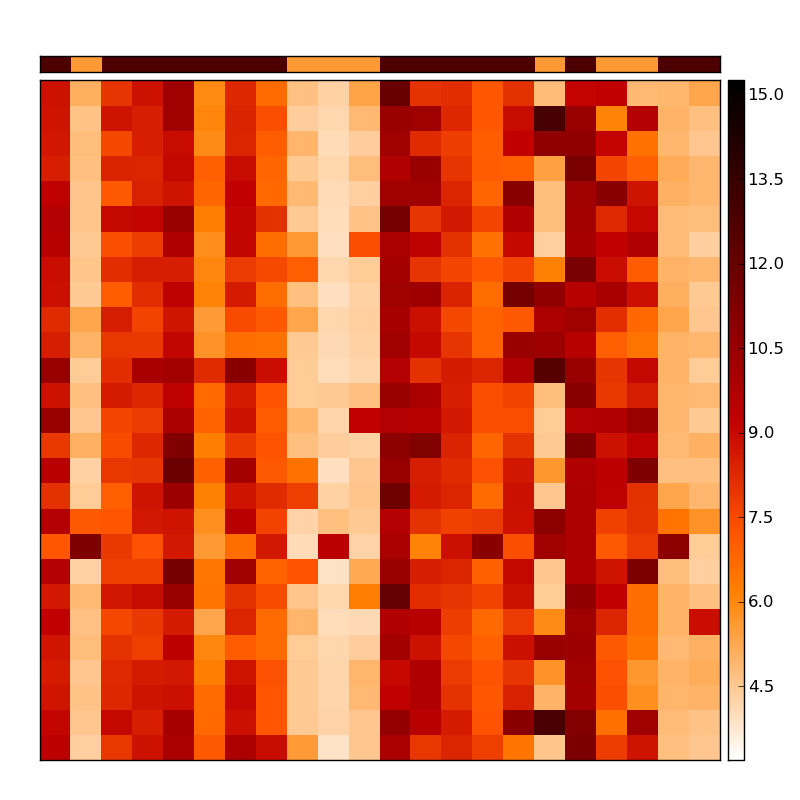

Supplement: Additional file 1 — Detailed information for multigene segments. [file 1471-2164-14-812-S1.zip › miniwebsite/heatmaps/chr2L_heatmap440.png]

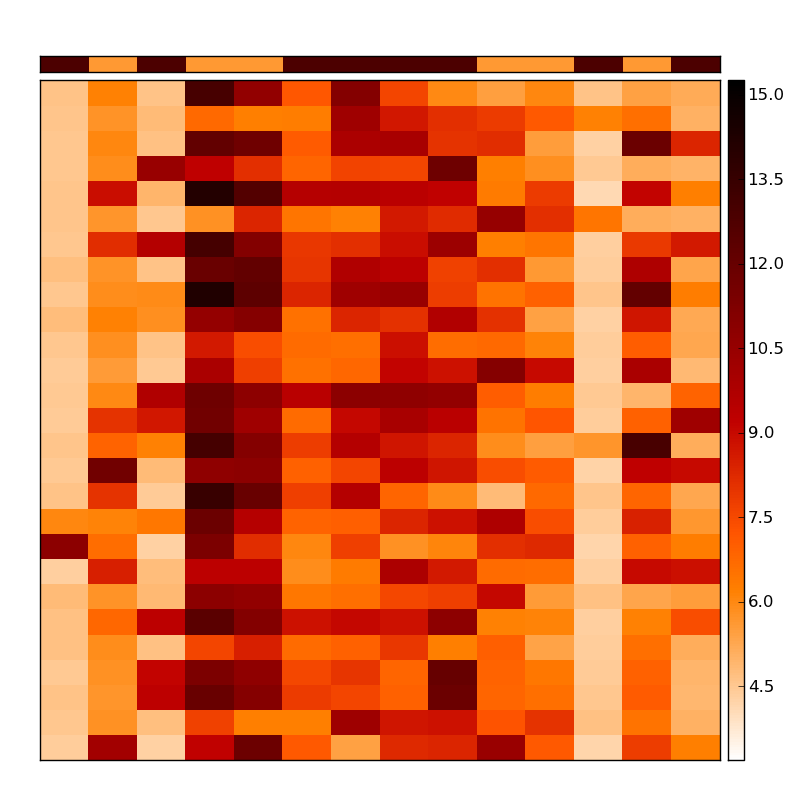

Supplement: Additional file 1 — Detailed information for multigene segments. [file 1471-2164-14-812-S1.zip › miniwebsite/heatmaps/chr2L_heatmap445.png]

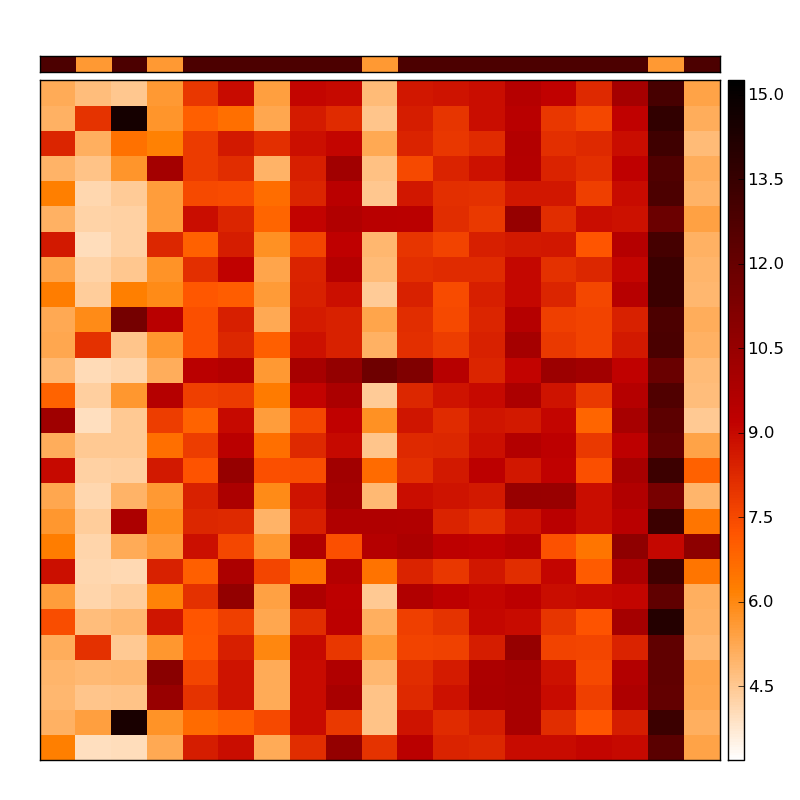

Supplement: Additional file 1 — Detailed information for multigene segments. [file 1471-2164-14-812-S1.zip › miniwebsite/heatmaps/chr2L_heatmap447.png]

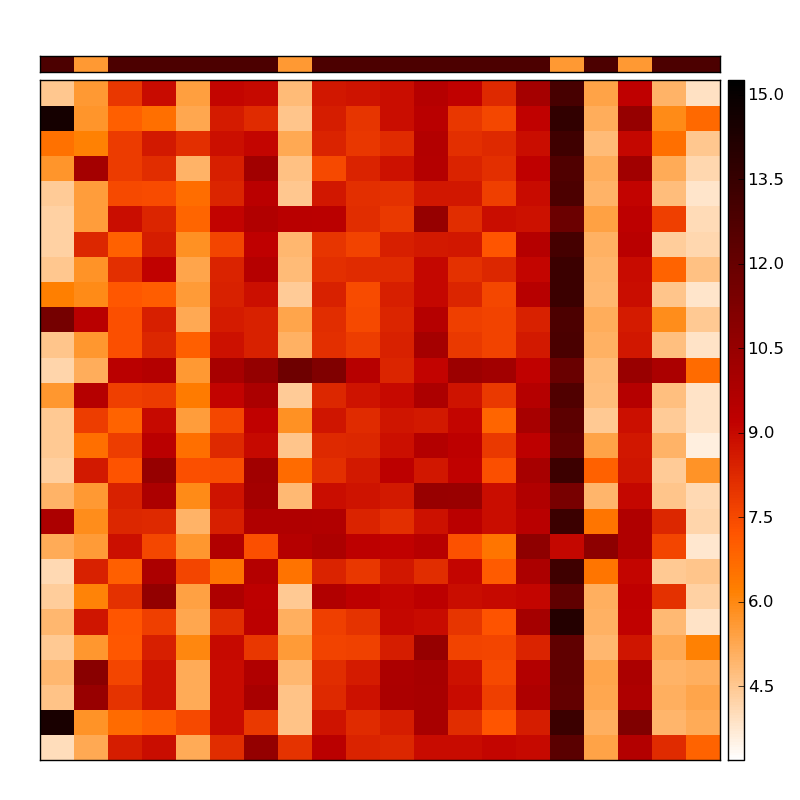

Supplement: Additional file 1 — Detailed information for multigene segments. [file 1471-2164-14-812-S1.zip › miniwebsite/heatmaps/chr2L_heatmap448.png]

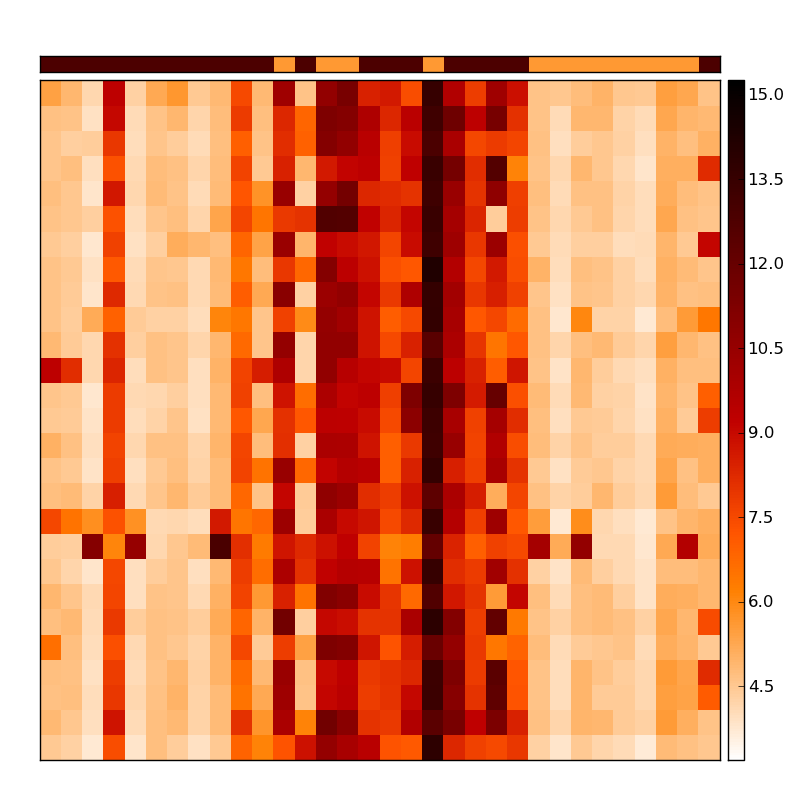

Supplement: Additional file 1 — Detailed information for multigene segments. [file 1471-2164-14-812-S1.zip › miniwebsite/heatmaps/chr2L_heatmap45.png]
