# Supplementary material for: Expression-based segmentation of the Drosophila genome
Source: BMC Genomics. 2013 Nov 20;14:812. doi: 10.1186/1471-2164-14-812 (PMC3909303; doi:10.1186/1471-2164-14-812)
Supplement: Additional file 2 — Supplemental figures. [file 1471-2164-14-812-S2.docx]

Supplemental Figures


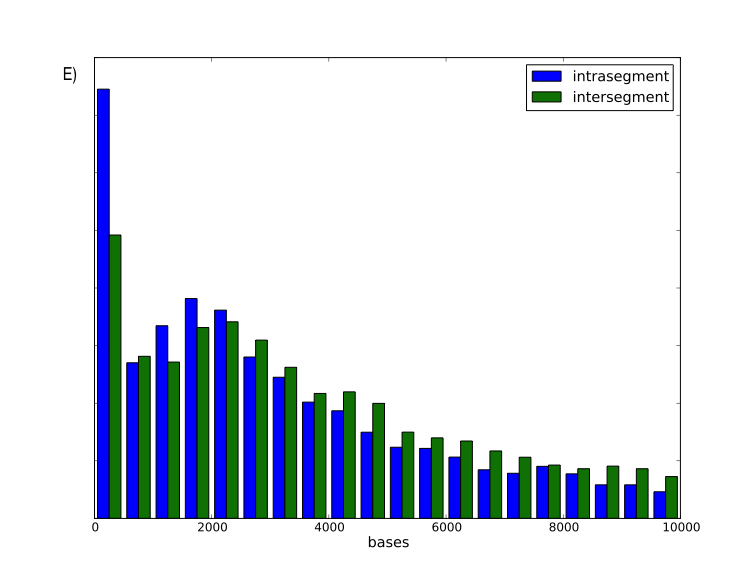

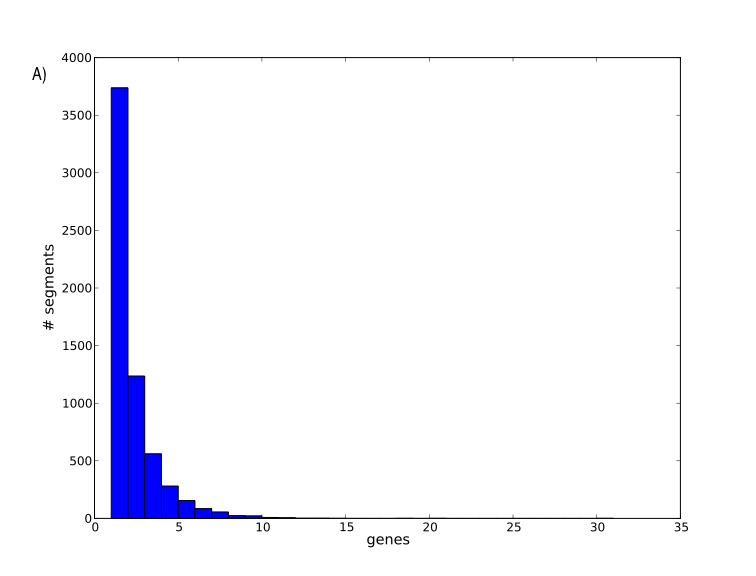

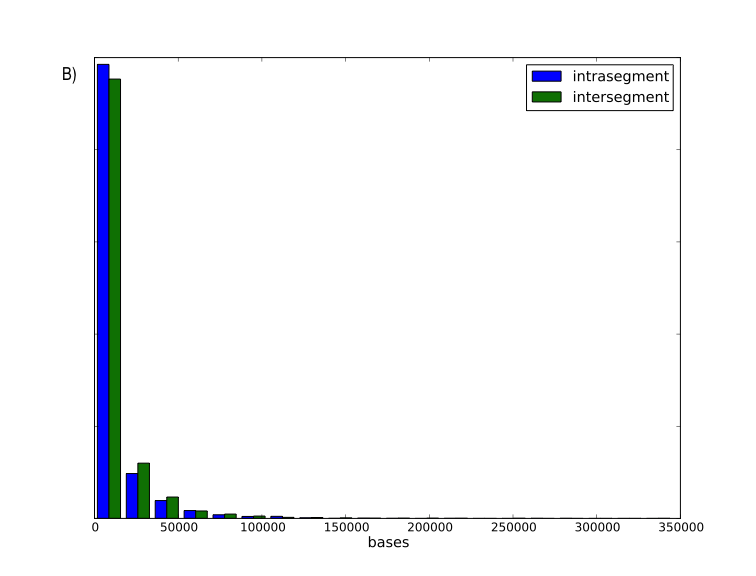

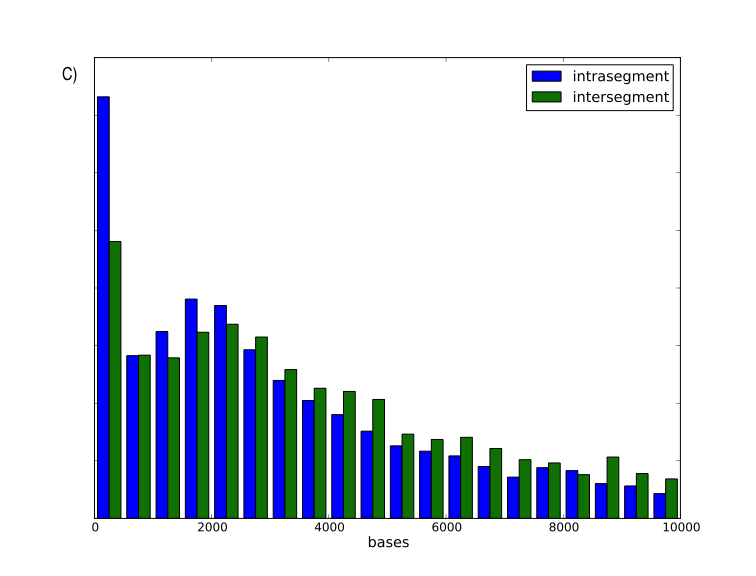

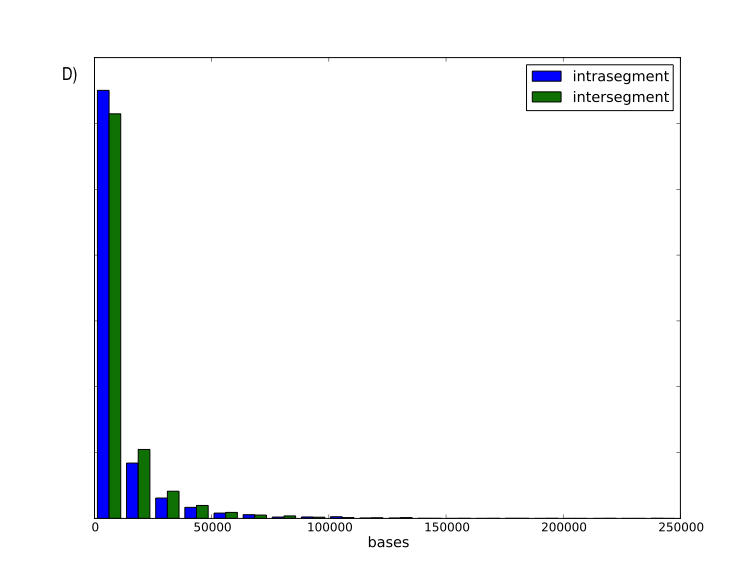


Fig. S1. Length distributions. (A) Histogram of segment lengths (# genes) for the best scoring segmentation pattern. (B and C) Normalized histogram of all intergenic lengths (in base pairs) within segments and between segments (B) and lengths below 10kb (C). Intersegment lengths are significantly longer than intergenic lengths within segments (P=1.39e-24). (D and E) Normalized histogram of all repeat-masked intergenic lengths (in base pairs) within segments and between segments (D) and lengths below 10kb (E). Repeat-masked intersegment lengths are significantly longer than repeat-masked intergenic lengths within segments (P=2.21e-24).


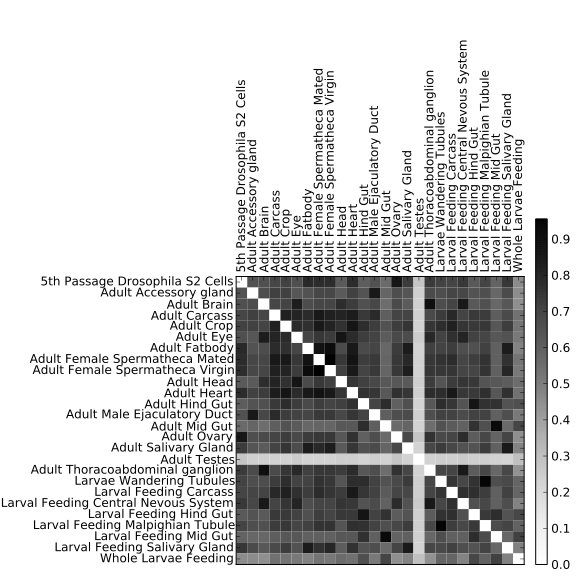


Fig. S2. Tissue coexpression. Cell color indicates ratio of the number of segments in which both tissues are expressed to the number of segments in which either tissue is expressed.


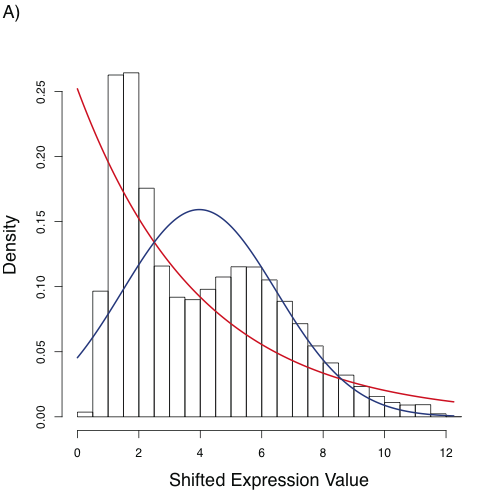

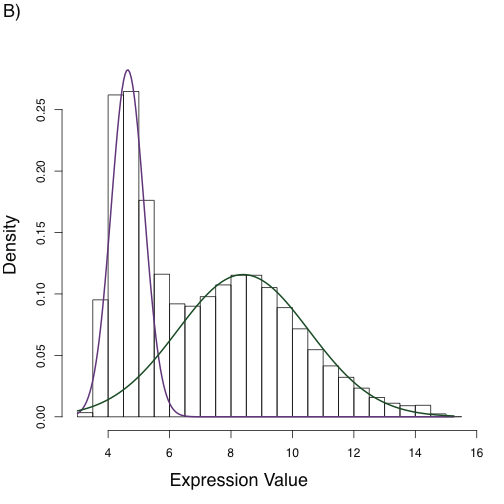


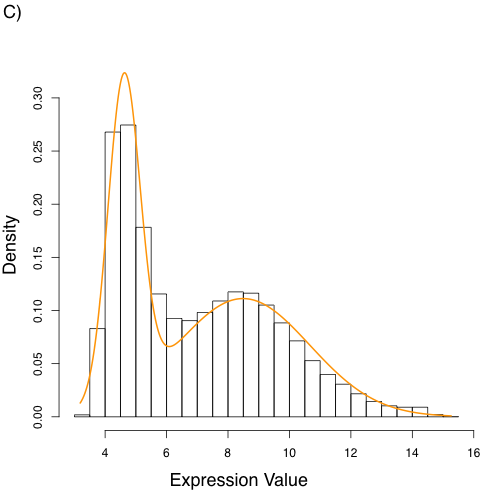


Fig. S3. FlyAtlas expression data, with best fitting distributions of various types. (A) Best fitting normal distribution (blue) and exponential distribution (red). To visualize the exponential fit, expression values are linearly shifted such that the smallest expression value is zero. (B, C) Mixture of two normal distributions (components: green and purple; sum: orange). This was taken as the segment effect distribution.


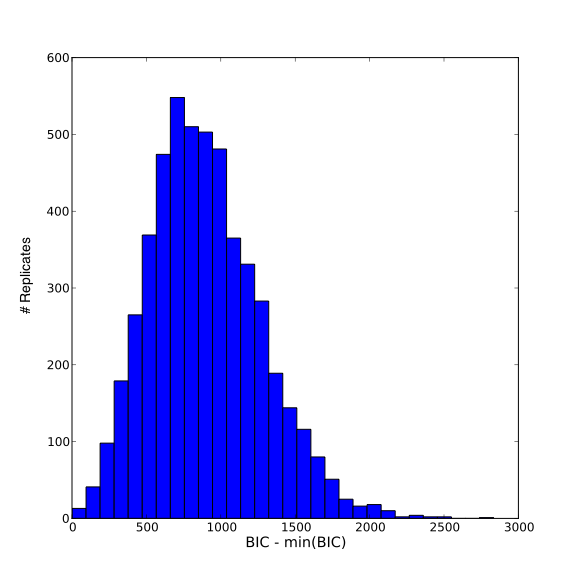


Fig. S4. Replicate score distribution. The scores of the optimal segmentations found by starting from 1024 random starting segmentations for each chromosome arm using the optimal parameter values are plotted. Horizontal axis indicates the difference between the replicate score and the best score found (lower is better).
