# Supplementary material for: Expression-based segmentation of the Drosophila genome
Source: BMC Genomics. 2013 Nov 20;14:812. doi: 10.1186/1471-2164-14-812 (PMC3909303; doi:10.1186/1471-2164-14-812)
Supplement: Additional file 1 — Detailed information for multigene segments. [file 1471-2164-14-812-S1.zip › index.html]

ExprSeg Report

#### Gene List

#### Segment Data (5.3 MB tab-separated file)

#### Chromosome Reports

- Chromosome 2L- Chromosome 2R- Chromosome 3L- Chromosome 3R- Chromosome X

#### Example heatmap

  

|  |  |  |
| --- | --- | --- |
| **Segments by length (genes)** | | |
| 1 | FBgn0051559-FBgn0037432 (3R) | 31 |
| 2 | FBgn0034829-FBgn0050409 (2R) | 20 |
| 3 | FBgn0030367-FBgn0030390 (X) | 18 |
| 4 | FBgn0032965-FBgn0032972 (2L) | 14 |
| 5 | FBgn0032577-FBgn0028893 (2L) | 13 |
| 6 | FBgn0039192-FBgn0039202 (3R) | 13 |
| 7 | FBgn0031462-FBgn0031473 (2L) | 12 |
| 8 | FBgn0037449-FBgn0051286 (3R) | 12 |
| 9 | FBgn0051926-FBgn0031351 (2L) | 11 |
| 10 | FBgn0028412-FBgn0032377 (2L) | 11 |
| 11 | FBgn0001222-FBgn0034313 (2R) | 11 |
| 12 | FBgn0035002-FBgn0035009 (2R) | 11 |
| 13 | FBgn0040816-FBgn0036224 (3L) | 11 |
| 14 | FBgn0040355-FBgn0029647 (X) | 11 |
| 15 | FBgn0031718-FBgn0031725 (2L) | 10 |
| 16 | FBgn0028853-FBgn0020371 (2L) | 10 |
| 17 | FBgn0023094-FBgn0036916 (3L) | 10 |
| 18 | FBgn0037168-FBgn0037179 (3L) | 10 |
| 19 | FBgn0085331-FBgn0053208 (3R) | 10 |
| 20 | FBgn0037876-FBgn0037884 (3R) | 10 |
| 21 | FBgn0029961-FBgn0029968 (X) | 10 |
| 22 | FBgn0031127-FBgn0031139 (X) | 10 |
| 23 | FBgn0031440-FBgn0031446 (2L) | 9 |
| 24 | FBgn0003386-FBgn0003430 (2L) | 9 |
| 25 | FBgn0051642-FBgn0053531 (2L) | 9 |
| 26 | FBgn0051816-FBgn0051807 (2L) | 9 |
| 27 | FBgn0033375-FBgn0027561 (2R) | 9 |
| 28 | FBgn0085213-FBgn0033933 (2R) | 9 |
| 29 | FBgn0034665-FBgn0054029 (2R) | 9 |
| 30 | FBgn0026384-FBgn0034870 (2R) | 9 |
| 31 | FBgn0010040-FBgn0053098 (3R) | 9 |
| 32 | FBgn0038471-FBgn0038478 (3R) | 9 |
| 33 | FBgn0051219-FBgn0038732 (3R) | 9 |
| 34 | FBgn0039075-FBgn0085318 (3R) | 9 |
| 35 | FBgn0039109-FBgn0039115 (3R) | 9 |
| 36 | FBgn0039140-FBgn0040283 (3R) | 9 |
| 37 | FBgn0039792-FBgn0039799 (3R) | 9 |
| 38 | FBgn0024288-FBgn0004389 (3R) | 9 |
| 39 | FBgn0025616-FBgn0029538 (X) | 9 |
| 40 | FBgn0040363-FBgn0040367 (X) | 9 |
| 41 | FBgn0029722-FBgn0029730 (X) | 9 |
| 42 | FBgn0030165-FBgn0030173 (X) | 9 |
| 43 | FBgn0030559-FBgn0030570 (X) | 9 |
| 44 | FBgn0030898-FBgn0004956 (X) | 9 |
| 45 | FBgn0024314-FBgn0031322 (2L) | 8 |
| 46 | FBgn0031364-FBgn0051933 (2L) | 8 |
| 47 | FBgn0004584-FBgn0025681 (2L) | 8 |
| 48 | FBgn0031601-FBgn0031607 (2L) | 8 |
| 49 | FBgn0031619-FBgn0031626 (2L) | 8 |
| 50 | FBgn0051876-FBgn0032171 (2L) | 8 |
| 51 | FBgn0028901-FBgn0051735 (2L) | 8 |
| 52 | FBgn0001202-FBgn0032748 (2L) | 8 |
| 53 | FBgn0026386-FBgn0033603 (2R) | 8 |
| 54 | FBgn0034329-FBgn0064237 (2R) | 8 |
| 55 | FBgn0027535-FBgn0010434 (2R) | 8 |
| 56 | FBgn0041237-FBgn0034684 (2R) | 8 |
| 57 | FBgn0035202-FBgn0035207 (3L) | 8 |
| 58 | FBgn0035569-FBgn0035577 (3L) | 8 |
| 59 | FBgn0020637-FBgn0020643 (3L) | 8 |
| 60 | FBgn0052369-FBgn0024187 (3L) | 8 |
| 61 | FBgn0036590-FBgn0036596 (3L) | 8 |
| 62 | FBgn0037074-FBgn0053288 (3L) | 8 |
| 63 | FBgn0039687-FBgn0039691 (3R) | 8 |
| 64 | FBgn0040370-FBgn0000137 (X) | 8 |
| 65 | FBgn0025628-FBgn0013432 (X) | 8 |
| 66 | FBgn0015818-FBgn0028685 (X) | 8 |
| 67 | FBgn0030661-FBgn0030667 (X) | 8 |
| 68 | FBgn0030766-FBgn0000152 (X) | 8 |
| 69 | FBgn0031390-FBgn0031395 (2L) | 7 |
| 70 | FBgn0031489-FBgn0031495 (2L) | 7 |
| 71 | FBgn0031657-FBgn0031664 (2L) | 7 |
| 72 | FBgn0011722-FBgn0031817 (2L) | 7 |
| 73 | FBgn0031853-FBgn0031858 (2L) | 7 |
| 74 | FBgn0031900-FBgn0002938 (2L) | 7 |
| 75 | FBgn0041247-FBgn0031923 (2L) | 7 |
| 76 | FBgn0032089-FBgn0032100 (2L) | 7 |
| 77 | FBgn0032193-FBgn0032197 (2L) | 7 |
| 78 | FBgn0032200-FBgn0032205 (2L) | 7 |
| 79 | FBgn0032247-FBgn0032251 (2L) | 7 |
| 80 | FBgn0032457-FBgn0032462 (2L) | 7 |
| 81 | FBgn0032525-FBgn0028941 (2L) | 7 |
| 82 | FBgn0051784-FBgn0032631 (2L) | 7 |
| 83 | FBgn0000183-FBgn0032644 (2L) | 7 |
| 84 | FBgn0032724-FBgn0032729 (2L) | 7 |
| 85 | FBgn0024371-FBgn0032926 (2L) | 7 |
| 86 | FBgn0033277-FBgn0050375 (2R) | 7 |
| 87 | FBgn0033587-FBgn0033592 (2R) | 7 |
| 88 | FBgn0034321-FBgn0034327 (2R) | 7 |
| 89 | FBgn0041240-FBgn0034551 (2R) | 7 |
| 90 | FBgn0034766-FBgn0050268 (2R) | 7 |
| 91 | FBgn0035023-FBgn0035028 (2R) | 7 |
| 92 | FBgn0035255-FBgn0035263 (3L) | 7 |
| 93 | FBgn0035376-FBgn0035382 (3L) | 7 |
| 94 | FBgn0010406-FBgn0026263 (3L) | 7 |
| 95 | FBgn0035995-FBgn0036000 (3L) | 7 |
| 96 | FBgn0052112-FBgn0036309 (3L) | 7 |
| 97 | FBgn0036437-FBgn0036443 (3L) | 7 |
| 98 | FBgn0085274-FBgn0036459 (3L) | 7 |
| 99 | FBgn0036479-FBgn0014002 (3L) | 7 |
| 100 | FBgn0052154-FBgn0036586 (3L) | 7 |
| 101 | FBgn0036703-FBgn0036709 (3L) | 7 |
| 102 | FBgn0037329-FBgn0051550 (3R) | 7 |
| 103 | FBgn0037368-FBgn0037374 (3R) | 7 |
| 104 | FBgn0037465-FBgn0037470 (3R) | 7 |
| 105 | FBgn0037506-FBgn0037512 (3R) | 7 |
| 106 | FBgn0024326-FBgn0037606 (3R) | 7 |
| 107 | FBgn0014023-FBgn0037710 (3R) | 7 |
| 108 | FBgn0038003-FBgn0003651 (3R) | 7 |
| 109 | FBgn0038156-FBgn0038160 (3R) | 7 |
| 110 | FBgn0038186-FBgn0038190 (3R) | 7 |
| 111 | FBgn0038260-FBgn0038268 (3R) | 7 |
| 112 | FBgn0051294-FBgn0038359 (3R) | 7 |
| 113 | FBgn0038525-FBgn0038529 (3R) | 7 |
| 114 | FBgn0046685-FBgn0020018 (3R) | 7 |
| 115 | FBgn0039434-FBgn0051080 (3R) | 7 |
| 116 | FBgn0039495-FBgn0039498 (3R) | 7 |
| 117 | FBgn0039623-FBgn0039627 (3R) | 7 |
| 118 | FBgn0039810-FBgn0053773 (3R) | 7 |
| 119 | FBgn0039838-FBgn0046332 (3R) | 7 |
| 120 | FBgn0023516-FBgn0029594 (X) | 7 |
| 121 | FBgn0040393-FBgn0003374 (X) | 7 |
| 122 | FBgn0029700-FBgn0040906 (X) | 7 |
| 123 | FBgn0030618-FBgn0030624 (X) | 7 |
| 124 | FBgn0011742-FBgn0025743 (X) | 7 |
| 125 | FBgn0031238-FBgn0031244 (2L) | 6 |
| 126 | FBgn0031264-FBgn0031268 (2L) | 6 |
| 127 | FBgn0031331-FBgn0031335 (2L) | 6 |
| 128 | FBgn0031384-FBgn0053516 (2L) | 6 |
| 129 | FBgn0031635-FBgn0031640 (2L) | 6 |
| 130 | FBgn0025742-FBgn0031769 (2L) | 6 |
| 131 | FBgn0031954-FBgn0031961 (2L) | 6 |
| 132 | FBgn0032029-FBgn0032034 (2L) | 6 |
| 133 | FBgn0052986-FBgn0085427 (2L) | 6 |
| 134 | FBgn0259225-FBgn0032385 (2L) | 6 |
| 135 | FBgn0028919-FBgn0015271 (2L) | 6 |
| 136 | FBgn0032553-FBgn0259213 (2L) | 6 |
| 137 | FBgn0051803-FBgn0032639 (2L) | 6 |
| 138 | FBgn0026756-FBgn0040998 (2L) | 6 |
| 139 | FBgn0032871-FBgn0032877 (2L) | 6 |
| 140 | FBgn0033041-FBgn0033046 (2R) | 6 |
| 141 | FBgn0033259-FBgn0005648 (2R) | 6 |
| 142 | FBgn0033359-FBgn0033365 (2R) | 6 |
| 143 | FBgn0050343-FBgn0033402 (2R) | 6 |
| 144 | FBgn0041242-FBgn0053475 (2R) | 6 |
| 145 | FBgn0033562-FBgn0033569 (2R) | 6 |
| 146 | FBgn0033572-FBgn0033579 (2R) | 6 |
| 147 | FBgn0033862-FBgn0033867 (2R) | 6 |
| 148 | FBgn0040752-FBgn0033900 (2R) | 6 |
| 149 | FBgn0034022-FBgn0050471 (2R) | 6 |
| 150 | FBgn0013983-FBgn0034351 (2R) | 6 |
| 151 | FBgn0050395-FBgn0034661 (2R) | 6 |
| 152 | FBgn0050272-FBgn0034785 (2R) | 6 |
| 153 | FBgn0034913-FBgn0034918 (2R) | 6 |
| 154 | FBgn0003008-FBgn0005612 (2R) | 6 |
| 155 | FBgn0034961-FBgn0034964 (2R) | 6 |
| 156 | FBgn0035370-FBgn0035375 (3L) | 6 |
| 157 | FBgn0035426-FBgn0035430 (3L) | 6 |
| 158 | FBgn0035453-FBgn0052266 (3L) | 6 |
| 159 | FBgn0035563-FBgn0035568 (3L) | 6 |
| 160 | FBgn0035733-FBgn0035741 (3L) | 6 |
| 161 | FBgn0035762-FBgn0035767 (3L) | 6 |
| 162 | FBgn0035898-FBgn0035902 (3L) | 6 |
| 163 | FBgn0036078-FBgn0052058 (3L) | 6 |
| 164 | FBgn0041775-FBgn0036301 (3L) | 6 |
| 165 | FBgn0036302-FBgn0014007 (3L) | 6 |
| 166 | FBgn0036325-FBgn0036330 (3L) | 6 |
| 167 | FBgn0036510-FBgn0036516 (3L) | 6 |
| 168 | FBgn0004593-FBgn0014851 (3L) | 6 |
| 169 | FBgn0000212-FBgn0036558 (3L) | 6 |
| 170 | FBgn0036676-FBgn0036681 (3L) | 6 |
| 171 | FBgn0036987-FBgn0036991 (3L) | 6 |
| 172 | FBgn0037018-FBgn0037022 (3L) | 6 |
| 173 | FBgn0003415-FBgn0026179 (3L) | 6 |
| 174 | FBgn0051501-FBgn0004172 (3R) | 6 |
| 175 | FBgn0037584-FBgn0037591 (3R) | 6 |
| 176 | FBgn0042110-FBgn0037941 (3R) | 6 |
| 177 | FBgn0038122-FBgn0038127 (3R) | 6 |
| 178 | FBgn0038316-FBgn0038321 (3R) | 6 |
| 179 | FBgn0026634-FBgn0038331 (3R) | 6 |
| 180 | FBgn0024491-FBgn0040237 (3R) | 6 |
| 181 | FBgn0038694-FBgn0051245 (3R) | 6 |
| 182 | FBgn0013812-FBgn0038850 (3R) | 6 |
| 183 | FBgn0038886-FBgn0011278 (3R) | 6 |
| 184 | FBgn0038922-FBgn0019957 (3R) | 6 |
| 185 | FBgn0039086-FBgn0039092 (3R) | 6 |
| 186 | FBgn0039116-FBgn0053111 (3R) | 6 |
| 187 | FBgn0039204-FBgn0039210 (3R) | 6 |
| 188 | FBgn0051102-FBgn0051436 (3R) | 6 |
| 189 | FBgn0039366-FBgn0039371 (3R) | 6 |
| 190 | FBgn0002592-FBgn0002633 (3R) | 6 |
| 191 | FBgn0051014-FBgn0039784 (3R) | 6 |
| 192 | FBgn0039874-FBgn0027620 (3R) | 6 |
| 193 | FBgn0025835-FBgn0024989 (X) | 6 |
| 194 | FBgn0025378-FBgn0029590 (X) | 6 |
| 195 | FBgn0023509-FBgn0023521 (X) | 6 |
| 196 | FBgn0000520-FBgn0040384 (X) | 6 |
| 197 | FBgn0029658-FBgn0029662 (X) | 6 |
| 198 | FBgn0029690-FBgn0029694 (X) | 6 |
| 199 | FBgn0025387-FBgn0011277 (X) | 6 |
| 200 | FBgn0029745-FBgn0029751 (X) | 6 |
| 201 | FBgn0029811-FBgn0029817 (X) | 6 |
| 202 | FBgn0030219-FBgn0030224 (X) | 6 |
| 203 | FBgn0030292-FBgn0027359 (X) | 6 |
| 204 | FBgn0030400-FBgn0030407 (X) | 6 |
| 205 | FBgn0030465-FBgn0085388 (X) | 6 |
| 206 | FBgn0086448-FBgn0010416 (X) | 6 |
| 207 | FBgn0030810-FBgn0030815 (X) | 6 |
| 208 | FBgn0030884-FBgn0013809 (X) | 6 |
| 209 | FBgn0030941-FBgn0030946 (X) | 6 |
| 210 | FBgn0031245-FBgn0020622 (2L) | 5 |
| 211 | FBgn0013323-FBgn0000497 (2L) | 5 |
| 212 | FBgn0051686-FBgn0031406 (2L) | 5 |
| 213 | FBgn0031423-FBgn0085477 (2L) | 5 |
| 214 | FBgn0031520-FBgn0031523 (2L) | 5 |
| 215 | FBgn0085204-FBgn0031533 (2L) | 5 |
| 216 | FBgn0031545-FBgn0031550 (2L) | 5 |
| 217 | FBgn0031695-FBgn0016076 (2L) | 5 |
| 218 | FBgn0031710-FBgn0031713 (2L) | 5 |
| 219 | FBgn0031802-FBgn0043854 (2L) | 5 |
| 220 | FBgn0000053-FBgn0022201 (2L) | 5 |
| 221 | FBgn0032050-FBgn0032054 (2L) | 5 |
| 222 | FBgn0051709-FBgn0051882 (2L) | 5 |
| 223 | FBgn0032178-FBgn0032184 (2L) | 5 |
| 224 | FBgn0032222-FBgn0032224 (2L) | 5 |
| 225 | FBgn0032311-FBgn0032315 (2L) | 5 |
| 226 | FBgn0023415-FBgn0032335 (2L) | 5 |
| 227 | FBgn0032446-FBgn0032450 (2L) | 5 |
| 228 | FBgn0032489-FBgn0032493 (2L) | 5 |
| 229 | FBgn0032512-FBgn0032516 (2L) | 5 |
| 230 | FBgn0027929-FBgn0028936 (2L) | 5 |
| 231 | FBgn0020258-FBgn0028844 (2L) | 5 |
| 232 | FBgn0032549-FBgn0053309 (2L) | 5 |
| 233 | FBgn0013810-FBgn0032650 (2L) | 5 |
| 234 | FBgn0032699-FBgn0032703 (2L) | 5 |
| 235 | FBgn0032749-FBgn0032753 (2L) | 5 |
| 236 | FBgn0010300-FBgn0010309 (2L) | 5 |
| 237 | FBgn0032811-FBgn0000464 (2L) | 5 |
| 238 | FBgn0032824-FBgn0053117 (2L) | 5 |
| 239 | FBgn0032880-FBgn0011202 (2L) | 5 |
| 240 | FBgn0032915-FBgn0032919 (2L) | 5 |
| 241 | FBgn0023091-FBgn0032945 (2L) | 5 |
| 242 | FBgn0033005-FBgn0033017 (2R) | 5 |
| 243 | FBgn0026761-FBgn0033081 (2R) | 5 |
| 244 | FBgn0033322-FBgn0050357 (2R) | 5 |
| 245 | FBgn0033340-FBgn0050349 (2R) | 5 |
| 246 | FBgn0033382-FBgn0050344 (2R) | 5 |
| 247 | FBgn0259234-FBgn0259246 (2R) | 5 |
| 248 | FBgn0005630-FBgn0033540 (2R) | 5 |
| 249 | FBgn0033605-FBgn0033609 (2R) | 5 |
| 250 | FBgn0043470-FBgn0011555 (2R) | 5 |
| 251 | FBgn0033636-FBgn0085257 (2R) | 5 |
| 252 | FBgn0033701-FBgn0033705 (2R) | 5 |
| 253 | FBgn0028683-FBgn0033762 (2R) | 5 |
| 254 | FBgn0033827-FBgn0033832 (2R) | 5 |
| 255 | FBgn0020269-FBgn0012042 (2R) | 5 |
| 256 | FBgn0034037-FBgn0053462 (2R) | 5 |
| 257 | FBgn0034096-FBgn0025519 (2R) | 5 |
| 258 | FBgn0034104-FBgn0050099 (2R) | 5 |
| 259 | FBgn0034120-FBgn0053960 (2R) | 5 |
| 260 | FBgn0034240-FBgn0040294 (2R) | 5 |
| 261 | FBgn0034251-FBgn0050106 (2R) | 5 |
| 262 | FBgn0034265-FBgn0034271 (2R) | 5 |
| 263 | FBgn0050325-FBgn0034286 (2R) | 5 |
| 264 | FBgn0034291-FBgn0034294 (2R) | 5 |
| 265 | FBgn0034460-FBgn0034464 (2R) | 5 |
| 266 | FBgn0034475-FBgn0034478 (2R) | 5 |
| 267 | FBgn0050295-FBgn0020617 (2R) | 5 |
| 268 | FBgn0050286-FBgn0053225 (2R) | 5 |
| 269 | FBgn0034820-FBgn0041236 (2R) | 5 |
| 270 | FBgn0034824-FBgn0034827 (2R) | 5 |
| 271 | FBgn0034904-FBgn0034906 (2R) | 5 |
| 272 | FBgn0041582-FBgn0015544 (2R) | 5 |
| 273 | FBgn0034990-FBgn0034996 (2R) | 5 |
| 274 | FBgn0028738-FBgn0000157 (2R) | 5 |
| 275 | FBgn0052845-FBgn0085293 (3L) | 5 |
| 276 | FBgn0035145-FBgn0035149 (3L) | 5 |
| 277 | FBgn0035155-FBgn0035160 (3L) | 5 |
| 278 | FBgn0035384-FBgn0004372 (3L) | 5 |
| 279 | FBgn0035388-FBgn0052485 (3L) | 5 |
| 280 | FBgn0035400-FBgn0035403 (3L) | 5 |
| 281 | FBgn0011653-FBgn0003710 (3L) | 5 |
| 282 | FBgn0035584-FBgn0035594 (3L) | 5 |
| 283 | FBgn0003984-FBgn0035645 (3L) | 5 |
| 284 | FBgn0035699-FBgn0020251 (3L) | 5 |
| 285 | FBgn0035906-FBgn0035911 (3L) | 5 |
| 286 | FBgn0035954-FBgn0035957 (3L) | 5 |
| 287 | FBgn0052061-FBgn0036089 (3L) | 5 |
| 288 | FBgn0052075-FBgn0036124 (3L) | 5 |
| 289 | FBgn0054032-FBgn0052117 (3L) | 5 |
| 290 | FBgn0036349-FBgn0036352 (3L) | 5 |
| 291 | FBgn0036377-FBgn0043550 (3L) | 5 |
| 292 | FBgn0052135-FBgn0036395 (3L) | 5 |
| 293 | FBgn0036396-FBgn0036402 (3L) | 5 |
| 294 | FBgn0041161-FBgn0013563 (3L) | 5 |
| 295 | FBgn0036448-FBgn0029148 (3L) | 5 |
| 296 | FBgn0036545-FBgn0036549 (3L) | 5 |
| 297 | FBgn0036564-FBgn0036569 (3L) | 5 |
| 298 | FBgn0036609-FBgn0053060 (3L) | 5 |
| 299 | FBgn0036661-FBgn0021768 (3L) | 5 |
| 300 | FBgn0004401-FBgn0036746 (3L) | 5 |
| 301 | FBgn0036794-FBgn0052199 (3L) | 5 |
| 302 | FBgn0036811-FBgn0036815 (3L) | 5 |
| 303 | FBgn0028695-FBgn0036921 (3L) | 5 |
| 304 | FBgn0036949-FBgn0036953 (3L) | 5 |
| 305 | FBgn0001323-FBgn0037015 (3L) | 5 |
| 306 | FBgn0037067-FBgn0037070 (3L) | 5 |
| 307 | FBgn0037129-FBgn0000560 (3L) | 5 |
| 308 | FBgn0037224-FBgn0041621 (3R) | 5 |
| 309 | FBgn0040684-FBgn0037296 (3R) | 5 |
| 310 | FBgn0037322-FBgn0037325 (3R) | 5 |
| 311 | FBgn0037339-FBgn0041191 (3R) | 5 |
| 312 | FBgn0037347-FBgn0044823 (3R) | 5 |
| 313 | FBgn0051481-FBgn0000166 (3R) | 5 |
| 314 | FBgn0037478-FBgn0037482 (3R) | 5 |
| 315 | FBgn0015770-FBgn0037503 (3R) | 5 |
| 316 | FBgn0042104-FBgn0037517 (3R) | 5 |
| 317 | FBgn0037548-FBgn0037551 (3R) | 5 |
| 318 | FBgn0027524-FBgn0037780 (3R) | 5 |
| 319 | FBgn0037826-FBgn0037829 (3R) | 5 |
| 320 | FBgn0037852-FBgn0037855 (3R) | 5 |
| 321 | FBgn0051358-FBgn0037988 (3R) | 5 |
| 322 | FBgn0038053-FBgn0038058 (3R) | 5 |
| 323 | FBgn0042207-FBgn0038070 (3R) | 5 |
| 324 | FBgn0038201-FBgn0038205 (3R) | 5 |
| 325 | FBgn0038208-FBgn0038210 (3R) | 5 |
| 326 | FBgn0038303-FBgn0015610 (3R) | 5 |
| 327 | FBgn0038424-FBgn0038428 (3R) | 5 |
| 328 | FBgn0051265-FBgn0051267 (3R) | 5 |
| 329 | FBgn0038535-FBgn0051249 (3R) | 5 |
| 330 | FBgn0038582-FBgn0038586 (3R) | 5 |
| 331 | FBgn0038666-FBgn0038673 (3R) | 5 |
| 332 | FBgn0038706-FBgn0038715 (3R) | 5 |
| 333 | FBgn0038765-FBgn0038769 (3R) | 5 |
| 334 | FBgn0043884-FBgn0019952 (3R) | 5 |
| 335 | FBgn0051109-FBgn0039270 (3R) | 5 |
| 336 | FBgn0039302-FBgn0039306 (3R) | 5 |
| 337 | FBgn0004509-FBgn0039338 (3R) | 5 |
| 338 | FBgn0051093-FBgn0039376 (3R) | 5 |
| 339 | FBgn0051081-FBgn0039448 (3R) | 5 |
| 340 | FBgn0039476-FBgn0039478 (3R) | 5 |
| 341 | FBgn0039505-FBgn0039509 (3R) | 5 |
| 342 | FBgn0039522-FBgn0046885 (3R) | 5 |
| 343 | FBgn0039596-FBgn0039599 (3R) | 5 |
| 344 | FBgn0039634-FBgn0039637 (3R) | 5 |
| 345 | FBgn0083969-FBgn0051036 (3R) | 5 |
| 346 | FBgn0025394-FBgn0023530 (X) | 5 |
| 347 | FBgn0040390-FBgn0001150 (X) | 5 |
| 348 | FBgn0086899-FBgn0000542 (X) | 5 |
| 349 | FBgn0029911-FBgn0029915 (X) | 5 |
| 350 | FBgn0003659-FBgn0027342 (X) | 5 |
| 351 | FBgn0085366-FBgn0040923 (X) | 5 |
| 352 | FBgn0014464-FBgn0000360 (X) | 5 |
| 353 | FBgn0030093-FBgn0030096 (X) | 5 |
| 354 | FBgn0052670-FBgn0030257 (X) | 5 |
| 355 | FBgn0030342-FBgn0030346 (X) | 5 |
| 356 | FBgn0030437-FBgn0030439 (X) | 5 |
| 357 | FBgn0030492-FBgn0052631 (X) | 5 |
| 358 | FBgn0030595-FBgn0052591 (X) | 5 |
| 359 | FBgn0030668-FBgn0030672 (X) | 5 |
| 360 | FBgn0030749-FBgn0054015 (X) | 5 |
| 361 | FBgn0030918-FBgn0052547 (X) | 5 |
| 362 | FBgn0030980-FBgn0030984 (X) | 5 |
| 363 | FBgn0027107-FBgn0031104 (X) | 5 |
| 364 | FBgn0083956-FBgn0052523 (X) | 5 |
| 365 | FBgn0031308-FBgn0031312 (2L) | 4 |
| 366 | FBgn0031360-FBgn0027597 (2L) | 4 |
| 367 | FBgn0031403-FBgn0051682 (2L) | 4 |
| 368 | FBgn0031419-FBgn0031422 (2L) | 4 |
| 369 | FBgn0031428-FBgn0011818 (2L) | 4 |
| 370 | FBgn0051694-FBgn0051950 (2L) | 4 |
| 371 | FBgn0031534-FBgn0031537 (2L) | 4 |
| 372 | FBgn0085205-FBgn0031581 (2L) | 4 |
| 373 | FBgn0031608-FBgn0031611 (2L) | 4 |
| 374 | FBgn0028694-FBgn0031682 (2L) | 4 |
| 375 | FBgn0031729-FBgn0054011 (2L) | 4 |
| 376 | FBgn0003980-FBgn0086265 (2L) | 4 |
| 377 | FBgn0031762-FBgn0031765 (2L) | 4 |
| 378 | FBgn0259749-FBgn0031820 (2L) | 4 |
| 379 | FBgn0031837-FBgn0051635 (2L) | 4 |
| 380 | FBgn0031871-FBgn0020616 (2L) | 4 |
| 381 | FBgn0032016-FBgn0032020 (2L) | 4 |
| 382 | FBgn0032039-FBgn0001084 (2L) | 4 |
| 383 | FBgn0032066-FBgn0032069 (2L) | 4 |
| 384 | FBgn0032110-FBgn0085395 (2L) | 4 |
| 385 | FBgn0032125-FBgn0032129 (2L) | 4 |
| 386 | FBgn0032140-FBgn0022720 (2L) | 4 |
| 387 | FBgn0024285-FBgn0032160 (2L) | 4 |
| 388 | FBgn0004419-FBgn0051717 (2L) | 4 |
| 389 | FBgn0032215-FBgn0032218 (2L) | 4 |
| 390 | FBgn0032236-FBgn0051716 (2L) | 4 |
| 391 | FBgn0032295-FBgn0027550 (2L) | 4 |
| 392 | FBgn0051869-FBgn0011676 (2L) | 4 |
| 393 | FBgn0032359-FBgn0003313 (2L) | 4 |
| 394 | FBgn0051862-FBgn0032428 (2L) | 4 |
| 395 | FBgn0085424-FBgn0032442 (2L) | 4 |
| 396 | FBgn0032453-FBgn0032455 (2L) | 4 |
| 397 | FBgn0032465-FBgn0028965 (2L) | 4 |
| 398 | FBgn0024291-FBgn0032477 (2L) | 4 |
| 399 | FBgn0032485-FBgn0032488 (2L) | 4 |
| 400 | FBgn0028540-FBgn0028539 (2L) | 4 |
| 401 | FBgn0028920-FBgn0028532 (2L) | 4 |
| 402 | FBgn0028949-FBgn0028946 (2L) | 4 |
| 403 | FBgn0051806-FBgn0032626 (2L) | 4 |
| 404 | FBgn0023096-FBgn0040989 (2L) | 4 |
| 405 | FBgn0051802-FBgn0032677 (2L) | 4 |
| 406 | FBgn0032754-FBgn0000075 (2L) | 4 |
| 407 | FBgn0032815-FBgn0032819 (2L) | 4 |
| 408 | FBgn0014127-FBgn0045064 (2L) | 4 |
| 409 | FBgn0051675-FBgn0032904 (2L) | 4 |
| 410 | FBgn0026577-FBgn0041244 (2L) | 4 |
| 411 | FBgn0058439-FBgn0000384 (2L) | 4 |
| 412 | FBgn0039969-FBgn0027507 (2R) | 4 |
| 413 | FBgn0033140-FBgn0033142 (2R) | 4 |
| 414 | FBgn0025885-FBgn0033179 (2R) | 4 |
| 415 | FBgn0033182-FBgn0033184 (2R) | 4 |
| 416 | FBgn0033224-FBgn0033226 (2R) | 4 |
| 417 | FBgn0050380-FBgn0033238 (2R) | 4 |
| 418 | FBgn0020279-FBgn0028563 (2R) | 4 |
| 419 | FBgn0033275-FBgn0010109 (2R) | 4 |
| 420 | FBgn0050362-FBgn0033287 (2R) | 4 |
| 421 | FBgn0002570-FBgn0033294 (2R) | 4 |
| 422 | FBgn0033349-FBgn0033352 (2R) | 4 |
| 423 | FBgn0033391-FBgn0033395 (2R) | 4 |
| 424 | FBgn0033404-FBgn0033405 (2R) | 4 |
| 425 | FBgn0033454-FBgn0027580 (2R) | 4 |
| 426 | FBgn0033471-FBgn0033474 (2R) | 4 |
| 427 | FBgn0033475-FBgn0033477 (2R) | 4 |
| 428 | FBgn0033737-FBgn0033739 (2R) | 4 |
| 429 | FBgn0033763-FBgn0050053 (2R) | 4 |
| 430 | FBgn0040754-FBgn0033812 (2R) | 4 |
| 431 | FBgn0013765-FBgn0033842 (2R) | 4 |
| 432 | FBgn0013733-FBgn0013770 (2R) | 4 |
| 433 | FBgn0033918-FBgn0028428 (2R) | 4 |
| 434 | FBgn0019938-FBgn0033945 (2R) | 4 |
| 435 | FBgn0033972-FBgn0022063 (2R) | 4 |
| 436 | FBgn0034005-FBgn0034007 (2R) | 4 |
| 437 | FBgn0034008-FBgn0013750 (2R) | 4 |
| 438 | FBgn0034057-FBgn0034060 (2R) | 4 |
| 439 | FBgn0034070-FBgn0034073 (2R) | 4 |
| 440 | FBgn0034087-FBgn0034091 (2R) | 4 |
| 441 | FBgn0034152-FBgn0053530 (2R) | 4 |
| 442 | FBgn0086350-FBgn0050460 (2R) | 4 |
| 443 | FBgn0021818-FBgn0025716 (2R) | 4 |
| 444 | FBgn0034230-FBgn0022085 (2R) | 4 |
| 445 | FBgn0259211-FBgn0003701 (2R) | 4 |
| 446 | FBgn0000566-FBgn0034371 (2R) | 4 |
| 447 | FBgn0053454-FBgn0003254 (2R) | 4 |
| 448 | FBgn0034456-FBgn0034459 (2R) | 4 |
| 449 | FBgn0011824-FBgn0002736 (2R) | 4 |
| 450 | FBgn0034617-FBgn0050394 (2R) | 4 |
| 451 | FBgn0034639-FBgn0034641 (2R) | 4 |
| 452 | FBgn0034646-FBgn0034650 (2R) | 4 |
| 453 | FBgn0085234-FBgn0029084 (2R) | 4 |
| 454 | FBgn0034748-FBgn0034750 (2R) | 4 |
| 455 | FBgn0034756-FBgn0034759 (2R) | 4 |
| 456 | FBgn0040658-FBgn0005631 (2R) | 4 |
| 457 | FBgn0034774-FBgn0259145 (2R) | 4 |
| 458 | FBgn0086408-FBgn0050274 (2R) | 4 |
| 459 | FBgn0034791-FBgn0034794 (2R) | 4 |
| 460 | FBgn0000241-FBgn0041234 (2R) | 4 |
| 461 | FBgn0050184-FBgn0034882 (2R) | 4 |
| 462 | FBgn0259209-FBgn0034974 (2R) | 4 |
| 463 | FBgn0034986-FBgn0034989 (2R) | 4 |
| 464 | FBgn0053527-FBgn0035031 (2R) | 4 |
| 465 | FBgn0035063-FBgn0041205 (2R) | 4 |
| 466 | FBgn0029514-FBgn0035216 (3L) | 4 |
| 467 | FBgn0028577-FBgn0035229 (3L) | 4 |
| 468 | FBgn0035270-FBgn0035272 (3L) | 4 |
| 469 | FBgn0035279-FBgn0035282 (3L) | 4 |
| 470 | FBgn0035283-FBgn0035285 (3L) | 4 |
| 471 | FBgn0015359-FBgn0000543 (3L) | 4 |
| 472 | FBgn0035336-FBgn0035347 (3L) | 4 |
| 473 | FBgn0053233-FBgn0035366 (3L) | 4 |
| 474 | FBgn0045479-FBgn0052255 (3L) | 4 |
| 475 | FBgn0035499-FBgn0004574 (3L) | 4 |
| 476 | FBgn0035507-FBgn0035510 (3L) | 4 |
| 477 | FBgn0035511-FBgn0035514 (3L) | 4 |
| 478 | FBgn0035523-FBgn0015829 (3L) | 4 |
| 479 | FBgn0052250-FBgn0035534 (3L) | 4 |
| 480 | FBgn0010894-FBgn0040239 (3L) | 4 |
| 481 | FBgn0040298-FBgn0086694 (3L) | 4 |
| 482 | FBgn0035690-FBgn0035691 (3L) | 4 |
| 483 | FBgn0035727-FBgn0035730 (3L) | 4 |
| 484 | FBgn0035746-FBgn0035751 (3L) | 4 |
| 485 | FBgn0024542-FBgn0035754 (3L) | 4 |
| 486 | FBgn0035770-FBgn0082598 (3L) | 4 |
| 487 | FBgn0035795-FBgn0035797 (3L) | 4 |
| 488 | FBgn0035855-FBgn0035857 (3L) | 4 |
| 489 | FBgn0035867-FBgn0087039 (3L) | 4 |
| 490 | FBgn0035876-FBgn0035879 (3L) | 4 |
| 491 | FBgn0020224-FBgn0010825 (3L) | 4 |
| 492 | FBgn0000357-FBgn0000356 (3L) | 4 |
| 493 | FBgn0035929-FBgn0035931 (3L) | 4 |
| 494 | FBgn0035970-FBgn0035978 (3L) | 4 |
| 495 | FBgn0053703-FBgn0036013 (3L) | 4 |
| 496 | FBgn0040823-FBgn0052054 (3L) | 4 |
| 497 | FBgn0052057-FBgn0052055 (3L) | 4 |
| 498 | FBgn0036159-FBgn0036162 (3L) | 4 |
| 499 | FBgn0015828-FBgn0036194 (3L) | 4 |
| 500 | FBgn0036196-FBgn0036199 (3L) | 4 |
| 501 | FBgn0036225-FBgn0036229 (3L) | 4 |
| 502 | FBgn0046296-FBgn0036257 (3L) | 4 |
| 503 | FBgn0036290-FBgn0027936 (3L) | 4 |
| 504 | FBgn0036343-FBgn0036346 (3L) | 4 |
| 505 | FBgn0052121-FBgn0036360 (3L) | 4 |
| 506 | FBgn0026376-FBgn0052137 (3L) | 4 |
| 507 | FBgn0036466-FBgn0036469 (3L) | 4 |
| 508 | FBgn0036500-FBgn0036502 (3L) | 4 |
| 509 | FBgn0036518-FBgn0036522 (3L) | 4 |
| 510 | FBgn0004588-FBgn0004591 (3L) | 4 |
| 511 | FBgn0052150-FBgn0036544 (3L) | 4 |
| 512 | FBgn0036570-FBgn0036571 (3L) | 4 |
| 513 | FBgn0036588-FBgn0040797 (3L) | 4 |
| 514 | FBgn0036747-FBgn0052182 (3L) | 4 |
| 515 | FBgn0036773-FBgn0002901 (3L) | 4 |
| 516 | FBgn0036781-FBgn0036783 (3L) | 4 |
| 517 | FBgn0036807-FBgn0036810 (3L) | 4 |
| 518 | FBgn0036826-FBgn0036828 (3L) | 4 |
| 519 | FBgn0036853-FBgn0017578 (3L) | 4 |
| 520 | FBgn0036878-FBgn0036881 (3L) | 4 |
| 521 | FBgn0001247-FBgn0011205 (3L) | 4 |
| 522 | FBgn0037008-FBgn0037011 (3L) | 4 |
| 523 | FBgn0037036-FBgn0037039 (3L) | 4 |
| 524 | FBgn0037040-FBgn0037042 (3L) | 4 |
| 525 | FBgn0037122-FBgn0037125 (3L) | 4 |
| 526 | FBgn0037149-FBgn0037151 (3L) | 4 |
| 527 | FBgn0037184-FBgn0052451 (3L) | 4 |
| 528 | FBgn0037195-FBgn0037197 (3L) | 4 |
| 529 | FBgn0037205-FBgn0052461 (3L) | 4 |
| 530 | FBgn0037234-FBgn0037236 (3R) | 4 |
| 531 | FBgn0037239-FBgn0037241 (3R) | 4 |
| 532 | FBgn0037242-FBgn0037245 (3R) | 4 |
| 533 | FBgn0037248-FBgn0037252 (3R) | 4 |
| 534 | FBgn0051526-FBgn0052945 (3R) | 4 |
| 535 | FBgn0051534-FBgn0026620 (3R) | 4 |
| 536 | FBgn0037283-FBgn0020280 (3R) | 4 |
| 537 | FBgn0037297-FBgn0037301 (3R) | 4 |
| 538 | FBgn0037312-FBgn0037315 (3R) | 4 |
| 539 | FBgn0037359-FBgn0010772 (3R) | 4 |
| 540 | FBgn0026563-FBgn0037464 (3R) | 4 |
| 541 | FBgn0037541-FBgn0010812 (3R) | 4 |
| 542 | FBgn0037608-FBgn0037611 (3R) | 4 |
| 543 | FBgn0037612-FBgn0037615 (3R) | 4 |
| 544 | FBgn0037617-FBgn0037620 (3R) | 4 |
| 545 | FBgn0083971-FBgn0037627 (3R) | 4 |
| 546 | FBgn0037646-FBgn0037648 (3R) | 4 |
| 547 | FBgn0014018-FBgn0037659 (3R) | 4 |
| 548 | FBgn0037660-FBgn0003165 (3R) | 4 |
| 549 | FBgn0037762-FBgn0037765 (3R) | 4 |
| 550 | FBgn0037797-FBgn0037802 (3R) | 4 |
| 551 | FBgn0037873-FBgn0086472 (3R) | 4 |
| 552 | FBgn0037892-FBgn0037894 (3R) | 4 |
| 553 | FBgn0037897-FBgn0037901 (3R) | 4 |
| 554 | FBgn0037918-FBgn0037922 (3R) | 4 |
| 555 | FBgn0037921-FBgn0037924 (3R) | 4 |
| 556 | FBgn0037926-FBgn0037931 (3R) | 4 |
| 557 | FBgn0037978-FBgn0037981 (3R) | 4 |
| 558 | FBgn0038090-FBgn0038097 (3R) | 4 |
| 559 | FBgn0038131-FBgn0038134 (3R) | 4 |
| 560 | FBgn0020510-FBgn0003276 (3R) | 4 |
| 561 | FBgn0051320-FBgn0038233 (3R) | 4 |
| 562 | FBgn0038280-FBgn0038285 (3R) | 4 |
| 563 | FBgn0038312-FBgn0027378 (3R) | 4 |
| 564 | FBgn0051291-FBgn0038386 (3R) | 4 |
| 565 | FBgn0038402-FBgn0038405 (3R) | 4 |
| 566 | FBgn0038447-FBgn0038448 (3R) | 4 |
| 567 | FBgn0038505-FBgn0038508 (3R) | 4 |
| 568 | FBgn0038547-FBgn0038550 (3R) | 4 |
| 569 | FBgn0038566-FBgn0038568 (3R) | 4 |
| 570 | FBgn0038588-FBgn0053520 (3R) | 4 |
| 571 | FBgn0024329-FBgn0038652 (3R) | 4 |
| 572 | FBgn0038654-FBgn0038656 (3R) | 4 |
| 573 | FBgn0038716-FBgn0038719 (3R) | 4 |
| 574 | FBgn0038854-FBgn0028468 (3R) | 4 |
| 575 | FBgn0038861-FBgn0025803 (3R) | 4 |
| 576 | FBgn0038868-FBgn0038871 (3R) | 4 |
| 577 | FBgn0259237-FBgn0038903 (3R) | 4 |
| 578 | FBgn0085406-FBgn0038942 (3R) | 4 |
| 579 | FBgn0038948-FBgn0038951 (3R) | 4 |
| 580 | FBgn0039055-FBgn0051457 (3R) | 4 |
| 581 | FBgn0039164-FBgn0026257 (3R) | 4 |
| 582 | FBgn0259685-FBgn0039182 (3R) | 4 |
| 583 | FBgn0039258-FBgn0039260 (3R) | 4 |
| 584 | FBgn0039271-FBgn0039274 (3R) | 4 |
| 585 | FBgn0039348-FBgn0027508 (3R) | 4 |
| 586 | FBgn0027865-FBgn0039385 (3R) | 4 |
| 587 | FBgn0039386-FBgn0051089 (3R) | 4 |
| 588 | FBgn0039395-FBgn0039398 (3R) | 4 |
| 589 | FBgn0259146-FBgn0002735 (3R) | 4 |
| 590 | FBgn0001139-FBgn0039405 (3R) | 4 |
| 591 | FBgn0039510-FBgn0039518 (3R) | 4 |
| 592 | FBgn0004387-FBgn0040080 (3R) | 4 |
| 593 | FBgn0005659-FBgn0039552 (3R) | 4 |
| 594 | FBgn0002865-FBgn0039577 (3R) | 4 |
| 595 | FBgn0039582-FBgn0051051 (3R) | 4 |
| 596 | FBgn0039639-FBgn0039642 (3R) | 4 |
| 597 | FBgn0015129-FBgn0039677 (3R) | 4 |
| 598 | FBgn0039698-FBgn0039702 (3R) | 4 |
| 599 | FBgn0039764-FBgn0039767 (3R) | 4 |
| 600 | FBgn0025633-FBgn0026879 (X) | 4 |
| 601 | FBgn0029587-FBgn0029588 (X) | 4 |
| 602 | FBgn0023527-FBgn0023526 (X) | 4 |
| 603 | FBgn0040356-FBgn0040354 (X) | 4 |
| 604 | FBgn0028369-FBgn0004647 (X) | 4 |
| 605 | FBgn0029667-FBgn0052792 (X) | 4 |
| 606 | FBgn0029685-FBgn0029689 (X) | 4 |
| 607 | FBgn0029696-FBgn0052774 (X) | 4 |
| 608 | FBgn0029710-FBgn0029713 (X) | 4 |
| 609 | FBgn0052772-FBgn0029738 (X) | 4 |
| 610 | FBgn0053980-FBgn0250904 (X) | 4 |
| 611 | FBgn0029946-FBgn0029949 (X) | 4 |
| 612 | FBgn0052719-FBgn0029952 (X) | 4 |
| 613 | FBgn0029999-FBgn0030004 (X) | 4 |
| 614 | FBgn0030014-FBgn0030017 (X) | 4 |
| 615 | FBgn0030018-FBgn0017566 (X) | 4 |
| 616 | FBgn0041629-FBgn0030063 (X) | 4 |
| 617 | FBgn0030081-FBgn0052708 (X) | 4 |
| 618 | FBgn0030088-FBgn0030092 (X) | 4 |
| 619 | FBgn0000077-FBgn0028292 (X) | 4 |
| 620 | FBgn0083940-FBgn0030182 (X) | 4 |
| 621 | FBgn0030200-FBgn0030204 (X) | 4 |
| 622 | FBgn0030240-FBgn0030243 (X) | 4 |
| 623 | FBgn0030277-FBgn0030283 (X) | 4 |
| 624 | FBgn0030296-FBgn0030298 (X) | 4 |
| 625 | FBgn0030322-FBgn0030323 (X) | 4 |
| 626 | FBgn0030327-FBgn0030330 (X) | 4 |
| 627 | FBgn0000259-FBgn0052662 (X) | 4 |
| 628 | FBgn0030528-FBgn0030530 (X) | 4 |
| 629 | FBgn0030532-FBgn0085375 (X) | 4 |
| 630 | FBgn0030552-FBgn0030554 (X) | 4 |
| 631 | FBgn0030673-FBgn0030676 (X) | 4 |
| 632 | FBgn0030743-FBgn0005411 (X) | 4 |
| 633 | FBgn0030774-FBgn0030777 (X) | 4 |
| 634 | FBgn0052570-FBgn0052574 (X) | 4 |
| 635 | FBgn0030787-FBgn0030790 (X) | 4 |
| 636 | FBgn0030829-FBgn0030832 (X) | 4 |
| 637 | FBgn0030869-FBgn0030872 (X) | 4 |
| 638 | FBgn0030963-FBgn0030966 (X) | 4 |
| 639 | FBgn0030970-FBgn0030974 (X) | 4 |
| 640 | FBgn0030995-FBgn0027603 (X) | 4 |
| 641 | FBgn0044030-FBgn0031021 (X) | 4 |
| 642 | FBgn0031082-FBgn0031086 (X) | 4 |
| 643 | FBgn0031114-FBgn0002641 (X) | 4 |
| 644 | FBgn0000709-FBgn0011710 (X) | 4 |
| 645 | FBgn0031187-FBgn0031190 (X) | 4 |
| 646 | FBgn0031208-FBgn0051973 (2L) | 3 |
| 647 | FBgn0031216-FBgn0026787 (2L) | 3 |
| 648 | FBgn0031227-FBgn0053635 (2L) | 3 |
| 649 | FBgn0031248-FBgn0053127 (2L) | 3 |
| 650 | FBgn0086130-FBgn0031282 (2L) | 3 |
| 651 | FBgn0029095-FBgn0053526 (2L) | 3 |
| 652 | FBgn0031294-FBgn0015905 (2L) | 3 |
| 653 | FBgn0031299-FBgn0031302 (2L) | 3 |
| 654 | FBgn0031337-FBgn0051928 (2L) | 3 |
| 655 | FBgn0031357-FBgn0031359 (2L) | 3 |
| 656 | FBgn0031410-FBgn0051949 (2L) | 3 |
| 657 | FBgn0031457-FBgn0026324 (2L) | 3 |
| 658 | FBgn0031498-FBgn0031500 (2L) | 3 |
| 659 | FBgn0015600-FBgn0011648 (2L) | 3 |
| 660 | FBgn0024244-FBgn0002985 (2L) | 3 |
| 661 | FBgn0031591-FBgn0010473 (2L) | 3 |
| 662 | FBgn0031596-FBgn0031597 (2L) | 3 |
| 663 | FBgn0031653-FBgn0020906 (2L) | 3 |
| 664 | FBgn0016075-FBgn0031673 (2L) | 3 |
| 665 | FBgn0016660-FBgn0031707 (2L) | 3 |
| 666 | FBgn0031738-FBgn0031740 (2L) | 3 |
| 667 | FBgn0031746-FBgn0002855 (2L) | 3 |
| 668 | FBgn0031749-FBgn0015381 (2L) | 3 |
| 669 | FBgn0031756-FBgn0031758 (2L) | 3 |
| 670 | FBgn0000308-FBgn0031759 (2L) | 3 |
| 671 | FBgn0031772-FBgn0031774 (2L) | 3 |
| 672 | FBgn0000392-FBgn0028990 (2L) | 3 |
| 673 | FBgn0042092-FBgn0011737 (2L) | 3 |
| 674 | FBgn0026196-FBgn0004838 (2L) | 3 |
| 675 | FBgn0031874-FBgn0031876 (2L) | 3 |
| 676 | FBgn0051630-FBgn0051907 (2L) | 3 |
| 677 | FBgn0031887-FBgn0085407 (2L) | 3 |
| 678 | FBgn0031897-FBgn0259111 (2L) | 3 |
| 679 | FBgn0031926-FBgn0040299 (2L) | 3 |
| 680 | FBgn0031947-FBgn0025687 (2L) | 3 |
| 681 | FBgn0031951-FBgn0027515 (2L) | 3 |
| 682 | FBgn0031968-FBgn0031969 (2L) | 3 |
| 683 | FBgn0031972-FBgn0031974 (2L) | 3 |
| 684 | FBgn0031996-FBgn0031998 (2L) | 3 |
| 685 | FBgn0032008-FBgn0003502 (2L) | 3 |
| 686 | FBgn0027780-FBgn0029173 (2L) | 3 |
| 687 | FBgn0052984-FBgn0032083 (2L) | 3 |
| 688 | FBgn0032105-FBgn0010314 (2L) | 3 |
| 689 | FBgn0032153-FBgn0010520 (2L) | 3 |
| 690 | FBgn0032163-FBgn0259713 (2L) | 3 |
| 691 | FBgn0032175-FBgn0053302 (2L) | 3 |
| 692 | FBgn0005322-FBgn0032214 (2L) | 3 |
| 693 | FBgn0032225-FBgn0032228 (2L) | 3 |
| 694 | FBgn0026431-FBgn0032259 (2L) | 3 |
| 695 | FBgn0032268-FBgn0032271 (2L) | 3 |
| 696 | FBgn0051872-FBgn0032275 (2L) | 3 |
| 697 | FBgn0032276-FBgn0040496 (2L) | 3 |
| 698 | FBgn0032280-FBgn0032282 (2L) | 3 |
| 699 | FBgn0024740-FBgn0032291 (2L) | 3 |
| 700 | FBgn0026148-FBgn0000146 (2L) | 3 |
| 701 | FBgn0021761-FBgn0250837 (2L) | 3 |
| 702 | FBgn0032339-FBgn0032343 (2L) | 3 |
| 703 | FBgn0032346-FBgn0032348 (2L) | 3 |
| 704 | FBgn0032361-FBgn0032363 (2L) | 3 |
| 705 | FBgn0032420-FBgn0032422 (2L) | 3 |
| 706 | FBgn0040972-FBgn0032473 (2L) | 3 |
| 707 | FBgn0051728-FBgn0051730 (2L) | 3 |
| 708 | FBgn0032503-FBgn0032504 (2L) | 3 |
| 709 | FBgn0032509-FBgn0051855 (2L) | 3 |
| 710 | FBgn0032522-FBgn0026760 (2L) | 3 |
| 711 | FBgn0028848-FBgn0028847 (2L) | 3 |
| 712 | FBgn0001961-FBgn0028509 (2L) | 3 |
| 713 | FBgn0028519-FBgn0028904 (2L) | 3 |
| 714 | FBgn0052971-FBgn0028531 (2L) | 3 |
| 715 | FBgn0028870-FBgn0028868 (2L) | 3 |
| 716 | FBgn0028896-FBgn0001992 (2L) | 3 |
| 717 | FBgn0028375-FBgn0028894 (2L) | 3 |
| 718 | FBgn0015338-FBgn0000339 (2L) | 3 |
| 719 | FBgn0000250-FBgn0001995 (2L) | 3 |
| 720 | FBgn0040984-FBgn0259151 (2L) | 3 |
| 721 | FBgn0032597-FBgn0032598 (2L) | 3 |
| 722 | FBgn0051741-FBgn0032664 (2L) | 3 |
| 723 | FBgn0032681-FBgn0032683 (2L) | 3 |
| 724 | FBgn0032693-FBgn0086681 (2L) | 3 |
| 725 | FBgn0032707-FBgn0045852 (2L) | 3 |
| 726 | FBgn0032713-FBgn0015808 (2L) | 3 |
| 727 | FBgn0051797-FBgn0032769 (2L) | 3 |
| 728 | FBgn0041789-FBgn0032782 (2L) | 3 |
| 729 | FBgn0003346-FBgn0003732 (2L) | 3 |
| 730 | FBgn0032790-FBgn0032793 (2L) | 3 |
| 731 | FBgn0051697-FBgn0032804 (2L) | 3 |
| 732 | FBgn0032845-FBgn0032847 (2L) | 3 |
| 733 | FBgn0032851-FBgn0040996 (2L) | 3 |
| 734 | FBgn0003475-FBgn0015803 (2L) | 3 |
| 735 | FBgn0051688-FBgn0032863 (2L) | 3 |
| 736 | FBgn0054007-FBgn0032867 (2L) | 3 |
| 737 | FBgn0014859-FBgn0032879 (2L) | 3 |
| 738 | FBgn0032974-FBgn0032979 (2L) | 3 |
| 739 | FBgn0046692-FBgn0040005 (2R) | 3 |
| 740 | FBgn0039994-FBgn0033000 (2R) | 3 |
| 741 | FBgn0085241-FBgn0033056 (2R) | 3 |
| 742 | FBgn0033074-FBgn0033076 (2R) | 3 |
| 743 | FBgn0033088-FBgn0033089 (2R) | 3 |
| 744 | FBgn0027066-FBgn0033101 (2R) | 3 |
| 745 | FBgn0033104-FBgn0028954 (2R) | 3 |
| 746 | FBgn0033107-FBgn0040674 (2R) | 3 |
| 747 | FBgn0066293-FBgn0053558 (2R) | 3 |
| 748 | FBgn0029507-FBgn0033127 (2R) | 3 |
| 749 | FBgn0033128-FBgn0033130 (2R) | 3 |
| 750 | FBgn0033135-FBgn0033137 (2R) | 3 |
| 751 | FBgn0033149-FBgn0026389 (2R) | 3 |
| 752 | FBgn0033153-FBgn0033155 (2R) | 3 |
| 753 | FBgn0003174-FBgn0033159 (2R) | 3 |
| 754 | FBgn0025185-FBgn0033186 (2R) | 3 |
| 755 | FBgn0050499-FBgn0033194 (2R) | 3 |
| 756 | FBgn0033204-FBgn0033206 (2R) | 3 |
| 757 | FBgn0040780-FBgn0050377 (2R) | 3 |
| 758 | FBgn0027548-FBgn0033244 (2R) | 3 |
| 759 | FBgn0027054-FBgn0033247 (2R) | 3 |
| 760 | FBgn0025360-FBgn0040778 (2R) | 3 |
| 761 | FBgn0028562-FBgn0033257 (2R) | 3 |
| 762 | FBgn0033272-FBgn0021995 (2R) | 3 |
| 763 | FBgn0050360-FBgn0033297 (2R) | 3 |
| 764 | FBgn0259226-FBgn0033308 (2R) | 3 |
| 765 | FBgn0033330-FBgn0050350 (2R) | 3 |
| 766 | FBgn0033353-FBgn0033355 (2R) | 3 |
| 767 | FBgn0027607-FBgn0033357 (2R) | 3 |
| 768 | FBgn0033388-FBgn0259728 (2R) | 3 |
| 769 | FBgn0033431-FBgn0050338 (2R) | 3 |
| 770 | FBgn0033451-FBgn0033453 (2R) | 3 |
| 771 | FBgn0011656-FBgn0033467 (2R) | 3 |
| 772 | FBgn0001291-FBgn0033486 (2R) | 3 |
| 773 | FBgn0033490-FBgn0033491 (2R) | 3 |
| 774 | FBgn0085250-FBgn0000448 (2R) | 3 |
| 775 | FBgn0033498-FBgn0033500 (2R) | 3 |
| 776 | FBgn0033543-FBgn0024956 (2R) | 3 |
| 777 | FBgn0053144-FBgn0033547 (2R) | 3 |
| 778 | FBgn0033554-FBgn0033557 (2R) | 3 |
| 779 | FBgn0016047-FBgn0003382 (2R) | 3 |
| 780 | FBgn0003863-FBgn0010357 (2R) | 3 |
| 781 | FBgn0001269-FBgn0000577 (2R) | 3 |
| 782 | FBgn0033650-FBgn0033651 (2R) | 3 |
| 783 | FBgn0026573-FBgn0050037 (2R) | 3 |
| 784 | FBgn0050203-FBgn0033712 (2R) | 3 |
| 785 | FBgn0033713-FBgn0033715 (2R) | 3 |
| 786 | FBgn0050047-FBgn0050043 (2R) | 3 |
| 787 | FBgn0053013-FBgn0033720 (2R) | 3 |
| 788 | FBgn0033726-FBgn0050048 (2R) | 3 |
| 789 | FBgn0050050-FBgn0033732 (2R) | 3 |
| 790 | FBgn0050056-FBgn0033753 (2R) | 3 |
| 791 | FBgn0011227-FBgn0086904 (2R) | 3 |
| 792 | FBgn0250842-FBgn0050486 (2R) | 3 |
| 793 | FBgn0028991-FBgn0033806 (2R) | 3 |
| 794 | FBgn0033808-FBgn0033810 (2R) | 3 |
| 795 | FBgn0004638-FBgn0024556 (2R) | 3 |
| 796 | FBgn0033848-FBgn0033851 (2R) | 3 |
| 797 | FBgn0033890-FBgn0002643 (2R) | 3 |
| 798 | FBgn0033903-FBgn0033905 (2R) | 3 |
| 799 | FBgn0033907-FBgn0050069 (2R) | 3 |
| 800 | FBgn0033935-FBgn0013725 (2R) | 3 |
| 801 | FBgn0033953-FBgn0033955 (2R) | 3 |
| 802 | FBgn0033987-FBgn0033989 (2R) | 3 |
| 803 | FBgn0034000-FBgn0034002 (2R) | 3 |
| 804 | FBgn0085394-FBgn0050470 (2R) | 3 |
| 805 | FBgn0050467-FBgn0034030 (2R) | 3 |
| 806 | FBgn0034032-FBgn0013762 (2R) | 3 |
| 807 | FBgn0034033-FBgn0034035 (2R) | 3 |
| 808 | FBgn0034084-FBgn0034086 (2R) | 3 |
| 809 | FBgn0034092-FBgn0034095 (2R) | 3 |
| 810 | FBgn0085428-FBgn0050096 (2R) | 3 |
| 811 | FBgn0034129-FBgn0053458 (2R) | 3 |
| 812 | FBgn0024232-FBgn0024319 (2R) | 3 |
| 813 | FBgn0011743-FBgn0034157 (2R) | 3 |
| 814 | FBgn0025827-FBgn0034165 (2R) | 3 |
| 815 | FBgn0085444-FBgn0034172 (2R) | 3 |
| 816 | FBgn0034179-FBgn0034181 (2R) | 3 |
| 817 | FBgn0034201-FBgn0050458 (2R) | 3 |
| 818 | FBgn0028953-FBgn0022987 (2R) | 3 |
| 819 | FBgn0034272-FBgn0034274 (2R) | 3 |
| 820 | FBgn0026309-FBgn0028744 (2R) | 3 |
| 821 | FBgn0053958-FBgn0000658 (2R) | 3 |
| 822 | FBgn0063494-FBgn0063491 (2R) | 3 |
| 823 | FBgn0034399-FBgn0034401 (2R) | 3 |
| 824 | FBgn0014269-FBgn0034410 (2R) | 3 |
| 825 | FBgn0012051-FBgn0040273 (2R) | 3 |
| 826 | FBgn0034481-FBgn0034484 (2R) | 3 |
| 827 | FBgn0085228-FBgn0034499 (2R) | 3 |
| 828 | FBgn0034505-FBgn0042198 (2R) | 3 |
| 829 | FBgn0034513-FBgn0034515 (2R) | 3 |
| 830 | FBgn0034527-FBgn0034529 (2R) | 3 |
| 831 | FBgn0250850-FBgn0034537 (2R) | 3 |
| 832 | FBgn0034568-FBgn0034570 (2R) | 3 |
| 833 | FBgn0001133-FBgn0034573 (2R) | 3 |
| 834 | FBgn0034583-FBgn0034585 (2R) | 3 |
| 835 | FBgn0050389-FBgn0034599 (2R) | 3 |
| 836 | FBgn0034602-FBgn0027073 (2R) | 3 |
| 837 | FBgn0085426-FBgn0050393 (2R) | 3 |
| 838 | FBgn0034622-FBgn0034624 (2R) | 3 |
| 839 | FBgn0034634-FBgn0050289 (2R) | 3 |
| 840 | FBgn0054040-FBgn0034664 (2R) | 3 |
| 841 | FBgn0034710-FBgn0034712 (2R) | 3 |
| 842 | FBgn0034726-FBgn0034728 (2R) | 3 |
| 843 | FBgn0034730-FBgn0034732 (2R) | 3 |
| 844 | FBgn0034734-FBgn0034736 (2R) | 3 |
| 845 | FBgn0050195-FBgn0034755 (2R) | 3 |
| 846 | FBgn0004896-FBgn0034789 (2R) | 3 |
| 847 | FBgn0085453-FBgn0034853 (2R) | 3 |
| 848 | FBgn0034893-FBgn0034897 (2R) | 3 |
| 849 | FBgn0010278-FBgn0028411 (2R) | 3 |
| 850 | FBgn0034911-FBgn0003401 (2R) | 3 |
| 851 | FBgn0025335-FBgn0025352 (2R) | 3 |
| 852 | FBgn0011236-FBgn0025336 (2R) | 3 |
| 853 | FBgn0034922-FBgn0034924 (2R) | 3 |
| 854 | FBgn0034938-FBgn0034940 (2R) | 3 |
| 855 | FBgn0025806-FBgn0034971 (2R) | 3 |
| 856 | FBgn0086129-FBgn0034975 (2R) | 3 |
| 857 | FBgn0034976-FBgn0034978 (2R) | 3 |
| 858 | FBgn0004101-FBgn0035001 (2R) | 3 |
| 859 | FBgn0029501-FBgn0035014 (2R) | 3 |
| 860 | FBgn0035035-FBgn0259742 (2R) | 3 |
| 861 | FBgn0085434-FBgn0035078 (2R) | 3 |
| 862 | FBgn0035107-FBgn0025592 (3L) | 3 |
| 863 | FBgn0027587-FBgn0035110 (3L) | 3 |
| 864 | FBgn0024806-FBgn0035122 (3L) | 3 |
| 865 | FBgn0004373-FBgn0052343 (3L) | 3 |
| 866 | FBgn0035210-FBgn0035213 (3L) | 3 |
| 867 | FBgn0035236-FBgn0035238 (3L) | 3 |
| 868 | FBgn0035333-FBgn0035335 (3L) | 3 |
| 869 | FBgn0035420-FBgn0035422 (3L) | 3 |
| 870 | FBgn0035436-FBgn0035437 (3L) | 3 |
| 871 | FBgn0035461-FBgn0052262 (3L) | 3 |
| 872 | FBgn0035477-FBgn0035479 (3L) | 3 |
| 873 | FBgn0042179-FBgn0052259 (3L) | 3 |
| 874 | FBgn0041630-FBgn0035529 (3L) | 3 |
| 875 | FBgn0035540-FBgn0035542 (3L) | 3 |
| 876 | FBgn0035545-FBgn0035547 (3L) | 3 |
| 877 | FBgn0052248-FBgn0035551 (3L) | 3 |
| 878 | FBgn0052237-FBgn0035581 (3L) | 3 |
| 879 | FBgn0016756-FBgn0035631 (3L) | 3 |
| 880 | FBgn0052413-FBgn0035648 (3L) | 3 |
| 881 | FBgn0035685-FBgn0035687 (3L) | 3 |
| 882 | FBgn0041625-FBgn0041623 (3L) | 3 |
| 883 | FBgn0035713-FBgn0035715 (3L) | 3 |
| 884 | FBgn0023001-FBgn0040837 (3L) | 3 |
| 885 | FBgn0035779-FBgn0035781 (3L) | 3 |
| 886 | FBgn0053278-FBgn0035786 (3L) | 3 |
| 887 | FBgn0035788-FBgn0035790 (3L) | 3 |
| 888 | FBgn0035799-FBgn0035802 (3L) | 3 |
| 889 | FBgn0035813-FBgn0035816 (3L) | 3 |
| 890 | FBgn0027549-FBgn0026252 (3L) | 3 |
| 891 | FBgn0042112-FBgn0010350 (3L) | 3 |
| 892 | FBgn0035873-FBgn0035875 (3L) | 3 |
| 893 | FBgn0052029-FBgn0035922 (3L) | 3 |
| 894 | FBgn0052024-FBgn0035933 (3L) | 3 |
| 895 | FBgn0035966-FBgn0004244 (3L) | 3 |
| 896 | FBgn0002528-FBgn0036020 (3L) | 3 |
| 897 | FBgn0036035-FBgn0036038 (3L) | 3 |
| 898 | FBgn0025866-FBgn0036059 (3L) | 3 |
| 899 | FBgn0036062-FBgn0036063 (3L) | 3 |
| 900 | FBgn0054050-FBgn0013469 (3L) | 3 |
| 901 | FBgn0036117-FBgn0052069 (3L) | 3 |
| 902 | FBgn0052076-FBgn0036142 (3L) | 3 |
| 903 | FBgn0036147-FBgn0064766 (3L) | 3 |
| 904 | FBgn0053490-FBgn0052086 (3L) | 3 |
| 905 | FBgn0036187-FBgn0052085 (3L) | 3 |
| 906 | FBgn0052088-FBgn0041231 (3L) | 3 |
| 907 | FBgn0052106-FBgn0036311 (3L) | 3 |
| 908 | FBgn0036335-FBgn0036337 (3L) | 3 |
| 909 | FBgn0036361-FBgn0036363 (3L) | 3 |
| 910 | FBgn0036364-FBgn0036366 (3L) | 3 |
| 911 | FBgn0052132-FBgn0085429 (3L) | 3 |
| 912 | FBgn0027375-FBgn0259175 (3L) | 3 |
| 913 | FBgn0047338-FBgn0025776 (3L) | 3 |
| 914 | FBgn0036487-FBgn0036489 (3L) | 3 |
| 915 | FBgn0036578-FBgn0036581 (3L) | 3 |
| 916 | FBgn0040794-FBgn0053257 (3L) | 3 |
| 917 | FBgn0085278-FBgn0036618 (3L) | 3 |
| 918 | FBgn0036626-FBgn0036628 (3L) | 3 |
| 919 | FBgn0036629-FBgn0004569 (3L) | 3 |
| 920 | FBgn0036637-FBgn0036639 (3L) | 3 |
| 921 | FBgn0036641-FBgn0250814 (3L) | 3 |
| 922 | FBgn0036643-FBgn0003741 (3L) | 3 |
| 923 | FBgn0036666-FBgn0036668 (3L) | 3 |
| 924 | FBgn0036684-FBgn0036686 (3L) | 3 |
| 925 | FBgn0036691-FBgn0036696 (3L) | 3 |
| 926 | FBgn0036729-FBgn0036731 (3L) | 3 |
| 927 | FBgn0036757-FBgn0052189 (3L) | 3 |
| 928 | FBgn0036759-FBgn0036761 (3L) | 3 |
| 929 | FBgn0015946-FBgn0036786 (3L) | 3 |
| 930 | FBgn0036789-FBgn0036790 (3L) | 3 |
| 931 | FBgn0036804-FBgn0036805 (3L) | 3 |
| 932 | FBgn0036838-FBgn0002945 (3L) | 3 |
| 933 | FBgn0036846-FBgn0036848 (3L) | 3 |
| 934 | FBgn0036851-FBgn0016797 (3L) | 3 |
| 935 | FBgn0036859-FBgn0036860 (3L) | 3 |
| 936 | FBgn0036871-FBgn0036874 (3L) | 3 |
| 937 | FBgn0036889-FBgn0010417 (3L) | 3 |
| 938 | FBgn0036896-FBgn0022774 (3L) | 3 |
| 939 | FBgn0036923-FBgn0036925 (3L) | 3 |
| 940 | FBgn0036936-FBgn0036938 (3L) | 3 |
| 941 | FBgn0005198-FBgn0036942 (3L) | 3 |
| 942 | FBgn0052228-FBgn0052227 (3L) | 3 |
| 943 | FBgn0036964-FBgn0052226 (3L) | 3 |
| 944 | FBgn0036969-FBgn0036970 (3L) | 3 |
| 945 | FBgn0036977-FBgn0036979 (3L) | 3 |
| 946 | FBgn0052428-FBgn0036999 (3L) | 3 |
| 947 | FBgn0037003-FBgn0037005 (3L) | 3 |
| 948 | FBgn0037026-FBgn0037028 (3L) | 3 |
| 949 | FBgn0053054-FBgn0053056 (3L) | 3 |
| 950 | FBgn0037081-FBgn0037082 (3L) | 3 |
| 951 | FBgn0014143-FBgn0037086 (3L) | 3 |
| 952 | FBgn0037099-FBgn0037101 (3L) | 3 |
| 953 | FBgn0014362-FBgn0037142 (3L) | 3 |
| 954 | FBgn0028500-FBgn0037156 (3L) | 3 |
| 955 | FBgn0052452-FBgn0016034 (3L) | 3 |
| 956 | FBgn0052944-FBgn0051525 (3R) | 3 |
| 957 | FBgn0037344-FBgn0014366 (3R) | 3 |
| 958 | FBgn0051548-FBgn0037358 (3R) | 3 |
| 959 | FBgn0037363-FBgn0037365 (3R) | 3 |
| 960 | FBgn0037382-FBgn0037384 (3R) | 3 |
| 961 | FBgn0029088-FBgn0037391 (3R) | 3 |
| 962 | FBgn0037397-FBgn0037399 (3R) | 3 |
| 963 | FBgn0014001-FBgn0037440 (3R) | 3 |
| 964 | FBgn0010282-FBgn0037443 (3R) | 3 |
| 965 | FBgn0002522-FBgn0004777 (3R) | 3 |
| 966 | FBgn0004778-FBgn0004781 (3R) | 3 |
| 967 | FBgn0000439-FBgn0001077 (3R) | 3 |
| 968 | FBgn0051493-FBgn0037477 (3R) | 3 |
| 969 | FBgn0037486-FBgn0037488 (3R) | 3 |
| 970 | FBgn0037489-FBgn0037491 (3R) | 3 |
| 971 | FBgn0037493-FBgn0259172 (3R) | 3 |
| 972 | FBgn0051473-FBgn0037525 (3R) | 3 |
| 973 | FBgn0037530-FBgn0014930 (3R) | 3 |
| 974 | FBgn0037563-FBgn0051463 (3R) | 3 |
| 975 | FBgn0037566-FBgn0037569 (3R) | 3 |
| 976 | FBgn0037570-FBgn0037572 (3R) | 3 |
| 977 | FBgn0037636-FBgn0037637 (3R) | 3 |
| 978 | FBgn0037652-FBgn0037653 (3R) | 3 |
| 979 | FBgn0037687-FBgn0037689 (3R) | 3 |
| 980 | FBgn0037697-FBgn0043791 (3R) | 3 |
| 981 | FBgn0014380-FBgn0003205 (3R) | 3 |
| 982 | FBgn0037718-FBgn0037720 (3R) | 3 |
| 983 | FBgn0051352-FBgn0016792 (3R) | 3 |
| 984 | FBgn0037743-FBgn0028401 (3R) | 3 |
| 985 | FBgn0037747-FBgn0037749 (3R) | 3 |
| 986 | FBgn0051410-FBgn0037783 (3R) | 3 |
| 987 | FBgn0037792-FBgn0037794 (3R) | 3 |
| 988 | FBgn0051391-FBgn0037824 (3R) | 3 |
| 989 | FBgn0037846-FBgn0037848 (3R) | 3 |
| 990 | FBgn0037857-FBgn0037860 (3R) | 3 |
| 991 | FBgn0037862-FBgn0040257 (3R) | 3 |
| 992 | FBgn0040250-FBgn0037870 (3R) | 3 |
| 993 | FBgn0025701-FBgn0037916 (3R) | 3 |
| 994 | FBgn0037958-FBgn0037960 (3R) | 3 |
| 995 | FBgn0038037-FBgn0038039 (3R) | 3 |
| 996 | FBgn0086134-FBgn0038049 (3R) | 3 |
| 997 | FBgn0051347-FBgn0038079 (3R) | 3 |
| 998 | FBgn0038106-FBgn0038108 (3R) | 3 |
| 999 | FBgn0038110-FBgn0038111 (3R) | 3 |
| 1000 | FBgn0038113-FBgn0003450 (3R) | 3 |
| 1001 | FBgn0040551-FBgn0038129 (3R) | 3 |
| 1002 | FBgn0038139-FBgn0045442 (3R) | 3 |
| 1003 | FBgn0038142-FBgn0259831 (3R) | 3 |
| 1004 | FBgn0004587-FBgn0038167 (3R) | 3 |
| 1005 | FBgn0038217-FBgn0038218 (3R) | 3 |
| 1006 | FBgn0038238-FBgn0038240 (3R) | 3 |
| 1007 | FBgn0025808-FBgn0051314 (3R) | 3 |
| 1008 | FBgn0038269-FBgn0067629 (3R) | 3 |
| 1009 | FBgn0038274-FBgn0038277 (3R) | 3 |
| 1010 | FBgn0038341-FBgn0019940 (3R) | 3 |
| 1011 | FBgn0038350-FBgn0053207 (3R) | 3 |
| 1012 | FBgn0038388-FBgn0052856 (3R) | 3 |
| 1013 | FBgn0038395-FBgn0038396 (3R) | 3 |
| 1014 | FBgn0038414-FBgn0038416 (3R) | 3 |
| 1015 | FBgn0027095-FBgn0038421 (3R) | 3 |
| 1016 | FBgn0038423-FBgn0051279 (3R) | 3 |
| 1017 | FBgn0038453-FBgn0015019 (3R) | 3 |
| 1018 | FBgn0063261-FBgn0015008 (3R) | 3 |
| 1019 | FBgn0038468-FBgn0038469 (3R) | 3 |
| 1020 | FBgn0038485-FBgn0038487 (3R) | 3 |
| 1021 | FBgn0259784-FBgn0038491 (3R) | 3 |
| 1022 | FBgn0038500-FBgn0038499 (3R) | 3 |
| 1023 | FBgn0053333-FBgn0038511 (3R) | 3 |
| 1024 | FBgn0038510-FBgn0038512 (3R) | 3 |
| 1025 | FBgn0038540-FBgn0038542 (3R) | 3 |
| 1026 | FBgn0038569-FBgn0038576 (3R) | 3 |
| 1027 | FBgn0051235-FBgn0038607 (3R) | 3 |
| 1028 | FBgn0051232-FBgn0038619 (3R) | 3 |
| 1029 | FBgn0038629-FBgn0038633 (3R) | 3 |
| 1030 | FBgn0038645-FBgn0038647 (3R) | 3 |
| 1031 | FBgn0015781-FBgn0038675 (3R) | 3 |
| 1032 | FBgn0038755-FBgn0038761 (3R) | 3 |
| 1033 | FBgn0051206-FBgn0045471 (3R) | 3 |
| 1034 | FBgn0038810-FBgn0023097 (3R) | 3 |
| 1035 | FBgn0038829-FBgn0027493 (3R) | 3 |
| 1036 | FBgn0038857-FBgn0038858 (3R) | 3 |
| 1037 | FBgn0016700-FBgn0038877 (3R) | 3 |
| 1038 | FBgn0038889-FBgn0038890 (3R) | 3 |
| 1039 | FBgn0038891-FBgn0038892 (3R) | 3 |
| 1040 | FBgn0038894-FBgn0054034 (3R) | 3 |
| 1041 | FBgn0051343-FBgn0051198 (3R) | 3 |
| 1042 | FBgn0041229-FBgn0045468 (3R) | 3 |
| 1043 | FBgn0067317-FBgn0051174 (3R) | 3 |
| 1044 | FBgn0025140-FBgn0038928 (3R) | 3 |
| 1045 | FBgn0051423-FBgn0038934 (3R) | 3 |
| 1046 | FBgn0038958-FBgn0038960 (3R) | 3 |
| 1047 | FBgn0038961-FBgn0038965 (3R) | 3 |
| 1048 | FBgn0038968-FBgn0085405 (3R) | 3 |
| 1049 | FBgn0028837-FBgn0038989 (3R) | 3 |
| 1050 | FBgn0051156-FBgn0051161 (3R) | 3 |
| 1051 | FBgn0039007-FBgn0039010 (3R) | 3 |
| 1052 | FBgn0086253-FBgn0039026 (3R) | 3 |
| 1053 | FBgn0039027-FBgn0039029 (3R) | 3 |
| 1054 | FBgn0039065-FBgn0039067 (3R) | 3 |
| 1055 | FBgn0039104-FBgn0047351 (3R) | 3 |
| 1056 | FBgn0051413-FBgn0039106 (3R) | 3 |
| 1057 | FBgn0053108-FBgn0020647 (3R) | 3 |
| 1058 | FBgn0039153-FBgn0027103 (3R) | 3 |
| 1059 | FBgn0039186-FBgn0039188 (3R) | 3 |
| 1060 | FBgn0083986-FBgn0045980 (3R) | 3 |
| 1061 | FBgn0039235-FBgn0039237 (3R) | 3 |
| 1062 | FBgn0039238-FBgn0013949 (3R) | 3 |
| 1063 | FBgn0039282-FBgn0039286 (3R) | 3 |
| 1064 | FBgn0083946-FBgn0039290 (3R) | 3 |
| 1065 | FBgn0039311-FBgn0039313 (3R) | 3 |
| 1066 | FBgn0051104-FBgn0039316 (3R) | 3 |
| 1067 | FBgn0039344-FBgn0039347 (3R) | 3 |
| 1068 | FBgn0011670-FBgn0011668 (3R) | 3 |
| 1069 | FBgn0039413-FBgn0051323 (3R) | 3 |
| 1070 | FBgn0039472-FBgn0039474 (3R) | 3 |
| 1071 | FBgn0039479-FBgn0039481 (3R) | 3 |
| 1072 | FBgn0039483-FBgn0039485 (3R) | 3 |
| 1073 | FBgn0039487-FBgn0039489 (3R) | 3 |
| 1074 | FBgn0046258-FBgn0039540 (3R) | 3 |
| 1075 | FBgn0045862-FBgn0039544 (3R) | 3 |
| 1076 | FBgn0053213-FBgn0039558 (3R) | 3 |
| 1077 | FBgn0039563-FBgn0039565 (3R) | 3 |
| 1078 | FBgn0039568-FBgn0085383 (3R) | 3 |
| 1079 | FBgn0026400-FBgn0039589 (3R) | 3 |
| 1080 | FBgn0005642-FBgn0039601 (3R) | 3 |
| 1081 | FBgn0039611-FBgn0039613 (3R) | 3 |
| 1082 | FBgn0039630-FBgn0039633 (3R) | 3 |
| 1083 | FBgn0014869-FBgn0041186 (3R) | 3 |
| 1084 | FBgn0039645-FBgn0039648 (3R) | 3 |
| 1085 | FBgn0004369-FBgn0039664 (3R) | 3 |
| 1086 | FBgn0039670-FBgn0051041 (3R) | 3 |
| 1087 | FBgn0001297-FBgn0039696 (3R) | 3 |
| 1088 | FBgn0039710-FBgn0039712 (3R) | 3 |
| 1089 | FBgn0039741-FBgn0039743 (3R) | 3 |
| 1090 | FBgn0039747-FBgn0039749 (3R) | 3 |
| 1091 | FBgn0026190-FBgn0051371 (3R) | 3 |
| 1092 | FBgn0051021-FBgn0051016 (3R) | 3 |
| 1093 | FBgn0039804-FBgn0039805 (3R) | 3 |
| 1094 | FBgn0051005-FBgn0039836 (3R) | 3 |
| 1095 | FBgn0039848-FBgn0039850 (3R) | 3 |
| 1096 | FBgn0039859-FBgn0039861 (3R) | 3 |
| 1097 | FBgn0039872-FBgn0039873 (3R) | 3 |
| 1098 | FBgn0020381-FBgn0025638 (X) | 3 |
| 1099 | FBgn0021764-FBgn0029532 (X) | 3 |
| 1100 | FBgn0040345-FBgn0040342 (X) | 3 |
| 1101 | FBgn0040397-FBgn0085349 (X) | 3 |
| 1102 | FBgn0024360-FBgn0024364 (X) | 3 |
| 1103 | FBgn0024986-FBgn0024985 (X) | 3 |
| 1104 | FBgn0027795-FBgn0027793 (X) | 3 |
| 1105 | FBgn0023519-FBgn0052803 (X) | 3 |
| 1106 | FBgn0023545-FBgn0000667 (X) | 3 |
| 1107 | FBgn0023541-FBgn0015036 (X) | 3 |
| 1108 | FBgn0003116-FBgn0040899 (X) | 3 |
| 1109 | FBgn0040900-FBgn0029603 (X) | 3 |
| 1110 | FBgn0000377-FBgn0029608 (X) | 3 |
| 1111 | FBgn0044047-FBgn0040389 (X) | 3 |
| 1112 | FBgn0040333-FBgn0011276 (X) | 3 |
| 1113 | FBgn0025838-FBgn0000092 (X) | 3 |
| 1114 | FBgn0024998-FBgn0024973 (X) | 3 |
| 1115 | FBgn0029664-FBgn0029666 (X) | 3 |
| 1116 | FBgn0026079-FBgn0026083 (X) | 3 |
| 1117 | FBgn0022942-FBgn0029720 (X) | 3 |
| 1118 | FBgn0029789-FBgn0027564 (X) | 3 |
| 1119 | FBgn0259149-FBgn0029809 (X) | 3 |
| 1120 | FBgn0029839-FBgn0029843 (X) | 3 |
| 1121 | FBgn0029858-FBgn0043796 (X) | 3 |
| 1122 | FBgn0062413-FBgn0025815 (X) | 3 |
| 1123 | FBgn0029892-FBgn0029894 (X) | 3 |
| 1124 | FBgn0029925-FBgn0027108 (X) | 3 |
| 1125 | FBgn0029931-FBgn0029930 (X) | 3 |
| 1126 | FBgn0029942-FBgn0029944 (X) | 3 |
| 1127 | FBgn0029958-FBgn0016041 (X) | 3 |
| 1128 | FBgn0025800-FBgn0030007 (X) | 3 |
| 1129 | FBgn0030010-FBgn0030012 (X) | 3 |
| 1130 | FBgn0030034-FBgn0052711 (X) | 3 |
| 1131 | FBgn0030037-FBgn0030039 (X) | 3 |
| 1132 | FBgn0030077-FBgn0030080 (X) | 3 |
| 1133 | FBgn0030086-FBgn0004856 (X) | 3 |
| 1134 | FBgn0002576-FBgn0054028 (X) | 3 |
| 1135 | FBgn0052685-FBgn0030178 (X) | 3 |
| 1136 | FBgn0030186-FBgn0085437 (X) | 3 |
| 1137 | FBgn0030192-FBgn0052679 (X) | 3 |
| 1138 | FBgn0052686-FBgn0052681 (X) | 3 |
| 1139 | FBgn0001206-FBgn0030218 (X) | 3 |
| 1140 | FBgn0030247-FBgn0030249 (X) | 3 |
| 1141 | FBgn0030258-FBgn0030260 (X) | 3 |
| 1142 | FBgn0020255-FBgn0030276 (X) | 3 |
| 1143 | FBgn0083980-FBgn0003411 (X) | 3 |
| 1144 | FBgn0030309-FBgn0030311 (X) | 3 |
| 1145 | FBgn0030314-FBgn0002948 (X) | 3 |
| 1146 | FBgn0030338-FBgn0030340 (X) | 3 |
| 1147 | FBgn0030351-FBgn0030354 (X) | 3 |
| 1148 | FBgn0030359-FBgn0030360 (X) | 3 |
| 1149 | FBgn0030364-FBgn0030366 (X) | 3 |
| 1150 | FBgn0259109-FBgn0030399 (X) | 3 |
| 1151 | FBgn0030412-FBgn0011837 (X) | 3 |
| 1152 | FBgn0259171-FBgn0052650 (X) | 3 |
| 1153 | FBgn0030433-FBgn0030435 (X) | 3 |
| 1154 | FBgn0052643-FBgn0030441 (X) | 3 |
| 1155 | FBgn0010303-FBgn0030448 (X) | 3 |
| 1156 | FBgn0030449-FBgn0250862 (X) | 3 |
| 1157 | FBgn0052638-FBgn0030452 (X) | 3 |
| 1158 | FBgn0030456-FBgn0030457 (X) | 3 |
| 1159 | FBgn0030480-FBgn0030481 (X) | 3 |
| 1160 | FBgn0030502-FBgn0030504 (X) | 3 |
| 1161 | FBgn0030518-FBgn0030520 (X) | 3 |
| 1162 | FBgn0040871-FBgn0030538 (X) | 3 |
| 1163 | FBgn0030542-FBgn0030544 (X) | 3 |
| 1164 | FBgn0030586-FBgn0030588 (X) | 3 |
| 1165 | FBgn0030590-FBgn0030592 (X) | 3 |
| 1166 | FBgn0030610-FBgn0030612 (X) | 3 |
| 1167 | FBgn0030628-FBgn0000028 (X) | 3 |
| 1168 | FBgn0030640-FBgn0030643 (X) | 3 |
| 1169 | FBgn0030646-FBgn0030648 (X) | 3 |
| 1170 | FBgn0030654-FBgn0030657 (X) | 3 |
| 1171 | FBgn0011741-FBgn0030679 (X) | 3 |
| 1172 | FBgn0030685-FBgn0030687 (X) | 3 |
| 1173 | FBgn0030704-FBgn0010315 (X) | 3 |
| 1174 | FBgn0030706-FBgn0010240 (X) | 3 |
| 1175 | FBgn0030718-FBgn0000611 (X) | 3 |
| 1176 | FBgn0030721-FBgn0030722 (X) | 3 |
| 1177 | FBgn0011571-FBgn0030734 (X) | 3 |
| 1178 | FBgn0030755-FBgn0030757 (X) | 3 |
| 1179 | FBgn0027528-FBgn0030786 (X) | 3 |
| 1180 | FBgn0030805-FBgn0030807 (X) | 3 |
| 1181 | FBgn0027602-FBgn0040877 (X) | 3 |
| 1182 | FBgn0026313-FBgn0030851 (X) | 3 |
| 1183 | FBgn0030855-FBgn0030858 (X) | 3 |
| 1184 | FBgn0030874-FBgn0052557 (X) | 3 |
| 1185 | FBgn0030876-FBgn0030878 (X) | 3 |
| 1186 | FBgn0030881-FBgn0030882 (X) | 3 |
| 1187 | FBgn0030891-FBgn0030892 (X) | 3 |
| 1188 | FBgn0030893-FBgn0022724 (X) | 3 |
| 1189 | FBgn0030914-FBgn0030915 (X) | 3 |
| 1190 | FBgn0030925-FBgn0030927 (X) | 3 |
| 1191 | FBgn0030933-FBgn0030937 (X) | 3 |
| 1192 | FBgn0030949-FBgn0030952 (X) | 3 |
| 1193 | FBgn0030959-FBgn0030960 (X) | 3 |
| 1194 | FBgn0031006-FBgn0031008 (X) | 3 |
| 1195 | FBgn0031011-FBgn0259834 (X) | 3 |
| 1196 | FBgn0031023-FBgn0052532 (X) | 3 |
| 1197 | FBgn0031047-FBgn0031049 (X) | 3 |
| 1198 | FBgn0031050-FBgn0031052 (X) | 3 |
| 1199 | FBgn0031060-FBgn0031062 (X) | 3 |
| 1200 | FBgn0052528-FBgn0031069 (X) | 3 |
| 1201 | FBgn0031078-FBgn0052529 (X) | 3 |
| 1202 | FBgn0031092-FBgn0031094 (X) | 3 |
| 1203 | FBgn0031109-FBgn0031111 (X) | 3 |
| 1204 | FBgn0031118-FBgn0026323 (X) | 3 |
| 1205 | FBgn0031155-FBgn0003950 (X) | 3 |
| 1206 | FBgn0031195-FBgn0031194 (X) | 3 |
| 1207 | FBgn0067779-FBgn0031213 (2L) | 2 |
| 1208 | FBgn0031214-FBgn0002931 (2L) | 2 |
| 1209 | FBgn0025683-FBgn0001142 (2L) | 2 |
| 1210 | FBgn0051975-FBgn0051976 (2L) | 2 |
| 1211 | FBgn0051974-FBgn0031224 (2L) | 2 |
| 1212 | FBgn0016977-FBgn0031231 (2L) | 2 |
| 1213 | FBgn0086902-FBgn0022246 (2L) | 2 |
| 1214 | FBgn0031239-FBgn0031240 (2L) | 2 |
| 1215 | FBgn0051920-FBgn0051921 (2L) | 2 |
| 1216 | FBgn0000061-FBgn0031251 (2L) | 2 |
| 1217 | FBgn0003278-FBgn0023444 (2L) | 2 |
| 1218 | FBgn0031254-FBgn0031255 (2L) | 2 |
| 1219 | FBgn0015924-FBgn0031256 (2L) | 2 |
| 1220 | FBgn0031257-FBgn0031258 (2L) | 2 |
| 1221 | FBgn0010602-FBgn0031260 (2L) | 2 |
| 1222 | FBgn0031276-FBgn0040725 (2L) | 2 |
| 1223 | FBgn0041250-FBgn0031279 (2L) | 2 |
| 1224 | FBgn0031284-FBgn0015621 (2L) | 2 |
| 1225 | FBgn0031286-FBgn0010583 (2L) | 2 |
| 1226 | FBgn0020304-FBgn0020545 (2L) | 2 |
| 1227 | FBgn0031288-FBgn0031289 (2L) | 2 |
| 1228 | FBgn0031295-FBgn0031296 (2L) | 2 |
| 1229 | FBgn0016926-FBgn0031304 (2L) | 2 |
| 1230 | FBgn0031314-FBgn0031315 (2L) | 2 |
| 1231 | FBgn0031324-FBgn0259229 (2L) | 2 |
| 1232 | FBgn0041097-FBgn0011294 (2L) | 2 |
| 1233 | FBgn0011570-FBgn0031356 (2L) | 2 |
| 1234 | FBgn0040719-FBgn0053673 (2L) | 2 |
| 1235 | FBgn0031375-FBgn0031377 (2L) | 2 |
| 1236 | FBgn0031378-FBgn0031379 (2L) | 2 |
| 1237 | FBgn0001125-FBgn0031381 (2L) | 2 |
| 1238 | FBgn0043539-FBgn0053543 (2L) | 2 |
| 1239 | FBgn0040718-FBgn0040717 (2L) | 2 |
| 1240 | FBgn0051668-FBgn0053124 (2L) | 2 |
| 1241 | FBgn0085203-FBgn0031398 (2L) | 2 |
| 1242 | FBgn0031399-FBgn0031401 (2L) | 2 |
| 1243 | FBgn0031407-FBgn0054049 (2L) | 2 |
| 1244 | FBgn0250841-FBgn0031409 (2L) | 2 |
| 1245 | FBgn0031414-FBgn0031413 (2L) | 2 |
| 1246 | FBgn0031430-FBgn0031431 (2L) | 2 |
| 1247 | FBgn0031432-FBgn0041337 (2L) | 2 |
| 1248 | FBgn0031434-FBgn0031435 (2L) | 2 |
| 1249 | FBgn0031436-FBgn0026479 (2L) | 2 |
| 1250 | FBgn0031449-FBgn0031450 (2L) | 2 |
| 1251 | FBgn0250906-FBgn0031453 (2L) | 2 |
| 1252 | FBgn0019830-FBgn0031459 (2L) | 2 |
| 1253 | FBgn0031460-FBgn0004242 (2L) | 2 |
| 1254 | FBgn0041111-FBgn0010263 (2L) | 2 |
| 1255 | FBgn0005779-FBgn0031504 (2L) | 2 |
| 1256 | FBgn0051698-FBgn0031512 (2L) | 2 |
| 1257 | FBgn0085422-FBgn0031513 (2L) | 2 |
| 1258 | FBgn0031515-FBgn0031516 (2L) | 2 |
| 1259 | FBgn0031518-FBgn0031519 (2L) | 2 |
| 1260 | FBgn0085423-FBgn0031528 (2L) | 2 |
| 1261 | FBgn0031529-FBgn0016698 (2L) | 2 |
| 1262 | FBgn0031540-FBgn0051776 (2L) | 2 |
| 1263 | FBgn0053123-FBgn0031544 (2L) | 2 |
| 1264 | FBgn0031558-FBgn0031559 (2L) | 2 |
| 1265 | FBgn0031560-FBgn0031561 (2L) | 2 |
| 1266 | FBgn0004567-FBgn0031574 (2L) | 2 |
| 1267 | FBgn0051957-FBgn0000256 (2L) | 2 |
| 1268 | FBgn0051773-FBgn0051774 (2L) | 2 |
| 1269 | FBgn0031585-FBgn0026394 (2L) | 2 |
| 1270 | FBgn0051961-FBgn0031589 (2L) | 2 |
| 1271 | FBgn0031590-FBgn0003941 (2L) | 2 |
| 1272 | FBgn0028482-FBgn0020762 (2L) | 2 |
| 1273 | FBgn0019982-FBgn0053002 (2L) | 2 |
| 1274 | FBgn0083938-FBgn0031617 (2L) | 2 |
| 1275 | FBgn0031627-FBgn0031628 (2L) | 2 |
| 1276 | FBgn0031631-FBgn0031632 (2L) | 2 |
| 1277 | FBgn0083961-FBgn0015834 (2L) | 2 |
| 1278 | FBgn0053113-FBgn0031670 (2L) | 2 |
| 1279 | FBgn0031677-FBgn0031678 (2L) | 2 |
| 1280 | FBgn0031690-FBgn0250847 (2L) | 2 |
| 1281 | FBgn0031697-FBgn0031698 (2L) | 2 |
| 1282 | FBgn0031708-FBgn0000318 (2L) | 2 |
| 1283 | FBgn0031715-FBgn0031716 (2L) | 2 |
| 1284 | FBgn0014189-FBgn0002525 (2L) | 2 |
| 1285 | FBgn0024191-FBgn0031736 (2L) | 2 |
| 1286 | FBgn0031735-FBgn0051913 (2L) | 2 |
| 1287 | FBgn0051644-FBgn0085410 (2L) | 2 |
| 1288 | FBgn0001128-FBgn0031752 (2L) | 2 |
| 1289 | FBgn0031753-FBgn0040949 (2L) | 2 |
| 1290 | FBgn0031776-FBgn0031777 (2L) | 2 |
| 1291 | FBgn0031792-FBgn0021873 (2L) | 2 |
| 1292 | FBgn0259714-FBgn0031811 (2L) | 2 |
| 1293 | FBgn0027496-FBgn0031822 (2L) | 2 |
| 1294 | FBgn0031832-FBgn0026585 (2L) | 2 |
| 1295 | FBgn0000351-FBgn0031848 (2L) | 2 |
| 1296 | FBgn0031850-FBgn0025595 (2L) | 2 |
| 1297 | FBgn0031851-FBgn0025777 (2L) | 2 |
| 1298 | FBgn0021944-FBgn0026170 (2L) | 2 |
| 1299 | FBgn0031879-FBgn0031880 (2L) | 2 |
| 1300 | FBgn0031894-FBgn0031895 (2L) | 2 |
| 1301 | FBgn0031929-FBgn0031930 (2L) | 2 |
| 1302 | FBgn0053296-FBgn0031935 (2L) | 2 |
| 1303 | FBgn0031936-FBgn0031937 (2L) | 2 |
| 1304 | FBgn0031939-FBgn0031940 (2L) | 2 |
| 1305 | FBgn0031944-FBgn0031943 (2L) | 2 |
| 1306 | FBgn0020618-FBgn0004177 (2L) | 2 |
| 1307 | FBgn0051902-FBgn0053121 (2L) | 2 |
| 1308 | FBgn0031977-FBgn0044323 (2L) | 2 |
| 1309 | FBgn0013531-FBgn0010287 (2L) | 2 |
| 1310 | FBgn0020240-FBgn0031985 (2L) | 2 |
| 1311 | FBgn0031987-FBgn0031988 (2L) | 2 |
| 1312 | FBgn0051607-FBgn0051900 (2L) | 2 |
| 1313 | FBgn0031990-FBgn0031992 (2L) | 2 |
| 1314 | FBgn0032000-FBgn0032001 (2L) | 2 |
| 1315 | FBgn0032003-FBgn0032004 (2L) | 2 |
| 1316 | FBgn0032005-FBgn0032006 (2L) | 2 |
| 1317 | FBgn0032014-FBgn0032015 (2L) | 2 |
| 1318 | FBgn0032035-FBgn0032036 (2L) | 2 |
| 1319 | FBgn0014417-FBgn0032042 (2L) | 2 |
| 1320 | FBgn0001137-FBgn0027490 (2L) | 2 |
| 1321 | FBgn0032057-FBgn0032058 (2L) | 2 |
| 1322 | FBgn0004914-FBgn0003209 (2L) | 2 |
| 1323 | FBgn0032074-FBgn0032075 (2L) | 2 |
| 1324 | FBgn0032078-FBgn0032079 (2L) | 2 |
| 1325 | FBgn0032080-FBgn0052985 (2L) | 2 |
| 1326 | FBgn0032085-FBgn0032086 (2L) | 2 |
| 1327 | FBgn0040964-FBgn0015316 (2L) | 2 |
| 1328 | FBgn0032087-FBgn0032088 (2L) | 2 |
| 1329 | FBgn0032117-FBgn0011232 (2L) | 2 |
| 1330 | FBgn0002973-FBgn0004868 (2L) | 2 |
| 1331 | FBgn0011207-FBgn0051710 (2L) | 2 |
| 1332 | FBgn0032142-FBgn0032143 (2L) | 2 |
| 1333 | FBgn0032145-FBgn0065108 (2L) | 2 |
| 1334 | FBgn0032147-FBgn0032149 (2L) | 2 |
| 1335 | FBgn0032161-FBgn0032162 (2L) | 2 |
| 1336 | FBgn0032170-FBgn0032172 (2L) | 2 |
| 1337 | FBgn0000180-FBgn0040965 (2L) | 2 |
| 1338 | FBgn0051755-FBgn0051875 (2L) | 2 |
| 1339 | FBgn0043456-FBgn0011584 (2L) | 2 |
| 1340 | FBgn0032188-FBgn0051874 (2L) | 2 |
| 1341 | FBgn0028675-FBgn0032189 (2L) | 2 |
| 1342 | FBgn0085188-FBgn0032191 (2L) | 2 |
| 1343 | FBgn0032229-FBgn0027568 (2L) | 2 |
| 1344 | FBgn0032242-FBgn0032243 (2L) | 2 |
| 1345 | FBgn0032244-FBgn0005683 (2L) | 2 |
| 1346 | FBgn0032246-FBgn0022268 (2L) | 2 |
| 1347 | FBgn0086347-FBgn0032261 (2L) | 2 |
| 1348 | FBgn0032266-FBgn0051721 (2L) | 2 |
| 1349 | FBgn0032286-FBgn0023496 (2L) | 2 |
| 1350 | FBgn0032292-FBgn0027868 (2L) | 2 |
| 1351 | FBgn0015320-FBgn0032298 (2L) | 2 |
| 1352 | FBgn0051867-FBgn0010197 (2L) | 2 |
| 1353 | FBgn0259750-FBgn0052830 (2L) | 2 |
| 1354 | FBgn0032358-FBgn0020270 (2L) | 2 |
| 1355 | FBgn0020309-FBgn0032378 (2L) | 2 |
| 1356 | FBgn0032390-FBgn0032391 (2L) | 2 |
| 1357 | FBgn0032393-FBgn0032394 (2L) | 2 |
| 1358 | FBgn0032406-FBgn0003145 (2L) | 2 |
| 1359 | FBgn0032407-FBgn0032408 (2L) | 2 |
| 1360 | FBgn0028707-FBgn0029137 (2L) | 2 |
| 1361 | FBgn0032412-FBgn0032413 (2L) | 2 |
| 1362 | FBgn0032414-FBgn0032416 (2L) | 2 |
| 1363 | FBgn0051860-FBgn0032419 (2L) | 2 |
| 1364 | FBgn0032429-FBgn0032430 (2L) | 2 |
| 1365 | FBgn0020443-FBgn0040286 (2L) | 2 |
| 1366 | FBgn0032436-FBgn0032437 (2L) | 2 |
| 1367 | FBgn0032444-FBgn0032445 (2L) | 2 |
| 1368 | FBgn0032470-FBgn0032471 (2L) | 2 |
| 1369 | FBgn0051848-FBgn0032498 (2L) | 2 |
| 1370 | FBgn0032499-FBgn0051729 (2L) | 2 |
| 1371 | FBgn0032519-FBgn0032520 (2L) | 2 |
| 1372 | FBgn0032521-FBgn0003983 (2L) | 2 |
| 1373 | FBgn0051813-FBgn0028541 (2L) | 2 |
| 1374 | FBgn0028538-FBgn0027844 (2L) | 2 |
| 1375 | FBgn0019890-FBgn0012037 (2L) | 2 |
| 1376 | FBgn0025115-FBgn0028938 (2L) | 2 |
| 1377 | FBgn0032538-FBgn0028544 (2L) | 2 |
| 1378 | FBgn0028430-FBgn0259896 (2L) | 2 |
| 1379 | FBgn0028939-FBgn0003255 (2L) | 2 |
| 1380 | FBgn0028906-FBgn0028905 (2L) | 2 |
| 1381 | FBgn0028530-FBgn0015791 (2L) | 2 |
| 1382 | FBgn0001970-FBgn0015546 (2L) | 2 |
| 1383 | FBgn0250844-FBgn0028913 (2L) | 2 |
| 1384 | FBgn0028855-FBgn0085194 (2L) | 2 |
| 1385 | FBgn0086711-FBgn0028926 (2L) | 2 |
| 1386 | FBgn0010422-FBgn0024183 (2L) | 2 |
| 1387 | FBgn0001978-FBgn0028888 (2L) | 2 |
| 1388 | FBgn0028880-FBgn0028370 (2L) | 2 |
| 1389 | FBgn0028945-FBgn0028518 (2L) | 2 |
| 1390 | FBgn0001987-FBgn0028507 (2L) | 2 |
| 1391 | FBgn0001990-FBgn0041627 (2L) | 2 |
| 1392 | FBgn0051823-FBgn0051821 (2L) | 2 |
| 1393 | FBgn0002673-FBgn0028897 (2L) | 2 |
| 1394 | FBgn0001994-FBgn0040079 (2L) | 2 |
| 1395 | FBgn0000307-FBgn0028506 (2L) | 2 |
| 1396 | FBgn0020416-FBgn0020415 (2L) | 2 |
| 1397 | FBgn0028523-FBgn0020414 (2L) | 2 |
| 1398 | FBgn0032586-FBgn0032587 (2L) | 2 |
| 1399 | FBgn0051782-FBgn0051812 (2L) | 2 |
| 1400 | FBgn0032595-FBgn0032596 (2L) | 2 |
| 1401 | FBgn0040262-FBgn0040261 (2L) | 2 |
| 1402 | FBgn0051809-FBgn0032615 (2L) | 2 |
| 1403 | FBgn0051805-FBgn0032620 (2L) | 2 |
| 1404 | FBgn0032632-FBgn0051804 (2L) | 2 |
| 1405 | FBgn0032634-FBgn0032635 (2L) | 2 |
| 1406 | FBgn0032654-FBgn0015609 (2L) | 2 |
| 1407 | FBgn0032660-FBgn0243486 (2L) | 2 |
| 1408 | FBgn0032670-FBgn0032671 (2L) | 2 |
| 1409 | FBgn0086707-FBgn0032679 (2L) | 2 |
| 1410 | FBgn0032690-FBgn0032691 (2L) | 2 |
| 1411 | FBgn0015553-FBgn0032698 (2L) | 2 |
| 1412 | FBgn0032704-FBgn0025608 (2L) | 2 |
| 1413 | FBgn0032720-FBgn0032721 (2L) | 2 |
| 1414 | FBgn0032731-FBgn0032732 (2L) | 2 |
| 1415 | FBgn0032733-FBgn0032734 (2L) | 2 |
| 1416 | FBgn0028569-FBgn0032740 (2L) | 2 |
| 1417 | FBgn0002036-FBgn0000422 (2L) | 2 |
| 1418 | FBgn0032771-FBgn0032772 (2L) | 2 |
| 1419 | FBgn0032773-FBgn0032774 (2L) | 2 |
| 1420 | FBgn0032775-FBgn0032779 (2L) | 2 |
| 1421 | FBgn0032788-FBgn0032789 (2L) | 2 |
| 1422 | FBgn0040268-FBgn0032796 (2L) | 2 |
| 1423 | FBgn0032797-FBgn0032798 (2L) | 2 |
| 1424 | FBgn0032801-FBgn0053116 (2L) | 2 |
| 1425 | FBgn0032808-FBgn0032809 (2L) | 2 |
| 1426 | FBgn0000529-FBgn0032821 (2L) | 2 |
| 1427 | FBgn0054051-FBgn0032834 (2L) | 2 |
| 1428 | FBgn0032835-FBgn0032836 (2L) | 2 |
| 1429 | FBgn0040995-FBgn0032838 (2L) | 2 |
| 1430 | FBgn0032848-FBgn0032850 (2L) | 2 |
| 1431 | FBgn0032868-FBgn0051680 (2L) | 2 |
| 1432 | FBgn0250785-FBgn0032886 (2L) | 2 |
| 1433 | FBgn0053322-FBgn0032888 (2L) | 2 |
| 1434 | FBgn0032889-FBgn0051673 (2L) | 2 |
| 1435 | FBgn0032897-FBgn0032899 (2L) | 2 |
| 1436 | FBgn0032906-FBgn0032907 (2L) | 2 |
| 1437 | FBgn0032929-FBgn0011829 (2L) | 2 |
| 1438 | FBgn0051624-FBgn0051988 (2L) | 2 |
| 1439 | FBgn0026255-FBgn0032934 (2L) | 2 |
| 1440 | FBgn0032940-FBgn0000370 (2L) | 2 |
| 1441 | FBgn0032955-FBgn0022893 (2L) | 2 |
| 1442 | FBgn0023416-FBgn0032957 (2L) | 2 |
| 1443 | FBgn0032964-FBgn0051612 (2L) | 2 |
| 1444 | FBgn0032981-FBgn0259683 (2L) | 2 |
| 1445 | FBgn0040010-FBgn0040011 (2L) | 2 |
| 1446 | FBgn0085229-FBgn0033038 (2R) | 2 |
| 1447 | FBgn0033047-FBgn0033048 (2R) | 2 |
| 1448 | FBgn0033050-FBgn0000043 (2R) | 2 |
| 1449 | FBgn0033059-FBgn0033060 (2R) | 2 |
| 1450 | FBgn0040502-FBgn0033067 (2R) | 2 |
| 1451 | FBgn0050432-FBgn0033069 (2R) | 2 |
| 1452 | FBgn0033083-FBgn0000054 (2R) | 2 |
| 1453 | FBgn0033090-FBgn0050446 (2R) | 2 |
| 1454 | FBgn0014009-FBgn0033095 (2R) | 2 |
| 1455 | FBgn0033096-FBgn0033097 (2R) | 2 |
| 1456 | FBgn0028579-FBgn0042083 (2R) | 2 |
| 1457 | FBgn0000473-FBgn0085421 (2R) | 2 |
| 1458 | FBgn0050157-FBgn0050156 (2R) | 2 |
| 1459 | FBgn0022960-FBgn0033122 (2R) | 2 |
| 1460 | FBgn0029508-FBgn0050159 (2R) | 2 |
| 1461 | FBgn0033134-FBgn0016032 (2R) | 2 |
| 1462 | FBgn0033138-FBgn0033139 (2R) | 2 |
| 1463 | FBgn0000352-FBgn0033160 (2R) | 2 |
| 1464 | FBgn0033164-FBgn0033165 (2R) | 2 |
| 1465 | FBgn0003460-FBgn0033168 (2R) | 2 |
| 1466 | FBgn0050491-FBgn0033199 (2R) | 2 |
| 1467 | FBgn0033202-FBgn0033203 (2R) | 2 |
| 1468 | FBgn0033208-FBgn0033209 (2R) | 2 |
| 1469 | FBgn0033210-FBgn0033212 (2R) | 2 |
| 1470 | FBgn0033215-FBgn0033216 (2R) | 2 |
| 1471 | FBgn0042135-FBgn0050497 (2R) | 2 |
| 1472 | FBgn0033221-FBgn0033222 (2R) | 2 |
| 1473 | FBgn0033232-FBgn0015509 (2R) | 2 |
| 1474 | FBgn0023171-FBgn0026722 (2R) | 2 |
| 1475 | FBgn0033248-FBgn0027788 (2R) | 2 |
| 1476 | FBgn0010504-FBgn0033269 (2R) | 2 |
| 1477 | FBgn0033273-FBgn0011659 (2R) | 2 |
| 1478 | FBgn0002533-FBgn0002534 (2R) | 2 |
| 1479 | FBgn0033310-FBgn0033312 (2R) | 2 |
| 1480 | FBgn0033316-FBgn0033317 (2R) | 2 |
| 1481 | FBgn0050354-FBgn0050355 (2R) | 2 |
| 1482 | FBgn0033320-FBgn0033321 (2R) | 2 |
| 1483 | FBgn0086784-FBgn0027585 (2R) | 2 |
| 1484 | FBgn0021825-FBgn0033339 (2R) | 2 |
| 1485 | FBgn0033347-FBgn0033348 (2R) | 2 |
| 1486 | FBgn0033371-FBgn0033372 (2R) | 2 |
| 1487 | FBgn0010220-FBgn0033373 (2R) | 2 |
| 1488 | FBgn0033413-FBgn0017558 (2R) | 2 |
| 1489 | FBgn0085436-FBgn0033427 (2R) | 2 |
| 1490 | FBgn0033428-FBgn0033429 (2R) | 2 |
| 1491 | FBgn0050000-FBgn0050005 (2R) | 2 |
| 1492 | FBgn0050001-FBgn0033447 (2R) | 2 |
| 1493 | FBgn0033449-FBgn0033450 (2R) | 2 |
| 1494 | FBgn0033463-FBgn0050007 (2R) | 2 |
| 1495 | FBgn0013435-FBgn0033481 (2R) | 2 |
| 1496 | FBgn0250840-FBgn0033512 (2R) | 2 |
| 1497 | FBgn0033527-FBgn0033528 (2R) | 2 |
| 1498 | FBgn0027525-FBgn0050015 (2R) | 2 |
| 1499 | FBgn0033570-FBgn0033571 (2R) | 2 |
| 1500 | FBgn0033580-FBgn0033581 (2R) | 2 |
| 1501 | FBgn0037084-FBgn0033584 (2R) | 2 |
| 1502 | FBgn0033594-FBgn0085254 (2R) | 2 |
| 1503 | FBgn0001276-FBgn0024188 (2R) | 2 |
| 1504 | FBgn0033628-FBgn0033629 (2R) | 2 |
| 1505 | FBgn0033631-FBgn0050022 (2R) | 2 |
| 1506 | FBgn0082585-FBgn0033633 (2R) | 2 |
| 1507 | FBgn0054054-FBgn0040503 (2R) | 2 |
| 1508 | FBgn0259832-FBgn0033656 (2R) | 2 |
| 1509 | FBgn0085259-FBgn0033658 (2R) | 2 |
| 1510 | FBgn0033663-FBgn0000556 (2R) | 2 |
| 1511 | FBgn0033667-FBgn0033668 (2R) | 2 |
| 1512 | FBgn0004839-FBgn0033674 (2R) | 2 |
| 1513 | FBgn0033677-FBgn0010339 (2R) | 2 |
| 1514 | FBgn0033680-FBgn0086677 (2R) | 2 |
| 1515 | FBgn0033686-FBgn0027504 (2R) | 2 |
| 1516 | FBgn0014184-FBgn0010621 (2R) | 2 |
| 1517 | FBgn0033692-FBgn0000426 (2R) | 2 |
| 1518 | FBgn0033696-FBgn0033697 (2R) | 2 |
| 1519 | FBgn0033698-FBgn0033699 (2R) | 2 |
| 1520 | FBgn0033716-FBgn0033717 (2R) | 2 |
| 1521 | FBgn0050042-FBgn0033723 (2R) | 2 |
| 1522 | FBgn0033724-FBgn0033725 (2R) | 2 |
| 1523 | FBgn0033729-FBgn0033730 (2R) | 2 |
| 1524 | FBgn0033734-FBgn0033735 (2R) | 2 |
| 1525 | FBgn0010238-FBgn0033741 (2R) | 2 |
| 1526 | FBgn0033748-FBgn0033749 (2R) | 2 |
| 1527 | FBgn0033751-FBgn0033752 (2R) | 2 |
| 1528 | FBgn0050055-FBgn0022764 (2R) | 2 |
| 1529 | FBgn0033770-FBgn0033773 (2R) | 2 |
| 1530 | FBgn0026741-FBgn0026619 (2R) | 2 |
| 1531 | FBgn0003326-FBgn0040755 (2R) | 2 |
| 1532 | FBgn0010488-FBgn0033781 (2R) | 2 |
| 1533 | FBgn0033783-FBgn0033784 (2R) | 2 |
| 1534 | FBgn0033785-FBgn0050487 (2R) | 2 |
| 1535 | FBgn0033786-FBgn0033787 (2R) | 2 |
| 1536 | FBgn0033788-FBgn0033789 (2R) | 2 |
| 1537 | FBgn0033794-FBgn0053007 (2R) | 2 |
| 1538 | FBgn0033818-FBgn0033819 (2R) | 2 |
| 1539 | FBgn0033820-FBgn0033821 (2R) | 2 |
| 1540 | FBgn0050060-FBgn0050065 (2R) | 2 |
| 1541 | FBgn0033855-FBgn0033856 (2R) | 2 |
| 1542 | FBgn0027581-FBgn0033859 (2R) | 2 |
| 1543 | FBgn0033882-FBgn0033883 (2R) | 2 |
| 1544 | FBgn0033887-FBgn0033888 (2R) | 2 |
| 1545 | FBgn0033893-FBgn0259184 (2R) | 2 |
| 1546 | FBgn0033911-FBgn0005613 (2R) | 2 |
| 1547 | FBgn0033915-FBgn0033916 (2R) | 2 |
| 1548 | FBgn0033921-FBgn0085408 (2R) | 2 |
| 1549 | FBgn0033924-FBgn0033925 (2R) | 2 |
| 1550 | FBgn0053469-FBgn0053468 (2R) | 2 |
| 1551 | FBgn0033948-FBgn0033949 (2R) | 2 |
| 1552 | FBgn0050479-FBgn0050480 (2R) | 2 |
| 1553 | FBgn0033961-FBgn0033962 (2R) | 2 |
| 1554 | FBgn0259112-FBgn0001319 (2R) | 2 |
| 1555 | FBgn0027783-FBgn0028434 (2R) | 2 |
| 1556 | FBgn0033990-FBgn0029082 (2R) | 2 |
| 1557 | FBgn0033993-FBgn0033994 (2R) | 2 |
| 1558 | FBgn0034011-FBgn0034012 (2R) | 2 |
| 1559 | FBgn0053465-FBgn0003130 (2R) | 2 |
| 1560 | FBgn0034045-FBgn0034046 (2R) | 2 |
| 1561 | FBgn0050085-FBgn0034049 (2R) | 2 |
| 1562 | FBgn0034061-FBgn0034063 (2R) | 2 |
| 1563 | FBgn0034065-FBgn0034067 (2R) | 2 |
| 1564 | FBgn0014020-FBgn0017549 (2R) | 2 |
| 1565 | FBgn0034076-FBgn0010052 (2R) | 2 |
| 1566 | FBgn0086676-FBgn0001124 (2R) | 2 |
| 1567 | FBgn0050094-FBgn0034083 (2R) | 2 |
| 1568 | FBgn0015925-FBgn0001308 (2R) | 2 |
| 1569 | FBgn0053017-FBgn0024249 (2R) | 2 |
| 1570 | FBgn0034109-FBgn0034110 (2R) | 2 |
| 1571 | FBgn0034117-FBgn0034118 (2R) | 2 |
| 1572 | FBgn0034132-FBgn0034133 (2R) | 2 |
| 1573 | FBgn0034135-FBgn0034136 (2R) | 2 |
| 1574 | FBgn0034154-FBgn0034156 (2R) | 2 |
| 1575 | FBgn0040743-FBgn0034159 (2R) | 2 |
| 1576 | FBgn0025830-FBgn0025832 (2R) | 2 |
| 1577 | FBgn0034177-FBgn0026402 (2R) | 2 |
| 1578 | FBgn0050456-FBgn0034194 (2R) | 2 |
| 1579 | FBgn0034198-FBgn0034199 (2R) | 2 |
| 1580 | FBgn0050457-FBgn0034204 (2R) | 2 |
| 1581 | FBgn0034205-FBgn0034206 (2R) | 2 |
| 1582 | FBgn0034217-FBgn0034218 (2R) | 2 |
| 1583 | FBgn0034219-FBgn0028956 (2R) | 2 |
| 1584 | FBgn0034221-FBgn0085221 (2R) | 2 |
| 1585 | FBgn0034225-FBgn0050104 (2R) | 2 |
| 1586 | FBgn0034238-FBgn0034239 (2R) | 2 |
| 1587 | FBgn0034245-FBgn0016701 (2R) | 2 |
| 1588 | FBgn0029079-FBgn0050105 (2R) | 2 |
| 1589 | FBgn0034259-FBgn0034261 (2R) | 2 |
| 1590 | FBgn0027572-FBgn0034277 (2R) | 2 |
| 1591 | FBgn0034279-FBgn0028740 (2R) | 2 |
| 1592 | FBgn0034295-FBgn0034296 (2R) | 2 |
| 1593 | FBgn0034299-FBgn0034300 (2R) | 2 |
| 1594 | FBgn0034301-FBgn0085225 (2R) | 2 |
| 1595 | FBgn0003520-FBgn0028983 (2R) | 2 |
| 1596 | FBgn0050115-FBgn0034360 (2R) | 2 |
| 1597 | FBgn0034361-FBgn0034362 (2R) | 2 |
| 1598 | FBgn0034366-FBgn0034367 (2R) | 2 |
| 1599 | FBgn0034374-FBgn0034377 (2R) | 2 |
| 1600 | FBgn0034379-FBgn0034380 (2R) | 2 |
| 1601 | FBgn0053998-FBgn0050121 (2R) | 2 |
| 1602 | FBgn0034387-FBgn0259682 (2R) | 2 |
| 1603 | FBgn0034396-FBgn0034397 (2R) | 2 |
| 1604 | FBgn0010053-FBgn0034405 (2R) | 2 |
| 1605 | FBgn0050126-FBgn0034413 (2R) | 2 |
| 1606 | FBgn0034416-FBgn0050125 (2R) | 2 |
| 1607 | FBgn0034426-FBgn0034427 (2R) | 2 |
| 1608 | FBgn0034430-FBgn0086358 (2R) | 2 |
| 1609 | FBgn0034433-FBgn0034434 (2R) | 2 |
| 1610 | FBgn0054045-FBgn0050127 (2R) | 2 |
| 1611 | FBgn0034440-FBgn0034441 (2R) | 2 |
| 1612 | FBgn0040730-FBgn0085227 (2R) | 2 |
| 1613 | FBgn0046880-FBgn0046879 (2R) | 2 |
| 1614 | FBgn0034486-FBgn0034487 (2R) | 2 |
| 1615 | FBgn0025720-FBgn0034491 (2R) | 2 |
| 1616 | FBgn0034495-FBgn0034496 (2R) | 2 |
| 1617 | FBgn0027529-FBgn0034500 (2R) | 2 |
| 1618 | FBgn0034501-FBgn0034502 (2R) | 2 |
| 1619 | FBgn0034503-FBgn0034504 (2R) | 2 |
| 1620 | FBgn0034507-FBgn0086604 (2R) | 2 |
| 1621 | FBgn0034509-FBgn0043535 (2R) | 2 |
| 1622 | FBgn0050151-FBgn0050154 (2R) | 2 |
| 1623 | FBgn0050145-FBgn0034520 (2R) | 2 |
| 1624 | FBgn0034539-FBgn0034541 (2R) | 2 |
| 1625 | FBgn0034552-FBgn0034553 (2R) | 2 |
| 1626 | FBgn0034563-FBgn0083951 (2R) | 2 |
| 1627 | FBgn0034565-FBgn0034566 (2R) | 2 |
| 1628 | FBgn0034578-FBgn0034579 (2R) | 2 |
| 1629 | FBgn0022700-FBgn0034582 (2R) | 2 |
| 1630 | FBgn0010415-FBgn0026369 (2R) | 2 |
| 1631 | FBgn0034631-FBgn0043070 (2R) | 2 |
| 1632 | FBgn0034643-FBgn0034644 (2R) | 2 |
| 1633 | FBgn0034651-FBgn0050263 (2R) | 2 |
| 1634 | FBgn0027360-FBgn0050290 (2R) | 2 |
| 1635 | FBgn0034688-FBgn0034691 (2R) | 2 |
| 1636 | FBgn0050280-FBgn0050281 (2R) | 2 |
| 1637 | FBgn0243516-FBgn0034697 (2R) | 2 |
| 1638 | FBgn0034703-FBgn0034704 (2R) | 2 |
| 1639 | FBgn0034705-FBgn0034707 (2R) | 2 |
| 1640 | FBgn0034708-FBgn0034709 (2R) | 2 |
| 1641 | FBgn0034718-FBgn0013272 (2R) | 2 |
| 1642 | FBgn0034737-FBgn0034739 (2R) | 2 |
| 1643 | FBgn0034802-FBgn0034803 (2R) | 2 |
| 1644 | FBgn0003900-FBgn0050194 (2R) | 2 |
| 1645 | FBgn0034814-FBgn0003062 (2R) | 2 |
| 1646 | FBgn0045483-FBgn0045482 (2R) | 2 |
| 1647 | FBgn0050410-FBgn0034849 (2R) | 2 |
| 1648 | FBgn0034854-FBgn0040660 (2R) | 2 |
| 1649 | FBgn0028497-FBgn0034859 (2R) | 2 |
| 1650 | FBgn0050185-FBgn0034876 (2R) | 2 |
| 1651 | FBgn0034878-FBgn0034879 (2R) | 2 |
| 1652 | FBgn0034883-FBgn0034884 (2R) | 2 |
| 1653 | FBgn0034931-FBgn0034933 (2R) | 2 |
| 1654 | FBgn0034956-FBgn0034957 (2R) | 2 |
| 1655 | FBgn0034958-FBgn0034959 (2R) | 2 |
| 1656 | FBgn0034966-FBgn0034965 (2R) | 2 |
| 1657 | FBgn0035011-FBgn0035012 (2R) | 2 |
| 1658 | FBgn0035016-FBgn0050169 (2R) | 2 |
| 1659 | FBgn0035050-FBgn0053988 (2R) | 2 |
| 1660 | FBgn0035056-FBgn0035057 (2R) | 2 |
| 1661 | FBgn0040666-FBgn0035059 (2R) | 2 |
| 1662 | FBgn0035070-FBgn0035073 (2R) | 2 |
| 1663 | FBgn0060296-FBgn0043792 (2R) | 2 |
| 1664 | FBgn0022343-FBgn0035082 (2R) | 2 |
| 1665 | FBgn0035085-FBgn0027599 (2R) | 2 |
| 1666 | FBgn0004919-FBgn0003715 (2R) | 2 |
| 1667 | FBgn0011694-FBgn0004181 (2R) | 2 |
| 1668 | FBgn0001325-FBgn0050429 (2R) | 2 |
| 1669 | FBgn0035097-FBgn0040688 (3L) | 2 |
| 1670 | FBgn0035099-FBgn0035101 (3L) | 2 |
| 1671 | FBgn0035131-FBgn0035132 (3L) | 2 |
| 1672 | FBgn0035134-FBgn0040291 (3L) | 2 |
| 1673 | FBgn0035136-FBgn0035137 (3L) | 2 |
| 1674 | FBgn0035138-FBgn0035139 (3L) | 2 |
| 1675 | FBgn0085296-FBgn0085298 (3L) | 2 |
| 1676 | FBgn0035140-FBgn0035141 (3L) | 2 |
| 1677 | FBgn0035143-FBgn0035144 (3L) | 2 |
| 1678 | FBgn0035150-FBgn0035151 (3L) | 2 |
| 1679 | FBgn0035152-FBgn0035153 (3L) | 2 |
| 1680 | FBgn0035154-FBgn0052483 (3L) | 2 |
| 1681 | FBgn0035162-FBgn0035164 (3L) | 2 |
| 1682 | FBgn0035167-FBgn0035168 (3L) | 2 |
| 1683 | FBgn0035170-FBgn0035171 (3L) | 2 |
| 1684 | FBgn0052479-FBgn0035173 (3L) | 2 |
| 1685 | FBgn0035178-FBgn0035179 (3L) | 2 |
| 1686 | FBgn0053966-FBgn0035192 (3L) | 2 |
| 1687 | FBgn0035199-FBgn0052333 (3L) | 2 |
| 1688 | FBgn0035217-FBgn0035218 (3L) | 2 |
| 1689 | FBgn0052319-FBgn0028567 (3L) | 2 |
| 1690 | FBgn0035233-FBgn0035232 (3L) | 2 |
| 1691 | FBgn0035234-FBgn0035235 (3L) | 2 |
| 1692 | FBgn0035243-FBgn0035244 (3L) | 2 |
| 1693 | FBgn0035247-FBgn0013753 (3L) | 2 |
| 1694 | FBgn0035248-FBgn0035249 (3L) | 2 |
| 1695 | FBgn0035251-FBgn0035252 (3L) | 2 |
| 1696 | FBgn0035253-FBgn0035254 (3L) | 2 |
| 1697 | FBgn0035264-FBgn0035265 (3L) | 2 |
| 1698 | FBgn0022702-FBgn0035268 (3L) | 2 |
| 1699 | FBgn0067864-FBgn0025820 (3L) | 2 |
| 1700 | FBgn0035293-FBgn0035294 (3L) | 2 |
| 1701 | FBgn0002872-FBgn0035295 (3L) | 2 |
| 1702 | FBgn0035298-FBgn0027547 (3L) | 2 |
| 1703 | FBgn0259703-FBgn0085288 (3L) | 2 |
| 1704 | FBgn0052311-FBgn0035308 (3L) | 2 |
| 1705 | FBgn0035309-FBgn0026570 (3L) | 2 |
| 1706 | FBgn0035315-FBgn0035316 (3L) | 2 |
| 1707 | FBgn0052301-FBgn0052305 (3L) | 2 |
| 1708 | FBgn0035325-FBgn0052302 (3L) | 2 |
| 1709 | FBgn0035348-FBgn0010905 (3L) | 2 |
| 1710 | FBgn0010909-FBgn0016794 (3L) | 2 |
| 1711 | FBgn0035358-FBgn0035359 (3L) | 2 |
| 1712 | FBgn0035392-FBgn0035393 (3L) | 2 |
| 1713 | FBgn0052486-FBgn0035397 (3L) | 2 |
| 1714 | FBgn0035405-FBgn0024194 (3L) | 2 |
| 1715 | FBgn0052278-FBgn0052281 (3L) | 2 |
| 1716 | FBgn0035412-FBgn0035413 (3L) | 2 |
| 1717 | FBgn0035415-FBgn0035416 (3L) | 2 |
| 1718 | FBgn0052271-FBgn0053159 (3L) | 2 |
| 1719 | FBgn0052282-FBgn0035434 (3L) | 2 |
| 1720 | FBgn0014388-FBgn0026259 (3L) | 2 |
| 1721 | FBgn0041164-FBgn0010317 (3L) | 2 |
| 1722 | FBgn0035449-FBgn0052267 (3L) | 2 |
| 1723 | FBgn0259224-FBgn0001254 (3L) | 2 |
| 1724 | FBgn0035469-FBgn0035470 (3L) | 2 |
| 1725 | FBgn0035475-FBgn0035476 (3L) | 2 |
| 1726 | FBgn0035481-FBgn0004880 (3L) | 2 |
| 1727 | FBgn0035482-FBgn0004895 (3L) | 2 |
| 1728 | FBgn0035483-FBgn0035484 (3L) | 2 |
| 1729 | FBgn0035488-FBgn0035489 (3L) | 2 |
| 1730 | FBgn0004516-FBgn0035495 (3L) | 2 |
| 1731 | FBgn0035497-FBgn0028484 (3L) | 2 |
| 1732 | FBgn0001257-FBgn0035515 (3L) | 2 |
| 1733 | FBgn0041171-FBgn0035517 (3L) | 2 |
| 1734 | FBgn0066365-FBgn0052251 (3L) | 2 |
| 1735 | FBgn0052241-FBgn0052249 (3L) | 2 |
| 1736 | FBgn0035554-FBgn0035555 (3L) | 2 |
| 1737 | FBgn0052243-FBgn0035558 (3L) | 2 |
| 1738 | FBgn0035593-FBgn0035592 (3L) | 2 |
| 1739 | FBgn0035591-FBgn0035590 (3L) | 2 |
| 1740 | FBgn0035589-FBgn0035588 (3L) | 2 |
| 1741 | FBgn0016031-FBgn0004380 (3L) | 2 |
| 1742 | FBgn0035600-FBgn0035601 (3L) | 2 |
| 1743 | FBgn0035604-FBgn0085420 (3L) | 2 |
| 1744 | FBgn0035619-FBgn0035620 (3L) | 2 |
| 1745 | FBgn0035627-FBgn0085447 (3L) | 2 |
| 1746 | FBgn0015806-FBgn0035639 (3L) | 2 |
| 1747 | FBgn0052409-FBgn0052412 (3L) | 2 |
| 1748 | FBgn0052407-FBgn0053523 (3L) | 2 |
| 1749 | FBgn0250815-FBgn0035665 (3L) | 2 |
| 1750 | FBgn0035666-FBgn0035667 (3L) | 2 |
| 1751 | FBgn0259164-FBgn0035673 (3L) | 2 |
| 1752 | FBgn0035674-FBgn0035675 (3L) | 2 |
| 1753 | FBgn0035676-FBgn0035677 (3L) | 2 |
| 1754 | FBgn0035679-FBgn0061492 (3L) | 2 |
| 1755 | FBgn0020645-FBgn0020765 (3L) | 2 |
| 1756 | FBgn0035692-FBgn0024921 (3L) | 2 |
| 1757 | FBgn0035695-FBgn0004513 (3L) | 2 |
| 1758 | FBgn0052392-FBgn0086680 (3L) | 2 |
| 1759 | FBgn0035722-FBgn0029117 (3L) | 2 |
| 1760 | FBgn0052391-FBgn0054030 (3L) | 2 |
| 1761 | FBgn0035742-FBgn0035743 (3L) | 2 |
| 1762 | FBgn0035760-FBgn0035761 (3L) | 2 |
| 1763 | FBgn0052380-FBgn0016983 (3L) | 2 |
| 1764 | FBgn0035777-FBgn0052379 (3L) | 2 |
| 1765 | FBgn0052383-FBgn0052382 (3L) | 2 |
| 1766 | FBgn0053276-FBgn0035805 (3L) | 2 |
| 1767 | FBgn0035838-FBgn0035839 (3L) | 2 |
| 1768 | FBgn0035842-FBgn0002899 (3L) | 2 |
| 1769 | FBgn0035845-FBgn0040290 (3L) | 2 |
| 1770 | FBgn0259232-FBgn0035861 (3L) | 2 |
| 1771 | FBgn0035871-FBgn0035872 (3L) | 2 |
| 1772 | FBgn0035886-FBgn0035887 (3L) | 2 |
| 1773 | FBgn0035888-FBgn0035890 (3L) | 2 |
| 1774 | FBgn0035891-FBgn0010431 (3L) | 2 |
| 1775 | FBgn0035915-FBgn0052351 (3L) | 2 |
| 1776 | FBgn0011509-FBgn0052022 (3L) | 2 |
| 1777 | FBgn0040828-FBgn0035924 (3L) | 2 |
| 1778 | FBgn0035942-FBgn0035943 (3L) | 2 |
| 1779 | FBgn0086706-FBgn0035947 (3L) | 2 |
| 1780 | FBgn0035948-FBgn0035949 (3L) | 2 |
| 1781 | FBgn0016070-FBgn0035953 (3L) | 2 |
| 1782 | FBgn0035959-FBgn0035960 (3L) | 2 |
| 1783 | FBgn0023479-FBgn0043806 (3L) | 2 |
| 1784 | FBgn0035964-FBgn0035965 (3L) | 2 |
| 1785 | FBgn0035981-FBgn0011769 (3L) | 2 |
| 1786 | FBgn0001224-FBgn0001226 (3L) | 2 |
| 1787 | FBgn0035986-FBgn0035987 (3L) | 2 |
| 1788 | FBgn0035989-FBgn0015296 (3L) | 2 |
| 1789 | FBgn0036004-FBgn0036005 (3L) | 2 |
| 1790 | FBgn0052036-FBgn0052037 (3L) | 2 |
| 1791 | FBgn0036016-FBgn0036017 (3L) | 2 |
| 1792 | FBgn0036023-FBgn0036024 (3L) | 2 |
| 1793 | FBgn0016081-FBgn0036028 (3L) | 2 |
| 1794 | FBgn0036030-FBgn0015321 (3L) | 2 |
| 1795 | FBgn0036031-FBgn0036032 (3L) | 2 |
| 1796 | FBgn0036044-FBgn0044051 (3L) | 2 |
| 1797 | FBgn0036046-FBgn0044050 (3L) | 2 |
| 1798 | FBgn0044049-FBgn0044328 (3L) | 2 |
| 1799 | FBgn0015618-FBgn0004390 (3L) | 2 |
| 1800 | FBgn0036052-FBgn0027567 (3L) | 2 |
| 1801 | FBgn0036070-FBgn0036072 (3L) | 2 |
| 1802 | FBgn0001179-FBgn0036090 (3L) | 2 |
| 1803 | FBgn0036093-FBgn0036094 (3L) | 2 |
| 1804 | FBgn0052066-FBgn0036096 (3L) | 2 |
| 1805 | FBgn0053493-FBgn0036101 (3L) | 2 |
| 1806 | FBgn0026160-FBgn0036104 (3L) | 2 |
| 1807 | FBgn0036107-FBgn0027615 (3L) | 2 |
| 1808 | FBgn0036108-FBgn0036109 (3L) | 2 |
| 1809 | FBgn0052079-FBgn0052081 (3L) | 2 |
| 1810 | FBgn0036128-FBgn0052072 (3L) | 2 |
| 1811 | FBgn0052071-FBgn0052073 (3L) | 2 |
| 1812 | FBgn0036131-FBgn0054012 (3L) | 2 |
| 1813 | FBgn0036134-FBgn0036135 (3L) | 2 |
| 1814 | FBgn0014368-FBgn0052074 (3L) | 2 |
| 1815 | FBgn0052082-FBgn0036139 (3L) | 2 |
| 1816 | FBgn0036145-FBgn0036146 (3L) | 2 |
| 1817 | FBgn0036152-FBgn0036153 (3L) | 2 |
| 1818 | FBgn0036155-FBgn0036156 (3L) | 2 |
| 1819 | FBgn0003378-FBgn0003377 (3L) | 2 |
| 1820 | FBgn0003292-FBgn0036179 (3L) | 2 |
| 1821 | FBgn0036205-FBgn0015278 (3L) | 2 |
| 1822 | FBgn0036207-FBgn0004381 (3L) | 2 |
| 1823 | FBgn0036230-FBgn0028573 (3L) | 2 |
| 1824 | FBgn0053265-FBgn0036232 (3L) | 2 |
| 1825 | FBgn0036233-FBgn0036234 (3L) | 2 |
| 1826 | FBgn0036235-FBgn0036236 (3L) | 2 |
| 1827 | FBgn0036248-FBgn0036249 (3L) | 2 |
| 1828 | FBgn0036259-FBgn0036260 (3L) | 2 |
| 1829 | FBgn0036263-FBgn0036264 (3L) | 2 |
| 1830 | FBgn0036266-FBgn0052100 (3L) | 2 |
| 1831 | FBgn0036271-FBgn0036272 (3L) | 2 |
| 1832 | FBgn0036274-FBgn0052105 (3L) | 2 |
| 1833 | FBgn0052103-FBgn0020655 (3L) | 2 |
| 1834 | FBgn0036285-FBgn0000625 (3L) | 2 |
| 1835 | FBgn0036287-FBgn0053725 (3L) | 2 |
| 1836 | FBgn0036320-FBgn0036321 (3L) | 2 |
| 1837 | FBgn0036331-FBgn0036332 (3L) | 2 |
| 1838 | FBgn0036338-FBgn0083978 (3L) | 2 |
| 1839 | FBgn0036341-FBgn0036342 (3L) | 2 |
| 1840 | FBgn0053262-FBgn0086916 (3L) | 2 |
| 1841 | FBgn0040814-FBgn0036348 (3L) | 2 |
| 1842 | FBgn0086708-FBgn0036372 (3L) | 2 |
| 1843 | FBgn0036373-FBgn0036374 (3L) | 2 |
| 1844 | FBgn0086785-FBgn0036376 (3L) | 2 |
| 1845 | FBgn0025874-FBgn0036389 (3L) | 2 |
| 1846 | FBgn0005592-FBgn0036410 (3L) | 2 |
| 1847 | FBgn0036411-FBgn0042630 (3L) | 2 |
| 1848 | FBgn0036416-FBgn0036417 (3L) | 2 |
| 1849 | FBgn0003459-FBgn0036423 (3L) | 2 |
| 1850 | FBgn0036428-FBgn0054039 (3L) | 2 |
| 1851 | FBgn0028418-FBgn0040318 (3L) | 2 |
| 1852 | FBgn0013263-FBgn0036446 (3L) | 2 |
| 1853 | FBgn0036460-FBgn0002778 (3L) | 2 |
| 1854 | FBgn0036474-FBgn0086690 (3L) | 2 |
| 1855 | FBgn0036476-FBgn0036478 (3L) | 2 |
| 1856 | FBgn0047178-FBgn0036484 (3L) | 2 |
| 1857 | FBgn0036491-FBgn0036492 (3L) | 2 |
| 1858 | FBgn0036496-FBgn0036498 (3L) | 2 |
| 1859 | FBgn0087035-FBgn0036509 (3L) | 2 |
| 1860 | FBgn0036527-FBgn0036528 (3L) | 2 |
| 1861 | FBgn0085481-FBgn0085480 (3L) | 2 |
| 1862 | FBgn0041160-FBgn0010105 (3L) | 2 |
| 1863 | FBgn0036531-FBgn0036532 (3L) | 2 |
| 1864 | FBgn0036534-FBgn0040230 (3L) | 2 |
| 1865 | FBgn0036537-FBgn0036538 (3L) | 2 |
| 1866 | FBgn0036552-FBgn0036553 (3L) | 2 |
| 1867 | FBgn0003076-FBgn0011016 (3L) | 2 |
| 1868 | FBgn0036575-FBgn0052152 (3L) | 2 |
| 1869 | FBgn0053795-FBgn0036577 (3L) | 2 |
| 1870 | FBgn0085275-FBgn0052155 (3L) | 2 |
| 1871 | FBgn0036601-FBgn0036602 (3L) | 2 |
| 1872 | FBgn0052160-FBgn0036603 (3L) | 2 |
| 1873 | FBgn0036605-FBgn0036606 (3L) | 2 |
| 1874 | FBgn0036607-FBgn0036608 (3L) | 2 |
| 1875 | FBgn0036614-FBgn0036615 (3L) | 2 |
| 1876 | FBgn0036619-FBgn0036620 (3L) | 2 |
| 1877 | FBgn0025558-FBgn0017572 (3L) | 2 |
| 1878 | FBgn0036652-FBgn0036654 (3L) | 2 |
| 1879 | FBgn0036655-FBgn0036656 (3L) | 2 |
| 1880 | FBgn0004108-FBgn0036660 (3L) | 2 |
| 1881 | FBgn0259794-FBgn0036670 (3L) | 2 |
| 1882 | FBgn0036689-FBgn0036690 (3L) | 2 |
| 1883 | FBgn0036711-FBgn0036712 (3L) | 2 |
| 1884 | FBgn0027660-FBgn0086475 (3L) | 2 |
| 1885 | FBgn0053051-FBgn0052176 (3L) | 2 |
| 1886 | FBgn0052185-FBgn0036734 (3L) | 2 |
| 1887 | FBgn0036735-FBgn0259174 (3L) | 2 |
| 1888 | FBgn0036741-FBgn0036742 (3L) | 2 |
| 1889 | FBgn0052181-FBgn0043025 (3L) | 2 |
| 1890 | FBgn0036754-FBgn0052187 (3L) | 2 |
| 1891 | FBgn0036763-FBgn0036764 (3L) | 2 |
| 1892 | FBgn0036766-FBgn0036767 (3L) | 2 |
| 1893 | FBgn0036771-FBgn0036772 (3L) | 2 |
| 1894 | FBgn0036784-FBgn0036785 (3L) | 2 |
| 1895 | FBgn0040322-FBgn0040323 (3L) | 2 |
| 1896 | FBgn0036818-FBgn0036819 (3L) | 2 |
| 1897 | FBgn0036824-FBgn0036825 (3L) | 2 |
| 1898 | FBgn0036836-FBgn0085285 (3L) | 2 |
| 1899 | FBgn0036842-FBgn0036843 (3L) | 2 |
| 1900 | FBgn0036861-FBgn0036862 (3L) | 2 |
| 1901 | FBgn0003089-FBgn0036868 (3L) | 2 |
| 1902 | FBgn0036875-FBgn0036876 (3L) | 2 |
| 1903 | FBgn0036887-FBgn0036888 (3L) | 2 |
| 1904 | FBgn0036892-FBgn0036893 (3L) | 2 |
| 1905 | FBgn0083952-FBgn0040786 (3L) | 2 |
| 1906 | FBgn0036928-FBgn0036929 (3L) | 2 |
| 1907 | FBgn0036959-FBgn0036958 (3L) | 2 |
| 1908 | FBgn0036960-FBgn0004366 (3L) | 2 |
| 1909 | FBgn0036975-FBgn0024887 (3L) | 2 |
| 1910 | FBgn0053969-FBgn0028978 (3L) | 2 |
| 1911 | FBgn0037017-FBgn0040634 (3L) | 2 |
| 1912 | FBgn0027364-FBgn0037023 (3L) | 2 |
| 1913 | FBgn0040636-FBgn0027055 (3L) | 2 |
| 1914 | FBgn0052432-FBgn0040637 (3L) | 2 |
| 1915 | FBgn0259239-FBgn0085290 (3L) | 2 |
| 1916 | FBgn0037059-FBgn0037060 (3L) | 2 |
| 1917 | FBgn0037061-FBgn0028427 (3L) | 2 |
| 1918 | FBgn0012034-FBgn0027945 (3L) | 2 |
| 1919 | FBgn0037071-FBgn0003042 (3L) | 2 |
| 1920 | FBgn0037087-FBgn0015239 (3L) | 2 |
| 1921 | FBgn0053214-FBgn0037092 (3L) | 2 |
| 1922 | FBgn0052442-FBgn0029152 (3L) | 2 |
| 1923 | FBgn0015283-FBgn0037097 (3L) | 2 |
| 1924 | FBgn0037105-FBgn0037106 (3L) | 2 |
| 1925 | FBgn0037116-FBgn0037117 (3L) | 2 |
| 1926 | FBgn0037120-FBgn0037121 (3L) | 2 |
| 1927 | FBgn0037127-FBgn0037128 (3L) | 2 |
| 1928 | FBgn0022936-FBgn0037134 (3L) | 2 |
| 1929 | FBgn0052448-FBgn0046301 (3L) | 2 |
| 1930 | FBgn0037137-FBgn0037138 (3L) | 2 |
| 1931 | FBgn0052450-FBgn0037162 (3L) | 2 |
| 1932 | FBgn0053170-FBgn0037199 (3L) | 2 |
| 1933 | FBgn0044324-FBgn0037202 (3L) | 2 |
| 1934 | FBgn0024733-FBgn0000258 (3L) | 2 |
| 1935 | FBgn0058470-FBgn0058053 (3L) | 2 |
| 1936 | FBgn0259697-FBgn0040045 (3L) | 2 |
| 1937 | FBgn0037215-FBgn0037218 (3R) | 2 |
| 1938 | FBgn0037220-FBgn0015331 (3R) | 2 |
| 1939 | FBgn0037261-FBgn0037262 (3R) | 2 |
| 1940 | FBgn0037265-FBgn0051531 (3R) | 2 |
| 1941 | FBgn0004913-FBgn0051536 (3R) | 2 |
| 1942 | FBgn0037291-FBgn0037292 (3R) | 2 |
| 1943 | FBgn0010313-FBgn0037293 (3R) | 2 |
| 1944 | FBgn0037305-FBgn0086689 (3R) | 2 |
| 1945 | FBgn0051538-FBgn0037310 (3R) | 2 |
| 1946 | FBgn0015324-FBgn0250753 (3R) | 2 |
| 1947 | FBgn0037352-FBgn0051551 (3R) | 2 |
| 1948 | FBgn0051549-FBgn0037354 (3R) | 2 |
| 1949 | FBgn0027608-FBgn0037376 (3R) | 2 |
| 1950 | FBgn0037377-FBgn0037378 (3R) | 2 |
| 1951 | FBgn0037388-FBgn0037389 (3R) | 2 |
| 1952 | FBgn0083949-FBgn0040679 (3R) | 2 |
| 1953 | FBgn0046878-FBgn0046876 (3R) | 2 |
| 1954 | FBgn0037435-FBgn0037436 (3R) | 2 |
| 1955 | FBgn0010355-FBgn0026566 (3R) | 2 |
| 1956 | FBgn0037483-FBgn0250845 (3R) | 2 |
| 1957 | FBgn0037492-FBgn0011282 (3R) | 2 |
| 1958 | FBgn0037498-FBgn0051496 (3R) | 2 |
| 1959 | FBgn0042101-FBgn0037521 (3R) | 2 |
| 1960 | FBgn0014931-FBgn0037531 (3R) | 2 |
| 1961 | FBgn0037536-FBgn0037537 (3R) | 2 |
| 1962 | FBgn0037538-FBgn0037539 (3R) | 2 |
| 1963 | FBgn0037540-FBgn0001255 (3R) | 2 |
| 1964 | FBgn0037555-FBgn0037556 (3R) | 2 |
| 1965 | FBgn0037561-FBgn0010433 (3R) | 2 |
| 1966 | FBgn0062412-FBgn0037576 (3R) | 2 |
| 1967 | FBgn0004908-FBgn0037578 (3R) | 2 |
| 1968 | FBgn0037579-FBgn0037581 (3R) | 2 |
| 1969 | FBgn0051454-FBgn0051259 (3R) | 2 |
| 1970 | FBgn0051450-FBgn0051460 (3R) | 2 |
| 1971 | FBgn0037623-FBgn0037624 (3R) | 2 |
| 1972 | FBgn0053191-FBgn0053189 (3R) | 2 |
| 1973 | FBgn0037633-FBgn0037634 (3R) | 2 |
| 1974 | FBgn0037655-FBgn0040534 (3R) | 2 |
| 1975 | FBgn0015014-FBgn0037657 (3R) | 2 |
| 1976 | FBgn0037665-FBgn0037666 (3R) | 2 |
| 1977 | FBgn0037669-FBgn0037670 (3R) | 2 |
| 1978 | FBgn0037680-FBgn0053936 (3R) | 2 |
| 1979 | FBgn0014022-FBgn0037703 (3R) | 2 |
| 1980 | FBgn0069242-FBgn0037731 (3R) | 2 |
| 1981 | FBgn0003886-FBgn0037736 (3R) | 2 |
| 1982 | FBgn0051415-FBgn0085438 (3R) | 2 |
| 1983 | FBgn0037739-FBgn0005777 (3R) | 2 |
| 1984 | FBgn0037751-FBgn0037753 (3R) | 2 |
| 1985 | FBgn0037759-FBgn0037760 (3R) | 2 |
| 1986 | FBgn0053631-FBgn0053784 (3R) | 2 |
| 1987 | FBgn0051477-FBgn0001321 (3R) | 2 |
| 1988 | FBgn0037808-FBgn0037809 (3R) | 2 |
| 1989 | FBgn0037812-FBgn0051406 (3R) | 2 |
| 1990 | FBgn0020379-FBgn0045443 (3R) | 2 |
| 1991 | FBgn0037814-FBgn0037815 (3R) | 2 |
| 1992 | FBgn0020385-FBgn0037822 (3R) | 2 |
| 1993 | FBgn0051390-FBgn0010421 (3R) | 2 |
| 1994 | FBgn0037836-FBgn0037837 (3R) | 2 |
| 1995 | FBgn0037842-FBgn0037843 (3R) | 2 |
| 1996 | FBgn0040253-FBgn0040255 (3R) | 2 |
| 1997 | FBgn0051441-FBgn0051388 (3R) | 2 |
| 1998 | FBgn0037890-FBgn0037891 (3R) | 2 |
| 1999 | FBgn0026064-FBgn0026063 (3R) | 2 |
| 2000 | FBgn0037895-FBgn0037896 (3R) | 2 |
| 2001 | FBgn0037902-FBgn0046225 (3R) | 2 |
| 2002 | FBgn0037908-FBgn0053512 (3R) | 2 |
| 2003 | FBgn0011290-FBgn0037933 (3R) | 2 |
| 2004 | FBgn0037935-FBgn0037936 (3R) | 2 |
| 2005 | FBgn0037943-FBgn0037944 (3R) | 2 |
| 2006 | FBgn0259139-FBgn0037955 (3R) | 2 |
| 2007 | FBgn0000147-FBgn0037970 (3R) | 2 |
| 2008 | FBgn0037989-FBgn0051361 (3R) | 2 |
| 2009 | FBgn0037992-FBgn0037993 (3R) | 2 |
| 2010 | FBgn0037994-FBgn0037995 (3R) | 2 |
| 2011 | FBgn0037998-FBgn0037999 (3R) | 2 |
| 2012 | FBgn0038000-FBgn0038001 (3R) | 2 |
| 2013 | FBgn0038011-FBgn0038012 (3R) | 2 |
| 2014 | FBgn0043043-FBgn0038051 (3R) | 2 |
| 2015 | FBgn0250910-FBgn0038063 (3R) | 2 |
| 2016 | FBgn0005671-FBgn0038065 (3R) | 2 |
| 2017 | FBgn0026207-FBgn0038076 (3R) | 2 |
| 2018 | FBgn0016693-FBgn0038080 (3R) | 2 |
| 2019 | FBgn0038100-FBgn0051342 (3R) | 2 |
| 2020 | FBgn0004666-FBgn0038118 (3R) | 2 |
| 2021 | FBgn0038135-FBgn0038136 (3R) | 2 |
| 2022 | FBgn0027610-FBgn0086371 (3R) | 2 |
| 2023 | FBgn0044511-FBgn0038145 (3R) | 2 |
| 2024 | FBgn0003060-FBgn0038161 (3R) | 2 |
| 2025 | FBgn0086897-FBgn0015778 (3R) | 2 |
| 2026 | FBgn0038166-FBgn0024555 (3R) | 2 |
| 2027 | FBgn0038170-FBgn0002937 (3R) | 2 |
| 2028 | FBgn0259721-FBgn0038183 (3R) | 2 |
| 2029 | FBgn0013998-FBgn0051495 (3R) | 2 |
| 2030 | FBgn0038191-FBgn0015270 (3R) | 2 |
| 2031 | FBgn0038195-FBgn0038196 (3R) | 2 |
| 2032 | FBgn0038211-FBgn0051326 (3R) | 2 |
| 2033 | FBgn0053329-FBgn0027563 (3R) | 2 |
| 2034 | FBgn0003862-FBgn0038220 (3R) | 2 |
| 2035 | FBgn0013767-FBgn0038223 (3R) | 2 |
| 2036 | FBgn0038234-FBgn0038235 (3R) | 2 |
| 2037 | FBgn0038241-FBgn0038242 (3R) | 2 |
| 2038 | FBgn0038244-FBgn0020299 (3R) | 2 |
| 2039 | FBgn0003169-FBgn0013981 (3R) | 2 |
| 2040 | FBgn0038247-FBgn0038248 (3R) | 2 |
| 2041 | FBgn0038256-FBgn0011217 (3R) | 2 |
| 2042 | FBgn0004597-FBgn0038272 (3R) | 2 |
| 2043 | FBgn0038290-FBgn0038291 (3R) | 2 |
| 2044 | FBgn0038293-FBgn0038294 (3R) | 2 |
| 2045 | FBgn0038295-FBgn0038296 (3R) | 2 |
| 2046 | FBgn0028984-FBgn0038299 (3R) | 2 |
| 2047 | FBgn0038300-FBgn0038301 (3R) | 2 |
| 2048 | FBgn0038306-FBgn0045035 (3R) | 2 |
| 2049 | FBgn0019925-FBgn0051301 (3R) | 2 |
| 2050 | FBgn0038325-FBgn0038326 (3R) | 2 |
| 2051 | FBgn0038365-FBgn0038366 (3R) | 2 |
| 2052 | FBgn0038387-FBgn0013334 (3R) | 2 |
| 2053 | FBgn0002783-FBgn0022787 (3R) | 2 |
| 2054 | FBgn0038397-FBgn0052855 (3R) | 2 |
| 2055 | FBgn0038398-FBgn0026059 (3R) | 2 |
| 2056 | FBgn0027053-FBgn0004087 (3R) | 2 |
| 2057 | FBgn0038439-FBgn0038440 (3R) | 2 |
| 2058 | FBgn0259244-FBgn0038445 (3R) | 2 |
| 2059 | FBgn0038449-FBgn0038450 (3R) | 2 |
| 2060 | FBgn0038451-FBgn0038452 (3R) | 2 |
| 2061 | FBgn0038462-FBgn0038463 (3R) | 2 |
| 2062 | FBgn0038479-FBgn0051269 (3R) | 2 |
| 2063 | FBgn0038492-FBgn0051262 (3R) | 2 |
| 2064 | FBgn0051418-FBgn0051419 (3R) | 2 |
| 2065 | FBgn0028992-FBgn0025456 (3R) | 2 |
| 2066 | FBgn0025571-FBgn0038519 (3R) | 2 |
| 2067 | FBgn0038523-FBgn0003375 (3R) | 2 |
| 2068 | FBgn0038545-FBgn0038546 (3R) | 2 |
| 2069 | FBgn0038551-FBgn0038552 (3R) | 2 |
| 2070 | FBgn0038558-FBgn0053547 (3R) | 2 |
| 2071 | FBgn0028499-FBgn0038581 (3R) | 2 |
| 2072 | FBgn0038611-FBgn0038612 (3R) | 2 |
| 2073 | FBgn0004652-FBgn0038626 (3R) | 2 |
| 2074 | FBgn0023083-FBgn0038627 (3R) | 2 |
| 2075 | FBgn0038628-FBgn0038630 (3R) | 2 |
| 2076 | FBgn0051224-FBgn0044871 (3R) | 2 |
| 2077 | FBgn0051229-FBgn0038638 (3R) | 2 |
| 2078 | FBgn0038639-FBgn0038640 (3R) | 2 |
| 2079 | FBgn0038643-FBgn0085311 (3R) | 2 |
| 2080 | FBgn0015323-FBgn0000303 (3R) | 2 |
| 2081 | FBgn0085313-FBgn0038658 (3R) | 2 |
| 2082 | FBgn0038680-FBgn0038681 (3R) | 2 |
| 2083 | FBgn0038683-FBgn0259704 (3R) | 2 |
| 2084 | FBgn0010768-FBgn0038685 (3R) | 2 |
| 2085 | FBgn0038690-FBgn0038691 (3R) | 2 |
| 2086 | FBgn0038700-FBgn0038701 (3R) | 2 |
| 2087 | FBgn0038702-FBgn0051244 (3R) | 2 |
| 2088 | FBgn0038704-FBgn0038705 (3R) | 2 |
| 2089 | FBgn0038720-FBgn0038721 (3R) | 2 |
| 2090 | FBgn0014135-FBgn0051459 (3R) | 2 |
| 2091 | FBgn0038735-FBgn0038737 (3R) | 2 |
| 2092 | FBgn0038738-FBgn0038739 (3R) | 2 |
| 2093 | FBgn0038740-FBgn0038741 (3R) | 2 |
| 2094 | FBgn0038742-FBgn0014006 (3R) | 2 |
| 2095 | FBgn0038746-FBgn0038747 (3R) | 2 |
| 2096 | FBgn0038753-FBgn0021776 (3R) | 2 |
| 2097 | FBgn0038763-FBgn0243511 (3R) | 2 |
| 2098 | FBgn0083975-FBgn0038775 (3R) | 2 |
| 2099 | FBgn0038783-FBgn0038784 (3R) | 2 |
| 2100 | FBgn0038787-FBgn0038788 (3R) | 2 |
| 2101 | FBgn0038796-FBgn0038797 (3R) | 2 |
| 2102 | FBgn0040575-FBgn0038803 (3R) | 2 |
| 2103 | FBgn0038805-FBgn0038808 (3R) | 2 |
| 2104 | FBgn0038809-FBgn0014029 (3R) | 2 |
| 2105 | FBgn0001169-FBgn0015279 (3R) | 2 |
| 2106 | FBgn0051201-FBgn0028422 (3R) | 2 |
| 2107 | FBgn0024944-FBgn0051205 (3R) | 2 |
| 2108 | FBgn0038821-FBgn0259222 (3R) | 2 |
| 2109 | FBgn0051199-FBgn0051200 (3R) | 2 |
| 2110 | FBgn0046689-FBgn0038827 (3R) | 2 |
| 2111 | FBgn0259215-FBgn0038837 (3R) | 2 |
| 2112 | FBgn0038838-FBgn0044809 (3R) | 2 |
| 2113 | FBgn0026056-FBgn0046763 (3R) | 2 |
| 2114 | FBgn0011672-FBgn0025865 (3R) | 2 |
| 2115 | FBgn0038851-FBgn0038852 (3R) | 2 |
| 2116 | FBgn0038873-FBgn0038874 (3R) | 2 |
| 2117 | FBgn0038879-FBgn0038880 (3R) | 2 |
| 2118 | FBgn0004110-FBgn0004862 (3R) | 2 |
| 2119 | FBgn0013984-FBgn0038893 (3R) | 2 |
| 2120 | FBgn0053092-FBgn0051465 (3R) | 2 |
| 2121 | FBgn0038915-FBgn0038917 (3R) | 2 |
| 2122 | FBgn0038918-FBgn0038919 (3R) | 2 |
| 2123 | FBgn0051164-FBgn0051424 (3R) | 2 |
| 2124 | FBgn0038944-FBgn0038945 (3R) | 2 |
| 2125 | FBgn0038953-FBgn0051169 (3R) | 2 |
| 2126 | FBgn0028688-FBgn0038972 (3R) | 2 |
| 2127 | FBgn0038978-FBgn0038979 (3R) | 2 |
| 2128 | FBgn0038996-FBgn0020278 (3R) | 2 |
| 2129 | FBgn0039000-FBgn0046114 (3R) | 2 |
| 2130 | FBgn0039003-FBgn0039004 (3R) | 2 |
| 2131 | FBgn0051151-FBgn0039013 (3R) | 2 |
| 2132 | FBgn0039017-FBgn0039018 (3R) | 2 |
| 2133 | FBgn0039020-FBgn0051139 (3R) | 2 |
| 2134 | FBgn0039022-FBgn0039023 (3R) | 2 |
| 2135 | FBgn0039033-FBgn0039034 (3R) | 2 |
| 2136 | FBgn0039041-FBgn0039042 (3R) | 2 |
| 2137 | FBgn0039044-FBgn0039045 (3R) | 2 |
| 2138 | FBgn0053193-FBgn0027526 (3R) | 2 |
| 2139 | FBgn0039051-FBgn0039052 (3R) | 2 |
| 2140 | FBgn0039059-FBgn0039060 (3R) | 2 |
| 2141 | FBgn0039070-FBgn0039071 (3R) | 2 |
| 2142 | FBgn0039094-FBgn0051145 (3R) | 2 |
| 2143 | FBgn0039101-FBgn0042106 (3R) | 2 |
| 2144 | FBgn0002922-FBgn0039108 (3R) | 2 |
| 2145 | FBgn0039125-FBgn0039126 (3R) | 2 |
| 2146 | FBgn0039128-FBgn0039129 (3R) | 2 |
| 2147 | FBgn0051140-FBgn0051451 (3R) | 2 |
| 2148 | FBgn0039135-FBgn0039136 (3R) | 2 |
| 2149 | FBgn0039149-FBgn0039150 (3R) | 2 |
| 2150 | FBgn0039152-FBgn0039151 (3R) | 2 |
| 2151 | FBgn0051133-FBgn0046322 (3R) | 2 |
| 2152 | FBgn0015765-FBgn0039156 (3R) | 2 |
| 2153 | FBgn0039158-FBgn0039159 (3R) | 2 |
| 2154 | FBgn0029503-FBgn0039163 (3R) | 2 |
| 2155 | FBgn0039169-FBgn0002863 (3R) | 2 |
| 2156 | FBgn0039190-FBgn0053340 (3R) | 2 |
| 2157 | FBgn0039224-FBgn0039225 (3R) | 2 |
| 2158 | FBgn0051118-FBgn0051357 (3R) | 2 |
| 2159 | FBgn0039233-FBgn0039234 (3R) | 2 |
| 2160 | FBgn0051121-FBgn0039244 (3R) | 2 |
| 2161 | FBgn0039246-FBgn0040601 (3R) | 2 |
| 2162 | FBgn0051120-FBgn0039249 (3R) | 2 |
| 2163 | FBgn0039252-FBgn0020626 (3R) | 2 |
| 2164 | FBgn0029157-FBgn0039254 (3R) | 2 |
| 2165 | FBgn0259178-FBgn0039264 (3R) | 2 |
| 2166 | FBgn0039265-FBgn0039266 (3R) | 2 |
| 2167 | FBgn0004897-FBgn0004898 (3R) | 2 |
| 2168 | FBgn0051108-FBgn0051510 (3R) | 2 |
| 2169 | FBgn0039324-FBgn0039325 (3R) | 2 |
| 2170 | FBgn0045761-FBgn0039328 (3R) | 2 |
| 2171 | FBgn0039329-FBgn0028647 (3R) | 2 |
| 2172 | FBgn0027376-FBgn0039331 (3R) | 2 |
| 2173 | FBgn0039354-FBgn0039355 (3R) | 2 |
| 2174 | FBgn0039356-FBgn0039357 (3R) | 2 |
| 2175 | FBgn0039358-FBgn0039359 (3R) | 2 |
| 2176 | FBgn0039360-FBgn0051092 (3R) | 2 |
| 2177 | FBgn0039408-FBgn0039411 (3R) | 2 |
| 2178 | FBgn0039421-FBgn0039424 (3R) | 2 |
| 2179 | FBgn0051084-FBgn0008649 (3R) | 2 |
| 2180 | FBgn0039451-FBgn0040607 (3R) | 2 |
| 2181 | FBgn0039453-FBgn0039454 (3R) | 2 |
| 2182 | FBgn0039459-FBgn0027889 (3R) | 2 |
| 2183 | FBgn0004903-FBgn0039461 (3R) | 2 |
| 2184 | FBgn0004359-FBgn0004510 (3R) | 2 |
| 2185 | FBgn0039464-FBgn0039465 (3R) | 2 |
| 2186 | FBgn0041224-FBgn0039466 (3R) | 2 |
| 2187 | FBgn0039469-FBgn0051077 (3R) | 2 |
| 2188 | FBgn0039470-FBgn0039471 (3R) | 2 |
| 2189 | FBgn0039490-FBgn0039491 (3R) | 2 |
| 2190 | FBgn0039492-FBgn0029155 (3R) | 2 |
| 2191 | FBgn0039500-FBgn0039501 (3R) | 2 |
| 2192 | FBgn0039503-FBgn0039504 (3R) | 2 |
| 2193 | FBgn0015737-FBgn0040609 (3R) | 2 |
| 2194 | FBgn0039519-FBgn0039520 (3R) | 2 |
| 2195 | FBgn0039531-FBgn0039532 (3R) | 2 |
| 2196 | FBgn0051058-FBgn0027494 (3R) | 2 |
| 2197 | FBgn0039538-FBgn0003330 (3R) | 2 |
| 2198 | FBgn0039554-FBgn0039555 (3R) | 2 |
| 2199 | FBgn0039561-FBgn0039562 (3R) | 2 |
| 2200 | FBgn0039566-FBgn0003890 (3R) | 2 |
| 2201 | FBgn0039591-FBgn0039592 (3R) | 2 |
| 2202 | FBgn0024273-FBgn0015589 (3R) | 2 |
| 2203 | FBgn0259220-FBgn0053203 (3R) | 2 |
| 2204 | FBgn0039628-FBgn0039629 (3R) | 2 |
| 2205 | FBgn0039651-FBgn0051044 (3R) | 2 |
| 2206 | FBgn0029176-FBgn0062442 (3R) | 2 |
| 2207 | FBgn0039656-FBgn0051427 (3R) | 2 |
| 2208 | FBgn0040623-FBgn0039665 (3R) | 2 |
| 2209 | FBgn0039681-FBgn0013972 (3R) | 2 |
| 2210 | FBgn0039686-FBgn0085325 (3R) | 2 |
| 2211 | FBgn0019972-FBgn0039697 (3R) | 2 |
| 2212 | FBgn0039703-FBgn0039704 (3R) | 2 |
| 2213 | FBgn0051040-FBgn0039727 (3R) | 2 |
| 2214 | FBgn0004622-FBgn0039728 (3R) | 2 |
| 2215 | FBgn0039730-FBgn0039733 (3R) | 2 |
| 2216 | FBgn0039737-FBgn0039738 (3R) | 2 |
| 2217 | FBgn0026597-FBgn0039740 (3R) | 2 |
| 2218 | FBgn0041102-FBgn0001281 (3R) | 2 |
| 2219 | FBgn0085346-FBgn0015542 (3R) | 2 |
| 2220 | FBgn0051031-FBgn0040625 (3R) | 2 |
| 2221 | FBgn0051025-FBgn0051029 (3R) | 2 |
| 2222 | FBgn0039756-FBgn0039757 (3R) | 2 |
| 2223 | FBgn0000278-FBgn0000279 (3R) | 2 |
| 2224 | FBgn0039758-FBgn0039759 (3R) | 2 |
| 2225 | FBgn0039760-FBgn0039761 (3R) | 2 |
| 2226 | FBgn0015221-FBgn0015222 (3R) | 2 |
| 2227 | FBgn0039768-FBgn0039769 (3R) | 2 |
| 2228 | FBgn0027654-FBgn0082582 (3R) | 2 |
| 2229 | FBgn0039800-FBgn0039801 (3R) | 2 |
| 2230 | FBgn0039806-FBgn0039807 (3R) | 2 |
| 2231 | FBgn0015032-FBgn0053483 (3R) | 2 |
| 2232 | FBgn0039828-FBgn0039829 (3R) | 2 |
| 2233 | FBgn0039832-FBgn0039833 (3R) | 2 |
| 2234 | FBgn0051204-FBgn0051002 (3R) | 2 |
| 2235 | FBgn0019990-FBgn0039846 (3R) | 2 |
| 2236 | FBgn0039851-FBgn0039852 (3R) | 2 |
| 2237 | FBgn0043900-FBgn0039854 (3R) | 2 |
| 2238 | FBgn0003870-FBgn0039868 (3R) | 2 |
| 2239 | FBgn0039869-FBgn0039870 (3R) | 2 |
| 2240 | FBgn0039879-FBgn0250755 (3R) | 2 |
| 2241 | FBgn0039882-FBgn0024891 (3R) | 2 |
| 2242 | FBgn0039886-FBgn0011224 (3R) | 2 |
| 2243 | FBgn0002780-FBgn0002645 (3R) | 2 |
| 2244 | FBgn0001337-FBgn0029522 (X) | 2 |
| 2245 | FBgn0029525-FBgn0023536 (X) | 2 |
| 2246 | FBgn0025621-FBgn0015799 (X) | 2 |
| 2247 | FBgn0040344-FBgn0040339 (X) | 2 |
| 2248 | FBgn0040346-FBgn0040347 (X) | 2 |
| 2249 | FBgn0042146-FBgn0029552 (X) | 2 |
| 2250 | FBgn0040337-FBgn0026143 (X) | 2 |
| 2251 | FBgn0040366-FBgn0026876 (X) | 2 |
| 2252 | FBgn0000826-FBgn0029573 (X) | 2 |
| 2253 | FBgn0024987-FBgn0023169 (X) | 2 |
| 2254 | FBgn0259108-FBgn0027796 (X) | 2 |
| 2255 | FBgn0028274-FBgn0026872 (X) | 2 |
| 2256 | FBgn0025383-FBgn0025382 (X) | 2 |
| 2257 | FBgn0053513-FBgn0025393 (X) | 2 |
| 2258 | FBgn0026090-FBgn0028969 (X) | 2 |
| 2259 | FBgn0026087-FBgn0023546 (X) | 2 |
| 2260 | FBgn0025632-FBgn0025625 (X) | 2 |
| 2261 | FBgn0029596-FBgn0025627 (X) | 2 |
| 2262 | FBgn0023550-FBgn0053548 (X) | 2 |
| 2263 | FBgn0003048-FBgn0001330 (X) | 2 |
| 2264 | FBgn0040392-FBgn0053218 (X) | 2 |
| 2265 | FBgn0003714-FBgn0004050 (X) | 2 |
| 2266 | FBgn0040066-FBgn0001404 (X) | 2 |
| 2267 | FBgn0028746-FBgn0040396 (X) | 2 |
| 2268 | FBgn0028961-FBgn0044046 (X) | 2 |
| 2269 | FBgn0023216-FBgn0023215 (X) | 2 |
| 2270 | FBgn0004832-FBgn0000221 (X) | 2 |
| 2271 | FBgn0026080-FBgn0000635 (X) | 2 |
| 2272 | FBgn0029704-FBgn0040907 (X) | 2 |
| 2273 | FBgn0029707-FBgn0052773 (X) | 2 |
| 2274 | FBgn0000179-FBgn0029708 (X) | 2 |
| 2275 | FBgn0003210-FBgn0029709 (X) | 2 |
| 2276 | FBgn0029714-FBgn0029715 (X) | 2 |
| 2277 | FBgn0029716-FBgn0029717 (X) | 2 |
| 2278 | FBgn0029718-FBgn0025615 (X) | 2 |
| 2279 | FBgn0003200-FBgn0022768 (X) | 2 |
| 2280 | FBgn0061196-FBgn0029740 (X) | 2 |
| 2281 | FBgn0052767-FBgn0086911 (X) | 2 |
| 2282 | FBgn0015617-FBgn0003449 (X) | 2 |
| 2283 | FBgn0029753-FBgn0029754 (X) | 2 |
| 2284 | FBgn0029755-FBgn0014024 (X) | 2 |
| 2285 | FBgn0029756-FBgn0026751 (X) | 2 |
| 2286 | FBgn0029764-FBgn0029763 (X) | 2 |
| 2287 | FBgn0052762-FBgn0029770 (X) | 2 |
| 2288 | FBgn0029783-FBgn0028474 (X) | 2 |
| 2289 | FBgn0052758-FBgn0029798 (X) | 2 |
| 2290 | FBgn0029819-FBgn0029820 (X) | 2 |
| 2291 | FBgn0003302-FBgn0029833 (X) | 2 |
| 2292 | FBgn0029834-FBgn0029835 (X) | 2 |
| 2293 | FBgn0052750-FBgn0052751 (X) | 2 |
| 2294 | FBgn0029849-FBgn0003514 (X) | 2 |
| 2295 | FBgn0029850-FBgn0029851 (X) | 2 |
| 2296 | FBgn0029867-FBgn0029868 (X) | 2 |
| 2297 | FBgn0029870-FBgn0003139 (X) | 2 |
| 2298 | FBgn0029873-FBgn0029874 (X) | 2 |
| 2299 | FBgn0029882-FBgn0029885 (X) | 2 |
| 2300 | FBgn0029939-FBgn0029940 (X) | 2 |
| 2301 | FBgn0004198-FBgn0029955 (X) | 2 |
| 2302 | FBgn0022786-FBgn0029957 (X) | 2 |
| 2303 | FBgn0029976-FBgn0029977 (X) | 2 |
| 2304 | FBgn0029979-FBgn0029980 (X) | 2 |
| 2305 | FBgn0004403-FBgn0004404 (X) | 2 |
| 2306 | FBgn0029986-FBgn0029987 (X) | 2 |
| 2307 | FBgn0010329-FBgn0029989 (X) | 2 |
| 2308 | FBgn0029992-FBgn0029993 (X) | 2 |
| 2309 | FBgn0030005-FBgn0030001 (X) | 2 |
| 2310 | FBgn0030008-FBgn0030009 (X) | 2 |
| 2311 | FBgn0026318-FBgn0030013 (X) | 2 |
| 2312 | FBgn0030030-FBgn0030029 (X) | 2 |
| 2313 | FBgn0030033-FBgn0040928 (X) | 2 |
| 2314 | FBgn0023506-FBgn0014032 (X) | 2 |
| 2315 | FBgn0030051-FBgn0030052 (X) | 2 |
| 2316 | FBgn0030053-FBgn0025864 (X) | 2 |
| 2317 | FBgn0030057-FBgn0027864 (X) | 2 |
| 2318 | FBgn0026411-FBgn0030065 (X) | 2 |
| 2319 | FBgn0030066-FBgn0030067 (X) | 2 |
| 2320 | FBgn0030071-FBgn0030074 (X) | 2 |
| 2321 | FBgn0030097-FBgn0030098 (X) | 2 |
| 2322 | FBgn0030099-FBgn0030100 (X) | 2 |
| 2323 | FBgn0030107-FBgn0030108 (X) | 2 |
| 2324 | FBgn0052703-FBgn0030120 (X) | 2 |
| 2325 | FBgn0030121-FBgn0030122 (X) | 2 |
| 2326 | FBgn0030137-FBgn0052702 (X) | 2 |
| 2327 | FBgn0030141-FBgn0030142 (X) | 2 |
| 2328 | FBgn0000233-FBgn0020378 (X) | 2 |
| 2329 | FBgn0052698-FBgn0019929 (X) | 2 |
| 2330 | FBgn0030161-FBgn0030162 (X) | 2 |
| 2331 | FBgn0030163-FBgn0030164 (X) | 2 |
| 2332 | FBgn0005391-FBgn0004045 (X) | 2 |
| 2333 | FBgn0001203-FBgn0085348 (X) | 2 |
| 2334 | FBgn0040942-FBgn0030189 (X) | 2 |
| 2335 | FBgn0030215-FBgn0085443 (X) | 2 |
| 2336 | FBgn0003204-FBgn0052676 (X) | 2 |
| 2337 | FBgn0030228-FBgn0030234 (X) | 2 |
| 2338 | FBgn0003321-FBgn0030239 (X) | 2 |
| 2339 | FBgn0030261-FBgn0003366 (X) | 2 |
| 2340 | FBgn0030268-FBgn0030269 (X) | 2 |
| 2341 | FBgn0052668-FBgn0026268 (X) | 2 |
| 2342 | FBgn0030286-FBgn0028421 (X) | 2 |
| 2343 | FBgn0030290-FBgn0030291 (X) | 2 |
| 2344 | FBgn0030299-FBgn0030301 (X) | 2 |
| 2345 | FBgn0030302-FBgn0030303 (X) | 2 |
| 2346 | FBgn0030305-FBgn0030306 (X) | 2 |
| 2347 | FBgn0030320-FBgn0030321 (X) | 2 |
| 2348 | FBgn0014133-FBgn0011754 (X) | 2 |
| 2349 | FBgn0030331-FBgn0002577 (X) | 2 |
| 2350 | FBgn0030349-FBgn0030350 (X) | 2 |
| 2351 | FBgn0030358-FBgn0005563 (X) | 2 |
| 2352 | FBgn0000808-FBgn0003865 (X) | 2 |
| 2353 | FBgn0030391-FBgn0259143 (X) | 2 |
| 2354 | FBgn0030408-FBgn0030409 (X) | 2 |
| 2355 | FBgn0030410-FBgn0030411 (X) | 2 |
| 2356 | FBgn0030420-FBgn0030421 (X) | 2 |
| 2357 | FBgn0052649-FBgn0030431 (X) | 2 |
| 2358 | FBgn0085440-FBgn0030459 (X) | 2 |
| 2359 | FBgn0030460-FBgn0005410 (X) | 2 |
| 2360 | FBgn0030485-FBgn0030486 (X) | 2 |
| 2361 | FBgn0030500-FBgn0030501 (X) | 2 |
| 2362 | FBgn0030506-FBgn0030507 (X) | 2 |
| 2363 | FBgn0030508-FBgn0030509 (X) | 2 |
| 2364 | FBgn0030510-FBgn0030511 (X) | 2 |
| 2365 | FBgn0030512-FBgn0030514 (X) | 2 |
| 2366 | FBgn0030524-FBgn0030525 (X) | 2 |
| 2367 | FBgn0030540-FBgn0030541 (X) | 2 |
| 2368 | FBgn0030571-FBgn0030572 (X) | 2 |
| 2369 | FBgn0030577-FBgn0030578 (X) | 2 |
| 2370 | FBgn0003301-FBgn0030581 (X) | 2 |
| 2371 | FBgn0030599-FBgn0000535 (X) | 2 |
| 2372 | FBgn0030605-FBgn0030606 (X) | 2 |
| 2373 | FBgn0030625-FBgn0004924 (X) | 2 |
| 2374 | FBgn0029090-FBgn0030636 (X) | 2 |
| 2375 | FBgn0030638-FBgn0004391 (X) | 2 |
| 2376 | FBgn0026433-FBgn0030645 (X) | 2 |
| 2377 | FBgn0027287-FBgn0022710 (X) | 2 |
| 2378 | FBgn0030659-FBgn0030660 (X) | 2 |
| 2379 | FBgn0030680-FBgn0030683 (X) | 2 |
| 2380 | FBgn0053172-FBgn0053173 (X) | 2 |
| 2381 | FBgn0030694-FBgn0003345 (X) | 2 |
| 2382 | FBgn0052582-FBgn0030697 (X) | 2 |
| 2383 | FBgn0002914-FBgn0065035 (X) | 2 |
| 2384 | FBgn0001105-FBgn0001092 (X) | 2 |
| 2385 | FBgn0053180-FBgn0045073 (X) | 2 |
| 2386 | FBgn0259110-FBgn0030715 (X) | 2 |
| 2387 | FBgn0030720-FBgn0003969 (X) | 2 |
| 2388 | FBgn0000459-FBgn0030729 (X) | 2 |
| 2389 | FBgn0000536-FBgn0030733 (X) | 2 |
| 2390 | FBgn0004432-FBgn0030740 (X) | 2 |
| 2391 | FBgn0030746-FBgn0030747 (X) | 2 |
| 2392 | FBgn0030763-FBgn0030764 (X) | 2 |
| 2393 | FBgn0030780-FBgn0052572 (X) | 2 |
| 2394 | FBgn0030792-FBgn0030791 (X) | 2 |
| 2395 | FBgn0030794-FBgn0030795 (X) | 2 |
| 2396 | FBgn0030799-FBgn0030800 (X) | 2 |
| 2397 | FBgn0030804-FBgn0053252 (X) | 2 |
| 2398 | FBgn0030808-FBgn0030809 (X) | 2 |
| 2399 | FBgn0030824-FBgn0052564 (X) | 2 |
| 2400 | FBgn0052563-FBgn0030826 (X) | 2 |
| 2401 | FBgn0030833-FBgn0030834 (X) | 2 |
| 2402 | FBgn0024238-FBgn0030838 (X) | 2 |
| 2403 | FBgn0030843-FBgn0030844 (X) | 2 |
| 2404 | FBgn0030853-FBgn0030854 (X) | 2 |
| 2405 | FBgn0030863-FBgn0030864 (X) | 2 |
| 2406 | FBgn0026058-FBgn0030868 (X) | 2 |
| 2407 | FBgn0030883-FBgn0027605 (X) | 2 |
| 2408 | FBgn0030928-FBgn0030929 (X) | 2 |
| 2409 | FBgn0030931-FBgn0010194 (X) | 2 |
| 2410 | FBgn0030938-FBgn0000242 (X) | 2 |
| 2411 | FBgn0030947-FBgn0004959 (X) | 2 |
| 2412 | FBgn0052541-FBgn0030979 (X) | 2 |
| 2413 | FBgn0030990-FBgn0030991 (X) | 2 |
| 2414 | FBgn0030998-FBgn0030999 (X) | 2 |
| 2415 | FBgn0031002-FBgn0031003 (X) | 2 |
| 2416 | FBgn0052537-FBgn0031010 (X) | 2 |
| 2417 | FBgn0040890-FBgn0027621 (X) | 2 |
| 2418 | FBgn0026430-FBgn0000257 (X) | 2 |
| 2419 | FBgn0031031-FBgn0031032 (X) | 2 |
| 2420 | FBgn0031033-FBgn0031034 (X) | 2 |
| 2421 | FBgn0043903-FBgn0031057 (X) | 2 |
| 2422 | FBgn0031059-FBgn0010341 (X) | 2 |
| 2423 | FBgn0040089-FBgn0031064 (X) | 2 |
| 2424 | FBgn0031070-FBgn0259789 (X) | 2 |
| 2425 | FBgn0026174-FBgn0031074 (X) | 2 |
| 2426 | FBgn0086782-FBgn0052821 (X) | 2 |
| 2427 | FBgn0031089-FBgn0031090 (X) | 2 |
| 2428 | FBgn0067861-FBgn0052823 (X) | 2 |
| 2429 | FBgn0031098-FBgn0031099 (X) | 2 |
| 2430 | FBgn0031106-FBgn0031107 (X) | 2 |
| 2431 | FBgn0031108-FBgn0040651 (X) | 2 |
| 2432 | FBgn0031116-FBgn0052506 (X) | 2 |
| 2433 | FBgn0259162-FBgn0083981 (X) | 2 |
| 2434 | FBgn0031141-FBgn0040648 (X) | 2 |
| 2435 | FBgn0031149-FBgn0052512 (X) | 2 |
| 2436 | FBgn0031168-FBgn0052521 (X) | 2 |
| 2437 | FBgn0003423-FBgn0001565 (X) | 2 |
| 2438 | FBgn0259245-FBgn0031181 (X) | 2 |
| 2439 | FBgn0027588-FBgn0024807 (X) | 2 |
| 2440 | FBgn0031196-FBgn0031197 (X) | 2 |
| 2441 | FBgn0069938-FBgn0069972 (X) | 2 |
| 2442 | FBgn0039966-FBgn0039965 (X) | 2 |

  

|  |  |  |
| --- | --- | --- |
| **Segments by length (bp)** | | |
| 1 | FBgn0030367-FBgn0030390 (X) | 426296 |
| 2 | FBgn0033074-FBgn0033076 (2R) | 375352 |
| 3 | FBgn0036377-FBgn0043550 (3L) | 324261 |
| 4 | FBgn0032577-FBgn0028893 (2L) | 294572 |
| 5 | FBgn0031127-FBgn0031139 (X) | 265755 |
| 6 | FBgn0033005-FBgn0033017 (2R) | 250405 |
| 7 | FBgn0033572-FBgn0033579 (2R) | 240102 |
| 8 | FBgn0030559-FBgn0030570 (X) | 229888 |
| 9 | FBgn0002576-FBgn0054028 (X) | 226876 |
| 10 | FBgn0063261-FBgn0015008 (3R) | 226407 |
| 11 | FBgn0037506-FBgn0037512 (3R) | 220042 |
| 12 | FBgn0085331-FBgn0053208 (3R) | 218999 |
| 13 | FBgn0259697-FBgn0040045 (3L) | 209223 |
| 14 | FBgn0032965-FBgn0032972 (2L) | 208991 |
| 15 | FBgn0028412-FBgn0032377 (2L) | 207251 |
| 16 | FBgn0032457-FBgn0032462 (2L) | 204910 |
| 17 | FBgn0032359-FBgn0003313 (2L) | 200587 |
| 18 | FBgn0028853-FBgn0020371 (2L) | 199742 |
| 19 | FBgn0034829-FBgn0050409 (2R) | 195570 |
| 20 | FBgn0032851-FBgn0040996 (2L) | 190645 |
| 21 | FBgn0036078-FBgn0052058 (3L) | 189400 |
| 22 | FBgn0037168-FBgn0037179 (3L) | 188863 |
| 23 | FBgn0051559-FBgn0037432 (3R) | 185776 |
| 24 | FBgn0058439-FBgn0000384 (2L) | 183568 |
| 25 | FBgn0029745-FBgn0029751 (X) | 175520 |
| 26 | FBgn0038706-FBgn0038715 (3R) | 175437 |
| 27 | FBgn0035453-FBgn0052266 (3L) | 175423 |
| 28 | FBgn0037660-FBgn0003165 (3R) | 172599 |
| 29 | FBgn0029722-FBgn0029730 (X) | 170362 |
| 30 | FBgn0039582-FBgn0051051 (3R) | 168274 |
| 31 | FBgn0028369-FBgn0004647 (X) | 166598 |
| 32 | FBgn0040370-FBgn0000137 (X) | 165539 |
| 33 | FBgn0037857-FBgn0037860 (3R) | 165423 |
| 34 | FBgn0052061-FBgn0036089 (3L) | 164669 |
| 35 | FBgn0036302-FBgn0014007 (3L) | 162428 |
| 36 | FBgn0035376-FBgn0035382 (3L) | 161405 |
| 37 | FBgn0051784-FBgn0032631 (2L) | 160334 |
| 38 | FBgn0038156-FBgn0038160 (3R) | 158657 |
| 39 | FBgn0039192-FBgn0039202 (3R) | 158453 |
| 40 | FBgn0025394-FBgn0023530 (X) | 156221 |
| 41 | FBgn0039568-FBgn0085383 (3R) | 156074 |
| 42 | FBgn0030884-FBgn0013809 (X) | 153140 |
| 43 | FBgn0037584-FBgn0037591 (3R) | 150197 |
| 44 | FBgn0032089-FBgn0032100 (2L) | 150166 |
| 45 | FBgn0051320-FBgn0038233 (3R) | 149954 |
| 46 | FBgn0035699-FBgn0020251 (3L) | 143291 |
| 47 | FBgn0035584-FBgn0035594 (3L) | 142006 |
| 48 | FBgn0039510-FBgn0039518 (3R) | 141865 |
| 49 | FBgn0051816-FBgn0051807 (2L) | 139857 |
| 50 | FBgn0035569-FBgn0035577 (3L) | 138252 |
| 51 | FBgn0013323-FBgn0000497 (2L) | 138073 |
| 52 | FBgn0034475-FBgn0034478 (2R) | 135880 |
| 53 | FBgn0005659-FBgn0039552 (3R) | 135851 |
| 54 | FBgn0015946-FBgn0036786 (3L) | 135524 |
| 55 | FBgn0052541-FBgn0030979 (X) | 135056 |
| 56 | FBgn0051642-FBgn0053531 (2L) | 134612 |
| 57 | FBgn0034665-FBgn0054029 (2R) | 132391 |
| 58 | FBgn0033041-FBgn0033046 (2R) | 131891 |
| 59 | FBgn0029690-FBgn0029694 (X) | 130061 |
| 60 | FBgn0030898-FBgn0004956 (X) | 129517 |
| 61 | FBgn0038694-FBgn0051245 (3R) | 129081 |
| 62 | FBgn0025835-FBgn0024989 (X) | 128924 |
| 63 | FBgn0050325-FBgn0034286 (2R) | 128009 |
| 64 | FBgn0051219-FBgn0038732 (3R) | 127893 |
| 65 | FBgn0024288-FBgn0004389 (3R) | 127868 |
| 66 | FBgn0052369-FBgn0024187 (3L) | 126139 |
| 67 | FBgn0046692-FBgn0040005 (2R) | 125154 |
| 68 | FBgn0031900-FBgn0002938 (2L) | 124947 |
| 69 | FBgn0036851-FBgn0016797 (3L) | 124606 |
| 70 | FBgn0052450-FBgn0037162 (3L) | 124596 |
| 71 | FBgn0041247-FBgn0031923 (2L) | 124391 |
| 72 | FBgn0039075-FBgn0085318 (3R) | 124353 |
| 73 | FBgn0031337-FBgn0051928 (2L) | 124194 |
| 74 | FBgn0035970-FBgn0035978 (3L) | 122284 |
| 75 | FBgn0027107-FBgn0031104 (X) | 121308 |
| 76 | FBgn0040823-FBgn0052054 (3L) | 120120 |
| 77 | FBgn0029961-FBgn0029968 (X) | 119679 |
| 78 | FBgn0054007-FBgn0032867 (2L) | 119513 |
| 79 | FBgn0000179-FBgn0029708 (X) | 117376 |
| 80 | FBgn0051926-FBgn0031351 (2L) | 115675 |
| 81 | FBgn0039838-FBgn0046332 (3R) | 114829 |
| 82 | FBgn0039792-FBgn0039799 (3R) | 113874 |
| 83 | FBgn0037449-FBgn0051286 (3R) | 113704 |
| 84 | FBgn0016660-FBgn0031707 (2L) | 113482 |
| 85 | FBgn0038350-FBgn0053207 (3R) | 113415 |
| 86 | FBgn0032654-FBgn0015609 (2L) | 113176 |
| 87 | FBgn0004369-FBgn0039664 (3R) | 112207 |
| 88 | FBgn0025378-FBgn0029590 (X) | 111988 |
| 89 | FBgn0028901-FBgn0051735 (2L) | 111453 |
| 90 | FBgn0030532-FBgn0085375 (X) | 110488 |
| 91 | FBgn0004198-FBgn0029955 (X) | 108781 |
| 92 | FBgn0003386-FBgn0003430 (2L) | 107494 |
| 93 | FBgn0033862-FBgn0033867 (2R) | 106318 |
| 94 | FBgn0041240-FBgn0034551 (2R) | 106058 |
| 95 | FBgn0025616-FBgn0029538 (X) | 105352 |
| 96 | FBgn0034033-FBgn0034035 (2R) | 104949 |
| 97 | FBgn0051806-FBgn0032626 (2L) | 102588 |
| 98 | FBgn0005630-FBgn0033540 (2R) | 102437 |
| 99 | FBgn0030661-FBgn0030667 (X) | 100083 |
| 100 | FBgn0083940-FBgn0030182 (X) | 99919 |
| 101 | FBgn0031384-FBgn0053516 (2L) | 99761 |
| 102 | FBgn0027535-FBgn0010434 (2R) | 99472 |
| 103 | FBgn0032824-FBgn0053117 (2L) | 98981 |
| 104 | FBgn0014362-FBgn0037142 (3L) | 98959 |
| 105 | FBgn0039810-FBgn0053773 (3R) | 98840 |
| 106 | FBgn0001323-FBgn0037015 (3L) | 98663 |
| 107 | FBgn0023094-FBgn0036916 (3L) | 97761 |
| 108 | FBgn0038186-FBgn0038190 (3R) | 95685 |
| 109 | FBgn0040816-FBgn0036224 (3L) | 94221 |
| 110 | FBgn0030618-FBgn0030624 (X) | 93944 |
| 111 | FBgn0083956-FBgn0052523 (X) | 93944 |
| 112 | FBgn0038796-FBgn0038797 (3R) | 93662 |
| 113 | FBgn0033827-FBgn0033832 (2R) | 93617 |
| 114 | FBgn0028961-FBgn0044046 (X) | 93507 |
| 115 | FBgn0035336-FBgn0035347 (3L) | 93176 |
| 116 | FBgn0032525-FBgn0028941 (2L) | 92772 |
| 117 | FBgn0052670-FBgn0030257 (X) | 92273 |
| 118 | FBgn0086899-FBgn0000542 (X) | 91923 |
| 119 | FBgn0051423-FBgn0038934 (3R) | 91640 |
| 120 | FBgn0032553-FBgn0259213 (2L) | 91025 |
| 121 | FBgn0031082-FBgn0031086 (X) | 90290 |
| 122 | FBgn0036781-FBgn0036783 (3L) | 89574 |
| 123 | FBgn0031887-FBgn0085407 (2L) | 88719 |
| 124 | FBgn0026080-FBgn0000635 (X) | 88649 |
| 125 | FBgn0010406-FBgn0026263 (3L) | 88119 |
| 126 | FBgn0003174-FBgn0033159 (2R) | 86562 |
| 127 | FBgn0031440-FBgn0031446 (2L) | 86292 |
| 128 | FBgn0051862-FBgn0032428 (2L) | 86035 |
| 129 | FBgn0036703-FBgn0036709 (3L) | 85734 |
| 130 | FBgn0034481-FBgn0034484 (2R) | 85699 |
| 131 | FBgn0085274-FBgn0036459 (3L) | 85694 |
| 132 | FBgn0051802-FBgn0032677 (2L) | 85668 |
| 133 | FBgn0034456-FBgn0034459 (2R) | 85624 |
| 134 | FBgn0053703-FBgn0036013 (3L) | 85195 |
| 135 | FBgn0039994-FBgn0033000 (2R) | 84890 |
| 136 | FBgn0037074-FBgn0053288 (3L) | 84092 |
| 137 | FBgn0041237-FBgn0034684 (2R) | 84049 |
| 138 | FBgn0038260-FBgn0038268 (3R) | 83135 |
| 139 | FBgn0031619-FBgn0031626 (2L) | 83056 |
| 140 | FBgn0030165-FBgn0030173 (X) | 82607 |
| 141 | FBgn0040684-FBgn0037296 (3R) | 81874 |
| 142 | FBgn0085424-FBgn0032442 (2L) | 81844 |
| 143 | FBgn0259162-FBgn0083981 (X) | 80717 |
| 144 | FBgn0052150-FBgn0036544 (3L) | 80395 |
| 145 | FBgn0039495-FBgn0039498 (3R) | 80110 |
| 146 | FBgn0032105-FBgn0010314 (2L) | 77652 |
| 147 | FBgn0028519-FBgn0028904 (2L) | 77645 |
| 148 | FBgn0030277-FBgn0030283 (X) | 77589 |
| 149 | FBgn0038566-FBgn0038568 (3R) | 77581 |
| 150 | FBgn0024491-FBgn0040237 (3R) | 77196 |
| 151 | FBgn0052698-FBgn0019929 (X) | 76737 |
| 152 | FBgn0025628-FBgn0013432 (X) | 76123 |
| 153 | FBgn0035746-FBgn0035751 (3L) | 75786 |
| 154 | FBgn0036811-FBgn0036815 (3L) | 75272 |
| 155 | FBgn0051473-FBgn0037525 (3R) | 74906 |
| 156 | FBgn0030018-FBgn0017566 (X) | 74513 |
| 157 | FBgn0029939-FBgn0029940 (X) | 73829 |
| 158 | FBgn0040356-FBgn0040354 (X) | 73753 |
| 159 | FBgn0038894-FBgn0054034 (3R) | 73533 |
| 160 | FBgn0052719-FBgn0029952 (X) | 73230 |
| 161 | FBgn0041097-FBgn0011294 (2L) | 72736 |
| 162 | FBgn0030810-FBgn0030815 (X) | 72543 |
| 163 | FBgn0000439-FBgn0001077 (3R) | 72489 |
| 164 | FBgn0031428-FBgn0011818 (2L) | 72148 |
| 165 | FBgn0032632-FBgn0051804 (2L) | 71295 |
| 166 | FBgn0031364-FBgn0051933 (2L) | 70983 |
| 167 | FBgn0034070-FBgn0034073 (2R) | 70644 |
| 168 | FBgn0015770-FBgn0037503 (3R) | 70462 |
| 169 | FBgn0085422-FBgn0031513 (2L) | 69818 |
| 170 | FBgn0020258-FBgn0028844 (2L) | 69753 |
| 171 | FBgn0026577-FBgn0041244 (2L) | 69399 |
| 172 | FBgn0034507-FBgn0086604 (2R) | 69283 |
| 173 | FBgn0041111-FBgn0010263 (2L) | 68811 |
| 174 | FBgn0259244-FBgn0038445 (3R) | 68716 |
| 175 | FBgn0024244-FBgn0002985 (2L) | 67498 |
| 176 | FBgn0035138-FBgn0035139 (3L) | 67470 |
| 177 | FBgn0259171-FBgn0052650 (X) | 67034 |
| 178 | FBgn0031718-FBgn0031725 (2L) | 66913 |
| 179 | FBgn0021764-FBgn0029532 (X) | 66814 |
| 180 | FBgn0034734-FBgn0034736 (2R) | 66760 |
| 181 | FBgn0036629-FBgn0004569 (3L) | 66537 |
| 182 | FBgn0001978-FBgn0028888 (2L) | 64839 |
| 183 | FBgn0038280-FBgn0038285 (3R) | 64516 |
| 184 | FBgn0037129-FBgn0000560 (3L) | 64440 |
| 185 | FBgn0029811-FBgn0029817 (X) | 64419 |
| 186 | FBgn0054050-FBgn0013469 (3L) | 64369 |
| 187 | FBgn0039094-FBgn0051145 (3R) | 64170 |
| 188 | FBgn0030918-FBgn0052547 (X) | 64029 |
| 189 | FBgn0032268-FBgn0032271 (2L) | 63764 |
| 190 | FBgn0035002-FBgn0035009 (2R) | 63536 |
| 191 | FBgn0052971-FBgn0028531 (2L) | 63446 |
| 192 | FBgn0051294-FBgn0038359 (3R) | 63221 |
| 193 | FBgn0038492-FBgn0051262 (3R) | 63127 |
| 194 | FBgn0259211-FBgn0003701 (2R) | 63104 |
| 195 | FBgn0050085-FBgn0034049 (2R) | 62785 |
| 196 | FBgn0030322-FBgn0030323 (X) | 62712 |
| 197 | FBgn0033893-FBgn0259184 (2R) | 62414 |
| 198 | FBgn0026384-FBgn0034870 (2R) | 62266 |
| 199 | FBgn0000520-FBgn0040384 (X) | 62243 |
| 200 | FBgn0051031-FBgn0040625 (3R) | 62050 |
| 201 | FBgn0032178-FBgn0032184 (2L) | 61901 |
| 202 | FBgn0033471-FBgn0033474 (2R) | 61740 |
| 203 | FBgn0036266-FBgn0052100 (3L) | 61532 |
| 204 | FBgn0040942-FBgn0030189 (X) | 61215 |
| 205 | FBgn0035145-FBgn0035149 (3L) | 60953 |
| 206 | FBgn0030071-FBgn0030074 (X) | 60566 |
| 207 | FBgn0030219-FBgn0030224 (X) | 60452 |
| 208 | FBgn0038423-FBgn0051279 (3R) | 60273 |
| 209 | FBgn0029514-FBgn0035216 (3L) | 59950 |
| 210 | FBgn0039086-FBgn0039092 (3R) | 59564 |
| 211 | FBgn0031792-FBgn0021873 (2L) | 59281 |
| 212 | FBgn0029700-FBgn0040906 (X) | 59103 |
| 213 | FBgn0034251-FBgn0050106 (2R) | 59030 |
| 214 | FBgn0033149-FBgn0026389 (2R) | 59028 |
| 215 | FBgn0004101-FBgn0035001 (2R) | 59010 |
| 216 | FBgn0051081-FBgn0039448 (3R) | 58650 |
| 217 | FBgn0032008-FBgn0003502 (2L) | 58414 |
| 218 | FBgn0026411-FBgn0030065 (X) | 58339 |
| 219 | FBgn0039421-FBgn0039424 (3R) | 58184 |
| 220 | FBgn0040780-FBgn0050377 (2R) | 57877 |
| 221 | FBgn0259234-FBgn0259246 (2R) | 57743 |
| 222 | FBgn0036789-FBgn0036790 (3L) | 57702 |
| 223 | FBgn0031926-FBgn0040299 (2L) | 57658 |
| 224 | FBgn0002973-FBgn0004868 (2L) | 57415 |
| 225 | FBgn0040333-FBgn0011276 (X) | 57149 |
| 226 | FBgn0039204-FBgn0039210 (3R) | 57058 |
| 227 | FBgn0011742-FBgn0025743 (X) | 56737 |
| 228 | FBgn0030721-FBgn0030722 (X) | 56709 |
| 229 | FBgn0050343-FBgn0033402 (2R) | 56664 |
| 230 | FBgn0040355-FBgn0029647 (X) | 56338 |
| 231 | FBgn0053980-FBgn0250904 (X) | 56188 |
| 232 | FBgn0029946-FBgn0029949 (X) | 55669 |
| 233 | FBgn0038090-FBgn0038097 (3R) | 55429 |
| 234 | FBgn0023415-FBgn0032335 (2L) | 55386 |
| 235 | FBgn0035563-FBgn0035568 (3L) | 55282 |
| 236 | FBgn0051675-FBgn0032904 (2L) | 55094 |
| 237 | FBgn0026087-FBgn0023546 (X) | 54845 |
| 238 | FBgn0035727-FBgn0035730 (3L) | 54361 |
| 239 | FBgn0035255-FBgn0035263 (3L) | 54307 |
| 240 | FBgn0013810-FBgn0032650 (2L) | 54299 |
| 241 | FBgn0001269-FBgn0000577 (2R) | 53746 |
| 242 | FBgn0039282-FBgn0039286 (3R) | 53724 |
| 243 | FBgn0050286-FBgn0053225 (2R) | 53592 |
| 244 | FBgn0037205-FBgn0052461 (3L) | 53369 |
| 245 | FBgn0040390-FBgn0001150 (X) | 53192 |
| 246 | FBgn0039360-FBgn0051092 (3R) | 53096 |
| 247 | FBgn0030358-FBgn0005563 (X) | 53054 |
| 248 | FBgn0029667-FBgn0052792 (X) | 52962 |
| 249 | FBgn0033770-FBgn0033773 (2R) | 52856 |
| 250 | FBgn0035906-FBgn0035911 (3L) | 52574 |
| 251 | FBgn0026756-FBgn0040998 (2L) | 52441 |
| 252 | FBgn0036325-FBgn0036330 (3L) | 52263 |
| 253 | FBgn0010300-FBgn0010309 (2L) | 52231 |
| 254 | FBgn0003984-FBgn0035645 (3L) | 51975 |
| 255 | FBgn0031208-FBgn0051973 (2L) | 51714 |
| 256 | FBgn0032470-FBgn0032471 (2L) | 51609 |
| 257 | FBgn0024329-FBgn0038652 (3R) | 51597 |
| 258 | FBgn0039116-FBgn0053111 (3R) | 51521 |
| 259 | FBgn0031601-FBgn0031607 (2L) | 51203 |
| 260 | FBgn0034205-FBgn0034206 (2R) | 51075 |
| 261 | FBgn0032707-FBgn0045852 (2L) | 50886 |
| 262 | FBgn0085394-FBgn0050470 (2R) | 50740 |
| 263 | FBgn0035348-FBgn0010905 (3L) | 50709 |
| 264 | FBgn0037397-FBgn0037399 (3R) | 50517 |
| 265 | FBgn0035426-FBgn0035430 (3L) | 50490 |
| 266 | FBgn0036807-FBgn0036810 (3L) | 50308 |
| 267 | FBgn0003089-FBgn0036868 (3L) | 50004 |
| 268 | FBgn0034730-FBgn0034732 (2R) | 49792 |
| 269 | FBgn0000459-FBgn0030729 (X) | 49761 |
| 270 | FBgn0030980-FBgn0030984 (X) | 49589 |
| 271 | FBgn0041242-FBgn0053475 (2R) | 48909 |
| 272 | FBgn0039969-FBgn0027507 (2R) | 48812 |
| 273 | FBgn0035604-FBgn0085420 (3L) | 48812 |
| 274 | FBgn0036437-FBgn0036443 (3L) | 48793 |
| 275 | FBgn0035155-FBgn0035160 (3L) | 48602 |
| 276 | FBgn0035733-FBgn0035741 (3L) | 48170 |
| 277 | FBgn0037283-FBgn0020280 (3R) | 48021 |
| 278 | FBgn0085204-FBgn0031533 (2L) | 47975 |
| 279 | FBgn0038471-FBgn0038478 (3R) | 47584 |
| 280 | FBgn0014023-FBgn0037710 (3R) | 47564 |
| 281 | FBgn0250910-FBgn0038063 (3R) | 47497 |
| 282 | FBgn0032974-FBgn0032979 (2L) | 47356 |
| 283 | FBgn0040995-FBgn0032838 (2L) | 47319 |
| 284 | FBgn0031390-FBgn0031395 (2L) | 47198 |
| 285 | FBgn0031954-FBgn0031961 (2L) | 47009 |
| 286 | FBgn0042135-FBgn0050497 (2R) | 46996 |
| 287 | FBgn0034990-FBgn0034996 (2R) | 46443 |
| 288 | FBgn0050295-FBgn0020617 (2R) | 45975 |
| 289 | FBgn0053795-FBgn0036577 (3L) | 45924 |
| 290 | FBgn0052250-FBgn0035534 (3L) | 45799 |
| 291 | FBgn0037368-FBgn0037374 (3R) | 45769 |
| 292 | FBgn0043884-FBgn0019952 (3R) | 45699 |
| 293 | FBgn0051709-FBgn0051882 (2L) | 45648 |
| 294 | FBgn0031414-FBgn0031413 (2L) | 45640 |
| 295 | FBgn0001222-FBgn0034313 (2R) | 45539 |
| 296 | FBgn0033359-FBgn0033365 (2R) | 45484 |
| 297 | FBgn0033391-FBgn0033395 (2R) | 45436 |
| 298 | FBgn0051803-FBgn0032639 (2L) | 45393 |
| 299 | FBgn0051526-FBgn0052945 (3R) | 45350 |
| 300 | FBgn0030628-FBgn0000028 (X) | 45015 |
| 301 | FBgn0023416-FBgn0032957 (2L) | 44932 |
| 302 | FBgn0016977-FBgn0031231 (2L) | 44751 |
| 303 | FBgn0036838-FBgn0002945 (3L) | 44724 |
| 304 | FBgn0038886-FBgn0011278 (3R) | 44504 |
| 305 | FBgn0011722-FBgn0031817 (2L) | 44393 |
| 306 | FBgn0037826-FBgn0037829 (3R) | 44387 |
| 307 | FBgn0039413-FBgn0051323 (3R) | 44162 |
| 308 | FBgn0039109-FBgn0039115 (3R) | 44089 |
| 309 | FBgn0259750-FBgn0052830 (2L) | 43894 |
| 310 | FBgn0039851-FBgn0039852 (3R) | 43794 |
| 311 | FBgn0052845-FBgn0085293 (3L) | 43715 |
| 312 | FBgn0013984-FBgn0038893 (3R) | 43638 |
| 313 | FBgn0046689-FBgn0038827 (3R) | 43601 |
| 314 | FBgn0026160-FBgn0036104 (3L) | 43508 |
| 315 | FBgn0051534-FBgn0026620 (3R) | 43320 |
| 316 | FBgn0031462-FBgn0031473 (2L) | 43313 |
| 317 | FBgn0035050-FBgn0053988 (2R) | 43106 |
| 318 | FBgn0030766-FBgn0000152 (X) | 42991 |
| 319 | FBgn0037908-FBgn0053512 (3R) | 42966 |
| 320 | FBgn0038666-FBgn0038673 (3R) | 42769 |
| 321 | FBgn0035954-FBgn0035957 (3L) | 42243 |
| 322 | FBgn0030200-FBgn0030204 (X) | 42192 |
| 323 | FBgn0030088-FBgn0030092 (X) | 42061 |
| 324 | FBgn0032110-FBgn0085395 (2L) | 42014 |
| 325 | FBgn0037478-FBgn0037482 (3R) | 41911 |
| 326 | FBgn0034774-FBgn0259145 (2R) | 41870 |
| 327 | FBgn0010313-FBgn0037293 (3R) | 41403 |
| 328 | FBgn0032586-FBgn0032587 (2L) | 41336 |
| 329 | FBgn0031853-FBgn0031858 (2L) | 41174 |
| 330 | FBgn0020240-FBgn0031985 (2L) | 41033 |
| 331 | FBgn0039027-FBgn0039029 (3R) | 41022 |
| 332 | FBgn0032915-FBgn0032919 (2L) | 40836 |
| 333 | FBgn0027375-FBgn0259175 (3L) | 40796 |
| 334 | FBgn0034129-FBgn0053458 (2R) | 40689 |
| 335 | FBgn0032311-FBgn0032315 (2L) | 40369 |
| 336 | FBgn0033701-FBgn0033705 (2R) | 39989 |
| 337 | FBgn0053958-FBgn0000658 (2R) | 39928 |
| 338 | FBgn0013983-FBgn0034351 (2R) | 39750 |
| 339 | FBgn0039804-FBgn0039805 (3R) | 39674 |
| 340 | FBgn0034005-FBgn0034007 (2R) | 39563 |
| 341 | FBgn0038122-FBgn0038127 (3R) | 39275 |
| 342 | FBgn0003475-FBgn0015803 (2L) | 39218 |
| 343 | FBgn0028418-FBgn0040318 (3L) | 39208 |
| 344 | FBgn0037797-FBgn0037802 (3R) | 39028 |
| 345 | FBgn0039596-FBgn0039599 (3R) | 38932 |
| 346 | FBgn0035966-FBgn0004244 (3L) | 38928 |
| 347 | FBgn0032981-FBgn0259683 (2L) | 38851 |
| 348 | FBgn0259108-FBgn0027796 (X) | 38826 |
| 349 | FBgn0083969-FBgn0051036 (3R) | 38740 |
| 350 | FBgn0029658-FBgn0029662 (X) | 38714 |
| 351 | FBgn0033140-FBgn0033142 (2R) | 38696 |
| 352 | FBgn0038889-FBgn0038890 (3R) | 38617 |
| 353 | FBgn0050184-FBgn0034882 (2R) | 38557 |
| 354 | FBgn0030949-FBgn0030952 (X) | 38442 |
| 355 | FBgn0259685-FBgn0039182 (3R) | 38237 |
| 356 | FBgn0031729-FBgn0054011 (2L) | 38182 |
| 357 | FBgn0051797-FBgn0032769 (2L) | 38174 |
| 358 | FBgn0034120-FBgn0053960 (2R) | 38022 |
| 359 | FBgn0034022-FBgn0050471 (2R) | 37989 |
| 360 | FBgn0029942-FBgn0029944 (X) | 37861 |
| 361 | FBgn0037493-FBgn0259172 (3R) | 37806 |
| 362 | FBgn0031735-FBgn0051913 (2L) | 37755 |
| 363 | FBgn0039476-FBgn0039478 (3R) | 37679 |
| 364 | FBgn0085250-FBgn0000448 (2R) | 37658 |
| 365 | FBgn0052643-FBgn0030441 (X) | 37642 |
| 366 | FBgn0033680-FBgn0086677 (2R) | 37597 |
| 367 | FBgn0031023-FBgn0052532 (X) | 37537 |
| 368 | FBgn0053262-FBgn0086916 (3L) | 37414 |
| 369 | FBgn0051869-FBgn0011676 (2L) | 37342 |
| 370 | FBgn0038891-FBgn0038892 (3R) | 36983 |
| 371 | FBgn0039395-FBgn0039398 (3R) | 36980 |
| 372 | FBgn0000259-FBgn0052662 (X) | 36883 |
| 373 | FBgn0034416-FBgn0050125 (2R) | 36754 |
| 374 | FBgn0029090-FBgn0030636 (X) | 36754 |
| 375 | FBgn0032175-FBgn0053302 (2L) | 36753 |
| 376 | FBgn0039348-FBgn0027508 (3R) | 36622 |
| 377 | FBgn0061196-FBgn0029740 (X) | 36540 |
| 378 | FBgn0259112-FBgn0001319 (2R) | 36150 |
| 379 | FBgn0053108-FBgn0020647 (3R) | 36141 |
| 380 | FBgn0033636-FBgn0085257 (2R) | 36047 |
| 381 | FBgn0052986-FBgn0085427 (2L) | 36029 |
| 382 | FBgn0025360-FBgn0040778 (2R) | 36020 |
| 383 | FBgn0027525-FBgn0050015 (2R) | 35999 |
| 384 | FBgn0032549-FBgn0053309 (2L) | 35808 |
| 385 | FBgn0039408-FBgn0039411 (3R) | 35701 |
| 386 | FBgn0014388-FBgn0026259 (3L) | 35668 |
| 387 | FBgn0000233-FBgn0020378 (X) | 35488 |
| 388 | FBgn0039344-FBgn0039347 (3R) | 35451 |
| 389 | FBgn0015818-FBgn0028685 (X) | 35436 |
| 390 | FBgn0050395-FBgn0034661 (2R) | 35376 |
| 391 | FBgn0036285-FBgn0000625 (3L) | 35354 |
| 392 | FBgn0027287-FBgn0022710 (X) | 35242 |
| 393 | FBgn0034087-FBgn0034091 (2R) | 35212 |
| 394 | FBgn0085366-FBgn0040923 (X) | 34956 |
| 395 | FBgn0051813-FBgn0028541 (2L) | 34948 |
| 396 | FBgn0013765-FBgn0033842 (2R) | 34912 |
| 397 | FBgn0031430-FBgn0031431 (2L) | 34808 |
| 398 | FBgn0035023-FBgn0035028 (2R) | 34615 |
| 399 | FBgn0028577-FBgn0035229 (3L) | 34552 |
| 400 | FBgn0030843-FBgn0030844 (X) | 34518 |
| 401 | FBgn0259109-FBgn0030399 (X) | 34219 |
| 402 | FBgn0028694-FBgn0031682 (2L) | 34206 |
| 403 | FBgn0037697-FBgn0043791 (3R) | 34164 |
| 404 | FBgn0025808-FBgn0051314 (3R) | 34144 |
| 405 | FBgn0040363-FBgn0040367 (X) | 34076 |
| 406 | FBgn0052057-FBgn0052055 (3L) | 34060 |
| 407 | FBgn0033763-FBgn0050053 (2R) | 33921 |
| 408 | FBgn0035898-FBgn0035902 (3L) | 33830 |
| 409 | FBgn0033330-FBgn0050350 (2R) | 33759 |
| 410 | FBgn0020269-FBgn0012042 (2R) | 33742 |
| 411 | FBgn0035236-FBgn0035238 (3L) | 33519 |
| 412 | FBgn0033375-FBgn0027561 (2R) | 33515 |
| 413 | FBgn0037918-FBgn0037922 (3R) | 33233 |
| 414 | FBgn0036274-FBgn0052105 (3L) | 33216 |
| 415 | FBgn0051235-FBgn0038607 (3R) | 33056 |
| 416 | FBgn0023545-FBgn0000667 (X) | 32969 |
| 417 | FBgn0051773-FBgn0051774 (2L) | 32873 |
| 418 | FBgn0038293-FBgn0038294 (3R) | 32815 |
| 419 | FBgn0033259-FBgn0005648 (2R) | 32757 |
| 420 | FBgn0030465-FBgn0085388 (X) | 32623 |
| 421 | FBgn0001297-FBgn0039696 (3R) | 32559 |
| 422 | FBgn0037548-FBgn0037551 (3R) | 32491 |
| 423 | FBgn0034737-FBgn0034739 (2R) | 32329 |
| 424 | FBgn0029685-FBgn0029689 (X) | 32314 |
| 425 | FBgn0036396-FBgn0036402 (3L) | 32184 |
| 426 | FBgn0032871-FBgn0032877 (2L) | 32177 |
| 427 | FBgn0031423-FBgn0085477 (2L) | 32174 |
| 428 | FBgn0023091-FBgn0032945 (2L) | 32123 |
| 429 | FBgn0086448-FBgn0010416 (X) | 32071 |
| 430 | FBgn0027528-FBgn0030786 (X) | 32045 |
| 431 | FBgn0023509-FBgn0023521 (X) | 32024 |
| 432 | FBgn0039645-FBgn0039648 (3R) | 31791 |
| 433 | FBgn0052112-FBgn0036309 (3L) | 31765 |
| 434 | FBgn0046685-FBgn0020018 (3R) | 31764 |
| 435 | FBgn0036187-FBgn0052085 (3L) | 31680 |
| 436 | FBgn0004914-FBgn0003209 (2L) | 31623 |
| 437 | FBgn0030192-FBgn0052679 (X) | 31578 |
| 438 | FBgn0040658-FBgn0005631 (2R) | 31507 |
| 439 | FBgn0035384-FBgn0004372 (3L) | 31342 |
| 440 | FBgn0011824-FBgn0002736 (2R) | 31277 |
| 441 | FBgn0051358-FBgn0037988 (3R) | 31188 |
| 442 | FBgn0004359-FBgn0004510 (3R) | 31153 |
| 443 | FBgn0032754-FBgn0000075 (2L) | 31132 |
| 444 | FBgn0031695-FBgn0016076 (2L) | 31131 |
| 445 | FBgn0036518-FBgn0036522 (3L) | 31080 |
| 446 | FBgn0036652-FBgn0036654 (3L) | 31054 |
| 447 | FBgn0004666-FBgn0038118 (3R) | 30893 |
| 448 | FBgn0051957-FBgn0000256 (2L) | 30403 |
| 449 | FBgn0037992-FBgn0037993 (3R) | 30399 |
| 450 | FBgn0052135-FBgn0036395 (3L) | 30378 |
| 451 | FBgn0051688-FBgn0032863 (2L) | 30369 |
| 452 | FBgn0000566-FBgn0034371 (2R) | 30308 |
| 453 | FBgn0031168-FBgn0052521 (X) | 30024 |
| 454 | FBgn0042110-FBgn0037941 (3R) | 30006 |
| 455 | FBgn0030331-FBgn0002577 (X) | 29934 |
| 456 | FBgn0031294-FBgn0015905 (2L) | 29928 |
| 457 | FBgn0030970-FBgn0030974 (X) | 29918 |
| 458 | FBgn0023527-FBgn0023526 (X) | 29907 |
| 459 | FBgn0026313-FBgn0030851 (X) | 29866 |
| 460 | FBgn0036155-FBgn0036156 (3L) | 29806 |
| 461 | FBgn0031596-FBgn0031597 (2L) | 29745 |
| 462 | FBgn0034096-FBgn0025519 (2R) | 29686 |
| 463 | FBgn0038402-FBgn0038405 (3R) | 29679 |
| 464 | FBgn0040397-FBgn0085349 (X) | 29519 |
| 465 | FBgn0052132-FBgn0085429 (3L) | 29430 |
| 466 | FBgn0052228-FBgn0052227 (3L) | 29318 |
| 467 | FBgn0038003-FBgn0003651 (3R) | 29294 |
| 468 | FBgn0036479-FBgn0014002 (3L) | 29089 |
| 469 | FBgn0040010-FBgn0040011 (2L) | 28951 |
| 470 | FBgn0000212-FBgn0036558 (3L) | 28944 |
| 471 | FBgn0036747-FBgn0052182 (3L) | 28922 |
| 472 | FBgn0033153-FBgn0033155 (2R) | 28913 |
| 473 | FBgn0033987-FBgn0033989 (2R) | 28910 |
| 474 | FBgn0052772-FBgn0029738 (X) | 28897 |
| 475 | FBgn0033404-FBgn0033405 (2R) | 28815 |
| 476 | FBgn0039522-FBgn0046885 (3R) | 28796 |
| 477 | FBgn0030400-FBgn0030407 (X) | 28733 |
| 478 | FBgn0041160-FBgn0010105 (3L) | 28706 |
| 479 | FBgn0036343-FBgn0036346 (3L) | 28576 |
| 480 | FBgn0026058-FBgn0030868 (X) | 28566 |
| 481 | FBgn0030492-FBgn0052631 (X) | 28460 |
| 482 | FBgn0029696-FBgn0052774 (X) | 28417 |
| 483 | FBgn0036661-FBgn0021768 (3L) | 28322 |
| 484 | FBgn0031460-FBgn0004242 (2L) | 28195 |
| 485 | FBgn0037322-FBgn0037325 (3R) | 28062 |
| 486 | FBgn0085296-FBgn0085298 (3L) | 27894 |
| 487 | FBgn0037498-FBgn0051496 (3R) | 27863 |
| 488 | FBgn0030412-FBgn0011837 (X) | 27743 |
| 489 | FBgn0039164-FBgn0026257 (3R) | 27720 |
| 490 | FBgn0066293-FBgn0053558 (2R) | 27649 |
| 491 | FBgn0031837-FBgn0051635 (2L) | 27389 |
| 492 | FBgn0259146-FBgn0002735 (3R) | 27287 |
| 493 | FBgn0037926-FBgn0037931 (3R) | 27126 |
| 494 | FBgn0259224-FBgn0001254 (3L) | 27036 |
| 495 | FBgn0052392-FBgn0086680 (3L) | 26907 |
| 496 | FBgn0037291-FBgn0037292 (3R) | 26800 |
| 497 | FBgn0030743-FBgn0005411 (X) | 26751 |
| 498 | FBgn0033667-FBgn0033668 (2R) | 26710 |
| 499 | FBgn0033275-FBgn0010109 (2R) | 26622 |
| 500 | FBgn0038654-FBgn0038656 (3R) | 26557 |
| 501 | FBgn0030625-FBgn0004924 (X) | 26460 |
| 502 | FBgn0031187-FBgn0031190 (X) | 26285 |
| 503 | FBgn0031375-FBgn0031377 (2L) | 26237 |
| 504 | FBgn0032811-FBgn0000464 (2L) | 26224 |
| 505 | FBgn0036637-FBgn0036639 (3L) | 26211 |
| 506 | FBgn0003326-FBgn0040755 (2R) | 26134 |
| 507 | FBgn0030749-FBgn0054015 (X) | 26087 |
| 508 | FBgn0036448-FBgn0029148 (3L) | 26047 |
| 509 | FBgn0035150-FBgn0035151 (3L) | 25962 |
| 510 | FBgn0035070-FBgn0035073 (2R) | 25922 |
| 511 | FBgn0004567-FBgn0031574 (2L) | 25840 |
| 512 | FBgn0041582-FBgn0015544 (2R) | 25815 |
| 513 | FBgn0034460-FBgn0034464 (2R) | 25777 |
| 514 | FBgn0030590-FBgn0030592 (X) | 25715 |
| 515 | FBgn0052154-FBgn0036586 (3L) | 25615 |
| 516 | FBgn0032775-FBgn0032779 (2L) | 25599 |
| 517 | FBgn0031591-FBgn0010473 (2L) | 25576 |
| 518 | FBgn0010329-FBgn0029989 (X) | 25568 |
| 519 | FBgn0032724-FBgn0032729 (2L) | 25552 |
| 520 | FBgn0029931-FBgn0029930 (X) | 25445 |
| 521 | FBgn0010282-FBgn0037443 (3R) | 25433 |
| 522 | FBgn0032276-FBgn0040496 (2L) | 25393 |
| 523 | FBgn0020224-FBgn0010825 (3L) | 25372 |
| 524 | FBgn0029925-FBgn0027108 (X) | 25254 |
| 525 | FBgn0031195-FBgn0031194 (X) | 25165 |
| 526 | FBgn0085213-FBgn0033933 (2R) | 25091 |
| 527 | FBgn0051093-FBgn0039376 (3R) | 25009 |
| 528 | FBgn0058470-FBgn0058053 (3L) | 24974 |
| 529 | FBgn0039566-FBgn0003890 (3R) | 24952 |
| 530 | FBgn0037040-FBgn0037042 (3L) | 24912 |
| 531 | FBgn0027588-FBgn0024807 (X) | 24892 |
| 532 | FBgn0036510-FBgn0036516 (3L) | 24756 |
| 533 | FBgn0040366-FBgn0026876 (X) | 24705 |
| 534 | FBgn0032503-FBgn0032504 (2L) | 24513 |
| 535 | FBgn0086350-FBgn0050460 (2R) | 24434 |
| 536 | FBgn0028906-FBgn0028905 (2L) | 24356 |
| 537 | FBgn0031011-FBgn0259834 (X) | 24346 |
| 538 | FBgn0023096-FBgn0040989 (2L) | 24208 |
| 539 | FBgn0000061-FBgn0031251 (2L) | 24066 |
| 540 | FBgn0024740-FBgn0032291 (2L) | 24066 |
| 541 | FBgn0053454-FBgn0003254 (2R) | 24061 |
| 542 | FBgn0032693-FBgn0086681 (2L) | 24015 |
| 543 | FBgn0038540-FBgn0038542 (3R) | 23962 |
| 544 | FBgn0051876-FBgn0032171 (2L) | 23928 |
| 545 | FBgn0032125-FBgn0032129 (2L) | 23873 |
| 546 | FBgn0033107-FBgn0040674 (2R) | 23868 |
| 547 | FBgn0032880-FBgn0011202 (2L) | 23838 |
| 548 | FBgn0035279-FBgn0035282 (3L) | 23809 |
| 549 | FBgn0032295-FBgn0027550 (2L) | 23803 |
| 550 | FBgn0037195-FBgn0037197 (3L) | 23720 |
| 551 | FBgn0004387-FBgn0040080 (3R) | 23666 |
| 552 | FBgn0030947-FBgn0004959 (X) | 23617 |
| 553 | FBgn0038365-FBgn0038366 (3R) | 23606 |
| 554 | FBgn0023001-FBgn0040837 (3L) | 23582 |
| 555 | FBgn0037363-FBgn0037365 (3R) | 23574 |
| 556 | FBgn0034986-FBgn0034989 (2R) | 23448 |
| 557 | FBgn0038316-FBgn0038321 (3R) | 23211 |
| 558 | FBgn0036411-FBgn0042630 (3L) | 23207 |
| 559 | FBgn0003870-FBgn0039868 (3R) | 23157 |
| 560 | FBgn0038629-FBgn0038633 (3R) | 23129 |
| 561 | FBgn0014001-FBgn0037440 (3R) | 23128 |
| 562 | FBgn0032522-FBgn0026760 (2L) | 23119 |
| 563 | FBgn0035097-FBgn0040688 (3L) | 23106 |
| 564 | FBgn0033918-FBgn0028428 (2R) | 22973 |
| 565 | FBgn0020379-FBgn0045443 (3R) | 22965 |
| 566 | FBgn0032815-FBgn0032819 (2L) | 22926 |
| 567 | FBgn0034911-FBgn0003401 (2R) | 22910 |
| 568 | FBgn0052088-FBgn0041231 (3L) | 22889 |
| 569 | FBgn0030673-FBgn0030676 (X) | 22845 |
| 570 | FBgn0000529-FBgn0032821 (2L) | 22834 |
| 571 | FBgn0037347-FBgn0044823 (3R) | 22690 |
| 572 | FBgn0033210-FBgn0033212 (2R) | 22539 |
| 573 | FBgn0030941-FBgn0030946 (X) | 22384 |
| 574 | FBgn0036320-FBgn0036321 (3L) | 22335 |
| 575 | FBgn0051538-FBgn0037310 (3R) | 22311 |
| 576 | FBgn0033454-FBgn0027580 (2R) | 22286 |
| 577 | FBgn0041775-FBgn0036301 (3L) | 22284 |
| 578 | FBgn0030755-FBgn0030757 (X) | 22266 |
| 579 | FBgn0039623-FBgn0039627 (3R) | 22249 |
| 580 | FBgn0039874-FBgn0027620 (3R) | 22237 |
| 581 | FBgn0031360-FBgn0027597 (2L) | 22171 |
| 582 | FBgn0026386-FBgn0033603 (2R) | 22147 |
| 583 | FBgn0031762-FBgn0031765 (2L) | 22086 |
| 584 | FBgn0037486-FBgn0037488 (3R) | 22056 |
| 585 | FBgn0041161-FBgn0013563 (3L) | 22001 |
| 586 | FBgn0030161-FBgn0030162 (X) | 21967 |
| 587 | FBgn0039747-FBgn0039749 (3R) | 21949 |
| 588 | FBgn0027602-FBgn0040877 (X) | 21840 |
| 589 | FBgn0032140-FBgn0022720 (2L) | 21694 |
| 590 | FBgn0037184-FBgn0052451 (3L) | 21671 |
| 591 | FBgn0031331-FBgn0031335 (2L) | 21656 |
| 592 | FBgn0032339-FBgn0032343 (2L) | 21622 |
| 593 | FBgn0036969-FBgn0036970 (3L) | 21618 |
| 594 | FBgn0035762-FBgn0035767 (3L) | 21553 |
| 595 | FBgn0083949-FBgn0040679 (3R) | 21463 |
| 596 | FBgn0042146-FBgn0029552 (X) | 21454 |
| 597 | FBgn0034602-FBgn0027073 (2R) | 21408 |
| 598 | FBgn0031951-FBgn0027515 (2L) | 21368 |
| 599 | FBgn0034230-FBgn0022085 (2R) | 21233 |
| 600 | FBgn0001291-FBgn0033486 (2R) | 21214 |
| 601 | FBgn0259245-FBgn0031181 (X) | 21212 |
| 602 | FBgn0030914-FBgn0030915 (X) | 21201 |
| 603 | FBgn0036676-FBgn0036681 (3L) | 21172 |
| 604 | FBgn0038569-FBgn0038576 (3R) | 21130 |
| 605 | FBgn0028939-FBgn0003255 (2L) | 21107 |
| 606 | FBgn0011653-FBgn0003710 (3L) | 21070 |
| 607 | FBgn0033650-FBgn0033651 (2R) | 21041 |
| 608 | FBgn0032193-FBgn0032197 (2L) | 20990 |
| 609 | FBgn0034240-FBgn0040294 (2R) | 20915 |
| 610 | FBgn0038996-FBgn0020278 (3R) | 20898 |
| 611 | FBgn0040250-FBgn0037870 (3R) | 20697 |
| 612 | FBgn0032465-FBgn0028965 (2L) | 20677 |
| 613 | FBgn0086782-FBgn0052821 (X) | 20644 |
| 614 | FBgn0040871-FBgn0030538 (X) | 20619 |
| 615 | FBgn0052432-FBgn0040637 (3L) | 20608 |
| 616 | FBgn0031070-FBgn0259789 (X) | 20606 |
| 617 | FBgn0001202-FBgn0032748 (2L) | 20587 |
| 618 | FBgn0047338-FBgn0025776 (3L) | 20586 |
| 619 | FBgn0030855-FBgn0030858 (X) | 20536 |
| 620 | FBgn0033935-FBgn0013725 (2R) | 20525 |
| 621 | FBgn0031897-FBgn0259111 (2L) | 20472 |
| 622 | FBgn0039687-FBgn0039691 (3R) | 20426 |
| 623 | FBgn0039140-FBgn0040283 (3R) | 20381 |
| 624 | FBgn0040752-FBgn0033900 (2R) | 20352 |
| 625 | FBgn0004584-FBgn0025681 (2L) | 20289 |
| 626 | FBgn0035788-FBgn0035790 (3L) | 20255 |
| 627 | FBgn0026761-FBgn0033081 (2R) | 20189 |
| 628 | FBgn0051140-FBgn0051451 (3R) | 20167 |
| 629 | FBgn0036159-FBgn0036162 (3L) | 20147 |
| 630 | FBgn0040814-FBgn0036348 (3L) | 20009 |
| 631 | FBgn0035795-FBgn0035797 (3L) | 19987 |
| 632 | FBgn0083971-FBgn0037627 (3R) | 19946 |
| 633 | FBgn0037234-FBgn0037236 (3R) | 19876 |
| 634 | FBgn0053527-FBgn0035031 (2R) | 19862 |
| 635 | FBgn0001325-FBgn0050429 (2R) | 19853 |
| 636 | FBgn0033322-FBgn0050357 (2R) | 19831 |
| 637 | FBgn0031116-FBgn0052506 (X) | 19813 |
| 638 | FBgn0030808-FBgn0030809 (X) | 19788 |
| 639 | FBgn0039000-FBgn0046114 (3R) | 19767 |
| 640 | FBgn0030261-FBgn0003366 (X) | 19720 |
| 641 | FBgn0083980-FBgn0003411 (X) | 19698 |
| 642 | FBgn0036496-FBgn0036498 (3L) | 19633 |
| 643 | FBgn0002592-FBgn0002633 (3R) | 19569 |
| 644 | FBgn0004401-FBgn0036746 (3L) | 19568 |
| 645 | FBgn0035199-FBgn0052333 (3L) | 19521 |
| 646 | FBgn0038139-FBgn0045442 (3R) | 19511 |
| 647 | FBgn0032681-FBgn0032683 (2L) | 19465 |
| 648 | FBgn0052185-FBgn0036734 (3L) | 19420 |
| 649 | FBgn0036871-FBgn0036874 (3L) | 19420 |
| 650 | FBgn0259225-FBgn0032385 (2L) | 19396 |
| 651 | FBgn0028738-FBgn0000157 (2R) | 19373 |
| 652 | FBgn0038110-FBgn0038111 (3R) | 19363 |
| 653 | FBgn0031141-FBgn0040648 (X) | 19271 |
| 654 | FBgn0029834-FBgn0029835 (X) | 19209 |
| 655 | FBgn0028683-FBgn0033762 (2R) | 19191 |
| 656 | FBgn0035481-FBgn0004880 (3L) | 19146 |
| 657 | FBgn0014009-FBgn0033095 (2R) | 19144 |
| 658 | FBgn0032005-FBgn0032006 (2L) | 19139 |
| 659 | FBgn0030704-FBgn0010315 (X) | 19077 |
| 660 | FBgn0005779-FBgn0031504 (2L) | 19049 |
| 661 | FBgn0051501-FBgn0004172 (3R) | 19018 |
| 662 | FBgn0259237-FBgn0038903 (3R) | 19015 |
| 663 | FBgn0038447-FBgn0038448 (3R) | 18987 |
| 664 | FBgn0037465-FBgn0037470 (3R) | 18958 |
| 665 | FBgn0033475-FBgn0033477 (2R) | 18884 |
| 666 | FBgn0032929-FBgn0011829 (2L) | 18805 |
| 667 | FBgn0030437-FBgn0030439 (X) | 18797 |
| 668 | FBgn0032188-FBgn0051874 (2L) | 18795 |
| 669 | FBgn0033562-FBgn0033569 (2R) | 18758 |
| 670 | FBgn0030528-FBgn0030530 (X) | 18616 |
| 671 | FBgn0037876-FBgn0037884 (3R) | 18575 |
| 672 | FBgn0013733-FBgn0013770 (2R) | 18550 |
| 673 | FBgn0030215-FBgn0085443 (X) | 18525 |
| 674 | FBgn0000709-FBgn0011710 (X) | 18481 |
| 675 | FBgn0030292-FBgn0027359 (X) | 18463 |
| 676 | FBgn0000308-FBgn0031759 (2L) | 18459 |
| 677 | FBgn0023479-FBgn0043806 (3L) | 18410 |
| 678 | FBgn0051630-FBgn0051907 (2L) | 18385 |
| 679 | FBgn0015014-FBgn0037657 (3R) | 18263 |
| 680 | FBgn0034583-FBgn0034585 (2R) | 18218 |
| 681 | FBgn0037612-FBgn0037615 (3R) | 18217 |
| 682 | FBgn0028919-FBgn0015271 (2L) | 18166 |
| 683 | FBgn0030595-FBgn0052591 (X) | 18100 |
| 684 | FBgn0031802-FBgn0043854 (2L) | 18085 |
| 685 | FBgn0051548-FBgn0037358 (3R) | 18047 |
| 686 | FBgn0027376-FBgn0039331 (3R) | 18034 |
| 687 | FBgn0030963-FBgn0030966 (X) | 17986 |
| 688 | FBgn0032361-FBgn0032363 (2L) | 17939 |
| 689 | FBgn0035202-FBgn0035207 (3L) | 17937 |
| 690 | FBgn0034820-FBgn0041236 (2R) | 17908 |
| 691 | FBgn0030342-FBgn0030346 (X) | 17899 |
| 692 | FBgn0033182-FBgn0033184 (2R) | 17876 |
| 693 | FBgn0037329-FBgn0051550 (3R) | 17740 |
| 694 | FBgn0030869-FBgn0030872 (X) | 17634 |
| 695 | FBgn0033990-FBgn0029082 (2R) | 17568 |
| 696 | FBgn0037862-FBgn0040257 (3R) | 17479 |
| 697 | FBgn0051481-FBgn0000166 (3R) | 17415 |
| 698 | FBgn0037989-FBgn0051361 (3R) | 17403 |
| 699 | FBgn0016075-FBgn0031673 (2L) | 17301 |
| 700 | FBgn0030460-FBgn0005410 (X) | 17294 |
| 701 | FBgn0030086-FBgn0004856 (X) | 17275 |
| 702 | FBgn0036757-FBgn0052189 (3L) | 17254 |
| 703 | FBgn0030518-FBgn0030520 (X) | 17246 |
| 704 | FBgn0037792-FBgn0037794 (3R) | 17155 |
| 705 | FBgn0024944-FBgn0051205 (3R) | 17091 |
| 706 | FBgn0037018-FBgn0037022 (3L) | 17012 |
| 707 | FBgn0030351-FBgn0030354 (X) | 16983 |
| 708 | FBgn0036152-FBgn0036153 (3L) | 16961 |
| 709 | FBgn0086408-FBgn0050274 (2R) | 16905 |
| 710 | FBgn0031585-FBgn0026394 (2L) | 16856 |
| 711 | FBgn0035499-FBgn0004574 (3L) | 16779 |
| 712 | FBgn0034708-FBgn0034709 (2R) | 16696 |
| 713 | FBgn0028991-FBgn0033806 (2R) | 16683 |
| 714 | FBgn0022942-FBgn0029720 (X) | 16677 |
| 715 | FBgn0015618-FBgn0004390 (3L) | 16630 |
| 716 | FBgn0037540-FBgn0001255 (3R) | 16622 |
| 717 | FBgn0001139-FBgn0039405 (3R) | 16514 |
| 718 | FBgn0025387-FBgn0011277 (X) | 16479 |
| 719 | FBgn0036570-FBgn0036571 (3L) | 16412 |
| 720 | FBgn0002780-FBgn0002645 (3R) | 16369 |
| 721 | FBgn0003048-FBgn0001330 (X) | 16328 |
| 722 | FBgn0050272-FBgn0034785 (2R) | 16305 |
| 723 | FBgn0027054-FBgn0033247 (2R) | 16288 |
| 724 | FBgn0039302-FBgn0039306 (3R) | 16251 |
| 725 | FBgn0014143-FBgn0037086 (3L) | 16218 |
| 726 | FBgn0031078-FBgn0052529 (X) | 16195 |
| 727 | FBgn0024314-FBgn0031322 (2L) | 16178 |
| 728 | FBgn0014133-FBgn0011754 (X) | 16118 |
| 729 | FBgn0250841-FBgn0031409 (2L) | 16056 |
| 730 | FBgn0038510-FBgn0038512 (3R) | 16049 |
| 731 | FBgn0040972-FBgn0032473 (2L) | 16038 |
| 732 | FBgn0033972-FBgn0022063 (2R) | 16003 |
| 733 | FBgn0038053-FBgn0038058 (3R) | 16003 |
| 734 | FBgn0051084-FBgn0008649 (3R) | 15974 |
| 735 | FBgn0034824-FBgn0034827 (2R) | 15958 |
| 736 | FBgn0036373-FBgn0036374 (3L) | 15937 |
| 737 | FBgn0051291-FBgn0038386 (3R) | 15931 |
| 738 | FBgn0035873-FBgn0035875 (3L) | 15910 |
| 739 | FBgn0028499-FBgn0038581 (3R) | 15830 |
| 740 | FBgn0033794-FBgn0053007 (2R) | 15826 |
| 741 | FBgn0034893-FBgn0034897 (2R) | 15795 |
| 742 | FBgn0036949-FBgn0036953 (3L) | 15774 |
| 743 | FBgn0014859-FBgn0032879 (2L) | 15721 |
| 744 | FBgn0031749-FBgn0015381 (2L) | 15649 |
| 745 | FBgn0034084-FBgn0034086 (2R) | 15647 |
| 746 | FBgn0035813-FBgn0035816 (3L) | 15631 |
| 747 | FBgn0035482-FBgn0004895 (3L) | 15623 |
| 748 | FBgn0038201-FBgn0038205 (3R) | 15560 |
| 749 | FBgn0067861-FBgn0052823 (X) | 15535 |
| 750 | FBgn0035315-FBgn0035316 (3L) | 15491 |
| 751 | FBgn0000473-FBgn0085421 (2R) | 15462 |
| 752 | FBgn0010238-FBgn0033741 (2R) | 15450 |
| 753 | FBgn0028848-FBgn0028847 (2L) | 15428 |
| 754 | FBgn0034399-FBgn0034401 (2R) | 15415 |
| 755 | FBgn0029999-FBgn0030004 (X) | 15344 |
| 756 | FBgn0035370-FBgn0035375 (3L) | 15277 |
| 757 | FBgn0004587-FBgn0038167 (3R) | 15250 |
| 758 | FBgn0039479-FBgn0039481 (3R) | 15241 |
| 759 | FBgn0035507-FBgn0035510 (3L) | 15186 |
| 760 | FBgn0039886-FBgn0011224 (3R) | 15149 |
| 761 | FBgn0052121-FBgn0036360 (3L) | 15136 |
| 762 | FBgn0035388-FBgn0052485 (3L) | 15132 |
| 763 | FBgn0031324-FBgn0259229 (2L) | 15123 |
| 764 | FBgn0036070-FBgn0036072 (3L) | 15060 |
| 765 | FBgn0034904-FBgn0034906 (2R) | 15058 |
| 766 | FBgn0053144-FBgn0033547 (2R) | 15047 |
| 767 | FBgn0020279-FBgn0028563 (2R) | 15024 |
| 768 | FBgn0015600-FBgn0011648 (2L) | 15018 |
| 769 | FBgn0015828-FBgn0036194 (3L) | 14961 |
| 770 | FBgn0027780-FBgn0029173 (2L) | 14921 |
| 771 | FBgn0034321-FBgn0034327 (2R) | 14890 |
| 772 | FBgn0004919-FBgn0003715 (2R) | 14846 |
| 773 | FBgn0036878-FBgn0036881 (3L) | 14816 |
| 774 | FBgn0034433-FBgn0034434 (2R) | 14798 |
| 775 | FBgn0039505-FBgn0039509 (3R) | 14792 |
| 776 | FBgn0030137-FBgn0052702 (X) | 14765 |
| 777 | FBgn0069938-FBgn0069972 (X) | 14756 |
| 778 | FBgn0052082-FBgn0036139 (3L) | 14737 |
| 779 | FBgn0030995-FBgn0027603 (X) | 14737 |
| 780 | FBgn0053278-FBgn0035786 (3L) | 14657 |
| 781 | FBgn0250844-FBgn0028913 (2L) | 14652 |
| 782 | FBgn0030286-FBgn0028421 (X) | 14623 |
| 783 | FBgn0039434-FBgn0051080 (3R) | 14612 |
| 784 | FBgn0040298-FBgn0086694 (3L) | 14605 |
| 785 | FBgn0034931-FBgn0034933 (2R) | 14513 |
| 786 | FBgn0032749-FBgn0032753 (2L) | 14500 |
| 787 | FBgn0037215-FBgn0037218 (3R) | 14477 |
| 788 | FBgn0030359-FBgn0030360 (X) | 14411 |
| 789 | FBgn0031155-FBgn0003950 (X) | 14302 |
| 790 | FBgn0032453-FBgn0032455 (2L) | 14297 |
| 791 | FBgn0038735-FBgn0038737 (3R) | 14295 |
| 792 | FBgn0004652-FBgn0038626 (3R) | 14284 |
| 793 | FBgn0036684-FBgn0036686 (3L) | 14281 |
| 794 | FBgn0031118-FBgn0026323 (X) | 14233 |
| 795 | FBgn0037359-FBgn0010772 (3R) | 14185 |
| 796 | FBgn0041229-FBgn0045468 (3R) | 14157 |
| 797 | FBgn0043539-FBgn0053543 (2L) | 14156 |
| 798 | FBgn0030706-FBgn0010240 (X) | 14077 |
| 799 | FBgn0034179-FBgn0034181 (2R) | 14076 |
| 800 | FBgn0036896-FBgn0022774 (3L) | 14068 |
| 801 | FBgn0000053-FBgn0022201 (2L) | 14059 |
| 802 | FBgn0004622-FBgn0039728 (3R) | 14006 |
| 803 | FBgn0026376-FBgn0052137 (3L) | 14005 |
| 804 | FBgn0026597-FBgn0039740 (3R) | 14003 |
| 805 | FBgn0054032-FBgn0052117 (3L) | 13942 |
| 806 | FBgn0031357-FBgn0031359 (2L) | 13926 |
| 807 | FBgn0025742-FBgn0031769 (2L) | 13910 |
| 808 | FBgn0035995-FBgn0036000 (3L) | 13900 |
| 809 | FBgn0029716-FBgn0029717 (X) | 13858 |
| 810 | FBgn0024232-FBgn0024319 (2R) | 13807 |
| 811 | FBgn0052311-FBgn0035308 (3L) | 13772 |
| 812 | FBgn0002528-FBgn0036020 (3L) | 13758 |
| 813 | FBgn0031403-FBgn0051682 (2L) | 13754 |
| 814 | FBgn0028695-FBgn0036921 (3L) | 13752 |
| 815 | FBgn0035545-FBgn0035547 (3L) | 13739 |
| 816 | FBgn0024291-FBgn0032477 (2L) | 13695 |
| 817 | FBgn0053465-FBgn0003130 (2R) | 13681 |
| 818 | FBgn0026148-FBgn0000146 (2L) | 13673 |
| 819 | FBgn0004108-FBgn0036660 (3L) | 13671 |
| 820 | FBgn0037897-FBgn0037901 (3R) | 13612 |
| 821 | FBgn0003423-FBgn0001565 (X) | 13600 |
| 822 | FBgn0024326-FBgn0037606 (3R) | 13570 |
| 823 | FBgn0037312-FBgn0037315 (3R) | 13559 |
| 824 | FBgn0038397-FBgn0052855 (3R) | 13487 |
| 825 | FBgn0033310-FBgn0033312 (2R) | 13479 |
| 826 | FBgn0010040-FBgn0053098 (3R) | 13464 |
| 827 | FBgn0030933-FBgn0030937 (X) | 13457 |
| 828 | FBgn0032512-FBgn0032516 (2L) | 13447 |
| 829 | FBgn0031149-FBgn0052512 (X) | 13418 |
| 830 | FBgn0086676-FBgn0001124 (2R) | 13370 |
| 831 | FBgn0036590-FBgn0036596 (3L) | 13365 |
| 832 | FBgn0038588-FBgn0053520 (3R) | 13286 |
| 833 | FBgn0086129-FBgn0034975 (2R) | 13276 |
| 834 | FBgn0085406-FBgn0038942 (3R) | 13275 |
| 835 | FBgn0002865-FBgn0039577 (3R) | 13268 |
| 836 | FBgn0037071-FBgn0003042 (3L) | 13260 |
| 837 | FBgn0038398-FBgn0026059 (3R) | 13257 |
| 838 | FBgn0039698-FBgn0039702 (3R) | 13254 |
| 839 | FBgn0029507-FBgn0033127 (2R) | 13159 |
| 840 | FBgn0031947-FBgn0025687 (2L) | 13102 |
| 841 | FBgn0034265-FBgn0034271 (2R) | 13094 |
| 842 | FBgn0030668-FBgn0030672 (X) | 13094 |
| 843 | FBgn0020637-FBgn0020643 (3L) | 13072 |
| 844 | FBgn0051206-FBgn0045471 (3R) | 13060 |
| 845 | FBgn0033277-FBgn0050375 (2R) | 13027 |
| 846 | FBgn0040794-FBgn0053257 (3L) | 13011 |
| 847 | FBgn0034135-FBgn0034136 (2R) | 12999 |
| 848 | FBgn0032200-FBgn0032205 (2L) | 12992 |
| 849 | FBgn0036545-FBgn0036549 (3L) | 12989 |
| 850 | FBgn0032414-FBgn0032416 (2L) | 12968 |
| 851 | FBgn0036205-FBgn0015278 (3L) | 12936 |
| 852 | FBgn0259149-FBgn0029809 (X) | 12909 |
| 853 | FBgn0037297-FBgn0037301 (3R) | 12879 |
| 854 | FBgn0039354-FBgn0039355 (3R) | 12873 |
| 855 | FBgn0016756-FBgn0035631 (3L) | 12862 |
| 856 | FBgn0030829-FBgn0030832 (X) | 12848 |
| 857 | FBgn0039366-FBgn0039371 (3R) | 12824 |
| 858 | FBgn0040393-FBgn0003374 (X) | 12820 |
| 859 | FBgn0038306-FBgn0045035 (3R) | 12798 |
| 860 | FBgn0032538-FBgn0028544 (2L) | 12783 |
| 861 | FBgn0010768-FBgn0038685 (3R) | 12780 |
| 862 | FBgn0001169-FBgn0015279 (3R) | 12766 |
| 863 | FBgn0033382-FBgn0050344 (2R) | 12763 |
| 864 | FBgn0036564-FBgn0036569 (3L) | 12759 |
| 865 | FBgn0062413-FBgn0025815 (X) | 12743 |
| 866 | FBgn0038525-FBgn0038529 (3R) | 12739 |
| 867 | FBgn0030093-FBgn0030096 (X) | 12717 |
| 868 | FBgn0051493-FBgn0037477 (3R) | 12697 |
| 869 | FBgn0031635-FBgn0031640 (2L) | 12644 |
| 870 | FBgn0032521-FBgn0003983 (2L) | 12643 |
| 871 | FBgn0024273-FBgn0015589 (3R) | 12616 |
| 872 | FBgn0024986-FBgn0024985 (X) | 12590 |
| 873 | FBgn0031657-FBgn0031664 (2L) | 12585 |
| 874 | FBgn0031520-FBgn0031523 (2L) | 12560 |
| 875 | FBgn0032066-FBgn0032069 (2L) | 12544 |
| 876 | FBgn0044030-FBgn0031021 (X) | 12507 |
| 877 | FBgn0085428-FBgn0050096 (2R) | 12506 |
| 878 | FBgn0030033-FBgn0040928 (X) | 12481 |
| 879 | FBgn0012051-FBgn0040273 (2R) | 12414 |
| 880 | FBgn0029501-FBgn0035014 (2R) | 12358 |
| 881 | FBgn0036196-FBgn0036199 (3L) | 12358 |
| 882 | FBgn0037646-FBgn0037648 (3R) | 12337 |
| 883 | FBgn0032029-FBgn0032034 (2L) | 12303 |
| 884 | FBgn0051741-FBgn0032664 (2L) | 12301 |
| 885 | FBgn0033907-FBgn0050069 (2R) | 12284 |
| 886 | FBgn0035400-FBgn0035403 (3L) | 12268 |
| 887 | FBgn0031489-FBgn0031495 (2L) | 12259 |
| 888 | FBgn0028569-FBgn0032740 (2L) | 12249 |
| 889 | FBgn0003659-FBgn0027342 (X) | 12240 |
| 890 | FBgn0034913-FBgn0034918 (2R) | 12210 |
| 891 | FBgn0031939-FBgn0031940 (2L) | 12174 |
| 892 | FBgn0038011-FBgn0038012 (3R) | 12171 |
| 893 | FBgn0011236-FBgn0025336 (2R) | 12157 |
| 894 | FBgn0032225-FBgn0032228 (2L) | 12139 |
| 895 | FBgn0024371-FBgn0032926 (2L) | 12100 |
| 896 | FBgn0027865-FBgn0039385 (3R) | 12058 |
| 897 | FBgn0038295-FBgn0038296 (3R) | 12025 |
| 898 | FBgn0026255-FBgn0032934 (2L) | 12008 |
| 899 | FBgn0030247-FBgn0030249 (X) | 11975 |
| 900 | FBgn0030309-FBgn0030311 (X) | 11971 |
| 901 | FBgn0031216-FBgn0026787 (2L) | 11967 |
| 902 | FBgn0028953-FBgn0022987 (2R) | 11952 |
| 903 | FBgn0034639-FBgn0034641 (2R) | 11950 |
| 904 | FBgn0032016-FBgn0032020 (2L) | 11885 |
| 905 | FBgn0038958-FBgn0038960 (3R) | 11876 |
| 906 | FBgn0037067-FBgn0037070 (3L) | 11796 |
| 907 | FBgn0050467-FBgn0034030 (2R) | 11768 |
| 908 | FBgn0036259-FBgn0036260 (3L) | 11748 |
| 909 | FBgn0013767-FBgn0038223 (3R) | 11747 |
| 910 | FBgn0034154-FBgn0034156 (2R) | 11737 |
| 911 | FBgn0036977-FBgn0036979 (3L) | 11731 |
| 912 | FBgn0031089-FBgn0031090 (X) | 11713 |
| 913 | FBgn0037239-FBgn0037241 (3R) | 11712 |
| 914 | FBgn0030552-FBgn0030554 (X) | 11699 |
| 915 | FBgn0033911-FBgn0005613 (2R) | 11689 |
| 916 | FBgn0040551-FBgn0038129 (3R) | 11670 |
| 917 | FBgn0000826-FBgn0029573 (X) | 11666 |
| 918 | FBgn0023519-FBgn0052803 (X) | 11640 |
| 919 | FBgn0036741-FBgn0036742 (3L) | 11624 |
| 920 | FBgn0250815-FBgn0035665 (3L) | 11618 |
| 921 | FBgn0020381-FBgn0025638 (X) | 11561 |
| 922 | FBgn0004897-FBgn0004898 (3R) | 11558 |
| 923 | FBgn0031238-FBgn0031244 (2L) | 11550 |
| 924 | FBgn0033527-FBgn0033528 (2R) | 11529 |
| 925 | FBgn0034643-FBgn0034644 (2R) | 11511 |
| 926 | FBgn0051848-FBgn0032498 (2L) | 11501 |
| 927 | FBgn0010303-FBgn0030448 (X) | 11499 |
| 928 | FBgn0003346-FBgn0003732 (2L) | 11467 |
| 929 | FBgn0035845-FBgn0040290 (3L) | 11447 |
| 930 | FBgn0032964-FBgn0051612 (2L) | 11433 |
| 931 | FBgn0004419-FBgn0051717 (2L) | 11412 |
| 932 | FBgn0025115-FBgn0028938 (2L) | 11411 |
| 933 | FBgn0029850-FBgn0029851 (X) | 11382 |
| 934 | FBgn0259226-FBgn0033308 (2R) | 11360 |
| 935 | FBgn0034008-FBgn0013750 (2R) | 11346 |
| 936 | FBgn0037852-FBgn0037855 (3R) | 11344 |
| 937 | FBgn0085426-FBgn0050393 (2R) | 11340 |
| 938 | FBgn0021818-FBgn0025716 (2R) | 11337 |
| 939 | FBgn0005198-FBgn0036942 (3L) | 11324 |
| 940 | FBgn0003008-FBgn0005612 (2R) | 11278 |
| 941 | FBgn0026174-FBgn0031074 (X) | 11271 |
| 942 | FBgn0000183-FBgn0032644 (2L) | 11264 |
| 943 | FBgn0023516-FBgn0029594 (X) | 11237 |
| 944 | FBgn0042101-FBgn0037521 (3R) | 11218 |
| 945 | FBgn0031545-FBgn0031550 (2L) | 11207 |
| 946 | FBgn0034617-FBgn0050394 (2R) | 11204 |
| 947 | FBgn0031264-FBgn0031268 (2L) | 11184 |
| 948 | FBgn0051109-FBgn0039270 (3R) | 11172 |
| 949 | FBgn0030034-FBgn0052711 (X) | 11156 |
| 950 | FBgn0037836-FBgn0037837 (3R) | 11139 |
| 951 | FBgn0032222-FBgn0032224 (2L) | 11132 |
| 952 | FBgn0083986-FBgn0045980 (3R) | 11122 |
| 953 | FBgn0027053-FBgn0004087 (3R) | 11099 |
| 954 | FBgn0034259-FBgn0034261 (2R) | 10955 |
| 955 | FBgn0025838-FBgn0000092 (X) | 10955 |
| 956 | FBgn0014464-FBgn0000360 (X) | 10950 |
| 957 | FBgn0031214-FBgn0002931 (2L) | 10937 |
| 958 | FBgn0052944-FBgn0051525 (3R) | 10918 |
| 959 | FBgn0041629-FBgn0030063 (X) | 10904 |
| 960 | FBgn0030302-FBgn0030303 (X) | 10895 |
| 961 | FBgn0031308-FBgn0031312 (2L) | 10847 |
| 962 | FBgn0038879-FBgn0038880 (3R) | 10802 |
| 963 | FBgn0003278-FBgn0023444 (2L) | 10784 |
| 964 | FBgn0030408-FBgn0030409 (X) | 10778 |
| 965 | FBgn0031410-FBgn0051949 (2L) | 10734 |
| 966 | FBgn0038208-FBgn0038210 (3R) | 10721 |
| 967 | FBgn0036004-FBgn0036005 (3L) | 10718 |
| 968 | FBgn0030763-FBgn0030764 (X) | 10650 |
| 969 | FBgn0032247-FBgn0032251 (2L) | 10608 |
| 970 | FBgn0050203-FBgn0033712 (2R) | 10605 |
| 971 | FBgn0010355-FBgn0026566 (3R) | 10598 |
| 972 | FBgn0016047-FBgn0003382 (2R) | 10588 |
| 973 | FBgn0040268-FBgn0032796 (2L) | 10582 |
| 974 | FBgn0039634-FBgn0039637 (3R) | 10563 |
| 975 | FBgn0032266-FBgn0051721 (2L) | 10560 |
| 976 | FBgn0035676-FBgn0035677 (3L) | 10548 |
| 977 | FBgn0014380-FBgn0003205 (3R) | 10511 |
| 978 | FBgn0031299-FBgn0031302 (2L) | 10483 |
| 979 | FBgn0025866-FBgn0036059 (3L) | 10468 |
| 980 | FBgn0034814-FBgn0003062 (2R) | 10457 |
| 981 | FBgn0032771-FBgn0032772 (2L) | 10453 |
| 982 | FBgn0040984-FBgn0259151 (2L) | 10442 |
| 983 | FBgn0039651-FBgn0051044 (3R) | 10430 |
| 984 | FBgn0051686-FBgn0031406 (2L) | 10414 |
| 985 | FBgn0029839-FBgn0029843 (X) | 10404 |
| 986 | FBgn0030364-FBgn0030366 (X) | 10385 |
| 987 | FBgn0031295-FBgn0031296 (2L) | 10379 |
| 988 | FBgn0026634-FBgn0038331 (3R) | 10286 |
| 989 | FBgn0030327-FBgn0030330 (X) | 10286 |
| 990 | FBgn0051961-FBgn0031589 (2L) | 10270 |
| 991 | FBgn0031968-FBgn0031969 (2L) | 10263 |
| 992 | FBgn0086711-FBgn0028926 (2L) | 10251 |
| 993 | FBgn0038961-FBgn0038965 (3R) | 10241 |
| 994 | FBgn0050380-FBgn0033238 (2R) | 10201 |
| 995 | FBgn0039258-FBgn0039260 (3R) | 10183 |
| 996 | FBgn0034646-FBgn0034650 (2R) | 10166 |
| 997 | FBgn0033605-FBgn0033609 (2R) | 10148 |
| 998 | FBgn0033848-FBgn0033851 (2R) | 10119 |
| 999 | FBgn0028538-FBgn0027844 (2L) | 10082 |
| 1000 | FBgn0031257-FBgn0031258 (2L) | 10065 |
| 1001 | FBgn0031697-FBgn0031698 (2L) | 10052 |
| 1002 | FBgn0032236-FBgn0051716 (2L) | 10048 |
| 1003 | FBgn0039055-FBgn0051457 (3R) | 10034 |
| 1004 | FBgn0038424-FBgn0038428 (3R) | 10027 |
| 1005 | FBgn0038720-FBgn0038721 (3R) | 10027 |
| 1006 | FBgn0038716-FBgn0038719 (3R) | 10007 |
| 1007 | FBgn0034766-FBgn0050268 (2R) | 9985 |
| 1008 | FBgn0044047-FBgn0040389 (X) | 9980 |
| 1009 | FBgn0023550-FBgn0053548 (X) | 9972 |
| 1010 | FBgn0052479-FBgn0035173 (3L) | 9971 |
| 1011 | FBgn0037224-FBgn0041621 (3R) | 9951 |
| 1012 | FBgn0027095-FBgn0038421 (3R) | 9951 |
| 1013 | FBgn0029858-FBgn0043796 (X) | 9921 |
| 1014 | FBgn0040754-FBgn0033812 (2R) | 9905 |
| 1015 | FBgn0004110-FBgn0004862 (3R) | 9905 |
| 1016 | FBgn0031449-FBgn0031450 (2L) | 9881 |
| 1017 | FBgn0030780-FBgn0052572 (X) | 9881 |
| 1018 | FBgn0034374-FBgn0034377 (2R) | 9860 |
| 1019 | FBgn0052984-FBgn0032083 (2L) | 9843 |
| 1020 | FBgn0034057-FBgn0034060 (2R) | 9842 |
| 1021 | FBgn0052075-FBgn0036124 (3L) | 9835 |
| 1022 | FBgn0039630-FBgn0039633 (3R) | 9822 |
| 1023 | FBgn0037149-FBgn0037151 (3L) | 9815 |
| 1024 | FBgn0026573-FBgn0050037 (2R) | 9812 |
| 1025 | FBgn0030014-FBgn0030017 (X) | 9810 |
| 1026 | FBgn0019990-FBgn0039846 (3R) | 9795 |
| 1027 | FBgn0004509-FBgn0039338 (3R) | 9772 |
| 1028 | FBgn0029892-FBgn0029894 (X) | 9734 |
| 1029 | FBgn0032215-FBgn0032218 (2L) | 9732 |
| 1030 | FBgn0034329-FBgn0064237 (2R) | 9714 |
| 1031 | FBgn0052758-FBgn0029798 (X) | 9711 |
| 1032 | FBgn0027066-FBgn0033101 (2R) | 9708 |
| 1033 | FBgn0038256-FBgn0011217 (3R) | 9703 |
| 1034 | FBgn0035799-FBgn0035802 (3L) | 9653 |
| 1035 | FBgn0039639-FBgn0039642 (3R) | 9600 |
| 1036 | FBgn0025701-FBgn0037916 (3R) | 9577 |
| 1037 | FBgn0030258-FBgn0030260 (X) | 9570 |
| 1038 | FBgn0010422-FBgn0024183 (2L) | 9564 |
| 1039 | FBgn0027548-FBgn0033244 (2R) | 9564 |
| 1040 | FBgn0042179-FBgn0052259 (3L) | 9564 |
| 1041 | FBgn0003460-FBgn0033168 (2R) | 9562 |
| 1042 | FBgn0034217-FBgn0034218 (2R) | 9554 |
| 1043 | FBgn0035770-FBgn0082598 (3L) | 9538 |
| 1044 | FBgn0035855-FBgn0035857 (3L) | 9495 |
| 1045 | FBgn0050060-FBgn0050065 (2R) | 9449 |
| 1046 | FBgn0032446-FBgn0032450 (2L) | 9442 |
| 1047 | FBgn0015324-FBgn0250753 (3R) | 9439 |
| 1048 | FBgn0003292-FBgn0036179 (3L) | 9435 |
| 1049 | FBgn0032489-FBgn0032493 (2L) | 9428 |
| 1050 | FBgn0038312-FBgn0027378 (3R) | 9427 |
| 1051 | FBgn0053490-FBgn0052086 (3L) | 9402 |
| 1052 | FBgn0003900-FBgn0050194 (2R) | 9389 |
| 1053 | FBgn0000392-FBgn0028990 (2L) | 9380 |
| 1054 | FBgn0036691-FBgn0036696 (3L) | 9366 |
| 1055 | FBgn0028482-FBgn0020762 (2L) | 9354 |
| 1056 | FBgn0036500-FBgn0036502 (3L) | 9325 |
| 1057 | FBgn0035436-FBgn0035437 (3L) | 9315 |
| 1058 | FBgn0025632-FBgn0025625 (X) | 9299 |
| 1059 | FBgn0052668-FBgn0026268 (X) | 9299 |
| 1060 | FBgn0085241-FBgn0033056 (2R) | 9273 |
| 1061 | FBgn0035210-FBgn0035213 (3L) | 9252 |
| 1062 | FBgn0039519-FBgn0039520 (3R) | 9251 |
| 1063 | FBgn0029710-FBgn0029713 (X) | 9251 |
| 1064 | FBgn0036225-FBgn0036229 (3L) | 9241 |
| 1065 | FBgn0034726-FBgn0034728 (2R) | 9233 |
| 1066 | FBgn0042207-FBgn0038070 (3R) | 9215 |
| 1067 | FBgn0060296-FBgn0043792 (2R) | 9201 |
| 1068 | FBgn0051014-FBgn0039784 (3R) | 9176 |
| 1069 | FBgn0036364-FBgn0036366 (3L) | 9167 |
| 1070 | FBgn0015129-FBgn0039677 (3R) | 9166 |
| 1071 | FBgn0039186-FBgn0039188 (3R) | 9137 |
| 1072 | FBgn0036853-FBgn0017578 (3L) | 9135 |
| 1073 | FBgn0029587-FBgn0029588 (X) | 9092 |
| 1074 | FBgn0035523-FBgn0015829 (3L) | 9075 |
| 1075 | FBgn0033713-FBgn0033715 (2R) | 9060 |
| 1076 | FBgn0051343-FBgn0051198 (3R) | 9056 |
| 1077 | FBgn0045479-FBgn0052255 (3L) | 9031 |
| 1078 | FBgn0036428-FBgn0054039 (3L) | 9030 |
| 1079 | FBgn0085434-FBgn0035078 (2R) | 9029 |
| 1080 | FBgn0033047-FBgn0033048 (2R) | 8982 |
| 1081 | FBgn0032406-FBgn0003145 (2L) | 8970 |
| 1082 | FBgn0087035-FBgn0036509 (3L) | 8947 |
| 1083 | FBgn0033543-FBgn0024956 (2R) | 8921 |
| 1084 | FBgn0030240-FBgn0030243 (X) | 8896 |
| 1085 | FBgn0011571-FBgn0030734 (X) | 8873 |
| 1086 | FBgn0034622-FBgn0034624 (2R) | 8860 |
| 1087 | FBgn0030638-FBgn0004391 (X) | 8851 |
| 1088 | FBgn0052237-FBgn0035581 (3L) | 8850 |
| 1089 | FBgn0000077-FBgn0028292 (X) | 8842 |
| 1090 | FBgn0024542-FBgn0035754 (3L) | 8832 |
| 1091 | FBgn0030893-FBgn0022724 (X) | 8812 |
| 1092 | FBgn0030720-FBgn0003969 (X) | 8808 |
| 1093 | FBgn0038829-FBgn0027493 (3R) | 8751 |
| 1094 | FBgn0086708-FBgn0036372 (3L) | 8746 |
| 1095 | FBgn0028949-FBgn0028946 (2L) | 8735 |
| 1096 | FBgn0085234-FBgn0029084 (2R) | 8726 |
| 1097 | FBgn0038857-FBgn0038858 (3R) | 8725 |
| 1098 | FBgn0029911-FBgn0029915 (X) | 8719 |
| 1099 | FBgn0039007-FBgn0039010 (3R) | 8680 |
| 1100 | FBgn0034961-FBgn0034964 (2R) | 8664 |
| 1101 | FBgn0036230-FBgn0028573 (3L) | 8641 |
| 1102 | FBgn0037137-FBgn0037138 (3L) | 8620 |
| 1103 | FBgn0031419-FBgn0031422 (2L) | 8611 |
| 1104 | FBgn0033948-FBgn0033949 (2R) | 8602 |
| 1105 | FBgn0036987-FBgn0036991 (3L) | 8592 |
| 1106 | FBgn0051102-FBgn0051436 (3R) | 8588 |
| 1107 | FBgn0036889-FBgn0010417 (3L) | 8573 |
| 1108 | FBgn0050145-FBgn0034520 (2R) | 8569 |
| 1109 | FBgn0000147-FBgn0037970 (3R) | 8565 |
| 1110 | FBgn0001247-FBgn0011205 (3L) | 8537 |
| 1111 | FBgn0052248-FBgn0035551 (3L) | 8525 |
| 1112 | FBgn0038868-FBgn0038871 (3R) | 8525 |
| 1113 | FBgn0013812-FBgn0038850 (3R) | 8497 |
| 1114 | FBgn0036794-FBgn0052199 (3L) | 8476 |
| 1115 | FBgn0031515-FBgn0031516 (2L) | 8440 |
| 1116 | FBgn0050389-FBgn0034599 (2R) | 8416 |
| 1117 | FBgn0036626-FBgn0036628 (3L) | 8377 |
| 1118 | FBgn0005642-FBgn0039601 (3R) | 8363 |
| 1119 | FBgn0034132-FBgn0034133 (2R) | 8330 |
| 1120 | FBgn0038922-FBgn0019957 (3R) | 8324 |
| 1121 | FBgn0031710-FBgn0031713 (2L) | 8319 |
| 1122 | FBgn0053333-FBgn0038511 (3R) | 8306 |
| 1123 | FBgn0025621-FBgn0015799 (X) | 8290 |
| 1124 | FBgn0030586-FBgn0030588 (X) | 8283 |
| 1125 | FBgn0032147-FBgn0032149 (2L) | 8278 |
| 1126 | FBgn0035283-FBgn0035285 (3L) | 8266 |
| 1127 | FBgn0082585-FBgn0033633 (2R) | 8257 |
| 1128 | FBgn0004896-FBgn0034789 (2R) | 8251 |
| 1129 | FBgn0053013-FBgn0033720 (2R) | 8225 |
| 1130 | FBgn0015032-FBgn0053483 (3R) | 8201 |
| 1131 | FBgn0031432-FBgn0041337 (2L) | 8200 |
| 1132 | FBgn0046258-FBgn0039540 (3R) | 8187 |
| 1133 | FBgn0035170-FBgn0035171 (3L) | 8158 |
| 1134 | FBgn0037081-FBgn0037082 (3L) | 8157 |
| 1135 | FBgn0015359-FBgn0000543 (3L) | 8143 |
| 1136 | FBgn0002522-FBgn0004777 (3R) | 8139 |
| 1137 | FBgn0035876-FBgn0035879 (3L) | 8111 |
| 1138 | FBgn0004373-FBgn0052343 (3L) | 8108 |
| 1139 | FBgn0030338-FBgn0030340 (X) | 8090 |
| 1140 | FBgn0037759-FBgn0037760 (3R) | 8078 |
| 1141 | FBgn0053969-FBgn0028978 (3L) | 8061 |
| 1142 | FBgn0030502-FBgn0030504 (X) | 8053 |
| 1143 | FBgn0037248-FBgn0037252 (3R) | 8052 |
| 1144 | FBgn0032699-FBgn0032703 (2L) | 8046 |
| 1145 | FBgn0029976-FBgn0029977 (X) | 8043 |
| 1146 | FBgn0038765-FBgn0038769 (3R) | 8030 |
| 1147 | FBgn0034104-FBgn0050099 (2R) | 8020 |
| 1148 | FBgn0001105-FBgn0001092 (X) | 8020 |
| 1149 | FBgn0051232-FBgn0038619 (3R) | 8016 |
| 1150 | FBgn0027608-FBgn0037376 (3R) | 7999 |
| 1151 | FBgn0043900-FBgn0039854 (3R) | 7979 |
| 1152 | FBgn0035690-FBgn0035691 (3L) | 7975 |
| 1153 | FBgn0000536-FBgn0030733 (X) | 7962 |
| 1154 | FBgn0052570-FBgn0052574 (X) | 7941 |
| 1155 | FBgn0035143-FBgn0035144 (3L) | 7936 |
| 1156 | FBgn0031996-FBgn0031998 (2L) | 7933 |
| 1157 | FBgn0037561-FBgn0010433 (3R) | 7911 |
| 1158 | FBgn0010488-FBgn0033781 (2R) | 7908 |
| 1159 | FBgn0053233-FBgn0035366 (3L) | 7904 |
| 1160 | FBgn0050094-FBgn0034083 (2R) | 7892 |
| 1161 | FBgn0037812-FBgn0051406 (3R) | 7884 |
| 1162 | FBgn0051607-FBgn0051900 (2L) | 7878 |
| 1163 | FBgn0031746-FBgn0002855 (2L) | 7877 |
| 1164 | FBgn0038505-FBgn0038508 (3R) | 7865 |
| 1165 | FBgn0025885-FBgn0033179 (2R) | 7864 |
| 1166 | FBgn0005592-FBgn0036410 (3L) | 7857 |
| 1167 | FBgn0050157-FBgn0050156 (2R) | 7854 |
| 1168 | FBgn0034037-FBgn0053462 (2R) | 7820 |
| 1169 | FBgn0038247-FBgn0038248 (3R) | 7811 |
| 1170 | FBgn0032078-FBgn0032079 (2L) | 7778 |
| 1171 | FBgn0016693-FBgn0038080 (3R) | 7773 |
| 1172 | FBgn0036861-FBgn0036862 (3L) | 7770 |
| 1173 | FBgn0033890-FBgn0002643 (2R) | 7708 |
| 1174 | FBgn0085453-FBgn0034853 (2R) | 7671 |
| 1175 | FBgn0050047-FBgn0050043 (2R) | 7661 |
| 1176 | FBgn0031006-FBgn0031008 (X) | 7659 |
| 1177 | FBgn0038628-FBgn0038630 (3R) | 7657 |
| 1178 | FBgn0031534-FBgn0031537 (2L) | 7644 |
| 1179 | FBgn0030805-FBgn0030807 (X) | 7615 |
| 1180 | FBgn0032713-FBgn0015808 (2L) | 7612 |
| 1181 | FBgn0037943-FBgn0037944 (3R) | 7603 |
| 1182 | FBgn0034092-FBgn0034095 (2R) | 7591 |
| 1183 | FBgn0026563-FBgn0037464 (3R) | 7590 |
| 1184 | FBgn0050050-FBgn0033732 (2R) | 7502 |
| 1185 | FBgn0030296-FBgn0030298 (X) | 7457 |
| 1186 | FBgn0037483-FBgn0250845 (3R) | 7446 |
| 1187 | FBgn0015553-FBgn0032698 (2L) | 7427 |
| 1188 | FBgn0051413-FBgn0039106 (3R) | 7398 |
| 1189 | FBgn0030804-FBgn0053252 (X) | 7365 |
| 1190 | FBgn0038468-FBgn0038469 (3R) | 7340 |
| 1191 | FBgn0014020-FBgn0017549 (2R) | 7330 |
| 1192 | FBgn0003980-FBgn0086265 (2L) | 7319 |
| 1193 | FBgn0036729-FBgn0036731 (3L) | 7305 |
| 1194 | FBgn0015925-FBgn0001308 (2R) | 7304 |
| 1195 | FBgn0003200-FBgn0022768 (X) | 7286 |
| 1196 | FBgn0038166-FBgn0024555 (3R) | 7283 |
| 1197 | FBgn0033340-FBgn0050349 (2R) | 7280 |
| 1198 | FBgn0029882-FBgn0029885 (X) | 7262 |
| 1199 | FBgn0035217-FBgn0035218 (3L) | 7241 |
| 1200 | FBgn0042104-FBgn0037517 (3R) | 7241 |
| 1201 | FBgn0030508-FBgn0030509 (X) | 7217 |
| 1202 | FBgn0026190-FBgn0051371 (3R) | 7211 |
| 1203 | FBgn0037099-FBgn0037101 (3L) | 7206 |
| 1204 | FBgn0037566-FBgn0037569 (3R) | 7181 |
| 1205 | FBgn0050126-FBgn0034413 (2R) | 7132 |
| 1206 | FBgn0003415-FBgn0026179 (3L) | 7116 |
| 1207 | FBgn0031850-FBgn0025595 (2L) | 7099 |
| 1208 | FBgn0036763-FBgn0036764 (3L) | 7070 |
| 1209 | FBgn0039059-FBgn0039060 (3R) | 7065 |
| 1210 | FBgn0034291-FBgn0034294 (2R) | 7046 |
| 1211 | FBgn0259749-FBgn0031820 (2L) | 7038 |
| 1212 | FBgn0001179-FBgn0036090 (3L) | 7027 |
| 1213 | FBgn0030314-FBgn0002948 (X) | 7018 |
| 1214 | FBgn0041625-FBgn0041623 (3L) | 6998 |
| 1215 | FBgn0033353-FBgn0033355 (2R) | 6993 |
| 1216 | FBgn0030646-FBgn0030648 (X) | 6991 |
| 1217 | FBgn0027929-FBgn0028936 (2L) | 6984 |
| 1218 | FBgn0038341-FBgn0019940 (3R) | 6966 |
| 1219 | FBgn0032346-FBgn0032348 (2L) | 6950 |
| 1220 | FBgn0030449-FBgn0250862 (X) | 6944 |
| 1221 | FBgn0035298-FBgn0027547 (3L) | 6933 |
| 1222 | FBgn0002570-FBgn0033294 (2R) | 6926 |
| 1223 | FBgn0051390-FBgn0010421 (3R) | 6915 |
| 1224 | FBgn0039483-FBgn0039485 (3R) | 6910 |
| 1225 | FBgn0051410-FBgn0037783 (3R) | 6900 |
| 1226 | FBgn0001206-FBgn0030218 (X) | 6899 |
| 1227 | FBgn0039737-FBgn0039738 (3R) | 6895 |
| 1228 | FBgn0029870-FBgn0003139 (X) | 6893 |
| 1229 | FBgn0039872-FBgn0039873 (3R) | 6887 |
| 1230 | FBgn0039386-FBgn0051089 (3R) | 6876 |
| 1231 | FBgn0031245-FBgn0020622 (2L) | 6873 |
| 1232 | FBgn0038861-FBgn0025803 (3R) | 6865 |
| 1233 | FBgn0026400-FBgn0039589 (3R) | 6859 |
| 1234 | FBgn0045862-FBgn0039544 (3R) | 6839 |
| 1235 | FBgn0053180-FBgn0045073 (X) | 6837 |
| 1236 | FBgn0024806-FBgn0035122 (3L) | 6833 |
| 1237 | FBgn0037026-FBgn0037028 (3L) | 6832 |
| 1238 | FBgn0036784-FBgn0036785 (3L) | 6816 |
| 1239 | FBgn0050360-FBgn0033297 (2R) | 6814 |
| 1240 | FBgn0033716-FBgn0033717 (2R) | 6812 |
| 1241 | FBgn0029707-FBgn0052773 (X) | 6780 |
| 1242 | FBgn0035178-FBgn0035179 (3L) | 6778 |
| 1243 | FBgn0033090-FBgn0050446 (2R) | 6771 |
| 1244 | FBgn0036936-FBgn0036938 (3L) | 6754 |
| 1245 | FBgn0053051-FBgn0052176 (3L) | 6741 |
| 1246 | FBgn0038854-FBgn0028468 (3R) | 6735 |
| 1247 | FBgn0032039-FBgn0001084 (2L) | 6730 |
| 1248 | FBgn0034688-FBgn0034691 (2R) | 6729 |
| 1249 | FBgn0259209-FBgn0034974 (2R) | 6720 |
| 1250 | FBgn0046296-FBgn0036257 (3L) | 6702 |
| 1251 | FBgn0028707-FBgn0029137 (2L) | 6700 |
| 1252 | FBgn0030863-FBgn0030864 (X) | 6698 |
| 1253 | FBgn0037680-FBgn0053936 (3R) | 6696 |
| 1254 | FBgn0038303-FBgn0015610 (3R) | 6694 |
| 1255 | FBgn0038500-FBgn0038499 (3R) | 6681 |
| 1256 | FBgn0038851-FBgn0038852 (3R) | 6677 |
| 1257 | FBgn0052029-FBgn0035922 (3L) | 6672 |
| 1258 | FBgn0037978-FBgn0037981 (3R) | 6671 |
| 1259 | FBgn0038131-FBgn0038134 (3R) | 6670 |
| 1260 | FBgn0003886-FBgn0037736 (3R) | 6665 |
| 1261 | FBgn0030694-FBgn0003345 (X) | 6648 |
| 1262 | FBgn0022786-FBgn0029957 (X) | 6626 |
| 1263 | FBgn0039065-FBgn0039067 (3R) | 6581 |
| 1264 | FBgn0037536-FBgn0037537 (3R) | 6560 |
| 1265 | FBgn0030524-FBgn0030525 (X) | 6535 |
| 1266 | FBgn0038170-FBgn0002937 (3R) | 6534 |
| 1267 | FBgn0031518-FBgn0031519 (2L) | 6526 |
| 1268 | FBgn0038753-FBgn0021776 (3R) | 6526 |
| 1269 | FBgn0035871-FBgn0035872 (3L) | 6523 |
| 1270 | FBgn0031894-FBgn0031895 (2L) | 6521 |
| 1271 | FBgn0037617-FBgn0037620 (3R) | 6521 |
| 1272 | FBgn0021761-FBgn0250837 (2L) | 6510 |
| 1273 | FBgn0040089-FBgn0031064 (X) | 6503 |
| 1274 | FBgn0052452-FBgn0016034 (3L) | 6496 |
| 1275 | FBgn0051872-FBgn0032275 (2L) | 6490 |
| 1276 | FBgn0032170-FBgn0032172 (2L) | 6481 |
| 1277 | FBgn0039591-FBgn0039592 (3R) | 6480 |
| 1278 | FBgn0037873-FBgn0086472 (3R) | 6467 |
| 1279 | FBgn0026056-FBgn0046763 (3R) | 6463 |
| 1280 | FBgn0035477-FBgn0035479 (3L) | 6456 |
| 1281 | FBgn0035063-FBgn0041205 (2R) | 6452 |
| 1282 | FBgn0051644-FBgn0085410 (2L) | 6450 |
| 1283 | FBgn0014931-FBgn0037531 (3R) | 6443 |
| 1284 | FBgn0036859-FBgn0036860 (3L) | 6441 |
| 1285 | FBgn0037743-FBgn0028401 (3R) | 6422 |
| 1286 | FBgn0033587-FBgn0033592 (2R) | 6411 |
| 1287 | FBgn0034854-FBgn0040660 (2R) | 6410 |
| 1288 | FBgn0024998-FBgn0024973 (X) | 6369 |
| 1289 | FBgn0051860-FBgn0032419 (2L) | 6346 |
| 1290 | FBgn0039464-FBgn0039465 (3R) | 6339 |
| 1291 | FBgn0028530-FBgn0015791 (2L) | 6330 |
| 1292 | FBgn0032955-FBgn0022893 (2L) | 6330 |
| 1293 | FBgn0042092-FBgn0011737 (2L) | 6321 |
| 1294 | FBgn0032597-FBgn0032598 (2L) | 6289 |
| 1295 | FBgn0259215-FBgn0038837 (3R) | 6285 |
| 1296 | FBgn0027572-FBgn0034277 (2R) | 6275 |
| 1297 | FBgn0038645-FBgn0038647 (3R) | 6268 |
| 1298 | FBgn0035888-FBgn0035890 (3L) | 6264 |
| 1299 | FBgn0040828-FBgn0035924 (3L) | 6264 |
| 1300 | FBgn0031050-FBgn0031052 (X) | 6262 |
| 1301 | FBgn0033737-FBgn0033739 (2R) | 6241 |
| 1302 | FBgn0039252-FBgn0020626 (3R) | 6221 |
| 1303 | FBgn0033128-FBgn0033130 (2R) | 6215 |
| 1304 | FBgn0036349-FBgn0036352 (3L) | 6207 |
| 1305 | FBgn0040900-FBgn0029603 (X) | 6205 |
| 1306 | FBgn0025185-FBgn0033186 (2R) | 6198 |
| 1307 | FBgn0037003-FBgn0037005 (3L) | 6197 |
| 1308 | FBgn0036474-FBgn0086690 (3L) | 6181 |
| 1309 | FBgn0041789-FBgn0032782 (2L) | 6170 |
| 1310 | FBgn0034791-FBgn0034794 (2R) | 6165 |
| 1311 | FBgn0003301-FBgn0030581 (X) | 6153 |
| 1312 | FBgn0035695-FBgn0004513 (3L) | 6148 |
| 1313 | FBgn0033224-FBgn0033226 (2R) | 6108 |
| 1314 | FBgn0029088-FBgn0037391 (3R) | 6101 |
| 1315 | FBgn0025571-FBgn0038519 (3R) | 6087 |
| 1316 | FBgn0034387-FBgn0259682 (2R) | 6078 |
| 1317 | FBgn0085436-FBgn0033427 (2R) | 6066 |
| 1318 | FBgn0032897-FBgn0032899 (2L) | 6059 |
| 1319 | FBgn0052391-FBgn0054030 (3L) | 6051 |
| 1320 | FBgn0053017-FBgn0024249 (2R) | 6047 |
| 1321 | FBgn0083946-FBgn0039290 (3R) | 6043 |
| 1322 | FBgn0039490-FBgn0039491 (3R) | 6043 |
| 1323 | FBgn0039741-FBgn0039743 (3R) | 6040 |
| 1324 | FBgn0035600-FBgn0035601 (3L) | 6036 |
| 1325 | FBgn0004778-FBgn0004781 (3R) | 6033 |
| 1326 | FBgn0039756-FBgn0039757 (3R) | 6028 |
| 1327 | FBgn0037814-FBgn0037815 (3R) | 6018 |
| 1328 | FBgn0050456-FBgn0034194 (2R) | 5981 |
| 1329 | FBgn0030938-FBgn0000242 (X) | 5977 |
| 1330 | FBgn0051265-FBgn0051267 (3R) | 5974 |
| 1331 | FBgn0032229-FBgn0027568 (2L) | 5961 |
| 1332 | FBgn0052686-FBgn0052681 (X) | 5941 |
| 1333 | FBgn0066365-FBgn0052251 (3L) | 5925 |
| 1334 | FBgn0027549-FBgn0026252 (3L) | 5916 |
| 1335 | FBgn0036271-FBgn0036272 (3L) | 5914 |
| 1336 | FBgn0037530-FBgn0014930 (3R) | 5912 |
| 1337 | FBgn0027524-FBgn0037780 (3R) | 5910 |
| 1338 | FBgn0043470-FBgn0011555 (2R) | 5909 |
| 1339 | FBgn0036575-FBgn0052152 (3L) | 5907 |
| 1340 | FBgn0002036-FBgn0000422 (2L) | 5900 |
| 1341 | FBgn0035779-FBgn0035781 (3L) | 5894 |
| 1342 | FBgn0032808-FBgn0032809 (2L) | 5884 |
| 1343 | FBgn0030654-FBgn0030657 (X) | 5883 |
| 1344 | FBgn0034225-FBgn0050104 (2R) | 5874 |
| 1345 | FBgn0025806-FBgn0034971 (2R) | 5858 |
| 1346 | FBgn0037008-FBgn0037011 (3L) | 5854 |
| 1347 | FBgn0030881-FBgn0030882 (X) | 5830 |
| 1348 | FBgn0001961-FBgn0028509 (2L) | 5829 |
| 1349 | FBgn0051151-FBgn0039013 (3R) | 5828 |
| 1350 | FBgn0020255-FBgn0030276 (X) | 5813 |
| 1351 | FBgn0033921-FBgn0085408 (2R) | 5810 |
| 1352 | FBgn0037036-FBgn0037039 (3L) | 5806 |
| 1353 | FBgn0001128-FBgn0031752 (2L) | 5803 |
| 1354 | FBgn0038547-FBgn0038550 (3R) | 5793 |
| 1355 | FBgn0035685-FBgn0035687 (3L) | 5785 |
| 1356 | FBgn0034718-FBgn0013272 (2R) | 5743 |
| 1357 | FBgn0030925-FBgn0030927 (X) | 5742 |
| 1358 | FBgn0029789-FBgn0027564 (X) | 5736 |
| 1359 | FBgn0024285-FBgn0032160 (2L) | 5730 |
| 1360 | FBgn0031851-FBgn0025777 (2L) | 5729 |
| 1361 | FBgn0037846-FBgn0037848 (3R) | 5725 |
| 1362 | FBgn0038704-FBgn0038705 (3R) | 5721 |
| 1363 | FBgn0037762-FBgn0037765 (3R) | 5717 |
| 1364 | FBgn0031114-FBgn0002641 (X) | 5702 |
| 1365 | FBgn0051352-FBgn0016792 (3R) | 5698 |
| 1366 | FBgn0030718-FBgn0000611 (X) | 5682 |
| 1367 | FBgn0050457-FBgn0034204 (2R) | 5677 |
| 1368 | FBgn0035107-FBgn0025592 (3L) | 5665 |
| 1369 | FBgn0259164-FBgn0035673 (3L) | 5650 |
| 1370 | FBgn0031098-FBgn0031099 (X) | 5642 |
| 1371 | FBgn0030874-FBgn0052557 (X) | 5640 |
| 1372 | FBgn0028497-FBgn0034859 (2R) | 5631 |
| 1373 | FBgn0034032-FBgn0013762 (2R) | 5628 |
| 1374 | FBgn0027364-FBgn0037023 (3L) | 5608 |
| 1375 | FBgn0032050-FBgn0032054 (2L) | 5603 |
| 1376 | FBgn0011743-FBgn0034157 (2R) | 5600 |
| 1377 | FBgn0030746-FBgn0030747 (X) | 5598 |
| 1378 | FBgn0086707-FBgn0032679 (2L) | 5592 |
| 1379 | FBgn0032868-FBgn0051680 (2L) | 5590 |
| 1380 | FBgn0032163-FBgn0259713 (2L) | 5587 |
| 1381 | FBgn0035627-FBgn0085447 (3L) | 5580 |
| 1382 | FBgn0027654-FBgn0082582 (3R) | 5576 |
| 1383 | FBgn0033628-FBgn0033629 (2R) | 5571 |
| 1384 | FBgn0043456-FBgn0011584 (2L) | 5562 |
| 1385 | FBgn0052319-FBgn0028567 (3L) | 5560 |
| 1386 | FBgn0030037-FBgn0030039 (X) | 5542 |
| 1387 | FBgn0001133-FBgn0034573 (2R) | 5528 |
| 1388 | FBgn0038582-FBgn0038586 (3R) | 5522 |
| 1389 | FBgn0011570-FBgn0031356 (2L) | 5512 |
| 1390 | FBgn0035497-FBgn0028484 (3L) | 5509 |
| 1391 | FBgn0033808-FBgn0033810 (2R) | 5493 |
| 1392 | FBgn0026431-FBgn0032259 (2L) | 5461 |
| 1393 | FBgn0004839-FBgn0033674 (2R) | 5447 |
| 1394 | FBgn0032690-FBgn0032691 (2L) | 5410 |
| 1395 | FBgn0014127-FBgn0045064 (2L) | 5396 |
| 1396 | FBgn0031436-FBgn0026479 (2L) | 5393 |
| 1397 | FBgn0038953-FBgn0051169 (3R) | 5392 |
| 1398 | FBgn0033349-FBgn0033352 (2R) | 5387 |
| 1399 | FBgn0038702-FBgn0051244 (3R) | 5378 |
| 1400 | FBgn0031608-FBgn0031611 (2L) | 5366 |
| 1401 | FBgn0052160-FBgn0036603 (3L) | 5346 |
| 1402 | FBgn0067779-FBgn0031213 (2L) | 5345 |
| 1403 | FBgn0033686-FBgn0027504 (2R) | 5332 |
| 1404 | FBgn0032420-FBgn0032422 (2L) | 5318 |
| 1405 | FBgn0029755-FBgn0014024 (X) | 5298 |
| 1406 | FBgn0030077-FBgn0030080 (X) | 5278 |
| 1407 | FBgn0035777-FBgn0052379 (3L) | 5277 |
| 1408 | FBgn0037339-FBgn0041191 (3R) | 5272 |
| 1409 | FBgn0051697-FBgn0032804 (2L) | 5246 |
| 1410 | FBgn0033204-FBgn0033206 (2R) | 5244 |
| 1411 | FBgn0029849-FBgn0003514 (X) | 5240 |
| 1412 | FBgn0030931-FBgn0010194 (X) | 5222 |
| 1413 | FBgn0031972-FBgn0031974 (2L) | 5211 |
| 1414 | FBgn0034756-FBgn0034759 (2R) | 5186 |
| 1415 | FBgn0039611-FBgn0039613 (3R) | 5181 |
| 1416 | FBgn0030640-FBgn0030643 (X) | 5177 |
| 1417 | FBgn0030787-FBgn0030790 (X) | 5177 |
| 1418 | FBgn0032889-FBgn0051673 (2L) | 5173 |
| 1419 | FBgn0036964-FBgn0052226 (3L) | 5168 |
| 1420 | FBgn0034710-FBgn0034712 (2R) | 5167 |
| 1421 | FBgn0037242-FBgn0037245 (3R) | 5163 |
| 1422 | FBgn0031871-FBgn0020616 (2L) | 5157 |
| 1423 | FBgn0011207-FBgn0051710 (2L) | 5149 |
| 1424 | FBgn0028870-FBgn0028868 (2L) | 5142 |
| 1425 | FBgn0034539-FBgn0034541 (2R) | 5141 |
| 1426 | FBgn0033371-FBgn0033372 (2R) | 5121 |
| 1427 | FBgn0010220-FBgn0033373 (2R) | 5117 |
| 1428 | FBgn0034568-FBgn0034570 (2R) | 5115 |
| 1429 | FBgn0034219-FBgn0028956 (2R) | 5112 |
| 1430 | FBgn0035511-FBgn0035514 (3L) | 5097 |
| 1431 | FBgn0036759-FBgn0036761 (3L) | 5097 |
| 1432 | FBgn0041164-FBgn0010317 (3L) | 5092 |
| 1433 | FBgn0034527-FBgn0034529 (2R) | 5072 |
| 1434 | FBgn0036527-FBgn0036528 (3L) | 5065 |
| 1435 | FBgn0035929-FBgn0035931 (3L) | 5052 |
| 1436 | FBgn0033924-FBgn0033925 (2R) | 5046 |
| 1437 | FBgn0036846-FBgn0036848 (3L) | 5038 |
| 1438 | FBgn0259703-FBgn0085288 (3L) | 5034 |
| 1439 | FBgn0038100-FBgn0051342 (3R) | 5019 |
| 1440 | FBgn0036233-FBgn0036234 (3L) | 5015 |
| 1441 | FBgn0034505-FBgn0042198 (2R) | 5009 |
| 1442 | FBgn0025633-FBgn0026879 (X) | 5007 |
| 1443 | FBgn0014869-FBgn0041186 (3R) | 5000 |
| 1444 | FBgn0024987-FBgn0023169 (X) | 5000 |
| 1445 | FBgn0053513-FBgn0025393 (X) | 4991 |
| 1446 | FBgn0083975-FBgn0038775 (3R) | 4983 |
| 1447 | FBgn0031288-FBgn0031289 (2L) | 4973 |
| 1448 | FBgn0039271-FBgn0039274 (3R) | 4956 |
| 1449 | FBgn0000357-FBgn0000356 (3L) | 4952 |
| 1450 | FBgn0243516-FBgn0034697 (2R) | 4950 |
| 1451 | FBgn0037751-FBgn0037753 (3R) | 4944 |
| 1452 | FBgn0033431-FBgn0050338 (2R) | 4942 |
| 1453 | FBgn0030141-FBgn0030142 (X) | 4933 |
| 1454 | FBgn0039469-FBgn0051077 (3R) | 4923 |
| 1455 | FBgn0030774-FBgn0030777 (X) | 4915 |
| 1456 | FBgn0036335-FBgn0036337 (3L) | 4912 |
| 1457 | FBgn0039246-FBgn0040601 (3R) | 4886 |
| 1458 | FBgn0025830-FBgn0025832 (2R) | 4881 |
| 1459 | FBgn0030081-FBgn0052708 (X) | 4880 |
| 1460 | FBgn0034379-FBgn0034380 (2R) | 4879 |
| 1461 | FBgn0034272-FBgn0034274 (2R) | 4835 |
| 1462 | FBgn0052076-FBgn0036142 (3L) | 4824 |
| 1463 | FBgn0036235-FBgn0036236 (3L) | 4771 |
| 1464 | FBgn0033135-FBgn0033137 (2R) | 4762 |
| 1465 | FBgn0037265-FBgn0051531 (3R) | 4756 |
| 1466 | FBgn0030891-FBgn0030892 (X) | 4752 |
| 1467 | FBgn0052528-FBgn0031069 (X) | 4744 |
| 1468 | FBgn0030542-FBgn0030544 (X) | 4734 |
| 1469 | FBgn0004638-FBgn0024556 (2R) | 4722 |
| 1470 | FBgn0036052-FBgn0027567 (3L) | 4718 |
| 1471 | FBgn0036147-FBgn0064766 (3L) | 4714 |
| 1472 | FBgn0016700-FBgn0038877 (3R) | 4707 |
| 1473 | FBgn0024360-FBgn0024364 (X) | 4687 |
| 1474 | FBgn0033490-FBgn0033491 (2R) | 4685 |
| 1475 | FBgn0038873-FBgn0038874 (3R) | 4676 |
| 1476 | FBgn0033631-FBgn0050022 (2R) | 4670 |
| 1477 | FBgn0030456-FBgn0030457 (X) | 4667 |
| 1478 | FBgn0037958-FBgn0037960 (3R) | 4631 |
| 1479 | FBgn0053322-FBgn0032888 (2L) | 4628 |
| 1480 | FBgn0038948-FBgn0038951 (3R) | 4627 |
| 1481 | FBgn0037892-FBgn0037894 (3R) | 4626 |
| 1482 | FBgn0034651-FBgn0050263 (2R) | 4622 |
| 1483 | FBgn0027795-FBgn0027793 (X) | 4614 |
| 1484 | FBgn0039235-FBgn0039237 (3R) | 4604 |
| 1485 | FBgn0031653-FBgn0020906 (2L) | 4591 |
| 1486 | FBgn0039153-FBgn0027103 (3R) | 4580 |
| 1487 | FBgn0035483-FBgn0035484 (3L) | 4571 |
| 1488 | FBgn0032057-FBgn0032058 (2L) | 4569 |
| 1489 | FBgn0036689-FBgn0036690 (3L) | 4560 |
| 1490 | FBgn0038535-FBgn0051249 (3R) | 4550 |
| 1491 | FBgn0036487-FBgn0036489 (3L) | 4542 |
| 1492 | FBgn0014018-FBgn0037659 (3R) | 4542 |
| 1493 | FBgn0034922-FBgn0034924 (2R) | 4541 |
| 1494 | FBgn0030833-FBgn0030834 (X) | 4531 |
| 1495 | FBgn0028579-FBgn0042083 (2R) | 4519 |
| 1496 | FBgn0051224-FBgn0044871 (3R) | 4502 |
| 1497 | FBgn0038485-FBgn0038487 (3R) | 4490 |
| 1498 | FBgn0039487-FBgn0039489 (3R) | 4484 |
| 1499 | FBgn0039563-FBgn0039565 (3R) | 4474 |
| 1500 | FBgn0035867-FBgn0087039 (3L) | 4470 |
| 1501 | FBgn0040392-FBgn0053218 (X) | 4470 |
| 1502 | FBgn0031047-FBgn0031049 (X) | 4453 |
| 1503 | FBgn0050001-FBgn0033447 (2R) | 4450 |
| 1504 | FBgn0052181-FBgn0043025 (3L) | 4445 |
| 1505 | FBgn0038810-FBgn0023097 (3R) | 4445 |
| 1506 | FBgn0039764-FBgn0039767 (3R) | 4434 |
| 1507 | FBgn0052442-FBgn0029152 (3L) | 4418 |
| 1508 | FBgn0051104-FBgn0039316 (3R) | 4417 |
| 1509 | FBgn0085481-FBgn0085480 (3L) | 4416 |
| 1510 | FBgn0037087-FBgn0015239 (3L) | 4416 |
| 1511 | FBgn0086130-FBgn0031282 (2L) | 4415 |
| 1512 | FBgn0036771-FBgn0036772 (3L) | 4415 |
| 1513 | FBgn0050362-FBgn0033287 (2R) | 4414 |
| 1514 | FBgn0025383-FBgn0025382 (X) | 4406 |
| 1515 | FBgn0032153-FBgn0010520 (2L) | 4405 |
| 1516 | FBgn0035099-FBgn0035101 (3L) | 4405 |
| 1517 | FBgn0038300-FBgn0038301 (3R) | 4395 |
| 1518 | FBgn0036923-FBgn0036925 (3L) | 4393 |
| 1519 | FBgn0038414-FBgn0038416 (3R) | 4388 |
| 1520 | FBgn0035131-FBgn0035132 (3L) | 4377 |
| 1521 | FBgn0037718-FBgn0037720 (3R) | 4373 |
| 1522 | FBgn0020309-FBgn0032378 (2L) | 4368 |
| 1523 | FBgn0038113-FBgn0003450 (3R) | 4358 |
| 1524 | FBgn0039017-FBgn0039018 (3R) | 4343 |
| 1525 | FBgn0039311-FBgn0039313 (3R) | 4337 |
| 1526 | FBgn0034201-FBgn0050458 (2R) | 4333 |
| 1527 | FBgn0085228-FBgn0034499 (2R) | 4329 |
| 1528 | FBgn0027607-FBgn0033357 (2R) | 4328 |
| 1529 | FBgn0034495-FBgn0034496 (2R) | 4311 |
| 1530 | FBgn0034440-FBgn0034441 (2R) | 4309 |
| 1531 | FBgn0036035-FBgn0036038 (3L) | 4284 |
| 1532 | FBgn0028562-FBgn0033257 (2R) | 4283 |
| 1533 | FBgn0036609-FBgn0053060 (3L) | 4279 |
| 1534 | FBgn0250906-FBgn0031453 (2L) | 4278 |
| 1535 | FBgn0039358-FBgn0039359 (3R) | 4274 |
| 1536 | FBgn0033748-FBgn0033749 (2R) | 4246 |
| 1537 | FBgn0051728-FBgn0051730 (2L) | 4234 |
| 1538 | FBgn0034748-FBgn0034750 (2R) | 4234 |
| 1539 | FBgn0035713-FBgn0035715 (3L) | 4234 |
| 1540 | FBgn0036643-FBgn0003741 (3L) | 4215 |
| 1541 | FBgn0039879-FBgn0250755 (3R) | 4194 |
| 1542 | FBgn0000377-FBgn0029608 (X) | 4150 |
| 1543 | FBgn0030010-FBgn0030012 (X) | 4145 |
| 1544 | FBgn0019938-FBgn0033945 (2R) | 4139 |
| 1545 | FBgn0062412-FBgn0037576 (3R) | 4133 |
| 1546 | FBgn0035162-FBgn0035164 (3L) | 4113 |
| 1547 | FBgn0024733-FBgn0000258 (3L) | 4112 |
| 1548 | FBgn0052301-FBgn0052305 (3L) | 4106 |
| 1549 | FBgn0036263-FBgn0036264 (3L) | 4105 |
| 1550 | FBgn0028920-FBgn0028532 (2L) | 4104 |
| 1551 | FBgn0050056-FBgn0033753 (2R) | 4103 |
| 1552 | FBgn0036875-FBgn0036876 (3L) | 4101 |
| 1553 | FBgn0038135-FBgn0038136 (3R) | 4098 |
| 1554 | FBgn0033316-FBgn0033317 (2R) | 4086 |
| 1555 | FBgn0038755-FBgn0038761 (3R) | 4084 |
| 1556 | FBgn0030540-FBgn0030541 (X) | 4084 |
| 1557 | FBgn0039104-FBgn0047351 (3R) | 4077 |
| 1558 | FBgn0054054-FBgn0040503 (2R) | 4073 |
| 1559 | FBgn0035420-FBgn0035422 (3L) | 4041 |
| 1560 | FBgn0050185-FBgn0034876 (2R) | 4040 |
| 1561 | FBgn0029753-FBgn0029754 (X) | 4030 |
| 1562 | FBgn0014135-FBgn0051459 (3R) | 4029 |
| 1563 | FBgn0067864-FBgn0025820 (3L) | 4027 |
| 1564 | FBgn0001257-FBgn0035515 (3L) | 4027 |
| 1565 | FBgn0037608-FBgn0037611 (3R) | 4027 |
| 1566 | FBgn0033134-FBgn0016032 (2R) | 4024 |
| 1567 | FBgn0000241-FBgn0041234 (2R) | 4018 |
| 1568 | FBgn0028837-FBgn0038989 (3R) | 4012 |
| 1569 | FBgn0030228-FBgn0030234 (X) | 4008 |
| 1570 | FBgn0039710-FBgn0039712 (3R) | 4007 |
| 1571 | FBgn0051694-FBgn0051950 (2L) | 4003 |
| 1572 | FBgn0038453-FBgn0015019 (3R) | 3985 |
| 1573 | FBgn0051021-FBgn0051016 (3R) | 3984 |
| 1574 | FBgn0030876-FBgn0030878 (X) | 3955 |
| 1575 | FBgn0030853-FBgn0030854 (X) | 3954 |
| 1576 | FBgn0004913-FBgn0051536 (3R) | 3931 |
| 1577 | FBgn0028540-FBgn0028539 (2L) | 3927 |
| 1578 | FBgn0036466-FBgn0036469 (3L) | 3925 |
| 1579 | FBgn0050151-FBgn0050154 (2R) | 3911 |
| 1580 | FBgn0039806-FBgn0039807 (3R) | 3907 |
| 1581 | FBgn0037579-FBgn0037581 (3R) | 3900 |
| 1582 | FBgn0053631-FBgn0053784 (3R) | 3899 |
| 1583 | FBgn0032848-FBgn0032850 (2L) | 3895 |
| 1584 | FBgn0036826-FBgn0036828 (3L) | 3882 |
| 1585 | FBgn0034513-FBgn0034515 (2R) | 3871 |
| 1586 | FBgn0032773-FBgn0032774 (2L) | 3869 |
| 1587 | FBgn0039686-FBgn0085325 (3R) | 3864 |
| 1588 | FBgn0051477-FBgn0001321 (3R) | 3861 |
| 1589 | FBgn0041630-FBgn0035529 (3L) | 3856 |
| 1590 | FBgn0041250-FBgn0031279 (2L) | 3855 |
| 1591 | FBgn0035981-FBgn0011769 (3L) | 3855 |
| 1592 | FBgn0004593-FBgn0014851 (3L) | 3848 |
| 1593 | FBgn0003116-FBgn0040899 (X) | 3847 |
| 1594 | FBgn0036491-FBgn0036492 (3L) | 3828 |
| 1595 | FBgn0037538-FBgn0037539 (3R) | 3828 |
| 1596 | FBgn0052537-FBgn0031010 (X) | 3810 |
| 1597 | FBgn0028430-FBgn0259896 (2L) | 3807 |
| 1598 | FBgn0033903-FBgn0033905 (2R) | 3802 |
| 1599 | FBgn0038195-FBgn0038196 (3R) | 3799 |
| 1600 | FBgn0003714-FBgn0004050 (X) | 3794 |
| 1601 | FBgn0038451-FBgn0038452 (3R) | 3792 |
| 1602 | FBgn0038838-FBgn0044809 (3R) | 3787 |
| 1603 | FBgn0028500-FBgn0037156 (3L) | 3783 |
| 1604 | FBgn0051058-FBgn0027494 (3R) | 3776 |
| 1605 | FBgn0004597-FBgn0038272 (3R) | 3761 |
| 1606 | FBgn0040743-FBgn0034159 (2R) | 3751 |
| 1607 | FBgn0036062-FBgn0036063 (3L) | 3750 |
| 1608 | FBgn0001137-FBgn0027490 (2L) | 3749 |
| 1609 | FBgn0030512-FBgn0030514 (X) | 3743 |
| 1610 | FBgn0053493-FBgn0036101 (3L) | 3740 |
| 1611 | FBgn0040337-FBgn0026143 (X) | 3740 |
| 1612 | FBgn0051201-FBgn0028422 (3R) | 3737 |
| 1613 | FBgn0035461-FBgn0052262 (3L) | 3726 |
| 1614 | FBgn0052448-FBgn0046301 (3L) | 3726 |
| 1615 | FBgn0259239-FBgn0085290 (3L) | 3720 |
| 1616 | FBgn0052106-FBgn0036311 (3L) | 3717 |
| 1617 | FBgn0027660-FBgn0086475 (3L) | 3701 |
| 1618 | FBgn0038395-FBgn0038396 (3R) | 3701 |
| 1619 | FBgn0051164-FBgn0051424 (3R) | 3700 |
| 1620 | FBgn0037747-FBgn0037749 (3R) | 3692 |
| 1621 | FBgn0052685-FBgn0030178 (X) | 3690 |
| 1622 | FBgn0033580-FBgn0033581 (2R) | 3689 |
| 1623 | FBgn0035035-FBgn0259742 (2R) | 3678 |
| 1624 | FBgn0040636-FBgn0027055 (3L) | 3676 |
| 1625 | FBgn0032292-FBgn0027868 (2L) | 3672 |
| 1626 | FBgn0011672-FBgn0025865 (3R) | 3669 |
| 1627 | FBgn0036773-FBgn0002901 (3L) | 3668 |
| 1628 | FBgn0037122-FBgn0037125 (3L) | 3651 |
| 1629 | FBgn0053170-FBgn0037199 (3L) | 3650 |
| 1630 | FBgn0010894-FBgn0040239 (3L) | 3638 |
| 1631 | FBgn0033692-FBgn0000426 (2R) | 3634 |
| 1632 | FBgn0024191-FBgn0031736 (2L) | 3620 |
| 1633 | FBgn0085205-FBgn0031581 (2L) | 3611 |
| 1634 | FBgn0033248-FBgn0027788 (2R) | 3606 |
| 1635 | FBgn0032244-FBgn0005683 (2L) | 3592 |
| 1636 | FBgn0050055-FBgn0022764 (2R) | 3590 |
| 1637 | FBgn0032246-FBgn0022268 (2L) | 3577 |
| 1638 | FBgn0003060-FBgn0038161 (3R) | 3576 |
| 1639 | FBgn0023541-FBgn0015036 (X) | 3572 |
| 1640 | FBgn0031631-FBgn0031632 (2L) | 3567 |
| 1641 | FBgn0026309-FBgn0028744 (2R) | 3556 |
| 1642 | FBgn0036588-FBgn0040797 (3L) | 3541 |
| 1643 | FBgn0038439-FBgn0038440 (3R) | 3541 |
| 1644 | FBgn0031092-FBgn0031094 (X) | 3541 |
| 1645 | FBgn0023506-FBgn0014032 (X) | 3522 |
| 1646 | FBgn0037636-FBgn0037637 (3R) | 3510 |
| 1647 | FBgn0036107-FBgn0027615 (3L) | 3509 |
| 1648 | FBgn0052071-FBgn0052073 (3L) | 3504 |
| 1649 | FBgn0036960-FBgn0004366 (3L) | 3497 |
| 1650 | FBgn0039848-FBgn0039850 (3R) | 3477 |
| 1651 | FBgn0031936-FBgn0031937 (2L) | 3476 |
| 1652 | FBgn0030186-FBgn0085437 (X) | 3474 |
| 1653 | FBgn0039044-FBgn0039045 (3R) | 3465 |
| 1654 | FBgn0030959-FBgn0030960 (X) | 3464 |
| 1655 | FBgn0015338-FBgn0000339 (2L) | 3462 |
| 1656 | FBgn0051867-FBgn0010197 (2L) | 3459 |
| 1657 | FBgn0035540-FBgn0035542 (3L) | 3449 |
| 1658 | FBgn0032286-FBgn0023496 (2L) | 3435 |
| 1659 | FBgn0032074-FBgn0032075 (2L) | 3427 |
| 1660 | FBgn0036476-FBgn0036478 (3L) | 3419 |
| 1661 | FBgn0015737-FBgn0040609 (3R) | 3419 |
| 1662 | FBgn0031874-FBgn0031876 (2L) | 3414 |
| 1663 | FBgn0039459-FBgn0027889 (3R) | 3410 |
| 1664 | FBgn0031399-FBgn0031401 (2L) | 3405 |
| 1665 | FBgn0036331-FBgn0036332 (3L) | 3398 |
| 1666 | FBgn0039758-FBgn0039759 (3R) | 3394 |
| 1667 | FBgn0034938-FBgn0034940 (2R) | 3389 |
| 1668 | FBgn0029873-FBgn0029874 (X) | 3389 |
| 1669 | FBgn0035056-FBgn0035057 (2R) | 3384 |
| 1670 | FBgn0036711-FBgn0036712 (3L) | 3381 |
| 1671 | FBgn0014417-FBgn0032042 (2L) | 3378 |
| 1672 | FBgn0020510-FBgn0003276 (3R) | 3374 |
| 1673 | FBgn0052241-FBgn0052249 (3L) | 3372 |
| 1674 | FBgn0032280-FBgn0032282 (2L) | 3361 |
| 1675 | FBgn0033677-FBgn0010339 (2R) | 3359 |
| 1676 | FBgn0052413-FBgn0035648 (3L) | 3354 |
| 1677 | FBgn0035152-FBgn0035153 (3L) | 3353 |
| 1678 | FBgn0011227-FBgn0086904 (2R) | 3350 |
| 1679 | FBgn0033724-FBgn0033725 (2R) | 3348 |
| 1680 | FBgn0250850-FBgn0034537 (2R) | 3346 |
| 1681 | FBgn0053172-FBgn0053173 (X) | 3346 |
| 1682 | FBgn0031227-FBgn0053635 (2L) | 3344 |
| 1683 | FBgn0036578-FBgn0036581 (3L) | 3341 |
| 1684 | FBgn0035760-FBgn0035761 (3L) | 3340 |
| 1685 | FBgn0034011-FBgn0034012 (2R) | 3337 |
| 1686 | FBgn0025800-FBgn0030007 (X) | 3334 |
| 1687 | FBgn0016031-FBgn0004380 (3L) | 3332 |
| 1688 | FBgn0037084-FBgn0033584 (2R) | 3331 |
| 1689 | FBgn0036361-FBgn0036363 (3L) | 3303 |
| 1690 | FBgn0019972-FBgn0039697 (3R) | 3301 |
| 1691 | FBgn0029867-FBgn0029868 (X) | 3297 |
| 1692 | FBgn0030824-FBgn0052564 (X) | 3295 |
| 1693 | FBgn0033726-FBgn0050048 (2R) | 3289 |
| 1694 | FBgn0026079-FBgn0026083 (X) | 3287 |
| 1695 | FBgn0037555-FBgn0037556 (3R) | 3281 |
| 1696 | FBgn0051025-FBgn0051029 (3R) | 3277 |
| 1697 | FBgn0038545-FBgn0038546 (3R) | 3262 |
| 1698 | FBgn0029176-FBgn0062442 (3R) | 3262 |
| 1699 | FBgn0036416-FBgn0036417 (3L) | 3256 |
| 1700 | FBgn0039768-FBgn0039769 (3R) | 3239 |
| 1701 | FBgn0031677-FBgn0031678 (2L) | 3238 |
| 1702 | FBgn0031109-FBgn0031111 (X) | 3235 |
| 1703 | FBgn0035085-FBgn0027599 (2R) | 3233 |
| 1704 | FBgn0039125-FBgn0039126 (3R) | 3230 |
| 1705 | FBgn0030053-FBgn0025864 (X) | 3230 |
| 1706 | FBgn0025335-FBgn0025352 (2R) | 3223 |
| 1707 | FBgn0038274-FBgn0038277 (3R) | 3221 |
| 1708 | FBgn0032499-FBgn0051729 (2L) | 3217 |
| 1709 | FBgn0035234-FBgn0035235 (3L) | 3217 |
| 1710 | FBgn0039500-FBgn0039501 (3R) | 3190 |
| 1711 | FBgn0037382-FBgn0037384 (3R) | 3179 |
| 1712 | FBgn0032801-FBgn0053116 (2L) | 3173 |
| 1713 | FBgn0031772-FBgn0031774 (2L) | 3169 |
| 1714 | FBgn0021944-FBgn0026170 (2L) | 3168 |
| 1715 | FBgn0053998-FBgn0050121 (2R) | 3167 |
| 1716 | FBgn0037435-FBgn0037436 (3R) | 3128 |
| 1717 | FBgn0037921-FBgn0037924 (3R) | 3118 |
| 1718 | FBgn0085278-FBgn0036618 (3L) | 3108 |
| 1719 | FBgn0038805-FBgn0038808 (3R) | 3106 |
| 1720 | FBgn0039472-FBgn0039474 (3R) | 3092 |
| 1721 | FBgn0036666-FBgn0036668 (3L) | 3088 |
| 1722 | FBgn0034177-FBgn0026402 (2R) | 3086 |
| 1723 | FBgn0037344-FBgn0014366 (3R) | 3084 |
| 1724 | FBgn0050479-FBgn0050480 (2R) | 3081 |
| 1725 | FBgn0028375-FBgn0028894 (2L) | 3078 |
| 1726 | FBgn0259139-FBgn0037955 (3R) | 3069 |
| 1727 | FBgn0052036-FBgn0052037 (3L) | 3061 |
| 1728 | FBgn0033882-FBgn0033883 (2R) | 3046 |
| 1729 | FBgn0033953-FBgn0033955 (2R) | 3041 |
| 1730 | FBgn0035838-FBgn0035839 (3L) | 3031 |
| 1731 | FBgn0000250-FBgn0001995 (2L) | 3028 |
| 1732 | FBgn0037895-FBgn0037896 (3R) | 3027 |
| 1733 | FBgn0039149-FBgn0039150 (3R) | 3022 |
| 1734 | FBgn0035591-FBgn0035590 (3L) | 3012 |
| 1735 | FBgn0063494-FBgn0063491 (2R) | 2999 |
| 1736 | FBgn0034295-FBgn0034296 (2R) | 2996 |
| 1737 | FBgn0032704-FBgn0025608 (2L) | 2990 |
| 1738 | FBgn0025558-FBgn0017572 (3L) | 2987 |
| 1739 | FBgn0010278-FBgn0028411 (2R) | 2979 |
| 1740 | FBgn0036290-FBgn0027936 (3L) | 2977 |
| 1741 | FBgn0028992-FBgn0025456 (3R) | 2975 |
| 1742 | FBgn0030680-FBgn0030683 (X) | 2971 |
| 1743 | FBgn0051809-FBgn0032615 (2L) | 2967 |
| 1744 | FBgn0001990-FBgn0041627 (2L) | 2964 |
| 1745 | FBgn0031276-FBgn0040725 (2L) | 2962 |
| 1746 | FBgn0038142-FBgn0259831 (3R) | 2962 |
| 1747 | FBgn0005322-FBgn0032214 (2L) | 2959 |
| 1748 | FBgn0030799-FBgn0030800 (X) | 2956 |
| 1749 | FBgn0039832-FBgn0039833 (3R) | 2946 |
| 1750 | FBgn0042112-FBgn0010350 (3L) | 2940 |
| 1751 | FBgn0038106-FBgn0038108 (3R) | 2939 |
| 1752 | FBgn0037994-FBgn0037995 (3R) | 2931 |
| 1753 | FBgn0028896-FBgn0001992 (2L) | 2924 |
| 1754 | FBgn0051040-FBgn0039727 (3R) | 2922 |
| 1755 | FBgn0033451-FBgn0033453 (2R) | 2910 |
| 1756 | FBgn0052409-FBgn0052412 (3L) | 2909 |
| 1757 | FBgn0053213-FBgn0039558 (3R) | 2907 |
| 1758 | FBgn0034152-FBgn0053530 (2R) | 2898 |
| 1759 | FBgn0043043-FBgn0038051 (3R) | 2892 |
| 1760 | FBgn0036641-FBgn0250814 (3L) | 2885 |
| 1761 | FBgn0046878-FBgn0046876 (3R) | 2885 |
| 1762 | FBgn0004588-FBgn0004591 (3L) | 2884 |
| 1763 | FBgn0035554-FBgn0035555 (3L) | 2880 |
| 1764 | FBgn0051668-FBgn0053124 (2L) | 2879 |
| 1765 | FBgn0053092-FBgn0051465 (3R) | 2875 |
| 1766 | FBgn0031196-FBgn0031197 (X) | 2875 |
| 1767 | FBgn0028688-FBgn0038972 (3R) | 2864 |
| 1768 | FBgn0010909-FBgn0016794 (3L) | 2863 |
| 1769 | FBgn0052103-FBgn0020655 (3L) | 2862 |
| 1770 | FBgn0051156-FBgn0051161 (3R) | 2860 |
| 1771 | FBgn0036975-FBgn0024887 (3L) | 2858 |
| 1772 | FBgn0037687-FBgn0037689 (3R) | 2856 |
| 1773 | FBgn0001970-FBgn0015546 (2L) | 2851 |
| 1774 | FBgn0031248-FBgn0053127 (2L) | 2848 |
| 1775 | FBgn0030433-FBgn0030435 (X) | 2848 |
| 1776 | FBgn0032790-FBgn0032793 (2L) | 2845 |
| 1777 | FBgn0053296-FBgn0031935 (2L) | 2843 |
| 1778 | FBgn0026090-FBgn0028969 (X) | 2842 |
| 1779 | FBgn0004432-FBgn0030740 (X) | 2838 |
| 1780 | FBgn0010053-FBgn0034405 (2R) | 2830 |
| 1781 | FBgn0054040-FBgn0034664 (2R) | 2824 |
| 1782 | FBgn0031990-FBgn0031992 (2L) | 2815 |
| 1783 | FBgn0039670-FBgn0051041 (3R) | 2807 |
| 1784 | FBgn0019982-FBgn0053002 (2L) | 2781 |
| 1785 | FBgn0086134-FBgn0038049 (3R) | 2778 |
| 1786 | FBgn0052024-FBgn0035933 (3L) | 2771 |
| 1787 | FBgn0034065-FBgn0034067 (2R) | 2762 |
| 1788 | FBgn0029704-FBgn0040907 (X) | 2762 |
| 1789 | FBgn0052638-FBgn0030452 (X) | 2745 |
| 1790 | FBgn0034221-FBgn0085221 (2R) | 2743 |
| 1791 | FBgn0037489-FBgn0037491 (3R) | 2741 |
| 1792 | FBgn0032407-FBgn0032408 (2L) | 2740 |
| 1793 | FBgn0039538-FBgn0003330 (3R) | 2734 |
| 1794 | FBgn0032509-FBgn0051855 (2L) | 2728 |
| 1795 | FBgn0034634-FBgn0050289 (2R) | 2728 |
| 1796 | FBgn0039152-FBgn0039151 (3R) | 2728 |
| 1797 | FBgn0050354-FBgn0050355 (2R) | 2724 |
| 1798 | FBgn0085313-FBgn0038658 (3R) | 2723 |
| 1799 | FBgn0085440-FBgn0030459 (X) | 2719 |
| 1800 | FBgn0038680-FBgn0038681 (3R) | 2712 |
| 1801 | FBgn0085229-FBgn0033038 (2R) | 2699 |
| 1802 | FBgn0034076-FBgn0010052 (2R) | 2696 |
| 1803 | FBgn0000278-FBgn0000279 (3R) | 2688 |
| 1804 | FBgn0029664-FBgn0029666 (X) | 2685 |
| 1805 | FBgn0014022-FBgn0037703 (3R) | 2671 |
| 1806 | FBgn0085423-FBgn0031528 (2L) | 2665 |
| 1807 | FBgn0035915-FBgn0052351 (3L) | 2654 |
| 1808 | FBgn0033855-FBgn0033856 (2R) | 2653 |
| 1809 | FBgn0034279-FBgn0028740 (2R) | 2647 |
| 1810 | FBgn0032670-FBgn0032671 (2L) | 2644 |
| 1811 | FBgn0085188-FBgn0032191 (2L) | 2642 |
| 1812 | FBgn0039022-FBgn0039023 (3R) | 2641 |
| 1813 | FBgn0038449-FBgn0038450 (3R) | 2640 |
| 1814 | FBgn0038000-FBgn0038001 (3R) | 2633 |
| 1815 | FBgn0259832-FBgn0033656 (2R) | 2631 |
| 1816 | FBgn0032485-FBgn0032488 (2L) | 2629 |
| 1817 | FBgn0033818-FBgn0033819 (2R) | 2629 |
| 1818 | FBgn0030794-FBgn0030795 (X) | 2628 |
| 1819 | FBgn0032845-FBgn0032847 (2L) | 2627 |
| 1820 | FBgn0032393-FBgn0032394 (2L) | 2623 |
| 1821 | FBgn0032014-FBgn0032015 (2L) | 2599 |
| 1822 | FBgn0015781-FBgn0038675 (3R) | 2593 |
| 1823 | FBgn0053329-FBgn0027563 (3R) | 2592 |
| 1824 | FBgn0035392-FBgn0035393 (3L) | 2589 |
| 1825 | FBgn0036341-FBgn0036342 (3L) | 2589 |
| 1826 | FBgn0039730-FBgn0039733 (3R) | 2589 |
| 1827 | FBgn0034238-FBgn0034239 (2R) | 2583 |
| 1828 | FBgn0038783-FBgn0038784 (3R) | 2583 |
| 1829 | FBgn0029503-FBgn0039163 (3R) | 2577 |
| 1830 | FBgn0051920-FBgn0051921 (2L) | 2575 |
| 1831 | FBgn0033388-FBgn0259728 (2R) | 2569 |
| 1832 | FBgn0031498-FBgn0031500 (2L) | 2566 |
| 1833 | FBgn0032788-FBgn0032789 (2L) | 2561 |
| 1834 | FBgn0035167-FBgn0035168 (3L) | 2551 |
| 1835 | FBgn0051347-FBgn0038079 (3R) | 2550 |
| 1836 | FBgn0038809-FBgn0014029 (3R) | 2550 |
| 1837 | FBgn0035964-FBgn0035965 (3L) | 2543 |
| 1838 | FBgn0028274-FBgn0026872 (X) | 2542 |
| 1839 | FBgn0031033-FBgn0031034 (X) | 2542 |
| 1840 | FBgn0036338-FBgn0083978 (3L) | 2528 |
| 1841 | FBgn0040066-FBgn0001404 (X) | 2525 |
| 1842 | FBgn0037541-FBgn0010812 (3R) | 2523 |
| 1843 | FBgn0034976-FBgn0034978 (2R) | 2520 |
| 1844 | FBgn0030510-FBgn0030511 (X) | 2512 |
| 1845 | FBgn0011694-FBgn0004181 (2R) | 2510 |
| 1846 | FBgn0050195-FBgn0034755 (2R) | 2506 |
| 1847 | FBgn0033734-FBgn0033735 (2R) | 2499 |
| 1848 | FBgn0039828-FBgn0039829 (3R) | 2497 |
| 1849 | FBgn0029958-FBgn0016041 (X) | 2488 |
| 1850 | FBgn0033783-FBgn0033784 (2R) | 2483 |
| 1851 | FBgn0031457-FBgn0026324 (2L) | 2481 |
| 1852 | FBgn0032519-FBgn0032520 (2L) | 2481 |
| 1853 | FBgn0051624-FBgn0051988 (2L) | 2480 |
| 1854 | FBgn0032085-FBgn0032086 (2L) | 2475 |
| 1855 | FBgn0030605-FBgn0030606 (X) | 2475 |
| 1856 | FBgn0033449-FBgn0033450 (2R) | 2471 |
| 1857 | FBgn0250840-FBgn0033512 (2R) | 2464 |
| 1858 | FBgn0050432-FBgn0033069 (2R) | 2454 |
| 1859 | FBgn0015283-FBgn0037097 (3L) | 2448 |
| 1860 | FBgn0037377-FBgn0037378 (3R) | 2446 |
| 1861 | FBgn0031715-FBgn0031716 (2L) | 2444 |
| 1862 | FBgn0028523-FBgn0020414 (2L) | 2443 |
| 1863 | FBgn0052649-FBgn0030431 (X) | 2442 |
| 1864 | FBgn0031879-FBgn0031880 (2L) | 2440 |
| 1865 | FBgn0037652-FBgn0037653 (3R) | 2435 |
| 1866 | FBgn0035325-FBgn0052302 (3L) | 2434 |
| 1867 | FBgn0035412-FBgn0035413 (3L) | 2432 |
| 1868 | FBgn0036046-FBgn0044050 (3L) | 2423 |
| 1869 | FBgn0039238-FBgn0013949 (3R) | 2420 |
| 1870 | FBgn0038700-FBgn0038701 (3R) | 2414 |
| 1871 | FBgn0031060-FBgn0031062 (X) | 2408 |
| 1872 | FBgn0031239-FBgn0031240 (2L) | 2401 |
| 1873 | FBgn0038683-FBgn0259704 (3R) | 2401 |
| 1874 | FBgn0004908-FBgn0037578 (3R) | 2398 |
| 1875 | FBgn0040623-FBgn0039665 (3R) | 2394 |
| 1876 | FBgn0030480-FBgn0030481 (X) | 2390 |
| 1877 | FBgn0033347-FBgn0033348 (2R) | 2386 |
| 1878 | FBgn0040262-FBgn0040261 (2L) | 2385 |
| 1879 | FBgn0050410-FBgn0034849 (2R) | 2383 |
| 1880 | FBgn0250842-FBgn0050486 (2R) | 2381 |
| 1881 | FBgn0029714-FBgn0029715 (X) | 2379 |
| 1882 | FBgn0027529-FBgn0034500 (2R) | 2375 |
| 1883 | FBgn0030571-FBgn0030572 (X) | 2375 |
| 1884 | FBgn0033413-FBgn0017558 (2R) | 2372 |
| 1885 | FBgn0033215-FBgn0033216 (2R) | 2361 |
| 1886 | FBgn0033088-FBgn0033089 (2R) | 2352 |
| 1887 | FBgn0038211-FBgn0051326 (3R) | 2340 |
| 1888 | FBgn0026207-FBgn0038076 (3R) | 2332 |
| 1889 | FBgn0033788-FBgn0033789 (2R) | 2331 |
| 1890 | FBgn0035293-FBgn0035294 (3L) | 2323 |
| 1891 | FBgn0039882-FBgn0024891 (3R) | 2322 |
| 1892 | FBgn0028984-FBgn0038299 (3R) | 2317 |
| 1893 | FBgn0038238-FBgn0038240 (3R) | 2309 |
| 1894 | FBgn0034198-FBgn0034199 (2R) | 2301 |
| 1895 | FBgn0035270-FBgn0035272 (3L) | 2299 |
| 1896 | FBgn0051005-FBgn0039836 (3R) | 2297 |
| 1897 | FBgn0038558-FBgn0053547 (3R) | 2296 |
| 1898 | FBgn0030685-FBgn0030687 (X) | 2294 |
| 1899 | FBgn0037570-FBgn0037572 (3R) | 2290 |
| 1900 | FBgn0028880-FBgn0028370 (2L) | 2273 |
| 1901 | FBgn0036766-FBgn0036767 (3L) | 2272 |
| 1902 | FBgn0036804-FBgn0036805 (3L) | 2270 |
| 1903 | FBgn0036460-FBgn0002778 (3L) | 2268 |
| 1904 | FBgn0035469-FBgn0035470 (3L) | 2257 |
| 1905 | FBgn0039760-FBgn0039761 (3R) | 2255 |
| 1906 | FBgn0031756-FBgn0031758 (2L) | 2240 |
| 1907 | FBgn0085444-FBgn0034172 (2R) | 2238 |
| 1908 | FBgn0052750-FBgn0052751 (X) | 2238 |
| 1909 | FBgn0038037-FBgn0038039 (3R) | 2236 |
| 1910 | FBgn0029596-FBgn0025627 (X) | 2236 |
| 1911 | FBgn0052582-FBgn0030697 (X) | 2230 |
| 1912 | FBgn0052703-FBgn0030120 (X) | 2228 |
| 1913 | FBgn0022700-FBgn0034582 (2R) | 2222 |
| 1914 | FBgn0038740-FBgn0038741 (3R) | 2220 |
| 1915 | FBgn0034552-FBgn0034553 (2R) | 2203 |
| 1916 | FBgn0259784-FBgn0038491 (3R) | 2198 |
| 1917 | FBgn0035405-FBgn0024194 (3L) | 2186 |
| 1918 | FBgn0001224-FBgn0001226 (3L) | 2182 |
| 1919 | FBgn0003862-FBgn0038220 (3R) | 2172 |
| 1920 | FBgn0034000-FBgn0034002 (2R) | 2171 |
| 1921 | FBgn0011741-FBgn0030679 (X) | 2171 |
| 1922 | FBgn0030928-FBgn0030929 (X) | 2169 |
| 1923 | FBgn0011670-FBgn0011668 (3R) | 2161 |
| 1924 | FBgn0037492-FBgn0011282 (3R) | 2159 |
| 1925 | FBgn0011656-FBgn0033467 (2R) | 2147 |
| 1926 | FBgn0035475-FBgn0035476 (3L) | 2144 |
| 1927 | FBgn0039070-FBgn0039071 (3R) | 2139 |
| 1928 | FBgn0036607-FBgn0036608 (3L) | 2135 |
| 1929 | FBgn0035154-FBgn0052483 (3L) | 2134 |
| 1930 | FBgn0022343-FBgn0035082 (2R) | 2132 |
| 1931 | FBgn0031944-FBgn0031943 (2L) | 2127 |
| 1932 | FBgn0029095-FBgn0053526 (2L) | 2113 |
| 1933 | FBgn0035886-FBgn0035887 (3L) | 2108 |
| 1934 | FBgn0032003-FBgn0032004 (2L) | 2103 |
| 1935 | FBgn0030008-FBgn0030009 (X) | 2096 |
| 1936 | FBgn0036537-FBgn0036538 (3L) | 2095 |
| 1937 | FBgn0039265-FBgn0039266 (3R) | 2093 |
| 1938 | FBgn0002922-FBgn0039108 (3R) | 2092 |
| 1939 | FBgn0033273-FBgn0011659 (2R) | 2090 |
| 1940 | FBgn0083961-FBgn0015834 (2L) | 2085 |
| 1941 | FBgn0035666-FBgn0035667 (3L) | 2077 |
| 1942 | FBgn0035593-FBgn0035592 (3L) | 2072 |
| 1943 | FBgn0036207-FBgn0004381 (3L) | 2067 |
| 1944 | FBgn0035140-FBgn0035141 (3L) | 2066 |
| 1945 | FBgn0020416-FBgn0020415 (2L) | 2059 |
| 1946 | FBgn0010602-FBgn0031260 (2L) | 2056 |
| 1947 | FBgn0031738-FBgn0031740 (2L) | 2052 |
| 1948 | FBgn0035264-FBgn0035265 (3L) | 2052 |
| 1949 | FBgn0032145-FBgn0065108 (2L) | 2049 |
| 1950 | FBgn0031314-FBgn0031315 (2L) | 2045 |
| 1951 | FBgn0031059-FBgn0010341 (X) | 2044 |
| 1952 | FBgn0039169-FBgn0002863 (3R) | 2039 |
| 1953 | FBgn0039051-FBgn0039052 (3R) | 2028 |
| 1954 | FBgn0052079-FBgn0052081 (3L) | 2019 |
| 1955 | FBgn0039033-FBgn0039034 (3R) | 2015 |
| 1956 | FBgn0033428-FBgn0033429 (2R) | 2009 |
| 1957 | FBgn0052407-FBgn0053523 (3L) | 2001 |
| 1958 | FBgn0085203-FBgn0031398 (2L) | 2000 |
| 1959 | FBgn0035248-FBgn0035249 (3L) | 1998 |
| 1960 | FBgn0259110-FBgn0030715 (X) | 1992 |
| 1961 | FBgn0033698-FBgn0033699 (2R) | 1991 |
| 1962 | FBgn0031031-FBgn0031032 (X) | 1990 |
| 1963 | FBgn0035589-FBgn0035588 (3L) | 1982 |
| 1964 | FBgn0028746-FBgn0040396 (X) | 1981 |
| 1965 | FBgn0038742-FBgn0014006 (3R) | 1972 |
| 1966 | FBgn0050042-FBgn0033723 (2R) | 1970 |
| 1967 | FBgn0051415-FBgn0085438 (3R) | 1969 |
| 1968 | FBgn0027610-FBgn0086371 (3R) | 1969 |
| 1969 | FBgn0039324-FBgn0039325 (3R) | 1969 |
| 1970 | FBgn0033554-FBgn0033557 (2R) | 1961 |
| 1971 | FBgn0015320-FBgn0032298 (2L) | 1960 |
| 1972 | FBgn0039859-FBgn0039861 (3R) | 1958 |
| 1973 | FBgn0029718-FBgn0025615 (X) | 1956 |
| 1974 | FBgn0259232-FBgn0035861 (3L) | 1955 |
| 1975 | FBgn0051118-FBgn0051357 (3R) | 1955 |
| 1976 | FBgn0026196-FBgn0004838 (2L) | 1953 |
| 1977 | FBgn0035358-FBgn0035359 (3L) | 1953 |
| 1978 | FBgn0013998-FBgn0051495 (3R) | 1945 |
| 1979 | FBgn0085259-FBgn0033658 (2R) | 1939 |
| 1980 | FBgn0037902-FBgn0046225 (3R) | 1936 |
| 1981 | FBgn0038918-FBgn0038919 (3R) | 1934 |
| 1982 | FBgn0053265-FBgn0036232 (3L) | 1927 |
| 1983 | FBgn0029979-FBgn0029980 (X) | 1926 |
| 1984 | FBgn0051454-FBgn0051259 (3R) | 1921 |
| 1985 | FBgn0035948-FBgn0035949 (3L) | 1916 |
| 1986 | FBgn0038944-FBgn0038945 (3R) | 1908 |
| 1987 | FBgn0040502-FBgn0033067 (2R) | 1907 |
| 1988 | FBgn0040253-FBgn0040255 (3R) | 1904 |
| 1989 | FBgn0038551-FBgn0038552 (3R) | 1903 |
| 1990 | FBgn0045761-FBgn0039328 (3R) | 1901 |
| 1991 | FBgn0051391-FBgn0037824 (3R) | 1900 |
| 1992 | FBgn0037935-FBgn0037936 (3R) | 1894 |
| 1993 | FBgn0051823-FBgn0051821 (2L) | 1891 |
| 1994 | FBgn0039128-FBgn0039129 (3R) | 1888 |
| 1995 | FBgn0039356-FBgn0039357 (3R) | 1873 |
| 1996 | FBgn0001203-FBgn0085348 (X) | 1872 |
| 1997 | FBgn0033050-FBgn0000043 (2R) | 1864 |
| 1998 | FBgn0028675-FBgn0032189 (2L) | 1861 |
| 1999 | FBgn0031407-FBgn0054049 (2L) | 1858 |
| 2000 | FBgn0035309-FBgn0026570 (3L) | 1834 |
| 2001 | FBgn0033729-FBgn0033730 (2R) | 1832 |
| 2002 | FBgn0034966-FBgn0034965 (2R) | 1828 |
| 2003 | FBgn0036248-FBgn0036249 (3L) | 1821 |
| 2004 | FBgn0040345-FBgn0040342 (X) | 1819 |
| 2005 | FBgn0031690-FBgn0250847 (2L) | 1804 |
| 2006 | FBgn0014269-FBgn0034410 (2R) | 1794 |
| 2007 | FBgn0031002-FBgn0031003 (X) | 1794 |
| 2008 | FBgn0037563-FBgn0051463 (3R) | 1787 |
| 2009 | FBgn0033498-FBgn0033500 (2R) | 1786 |
| 2010 | FBgn0021825-FBgn0033339 (2R) | 1785 |
| 2011 | FBgn0032358-FBgn0020270 (2L) | 1780 |
| 2012 | FBgn0037998-FBgn0037999 (3R) | 1774 |
| 2013 | FBgn0036552-FBgn0036553 (3L) | 1773 |
| 2014 | FBgn0051204-FBgn0051002 (3R) | 1772 |
| 2015 | FBgn0036601-FBgn0036602 (3L) | 1769 |
| 2016 | FBgn0051805-FBgn0032620 (2L) | 1759 |
| 2017 | FBgn0046880-FBgn0046879 (2R) | 1752 |
| 2018 | FBgn0039041-FBgn0039042 (3R) | 1750 |
| 2019 | FBgn0030998-FBgn0030999 (X) | 1748 |
| 2020 | FBgn0036108-FBgn0036109 (3L) | 1747 |
| 2021 | FBgn0030305-FBgn0030306 (X) | 1746 |
| 2022 | FBgn0036023-FBgn0036024 (3L) | 1744 |
| 2023 | FBgn0038234-FBgn0038235 (3R) | 1738 |
| 2024 | FBgn0027496-FBgn0031822 (2L) | 1737 |
| 2025 | FBgn0039158-FBgn0039159 (3R) | 1735 |
| 2026 | FBgn0037388-FBgn0037389 (3R) | 1734 |
| 2027 | FBgn0033096-FBgn0033097 (2R) | 1727 |
| 2028 | FBgn0035333-FBgn0035335 (3L) | 1719 |
| 2029 | FBgn0035942-FBgn0035943 (3L) | 1712 |
| 2030 | FBgn0083938-FBgn0031617 (2L) | 1703 |
| 2031 | FBgn0037665-FBgn0037666 (3R) | 1693 |
| 2032 | FBgn0039656-FBgn0051427 (3R) | 1692 |
| 2033 | FBgn0036145-FBgn0036146 (3L) | 1689 |
| 2034 | FBgn0030107-FBgn0030108 (X) | 1682 |
| 2035 | FBgn0032412-FBgn0032413 (2L) | 1677 |
| 2036 | FBgn0033887-FBgn0033888 (2R) | 1675 |
| 2037 | FBgn0020304-FBgn0020545 (2L) | 1672 |
| 2038 | FBgn0032906-FBgn0032907 (2L) | 1672 |
| 2039 | FBgn0036928-FBgn0036929 (3L) | 1672 |
| 2040 | FBgn0002914-FBgn0065035 (X) | 1667 |
| 2041 | FBgn0030420-FBgn0030421 (X) | 1664 |
| 2042 | FBgn0036836-FBgn0085285 (3L) | 1658 |
| 2043 | FBgn0031254-FBgn0031255 (2L) | 1653 |
| 2044 | FBgn0036754-FBgn0052187 (3L) | 1653 |
| 2045 | FBgn0040719-FBgn0053673 (2L) | 1651 |
| 2046 | FBgn0036030-FBgn0015321 (3L) | 1650 |
| 2047 | FBgn0045483-FBgn0045482 (2R) | 1643 |
| 2048 | FBgn0031929-FBgn0031930 (2L) | 1627 |
| 2049 | FBgn0039628-FBgn0039629 (3R) | 1625 |
| 2050 | FBgn0036093-FBgn0036094 (3L) | 1618 |
| 2051 | FBgn0034301-FBgn0085225 (2R) | 1615 |
| 2052 | FBgn0034509-FBgn0043535 (2R) | 1612 |
| 2053 | FBgn0032087-FBgn0032088 (2L) | 1608 |
| 2054 | FBgn0039224-FBgn0039225 (3R) | 1608 |
| 2055 | FBgn0040730-FBgn0085227 (2R) | 1601 |
| 2056 | FBgn0053966-FBgn0035192 (3L) | 1592 |
| 2057 | FBgn0032797-FBgn0032798 (2L) | 1587 |
| 2058 | FBgn0039470-FBgn0039471 (3R) | 1587 |
| 2059 | FBgn0038643-FBgn0085311 (3R) | 1572 |
| 2060 | FBgn0031776-FBgn0031777 (2L) | 1568 |
| 2061 | FBgn0035134-FBgn0040291 (3L) | 1560 |
| 2062 | FBgn0039101-FBgn0042106 (3R) | 1555 |
| 2063 | FBgn0036818-FBgn0036819 (3L) | 1553 |
| 2064 | FBgn0259794-FBgn0036670 (3L) | 1543 |
| 2065 | FBgn0086902-FBgn0022246 (2L) | 1537 |
| 2066 | FBgn0025827-FBgn0034165 (2R) | 1530 |
| 2067 | FBgn0051441-FBgn0051388 (3R) | 1530 |
| 2068 | FBgn0029079-FBgn0050105 (2R) | 1529 |
| 2069 | FBgn0052563-FBgn0030826 (X) | 1526 |
| 2070 | FBgn0051229-FBgn0038638 (3R) | 1520 |
| 2071 | FBgn0050499-FBgn0033194 (2R) | 1517 |
| 2072 | FBgn0086784-FBgn0027585 (2R) | 1516 |
| 2073 | FBgn0067317-FBgn0051174 (3R) | 1512 |
| 2074 | FBgn0030066-FBgn0030067 (X) | 1512 |
| 2075 | FBgn0038968-FBgn0085405 (3R) | 1509 |
| 2076 | FBgn0034563-FBgn0083951 (2R) | 1504 |
| 2077 | FBgn0038388-FBgn0052856 (3R) | 1503 |
| 2078 | FBgn0035415-FBgn0035416 (3L) | 1502 |
| 2079 | FBgn0039020-FBgn0051139 (3R) | 1501 |
| 2080 | FBgn0032835-FBgn0032836 (2L) | 1496 |
| 2081 | FBgn0034883-FBgn0034884 (2R) | 1493 |
| 2082 | FBgn0038915-FBgn0038917 (3R) | 1492 |
| 2083 | FBgn0014368-FBgn0052074 (3L) | 1486 |
| 2084 | FBgn0034878-FBgn0034879 (2R) | 1484 |
| 2085 | FBgn0030610-FBgn0030612 (X) | 1484 |
| 2086 | FBgn0032161-FBgn0032162 (2L) | 1479 |
| 2087 | FBgn0038821-FBgn0259222 (3R) | 1477 |
| 2088 | FBgn0001987-FBgn0028507 (2L) | 1476 |
| 2089 | FBgn0051199-FBgn0051200 (3R) | 1470 |
| 2090 | FBgn0022936-FBgn0037134 (3L) | 1465 |
| 2091 | FBgn0085346-FBgn0015542 (3R) | 1463 |
| 2092 | FBgn0039190-FBgn0053340 (3R) | 1450 |
| 2093 | FBgn0033785-FBgn0050487 (2R) | 1449 |
| 2094 | FBgn0020645-FBgn0020765 (3L) | 1442 |
| 2095 | FBgn0035891-FBgn0010431 (3L) | 1441 |
| 2096 | FBgn0051549-FBgn0037354 (3R) | 1433 |
| 2097 | FBgn0034396-FBgn0034397 (2R) | 1419 |
| 2098 | FBgn0030577-FBgn0030578 (X) | 1416 |
| 2099 | FBgn0259721-FBgn0038183 (3R) | 1412 |
| 2100 | FBgn0050280-FBgn0050281 (2R) | 1409 |
| 2101 | FBgn0051902-FBgn0053121 (2L) | 1402 |
| 2102 | FBgn0028945-FBgn0028518 (2L) | 1401 |
| 2103 | FBgn0036117-FBgn0052069 (3L) | 1396 |
| 2104 | FBgn0033202-FBgn0033203 (2R) | 1395 |
| 2105 | FBgn0034426-FBgn0034427 (2R) | 1380 |
| 2106 | FBgn0033272-FBgn0021995 (2R) | 1373 |
| 2107 | FBgn0038244-FBgn0020299 (3R) | 1372 |
| 2108 | FBgn0036128-FBgn0052072 (3L) | 1349 |
| 2109 | FBgn0039703-FBgn0039704 (3R) | 1349 |
| 2110 | FBgn0053113-FBgn0031670 (2L) | 1338 |
| 2111 | FBgn0039453-FBgn0039454 (3R) | 1331 |
| 2112 | FBgn0052383-FBgn0052382 (3L) | 1329 |
| 2113 | FBgn0050115-FBgn0034360 (2R) | 1328 |
| 2114 | FBgn0033961-FBgn0033962 (2R) | 1309 |
| 2115 | FBgn0033820-FBgn0033821 (2R) | 1304 |
| 2116 | FBgn0050000-FBgn0050005 (2R) | 1303 |
| 2117 | FBgn0030792-FBgn0030791 (X) | 1294 |
| 2118 | FBgn0052271-FBgn0053159 (3L) | 1287 |
| 2119 | FBgn0051133-FBgn0046322 (3R) | 1281 |
| 2120 | FBgn0036655-FBgn0036656 (3L) | 1279 |
| 2121 | FBgn0033104-FBgn0028954 (2R) | 1274 |
| 2122 | FBgn0030099-FBgn0030100 (X) | 1274 |
| 2123 | FBgn0033786-FBgn0033787 (2R) | 1271 |
| 2124 | FBgn0025683-FBgn0001142 (2L) | 1270 |
| 2125 | FBgn0054051-FBgn0032834 (2L) | 1269 |
| 2126 | FBgn0040718-FBgn0040717 (2L) | 1268 |
| 2127 | FBgn0031977-FBgn0044323 (2L) | 1260 |
| 2128 | FBgn0012034-FBgn0027945 (3L) | 1259 |
| 2129 | FBgn0031560-FBgn0031561 (2L) | 1256 |
| 2130 | FBgn0034045-FBgn0034046 (2R) | 1247 |
| 2131 | FBgn0036131-FBgn0054012 (3L) | 1243 |
| 2132 | FBgn0038387-FBgn0013334 (3R) | 1237 |
| 2133 | FBgn0037127-FBgn0037128 (3L) | 1231 |
| 2134 | FBgn0027587-FBgn0035110 (3L) | 1226 |
| 2135 | FBgn0003863-FBgn0010357 (2R) | 1225 |
| 2136 | FBgn0005391-FBgn0004045 (X) | 1217 |
| 2137 | FBgn0038217-FBgn0038218 (3R) | 1206 |
| 2138 | FBgn0053276-FBgn0035805 (3L) | 1201 |
| 2139 | FBgn0259178-FBgn0039264 (3R) | 1191 |
| 2140 | FBgn0038978-FBgn0038979 (3R) | 1181 |
| 2141 | FBgn0033221-FBgn0033222 (2R) | 1178 |
| 2142 | FBgn0033570-FBgn0033571 (2R) | 1176 |
| 2143 | FBgn0052282-FBgn0035434 (3L) | 1173 |
| 2144 | FBgn0000808-FBgn0003865 (X) | 1167 |
| 2145 | FBgn0038241-FBgn0038242 (3R) | 1165 |
| 2146 | FBgn0083952-FBgn0040786 (3L) | 1161 |
| 2147 | FBgn0004403-FBgn0004404 (X) | 1159 |
| 2148 | FBgn0033594-FBgn0085254 (2R) | 1155 |
| 2149 | FBgn0051418-FBgn0051419 (3R) | 1116 |
| 2150 | FBgn0028855-FBgn0085194 (2L) | 1113 |
| 2151 | FBgn0034565-FBgn0034566 (2R) | 1108 |
| 2152 | FBgn0052767-FBgn0086911 (X) | 1107 |
| 2153 | FBgn0038690-FBgn0038691 (3R) | 1097 |
| 2154 | FBgn0035011-FBgn0035012 (2R) | 1094 |
| 2155 | FBgn0030097-FBgn0030098 (X) | 1093 |
| 2156 | FBgn0032595-FBgn0032596 (2L) | 1091 |
| 2157 | FBgn0033164-FBgn0033165 (2R) | 1082 |
| 2158 | FBgn0036619-FBgn0036620 (3L) | 1082 |
| 2159 | FBgn0036531-FBgn0036532 (3L) | 1081 |
| 2160 | FBgn0033663-FBgn0000556 (2R) | 1078 |
| 2161 | FBgn0036031-FBgn0036032 (3L) | 1076 |
| 2162 | FBgn0036016-FBgn0036017 (3L) | 1067 |
| 2163 | FBgn0011509-FBgn0052022 (3L) | 1056 |
| 2164 | FBgn0003076-FBgn0011016 (3L) | 1045 |
| 2165 | FBgn0039800-FBgn0039801 (3R) | 1028 |
| 2166 | FBgn0085275-FBgn0052155 (3L) | 1021 |
| 2167 | FBgn0051755-FBgn0051875 (2L) | 1014 |
| 2168 | FBgn0259714-FBgn0031811 (2L) | 1010 |
| 2169 | FBgn0041102-FBgn0001281 (3R) | 1009 |
| 2170 | FBgn0013263-FBgn0036446 (3L) | 997 |
| 2171 | FBgn0030391-FBgn0259143 (X) | 992 |
| 2172 | FBgn0032390-FBgn0032391 (2L) | 990 |
| 2173 | FBgn0019890-FBgn0012037 (2L) | 987 |
| 2174 | FBgn0038523-FBgn0003375 (3R) | 977 |
| 2175 | FBgn0000351-FBgn0031848 (2L) | 972 |
| 2176 | FBgn0039233-FBgn0039234 (3R) | 954 |
| 2177 | FBgn0030290-FBgn0030291 (X) | 935 |
| 2178 | FBgn0032660-FBgn0243486 (2L) | 933 |
| 2179 | FBgn0039451-FBgn0040607 (3R) | 927 |
| 2180 | FBgn0031378-FBgn0031379 (2L) | 925 |
| 2181 | FBgn0259220-FBgn0053203 (3R) | 925 |
| 2182 | FBgn0036044-FBgn0044051 (3L) | 909 |
| 2183 | FBgn0040964-FBgn0015316 (2L) | 897 |
| 2184 | FBgn0004903-FBgn0039461 (3R) | 892 |
| 2185 | FBgn0002533-FBgn0002534 (2R) | 870 |
| 2186 | FBgn0053191-FBgn0053189 (3R) | 867 |
| 2187 | FBgn0030268-FBgn0030269 (X) | 860 |
| 2188 | FBgn0033138-FBgn0033139 (2R) | 851 |
| 2189 | FBgn0038611-FBgn0038612 (3R) | 828 |
| 2190 | FBgn0032733-FBgn0032734 (2L) | 825 |
| 2191 | FBgn0032940-FBgn0000370 (2L) | 823 |
| 2192 | FBgn0052243-FBgn0035558 (3L) | 814 |
| 2193 | FBgn0036824-FBgn0036825 (3L) | 785 |
| 2194 | FBgn0039869-FBgn0039870 (3R) | 782 |
| 2195 | FBgn0035449-FBgn0052267 (3L) | 776 |
| 2196 | FBgn0052428-FBgn0036999 (3L) | 766 |
| 2197 | FBgn0040575-FBgn0038803 (3R) | 750 |
| 2198 | FBgn0038191-FBgn0015270 (3R) | 749 |
| 2199 | FBgn0010415-FBgn0026369 (2R) | 745 |
| 2200 | FBgn0036287-FBgn0053725 (3L) | 737 |
| 2201 | FBgn0030051-FBgn0030052 (X) | 724 |
| 2202 | FBgn0034061-FBgn0034063 (2R) | 718 |
| 2203 | FBgn0003302-FBgn0029833 (X) | 710 |
| 2204 | FBgn0003459-FBgn0036423 (3L) | 704 |
| 2205 | FBgn0031106-FBgn0031107 (X) | 699 |
| 2206 | FBgn0037633-FBgn0037634 (3R) | 695 |
| 2207 | FBgn0053469-FBgn0053468 (2R) | 693 |
| 2208 | FBgn0037220-FBgn0015331 (3R) | 689 |
| 2209 | FBgn0250785-FBgn0032886 (2L) | 678 |
| 2210 | FBgn0029764-FBgn0029763 (X) | 668 |
| 2211 | FBgn0031434-FBgn0031435 (2L) | 666 |
| 2212 | FBgn0086897-FBgn0015778 (3R) | 662 |
| 2213 | FBgn0031832-FBgn0026585 (2L) | 659 |
| 2214 | FBgn0039561-FBgn0039562 (3R) | 650 |
| 2215 | FBgn0052762-FBgn0029770 (X) | 647 |
| 2216 | FBgn0035742-FBgn0035743 (3L) | 644 |
| 2217 | FBgn0033320-FBgn0033321 (2R) | 631 |
| 2218 | FBgn0035842-FBgn0002899 (3L) | 602 |
| 2219 | FBgn0031558-FBgn0031559 (2L) | 595 |
| 2220 | FBgn0034486-FBgn0034487 (2R) | 589 |
| 2221 | FBgn0040344-FBgn0040339 (X) | 575 |
| 2222 | FBgn0039492-FBgn0029155 (3R) | 573 |
| 2223 | FBgn0001994-FBgn0040079 (2L) | 565 |
| 2224 | FBgn0031108-FBgn0040651 (X) | 564 |
| 2225 | FBgn0029756-FBgn0026751 (X) | 562 |
| 2226 | FBgn0001337-FBgn0029522 (X) | 556 |
| 2227 | FBgn0031590-FBgn0003941 (2L) | 546 |
| 2228 | FBgn0001276-FBgn0024188 (2R) | 546 |
| 2229 | FBgn0033059-FBgn0033060 (2R) | 534 |
| 2230 | FBgn0001125-FBgn0031381 (2L) | 532 |
| 2231 | FBgn0030121-FBgn0030122 (X) | 529 |
| 2232 | FBgn0040890-FBgn0027621 (X) | 528 |
| 2233 | FBgn0037352-FBgn0051551 (3R) | 526 |
| 2234 | FBgn0014184-FBgn0010621 (2R) | 515 |
| 2235 | FBgn0025874-FBgn0036389 (3L) | 513 |
| 2236 | FBgn0031627-FBgn0031628 (2L) | 510 |
| 2237 | FBgn0024238-FBgn0030838 (X) | 499 |
| 2238 | FBgn0022702-FBgn0035268 (3L) | 497 |
| 2239 | FBgn0020618-FBgn0004177 (2L) | 492 |
| 2240 | FBgn0030299-FBgn0030301 (X) | 490 |
| 2241 | FBgn0003520-FBgn0028983 (2R) | 474 |
| 2242 | FBgn0031284-FBgn0015621 (2L) | 473 |
| 2243 | FBgn0037669-FBgn0037670 (3R) | 469 |
| 2244 | FBgn0003378-FBgn0003377 (3L) | 468 |
| 2245 | FBgn0052066-FBgn0036096 (3L) | 467 |
| 2246 | FBgn0041171-FBgn0035517 (3L) | 466 |
| 2247 | FBgn0032242-FBgn0032243 (2L) | 463 |
| 2248 | FBgn0034503-FBgn0034504 (2R) | 462 |
| 2249 | FBgn0039681-FBgn0013972 (3R) | 462 |
| 2250 | FBgn0036534-FBgn0040230 (3L) | 461 |
| 2251 | FBgn0038787-FBgn0038788 (3R) | 461 |
| 2252 | FBgn0041224-FBgn0039466 (3R) | 459 |
| 2253 | FBgn0035247-FBgn0013753 (3L) | 454 |
| 2254 | FBgn0027360-FBgn0050290 (2R) | 453 |
| 2255 | FBgn0032731-FBgn0032732 (2L) | 452 |
| 2256 | FBgn0032436-FBgn0032437 (2L) | 435 |
| 2257 | FBgn0026430-FBgn0000257 (X) | 424 |
| 2258 | FBgn0034501-FBgn0034502 (2R) | 414 |
| 2259 | FBgn0013435-FBgn0033481 (2R) | 409 |
| 2260 | FBgn0003321-FBgn0030239 (X) | 405 |
| 2261 | FBgn0030883-FBgn0027605 (X) | 404 |
| 2262 | FBgn0029992-FBgn0029993 (X) | 403 |
| 2263 | FBgn0035959-FBgn0035960 (3L) | 400 |
| 2264 | FBgn0003204-FBgn0052676 (X) | 400 |
| 2265 | FBgn0033232-FBgn0015509 (2R) | 394 |
| 2266 | FBgn0030005-FBgn0030001 (X) | 390 |
| 2267 | FBgn0030030-FBgn0030029 (X) | 390 |
| 2268 | FBgn0040322-FBgn0040323 (3L) | 388 |
| 2269 | FBgn0038763-FBgn0243511 (3R) | 388 |
| 2270 | FBgn0029525-FBgn0023536 (X) | 386 |
| 2271 | FBgn0002783-FBgn0022787 (3R) | 385 |
| 2272 | FBgn0039554-FBgn0039555 (3R) | 383 |
| 2273 | FBgn0037261-FBgn0037262 (3R) | 380 |
| 2274 | FBgn0030599-FBgn0000535 (X) | 378 |
| 2275 | FBgn0032117-FBgn0011232 (2L) | 375 |
| 2276 | FBgn0037655-FBgn0040534 (3R) | 375 |
| 2277 | FBgn0030320-FBgn0030321 (X) | 372 |
| 2278 | FBgn0036842-FBgn0036843 (3L) | 371 |
| 2279 | FBgn0032429-FBgn0032430 (2L) | 370 |
| 2280 | FBgn0034430-FBgn0086358 (2R) | 366 |
| 2281 | FBgn0038462-FBgn0038463 (3R) | 365 |
| 2282 | FBgn0030163-FBgn0030164 (X) | 364 |
| 2283 | FBgn0037116-FBgn0037117 (3L) | 356 |
| 2284 | FBgn0022960-FBgn0033122 (2R) | 355 |
| 2285 | FBgn0053054-FBgn0053056 (3L) | 355 |
| 2286 | FBgn0039531-FBgn0039532 (3R) | 353 |
| 2287 | FBgn0034802-FBgn0034803 (2R) | 350 |
| 2288 | FBgn0031708-FBgn0000318 (2L) | 347 |
| 2289 | FBgn0037739-FBgn0005777 (3R) | 344 |
| 2290 | FBgn0037305-FBgn0086689 (3R) | 341 |
| 2291 | FBgn0031753-FBgn0040949 (2L) | 340 |
| 2292 | FBgn0086347-FBgn0032261 (2L) | 340 |
| 2293 | FBgn0036605-FBgn0036606 (3L) | 340 |
| 2294 | FBgn0000307-FBgn0028506 (2L) | 337 |
| 2295 | FBgn0052380-FBgn0016983 (3L) | 337 |
| 2296 | FBgn0051974-FBgn0031224 (2L) | 332 |
| 2297 | FBgn0086253-FBgn0039026 (3R) | 332 |
| 2298 | FBgn0038639-FBgn0038640 (3R) | 328 |
| 2299 | FBgn0038325-FBgn0038326 (3R) | 327 |
| 2300 | FBgn0044324-FBgn0037202 (3L) | 326 |
| 2301 | FBgn0035989-FBgn0015296 (3L) | 324 |
| 2302 | FBgn0031529-FBgn0016698 (2L) | 323 |
| 2303 | FBgn0034245-FBgn0016701 (2R) | 321 |
| 2304 | FBgn0047178-FBgn0036484 (3L) | 320 |
| 2305 | FBgn0027783-FBgn0028434 (2R) | 316 |
| 2306 | FBgn0034705-FBgn0034707 (2R) | 316 |
| 2307 | FBgn0033463-FBgn0050007 (2R) | 314 |
| 2308 | FBgn0027581-FBgn0033859 (2R) | 314 |
| 2309 | FBgn0030410-FBgn0030411 (X) | 313 |
| 2310 | FBgn0034299-FBgn0034300 (2R) | 312 |
| 2311 | FBgn0015617-FBgn0003449 (X) | 311 |
| 2312 | FBgn0053214-FBgn0037092 (3L) | 310 |
| 2313 | FBgn0037890-FBgn0037891 (3R) | 307 |
| 2314 | FBgn0033696-FBgn0033697 (2R) | 306 |
| 2315 | FBgn0032000-FBgn0032001 (2L) | 299 |
| 2316 | FBgn0032035-FBgn0032036 (2L) | 299 |
| 2317 | FBgn0035692-FBgn0024921 (3L) | 299 |
| 2318 | FBgn0035674-FBgn0035675 (3L) | 298 |
| 2319 | FBgn0015221-FBgn0015222 (3R) | 298 |
| 2320 | FBgn0086706-FBgn0035947 (3L) | 295 |
| 2321 | FBgn0032634-FBgn0032635 (2L) | 290 |
| 2322 | FBgn0052486-FBgn0035397 (3L) | 290 |
| 2323 | FBgn0030659-FBgn0030660 (X) | 290 |
| 2324 | FBgn0026318-FBgn0030013 (X) | 289 |
| 2325 | FBgn0051120-FBgn0039249 (3R) | 287 |
| 2326 | FBgn0030057-FBgn0027864 (X) | 287 |
| 2327 | FBgn0044511-FBgn0038145 (3R) | 286 |
| 2328 | FBgn0038269-FBgn0067629 (3R) | 285 |
| 2329 | FBgn0016926-FBgn0031304 (2L) | 283 |
| 2330 | FBgn0029157-FBgn0039254 (3R) | 282 |
| 2331 | FBgn0035619-FBgn0035620 (3L) | 275 |
| 2332 | FBgn0037105-FBgn0037106 (3L) | 275 |
| 2333 | FBgn0037120-FBgn0037121 (3L) | 275 |
| 2334 | FBgn0003210-FBgn0029709 (X) | 275 |
| 2335 | FBgn0034109-FBgn0034110 (2R) | 273 |
| 2336 | FBgn0038746-FBgn0038747 (3R) | 273 |
| 2337 | FBgn0025720-FBgn0034491 (2R) | 272 |
| 2338 | FBgn0034578-FBgn0034579 (2R) | 271 |
| 2339 | FBgn0051450-FBgn0051460 (3R) | 270 |
| 2340 | FBgn0036892-FBgn0036893 (3L) | 268 |
| 2341 | FBgn0032080-FBgn0052985 (2L) | 264 |
| 2342 | FBgn0035679-FBgn0061492 (3L) | 258 |
| 2343 | FBgn0004516-FBgn0035495 (3L) | 257 |
| 2344 | FBgn0040346-FBgn0040347 (X) | 257 |
| 2345 | FBgn0039966-FBgn0039965 (X) | 252 |
| 2346 | FBgn0002673-FBgn0028897 (2L) | 249 |
| 2347 | FBgn0036959-FBgn0036958 (3L) | 248 |
| 2348 | FBgn0033915-FBgn0033916 (2R) | 240 |
| 2349 | FBgn0020443-FBgn0040286 (2L) | 234 |
| 2350 | FBgn0035722-FBgn0029117 (3L) | 233 |
| 2351 | FBgn0037808-FBgn0037809 (3R) | 233 |
| 2352 | FBgn0023216-FBgn0023215 (X) | 233 |
| 2353 | FBgn0043903-FBgn0031057 (X) | 233 |
| 2354 | FBgn0033751-FBgn0033752 (2R) | 232 |
| 2355 | FBgn0035986-FBgn0035987 (3L) | 229 |
| 2356 | FBgn0069242-FBgn0037731 (3R) | 229 |
| 2357 | FBgn0053193-FBgn0027526 (3R) | 227 |
| 2358 | FBgn0034956-FBgn0034957 (2R) | 226 |
| 2359 | FBgn0030990-FBgn0030991 (X) | 224 |
| 2360 | FBgn0035243-FBgn0035244 (3L) | 223 |
| 2361 | FBgn0034366-FBgn0034367 (2R) | 220 |
| 2362 | FBgn0035233-FBgn0035232 (3L) | 220 |
| 2363 | FBgn0014189-FBgn0002525 (2L) | 218 |
| 2364 | FBgn0030506-FBgn0030507 (X) | 216 |
| 2365 | FBgn0005671-FBgn0038065 (3R) | 213 |
| 2366 | FBgn0036735-FBgn0259174 (3L) | 208 |
| 2367 | FBgn0030485-FBgn0030486 (X) | 205 |
| 2368 | FBgn0054045-FBgn0050127 (2R) | 202 |
| 2369 | FBgn0034117-FBgn0034118 (2R) | 199 |
| 2370 | FBgn0016070-FBgn0035953 (3L) | 198 |
| 2371 | FBgn0038479-FBgn0051269 (3R) | 189 |
| 2372 | FBgn0025140-FBgn0038928 (3R) | 185 |
| 2373 | FBgn0050491-FBgn0033199 (2R) | 184 |
| 2374 | FBgn0044049-FBgn0044328 (3L) | 183 |
| 2375 | FBgn0038290-FBgn0038291 (3R) | 183 |
| 2376 | FBgn0031540-FBgn0051776 (2L) | 176 |
| 2377 | FBgn0034703-FBgn0034704 (2R) | 171 |
| 2378 | FBgn0051698-FBgn0031512 (2L) | 164 |
| 2379 | FBgn0037623-FBgn0037624 (3R) | 157 |
| 2380 | FBgn0033083-FBgn0000054 (2R) | 152 |
| 2381 | FBgn0039003-FBgn0039004 (3R) | 151 |
| 2382 | FBgn0031987-FBgn0031988 (2L) | 149 |
| 2383 | FBgn0032142-FBgn0032143 (2L) | 148 |
| 2384 | FBgn0086785-FBgn0036376 (3L) | 148 |
| 2385 | FBgn0004832-FBgn0000221 (X) | 148 |
| 2386 | FBgn0037059-FBgn0037060 (3L) | 146 |
| 2387 | FBgn0051782-FBgn0051812 (2L) | 143 |
| 2388 | FBgn0036614-FBgn0036615 (3L) | 143 |
| 2389 | FBgn0051121-FBgn0039244 (3R) | 140 |
| 2390 | FBgn0030500-FBgn0030501 (X) | 139 |
| 2391 | FBgn0016081-FBgn0036028 (3L) | 127 |
| 2392 | FBgn0020385-FBgn0037822 (3R) | 126 |
| 2393 | FBgn0029783-FBgn0028474 (X) | 126 |
| 2394 | FBgn0030349-FBgn0030350 (X) | 122 |
| 2395 | FBgn0034958-FBgn0034959 (2R) | 118 |
| 2396 | FBgn0034631-FBgn0043070 (2R) | 117 |
| 2397 | FBgn0035016-FBgn0050169 (2R) | 107 |
| 2398 | FBgn0037017-FBgn0040634 (3L) | 105 |
| 2399 | FBgn0031286-FBgn0010583 (2L) | 104 |
| 2400 | FBgn0000352-FBgn0033160 (2R) | 103 |
| 2401 | FBgn0035136-FBgn0035137 (3L) | 96 |
| 2402 | FBgn0015924-FBgn0031256 (2L) | 89 |
| 2403 | FBgn0052278-FBgn0052281 (3L) | 89 |
| 2404 | FBgn0035251-FBgn0035252 (3L) | 88 |
| 2405 | FBgn0036134-FBgn0036135 (3L) | 85 |
| 2406 | FBgn0035253-FBgn0035254 (3L) | 84 |
| 2407 | FBgn0040666-FBgn0035059 (2R) | 83 |
| 2408 | FBgn0026433-FBgn0030645 (X) | 81 |
| 2409 | FBgn0037842-FBgn0037843 (3R) | 78 |
| 2410 | FBgn0053123-FBgn0031544 (2L) | 76 |
| 2411 | FBgn0029986-FBgn0029987 (X) | 75 |
| 2412 | FBgn0011290-FBgn0037933 (3R) | 74 |
| 2413 | FBgn0015323-FBgn0000303 (3R) | 72 |
| 2414 | FBgn0039135-FBgn0039136 (3R) | 72 |
| 2415 | FBgn0019830-FBgn0031459 (2L) | 71 |
| 2416 | FBgn0037061-FBgn0028427 (3L) | 71 |
| 2417 | FBgn0034361-FBgn0034362 (2R) | 67 |
| 2418 | FBgn0013531-FBgn0010287 (2L) | 54 |
| 2419 | FBgn0033993-FBgn0033994 (2R) | 54 |
| 2420 | FBgn0035488-FBgn0035489 (3L) | 53 |
| 2421 | FBgn0002872-FBgn0035295 (3L) | 52 |
| 2422 | FBgn0032444-FBgn0032445 (2L) | 50 |
| 2423 | FBgn0019925-FBgn0051301 (3R) | 49 |
| 2424 | FBgn0033208-FBgn0033209 (2R) | 44 |
| 2425 | FBgn0023171-FBgn0026722 (2R) | 41 |
| 2426 | FBgn0003169-FBgn0013981 (3R) | 41 |
| 2427 | FBgn0038738-FBgn0038739 (3R) | 38 |
| 2428 | FBgn0036887-FBgn0036888 (3L) | 28 |
| 2429 | FBgn0039329-FBgn0028647 (3R) | 28 |
| 2430 | FBgn0039503-FBgn0039504 (3R) | 28 |
| 2431 | FBgn0029819-FBgn0029820 (X) | 26 |
| 2432 | FBgn0032720-FBgn0032721 (2L) | 23 |
| 2433 | FBgn0015806-FBgn0035639 (3L) | 17 |
| 2434 | FBgn0015765-FBgn0039156 (3R) | 14 |
| 2435 | FBgn0026741-FBgn0026619 (2R) | 12 |
| 2436 | FBgn0000180-FBgn0040965 (2L) | 11 |
| 2437 | FBgn0029508-FBgn0050159 (2R) | 11 |
| 2438 | FBgn0010504-FBgn0033269 (2R) | 3 |
| 2439 | FBgn0051975-FBgn0051976 (2L) | 1 |
| 2440 | FBgn0026064-FBgn0026063 (3R) | 1 |
| 2441 | FBgn0023083-FBgn0038627 (3R) | 1 |
| 2442 | FBgn0051108-FBgn0051510 (3R) | 1 |

  

|  |  |  |
| --- | --- | --- |
| **Segments by likelihood ratio** | | |
| 1 | FBgn0051559-FBgn0037432 (3R) | 1068.439046 |
| 2 | FBgn0030367-FBgn0030390 (X) | 498.113078 |
| 3 | FBgn0039192-FBgn0039202 (3R) | 483.258411 |
| 4 | FBgn0032965-FBgn0032972 (2L) | 475.371529 |
| 5 | FBgn0034829-FBgn0050409 (2R) | 470.653167 |
| 6 | FBgn0023094-FBgn0036916 (3L) | 434.606376 |
| 7 | FBgn0037449-FBgn0051286 (3R) | 387.702084 |
| 8 | FBgn0040355-FBgn0029647 (X) | 370.746229 |
| 9 | FBgn0023516-FBgn0029594 (X) | 364.260361 |
| 10 | FBgn0037876-FBgn0037884 (3R) | 347.801103 |
| 11 | FBgn0038922-FBgn0019957 (3R) | 332.181204 |
| 12 | FBgn0030898-FBgn0004956 (X) | 322.114395 |
| 13 | FBgn0024371-FBgn0032926 (2L) | 303.838219 |
| 14 | FBgn0001202-FBgn0032748 (2L) | 297.904024 |
| 15 | FBgn0029961-FBgn0029968 (X) | 296.089524 |
| 16 | FBgn0039140-FBgn0040283 (3R) | 293.871922 |
| 17 | FBgn0031601-FBgn0031607 (2L) | 293.045099 |
| 18 | FBgn0029722-FBgn0029730 (X) | 293.008245 |
| 19 | FBgn0085213-FBgn0033933 (2R) | 290.194890 |
| 20 | FBgn0033375-FBgn0027561 (2R) | 287.122628 |
| 21 | FBgn0034665-FBgn0054029 (2R) | 284.493272 |
| 22 | FBgn0039109-FBgn0039115 (3R) | 283.183217 |
| 23 | FBgn0037168-FBgn0037179 (3L) | 279.439114 |
| 24 | FBgn0035202-FBgn0035207 (3L) | 278.485128 |
| 25 | FBgn0032577-FBgn0028893 (2L) | 278.303890 |
| 26 | FBgn0000183-FBgn0032644 (2L) | 277.637729 |
| 27 | FBgn0034329-FBgn0064237 (2R) | 276.039574 |
| 28 | FBgn0034913-FBgn0034918 (2R) | 265.189321 |
| 29 | FBgn0028412-FBgn0032377 (2L) | 264.069625 |
| 30 | FBgn0041237-FBgn0034684 (2R) | 263.485339 |
| 31 | FBgn0004593-FBgn0014851 (3L) | 262.806673 |
| 32 | FBgn0041242-FBgn0053475 (2R) | 262.207062 |
| 33 | FBgn0037329-FBgn0051550 (3R) | 261.837455 |
| 34 | FBgn0031657-FBgn0031664 (2L) | 257.093469 |
| 35 | FBgn0040816-FBgn0036224 (3L) | 254.670693 |
| 36 | FBgn0051784-FBgn0032631 (2L) | 251.183203 |
| 37 | FBgn0024326-FBgn0037606 (3R) | 250.900802 |
| 38 | FBgn0033005-FBgn0033017 (2R) | 250.305369 |
| 39 | FBgn0029700-FBgn0040906 (X) | 247.753770 |
| 40 | FBgn0036590-FBgn0036596 (3L) | 246.921146 |
| 41 | FBgn0040363-FBgn0040367 (X) | 246.392683 |
| 42 | FBgn0034321-FBgn0034327 (2R) | 241.861451 |
| 43 | FBgn0025628-FBgn0013432 (X) | 241.858592 |
| 44 | FBgn0037368-FBgn0037374 (3R) | 240.112050 |
| 45 | FBgn0004584-FBgn0025681 (2L) | 233.963139 |
| 46 | FBgn0031364-FBgn0051933 (2L) | 231.786515 |
| 47 | FBgn0000212-FBgn0036558 (3L) | 230.342468 |
| 48 | FBgn0040752-FBgn0033900 (2R) | 227.861474 |
| 49 | FBgn0031440-FBgn0031446 (2L) | 225.110419 |
| 50 | FBgn0041240-FBgn0034551 (2R) | 221.239258 |
| 51 | FBgn0031238-FBgn0031244 (2L) | 218.970243 |
| 52 | FBgn0034022-FBgn0050471 (2R) | 217.567347 |
| 53 | FBgn0014023-FBgn0037710 (3R) | 215.310390 |
| 54 | FBgn0025616-FBgn0029538 (X) | 214.463039 |
| 55 | FBgn0033259-FBgn0005648 (2R) | 214.076596 |
| 56 | FBgn0028901-FBgn0051735 (2L) | 213.798270 |
| 57 | FBgn0031127-FBgn0031139 (X) | 213.693582 |
| 58 | FBgn0051294-FBgn0038359 (3R) | 212.901508 |
| 59 | FBgn0001222-FBgn0034313 (2R) | 211.470840 |
| 60 | FBgn0026384-FBgn0034870 (2R) | 210.984555 |
| 61 | FBgn0035770-FBgn0082598 (3L) | 209.774109 |
| 62 | FBgn0039810-FBgn0053773 (3R) | 209.312261 |
| 63 | FBgn0052112-FBgn0036309 (3L) | 207.119305 |
| 64 | FBgn0039366-FBgn0039371 (3R) | 204.168133 |
| 65 | FBgn0035002-FBgn0035009 (2R) | 202.867142 |
| 66 | FBgn0035023-FBgn0035028 (2R) | 201.871380 |
| 67 | FBgn0032193-FBgn0032197 (2L) | 201.120207 |
| 68 | FBgn0036794-FBgn0052199 (3L) | 200.575667 |
| 69 | FBgn0031619-FBgn0031626 (2L) | 199.430721 |
| 70 | FBgn0038156-FBgn0038160 (3R) | 197.794038 |
| 71 | FBgn0034766-FBgn0050268 (2R) | 197.554931 |
| 72 | FBgn0038471-FBgn0038478 (3R) | 197.214213 |
| 73 | FBgn0036703-FBgn0036709 (3L) | 196.743732 |
| 74 | FBgn0040393-FBgn0003374 (X) | 196.575924 |
| 75 | FBgn0041775-FBgn0036301 (3L) | 195.550408 |
| 76 | FBgn0032200-FBgn0032205 (2L) | 195.293873 |
| 77 | FBgn0030559-FBgn0030570 (X) | 194.038154 |
| 78 | FBgn0037465-FBgn0037470 (3R) | 191.618354 |
| 79 | FBgn0032457-FBgn0032462 (2L) | 190.479398 |
| 80 | FBgn0039505-FBgn0039509 (3R) | 188.781844 |
| 81 | FBgn0031331-FBgn0031335 (2L) | 188.730385 |
| 82 | FBgn0031718-FBgn0031725 (2L) | 186.320713 |
| 83 | FBgn0036987-FBgn0036991 (3L) | 185.677193 |
| 84 | FBgn0036325-FBgn0036330 (3L) | 185.574738 |
| 85 | FBgn0037584-FBgn0037591 (3R) | 185.494334 |
| 86 | FBgn0031264-FBgn0031268 (2L) | 184.315990 |
| 87 | FBgn0032050-FBgn0032054 (2L) | 184.083457 |
| 88 | FBgn0003415-FBgn0026179 (3L) | 183.483714 |
| 89 | FBgn0030941-FBgn0030946 (X) | 182.311672 |
| 90 | FBgn0033349-FBgn0033352 (2R) | 182.210519 |
| 91 | FBgn0032699-FBgn0032703 (2L) | 182.086488 |
| 92 | FBgn0026761-FBgn0033081 (2R) | 182.065967 |
| 93 | FBgn0050343-FBgn0033402 (2R) | 181.284573 |
| 94 | FBgn0039522-FBgn0046885 (3R) | 181.184564 |
| 95 | FBgn0030661-FBgn0030667 (X) | 181.061422 |
| 96 | FBgn0051501-FBgn0004172 (3R) | 180.875943 |
| 97 | FBgn0030668-FBgn0030672 (X) | 180.775905 |
| 98 | FBgn0032029-FBgn0032034 (2L) | 180.702876 |
| 99 | FBgn0034824-FBgn0034827 (2R) | 180.481819 |
| 100 | FBgn0038186-FBgn0038190 (3R) | 180.384533 |
| 101 | FBgn0036437-FBgn0036443 (3L) | 179.721116 |
| 102 | FBgn0029658-FBgn0029662 (X) | 179.410339 |
| 103 | FBgn0052986-FBgn0085427 (2L) | 179.246001 |
| 104 | FBgn0030618-FBgn0030624 (X) | 177.969724 |
| 105 | FBgn0052154-FBgn0036586 (3L) | 177.779752 |
| 106 | FBgn0033041-FBgn0033046 (2R) | 176.798179 |
| 107 | FBgn0010040-FBgn0053098 (3R) | 176.461255 |
| 108 | FBgn0035388-FBgn0052485 (3L) | 176.118695 |
| 109 | FBgn0041161-FBgn0013563 (3L) | 173.465564 |
| 110 | FBgn0031390-FBgn0031395 (2L) | 172.699303 |
| 111 | FBgn0036078-FBgn0052058 (3L) | 171.312343 |
| 112 | FBgn0038525-FBgn0038529 (3R) | 171.056056 |
| 113 | FBgn0033587-FBgn0033592 (2R) | 170.749238 |
| 114 | FBgn0085274-FBgn0036459 (3L) | 169.412616 |
| 115 | FBgn0037018-FBgn0037022 (3L) | 169.180508 |
| 116 | FBgn0086899-FBgn0000542 (X) | 169.059631 |
| 117 | FBgn0003008-FBgn0005612 (2R) | 168.554796 |
| 118 | FBgn0040754-FBgn0033812 (2R) | 167.984435 |
| 119 | FBgn0035499-FBgn0004574 (3L) | 167.258512 |
| 120 | FBgn0030437-FBgn0030439 (X) | 166.555312 |
| 121 | FBgn0038854-FBgn0028468 (3R) | 166.495734 |
| 122 | FBgn0004401-FBgn0036746 (3L) | 165.921640 |
| 123 | FBgn0015221-FBgn0015222 (3R) | 165.654412 |
| 124 | FBgn0030718-FBgn0000611 (X) | 165.516067 |
| 125 | FBgn0030342-FBgn0030346 (X) | 165.300541 |
| 126 | FBgn0035400-FBgn0035403 (3L) | 165.030070 |
| 127 | FBgn0039302-FBgn0039306 (3R) | 164.741869 |
| 128 | FBgn0033322-FBgn0050357 (2R) | 163.654199 |
| 129 | FBgn0039075-FBgn0085318 (3R) | 163.442163 |
| 130 | FBgn0039874-FBgn0027620 (3R) | 163.264682 |
| 131 | FBgn0030165-FBgn0030173 (X) | 162.727766 |
| 132 | FBgn0034230-FBgn0022085 (2R) | 162.712212 |
| 133 | FBgn0259234-FBgn0259246 (2R) | 162.211997 |
| 134 | FBgn0032553-FBgn0259213 (2L) | 162.040410 |
| 135 | FBgn0034791-FBgn0034794 (2R) | 161.825760 |
| 136 | FBgn0024285-FBgn0032160 (2L) | 161.478816 |
| 137 | FBgn0037506-FBgn0037512 (3R) | 160.112818 |
| 138 | FBgn0035255-FBgn0035263 (3L) | 160.087586 |
| 139 | FBgn0038208-FBgn0038210 (3R) | 159.483046 |
| 140 | FBgn0015359-FBgn0000543 (3L) | 159.182028 |
| 141 | FBgn0028853-FBgn0020371 (2L) | 158.789429 |
| 142 | FBgn0036510-FBgn0036516 (3L) | 158.275353 |
| 143 | FBgn0039792-FBgn0039799 (3R) | 156.937806 |
| 144 | FBgn0015818-FBgn0028685 (X) | 156.862226 |
| 145 | FBgn0032512-FBgn0032516 (2L) | 156.266034 |
| 146 | FBgn0038303-FBgn0015610 (3R) | 155.329379 |
| 147 | FBgn0035995-FBgn0036000 (3L) | 154.890350 |
| 148 | FBgn0038424-FBgn0038428 (3R) | 154.688872 |
| 149 | FBgn0051686-FBgn0031406 (2L) | 154.579288 |
| 150 | FBgn0013810-FBgn0032650 (2L) | 154.417606 |
| 151 | FBgn0038003-FBgn0003651 (3R) | 153.675823 |
| 152 | FBgn0046685-FBgn0020018 (3R) | 153.671100 |
| 153 | FBgn0030749-FBgn0054015 (X) | 153.460512 |
| 154 | FBgn0033277-FBgn0050375 (2R) | 153.407127 |
| 155 | FBgn0030743-FBgn0005411 (X) | 152.631315 |
| 156 | FBgn0032465-FBgn0028965 (2L) | 152.288692 |
| 157 | FBgn0036570-FBgn0036571 (3L) | 151.712757 |
| 158 | FBgn0038666-FBgn0038673 (3R) | 151.656916 |
| 159 | FBgn0025378-FBgn0029590 (X) | 150.687647 |
| 160 | FBgn0050325-FBgn0034286 (2R) | 150.452847 |
| 161 | FBgn0033862-FBgn0033867 (2R) | 149.616609 |
| 162 | FBgn0027524-FBgn0037780 (3R) | 149.180019 |
| 163 | FBgn0051014-FBgn0039784 (3R) | 148.620673 |
| 164 | FBgn0020510-FBgn0003276 (3R) | 148.472536 |
| 165 | FBgn0036676-FBgn0036681 (3L) | 148.077359 |
| 166 | FBgn0051093-FBgn0039376 (3R) | 147.971671 |
| 167 | FBgn0000357-FBgn0000356 (3L) | 147.917434 |
| 168 | FBgn0036661-FBgn0021768 (3L) | 147.627434 |
| 169 | FBgn0028695-FBgn0036921 (3L) | 147.494018 |
| 170 | FBgn0035370-FBgn0035375 (3L) | 147.448130 |
| 171 | FBgn0035376-FBgn0035382 (3L) | 147.195107 |
| 172 | FBgn0032525-FBgn0028941 (2L) | 146.833305 |
| 173 | FBgn0033562-FBgn0033569 (2R) | 146.459770 |
| 174 | FBgn0027107-FBgn0031104 (X) | 145.904658 |
| 175 | FBgn0019938-FBgn0033945 (2R) | 145.468775 |
| 176 | FBgn0032215-FBgn0032218 (2L) | 145.225022 |
| 177 | FBgn0004509-FBgn0039338 (3R) | 144.076147 |
| 178 | FBgn0015770-FBgn0037503 (3R) | 143.955476 |
| 179 | FBgn0032222-FBgn0032224 (2L) | 143.855892 |
| 180 | FBgn0039055-FBgn0051457 (3R) | 143.819788 |
| 181 | FBgn0037339-FBgn0041191 (3R) | 143.283028 |
| 182 | FBgn0052061-FBgn0036089 (3L) | 143.122470 |
| 183 | FBgn0034152-FBgn0053530 (2R) | 142.281927 |
| 184 | FBgn0045479-FBgn0052255 (3L) | 141.828252 |
| 185 | FBgn0034265-FBgn0034271 (2R) | 141.708623 |
| 186 | FBgn0002592-FBgn0002633 (3R) | 141.403690 |
| 187 | FBgn0052250-FBgn0035534 (3L) | 141.232826 |
| 188 | FBgn0086448-FBgn0010416 (X) | 140.471113 |
| 189 | FBgn0033224-FBgn0033226 (2R) | 140.467465 |
| 190 | FBgn0035569-FBgn0035577 (3L) | 139.881301 |
| 191 | FBgn0052075-FBgn0036124 (3L) | 139.812495 |
| 192 | FBgn0051265-FBgn0051267 (3R) | 139.616027 |
| 193 | FBgn0051481-FBgn0000166 (3R) | 139.437921 |
| 194 | FBgn0031114-FBgn0002641 (X) | 138.454707 |
| 195 | FBgn0038582-FBgn0038586 (3R) | 137.599912 |
| 196 | FBgn0038201-FBgn0038205 (3R) | 137.107988 |
| 197 | FBgn0038535-FBgn0051249 (3R) | 137.013896 |
| 198 | FBgn0032311-FBgn0032315 (2L) | 136.959603 |
| 199 | FBgn0033636-FBgn0085257 (2R) | 135.810683 |
| 200 | FBgn0039634-FBgn0039637 (3R) | 135.483018 |
| 201 | FBgn0031489-FBgn0031495 (2L) | 135.385969 |
| 202 | FBgn0024314-FBgn0031322 (2L) | 135.358870 |
| 203 | FBgn0037978-FBgn0037981 (3R) | 135.337936 |
| 204 | FBgn0029696-FBgn0052774 (X) | 135.249613 |
| 205 | FBgn0031710-FBgn0031713 (2L) | 134.451288 |
| 206 | FBgn0001247-FBgn0011205 (3L) | 134.329431 |
| 207 | FBgn0039969-FBgn0027507 (2R) | 134.088651 |
| 208 | FBgn0050362-FBgn0033287 (2R) | 133.597555 |
| 209 | FBgn0037248-FBgn0037252 (3R) | 133.035007 |
| 210 | FBgn0037242-FBgn0037245 (3R) | 132.986960 |
| 211 | FBgn0004588-FBgn0004591 (3L) | 132.815571 |
| 212 | FBgn0051081-FBgn0039448 (3R) | 132.535927 |
| 213 | FBgn0053703-FBgn0036013 (3L) | 132.300863 |
| 214 | FBgn0027535-FBgn0010434 (2R) | 132.240311 |
| 215 | FBgn0032089-FBgn0032100 (2L) | 132.119748 |
| 216 | FBgn0040390-FBgn0001150 (X) | 131.894442 |
| 217 | FBgn0035795-FBgn0035797 (3L) | 131.878655 |
| 218 | FBgn0031384-FBgn0053516 (2L) | 131.149703 |
| 219 | FBgn0015338-FBgn0000339 (2L) | 130.675777 |
| 220 | FBgn0035563-FBgn0035568 (3L) | 130.570124 |
| 221 | FBgn0259685-FBgn0039182 (3R) | 130.377755 |
| 222 | FBgn0030309-FBgn0030311 (X) | 130.206688 |
| 223 | FBgn0037312-FBgn0037315 (3R) | 130.156355 |
| 224 | FBgn0037762-FBgn0037765 (3R) | 130.107837 |
| 225 | FBgn0035511-FBgn0035514 (3L) | 130.038008 |
| 226 | FBgn0036302-FBgn0014007 (3L) | 130.022959 |
| 227 | FBgn0021818-FBgn0025716 (2R) | 129.831738 |
| 228 | FBgn0025866-FBgn0036059 (3L) | 129.421141 |
| 229 | FBgn0037347-FBgn0044823 (3R) | 129.064195 |
| 230 | FBgn0058439-FBgn0000384 (2L) | 128.662814 |
| 231 | FBgn0032236-FBgn0051716 (2L) | 128.355526 |
| 232 | FBgn0051802-FBgn0032677 (2L) | 127.680818 |
| 233 | FBgn0028920-FBgn0028532 (2L) | 127.496616 |
| 234 | FBgn0030400-FBgn0030407 (X) | 127.411829 |
| 235 | FBgn0035507-FBgn0035510 (3L) | 127.282287 |
| 236 | FBgn0035584-FBgn0035594 (3L) | 126.569337 |
| 237 | FBgn0033663-FBgn0000556 (2R) | 126.464486 |
| 238 | FBgn0028577-FBgn0035229 (3L) | 126.003069 |
| 239 | FBgn0024491-FBgn0040237 (3R) | 125.682337 |
| 240 | FBgn0014464-FBgn0000360 (X) | 125.402608 |
| 241 | FBgn0032489-FBgn0032493 (2L) | 125.316185 |
| 242 | FBgn0035384-FBgn0004372 (3L) | 125.116008 |
| 243 | FBgn0037541-FBgn0010812 (3R) | 124.422455 |
| 244 | FBgn0029911-FBgn0029915 (X) | 124.148282 |
| 245 | FBgn0032824-FBgn0053117 (2L) | 123.751220 |
| 246 | FBgn0051694-FBgn0051950 (2L) | 123.686644 |
| 247 | FBgn0016756-FBgn0035631 (3L) | 123.561631 |
| 248 | FBgn0039258-FBgn0039260 (3R) | 123.489006 |
| 249 | FBgn0032815-FBgn0032819 (2L) | 123.468720 |
| 250 | FBgn0032453-FBgn0032455 (2L) | 123.069292 |
| 251 | FBgn0035333-FBgn0035335 (3L) | 122.983839 |
| 252 | FBgn0027549-FBgn0026252 (3L) | 122.610881 |
| 253 | FBgn0031308-FBgn0031312 (2L) | 122.426895 |
| 254 | FBgn0035336-FBgn0035347 (3L) | 122.422066 |
| 255 | FBgn0051816-FBgn0051807 (2L) | 122.194700 |
| 256 | FBgn0037040-FBgn0037042 (3L) | 121.892757 |
| 257 | FBgn0024542-FBgn0035754 (3L) | 121.850200 |
| 258 | FBgn0043470-FBgn0011555 (2R) | 121.792545 |
| 259 | FBgn0031462-FBgn0031473 (2L) | 121.704318 |
| 260 | FBgn0051358-FBgn0037988 (3R) | 121.462313 |
| 261 | FBgn0027865-FBgn0039385 (3R) | 121.367942 |
| 262 | FBgn0032549-FBgn0053309 (2L) | 121.063689 |
| 263 | FBgn0030327-FBgn0030330 (X) | 120.730457 |
| 264 | FBgn0015129-FBgn0039677 (3R) | 120.691467 |
| 265 | FBgn0031245-FBgn0020622 (2L) | 120.328739 |
| 266 | FBgn0034057-FBgn0034060 (2R) | 119.783558 |
| 267 | FBgn0010894-FBgn0040239 (3L) | 119.691443 |
| 268 | FBgn0032974-FBgn0032979 (2L) | 119.508665 |
| 269 | FBgn0020224-FBgn0010825 (3L) | 119.458355 |
| 270 | FBgn0030970-FBgn0030974 (X) | 118.982143 |
| 271 | FBgn0024291-FBgn0032477 (2L) | 118.963459 |
| 272 | FBgn0000077-FBgn0028292 (X) | 118.767012 |
| 273 | FBgn0030492-FBgn0052631 (X) | 118.713292 |
| 274 | FBgn0030292-FBgn0027359 (X) | 118.670473 |
| 275 | FBgn0005630-FBgn0033540 (2R) | 118.628573 |
| 276 | FBgn0051876-FBgn0032171 (2L) | 118.227556 |
| 277 | FBgn0035699-FBgn0020251 (3L) | 117.780063 |
| 278 | FBgn0025742-FBgn0031769 (2L) | 117.737321 |
| 279 | FBgn0259749-FBgn0031820 (2L) | 117.331031 |
| 280 | FBgn0032871-FBgn0032877 (2L) | 117.276420 |
| 281 | FBgn0035420-FBgn0035422 (3L) | 117.187884 |
| 282 | FBgn0035155-FBgn0035160 (3L) | 117.049500 |
| 283 | FBgn0037008-FBgn0037011 (3L) | 116.941560 |
| 284 | FBgn0005391-FBgn0004045 (X) | 116.912413 |
| 285 | FBgn0051109-FBgn0039270 (3R) | 116.511324 |
| 286 | FBgn0035523-FBgn0015829 (3L) | 116.321753 |
| 287 | FBgn0050295-FBgn0020617 (2R) | 116.016913 |
| 288 | FBgn0036349-FBgn0036352 (3L) | 115.999990 |
| 289 | FBgn0259225-FBgn0032385 (2L) | 115.745534 |
| 290 | FBgn0004419-FBgn0051717 (2L) | 115.729572 |
| 291 | FBgn0034748-FBgn0034750 (2R) | 115.703042 |
| 292 | FBgn0033972-FBgn0022063 (2R) | 115.660279 |
| 293 | FBgn0000566-FBgn0034371 (2R) | 115.529370 |
| 294 | FBgn0036448-FBgn0029148 (3L) | 115.353826 |
| 295 | FBgn0038868-FBgn0038871 (3R) | 114.322512 |
| 296 | FBgn0027587-FBgn0035110 (3L) | 114.257184 |
| 297 | FBgn0051806-FBgn0032626 (2L) | 114.067040 |
| 298 | FBgn0027095-FBgn0038421 (3R) | 114.007298 |
| 299 | FBgn0032247-FBgn0032251 (2L) | 113.947108 |
| 300 | FBgn0039687-FBgn0039691 (3R) | 113.888944 |
| 301 | FBgn0000250-FBgn0001995 (2L) | 113.603116 |
| 302 | FBgn0037826-FBgn0037829 (3R) | 113.554930 |
| 303 | FBgn0023509-FBgn0023521 (X) | 113.417679 |
| 304 | FBgn0031534-FBgn0031537 (2L) | 113.408668 |
| 305 | FBgn0039434-FBgn0051080 (3R) | 113.001873 |
| 306 | FBgn0029958-FBgn0016041 (X) | 112.953474 |
| 307 | FBgn0038053-FBgn0038058 (3R) | 112.582527 |
| 308 | FBgn0033340-FBgn0050349 (2R) | 112.233777 |
| 309 | FBgn0029745-FBgn0029751 (X) | 112.046634 |
| 310 | FBgn0038106-FBgn0038108 (3R) | 111.848050 |
| 311 | FBgn0037234-FBgn0037236 (3R) | 111.761361 |
| 312 | FBgn0030774-FBgn0030777 (X) | 111.693224 |
| 313 | FBgn0038948-FBgn0038951 (3R) | 111.461312 |
| 314 | FBgn0083940-FBgn0030182 (X) | 111.067934 |
| 315 | FBgn0038131-FBgn0038134 (3R) | 110.657185 |
| 316 | FBgn0030810-FBgn0030815 (X) | 110.301833 |
| 317 | FBgn0037636-FBgn0037637 (3R) | 110.156433 |
| 318 | FBgn0051219-FBgn0038732 (3R) | 109.977936 |
| 319 | FBgn0038505-FBgn0038508 (3R) | 109.799579 |
| 320 | FBgn0036853-FBgn0017578 (3L) | 109.768222 |
| 321 | FBgn0035600-FBgn0035601 (3L) | 109.660225 |
| 322 | FBgn0014018-FBgn0037659 (3R) | 109.174108 |
| 323 | FBgn0034104-FBgn0050099 (2R) | 108.975094 |
| 324 | FBgn0020279-FBgn0028563 (2R) | 108.666157 |
| 325 | FBgn0034008-FBgn0013750 (2R) | 108.561223 |
| 326 | FBgn0051548-FBgn0037358 (3R) | 108.386345 |
| 327 | FBgn0036609-FBgn0053060 (3L) | 107.992955 |
| 328 | FBgn0035876-FBgn0035879 (3L) | 107.476034 |
| 329 | FBgn0035855-FBgn0035857 (3L) | 107.475297 |
| 330 | FBgn0036807-FBgn0036810 (3L) | 107.357899 |
| 331 | FBgn0023096-FBgn0040989 (2L) | 106.986290 |
| 332 | FBgn0035107-FBgn0025592 (3L) | 106.932783 |
| 333 | FBgn0042207-FBgn0038070 (3R) | 106.917677 |
| 334 | FBgn0040684-FBgn0037296 (3R) | 106.858245 |
| 335 | FBgn0011653-FBgn0003710 (3L) | 106.566233 |
| 336 | FBgn0029710-FBgn0029713 (X) | 106.348110 |
| 337 | FBgn0037608-FBgn0037611 (3R) | 106.314193 |
| 338 | FBgn0036691-FBgn0036696 (3L) | 106.143485 |
| 339 | FBgn0036225-FBgn0036229 (3L) | 105.660779 |
| 340 | FBgn0034904-FBgn0034906 (2R) | 105.411994 |
| 341 | FBgn0004403-FBgn0004404 (X) | 105.351515 |
| 342 | FBgn0030277-FBgn0030283 (X) | 105.298179 |
| 343 | FBgn0035733-FBgn0035741 (3L) | 105.117960 |
| 344 | FBgn0039596-FBgn0039599 (3R) | 104.852392 |
| 345 | FBgn0053454-FBgn0003254 (2R) | 104.325818 |
| 346 | FBgn0041630-FBgn0035529 (3L) | 104.139031 |
| 347 | FBgn0037122-FBgn0037125 (3L) | 104.023445 |
| 348 | FBgn0030296-FBgn0030298 (X) | 103.937140 |
| 349 | FBgn0027066-FBgn0033101 (2R) | 103.879708 |
| 350 | FBgn0014869-FBgn0041186 (3R) | 103.203883 |
| 351 | FBgn0034726-FBgn0034728 (2R) | 103.062899 |
| 352 | FBgn0036643-FBgn0003741 (3L) | 102.613251 |
| 353 | FBgn0035746-FBgn0035751 (3L) | 102.553178 |
| 354 | FBgn0046692-FBgn0040005 (2R) | 102.255842 |
| 355 | FBgn0050499-FBgn0033194 (2R) | 102.018336 |
| 356 | FBgn0028500-FBgn0037156 (3L) | 101.606393 |
| 357 | FBgn0032485-FBgn0032488 (2L) | 101.555719 |
| 358 | FBgn0038829-FBgn0027493 (3R) | 101.508008 |
| 359 | FBgn0038861-FBgn0025803 (3R) | 101.436703 |
| 360 | FBgn0033275-FBgn0010109 (2R) | 101.089517 |
| 361 | FBgn0013765-FBgn0033842 (2R) | 101.084278 |
| 362 | FBgn0042104-FBgn0037517 (3R) | 100.810979 |
| 363 | FBgn0036846-FBgn0036848 (3L) | 100.763218 |
| 364 | FBgn0036964-FBgn0052226 (3L) | 100.752846 |
| 365 | FBgn0031635-FBgn0031640 (2L) | 100.652250 |
| 366 | FBgn0052670-FBgn0030257 (X) | 100.596828 |
| 367 | FBgn0034938-FBgn0034940 (2R) | 100.571217 |
| 368 | FBgn0030673-FBgn0030676 (X) | 100.553555 |
| 369 | FBgn0036290-FBgn0027936 (3L) | 99.671138 |
| 370 | FBgn0010282-FBgn0037443 (3R) | 99.396468 |
| 371 | FBgn0250906-FBgn0031453 (2L) | 99.380204 |
| 372 | FBgn0034087-FBgn0034091 (2R) | 99.224899 |
| 373 | FBgn0030456-FBgn0030457 (X) | 99.183314 |
| 374 | FBgn0025806-FBgn0034971 (2R) | 99.077059 |
| 375 | FBgn0005642-FBgn0039601 (3R) | 99.037393 |
| 376 | FBgn0025633-FBgn0026879 (X) | 98.916133 |
| 377 | FBgn0030219-FBgn0030224 (X) | 98.760944 |
| 378 | FBgn0030351-FBgn0030354 (X) | 98.576615 |
| 379 | FBgn0033088-FBgn0033089 (2R) | 98.539100 |
| 380 | FBgn0010406-FBgn0026263 (3L) | 98.455810 |
| 381 | FBgn0034986-FBgn0034989 (2R) | 98.392317 |
| 382 | FBgn0002570-FBgn0033294 (2R) | 98.277920 |
| 383 | FBgn0039186-FBgn0039188 (3R) | 98.221823 |
| 384 | FBgn0003659-FBgn0027342 (X) | 98.078358 |
| 385 | FBgn0031729-FBgn0054011 (2L) | 97.941007 |
| 386 | FBgn0036826-FBgn0036828 (3L) | 97.882851 |
| 387 | FBgn0032811-FBgn0000464 (2L) | 97.496574 |
| 388 | FBgn0000053-FBgn0022201 (2L) | 97.264017 |
| 389 | FBgn0039204-FBgn0039210 (3R) | 97.227544 |
| 390 | FBgn0041247-FBgn0031923 (2L) | 96.967388 |
| 391 | FBgn0026634-FBgn0038331 (3R) | 96.800300 |
| 392 | FBgn0035762-FBgn0035767 (3L) | 96.483088 |
| 393 | FBgn0053490-FBgn0052086 (3L) | 96.206972 |
| 394 | FBgn0039116-FBgn0053111 (3R) | 96.060466 |
| 395 | FBgn0052150-FBgn0036544 (3L) | 95.817815 |
| 396 | FBgn0034032-FBgn0013762 (2R) | 95.467092 |
| 397 | FBgn0003863-FBgn0010357 (2R) | 95.452057 |
| 398 | FBgn0033359-FBgn0033365 (2R) | 95.411438 |
| 399 | FBgn0011742-FBgn0025743 (X) | 95.302431 |
| 400 | FBgn0038316-FBgn0038321 (3R) | 95.183375 |
| 401 | FBgn0001139-FBgn0039405 (3R) | 95.169764 |
| 402 | FBgn0038260-FBgn0038268 (3R) | 95.001643 |
| 403 | FBgn0030829-FBgn0030832 (X) | 94.996562 |
| 404 | FBgn0039271-FBgn0039274 (3R) | 94.849100 |
| 405 | FBgn0035690-FBgn0035691 (3L) | 94.838915 |
| 406 | FBgn0036545-FBgn0036549 (3L) | 94.823751 |
| 407 | FBgn0035283-FBgn0035285 (3L) | 94.673661 |
| 408 | FBgn0041229-FBgn0045468 (3R) | 94.381955 |
| 409 | FBgn0037548-FBgn0037551 (3R) | 94.297631 |
| 410 | FBgn0038765-FBgn0038769 (3R) | 94.247973 |
| 411 | FBgn0036878-FBgn0036881 (3L) | 94.146327 |
| 412 | FBgn0025140-FBgn0038928 (3R) | 94.116192 |
| 413 | FBgn0030412-FBgn0011837 (X) | 94.068473 |
| 414 | FBgn0043884-FBgn0019952 (3R) | 94.004443 |
| 415 | FBgn0031520-FBgn0031523 (2L) | 93.935399 |
| 416 | FBgn0025387-FBgn0011277 (X) | 93.912247 |
| 417 | FBgn0032845-FBgn0032847 (2L) | 93.879848 |
| 418 | FBgn0030433-FBgn0030435 (X) | 93.851417 |
| 419 | FBgn0014269-FBgn0034410 (2R) | 93.724072 |
| 420 | FBgn0031195-FBgn0031194 (X) | 93.235621 |
| 421 | FBgn0033737-FBgn0033739 (2R) | 92.983084 |
| 422 | FBgn0250850-FBgn0034537 (2R) | 92.913120 |
| 423 | FBgn0011571-FBgn0030734 (X) | 92.798601 |
| 424 | FBgn0033382-FBgn0050344 (2R) | 92.757160 |
| 425 | FBgn0034399-FBgn0034401 (2R) | 92.464299 |
| 426 | FBgn0085424-FBgn0032442 (2L) | 92.266859 |
| 427 | FBgn0039764-FBgn0039767 (3R) | 92.101195 |
| 428 | FBgn0051021-FBgn0051016 (3R) | 92.029429 |
| 429 | FBgn0001961-FBgn0028509 (2L) | 91.962230 |
| 430 | FBgn0053213-FBgn0039558 (3R) | 91.913160 |
| 431 | FBgn0013812-FBgn0038850 (3R) | 91.866545 |
| 432 | FBgn0025335-FBgn0025352 (2R) | 91.824505 |
| 433 | FBgn0085205-FBgn0031581 (2L) | 91.750802 |
| 434 | FBgn0029587-FBgn0029588 (X) | 91.370574 |
| 435 | FBgn0029858-FBgn0043796 (X) | 91.233787 |
| 436 | FBgn0054032-FBgn0052117 (3L) | 91.035077 |
| 437 | FBgn0052237-FBgn0035581 (3L) | 91.001378 |
| 438 | FBgn0031006-FBgn0031008 (X) | 90.915953 |
| 439 | FBgn0035145-FBgn0035149 (3L) | 90.736813 |
| 440 | FBgn0031403-FBgn0051682 (2L) | 90.562422 |
| 441 | FBgn0030081-FBgn0052708 (X) | 90.457225 |
| 442 | FBgn0000259-FBgn0052662 (X) | 90.418110 |
| 443 | FBgn0039710-FBgn0039712 (3R) | 90.273830 |
| 444 | FBgn0035270-FBgn0035272 (3L) | 90.165381 |
| 445 | FBgn0036588-FBgn0040797 (3L) | 90.096760 |
| 446 | FBgn0040356-FBgn0040354 (X) | 90.088301 |
| 447 | FBgn0032110-FBgn0085395 (2L) | 90.041569 |
| 448 | FBgn0037344-FBgn0014366 (3R) | 89.484223 |
| 449 | FBgn0002865-FBgn0039577 (3R) | 89.271413 |
| 450 | FBgn0034527-FBgn0034529 (2R) | 88.894219 |
| 451 | FBgn0030086-FBgn0004856 (X) | 88.677864 |
| 452 | FBgn0021761-FBgn0250837 (2L) | 88.596741 |
| 453 | FBgn0051872-FBgn0032275 (2L) | 88.444109 |
| 454 | FBgn0030480-FBgn0030481 (X) | 87.955555 |
| 455 | FBgn0037067-FBgn0037070 (3L) | 87.951654 |
| 456 | FBgn0083980-FBgn0003411 (X) | 87.792378 |
| 457 | FBgn0037921-FBgn0037924 (3R) | 87.620197 |
| 458 | FBgn0031216-FBgn0026787 (2L) | 87.607403 |
| 459 | FBgn0039065-FBgn0039067 (3R) | 87.502272 |
| 460 | FBgn0033272-FBgn0021995 (2R) | 87.465442 |
| 461 | FBgn0036666-FBgn0036668 (3L) | 87.366618 |
| 462 | FBgn0034820-FBgn0041236 (2R) | 87.326806 |
| 463 | FBgn0044030-FBgn0031021 (X) | 87.162147 |
| 464 | FBgn0037195-FBgn0037197 (3L) | 87.088241 |
| 465 | FBgn0053980-FBgn0250904 (X) | 86.927596 |
| 466 | FBgn0030088-FBgn0030092 (X) | 86.844998 |
| 467 | FBgn0051343-FBgn0051198 (3R) | 86.691182 |
| 468 | FBgn0036757-FBgn0052189 (3L) | 86.660299 |
| 469 | FBgn0032016-FBgn0032020 (2L) | 86.520907 |
| 470 | FBgn0039282-FBgn0039286 (3R) | 86.512795 |
| 471 | FBgn0035063-FBgn0041205 (2R) | 86.320061 |
| 472 | FBgn0031746-FBgn0002855 (2L) | 86.224015 |
| 473 | FBgn0041625-FBgn0041623 (3L) | 85.913298 |
| 474 | FBgn0030014-FBgn0030017 (X) | 85.785507 |
| 475 | FBgn0035713-FBgn0035715 (3L) | 85.618466 |
| 476 | FBgn0040658-FBgn0005631 (2R) | 85.617704 |
| 477 | FBgn0013733-FBgn0013770 (2R) | 85.382977 |
| 478 | FBgn0036377-FBgn0043550 (3L) | 85.374409 |
| 479 | FBgn0029176-FBgn0062442 (3R) | 85.373110 |
| 480 | FBgn0037382-FBgn0037384 (3R) | 85.322521 |
| 481 | FBgn0034120-FBgn0053960 (2R) | 85.199015 |
| 482 | FBgn0036977-FBgn0036979 (3L) | 85.187819 |
| 483 | FBgn0034005-FBgn0034007 (2R) | 85.186603 |
| 484 | FBgn0052685-FBgn0030178 (X) | 85.001059 |
| 485 | FBgn0039838-FBgn0046332 (3R) | 84.981923 |
| 486 | FBgn0052643-FBgn0030441 (X) | 84.978799 |
| 487 | FBgn0013983-FBgn0034351 (2R) | 84.890565 |
| 488 | FBgn0052719-FBgn0029952 (X) | 84.839985 |
| 489 | FBgn0038569-FBgn0038576 (3R) | 84.496652 |
| 490 | FBgn0030552-FBgn0030554 (X) | 84.374972 |
| 491 | FBgn0038961-FBgn0038965 (3R) | 84.334242 |
| 492 | FBgn0038857-FBgn0038858 (3R) | 84.159093 |
| 493 | FBgn0028949-FBgn0028946 (2L) | 83.849595 |
| 494 | FBgn0014380-FBgn0003205 (3R) | 83.797429 |
| 495 | FBgn0020637-FBgn0020643 (3L) | 83.559428 |
| 496 | FBgn0036487-FBgn0036489 (3L) | 83.347884 |
| 497 | FBgn0031695-FBgn0016076 (2L) | 83.097742 |
| 498 | FBgn0037873-FBgn0086472 (3R) | 82.985884 |
| 499 | FBgn0025185-FBgn0033186 (2R) | 82.889125 |
| 500 | FBgn0035685-FBgn0035687 (3L) | 82.867775 |
| 501 | FBgn0034460-FBgn0034464 (2R) | 82.846642 |
| 502 | FBgn0033104-FBgn0028954 (2R) | 82.642940 |
| 503 | FBgn0053144-FBgn0033547 (2R) | 82.631473 |
| 504 | FBgn0004778-FBgn0004781 (3R) | 82.537065 |
| 505 | FBgn0033827-FBgn0033832 (2R) | 82.512468 |
| 506 | FBgn0032880-FBgn0011202 (2L) | 82.473197 |
| 507 | FBgn0036343-FBgn0036346 (3L) | 82.439551 |
| 508 | FBgn0020381-FBgn0025638 (X) | 82.368130 |
| 509 | FBgn0030018-FBgn0017566 (X) | 82.326953 |
| 510 | FBgn0031419-FBgn0031422 (2L) | 82.191480 |
| 511 | FBgn0038217-FBgn0038218 (3R) | 82.036416 |
| 512 | FBgn0052570-FBgn0052574 (X) | 81.984247 |
| 513 | FBgn0032246-FBgn0022268 (2L) | 81.896034 |
| 514 | FBgn0052686-FBgn0052681 (X) | 81.833518 |
| 515 | FBgn0051803-FBgn0032639 (2L) | 81.571685 |
| 516 | FBgn0063261-FBgn0015008 (3R) | 81.485167 |
| 517 | FBgn0024986-FBgn0024985 (X) | 81.421342 |
| 518 | FBgn0036159-FBgn0036162 (3L) | 81.358009 |
| 519 | FBgn0016047-FBgn0003382 (2R) | 81.227722 |
| 520 | FBgn0034774-FBgn0259145 (2R) | 81.203213 |
| 521 | FBgn0019830-FBgn0031459 (2L) | 81.122937 |
| 522 | FBgn0016075-FBgn0031673 (2L) | 80.765545 |
| 523 | FBgn0031837-FBgn0051635 (2L) | 80.563043 |
| 524 | FBgn0000241-FBgn0041234 (2R) | 80.517755 |
| 525 | FBgn0005659-FBgn0039552 (3R) | 80.306681 |
| 526 | FBgn0034617-FBgn0050394 (2R) | 80.255963 |
| 527 | FBgn0004373-FBgn0052343 (3L) | 80.201191 |
| 528 | FBgn0050184-FBgn0034882 (2R) | 80.131473 |
| 529 | FBgn0039348-FBgn0027508 (3R) | 79.924750 |
| 530 | FBgn0030314-FBgn0002948 (X) | 79.871258 |
| 531 | FBgn0035426-FBgn0035430 (3L) | 79.834401 |
| 532 | FBgn0033454-FBgn0027580 (2R) | 79.827237 |
| 533 | FBgn0051391-FBgn0037824 (3R) | 79.811807 |
| 534 | FBgn0031050-FBgn0031052 (X) | 79.801663 |
| 535 | FBgn0038716-FBgn0038719 (3R) | 79.503421 |
| 536 | FBgn0039311-FBgn0039313 (3R) | 79.338203 |
| 537 | FBgn0030034-FBgn0052711 (X) | 79.253043 |
| 538 | FBgn0083956-FBgn0052523 (X) | 79.170846 |
| 539 | FBgn0028837-FBgn0038989 (3R) | 79.101826 |
| 540 | FBgn0039994-FBgn0033000 (2R) | 78.886824 |
| 541 | FBgn0085406-FBgn0038942 (3R) | 78.791757 |
| 542 | FBgn0030891-FBgn0030892 (X) | 78.740268 |
| 543 | FBgn0036729-FBgn0036731 (3L) | 78.733999 |
| 544 | FBgn0015324-FBgn0250753 (3R) | 78.146564 |
| 545 | FBgn0035236-FBgn0035238 (3L) | 78.129384 |
| 546 | FBgn0086130-FBgn0031282 (2L) | 78.087159 |
| 547 | FBgn0005671-FBgn0038065 (3R) | 78.007431 |
| 548 | FBgn0035692-FBgn0024921 (3L) | 77.884810 |
| 549 | FBgn0031457-FBgn0026324 (2L) | 77.784681 |
| 550 | FBgn0053113-FBgn0031670 (2L) | 77.505476 |
| 551 | FBgn0001133-FBgn0034573 (2R) | 77.249070 |
| 552 | FBgn0041582-FBgn0015544 (2R) | 77.105913 |
| 553 | FBgn0034505-FBgn0042198 (2R) | 76.983610 |
| 554 | FBgn0034893-FBgn0034897 (2R) | 76.923717 |
| 555 | FBgn0038312-FBgn0027378 (3R) | 76.824235 |
| 556 | FBgn0036949-FBgn0036953 (3L) | 76.709053 |
| 557 | FBgn0038645-FBgn0038647 (3R) | 76.550728 |
| 558 | FBgn0034456-FBgn0034459 (2R) | 76.546140 |
| 559 | FBgn0051862-FBgn0032428 (2L) | 76.486775 |
| 560 | FBgn0036062-FBgn0036063 (3L) | 76.325625 |
| 561 | FBgn0034990-FBgn0034996 (2R) | 76.125047 |
| 562 | FBgn0030980-FBgn0030984 (X) | 75.988176 |
| 563 | FBgn0033650-FBgn0033651 (2R) | 75.713161 |
| 564 | FBgn0010602-FBgn0031260 (2L) | 75.626636 |
| 565 | FBgn0038110-FBgn0038111 (3R) | 75.535106 |
| 566 | FBgn0050360-FBgn0033297 (2R) | 75.452320 |
| 567 | FBgn0036147-FBgn0064766 (3L) | 75.351750 |
| 568 | FBgn0036537-FBgn0036538 (3L) | 75.210213 |
| 569 | FBgn0051709-FBgn0051882 (2L) | 75.121073 |
| 570 | FBgn0042112-FBgn0010350 (3L) | 75.104859 |
| 571 | FBgn0030532-FBgn0085375 (X) | 74.938825 |
| 572 | FBgn0014001-FBgn0037440 (3R) | 74.869671 |
| 573 | FBgn0026431-FBgn0032259 (2L) | 74.799932 |
| 574 | FBgn0005322-FBgn0032214 (2L) | 74.701029 |
| 575 | FBgn0085250-FBgn0000448 (2R) | 74.668015 |
| 576 | FBgn0040823-FBgn0052054 (3L) | 74.667159 |
| 577 | FBgn0031357-FBgn0031359 (2L) | 74.651794 |
| 578 | FBgn0038566-FBgn0038568 (3R) | 74.650194 |
| 579 | FBgn0030200-FBgn0030204 (X) | 74.649086 |
| 580 | FBgn0051232-FBgn0038619 (3R) | 74.568183 |
| 581 | FBgn0031900-FBgn0002938 (2L) | 74.530407 |
| 582 | FBgn0029811-FBgn0029817 (X) | 74.460202 |
| 583 | FBgn0023527-FBgn0023526 (X) | 74.430157 |
| 584 | FBgn0038122-FBgn0038127 (3R) | 74.373042 |
| 585 | FBgn0037239-FBgn0037241 (3R) | 74.008161 |
| 586 | FBgn0025800-FBgn0030007 (X) | 74.006537 |
| 587 | FBgn0035540-FBgn0035542 (3L) | 73.750809 |
| 588 | FBgn0037897-FBgn0037901 (3R) | 73.696374 |
| 589 | FBgn0051005-FBgn0039836 (3R) | 73.486062 |
| 590 | FBgn0031871-FBgn0020616 (2L) | 73.359995 |
| 591 | FBgn0052528-FBgn0031069 (X) | 73.308287 |
| 592 | FBgn0259149-FBgn0029809 (X) | 73.300553 |
| 593 | FBgn0001125-FBgn0031381 (2L) | 73.201663 |
| 594 | FBgn0031996-FBgn0031998 (2L) | 73.105456 |
| 595 | FBgn0031653-FBgn0020906 (2L) | 73.040487 |
| 596 | FBgn0037926-FBgn0037931 (3R) | 72.764681 |
| 597 | FBgn0039495-FBgn0039498 (3R) | 72.735368 |
| 598 | FBgn0067317-FBgn0051174 (3R) | 72.569287 |
| 599 | FBgn0036887-FBgn0036888 (3L) | 72.423872 |
| 600 | FBgn0001291-FBgn0033486 (2R) | 72.410025 |
| 601 | FBgn0031738-FBgn0031740 (2L) | 72.320279 |
| 602 | FBgn0037687-FBgn0037689 (3R) | 72.223277 |
| 603 | FBgn0032175-FBgn0053302 (2L) | 72.193768 |
| 604 | FBgn0003346-FBgn0003732 (2L) | 71.966823 |
| 605 | FBgn0020443-FBgn0040286 (2L) | 71.890068 |
| 606 | FBgn0052057-FBgn0052055 (3L) | 71.679840 |
| 607 | FBgn0052845-FBgn0085293 (3L) | 71.634979 |
| 608 | FBgn0250815-FBgn0035665 (3L) | 71.624668 |
| 609 | FBgn0037489-FBgn0037491 (3R) | 71.370569 |
| 610 | FBgn0032509-FBgn0051855 (2L) | 71.029308 |
| 611 | FBgn0015283-FBgn0037097 (3L) | 70.906536 |
| 612 | FBgn0003076-FBgn0011016 (3L) | 70.844192 |
| 613 | FBgn0039476-FBgn0039478 (3R) | 70.639613 |
| 614 | FBgn0034583-FBgn0034585 (2R) | 70.608511 |
| 615 | FBgn0054050-FBgn0013469 (3L) | 70.448311 |
| 616 | FBgn0030787-FBgn0030790 (X) | 70.407077 |
| 617 | FBgn0037074-FBgn0053288 (3L) | 70.387102 |
| 618 | FBgn0052024-FBgn0035933 (3L) | 70.131051 |
| 619 | FBgn0036859-FBgn0036860 (3L) | 70.114323 |
| 620 | FBgn0032178-FBgn0032184 (2L) | 70.102789 |
| 621 | FBgn0050195-FBgn0034755 (2R) | 69.935314 |
| 622 | FBgn0033605-FBgn0033609 (2R) | 69.889775 |
| 623 | FBgn0036500-FBgn0036502 (3L) | 69.859571 |
| 624 | FBgn0029664-FBgn0029666 (X) | 69.764562 |
| 625 | FBgn0020258-FBgn0028844 (2L) | 69.673593 |
| 626 | FBgn0030093-FBgn0030096 (X) | 69.611764 |
| 627 | FBgn0039265-FBgn0039266 (3R) | 69.397971 |
| 628 | FBgn0037478-FBgn0037482 (3R) | 69.393729 |
| 629 | FBgn0031118-FBgn0026323 (X) | 68.910707 |
| 630 | FBgn0026563-FBgn0037464 (3R) | 68.900613 |
| 631 | FBgn0086129-FBgn0034975 (2R) | 68.812428 |
| 632 | FBgn0038809-FBgn0014029 (3R) | 68.759790 |
| 633 | FBgn0033701-FBgn0033705 (2R) | 68.749841 |
| 634 | FBgn0039639-FBgn0039642 (3R) | 68.745812 |
| 635 | FBgn0004896-FBgn0034789 (2R) | 68.727622 |
| 636 | FBgn0031155-FBgn0003950 (X) | 68.683103 |
| 637 | FBgn0000308-FBgn0031759 (2L) | 68.429368 |
| 638 | FBgn0034646-FBgn0034650 (2R) | 68.343108 |
| 639 | FBgn0024244-FBgn0002985 (2L) | 68.225740 |
| 640 | FBgn0011722-FBgn0031817 (2L) | 67.964394 |
| 641 | FBgn0027054-FBgn0033247 (2R) | 67.918145 |
| 642 | FBgn0029946-FBgn0029949 (X) | 67.815936 |
| 643 | FBgn0031227-FBgn0053635 (2L) | 67.794089 |
| 644 | FBgn0030654-FBgn0030657 (X) | 67.680956 |
| 645 | FBgn0030918-FBgn0052547 (X) | 67.667932 |
| 646 | FBgn0016700-FBgn0038877 (3R) | 67.590306 |
| 647 | FBgn0052772-FBgn0029738 (X) | 67.515793 |
| 648 | FBgn0050389-FBgn0034599 (2R) | 67.450791 |
| 649 | FBgn0038510-FBgn0038512 (3R) | 67.404449 |
| 650 | FBgn0034201-FBgn0050458 (2R) | 67.391731 |
| 651 | FBgn0086408-FBgn0050274 (2R) | 67.331463 |
| 652 | FBgn0031498-FBgn0031500 (2L) | 67.324734 |
| 653 | FBgn0001105-FBgn0001092 (X) | 67.293269 |
| 654 | FBgn0037322-FBgn0037325 (3R) | 67.276841 |
| 655 | FBgn0034272-FBgn0034274 (2R) | 66.976295 |
| 656 | FBgn0042110-FBgn0037941 (3R) | 66.873075 |
| 657 | FBgn0031423-FBgn0085477 (2L) | 66.867067 |
| 658 | FBgn0031954-FBgn0031961 (2L) | 66.805618 |
| 659 | FBgn0039859-FBgn0039861 (3R) | 66.653108 |
| 660 | FBgn0087035-FBgn0036509 (3L) | 66.532677 |
| 661 | FBgn0028870-FBgn0028868 (2L) | 66.421066 |
| 662 | FBgn0032346-FBgn0032348 (2L) | 66.351333 |
| 663 | FBgn0026400-FBgn0039589 (3R) | 66.348104 |
| 664 | FBgn0011570-FBgn0031356 (2L) | 66.301538 |
| 665 | FBgn0038350-FBgn0053207 (3R) | 66.216726 |
| 666 | FBgn0034718-FBgn0013272 (2R) | 66.211062 |
| 667 | FBgn0035593-FBgn0035592 (3L) | 66.166259 |
| 668 | FBgn0085278-FBgn0036618 (3L) | 66.136125 |
| 669 | FBgn0038706-FBgn0038715 (3R) | 66.107145 |
| 670 | FBgn0037958-FBgn0037960 (3R) | 66.047114 |
| 671 | FBgn0033204-FBgn0033206 (2R) | 65.861620 |
| 672 | FBgn0031187-FBgn0031190 (X) | 65.689516 |
| 673 | FBgn0038037-FBgn0038039 (3R) | 65.683196 |
| 674 | FBgn0053958-FBgn0000658 (2R) | 65.681029 |
| 675 | FBgn0085188-FBgn0032191 (2L) | 65.677329 |
| 676 | FBgn0034475-FBgn0034478 (2R) | 65.398274 |
| 677 | FBgn0042092-FBgn0011737 (2L) | 65.378481 |
| 678 | FBgn0051797-FBgn0032769 (2L) | 65.217967 |
| 679 | FBgn0024360-FBgn0024364 (X) | 65.108583 |
| 680 | FBgn0037890-FBgn0037891 (3R) | 64.748974 |
| 681 | FBgn0085453-FBgn0034853 (2R) | 64.710642 |
| 682 | FBgn0037297-FBgn0037301 (3R) | 64.699485 |
| 683 | FBgn0086706-FBgn0035947 (3L) | 64.646769 |
| 684 | FBgn0037036-FBgn0037039 (3L) | 64.468133 |
| 685 | FBgn0032707-FBgn0045852 (2L) | 64.369493 |
| 686 | FBgn0014184-FBgn0010621 (2R) | 64.309577 |
| 687 | FBgn0033987-FBgn0033989 (2R) | 64.160010 |
| 688 | FBgn0051642-FBgn0053531 (2L) | 63.964476 |
| 689 | FBgn0041789-FBgn0032782 (2L) | 63.891910 |
| 690 | FBgn0031802-FBgn0043854 (2L) | 63.845149 |
| 691 | FBgn0029514-FBgn0035216 (3L) | 63.796149 |
| 692 | FBgn0030586-FBgn0030588 (X) | 63.793492 |
| 693 | FBgn0035727-FBgn0035730 (3L) | 63.665640 |
| 694 | FBgn0025701-FBgn0037916 (3R) | 63.584368 |
| 695 | FBgn0086134-FBgn0038049 (3R) | 63.147828 |
| 696 | FBgn0028738-FBgn0000157 (2R) | 63.117569 |
| 697 | FBgn0036684-FBgn0036686 (3L) | 63.083514 |
| 698 | FBgn0000709-FBgn0011710 (X) | 63.010896 |
| 699 | FBgn0037918-FBgn0037922 (3R) | 62.908576 |
| 700 | FBgn0034037-FBgn0053462 (2R) | 62.512908 |
| 701 | FBgn0036896-FBgn0022774 (3L) | 62.476796 |
| 702 | FBgn0031853-FBgn0031858 (2L) | 62.458357 |
| 703 | FBgn0035453-FBgn0052266 (3L) | 62.358398 |
| 704 | FBgn0024232-FBgn0024319 (2R) | 62.337646 |
| 705 | FBgn0035035-FBgn0259742 (2R) | 62.212150 |
| 706 | FBgn0024733-FBgn0000258 (3L) | 62.059466 |
| 707 | FBgn0051549-FBgn0037354 (3R) | 61.964646 |
| 708 | FBgn0038889-FBgn0038890 (3R) | 61.943997 |
| 709 | FBgn0029501-FBgn0035014 (2R) | 61.753482 |
| 710 | FBgn0021825-FBgn0033339 (2R) | 61.690628 |
| 711 | FBgn0053278-FBgn0035786 (3L) | 61.657131 |
| 712 | FBgn0031608-FBgn0031611 (2L) | 61.607279 |
| 713 | FBgn0027929-FBgn0028936 (2L) | 61.464750 |
| 714 | FBgn0038280-FBgn0038285 (3R) | 61.357851 |
| 715 | FBgn0036773-FBgn0002901 (3L) | 61.242962 |
| 716 | FBgn0034639-FBgn0034641 (2R) | 61.152723 |
| 717 | FBgn0041629-FBgn0030063 (X) | 60.992525 |
| 718 | FBgn0038886-FBgn0011278 (3R) | 60.929473 |
| 719 | FBgn0000439-FBgn0001077 (3R) | 60.845243 |
| 720 | FBgn0039344-FBgn0039347 (3R) | 60.833733 |
| 721 | FBgn0014127-FBgn0045064 (2L) | 60.758631 |
| 722 | FBgn0029942-FBgn0029944 (X) | 60.724142 |
| 723 | FBgn0031990-FBgn0031992 (2L) | 60.650608 |
| 724 | FBgn0023519-FBgn0052803 (X) | 60.623512 |
| 725 | FBgn0029819-FBgn0029820 (X) | 60.559568 |
| 726 | FBgn0035251-FBgn0035252 (3L) | 60.481115 |
| 727 | FBgn0002522-FBgn0004777 (3R) | 60.302545 |
| 728 | FBgn0035898-FBgn0035902 (3L) | 60.251029 |
| 729 | FBgn0030258-FBgn0030260 (X) | 60.203252 |
| 730 | FBgn0037305-FBgn0086689 (3R) | 60.139458 |
| 731 | FBgn0037397-FBgn0037399 (3R) | 60.133099 |
| 732 | FBgn0033413-FBgn0017558 (2R) | 60.117162 |
| 733 | FBgn0034033-FBgn0034035 (2R) | 60.051571 |
| 734 | FBgn0040984-FBgn0259151 (2L) | 59.919792 |
| 735 | FBgn0032889-FBgn0051673 (2L) | 59.901301 |
| 736 | FBgn0024998-FBgn0024973 (X) | 59.883304 |
| 737 | FBgn0036641-FBgn0250814 (3L) | 59.823392 |
| 738 | FBgn0030186-FBgn0085437 (X) | 59.724281 |
| 739 | FBgn0052088-FBgn0041231 (3L) | 59.661507 |
| 740 | FBgn0036871-FBgn0036874 (3L) | 59.591673 |
| 741 | FBgn0032163-FBgn0259713 (2L) | 59.590799 |
| 742 | FBgn0053013-FBgn0033720 (2R) | 59.580381 |
| 743 | FBgn0016660-FBgn0031707 (2L) | 59.552709 |
| 744 | FBgn0025827-FBgn0034165 (2R) | 59.373679 |
| 745 | FBgn0035799-FBgn0035802 (3L) | 59.312461 |
| 746 | FBgn0052413-FBgn0035648 (3L) | 59.120423 |
| 747 | FBgn0015828-FBgn0036194 (3L) | 59.064734 |
| 748 | FBgn0259209-FBgn0034974 (2R) | 59.011032 |
| 749 | FBgn0030949-FBgn0030952 (X) | 58.987099 |
| 750 | FBgn0259109-FBgn0030399 (X) | 58.938876 |
| 751 | FBgn0026090-FBgn0028969 (X) | 58.889588 |
| 752 | FBgn0033498-FBgn0033500 (2R) | 58.756917 |
| 753 | FBgn0039670-FBgn0051041 (3R) | 58.602418 |
| 754 | FBgn0250842-FBgn0050486 (2R) | 58.457250 |
| 755 | FBgn0032268-FBgn0032271 (2L) | 58.397048 |
| 756 | FBgn0030590-FBgn0030592 (X) | 58.387874 |
| 757 | FBgn0033543-FBgn0024956 (2R) | 58.345000 |
| 758 | FBgn0030990-FBgn0030991 (X) | 58.337039 |
| 759 | FBgn0038588-FBgn0053520 (3R) | 58.102892 |
| 760 | FBgn0026756-FBgn0040998 (2L) | 58.019223 |
| 761 | FBgn0037655-FBgn0040534 (3R) | 58.017551 |
| 762 | FBgn0037149-FBgn0037151 (3L) | 58.016028 |
| 763 | FBgn0052121-FBgn0036360 (3L) | 57.991016 |
| 764 | FBgn0035929-FBgn0035931 (3L) | 57.868004 |
| 765 | FBgn0032522-FBgn0026760 (2L) | 57.838532 |
| 766 | FBgn0039582-FBgn0051051 (3R) | 57.813768 |
| 767 | FBgn0037566-FBgn0037569 (3R) | 57.776752 |
| 768 | FBgn0040397-FBgn0085349 (X) | 57.760500 |
| 769 | FBgn0032897-FBgn0032899 (2L) | 57.732939 |
| 770 | FBgn0031011-FBgn0259834 (X) | 57.712768 |
| 771 | FBgn0034730-FBgn0034732 (2R) | 57.637798 |
| 772 | FBgn0029718-FBgn0025615 (X) | 57.601609 |
| 773 | FBgn0011656-FBgn0033467 (2R) | 57.547963 |
| 774 | FBgn0002780-FBgn0002645 (3R) | 57.479058 |
| 775 | FBgn0086676-FBgn0001124 (2R) | 57.433713 |
| 776 | FBgn0050286-FBgn0053225 (2R) | 57.423703 |
| 777 | FBgn0085228-FBgn0034499 (2R) | 57.292763 |
| 778 | FBgn0037570-FBgn0037572 (3R) | 57.280858 |
| 779 | FBgn0011694-FBgn0004181 (2R) | 57.207244 |
| 780 | FBgn0034129-FBgn0053458 (2R) | 57.017851 |
| 781 | FBgn0030528-FBgn0030530 (X) | 56.996607 |
| 782 | FBgn0032000-FBgn0032001 (2L) | 56.969986 |
| 783 | FBgn0023545-FBgn0000667 (X) | 56.967919 |
| 784 | FBgn0030465-FBgn0085388 (X) | 56.865032 |
| 785 | FBgn0044511-FBgn0038145 (3R) | 56.785213 |
| 786 | FBgn0031410-FBgn0051949 (2L) | 56.667551 |
| 787 | FBgn0030077-FBgn0030080 (X) | 56.592976 |
| 788 | FBgn0038269-FBgn0067629 (3R) | 56.588894 |
| 789 | FBgn0038462-FBgn0038463 (3R) | 56.557134 |
| 790 | FBgn0033490-FBgn0033491 (2R) | 56.415050 |
| 791 | FBgn0035210-FBgn0035213 (3L) | 56.347396 |
| 792 | FBgn0036134-FBgn0036135 (3L) | 55.952232 |
| 793 | FBgn0085426-FBgn0050393 (2R) | 55.897609 |
| 794 | FBgn0030364-FBgn0030366 (X) | 55.810173 |
| 795 | FBgn0039386-FBgn0051089 (3R) | 55.783439 |
| 796 | FBgn0034361-FBgn0034362 (2R) | 55.626449 |
| 797 | FBgn0028540-FBgn0028539 (2L) | 55.624953 |
| 798 | FBgn0031047-FBgn0031049 (X) | 55.609222 |
| 799 | FBgn0034245-FBgn0016701 (2R) | 55.605713 |
| 800 | FBgn0050272-FBgn0034785 (2R) | 55.461558 |
| 801 | FBgn0030322-FBgn0030323 (X) | 55.430722 |
| 802 | FBgn0039135-FBgn0039136 (3R) | 55.382616 |
| 803 | FBgn0031248-FBgn0053127 (2L) | 55.344044 |
| 804 | FBgn0040250-FBgn0037870 (3R) | 55.324576 |
| 805 | FBgn0033726-FBgn0050048 (2R) | 55.306478 |
| 806 | FBgn0083986-FBgn0045980 (3R) | 55.267158 |
| 807 | FBgn0053333-FBgn0038511 (3R) | 55.062780 |
| 808 | FBgn0035545-FBgn0035547 (3L) | 55.047293 |
| 809 | FBgn0031436-FBgn0026479 (2L) | 55.043071 |
| 810 | FBgn0020304-FBgn0020545 (2L) | 54.897290 |
| 811 | FBgn0037852-FBgn0037855 (3R) | 54.866561 |
| 812 | FBgn0029685-FBgn0029689 (X) | 54.819818 |
| 813 | FBgn0034295-FBgn0034296 (2R) | 54.801769 |
| 814 | FBgn0036605-FBgn0036606 (3L) | 54.768862 |
| 815 | FBgn0038742-FBgn0014006 (3R) | 54.764316 |
| 816 | FBgn0038402-FBgn0038405 (3R) | 54.653451 |
| 817 | FBgn0032704-FBgn0025608 (2L) | 54.536637 |
| 818 | FBgn0035666-FBgn0035667 (3L) | 54.497514 |
| 819 | FBgn0019982-FBgn0053002 (2L) | 54.374303 |
| 820 | FBgn0029931-FBgn0029930 (X) | 54.277313 |
| 821 | FBgn0034096-FBgn0025519 (2R) | 54.256025 |
| 822 | FBgn0031082-FBgn0031086 (X) | 54.243986 |
| 823 | FBgn0085434-FBgn0035078 (2R) | 54.219110 |
| 824 | FBgn0035477-FBgn0035479 (3L) | 54.178441 |
| 825 | FBgn0016977-FBgn0031231 (2L) | 54.174313 |
| 826 | FBgn0052486-FBgn0035397 (3L) | 54.052848 |
| 827 | FBgn0067864-FBgn0025820 (3L) | 54.039678 |
| 828 | FBgn0037224-FBgn0041621 (3R) | 53.971273 |
| 829 | FBgn0039395-FBgn0039398 (3R) | 53.866223 |
| 830 | FBgn0022343-FBgn0035082 (2R) | 53.813198 |
| 831 | FBgn0030893-FBgn0022724 (X) | 53.801373 |
| 832 | FBgn0004914-FBgn0003209 (2L) | 53.772628 |
| 833 | FBgn0029849-FBgn0003514 (X) | 53.640673 |
| 834 | FBgn0015014-FBgn0037657 (3R) | 53.621160 |
| 835 | FBgn0031772-FBgn0031774 (2L) | 53.601822 |
| 836 | FBgn0038996-FBgn0020278 (3R) | 53.536855 |
| 837 | FBgn0033208-FBgn0033209 (2R) | 53.462773 |
| 838 | FBgn0052448-FBgn0046301 (3L) | 53.403621 |
| 839 | FBgn0038683-FBgn0259704 (3R) | 53.318257 |
| 840 | FBgn0039027-FBgn0039029 (3R) | 53.248120 |
| 841 | FBgn0027588-FBgn0024807 (X) | 53.173179 |
| 842 | FBgn0050395-FBgn0034661 (2R) | 52.850795 |
| 843 | FBgn0035970-FBgn0035978 (3L) | 52.800481 |
| 844 | FBgn0030959-FBgn0030960 (X) | 52.796208 |
| 845 | FBgn0085444-FBgn0034172 (2R) | 52.728633 |
| 846 | FBgn0023091-FBgn0032945 (2L) | 52.497881 |
| 847 | FBgn0033572-FBgn0033579 (2R) | 52.484632 |
| 848 | FBgn0053233-FBgn0035366 (3L) | 52.481610 |
| 849 | FBgn0053051-FBgn0052176 (3L) | 52.338347 |
| 850 | FBgn0039153-FBgn0027103 (3R) | 52.112813 |
| 851 | FBgn0243516-FBgn0034697 (2R) | 51.731974 |
| 852 | FBgn0034366-FBgn0034367 (2R) | 51.645885 |
| 853 | FBgn0034854-FBgn0040660 (2R) | 51.604654 |
| 854 | FBgn0053969-FBgn0028978 (3L) | 51.517595 |
| 855 | FBgn0039252-FBgn0020626 (3R) | 51.425416 |
| 856 | FBgn0037120-FBgn0037121 (3L) | 51.350384 |
| 857 | FBgn0030502-FBgn0030504 (X) | 51.221802 |
| 858 | FBgn0032039-FBgn0001084 (2L) | 51.207730 |
| 859 | FBgn0040972-FBgn0032473 (2L) | 51.152617 |
| 860 | FBgn0016693-FBgn0038080 (3R) | 51.148207 |
| 861 | FBgn0037081-FBgn0037082 (3L) | 51.063263 |
| 862 | FBgn0030876-FBgn0030878 (X) | 51.019789 |
| 863 | FBgn0038978-FBgn0038979 (3R) | 51.012132 |
| 864 | FBgn0035415-FBgn0035416 (3L) | 50.905817 |
| 865 | FBgn0053123-FBgn0031544 (2L) | 50.807811 |
| 866 | FBgn0036335-FBgn0036337 (3L) | 50.787042 |
| 867 | FBgn0259139-FBgn0037955 (3R) | 50.778381 |
| 868 | FBgn0026573-FBgn0050037 (2R) | 50.496505 |
| 869 | FBgn0052380-FBgn0016983 (3L) | 50.439705 |
| 870 | FBgn0036396-FBgn0036402 (3L) | 50.347577 |
| 871 | FBgn0024238-FBgn0030838 (X) | 50.265170 |
| 872 | FBgn0004908-FBgn0037578 (3R) | 50.263095 |
| 873 | FBgn0033074-FBgn0033076 (2R) | 50.218267 |
| 874 | FBgn0036527-FBgn0036528 (3L) | 50.188081 |
| 875 | FBgn0031887-FBgn0085407 (2L) | 50.156640 |
| 876 | FBgn0030914-FBgn0030915 (X) | 50.147830 |
| 877 | FBgn0038274-FBgn0038277 (3R) | 50.147323 |
| 878 | FBgn0038139-FBgn0045442 (3R) | 50.136918 |
| 879 | FBgn0038500-FBgn0038499 (3R) | 50.123417 |
| 880 | FBgn0034878-FBgn0034879 (2R) | 50.120619 |
| 881 | FBgn0034379-FBgn0034380 (2R) | 50.101698 |
| 882 | FBgn0035011-FBgn0035012 (2R) | 50.088792 |
| 883 | FBgn0038234-FBgn0038235 (3R) | 50.028327 |
| 884 | FBgn0086707-FBgn0032679 (2L) | 49.961019 |
| 885 | FBgn0051390-FBgn0010421 (3R) | 49.899637 |
| 886 | FBgn0030855-FBgn0030858 (X) | 49.799933 |
| 887 | FBgn0039479-FBgn0039481 (3R) | 49.787660 |
| 888 | FBgn0052029-FBgn0035922 (3L) | 49.742169 |
| 889 | FBgn0028896-FBgn0001992 (2L) | 49.685584 |
| 890 | FBgn0020240-FBgn0031985 (2L) | 49.599112 |
| 891 | FBgn0259237-FBgn0038903 (3R) | 49.505112 |
| 892 | FBgn0259703-FBgn0085288 (3L) | 49.472354 |
| 893 | FBgn0035481-FBgn0004880 (3L) | 49.386436 |
| 894 | FBgn0038395-FBgn0038396 (3R) | 49.346710 |
| 895 | FBgn0035152-FBgn0035153 (3L) | 49.325172 |
| 896 | FBgn0250840-FBgn0033512 (2R) | 49.306294 |
| 897 | FBgn0085394-FBgn0050470 (2R) | 49.281037 |
| 898 | FBgn0034179-FBgn0034181 (2R) | 49.250513 |
| 899 | FBgn0037087-FBgn0015239 (3L) | 49.247791 |
| 900 | FBgn0031078-FBgn0052529 (X) | 49.150325 |
| 901 | FBgn0028688-FBgn0038972 (3R) | 49.109135 |
| 902 | FBgn0052106-FBgn0036311 (3L) | 49.077334 |
| 903 | FBgn0037530-FBgn0014930 (3R) | 49.031098 |
| 904 | FBgn0050457-FBgn0034204 (2R) | 49.020099 |
| 905 | FBgn0037026-FBgn0037028 (3L) | 48.931690 |
| 906 | FBgn0037857-FBgn0037860 (3R) | 48.900507 |
| 907 | FBgn0002783-FBgn0022787 (3R) | 48.762321 |
| 908 | FBgn0003116-FBgn0040899 (X) | 48.749340 |
| 909 | FBgn0034291-FBgn0034294 (2R) | 48.748375 |
| 910 | FBgn0036747-FBgn0052182 (3L) | 48.696526 |
| 911 | FBgn0031591-FBgn0010473 (2L) | 48.679248 |
| 912 | FBgn0042135-FBgn0050497 (2R) | 48.673992 |
| 913 | FBgn0032170-FBgn0032172 (2L) | 48.662784 |
| 914 | FBgn0015781-FBgn0038675 (3R) | 48.604638 |
| 915 | FBgn0037563-FBgn0051463 (3R) | 48.575917 |
| 916 | FBgn0034070-FBgn0034073 (2R) | 48.500898 |
| 917 | FBgn0029095-FBgn0053526 (2L) | 48.486599 |
| 918 | FBgn0032731-FBgn0032732 (2L) | 48.436834 |
| 919 | FBgn0027496-FBgn0031822 (2L) | 48.424094 |
| 920 | FBgn0035136-FBgn0035137 (3L) | 48.420309 |
| 921 | FBgn0015924-FBgn0031256 (2L) | 48.354238 |
| 922 | FBgn0052103-FBgn0020655 (3L) | 48.207035 |
| 923 | FBgn0037493-FBgn0259172 (3R) | 48.200278 |
| 924 | FBgn0259721-FBgn0038183 (3R) | 48.154476 |
| 925 | FBgn0037612-FBgn0037615 (3R) | 48.123125 |
| 926 | FBgn0026597-FBgn0039740 (3R) | 47.996994 |
| 927 | FBgn0037718-FBgn0037720 (3R) | 47.994493 |
| 928 | FBgn0026741-FBgn0026619 (2R) | 47.950502 |
| 929 | FBgn0052228-FBgn0052227 (3L) | 47.915970 |
| 930 | FBgn0030721-FBgn0030722 (X) | 47.803982 |
| 931 | FBgn0031596-FBgn0031597 (2L) | 47.752927 |
| 932 | FBgn0040718-FBgn0040717 (2L) | 47.726866 |
| 933 | FBgn0039698-FBgn0039702 (3R) | 47.601494 |
| 934 | FBgn0032720-FBgn0032721 (2L) | 47.546216 |
| 935 | FBgn0051688-FBgn0032863 (2L) | 47.454250 |
| 936 | FBgn0259245-FBgn0031181 (X) | 47.371540 |
| 937 | FBgn0035788-FBgn0035790 (3L) | 47.369310 |
| 938 | FBgn0051675-FBgn0032904 (2L) | 47.363103 |
| 939 | FBgn0037536-FBgn0037537 (3R) | 47.264503 |
| 940 | FBgn0259211-FBgn0003701 (2R) | 47.190577 |
| 941 | FBgn0259832-FBgn0033656 (2R) | 47.166242 |
| 942 | FBgn0027053-FBgn0004087 (3R) | 47.088318 |
| 943 | FBgn0030925-FBgn0030927 (X) | 47.015838 |
| 944 | FBgn0038643-FBgn0085311 (3R) | 47.013022 |
| 945 | FBgn0035722-FBgn0029117 (3L) | 46.964069 |
| 946 | FBgn0000520-FBgn0040384 (X) | 46.955859 |
| 947 | FBgn0032393-FBgn0032394 (2L) | 46.915820 |
| 948 | FBgn0031951-FBgn0027515 (2L) | 46.867376 |
| 949 | FBgn0024740-FBgn0032291 (2L) | 46.842696 |
| 950 | FBgn0039487-FBgn0039489 (3R) | 46.814920 |
| 951 | FBgn0033475-FBgn0033477 (2R) | 46.795551 |
| 952 | FBgn0032066-FBgn0032069 (2L) | 46.723820 |
| 953 | FBgn0030518-FBgn0030520 (X) | 46.682643 |
| 954 | FBgn0051224-FBgn0044871 (3R) | 46.616273 |
| 955 | FBgn0032225-FBgn0032228 (2L) | 46.573592 |
| 956 | FBgn0010909-FBgn0016794 (3L) | 46.385199 |
| 957 | FBgn0050380-FBgn0033238 (2R) | 46.311490 |
| 958 | FBgn0040370-FBgn0000137 (X) | 46.177215 |
| 959 | FBgn0029704-FBgn0040907 (X) | 46.149586 |
| 960 | FBgn0062412-FBgn0037576 (3R) | 46.089632 |
| 961 | FBgn0039730-FBgn0039733 (3R) | 45.943805 |
| 962 | FBgn0038300-FBgn0038301 (3R) | 45.864378 |
| 963 | FBgn0004597-FBgn0038272 (3R) | 45.848932 |
| 964 | FBgn0030640-FBgn0030643 (X) | 45.835696 |
| 965 | FBgn0011741-FBgn0030679 (X) | 45.798574 |
| 966 | FBgn0036804-FBgn0036805 (3L) | 45.775753 |
| 967 | FBgn0036936-FBgn0036938 (3L) | 45.750824 |
| 968 | FBgn0037792-FBgn0037794 (3R) | 45.644216 |
| 969 | FBgn0004369-FBgn0039664 (3R) | 45.564009 |
| 970 | FBgn0036735-FBgn0259174 (3L) | 45.519459 |
| 971 | FBgn0053054-FBgn0053056 (3L) | 45.504056 |
| 972 | FBgn0037669-FBgn0037670 (3R) | 45.479170 |
| 973 | FBgn0032868-FBgn0051680 (2L) | 45.432709 |
| 974 | FBgn0031529-FBgn0016698 (2L) | 45.402018 |
| 975 | FBgn0036959-FBgn0036958 (3L) | 45.374325 |
| 976 | FBgn0039966-FBgn0039965 (X) | 45.350261 |
| 977 | FBgn0035233-FBgn0035232 (3L) | 45.308909 |
| 978 | FBgn0003378-FBgn0003377 (3L) | 45.239658 |
| 979 | FBgn0051450-FBgn0051460 (3R) | 45.214361 |
| 980 | FBgn0051120-FBgn0039249 (3R) | 45.206222 |
| 981 | FBgn0050491-FBgn0033199 (2R) | 45.162722 |
| 982 | FBgn0039851-FBgn0039852 (3R) | 45.160414 |
| 983 | FBgn0052181-FBgn0043025 (3L) | 45.087769 |
| 984 | FBgn0036474-FBgn0086690 (3L) | 45.080531 |
| 985 | FBgn0038414-FBgn0038416 (3R) | 45.074202 |
| 986 | FBgn0036889-FBgn0010417 (3L) | 45.064800 |
| 987 | FBgn0047338-FBgn0025776 (3L) | 45.026132 |
| 988 | FBgn0026190-FBgn0051371 (3R) | 44.998884 |
| 989 | FBgn0031708-FBgn0000318 (2L) | 44.968369 |
| 990 | FBgn0028746-FBgn0040396 (X) | 44.959665 |
| 991 | FBgn0000529-FBgn0032821 (2L) | 44.897260 |
| 992 | FBgn0036614-FBgn0036615 (3L) | 44.808292 |
| 993 | FBgn0039003-FBgn0039004 (3R) | 44.789731 |
| 994 | FBgn0036564-FBgn0036569 (3L) | 44.758617 |
| 995 | FBgn0024329-FBgn0038652 (3R) | 44.666355 |
| 996 | FBgn0036341-FBgn0036342 (3L) | 44.665447 |
| 997 | FBgn0259697-FBgn0040045 (3L) | 44.635363 |
| 998 | FBgn0085234-FBgn0029084 (2R) | 44.604253 |
| 999 | FBgn0038755-FBgn0038761 (3R) | 44.541492 |
| 1000 | FBgn0030694-FBgn0003345 (X) | 44.534228 |
| 1001 | FBgn0035591-FBgn0035590 (3L) | 44.497685 |
| 1002 | FBgn0054007-FBgn0032867 (2L) | 44.491721 |
| 1003 | FBgn0030646-FBgn0030648 (X) | 44.471552 |
| 1004 | FBgn0029892-FBgn0029894 (X) | 44.397328 |
| 1005 | FBgn0050185-FBgn0034876 (2R) | 44.321804 |
| 1006 | FBgn0037538-FBgn0037539 (3R) | 44.309147 |
| 1007 | FBgn0036496-FBgn0036498 (3L) | 44.143694 |
| 1008 | FBgn0038485-FBgn0038487 (3R) | 44.131378 |
| 1009 | FBgn0035915-FBgn0052351 (3L) | 44.130210 |
| 1010 | FBgn0030286-FBgn0028421 (X) | 44.121373 |
| 1011 | FBgn0030163-FBgn0030164 (X) | 44.088312 |
| 1012 | FBgn0035449-FBgn0052267 (3L) | 44.067667 |
| 1013 | FBgn0030542-FBgn0030544 (X) | 44.060242 |
| 1014 | FBgn0039747-FBgn0039749 (3R) | 44.021589 |
| 1015 | FBgn0012034-FBgn0027945 (3L) | 43.977968 |
| 1016 | FBgn0038142-FBgn0259831 (3R) | 43.958618 |
| 1017 | FBgn0086897-FBgn0015778 (3R) | 43.928164 |
| 1018 | FBgn0032420-FBgn0032422 (2L) | 43.920188 |
| 1019 | FBgn0051418-FBgn0051419 (3R) | 43.869866 |
| 1020 | FBgn0037215-FBgn0037218 (3R) | 43.862182 |
| 1021 | FBgn0041102-FBgn0001281 (3R) | 43.789226 |
| 1022 | FBgn0052243-FBgn0035558 (3L) | 43.729419 |
| 1023 | FBgn0033677-FBgn0010339 (2R) | 43.725444 |
| 1024 | FBgn0038241-FBgn0038242 (3R) | 43.700960 |
| 1025 | FBgn0050060-FBgn0050065 (2R) | 43.670218 |
| 1026 | FBgn0052383-FBgn0052382 (3L) | 43.624768 |
| 1027 | FBgn0026174-FBgn0031074 (X) | 43.597763 |
| 1028 | FBgn0003870-FBgn0039868 (3R) | 43.596029 |
| 1029 | FBgn0051164-FBgn0051424 (3R) | 43.549108 |
| 1030 | FBgn0033818-FBgn0033819 (2R) | 43.512768 |
| 1031 | FBgn0030874-FBgn0052557 (X) | 43.485301 |
| 1032 | FBgn0030247-FBgn0030249 (X) | 43.428494 |
| 1033 | FBgn0037812-FBgn0051406 (3R) | 43.379867 |
| 1034 | FBgn0019925-FBgn0051301 (3R) | 43.346991 |
| 1035 | FBgn0052442-FBgn0029152 (3L) | 43.342680 |
| 1036 | FBgn0030524-FBgn0030525 (X) | 43.310105 |
| 1037 | FBgn0037665-FBgn0037666 (3R) | 43.244476 |
| 1038 | FBgn0031031-FBgn0031032 (X) | 43.224362 |
| 1039 | FBgn0050055-FBgn0022764 (2R) | 43.186326 |
| 1040 | FBgn0037814-FBgn0037815 (3R) | 43.089209 |
| 1041 | FBgn0038805-FBgn0038808 (3R) | 43.086660 |
| 1042 | FBgn0051206-FBgn0045471 (3R) | 43.065872 |
| 1043 | FBgn0034911-FBgn0003401 (2R) | 42.982151 |
| 1044 | FBgn0033734-FBgn0033735 (2R) | 42.911866 |
| 1045 | FBgn0027610-FBgn0086371 (3R) | 42.888359 |
| 1046 | FBgn0033924-FBgn0033925 (2R) | 42.861401 |
| 1047 | FBgn0030577-FBgn0030578 (X) | 42.847725 |
| 1048 | FBgn0052301-FBgn0052305 (3L) | 42.836030 |
| 1049 | FBgn0029508-FBgn0050159 (2R) | 42.798271 |
| 1050 | FBgn0035674-FBgn0035675 (3L) | 42.796894 |
| 1051 | FBgn0039623-FBgn0039627 (3R) | 42.731017 |
| 1052 | FBgn0039149-FBgn0039150 (3R) | 42.718353 |
| 1053 | FBgn0020255-FBgn0030276 (X) | 42.716382 |
| 1054 | FBgn0045483-FBgn0045482 (2R) | 42.675571 |
| 1055 | FBgn0036108-FBgn0036109 (3L) | 42.646495 |
| 1056 | FBgn0019990-FBgn0039846 (3R) | 42.565511 |
| 1057 | FBgn0025720-FBgn0034491 (2R) | 42.549496 |
| 1058 | FBgn0032632-FBgn0051804 (2L) | 42.500503 |
| 1059 | FBgn0033893-FBgn0259184 (2R) | 42.488196 |
| 1060 | FBgn0038195-FBgn0038196 (3R) | 42.477846 |
| 1061 | FBgn0026196-FBgn0004838 (2L) | 42.457462 |
| 1062 | FBgn0033138-FBgn0033139 (2R) | 42.339003 |
| 1063 | FBgn0051698-FBgn0031512 (2L) | 42.329572 |
| 1064 | FBgn0029925-FBgn0027108 (X) | 42.312923 |
| 1065 | FBgn0034503-FBgn0034504 (2R) | 42.311290 |
| 1066 | FBgn0034299-FBgn0034300 (2R) | 42.299500 |
| 1067 | FBgn0067861-FBgn0052823 (X) | 42.299026 |
| 1068 | FBgn0031756-FBgn0031758 (2L) | 42.281762 |
| 1069 | FBgn0050467-FBgn0034030 (2R) | 42.274307 |
| 1070 | FBgn0037283-FBgn0020280 (3R) | 42.235929 |
| 1071 | FBgn0025571-FBgn0038519 (3R) | 42.184434 |
| 1072 | FBgn0016926-FBgn0031304 (2L) | 42.182644 |
| 1073 | FBgn0036044-FBgn0044051 (3L) | 42.181341 |
| 1074 | FBgn0027376-FBgn0039331 (3R) | 42.160273 |
| 1075 | FBgn0038449-FBgn0038450 (3R) | 42.139446 |
| 1076 | FBgn0033594-FBgn0085254 (2R) | 42.110497 |
| 1077 | FBgn0039768-FBgn0039769 (3R) | 42.108396 |
| 1078 | FBgn0036274-FBgn0052105 (3L) | 41.997684 |
| 1079 | FBgn0030410-FBgn0030411 (X) | 41.986478 |
| 1080 | FBgn0001269-FBgn0000577 (2R) | 41.885629 |
| 1081 | FBgn0001323-FBgn0037015 (3L) | 41.871163 |
| 1082 | FBgn0032634-FBgn0032635 (2L) | 41.865041 |
| 1083 | FBgn0022960-FBgn0033122 (2R) | 41.841517 |
| 1084 | FBgn0038388-FBgn0052856 (3R) | 41.806247 |
| 1085 | FBgn0030141-FBgn0030142 (X) | 41.780384 |
| 1086 | FBgn0031284-FBgn0015621 (2L) | 41.778978 |
| 1087 | FBgn0038763-FBgn0243511 (3R) | 41.769435 |
| 1088 | FBgn0031023-FBgn0052532 (X) | 41.755637 |
| 1089 | FBgn0036117-FBgn0052069 (3L) | 41.719081 |
| 1090 | FBgn0038479-FBgn0051269 (3R) | 41.715189 |
| 1091 | FBgn0030161-FBgn0030162 (X) | 41.662002 |
| 1092 | FBgn0025683-FBgn0001142 (2L) | 41.654353 |
| 1093 | FBgn0011743-FBgn0034157 (2R) | 41.651259 |
| 1094 | FBgn0050000-FBgn0050005 (2R) | 41.589501 |
| 1095 | FBgn0030706-FBgn0010240 (X) | 41.585817 |
| 1096 | FBgn0032229-FBgn0027568 (2L) | 41.575327 |
| 1097 | FBgn0050126-FBgn0034413 (2R) | 41.565762 |
| 1098 | FBgn0038838-FBgn0044809 (3R) | 41.507209 |
| 1099 | FBgn0039086-FBgn0039092 (3R) | 41.486843 |
| 1100 | FBgn0053998-FBgn0050121 (2R) | 41.458077 |
| 1101 | FBgn0038100-FBgn0051342 (3R) | 41.425788 |
| 1102 | FBgn0035217-FBgn0035218 (3L) | 41.383164 |
| 1103 | FBgn0052391-FBgn0054030 (3L) | 41.359060 |
| 1104 | FBgn0033096-FBgn0033097 (2R) | 41.343473 |
| 1105 | FBgn0031060-FBgn0031062 (X) | 41.329860 |
| 1106 | FBgn0015323-FBgn0000303 (3R) | 41.300190 |
| 1107 | FBgn0038397-FBgn0052855 (3R) | 41.288235 |
| 1108 | FBgn0039007-FBgn0039010 (3R) | 41.264936 |
| 1109 | FBgn0051526-FBgn0052945 (3R) | 41.262365 |
| 1110 | FBgn0037994-FBgn0037995 (3R) | 41.208519 |
| 1111 | FBgn0024191-FBgn0031736 (2L) | 41.205752 |
| 1112 | FBgn0030358-FBgn0005563 (X) | 41.173149 |
| 1113 | FBgn0032358-FBgn0020270 (2L) | 41.163728 |
| 1114 | FBgn0035554-FBgn0035555 (3L) | 41.139371 |
| 1115 | FBgn0040322-FBgn0040323 (3L) | 41.111695 |
| 1116 | FBgn0022786-FBgn0029957 (X) | 41.104090 |
| 1117 | FBgn0040344-FBgn0040339 (X) | 41.002930 |
| 1118 | FBgn0029079-FBgn0050105 (2R) | 40.946092 |
| 1119 | FBgn0053191-FBgn0053189 (3R) | 40.913275 |
| 1120 | FBgn0004832-FBgn0000221 (X) | 40.863478 |
| 1121 | FBgn0039358-FBgn0039359 (3R) | 40.853367 |
| 1122 | FBgn0053465-FBgn0003130 (2R) | 40.842545 |
| 1123 | FBgn0029525-FBgn0023536 (X) | 40.829160 |
| 1124 | FBgn0032057-FBgn0032058 (2L) | 40.771319 |
| 1125 | FBgn0052428-FBgn0036999 (3L) | 40.710455 |
| 1126 | FBgn0054045-FBgn0050127 (2R) | 40.623211 |
| 1127 | FBgn0033848-FBgn0033851 (2R) | 40.621682 |
| 1128 | FBgn0037003-FBgn0037005 (3L) | 40.611008 |
| 1129 | FBgn0039872-FBgn0039873 (3R) | 40.608069 |
| 1130 | FBgn0010053-FBgn0034405 (2R) | 40.586355 |
| 1131 | FBgn0036107-FBgn0027615 (3L) | 40.500852 |
| 1132 | FBgn0038787-FBgn0038788 (3R) | 40.470828 |
| 1133 | FBgn0034205-FBgn0034206 (2R) | 40.450619 |
| 1134 | FBgn0040890-FBgn0027621 (X) | 40.444036 |
| 1135 | FBgn0026577-FBgn0041244 (2L) | 40.411722 |
| 1136 | FBgn0052649-FBgn0030431 (X) | 40.270200 |
| 1137 | FBgn0036578-FBgn0036581 (3L) | 40.262554 |
| 1138 | FBgn0032503-FBgn0032504 (2L) | 40.239200 |
| 1139 | FBgn0250910-FBgn0038063 (3R) | 40.178237 |
| 1140 | FBgn0036842-FBgn0036843 (3L) | 40.174518 |
| 1141 | FBgn0051624-FBgn0051988 (2L) | 40.162527 |
| 1142 | FBgn0033915-FBgn0033916 (2R) | 40.145291 |
| 1143 | FBgn0011509-FBgn0052022 (3L) | 40.139612 |
| 1144 | FBgn0023171-FBgn0026722 (2R) | 40.135275 |
| 1145 | FBgn0003862-FBgn0038220 (3R) | 40.010383 |
| 1146 | FBgn0086782-FBgn0052821 (X) | 39.998793 |
| 1147 | FBgn0039408-FBgn0039411 (3R) | 39.989120 |
| 1148 | FBgn0030057-FBgn0027864 (X) | 39.959335 |
| 1149 | FBgn0035243-FBgn0035244 (3L) | 39.946009 |
| 1150 | FBgn0030799-FBgn0030800 (X) | 39.812861 |
| 1151 | FBgn0031545-FBgn0031550 (2L) | 39.797267 |
| 1152 | FBgn0029986-FBgn0029987 (X) | 39.748567 |
| 1153 | FBgn0028991-FBgn0033806 (2R) | 39.732458 |
| 1154 | FBgn0039233-FBgn0039234 (3R) | 39.642155 |
| 1155 | FBgn0043456-FBgn0011584 (2L) | 39.602728 |
| 1156 | FBgn0029707-FBgn0052773 (X) | 39.583463 |
| 1157 | FBgn0030685-FBgn0030687 (X) | 39.577513 |
| 1158 | FBgn0030302-FBgn0030303 (X) | 39.553010 |
| 1159 | FBgn0051025-FBgn0051029 (3R) | 39.535116 |
| 1160 | FBgn0026080-FBgn0000635 (X) | 39.521082 |
| 1161 | FBgn0046296-FBgn0036257 (3L) | 39.511343 |
| 1162 | FBgn0054040-FBgn0034664 (2R) | 39.510471 |
| 1163 | FBgn0010422-FBgn0024183 (2L) | 39.494458 |
| 1164 | FBgn0029503-FBgn0039163 (3R) | 39.481899 |
| 1165 | FBgn0031286-FBgn0010583 (2L) | 39.403362 |
| 1166 | FBgn0037359-FBgn0010772 (3R) | 39.401206 |
| 1167 | FBgn0083952-FBgn0040786 (3L) | 39.351310 |
| 1168 | FBgn0039125-FBgn0039126 (3R) | 39.338604 |
| 1169 | FBgn0086350-FBgn0050460 (2R) | 39.274319 |
| 1170 | FBgn0039022-FBgn0039023 (3R) | 39.257366 |
| 1171 | FBgn0039101-FBgn0042106 (3R) | 39.252122 |
| 1172 | FBgn0029157-FBgn0039254 (3R) | 39.250260 |
| 1173 | FBgn0053214-FBgn0037092 (3L) | 39.240428 |
| 1174 | FBgn0034643-FBgn0034644 (2R) | 39.238802 |
| 1175 | FBgn0085204-FBgn0031533 (2L) | 39.234259 |
| 1176 | FBgn0037105-FBgn0037106 (3L) | 39.219090 |
| 1177 | FBgn0050432-FBgn0033069 (2R) | 39.208210 |
| 1178 | FBgn0024987-FBgn0023169 (X) | 39.203113 |
| 1179 | FBgn0036759-FBgn0036761 (3L) | 39.185295 |
| 1180 | FBgn0010355-FBgn0026566 (3R) | 39.181380 |
| 1181 | FBgn0032595-FBgn0032596 (2L) | 39.130423 |
| 1182 | FBgn0037352-FBgn0051551 (3R) | 39.061076 |
| 1183 | FBgn0053795-FBgn0036577 (3L) | 39.036055 |
| 1184 | FBgn0034634-FBgn0050289 (2R) | 39.034796 |
| 1185 | FBgn0051040-FBgn0039727 (3R) | 39.026177 |
| 1186 | FBgn0030192-FBgn0052679 (X) | 39.019048 |
| 1187 | FBgn0038295-FBgn0038296 (3R) | 38.995348 |
| 1188 | FBgn0050280-FBgn0050281 (2R) | 38.981886 |
| 1189 | FBgn0033221-FBgn0033222 (2R) | 38.979112 |
| 1190 | FBgn0030595-FBgn0052591 (X) | 38.965696 |
| 1191 | FBgn0036892-FBgn0036893 (3L) | 38.964190 |
| 1192 | FBgn0032519-FBgn0032520 (2L) | 38.920611 |
| 1193 | FBgn0038113-FBgn0003450 (3R) | 38.912286 |
| 1194 | FBgn0031106-FBgn0031107 (X) | 38.885905 |
| 1195 | FBgn0053108-FBgn0020647 (3R) | 38.856606 |
| 1196 | FBgn0040502-FBgn0033067 (2R) | 38.820455 |
| 1197 | FBgn0030843-FBgn0030844 (X) | 38.781579 |
| 1198 | FBgn0038439-FBgn0038440 (3R) | 38.756116 |
| 1199 | FBgn0029088-FBgn0037391 (3R) | 38.744997 |
| 1200 | FBgn0034802-FBgn0034803 (2R) | 38.726463 |
| 1201 | FBgn0034440-FBgn0034441 (2R) | 38.721742 |
| 1202 | FBgn0051860-FBgn0032419 (2L) | 38.661505 |
| 1203 | FBgn0029882-FBgn0029885 (X) | 38.653150 |
| 1204 | FBgn0083938-FBgn0031617 (2L) | 38.573925 |
| 1205 | FBgn0029090-FBgn0030636 (X) | 38.571712 |
| 1206 | FBgn0039879-FBgn0250755 (3R) | 38.548389 |
| 1207 | FBgn0010278-FBgn0028411 (2R) | 38.510534 |
| 1208 | FBgn0035154-FBgn0052483 (3L) | 38.506063 |
| 1209 | FBgn0259162-FBgn0083981 (X) | 38.476909 |
| 1210 | FBgn0030780-FBgn0052572 (X) | 38.370155 |
| 1211 | FBgn0036287-FBgn0053725 (3L) | 38.361320 |
| 1212 | FBgn0038523-FBgn0003375 (3R) | 38.358881 |
| 1213 | FBgn0035178-FBgn0035179 (3L) | 38.341193 |
| 1214 | FBgn0052638-FBgn0030452 (X) | 38.277667 |
| 1215 | FBgn0259108-FBgn0027796 (X) | 38.227744 |
| 1216 | FBgn0039882-FBgn0024891 (3R) | 38.183810 |
| 1217 | FBgn0040794-FBgn0053257 (3L) | 38.144117 |
| 1218 | FBgn0036233-FBgn0036234 (3L) | 38.121158 |
| 1219 | FBgn0039503-FBgn0039504 (3R) | 38.110206 |
| 1220 | FBgn0043903-FBgn0031057 (X) | 38.050571 |
| 1221 | FBgn0047178-FBgn0036484 (3L) | 38.017216 |
| 1222 | FBgn0038690-FBgn0038691 (3R) | 38.016689 |
| 1223 | FBgn0036070-FBgn0036072 (3L) | 38.016090 |
| 1224 | FBgn0030610-FBgn0030612 (X) | 37.989595 |
| 1225 | FBgn0039070-FBgn0039071 (3R) | 37.972856 |
| 1226 | FBgn0259146-FBgn0002735 (3R) | 37.957840 |
| 1227 | FBgn0040719-FBgn0053673 (2L) | 37.947263 |
| 1228 | FBgn0036230-FBgn0028573 (3L) | 37.943680 |
| 1229 | FBgn0003321-FBgn0030239 (X) | 37.906379 |
| 1230 | FBgn0030947-FBgn0004959 (X) | 37.898877 |
| 1231 | FBgn0005779-FBgn0031504 (2L) | 37.897833 |
| 1232 | FBgn0012051-FBgn0040273 (2R) | 37.880849 |
| 1233 | FBgn0037116-FBgn0037117 (3L) | 37.878225 |
| 1234 | FBgn0036711-FBgn0036712 (3L) | 37.875404 |
| 1235 | FBgn0038700-FBgn0038701 (3R) | 37.863888 |
| 1236 | FBgn0039470-FBgn0039471 (3R) | 37.856084 |
| 1237 | FBgn0259110-FBgn0030715 (X) | 37.829595 |
| 1238 | FBgn0039568-FBgn0085383 (3R) | 37.829305 |
| 1239 | FBgn0031929-FBgn0031930 (2L) | 37.744628 |
| 1240 | FBgn0051199-FBgn0051200 (3R) | 37.705031 |
| 1241 | FBgn0085346-FBgn0015542 (3R) | 37.663335 |
| 1242 | FBgn0035488-FBgn0035489 (3L) | 37.659101 |
| 1243 | FBgn0035954-FBgn0035957 (3L) | 37.584884 |
| 1244 | FBgn0020309-FBgn0032378 (2L) | 37.553444 |
| 1245 | FBgn0036196-FBgn0036199 (3L) | 37.542271 |
| 1246 | FBgn0032161-FBgn0032162 (2L) | 37.522375 |
| 1247 | FBgn0026087-FBgn0023546 (X) | 37.484471 |
| 1248 | FBgn0039356-FBgn0039357 (3R) | 37.471655 |
| 1249 | FBgn0044047-FBgn0040389 (X) | 37.449424 |
| 1250 | FBgn0051031-FBgn0040625 (3R) | 37.448676 |
| 1251 | FBgn0002922-FBgn0039108 (3R) | 37.431081 |
| 1252 | FBgn0031196-FBgn0031197 (X) | 37.352042 |
| 1253 | FBgn0032142-FBgn0032143 (2L) | 37.341385 |
| 1254 | FBgn0051697-FBgn0032804 (2L) | 37.320884 |
| 1255 | FBgn0033580-FBgn0033581 (2R) | 37.298583 |
| 1256 | FBgn0035619-FBgn0035620 (3L) | 37.282956 |
| 1257 | FBgn0052703-FBgn0030120 (X) | 37.263169 |
| 1258 | FBgn0039017-FBgn0039018 (3R) | 37.255334 |
| 1259 | FBgn0029783-FBgn0028474 (X) | 37.191156 |
| 1260 | FBgn0015765-FBgn0039156 (3R) | 37.174603 |
| 1261 | FBgn0038423-FBgn0051279 (3R) | 37.169781 |
| 1262 | FBgn0052135-FBgn0036395 (3L) | 37.157123 |
| 1263 | FBgn0037184-FBgn0052451 (3L) | 37.150141 |
| 1264 | FBgn0039611-FBgn0039613 (3R) | 37.136461 |
| 1265 | FBgn0051902-FBgn0053121 (2L) | 37.132344 |
| 1266 | FBgn0035679-FBgn0061492 (3L) | 37.130960 |
| 1267 | FBgn0001179-FBgn0036090 (3L) | 37.122490 |
| 1268 | FBgn0015618-FBgn0004390 (3L) | 37.093572 |
| 1269 | FBgn0051347-FBgn0038079 (3R) | 37.091412 |
| 1270 | FBgn0039628-FBgn0039629 (3R) | 37.067969 |
| 1271 | FBgn0035676-FBgn0035677 (3L) | 37.032350 |
| 1272 | FBgn0036145-FBgn0036146 (3L) | 37.019742 |
| 1273 | FBgn0032188-FBgn0051874 (2L) | 37.017527 |
| 1274 | FBgn0051920-FBgn0051921 (2L) | 36.952140 |
| 1275 | FBgn0016081-FBgn0036028 (3L) | 36.905790 |
| 1276 | FBgn0014022-FBgn0037703 (3R) | 36.845829 |
| 1277 | FBgn0051352-FBgn0016792 (3R) | 36.845805 |
| 1278 | FBgn0000808-FBgn0003865 (X) | 36.816997 |
| 1279 | FBgn0033140-FBgn0033142 (2R) | 36.764655 |
| 1280 | FBgn0025874-FBgn0036389 (3L) | 36.681256 |
| 1281 | FBgn0003174-FBgn0033159 (2R) | 36.668041 |
| 1282 | FBgn0052271-FBgn0053159 (3L) | 36.666500 |
| 1283 | FBgn0051291-FBgn0038386 (3R) | 36.639959 |
| 1284 | FBgn0034931-FBgn0034933 (2R) | 36.630192 |
| 1285 | FBgn0035050-FBgn0053988 (2R) | 36.547248 |
| 1286 | FBgn0045862-FBgn0039544 (3R) | 36.531486 |
| 1287 | FBgn0032538-FBgn0028544 (2L) | 36.519444 |
| 1288 | FBgn0032280-FBgn0032282 (2L) | 36.481511 |
| 1289 | FBgn0030121-FBgn0030122 (X) | 36.480709 |
| 1290 | FBgn0038746-FBgn0038747 (3R) | 36.447913 |
| 1291 | FBgn0031108-FBgn0040651 (X) | 36.444538 |
| 1292 | FBgn0033953-FBgn0033955 (2R) | 36.409247 |
| 1293 | FBgn0039538-FBgn0003330 (3R) | 36.407597 |
| 1294 | FBgn0034622-FBgn0034624 (2R) | 36.406712 |
| 1295 | FBgn0038306-FBgn0045035 (3R) | 36.373688 |
| 1296 | FBgn0051235-FBgn0038607 (3R) | 36.310937 |
| 1297 | FBgn0050042-FBgn0033723 (2R) | 36.306295 |
| 1298 | FBgn0039451-FBgn0040607 (3R) | 36.270998 |
| 1299 | FBgn0035279-FBgn0035282 (3L) | 36.217738 |
| 1300 | FBgn0001994-FBgn0040079 (2L) | 36.201903 |
| 1301 | FBgn0032005-FBgn0032006 (2L) | 36.197748 |
| 1302 | FBgn0052036-FBgn0052037 (3L) | 36.191290 |
| 1303 | FBgn0034688-FBgn0034691 (2R) | 36.189805 |
| 1304 | FBgn0031874-FBgn0031876 (2L) | 36.179462 |
| 1305 | FBgn0031098-FBgn0031099 (X) | 36.152581 |
| 1306 | FBgn0046880-FBgn0046879 (2R) | 36.149824 |
| 1307 | FBgn0051156-FBgn0051161 (3R) | 36.138144 |
| 1308 | FBgn0004897-FBgn0004898 (3R) | 36.130501 |
| 1309 | FBgn0259714-FBgn0031811 (2L) | 36.033114 |
| 1310 | FBgn0036784-FBgn0036785 (3L) | 36.023865 |
| 1311 | FBgn0085241-FBgn0033056 (2R) | 36.019861 |
| 1312 | FBgn0031430-FBgn0031431 (2L) | 36.016278 |
| 1313 | FBgn0003459-FBgn0036423 (3L) | 35.992541 |
| 1314 | FBgn0085313-FBgn0038658 (3R) | 35.961929 |
| 1315 | FBgn0066293-FBgn0053558 (2R) | 35.953554 |
| 1316 | FBgn0034539-FBgn0034541 (2R) | 35.875090 |
| 1317 | FBgn0037017-FBgn0040634 (3L) | 35.866622 |
| 1318 | FBgn0031116-FBgn0052506 (X) | 35.785187 |
| 1319 | FBgn0030349-FBgn0030350 (X) | 35.769800 |
| 1320 | FBgn0035777-FBgn0052379 (3L) | 35.713195 |
| 1321 | FBgn0037061-FBgn0028427 (3L) | 35.546118 |
| 1322 | FBgn0040253-FBgn0040255 (3R) | 35.533577 |
| 1323 | FBgn0085481-FBgn0085480 (3L) | 35.522403 |
| 1324 | FBgn0052944-FBgn0051525 (3R) | 35.494993 |
| 1325 | FBgn0032085-FBgn0032086 (2L) | 35.462171 |
| 1326 | FBgn0085331-FBgn0053208 (3R) | 35.388223 |
| 1327 | FBgn0031407-FBgn0054049 (2L) | 35.366476 |
| 1328 | FBgn0030540-FBgn0030541 (X) | 35.353620 |
| 1329 | FBgn0010313-FBgn0037293 (3R) | 35.343439 |
| 1330 | FBgn0052076-FBgn0036142 (3L) | 35.336354 |
| 1331 | FBgn0027602-FBgn0040877 (X) | 35.326142 |
| 1332 | FBgn0037836-FBgn0037837 (3R) | 35.273115 |
| 1333 | FBgn0036266-FBgn0052100 (3L) | 35.226398 |
| 1334 | FBgn0033628-FBgn0033629 (2R) | 35.180274 |
| 1335 | FBgn0038468-FBgn0038469 (3R) | 35.178223 |
| 1336 | FBgn0053469-FBgn0053468 (2R) | 35.177364 |
| 1337 | FBgn0030704-FBgn0010315 (X) | 35.162798 |
| 1338 | FBgn0023001-FBgn0040837 (3L) | 35.160529 |
| 1339 | FBgn0026160-FBgn0036104 (3L) | 35.155177 |
| 1340 | FBgn0039832-FBgn0039833 (3R) | 35.138905 |
| 1341 | FBgn0030305-FBgn0030306 (X) | 35.093445 |
| 1342 | FBgn0031977-FBgn0044323 (2L) | 35.091248 |
| 1343 | FBgn0051728-FBgn0051730 (2L) | 35.045289 |
| 1344 | FBgn0040575-FBgn0038803 (3R) | 35.033914 |
| 1345 | FBgn0033820-FBgn0033821 (2R) | 34.966624 |
| 1346 | FBgn0035138-FBgn0035139 (3L) | 34.965614 |
| 1347 | FBgn0034426-FBgn0034427 (2R) | 34.948484 |
| 1348 | FBgn0031314-FBgn0031315 (2L) | 34.933020 |
| 1349 | FBgn0031092-FBgn0031094 (X) | 34.919398 |
| 1350 | FBgn0037555-FBgn0037556 (3R) | 34.850427 |
| 1351 | FBgn0034486-FBgn0034487 (2R) | 34.708046 |
| 1352 | FBgn0036476-FBgn0036478 (3L) | 34.652600 |
| 1353 | FBgn0028683-FBgn0033762 (2R) | 34.649031 |
| 1354 | FBgn0011672-FBgn0025865 (3R) | 34.598171 |
| 1355 | FBgn0027783-FBgn0028434 (2R) | 34.583847 |
| 1356 | FBgn0034177-FBgn0026402 (2R) | 34.574820 |
| 1357 | FBgn0036771-FBgn0036772 (3L) | 34.561342 |
| 1358 | FBgn0051493-FBgn0037477 (3R) | 34.537280 |
| 1359 | FBgn0030053-FBgn0025864 (X) | 34.529711 |
| 1360 | FBgn0050050-FBgn0033732 (2R) | 34.503501 |
| 1361 | FBgn0035482-FBgn0004895 (3L) | 34.497047 |
| 1362 | FBgn0051201-FBgn0028422 (3R) | 34.415477 |
| 1363 | FBgn0033164-FBgn0033165 (2R) | 34.353696 |
| 1364 | FBgn0002533-FBgn0002534 (2R) | 34.322055 |
| 1365 | FBgn0036023-FBgn0036024 (3L) | 34.312895 |
| 1366 | FBgn0030808-FBgn0030809 (X) | 34.298932 |
| 1367 | FBgn0062413-FBgn0025815 (X) | 34.290055 |
| 1368 | FBgn0250844-FBgn0028913 (2L) | 34.287729 |
| 1369 | FBgn0014368-FBgn0052074 (3L) | 34.282310 |
| 1370 | FBgn0030099-FBgn0030100 (X) | 34.254391 |
| 1371 | FBgn0004516-FBgn0035495 (3L) | 34.244615 |
| 1372 | FBgn0014009-FBgn0033095 (2R) | 34.219903 |
| 1373 | FBgn0030107-FBgn0030108 (X) | 34.152391 |
| 1374 | FBgn0031254-FBgn0031255 (2L) | 34.149310 |
| 1375 | FBgn0036248-FBgn0036249 (3L) | 34.131315 |
| 1376 | FBgn0040995-FBgn0032838 (2L) | 34.130618 |
| 1377 | FBgn0035150-FBgn0035151 (3L) | 34.094399 |
| 1378 | FBgn0030933-FBgn0030937 (X) | 34.052366 |
| 1379 | FBgn0033149-FBgn0026389 (2R) | 34.020501 |
| 1380 | FBgn0035886-FBgn0035887 (3L) | 34.016469 |
| 1381 | FBgn0015806-FBgn0035639 (3L) | 34.000915 |
| 1382 | FBgn0015553-FBgn0032698 (2L) | 33.916347 |
| 1383 | FBgn0039554-FBgn0039555 (3R) | 33.911058 |
| 1384 | FBgn0032499-FBgn0051729 (2L) | 33.909883 |
| 1385 | FBgn0034495-FBgn0034496 (2R) | 33.869243 |
| 1386 | FBgn0039224-FBgn0039225 (3R) | 33.821014 |
| 1387 | FBgn0038639-FBgn0038640 (3R) | 33.802011 |
| 1388 | FBgn0034084-FBgn0034086 (2R) | 33.748383 |
| 1389 | FBgn0031276-FBgn0040725 (2L) | 33.743824 |
| 1390 | FBgn0033855-FBgn0033856 (2R) | 33.733517 |
| 1391 | FBgn0031399-FBgn0031401 (2L) | 33.645551 |
| 1392 | FBgn0027360-FBgn0050290 (2R) | 33.619644 |
| 1393 | FBgn0052082-FBgn0036139 (3L) | 33.609838 |
| 1394 | FBgn0033763-FBgn0050053 (2R) | 33.573482 |
| 1395 | FBgn0030792-FBgn0030791 (X) | 33.556928 |
| 1396 | FBgn0052241-FBgn0052249 (3L) | 33.541111 |
| 1397 | FBgn0038720-FBgn0038721 (3R) | 33.518988 |
| 1398 | FBgn0025830-FBgn0025832 (2R) | 33.451422 |
| 1399 | FBgn0001990-FBgn0041627 (2L) | 33.423064 |
| 1400 | FBgn0036607-FBgn0036608 (3L) | 33.408483 |
| 1401 | FBgn0052160-FBgn0036603 (3L) | 33.396762 |
| 1402 | FBgn0086347-FBgn0032261 (2L) | 33.277344 |
| 1403 | FBgn0033890-FBgn0002643 (2R) | 33.224997 |
| 1404 | FBgn0039869-FBgn0039870 (3R) | 33.184160 |
| 1405 | FBgn0033059-FBgn0033060 (2R) | 33.164732 |
| 1406 | FBgn0034552-FBgn0034553 (2R) | 33.162215 |
| 1407 | FBgn0039190-FBgn0053340 (3R) | 33.092436 |
| 1408 | FBgn0026058-FBgn0030868 (X) | 33.074797 |
| 1409 | FBgn0040814-FBgn0036348 (3L) | 32.811236 |
| 1410 | FBgn0083946-FBgn0039290 (3R) | 32.740870 |
| 1411 | FBgn0032292-FBgn0027868 (2L) | 32.686174 |
| 1412 | FBgn0035842-FBgn0002899 (3L) | 32.677579 |
| 1413 | FBgn0034238-FBgn0034239 (2R) | 32.649956 |
| 1414 | FBgn0032125-FBgn0032129 (2L) | 32.642227 |
| 1415 | FBgn0013263-FBgn0036446 (3L) | 32.594922 |
| 1416 | FBgn0036601-FBgn0036602 (3L) | 32.592034 |
| 1417 | FBgn0029789-FBgn0027564 (X) | 32.575902 |
| 1418 | FBgn0026313-FBgn0030851 (X) | 32.534286 |
| 1419 | FBgn0027607-FBgn0033357 (2R) | 32.491390 |
| 1420 | FBgn0050479-FBgn0050480 (2R) | 32.421063 |
| 1421 | FBgn0036030-FBgn0015321 (3L) | 32.415500 |
| 1422 | FBgn0032724-FBgn0032729 (2L) | 32.400879 |
| 1423 | FBgn0031033-FBgn0031034 (X) | 32.354086 |
| 1424 | FBgn0038166-FBgn0024555 (3R) | 32.343138 |
| 1425 | FBgn0037747-FBgn0037749 (3R) | 32.315806 |
| 1426 | FBgn0032035-FBgn0032036 (2L) | 32.274478 |
| 1427 | FBgn0010303-FBgn0030448 (X) | 32.146872 |
2R) 11.226134 || 1977 | FBgn0034922-FBgn0034924 (2R) | 11.208218 |
| 1978 | FBgn0032670-FBgn0032671 (2L) | 11.120221 |
| 1979 | FBgn0002914-FBgn0065035 (X) | 11.040183 |
| 1980 | FBgn0013984-FBgn0038893 (3R) | 10.956238 |
| 1981 | FBgn0033921-FBgn0085408 (2R) | 10.875436 |
| 1982 | FBgn0038256-FBgn0011217 (3R) | 10.873494 |
| 1983 | FBgn0051204-FBgn0051002 (3R) | 10.762726 |
| 1984 | FBgn0032955-FBgn0022893 (2L) | 10.756391 |
| 1985 | FBgn0044049-FBgn0044328 (3L) | 10.569008 |
| 1986 | FBgn0037099-FBgn0037101 (3L) | 10.482881 |
| 1987 | FBgn0035461-FBgn0052262 (3L) | 10.468714 |
| 1988 | FBgn0038000-FBgn0038001 (3R) | 10.428685 |
| 1989 | FBgn0051413-FBgn0039106 (3R) | 10.375706 |
| 1990 | FBgn0031288-FBgn0031289 (2L) | 10.364360 |
| 1991 | FBgn0030506-FBgn0030507 (X) | 10.302999 |
| 1992 | FBgn0037992-FBgn0037993 (3R) | 10.301365 |
| 1993 | FBgn0036619-FBgn0036620 (3L) | 10.290662 |
| 1994 | FBgn0031518-FBgn0031519 (2L) | 10.279568 |
| 1995 | FBgn0086253-FBgn0039026 (3R) | 10.260590 |
| 1996 | FBgn0015617-FBgn0003449 (X) | 10.244177 |
| 1997 | FBgn0034109-FBgn0034110 (2R) | 10.237216 |
| 1998 | FBgn0039737-FBgn0039738 (3R) | 10.219480 |
| 1999 | FBgn0030460-FBgn0005410 (X) | 10.164667 |
| 2000 | FBgn0028538-FBgn0027844 (2L) | 10.070574 |
| 2001 | FBgn0034279-FBgn0028740 (2R) | 10.033656 |
| 2002 | FBgn0051104-FBgn0039316 (3R) | 9.946668 |
| 2003 | FBgn0030755-FBgn0030757 (X) | 9.916582 |
| 2004 | FBgn0003048-FBgn0001330 (X) | 9.830810 |
| 2005 | FBgn0033729-FBgn0033730 (2R) | 9.811850 |
| 2006 | FBgn0033751-FBgn0033752 (2R) | 9.645562 |
| 2007 | FBgn0037935-FBgn0037936 (3R) | 9.587752 |
| 2008 | FBgn0038740-FBgn0038741 (3R) | 9.566783 |
| 2009 | FBgn0025835-FBgn0024989 (X) | 9.535223 |
| 2010 | FBgn0036741-FBgn0036742 (3L) | 9.479982 |
| 2011 | FBgn0003520-FBgn0028983 (2R) | 9.467812 |
| 2012 | FBgn0042179-FBgn0052259 (3L) | 9.316933 |
| 2013 | FBgn0033471-FBgn0033474 (2R) | 9.256310 |
| 2014 | FBgn0033463-FBgn0050007 (2R) | 9.237696 |
| 2015 | FBgn0033248-FBgn0027788 (2R) | 9.229740 |
| 2016 | FBgn0033134-FBgn0016032 (2R) | 9.207441 |
| 2017 | FBgn0003292-FBgn0036179 (3L) | 9.070164 |
| 2018 | FBgn0034132-FBgn0034133 (2R) | 8.969372 |
| 2019 | FBgn0041171-FBgn0035517 (3L) | 8.962567 |
| 2020 | FBgn0038821-FBgn0259222 (3R) | 8.930981 |
| 2021 | FBgn0031239-FBgn0031240 (2L) | 8.778194 |
| 2022 | FBgn0039246-FBgn0040601 (3R) | 8.760735 |
| 2023 | FBgn0033202-FBgn0033203 (2R) | 8.740297 |
| 2024 | FBgn0038783-FBgn0038784 (3R) | 8.654624 |
| 2025 | FBgn0030746-FBgn0030747 (X) | 8.549406 |
| 2026 | FBgn0031002-FBgn0031003 (X) | 8.517194 |
| 2027 | FBgn0030137-FBgn0052702 (X) | 8.419687 |
| 2028 | FBgn0035845-FBgn0040290 (3L) | 8.417790 |
| 2029 | FBgn0030485-FBgn0030486 (X) | 8.196036 |
| 2030 | FBgn0036975-FBgn0024887 (3L) | 8.157927 |
| 2031 | FBgn0051813-FBgn0028541 (2L) | 8.122783 |
| 2032 | FBgn0031560-FBgn0031561 (2L) | 8.082738 |
| 2033 | FBgn0037943-FBgn0037944 (3R) | 8.081726 |
| 2034 | FBgn0034061-FBgn0034063 (2R) | 8.079887 |
| 2035 | FBgn0038453-FBgn0015019 (3R) | 8.033336 |
| 2036 | FBgn0051477-FBgn0001321 (3R) | 8.016396 |
| 2037 | FBgn0032586-FBgn0032587 (2L) | 7.956465 |
| 2038 | FBgn0039469-FBgn0051077 (3R) | 7.928680 |
| 2039 | FBgn0015737-FBgn0040609 (3R) | 7.880629 |
| 2040 | FBgn0036824-FBgn0036825 (3L) | 7.857487 |
| 2041 | FBgn0032470-FBgn0032471 (2L) | 7.833715 |
| 2042 | FBgn0039500-FBgn0039501 (3R) | 7.821253 |
| 2043 | FBgn0038558-FBgn0053547 (3R) | 7.808873 |
| 2044 | FBgn0000473-FBgn0085421 (2R) | 7.800461 |
| 2045 | FBgn0001137-FBgn0027490 (2L) | 7.785039 |
| 2046 | FBgn0003301-FBgn0030581 (X) | 7.663099 |
| 2047 | FBgn0032446-FBgn0032450 (2L) | 7.648233 |
| 2048 | FBgn0034737-FBgn0034739 (2R) | 7.637387 |
| 2049 | FBgn0035964-FBgn0035965 (3L) | 7.593297 |
| 2050 | FBgn0050056-FBgn0033753 (2R) | 7.550150 |
| 2051 | FBgn0032078-FBgn0032079 (2L) | 7.471060 |
| 2052 | FBgn0052278-FBgn0052281 (3L) | 7.401974 |
| 2053 | FBgn0033232-FBgn0015509 (2R) | 7.400643 |
| 2054 | FBgn0031360-FBgn0027597 (2L) | 7.367493 |
| 2055 | FBgn0054051-FBgn0032834 (2L) | 7.328424 |
| 2056 | FBgn0016031-FBgn0004380 (3L) | 7.292445 |
| 2057 | FBgn0031089-FBgn0031090 (X) | 7.266723 |
| 2058 | FBgn0028482-FBgn0020762 (2L) | 7.161865 |
| 2059 | FBgn0037846-FBgn0037848 (3R) | 7.160262 |
| 2060 | FBgn0038738-FBgn0038739 (3R) | 7.151515 |
| 2061 | FBgn0039453-FBgn0039454 (3R) | 7.146169 |
| 2062 | FBgn0040828-FBgn0035924 (3L) | 7.140108 |
| 2063 | FBgn0030863-FBgn0030864 (X) | 7.059513 |
| 2064 | FBgn0033724-FBgn0033725 (2R) | 6.991881 |
| 2065 | FBgn0010300-FBgn0010309 (2L) | 6.943650 |
| 2066 | FBgn0032681-FBgn0032683 (2L) | 6.937617 |
| 2067 | FBgn0051974-FBgn0031224 (2L) | 6.931687 |
| 2068 | FBgn0031590-FBgn0003941 (2L) | 6.918147 |
| 2069 | FBgn0032361-FBgn0032363 (2L) | 6.889530 |
| 2070 | FBgn0033107-FBgn0040674 (2R) | 6.802163 |
| 2071 | FBgn0035779-FBgn0035781 (3L) | 6.798898 |
| 2072 | FBgn0032848-FBgn0032850 (2L) | 6.777425 |
| 2073 | FBgn0014417-FBgn0032042 (2L) | 6.777414 |
| 2074 | FBgn0028707-FBgn0029137 (2L) | 6.764452 |
| 2075 | FBgn0029507-FBgn0033127 (2R) | 6.710889 |
| 2076 | FBgn0032940-FBgn0000370 (2L) | 6.701485 |
| 2077 | FBgn0001325-FBgn0050429 (2R) | 6.677287 |
| 2078 | FBgn0033911-FBgn0005613 (2R) | 6.604508 |
| 2079 | FBgn0039800-FBgn0039801 (3R) | 6.565978 |
| 2080 | FBgn0036128-FBgn0052072 (3L) | 6.535438 |
| 2081 | FBgn0034433-FBgn0034434 (2R) | 6.511028 |
| 2082 | FBgn0259178-FBgn0039264 (3R) | 6.431540 |
| 2083 | FBgn0039059-FBgn0039060 (3R) | 6.422674 |
| 2084 | FBgn0033716-FBgn0033717 (2R) | 6.386680 |
| 2085 | FBgn0069938-FBgn0069972 (X) | 6.332799 |
| 2086 | FBgn0052432-FBgn0040637 (3L) | 6.316720 |
| 2087 | FBgn0034387-FBgn0259682 (2R) | 6.312655 |
| 2088 | FBgn0004387-FBgn0040080 (3R) | 6.230924 |
| 2089 | FBgn0085428-FBgn0050096 (2R) | 6.100785 |
| 2090 | FBgn0037205-FBgn0052461 (3L) | 6.030634 |
| 2091 | FBgn0030010-FBgn0030012 (X) | 6.018848 |
| 2092 | FBgn0011207-FBgn0051710 (2L) | 5.949296 |
| 2093 | FBgn0039324-FBgn0039325 (3R) | 5.930804 |
| 2094 | FBgn0002673-FBgn0028897 (2L) | 5.794399 |
| 2095 | FBgn0038170-FBgn0002937 (3R) | 5.787955 |
| 2096 | FBgn0051133-FBgn0046322 (3R) | 5.676368 |
| 2097 | FBgn0037435-FBgn0037436 (3R) | 5.579466 |
| 2098 | FBgn0004432-FBgn0030740 (X) | 5.550013 |
| 2099 | FBgn0030290-FBgn0030291 (X) | 5.542430 |
| 2100 | FBgn0031850-FBgn0025595 (2L) | 5.539828 |
| 2101 | FBgn0028675-FBgn0032189 (2L) | 5.480798 |
| 2102 | FBgn0032597-FBgn0032598 (2L) | 5.439707 |
| 2103 | FBgn0050151-FBgn0050154 (2R) | 5.378556 |
| 2104 | FBgn0031449-FBgn0031450 (2L) | 5.378097 |
| 2105 | FBgn0033686-FBgn0027504 (2R) | 5.364114 |
| 2106 | FBgn0004110-FBgn0004862 (3R) | 5.346377 |
| 2107 | FBgn0032286-FBgn0023496 (2L) | 5.344242 |
| 2108 | FBgn0033785-FBgn0050487 (2R) | 5.230212 |
| 2109 | FBgn0039152-FBgn0039151 (3R) | 5.099366 |
| 2110 | FBgn0019972-FBgn0039697 (3R) | 5.091153 |
| 2111 | FBgn0041097-FBgn0011294 (2L) | 5.014794 |
| 2112 | FBgn0037680-FBgn0053936 (3R) | 5.010115 |
| 2113 | FBgn0038958-FBgn0038960 (3R) | 5.001689 |
| 2114 | FBgn0023479-FBgn0043806 (3L) | 4.963273 |
| 2115 | FBgn0034734-FBgn0034736 (2R) | 4.958000 |
| 2116 | FBgn0052079-FBgn0052081 (3L) | 4.957778 |
| 2117 | FBgn0036811-FBgn0036815 (3L) | 4.891289 |
| 2118 | FBgn0030033-FBgn0040928 (X) | 4.873343 |
| 2119 | FBgn0035948-FBgn0035949 (3L) | 4.853588 |
| 2120 | FBgn0025808-FBgn0051314 (3R) | 4.836441 |
| 2121 | FBgn0033783-FBgn0033784 (2R) | 4.778073 |
| 2122 | FBgn0031168-FBgn0052521 (X) | 4.688725 |
| 2123 | FBgn0030037-FBgn0030039 (X) | 4.669984 |
| 2124 | FBgn0025394-FBgn0023530 (X) | 4.637166 |
| 2125 | FBgn0037377-FBgn0037378 (3R) | 4.547004 |
| 2126 | FBgn0026386-FBgn0033603 (2R) | 4.539409 |
| 2127 | FBgn0050085-FBgn0034049 (2R) | 4.452401 |
| 2128 | FBgn0003460-FBgn0033168 (2R) | 4.385764 |
| 2129 | FBgn0000061-FBgn0031251 (2L) | 4.297724 |
| 2130 | FBgn0030508-FBgn0030509 (X) | 4.231182 |
| 2131 | FBgn0039531-FBgn0039532 (3R) | 4.211582 |
| 2132 | FBgn0052452-FBgn0016034 (3L) | 4.198087 |
| 2133 | FBgn0259750-FBgn0052830 (2L) | 4.131964 |
| 2134 | FBgn0083961-FBgn0015834 (2L) | 4.101763 |
| 2135 | FBgn0031753-FBgn0040949 (2L) | 4.090168 |
| 2136 | FBgn0033047-FBgn0033048 (2R) | 4.056865 |
| 2137 | FBgn0035873-FBgn0035875 (3L) | 4.034214 |
| 2138 | FBgn0040551-FBgn0038129 (3R) | 3.978161 |
| 2139 | FBgn0036416-FBgn0036417 (3L) | 3.888040 |
| 2140 | FBgn0032266-FBgn0051721 (2L) | 3.874287 |
| 2141 | FBgn0029764-FBgn0029763 (X) | 3.857812 |
| 2142 | FBgn0030638-FBgn0004391 (X) | 3.839237 |
| 2143 | FBgn0039566-FBgn0003890 (3R) | 3.708948 |
| 2144 | FBgn0036629-FBgn0004569 (3L) | 3.570199 |
| 2145 | FBgn0038968-FBgn0085405 (3R) | 3.486382 |
| 2146 | FBgn0082585-FBgn0033633 (2R) | 3.472579 |
| 2147 | FBgn0038944-FBgn0038945 (3R) | 3.446589 |
| 2148 | FBgn0035891-FBgn0010431 (3L) | 3.360210 |
| 2149 | FBgn0030625-FBgn0004924 (X) | 3.257407 |
| 2150 | FBgn0015032-FBgn0053483 (3R) | 3.243015 |
| 2151 | FBgn0032929-FBgn0011829 (2L) | 3.172249 |
| 2152 | FBgn0034217-FBgn0034218 (2R) | 3.094373 |
| 2153 | FBgn0051773-FBgn0051774 (2L) | 3.063459 |
| 2154 | FBgn0025885-FBgn0033179 (2R) | 3.060066 |
| 2155 | FBgn0004198-FBgn0029955 (X) | 3.021478 |
| 2156 | FBgn0037989-FBgn0051361 (3R) | 2.872880 |
| 2157 | FBgn0035143-FBgn0035144 (3L) | 2.731338 |
| 2158 | FBgn0029992-FBgn0029993 (X) | 2.729452 |
| 2159 | FBgn0002036-FBgn0000422 (2L) | 2.628657 |
| 2160 | FBgn0024944-FBgn0051205 (3R) | 2.574948 |
| 2161 | FBgn0036875-FBgn0036876 (3L) | 2.565404 |
| 2162 | FBgn0051473-FBgn0037525 (3R) | 2.546754 |
| 2163 | FBgn0259784-FBgn0038491 (3R) | 2.524066 |
| 2164 | FBgn0034565-FBgn0034566 (2R) | 2.521861 |
| 2165 | FBgn0034396-FBgn0034397 (2R) | 2.371616 |
| 2166 | FBgn0067779-FBgn0031213 (2L) | 2.357249 |
| 2167 | FBgn0039041-FBgn0039042 (3R) | 2.351419 |
| 2168 | FBgn0052450-FBgn0037162 (3L) | 2.324923 |
| 2169 | FBgn0052668-FBgn0026268 (X) | 2.295682 |
| 2170 | FBgn0039563-FBgn0039565 (3R) | 2.259543 |
| 2171 | FBgn0031214-FBgn0002931 (2L) | 2.256944 |
| 2172 | FBgn0040066-FBgn0001404 (X) | 2.135917 |
| 2173 | FBgn0024806-FBgn0035122 (3L) | 2.111619 |
| 2174 | FBgn0259244-FBgn0038445 (3R) | 1.827061 |
| 2175 | FBgn0026207-FBgn0038076 (3R) | 1.823016 |
| 2176 | FBgn0005592-FBgn0036410 (3L) | 1.807589 |
| 2177 | FBgn0028430-FBgn0259896 (2L) | 1.741684 |
| 2178 | FBgn0030331-FBgn0002577 (X) | 1.658546 |
| 2179 | FBgn0028562-FBgn0033257 (2R) | 1.652748 |
| 2180 | FBgn0014135-FBgn0051459 (3R) | 1.578270 |
| 2181 | FBgn0020385-FBgn0037822 (3R) | 1.522122 |
| 2182 | FBgn0030338-FBgn0030340 (X) | 1.489718 |
| 2183 | FBgn0034154-FBgn0034156 (2R) | 1.421430 |
| 2184 | FBgn0030005-FBgn0030001 (X) | 1.339451 |
| 2185 | FBgn0040392-FBgn0053218 (X) | 1.301298 |
| 2186 | FBgn0031540-FBgn0051776 (2L) | 1.164654 |
| 2187 | FBgn0030228-FBgn0030234 (X) | 1.113301 |
| 2188 | FBgn0031375-FBgn0031377 (2L) | 1.081270 |
| 2189 | FBgn0038293-FBgn0038294 (3R) | 1.062453 |
| 2190 | FBgn0033680-FBgn0086677 (2R) | 1.054242 |
| 2191 | FBgn0051961-FBgn0031589 (2L) | 0.960048 |
| 2192 | FBgn0038628-FBgn0038630 (3R) | 0.952150 |
| 2193 | FBgn0051809-FBgn0032615 (2L) | 0.903018 |
| 2194 | FBgn0039094-FBgn0051145 (3R) | 0.850904 |
| 2195 | FBgn0033431-FBgn0050338 (2R) | 0.792582 |
| 2196 | FBgn0032713-FBgn0015808 (2L) | 0.708249 |
| 2197 | FBgn0035248-FBgn0035249 (3L) | 0.663821 |
| 2198 | FBgn0030998-FBgn0030999 (X) | 0.604514 |
| 2199 | FBgn0033570-FBgn0033571 (2R) | 0.588659 |
| 2200 | FBgn0032797-FBgn0032798 (2L) | 0.529522 |
| 2201 | FBgn0027548-FBgn0033244 (2R) | 0.517009 |
| 2202 | FBgn0027660-FBgn0086475 (3L) | 0.460177 |
| 2203 | FBgn0039630-FBgn0039633 (3R) | 0.314472 |
| 2204 | FBgn0032654-FBgn0015609 (2L) | 0.284936 |
| 2205 | FBgn0026255-FBgn0032934 (2L) | 0.087528 |
| 2206 | FBgn0028919-FBgn0015271 (2L) | 0.033290 |
| 2207 | FBgn0040942-FBgn0030189 (X) | -0.020026 |
| 2208 | FBgn0003204-FBgn0052676 (X) | -0.249925 |
| 2209 | FBgn0027287-FBgn0022710 (X) | -0.260181 |
| 2210 | FBgn0083949-FBgn0040679 (3R) | -0.261846 |
| 2211 | FBgn0038244-FBgn0020299 (3R) | -0.415842 |
| 2212 | FBgn0054054-FBgn0040503 (2R) | -0.529160 |
| 2213 | FBgn0030240-FBgn0030243 (X) | -0.599871 |
| 2214 | FBgn0037220-FBgn0015331 (3R) | -0.612159 |
| 2215 | FBgn0035742-FBgn0035743 (3L) | -0.781236 |
| 2216 | FBgn0041164-FBgn0010317 (3L) | -0.811536 |
| 2217 | FBgn0052282-FBgn0035434 (3L) | -0.921194 |
| 2218 | FBgn0027654-FBgn0082582 (3R) | -0.922288 |
| 2219 | FBgn0031432-FBgn0041337 (2L) | -0.951998 |
| 2220 | FBgn0038365-FBgn0038366 (3R) | -0.959836 |
| 2221 | FBgn0037540-FBgn0001255 (3R) | -0.971468 |
| 2222 | FBgn0086902-FBgn0022246 (2L) | -1.007456 |
| 2223 | FBgn0085259-FBgn0033658 (2R) | -1.107528 |
| 2224 | FBgn0002576-FBgn0054028 (X) | -1.269454 |
| 2225 | FBgn0034117-FBgn0034118 (2R) | -1.326538 |
| 2226 | FBgn0085366-FBgn0040923 (X) | -1.554315 |
| 2227 | FBgn0052392-FBgn0086680 (3L) | -1.592035 |
| 2228 | FBgn0038247-FBgn0038248 (3R) | -1.648337 |
| 2229 | FBgn0035483-FBgn0035484 (3L) | -1.718335 |
| 2230 | FBgn0039848-FBgn0039850 (3R) | -1.726638 |
| 2231 | FBgn0011670-FBgn0011668 (3R) | -1.743892 |
| 2232 | FBgn0050145-FBgn0034520 (2R) | -1.782887 |
| 2233 | FBgn0037498-FBgn0051496 (3R) | -1.882709 |
| 2234 | FBgn0051644-FBgn0085410 (2L) | -1.900242 |
| 2235 | FBgn0036411-FBgn0042630 (3L) | -1.906746 |
| 2236 | FBgn0035099-FBgn0035101 (3L) | -1.933928 |
| 2237 | FBgn0035247-FBgn0013753 (3L) | -1.969521 |
| 2238 | FBgn0035162-FBgn0035164 (3L) | -2.083970 |
| 2239 | FBgn0026411-FBgn0030065 (X) | -2.086859 |
| 2240 | FBgn0035412-FBgn0035413 (3L) | -2.104927 |
| 2241 | FBgn0031460-FBgn0004242 (2L) | -2.234514 |
| 2242 | FBgn0051607-FBgn0051900 (2L) | -2.239295 |
| 2243 | FBgn0035959-FBgn0035960 (3L) | -2.271390 |
| 2244 | FBgn0039741-FBgn0039743 (3R) | -2.291769 |
| 2245 | FBgn0035199-FBgn0052333 (3L) | -2.301568 |
| 2246 | FBgn0039656-FBgn0051427 (3R) | -2.351001 |
| 2247 | FBgn0028418-FBgn0040318 (3L) | -2.385534 |
| 2248 | FBgn0034814-FBgn0003062 (2R) | -2.521287 |
| 2249 | FBgn0053966-FBgn0035192 (3L) | -2.522785 |
| 2250 | FBgn0085436-FBgn0033427 (2R) | -2.542185 |
| 2251 | FBgn0035942-FBgn0035943 (3L) | -2.614340 |
| 2252 | FBgn0031149-FBgn0052512 (X) | -2.660765 |
| 2253 | FBgn0033215-FBgn0033216 (2R) | -2.861189 |
| 2254 | FBgn0036205-FBgn0015278 (3L) | -2.901742 |
| 2255 | FBgn0000278-FBgn0000279 (3R) | -2.914951 |
| 2256 | FBgn0003210-FBgn0029709 (X) | -2.956964 |
| 2257 | FBgn0035760-FBgn0035761 (3L) | -3.021132 |
| 2258 | FBgn0010488-FBgn0033781 (2R) | -3.030424 |
| 2259 | FBgn0035813-FBgn0035816 (3L) | -3.183952 |
| 2260 | FBgn0023083-FBgn0038627 (3R) | -3.275582 |
| 2261 | FBgn0032801-FBgn0053116 (2L) | -3.281552 |
| 2262 | FBgn0027608-FBgn0037376 (3R) | -3.336959 |
| 2263 | FBgn0037743-FBgn0028401 (3R) | -3.439883 |
| 2264 | FBgn0003423-FBgn0001565 (X) | -3.517373 |
| 2265 | FBgn0051140-FBgn0051451 (3R) | -3.693925 |
| 2266 | FBgn0040337-FBgn0026143 (X) | -3.710151 |
| 2267 | FBgn0086711-FBgn0028926 (2L) | -3.729829 |
| 2268 | FBgn0015320-FBgn0032298 (2L) | -3.779393 |
| 2269 | FBgn0051084-FBgn0008649 (3R) | -3.793330 |
| 2270 | FBgn0036766-FBgn0036767 (3L) | -3.817559 |
| 2271 | FBgn0035085-FBgn0027599 (2R) | -3.925970 |
| 2272 | FBgn0035293-FBgn0035294 (3L) | -3.956740 |
| 2273 | FBgn0030097-FBgn0030098 (X) | -3.980444 |
| 2274 | FBgn0085296-FBgn0085298 (3L) | -4.013490 |
| 2275 | FBgn0032749-FBgn0032753 (2L) | -4.049615 |
| 2276 | FBgn0039051-FBgn0039052 (3R) | -4.076188 |
| 2277 | FBgn0036851-FBgn0016797 (3L) | -4.083265 |
| 2278 | FBgn0040964-FBgn0015316 (2L) | -4.129464 |
| 2279 | FBgn0033273-FBgn0011659 (2R) | -4.221978 |
| 2280 | FBgn0028375-FBgn0028894 (2L) | -4.235335 |
| 2281 | FBgn0004587-FBgn0038167 (3R) | -4.235412 |
| 2282 | FBgn0034710-FBgn0034712 (2R) | -4.313081 |
| 2283 | FBgn0032008-FBgn0003502 (2L) | -4.325434 |
| 2284 | FBgn0034631-FBgn0043070 (2R) | -4.344932 |
| 2285 | FBgn0032074-FBgn0032075 (2L) | -4.349852 |
| 2286 | FBgn0052537-FBgn0031010 (X) | -4.368511 |
| 2287 | FBgn0033128-FBgn0033130 (2R) | -4.708093 |
| 2288 | FBgn0000147-FBgn0037970 (3R) | -4.836211 |
| 2289 | FBgn0033882-FBgn0033883 (2R) | -4.875323 |
| 2290 | FBgn0001169-FBgn0015279 (3R) | -5.014270 |
| 2291 | FBgn0043539-FBgn0053543 (2L) | -5.042877 |
| 2292 | FBgn0031428-FBgn0011818 (2L) | -5.071460 |
| 2293 | FBgn0033210-FBgn0033212 (2R) | -5.118826 |
| 2294 | FBgn0004622-FBgn0039728 (3R) | -5.155385 |
| 2295 | FBgn0025838-FBgn0000092 (X) | -5.224191 |
| 2296 | FBgn0038135-FBgn0038136 (3R) | -5.255851 |
| 2297 | FBgn0037084-FBgn0033584 (2R) | -5.332534 |
| 2298 | FBgn0031715-FBgn0031716 (2L) | -5.365655 |
| 2299 | FBgn0051805-FBgn0032620 (2L) | -5.467675 |
| 2300 | FBgn0053172-FBgn0053173 (X) | -5.497124 |
| 2301 | FBgn0036838-FBgn0002945 (3L) | -5.730448 |
| 2302 | FBgn0030881-FBgn0030882 (X) | -5.739023 |
| 2303 | FBgn0040268-FBgn0032796 (2L) | -5.814626 |
| 2304 | FBgn0034221-FBgn0085221 (2R) | -5.816898 |
| 2305 | FBgn0031749-FBgn0015381 (2L) | -5.833305 |
| 2306 | FBgn0053296-FBgn0031935 (2L) | -5.835042 |
| 2307 | FBgn0032521-FBgn0003983 (2L) | -5.841633 |
| 2308 | FBgn0036552-FBgn0036553 (3L) | -5.947221 |
| 2309 | FBgn0001276-FBgn0024188 (2R) | -5.952162 |
| 2310 | FBgn0022942-FBgn0029720 (X) | -6.382225 |
| 2311 | FBgn0039651-FBgn0051044 (3R) | -6.494076 |
| 2312 | FBgn0036763-FBgn0036764 (3L) | -6.699503 |
| 2313 | FBgn0036861-FBgn0036862 (3L) | -6.709533 |
| 2314 | FBgn0051926-FBgn0031351 (2L) | -6.757892 |
| 2315 | FBgn0032105-FBgn0010314 (2L) | -6.786891 |
| 2316 | FBgn0037059-FBgn0037060 (3L) | -6.862325 |
| 2317 | FBgn0026056-FBgn0046763 (3R) | -7.249936 |
| 2318 | FBgn0029873-FBgn0029874 (X) | -7.268561 |
| 2319 | FBgn0034045-FBgn0034046 (2R) | -7.283069 |
| 2320 | FBgn0010504-FBgn0033269 (2R) | -7.316964 |
| 2321 | FBgn0030391-FBgn0259143 (X) | -7.366365 |
| 2322 | FBgn0014388-FBgn0026259 (3L) | -7.466678 |
| 2323 | FBgn0259220-FBgn0053203 (3R) | -7.473810 |
| 2324 | FBgn0029976-FBgn0029977 (X) | -7.489779 |
| 2325 | FBgn0039886-FBgn0011224 (3R) | -7.548097 |
| 2326 | FBgn0039645-FBgn0039648 (3R) | -7.564158 |
| 2327 | FBgn0035134-FBgn0040291 (3L) | -7.573636 |
| 2328 | FBgn0060296-FBgn0043792 (2R) | -7.592019 |
| 2329 | FBgn0029939-FBgn0029940 (X) | -7.616624 |
| 2330 | FBgn0001203-FBgn0085348 (X) | -7.668809 |
| 2331 | FBgn0035627-FBgn0085447 (3L) | -7.680523 |
| 2332 | FBgn0038387-FBgn0013334 (3R) | -7.751342 |
| 2333 | FBgn0042101-FBgn0037521 (3R) | -7.826948 |
| 2334 | FBgn0066365-FBgn0052251 (3L) | -7.934714 |
| 2335 | FBgn0046689-FBgn0038827 (3R) | -7.990804 |
| 2336 | FBgn0023550-FBgn0053548 (X) | -8.089746 |
| 2337 | FBgn0031257-FBgn0031258 (2L) | -8.145153 |
| 2338 | FBgn0015925-FBgn0001308 (2R) | -8.157776 |
| 2339 | FBgn0034416-FBgn0050125 (2R) | -8.172637 |
| 2340 | FBgn0036960-FBgn0004366 (3L) | -8.197120 |
| 2341 | FBgn0036093-FBgn0036094 (3L) | -8.238945 |
| 2342 | FBgn0051823-FBgn0051821 (2L) | -8.242745 |
| 2343 | FBgn0030605-FBgn0030606 (X) | -8.265751 |
| 2344 | FBgn0039703-FBgn0039704 (3R) | -8.296530 |
| 2345 | FBgn0035469-FBgn0035470 (3L) | -8.313857 |
| 2346 | FBgn0034135-FBgn0034136 (2R) | -8.352727 |
| 2347 | FBgn0027529-FBgn0034500 (2R) | -8.373102 |
| 2348 | FBgn0038753-FBgn0021776 (3R) | -8.443410 |
| 2349 | FBgn0034225-FBgn0050104 (2R) | -8.480640 |
| 2350 | FBgn0041160-FBgn0010105 (3L) | -8.490304 |
| 2351 | FBgn0040089-FBgn0031064 (X) | -8.542254 |
| 2352 | FBgn0037895-FBgn0037896 (3R) | -8.542696 |
| 2353 | FBgn0040346-FBgn0040347 (X) | -8.613260 |
| 2354 | FBgn0035131-FBgn0035132 (3L) | -8.666753 |
| 2355 | FBgn0028984-FBgn0038299 (3R) | -8.677146 |
| 2356 | FBgn0035906-FBgn0035911 (3L) | -8.763754 |
| 2357 | FBgn0000377-FBgn0029608 (X) | -8.772772 |
| 2358 | FBgn0010768-FBgn0038685 (3R) | -8.785765 |
| 2359 | FBgn0036428-FBgn0054039 (3L) | -8.787966 |
| 2360 | FBgn0039360-FBgn0051092 (3R) | -8.812485 |
| 2361 | FBgn0038398-FBgn0026059 (3R) | -8.876834 |
| 2362 | FBgn0038702-FBgn0051244 (3R) | -8.964780 |
| 2363 | FBgn0001337-FBgn0029522 (X) | -8.969428 |
| 2364 | FBgn0031677-FBgn0031678 (2L) | -9.122257 |
| 2365 | FBgn0031939-FBgn0031940 (2L) | -9.129535 |
| 2366 | FBgn0034883-FBgn0034884 (2R) | -9.271690 |
| 2367 | FBgn0030268-FBgn0030269 (X) | -9.342662 |
| 2368 | FBgn0003886-FBgn0037736 (3R) | -9.380427 |
| 2369 | FBgn0029834-FBgn0029835 (X) | -9.668007 |
| 2370 | FBgn0031070-FBgn0259789 (X) | -9.682256 |
| 2371 | FBgn0004919-FBgn0003715 (2R) | -9.718781 |
| 2372 | FBgn0022702-FBgn0035268 (3L) | -9.763920 |
| 2373 | FBgn0033347-FBgn0033348 (2R) | -9.919699 |
| 2374 | FBgn0039519-FBgn0039520 (3R) | -9.922911 |
| 2375 | FBgn0053513-FBgn0025393 (X) | -9.958606 |
| 2376 | FBgn0025360-FBgn0040778 (2R) | -10.086220 |
| 2377 | FBgn0028945-FBgn0028518 (2L) | -10.130920 |
| 2378 | FBgn0041111-FBgn0010263 (2L) | -10.187675 |
| 2379 | FBgn0033948-FBgn0033949 (2R) | -10.195480 |
| 2380 | FBgn0031558-FBgn0031559 (2L) | -10.265182 |
| 2381 | FBgn0036031-FBgn0036032 (3L) | -10.290771 |
| 2382 | FBgn0063494-FBgn0063491 (2R) | -10.359598 |
| 2383 | FBgn0052311-FBgn0035308 (3L) | -10.393705 |
| 2384 | FBgn0031414-FBgn0031413 (2L) | -10.469117 |
| 2385 | FBgn0034956-FBgn0034957 (2R) | -10.515269 |
| 2386 | FBgn0013767-FBgn0038223 (3R) | -10.572442 |
| 2387 | FBgn0051229-FBgn0038638 (3R) | -10.583571 |
| 2388 | FBgn0053493-FBgn0036101 (3L) | -10.585661 |
| 2389 | FBgn0035358-FBgn0035359 (3L) | -10.596622 |
| 2390 | FBgn0033428-FBgn0033429 (2R) | -10.642574 |
| 2391 | FBgn0028497-FBgn0034859 (2R) | -10.643113 |
| 2392 | FBgn0038851-FBgn0038852 (3R) | -10.666604 |
| 2393 | FBgn0028523-FBgn0020414 (2L) | -10.718955 |
| 2394 | FBgn0036479-FBgn0014002 (3L) | -10.784340 |
| 2395 | FBgn0039238-FBgn0013949 (3R) | -11.207319 |
| 2396 | FBgn0040623-FBgn0039665 (3R) | -11.226082 |
| 2397 | FBgn0011236-FBgn0025336 (2R) | -12.627962 |
| 2398 | FBgn0029839-FBgn0029843 (X) | -15.357274 |
| 2399 | FBgn0038090-FBgn0038097 (3R) | -15.789119 |
| 2400 | FBgn0034602-FBgn0027073 (2R) | -16.154816 |
| 2401 | FBgn0037797-FBgn0037802 (3R) | -16.434791 |
| 2402 | FBgn0037697-FBgn0043791 (3R) | -16.555707 |
| 2403 | FBgn0046258-FBgn0039540 (3R) | -17.174946 |
| 2404 | FBgn0039483-FBgn0039485 (3R) | -17.409087 |
| 2405 | FBgn0011227-FBgn0086904 (2R) | -17.878196 |
| 2406 | FBgn0032851-FBgn0040996 (2L) | -18.630042 |
| 2407 | FBgn0038341-FBgn0019940 (3R) | -19.663430 |
| 2408 | FBgn0034092-FBgn0034095 (2R) | -21.023723 |
| 2409 | FBgn0033153-FBgn0033155 (2R) | -21.227495 |
| 2410 | FBgn0033935-FBgn0013725 (2R) | -21.586055 |
| 2411 | FBgn0031762-FBgn0031765 (2L) | -22.459147 |
| 2412 | FBgn0051102-FBgn0051436 (3R) | -23.058502 |
| 2413 | FBgn0036969-FBgn0036970 (3L) | -23.476857 |
| 2414 | FBgn0004638-FBgn0024556 (2R) | -24.571777 |
| 2415 | FBgn0034961-FBgn0034964 (2R) | -25.126281 |
| 2416 | FBgn0030766-FBgn0000152 (X) | -25.189583 |
| 2417 | FBgn0030628-FBgn0000028 (X) | -26.738685 |
| 2418 | FBgn0052971-FBgn0028531 (2L) | -30.339598 |
| 2419 | FBgn0037129-FBgn0000560 (3L) | -30.981532 |
| 2420 | FBgn0033918-FBgn0028428 (2R) | -31.727577 |
| 2421 | FBgn0038894-FBgn0054034 (3R) | -31.883939 |
| 2422 | FBgn0036626-FBgn0036628 (3L) | -32.902466 |
| 2423 | FBgn0023541-FBgn0015036 (X) | -33.135237 |
| 2424 | FBgn0053527-FBgn0035031 (2R) | -33.748757 |
| 2425 | FBgn0037660-FBgn0003165 (3R) | -33.825706 |
| 2426 | FBgn0039472-FBgn0039474 (3R) | -35.132885 |
| 2427 | FBgn0005198-FBgn0036942 (3L) | -36.476924 |
| 2428 | FBgn0011824-FBgn0002736 (2R) | -38.485381 |
| 2429 | FBgn0033554-FBgn0033557 (2R) | -38.944656 |
| 2430 | FBgn0030995-FBgn0027603 (X) | -39.274723 |
| 2431 | FBgn0038654-FBgn0038656 (3R) | -43.858383 |
| 2432 | FBgn0039104-FBgn0047351 (3R) | -46.683186 |
| 2433 | FBgn0036789-FBgn0036790 (3L) | -48.798012 |
| 2434 | FBgn0031337-FBgn0051928 (2L) | -55.416141 |
| 2435 | FBgn0004101-FBgn0035001 (2R) | -60.686349 |
| 2436 | FBgn0040298-FBgn0086694 (3L) | -73.831776 |
| 2437 | FBgn0031968-FBgn0031969 (2L) | -77.334679 |
| 2438 | FBgn0032140-FBgn0022720 (2L) | -80.785021 |
| 2439 | FBgn0038447-FBgn0038448 (3R) | -84.466413 |
| 2440 | FBgn0052369-FBgn0024187 (3L) | -101.724631 |
| 2441 | FBgn0024288-FBgn0004389 (3R) | -125.887856 |
| 2442 | FBgn0033182-FBgn0033184 (2R) | -126.745074 |
